# Regional gene expression patterns are associated with task‐specific brain activation during reward and emotion processing measured with functional MRI

**DOI:** 10.1002/hbm.26001

**Published:** 2022-07-07

**Authors:** Arkadiusz Komorowski, Matej Murgaš, Ramon Vidal, Aditya Singh, Gregor Gryglewski, Siegfried Kasper, Jens Wiltfang, Rupert Lanzenberger, Roberto Goya‐Maldonado

**Affiliations:** ^1^ Department of Psychiatry and Psychotherapy, Comprehensive Center for Clinical Neurosciences and Mental Health (C3NMH) Medical University of Vienna Vienna; ^2^ Max Delbrück Center for Molecular Medicine Berlin Germany; ^3^ Laboratory of Systems Neuroscience and Imaging in Psychiatry (SNIP‐Lab), Department of Psychiatry and Psychotherapy, University Medical Center Goettingen (UMG) Georg‐August University Goettingen Germany; ^4^ Child Study Center Yale University New Haven Connecticut USA; ^5^ Center for Brain Research Medical University of Vienna Vienna Austria; ^6^ Department of Psychiatry and Psychotherapy University Medical Center Goettingen (UMG), Georg‐August University Goettingen Germany; ^7^ German Center for Neurodegenerative Diseases (DZNE) Goettingen Germany; ^8^ Neurosciences and Signalling Group, Institute of Biomedicine (iBiMED), Department of Medical Sciences University of Aveiro Aveiro Portugal

**Keywords:** depression, emotions, fMRI, mRNA, reward

## Abstract

The exploration of the spatial relationship between gene expression profiles and task‐evoked response patterns known to be altered in neuropsychiatric disorders, for example depression, can guide the development of more targeted therapies. Here, we estimated the correlation between human transcriptome data and two different brain activation maps measured with functional magnetic resonance imaging (fMRI) in healthy subjects. Whole‐brain activation patterns evoked during an emotional face recognition task were associated with topological mRNA expression of genes involved in cellular transport. In contrast, fMRI activation patterns related to the acceptance of monetary rewards were associated with genes implicated in cellular localization processes, metabolism, translation, and synapse regulation. An overlap of these genes with risk genes from major depressive disorder genome‐wide association studies revealed the involvement of the master regulators TCF4 and PAX6 in emotion and reward processing. Overall, the identification of stable relationships between spatial gene expression profiles and fMRI data may reshape the prospects for imaging transcriptomics studies.

## Graphical abstract

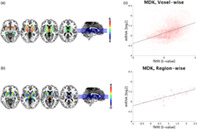

## 1 INTRODUCTION

Over the last decades, genetic and neuroimaging studies have significantly contributed to current knowledge about human neural functions. While individual neuroscientific methods facilitated the comprehension of physiological processes, as well as pathological alterations in psychiatric disorders, a multimodal integration of large‐scale data has proven to be even more conducive for in‐depth understanding (Kitchen et al., 2014; Medland et al., 2014). In general, neural correlates of common psychological processes can be quantified by functional magnetic resonance imaging (fMRI) with high spatial resolution. The fact that dynamic in vivo signal patterns are to some extent susceptible to genotype variations (Rose & Donohoe, 2013; Wolf et al., 2015) justifies the conceptualization of studies linking data across disparate scales of biological resolution. Albeit investigating the influence of the genome on quantitative traits at a cognition‐relevant timescale, imaging genetics studies mostly ignore transcriptional regulation. In the light of the complexity of the human brain, however, it appears beneficial to integrate the transcriptome within the realms of neuroimaging studies. For instance, post‐mortem gene expression data from the Allen Human Brain Atlas (AHBA) can be applied to investigate the relationship between the transcriptome and protein distribution (Komorowski et al., 2017), brain morphology (Shin et al., 2018), or functional connectivity (Richiardi et al., 2015). Focusing on single genes, previous studies also integrated characteristic imaging findings to assess the influence of static gene expression profiles on neurological and psychiatric disorders (Freeze et al., 2018; Romme et al., 2017). It can be argued that various differentially expressed genes impact on regional fMRI activation due to their underlying biological functions that affect resulting neuronal signaling. In this line, a promising approach to contrast in vivo regional blood oxygenation level dependent (BOLD) signaling and the transcriptome was presented by Fox et al. (2014), but limitations such as a low spatial resolution and absence of potential disease‐related master regulators prevented clinical translation.

From all psychiatric disorders, major depressive disorder (MDD) is now the leading cause of disability worldwide (James et al., 2018). MDD strongly contributes to the overall global burden of disease with an increasing prevalence over the years, whereby additive genetic effects attribute to approximately 9% of the variation in liability of this disorder (Wray et al., 2018). Notably, the differential expression of genes between depressed individuals and the general population highlights the relevance of specific transcriptomic signatures for human brain function (Ciobanu et al., 2016; Mehta et al., 2010). Paradigms examining prominent behavioral elements such as impaired affect modulation or loss of interest and pleasure in common experiences are amongst the best‐established in neuroimaging studies, which justifies their application to investigate core depressive symptoms (Foland‐Ross & Gotlib, 2012). Alterations of BOLD reactivity during processing of negatively valenced information or incentive‐based learning thereby drive the conceptualization of major domains of functioning within the realms of the Research Domain Criteria (RDoC) framework (Sanislow et al., 2019), spanning from a physiological to a more critical pathological state. Consequently, spatial gene expression patterns of disease‐related risk genes may affect different types of psychological processes and corresponding neuronal activation. In addition, a modulatory role of disease‐related master regulators is further assumed due to the notion that BOLD signaling elicited by emotion and reward processing can be altered in depressive patients.

The goal of this study was to explore the spatial relationship between whole‐transcriptome expression maps and specific brain activation patterns measured in healthy human subjects during emotion and reward processing, in order to evaluate associations of task‐based fMRI data with biological processes according to the Gene Ontology (GO) database. In addition, static gene expression profiles of risk genes implicated in MDD were analyzed to assess potential effects of disease‐related genes in imaging transcriptomics studies.

## 2 MATERIALS AND METHODS

### 2.1 Participants

Healthy subjects were recruited from the university environment and gave written informed consent to the study procedures previously approved by the Ethics Committee of the University Medical Center Göttingen. Included participants (aged between 22 and 52 years; *M* = 40.5, SD = 14.37) were of Caucasian European ethnicity and fluent in German language. Exclusion criteria comprised contraindications to MRI, past or present psychiatric, neurological, or medical disorders, consumption of psychotropic drugs, and a positive family history of psychiatric disorders. In total, 26 men and 22 women completed two fMRI paradigms related to emotional face recognition and reward processing. Excessive movement in any of the three translation (>2 mm) or rotation (>2°) planes resulted in exclusion of four participants.

### 2.2 Functional brain imaging

Functional imaging data was acquired using a 3 T scanner (Siemens Magnetom TRIO, Siemens Healthcare, Erlangen, Germany) and a 32‐channel head coil with a 2 × 2 × 2 mm voxel size, TR 2500 ms, TE 33 ms, 70° flip angle, 10% distance factor, FOV 256 mm and 60 slices with multiband factor of 3 for the acquisition of T2*‐weighted images. Imaging data analysis was performed using Statistical Parameter Mapping (SPM12; Wellcome Department of Imaging Neuroscience, Institute of Neurology, London, UK) and Matlab R2015b (The Mathworks Inc., Natick, MA). First, echo planar imaging (EPI) images were standardly preprocessed with slice time correction, realignment, and normalization into the Montreal Neurological Institute (MNI) space, as well as smoothing with an 8 × 8 × 8 mm FWHM Gaussian kernel.

Null hypotheses relating to random fMRI activation were tested for both imaging paradigms. First, specific activation maps reflected brain activation during performance of the tasks. In contrast, corresponding control conditions represented non‐specific hemodynamic activity inherent to any task performance during fMRI measurement, caused by unspecific physiological activation, for example, related to visual, auditory, attentional or motor functions. Estimates of task‐evoked response patterns were initially computed with a general linear model (GLM) for each subject individually (first‐level analysis) with nuisance movement parameters regressed as covariates‐of‐no‐interest. Later, experimental and control conditions were evaluated at group level (second‐level analysis) and resulting activation maps that represented task‐specific brain activation were used for further analyses.

### 2.3 Emotional face recognition

The paradigm of implicit emotional face recognition contained two different contexts: human faces and geometric objects. Pictures of males and females with negative face expressions obtained from the Radboud database (Langner et al., 2010) were presented for 17 s, during which participants responded to the gender of the presented person with a button press. Thereby, perception of emotions was rather implicit, which has been shown to enhance the activation of emotional correlates (Keightley et al., 2003). For the control condition, participants were instructed to respond analogously to the shape of an object, either an ellipse or a rectangle, positioned in the face area and made from scrambling original face trials. All trials were controlled for brightness, contrast, and presented in a very similar composition. The activation patterns representing the experimental and control conditions were computed using first‐level (single‐subject) contrasts of the trials from emotional faces and object blocks, respectively. Resulting data were then used for second‐level (group) analysis, as standardly performed for random effects models.

### 2.4 Reward processing

For this study, a previously established fMRI paradigm was implemented, which has been broadly used to investigate physiological and pathological reward mechanisms (Diekhof & Gruber, 2010; Goya‐Maldonado et al., 2015). Briefly, participants performed a modified delayed match to sample task, including two contexts involving previously conditioned stimuli to monetary rewards: acceptance or rejection of rewards, that is, pressing a button when squares are shown. Subjects were instructed they would receive 30€ for their participation and that they were able to double this amount according to their task performance. During the control trials, subjects responded with a button press to stimuli that required motor performance as well as attentional and memory resources, but were not conditioned to monetary reward. To compute the control condition, first‐level (single‐subject) contrasts of correctly matched sample trials within the same experimental block of reward trials were used. For experimental conditions, first‐level experimental contrasts were calculated from brain activation elicited during acceptance of previously conditioned stimuli. At group‐level, activations related to experimental and control trials were contrasted to obtain functional activation patterns.

### 2.5 Meta‐analytic functional brain activation

Besides fMRI data obtained from participants performing two different tasks at our institution, we evaluated large‐scale meta‐analytic imaging data from the Neurosynth platform (https://neurosynth.org/), which provides probabilistic brain activation maps computed from an automated meta‐analysis based on published fMRI studies. This online database combines text‐mining and machine‐learning techniques to generate statistical inference maps of currently 1335 imaging terms from 14,371 fMRI studies including male and female participants (Yarkoni et al., 2011). Within the framework of the Neurosynth database, particular psychological processes are labeled with terms of interest and represented by uniformity test maps. For this study, whole‐brain activation maps were downloaded in MNI152 space with 2 mm resolution to validate fMRI data obtained at our institution. The Neurosynth maps depicted specific brain areas that were consistently reported in studies investigating fMRI activation for emotion (“fearful faces”) and reward (“rewards”) processing, respectively.

**FIGURE 1:**
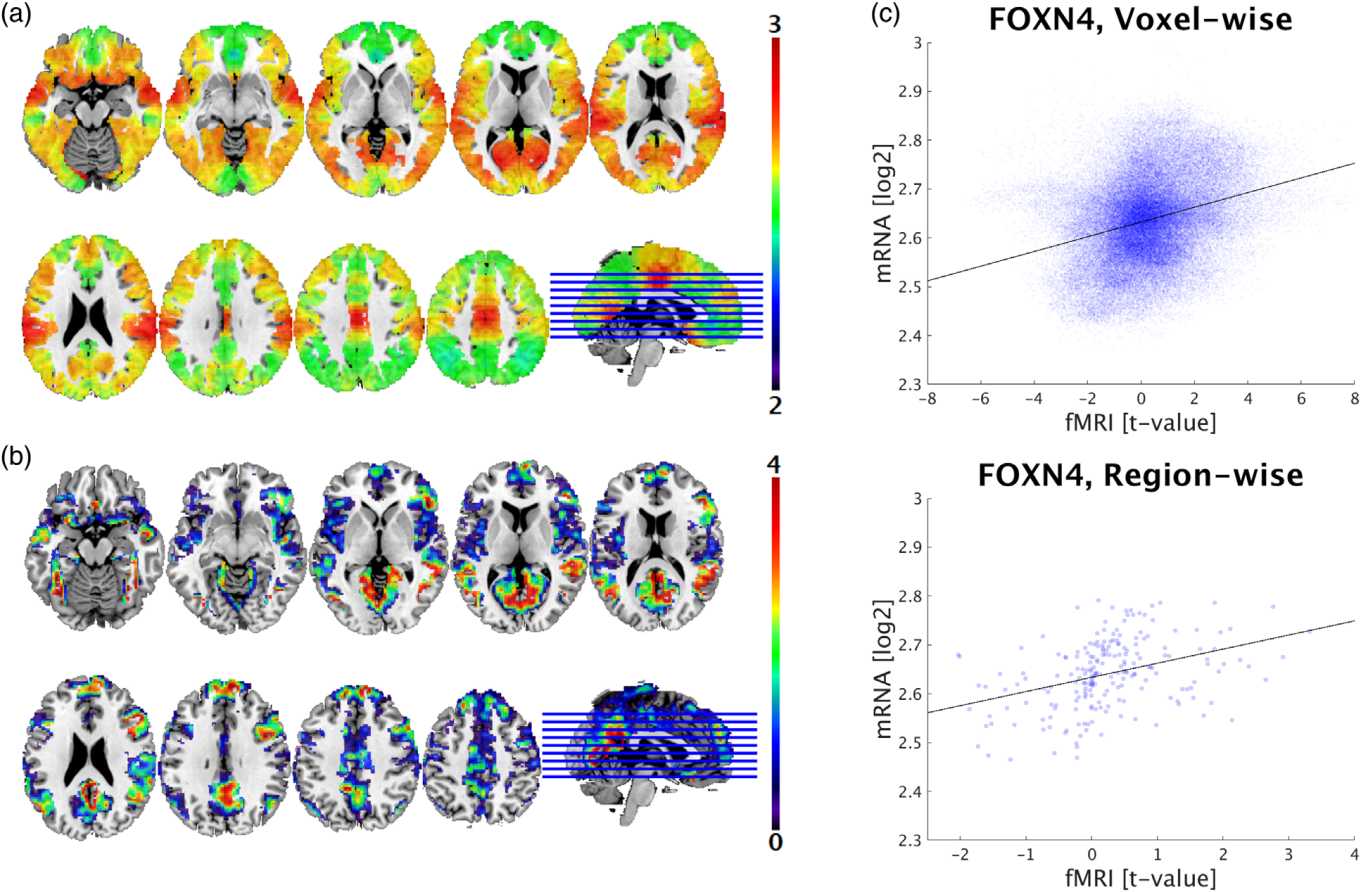
Comparison of task‐specific functional brain activation and mRNA expression in cortical regions. (a) Single‐site functional magnetic resonance imaging data (*t*‐value) during emotion processing is visualized in MNI space (activation maps are thresholded above 0 for visualization purposes only). (b) FOXN4 gene expression is based on cortical transcriptome maps (log_2_) by Gryglewski et al. (2018). (c) The scatter plots depict correlations between cortical mRNA levels of FOXN4 and imaging data (emotional face recognition) for voxel‐wise (rho = 0.286; 129,817 voxels) and region‐wise (rho = 0.429; 210 regions; *p*_cor_ <.001) analyses. Each dot represents expression values and corresponding imaging parameters at target coordinates or within anatomical regions, respectively

In a first step, activation maps, with *z*‐scores representing the results from studies that related to the chosen term, were generated from the Neurosynth online database. Since the online user interface only provides thresholded maps, we used the Neurosynth toolbox (https://github.com/neurosynth/neurosynth) for python to download unthresholded maps that were further smoothed using 8 × 8 × 8 mm FWHM to match the kernel size of single‐site data. Assessment of conformity between meta‐analytic data and measured fMRI maps was performed qualitatively (Figures 1a, b and S1a,b). The Neurosynth maps matched well with task‐evoked response patterns related to recognition of negative faces and acceptance of monetary rewards, respectively. In contrast to single‐site fMRI data, meta‐analytic information comprised rather positive values due to the sparse reporting of brain regions showing negative activation in most neuroimaging studies. Hence, when processing unthresholded data from the uniformity test maps, mainly positive values determined the association analysis between single‐site and Neurosynth data (Figure S1a,b).

### 2.6 Whole‐brain gene expression

The AHBA (human.brain-map.org) consists of microarray assessments from 3702 brain tissue samples collected across six human donors (one female, mean age = 42.5, SD = 13.4) derived from diverse regions of the brain, extensively described in the original publications (Hawrylycz et al., 2012; Shen et al., 2012). While normalization for inter‐individual differences between donor brains was applied, sex‐specific differences were not considered due to the limited availability of female transcriptome data. Interpolated high‐resolution transcriptome maps were obtained from a publicly accessible database for all analyses (available for download at www.meduniwien.ac.at/neuroimaging/mRNA.html). The methodology regarding the creation of these maps was described extensively by Gryglewski et al. (2018). Briefly, seamless gene expression values (log_2_) were predicted by the authors across the whole brain to compensate for the sparse anatomical sampling of the AHBA. Using Gaussian process regression, missing expression values were inferred at cortical, subcortical, and cerebellar structures for all available genes. Compliant with data processing steps recommended by Arnatkevic̆iūtė et al. (2019), probe selection and sample assignment were performed to enable correlation analyses between gene expression and neuroimaging data. Probes with a mean signal indistinguishable from the background noise were excluded and representative probes for each gene were selected based on their autocorrelation as well as relative structured variability assessed with variogram modelling. Signal intensity normalization of gene expression was performed for each donor brain of the AHBA to correct for batch‐effects and inter‐individual variation. Additionally, expression values from both hemispheres were mirrored to generate bilaterally symmetric data, which attenuated sampling bias. The interpolated expression maps were associated with Entrez Gene IDs and registered to MNI space. In contrast to the original data including 18,686 genes (Gryglewski et al., 2018), multiple genes were excluded in this study as their annotations were removed from the NCBI database in the meantime, which resulted in a reduced number of 18,179 transcriptome maps.

### 2.7 Spatial correlation between gene expression and brain activity

All available transcriptome maps were aligned with fMRI activation maps in order to conduct region‐wise association analyses for each paradigm and Neurosynth term, respectively. Since conventional parametric tests fail to account for the autocorrelated properties of structural and functional brain maps, spatially constrained null models were applied according to current standards (Markello & Misic, 2021). Compiled gene lists included Spearman's correlation coefficients to account for partly non‐symmetrical distribution of mRNA data and existence of outliers, whereby the ranking of each gene depended on its correlation strength with task‐specific BOLD signaling. Complementary, voxel‐wise analyses were performed (total number of voxels was 129,817 in the cortex and 10,863 in subcortex; zero values outside of the investigated area were excluded).

On the basis of known differences in gene expression between broad anatomical areas (Chen et al., 2016; Hawrylycz et al., 2012), correlations were evaluated within cortical and subcortical regions, separately (Figure S2a,b). After initial inspection, the cerebellum was excluded from further analysis due to marginal activation during both fMRI paradigms. Each statistical map was parcellated according to the Brainnetome atlas, because it labels a sufficient number of subcortical (*n* = 36) and cortical (*n* = 210) regions‐of‐interest (ROIs; Fan et al., 2016). An additional analysis with fewer brain regions (12 subcortical and 78 cortical ROIs) was done using the automated anatomical labeling (AAL) brain atlas (Tzourio‐Mazoyer et al., 2002) to evaluate influences of different parcellation methods. Both atlases were aligned with fMRI and transcriptome data in MNI space using SPM12, while extraction of ROIs and correlation analyses were performed in MATLAB2018a (www.mathworks.com). Region‐wise correlations were assessed using the generative null modeling framework presented by Burt et al. (2020), because it can be applied to cortical and subcortical data. For each gene, 1000 parcellated spatial autocorrelation‐preserving surrogate transcriptome maps were generated in the cortex (keeping 20 nearest neighbors) and subcortex (keeping 5 nearest neighbors). Two‐sided *p* values were defined as the proportion of surrogate maps, where the absolute value of correlation was greater than the true correlation coefficient (empirical mRNA data).

### 2.8 Identification of overlap between analyzed datasets

To compare various sets of mRNA–fMRI correlations we used Rank–Rank Hypergeometric Overlap (RRHO) package (version 1.26.0) in R (https://www.bioconductor.org/packages/release/bioc/html/RRHO.html), which allows statistical testing of the extent of overlap between two ranked lists. RRHO determines the degree of differential expression observed in profiling experiments using the hypergeometric distribution. While originally applied for the comparison of expression profiles between different microarray platforms, we used RRHO to compare gene lists ranked according to relevant measures of differential information (in this case the correlation strength with fMRI data). The similarity of two datasets was assessed by means of a matrix where the indices related to the ranks in each list. We provide both a graphical representation of the characteristics of analyzed data as well as a statistical measure of overlap (rho_RRHO_). The color scale depicts the degree of statistically significant overlap (log_10_‐transformed *p* values) between two gene lists from that point on the graphical map to the bottom left corner. Applying this method offered the advantage of using the whole continuum of previously ranked genes for data visualization.

### 2.9 Analysis of biological processes

Making use of the GO knowledgebase (Ashburner et al., 2000; The Gene Ontology Consortium, 2018), we explored associations of functional gene categories with single‐site imaging data. Given that only larger biological programs generally reflect selected effect functionality, categories referring to biological processes were included in this study. Enriched GO terms including annotated genes were identified by means of gene‐category enrichment analysis (GCEA). Gene expression patterns that were strongly correlated (region‐wise) with fMRI maps yielded high gene scores according to gene‐score resampling (Gillis et al., 2010). To minimize non‐specific spatial effects related to gene expression differences between broad anatomical areas, GCEA was performed separately in cortical and subcortical regions. The Matlab toolbox presented by Fulcher et al. (2021) (available at https://github.com/benfulcher/GeneCategoryEnrichmentAnalysis) was used to compute ensemble‐based nulls (default parameters). GCEA comprised spatial autocorrelation‐preserving brain maps and all GO terms that were available at the time of analysis (28,428 biological process terms; February 2, 2022). Values of *p* were estimated for categories with 5–200 gene annotations according to a permutation‐based approach and adjusted for multiple testing by applying the Benjamini–Hochberg procedure (*p*_cor_).

### 2.10 Association of Risk Genes Implicated in major depression with functional imaging data

Regarding genetic risks for depressive disorders, recently 42 functional and 27 non‐functional risk genes implicated in major depression were identified in a genome‐wide association meta‐analysis, which included 135,458 cases and 344,901 controls (Wray et al., 2018). The role of master regulators among the 42 functional risk genes (Table S1) was evaluated for each measured fMRI paradigm and uniformity test map. The analysis was performed by means of the R package RcisTarget that identifies master regulators over‐represented on diverse types of gene lists (Aibar et al., 2017). Applying a normalized enrichment score (NES) above 3, master regulatory genes and their direct transcriptional targets were identified using motif discovery within each set of correlated genes based on previously ranked lists. We used the cisTarget function with default parameters and the motif annotation “hg19_500bpUpstream_motifRanking_cispbOnly” that contains motifs with a distance up to 500 bp from TSS. Corresponding master regulators for each predicted regulon of the input gene sets were compared with the risk genes implicated in major depression (Wray et al., 2018). All computations were performed separately for positive and negative region‐wise mRNA–fMRI associations above rho = 0.5 and below rho = −0.5, respectively. These thresholds were set to retain solely correlations with a large effect size (Cohen, 1992).

Additionally, distributions of rankings for risk genes implicated in major depression were assessed for each fMRI paradigm by means of gene set enrichment analysis (GSEA; Mootha et al., 2003). Corresponding to a weighted Kolmogorov–Smirnov‐like statistic, the GSEA enrichment score (ES) was calculated by a stepwise increase or decrease of the total sum statistic of a ranked list as described by Subramanian et al. (2005). Here, the implementation in R available in the package clusterProfiler was utilized for the analysis (Yu et al., 2012). Based on initially compiled gene lists ranked by correlation strengths with fMRI data, the position of each MDD risk gene was compared to the position of all other genes. We tested, whether the risk genes were randomly distributed throughout each ranked list or primarily found at the top (showing positive correlations with fMRI data) and bottom (showing negative correlations). Ranked correlation coefficients and their corresponding *p* values were defined as input factors for the GSEA, which required a summarized biological value for each included gene. Risk genes ranked in higher positions contributed more to the resulting significance of the ES than lower ranked genes. The maximum ES (with positive or negative values) represented the maximum deviation from zero, whereby statistical significance was tested against an ES referring to a null distribution of permuted data. Since distribution of rankings was evaluated for only one gene set, adjustment of the estimated significance level (*p* < .05) for multiple hypothesis testing was dispensable.

## 3 RESULTS

### 3.1 Topological specificity of emotion and reward processing

In conjunction with known abnormalities in social interaction and reward responsiveness of patients with depressive disorders, fMRI activation within the social processes and positive valence systems domains of the RDoC framework was evaluated. Unspecific signal variations related to visual, auditory, attentional, and executive processing were minimized by contrasting brain activation elicited by the experimental condition with task control conditions in each participant. Second level analyses provided specific activation patterns elicited by emotion and reward processing (full acquisition and analysis pipeline described in Methods section). Resulting signal clusters above statistical threshold were similar to those identified in fMRI literature (Figure S1 and Table S2). The spatial activation clusters detected at our institution were validated using matching uniformity maps derived from 91 and 246 studies associated with the terms “fearful faces” and “rewards”, respectively. Both meta‐analytic maps were representative of the expected neural correlates elicited by emotion recognition and reward tasks and can be obtained online (https://neurosynth.org).

### 3.2 Associations between the transcriptome and functional brain activation

For each paradigm, correlation analyses yielded spatial associations between functional brain activation and mRNA expression for 18,179 individual genes (rankings of genes as well as corresponding Spearman's correlation coefficients for both datasets are reported in Table S3). Correlation analyses were performed separately for cortical and subcortical regions to prevent bias arising from expression differences between broad anatomical areas. Resulting correlations were highly specific for each paradigm, accounted for by the weak overlap of compiled correlation lists between the two psychological processes, that is, emotion and reward processing (subcortex: rho_RRHO_ = −0.267, *p* < .001; cortex: rho_RRHO_ = 0.063, *p* < .001; Figure S2a,b). Notably, high agreement of ranked mRNA–fMRI correlations between both applied brain parcellation atlases was observed for emotional face recognition (subcortex: rho_RRHO_ = 0.710, *p* < .001; cortex: rho_RRHO_ = 0.830, *p* < .001) and acceptance of monetary rewards (subcortex: rho_RRHO_ = 0.746, *p* < .001; cortex: rho_RRHO_ = 0.914, *p* < .001; Figure S4a–c).

Regarding emotion processing, single‐site brain activity patterns correlated positively as well as negatively with whole‐brain transcriptome maps (Figure 1a,b). Significant region‐wise correlations yielded similar results compared to voxel‐wise analyses, ranging from rho = −0.74 to rho = 0.865 for subcortical and from rho = −0.449 to rho = 0.429 for cortical regions (Figure S5a,b). Due to statistical dependence of spatially autocorrelated data points, *p* values were markedly lower applying the voxel‐wise approach. Out of 18,179 spatial associations between gene expression and brain function the 15 highest positive correlating genes are listed in Table 1 (*p* ≤ .001, corrected for spatial autocorrelation). In subcortical regions, MALL showed the strongest voxel‐wise correlation (rho = 0.635), while C10orf125 showed the highest region‐wise correlation (rho = 0.865). In the cortex, SPDYA yielded strongest voxel‐wise (rho = 0.328) and FOXN4 strongest region‐wise (rho = 0.429) correlation (Figure 1c).

**TABLE 1:** Ranking of Spearman's correlation coefficients for genes with expression patterns showing highest positive associations with single‐site imaging data (emotion processing)

Subcortex				Cortex			
Voxel‐wise correlations		Region‐wise correlations		Voxel‐wise correlations		Region‐wise correlations	
rho	Gene name	rho	Gene name	rho	Gene name	rho	Gene name
**0.635**	**MALL**	0.865	C10orf125	0.328	SPDYA	**0.429**	**FOXN4**
0.619	HRASLS5	0.841	PTRH1	**0.299**	**CCDC62**	0.393	PIK3R6
0.618	FAT4	**0.818**	**AC022098.3**	0.296	PYGO2	0.390	RYBP
0.614	SCARA5	0.817	GHRLOS	0.290	CPZ	0.388	STC1
0.613	LINC00260	0.817	ZNF280C	**0.286**	**FOXN4**	**0.384**	**CCDC62**
0.613	MESP1	0.816	SLC24A4	0.283	FRMD3	0.379	FUBP1
0.605	SCPEP1	0.807	NLE1	0.282	PHOX2B	0.378	SMYD1
0.604	SKAP2	0.806	FUT1	**0.281**	**ATXN10**	0.355	TMPRSS4
0.599	RAB3GAP1	0.803	FBP1	0.276	XAGE3	0.349	DLG3
0.598	CRHBP	0.803	C16orf55	0.273	KIAA1328	0.346	HSPA1A
0.597	POLB	**0.800**	**MALL**	0.273	EGFL6	0.346	CSTA
0.593	LOC642852	0.797	CELSR1	0.273	RNF215	0.343	NR4A2
0.591	SNAP29	0.795	B4GALT5	0.272	ATP6V1D	**0.341**	**ATXN10**
0.591	KIAA0947	0.795	PDK1	0.272	LINC00158	0.339	AC008026.2
**0.589**	**AC022098.3**	0.792	RASGEF1C	0.272	ERC2‐IT1	0.334	C7

*Note*: All listed region‐wise correlation coefficients were significant (*p* values ≤ .001, corrected for spatial autocorrelation). Genes marked in bold ranked within the 15 highest positively correlating genes in both voxel‐wise and region‐wise analyses.

**FIGURE 2:**
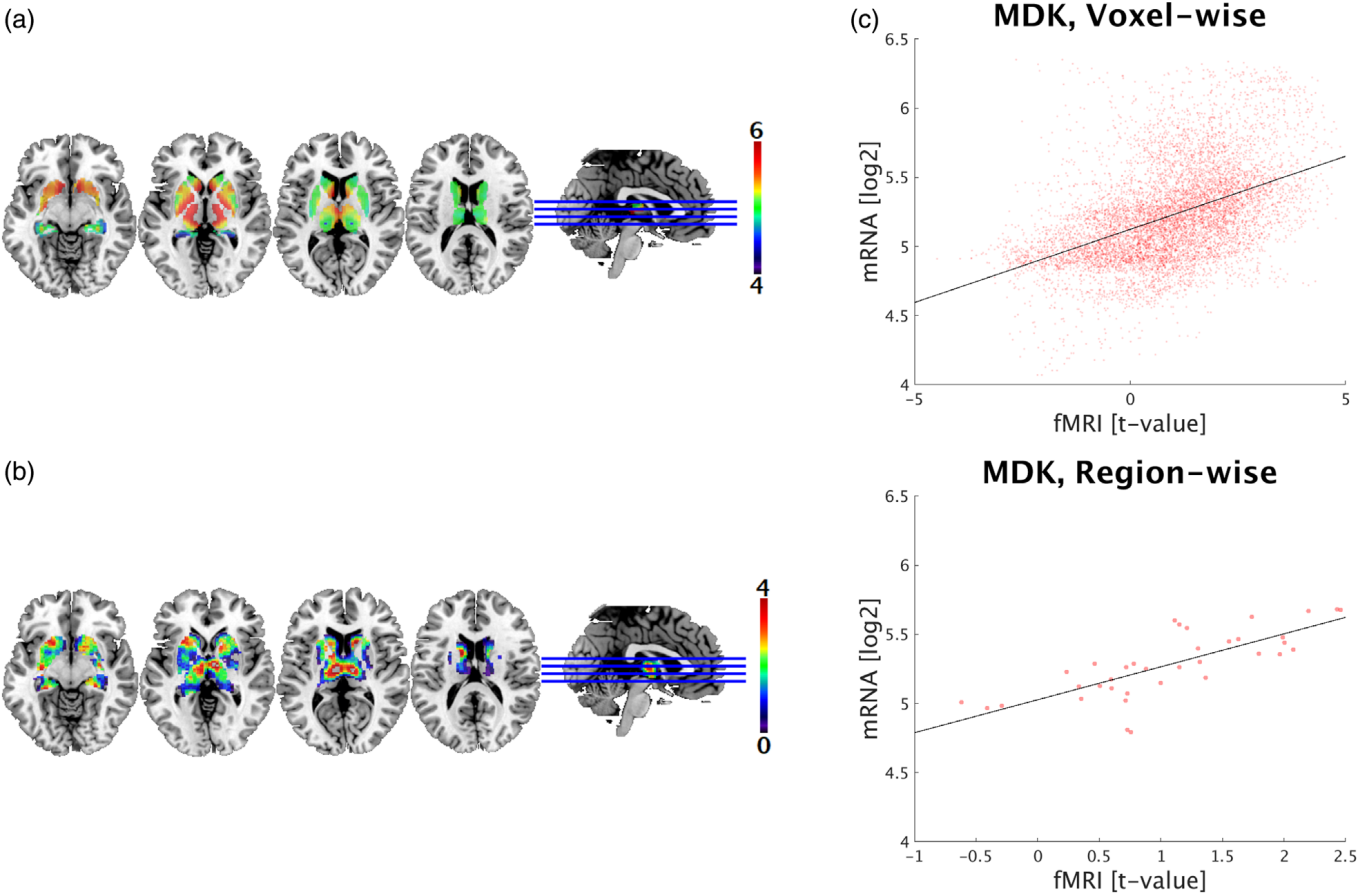
Comparison of task‐specific functional brain activation and mRNA expression in subcortical regions. (a) Single‐site functional magnetic resonance imaging data (*t*‐value) during reward processing is visualized in MNI space (activation maps are thresholded above 0 for visualization purposes only). (b) MDK gene expression is based on subcortical transcriptome maps (log_2_) by Gryglewski et al. (2018). (c) The scatter plots depict correlations between subcortical mRNA levels of MDK and imaging data (acceptance of monetary rewards) for voxel‐wise (rho = 0.49; 10,863 voxels) and region‐wise (rho = 0.803; 36 regions, *p*_cor_ <.001) analyses. Each dot represents expression values and corresponding imaging parameters at target coordinates or within anatomical regions, respectively

Analogous to the emotion task, ranked lists with gene expression patterns spatially associated with measured imaging data were compiled for the reward system, whereby the 15 highest positive correlating genes are listed in Table 2 (*p* < .001, corrected for spatial autocorrelation). Region‐wise analyses yielded higher correlation coefficients than the voxel‐wise approach with less prominent associations in the cortex (rho = −0.639 to rho = 0.698) compared to subcortical regions (rho = −0.793 to rho = 0.811). In the subcortex, MDK showed the strongest voxel‐wise correlation (rho = 0.49) of all 18,179 genes and also a high region‐wise correlation coefficient (rho = 0.803; Figure 2a–c). Comparing strongest voxel‐wise versus region‐wise correlations in the cortex, 11 out of 15 genes were congruent (DUSP3, PIK3CD, CA10, HDAC9, LASS6, GRB14, OLFM3, SHC1, NT5DC2, ASS1, and SPRN), indicating high agreement between both approaches (Figure S6a,b). Notably, gene expression of DUSP3 yielded strongest cortical correlations with reward processing both in the voxel‐wise (rho = 0.549) and region‐wise analysis (rho = 0.698; Figure S7).

**TABLE 2:** Ranking of Spearman's correlation coefficients for genes with expression patterns showing highest positive associations with single‐site imaging data (reward processing)

Subcortex				Cortex			
Voxel‐wise correlations		Region‐wise correlations		Voxel‐wise correlations		Region‐wise correlations	
rho	Gene name	rho	Gene name	rho	Gene name	rho	Gene name
**0.490**	**MDK**	0.811	VMO1	**0.549**	**DUSP3**	**0.698**	**DUSP3**
0.484	HELLS	0.807	OSTM1	**0.548**	**CA10**	**0.681**	**PIK3CD**
0.480	RBBP8	**0.803**	**MDK**	**0.543**	**PIK3CD**	**0.681**	**CA10**
**0.457**	**ATF1**	**0.795**	**KRT18P19**	**0.534**	**GRB14**	**0.653**	**HDAC9**
**0.455**	**KRT18P19**	0.788	NEK1	**0.518**	**ASS1**	**0.640**	**LASS6**
0.453	CD274	**0.785**	**CD99**	**0.517**	**LASS6**	0.639	CCNYL1
0.449	C8orf22	**0.784**	**USP24**	**0.506**	**HDAC9**	**0.636**	**GRB14**
0.446	SALL4	0.784	RCBTB2	0.505	FBXL2	**0.626**	**OLFM**
0.444	SFRP5	0.780	PCBD2	**0.497**	**OLFM3**	**0.608**	**SHC1**
0.443	PMCH	0.777	IMPACT	0.493	TMEM150C	**0.607**	**NT5DC2**
**0.442**	**USP24**	0.773	TRIM34	**0.478**	**SHC1**	0.606	ASS1
**0.440**	**CD99**	0.764	WRB	**0.475**	**SPRN**	0.605	GOLPH3L
0.436	C12ORF75	**0.760**	**ATF1**	**0.474**	**NT5DC2**	0.602	MYBPC2
0.433	TRIM42	0.760	ADAL	0.470	MUM1L1	0.594	MPO
0.433	ELAC1	0.759	ARF4	0.464	ARHGAP8	**0.592**	**SPRN**

*Note*: All listed region‐wise correlation coefficients were significant (*p* values < .001, corrected for spatial autocorrelation). Genes marked in bold ranked within the 15 highest positively correlating genes in both voxel‐wise and region‐wise analyses.

### 3.3 Ontological analysis of task‐specific biological processes

**FIGURE 3:**
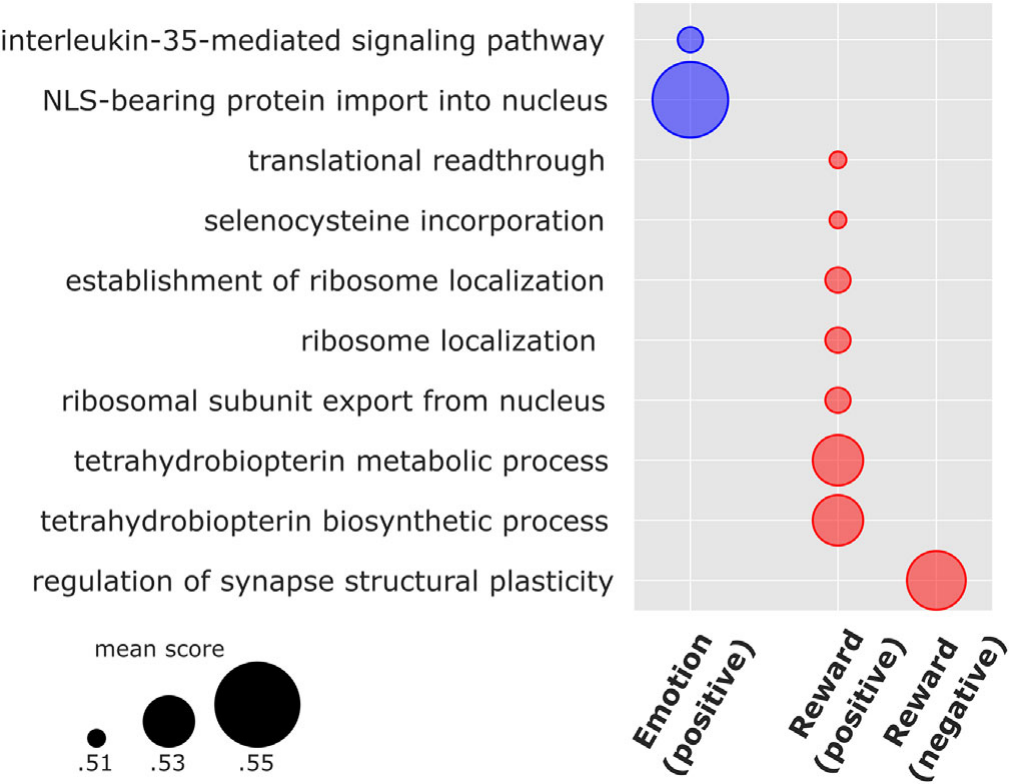
Enriched gene categories for emotion and reward processing based on ontological structure. In the subcortex, multiple specific biological processes (*y*‐axis) were associated with imaging data. Regarding genes with expression patterns positively correlated with emotion processing (blue circles), two biological programs yielded significance (*p*_cor_ <.001). Focusing on the reward system (red circles), altogether seven categories were associated with genes that correlated positively with imaging data (*p*_cor_ <.001). Further, one category was associated with negatively correlated genes (*p*_cor_ <.001). For illustrative purposes, the depicted terms are limited to categories with a mean gene score >0.5

GCEA of previously compiled mRNA‐fMRI correlations revealed multiple associations with gene categories listed in the GO knowledgebase. Several task‐specific biological process terms were enriched in subcortical regions after correction for multiple testing (Figure 3). In contrast, significance levels and associations with imaging data were markedly lower in the cortex. All enriched categories including mean gene scores and *p* values (raw as well as corrected) are listed in Table S4.

Regarding task‐evoked responses during emotional face recognition in subcortical regions, enriched biological programs were related to cellular transport processes and included genes positively correlated with imaging data. Highest gene scores were observed for the GO terms “NLS‐bearing protein import into nucleus” (GO:0006607; 0.55, *p*_cor_ <.001) and “interleukin‐35‐mediated signaling pathway” (GO:0070757; 0.52, *p*_cor_ <.001). In the cortex, GCEA yielded no meaningful associations. Biological programs associated with subcortical brain activation during acceptance of monetary rewards primary affected the regulation of cellular components, metabolism, and translation. Genes positively correlated with imaging data were annotated to the categories “tetrahydrobiopterin biosynthetic process” (GO:0006729, mean gene score: 0.54, *p*_cor_ < .001), “tetrahydrobiopterin metabolic process” (GO:0046146, 0.54, *p*_cor_ < .001), “ribosomal subunit export from nucleus” (GO:0000054, 0.52, *p*_cor_ < .001), “ribosome localization” (GO:0033750, 0.52, *p*_cor_ < .001), “establishment of ribosome localization” (GO:0033753, 0.52, *p*_cor_ < .001), “selenocysteine incorporation” (GO:0001514, 0.51, *p*_cor_ <.001), and “translational readthrough” (GO:0006451, 0.51, *p*_cor_ < .001). GO terms associated with genes negatively correlated with the acceptance of monetary rewards mainly related to synaptic processes, that is, “regulation of synapse structural plasticity” (GO:0051823, 0.54, *p*_cor_ < .001). In cortical regions, no gene category exceeded a mean gene score of 0.5 after correction for multiple testing.

### 3.4 The role of risk genes implicated in major depression

The relationship between task‐evoked response patterns and 42 functional genes associated with MDD obtained from a pre‐defined gene set was investigated to evaluate the superordinate role of these risk genes on BOLD activation. The analysis of transcription factor binding motifs yielded individual candidates regulating genes correlated spatially with imaging data (NES >3; Table 3). Regarding the cortex, two regulators, TCF4 and PAX6, were revealed for reward processing in the single‐site dataset. TCF4 was associated with 12 positively correlated genes (out of 158 correlations with a large effect size) and PAX6 was associated with seven negatively correlated genes (34 possible targets). In the subcortex, TCF4 further regulated positive gene correlations in emotional face recognition (24/707 targets). The fact that TCF4 was associated with both imaging paradigms indicated a rather superordinate role of this master regulator in MDD, regardless of cognitive system (Table S5). While multiple master regulators were also identified in the meta‐analytic dataset, only SOX5 matched with the MDD risk genes presented by Wray et al. (2018). Although single‐site analysis did not yield SOX5, it was associated with genes positively correlated with the Neurosynth term “rewards” (20/332 targets; Table S6).

**TABLE 3:** Master regulators associated with major depression and functional brain imaging

	Emotion processing		Reward processing	
	Negative gene associations	Positive gene associations	Negative gene associations	Positive gene associations
Cortex	–	–	PAX6 (7/34)	TCF4 (12/158)
Subcortex	–	TCF4 (24/707)	–	–

*Note*: Master regulators were evaluated by means of transcription factor binding motifs identified with RcisTarget (Aibar et al., 2017). Ranked genes that were associated with single‐site activation maps and 42 functional risk genes implicated in major depression were used as input data. Values in parenthesis represent the number of targets of each regulatory gene and the total number of possible targets.

Complementing the findings from gene ontology and master regulator analyses, GSEA assessed the distribution of rankings for genes associated with MDD. For both imaging paradigms, aggregation of the risk genes within positively or negatively correlated genes were insignificant. Regarding emotion processing in the cortex, a larger portion of the genes showed positive correlations (maximum ES: 0.284, *p* = .159). In contrast, more risk genes were negatively correlated with reward processing (maximum ES: −0.253, *p* = .247; Figure S8). Similarly, the maximum ES yielded 0.285 (*p* = .464) for emotion and −0.201 for reward processing (*p* = .466) in subcortical regions.

## 4 DISCUSSION

### 4.1 Integration of human gene expression and functional imaging data

Here, we applied a comprehensive and integrative methodological approach to investigate the relationship between regional gene expression patterns and macroscopic BOLD responses elicited by emotional face recognition and the acceptance of monetary rewards, under the assumption that strongly correlated genes would coincide with distinct biological programs. Large‐scale screening for spatial associations between mRNA expression and functional brain activation resulted in ranked lists of 18,179 genes positively and negatively correlated with BOLD signaling in healthy subjects. Similar distributions of region‐wise Spearman's correlation coefficients were present for emotion (ranging from rho = −0.74 to rho = 0.865 in subcortical and from rho = −0.449 to rho = 0.429 in cortical areas) and reward processing (from rho = −0.793 to rho = 0.811 in the subcortex and from rho = −0.639 to rho = 0.698 in the cortex). Control conditions were implemented for each paradigm to minimize fMRI activation elicited by superimposed executive functions and to ensure specificity of the performed tasks. Since fMRI contrasts (experimental > control conditions) were positive, negative correlations implied lower gene expression in strongly activated brain regions. A validation sample including over 90 fMRI studies was obtained from the Neurosynth framework, which corroborated the findings originating from single‐site fMRI measurements.

Exploring the GO knowledgebase, we detected a task‐specific enrichment of gene categories related to cellular transport and cellular localization processes, metabolism, as well as translation for emotion and reward processing, respectively. Regarding genes with lower expression values in regions with stronger brain activation during the acceptance of monetary rewards, GCEA yielded an association with synapse regulation. Given the relevance of imaging paradigms examining subthreshold depressive symptoms within the general population (Lewinsohn et al., 2000), MDD risk genes were also analyzed with regard to physiological brain activation. We identified two master regulators associated with MDD and task‐specific functional brain activation, TCF4 and PAX6. While TCF4 emerged as a regulator for genes showing positive correlations with both paradigms, PAX6 was associated solely with reward processing. Given that both genes were previously implicated in MDD highlights their potential relevance for targeted pharmacotherapy in psychiatry.

### 4.2 Implications for imaging Transcriptomics and major depressive disorder

Regarding imaging genetics studies, common candidate genes may exert distinct effects on brain structure or function (Rose & Donohoe, 2013). Since numerous individual and environmental factors interact with potential genotype effects, sufficiently powered sample sizes are required to detect a significant impact on neuroimaging correlates. However, imaging genetics studies commonly neglect transcriptional and post‐transcriptional mechanisms that impact on the actual expression of disorder‐related genes (Maričić et al., 2020). Genetic influences on emotional face recognition or adaptive reward‐based decision‐making are usually evaluated for the mere presence of single gene variants of functional proteins, irrespective of their topological distribution across the brain (Rose & Donohoe, 2013; Wolf et al., 2015). By including whole‐brain gene expression patterns, our approach can be discriminated from previous imaging genetics studies investigating effects of individual disease‐related single‐nucleotide polymorphisms or environmental factors. This study adds up to the wide‐ranging research findings combining topological mRNA expression with neuroimaging properties (Richiardi et al., 2015; Shin et al., 2018), partially offering toolboxes for an integrative data analysis (Fulcher et al., 2021; Rizzo et al., 2016; Unterholzner et al., 2020). We show associations between functional imaging data and biological processes listed within the hierarchically structured GO database, which implies a prominent role of individual gene categories for regional fMRI activation (Ashburner et al., 2000).

Additive genetic effects may attribute to individual imaging phenotypes, which highlights the importance of large‐scale data in systems medicine to resolve unsettled genetic influences on fMRI paradigms (Fabbri et al., 2017). The differentiation between short‐ and long‐term signal variations during emotion and reward processing appears particularly relevant for personalized treatment of depressed patients. Still, topological expression patterns of MDD risk genes and their impact on imaging properties have not yet been investigated. While over 322 million people worldwide suffer from depressive disorders, a number that increased by 18.4% between 2005 and 2015 (James et al., 2018), a significant part of the population is also affected by subthreshold depressive symptoms, potentially originating from different levels of genetic susceptibility in relevant neuronal systems. Our findings endorse the characterization of neuropsychiatric disorders in terms of functions rather than diagnoses, which was recently highlighted within the much‐noticed RDoC framework (Sanislow et al., 2019). In line with the debilitating symptoms of depressive disorders, we investigated paradigms reflecting principal functions of the reward responsiveness construct within the positive valence systems domain and the social communication construct that is part of the systems for social processes domain. Hyper‐ as well as hypoactivations of brain regions involved in the integration of social information together with a reduced reward sensitivity suggest a polygenic nature of depressive symptoms with distinct imaging features (Ghaemi & Vohringer, 2011; Knutson et al., 2008; Luijten et al., 2017).

Generally, identifying a core set of risk genes implicated in subthreshold neuropsychiatric disorders is complicated due to widespread and disease‐specific network interactions. A potential solution to this key challenge in systems biology might be the analysis of master regulatory genes as well as their corresponding regulons (Aibar et al., 2017). Hereof, we present two transcription factors that regulate downstream networks formed by genes strongly correlated with imaging data for two major domains of brain functioning.

### 4.3 Molecular mechanisms associated with neuroimaging properties

Emotion and reward processes have been linked to core symptoms of depression, which provides the basis for studies investigating disease‐related traits in the healthy population. The results from this study support findings in neural tissue, suggesting altered cellular localization processes in anxiety pathways (Panayotis et al., 2018) and molecular associations between synapse regulation and the reward system (Calabresi et al., 2007; Iino et al., 2020). Since enriched biological programs reflect selected effect functions, the identification of associated genes may facilitate a more targeted drug development in the future. However, interpretation of enriched GO categories in both circuits appears complex due to gene–gene interactions and interconnected signaling pathways. Up to now, clinical applications of imaging transcriptomic findings have not been established in psychiatry. In line with recent recommendations for enrichment analyses (Fulcher et al., 2021), we therefore provide the full output of the GCEA as Supporting Information.

Findings from the master regulator analyses affirm previously reported relationships between neuropsychiatric disorders and mutations of TCF4 that have been implicated not only in depression, but also in schizophrenia and autism (Amare et al., 2019; Li et al., 2019). This transcription factor is mainly characterized by its regulatory role for the proliferation and differentiation of neuronal and glial progenitor cells (Ross et al., 2003). Besides impaired emotion processing, mutations in TCF4 may lead to Pitt‐Hopkins syndrome, characterized by intellectual disabilities as well as altered brain morphology (Kirikae et al., 2021; Liu et al., 2018). The regulatory role of TCF4 was further demonstrated in schizophrenia by Torshizi et al. (2019) in two independent datasets by means of transcriptional network analysis. In contrast, literature is sparse for the other master regulatory genes identified in this study. While PAX6 was previously associated with functional brain alterations and deficits in cognitive processing (Berntsson et al., 2020; Grant et al., 2020), SOX5 was not linked with imaging findings yet.

Since the master regulators reported in this study exhibit their effects by numerous molecular mechanisms, a closer investigation of the genes strongly associated with imaging parameters will prospectively allow elaborated statements about regional protein biosynthesis and the allocation of resulting proteins to cellular compartments. For example, DNA‐binding transcription factor FOXN4 (Forkhead Box N4), belonging to the Forkhead Box (FOX) superfamily, showed highest correlation with the emotional face recognition paradigm in the cortex. FOX transcription factors are involved in regulatory biological processes and mutations in forkhead genes have been linked to developmental disorders in humans due to substitutions or frameshifts that disable or remove the DNA binding domain (Carlsson & Mahlapuu, 2002). The subtype FOXN4 thereby expresses developmental functions in neural and non‐neural tissues, particularly during spinal neurogenesis by modulating a specific expression mosaic of other proneural factors (Misra et al., 2014). Further relevance for neural development was shown by Chen et al. (2014), who demonstrated the location of FOXN4 on neurons and astrocytes as well as an increased expression after spinal cord injury lesions. Although associations with depressive or other neuropsychiatric disorders have not been published, the role of FOXN4 as a key transcriptional regulator during developmental processes demands further research, especially since the full set of its targets in the CNS are not known yet. Accordingly, the C10orf125 gene (Fucose Mutarotase, FUOM), expressed in the brain and other tissues, showed highest correlation with emotional face recognition in the subcortex. The corresponding gene transcript, fucose mutarotase, is an enzyme of the fucose‐utilization pathway performing the interconversion between α‐l‐fucose and β‐l‐fucose on human cell surfaces. Hereof, besides one animal study demonstrating male‐like sexual behavior in FUOM knock‐out mice, presumably resulting from reduced fucosylation during neurodevelopment (Park et al., 2010), further associations with pathological states in mammals have not been published for this gene. Regarding reward processing, the protein coding DUSP3 (Dual‐specificity phosphatase 3) gene, member of the dual‐specificity protein phosphatase subfamily, showed strongest correlation with measured fMRI data in the cortex. Members of these protein tyrosine phosphatases (PTPs) regulate the phosphorylation of the mitogen‐activated protein (MAP) kinase signaling pathway and control cell signaling, especially in regard to cytoskeleton reorganization, apoptosis and RNA metabolism (Tonks, 2013). DUSP3 shows a wide expression in different tissues as an opposing factor of protein tyrosine kinases (PTKs) and acts as a central mediator of cellular proliferation and differentiation. Whereas a role in neoplastic disorders, pathologies related to immunology, angiogenesis as well as Parkinson's disease (PD) have been related to anomalous tyrosine phosphorylation (Cohen & Alessi, 2013; Russo et al., 2018), associations with psychiatric disorders have not been described. In subcortical regions, MDK (midkine) was among the highest correlating genes for the reward paradigm. MDK transcribes one of two growth factors from the heparin‐binding cytokine family and plays a role during differentiation of neurons, especially within dopaminergic pathways (Alguacil & Herradón, 2015). Notably, it facilitates neuroprotective effects in neurodegenerative disorders, drug‐induced neurotoxicity in the striatum, or after neural injury (Yoshida et al., 2014). Disease‐related publications suggest accumulation of midkine in senile plaques and increased serum levels in patients with Alzheimer's disease (Salama et al., 2005), genetic variations associated with PD (Prediger et al., 2011), and an influence on addictive behaviors (Gramage et al., 2013). In line with proposed deficits in emotion recognition in autism, elevated serum levels of this neurotrophic factor were also associated with autism spectrum disorder (Esnafoglu & Cirrik, 2018).

### 4.4 Limitations of the study

In general, imaging transcriptomics findings reflect rather subtle genetic influences on psychological processes, disregarding the dynamic nature of short‐term regulatory mechanisms, environmental factors, and individual variations due to genetic ancestry. Human gene expression levels do not necessarily reflect in vivo protein densities (Komorowski et al., 2020) and BOLD signaling can be impaired by confounding variables related to structural and functional imaging measures. Inclusion of meta‐analytic maps may outweigh potential biases originating from small population sizes, however, the Neurosynth framework lacks specificity due to rather broadly defined terms of interest. Lower mRNA‐fMRI correlations in the cortex compared to the subcortex may be ascribed to data analysis in volume space, which is a less precise representation of the cortical sheet than the surface space (Coalson et al., 2018). However, considering strongest activation in ventral striatum, amygdala, ventral tegmental area, fusiform gyri, insula, and medial prefrontal cortex, in that order, it seems plausible that higher correlation levels were observed in subcortical regions. When testing reward responsiveness after acceptance of prior conditioned stimuli, functional activation was likewise more prominent in the subcortex, particularly in the mesolimbic reward system (Goya‐Maldonado et al., 2015).

Further limitations pertain to locally regulated epigenetic and epitranscriptomic modifications that affect actual protein distribution (Maier et al., 2009). Inter‐individual differences related to age, gender, or genotype are disregarded, when performing integrative analyses on the basis of the AHBA that derives expression values from one female and five male post‐mortem brains (Hawrylycz et al., 2012; Shen et al., 2012). The distance‐dependent structure of gene expression data based on only 3702 mRNA samples further complicates association analyses with functional imaging maps. Additionally, outdated annotation information, inaccurate sample assignment, and ubiquitous noise due to expression of genes with a low spatial dependence limit data analysis. These issues were thoroughly addressed by Gryglewski et al. (2018) prior to the creation of interpolated transcriptome maps that allow for voxel‐ and region‐wise integration with other neuroimaging modalities. In contrast to a previous study that identified gene–cognition associations based on the Neurosynth framework (Fox et al., 2014), we increased spatial resolution and advanced probe selection of gene expression data. To correct for potentially inflated *p* values in parcellated brain maps, the open‐access software platform provided by Burt et al. (2020) was utilized. Spatial autocorrelation was reintroduced in randomized gene expression data for all region‐wise analyses to generate surrogate brain maps.

Likewise, associations between imaging data and biological processes are affected by false‐positive rates, limiting overall explanatory power of GCEA. Although Fulcher et al. (2021) acknowledge that the majority of significantly enriched GO terms could be plausibly linked to human brain activation, the authors delineate potential statistical biases due to gene–gene coexpression within certain categories referring to metabolic, neuronal, or generic biological processes. Therefore, *p* values were assessed by means of adjusted null models that randomize imaging rather than gene expression data. Separate analyses in cortical and subcortical brain regions further minimized potential false‐positive rates (Fulcher et al., 2021). In contrast, insignificant results of the GSEA were partly hampered by the small number of included risk genes and the occurrence of master regulators modulating upregulation and downregulation. In line with core symptoms of depression, TCF4 affected subordinate genes positively and negatively correlated with emotional face recognition and PAX6 mainly affected those genes that were negatively correlated with reward processing. Presumably, these master regulators were not present in the Neurosynth dataset due to differing correlation strengths of the analyzed mRNA–fMRI associations. While cortical correlation coefficients for emotion processing were below 0.5 in both datasets, the total number of subcortical above‐threshold correlations varied between single‐site and meta‐analytic data. However, given the exploratory nature of this study, thresholds were kept consistent in all analyses to exclude correlations with low effect sizes (Cohen, 1992). Overall, the GWAS performed by Wray et al. (2018) is among the largest ever conducted in psychiatric genetics and provides a solid basis for research about the genetic architecture of MDD.

Within the framework of future studies, the vast potential of the AHBA might even be increased by re‐assigning available mRNA probes to corresponding genes on the basis of the latest sequencing information to increase the number of specifically annotated genes. Harmonized data processing pipelines and methodological guidelines instead of rather unique approaches to data integration and corresponding statistical measures could further enhance comparability between studies (Arnatkevic̆iūtė et al., 2019; Fornito et al., 2019; Keil et al., 2018; Kim et al., 2014; Markello & Misic, 2021).

## 5 CONCLUSION

This multimodal investigation highlights the advantages of a comprehensive approach to reveal genetic influences on functional brain imaging by integrating imaging and large‐scale transcriptome data with sufficient power. Despite advances and decreased costs of high‐throughput gene expression profiling, the necessity for large cohorts in genetic studies calls for collaborative approaches. In line with recent insights from the RDoC framework, this study highlights the relevance of functional brain activation related to social interaction and the experience of reward for systems medicine. Both, the emotion and the reward system seem to be associated with specific biological programs like cellular transport, cellular localization processes, metabolism, translation, and synapse regulation. The identification of regulatory genes TCF4 and PAX6 thereby implies a potential role of master regulators for functional brain imaging in major depression. Moreover, the analysis of the whole transcriptome provides superior information that are needed for the understanding of neuropsychiatric disorders and targeted pharmacotherapy. This work exemplifies an integrative approach including complementary information from multiscale data, which becomes increasingly relevant in the big data era.

## AUTHOR CONTRIBUTIONS

Roberto Goya‐Maldonado and Rupert Lanzenberger conceived the study; Arkadiusz Komorowski and Roberto Goya‐Maldonado designed the study, primarily interpreted results, and drafted the article; Roberto Goya‐Maldonado, Ramon Vidal, Aditya Singh, Matej Murgaš, and Gregor Gryglewski made significant contributions to data collection, including quality control, data processing, and statistical analysis; Siegfried Kasper and Jens Wiltfang revised the study and contributed to the intellectual content. All authors have critically revised the article and approved it for publication.

## CONFLICT OF INTEREST

Siegfried Kasper received grants/research support, consulting fees and/or honoraria within the last 3 years from Angelini, AOP Orphan Pharmaceuticals AG, Celegne GmbH, Eli Lilly, Janssen‐Cilag Pharma GmbH, KRKA‐Pharma, Lundbeck A/S, Mundipharma, Neuraxpharm, Pfizer, Sanofi, Schwabe, Servier, Shire, Sumitomo Dainippon Pharma Co. Ltd. and Takeda. Rupert Lanzenberger received travel grants and/or conference speaker honoraria within the last 3 years from Bruker BioSpin MR, Heel, and support from Siemens Healthcare regarding clinical research using PET/MR; he is shareholder of BM Health GmbH since 2019. The remaining authors declare no competing interests.

## Supporting information


**FIGURE S1** Meta‐analytical functional magnetic resonance imaging data (*z*‐score) obtained from the Neurosynth database is visualized in MNI space (activation maps are thresholded above 0 for visualization purposes only; Yarkoni et al., 2011). (a) The uniformity test map related to the term “fearful faces” matched with single‐site fMRI data measured during recognition of negative faces. (b) The uniformity test map related to the term “rewards” matched with single‐site fMRI data measured during the acceptance of monetary rewards.
**FIGURE S2**: Gene expression differences in cortical, subcortical, and cerebellar structures for emotion and reward processing. (a) The scatter plots depict voxel‐wise correlations (subcortex: 10,863 voxels, cortex: 129,817 voxels, cerebellum: 24,415 voxels) between whole‐brain transcriptome maps and single‐site imaging data for emotional face recognition (RNF215) and reward processing (ASS1). (b) Histograms show distributions of correlation coefficients of 18,179 genes for region‐wise analyses using the Brainnetome atlas for emotional face recognition and reward processing. Markedly differing expression levels justified separate analyses for each brain structure.
**FIGURE S3**: Rank–rank hypergeometric overlap (RRHO) visual representation of single‐site imaging data for reward versus emotion processing. Genes with low agreement of correlation coefficients between both lists (either positive or negative) show lower statistical significance in the bottom left and top right corner. Region‐wise RRHO comparing ranked lists including 18,179 genes indicated low congruence between both paradigms in (a) subcortical (rho_RRHO_ = −0.267, *p* < .001) and (b) cortical structures (rho_RRHO_ = 0.063, *p* < .001).
**FIGURE S4**: Comparison of single‐site imaging data applying two different parcellation schemes. (a) Visualization of cortical and subcortical regions of interest according to automated anatomical labeling (left) and the Brainnetome atlas (right; transversal plane in MNI standard space; *z* = 8). (b) Region‐wise Rank–rank hypergeometric overlap (RRHO) comparing ranked lists including 18,179 genes indicated high agreement between both atlases for emotional face recognition in the subcortex (rho_RRHO_ = 0.710, *p* < .001) and cortex (rho_RRHO_ = 0.830, *p* < .001). Genes with congruent correlation coefficients (either positive or negative) showed higher statistical significance in the bottom left and top right corner. (c) Likewise, RRHO of both parcellation methods was performed for reward processing in subcortical (rho_RRHO_ = 0.746, *p* < .001) and cortical regions (rho_RRHO_ = 0.914, *p* < .001).
**FIGURE S5**: Voxel‐wise versus region‐wise correlation analyses of single‐site imaging data during emotion processing. (a) Agreement between compiled lists including 18,179 genes was compared by means of rank–rank hypergeometric overlap (RRHO), which indicated a fairly high congruence for the emotional face recognition paradigm. Visual representations of RRHO depict significance of overlap between ranked lists (warmer colors correspond to lower *p* values), comparing the voxel‐wise vs. region‐wise approach for the subcortex (rho_RRHO_ = 0.799, *p* < .001) and the cortex (rho_RRHO_ = 0.736, *p* < .001). (b) Histograms of Spearman's correlation coefficients applying a voxel‐wise as well as a region‐wise approach are provided for subcortical and cortical regions.
**FIGURE S6**: Voxel‐wise versus region‐wise correlation analyses for single‐site imaging data during reward processing. (a) Agreement between compiled lists including 18,179 genes was compared by means of rank–rank hypergeometric overlap (RRHO), which indicated a fairly high congruence for the reward paradigm. Visual representations of RRHO depict significance of overlap between ranked lists (warmer colors correspond to lower *p* values), comparing the voxel‐wise versus region‐wise approach for subcortex (rho_RRHO_ = 0.871, *p* < .001) and cortex (rho_RRHO_ = 0.954, *p* < .001). (b) Histograms of Spearman's correlation coefficients applying a voxel‐wise as well as a region‐wise approach are provided for subcortical and cortical regions.
**FIGURE S7**: Comparison of functional brain activation during reward processing and mRNA expression of DUSP3 in cortical regions. The scatter plots depict correlations between mRNA levels and single‐site imaging data (acceptance of monetary rewards) for voxel‐wise (rho = 0.549; 129,817 voxels) and region‐wise (rho = 0.698; 210 regions, *p*_corr_ <.001) analyses. Each dot represents expression values and corresponding imaging parameters at target coordinates or within anatomical regions, respectively.
**FIGURE S8**: Gene Set Enrichment Analysis (GSEA) for emotion and reward processing, including risk genes implicated in major depression. Vertical lines on the *x*‐axis represent positions of 42 functional risk genes within each ranked list including 18,179 genes; dashed lines mark the locations of the maximum enrichment score (ES). Analyzing single‐site data, GSEA showed an inversed relationship within cortical structures, yielding maximum ES for emotion processing of 0.284 (*p* = .159, blue graph) and − 0.253 for reward processing (*p* = .247, red graph), respectively.
**TABLE S1**: Previously published risk genes associated with major depression. The gene set composed of 69 published functional and non‐functional risk genes; bold names correspond to 42 functional genes that were included for gene set enrichment and master regulator analyses.
**TABLE S2**: Regions showing functional brain activation during emotional face recognition and acceptance of monetary rewards. Single‐site emotion (sad > object) and reward (reward > attention) contrasts are reported at a collection threshold *p* < .001 with *k* > 10.
**TABLE S5**: Relationship between TCF4 target genes and imaging data.

**TABLE S3:** Associations between functional imaging and transcriptome data. Correlation analyses yielded comparable results between single‐site measurements and the Neurosynth uniformity maps “fearful faces” as well as “rewards”. For both datasets, ranked genes with corresponding Spearman's correlation coefficients are reported separately in cortical as well as subcortical brain regions; *p* values adjusted for spatial autocorrelation are provided for region‐wise correlations (excel‐file)

**TABLE S4:** Enriched biological programs for emotion and reward processing based on ontological structure. Specific gene categories listed within the gene ontology (GO) knowledgebase were significantly enriched for genes showing expression patterns strongly correlated with single‐site imaging data in the subcortex. Resulting GO categories and corresponding *p* values are provided for both paradigms (excel‐file)

**TABLE S6**: Master regulators associated with Major Depressive Disorder and task‐specific functional brain activation. Transcription factor binding motifs identified with RcisTarget (Aibar et al., 2017), corresponding target genes, normalized enrichment scores and AUC values are provided for single‐site and meta‐analytical imaging data. (excel‐file)

## Data Availability

The functional imaging and gene expression data generated during the current study, including surrogate transcriptome maps are available from the corresponding author upon reasonable request. The correlation lists generated in this study, the associated biological processes, as well as the analyzed risk genes are included in the supplementary information files. All meta‐analytic imaging datasets analyzed are available in the Neurosynth repository, https://neurosynth.org/; whole‐brain transcriptome maps are available from the Neuroimaging Labs (NIL), www.meduniwien.ac.at/neuroimaging/mRNA.html; and gene ontology data are available from the Gene Ontology Consortium, http://geneontology.org/. The algorithms and codes that were used in this study are available from the corresponding authors upon reasonable request. To process data, licensed software as well as freely accessible web‐based tools were used, whereby the authors presume further availability without restrictions on existing code or algorithm availability in the foreseeable future. The Python‐based open‐access software package, BrainSMASH: Brain Surrogate Maps with Autocorrelated Spatial Heterogeneity (https://github.com/murraylab/brainsmash) was utilized to generate surrogate transcriptome maps (Burt et al., 2020). Ensemble‐based gene set enrichment analysis was performed by means of the Matlab toolbox presented by Fulcher et al. (2021), available at https://github.com/benfulcher/GeneCategoryEnrichmentAnalysis.
