# Supplementary material for: Regional gene expression patterns are associated with task‐specific brain activation during reward and emotion processing measured with functional MRI
Source: Hum Brain Mapp. 2022 Jul 7;43(17):5266–80. doi: 10.1002/hbm.26001 (PMC9812247; doi:10.1002/hbm.26001)
Supplement: Supplementary file 2 — TABLE S3: Associations between functional imaging and transcriptome data. Correlation analyses yielded comparable results between single‐site measurements and the Neurosynth uniformity maps “fearful faces” as well as “rewards”. For both datasets, ranked genes with corresponding Spearman's correlation coefficients are reported separately in cortical as well as subcortical brain regions; p values adjusted for spatial autocorrelation are provided for region‐wise correlations (excel‐file) [file HBM-43-5266-s003.pdf]

Emotion (single-site) Subcortex

voxel-wise, subcortex			
Spearman's corr coef	p-val	GenID	Gene
0.63544184	< 0.001	7851	MALL
0.618690431	< 0.001	117245	HRASLS5
0.618205607	< 0.001	79633	FAT4
0.614138007	< 0.001	286133	SCARA5
0.61343956	< 0.001	84719	LINC00260
0.612739086	< 0.001	55897	MESP1
0.604625404	< 0.001	59342	SCPEP1
0.604032457	< 0.001	8935	SKAP2
0.598935783	< 0.001	22930	RAB3GAP1
0.597995698	< 0.001	1393	CRHBP
0.596665025	< 0.001	5423	POLB
0.592611015	< 0.001	642852	LOC642852
0.591069698	< 0.001	9342	SNAP29
0.59097296	< 0.001	23379	KIAA0947
0.589225054	< 0.001	342979	AC022098.3
0.584952354	< 0.001	54475	NLE1
0.583401978	< 0.001	3957	LGALS2
0.583095253	< 0.001	340419	RSPO2
0.582629979	< 0.001	5136	PDE1A
0.581990659	< 0.001	3990	LIPC
0.581290245	< 0.001	117581	TWIST2
0.58062005	< 0.001	27202	GPR77
0.580504298	< 0.001	253582	C6orf191
0.580268919	< 0.001	902	CCNH
0.578025222	< 0.001	51538	ZCCHC17
0.57534647	< 0.001	56990	CDC42SE2
0.574061334	< 0.001	5733	PTGER3
0.569032371	< 0.001	91663	MYADM
0.568460464	< 0.001	10534	SSSCA1
0.568352282	< 0.001	282969	C10orf125
0.567745388	< 0.001	139221	MUM1L1
0.56400013	< 0.001	342184	FMN1
0.563577592	< 0.001	154075	SAMD3
0.563183486	< 0.001	5734	PTGER4
0.562810957	< 0.001	55253	TYW1
0.559732258	< 0.001	91368	CDKN2AIPNL
0.558957458	< 0.001	26007	DAK
0.558184564	< 0.001	10535	RNASEH2A
0.556302667	< 0.001	5624	PROC
0.555503428	< 0.001	5865	RAB3B
0.555376053	< 0.001	374969	CCDC23
0.555271029	< 0.001	255022	CALHM1
0.554882348	< 0.001	255426	RASGEF1C
0.554464936	< 0.001	23643	LY96
0.553847313	< 0.001	80816	ASXL3
0.553043783	< 0.001	8704	B4GALT2
0.551643729	< 0.001	114798	SLITRK1
0.551535487	< 0.001	64860	ARMCX5
0.551389575	< 0.001	55907	CMAS
0.551014006	< 0.001	29986	SLC39A2
0.550206184	< 0.001	2764	GMFB
0.549906135	< 0.001	338761	C1QL4
0.549808621	< 0.001	81617	CAB39L
0.548884034	< 0.001	10361	NPM2
0.548569322	< 0.001	23151	GRAMD4
0.548013628	< 0.001	54328	GPR173
0.547345102	< 0.001	50810	AC024270.1
0.547110558	< 0.001	6304	SATB1
0.545974195	< 0.001	4861	NPAS1
0.545728087	< 0.001	881	CCIN
0.544784188	< 0.001	5921	RASA1
0.544021904	< 0.001	4707	NDUFB1
0.542921662	< 0.001	7280	TUBB2A
0.541979551	< 0.001	10947	AP3M2
0.541659355	< 0.001	126353	C19orf21
0.541431546	< 0.001	80086	TUBA4B
0.541377485	< 0.001	25809	TTLL1
0.541348994	< 0.001	5961	PRPH2
0.541256249	< 0.001	9759	HDAC4
0.541012406	< 0.001	114801	TMEM200A
0.540145755	< 0.001	6578	SLCO2A1
0.539928615	< 0.001	79931	TNIP3
0.539462566	< 0.001	116113	FOXP4
0.539075613	< 0.001	440895	AC013271.3
0.538272142	< 0.001	65990	FAM173A
0.537954628	< 0.001	123355	LRRC28
0.537194192	< 0.001	9465	AKAP7

region-wise, subcortex					
Spearman's corr coef	p-val	p-val, SA	p-val, SA (BH- correction)	GenID	Gene
0.865122259	1.3834E-09	< 0.001	< 0.001	282969	C10orf125
0.840926588	5.87854E-08	< 0.001	< 0.001	138428	PTRH1
0.818018019	1.40611E-07	< 0.001	< 0.001	342979	AC022098.3
0.817245781	1.43868E-07	< 0.001	< 0.001	100126793	GHRLOS
0.816988409	1.44964E-07	< 0.001	< 0.001	55609	ZNF280C
0.816216171	1.48266E-07	< 0.001	< 0.001	123041	SLC24A4
0.806949794	1.90778E-07	< 0.001	< 0.001	54475	NLE1
0.805920184	1.95851E-07	< 0.001	< 0.001	2523	FUT1
0.803346157	2.0886E-07	< 0.001	< 0.001	2203	FBP1
0.802831411	2.1152E-07	< 0.001	< 0.001	124045	C16orf55
0.799742579	2.27924E-07	< 0.001	< 0.001	7851	MALL
0.797425985	2.40751E-07	< 0.001	< 0.001	9620	CELSR1
0.795366764	2.52558E-07	< 0.001	< 0.001	9334	B4GALT5
0.795109391	2.54061E-07	< 0.001	< 0.001	5163	PDK1
0.791763186	2.74232E-07	< 0.001	< 0.001	255426	RASGEF1C
0.790476203	2.82318E-07	< 0.001	< 0.001	51538	ZCCHC17
0.788674355	2.93972E-07	< 0.001	< 0.001	819	CAMLG
0.788159609	2.97372E-07	< 0.001	< 0.001	126402	CCDC105
0.785842955	3.1312E-07	< 0.001	< 0.001	256281	NUDT14
0.784555972	3.22199E-07	< 0.001	< 0.001	9166	EBAG9
0.783783793	3.27772E-07	< 0.001	< 0.001	388581	FAM132A
0.782239377	3.3919E-07	< 0.001	< 0.001	51031	GLOD4
0.780180156	3.55058E-07	< 0.001	< 0.001	2779	GNAT1
0.779150546	3.63281E-07	< 0.001	< 0.001	5423	POLB
0.778893173	3.65366E-07	< 0.001	< 0.001	113452	TMEM54
0.778378367	3.69584E-07	< 0.001	< 0.001	80213	TM2D3
0.775546908	3.93777E-07	< 0.001	< 0.001	91663	MYADM
0.775032163	3.98373E-07	< 0.001	< 0.001	23379	KIAA0947
0.774002552	4.07751E-07	< 0.001	< 0.001	55591	VEZT
0.771171212	4.34954E-07	< 0.001	< 0.001	79931	TNIP3
0.769369364	4.53462E-07	< 0.001	< 0.001	4938	OAS1
0.767567575	4.72993E-07	< 0.001	< 0.001	642852	LOC642852
0.765765786	4.93641E-07	< 0.001	< 0.001	8935	SKAP2
0.765508354	4.96695E-07	< 0.001	< 0.001	10121	ACTR1A
0.763963938	5.1553E-07	< 0.001	< 0.001	340542	BEX5
0.763449192	5.22019E-07	< 0.001	< 0.001	54843	SYTL2
0.761389971	5.49146E-07	< 0.001	< 0.001	5441	POLR2L
0.760875165	5.56221E-07	< 0.001	< 0.001	55733	HHAT
0.758558512	5.89716E-07	< 0.001	< 0.001	55003	PAK1IP1
0.758558512	5.89716E-07	< 0.001	< 0.001	340419	RSPO2
0.757014155	6.13632E-07	< 0.001	< 0.001	80274	SCUBE1
0.755469739	6.38929E-07	< 0.001	< 0.001	26232	FBXO2
0.754954934	6.47678E-07	< 0.001	< 0.001	10133	OPTN
0.753410518	6.74993E-07	< 0.001	< 0.001	84837	RP11-431H16.2
0.751608729	7.08922E-07	< 0.001	< 0.001	84668	FAM126A
0.750064313	7.39944E-07	< 0.001	< 0.001	10534	SSSCA1
0.750064313	7.39944E-07	< 0.001	< 0.001	84958	SYTL1
0.749549568	7.50708E-07	< 0.001	< 0.001	5624	PROC
0.749549568	7.50708E-07	< 0.001	< 0.001	55907	CMAS
0.745431185	8.45129E-07	< 0.001	< 0.001	154075	SAMD3
0.74491632	8.58053E-07	< 0.001	< 0.001	64860	ARMCX5
0.744401574	8.7125E-07	< 0.001	< 0.001	57670	KIAA1549
0.744144142	8.77965E-07	< 0.001	< 0.001	10653	AC011479.2
0.743629336	8.91589E-07	< 0.001	< 0.001	59345	GNB4
0.742857099	9.12552E-07	< 0.001	< 0.001	151507	MSL3P1
0.741055369	9.64136E-07	< 0.001	< 0.001	3718	JAK3
0.740283072	9.8743E-07	< 0.001	< 0.001	902	CCNH
0.740283072	9.8743E-07	< 0.001	< 0.001	1393	CRHBP
0.740283072	9.8743E-07	< 0.001	< 0.001	1491	CTH
0.737451732	1.07951E-06	< 0.001	< 0.001	64769	MEAF6
0.736679554	1.10654E-06	< 0.001	< 0.001	6659	SOX4
0.736164689	1.12507E-06	< 0.001	< 0.001	22950	SLC4A1AP
0.735135078	1.16332E-06	< 0.001	< 0.001	148479	PHF13
0.734620333	1.18307E-06	< 0.001	< 0.001	53353	LRP1B
0.733333349	1.23434E-06	< 0.001	< 0.001	23643	LY96
0.731274128	1.32237E-06	< 0.001	< 0.001	10200	MPHOSPH6
0.730759323	1.34559E-06	< 0.001	< 0.001	59342	SCPEP1
0.730759323	1.34559E-06	< 0.001	< 0.001	221074	SLC39A12
0.73050195	1.35736E-06	< 0.001	< 0.001	81617	CAB39L
0.730244517	1.36932E-06	< 0.001	< 0.001	57823	SLAMF7
0.729987085	1.38135E-06	< 0.001	< 0.001	56063	C1orf91
0.729472339	1.40588E-06	< 0.001	< 0.001	440435	GPR179
0.728957534	1.43092E-06	< 0.001	< 0.001	27253	PCDH17
0.727927923	1.48266E-06	< 0.001	< 0.001	26094	DCAF4
0.727670491	1.49595E-06	< 0.001	< 0.001	5917	RARS
0.727670491	1.49595E-06	< 0.001	< 0.001	10965	ACOT2
0.727413118	1.50936E-06	< 0.001	< 0.001	51715	RAB23

voxel-wise, subcortex			
Spearman's corr coef	p-val	GenID	Gene
0.536276817	< 0.001	1264	CNN1
0.53621316	< 0.001	1950	EGF
0.534398437	< 0.001	493	ATP2B4
0.533013165	< 0.001	401232	RP1-90J20.7
0.532896817	< 0.001	148014	TTC9B
0.532428682	< 0.001	93082	NEURL3
0.532357514	< 0.001	56950	SMYD2
0.532067299	< 0.001	259236	TMIE
0.531567872	< 0.001	10882	AC015936.3
0.531279325	< 0.001	59269	HIVEP3
0.531278551	< 0.001	54843	SYTL2
0.531230032	< 0.001	151507	MSL3P1
0.531156123	< 0.001	126567	FAM148C
0.531143308	< 0.001	401472	FLJ45248
0.530109823	< 0.001	4325	MMP16
0.53002882	< 0.001	51291	GMIP
0.529702842	< 0.001	8663	EIF3S8
0.529260099	< 0.001	84628	NTNG2
0.528217673	< 0.001	221491	C6orf1
0.52780968	< 0.001	6660	SOX5
0.5267452	< 0.001	54331	GNG2
0.526495814	< 0.001	84656	AC020663.1
0.526323259	< 0.001	53353	LRP1B
0.526319742	< 0.001	25945	PVRL3
0.525725305	< 0.001	10200	MPHOSPH6
0.525475085	< 0.001	22898	DENND3
0.524845719	< 0.001	27338	UBE2S
0.524410725	< 0.001	8499	PPFIA2
0.523271084	< 0.001	819	CAMLG
0.522161901	< 0.001	29844	TFPT
0.521997869	< 0.001	2520	GAST
0.521640718	< 0.001	55591	VEZT
0.521421313	< 0.001	148479	PHF13
0.521192908	< 0.001	388581	FAM132A
0.520679891	< 0.001	26256	CABYR
0.520412862	< 0.001	54958	TMEM160
0.519973755	< 0.001	389658	FAM150A
0.51985997	< 0.001	203068	TUBB
0.51954186	< 0.001	740	MRPL49
0.518176496	< 0.001	79712	GTDC1
0.518066585	< 0.001	2523	FUT1
0.517959893	< 0.001	23467	NPTXR
0.517800093	< 0.001	758	MPPED1
0.517506838	< 0.001	654433	AC016683.6
0.517327249	< 0.001	4155	MBP
0.517255485	< 0.001	80790	AC099524.1
0.51688683	< 0.001	23046	KIF21B
0.515969217	< 0.001	1008	CDH10
0.515855849	< 0.001	161829	EXD1
0.515333593	< 0.001	100191040	AL589765.4
0.515237927	< 0.001	4355	MPP2
0.514707625	< 0.001	79814	AGMAT
0.514455438	< 0.001	138428	PTRH1
0.513513744	< 0.001	2203	FBP1
0.513420999	< 0.001	9537	TP53I11
0.513328373	< 0.001	114884	OSBPL10
0.512639225	< 0.001	1025	CDK9
0.512224853	< 0.001	80213	TM2D3
0.511802137	< 0.001	26038	CHD5
0.510605693	< 0.001	57182	ANKRD50
0.509865403	< 0.001	5294	PIK3CG
0.509731054	< 0.001	81563	C1orf21
0.509702981	< 0.001	285761	DCBLD1
0.509673357	< 0.001	51701	NLK
0.509639561	< 0.001	404636	FAM45A
0.509624004	< 0.001	148808	MFSD4
0.509365261	< 0.001	92	ACVR2A
0.509078681	< 0.001	9166	EBAG9
0.508979917	< 0.001	8735	MYH13
0.508669496	< 0.001	23026	MYO16
0.508646786	< 0.001	165257	C1QL2
0.508407593	< 0.001	9823	ARMCX2
0.508300424	< 0.001	91010	FMNL3
0.50797689	< 0.001	4097	MAFG
0.507870674	< 0.001	121268	RHEBL1
0.507724106	< 0.001	57495	KIAA1239
0.507364035	< 0.001	1488	CTBP2
0.507068336	< 0.001	347688	TUBB8
0.50706768	< 0.001	7533	YWHAH

region-wise, subcortex					
Spearman's corr coef	p-val	p-val, SA	p-val, SA (BH- correction)	GenID	Gene
0.726126134	1.57875E-06	< 0.001	< 0.001	9823	ARMCX2
0.726126134	1.57875E-06	< 0.001	< 0.001	80381	CD276
0.725353897	1.62225E-06	< 0.001	< 0.001	51282	SCAND1
0.725353897	1.62225E-06	< 0.001	< 0.001	56990	CDC42SE2
0.725096524	1.63706E-06	< 0.001	< 0.001	29081	METTL5
0.724839091	1.65201E-06	< 0.001	< 0.001	51458	RHCG
0.72380954	1.71355E-06	< 0.001	< 0.001	80816	ASXL3
0.722522497	1.79428E-06	< 0.001	< 0.001	9014	TAF1B
0.722522497	1.79428E-06	< 0.001	< 0.001	200879	LIPH
0.720978141	1.89709E-06	< 0.001	< 0.001	1675	CFD
0.720978141	1.89709E-06	< 0.001	< 0.001	112812	FDX1L
0.719948471	1.9694E-06	< 0.001	< 0.001	10361	NPM2
0.719691098	1.98799E-06	< 0.001	< 0.001	5031	P2RY6
0.719691098	1.98799E-06	< 0.001	< 0.001	10055	SAE1
0.719691098	1.98799E-06	< 0.001	< 0.001	387849	REP15
0.718404114	2.08391E-06	< 0.001	< 0.001	8655	DYNLL1
0.718404114	2.08391E-06	< 0.001	< 0.001	93082	NEURL3
0.718146682	2.10368E-06	< 0.001	< 0.001	90480	GADD45GIP1
0.717889309	2.12375E-06	< 0.001	< 0.001	55105	GPATCH2
0.717889309	2.12375E-06	< 0.001	< 0.001	401097	AC112641.2
0.717631936	2.14397E-06	< 0.001	< 0.001	10772	FUSIP1
0.717374504	2.16442E-06	< 0.001	< 0.001	138046	RALYL
0.716859698	2.20599E-06	< 0.001	< 0.001	705	BYSL
0.71608752	2.27007E-06	< 0.001	< 0.001	57182	ANKRD50
0.715830088	2.2919E-06	< 0.001	< 0.001	286207	C9orf117
0.715572715	2.31393E-06	< 0.001	< 0.001	6675	UAP1
0.713770866	2.47504E-06	< 0.001	< 0.001	65059	RAPH1
0.713770866	2.47504E-06	< 0.001	< 0.001	375607	C7orf52
0.713513553	2.49906E-06	< 0.001	< 0.001	27338	UBE2S
0.713256121	2.52333E-06	< 0.001	< 0.001	285753	C6orf182
0.711454272	2.70062E-06	< 0.001	< 0.001	7421	VDR
0.711196899	2.72705E-06	< 0.001	< 0.001	90411	MCFD2
0.710682094	2.78071E-06	< 0.001	< 0.001	84628	NTNG2
0.709909916	2.86341E-06	< 0.001	< 0.001	55897	MESP1
0.709909916	2.86341E-06	< 0.001	< 0.001	728586	AC068658.1
0.709652483	2.89152E-06	< 0.001	< 0.001	22898	DENND3
0.709137738	2.94873E-06	< 0.001	< 0.001	139221	MUM1L1
0.708880305	2.97777E-06	< 0.001	< 0.001	7158	TP53BP1
0.708880305	2.97777E-06	< 0.001	< 0.001	55840	EAF2
0.708622873	3.00714E-06	< 0.001	< 0.001	23151	GRAMD4
0.7083655	3.03683E-06	< 0.001	< 0.001	64805	P2RY12
0.7083655	3.03683E-06	< 0.001	< 0.001	84959	UBASH3B
0.708108127	3.06678E-06	< 0.001	< 0.001	6578	SLCO2A1
0.708108127	3.06678E-06	< 0.001	< 0.001	126789	PUSL1
0.707593322	3.12771E-06	< 0.001	< 0.001	5294	PIK3CG
0.707593322	3.12771E-06	< 0.001	< 0.001	5877	RABIF
0.707335889	3.15865E-06	< 0.001	< 0.001	11091	WDR5
0.706306279	3.28569E-06	< 0.001	< 0.001	51569	UFM1
0.706048846	3.31825E-06	< 0.001	< 0.001	6304	SATB1
0.705791533	3.3512E-06	< 0.001	< 0.001	10882	AC015936.3
0.705534101	3.38449E-06	< 0.001	< 0.001	342184	FMN1
0.70450449	3.52115E-06	< 0.001	< 0.001	55312	RFK
0.704247057	3.55625E-06	< 0.001	< 0.001	5645	PRSS2
0.704247057	3.55625E-06	< 0.001	< 0.001	123355	LRRC28
0.704247057	3.55625E-06	< 0.001	< 0.001	389658	FAM150A
0.703732312	3.62746E-06	< 0.001	< 0.001	79016	DDA1
0.703732312	3.62746E-06	< 0.001	< 0.001	286133	SCARA5
0.701158285	4.00659E-06	< 0.001	< 0.001	22930	RAB3GAP1
0.700386107	4.12813E-06	< 0.001	< 0.001	2044	EPHA5
0.700128675	4.1695E-06	< 0.001	< 0.001	25809	TTLL1
0.699613929	4.25352E-06	< 0.001	< 0.001	4074	M6PR
0.698841691	4.38278E-06	< 0.001	< 0.001	6509	SLC1A4
0.698069513	4.51611E-06	< 0.001	< 0.001	9938	ARHGAP25
0.698069513	4.51611E-06	< 0.001	< 0.001	83741	TFAP2D
0.697554648	4.60726E-06	< 0.001	< 0.001	3938	LCT
0.69678247	4.74753E-06	< 0.001	< 0.001	253582	C6orf191
0.696525037	4.79522E-06	< 0.001	< 0.001	23254	RP1-21O18.1
0.696267724	4.84335E-06	< 0.001	< 0.001	135154	C6orf57
0.695752859	4.94125E-06	< 0.001	< 0.001	642909	LOC642909
0.694980681	5.09177E-06	< 0.001	< 0.001	4664	NAB1
0.694465876	5.19476E-06	< 0.001	< 0.001	29883	CNOT7
0.694465876	5.19476E-06	< 0.001	< 0.001	81573	ANKRD13C
0.69395107	5.29977E-06	< 0.001	< 0.001	2764	GMFB
0.693178892	5.46131E-06	< 0.001	< 0.001	9891	NUAK1
0.692921519	5.51625E-06	< 0.001	< 0.001	51246	SHISA5
0.692406654	5.62775E-06	< 0.001	< 0.001	3840	KPNA4
0.691891909	5.74154E-06	< 0.001	< 0.001	6444	SGCD
0.691119671	5.91651E-06	< 0.001	< 0.001	387103	C6orf173
0.690604866	6.03613E-06	< 0.001	< 0.001	92	ACVR2A

voxel-wise, subcortex			
Spearman's corr coef	p-val	GenID	Gene
0.507060945	< 0.001	728568	C12orf73
0.505816877	< 0.001	10121	ACTR1A
0.505544543	< 0.001	200879	LIPH
0.505264878	< 0.001	441027	TMEM150C
0.504761219	< 0.001	51715	RAB23
0.50458616	< 0.001	5918	RARRES1
0.504584372	< 0.001	55105	GPATCH2
0.504262269	< 0.001	89795	NAV3
0.504208684	< 0.001	29951	PDZRN4
0.504151106	< 0.001	344787	ZNF860
0.504021406	< 0.001	79876	UBA5
0.503634334	< 0.001	65059	RAPH1
0.502955377	< 0.001	1756	DMD
0.50286293	< 0.001	257019	FRMD3
0.502783418	< 0.001	64131	XYLT1
0.501924396	< 0.001	409	ARRB2
0.501546085	< 0.001	5076	PAX2
0.501179993	< 0.001	85409	NKD2
0.501152515	< 0.001	79180	EFHD2
0.501092851	< 0.001	2779	GNAT1
0.500593841	< 0.001	3350	HTR1A
0.500021458	< 0.001	84464	BTBD12
0.500005126	< 0.001	54809	SAMD9
0.499721408	< 0.001	25934	NIPSNAP3A
0.499476016	< 0.001	90835	C16orf93
0.499456316	< 0.001	80206	FHOD3
0.499364227	< 0.001	7421	VDR
0.499305785	< 0.001	55521	TRIM36
0.499275595	< 0.001	10313	RTN3
0.498934358	< 0.001	11091	WDR5
0.497715592	< 0.001	8036	SHOC2
0.497115493	< 0.001	374470	C12orf42
0.496800065	< 0.001	1386	ATF2
0.496733487	< 0.001	342977	NANOS3
0.496428698	< 0.001	51162	EGFL7
0.496208459	< 0.001	200407	CREG2
0.49596566	< 0.001	79016	DDA1
0.495246291	< 0.001	10965	ACOT2
0.494705528	< 0.001	23002	DAAM1
0.494516343	< 0.001	4335	MNT
0.494244128	< 0.001	57136	C20orf3
0.494024068	< 0.001	137362	GOT1L1
0.493875533	< 0.001	642909	LOC642909
0.493835151	< 0.001	285313	IGSF10
0.493703634	< 0.001	64841	GNPNAT1
0.493301213	< 0.001	138882	OR1N2
0.49302423	< 0.001	8682	PEA15
0.492633879	< 0.001	401097	AC112641.2
0.491627812	< 0.001	29970	IQCJ-SCHIP1
0.491557121	< 0.001	7293	TNFRSF4
0.491307735	< 0.001	57181	SLC39A10
0.4909105	< 0.001	2629	GBA
0.490581393	< 0.001	6444	SGCD
0.490509093	< 0.001	51393	TRPV2
0.49041307	< 0.001	285753	C6orf182
0.49031049	< 0.001	2044	EPHA5
0.490204364	< 0.001	84536	C21orf67
0.489621907	< 0.001	1491	CTH
0.489453822	< 0.001	161	AP2A2
0.489334017	< 0.001	808	CALM3
0.489011317	< 0.001	441461	RP11-60I3.5
0.488879114	< 0.001	1845	DUSP3
0.488206893	< 0.001	6045	RNF2
0.487772852	< 0.001	84334	APOPT1
0.487744182	< 0.001	6675	UAP1
0.487717181	< 0.001	408	ARRB1
0.486872524	< 0.001	84959	UBASH3B
0.48662594	< 0.001	9871	SEC24D
0.486610591	< 0.001	10204	NUTF2
0.486320049	< 0.001	23514	KIAA0146
0.485677183	< 0.001	79000	C1orf135
0.485261977	< 0.001	653145	ANXA8
0.485123515	< 0.001	2770	GNAI1
0.485117704	< 0.001	121053	C12orf45
0.485022187	< 0.001	56604	TUBB7P
0.485008597	< 0.001	114787	GPRIN1
0.484739929	< 0.001	55854	ZC3H15
0.484563202	< 0.001	25999	CLIP3
0.484420061	< 0.001	51280	GOLM1

region-wise, subcortex					
Spearman's corr coef	p-val	p-val, SA	p-val, SA (BH- correction)	GenID	Gene
0.690604866	6.03613E-06	< 0.001	< 0.001	8682	PEA15
0.690347493	6.09687E-06	< 0.001	< 0.001	221044	UCMA
0.69009006	6.15817E-06	< 0.001	< 0.001	134957	STXBP5
0.689832687	6.22008E-06	< 0.001	< 0.001	9342	SNAP29
0.689317882	6.34574E-06	< 0.001	< 0.001	2953	GSTT2
0.689317882	6.34574E-06	< 0.001	< 0.001	192668	CYS1
0.688803077	6.47394E-06	< 0.001	< 0.001	728695	SPANXB1
0.688545704	6.53899E-06	< 0.001	< 0.001	25945	PVRL3
0.688288271	6.60462E-06	< 0.001	< 0.001	9100	USP10
0.688030839	6.671E-06	< 0.001	< 0.001	256380	SCML4
0.687258661	6.87403E-06	< 0.001	< 0.001	1488	CTBP2
0.687258661	6.87403E-06	< 0.001	< 0.001	23760	PITPNB
0.687001228	6.94302E-06	< 0.001	< 0.001	51451	LCMT1
0.687001228	6.94302E-06	< 0.001	< 0.001	100113407	TMEM170B
0.686486483	7.08305E-06	< 0.001	< 0.001	8663	EIF3S8
0.686486483	7.08305E-06	< 0.001	< 0.001	79712	GTDC1
0.685971677	7.22591E-06	< 0.001	< 0.001	5136	PDE1A
0.685714304	7.29831E-06	< 0.001	< 0.001	26086	GPSM1
0.685714304	7.29831E-06	< 0.001	< 0.001	54332	GDAP1
0.684684634	7.59539E-06	< 0.001	< 0.001	3957	LGALS2
0.684427321	7.67146E-06	< 0.001	< 0.001	84303	CHCHD6
0.684427321	7.67146E-06	< 0.001	< 0.001	728568	C12orf73
0.684169888	7.74829E-06	< 0.001	< 0.001	440895	AC013271.3
0.683912456	7.82584E-06	< 0.001	< 0.001	79631	EFTUD1
0.683655024	7.90418E-06	< 0.001	< 0.001	27202	GPR77
0.683655024	7.90418E-06	< 0.001	< 0.001	57617	VPS18
0.68339771	7.98328E-06	< 0.001	< 0.001	91368	CDKN2AIPNL
0.68339771	7.98328E-06	< 0.001	< 0.001	127281	C1orf93
0.683140278	8.06315E-06	< 0.001	< 0.001	116113	FOXP4
0.682625473	8.22513E-06	< 0.001	< 0.001	23284	LPHN3
0.682625473	8.22513E-06	< 0.001	< 0.001	84719	LINC00260
0.6823681	8.30736E-06	< 0.001	< 0.001	57495	KIAA1239
0.682110667	8.39033E-06	< 0.001	< 0.001	6045	RNF2
0.682110667	8.39033E-06	< 0.001	< 0.001	11017	SNRNP27
0.681853235	8.47423E-06	< 0.001	< 0.001	9759	HDAC4
0.681853235	8.47423E-06	< 0.001	< 0.001	84315	MON1A
0.680566251	8.90557E-06	< 0.001	< 0.001	1264	CNN1
0.680566251	8.90557E-06	< 0.001	< 0.001	5734	PTGER4
0.680566251	8.90557E-06	< 0.001	< 0.001	80097	FAM128B
0.679794073	9.17448E-06	< 0.001	< 0.001	1816	DRD5
0.679021895	9.45111E-06	< 0.001	< 0.001	201475	RAB12
0.67850703	9.64002E-06	< 0.001	< 0.001	4325	MMP16
0.678249657	9.73586E-06	< 0.001	< 0.001	126567	FAM148C
0.677734852	9.93027E-06	< 0.001	< 0.001	2516	NR5A1
0.677220047	1.01284E-05	< 0.001	< 0.001	317	APAF1
0.677220047	1.01284E-05	< 0.001	< 0.001	3727	JUND
0.676962674	1.02288E-05	< 0.001	< 0.001	8000	PSCA
0.676705241	1.03303E-05	< 0.001	< 0.001	158399	ZNF483
0.676447868	1.04327E-05	< 0.001	< 0.001	10898	CPSF4
0.676447868	1.04327E-05	< 0.001	< 0.001	260434	PYDC1
0.676190436	1.0536E-05	< 0.001	< 0.001	90835	C16orf93
0.675933063	1.06403E-05	< 0.001	< 0.001	84918	LRP11
0.675418258	1.08519E-05	< 0.001	< 0.001	5733	PTGER3
0.675418258	1.08519E-05	0.001	1	64792	RABL5
0.675160825	1.09593E-05	< 0.001	< 0.001	113	ADCY7
0.675160825	1.09593E-05	< 0.001	< 0.001	11077	HSF2BP
0.674131215	1.13988E-05	< 0.001	< 0.001	79697	C14orf169
0.674131215	1.13988E-05	< 0.001	< 0.001	114801	TMEM200A
0.673873901	1.15112E-05	< 0.001	< 0.001	4861	NPAS1
0.673616469	1.16248E-05	< 0.001	< 0.001	129049	SGSM1
0.673359036	1.17393E-05	< 0.001	< 0.001	148014	TTC9B
0.672844291	1.19717E-05	< 0.001	< 0.001	114335	CGB1
0.672072053	1.23285E-05	< 0.001	< 0.001	29089	UBE2T
0.671557248	1.2572E-05	< 0.001	< 0.001	808	CALM3
0.671557248	1.2572E-05	< 0.001	< 0.001	84334	APOPT1
0.671557248	1.2572E-05	< 0.001	< 0.001	140597	TCEAL2
0.671299875	1.26954E-05	0.001	1	2629	GBA
0.67078501	1.29457E-05	< 0.001	< 0.001	51605	TRMT6
0.670270264	1.32007E-05	< 0.001	< 0.001	11245	GPR176
0.670012832	1.333E-05	< 0.001	< 0.001	11231	SEC63
0.669755459	1.34604E-05	< 0.001	< 0.001	1947	EFNB1
0.669498086	1.35921E-05	< 0.001	< 0.001	64895	PAPOLG
0.669498086	1.35921E-05	< 0.001	< 0.001	79908	BTNL8
0.669240654	1.3725E-05	< 0.001	< 0.001	8499	PPFIA2
0.669240654	1.3725E-05	< 0.001	< 0.001	118788	PIK3AP1
0.669240654	1.3725E-05	< 0.001	< 0.001	404636	FAM45A
0.668983221	1.38591E-05	< 0.001	< 0.001	29951	PDZRN4
0.668983221	1.38591E-05	< 0.001	< 0.001	390079	OR52E8
0.668468475	1.41311E-05	< 0.001	< 0.001	740	MRPL49

voxel-wise, subcortex			
Spearman's corr coef	p-val	GenID	Gene
0.484128833	< 0.001	340542	BEX5
0.484015763	< 0.001	84779	NAA11
0.483836174	< 0.001	23139	MAST2
0.48382014	< 0.001	9317	PTER
0.483671248	< 0.001	988	CDC5L
0.483646065	< 0.001	91050	CCDC149
0.483631909	< 0.001	78991	PCYOX1L
0.483536035	< 0.001	23167	EFR3A
0.483367413	< 0.001	383	ARG1
0.483243406	< 0.001	26994	RNF11
0.483080506	< 0.001	65244	SPATS2
0.483072072	< 0.001	387787	AP001372.1
0.482578605	< 0.001	56978	PRDM8
0.482547879	< 0.001	55609	ZNF280C
0.482313722	< 0.001	2996	GYPE
0.482241988	< 0.001	51760	SYT17
0.48213318	< 0.001	25766	PRPF40B
0.481980383	< 0.001	2170	FABP3
0.481798321	< 0.001	152940	C4orf45
0.481563926	< 0.001	64854	USP46
0.481535971	< 0.001	5441	POLR2L
0.481340259	< 0.001	1577	CYP3A5
0.481163859	< 0.001	728695	SPANXB1
0.481139421	< 0.001	26094	DCAF4
0.481075406	< 0.001	5031	P2RY6
0.480773956	< 0.001	283209	PGM2L1
0.480032295	< 0.001	25851	TECPR1
0.480013907	< 0.001	2847	MCHR1
0.479888886	< 0.001	627	BDNF
0.479653537	< 0.001	57805	KIAA1967
0.4795928	< 0.001	9479	MAPK8IP1
0.479505539	< 0.001	7295	TXN
0.479370981	< 0.001	80212	CCDC92
0.479133099	< 0.001	284353	NKPD1
0.479104221	< 0.001	203102	ADAM32
0.479069114	< 0.001	25759	SHC2
0.478955954	< 0.001	389941	C1QL1
0.47890237	< 0.001	90050	FAM181A
0.47886008	< 0.001	399668	LINC00086
0.478817165	< 0.001	10073	SNUPN
0.478662878	< 0.001	127281	C1orf93
0.478611797	< 0.001	9612	NCOR2
0.478473842	< 0.001	84958	SYTL1
0.478422493	< 0.001	2319	FLOT2
0.478119969	< 0.001	10656	KHDRBS3
0.477786601	< 0.001	728858	C12orf71
0.477732956	< 0.001	126282	TNFAIP8L1
0.477699071	< 0.001	6256	RXRA
0.47759074	< 0.001	9891	NUAK1
0.477542907	< 0.001	10318	TNIP1
0.477322698	< 0.001	3381	IBSP
0.477278054	< 0.001	8767	RIPK2
0.477272958	< 0.001	23180	RFTN1
0.477091968	< 0.001	148066	ZNRF4
0.476275444	< 0.001	5784	PTPN14
0.476050228	< 0.001	140688	C20orf112
0.475821078	< 0.001	90411	MCFD2
0.475539505	< 0.001	6307	SC4MOL
0.475256324	< 0.001	22821	RASA3
0.474954635	< 0.001	64084	CLSTN2
0.474826962	< 0.001	7277	TUBA4A
0.474820673	< 0.001	5119	CHMP1A
0.474214584	< 0.001	1139	CHRNA7
0.474098802	< 0.001	6751	SSTR1
0.47364834	< 0.001	3486	IGFBP3
0.473313153	< 0.001	5917	RARS
0.473290205	< 0.001	56660	KCNK12
0.473159999	< 0.001	146439	CCDC64B
0.473068118	< 0.001	83445	GSG1
0.473021686	< 0.001	79631	EFTUD1
0.472525001	< 0.001	6950	TCP1
0.472358495	< 0.001	5690	PSMB2
0.472326159	< 0.001	103910	MYL12B
0.472291768	< 0.001	130951	C2orf65
0.47199598	< 0.001	400553	AC134312.1
0.471606225	< 0.001	79654	HECTD3
0.471470445	< 0.001	100133234	SBF1P1
0.47141245	< 0.001	25819	CCRN4L
0.471168399	< 0.001	54332	GDAP1

region-wise, subcortex					
Spearman's corr coef	p-val	p-val, SA	p-val, SA (BH- correction)	GenID	Gene
0.668468475	1.41311E-05	< 0.001	< 0.001	152185	CCDC52
0.668468475	1.41311E-05	< 0.001	< 0.001	339804	C2orf74
0.668211043	1.42688E-05	< 0.001	< 0.001	51218	GLRX5
0.668211043	1.42688E-05	< 0.001	< 0.001	55466	DNAJA4
0.66795367	1.44079E-05	< 0.001	< 0.001	79178	THTPA
0.66795367	1.44079E-05	< 0.001	< 0.001	400451	FAM174B
0.667181492	1.4833E-05	< 0.001	< 0.001	340481	ZDHHC21
0.666924059	1.49773E-05	< 0.001	< 0.001	117245	HRASLS5
0.666151881	1.54181E-05	< 0.001	< 0.001	50810	AC024270.1
0.665637016	1.57187E-05	< 0.001	< 0.001	7410	VAV2
0.665379703	1.5871E-05	< 0.001	< 0.001	11034	DSTN
0.665379703	1.5871E-05	< 0.001	< 0.001	154150	HDGFL1
0.665122271	1.60249E-05	0.001	1	647476	FLJ20518
0.664864838	1.618E-05	< 0.001	< 0.001	6751	SSTR1
0.664607465	1.63365E-05	< 0.001	< 0.001	27018	NGFRAP1
0.66409266	1.6654E-05	< 0.001	< 0.001	5707	PSMD1
0.663835227	1.68147E-05	< 0.001	< 0.001	439915	KRTAP5-5
0.663320422	1.71408E-05	< 0.001	< 0.001	10479	SLC9A6
0.663320422	1.71408E-05	< 0.001	< 0.001	606551	UBE2MP1
0.663063049	1.73061E-05	< 0.001	< 0.001	123263	MTFMT
0.662805676	1.74728E-05	< 0.001	< 0.001	10947	AP3M2
0.662548244	1.76411E-05	< 0.001	< 0.001	22885	ABLIM3
0.662548244	1.76411E-05	< 0.001	< 0.001	140807	KRT72
0.662290812	1.78108E-05	< 0.001	< 0.001	9871	SEC24D
0.661003888	1.86827E-05	< 0.001	< 0.001	5918	RARRES1
0.661003888	1.86827E-05	< 0.001	< 0.001	51382	ATP6V1D
0.661003888	1.86827E-05	< 0.001	< 0.001	65990	FAM173A
0.660746455	1.88618E-05	< 0.001	< 0.001	1176	AP3S1
0.660489023	1.90425E-05	< 0.001	< 0.001	171483	FAM9B
0.66023165	1.92248E-05	< 0.001	< 0.001	51693	TRAPPC2L
0.659202039	1.99703E-05	< 0.001	< 0.001	55071	C9orf40
0.658944666	2.0161E-05	< 0.001	< 0.001	7293	TNFRSF4
0.658429861	2.05472E-05	< 0.001	< 0.001	5690	PSMB2
0.658429861	2.05472E-05	< 0.001	< 0.001	387787	AP001372.1
0.657657683	2.11394E-05	< 0.001	< 0.001	5921	RASA1
0.657657683	2.11394E-05	< 0.001	< 0.001	6950	TCP1
0.65740025	2.13404E-05	0.001	1	151477	LINC00471
0.656885445	2.17476E-05	< 0.001	< 0.001	4647	MYO7A
0.656628072	2.19537E-05	< 0.001	< 0.001	25759	SHC2
0.656628072	2.19537E-05	< 0.001	< 0.001	54958	TMEM160
0.65637064	2.21619E-05	< 0.001	< 0.001	1845	DUSP3
0.65637064	2.21619E-05	< 0.001	< 0.001	55854	ZC3H15
0.656113207	2.23718E-05	< 0.001	< 0.001	23139	MAST2
0.655341029	2.30127E-05	< 0.001	< 0.001	2243	FGA
0.655083656	2.32301E-05	0.001	1	126003	TRAPPC5
0.654826224	2.34494E-05	< 0.001	< 0.001	26007	DAK
0.654568851	2.36705E-05	< 0.001	< 0.001	89870	TRIM15
0.654311478	2.38937E-05	< 0.001	< 0.001	56950	SMYD2
0.654311478	2.38937E-05	< 0.001	< 0.001	57805	KIAA1967
0.653796613	2.43459E-05	< 0.001	< 0.001	79633	FAT4
0.65353924	2.45748E-05	< 0.001	< 0.001	129025	ZNF280A
0.653281868	2.48058E-05	< 0.001	< 0.001	23172	FAM175B
0.653024435	2.50388E-05	< 0.001	< 0.001	10204	NUTF2
0.652252257	2.57502E-05	< 0.001	< 0.001	11179	ZNF277
0.651994824	2.59913E-05	< 0.001	< 0.001	55327	LIN7C
0.651737452	2.62348E-05	< 0.001	< 0.001	7137	TNNI3
0.651737452	2.62348E-05	< 0.001	< 0.001	11163	NUDT4
0.651737452	2.62348E-05	< 0.001	< 0.001	23180	RFTN1
0.651737452	2.62348E-05	< 0.001	< 0.001	219621	C10orf107
0.651222646	2.67276E-05	< 0.001	< 0.001	112597	LINC00152
0.650450408	2.7483E-05	< 0.001	< 0.001	10073	SNUPN
0.649935663	2.79975E-05	< 0.001	< 0.001	10682	EBP
0.649935663	2.79975E-05	< 0.001	< 0.001	10907	TXNL4A
0.649935663	2.79975E-05	< 0.001	< 0.001	285780	LY86-AS1
0.649420798	2.85208E-05	< 0.001	< 0.001	441381	LRRC24
0.648391187	2.95948E-05	< 0.001	< 0.001	10687	PNMA2
0.648391187	2.95948E-05	< 0.001	< 0.001	51253	MRPL37
0.647619009	3.04245E-05	< 0.001	< 0.001	91050	CCDC149
0.647361636	3.07057E-05	< 0.001	< 0.001	9465	AKAP7
0.647361636	3.07057E-05	< 0.001	< 0.001	103910	MYL12B
0.646846831	3.12755E-05	< 0.001	< 0.001	9908	G3BP2
0.646846831	3.12755E-05	< 0.001	< 0.001	51162	EGFL7
0.646332026	3.1855E-05	< 0.001	< 0.001	55500	ETNK1
0.646074653	3.21486E-05	< 0.001	< 0.001	51280	GOLM1
0.64581722	3.24445E-05	< 0.001	< 0.001	4335	MNT
0.64581722	3.24445E-05	< 0.001	< 0.001	29944	PNMA3
0.64581722	3.24445E-05	< 0.001	< 0.001	152940	C4orf45
0.645559847	3.27429E-05	< 0.001	< 0.001	23077	MYCBP2
0.645302415	3.30438E-05	< 0.001	< 0.001	254528	C16orf73

voxel-wise, subcortex			
Spearman's corr coef	p-val	GenID	Gene
0.470728397	< 0.001	8707	B3GALT2
0.470574707	< 0.001	51582	AZIN1
0.470547974	< 0.001	353189	SLCO4C1
0.470540345	< 0.001	3718	JAK3
0.470261186	< 0.001	644596	LINC00087
0.469254255	< 0.001	54033	RBM11
0.468605727	< 0.001	10326	SIRPB1
0.468548417	< 0.001	1820	ARID3A
0.468472868	< 0.001	284611	FAM102B
0.468399227	< 0.001	65264	UBE2Z
0.467877001	< 0.001	127703	C1orf216
0.46767354	< 0.001	388121	TNFAIP8L3
0.467466176	< 0.001	54434	SSH1
0.467423081	< 0.001	127933	UHMK1
0.465778887	< 0.001	116443	GRIN3A
0.465544075	< 0.001	25924	MYRIP
0.465424269	< 0.001	4951	OCM2
0.465179443	< 0.001	7173	TPO
0.464589417	< 0.001	7837	PXDN
0.464315176	< 0.001	2038	EPB42
0.46424219	< 0.001	7982	ST7
0.464021295	< 0.001	26290	GALNT8
0.463973045	< 0.001	23498	HAAO
0.463918269	< 0.001	10160	FARP1
0.46390748	< 0.001	11231	SEC63
0.463904798	< 0.001	157	ADRBK2
0.463821769	< 0.001	11022	TDRKH
0.463298529	< 0.001	23603	CORO1C
0.463097066	< 0.001	64859	OBFC2A
0.462987751	< 0.001	11128	POLR3A
0.462932229	< 0.001	348980	HCN1
0.462656349	< 0.001	203054	ADCK5
0.462267846	< 0.001	7201	TRHR
0.461541265	< 0.001	78995	C17orf53
0.461499006	< 0.001	56477	CCL28
0.461281508	< 0.001	4761	NEUROD2
0.461211741	< 0.001	1978	EIF4EBP1
0.461203665	< 0.001	79862	ZNF669
0.461080194	< 0.001	541471	AC068491.1
0.460622162	< 0.001	528	ATP6V1C1
0.460611314	< 0.001	25939	SAMHD1
0.460400373	< 0.001	6545	SLC7A4
0.460321695	< 0.001	389649	C8orf86
0.460226148	< 0.001	128239	IQGAP3
0.460165977	< 0.001	57030	SLC17A7
0.460107237	< 0.001	10025	MED16
0.460005373	< 0.001	261726	TIPRL
0.45999831	< 0.001	729806	ZNF731P
0.459416956	< 0.001	5719	PSMD13
0.459294319	< 0.001	23671	TMEFF2
0.459218353	< 0.001	7187	TRAF3
0.459176481	< 0.001	116150	NUS1
0.459128708	< 0.001	80262	C16orf70
0.459026933	< 0.001	59349	KLHL12
0.458838969	< 0.001	2925	GRPR
0.458691776	< 0.001	285780	LY86-AS1
0.458553672	< 0.001	27300	ZNF544
0.458384186	< 0.001	93974	ATPIF1
0.458350122	< 0.001	134957	STXBP5
0.457927614	< 0.001	26872	STEAP1
0.457847238	< 0.001	25859	AC109486.1
0.457784563	< 0.001	10665	C6orf10
0.457533598	< 0.001	63908	NAPB
0.457466722	< 0.001	4938	OAS1
0.457343727	< 0.001	57709	SLC7A14
0.457270265	< 0.001	10799	RPP40
0.457134902	< 0.001	1543	CYP1A1
0.456960708	< 0.001	56934	CA10
0.456872016	< 0.001	1272	CNTN1
0.456726432	< 0.001	2909	GRLF1
0.456339896	< 0.001	92002	FAM58A
0.456275821	< 0.001	10622	POLR3G
0.456119925	< 0.001	51458	RHCG
0.456055492	< 0.001	10653	AC011479.2
0.456011653	< 0.001	100137047	JMJD7
0.455967367	< 0.001	11034	DSTN
0.45594874	< 0.001	23014	FBXO21
0.455748856	< 0.001	11077	HSF2BP
0.455569714	< 0.001	4356	MPP3

region-wise, subcortex					
Spearman's corr coef	p-val	p-val, SA	p-val, SA (BH- correction)	GenID	Gene
0.645045042	3.33474E-05	< 0.001	< 0.001	3156	HMGCR
0.644787669	3.36533E-05	< 0.001	< 0.001	374969	CCDC23
0.644272804	3.42731E-05	< 0.001	< 0.001	8704	B4GALT2
0.644272804	3.42731E-05	< 0.001	< 0.001	51309	ARMCX1
0.644015491	3.45872E-05	< 0.001	< 0.001	7837	PXDN
0.644015491	3.45872E-05	< 0.001	< 0.001	8799	PEX11B
0.644015491	3.45872E-05	< 0.001	< 0.001	54908	CCDC99
0.643758059	3.49035E-05	< 0.001	< 0.001	51701	NLK
0.643500626	3.52227E-05	< 0.001	< 0.001	23637	RABGAP1
0.643243194	3.55444E-05	< 0.001	< 0.001	8727	CTNNAL1
0.643243194	3.55444E-05	< 0.001	< 0.001	10325	RRAGB
0.642985821	3.58689E-05	< 0.001	< 0.001	282890	ZNF311
0.642471015	3.65262E-05	< 0.001	< 0.001	5865	RAB3B
0.64195621	3.71941E-05	0.001	1	128239	IQGAP3
0.641184032	3.82176E-05	< 0.001	< 0.001	80790	AC099524.1
0.6409266	3.85644E-05	< 0.001	< 0.001	255022	CALHM1
0.640669227	3.8914E-05	< 0.001	< 0.001	57136	C20orf3
0.640154421	3.96222E-05	< 0.001	< 0.001	8938	BAIAP3
0.640154421	3.96222E-05	0.001	1	116150	NUS1
0.639896989	3.99804E-05	< 0.001	< 0.001	393	ARHGAP4
0.639896989	3.99804E-05	< 0.001	< 0.001	10535	RNASEH2A
0.639382243	4.07063E-05	< 0.001	< 0.001	11052	CPSF6
0.639124811	4.10737E-05	< 0.001	< 0.001	2870	GRK6
0.638867438	4.14441E-05	< 0.001	< 0.001	149708	WFDC5
0.638610065	4.18176E-05	< 0.001	< 0.001	85409	NKD2
0.637580454	4.33426E-05	< 0.001	< 0.001	2959	GTF2B
0.637323022	4.37314E-05	< 0.001	< 0.001	9240	PNMA1
0.637323022	4.37314E-05	< 0.001	< 0.001	10299	MARCHF6
0.637323022	4.37314E-05	< 0.001	< 0.001	11212	PROSC
0.637065649	4.41239E-05	< 0.001	< 0.001	5621	PRNP
0.637065649	4.41239E-05	< 0.001	< 0.001	10197	PSME3
0.637065649	4.41239E-05	< 0.001	< 0.001	26091	HERC4
0.637065649	4.41239E-05	< 0.001	< 0.001	27300	ZNF544
0.636808217	4.45193E-05	< 0.001	< 0.001	8735	MYH13
0.636808217	4.45193E-05	< 0.001	< 0.001	26100	WIPI2
0.636293471	4.532E-05	< 0.001	< 0.001	9532	BAG2
0.635521173	4.65459E-05	< 0.001	< 0.001	114884	OSBPL10
0.63526386	4.69609E-05	< 0.001	< 0.001	161	AP2A2
0.635006428	4.73799E-05	< 0.001	< 0.001	121053	C12orf45
0.634748995	4.78017E-05	< 0.001	< 0.001	253782	LASS6
0.634491622	4.82272E-05	< 0.001	< 0.001	10857	PGRMC1
0.634491622	4.82272E-05	< 0.001	< 0.001	254173	TTLL10
0.633976817	4.90884E-05	< 0.001	< 0.001	2675	GFRA2
0.633719444	4.95244E-05	< 0.001	< 0.001	3096	HIVEP1
0.633204639	5.04066E-05	0.001	1	5695	PSMB7
0.632947206	5.08533E-05	< 0.001	< 0.001	57415	C3orf14
0.632432401	5.17573E-05	< 0.001	< 0.001	65260	SELRC1
0.632432401	5.17573E-05	< 0.001	< 0.001	79876	UBA5
0.631917655	5.26757E-05	< 0.001	< 0.001	4175	MCM6
0.631917655	5.26757E-05	< 0.001	< 0.001	64841	GNPNAT1
0.631917655	5.26757E-05	< 0.001	< 0.001	83852	SETDB2
0.631917655	5.26757E-05	< 0.001	< 0.001	158798	AKAP14
0.631660223	5.31407E-05	< 0.001	< 0.001	154754	PRSS23P2
0.631402791	5.36093E-05	< 0.001	< 0.001	338761	C1QL4
0.631145477	5.40815E-05	< 0.001	< 0.001	29106	SCG3
0.631145477	5.40815E-05	< 0.001	< 0.001	56917	MEIS3P2
0.630630612	5.50372E-05	< 0.001	< 0.001	84539	MCHR2
0.630630612	5.50372E-05	< 0.001	< 0.001	152006	RNF38
0.629343629	5.74953E-05	< 0.001	< 0.001	114798	SLITRK1
0.629086196	5.79985E-05	< 0.001	< 0.001	1950	EGF
0.628571391	5.90173E-05	0.001	1	2191	FAP
0.628571391	5.90173E-05	< 0.001	< 0.001	7982	ST7
0.628314018	5.95329E-05	< 0.001	< 0.001	3093	UBE2K
0.628314018	5.95329E-05	< 0.001	< 0.001	8260	NAA10
0.628314018	5.95329E-05	< 0.001	< 0.001	58530	LY6G6D
0.628056586	6.00522E-05	< 0.001	< 0.001	51668	HSPB11
0.627026975	6.21721E-05	< 0.001	< 0.001	401331	RASA4CP
0.626254797	6.38061E-05	< 0.001	< 0.001	150244	ZDHHC8P1
0.625997365	6.43596E-05	< 0.001	< 0.001	23248	RPRD2
0.625482619	6.54791E-05	< 0.001	< 0.001	10160	FARP1
0.625482619	6.54791E-05	< 0.001	< 0.001	54509	RHOF
0.625482619	6.54791E-05	< 0.001	< 0.001	148808	MFSD4
0.625225186	6.60458E-05	< 0.001	< 0.001	3836	KPNA1
0.625225186	6.60458E-05	< 0.001	< 0.001	203447	NRK
0.624967813	6.66166E-05	< 0.001	< 0.001	2590	GALNT2
0.624967813	6.66166E-05	< 0.001	< 0.001	11022	TDRKH
0.624967813	6.66166E-05	0.002	1	56271	BEX4
0.624710441	6.71922E-05	< 0.001	< 0.001	2664	GDI1
0.624710441	6.71922E-05	< 0.001	< 0.001	5504	PPP1R2

voxel-wise, subcortex			
Spearman's corr coef	p-val	GenID	Gene
0.455379605	< 0.001	711	ERC2-IT1
0.455159009	< 0.001	5717	PSMD11
0.455149502	< 0.001	8417	STX7
0.455127805	< 0.001	7532	YWHAG
0.455122977	< 0.001	442308	LOC442308
0.454864532	< 0.001	286207	C9orf117
0.454863638	< 0.001	153642	ARSK
0.454790413	< 0.001	221935	SDK1
0.45459941	< 0.001	80223	RAB11FIP1
0.454521894	< 0.001	79442	LRRC2
0.454332173	< 0.001	79863	RBFA
0.454010338	< 0.001	221074	SLC39A12
0.453830689	< 0.001	10907	TXNL4A
0.453719586	< 0.001	126433	FBXO27
0.453376085	< 0.001	5650	KLK7
0.452994078	< 0.001	401647	GOLGA7B
0.452789217	< 0.001	54472	TOLLIP
0.452702284	< 0.001	79895	ATP8B4
0.452683151	< 0.001	642778	AC136443.1
0.452592224	< 0.001	51253	MRPL37
0.452367216	< 0.001	29985	SLC39A3
0.452363193	< 0.001	321	APBA2
0.452337146	< 0.001	2781	GNAZ
0.452314049	< 0.001	408029	C2orf27B
0.452230334	< 0.001	4647	MYO7A
0.451939344	< 0.001	2016	EMX1
0.451938093	< 0.001	81794	ADAMTS10
0.45176369	< 0.001	345079	ANKRD56
0.451754302	< 0.001	206358	SLC36A1
0.451744199	< 0.001	9334	B4GALT5
0.451640636	< 0.001	112812	FDX1L
0.451586127	< 0.001	6812	STXBP1
0.451188892	< 0.001	393	ARHGAP4
0.450830191	< 0.001	4314	MMP3
0.450802892	< 0.001	200845	KCTD6
0.450719267	< 0.001	6252	RTN1
0.450400054	< 0.001	54873	PALMD
0.450267673	< 0.001	523	ATP6V1A
0.449851006	< 0.001	9373	PLAA
0.449552655	< 0.001	51031	GLOD4
0.449536026	< 0.001	10744	PTTG2
0.449350476	< 0.001	10682	EBP
0.449313104	< 0.001	23299	BICD2
0.449308276	< 0.001	50944	SHANK1
0.449290931	< 0.001	84301	DDI2
0.449248224	< 0.001	10716	TBR1
0.449116528	< 0.001	8567	MADD
0.449009985	< 0.001	434	ASIP
0.449003845	< 0.001	11163	NUDT4
0.448902488	< 0.001	9647	PPM1F
0.448819637	< 0.001	11261	AC012652.1
0.448663592	< 0.001	78997	GDAP1L1
0.448598951	< 0.001	29906	ST8SIA5
0.448593646	< 0.001	729603	LOC729603
0.448513895	< 0.001	10055	SAE1
0.448469073	< 0.001	10325	RRAGB
0.448408723	< 0.001	29106	SCG3
0.44829908	< 0.001	494197	SPANXN5
0.448194295	< 0.001	80115	BAIAP2L2
0.448137879	< 0.001	154807	VKORC1L1
0.448101342	< 0.001	57469	PNMAL2
0.448028386	< 0.001	9221	NOLC1
0.448019981	< 0.001	387849	REP15
0.447972327	< 0.001	29944	PNMA3
0.44795543	< 0.001	284403	WDR62
0.447718173	< 0.001	84303	CHCHD6
0.44756493	< 0.001	3836	KPNA1
0.447249562	< 0.001	284415	VSTM1
0.447136968	< 0.001	7143	TNR
0.447037518	< 0.001	400120	C13orf36
0.446845889	< 0.001	192668	CYS1
0.446812361	< 0.001	6706	SPRR2G
0.446810037	< 0.001	57521	RPTOR
0.446683347	< 0.001	256281	NUDT14
0.446638405	< 0.001	7158	TP53BP1
0.446573228	< 0.001	4685	NCAM2
0.446419716	< 0.001	400451	FAM174B
0.446368545	< 0.001	8000	PSCA
0.446283758	< 0.001	84790	TUBA1C

region-wise, subcortex					
Spearman's corr coef	p-val	p-val, SA	p-val, SA (BH- correction)	GenID	Gene
0.624710441	6.71922E-05	< 0.001	< 0.001	26872	STEAP1
0.624453008	6.77719E-05	< 0.001	< 0.001	3301	DNAJA1
0.624453008	6.77719E-05	< 0.001	< 0.001	3717	JAK2
0.624195635	6.83564E-05	< 0.001	< 0.001	79822	ARHGAP28
0.623938203	6.89453E-05	< 0.001	< 0.001	84779	NAA11
0.62368083	6.95392E-05	< 0.001	< 0.001	26511	CHIC2
0.62368083	6.95392E-05	< 0.001	< 0.001	79895	ATP8B4
0.623423398	7.01373E-05	< 0.001	< 0.001	84246	MED10
0.623166025	7.07403E-05	< 0.001	< 0.001	54328	GPR173
0.622908592	7.13476E-05	< 0.001	< 0.001	26297	SERGEF
0.622651219	7.19604E-05	< 0.001	< 0.001	56683	C21orf59
0.622651219	7.19604E-05	< 0.001	< 0.001	729141	LOC729141
0.622136414	7.31994E-05	< 0.001	< 0.001	10923	SUB1
0.621878982	7.38258E-05	< 0.001	< 0.001	221833	SP8
0.621621609	7.44577E-05	< 0.001	< 0.001	395	ARHGAP6
0.621621609	7.44577E-05	< 0.001	< 0.001	90417	C15orf23
0.620591998	7.70332E-05	< 0.001	< 0.001	4856	NOV
0.620591998	7.70332E-05	< 0.001	< 0.001	79172	CENPO
0.620334625	7.76896E-05	< 0.001	< 0.001	9686	VGLL4
0.619562387	7.96891E-05	< 0.001	< 0.001	257019	FRMD3
0.619047642	8.10484E-05	< 0.001	< 0.001	203054	ADCK5
0.619047642	8.10484E-05	0.001	1	257313	UTS2D
0.618790209	8.17356E-05	0.001	1	56925	LXN
0.618532777	8.24283E-05	< 0.001	< 0.001	2630	GBAP
0.618532777	8.24283E-05	< 0.001	< 0.001	729806	ZNF731P
0.618018031	8.38296E-05	< 0.001	< 0.001	3098	HK1
0.617760599	8.45379E-05	< 0.001	< 0.001	2038	EPB42
0.617760599	8.45379E-05	< 0.001	< 0.001	55049	C19orf60
0.617503166	8.52518E-05	< 0.001	< 0.001	10951	CBX1
0.617245853	8.59713E-05	0.001	1	7295	TXN
0.616216242	8.89048E-05	< 0.001	< 0.001	2925	GRPR
0.616216242	8.89048E-05	< 0.001	< 0.001	3298	HSF2
0.616216242	8.89048E-05	< 0.001	< 0.001	51725	FBXO40
0.615701377	9.04053E-05	0.001	1	55253	TYW1
0.615701377	9.04053E-05	< 0.001	< 0.001	56978	PRDM8
0.615444005	9.11636E-05	< 0.001	< 0.001	527	ATP6V0C
0.615444005	9.11636E-05	< 0.001	< 0.001	25851	TECPR1
0.615186572	9.19281E-05	< 0.001	< 0.001	4356	MPP3
0.614929199	9.26984E-05	< 0.001	< 0.001	4707	NDUFB1
0.614929199	9.26984E-05	< 0.001	< 0.001	9666	DZIP3
0.614929199	9.26984E-05	< 0.001	< 0.001	79148	MMP28
0.614929199	9.26984E-05	< 0.001	< 0.001	84261	FBXW9
0.614929199	9.26984E-05	< 0.001	< 0.001	286319	TUSC1
0.614414394	9.42566E-05	< 0.001	< 0.001	7993	UBXN8
0.614414394	9.42566E-05	< 0.001	< 0.001	91010	FMNL3
0.614414394	9.42566E-05	< 0.001	< 0.001	391722	LOC391722
0.614156961	9.50445E-05	0.001	1	203068	TUBB
0.613899648	9.58384E-05	< 0.001	< 0.001	150572	SMYD1
0.613642216	9.66384E-05	< 0.001	< 0.001	4312	MMP1
0.613642216	9.66384E-05	< 0.001	< 0.001	6400	SEL1L
0.613642216	9.66384E-05	0.001	1	23033	DOPEY1
0.613384783	9.74443E-05	< 0.001	< 0.001	881	CCIN
0.613127351	9.82561E-05	< 0.001	< 0.001	3251	HPRT1
0.613127351	9.82561E-05	< 0.001	< 0.001	6660	SOX5
0.613127351	9.82561E-05	< 0.001	< 0.001	9577	BRE
0.612870038	9.90737E-05	< 0.001	< 0.001	6728	SRP19
0.612612605	9.98983E-05	< 0.001	< 0.001	4151	MB
0.612612605	9.98983E-05	0.001	1	644242	LOC644242
0.612355173	0.000100728	< 0.001	< 0.001	79814	AGMAT
0.6120978	0.000101565	< 0.001	< 0.001	2742	GLRA2
0.6120978	0.000101565	< 0.001	< 0.001	401647	GOLGA7B
0.611840427	0.000102408	< 0.001	< 0.001	54495	TMX3
0.611840427	0.000102408	< 0.001	< 0.001	152641	C4ORF38
0.611840427	0.000102408	< 0.001	< 0.001	644189	LOC644189
0.611582994	0.000103257	0.001	1	168455	C7ORF74
0.611068189	0.000104975	< 0.001	< 0.001	1978	EIF4EBP1
0.610810816	0.000105843	< 0.001	< 0.001	4929	NR4A2
0.610553384	0.000106718	< 0.001	< 0.001	1386	ATF2
0.610553384	0.000106718	0.001	1	138009	DCAF4L2
0.610296011	0.000107599	< 0.001	< 0.001	10943	MSL3
0.610296011	0.000107599	< 0.001	< 0.001	51251	NT5C3
0.610296011	0.000107599	< 0.001	< 0.001	221491	C6orf1
0.609523833	0.000110283	< 0.001	< 0.001	5644	PRSS1
0.609523833	0.000110283	< 0.001	< 0.001	6398	SECTM1
0.6092664	0.000111191	< 0.001	< 0.001	29844	TFPT
0.609008968	0.000112106	< 0.001	< 0.001	5601	MAPK9
0.609008968	0.000112106	0.001	1	10007	GNPDA1
0.608751655	0.000113027	0.001	1	54566	EPB41L4B
0.608494222	0.000113955	< 0.001	< 0.001	389072	PLEKHM3

voxel-wise, subcortex			
Spearman's corr coef	p-val	GenID	Gene
0.445821106	< 0.001	221078	NSUN6
0.445693463	< 0.001	55013	CCDC109B
0.44566521	< 0.001	54509	RHOF
0.445519924	< 0.001	64283	AC093283.3
0.445476949	< 0.001	26297	SERGEF
0.445321947	< 0.001	644189	LOC644189
0.445230186	< 0.001	51256	TBC1D7
0.444995433	< 0.001	389634	RP11-90D4.2
0.444639206	< 0.001	2994	GYPB
0.444635123	< 0.001	1759	DNM1
0.443641365	< 0.001	5110	PCMT1
0.443594307	< 0.001	152006	RNF38
0.443497121	< 0.001	492303	GEMIN8P4
0.443491876	< 0.001	5575	PRKAR1B
0.443342835	< 0.001	64208	POPDC3
0.443074584	< 0.001	90523	C6orf142
0.442805469	< 0.001	11127	KIF3A
0.442599475	< 0.001	4887	NPY2R
0.442532122	< 0.001	374868	ATP9B
0.442286611	< 0.001	58491	ZNF71
0.44221729	< 0.001	5880	RAC2
0.442213476	< 0.001	221421	RSPH9
0.442196459	< 0.001	100507055	LRCOL1
0.442044765	< 0.001	10943	MSL3
0.442015648	< 0.001	3838	KPNA2
0.441862226	< 0.001	3098	HK1
0.441782951	< 0.001	11179	ZNF277
0.441720515	< 0.001	168455	C7ORF74
0.441652626	< 0.001	3156	HMGCR
0.441648275	< 0.001	360	AQP3
0.441541702	< 0.001	23254	RP1-21O18.1
0.441149026	< 0.001	645426	TMEM191C
0.441111863	< 0.001	1859	DYRK1A
0.440787613	< 0.001	10673	TNFSF13B
0.440766394	< 0.001	5899	RALB
0.440600365	< 0.001	23309	SIN3B
0.440588415	< 0.001	8739	HRK
0.440472215	< 0.001	7220	TRPC1
0.440387279	< 0.001	2709	GJB5
0.440051854	< 0.001	256380	SCML4
0.440012425	< 0.001	407738	FAM19A1
0.439882696	< 0.001	5619	PRM1
0.439430356	< 0.001	389072	PLEKHM3
0.439399511	< 0.001	282890	ZNF311
0.439390302	< 0.001	10955	SERINC3
0.43930763	< 0.001	84664	CSPG4P2Y
0.43903631	< 0.001	100288413	ERVMER34-1
0.43890965	< 0.001	56063	C1orf91
0.438808709	< 0.001	3727	JUND
0.438625276	< 0.001	84315	MON1A
0.438566864	< 0.001	3479	IGF1
0.438348442	< 0.001	27304	MOCS3
0.43818891	< 0.001	1176	AP3S1
0.438176095	< 0.001	644242	LOC644242
0.438092321	< 0.001	7301	TYRO3
0.437809825	< 0.001	79160	MGC4294
0.437786579	< 0.001	57465	TBC1D24
0.437731594	< 0.001	885	CCK
0.437561512	< 0.001	4062	LY6H
0.437533736	< 0.001	9901	SRGAP3
0.43728742	< 0.001	80728	AC084125.1
0.437102735	< 0.001	57823	SLAMF7
0.436622202	< 0.001	5746	PTH2R
0.436421335	< 0.001	1816	DRD5
0.436403841	< 0.001	138046	RALYL
0.436184406	< 0.001	1033	CDKN3
0.43609497	< 0.001	4232	MEST
0.436063021	< 0.001	3938	LCT
0.435957164	< 0.001	26262	TSPAN17
0.435880721	< 0.001	83852	SETDB2
0.435853243	< 0.001	8698	S1PR4
0.435807168	< 0.001	378805	AC058791.2
0.435704827	< 0.001	10133	OPTN
0.435548812	< 0.001	56907	SPIRE1
0.435524106	< 0.001	25927	CNRIP1
0.435433537	< 0.001	5328	PLAU
0.435276031	< 0.001	1434	CSE1L
0.435138643	< 0.001	2239	GPC4
0.435097396	< 0.001	2922	GRP

region-wise, subcortex					
Spearman's corr coef	p-val	p-val, SA	p-val, SA (BH- correction)	GenID	Gene
0.607979357	0.000115832	< 0.001	< 0.001	348980	HCN1
0.607721984	0.000116781	0.001	1	2098	ESD
0.607721984	0.000116781	< 0.001	< 0.001	3839	KPNA3
0.607721984	0.000116781	< 0.001	< 0.001	25901	CCDC28A
0.607464612	0.000117737	< 0.001	< 0.001	170685	NUDT10
0.607207179	0.0001187	< 0.001	< 0.001	402055	SRRD
0.606949806	0.000119671	< 0.001	< 0.001	51582	AZIN1
0.606692374	0.000120648	< 0.001	< 0.001	78997	GDAP1L1
0.606435001	0.000121633	< 0.001	< 0.001	126014	OSCAR
0.606435001	0.000121633	< 0.001	< 0.001	442308	LOC442308
0.606177628	0.000122624	< 0.001	< 0.001	758	MPPED1
0.605920196	0.000123624	0.001	1	1459	CSNK2A2
0.605920196	0.000123624	< 0.001	< 0.001	7173	TPO
0.605920196	0.000123624	< 0.001	< 0.001	255743	NPNT
0.604890585	0.000127693	< 0.001	< 0.001	6622	SNCA
0.604890585	0.000127693	< 0.001	< 0.001	643783	USP46-AS1
0.604633152	0.00012873	< 0.001	< 0.001	23467	NPTXR
0.603603601	0.00013295	< 0.001	< 0.001	3995	FADS3
0.603088796	0.000135107	< 0.001	< 0.001	9221	NOLC1
0.602831364	0.000136198	< 0.001	< 0.001	9218	VAPA
0.602831364	0.000136198	0.001	1	84656	AC020663.1
0.602831364	0.000136198	0.001	1	344787	ZNF860
0.602573991	0.000137296	< 0.001	< 0.001	85459	KIAA1731
0.602316618	0.000138402	0.001	1	100144602	RP11-807H7.1
0.602059186	0.000139516	< 0.001	< 0.001	8532	CPZ
0.601801813	0.000140638	< 0.001	< 0.001	123346	HIGD2B
0.601286948	0.000142908	0.001	1	885	CCK
0.601029634	0.000144054	< 0.001	< 0.001	4698	NDUFA5
0.601029634	0.000144054	0.001	1	5119	CHMP1A
0.600772202	0.000145209	0.001	1	670	BPHL
0.600257337	0.000147544	0.001	1	80086	TUBA4B
0.600000024	0.000148724	< 0.001	< 0.001	1008	CDH10
0.600000024	0.000148724	< 0.001	< 0.001	56654	NPDC1
0.600000024	0.000148724	0.001	1	79863	RBFA
0.599742591	0.000149913	< 0.001	< 0.001	2534	FYN
0.599742591	0.000149913	< 0.001	< 0.001	23266	LPHN2
0.599485159	0.00015111	0.001	1	2275	FHL3
0.599485159	0.00015111	0.001	1	4685	NCAM2
0.599485159	0.00015111	< 0.001	< 0.001	65264	UBE2Z
0.599485159	0.00015111	< 0.001	< 0.001	441027	TMEM150C
0.599227786	0.000152316	< 0.001	< 0.001	259236	TMIE
0.598970413	0.00015353	< 0.001	< 0.001	2747	GLUD2
0.598970413	0.00015353	0.001	1	4232	MEST
0.598712981	0.000154754	< 0.001	< 0.001	131375	LYZL4
0.598455608	0.000155985	0.001	1	1809	DPYSL3
0.598455608	0.000155985	< 0.001	< 0.001	79180	EFHD2
0.598455608	0.000155985	< 0.001	< 0.001	80725	AC115090.1
0.598198175	0.000157226	< 0.001	< 0.001	3398	ID2
0.598198175	0.000157226	< 0.001	< 0.001	80344	WDR23
0.59768337	0.000159734	< 0.001	< 0.001	57491	AHRR
0.59768337	0.000159734	< 0.001	< 0.001	64131	XYLT1
0.59768337	0.000159734	< 0.001	< 0.001	79595	SAP130
0.597168565	0.000162278	< 0.001	< 0.001	2626	GATA4
0.596911192	0.000163564	< 0.001	< 0.001	550631	CCDC157
0.596653819	0.000164859	< 0.001	< 0.001	100288413	ERVMER34-1
0.596396387	0.000166162	< 0.001	< 0.001	1175	AP2S1
0.596396387	0.000166162	< 0.001	< 0.001	130497	OSR1
0.596396387	0.000166162	< 0.001	< 0.001	284353	NKPD1
0.596138954	0.000167476	< 0.001	< 0.001	116988	AGAP3
0.595881641	0.000168798	0.001	1	78995	C17orf53
0.595624208	0.000170131	< 0.001	< 0.001	55335	NIPSNAP3B
0.595366776	0.000171472	< 0.001	< 0.001	10744	PTTG2
0.595366776	0.000171472	< 0.001	< 0.001	10888	GPR83
0.595366776	0.000171472	< 0.001	< 0.001	23014	FBXO21
0.595109344	0.000172823	< 0.001	< 0.001	988	CDC5L
0.59485203	0.000174183	< 0.001	< 0.001	9698	PUM1
0.594594598	0.000175554	< 0.001	< 0.001	56604	TUBB7P
0.594594598	0.000175554	0.001	1	140883	ZNF280B
0.594337165	0.000176934	0.001	1	8840	WISP1
0.594079792	0.000178323	0.001	1	1446	CSN1S1
0.594079792	0.000178323	0.001	1	8832	CD84
0.594079792	0.000178323	< 0.001	< 0.001	79161	C7orf23
0.593564987	0.000181132	< 0.001	< 0.001	2239	GPC4
0.593564987	0.000181132	< 0.001	< 0.001	84955	NUDCD1
0.593564987	0.000181132	< 0.001	< 0.001	203523	ZNF449
0.593564987	0.000181132	< 0.001	< 0.001	286514	MAGEB18
0.593564987	0.000181132	< 0.001	< 0.001	441461	RP11-60I3.5
0.593307614	0.000182551	< 0.001	< 0.001	635	BHMT
0.593307614	0.000182551	0.002	1	56477	CCL28

voxel-wise, subcortex			
Spearman's corr coef	p-val	GenID	Gene
0.434904695	< 0.001	84668	FAM126A
0.434889793	< 0.001	3223	HOXC6
0.434771121	< 0.001	51246	SHISA5
0.434520364	< 0.001	10197	PSME3
0.4344486	< 0.001	25818	KLK5
0.434351861	< 0.001	51251	NT5C3
0.43405354	< 0.001	273	AMPH
0.434038728	< 0.001	260434	PYDC1
0.433852762	< 0.001	79183	TTPAL
0.433802396	< 0.001	2566	GABRG2
0.433645546	< 0.001	4908	NTF3
0.433532983	< 0.001	3781	KCNN2
0.433472186	< 0.001	83661	MS4A8B
0.433461428	< 0.001	55117	SLC6A15
0.43321228	< 0.001	200734	SPRED2
0.433194429	< 0.001	375607	C7orf52
0.433094501	< 0.001	57369	GJD2
0.433006883	< 0.001	79595	SAP130
0.432854682	< 0.001	91442	C19orf40
0.432375073	< 0.001	3840	KPNA4
0.432369173	< 0.001	57586	SYT13
0.432340801	< 0.001	129049	SGSM1
0.432319999	< 0.001	286102	TMED10P1
0.432285368	< 0.001	9899	SV2B
0.43222937	< 0.001	64769	MEAF6
0.431816727	< 0.001	9014	TAF1B
0.431562841	< 0.001	9536	PTGES
0.431392312	< 0.001	53826	FXYD6
0.431308657	< 0.001	5834	PYGB
0.431209326	< 0.001	11187	PKP3
0.430978239	< 0.001	145483	FAM161B
0.430958539	< 0.001	126789	PUSL1
0.430906922	< 0.001	100288332	NPIPA5
0.430900544	< 0.001	10053	AP1M2
0.430747181	< 0.001	132332	TMEM155
0.43047291	< 0.001	55959	SULF2
0.430395961	< 0.001	5796	PTPRK
0.430349618	< 0.001	9938	ARHGAP25
0.430198222	< 0.001	9177	HTR3B
0.430158615	< 0.001	23035	PHLPP2
0.430151552	< 0.001	3619	INCENP
0.430120111	< 0.001	55742	PARVA
0.430050731	< 0.001	339965	CCDC158
0.429783762	< 0.001	3839	KPNA3
0.429597378	< 0.001	157567	ANKRD46
0.429475158	< 0.001	257313	UTS2D
0.429443449	< 0.001	3251	HPRT1
0.429317921	< 0.001	670	BPHL
0.42928046	< 0.001	391722	LOC391722
0.429270715	< 0.001	11073	TOPBP1
0.429235518	< 0.001	57526	PCDH19
0.429183543	< 0.001	150244	ZDHHC8P1
0.428843111	< 0.001	131375	LYZL4
0.428805083	< 0.001	8891	EIF2B3
0.428765714	< 0.001	9821	RB1CC1
0.428759485	< 0.001	57617	VPS18
0.428724289	< 0.001	650655	ABCA17P
0.428680867	< 0.001	440435	GPR179
0.428452104	< 0.001	6855	SYP
0.428411424	< 0.001	84216	TMEM117
0.42839843	< 0.001	84837	RP11-431H16.2
0.428301871	< 0.001	9218	VAPA
0.428289354	< 0.001	51693	TRAPPC2L
0.428283542	< 0.001	54749	EPDR1
0.4282718	< 0.001	1175	AP2S1
0.428164124	< 0.001	2534	FYN
0.428112209	< 0.001	5529	PPP2R5E
0.428042173	< 0.001	5922	RASA2
0.427972525	< 0.001	6482	ST3GAL1
0.427769214	< 0.001	56980	PRDM10
0.427742332	< 0.001	2767	GNA11
0.427455723	< 0.001	5874	RAB27B
0.427169561	< 0.001	89927	C16orf45
0.427145571	< 0.001	80381	CD276
0.427037179	< 0.001	83931	STK40
0.426957786	< 0.001	10439	OLFM1
0.426841885	< 0.001	196394	AMN1
0.426683187	< 0.001	56936	C14orf162
0.426622212	< 0.001	3157	HMGCS1

region-wise, subcortex					
Spearman's corr coef	p-val	p-val, SA	p-val, SA (BH- correction)	GenID	Gene
0.593307614	0.000182551	0.001	1	84691	FAM71F1
0.593307614	0.000182551	< 0.001	< 0.001	91574	C12orf65
0.593050182	0.00018398	< 0.001	< 0.001	55013	CCDC109B
0.593050182	0.00018398	< 0.001	< 0.001	55214	LEPREL1
0.592792749	0.000185419	< 0.001	< 0.001	9958	USP15
0.592792749	0.000185419	< 0.001	< 0.001	90874	ZNF697
0.592535377	0.000186868	< 0.001	< 0.001	79038	ZFYVE21
0.592020571	0.000189798	0.001	1	23002	DAAM1
0.592020571	0.000189798	0.001	1	27151	CPAMD8
0.592020571	0.000189798	< 0.001	< 0.001	390162	OR5M9
0.591763139	0.000191278	0.002	1	149951	COMMD7
0.591763139	0.000191278	< 0.001	< 0.001	387357	THEMIS
0.590990961	0.00019578	< 0.001	< 0.001	9373	PLAA
0.590990961	0.00019578	< 0.001	< 0.001	389043	LOC389043
0.590476215	0.000198834	0.001	1	8204	NRIP1
0.590476215	0.000198834	< 0.001	< 0.001	205327	C2orf69
0.590218782	0.000200377	< 0.001	< 0.001	2241	FER
0.590218782	0.000200377	< 0.001	< 0.001	6482	ST3GAL1
0.590218782	0.000200377	< 0.001	< 0.001	10046	MAMLD1
0.590218782	0.000200377	< 0.001	< 0.001	60558	GUF1
0.590218782	0.000200377	< 0.001	< 0.001	65123	INTS3
0.58996135	0.000201931	< 0.001	< 0.001	5796	PTPRK
0.589446604	0.000205071	0.002	1	89927	C16orf45
0.589446604	0.000205071	< 0.001	< 0.001	114787	GPRIN1
0.589189172	0.000206657	< 0.001	< 0.001	4810	NHS
0.589189172	0.000206657	0.001	1	25859	AC109486.1
0.588931799	0.000208255	< 0.001	< 0.001	55697	VAC14
0.588674366	0.000209863	< 0.001	< 0.001	23046	KIF21B
0.588674366	0.000209863	< 0.001	< 0.001	23673	STX12
0.588674366	0.000209863	< 0.001	< 0.001	65018	PINK1
0.588674366	0.000209863	< 0.001	< 0.001	145483	FAM161B
0.588416994	0.000211483	< 0.001	< 0.001	1577	CYP3A5
0.588159621	0.000213114	< 0.001	< 0.001	7042	TGFB2
0.588159621	0.000213114	< 0.001	< 0.001	8491	MAP4K3
0.587644756	0.00021641	< 0.001	< 0.001	1456	CSNK1G3
0.587644756	0.00021641	0.001	1	5076	PAX2
0.587644756	0.00021641	< 0.001	< 0.001	9537	TP53I11
0.587644756	0.00021641	< 0.001	< 0.001	10457	GPNMB
0.587644756	0.00021641	< 0.001	< 0.001	203547	VMA21
0.587387323	0.000218075	0.001	1	10038	PARP2
0.587387323	0.000218075	< 0.001	< 0.001	80000	KIAA1772
0.586872578	0.00022144	0.001	1	65057	ACD
0.586615145	0.000223139	< 0.001	< 0.001	6545	SLC7A4
0.586615145	0.000223139	< 0.001	< 0.001	80823	BHLHB9
0.586357772	0.00022485	0.001	1	1859	DYRK1A
0.586357772	0.00022485	0.001	1	7223	TRPC4
0.586357772	0.00022485	0.002	1	93974	ATPIF1
0.586357772	0.00022485	< 0.001	< 0.001	114824	PNMA5
0.586357772	0.00022485	0.004	1	196383	RILPL2
0.586357772	0.00022485	< 0.001	< 0.001	255061	TAC4
0.585842967	0.000228307	< 0.001	< 0.001	56776	FMN2
0.585585594	0.000230055	< 0.001	< 0.001	145837	LOC145837
0.585328162	0.000231813	< 0.001	< 0.001	3990	LIPC
0.585328162	0.000231813	< 0.001	< 0.001	4983	OPHN1
0.585328162	0.000231813	< 0.001	< 0.001	55228	PNMAL1
0.585070789	0.000233584	< 0.001	< 0.001	11073	TOPBP1
0.584813356	0.000235366	< 0.001	< 0.001	10927	SPIN1
0.584813356	0.000235366	< 0.001	< 0.001	11187	PKP3
0.584813356	0.000235366	< 0.001	< 0.001	93487	MAPK1IP1L
0.584813356	0.000235366	< 0.001	< 0.001	117154	DACH2
0.584813356	0.000235366	< 0.001	< 0.001	200407	CREG2
0.584555984	0.000237163	< 0.001	< 0.001	23439	ATP1B4
0.584555984	0.000237163	0.002	1	25939	SAMHD1
0.584555984	0.000237163	0.001	1	127703	C1orf216
0.584298551	0.000238969	0.001	1	79442	LRRC2
0.584298551	0.000238969	0.001	1	79969	C6orf134
0.584041178	0.00024079	< 0.001	< 0.001	26010	SPATS2L
0.583783805	0.000242621	0.001	1	5805	PTS
0.583783805	0.000242621	< 0.001	< 0.001	10695	CNPY3
0.583783805	0.000242621	< 0.001	< 0.001	55634	ZNF673
0.583783805	0.000242621	0.001	1	401232	RP1-90J20.7
0.583526373	0.000244467	0.001	1	4062	LY6H
0.583526373	0.000244467	< 0.001	< 0.001	90956	ADCK2
0.583526373	0.000244467	< 0.001	< 0.001	117581	TWIST2
0.583011627	0.000248194	< 0.001	< 0.001	8417	STX7
0.583011627	0.000248194	< 0.001	< 0.001	29985	SLC39A3
0.583011627	0.000248194	< 0.001	< 0.001	93973	ACTR8
0.582754195	0.000250076	0.001	1	8029	CUBN
0.582754195	0.000250076	< 0.001	< 0.001	8036	SHOC2

voxel-wise, subcortex			
Spearman's corr coef	p-val	GenID	Gene
0.426572621	< 0.001	79038	ZFYVE21
0.426223963	< 0.001	152641	C4ORF38
0.426140279	< 0.001	9917	FAM20B
0.426124185	< 0.001	10376	TUBA1B
0.426016927	< 0.001	6416	MAP2K4
0.425981164	< 0.001	27151	CPAMD8
0.42592302	< 0.001	643783	USP46-AS1
0.42587173	< 0.001	90874	ZNF697
0.425857693	< 0.001	23637	RABGAP1
0.425771803	< 0.001	26276	VPS33B
0.425731778	< 0.001	1012	CDH13
0.425726205	< 0.001	79644	SRD5A3
0.425274312	< 0.001	4664	NAB1
0.424999535	< 0.001	159	ADSS
0.424954385	< 0.001	22932	POMZP3
0.42491585	< 0.001	100113407	TMEM170B
0.424868315	< 0.001	51102	MECR
0.424699098	< 0.001	23760	PITPNB
0.424556881	< 0.001	56107	PCDHGA9
0.424545914	< 0.001	3096	HIVEP1
0.424289435	< 0.001	387103	C6orf173
0.42428872	< 0.001	10202	DHRS2
0.424288541	< 0.001	123041	SLC24A4
0.424254328	< 0.001	9948	WDR1
0.424166977	< 0.001	317	APAF1
0.424137205	< 0.001	1675	CFD
0.424103796	< 0.001	51569	UFM1
0.42406103	< 0.001	9908	G3BP2
0.424012035	< 0.001	23312	DMXL2
0.423781544	< 0.001	27346	TMEM97
0.42357251	< 0.001	3052	HCCS
0.423499644	< 0.001	84955	NUDCD1
0.423384756	< 0.001	113452	TMEM54
0.423174113	< 0.001	349667	RTN4RL2
0.423117369	< 0.001	4133	MAP2
0.423097342	< 0.001	917	CD3G
0.422896117	< 0.001	705	BYSL
0.422869354	< 0.001	338811	FAM19A2
0.422831744	< 0.001	5983	RFC3
0.422813743	< 0.001	79705	LRRK1
0.422748774	< 0.001	5987	TRIM27
0.422342539	< 0.001	9693	RAPGEF2
0.422308832	< 0.001	140597	TCEAL2
0.42221716	< 0.001	135154	C6orf57
0.422181875	< 0.001	55733	HHAT
0.422024339	< 0.001	6509	SLC1A4
0.421994239	< 0.001	339044	PKD1P1
0.421874762	< 0.001	8809	IL18R1
0.421861947	< 0.001	79637	ARMC7
0.421799541	< 0.001	635	BHMT
0.421778411	< 0.001	65997	RASL11B
0.42159161	< 0.001	55697	VAC14
0.421572059	< 0.001	9722	NOS1AP
0.42150712	< 0.001	5361	PLXNA1
0.42140612	< 0.001	80823	BHLHB9
0.421354622	< 0.001	10650	SLMO1
0.421344608	< 0.001	7224	TRPC5
0.420475274	< 0.001	153770	PLAC8L1
0.420371175	< 0.001	5214	PFKP
0.420199335	< 0.001	80114	BICC1
0.42005384	< 0.001	4983	OPHN1
0.419966131	< 0.001	1996	ELAVL4
0.419837713	< 0.001	6622	SNCA
0.419827968	< 0.001	79178	THTPA
0.419747174	< 0.001	84618	NT5C1A
0.419730037	< 0.001	11063	SOX30
0.41972363	< 0.001	11017	SNRNP27
0.419635534	< 0.001	728689	EIF3CL
0.419397056	< 0.001	63979	FIGNL1
0.41927281	< 0.001	2168	FABP1
0.419217587	< 0.001	283987	C17orf28
0.419202745	< 0.001	57670	KIAA1549
0.419181466	< 0.001	92235	DUSP27
0.419012189	< 0.001	54996	MOSC2
0.41881302	< 0.001	79838	TMC5
0.418723375	< 0.001	25901	CCDC28A
0.418562293	< 0.001	54862	CC2D1A
0.418052405	< 0.001	832	CAPZB
0.41785273	< 0.001	9424	KCNK6

region-wise, subcortex					
Spearman's corr coef	p-val	p-val, SA	p-val, SA (BH- correction)	GenID	Gene
0.582754195	0.000250076	< 0.001	< 0.001	10336	PCGF3
0.582496762	0.000251971	0.001	1	7345	UCHL1
0.582496762	0.000251971	0.001	1	22932	POMZP3
0.582496762	0.000251971	0.001	1	27285	TEKT2
0.582496762	0.000251971	0.002	1	140688	C20orf112
0.58223933	0.00025388	< 0.001	< 0.001	7533	YWHAH
0.581724584	0.000257735	< 0.001	< 0.001	5650	KLK7
0.581467152	0.000259682	0.001	1	5646	PRSS3
0.581467152	0.000259682	0.001	1	83445	GSG1
0.581467152	0.000259682	< 0.001	< 0.001	119032	C10orf32
0.580952406	0.000263616	< 0.001	< 0.001	3674	ITGA2B
0.580952406	0.000263616	< 0.001	< 0.001	64284	RAB17
0.580952406	0.000263616	0.001	1	339231	ARL16
0.580694973	0.000265603	< 0.001	< 0.001	153743	PPP1R2P3
0.580437601	0.000267604	< 0.001	< 0.001	167227	DCP2
0.579665363	0.000273686	< 0.001	< 0.001	57414	RHBDD2
0.57940799	0.000275742	0.001	1	1208	CLPS
0.579150558	0.000277809	< 0.001	< 0.001	83959	SLC4A11
0.578378379	0.0002841	< 0.001	< 0.001	7280	TUBB2A
0.578378379	0.0002841	0.003	1	54331	GNG2
0.578378379	0.0002841	0.001	1	81794	ADAMTS10
0.577863514	0.000288364	< 0.001	< 0.001	645426	TMEM191C
0.577606201	0.000290517	< 0.001	< 0.001	10318	TNIP1
0.577091336	0.000294866	< 0.001	< 0.001	7301	TYRO3
0.577091336	0.000294866	0.001	1	8572	PDLIM4
0.577091336	0.000294866	0.001	1	8742	TNFSF12
0.577091336	0.000294866	< 0.001	< 0.001	284656	RP4-783C10.1
0.576576591	0.000299274	< 0.001	< 0.001	2157	F8
0.576576591	0.000299274	0.001	1	9645	MICAL2
0.576576591	0.000299274	< 0.001	< 0.001	81706	PPP1R14C
0.576061785	0.000303741	< 0.001	< 0.001	84216	TMEM117
0.575804353	0.000305997	0.001	1	84276	NICN1
0.57554698	0.000308268	< 0.001	< 0.001	4636	MYL5
0.575289607	0.000310554	0.002	1	9922	IQSEC1
0.575032175	0.000312854	< 0.001	< 0.001	155006	TMEM213
0.574774742	0.000315171	< 0.001	< 0.001	5328	PLAU
0.574774742	0.000315171	0.001	1	55116	TMEM39B
0.574774742	0.000315171	< 0.001	< 0.001	80313	LRRC27
0.574517369	0.000317502	< 0.001	< 0.001	10523	CHERP
0.574517369	0.000317502	0.001	1	317648	C4ORF10
0.574259996	0.00031985	0.001	1	4718	NDUFC2
0.574259996	0.00031985	< 0.001	< 0.001	54522	ANKRD16
0.573745131	0.000324589	0.001	1	10444	ZER1
0.573487759	0.000326983	< 0.001	< 0.001	8556	CDC14A
0.573487759	0.000326983	< 0.001	< 0.001	57181	SLC39A10
0.573230386	0.000329393	0.001	1	6706	SPRR2G
0.573230386	0.000329393	< 0.001	< 0.001	8567	MADD
0.572972953	0.000331818	0.001	1	221421	RSPH9
0.572715521	0.00033426	< 0.001	< 0.001	55231	CCDC87
0.572715521	0.00033426	0.001	1	60312	AFAP1
0.571943343	0.00034168	< 0.001	< 0.001	283638	KIAA0284
0.571943343	0.00034168	< 0.001	< 0.001	347688	TUBB8
0.57168597	0.000344187	< 0.001	< 0.001	2170	FABP3
0.57168597	0.000344187	0.001	1	27000	DNAJC2
0.57168597	0.000344187	0.001	1	51154	MRTO4
0.57168597	0.000344187	< 0.001	< 0.001	57863	CADM3
0.571428537	0.000346709	0.002	1	49855	SCAPER
0.571428537	0.000346709	< 0.001	< 0.001	55790	CSGALNACT1
0.571428537	0.000346709	0.001	1	64854	USP46
0.571171165	0.000349249	< 0.001	< 0.001	1025	CDK9
0.571171165	0.000349249	0.001	1	79047	KCTD15
0.570656359	0.000354376	< 0.001	< 0.001	54809	SAMD9
0.570656359	0.000354376	0.001	1	57226	LYRM2
0.570141613	0.000359571	< 0.001	< 0.001	396	ARHGDIA
0.570141613	0.000359571	0.001	1	10665	C6orf10
0.570141613	0.000359571	< 0.001	< 0.001	25819	CCRN4L
0.570141613	0.000359571	0.001	1	114928	GPRASP2
0.569884181	0.000362195	0.001	1	3559	IL2RA
0.569884181	0.000362195	0.001	1	121665	AC069234.1
0.569884181	0.000362195	< 0.001	< 0.001	221078	NSUN6
0.569626749	0.000364835	0.001	1	2876	GPX1
0.569626749	0.000364835	< 0.001	< 0.001	4155	MBP
0.569626749	0.000364835	< 0.001	< 0.001	57521	RPTOR
0.569369316	0.000367491	0.001	1	25934	NIPSNAP3A
0.568597138	0.000375569	0.001	1	127933	UHMK1
0.568597138	0.000375569	0.001	1	145773	FAM81A
0.568339765	0.000378295	< 0.001	< 0.001	2731	GLDC
0.568339765	0.000378295	0.002	1	22873	DZIP1
0.568339765	0.000378295	< 0.001	< 0.001	57030	SLC17A7

voxel-wise, subcortex			
Spearman's corr coef	p-val	GenID	Gene
0.417659342	< 0.001	136853	SRCRB4D
0.417625874	< 0.001	2846	LPAR4
0.417466134	< 0.001	55816	DOK5
0.417426527	< 0.001	93664	CADPS2
0.417291731	< 0.001	4233	MET
0.417242885	< 0.001	9037	SEMA5A
0.416838825	< 0.001	8974	P4HA2
0.416809797	< 0.001	113	ADCY7
0.416788399	< 0.001	284996	RNF149
0.416781157	< 0.001	5025	P2RX4
0.416625708	< 0.001	8491	MAP4K3
0.416522533	< 0.001	9837	GINS1
0.416345716	< 0.001	124045	C16orf55
0.416319013	< 0.001	80020	FOXRED2
0.416135609	< 0.001	7428	VHL
0.416107565	< 0.001	51605	TRMT6
0.416064471	< 0.001	5805	PTS
0.415955156	< 0.001	3797	KIF3C
0.415704519	< 0.001	134111	UBE2QL1
0.415626973	< 0.001	4745	NELL1
0.415578604	< 0.001	81706	PPP1R14C
0.415513963	< 0.001	342850	ANKRD62
0.41549167	< 0.001	2048	EPHB2
0.415154397	< 0.001	10921	RNPS1
0.415131032	< 0.001	9114	ATP6V0D1
0.414970875	< 0.001	2630	GBAP
0.414725333	< 0.001	6456	SH3GL2
0.414704353	< 0.001	5176	SERPINF1
0.414688438	< 0.001	57622	LRFN1
0.414674163	< 0.001	9609	RAB36
0.414669961	< 0.001	127943	FCRLB
0.414669573	< 0.001	338879	RNASE10
0.414629728	< 0.001	5645	PRSS2
0.414468348	< 0.001	57120	GOPC
0.414409101	< 0.001	9399	STOML1
0.41435346	< 0.001	8260	NAA10
0.414192617	< 0.001	2959	GTF2B
0.414121807	< 0.001	728586	AC068658.1
0.414084256	< 0.001	4150	MAZ
0.413805604	< 0.001	2556	GABRA3
0.41372022	< 0.001	155	ADRB3
0.413687557	< 0.001	55706	TMEM48
0.413520217	< 0.001	55228	PNMAL1
0.413456202	< 0.001	54932	EXD3
0.41306749	< 0.001	80350	LPAL2
0.412791312	< 0.001	55294	FBXW7
0.412697792	< 0.001	51299	NRN1
0.412514329	< 0.001	613038	RP11-347C12.3
0.412487686	< 0.001	8120	AP3B2
0.412229717	< 0.001	7134	TNNC1
0.412225425	< 0.001	128553	TSHZ2
0.412219733	< 0.001	80274	SCUBE1
0.412149191	< 0.001	9575	CLOCK
0.412056416	< 0.001	4135	MAP6
0.411993742	< 0.001	51555	PEX5L
0.411886394	< 0.001	83877	TM2D2
0.411771566	< 0.001	10409	BASP1
0.411761105	< 0.001	40	ACCN1
0.411699384	< 0.001	23077	MYCBP2
0.411697954	< 0.001	7993	UBXN8
0.411516458	< 0.001	117157	SH2D1B
0.411500424	< 0.001	51218	GLRX5
0.411215514	< 0.001	401331	RASA4CP
0.411088854	< 0.001	440224	CXADRP3
0.411045015	< 0.001	114824	PNMA5
0.410830855	< 0.001	64848	YTHDC2
0.410617262	< 0.001	9223	MAGI1
0.410553336	< 0.001	285598	ARL10
0.410465211	< 0.001	149111	CNIH3
0.410384566	< 0.001	56271	BEX4
0.41036123	< 0.001	2098	ESD
0.410221308	< 0.001	23600	AMACR
0.410221189	< 0.001	29890	RBM15B
0.410194188	< 0.001	29919	C18orf8
0.410125822	< 0.001	134353	LSM11
0.40999195	< 0.001	80725	AC115090.1
0.409947634	< 0.001	33	ACADL
0.409836471	< 0.001	340204	C6orf127
0.409789175	< 0.001	154150	HDGFL1

region-wise, subcortex					
Spearman's corr coef	p-val	p-val, SA	p-val, SA (BH- correction)	GenID	Gene
0.568339765	0.000378295	< 0.001	< 0.001	59269	HIVEP3
0.568339765	0.000378295	0.003	1	338322	NLRP10
0.568082392	0.00038104	0.001	1	1589	CYP21A2
0.568082392	0.00038104	< 0.001	< 0.001	78991	PCYOX1L
0.56782496	0.000383803	0.001	1	1434	CSE1L
0.567567527	0.000386584	0.002	1	10650	SLMO1
0.567567527	0.000386584	< 0.001	< 0.001	23648	SSBP3
0.567567527	0.000386584	< 0.001	< 0.001	56829	ZC3HAV1
0.567310154	0.000389381	0.001	1	51177	PLEKHO1
0.567052782	0.000392198	< 0.001	< 0.001	7089	TLE2
0.566795349	0.000395032	< 0.001	< 0.001	9647	PPM1F
0.566795349	0.000395032	< 0.001	< 0.001	145282	MIPOL1
0.566537976	0.000397887	0.002	1	9612	NCOR2
0.566280544	0.000400757	0.001	1	8030	CCDC6
0.566280544	0.000400757	< 0.001	< 0.001	8809	IL18R1
0.566280544	0.000400757	0.001	1	54585	LZTFL1
0.566280544	0.000400757	< 0.001	< 0.001	80335	WDR82
0.566023111	0.000403647	< 0.001	< 0.001	22864	R3HDM2
0.566023111	0.000403647	0.001	1	54873	PALMD
0.566023111	0.000403647	< 0.001	< 0.001	65997	RASL11B
0.565765798	0.000406555	< 0.001	< 0.001	25766	PRPF40B
0.565765798	0.000406555	0.001	1	112858	TP53RK
0.565508366	0.000409483	0.002	1	3925	STMN1
0.565508366	0.000409483	< 0.001	< 0.001	7170	TPM3
0.565508366	0.000409483	< 0.001	< 0.001	10466	COG5
0.565508366	0.000409483	0.001	1	284611	FAM102B
0.565250933	0.000412429	0.003	1	138882	OR1N2
0.564993501	0.000415394	0.002	1	285855	RPL7L1
0.564736187	0.000418377	0.001	1	7337	UBE3A
0.564736187	0.000418377	< 0.001	< 0.001	26256	CABYR
0.564736187	0.000418377	< 0.001	< 0.001	342977	NANOS3
0.564478755	0.000421382	0.002	1	26281	FGF20
0.56396395	0.000427444	0.001	1	7187	TRAF3
0.563706577	0.000430505	0.003	1	9399	STOML1
0.563191772	0.000436687	< 0.001	< 0.001	84172	POLR1B
0.563191772	0.000436687	< 0.001	< 0.001	112942	CCDC104
0.563191772	0.000436687	< 0.001	< 0.001	138050	HGSNAT
0.563191772	0.000436687	0.001	1	196394	AMN1
0.562934339	0.000439806	0.001	1	10622	POLR3G
0.562934339	0.000439806	< 0.001	< 0.001	135935	NOBOX
0.562934339	0.000439806	0.001	1	100137047	JMJD7
0.562676966	0.000442946	< 0.001	< 0.001	2847	MCHR1
0.562676966	0.000442946	0.001	1	126272	EID2B
0.562419534	0.000446105	0.002	1	7014	TERF2
0.562419534	0.000446105	0.002	1	10202	DHRS2
0.562419534	0.000446105	0.001	1	26521	TIMM8B
0.562419534	0.000446105	0.001	1	286102	TMED10P1
0.562162161	0.000449286	0.001	1	7727	ZNF174
0.561647356	0.000455706	< 0.001	< 0.001	84707	BEX2
0.561647356	0.000455706	0.002	1	728689	EIF3CL
0.561389983	0.000458947	0.001	1	3692	EIF6
0.561389983	0.000458947	0.001	1	646174	C16orf90
0.560875118	0.000465492	0.002	1	284801	RP3-410C9.1
0.560617805	0.000468795	0.001	1	56980	PRDM10
0.560617805	0.000468795	< 0.001	< 0.001	92002	FAM58A
0.56010294	0.000475463	< 0.001	< 0.001	84247	LDOC1L
0.559845507	0.000478829	< 0.001	< 0.001	3049	HBQ1
0.559845507	0.000478829	0.002	1	55084	SOBP
0.559845507	0.000478829	0.001	1	147968	CAPN12
0.559845507	0.000478829	0.004	1	286023	AC009365.1
0.559845507	0.000478829	0.002	1	388121	TNFAIP8L3
0.559588194	0.000482216	< 0.001	< 0.001	10360	NPM3
0.559588194	0.000482216	0.001	1	51379	CRLF3
0.559588194	0.000482216	0.001	1	55010	C12orf48
0.559330761	0.000485624	0.001	1	2319	FLOT2
0.559330761	0.000485624	< 0.001	< 0.001	9172	MYOM2
0.559073329	0.000489054	< 0.001	< 0.001	6252	RTN1
0.559073329	0.000489054	0.001	1	9479	MAPK8IP1
0.558815956	0.000492507	< 0.001	< 0.001	100133234	SBF1P1
0.558558524	0.00049598	0.001	1	27289	RND1
0.558558524	0.00049598	0.002	1	79363	C1orf89
0.558558524	0.00049598	0.001	1	100130311	AC233723.1
0.558301151	0.000499475	< 0.001	< 0.001	29935	RPA4
0.558043778	0.000502992	< 0.001	< 0.001	100128553	CTAGE4
0.557786345	0.000506533	< 0.001	< 0.001	51291	GMIP
0.557786345	0.000506533	0.001	1	64788	LMF1
0.557528913	0.000510094	0.001	1	23603	CORO1C
0.557528913	0.000510094	0.003	1	57505	AARS2
0.557528913	0.000510094	< 0.001	< 0.001	83638	C11orf68

voxel-wise, subcortex			
Spearman's corr coef	p-val	GenID	Gene
0.409697771	< 0.001	55681	SCYL2
0.409667909	< 0.001	92912	UBE2Q2
0.409656763	< 0.001	5067	CNTN3
0.409646928	< 0.001	57491	AHRR
0.40961808	< 0.001	10479	SLC9A6
0.409547836	< 0.001	4139	MARK1
0.409531295	< 0.001	56776	FMN2
0.409409195	< 0.001	2675	GFRA2
0.409284234	< 0.001	51309	ARMCX1
0.409086347	< 0.001	56917	MEIS3P2
0.409001261	< 0.001	402509	SLC29A4P1
0.408925444	< 0.001	10076	PTPRU
0.408906221	< 0.001	1947	EFNB1
0.408808947	< 0.001	4486	MST1R
0.408619463	< 0.001	23673	STX12
0.408610523	< 0.001	55423	SIRPG
0.408540606	< 0.001	22885	ABLIM3
0.408422977	< 0.001	10299	MARCHF6
0.408366889	< 0.001	55879	GABRQ
0.408168823	< 0.001	54968	TMEM70
0.407992184	< 0.001	80097	FAM128B
0.40789184	< 0.001	643834	PGA3
0.407698691	< 0.001	63967	CLSPN
0.407588512	< 0.001	2664	GDI1
0.407545537	< 0.001	316	AOX1
0.407442659	< 0.001	10554	AGPAT1
0.40739882	< 0.001	10695	CNPY3
0.407156587	< 0.001	64284	RAB17
0.407103688	< 0.001	128434	VSTM2L
0.406946838	< 0.001	25940	FAM98A
0.406936169	< 0.001	10962	MLLT11
0.406904459	< 0.001	55973	BCAP29
0.406831443	< 0.001	401127	AC021148.2
0.40680939	< 0.001	57497	LRFN2
0.406737983	< 0.001	51343	FZR1
0.40667206	< 0.001	1446	CSN1S1
0.406592935	< 0.001	55565	ZNF821
0.406448722	< 0.001	921	CD5
0.40623799	< 0.001	83902	KRTAP17-1
0.406220108	< 0.001	55231	CCDC87
0.406096935	< 0.001	4856	NOV
0.406084388	< 0.001	10772	FUSIP1
0.405892342	< 0.001	5680	PSG11
0.405891746	< 0.001	283310	C12orf64
0.4057284	< 0.001	54555	DDX49
0.405706882	< 0.001	11124	FAF1
0.405552655	< 0.001	55662	HIF1AN
0.40528506	< 0.001	340385	ZNF517
0.405266851	< 0.001	152185	CCDC52
0.405226827	< 0.001	171177	RHOV
0.404717505	< 0.001	9578	AL117209.1
0.404479265	< 0.001	132789	GNPDA2
0.404292762	< 0.001	100288198	LOC100288198
0.404188961	< 0.001	55049	C19orf60
0.404180169	< 0.001	2802	GOLGA3
0.404141217	< 0.001	8572	PDLIM4
0.40409252	< 0.001	6853	SYN1
0.404023737	< 0.001	10056	FARSB
0.404006064	< 0.001	51207	DUSP13
0.403993517	< 0.001	5714	PSMD8
0.403865725	< 0.001	10927	SPIN1
0.403793216	< 0.001	10920	COPS8
0.403733343	< 0.001	3093	UBE2K
0.403679073	< 0.001	389384	C6orf222
0.4036403	< 0.001	645166	RP11-14N7.2
0.403563827	< 0.001	10631	POSTN
0.403499424	< 0.001	503639	DUXAP10
0.403259039	< 0.001	116328	C8orf34
0.403193742	< 0.001	5707	PSMD1
0.403151721	< 0.001	25830	SULT4A1
0.402898848	< 0.001	2222	FDFT1
0.402896941	< 0.001	26013	L3MBTL
0.402813375	< 0.001	10728	PTGES3
0.402775168	< 0.001	56925	LXN
0.40264079	< 0.001	10556	RPP30
0.402382374	< 0.001	158399	ZNF483
0.402203053	< 0.001	84261	FBXW9
0.402021229	< 0.001	55915	LANCL2
0.401894897	< 0.001	134430	WDR36

region-wise, subcortex					
Spearman's corr coef	p-val	p-val, SA	p-val, SA (BH- correction)	GenID	Gene
0.55727154	0.000513676	0.001	1	6305	SBF1
0.55727154	0.000513676	0.002	1	7428	VHL
0.55727154	0.000513676	< 0.001	< 0.001	26038	CHD5
0.556499302	0.000524563	0.001	1	3423	IDS
0.556499302	0.000524563	0.003	1	8270	LAGE3
0.556241989	0.000528237	< 0.001	< 0.001	56888	KCMF1
0.556241989	0.000528237	< 0.001	< 0.001	79703	C11orf80
0.556241989	0.000528237	0.001	1	115557	GEFT
0.555984557	0.000531934	< 0.001	< 0.001	392145	LOC392145
0.555469751	0.000539398	0.001	1	2996	GYPE
0.555469751	0.000539398	< 0.001	< 0.001	152098	ZCWPW2
0.555469751	0.000539398	0.001	1	100131482	LOC100131482
0.555212379	0.000543164	0.002	1	203238	C9orf93
0.554954946	0.000546955	< 0.001	< 0.001	445	ASS1
0.554954946	0.000546955	0.001	1	386672	KRTAP10-4
0.554697514	0.000550767	0.002	1	25999	CLIP3
0.554440141	0.000554604	0.001	1	3595	IL12RB2
0.554440141	0.000554604	0.001	1	6512	SLC1A7
0.553925335	0.000562351	0.001	1	1543	CYP1A1
0.553925335	0.000562351	< 0.001	< 0.001	51260	CXorf26
0.55341053	0.000570193	0.002	1	2863	GPR39
0.55341053	0.000570193	< 0.001	< 0.001	54972	TMEM132A
0.552895784	0.000578133	0.001	1	252969	NEIL2
0.552380919	0.00058617	0.001	1	284573	C1orf157
0.552123487	0.000590227	0.001	1	51102	MECR
0.551866174	0.000594308	0.001	1	8767	RIPK2
0.551866174	0.000594308	0.001	1	29970	IQCJ-SCHIP1
0.551866174	0.000594308	< 0.001	< 0.001	340267	COL28A1
0.551608741	0.000598413	0.001	1	57102	C12orf4
0.551608741	0.000598413	< 0.001	< 0.001	83636	C19orf12
0.551351309	0.000602545	0.002	1	523	ATP6V1A
0.551351309	0.000602545	0.001	1	795	S100G
0.551351309	0.000602545	0.001	1	1745	DLX1
0.551351309	0.000602545	< 0.001	< 0.001	5584	PRKCI
0.551093936	0.000606701	0.002	1	2898	GRIK2
0.551093936	0.000606701	0.002	1	10025	MED16
0.551093936	0.000606701	0.001	1	27072	VPS41
0.551093936	0.000606701	0.001	1	51646	YPEL5
0.550836563	0.000610885	< 0.001	< 0.001	129642	MBOAT2
0.550579131	0.000615091	0.001	1	51182	HSPA14
0.550579131	0.000615091	0.002	1	64326	RFWD2
0.550579131	0.000615091	0.005	1	165257	C1QL2
0.550321758	0.000619326	0.001	1	51207	DUSP13
0.550321758	0.000619326	< 0.001	< 0.001	54996	MOSC2
0.550321758	0.000619326	0.001	1	64963	MRPS11
0.549806952	0.000627873	< 0.001	< 0.001	7480	WNT10B
0.549806952	0.000627873	0.001	1	9442	MED27
0.549806952	0.000627873	0.001	1	200734	SPRED2
0.549806952	0.000627873	0.002	1	400553	AC134312.1
0.54954952	0.000632183	0.001	1	653145	ANXA8
0.549292147	0.000636521	< 0.001	< 0.001	9468	PCYT1B
0.549292147	0.000636521	< 0.001	< 0.001	54944	AC073548.1
0.549034715	0.000640885	0.001	1	390	RND3
0.549034715	0.000640885	0.001	1	84524	ZC3H8
0.548777342	0.000645278	0.004	1	124583	CANT1
0.548777342	0.000645278	0.002	1	345079	ANKRD56
0.548519969	0.000649696	0.001	1	25827	FBXL2
0.548262537	0.000654141	0.001	1	4097	MAFG
0.548262537	0.000654141	< 0.001	< 0.001	23314	SATB2
0.548005104	0.000658611	< 0.001	< 0.001	9615	GDA
0.548005104	0.000658611	0.001	1	9948	WDR1
0.548005104	0.000658611	0.002	1	26127	FGFR1OP2
0.548005104	0.000658611	0.001	1	126433	FBXO27
0.547747791	0.000663112	< 0.001	< 0.001	2677	GGCX
0.547747791	0.000663112	< 0.001	< 0.001	3781	KCNN2
0.547490358	0.000667639	0.001	1	57465	TBC1D24
0.547232926	0.000672194	< 0.001	< 0.001	3797	KIF3C
0.547232926	0.000672194	0.001	1	157567	ANKRD46
0.546975493	0.000676774	0.002	1	53917	RAB24
0.546975493	0.000676774	< 0.001	< 0.001	55565	ZNF821
0.546975493	0.000676774	0.001	1	64968	MRPS6
0.54671818	0.000681383	0.002	1	116328	C8orf34
0.54671818	0.000681383	0.003	1	283209	PGM2L1
0.546460748	0.000686022	< 0.001	< 0.001	6404	SELPLG
0.546460748	0.000686022	0.003	1	54584	GNB1L
0.546460748	0.000686022	0.003	1	57369	GJD2
0.546460748	0.000686022	0.002	1	200845	KCTD6
0.546203315	0.000690687	0.001	1	4887	NPY2R
0.54568857	0.000700102	0.003	1	108	ADCY2

voxel-wise, subcortex			
Spearman's corr coef	p-val	GenID	Gene
0.401879251	< 0.001	5125	PCSK5
0.401731163	< 0.001	10098	TSPAN5
0.401531726	< 0.001	23367	LARP1
0.40150708	< 0.001	55084	SOBP
0.40144524	< 0.001	58524	DMRT3
0.401322037	< 0.001	10242	KCNMB2
0.401046515	< 0.001	56651	LINC00470
0.400908232	< 0.001	55171	TBCCD1
0.400555789	< 0.001	1861	TOR1A
0.400518835	< 0.001	84246	MED10
0.400433779	< 0.001	83546	RTBDN
0.400315613	< 0.001	158798	AKAP14
0.400274962	< 0.001	85459	KIAA1731
0.399977148	< 0.001	123346	HIGD2B
0.399812222	< 0.001	254528	C16orf73
0.399770796	< 0.001	2067	ERCC1
0.3995848	< 0.001	79720	VPS37B
0.399443179	< 0.001	3749	KCNC4
0.399359405	< 0.001	79908	BTNL8
0.399344414	< 0.001	11212	PROSC
0.399280578	< 0.001	7334	UBE2N
0.399077058	< 0.001	25827	FBXL2
0.399024397	< 0.001	390162	OR5M9
0.398958325	< 0.001	56061	UBFD1
0.398636401	< 0.001	8554	PIAS1
0.398614705	< 0.001	91408	BTF3L4
0.398404002	< 0.001	54847	SIDT1
0.398391753	< 0.001	2744	GLS
0.398302317	< 0.001	285855	RPL7L1
0.398096442	< 0.001	83636	C19orf12
0.397877783	< 0.001	57226	LYRM2
0.397639126	< 0.001	3082	HGF
0.397488445	< 0.001	163	AP2B1
0.397426307	< 0.001	157753	TMEM74
0.397402763	< 0.001	4175	MCM6
0.397293389	< 0.001	23648	SSBP3
0.397164255	< 0.001	55210	ATAD3A
0.397156179	< 0.001	79414	LRFN3
0.397104383	< 0.001	3964	LGALS8
0.39710328	< 0.001	55500	ETNK1
0.396996528	< 0.001	284486	THEM5
0.39685455	< 0.001	80025	PANK2
0.396785438	< 0.001	81889	FAHD1
0.39677301	< 0.001	398	ARHGDIG
0.396748483	< 0.001	9620	CELSR1
0.396395504	< 0.001	646548	LOC646548
0.39625445	< 0.001	9378	NRXN1
0.396182477	< 0.001	374879	ZNF699
0.396012753	< 0.001	84792	C7orf70
0.395847589	< 0.001	21	ABCA3
0.395698965	< 0.001	3692	EIF6
0.395664543	< 0.001	1212	CLTB
0.395647705	< 0.001	84717	AC011498.3
0.395617425	< 0.001	124801	LSM12
0.395488381	< 0.001	9232	PTTG1
0.395474374	< 0.001	390079	OR52E8
0.395395041	< 0.001	7025	NR2F1
0.395310789	< 0.001	1589	CYP21A2
0.395239025	< 0.001	6248	RSC1A1
0.395219535	< 0.001	9686	VGLL4
0.395148456	< 0.001	54495	TMX3
0.39452675	< 0.001	399942	TUBAP2
0.394354761	< 0.001	57179	KIAA1191
0.394349605	< 0.001	10633	RASL10A
0.394038379	< 0.001	8655	DYNLL1
0.394033313	< 0.001	8938	BAIAP3
0.393892109	< 0.001	10898	CPSF4
0.393791199	< 0.001	23335	WDR7
0.393744737	< 0.001	6772	STAT1
0.393705904	< 0.001	114335	CGB1
0.393695295	< 0.001	284801	RP3-410C9.1
0.393625855	< 0.001	29767	TMOD2
0.393622071	< 0.001	113828	FAM83F
0.393575639	< 0.001	22906	TRAK1
0.39356035	< 0.001	113457	TUBA3D
0.393277884	< 0.001	1114	CHGB
0.393135756	< 0.001	84557	MAP1LC3A
0.393012911	< 0.001	200312	RNF215
0.393000513	< 0.001	10923	SUB1

region-wise, subcortex					
Spearman's corr coef	p-val	p-val, SA	p-val, SA (BH- correction)	GenID	Gene
0.545431137	0.000704852	0.002	1	23008	KLHDC10
0.545431137	0.000704852	0.001	1	64928	MRPL14
0.545173764	0.000709632	0.001	1	10093	ARPC4
0.544658899	0.000719275	0.001	1	7262	PHLDA2
0.544658899	0.000719275	< 0.001	< 0.001	89795	NAV3
0.544401526	0.00072414	0.001	1	7625	ZNF74
0.544401526	0.00072414	0.001	1	10799	RPP40
0.544144154	0.000729037	0.002	1	9609	RAB36
0.544144154	0.000729037	0.002	1	9735	KNTC1
0.544144154	0.000729037	0.003	1	285148	IAH1
0.543886721	0.000733961	0.006	1	90637	ZFAND2A
0.543629289	0.000738915	0.001	1	408	ARRB1
0.543629289	0.000738915	< 0.001	< 0.001	26262	TSPAN17
0.543629289	0.000738915	0.002	1	79862	ZNF669
0.543629289	0.000738915	0.002	1	93664	CADPS2
0.543629289	0.000738915	0.001	1	115290	FBXO17
0.543629289	0.000738915	0.001	1	116447	TOP1MT
0.543629289	0.000738915	0.001	1	360200	TMPRSS9
0.543371975	0.000743898	< 0.001	< 0.001	9016	SLC25A14
0.543371975	0.000743898	0.001	1	22911	WDR47
0.543371975	0.000743898	0.001	1	92344	GORAB
0.543114543	0.000748914	0.002	1	26040	SETBP1
0.543114543	0.000748914	0.002	1	51441	YTHDF2
0.543114543	0.000748914	0.001	1	203102	ADAM32
0.542599738	0.000759031	< 0.001	< 0.001	206358	SLC36A1
0.542599738	0.000759031	< 0.001	< 0.001	283310	C12orf64
0.542599738	0.000759031	< 0.001	< 0.001	728024	LOC728024
0.542342365	0.000764133	0.001	1	155382	VPS37D
0.542084932	0.000769269	0.001	1	1033	CDKN3
0.542084932	0.000769269	< 0.001	< 0.001	2770	GNAI1
0.542084932	0.000769269	0.002	1	5125	PCSK5
0.542084932	0.000769269	0.001	1	9693	RAPGEF2
0.542084932	0.000769269	< 0.001	< 0.001	55959	SULF2
0.542084932	0.000769269	0.004	1	83902	KRTAP17-1
0.5418275	0.000774434	0.003	1	4150	MAZ
0.5418275	0.000774434	0.003	1	56107	PCDHGA9
0.5418275	0.000774434	0.002	1	56955	MEPE
0.541570127	0.00077963	0.004	1	4282	MIF
0.541570127	0.00077963	0.001	1	4951	OCM2
0.541312754	0.000784856	0.001	1	2520	GAST
0.541312754	0.000784856	0.001	1	374868	ATP9B
0.541055322	0.000790114	< 0.001	< 0.001	10439	OLFM1
0.541055322	0.000790114	0.004	1	113802	C1orf59
0.541055322	0.000790114	< 0.001	< 0.001	645166	RP11-14N7.2
0.540797949	0.000795404	0.001	1	163	AP2B1
0.540797949	0.000795404	0.002	1	9929	JOSD1
0.540540516	0.000800726	0.003	1	143098	MPP7
0.540540516	0.000800726	0.001	1	389634	RP11-90D4.2
0.540283144	0.000806076	0.002	1	57692	MAGEE1
0.540283144	0.000806076	0.001	1	80728	AC084125.1
0.539768338	0.000816878	0.001	1	2029	ENSA
0.539768338	0.000816878	0.001	1	7355	SLC35A2
0.539768338	0.000816878	0.001	1	22906	TRAK1
0.539768338	0.000816878	< 0.001	< 0.001	64848	YTHDC2
0.539768338	0.000816878	0.003	1	79017	GGCT
0.539768338	0.000816878	0.001	1	132332	TMEM155
0.539510906	0.000822326	0.001	1	9382	COG1
0.539510906	0.000822326	0.001	1	27294	DHDH
0.539510906	0.000822326	0.001	1	84560	MT4
0.539253533	0.000827806	< 0.001	< 0.001	5110	PCMT1
0.539253533	0.000827806	0.001	1	26998	FETUB
0.539253533	0.000827806	0.003	1	54677	CROT
0.53899616	0.000833318	0.001	1	4355	MPP2
0.53899616	0.000833318	0.002	1	6870	TACR3
0.53899616	0.000833318	0.001	1	51373	MRPS17
0.53899616	0.000833318	0.001	1	81563	C1orf21
0.538738728	0.000838865	0.002	1	55593	OTUD5
0.538481295	0.000844443	0.002	1	51760	SYT17
0.538481295	0.000844443	0.001	1	389874	ZCCHC13
0.538223922	0.000850052	< 0.001	< 0.001	409	ARRB2
0.538223922	0.000850052	< 0.001	< 0.001	5496	PPM1G
0.538223922	0.000850052	0.002	1	80114	BICC1
0.538223922	0.000850052	0.004	1	123036	TC2N
0.537966549	0.000855696	0.002	1	5791	PTPRE
0.537966549	0.000855696	0.001	1	6613	SUMO2
0.537966549	0.000855696	0.002	1	11044	POLS
0.537966549	0.000855696	0.002	1	57489	ODF2L
0.537709117	0.000861373	0.003	1	9466	IL27RA
0.537451744	0.000867085	0.001	1	3213	HOXB3

voxel-wise, subcortex			
Spearman's corr coef	p-val	GenID	Gene
0.392998874	< 0.001	344901	OSTN
0.392966151	< 0.001	56683	C21orf59
0.392915905	< 0.001	7137	TNNI3
0.392884135	< 0.001	114960	TSGA13
0.39284417	< 0.001	8395	PIP5K1B
0.392788112	< 0.001	114818	KLHL29
0.392617285	< 0.001	390113	OR4X1
0.392587751	< 0.001	85460	ZNF518B
0.392534792	< 0.001	285315	C3orf33
0.392510265	< 0.001	11237	RNF24
0.392397583	< 0.001	89870	TRIM15
0.392388046	< 0.001	55757	UGCGL2
0.392357647	< 0.001	64805	P2RY12
0.392172456	< 0.001	286514	MAGEB18
0.392121375	< 0.001	57795	FAM5B
0.392052144	< 0.001	7477	WNT7B
0.392019004	< 0.001	997	CDC34
0.391931862	< 0.001	114548	NLRP3
0.391822278	< 0.001	444882	IGFL4
0.391550392	< 0.001	5802	PTPRS
0.39119193	< 0.001	727803	LOC727803
0.39116928	< 0.001	60676	PAPPA2
0.391083091	< 0.001	5493	PPL
0.390897125	< 0.001	11230	PRAF2
0.390830398	< 0.001	22881	ANKRD6
0.390656054	< 0.001	148	ADRA1A
0.390606105	< 0.001	51382	ATP6V1D
0.390202165	< 0.001	3355	HTR1F
0.39010182	< 0.001	114928	GPRASP2
0.390084326	< 0.001	284417	TMEM150B
0.390074998	< 0.001	9473	C1orf38
0.390025079	< 0.001	1600	DAB1
0.389651805	< 0.001	10007	GNPDA1
0.389649808	< 0.001	5787	PTPRB
0.389606476	< 0.001	157697	ERICH1
0.389593154	< 0.001	5271	SERPINB8
0.389406025	< 0.001	10093	ARPC4
0.389384568	< 0.001	23414	ZFPM2
0.38926962	< 0.001	152579	SCFD2
0.389232427	< 0.001	138009	DCAF4L2
0.389205992	< 0.001	90362	FAM110B
0.38912493	< 0.001	51282	SCAND1
0.388945609	< 0.001	7140	TNNT3
0.388889611	< 0.001	55545	MSX2P1
0.38886857	< 0.001	100288929	LOC100288929
0.38885957	< 0.001	138715	ARID3C
0.388848752	< 0.001	26086	GPSM1
0.388848037	< 0.001	79173	C19orf57
0.388596237	< 0.001	9643	MORF4L2
0.388580352	< 0.001	84850	GLIS3-AS1
0.388303667	< 0.001	84918	LRP11
0.388085186	< 0.001	154215	NKAIN2
0.388067663	< 0.001	1525	CXADR
0.388006449	< 0.001	3326	HSP90AB1
0.387943536	< 0.001	51276	ZNF571
0.387789696	< 0.001	1806	DPYD
0.387627989	< 0.001	84259	DCUN1D5
0.387452871	< 0.001	3717	JAK2
0.387228847	< 0.001	79717	PPCS
0.386880308	< 0.001	65260	SELRC1
0.386696339	< 0.001	7355	SLC35A2
0.386690438	< 0.001	2769	GNA15
0.386665732	< 0.001	26047	CNTNAP2
0.386615127	< 0.001	55088	C10orf118
0.38656202	< 0.001	219970	GLYATL2
0.386414111	< 0.001	4087	SMAD2
0.386412859	< 0.001	49855	SCAPER
0.386297911	< 0.001	130497	OSR1
0.386293441	< 0.001	9766	KIAA0247
0.386253804	< 0.001	10857	PGRMC1
0.386192739	< 0.001	3755	KCNG1
0.386178464	< 0.001	10040	TOM1L1
0.385990798	< 0.001	83638	C11orf68
0.385864228	< 0.001	8511	MMP23A
0.385795772	< 0.001	28988	DBNL
0.385759413	< 0.001	112858	TP53RK
0.385628611	< 0.001	26255	PTTG3
0.385624111	< 0.001	59345	GNB4
0.385573328	< 0.001	2103	ESRRB

region-wise, subcortex					
Spearman's corr coef	p-val	p-val, SA	p-val, SA (BH- correction)	GenID	Gene
0.537451744	0.000867085	0.002	1	131450	CD200R1
0.537194312	0.000872829	0.001	1	23167	EFR3A
0.537194312	0.000872829	0.003	1	645369	AP005433.1
0.536936939	0.000878607	0.001	1	1139	CHRNA7
0.536936939	0.000878607	0.001	1	339044	PKD1P1
0.536679506	0.000884419	0.001	1	8480	RAE1
0.536679506	0.000884419	0.001	1	10716	TBR1
0.536679506	0.000884419	0.002	1	66005	CHID1
0.536422133	0.000890267	< 0.001	< 0.001	57504	MTA3
0.536422133	0.000890267	0.001	1	100288332	NPIPA5
0.535907328	0.000902061	0.002	1	79173	C19orf57
0.535907328	0.000902061	0.001	1	161829	EXD1
0.535649955	0.000908009	0.004	1	284485	C1orf230
0.535392523	0.000913997	0.001	1	115353	LRRC42
0.53513509	0.000920014	0.003	1	374470	C12orf42
0.534877777	0.000926068	0.001	1	29765	TMOD4
0.534877777	0.000926068	< 0.001	< 0.001	51003	MED31
0.534877777	0.000926068	< 0.001	< 0.001	55117	SLC6A15
0.534877777	0.000926068	0.001	1	56907	SPIRE1
0.534620345	0.000932157	0.004	1	25814	ATXN10
0.534620345	0.000932157	0.002	1	116832	RPL39L
0.53410548	0.000944445	0.003	1	7201	TRHR
0.53410548	0.000944445	0.004	1	10633	RASL10A
0.533848166	0.000950641	0.004	1	3048	HBG2
0.533848166	0.000950641	0.001	1	9474	ATG5
0.533848166	0.000950641	< 0.001	< 0.001	10056	FARSB
0.533848166	0.000950641	0.004	1	55742	PARVA
0.533848166	0.000950641	0.001	1	79921	TCEAL4
0.533848166	0.000950641	0.002	1	91156	IGFN1
0.533075929	0.000969448	< 0.001	< 0.001	4014	LOR
0.533075929	0.000969448	< 0.001	< 0.001	9232	PTTG1
0.532818556	0.000975789	0.002	1	923	CD6
0.532818556	0.000975789	< 0.001	< 0.001	23141	ANKLE2
0.532818556	0.000975789	0.004	1	90865	IL33
0.532303751	0.000988583	0.001	1	80262	C16orf70
0.532046318	0.000995035	0.001	1	5719	PSMD13
0.532046318	0.000995035	0.001	1	374879	ZNF699
0.531788945	0.001001528	0.003	1	7247	TSN
0.531788945	0.001001528	0.003	1	8739	HRK
0.531788945	0.001001528	0.002	1	51166	AADAT
0.531531513	0.001008054	0.003	1	6772	STAT1
0.531531513	0.001008054	0.001	1	10868	USP20
0.531531513	0.001008054	0.001	1	23349	KIAA1045
0.531531513	0.001008054	0.001	1	55129	ANO10
0.531531513	0.001008054	0.006	1	164832	LONRF2
0.531531513	0.001008054	< 0.001	< 0.001	378925	RNF148
0.53127414	0.001014617	0.002	1	3223	HOXC6
0.531016707	0.001021222	0.003	1	23646	PLD3
0.530759275	0.001027864	< 0.001	< 0.001	55720	TSR1
0.530244529	0.00104126	0.002	1	162968	ZNF497
0.529987097	0.001048016	0.002	1	51586	MED15
0.529729724	0.001054814	0.002	1	55850	USE1
0.529729724	0.001054814	< 0.001	< 0.001	59349	KLHL12
0.529472351	0.001061648	0.003	1	59285	CACNG6
0.529472351	0.001061648	< 0.001	< 0.001	283358	B4GALNT3
0.529214919	0.001068519	0.003	1	79717	PPCS
0.528957486	0.001075433	0.004	1	11101	ATE1
0.528957486	0.001075433	0.001	1	154807	VKORC1L1
0.528957486	0.001075433	0.001	1	100507055	LRCOL1
0.528700113	0.001082384	0.003	1	5050	PAFAH1B3
0.528700113	0.001082384	0.002	1	80020	FOXRED2
0.528700113	0.001082384	0.001	1	80777	CYB5B
0.528700113	0.001082384	0.002	1	157697	ERICH1
0.52844274	0.00108938	0.004	1	1756	DMD
0.52844274	0.00108938	0.001	1	4884	NPTX1
0.52844274	0.00108938	< 0.001	< 0.001	126328	NDUFA11
0.528185308	0.00109641	0.005	1	374	AREG
0.528185308	0.00109641	0.002	1	1820	ARID3A
0.528185308	0.00109641	0.003	1	90050	FAM181A
0.527927935	0.001103482	< 0.001	< 0.001	57120	GOPC
0.527670503	0.001110595	0.002	1	58155	PTBP2
0.527670503	0.001110595	0.002	1	116028	C16orf75
0.52741313	0.001117751	0.003	1	7010	TEK
0.52741313	0.001117751	0.001	1	79654	HECTD3
0.52741313	0.001117751	0.003	1	126792	B3GALT6
0.52741313	0.001117751	0.001	1	170954	KIAA1949
0.527155757	0.001124945	0.001	1	948	CD36
0.527155757	0.001124945	0.001	1	4515	MTCP1
0.527155757	0.001124945	0.002	1	9626	GUCA1C

voxel-wise, subcortex			
Spearman's corr coef	p-val	GenID	Gene
0.385464251	< 0.001	3995	FADS3
0.385454267	< 0.001	80328	ULBP2
0.385395795	< 0.001	7345	UCHL1
0.38518551	< 0.001	9519	TBPL1
0.385158986	< 0.001	90480	GADD45GIP1
0.384975851	< 0.001	84153	RNASEH2C
0.384970754	< 0.001	3356	HTR2A
0.38494119	< 0.001	10626	TRIM16
0.384901762	< 0.001	387522	TMEM189-UBE2V1
0.384776622	< 0.001	57489	ODF2L
0.384771734	< 0.001	2845	GPR22
0.384644657	< 0.001	400359	C15orf53
0.384547979	< 0.001	1429	CRYZ
0.384444118	< 0.001	3763	KCNJ6
0.384418398	< 0.001	52	ACP1
0.384336472	< 0.001	200933	FBXO45
0.384236038	< 0.001	9829	DNAJC6
0.38422364	< 0.001	50614	GALNT9
0.384146929	< 0.001	65078	RTN4R
0.384061724	< 0.001	401027	C2orf66
0.383905023	< 0.001	26127	FGFR1OP2
0.383705676	< 0.001	9645	MICAL2
0.383660197	< 0.001	112	ADCY6
0.383660048	< 0.001	93487	MAPK1IP1L
0.383604497	< 0.001	255374	MBLAC1
0.383502543	< 0.001	56603	CYP26B1
0.383495659	< 0.001	57707	KIAA1609
0.383454382	< 0.001	26010	SPATS2L
0.383301735	< 0.001	55720	TSR1
0.383217037	< 0.001	9735	KNTC1
0.38321346	< 0.001	128308	MRPL55
0.383058935	< 0.001	401577	CD99P1
0.383015752	< 0.001	23266	LPHN2
0.382995248	< 0.001	9442	MED27
0.382813811	< 0.001	1608	DGKG
0.382771581	< 0.001	9532	BAG2
0.382729352	< 0.001	55964	SEPTIN3
0.382602721	< 0.001	79143	LENG4
0.382546544	< 0.001	129025	ZNF280A
0.382519662	< 0.001	221833	SP8
0.382205635	< 0.001	64426	SUDS3
0.382170528	< 0.001	643226	GRXCR2
0.382162452	< 0.001	57863	CADM3
0.382114202	< 0.001	89885	FATE1
0.382006109	< 0.001	5563	PRKAA2
0.381622851	< 0.001	2173	FABP7
0.381478906	< 0.001	4312	MMP1
0.381411016	< 0.001	22884	WDR37
0.381275207	< 0.001	286002	AC078937.4
0.381142348	< 0.001	923	CD6
0.380884081	< 0.001	112849	C14orf149
0.380837202	< 0.001	149473	CCDC24
0.38074702	< 0.001	79815	NIPAL2
0.380664945	< 0.001	29841	GRHL1
0.380482435	< 0.001	6620	SNCB
0.380416691	< 0.001	10505	SEMA4F
0.38014251	< 0.001	374	AREG
0.379990458	< 0.001	222658	KCTD20
0.379968166	< 0.001	283267	LINC00294
0.37992534	< 0.001	5340	PLG
0.379861236	< 0.001	23053	KIAA0913
0.379847705	< 0.001	201475	RAB12
0.379518628	< 0.001	378925	RNF148
0.379467189	< 0.001	7410	VAV2
0.37945202	< 0.001	57579	FAM135A
0.379419774	< 0.001	5700	PSMC1
0.379129887	< 0.001	3269	HRH1
0.379123688	< 0.001	3299	HSF4
0.378982931	< 0.001	124220	ZG16B
0.3787902	< 0.001	10141	C4orf6
0.378689855	< 0.001	27098	CLUL1
0.378579527	< 0.001	90379	DCAF15
0.378562987	< 0.001	11001	SLC27A2
0.37854746	< 0.001	3925	STMN1
0.378481746	< 0.001	10687	PNMA2
0.37837857	< 0.001	5791	PTPRE
0.378084421	< 0.001	389898	UBE2NL
0.377928555	< 0.001	64895	PAPOLG
0.377924502	< 0.001	253782	LASS6

region-wise, subcortex					
Spearman's corr coef	p-val	p-val, SA	p-val, SA (BH- correction)	GenID	Gene
0.527155757	0.001124945	< 0.001	< 0.001	143384	C10orf46
0.526640892	0.001139454	0.001	1	5987	TRIM27
0.526640892	0.001139454	< 0.001	< 0.001	8310	ACOX3
0.526383519	0.001146776	0.005	1	55879	GABRQ
0.525868714	0.001161535	0.001	1	1007	CDH9
0.525868714	0.001161535	0.001	1	5880	RAC2
0.525868714	0.001161535	0.002	1	10376	TUBA1B
0.525868714	0.001161535	0.001	1	221935	SDK1
0.525611281	0.001168976	0.002	1	642778	AC136443.1
0.525353968	0.001176461	0.002	1	4953	ODC1
0.525353968	0.001176461	0.004	1	654433	AC016683.6
0.525096536	0.001183991	0.003	1	7220	TRPC1
0.525096536	0.001183991	0.003	1	26255	PTTG3
0.524839103	0.001191561	0.004	1	23787	MTCH1
0.52458173	0.001199174	0.005	1	5595	MAPK3
0.52458173	0.001199174	0.002	1	6439	SFTPB
0.52458173	0.001199174	0.003	1	54862	CC2D1A
0.52458173	0.001199174	0.001	1	55206	SBNO1
0.52458173	0.001199174	0.002	1	151242	PPP1R1C
0.524324298	0.001206829	< 0.001	< 0.001	55860	ACTR10
0.524324298	0.001206829	0.002	1	117143	TADA1L
0.524066925	0.00121453	0.003	1	5932	RBBP8
0.524066925	0.00121453	0.005	1	6565	SLC15A2
0.524066925	0.00121453	0.001	1	51256	TBC1D7
0.523809493	0.001222274	0.003	1	153364	MBLAC2
0.523809493	0.001222274	0.001	1	401027	C2orf66
0.52355212	0.001230058	0.002	1	9578	AL117209.1
0.52355212	0.001230058	0.003	1	55816	DOK5
0.52355212	0.001230058	0.003	1	126353	C19orf21
0.523294687	0.00123789	0.002	1	80237	ELL3
0.523037314	0.001245765	0.001	1	5985	RFC5
0.523037314	0.001245765	0.001	1	23507	LRRC8B
0.522779942	0.001253683	0.002	1	51633	OTUD6B
0.522779942	0.001253683	0.002	1	283078	MKX
0.522522509	0.001261646	0.001	1	10248	POP7
0.522265077	0.001269653	0.001	1	140606	SELM
0.522007763	0.001277705	0.003	1	10505	SEMA4F
0.522007763	0.001277705	0.004	1	54662	TBC1D13
0.521492898	0.001293947	0.001	1	2786	GNG4
0.521492898	0.001293947	0.003	1	4761	NEUROD2
0.521492898	0.001293947	0.002	1	29108	PYCARD
0.521492898	0.001293947	0.002	1	56936	C14orf162
0.521492898	0.001293947	< 0.001	< 0.001	79637	ARMC7
0.521235466	0.001302138	0.007	1	711	ERC2-IT1
0.521235466	0.001302138	0.001	1	84222	TMEM191A
0.520978153	0.00131037	0.002	1	100192379	AC079341.1
0.520463288	0.00132698	0.002	1	3800	KIF5C
0.520463288	0.00132698	0.001	1	6709	SPTAN1
0.520205915	0.001335353	0.003	1	10141	C4orf6
0.520205915	0.001335353	0.002	1	56061	UBFD1
0.519948542	0.001343772	0.001	1	3350	HTR1A
0.51969111	0.001352242	0.002	1	9659	PDE4DIP
0.519433737	0.001360756	0.001	1	7552	ZNF711
0.519433737	0.001360756	0.001	1	8895	CPNE3
0.519433737	0.001360756	0.001	1	9319	TRIP13
0.519433737	0.001360756	0.003	1	9858	KIAA0649
0.519433737	0.001360756	0.002	1	25977	NECAP1
0.519176304	0.001369317	0.003	1	11128	POLR3A
0.519176304	0.001369317	0.004	1	55351	STK32B
0.519176304	0.001369317	0.001	1	84253	GARNL3
0.518918931	0.001377923	0.003	1	11063	SOX30
0.518918931	0.001377923	0.004	1	84277	DNAJC30
0.518661499	0.001386582	0.001	1	8554	PIAS1
0.518661499	0.001386582	0.002	1	28986	MAGEH1
0.518661499	0.001386582	< 0.001	< 0.001	55812	SPATA7
0.518404126	0.001395288	0.001	1	85460	ZNF518B
0.517889321	0.001412841	< 0.001	< 0.001	222658	KCTD20
0.517889321	0.001412841	0.001	1	378884	NHLRC1
0.517631948	0.001421691	0.001	1	4135	MAP6
0.517631948	0.001421691	0.002	1	64420	SUSD1
0.517631948	0.001421691	0.003	1	84536	C21orf67
0.517117083	0.001439542	< 0.001	< 0.001	10955	SERINC3
0.517117083	0.001439542	0.002	1	57150	C6orf162
0.516859651	0.001448539	0.002	1	5834	PYGB
0.516859651	0.001448539	0.001	1	58491	ZNF71
0.516859651	0.001448539	0.003	1	79980	DSN1
0.516859651	0.001448539	0.002	1	317781	DDX51
0.516602337	0.001457586	0.003	1	9071	CLDN10
0.516344905	0.001466685	0.002	1	84058	WDR54

voxel-wise, subcortex			
Spearman's corr coef	p-val	GenID	Gene
0.377845615	< 0.001	161882	ZFPM1
0.377711326	< 0.001	121273	C12orf54
0.377683461	< 0.001	7223	TRPC4
0.377644509	< 0.001	84639	IL1F10
0.377624065	< 0.001	56938	ARNTL2
0.377154976	< 0.001	29765	TMOD4
0.377144694	< 0.001	64393	ZMAT3
0.376975	< 0.001	2785	GNG3
0.376918584	< 0.001	387893	SETD8
0.376888454	< 0.001	4885	NPTX2
0.376745999	< 0.001	2516	NR5A1
0.376700252	< 0.001	79822	ARHGAP28
0.376686454	< 0.001	606551	UBE2MP1
0.376552373	< 0.001	55631	LRRC40
0.375990838	< 0.001	23075	SWAP70
0.375989228	< 0.001	134829	CLVS2
0.375807285	< 0.001	203447	NRK
0.375786155	< 0.001	22820	COPG
0.375688195	< 0.001	219621	C10orf107
0.375677466	< 0.001	202500	TCTE1
0.375672579	< 0.001	145581	LRFN5
0.375492215	< 0.001	54480	CSGLCA-T
0.375488758	< 0.001	5889	RAD51C
0.375477523	< 0.001	386672	KRTAP10-4
0.375470757	< 0.001	7026	NR2F2
0.375450641	< 0.001	283767	AC116165.2
0.375362337	< 0.001	8131	C16orf35
0.375190169	< 0.001	9698	PUM1
0.374964446	< 0.001	817	CAMK2D
0.374678135	< 0.001	55176	SEC61A2
0.37462762	< 0.001	2590	GALNT2
0.374441981	< 0.001	57159	TRIM54
0.374382257	< 0.001	7552	ZNF711
0.374337226	< 0.001	79729	C1orf113
0.374312192	< 0.001	84966	IGSF21
0.374253929	< 0.001	5099	PCDH7
0.374137551	< 0.001	392288	LOC392288
0.374012947	< 0.001	1000	CDH2
0.373919994	< 0.001	4147	MATN2
0.373917699	< 0.001	7846	TUBA1A
0.373802304	< 0.001	55219	TMEM57
0.373788148	< 0.001	728613	LOC728613
0.373777151	< 0.001	387890	AC002563.2
0.373487741	< 0.001	64788	LMF1
0.373426199	< 0.001	219870	OR10G9
0.373261005	< 0.001	80023	NRSN2
0.373211563	< 0.001	440799	P2RX6P
0.373078257	< 0.001	2778	GNAS
0.372955203	< 0.001	65263	PYCRL
0.372871011	< 0.001	283316	CD163L1
0.372869015	< 0.001	486	FXYD2
0.372822642	< 0.001	9892	SNAP91
0.372662246	< 0.001	199920	C1orf168
0.372514725	< 0.001	10518	CIB2
0.372490495	< 0.001	93973	ACTR8
0.37240535	< 0.001	1284	COL4A2
0.372295469	< 0.001	63939	C20orf177
0.372293442	< 0.001	10044	SH2D3C
0.372265726	< 0.001	84871	AGBL4
0.372089118	< 0.001	122970	ACOT4
0.371972024	< 0.001	647476	FLJ20518
0.371951222	< 0.001	91574	C12orf65
0.371901691	< 0.001	23326	USP22
0.371889174	< 0.001	474343	SPIN2B
0.371789694	< 0.001	4878	NPPA
0.371626109	< 0.001	2898	GRIK2
0.37162444	< 0.001	57679	ALS2
0.371607482	< 0.001	55003	PAK1IP1
0.37149781	< 0.001	220042	C11orf82
0.371474922	< 0.001	360200	TMPRSS9
0.371439159	< 0.001	11202	KLK8
0.371393412	< 0.001	23476	BRD4
0.371374249	< 0.001	3298	HSF2
0.371294618	< 0.001	340481	ZDHHC21
0.371278584	< 0.001	5985	RFC5
0.371278256	< 0.001	116988	AGAP3
0.371269226	< 0.001	23185	LARP4B
0.371222138	< 0.001	10652	YKT6
0.371143609	< 0.001	55215	FANCI

region-wise, subcortex					
Spearman's corr coef	p-val	p-val, SA	p-val, SA (BH- correction)	GenID	Gene
0.5158301	0.001485029	< 0.001	< 0.001	1572	CYP2F1
0.5158301	0.001485029	0.003	1	5911	RAP2A
0.515572727	0.001494277	0.004	1	9167	COX7A2L
0.515572727	0.001494277	0.005	1	56171	DNAH7
0.515315294	0.001503575	0.002	1	990	CDC6
0.515315294	0.001503575	0.002	1	91408	BTF3L4
0.515315294	0.001503575	0.003	1	114571	SLC22A9
0.515315294	0.001503575	0.008	1	126374	WTIP
0.515315294	0.001503575	0.002	1	414918	FAM116B
0.515057921	0.001512929	0.002	1	55190	NUDT11
0.515057921	0.001512929	0.001	1	344387	CDKL4
0.514800489	0.00152233	0.004	1	157753	TMEM74
0.514543116	0.001531783	0.005	1	57586	SYT13
0.514285743	0.001541287	0.001	1	4094	MAF
0.514285743	0.001541287	0.003	1	51393	TRPV2
0.514285743	0.001541287	< 0.001	< 0.001	54869	EPS8L1
0.514285743	0.001541287	0.001	1	255374	MBLAC1
0.514028311	0.001550848	0.001	1	54904	WHSC1L1
0.514028311	0.001550848	0.003	1	148979	GLIS1
0.513770878	0.001560454	0.003	1	149111	CNIH3
0.513513505	0.001570116	0.006	1	199920	C1orf168
0.513513505	0.001570116	< 0.001	< 0.001	728734	LOC728734
0.513256133	0.00157983	0.005	1	8395	PIP5K1B
0.513256133	0.00157983	< 0.001	< 0.001	201595	STT3B
0.5129987	0.001589601	0.003	1	4087	SMAD2
0.5129987	0.001589601	0.003	1	10817	FRS3
0.5129987	0.001589601	0.001	1	23075	SWAP70
0.5129987	0.001589601	0.003	1	90780	PYGO2
0.5129987	0.001589601	0.003	1	317772	HIST2H2AB
0.512741327	0.001599421	0.003	1	3845	KRAS
0.512741327	0.001599421	0.001	1	26276	VPS33B
0.512741327	0.001599421	0.001	1	128854	TSPY26P
0.512741327	0.001599421	0.003	1	286002	AC078937.4
0.512483895	0.001609295	0.005	1	921	CD5
0.512226522	0.001619223	0.001	1	892	CCNC
0.512226522	0.001619223	0.002	1	5717	PSMD11
0.51196909	0.001629204	0.003	1	8511	MMP23A
0.51196909	0.001629204	0.005	1	23171	GPD1L
0.511711717	0.001639244	0.001	1	493	ATP2B4
0.511711717	0.001639244	0.001	1	2994	GYPB
0.511711717	0.001639244	0.008	1	5612	PRKRIR
0.511711717	0.001639244	0.004	1	6585	SLIT1
0.511711717	0.001639244	0.002	1	55532	SLC30A10
0.511454284	0.001649335	0.002	1	3843	IPO5
0.511454284	0.001649335	0.001	1	387715	ARMS2
0.511196911	0.001659477	0.004	1	4046	LSP1
0.511196911	0.001659477	0.004	1	80350	LPAL2
0.511196911	0.001659477	< 0.001	< 0.001	440917	LOC440917
0.510939479	0.001669677	0.001	1	4082	MARCKS
0.510939479	0.001669677	0.002	1	5273	SERPINB10
0.510939479	0.001669677	0.003	1	10313	RTN3
0.510939479	0.001669677	0.003	1	51606	ATP6V1H
0.510682106	0.001679937	0.001	1	5700	PSMC1
0.510682106	0.001679937	0.009	1	6307	SC4MOL
0.510424674	0.001690248	0.004	1	113828	FAM83F
0.510424674	0.001690248	< 0.001	< 0.001	344901	OSTN
0.510167301	0.001700615	0.003	1	1193	CLIC2
0.510167301	0.001700615	0.003	1	58525	WIZ
0.510167301	0.001700615	0.001	1	84954	MPND
0.509909928	0.001711038	0.002	1	58475	MS4A7
0.509909928	0.001711038	0.003	1	80731	THSD7B
0.509909928	0.001711038	0.002	1	81929	SEH1L
0.509909928	0.001711038	0.001	1	90525	SHF
0.509652495	0.001721516	0.005	1	1453	CSNK1D
0.509652495	0.001721516	0.001	1	192683	SCAMP5
0.509652495	0.001721516	0.001	1	196743	PAOX
0.509395123	0.001732056	0.002	1	6248	RSC1A1
0.509395123	0.001732056	0.004	1	201283	AMZ2P1
0.50913769	0.001742649	0.005	1	222553	SLC35F1
0.508880317	0.001753296	0.003	1	27304	MOCS3
0.508880317	0.001753296	0.009	1	56852	RAD18
0.508622885	0.001764002	0.003	1	7738	ZNF184
0.508622885	0.001764002	0.001	1	51329	ARL6IP4
0.508365512	0.001774771	0.003	1	10019	SH2B3
0.508365512	0.001774771	0.003	1	63827	BCAN
0.508365512	0.001774771	0.006	1	91442	C19orf40
0.508365512	0.001774771	0.005	1	503693	LOH12CR2
0.508365512	0.001774771	0.006	1	100126784	LOC100126784
0.508108079	0.001785594	0.001	1	5274	SERPINI1

voxel-wise, subcortex			
Spearman's corr coef	p-val	GenID	Gene
0.371057063	< 0.001	255061	TAC4
0.371052504	< 0.001	647288	CTAGE11P
0.370954126	< 0.001	23670	TMEM2
0.370952636	< 0.001	80000	KIAA1772
0.37089932	< 0.001	8988	HSPB3
0.370890051	< 0.001	460	ASTN1
0.370859295	< 0.001	8900	CCNA1
0.370812863	< 0.001	54492	NEURL1B
0.370792776	< 0.001	145957	NRG4
0.37068221	< 0.001	84222	TMEM191A
0.370440096	< 0.001	56852	RAD18
0.370377272	< 0.001	79921	TCEAL4
0.370338172	< 0.001	7337	UBE3A
0.370288312	< 0.001	286887	KRT6C
0.370208025	< 0.001	83539	CHST9
0.370062143	< 0.001	729515	TMEM242
0.370018274	< 0.001	100126784	LOC100126784
0.369915783	< 0.001	7504	XK
0.369744718	< 0.001	200523	C2orf51
0.369717479	< 0.001	23162	MAPK8IP3
0.369709909	< 0.001	5601	MAPK9
0.369627655	< 0.001	91137	SLC25A46
0.369617313	< 0.001	112611	RWDD2A
0.369596362	< 0.001	778	CACNA1F
0.369392335	< 0.001	81573	ANKRD13C
0.369248271	< 0.001	357	SHROOM2
0.369221002	< 0.001	7306	TYRP1
0.369220912	< 0.001	196743	PAOX
0.369063139	< 0.001	63876	PKNOX2
0.368998319	< 0.001	65094	JMJD4
0.368981183	< 0.001	554206	LOC554206
0.368939638	< 0.001	256536	TCERG1L
0.368836761	< 0.001	285848	PNPLA1
0.36881119	< 0.001	26985	AP3M1
0.368801802	< 0.001	338322	NLRP10
0.368685454	< 0.001	7529	YWHAB
0.36862132	< 0.001	151477	LINC00471
0.368510991	< 0.001	64218	SEMA4A
0.36846754	< 0.001	338667	LOC338667
0.368438065	< 0.001	392617	PPP1R28
0.368313372	< 0.001	7411	VBP1
0.368162662	< 0.001	4014	LOR
0.368066639	< 0.001	166752	FREM3
0.368060142	< 0.001	642273	FAM110C
0.367987424	< 0.001	56654	NPDC1
0.367862284	< 0.001	25977	NECAP1
0.367772311	< 0.001	245806	VGLL2
0.367772162	< 0.001	8239	USP9X
0.367647916	< 0.001	57134	MAN1C1
0.367568552	< 0.001	9551	ATP5J2
0.367383331	< 0.001	201229	C17orf108
0.367370784	< 0.001	4074	M6PR
0.367350817	< 0.001	396	ARHGDIA
0.367274016	< 0.001	340267	COL28A1
0.367269933	< 0.001	6515	SLC2A3
0.367209762	< 0.001	23172	FAM175B
0.367111236	< 0.001	23439	ATP1B4
0.367074192	< 0.001	27253	PCDH17
0.366977781	< 0.001	55071	C9orf40
0.366969049	< 0.001	246243	RNASEH1
0.36690563	< 0.001	84253	GARNL3
0.366868466	< 0.001	85369	FAM40A
0.366821498	< 0.001	729141	LOC729141
0.366773844	< 0.001	5274	SERPINI1
0.366627157	< 0.001	55670	PEX26
0.366549492	< 0.001	10612	TRIM3
0.366537422	< 0.001	9466	IL27RA
0.366483748	< 0.001	2626	GATA4
0.366369426	< 0.001	112597	LINC00152
0.366354644	< 0.001	57210	SLC45A4
0.366351396	< 0.001	9666	DZIP3
0.366181701	< 0.001	395	ARHGAP6
0.366076529	< 0.001	388387	AC100793.3
0.366052151	< 0.001	199964	TMEM61
0.366011828	< 0.001	5786	PTPRA
0.366011411	< 0.001	80777	CYB5B
0.365963876	< 0.001	151987	PPP4R2
0.365962803	< 0.001	10523	CHERP
0.365836054	< 0.001	6721	SREBF2

region-wise, subcortex					
Spearman's corr coef	p-val	p-val, SA	p-val, SA (BH- correction)	GenID	Gene
0.508108079	0.001785594	0.004	1	30818	KCNIP3
0.507850707	0.001796474	0.003	1	4725	NDUFS5
0.507850707	0.001796474	0.004	1	29986	SLC39A2
0.507593274	0.001807413	0.001	1	11165	NUDT3
0.507593274	0.001807413	0.004	1	84618	NT5C1A
0.507078469	0.001829471	0.005	1	434	ASIP
0.507078469	0.001829471	0.003	1	2580	GAK
0.507078469	0.001829471	0.001	1	7037	TFRC
0.507078469	0.001829471	0.005	1	54555	DDX49
0.507078469	0.001829471	0.005	1	84629	TNRC18B
0.506821096	0.001840584	< 0.001	< 0.001	7432	VIP
0.506821096	0.001840584	0.001	1	201191	SAMD14
0.506563663	0.001851759	0.005	1	7277	TUBA4A
0.506563663	0.001851759	0.002	1	65244	SPATS2
0.506306291	0.001862994	0.004	1	185	AGTR1
0.506306291	0.001862994	0.006	1	55973	BCAP29
0.506306291	0.001862994	0.005	1	84259	DCUN1D5
0.506048918	0.001874294	0.004	1	7140	TNNT3
0.505791485	0.001885648	0.006	1	11261	AC012652.1
0.505791485	0.001885648	0.002	1	83259	PCDH11Y
0.505791485	0.001885648	0.002	1	343450	KCNT2
0.505791485	0.001885648	0.003	1	440224	CXADRP3
0.505534112	0.001897064	0.003	1	967	CD63
0.505534112	0.001897064	< 0.001	< 0.001	22826	DNAJC8
0.505534112	0.001897064	0.003	1	84285	EIF1AD
0.505534112	0.001897064	0.001	1	347516	DGAT2L6
0.504761934	0.001931679	0.003	1	23476	BRD4
0.504761934	0.001931679	0.003	1	84934	C12orf52
0.504761934	0.001931679	0.004	1	90379	DCAF15
0.504504502	0.001943343	0.004	1	3326	HSP90AB1
0.504504502	0.001943343	0.003	1	137970	UNC5D
0.504247129	0.001955062	0.003	1	10497	UNC13B
0.504247129	0.001955062	0.006	1	81873	ARPC5L
0.504247129	0.001955062	0.006	1	199964	TMEM61
0.504247129	0.001955062	0.001	1	221656	AOF1
0.503732324	0.001978699	0.004	1	357	SHROOM2
0.503732324	0.001978699	0.002	1	3904	LAIR2
0.503732324	0.001978699	0.004	1	56603	CYP26B1
0.503732324	0.001978699	0.006	1	57728	WDR19
0.503474891	0.00199061	0.001	1	528	ATP6V1C1
0.503474891	0.00199061	0.005	1	5905	RANGAP1
0.503474891	0.00199061	0.003	1	84324	SARNP
0.503474891	0.00199061	0.002	1	150538	AC017096.1
0.503217459	0.002002583	0.002	1	23026	MYO16
0.503217459	0.002002583	0.003	1	79000	C1orf135
0.503217459	0.002002583	0.004	1	140576	S100A16
0.502960086	0.002014619	0.002	1	8897	MTMR3
0.502960086	0.002014619	0.006	1	11127	KIF3A
0.502960086	0.002014619	0.004	1	51496	CTDSPL2
0.502702653	0.002026725	0.007	1	7071	KLF10
0.502702653	0.002026725	0.004	1	403340	MGC70870
0.502702653	0.002026725	0.002	1	444882	IGFL4
0.502445281	0.002038889	0.009	1	128308	MRPL55
0.502187908	0.002051114	0.007	1	2175	FANCA
0.502187908	0.002051114	0.004	1	55207	ARL8B
0.502187908	0.002051114	0.002	1	55909	BIN3
0.501930475	0.00206341	0.006	1	64283	AC093283.3
0.501930475	0.00206341	0.005	1	84717	AC011498.3
0.501673102	0.00207577	0.003	1	22881	ANKRD6
0.501673102	0.00207577	0.003	1	84134	TOMM40L
0.501673102	0.00207577	0.005	1	145407	C14orf37
0.50141567	0.002088192	0.006	1	3082	HGF
0.50141567	0.002088192	0.001	1	5619	PRM1
0.50141567	0.002088192	0.003	1	8815	BANF1
0.50141567	0.002088192	0.003	1	223117	SEMA3D
0.501158297	0.002100681	0.004	1	8237	USP11
0.501158297	0.002100681	0.002	1	9830	TRIM14
0.501158297	0.002100681	0.002	1	10786	SLC17A3
0.500900924	0.002113234	0.003	1	8425	LTBP4
0.500900924	0.002113234	0.003	1	10921	RNPS1
0.500900924	0.002113234	0.006	1	29890	RBM15B
0.500900924	0.002113234	0.004	1	63908	NAPB
0.500900924	0.002113234	0.002	1	81501	TM7SF4
0.500900924	0.002113234	0.008	1	255758	TCTEX1D2
0.500900924	0.002113234	0.003	1	283267	LINC00294
0.500643492	0.002125859	0.004	1	9500	MAGED1
0.500643492	0.002125859	0.004	1	79720	VPS37B
0.500643492	0.002125859	0.001	1	285761	DCBLD1
0.500386119	0.002138545	0.001	1	23705	CADM1

voxel-wise, subcortex			
Spearman's corr coef	p-val	GenID	Gene
0.365827173	< 0.001	170690	ADAMTS16
0.365781397	< 0.001	203547	VMA21
0.365487933	< 0.001	171483	FAM9B
0.365351051	< 0.001	51725	FBXO40
0.365243524	< 0.001	66036	MTMR9
0.36521399	< 0.001	225689	MAPK15
0.36505577	< 0.001	55142	HAUS2
0.36501807	< 0.001	810	CALML3
0.36500439	< 0.001	117154	DACH2
0.364991158	< 0.001	7781	SLC30A3
0.364977807	< 0.001	2517	FUCA1
0.364965737	< 0.001	400673	VMAC
0.364952475	< 0.001	9128	PRPF4
0.364940107	< 0.001	2776	GNAQ
0.364704341	< 0.001	5877	RABIF
0.364665359	< 0.001	665	BNIP3L
0.364429623	< 0.001	57662	KIAA1543
0.364345729	< 0.001	3487	IGFBP4
0.364320427	< 0.001	256158	HMCN2
0.364305019	< 0.001	4760	NEUROD1
0.364298493	< 0.001	6654	SOS1
0.364287913	< 0.001	23646	PLD3
0.364278734	< 0.001	8840	WISP1
0.364272863	< 0.001	339448	C1orf174
0.364270568	< 0.001	55116	TMEM39B
0.364261687	< 0.001	3559	IL2RA
0.364231318	< 0.001	54908	CCDC99
0.364195287	< 0.001	55207	ARL8B
0.364186227	< 0.001	84830	C6orf105
0.364163578	< 0.001	100144602	RP11-807H7.1
0.36404711	< 0.001	26245	OR2M4
0.363993853	< 0.001	374739	TEPP
0.363980204	< 0.001	143187	VTI1A
0.363909036	< 0.001	55346	TCP11L1
0.363736063	< 0.001	728392	LOC728392
0.363620549	< 0.001	55039	TRMT12
0.36352995	< 0.001	10558	SPTLC1
0.363495588	< 0.001	11329	STK38
0.363378167	< 0.001	55840	EAF2
0.363272905	< 0.001	81577	GFOD2
0.363202721	< 0.001	4301	MLLT4
0.363189489	< 0.001	4674	NAP1L2
0.363121003	< 0.001	51380	CSAD
0.363031626	< 0.001	83719	YPEL3
0.362873852	< 0.001	55327	LIN7C
0.362258881	< 0.001	5655	KLK10
0.362011164	< 0.001	9133	CCNB2
0.361985713	< 0.001	84811	BUD13
0.361959308	< 0.001	100288346	AP000880.1
0.361777484	< 0.001	10444	ZER1
0.36174503	< 0.001	150572	SMYD1
0.361744404	< 0.001	57559	STAMBPL1
0.36167267	< 0.001	886	CCKAR
0.361543834	< 0.001	345557	PLCXD3
0.361431926	< 0.001	8832	CD84
0.361422479	< 0.001	64897	C12orf43
0.361418307	< 0.001	1555	CYP2B7P1
0.361357868	< 0.001	140885	SIRPA
0.361280739	< 0.001	51633	OTUD6B
0.361231714	< 0.001	9055	PRC1
0.361071318	< 0.001	2275	FHL3
0.361062229	< 0.001	57761	TRIB3
0.360980779	< 0.001	154754	PRSS23P2
0.360902995	< 0.001	5602	MAPK10
0.360896766	< 0.001	5218	PFTK1
0.360798389	< 0.001	1453	CSNK1D
0.360646456	< 0.001	57646	USP28
0.360617578	< 0.001	266655	LINC00094
0.360577166	< 0.001	27239	GPR162
0.36053884	< 0.001	3595	IL12RB2
0.360385984	< 0.001	152098	ZCWPW2
0.360257119	< 0.001	1621	DBH
0.360197216	< 0.001	80313	LRRC27
0.360064238	< 0.001	131540	ZDHHC19
0.359994531	< 0.001	26511	CHIC2
0.359966248	< 0.001	100126793	GHRLOS
0.359852552	< 0.001	440101	RP11-834C11.3
0.3598167	< 0.001	8541	PPFIA3
0.359705538	< 0.001	285126	DNAJC5G

region-wise, subcortex					
Spearman's corr coef	p-val	p-val, SA	p-val, SA (BH- correction)	GenID	Gene
0.500386119	0.002138545	0.002	1	29953	TRHDE
0.500386119	0.002138545	0.003	1	92552	ATXN3L
0.500128686	0.002151295	0.002	1	3838	KPNA2
0.500128686	0.002151295	0.005	1	55219	TMEM57
0.500128686	0.002151295	0.002	1	56954	NIT2
0.500128686	0.002151295	0.002	1	83658	DYNLRB1
0.500128686	0.002151295	0.002	1	146059	CDAN1
0.499871314	0.002164117	0.002	1	799	CALCR
0.499871314	0.002164117	0.003	1	5172	SLC26A4
0.499871314	0.002164117	0.006	1	650655	ABCA17P
0.499613881	0.002177	0.003	1	3355	HTR1F
0.499613881	0.002177	0.002	1	27328	PCDH11X
0.499356508	0.002189963	0.001	1	10631	POSTN
0.499356508	0.002189963	0.004	1	23552	CCRK
0.499356508	0.002189963	0.002	1	26290	GALNT8
0.499356508	0.002189963	0.005	1	114548	NLRP3
0.499356508	0.002189963	0.002	1	643834	PGA3
0.499099076	0.002202981	0.003	1	23600	AMACR
0.498841703	0.002216071	0.004	1	7341	SUMO1
0.498841703	0.002216071	0.01	1	8445	DYRK2
0.498841703	0.002216071	0.001	1	54434	SSH1
0.498841703	0.002216071	0.003	1	55852	TEX2
0.498841703	0.002216071	0.003	1	81566	CSRNP2
0.498841703	0.002216071	0.005	1	728613	LOC728613
0.4985843	0.002229229	0.002	1	5025	P2RX4
0.4985843	0.002229229	0.005	1	51134	CCDC41
0.4985843	0.002229229	0.004	1	221002	RASGEF1A
0.498069495	0.002255757	0.007	1	7374	UNG
0.498069495	0.002255757	0.002	1	8394	PIP5K1A
0.497812092	0.002269122	0.005	1	5883	RAD9A
0.497812092	0.002269122	< 0.001	< 0.001	57152	SLURP1
0.497812092	0.002269122	0.005	1	246243	RNASEH1
0.49755469	0.002282553	0.004	1	383	ARG1
0.49755469	0.002282553	0.003	1	51335	NGRN
0.497297317	0.002296066	0.006	1	8220	DGCR14
0.497297317	0.002296066	0.001	1	10556	RPP30
0.497297317	0.002296066	0.002	1	442213	C6orf138
0.497039884	0.002309637	0.006	1	25924	MYRIP
0.497039884	0.002309637	0.002	1	80328	ULBP2
0.496782511	0.002323285	0.004	1	2767	GNA11
0.496782511	0.002323285	0.001	1	57469	PNMAL2
0.496782511	0.002323285	0.005	1	64699	TMPRSS3
0.496782511	0.002323285	0.004	1	441666	LOC441666
0.496525079	0.002336998	0.007	1	11344	TWF2
0.496525079	0.002336998	0.001	1	79084	WDR77
0.496525079	0.002336998	0.006	1	79183	TTPAL
0.496525079	0.002336998	0.006	1	649330	LOC649330
0.496267676	0.002350784	0.006	1	6616	SNAP25
0.496267676	0.002350784	0.005	1	113220	KIF12
0.496267676	0.002350784	0.002	1	137362	GOT1L1
0.496267676	0.002350784	0.006	1	647288	CTAGE11P
0.496010303	0.002364649	0.007	1	5983	RFC3
0.496010303	0.002364649	0.011	1	6885	MAP3K7
0.496010303	0.002364649	0.003	1	8805	TRIM24
0.495752871	0.002378579	0.006	1	9917	FAM20B
0.495752871	0.002378579	0.003	1	55621	TRMT1
0.495752871	0.002378579	0.002	1	347168	OR1J1
0.495495498	0.002392581	0.007	1	5563	PRKAA2
0.495495498	0.002392581	0.006	1	10189	THOC4
0.495495498	0.002392581	0.003	1	55079	FEZF2
0.495495498	0.002392581	0.004	1	284486	THEM5
0.495238066	0.002406655	0.004	1	3606	IL18
0.495238066	0.002406655	0.003	1	4620	MYH2
0.494980693	0.002420804	0.003	1	4201	MEA1
0.494980693	0.002420804	0.005	1	6532	SLC6A4
0.494980693	0.002420804	0.002	1	63876	PKNOX2
0.49472329	0.002435028	0.002	1	84984	C3orf34
0.49472329	0.002435028	0.004	1	122970	ACOT4
0.494465888	0.002449318	0.003	1	4882	NPR2
0.494465888	0.002449318	0.004	1	8891	EIF2B3
0.494465888	0.002449318	0.007	1	54762	GRAMD1C
0.494465888	0.002449318	0.003	1	123264	AC013553.1
0.494208485	0.002463688	0.001	1	9570	GOSR2
0.494208485	0.002463688	0.005	1	10974	C10orf116
0.493951082	0.002478132	0.006	1	23514	KIAA0146
0.493951082	0.002478132	0.007	1	79705	LRRK1
0.49369368	0.002492647	0.003	1	51744	CD244
0.49369368	0.002492647	0.006	1	56666	PANX2
0.493436307	0.002507237	0.006	1	8925	HERC1

voxel-wise, subcortex			
Spearman's corr coef	p-val	GenID	Gene
0.359603018	< 0.001	57706	DENND1A
0.359588385	< 0.001	345462	ZNF879
0.359557778	< 0.001	3889	KRT83
0.359525055	< 0.001	26091	HERC4
0.359396189	< 0.001	100287114	LINC00421
0.359395921	< 0.001	84698	CAPS2
0.359357715	< 0.001	150	ADRA2A
0.359292239	< 0.001	9468	PCYT1B
0.359223902	< 0.001	10360	NPM3
0.359015375	< 0.001	401565	FAM166A
0.358933359	< 0.001	7738	ZNF184
0.35889864	< 0.001	22920	KIFAP3
0.358786762	< 0.001	149345	SHISA4
0.358781189	< 0.001	30818	KCNIP3
0.358636826	< 0.001	7042	TGFB2
0.358590037	< 0.001	23515	MORC3
0.358487934	< 0.001	153743	PPP1R2P3
0.358486712	< 0.001	26998	FETUB
0.3584342	< 0.001	9100	USP10
0.358402431	< 0.001	203523	ZNF449
0.358291745	< 0.001	645369	AP005433.1
0.358236969	< 0.001	29797	POM121L8P
0.358219594	< 0.001	84626	KRBA1
0.358203709	< 0.001	5902	RANBP1
0.358186543	< 0.001	133957	CCDC127
0.358093768	< 0.001	7432	VIP
0.358065814	< 0.001	9577	BRE
0.358029485	< 0.001	145773	FAM81A
0.357974797	< 0.001	6500	SKP1
0.357910395	< 0.001	55335	NIPSNAP3B
0.35786885	< 0.001	79969	C6orf134
0.357687145	< 0.001	245940	AC144830.1
0.357653052	< 0.001	7291	TWIST1
0.35756883	< 0.001	8785	MATN4
0.357486844	< 0.001	171484	FAM9C
0.357424766	< 0.001	439915	KRTAP5-5
0.357273012	< 0.001	128854	TSPY26P
0.357184708	< 0.001	79837	PIP4K2C
0.357139379	< 0.001	260425	MAGI3
0.357112944	< 0.001	414918	FAM116B
0.357054323	< 0.001	80335	WDR82
0.357031941	< 0.001	377677	CA13
0.357020736	< 0.001	4094	MAF
0.356942087	< 0.001	51669	TMEM66
0.356758147	< 0.001	84629	TNRC18B
0.356723338	< 0.001	27319	BHLHE22
0.356569558	< 0.001	51523	CXXC5
0.356311649	< 0.001	23171	GPD1L
0.356239676	< 0.001	552	AVPR1A
0.356023252	< 0.001	54622	ARL15
0.356014222	< 0.001	124583	CANT1
0.356006414	< 0.001	11334	TUSC2
0.355887413	< 0.001	644054	FAM25C
0.35584873	< 0.001	94160	ABCC12
0.355842739	< 0.001	123263	MTFMT
0.355825692	< 0.001	84343	HPS3
0.355816603	< 0.001	2021	ENDOG
0.355781108	< 0.001	3241	HPCAL1
0.35570997	< 0.001	51560	RAB6B
0.355487764	< 0.001	132864	CPEB2
0.355485559	< 0.001	91283	MSANTD3
0.35544762	< 0.001	3790	KCNS3
0.355377018	< 0.001	3679	ITGA7
0.355369717	< 0.001	51379	CRLF3
0.355249822	< 0.001	6051	RNPEP
0.355194718	< 0.001	2848	GPR25
0.355079979	< 0.001	65018	PINK1
0.355075657	< 0.001	121278	TPH2
0.355037063	< 0.001	100192379	AC079341.1
0.354982316	< 0.001	118672	PSTK
0.354931712	< 0.001	7447	VSNL1
0.354903162	< 0.001	126321	C19orf28
0.354880184	< 0.001	23321	TRIM2
0.354853749	< 0.001	83658	DYNLRB1
0.354814231	< 0.001	5737	PTGFR
0.35452956	< 0.001	8396	PIP5K2B
0.354470044	< 0.001	55214	LEPREL1
0.354370356	< 0.001	221895	JAZF1
0.354278177	< 0.001	83989	FAM172A

region-wise, subcortex					
Spearman's corr coef	p-val	p-val, SA	p-val, SA (BH- correction)	GenID	Gene
0.493178874	0.002521902	0.009	1	7134	TNNC1
0.493178874	0.002521902	0.005	1	10229	COQ7
0.492921501	0.002536641	0.001	1	11329	STK38
0.492921501	0.002536641	0.002	1	29989	OBP2B
0.492664069	0.002551463	0.002	1	26002	MOXD1
0.492664069	0.002551463	0.005	1	340508	RP11-520B13.4
0.492406696	0.002566352	0.005	1	3460	IFNGR2
0.492406696	0.002566352	0.002	1	9076	CLDN1
0.492406696	0.002566352	0.004	1	283848	CES8
0.492149293	0.002581322	0.004	1	5924	RASGRF2
0.492149293	0.002581322	0.003	1	10076	PTPRU
0.492149293	0.002581322	0.001	1	149473	CCDC24
0.491891891	0.002596363	0.007	1	2790	GNG10
0.491891891	0.002596363	0.003	1	6526	SLC5A3
0.491891891	0.002596363	0.005	1	8446	DUSP11
0.491891891	0.002596363	0.004	1	10673	TNFSF13B
0.491634488	0.002611494	0.003	1	51535	PPHLN1
0.491634488	0.002611494	0.004	1	57559	STAMBPL1
0.491377085	0.002626689	0.004	1	129080	EMID1
0.491119683	0.002641965	0.005	1	285313	IGSF10
0.49086231	0.002657318	0.002	1	1562	CYP2C18
0.49086231	0.002657318	0.003	1	9201	DCLK1
0.49086231	0.002657318	0.004	1	9223	MAGI1
0.49086231	0.002657318	0.001	1	80063	ATF7IP2
0.490604877	0.002672757	0.004	1	4809	NHP2L1
0.490604877	0.002672757	0.004	1	6750	SST
0.490604877	0.002672757	0.003	1	55911	AC138894.3
0.490604877	0.002672757	0.003	1	120400	FAM55A
0.490604877	0.002672757	0.006	1	100129361	LOC100129361
0.490347505	0.002688267	0.003	1	2744	GLS
0.490347505	0.002688267	0.005	1	4923	NTSR1
0.490347505	0.002688267	0.007	1	10124	ARL4A
0.490347505	0.002688267	0.007	1	10526	IPO8
0.490347505	0.002688267	0.003	1	541471	AC068491.1
0.490090072	0.002703857	0.003	1	25992	SNED1
0.490090072	0.002703857	0.005	1	51400	PPME1
0.490090072	0.002703857	0.002	1	54033	RBM11
0.489832699	0.002719522	0.004	1	1746	DLX2
0.489832699	0.002719522	0.003	1	7477	WNT7B
0.489575297	0.00273527	0.006	1	4133	MAP2
0.489575297	0.00273527	0.005	1	10533	ATG7
0.489575297	0.00273527	0.006	1	387522	TMEM189-UBE2V1
0.489317864	0.002751105	0.008	1	9791	PTDSS1
0.489317864	0.002751105	0.006	1	51278	IER5
0.489317864	0.002751105	0.007	1	54386	TERF2IP
0.489060491	0.002767013	0.003	1	624	BDKRB2
0.489060491	0.002767013	0.005	1	51380	CSAD
0.489060491	0.002767013	0.004	1	116349	C5orf55
0.488803059	0.002783002	0.003	1	23708	GSPT2
0.488803059	0.002783002	0.007	1	399726	C10orf114
0.488545686	0.002799073	0.005	1	80818	ZNF436
0.488288254	0.002815231	0.005	1	40	ACCN1
0.488288254	0.002815231	0.004	1	398	ARHGDIG
0.488030881	0.002831464	0.006	1	1212	CLTB
0.488030881	0.002831464	0.002	1	54065	C21orf51
0.488030881	0.002831464	0.008	1	170679	PSORS1C1
0.488030881	0.002831464	< 0.001	< 0.001	728392	LOC728392
0.487773478	0.002847779	0.004	1	2222	FDFT1
0.487773478	0.002847779	0.005	1	23670	TMEM2
0.487516075	0.002864176	0.007	1	3269	HRH1
0.487516075	0.002864176	0.003	1	11188	NISCH
0.487516075	0.002864176	0.002	1	127018	LYPLAL1
0.487516075	0.002864176	0.006	1	100132247	NPIPL3
0.487258673	0.002880652	0.001	1	51095	TRNT1
0.4870013	0.002897226	0.009	1	284021	MILR1
0.4870013	0.002897226	0.002	1	285016	FAM150B
0.486743867	0.002913869	0.004	1	261726	TIPRL
0.486486495	0.002930598	0.005	1	3008	HIST1H1E
0.486486495	0.002930598	0.004	1	25873	RPL36
0.486486495	0.002930598	0.007	1	25897	RNF19A
0.486486495	0.002930598	0.005	1	55290	BRF2
0.486486495	0.002930598	0.004	1	56979	PRDM9
0.486486495	0.002930598	0.005	1	79414	LRFN3
0.486486495	0.002930598	0.006	1	84457	PHYHIP
0.486486495	0.002930598	0.005	1	200523	C2orf51
0.486229062	0.002947413	0.003	1	4878	NPPA
0.485971689	0.002964319	0.005	1	22887	FOXJ3
0.485971689	0.002964319	0.003	1	23177	CEP68
0.485714257	0.002981302	0.008	1	8667	EIF3H

voxel-wise, subcortex			
Spearman's corr coef	p-val	GenID	Gene
0.354073137	< 0.001	948	CD36
0.354055434	< 0.001	8717	TRADD
0.353947759	< 0.001	3742	KCNA6
0.353899479	< 0.001	222962	SLC29A4
0.353884369	< 0.001	255252	LRRC57
0.353850901	< 0.001	6647	SOD1
0.353776544	< 0.001	146223	CMTM4
0.353756398	< 0.001	220388	CCDC89
0.353645235	< 0.001	7474	WNT5A
0.353638411	< 0.001	84525	HOPX
0.353580743	< 0.001	7066	THPO
0.353577048	< 0.001	192111	PGAM5
0.353554606	< 0.001	84722	PSRC1
0.353548884	< 0.001	56940	DUSP22
0.353489012	< 0.001	254778	C8orf46
0.353407174	< 0.001	2157	F8
0.353297561	< 0.001	79656	BEND5
0.353226244	< 0.001	27101	CACYBP
0.353202939	< 0.001	63974	NEUROD6
0.353200823	< 0.001	1282	COL4A1
0.353117168	< 0.001	22983	MAST1
0.353019744	< 0.001	23780	APOL2
0.352961242	< 0.001	55617	TASP1
0.352868974	< 0.001	6278	S100A7
0.352773547	< 0.001	2529	FUT7
0.352754146	< 0.001	7089	TLE2
0.352524042	< 0.001	2029	ENSA
0.352486312	< 0.001	118788	PIK3AP1
0.352283001	< 0.001	25789	TMEM59L
0.352235585	< 0.001	22903	BTBD3
0.352223098	< 0.001	8364	HIST1H4C
0.352055877	< 0.001	27294	DHDH
0.352006525	< 0.001	57628	DPP10
0.351784438	< 0.001	150771	ITPRIPL1
0.35152173	< 0.001	11069	RAPGEF4
0.351466894	< 0.001	388762	OR2M1P
0.351384938	< 0.001	9208	LRRFIP1
0.351332039	< 0.001	84894	LINGO1
0.351320565	< 0.001	135935	NOBOX
0.351298749	< 0.001	3927	LASP1
0.351286352	< 0.001	79148	MMP28
0.351239145	< 0.001	996	CDC27
0.35121271	< 0.001	100268168	AC008429.1
0.350864619	< 0.001	9615	GDA
0.350687683	< 0.001	339804	C2orf74
0.350597531	< 0.001	23314	SATB2
0.350561023	< 0.001	84225	ZMYND15
0.350548178	< 0.001	30811	HUNK
0.350467592	< 0.001	5833	PCYT2
0.350396782	< 0.001	646936	LOC646936
0.350216806	< 0.001	4216	MAP3K4
0.349876702	< 0.001	54522	ANKRD16
0.34986499	< 0.001	967	CD63
0.349698484	< 0.001	51761	ATP8A2
0.349667013	< 0.001	387715	ARMS2
0.349587381	< 0.001	3845	KRAS
0.349572092	< 0.001	57583	TMEM181
0.349536896	< 0.001	23504	RIMBP2
0.34942022	< 0.001	1416	CRYBB2P1
0.349411845	< 0.001	126170	LOC126170
0.349213451	< 0.001	51097	SCCPDH
0.349176943	< 0.001	164633	CABP7
0.349174172	< 0.001	56849	TCEAL7
0.348822981	< 0.001	55197	RPRD1A
0.348658293	< 0.001	29979	UBQLN1
0.348535717	< 0.001	6611	SMS
0.348475218	< 0.001	8437	RASAL1
0.348280936	< 0.001	79084	WDR77
0.348118007	< 0.001	149708	WFDC5
0.348080218	< 0.001	64963	MRPS11
0.348017991	< 0.001	8480	RAE1
0.347866982	< 0.001	91522	COL23A1
0.347828895	< 0.001	5905	RANGAP1
0.347651631	< 0.001	9570	GOSR2
0.347514898	< 0.001	54904	WHSC1L1
0.347511768	< 0.001	64207	C14orf4
0.347498864	< 0.001	4218	RAB8A
0.347485304	< 0.001	339240	LOC339240
0.347393364	< 0.001	1414	CRYBB1

region-wise, subcortex					
Spearman's corr coef	p-val	p-val, SA	p-val, SA (BH- correction)	GenID	Gene
0.485714257	0.002981302	0.011	1	26526	TSPAN16
0.485714257	0.002981302	0.004	1	51263	MRPL30
0.485456884	0.002998371	0.008	1	6456	SH3GL2
0.485456884	0.002998371	0.004	1	25822	DNAJB5
0.485456884	0.002998371	0.012	1	55885	LMO3
0.485456884	0.002998371	0.006	1	56943	ENY2
0.485456884	0.002998371	0.004	1	349136	WDR86
0.485199481	0.003015525	0.005	1	4624	MYH6
0.485199481	0.003015525	0.004	1	10554	AGPAT1
0.484942079	0.003032772	0.004	1	375	ARF1
0.484942079	0.003032772	0.004	1	170302	ARX
0.484684676	0.003050095	0.006	1	5527	PPP2R5C
0.484684676	0.003050095	0.009	1	26985	AP3M1
0.484427303	0.003067508	0.004	1	5743	PTGS2
0.484427303	0.003067508	0.004	1	143282	FGFBP3
0.484169871	0.003085007	0.004	1	6118	RPA2
0.484169871	0.003085007	0.005	1	10728	PTGES3
0.483912498	0.003102595	0.006	1	5471	PPAT
0.483655065	0.003120278	0.008	1	203	AK1
0.483655065	0.003120278	0.011	1	23047	PDS5B
0.483397692	0.003138041	0.004	1	220042	C11orf82
0.48314026	0.003155892	0.004	1	2785	GNG3
0.48314026	0.003155892	0.003	1	4905	NSF
0.48314026	0.003155892	0.01	1	55884	WSB2
0.48314026	0.003155892	0.008	1	84622	ZNF594
0.482882887	0.003173829	0.007	1	55153	SDAD1
0.482882887	0.003173829	0.005	1	56475	RPRM
0.482882887	0.003173829	0.008	1	201229	C17orf108
0.482882887	0.003173829	0.005	1	100130386	LINC00552
0.482368052	0.003209985	0.002	1	9821	RB1CC1
0.482110679	0.003228194	0.004	1	5999	RGS4
0.482110679	0.003228194	0.011	1	6515	SLC2A3
0.482110679	0.003228194	0.006	1	63947	DMRTC1B
0.481853247	0.003246494	0.004	1	112	ADCY6
0.481853247	0.003246494	0.005	1	27290	SPINK4
0.481853247	0.003246494	0.003	1	57706	DENND1A
0.481853247	0.003246494	0.004	1	57709	SLC7A14
0.481853247	0.003246494	0.005	1	83661	MS4A8B
0.481853247	0.003246494	0.005	1	266655	LINC00094
0.481595874	0.003264892	0.004	1	55132	LARP1B
0.481595874	0.003264892	0.009	1	55681	SCYL2
0.481338471	0.003283373	0.004	1	162239	ZFP1
0.481338471	0.003283373	0.008	1	100133211	LOC100133211
0.481081069	0.003301946	0.006	1	8514	KCNAB2
0.481081069	0.003301946	0.011	1	11334	TUSC2
0.481081069	0.003301946	0.007	1	79018	C17orf39
0.481081069	0.003301946	0.007	1	84993	UBL7
0.480823666	0.00332061	0.005	1	26468	LHX6
0.480566263	0.003339367	0.001	1	1740	DLG2
0.480566263	0.003339367	0.006	1	81577	GFOD2
0.480308861	0.003358224	0.006	1	4908	NTF3
0.480308861	0.003358224	0.002	1	84664	CSPG4P2Y
0.480051488	0.003377167	0.003	1	4901	NRL
0.480051488	0.003377167	0.007	1	23199	KIAA0182
0.480051488	0.003377167	0.01	1	84790	TUBA1C
0.480051488	0.003377167	0.006	1	727803	LOC727803
0.479794055	0.003396197	0.004	1	6881	TAF10
0.479536682	0.003415327	0.002	1	8706	B3GALNT1
0.479536682	0.003415327	0.009	1	55022	PID1
0.479536682	0.003415327	0.011	1	148066	ZNRF4
0.479021877	0.003453877	0.009	1	55026	FAM70A
0.478764474	0.00347329	0.01	1	6812	STXBP1
0.478764474	0.00347329	0.007	1	140461	ASB8
0.478507072	0.003492798	0.008	1	6399	TRAPPC2
0.478507072	0.003492798	0.004	1	80206	FHOD3
0.478249669	0.003512411	0.001	1	3094	HINT1
0.477992266	0.003532111	0.006	1	8434	RECK
0.477992266	0.003532111	0.003	1	23299	BICD2
0.477992266	0.003532111	0.005	1	56158	TEX12
0.477992266	0.003532111	0.004	1	64757	MOSC1
0.477992266	0.003532111	0.007	1	91137	SLC25A46
0.477734864	0.003551905	0.009	1	2209	FCGR1A
0.477734864	0.003551905	0.007	1	23253	ANKRD12
0.477734864	0.003551905	0.004	1	84301	DDI2
0.477734864	0.003551905	0.007	1	90410	IFT20
0.477734864	0.003551905	0.007	1	134430	WDR36
0.477477491	0.003571798	0.006	1	2566	GABRG2
0.477477491	0.003571798	0.005	1	2746	GLUD1
0.477477491	0.003571798	0.009	1	30811	HUNK

voxel-wise, subcortex			
Spearman's corr coef	p-val	GenID	Gene
0.347336501	< 0.001	55312	RFK
0.347074836	< 0.001	54754	FAM22F
0.347032756	< 0.001	23204	ARL6IP1
0.346922338	< 0.001	2853	GPR31
0.346920252	< 0.001	8764	TNFRSF14
0.346821547	< 0.001	134526	ACOT12
0.346725851	< 0.001	3909	LAMA3
0.346655637	< 0.001	51649	MRPS23
0.34651643	< 0.001	7325	UBE2E2
0.346512705	< 0.001	4897	NRCAM
0.346465826	< 0.001	9819	TSC22D2
0.346370548	< 0.001	95681	TSGA14
0.346341997	< 0.001	8581	LY6D
0.34616968	< 0.001	55351	STK32B
0.346053183	< 0.001	8811	GALR2
0.346013665	< 0.001	174	AFP
0.345997512	< 0.001	50512	PODXL2
0.345915467	< 0.001	7247	TSN
0.34572053	< 0.001	6947	TCN1
0.34568733	< 0.001	10924	SMPDL3A
0.345684022	< 0.001	4718	NDUFC2
0.345523596	< 0.001	158326	FREM1
0.345517755	< 0.001	6804	STX1A
0.345457196	< 0.001	26281	FGF20
0.345410854	< 0.001	6305	SBF1
0.345278442	< 0.001	283638	KIAA0284
0.345263392	< 0.001	56943	ENY2
0.345235527	< 0.001	92335	STRADA
0.345226765	< 0.001	339287	MSL1
0.34518519	< 0.001	286077	FAM83H
0.345141739	< 0.001	23616	SH3BP1
0.344922662	< 0.001	79570	NKAIN1
0.344900459	< 0.001	6439	SFTPB
0.344826937	< 0.001	324	APC
0.344801754	< 0.001	51622	C7orf28A
0.3447043	< 0.001	56977	STOX2
0.344632	< 0.001	3633	INPP5B
0.344626069	< 0.001	3242	HPD
0.344602525	< 0.001	57221	KIAA1244
0.344586641	< 0.001	403340	MGC70870
0.344565213	< 0.001	4929	NR4A2
0.344309181	< 0.001	11165	NUDT3
0.344264716	< 0.001	29953	TRHDE
0.344254583	< 0.001	441728	GOLGA6B
0.344250262	< 0.001	9502	XAGE2B
0.344173044	< 0.001	2224	FDPS
0.344136924	< 0.001	56981	PRDM11
0.344042122	< 0.001	3301	DNAJA1
0.343883485	< 0.001	51451	LCMT1
0.343832552	< 0.001	201140	DHRS7C
0.343818307	< 0.001	55738	ARFGAP1
0.343784124	< 0.001	5644	PRSS1
0.343721777	< 0.001	51586	MED15
0.343676955	< 0.001	27160	INGX
0.343639433	< 0.001	23094	SIPA1L3
0.343572825	< 0.001	122876	GPHB5
0.343446374	< 0.001	27087	B3GAT1
0.343396008	< 0.001	117143	TADA1L
0.343315959	< 0.001	55669	MFN1
0.343315482	< 0.001	55789	DEPDC1B
0.343252838	< 0.001	10211	FLOT1
0.342998564	< 0.001	6999	TDO2
0.342889547	< 0.001	2870	GRK6
0.34279874	< 0.001	128408	BHLHE23
0.342542738	< 0.001	2965	GTF2H1
0.34245196	< 0.001	7077	TIMP2
0.34240678	< 0.001	79586	CHPF
0.342357665	< 0.001	6404	SELPLG
0.342273444	< 0.001	219539	YPEL4
0.342248678	< 0.001	375686	SPATC1
0.342161536	< 0.001	160428	ALDH1L2
0.342124522	< 0.001	5768	QSOX1
0.342053294	< 0.001	8204	NRIP1
0.342030972	< 0.001	108	ADCY2
0.342021734	< 0.001	84726	BAT2L
0.341977507	< 0.001	7849	PAX8
0.341919839	< 0.001	79017	GGCT
0.341889113	< 0.001	5504	PPP1R2
0.341795027	< 0.001	6585	SLIT1

region-wise, subcortex					
Spearman's corr coef	p-val	p-val, SA	p-val, SA (BH- correction)	GenID	Gene
0.477220058	0.003591789	0.006	1	5961	PRPH2
0.477220058	0.003591789	0.012	1	118672	PSTK
0.477220058	0.003591789	0.007	1	440400	RNASEK
0.477220058	0.003591789	0.006	1	643226	GRXCR2
0.476962686	0.003611884	0.007	1	220001	VWCE
0.476962686	0.003611884	0.009	1	338707	B4GALNT4
0.476705253	0.003632073	0.006	1	9575	CLOCK
0.476705253	0.003632073	0.005	1	53346	TM6SF1
0.47644788	0.003652356	0.014	1	460	ASTN1
0.47644788	0.003652356	0.008	1	50801	KCNK4
0.476190478	0.003672739	0.006	1	5922	RASA2
0.476190478	0.003672739	0.007	1	7884	SLBP
0.476190478	0.003672739	0.007	1	8683	SFRS9
0.476190478	0.003672739	0.009	1	100132147	LOC100132147
0.475933075	0.003693231	0.006	1	9837	GINS1
0.475933075	0.003693231	0.013	1	399668	LINC00086
0.475675672	0.003713812	0.008	1	321	APBA2
0.475675672	0.003713812	0.008	1	3487	IGFBP4
0.475675672	0.003713812	0.007	1	28983	TMPRSS11E2
0.475675672	0.003713812	0.006	1	140628	GATA5
0.47541824	0.003734495	0.005	1	8945	BTRC
0.47541824	0.003734495	0.008	1	53340	SPA17
0.47541824	0.003734495	0.003	1	57619	SHROOM3
0.475160867	0.003755278	0.011	1	79644	SRD5A3
0.475160867	0.003755278	0.005	1	93145	OLFM2
0.475160867	0.003755278	0.003	1	200810	ALG1L
0.475160867	0.003755278	0.006	1	219479	OR5R1
0.475160867	0.003755278	0.006	1	386618	KCTD4
0.474903464	0.003776169	< 0.001	< 0.001	4986	OPRK1
0.474903464	0.003776169	0.005	1	5706	PSMC6
0.474903464	0.003776169	0.01	1	6175	RPLP0P3
0.474903464	0.003776169	0.009	1	9037	SEMA5A
0.474903464	0.003776169	0.009	1	10005	ACOT8
0.474903464	0.003776169	0.007	1	23741	EID1
0.474646062	0.003797158	0.008	1	339524	LOC339524
0.474388659	0.003818243	0.004	1	5630	PRPH
0.474388659	0.003818243	0.008	1	8437	RASAL1
0.474388659	0.003818243	0.007	1	57679	ALS2
0.474388659	0.003818243	0.004	1	112849	C14orf149
0.474388659	0.003818243	0.005	1	114905	C1QTNF7
0.474388659	0.003818243	0.008	1	158763	RP13-102H20.1
0.474388659	0.003818243	0.011	1	343702	XKR7
0.474131256	0.003839432	0.011	1	4361	MRE11A
0.474131256	0.003839432	0.006	1	7567	ZNF19
0.474131256	0.003839432	0.003	1	84456	L3MBTL3
0.473873854	0.003860724	0.006	1	64084	CLSTN2
0.473873854	0.003860724	0.006	1	149986	LSM14B
0.473359048	0.003903627	0.004	1	51372	CCDC72
0.473101676	0.003925227	0.005	1	781	CACNA2D1
0.473101676	0.003925227	0.003	1	7334	UBE2N
0.472329468	0.003990668	0.003	1	84726	BAT2L
0.472329468	0.003990668	0.007	1	117157	SH2D1B
0.472072065	0.00401269	0.01	1	79095	C9orf16
0.471814662	0.004034818	0.01	1	10652	YKT6
0.471814662	0.004034818	0.008	1	27067	STAU2
0.471814662	0.004034818	0.009	1	152926	PPM1K
0.471814662	0.004034818	0.009	1	260293	CYP4X1
0.471814662	0.004034818	0.01	1	440672	NUDT4P1
0.47155726	0.004057061	0.009	1	57573	ZNF471
0.47155726	0.004057061	0.005	1	149175	MANEAL
0.471299857	0.004079402	0.005	1	4139	MARK1
0.471299857	0.004079402	0.007	1	23567	ZNF346
0.471299857	0.004079402	0.011	1	116068	AC027323.1
0.471299857	0.004079402	0.01	1	221302	ZUFSP
0.471042484	0.004101848	0.008	1	311	ANXA11
0.471042484	0.004101848	0.011	1	2820	GPD2
0.470785052	0.004124403	0.003	1	5099	PCDH7
0.470785052	0.004124403	0.005	1	10969	EBNA1BP2
0.470785052	0.004124403	0.012	1	58189	WFDC1
0.470527679	0.004147067	0.009	1	2845	GPR22
0.470527679	0.004147067	0.007	1	8361	HIST1H4F
0.470527679	0.004147067	0.008	1	51193	ZNF639
0.470527679	0.004147067	0.009	1	401127	AC021148.2
0.470270246	0.004169849	0.006	1	400578	KRT16P2
0.470270246	0.004169849	0.01	1	414260	C10orf136
0.470012873	0.00419273	0.005	1	3486	IGFBP3
0.470012873	0.00419273	0.012	1	9867	PJA2
0.470012873	0.00419273	0.008	1	89885	FATE1
0.469755471	0.004215718	0.004	1	2669	GEM

voxel-wise, subcortex			
Spearman's corr coef	p-val	GenID	Gene
0.341765493	< 0.001	440173	LOC440173
0.341748506	< 0.001	53346	TM6SF1
0.341584682	< 0.001	5630	PRPH
0.341558188	< 0.001	4705	NDUFA10
0.34133628	< 0.001	728734	LOC728734
0.341194659	< 0.001	56475	RPRM
0.341108143	< 0.001	4101	MAGEA2
0.341012359	< 0.001	374286	CDRT1
0.340889275	< 0.001	55571	C2orf29
0.340873539	< 0.001	57103	C12orf5
0.34085691	< 0.001	6857	SYT1
0.340443224	< 0.001	8825	LIN7A
0.340354204	< 0.001	8897	MTMR3
0.340297252	< 0.001	8880	FUBP1
0.340110272	< 0.001	284067	C17orf105
0.340034068	< 0.001	5911	RAP2A
0.339981824	< 0.001	8774	NAPG
0.339957952	< 0.001	9127	P2RX6
0.339935482	< 0.001	126792	B3GALT6
0.339751691	< 0.001	4208	MEF2C
0.33970958	< 0.001	39	ACAT2
0.339609146	< 0.001	8706	B3GALNT1
0.339606136	< 0.001	56156	TEX13B
0.339511275	< 0.001	145837	LOC145837
0.339421332	< 0.001	4209	MEF2D
0.339413941	< 0.001	285704	RGMB
0.339398772	< 0.001	526	ATP6V1B2
0.339296043	< 0.001	26974	ZNF285
0.339126378	< 0.001	80273	GRPEL1
0.339120954	< 0.001	91461	SGK493
0.339035183	< 0.001	51134	CCDC41
0.338843524	< 0.001	29966	STRN3
0.338813305	< 0.001	7804	LRP8
0.338787556	< 0.001	79690	GAL3ST4
0.338763803	< 0.001	23024	PDZRN3
0.338737518	< 0.001	8895	CPNE3
0.338653207	< 0.001	51087	YBX2
0.338478595	< 0.001	100129361	LOC100129361
0.338473827	< 0.001	5584	PRKCI
0.338451862	< 0.001	100289341	LOC100289341
0.33844769	< 0.001	9319	TRIP13
0.338341057	< 0.001	27067	STAU2
0.338298202	< 0.001	320	APBA1
0.338275373	< 0.001	5471	PPAT
0.338051617	< 0.001	26953	RANBP6
0.337949872	< 0.001	22883	CLSTN1
0.337941229	< 0.001	9978	RBX1
0.337752581	< 0.001	154881	KCTD7
0.337699503	< 0.001	83741	TFAP2D
0.337625027	< 0.001	644794	LOC644794
0.337559372	< 0.001	120227	CYP2R1
0.337547898	< 0.001	9672	SDC3
0.337528676	< 0.001	387911	RP11-45B20.3
0.33745268	< 0.001	1562	CYP2C18
0.337365419	< 0.001	3933	LCN1
0.337357432	< 0.001	401548	SNX30
0.337174773	< 0.001	51535	PPHLN1
0.337045968	< 0.001	201895	C4orf34
0.336942285	< 0.001	81929	SEH1L
0.336932838	< 0.001	5273	SERPINB10
0.33691299	< 0.001	285456	AC084209.1
0.336856425	< 0.001	5646	PRSS3
0.336702734	< 0.001	57505	AARS2
0.336684585	< 0.001	607	BCL9
0.336560279	< 0.001	11344	TWF2
0.336385787	< 0.001	84993	UBL7
0.33638522	< 0.001	23542	MAPK8IP2
0.336378813	< 0.001	65217	PCDH15
0.336213738	< 0.001	92344	GORAB
0.336159796	< 0.001	7366	AC147055.1
0.336150169	< 0.001	2134	EXTL1
0.336060613	< 0.001	55206	SBNO1
0.336048663	< 0.001	65080	MRPL44
0.336039335	< 0.001	649330	LOC649330
0.335833788	< 0.001	140458	ASB5
0.3354761	< 0.001	100132304	RP1-172N19.1
0.335448712	< 0.001	129642	MBOAT2
0.335428268	< 0.001	56267	CCBL2
0.335364521	< 0.001	79605	PGBD5

region-wise, subcortex					
Spearman's corr coef	p-val	p-val, SA	p-val, SA (BH- correction)	GenID	Gene
0.469755471	0.004215718	0.003	1	25927	CNRIP1
0.469755471	0.004215718	0.006	1	79803	HPS6
0.469755471	0.004215718	0.007	1	284422	AC005551.1
0.469498068	0.004238818	0.007	1	23326	USP22
0.469498068	0.004238818	0.013	1	23541	SEC14L2
0.469498068	0.004238818	0.007	1	26533	OR10G3
0.469498068	0.004238818	0.003	1	55631	LRRC40
0.469498068	0.004238818	0.006	1	494197	SPANXN5
0.469240665	0.004262039	0.01	1	33	ACADL
0.469240665	0.004262039	0.011	1	9832	JAKMIP2
0.469240665	0.004262039	0.009	1	125972	CALR3
0.469240665	0.004262039	0.008	1	154215	NKAIN2
0.469240665	0.004262039	0.007	1	159091	FAM122C
0.468983263	0.00428536	0.006	1	8448	DOC2A
0.468983263	0.00428536	0.005	1	23170	TTLL12
0.468983263	0.00428536	0.005	1	57626	KLHL1
0.468983263	0.00428536	0.008	1	84525	HOPX
0.46872586	0.00430879	0.004	1	23426	GRIP1
0.46872586	0.00430879	0.008	1	83989	FAM172A
0.46872586	0.00430879	0.011	1	84937	ZNRF1
0.468468457	0.004332334	0.006	1	11124	FAF1
0.468468457	0.004332334	0.012	1	57599	WDR48
0.468468457	0.004332334	0.005	1	284018	C17orf58
0.468211055	0.004356	0.007	1	1114	CHGB
0.468211055	0.004356	0.013	1	64091	POPDC2
0.468211055	0.004356	0.004	1	80169	CTC1
0.468211055	0.004356	0.005	1	84464	BTBD12
0.468211055	0.004356	0.005	1	283450	C12orf51
0.467953652	0.004379768	0.007	1	4234	METTL1
0.467953652	0.004379768	0.011	1	23309	SIN3B
0.467953652	0.004379768	0.005	1	130951	C2orf65
0.467953652	0.004379768	0.006	1	144809	C13orf30
0.467953652	0.004379768	0.011	1	338440	ANO9
0.467696249	0.004403649	0.007	1	123688	AGPHD1
0.467696249	0.004403649	0.007	1	126282	TNFAIP8L1
0.467696249	0.004403649	0.01	1	154822	AC004863.6
0.467438847	0.004427641	0.009	1	3671	ISLR
0.467438847	0.004427641	0.013	1	65981	CAPRIN2
0.467438847	0.004427641	0.007	1	245806	VGLL2
0.467438847	0.004427641	0.007	1	284403	WDR62
0.467181474	0.004451749	0.007	1	55222	LRRC20
0.467181474	0.004451749	0.008	1	84925	DIRC2
0.467181474	0.004451749	0.009	1	284415	VSTM1
0.467181474	0.004451749	0.007	1	440345	61E3.4
0.466924042	0.004475982	0.01	1	2517	FUCA1
0.466666669	0.00450032	0.007	1	2846	LPAR4
0.466409236	0.004524772	0.01	1	1571	CYP2E1
0.466409236	0.004524772	0.007	1	6278	S100A7
0.466409236	0.004524772	0.009	1	51138	COPS4
0.466409236	0.004524772	0.008	1	91298	C12orf29
0.466151863	0.004549338	0.008	1	5001	ORC5L
0.466151863	0.004549338	0.006	1	51303	FKBP11
0.466151863	0.004549338	0.007	1	54968	TMEM70
0.466151863	0.004549338	0.015	1	55471	C2orf56
0.466151863	0.004549338	0.003	1	85364	ZCCHC3
0.465894461	0.004574033	0.004	1	6335	SCN9A
0.465894461	0.004574033	0.009	1	55584	CHRNA9
0.465894461	0.004574033	0.007	1	92235	DUSP27
0.465894461	0.004574033	0.01	1	146223	CMTM4
0.465637058	0.004598832	0.006	1	360	AQP3
0.465637058	0.004598832	0.008	1	136853	SRCRB4D
0.465637058	0.004598832	0.009	1	283316	CD163L1
0.465379655	0.004623747	0.005	1	205717	KIAA2018
0.465122253	0.004648785	0.011	1	9317	PTER
0.465122253	0.004648785	0.009	1	55181	C17orf71
0.465122253	0.004648785	0.007	1	127943	FCRLB
0.465122253	0.004648785	0.007	1	388341	C17orf76
0.464350045	0.004724606	0.007	1	1948	EFNB2
0.464350045	0.004724606	0.005	1	5862	RAB2A
0.464350045	0.004724606	0.007	1	6925	TCF4
0.464350045	0.004724606	0.01	1	25930	PTPN23
0.464350045	0.004724606	0.012	1	137392	FAM92A1
0.464092672	0.004750113	0.01	1	134829	CLVS2
0.464092672	0.004750113	0.013	1	441481	GPX1P1
0.464092672	0.004750113	0.004	1	647979	LOC647979
0.463835239	0.004775744	0.005	1	159	ADSS
0.463835239	0.004775744	0.012	1	5067	CNTN3
0.463835239	0.004775744	0.006	1	25987	TSKU
0.463577867	0.004801502	0.007	1	83877	TM2D2

voxel-wise, subcortex			
Spearman's corr coef	p-val	GenID	Gene
0.335298747	< 0.001	10718	NRG3
0.335223287	< 0.001	205564	SENP5
0.335140437	< 0.001	440	ASNS
0.335134268	< 0.001	387357	THEMIS
0.335099399	< 0.001	130733	TMEM178
0.335022271	< 0.001	10466	COG5
0.334948868	< 0.001	10229	COQ7
0.33481583	< 0.001	4923	NTSR1
0.334771812	< 0.001	26093	CCDC9
0.334701359	< 0.001	51517	NCKIPSD
0.334693581	< 0.001	23541	SEC14L2
0.334679037	< 0.001	2642	GCGR
0.334579587	< 0.001	23705	CADM1
0.33447659	< 0.001	23627	PRND
0.334439963	< 0.001	10295	BCKDK
0.334437728	< 0.001	63932	CXorf56
0.334355503	< 0.001	729313	LOC729313
0.334340125	< 0.001	152189	CMTM8
0.334237009	< 0.001	84277	DNAJC30
0.334205836	< 0.001	5695	PSMB7
0.33383745	< 0.001	79057	PRRG3
0.333723485	< 0.001	7216	TRO
0.333707184	< 0.001	2876	GPX1
0.333631098	< 0.001	132720	C4orf32
0.333589494	< 0.001	5924	RASGRF2
0.3335886	< 0.001	442517	LOC442517
0.333587855	< 0.001	3195	TLX1
0.333535343	< 0.001	23033	DOPEY1
0.333535075	< 0.001	126014	OSCAR
0.33348766	< 0.001	153364	MBLAC2
0.333475649	< 0.001	27134	TJP3
0.333402842	< 0.001	221223	CES7
0.333186954	< 0.001	54440	SASH3
0.333158553	< 0.001	49861	CLDN20
0.333146691	< 0.001	3116	HLA-DPB2
0.333034903	< 0.001	3892	KRT86
0.33291629	< 0.001	81873	ARPC5L
0.332851678	< 0.001	192683	SCAMP5
0.332829386	< 0.001	10189	THOC4
0.332818985	< 0.001	3674	ITGA2B
0.332750738	< 0.001	92255	LMBRD2
0.332598239	< 0.001	8646	CHRD
0.332541019	< 0.001	1510	CTSE
0.332387328	< 0.001	10402	ST3GAL6
0.332342118	< 0.001	5055	SERPINB2
0.332273215	< 0.001	728024	LOC728024
0.332094848	< 0.001	252969	NEIL2
0.331979573	< 0.001	51440	HPCAL4
0.331944287	< 0.001	10336	PCGF3
0.331850946	< 0.001	83729	INHBE
0.331740141	< 0.001	8425	LTBP4
0.331711799	< 0.001	6709	SPTAN1
0.331706852	< 0.001	8814	CDKL1
0.33168143	< 0.001	25804	LSM4
0.331637293	< 0.001	140459	ASB6
0.331626177	< 0.001	55715	DOK4
0.331531852	< 0.001	822	CAPG
0.331480861	< 0.001	5295	PIK3R1
0.331424445	< 0.001	8434	RECK
0.331420779	< 0.001	284194	AC025627.16
0.331359684	< 0.001	8361	HIST1H4F
0.331335396	< 0.001	119032	C10orf32
0.331328839	< 0.001	79703	C11orf80
0.331211716	< 0.001	1340	COX6B1
0.331209064	< 0.001	54510	PCDH18
0.331195861	< 0.001	1952	CELSR2
0.331176788	< 0.001	84870	RSPO3
0.330884486	< 0.001	56967	C14orf132
0.330875337	< 0.001	100132147	LOC100132147
0.330753267	< 0.001	5715	PSMD9
0.330734968	< 0.001	3681	ITGAD
0.33051005	< 0.001	126374	WTIP
0.330383927	< 0.001	374875	HSD11B1L
0.330359697	< 0.001	2688	GH1
0.330351144	< 0.001	2819	GPD1
0.330278963	< 0.001	8867	SYNJ1
0.330213308	< 0.001	54733	SLC35F2
0.330087751	< 0.001	2182	ACSL4
0.330029249	< 0.001	201595	STT3B

region-wise, subcortex					
Spearman's corr coef	p-val	p-val, SA	p-val, SA (BH- correction)	GenID	Gene
0.463320464	0.004827368	0.006	1	10040	TOM1L1
0.463320464	0.004827368	0.011	1	79160	MGC4294
0.463063061	0.004853362	0.004	1	3787	KCNS1
0.463063061	0.004853362	0.017	1	23531	MMD
0.463063061	0.004853362	0.004	1	92400	RBM18
0.462805659	0.004879472	0.008	1	11196	SEC23IP
0.462805659	0.004879472	0.007	1	644684	LOC644684
0.462548256	0.00490572	0.004	1	8880	FUBP1
0.462548256	0.00490572	0.012	1	55859	BEX1
0.462290853	0.004932074	0.011	1	3381	IBSP
0.462290853	0.004932074	0.014	1	5903	RANBP2
0.462290853	0.004932074	0.011	1	27042	C1orf107
0.462290853	0.004932074	0.005	1	29121	CLEC2D
0.462290853	0.004932074	0.008	1	79948	AC006273.1
0.46203348	0.004958551	0.008	1	51005	AMDHD2
0.46203348	0.004958551	0.009	1	338667	LOC338667
0.461776048	0.004985158	0.008	1	9684	LRRC14
0.461776048	0.004985158	0.013	1	389941	C1QL1
0.461518645	0.005011882	0.008	1	6853	SYN1
0.461261243	0.005038749	0.007	1	8294	HIST1H4I
0.461261243	0.005038749	0.012	1	9630	GNA14
0.461261243	0.005038749	0.006	1	254778	C8orf46
0.46100384	0.005065723	0.006	1	5957	RCVRN
0.46100384	0.005065723	0.01	1	9899	SV2B
0.460746467	0.005092822	0.008	1	2709	GJB5
0.460746467	0.005092822	0.011	1	27232	GNMT
0.460746467	0.005092822	0.014	1	51626	DYNC2LI1
0.460746467	0.005092822	0.005	1	64743	WDR13
0.460746467	0.005092822	0.011	1	84460	ZMAT1
0.460746467	0.005092822	0.009	1	90362	FAM110B
0.460746467	0.005092822	0.015	1	202181	LOC202181
0.460746467	0.005092822	0.008	1	642273	FAM110C
0.460489035	0.005120053	0.007	1	22800	RRAS2
0.460489035	0.005120053	0.01	1	55226	NAT10
0.460489035	0.005120053	0.006	1	57690	TNRC6C
0.460489035	0.005120053	0.005	1	284996	RNF149
0.460489035	0.005120053	0.01	1	100289341	LOC100289341
0.460231662	0.005147417	0.012	1	84319	C3orf26
0.460231662	0.005147417	0.01	1	644596	LINC00087
0.459974229	0.005174901	0.01	1	10326	SIRPB1
0.459974229	0.005174901	0.007	1	408029	C2orf27B
0.459716856	0.005202507	0.012	1	8900	CCNA1
0.459716856	0.005202507	0.009	1	26996	GPR160
0.459459454	0.005230241	0.006	1	9128	PRPF4
0.459202051	0.005258121	0.009	1	2909	GRLF1
0.459202051	0.005258121	0.012	1	5603	MAPK13
0.459202051	0.005258121	0.007	1	80115	BAIAP2L2
0.459202051	0.005258121	0.008	1	138724	C9orf131
0.459202051	0.005258121	0.009	1	150590	C2orf15
0.458944649	0.005286111	0.01	1	23176	SEPTIN8
0.458944649	0.005286111	0.009	1	54749	EPDR1
0.458944649	0.005286111	0.004	1	64921	CASD1
0.458944649	0.005286111	0.01	1	122830	NAA30
0.458944649	0.005286111	0.008	1	728858	C12orf71
0.458687246	0.005314238	0.005	1	22821	RASA3
0.458687246	0.005314238	0.008	1	27087	B3GAT1
0.458687246	0.005314238	0.01	1	29906	ST8SIA5
0.458429843	0.005342488	0.015	1	694	BTG1
0.458429843	0.005342488	0.003	1	1436	CSF1R
0.458429843	0.005342488	0.009	1	1977	EIF4E
0.458429843	0.005342488	0.012	1	9141	PDCD5
0.458429843	0.005342488	0.01	1	79882	ZC3H14
0.45817247	0.005370868	0.008	1	2067	ERCC1
0.45817247	0.005370868	0.006	1	4199	ME1
0.45817247	0.005370868	0.005	1	79078	C1orf50
0.457915038	0.005399398	0.009	1	6810	STX4
0.457915038	0.005399398	0.006	1	57221	KIAA1244
0.457915038	0.005399398	0.014	1	613038	RP11-347C12.3
0.457657665	0.005428039	0.01	1	5768	QSOX1
0.457657665	0.005428039	0.01	1	133957	CCDC127
0.457657665	0.005428039	0.013	1	140458	ASB5
0.457400233	0.00545682	0.008	1	5787	PTPRB
0.457400233	0.00545682	0.009	1	57497	LRFN2
0.457400233	0.00545682	0.005	1	203522	DDX26B
0.457400233	0.00545682	0.012	1	339479	FAM5C
0.45714286	0.005485726	0.01	1	1838	DTNB
0.45714286	0.005485726	0.015	1	84067	FAM160A2
0.45714286	0.005485726	0.011	1	399697	CTXN2
0.45714286	0.005485726	0.006	1	440101	RP11-834C11.3

voxel-wise, subcortex			
Spearman's corr coef	p-val	GenID	Gene
0.329946309	< 0.001	5612	PRKRIR
0.329876333	< 0.001	81616	ACSBG2
0.32982859	< 0.001	83959	SLC4A11
0.329826087	< 0.001	2155	F7
0.329797298	< 0.001	722	C4BPA
0.329771399	< 0.001	5795	PTPRJ
0.329708993	< 0.001	25928	SOSTDC1
0.329684108	< 0.001	5013	OTX1
0.329605073	< 0.001	6038	RNASE4
0.329583704	< 0.001	6503	SLA
0.329516023	< 0.001	66004	LYNX1
0.329472423	< 0.001	7262	PHLDA2
0.329392165	< 0.001	11264	PXMP4
0.32926482	< 0.001	266812	NAP1L5
0.329231024	< 0.001	646174	C16orf90
0.329208732	< 0.001	10526	IPO8
0.329016358	< 0.001	84456	L3MBTL3
0.329004496	< 0.001	84769	MPV17L2
0.328896463	< 0.001	22916	NCBP2
0.328850359	< 0.001	283358	B4GALNT3
0.328767002	< 0.001	51170	HSD17B11
0.328679025	< 0.001	137970	UNC5D
0.328406274	< 0.001	80184	CEP290
0.328110993	< 0.001	653505	PPIAL4B
0.328105658	< 0.001	140612	ZFP28
0.328021109	< 0.001	204962	SLC44A5
0.327977955	< 0.001	219869	OR10G8
0.32793349	< 0.001	94033	FTMT
0.327924579	< 0.001	8660	IRS2
0.32791996	< 0.001	4277	MICB
0.327668637	< 0.001	4435	CITED1
0.32758376	< 0.001	55466	DNAJA4
0.327535778	< 0.001	5293	PIK3CD
0.327533334	< 0.001	9626	GUCA1C
0.327475756	< 0.001	10005	ACOT8
0.327411622	< 0.001	440672	NUDT4P1
0.32728824	< 0.001	4185	ADAM11
0.32715416	< 0.001	170261	ZCCHC12
0.327092111	< 0.001	266675	BEST4
0.326895803	< 0.001	9956	HS3ST2
0.326889485	< 0.001	10528	NOP56
0.3267304	< 0.001	55203	LGI2
0.326725721	< 0.001	10383	TUBB2C
0.326718479	< 0.001	2742	GLRA2
0.32616666	< 0.001	54207	KCNK10
0.326139301	< 0.001	2191	FAP
0.326006413	< 0.001	64921	CASD1
0.325925529	< 0.001	51186	WBP5
0.325917095	< 0.001	285033	LOC285033
0.325910479	< 0.001	84937	ZNRF1
0.325900376	< 0.001	100129291	LOC100129291
0.325728983	< 0.001	79172	CENPO
0.32553944	< 0.001	79697	C14orf169
0.325432509	< 0.001	55437	STRADB
0.325422496	< 0.001	7380	UPK3A
0.325291634	< 0.001	6925	TCF4
0.325283229	< 0.001	57504	MTA3
0.325223058	< 0.001	51032	CELA2B
0.325147182	< 0.001	84954	MPND
0.325119406	< 0.001	27178	IL1F7
0.325003296	< 0.001	79161	C7orf23
0.324972123	< 0.001	3024	HIST1H1A
0.324961901	< 0.001	51098	IFT52
0.324945509	< 0.001	10745	PHTF1
0.324835569	< 0.001	5566	PRKACA
0.324827582	< 0.001	90410	IFT20
0.324802607	< 0.001	29886	SNX8
0.324772388	< 0.001	55801	IL26
0.324640274	< 0.001	79363	C1orf89
0.324624777	< 0.001	152992	C4orf23
0.324572951	< 0.001	8444	DYRK3
0.324525595	< 0.001	63941	NECAB3
0.32449922	< 0.001	8775	NAPA
0.324466139	< 0.001	29883	CNOT7
0.324339658	< 0.001	57213	C13orf1
0.324317515	< 0.001	100131482	LOC100131482
0.324193269	< 0.001	140883	ZNF280B
0.32416749	< 0.001	4751	NEK2
0.324111879	< 0.001	2979	GUCA1B

region-wise, subcortex					
Spearman's corr coef	p-val	p-val, SA	p-val, SA (BH- correction)	GenID	Gene
0.45714286	0.005485726	0.007	1	646548	LOC646548
0.456885457	0.005514778	0.01	1	6833	ABCC8
0.456885457	0.005514778	0.015	1	6940	ZNF354A
0.456885457	0.005514778	0.006	1	10979	PLEKHC1
0.456885457	0.005514778	0.01	1	84289	ING5
0.456885457	0.005514778	0.002	1	388242	RP11-345J4.3
0.456628054	0.005543957	0.006	1	2971	GTF3A
0.456628054	0.005543957	0.016	1	55610	CCDC132
0.456370652	0.005573262	0.008	1	3059	HCLS1
0.456370652	0.005573262	0.013	1	8208	CHAF1B
0.456370652	0.005573262	0.009	1	11170	FAM107A
0.456370652	0.005573262	0.009	1	84203	TXNDC2
0.456370652	0.005573262	0.006	1	100191040	AL589765.4
0.456113249	0.00560271	0.011	1	29960	FTSJ2
0.455855846	0.005632298	0.01	1	5926	ARID4A
0.455855846	0.005632298	0.009	1	57689	LRRC4C
0.455598474	0.005662009	0.01	1	51086	TNNI3K
0.455598474	0.005662009	0.007	1	51343	FZR1
0.455598474	0.005662009	0.01	1	51729	WBP11
0.455598474	0.005662009	0.006	1	219736	STOX1
0.455341041	0.005691862	0.009	1	53615	MBD3
0.455341041	0.005691862	0.008	1	64897	C12orf43
0.455341041	0.005691862	0.006	1	401067	IQCF3
0.455083668	0.005721845	0.011	1	4681	NBL1
0.455083668	0.005721845	0.012	1	23022	PALLD
0.455083668	0.005721845	0.008	1	161882	ZFPM1
0.454826236	0.005751972	0.011	1	9820	CUL7
0.454826236	0.005751972	0.009	1	124220	ZG16B
0.454826236	0.005751972	0.013	1	286887	KRT6C
0.454826236	0.005751972	0.005	1	346389	MACC1
0.454826236	0.005751972	0.017	1	353189	SLCO4C1
0.454826236	0.005751972	0.011	1	389383	C6orf126
0.45431146	0.005812637	0.014	1	6755	SSTR5
0.45431146	0.005812637	0.012	1	9847	KIAA0528
0.45431146	0.005812637	0.008	1	100287114	LINC00421
0.454054028	0.005843177	0.012	1	4659	PPP1R12A
0.453796655	0.005873849	0.003	1	283767	AC116165.2
0.453796655	0.005873849	0.013	1	388564	TMEM238
0.453539222	0.005904682	0.007	1	7138	TNNT1
0.453539222	0.005904682	0.009	1	221895	JAZF1
0.45328185	0.005935633	0.008	1	5048	PAFAH1B1
0.45328185	0.005935633	0.006	1	6752	SSTR2
0.45328185	0.005935633	0.007	1	29070	CCDC113
0.45328185	0.005935633	0.009	1	80212	CCDC92
0.45328185	0.005935633	0.015	1	374875	HSD11B1L
0.45328185	0.005935633	0.009	1	389384	C6orf222
0.453024447	0.005966726	0.015	1	23564	DDAH2
0.453024447	0.005966726	0.014	1	84063	KIRREL2
0.452767044	0.005997966	0.011	1	2555	GABRA2
0.452767044	0.005997966	0.007	1	51514	DTL
0.452509642	0.006029354	0.011	1	5784	PTPN14
0.452509642	0.006029354	0.01	1	51098	IFT52
0.452509642	0.006029354	0.009	1	317701	VN1R2
0.452252239	0.006060879	0.012	1	516	ATP5G1
0.452252239	0.006060879	0.008	1	23168	RTF1
0.452252239	0.006060879	0.004	1	257194	NEGR1
0.451994836	0.006092538	0.014	1	60	ACTB
0.451994836	0.006092538	0.012	1	64595	TTTY15
0.451994836	0.006092538	0.011	1	151987	PPP4R2
0.451994836	0.006092538	0.009	1	153770	PLAC8L1
0.451737463	0.00612434	0.011	1	22948	CCT5
0.451480031	0.006156294	0.007	1	4781	NFIB
0.451480031	0.006156294	0.008	1	79033	ERI3
0.451222658	0.006188399	0.007	1	3216	HOXB6
0.451222658	0.006188399	0.008	1	55670	PEX26
0.451222658	0.006188399	0.01	1	79158	GNPTAB
0.451222658	0.006188399	0.009	1	84557	MAP1LC3A
0.450965226	0.006220642	0.009	1	10253	SPRY2
0.450965226	0.006220642	0.011	1	81688	C6orf62
0.450965226	0.006220642	0.011	1	134121	C5orf49
0.450965226	0.006220642	0.014	1	100129291	LOC100129291
0.450707853	0.006253023	0.01	1	25893	TRIM58
0.450707853	0.006253023	0.007	1	55088	C10orf118
0.450707853	0.006253023	0.015	1	55273	TMEM100
0.450707853	0.006253023	0.008	1	114915	EPB41L4A-AS1
0.45045045	0.006285549	0.014	1	3586	IL10
0.450193048	0.006318243	0.016	1	1012	CDH13
0.450193048	0.006318243	0.014	1	8525	DGKZ
0.450193048	0.006318243	0.017	1	54510	PCDH18

voxel-wise, subcortex			
Spearman's corr coef	p-val	GenID	Gene
0.324065536	< 0.001	8907	AP1M1
0.324060738	< 0.001	10552	ARPC1A
0.323931783	< 0.001	10911	UTS2
0.323907614	< 0.001	343477	HSP90B3P
0.323860765	< 0.001	8237	USP11
0.323771983	< 0.001	5887	RAD23B
0.323729992	< 0.001	85442	KNDC1
0.323722094	< 0.001	26521	TIMM8B
0.323620558	< 0.001	11245	GPR176
0.323597431	< 0.001	65268	WNK2
0.323478341	< 0.001	5000	ORC4
0.323343664	< 0.001	7332	UBE2L3
0.323301226	< 0.001	727800	RNF208
0.323284894	< 0.001	80007	C10orf88
0.323108822	< 0.001	2312	FLG
0.322998345	< 0.001	8799	PEX11B
0.322954327	< 0.001	892	CCNC
0.322944343	< 0.001	4698	NDUFA5
0.322919011	< 0.001	3049	HBQ1
0.322896302	< 0.001	53339	BTBD1
0.322784215	< 0.001	3787	KCNS1
0.32259208	< 0.001	146861	AMAC1
0.322558224	< 0.001	2953	GSTT2
0.322533458	< 0.001	151242	PPP1R1C
0.322453827	< 0.001	344558	SH3RF3
0.32235086	< 0.001	339231	ARL16
0.322350323	< 0.001	79827	AP000926.2
0.322335154	< 0.001	285659	OR2V2
0.322241157	< 0.001	2241	FER
0.32212615	< 0.001	10990	LILRB5
0.322123975	< 0.001	84560	MT4
0.322119802	< 0.001	245711	SPDYA
0.322007418	< 0.001	8626	TP63
0.321955413	< 0.001	9158	FIBP
0.321785569	< 0.001	1572	CYP2F1
0.321761847	< 0.001	126328	NDUFA11
0.321750015	< 0.001	54529	ASNSD1
0.32169205	< 0.001	8754	ADAM9
0.321610332	< 0.001	139599	MAGEE2
0.321607322	< 0.001	6903	TBCC
0.321549892	< 0.001	401913	LOC401913
0.321432322	< 0.001	1016	CDH18
0.321330309	< 0.001	125488	TTC39C
0.321220666	< 0.001	389812	LCN15
0.321045935	< 0.001	157313	CDCA2
0.320947886	< 0.001	90417	C15orf23
0.320924759	< 0.001	80762	NDFIP1
0.320908457	< 0.001	6988	TCTA
0.320880175	< 0.001	347454	ANKRD58
0.320873022	< 0.001	3760	KCNJ3
0.320786327	< 0.001	22911	WDR47
0.320691377	< 0.001	445	ASS1
0.32066083	< 0.001	529	ATP6V1E1
0.32064417	< 0.001	6623	SNCG
0.32057786	< 0.001	3796	KIF2A
0.320491284	< 0.001	160	AP2A1
0.320485294	< 0.001	343450	KCNT2
0.320482045	< 0.001	84289	ING5
0.320463538	< 0.001	8732	RNGTT
0.320362717	< 0.001	1740	DLG2
0.320222229	< 0.001	170393	C10orf91
0.320198596	< 0.001	22953	P2RX2
0.320099622	< 0.001	4151	MB
0.319941908	< 0.001	342541	LOC342541
0.319938838	< 0.001	8618	CADPS
0.319929183	< 0.001	8506	CNTNAP1
0.319919527	< 0.001	8723	SNX4
0.319891572	< 0.001	100506084	AC005829.1
0.319807142	< 0.001	26533	OR10G3
0.319787085	< 0.001	7415	VCP
0.319774479	< 0.001	1262	CNGA4
0.319768578	< 0.001	57600	FNIP2
0.319601685	< 0.001	51166	AADAT
0.319583863	< 0.001	54897	CASZ1
0.319580048	< 0.001	221044	UCMA
0.319401562	< 0.001	4624	MYH6
0.319143355	< 0.001	1609	DGKQ
0.319127709	< 0.001	727830	FAM75A3
0.319052756	< 0.001	84923	FAM104A

region-wise, subcortex					
Spearman's corr coef	p-val	p-val, SA	p-val, SA (BH- correction)	GenID	Gene
0.450193048	0.006318243	0.007	1	64130	LIN7B
0.450193048	0.006318243	0.011	1	64859	OBFC2A
0.450193048	0.006318243	0.015	1	340307	CTAGE6P
0.449935645	0.006351063	0.011	1	2048	EPHB2
0.449935645	0.006351063	0.007	1	79690	GAL3ST4
0.449678242	0.006384034	0.011	1	9551	ATP5J2
0.449678242	0.006384034	0.009	1	29924	EPN1
0.449678242	0.006384034	0.01	1	56651	LINC00470
0.449678242	0.006384034	0.01	1	389206	BEND4
0.44942084	0.006417152	0.013	1	10053	AP1M2
0.44942084	0.006417152	0.015	1	25794	FSCN2
0.44942084	0.006417152	0.003	1	339287	MSL1
0.449163467	0.006450431	0.015	1	1448	CSN3
0.448906034	0.006483852	0.01	1	1576	CYP3A4
0.448906034	0.006483852	0.012	1	2802	GOLGA3
0.448906034	0.006483852	0.007	1	3109	HLA-DMB
0.448906034	0.006483852	0.005	1	378805	AC058791.2
0.448391229	0.006551131	0.013	1	680	BRS3
0.448391229	0.006551131	0.003	1	64219	PJA1
0.448133856	0.006584994	0.008	1	29993	PACSIN1
0.448133856	0.006584994	0.008	1	79570	NKAIN1
0.447876453	0.006619029	0.007	1	53829	P2RY13
0.447876453	0.006619029	0.005	1	727800	RNF208
0.447619021	0.006653198	0.012	1	56981	PRDM11
0.447104216	0.006721998	0.006	1	1965	EIF2S1
0.446846843	0.006756642	0.017	1	4257	MGST1
0.446846843	0.006756642	0.007	1	65008	MRPL1
0.446846843	0.006756642	0.012	1	128434	VSTM2L
0.446846843	0.006756642	0.012	1	727910	TLCD2
0.44658944	0.006791422	0.009	1	2888	GRB14
0.44658944	0.006791422	0.008	1	63979	FIGNL1
0.446332037	0.00682636	0.008	1	2922	GRP
0.446332037	0.00682636	0.013	1	57551	TAOK1
0.446332037	0.00682636	0.01	1	283152	CCDC153
0.446074635	0.006861453	0.013	1	26039	SS18L1
0.446074635	0.006861453	0.013	1	84126	ATRIP
0.446074635	0.006861453	0.014	1	126170	LOC126170
0.445817232	0.006896718	0.013	1	60676	PAPPA2
0.445817232	0.006896718	0.005	1	391104	VHLL
0.445559829	0.006932124	0.009	1	9535	GMFG
0.445559829	0.006932124	0.012	1	388882	AP000345.1
0.445302457	0.006967682	0.019	1	8508	NIPSNAP1
0.445302457	0.006967682	0.007	1	55423	SIRPG
0.445045024	0.007003402	0.01	1	813	CALU
0.445045024	0.007003402	0.008	1	1089	CEACAM4
0.445045024	0.007003402	0.014	1	4338	MOCS2
0.444787651	0.007039277	0.015	1	2781	GNAZ
0.444787651	0.007039277	0.016	1	9992	KCNE2
0.444787651	0.007039277	0.019	1	53826	FXYD6
0.444787651	0.007039277	0.01	1	55757	UGCGL2
0.444787651	0.007039277	0.01	1	254102	EHBP1L1
0.444530219	0.007075333	0.006	1	23518	R3HDM1
0.444272846	0.007111525	0.009	1	3679	ITGA7
0.444272846	0.007111525	0.02	1	6595	SMARCA2
0.444272846	0.007111525	0.015	1	9249	DHRS3
0.444272846	0.007111525	0.015	1	51669	TMEM66
0.444272846	0.007111525	0.005	1	54946	SLC41A3
0.444272846	0.007111525	0.02	1	55028	C17orf80
0.444015443	0.00714788	0.011	1	29	ABR
0.444015443	0.00714788	0.015	1	148	ADRA1A
0.444015443	0.00714788	0.009	1	8721	EDF1
0.444015443	0.00714788	0.012	1	284194	AC025627.16
0.443758041	0.007184397	0.019	1	5746	PTH2R
0.443500638	0.007221091	0.012	1	23369	PUM2
0.443500638	0.007221091	0.013	1	57583	TMEM181
0.443500638	0.007221091	0.011	1	64744	SMAP2
0.443500638	0.007221091	0.013	1	121268	RHEBL1
0.443500638	0.007221091	0.014	1	374387	AC044839.2
0.443243235	0.007257929	0.007	1	132160	PPM1M
0.443243235	0.007257929	0.009	1	155368	WBSCR27
0.443243235	0.007257929	0.006	1	255252	LRRC57
0.442985833	0.007294925	0.013	1	84626	KRBA1
0.44272846	0.007332089	0.017	1	23035	PHLPP2
0.442471027	0.007369416	0.014	1	7325	UBE2E2
0.442471027	0.007369416	0.013	1	140612	ZFP28
0.442471027	0.007369416	0.014	1	285598	ARL10
0.442471027	0.007369416	0.012	1	653513	LOC653513
0.442213655	0.007406923	0.012	1	51186	WBP5
0.442213655	0.007406923	0.012	1	401472	FLJ45248

voxel-wise, subcortex			
Spearman's corr coef	p-val	GenID	Gene
0.319027066	< 0.001	389538	MGC72080
0.319024771	< 0.001	55790	CSGALNACT1
0.318976372	< 0.001	1838	DTNB
0.318929434	< 0.001	51182	HSPA14
0.318892658	< 0.001	6689	SPIB
0.318842053	< 0.001	64968	MRPS6
0.318761915	< 0.001	401992	OR2T2
0.318658948	< 0.001	55737	VPS35
0.318624377	< 0.001	56160	NDNL2
0.318575382	< 0.001	3512	IGJ
0.318557054	< 0.001	64282	PAPD5
0.318532467	< 0.001	54842	MFSD6
0.318444431	< 0.001	8111	GPR68
0.31829083	< 0.001	10640	EXOC5
0.318194568	< 0.001	113730	KLHDC7B
0.318176836	< 0.001	79139	DERL1
0.318167418	< 0.001	50487	PLA2G3
0.317984849	< 0.001	84648	LCE3D
0.317911446	< 0.001	168374	ZNF92
0.317895263	< 0.001	3050	HBZ
0.31788817	< 0.001	644578	LARP1BP1
0.317789137	< 0.001	57551	TAOK1
0.31777367	< 0.001	56666	PANX2
0.317548096	< 0.001	1984	EIF5A
0.317476869	< 0.001	149175	MANEAL
0.317419231	< 0.001	84944	MAEL
0.317377508	< 0.001	2926	GRSF1
0.317372948	< 0.001	59285	CACNG6
0.317313373	< 0.001	645455	CEP170L
0.317276925	< 0.001	10424	PGRMC2
0.317268521	< 0.001	254173	TTLL10
0.317131698	< 0.001	79980	DSN1
0.317075789	< 0.001	26512	INTS6
0.31688723	< 0.001	8542	APOL1
0.316839248	< 0.001	23300	ATMIN
0.316821277	< 0.001	550631	CCDC157
0.316796869	< 0.001	7975	MAFK
0.316750288	< 0.001	54869	EPS8L1
0.316738486	< 0.001	6683	SPAST
0.316659242	< 0.001	6532	SLC6A4
0.316649765	< 0.001	1186	CLCN7
0.316615254	< 0.001	29089	UBE2T
0.316607326	< 0.001	51373	MRPS17
0.316428006	< 0.001	146227	AC132186.2
0.316425711	< 0.001	440917	LOC440917
0.31642136	< 0.001	3954	LETM1
0.316406488	< 0.001	90809	TMEM55B
0.316294163	< 0.001	9352	TXNL1
0.316289961	< 0.001	6854	SYN2
0.316269577	< 0.001	3008	HIST1H1E
0.316245317	< 0.001	9779	TBC1D5
0.316186637	< 0.001	56606	SLC2A9
0.316144943	< 0.001	114571	SLC22A9
0.316113025	< 0.001	344387	CDKL4
0.316105992	< 0.001	8220	DGCR14
0.316091686	< 0.001	148979	GLIS1
0.315994918	< 0.001	100134934	C17ORF106
0.315917969	< 0.001	84984	C3orf34
0.315884173	< 0.001	162993	ZNF846
0.315866768	< 0.001	57731	SPTBN4
0.315788835	< 0.001	80309	SPHKAP
0.315700948	< 0.001	23741	EID1
0.315574825	< 0.001	8022	LHX3
0.315478772	< 0.001	283848	CES8
0.315458804	< 0.001	8792	TNFRSF11A
0.315412492	< 0.001	84707	BEX2
0.315340102	< 0.001	80063	ATF7IP2
0.315320015	< 0.001	3214	HOXB4
0.315277189	< 0.001	55922	NKRF
0.315272838	< 0.001	6185	RPN2
0.315195948	< 0.001	5197	PF4V1
0.315165699	< 0.001	84661	DPY30
0.315152258	< 0.001	283177	LOC283177
0.315138221	< 0.001	55336	FBXL8
0.315018088	< 0.001	7108	TM7SF2
0.314886451	< 0.001	1803	DPP4
0.314874202	< 0.001	81871	KRTAP4-6
0.31486237	< 0.001	140461	ASB8
0.314792961	< 0.001	27289	RND1

region-wise, subcortex					
Spearman's corr coef	p-val	p-val, SA	p-val, SA (BH- correction)	GenID	Gene
0.441956222	0.007444578	0.011	1	8974	P4HA2
0.441956222	0.007444578	0.008	1	9394	HS6ST1
0.441956222	0.007444578	0.013	1	84960	KIAA1984
0.441956222	0.007444578	0.015	1	92370	ACPL2
0.441956222	0.007444578	0.008	1	148137	C19orf55
0.441698849	0.007482394	0.019	1	5691	PSMB3
0.441698849	0.007482394	0.013	1	55034	MOCOS
0.441441447	0.007520374	0.011	1	5295	PIK3R1
0.441441447	0.007520374	0.009	1	6721	SREBF2
0.441441447	0.007520374	0.014	1	65267	WNK3
0.441184044	0.007558549	0.016	1	917	CD3G
0.441184044	0.007558549	0.007	1	7414	VCL
0.441184044	0.007558549	0.016	1	152992	C4orf23
0.440669239	0.007635354	0.013	1	10286	BCAS2
0.440669239	0.007635354	0.018	1	200312	RNF215
0.440411836	0.007674003	0.014	1	23031	MAST3
0.440411836	0.007674003	0.008	1	64430	C14orf135
0.440411836	0.007674003	0.011	1	79707	NOL9
0.440411836	0.007674003	0.011	1	95681	TSGA14
0.440411836	0.007674003	0.016	1	118424	UBE2J2
0.440154433	0.007712843	0.01	1	120227	CYP2R1
0.440154433	0.007712843	0.011	1	166752	FREM3
0.440154433	0.007712843	0.006	1	222068	TMED4
0.440154433	0.007712843	0.009	1	441308	OR4F21
0.439897031	0.007751835	0.009	1	9289	GPR56
0.439382225	0.007830329	0.012	1	2890	GRIA1
0.439382225	0.007830329	0.016	1	54959	ODAM
0.439124823	0.007869828	0.014	1	84268	RPAIN
0.439124823	0.007869828	0.011	1	135295	RP11-63L7.1
0.43886745	0.007909521	0.014	1	1564	CYP2D7P1
0.43886745	0.007909521	0.015	1	54438	GFOD1
0.438610017	0.007949368	0.018	1	284	ANGPT1
0.438352644	0.007989384	0.01	1	4147	MATN2
0.438352644	0.007989384	0.013	1	10724	MGEA5
0.438352644	0.007989384	0.014	1	22801	ITGA11
0.438352644	0.007989384	0.011	1	349667	RTN4RL2
0.438095212	0.008029581	0.007	1	3214	HOXB4
0.438095212	0.008029581	0.015	1	6503	SLA
0.438095212	0.008029581	0.014	1	10940	POP1
0.438095212	0.008029581	0.012	1	24145	PANX1
0.438095212	0.008029581	0.013	1	29841	GRHL1
0.438095212	0.008029581	0.012	1	55615	PRR5
0.438095212	0.008029581	0.014	1	80131	LRRC8E
0.437837839	0.008069962	0.011	1	9921	RNF10
0.437837839	0.008069962	0.019	1	83643	CCDC3
0.437837839	0.008069962	0.017	1	221468	TMEM217
0.437837839	0.008069962	0.013	1	353500	BMP8A
0.437837839	0.008069962	0.014	1	389493	RP4-725G10.1
0.437580436	0.0081105	0.01	1	64407	RGS18
0.437580436	0.0081105	0.019	1	388963	C2orf81
0.437323034	0.008151222	0.01	1	9114	ATP6V0D1
0.437323034	0.008151222	0.017	1	23092	ARHGAP26
0.437323034	0.008151222	0.012	1	388165	UBE2Q2P1
0.437065631	0.008192111	0.012	1	1525	CXADR
0.437065631	0.008192111	0.023	1	3619	INCENP
0.437065631	0.008192111	0.005	1	4277	MICB
0.437065631	0.008192111	0.011	1	64946	CENPH
0.437065631	0.008192111	0.011	1	115098	CCDC124
0.436808228	0.008233199	0.016	1	997	CDC34
0.436808228	0.008233199	0.012	1	6185	RPN2
0.436808228	0.008233199	0.012	1	550643	LOC550643
0.436550826	0.008274446	0.012	1	2821	GPI
0.436550826	0.008274446	0.013	1	23671	TMEFF2
0.436550826	0.008274446	0.016	1	26994	RNF11
0.436550826	0.008274446	0.013	1	346157	ZNF391
0.436550826	0.008274446	0.016	1	400410	ST20
0.436036021	0.008357475	0.012	1	25799	ZNF324
0.436036021	0.008357475	0.012	1	57707	KIAA1609
0.436036021	0.008357475	0.014	1	83696	TRAPPC9
0.436036021	0.008357475	0.009	1	440073	IQSEC3
0.435778648	0.008399253	0.01	1	9637	FEZ2
0.435521215	0.008441228	0.018	1	1363	CPE
0.435521215	0.008441228	0.016	1	57544	TXNDC16
0.435521215	0.008441228	0.014	1	90678	LRSAM1
0.435263842	0.008483375	0.015	1	8764	TNFRSF14
0.435263842	0.008483375	0.017	1	245711	SPDYA
0.43500644	0.008525694	0.012	1	5297	PI4KA
0.43500644	0.008525694	0.009	1	55521	TRIM36
0.43500644	0.008525694	0.011	1	55915	LANCL2

voxel-wise, subcortex			
Spearman's corr coef	p-val	GenID	Gene
0.314676762	< 0.001	348094	ANKDD1A
0.314626902	< 0.001	29	ABR
0.314602077	< 0.001	57573	ZNF471
0.314576805	< 0.001	654434	LINC00338
0.314559311	< 0.001	65108	MARCKSL1
0.314498901	< 0.001	25814	ATXN10
0.314427048	< 0.001	27018	NGFRAP1
0.31439209	< 0.001	27010	TPK1
0.314348966	< 0.001	57591	MKL1
0.314208835	< 0.001	222642	BZRPL1
0.31417799	< 0.001	400578	KRT16P2
0.314095765	< 0.001	9382	COG1
0.313986212	< 0.001	3423	IDS
0.313930303	< 0.001	142940	TRUB1
0.313834488	< 0.001	54584	GNB1L
0.313783675	< 0.001	56255	TMX4
0.313732564	< 0.001	55588	MED29
0.313728899	< 0.001	730101	RP1-152L7.5
0.313701212	< 0.001	1173	AP2M1
0.313646942	< 0.001	388882	AP000345.1
0.31361866	< 0.001	51400	PPME1
0.313559592	< 0.001	23284	LPHN3
0.313396394	< 0.001	116519	APOA5
0.313361049	< 0.001	4843	NOS2
0.313245118	< 0.001	51132	RLIM
0.313113093	< 0.001	6616	SNAP25
0.313077301	< 0.001	435	ASL
0.313073069	< 0.001	440072	LINC00167
0.313023865	< 0.001	79072	FASTKD3
0.312990546	< 0.001	3361	HTR5A
0.312969148	< 0.001	9867	PJA2
0.312934428	< 0.001	55175	KLHL11
0.312915891	< 0.001	92558	CCDC64
0.312806785	< 0.001	3188	HNRNPH2
0.312791884	< 0.001	3048	HBG2
0.312787175	< 0.001	6512	SLC1A7
0.312745482	< 0.001	79047	KCTD15
0.312732995	< 0.001	1063	CENPF
0.312676728	< 0.001	205717	KIAA2018
0.31265229	< 0.001	9895	TECPR2
0.312613338	< 0.001	2967	GTF2H3
0.312601447	< 0.001	282991	BLOC1S2
0.312588036	< 0.001	5688	PSMA7
0.31257242	< 0.001	90780	PYGO2
0.31246841	< 0.001	100132247	NPIPL3
0.312274873	< 0.001	64407	RGS18
0.312269598	< 0.001	1819	DRG2
0.312236995	< 0.001	57410	SCYL1
0.312221527	< 0.001	79751	SLC25A22
0.31188184	< 0.001	23092	ARHGAP26
0.311824173	< 0.001	8851	CDK5R1
0.311594278	< 0.001	4675	NAP1L3
0.311572313	< 0.001	79668	PARP8
0.31157124	< 0.001	4850	CNOT4
0.311568826	< 0.001	80071	CCDC15
0.311514586	< 0.001	646870	LOC646870
0.311488867	< 0.001	219738	C10orf35
0.311367482	< 0.001	79026	AHNAK
0.31135115	< 0.001	3843	IPO5
0.311260521	< 0.001	389206	BEND4
0.311190963	< 0.001	378884	NHLRC1
0.310926229	< 0.001	123036	TC2N
0.310492098	< 0.001	221302	ZUFSP
0.310378015	< 0.001	9777	TM9SF4
0.31025064	< 0.001	283431	GAS2L3
0.310195476	< 0.001	5058	PAK1
0.310132504	< 0.001	143384	C10orf46
0.310121566	< 0.001	23008	KLHDC10
0.310051084	< 0.001	440292	LOC440292
0.310047835	< 0.001	7879	RAB7A
0.310023218	< 0.001	10868	USP20
0.309947014	< 0.001	56886	UGCGL1
0.3098647	< 0.001	390	RND3
0.309852719	< 0.001	51606	ATP6V1H
0.309749961	< 0.001	83855	KLF16
0.309719294	< 0.001	4320	MMP11
0.309687883	< 0.001	9506	PAGE4
0.309544355	< 0.001	23248	RPRD2
0.309505016	< 0.001	83696	TRAPPC9

region-wise, subcortex					
Spearman's corr coef	p-val	p-val, SA	p-val, SA (BH- correction)	GenID	Gene
0.43500644	0.008525694	0.022	1	55964	SEPTIN3
0.434749037	0.008568203	0.018	1	316	AOX1
0.434749037	0.008568203	0.017	1	8543	LMO4
0.434749037	0.008568203	0.013	1	23066	CAND2
0.434749037	0.008568203	0.017	1	30062	RAX
0.434749037	0.008568203	0.012	1	54938	SARS2
0.434491634	0.008610904	0.012	1	2960	GTF2E1
0.434491634	0.008610904	0.019	1	55295	KLHL26
0.434491634	0.008610904	0.012	1	646808	LOC646808
0.434234232	0.008653768	0.012	1	3116	HLA-DPB2
0.434234232	0.008653768	0.018	1	3964	LGALS8
0.434234232	0.008653768	0.015	1	8367	HIST1H4E
0.434234232	0.008653768	0.016	1	26013	L3MBTL
0.434234232	0.008653768	0.013	1	29919	C18orf8
0.434234232	0.008653768	0.01	1	64282	PAPD5
0.434234232	0.008653768	0.011	1	83468	GLT8D2
0.433976829	0.008696826	0.012	1	26043	UBXN7
0.433976829	0.008696826	0.015	1	64840	PORCN
0.433719456	0.008740058	0.012	1	57821	C1orf114
0.433719456	0.008740058	0.014	1	139599	MAGEE2
0.433204621	0.008827107	0.016	1	3790	KCNS3
0.433204621	0.008827107	0.021	1	64426	SUDS3
0.432947218	0.008870896	0.014	1	6647	SOD1
0.432947218	0.008870896	0.017	1	57621	ZBTB2
0.432947218	0.008870896	0.01	1	116519	APOA5
0.432947218	0.008870896	0.007	1	140689	CBLN4
0.432689816	0.00891488	0.014	1	381	ARF5
0.432689816	0.00891488	0.012	1	79184	BRCC3
0.432432443	0.008959042	0.015	1	759	CA1
0.432432443	0.008959042	0.011	1	63974	NEUROD6
0.432432443	0.008959042	0.008	1	80343	SEL1L2
0.432432443	0.008959042	0.009	1	84153	RNASEH2C
0.432432443	0.008959042	0.014	1	84769	MPV17L2
0.43217501	0.009003422	0.016	1	10752	CHL1
0.43217501	0.009003422	0.015	1	100129321	LOC100129321
0.431917638	0.009047961	0.014	1	57159	TRIM54
0.431917638	0.009047961	0.018	1	283489	ZNF828
0.431660205	0.009092689	0.011	1	1063	CENPF
0.431660205	0.009092689	0.019	1	25903	OLFML2B
0.431660205	0.009092689	0.016	1	51170	HSD17B11
0.431660205	0.009092689	0.019	1	285659	OR2V2
0.431402832	0.009137617	0.013	1	5604	MAP2K1
0.431402832	0.009137617	0.014	1	83931	STK40
0.431402832	0.009137617	0.013	1	348654	GEN1
0.4311454	0.009182745	0.025	1	5569	PKIA
0.4311454	0.009182745	0.014	1	7068	THRB
0.4311454	0.009182745	0.015	1	90557	CCDC74A
0.4311454	0.009182745	0.018	1	91860	CALML4
0.4311454	0.009182745	0.015	1	167555	FAM151B
0.4311454	0.009182745	0.014	1	474343	SPIN2B
0.430888027	0.009228054	0.013	1	2255	FGF10
0.430888027	0.009228054	0.013	1	7799	PRDM2
0.430888027	0.009228054	0.011	1	8365	HIST1H4H
0.430888027	0.009228054	0.016	1	23491	CES3
0.430888027	0.009228054	0.012	1	27012	KCNV1
0.430888027	0.009228054	0.014	1	58524	DMRT3
0.430630624	0.009273546	0.017	1	2134	EXTL1
0.430630624	0.009273546	0.011	1	9203	ZMYM3
0.430630624	0.009273546	0.017	1	22808	MRAS
0.430373222	0.00931923	0.022	1	55630	AF205589.2
0.430373222	0.00931923	0.013	1	89932	PAPLN
0.430373222	0.00931923	0.008	1	92255	LMBRD2
0.430373222	0.00931923	0.013	1	146439	CCDC64B
0.430373222	0.00931923	0.018	1	256158	HMCN2
0.430115819	0.009365137	0.015	1	1840	DTX1
0.430115819	0.009365137	0.019	1	7268	TTC4
0.430115819	0.009365137	0.018	1	10092	ARPC5
0.429858446	0.009411207	0.013	1	529	ATP6V1E1
0.429858446	0.009411207	0.012	1	8785	MATN4
0.429858446	0.009411207	0.017	1	23504	RIMBP2
0.429858446	0.009411207	0.02	1	84689	MS4A14
0.429858446	0.009411207	0.014	1	402509	SLC29A4P1
0.429601014	0.009457483	0.018	1	886	CCKAR
0.429601014	0.009457483	0.013	1	116443	GRIN3A
0.429601014	0.009457483	0.014	1	389898	UBE2NL
0.429343641	0.009503943	0.016	1	55620	STAP2
0.429343641	0.009503943	0.014	1	57646	USP28
0.429343641	0.009503943	0.018	1	83756	TAS1R3
0.429086208	0.009550599	0.017	1	196792	FAM24B

voxel-wise, subcortex			
Spearman's corr coef	p-val	GenID	Gene
0.309427708	< 0.001	22843	PPM1E
0.309373349	< 0.001	56955	MEPE
0.309372932	< 0.001	92292	GLYATL1
0.309197724	< 0.001	10219	KLRG1
0.30918774	< 0.001	79829	NAT11
0.309163749	< 0.001	89790	SIGLEC10
0.309120148	< 0.001	57405	SPC25
0.309116006	< 0.001	84973	SNHG7
0.309066474	< 0.001	91582	RPS19BP1
0.308903605	< 0.001	8672	EIF4G3
0.308877856	< 0.001	127018	LYPLAL1
0.308686256	< 0.001	53833	IL20RB
0.308632135	< 0.001	4830	NME1
0.308573723	< 0.001	55095	SAMD4B
0.308571666	< 0.001	80818	ZNF436
0.308433294	< 0.001	51194	IPO11
0.308398664	< 0.001	8683	SFRS9
0.308279783	< 0.001	1452	CSNK1A1
0.308260649	< 0.001	1557	CYP2C19
0.308228284	< 0.001	23265	EXOC7
0.308191001	< 0.001	149647	FAM71A
0.308068395	< 0.001	4636	MYL5
0.308044225	< 0.001	10855	HPSE
0.308042645	< 0.001	23107	MRPS27
0.30800277	< 0.001	10621	POLR3F
0.307982653	< 0.001	55812	SPATA7
0.307960749	< 0.001	9453	GGPS1
0.30792439	< 0.001	440107	PLEKHG7
0.307796091	< 0.001	643988	RP11-345P4.1
0.307662785	< 0.001	84083	ZRANB3
0.307620168	< 0.001	5980	REV3L
0.307381928	< 0.001	6754	SSTR4
0.307363749	< 0.001	140902	R3HDML
0.307329446	< 0.001	6400	SEL1L
0.307269096	< 0.001	170954	KIAA1949
0.307245344	< 0.001	117579	RLN3
0.30723393	< 0.001	5210	PFKFB4
0.307208687	< 0.001	51263	MRPL30
0.307104707	< 0.001	2960	GTF2E1
0.30707407	< 0.001	8727	CTNNAL1
0.306996852	< 0.001	23481	PES1
0.306966841	< 0.001	55852	TEX2
0.30694142	< 0.001	55974	RAG1AP1
0.306901753	< 0.001	8516	ITGA8
0.306864977	< 0.001	3984	LIMK1
0.306838095	< 0.001	90956	ADCK2
0.306774259	< 0.001	401944	LDLRAD2
0.306720793	< 0.001	84072	HORMAD1
0.306695729	< 0.001	8815	BANF1
0.306523144	< 0.001	83544	DNAL1
0.306422204	< 0.001	23047	PDS5B
0.306381226	< 0.001	10752	CHL1
0.306331933	< 0.001	4753	NELL2
0.306204706	< 0.001	84622	ZNF594
0.306108177	< 0.001	10888	GPR83
0.306036532	< 0.001	84449	ZNF333
0.306000531	< 0.001	56949	XAB2
0.30598852	< 0.001	338442	NIACR2
0.305975795	< 0.001	100128553	CTAGE4
0.305799067	< 0.001	58475	MS4A7
0.305786908	< 0.001	317648	C4ORF10
0.305740565	< 0.001	3656	IRAK2
0.305723369	< 0.001	64326	RFWD2
0.305680811	< 0.001	8030	CCDC6
0.305650979	< 0.001	58531	PRM3
0.305634081	< 0.001	55074	OXR1
0.305626333	< 0.001	8448	DOC2A
0.305615276	< 0.001	11051	NUDT21
0.305598766	< 0.001	5431	POLR2B
0.305593073	< 0.001	653499	LGALS7B
0.305592895	< 0.001	100129201	LOC100129201
0.305532366	< 0.001	10620	ARID3B
0.30547756	< 0.001	337880	KRTAP11-1
0.305367053	< 0.001	154865	IQUB
0.305319071	< 0.001	138724	C9orf131
0.305224001	< 0.001	147968	CAPN12
0.305107296	< 0.001	3109	HLA-DMB
0.304962188	< 0.001	26984	SEC22A
0.304904312	< 0.001	123228	SENP8

region-wise, subcortex					
Spearman's corr coef	p-val	p-val, SA	p-val, SA (BH- correction)	GenID	Gene
0.428828835	0.009597483	0.019	1	10409	BASP1
0.428828835	0.009597483	0.01	1	54518	APBB1IP
0.428828835	0.009597483	0.021	1	58510	PRODH2
0.428571403	0.009644533	0.018	1	4486	MST1R
0.428571403	0.009644533	0.021	1	132671	SPATA18
0.42831403	0.00969179	0.016	1	9766	KIAA0247
0.42831403	0.00969179	0.02	1	387890	AC002563.2
0.428056628	0.009739236	0.017	1	4830	NME1
0.428056628	0.009739236	0.017	1	23613	ZMYND8
0.428056628	0.009739236	0.019	1	91283	MSANTD3
0.428056628	0.009739236	0.017	1	91300	C19orf22
0.427799225	0.009786902	0.013	1	137902	PXDNL
0.427541822	0.009834757	0.015	1	11142	PKIG
0.427541822	0.009834757	0.022	1	79143	LENG4
0.427541822	0.009834757	0.012	1	128646	SIRPD
0.427284449	0.009882802	0.018	1	8317	CDC7
0.427027017	0.00993106	0.015	1	83648	FAM167A
0.427027017	0.00993106	0.018	1	134111	UBE2QL1
0.427027017	0.00993106	0.022	1	148534	TMEM56
0.426769644	0.00997953	0.009	1	1002	CDH4
0.426769644	0.00997953	0.008	1	84632	AFAP1L2
0.426769644	0.00997953	0.014	1	554206	LOC554206
0.426512212	0.010028181	0.019	1	8988	HSPB3
0.426254809	0.010077045	0.016	1	653140	AC008073.2
0.425997406	0.010126103	0.017	1	1606	DGKA
0.425997406	0.010126103	0.019	1	23108	GARNL4
0.425740004	0.010175371	0.015	1	5898	RALA
0.425740004	0.010175371	0.019	1	8854	ALDH1A2
0.425740004	0.010175371	0.016	1	55545	MSX2P1
0.425740004	0.010175371	0.009	1	57616	TSHZ3
0.425740004	0.010175371	0.017	1	84871	AGBL4
0.425482631	0.010224866	0.027	1	4061	LY6E
0.425482631	0.010224866	0.013	1	80223	RAB11FIP1
0.425225198	0.010274539	0.017	1	4200	ME2
0.425225198	0.010274539	0.012	1	5529	PPP2R5E
0.424967825	0.010324431	0.019	1	11264	PXMP4
0.424967825	0.010324431	0.017	1	100288540	POM121B
0.424710393	0.010374518	0.018	1	197	AHSG
0.424710393	0.010374518	0.022	1	8600	TNFSF11
0.424710393	0.010374518	0.015	1	51010	EXOSC3
0.424710393	0.010374518	0.017	1	57190	SEPN1
0.424710393	0.010374518	0.016	1	84321	THOC3
0.42445302	0.010424848	0.012	1	92335	STRADA
0.424195617	0.010475351	0.007	1	10870	HCST
0.424195617	0.010475351	0.022	1	11033	ADAP1
0.424195617	0.010475351	0.016	1	23657	SLC7A11
0.424195617	0.010475351	0.027	1	55215	FANCI
0.424195617	0.010475351	0.015	1	253143	C22orf30
0.423938215	0.010526065	0.012	1	10098	TSPAN5
0.423938215	0.010526065	0.018	1	92912	UBE2Q2
0.423938215	0.010526065	0.017	1	100131801	PET100
0.423423409	0.010628135	0.022	1	8131	C16orf35
0.423423409	0.010628135	0.013	1	112755	STX1B
0.423166007	0.010679515	0.017	1	220202	ATOH7
0.422908634	0.010731074	0.023	1	10006	ABI1
0.422908634	0.010731074	0.02	1	57498	KIDINS220
0.422908634	0.010731074	0.025	1	83544	DNAL1
0.422908634	0.010731074	0.017	1	100288198	LOC100288198
0.422651201	0.010782844	0.016	1	10424	PGRMC2
0.422651201	0.010782844	0.022	1	54472	TOLLIP
0.422393829	0.010834835	0.013	1	23581	CASP14
0.422393829	0.010834835	0.02	1	27345	KCNMB4
0.422393829	0.010834835	0.015	1	57167	SALL4
0.422393829	0.010834835	0.022	1	60559	SPCS3
0.422393829	0.010834835	0.015	1	286183	NKAIN3
0.422136396	0.010887064	0.012	1	826	CAPNS1
0.422136396	0.010887064	0.011	1	9672	SDC3
0.421879023	0.010939483	0.015	1	3954	LETM1
0.421879023	0.010939483	0.016	1	4301	MLLT4
0.421879023	0.010939483	0.019	1	28988	DBNL
0.421879023	0.010939483	0.018	1	134353	LSM11
0.421879023	0.010939483	0.019	1	338699	ANKRD42
0.421621621	0.01099212	0.02	1	100128511	RP11-565H13.2
0.421364218	0.011044967	0.021	1	3241	HPCAL1
0.421364218	0.011044967	0.02	1	54932	EXD3
0.421364218	0.011044967	0.012	1	55617	TASP1
0.421364218	0.011044967	0.025	1	401577	CD99P1
0.421106815	0.011098064	0.015	1	11202	KLK8
0.420849413	0.011151346	0.011	1	53616	ADAM22

voxel-wise, subcortex			
Spearman's corr coef	p-val	GenID	Gene
0.30489549	< 0.001	4288	MKI67
0.304806799	< 0.001	29081	METTL5
0.30477652	< 0.001	341568	OR8S1
0.304749638	< 0.001	7567	ZNF19
0.304735839	< 0.001	25797	QPCT
0.304719537	< 0.001	494143	CHAC2
0.304618865	< 0.001	57414	RHBDD2
0.304601043	< 0.001	2849	GPR26
0.30459553	< 0.001	167838	TXLNB
0.304555357	< 0.001	644128	RPL23AP53
0.304546118	< 0.001	5511	PPP1R8
0.304453433	< 0.001	51552	RAB14
0.304395348	< 0.001	100169851	PATE3
0.304355085	< 0.001	50508	NOX3
0.304275841	< 0.001	25930	PTPN23
0.304207206	< 0.001	26100	WIPI2
0.304038078	< 0.001	57565	KLHL14
0.303803891	< 0.001	7621	ZNF70
0.303683341	< 0.001	64376	IKZF5
0.303585172	< 0.001	85300	ATCAY
0.303567588	< 0.001	6688	SPI1
0.303547531	< 0.001	5793	PTPRG
0.303517908	< 0.001	25903	OLFML2B
0.303516656	< 0.001	81570	CLPB
0.303435445	< 0.001	813	CALU
0.303204387	< 0.001	283991	FAM100B
0.303145915	< 0.001	440043	PGAM1P8
0.303108007	< 0.001	9651	PLCH2
0.303052276	< 0.001	6881	TAF10
0.302998215	< 0.001	1089	CEACAM4
0.302925408	< 0.001	30845	EHD3
0.302919298	< 0.001	54944	AC073548.1
0.30277878	< 0.001	10940	POP1
0.302721888	< 0.001	2271	FH
0.302696645	< 0.001	337972	KRTAP19-5
0.302693069	< 0.001	4104	MAGEA5
0.30265379	< 0.001	283489	ZNF828
0.302596629	< 0.001	8882	ZNF259
0.302560508	< 0.001	79873	NUDT18
0.302455038	< 0.001	4884	NPTX1
0.302443773	< 0.001	6821	SUOX
0.302395165	< 0.001	23144	ZC3H3
0.302360505	< 0.001	146433	IL34
0.302221954	< 0.001	80345	ZNF435
0.302204132	< 0.001	390637	C15orf58
0.302123785	< 0.001	23369	PUM2
0.30207625	< 0.001	51388	NIP7
0.301899344	< 0.001	51222	ZNF219
0.301810741	< 0.001	56888	KCMF1
0.301677287	< 0.001	8504	PEX3
0.301529408	< 0.001	50486	G0S2
0.301481545	< 0.001	91156	IGFN1
0.301475823	< 0.001	200942	KLHDC8B
0.301463157	< 0.001	150590	C2orf15
0.30135107	< 0.001	6941	TCF19
0.301300496	< 0.001	392145	LOC392145
0.301252633	< 0.001	144448	TSPAN19
0.301252007	< 0.001	6526	SLC5A3
0.301186323	< 0.001	100130386	LINC00552
0.301175267	< 0.001	387775	SLC22A10
0.301089406	< 0.001	10135	PBEF1
0.301065177	< 0.001	220992	ZNF485
0.301022768	< 0.001	92691	TMEM169
0.300986528	< 0.001	4361	MRE11A
0.300817639	< 0.001	54938	SARS2
0.300773263	< 0.001	6713	SQLE
0.300745428	< 0.001	4285	MIPEP
0.300655782	< 0.001	4130	MAP1A
0.30058983	< 0.001	254359	ZDHHC24
0.30057174	< 0.001	84524	ZC3H8
0.300571531	< 0.001	10533	ATG7
0.300561756	< 0.001	29099	COMMD9
0.300514251	< 0.001	11052	CPSF6
0.300420552	< 0.001	285148	IAH1
0.300378114	< 0.001	83756	TAS1R3
0.300361633	< 0.001	10408	MYCNOS
0.300339878	< 0.001	65123	INTS3
0.3003079	< 0.001	317662	FAM149B1
0.30017361	< 0.001	23199	KIAA0182

region-wise, subcortex					
Spearman's corr coef	p-val	p-val, SA	p-val, SA (BH- correction)	GenID	Gene
0.420849413	0.011151346	0.02	1	100288346	AP000880.1
0.42059201	0.011204853	0.014	1	2769	GNA15
0.42059201	0.011204853	0.022	1	9208	LRRFIP1
0.42059201	0.011204853	0.025	1	29789	OLA1
0.42059201	0.011204853	0.019	1	220388	CCDC89
0.420334637	0.011258571	0.013	1	55692	LUC7L
0.420334637	0.011258571	0.016	1	147965	FAM98C
0.420077205	0.011312517	0.016	1	9502	XAGE2B
0.420077205	0.011312517	0.012	1	199800	C19orf76
0.419819832	0.011366708	0.021	1	9643	MORF4L2
0.419819832	0.011366708	0.018	1	57001	ACN9
0.419819832	0.011366708	0.017	1	65982	ZSCAN18
0.419819832	0.011366708	0.02	1	112611	RWDD2A
0.419819832	0.011366708	0.02	1	170261	ZCCHC12
0.419562399	0.011421096	0.017	1	8507	ENC1
0.419562399	0.011421096	0.021	1	54602	NDFIP2
0.419562399	0.011421096	0.013	1	154865	IQUB
0.419304997	0.011475705	0.022	1	155	ADRB3
0.419304997	0.011475705	0.013	1	5824	PEX19
0.419304997	0.011475705	0.02	1	51155	HN1
0.419304997	0.011475705	0.023	1	225689	MAPK15
0.419304997	0.011475705	0.028	1	642943	LOC642943
0.419047624	0.011530533	0.017	1	3780	KCNN1
0.419047624	0.011530533	0.008	1	9536	PTGES
0.419047624	0.011530533	0.008	1	23074	UHRF1BP1L
0.419047624	0.011530533	0.019	1	25940	FAM98A
0.419047624	0.011530533	0.017	1	57542	KLHDC5
0.419047624	0.011530533	0.024	1	84830	C6orf105
0.419047624	0.011530533	0.022	1	128408	BHLHE23
0.419047624	0.011530533	0.015	1	130733	TMEM178
0.418790191	0.011585613	0.019	1	3050	HBZ
0.418790191	0.011585613	0.02	1	3909	LAMA3
0.418790191	0.011585613	0.022	1	11001	SLC27A2
0.418790191	0.011585613	0.019	1	22924	MAPRE3
0.418790191	0.011585613	0.017	1	79672	FN3KRP
0.418790191	0.011585613	0.022	1	200933	FBXO45
0.418532819	0.011640895	0.016	1	10783	NEK6
0.418532819	0.011640895	0.024	1	374739	TEPP
0.418275386	0.011696401	0.008	1	21	ABCA3
0.418275386	0.011696401	0.023	1	4288	MKI67
0.418275386	0.011696401	0.017	1	25942	SIN3A
0.418275386	0.011696401	0.016	1	29087	THYN1
0.418275386	0.011696401	0.026	1	91461	SGK493
0.418018013	0.011752132	0.015	1	55738	ARFGAP1
0.418018013	0.011752132	0.014	1	100170841	C17orf96
0.417760611	0.011808117	0.021	1	65992	DDRGK1
0.417760611	0.011808117	0.016	1	79589	RNF128
0.417760611	0.011808117	0.019	1	730101	RP1-152L7.5
0.417503208	0.011864296	0.028	1	1414	CRYBB1
0.417503208	0.011864296	0.02	1	51313	C4orf18
0.417503208	0.011864296	0.023	1	55752	SEPTIN11
0.417503208	0.011864296	0.018	1	126382	NR2C2AP
0.417245805	0.01192071	0.008	1	1643	DDB2
0.417245805	0.01192071	0.022	1	3775	KCNK1
0.417245805	0.01192071	0.026	1	6038	RNASE4
0.416988403	0.011977348	0.018	1	810	CALML3
0.416988403	0.011977348	0.023	1	54536	EXOC6
0.416988403	0.011977348	0.013	1	64208	POPDC3
0.416731	0.012034226	0.032	1	83693	HSDL1
0.416731	0.012034226	0.019	1	503639	DUXAP10
0.416473627	0.012091348	0.013	1	57134	MAN1C1
0.416473627	0.012091348	0.024	1	79677	SMC6
0.416216195	0.012148682	0.012	1	7182	NR2C2
0.416216195	0.012148682	0.019	1	9935	MAFB
0.415958822	0.012206248	0.022	1	8707	B3GALT2
0.415958822	0.012206248	0.022	1	94030	LRRC4B
0.415701389	0.012264041	0.023	1	5431	POLR2B
0.415701389	0.012264041	0.014	1	9556	C14orf2
0.415701389	0.012264041	0.022	1	9895	TECPR2
0.415701389	0.012264041	0.019	1	79632	FAM184A
0.415701389	0.012264041	0.024	1	125875	CLDND2
0.415186614	0.012380364	0.016	1	2986	GUCY2F
0.415186614	0.012380364	0.024	1	9901	SRGAP3
0.415186614	0.012380364	0.021	1	55814	BDP1
0.415186614	0.012380364	0.026	1	252839	TMEM9
0.414929211	0.012438864	0.021	1	6633	SNRPD2
0.414929211	0.012438864	0.026	1	11311	VPS45
0.414929211	0.012438864	0.011	1	619208	C6orf225
0.414671808	0.0124976	0.015	1	139716	GAB3

voxel-wise, subcortex			
Spearman's corr coef	p-val	GenID	Gene
0.300165027	< 0.001	220213	OTUD1
0.300161958	< 0.001	344382	LOC344382
0.300148308	< 0.001	203259	C9orf25
0.300143898	< 0.001	339524	LOC339524
0.300124347	< 0.001	79982	DNAJB14
0.300123096	< 0.001	112950	MED8
0.300090164	< 0.001	7036	TFR2
0.300036222	< 0.001	23262	HISPPD1
0.300035954	< 0.001	22943	DKK1
0.299961865	< 0.001	286023	AC009365.1
0.299917996	< 0.001	266977	GPR110
0.299909592	< 0.001	768	CA9
0.299767971	< 0.001	131055	LOC131055
0.299767315	< 0.001	6659	SOX4
0.299722463	< 0.001	2209	FCGR1A
0.299651831	< 0.001	51155	HN1
0.299583077	< 0.001	55034	MOCOS
0.299559593	< 0.001	64946	CENPH
0.299434215	< 0.001	7090	TLE3
0.299389631	< 0.001	8808	IL1RL2
0.299238443	< 0.001	2243	FGA
0.299188584	< 0.001	29995	LMCD1
0.299034417	< 0.001	9194	SLC16A7
0.298822045	< 0.001	84203	TXNDC2
0.298758566	< 0.001	51022	GLRX2
0.298700005	< 0.001	55450	CAMK2N1
0.298568249	< 0.001	9474	ATG5
0.298321486	< 0.001	155382	VPS37D
0.29827112	< 0.001	126695	C1orf172
0.298262984	< 0.001	80271	ITPKC
0.298145324	< 0.001	51086	TNNI3K
0.298047811	< 0.001	79898	ZNF613
0.298027396	< 0.001	27099	SND1-IT1
0.297958106	< 0.001	63893	UBE2O
0.297798574	< 0.001	5172	SLC26A4
0.29779458	< 0.001	28992	MACROD1
0.297788709	< 0.001	4709	NDUFB3
0.297619879	< 0.001	1400	CRMP1
0.297528595	< 0.001	5685	PSMA4
0.297502458	< 0.001	3425	IDUA
0.297328651	< 0.001	64396	GMCL1P1
0.297294527	< 0.001	6358	CCL14
0.297126532	< 0.001	147906	DACT3
0.297116548	< 0.001	55028	C17orf80
0.29704845	< 0.001	2865	AC002511.1
0.296858847	< 0.001	5432	POLR2C
0.296809256	< 0.001	127602	DNAH14
0.296743691	< 0.001	8744	TNFSF9
0.296742767	< 0.001	8446	DUSP11
0.296449035	< 0.001	3062	HCRTR2
0.296397775	< 0.001	9992	KCNE2
0.296387017	< 0.001	158763	RP13-102H20.1
0.296379954	< 0.001	9597	SMAD5OS
0.296307981	< 0.001	728224	KRTAP4-8
0.296285868	< 0.001	27245	AHDC1
0.29627046	< 0.001	659	BMPR2
0.296214491	< 0.001	55699	IARS2
0.296169043	< 0.001	9071	CLDN10
0.296155959	< 0.001	126402	CCDC105
0.296119273	< 0.001	728215	FAM155A
0.295909494	< 0.001	196792	FAM24B
0.295818239	< 0.001	137886	UBXN2B
0.295757413	< 0.001	197	AHSG
0.295710385	< 0.001	51062	ATL1
0.295642555	< 0.001	3216	HOXB6
0.295639515	< 0.001	8363	HIST1H4J
0.295624942	< 0.001	387597	ILDR2
0.295619458	< 0.001	80344	WDR23
0.295586854	< 0.001	220965	FAM13C
0.295575321	< 0.001	26232	FBXO2
0.295540214	< 0.001	28512	NKIRAS1
0.295530885	< 0.001	129868	TRIM43
0.295400679	< 0.001	3176	HNMT
0.295330614	< 0.001	10564	ARFGEF2
0.295313001	< 0.001	9742	IFT140
0.295309007	< 0.001	6335	SCN9A
0.295217723	< 0.001	84896	ATAD1
0.295157045	< 0.001	6445	SGCG
0.295065969	< 0.001	4200	ME2

region-wise, subcortex					
Spearman's corr coef	p-val	p-val, SA	p-val, SA (BH- correction)	GenID	Gene
0.414671808	0.0124976	0.022	1	729313	LOC729313
0.414414406	0.012556599	0.012	1	939	CD27
0.414414406	0.012556599	0.023	1	54733	SLC35F2
0.414414406	0.012556599	0.015	1	63877	FAM204A
0.414157003	0.012615808	0.02	1	7251	TSG101
0.414157003	0.012615808	0.023	1	149647	FAM71A
0.41389963	0.01267525	0.018	1	140459	ASB6
0.41389963	0.01267525	0.033	1	167838	TXLNB
0.41389963	0.01267525	0.029	1	284352	LRRC68
0.41389963	0.01267525	0.019	1	402381	SOHLH1
0.413642198	0.012734929	0.017	1	55294	FBXW7
0.413384825	0.012794862	0.018	1	55039	TRMT12
0.413384825	0.012794862	0.02	1	83855	KLF16
0.413127393	0.012855047	0.017	1	1000	CDH2
0.413127393	0.012855047	0.028	1	79072	FASTKD3
0.413127393	0.012855047	0.013	1	80184	CEP290
0.413127393	0.012855047	0.022	1	138715	ARID3C
0.41287002	0.012915459	0.027	1	84072	HORMAD1
0.412612617	0.012976101	0.026	1	6462	SHBG
0.412612617	0.012976101	0.023	1	116372	LYPD1
0.412355185	0.013036985	0.023	1	7529	YWHAB
0.412355185	0.013036985	0.03	1	123624	AGBL1
0.412355185	0.013036985	0.02	1	341277	OVCH2
0.412355185	0.013036985	0.024	1	100288929	LOC100288929
0.412097812	0.013098152	0.019	1	4843	NOS2
0.411840379	0.013159524	0.017	1	337985	KRTAP20-3
0.411583006	0.013221152	0.022	1	894	CCND2
0.411583006	0.013221152	0.027	1	9777	TM9SF4
0.411583006	0.013221152	0.021	1	80152	CENPT
0.411325604	0.013283013	0.022	1	388567	ZNF749
0.411068201	0.013345155	0.018	1	23312	DMXL2
0.410810798	0.013407516	0.028	1	8848	TSC22D1
0.410810798	0.013407516	0.024	1	11218	DDX20
0.410810798	0.013407516	0.026	1	51121	RPL26L1
0.410810798	0.013407516	0.022	1	90416	C15orf57
0.410810798	0.013407516	0.024	1	340811	AKR1CL1
0.410553396	0.013470119	0.02	1	51440	HPCAL4
0.410553396	0.013470119	0.024	1	51499	TRIAP1
0.410553396	0.013470119	0.015	1	152302	CIDECP
0.410553396	0.013470119	0.027	1	389649	C8orf86
0.410295993	0.013532983	0.025	1	273	AMPH
0.410295993	0.013532983	0.012	1	7066	THPO
0.410295993	0.013532983	0.016	1	55142	HAUS2
0.410295993	0.013532983	0.018	1	143187	VTI1A
0.410295993	0.013532983	0.027	1	152189	CMTM8
0.41003862	0.013596083	0.023	1	7139	TNNT2
0.41003862	0.013596083	0.019	1	64168	NECAB1
0.409781188	0.013659461	0.03	1	5833	PCYT2
0.409781188	0.013659461	0.022	1	79722	ANKRD55
0.409266382	0.013786931	0.019	1	57526	PCDH19
0.40900901	0.013851049	0.023	1	822	CAPG
0.40900901	0.013851049	0.023	1	2642	GCGR
0.40900901	0.013851049	0.04	1	6557	SLC12A1
0.40900901	0.013851049	0.026	1	10827	FAM114A2
0.40900901	0.013851049	0.014	1	54431	DNAJC10
0.40900901	0.013851049	0.024	1	63934	ZNF667
0.40900901	0.013851049	0.029	1	100133941	CD24
0.408751607	0.013915436	0.018	1	9764	KIAA0513
0.408751607	0.013915436	0.02	1	83719	YPEL3
0.408751607	0.013915436	0.021	1	90135	BTBD6
0.408751607	0.013915436	0.016	1	140456	ASB11
0.408751607	0.013915436	0.022	1	374654	KIF7
0.408494204	0.013980049	0.023	1	1759	DNM1
0.408494204	0.013980049	0.02	1	80204	FBXO11
0.408494204	0.013980049	0.029	1	285676	ZNF454
0.408236802	0.014044932	0.022	1	51097	SCCPDH
0.407979399	0.014110055	0.015	1	7781	SLC30A3
0.407979399	0.014110055	0.021	1	54457	TAF7L
0.407979399	0.014110055	0.029	1	285704	RGMB
0.407721996	0.014175464	0.019	1	84239	ATP13A4
0.407721996	0.014175464	0.022	1	260425	MAGI3
0.407721996	0.014175464	0.025	1	653505	PPIAL4B
0.407464623	0.014241118	0.022	1	27147	DENND2A
0.407464623	0.014241118	0.032	1	729603	LOC729603
0.407207191	0.014307013	0.02	1	6597	SMARCA4
0.407207191	0.014307013	0.018	1	55210	ATAD3A
0.406949818	0.014373181	0.019	1	2312	FLG
0.406949818	0.014373181	0.023	1	10518	CIB2
0.406949818	0.014373181	0.02	1	55203	LGI2

voxel-wise, subcortex			
Spearman's corr coef	p-val	GenID	Gene
0.295012623	< 0.001	7170	TPM3
0.295005709	< 0.001	140628	GATA5
0.295004427	< 0.001	54566	EPB41L4B
0.295001626	< 0.001	8822	FGF17
0.294979423	< 0.001	2903	GRIN2A
0.294948727	< 0.001	79810	PTCD2
0.294920176	< 0.001	388567	ZNF749
0.294843286	< 0.001	4645	MYO5B
0.294530064	< 0.001	79882	ZC3H14
0.29451403	< 0.001	4817	NIT1
0.294469863	< 0.001	286006	C7orf53
0.294427723	< 0.001	55610	CCDC132
0.294314057	< 0.001	29934	SNX12
0.294292033	< 0.001	58504	ARHGAP22
0.294240862	< 0.001	51491	NOP16
0.294224024	< 0.001	9830	TRIM14
0.294184357	< 0.001	283970	PDXDC2
0.294160068	< 0.001	203	AK1
0.294063717	< 0.001	57449	PLEKHG5
0.293956697	< 0.001	286234	C9orf79
0.293883443	< 0.001	6711	SPTBN1
0.293830156	< 0.001	80204	FBXO11
0.29380694	< 0.001	11075	STMN2
0.29372552	< 0.001	200810	ALG1L
0.293541461	< 0.001	60312	AFAP1
0.293499649	< 0.001	55319	C4orf43
0.293475419	< 0.001	130535	KCTD18
0.29344663	< 0.001	5599	MAPK8
0.293439627	< 0.001	1745	DLX1
0.293402463	< 0.001	54677	CROT
0.293291956	< 0.001	10092	ARPC5
0.293284804	< 0.001	254102	EHBP1L1
0.29328388	< 0.001	54585	LZTFL1
0.293254912	< 0.001	11021	RAB35
0.293247074	< 0.001	128977	C22orf39
0.293235421	< 0.001	170691	ADAMTS17
0.293232113	< 0.001	84302	TMEM246
0.293141484	< 0.001	51005	AMDHD2
0.293111891	< 0.001	23108	GARNL4
0.293049961	< 0.001	55132	LARP1B
0.293025672	< 0.001	284352	LRRC68
0.29297635	< 0.001	55860	ACTR10
0.292969584	< 0.001	645453	RLIMP3
0.292958766	< 0.001	90324	CCDC97
0.292934954	< 0.001	124739	USP43
0.292899042	< 0.001	148231	AC114273.1
0.292837054	< 0.001	126272	EID2B
0.292768806	< 0.001	284573	C1orf157
0.292709917	< 0.001	440400	RNASEK
0.292564869	< 0.001	7341	SUMO1
0.292558312	< 0.001	115098	CCDC124
0.292451978	< 0.001	51377	UCHL5
0.29236415	< 0.001	100128139	LOC100128139
0.292363226	< 0.001	9807	IP6K1
0.292358428	< 0.001	347516	DGAT2L6
0.292341024	< 0.001	401993	OR2T5
0.29227528	< 0.001	8514	KCNAB2
0.292197257	< 0.001	257397	MAP3K7IP3
0.292182177	< 0.001	8310	ACOX3
0.292178363	< 0.001	732253	RP11-535K1.1
0.292102128	< 0.001	2673	GFPT1
0.29210034	< 0.001	219968	OR5B21
0.292098463	< 0.001	1007	CDH9
0.29207778	< 0.001	10908	PNPLA6
0.292070955	< 0.001	23787	MTCH1
0.29204914	< 0.001	26173	INTS1
0.291962415	< 0.001	26043	UBXN7
0.291956723	< 0.001	55634	ZNF673
0.291875839	< 0.001	64748	AC024575.2
0.291816592	< 0.001	140807	KRT72
0.291767836	< 0.001	51441	YTHDF2
0.291717649	< 0.001	27290	SPINK4
0.291508019	< 0.001	118471	PRAP1
0.291440964	< 0.001	83481	EPPK1
0.29142797	< 0.001	8747	ADAM21
0.291156501	< 0.001	113691	AC002472.8
0.291150302	< 0.001	23333	DPY19L1
0.291148663	< 0.001	79095	C9orf16
0.291120529	< 0.001	83982	IFI27L2

region-wise, subcortex					
Spearman's corr coef	p-val	p-val, SA	p-val, SA (BH- correction)	GenID	Gene
0.406949818	0.014373181	0.025	1	55558	PLXNA3
0.406949818	0.014373181	0.021	1	56949	XAB2
0.406692386	0.014439595	0.015	1	1621	DBH
0.406692386	0.014439595	0.019	1	2647	BLOC1S1
0.406692386	0.014439595	0.024	1	25830	SULT4A1
0.406692386	0.014439595	0.018	1	91409	CCDC74B
0.406692386	0.014439595	0.024	1	136051	ZNF786
0.406435013	0.0145063	0.02	1	51232	CRIM1
0.406435013	0.0145063	0.018	1	63939	C20orf177
0.406435013	0.0145063	0.018	1	148231	AC114273.1
0.40617761	0.01457325	0.017	1	6051	RNPEP
0.40617761	0.01457325	0.024	1	57761	TRIB3
0.40617761	0.01457325	0.022	1	145814	AC069029.1
0.405920208	0.014640447	0.019	1	1893	ECM1
0.405920208	0.014640447	0.028	1	4314	MMP3
0.405920208	0.014640447	0.016	1	55176	SEC61A2
0.405920208	0.014640447	0.023	1	90523	C6orf142
0.405662805	0.014707925	0.037	1	1608	DGKG
0.405662805	0.014707925	0.024	1	1984	EIF5A
0.405662805	0.014707925	0.02	1	51510	CHMP5
0.405662805	0.014707925	0.021	1	80025	PANK2
0.405662805	0.014707925	0.018	1	389073	C2orf80
0.405405372	0.014775679	0.016	1	8761	PABPC4
0.405405372	0.014775679	0.032	1	81849	ST6GALNAC5
0.405148	0.014843669	0.03	1	5798	PTPRN
0.405148	0.014843669	0.028	1	57648	KIAA1522
0.405148	0.014843669	0.037	1	64320	RNF25
0.405148	0.014843669	0.031	1	84832	ANKRD36BL1
0.405148	0.014843669	0.019	1	126306	JSRP1
0.405148	0.014843669	0.021	1	284355	TPRX1
0.404633194	0.014980458	0.029	1	6611	SMS
0.404633194	0.014980458	0.022	1	9453	GGPS1
0.404633194	0.014980458	0.025	1	23498	HAAO
0.404633194	0.014980458	0.025	1	51388	NIP7
0.404633194	0.014980458	0.013	1	93627	AC004053.1
0.404375792	0.015049292	0.017	1	23129	PLXND1
0.404375792	0.015049292	0.021	1	57132	CHMP1B
0.404118389	0.015118347	0.025	1	158293	FAM120AOS
0.403860986	0.015187671	0.026	1	23271	CAMSAP1L1
0.403860986	0.015187671	0.02	1	57157	PHTF2
0.403860986	0.015187671	0.027	1	114900	C1QTNF4
0.403860986	0.015187671	0.025	1	149603	RNF187
0.403860986	0.015187671	0.027	1	339210	C17orf67
0.403603613	0.01525728	0.027	1	1501	CTNND2
0.403603613	0.01525728	0.024	1	219333	USP12
0.403603613	0.01525728	0.02	1	442517	LOC442517
0.403346181	0.015327144	0.023	1	2823	GPM6A
0.403346181	0.015327144	0.031	1	6654	SOS1
0.403346181	0.015327144	0.024	1	7143	TNR
0.403346181	0.015327144	0.03	1	122876	GPHB5
0.403346181	0.015327144	0.022	1	132001	C3orf31
0.403088808	0.015397327	0.014	1	839	CASP6
0.403088808	0.015397327	0.023	1	4915	NTRK2
0.403088808	0.015397327	0.016	1	8822	FGF17
0.403088808	0.015397327	0.017	1	79798	ARMC5
0.403088808	0.015397327	0.026	1	125488	TTC39C
0.403088808	0.015397327	0.027	1	282991	BLOC1S2
0.402831376	0.015467734	0.024	1	5395	PMS2
0.402831376	0.015467734	0.017	1	8315	BRAP
0.402831376	0.015467734	0.025	1	84102	SLC41A2
0.402574003	0.015538413	0.02	1	377	ARF3
0.402574003	0.015538413	0.02	1	84923	FAM104A
0.402574003	0.015538413	0.017	1	192111	PGAM5
0.4023166	0.015609383	0.024	1	9033	PKD2L1
0.4023166	0.015609383	0.016	1	54756	IL17RD
0.4023166	0.015609383	0.023	1	90488	C12orf23
0.402059197	0.015680641	0.012	1	662	BNIP1
0.402059197	0.015680641	0.02	1	89837	FAM7A3
0.401801795	0.015752161	0.029	1	100131187	RP11-544M22.1
0.401544392	0.01582394	0.022	1	3714	JAG2
0.401544392	0.01582394	0.025	1	84620	ST6GAL2
0.401544392	0.01582394	0.023	1	222865	TMEM130
0.401286989	0.015895996	0.025	1	5575	PRKAR1B
0.401286989	0.015895996	0.027	1	5714	PSMD8
0.401286989	0.015895996	0.023	1	10203	CALCRL
0.401286989	0.015895996	0.019	1	84140	FAM161A
0.401029617	0.015968347	0.033	1	6855	SYP
0.401029617	0.015968347	0.02	1	9819	TSC22D2
0.401029617	0.015968347	0.027	1	80055	PGAP1

voxel-wise, subcortex			
Spearman's corr coef	p-val	GenID	Gene
0.291014194	< 0.001	84128	WDR75
0.290992558	< 0.001	23317	DNAJC13
0.290935665	< 0.001	53942	CNTN5
0.290925145	< 0.001	310	ANXA7
0.290810823	< 0.001	9201	DCLK1
0.290784955	< 0.001	9097	USP14
0.290691167	< 0.001	10116	FEM1B
0.290600896	< 0.001	147381	CBLN2
0.290572107	< 0.001	147841	SPC24
0.290567875	< 0.001	55471	C2orf56
0.290548205	< 0.001	55584	CHRNA9
0.290464103	< 0.001	3725	JUN
0.290421516	< 0.001	57150	C6orf162
0.290419906	< 0.001	5217	PFN2
0.29039228	< 0.001	55170	PRMT6
0.290347606	< 0.001	29887	SNX10
0.290303439	< 0.001	8562	DENR
0.290298283	< 0.001	10059	DNM1L
0.290260226	< 0.001	284355	TPRX1
0.290080369	< 0.001	862	RUNX1T1
0.289995313	< 0.001	1622	DBI
0.289827824	< 0.001	55859	BEX1
0.289815992	< 0.001	90525	SHF
0.289806575	< 0.001	64320	RNF25
0.289772063	< 0.001	245973	ATP6V1C2
0.289723128	< 0.001	11047	ADRM1
0.289705843	< 0.001	126370	OR1I1
0.289663583	< 0.001	2056	EPO
0.289433241	< 0.001	81696	OR5V1
0.289430231	< 0.001	642696	AC007601.1
0.2894288	< 0.001	338645	LUZP2
0.289380908	< 0.001	171024	SYNPO2
0.289379328	< 0.001	54432	YIPF1
0.289364338	< 0.001	400456	CTD-2536I1.1
0.289340436	< 0.001	1193	CLIC2
0.289027095	< 0.001	387104	AL096711.2
0.289008051	< 0.001	5201	PFDN1
0.288812906	< 0.001	10951	CBX1
0.288732141	< 0.001	57402	S100A14
0.288651228	< 0.001	8462	KLF11
0.28864941	< 0.001	4781	NFIB
0.288644314	< 0.001	10190	TXNDC9
0.288552701	< 0.001	7454	WAS
0.288545996	< 0.001	58530	LY6G6D
0.288459361	< 0.001	8908	GYG2
0.288389206	< 0.001	25897	RNF19A
0.288290888	< 0.001	203859	ANO5
0.288167298	< 0.001	22873	DZIP1
0.287975103	< 0.001	9853	RUSC2
0.287949532	< 0.001	66000	TMEM108
0.287701666	< 0.001	57692	MAGEE1
0.287669897	< 0.001	80152	CENPT
0.287611395	< 0.001	9154	SLC28A1
0.287590444	< 0.001	23530	NNT
0.287551731	< 0.001	9521	EEF1E1
0.287540317	< 0.001	145282	MIPOL1
0.287513226	< 0.001	140775	SMCR8
0.287405938	< 0.001	114905	C1QTNF7
0.287390411	< 0.001	63950	DMRTA2
0.287293613	< 0.001	126003	TRAPPC5
0.287145168	< 0.001	3620	IDO1
0.287076622	< 0.001	89872	AQP10
0.287042886	< 0.001	441666	LOC441666
0.287015289	< 0.001	8925	HERC1
0.286985159	< 0.001	440345	61E3.4
0.286977112	< 0.001	887	CCKBR
0.286936164	< 0.001	146723	C17orf77
0.286923945	< 0.001	83930	STARD3NL
0.286922067	< 0.001	60682	SMAP1
0.286846519	< 0.001	163778	SPRR4
0.286712825	< 0.001	4883	NPR3
0.286661893	< 0.001	55621	TRMT1
0.2865569	< 0.001	7480	WNT10B
0.286477357	< 0.001	9568	GABBR2
0.286441296	< 0.001	1551	CYP3A7
0.286440164	< 0.001	9229	DLGAP1
0.286392152	< 0.001	60559	SPCS3
0.286358953	< 0.001	51026	GOLT1B
0.286346108	< 0.001	9603	NFE2L3

region-wise, subcortex					
Spearman's corr coef	p-val	p-val, SA	p-val, SA (BH- correction)	GenID	Gene
0.400772184	0.016040992	0.02	1	52	ACP1
0.400772184	0.016040992	0.035	1	10920	COPS8
0.400257379	0.01618707	0.023	1	5140	PDE3B
0.400257379	0.01618707	0.023	1	10886	NPFFR2
0.400257379	0.01618707	0.024	1	26059	ERC2
0.400257379	0.01618707	0.026	1	55706	TMEM48
0.400257379	0.01618707	0.022	1	84911	ZNF382
0.400257379	0.01618707	0.027	1	128371	OR6K6
0.400000006	0.016260525	0.017	1	5980	REV3L
0.400000006	0.016260525	0.02	1	6804	STX1A
0.399742603	0.01633431	0.021	1	5342	PLGLB2
0.399742603	0.01633431	0.032	1	55173	MRPS10
0.399742603	0.01633431	0.036	1	199720	GGN
0.399742603	0.01633431	0.029	1	340385	ZNF517
0.399485201	0.016408328	0.028	1	3159	HMGA1
0.399485201	0.016408328	0.035	1	3242	HPD
0.399485201	0.016408328	0.03	1	9947	MAGEC1
0.399485201	0.016408328	0.029	1	54545	MTMR12
0.399485201	0.016408328	0.037	1	65080	MRPL44
0.399485201	0.016408328	0.025	1	85300	ATCAY
0.399227798	0.016482646	0.023	1	56660	KCNK12
0.399227798	0.016482646	0.024	1	157376	FER1L6-AS2
0.398970395	0.016557232	0.03	1	1404	HAPLN1
0.398970395	0.016557232	0.024	1	54998	AURKAIP1
0.398970395	0.016557232	0.03	1	149628	PYHIN1
0.398970395	0.016557232	0.024	1	727837	SSX2B
0.398712993	0.016632138	0.023	1	57631	LRCH2
0.398712993	0.016632138	0.026	1	124801	LSM12
0.39845559	0.016707312	0.028	1	8120	AP3B2
0.39845559	0.016707312	0.031	1	29798	C2orf27A
0.39845559	0.016707312	0.024	1	59350	RXFP1
0.39845559	0.016707312	0.026	1	152138	PYDC2
0.398198187	0.016782759	0.032	1	2232	FDXR
0.398198187	0.016782759	0.024	1	5912	RAP2B
0.398198187	0.016782759	0.031	1	11074	TRIM31
0.398198187	0.016782759	0.027	1	50944	SHANK1
0.397940785	0.016858498	0.019	1	6455	SH3GL1
0.397940785	0.016858498	0.025	1	27319	BHLHE22
0.397940785	0.016858498	0.022	1	55845	BRK1
0.397940785	0.016858498	0.025	1	79605	PGBD5
0.397940785	0.016858498	0.022	1	339965	CCDC158
0.397683382	0.016934531	0.027	1	55662	HIF1AN
0.397683382	0.016934531	0.023	1	338879	RNASE10
0.397683382	0.016934531	0.023	1	341640	FREM2
0.397425979	0.017010875	0.027	1	4295	MLN
0.397425979	0.017010875	0.036	1	146177	VWA3A
0.397425979	0.017010875	0.03	1	553158	ARHGAP8
0.397168607	0.017087493	0.031	1	26512	INTS6
0.397168607	0.017087493	0.033	1	28968	SLC6A16
0.397168607	0.017087493	0.021	1	29123	ANKRD11
0.397168607	0.017087493	0.027	1	91966	CXorf40A
0.396911174	0.017164389	0.02	1	4883	NPR3
0.396911174	0.017164389	0.024	1	60487	TRMT11
0.396653801	0.017241584	0.033	1	832	CAPZB
0.396653801	0.017241584	0.02	1	3742	KCNA6
0.396653801	0.017241584	0.018	1	9228	DLGAP2
0.396396369	0.017319106	0.028	1	153642	ARSK
0.396138996	0.017396878	0.029	1	1861	TOR1A
0.396138996	0.017396878	0.026	1	9326	ZNHIT3
0.396138996	0.017396878	0.035	1	79729	C1orf113
0.395881593	0.017474962	0.034	1	55315	SLC29A3
0.395881593	0.017474962	0.028	1	151009	LOC151009
0.395881593	0.017474962	0.038	1	375790	AGRN
0.395624191	0.017553328	0.033	1	7342	UBP1
0.395624191	0.017553328	0.025	1	64375	IKZF4
0.395624191	0.017553328	0.031	1	646870	LOC646870
0.395366788	0.017632039	0.02	1	11047	ADRM1
0.395366788	0.017632039	0.024	1	60412	EXOC4
0.395366788	0.017632039	0.023	1	170690	ADAMTS16
0.395109385	0.017710999	0.025	1	4208	MEF2C
0.395109385	0.017710999	0.031	1	84708	LNX1
0.395109385	0.017710999	0.029	1	401992	OR2T2
0.394851983	0.017790254	0.028	1	3299	HSF4
0.394851983	0.017790254	0.03	1	10345	TRDN
0.394851983	0.017790254	0.025	1	56940	DUSP22
0.394851983	0.017790254	0.035	1	161142	C14orf54
0.39459461	0.017869817	0.035	1	10528	NOP56
0.39459461	0.017869817	0.041	1	23204	ARL6IP1
0.39459461	0.017869817	0.026	1	54957	TXNL4B

voxel-wise, subcortex			
Spearman's corr coef	p-val	GenID	Gene
0.286318839	< 0.001	6870	TACR3
0.286229283	< 0.001	7762	ZNF215
0.286206096	< 0.001	441381	LRRC24
0.286176234	< 0.001	84671	ZNF347
0.286064893	< 0.001	375519	GJB7
0.286062121	< 0.001	3607	FOXK2
0.286007196	< 0.001	150538	AC017096.1
0.285962552	< 0.001	25794	FSCN2
0.285955876	< 0.001	140689	CBLN4
0.285948992	< 0.001	55129	ANO10
0.28593725	< 0.001	643446	RNPS1P1
0.285900205	< 0.001	11281	POU6F2
0.28574276	< 0.001	57478	USP31
0.285672814	< 0.001	55079	FEZF2
0.285655886	< 0.001	1571	CYP2E1
0.285632133	< 0.001	80831	APOL5
0.28542766	< 0.001	343263	MYBPHL
0.285389066	< 0.001	10971	YWHAQ
0.285388976	< 0.001	203238	C9orf93
0.285306424	< 0.001	23396	PIP5K1C
0.285228699	< 0.001	10046	MAMLD1
0.285208762	< 0.001	3202	HOXA5
0.28520149	< 0.001	990	CDC6
0.285172492	< 0.001	51686	OAZ3
0.285125285	< 0.001	56136	PCDHA13
0.28504923	< 0.001	51329	ARL6IP4
0.284999043	< 0.001	266811	NPSA
0.284970343	< 0.001	4620	MYH2
0.284858346	< 0.001	140706	C20orf160
0.284843624	< 0.001	1303	COL12A1
0.284823418	< 0.001	6885	MAP3K7
0.284811825	< 0.001	146325	C16orf11
0.284796119	< 0.001	4012	LNPEP
0.284778982	< 0.001	9622	KLK4
0.284679562	< 0.001	283219	KCTD21
0.284642786	< 0.001	79187	FSD1
0.284561217	< 0.001	1418	CRYGA
0.284542233	< 0.001	116349	C5orf55
0.284493297	< 0.001	84102	SLC41A2
0.284479171	< 0.001	389073	C2orf80
0.28447184	< 0.001	146547	PRSS36
0.284353763	< 0.001	7514	XPO1
0.284192592	< 0.001	23767	FLRT3
0.284036994	< 0.001	23349	KIAA1045
0.284006029	< 0.001	50801	KCNK4
0.283941418	< 0.001	5001	ORC5L
0.283899456	< 0.001	84069	PLEKHN1
0.283870101	< 0.001	80727	TTYH3
0.283821106	< 0.001	23518	R3HDM1
0.283791959	< 0.001	10447	FAM3C
0.283788532	< 0.001	4142	MAS1
0.283761978	< 0.001	10213	PSMD14
0.283751935	< 0.001	1154	CISH
0.283537596	< 0.001	29989	OBP2B
0.283456624	< 0.001	9652	TTC37
0.283361018	< 0.001	64333	ARHGAP9
0.283354461	< 0.001	431705	ASTL
0.283266723	< 0.001	553158	ARHGAP8
0.283258438	< 0.001	1291	COL6A1
0.283237964	< 0.001	23510	KCTD2
0.283083171	< 0.001	80055	PGAP1
0.282909453	< 0.001	57616	TSHZ3
0.282906353	< 0.001	84312	BRMS1L
0.282816261	< 0.001	402778	AC068580.3
0.282630354	< 0.001	27287	VENTX
0.282585263	< 0.001	9493	KIF23
0.282554656	< 0.001	10982	MAPRE2
0.282497793	< 0.001	55908	AC011472.2
0.282404065	< 0.001	8368	HIST1H4L
0.282403976	< 0.001	10737	RFPL3S
0.282209158	< 0.001	346389	MACC1
0.282178879	< 0.001	23214	XPO6
0.282176316	< 0.001	118424	UBE2J2
0.282146245	< 0.001	4776	NFATC4
0.282081842	< 0.001	26102	DKFZP434A062
0.282048345	< 0.001	9140	ATG12
0.282026291	< 0.001	113802	C1orf59
0.282021016	< 0.001	80179	MYO19
0.28197217	< 0.001	57547	ZNF624

region-wise, subcortex					
Spearman's corr coef	p-val	p-val, SA	p-val, SA (BH- correction)	GenID	Gene
0.39459461	0.017869817	0.022	1	55968	NSFL1C
0.39459461	0.017869817	0.027	1	133686	C5orf33
0.39459461	0.017869817	0.029	1	375484	C5orf25
0.394337177	0.017949682	0.03	1	9478	CABP1
0.394337177	0.017949682	0.031	1	114769	CARD16
0.394337177	0.017949682	0.022	1	400954	EML6
0.394079804	0.018029882	0.032	1	9215	LARGE
0.393822372	0.018110352	0.031	1	1996	ELAVL4
0.393822372	0.018110352	0.033	1	22931	RAB18
0.393822372	0.018110352	0.024	1	60560	MAK10
0.393822372	0.018110352	0.023	1	285190	RGPD4
0.393564999	0.018191112	0.026	1	5080	PAX6
0.393564999	0.018191112	0.024	1	8882	ZNF259
0.393564999	0.018191112	0.037	1	57179	KIAA1191
0.393564999	0.018191112	0.032	1	81031	SLC2A10
0.393307596	0.018272186	0.018	1	5437	POLR2H
0.393307596	0.018272186	0.024	1	27339	PRPF19
0.393307596	0.018272186	0.02	1	284309	ZNF776
0.393307596	0.018272186	0.029	1	375449	MAST4
0.392792791	0.018435284	0.027	1	2350	FOLR2
0.392792791	0.018435284	0.02	1	65012	SLC26A10
0.392792791	0.018435284	0.03	1	255631	COL24A1
0.392535388	0.018517267	0.022	1	129285	KLRAQ1
0.392277986	0.018599564	0.023	1	492303	GEMIN8P4
0.392020613	0.018682206	0.023	1	4674	NAP1L2
0.392020613	0.018682206	0.023	1	57403	RAB22A
0.39176318	0.018765122	0.028	1	9887	SMG7
0.39176318	0.018765122	0.03	1	23365	ARHGEF12
0.39176318	0.018765122	0.034	1	139886	SPIN4
0.391505778	0.018848347	0.036	1	25789	TMEM59L
0.391505778	0.018848347	0.017	1	343990	C2orf55
0.391248375	0.018931873	0.034	1	80232	WDR26
0.391248375	0.018931873	0.025	1	83637	ZMIZ2
0.391248375	0.018931873	0.026	1	161931	ADAD2
0.391248375	0.018931873	0.024	1	730153	LOC730153
0.3907336	0.019099917	0.022	1	2885	GRB2
0.3907336	0.019099917	0.041	1	54922	RASIP1
0.3907336	0.019099917	0.023	1	55016	MARCHF1
0.3907336	0.019099917	0.032	1	58497	PRUNE
0.3907336	0.019099917	0.025	1	64223	MLST8
0.3907336	0.019099917	0.025	1	137695	TMEM68
0.390476167	0.01918439	0.027	1	5889	RAD51C
0.390476167	0.01918439	0.022	1	728039	SSR4P1
0.390218794	0.01926918	0.029	1	23214	XPO6
0.390218794	0.01926918	0.042	1	387775	SLC22A10
0.389961362	0.019354273	0.021	1	4067	LYN
0.389961362	0.019354273	0.029	1	5715	PSMD9
0.389961362	0.019354273	0.029	1	134526	ACOT12
0.389961362	0.019354273	0.036	1	200728	TMEM17
0.389961362	0.019354273	0.032	1	390637	C15orf58
0.389703989	0.019439729	0.027	1	8650	NUMB
0.389446586	0.019525468	0.026	1	4233	MET
0.389446586	0.019525468	0.028	1	6331	SCN5A
0.389446586	0.019525468	0.04	1	171177	RHOV
0.389189184	0.019611511	0.024	1	238	ALK
0.389189184	0.019611511	0.035	1	4108	MAGEA9
0.389189184	0.019611511	0.034	1	29958	DMGDH
0.389189184	0.019611511	0.027	1	85359	DGCR6L
0.389189184	0.019611511	0.03	1	440915	POTEKP
0.388931781	0.019697895	0.027	1	93	ACVR2B
0.388931781	0.019697895	0.024	1	2016	EMX1
0.388931781	0.019697895	0.03	1	3376	IARS
0.388931781	0.019697895	0.03	1	100129792	CCDC152
0.388674378	0.019784618	0.028	1	1272	CNTN1
0.388674378	0.019784618	0.036	1	6500	SKP1
0.388674378	0.019784618	0.027	1	92597	MOBKL1A
0.388674378	0.019784618	0.031	1	114960	TSGA13
0.388674378	0.019784618	0.032	1	100288911	LOC100288911
0.388416976	0.019871632	0.026	1	5607	MAP2K5
0.388416976	0.019871632	0.032	1	6328	SCN3A
0.388416976	0.019871632	0.031	1	9168	TMSB10
0.388416976	0.019871632	0.033	1	57628	DPP10
0.388416976	0.019871632	0.03	1	81889	FAHD1
0.388416976	0.019871632	0.034	1	344558	SH3RF3
0.388416976	0.019871632	0.032	1	100129272	LOC100129272
0.388159603	0.019958969	0.029	1	1808	DPYSL2
0.388159603	0.019958969	0.033	1	6988	TCTA
0.388159603	0.019958969	0.038	1	22992	KDM2A
0.388159603	0.019958969	0.031	1	442862	PRY

voxel-wise, subcortex			
Spearman's corr coef	p-val	GenID	Gene
0.28192094	< 0.001	51094	ADIPOR1
0.281910598	< 0.001	2553	GABPB1
0.281706601	< 0.001	1362	CPD
0.281695962	< 0.001	80129	C6orf97
0.281621873	< 0.001	2863	GPR39
0.281617403	< 0.001	7265	TTC1
0.281584173	< 0.001	54531	MIER2
0.281564295	< 0.001	6574	SLC20A1
0.281497061	< 0.001	80339	PNPLA3
0.281486005	< 0.001	389114	ZNF662
0.281403869	< 0.001	10970	CKAP4
0.281375021	< 0.001	5154	PDGFA
0.281223655	< 0.001	140707	BRI3BP
0.281051397	< 0.001	1417	CRYBB3
0.280979425	< 0.001	474149	TTTY4B
0.280828983	< 0.001	4986	OPRK1
0.280807346	< 0.001	26258	PLDN
0.280787557	< 0.001	223117	SEMA3D
0.280602902	< 0.001	199953	TMEM201
0.280589014	< 0.001	221960	CCZ1B
0.280531704	< 0.001	64223	MLST8
0.280441314	< 0.001	5568	PRKACG
0.280425072	< 0.001	55911	AC138894.3
0.28035894	< 0.001	28986	MAGEH1
0.280335665	< 0.001	634	CEACAM1
0.280331492	< 0.001	8394	PIP5K1A
0.28025344	< 0.001	51372	CCDC72
0.280188859	< 0.001	113220	KIF12
0.280126125	< 0.001	26289	AK5
0.280105591	< 0.001	8027	STAM
0.280041248	< 0.001	135112	NCOA7
0.279990315	< 0.001	2862	MLNR
0.279956311	< 0.001	283373	ANKRD52
0.279874235	< 0.001	280660	SSX9
0.279794812	< 0.001	205327	C2orf69
0.279785901	< 0.001	7010	TEK
0.27975741	< 0.001	4836	NMT1
0.279728502	< 0.001	6398	SECTM1
0.279644161	< 0.001	9958	USP15
0.27953127	< 0.001	8609	KLF7
0.279457241	< 0.001	392392	OR1K1
0.279361129	< 0.001	2264	FGFR4
0.279292524	< 0.001	7071	KLF10
0.279291242	< 0.001	27445	PCLO
0.279190212	< 0.001	1072	CFL1
0.279127777	< 0.001	9530	BAG4
0.2790896	< 0.001	29760	BLNK
0.278945386	< 0.001	9478	CABP1
0.278903484	< 0.001	2081	ERN1
0.278899223	< 0.001	8933	FAM127A
0.278777391	< 0.001	54102	CLIC6
0.278731138	< 0.001	51054	PLEKHA9
0.278694451	< 0.001	5576	PRKAR2A
0.278667003	< 0.001	818	CAMK2G
0.278629899	< 0.001	6288	SAA1
0.278584123	< 0.001	84342	COG8
0.27854684	< 0.001	51042	ZNF593
0.278515875	< 0.001	7625	ZNF74
0.278500885	< 0.001	266697	POM121L4P
0.27847752	< 0.001	694	BTG1
0.278393805	< 0.001	441228	LOC441228
0.278366297	< 0.001	653548	DUX4L3
0.278354317	< 0.001	22928	SEPHS2
0.278336078	< 0.001	221472	FGD2
0.278301388	< 0.001	1144	CHRND
0.278245866	< 0.001	256329	C11orf35
0.278241813	< 0.001	219333	USP12
0.278109014	< 0.001	84530	KIAA1853
0.278102249	< 0.001	84539	MCHR2
0.277966708	< 0.001	5251	PHEX
0.277958632	< 0.001	4835	NQO2
0.277944386	< 0.001	84542	KIAA1841
0.277939856	< 0.001	378807	CATSPER4
0.277827561	< 0.001	5223	PGAM1
0.277787745	< 0.001	22950	SLC4A1AP
0.277780294	< 0.001	125170	SMCR7
0.277736634	< 0.001	5163	PDK1
0.277620137	< 0.001	57456	KIAA1143
0.277596861	< 0.001	160418	TMTC3

region-wise, subcortex					
Spearman's corr coef	p-val	p-val, SA	p-val, SA (BH- correction)	GenID	Gene
0.38790217	0.020046618	0.025	1	51750	RTEL1
0.38790217	0.020046618	0.028	1	54675	CRLS1
0.38790217	0.020046618	0.029	1	55135	WRAP53
0.387644798	0.020134611	0.033	1	4207	MEF2BNB-MEF2B
0.387644798	0.020134611	0.033	1	84144	SYDE2
0.387387365	0.020222949	0.036	1	83856	FSD1L
0.387387365	0.020222949	0.024	1	166614	DCLK2
0.387387365	0.020222949	0.017	1	392617	PPP1R28
0.387129992	0.020311579	0.036	1	150	ADRA2A
0.387129992	0.020311579	0.03	1	2565	GABRG1
0.387129992	0.020311579	0.031	1	8881	CDC16
0.387129992	0.020311579	0.032	1	339448	C1orf174
0.38687259	0.020400539	0.037	1	3024	HIST1H1A
0.38687259	0.020400539	0.019	1	10255	HCG9
0.38687259	0.020400539	0.037	1	81871	KRTAP4-6
0.38687259	0.020400539	0.028	1	340204	C6orf127
0.386615187	0.020489816	0.037	1	3052	HCCS
0.386615187	0.020489816	0.028	1	65117	RSRC2
0.386615187	0.020489816	0.024	1	84503	ZNF527
0.386615187	0.020489816	0.037	1	90249	UNC5A
0.386357784	0.020579478	0.019	1	131055	LOC131055
0.386100382	0.020669412	0.035	1	11133	KPTN
0.385842979	0.020759676	0.036	1	6769	STAC
0.385842979	0.020759676	0.03	1	23335	WDR7
0.385842979	0.020759676	0.029	1	375133	PI1KAP2
0.385585606	0.02085029	0.028	1	7846	TUBA1A
0.385585606	0.02085029	0.022	1	9827	RGP1
0.385585606	0.02085029	0.032	1	64218	SEMA4A
0.385328174	0.020941256	0.031	1	23185	LARP4B
0.385328174	0.020941256	0.035	1	84306	PDCD2L
0.385328174	0.020941256	0.037	1	93185	IGSF8
0.384813368	0.021124128	0.04	1	3730	KAL1
0.384813368	0.021124128	0.039	1	53343	NUDT9
0.384813368	0.021124128	0.034	1	54107	POLE3
0.384813368	0.021124128	0.036	1	84833	USMG5
0.384813368	0.021124128	0.029	1	201140	DHRS7C
0.384555966	0.021216057	0.019	1	8396	PIP5K2B
0.384555966	0.021216057	0.039	1	150921	TCF23
0.384298593	0.02130834	0.032	1	1994	ELAVL1
0.384298593	0.02130834	0.028	1	127262	TPRG1L
0.38404116	0.021400981	0.035	1	9424	KCNK6
0.38404116	0.021400981	0.037	1	22916	NCBP2
0.38404116	0.021400981	0.034	1	29797	POM121L8P
0.38404116	0.021400981	0.029	1	55810	FOXJ2
0.38404116	0.021400981	0.029	1	93210	PGAP3
0.383526355	0.021587214	0.036	1	4692	NDN
0.383526355	0.021587214	0.033	1	7320	UBE2B
0.383526355	0.021587214	0.039	1	10740	RFPL1-AS1
0.383526355	0.021587214	0.029	1	54802	TRIT1
0.383268982	0.021680828	0.034	1	5368	PNOC
0.383268982	0.021680828	0.034	1	79042	TSEN34
0.383268982	0.021680828	0.03	1	220164	DOK6
0.383268982	0.021680828	0.026	1	441089	CRSP8P
0.38301158	0.021774847	0.036	1	5382	PMS2P4
0.38301158	0.021774847	0.029	1	9933	KIAA0020
0.38301158	0.021774847	0.025	1	283431	GAS2L3
0.382754177	0.021869145	0.032	1	5600	MAPK11
0.382754177	0.021869145	0.029	1	11230	PRAF2
0.382754177	0.021869145	0.035	1	23265	EXOC7
0.382754177	0.021869145	0.05	1	65985	AACS
0.382496774	0.021963805	0.042	1	55201	MAP1S
0.382496774	0.021963805	0.03	1	55837	EAPP
0.382496774	0.021963805	0.033	1	64881	PCDH20
0.382239372	0.022058787	0.03	1	50640	PNPLA8
0.382239372	0.022058787	0.031	1	53635	PTOV1
0.382239372	0.022058787	0.027	1	55346	TCP11L1
0.382239372	0.022058787	0.03	1	56934	CA10
0.382239372	0.022058787	0.048	1	85439	STON2
0.382239372	0.022058787	0.041	1	149934	NCOR1P1
0.381981969	0.022154156	0.03	1	11237	RNF24
0.381981969	0.022154156	0.028	1	26063	DECR2
0.381981969	0.022154156	0.032	1	54480	CSGLCA-T
0.381724596	0.02224985	0.028	1	6662	SOX9
0.381724596	0.02224985	0.032	1	57669	EPB41L5
0.381724596	0.02224985	0.04	1	79786	KLHL36
0.381724596	0.02224985	0.027	1	89790	SIGLEC10
0.381724596	0.02224985	0.033	1	140462	ASB9
0.381724596	0.02224985	0.035	1	653548	DUX4L3
0.381467164	0.022345865	0.03	1	5138	PDE2A

voxel-wise, subcortex			
Spearman's corr coef	p-val	GenID	Gene
0.277571857	< 0.001	23550	PSD4
0.277546018	< 0.001	5824	PEX19
0.277544767	< 0.001	84084	RAB6C
0.27751857	< 0.001	58538	MPP4
0.27744931	< 0.001	1261	CNGA3
0.277422756	< 0.001	55851	PSENEN
0.277419358	< 0.001	90678	LRSAM1
0.277371675	< 0.001	11282	MGAT4B
0.2773633	< 0.001	53831	GPR84
0.277334154	< 0.001	83693	HSDL1
0.277331471	< 0.001	337979	KRTAP22-1
0.277304709	< 0.001	143379	C10orf82
0.277267814	< 0.001	54103	PION
0.277250111	< 0.001	132989	C4orf36
0.277206361	< 0.001	6949	TCOF1
0.277138025	< 0.001	7189	TRAF6
0.277035117	< 0.001	7531	YWHAE
0.277021855	< 0.001	6794	STK11
0.276999503	< 0.001	5923	RASGRF1
0.276942253	< 0.001	9630	GNA14
0.276867121	< 0.001	55315	SLC29A3
0.276642263	< 0.001	7268	TTC4
0.276605755	< 0.001	4905	NSF
0.276560843	< 0.001	339210	C17orf67
0.276512206	< 0.001	81855	SFXN3
0.276488096	< 0.001	5527	PPP2R5C
0.276466727	< 0.001	54503	ZDHHC13
0.276466519	< 0.001	662	BNIP1
0.276421279	< 0.001	29123	ANKRD11
0.276407808	< 0.001	5007	OSBP
0.276365966	< 0.001	64175	LEPRE1
0.276359349	< 0.001	8078	USP5
0.276269078	< 0.001	155185	AMZ1
0.276252925	< 0.001	11196	SEC23IP
0.276212603	< 0.001	375	ARF1
0.276208103	< 0.001	4882	NPR2
0.276148677	< 0.001	23558	WBP2
0.276138842	< 0.001	94137	RP1L1
0.27601105	< 0.001	57520	HECW2
0.275978386	< 0.001	161142	C14orf54
0.275825351	< 0.001	84276	NICN1
0.275697261	< 0.001	9404	LPXN
0.275692374	< 0.001	64400	AKTIP
0.275670886	< 0.001	115557	GEFT
0.275632054	< 0.001	5871	MAP4K2
0.275614381	< 0.001	100133211	LOC100133211
0.275510222	< 0.001	4920	ROR2
0.275433272	< 0.001	26059	ERC2
0.27541396	< 0.001	441094	AC106818.2
0.275262505	< 0.001	2874	GPS2
0.275252551	< 0.001	84457	PHYHIP
0.275221407	< 0.001	344892	RTP2
0.275138617	< 0.001	1576	CYP3A4
0.275124818	< 0.001	55585	UBE2Q1
0.275123	< 0.001	7014	TERF2
0.274957329	< 0.001	29964	PRICKLE4
0.274929076	< 0.001	442459	LOC442459
0.274917305	< 0.001	391334	LOC391334
0.274915546	< 0.001	4207	MEF2BNB-MEF2B
0.274843663	< 0.001	55847	CISD1
0.274815947	< 0.001	84662	GLIS2
0.274803102	< 0.001	9925	ZBTB5
0.274742037	< 0.001	3841	KPNA5
0.274607003	< 0.001	123775	C16orf46
0.274564296	< 0.001	196410	METTL7B
0.274513245	< 0.001	6399	TRAPPC2
0.274502307	< 0.001	1717	DHCR7
0.274474353	< 0.001	29924	EPN1
0.274452895	< 0.001	51304	ZDHHC3
0.274414033	< 0.001	6276	S100A5
0.274145454	< 0.001	100133941	CD24
0.273981512	< 0.001	162979	ZNF296
0.273964614	< 0.001	79640	Z83840.4
0.273936361	< 0.001	55290	BRF2
0.273887485	< 0.001	51118	UTP11L
0.273880094	< 0.001	399979	SNX19
0.273860455	< 0.001	219402	MTIF3
0.273777664	< 0.001	10381	RP11-566K11.2
0.273771852	< 0.001	4035	LRP1

region-wise, subcortex					
Spearman's corr coef	p-val	p-val, SA	p-val, SA (BH- correction)	GenID	Gene
0.381467164	0.022345865	0.03	1	78999	LRFN4
0.381209791	0.022442251	0.036	1	8784	TNFRSF18
0.381209791	0.022442251	0.029	1	55588	MED29
0.380694985	0.022636071	0.034	1	25911	RP11-529I10.1
0.380694985	0.022636071	0.034	1	222962	SLC29A4
0.380694985	0.022636071	0.042	1	283987	C17orf28
0.380437583	0.022733504	0.028	1	6854	SYN2
0.380437583	0.022733504	0.041	1	9737	GPRASP1
0.380437583	0.022733504	0.038	1	84792	C7orf70
0.380437583	0.022733504	0.037	1	390199	OR4D9
0.380437583	0.022733504	0.029	1	400359	C15orf53
0.38018018	0.022831265	0.037	1	4837	NNMT
0.38018018	0.022831265	0.038	1	7701	ZNF142
0.38018018	0.022831265	0.036	1	10656	KHDRBS3
0.38018018	0.022831265	0.024	1	63826	SRR
0.38018018	0.022831265	0.041	1	84326	C16orf13
0.379922777	0.0229294	0.024	1	51016	FAM158A
0.379922777	0.0229294	0.039	1	51332	SPTBN5
0.379922777	0.0229294	0.031	1	283970	PDXDC2
0.379665375	0.023027912	0.036	1	8698	S1PR4
0.379407972	0.023126734	0.022	1	23542	MAPK8IP2
0.378893167	0.023325462	0.027	1	2168	FABP1
0.378893167	0.023325462	0.029	1	57492	ARID1B
0.378893167	0.023325462	0.045	1	348303	AC011500.2
0.378635794	0.023425372	0.03	1	2776	GNAQ
0.378635794	0.023425372	0.029	1	8216	LZTR1
0.378635794	0.023425372	0.027	1	10368	CACNG3
0.378635794	0.023425372	0.035	1	23368	PPP1R13B
0.378635794	0.023425372	0.039	1	55957	LIN37
0.378378361	0.023525663	0.04	1	631	BFSP1
0.378378361	0.023525663	0.031	1	10138	YAF2
0.378378361	0.023525663	0.024	1	11005	SPINK5
0.378378361	0.023525663	0.035	1	84083	ZRANB3
0.378120989	0.023626266	0.031	1	145957	NRG4
0.378120989	0.023626266	0.036	1	284129	SLC26A11
0.377863586	0.023727253	0.025	1	56938	ARNTL2
0.377863586	0.023727253	0.03	1	81624	DIAPH3
0.377863586	0.023727253	0.034	1	126969	SLC44A3
0.377606153	0.023828575	0.038	1	7415	VCP
0.377348781	0.02393033	0.023	1	3356	HTR2A
0.377348781	0.02393033	0.035	1	64396	GMCL1P1
0.377348781	0.02393033	0.04	1	124402	FAM100A
0.377091348	0.024032379	0.036	1	5737	PTGFR
0.377091348	0.024032379	0.04	1	84902	CCDC123
0.377091348	0.024032379	0.026	1	131408	FAM131A
0.376833975	0.024134791	0.038	1	29086	C19orf62
0.376833975	0.024134791	0.038	1	115350	FCRL1
0.376576573	0.02423759	0.031	1	26270	FBXO6
0.376576573	0.02423759	0.039	1	27346	TMEM97
0.376576573	0.02423759	0.027	1	55699	IARS2
0.37631917	0.024340777	0.04	1	7485	WRB
0.37631917	0.024340777	0.038	1	84221	C21orf56
0.37631917	0.024340777	0.035	1	221955	DAGLB
0.376061767	0.024444308	0.035	1	5432	POLR2C
0.376061767	0.024444308	0.043	1	7762	ZNF215
0.376061767	0.024444308	0.034	1	8715	NOL4
0.376061767	0.024444308	0.032	1	65078	RTN4R
0.376061767	0.024444308	0.036	1	114781	BTBD9
0.375804365	0.024548186	0.03	1	5687	PSMA6
0.375804365	0.024548186	0.035	1	647012	LOC647012
0.375546962	0.024652431	0.037	1	81572	PDRG1
0.375289589	0.024757067	0.039	1	2560	GABRB1
0.375289589	0.024757067	0.028	1	51188	SS18L2
0.375032157	0.024862099	0.039	1	8609	KLF7
0.375032157	0.024862099	0.032	1	9804	TOMM20
0.375032157	0.024862099	0.038	1	389119	C3orf54
0.374774784	0.024967477	0.026	1	51255	RNF181
0.374517351	0.025073206	0.042	1	2171	FABP5
0.374517351	0.025073206	0.035	1	4850	CNOT4
0.374517351	0.025073206	0.026	1	7532	YWHAG
0.374517351	0.025073206	0.031	1	10962	MLLT11
0.374517351	0.025073206	0.04	1	114294	LACTB
0.374517351	0.025073206	0.035	1	140545	RNF32
0.374259979	0.025179308	0.034	1	3607	FOXK2
0.374259979	0.025179308	0.036	1	6229	RPS24
0.374002546	0.025285855	0.024	1	5688	PSMA7
0.374002546	0.025285855	0.041	1	253018	HCG27
0.373745173	0.025392706	0.048	1	5602	MAPK10
0.373487771	0.025499946	0.045	1	2891	GRIA2

voxel-wise, subcortex			
Spearman's corr coef	p-val	GenID	Gene
0.27373296	< 0.001	65008	MRPL1
0.273678422	< 0.001	5127	PCTK1
0.273604691	< 0.001	54491	FAM105A
0.273530871	< 0.001	29982	NRBF2
0.273464233	< 0.001	1448	CSN3
0.273437649	< 0.001	5524	PPP2R4
0.273433656	< 0.001	2132	EXT2
0.273421526	< 0.001	6728	SRP19
0.273380011	< 0.001	2060	EPS15
0.273315102	< 0.001	8270	LAGE3
0.273313105	< 0.001	92400	RBM18
0.273212373	< 0.001	22902	RUFY3
0.273180813	< 0.001	10692	RRH
0.273160934	< 0.001	9076	CLDN1
0.273109257	< 0.001	128646	SIRPD
0.273004919	< 0.001	1808	DPYSL2
0.272994399	< 0.001	79735	TBC1D17
0.27292487	< 0.001	51335	NGRN
0.272915393	< 0.001	26000	TBC1D10B
0.272884756	< 0.001	23507	LRRC8B
0.272839427	< 0.001	157570	ESCO2
0.27271077	< 0.001	220202	ATOH7
0.272702634	< 0.001	100288540	POM121B
0.272680402	< 0.001	51350	KRT76
0.272645444	< 0.001	1809	DPYSL3
0.272608668	< 0.001	25891	PAMR1
0.27258265	< 0.001	2890	GRIA1
0.272542	< 0.001	51339	DACT1
0.272540599	< 0.001	9095	TBX19
0.272504091	< 0.001	283459	GATC
0.272500902	< 0.001	84520	C14orf142
0.272465527	< 0.001	57542	KLHDC5
0.272463799	< 0.001	390243	FOLR4
0.27246058	< 0.001	285331	CCDC66
0.272405386	< 0.001	646719	LOC646719
0.2723836	< 0.001	5732	PTGER2
0.272372037	< 0.001	5555	PRH2
0.272305727	< 0.001	154313	C6orf165
0.272301286	< 0.001	22998	LIMCH1
0.272246867	< 0.001	167227	DCP2
0.272240728	< 0.001	84172	POLR1B
0.272221744	< 0.001	84691	FAM71F1
0.272173405	< 0.001	112770	C1orf85
0.272140682	< 0.001	346606	MOGAT3
0.272094101	< 0.001	57663	USP29
0.272029221	< 0.001	347744	C6orf52
0.272012919	< 0.001	729475	RAD51AP2
0.272002667	< 0.001	1564	CYP2D7P1
0.271966487	< 0.001	57102	C12orf4
0.271900743	< 0.001	441273	SPDYE2
0.271786243	< 0.001	4363	ABCC1
0.271768332	< 0.001	113146	AHNAK2
0.271768123	< 0.001	286128	ZFP41
0.271767586	< 0.001	29931	LINC00312
0.271659434	< 0.001	7178	TPT1
0.27161932	< 0.001	394	ARHGAP5
0.271612495	< 0.001	1595	CYP51A1
0.271560729	< 0.001	27183	VPS4A
0.27151829	< 0.001	4633	MYL2
0.271474779	< 0.001	8065	CUL5
0.271443248	< 0.001	3833	KIFC1
0.271352053	< 0.001	388569	ZNF324B
0.271335274	< 0.001	645	BLVRB
0.271333337	< 0.001	79643	CHMP6
0.271322101	< 0.001	1208	CLPS
0.271280974	< 0.001	27036	SIGLEC7
0.271271825	< 0.001	10401	PIAS3
0.271212012	< 0.001	56829	ZC3HAV1
0.27119869	< 0.001	84262	PSMG3
0.271139532	< 0.001	10651	MTX2
0.271103829	< 0.001	29108	PYCARD
0.271011919	< 0.001	23581	CASP14
0.270945519	< 0.001	7850	IL1R2
0.270940155	< 0.001	9972	NUP153
0.270937085	< 0.001	440738	MAP1LC3C
0.270704031	< 0.001	1459	CSNK2A2
0.270695865	< 0.001	2334	AFF2
0.270666927	< 0.001	23623	RUSC1
0.270641208	< 0.001	25847	ANAPC13

region-wise, subcortex					
Spearman's corr coef	p-val	p-val, SA	p-val, SA (BH- correction)	GenID	Gene
0.373487771	0.025499946	0.037	1	219348	PLAC9
0.373230368	0.025607564	0.036	1	132851	SPATA4
0.372972965	0.025715595	0.046	1	1487	CTBP1
0.372972965	0.025715595	0.037	1	84318	CCDC77
0.372972965	0.025715595	0.044	1	441061	MARCHF11
0.372715592	0.025823982	0.047	1	1734	DIO2
0.37245816	0.025932726	0.053	1	7447	VSNL1
0.37245816	0.025932726	0.044	1	27330	RPS6KA6
0.372200787	0.026041863	0.051	1	755	C21orf2
0.372200787	0.026041863	0.049	1	23416	KCNH3
0.372200787	0.026041863	0.032	1	57576	KIF17
0.372200787	0.026041863	0.034	1	133690	CAPSL
0.372200787	0.026041863	0.034	1	148423	C1orf52
0.371943355	0.026151383	0.045	1	119358	SNRPD2P1
0.371943355	0.026151383	0.033	1	138307	LCN8
0.371685982	0.026261322	0.046	1	6604	SMARCD3
0.371685982	0.026261322	0.036	1	22883	CLSTN1
0.371428549	0.026371622	0.038	1	27099	SND1-IT1
0.371428549	0.026371622	0.041	1	114132	SIGLEC11
0.371171176	0.026482284	0.055	1	25864	ABHD14A
0.370913774	0.026593344	0.042	1	9807	IP6K1
0.370656341	0.02670484	0.045	1	551	AVP
0.370656341	0.02670484	0.04	1	55669	MFN1
0.370656341	0.02670484	0.034	1	219870	OR10G9
0.370398968	0.026816666	0.04	1	5899	RALB
0.370398968	0.026816666	0.032	1	57795	FAM5B
0.370141536	0.02692889	0.038	1	55320	C14orf106
0.370141536	0.02692889	0.035	1	133015	PACRGL
0.369884163	0.027041508	0.051	1	53358	SHC3
0.369884163	0.027041508	0.04	1	57479	PRR12
0.369884163	0.027041508	0.031	1	79935	CNTD2
0.36962676	0.027154554	0.037	1	5000	ORC4
0.36962676	0.027154554	0.037	1	27039	PKD2L2
0.36962676	0.027154554	0.041	1	80310	PDGFD
0.369111955	0.027381748	0.031	1	3157	HMGCS1
0.369111955	0.027381748	0.048	1	50512	PODXL2
0.369111955	0.027381748	0.037	1	57534	MIB1
0.369111955	0.027381748	0.044	1	387893	SETD8
0.368854553	0.027495936	0.047	1	1952	CELSR2
0.368854553	0.027495936	0.045	1	9369	NRXN3
0.36859715	0.027610522	0.038	1	94160	ABCC12
0.36859715	0.027610522	0.032	1	146206	RLTPR
0.36859715	0.027610522	0.036	1	374286	CDRT1
0.368339777	0.027725542	0.027	1	80023	NRSN2
0.368082345	0.027840935	0.042	1	23162	MAPK8IP3
0.368082345	0.027840935	0.055	1	146198	ZFP90
0.367824972	0.0279567	0.037	1	9805	SCRN1
0.367824972	0.0279567	0.041	1	26047	CNTNAP2
0.367824972	0.0279567	0.04	1	51699	VPS29
0.367567539	0.028072879	0.049	1	1877	E4F1
0.367567539	0.028072879	0.046	1	6289	SAA2
0.367567539	0.028072879	0.043	1	58495	OVOL2
0.367567539	0.028072879	0.041	1	387911	RP11-45B20.3
0.367310166	0.028189508	0.027	1	1806	DPYD
0.367310166	0.028189508	0.038	1	6717	SRI
0.367310166	0.028189508	0.032	1	8723	SNX4
0.367052764	0.028306477	0.039	1	23367	LARP1
0.367052764	0.028306477	0.043	1	57452	GALNTL1
0.367052764	0.028306477	0.043	1	83660	TLN2
0.367052764	0.028306477	0.036	1	727830	FAM75A3
0.366795361	0.028423861	0.043	1	150290	DUSP18
0.366795361	0.028423861	0.043	1	644172	LOC644172
0.366537958	0.028541651	0.041	1	29115	SAP30BP
0.366537958	0.028541651	0.035	1	79838	TMC5
0.366280556	0.028659895	0.034	1	147699	PPM1N
0.366280556	0.028659895	0.054	1	441728	GOLGA6B
0.366023153	0.028778495	0.038	1	55847	CISD1
0.366023153	0.028778495	0.048	1	80273	GRPEL1
0.366023153	0.028778495	0.036	1	768206	PRCD
0.36576578	0.028897488	0.043	1	7306	TYRP1
0.365508348	0.029016903	0.042	1	25852	ARMC8
0.365508348	0.029016903	0.041	1	55030	FBXO34
0.365508348	0.029016903	0.03	1	200844	C3orf67
0.365250975	0.029136727	0.044	1	10605	PAIP1
0.365250975	0.029136727	0.043	1	22980	TCF25
0.365250975	0.029136727	0.046	1	51275	C12ORF47
0.365250975	0.029136727	0.04	1	84342	COG8
0.365250975	0.029136727	0.041	1	197187	MGC23284
0.364993542	0.029257014	0.056	1	2103	ESRRB

voxel-wise, subcortex			
Spearman's corr coef	p-val	GenID	Gene
0.270630628	< 0.001	285759	RP11-544L8__B.4
0.27061373	< 0.001	80731	THSD7B
0.270591795	< 0.001	28983	TMPRSS11E2
0.270573318	< 0.001	140606	SELM
0.270572484	< 0.001	30836	DNTTIP2
0.270564139	< 0.001	401777	LOC401777
0.270546287	< 0.001	4659	PPP1R12A
0.270476609	< 0.001	388722	C1orf53
0.270466328	< 0.001	5216	PFN1
0.270384073	< 0.001	3654	IRAK1
0.270334393	< 0.001	92609	TIMM50
0.270280242	< 0.001	23615	PYY2
0.270275235	< 0.001	255313	CT47A11
0.270258427	< 0.001	10045	SH2D3A
0.27023074	< 0.001	1118	CHIT1
0.270191461	< 0.001	81631	MAP1LC3B
0.270118743	< 0.001	133308	NHEDC2
0.27008605	< 0.001	7167	TPI1
0.270050764	< 0.001	128864	C20orf144
0.270006061	< 0.001	126068	ZNF441
0.269998014	< 0.001	8784	TNFRSF18
0.269967467	< 0.001	2295	FOXF2
0.269933611	< 0.001	220001	VWCE
0.269875616	< 0.001	25873	RPL36
0.269870609	< 0.001	7371	UCK2
0.269856483	< 0.001	9107	MTMR6
0.269828826	< 0.001	158062	LCN6
0.269799292	< 0.001	199882	LOC199882
0.269762754	< 0.001	22989	MYH15
0.269627213	< 0.001	54662	TBC1D13
0.269613087	< 0.001	2743	GLRB
0.269608349	< 0.001	79647	AKIRIN1
0.269515246	< 0.001	135295	RP11-63L7.1
0.269497573	< 0.001	388341	C17orf76
0.269494593	< 0.001	57690	TNRC6C
0.269420564	< 0.001	3752	KCND3
0.269400239	< 0.001	55884	WSB2
0.269383013	< 0.001	389668	XKR9
0.269338161	< 0.001	128371	OR6K6
0.269264132	< 0.001	9931	HELZ
0.269238323	< 0.001	27247	NFU1
0.269139439	< 0.001	10890	RAB10
0.269105196	< 0.001	389043	LOC389043
0.269023657	< 0.001	81688	C6orf62
0.26880312	< 0.001	6361	CCL17
0.268699944	< 0.001	7534	YWHAZ
0.2686795	< 0.001	84215	ZNF541
0.268625468	< 0.001	84925	DIRC2
0.268607527	< 0.001	80772	GLTPD1
0.268600255	< 0.001	440387	CTRB2
0.26851055	< 0.001	23552	CCRK
0.268457681	< 0.001	2790	GNG10
0.26844427	< 0.001	646517	LOC646517
0.268418819	< 0.001	169044	COL22A1
0.268375367	< 0.001	149603	RNF187
0.268299162	< 0.001	1826	DSCAM
0.268257111	< 0.001	135114	HINT3
0.268140107	< 0.001	342931	RFPL4A
0.268084317	< 0.001	10018	BCL2L11
0.26801157	< 0.001	100129250	C9ORF133
0.26796338	< 0.001	6809	STX3
0.267906666	< 0.001	9898	UBAP2L
0.267880529	< 0.001	338440	ANO9
0.26787284	< 0.001	84666	RETNLB
0.267837107	< 0.001	3589	IL11
0.267778575	< 0.001	729595	HMGB3P22
0.267596781	< 0.001	152002	C3orf21
0.267540693	< 0.001	10956	OS9
0.267522663	< 0.001	388242	RP11-345J4.3
0.267518044	< 0.001	57001	ACN9
0.26751247	< 0.001	27324	TOX3
0.267478883	< 0.001	728409	C21orf54
0.267457634	< 0.001	57403	RAB22A
0.267418623	< 0.001	57216	VANGL2
0.26734376	< 0.001	3897	L1CAM
0.267285585	< 0.001	333929	SNAI3
0.267206848	< 0.001	54888	NSUN2
0.267184556	< 0.001	65057	ACD
0.267183155	< 0.001	8667	EIF3H

region-wise, subcortex					
Spearman's corr coef	p-val	p-val, SA	p-val, SA (BH- correction)	GenID	Gene
0.364993542	0.029257014	0.046	1	5592	PRKG1
0.364993542	0.029257014	0.045	1	9793	CKAP5
0.364993542	0.029257014	0.045	1	10787	NCKAP1
0.36473617	0.029377645	0.039	1	5217	PFN2
0.36473617	0.029377645	0.036	1	8626	TP63
0.36473617	0.029377645	0.033	1	11281	POU6F2
0.364478767	0.029498704	0.042	1	3574	IL7
0.364478767	0.029498704	0.043	1	63967	CLSPN
0.364478767	0.029498704	0.038	1	64850	AGXT2L1
0.364478767	0.029498704	0.04	1	84280	BTBD10
0.364221364	0.029620174	0.036	1	1635	DCTD
0.364221364	0.029620174	0.047	1	2199	FBLN2
0.363963962	0.029742114	0.05	1	3755	KCNG1
0.363963962	0.029742114	0.048	1	4885	NPTX2
0.363963962	0.029742114	0.034	1	9177	HTR3B
0.363449156	0.029987121	0.047	1	4886	NPY1R
0.363449156	0.029987121	0.045	1	23207	PLEKHM2
0.363449156	0.029987121	0.035	1	149992	ANKRD30BP2
0.363449156	0.029987121	0.042	1	100128771	EIF4BP3
0.363191754	0.030110257	0.034	1	5537	PPP6C
0.363191754	0.030110257	0.04	1	8933	FAM127A
0.363191754	0.030110257	0.06	1	347454	ANKRD58
0.363191754	0.030110257	0.043	1	399942	TUBAP2
0.362934351	0.030233864	0.044	1	4016	LOXL1
0.362934351	0.030233864	0.047	1	50628	GEMIN4
0.362934351	0.030233864	0.048	1	113730	KLHDC7B
0.362934351	0.030233864	0.047	1	162979	ZNF296
0.362676948	0.03035784	0.049	1	435	ASL
0.362676948	0.03035784	0.038	1	51020	HDDC2
0.362419546	0.030482236	0.05	1	3589	IL11
0.362419546	0.030482236	0.037	1	392288	LOC392288
0.362162143	0.030607035	0.051	1	1128	CHRM1
0.362162143	0.030607035	0.052	1	3203	HOXA6
0.362162143	0.030607035	0.041	1	25818	KLK5
0.362162143	0.030607035	0.044	1	55789	DEPDC1B
0.362162143	0.030607035	0.048	1	387882	C12ORF75
0.36190477	0.030732265	0.046	1	7534	YWHAZ
0.36190477	0.030732265	0.045	1	84215	ZNF541
0.361389965	0.030984072	0.043	1	29035	C16orf72
0.361389965	0.030984072	0.05	1	29761	USP25
0.361132532	0.0311106	0.05	1	9597	SMAD5OS
0.361132532	0.0311106	0.049	1	54866	PPP1R14D
0.361132532	0.0311106	0.03	1	55851	PSENEN
0.361132532	0.0311106	0.051	1	84698	CAPS2
0.361132532	0.0311106	0.045	1	643311	CT47B1
0.36087516	0.031237528	0.041	1	2290	FOXG1B
0.36087516	0.031237528	0.046	1	10522	DEAF1
0.360617757	0.031364955	0.05	1	1118	CHIT1
0.360617757	0.031364955	0.051	1	10213	PSMD14
0.360617757	0.031364955	0.047	1	11321	GPN1
0.360617757	0.031364955	0.034	1	54785	C17orf59
0.360617757	0.031364955	0.059	1	64376	IKZF5
0.360617757	0.031364955	0.047	1	84059	GPR98
0.360360354	0.031492755	0.061	1	147841	SPC24
0.360102952	0.031620972	0.049	1	665	BNIP3L
0.360102952	0.031620972	0.044	1	7461	CLIP2
0.360102952	0.031620972	0.049	1	55779	WDR52
0.360102952	0.031620972	0.048	1	85313	PPIL4
0.359845549	0.031749647	0.05	1	1032	CDKN2D
0.359845549	0.031749647	0.041	1	6256	RXRA
0.359845549	0.031749647	0.036	1	91582	RPS19BP1
0.359588146	0.031878781	0.039	1	55450	CAMK2N1
0.359588146	0.031878781	0.054	1	57591	MKL1
0.359588146	0.031878781	0.036	1	64092	SAMSN1
0.359588146	0.031878781	0.049	1	123228	SENP8
0.359588146	0.031878781	0.053	1	152579	SCFD2
0.359073341	0.032138273	0.046	1	2567	GABRG3
0.359073341	0.032138273	0.05	1	57585	CRAMP1L
0.359073341	0.032138273	0.04	1	84305	WIBG
0.359073341	0.032138273	0.048	1	343035	RD3
0.359073341	0.032138273	0.046	1	440107	PLEKHG7
0.358815968	0.032268658	0.059	1	5641	LGMN
0.358815968	0.032268658	0.049	1	7443	VRK1
0.358815968	0.032268658	0.044	1	8541	PPFIA3
0.358815968	0.032268658	0.058	1	51560	RAB6B
0.358815968	0.032268658	0.045	1	79057	PRRG3
0.358558536	0.032399505	0.035	1	1538	CYLC1
0.358558536	0.032399505	0.038	1	55715	DOK4
0.358558536	0.032399505	0.04	1	643836	ZFP62

voxel-wise, subcortex			
Spearman's corr coef	p-val	GenID	Gene
0.267136902	< 0.001	283871	PGP
0.26711908	< 0.001	155006	TMEM213
0.267088622	< 0.001	7267	TTC3
0.267053574	< 0.001	196383	RILPL2
0.266875297	< 0.001	3044	HBBP1
0.266871125	< 0.001	1107	CHD3
0.266843915	< 0.001	5209	PFKFB3
0.266808063	< 0.001	353500	BMP8A
0.266796887	< 0.001	56145	PCDHA3
0.266718626	< 0.001	26526	TSPAN16
0.266703606	< 0.001	132671	SPATA18
0.266676664	< 0.001	1066	CES1
0.266627491	< 0.001	339479	FAM5C
0.266593814	< 0.001	2220	FCN2
0.266458482	< 0.001	83983	TSSK6
0.266435117	< 0.001	9451	EIF2AK3
0.266382188	< 0.001	55968	NSFL1C
0.266362965	< 0.001	253143	C22orf30
0.266355336	< 0.001	90249	UNC5A
0.266335666	< 0.001	10746	MAP3K2
0.266272038	< 0.001	5806	PTX3
0.266252726	< 0.001	83401	ELOVL3
0.266245902	< 0.001	116832	RPL39L
0.266230494	< 0.001	3386	ICAM4
0.266214281	< 0.001	91694	LONRF1
0.26606226	< 0.001	84947	SERAC1
0.266006976	< 0.001	2116	ETV2
0.265908301	< 0.001	3963	LGALS7
0.265863776	< 0.001	158038	LINGO2
0.265820473	< 0.001	29935	RPA4
0.265787095	< 0.001	79800	ALS2CR8
0.265681565	< 0.001	92675	DTD1
0.265524745	< 0.001	55758	RCOR3
0.265496701	< 0.001	595101	LOC595101
0.265341014	< 0.001	170302	ARX
0.265280485	< 0.001	1211	CLTA
0.265249401	< 0.001	84934	C12orf52
0.265230358	< 0.001	55022	PID1
0.265165329	< 0.001	1585	CYP11B2
0.265108258	< 0.001	80094	FLJ14107
0.265040874	< 0.001	286319	TUSC1
0.264911145	< 0.001	89845	ABCC10
0.264882684	< 0.001	4588	MUC6
0.264853328	< 0.001	23034	SAMD4A
0.2648471	< 0.001	730153	LOC730153
0.264766216	< 0.001	8543	LMO4
0.264731586	< 0.001	84070	FAM186B
0.264727503	< 0.001	23614	PPY2
0.264688045	< 0.001	23270	TSPYL5
0.264675498	< 0.001	117159	DCD
0.264662415	< 0.001	10827	FAM114A2
0.264617354	< 0.001	11119	BTN3A1
0.264577836	< 0.001	339834	CCDC36
0.2644732	< 0.001	26071	FAM127B
0.264472246	< 0.001	148423	C1orf52
0.264470577	< 0.001	11078	TRIOBP
0.264469892	< 0.001	3638	INSIG1
0.264403343	< 0.001	113091	PTH2
0.264393866	< 0.001	11249	NXPH2
0.264387578	< 0.001	116447	TOP1MT
0.26436156	< 0.001	7117	TMSB4XP8
0.264314711	< 0.001	10974	C10orf116
0.264292389	< 0.001	29998	GLTSCR1
0.264236063	< 0.001	11259	FILIP1L
0.264208525	< 0.001	27189	IL17C
0.26411894	< 0.001	54478	FAM64A
0.264117748	< 0.001	400	ARL1
0.264037818	< 0.001	2299	FOXI1
0.264012516	< 0.001	22999	RIMS1
0.263992935	< 0.001	53	ACP2
0.263961464	< 0.001	9858	KIAA0649
0.26390925	< 0.001	80195	RP11-369J21.1
0.263902754	< 0.001	65084	TMEM135
0.263901919	< 0.001	9392	TGFBRAP1
0.26383701	< 0.001	55026	FAM70A
0.263779372	< 0.001	8854	ALDH1A2
0.263758868	< 0.001	285943	AC004080.12
0.263728499	< 0.001	10527	IPO7
0.263703942	< 0.001	30844	EHD4

region-wise, subcortex					
Spearman's corr coef	p-val	p-val, SA	p-val, SA (BH- correction)	GenID	Gene
0.358301163	0.032530826	0.041	1	10211	FLOT1
0.358301163	0.032530826	0.044	1	10488	CREB3
0.358301163	0.032530826	0.053	1	10559	SLC35A1
0.358301163	0.032530826	0.048	1	23373	CRTC1
0.35804376	0.032662522	0.04	1	4665	NAB2
0.35804376	0.032662522	0.044	1	158326	FREM1
0.35804376	0.032662522	0.047	1	161835	FSIP1
0.35804376	0.032662522	0.047	1	245940	AC144830.1
0.357786357	0.032794688	0.051	1	1600	DAB1
0.357786357	0.032794688	0.038	1	200424	TET3
0.357528955	0.032927267	0.045	1	3798	KIF5A
0.357271552	0.033060376	0.064	1	3875	KRT18P19
0.357014149	0.033193842	0.047	1	8646	CHRD
0.357014149	0.033193842	0.048	1	8816	DCAF5
0.357014149	0.033193842	0.053	1	155051	CRYGN
0.356756777	0.033327751	0.052	1	3087	HHEX
0.356756777	0.033327751	0.043	1	10408	MYCNOS
0.356756777	0.033327751	0.05	1	79666	PLEKHF2
0.356756777	0.033327751	0.052	1	140885	SIRPA
0.356756777	0.033327751	0.051	1	222967	RSPH10B
0.356756777	0.033327751	0.045	1	440173	LOC440173
0.356499344	0.033462133	0.044	1	6945	MLX
0.356499344	0.033462133	0.049	1	9392	TGFBRAP1
0.356241941	0.033596937	0.045	1	3622	ING2
0.356241941	0.033596937	0.054	1	10001	MED6
0.356241941	0.033596937	0.051	1	29118	DDX25
0.356241941	0.033596937	0.05	1	58489	FAM108C1
0.355984539	0.033732273	0.048	1	165215	FAM171B
0.355984539	0.033732273	0.039	1	222029	DKFZp434L192
0.355984539	0.033732273	0.057	1	401474	SAMD12
0.355727136	0.03386797	0.051	1	4760	NEUROD1
0.355727136	0.03386797	0.051	1	8905	AP1S2
0.355727136	0.03386797	0.046	1	79986	ZNF702P
0.355469763	0.034004118	0.054	1	1416	CRYBB2P1
0.355469763	0.034004118	0.058	1	22823	MTF2
0.355469763	0.034004118	0.048	1	56729	RETN
0.355212331	0.034140743	0.044	1	627	BDNF
0.355212331	0.034140743	0.038	1	4253	CTAGE5
0.355212331	0.034140743	0.054	1	23163	GGA3
0.354954958	0.034277856	0.064	1	3479	IGF1
0.354954958	0.034277856	0.049	1	10908	PNPLA6
0.354697526	0.03441539	0.054	1	2778	GNAS
0.354697526	0.03441539	0.055	1	57731	SPTBN4
0.354697526	0.03441539	0.042	1	113174	SAAL1
0.354440153	0.034553345	0.05	1	324	APC
0.354440153	0.034553345	0.053	1	51649	MRPS23
0.35418275	0.034691758	0.059	1	4192	MDK
0.35418275	0.034691758	0.048	1	11137	PWP1
0.35418275	0.034691758	0.041	1	57325	CSRP2BP
0.35418275	0.034691758	0.061	1	65268	WNK2
0.353925347	0.034830723	0.047	1	79762	C1orf115
0.353667945	0.034970045	0.062	1	778	CACNA1F
0.353667945	0.034970045	0.057	1	51122	COMMD2
0.353667945	0.034970045	0.051	1	55319	C4orf43
0.353667945	0.034970045	0.054	1	122553	TRAPPC6B
0.353667945	0.034970045	0.046	1	148930	KNCN
0.353410542	0.035109863	0.057	1	57622	LRFN1
0.353410542	0.035109863	0.059	1	116987	AGAP1
0.353410542	0.035109863	0.055	1	285943	AC004080.12
0.353410542	0.035109863	0.054	1	728224	KRTAP4-8
0.353153139	0.035250109	0.058	1	7267	TTC3
0.353153139	0.035250109	0.057	1	9978	RBX1
0.353153139	0.035250109	0.048	1	57333	RCN3
0.352895766	0.035390813	0.046	1	8239	USP9X
0.352895766	0.035390813	0.051	1	8814	CDKL1
0.352895766	0.035390813	0.044	1	9040	UBE2M
0.352895766	0.035390813	0.053	1	26532	OR10H3
0.352895766	0.035390813	0.052	1	63932	CXorf56
0.352895766	0.035390813	0.03	1	84310	C7orf50
0.352895766	0.035390813	0.04	1	144108	SPTY2D1
0.352638334	0.035532076	0.059	1	1662	DDX10
0.352638334	0.035532076	0.049	1	54914	KIAA1797
0.352638334	0.035532076	0.051	1	55683	KIAA1310
0.352638334	0.035532076	0.05	1	64478	CSMD1
0.352638334	0.035532076	0.046	1	220832	FABP5P3
0.352638334	0.035532076	0.044	1	391191	OR2AK2
0.352638334	0.035532076	0.044	1	100506084	AC005829.1
0.352380961	0.035673708	0.043	1	4493	MT1E
0.352123529	0.035815831	0.066	1	1123	CHN1

voxel-wise, subcortex			
Spearman's corr coef	p-val	GenID	Gene
0.263650864	< 0.001	51719	CAB39
0.263643444	< 0.001	343702	XKR7
0.263640553	< 0.001	146177	VWA3A
0.263639331	< 0.001	391196	OR2M7
0.263617933	< 0.001	119678	OR52E2
0.26353845	< 0.001	3851	KRT4
0.263465077	< 0.001	2835	GPR12
0.263378382	< 0.001	26189	OR1A2
0.263374269	< 0.001	535	ATP6V0A1
0.263354301	< 0.001	55837	EAPP
0.263326526	< 0.001	114112	TXNRD3
0.263288021	< 0.001	57558	USP35
0.263275266	< 0.001	317701	VN1R2
0.263253719	< 0.001	11321	GPN1
0.263227671	< 0.001	57492	ARID1B
0.263100117	< 0.001	84632	AFAP1L2
0.263095349	< 0.001	4082	MARCKS
0.262986183	< 0.001	269	AMHR2
0.262923032	< 0.001	200424	TET3
0.262885749	< 0.001	167555	FAM151B
0.262876272	< 0.001	57476	GRAMD1B
0.26286611	< 0.001	7695	ZNF136
0.262847334	< 0.001	29789	OLA1
0.262827456	< 0.001	8530	CST7
0.262792766	< 0.001	3569	IL6
0.262593627	< 0.001	89796	NAV1
0.26254198	< 0.001	54534	MRPL50
0.262540311	< 0.001	219770	GJD4
0.262380451	< 0.001	1456	CSNK1G3
0.2622419	< 0.001	259232	NALCN
0.262175739	< 0.001	27120	DKKL1
0.262168199	< 0.001	100131360	LOC100131360
0.26214397	< 0.001	29115	SAP30BP
0.262132257	< 0.001	284904	SEC14L4
0.262064248	< 0.001	118813	ZFYVE27
0.262038112	< 0.001	4108	MAGEA9
0.261996299	< 0.001	353143	LCE3B
0.261958003	< 0.001	64377	CHST8
0.261942446	< 0.001	4605	MYBL2
0.261857331	< 0.001	26002	MOXD1
0.261823803	< 0.001	3482	IGF2R
0.261794239	< 0.001	3203	HOXA6
0.261698633	< 0.001	284021	MILR1
0.261658937	< 0.001	257194	NEGR1
0.261633843	< 0.001	10463	SLC30A9
0.261604369	< 0.001	100129583	FAM47E
0.261563987	< 0.001	7767	ZNF224
0.261516929	< 0.001	84665	MYPN
0.261449099	< 0.001	51095	TRNT1
0.261444688	< 0.001	10019	SH2B3
0.261381954	< 0.001	6329	SCN4A
0.261342853	< 0.001	90637	ZFAND2A
0.26127243	< 0.001	220032	GDPD4
0.261242062	< 0.001	4597	MVD
0.261206061	< 0.001	100129935	LOC100129935
0.261144221	< 0.001	55010	C12orf48
0.261095226	< 0.001	1837	DTNA
0.261073053	< 0.001	1457	CSNK2A1
0.26097855	< 0.001	84197	AC113191.1
0.260961771	< 0.001	10000	AKT3
0.260823965	< 0.001	27199	OXGR1
0.260823429	< 0.001	5478	PPIA
0.260791093	< 0.001	149018	LELP1
0.260738313	< 0.001	340075	ARSI
0.260551512	< 0.001	128387	TATDN3
0.26054889	< 0.001	29070	CCDC113
0.260537952	< 0.001	139716	GAB3
0.260534227	< 0.001	284069	FAM171A2
0.260517687	< 0.001	8216	LZTR1
0.26051569	< 0.001	284	ANGPT1
0.260487705	< 0.001	94030	LRRC4B
0.260443926	< 0.001	51085	MLXIPL
0.260434628	< 0.001	23677	SH3BP4
0.260298997	< 0.001	53616	ADAM22
0.260296583	< 0.001	22992	KDM2A
0.260260731	< 0.001	55577	NAGK
0.260240078	< 0.001	1444	CSHL1
0.260217011	< 0.001	260293	CYP4X1
0.260156184	< 0.001	642943	LOC642943

region-wise, subcortex					
Spearman's corr coef	p-val	p-val, SA	p-val, SA (BH- correction)	GenID	Gene
0.352123529	0.035815831	0.045	1	7763	ZFAND5
0.352123529	0.035815831	0.051	1	10572	SIVA1
0.352123529	0.035815831	0.055	1	50509	COL5A3
0.352123529	0.035815831	0.043	1	80331	DNAJC5
0.352123529	0.035815831	0.041	1	84520	C14orf142
0.351866156	0.035958394	0.049	1	5662	PSD
0.351866156	0.035958394	0.052	1	50861	STMN3
0.351866156	0.035958394	0.056	1	134147	CMBL
0.351866156	0.035958394	0.061	1	157570	ESCO2
0.351351351	0.036245011	0.059	1	154313	C6orf165
0.351351351	0.036245011	0.054	1	353116	RILPL1
0.351093948	0.036388971	0.054	1	92154	MTSS1L
0.350836545	0.036533434	0.054	1	904	CCNT1
0.350836545	0.036533434	0.058	1	9070	ASH2L
0.350836545	0.036533434	0.044	1	10221	TRIB1
0.350836545	0.036533434	0.054	1	57496	MKL2
0.350579143	0.036678404	0.042	1	23107	MRPS27
0.350579143	0.036678404	0.032	1	23480	SEC61G
0.350579143	0.036678404	0.048	1	83546	RTBDN
0.35032177	0.036823779	0.05	1	3140	MR1
0.35032177	0.036823779	0.052	1	11069	RAPGEF4
0.35032177	0.036823779	0.061	1	23321	TRIM2
0.35032177	0.036823779	0.049	1	57834	CYP4F11
0.35032177	0.036823779	0.051	1	205428	C3orf58
0.35032177	0.036823779	0.062	1	404550	C16orf74
0.35032177	0.036823779	0.047	1	729991	MEF2BNB
0.350064337	0.036969658	0.046	1	644192	RP11-327J17.3
0.349806964	0.03711598	0.055	1	1837	DTNA
0.349806964	0.03711598	0.063	1	8748	ADAM20
0.349806964	0.03711598	0.056	1	133746	JMY
0.349549532	0.037262797	0.056	1	2210	FCGR1B
0.349549532	0.037262797	0.051	1	3749	KCNC4
0.349549532	0.037262797	0.056	1	27352	SGSM3
0.349549532	0.037262797	0.043	1	51222	ZNF219
0.349549532	0.037262797	0.048	1	646627	LOC646627
0.349292129	0.037410151	0.065	1	6809	STX3
0.349292129	0.037410151	0.048	1	23759	PPIL2
0.349292129	0.037410151	0.047	1	54847	SIDT1
0.349292129	0.037410151	0.063	1	375190	C2orf84
0.349034756	0.037557904	0.052	1	821	CANX
0.349034756	0.037557904	0.043	1	5902	RANBP1
0.349034756	0.037557904	0.063	1	23466	CBX6
0.349034756	0.037557904	0.045	1	60685	ZFAND3
0.349034756	0.037557904	0.066	1	113457	TUBA3D
0.349034756	0.037557904	0.059	1	114796	AC093734.11
0.348777324	0.037706167	0.059	1	1678	TIMM8A
0.348777324	0.037706167	0.054	1	6713	SQLE
0.348777324	0.037706167	0.045	1	64755	C16orf58
0.348519951	0.037854876	0.059	1	1400	CRMP1
0.348519951	0.037854876	0.061	1	5493	PPL
0.348519951	0.037854876	0.048	1	9506	PAGE4
0.348519951	0.037854876	0.055	1	10126	DNAL4
0.348519951	0.037854876	0.051	1	26289	AK5
0.348519951	0.037854876	0.044	1	219402	MTIF3
0.348262519	0.038004167	0.058	1	10059	DNM1L
0.348262519	0.038004167	0.053	1	55813	UTP6
0.348005146	0.038153838	0.063	1	4435	CITED1
0.348005146	0.038153838	0.076	1	286077	FAM83H
0.347747743	0.038303997	0.057	1	28958	CCDC56
0.347747743	0.038303997	0.048	1	342850	ANKRD62
0.34749034	0.038454667	0.063	1	310	ANXA7
0.34749034	0.038454667	0.059	1	3425	IDUA
0.34749034	0.038454667	0.058	1	6457	SH3GL3
0.34749034	0.038454667	0.066	1	8997	KALRN
0.34749034	0.038454667	0.054	1	10855	HPSE
0.34749034	0.038454667	0.053	1	51529	ANAPC11
0.34749034	0.038454667	0.05	1	81616	ACSBG2
0.347232938	0.038605861	0.05	1	80028	FBXL18
0.347232938	0.038605861	0.061	1	114926	C8orf40
0.346975535	0.038757507	0.058	1	11214	AKAP13
0.346718132	0.038909607	0.059	1	320	APBA1
0.346718132	0.038909607	0.043	1	84320	ACBD6
0.346718132	0.038909607	0.061	1	159989	CCDC67
0.34646076	0.039062198	0.063	1	4142	MAS1
0.34646076	0.039062198	0.056	1	5887	RAD23B
0.34646076	0.039062198	0.058	1	644054	FAM25C
0.34646076	0.039062198	0.056	1	100129492	LOC100129492
0.346203327	0.039215293	0.054	1	1824	DSC2
0.346203327	0.039215293	0.05	1	4712	NDUFB6

voxel-wise, subcortex			
Spearman's corr coef	p-val	GenID	Gene
0.260114849	< 0.001	146853	C17orf50
0.259953678	< 0.001	126526	C19orf47
0.259938389	< 0.001	4105	MAGEA6
0.259896785	< 0.001	493856	CISD2
0.259883344	< 0.001	4199	ME1
0.259826362	< 0.001	64792	RABL5
0.259763449	< 0.001	8509	NDST2
0.259710193	< 0.001	54065	C21orf51
0.259633154	< 0.001	647309	RP11-332P22.1
0.25962168	< 0.001	9276	COPB2
0.259488642	< 0.001	548593	GIYD2
0.259409249	< 0.001	5861	RAB1A
0.259405494	< 0.001	79135	APOO
0.259395331	< 0.001	144165	PRICKLE1
0.259359092	< 0.001	51168	MYO15A
0.259357721	< 0.001	64375	IKZF4
0.259207487	< 0.001	55234	SMU1
0.259202242	< 0.001	10180	RBM6
0.259176254	< 0.001	337971	KRTAP19-4
0.259024888	< 0.001	153396	TMEM161B
0.259019971	< 0.001	23461	ABCA5
0.258989483	< 0.001	90271	LINC00263
0.258944184	< 0.001	84435	GPR123
0.258871734	< 0.001	115290	FBXO17
0.258810401	< 0.001	27012	KCNV1
0.258732021	< 0.001	6528	SLC5A5
0.258683443	< 0.001	4779	NFE2L1
0.258667588	< 0.001	1800	DPEP1
0.258666337	< 0.001	51203	NUSAP1
0.258644402	< 0.001	84134	TOMM40L
0.258638233	< 0.001	7453	WARS
0.258633316	< 0.001	56203	LMOD3
0.258626014	< 0.001	284656	RP4-783C10.1
0.25854969	< 0.001	400236	RP11-33N16.1
0.258547783	< 0.001	80856	KIAA1715
0.258526146	< 0.001	8681	JMJD7-PLA2G4B
0.258506745	< 0.001	150786	WTH3DI
0.258430481	< 0.001	54959	ODAM
0.258427739	< 0.001	57231	SNX14
0.25839442	< 0.001	3149	HMGB3
0.258379102	< 0.001	147	ADRA1B
0.258290917	< 0.001	64359	NXN
0.258283436	< 0.001	222950	C7orf51
0.258243889	< 0.001	146198	ZFP90
0.258223683	< 0.001	25822	DNAJB5
0.258157969	< 0.001	23708	GSPT2
0.258155704	< 0.001	64240	ABCG5
0.258154809	< 0.001	246704	LINC00315
0.25813207	< 0.001	23491	CES3
0.258106023	< 0.001	7727	ZNF174
0.25806585	< 0.001	9455	HOMER2
0.258031875	< 0.001	57282	SLC4A10
0.258014649	< 0.001	23316	CUX2
0.257869333	< 0.001	57705	WDFY4
0.257797331	< 0.001	388566	ZNF470
0.257777542	< 0.001	729627	AC130416.2
0.257774472	< 0.001	83732	RIOK1
0.25773567	< 0.001	3606	IL18
0.257693261	< 0.001	2677	GGCX
0.257626027	< 0.001	10255	HCG9
0.257575661	< 0.001	9159	PCSK7
0.257475793	< 0.001	5864	RAB3A
0.257467151	< 0.001	636	BICD1
0.257466167	< 0.001	23764	MAFF
0.257458419	< 0.001	116379	IL22RA2
0.257341176	< 0.001	220164	DOK6
0.257316619	< 0.001	22826	DNAJC8
0.257311046	< 0.001	89874	SLC25A21
0.257089704	< 0.001	81501	TM7SF4
0.256896526	< 0.001	91966	CXorf40A
0.256747901	< 0.001	9437	NCR1
0.25669989	< 0.001	4747	NEFL
0.256672174	< 0.001	51260	CXorf26
0.256609887	< 0.001	54922	RASIP1
0.256560445	< 0.001	64577	ALDH8A1
0.256471425	< 0.001	124997	WDR81
0.256389886	< 0.001	25829	TMEM184B
0.256296098	< 0.001	727957	HEATR7A
0.256189436	< 0.001	5203	PFDN4

region-wise, subcortex					
Spearman's corr coef	p-val	p-val, SA	p-val, SA (BH- correction)	GenID	Gene
0.346203327	0.039215293	0.054	1	27239	GPR162
0.345945954	0.039368946	0.046	1	8754	ADAM9
0.345945954	0.039368946	0.065	1	170691	ADAMTS17
0.345945954	0.039368946	0.051	1	284677	C1orf204
0.345688522	0.039523024	0.046	1	3933	LCN1
0.345688522	0.039523024	0.048	1	55803	ADAP2
0.345688522	0.039523024	0.061	1	80195	RP11-369J21.1
0.345431149	0.039677598	0.054	1	4852	NPY
0.345431149	0.039677598	0.049	1	9024	BRSK2
0.345431149	0.039677598	0.047	1	27344	PCSK1N
0.345431149	0.039677598	0.054	1	728591	C13orf38
0.345173746	0.039832648	0.056	1	9750	FAM65B
0.345173746	0.039832648	0.051	1	50650	ARHGEF3
0.344916344	0.039988298	0.056	1	54986	ULK4
0.344916344	0.039988298	0.057	1	83930	STARD3NL
0.344916344	0.039988298	0.063	1	340156	MYLK4
0.344658941	0.040144343	0.063	1	8569	MKNK1
0.344658941	0.040144343	0.051	1	27341	RRP7A
0.344658941	0.040144343	0.054	1	57600	FNIP2
0.344401538	0.040300898	0.056	1	138255	C9orf135
0.344401538	0.040300898	0.059	1	654434	LINC00338
0.344144136	0.040457953	0.06	1	5097	PCDH1
0.343886763	0.040615566	0.04	1	2515	ADAM2
0.343886763	0.040615566	0.044	1	26974	ZNF285
0.34362933	0.040773641	0.05	1	5340	PLG
0.343371958	0.040932182	0.07	1	5874	RAB27B
0.343371958	0.040932182	0.077	1	7076	TIMP1
0.343371958	0.040932182	0.057	1	51066	C3orf32
0.343371958	0.040932182	0.067	1	84449	ZNF333
0.343114525	0.041091245	0.073	1	127254	C1orf173
0.342857152	0.041250799	0.052	1	23641	LDOC1
0.342857152	0.041250799	0.054	1	23764	MAFF
0.342857152	0.041250799	0.062	1	80271	ITPKC
0.34259975	0.041410945	0.068	1	3899	AFF3
0.34259975	0.041410945	0.051	1	90134	KCNH7
0.34259975	0.041410945	0.061	1	170850	KCNG3
0.34259975	0.041410945	0.068	1	219749	ZNF25
0.342342317	0.041571528	0.062	1	1043	CD52
0.342342317	0.041571528	0.06	1	57393	TMEM27
0.342342317	0.041571528	0.071	1	169166	SNX31
0.342084944	0.041732617	0.058	1	3927	LASP1
0.342084944	0.041732617	0.05	1	5393	EXOSC9
0.342084944	0.041732617	0.057	1	11151	CORO1A
0.342084944	0.041732617	0.055	1	118472	ZNF511
0.342084944	0.041732617	0.071	1	338811	FAM19A2
0.341827512	0.041894216	0.064	1	9920	KBTBD11
0.341827512	0.041894216	0.065	1	10675	CSPG5
0.341827512	0.041894216	0.063	1	26286	ARFGAP3
0.341827512	0.041894216	0.057	1	91584	PLXNA4
0.341570139	0.042056397	0.061	1	23339	VPS39
0.341570139	0.042056397	0.063	1	57630	SH3RF1
0.341570139	0.042056397	0.069	1	84314	TMEM107
0.341312736	0.042219002	0.055	1	4145	MATK
0.341312736	0.042219002	0.052	1	125988	C19orf70
0.341055334	0.042382136	0.063	1	1490	CTGF
0.341055334	0.042382136	0.057	1	5786	PTPRA
0.340797931	0.042545784	0.063	1	55336	FBXL8
0.340797931	0.042545784	0.063	1	81557	MAGED4B
0.340540528	0.042710006	0.062	1	2267	FGL1
0.340540528	0.042710006	0.075	1	5330	PLCB2
0.340540528	0.042710006	0.079	1	23024	PDZRN3
0.340540528	0.042710006	0.049	1	55505	NOP10
0.340283126	0.042874705	0.06	1	6019	RLN2
0.340283126	0.042874705	0.059	1	51622	C7orf28A
0.340025753	0.043039881	0.047	1	100288870	ERLEC1P1
0.33976832	0.043205597	0.051	1	5361	PLXNA1
0.33976832	0.043205597	0.072	1	8453	CUL2
0.33976832	0.043205597	0.05	1	55352	C17orf79
0.339510947	0.043371815	0.05	1	57007	CXCR7
0.339510947	0.043371815	0.059	1	79898	ZNF613
0.339253515	0.043538649	0.075	1	6867	TACC1
0.339253515	0.043538649	0.067	1	22953	P2RX2
0.339253515	0.043538649	0.058	1	130026	ICA1L
0.339253515	0.043538649	0.054	1	401548	SNX30
0.339253515	0.043538649	0.069	1	646719	LOC646719
0.338996142	0.043705929	0.064	1	85369	FAM40A
0.338738739	0.043873727	0.054	1	5478	PPIA
0.338738739	0.043873727	0.076	1	8927	BSN
0.338738739	0.043873727	0.048	1	55122	AKIRIN2

voxel-wise, subcortex			
Spearman's corr coef	p-val	GenID	Gene
0.256179273	< 0.001	83988	NCALD
0.256170839	< 0.001	8929	PHOX2B
0.256139725	< 0.001	79050	NOC4L
0.256106973	< 0.001	83468	GLT8D2
0.256085753	< 0.001	27232	GNMT
0.256055593	< 0.001	23368	PPP1R13B
0.256027579	< 0.001	100130311	AC233723.1
0.255961031	< 0.001	51289	RXFP3
0.255677074	< 0.001	11283	CYP4F8
0.255616665	< 0.001	259173	ALS2CL
0.255534232	< 0.001	122509	IFI27L1
0.255442291	< 0.001	10800	CYSLTR1
0.255250663	< 0.001	7092	TLL1
0.255235255	< 0.001	78999	LRFN4
0.25517568	< 0.001	229	ALDOB
0.255169839	< 0.001	353355	ZNF233
0.255144387	< 0.001	11094	C9orf7
0.25511995	< 0.001	55586	MIOX
0.255025327	< 0.001	79078	C1orf50
0.25489229	< 0.001	8315	BRAP
0.254882216	< 0.001	4952	OCRL
0.2548154	< 0.001	5671	PSG3
0.254806161	< 0.001	6752	SSTR2
0.254803002	< 0.001	55647	RAB20
0.254784286	< 0.001	83660	TLN2
0.254656136	< 0.001	9587	MAD2L1BP
0.25463298	< 0.001	9827	RGP1
0.254620165	< 0.001	440044	SLC22A20
0.254603714	< 0.001	4591	TRIM37
0.254573941	< 0.001	51438	MAGEC2
0.254535884	< 0.001	652276	AC141586.5
0.254525602	< 0.001	54675	CRLS1
0.254514486	< 0.001	3087	HHEX
0.254412889	< 0.001	54762	GRAMD1C
0.25438121	< 0.001	54442	KCTD5
0.254360735	< 0.001	2867	FFAR2
0.254196644	< 0.001	26012	NELF
0.25414142	< 0.001	8507	ENC1
0.254133731	< 0.001	8525	DGKZ
0.254124522	< 0.001	10253	SPRY2
0.254056633	< 0.001	148268	ZNF570
0.254055262	< 0.001	7204	TRIO
0.25402832	< 0.001	1404	HAPLN1
0.253967315	< 0.001	9545	RAB3D
0.253948271	< 0.001	5932	RBBP8
0.253811479	< 0.001	441089	CRSP8P
0.253700882	< 0.001	124773	C17orf64
0.253656387	< 0.001	7772	ZNF229
0.253498018	< 0.001	54431	DNAJC10
0.253492713	< 0.001	56171	DNAH7
0.253478259	< 0.001	2071	ERCC3
0.25342375	< 0.001	7582	ZNF33B
0.253340542	< 0.001	25843	MOBKL3
0.253334612	< 0.001	10124	ARL4A
0.253315836	< 0.001	10605	PAIP1
0.253274798	< 0.001	496	ATP4B
0.253229588	< 0.001	8742	TNFSF12
0.253219515	< 0.001	92552	ATXN3L
0.253164768	< 0.001	64432	MRPS25
0.253091872	< 0.001	201294	UNC13D
0.25308767	< 0.001	23567	ZNF346
0.25304088	< 0.001	115353	LRRC42
0.252860308	< 0.001	23594	ORC6L
0.252800822	< 0.001	10936	GPR75
0.252702743	< 0.001	954	ENTPD2
0.252686858	< 0.001	90273	CEACAM21
0.252661645	< 0.001	120400	FAM55A
0.252622694	< 0.001	9139	CBFA2T2
0.252598494	< 0.001	340665	CYP26C1
0.252510875	< 0.001	283130	SLC25A45
0.252496868	< 0.001	64711	HS3ST6
0.25236699	< 0.001	286359	LOC286359
0.252350092	< 0.001	219927	MRPL21
0.252293587	< 0.001	7001	PRDX2
0.252250314	< 0.001	222553	SLC35F1
0.252193511	< 0.001	8454	CUL1
0.252193004	< 0.001	4109	MAGEA10
0.2520096	< 0.001	400765	MIR2682
0.251936227	< 0.001	341416	OR6C2

region-wise, subcortex					
Spearman's corr coef	p-val	p-val, SA	p-val, SA (BH- correction)	GenID	Gene
0.338738739	0.043873727	0.056	1	55437	STRADB
0.338738739	0.043873727	0.056	1	79586	CHPF
0.338738739	0.043873727	0.066	1	83874	TBC1D10A
0.338738739	0.043873727	0.067	1	117166	WFIKKN1
0.338738739	0.043873727	0.06	1	285596	FAM153A
0.338481337	0.044042055	0.057	1	4216	MAP3K4
0.338481337	0.044042055	0.055	1	27245	AHDC1
0.338481337	0.044042055	0.06	1	55777	MBD5
0.338481337	0.044042055	0.068	1	65263	PYCRL
0.338481337	0.044042055	0.056	1	393046	OR2A5
0.338223934	0.044210982	0.061	1	10219	KLRG1
0.338223934	0.044210982	0.06	1	10525	HYOU1
0.338223934	0.044210982	0.058	1	25994	HIGD1A
0.338223934	0.044210982	0.073	1	79026	AHNAK
0.338223934	0.044210982	0.06	1	221960	CCZ1B
0.337966532	0.044380344	0.074	1	8454	CUL1
0.337966532	0.044380344	0.063	1	23219	FBXO28
0.337966532	0.044380344	0.078	1	27101	CACYBP
0.337966532	0.044380344	0.062	1	123811	C16orf63
0.337966532	0.044380344	0.054	1	283951	C16orf91
0.337709129	0.044550251	0.066	1	1262	CNGA4
0.337709129	0.044550251	0.065	1	5591	PRKDC
0.337709129	0.044550251	0.065	1	5625	PRODH
0.337709129	0.044550251	0.068	1	10081	PDCD7
0.337709129	0.044550251	0.056	1	221424	C6orf154
0.337451756	0.044720683	0.047	1	3851	KRT4
0.337451756	0.044720683	0.07	1	7224	TRPC5
0.337194324	0.044891726	0.067	1	8818	DPM2
0.337194324	0.044891726	0.069	1	79902	NUP85
0.336936951	0.045063205	0.066	1	486	FXYD2
0.336936951	0.045063205	0.059	1	55284	UBE2W
0.336936951	0.045063205	0.071	1	388387	AC100793.3
0.336679518	0.045235235	0.058	1	5164	PDK2
0.336679518	0.045235235	0.065	1	9774	BCLAF1
0.336422145	0.045407802	0.068	1	5801	PTPRR
0.336422145	0.045407802	0.06	1	7874	USP7
0.336164743	0.045580897	0.073	1	401237	LINC00340
0.33590734	0.045754611	0.06	1	1	A1BG
0.335649937	0.045928765	0.068	1	3275	PRMT2
0.335649937	0.045928765	0.075	1	8777	MPDZ
0.335649937	0.045928765	0.055	1	55082	ARGLU1
0.335649937	0.045928765	0.072	1	57520	HECW2
0.335649937	0.045928765	0.065	1	85019	C18orf45
0.335649937	0.045928765	0.062	1	254863	C17orf61
0.335392505	0.046103474	0.055	1	8407	TAGLN2
0.334877729	0.046454594	0.066	1	2483	FRG1
0.334877729	0.046454594	0.058	1	140707	BRI3BP
0.334877729	0.046454594	0.064	1	254887	ZDHHC23
0.334620327	0.046630908	0.081	1	1803	DPP4
0.334620327	0.046630908	0.058	1	91752	ZNF804A
0.334362924	0.046807781	0.06	1	5929	RBBP5
0.334362924	0.046807781	0.061	1	10044	SH2D3C
0.334362924	0.046807781	0.073	1	54674	LRRN3
0.334362924	0.046807781	0.069	1	57128	LYRM4
0.334362924	0.046807781	0.063	1	116154	PHACTR3
0.334362924	0.046807781	0.053	1	343263	MYBPHL
0.334105521	0.046985202	0.067	1	1443	CSH2
0.333848119	0.04716317	0.071	1	10801	SEPTIN9
0.333848119	0.04716317	0.063	1	23089	PEG10
0.333848119	0.04716317	0.073	1	28316	CDH20
0.333848119	0.04716317	0.071	1	29978	UBQLN2
0.333848119	0.04716317	0.054	1	54927	CHCHD3
0.333848119	0.04716317	0.072	1	129868	TRIM43
0.333590746	0.047341757	0.069	1	887	CCKBR
0.333590746	0.047341757	0.063	1	9657	IQCB1
0.333590746	0.047341757	0.065	1	150864	FAM117B
0.333333313	0.047520798	0.08	1	79837	PIP4K2C
0.333075941	0.047700409	0.067	1	5218	PFTK1
0.333075941	0.047700409	0.06	1	6416	MAP2K4
0.333075941	0.047700409	0.07	1	26084	RP11-23D24.1
0.333075941	0.047700409	0.081	1	55337	C19orf66
0.333075941	0.047700409	0.065	1	92595	ZNF764
0.332818508	0.047880568	0.071	1	1650	DDOST
0.332818508	0.047880568	0.079	1	5892	RAD51L3
0.332818508	0.047880568	0.064	1	6041	RNASEL
0.332818508	0.047880568	0.071	1	7101	NR2E1
0.332818508	0.047880568	0.086	1	9956	HS3ST2
0.332818508	0.047880568	0.077	1	126731	C1orf96
0.332561135	0.04806136	0.056	1	1303	COL12A1

voxel-wise, subcortex			
Spearman's corr coef	p-val	GenID	Gene
0.251810253	< 0.001	126326	GIPC3
0.251785427	< 0.001	5610	EIF2AK2
0.251664609	< 0.001	440153	OR11H12
0.251663566	< 0.001	284612	SYPL2
0.251546949	< 0.001	84823	LMNB2
0.2515091	< 0.001	55280	CWF19L1
0.251450121	< 0.001	201255	LRRC45
0.251338303	< 0.001	939	CD27
0.251311809	< 0.001	57084	SLC17A6
0.251175255	< 0.001	10001	MED6
0.251081228	< 0.001	153733	CCDC112
0.251049131	< 0.001	6344	SCTR
0.250660777	< 0.001	3275	PRMT2
0.250573695	< 0.001	1870	E2F2
0.250557691	< 0.001	2739	GLO1
0.250286281	< 0.001	53829	P2RY13
0.250277728	< 0.001	64579	NDST4
0.250202805	< 0.001	64478	CSMD1
0.250194609	< 0.001	3084	NRG1
0.250171602	< 0.001	122830	NAA30
0.250040323	< 0.001	149837	LOC149837
0.250006199	< 0.001	5045	FURIN
0.250003546	< 0.001	3747	KCNC2
0.24998568	< 0.001	146206	RLTPR
0.24987559	< 0.001	259197	NCR3
0.249862626	< 0.001	5536	PPP5C
0.249828503	< 0.001	8128	ST8SIA2
0.249750674	< 0.001	27255	CNTN6
0.249682277	< 0.001	51651	PTRH2
0.249594659	< 0.001	3503	IGHG4
0.249552041	< 0.001	8835	SOCS2
0.249519065	< 0.001	9921	RNF10
0.249343738	< 0.001	8266	UBL4A
0.249305889	< 0.001	342897	NCCRP1
0.24926351	< 0.001	79798	ARMC5
0.249176532	< 0.001	2114	ETS2
0.249152616	< 0.001	4359	MPZ
0.24911125	< 0.001	55240	STEAP3
0.24910298	< 0.001	91584	PLXNA4
0.249101266	< 0.001	29035	C16orf72
0.249091834	< 0.001	114786	XKR4
0.249048427	< 0.001	5898	RALA
0.249032691	< 0.001	7317	UBA1
0.248947948	< 0.001	80231	CXorf21
0.248940229	< 0.001	4210	MEFV
0.248850569	< 0.001	10426	TUBGCP3
0.248822674	< 0.001	26532	OR10H3
0.24882035	< 0.001	93627	AC004053.1
0.248815656	< 0.001	8560	DEGS1
0.248791412	< 0.001	56681	SAR1A
0.248781115	< 0.001	90353	ATPBD3
0.248751715	< 0.001	899	CCNF
0.248740718	< 0.001	9689	BZW1
0.248610973	< 0.001	247	ALOX15B
0.24853529	< 0.001	10048	RANBP9
0.248461142	< 0.001	85364	ZCCHC3
0.248424321	< 0.001	56993	TOMM22
0.248410389	< 0.001	5798	PTPRN
0.248395994	< 0.001	6059	ABCE1
0.248251364	< 0.001	5290	PIK3CA
0.247966304	< 0.001	3270	HRC
0.247888163	< 0.001	359710	C20orf185
0.247876868	< 0.001	55181	C17orf71
0.247874558	< 0.001	55016	MARCHF1
0.247801334	< 0.001	27039	PKD2L2
0.247708321	< 0.001	23397	NCAPH
0.247622654	< 0.001	23158	TBC1D9
0.247546643	< 0.001	387882	C12ORF75
0.247488141	< 0.001	5743	PTGS2
0.247476071	< 0.001	83483	PLVAP
0.247475788	< 0.001	222068	TMED4
0.247397035	< 0.001	147199	SCGB1C1
0.247327805	< 0.001	27072	VPS41
0.247255176	< 0.001	90416	C15orf57
0.247250035	< 0.001	551	AVP
0.247197241	< 0.001	152518	NFXL1
0.247180015	< 0.001	55226	NAT10
0.247132137	< 0.001	284600	RP11-54O7.1
0.247100383	< 0.001	54965	PIGX

region-wise, subcortex					
Spearman's corr coef	p-val	p-val, SA	p-val, SA (BH- correction)	GenID	Gene
0.332561135	0.04806136	0.059	1	64711	HS3ST6
0.332561135	0.04806136	0.074	1	118471	PRAP1
0.332303733	0.048242621	0.061	1	25771	TBC1D22A
0.332303733	0.048242621	0.067	1	60626	RIC8A
0.332303733	0.048242621	0.058	1	339559	ZNF642
0.33204633	0.048424415	0.069	1	1240	CMKLR1
0.33204633	0.048424415	0.067	1	9107	MTMR6
0.33204633	0.048424415	0.063	1	10574	CCT7
0.33204633	0.048424415	0.072	1	11346	SYNPO
0.33204633	0.048424415	0.082	1	65217	PCDH15
0.33204633	0.048424415	0.072	1	646517	LOC646517
0.331788927	0.048606791	0.064	1	23396	PIP5K1C
0.331788927	0.048606791	0.072	1	29966	STRN3
0.331788927	0.048606791	0.062	1	51377	UCHL5
0.331788927	0.048606791	0.072	1	284013	VMO1
0.331531525	0.048789795	0.073	1	4753	NELL2
0.331531525	0.048789795	0.069	1	407738	FAM19A1
0.331274122	0.048973285	0.073	1	4018	LPA
0.331274122	0.048973285	0.073	1	9473	C1orf38
0.331274122	0.048973285	0.058	1	84766	EFCAB4B
0.331274122	0.048973285	0.067	1	153733	CCDC112
0.331274122	0.048973285	0.067	1	284417	TMEM150B
0.330759317	0.04934191	0.071	1	2132	EXT2
0.330759317	0.04934191	0.06	1	5680	PSG11
0.330759317	0.04934191	0.069	1	22974	TPX2
0.330759317	0.04934191	0.077	1	27010	TPK1
0.330759317	0.04934191	0.065	1	128611	ZNF831
0.330501944	0.049527071	0.071	1	157	ADRBK2
0.330501944	0.049527071	0.07	1	2556	GABRA3
0.330501944	0.049527071	0.062	1	645212	LOC645212
0.330501944	0.049527071	0.077	1	729515	TMEM242
0.330244511	0.049712881	0.073	1	9743	AP000751.3
0.330244511	0.049712881	0.055	1	25943	C20orf194
0.330244511	0.049712881	0.063	1	55359	STYK1
0.330244511	0.049712881	0.085	1	199882	LOC199882
0.329987139	0.049899168	0.069	1	23635	SSBP2
0.329729736	0.050086025	0.067	1	2021	ENDOG
0.329729736	0.050086025	0.079	1	27258	LSM3
0.329729736	0.050086025	0.068	1	28512	NKIRAS1
0.329729736	0.050086025	0.064	1	55530	SVOP
0.329729736	0.050086025	0.07	1	57449	PLEKHG5
0.329729736	0.050086025	0.064	1	124637	CYB5D1
0.329729736	0.050086025	0.077	1	145864	HAPLN3
0.329729736	0.050086025	0.068	1	728739	LOC728739
0.329472333	0.050273418	0.083	1	2039	EPB49
0.329472333	0.050273418	0.07	1	26960	NBEA
0.329472333	0.050273418	0.077	1	128387	TATDN3
0.329472333	0.050273418	0.051	1	348180	C16orf84
0.329214931	0.05046149	0.067	1	659	BMPR2
0.329214931	0.05046149	0.084	1	2867	FFAR2
0.329214931	0.05046149	0.074	1	3980	LIG3
0.329214931	0.05046149	0.076	1	5214	PFKP
0.329214931	0.05046149	0.077	1	83539	CHST9
0.328957528	0.050650042	0.068	1	1107	CHD3
0.328957528	0.050650042	0.086	1	3763	KCNJ6
0.328957528	0.050650042	0.068	1	5567	PRKACB
0.328957528	0.050650042	0.087	1	11250	GPR45
0.328957528	0.050650042	0.08	1	23094	SIPA1L3
0.328957528	0.050650042	0.065	1	51241	COX16
0.328957528	0.050650042	0.071	1	54769	DIRAS2
0.328957528	0.050650042	0.067	1	400456	CTD-2536I1.1
0.328700125	0.050839163	0.06	1	400120	C13orf36
0.328442723	0.051028855	0.074	1	3361	HTR5A
0.328442723	0.051028855	0.061	1	8976	WASL
0.328442723	0.051028855	0.076	1	9653	HS2ST1
0.328442723	0.051028855	0.068	1	22884	WDR37
0.328442723	0.051028855	0.064	1	23053	KIAA0913
0.328442723	0.051028855	0.093	1	57662	KIAA1543
0.32818532	0.051219184	0.073	1	2224	FDPS
0.32818532	0.051219184	0.076	1	79101	TAF1D
0.327927917	0.051410023	0.071	1	10094	ARPC3
0.327670515	0.051601432	0.065	1	552	AVPR1A
0.327670515	0.051601432	0.065	1	4209	MEF2D
0.327413112	0.051793419	0.09	1	5578	PRKCA
0.327413112	0.051793419	0.065	1	6634	SNRPD3
0.327413112	0.051793419	0.063	1	25854	FAM149A
0.327155739	0.05198596	0.065	1	3283	HSD3B1
0.327155739	0.05198596	0.081	1	10788	IQGAP2
0.327155739	0.05198596	0.07	1	114784	CSMD2

voxel-wise, subcortex			
Spearman's corr coef	p-val	GenID	Gene
0.247061744	< 0.001	285237	C3orf38
0.246905029	< 0.001	140685	ZBTB46
0.246834636	< 0.001	624	BDKRB2
0.24678719	< 0.001	140456	ASB11
0.246733502	< 0.001	6810	STX4
0.246706396	< 0.001	6093	ROCK1
0.246658847	< 0.001	142913	CFL1P1
0.246620581	< 0.001	5009	OTC
0.246536821	< 0.001	7257	TSNAX
0.246477947	< 0.001	5292	PIM1
0.246435136	< 0.001	10256	CNKSR1
0.246434942	< 0.001	79674	VEPH1
0.246350989	< 0.001	80832	APOL4
0.246309385	< 0.001	10154	PLXNC1
0.246221021	< 0.001	1140	CHRNB1
0.246187359	< 0.001	91948	RP11-82I10.1
0.246162474	< 0.001	55573	CDV3
0.246084377	< 0.001	60314	C12orf10
0.24603571	< 0.001	55127	HEATR1
0.245991692	< 0.001	339230	CCDC137
0.245970711	< 0.001	57560	IFT80
0.245871902	< 0.001	79683	ZDHHC14
0.2458653	< 0.001	122416	ANKRD9
0.245740265	< 0.001	54901	CDKAL1
0.245715514	< 0.001	11166	SOX21
0.245647073	< 0.001	60558	GUF1
0.245623067	< 0.001	8624	PSMG1
0.245601192	< 0.001	8871	SYNJ2
0.245561078	< 0.001	150921	TCF23
0.245558292	< 0.001	9338	TCEAL1
0.245534882	< 0.001	11171	STRAP
0.24551706	< 0.001	729830	FAM160A1
0.245516315	< 0.001	5870	RAB6A
0.245500669	< 0.001	55763	EXOC1
0.245369121	< 0.001	113174	SAAL1
0.245360851	< 0.001	60685	ZFAND3
0.245286286	< 0.001	10606	PAICS
0.245244682	< 0.001	56729	RETN
0.245212868	< 0.001	55770	EXOC2
0.245184824	< 0.001	2273	FHL1
0.245175868	< 0.001	11284	PNKP
0.245127618	< 0.001	29881	NPC1L1
0.245110169	< 0.001	6472	SHMT2
0.245082259	< 0.001	10077	TSPAN32
0.245014608	< 0.001	51003	MED31
0.244979784	< 0.001	29121	CLEC2D
0.244941607	< 0.001	2255	FGF10
0.244918868	< 0.001	9168	TMSB10
0.244907394	< 0.001	905	CCNT2
0.244880512	< 0.001	2175	FANCA
0.244827807	< 0.001	2932	GSK3B
0.24467203	< 0.001	143162	FRMPD2
0.244671702	< 0.001	781	CACNA2D1
0.244668081	< 0.001	23332	CLASP1
0.244514614	< 0.001	440132	LINC00545
0.244354561	< 0.001	2730	GCLM
0.244353637	< 0.001	149095	DCST1
0.244275481	< 0.001	3780	KCNN1
0.244247764	< 0.001	284613	CYB561D1
0.244233787	< 0.001	442213	C6orf138
0.244230628	< 0.001	347168	OR1J1
0.244226873	< 0.001	799	CALCR
0.244212657	< 0.001	8532	CPZ
0.244208619	< 0.001	94039	ZNF101
0.244201794	< 0.001	29933	GPR132
0.244131595	< 0.001	148266	ZNF569
0.244108468	< 0.001	1356	CP
0.244102284	< 0.001	142891	SAMD8
0.244078323	< 0.001	80108	ZFP2
0.244065747	< 0.001	94009	SERHL
0.244015694	< 0.001	100287016	DSTNP3
0.243992433	< 0.001	152065	C3orf22
0.243870452	< 0.001	7538	ZFP36
0.24379918	< 0.001	50650	ARHGEF3
0.243792072	< 0.001	125875	CLDND2
0.243737787	< 0.001	154743	C7orf60
0.243723124	< 0.001	197335	WDR90
0.243678123	< 0.001	29801	ZDHHC8
0.243673906	< 0.001	55593	OTUD5

region-wise, subcortex					
Spearman's corr coef	p-val	p-val, SA	p-val, SA (BH- correction)	GenID	Gene
0.327155739	0.05198596	0.078	1	353355	ZNF233
0.326898307	0.052179184	0.071	1	5251	PHEX
0.326898307	0.052179184	0.077	1	23237	ARC
0.326898307	0.052179184	0.097	1	374860	C21orf110
0.326640934	0.052372903	0.065	1	10242	KCNMB2
0.326640934	0.052372903	0.056	1	11092	C9orf9
0.326383501	0.052567199	0.074	1	23034	SAMD4A
0.326383501	0.052567199	0.079	1	51534	VTA1
0.326383501	0.052567199	0.081	1	54557	SGTB
0.326383501	0.052567199	0.064	1	219970	GLYATL2
0.326126128	0.05276208	0.066	1	9753	ZSCAN12
0.326126128	0.05276208	0.069	1	10295	BCKDK
0.326126128	0.05276208	0.074	1	10620	ARID3B
0.326126128	0.05276208	0.072	1	63925	ZNF335
0.326126128	0.05276208	0.075	1	121355	GTSF1
0.325868726	0.052957606	0.07	1	10911	UTS2
0.325868726	0.052957606	0.072	1	246213	SLC17A8
0.325611323	0.053153649	0.07	1	205	AK3L1
0.325611323	0.053153649	0.067	1	1973	EIF4A1
0.325611323	0.053153649	0.079	1	3598	IL13RA2
0.325611323	0.053153649	0.075	1	10570	DPYSL4
0.325611323	0.053153649	0.076	1	340543	TCEAL5
0.325611323	0.053153649	0.078	1	342541	LOC342541
0.325611323	0.053153649	0.068	1	100287728	LOC100287728
0.32535392	0.053350281	0.079	1	51310	SLC22A17
0.325096518	0.053547494	0.079	1	2853	GPR31
0.325096518	0.053547494	0.085	1	132789	GNPDA2
0.324839115	0.053745385	0.074	1	1284	COL4A2
0.324839115	0.053745385	0.066	1	9229	DLGAP1
0.324581742	0.053943753	0.088	1	10004	NAALADL1
0.324581742	0.053943753	0.079	1	246182	AKR7A2P1
0.324581742	0.053943753	0.078	1	387836	CLEC2A
0.32432431	0.054142732	0.068	1	1432	MAPK14
0.32432431	0.054142732	0.069	1	2931	GSK3A
0.32432431	0.054142732	0.069	1	162632	AC022596.6
0.32432431	0.054142732	0.074	1	200942	KLHDC8B
0.32432431	0.054142732	0.085	1	644353	ZCCHC18
0.324066937	0.054342303	0.083	1	22983	MAST1
0.324066937	0.054342303	0.071	1	56034	PDGFC
0.324066937	0.054342303	0.076	1	389762	FAM75D3
0.323809505	0.054542467	0.073	1	5710	PSMD4
0.323809505	0.054542467	0.064	1	221786	C7orf38
0.323552132	0.054743312	0.066	1	865	CBFB
0.323552132	0.054743312	0.079	1	4920	ROR2
0.323552132	0.054743312	0.074	1	8471	IRS4
0.323552132	0.054743312	0.072	1	27098	CLUL1
0.323552132	0.054743312	0.07	1	57037	ANKMY2
0.323552132	0.054743312	0.074	1	81579	PLA2G12A
0.323552132	0.054743312	0.074	1	168391	GALNTL5
0.323294729	0.054944638	0.085	1	5802	PTPRS
0.323294729	0.054944638	0.072	1	6360	CCL16
0.323294729	0.054944638	0.096	1	6861	SYT5
0.323294729	0.054944638	0.068	1	10691	GMEB1
0.323294729	0.054944638	0.062	1	56136	PCDHA13
0.323294729	0.054944638	0.072	1	84461	NEURL4
0.323294729	0.054944638	0.067	1	142940	TRUB1
0.323037326	0.055146586	0.078	1	5713	PSMD7
0.323037326	0.055146586	0.073	1	6607	SMN2
0.323037326	0.055146586	0.073	1	23418	CRB1
0.323037326	0.055146586	0.066	1	29933	GPR132
0.323037326	0.055146586	0.069	1	145553	MDP1
0.322779924	0.055349123	0.087	1	116238	TLCD1
0.322522521	0.055552356	0.068	1	23414	ZFPM2
0.322522521	0.055552356	0.066	1	26009	ZZZ3
0.322522521	0.055552356	0.079	1	284123	FAM27L
0.322265118	0.055756092	0.079	1	27075	TSPAN13
0.322265118	0.055756092	0.077	1	54714	CNGB3
0.322007746	0.055960406	0.079	1	7786	MAP3K12
0.322007746	0.055960406	0.064	1	55924	C1orf183
0.322007746	0.055960406	0.07	1	64926	RASAL3
0.322007746	0.055960406	0.063	1	116138	KLHDC3
0.322007746	0.055960406	0.082	1	200035	NUDT17
0.322007746	0.055960406	0.085	1	285759	RP11-544L8__B.4
0.321750313	0.056165349	0.082	1	2118	ETV4
0.321750313	0.056165349	0.072	1	5331	PLCB3
0.321750313	0.056165349	0.084	1	5991	RFX3
0.321750313	0.056165349	0.082	1	51019	CCDC53
0.321750313	0.056165349	0.091	1	51466	EVL
0.321750313	0.056165349	0.067	1	56947	MFF

voxel-wise, subcortex			
Spearman's corr coef	p-val	GenID	Gene
0.243647575	< 0.001	146167	SLC38A8
0.243551403	< 0.001	57665	RDH14
0.24352406	< 0.001	346157	ZNF391
0.243522063	< 0.001	55300	PI4K2B
0.243493035	< 0.001	54998	AURKAIP1
0.243473679	< 0.001	8578	SCARF1
0.2434728	< 0.001	611	OPN1SW
0.243422329	< 0.001	2627	GATA6
0.243382066	< 0.001	6360	CCL16
0.243371293	< 0.001	4893	NRAS
0.243353143	< 0.001	23253	ANKRD12
0.243350208	< 0.001	7038	TG
0.243336514	< 0.001	375484	C5orf25
0.243274108	< 0.001	23190	UBXN4
0.243085638	< 0.001	340533	KIAA2022
0.243082806	< 0.001	100288870	ERLEC1P1
0.243063956	< 0.001	10159	ATP6AP2
0.243047848	< 0.001	1364	CLDN4
0.243041113	< 0.001	442361	OR2A2
0.243008196	< 0.001	4111	MAGEA12
0.242988452	< 0.001	7084	TK2
0.242976248	< 0.001	100131315	LOC100131315
0.242930278	< 0.001	116028	C16orf75
0.242898613	< 0.001	135941	OR2A14
0.242883697	< 0.001	9947	MAGEC1
0.242847815	< 0.001	3748	KCNC3
0.242819861	< 0.001	285231	FBXW12
0.242772117	< 0.001	10817	FRS3
0.242759779	< 0.001	440073	IQSEC3
0.242748901	< 0.001	339803	AC016747.3
0.242715985	< 0.001	421	ARVCF
0.242633805	< 0.001	51193	ZNF639
0.242608964	< 0.001	4141	MARS
0.242595911	< 0.001	54984	PINX1
0.242584959	< 0.001	10870	HCST
0.242397591	< 0.001	4898	NRD1
0.242319629	< 0.001	9657	IQCB1
0.242286295	< 0.001	128826	C20orf166
0.242242187	< 0.001	152118	RP11-292E2.1
0.242222175	< 0.001	55620	STAP2
0.242218226	< 0.001	9125	RQCD1
0.242109895	< 0.001	5244	ABCB4
0.242096528	< 0.001	7031	TFF1
0.242006719	< 0.001	57689	LRRC4C
0.24195829	< 0.001	4809	NHP2L1
0.241880059	< 0.001	113130	CDCA5
0.241736293	< 0.001	6309	SC5DL
0.241669208	< 0.001	10014	HDAC5
0.241653129	< 0.001	50628	GEMIN4
0.241641715	< 0.001	5578	PRKCA
0.241630808	< 0.001	64417	C5orf28
0.24162513	< 0.001	338707	B4GALNT4
0.241583765	< 0.001	4915	NTRK2
0.241531208	< 0.001	1662	DDX10
0.241510674	< 0.001	84516	DCTN5
0.241432533	< 0.001	7136	TNNI2
0.241409495	< 0.001	344805	TMPRSS7
0.24126853	< 0.001	8445	DYRK2
0.241236448	< 0.001	6844	VAMP2
0.24122034	< 0.001	9278	ZBTB22
0.241214022	< 0.001	90843	TCEAL8
0.241191864	< 0.001	10471	PFDN6
0.241164938	< 0.001	152138	PYDC2
0.241152734	< 0.001	222	ALDH3B2
0.241104245	< 0.001	9607	CARTPT
0.241090596	< 0.001	314	AOC2
0.241086513	< 0.001	85363	TRIM5
0.240999311	< 0.001	92949	ADAMTSL1
0.240994111	< 0.001	503693	LOH12CR2
0.240993828	< 0.001	84322	C18orf12
0.240984589	< 0.001	55269	PSPC1
0.240966991	< 0.001	1501	CTNND2
0.240909651	< 0.001	51729	WBP11
0.240877375	< 0.001	644	BLVRA
0.24086763	< 0.001	80139	ZNF703
0.24085778	< 0.001	7399	USH2A
0.240847051	< 0.001	79315	OR7E91P
0.240846545	< 0.001	1718	DHCR24
0.240839168	< 0.001	51478	HSD17B7

region-wise, subcortex					
Spearman's corr coef	p-val	p-val, SA	p-val, SA (BH- correction)	GenID	Gene
0.321750313	0.056165349	0.079	1	84926	SPRYD3
0.321750313	0.056165349	0.071	1	151651	EFHB
0.32149291	0.056370981	0.071	1	27445	PCLO
0.32149291	0.056370981	0.076	1	30836	DNTTIP2
0.32149291	0.056370981	0.072	1	57605	PITPNM2
0.32149291	0.056370981	0.065	1	219902	TMEM136
0.32149291	0.056370981	0.073	1	401994	OR14I1
0.321235508	0.056577131	0.081	1	79751	SLC25A22
0.321235508	0.056577131	0.093	1	139728	PNCK
0.320978105	0.056783881	0.089	1	2355	FOSL2
0.320978105	0.056783881	0.074	1	2967	GTF2H3
0.320978105	0.056783881	0.095	1	84648	LCE3D
0.320720732	0.056991216	0.084	1	284346	ZNF575
0.3204633	0.05719918	0.082	1	9519	TBPL1
0.3204633	0.05719918	0.07	1	10302	SNAPC5
0.320205927	0.057407849	0.074	1	862	RUNX1T1
0.320205927	0.057407849	0.066	1	7099	TLR4
0.320205927	0.057407849	0.089	1	55259	CASC1
0.320205927	0.057407849	0.068	1	85028	SNHG12
0.320205927	0.057407849	0.085	1	149297	FAM78B
0.320205927	0.057407849	0.079	1	206338	AC034236.3
0.319948494	0.057617031	0.078	1	4597	MVD
0.319948494	0.057617031	0.09	1	4675	NAP1L3
0.319948494	0.057617031	0.096	1	84850	GLIS3-AS1
0.319948494	0.057617031	0.07	1	146279	TEKT5
0.319948494	0.057617031	0.078	1	146861	AMAC1
0.319948494	0.057617031	0.093	1	253635	CCDC75
0.319691122	0.057826828	0.058	1	1173	AP2M1
0.319691122	0.057826828	0.076	1	8214	DGCR6
0.319691122	0.057826828	0.083	1	124359	CDYL2
0.319691122	0.057826828	0.073	1	140710	C20orf117
0.319691122	0.057826828	0.091	1	150275	CCDC117
0.319433719	0.05803721	0.062	1	6620	SNCB
0.319433719	0.05803721	0.104	1	6712	SPTBN2
0.319433719	0.05803721	0.089	1	27145	FILIP1
0.319433719	0.05803721	0.074	1	81608	FIP1L1
0.319176316	0.058248326	0.088	1	6857	SYT1
0.319176316	0.058248326	0.075	1	79735	TBC1D17
0.318918914	0.05845996	0.076	1	345193	LRIT3
0.318661511	0.058672212	0.077	1	66036	MTMR9
0.318661511	0.058672212	0.074	1	131965	METTL6
0.318661511	0.058672212	0.074	1	221223	CES7
0.318404108	0.058885057	0.074	1	634	CEACAM1
0.318404108	0.058885057	0.082	1	65056	GPBP1
0.318404108	0.058885057	0.063	1	146779	AC008026.2
0.318404108	0.058885057	0.074	1	474149	TTTY4B
0.318146735	0.059098639	0.088	1	623	BDKRB1
0.318146735	0.059098639	0.088	1	5532	PPP3CB
0.317889303	0.059312742	0.085	1	5901	RAN
0.31763193	0.059527472	0.082	1	2026	ENO2
0.31763193	0.059527472	0.078	1	2848	GPR25
0.31763193	0.059527472	0.09	1	4826	NNAT
0.31763193	0.059527472	0.088	1	9068	ANGPTL1
0.317374498	0.059742823	0.099	1	8643	PTCH2
0.317374498	0.059742823	0.101	1	23295	MGRN1
0.317374498	0.059742823	0.082	1	27044	SND1
0.317117125	0.059958767	0.101	1	10458	BAIAP2
0.317117125	0.059958767	0.094	1	51250	C6orf203
0.316859722	0.060175464	0.075	1	10473	HMGN4
0.316859722	0.060175464	0.099	1	10900	RUNDC3A
0.316859722	0.060175464	0.091	1	29767	TMOD2
0.316602319	0.060392685	0.074	1	4128	MAOA
0.316602319	0.060392685	0.086	1	6590	SLPI
0.316602319	0.060392685	0.081	1	79946	C10orf95
0.316602319	0.060392685	0.091	1	84916	CIRH1A
0.316344917	0.060610536	0.088	1	3064	HTT
0.316344917	0.060610536	0.088	1	7849	PAX8
0.316344917	0.060610536	0.084	1	11178	LZTS1
0.316344917	0.060610536	0.089	1	51334	PRR16
0.316344917	0.060610536	0.102	1	54700	RRN3
0.316344917	0.060610536	0.073	1	55577	NAGK
0.316087514	0.060828988	0.082	1	5727	PTCH1
0.316087514	0.060828988	0.081	1	83940	TATDN1
0.316087514	0.060828988	0.091	1	119678	OR52E2
0.316087514	0.060828988	0.074	1	139065	SLITRK4
0.316087514	0.060828988	0.085	1	645752	LOC645752
0.315830112	0.061048191	0.098	1	81470	OR2G2
0.315830112	0.061048191	0.082	1	346653	FAM71F2
0.315572739	0.061267931	0.087	1	2194	FASN

voxel-wise, subcortex			
Spearman's corr coef	p-val	GenID	Gene
0.240747929	< 0.001	57446	NDRG3
0.240735158	< 0.001	149461	CLDN19
0.240687236	< 0.001	574406	C1orf138
0.24068208	< 0.001	51696	HECA
0.240642667	< 0.001	221060	C10orf111
0.240595907	< 0.001	100129478	LOC100129478
0.240560979	< 0.001	65981	CAPRIN2
0.240537167	< 0.001	79977	GRHL2
0.240417778	< 0.001	121643	FOXN4
0.240377486	< 0.001	146760	RTN4RL1
0.240356117	< 0.001	64744	SMAP2
0.240352497	< 0.001	729583	C6orf100
0.240343913	< 0.001	3422	IDI1
0.240335837	< 0.001	643836	ZFP62
0.240291864	< 0.001	283129	C11orf85
0.240288243	< 0.001	63934	ZNF667
0.240198523	< 0.001	8569	MKNK1
0.240167141	< 0.001	6277	S100A6
0.240167007	< 0.001	6311	ATXN2
0.240165278	< 0.001	79831	JMJD5
0.240075901	< 0.001	25992	SNED1
0.240074649	< 0.001	84305	WIBG
0.239996493	< 0.001	81849	ST6GALNAC5
0.239917234	< 0.001	6531	SLC6A3
0.23971042	< 0.001	126306	JSRP1
0.23966445	< 0.001	57549	IGSF9
0.239614591	< 0.001	283375	SLC39A5
0.239612401	< 0.001	6272	SORT1
0.239601821	< 0.001	728175	AC103540.1
0.239483088	< 0.001	2237	FEN1
0.23948285	< 0.001	93	ACVR2B
0.239404008	< 0.001	200844	C3orf67
0.23938641	< 0.001	51534	VTA1
0.239382789	< 0.001	221656	AOF1
0.239247352	< 0.001	2617	GARS
0.23913224	< 0.001	644844	AC013356.1
0.23911719	< 0.001	400752	LOC400752
0.23907271	< 0.001	10959	TMED2
0.239057109	< 0.001	9202	ZMYM4
0.238944843	< 0.001	55009	C19orf24
0.238936484	< 0.001	7037	TFRC
0.238908991	< 0.001	164091	PAQR7
0.238882527	< 0.001	116449	CLNK
0.238803864	< 0.001	2289	FKBP5
0.238729671	< 0.001	1436	CSF1R
0.238715976	< 0.001	338699	ANKRD42
0.238714993	< 0.001	374387	AC044839.2
0.238627329	< 0.001	727837	SSX2B
0.238595665	< 0.001	403239	OR2T27
0.2385014	< 0.001	116729	DYSFIP1
0.238442391	< 0.001	51816	CECR1
0.238439515	< 0.001	79981	FRMD1
0.238438398	< 0.001	9993	DGCR2
0.238419816	< 0.001	1359	CPA3
0.238407284	< 0.001	8828	NRP2
0.238381699	< 0.001	5903	RANBP2
0.238379568	< 0.001	124538	OR4D2
0.238376632	< 0.001	10126	DNAL4
0.238299146	< 0.001	84193	SETD3
0.238288417	< 0.001	51069	MRPL2
0.238220572	< 0.001	84126	ATRIP
0.238199964	< 0.001	1810	DR1
0.238193884	< 0.001	130813	C2orf50
0.238106161	< 0.001	375567	VWC2
0.238098919	< 0.001	5993	RFX5
0.238021418	< 0.001	4731	NDUFV3
0.237980515	< 0.001	55532	SLC30A10
0.237973884	< 0.001	374491	TPTE2P6
0.237896293	< 0.001	392335	MYO5BP2
0.2378674	< 0.001	83699	SH3BGRL2
0.237850368	< 0.001	121665	AC069234.1
0.237729177	< 0.001	391104	VHLL
0.237669602	< 0.001	162239	ZFP1
0.237657756	< 0.001	389816	LRRC26
0.23765409	< 0.001	158055	C9orf163
0.237616852	< 0.001	54511	HMGCLL1
0.237601161	< 0.001	6712	SPTBN2
0.237592205	< 0.001	25778	DSTYK
0.237589002	< 0.001	84258	SYT3

region-wise, subcortex					
Spearman's corr coef	p-val	p-val, SA	p-val, SA (BH- correction)	GenID	Gene
0.315572739	0.061267931	0.087	1	7433	VIPR1
0.315572739	0.061267931	0.076	1	54707	GPN2
0.315572739	0.061267931	0.079	1	91694	LONRF1
0.315572739	0.061267931	0.062	1	375035	SFT2D2
0.315315306	0.061488297	0.073	1	3475	IFRD1
0.315315306	0.061488297	0.067	1	23317	DNAJC13
0.315315306	0.061488297	0.076	1	84894	LINGO1
0.315315306	0.061488297	0.097	1	148870	CCDC27
0.315057933	0.061709274	0.086	1	6894	TARBP1
0.315057933	0.061709274	0.098	1	8505	PARG
0.315057933	0.061709274	0.087	1	347744	C6orf52
0.314800501	0.061930913	0.091	1	4868	NPHS1
0.314800501	0.061930913	0.087	1	10990	LILRB5
0.314800501	0.061930913	0.087	1	56897	WRNIP1
0.314543098	0.062153284	0.092	1	83851	SYT16
0.314543098	0.062153284	0.097	1	140465	MYL6B
0.314285696	0.06237619	0.079	1	641	BLM
0.314285696	0.06237619	0.078	1	10096	ACTR3
0.314285696	0.06237619	0.091	1	25884	CHRDL2
0.314285696	0.06237619	0.091	1	26093	CCDC9
0.314285696	0.06237619	0.088	1	80071	CCDC15
0.314285696	0.06237619	0.085	1	84671	ZNF347
0.314028293	0.062599711	0.093	1	656	BMP8B
0.314028293	0.062599711	0.071	1	23039	XPO7
0.314028293	0.062599711	0.073	1	25928	SOSTDC1
0.314028293	0.062599711	0.06	1	27134	TJP3
0.314028293	0.062599711	0.075	1	51491	NOP16
0.31377092	0.062823892	0.078	1	80235	PIGZ
0.313513488	0.063048817	0.089	1	1340	COX6B1
0.313513488	0.063048817	0.095	1	9515	STXBP5L
0.313513488	0.063048817	0.063	1	9701	SAPS2
0.313513488	0.063048817	0.076	1	23013	SPEN
0.313513488	0.063048817	0.087	1	59277	NTN4
0.313513488	0.063048817	0.074	1	83592	AKR1CL2
0.313513488	0.063048817	0.094	1	168374	ZNF92
0.313513488	0.063048817	0.081	1	338442	NIACR2
0.313256115	0.063274279	0.094	1	617	BCS1L
0.313256115	0.063274279	0.084	1	9729	KIAA0408
0.313256115	0.063274279	0.089	1	57595	PDZD4
0.312998682	0.063500389	0.077	1	10494	STK25
0.312741309	0.063727126	0.087	1	1949	EFNB3
0.312741309	0.063727126	0.072	1	3047	HBG1
0.312741309	0.063727126	0.079	1	57026	PDXP
0.312741309	0.063727126	0.104	1	64748	AC024575.2
0.312741309	0.063727126	0.069	1	84973	SNHG7
0.312483907	0.063954644	0.079	1	9046	DOK2
0.312483907	0.063954644	0.085	1	348262	AC174470.1
0.312226504	0.064182661	0.115	1	285231	FBXW12
0.312226504	0.064182661	0.089	1	377677	CA13
0.311969101	0.06441135	0.095	1	4496	MT1H
0.311969101	0.06441135	0.096	1	8835	SOCS2
0.311969101	0.06441135	0.084	1	23173	METAP1
0.311969101	0.06441135	0.09	1	25829	TMEM184B
0.311969101	0.06441135	0.082	1	27178	IL1F7
0.311969101	0.06441135	0.087	1	54437	SEMA5B
0.311969101	0.06441135	0.091	1	80315	CPEB4
0.311969101	0.06441135	0.086	1	221143	N6AMT2
0.311711699	0.064640664	0.084	1	1452	CSNK1A1
0.311711699	0.064640664	0.088	1	2274	FHL2
0.311711699	0.064640664	0.084	1	10109	ARPC2
0.311711699	0.064640664	0.078	1	50488	MINK1
0.311711699	0.064640664	0.092	1	55011	PIH1D1
0.311454296	0.064870656	0.096	1	8677	STX10
0.311454296	0.064870656	0.077	1	9437	NCR1
0.311454296	0.064870656	0.087	1	26149	ZNF658
0.311454296	0.064870656	0.09	1	169044	COL22A1
0.311454296	0.064870656	0.075	1	100188893	TOMM6
0.311196923	0.0651014	0.085	1	4101	MAGEA2
0.311196923	0.0651014	0.097	1	26220	DGCR5
0.311196923	0.0651014	0.08	1	171391	NS3BP
0.310939491	0.065332688	0.11	1	1871	E2F3
0.310939491	0.065332688	0.082	1	2005	ELK4
0.310939491	0.065332688	0.099	1	83597	RTP3
0.310939491	0.065332688	0.092	1	284069	FAM171A2
0.310939491	0.065332688	0.092	1	400073	C12orf76
0.310682118	0.065564618	0.086	1	1418	CRYGA
0.310682118	0.065564618	0.123	1	129450	C2orf60
0.310682118	0.065564618	0.065	1	151230	KLHL23
0.310424685	0.065797217	0.083	1	8971	H1FX

voxel-wise, subcortex			
Spearman's corr coef	p-val	GenID	Gene
0.23740828	< 0.001	23509	POFUT1
0.237407714	< 0.001	5048	PAFAH1B1
0.237346098	< 0.001	50485	SMARCAL1
0.237260103	< 0.001	340307	CTAGE6P
0.237254217	< 0.001	55692	LUC7L
0.237247616	< 0.001	23277	KIAA0664
0.237099454	< 0.001	2199	FBLN2
0.237067923	< 0.001	54905	CYP2W1
0.237065375	< 0.001	56624	ASAH2
0.237051114	< 0.001	84660	CCDC62
0.236926123	< 0.001	641702	FAM138E
0.236911163	< 0.001	9247	GCM2
0.236823365	< 0.001	84144	SYDE2
0.236812443	< 0.001	1678	TIMM8A
0.236785144	< 0.001	57152	SLURP1
0.236775115	< 0.001	8139	GAN
0.236692935	< 0.001	85865	GTPBP10
0.236683086	< 0.001	1846	DUSP4
0.236530513	< 0.001	337976	KRTAP20-2
0.236496285	< 0.001	85441	RP4-697K14.1
0.236477852	< 0.001	151742	PPM1L
0.236454308	< 0.001	8570	KHSRP
0.236382753	< 0.001	100130746	LOC100130746
0.23625581	< 0.001	126767	AADACL3
0.236207515	< 0.001	11151	CORO1A
0.236168101	< 0.001	221409	SPATS1
0.236107543	< 0.001	246705	LINC00314
0.236068264	< 0.001	6940	ZNF354A
0.236011103	< 0.001	10472	ZNF238
0.235995695	< 0.001	57088	PLSCR4
0.235909924	< 0.001	266727	MDGA1
0.235888615	< 0.001	57824	HMHB1
0.235836238	< 0.001	157378	TMEM65
0.235825509	< 0.001	89953	KLC4
0.23577109	< 0.001	286238	LOC286238
0.235689327	< 0.001	2823	GPM6A
0.235664204	< 0.001	27293	SMPDL3B
0.235646471	< 0.001	11218	DDX20
0.235544264	< 0.001	4093	SMAD9
0.235531867	< 0.001	282617	IL28B
0.235510051	< 0.001	386618	KCTD4
0.235468358	< 0.001	126006	PCP2
0.235462546	< 0.001	64601	VPS16A
0.235406548	< 0.001	55779	WDR52
0.235404328	< 0.001	164	AP1G1
0.235370755	< 0.001	795	S100G
0.2353383	< 0.001	9329	GTF3C4
0.235322133	< 0.001	805	CALM2
0.235301286	< 0.001	9704	DHX34
0.235280618	< 0.001	23161	SNX13
0.235241622	< 0.001	10123	ARL4C
0.235154286	< 0.001	5395	PMS2
0.235106915	< 0.001	10525	HYOU1
0.23508212	< 0.001	54386	TERF2IP
0.235074282	< 0.001	283237	TTC9C
0.235052869	< 0.001	5498	PPOX
0.234998405	< 0.001	9764	KIAA0513
0.234910175	< 0.001	78990	OTUB2
0.234854698	< 0.001	3630	INS
0.234818339	< 0.001	79699	ZYG11B
0.234800786	< 0.001	25885	POLR1A
0.234795868	< 0.001	50615	IL21R
0.23476252	< 0.001	6920	TCEA3
0.234692976	< 0.001	400027	LOC400027
0.234606877	< 0.001	8492	PRSS12
0.234587938	< 0.001	9070	ASH2L
0.234569088	< 0.001	1001	CDH3
0.234287143	< 0.001	405	ARNT
0.234210715	< 0.001	5097	PCDH1
0.234183416	< 0.001	156	ADRBK1
0.234142646	< 0.001	6332	SCN7A
0.234132707	< 0.001	81876	RAB1B
0.234097928	< 0.001	149992	ANKRD30BP2
0.234091938	< 0.001	10262	SF3B4
0.23409009	< 0.001	9472	AKAP6
0.234080896	< 0.001	55322	C5orf22
0.234043136	< 0.001	100506049	AC005014.1
0.233941406	< 0.001	6715	SRD5A1
0.23390612	< 0.001	92014	MCART1

region-wise, subcortex					
Spearman's corr coef	p-val	p-val, SA	p-val, SA (BH- correction)	GenID	Gene
0.310424685	0.065797217	0.104	1	151354	FAM84A
0.310167313	0.066030577	0.082	1	4900	NRGN
0.310167313	0.066030577	0.096	1	114036	LINC00310
0.310167313	0.066030577	0.082	1	197320	ZNF778
0.30990991	0.066264495	0.093	1	2965	GTF2H1
0.30990991	0.066264495	0.089	1	9892	SNAP91
0.309652507	0.066499062	0.089	1	1266	CNN3
0.309652507	0.066499062	0.096	1	3897	L1CAM
0.309652507	0.066499062	0.095	1	57198	ATP8B2
0.309652507	0.066499062	0.088	1	80145	THOC7
0.309652507	0.066499062	0.098	1	154881	KCTD7
0.309395105	0.066734269	0.079	1	355	FAS
0.309395105	0.066734269	0.099	1	5188	PET112L
0.309395105	0.066734269	0.082	1	6445	SGCG
0.309395105	0.066734269	0.085	1	7329	UBE2I
0.309395105	0.066734269	0.092	1	23780	APOL2
0.309395105	0.066734269	0.101	1	131616	TMEM42
0.309395105	0.066734269	0.089	1	283464	GLT8D3
0.309137702	0.066970266	0.089	1	1346	COX7A1
0.309137702	0.066970266	0.082	1	3188	HNRNPH2
0.309137702	0.066970266	0.098	1	5721	PSME2
0.309137702	0.066970266	0.084	1	6711	SPTBN1
0.309137702	0.066970266	0.079	1	7117	TMSB4XP8
0.309137702	0.066970266	0.096	1	7216	TRO
0.309137702	0.066970266	0.084	1	9378	NRXN1
0.309137702	0.066970266	0.066	1	56977	STOX2
0.309137702	0.066970266	0.089	1	221711	SYCP2L
0.308880299	0.067206822	0.1	1	55908	AC011472.2
0.308365494	0.067681931	0.088	1	1268	CNR1
0.308365494	0.067681931	0.081	1	5176	SERPINF1
0.308108121	0.067920446	0.097	1	653499	LGALS7B
0.307850689	0.068159774	0.09	1	2932	GSK3B
0.307850689	0.068159774	0.08	1	345456	PFN3
0.307593286	0.06839966	0.108	1	440	ASNS
0.307593286	0.06839966	0.095	1	7259	TSPYL1
0.307335913	0.06864021	0.089	1	10791	VAMP5
0.307335913	0.06864021	0.077	1	55917	CTTNBP2NL
0.307335913	0.06864021	0.085	1	56655	POLE4
0.307078481	0.06888143	0.088	1	9253	NUMBL
0.306821108	0.069123417	0.09	1	782	CACNB1
0.306821108	0.069123417	0.103	1	340554	ZC3H12B
0.306563675	0.069365986	0.104	1	56970	ATXN7L3
0.306563675	0.069365986	0.099	1	65977	PLEKHA3
0.306563675	0.069365986	0.083	1	113540	CMTM1
0.306563675	0.069365986	0.086	1	339230	CCDC137
0.306306303	0.069609225	0.086	1	1397	CRIP2
0.306306303	0.069609225	0.113	1	2064	ERBB2
0.306306303	0.069609225	0.091	1	8660	IRS2
0.306306303	0.069609225	0.105	1	11147	HHLA3
0.3060489	0.069853142	0.097	1	5064	PALM
0.3060489	0.069853142	0.105	1	5447	POR
0.3060489	0.069853142	0.091	1	79369	B3GNT4
0.3060489	0.069853142	0.096	1	284424	MIR7-3HG
0.305791497	0.070097849	0.101	1	57216	VANGL2
0.305791497	0.070097849	0.103	1	112476	PRRT2
0.305791497	0.070097849	0.082	1	132112	RTP1
0.305791497	0.070097849	0.11	1	442319	ZNF727
0.305534095	0.0703431	0.103	1	4100	MAGEA1
0.305534095	0.0703431	0.092	1	64800	EFCAB6
0.305276692	0.070589051	0.109	1	4794	NFKBIE
0.305276692	0.070589051	0.088	1	27257	LSM1
0.305276692	0.070589051	0.1	1	51339	DACT1
0.305019289	0.07083568	0.097	1	55008	HERC6
0.305019289	0.07083568	0.11	1	135112	NCOA7
0.304761916	0.071083002	0.084	1	540	ATP7B
0.304761916	0.071083002	0.083	1	6920	TCEA3
0.304761916	0.071083002	0.107	1	10228	STX6
0.304761916	0.071083002	0.112	1	30000	TNPO2
0.304761916	0.071083002	0.12	1	54754	FAM22F
0.304504484	0.071331106	0.107	1	57536	KIAA1328
0.304504484	0.071331106	0.088	1	391334	LOC391334
0.304247111	0.071579754	0.086	1	54529	ASNSD1
0.303989679	0.071829125	0.11	1	11282	MGAT4B
0.303732306	0.072079182	0.097	1	23468	CBX5
0.303732306	0.072079182	0.094	1	26523	EIF2C1
0.303474903	0.072330035	0.101	1	9497	SLC4A7
0.303474903	0.072330035	0.08	1	11258	DCTN3
0.303474903	0.072330035	0.088	1	23710	GABARAPL1
0.303474903	0.072330035	0.089	1	114788	CSMD3

voxel-wise, subcortex			
Spearman's corr coef	p-val	GenID	Gene
0.233897522	< 0.001	648987	AC008875.1
0.233692482	< 0.001	100129492	LOC100129492
0.233653426	< 0.001	2266	FGG
0.233647481	< 0.001	126549	ANKLE1
0.233603001	< 0.001	157556	C8orf56
0.233562976	< 0.001	84298	LLPH
0.233547553	< 0.001	66005	CHID1
0.233474806	< 0.001	9409	PEX16
0.233419076	< 0.001	284424	MIR7-3HG
0.233229965	< 0.001	60	ACTB
0.233217657	< 0.001	119764	OR4X2
0.233186677	< 0.001	55850	USE1
0.233180925	< 0.001	4810	NHS
0.233165696	< 0.001	23682	RAB38
0.233149305	< 0.001	503542	SPRN
0.233144104	< 0.001	89890	KBTBD6
0.233121723	< 0.001	93655	ST7-OT3
0.233114943	< 0.001	3899	AFF3
0.233100742	< 0.001	114609	TIRAP
0.233047843	< 0.001	642585	VDAC1P1
0.233009547	< 0.001	10102	TSFM
0.232985169	< 0.001	84503	ZNF527
0.232974812	< 0.001	9874	TLK1
0.232918859	< 0.001	4668	NAGA
0.23286593	< 0.001	392391	OR5C1
0.232836664	< 0.001	7321	UBE2D1
0.2328206	< 0.001	3615	IMPDH2
0.232801527	< 0.001	7068	THRB
0.232778028	< 0.001	708	C1QBP
0.23275885	< 0.001	254187	TSGA10IP
0.232749522	< 0.001	50855	PARD6A
0.232740045	< 0.001	80739	C6orf25
0.2326352	< 0.001	5358	PLS3
0.232540339	< 0.001	1415	CRYBB2
0.232526407	< 0.001	79020	C7orf25
0.232408464	< 0.001	376497	SLC27A1
0.232395276	< 0.001	5607	MAP2K5
0.232269078	< 0.001	9058	SLC13A2
0.232244968	< 0.001	22941	SHANK2
0.232199013	< 0.001	83884	SLC25A2
0.232128203	< 0.001	9684	LRRC14
0.232097968	< 0.001	4295	MLN
0.232043356	< 0.001	3227	HOXC11
0.231999576	< 0.001	55190	NUDT11
0.231867105	< 0.001	4515	MTCP1
0.231775299	< 0.001	2117	ETV3
0.231712341	< 0.001	28982	FLVCR1
0.231711924	< 0.001	100506581	C16orf95
0.231682897	< 0.001	4828	NMB
0.231638148	< 0.001	3574	IL7
0.231543884	< 0.001	81793	TLR10
0.231342316	< 0.001	285676	ZNF454
0.231290683	< 0.001	246721	POLR2J2
0.231219321	< 0.001	64122	FN3K
0.231203526	< 0.001	3228	HOXC12
0.231182545	< 0.001	79087	ALG12
0.231138065	< 0.001	8029	CUBN
0.23110202	< 0.001	221002	RASGEF1A
0.231068045	< 0.001	58508	MLL3
0.230889365	< 0.001	55153	SDAD1
0.230886206	< 0.001	146849	CCDC42
0.230858818	< 0.001	727910	TLCD2
0.230796456	< 0.001	6902	TBCA
0.230781823	< 0.001	59353	TMEM35
0.230759025	< 0.001	54931	RG9MTD1
0.230742216	< 0.001	6814	STXBP3
0.230714083	< 0.001	138474	TAF1L
0.230689794	< 0.001	23547	LILRA4
0.230686262	< 0.001	326625	MMAB
0.23066546	< 0.001	339742	FAM201B
0.230599478	< 0.001	57099	AVEN
0.230586901	< 0.001	6619	SNAPC3
0.230551139	< 0.001	79589	RNF128
0.230517209	< 0.001	9315	C5orf13
0.230499327	< 0.001	147700	KLC3
0.230497092	< 0.001	116987	AGAP1
0.230437204	< 0.001	5155	PDGFB
0.230416492	< 0.001	3171	FOXA3
0.230284706	< 0.001	84620	ST6GAL2

region-wise, subcortex					
Spearman's corr coef	p-val	p-val, SA	p-val, SA (BH- correction)	GenID	Gene
0.303474903	0.072330035	0.1	1	646903	LOC646903
0.3032175	0.072581463	0.098	1	1875	E2F5
0.3032175	0.072581463	0.104	1	55722	CEP72
0.3032175	0.072581463	0.085	1	79959	CEP76
0.302960098	0.072833553	0.099	1	1455	CSNK1G2
0.302960098	0.072833553	0.102	1	6567	SLC16A2
0.302960098	0.072833553	0.09	1	8776	MTMR1
0.302960098	0.072833553	0.084	1	9364	RAB28
0.302960098	0.072833553	0.099	1	11054	OGFR
0.302702695	0.073086366	0.103	1	9722	NOS1AP
0.302445292	0.073339984	0.114	1	57535	KIAA1324
0.302445292	0.073339984	0.092	1	84661	DPY30
0.302445292	0.073339984	0.107	1	400765	MIR2682
0.30218792	0.073594183	0.104	1	55107	ANO1
0.30218792	0.073594183	0.114	1	55321	C20orf46
0.30218792	0.073594183	0.102	1	91862	MARVELD3
0.30218792	0.073594183	0.104	1	205564	SENP5
0.30218792	0.073594183	0.076	1	256933	NPB
0.301930487	0.073849075	0.103	1	85456	TNKS1BP1
0.301673114	0.074104637	0.098	1	3752	KCND3
0.301673114	0.074104637	0.111	1	6093	ROCK1
0.301673114	0.074104637	0.083	1	7429	VIL1
0.301673114	0.074104637	0.106	1	22859	LPHN1
0.301673114	0.074104637	0.095	1	63891	RNF123
0.301673114	0.074104637	0.09	1	220074	AP000812.2
0.301415682	0.074360922	0.086	1	51156	SERPINA10
0.301415682	0.074360922	0.106	1	55095	SAMD4B
0.301415682	0.074360922	0.09	1	65996	AC016629.3
0.301415682	0.074360922	0.099	1	286187	LRRC67
0.301158309	0.074618027	0.104	1	501	ALDH7A1
0.301158309	0.074618027	0.094	1	1142	CHRNB3
0.301158309	0.074618027	0.115	1	9829	DNAJC6
0.301158309	0.074618027	0.102	1	29068	ZBTB44
0.300900906	0.074875712	0.093	1	283991	FAM100B
0.300643474	0.075134091	0.095	1	8718	TNFRSF25
0.300643474	0.075134091	0.1	1	9355	LHX2
0.300643474	0.075134091	0.099	1	388762	OR2M1P
0.300386101	0.075393155	0.114	1	9262	STK17B
0.300386101	0.075393155	0.087	1	146433	IL34
0.300386101	0.075393155	0.096	1	167359	AC114947.2
0.300128669	0.075653061	0.112	1	5498	PPOX
0.300128669	0.075653061	0.089	1	54984	PINX1
0.300128669	0.075653061	0.095	1	55901	THSD1
0.299871296	0.075913556	0.101	1	5780	PTPN9
0.299871296	0.075913556	0.093	1	144448	TSPAN19
0.299613893	0.076174766	0.096	1	6839	SUV39H1
0.299613893	0.076174766	0.088	1	7637	ZNF84
0.299613893	0.076174766	0.104	1	8452	CUL3
0.299613893	0.076174766	0.084	1	51301	GCNT4
0.299613893	0.076174766	0.084	1	391356	PTRHD1
0.29935649	0.076436669	0.089	1	22949	PTGR1
0.29935649	0.076436669	0.105	1	29114	TAGLN3
0.29935649	0.076436669	0.105	1	202134	FAM153B
0.299099088	0.076699376	0.113	1	9055	PRC1
0.299099088	0.076699376	0.093	1	9939	RBM8A
0.299099088	0.076699376	0.082	1	54456	MOV10L1
0.299099088	0.076699376	0.103	1	79953	C20orf39
0.298841685	0.076962702	0.1	1	1869	E2F1
0.298841685	0.076962702	0.129	1	2892	GRIA3
0.298841685	0.076962702	0.114	1	7504	XK
0.298841685	0.076962702	0.11	1	22820	COPG
0.298841685	0.076962702	0.1	1	57565	KLHL14
0.298841685	0.076962702	0.11	1	123803	NTAN1
0.298584282	0.077226743	0.096	1	56267	CCBL2
0.29832691	0.077491492	0.108	1	5352	PLOD2
0.29832691	0.077491492	0.112	1	6688	SPI1
0.29832691	0.077491492	0.086	1	8924	HERC2
0.29832691	0.077491492	0.095	1	63924	CIDEC
0.29832691	0.077491492	0.105	1	201254	STRA13
0.298069477	0.077756964	0.112	1	2074	ERCC6
0.298069477	0.077756964	0.09	1	9649	RALGPS1
0.298069477	0.077756964	0.096	1	10014	HDAC5
0.298069477	0.077756964	0.106	1	51062	ATL1
0.298069477	0.077756964	0.103	1	130813	C2orf50
0.297812104	0.078023225	0.109	1	3199	HOXA2
0.297812104	0.078023225	0.09	1	4858	NOVA2
0.297812104	0.078023225	0.107	1	8065	CUL5
0.297812104	0.078023225	0.117	1	29945	ANAPC4
0.297554672	0.07829012	0.106	1	8363	HIST1H4J

voxel-wise, subcortex			
Spearman's corr coef	p-val	GenID	Gene
0.23026596	< 0.001	2330	FMO5
0.23024492	< 0.001	200959	GABRR3
0.230231553	< 0.001	653657	AC010606.4
0.230218679	< 0.001	124872	B4GALNT2
0.230193615	< 0.001	199745	THAP8
0.230183303	< 0.001	113540	CMTM1
0.230177179	< 0.001	11074	TRIM31
0.230151355	< 0.001	9665	KIAA0430
0.230140731	< 0.001	56675	NRIP3
0.230134174	< 0.001	79629	OCEL1
0.230124384	< 0.001	57482	KIAA1211
0.230108365	< 0.001	80131	LRRC8E
0.230107471	< 0.001	643668	PSMC1P9
0.229993254	< 0.001	10961	ERP29
0.229838997	< 0.001	282616	IL28A
0.229828373	< 0.001	64757	MOSC1
0.229764432	< 0.001	23234	DNAJC9
0.229742378	< 0.001	53340	SPA17
0.229701325	< 0.001	125972	CALR3
0.229669616	< 0.001	4681	NBL1
0.229657039	< 0.001	59340	HRH4
0.22962597	< 0.001	4128	MAOA
0.229603916	< 0.001	9132	KCNQ4
0.229550466	< 0.001	3175	ONECUT1
0.229543418	< 0.001	151651	EFHB
0.229468882	< 0.001	1761	DMRT1
0.229456723	< 0.001	6643	SNX2
0.229454026	< 0.001	57713	SFMBT2
0.22944288	< 0.001	669	BPGM
0.229432806	< 0.001	133688	UGT3A1
0.2293697	< 0.001	3120	HLA-DQB2
0.229352519	< 0.001	387263	C6orf120
0.229297921	< 0.001	144809	C13orf30
0.229242802	< 0.001	23217	ZFR2
0.22922419	< 0.001	3430	IFI35
0.229221866	< 0.001	6175	RPLP0P3
0.229148597	< 0.001	140564	APOBEC3D
0.229132101	< 0.001	344967	AC098591.2
0.229123235	< 0.001	57728	WDR19
0.229033157	< 0.001	6833	ABCC8
0.229020208	< 0.001	60592	SCOC
0.228919029	< 0.001	402381	SOHLH1
0.228899896	< 0.001	92591	ASB16
0.228871346	< 0.001	100130331	RP11-193H5.1
0.228825271	< 0.001	6860	SYT4
0.228772298	< 0.001	51332	SPTBN5
0.228714809	< 0.001	8110	DPF3
0.22871092	< 0.001	143282	FGFBP3
0.228706643	< 0.001	23080	AVL9
0.228696749	< 0.001	1643	DDB2
0.228678405	< 0.001	79816	TLE6
0.228675663	< 0.001	10460	TACC3
0.22867015	< 0.001	8538	BARX2
0.22855787	< 0.001	2584	GALK1
0.228528529	< 0.001	1399	CRKL
0.228522867	< 0.001	2660	MSTN
0.228501365	< 0.001	3266	ERAS
0.228444114	< 0.001	64840	PORCN
0.228322089	< 0.001	55924	C1orf183
0.228297472	< 0.001	23074	UHRF1BP1L
0.228257686	< 0.001	9859	CEP170
0.228195325	< 0.001	115350	FCRL1
0.22814931	< 0.001	375791	C9orf169
0.228107885	< 0.001	388324	INCA1
0.228056625	< 0.001	9791	PTDSS1
0.227973416	< 0.001	80737	C6orf27
0.227930188	< 0.001	5639	PRRG2
0.227907896	< 0.001	9057	SLC7A6
0.227877021	< 0.001	64089	SNX16
0.227789536	< 0.001	146771	TCAM1
0.227778539	< 0.001	5868	RAB5A
0.227725655	< 0.001	6755	SSTR5
0.227659672	< 0.001	9500	MAGED1
0.22763817	< 0.001	145873	MESP2
0.22759299	< 0.001	222029	DKFZp434L192
0.227510244	< 0.001	4067	LYN
0.227358222	< 0.001	114915	EPB41L4A-AS1
0.227303803	< 0.001	4802	NFYC
0.227281705	< 0.001	24149	ZNF318

region-wise, subcortex					
Spearman's corr coef	p-val	p-val, SA	p-val, SA (BH- correction)	GenID	Gene
0.297554672	0.07829012	0.102	1	79622	SNRNP25
0.297297299	0.07855773	0.087	1	1983	EIF5
0.297039896	0.078826062	0.12	1	577	BAI3
0.297039896	0.078826062	0.102	1	7025	NR2F1
0.297039896	0.078826062	0.106	1	8338	HIST2H2AC
0.297039896	0.078826062	0.108	1	9148	NEURL
0.297039896	0.078826062	0.084	1	51132	RLIM
0.297039896	0.078826062	0.107	1	159090	FAM122B
0.296782494	0.079095192	0.094	1	80345	ZNF435
0.296525091	0.07936497	0.11	1	2247	FGF2
0.296525091	0.07936497	0.112	1	10294	DNAJA2
0.296525091	0.07936497	0.092	1	85406	DNAJC14
0.296267688	0.079635456	0.115	1	8424	BBOX1
0.296267688	0.079635456	0.105	1	8930	MBD4
0.296267688	0.079635456	0.089	1	80218	NAA50
0.296010286	0.079906672	0.12	1	5087	PBX1
0.296010286	0.079906672	0.102	1	7274	TTPA
0.296010286	0.079906672	0.113	1	163255	ZNF540
0.295752913	0.08017873	0.1	1	2152	F3
0.295752913	0.08017873	0.106	1	150465	TTL
0.29549548	0.080451362	0.119	1	6507	SLC1A3
0.29549548	0.080451362	0.11	1	8450	CUL4B
0.29549548	0.080451362	0.106	1	121273	C12orf54
0.295238107	0.080724746	0.113	1	5156	PDGFRA
0.295238107	0.080724746	0.102	1	56997	CABC1
0.294980675	0.080998868	0.098	1	29781	NCAPH2
0.294980675	0.080998868	0.124	1	149018	LELP1
0.294723302	0.081273712	0.114	1	5793	PTPRG
0.294723302	0.081273712	0.102	1	54622	ARL15
0.294723302	0.081273712	0.106	1	79680	C22orf29
0.294723302	0.081273712	0.112	1	131540	ZDHHC19
0.294723302	0.081273712	0.132	1	653121	ZBTB8A
0.294465899	0.081549414	0.115	1	6621	SNAPC4
0.294465899	0.081549414	0.101	1	7574	ZNF26
0.294465899	0.081549414	0.105	1	246181	AKR7L
0.294208497	0.081825696	0.128	1	2978	GUCA1A
0.294208497	0.081825696	0.12	1	5201	PFDN1
0.294208497	0.081825696	0.112	1	51592	TRIM33
0.294208497	0.081825696	0.096	1	55691	FRMD4A
0.294208497	0.081825696	0.1	1	64207	C14orf4
0.294208497	0.081825696	0.109	1	285126	DNAJC5G
0.293951094	0.082102731	0.101	1	8857	FCGBP
0.293951094	0.082102731	0.105	1	9564	BCAR1
0.293951094	0.082102731	0.117	1	54535	CCHCR1
0.293951094	0.082102731	0.1	1	122616	C14orf79
0.293951094	0.082102731	0.11	1	132200	C3orf49
0.293693662	0.082380503	0.099	1	2491	CENPI
0.293436289	0.08265914	0.102	1	9247	GCM2
0.293436289	0.08265914	0.098	1	56892	C8orf4
0.293178886	0.082938388	0.099	1	6001	RGS10
0.293178886	0.082938388	0.136	1	29886	SNX8
0.293178886	0.082938388	0.094	1	80301	PLEKHO2
0.292921484	0.083218336	0.099	1	8811	GALR2
0.292921484	0.083218336	0.106	1	54915	YTHDF1
0.292921484	0.083218336	0.098	1	79873	NUDT18
0.292664081	0.083499067	0.111	1	9547	CXCL14
0.292664081	0.083499067	0.12	1	10480	EIF3M
0.292664081	0.083499067	0.111	1	54069	C21orf45
0.292664081	0.083499067	0.12	1	55638	AC079061.1
0.292664081	0.083499067	0.096	1	56062	KLHL4
0.292664081	0.083499067	0.113	1	56160	NDNL2
0.292664081	0.083499067	0.109	1	79139	DERL1
0.292406678	0.083780527	0.111	1	1849	DUSP7
0.292406678	0.083780527	0.11	1	8570	KHSRP
0.292406678	0.083780527	0.1	1	9145	SYNGR1
0.292406678	0.083780527	0.097	1	81570	CLPB
0.292406678	0.083780527	0.109	1	158067	C9orf98
0.292149276	0.084062852	0.115	1	4733	DRG1
0.292149276	0.084062852	0.122	1	5820	PVT1
0.292149276	0.084062852	0.118	1	23230	VPS13A
0.292149276	0.084062852	0.132	1	80700	UBXN6
0.292149276	0.084062852	0.095	1	84225	ZMYND15
0.292149276	0.084062852	0.106	1	91057	CCDC34
0.292149276	0.084062852	0.129	1	100134934	C17ORF106
0.291891903	0.084345795	0.107	1	3788	KCNS2
0.291891903	0.084345795	0.113	1	29109	FHOD1
0.291891903	0.084345795	0.124	1	51195	RAPGEFL1
0.291891903	0.084345795	0.113	1	574029	DUSP5P
0.291891903	0.084345795	0.103	1	100132304	RP1-172N19.1

voxel-wise, subcortex			
Spearman's corr coef	p-val	GenID	Gene
0.227209449	< 0.001	201965	RWDD4A
0.227098674	< 0.001	81034	SLC25A32
0.227019683	< 0.001	128989	C22orf25
0.226894274	< 0.001	127074	OR2T4
0.226797417	< 0.001	88745	C6orf153
0.226781249	< 0.001	2205	FCER1A
0.226759523	< 0.001	10585	POMT1
0.22671847	< 0.001	90993	CREB3L1
0.226666242	< 0.001	83953	FCAMR
0.226610467	< 0.001	9564	BCAR1
0.226597384	< 0.001	27034	ACAD8
0.226569742	< 0.001	92703	TMEM183A
0.226427987	< 0.001	54734	RAB39
0.226422742	< 0.001	80218	NAA50
0.226357922	< 0.001	3308	HSPA4
0.226341069	< 0.001	154822	AC004863.6
0.226317659	< 0.001	826	CAPNS1
0.226315856	< 0.001	114794	LRRC62
0.226236969	< 0.001	5709	PSMD3
0.22615692	< 0.001	129684	CNTNAP5
0.226145208	< 0.001	728833	FAM72D
0.226115867	< 0.001	685	BTC
0.225911841	< 0.001	441308	OR4F21
0.225889832	< 0.001	5970	RELA
0.225887045	< 0.001	55288	RHOT1
0.225833043	< 0.001	1054	CEBPG
0.225820556	< 0.001	8715	NOL4
0.225739986	< 0.001	26539	OR10H1
0.225662455	< 0.001	440279	UNC13C
0.225591317	< 0.001	9922	IQSEC1
0.225583971	< 0.001	8643	PTCH2
0.22550787	< 0.001	644172	LOC644172
0.225487575	< 0.001	57821	C1orf114
0.225422069	< 0.001	84694	GJA10
0.225321397	< 0.001	10512	SEMA3C
0.225195006	< 0.001	26032	SUSD5
0.22516194	< 0.001	255631	COL24A1
0.225138351	< 0.001	26009	ZZZ3
0.225116119	< 0.001	284593	FAM41C
0.225087374	< 0.001	9929	JOSD1
0.225034669	< 0.001	1014	CDH16
0.22496368	< 0.001	402117	VWC2L
0.224955231	< 0.001	79825	CCDC48
0.224922955	< 0.001	100131289	LOC100131289
0.22490035	< 0.001	729991	MEF2BNB
0.224849075	< 0.001	54707	GPN2
0.224805668	< 0.001	84929	FIBCD1
0.224760205	< 0.001	84272	YIPF4
0.224749625	< 0.001	80331	DNAJC5
0.224681601	< 0.001	401237	LINC00340
0.2246719	< 0.001	221786	C7orf38
0.22464022	< 0.001	58497	PRUNE
0.224551499	< 0.001	283464	GLT8D3
0.224524423	< 0.001	6328	SCN3A
0.224499807	< 0.001	1018	CDK3
0.224452272	< 0.001	399726	C10orf114
0.22442013	< 0.001	138804	OR13C4
0.224405885	< 0.001	57157	PHTF2
0.224380895	< 0.001	55014	STX17
0.224367663	< 0.001	9032	TM4SF5
0.224313617	< 0.001	64092	SAMSN1
0.224302053	< 0.001	4654	MYOD1
0.224242851	< 0.001	23544	SEZ6L
0.224191397	< 0.001	158293	FAM120AOS
0.224120542	< 0.001	3213	HOXB3
0.224068448	< 0.001	11253	MAN1B1
0.223989472	< 0.001	84885	ZDHHC12
0.223899707	< 0.001	11285	B4GALT7
0.223868281	< 0.001	6367	CCL22
0.223834991	< 0.001	4606	MYBPC2
0.223830521	< 0.001	9949	AMMECR1
0.22378771	< 0.001	7407	VARS
0.22377564	< 0.001	9362	CPNE6
0.223756284	< 0.001	3904	LAIR2
0.223636523	< 0.001	162632	AC022596.6
0.223528385	< 0.001	3982	LIM2
0.223451674	< 0.001	3475	IFRD1
0.223379478	< 0.001	4534	MTM1
0.223368749	< 0.001	55680	RUFY2

region-wise, subcortex					
Spearman's corr coef	p-val	p-val, SA	p-val, SA (BH- correction)	GenID	Gene
0.29163447	0.084629454	0.126	1	64101	LRRC4
0.29163447	0.084629454	0.109	1	145581	LRFN5
0.29163447	0.084629454	0.085	1	163126	EID2
0.291377097	0.084913887	0.104	1	2529	FUT7
0.291377097	0.084913887	0.101	1	9329	GTF3C4
0.291377097	0.084913887	0.108	1	55890	GPRC5C
0.291377097	0.084913887	0.116	1	92345	NAF1
0.291119665	0.085199192	0.112	1	6247	RS1
0.291119665	0.085199192	0.11	1	8193	DPF1
0.291119665	0.085199192	0.111	1	91875	TTC5
0.290862292	0.085485116	0.118	1	174	AFP
0.290862292	0.085485116	0.105	1	11168	PSIP1
0.290862292	0.085485116	0.089	1	219957	OR9Q2
0.290604889	0.085771762	0.115	1	80851	SH3BP5L
0.290347487	0.08605919	0.109	1	2036	EPB41L1
0.290347487	0.08605919	0.107	1	3195	TLX1
0.290090084	0.086347491	0.102	1	2775	GNAO1
0.290090084	0.086347491	0.115	1	8929	PHOX2B
0.289832681	0.086636424	0.097	1	90378	SAMD1
0.289832681	0.086636424	0.124	1	146542	ZNF688
0.289575279	0.086926095	0.123	1	4745	NELL1
0.289575279	0.086926095	0.109	1	10245	TIMM17B
0.289317906	0.087216504	0.103	1	84197	AC113191.1
0.289060473	0.087507695	0.107	1	5155	PDGFB
0.289060473	0.087507695	0.121	1	7468	WHSC1
0.289060473	0.087507695	0.122	1	10904	BLCAP
0.289060473	0.087507695	0.13	1	51299	NRN1
0.289060473	0.087507695	0.099	1	114659	LRRC37B
0.289060473	0.087507695	0.109	1	123016	TTC8
0.289060473	0.087507695	0.113	1	130162	C2orf63
0.288803101	0.087799765	0.125	1	4771	NF2
0.288803101	0.087799765	0.115	1	7084	TK2
0.288803101	0.087799765	0.124	1	23331	TTC28
0.288803101	0.087799765	0.1	1	55922	NKRF
0.288803101	0.087799765	0.109	1	114818	KLHL29
0.288803101	0.087799765	0.107	1	143279	HECTD2
0.288803101	0.087799765	0.119	1	163115	ZNF781
0.288803101	0.087799765	0.113	1	282808	RAB40AL
0.288803101	0.087799765	0.123	1	389538	MGC72080
0.288803101	0.087799765	0.117	1	645676	RP11-29H23.1
0.288545668	0.088092476	0.119	1	361	AQP4
0.288545668	0.088092476	0.112	1	10944	C11orf58
0.288545668	0.088092476	0.124	1	51650	MRPS33
0.288545668	0.088092476	0.109	1	54466	SPIN2A
0.288545668	0.088092476	0.111	1	64210	MMS19
0.288545668	0.088092476	0.115	1	84332	DYDC2
0.288030893	0.088680126	0.115	1	6645	SNTB2
0.288030893	0.088680126	0.12	1	9061	PAPSS1
0.28777349	0.088975251	0.112	1	8681	JMJD7-PLA2G4B
0.28777349	0.088975251	0.091	1	9154	SLC28A1
0.28777349	0.088975251	0.113	1	24149	ZNF318
0.287516087	0.089270994	0.122	1	9112	MTA1
0.287516087	0.089270994	0.13	1	29855	UBN1
0.287516087	0.089270994	0.109	1	55300	PI4K2B
0.287516087	0.089270994	0.115	1	401720	FIGNL2
0.287258685	0.089567475	0.112	1	6157	RPL27A
0.287258685	0.089567475	0.11	1	8732	RNGTT
0.287258685	0.089567475	0.114	1	643988	RP11-345P4.1
0.287001282	0.089864753	0.129	1	10486	CAP2
0.286743879	0.090162925	0.109	1	2002	ELK1
0.286743879	0.090162925	0.112	1	4948	OCA2
0.286743879	0.090162925	0.117	1	150726	FBXO41
0.286486477	0.090461738	0.109	1	2926	GRSF1
0.286486477	0.090461738	0.121	1	55269	PSPC1
0.286486477	0.090461738	0.123	1	338651	AP006285.4
0.286229074	0.090761289	0.133	1	285315	C3orf33
0.285971671	0.091061637	0.118	1	378	ARF4
0.285971671	0.091061637	0.116	1	10465	PPIH
0.285971671	0.091061637	0.117	1	440799	P2RX6P
0.285714269	0.091362759	0.107	1	475	ATOX1
0.285714269	0.091362759	0.125	1	137886	UBXN2B
0.285456896	0.091664784	0.128	1	5271	SERPINB8
0.285456896	0.091664784	0.111	1	9761	MLEC
0.285456896	0.091664784	0.125	1	26015	RPAP1
0.285456896	0.091664784	0.12	1	28992	MACROD1
0.285456896	0.091664784	0.118	1	56143	PCDHA5
0.285456896	0.091664784	0.134	1	57149	LYRM1
0.285456896	0.091664784	0.122	1	200931	Osta
0.285199463	0.091967456	0.119	1	6441	SFTPD

voxel-wise, subcortex			
Spearman's corr coef	p-val	GenID	Gene
0.223366573	< 0.001	147372	CCBE1
0.223313585	< 0.001	8761	PABPC4
0.223237306	< 0.001	7049	TGFBR3
0.223217756	< 0.001	139065	SLITRK4
0.223212093	< 0.001	79719	AAGAB
0.223196432	< 0.001	283078	MKX
0.223111182	< 0.001	23122	CLASP2
0.223092422	< 0.001	11222	MRPL3
0.223037496	< 0.001	54521	WDR44
0.223002255	< 0.001	25799	ZNF324
0.222943425	< 0.001	10531	PITRM1
0.222934052	< 0.001	3949	LDLR
0.222877443	< 0.001	4131	MAP1B
0.222851992	< 0.001	51307	FAM53C
0.222843736	< 0.001	130827	TMEM182
0.222838581	< 0.001	6547	SLC8A3
0.222779244	< 0.001	4018	LPA
0.222774729	< 0.001	8175	SF3A2
0.222734481	< 0.001	5481	PPID
0.222700074	< 0.001	404266	RP11-357H14.1
0.222665384	< 0.001	119710	C11orf74
0.222598702	< 0.001	122616	C14orf79
0.222567692	< 0.001	646643	SBK2
0.222555473	< 0.001	387804	VSTM5
0.222464383	< 0.001	4154	MBNL1
0.222356558	< 0.001	22801	ITGA11
0.222125739	< 0.001	222696	ZSCAN23
0.2221255	< 0.001	22849	CPEB3
0.222080752	< 0.001	719	C3AR1
0.222052798	< 0.001	130888	FBXO36
0.222008362	< 0.001	54714	CNGB3
0.221962109	< 0.001	4833	NME4
0.221952781	< 0.001	10206	TRIM13
0.221944839	< 0.001	8208	CHAF1B
0.221940473	< 0.001	6457	SH3GL3
0.221925616	< 0.001	23168	RTF1
0.221919537	< 0.001	339896	GADL1
0.22190477	< 0.001	4049	LTA
0.221673384	< 0.001	29922	NME7
0.221653476	< 0.001	90550	CCDC109A
0.221624121	< 0.001	149628	PYHIN1
0.221575692	< 0.001	389119	C3orf54
0.22144717	< 0.001	285349	ZNF660
0.221430033	< 0.001	401036	ASB18
0.221395954	< 0.001	57333	RCN3
0.221368	< 0.001	51750	RTEL1
0.221367493	< 0.001	256586	LYSMD2
0.221367449	< 0.001	6565	SLC15A2
0.221358299	< 0.001	201191	SAMD14
0.221327364	< 0.001	23595	ORC3L
0.221309766	< 0.001	23001	WDFY3
0.221285343	< 0.001	1780	DYNC1I1
0.221272722	< 0.001	57016	AKR1B10
0.221225917	< 0.001	441525	SPANXN4
0.221206784	< 0.001	2525	FUT3
0.221174553	< 0.001	8317	CDC7
0.221118286	< 0.001	80270	HSD3B7
0.221054018	< 0.001	9167	COX7A2L
0.221043989	< 0.001	79953	C20orf39
0.221032336	< 0.001	54751	FBLIM1
0.221026391	< 0.001	51646	YPEL5
0.220952138	< 0.001	5986	RFNG
0.220882595	< 0.001	1182	CLCN3
0.220857888	< 0.001	100131733	USP30-AS1
0.220778957	< 0.001	7051	TGM1
0.220778018	< 0.001	147912	SIX5
0.220736787	< 0.001	56931	DUS3L
0.220676333	< 0.001	7286	TUFT1
0.220666394	< 0.001	119687	OR51A7
0.220642433	< 0.001	3191	HNRNPL
0.220638722	< 0.001	57621	ZBTB2
0.220610484	< 0.001	5515	PPP2CA
0.220602602	< 0.001	339778	C2orf70
0.22059916	< 0.001	58155	PTBP2
0.220580131	< 0.001	253635	CCDC75
0.220530808	< 0.001	8786	RGS11
0.220521986	< 0.001	8721	EDF1
0.220515773	< 0.001	2691	GHRH
0.22046636	< 0.001	84961	FBXL20

region-wise, subcortex					
Spearman's corr coef	p-val	p-val, SA	p-val, SA (BH- correction)	GenID	Gene
0.285199463	0.091967456	0.12	1	22943	DKK1
0.285199463	0.091967456	0.12	1	84639	IL1F10
0.284684658	0.092575073	0.131	1	1508	CTSB
0.284684658	0.092575073	0.141	1	2354	FOSB
0.284684658	0.092575073	0.139	1	6002	RGS12
0.284684658	0.092575073	0.127	1	10859	LILRB1
0.284684658	0.092575073	0.116	1	64393	ZMAT3
0.284684658	0.092575073	0.134	1	128977	C22orf39
0.284169883	0.093185984	0.115	1	4107	MAGEA8
0.284169883	0.093185984	0.11	1	6476	SI
0.284169883	0.093185984	0.129	1	51276	ZNF571
0.284169883	0.093185984	0.126	1	202500	TCTE1
0.28391248	0.0934925	0.122	1	22920	KIFAP3
0.28391248	0.0934925	0.118	1	166647	GPR125
0.283655077	0.093799837	0.118	1	4713	NDUFB7
0.283655077	0.093799837	0.127	1	375196	LOC375196
0.283140272	0.094416983	0.116	1	160	AP2A1
0.283140272	0.094416983	0.121	1	1534	CYB561
0.283140272	0.094416983	0.117	1	65243	ZNF643
0.283140272	0.094416983	0.134	1	84065	TMEM222
0.283140272	0.094416983	0.121	1	171484	FAM9C
0.282625467	0.095037095	0.126	1	6586	SLIT3
0.282625467	0.095037095	0.122	1	56097	PCDHGC5
0.282625467	0.095037095	0.119	1	57510	XPO5
0.282368094	0.095348351	0.129	1	2762	GMDS
0.282368094	0.095348351	0.146	1	5886	RAD23A
0.282368094	0.095348351	0.122	1	9991	ROD1
0.282368094	0.095348351	0.124	1	170960	ZNF721
0.282368094	0.095348351	0.129	1	284257	AC100775.1
0.282368094	0.095348351	0.126	1	553115	PEF1
0.282110661	0.09566053	0.123	1	896	CCND3
0.282110661	0.09566053	0.127	1	2941	GSTA4
0.282110661	0.09566053	0.109	1	4072	EPCAM
0.282110661	0.09566053	0.113	1	23350	SR140
0.282110661	0.09566053	0.124	1	25820	ARIH1
0.282110661	0.09566053	0.12	1	152518	NFXL1
0.281853288	0.095973358	0.107	1	3149	HMGB3
0.281853288	0.095973358	0.123	1	7291	TWIST1
0.281853288	0.095973358	0.136	1	10322	SMYD5
0.281853288	0.095973358	0.127	1	11119	BTN3A1
0.281853288	0.095973358	0.112	1	54020	SLC37A1
0.281853288	0.095973358	0.134	1	154214	RNF217
0.281853288	0.095973358	0.109	1	440823	MIAT
0.281595886	0.096286982	0.142	1	9409	PEX16
0.281595886	0.096286982	0.136	1	255928	SYT14
0.281595886	0.096286982	0.128	1	100288367	LOC100288367
0.281338483	0.096601352	0.113	1	996	CDC27
0.281338483	0.096601352	0.119	1	25804	LSM4
0.28108108	0.096916698	0.134	1	26156	RSL1D1
0.28108108	0.096916698	0.142	1	55171	TBCCD1
0.28108108	0.096916698	0.117	1	114795	TMEM132B
0.28108108	0.096916698	0.127	1	116236	ABHD15
0.28108108	0.096916698	0.144	1	126370	OR1I1
0.280823678	0.097232692	0.128	1	4012	LNPEP
0.280823678	0.097232692	0.124	1	26050	SLITRK5
0.280823678	0.097232692	0.117	1	55239	OGFOD1
0.280823678	0.097232692	0.123	1	79896	THNSL1
0.280823678	0.097232692	0.125	1	100288713	PMS2P10
0.280566275	0.097549483	0.123	1	126129	CPT1C
0.280308902	0.097867072	0.125	1	611	OPN1SW
0.280308902	0.097867072	0.112	1	867	CBL
0.280308902	0.097867072	0.15	1	2220	FCN2
0.280308902	0.097867072	0.159	1	6448	SGSH
0.280308902	0.097867072	0.128	1	9422	ZNF264
0.28005147	0.098185562	0.109	1	2949	GSTM5
0.28005147	0.098185562	0.126	1	4897	NRCAM
0.28005147	0.098185562	0.125	1	390113	OR4X1
0.279794097	0.098504752	0.139	1	1036	CDO1
0.279794097	0.098504752	0.138	1	9344	TAOK2
0.279794097	0.098504752	0.129	1	9552	SPAG7
0.279794097	0.098504752	0.145	1	283106	CSNK2A1P
0.279536664	0.098824732	0.127	1	2695	GIP
0.279536664	0.098824732	0.137	1	92703	TMEM183A
0.279536664	0.098824732	0.139	1	162333	MARCHF10
0.279536664	0.098824732	0.14	1	284040	CDRT4
0.279279262	0.099145517	0.14	1	11140	CDC37
0.279279262	0.099145517	0.115	1	57231	SNX14
0.279279262	0.099145517	0.129	1	79022	TMEM106C
0.279279262	0.099145517	0.146	1	199974	CYP4Z1

voxel-wise, subcortex			
Spearman's corr coef	p-val	GenID	Gene
0.220451325	< 0.001	8756	ADAM7
0.220448807	< 0.001	166336	PRICKLE2
0.220314801	< 0.001	353142	LCE3A
0.220216736	< 0.001	64746	ACBD3
0.220189989	< 0.001	602	BCL3
0.220152944	< 0.001	2690	GHR
0.220139295	< 0.001	9832	JAKMIP2
0.22013855	< 0.001	27248	C2orf30
0.220094621	< 0.001	646508	AC092070.5
0.21989505	< 0.001	1421	CRYGD
0.219850108	< 0.001	401115	C4orf48
0.219809994	< 0.001	64091	POPDC2
0.21978046	< 0.001	1990	CELA1
0.219766527	< 0.001	22837	COBLL1
0.219759658	< 0.001	57599	WDR48
0.219738483	< 0.001	23339	VPS39
0.219671801	< 0.001	168433	RNF133
0.219606519	< 0.001	1155	TBCB
0.219573826	< 0.001	58525	WIZ
0.219572246	< 0.001	57171	DOLPP1
0.219539925	< 0.001	23129	PLXND1
0.219499901	< 0.001	134549	SHROOM1
0.219457656	< 0.001	3219	HOXB9
0.219439268	< 0.001	55869	HDAC8
0.219414756	< 0.001	441869	ANKRD65
0.21939525	< 0.001	4837	NNMT
0.219385594	< 0.001	90060	CCDC120
0.219356239	< 0.001	3417	IDH1
0.219352901	< 0.001	3159	HMGA1
0.219341382	< 0.001	4608	MYBPH
0.219340369	< 0.001	728716	LOC728716
0.219294026	< 0.001	60412	EXOC4
0.219207942	< 0.001	4353	MPO
0.21909529	< 0.001	2232	FDXR
0.218983203	< 0.001	1124	CHN2
0.218904361	< 0.001	22954	TRIM32
0.218903616	< 0.001	60677	BRUNOL6
0.21889405	< 0.001	7296	TXNRD1
0.218843624	< 0.001	387856	C12orf68
0.218789205	< 0.001	475	ATOX1
0.218740538	< 0.001	79849	PDZD3
0.218705624	< 0.001	768206	PRCD
0.21859251	< 0.001	2218	FKTN
0.218558893	< 0.001	93145	OLFM2
0.218554348	< 0.001	80237	ELL3
0.218549237	< 0.001	8433	UTF1
0.218510792	< 0.001	93663	ARHGAP18
0.218510613	< 0.001	28904	IGKV1D-8
0.218301103	< 0.001	11250	GPR45
0.218183354	< 0.001	392255	GDF6
0.218169793	< 0.001	134359	C5orf37
0.218027979	< 0.001	80320	SP6
0.21797727	< 0.001	56964	WDR93
0.217961669	< 0.001	9038	TAAR5
0.217904463	< 0.001	145567	TTC7B
0.217891753	< 0.001	389137	ARMC10P1
0.217851356	< 0.001	5926	ARID4A
0.2178417	< 0.001	400406	LOC400406
0.217833057	< 0.001	55902	ACSS2
0.217816621	< 0.001	7251	TSG101
0.217779756	< 0.001	51010	EXOSC3
0.217729419	< 0.001	51143	DYNC1LI1
0.217690319	< 0.001	56180	MOSPD1
0.217681319	< 0.001	54962	TIPIN
0.217638031	< 0.001	23380	SRGAP2
0.217636093	< 0.001	4046	LSP1
0.217582136	< 0.001	5818	PVRL1
0.217541084	< 0.001	9172	MYOM2
0.217484713	< 0.001	55083	KIF26B
0.217464566	< 0.001	3283	HSD3B1
0.21736148	< 0.001	158158	RASEF
0.217288673	< 0.001	116224	FAM122A
0.217239648	< 0.001	1113	CHGA
0.217234448	< 0.001	1394	CRHR1
0.217177615	< 0.001	57121	LPAR5
0.217104703	< 0.001	56262	LRRC8A
0.217089206	< 0.001	81031	SLC2A10
0.217042297	< 0.001	373856	USP41
0.217036635	< 0.001	392188	USP17L8

region-wise, subcortex					
Spearman's corr coef	p-val	p-val, SA	p-val, SA (BH- correction)	GenID	Gene
0.279279262	0.099145517	0.141	1	441452	FAM75C1
0.279021889	0.099467099	0.11	1	107	ADCY1
0.278764457	0.099789605	0.14	1	9391	CIAO1
0.278764457	0.099789605	0.112	1	79147	FKRP
0.278764457	0.099789605	0.139	1	353143	LCE3B
0.278507084	0.100112788	0.136	1	6132	RPL8
0.278507084	0.100112788	0.137	1	6588	SLN
0.278507084	0.100112788	0.136	1	8364	HIST1H4C
0.278507084	0.100112788	0.134	1	283385	MORN3
0.278249651	0.100436792	0.127	1	23300	ATMIN
0.277992278	0.100761592	0.13	1	5770	PTPN1
0.277992278	0.100761592	0.12	1	7067	THRA
0.277992278	0.100761592	0.124	1	80014	WWC2
0.277992278	0.100761592	0.128	1	89970	RSPRY1
0.277992278	0.100761592	0.12	1	119467	CLRN3
0.277992278	0.100761592	0.137	1	128414	NKAIN4
0.277992278	0.100761592	0.13	1	390407	EIF4A1P6
0.277734876	0.101087317	0.106	1	10641	TUSC4
0.277477473	0.101413734	0.132	1	10683	DLL3
0.277477473	0.101413734	0.123	1	65094	JMJD4
0.277477473	0.101413734	0.136	1	163720	CYP4Z2P
0.27722007	0.101740964	0.136	1	1855	DVL1
0.27722007	0.101740964	0.138	1	8775	NAPA
0.27722007	0.101740964	0.123	1	89780	WNT3A
0.276962668	0.102069005	0.135	1	6636	SNRPF
0.276962668	0.102069005	0.142	1	10573	MRPL28
0.276962668	0.102069005	0.13	1	79656	BEND5
0.276705265	0.102398008	0.121	1	5440	AC025647.1
0.276705265	0.102398008	0.145	1	10737	RFPL3S
0.276705265	0.102398008	0.135	1	23479	ISCU
0.276705265	0.102398008	0.127	1	51194	IPO11
0.276705265	0.102398008	0.131	1	51555	PEX5L
0.276705265	0.102398008	0.14	1	126321	C19orf28
0.276705265	0.102398008	0.136	1	100129201	LOC100129201
0.276447892	0.102727637	0.131	1	9898	UBAP2L
0.276447892	0.102727637	0.135	1	54737	MPHOSPH8
0.276447892	0.102727637	0.136	1	55571	C2orf29
0.27619046	0.103058122	0.12	1	7070	THY1
0.27619046	0.103058122	0.119	1	7087	ICAM5
0.27619046	0.103058122	0.123	1	9890	RP4-788L13.1
0.27619046	0.103058122	0.112	1	284802	FRG1B
0.275933087	0.10338942	0.123	1	1551	CYP3A7
0.275933087	0.10338942	0.13	1	123591	C15orf27
0.275933087	0.10338942	0.125	1	345462	ZNF879
0.275675654	0.103721537	0.115	1	57088	PLSCR4
0.275675654	0.103721537	0.118	1	84193	SETD3
0.275675654	0.103721537	0.122	1	339778	C2orf70
0.275418282	0.104054615	0.136	1	10238	DCAF7
0.275418282	0.104054615	0.125	1	64061	TSPYL2
0.275418282	0.104054615	0.126	1	150962	PUS10
0.275160879	0.104388334	0.123	1	641455	AL589182.2
0.274903476	0.104722902	0.128	1	5346	PLIN
0.274903476	0.104722902	0.131	1	7064	THOP1
0.274903476	0.104722902	0.14	1	140685	ZBTB46
0.274646074	0.105058305	0.148	1	7136	TNNI2
0.274646074	0.105058305	0.125	1	57103	C12orf5
0.274646074	0.105058305	0.142	1	80034	CSRNP3
0.274388671	0.105394669	0.148	1	6276	S100A5
0.274388671	0.105394669	0.121	1	6311	ATXN2
0.274388671	0.105394669	0.144	1	10529	NEBL
0.274388671	0.105394669	0.13	1	10949	HNRNPA0
0.274388671	0.105394669	0.134	1	23732	FRRS1L
0.274131268	0.105731703	0.139	1	6778	STAT6
0.274131268	0.105731703	0.13	1	51289	RXFP3
0.274131268	0.105731703	0.146	1	440956	IQCF6
0.273873895	0.106069535	0.127	1	9127	P2RX6
0.273873895	0.106069535	0.133	1	9678	PHF14
0.273873895	0.106069535	0.124	1	10154	PLXNC1
0.273873895	0.106069535	0.117	1	57794	SF4
0.273873895	0.106069535	0.141	1	93426	SYCE1
0.273616463	0.106408231	0.133	1	3833	KIFC1
0.273616463	0.106408231	0.155	1	390748	AC092384.2
0.27335909	0.106747895	0.123	1	6449	SGTA
0.27335909	0.106747895	0.161	1	7077	TIMP2
0.27335909	0.106747895	0.116	1	26121	PRPF31
0.273101658	0.107088245	0.139	1	5212	VIT
0.273101658	0.107088245	0.134	1	10885	WDR3
0.273101658	0.107088245	0.134	1	22797	TFEC
0.273101658	0.107088245	0.109	1	154743	C7orf60

voxel-wise, subcortex			
Spearman's corr coef	p-val	GenID	Gene
0.217026979	< 0.001	64714	PDIA2
0.21699281	< 0.001	85474	LBX2
0.216910526	< 0.001	5718	PSMD12
0.216869473	< 0.001	10565	ARFGEF1
0.21685797	< 0.001	80169	CTC1
0.216802984	< 0.001	1133	CHRM5
0.216793701	< 0.001	348654	GEN1
0.216746986	< 0.001	10791	VAMP5
0.216742069	< 0.001	63036	CELA2A
0.216713652	< 0.001	8092	ALX1
0.216677338	< 0.001	1977	EIF4E
0.216572866	< 0.001	10480	EIF3M
0.216570541	< 0.001	55204	GOLPH3L
0.216534138	< 0.001	728045	PPBPP1
0.216516957	< 0.001	7587	ZNF37A
0.216431901	< 0.001	642587	MIR205HG
0.2162835	< 0.001	11153	FICD
0.216274202	< 0.001	23560	GTPBP4
0.216266587	< 0.001	2820	GPD2
0.216243565	< 0.001	170685	NUDT10
0.216222674	< 0.001	998	CDC42
0.216168523	< 0.001	27147	DENND2A
0.216131747	< 0.001	51072	MEMO1
0.216112614	< 0.001	23641	LDOC1
0.216105849	< 0.001	5955	RCN2
0.216070369	< 0.001	375298	CERKL
0.216065913	< 0.001	339745	SPOPL
0.216062471	< 0.001	51499	TRIAP1
0.216026261	< 0.001	1102	RCBTB2
0.215993941	< 0.001	11137	PWP1
0.215990961	< 0.001	162466	PHOSPHO1
0.215955302	< 0.001	57523	KIAA1305
0.215952337	< 0.001	22924	MAPRE3
0.215928003	< 0.001	9826	ARHGEF11
0.215920478	< 0.001	84962	JUB
0.215851203	< 0.001	466	ATF1
0.215801433	< 0.001	140890	SFRS12
0.215800032	< 0.001	375196	LOC375196
0.215799198	< 0.001	23142	DCUN1D4
0.215779692	< 0.001	81492	AC011530.1
0.215672061	< 0.001	388021	TMEM179
0.215615794	< 0.001	9242	MSC
0.215543717	< 0.001	84248	FYTTD1
0.215467021	< 0.001	57657	HCN3
0.215453267	< 0.001	83592	AKR1CL2
0.215443522	< 0.001	2257	FGF12
0.215433583	< 0.001	126868	C1orf161
0.215387523	< 0.001	4713	NDUFB7
0.215343416	< 0.001	55909	BIN3
0.215333387	< 0.001	64506	CPEB1
0.215263262	< 0.001	169166	SNX31
0.215261042	< 0.001	200931	Osta
0.215226486	< 0.001	11130	ZWINT
0.215206623	< 0.001	924	CD7
0.215181261	< 0.001	399844	FLJ45445
0.215172917	< 0.001	8841	HDAC3
0.215168074	< 0.001	83873	GPR61
0.215129361	< 0.001	23400	ATP13A2
0.21505478	< 0.001	79707	NOL9
0.214952171	< 0.001	6613	SUMO2
0.214916408	< 0.001	337879	KRTAP8-1
0.21486868	< 0.001	4771	NF2
0.214813143	< 0.001	6541	SLC7A1
0.214807302	< 0.001	118881	COMTD1
0.214770421	< 0.001	2885	GRB2
0.214732453	< 0.001	4259	MGST3
0.214727983	< 0.001	7803	PTP4A1
0.214710712	< 0.001	129852	C2orf73
0.214710072	< 0.001	970	CD70
0.21468769	< 0.001	158866	ZDHHC15
0.214657784	< 0.001	5459	POU4F3
0.214632794	< 0.001	5002	SLC22A18
0.214631498	< 0.001	283897	C16orf54
0.214574948	< 0.001	64220	STRA6
0.214519322	< 0.001	204801	NLRP11
0.214514762	< 0.001	864	RUNX3
0.214476973	< 0.001	388536	ZNF790
0.214442983	< 0.001	6455	SH3GL1
0.214412525	< 0.001	15	AANAT

region-wise, subcortex					
Spearman's corr coef	p-val	p-val, SA	p-val, SA (BH- correction)	GenID	Gene
0.272844285	0.10742943	0.131	1	4490	MT1B
0.272844285	0.10742943	0.138	1	283455	KSR2
0.272586882	0.107771397	0.157	1	2280	FKBP1A
0.272586882	0.107771397	0.129	1	9125	RQCD1
0.27232945	0.108114235	0.147	1	5360	PLTP
0.27232945	0.108114235	0.128	1	7332	UBE2L3
0.27232945	0.108114235	0.121	1	54207	KCNK10
0.27232945	0.108114235	0.129	1	54888	NSUN2
0.27232945	0.108114235	0.132	1	57095	C1orf128
0.27232945	0.108114235	0.132	1	254359	ZDHHC24
0.27232945	0.108114235	0.145	1	400673	VMAC
0.27232945	0.108114235	0.13	1	552891	DNAJC25-GNG10
0.272072077	0.108458057	0.141	1	9242	MSC
0.272072077	0.108458057	0.129	1	23025	UNC13A
0.271814644	0.108802564	0.142	1	4184	SMCP
0.271814644	0.108802564	0.15	1	8808	IL1RL2
0.271814644	0.108802564	0.141	1	11169	WDHD1
0.271814644	0.108802564	0.123	1	25929	GEMIN5
0.271814644	0.108802564	0.115	1	58538	MPP4
0.271814644	0.108802564	0.131	1	100130967	C6orf99
0.271557271	0.109147906	0.153	1	5524	PPP2R4
0.271557271	0.109147906	0.14	1	7704	ZBTB16
0.271557271	0.109147906	0.137	1	27315	PGAP2
0.271557271	0.109147906	0.142	1	29982	NRBF2
0.271557271	0.109147906	0.131	1	56138	PCDHA11
0.271557271	0.109147906	0.155	1	256329	C11orf35
0.271299869	0.109494053	0.15	1	388753	C1orf31
0.271042466	0.10984122	0.146	1	7514	XPO1
0.271042466	0.10984122	0.14	1	9077	DIRAS3
0.271042466	0.10984122	0.14	1	143630	UBQLNL
0.270785064	0.11018908	0.129	1	9993	DGCR2
0.270785064	0.11018908	0.147	1	23080	AVL9
0.270785064	0.11018908	0.129	1	84272	YIPF4
0.270785064	0.11018908	0.12	1	345557	PLCXD3
0.270527661	0.110537775	0.143	1	1186	CLCN7
0.270527661	0.110537775	0.142	1	6817	SULT1A1
0.270527661	0.110537775	0.16	1	6923	TCEB2
0.270527661	0.110537775	0.139	1	59353	TMEM35
0.270527661	0.110537775	0.133	1	81854	MGC3771
0.270527661	0.110537775	0.152	1	137868	SGCZ
0.270270258	0.110887319	0.121	1	9467	SH3BP5
0.270270258	0.110887319	0.155	1	124872	B4GALNT2
0.270012885	0.111237653	0.146	1	4709	NDUFB3
0.269755453	0.111589029	0.133	1	318	NUDT2
0.269755453	0.111589029	0.152	1	2257	FGF12
0.269755453	0.111589029	0.145	1	5210	PFKFB4
0.269755453	0.111589029	0.126	1	8851	CDK5R1
0.269755453	0.111589029	0.135	1	10472	ZNF238
0.269755453	0.111589029	0.15	1	23160	WDR43
0.26949808	0.111941099	0.151	1	726	CAPN5
0.26949808	0.111941099	0.146	1	4705	NDUFA10
0.26949808	0.111941099	0.149	1	54977	SLC25A38
0.26949808	0.111941099	0.147	1	286256	LCN12
0.269240648	0.112294003	0.159	1	526	ATP6V1B2
0.269240648	0.112294003	0.133	1	5296	PIK3R2
0.269240648	0.112294003	0.151	1	92906	HNRPLL
0.268983275	0.112647764	0.112	1	8867	SYNJ1
0.268983275	0.112647764	0.138	1	10236	HNRNPR
0.268983275	0.112647764	0.136	1	91133	L3MBTL4
0.268983275	0.112647764	0.166	1	128553	TSHZ2
0.268983275	0.112647764	0.132	1	376267	RAB15
0.268725872	0.113002494	0.146	1	144402	CPNE8
0.268725872	0.113002494	0.129	1	196446	C12orf28
0.268725872	0.113002494	0.165	1	389812	LCN15
0.268725872	0.113002494	0.131	1	440792	LOC440792
0.268725872	0.113002494	0.131	1	474148	TTTY3B
0.268468469	0.113357946	0.128	1	3914	LAMB3
0.268468469	0.113357946	0.117	1	57470	LRRC47
0.268468469	0.113357946	0.143	1	112936	VPS26B
0.268468469	0.113357946	0.152	1	146771	TCAM1
0.268211067	0.113714248	0.15	1	222008	VSTM2A
0.267953664	0.114071406	0.145	1	5209	PFKFB3
0.267953664	0.114071406	0.152	1	54937	SOHLH2
0.267953664	0.114071406	0.16	1	219938	SPATA19
0.267953664	0.114071406	0.151	1	340533	KIAA2022
0.267696261	0.114429578	0.157	1	3778	KCNMA1
0.267696261	0.114429578	0.144	1	84861	KLHL22
0.267696261	0.114429578	0.135	1	118812	MORN4
0.267438889	0.114788406	0.161	1	2583	B4GALNT1

voxel-wise, subcortex			
Spearman's corr coef	p-val	GenID	Gene
0.214365527	< 0.001	714	C1QC
0.214329779	< 0.001	8778	SIGLEC5
0.214323089	< 0.001	6865	TACR2
0.214298025	< 0.001	11019	LIAS
0.214296818	< 0.001	6633	SNRPD2
0.21429421	< 0.001	8662	EIF3B
0.214285016	< 0.001	57139	RGL3
0.214188442	< 0.001	138802	OR13C8
0.214144513	< 0.001	85458	DIXDC1
0.214101985	< 0.001	10567	RABAC1
0.214078665	< 0.001	7320	UBE2B
0.21407263	< 0.001	55186	SLC25A36
0.21398747	< 0.001	10522	DEAF1
0.213949218	< 0.001	1496	CTNNA2
0.213899434	< 0.001	124637	CYB5D1
0.213889599	< 0.001	1995	ELAVL3
0.213855043	< 0.001	9636	ISG15
0.213834807	< 0.001	147463	ANKRD29
0.213744074	< 0.001	64430	C14orf135
0.21372503	< 0.001	85352	KIAA1644
0.213701442	< 0.001	55625	ZDHHC7
0.213583782	< 0.001	728012	RP11-14I4.3
0.213519529	< 0.001	54927	CHCHD3
0.213423222	< 0.001	100129434	AC007743.1
0.213411063	< 0.001	22848	AAK1
0.213403806	< 0.001	387836	CLEC2A
0.213367403	< 0.001	25820	ARIH1
0.213363975	< 0.001	79188	TMEM43
0.213363051	< 0.001	91289	LMF2
0.213329419	< 0.001	10801	SEPTIN9
0.213326827	< 0.001	51225	ABI3
0.213312805	< 0.001	5467	PPARD
0.213302121	< 0.001	84443	FRMPD3
0.213293672	< 0.001	222826	C6orf146
0.213178426	< 0.001	400935	IL17REL
0.213177398	< 0.001	26031	OSBPL3
0.213117048	< 0.001	5591	PRKDC
0.213100329	< 0.001	100129792	CCDC152
0.213095874	< 0.001	55630	AF205589.2
0.212965399	< 0.001	138255	C9orf135
0.212946907	< 0.001	79094	CHAC1
0.212813184	< 0.001	390003	GUCY2GP
0.21280019	< 0.001	285782	CAGE1
0.21278505	< 0.001	8496	PPFIBP1
0.212765828	< 0.001	116843	C6orf192
0.212753758	< 0.001	51185	CRBN
0.212746128	< 0.001	23271	CAMSAP1L1
0.212676957	< 0.001	57415	C3orf14
0.212346718	< 0.001	4953	ODC1
0.212331399	< 0.001	221143	N6AMT2
0.212330937	< 0.001	9146	HGS
0.212176874	< 0.001	345757	FAM174A
0.212170273	< 0.001	127829	ARL8A
0.212039515	< 0.001	122553	TRAPPC6B
0.21201992	< 0.001	10999	SLC27A4
0.211984724	< 0.001	389434	IYD
0.211871803	< 0.001	25803	SPDEF
0.21185945	< 0.001	10863	ADAM28
0.211708635	< 0.001	25943	C20orf194
0.211674735	< 0.001	90632	LINC00473
0.211612523	< 0.001	2998	GYS2
0.211592093	< 0.001	92906	HNRPLL
0.211578622	< 0.001	80161	ASMTL-AS1
0.211532012	< 0.001	159090	FAM122B
0.211456507	< 0.001	219	ALDH1B1
0.211430132	< 0.001	4978	AP000843.1
0.211320728	< 0.001	157376	FER1L6-AS2
0.211313218	< 0.001	388552	BLOC1S3
0.211256281	< 0.001	9562	MINPP1
0.21124728	< 0.001	2636	GBX1
0.211193323	< 0.001	11007	CCDC85B
0.211190537	< 0.001	83723	FAM57B
0.211086527	< 0.001	623	BDKRB1
0.211052164	< 0.001	8318	CDC45L
0.211039171	< 0.001	823	CAPN1
0.210981965	< 0.001	10096	ACTR3
0.210884169	< 0.001	63933	CCDC90A
0.210831419	< 0.001	134121	C5orf49
0.210794747	< 0.001	1642	DDB1

region-wise, subcortex					
Spearman's corr coef	p-val	p-val, SA	p-val, SA (BH- correction)	GenID	Gene
0.267438889	0.114788406	0.158	1	3297	HSF1
0.267438889	0.114788406	0.145	1	646936	LOC646936
0.267438889	0.114788406	0.144	1	100290481	CYAT1
0.267181456	0.115148112	0.152	1	5803	PTPRZ1
0.267181456	0.115148112	0.157	1	83871	RAB34
0.266924083	0.115508683	0.144	1	79699	ZYG11B
0.266666651	0.115870111	0.144	1	2173	FABP7
0.266666651	0.115870111	0.146	1	4085	MAD2L1
0.266666651	0.115870111	0.146	1	5007	OSBP
0.266666651	0.115870111	0.123	1	23386	NUDCD3
0.266666651	0.115870111	0.148	1	29760	BLNK
0.266666651	0.115870111	0.133	1	64175	LEPRE1
0.266409278	0.116232559	0.147	1	5657	PRTN3
0.266409278	0.116232559	0.138	1	6628	SNRPB
0.266409278	0.116232559	0.163	1	23627	PRND
0.266409278	0.116232559	0.135	1	94097	SFXN5
0.266409278	0.116232559	0.164	1	116983	ACAP3
0.266409278	0.116232559	0.13	1	124773	C17orf64
0.266151875	0.116595671	0.158	1	1102	RCBTB2
0.266151875	0.116595671	0.15	1	2660	MSTN
0.266151875	0.116595671	0.156	1	8538	BARX2
0.266151875	0.116595671	0.138	1	59335	PRDM12
0.265894473	0.116959676	0.152	1	3200	HOXA3
0.265894473	0.116959676	0.132	1	85465	AC010896.1
0.265894473	0.116959676	0.164	1	245812	CNPY4
0.26563707	0.117324546	0.126	1	5686	PSMA5
0.26563707	0.117324546	0.128	1	10079	ATP9A
0.265379637	0.117690429	0.163	1	2258	FGF13
0.265379637	0.117690429	0.162	1	6580	SLC22A1
0.265379637	0.117690429	0.148	1	56999	ADAMTS9
0.265122265	0.118057035	0.149	1	5223	PGAM1
0.265122265	0.118057035	0.148	1	155054	ZNF425
0.265122265	0.118057035	0.164	1	170626	XAGE3
0.265122265	0.118057035	0.164	1	100129583	FAM47E
0.264864862	0.118424445	0.14	1	4130	MAP1A
0.264864862	0.118424445	0.154	1	10075	HUWE1
0.264864862	0.118424445	0.138	1	51094	ADIPOR1
0.264864862	0.118424445	0.159	1	375444	C5orf34
0.264607459	0.118792765	0.155	1	817	CAMK2D
0.264607459	0.118792765	0.154	1	9931	HELZ
0.264607459	0.118792765	0.149	1	10626	TRIM16
0.264607459	0.118792765	0.164	1	50614	GALNT9
0.264350057	0.11916212	0.128	1	3681	ITGAD
0.264350057	0.11916212	0.168	1	55800	SCN3B
0.264092654	0.119532183	0.121	1	9973	CCS
0.264092654	0.119532183	0.14	1	114786	XKR4
0.264092654	0.119532183	0.147	1	440072	LINC00167
0.264092654	0.119532183	0.154	1	100131827	ZNF717
0.263835251	0.119903065	0.149	1	3725	JUN
0.263835251	0.119903065	0.164	1	4943	TBC1D25
0.263835251	0.119903065	0.137	1	10300	KATNB1
0.263835251	0.119903065	0.166	1	53349	ZFYVE1
0.263577878	0.120274864	0.169	1	2591	GALNT3
0.263577878	0.120274864	0.161	1	23616	SH3BP1
0.263320446	0.12064749	0.149	1	23587	C17orf81
0.263320446	0.12064749	0.144	1	23654	PLXNB2
0.263320446	0.12064749	0.18	1	26517	TIMM13
0.263320446	0.12064749	0.148	1	162989	DEDD2
0.263320446	0.12064749	0.174	1	256536	TCERG1L
0.263063073	0.121021233	0.155	1	10746	MAP3K2
0.263063073	0.121021233	0.151	1	55178	RNMTL1
0.263063073	0.121021233	0.129	1	652968	GATSL3
0.262805641	0.121395603	0.159	1	83394	PITPNM3
0.262805641	0.121395603	0.146	1	280636	C11orf31
0.262805641	0.121395603	0.147	1	401565	FAM166A
0.262548268	0.121770896	0.151	1	9913	SUPT7L
0.262290865	0.122147053	0.158	1	7411	VBP1
0.262290865	0.122147053	0.167	1	80059	LRRTM4
0.262290865	0.122147053	0.169	1	729627	AC130416.2
0.262033463	0.122524261	0.148	1	1832	DSP
0.262033463	0.122524261	0.193	1	4482	MSRA
0.262033463	0.122524261	0.165	1	146556	C16orf89
0.262033463	0.122524261	0.162	1	404672	GTF2H5
0.26177606	0.122902177	0.151	1	2023	ENO1
0.26177606	0.122902177	0.147	1	9853	RUSC2
0.26177606	0.122902177	0.158	1	343477	HSP90B3P
0.26177606	0.122902177	0.168	1	652276	AC141586.5
0.261261255	0.123660602	0.166	1	4857	NOVA1
0.261261255	0.123660602	0.156	1	26231	LRRC29

voxel-wise, subcortex			
Spearman's corr coef	p-val	GenID	Gene
0.2107784	< 0.001	287	ANK2
0.210715532	< 0.001	9685	CLINT1
0.210661113	< 0.001	81614	NIPA2
0.210654229	< 0.001	91752	ZNF804A
0.210617185	< 0.001	54914	KIAA1797
0.210606962	< 0.001	9024	BRSK2
0.210594714	< 0.001	5705	PSMC5
0.210554212	< 0.001	346525	OR2A12
0.210481882	< 0.001	6750	SST
0.210384652	< 0.001	400410	ST20
0.210368395	< 0.001	25974	MMACHC
0.210341841	< 0.001	8737	RIPK1
0.21027182	< 0.001	57190	SEPN1
0.210267916	< 0.001	3004	GZMM
0.210257053	< 0.001	1443	CSH2
0.210157499	< 0.001	5935	RBM3
0.210106879	< 0.001	651746	ANKRD33B
0.210103348	< 0.001	2331	FMOD
0.21005933	< 0.001	5306	PITPNA
0.210030824	< 0.001	7485	WRB
0.210027292	< 0.001	136371	ASB10
0.210004181	< 0.001	81537	SGPP1
0.209925607	< 0.001	84616	KRTAP4-4
0.209888652	< 0.001	389383	C6orf126
0.209854573	< 0.001	81557	MAGED4B
0.209802285	< 0.001	79786	KLHL36
0.209782496	< 0.001	5581	PRKCE
0.209782228	< 0.001	8927	BSN
0.209745914	< 0.001	6449	SGTA
0.209732726	< 0.001	65061	PFTK2
0.209720939	< 0.001	152926	PPM1K
0.209644139	< 0.001	161835	FSIP1
0.209601045	< 0.001	27133	KCNH5
0.20950985	< 0.001	55800	SCN3B
0.209504455	< 0.001	89846	FGD3
0.209452271	< 0.001	3419	IDH3A
0.209434628	< 0.001	284912	LOC284912
0.209421247	< 0.001	9138	ARHGEF1
0.209384486	< 0.001	116412	ZNF837
0.209381878	< 0.001	64600	PLA2G2F
0.209330156	< 0.001	388165	UBE2Q2P1
0.209330067	< 0.001	84569	LYZL1
0.209248245	< 0.001	51340	CRNKL1
0.209185928	< 0.001	91464	ISX
0.209156215	< 0.001	904	CCNT1
0.209129289	< 0.001	115509	ZNF689
0.209129095	< 0.001	8915	BCL10
0.209122226	< 0.001	28316	CDH20
0.209047377	< 0.001	3586	IL10
0.209017292	< 0.001	64595	TTTY15
0.209014416	< 0.001	221264	AKD1
0.208989426	< 0.001	63827	BCAN
0.20898442	< 0.001	85021	REPS1
0.208941132	< 0.001	65267	WNK3
0.208883554	< 0.001	30062	RAX
0.208875105	< 0.001	100101267	POM121C
0.208849758	< 0.001	8924	HERC2
0.208845302	< 0.001	114800	CCDC85A
0.208781406	< 0.001	646903	LOC646903
0.208765343	< 0.001	57592	ZNF687
0.208717793	< 0.001	2049	EPHB3
0.208716959	< 0.001	23613	ZMYND8
0.208683521	< 0.001	3059	HCLS1
0.20864889	< 0.001	27075	TSPAN13
0.208641186	< 0.001	7520	XRCC5
0.208625302	< 0.001	9046	DOK2
0.208611369	< 0.001	642946	FLVCR1-AS1
0.208433807	< 0.001	124402	FAM100A
0.208421826	< 0.001	9110	MTMR4
0.208310112	< 0.001	441481	GPX1P1
0.208299428	< 0.001	643853	TMPPE
0.208274171	< 0.001	327	APEH
0.20826897	< 0.001	54769	DIRAS2
0.208266377	< 0.001	390077	OR52N2
0.208201826	< 0.001	150147	C21orf128
0.208169609	< 0.001	83446	CCDC70
0.20814082	< 0.001	64180	DPEP3
0.208137751	< 0.001	10629	TAF6L
0.208122984	< 0.001	285596	FAM153A

region-wise, subcortex					
Spearman's corr coef	p-val	p-val, SA	p-val, SA (BH- correction)	GenID	Gene
0.261261255	0.123660602	0.168	1	341568	OR8S1
0.261261255	0.123660602	0.145	1	100128588	LOC100128588
0.261003882	0.124041326	0.154	1	128	ADH5
0.261003882	0.124041326	0.173	1	1960	EGR3
0.261003882	0.124041326	0.161	1	9244	CRLF1
0.261003882	0.124041326	0.152	1	285848	PNPLA1
0.260746449	0.124422774	0.159	1	211	ALAS1
0.260746449	0.124422774	0.159	1	6832	SUPV3L1
0.260746449	0.124422774	0.159	1	7088	TLE1
0.260746449	0.124422774	0.152	1	8241	RBM10
0.260746449	0.124422774	0.165	1	54991	C1orf159
0.260746449	0.124422774	0.168	1	127602	DNAH14
0.260746449	0.124422774	0.164	1	285987	DLX6-AS1
0.260746449	0.124422774	0.176	1	353332	KRTAP12-1
0.260489076	0.124805048	0.126	1	834	CASP1
0.260489076	0.124805048	0.172	1	8991	SELENBP1
0.260489076	0.124805048	0.162	1	253980	KCTD13
0.260231644	0.125188261	0.145	1	226	ALDOA
0.260231644	0.125188261	0.171	1	1282	COL4A1
0.260231644	0.125188261	0.13	1	55902	ACSS2
0.260231644	0.125188261	0.154	1	388272	C16orf87
0.259974271	0.125572339	0.153	1	160428	ALDH1L2
0.259974271	0.125572339	0.171	1	389857	CENPVL1
0.259716868	0.125957489	0.176	1	23768	FLRT2
0.259459466	0.12634331	0.174	1	7511	XPNPEP1
0.259459466	0.12634331	0.154	1	55345	C4orf21
0.259459466	0.12634331	0.17	1	55785	FGD6
0.259459466	0.12634331	0.157	1	220972	MARCHF8
0.259459466	0.12634331	0.159	1	387758	FIBIN
0.259202063	0.12673007	0.152	1	5709	PSMD3
0.25894466	0.127117708	0.162	1	25890	ABI3BP
0.25894466	0.127117708	0.162	1	51761	ATP8A2
0.25894466	0.127117708	0.164	1	55198	APPL2
0.258687258	0.12750642	0.135	1	84179	MFSD7
0.258172452	0.128286108	0.155	1	100289274	DNAJC3-AS1
0.25791505	0.128677323	0.163	1	685	BTC
0.257657647	0.129069611	0.155	1	10966	RAB40B
0.257657647	0.129069611	0.162	1	56203	LMOD3
0.257657647	0.129069611	0.151	1	154467	C6orf129
0.257400244	0.129462615	0.151	1	57210	SLC45A4
0.256885439	0.130251259	0.157	1	1974	EIF4A2
0.256885439	0.130251259	0.178	1	2295	FOXF2
0.256885439	0.130251259	0.172	1	10591	C6orf108
0.256885439	0.130251259	0.162	1	83733	SLC25A18
0.256885439	0.130251259	0.175	1	84722	PSRC1
0.256628066	0.130646944	0.153	1	5655	KLK10
0.256628066	0.130646944	0.153	1	54492	NEURL1B
0.256628066	0.130646944	0.163	1	57876	MUC3B
0.256628066	0.130646944	0.166	1	58504	ARHGAP22
0.256628066	0.130646944	0.182	1	83943	IMMP2L
0.256628066	0.130646944	0.165	1	286234	C9orf79
0.256628066	0.130646944	0.181	1	439953	LOC439953
0.256370664	0.131043717	0.159	1	4946	OAZ1
0.256370664	0.131043717	0.182	1	5100	PCDH8
0.256370664	0.131043717	0.162	1	440699	LRRC52
0.256113261	0.131441176	0.159	1	330	BIRC3
0.256113261	0.131441176	0.157	1	1415	CRYBB2
0.256113261	0.131441176	0.164	1	114112	TXNRD3
0.256113261	0.131441176	0.176	1	347733	TUBB2B
0.255855858	0.131839573	0.194	1	1641	DCX
0.255855858	0.131839573	0.153	1	9652	TTC37
0.255598456	0.132238865	0.175	1	57547	ZNF624
0.255598456	0.132238865	0.152	1	388015	RTL1
0.255341053	0.132639259	0.157	1	10775	POP4
0.255341053	0.132639259	0.139	1	27293	SMPDL3B
0.255341053	0.132639259	0.176	1	84243	ZDHHC18
0.255341053	0.132639259	0.16	1	144423	GLT1D1
0.25508365	0.133040369	0.171	1	1010	CDH12
0.25508365	0.133040369	0.176	1	7621	ZNF70
0.25508365	0.133040369	0.175	1	22941	SHANK2
0.25508365	0.133040369	0.176	1	55218	EXD2
0.254826248	0.133442342	0.178	1	9751	SNPH
0.254568845	0.13384527	0.172	1	22999	RIMS1
0.254311442	0.13424927	0.183	1	57528	KCTD16
0.254311442	0.13424927	0.166	1	493861	EID3
0.25405404	0.134654015	0.173	1	1337	COX6A1
0.25405404	0.134654015	0.16	1	4185	ADAM11
0.25405404	0.134654015	0.182	1	51411	BIN2
0.25405404	0.134654015	0.173	1	153657	TTC23L

voxel-wise, subcortex			
Spearman's corr coef	p-val	GenID	Gene
0.208064571	< 0.001	726	CAPN5
0.208042011	< 0.001	55257	C20orf20
0.208037928	< 0.001	100132062	LOC100132062
0.20798777	< 0.001	3237	HOXD11
0.20796214	< 0.001	285905	INTS4L1
0.207910419	< 0.001	11170	FAM107A
0.207905129	< 0.001	8362	HIST1H4K
0.207848102	< 0.001	126259	TMIGD2
0.207779944	< 0.001	2194	FASN
0.207730904	< 0.001	9145	SYNGR1
0.207689583	< 0.001	414318	C9orf106
0.207676217	< 0.001	286097	EFHA2
0.207653552	< 0.001	117156	SCGB3A2
0.207642123	< 0.001	140710	C20orf117
0.207628012	< 0.001	127391	TMCO2
0.207578793	< 0.001	58484	NLRC4
0.207527712	< 0.001	2731	GLDC
0.207488716	< 0.001	3775	KCNK1
0.20740588	< 0.001	116442	RAB39B
0.20738247	< 0.001	56994	CHPT1
0.207365945	< 0.001	242	ALOX12B
0.207326204	< 0.001	2171	FABP5
0.207316443	< 0.001	259307	IL4I1
0.207094222	< 0.001	51550	AL137229.4
0.206985012	< 0.001	23759	PPIL2
0.206967175	< 0.001	11311	VPS45
0.206960857	< 0.001	79042	TSEN34
0.206846356	< 0.001	131450	CD200R1
0.206767514	< 0.001	5205	ATP8B1
0.206586063	< 0.001	51744	CD244
0.206443131	< 0.001	57613	KIAA1467
0.206435427	< 0.001	84970	C1orf94
0.206321761	< 0.001	79890	RIN3
0.206314147	< 0.001	533	ATP6V0B
0.206308946	< 0.001	219537	SMTNL1
0.206253037	< 0.001	2821	GPI
0.206232056	< 0.001	90485	ZNF835
0.206224903	< 0.001	79444	BIRC7
0.2062172	< 0.001	64837	KLC2
0.206206694	< 0.001	6677	SPAM1
0.206182092	< 0.001	100130733	LRRC70
0.206133246	< 0.001	1122	CHML
0.206107616	< 0.001	22797	TFEC
0.206098139	< 0.001	441554	DUSPP
0.206078544	< 0.001	223082	ZNRF2
0.206064701	< 0.001	387778	SPDYC
0.206044376	< 0.001	390594	AC013553.2
0.206037864	< 0.001	6502	SKP2
0.206020966	< 0.001	7003	TEAD1
0.206010282	< 0.001	7763	ZFAND5
0.205982059	< 0.001	4888	NPY6R
0.20596227	< 0.001	9782	MATR3
0.205933213	< 0.001	84321	THOC3
0.205903396	< 0.001	55154	MSTO1
0.205883801	< 0.001	58495	OVOL2
0.205867827	< 0.001	7419	VDAC3
0.205781564	< 0.001	219957	OR9Q2
0.20577459	< 0.001	25902	MTHFD1L
0.205758899	< 0.001	10368	CACNG3
0.205746919	< 0.001	399670	RPL13AP17
0.205737635	< 0.001	54760	PCSK4
0.205666035	< 0.001	22931	RAB18
0.205610588	< 0.001	57198	ATP8B2
0.205572888	< 0.001	54413	NLGN3
0.205562666	< 0.001	55236	UBA6
0.205533698	< 0.001	11173	ADAMTS7
0.205441013	< 0.001	1734	DIO2
0.205426902	< 0.001	4924	NUCB1
0.20534119	< 0.001	339977	LRRC66
0.205323637	< 0.001	79574	EPS8L3
0.205308691	< 0.001	388564	TMEM238
0.205261976	< 0.001	84324	SARNP
0.205228359	< 0.001	10347	ABCA7
0.205227703	< 0.001	92597	MOBKL1A
0.205194652	< 0.001	26049	FAM169A
0.205184057	< 0.001	6019	RLN2
0.205156296	< 0.001	3046	HBE1
0.205137908	< 0.001	158160	HSD17B7P2
0.205124989	< 0.001	9734	HDAC9

region-wise, subcortex					
Spearman's corr coef	p-val	p-val, SA	p-val, SA (BH- correction)	GenID	Gene
0.253796637	0.13505967	0.153	1	5885	RAD21
0.253796637	0.13505967	0.175	1	10371	SEMA3A
0.253796637	0.13505967	0.146	1	201164	PLD6
0.253539234	0.135466188	0.165	1	2935	GSPT1
0.253539234	0.135466188	0.157	1	27152	INTU
0.253281832	0.13587366	0.176	1	23220	DTX4
0.253281832	0.13587366	0.162	1	26054	SENP6
0.253281832	0.13587366	0.149	1	728361	OVOL3
0.252767056	0.13669154	0.182	1	576	BAI2
0.252767056	0.13669154	0.157	1	283149	BCL9L
0.252509654	0.137101769	0.186	1	6326	SCN2A
0.252509654	0.137101769	0.171	1	8534	CHST1
0.252252251	0.137512848	0.167	1	127	ADH4
0.252252251	0.137512848	0.194	1	5013	OTX1
0.252252251	0.137512848	0.176	1	132864	CPEB2
0.252252251	0.137512848	0.18	1	144195	SLC2A14
0.252252251	0.137512848	0.177	1	401913	LOC401913
0.251994848	0.137925088	0.185	1	440503	AC011498.2
0.251737446	0.138338059	0.174	1	51655	RASD1
0.251737446	0.138338059	0.168	1	159686	CCDC147
0.251480043	0.138751969	0.192	1	5716	PSMD10
0.251480043	0.138751969	0.2	1	25886	WDR51A
0.251480043	0.138751969	0.176	1	78994	PRR14
0.251480043	0.138751969	0.175	1	83879	CDCA7
0.25122264	0.139166772	0.178	1	84660	CCDC62
0.25122264	0.139166772	0.181	1	134548	ANKRD43
0.25122264	0.139166772	0.182	1	157680	VPS13B
0.250965238	0.139582649	0.185	1	1427	CRYGS
0.250965238	0.139582649	0.188	1	23530	NNT
0.250965238	0.139582649	0.177	1	26071	FAM127B
0.250965238	0.139582649	0.162	1	79815	NIPAL2
0.250965238	0.139582649	0.172	1	79849	PDZD3
0.250707835	0.1399993	0.204	1	784	CACNB3
0.250707835	0.1399993	0.177	1	51087	YBX2
0.250707835	0.1399993	0.165	1	78986	DUSP26
0.250450462	0.140416875	0.188	1	3476	IGBP1
0.250450462	0.140416875	0.171	1	8831	SYNGAP1
0.250450462	0.140416875	0.172	1	552900	BOLA2
0.250193059	0.140835389	0.171	1	5720	PSME1
0.250193059	0.140835389	0.177	1	11249	NXPH2
0.250193059	0.140835389	0.167	1	54857	GDPD2
0.249935657	0.141254812	0.172	1	2079	ERH
0.249935657	0.141254812	0.19	1	4191	MDH2
0.249935657	0.141254812	0.175	1	6294	SAFB
0.249935657	0.141254812	0.18	1	27183	VPS4A
0.249678254	0.141675323	0.2	1	5075	PAX1
0.249678254	0.141675323	0.171	1	23515	MORC3
0.249678254	0.141675323	0.177	1	157313	CDCA2
0.249420851	0.142096594	0.193	1	6017	RLBP1
0.249420851	0.142096594	0.159	1	7038	TG
0.249420851	0.142096594	0.166	1	8581	LY6D
0.249163449	0.142518818	0.171	1	10640	EXOC5
0.249163449	0.142518818	0.183	1	55148	UBR7
0.249163449	0.142518818	0.168	1	93655	ST7-OT3
0.249163449	0.142518818	0.179	1	122664	TPPP2
0.249163449	0.142518818	0.181	1	729952	HMGB3P6
0.248906046	0.142941967	0.179	1	5955	RCN2
0.248906046	0.142941967	0.173	1	54897	CASZ1
0.248648658	0.143366188	0.194	1	6866	TAC3
0.248648658	0.143366188	0.16	1	59286	UBL5
0.248648658	0.143366188	0.153	1	161582	DYX1C1-CCPG1
0.248391256	0.143791184	0.174	1	39	ACAT2
0.248391256	0.143791184	0.17	1	4869	NPM1
0.248391256	0.143791184	0.179	1	374864	C18orf34
0.248391256	0.143791184	0.194	1	401944	LDLRAD2
0.248391256	0.143791184	0.182	1	441525	SPANXN4
0.248133838	0.144217119	0.185	1	6683	SPAST
0.248133838	0.144217119	0.19	1	285074	AC125232.1
0.247876436	0.144643992	0.173	1	1364	CLDN4
0.247876436	0.144643992	0.19	1	80224	NUBPL
0.247876436	0.144643992	0.17	1	337880	KRTAP11-1
0.247876436	0.144643992	0.176	1	643037	LOC643037
0.247619033	0.145071805	0.168	1	3512	IGJ
0.247619033	0.145071805	0.174	1	6498	SKIL
0.247619033	0.145071805	0.183	1	9296	ATP6V1F
0.247619033	0.145071805	0.18	1	53833	IL20RB
0.247619033	0.145071805	0.17	1	84789	MGC2889
0.247361645	0.14550069	0.161	1	648740	ACTG1P4
0.247104242	0.145930365	0.165	1	25758	C11orf41

voxel-wise, subcortex			
Spearman's corr coef	p-val	GenID	Gene
0.20508191	< 0.001	10771	ZMYND11
0.20506835	< 0.001	84930	MASTL
0.205053031	< 0.001	54457	TAF7L
0.205017835	< 0.001	200159	C1orf100
0.204916134	< 0.001	5516	PPP2CB
0.204914287	< 0.001	54853	WDR55
0.204883724	< 0.001	5546	PRCC
0.20486182	< 0.001	79924	ADM2
0.204829589	< 0.001	9556	C14orf2
0.204778507	< 0.001	375346	TMEM110
0.204761893	< 0.001	54928	IMPAD1
0.204742104	< 0.001	118427	OLFM3
0.204734862	< 0.001	92154	MTSS1L
0.204681009	< 0.001	167826	OLIG3
0.204653054	< 0.001	57648	KIAA1522
0.204593152	< 0.001	57167	SALL4
0.204522759	< 0.001	51668	HSPB11
0.204495832	< 0.001	3628	INPP1
0.20446676	< 0.001	23647	ARFIP2
0.204386339	< 0.001	206338	AC034236.3
0.204378068	< 0.001	993	CDC25A
0.20437111	< 0.001	10436	EMG1
0.20417887	< 0.001	342510	CD300E
0.204064369	< 0.001	83850	ESYT3
0.204054341	< 0.001	692312	PPAN-P2RY11
0.203989238	< 0.001	2561	GABRB2
0.20398432	< 0.001	26470	SEZ6L2
0.203840852	< 0.001	63910	SLC17A9
0.203829512	< 0.001	11336	EXOC3
0.203816056	< 0.001	2746	GLUD1
0.203813821	< 0.001	9320	TRIP12
0.20377937	< 0.001	340980	OR52B6
0.203762934	< 0.001	11101	ATE1
0.203734145	< 0.001	3094	HINT1
0.20372355	< 0.001	337985	KRTAP20-3
0.203717604	< 0.001	401546	C9orf152
0.203698114	< 0.001	10384	BTN3A3
0.203691706	< 0.001	29850	TRPM5
0.203689188	< 0.001	2005	ELK4
0.203688264	< 0.001	5270	SERPINE2
0.203657061	< 0.001	9637	FEZ2
0.203618333	< 0.001	78992	YIPF2
0.203613043	< 0.001	85446	ZFHX2
0.203611091	< 0.001	28996	HIPK2
0.203555554	< 0.001	10164	CHST4
0.203510523	< 0.001	127707	KLHDC7A
0.203488976	< 0.001	51449	PCYOX1
0.203462601	< 0.001	5774	PTPN3
0.203451738	< 0.001	79896	THNSL1
0.203425646	< 0.001	23659	PLA2G15
0.203423038	< 0.001	149466	C1ORF210
0.203418672	< 0.001	1435	CSF1
0.203396484	< 0.001	27339	PRPF19
0.203334451	< 0.001	402665	IGLON5
0.203312531	< 0.001	27145	FILIP1
0.203293771	< 0.001	4107	MAGEA8
0.203275383	< 0.001	388611	CD164L2
0.203257084	< 0.001	10241	CALCOCO2
0.20325467	< 0.001	92960	PEX11G
0.203129113	< 0.001	64241	ABCG8
0.20309402	< 0.001	8987	STBD1
0.203012764	< 0.001	53349	ZFYVE1
0.202811882	< 0.001	55	ACPP
0.202791646	< 0.001	5435	POLR2F
0.202756628	< 0.001	55534	MAML3
0.20275645	< 0.001	25852	ARMC8
0.202750131	< 0.001	22846	VASH1
0.202700168	< 0.001	22899	ARHGEF15
0.202685133	< 0.001	84981	MIR22HG
0.202642038	< 0.001	124976	SPNS2
0.202621147	< 0.001	527	ATP6V0C
0.202417955	< 0.001	84064	HDHD2
0.202377364	< 0.001	8573	CASK
0.202329546	< 0.001	338398	TAS2R60
0.202268347	< 0.001	25948	KBTBD2
0.202263325	< 0.001	64582	GPR135
0.20225516	< 0.001	114823	LENG8
0.202168792	< 0.001	79147	FKRP
0.202158943	< 0.001	9774	BCLAF1

region-wise, subcortex					
Spearman's corr coef	p-val	p-val, SA	p-val, SA (BH- correction)	GenID	Gene
0.247104242	0.145930365	0.175	1	284111	SLC13A5
0.24684684	0.146360993	0.183	1	5580	PRKCD
0.24684684	0.146360993	0.189	1	8362	HIST1H4K
0.24684684	0.146360993	0.18	1	116135	LRRC3B
0.246589437	0.146792531	0.186	1	51072	MEMO1
0.246589437	0.146792531	0.178	1	112950	MED8
0.246589437	0.146792531	0.173	1	388552	BLOC1S3
0.246332034	0.147225231	0.23	1	7431	VIM
0.246332034	0.147225231	0.158	1	57533	TBC1D14
0.246332034	0.147225231	0.18	1	136319	MTPN
0.246074647	0.147658616	0.187	1	1642	DDB1
0.245817244	0.148092985	0.191	1	55365	TMEM176A
0.245817244	0.148092985	0.186	1	158038	LINGO2
0.245559841	0.148528308	0.175	1	3892	KRT86
0.245559841	0.148528308	0.173	1	6018	RLF
0.245559841	0.148528308	0.195	1	7178	TPT1
0.245559841	0.148528308	0.203	1	8022	LHX3
0.245559841	0.148528308	0.195	1	8861	LDB1
0.245559841	0.148528308	0.183	1	23332	CLASP1
0.245559841	0.148528308	0.185	1	90634	N4BP2L1
0.245559841	0.148528308	0.193	1	219956	OR9Q1
0.245302439	0.148964763	0.19	1	6288	SAA1
0.245302439	0.148964763	0.202	1	7416	VDAC1
0.245302439	0.148964763	0.208	1	8536	CAMK1
0.245302439	0.148964763	0.177	1	9715	FAM131B
0.245045036	0.149401963	0.196	1	7026	NR2F2
0.245045036	0.149401963	0.162	1	8336	HIST1H2AM
0.244787648	0.149840072	0.194	1	4751	NEK2
0.244787648	0.149840072	0.197	1	11040	PIM2
0.244787648	0.149840072	0.182	1	22928	SEPHS2
0.244787648	0.149840072	0.191	1	28981	IFT81
0.244787648	0.149840072	0.167	1	221409	SPATS1
0.244787648	0.149840072	0.195	1	285456	AC084209.1
0.244787648	0.149840072	0.185	1	595101	LOC595101
0.244530246	0.150279179	0.179	1	9158	FIBP
0.244530246	0.150279179	0.202	1	120863	DEPDC4
0.244272843	0.150719211	0.184	1	328	APEX1
0.244272843	0.150719211	0.192	1	1557	CYP2C19
0.244272843	0.150719211	0.216	1	9095	TBX19
0.244272843	0.150719211	0.193	1	445577	C9orf129
0.24401544	0.151160404	0.168	1	8289	ARID1A
0.24401544	0.151160404	0.151	1	121278	TPH2
0.24401544	0.151160404	0.193	1	283130	SLC25A45
0.243758038	0.151602343	0.182	1	768	CA9
0.243758038	0.151602343	0.206	1	5982	RFC2
0.243758038	0.151602343	0.205	1	116225	ZMYND19
0.24350065	0.15204519	0.199	1	10785	WDR4
0.24350065	0.15204519	0.165	1	27177	IL1F8
0.24350065	0.15204519	0.196	1	57282	SLC4A10
0.24350065	0.15204519	0.199	1	146760	RTN4RL1
0.243243247	0.152489036	0.184	1	7695	ZNF136
0.243243247	0.152489036	0.18	1	9217	VAPB
0.243243247	0.152489036	0.2	1	9915	ARNT2
0.243243247	0.152489036	0.187	1	56965	PARP6
0.243243247	0.152489036	0.2	1	121536	AEBP2
0.243243247	0.152489036	0.182	1	147199	SCGB1C1
0.243243247	0.152489036	0.189	1	284615	ANKRD34A
0.242985845	0.15293403	0.184	1	2562	GABRB3
0.242985845	0.15293403	0.18	1	222642	BZRPL1
0.242728442	0.153379768	0.199	1	269	AMHR2
0.242728442	0.153379768	0.177	1	8497	PPFIA4
0.242728442	0.153379768	0.194	1	25858	C11orf20
0.242728442	0.153379768	0.219	1	29881	NPC1L1
0.242728442	0.153379768	0.211	1	29998	GLTSCR1
0.242728442	0.153379768	0.18	1	54898	ELOVL2
0.242471039	0.153826475	0.21	1	23594	ORC6L
0.242471039	0.153826475	0.169	1	55146	ZDHHC4
0.242471039	0.153826475	0.2	1	90639	COX19
0.242213652	0.154274076	0.199	1	156	ADRBK1
0.242213652	0.154274076	0.187	1	3488	IGFBP5
0.242213652	0.154274076	0.185	1	9779	TBC1D5
0.242213652	0.154274076	0.192	1	23324	MAN2B2
0.242213652	0.154274076	0.189	1	54921	CHTF8
0.242213652	0.154274076	0.193	1	84501	SPIRE2
0.242213652	0.154274076	0.172	1	554226	NCRNA00164
0.241956249	0.154722884	0.185	1	388394	RPRML
0.241956249	0.154722884	0.184	1	729583	C6orf100
0.241698846	0.155172467	0.212	1	5594	MAPK1
0.241698846	0.155172467	0.183	1	6722	SRF

voxel-wise, subcortex			
Spearman's corr coef	p-val	GenID	Gene
0.202144459	< 0.001	55530	SVOP
0.20212701	< 0.001	90639	COX19
0.202099234	< 0.001	2118	ETV4
0.202090412	< 0.001	8460	TPST1
0.202061161	< 0.001	23350	SR140
0.202012777	< 0.001	3501	IGHG2
0.201940715	< 0.001	2623	GATA1
0.201919824	< 0.001	115704	EVI5L
0.201890931	< 0.001	402055	SRRD
0.20178099	< 0.001	65055	REEP1
0.201737702	< 0.001	3875	KRT18P19
0.201717377	< 0.001	645367	GGT8P
0.201589495	< 0.001	51241	COX16
0.20152244	< 0.001	8976	WASL
0.201452047	< 0.001	360132	FKBP9L
0.201437384	< 0.001	127	ADH4
0.201418057	< 0.001	5780	PTPN9
0.201416954	< 0.001	168620	BHLHA15
0.201363921	< 0.001	8812	CCNK
0.201356858	< 0.001	9061	PAPSS1
0.201301605	< 0.001	3608	ILF2
0.201275796	< 0.001	27043	PELP1
0.201217845	< 0.001	8997	KALRN
0.201164722	< 0.001	129080	EMID1
0.201124787	< 0.001	114	ADCY8
0.201117456	< 0.001	148870	CCDC27
0.201115042	< 0.001	6595	SMARCA2
0.201033041	< 0.001	91782	CHMP7
0.201020882	< 0.001	1553	CYP2A13
0.201017439	< 0.001	23230	VPS13A
0.200956151	< 0.001	97	ACYP1
0.200812668	< 0.001	55249	YY1AP1
0.200806633	< 0.001	286151	FBXO43
0.200626478	< 0.001	9002	F2RL3
0.200613752	< 0.001	140881	DEFB129
0.20055905	< 0.001	79759	ZNF668
0.200556636	< 0.001	1427	CRYGS
0.200523853	< 0.001	254863	C17orf61
0.200510859	< 0.001	9583	ENTPD4
0.20043312	< 0.001	404550	C16orf74
0.200290203	< 0.001	91703	ACY3
0.200283155	< 0.001	149951	COMMD7
0.200250551	< 0.001	10462	CLEC10A
0.200250268	< 0.001	80789	INTS5
0.200226218	< 0.001	10128	LRPPRC
0.200139955	< 0.001	11140	CDC37
0.20002155	< 0.001	64064	OXCT2
0.199932113	< 0.001	10979	PLEKHC1
0.199906856	< 0.001	55754	TMEM30A
0.199906573	< 0.001	64109	CRLF2
0.199900448	< 0.001	2747	GLUD2
0.199897662	< 0.001	399697	CTXN2
0.199892282	< 0.001	4794	NFKBIE
0.199856058	< 0.001	2893	GRIA4
0.199855313	< 0.001	439953	LOC439953
0.199834138	< 0.001	3641	INSL4
0.199799687	< 0.001	6477	SIAH1
0.199776664	< 0.001	81602	CDADC1
0.199760586	< 0.001	27242	TNFRSF21
0.199752882	< 0.001	51255	RNF181
0.199690476	< 0.001	10149	GPR64
0.199690193	< 0.001	83901	KRTAP9-8
0.199668005	< 0.001	6704	SPRR2E
0.199614882	< 0.001	54165	DCUN1D1
0.19955954	< 0.001	10099	TSPAN3
0.199547559	< 0.001	81552	VOPP1
0.19954069	< 0.001	10486	CAP2
0.199496672	< 0.001	54549	SDK2
0.199493691	< 0.001	58498	MYL7
0.19948478	< 0.001	147710	AC138472.2
0.19947587	< 0.001	145741	FAM148A
0.199443176	< 0.001	89801	PPP1R3F
0.199433237	< 0.001	27130	INVS
0.19941847	< 0.001	79935	CNTD2
0.199374646	< 0.001	7135	TNNI1
0.199331924	< 0.001	8760	CDS2
0.199275464	< 0.001	2931	GSK3A
0.199195966	< 0.001	339105	AC135050.2
0.199194103	< 0.001	387820	LOC387820

region-wise, subcortex					
Spearman's corr coef	p-val	p-val, SA	p-val, SA (BH- correction)	GenID	Gene
0.241698846	0.155172467	0.18	1	51759	C9orf78
0.241698846	0.155172467	0.201	1	285331	CCDC66
0.241441444	0.155622989	0.193	1	3359	HTR3A
0.241441444	0.155622989	0.206	1	10000	AKT3
0.241441444	0.155622989	0.178	1	23001	WDFY3
0.241441444	0.155622989	0.215	1	50508	NOX3
0.241441444	0.155622989	0.209	1	51172	NAGPA
0.241441444	0.155622989	0.195	1	54716	SLC6A20
0.241441444	0.155622989	0.2	1	57563	KLHL8
0.241441444	0.155622989	0.2	1	131831	C3orf44
0.241441444	0.155622989	0.187	1	146713	AC021534.2
0.241184026	0.156074479	0.183	1	23639	LRRC6
0.241184026	0.156074479	0.172	1	54665	RSBN1
0.240926623	0.156526864	0.181	1	7879	RAB7A
0.240669236	0.156980455	0.189	1	6347	CCL2
0.240669236	0.156980455	0.205	1	10476	ATP5H
0.240669236	0.156980455	0.199	1	29979	UBQLN1
0.240669236	0.156980455	0.204	1	55175	KLHL11
0.240411833	0.157434836	0.161	1	8812	CCNK
0.240411833	0.157434836	0.164	1	56245	C21orf62
0.240411833	0.157434836	0.178	1	137682	C8orf38
0.240411833	0.157434836	0.182	1	401777	LOC401777
0.240411833	0.157434836	0.213	1	494118	SPANXN1
0.24015443	0.157890156	0.191	1	844	CASQ1
0.24015443	0.157890156	0.203	1	1428	CRYM
0.24015443	0.157890156	0.175	1	11078	TRIOBP
0.24015443	0.157890156	0.203	1	51026	GOLT1B
0.239897028	0.158346385	0.177	1	7172	TPMT
0.239897028	0.158346385	0.23	1	51594	NBAS
0.239639625	0.15880385	0.172	1	4644	MYO5A
0.239382237	0.159262061	0.179	1	92558	CCDC64
0.239124835	0.159721226	0.177	1	84896	ATAD1
0.239124835	0.159721226	0.199	1	116986	AGAP2
0.239124835	0.159721226	0.188	1	151871	DPPA2
0.239124835	0.159721226	0.175	1	285237	C3orf38
0.239124835	0.159721226	0.185	1	388327	C17orf100
0.238867432	0.160181388	0.195	1	54732	TMED9
0.238867432	0.160181388	0.214	1	60528	ELAC2
0.238610029	0.160642698	0.182	1	10608	MXD4
0.238610029	0.160642698	0.181	1	10725	NFAT5
0.238610029	0.160642698	0.211	1	64359	NXN
0.238610029	0.160642698	0.193	1	84856	RP11-178A10.1
0.238610029	0.160642698	0.199	1	166336	PRICKLE2
0.238352627	0.161104769	0.186	1	284067	C17orf105
0.238095239	0.161567792	0.219	1	1131	CHRM3
0.238095239	0.161567792	0.206	1	132720	C4orf32
0.237837836	0.162031814	0.209	1	344382	LOC344382
0.237580433	0.162496805	0.213	1	752	FMNL1
0.237580433	0.162496805	0.206	1	4495	MT1G
0.237580433	0.162496805	0.174	1	10718	NRG3
0.237580433	0.162496805	0.209	1	23057	NMNAT2
0.237580433	0.162496805	0.197	1	100128139	LOC100128139
0.237323031	0.162962988	0.218	1	5468	PPARG
0.237323031	0.162962988	0.219	1	124930	ANKRD13B
0.237323031	0.162962988	0.184	1	151195	CCNYL1
0.237065628	0.163429871	0.193	1	57405	SPC25
0.237065628	0.163429871	0.191	1	121256	TMEM132D
0.23680824	0.163897797	0.188	1	5036	PA2G4
0.23680824	0.163897797	0.199	1	8908	GYG2
0.23680824	0.163897797	0.207	1	84930	MASTL
0.23680824	0.163897797	0.198	1	440292	LOC440292
0.236550838	0.164366677	0.202	1	5413	SEPTIN5
0.236550838	0.164366677	0.202	1	283897	C16orf54
0.236293435	0.164836764	0.169	1	1717	DHCR7
0.236293435	0.164836764	0.209	1	2266	FGG
0.236293435	0.164836764	0.214	1	10447	FAM3C
0.236293435	0.164836764	0.193	1	84451	MAP3K19
0.236036032	0.165307596	0.201	1	23521	RPL13A
0.236036032	0.165307596	0.214	1	91695	RRP7B
0.23577863	0.165779367	0.197	1	1310	COL19A1
0.23577863	0.165779367	0.201	1	6423	SFRP2
0.23577863	0.165779367	0.214	1	56548	CHST7
0.23577863	0.165779367	0.176	1	89846	FGD3
0.235521242	0.166252166	0.217	1	4683	NBN
0.235521242	0.166252166	0.19	1	9185	REPS2
0.235521242	0.166252166	0.179	1	10602	CDC42EP3
0.235521242	0.166252166	0.192	1	64405	CDH22
0.235521242	0.166252166	0.162	1	138162	C9orf116
0.235263839	0.166726142	0.177	1	10558	SPTLC1

voxel-wise, subcortex			
Spearman's corr coef	p-val	GenID	Gene
0.19913727	< 0.001	81928	CABLES2
0.199127987	< 0.001	752	FMNL1
0.19911629	< 0.001	283849	EXOC3L
0.199099392	< 0.001	143678	AC068385.1
0.198953956	< 0.001	1894	ECT2
0.198892474	< 0.001	150290	DUSP18
0.198857188	< 0.001	79986	ZNF702P
0.198853016	< 0.001	23587	C17orf81
0.198787168	< 0.001	100131801	PET100
0.198718444	< 0.001	25771	TBC1D22A
0.198717713	< 0.001	29800	ZDHHC1
0.198701546	< 0.001	145864	HAPLN3
0.198699325	< 0.001	8366	HIST1H4B
0.198687717	< 0.001	55218	EXD2
0.198648423	< 0.001	317781	DDX51
0.198624656	< 0.001	7283	TUBG1
0.198584542	< 0.001	400073	C12orf76
0.198552221	< 0.001	79625	C4orf31
0.198473558	< 0.001	284085	KRT18P55
0.198382095	< 0.001	1487	CTBP1
0.198258668	< 0.001	9543	IGDCC3
0.198196828	< 0.001	2678	GGT1
0.198187158	< 0.001	219952	OR6Q1
0.198152333	< 0.001	144097	C11orf84
0.198109627	< 0.001	6289	SAA2
0.198053524	< 0.001	5881	RAC3
0.197930202	< 0.001	23275	POFUT2
0.197870672	< 0.001	4282	MIF
0.197828799	< 0.001	6462	SHBG
0.197826385	< 0.001	54972	TMEM132A
0.197823778	< 0.001	894	CCND2
0.197773725	< 0.001	341640	FREM2
0.19775933	< 0.001	123876	ACSM2A
0.197740197	< 0.001	7461	CLIP2
0.197595417	< 0.001	80781	COL18A1
0.197582975	< 0.001	25806	VAX2
0.197570711	< 0.001	51156	SERPINA10
0.19756636	< 0.001	9462	RASAL2
0.197456494	< 0.001	340529	PABPC1L2A
0.197436616	< 0.001	10075	HUWE1
0.197389722	< 0.001	9389	SLC22A14
0.197298244	< 0.001	238	ALK
0.197268814	< 0.001	57448	BIRC6
0.19724077	< 0.001	1893	ECM1
0.197231665	< 0.001	57698	KIAA1598
0.197229251	< 0.001	55334	SLC39A9
0.197167963	< 0.001	81578	COL21A1
0.197154954	< 0.001	23624	CBLC
0.197142228	< 0.001	8508	NIPSNAP1
0.197112337	< 0.001	126308	MOBKL2A
0.197104067	< 0.001	3801	KIFC3
0.197006091	< 0.001	1981	EIF4G1
0.196991146	< 0.001	55554	KLK15
0.196961328	< 0.001	92170	MTG1
0.1969136	< 0.001	84467	FBN3
0.196886763	< 0.001	26263	FBXO22
0.196771786	< 0.001	162515	SLC16A11
0.196737707	< 0.001	5892	RAD51L3
0.196682915	< 0.001	114036	LINC00310
0.19664754	< 0.001	9628	RGS6
0.196498111	< 0.001	646019	CBY3
0.196488366	< 0.001	55194	FAM176B
0.196472585	< 0.001	54602	NDFIP2
0.196301803	< 0.001	3992	FADS1
0.196251839	< 0.001	51807	TUBA8
0.196225554	< 0.001	79677	SMC6
0.196176335	< 0.001	125988	C19orf70
0.196145326	< 0.001	222234	FAM185A
0.196096465	< 0.001	339366	ADAMTSL5
0.196094707	< 0.001	100128771	EIF4BP3
0.196010113	< 0.001	9135	RABEP1
0.195995435	< 0.001	333926	PPM1J
0.195850566	< 0.001	1591	CYP24A1
0.195811182	< 0.001	1993	ELAVL2
0.195759743	< 0.001	9231	DLG5
0.195713684	< 0.001	200373	AC069154.2
0.195686281	< 0.001	55843	ARHGAP15
0.195674673	< 0.001	26011	ODZ4
0.195657864	< 0.001	57498	KIDINS220

region-wise, subcortex					
Spearman's corr coef	p-val	p-val, SA	p-val, SA (BH- correction)	GenID	Gene
0.235263839	0.166726142	0.211	1	25847	ANAPC13
0.235263839	0.166726142	0.222	1	63875	MRPL17
0.235263839	0.166726142	0.187	1	79800	ALS2CR8
0.235263839	0.166726142	0.173	1	79810	PTCD2
0.235006437	0.167200893	0.19	1	29099	COMMD9
0.235006437	0.167200893	0.232	1	164633	CABP7
0.235006437	0.167200893	0.181	1	201965	RWDD4A
0.234749034	0.167676628	0.214	1	5682	PSMA1
0.234749034	0.167676628	0.214	1	6450	SH3BGR
0.234749034	0.167676628	0.202	1	6782	HSPA13
0.234749034	0.167676628	0.191	1	266697	POM121L4P
0.234749034	0.167676628	0.212	1	266812	NAP1L5
0.234749034	0.167676628	0.194	1	400624	FLJ45079
0.234491631	0.168153301	0.196	1	6453	ITSN1
0.234491631	0.168153301	0.21	1	9862	MED24
0.234491631	0.168153301	0.198	1	89849	ATG16L2
0.234491631	0.168153301	0.202	1	201895	C4orf34
0.234234229	0.168630987	0.215	1	3848	KRT1
0.234234229	0.168630987	0.213	1	5792	PTPRF
0.234234229	0.168630987	0.215	1	8999	CDKL2
0.234234229	0.168630987	0.212	1	25950	RWDD3
0.234234229	0.168630987	0.224	1	26037	SIPA1L1
0.233976826	0.169109881	0.199	1	6150	MRPL23
0.233976826	0.169109881	0.216	1	6300	MAPK12
0.233976826	0.169109881	0.209	1	79850	FAM57A
0.233976826	0.169109881	0.217	1	117854	TRIM6
0.233976826	0.169109881	0.178	1	155185	AMZ1
0.233976826	0.169109881	0.183	1	219539	YPEL4
0.233719423	0.169589564	0.212	1	3656	IRAK2
0.233719423	0.169589564	0.19	1	6358	CCL14
0.233719423	0.169589564	0.243	1	23042	PDXDC1
0.233719423	0.169589564	0.193	1	57574	MARCHF4
0.233719423	0.169589564	0.209	1	89874	SLC25A21
0.233719423	0.169589564	0.213	1	115399	LRRC56
0.233719423	0.169589564	0.196	1	142913	CFL1P1
0.233719423	0.169589564	0.19	1	389831	LOC389831
0.233462021	0.170070201	0.202	1	56967	C14orf132
0.233462021	0.170070201	0.229	1	389792	IER5L
0.233204618	0.170551792	0.215	1	79748	LMAN1L
0.233204618	0.170551792	0.181	1	80024	SLC24A6
0.23294723	0.171034634	0.227	1	51428	DDX41
0.23294723	0.171034634	0.222	1	81543	LRRC3
0.232689828	0.171518236	0.198	1	56884	FSTL5
0.232432425	0.172002837	0.219	1	23196	FAM120A
0.232432425	0.172002837	0.177	1	132989	C4orf36
0.232175022	0.172488391	0.196	1	83640	FAM103A1
0.232175022	0.172488391	0.18	1	100130331	RP11-193H5.1
0.23191762	0.172975168	0.205	1	26098	C10orf137
0.23191762	0.172975168	0.21	1	56145	PCDHA3
0.23191762	0.172975168	0.19	1	93474	ZNF670
0.23191762	0.172975168	0.207	1	284085	KRT18P55
0.23191762	0.172975168	0.206	1	348021	LINC00442
0.231660232	0.173462734	0.201	1	59339	PLEKHA2
0.231660232	0.173462734	0.23	1	83401	ELOVL3
0.231402829	0.173951298	0.202	1	6543	SLC8A2
0.231402829	0.173951298	0.195	1	503542	SPRN
0.231145427	0.174440831	0.199	1	6128	RPL6P10
0.231145427	0.174440831	0.218	1	221150	C13orf3
0.230888024	0.174931362	0.22	1	10135	PBEF1
0.230888024	0.174931362	0.177	1	11253	MAN1B1
0.230888024	0.174931362	0.22	1	28227	PPP2R3B
0.230888024	0.174931362	0.19	1	79441	HAUS3
0.230630621	0.175423086	0.186	1	84866	TMEM25
0.230630621	0.175423086	0.211	1	359710	C20orf185
0.230630621	0.175423086	0.216	1	645832	SEBOX
0.230373234	0.175915629	0.223	1	2056	EPO
0.230373234	0.175915629	0.198	1	10180	RBM6
0.230373234	0.175915629	0.208	1	57410	SCYL1
0.230373234	0.175915629	0.208	1	84057	MND1
0.230373234	0.175915629	0.224	1	728294	D2HGDH
0.230115831	0.176409125	0.202	1	11041	AP001107.1
0.230115831	0.176409125	0.202	1	23174	ZCCHC14
0.230115831	0.176409125	0.227	1	29083	GTPBP8
0.230115831	0.176409125	0.184	1	57663	USP29
0.230115831	0.176409125	0.216	1	79645	EFCAB1
0.229858428	0.176903665	0.214	1	3183	HNRNPC
0.229858428	0.176903665	0.204	1	3889	KRT83
0.229601026	0.177399367	0.22	1	6780	STAU1
0.229601026	0.177399367	0.226	1	8717	TRADD

voxel-wise, subcortex			
Spearman's corr coef	p-val	GenID	Gene
0.19564496	< 0.001	56916	SMARCAD1
0.195638642	< 0.001	8407	TAGLN2
0.195527017	< 0.001	391191	OR2AK2
0.195519209	< 0.001	84075	FSCB
0.195489496	< 0.001	166012	CHST13
0.195460707	< 0.001	116085	SLC22A12
0.195427746	< 0.001	58510	PRODH2
0.19540517	< 0.001	84519	ACRBP
0.195364773	< 0.001	165082	GPR113
0.195314452	< 0.001	1762	DMWD
0.195307672	< 0.001	6915	TBXA2R
0.19527182	< 0.001	22926	ATF6
0.195233837	< 0.001	57546	PDP2
0.195142463	< 0.001	23011	RAB21
0.195136234	< 0.001	65012	SLC26A10
0.195116356	< 0.001	23208	SYT11
0.195064455	< 0.001	55133	SRBD1
0.19501923	< 0.001	8600	TNFSF11
0.195004553	< 0.001	4583	MUC2
0.194955155	< 0.001	55239	OGFOD1
0.194913551	< 0.001	199974	CYP4Z1
0.194865346	< 0.001	55157	DARS2
0.194743231	< 0.001	162968	ZNF497
0.194723815	< 0.001	401498	TMEM215
0.194685832	< 0.001	7163	TPD52
0.194511801	< 0.001	5869	RAB5B
0.194493786	< 0.001	5104	SERPINA5
0.194483206	< 0.001	55135	WRAP53
0.194476143	< 0.001	222865	TMEM130
0.194454044	< 0.001	8677	STX10
0.19443436	< 0.001	11005	SPINK5
0.194402412	< 0.001	65266	WNK4
0.194397852	< 0.001	79803	HPS6
0.194332764	< 0.001	2917	GRM7
0.194245934	< 0.001	58477	SRPRB
0.194240734	< 0.001	253314	EIF4E1B
0.194207951	< 0.001	205428	C3orf58
0.194036976	< 0.001	203245	NAIF1
0.194033548	< 0.001	401335	C7orf65
0.194014594	< 0.001	2596	GAP43
0.193996117	< 0.001	128372	OR6N1
0.193978295	< 0.001	114788	CSMD3
0.19397597	< 0.001	8843	HCAR3
0.193913013	< 0.001	11147	HHLA3
0.193895087	< 0.001	56890	MDM1
0.193771482	< 0.001	51634	RBMX2
0.193733782	< 0.001	114804	RNF157
0.193610907	< 0.001	23072	HECW1
0.193603769	< 0.001	89970	RSPRY1
0.193575248	< 0.001	55259	CASC1
0.193560109	< 0.001	23484	LEPROTL1
0.193552494	< 0.001	8848	TSC22D1
0.193491116	< 0.001	79760	GEMIN7
0.193462238	< 0.001	56901	NDUFA4L2
0.193397596	< 0.001	9184	BUB3
0.193397135	< 0.001	8668	EIF3I
0.193311051	< 0.001	5652	PRSS8
0.193251237	< 0.001	197131	UBR1
0.193247527	< 0.001	23186	RCOR1
0.193243355	< 0.001	64926	RASAL3
0.193154469	< 0.001	85313	PPIL4
0.193149835	< 0.001	221687	RNF182
0.193117335	< 0.001	2970	GTF2IP1
0.193092346	< 0.001	123264	AC013553.1
0.193089291	< 0.001	727851	RGPD8
0.193001062	< 0.001	6745	SSR1
0.192976549	< 0.001	57654	KIAA1530
0.192921937	< 0.001	55237	C14orf115
0.192848951	< 0.001	79746	ECHDC3
0.192744106	< 0.001	389199	LOC389199
0.192680299	< 0.001	55295	KLHL26
0.19267714	< 0.001	8540	AGPS
0.192637026	< 0.001	146779	AC008026.2
0.192604154	< 0.001	284358	MAMSTR
0.192570075	< 0.001	8468	FKBP6
0.192559302	< 0.001	10367	CBARA1
0.192527637	< 0.001	64710	NUCKS1
0.192525119	< 0.001	619208	C6orf225
0.192358807	< 0.001	11219	TREX2

region-wise, subcortex					
Spearman's corr coef	p-val	p-val, SA	p-val, SA (BH- correction)	GenID	Gene
0.229601026	0.177399367	0.217	1	54764	ZRANB1
0.229601026	0.177399367	0.219	1	219738	C10orf35
0.229343623	0.177895904	0.204	1	535	ATP6V0A1
0.229343623	0.177895904	0.234	1	9818	NUPL1
0.229343623	0.177895904	0.199	1	146909	KIF18B
0.229343623	0.177895904	0.215	1	349149	GJC3
0.228828833	0.178891912	0.221	1	5581	PRKCE
0.228828833	0.178891912	0.205	1	10471	PFDN6
0.228828833	0.178891912	0.201	1	255783	PRR24
0.22857143	0.179391682	0.202	1	92675	DTD1
0.228314027	0.179892153	0.224	1	8559	PRPF18
0.228314027	0.179892153	0.21	1	56704	JPH1
0.228056625	0.180393681	0.231	1	1555	CYP2B7P1
0.228056625	0.180393681	0.207	1	7803	PTP4A1
0.228056625	0.180393681	0.217	1	286205	SCAI
0.227799237	0.180896163	0.209	1	122740	OR4K14
0.227541834	0.181399688	0.188	1	214	ALCAM
0.227541834	0.181399688	0.208	1	9389	SLC22A14
0.227541834	0.181399688	0.226	1	22866	CNKSR2
0.227541834	0.181399688	0.204	1	282974	STK32C
0.227284417	0.18190445	0.217	1	79829	NAT11
0.227284417	0.18190445	0.232	1	151313	FAHD2B
0.227027014	0.182409972	0.236	1	6742	SSBP1
0.227027014	0.182409972	0.218	1	51364	ZMYND10
0.227027014	0.182409972	0.216	1	90161	HS6ST2
0.227027014	0.182409972	0.21	1	440279	UNC13C
0.227027014	0.182409972	0.235	1	729595	HMGB3P22
0.226769611	0.182916492	0.223	1	1510	CTSE
0.226769611	0.182916492	0.205	1	2114	ETS2
0.226769611	0.182916492	0.241	1	5205	ATP8B1
0.226769611	0.182916492	0.217	1	7090	TLE3
0.226769611	0.182916492	0.243	1	7257	TSNAX
0.226769611	0.182916492	0.215	1	9063	PIAS2
0.226769611	0.182916492	0.22	1	56853	BRUNOL4
0.226769611	0.182916492	0.233	1	60509	AGBL5
0.226769611	0.182916492	0.187	1	283219	KCTD21
0.226769611	0.182916492	0.221	1	100131360	LOC100131360
0.226512223	0.183424041	0.212	1	7108	TM7SF2
0.226512223	0.183424041	0.207	1	11279	KLF8
0.226254821	0.183932826	0.227	1	9140	ATG12
0.226254821	0.183932826	0.237	1	128240	APOA1BP
0.225997418	0.184442386	0.253	1	677	ZFP36L1
0.225997418	0.184442386	0.232	1	8404	SPARCL1
0.225997418	0.184442386	0.214	1	9352	TXNL1
0.225997418	0.184442386	0.231	1	11158	RABL2B
0.225997418	0.184442386	0.208	1	23262	HISPPD1
0.225997418	0.184442386	0.194	1	90843	TCEAL8
0.225740016	0.184952945	0.208	1	2537	IFI6
0.225740016	0.184952945	0.229	1	6415	SEPW1
0.225740016	0.184952945	0.222	1	9256	BZRAP1
0.225740016	0.184952945	0.215	1	93408	MYL10
0.225482613	0.185464486	0.22	1	1795	DOCK3
0.225482613	0.185464486	0.209	1	2081	ERN1
0.225482613	0.185464486	0.224	1	6196	RPS6KA2
0.225482613	0.185464486	0.211	1	63905	MANBAL
0.225482613	0.185464486	0.219	1	84248	FYTTD1
0.225225225	0.185977086	0.227	1	5671	PSG3
0.225225225	0.185977086	0.224	1	23028	KDM1
0.224967822	0.186490923	0.219	1	3654	IRAK1
0.224967822	0.186490923	0.215	1	8841	HDAC3
0.224967822	0.186490923	0.219	1	10928	RALBP1
0.224967822	0.186490923	0.222	1	23568	ARL2BP
0.224967822	0.186490923	0.218	1	56926	NCLN
0.224967822	0.186490923	0.199	1	89890	KBTBD6
0.22471042	0.187005535	0.222	1	8266	UBL4A
0.22471042	0.187005535	0.225	1	338645	LUZP2
0.22471042	0.187005535	0.213	1	100131390	SP9
0.224453017	0.1875211	0.205	1	23592	LEMD3
0.224453017	0.1875211	0.246	1	50487	PLA2G3
0.224453017	0.1875211	0.212	1	85478	CCDC65
0.224453017	0.1875211	0.21	1	199953	TMEM201
0.224195614	0.188037768	0.207	1	26245	OR2M4
0.224195614	0.188037768	0.247	1	58527	C6orf115
0.224195614	0.188037768	0.242	1	160418	TMTC3
0.223938227	0.188555643	0.231	1	8562	DENR
0.223938227	0.188555643	0.234	1	84439	HHIPL1
0.223680824	0.189074323	0.224	1	6953	TCP10
0.223680824	0.189074323	0.216	1	100287482	RP11-305M3.3
0.223423421	0.189594015	0.245	1	10950	BTG3

voxel-wise, subcortex			
Spearman's corr coef	p-val	GenID	Gene
0.192262501	< 0.001	54458	PRR13
0.192099154	< 0.001	54866	PPP1R14D
0.191978425	< 0.001	134083	OR2Y1
0.191969141	< 0.001	8001	GLRA3
0.191955209	< 0.001	728470	LOC728470
0.191949069	< 0.001	84964	ALKBH6
0.191877201	< 0.001	51559	NT5DC3
0.19182454	< 0.001	10607	TBL3
0.191785067	< 0.001	10107	TRIM10
0.191765845	< 0.001	100133204	C9orf147
0.191744402	< 0.001	283152	CCDC153
0.191661283	< 0.001	149371	EXOC8
0.191620231	< 0.001	376267	RAB15
0.191584677	< 0.001	122706	PSMB11
0.191579103	< 0.001	5716	PSMD10
0.191575482	< 0.001	22864	R3HDM2
0.191459954	< 0.001	494118	SPANXN1
0.191397175	< 0.001	1455	CSNK1G2
0.191326231	< 0.001	51138	COPS4
0.19128406	< 0.001	7780	SLC30A2
0.191215992	< 0.001	1659	DHX8
0.191211998	< 0.001	100131785	LOC100131785
0.191203639	< 0.001	51762	RAB8B
0.191167429	< 0.001	4994	OR3A1
0.19115442	< 0.001	1948	EFNB2
0.191110313	< 0.001	51496	CTDSPL2
0.19106406	< 0.001	90865	IL33
0.19106397	< 0.001	2745	GLRX
0.191041499	< 0.001	79948	AC006273.1
0.191040292	< 0.001	5537	PPP6C
0.191015959	< 0.001	642757	FLJ32756
0.190991446	< 0.001	9820	CUL7
0.190963671	< 0.001	9391	CIAO1
0.190893754	< 0.001	84167	C19orf44
0.190892071	< 0.001	100128927	ZBTB42
0.190861061	< 0.001	91039	DPP9
0.19085066	< 0.001	54438	GFOD1
0.190625742	< 0.001	23251	KIAA1024
0.190575585	< 0.001	6294	SAFB
0.190573364	< 0.001	10286	BCAS2
0.190448642	< 0.001	51316	PLAC8
0.190446138	< 0.001	29948	OSGIN1
0.190423474	< 0.001	23416	KCNH3
0.190418452	< 0.001	4600	MX2
0.190286398	< 0.001	1038	CDR1
0.190254644	< 0.001	81300	OR4P4
0.19023867	< 0.001	79999	AC007952.2
0.190229565	< 0.001	25833	POU2F3
0.190164745	< 0.001	2307	FOXS1
0.19015649	< 0.001	10900	RUNDC3A
0.190115437	< 0.001	1161	ERCC8
0.190077737	< 0.001	3064	HTT
0.19007653	< 0.001	23287	AGTPBP1
0.19000484	< 0.001	5706	PSMC6
0.189956263	< 0.001	10050	SLC17A4
0.189872041	< 0.001	734	OSGIN2
0.189827919	< 0.001	55163	PNPO
0.189779535	< 0.001	3817	KLK2
0.189778611	< 0.001	90673	PPP1R3E
0.189763561	< 0.001	286343	LURAP1L
0.189711198	< 0.001	23517	SKIV2L2
0.189625204	< 0.001	7112	TMPO
0.189604118	< 0.001	116068	AC027323.1
0.189590007	< 0.001	388692	LOC388692
0.189559639	< 0.001	128653	C20ORF141
0.189489618	< 0.001	865	CBFB
0.189481348	< 0.001	284383	OR2Z1
0.189379096	< 0.001	9033	PKD2L1
0.189372972	< 0.001	2395	FXN
0.189368144	< 0.001	54850	FBXL12
0.189361453	< 0.001	284349	ZNF283
0.18935357	< 0.001	282973	JAKMIP3
0.189286694	< 0.001	1945	EFNA4
0.189281493	< 0.001	54831	BEST2
0.189227358	< 0.001	5020	OXT
0.189174145	< 0.001	345651	ACTBL2
0.189162359	< 0.001	140701	C20orf135
0.189153343	< 0.001	134510	UBLCP1
0.18914713	< 0.001	63877	FAM204A

region-wise, subcortex					
Spearman's corr coef	p-val	p-val, SA	p-val, SA (BH- correction)	GenID	Gene
0.223423421	0.189594015	0.22	1	23255	KIAA0802
0.223423421	0.189594015	0.232	1	26260	FBXO25
0.223423421	0.189594015	0.23	1	55791	C1orf103
0.223423421	0.189594015	0.24	1	158046	NXNL2
0.223423421	0.189594015	0.22	1	378708	APITD1
0.223166019	0.190114662	0.224	1	2998	GYS2
0.223166019	0.190114662	0.225	1	4801	NFYB
0.223166019	0.190114662	0.223	1	10446	LRRN2
0.223166019	0.190114662	0.225	1	64064	OXCT2
0.222908616	0.19063662	0.223	1	525	ATP6V1B1
0.222908616	0.19063662	0.237	1	2117	ETV3
0.222908616	0.19063662	0.222	1	7750	ZMYM2
0.222908616	0.19063662	0.235	1	9545	RAB3D
0.222908616	0.19063662	0.217	1	10956	OS9
0.222908616	0.19063662	0.194	1	51637	C14orf166
0.222651228	0.191159353	0.215	1	5372	PMM1
0.222651228	0.191159353	0.217	1	10402	ST3GAL6
0.222651228	0.191159353	0.237	1	25920	COBRA1
0.222651228	0.191159353	0.226	1	55765	C1orf106
0.222651228	0.191159353	0.22	1	84966	IGSF21
0.222651228	0.191159353	0.194	1	130612	TMEM198
0.222651228	0.191159353	0.222	1	197131	UBR1
0.222393826	0.191683114	0.236	1	2547	XRCC6
0.222393826	0.191683114	0.201	1	54503	ZDHHC13
0.222393826	0.191683114	0.22	1	79587	CARS2
0.222393826	0.191683114	0.196	1	79857	AC024940.3
0.222393826	0.191683114	0.224	1	344595	RP11-446H18.3
0.222393826	0.191683114	0.233	1	401262	CRIP3
0.222136423	0.192207888	0.232	1	7710	ZNF154
0.222136423	0.192207888	0.249	1	9419	CRIPT
0.222136423	0.192207888	0.225	1	57154	SMURF1
0.22187902	0.192733631	0.241	1	605	BCL7A
0.22187902	0.192733631	0.242	1	5950	RBP4
0.22187902	0.192733631	0.245	1	10713	USP39
0.22187902	0.192733631	0.215	1	27128	CYTH4
0.221621618	0.193260685	0.244	1	8462	KLF11
0.221621618	0.193260685	0.239	1	55843	ARHGAP15
0.221621618	0.193260685	0.221	1	56924	PAK6
0.22136423	0.193788514	0.206	1	411	ARSB
0.22136423	0.193788514	0.213	1	3608	ILF2
0.22136423	0.193788514	0.222	1	92105	INTS4
0.22136423	0.193788514	0.223	1	283459	GATC
0.221106827	0.194317386	0.251	1	6361	CCL17
0.221106827	0.194317386	0.226	1	6689	SPIB
0.221106827	0.194317386	0.234	1	29888	STRN4
0.221106827	0.194317386	0.252	1	54894	RNF43
0.221106827	0.194317386	0.239	1	55611	OTUB1
0.221106827	0.194317386	0.232	1	57466	SFRS15
0.220849425	0.194847226	0.244	1	2688	GH1
0.220849425	0.194847226	0.237	1	4627	MYH9
0.220849425	0.194847226	0.264	1	5318	PKP2
0.220849425	0.194847226	0.218	1	7040	TGFB1
0.220849425	0.194847226	0.207	1	9462	RASAL2
0.220849425	0.194847226	0.243	1	10576	CCT2
0.220849425	0.194847226	0.249	1	374946	C1orf187
0.220592022	0.195378348	0.248	1	405	ARNT
0.220592022	0.195378348	0.241	1	1609	DGKQ
0.220592022	0.195378348	0.242	1	4363	ABCC1
0.220592022	0.195378348	0.241	1	6574	SLC20A1
0.220592022	0.195378348	0.225	1	51634	RBMX2
0.220592022	0.195378348	0.259	1	54102	CLIC6
0.220592022	0.195378348	0.26	1	340562	SATL1
0.220334619	0.195910275	0.218	1	4927	NUP88
0.220334619	0.195910275	0.231	1	79674	VEPH1
0.220334619	0.195910275	0.223	1	89796	NAV1
0.220334619	0.195910275	0.246	1	143872	AP000872.1
0.220334619	0.195910275	0.216	1	146325	C16orf11
0.220334619	0.195910275	0.228	1	340719	NANOS1
0.220077217	0.19644323	0.252	1	2771	GNAI2
0.220077217	0.19644323	0.232	1	202781	LOC202781
0.220077217	0.19644323	0.252	1	339240	LOC339240
0.220077217	0.19644323	0.218	1	340152	ZC3H12D
0.220077217	0.19644323	0.236	1	100131289	LOC100131289
0.219819814	0.196977213	0.225	1	6821	SUOX
0.219819814	0.196977213	0.201	1	11222	MRPL3
0.219819814	0.196977213	0.222	1	53831	GPR84
0.219819814	0.196977213	0.242	1	64577	ALDH8A1
0.219819814	0.196977213	0.234	1	66004	LYNX1
0.219819814	0.196977213	0.233	1	399818	METTL10

voxel-wise, subcortex			
Spearman's corr coef	p-val	GenID	Gene
0.189144239	< 0.001	149281	METTL11B
0.18914257	< 0.001	493861	EID3
0.18912892	< 0.001	1548	CYP2A6
0.189093262	< 0.001	114132	SIGLEC11
0.189074963	< 0.001	84056	KATNAL1
0.189004108	< 0.001	219293	ATAD3C
0.188984707	< 0.001	9354	UBE4A
0.188940778	< 0.001	131583	FAM43A
0.188933805	< 0.001	541565	C8orf58
0.188912079	< 0.001	139425	WDR42B
0.188848287	< 0.001	7566	ZNF18
0.188830629	< 0.001	11142	PKIG
0.188824326	< 0.001	55803	ADAP2
0.188802689	< 0.001	754	PTTG1IP
0.188788667	< 0.001	2971	GTF3A
0.188774452	< 0.001	4660	PPP1R12B
0.188629672	< 0.001	6785	ELOVL4
0.188612863	< 0.001	132112	RTP1
0.1885629	< 0.001	1778	DYNC1H1
0.18854025	< 0.001	196074	METT5D1
0.18853569	< 0.001	11335	CBX3
0.188516662	< 0.001	128077	LIX1L
0.188467711	< 0.001	91319	DERL3
0.188423693	< 0.001	58488	PCTP
0.18838878	< 0.001	340485	ACER2
0.188358501	< 0.001	79917	MAGIX
0.188357949	< 0.001	5592	PRKG1
0.188346997	< 0.001	80230	RUFY1
0.188344672	< 0.001	57568	SIPA1L2
0.188343465	< 0.001	164284	APCDD1L
0.18833454	< 0.001	205	AK3L1
0.188275665	< 0.001	348303	AC011500.2
0.188257098	< 0.001	389692	MAFA
0.188166082	< 0.001	5140	PDE3B
0.188061148	< 0.001	5862	RAB2A
0.188011006	< 0.001	54518	APBB1IP
0.187999114	< 0.001	23118	MAP3K7IP2
0.187970042	< 0.001	6839	SUV39H1
0.187886462	< 0.001	4598	MVK
0.187875226	< 0.001	4990	SIX6
0.187871695	< 0.001	79412	KREMEN2
0.187851265	< 0.001	80124	VCPIP1
0.187781245	< 0.001	393046	OR2A5
0.187757298	< 0.001	2891	GRIA2
0.187732399	< 0.001	599	BCL2L2
0.187703058	< 0.001	388662	SLC6A17
0.187695533	< 0.001	55891	LENEP
0.18765077	< 0.001	11026	LILRA3
0.187640101	< 0.001	4607	MYBPC3
0.18759793	< 0.001	221336	BEND6
0.187571377	< 0.001	56970	ATXN7L3
0.187488347	< 0.001	219527	LRRC55
0.187422976	< 0.001	60385	TSKS
0.187410995	< 0.001	5261	PHKG2
0.187339947	< 0.001	3948	LDHC
0.187328622	< 0.001	144110	TMEM86A
0.187272996	< 0.001	26286	ARFGAP3
0.187208548	< 0.001	503834	DPRXP4
0.187144473	< 0.001	6780	STAU1
0.187054113	< 0.001	8911	CACNA1I
0.187039629	< 0.001	54995	OXSM
0.186979443	< 0.001	8174	MADCAM1
0.186962172	< 0.001	115650	TNFRSF13C
0.186916858	< 0.001	284366	KLK9
0.186912403	< 0.001	131408	FAM131A
0.186902374	< 0.001	84969	C20orf100
0.18689847	< 0.001	1746	DLX2
0.186885282	< 0.001	80003	PCNXL2
0.18688342	< 0.001	57710	KIAA1614
0.186871067	< 0.001	728327	LOC728327
0.186808765	< 0.001	5101	PCDH9
0.186793715	< 0.001	5801	PTPRR
0.18662934	< 0.001	148213	ZNF681
0.18659842	< 0.001	375133	PI1KAP2
0.186544746	< 0.001	4007	PRICKLE3
0.186534524	< 0.001	10500	SEMA6C
0.186378971	< 0.001	1606	DGKA
0.186340615	< 0.001	3868	KRT16
0.186313868	< 0.001	9939	RBM8A

region-wise, subcortex					
Spearman's corr coef	p-val	p-val, SA	p-val, SA (BH- correction)	GenID	Gene
0.219562411	0.197512463	0.247	1	6903	TBCC
0.219562411	0.197512463	0.238	1	55057	AIM1L
0.219562411	0.197512463	0.227	1	60385	TSKS
0.219562411	0.197512463	0.219	1	100268168	AC008429.1
0.219305009	0.198048443	0.243	1	366	AQP9
0.219305009	0.198048443	0.243	1	10498	CARM1
0.219305009	0.198048443	0.255	1	24142	NAT6
0.219305009	0.198048443	0.228	1	57143	ADCK1
0.219305009	0.198048443	0.229	1	63922	CHTF18
0.219305009	0.198048443	0.259	1	63950	DMRTA2
0.219305009	0.198048443	0.221	1	89953	KLC4
0.219047606	0.198585495	0.25	1	8635	RNASET2
0.219047606	0.198585495	0.216	1	10527	IPO7
0.219047606	0.198585495	0.226	1	11154	AP4S1
0.219047606	0.198585495	0.246	1	83863	TTTY5
0.219047606	0.198585495	0.216	1	90485	ZNF835
0.218790218	0.199123591	0.231	1	54760	PCSK4
0.218532816	0.199662685	0.241	1	8318	CDC45L
0.218532816	0.199662685	0.245	1	9097	USP14
0.218532816	0.199662685	0.24	1	10262	SF3B4
0.218532816	0.199662685	0.232	1	283284	IGSF22
0.218532816	0.199662685	0.236	1	441168	FAM26F
0.218532816	0.199662685	0.23	1	728673	LOC728673
0.218275413	0.200203106	0.238	1	1789	DNMT3B
0.218275413	0.200203106	0.243	1	2947	GSTM3
0.218275413	0.200203106	0.256	1	4736	RPL10A
0.218275413	0.200203106	0.225	1	10152	ABI2
0.218275413	0.200203106	0.256	1	51621	KLF13
0.218275413	0.200203106	0.223	1	81618	ITM2C
0.218275413	0.200203106	0.24	1	474382	H2AFB1
0.21801801	0.200744227	0.208	1	10463	SLC30A9
0.21801801	0.200744227	0.237	1	56624	ASAH2
0.21801801	0.200744227	0.251	1	57553	MICAL3
0.21801801	0.200744227	0.236	1	157574	FBXO16
0.217760608	0.20128642	0.239	1	2273	FHL1
0.217760608	0.20128642	0.266	1	4294	MAP3K10
0.217760608	0.20128642	0.246	1	55280	CWF19L1
0.217760608	0.20128642	0.244	1	80173	IFT74
0.21750322	0.201829627	0.252	1	1046	CDX4
0.21750322	0.201829627	0.272	1	23109	DDN
0.21750322	0.201829627	0.257	1	57118	CAMK1D
0.21750322	0.201829627	0.236	1	81027	TUBB1
0.217245817	0.202374175	0.25	1	2849	GPR26
0.217245817	0.202374175	0.243	1	54850	FBXL12
0.217245817	0.202374175	0.234	1	130888	FBXO36
0.216988415	0.202919468	0.271	1	9905	SGSM2
0.216988415	0.202919468	0.224	1	84070	FAM186B
0.216988415	0.202919468	0.238	1	148898	C1ORF213
0.216731012	0.203465775	0.244	1	5616	PRKY
0.216731012	0.203465775	0.238	1	58531	PRM3
0.216731012	0.203465775	0.25	1	728327	LOC728327
0.216473609	0.204013109	0.222	1	5028	P2RY1
0.216473609	0.204013109	0.249	1	7041	TGFB1I1
0.216473609	0.204013109	0.246	1	10612	TRIM3
0.216473609	0.204013109	0.226	1	79869	CPSF7
0.216216221	0.2045618	0.251	1	9702	CEP57
0.216216221	0.2045618	0.25	1	57213	C13orf1
0.216216221	0.2045618	0.228	1	84706	GPT2
0.216216221	0.2045618	0.272	1	348094	ANKDD1A
0.215958819	0.205111206	0.243	1	2874	GPS2
0.215958819	0.205111206	0.255	1	5019	OXCT1
0.215958819	0.205111206	0.241	1	29934	SNX12
0.215958819	0.205111206	0.247	1	92691	TMEM169
0.215958819	0.205111206	0.227	1	342898	SYCN
0.215958819	0.205111206	0.224	1	645323	LINC00461
0.215701416	0.205661684	0.26	1	9045	RPL14
0.215701416	0.205661684	0.239	1	11194	ABCB8
0.215444013	0.206213161	0.232	1	815	CAMK2A
0.215186611	0.206765696	0.259	1	607	BCL9
0.215186611	0.206765696	0.234	1	9133	CCNB2
0.215186611	0.206765696	0.255	1	22846	VASH1
0.214929223	0.207319558	0.253	1	119	ADD2
0.214929223	0.207319558	0.234	1	8506	CNTNAP1
0.214929223	0.207319558	0.253	1	219981	OR5A2
0.214929223	0.207319558	0.255	1	392255	GDF6
0.21467182	0.207874149	0.245	1	4726	NDUFS6
0.21467182	0.207874149	0.24	1	9562	MINPP1
0.21467182	0.207874149	0.239	1	10613	ERLIN1
0.21467182	0.207874149	0.266	1	119369	NUDT9P1

voxel-wise, subcortex			
Spearman's corr coef	p-val	GenID	Gene
0.186237261	< 0.001	5049	PAFAH1B2
0.186084211	< 0.001	390110	ACCSL
0.186074838	< 0.001	9760	TOX
0.186064258	< 0.001	1050	CEBPA
0.186055422	< 0.001	139886	SPIN4
0.18605338	< 0.001	782	CACNB1
0.186028212	< 0.001	6519	SLC3A1
0.186004356	< 0.001	389842	LOC389842
0.185939342	< 0.001	22829	NLGN4Y
0.185930714	< 0.001	2527	FUT5
0.185850099	< 0.001	343171	OR2W3
0.185828924	< 0.001	79667	AC021860.1
0.185736626	< 0.001	10602	CDC42EP3
0.185732812	< 0.001	55220	KLHDC8A
0.185715348	< 0.001	28991	COMMD5
0.185686558	< 0.001	6652	SORD
0.185644776	< 0.001	1240	CMKLR1
0.185617104	< 0.001	54986	ULK4
0.185571045	< 0.001	63826	SRR
0.185535386	< 0.001	92749	C2orf39
0.185533151	< 0.001	6319	SCD
0.185515881	< 0.001	136227	EMID2
0.185507894	< 0.001	56244	BTNL2
0.185487837	< 0.001	197257	LDHD
0.185414463	< 0.001	5050	PAFAH1B3
0.185358003	< 0.001	3560	IL2RB
0.185251951	< 0.001	81615	TMEM163
0.185199484	< 0.001	10446	LRRN2
0.185182855	< 0.001	1213	CLTC
0.184991375	< 0.001	151531	UPP2
0.184889406	< 0.001	64168	NECAB1
0.184859216	< 0.001	27285	TEKT2
0.184855416	< 0.001	55723	ASF1B
0.184796169	< 0.001	7292	TNFSF4
0.184756324	< 0.001	200172	SLFNL1
0.184676647	< 0.001	6583	SLC22A4
0.184657246	< 0.001	10390	CEPT1
0.184646741	< 0.001	27042	C1orf107
0.184576452	< 0.001	10069	RWDD2B
0.184555277	< 0.001	84911	ZNF382
0.184509948	< 0.001	182	JAG1
0.184501231	< 0.001	10539	GLRX3
0.184497595	< 0.001	84547	PGBD1
0.184496209	< 0.001	449520	GGNBP1
0.184483394	< 0.001	10112	KIF20A
0.184410498	< 0.001	339168	TMEM95
0.184366018	< 0.001	51195	RAPGEFL1
0.184307322	< 0.001	56103	PCDHGB2
0.184283733	< 0.001	83463	MXD3
0.18427676	< 0.001	130162	C2orf63
0.184225038	< 0.001	750	C16orf3
0.18411611	< 0.001	91409	CCDC74B
0.184072003	< 0.001	84552	PARD6G
0.184025928	< 0.001	9287	TAAR2
0.184018314	< 0.001	51028	VPS36
0.183990091	< 0.001	7032	TFF2
0.183908001	< 0.001	8996	NOL3
0.18388468	< 0.001	29100	TMEM208
0.183859155	< 0.001	5309	PITX3
0.183774829	< 0.001	83643	CCDC3
0.183736652	< 0.001	7391	USF1
0.183713347	< 0.001	7182	NR2C2
0.183555946	< 0.001	4482	MSRA
0.183548793	< 0.001	6239	RREB1
0.183505148	< 0.001	51514	DTL
0.183451191	< 0.001	22948	CCT5
0.183332965	< 0.001	4063	LY9
0.183318108	< 0.001	340156	MYLK4
0.183285058	< 0.001	440915	POTEKP
0.183273539	< 0.001	30012	TLX3
0.183234721	< 0.001	26040	SETBP1
0.183216423	< 0.001	1580	CYP4B1
0.183200911	< 0.001	29068	ZBTB44
0.183199614	< 0.001	513	ATP5D
0.183181778	< 0.001	3675	ITGA3
0.183158472	< 0.001	1269	CNR2
0.183134049	< 0.001	655	BMP7
0.183035523	< 0.001	5777	PTPN6
0.182994202	< 0.001	25941	C18orf10

region-wise, subcortex					
Spearman's corr coef	p-val	p-val, SA	p-val, SA (BH- correction)	GenID	Gene
0.214414418	0.208429813	0.27	1	115703	SNX26
0.214157015	0.208986476	0.245	1	28956	ROBLD3
0.214157015	0.208986476	0.236	1	54521	WDR44
0.214157015	0.208986476	0.248	1	54531	MIER2
0.214157015	0.208986476	0.233	1	56994	CHPT1
0.214157015	0.208986476	0.266	1	85442	KNDC1
0.213899612	0.209544495	0.246	1	6947	TCN1
0.213899612	0.209544495	0.241	1	26953	RANBP6
0.213899612	0.209544495	0.265	1	55650	PIGV
0.213899612	0.209544495	0.246	1	284613	CYB561D1
0.213642225	0.210103244	0.263	1	1990	CELA1
0.213642225	0.210103244	0.225	1	3621	ING1
0.213642225	0.210103244	0.262	1	6286	S100P
0.213642225	0.210103244	0.255	1	64432	MRPS25
0.213384822	0.210663065	0.227	1	5371	PML
0.213384822	0.210663065	0.244	1	6351	CCL4
0.213384822	0.210663065	0.265	1	8672	EIF4G3
0.213384822	0.210663065	0.264	1	80003	PCNXL2
0.213384822	0.210663065	0.234	1	285696	AC091878.1
0.213384822	0.210663065	0.233	1	286016	TPI1P2
0.213127404	0.211223945	0.253	1	2903	GRIN2A
0.213127404	0.211223945	0.234	1	9015	TAF1A
0.213127404	0.211223945	0.248	1	222826	C6orf146
0.213127404	0.211223945	0.224	1	348825	TPRXL
0.212870002	0.211786076	0.252	1	1325	CORT
0.212870002	0.211786076	0.253	1	220382	FAM181B
0.212612599	0.212348998	0.246	1	1072	CFL1
0.212612599	0.212348998	0.265	1	4602	MYB
0.212612599	0.212348998	0.249	1	11193	WBP4
0.212612599	0.212348998	0.262	1	54965	PIGX
0.212355196	0.212913007	0.243	1	1144	CHRND
0.212355196	0.212913007	0.248	1	2857	GPR34
0.212355196	0.212913007	0.257	1	6687	SPG7
0.212355196	0.212913007	0.266	1	8079	MLF2
0.212355196	0.212913007	0.251	1	54542	RC3H2
0.212355196	0.212913007	0.249	1	55236	UBA6
0.212097809	0.213478044	0.255	1	384	ARG2
0.212097809	0.213478044	0.247	1	8823	FGF16
0.212097809	0.213478044	0.229	1	51552	RAB14
0.212097809	0.213478044	0.224	1	780776	FAM18A
0.211840406	0.214044139	0.262	1	1207	CLNS1A
0.211840406	0.214044139	0.249	1	51025	AC009171.1
0.211840406	0.214044139	0.245	1	51123	ZNF706
0.211840406	0.214044139	0.231	1	136227	EMID2
0.211583003	0.214611501	0.239	1	722	C4BPA
0.211583003	0.214611501	0.247	1	5879	RAC1
0.211583003	0.214611501	0.241	1	10022	INSL5
0.211583003	0.214611501	0.224	1	25778	DSTYK
0.211583003	0.214611501	0.247	1	202051	AC135457.3
0.211325601	0.215179637	0.285	1	59336	PRDM13
0.211068198	0.215748876	0.232	1	714	C1QC
0.211068198	0.215748876	0.249	1	4776	NFATC4
0.211068198	0.215748876	0.239	1	51130	ASB3
0.211068198	0.215748876	0.252	1	219927	MRPL21
0.21081081	0.216319159	0.238	1	22853	LMTK2
0.21081081	0.216319159	0.26	1	51149	C5orf45
0.21081081	0.216319159	0.273	1	124540	MSI2
0.210553408	0.216890693	0.245	1	8558	CDK10
0.210553408	0.216890693	0.261	1	26284	ERAL1
0.210553408	0.216890693	0.26	1	55422	ZNF331
0.210553408	0.216890693	0.261	1	57705	WDFY4
0.210296005	0.217463046	0.265	1	6346	CCL1
0.210296005	0.217463046	0.233	1	55234	SMU1
0.210296005	0.217463046	0.266	1	58484	NLRC4
0.210038602	0.218036473	0.261	1	4817	NIT1
0.210038602	0.218036473	0.271	1	8574	AKR7A2
0.210038602	0.218036473	0.235	1	9910	RABGAP1L
0.210038602	0.218036473	0.252	1	10225	CD96
0.2097812	0.218610942	0.265	1	9727	RAB11FIP3
0.2097812	0.218610942	0.258	1	9949	AMMECR1
0.209523812	0.219186708	0.245	1	83483	PLVAP
0.209523812	0.219186708	0.245	1	84298	LLPH
0.209266409	0.219763219	0.248	1	285958	C7orf40
0.209266409	0.219763219	0.263	1	286097	EFHA2
0.209266409	0.219763219	0.261	1	401399	RP11-155G14.1
0.209009007	0.220340848	0.263	1	4779	NFE2L1
0.209009007	0.220340848	0.266	1	5936	RBM4
0.209009007	0.220340848	0.249	1	10490	VTI1B
0.209009007	0.220340848	0.242	1	81875	ISG20L2

voxel-wise, subcortex			
Spearman's corr coef	p-val	GenID	Gene
0.182964563	< 0.001	337882	KRTAP19-1
0.182883129	< 0.001	130589	GALM
0.18283911	< 0.001	9205	ZMYM5
0.182835758	< 0.001	9230	RAB11B
0.18278116	< 0.001	23508	TTC9
0.182768166	< 0.001	26284	ERAL1
0.182742342	< 0.001	116154	PHACTR3
0.182737142	< 0.001	645212	LOC645212
0.182725444	< 0.001	79184	BRCC3
0.182710215	< 0.001	121981	PPIAP26
0.182706967	< 0.001	4921	DDR2
0.182624489	< 0.001	10006	ABI1
0.182560697	< 0.001	341277	OVCH2
0.182496995	< 0.001	91298	C12orf29
0.182496533	< 0.001	389874	ZCCHC13
0.182478786	< 0.001	57590	WDFY1
0.182435602	< 0.001	5087	PBX1
0.182421207	< 0.001	55057	AIM1L
0.18235305	< 0.001	5979	RET
0.182287484	< 0.001	946	SIGLEC6
0.182273373	< 0.001	54978	C2orf18
0.182225913	< 0.001	7258	TSPY1
0.182217643	< 0.001	6665	SOX15
0.182169259	< 0.001	4689	NCF4
0.182146981	< 0.001	4783	NFIL3
0.182145864	< 0.001	129831	RBM45
0.182041943	< 0.001	2559	GABRA6
0.182039529	< 0.001	65985	AACS
0.182038695	< 0.001	23531	MMD
0.181965142	< 0.001	23366	KIAA0895
0.181906268	< 0.001	83896	KRTAP3-1
0.181898847	< 0.001	6717	SRI
0.181869954	< 0.001	7571	ZNF23
0.181801423	< 0.001	79778	MICALL2
0.181755915	< 0.001	285097	FLJ38379
0.181724444	< 0.001	55734	ZFP64
0.181667417	< 0.001	3215	HOXB5
0.181481034	< 0.001	390199	OR4D9
0.181456894	< 0.001	285501	AC021850.1
0.181366816	< 0.001	5457	POU4F1
0.181366071	< 0.001	80343	SEL1L2
0.181360409	< 0.001	27340	UTP20
0.181346387	< 0.001	23160	WDR43
0.181344062	< 0.001	10345	TRDN
0.181337193	< 0.001	429	ASCL1
0.181299761	< 0.001	7097	TLR2
0.181287691	< 0.001	9935	MAFB
0.181219429	< 0.001	1142	CHRNB3
0.181205779	< 0.001	123970	C16orf78
0.181203276	< 0.001	219479	OR5R1
0.181184977	< 0.001	80333	KCNIP4
0.181138918	< 0.001	51371	POMP
0.181136683	< 0.001	3887	KRT81
0.181125179	< 0.001	170961	ANKRD24
0.18112053	< 0.001	124359	CDYL2
0.181056455	< 0.001	6123	RPL3L
0.18097055	< 0.001	55752	SEPTIN11
0.180942506	< 0.001	440712	C1orf186
0.180881679	< 0.001	220832	FABP5P3
0.180822611	< 0.001	284756	C20orf197
0.180799767	< 0.001	4316	MMP7
0.180774599	< 0.001	112755	STX1B
0.180651471	< 0.001	136051	ZNF786
0.180644125	< 0.001	957	ENTPD5
0.180573463	< 0.001	26219	OR1J4
0.180490896	< 0.001	8881	CDC16
0.180418462	< 0.001	650	BMP2
0.180415586	< 0.001	7264	TSTA3
0.180397108	< 0.001	353	APRT
0.180353463	< 0.001	386683	KRTAP12-3
0.180350676	< 0.001	8798	DYRK4
0.180347517	< 0.001	83593	RASSF5
0.180292264	< 0.001	26515	FXC1
0.180256322	< 0.001	222967	RSPH10B
0.180242762	< 0.001	29092	LINC00339
0.180222243	< 0.001	10945	KDELR1
0.180139124	< 0.001	9890	RP4-788L13.1
0.180093616	< 0.001	728294	D2HGDH
0.18009083	< 0.001	23013	SPEN

region-wise, subcortex					
Spearman's corr coef	p-val	p-val, SA	p-val, SA (BH- correction)	GenID	Gene
0.209009007	0.220340848	0.253	1	114770	PGLYRP2
0.209009007	0.220340848	0.255	1	116369	SLC26A8
0.209009007	0.220340848	0.278	1	200726	LOC200726
0.209009007	0.220340848	0.246	1	401145	FAM190A
0.208751604	0.22091952	0.263	1	26189	OR1A2
0.208751604	0.22091952	0.238	1	55534	MAML3
0.208751604	0.22091952	0.251	1	80108	ZFP2
0.208494201	0.221499234	0.29	1	23276	KLHL18
0.208236814	0.222080275	0.273	1	11153	FICD
0.208236814	0.222080275	0.25	1	51773	RSF1
0.207722008	0.22324492	0.278	1	4696	NDUFA3
0.207722008	0.22324492	0.245	1	8539	API5L1
0.207722008	0.22324492	0.258	1	56731	SLC2A4RG
0.207722008	0.22324492	0.266	1	65108	MARCKSL1
0.207722008	0.22324492	0.254	1	345778	MTX3
0.207464606	0.223828852	0.258	1	9752	PCDHA9
0.207464606	0.223828852	0.272	1	53919	SLCO1C1
0.207464606	0.223828852	0.272	1	100128327	BET3L
0.207207203	0.22441411	0.273	1	1741	DLG3
0.207207203	0.22441411	0.272	1	55805	LRP2BP
0.207207203	0.22441411	0.266	1	375287	RBM43
0.206949815	0.225000173	0.24	1	29968	PSAT1
0.206949815	0.225000173	0.245	1	54881	TEX10
0.206692412	0.225587204	0.248	1	10589	DRAP1
0.206692412	0.225587204	0.27	1	23101	MCF2L2
0.206692412	0.225587204	0.255	1	81892	C14orf156
0.206692412	0.225587204	0.281	1	84878	ZBTB45
0.206692412	0.225587204	0.268	1	337979	KRTAP22-1
0.20643501	0.226175368	0.273	1	51196	PLCE1
0.20643501	0.226175368	0.29	1	643668	PSMC1P9
0.206177592	0.226764575	0.253	1	7916	BAT2
0.206177592	0.226764575	0.271	1	8509	NDST2
0.206177592	0.226764575	0.277	1	51067	YARS2
0.206177592	0.226764575	0.282	1	389895	LOC389895
0.20592019	0.227355123	0.239	1	6598	SMARCB1
0.20592019	0.227355123	0.248	1	9972	NUP153
0.20592019	0.227355123	0.263	1	22856	CHSY1
0.20592019	0.227355123	0.284	1	100093630	SNHG8
0.205662802	0.227946445	0.293	1	342933	ZSCAN5B
0.205405399	0.228538767	0.256	1	4345	CD200
0.205405399	0.228538767	0.241	1	6146	RPL22
0.205405399	0.228538767	0.27	1	51118	UTP11L
0.205405399	0.228538767	0.252	1	79065	ATG9A
0.205405399	0.228538767	0.293	1	84935	C13orf33
0.205405399	0.228538767	0.273	1	643161	FAM25A
0.205147997	0.229132235	0.27	1	51504	TRMT112
0.204890594	0.229727	0.304	1	22904	SBNO2
0.204890594	0.229727	0.28	1	56156	TEX13B
0.204890594	0.229727	0.257	1	57113	TRPC7
0.204890594	0.229727	0.271	1	121793	C13orf16
0.204633191	0.23032257	0.248	1	3680	ITGA9
0.204633191	0.23032257	0.258	1	6602	SMARCD1
0.204633191	0.23032257	0.273	1	51015	ISOC1
0.204633191	0.23032257	0.305	1	89832	CHRFAM7A
0.204633191	0.23032257	0.273	1	170463	SSBP4
0.204375803	0.230919167	0.284	1	196	AHR
0.204375803	0.230919167	0.29	1	6868	ADAM17
0.204375803	0.230919167	0.266	1	11051	NUDT21
0.204375803	0.230919167	0.283	1	23216	TBC1D1
0.204375803	0.230919167	0.268	1	284382	ACTL9
0.204375803	0.230919167	0.286	1	100131755	ARMCX4
0.204118401	0.231516823	0.268	1	55666	NPLOC4
0.204118401	0.231516823	0.28	1	388743	CAPN8
0.204118401	0.231516823	0.291	1	392392	OR1K1
0.203860998	0.232115835	0.256	1	4306	NR3C2
0.203860998	0.232115835	0.28	1	26986	PABPCP5
0.203860998	0.232115835	0.277	1	50626	CYHR1
0.203603595	0.232715636	0.3	1	5576	PRKAR2A
0.203603595	0.232715636	0.272	1	9588	PRDX6
0.203603595	0.232715636	0.296	1	51728	POLR3K
0.203603595	0.232715636	0.28	1	196403	DTX3
0.203603595	0.232715636	0.242	1	642585	VDAC1P1
0.203346193	0.233316481	0.266	1	6184	RPN1
0.203346193	0.233316481	0.258	1	51619	UBE2D4
0.203346193	0.233316481	0.255	1	55647	RAB20
0.203346193	0.233316481	0.277	1	221662	RBM24
0.203088805	0.233918414	0.271	1	3073	HEXA
0.203088805	0.233918414	0.278	1	10621	POLR3F
0.203088805	0.233918414	0.28	1	92749	C2orf39

voxel-wise, subcortex			
Spearman's corr coef	p-val	GenID	Gene
0.18005006	< 0.001	56947	MFF
0.179994717	< 0.001	159091	FAM122C
0.179971308	< 0.001	64386	MMP25
0.179913461	< 0.001	29785	CYP2S1
0.179904178	< 0.001	56971	CEACAM19
0.179899707	< 0.001	63905	MANBAL
0.179805174	< 0.001	23363	OBSL1
0.179734141	< 0.001	6347	CCL2
0.179649726	< 0.001	4004	LMO1
0.179520071	< 0.001	440087	C12orf69
0.179505691	< 0.001	554251	FBXO48
0.179453313	< 0.001	51154	MRTO4
0.179375201	< 0.001	6733	SRPK2
0.17933704	< 0.001	285016	FAM150B
0.179319113	< 0.001	8790	FPGT
0.179303333	< 0.001	55005	RMND1
0.179170161	< 0.001	10642	IGF2BP1
0.179130226	< 0.001	6430	SFRS5
0.179046363	< 0.001	401067	IQCF3
0.179034665	< 0.001	282679	AQP11
0.179023892	< 0.001	10641	TUSC4
0.178969666	< 0.001	55768	NGLY1
0.178954333	< 0.001	5593	PRKG2
0.178945616	< 0.001	29895	MYLPF
0.178914413	< 0.001	5475	PPEF1
0.178896949	< 0.001	5957	RCVRN
0.178796366	< 0.001	128	ADH5
0.17879191	< 0.001	150372	NFAM1
0.178763315	< 0.001	442444	FAM47C
0.178744376	< 0.001	83648	FAM167A
0.178738058	< 0.001	114131	UCN3
0.178718463	< 0.001	10592	SMC2
0.178713262	< 0.001	7095	SEC62
0.178691626	< 0.001	256297	PTF1A
0.178664044	< 0.001	84285	EIF1AD
0.178598017	< 0.001	55605	KIF21A
0.178555474	< 0.001	11273	ATXN2L
0.178528458	< 0.001	388228	SBK1
0.178505704	< 0.001	1185	CLCN6
0.178477377	< 0.001	83444	INO80B
0.178431869	< 0.001	440498	AC090360.1
0.178423613	< 0.001	151174	AC016757.1
0.178410426	< 0.001	27314	RAB30
0.178401783	< 0.001	6634	SNRPD3
0.178379133	< 0.001	10570	DPYSL4
0.178352565	< 0.001	566	AZU1
0.178338364	< 0.001	4841	NONO
0.178322479	< 0.001	96459	FNIP1
0.178274378	< 0.001	83856	FSD1L
0.178272799	< 0.001	6464	SHC1
0.178262487	< 0.001	84206	MEX3B
0.178247631	< 0.001	80256	KIAA1539
0.178142875	< 0.001	414260	C10orf136
0.178124204	< 0.001	221468	TMEM217
0.178059489	< 0.001	56143	PCDHA5
0.178014994	< 0.001	390916	NUDT19
0.177999213	< 0.001	51076	CUTC
0.177938014	< 0.001	3183	HNRNPC
0.177909136	< 0.001	5148	PDE6G
0.177891865	< 0.001	57576	KIF17
0.177834287	< 0.001	677	ZFP36L1
0.177769095	< 0.001	22800	RRAS2
0.177766487	< 0.001	5711	PSMD5
0.177739099	< 0.001	509	ATP5C1
0.1777208	< 0.001	23141	ANKLE2
0.17768839	< 0.001	7905	REEP5
0.177682996	< 0.001	285190	RGPD4
0.177640289	< 0.001	388199	AL023882.1
0.177633882	< 0.001	6891	TAP2
0.177624583	< 0.001	94234	FOXQ1
0.177461982	< 0.001	6656	SOX1
0.177452505	< 0.001	11116	FGFR1OP
0.177415088	< 0.001	65987	KCTD14
0.177402824	< 0.001	9957	HS3ST1
0.177391216	< 0.001	79727	LIN28
0.177380532	< 0.001	187	APLNR
0.177378684	< 0.001	6898	TAT
0.177336708	< 0.001	54806	AHI1
0.177324906	< 0.001	153579	BTNL9

region-wise, subcortex					
Spearman's corr coef	p-val	p-val, SA	p-val, SA (BH- correction)	GenID	Gene
0.203088805	0.233918414	0.277	1	154796	AMOT
0.203088805	0.233918414	0.264	1	160622	GRASP
0.202831402	0.234521344	0.279	1	10477	UBE2E3
0.202831402	0.234521344	0.251	1	51281	ANKMY1
0.202574	0.235125706	0.276	1	10564	ARFGEF2
0.202574	0.235125706	0.252	1	23517	SKIV2L2
0.202574	0.235125706	0.276	1	23588	KLHDC2
0.202574	0.235125706	0.277	1	54103	PION
0.202574	0.235125706	0.296	1	219771	CCNY
0.202316597	0.235730797	0.267	1	5023	P2RX1
0.202316597	0.235730797	0.268	1	146845	WDR16
0.202316597	0.235730797	0.291	1	440356	AC120114.2
0.202059194	0.236336991	0.252	1	55801	IL26
0.202059194	0.236336991	0.255	1	147710	AC138472.2
0.201801807	0.236944243	0.287	1	3358	HTR2C
0.201801807	0.236944243	0.271	1	4651	MYO10
0.201801807	0.236944243	0.286	1	57579	FAM135A
0.201544404	0.237552807	0.284	1	1356	CP
0.201544404	0.237552807	0.256	1	3868	KRT16
0.201544404	0.237552807	0.263	1	23082	PPRC1
0.201544404	0.237552807	0.266	1	57596	BEGAIN
0.201287001	0.238162175	0.283	1	5154	PDGFA
0.201287001	0.238162175	0.279	1	9079	LDB2
0.201287001	0.238162175	0.295	1	9703	KIAA0100
0.201029599	0.238772601	0.291	1	10293	TRAIP
0.201029599	0.238772601	0.307	1	169981	SPIN3
0.200772196	0.239384115	0.278	1	4645	MYO5B
0.200772196	0.239384115	0.274	1	6334	SCN8A
0.200772196	0.239384115	0.26	1	9263	STK17A
0.200772196	0.239384115	0.292	1	9343	EFTUD2
0.200772196	0.239384115	0.293	1	9941	EXOG
0.200772196	0.239384115	0.265	1	84750	FUT10
0.200772196	0.239384115	0.31	1	167681	PRSS23
0.200772196	0.239384115	0.28	1	200420	ALMS1P
0.200514808	0.23999697	0.306	1	2782	GNB1
0.200514808	0.23999697	0.31	1	6447	SCG5
0.200514808	0.23999697	0.288	1	9611	NCOR1
0.200514808	0.23999697	0.266	1	81691	AC004381.2
0.200257406	0.24061057	0.298	1	2182	ACSL4
0.200257406	0.24061057	0.312	1	7556	ZNF10
0.200257406	0.24061057	0.291	1	10487	CAP1
0.200257406	0.24061057	0.269	1	25797	QPCT
0.200257406	0.24061057	0.28	1	59338	PLEKHA1
0.200257406	0.24061057	0.292	1	219968	OR5B21
0.200000003	0.241225272	0.295	1	2495	FTHL16
0.200000003	0.241225272	0.273	1	8774	NAPG
0.200000003	0.241225272	0.286	1	23165	NUP205
0.200000003	0.241225272	0.287	1	60492	CCDC90B
0.1997426	0.241841048	0.272	1	6640	SNTA1
0.1997426	0.241841048	0.282	1	125150	ZSWIM7
0.1997426	0.241841048	0.296	1	442249	LOC442249
0.199485198	0.242457896	0.293	1	64919	BCL11B
0.199227795	0.243076101	0.258	1	10250	SRRM1
0.198970392	0.243695021	0.287	1	4285	MIPEP
0.198970392	0.243695021	0.281	1	9320	TRIP12
0.198970392	0.243695021	0.284	1	23209	MLC1
0.198970392	0.243695021	0.291	1	55998	NXF5
0.198970392	0.243695021	0.284	1	57538	ALPK3
0.198970392	0.243695021	0.291	1	317751	MESTIT1
0.198970392	0.243695021	0.303	1	100506049	AC005014.1
0.19871299	0.244315073	0.236	1	58487	CREBZF
0.198455587	0.244936213	0.286	1	56255	TMX4
0.198198184	0.245558679	0.291	1	1652	DDT
0.198198184	0.245558679	0.276	1	6749	SSRP1
0.198198184	0.245558679	0.294	1	8790	FPGT
0.198198184	0.245558679	0.267	1	9945	GFPT2
0.198198184	0.245558679	0.288	1	90809	TMEM55B
0.198198184	0.245558679	0.283	1	387923	SERP2
0.198198184	0.245558679	0.276	1	728833	FAM72D
0.197940797	0.24618195	0.254	1	84148	MYST1
0.197683394	0.246806219	0.301	1	3631	INPP4A
0.197683394	0.246806219	0.277	1	57799	RAB40C
0.197683394	0.246806219	0.29	1	80757	TMEM121
0.197683394	0.246806219	0.277	1	100129566	LOC100129566
0.197425991	0.247431636	0.281	1	1479	CSTF3
0.197425991	0.247431636	0.268	1	51105	PHF20L1
0.197168589	0.248058408	0.292	1	1961	EGR4
0.197168589	0.248058408	0.305	1	8671	SLC4A4
0.197168589	0.248058408	0.262	1	10609	AC130686.2

voxel-wise, subcortex			
Spearman's corr coef	p-val	GenID	Gene
0.177314416	< 0.001	3484	IGFBP1
0.177295566	< 0.001	286183	NKAIN3
0.17724672	< 0.001	85406	DNAJC14
0.177216068	< 0.001	90161	HS6ST2
0.177212909	< 0.001	201243	C17orf74
0.17715849	< 0.001	29124	LGALS13
0.177126557	< 0.001	115273	RAB42
0.177084953	< 0.001	10740	RFPL1-AS1
0.177054018	< 0.001	54808	DYM
0.176978335	< 0.001	100652929	AC105337.1
0.176924661	< 0.001	79748	LMAN1L
0.176896617	< 0.001	2251	FGF6
0.176889002	< 0.001	389873	SEPHS1P4
0.176845074	< 0.001	54737	MPHOSPH8
0.176773652	< 0.001	388015	RTL1
0.176755831	< 0.001	5604	MAP2K1
0.176715612	< 0.001	64781	CERK
0.176702052	< 0.001	391109	OR10K1
0.176686645	< 0.001	347365	ITIH5L
0.176664636	< 0.001	84181	CHD6
0.176626191	< 0.001	64699	TMPRSS3
0.176482618	< 0.001	481	ATP1B1
0.17644529	< 0.001	57733	GBA3
0.17642644	< 0.001	131076	CCDC58
0.176362634	< 0.001	728431	RP11-422J8.1
0.176353633	< 0.001	9793	CKAP5
0.17624405	< 0.001	57758	SCUBE2
0.176109478	< 0.001	27124	INPP5J
0.17610772	< 0.001	127665	ZNF648
0.176071599	< 0.001	442038	SULT1C3
0.176061198	< 0.001	5651	PRSS7
0.176026002	< 0.001	26112	CCDC69
0.175875455	< 0.001	119467	CLRN3
0.175862268	< 0.001	2707	GJB3
0.175861344	< 0.001	4627	MYH9
0.175849825	< 0.001	100132911	DPH3P1
0.175830513	< 0.001	440956	IQCF6
0.175808504	< 0.001	730051	ZNF814
0.175807759	< 0.001	7429	VIL1
0.17577675	< 0.001	4901	NRL
0.175768659	< 0.001	5770	PTPN1
0.17576021	< 0.001	286467	RP11-453F18__B.1
0.175750643	< 0.001	140739	UBE2F
0.175736532	< 0.001	23039	XPO7
0.175682768	< 0.001	100131434	LOC100131434
0.17567496	< 0.001	3375	IAPP
0.1756576	< 0.001	26279	PLA2G2D
0.175580516	< 0.001	84968	PNMA6A
0.175539836	< 0.001	2560	GABRB1
0.175524607	< 0.001	84905	ZNF341
0.175508738	< 0.001	84247	LDOC1L
0.175431743	< 0.001	54540	AC145098.2
0.175363585	< 0.001	101	ADAM8
0.175313711	< 0.001	23065	KIAA0090
0.175307497	< 0.001	144124	OR10A5
0.175304338	< 0.001	10590	SCGN
0.175272107	< 0.001	151613	TTC14
0.175259382	< 0.001	9355	LHX2
0.175240532	< 0.001	114294	LACTB
0.175237745	< 0.001	222171	PRR15
0.175233841	< 0.001	197358	NLRC3
0.175218895	< 0.001	389762	FAM75D3
0.175157785	< 0.001	286333	LINC00256A
0.175133094	< 0.001	51308	REEP2
0.175101519	< 0.001	79102	RNF26
0.175094828	< 0.001	5723	PSPH
0.175087392	< 0.001	84960	KIAA1984
0.175036132	< 0.001	163720	CYP4Z2P
0.175024256	< 0.001	79622	SNRNP25
0.174951077	< 0.001	10987	COPS5
0.174928412	< 0.001	8471	IRS4
0.174890622	< 0.001	54536	EXOC6
0.174870923	< 0.001	121599	SPIC
0.174870923	< 0.001	390326	OR6C76
0.174756795	< 0.001	829	CAPZA1
0.174671724	< 0.001	8712	PAGE1
0.174656779	< 0.001	133690	CAPSL
0.174628362	< 0.001	7005	TEAD3
0.174577743	< 0.001	389856	USP27X

region-wise, subcortex					
Spearman's corr coef	p-val	p-val, SA	p-val, SA (BH- correction)	GenID	Gene
0.197168589	0.248058408	0.273	1	83862	TMEM120A
0.197168589	0.248058408	0.311	1	84084	RAB6C
0.197168589	0.248058408	0.285	1	202151	RANBP3L
0.196911186	0.248685941	0.271	1	10440	TIMM17A
0.196653798	0.249314591	0.33	1	2071	ERCC3
0.196653798	0.249314591	0.304	1	2865	AC002511.1
0.196653798	0.249314591	0.275	1	55773	TBC1D23
0.196396396	0.24994421	0.249	1	22827	PUF60
0.196396396	0.24994421	0.3	1	644578	LARP1BP1
0.196138993	0.250574976	0.265	1	10539	GLRX3
0.196138993	0.250574976	0.298	1	25902	MTHFD1L
0.196138993	0.250574976	0.292	1	54504	CPVL
0.196138993	0.250574976	0.273	1	204801	NLRP11
0.19588159	0.251207113	0.276	1	2161	F12
0.19588159	0.251207113	0.287	1	10765	KDM5B
0.195624188	0.251840025	0.301	1	57016	AKR1B10
0.195624188	0.251840025	0.31	1	57484	RNF150
0.195624188	0.251840025	0.274	1	401115	C4orf48
0.1953668	0.25247401	0.292	1	28951	TRIB2
0.1953668	0.25247401	0.294	1	55012	PPP2R3C
0.195109397	0.253109038	0.288	1	3615	IMPDH2
0.195109397	0.253109038	0.294	1	5534	PPP3R1
0.195109397	0.253109038	0.289	1	374928	ZNF773
0.195109397	0.253109038	0.273	1	375519	GJB7
0.195109397	0.253109038	0.298	1	728492	SERF1B
0.194851995	0.253745437	0.295	1	1020	CDK5
0.194851995	0.253745437	0.298	1	23242	COBL
0.194851995	0.253745437	0.289	1	642929	LOC642929
0.194594592	0.25438267	0.3	1	3062	HCRTR2
0.194594592	0.25438267	0.309	1	3939	LDHA
0.194594592	0.25438267	0.295	1	50485	SMARCAL1
0.194337189	0.255020976	0.299	1	3354	HTR1E
0.194337189	0.255020976	0.283	1	118813	ZFYVE27
0.194337189	0.255020976	0.298	1	127003	C1orf194
0.194337189	0.255020976	0.288	1	645455	CEP170L
0.194079801	0.255660295	0.289	1	2281	FKBP1B
0.194079801	0.255660295	0.306	1	5303	PIN4
0.194079801	0.255660295	0.313	1	6059	ABCE1
0.194079801	0.255660295	0.323	1	140838	NANP
0.193822399	0.256300986	0.309	1	3508	IGHMBP2
0.193822399	0.256300986	0.282	1	120775	OR2D3
0.193822399	0.256300986	0.299	1	147179	WIPF2
0.193564996	0.256942511	0.264	1	2010	EMD
0.193564996	0.256942511	0.295	1	3575	IL7R
0.193564996	0.256942511	0.285	1	10726	NUDC
0.193564996	0.256942511	0.29	1	127829	ARL8A
0.193564996	0.256942511	0.287	1	128178	EDARADD
0.193307593	0.257585108	0.319	1	901	CCNG2
0.193307593	0.257585108	0.299	1	50853	VILL
0.193307593	0.257585108	0.293	1	221322	C6orf170
0.192792803	0.258873492	0.33	1	2271	FH
0.192792803	0.258873492	0.322	1	4084	MXD1
0.192792803	0.258873492	0.3	1	9914	ATP2C2
0.192792803	0.258873492	0.295	1	23481	PES1
0.192792803	0.258873492	0.294	1	65250	C5orf42
0.192792803	0.258873492	0.316	1	100505741	SPATA1
0.1925354	0.259519547	0.31	1	3670	ISL1
0.192277983	0.260166466	0.291	1	1890	TYMP
0.192277983	0.260166466	0.275	1	6158	RPL28
0.192277983	0.260166466	0.305	1	84128	WDR75
0.192277983	0.260166466	0.284	1	119774	OR52K2
0.192277983	0.260166466	0.293	1	344657	LRRIQ4
0.192277983	0.260166466	0.279	1	100286979	ANAPC1P1
0.19202058	0.260814458	0.297	1	5199	CFP
0.19202058	0.260814458	0.298	1	25796	PGLS
0.19202058	0.260814458	0.309	1	27316	RBMX
0.19202058	0.260814458	0.3	1	51085	MLXIPL
0.19202058	0.260814458	0.295	1	94033	FTMT
0.19202058	0.260814458	0.319	1	132884	EVC2
0.191763178	0.261463493	0.316	1	5568	PRKACG
0.191763178	0.261463493	0.314	1	8907	AP1M1
0.191763178	0.261463493	0.335	1	10924	SMPDL3A
0.191763178	0.261463493	0.289	1	51517	NCKIPSD
0.191763178	0.261463493	0.295	1	55737	VPS35
0.191763178	0.261463493	0.308	1	149345	SHISA4
0.19150579	0.262113869	0.298	1	245	ALOX12P2
0.19150579	0.262113869	0.309	1	126068	ZNF441
0.191248387	0.26276508	0.317	1	84262	PSMG3
0.191248387	0.26276508	0.292	1	284307	ZIK1

voxel-wise, subcortex			
Spearman's corr coef	p-val	GenID	Gene
0.174574316	< 0.001	29034	CPS1-IT1
0.17449677	< 0.001	2555	GABRA2
0.174485996	< 0.001	6586	SLIT3
0.174445137	< 0.001	163126	EID2
0.174435481	< 0.001	6157	RPL27A
0.174365461	< 0.001	9887	SMG7
0.174362674	< 0.001	27345	KCNMB4
0.174353018	< 0.001	654466	KGFLP2
0.174351528	< 0.001	11252	PACSIN2
0.174322188	< 0.001	283989	TSEN54
0.174294323	< 0.001	120787	OR52W1
0.174292654	< 0.001	6615	SNAI1
0.174201831	< 0.001	81606	LBH
0.174149632	< 0.001	22908	SACM1L
0.174086019	< 0.001	27185	DISC1
0.174073771	< 0.001	84239	ATP13A4
0.174055845	< 0.001	100290481	CYAT1
0.174012572	< 0.001	5075	PAX1
0.173948124	< 0.001	23543	RBM9
0.173938096	< 0.001	26224	FBXL3
0.173905402	< 0.001	3199	HOXA2
0.173869178	< 0.001	55664	CDC37L1
0.173826098	< 0.001	26492	OR8G2
0.173759595	< 0.001	140771	AL135932.1
0.17374827	< 0.001	2150	F2RL1
0.17373769	< 0.001	100128511	RP11-565H13.2
0.17368196	< 0.001	55823	VPS11
0.173600614	< 0.001	23023	TMCC1
0.173592165	< 0.001	116039	OSR2
0.173551217	< 0.001	4100	MAGEA1
0.173538759	< 0.001	50640	PNPLA8
0.173503295	< 0.001	8777	MPDZ
0.173490658	< 0.001	145407	C14orf37
0.173393056	< 0.001	22887	FOXJ3
0.173371971	< 0.001	148753	FAM163A
0.173098773	< 0.001	55201	MAP1S
0.173060685	< 0.001	5912	RAP2B
0.172973394	< 0.001	57538	ALPK3
0.172919348	< 0.001	9262	STK17B
0.172884986	< 0.001	2892	GRIA3
0.172850728	< 0.001	284390	ZNF763
0.172844127	< 0.001	386679	KRTAP10-2
0.172829643	< 0.001	664	BNIP3
0.172821462	< 0.001	22859	LPHN1
0.172761381	< 0.001	57452	GALNTL1
0.172761008	< 0.001	285600	C5orf36
0.172748655	< 0.001	26579	MYEOV
0.172744483	< 0.001	140576	S100A16
0.172741696	< 0.001	51778	MYOZ2
0.172707796	< 0.001	9852	EPM2AIP1
0.172695175	< 0.001	7288	TULP2
0.172650039	< 0.001	50861	STMN3
0.172613174	< 0.001	5828	PXMP3
0.172462076	< 0.001	400668	PRSSL1
0.172451392	< 0.001	3460	IFNGR2
0.17243357	< 0.001	51248	PDZD11
0.172357231	< 0.001	5965	RECQL
0.172329277	< 0.001	11044	POLS
0.172307551	< 0.001	5989	RFX1
0.172215328	< 0.001	29058	C20orf30
0.172124788	< 0.001	392376	OR13C2
0.172103986	< 0.001	353116	RILPL1
0.172086626	< 0.001	55735	DNAJC11
0.172074914	< 0.001	151254	ALS2CR11
0.17202951	< 0.001	125950	RAVER1
0.171920851	< 0.001	84065	TMEM222
0.171896055	< 0.001	1805	DPT
0.171861976	< 0.001	80775	TMEM177
0.17181462	< 0.001	9318	COPS2
0.171801984	< 0.001	121355	GTSF1
0.171708196	< 0.001	57479	PRR12
0.171702623	< 0.001	390431	OR4K2
0.171602502	< 0.001	10440	TIMM17A
0.171592295	< 0.001	148930	KNCN
0.171579942	< 0.001	57630	SH3RF1
0.171573535	< 0.001	645104	CLRN2
0.171570003	< 0.001	10298	PAK4
0.17156592	< 0.001	150368	FAM109B
0.171492651	< 0.001	9649	RALGPS1

region-wise, subcortex					
Spearman's corr coef	p-val	p-val, SA	p-val, SA (BH- correction)	GenID	Gene
0.190990984	0.263417393	0.301	1	158358	KIAA2026
0.190990984	0.263417393	0.294	1	440043	PGAM1P8
0.190733582	0.264070719	0.298	1	9406	ZRANB2
0.190733582	0.264070719	0.305	1	10016	PDCD6
0.190733582	0.264070719	0.287	1	10190	TXNDC9
0.190733582	0.264070719	0.303	1	25975	EGFL6
0.190733582	0.264070719	0.299	1	170393	C10orf91
0.190733582	0.264070719	0.272	1	389668	XKR9
0.190476179	0.264725506	0.286	1	23397	NCAPH
0.190476179	0.264725506	0.301	1	93611	FBXO44
0.190218791	0.265380949	0.303	1	8504	PEX3
0.190218791	0.265380949	0.321	1	79758	DHRS12
0.189961389	0.266037583	0.307	1	7476	WNT7A
0.189961389	0.266037583	0.315	1	23229	ARHGEF9
0.189961389	0.266037583	0.293	1	221477	C6orf89
0.189961389	0.266037583	0.288	1	342357	ZKSCAN2
0.189703986	0.266695231	0.321	1	3852	KRT5
0.189703986	0.266695231	0.301	1	9349	RPL23
0.189703986	0.266695231	0.299	1	55092	TMEM51
0.189446583	0.267354012	0.316	1	3841	KPNA5
0.189189181	0.268014163	0.324	1	400	ARL1
0.189189181	0.268014163	0.32	1	51350	KRT76
0.188931793	0.268675059	0.321	1	658	BMPR1B
0.18867439	0.269337058	0.315	1	79144	PPDPF
0.18867439	0.269337058	0.321	1	84254	CAMKK1
0.18867439	0.269337058	0.35	1	84944	MAEL
0.188159585	0.270664603	0.322	1	10436	EMG1
0.188159585	0.270664603	0.331	1	22998	LIMCH1
0.187902182	0.27132988	0.312	1	466	ATF1
0.187902182	0.27132988	0.31	1	3745	KCNB1
0.187902182	0.27132988	0.318	1	10018	BCL2L11
0.187902182	0.27132988	0.294	1	80094	FLJ14107
0.187902182	0.27132988	0.318	1	440278	CATSPER2P1
0.187644795	0.27199617	0.299	1	1154	CISH
0.187644795	0.27199617	0.339	1	1211	CLTA
0.187644795	0.27199617	0.308	1	6499	SKIV2L
0.187387392	0.272663563	0.31	1	874	CBR3
0.187387392	0.272663563	0.31	1	9984	THOC1
0.186872587	0.274001956	0.309	1	90627	STARD13
0.186872587	0.274001956	0.287	1	117177	RAB3IP
0.186872587	0.274001956	0.295	1	118429	ANTXR2
0.186872587	0.274001956	0.315	1	220064	ORAOV1
0.186615184	0.274672627	0.279	1	545	ATR
0.186615184	0.274672627	0.335	1	51481	VCX3A
0.186615184	0.274672627	0.339	1	54478	FAM64A
0.186615184	0.274672627	0.303	1	347365	ITIH5L
0.186357796	0.275344312	0.323	1	6259	RYK
0.186357796	0.275344312	0.3	1	6754	SSTR4
0.186357796	0.275344312	0.329	1	6794	STK11
0.186357796	0.275344312	0.337	1	8703	B4GALT3
0.186357796	0.275344312	0.328	1	9146	HGS
0.186357796	0.275344312	0.324	1	23333	DPY19L1
0.186357796	0.275344312	0.307	1	56131	PCDHB4
0.186357796	0.275344312	0.362	1	57451	ODZ2
0.186357796	0.275344312	0.279	1	163404	PPAPR5
0.186357796	0.275344312	0.325	1	219770	GJD4
0.186100394	0.276017159	0.301	1	51347	TAOK3
0.186100394	0.276017159	0.319	1	56260	C8orf44
0.186100394	0.276017159	0.327	1	338917	VSX2
0.186100394	0.276017159	0.311	1	100129482	ZNF37BP
0.185842991	0.276691377	0.305	1	8314	BAP1
0.185842991	0.276691377	0.323	1	11190	CEP250
0.185842991	0.276691377	0.318	1	55083	KIF26B
0.185842991	0.276691377	0.314	1	146754	DNAH2
0.185842991	0.276691377	0.324	1	728716	LOC728716
0.185585588	0.2773664	0.318	1	10172	ZNF256
0.185585588	0.2773664	0.312	1	79667	AC021860.1
0.185328171	0.278042465	0.303	1	7311	RPS27AP11
0.185328171	0.278042465	0.313	1	91689	C22orf32
0.185328171	0.278042465	0.324	1	404281	YY2
0.185070768	0.278719574	0.315	1	4358	MPV17
0.185070768	0.278719574	0.308	1	57494	AC092490.2
0.185070768	0.278719574	0.301	1	342538	NACA2
0.18481338	0.279398113	0.282	1	1476	CSTB
0.18481338	0.279398113	0.302	1	29101	SSU72
0.18481338	0.279398113	0.323	1	54995	OXSM
0.18481338	0.279398113	0.288	1	56478	EIF4ENIF1
0.18481338	0.279398113	0.332	1	138649	ANKRD19P
0.18481338	0.279398113	0.314	1	284306	ZNF547

voxel-wise, subcortex			
Spearman's corr coef	p-val	GenID	Gene
0.171454296	< 0.001	10874	NMU
0.171435446	< 0.001	93408	MYL10
0.171352148	< 0.001	23355	VPS8
0.171262249	< 0.001	200728	TMEM17
0.171152115	< 0.001	10865	ARID5A
0.171133623	< 0.001	116969	ART5
0.171113849	< 0.001	100131390	SP9
0.171076328	< 0.001	645431	LOC645431
0.171072155	< 0.001	56926	NCLN
0.171049774	< 0.001	256714	MAP7D2
0.171037421	< 0.001	340508	RP11-520B13.4
0.171008542	< 0.001	6118	RPA2
0.17097047	< 0.001	6184	RPN1
0.17095533	< 0.001	644422	LOC644422
0.170891717	< 0.001	8556	CDC14A
0.17084454	< 0.001	8087	FXR1
0.170841381	< 0.001	2554	GABRA1
0.170807391	< 0.001	81569	ACTL8
0.170732543	< 0.001	51305	KCNK9
0.170584321	< 0.001	7818	DAP3
0.170505762	< 0.001	6768	ST14
0.170468807	< 0.001	373	TRIM23
0.170417175	< 0.001	114784	CSMD2
0.170397207	< 0.001	51390	AIG1
0.170388564	< 0.001	92521	CYTSB
0.170327082	< 0.001	80024	SLC24A6
0.170318261	< 0.001	400793	C1orf226
0.170244068	< 0.001	442862	PRY
0.170189276	< 0.001	9289	GPR56
0.170180917	< 0.001	5514	PPP1R10
0.170167729	< 0.001	114880	OSBPL6
0.170105144	< 0.001	9152	SLC6A5
0.170102999	< 0.001	81470	OR2G2
0.170091957	< 0.001	100130776	RP11-571M6.6
0.170082659	< 0.001	114769	CARD16
0.169997692	< 0.001	148223	C19orf25
0.169902503	< 0.001	940	CD28
0.169893399	< 0.001	80146	UXS1
0.169881523	< 0.001	43847	KLK14
0.169811308	< 0.001	6366	CCL21
0.169747606	< 0.001	56134	PCDHAC2
0.169736281	< 0.001	125206	SLC5A10
0.169638395	< 0.001	728739	LOC728739
0.169605896	< 0.001	442721	LMOD2
0.169508845	< 0.001	4734	NEDD4
0.16950132	< 0.001	65117	RSRC2
0.169475883	< 0.001	79843	FAM124B
0.169448018	< 0.001	23301	EHBP1
0.169444114	< 0.001	8326	FZD9
0.169436589	< 0.001	644684	LOC644684
0.169332489	< 0.001	339221	ENPP7
0.169329807	< 0.001	100128998	C20orf181
0.169320971	< 0.001	55777	MBD5
0.169277325	< 0.001	390424	ATP5G1P5
0.169276595	< 0.001	51018	RRP15
0.169268236	< 0.001	116372	LYPD1
0.16919969	< 0.001	340390	KIAA1875
0.169128373	< 0.001	22888	UBOX5
0.169041082	< 0.001	54898	ELOVL2
0.169001892	< 0.001	120775	OR2D3
0.168997332	< 0.001	27123	DKK2
0.168994188	< 0.001	51504	TRMT112
0.168957129	< 0.001	64318	NOC3L
0.168909207	< 0.001	23586	DDX58
0.168861389	< 0.001	53635	PTOV1
0.168851361	< 0.001	440026	TMEM41B
0.16884847	< 0.001	113835	ZNF257
0.16882284	< 0.001	1207	CLNS1A
0.168707415	< 0.001	5550	PREP
0.168665528	< 0.001	29798	C2orf27A
0.168643236	< 0.001	100130522	PARD6G-AS1
0.168611482	< 0.001	79623	GALNT14
0.168551862	< 0.001	8428	STK24
0.168551207	< 0.001	23440	OTP
0.168545455	< 0.001	9369	NRXN3
0.168464571	< 0.001	388531	RGS9BP
0.168449342	< 0.001	390598	SKOR1
0.168445989	< 0.001	84640	USP38
0.168439403	< 0.001	5342	PLGLB2

region-wise, subcortex					
Spearman's corr coef	p-val	p-val, SA	p-val, SA (BH- correction)	GenID	Gene
0.18481338	0.279398113	0.314	1	552889	ATXN7L3B
0.18481338	0.279398113	0.299	1	648771	LOC648771
0.184555978	0.280077457	0.311	1	10579	TACC2
0.184555978	0.280077457	0.304	1	57381	RHOJ
0.184555978	0.280077457	0.334	1	337972	KRTAP19-5
0.184555978	0.280077457	0.337	1	100128239	AP000911.1
0.184298575	0.280757874	0.32	1	4302	MLLT6
0.184298575	0.280757874	0.342	1	7264	TSTA3
0.184298575	0.280757874	0.319	1	7366	AC147055.1
0.184298575	0.280757874	0.325	1	8540	AGPS
0.184298575	0.280757874	0.35	1	9227	LRAT
0.184298575	0.280757874	0.326	1	51371	POMP
0.184298575	0.280757874	0.315	1	79187	FSD1
0.184298575	0.280757874	0.331	1	126695	C1orf172
0.184041172	0.281439394	0.338	1	2066	ERBB4
0.184041172	0.281439394	0.339	1	6006	RHCE
0.18378377	0.282121897	0.319	1	6907	TBL1X
0.18378377	0.282121897	0.309	1	27036	SIGLEC7
0.183526382	0.28280586	0.324	1	3638	INSIG1
0.183526382	0.28280586	0.332	1	54072	LINC00158
0.183526382	0.28280586	0.287	1	55628	ZNF407
0.183526382	0.28280586	0.344	1	90121	TSR2
0.183268979	0.283490628	0.321	1	2752	GLUL
0.183268979	0.283490628	0.329	1	166348	KLHDC6
0.183268979	0.283490628	0.339	1	285180	RUFY4
0.183011577	0.284176469	0.333	1	3785	KCNQ2
0.183011577	0.284176469	0.319	1	7260	TSSC1
0.183011577	0.284176469	0.285	1	57110	HRASLS
0.182754174	0.284863412	0.332	1	8027	STAM
0.182754174	0.284863412	0.322	1	60526	C2orf43
0.182754174	0.284863412	0.344	1	63973	NEUROG2
0.182754174	0.284863412	0.305	1	84316	LSMD1
0.182754174	0.284863412	0.324	1	123722	FSD2
0.182754174	0.284863412	0.308	1	346329	LOC346329
0.182496771	0.285551667	0.298	1	9925	ZBTB5
0.182496771	0.285551667	0.332	1	23263	MCF2L
0.182239383	0.286240727	0.32	1	2205	FCER1A
0.182239383	0.286240727	0.332	1	6671	SP4
0.182239383	0.286240727	0.315	1	10401	PIAS3
0.182239383	0.286240727	0.337	1	22977	AKR7A3
0.182239383	0.286240727	0.337	1	55317	C20orf29
0.182239383	0.286240727	0.337	1	387104	AL096711.2
0.181981981	0.286930919	0.341	1	5514	PPP1R10
0.181981981	0.286930919	0.342	1	8470	SORBS2
0.181981981	0.286930919	0.34	1	9742	IFT140
0.181981981	0.286930919	0.315	1	55131	RBM28
0.181981981	0.286930919	0.31	1	285193	DUSP28
0.181981981	0.286930919	0.341	1	392390	OR1L6
0.181724578	0.287622213	0.335	1	10252	SPRY1
0.181724578	0.287622213	0.313	1	10393	ANAPC10
0.181724578	0.287622213	0.327	1	142891	SAMD8
0.181724578	0.287622213	0.337	1	150771	ITPRIPL1
0.181467175	0.288314849	0.348	1	1200	TPP1
0.181467175	0.288314849	0.335	1	23409	SIRT4
0.181467175	0.288314849	0.346	1	29100	TMEM208
0.181467175	0.288314849	0.33	1	80119	PIF1
0.181467175	0.288314849	0.328	1	548593	GIYD2
0.181209773	0.28900823	0.334	1	4218	RAB8A
0.181209773	0.28900823	0.33	1	8530	CST7
0.181209773	0.28900823	0.342	1	10114	HIPK3
0.180952385	0.289702773	0.354	1	5445	PON2
0.180952385	0.289702773	0.304	1	5920	RARRES3
0.180952385	0.289702773	0.357	1	146849	CCDC42
0.180952385	0.289702773	0.346	1	326625	MMAB
0.180694982	0.290398359	0.361	1	2918	GRM8
0.18043758	0.291094989	0.343	1	10316	NMUR1
0.18043758	0.291094989	0.343	1	55286	C4orf19
0.18043758	0.291094989	0.327	1	91869	RFT1
0.18043758	0.291094989	0.333	1	124944	C17ORF49
0.180180177	0.291793138	0.318	1	5292	PIM1
0.179922774	0.292491913	0.319	1	4898	NRD1
0.179922774	0.292491913	0.316	1	7109	TRAPPC10
0.179665387	0.29319188	0.33	1	1901	S1PR1
0.179665387	0.29319188	0.341	1	56904	SH3GLB2
0.179407984	0.29389286	0.339	1	6227	RPS21
0.179407984	0.29389286	0.329	1	9675	KIAA0406
0.179407984	0.29389286	0.348	1	55573	CDV3
0.179407984	0.29389286	0.358	1	80230	RUFY1
0.179150581	0.294595361	0.327	1	7528	YY1

voxel-wise, subcortex			
Spearman's corr coef	p-val	GenID	Gene
0.168434575	< 0.001	11040	PIM2
0.168353125	< 0.001	84465	MEGF11
0.168352008	< 0.001	341152	OR2AT4
0.168320253	< 0.001	2515	ADAM2
0.168267414	< 0.001	29071	C1GALT1C1
0.168234721	< 0.001	56984	PSMG2
0.168188751	< 0.001	59284	CACNG7
0.168166101	< 0.001	55068	ENOX1
0.168038219	< 0.001	285962	RP5-894A10.1
0.167972475	< 0.001	377	ARF3
0.167943403	< 0.001	5551	PRF1
0.167940244	< 0.001	339229	C17orf90
0.167920843	< 0.001	7549	ZNF2
0.167899206	< 0.001	334	APLP2
0.167885825	< 0.001	390892	OR7A10
0.167872176	< 0.001	540	ATP7B
0.167816922	< 0.001	81551	STMN4
0.167814046	< 0.001	388512	CLEC17A
0.167812929	< 0.001	10724	MGEA5
0.167794347	< 0.001	10266	RAMP2
0.167769372	< 0.001	29991	OBP2A
0.167727023	< 0.001	51385	ZNF589
0.167692766	< 0.001	51533	PHF7
0.167668059	< 0.001	2210	FCGR1B
0.167623013	< 0.001	1877	E4F1
0.167578995	< 0.001	388336	AC007510.1
0.167563021	< 0.001	256691	MAMDC2
0.167491615	< 0.001	5411	PNN
0.167439878	< 0.001	284618	RUSC1-AS1
0.167437658	< 0.001	2583	B4GALNT1
0.167435527	< 0.001	10555	AGPAT2
0.167389736	< 0.001	5382	PMS2P4
0.167307451	< 0.001	154467	C6orf129
0.167283595	< 0.001	3305	HSPA1L
0.167273566	< 0.001	90557	CCDC74A
0.167242825	< 0.001	23157	SEPTIN6
0.16717419	< 0.001	8776	MTMR1
0.167151079	< 0.001	6767	ST13
0.167092934	< 0.001	653635	WASH5P
0.167008981	< 0.001	53354	PANK1
0.166971475	< 0.001	1442	CSH1
0.166965529	< 0.001	3073	HEXA
0.166934133	< 0.001	9755	TBKBP1
0.166930705	< 0.001	85294	KRTAP2-4
0.166887894	< 0.001	56254	RNF20
0.166875452	< 0.001	64718	C16orf28
0.166875258	< 0.001	28968	SLC6A16
0.166861236	< 0.001	9797	TATDN2
0.166784063	< 0.001	64405	CDH22
0.166773662	< 0.001	548596	CKMT1A
0.166709125	< 0.001	100130691	AC074286.1
0.166704014	< 0.001	9728	SECISBP2L
0.166636407	< 0.001	6473	SHOX
0.166626289	< 0.001	23197	FAF2
0.166623592	< 0.001	126133	ALDH16A1
0.166618019	< 0.001	57631	LRCH2
0.166548833	< 0.001	341346	C12orf70
0.166543454	< 0.001	55030	FBXO34
0.166487262	< 0.001	347853	TBX10
0.166452348	< 0.001	10457	GPNMB
0.166450396	< 0.001	139596	UPRT
0.166441202	< 0.001	51510	CHMP5
0.166333482	< 0.001	53343	NUDT9
0.166305989	< 0.001	4733	DRG1
0.166305155	< 0.001	349149	GJC3
0.166269302	< 0.001	23529	CLCF1
0.166253433	< 0.001	127544	RNF19B
0.166153789	< 0.001	286204	CRB2
0.166130841	< 0.001	60493	FASTKD5
0.166128621	< 0.001	22980	TCF25
0.166119695	< 0.001	54469	ZFAND6
0.166091844	< 0.001	645974	PABPC1L2B
0.166052192	< 0.001	202181	LOC202181
0.166044295	< 0.001	641	BLM
0.165981427	< 0.001	284123	FAM27L
0.165796161	< 0.001	390616	ANKRD34C
0.165747955	< 0.001	100287049	RP11-574F21.2
0.165734679	< 0.001	81847	RNF146
0.165729672	< 0.001	27328	PCDH11X

region-wise, subcortex					
Spearman's corr coef	p-val	p-val, SA	p-val, SA (BH- correction)	GenID	Gene
0.179150581	0.294595361	0.361	1	645121	CCNI2
0.178893179	0.295298487	0.32	1	171568	POLR3H
0.178893179	0.295298487	0.342	1	474381	H2AFB2
0.178635776	0.296002775	0.315	1	3184	HNRNPD
0.178635776	0.296002775	0.341	1	7915	ALDH5A1
0.178635776	0.296002775	0.325	1	55425	KIAA1704
0.178635776	0.296002775	0.334	1	166379	BBS12
0.178378388	0.296708167	0.323	1	2125	EVPL
0.178378388	0.296708167	0.342	1	7409	VAV1
0.178378388	0.296708167	0.349	1	51611	DPH5
0.178120971	0.297414929	0.34	1	27164	SALL3
0.178120971	0.297414929	0.334	1	64778	FNDC3B
0.178120971	0.297414929	0.313	1	113263	GLCCI1
0.178120971	0.297414929	0.335	1	256586	LYSMD2
0.178120971	0.297414929	0.344	1	266977	GPR110
0.177863568	0.298122495	0.366	1	5173	PDYN
0.177863568	0.298122495	0.336	1	7391	USF1
0.177863568	0.298122495	0.339	1	7571	ZNF23
0.177863568	0.298122495	0.346	1	10149	GPR64
0.177863568	0.298122495	0.321	1	23389	MED13L
0.177863568	0.298122495	0.355	1	27077	B9D1
0.177863568	0.298122495	0.343	1	125476	INO80C
0.177606165	0.298831046	0.325	1	56886	UGCGL1
0.177606165	0.298831046	0.34	1	85290	KRTAP4-3
0.177348763	0.299540758	0.331	1	9683	N4BP1
0.177348763	0.299540758	0.339	1	23243	ANKRD28
0.177348763	0.299540758	0.34	1	57338	JPH3
0.177348763	0.299540758	0.33	1	79077	DCTPP1
0.177091375	0.300251573	0.344	1	22986	SORCS3
0.177091375	0.300251573	0.322	1	124454	EARS2
0.177091375	0.300251573	0.347	1	136288	C7orf57
0.176833972	0.300963819	0.332	1	1362	CPD
0.176833972	0.300963819	0.306	1	2121	EVC
0.176833972	0.300963819	0.334	1	10953	TOMM34
0.176833972	0.300963819	0.357	1	84131	CEP78
0.17657657	0.30167675	0.363	1	10426	TUBGCP3
0.17657657	0.30167675	0.328	1	27165	GLS2
0.176319167	0.302390754	0.329	1	26012	NELF
0.176319167	0.302390754	0.341	1	54758	KLHDC4
0.176061764	0.303105891	0.33	1	8728	ADAM19
0.176061764	0.303105891	0.356	1	55970	GNG12
0.176061764	0.303105891	0.353	1	79050	NOC4L
0.176061764	0.303105891	0.373	1	126326	GIPC3
0.176061764	0.303105891	0.366	1	285033	LOC285033
0.175804377	0.303822488	0.353	1	770	CA11
0.175804377	0.303822488	0.361	1	51559	NT5DC3
0.175804377	0.303822488	0.343	1	54534	MRPL50
0.175804377	0.303822488	0.357	1	55069	C7orf42
0.175804377	0.303822488	0.329	1	79747	C6orf103
0.175804377	0.303822488	0.355	1	121457	AC013283.1
0.175546974	0.30453977	0.345	1	1385	CREB1
0.175546974	0.30453977	0.334	1	4121	MAN1A1
0.175546974	0.30453977	0.327	1	54432	YIPF1
0.175546974	0.30453977	0.345	1	151531	UPP2
0.175546974	0.30453977	0.353	1	401993	OR2T5
0.175289571	0.305258185	0.36	1	1844	DUSP2
0.175289571	0.305258185	0.328	1	3428	IFI16
0.175289571	0.305258185	0.343	1	26519	TIMM10
0.175289571	0.305258185	0.32	1	90271	LINC00263
0.174774766	0.306698561	0.347	1	3645	INSRR
0.174774766	0.306698561	0.378	1	6531	SLC6A3
0.174774766	0.306698561	0.352	1	23413	FREQ
0.174774766	0.306698561	0.335	1	54902	TTC19
0.174774766	0.306698561	0.34	1	55283	MCOLN3
0.174774766	0.306698561	0.351	1	286499	FAM133A
0.174517378	0.307420194	0.352	1	26272	FBXO4
0.174517378	0.307420194	0.363	1	56850	GRIPAP1
0.174259976	0.30814293	0.352	1	1291	COL6A1
0.174259976	0.30814293	0.367	1	1951	CELSR3
0.174259976	0.30814293	0.341	1	6874	TAF4
0.174259976	0.30814293	0.359	1	85865	GTPBP10
0.174259976	0.30814293	0.338	1	90701	SEC11C
0.174259976	0.30814293	0.371	1	256471	MFSD8
0.174259976	0.30814293	0.346	1	390424	ATP5G1P5
0.174002573	0.308866799	0.351	1	7976	FZD3
0.174002573	0.308866799	0.34	1	9942	XYLB
0.17374517	0.309591651	0.348	1	10499	NCOA2
0.17374517	0.309591651	0.334	1	23095	KIF1B
0.17374517	0.309591651	0.356	1	84418	CYSTM1

voxel-wise, subcortex			
Spearman's corr coef	p-val	GenID	Gene
0.165727243	< 0.001	51164	DCTN4
0.165724739	< 0.001	2017	CTTN
0.165654257	< 0.001	83259	PCDH11Y
0.165645719	< 0.001	11120	BTN2A1
0.165593341	< 0.001	56910	STARD7
0.165592685	< 0.001	5468	PPARG
0.165579587	< 0.001	2274	FHL2
0.165563434	< 0.001	56413	LTB4R2
0.165548116	< 0.001	55615	PRR5
0.165537894	< 0.001	163175	LGI4
0.165473908	< 0.001	284086	NEK8
0.165430829	< 0.001	219982	OR5A1
0.16535458	< 0.001	84975	MFSD5
0.165319666	< 0.001	10788	IQGAP2
0.165285021	< 0.001	11041	AP001107.1
0.16522865	< 0.001	442117	GALNTL6
0.165182769	< 0.001	26130	GAPVD1
0.165172562	< 0.001	64328	XPO4
0.165172189	< 0.001	152302	CIDECP
0.165163651	< 0.001	6758	SSX5
0.165157422	< 0.001	23562	CLDN14
0.165139034	< 0.001	116285	ACSM1
0.165131792	< 0.001	163404	PPAPR5
0.165113226	< 0.001	6372	CXCL6
0.165030196	< 0.001	8498	RANBP3
0.16500847	< 0.001	119695	OR52R1
0.164984509	< 0.001	138050	HGSNAT
0.164981723	< 0.001	23568	ARL2BP
0.164953962	< 0.001	55966	AJAP1
0.164951265	< 0.001	57669	EPB41L5
0.164897591	< 0.001	80122	YSK4
0.164895356	< 0.001	5357	PLS1
0.164883003	< 0.001	7564	ZNF16
0.16487585	< 0.001	163154	PRR22
0.164853841	< 0.001	3972	LHB
0.164845675	< 0.001	2647	BLOC1S1
0.164801285	< 0.001	4677	NARS
0.164701641	< 0.001	1141	CHRNB2
0.164675817	< 0.001	390151	OR8H2
0.164672852	< 0.001	56062	KLHL4
0.164653167	< 0.001	150864	FAM117B
0.164577663	< 0.001	23263	MCF2L
0.164574414	< 0.001	27112	FAM155B
0.164568841	< 0.001	220429	CTAGE10P
0.164424524	< 0.001	124935	SLC43A2
0.164385527	< 0.001	728361	OVOL3
0.164371595	< 0.001	150465	TTL
0.164304271	< 0.001	10661	KLF1
0.164267674	< 0.001	337966	KRTAP6-1
0.164252162	< 0.001	3906	LALBA
0.164126977	< 0.001	767846	PFN1P2
0.164116398	< 0.001	651	BMP3
0.164110914	< 0.001	9749	PHACTR2
0.164076284	< 0.001	64067	NPAS3
0.164065689	< 0.001	79018	C17orf39
0.164039403	< 0.001	57127	RHBG
0.163970038	< 0.001	2735	GLI1
0.16392082	< 0.001	55856	ACOT13
0.163871139	< 0.001	340543	TCEAL5
0.16380623	< 0.001	56891	LGALS14
0.163766667	< 0.001	10894	LYVE1
0.163508311	< 0.001	6580	SLC22A1
0.163480639	< 0.001	27132	CPNE7
0.163467169	< 0.001	200081	TXLNA
0.16344367	< 0.001	1134	CHRNA1
0.163431227	< 0.001	199800	C19orf76
0.163390189	< 0.001	5595	MAPK3
0.163387403	< 0.001	9955	HS3ST3A1
0.163352847	< 0.001	867	CBL
0.163346902	< 0.001	165829	GPR156
0.163282081	< 0.001	90139	TSPAN18
0.163271964	< 0.001	221710	C6ORF228
0.163270757	< 0.001	116985	ARAP1
0.163241789	< 0.001	4192	MDK
0.163234919	< 0.001	51378	ANGPT4
0.163234636	< 0.001	10022	INSL5
0.163214207	< 0.001	312	ANXA13
0.16317603	< 0.001	390212	GPR152
0.16305846	< 0.001	5005	ORM2

region-wise, subcortex					
Spearman's corr coef	p-val	p-val, SA	p-val, SA (BH- correction)	GenID	Gene
0.17374517	0.309591651	0.349	1	390598	SKOR1
0.173487768	0.310318023	0.372	1	2961	GTF2E2
0.173487768	0.310318023	0.335	1	7167	TPI1
0.173487768	0.310318023	0.334	1	22955	SCMH1
0.173487768	0.310318023	0.349	1	53339	BTBD1
0.173487768	0.310318023	0.376	1	146434	ZNF597
0.17323038	0.311045051	0.34	1	48	ACO1
0.17323038	0.311045051	0.364	1	4795	NFKBIL1
0.17323038	0.311045051	0.342	1	54828	BCAS3
0.172972977	0.311773241	0.368	1	5522	PPP2R2C
0.172972977	0.311773241	0.369	1	10137	RBM12
0.172972977	0.311773241	0.359	1	55833	UBAP2
0.172715575	0.312502503	0.349	1	27286	SRPX2
0.172715575	0.312502503	0.345	1	57611	ISLR2
0.172458172	0.313233137	0.353	1	3760	KCNJ3
0.172458172	0.313233137	0.337	1	8825	LIN7A
0.172458172	0.313233137	0.349	1	10776	ARPP19
0.172200769	0.313964546	0.373	1	3992	FADS1
0.172200769	0.313964546	0.352	1	7549	ZNF2
0.172200769	0.313964546	0.349	1	9354	UBE4A
0.172200769	0.313964546	0.355	1	64789	DEM1
0.172200769	0.313964546	0.374	1	80216	ALPK1
0.172200769	0.313964546	0.38	1	266675	BEST4
0.171943381	0.314697087	0.332	1	3796	KIF2A
0.171943381	0.314697087	0.349	1	4524	MTHFR
0.171943381	0.314697087	0.365	1	27230	SERP1
0.171943381	0.314697087	0.348	1	140739	UBE2F
0.171685979	0.315430701	0.361	1	55068	ENOX1
0.171685979	0.315430701	0.359	1	146227	AC132186.2
0.171685979	0.315430701	0.366	1	392264	LOC392264
0.171428576	0.316165745	0.366	1	26258	PLDN
0.171171159	0.316901445	0.372	1	1478	CSTF2
0.171171159	0.316901445	0.343	1	2983	GUCY1B3
0.171171159	0.316901445	0.369	1	7296	TXNRD1
0.171171159	0.316901445	0.328	1	10552	ARPC1A
0.171171159	0.316901445	0.359	1	254263	CNIH2
0.171171159	0.316901445	0.342	1	337971	KRTAP19-4
0.170913756	0.317638278	0.351	1	10367	CBARA1
0.170913756	0.317638278	0.354	1	26224	FBXL3
0.170913756	0.317638278	0.359	1	80774	LIMD2
0.170656368	0.318376243	0.386	1	547	KIF1A
0.170656368	0.318376243	0.377	1	2739	GLO1
0.170656368	0.318376243	0.358	1	7347	UCHL3
0.170656368	0.318376243	0.366	1	51523	CXXC5
0.170656368	0.318376243	0.342	1	340595	ZCCHC16
0.170398965	0.319115281	0.35	1	7975	MAFK
0.170398965	0.319115281	0.339	1	81545	FBXO38
0.170398965	0.319115281	0.364	1	124739	USP43
0.170398965	0.319115281	0.368	1	128866	CHMP4B
0.170398965	0.319115281	0.343	1	100129503	PP13
0.170141563	0.31985572	0.379	1	92092	ZC3HAV1L
0.169626758	0.32133913	0.359	1	599	BCL2L2
0.169626758	0.32133913	0.349	1	1408	CRY2
0.169626758	0.32133913	0.36	1	79868	ALG13
0.16936937	0.32208252	0.359	1	10952	SEC61B
0.16936937	0.32208252	0.355	1	54663	WDR74
0.169111967	0.322827309	0.368	1	3069	HDLBP
0.169111967	0.322827309	0.389	1	8564	KMO
0.169111967	0.322827309	0.343	1	9451	EIF2AK3
0.169111967	0.322827309	0.358	1	10459	MAD2L2
0.169111967	0.322827309	0.394	1	23784	POTEH
0.169111967	0.322827309	0.346	1	55585	UBE2Q1
0.169111967	0.322827309	0.359	1	150786	WTH3DI
0.168854564	0.323572785	0.352	1	5699	PSMB10
0.168854564	0.323572785	0.369	1	11266	DUSP12
0.168854564	0.323572785	0.365	1	80762	NDFIP1
0.168854564	0.323572785	0.357	1	94009	SERHL
0.168597162	0.324319422	0.358	1	2119	ETV5
0.168597162	0.324319422	0.364	1	2944	GSTM1
0.168597162	0.324319422	0.357	1	3009	HIST1H1B
0.168339759	0.325067103	0.382	1	9353	SLIT2
0.168082371	0.325816214	0.353	1	419	ART3
0.168082371	0.325816214	0.37	1	9969	MED13
0.168082371	0.325816214	0.36	1	10769	PLK2
0.168082371	0.325816214	0.381	1	54865	GPATCH4
0.168082371	0.325816214	0.38	1	285905	INTS4L1
0.167824969	0.3265661	0.372	1	9534	ZNF254
0.167824969	0.3265661	0.358	1	11224	RPL35
0.167824969	0.3265661	0.368	1	23016	EXOSC7

voxel-wise, subcortex			
Spearman's corr coef	p-val	GenID	Gene
0.16305846	< 0.001	29888	STRN4
0.163048998	< 0.001	130612	TMEM198
0.162877664	< 0.001	147804	CTD-2224J9.2
0.162859172	< 0.001	11272	PRR4
0.162825286	< 0.001	11251	GPR44
0.162824258	< 0.001	201283	AMZ2P1
0.16279909	< 0.001	5081	PAX7
0.162793517	< 0.001	151009	LOC151009
0.162755534	< 0.001	10287	RGS19
0.162696287	< 0.001	8539	API5L1
0.162651435	< 0.001	185	AGTR1
0.162632674	< 0.001	64343	AZI2
0.162622273	< 0.001	117196	MRGPRX4
0.162579283	< 0.001	81566	CSRNP2
0.162568688	< 0.001	1159	CKMT1B
0.162565067	< 0.001	8534	CHST1
0.162546217	< 0.001	201292	TRIM65
0.162525788	< 0.001	56005	C19orf10
0.162520871	< 0.001	55082	ARGLU1
0.162494957	< 0.001	3118	HLA-DQA2
0.162434414	< 0.001	349136	WDR86
0.162433013	< 0.001	147694	ZNF548
0.16238983	< 0.001	29960	FTSJ2
0.162362993	< 0.001	55291	SAPS3
0.162284523	< 0.001	284361	C19orf63
0.162243292	< 0.001	23032	USP33
0.162214965	< 0.001	90826	PRMT10
0.162195563	< 0.001	8497	PPFIA4
0.16214931	< 0.001	388555	IGFL3
0.162145033	< 0.001	891	CCNB1
0.162139282	< 0.001	83755	KRTAP4-12
0.162078366	< 0.001	286	ANK1
0.162072599	< 0.001	9768	KIAA0101
0.162068695	< 0.001	340371	NRBP2
0.162056074	< 0.001	5370	PMCHL2
0.162041962	< 0.001	1849	DUSP7
0.161967665	< 0.001	23408	SIRT5
0.161902651	< 0.001	27091	CACNG5
0.161894396	< 0.001	25994	HIGD1A
0.161865324	< 0.001	401563	C9orf139
0.161784813	< 0.001	65999	LRRC61
0.161776826	< 0.001	59335	PRDM12
0.161741346	< 0.001	2888	GRB14
0.161644682	< 0.001	79780	CCDC82
0.161631495	< 0.001	221914	GPC2
0.161572799	< 0.001	6476	SI
0.161568344	< 0.001	63947	DMRTC1B
0.161546975	< 0.001	131616	TMEM42
0.161451057	< 0.001	79833	GEMIN6
0.161412969	< 0.001	375035	SFT2D2
0.161407769	< 0.001	283345	RPL13P5
0.161407307	< 0.001	4284	MIP
0.161387622	< 0.001	27257	LSM1
0.161354557	< 0.001	148534	TMEM56
0.161325127	< 0.001	164714	TTLL8
0.161317691	< 0.001	10886	NPFFR2
0.161316201	< 0.001	5440	AC025647.1
0.16131556	< 0.001	26330	GAPDHS
0.161252692	< 0.001	150383	C22orf40
0.161243305	< 0.001	9343	EFTUD2
0.161144689	< 0.001	100049587	SIGLEC14
0.161117017	< 0.001	84460	ZMAT1
0.160971209	< 0.001	8467	SMARCA5
0.160969719	< 0.001	27143	KIAA1274
0.160895243	< 0.001	23348	DOCK9
0.160886616	< 0.001	643418	LIPN
0.160869151	< 0.001	55284	UBE2W
0.160863578	< 0.001	51495	PTPLAD1
0.16086182	< 0.001	57010	CABP4
0.160840452	< 0.001	133015	PACRGL
0.160826519	< 0.001	10499	NCOA2
0.160819933	< 0.001	53344	CHIC1
0.160800621	< 0.001	4801	NFYB
0.160770521	< 0.001	63923	TNN
0.160768211	< 0.001	10162	U47924.1
0.160753161	< 0.001	9653	HS2ST1
0.160720468	< 0.001	30009	TBX21
0.160701156	< 0.001	51149	C5orf45
0.160661504	< 0.001	29958	DMGDH

region-wise, subcortex					
Spearman's corr coef	p-val	p-val, SA	p-val, SA (BH- correction)	GenID	Gene
0.167824969	0.3265661	0.361	1	23620	NTSR2
0.167824969	0.3265661	0.353	1	51069	MRPL2
0.167824969	0.3265661	0.353	1	59307	SIGIRR
0.167824969	0.3265661	0.356	1	79596	RNF219
0.167824969	0.3265661	0.365	1	80336	PABPC1L
0.167824969	0.3265661	0.365	1	154288	C6orf221
0.167824969	0.3265661	0.381	1	283871	PGP
0.167824969	0.3265661	0.405	1	285989	ZNF789
0.167824969	0.3265661	0.39	1	100652929	AC105337.1
0.167567566	0.327316999	0.381	1	182	JAG1
0.167567566	0.327316999	0.379	1	4666	NACA
0.167567566	0.327316999	0.372	1	5058	PAK1
0.167567566	0.327316999	0.341	1	8444	DYRK3
0.167567566	0.327316999	0.343	1	10863	ADAM28
0.167567566	0.327316999	0.375	1	55288	RHOT1
0.167567566	0.327316999	0.368	1	374819	LRRC37A3
0.167310163	0.328069061	0.371	1	83876	MRO
0.167310163	0.328069061	0.356	1	256130	TMEM196
0.167052761	0.328822136	0.378	1	2651	GCNT2
0.167052761	0.328822136	0.354	1	2681	GGTA1
0.167052761	0.328822136	0.381	1	5358	PLS3
0.167052761	0.328822136	0.341	1	8744	TNFSF9
0.167052761	0.328822136	0.367	1	56910	STARD7
0.167052761	0.328822136	0.357	1	201626	PDE12
0.167052761	0.328822136	0.373	1	219743	TYSND1
0.166795373	0.329576671	0.411	1	3270	HRC
0.166795373	0.329576671	0.372	1	9156	EXO1
0.166795373	0.329576671	0.363	1	9371	KIF3B
0.166795373	0.329576671	0.403	1	10125	RASGRP1
0.166795373	0.329576671	0.372	1	10134	BCAP31
0.166795373	0.329576671	0.372	1	23152	CIC
0.166795373	0.329576671	0.365	1	57142	RTN4
0.166795373	0.329576671	0.37	1	79925	SPEF2
0.166795373	0.329576671	0.36	1	84665	MYPN
0.166795373	0.329576671	0.351	1	222950	C7orf51
0.16653797	0.330331951	0.365	1	4976	OPA1
0.16653797	0.330331951	0.369	1	5475	PPEF1
0.16653797	0.330331951	0.378	1	23054	NCOA6
0.16653797	0.330331951	0.406	1	23523	CABIN1
0.166280568	0.331088305	0.418	1	7531	YWHAE
0.166280568	0.331088305	0.382	1	9447	AIM2
0.166280568	0.331088305	0.375	1	84251	SGIP1
0.166023165	0.331845701	0.376	1	10971	YWHAQ
0.166023165	0.331845701	0.347	1	51060	TXNDC12
0.166023165	0.331845701	0.365	1	53840	TRIM34
0.166023165	0.331845701	0.412	1	404217	CTXN1
0.165765762	0.332604587	0.374	1	25875	LETMD1
0.165765762	0.332604587	0.391	1	27034	ACAD8
0.165765762	0.332604587	0.388	1	51652	VPS24
0.165765762	0.332604587	0.38	1	55048	VPS37C
0.165765762	0.332604587	0.382	1	64333	ARHGAP9
0.165250972	0.334124833	0.375	1	3756	KCNH1
0.165250972	0.334124833	0.377	1	29094	AC008074.2
0.165250972	0.334124833	0.356	1	55322	C5orf22
0.165250972	0.334124833	0.368	1	59067	IL21
0.164993569	0.33488664	0.387	1	339803	AC016747.3
0.164993569	0.33488664	0.398	1	375323	LHFPL4
0.164993569	0.33488664	0.36	1	375686	SPATC1
0.164736167	0.335649818	0.363	1	4990	SIX6
0.164736167	0.335649818	0.38	1	23625	FAM89B
0.164736167	0.335649818	0.386	1	27005	USP21
0.164736167	0.335649818	0.395	1	51503	CWC15
0.164736167	0.335649818	0.382	1	80007	C10orf88
0.164736167	0.335649818	0.349	1	254956	MORN5
0.164736167	0.335649818	0.377	1	728409	C21orf54
0.164478764	0.336413711	0.393	1	5502	PPP1R1A
0.164478764	0.336413711	0.369	1	6209	RPS15P5
0.164478764	0.336413711	0.383	1	23434	C3orf27
0.164478764	0.336413711	0.361	1	28964	GIT1
0.164478764	0.336413711	0.382	1	57623	ZFAT
0.164221361	0.337178737	0.372	1	10970	CKAP4
0.164221361	0.337178737	0.371	1	317662	FAM149B1
0.163963959	0.337944806	0.361	1	2155	F7
0.163963959	0.337944806	0.378	1	54436	SH3TC1
0.163963959	0.337944806	0.364	1	56986	DTWD1
0.163963959	0.337944806	0.351	1	60673	C12orf44
0.163963959	0.337944806	0.353	1	84967	LSM10
0.163963959	0.337944806	0.371	1	220323	OAF
0.163963959	0.337944806	0.391	1	284406	ZFP82

voxel-wise, subcortex			
Spearman's corr coef	p-val	GenID	Gene
0.160639778	< 0.001	7730	ZNF177
0.160627797	< 0.001	25893	TRIM58
0.160599098	< 0.001	118472	ZNF511
0.160597801	< 0.001	11070	TMEM115
0.160573557	< 0.001	8650	NUMB
0.160514772	< 0.001	4832	NME3
0.160468996	< 0.001	64409	WBSCR17
0.160432309	< 0.001	10675	CSPG5
0.160427019	< 0.001	1131	CHRM3
0.160338148	< 0.001	9498	SLC4A8
0.160280377	< 0.001	643311	CT47B1
0.16027379	< 0.001	57534	MIB1
0.160231069	< 0.001	161725	OTUD7A
0.160229579	< 0.001	84818	IL17RC
0.160227448	< 0.001	92595	ZNF764
0.160163283	< 0.001	1611	DAP
0.160144791	< 0.001	6744	SSFA2
0.160102084	< 0.001	2935	GSPT1
0.160076171	< 0.001	23464	GCAT
0.160074502	< 0.001	56673	C11orf16
0.160048872	< 0.001	137902	PXDNL
0.159993336	< 0.001	1017	CDK2
0.159981638	< 0.001	155051	CRYGN
0.159926847	< 0.001	9497	SLC4A7
0.159886912	< 0.001	448835	LCE6A
0.159845948	< 0.001	9501	RPH3AL
0.159827754	< 0.001	149076	ZNF362
0.159823388	< 0.001	162394	SLFN5
0.159820512	< 0.001	146850	PIK3R6
0.15976581	< 0.001	5901	RAN
0.159709722	< 0.001	22879	MON1B
0.159642488	< 0.001	28232	SLCO3A1
0.159621596	< 0.001	4692	NDN
0.159610912	< 0.001	3676	ITGA4
0.159555197	< 0.001	550643	LOC550643
0.159486845	< 0.001	733	C8G
0.159446999	< 0.001	29802	VPREB3
0.159428895	< 0.001	7336	UBE2V2
0.159216508	< 0.001	570	BAAT
0.159199983	< 0.001	389813	AL355987.1
0.159137666	< 0.001	441795	HMGB3P27
0.159105167	< 0.001	401250	MCCD1
0.15902622	< 0.001	57460	PPM1H
0.158982769	< 0.001	80072	HEXA-AS1
0.15892899	< 0.001	29125	C11orf21
0.158919245	< 0.001	9094	UNC119
0.158859909	< 0.001	51281	ANKMY1
0.158830836	< 0.001	84364	ARFGAP2
0.158776328	< 0.001	8411	EEA1
0.158685967	< 0.001	3713	IVL
0.158676863	< 0.001	9464	HAND2
0.1586698	< 0.001	4858	NOVA2
0.158664048	< 0.001	731789	RP13-16H11.1
0.158663675	< 0.001	27301	APEX2
0.158651143	< 0.001	716	C1S
0.158636838	< 0.001	9200	PTPLA
0.158616692	< 0.001	90355	C5orf30
0.158609077	< 0.001	283687	AC015871.2
0.158564687	< 0.001	5832	ALDH18A1
0.158504784	< 0.001	6009	RHEB
0.158497825	< 0.001	22809	ATF5
0.158483148	< 0.001	84318	CCDC77
0.158447295	< 0.001	5641	LGMN
0.158416659	< 0.001	83874	TBC1D10A
0.158400863	< 0.001	55365	TMEM176A
0.15837802	< 0.001	64101	LRRC4
0.15837431	< 0.001	65996	AC016629.3
0.158363625	< 0.001	8050	PDHX
0.158355922	< 0.001	387755	INSC
0.158327416	< 0.001	28977	MRPL42
0.158264443	< 0.001	445815	PALM2-AKAP2
0.158256546	< 0.001	57048	PLSCR3
0.158254042	< 0.001	100134713	RP4-726N20.2
0.1582378	< 0.001	10742	RAI2
0.1582077	< 0.001	6672	SP100
0.158186898	< 0.001	26517	TIMM13
0.158176228	< 0.001	399473	SPRED3
0.158174187	< 0.001	9203	ZMYM3
0.158165827	< 0.001	90957	DHX57

region-wise, subcortex					
Spearman's corr coef	p-val	p-val, SA	p-val, SA (BH- correction)	GenID	Gene
0.163706556	0.338711947	0.42	1	1592	CYP26A1
0.163706556	0.338711947	0.37	1	3202	HOXA5
0.163706556	0.338711947	0.368	1	5507	PPP1R3C
0.163706556	0.338711947	0.398	1	22903	BTBD3
0.163706556	0.338711947	0.388	1	54583	EGLN1
0.163706556	0.338711947	0.393	1	144406	WDR66
0.163449153	0.339480609	0.388	1	2262	GPC5
0.163449153	0.339480609	0.378	1	55553	SOX6
0.163449153	0.339480609	0.361	1	342945	ZSCAN22
0.163191751	0.340249896	0.384	1	10329	TMEM5
0.163191751	0.340249896	0.394	1	27111	SDCBP2
0.163191751	0.340249896	0.377	1	57223	SMEK2
0.163191751	0.340249896	0.338	1	161725	OTUD7A
0.162934363	0.341020256	0.372	1	4862	NPAS2
0.162934363	0.341020256	0.367	1	6643	SNX2
0.162934363	0.341020256	0.383	1	7008	TEF
0.162934363	0.341020256	0.393	1	7159	TP53BP2
0.162934363	0.341020256	0.378	1	7699	ZNF140
0.162934363	0.341020256	0.399	1	22990	PCNX
0.162934363	0.341020256	0.366	1	83481	EPPK1
0.162934363	0.341020256	0.364	1	441543	TTTY6B
0.162934363	0.341020256	0.38	1	728927	RP11-468B6.3
0.16267696	0.341791719	0.371	1	2636	GBX1
0.162419558	0.342564672	0.351	1	25981	DNAH1
0.162419558	0.342564672	0.428	1	64750	SMURF2
0.162419558	0.342564672	0.372	1	84330	ZNF414
0.162162155	0.343338281	0.386	1	23096	IQSEC2
0.162162155	0.343338281	0.376	1	54784	ALKBH4
0.162162155	0.343338281	0.374	1	126526	C19orf47
0.162162155	0.343338281	0.376	1	146225	CMTM2
0.161904752	0.344112962	0.376	1	2299	FOXI1
0.161904752	0.344112962	0.399	1	4668	NAGA
0.161904752	0.344112962	0.368	1	51562	MBIP
0.161904752	0.344112962	0.364	1	55163	PNPO
0.161647364	0.344888806	0.4	1	1261	CNGA3
0.161647364	0.344888806	0.415	1	6191	RPS4X
0.161647364	0.344888806	0.369	1	55276	PGM2
0.161647364	0.344888806	0.384	1	57642	COL20A1
0.161647364	0.344888806	0.373	1	118425	RP11-610O8.1
0.161647364	0.344888806	0.375	1	140890	SFRS12
0.161389962	0.345665634	0.409	1	1774	DNASE1L1
0.161389962	0.345665634	0.384	1	9057	SLC7A6
0.161389962	0.345665634	0.363	1	29942	PURG
0.161132559	0.346443951	0.396	1	4173	MCM4
0.160875157	0.347223014	0.398	1	158	ADSL
0.160875157	0.347223014	0.405	1	779	CACNA1S
0.160875157	0.347223014	0.37	1	3984	LIMK1
0.160875157	0.347223014	0.375	1	5777	PTPN6
0.160875157	0.347223014	0.385	1	6401	SELE
0.160875157	0.347223014	0.395	1	10988	METAP2
0.160875157	0.347223014	0.372	1	54851	ANKRD49
0.160875157	0.347223014	0.399	1	79080	CCDC86
0.160875157	0.347223014	0.402	1	79145	CHCHD7
0.160875157	0.347223014	0.373	1	145389	SLC38A6
0.160875157	0.347223014	0.404	1	163259	DENND2C
0.160875157	0.347223014	0.384	1	167410	LIX1
0.160617754	0.348003089	0.405	1	3706	ITPKA
0.160617754	0.348003089	0.371	1	51032	CELA2B
0.160617754	0.348003089	0.371	1	119710	C11orf74
0.160617754	0.348003089	0.377	1	150737	TTC30B
0.160360366	0.348784268	0.373	1	3783	KCNN4
0.160360366	0.348784268	0.374	1	5189	PEX1
0.160360366	0.348784268	0.371	1	7322	UBE2D2
0.160360366	0.348784268	0.385	1	79036	C19orf50
0.160360366	0.348784268	0.382	1	79893	GGNBP2
0.160360366	0.348784268	0.372	1	347741	OTOP3
0.160102963	0.349566877	0.379	1	136	ADORA2B
0.160102963	0.349566877	0.391	1	1444	CSHL1
0.160102963	0.349566877	0.387	1	4706	NDUFAB1
0.160102963	0.349566877	0.37	1	79573	TTC13
0.160102963	0.349566877	0.376	1	400966	RGPD1
0.159845561	0.350350201	0.355	1	147686	ZNF418
0.159845561	0.350350201	0.385	1	285501	AC021850.1
0.159588158	0.351134628	0.383	1	5481	PPID
0.159330755	0.351920068	0.384	1	31	ACACA
0.159330755	0.351920068	0.373	1	673	BRAF
0.159330755	0.351920068	0.396	1	7168	TPM1
0.159330755	0.351920068	0.393	1	64780	MICAL1
0.159330755	0.351920068	0.384	1	135138	PACRG

voxel-wise, subcortex			
Spearman's corr coef	p-val	GenID	Gene
0.158129603	< 0.001	3161	HMMR
0.158088923	< 0.001	11247	NXPH4
0.158080101	< 0.001	4976	OPA1
0.158046395	< 0.001	79639	TMEM53
0.15800488	< 0.001	27286	SRPX2
0.157999679	< 0.001	473	RERE
0.157981202	< 0.001	8766	RAB11A
0.157974884	< 0.001	7556	ZNF10
0.157941923	< 0.001	6492	SIM1
0.157912388	< 0.001	160364	CLEC12A
0.157810986	< 0.001	83933	HDAC10
0.15778777	< 0.001	165679	SPTSSB
0.15777272	< 0.001	27434	POLM
0.157771334	< 0.001	1384	CRAT
0.157748669	< 0.001	91	ACVR1B
0.157511577	< 0.001	8862	APLN
0.157455206	< 0.001	5539	PPY
0.157451779	< 0.001	2671	GFER
0.15737915	< 0.001	64112	MOAP1
0.157347202	< 0.001	135927	C7orf34
0.157342374	< 0.001	1673	DEFB4
0.157321677	< 0.001	79168	LILRA6
0.157318607	< 0.001	219541	MED19
0.15731591	< 0.001	6795	AURKC
0.15730755	< 0.001	7874	USP7
0.157274127	< 0.001	84948	TIGD5
0.157215983	< 0.001	59341	TRPV4
0.15720661	< 0.001	353137	LCE1F
0.157159254	< 0.001	81689	ISCA1
0.1571365	< 0.001	286753	TUSC5
0.157115966	< 0.001	6251	RSU1
0.157115787	< 0.001	84058	WDR54
0.157075763	< 0.001	339906	RP11-427P5.1
0.15701437	< 0.001	9150	CTDP1
0.157012701	< 0.001	51294	PCDH12
0.156893373	< 0.001	27330	RPS6KA6
0.156842485	< 0.001	114990	VASN
0.156840622	< 0.001	266	AMELY
0.156804129	< 0.001	10618	TGOLN2
0.156788245	< 0.001	2217	FCGRT
0.156781837	< 0.001	165100	C2orf57
0.156761691	< 0.001	2046	EPHA8
0.156759828	< 0.001	4543	MTNR1A
0.156755283	< 0.001	51375	SNX7
0.156510577	< 0.001	25929	GEMIN5
0.15650101	< 0.001	389705	LOC389705
0.156455606	< 0.001	10248	POP7
0.156419471	< 0.001	9805	SCRN1
0.156405553	< 0.001	3837	KPNB1
0.156273961	2.8026E-45	7704	ZBTB16
0.156248137	2.8026E-45	55629	PNRC2
0.156185642	2.8026E-45	126382	NR2C2AP
0.156154439	2.8026E-45	27090	ST6GALNAC4
0.156153232	2.8026E-45	29993	PACSIN1
0.156142458	2.8026E-45	65003	MRPL11
0.156128615	2.8026E-45	339318	ZNF181
0.156120628	2.8026E-45	401533	LOC401533
0.156014308	2.8026E-45	22974	TPX2
0.156007245	2.8026E-45	161635	CSNK1A1P1
0.156004459	2.8026E-45	23434	C3orf27
0.155905843	2.8026E-45	203069	R3HCC1
0.15587537	5.60519E-45	80332	ADAM33
0.155865803	5.60519E-45	83932	C1orf124
0.155811384	5.60519E-45	1123	CHN1
0.155792266	5.60519E-45	441061	MARCHF11
0.155657604	5.60519E-45	79101	TAF1D
0.155633926	5.60519E-45	55704	CCDC88A
0.155632347	5.60519E-45	25917	THUMPD3
0.155507818	5.60519E-45	9422	ZNF264
0.155502424	5.60519E-45	51088	KLHL5
0.155492112	5.60519E-45	2806	GOT2
0.155468434	5.60519E-45	43849	KLK12
0.155450702	5.60519E-45	155368	WBSCR27
0.155436307	5.60519E-45	8844	KSR1
0.155403897	5.60519E-45	9701	SAPS2
0.155326635	5.60519E-45	51275	C12ORF47
0.155308247	5.60519E-45	219749	ZNF25
0.15529339	5.60519E-45	57325	CSRP2BP
0.155277416	8.40779E-45	127262	TPRG1L

region-wise, subcortex					
Spearman's corr coef	p-val	p-val, SA	p-val, SA (BH- correction)	GenID	Gene
0.159330755	0.351920068	0.385	1	401431	RP4-751H13.1
0.159073368	0.352707028	0.386	1	8909	AC004241.1
0.159073368	0.352707028	0.415	1	9889	ZBED4
0.159073368	0.352707028	0.398	1	10170	DHRS9
0.159073368	0.352707028	0.378	1	55929	DMAP1
0.159073368	0.352707028	0.427	1	57695	USP37
0.159073368	0.352707028	0.414	1	57862	ZNF410
0.159073368	0.352707028	0.381	1	220213	OTUD1
0.158815965	0.353494585	0.382	1	6281	S100A10
0.158815965	0.353494585	0.374	1	7582	ZNF33B
0.158815965	0.353494585	0.42	1	55197	RPRD1A
0.158815965	0.353494585	0.398	1	64409	WBSCR17
0.158558562	0.354283333	0.357	1	513	ATP5D
0.158558562	0.354283333	0.411	1	3633	INPP5B
0.158558562	0.354283333	0.384	1	4836	NMT1
0.158558562	0.354283333	0.381	1	5149	PDE6H
0.158558562	0.354283333	0.42	1	8792	TNFRSF11A
0.158558562	0.354283333	0.381	1	11146	GLMN
0.158558562	0.354283333	0.386	1	23461	ABCA5
0.158558562	0.354283333	0.393	1	114794	LRRC62
0.15830116	0.355073154	0.423	1	11160	ERLIN2
0.15830116	0.355073154	0.392	1	646934	LOC646934
0.158043757	0.355863959	0.383	1	57156	TMEM63C
0.158043757	0.355863959	0.375	1	201161	CENPV
0.157786369	0.356656253	0.41	1	2719	GPC3
0.157786369	0.356656253	0.402	1	56647	BCCIP
0.157786369	0.356656253	0.394	1	147727	LOC147727
0.157786369	0.356656253	0.419	1	389903	CSAG3
0.157786369	0.356656253	0.397	1	729873	TBC1D3E
0.157528967	0.357449234	0.402	1	7203	CCT3
0.157528967	0.357449234	0.428	1	29967	LRP12
0.157528967	0.357449234	0.406	1	55001	TTC22
0.157528967	0.357449234	0.409	1	130271	PLEKHH2
0.157271549	0.358243316	0.385	1	9711	KIAA0226
0.157271549	0.358243316	0.388	1	26747	NUFIP1
0.157271549	0.358243316	0.384	1	51386	EIF3L
0.157271549	0.358243316	0.409	1	374491	TPTE2P6
0.157014146	0.359038472	0.424	1	30001	ERO1L
0.157014146	0.359038472	0.403	1	55735	DNAJC11
0.157014146	0.359038472	0.367	1	84265	POLR3GL
0.156756744	0.359834999	0.393	1	4048	LTA4H
0.156499341	0.36063233	0.405	1	10519	CIB1
0.156499341	0.36063233	0.388	1	10869	USP19
0.156499341	0.36063233	0.387	1	22934	RPIA
0.156499341	0.36063233	0.413	1	55728	N4BP2
0.156499341	0.36063233	0.383	1	284612	SYPL2
0.156499341	0.36063233	0.396	1	100130733	LRRC70
0.156241953	0.361430645	0.377	1	1359	CPA3
0.156241953	0.361430645	0.436	1	2938	GSTA1
0.156241953	0.361430645	0.379	1	54413	NLGN3
0.156241953	0.361430645	0.408	1	56270	WDR45L
0.156241953	0.361430645	0.395	1	152789	JAKMIP1
0.155984551	0.362230092	0.39	1	2261	FGFR3
0.155984551	0.362230092	0.389	1	4353	MPO
0.155984551	0.362230092	0.404	1	9340	GLP2R
0.155727148	0.36303091	0.43	1	57348	TTYH1
0.155727148	0.36303091	0.398	1	126133	ALDH16A1
0.155727148	0.36303091	0.418	1	494470	RNF165
0.155727148	0.36303091	0.419	1	643394	SPINK9
0.155469745	0.363832504	0.376	1	247	ALOX15B
0.155469745	0.363832504	0.426	1	1846	DUSP4
0.155469745	0.363832504	0.387	1	4154	MBNL1
0.155469745	0.363832504	0.399	1	8621	CDC2L5
0.155469745	0.363832504	0.414	1	85378	TUBGCP6
0.155212343	0.36463517	0.424	1	11122	PTPRT
0.155212343	0.36463517	0.392	1	55827	IQWD1
0.155212343	0.36463517	0.409	1	55974	RAG1AP1
0.155212343	0.36463517	0.359	1	112464	PRKCDBP
0.154954955	0.365438849	0.402	1	2197	FAU
0.154954955	0.365438849	0.393	1	2824	GPM6B
0.154954955	0.365438849	0.408	1	7380	UPK3A
0.154954955	0.365438849	0.395	1	23118	MAP3K7IP2
0.154954955	0.365438849	0.407	1	79839	CCDC102B
0.154954955	0.365438849	0.386	1	84687	PPP1R9B
0.154697552	0.366243631	0.393	1	91	ACVR1B
0.154697552	0.366243631	0.442	1	7343	UBTF
0.154697552	0.366243631	0.428	1	23510	KCTD2
0.154697552	0.366243631	0.418	1	27287	VENTX
0.154697552	0.366243631	0.394	1	80039	FAM106A

voxel-wise, subcortex			
Spearman's corr coef	p-val	GenID	Gene
0.15527685	8.40779E-45	80317	ZKSCAN3
0.155260608	8.40779E-45	149069	DCDC2B
0.155232087	8.40779E-45	11317	RBPJL
0.155206084	8.40779E-45	79568	C2orf47
0.155196249	8.40779E-45	118932	ANKRD22
0.155161053	1.12104E-44	387601	SLC22A25
0.155151114	1.12104E-44	585	BBS4
0.155137271	1.12104E-44	113278	C20orf54
0.155126691	1.12104E-44	23426	GRIP1
0.155078024	1.12104E-44	84832	ANKRD36BL1
0.15501228	1.12104E-44	311	ANXA11
0.154997885	1.12104E-44	63914	C6orf164
0.154957578	1.12104E-44	58191	CXCL16
0.154897779	1.12104E-44	266722	HS6ST3
0.154853567	1.4013E-44	221303	FAM162B
0.154837593	1.4013E-44	6489	ST8SIA1
0.154769897	1.68156E-44	51177	PLEKHO1
0.154763311	1.68156E-44	114042	LINC00334
0.154758483	1.68156E-44	151449	GDF7
0.154632822	1.68156E-44	255349	TMEM211
0.154567078	1.96182E-44	91750	LIN52
0.154500499	2.24208E-44	54460	MRPS21
0.154480904	2.24208E-44	6006	RHCE
0.154471979	2.24208E-44	4585	MUC4
0.154405221	2.24208E-44	4803	NGF
0.154402614	2.24208E-44	259217	HSPA12A
0.15436621	2.52234E-44	51109	RDH11
0.154334262	2.8026E-44	2054	STX2
0.154304728	2.8026E-44	10691	GMEB1
0.154281512	2.8026E-44	11182	SLC2A6
0.154252082	2.8026E-44	392390	OR1L6
0.154230252	3.08286E-44	81622	UNC93B3
0.154206112	3.08286E-44	25942	SIN3A
0.154198676	3.36312E-44	9604	RNF14
0.154191345	3.36312E-44	547	KIF1A
0.154163212	3.36312E-44	387712	C10orf134
0.154146492	3.36312E-44	54867	TMEM214
0.154090211	3.64338E-44	55635	DEPDC1
0.153915063	4.48416E-44	5620	PRM2
0.153883487	4.76441E-44	22905	EPN2
0.153869569	4.76441E-44	23200	ATP11B
0.153861761	5.04467E-44	285679	AC136604.1
0.153824612	5.04467E-44	574029	DUSP5P
0.15374364	5.60519E-44	147664	ERVV-1
0.153727472	5.60519E-44	164832	LONRF2
0.15369758	6.16571E-44	255809	C19orf38
0.153567001	7.28675E-44	4644	MYO5A
0.153469399	7.84727E-44	83940	TATDN1
0.153405607	8.68805E-44	7041	TGFB1I1
0.153361961	9.24857E-44	348158	ACSM2
0.153301969	1.00893E-43	28951	TRIB2
0.153293788	1.00893E-43	256933	NPB
0.15328005	1.00893E-43	147929	ZNF565
0.153277636	1.00893E-43	3953	LEPR
0.153160632	1.17709E-43	170589	GPHA2
0.153074354	1.34525E-43	6843	VAMP1
0.152929947	1.59748E-43	56979	PRDM9
0.152922139	1.62551E-43	9950	GOLGA5
0.152884439	1.68156E-43	10081	PDCD7
0.152876824	1.70958E-43	410	ARSA
0.152843669	1.79366E-43	5569	PKIA
0.152828813	1.82169E-43	100128496	C20orf78
0.152761489	1.96182E-43	47	ACLY
0.152758241	1.98984E-43	81608	FIP1L1
0.152754799	1.98984E-43	65977	PLEKHA3
0.152666211	2.24208E-43	4712	NDUFB6
0.152649209	2.29813E-43	85014	TMEM141
0.152643725	2.29813E-43	126075	AC024575.3
0.152640671	2.29813E-43	171568	POLR3H
0.152555227	2.57839E-43	359	AQP2
0.152547434	2.57839E-43	5820	PVT1
0.15247713	2.85865E-43	90655	TGIF2LY
0.152452156	2.9147E-43	8459	TPST2
0.152371258	3.25101E-43	170679	PSORS1C1
0.152366802	3.25101E-43	4045	LSAMP
0.152359843	3.30706E-43	56131	PCDHB4
0.152279228	3.64338E-43	5346	PLIN
0.152275607	3.6714E-43	374569	ASPG
0.152266413	3.69943E-43	387	RHOA

region-wise, subcortex					
Spearman's corr coef	p-val	p-val, SA	p-val, SA (BH- correction)	GenID	Gene
0.154697552	0.366243631	0.421	1	285172	FAM126B
0.15444015	0.367049783	0.386	1	3983	ABLIM1
0.15444015	0.367049783	0.413	1	6594	SMARCA1
0.15444015	0.367049783	0.407	1	10800	CYSLTR1
0.15444015	0.367049783	0.413	1	23398	PPWD1
0.15444015	0.367049783	0.401	1	56995	TULP4
0.15444015	0.367049783	0.407	1	60686	C14orf93
0.15444015	0.367049783	0.44	1	79847	TMEM180
0.154182747	0.367856711	0.422	1	10495	ENOX2
0.154182747	0.367856711	0.414	1	23144	ZC3H3
0.154182747	0.367856711	0.428	1	50649	ARHGEF4
0.154182747	0.367856711	0.425	1	56001	NXF2
0.154182747	0.367856711	0.42	1	81533	ITFG1
0.153925344	0.368664712	0.427	1	83873	GPR61
0.153667957	0.369473726	0.383	1	1716	DGUOK
0.153667957	0.369473726	0.375	1	3552	IL1A
0.153667957	0.369473726	0.374	1	6860	SYT4
0.153667957	0.369473726	0.395	1	10485	C1orf61
0.153667957	0.369473726	0.404	1	23586	DDX58
0.153667957	0.369473726	0.415	1	56931	DUS3L
0.153667957	0.369473726	0.415	1	80243	PREX2
0.153667957	0.369473726	0.393	1	440153	OR11H12
0.153667957	0.369473726	0.438	1	731275	LOC731275
0.153410554	0.3702842	0.382	1	2987	GUK1
0.153410554	0.3702842	0.403	1	26128	KIAA1279
0.153410554	0.3702842	0.393	1	79843	FAM124B
0.153410554	0.3702842	0.412	1	84649	DGAT2
0.153153151	0.371095359	0.405	1	4504	MT3
0.153153151	0.371095359	0.402	1	10643	IGF2BP3
0.153153151	0.371095359	0.409	1	57582	KCNT1
0.153153151	0.371095359	0.434	1	84695	LOXL3
0.153153151	0.371095359	0.419	1	90826	PRMT10
0.152895749	0.371907622	0.4	1	1780	DYNC1I1
0.152895749	0.371907622	0.411	1	4437	MSH3
0.152895749	0.371907622	0.401	1	11197	WIF1
0.152895749	0.371907622	0.405	1	64145	ZFYVE20
0.152895749	0.371907622	0.404	1	91768	CABLES1
0.152895749	0.371907622	0.433	1	222234	FAM185A
0.152895749	0.371907622	0.408	1	374786	EFCAB5
0.152895749	0.371907622	0.395	1	100169750	PRINS
0.152638346	0.372720897	0.391	1	1595	CYP51A1
0.152638346	0.372720897	0.406	1	7164	TPD52L1
0.152638346	0.372720897	0.439	1	554251	FBXO48
0.152380958	0.373535663	0.405	1	1052	CEBPD
0.152123556	0.374351114	0.423	1	1457	CSNK2A1
0.152123556	0.374351114	0.397	1	3963	LGALS7
0.152123556	0.374351114	0.414	1	9720	CCDC144A
0.152123556	0.374351114	0.414	1	10467	ZNHIT1
0.152123556	0.374351114	0.413	1	51385	ZNF589
0.152123556	0.374351114	0.399	1	286128	ZFP41
0.151866153	0.375167578	0.415	1	7141	TNP1
0.151866153	0.375167578	0.422	1	26019	UPF2
0.151866153	0.375167578	0.442	1	81562	LMAN2L
0.151866153	0.375167578	0.396	1	100129034	LOC100129034
0.15160875	0.375985116	0.424	1	4833	NME4
0.15160875	0.375985116	0.421	1	6610	SMPD2
0.15160875	0.375985116	0.426	1	51678	MPP6
0.15160875	0.375985116	0.403	1	339896	GADL1
0.151351348	0.376803726	0.42	1	4633	MYL2
0.151351348	0.376803726	0.419	1	115286	SLC25A26
0.151351348	0.376803726	0.398	1	151056	PLB1
0.15109396	0.377623826	0.416	1	2743	GLRB
0.15109396	0.377623826	0.44	1	8128	ST8SIA2
0.15109396	0.377623826	0.416	1	9404	LPXN
0.15109396	0.377623826	0.384	1	51507	C20orf43
0.15109396	0.377623826	0.422	1	55366	LGR4
0.15109396	0.377623826	0.418	1	84343	HPS3
0.15109396	0.377623826	0.406	1	286467	RP11-453F18__B.1
0.15109396	0.377623826	0.43	1	442038	SULT1C3
0.150836557	0.378444523	0.4	1	2844	GPR21
0.150836557	0.378444523	0.402	1	56849	TCEAL7
0.150836557	0.378444523	0.425	1	143689	PIWIL4
0.150836557	0.378444523	0.427	1	160065	PATE1
0.150836557	0.378444523	0.398	1	340529	PABPC1L2A
0.150836557	0.378444523	0.414	1	344405	NCRNA00117
0.150836557	0.378444523	0.401	1	353088	ZNF429
0.150579154	0.379266351	0.421	1	55754	TMEM30A
0.150579154	0.379266351	0.409	1	57172	CAMK1G
0.150579154	0.379266351	0.411	1	340665	CYP26C1

voxel-wise, subcortex			
Spearman's corr coef	p-val	GenID	Gene
0.152254254	3.75548E-43	29968	PSAT1
0.152192965	4.06377E-43	2939	GSTA2
0.152132511	4.40008E-43	267002	PGBD2
0.152129248	4.4281E-43	23597	ACOT9
0.152106881	4.54021E-43	151050	C2orf67
0.152089417	4.65231E-43	85320	ABCC11
0.152062759	4.82047E-43	9263	STK17A
0.151952997	5.52112E-43	388325	SCIMP
0.151877776	6.05361E-43	57647	DHX37
0.151835427	6.38992E-43	64763	ZNF574
0.1517355	7.25873E-43	9326	ZNHIT3
0.151699096	7.59504E-43	374899	ZNF829
0.151667252	7.90332E-43	9550	ATP6V1G1
0.151631489	8.29569E-43	283171	C11orf44
0.151626483	8.32371E-43	140832	WFDC10A
0.151583761	8.80015E-43	219409	GSX1
0.151576057	8.85621E-43	2128	EVX1
0.151548102	9.19252E-43	125931	CEACAM20
0.151493862	9.86514E-43	25979	DHRS7B
0.151489779	9.89317E-43	53358	SHC3
0.151458755	1.02855E-42	131377	KBTBD5
0.1513111	1.23875E-42	50940	PDE11A
0.151255846	1.32843E-42	3981	LIG4
0.151249349	1.33964E-42	10939	AFG3L2
0.151182771	1.45735E-42	56889	TM9SF3
0.151153415	1.5134E-42	55740	ENAH
0.151063889	1.69277E-42	29110	TBK1
0.151059717	1.69837E-42	27121	DKK4
0.151033536	1.75723E-42	399881	HNRNPKP3
0.150990158	1.85532E-42	2877	GPX2
0.150825977	2.28131E-42	27349	MCAT
0.150788262	2.39062E-42	284485	C1orf230
0.150779441	2.41584E-42	153478	PLEKHG4B
0.15074648	2.51953E-42	54545	MTMR12
0.150684252	2.72412E-42	337969	KRTAP19-2
0.150520161	3.3463E-42	9582	APOBEC3B
0.150483206	3.50325E-42	1620	DBC1
0.150482833	3.50605E-42	80714	PBX4
0.150478557	3.52567E-42	2591	GALNT3
0.150450423	3.64898E-42	196993	AC068181.1
0.150423393	3.7779E-42	51052	PRLH
0.150381789	3.97969E-42	340811	AKR1CL1
0.150380224	3.98529E-42	127579	DCST2
0.150331274	4.23753E-42	27022	FOXD3
0.150308892	4.35804E-42	283455	KSR2
0.150206	4.95499E-42	84766	EFCAB4B
0.150195509	5.02225E-42	4640	MYO1A
0.150134772	5.41462E-42	100131816	RP1-215K18.2
0.150111839	5.57437E-42	55814	BDP1
0.150090382	5.72571E-42	54466	SPIN2A
0.150048688	6.03119E-42	10134	BCAP31
0.150010511	6.32546E-42	53615	MBD3
0.149899453	7.26433E-42	4134	MAP4
0.149812713	8.0939E-42	9989	PPP4R1
0.14980343	8.18919E-42	59350	RXFP1
0.149764508	8.59556E-42	729739	LOC729739
0.149733871	8.92907E-42	150353	DNAJB7
0.149709821	9.20093E-42	4863	NPAT
0.149667844	9.69699E-42	51804	SIX4
0.149611101	1.04032E-41	51637	C14orf166
0.149536625	1.14122E-41	2074	ERCC6
0.149536341	1.14178E-41	9914	ATP2C2
0.149511456	1.17765E-41	388886	C22orf36
0.149395838	1.35982E-41	90701	SEC11C
0.149394259	1.36234E-41	9856	KIAA0319
0.149339557	1.45819E-41	26995	TRUB2
0.149236858	1.65633E-41	131034	CPNE4
0.149207592	1.71771E-41	351	APP
0.149132937	1.88447E-41	56344	CABP5
0.149118632	1.9181E-41	79750	ZNF385D
0.149072483	2.03104E-41	138652	PRSS47
0.149055675	2.07392E-41	51463	GPR89B
0.149040252	2.11372E-41	9253	NUMBL
0.148978874	2.28075E-41	347468	OR13H1
0.148970053	2.30598E-41	5710	PSMD4
0.148884326	2.5641E-41	25920	COBRA1
0.148841977	2.70226E-41	84221	C21orf56
0.14878571	2.89704E-41	10231	RCAN2
0.148769364	2.95618E-41	132851	SPATA4

region-wise, subcortex					
Spearman's corr coef	p-val	p-val, SA	p-val, SA (BH- correction)	GenID	Gene
0.150579154	0.379266351	0.421	1	392196	RP11-1236K1.8
0.150321737	0.380089194	0.454	1	2014	EMP3
0.150321737	0.380089194	0.434	1	4259	MGST3
0.150321737	0.380089194	0.398	1	51506	UFC1
0.150321737	0.380089194	0.439	1	121599	SPIC
0.150064334	0.380913496	0.416	1	2011	MARK2
0.150064334	0.380913496	0.444	1	8061	FOSL1
0.150064334	0.380913496	0.428	1	8460	TPST1
0.150064334	0.380913496	0.428	1	92979	MARCHF9
0.150064334	0.380913496	0.432	1	400798	RP5-990P15.1
0.150064334	0.380913496	0.432	1	644128	RPL23AP53
0.149806947	0.381738514	0.44	1	2830	GPR6
0.149806947	0.381738514	0.44	1	4605	MYBL2
0.149806947	0.381738514	0.411	1	10714	POLD3
0.149806947	0.381738514	0.423	1	27153	ZNF777
0.149806947	0.381738514	0.408	1	55589	BMP2K
0.149806947	0.381738514	0.423	1	59348	ZNF350
0.149806947	0.381738514	0.42	1	91851	CHRDL1
0.149549544	0.382564515	0.4	1	2018	EMX2
0.149292141	0.383391589	0.449	1	199	AIF1
0.149292141	0.383391589	0.422	1	847	CAT
0.149292141	0.383391589	0.417	1	4968	OGG1
0.149292141	0.383391589	0.406	1	5800	PTPRO
0.149292141	0.383391589	0.417	1	10256	CNKSR1
0.149292141	0.383391589	0.435	1	10998	SLC27A5
0.149292141	0.383391589	0.436	1	11075	STMN2
0.149292141	0.383391589	0.402	1	30837	SOCS7
0.149034739	0.384220213	0.435	1	3853	KRT6A
0.149034739	0.384220213	0.424	1	5349	FXYD3
0.149034739	0.384220213	0.405	1	112770	C1orf85
0.148777336	0.385049373	0.419	1	164	AP1G1
0.148777336	0.385049373	0.428	1	267	AMFR
0.148777336	0.385049373	0.432	1	6249	CLIP1
0.148777336	0.385049373	0.405	1	7818	DAP3
0.148777336	0.385049373	0.437	1	8936	WASF1
0.148777336	0.385049373	0.411	1	23543	RBM9
0.148777336	0.385049373	0.438	1	51076	CUTC
0.148777336	0.385049373	0.404	1	55604	LRRC16A
0.148777336	0.385049373	0.424	1	645974	PABPC1L2B
0.148519948	0.385879636	0.444	1	64417	C5orf28
0.148519948	0.385879636	0.443	1	126308	MOBKL2A
0.148262545	0.386711001	0.428	1	5308	PITX2
0.148262545	0.386711001	0.436	1	7772	ZNF229
0.148262545	0.386711001	0.416	1	10975	UQCR11
0.148262545	0.386711001	0.453	1	51501	C11orf73
0.148262545	0.386711001	0.455	1	122402	TDRD9
0.148005143	0.38754335	0.434	1	1463	NCAN
0.148005143	0.38754335	0.428	1	54439	RBM27
0.148005143	0.38754335	0.409	1	64777	RMND5B
0.148005143	0.38754335	0.399	1	340075	ARSI
0.14774774	0.38837716	0.425	1	1460	CSNK2B
0.14774774	0.38837716	0.421	1	2050	EPHB4
0.14774774	0.38837716	0.44	1	29991	OBP2A
0.14774774	0.38837716	0.421	1	79611	ACSS3
0.14774774	0.38837716	0.434	1	91703	ACY3
0.147490337	0.389211655	0.409	1	6638	SNRPN
0.147490337	0.389211655	0.422	1	7390	UROS
0.147490337	0.389211655	0.422	1	79781	IQCA1
0.147490337	0.389211655	0.456	1	150280	HORMAD2
0.14723295	0.390047222	0.435	1	864	RUNX3
0.14723295	0.390047222	0.379	1	1087	CEACAM7
0.14723295	0.390047222	0.428	1	3081	HGD
0.14723295	0.390047222	0.44	1	8693	GALNT4
0.14723295	0.390047222	0.439	1	54440	SASH3
0.146975547	0.390883863	0.415	1	2783	GNB2
0.146975547	0.390883863	0.426	1	5783	PTPN13
0.146975547	0.390883863	0.429	1	10555	AGPAT2
0.146975547	0.390883863	0.432	1	51647	FAM96B
0.146718144	0.391721845	0.438	1	4534	MTM1
0.146718144	0.391721845	0.449	1	9265	CYTH3
0.146718144	0.391721845	0.431	1	23608	MKRN1
0.146718144	0.391721845	0.406	1	26279	PLA2G2D
0.146718144	0.391721845	0.429	1	53834	FGFRL1
0.146718144	0.391721845	0.436	1	79368	FCRL2
0.146718144	0.391721845	0.446	1	378807	CATSPER4
0.146460742	0.392560571	0.448	1	5053	PAH
0.146460742	0.392560571	0.448	1	50613	UBQLN3
0.146460742	0.392560571	0.445	1	51651	PTRH2
0.146460742	0.392560571	0.455	1	494143	CHAC2

voxel-wise, subcortex			
Spearman's corr coef	p-val	GenID	Gene
0.148750886	3.02456E-41	113746	ODF3
0.148732215	3.09519E-41	23521	RPL13A
0.148701295	3.2157E-41	81025	GJA9
0.148641765	3.46121E-41	51635	DHRS7
0.148599237	3.64786E-41	841	CASP8
0.148515001	4.04807E-41	79836	LONRF3
0.148505807	4.09431E-41	149297	FAM78B
0.148463741	4.31264E-41	1896	EDA
0.148430958	4.4906E-41	375759	C9orf50
0.148397535	4.67978E-41	10694	CCT8
0.1483327	5.06934E-41	1238	CCBP2
0.148305029	5.24534E-41	51286	CEND1
0.14819099	6.03679E-41	146057	TTBK2
0.148094878	6.79546E-41	29978	UBQLN2
0.148084387	6.88374E-41	84188	FAR1
0.148084104	6.88626E-41	138311	FAM69B
0.148068041	7.02387E-41	729196	UNC93B5
0.148037851	7.28955E-41	58513	EPS15L1
0.147948802	8.1337E-41	346653	FAM71F2
0.147940069	8.22142E-41	2491	CENPI
0.147929668	8.32708E-41	10393	ANAPC10
0.147910535	8.5255E-41	6571	SLC18A2
0.147900969	8.62639E-41	54937	SOHLH2
0.147893548	8.70543E-41	121340	SP7
0.147867635	8.98737E-41	6150	MRPL23
0.147813499	9.60562E-41	85476	GFM1
0.147786841	9.9254E-41	55813	UTP6
0.147753969	1.03343E-40	56998	CTNNBIP1
0.147707716	1.09385E-40	372	ARCN1
0.147694528	1.11168E-40	84085	FBXO30
0.147661656	1.15747E-40	4234	METTL1
0.147586346	1.26952E-40	4188	MDFI
0.147476479	1.45264E-40	146059	CDAN1
0.14746654	1.47047E-40	342945	ZSCAN22
0.147465155	1.47293E-40	199699	DAND5
0.147441655	1.51598E-40	6273	S100A2
0.14743042	1.537E-40	29094	AC008074.2
0.147263169	1.8864E-40	401375	GTF2IRD2P1
0.147251651	1.91319E-40	26973	CHORDC1
0.147242084	1.93575E-40	78986	DUSP26
0.147232622	1.95829E-40	54600	UGT1A9
0.147231311	1.96143E-40	54101	RIPK4
0.147163332	2.13154E-40	442425	FOXB2
0.147145882	2.17751E-40	6877	TAF5
0.147063315	2.4088E-40	152330	CNTN4
0.147025898	2.5215E-40	55320	C14orf106
0.146968976	2.7031E-40	100288713	PMS2P10
0.146945566	2.78155E-40	839	CASP6
0.146920308	2.86863E-40	9984	THOC1
0.146911398	2.90007E-40	642597	C18orf42
0.146888644	2.98174E-40	151246	SGOL2
0.146775156	3.42466E-40	284013	VMO1
0.146745622	3.55025E-40	552900	BOLA2
0.14666456	3.91904E-40	7139	TNNT2
0.146622956	4.1229E-40	79611	ACSS3
0.146591097	4.28607E-40	317772	HIST2H2AB
0.146567702	4.41E-40	57502	NLGN4X
0.146560088	4.45103E-40	84376	HOOK3
0.146531388	4.60938E-40	653140	AC008073.2
0.146451518	5.08016E-40	91012	LASS5
0.146448269	5.10022E-40	2703	GJA8
0.146405652	5.37168E-40	7760	ZNF213
0.146403328	5.38701E-40	84689	MS4A14
0.146382794	5.52316E-40	643997	LOC643997
0.146376759	5.56388E-40	8829	NRP1
0.146350756	5.74263E-40	285966	FAM115C
0.146343604	5.79286E-40	60487	TRMT11
0.146323457	5.93652E-40	1538	CYLC1
0.146156296	7.27372E-40	11318	GPR182
0.146126583	7.54089E-40	29113	C6orf15
0.146126583	7.54089E-40	93185	IGSF8
0.145981714	8.99051E-40	6607	SMN2
0.145966113	9.16225E-40	2864	FFAR1
0.145955518	9.28086E-40	643161	FAM25A
0.145922184	9.66363E-40	100130613	CXorf64
0.14590463	9.87153E-40	6591	SNAI2
0.145899996	9.92697E-40	7152	TOP1P2
0.145897016	9.96284E-40	27173	SLC39A1
0.145787522	1.13766E-39	338674	OR5F1

region-wise, subcortex					
Spearman's corr coef	p-val	p-val, SA	p-val, SA (BH- correction)	GenID	Gene
0.146460742	0.392560571	0.438	1	728175	AC103540.1
0.146203339	0.393400311	0.474	1	65084	TMEM135
0.146203339	0.393400311	0.431	1	147906	DACT3
0.145945951	0.394241154	0.445	1	5515	PPP2CA
0.145945951	0.394241154	0.449	1	5864	RAB3A
0.145945951	0.394241154	0.409	1	6169	RPL38
0.145945951	0.394241154	0.425	1	8987	STBD1
0.145945951	0.394241154	0.398	1	9191	DEDD
0.145945951	0.394241154	0.439	1	64579	NDST4
0.145945951	0.394241154	0.429	1	92806	AC092143.3
0.145945951	0.394241154	0.429	1	255043	TMEM86B
0.145945951	0.394241154	0.399	1	283177	LOC283177
0.145688549	0.395083398	0.422	1	10469	TIMM44
0.145688549	0.395083398	0.42	1	10562	OLFM4
0.145688549	0.395083398	0.463	1	10629	TAF6L
0.145688549	0.395083398	0.43	1	51808	PHAX
0.145688549	0.395083398	0.436	1	56606	SLC2A9
0.145688549	0.395083398	0.462	1	387082	SUMO4
0.145431146	0.395926267	0.45	1	1819	DRG2
0.145431146	0.395926267	0.413	1	5321	PLA2G4A
0.145431146	0.395926267	0.447	1	56134	PCDHAC2
0.145431146	0.395926267	0.46	1	119687	OR51A7
0.145173743	0.396770269	0.457	1	3482	IGF2R
0.145173743	0.396770269	0.436	1	57608	KIAA1462
0.145173743	0.396770269	0.415	1	63941	NECAB3
0.145173743	0.396770269	0.417	1	83982	IFI27L2
0.145173743	0.396770269	0.425	1	84376	HOOK3
0.145173743	0.396770269	0.435	1	118432	RPL29P2
0.145173743	0.396770269	0.455	1	339302	CPLX4
0.144916341	0.397615254	0.442	1	1565	CYP2D6
0.144916341	0.397615254	0.446	1	11336	EXOC3
0.144658953	0.398461342	0.423	1	191	AHCY
0.144658953	0.398461342	0.461	1	1629	DBT
0.144658953	0.398461342	0.455	1	8578	SCARF1
0.144658953	0.398461342	0.436	1	11037	STON1
0.144658953	0.398461342	0.448	1	23072	HECW1
0.144658953	0.398461342	0.455	1	51209	RAB9B
0.144658953	0.398461342	0.442	1	51657	STYXL1
0.144658953	0.398461342	0.462	1	140775	SMCR8
0.14440155	0.39930883	0.45	1	496	ATP4B
0.14440155	0.39930883	0.444	1	55204	GOLPH3L
0.14440155	0.39930883	0.466	1	729973	LOC729973
0.144144148	0.400156945	0.454	1	5367	PMCH
0.144144148	0.400156945	0.402	1	5428	POLG
0.144144148	0.400156945	0.47	1	9834	KIAA0125
0.144144148	0.400156945	0.432	1	9990	SLC12A6
0.144144148	0.400156945	0.439	1	57418	WDR18
0.143886745	0.401006162	0.433	1	4842	NOS1
0.143886745	0.401006162	0.475	1	6301	SARS
0.143886745	0.401006162	0.435	1	10443	N4BP2L2
0.143886745	0.401006162	0.446	1	55625	ZDHHC7
0.143886745	0.401006162	0.44	1	57683	ZDBF2
0.143886745	0.401006162	0.469	1	196410	METTL7B
0.143886745	0.401006162	0.447	1	343099	CCDC18
0.143886745	0.401006162	0.448	1	401335	C7orf65
0.143886745	0.401006162	0.473	1	100289410	MCF2L-AS1
0.143629342	0.401856393	0.444	1	1429	CRYZ
0.143629342	0.401856393	0.435	1	6921	TCEB1
0.143629342	0.401856393	0.416	1	30820	KCNIP1
0.143629342	0.401856393	0.467	1	122509	IFI27L1
0.14337194	0.402708083	0.442	1	3454	IFNAR1
0.14337194	0.402708083	0.413	1	5270	SERPINE2
0.14337194	0.402708083	0.427	1	6789	STK4
0.14337194	0.402708083	0.428	1	27020	NPTN
0.143114537	0.40356046	0.397	1	8456	FOXN1
0.143114537	0.40356046	0.455	1	30845	EHD3
0.143114537	0.40356046	0.426	1	50511	SYCP3
0.142857134	0.40441379	0.471	1	2962	GTF2F1
0.142857134	0.40441379	0.443	1	23113	CUL9
0.142857134	0.40441379	0.47	1	55103	RALGPS2
0.142857134	0.40441379	0.429	1	80863	PRRT1
0.142599732	0.405268222	0.465	1	1749	DLX5
0.142599732	0.405268222	0.453	1	4043	LRPAP1
0.142599732	0.405268222	0.479	1	6904	TBCD
0.142599732	0.405268222	0.466	1	63976	PRDM16
0.142342329	0.406124055	0.439	1	23682	RAB38
0.142342329	0.406124055	0.471	1	254122	SNX32
0.142342329	0.406124055	0.445	1	285349	ZNF660
0.142084941	0.406980574	0.463	1	3761	KCNJ4

voxel-wise, subcortex			
Spearman's corr coef	p-val	GenID	Gene
0.145782977	1.14396E-39	5908	RAP1B
0.145760313	1.1758E-39	1346	COX7A1
0.145746395	1.19578E-39	8755	ADAM6
0.14571026	1.24927E-39	26103	LRIT1
0.145691141	1.27851E-39	343472	BARHL2
0.14566727	1.31598E-39	1137	CHRNA4
0.145638287	1.36298E-39	7637	ZNF84
0.145542085	1.53118E-39	3069	HDLBP
0.145516545	1.5792E-39	54516	MTRF1L
0.145514965	1.58222E-39	64319	FBRS
0.145444021	1.72387E-39	329	BIRC2
0.14543584	1.74102E-39	84501	SPIRE2
0.145341128	1.95207E-39	10786	SLC17A3
0.145305276	2.0384E-39	386674	KRTAP10-6
0.14530082	2.04939E-39	283537	SLC46A3
0.145274073	2.11664E-39	254827	NAALADL2
0.145270824	2.12493E-39	248	ALPI
0.145202011	2.30897E-39	130814	PQLC3
0.145184636	2.35779E-39	100287932	TIMM23
0.145181492	2.3668E-39	116115	ZNF526
0.145131707	2.51324E-39	80036	TRPM3
0.145128638	2.52256E-39	9700	ESPL1
0.144996122	2.95937E-39	56158	TEX12
0.144929171	3.20791E-39	390201	OR10V1
0.144916162	3.25847E-39	3860	KRT13
0.144858316	3.49342E-39	23030	KDM4B
0.144852281	3.51892E-39	7079	TIMP4
0.144834071	3.59688E-39	7627	ZNF75A
0.144817725	3.66834E-39	124944	C17ORF49
0.14473629	4.04577E-39	5616	PRKY
0.144648716	4.49484E-39	80724	ACAD10
0.144552603	5.04492E-39	60560	MAK10
0.144423977	5.88716E-39	8501	SLC43A1
0.144416913	5.9372E-39	6229	RPS24
0.144391283	6.12256E-39	57720	GPR107
0.144375131	6.24232E-39	117285	DEFB118
0.14434588	6.46531E-39	653513	LOC653513
0.144326463	6.61752E-39	129285	KLRAQ1
0.144282266	6.97749E-39	7087	ICAM5
0.144221619	7.50337E-39	84270	C9orf89
0.144119188	8.48268E-39	151871	DPPA2
0.144103214	8.64645E-39	10294	DNAJA2
0.14407517	8.94159E-39	9746	CLSTN3
0.144051582	9.19751E-39	5144	PDE4D
0.144037932	9.34894E-39	23468	CBX5
0.14402011	9.55039E-39	8805	TRIM24
0.144013137	9.63036E-39	5347	PLK1
0.143997729	9.80953E-39	3577	IL8RA
0.143957332	1.02951E-38	83452	RAB33B
0.143885165	1.12227E-38	79165	LENG1
0.143850535	1.16967E-38	4289	MKLN1
0.143800661	1.24148E-38	79075	DSCC1
0.143748477	1.32129E-38	5420	PODXL
0.143748283	1.32161E-38	283093	OR4C12
0.143722191	1.36343E-38	253868	C20orf200
0.143693775	1.41045E-38	130576	LYPD6B
0.143678084	1.43711E-38	51764	GNG13
0.143526435	1.722E-38	4943	TBC1D25
0.143511295	1.7534E-38	648740	ACTG1P4
0.143467546	1.84722E-38	1010	CDH12
0.143458351	1.86755E-38	3434	IFIT1
0.143408209	1.98253E-38	2972	BRF1
0.14333865	2.15372E-38	65082	VPS33A
0.143329829	2.17642E-38	285641	SLC36A3
0.143327236	2.18317E-38	85290	KRTAP4-3
0.143315718	2.2133E-38	9050	PSTPIP2
0.143301964	2.24982E-38	51621	KLF13
0.143283591	2.29957E-38	92070	CTBP1-AS1
0.143282279	2.30315E-38	56850	GRIPAP1
0.143276438	2.3192E-38	26689	AC005962.2
0.143258974	2.36792E-38	54902	TTC19
0.143252015	2.38761E-38	23612	PHLDA3
0.143230096	2.45068E-38	202051	AC135457.3
0.143221825	2.47491E-38	286016	TPI1P2
0.143211335	2.50598E-38	55655	NLRP2
0.143196568	2.55038E-38	84517	AC078802.1
0.143177345	2.60932E-38	3398	ID2
0.143133789	2.74801E-38	3800	KIF5C
0.143119678	2.7945E-38	23090	ZNF423

region-wise, subcortex					
Spearman's corr coef	p-val	p-val, SA	p-val, SA (BH- correction)	GenID	Gene
0.142084941	0.406980574	0.429	1	4500	MT1L
0.142084941	0.406980574	0.466	1	5127	PCTK1
0.141827539	0.407838136	0.429	1	29079	MED4
0.141827539	0.407838136	0.446	1	56681	SAR1A
0.141827539	0.407838136	0.443	1	80054	LOC80054
0.141570136	0.408696711	0.425	1	6218	RPS17
0.141570136	0.408696711	0.451	1	114783	LMTK3
0.141312733	0.409556359	0.406	1	6155	RPL27
0.141312733	0.409556359	0.471	1	10936	GPR75
0.141312733	0.409556359	0.465	1	11171	STRAP
0.141312733	0.409556359	0.458	1	80776	B9D2
0.141312733	0.409556359	0.443	1	285672	SREK1IP1
0.141312733	0.409556359	0.436	1	376497	SLC27A1
0.141312733	0.409556359	0.445	1	440087	C12orf69
0.141055331	0.410417348	0.452	1	54952	TRNAU1AP
0.141055331	0.410417348	0.445	1	92292	GLYATL1
0.140797943	0.411279082	0.436	1	10396	ATP8A1
0.140797943	0.411279082	0.449	1	23005	MAPKBP1
0.140797943	0.411279082	0.457	1	24141	C20orf103
0.140797943	0.411279082	0.458	1	84929	FIBCD1
0.140797943	0.411279082	0.43	1	285051	C2orf61
0.140797943	0.411279082	0.428	1	644591	PPIAL4G
0.14054054	0.41214186	0.434	1	11144	DMC1
0.14054054	0.41214186	0.479	1	341416	OR6C2
0.14054054	0.41214186	0.443	1	349565	NMNAT3
0.140283138	0.41300559	0.478	1	5724	PTAFR
0.140283138	0.41300559	0.44	1	23306	TMEM194A
0.140283138	0.41300559	0.485	1	27297	CRCP
0.140283138	0.41300559	0.435	1	56890	MDM1
0.140283138	0.41300559	0.444	1	374378	GALNTL4
0.140025735	0.413870811	0.46	1	8618	CADPS
0.140025735	0.413870811	0.452	1	10116	FEM1B
0.139768332	0.414736629	0.458	1	81793	TLR10
0.139510944	0.415603518	0.441	1	4660	PPP1R12B
0.139510944	0.415603518	0.481	1	11283	CYP4F8
0.139510944	0.415603518	0.464	1	79802	HHIPL2
0.139510944	0.415603518	0.479	1	220032	GDPD4
0.139510944	0.415603518	0.463	1	344892	RTP2
0.139253542	0.416471422	0.476	1	3872	KRT17
0.139253542	0.416471422	0.463	1	23067	SETD1B
0.139253542	0.416471422	0.428	1	60386	SLC25A19
0.139253542	0.416471422	0.461	1	63923	TNN
0.139253542	0.416471422	0.466	1	91749	KIAA1919
0.139253542	0.416471422	0.46	1	221692	PHACTR1
0.139253542	0.416471422	0.474	1	286257	C9orf142
0.139253542	0.416471422	0.474	1	389873	SEPHS1P4
0.139253542	0.416471422	0.458	1	440738	MAP1LC3C
0.138996139	0.417340338	0.448	1	11015	KDELR3
0.138996139	0.417340338	0.463	1	154091	SLC2A12
0.138738737	0.418210804	0.438	1	639	PRDM1
0.138738737	0.418210804	0.454	1	10383	TUBB2C
0.138738737	0.418210804	0.454	1	266811	NPSA
0.138738737	0.418210804	0.444	1	399959	MIR100HG
0.138481334	0.419081777	0.468	1	5546	PRCC
0.138481334	0.419081777	0.441	1	79982	DNAJB14
0.138481334	0.419081777	0.449	1	168620	BHLHA15
0.138223946	0.419953793	0.46	1	26999	CYFIP2
0.138223946	0.419953793	0.412	1	347918	EP400NL
0.137966543	0.420826912	0.452	1	8864	PER2
0.137966543	0.420826912	0.479	1	26168	SENP3
0.137966543	0.420826912	0.464	1	80309	SPHKAP
0.137709141	0.421701401	0.447	1	4070	TACSTD2
0.137709141	0.421701401	0.455	1	5383	PMS2L5
0.137709141	0.421701401	0.46	1	22832	KIAA1009
0.137709141	0.421701401	0.444	1	23049	SMG1
0.137709141	0.421701401	0.431	1	55329	MNS1
0.137709141	0.421701401	0.442	1	64395	GMCL1
0.137709141	0.421701401	0.453	1	149095	DCST1
0.137709141	0.421701401	0.462	1	221914	GPC2
0.137709141	0.421701401	0.463	1	283008	FAM22E
0.137709141	0.421701401	0.459	1	643201	RP11-844P9.2
0.137709141	0.421701401	0.479	1	100131176	RP4-555L14.5
0.137451738	0.422576487	0.466	1	3835	KIF22
0.137451738	0.422576487	0.467	1	3960	LGALS4
0.137451738	0.422576487	0.483	1	9796	AC105206.1
0.137451738	0.422576487	0.473	1	23345	SYNE1
0.137451738	0.422576487	0.454	1	79581	GPR172A
0.137451738	0.422576487	0.484	1	91782	CHMP7
0.137451738	0.422576487	0.436	1	100101267	POM121C

voxel-wise, subcortex			
Spearman's corr coef	p-val	GenID	Gene
0.143113732	2.81432E-38	55122	AKIRIN2
0.143101931	2.85404E-38	81693	AMN
0.143077984	2.93646E-38	10509	SEMA4B
0.143016309	3.15962E-38	55173	MRPS10
0.143010929	3.17985E-38	815	CAMK2A
0.14300926	3.1862E-38	4857	NOVA1
0.142936826	3.47231E-38	6326	SCN2A
0.142873853	3.74174E-38	114876	OSBPL1A
0.14283514	3.91752E-38	2208	FCER2
0.142796323	4.10215E-38	57722	IGDCC4
0.142669737	4.76615E-38	3054	HCFC1
0.142645776	4.90336E-38	7172	TPMT
0.142638355	4.94666E-38	23657	SLC7A11
0.142625809	5.0207E-38	22870	SAPS1
0.142579466	5.30395E-38	50846	DHH
0.142565921	5.38958E-38	10787	NCKAP1
0.142546594	5.51432E-38	54345	SOX18
0.142503783	5.80101E-38	148709	RP11-480I12.7
0.142485872	5.92523E-38	57451	ODZ2
0.142420486	6.40171E-38	137695	TMEM68
0.142392814	6.61468E-38	388963	C2orf81
0.142385855	6.66922E-38	617	BCS1L
0.142382592	6.69495E-38	128229	C1orf182
0.142380282	6.71331E-38	7252	TSHB
0.142373875	6.76432E-38	79877	DCAKD
0.142340258	7.03849E-38	144699	FBXL14
0.142230853	8.0097E-38	9063	PIAS2
0.142215803	8.1533E-38	10864	SLC22A7
0.142178565	8.51971E-38	151306	GPBAR1
0.142122015	9.10778E-38	1032	CDKN2D
0.14210251	9.31986E-38	54753	AC073343.8
0.142052367	9.88756E-38	2799	GNS
0.141995341	1.05754E-37	8945	BTRC
0.141994879	1.05813E-37	83442	SH3BGRL3
0.141951978	1.113E-37	80305	TRABD
0.141943336	1.12441E-37	83899	KRTAP9-2
0.141929418	1.14297E-37	2995	GYPC
0.141917616	1.159E-37	10928	RALBP1
0.141904712	1.17676E-37	2350	FOLR2
0.141846389	1.26044E-37	89837	FAM7A3
0.14179568	1.33797E-37	728373	AC116655.15
0.141761974	1.39214E-37	203228	C9orf72
0.141741365	1.42631E-37	5884	RAD17
0.141707927	1.48354E-37	90134	KCNH7
0.141636416	1.61371E-37	4494	MT1F
0.141627312	1.63108E-37	274	BIN1
0.141618311	1.64842E-37	284367	SIGLECP3
0.141563892	1.75733E-37	119	ADD2
0.141547084	1.7924E-37	5608	MAP2K6
0.141542166	1.80275E-37	84140	FAM161A
0.141540393	1.80653E-37	8718	TNFRSF25
0.141504645	1.88402E-37	26960	NBEA
0.141485795	1.92619E-37	5540	PPYR1
0.141447157	2.01559E-37	126364	LRRC25
0.141397655	2.13623E-37	2215	FCGR3B
0.141296342	2.4059E-37	219981	OR5A2
0.141241744	2.56493E-37	3207	HOXA11
0.141145721	2.8705E-37	100130274	CCDC166
0.141123056	2.94777E-37	27000	DNAJC2
0.141115159	2.97515E-37	55273	TMEM100
0.140960351	3.56632E-37	10342	TFG
0.140923858	3.72185E-37	9240	PNMA1
0.140841767	4.09679E-37	680	BRS3
0.140819103	4.20684E-37	8728	ADAM19
0.140730143	4.66767E-37	81872	KRTAP2-3
0.140723452	4.7043E-37	6407	SEMG2
0.140609235	5.37542E-37	93210	PGAP3
0.140608862	5.3777E-37	79762	C1orf115
0.140598923	5.44046E-37	9394	HS6ST1
0.14059037	5.49503E-37	84814	PPAPDC3
0.140503183	6.0833E-37	728262	FAM157A
0.140478656	6.25984E-37	4982	TNFRSF11B
0.140462786	6.37676E-37	283742	FAM98B
0.140433893	6.59518E-37	10370	CITED2
0.140405387	6.81792E-37	1991	ELANE
0.140404925	6.82171E-37	344167	FOXI3
0.14037846	7.03527E-37	54785	C17orf59
0.140374377	7.0688E-37	25825	BACE2
0.140313074	7.59207E-37	399909	PCNXL3

region-wise, subcortex					
Spearman's corr coef	p-val	p-val, SA	p-val, SA (BH- correction)	GenID	Gene
0.137194335	0.423452675	0.467	1	514	ATP5E
0.137194335	0.423452675	0.475	1	10531	PITRM1
0.137194335	0.423452675	0.467	1	64343	AZI2
0.137194335	0.423452675	0.454	1	79574	EPS8L3
0.136936948	0.424329847	0.446	1	645	BLVRB
0.136936948	0.424329847	0.435	1	1030	CDKN2B
0.136936948	0.424329847	0.46	1	7730	ZNF177
0.136936948	0.424329847	0.43	1	89845	ABCC10
0.136936948	0.424329847	0.464	1	117159	DCD
0.136936948	0.424329847	0.472	1	337876	CHSY3
0.136679545	0.425208479	0.471	1	3422	IDI1
0.136679545	0.425208479	0.451	1	3855	KRT7
0.136679545	0.425208479	0.476	1	56479	KCNQ5
0.136679545	0.425208479	0.478	1	158427	C9orf97
0.136679545	0.425208479	0.454	1	282775	OR5J2
0.136679545	0.425208479	0.475	1	390705	LOC390705
0.136679545	0.425208479	0.486	1	403315	FAM92A3
0.136422142	0.426087707	0.448	1	313	AOAH
0.136422142	0.426087707	0.464	1	322	APBB1
0.136422142	0.426087707	0.456	1	6130	RPL7A
0.136422142	0.426087707	0.455	1	10590	SCGN
0.136422142	0.426087707	0.482	1	55256	ADI1
0.136422142	0.426087707	0.462	1	55586	MIOX
0.136164725	0.426967978	0.452	1	11070	TMEM115
0.136164725	0.426967978	0.445	1	51361	HOOK1
0.136164725	0.426967978	0.447	1	124808	CCDC43
0.135907322	0.427849233	0.471	1	5909	RAP1GAP
0.135907322	0.427849233	0.474	1	9610	RIN1
0.135907322	0.427849233	0.478	1	56147	PCDHA1
0.135907322	0.427849233	0.462	1	57473	ZNF512B
0.135907322	0.427849233	0.485	1	80351	TNKS2
0.135907322	0.427849233	0.447	1	84811	BUD13
0.135907322	0.427849233	0.445	1	727851	RGPD8
0.135649934	0.428731561	0.496	1	6492	SIM1
0.135649934	0.428731561	0.503	1	8013	NR4A3
0.135649934	0.428731561	0.452	1	54539	NDUFB11
0.135649934	0.428731561	0.436	1	79647	AKIRIN1
0.135392532	0.429615289	0.456	1	993	CDC25A
0.135392532	0.429615289	0.49	1	53947	A4GALT
0.135392532	0.429615289	0.489	1	81846	SBF2
0.135135129	0.430499643	0.467	1	8577	TMEFF1
0.135135129	0.430499643	0.469	1	51340	CRNKL1
0.135135129	0.430499643	0.495	1	729956	AC135592.2
0.134877726	0.43138501	0.445	1	71	ACTG1
0.134877726	0.43138501	0.46	1	51412	ACTL6B
0.134877726	0.43138501	0.464	1	92714	ARRDC1
0.134877726	0.43138501	0.425	1	117579	RLN3
0.134620324	0.432271421	0.474	1	5555	PRH2
0.134620324	0.432271421	0.459	1	5705	PSMC5
0.134620324	0.432271421	0.485	1	25974	MMACHC
0.134362936	0.433159232	0.465	1	6915	TBXA2R
0.134362936	0.433159232	0.501	1	8786	RGS11
0.134362936	0.433159232	0.467	1	9338	TCEAL1
0.134362936	0.433159232	0.482	1	503834	DPRXP4
0.134105533	0.434047639	0.502	1	97	ACYP1
0.134105533	0.434047639	0.471	1	27243	CHMP2A
0.134105533	0.434047639	0.464	1	84975	MFSD5
0.134105533	0.434047639	0.49	1	168433	RNF133
0.133848131	0.434937119	0.454	1	1358	CPA2
0.133848131	0.434937119	0.467	1	10645	CAMKK2
0.133848131	0.434937119	0.467	1	51333	ZNF771
0.133848131	0.434937119	0.477	1	55330	CNO
0.133848131	0.434937119	0.486	1	84240	ZCCHC9
0.133590728	0.435827583	0.509	1	57448	BIRC6
0.133590728	0.435827583	0.472	1	84530	KIAA1853
0.133590728	0.435827583	0.448	1	144577	C12orf66
0.133333325	0.436719447	0.483	1	152	ADRA2C
0.133333325	0.436719447	0.49	1	2593	GAMT
0.133333325	0.436719447	0.46	1	9032	TM4SF5
0.133333325	0.436719447	0.477	1	54494	C11orf71
0.133333325	0.436719447	0.461	1	83442	SH3BGRL3
0.133333325	0.436719447	0.465	1	84908	FAM136A
0.133333325	0.436719447	0.456	1	114814	GNRHR2
0.133333325	0.436719447	0.469	1	340371	NRBP2
0.133075938	0.437611967	0.494	1	290	ANPEP
0.133075938	0.437611967	0.463	1	7739	ZNF185
0.133075938	0.437611967	0.457	1	7991	TUSC3
0.133075938	0.437611967	0.466	1	51160	VPS28
0.133075938	0.437611967	0.459	1	54926	UBE2R2

voxel-wise, subcortex			
Spearman's corr coef	p-val	GenID	Gene
0.140184	8.82276E-37	26577	PCOLCE2
0.140180647	8.85727E-37	100287728	LOC100287728
0.140152141	9.15589E-37	4909	AC008687.3
0.14012669	9.43106E-37	200765	TIGD1
0.139989063	1.10671E-36	55359	STYK1
0.13993381	1.18005E-36	159989	CCDC67
0.139928713	1.18705E-36	51157	ZNF580
0.139919415	1.19996E-36	57804	POLD4
0.139880702	1.25513E-36	344905	ATP13A5
0.139875218	1.26313E-36	389690	AC100803.1
0.139849961	1.30071E-36	27429	HTRA2
0.139820337	1.34619E-36	9729	KIAA0408
0.139801294	1.37629E-36	9737	GPRASP1
0.139799625	1.37895E-36	440097	DBX2
0.139795721	1.38521E-36	130574	LYPD6
0.139786154	1.40066E-36	54802	TRIT1
0.139760345	1.44323E-36	137994	LETM2
0.139752269	1.45681E-36	92659	MAFG-AS1
0.139736474	1.48375E-36	100132249	LOC100132249
0.139692456	1.56143E-36	55244	SLC47A1
0.139690787	1.56445E-36	138799	OR13C5
0.139687434	1.57054E-36	84650	EBPL
0.139577866	1.78317E-36	145553	MDP1
0.13956216	1.81589E-36	91653	BOC
0.139435589	2.10246E-36	388595	TMEM82
0.139428437	2.1199E-36	84250	ANKRD32
0.139312819	2.4232E-36	401149	FLJ14186
0.139287561	2.49499E-36	7441	VPREB1
0.139244378	2.62265E-36	3594	IL12RB1
0.139156431	2.90302E-36	22808	MRAS
0.139147237	2.93403E-36	24140	FTSJ1
0.139126524	3.00505E-36	57224	NHSL1
0.139097273	3.10827E-36	55668	C14orf118
0.139068112	3.2146E-36	202243	CCDC125
0.139062077	3.23709E-36	340554	ZC3H12B
0.139051303	3.27756E-36	85416	ZIC5
0.139006734	3.45048E-36	22818	COPZ1
0.138926312	3.78578E-36	8823	FGF16
0.138774291	4.51039E-36	386685	KRTAP10-12
0.138726652	4.76472E-36	100132594	PGAM1P5
0.138518438	6.05398E-36	4967	OGDH
0.138394102	6.98341E-36	79906	MORN1
0.138387412	7.0372E-36	353139	LCE2A
0.138332158	7.49801E-36	24145	PANX1
0.138113648	9.63319E-36	57026	PDXP
0.13810277	9.75399E-36	146909	KIF18B
0.138026342	1.06462E-35	9459	ARHGEF6
0.137970254	1.13523E-35	123688	AGPHD1
0.13794221	1.17225E-35	57470	LRRC47
0.13792856	1.19071E-35	222662	LHFPL5
0.137915462	1.20869E-35	338773	TMEM119
0.137833089	1.3281E-35	10279	PRSS16
0.137802452	1.37547E-35	253018	HCG27
0.137749135	1.46185E-35	442319	ZNF727
0.137745336	1.46821E-35	148252	DIRAS1
0.137710512	1.52782E-35	36	ACADSB
0.137654141	1.62943E-35	402360	LOC402360
0.137626842	1.68101E-35	8034	SLC25A16
0.137614295	1.70527E-35	1657	DMXL1
0.137458384	2.03723E-35	1439	CSF2RB
0.137311742	2.40777E-35	124936	CYB5D2
0.137295023	2.45408E-35	94032	CAMK2N2
0.137283519	2.48643E-35	7040	TGFB1
0.13728036	2.49541E-35	375704	ENHO
0.137266517	2.53503E-35	9118	INA
0.13724637	2.59385E-35	254439	C11orf86
0.137120441	2.99351E-35	140838	NANP
0.137119979	2.99511E-35	6670	SP3
0.137069732	3.17118E-35	341405	ANKRD33
0.137067318	3.17989E-35	286187	LRRC67
0.137061	3.20279E-35	440955	TMEM89
0.137005746	3.41041E-35	50807	ASAP1
0.136996552	3.44624E-35	23138	AC136632.1
0.136909455	3.80464E-35	79071	ELOVL6
0.136864409	4.00425E-35	9716	AQR
0.136811748	4.25079E-35	3671	ISLR
0.136780545	4.404E-35	134266	GRPEL2
0.136761785	4.49876E-35	728591	C13orf38
0.136716381	4.73651E-35	474148	TTTY3B

region-wise, subcortex					
Spearman's corr coef	p-val	p-val, SA	p-val, SA (BH- correction)	GenID	Gene
0.133075938	0.437611967	0.476	1	80221	ACSF2
0.133075938	0.437611967	0.454	1	84100	ARL6
0.133075938	0.437611967	0.474	1	246175	CNOT6L
0.132818535	0.438505441	0.498	1	636	BICD1
0.132818535	0.438505441	0.496	1	51608	C7orf20
0.132818535	0.438505441	0.485	1	84516	DCTN5
0.132818535	0.438505441	0.464	1	220594	AC107983.3
0.132818535	0.438505441	0.451	1	337978	KRTAP21-2
0.132561132	0.439399987	0.492	1	1140	CHRNB1
0.132561132	0.439399987	0.483	1	1729	DIAPH1
0.132561132	0.439399987	0.478	1	2213	FCGR2B
0.132561132	0.439399987	0.453	1	29097	CNIH4
0.13230373	0.440295547	0.447	1	637	BID
0.13230373	0.440295547	0.454	1	7478	WNT8A
0.13230373	0.440295547	0.482	1	8467	SMARCA5
0.13230373	0.440295547	0.46	1	23158	TBC1D9
0.13230373	0.440295547	0.49	1	80222	TARS2
0.132046327	0.441192478	0.462	1	6277	S100A6
0.132046327	0.441192478	0.49	1	23197	FAF2
0.132046327	0.441192478	0.471	1	55770	EXOC2
0.132046327	0.441192478	0.461	1	728661	RP11-345P4.2
0.131788939	0.442090064	0.474	1	53942	CNTN5
0.131788939	0.442090064	0.48	1	79568	C2orf47
0.131788939	0.442090064	0.475	1	90024	AP001816.1
0.131788939	0.442090064	0.483	1	121643	FOXN4
0.131788939	0.442090064	0.481	1	221823	PRPS1L1
0.131788939	0.442090064	0.488	1	100129434	AC007743.1
0.131531537	0.442988575	0.499	1	327	APEH
0.131531537	0.442988575	0.44	1	353	APRT
0.131531537	0.442988575	0.467	1	9318	COPS2
0.131531537	0.442988575	0.497	1	170591	S100Z
0.131531537	0.442988575	0.461	1	246721	POLR2J2
0.131531537	0.442988575	0.5	1	390940	AC026369.2
0.131274134	0.443888187	0.484	1	2587	GALR1
0.131274134	0.443888187	0.504	1	7270	TTF1
0.131274134	0.443888187	0.495	1	129831	RBM45
0.131274134	0.443888187	0.507	1	219844	HYLS1
0.131016731	0.444789141	0.507	1	925	CD8A
0.131016731	0.444789141	0.479	1	7753	ZNF202
0.131016731	0.444789141	0.498	1	27125	AFF4
0.131016731	0.444789141	0.488	1	285362	SUMF1
0.131016731	0.444789141	0.494	1	346606	MOGAT3
0.131016731	0.444789141	0.481	1	100129250	C9ORF133
0.130759329	0.445690751	0.471	1	249	ALPL
0.130759329	0.445690751	0.479	1	602	BCL3
0.130759329	0.445690751	0.481	1	5055	SERPINB2
0.130759329	0.445690751	0.47	1	26578	OSTF1
0.130759329	0.445690751	0.465	1	54901	CDKAL1
0.130759329	0.445690751	0.477	1	84666	RETNLB
0.130501941	0.446593404	0.47	1	6137	RPL13
0.130501941	0.446593404	0.46	1	7319	UBE2A
0.130501941	0.446593404	0.457	1	8943	AP3D1
0.130501941	0.446593404	0.478	1	23029	RBM34
0.130501941	0.446593404	0.501	1	135114	HINT3
0.130501941	0.446593404	0.479	1	221472	FGD2
0.130501941	0.446593404	0.49	1	100506581	C16orf95
0.130244538	0.447496951	0.519	1	2289	FKBP5
0.130244538	0.447496951	0.488	1	6154	RPL26
0.130244538	0.447496951	0.483	1	9665	KIAA0430
0.130244538	0.447496951	0.498	1	83716	CRISPLD2
0.130244538	0.447496951	0.52	1	127700	C1orf102
0.130244538	0.447496951	0.496	1	401805	LOC401805
0.130244538	0.447496951	0.465	1	440577	LOC440577
0.129987136	0.448401988	0.491	1	9919	SEC16A
0.129987136	0.448401988	0.498	1	54840	APTX
0.129987136	0.448401988	0.478	1	81696	OR5V1
0.129987136	0.448401988	0.49	1	341359	SYT10
0.129729733	0.449307591	0.475	1	2963	GTF2F2
0.129729733	0.449307591	0.533	1	4978	AP000843.1
0.129729733	0.449307591	0.484	1	26057	ANKRD17
0.129729733	0.449307591	0.462	1	51011	FAHD2A
0.129729733	0.449307591	0.508	1	54905	CYP2W1
0.129729733	0.449307591	0.482	1	64428	NARFL
0.129729733	0.449307591	0.458	1	148268	ZNF570
0.129729733	0.449307591	0.489	1	157773	C8orf48
0.12947233	0.450214207	0.504	1	28974	C19orf53
0.12947233	0.450214207	0.471	1	116729	DYSFIP1
0.12947233	0.450214207	0.482	1	729220	FLJ45513
0.129214913	0.451121837	0.484	1	10844	TUBGCP2

voxel-wise, subcortex			
Spearman's corr coef	p-val	GenID	Gene
0.136712104	4.75954E-35	1296	COL8A2
0.136682481	4.92206E-35	2937	GSS
0.136478081	6.20443E-35	375790	AGRN
0.13647595	6.2194E-35	27336	HTATSF1
0.13645412	6.37499E-35	85318	BAGE3
0.1364467	6.42874E-35	777	CACNA1E
0.136384755	6.89536E-35	90668	LRRC16B
0.136279166	7.76984E-35	5573	PRKAR1A
0.136241376	8.10877E-35	83786	FRMD8
0.136174873	8.74135E-35	390748	AC092384.2
0.136100397	9.50823E-35	25858	C11orf20
0.136094555	9.57115E-35	5533	PPP3CC
0.13603279	1.0262E-34	6817	SULT1A1
0.135987282	1.08025E-34	79947	DHDDS
0.135946617	1.13093E-34	1258	CNGB1
0.135935649	1.145E-34	64219	PJA1
0.135900453	1.19133E-34	28227	PPP2R3B
0.135830805	1.28858E-34	64782	AEN
0.135814741	1.31211E-34	3716	JAK1
0.135799706	1.3345E-34	23480	SEC61G
0.135709986	1.47635E-34	340591	CA5BP
0.135675535	1.53471E-34	516	ATP5G1
0.135669127	1.54581E-34	3658	IREB2
0.135638118	1.60066E-34	4744	NEFH
0.135617122	1.6389E-34	79158	GNPTAB
0.135603935	1.66339E-34	23344	ESYT1
0.135597438	1.67559E-34	5509	PPP1R3D
0.135596976	1.67648E-34	55810	FOXJ2
0.135579512	1.7097E-34	142680	SLC34A3
0.135567069	1.73377E-34	10802	SEC24A
0.135550633	1.76614E-34	85456	TNKS1BP1
0.13553299	1.80148E-34	22955	SCMH1
0.135467514	1.93902E-34	3773	KCNJ16
0.135414764	2.05737E-34	140767	NRSN1
0.135267392	2.42736E-34	284339	TMEM145
0.135224953	2.54569E-34	9390	SLC22A13
0.135214463	2.57579E-34	51655	RASD1
0.13518855	2.65171E-34	3728	JUP
0.135178328	2.6823E-34	22872	SEC31A
0.135178238	2.68257E-34	9780	FAM38A
0.135017857	3.21047E-34	5479	PPIB
0.134994462	3.29567E-34	4351	MPI
0.134900659	3.66035E-34	257068	PLCXD2
0.134889796	3.70505E-34	202134	FAM153B
0.134870574	3.78556E-34	11024	LILRA1
0.1348598	3.83149E-34	440823	MIAT
0.134848565	3.8799E-34	1158	CKM
0.134831667	3.95394E-34	2267	FGL1
0.13482517	3.98274E-34	404037	HAPLN4
0.134824231	3.98693E-34	55975	KLHL7
0.134823486	3.99025E-34	64210	MMS19
0.13480185	4.0879E-34	100132306	LOC100132306
0.134736389	4.39812E-34	729420	AL359392.2
0.134696633	4.59782E-34	5532	PPP3CB
0.134679273	4.6878E-34	113026	PLCD3
0.134659767	4.79096E-34	50636	ANO7
0.134584457	5.21115E-34	3234	HOXD8
0.134517968	5.61236E-34	96597	TBC1D27
0.134383962	6.51649E-34	80705	TSGA10
0.134373188	6.5952E-34	126731	C1orf96
0.134371042	6.611E-34	161502	C15orf26
0.134299815	7.15678E-34	6741	SSB
0.134298056	7.17077E-34	55906	ZC4H2
0.134296298	7.18496E-34	3201	HOXA4
0.134244189	7.61383E-34	60488	MRPS35
0.134214476	7.86987E-34	4759	NEU2
0.134206116	7.94343E-34	8455	ATRN
0.134197578	8.01929E-34	284114	TMEM102
0.134171203	8.25805E-34	441864	TARM1
0.134137869	8.56989E-34	338321	NLRP9
0.134134799	8.59919E-34	253650	ANKRD18A
0.134074345	9.19699E-34	196463	PLBD2
0.134052888	9.41896E-34	56138	PCDHA11
0.134045646	9.49497E-34	157848	NKX6-3
0.133972093	1.03035E-33	58506	SCAF1
0.133972004	1.03045E-33	8724	SNX3
0.133922502	1.08867E-33	7358	UGDH
0.133882299	1.13836E-33	7168	TPM1
0.133813754	1.22831E-33	3110	MNX1

region-wise, subcortex					
Spearman's corr coef	p-val	p-val, SA	p-val, SA (BH- correction)	GenID	Gene
0.129214913	0.451121837	0.498	1	10894	LYVE1
0.128957525	0.45203042	0.491	1	10745	PHTF1
0.128957525	0.45203042	0.489	1	134359	C5orf37
0.128957525	0.45203042	0.484	1	729009	FTH1P20
0.128700122	0.452940434	0.498	1	8928	FOXH1
0.128700122	0.452940434	0.476	1	113091	PTH2
0.12844272	0.453851044	0.468	1	1001	CDH3
0.12844272	0.453851044	0.478	1	4774	NFIA
0.12844272	0.453851044	0.474	1	7482	WNT2B
0.12844272	0.453851044	0.48	1	80129	C6orf97
0.12844272	0.453851044	0.474	1	81631	MAP1LC3B
0.12844272	0.453851044	0.485	1	96459	FNIP1
0.12844272	0.453851044	0.487	1	401036	ASB18
0.128185332	0.454762697	0.5	1	3118	HLA-DQA2
0.128185332	0.454762697	0.485	1	11315	PARK7
0.128185332	0.454762697	0.5	1	79625	C4orf31
0.128185332	0.454762697	0.511	1	146723	C17orf77
0.128185332	0.454762697	0.475	1	153396	TMEM161B
0.127927929	0.455675244	0.491	1	5020	OXT
0.127927929	0.455675244	0.501	1	55127	HEATR1
0.127927929	0.455675244	0.505	1	129138	ANKRD54
0.127927929	0.455675244	0.474	1	388585	HES5
0.127670527	0.456589252	0.492	1	25885	POLR1A
0.127670527	0.456589252	0.505	1	64710	NUCKS1
0.127670527	0.456589252	0.497	1	78987	CRELD1
0.127670527	0.456589252	0.525	1	85474	LBX2
0.127670527	0.456589252	0.46	1	94039	ZNF101
0.127413124	0.457503915	0.511	1	2730	GCLM
0.127413124	0.457503915	0.511	1	11094	C9orf7
0.127155721	0.458419472	0.514	1	4893	NRAS
0.127155721	0.458419472	0.484	1	5460	POU5F1
0.127155721	0.458419472	0.474	1	5694	PSMB6
0.127155721	0.458419472	0.506	1	9851	KIAA0753
0.127155721	0.458419472	0.517	1	153830	RNF145
0.126898319	0.459336072	0.485	1	4088	SMAD3
0.126898319	0.459336072	0.508	1	51654	CDK5RAP1
0.126898319	0.459336072	0.459	1	441263	DTX2P1-UPK3BP1-PMS2P11
0.126640916	0.460254073	0.478	1	7566	ZNF18
0.126640916	0.460254073	0.524	1	23215	BAT2D1
0.126640916	0.460254073	0.487	1	25949	SYF2
0.126640916	0.460254073	0.499	1	57620	STIM2
0.126640916	0.460254073	0.48	1	83448	PUS7L
0.126640916	0.460254073	0.478	1	84619	ZGPAT
0.126640916	0.460254073	0.508	1	359821	MRPL42P5
0.126383528	0.461172581	0.483	1	6497	SKI
0.126383528	0.461172581	0.51	1	9687	AC110754.1
0.126383528	0.461172581	0.492	1	51073	MRPL4
0.126383528	0.461172581	0.469	1	64601	VPS16A
0.126126125	0.462092161	0.469	1	138799	OR13C5
0.125868723	0.463012695	0.502	1	5582	PRKCG
0.125868723	0.463012695	0.517	1	5631	PRPS1
0.125868723	0.463012695	0.485	1	84624	FNDC1
0.125868723	0.463012695	0.494	1	90665	TBL1Y
0.12561132	0.463934183	0.497	1	63943	FKBPL
0.12561132	0.463934183	0.506	1	116224	FAM122A
0.12561132	0.463934183	0.485	1	196047	EMX2OS
0.12561132	0.463934183	0.522	1	389787	LOC389787
0.12561132	0.463934183	0.489	1	442245	GSTM2P1
0.125353917	0.464857221	0.503	1	776	CACNA1D
0.125353917	0.464857221	0.521	1	2334	AFF2
0.125353917	0.464857221	0.514	1	8601	RGS20
0.125353917	0.464857221	0.486	1	10567	RABAC1
0.125353917	0.464857221	0.492	1	54741	LEPROT
0.125353917	0.464857221	0.486	1	59340	HRH4
0.125353917	0.464857221	0.484	1	84078	KBTBD7
0.125353917	0.464857221	0.509	1	388588	RP1-286D6.2
0.125353917	0.464857221	0.525	1	643837	RP11-206L10.11
0.12509653	0.465780675	0.522	1	3675	ITGA3
0.12509653	0.465780675	0.506	1	4205	MEF2A
0.12509653	0.465780675	0.513	1	7386	UQCRFS1
0.12509653	0.465780675	0.5	1	7474	WNT5A
0.12509653	0.465780675	0.491	1	8575	PRKRA
0.12509653	0.465780675	0.49	1	85453	AP003115.1
0.124839127	0.466705143	0.536	1	8630	HSD17B6
0.124839127	0.466705143	0.504	1	114625	ERMAP
0.124581724	0.467630595	0.489	1	6173	RPL36A
0.124324329	0.468557507	0.51	1	4093	SMAD9
0.124324329	0.468557507	0.508	1	9456	HOMER1
0.124324329	0.468557507	0.495	1	28952	CCDC22

voxel-wise, subcortex			
Spearman's corr coef	p-val	GenID	Gene
0.133783206	1.27064E-33	54107	POLE3
0.133769467	1.29014E-33	220070	SHANK2-AS3
0.133766681	1.29415E-33	84651	SPINK7
0.133764267	1.29761E-33	83882	TSPAN10
0.133755624	1.31011E-33	1056	CEL
0.133726656	1.35284E-33	23269	MGA
0.133719593	1.36348E-33	1188	CLCNKB
0.133718297	1.36547E-33	55901	THSD1
0.133713648	1.37253E-33	3959	LGALS3BP
0.133667126	1.44513E-33	23365	ARHGEF12
0.133640379	1.4886E-33	154790	CLEC2L
0.133550853	1.64373E-33	345193	LRIT3
0.133486778	1.76454E-33	83667	SESN2
0.133464217	1.80916E-33	51507	C20orf43
0.133453712	1.83029E-33	9839	ZEB2
0.133440346	1.85755E-33	78994	PRR14
0.133435234	1.86806E-33	57626	KLHL1
0.133424193	1.89103E-33	100131138	AC002351.1
0.133419722	1.9004E-33	89780	WNT3A
0.133406356	1.9287E-33	133686	C5orf33
0.133393079	1.95723E-33	163859	C1orf55
0.133365303	2.01826E-33	26523	EIF2C1
0.133364573	2.01988E-33	2906	GRIN2D
0.133350357	2.05188E-33	646405	TPTE2P1
0.133345619	2.06267E-33	647012	LOC647012
0.133341819	2.07135E-33	51676	ASB2
0.133337259	2.0818E-33	10467	ZNHIT1
0.133311629	2.14157E-33	4791	NFKB2
0.133311167	2.14271E-33	116534	MRGPRE
0.133265391	2.25384E-33	58189	WFDC1
0.133237243	2.32502E-33	11056	DDX52
0.133185148	2.46269E-33	123722	FSD2
0.133143172	2.57946E-33	51310	SLC22A17
0.133119866	2.64666E-33	3200	HOXA3
0.133047521	2.86651E-33	3777	KCNK3
0.133044735	2.87535E-33	27302	BMP10
0.132983252	3.07703E-33	114898	C1QTNF2
0.132925034	3.2809E-33	54943	DNAJC28
0.132849813	3.56429E-33	9547	CXCL14
0.132765114	3.91254E-33	85445	CNTNAP4
0.132674113	4.32445E-33	51060	TXNDC12
0.13267383	4.32582E-33	26039	SS18L1
0.132660553	4.38942E-33	4257	MGST1
0.13263993	4.49003E-33	3556	IL1RAP
0.132596567	4.70929E-33	84159	ARID5B
0.132568896	4.85471E-33	64395	GMCL1
0.132561371	4.89501E-33	646938	AC022748.1
0.132532209	5.0543E-33	54841	BIVM
0.13247937	5.35627E-33	55101	ATP5SL
0.132434145	5.62889E-33	64431	ACTR6
0.132409722	5.78182E-33	2131	EXT1
0.132400334	5.84171E-33	284266	SIGLEC15
0.132381946	5.96081E-33	7425	VGF
0.132376015	5.99958E-33	90070	LACRT
0.1323241	6.3512E-33	340152	ZC3H12D
0.132314995	6.41495E-33	7151	TOP1P1
0.132310718	6.44507E-33	3297	HSF1
0.132248878	6.89713E-33	1604	CD55
0.132245719	6.92107E-33	5296	PIK3R2
0.132170126	7.51871E-33	123745	PLA2G4E
0.13216427	7.56714E-33	56951	C5orf15
0.132154241	7.65065E-33	2826	CCR10
0.132129908	7.85729E-33	387338	NSUN4
0.132048383	8.5908E-33	150094	SIK1
0.1320429	8.64254E-33	3750	KCND1
0.132038161	8.68735E-33	10249	GLYAT
0.131966472	9.3959E-33	51278	IER5
0.131933033	9.74592E-33	1982	EIF4G2
0.131931365	9.76383E-33	284359	IZUMO1
0.131918177	9.90562E-33	100128588	LOC100128588
0.131643757	1.33667E-32	26054	SENP6
0.131605685	1.39334E-32	9534	ZNF254
0.131591663	1.41479E-32	11193	WBP4
0.131558508	1.46689E-32	57515	SERINC1
0.131553501	1.47492E-32	10363	HMG20A
0.131552652	1.4763E-32	2771	GNAI2
0.131540209	1.49645E-32	253959	GARNL1
0.131535754	1.50372E-32	79886	C9orf82
0.131505013	1.55496E-32	6097	RORC

region-wise, subcortex					
Spearman's corr coef	p-val	p-val, SA	p-val, SA (BH- correction)	GenID	Gene
0.124324329	0.468557507	0.53	1	63894	C14orf133
0.124324329	0.468557507	0.505	1	157627	RP11-403C10.2
0.124066919	0.469484925	0.51	1	2970	GTF2IP1
0.124066919	0.469484925	0.525	1	54517	PUS7
0.123809516	0.470413327	0.476	1	4172	MCM3
0.123809516	0.470413327	0.495	1	10771	ZMYND11
0.123809516	0.470413327	0.465	1	79159	NOL12
0.123809516	0.470413327	0.527	1	80772	GLTPD1
0.123809516	0.470413327	0.515	1	84069	PLEKHN1
0.123809516	0.470413327	0.52	1	85440	DOCK7
0.123552121	0.471342742	0.492	1	7784	ZP3
0.123552121	0.471342742	0.495	1	26984	SEC22A
0.123552121	0.471342742	0.511	1	57700	FAM160B1
0.123552121	0.471342742	0.519	1	120071	GYLTL1B
0.123552121	0.471342742	0.511	1	338094	FAM151A
0.123552121	0.471342742	0.504	1	442459	LOC442459
0.123552121	0.471342742	0.515	1	493856	CISD2
0.123294719	0.472273529	0.508	1	644	BLVRA
0.123294719	0.472273529	0.506	1	924	CD7
0.123294719	0.472273529	0.494	1	4598	MVK
0.123294719	0.472273529	0.525	1	5535	PPP3R2
0.123294719	0.472273529	0.517	1	7940	LST1
0.123294719	0.472273529	0.496	1	8542	APOL1
0.123294719	0.472273529	0.536	1	29799	YPEL1
0.123294719	0.472273529	0.494	1	51103	NDUFAF1
0.123294719	0.472273529	0.505	1	146547	PRSS36
0.123037323	0.473204881	0.531	1	5126	PCSK2
0.123037323	0.473204881	0.512	1	5795	PTPRJ
0.123037323	0.473204881	0.521	1	9450	LY86
0.123037323	0.473204881	0.507	1	10243	GPHN
0.123037323	0.473204881	0.504	1	151525	WDSUB1
0.123037323	0.473204881	0.514	1	162514	TRPV3
0.123037323	0.473204881	0.533	1	283458	NME2P1
0.123037323	0.473204881	0.512	1	641311	RPL31P11
0.122779921	0.474137217	0.479	1	10580	SORBS1
0.122522518	0.475070477	0.509	1	4159	MC3R
0.122522518	0.475070477	0.5	1	5995	RGR
0.122522518	0.475070477	0.505	1	6201	RPS7
0.122522518	0.475070477	0.509	1	7767	ZNF224
0.122522518	0.475070477	0.516	1	9362	CPNE6
0.122522518	0.475070477	0.521	1	94122	SYTL5
0.122265123	0.476004809	0.496	1	387	RHOA
0.122265123	0.476004809	0.494	1	10381	RP11-566K11.2
0.122265123	0.476004809	0.483	1	26751	SH3YL1
0.122265123	0.476004809	0.537	1	51018	RRP15
0.122265123	0.476004809	0.522	1	56673	C11orf16
0.122265123	0.476004809	0.486	1	58191	CXCL16
0.12200772	0.476940513	0.52	1	50855	PARD6A
0.12200772	0.476940513	0.529	1	63982	ANO3
0.12200772	0.476940513	0.497	1	84961	FBXL20
0.121750325	0.477876663	0.515	1	1307	COL16A1
0.121750325	0.477876663	0.512	1	1981	EIF4G1
0.121750325	0.477876663	0.479	1	4693	NDP
0.121750325	0.477876663	0.475	1	10961	ERP29
0.121750325	0.477876663	0.521	1	11173	ADAMTS7
0.121750325	0.477876663	0.503	1	11189	CELF3
0.121750325	0.477876663	0.507	1	23411	SIRT1
0.121750325	0.477876663	0.501	1	27085	MTBP
0.121750325	0.477876663	0.524	1	51686	OAZ3
0.121750325	0.477876663	0.524	1	146923	RUNDC1
0.121750325	0.477876663	0.504	1	399979	SNX19
0.121492922	0.478813857	0.505	1	1390	CREM
0.121492922	0.478813857	0.498	1	2189	FANCG
0.121492922	0.478813857	0.548	1	27189	IL17C
0.121492922	0.478813857	0.52	1	64754	SMYD3
0.121492922	0.478813857	0.512	1	84270	C9orf89
0.121492922	0.478813857	0.502	1	284593	FAM41C
0.121492922	0.478813857	0.532	1	339745	SPOPL
0.121492922	0.478813857	0.523	1	391123	VSIG8
0.121492922	0.478813857	0.527	1	399948	C11orf92
0.12123552	0.479752034	0.531	1	5438	POLR2I
0.12123552	0.479752034	0.492	1	6344	SCTR
0.12123552	0.479752034	0.516	1	8487	SIP1
0.12123552	0.479752034	0.499	1	11159	RABL2A
0.12123552	0.479752034	0.524	1	23376	KIAA0776
0.12123552	0.479752034	0.511	1	29959	NRBP1
0.120978124	0.480691612	0.524	1	223	ALDH9A1
0.120978124	0.480691612	0.505	1	8111	GPR68
0.120978124	0.480691612	0.503	1	23647	ARFIP2

voxel-wise, subcortex			
Spearman's corr coef	p-val	GenID	Gene
0.131485701	1.58803E-32	1890	TYMP
0.131451905	1.64759E-32	4191	MDH2
0.131419867	1.70608E-32	4253	CTAGE5
0.131398499	1.74627E-32	220107	DLEU7
0.131215841	2.1302E-32	10468	FST
0.131170332	2.23822E-32	347088	GPR144
0.131137177	2.32033E-32	100128062	SLC2A3P1
0.131119534	2.36527E-32	9191	DEDD
0.131084055	2.45819E-32	85028	SNHG12
0.130817249	3.28345E-32	55100	WDR70
0.130719185	3.65157E-32	5570	PKIB
0.130710915	3.68443E-32	29118	DDX25
0.130664304	3.87513E-32	284040	CDRT4
0.130580261	4.2441E-32	83881	MIXL1
0.130537912	4.44305E-32	8031	NCOA4
0.13050662	4.59595E-32	83853	ROPN1L
0.130506337	4.59737E-32	554	AVPR2
0.130477265	4.74415E-32	148137	C19orf55
0.130472064	4.77091E-32	89866	SEC16B
0.130465567	4.80449E-32	27004	TCL6
0.130419418	5.05023E-32	80173	IFT74
0.130403534	5.13768E-32	2697	GJA1
0.130370468	5.3245E-32	63035	BCORL1
0.130352557	5.42845E-32	401176	RP11-19O2.2
0.13029924	5.75017E-32	337	APOA4
0.130194873	6.43567E-32	2805	GOT1
0.130180195	6.53832E-32	5371	PML
0.130136728	6.85203E-32	115727	RASGRP4
0.130101815	7.11483E-32	57466	SFRS15
0.130100518	7.12472E-32	221	ALDH3B1
0.130083799	7.25435E-32	25807	RHBDD3
0.130061135	7.43357E-32	284412	LOC284412
0.129952118	8.35986E-32	3683	ITGAL
0.129950806	8.37168E-32	7791	ZYX
0.129889801	8.93965E-32	6338	SCNN1B
0.129814297	9.69623E-32	2980	GUCA2A
0.129801214	9.83365E-32	10942	PRSS21
0.129788026	9.97401E-32	3980	LIG3
0.129786074	9.99488E-32	7589	ZSCAN21
0.129784495	1.00119E-31	64847	SPATA20
0.129698306	1.09836E-31	10914	PAPOLA
0.129698128	1.09857E-31	1478	CSTF2
0.129663676	1.13999E-31	123624	AGBL1
0.129662737	1.14115E-31	10225	CD96
0.129631162	1.18049E-31	401994	OR14I1
0.129606649	1.21199E-31	2068	ERCC2
0.129595414	1.2267E-31	54915	YTHDF1
0.129556686	1.27876E-31	83551	TAAR8
0.129556134	1.27952E-31	1503	CTPS
0.129555762	1.28003E-31	10989	IMMT
0.129482299	1.385E-31	100131943	LOC100131943
0.129457876	1.42177E-31	3009	HIST1H1B
0.129440978	1.44775E-31	1046	CDX4
0.12943086	1.46354E-31	2212	FCGR2A
0.129404485	1.50553E-31	10394	PRG3
0.129365012	1.57058E-31	50484	RRM2B
0.129277542	1.72487E-31	55198	APPL2
0.12921828	1.83786E-31	79594	MUL1
0.12920092	1.87231E-31	10146	G3BP1
0.129150122	1.9769E-31	8939	FUBP3
0.129149005	1.97926E-31	5603	MAPK13
0.129146323	1.98497E-31	6003	RGS13
0.129128858	2.02237E-31	128611	ZNF831
0.129064322	2.16688E-31	114827	FHAD1
0.129050285	2.19961E-31	161424	C14orf21
0.129041195	2.22111E-31	147183	KRT25
0.129024565	2.26094E-31	7555	CNBP
0.12900804	2.30124E-31	65056	GPBP1
0.128969774	2.39724E-31	11148	HHLA2
0.128871992	2.66109E-31	347741	OTOP3
0.128821746	2.80771E-31	100101467	ZNF397OS
0.128789157	2.90707E-31	646424	SPINK8
0.128729254	3.09883E-31	55276	PGM2
0.128620699	3.4789E-31	92579	G6PC3
0.128616139	3.49585E-31	54557	SGTB
0.128495887	3.97341E-31	54437	SEMA5B
0.128463015	4.1148E-31	55111	PLEKHJ1
0.128462359	4.11765E-31	6567	SLC16A2
0.128449544	4.17419E-31	57701	KIAA1602

region-wise, subcortex					
Spearman's corr coef	p-val	p-val, SA	p-val, SA (BH- correction)	GenID	Gene
0.120978124	0.480691612	0.535	1	55784	MCTP2
0.120978124	0.480691612	0.509	1	84552	PARD6G
0.120720722	0.481631666	0.503	1	4137	MAPT
0.120720722	0.481631666	0.511	1	4831	NME2
0.120463312	0.482572794	0.527	1	4802	NFYC
0.120463312	0.482572794	0.533	1	7372	UMPS
0.120463312	0.482572794	0.516	1	11026	LILRA3
0.120463312	0.482572794	0.488	1	64089	SNX16
0.120463312	0.482572794	0.555	1	142679	DUSP19
0.120463312	0.482572794	0.532	1	284323	ZNF780A
0.120463312	0.482572794	0.522	1	541565	C8orf58
0.120205916	0.483514786	0.479	1	3005	H1F0
0.120205916	0.483514786	0.534	1	6124	RPL4
0.120205916	0.483514786	0.547	1	7044	LEFTY2
0.120205916	0.483514786	0.505	1	80975	TMPRSS5
0.120205916	0.483514786	0.506	1	200634	KRTCAP3
0.120205916	0.483514786	0.501	1	414328	IDNK
0.119948514	0.484458208	0.515	1	1130	LYST
0.119948514	0.484458208	0.533	1	6902	TBCA
0.119948514	0.484458208	0.547	1	23549	DNPEP
0.119948514	0.484458208	0.543	1	23770	FKBP8
0.119948514	0.484458208	0.505	1	79954	NOL10
0.119691119	0.485402197	0.522	1	5146	PDE6C
0.119691119	0.485402197	0.533	1	25979	DHRS7B
0.119691119	0.485402197	0.512	1	80179	MYO19
0.119691119	0.485402197	0.522	1	644246	AC217773.2
0.119433716	0.486347079	0.535	1	394	ARHGAP5
0.119433716	0.486347079	0.517	1	23558	WBP2
0.119433716	0.486347079	0.514	1	29965	C16orf5
0.119433716	0.486347079	0.541	1	80333	KCNIP4
0.119433716	0.486347079	0.513	1	84749	USP30
0.119433716	0.486347079	0.521	1	150568	AC013402.2
0.119176313	0.487293005	0.537	1	9521	EEF1E1
0.119176313	0.487293005	0.52	1	10840	ALDH1L1
0.119176313	0.487293005	0.538	1	23597	ACOT9
0.119176313	0.487293005	0.545	1	27247	NFU1
0.119176313	0.487293005	0.509	1	55554	KLK15
0.118918918	0.488239825	0.517	1	1622	DBI
0.118918918	0.488239825	0.549	1	7561	ZNF14
0.118918918	0.488239825	0.523	1	10384	BTN3A3
0.118918918	0.488239825	0.524	1	80727	TTYH3
0.118918918	0.488239825	0.481	1	90120	C9ORF69
0.118918918	0.488239825	0.517	1	90987	ZNF251
0.118918918	0.488239825	0.53	1	389332	LOC389332
0.118918918	0.488239825	0.533	1	692312	PPAN-P2RY11
0.118661515	0.489188045	0.52	1	495	ATP4A
0.118661515	0.489188045	0.541	1	6208	RPS14
0.118661515	0.489188045	0.507	1	9555	H2AFY
0.118661515	0.489188045	0.51	1	51527	C14orf129
0.118661515	0.489188045	0.511	1	57654	KIAA1530
0.118661515	0.489188045	0.528	1	89872	AQP10
0.118661515	0.489188045	0.516	1	282973	JAKMIP3
0.118661515	0.489188045	0.525	1	342667	STAC2
0.11840412	0.490136832	0.546	1	1069	CETN2
0.11840412	0.490136832	0.549	1	2091	AC005393.1
0.11840412	0.490136832	0.51	1	60496	AASDHPPT
0.118146718	0.491086543	0.513	1	26769	SNORD81
0.118146718	0.491086543	0.508	1	128864	C20orf144
0.118146718	0.491086543	0.506	1	130535	KCTD18
0.118146718	0.491086543	0.525	1	140823	ROMO1
0.117889315	0.492037207	0.544	1	688	KLF5
0.117889315	0.492037207	0.514	1	1329	COX5B
0.117889315	0.492037207	0.532	1	374887	YJEFN3
0.11763192	0.492989242	0.517	1	5192	PEX10
0.11763192	0.492989242	0.519	1	26166	RGS22
0.11763192	0.492989242	0.521	1	64837	KLC2
0.11763192	0.492989242	0.523	1	94233	OPN4
0.11763192	0.492989242	0.528	1	219873	OR10S1
0.11763192	0.492989242	0.536	1	401533	LOC401533
0.117374517	0.493941814	0.505	1	6827	SUPT4H1
0.117374517	0.493941814	0.52	1	8794	TNFRSF10C
0.117374517	0.493941814	0.54	1	440387	CTRB2
0.117117114	0.494895399	0.522	1	818	CAMK2G
0.117117114	0.494895399	0.563	1	6641	SNTB1
0.117117114	0.494895399	0.533	1	8366	HIST1H4B
0.117117114	0.494895399	0.522	1	10492	SYNCRIP
0.117117114	0.494895399	0.518	1	10982	MAPRE2
0.117117114	0.494895399	0.55	1	54884	RETSAT
0.117117114	0.494895399	0.523	1	55454	CSGALNACT2

voxel-wise, subcortex			
Spearman's corr coef	p-val	GenID	Gene
0.128439233	4.22026E-31	139604	MAGEB16
0.128383145	4.47969E-31	345456	PFN3
0.128372461	4.53085E-31	84063	KIRREL2
0.128342092	4.67953E-31	25854	FAM149A
0.128317863	4.80165E-31	10844	TUBGCP2
0.128247738	5.17298E-31	79758	DHRS12
0.128237709	5.22838E-31	80835	TAS1R1
0.128127292	5.8786E-31	54664	TMEM106B
0.128055796	6.34175E-31	9415	FADS2
0.128042415	6.4325E-31	6356	CCL11
0.128038049	6.46234E-31	348932	SLC6A18
0.127949268	7.10008E-31	644353	ZCCHC18
0.127907574	7.42068E-31	162655	ZNF519
0.127895415	7.51694E-31	2940	GSTA3
0.127845451	7.92531E-31	10778	ZNF271
0.127787039	8.43077E-31	83637	ZMIZ2
0.127749518	8.77218E-31	80021	TMEM62
0.127735585	8.90264E-31	4593	MUSK
0.127690077	9.34158E-31	4250	SCGB2A2
0.127673745	9.50415E-31	84572	GNPTG
0.127588853	1.03965E-30	79007	DBNDD1
0.127495438	1.14746E-30	50805	IRX4
0.127458662	1.19286E-30	94134	ARHGAP12
0.127309054	1.3968E-30	353134	LCE1D
0.127289653	1.42567E-30	92104	TTC30A
0.127280354	1.43971E-30	656	BMP8B
0.127268851	1.45727E-30	136541	PRSS58
0.127252132	1.48317E-30	4025	LPO
0.127193719	1.57732E-30	2181	ACSL3
0.127163723	1.62792E-30	51121	RPL26L1
0.127139941	1.66924E-30	57544	TXNDC16
0.127098799	1.74309E-30	26298	EHF
0.127089337	1.76052E-30	100038246	TLX1NB
0.127087668	1.76365E-30	54097	FAM3B
0.126999632	1.93477E-30	51754	TMEM8B
0.12695004	2.03834E-30	6369	CCL24
0.126945108	2.04892E-30	645752	LOC645752
0.126783147	2.42895E-30	2562	GABRB3
0.126746386	2.52453E-30	5291	PIK3CB
0.126687601	2.68512E-30	64778	FNDC3B
0.126641065	2.81942E-30	84068	SLC10A7
0.126532599	3.15892E-30	283576	ZDHHC22
0.126521274	3.19661E-30	57037	ANKMY2
0.12649481	3.28644E-30	10477	UBE2E3
0.12647289	3.36276E-30	11346	SYNPO
0.126458496	3.41384E-30	8634	RTCD1
0.126432955	3.50637E-30	80207	OPA3
0.126365259	3.76379E-30	117	ADCYAP1R1
0.126362473	3.77477E-30	23019	CNOT1
0.126360714	3.78174E-30	8816	DCAF5
0.126333311	3.89176E-30	8821	INPP4B
0.126293287	4.05808E-30	57862	ZNF410
0.126291335	4.06638E-30	55008	HERC6
0.126258925	4.20652E-30	646627	LOC646627
0.126209795	4.42813E-30	10369	CACNG2
0.126209423	4.42991E-30	9340	GLP2R
0.126175433	4.59007E-30	741	ZNHIT2
0.126174599	4.59407E-30	54954	FAM120C
0.12616235	4.65318E-30	64951	MRPS24
0.125964075	5.72307E-30	128954	GAB4
0.125931948	5.91807E-30	9450	LY86
0.125885785	6.20978E-30	51257	MARCHF2
0.125822544	6.63281E-30	56659	KCNK13
0.125816137	6.67725E-30	26999	CYFIP2
0.125747696	7.17045E-30	1652	DDT
0.125744268	7.19609E-30	5885	RAD21
0.125681862	7.67902E-30	54805	CNNM2
0.125574321	8.58785E-30	10492	SYNCRIP
0.125567168	8.65205E-30	64130	LIN7B
0.125510246	9.1793E-30	2213	FCGR2B
0.125505418	9.22542E-30	5030	P2RY4
0.12549223	9.35269E-30	6341	SCO1
0.125436038	9.91482E-30	2039	EPB49
0.12538524	1.04517E-29	7743	ZNF189
0.125382736	1.04789E-29	1534	CYB561
0.125231639	1.22572E-29	23460	ABCA6
0.12506941	1.45006E-29	492307	C8orf22
0.125030592	1.50952E-29	8831	SYNGAP1
0.124982484	1.58658E-29	246181	AKR7L

region-wise, subcortex					
Spearman's corr coef	p-val	p-val, SA	p-val, SA (BH- correction)	GenID	Gene
0.117117114	0.494895399	0.534	1	375298	CERKL
0.116859712	0.495849818	0.521	1	5884	RAD17
0.116859712	0.495849818	0.495	1	23408	SIRT5
0.116602309	0.49680528	0.558	1	5652	PRSS8
0.116602309	0.49680528	0.517	1	23352	UBR4
0.116602309	0.49680528	0.535	1	55249	YY1AP1
0.116602309	0.49680528	0.529	1	58528	RRAGD
0.116602309	0.49680528	0.571	1	79575	ABHD8
0.116344914	0.497762054	0.521	1	1857	DVL3
0.116344914	0.497762054	0.537	1	2553	GABPB1
0.116344914	0.497762054	0.518	1	10084	PQBP1
0.116344914	0.497762054	0.557	1	10280	SIGMAR1
0.116344914	0.497762054	0.518	1	51422	PRKAG2
0.116344914	0.497762054	0.542	1	440132	LINC00545
0.116087511	0.498719394	0.543	1	6949	TCOF1
0.116087511	0.498719394	0.518	1	57062	DDX24
0.116087511	0.498719394	0.524	1	440498	AC090360.1
0.115830116	0.499677658	0.525	1	55009	C19orf24
0.115830116	0.499677658	0.517	1	79744	ZNF419
0.115830116	0.499677658	0.548	1	91687	CENPL
0.115830116	0.499677658	0.538	1	643446	RNPS1P1
0.115830116	0.499677658	0.534	1	653073	AC120045.5
0.115830116	0.499677658	0.543	1	732253	RP11-535K1.1
0.115572713	0.500636876	0.536	1	8347	HIST1H2BC
0.115572713	0.500636876	0.53	1	100287016	DSTNP3
0.115315311	0.501597464	0.512	1	2917	GRM7
0.115315311	0.501597464	0.538	1	55090	MED9
0.115315311	0.501597464	0.562	1	283143	LOC283143
0.115057915	0.502558589	0.551	1	1725	DHPS
0.115057915	0.502558589	0.548	1	4952	OCRL
0.115057915	0.502558589	0.546	1	5728	PTEN
0.115057915	0.502558589	0.576	1	10980	COPS6
0.115057915	0.502558589	0.54	1	26033	ATRNL1
0.115057915	0.502558589	0.561	1	165082	GPR113
0.115057915	0.502558589	0.538	1	284904	SEC14L4
0.114800513	0.503520608	0.535	1	1230	CCR1
0.114800513	0.503520608	0.522	1	2116	ETV2
0.114800513	0.503520608	0.536	1	4359	MPZ
0.114800513	0.503520608	0.533	1	7743	ZNF189
0.114800513	0.503520608	0.55	1	145567	TTC7B
0.114543118	0.504483581	0.54	1	1870	E2F2
0.114543118	0.504483581	0.555	1	2997	GYS1
0.114543118	0.504483581	0.524	1	5452	POU2F2
0.114543118	0.504483581	0.524	1	23612	PHLDA3
0.114543118	0.504483581	0.519	1	57523	KIAA1305
0.114543118	0.504483581	0.551	1	125170	SMCR7
0.114543118	0.504483581	0.545	1	253152	EPHX4
0.114543118	0.504483581	0.542	1	360132	FKBP9L
0.114543118	0.504483581	0.561	1	645811	CCDC154
0.114285715	0.505447924	0.543	1	2584	GALK1
0.114285715	0.505447924	0.527	1	64600	PLA2G2F
0.114285715	0.505447924	0.549	1	65266	WNK4
0.114285715	0.505447924	0.569	1	152217	LOC152217
0.114028312	0.506412804	0.524	1	2673	GFPT1
0.114028312	0.506412804	0.536	1	6262	RYR2
0.114028312	0.506412804	0.527	1	8664	EIF3D
0.114028312	0.506412804	0.553	1	9194	SLC16A7
0.114028312	0.506412804	0.533	1	134266	GRPEL2
0.114028312	0.506412804	0.556	1	163589	TDRD5
0.113770917	0.507378578	0.53	1	6632	AC106037.2
0.113770917	0.507378578	0.542	1	7371	UCK2
0.113770917	0.507378578	0.556	1	22871	NLGN1
0.113770917	0.507378578	0.567	1	23344	ESYT1
0.113770917	0.507378578	0.544	1	56983	KTELC1
0.113770917	0.507378578	0.559	1	131377	KBTBD5
0.113513507	0.508345306	0.54	1	1384	CRAT
0.113513507	0.508345306	0.556	1	57097	PARP11
0.113513507	0.508345306	0.561	1	65055	REEP1
0.113256112	0.509312928	0.537	1	25775	C22orf24
0.113256112	0.509312928	0.544	1	56181	FAM54B
0.112998709	0.51028198	0.559	1	3641	INSL4
0.112998709	0.51028198	0.526	1	9472	AKAP6
0.112998709	0.51028198	0.546	1	84885	ZDHHC12
0.112998709	0.51028198	0.531	1	388695	LYSMD1
0.112741306	0.511251509	0.515	1	2043	EPHA4
0.112741306	0.511251509	0.534	1	2819	GPD1
0.112741306	0.511251509	0.542	1	5216	PFN1
0.112741306	0.511251509	0.567	1	6165	RPL35A
0.112741306	0.511251509	0.553	1	10742	RAI2

voxel-wise, subcortex			
Spearman's corr coef	p-val	GenID	Gene
0.124956667	1.62952E-29	2249	FGF4
0.124947563	1.64495E-29	56894	AGPAT3
0.124737598	2.04357E-29	1366	CLDN7
0.124613807	2.32208E-29	10497	UNC13B
0.12448974	2.63896E-29	284459	AC008806.1
0.124371983	2.97922E-29	6632	AC106037.2
0.124359168	3.01882E-29	3091	HIF1A
0.124342263	3.07179E-29	790955	C11orf83
0.124241784	3.40633E-29	134147	CMBL
0.124207236	3.52955E-29	388610	TRNP1
0.124145202	3.76189E-29	54718	BTN2A3
0.124105364	3.91909E-29	961	CD47
0.124024667	4.25772E-29	55616	ASAP3
0.123991415	4.40552E-29	64925	CCDC71
0.123979434	4.46005E-29	25782	RAB3GAP2
0.123979345	4.46045E-29	100130958	AC009139.1
0.123953432	4.5806E-29	3313	HSPA9
0.123933837	4.67364E-29	55425	KIAA1704
0.123923257	4.72464E-29	89886	SLAMF9
0.123911649	4.78121E-29	51806	CALML5
0.123887777	4.89972E-29	7054	TH
0.123883411	4.92169E-29	3664	IRF6
0.12382862	5.20607E-29	283460	HNF1A-AS1
0.123814598	5.28146E-29	22845	DOLK
0.123814046	5.28445E-29	113179	ADAT3
0.123767242	5.54405E-29	255743	NPNT
0.123757951	5.59708E-29	92737	DNER
0.123717554	5.83347E-29	26056	RAB11FIP5
0.123661555	6.17767E-29	3428	IFI16
0.123595811	6.60765E-29	8559	PRPF18
0.123594135	6.61889E-29	91978	C19orf20
0.123524114	7.11027E-29	27136	MORC1
0.123453908	7.6392E-29	5023	P2RX1
0.12331944	8.76368E-29	151300	LINC00608
0.123313121	8.82043E-29	221823	PRPS1L1
0.12321803	9.71895E-29	150737	TTC30B
0.123208374	9.81509E-29	50674	NEUROG3
0.123199828	9.90103E-29	257101	ZNF683
0.123135097	1.05765E-28	63951	DMRTA1
0.123121448	1.07246E-28	441452	FAM75C1
0.123091549	1.10562E-28	645181	LOC645181
0.123076871	1.12229E-28	55972	SLC25A40
0.123062015	1.13939E-28	112479	ERI2
0.123038799	1.16667E-28	2258	FGF13
0.123024128	1.18422E-28	90850	ZNF598
0.122994684	1.22026E-28	251	ALPPL2
0.122921228	1.31497E-28	6923	TCEB2
0.122894481	1.35124E-28	347475	AC004409.1
0.122743301	1.57568E-28	83737	ITCH
0.122685075	1.67169E-28	4741	NEFM
0.122669376	1.69851E-28	284129	SLC26A11
0.122595176	1.83136E-28	79657	RPAP3
0.122587748	1.84523E-28	83897	KRTAP3-2
0.122558124	1.90151E-28	22824	HSPA4L
0.122514293	1.98796E-28	6320	CLEC11A
0.122507699	2.00127E-28	259266	ASPM
0.122505933	2.00488E-28	220	ALDH1A3
0.122491628	2.03414E-28	54497	HEATR5B
0.122444458	2.13378E-28	283106	CSNK2A1P
0.122430339	2.1645E-28	283518	KCNRG
0.122407399	2.21537E-28	8740	TNFSF14
0.122389108	2.25684E-28	285362	SUMF1
0.122337192	2.37865E-28	8517	IKBKG
0.122276552	2.52922E-28	613209	DEFB136
0.122238755	2.62784E-28	23516	SLC39A14
0.122194737	2.74746E-28	161753	ODF3L1
0.122125	2.94813E-28	643637	GLYATL1P4
0.122079305	3.08744E-28	245934	DEFB121
0.12203566	3.22659E-28	10296	MAEA
0.122008726	3.31557E-28	8505	PARG
0.121953934	3.50407E-28	5929	RBBP5
0.121899985	3.70014E-28	51122	COMMD2
0.121815473	4.02929E-28	84109	QRFPR
0.121809065	4.05538E-28	7423	VEGFB
0.121700041	4.52618E-28	57496	MKL2
0.1215197	5.42692E-28	11277	TREX1
0.121515609	5.4493E-28	32	ACACB
0.121454135	5.79666E-28	641384	TMEM75
0.121442616	5.86417E-28	10473	HMGN4

region-wise, subcortex					
Spearman's corr coef	p-val	p-val, SA	p-val, SA (BH- correction)	GenID	Gene
0.112741306	0.511251509	0.531	1	28991	COMMD5
0.112741306	0.511251509	0.539	1	79937	CNTNAP3
0.112741306	0.511251509	0.566	1	143888	KDELC2
0.112741306	0.511251509	0.565	1	282679	AQP11
0.112483911	0.512221932	0.516	1	11108	PRDM4
0.112483911	0.512221932	0.542	1	64746	ACBD3
0.112226509	0.513193309	0.56	1	669	BPGM
0.112226509	0.513193309	0.575	1	51225	ABI3
0.112226509	0.513193309	0.55	1	112840	WDR89
0.112226509	0.513193309	0.552	1	283298	OLFML1
0.112226509	0.513193309	0.512	1	440515	ZNF506
0.111969113	0.514166057	0.533	1	572	BAD
0.111969113	0.514166057	0.576	1	7225	TRPC6
0.111969113	0.514166057	0.557	1	10206	TRIM13
0.111969113	0.514166057	0.546	1	56262	LRRC8A
0.111969113	0.514166057	0.593	1	284441	LOC284441
0.111711711	0.515139282	0.578	1	4320	MMP11
0.111711711	0.515139282	0.559	1	9622	KLK4
0.111711711	0.515139282	0.563	1	51449	PCYOX1
0.111711711	0.515139282	0.584	1	57718	PPP4R4
0.111711711	0.515139282	0.543	1	89978	ATPBD4
0.111711711	0.515139282	0.556	1	153527	ZMAT2
0.111454308	0.51611346	0.548	1	22836	RHOBTB3
0.111196913	0.517088532	0.571	1	9736	USP34
0.111196913	0.517088532	0.538	1	57180	ACTR3B
0.111196913	0.517088532	0.532	1	124936	CYB5D2
0.11093951	0.518064916	0.573	1	6649	SOD3
0.11093951	0.518064916	0.554	1	6814	STXBP3
0.11093951	0.518064916	0.535	1	9839	ZEB2
0.11093951	0.518064916	0.548	1	11056	DDX52
0.11093951	0.518064916	0.53	1	23161	SNX13
0.11093951	0.518064916	0.551	1	84233	TMEM126A
0.110682115	0.519041836	0.562	1	29124	LGALS13
0.110682115	0.519041836	0.557	1	81553	FAM49A
0.110682115	0.519041836	0.564	1	146456	TMED6
0.110682115	0.519041836	0.538	1	641384	TMEM75
0.110424712	0.52001977	0.545	1	5610	EIF2AK2
0.110424712	0.52001977	0.548	1	8492	PRSS12
0.110424712	0.52001977	0.573	1	10890	RAB10
0.110424712	0.52001977	0.521	1	26160	IFT172
0.110424712	0.52001977	0.575	1	170961	ANKRD24
0.110424712	0.52001977	0.553	1	219482	OR5M3
0.11016731	0.520998478	0.564	1	9951	HS3ST4
0.11016731	0.520998478	0.55	1	131405	TRIM71
0.11016731	0.520998478	0.582	1	162655	ZNF519
0.11016731	0.520998478	0.553	1	374973	ATPAF1-AS1
0.109909907	0.52197814	0.551	1	2395	FXN
0.109909907	0.52197814	0.559	1	6744	SSFA2
0.109909907	0.52197814	0.554	1	7442	TRPV1
0.109909907	0.52197814	0.56	1	9348	NDST3
0.109652504	0.522959173	0.543	1	9852	EPM2AIP1
0.109652504	0.522959173	0.543	1	94121	SYTL4
0.109395109	0.523940682	0.571	1	3430	IFI35
0.109395109	0.523940682	0.557	1	5832	ALDH18A1
0.109395109	0.523940682	0.561	1	6838	SURF6
0.109395109	0.523940682	0.561	1	28982	FLVCR1
0.109395109	0.523940682	0.571	1	79924	ADM2
0.109395109	0.523940682	0.551	1	127391	TMCO2
0.109395109	0.523940682	0.573	1	403239	OR2T27
0.109137706	0.524923146	0.565	1	1124	CHN2
0.109137706	0.524923146	0.562	1	1995	ELAVL3
0.109137706	0.524923146	0.543	1	51203	NUSAP1
0.109137706	0.524923146	0.59	1	55170	PRMT6
0.108880304	0.525906384	0.566	1	27154	BRPF3
0.108880304	0.525906384	0.574	1	56120	PCDHGB8P
0.108880304	0.525906384	0.559	1	84125	LRRIQ1
0.108880304	0.525906384	0.554	1	127002	ATXN7L2
0.108880304	0.525906384	0.556	1	644890	MEIG1
0.108622909	0.526891112	0.534	1	7919	BAT1
0.108622909	0.526891112	0.557	1	84874	ZNF514
0.108622909	0.526891112	0.563	1	116115	ZNF526
0.108622909	0.526891112	0.572	1	118460	EXOSC6
0.108622909	0.526891112	0.567	1	124446	TMEM219
0.108365506	0.527876258	0.556	1	5566	PRKACA
0.108365506	0.527876258	0.576	1	9495	AKAP5
0.108365506	0.527876258	0.575	1	51601	LIPT1
0.108365506	0.527876258	0.541	1	55643	BTBD2
0.108365506	0.527876258	0.534	1	64240	ABCG5
0.108365506	0.527876258	0.589	1	93594	WDR67

voxel-wise, subcortex			
Spearman's corr coef	p-val	GenID	Gene
0.121437326	5.89537E-28	23305	ACSL6
0.121382251	6.23082E-28	345645	PSMC1P4
0.121103846	8.23841E-28	353145	LCE3E
0.121012367	9.02896E-28	6448	SGSH
0.120974757	9.37547E-28	122740	OR4K14
0.120973364	9.38852E-28	51650	MRPS33
0.120872788	1.03825E-27	5994	RFXAP
0.120867409	1.04385E-27	6702	SPRR2C
0.120858863	1.05282E-27	10178	ODZ1
0.12083295	1.08045E-27	9141	PDCD5
0.120817818	1.09692E-27	339302	CPLX4
0.120781042	1.13798E-27	54832	VPS13C
0.120749094	1.17489E-27	9894	TELO2
0.120743707	1.18124E-27	10223	GPA33
0.120732561	1.19446E-27	347404	LANCL3
0.120714359	1.21638E-27	155061	ZNF746
0.12069384	1.24157E-27	64221	ROBO3
0.120649636	1.29759E-27	2741	GLRA1
0.120614998	1.34322E-27	10131	TRAP1
0.120596513	1.36823E-27	10350	ABCA9
0.120575339	1.39746E-27	5789	PTPRD
0.120560944	1.41766E-27	138307	LCN8
0.120546088	1.43884E-27	165215	FAM171B
0.120471239	1.55031E-27	84437	KIAA1826
0.120444588	1.59206E-27	83858	ATAD3B
0.120386913	1.68622E-27	1310	COL19A1
0.120358869	1.73397E-27	150280	HORMAD2
0.120348379	1.75217E-27	26095	PTPN20B
0.12033473	1.77615E-27	55331	ACER3
0.120238148	1.95535E-27	137392	FAM92A1
0.120226353	1.97843E-27	80034	CSRNP3
0.120161161	2.11095E-27	23432	GPR161
0.120141938	2.15166E-27	84290	CAPNS2
0.120112032	2.21656E-27	51303	FKBP11
0.120069407	2.31249E-27	158314	LINC00475
0.120047957	2.36229E-27	201725	C4orf46
0.120022327	2.42316E-27	114900	C1QTNF4
0.120021679	2.42472E-27	6991	TCTE3
0.119996786	2.4854E-27	23219	FBXO28
0.119967073	2.55983E-27	400830	DEFB132
0.119966887	2.56027E-27	133418	EMB
0.119727567	3.24593E-27	56905	C15orf39
0.119725898	3.25133E-27	112487	C14orf126
0.119712152	3.29587E-27	63940	GPSM3
0.119617335	3.62025E-27	143098	MPP7
0.119595513	3.6993E-27	221357	GSTA5
0.119414702	4.42337E-27	7442	TRPV1
0.119392321	4.52227E-27	89792	GAL3ST3
0.119388238	4.54053E-27	4061	LY6E
0.119377002	4.5912E-27	57532	NUFIP2
0.11937096	4.61865E-27	10748	RP11-904M10.1
0.119348861	4.72059E-27	127623	OR2B11
0.119319141	4.8611E-27	51545	ZNF581
0.119314872	4.88165E-27	55666	NPLOC4
0.1192794	5.0556E-27	132200	C3orf49
0.119269736	5.10406E-27	284827	KRTAP13-4
0.119229063	5.31299E-27	163590	TOR1AIP2
0.119203158	5.45047E-27	80851	SH3BP5L
0.119061716	6.26574E-27	189	AGXT
0.119028009	6.47722E-27	1385	CREB1
0.118947588	7.01085E-27	9535	GMFG
0.118921585	7.19252E-27	3196	TLX2
0.118912205	7.25916E-27	23479	ISCU
0.118823983	7.91705E-27	79170	ATAD4
0.118718207	8.78412E-27	80045	GPR157
0.118656449	9.33323E-27	1955	MEGF9
0.118628591	9.59195E-27	256148	OR4S1
0.118604444	9.82193E-27	23786	BCL2L13
0.118595622	9.90728E-27	6231	RPS26P20
0.11857371	1.01225E-26	55638	AC079061.1
0.118487254	1.10181E-26	5567	PRKACB
0.118453167	1.13924E-26	375057	C1orf95
0.118432552	1.16248E-26	55958	KLHL9
0.118332349	1.28233E-26	405753	DUOXA2
0.118328452	1.28724E-26	6990	DYNLT3
0.11831136	1.30895E-26	3667	IRS1
0.118227415	1.42099E-26	729409	LOC729409
0.118216828	1.43577E-26	8857	FCGBP
0.118156463	1.52305E-26	124460	SNX20

region-wise, subcortex					
Spearman's corr coef	p-val	p-val, SA	p-val, SA (BH- correction)	GenID	Gene
0.108365506	0.527876258	0.564	1	134549	SHROOM1
0.108108111	0.528862298	0.554	1	11340	EXOSC8
0.108108111	0.528862298	0.564	1	23527	ACAP2
0.108108111	0.528862298	0.535	1	26229	B3GAT3
0.107850708	0.529849291	0.58	1	2691	GHRH
0.107850708	0.529849291	0.545	1	55760	DHX32
0.107850708	0.529849291	0.573	1	57650	KIAA1524
0.107593305	0.530837536	0.572	1	5204	PFDN5
0.107593305	0.530837536	0.538	1	10905	MAN1A2
0.107593305	0.530837536	0.537	1	29882	ANAPC2
0.107593305	0.530837536	0.548	1	84962	JUB
0.107593305	0.530837536	0.537	1	161742	SPRED1
0.107593305	0.530837536	0.547	1	339834	CCDC36
0.10733591	0.531826317	0.553	1	863	CBFA2T3
0.10733591	0.531826317	0.546	1	130940	CCDC148
0.107078508	0.532815993	0.564	1	942	CD86
0.107078508	0.532815993	0.589	1	4485	MST1
0.107078508	0.532815993	0.571	1	5693	PSMB5
0.107078508	0.532815993	0.559	1	7189	TRAF6
0.107078508	0.532815993	0.581	1	26173	INTS1
0.107078508	0.532815993	0.574	1	30815	ST6GALNAC6
0.107078508	0.532815993	0.589	1	51168	MYO15A
0.107078508	0.532815993	0.553	1	57455	REXO1
0.107078508	0.532815993	0.58	1	89765	RSPH1
0.106821112	0.533806503	0.571	1	6786	STIM1
0.106821112	0.533806503	0.577	1	6811	STX5
0.106821112	0.533806503	0.561	1	6929	TCF3
0.106821112	0.533806503	0.588	1	340591	CA5BP
0.106563702	0.534798026	0.546	1	3600	IL15
0.1063063	0.535790801	0.55	1	5459	POU4F3
0.1063063	0.535790801	0.564	1	7049	TGFBR3
0.1063063	0.535790801	0.569	1	7694	ZNF135
0.106048904	0.536784053	0.566	1	5689	PSMB1
0.106048904	0.536784053	0.579	1	11184	MAP4K1
0.106048904	0.536784053	0.549	1	54497	HEATR5B
0.106048904	0.536784053	0.563	1	56915	EXOSC5
0.106048904	0.536784053	0.551	1	339541	C1orf228
0.106048904	0.536784053	0.579	1	375346	TMEM110
0.105791502	0.537778258	0.553	1	1620	DBC1
0.105791502	0.537778258	0.58	1	4916	NTRK3
0.105791502	0.537778258	0.578	1	7520	XRCC5
0.105791502	0.537778258	0.552	1	10861	SLC26A1
0.105791502	0.537778258	0.571	1	10972	TMED10
0.105791502	0.537778258	0.568	1	26502	NARF
0.105791502	0.537778258	0.579	1	27439	CECR6
0.105791502	0.537778258	0.555	1	55893	ZNF395
0.105791502	0.537778258	0.558	1	79753	SNIP1
0.105791502	0.537778258	0.563	1	140460	ASB7
0.105791502	0.537778258	0.57	1	284297	AC008735.3
0.105791502	0.537778258	0.6	1	390259	BSX
0.105534099	0.538773239	0.564	1	998	CDC42
0.105534099	0.538773239	0.58	1	3503	IGHG4
0.105534099	0.538773239	0.563	1	147138	TMC8
0.105534099	0.538773239	0.554	1	648987	AC008875.1
0.105276704	0.53976965	0.592	1	1155	TBCB
0.105276704	0.53976965	0.603	1	3046	HBE1
0.105276704	0.53976965	0.598	1	79039	DDX54
0.105276704	0.53976965	0.604	1	129787	TMEM18
0.105276704	0.53976965	0.587	1	387820	LOC387820
0.105019301	0.540766478	0.597	1	54596	L1TD1
0.105019301	0.540766478	0.574	1	155435	RBM33
0.104761906	0.541764259	0.566	1	4052	LTBP1
0.104761906	0.541764259	0.56	1	5521	PPP2R2B
0.104761906	0.541764259	0.564	1	57089	ENTPD7
0.104761906	0.541764259	0.556	1	57402	S100A14
0.104761906	0.541764259	0.579	1	100240735	LOC100240735
0.104761906	0.541764259	0.567	1	100288570	AC108938.1
0.104504503	0.542762816	0.582	1	7286	TUFT1
0.104504503	0.542762816	0.58	1	84312	BRMS1L
0.104247101	0.543762803	0.579	1	2558	GABRA5
0.104247101	0.543762803	0.576	1	146330	FBXL16
0.104247101	0.543762803	0.591	1	440275	EIF2AK4
0.104247101	0.543762803	0.568	1	730098	RP11-195F19.5
0.103989705	0.544763148	0.583	1	6430	SFRS5
0.103989705	0.544763148	0.536	1	10273	STUB1
0.103989705	0.544763148	0.573	1	55671	SMEK1
0.103989705	0.544763148	0.589	1	441273	SPDYE2
0.103732303	0.545764446	0.599	1	3386	ICAM4
0.103732303	0.545764446	0.596	1	6623	SNCG

voxel-wise, subcortex			
Spearman's corr coef	p-val	GenID	Gene
0.118096009	1.61574E-26	143425	SYT9
0.118057281	1.67803E-26	55296	TBC1D19
0.118038893	1.70842E-26	374986	FAM73A
0.118028119	1.72651E-26	55262	C7orf43
0.118008524	1.75984E-26	283685	AC100756.1
0.118000261	1.77408E-26	84752	B3GNT9
0.117997937	1.77812E-26	137797	LYPD2
0.117992833	1.787E-26	284098	PIGW
0.117932469	1.89539E-26	79722	ANKRD55
0.117918633	1.92117E-26	4076	CAPRIN1
0.117861427	2.03137E-26	322	APBB1
0.117845178	2.06382E-26	8748	ADAM20
0.117736615	2.29409E-26	284716	RIMKLA
0.117693707	2.39198E-26	10257	ABCC4
0.117692783	2.39411E-26	115861	NXNL1
0.117613569	2.58596E-26	140686	WFDC3
0.117561378	2.72063E-26	5165	PDK3
0.117498875	2.89106E-26	57211	GPR126
0.117469907	2.9736E-26	6879	TAF7
0.117443249	3.05164E-26	725	C4BPB
0.117438607	3.06544E-26	149986	LSM14B
0.117433965	3.07924E-26	165140	OXER1
0.117367655	3.28405E-26	9219	MTA2
0.117331252	3.40216E-26	23066	CAND2
0.117328286	3.41199E-26	344595	RP11-446H18.3
0.117320389	3.43825E-26	84647	PLA2G12B
0.117275722	3.59048E-26	1964	EIF1AX
0.11726848	3.61579E-26	92105	INTS4
0.116995819	4.70901E-26	28956	ROBLD3
0.11695125	4.91651E-26	51144	HSD17B12
0.116928957	5.02368E-26	145814	AC069029.1
0.116886429	5.23453E-26	150696	PROM2
0.116885588	5.23877E-26	317751	MESTIT1
0.116871193	5.31222E-26	53917	RAB24
0.116857454	5.38323E-26	3552	IL1A
0.116813064	5.6192E-26	5999	RGS4
0.116812043	5.62472E-26	375444	C5orf34
0.116670795	6.44658E-26	401021	LOC401021
0.116639592	6.64359E-26	2936	GSR
0.116639219	6.646E-26	124783	C17orf46
0.11661043	6.83318E-26	26121	PRPF31
0.116557121	7.19357E-26	55012	PPP2R3C
0.11655657	7.19739E-26	375033	PEAR1
0.116542079	7.29861E-26	56124	PCDHB12
0.116524808	7.42111E-26	1052	CEBPD
0.116522945	7.4345E-26	70	ACTC1
0.116510786	7.52211E-26	100144596	LINC00029
0.116431758	8.11707E-26	3014	H2AFX
0.116381511	8.51942E-26	23564	DDAH2
0.116365544	8.65135E-26	27283	TINAG
0.116358668	8.70888E-26	338651	AP006285.4
0.116256334	9.60963E-26	130271	PLEKHH2
0.116243333	9.73056E-26	4942	OAT
0.116185099	1.02907E-25	84902	CCDC123
0.116178416	1.0357E-25	57089	ENTPD7
0.116169408	1.0447E-25	401474	SAMD12
0.116133466	1.0814E-25	6820	SULT2B1
0.116124742	1.09051E-25	223075	CCDC129
0.11603494	1.18869E-25	6658	SOX3
0.115987301	1.24429E-25	2043	EPHA4
0.115983859	1.2484E-25	11012	KLK11
0.115952939	1.28598E-25	5279	PIGC
0.115919136	1.32834E-25	3857	KRT9
0.115913652	1.33534E-25	26098	C10orf137
0.115884028	1.3738E-25	146880	AC132812.1
0.115835555	1.43911E-25	6499	SKIV2L
0.115749374	1.56293E-25	9515	STXBP5L
0.115737863	1.58023E-25	51020	HDDC2
0.115716875	1.6123E-25	4221	MEN1
0.115638122	1.73847E-25	386675	KRTAP10-7
0.115633197	1.74667E-25	5228	PGF
0.115629576	1.75273E-25	441177	LINC00602
0.115556121	1.88024E-25	84700	MYO18B
0.115521021	1.94435E-25	64420	SUSD1
0.115482569	2.01709E-25	3047	HBG1
0.115389057	2.20547E-25	55212	BBS7
0.11532145	2.35239E-25	222008	VSTM2A
0.115297675	2.40633E-25	144423	GLT1D1
0.115274832	2.45932E-25	153657	TTC23L

region-wise, subcortex					
Spearman's corr coef	p-val	p-val, SA	p-val, SA (BH- correction)	GenID	Gene
0.103732303	0.545764446	0.554	1	79659	DYNC2H1
0.103732303	0.545764446	0.568	1	493829	TRIM72
0.103474908	0.546766579	0.605	1	1585	CYP11B2
0.103474908	0.546766579	0.569	1	4217	MAP3K5
0.103474908	0.546766579	0.557	1	5098	PCDHGC3
0.103474908	0.546766579	0.587	1	6489	ST8SIA1
0.103474908	0.546766579	0.559	1	8613	PPAP2B
0.103474908	0.546766579	0.566	1	10146	G3BP1
0.103474908	0.546766579	0.559	1	55086	CXorf57
0.103474908	0.546766579	0.62	1	58490	RPRD1B
0.103474908	0.546766579	0.583	1	388324	INCA1
0.103474908	0.546766579	0.588	1	399844	FLJ45445
0.103217505	0.547769547	0.573	1	8001	GLRA3
0.103217505	0.547769547	0.581	1	23125	CAMTA2
0.102960095	0.548773944	0.575	1	6309	SC5DL
0.102960095	0.548773944	0.586	1	10585	POMT1
0.102960095	0.548773944	0.583	1	10902	BRD8
0.102960095	0.548773944	0.582	1	140701	C20orf135
0.1027027	0.549778759	0.583	1	1399	CRKL
0.1027027	0.549778759	0.603	1	1438	CSF2RA
0.1027027	0.549778759	0.604	1	3837	KPNB1
0.102445297	0.550784349	0.634	1	9332	CD163
0.102445297	0.550784349	0.56	1	55635	DEPDC1
0.102187902	0.551790893	0.583	1	1826	DSCAM
0.102187902	0.551790893	0.605	1	10120	ACTR1B
0.102187902	0.551790893	0.607	1	53342	IL17D
0.102187902	0.551790893	0.576	1	84186	ZCCHC7
0.102187902	0.551790893	0.556	1	100049716	AC021054.1
0.101930499	0.552798748	0.602	1	4326	MMP17
0.101930499	0.552798748	0.568	1	6322	SCML1
0.101930499	0.552798748	0.56	1	10394	PRG3
0.101930499	0.552798748	0.582	1	11341	SCRG1
0.101930499	0.552798748	0.593	1	23252	OTUD3
0.101930499	0.552798748	0.578	1	147081	AC126544.1
0.101930499	0.552798748	0.563	1	389434	IYD
0.101673096	0.55380702	0.602	1	7580	ZNF32
0.101673096	0.55380702	0.589	1	8287	USP9Y
0.101673096	0.55380702	0.592	1	57048	PLSCR3
0.101673096	0.55380702	0.595	1	63893	UBE2O
0.101673096	0.55380702	0.582	1	65983	GRAMD3
0.101673096	0.55380702	0.604	1	84502	JPH4
0.101673096	0.55380702	0.593	1	92609	TIMM50
0.101673096	0.55380702	0.609	1	339105	AC135050.2
0.101673096	0.55380702	0.587	1	347240	KIF24
0.101415701	0.554816127	0.625	1	23190	UBXN4
0.101415701	0.554816127	0.597	1	88745	C6orf153
0.101415701	0.554816127	0.589	1	91653	BOC
0.101415701	0.554816127	0.585	1	139596	UPRT
0.101415701	0.554816127	0.6	1	196500	C12orf53
0.101158299	0.555826128	0.562	1	8087	FXR1
0.101158299	0.555826128	0.609	1	10123	ARL4C
0.101158299	0.555826128	0.58	1	84881	RPUSD4
0.101158299	0.555826128	0.569	1	89887	ZNF628
0.101158299	0.555826128	0.579	1	257397	MAP3K7IP3
0.100900903	0.556837559	0.563	1	2904	GRIN2B
0.100900903	0.556837559	0.573	1	5348	FXYD1
0.100900903	0.556837559	0.613	1	8843	HCAR3
0.100900903	0.556837559	0.563	1	60674	GAS5
0.100643501	0.557849288	0.585	1	801	CALM1
0.100643501	0.557849288	0.588	1	2745	GLRX
0.100643501	0.557849288	0.587	1	5685	PSMA4
0.100643501	0.557849288	0.603	1	9205	ZMYM5
0.100643501	0.557849288	0.578	1	10389	SCML2
0.100643501	0.557849288	0.571	1	28962	OSTM1
0.100643501	0.557849288	0.603	1	353149	TBC1D26
0.100386098	0.558861852	0.595	1	1659	DHX8
0.100386098	0.558861852	0.565	1	5316	PKNOX1
0.100386098	0.558861852	0.598	1	50837	TAS2R7
0.100386098	0.558861852	0.591	1	202559	KHDRBS2
0.100386098	0.558861852	0.583	1	401546	C9orf152
0.100386098	0.558861852	0.581	1	728769	CTD-2037K23.2
0.100128703	0.559875369	0.597	1	2873	GPS1
0.100128703	0.559875369	0.586	1	27185	DISC1
0.100128703	0.559875369	0.591	1	51816	CECR1
0.100128703	0.559875369	0.586	1	55665	URG4
0.100128703	0.559875369	0.599	1	126006	PCP2
0.0998713	0.560889661	0.622	1	84947	SERAC1
0.0998713	0.560889661	0.588	1	286753	TUSC5
0.099356495	0.562921405	0.618	1	2895	GRID2

voxel-wise, subcortex			
Spearman's corr coef	p-val	GenID	Gene
0.115244001	2.53265E-25	26257	NKX2-8
0.11518763	2.67238E-25	81619	TSPAN14
0.115180664	2.6902E-25	100131454	AC087392.3
0.115160421	2.74255E-25	59067	IL21
0.115116119	2.86071E-25	84317	CCDC115
0.115110546	2.87595E-25	54946	SLC41A3
0.115107946	2.88308E-25	83863	TTTY5
0.114962429	3.31113E-25	195814	SDR16C5
0.114908755	3.48442E-25	23207	PLEKHM2
0.114905313	3.49582E-25	4763	NF1
0.114900485	3.5119E-25	51184	GPN3
0.114894547	3.53178E-25	63875	MRPL17
0.114891849	3.54084E-25	65992	DDRGK1
0.114834271	3.73989E-25	79812	MMRN2
0.114815883	3.80572E-25	4201	MEA1
0.114789233	3.90326E-25	57095	C1orf128
0.114782639	3.92776E-25	56956	LHX9
0.114763044	4.0015E-25	5367	PMCH
0.114702217	4.23923E-25	23016	EXOSC7
0.114688285	4.29561E-25	219956	OR9Q1
0.114659868	4.41295E-25	113000	RPUSD1
0.114609815	4.62739E-25	56120	PCDHGB8P
0.114599884	4.67114E-25	5496	PPM1G
0.1145996	4.67242E-25	54463	FAM134B
0.114587992	4.72409E-25	283932	FBXL19-AS1
0.114580378	4.75829E-25	7161	TP73
0.114569888	4.80583E-25	4205	MEF2A
0.1145541	4.87827E-25	27085	MTBP
0.114513047	5.07167E-25	2565	GABRG1
0.114469685	5.28425E-25	10349	ABCA10
0.114442661	5.4211E-25	2786	GNG4
0.114391677	5.68897E-25	5083	PAX9
0.114389166	5.70255E-25	93650	ACPT
0.114384241	5.72918E-25	11266	DUSP12
0.114325739	6.05506E-25	51490	C9orf114
0.114322953	6.07099E-25	375449	MAST4
0.114316642	6.10733E-25	1757	SARDH
0.114299364	6.20788E-25	223	ALDH9A1
0.114298806	6.21114E-25	6132	RPL8
0.114241697	6.55554E-25	10599	SLCO1B1
0.114239559	6.56886E-25	4145	MATK
0.114218757	6.69918E-25	56478	EIF4ENIF1
0.114163317	7.0592E-25	100129482	ZNF37BP
0.114154398	7.11884E-25	7329	UBE2I
0.114132024	7.27079E-25	140596	DEFB104A
0.114128307	7.2964E-25	201266	SLC39A11
0.114125334	7.31688E-25	7464	CORO2A
0.114124037	7.32587E-25	85439	STON2
0.114111125	7.41565E-25	55337	C19orf66
0.114077784	7.65264E-25	81572	PDRG1
0.114012964	8.13497E-25	120224	TMEM45B
0.114012778	8.13644E-25	112970	KTI12
0.113920748	8.87365E-25	23304	UBR2
0.113859549	9.40007E-25	199720	GGN
0.113798536	9.95569E-25	245812	CNPY4
0.113784328	1.00896E-24	388695	LYSMD1
0.113757677	1.03459E-24	3140	MR1
0.113750525	1.04157E-24	57486	NLN
0.113747559	1.04448E-24	55223	TRIM62
0.113712639	1.07935E-24	219431	OR4S2
0.113687843	1.10481E-24	123103	KLHL33
0.113687471	1.10518E-24	23031	MAST3
0.113681436	1.11147E-24	7076	TIMP1
0.113664165	1.12966E-24	127733	UBXN10
0.113548823	1.25894E-24	6916	TBXAS1
0.113508701	1.30727E-24	5883	RAD9A
0.113469519	1.35623E-24	196872	RP11-477I4.3
0.113443695	1.3895E-24	6662	SOX9
0.113442674	1.39081E-24	140691	TRIM69
0.113425128	1.4139E-24	64864	RFX7
0.113405719	1.43986E-24	11164	NUDT5
0.113389187	1.46237E-24	55863	TMEM126B
0.113356128	1.50838E-24	83715	ESPN
0.113203824	1.73968E-24	9997	SCO2
0.113165654	1.80294E-24	10783	NEK6
0.113148011	1.83297E-24	1441	CSF3R
0.113077432	1.95803E-24	834	CASP1
0.113029331	2.04808E-24	5052	PRDX1
0.113027565	2.05148E-24	56848	SPHK2

region-wise, subcortex					
Spearman's corr coef	p-val	p-val, SA	p-val, SA (BH- correction)	GenID	Gene
0.099356495	0.562921405	0.573	1	4351	MPI
0.099356495	0.562921405	0.596	1	6799	SULT1A2
0.099356495	0.562921405	0.614	1	9986	RCE1
0.099356495	0.562921405	0.59	1	57215	THAP11
0.099356495	0.562921405	0.606	1	80856	KIAA1715
0.099356495	0.562921405	0.614	1	84085	FBXO30
0.099356495	0.562921405	0.599	1	441294	CTAGE15P
0.099099092	0.56393832	0.57	1	23560	GTPBP4
0.099099092	0.56393832	0.594	1	29931	LINC00312
0.099099092	0.56393832	0.59	1	57558	USP35
0.098841697	0.564956129	0.605	1	8092	ALX1
0.098841697	0.564956129	0.603	1	10051	SMC4
0.098584294	0.56597513	0.599	1	4109	MAGEA10
0.098584294	0.56597513	0.593	1	5370	PMCHL2
0.098584294	0.56597513	0.594	1	10382	TUBB4
0.098584294	0.56597513	0.593	1	58492	ZNF77
0.098584294	0.56597513	0.613	1	84322	C18orf12
0.098326899	0.566994607	0.616	1	1182	CLCN3
0.098326899	0.566994607	0.607	1	1213	CLTC
0.098326899	0.566994607	0.59	1	2196	FAT2
0.098326899	0.566994607	0.591	1	6519	SLC3A1
0.098326899	0.566994607	0.562	1	9482	STX8
0.098326899	0.566994607	0.606	1	10217	CTDSPL
0.098326899	0.566994607	0.616	1	24140	FTSJ1
0.098326899	0.566994607	0.557	1	51322	WAC
0.098326899	0.566994607	0.589	1	54872	PIGG
0.098326899	0.566994607	0.59	1	57446	NDRG3
0.098326899	0.566994607	0.59	1	79780	CCDC82
0.098326899	0.566994607	0.582	1	125144	C17orf45
0.098069496	0.56801492	0.614	1	8512	MBL1P
0.098069496	0.56801492	0.598	1	10491	CRTAP
0.098069496	0.56801492	0.594	1	29995	LMCD1
0.098069496	0.56801492	0.621	1	57540	PTCHD2
0.098069496	0.56801492	0.627	1	267004	PGBD3
0.097812094	0.569036067	0.581	1	3099	HK2
0.097812094	0.569036067	0.595	1	5518	PPP2R1A
0.097812094	0.569036067	0.601	1	5891	RAGE
0.097812094	0.569036067	0.603	1	8668	EIF3I
0.097812094	0.569036067	0.593	1	9797	TATDN2
0.097812094	0.569036067	0.554	1	51184	GPN3
0.097812094	0.569036067	0.635	1	51390	AIG1
0.097812094	0.569036067	0.595	1	158160	HSD17B7P2
0.097554699	0.570058048	0.595	1	6136	RPL12
0.097554699	0.570058048	0.618	1	7804	LRP8
0.097554699	0.570058048	0.603	1	84988	PPP1R16A
0.097554699	0.570058048	0.597	1	91120	ZNF682
0.097297296	0.5710814	0.602	1	8110	DPF3
0.097297296	0.5710814	0.609	1	9648	GCC2
0.097297296	0.5710814	0.612	1	91056	AP5B1
0.097297296	0.5710814	0.636	1	128372	OR6N1
0.097297296	0.5710814	0.614	1	641702	FAM138E
0.097039901	0.572105169	0.598	1	521	ATP5I
0.097039901	0.572105169	0.597	1	55194	FAM176B
0.097039901	0.572105169	0.602	1	219869	OR10G8
0.097039901	0.572105169	0.601	1	790955	C11orf83
0.096782498	0.573129714	0.593	1	3150	HMGN1
0.096782498	0.573129714	0.594	1	3417	IDH1
0.096782498	0.573129714	0.596	1	83894	TTC29
0.096782498	0.573129714	0.575	1	84870	RSPO3
0.096782498	0.573129714	0.61	1	283373	ANKRD52
0.096782498	0.573129714	0.589	1	391196	OR2M7
0.096525095	0.574155033	0.605	1	10536	LEPREL2
0.096525095	0.574155033	0.603	1	10569	SLU7
0.096525095	0.574155033	0.61	1	10738	RFPL3
0.096525095	0.574155033	0.622	1	23450	SF3B3
0.096525095	0.574155033	0.574	1	140564	APOBEC3D
0.0962677	0.575181782	0.599	1	175	AGA
0.0962677	0.575181782	0.581	1	57549	IGSF9
0.09601029	0.57620877	0.602	1	2835	GPR12
0.09601029	0.57620877	0.587	1	6583	SLC22A4
0.09601029	0.57620877	0.59	1	27071	DAPP1
0.09601029	0.57620877	0.637	1	79890	RIN3
0.095752895	0.577236712	0.601	1	55629	PNRC2
0.095752895	0.577236712	0.6	1	201243	C17orf74
0.095752895	0.577236712	0.612	1	389842	LOC389842
0.095495492	0.578265488	0.58	1	6303	SAT1
0.095495492	0.578265488	0.608	1	22917	ZP1
0.095495492	0.578265488	0.619	1	57713	SFMBT2
0.095495492	0.578265488	0.58	1	64949	MRPS26

voxel-wise, subcortex			
Spearman's corr coef	p-val	GenID	Gene
0.113002956	2.0992E-24	26036	ZNF451
0.112941571	2.22308E-24	54857	GDPD2
0.112906747	2.29655E-24	151295	SLC23A3
0.112824656	2.4794E-24	29079	MED4
0.112816386	2.49859E-24	84879	MFSD2
0.11278639	2.56947E-24	7884	SLBP
0.112764016	2.62368E-24	11076	TPPP
0.112664364	2.879E-24	56954	NIT2
0.112610228	3.02789E-24	26060	APPL1
0.112606511	3.03836E-24	54877	ZCCHC2
0.112531103	3.25929E-24	3177	SLC29A2
0.11252813	3.26833E-24	54764	ZRANB1
0.112523951	3.28107E-24	130026	ICA1L
0.112380013	3.75091E-24	3483	IGFALS
0.112345189	3.87424E-24	1	A1BG
0.112332277	3.92097E-24	6334	SCN8A
0.112263277	4.18041E-24	83851	SYT16
0.112258911	4.19733E-24	5988	RFPL1
0.112168558	4.56439E-24	58527	C6orf115
0.112164564	4.58132E-24	255783	PRR24
0.11216215	4.5916E-24	646023	C22ORF45
0.112140976	4.68265E-24	120237	DBX1
0.112042256	5.13125E-24	639	PRDM1
0.112002045	5.32595E-24	60598	KCNK15
0.111867949	6.02973E-24	441818	WBP11P1
0.111847147	6.14686E-24	11337	GABARAP
0.11177963	6.5427E-24	57175	CORO1B
0.111734502	6.82129E-24	3108	HLA-DMA
0.111731805	6.83828E-24	150677	OTOS
0.111719735	6.915E-24	80329	ULBP1
0.111645997	7.4022E-24	50488	MINK1
0.111609779	7.6538E-24	326624	RAB37
0.111597151	7.74349E-24	6183	MRPS12
0.111591578	7.78345E-24	5664	PSEN2
0.11154858	8.09833E-24	23025	UNC13A
0.111532703	8.21775E-24	80741	LY6G5C
0.1113986	9.29881E-24	2986	GUCY2F
0.111303233	1.01521E-23	100130449	AC110619.2
0.111301281	1.01704E-23	147965	FAM98C
0.111300536	1.01773E-23	91523	FAM113B
0.111292645	1.02516E-23	90226	UCN2
0.111180365	1.13667E-23	2483	FRG1
0.111174516	1.14279E-23	64083	GOLPH3
0.111143224	1.17614E-23	51727	CMPK1
0.111090474	1.23453E-23	150962	PUS10
0.111052029	1.27889E-23	23215	BAT2D1
0.111049242	1.28218E-23	646024	RAET1K
0.111031689	1.303E-23	55558	PLXNA3
0.111031041	1.30378E-23	55432	YOD1
0.110964552	1.38579E-23	54542	RC3H2
0.110887282	1.48758E-23	129293	AC010087.3
0.110879019	1.49888E-23	3222	HOXC5
0.110865369	1.51776E-23	1438	CSF2RA
0.110851809	1.53673E-23	29104	N6AMT1
0.110825621	1.57406E-23	5728	PTEN
0.110803984	1.60557E-23	126123	C19orf41
0.110678241	1.80147E-23	84340	GFM2
0.110617042	1.90519E-23	25978	CHMP2B
0.110606365	1.92389E-23	2620	GAS2
0.110592902	1.94772E-23	54456	MOV10L1
0.110558167	2.01055E-23	219873	OR10S1
0.110549994	2.02562E-23	9985	REC8
0.110504858	2.11092E-23	287015	TRIM42
0.110484242	2.15104E-23	337977	KRTAP21-1
0.11047329	2.17268E-23	9703	KIAA0100
0.11040587	2.31062E-23	114795	TMEM132B
0.110405028	2.31239E-23	441543	TTTY6B
0.110355534	2.41924E-23	221150	C13orf3
0.110310957	2.51962E-23	8642	DCHS1
0.110285051	2.57985E-23	5701	PSMC2
0.110264525	2.62857E-23	11108	PRDM4
0.110245951	2.67348E-23	161931	ADAD2
0.110152341	2.91147E-23	123811	C16orf63
0.11014612	2.92799E-23	9875	URB1
0.110141106	2.9414E-23	8898	MTMR2
0.110090122	3.08114E-23	4985	OPRD1
0.110070065	3.1379E-23	647979	LOC647979
0.110034399	3.2414E-23	5393	EXOSC9
0.109956764	3.47849E-23	10734	STAG3

region-wise, subcortex					
Spearman's corr coef	p-val	p-val, SA	p-val, SA (BH- correction)	GenID	Gene
0.095495492	0.578265488	0.601	1	375748	C9orf102
0.095495492	0.578265488	0.644	1	574406	C1orf138
0.09523809	0.579295456	0.624	1	187	APLNR
0.09523809	0.579295456	0.612	1	50940	PDE11A
0.09523809	0.579295456	0.623	1	55668	C14orf118
0.09523809	0.579295456	0.608	1	55862	ECHDC1
0.09523809	0.579295456	0.583	1	64318	NOC3L
0.09523809	0.579295456	0.596	1	64344	HIF3A
0.09523809	0.579295456	0.59	1	164781	WDR69
0.09523809	0.579295456	0.627	1	388886	C22orf36
0.09523809	0.579295456	0.652	1	644794	LOC644794
0.094980694	0.580325902	0.618	1	3569	IL6
0.094980694	0.580325902	0.597	1	5203	PFDN4
0.094980694	0.580325902	0.61	1	11209	RP5-1182A14.1
0.094980694	0.580325902	0.632	1	23270	TSPYL5
0.094980694	0.580325902	0.608	1	84292	AC010422.1
0.094980694	0.580325902	0.594	1	158062	LCN6
0.094980694	0.580325902	0.589	1	253314	EIF4E1B
0.094980694	0.580325902	0.626	1	254048	UBN2
0.094723292	0.581357121	0.62	1	9950	GOLGA5
0.094723292	0.581357121	0.613	1	10099	TSPAN3
0.094723292	0.581357121	0.631	1	57176	VARS2
0.094723292	0.581357121	0.603	1	83698	CALN1
0.094465896	0.582389176	0.615	1	954	ENTPD2
0.094465896	0.582389176	0.638	1	4600	MX2
0.094465896	0.582389176	0.623	1	4828	NMB
0.094465896	0.582389176	0.613	1	79176	FBXL15
0.094465896	0.582389176	0.615	1	155060	RP4-800G7.1
0.094465896	0.582389176	0.604	1	221264	AKD1
0.094208494	0.583422065	0.6	1	1917	EEF1A2
0.094208494	0.583422065	0.635	1	2940	GSTA3
0.094208494	0.583422065	0.624	1	25891	PAMR1
0.093951091	0.584456265	0.618	1	3623	INHA
0.093951091	0.584456265	0.603	1	6376	CX3CL1
0.093951091	0.584456265	0.608	1	7454	WAS
0.093951091	0.584456265	0.644	1	9859	CEP170
0.093951091	0.584456265	0.627	1	27160	INGX
0.093951091	0.584456265	0.602	1	50615	IL21R
0.093951091	0.584456265	0.641	1	143379	C10orf82
0.093951091	0.584456265	0.627	1	143570	XRRA1
0.093693696	0.585490823	0.614	1	5993	RFX5
0.093693696	0.585490823	0.596	1	10048	RANBP9
0.093693696	0.585490823	0.636	1	51663	ZFR
0.093693696	0.585490823	0.622	1	203197	C9orf91
0.093436293	0.586526096	0.632	1	6745	SSR1
0.093436293	0.586526096	0.621	1	10270	AKAP8
0.093436293	0.586526096	0.615	1	51022	GLRX2
0.093436293	0.586526096	0.597	1	80122	YSK4
0.093436293	0.586526096	0.624	1	149134	LOC149134
0.093436293	0.586526096	0.616	1	200150	PLD5
0.093436293	0.586526096	0.608	1	201456	FBXO15
0.093178898	0.587562323	0.638	1	6284	S100A13
0.093178898	0.587562323	0.64	1	23362	PSD3
0.093178898	0.587562323	0.666	1	25837	RAB26
0.093178898	0.587562323	0.602	1	28970	C11orf54
0.093178898	0.587562323	0.616	1	84056	KATNAL1
0.093178898	0.587562323	0.648	1	120796	OR56A1
0.092921495	0.588599801	0.628	1	84667	HES7
0.092921495	0.588599801	0.644	1	157556	C8orf56
0.092921495	0.588599801	0.625	1	220992	ZNF485
0.092921495	0.588599801	0.583	1	440023	KRTAP5-6
0.092664085	0.589637578	0.624	1	3036	HAS1
0.092664085	0.589637578	0.591	1	6159	RPL29
0.092664085	0.589637578	0.603	1	6160	RPL31
0.092664085	0.589637578	0.626	1	79827	AP000926.2
0.092664085	0.589637578	0.619	1	84328	LZIC
0.092664085	0.589637578	0.602	1	266629	SEC14L3
0.09240669	0.590676188	0.632	1	9989	PPP4R1
0.09240669	0.590676188	0.624	1	26750	RPS6KC1
0.09240669	0.590676188	0.632	1	163081	ZNF567
0.09240669	0.590676188	0.604	1	285962	RP5-894A10.1
0.092149287	0.591715634	0.613	1	90871	C9orf123
0.092149287	0.591715634	0.647	1	147429	CHST9-AS1
0.092149287	0.591715634	0.629	1	201299	RDM1
0.091891885	0.592756331	0.654	1	9771	RAPGEF5
0.091891885	0.592756331	0.627	1	55347	ABHD10
0.091891885	0.592756331	0.613	1	284756	C20orf197
0.09163449	0.593797386	0.62	1	558	AXL
0.09163449	0.593797386	0.628	1	2617	GARS

voxel-wise, subcortex			
Spearman's corr coef	p-val	GenID	Gene
0.109854519	3.81714E-23	8803	SUCLA2
0.109727941	4.28187E-23	59084	ENPP5
0.109684482	4.454E-23	284521	OR2L13
0.109654583	4.57634E-23	9185	REPS2
0.109609447	4.76747E-23	7126	TNFAIP1
0.109569982	4.94104E-23	5626	PROP1
0.109519556	5.17188E-23	140893	C20orf151
0.109500609	5.2614E-23	389493	RP4-725G10.1
0.109483339	5.34421E-23	7511	XPNPEP1
0.10944628	5.52651E-23	286527	TMSB15B
0.109440058	5.5577E-23	9525	VPS4B
0.109424643	5.63578E-23	9077	DIRAS3
0.109307632	6.26464E-23	5188	PET112L
0.109285809	6.38941E-23	1300	COL10A1
0.109238915	6.6658E-23	6691	SPINK2
0.109154031	7.19676E-23	54187	NANS
0.109104812	7.52344E-23	2260	FGFR1
0.10907983	7.69491E-23	55149	MTPAP
0.109049648	7.90715E-23	57619	SHROOM3
0.109029219	8.05411E-23	340527	NHSL2
0.108968675	8.50568E-23	284382	ACTL9
0.108957991	8.58796E-23	83887	TTLL2
0.108948238	8.66374E-23	10427	SEC24B
0.108947404	8.67024E-23	4358	MPV17
0.108936541	8.7555E-23	55140	ELP3
0.108909793	8.96895E-23	55539	KCNQ1DN
0.108831696	9.622E-23	7142	TNP2
0.108817115	9.74903E-23	53841	MUPCDH
0.108772449	1.01486E-22	1358	CPA2
0.108714961	1.06868E-22	353174	ZACN
0.10864123	1.14188E-22	84451	MAP3K19
0.108590335	1.19527E-22	92249	LOC92249
0.10856656	1.22105E-22	23475	QPRT
0.108521149	1.27184E-22	27032	ATP2C1
0.10848967	1.30825E-22	84263	HSDL2
0.108475648	1.3248E-22	27235	COQ2
0.108474627	1.32602E-22	54952	TRNAU1AP
0.108451597	1.35369E-22	400624	FLJ45079
0.10843692	1.37163E-22	55827	IQWD1
0.108373493	1.45183E-22	64753	CCDC136
0.108312853	1.53288E-22	171392	ZNF675
0.108184792	1.71897E-22	131870	NUDT16
0.108152382	1.76953E-22	79784	MYH14
0.108047813	1.94285E-22	140823	ROMO1
0.107966833	2.08853E-22	325	APCS
0.107921794	2.17417E-22	177	AGER
0.107895233	2.2263E-22	51763	INPP5K
0.107884556	2.24761E-22	9212	AURKB
0.10786134	2.29462E-22	100288797	RP5-860F19.1
0.107815832	2.38962E-22	339761	CYP27C1
0.107780918	2.46515E-22	22871	NLGN1
0.107779801	2.4676E-22	9411	ARHGAP29
0.107779801	2.4676E-22	55361	PI4K2A
0.107774138	2.48007E-22	2537	IFI6
0.107771255	2.48645E-22	65124	ANKRD57
0.107676908	2.70436E-22	11158	RABL2B
0.107636884	2.80244E-22	55137	FIGN
0.107561193	2.99759E-22	430	ASCL2
0.107521728	3.10464E-22	6753	SSTR3
0.107504174	3.15347E-22	57148	KIAA1219
0.107502133	3.15919E-22	10168	ZNF197
0.107478358	3.22663E-22	1293	COL6A3
0.107452728	3.30093E-22	1365	CLDN3
0.107439168	3.34093E-22	2524	FUT2
0.107426353	3.37918E-22	64102	TNMD
0.10742598	3.3803E-22	495	ATP4A
0.107341938	3.64214E-22	81697	OR2B2
0.107317053	3.7234E-22	4725	NDUFS5
0.107253067	3.94079E-22	9074	CLDN6
0.107251391	3.94669E-22	84908	FAM136A
0.107157879	4.28766E-22	55811	ADCY10
0.107144415	4.3391E-22	29761	USP25
0.107052751	4.70592E-22	441151	TMEM151B
0.107049689	4.7187E-22	220972	MARCHF8
0.107004091	4.91291E-22	5051	PAFAH2
0.106875658	5.50363E-22	80006	C5orf44
0.106830895	5.72556E-22	2303	FOXC2
0.106805079	5.85752E-22	64924	SLC30A5
0.106717974	6.32562E-22	55187	VPS13D

region-wise, subcortex					
Spearman's corr coef	p-val	p-val, SA	p-val, SA (BH- correction)	GenID	Gene
0.09163449	0.593797386	0.651	1	2972	BRF1
0.09163449	0.593797386	0.592	1	4800	NFYA
0.09163449	0.593797386	0.613	1	8106	PABPN1
0.09163449	0.593797386	0.613	1	57461	ISY1
0.09163449	0.593797386	0.613	1	85302	FBF1
0.09163449	0.593797386	0.633	1	116841	SNAP47
0.091377087	0.594839275	0.639	1	4171	MCM2
0.091377087	0.594839275	0.66	1	51108	METTL9
0.091377087	0.594839275	0.629	1	84627	ZNF469
0.091377087	0.594839275	0.633	1	90655	TGIF2LY
0.091377087	0.594839275	0.619	1	348751	AC073043.2
0.091377087	0.594839275	0.633	1	441455	LOC441455
0.091377087	0.594839275	0.623	1	641339	ZNF674
0.091119692	0.595881999	0.63	1	5718	PSMD12
0.091119692	0.595881999	0.63	1	6656	SOX1
0.091119692	0.595881999	0.634	1	55798	METTL2B
0.091119692	0.595881999	0.606	1	57463	AMIGO1
0.091119692	0.595881999	0.602	1	254910	LCE5A
0.090862289	0.596925437	0.616	1	8573	CASK
0.090862289	0.596925437	0.649	1	55870	ASH1L
0.090604886	0.597970247	0.627	1	983	CDC2
0.090604886	0.597970247	0.631	1	23186	RCOR1
0.090604886	0.597970247	0.609	1	27283	TINAG
0.090604886	0.597970247	0.632	1	55056	FLJ10038
0.090604886	0.597970247	0.607	1	55509	BATF3
0.090604886	0.597970247	0.588	1	126549	ANKLE1
0.090604886	0.597970247	0.623	1	283093	OR4C12
0.090347491	0.599015236	0.664	1	2512	FTL
0.090347491	0.599015236	0.639	1	9704	DHX34
0.090347491	0.599015236	0.658	1	55559	HAUS7
0.090347491	0.599015236	0.631	1	389549	FEZF1
0.090090089	0.600061238	0.632	1	1975	EIF4B
0.090090089	0.600061238	0.627	1	4439	MSH5
0.090090089	0.600061238	0.615	1	6919	TCEA2
0.089832693	0.601107895	0.658	1	6258	RXRG
0.089832693	0.601107895	0.622	1	55070	DET1
0.089832693	0.601107895	0.653	1	64682	ANAPC1
0.089832693	0.601107895	0.627	1	65998	C11orf95
0.089832693	0.601107895	0.628	1	126123	C19orf41
0.089832693	0.601107895	0.61	1	440068	CARD17
0.089575291	0.602155805	0.61	1	6879	TAF7
0.089575291	0.602155805	0.642	1	9202	ZMYM4
0.089575291	0.602155805	0.614	1	9470	EIF4E2
0.089575291	0.602155805	0.614	1	55174	INTS10
0.089575291	0.602155805	0.619	1	56901	NDUFA4L2
0.089317888	0.603204131	0.612	1	4820	NKTR
0.089317888	0.603204131	0.618	1	5252	PHF1
0.089317888	0.603204131	0.645	1	5587	PRKD1
0.089317888	0.603204131	0.63	1	7059	THBS3
0.089317888	0.603204131	0.636	1	7430	VIL2
0.089317888	0.603204131	0.628	1	26263	FBXO22
0.089317888	0.603204131	0.636	1	29116	MYLIP
0.089317888	0.603204131	0.628	1	55183	RIF1
0.089317888	0.603204131	0.662	1	55680	RUFY2
0.089317888	0.603204131	0.625	1	91151	TIGD7
0.089317888	0.603204131	0.656	1	91544	UBXN11
0.089060485	0.604253113	0.633	1	2150	F2RL1
0.089060485	0.604253113	0.655	1	2812	GP1BB
0.089060485	0.604253113	0.624	1	23615	PYY2
0.089060485	0.604253113	0.618	1	84986	ARHGAP19
0.088803083	0.605302989	0.657	1	172	AFG3L1P
0.088803083	0.605302989	0.635	1	597	BCL2A1
0.088803083	0.605302989	0.638	1	1496	CTNNA2
0.088803083	0.605302989	0.625	1	54491	FAM105A
0.088545687	0.606354117	0.626	1	64400	AKTIP
0.088545687	0.606354117	0.652	1	90850	ZNF598
0.088545687	0.606354117	0.648	1	147804	CTD-2224J9.2
0.088288285	0.607405543	0.609	1	3953	LEPR
0.088288285	0.607405543	0.654	1	6181	RPLP2
0.088288285	0.607405543	0.643	1	26060	APPL1
0.088288285	0.607405543	0.632	1	57099	AVEN
0.088288285	0.607405543	0.657	1	254973	OR1L4
0.088288285	0.607405543	0.656	1	729535	FAM207BP
0.088030882	0.608457804	0.611	1	22829	NLGN4Y
0.088030882	0.608457804	0.626	1	79626	TNFAIP8L2
0.088030882	0.608457804	0.645	1	163688	CALML6
0.087773487	0.609510779	0.665	1	4867	NPHP1
0.087773487	0.609510779	0.614	1	253959	GARNL1
0.087516084	0.61056459	0.622	1	1054	CEBPG

voxel-wise, subcortex			
Spearman's corr coef	p-val	GenID	Gene
0.10659799	7.03136E-22	482	ATP1B2
0.106586844	7.10083E-22	2099	ESR1
0.106584057	7.11826E-22	5092	PCBD1
0.106543846	7.37491E-22	57507	ZNF608
0.106512085	7.58409E-22	440335	LOC440335
0.106505774	7.62633E-22	2207	FCER1G
0.106500112	7.66441E-22	150365	MEI1
0.106441416	8.07073E-22	8140	SLC7A5
0.106410213	8.29543E-22	8289	ARID1A
0.106385142	8.48032E-22	1103	CHAT
0.106365547	8.62773E-22	55731	C17orf63
0.106336482	8.85102E-22	6240	RRM1
0.106331095	8.89302E-22	29927	SEC61A1
0.106329516	8.90535E-22	5684	PSMA3
0.106320225	8.97836E-22	137682	C8orf38
0.106312707	9.03789E-22	221188	GPR114
0.10629905	9.14698E-22	2585	GALK2
0.106248349	9.5637E-22	9344	TAOK2
0.106064752	1.12355E-21	26301	GBGT1
0.10606382	1.12447E-21	154214	RNF217
0.106022038	1.16641E-21	80039	FAM106A
0.106005505	1.18343E-21	4547	MTTP
0.105967335	1.22368E-21	401105	AC022498.2
0.105918951	1.27664E-21	1645	AKR1C1
0.105905212	1.29209E-21	2597	GAPDH
0.105836861	1.37172E-21	79777	ACBD4
0.105827853	1.38259E-21	4637	MYL6
0.10582386	1.38742E-21	252839	TMEM9
0.105776496	1.4461E-21	9296	ATP6V1F
0.105752632	1.47658E-21	342527	SMTNL2
0.105749846	1.48019E-21	79933	SYNPO2L
0.105712883	1.5288E-21	391013	PLA2G2C
0.105670631	1.58626E-21	9376	SLC22A8
0.105661899	1.5984E-21	3099	HK2
0.105644815	1.62244E-21	389903	CSAG3
0.105640449	1.62864E-21	7701	ZNF142
0.105638683	1.63116E-21	175	AGA
0.105630323	1.64309E-21	5310	PKD1
0.105576091	1.72275E-21	4009	LMX1A
0.105469108	1.89127E-21	7703	PCGF2
0.105433546	1.95079E-21	7056	THBD
0.105316162	2.16083E-21	3842	TNPO1
0.105310127	2.17219E-21	100128327	BET3L
0.105288856	2.21279E-21	50835	TAS2R9
0.105279475	2.23092E-21	116840	CNTROB
0.105266944	2.25539E-21	221184	CPNE2
0.105201006	2.38853E-21	4586	MUC5AC
0.105086319	2.63887E-21	2587	GALR1
0.105055764	2.70985E-21	26341	OR5H1
0.105034404	2.76057E-21	10038	PARP2
0.10497915	2.89618E-21	6598	SMARCB1
0.104955003	2.95752E-21	8690	JRKL
0.104929835	3.02277E-21	10693	CCT6B
0.104925655	3.03377E-21	142678	MIB2
0.104907177	3.08278E-21	2580	GAK
0.104899652	3.10297E-21	254783	OR6C74
0.104875974	3.16734E-21	25987	TSKU
0.104821831	3.31947E-21	784	CACNB3
0.104784966	3.42717E-21	9312	KCNB2
0.104738347	3.56837E-21	5720	PSME1
0.10473454	3.58018E-21	3169	FOXA1
0.104712717	3.64843E-21	11235	PDCD10
0.104607403	3.99644E-21	3866	KRT15
0.104597747	4.02996E-21	440792	LOC440792
0.104571186	4.12356E-21	123872	LRRC50
0.104548343	4.20578E-21	11260	XPOT
0.10448724	4.4338E-21	83597	RTP3
0.104447581	4.58823E-21	7620	ZNF69
0.104434215	4.6415E-21	55683	KIAA1310
0.10441044	4.73775E-21	57595	PDZD4
0.104395114	4.80082E-21	6401	SELE
0.104366235	4.92192E-21	219793	C10orf27
0.104284696	5.28044E-21	4854	NOTCH3
0.104247548	5.45226E-21	23136	EPB41L3
0.104237989	5.49734E-21	23295	MGRN1
0.104232505	5.52338E-21	162989	DEDD2
0.104203627	5.66251E-21	78988	MRP63
0.104192205	5.71847E-21	10488	CREB3
0.10418617	5.74829E-21	54981	C9orf95

region-wise, subcortex					
Spearman's corr coef	p-val	p-val, SA	p-val, SA (BH- correction)	GenID	Gene
0.087516084	0.61056459	0.654	1	3400	ID4
0.087516084	0.61056459	0.635	1	114609	TIRAP
0.087516084	0.61056459	0.644	1	114876	OSBPL1A
0.087516084	0.61056459	0.648	1	162993	ZNF846
0.087516084	0.61056459	0.659	1	199870	FAM76A
0.087516084	0.61056459	0.62	1	414777	HCG18
0.087516084	0.61056459	0.651	1	729739	LOC729739
0.087258689	0.611619651	0.666	1	1296	COL8A2
0.087258689	0.611619651	0.628	1	1812	DRD1
0.087258689	0.611619651	0.646	1	2542	SLC37A4
0.087258689	0.611619651	0.618	1	4105	MAGEA6
0.087258689	0.611619651	0.635	1	7425	VGF
0.087258689	0.611619651	0.618	1	51004	COQ6
0.087258689	0.611619651	0.64	1	54956	PARP16
0.087258689	0.611619651	0.641	1	84680	ACCS
0.087258689	0.611619651	0.633	1	112885	PHF21B
0.087258689	0.611619651	0.621	1	729683	LOC729683
0.087001286	0.612675011	0.65	1	733	C8G
0.087001286	0.612675011	0.669	1	8622	PDE8B
0.087001286	0.612675011	0.611	1	199745	THAP8
0.086743884	0.613731146	0.64	1	11326	VSIG4
0.086743884	0.613731146	0.671	1	25906	C11orf51
0.086743884	0.613731146	0.633	1	56103	PCDHGB2
0.086743884	0.613731146	0.647	1	57577	KIAA1407
0.086743884	0.613731146	0.637	1	64776	C11orf1
0.086743884	0.613731146	0.64	1	79731	NARS2
0.086743884	0.613731146	0.638	1	85464	SSH2
0.086743884	0.613731146	0.648	1	374882	TMEM205
0.086486489	0.614787996	0.65	1	4591	TRIM37
0.086486489	0.614787996	0.625	1	23550	PSD4
0.086486489	0.614787996	0.649	1	23640	HSPBP1
0.086486489	0.614787996	0.636	1	80021	TMEM62
0.086486489	0.614787996	0.619	1	80831	APOL5
0.086486489	0.614787996	0.639	1	283232	TMEM80
0.086486489	0.614787996	0.662	1	390003	GUCY2GP
0.086229086	0.615846217	0.637	1	734	OSGIN2
0.086229086	0.615846217	0.641	1	10563	CXCL13
0.086229086	0.615846217	0.643	1	10915	TCERG1
0.086229086	0.615846217	0.614	1	163131	ZNF780B
0.086229086	0.615846217	0.648	1	257169	C9orf43
0.085971691	0.616904616	0.66	1	8760	CDS2
0.085971691	0.616904616	0.637	1	10743	RAI1
0.085971691	0.616904616	0.634	1	51257	MARCHF2
0.085971691	0.616904616	0.643	1	84294	UTP23
0.085971691	0.616904616	0.599	1	147947	ZNF542
0.085714288	0.61796385	0.66	1	22841	RAB11FIP2
0.085714288	0.61796385	0.646	1	23231	AC092436.3
0.085714288	0.61796385	0.658	1	26122	EPC2
0.085714288	0.61796385	0.651	1	84337	ELOF1
0.085714288	0.61796385	0.649	1	92211	PCDH21
0.085456878	0.619023919	0.649	1	421	ARVCF
0.085456878	0.619023919	0.665	1	728012	RP11-14I4.3
0.085199483	0.620085061	0.649	1	6187	RPS2
0.085199483	0.620085061	0.627	1	115196	ZNF554
0.085199483	0.620085061	0.646	1	445329	SULT1A4
0.085199483	0.620085061	0.636	1	653635	WASH5P
0.08494208	0.621146619	0.643	1	2979	GUCA1B
0.08494208	0.621146619	0.64	1	10357	HMGB1P1
0.08494208	0.621146619	0.638	1	23508	TTC9
0.08494208	0.621146619	0.638	1	27175	TUBG2
0.08494208	0.621146619	0.641	1	60677	BRUNOL6
0.08494208	0.621146619	0.663	1	343069	HNRNPCL1
0.08494208	0.621146619	0.639	1	645453	RLIMP3
0.084684685	0.622208893	0.642	1	2264	FGFR4
0.084684685	0.622208893	0.637	1	3176	HNMT
0.084684685	0.622208893	0.655	1	10501	SEMA6B
0.084684685	0.622208893	0.646	1	57610	RANBP10
0.084427282	0.623271942	0.649	1	23011	RAB21
0.084427282	0.623271942	0.666	1	27433	TOR2A
0.08416988	0.624335706	0.636	1	6098	ROS1
0.08416988	0.624335706	0.683	1	374462	PTPRQ
0.083912484	0.625400782	0.645	1	4583	MUC2
0.083912484	0.625400782	0.663	1	5935	RBM3
0.083912484	0.625400782	0.661	1	7001	PRDX2
0.083912484	0.625400782	0.646	1	55705	IPO9
0.083912484	0.625400782	0.659	1	79966	SCD5
0.083655082	0.626466155	0.64	1	145853	C15orf61
0.083655082	0.626466155	0.648	1	259197	NCR3
0.083397686	0.627532184	0.669	1	3084	NRG1

voxel-wise, subcortex			
Spearman's corr coef	p-val	GenID	Gene
0.104184404	5.75703E-21	57524	CASKIN1
0.104125157	6.05828E-21	54884	RETSAT
0.104115121	6.11091E-21	11330	CTRC
0.104084104	6.27616E-21	116511	MAS1L
0.104055315	6.4335E-21	23636	NUP62
0.103981398	6.85572E-21	192134	B3GNT6
0.103943139	7.08479E-21	6884	TAF13
0.103923261	7.20686E-21	245939	DEFB128
0.103912957	7.27097E-21	1081	CGA
0.1038967	7.37313E-21	127062	OR2M3
0.10382928	7.81253E-21	23170	TTLL12
0.10379789	8.02583E-21	23177	CEP68
0.10374561	8.39392E-21	54947	LPCAT2
0.103732981	8.48526E-21	51298	THEG
0.103725828	8.53748E-21	59336	PRDM13
0.103705212	8.68976E-21	100130890	LOC100130890
0.103665002	8.9944E-21	3002	GZMB
0.103644945	9.15039E-21	266629	SEC14L3
0.103595167	9.54889E-21	60528	ELAC2
0.103589408	9.59609E-21	390927	ZNF793
0.1035804	9.6704E-21	79650	C16orf57
0.103528209	1.01122E-20	337975	KRTAP20-1
0.103453085	1.07834E-20	201254	STRA13
0.103451319	1.07997E-20	55178	RNMTL1
0.103437111	1.09317E-20	353141	LCE2D
0.103417978	1.11119E-20	54361	WNT4
0.103409715	1.11908E-20	57332	CBX8
0.103389747	1.13833E-20	2829	XCR1
0.103245713	1.28734E-20	6406	SEMG1
0.103204384	1.33354E-20	55324	ABCF3
0.103131764	1.41874E-20	7259	TSPYL1
0.103130743	1.41996E-20	440352	SNX29P2
0.103117004	1.43671E-20	23406	COTL1
0.103107154	1.44881E-20	93474	ZNF670
0.103067778	1.49825E-20	153571	C5orf38
0.10306713	1.49908E-20	3975	LHX1
0.10306704	1.49918E-20	2854	GPR32
0.103046611	1.52551E-20	125228	C18orf19
0.103005193	1.58027E-20	7138	TNNT1
0.102984481	1.60839E-20	115201	ATG4A
0.102958016	1.64503E-20	84771	DDX11L2
0.10292384	1.69358E-20	80143	SIKE1
0.102868117	1.77578E-20	100129726	LOC100129726
0.102786772	1.90289E-20	65010	SLC26A6
0.102664746	2.11064E-20	54854	FAM83E
0.102651559	2.13439E-20	139231	CXorf39
0.102651089	2.13522E-20	337978	KRTAP21-2
0.102609396	2.21212E-20	337968	KRTAP6-3
0.102570392	2.28649E-20	374977	C1orf175
0.102546528	2.33322E-20	10017	BCL2L10
0.102490716	2.44624E-20	10465	PPIH
0.10248746	2.453E-20	80032	ZNF556
0.102473348	2.48248E-20	80764	THAP7
0.10246072	2.50918E-20	3489	IGFBP6
0.102389857	2.66431E-20	4338	MOCS2
0.102382615	2.6807E-20	2669	GEM
0.102369897	2.70972E-20	23466	CBX6
0.102348626	2.75893E-20	4026	LPP
0.102205612	3.11351E-20	55660	PRPF40A
0.102117762	3.35327E-20	23471	TRAM1
0.102113865	3.36434E-20	27159	CHIA
0.102110706	3.3733E-20	57605	PITPNM2
0.102076717	3.47146E-20	1824	DSC2
0.10206455	3.50727E-20	3884	KRT33B
0.102029264	3.61322E-20	84812	PLCD4
0.10193073	3.92616E-20	386653	IL31
0.101803042	4.37185E-20	94056	SYAP1
0.101781219	4.45284E-20	11092	C9orf9
0.101769976	4.49519E-20	9215	LARGE
0.101710267	4.72666E-20	80301	PLEKHO2
0.101701446	4.76185E-20	116512	MRGPRD
0.101698749	4.77264E-20	1380	CR2
0.101582669	5.26157E-20	55671	SMEK1
0.101562329	5.35219E-20	23513	SCRIB
0.101535305	5.47498E-20	114336	CGB2
0.101534009	5.48094E-20	100170841	C17orf96
0.101518407	5.55317E-20	5266	PI3
0.101492032	5.6774E-20	353131	LCE1A
0.101393409	6.16686E-20	56853	BRUNOL4

region-wise, subcortex					
Spearman's corr coef	p-val	p-val, SA	p-val, SA (BH- correction)	GenID	Gene
0.083397686	0.627532184	0.634	1	286527	TMSB15B
0.083397686	0.627532184	0.642	1	441502	RPS26P11
0.083397686	0.627532184	0.647	1	493753	C2orf64
0.083397686	0.627532184	0.666	1	653583	PHLDB3
0.083140284	0.628598988	0.678	1	7265	TTC1
0.083140284	0.628598988	0.654	1	9854	C2CD2L
0.083140284	0.628598988	0.67	1	25907	TMEM158
0.083140284	0.628598988	0.653	1	26061	HACL1
0.083140284	0.628598988	0.638	1	26575	RSG17
0.083140284	0.628598988	0.66	1	29926	GMPPA
0.083140284	0.628598988	0.645	1	56889	TM9SF3
0.083140284	0.628598988	0.653	1	56993	TOMM22
0.083140284	0.628598988	0.636	1	79640	Z83840.4
0.083140284	0.628598988	0.641	1	123775	C16orf46
0.083140284	0.628598988	0.626	1	144486	LOC144486
0.083140284	0.628598988	0.624	1	223082	ZNRF2
0.082882881	0.629667103	0.673	1	1435	CSF1
0.082882881	0.629667103	0.666	1	5664	PSEN2
0.082882881	0.629667103	0.656	1	8438	RAD54L
0.082882881	0.629667103	0.652	1	8871	SYNJ2
0.082882881	0.629667103	0.652	1	79648	MCPH1
0.082882881	0.629667103	0.642	1	81855	SFXN3
0.082882881	0.629667103	0.632	1	386617	KCTD8
0.082625486	0.630735397	0.67	1	8878	SQSTM1
0.082625486	0.630735397	0.669	1	9060	PAPSS2
0.082625486	0.630735397	0.647	1	10811	NOXA1
0.082625486	0.630735397	0.646	1	54460	MRPS21
0.082625486	0.630735397	0.682	1	55795	PCID2
0.082625486	0.630735397	0.652	1	85458	DIXDC1
0.082625486	0.630735397	0.645	1	100169851	PATE3
0.082368083	0.631804466	0.66	1	1802	DPH2
0.082368083	0.631804466	0.646	1	8195	MKKS
0.082368083	0.631804466	0.66	1	8766	RAB11A
0.082368083	0.631804466	0.661	1	152404	IGSF11
0.082110681	0.63287431	0.655	1	3044	HBBP1
0.082110681	0.63287431	0.673	1	4547	MTTP
0.082110681	0.63287431	0.658	1	10102	TSFM
0.082110681	0.63287431	0.678	1	54458	PRR13
0.082110681	0.63287431	0.652	1	90324	CCDC97
0.082110681	0.63287431	0.66	1	90806	ANGEL2
0.082110681	0.63287431	0.662	1	116449	CLNK
0.082110681	0.63287431	0.639	1	199746	U2AF1L4
0.081853278	0.633945346	0.631	1	3175	ONECUT1
0.081853278	0.633945346	0.666	1	7407	VARS
0.081853278	0.633945346	0.675	1	54487	DGCR8
0.081853278	0.633945346	0.644	1	56252	YLPM1
0.081595875	0.63501668	0.653	1	6727	SRP14
0.081595875	0.63501668	0.678	1	10777	ARPP-21
0.081595875	0.63501668	0.644	1	158866	ZDHHC15
0.081595875	0.63501668	0.672	1	349075	ZNF713
0.081595875	0.63501668	0.618	1	390201	OR10V1
0.081595875	0.63501668	0.674	1	390326	OR6C76
0.08133848	0.636088729	0.685	1	1852	DUSP9
0.08133848	0.636088729	0.653	1	2527	FUT5
0.08133848	0.636088729	0.638	1	11238	CA5B
0.08133848	0.636088729	0.648	1	79020	C7orf25
0.08133848	0.636088729	0.665	1	84513	PPAPDC1B
0.08133848	0.636088729	0.663	1	642280	ZNF876P
0.081081077	0.637161493	0.67	1	1739	DLG1
0.081081077	0.637161493	0.671	1	2862	MLNR
0.081081077	0.637161493	0.678	1	5356	PLRG1
0.081081077	0.637161493	0.671	1	6224	RPS20
0.081081077	0.637161493	0.679	1	23539	SLC16A8
0.081081077	0.637161493	0.626	1	64783	RBM15
0.081081077	0.637161493	0.648	1	118426	LOH12CR1
0.080823682	0.638235033	0.675	1	539	ATP5O
0.080823682	0.638235033	0.668	1	51054	PLEKHA9
0.080823682	0.638235033	0.666	1	51478	HSD17B7
0.080823682	0.638235033	0.656	1	55778	ZNF839
0.080823682	0.638235033	0.679	1	145978	NCRNA00052
0.08056628	0.639309764	0.633	1	3906	LALBA
0.08056628	0.639309764	0.658	1	5309	PITX3
0.08056628	0.639309764	0.687	1	55758	RCOR3
0.08056628	0.639309764	0.657	1	440044	SLC22A20
0.080308877	0.640384734	0.69	1	2703	GJA8
0.080308877	0.640384734	0.639	1	8516	ITGA8
0.080308877	0.640384734	0.688	1	100289230	LOC100289230
0.080051482	0.641460419	0.682	1	2901	GRIK5
0.080051482	0.641460419	0.674	1	5228	PGF

voxel-wise, subcortex			
Spearman's corr coef	p-val	GenID	Gene
0.101318836	6.56448E-20	63973	NEUROG2
0.101318277	6.56751E-20	158931	TCEAL6
0.101311684	6.60391E-20	9834	KIAA0125
0.101307787	6.62551E-20	53840	TRIM34
0.101106733	7.83914E-20	3788	KCNS2
0.101096421	7.90696E-20	9726	ZNF646
0.100986101	8.67018E-20	1869	E2F1
0.100985356	8.67559E-20	7349	UCN
0.100982472	8.69645E-20	6836	SURF4
0.10098201	8.69987E-20	85455	DISP2
0.100790426	1.0207E-19	11228	RASSF8
0.100765266	1.04231E-19	440050	KRTAP5-7
0.100754678	1.05155E-19	57666	FBRSL1
0.100725606	1.07732E-19	8193	DPF1
0.100646861	1.15028E-19	11086	ADAM29
0.100614265	1.1819E-19	390928	AC011443.1
0.100552507	1.24417E-19	152137	CCDC50
0.100548238	1.2486E-19	81579	PLA2G12A
0.100496322	1.30364E-19	100130967	C6orf99
0.10049326	1.30696E-19	30837	SOCS7
0.100409679	1.40089E-19	25831	HECTD1
0.100384697	1.43023E-19	51066	C3orf32
0.100373738	1.4433E-19	55718	POLR3E
0.100344948	1.4782E-19	146279	TEKT5
0.100328051	1.49905E-19	23022	PALLD
0.100325078	1.50274E-19	27128	CYTH4
0.100249857	1.59944E-19	100128124	HGC6.3
0.100061156	1.8699E-19	219960	OR10Q1
0.100038864	1.90469E-19	5250	SLC25A3
0.10000135	1.96469E-19	9079	LDB2
0.099972002	2.01295E-19	2567	GABRG3
0.099959746	2.03343E-19	7324	UBE2E1
0.099956587	2.03874E-19	119692	OR51S1
0.099946	2.05665E-19	55686	MREG
0.099889167	2.15549E-19	399664	MEX3D
0.099882662	2.16709E-19	5600	MAPK11
0.099858336	2.21108E-19	10103	TSPAN1
0.099857591	2.21244E-19	27071	DAPP1
0.099784136	2.35068E-19	170082	TCEANC
0.09968207	2.55705E-19	284654	RSPO1
0.099676684	2.56843E-19	796	CALCA
0.099665448	2.59231E-19	5192	PEX10
0.09965802	2.60822E-19	256643	CXorf23
0.099587724	2.76365E-19	3956	LGALS1
0.099534042	2.88851E-19	951	CD37
0.099532649	2.89181E-19	4762	NEUROG1
0.099531077	2.89554E-19	23120	ATP10B
0.099400595	3.22347E-19	92421	CHMP4C
0.099346176	3.37087E-19	65258	MPPE1
0.099228419	3.71292E-19	7379	UPK2
0.09919063	3.8298E-19	23165	NUP205
0.099169642	3.89627E-19	79091	C16orf68
0.099118561	4.06284E-19	390980	ZNF805
0.099102221	4.1176E-19	10277	UBE4B
0.099101849	4.11884E-19	5437	POLR2H
0.099100173	4.12452E-19	493753	C2orf64
0.099077329	4.20241E-19	8824	CES2
0.09904696	4.30824E-19	7132	TNFRSF1A
0.099018827	4.4086E-19	10659	CUGBP2
0.098994307	4.49798E-19	10280	SIGMAR1
0.098992825	4.50345E-19	374654	KIF7
0.098991521	4.50825E-19	253832	ZDHHC20
0.09898781	4.52197E-19	7809	BSND
0.09889067	4.89589E-19	100134229	LOC100134229
0.098873399	4.96551E-19	3055	HCK
0.098850548	5.05911E-19	84240	ZCCHC9
0.098797619	5.28263E-19	4869	NPM1
0.09878666	5.33014E-19	84214	RP13-254B10.1
0.098679774	5.81608E-19	10984	KCNQ1OT1
0.098662034	5.90083E-19	6046	BRD2
0.098615229	6.13038E-19	50939	IMPG2
0.098590709	6.25414E-19	51667	NUB1
0.098587275	6.27169E-19	51526	C20orf111
0.098556906	6.42877E-19	55454	CSGALNACT2
0.098556168	6.43268E-19	147339	C18orf25
0.09845791	6.96856E-19	79966	SCD5
0.09841761	7.20087E-19	10138	YAF2
0.098261036	8.17837E-19	5316	PKNOX1
0.098205693	8.55435E-19	4342	MOS

region-wise, subcortex					
Spearman's corr coef	p-val	p-val, SA	p-val, SA (BH- correction)	GenID	Gene
0.080051482	0.641460419	0.645	1	55605	KIF21A
0.080051482	0.641460419	0.685	1	117583	PARD3B
0.080051482	0.641460419	0.65	1	259232	NALCN
0.080051482	0.641460419	0.687	1	400752	LOC400752
0.079794079	0.642536819	0.665	1	2139	EYA2
0.079794079	0.642536819	0.665	1	23359	FAM189A1
0.079794079	0.642536819	0.674	1	23585	TMEM50A
0.079794079	0.642536819	0.672	1	51248	PDZD11
0.079794079	0.642536819	0.655	1	60684	C4orf41
0.079794079	0.642536819	0.673	1	84448	ABLIM2
0.079794079	0.642536819	0.668	1	113278	C20orf54
0.079794079	0.642536819	0.667	1	202243	CCDC125
0.079536684	0.643614531	0.687	1	3074	HEXB
0.079536684	0.643614531	0.681	1	58478	ENOPH1
0.079536684	0.643614531	0.699	1	64081	PBLD
0.079536684	0.643614531	0.68	1	124961	ZFP3
0.079536684	0.643614531	0.664	1	391358	TRMT112P6
0.079279281	0.644692421	0.663	1	3097	HIVEP2
0.079279281	0.644692421	0.653	1	6222	RPS18
0.079279281	0.644692421	0.675	1	29887	SNX10
0.079021879	0.645771027	0.662	1	2893	GRIA4
0.079021879	0.645771027	0.659	1	5197	PF4V1
0.079021879	0.645771027	0.661	1	56521	DNAJC12
0.079021879	0.645771027	0.669	1	78998	C8ORF51
0.079021879	0.645771027	0.681	1	79877	DCAKD
0.078764483	0.646850407	0.668	1	1147	CHUK
0.078764483	0.646850407	0.674	1	7905	REEP5
0.078764483	0.646850407	0.69	1	8560	DEGS1
0.078764483	0.646850407	0.674	1	23175	LPIN1
0.078764483	0.646850407	0.692	1	81537	SGPP1
0.078507073	0.64793098	0.671	1	3735	KARS
0.078507073	0.64793098	0.672	1	10097	ACTR2
0.078507073	0.64793098	0.663	1	51042	ZNF593
0.078507073	0.64793098	0.679	1	51163	DBR1
0.078507073	0.64793098	0.669	1	55713	ZNF334
0.078507073	0.64793098	0.682	1	84260	TCHP
0.078507073	0.64793098	0.661	1	131076	CCDC58
0.078507073	0.64793098	0.673	1	392188	USP17L8
0.078249671	0.649011731	0.659	1	6650	SOLH
0.078249671	0.649011731	0.698	1	9836	LCMT2
0.078249671	0.649011731	0.679	1	10406	WFDC2
0.078249671	0.649011731	0.673	1	79188	TMEM43
0.078249671	0.649011731	0.699	1	94005	PIGS
0.078249671	0.649011731	0.669	1	115650	TNFRSF13C
0.078249671	0.649011731	0.695	1	221060	C10orf111
0.077992275	0.650093257	0.664	1	2805	GOT1
0.077992275	0.650093257	0.672	1	6261	RYR1
0.077992275	0.650093257	0.7	1	7741	ZNF187
0.077992275	0.650093257	0.672	1	54812	AFTPH
0.077992275	0.650093257	0.668	1	55811	ADCY10
0.077992275	0.650093257	0.681	1	57826	RAP2C
0.077992275	0.650093257	0.687	1	147912	SIX5
0.077992275	0.650093257	0.67	1	401494	PTPLAD2
0.077992275	0.650093257	0.671	1	442425	FOXB2
0.077734873	0.651175439	0.682	1	8329	HIST1H2AI
0.077734873	0.651175439	0.677	1	10960	LMAN2
0.077734873	0.651175439	0.69	1	22926	ATF6
0.077734873	0.651175439	0.679	1	55930	MYO5C
0.077734873	0.651175439	0.702	1	57168	ASPHD2
0.077734873	0.651175439	0.665	1	64770	CCDC14
0.077734873	0.651175439	0.663	1	80142	PTGES2
0.077734873	0.651175439	0.667	1	392490	FLJ44635
0.077477477	0.652258277	0.669	1	5799	PTPRN2
0.077477477	0.652258277	0.694	1	8360	HIST1H4D
0.077477477	0.652258277	0.673	1	152002	C3orf21
0.077477477	0.652258277	0.679	1	168090	C6orf118
0.077477477	0.652258277	0.643	1	390928	AC011443.1
0.077220075	0.653342485	0.686	1	5558	PRIM2
0.077220075	0.653342485	0.699	1	6139	RPL17P20
0.077220075	0.653342485	0.675	1	84694	GJA10
0.076962672	0.654426754	0.694	1	1848	DUSP6
0.076962672	0.654426754	0.671	1	7405	UVRAG
0.076962672	0.654426754	0.673	1	50632	CALY
0.076962672	0.654426754	0.688	1	201294	UNC13D
0.076962672	0.654426754	0.696	1	285741	LOC285741
0.076962672	0.654426754	0.691	1	390594	AC013553.2
0.076962672	0.654426754	0.684	1	644762	LOC644762
0.076705277	0.655511796	0.687	1	1134	CHRNA1
0.076705277	0.655511796	0.682	1	8607	RUVBL1

voxel-wise, subcortex			
Spearman's corr coef	p-val	GenID	Gene
0.098196313	8.6197E-19	25895	FAM119B
0.098194264	8.63405E-19	222484	LNX2
0.098119602	9.17332E-19	9529	BAG5
0.098081157	9.46379E-19	55691	FRMD4A
0.098061189	9.6182E-19	134637	ADAT2
0.098050043	9.70557E-19	378	ARF4
0.098040201	9.78333E-19	54682	MANSC1
0.098037414	9.80539E-19	388327	C17orf100
0.098030269	9.86232E-19	65980	BRD9
0.098012529	1.00052E-18	1460	CSNK2B
0.097997576	1.01272E-18	6769	STAC
0.097960152	1.04388E-18	10139	ARFRP1
0.097953282	1.0497E-18	84280	BTBD10
0.097915299	1.08248E-18	55728	N4BP2
0.097907685	1.08917E-18	139760	GPR119
0.097885676	1.10875E-18	79290	OR13A1
0.09762416	1.36969E-18	203522	DDX26B
0.097619802	1.37451E-18	154	ADRB2
0.097599924	1.39673E-18	348487	FAM131C
0.09759324	1.40429E-18	9467	SH3BP5
0.097584046	1.41475E-18	683	BST1
0.097575501	1.42453E-18	25800	SLC39A6
0.097575128	1.42496E-18	389791	LOC389791
0.097558603	1.44408E-18	149041	RC3H1
0.097511798	1.49962E-18	116496	FAM129A
0.097492479	1.52316E-18	10476	ATP5H
0.097483195	1.5346E-18	641455	AL589182.2
0.097460903	1.56242E-18	79626	TNFAIP8L2
0.097449943	1.57627E-18	6534	SLC6A7
0.097432584	1.59846E-18	1431	CS
0.097375281	1.67396E-18	25824	PRDX5
0.097354107	1.70273E-18	65982	ZSCAN18
0.097309902	1.76439E-18	9690	UBE3C
0.097281024	1.80584E-18	2713	GK3P
0.097227998	1.8845E-18	10024	TROAP
0.097202182	1.92402E-18	7741	ZNF187
0.097115353	2.06301E-18	9912	AC005277.1
0.097027592	2.21355E-18	10097	ACTR2
0.096998058	2.26659E-18	114781	BTBD9
0.096987195	2.28643E-18	2593	GAMT
0.096972056	2.31434E-18	56935	C11orf75
0.096972056	2.31434E-18	221527	ZBTB12
0.096942157	2.37049E-18	79752	ZFAND1
0.09693519	2.38376E-18	27102	EIF2AK1
0.096885696	2.4802E-18	57673	BEND3
0.096871853	2.50782E-18	80232	WDR26
0.096856065	2.53974E-18	5691	PSMB3
0.096782424	2.69398E-18	140886	PABPC5
0.096736178	2.79549E-18	6770	STAR
0.096687332	2.90685E-18	348793	WDR53
0.096655756	2.98112E-18	6098	ROS1
0.096621491	3.06386E-18	286101	ZNF252P
0.096579142	3.16927E-18	25960	GPR124
0.096575059	3.17961E-18	2026	ENO2
0.096567161	3.19971E-18	79772	MCTP1
0.096548215	3.24848E-18	90231	KIAA2013
0.096470952	3.45506E-18	26018	LRIG1
0.096385978	3.69724E-18	79155	TNIP2
0.096367128	3.75317E-18	55954	ZMAT5
0.096333049	3.85647E-18	9605	C16orf7
0.096314289	3.91451E-18	84734	FAM167B
0.096266463	4.06642E-18	1314	COPA
0.096245661	4.13435E-18	23650	TRIM29
0.096225232	4.20207E-18	197320	ZNF778
0.096194588	4.30575E-18	440503	AC011498.2
0.096074976	4.7352E-18	411	ARSB
0.096059375	4.79425E-18	11033	ADAP1
0.095956102	5.20384E-18	117286	CIB3
0.095938735	5.27601E-18	285051	C2orf61
0.095912829	5.38554E-18	84895	FAM73B
0.095880605	5.52491E-18	728882	FAM182B
0.095846154	5.67787E-18	8934	RAB7L1
0.095842622	5.69378E-18	203197	C9orf91
0.095821261	5.79095E-18	6346	CCL1
0.095712885	6.30983E-18	6451	SH3BGRL
0.09570732	6.33773E-18	5727	PTCH1
0.095702484	6.362E-18	116	ADCYAP1
0.095694967	6.39991E-18	8450	CUL4B
0.095693015	6.40983E-18	151194	FAM119A

region-wise, subcortex					
Spearman's corr coef	p-val	p-val, SA	p-val, SA (BH- correction)	GenID	Gene
0.076705277	0.655511796	0.662	1	10880	ACTL7B
0.076705277	0.655511796	0.666	1	11275	KLHL2
0.076705277	0.655511796	0.676	1	64795	RMND5A
0.076705277	0.655511796	0.662	1	84968	PNMA6A
0.076705277	0.655511796	0.678	1	388199	AL023882.1
0.076705277	0.655511796	0.67	1	440955	TMEM89
0.076447874	0.656597495	0.66	1	4606	MYBPC2
0.076447874	0.656597495	0.698	1	4994	OR3A1
0.076447874	0.656597495	0.669	1	6372	CXCL6
0.076447874	0.656597495	0.692	1	84823	LMNB2
0.076190479	0.657684445	0.66	1	5510	PPP1R7
0.076190479	0.657684445	0.69	1	9616	RNF7
0.076190479	0.657684445	0.68	1	26240	FAM50B
0.076190479	0.657684445	0.681	1	51430	C1orf9
0.076190479	0.657684445	0.695	1	84688	C9orf24
0.075933076	0.658771634	0.674	1	6138	RPL15
0.075933076	0.658771634	0.696	1	10131	TRAP1
0.075933076	0.658771634	0.654	1	51635	DHRS7
0.075933076	0.658771634	0.673	1	54877	ZCCHC2
0.075933076	0.658771634	0.681	1	440184	LINC00238
0.075675674	0.659859419	0.678	1	5052	PRDX1
0.075675674	0.659859419	0.698	1	79643	CHMP6
0.075675674	0.659859419	0.676	1	643853	TMPPE
0.075418279	0.660948038	0.697	1	5511	PPP1R8
0.075418279	0.660948038	0.705	1	26051	PPP1R16B
0.075418279	0.660948038	0.681	1	51550	AL137229.4
0.075418279	0.660948038	0.678	1	79607	FAM118B
0.075418279	0.660948038	0.702	1	79981	FRMD1
0.075160868	0.662037253	0.711	1	351	APP
0.075160868	0.662037253	0.689	1	3576	IL8
0.075160868	0.662037253	0.672	1	4329	ALDH6A1
0.075160868	0.662037253	0.711	1	5651	PRSS7
0.075160868	0.662037253	0.672	1	9681	DEPDC5
0.075160868	0.662037253	0.702	1	10241	CALCOCO2
0.075160868	0.662037253	0.72	1	30010	NXPH1
0.075160868	0.662037253	0.697	1	55186	SLC25A36
0.075160868	0.662037253	0.693	1	64222	TOR3A
0.075160868	0.662037253	0.707	1	284912	LOC284912
0.074903473	0.663127661	0.694	1	51187	RSL24D1
0.074903473	0.663127661	0.702	1	140706	C20orf160
0.074903473	0.663127661	0.712	1	157807	RLBP1L1
0.074903473	0.663127661	0.678	1	100272147	MTCP1NB
0.074646071	0.664218247	0.652	1	314	AOC2
0.074646071	0.664218247	0.692	1	5034	P4HB
0.074646071	0.664218247	0.676	1	6612	SUMO3
0.074646071	0.664218247	0.701	1	7941	PLA2G7
0.074646071	0.664218247	0.684	1	9903	KLHL21
0.074646071	0.664218247	0.68	1	10199	MPHOSPH10
0.074646071	0.664218247	0.693	1	54097	FAM3B
0.074646071	0.664218247	0.681	1	54870	QRICH1
0.074388668	0.665309548	0.68	1	7776	ZNF236
0.074388668	0.665309548	0.693	1	79774	GRTP1
0.074388668	0.665309548	0.702	1	152110	NEK10
0.074388668	0.665309548	0.681	1	166785	MMAA
0.074388668	0.665309548	0.672	1	203259	C9orf25
0.074388668	0.665309548	0.664	1	100129478	LOC100129478
0.074131273	0.666401565	0.714	1	6572	SLC18A3
0.074131273	0.666401565	0.701	1	7150	TOP1
0.074131273	0.666401565	0.688	1	83861	RSPH3
0.074131273	0.666401565	0.714	1	149069	DCDC2B
0.074131273	0.666401565	0.7	1	337879	KRTAP8-1
0.074131273	0.666401565	0.704	1	441478	NRARP
0.07387387	0.667494774	0.695	1	708	C1QBP
0.07387387	0.667494774	0.699	1	4247	MGAT2
0.07387387	0.667494774	0.682	1	4811	NID1
0.07387387	0.667494774	0.696	1	91893	FDXACB1
0.073616475	0.668588102	0.706	1	5439	POLR2J
0.073616475	0.668588102	0.683	1	7114	TMSB4X
0.073359072	0.669682086	0.706	1	30846	EHD2
0.073359072	0.669682086	0.672	1	57139	RGL3
0.073359072	0.669682086	0.681	1	57456	KIAA1143
0.073359072	0.669682086	0.703	1	80128	TRIM46
0.073359072	0.669682086	0.7	1	118881	COMTD1
0.073359072	0.669682086	0.677	1	728045	PPBPP1
0.07310167	0.670776844	0.713	1	59341	TRPV4
0.07310167	0.670776844	0.735	1	85363	TRIM5
0.07310167	0.670776844	0.696	1	162073	ITPRIPL2
0.072844274	0.671872735	0.711	1	9276	COPB2
0.072844274	0.671872735	0.73	1	60314	C12orf10

voxel-wise, subcortex			
Spearman's corr coef	p-val	GenID	Gene
0.095648348	6.64038E-18	218	ALDH3A1
0.095618255	6.80022E-18	83479	DDX59
0.095580928	7.00387E-18	6557	SLC12A1
0.095541365	7.22627E-18	5272	SERPINB9
0.095499948	7.46662E-18	389123	IQCF2
0.09546949	7.6483E-18	23352	UBR4
0.095452771	7.74988E-18	51368	TEX264
0.095398165	8.09114E-18	56647	BCCIP
0.095387951	8.15655E-18	57092	PCNP
0.095273539	8.9263E-18	10887	PROKR1
0.095263138	8.9997E-18	9733	SART3
0.095213644	9.35748E-18	113622	ADPRHL1
0.095193021	9.51054E-18	26354	GNL3
0.095156439	9.78843E-18	51337	C8orf55
0.095113717	1.0123E-17	25975	EGFL6
0.095105171	1.01912E-17	117608	ZNF354B
0.095072389	1.04573E-17	160728	SLC5A8
0.095060878	1.05523E-17	56999	ADAMTS9
0.095025122	1.0853E-17	100130717	CECR5-AS1
0.094963364	1.13925E-17	9143	SYNGR3
0.094956867	1.14507E-17	6835	SURF2
0.094934024	1.1658E-17	27175	TUBG2
0.094912939	1.18525E-17	3945	LDHB
0.094899848	1.19748E-17	441631	TSPAN11
0.094897151	1.20003E-17	285696	AC091878.1
0.094813757	1.28114E-17	80254	CEP63
0.094793141	1.30202E-17	10148	EBI3
0.094749406	1.34741E-17	7099	TLR4
0.094681241	1.42129E-17	285074	AC125232.1
0.094679937	1.42275E-17	23176	SEPTIN8
0.094655141	1.45064E-17	169792	GLIS3
0.094633788	1.47507E-17	340205	TREML1
0.094611593	1.50092E-17	10559	SLC35A1
0.094510831	1.62396E-17	249	ALPL
0.094485015	1.65706E-17	222894	FERD3L
0.094471455	1.6747E-17	3626	INHBC
0.094450466	1.70237E-17	79783	C7orf10
0.094363637	1.82177E-17	93979	CPA5
0.094349056	1.84261E-17	90187	EMILIN3
0.094316185	1.89047E-17	255031	FLJ35390
0.094278477	1.94687E-17	6928	HNF1B
0.094266497	1.96516E-17	283683	LOC283683
0.094229817	2.02214E-17	245937	DEFB124
0.094226755	2.02696E-17	84699	CREB3L3
0.094147071	2.15674E-17	143872	AP000872.1
0.09397722	2.46137E-17	10289	EIF1B
0.093920574	2.57212E-17	389541	C7orf59
0.093891881	2.63007E-17	139341	FUNDC1
0.093887053	2.63994E-17	55917	CTTNBP2NL
0.093872838	2.66923E-17	3853	KRT6A
0.093844607	2.72837E-17	2997	GYS1
0.093843587	2.73052E-17	197187	MGC23284
0.093778767	2.87134E-17	23710	GABARAPL1
0.093730666	2.98046E-17	219986	OR4D11
0.093718588	3.00847E-17	84319	C3orf26
0.0936286	3.22568E-17	338917	VSX2
0.093597956	3.30311E-17	390705	LOC390705
0.093544185	3.44345E-17	353133	LCE1C
0.093523107	3.50005E-17	27315	PGAP2
0.093480393	3.61754E-17	2357	FPR1
0.093460053	3.67489E-17	25950	RWDD3
0.093447976	3.70936E-17	7405	UVRAG
0.093420304	3.78952E-17	5033	P4HA1
0.093417704	3.79713E-17	22823	MTF2
0.093379721	3.91019E-17	24138	IFIT5
0.093355112	3.98517E-17	9601	PDIA4
0.093337655	4.03925E-17	11316	COPE
0.093306355	4.13804E-17	3376	IARS
0.09325593	4.30218E-17	759	CA1
0.09316697	4.60751E-17	645832	SEBOX
0.093089148	4.89213E-17	644139	PIRT
0.093057945	5.01106E-17	493829	TRIM72
0.093031757	5.11305E-17	7148	TNXB
0.092996836	5.25229E-17	23175	LPIN1
0.092878714	5.75144E-17	79869	CPSF7
0.092866734	5.8046E-17	400657	LOC400657
0.092862368	5.8241E-17	389831	LOC389831
0.092779905	6.20467E-17	7576	ZNF28
0.092730589	6.44394E-17	374378	GALNTL4

region-wise, subcortex					
Spearman's corr coef	p-val	p-val, SA	p-val, SA (BH- correction)	GenID	Gene
0.072844274	0.671872735	0.689	1	84547	PGBD1
0.072844274	0.671872735	0.696	1	388939	C2orf71
0.072844274	0.671872735	0.702	1	728047	AC135983.8
0.072586872	0.672968805	0.696	1	3416	IDE
0.072586872	0.672968805	0.699	1	4436	MSH2
0.072586872	0.672968805	0.703	1	8996	NOL3
0.072586872	0.672968805	0.682	1	11259	FILIP1L
0.072586872	0.672968805	0.724	1	114823	LENG8
0.072329476	0.67406553	0.711	1	5869	RAB5B
0.072329476	0.67406553	0.711	1	55052	MRPL20
0.072329476	0.67406553	0.71	1	100128775	LOC100128775
0.072072074	0.675162971	0.661	1	6272	SORT1
0.072072074	0.675162971	0.686	1	7321	UBE2D1
0.072072074	0.675162971	0.712	1	51513	ETV7
0.072072074	0.675162971	0.728	1	79135	APOO
0.072072074	0.675162971	0.705	1	84317	CCDC115
0.072072074	0.675162971	0.695	1	128872	HMGB3P1
0.071814671	0.676261008	0.707	1	3131	HLF
0.071814671	0.676261008	0.699	1	3146	HMGB1
0.071814671	0.676261008	0.701	1	8368	HIST1H4L
0.071814671	0.676261008	0.7	1	221481	C6orf81
0.071814671	0.676261008	0.685	1	730268	LOC730268
0.071557269	0.677360237	0.693	1	2826	CCR10
0.071557269	0.677360237	0.725	1	3352	HTR1D
0.071557269	0.677360237	0.7	1	3948	LDHC
0.071557269	0.677360237	0.683	1	5300	PIN1
0.071557269	0.677360237	0.708	1	10111	RAD50
0.071557269	0.677360237	0.689	1	10261	IGSF6
0.071557269	0.677360237	0.722	1	54550	NECAB2
0.071557269	0.677360237	0.702	1	133688	UGT3A1
0.071299866	0.678459704	0.721	1	1174	AP1S1
0.071299866	0.678459704	0.707	1	1810	DR1
0.071299866	0.678459704	0.71	1	4888	NPY6R
0.071299866	0.678459704	0.722	1	28976	ACAD9
0.071042471	0.679559708	0.702	1	1081	CGA
0.071042471	0.679559708	0.715	1	7419	VDAC3
0.071042471	0.679559708	0.685	1	92521	CYTSB
0.071042471	0.679559708	0.698	1	440354	AC009086.2
0.070785068	0.680660427	0.71	1	55809	TRERF1
0.070527665	0.681762338	0.679	1	1269	CNR2
0.070527665	0.681762338	0.703	1	3728	JUP
0.070527665	0.681762338	0.713	1	4001	LMNB1
0.070527665	0.681762338	0.71	1	7097	TLR2
0.070527665	0.681762338	0.695	1	57476	GRAMD1B
0.070527665	0.681762338	0.691	1	79315	OR7E91P
0.070527665	0.681762338	0.707	1	79444	BIRC7
0.070527665	0.681762338	0.715	1	84631	SLITRK2
0.070527665	0.681762338	0.694	1	113691	AC002472.8
0.070527665	0.681762338	0.706	1	147700	KLC3
0.07027027	0.682864368	0.694	1	645104	CLRN2
0.070012867	0.683967054	0.713	1	2690	GHR
0.070012867	0.683967054	0.703	1	4013	VWA5A
0.070012867	0.683967054	0.717	1	4548	MTR
0.070012867	0.683967054	0.701	1	5868	RAB5A
0.070012867	0.683967054	0.709	1	10946	SF3A3
0.070012867	0.683967054	0.717	1	80737	C6orf27
0.070012867	0.683967054	0.68	1	390354	RPL18AP3
0.069755472	0.685070455	0.694	1	1630	DCC
0.069755472	0.685070455	0.714	1	2902	GRIN1
0.069755472	0.685070455	0.722	1	84263	HSDL2
0.069755472	0.685070455	0.683	1	115509	ZNF689
0.069755472	0.685070455	0.711	1	645683	RPL13AP3
0.06949807	0.686174929	0.704	1	217	ALDH2
0.06949807	0.686174929	0.708	1	51398	WDR83OS
0.06949807	0.686174929	0.71	1	54435	HCG4
0.06949807	0.686174929	0.722	1	80231	CXorf21
0.06949807	0.686174929	0.709	1	80254	CEP63
0.069240667	0.687279582	0.739	1	83538	TTC25
0.069240667	0.687279582	0.71	1	386675	KRTAP10-7
0.068983272	0.688384891	0.717	1	10193	RNF41
0.068983272	0.688384891	0.708	1	23636	NUP62
0.068983272	0.688384891	0.731	1	27440	CECR5
0.068983272	0.688384891	0.729	1	63977	PRDM15
0.068983272	0.688384891	0.702	1	64425	POLR1E
0.068983272	0.688384891	0.715	1	83953	FCAMR
0.068983272	0.688384891	0.74	1	246705	LINC00314
0.068983272	0.688384891	0.716	1	653319	KIAA0895L
0.068725869	0.689490855	0.737	1	3083	HGFAC
0.068725869	0.689490855	0.717	1	4593	MUSK

voxel-wise, subcortex			
Spearman's corr coef	p-val	GenID	Gene
0.092600949	7.11702E-17	259294	TAS2R48
0.092600115	7.12159E-17	100133920	LOC100133920
0.092595562	7.14646E-17	10603	SH2B2
0.092587948	7.18822E-17	23221	RHOBTB2
0.092509106	7.63538E-17	8402	SLC25A11
0.092507899	7.64241E-17	387758	FIBIN
0.092503347	7.6691E-17	9482	STX8
0.092433237	8.09152E-17	729992	ST13P1
0.092430726	8.10708E-17	3217	HOXB7
0.092417538	8.18923E-17	55650	PIGV
0.092279449	9.1001E-17	24142	NAT6
0.092256695	9.25947E-17	2568	GABRP
0.092242211	9.36242E-17	3709	ITPR2
0.092230044	9.44973E-17	118426	LOH12CR1
0.092181288	9.80769E-17	9443	MED7
0.092151202	1.00352E-16	755	C21orf2
0.092076346	1.06242E-16	136647	C7orf11
0.092070684	1.06701E-16	286075	ZNF707
0.092048399	1.08526E-16	347344	ZNF81
0.092037901	1.09398E-16	4868	NPHS1
0.092032328	1.09863E-16	26538	OR10H2
0.092017472	1.11111E-16	56108	PCDHGA7
0.09201283	1.11505E-16	132160	PPM1M
0.091980323	1.14298E-16	57563	KLHL8
0.09194281	1.17606E-16	284532	OR14A16
0.09193185	1.1859E-16	1363	CPE
0.091877431	1.23598E-16	9439	MED23
0.091861919	1.25064E-16	151325	MYADML
0.091830999	1.28037E-16	3670	ISL1
0.091778435	1.3325E-16	4706	NDUFAB1
0.091764316	1.34686E-16	401612	MCART6
0.091762744	1.34847E-16	100129321	LOC100129321
0.091748998	1.36261E-16	126299	ZNF428
0.091608301	1.51602E-16	9847	KIAA0528
0.091567069	1.56412E-16	84441	MAML2
0.091543853	1.59187E-16	23038	WDTC1
0.09151005	1.63312E-16	100128108	RP11-89K11.1
0.091503456	1.6413E-16	728743	LOC728743
0.091492593	1.65486E-16	441108	C5orf56
0.091410503	1.76087E-16	157769	FAM91A1
0.091383383	1.79734E-16	256472	TMEM151A
0.0913155	1.89194E-16	112399	EGLN3
0.091295905	1.92015E-16	1584	CYP11B1
0.091275193	1.95041E-16	55663	ZNF446
0.091269344	1.95905E-16	80183	C13orf18
0.091204986	2.05655E-16	353135	LCE1E
0.091170259	2.11113E-16	285172	FAM126B
0.091107108	2.21406E-16	2797	GNRH2
0.091034576	2.3384E-16	163351	GBP6
0.091012381	2.37782E-16	3416	IDE
0.090993717	2.41145E-16	6818	SULT1A3
0.090965487	2.46322E-16	1197	CLK2P
0.090908282	2.57156E-16	7468	WHSC1
0.090895839	2.59571E-16	2178	FANCE
0.090894535	2.59827E-16	29945	ANAPC4
0.090788946	2.81283E-16	51068	NMD3
0.090765454	2.86288E-16	8372	HYAL3
0.090749204	2.89802E-16	64901	RANBP17
0.090730719	2.93852E-16	283551	C14orf182
0.090687446	3.03551E-16	9715	FAM131B
0.090640828	3.1435E-16	5308	PITX2
0.090601452	3.2377E-16	81448	OR6K2
0.090510346	3.46635E-16	118433	RPL23AP7
0.090498559	3.49707E-16	55209	SETD5
0.090424359	3.69676E-16	9806	SPOCK2
0.090378575	3.82551E-16	730094	C16ORF52
0.090247542	4.21901E-16	728661	RP11-345P4.2
0.090214573	4.32409E-16	51529	ANAPC11
0.09016823	4.47622E-16	165186	FAM179A
0.090154395	4.52266E-16	80222	TARS2
0.090083256	4.76899E-16	58478	ENOPH1
0.090051502	4.8832E-16	92806	AC092143.3
0.089947306	5.27712E-16	342865	VSTM2B
0.089903288	5.45278E-16	90378	SAMD1
0.089894556	5.48834E-16	91300	C19orf22
0.089866325	5.60472E-16	9865	TRIL
0.089779682	5.97749E-16	93010	B3GNT7
0.089763425	6.0501E-16	56897	WRNIP1
0.089757115	6.07848E-16	4582	MUC1

region-wise, subcortex					
Spearman's corr coef	p-val	p-val, SA	p-val, SA (BH- correction)	GenID	Gene
0.068725869	0.689490855	0.696	1	6141	RPL18
0.068725869	0.689490855	0.73	1	51029	PPPDE1
0.068725869	0.689490855	0.725	1	79668	PARP8
0.068725869	0.689490855	0.716	1	114827	FHAD1
0.068468474	0.690597415	0.717	1	5310	PKD1
0.068468474	0.690597415	0.746	1	9324	HMGN3
0.068468474	0.690597415	0.726	1	55014	STX17
0.068468474	0.690597415	0.721	1	65993	MRPS34
0.068468474	0.690597415	0.732	1	83892	KCTD10
0.068468474	0.690597415	0.69	1	112479	ERI2
0.068468474	0.690597415	0.707	1	283171	C11orf44
0.068211071	0.691705108	0.7	1	4141	MARS
0.068211071	0.691705108	0.727	1	23404	EXOSC2
0.068211071	0.691705108	0.719	1	137835	TMEM71
0.068211071	0.691705108	0.731	1	197257	LDHD
0.067953661	0.692812979	0.729	1	53	ACP2
0.067953661	0.692812979	0.728	1	5179	PENK
0.067953661	0.692812979	0.731	1	8522	GAS7
0.067953661	0.692812979	0.724	1	51315	KRCC1
0.067953661	0.692812979	0.707	1	79917	MAGIX
0.067953661	0.692812979	0.709	1	81622	UNC93B3
0.067953661	0.692812979	0.727	1	84467	FBN3
0.067953661	0.692812979	0.71	1	375567	VWC2
0.067953661	0.692812979	0.742	1	728855	BX248398.1
0.067696266	0.693921447	0.721	1	11072	DUSP14
0.067696266	0.693921447	0.714	1	27248	C2orf30
0.067696266	0.693921447	0.737	1	54997	TESC
0.067696266	0.693921447	0.718	1	221154	EFHA1
0.067696266	0.693921447	0.717	1	284184	C17orf89
0.067438863	0.69503063	0.708	1	1718	DHCR24
0.067438863	0.69503063	0.711	1	79629	OCEL1
0.067438863	0.69503063	0.724	1	114800	CCDC85A
0.067438863	0.69503063	0.711	1	388569	ZNF324B
0.067181468	0.696140885	0.71	1	23677	SH3BP4
0.067181468	0.696140885	0.729	1	57214	KIAA1199
0.067181468	0.696140885	0.704	1	80032	ZNF556
0.067181468	0.696140885	0.697	1	284600	RP11-54O7.1
0.067181468	0.696140885	0.704	1	340947	LOC340947
0.066924065	0.69725126	0.697	1	55651	NHP2
0.066924065	0.69725126	0.703	1	116969	ART5
0.066924065	0.69725126	0.712	1	254559	LOC254559
0.066666663	0.698362231	0.724	1	130540	ALS2CR12
0.066666663	0.698362231	0.705	1	200197	C1orf126
0.066666663	0.698362231	0.718	1	221938	MMD2
0.066666663	0.698362231	0.726	1	400236	RP11-33N16.1
0.066409267	0.699473917	0.735	1	2218	FKTN
0.066409267	0.699473917	0.725	1	2561	GABRB2
0.066409267	0.699473917	0.73	1	85013	TMEM128
0.066151865	0.700586677	0.726	1	6863	TAC1
0.066151865	0.700586677	0.718	1	23358	USP24
0.066151865	0.700586677	0.728	1	84187	TMEM164
0.066151865	0.700586677	0.717	1	337867	UBAC2
0.066151865	0.700586677	0.717	1	400720	ZNF772
0.066151865	0.700586677	0.724	1	100132911	DPH3P1
0.06589447	0.701699555	0.732	1	1906	EDN1
0.06589447	0.701699555	0.725	1	6176	RPLP1
0.06589447	0.701699555	0.693	1	10989	IMMT
0.06589447	0.701699555	0.732	1	78990	OTUB2
0.06589447	0.701699555	0.734	1	91522	COL23A1
0.06589447	0.701699555	0.747	1	100133920	LOC100133920
0.065637067	0.702813089	0.756	1	27314	RAB30
0.065637067	0.702813089	0.717	1	81928	CABLES2
0.065637067	0.702813089	0.706	1	285550	FAM200B
0.065379664	0.703927219	0.747	1	4037	LRP3
0.065379664	0.703927219	0.736	1	54753	AC073343.8
0.065379664	0.703927219	0.726	1	116541	MRPL54
0.065379664	0.703927219	0.742	1	157695	C8orf42
0.065379664	0.703927219	0.738	1	283742	FAM98B
0.065379664	0.703927219	0.734	1	389541	C7orf59
0.065379664	0.703927219	0.723	1	100133204	C9orf147
0.065122269	0.705041945	0.741	1	1370	CPN2
0.065122269	0.705041945	0.716	1	2193	FARSA
0.065122269	0.705041945	0.713	1	2331	FMOD
0.065122269	0.705041945	0.732	1	8662	EIF3B
0.065122269	0.705041945	0.714	1	55108	BSDC1
0.065122269	0.705041945	0.715	1	84897	TBRG1
0.065122269	0.705041945	0.719	1	85236	HIST1H2BK
0.065122269	0.705041945	0.707	1	286006	C7orf53
0.064864866	0.706157804	0.773	1	1768	DNAH6

voxel-wise, subcortex			
Spearman's corr coef	p-val	GenID	Gene
0.089735381	6.17738E-16	7512	XPNPEP2
0.08966852	6.49164E-16	51673	TPPP3
0.089566462	7.00194E-16	643904	RNF222
0.089499228	7.35949E-16	5621	PRNP
0.089476563	7.48401E-16	2332	FMR1
0.089471176	7.51394E-16	285601	GPR150
0.089441463	7.68099E-16	146691	TOM1L2
0.089397632	7.93415E-16	254887	ZDHHC23
0.08939568	7.94562E-16	5422	POLA1
0.089363918	8.13451E-16	6490	SILV
0.089362994	8.14002E-16	10972	TMED10
0.089351751	8.20795E-16	81833	SPACA1
0.089342371	8.26507E-16	5998	RGS3
0.08926715	8.73749E-16	85569	GALP
0.089208089	9.12685E-16	10767	HBS1L
0.089180134	9.31715E-16	60492	CCDC90B
0.08917354	9.36256E-16	51274	KLF3
0.089111418	9.80156E-16	5724	PTAFR
0.08910501	9.84793E-16	219902	TMEM136
0.089064054	1.01497E-15	9294	S1PR2
0.089055695	1.02125E-15	83706	FERMT3
0.088900425	1.14492E-15	2912	GRM2
0.088845357	1.19224E-15	128486	FITM2
0.088811181	1.22257E-15	255057	C19orf26
0.088793069	1.23895E-15	83862	TMEM120A
0.088715345	1.31172E-15	2494	NR5A2
0.088621266	1.40547E-15	10333	TLR6
0.088569544	1.45977E-15	55502	HES6
0.088556081	1.47425E-15	729952	HMGB3P6
0.088474914	1.56455E-15	158046	NXNL2
0.088364214	1.69654E-15	196051	PPAPDC1A
0.088256583	1.83537E-15	253190	SERHL2
0.088252224	1.84123E-15	10553	HTATIP2
0.088185355	1.93333E-15	1840	DTX1
0.088183314	1.93622E-15	339855	KY
0.08815629	1.97478E-15	8386	OR1D5
0.088092402	2.06896E-15	5775	PTPN4
0.088079304	2.08881E-15	9678	PHF14
0.088059522	2.11913E-15	79096	C11orf49
0.088029154	2.16655E-15	55773	TBC1D23
0.087957554	2.28252E-15	3598	IL13RA2
0.087925054	2.33715E-15	8930	MBD4
0.087882519	2.41059E-15	162514	TRPV3
0.087838687	2.48865E-15	222166	C7orf41
0.087810643	2.5399E-15	28974	C19orf53
0.087671161	2.81061E-15	6945	MLX
0.087619245	2.91848E-15	100188893	TOMM6
0.087576531	3.01027E-15	2288	FKBP4
0.087484129	3.21865E-15	84365	MKI67IP
0.087443449	3.31484E-15	9933	KIAA0020
0.087430634	3.34573E-15	1520	CTSS
0.087356716	3.52948E-15	100131755	ARMCX4
0.087315015	3.63747E-15	6636	SNRPF
0.087284371	3.71891E-15	112942	CCDC104
0.08728233	3.7244E-15	441531	PGAM4
0.087248616	3.8162E-15	84286	TMEM175
0.087202184	3.94627E-15	137814	NKX2-6
0.087198101	3.95792E-15	340745	LRIT2
0.087197632	3.95926E-15	6597	SMARCA4
0.087175809	4.02212E-15	3663	IRF5
0.087172098	4.03287E-15	60386	SLC25A19
0.087059915	4.37266E-15	140680	C20orf96
0.087057874	4.37909E-15	55885	LMO3
0.086975962	4.6452E-15	403282	OR6C65
0.086964265	4.68451E-15	1412	CRYBA2
0.086846232	5.09961E-15	3762	KCNJ5
0.086825058	5.17781E-15	1903	S1PR3
0.086812794	5.2236E-15	10232	MSLN
0.086799614	5.27333E-15	5879	RAC1
0.086718164	5.59095E-15	85280	KRTAP9-4
0.086642854	5.90135E-15	27125	AFF4
0.086640157	5.91281E-15	4261	CIITA
0.086583324	6.15868E-15	5164	PDK2
0.086526029	6.41676E-15	29066	ZC3H7A
0.086498171	6.54602E-15	3611	ILK
0.086484142	6.61213E-15	23409	SIRT4
0.086467803	6.68993E-15	84900	RNFT2
0.086440496	6.82195E-15	128872	HMGB3P1
0.086315967	7.45721E-15	85012	TCEAL3

region-wise, subcortex					
Spearman's corr coef	p-val	p-val, SA	p-val, SA (BH- correction)	GenID	Gene
0.064864866	0.706157804	0.734	1	8813	DPM1
0.064864866	0.706157804	0.727	1	57146	TMEM159
0.064864866	0.706157804	0.7	1	118487	CHCHD1
0.064864866	0.706157804	0.727	1	285755	PPIL6
0.064607456	0.707273722	0.733	1	9493	KIF23
0.064607456	0.707273722	0.747	1	55072	RNF31
0.064607456	0.707273722	0.724	1	59274	MESDC1
0.064607456	0.707273722	0.735	1	116843	C6orf192
0.064350061	0.708390355	0.749	1	2650	GCNT1
0.064350061	0.708390355	0.727	1	10036	CHAF1A
0.064350061	0.708390355	0.738	1	11130	ZWINT
0.064350061	0.708390355	0.734	1	64386	MMP25
0.064350061	0.708390355	0.754	1	84561	SLC12A8
0.064350061	0.708390355	0.732	1	84640	USP38
0.064092666	0.709507525	0.714	1	9310	ZNF235
0.064092666	0.709507525	0.739	1	11076	TPPP
0.064092666	0.709507525	0.728	1	388403	YPEL2
0.063835263	0.710625768	0.736	1	7107	GPR137B
0.063835263	0.710625768	0.738	1	134083	OR2Y1
0.063835263	0.710625768	0.736	1	280660	SSX9
0.063835263	0.710625768	0.711	1	337968	KRTAP6-3
0.063835263	0.710625768	0.748	1	390110	ACCSL
0.063577861	0.711744189	0.738	1	968	CD68
0.063577861	0.711744189	0.755	1	3613	IMPA2
0.063577861	0.711744189	0.752	1	5104	SERPINA5
0.063577861	0.711744189	0.749	1	8970	HIST1H2BJ
0.063577861	0.711744189	0.708	1	55291	SAPS3
0.063320458	0.712863147	0.724	1	147	ADRA1B
0.063320458	0.712863147	0.739	1	10651	MTX2
0.063320458	0.712863147	0.718	1	51321	AMZ2
0.063320458	0.712863147	0.735	1	54165	DCUN1D1
0.063320458	0.712863147	0.741	1	79791	FBXO31
0.063320458	0.712863147	0.764	1	220115	TPTE2P3
0.063320458	0.712863147	0.716	1	727957	HEATR7A
0.063063063	0.713982701	0.73	1	1018	CDK3
0.063063063	0.713982701	0.755	1	1730	DIAPH2
0.063063063	0.713982701	0.752	1	6506	SLC1A2
0.063063063	0.713982701	0.727	1	7936	RDBP
0.063063063	0.713982701	0.721	1	8800	PEX11A
0.063063063	0.713982701	0.753	1	51061	TXNDC11
0.063063063	0.713982701	0.735	1	113246	C12orf57
0.063063063	0.713982701	0.73	1	253512	SLC25A30
0.063063063	0.713982701	0.734	1	337966	KRTAP6-1
0.063063063	0.713982701	0.724	1	339742	FAM201B
0.06280566	0.715103447	0.726	1	88	ACTN2
0.06280566	0.715103447	0.742	1	4731	NDUFV3
0.06280566	0.715103447	0.725	1	23595	ORC3L
0.06280566	0.715103447	0.732	1	54543	TOMM7
0.06280566	0.715103447	0.736	1	114815	SORCS1
0.06280566	0.715103447	0.74	1	339184	CCDC144NL
0.062548265	0.716224253	0.74	1	1062	CENPE
0.062548265	0.716224253	0.723	1	1656	DDX6
0.062548265	0.716224253	0.756	1	9841	ZBTB24
0.062548265	0.716224253	0.75	1	23582	CCNDBP1
0.062290862	0.717345595	0.752	1	4848	CNOT2
0.062290862	0.717345595	0.749	1	9695	EDEM1
0.062290862	0.717345595	0.744	1	29903	CCDC106
0.062290862	0.717345595	0.722	1	57453	DSCAML1
0.062290862	0.717345595	0.743	1	79068	FTO
0.062290862	0.717345595	0.763	1	112495	GTF3C6
0.062290862	0.717345595	0.736	1	374899	ZNF829
0.06203346	0.718467534	0.737	1	1800	DPEP1
0.06203346	0.718467534	0.746	1	1959	EGR2
0.06203346	0.718467534	0.753	1	2907	GRINA
0.06203346	0.718467534	0.736	1	3004	GZMM
0.06203346	0.718467534	0.756	1	3658	IREB2
0.06203346	0.718467534	0.723	1	4246	SCGB2A1
0.06203346	0.718467534	0.726	1	23142	DCUN1D4
0.06203346	0.718467534	0.748	1	55764	IFT122
0.06203346	0.718467534	0.754	1	90580	C19orf52
0.061776061	0.719590068	0.728	1	6161	RPL32
0.061776061	0.719590068	0.724	1	6758	SSX5
0.061776061	0.719590068	0.747	1	51052	PRLH
0.061776061	0.719590068	0.755	1	79977	GRHL2
0.061776061	0.719590068	0.742	1	84311	MRPL45
0.061776061	0.719590068	0.734	1	115024	NT5C3L
0.061776061	0.719590068	0.735	1	100129128	RP11-257K9.7
0.061518662	0.720713735	0.714	1	2596	GAP43
0.061518662	0.720713735	0.74	1	5244	ABCB4

voxel-wise, subcortex			
Spearman's corr coef	p-val	GenID	Gene
0.086310856	7.48449E-15	23546	SYNGR4
0.086263493	7.74204E-15	6992	PPP1R11
0.086180381	8.21523E-15	23359	FAM189A1
0.086163014	8.31759E-15	2938	GSTA1
0.086126983	8.53406E-15	1369	CPN1
0.085969388	9.54796E-15	219477	OR8J1
0.085951559	9.66986E-15	1121	CHM
0.085892685	1.00834E-14	81620	CDT1
0.085852474	1.03758E-14	55957	LIN37
0.085830182	1.05414E-14	64682	ANAPC1
0.08580938	1.06985E-14	285180	RUFY4
0.085723013	1.1375E-14	5977	DPF2
0.085656524	1.19244E-14	10912	GADD45G
0.085627273	1.21743E-14	80821	DDHD1
0.085618727	1.22483E-14	4795	NFKBIL1
0.085581861	1.25725E-14	5991	RFX3
0.085552417	1.28375E-14	89839	ARHGAP11B
0.085540995	1.29418E-14	51322	WAC
0.085508585	1.32422E-14	5920	RARRES3
0.085504591	1.32797E-14	5613	PRKX
0.085501626	1.33076E-14	8192	CLPP
0.085480727	1.35059E-14	51008	ASCC1
0.085424356	1.40554E-14	5582	PRKCG
0.085408293	1.4216E-14	22866	CNKSR2
0.085360467	1.47048E-14	58493	C9orf80
0.085346632	1.48493E-14	283303	C11ORF36
0.085322298	1.51068E-14	7638	ZNF221
0.085318029	1.51525E-14	11079	RER1
0.085271038	1.56636E-14	6575	SLC20A2
0.085239649	1.60145E-14	10469	TIMM44
0.085217357	1.62685E-14	7678	ZNF124
0.085118085	1.74482E-14	55341	LSG1
0.085081309	1.79062E-14	196446	C12orf28
0.085062832	1.81407E-14	9282	MED14
0.085046209	1.83544E-14	5713	PSMD7
0.085043885	1.83844E-14	79666	PLEKHF2
0.085026518	1.86106E-14	129804	FBLN7
0.085019834	1.86983E-14	114659	LRRC37B
0.084973119	1.93234E-14	114821	SCAND3
0.084949531	1.96467E-14	79959	CEP76
0.084931798	1.98932E-14	84675	TRIM55
0.084879145	2.06434E-14	55146	ZDHHC4
0.08487422	2.0715E-14	51313	C4orf18
0.084748015	2.26347E-14	220441	RNF152
0.084739283	2.27737E-14	26468	LHX6
0.084720619	2.3074E-14	9217	VAPB
0.084699444	2.34192E-14	8392	OR3A3
0.084615499	2.48387E-14	4488	MSX2
0.084574632	2.556E-14	10013	HDAC6
0.084567949	2.56799E-14	9308	CD83
0.084520213	2.65526E-14	9047	SH2D2A
0.084514089	2.66667E-14	9942	XYLB
0.08448223	2.72678E-14	6978	TRGV5
0.084474802	2.74098E-14	222487	GPR97
0.084315352	3.0641E-14	2041	EPHA1
0.084294178	3.10972E-14	3067	HDC
0.08427681	3.14766E-14	255101	CCDC108
0.084257215	3.19099E-14	124599	CD300LB
0.084216356	3.28324E-14	8536	CAMK1
0.084191099	3.34159E-14	57585	CRAMP1L
0.084183574	3.35916E-14	55172	C14orf104
0.084161937	3.41021E-14	10272	FSTL3
0.084112346	3.53008E-14	118738	ZNF488
0.084064521	3.64966E-14	1109	AKR1C4
0.084036194	3.72233E-14	2036	EPB41L1
0.083998308	3.82175E-14	8973	CHRNA6
0.083834216	4.28341E-14	49860	CRNN
0.083774596	4.46437E-14	597	BCL2A1
0.083745621	4.55502E-14	124912	SPACA3
0.083740607	4.57087E-14	132884	EVC2
0.083717018	4.64628E-14	148113	CILP2
0.083697237	4.71044E-14	388650	FAM69A
0.083694912	4.71803E-14	57528	KCTD16
0.083692409	4.72621E-14	10584	COLEC10
0.083680056	4.76687E-14	6343	SCT
0.083659068	4.83671E-14	57826	RAP2C
0.083536766	5.2641E-14	199786	FAM129C
0.083481789	5.46813E-14	1339	COX6A2
0.083453558	5.57589E-14	51808	PHAX

region-wise, subcortex					
Spearman's corr coef	p-val	p-val, SA	p-val, SA (BH- correction)	GenID	Gene
0.061518662	0.720713735	0.745	1	5409	PNMT
0.061518662	0.720713735	0.751	1	6039	RNASE6
0.061518662	0.720713735	0.749	1	7587	ZNF37A
0.061518662	0.720713735	0.755	1	25792	CIZ1
0.061518662	0.720713735	0.753	1	54499	TMCO1
0.061518662	0.720713735	0.745	1	261729	STEAP2
0.061518662	0.720713735	0.729	1	286140	RNF5P1
0.061518662	0.720713735	0.711	1	728621	CCDC30
0.061261259	0.721837342	0.745	1	1439	CSF2RB
0.061261259	0.721837342	0.749	1	4267	CD99
0.061261259	0.721837342	0.745	1	10087	COL4A3BP
0.061261259	0.721837342	0.765	1	26049	FAM169A
0.061261259	0.721837342	0.731	1	27143	KIAA1274
0.061261259	0.721837342	0.726	1	60682	SMAP1
0.061261259	0.721837342	0.753	1	401375	GTF2IRD2P1
0.06100386	0.722961724	0.746	1	2526	FUT4
0.06100386	0.722961724	0.73	1	8405	SPOP
0.06100386	0.722961724	0.766	1	51754	TMEM8B
0.06100386	0.722961724	0.746	1	55823	VPS11
0.06100386	0.722961724	0.743	1	81789	TIGD6
0.06100386	0.722961724	0.713	1	343171	OR2W3
0.060746461	0.724086583	0.73	1	4008	LMO7
0.060746461	0.724086583	0.745	1	8894	EIF2S2
0.060746461	0.724086583	0.724	1	10734	STAG3
0.060746461	0.724086583	0.745	1	55114	ARHGAP17
0.060746461	0.724086583	0.763	1	286336	FAM78A
0.060746461	0.724086583	0.741	1	442361	OR2A2
0.060489062	0.725212514	0.752	1	5577	PRKAR2B
0.060489062	0.725212514	0.738	1	6338	SCNN1B
0.060489062	0.725212514	0.729	1	23122	CLASP2
0.060489062	0.725212514	0.728	1	64121	RRAGC
0.060489062	0.725212514	0.729	1	414149	ACBD7
0.060489062	0.725212514	0.738	1	414318	C9orf106
0.060231656	0.726338565	0.752	1	6571	SLC18A2
0.060231656	0.726338565	0.733	1	84181	CHD6
0.059974257	0.727465153	0.748	1	820	CAMP
0.059974257	0.727465153	0.731	1	6677	SPAM1
0.059974257	0.727465153	0.722	1	6999	TDO2
0.059974257	0.727465153	0.763	1	7643	ZNF90
0.059974257	0.727465153	0.756	1	9135	RABEP1
0.059974257	0.727465153	0.74	1	90353	ATPBD3
0.059716858	0.728592277	0.731	1	2627	GATA6
0.059716858	0.728592277	0.737	1	23032	USP33
0.059716858	0.728592277	0.721	1	65061	PFTK2
0.059716858	0.728592277	0.753	1	283849	EXOC3L
0.059716858	0.728592277	0.746	1	375056	MIA3
0.059716858	0.728592277	0.719	1	389114	ZNF662
0.059459459	0.729720533	0.758	1	5908	RAP1B
0.059459459	0.729720533	0.772	1	22839	DLGAP4
0.059459459	0.729720533	0.731	1	23624	CBLC
0.059459459	0.729720533	0.758	1	55664	CDC37L1
0.059459459	0.729720533	0.743	1	728698	EIF4A1P4
0.059459459	0.729720533	0.741	1	100287497	RP11-504P24.4
0.05920206	0.730848849	0.724	1	5092	PCBD1
0.05920206	0.730848849	0.767	1	5881	RAC3
0.05920206	0.730848849	0.772	1	9379	NRXN2
0.05920206	0.730848849	0.774	1	79915	ATAD5
0.05920206	0.730848849	0.728	1	119764	OR4X2
0.05920206	0.730848849	0.748	1	200558	APLF
0.05920206	0.730848849	0.74	1	253738	EBF3
0.05920206	0.730848849	0.761	1	646643	SBK2
0.058944657	0.731977761	0.754	1	2548	GAA
0.058944657	0.731977761	0.759	1	3120	HLA-DQB2
0.058944657	0.731977761	0.745	1	25849	AC110760.2
0.058944657	0.731977761	0.743	1	51526	C20orf111
0.058944657	0.731977761	0.757	1	100128062	SLC2A3P1
0.058687259	0.73310715	0.766	1	54981	C9orf95
0.058687259	0.73310715	0.738	1	388611	CD164L2
0.058429856	0.734237075	0.764	1	3484	IGFBP1
0.058429856	0.734237075	0.75	1	3652	IPP
0.058429856	0.734237075	0.747	1	7036	TFR2
0.058429856	0.734237075	0.746	1	8624	PSMG1
0.058429856	0.734237075	0.756	1	26273	FBXO3
0.058429856	0.734237075	0.753	1	55937	APOM
0.058429856	0.734237075	0.757	1	64901	RANBP17
0.058429856	0.734237075	0.748	1	388536	ZNF790
0.058172457	0.735368133	0.769	1	29080	CCDC59
0.058172457	0.735368133	0.765	1	56005	C19orf10
0.058172457	0.735368133	0.758	1	127540	HMGB4

voxel-wise, subcortex			
Spearman's corr coef	p-val	GenID	Gene
0.083435357	5.64648E-14	100505929	C21orf37
0.083380938	5.86281E-14	3931	LCAT
0.083272465	6.31858E-14	9712	USP6NL
0.083260484	6.37103E-14	220416	LRRC63
0.083194092	6.66938E-14	64743	WDR13
0.083192885	6.67494E-14	23483	TGDS
0.083176441	6.75098E-14	79672	FN3KRP
0.083146915	6.8897E-14	25792	CIZ1
0.083116919	7.03348E-14	2775	GNAO1
0.083095461	7.13818E-14	53904	MYO3A
0.083081812	7.20554E-14	1030	CDKN2B
0.082982726	7.7137E-14	9388	LIPG
0.082962483	7.82179E-14	54988	ACSM5
0.082960621	7.83183E-14	84878	ZBTB45
0.082929976	7.99845E-14	1117	CHI3L2
0.082840547	8.50496E-14	5936	RBM4
0.082793556	8.78358E-14	53916	RAB4B
0.08275409	9.02458E-14	5866	RAB3IL1
0.082745455	9.07811E-14	201626	PDE12
0.082725674	9.20202E-14	23029	RBM34
0.08270552	9.32996E-14	3281	HSBP1
0.08264599	9.71827E-14	2019	EN1
0.082594916	1.0064E-13	92370	ACPL2
0.08258684	1.01198E-13	51083	GAL
0.082573183	1.02148E-13	2923	PDIA3
0.082470477	1.09578E-13	143941	TTC36
0.082458407	1.10485E-13	54741	LEPROT
0.082442991	1.11654E-13	63943	FKBPL
0.082410485	1.1416E-13	401145	FAM190A
0.082400359	1.14952E-13	54823	C1orf26
0.08237882	1.16654E-13	23774	BRD1
0.082346126	1.19287E-13	219348	PLAC9
0.082286231	1.24259E-13	165055	CCDC138
0.082278989	1.24875E-13	374786	EFCAB5
0.0822189	1.30094E-13	11216	AKAP10
0.082174793	1.34061E-13	54977	SLC25A38
0.082106255	1.40462E-13	7221	TRPC2
0.082102261	1.40844E-13	116236	ABHD15
0.082079791	1.43013E-13	100131471	LOC100131471
0.082056575	1.45289E-13	79001	VKORC1
0.082033545	1.47582E-13	645676	RP11-29H23.1
0.082004756	1.50497E-13	94235	GNG8
0.08199092	1.51918E-13	219482	OR5M3
0.081985347	1.52494E-13	115749	C12orf56
0.081917927	1.59638E-13	374407	DNAJB13
0.081874274	1.64437E-13	29083	GTPBP8
0.081834346	1.68951E-13	728673	LOC728673
0.081811778	1.71556E-13	171423	PDIA3P
0.081765249	1.77052E-13	27097	TAF5L
0.081746124	1.7936E-13	79731	NARS2
0.08171501	1.8318E-13	171391	NS3BP
0.081713527	1.83364E-13	286530	P2RY8
0.08168149	1.87384E-13	9736	USP34
0.081647128	1.91792E-13	8795	TNFRSF10B
0.081502996	2.11419E-13	81555	YIPF5
0.081482291	2.14395E-13	5687	PSMA6
0.081478484	2.14948E-13	220594	AC107983.3
0.081430562	2.22013E-13	710	SERPING1
0.081374191	2.30619E-13	335	APOA1
0.081363976	2.32213E-13	11188	NISCH
0.081239723	2.52489E-13	51497	TH1L
0.081227556	2.54565E-13	83892	KCTD10
0.081177041	2.63368E-13	79870	BAALC
0.081098936	2.77568E-13	9363	RAB33A
0.080999292	2.96782E-13	147699	PPM1N
0.080973759	3.01912E-13	2314	FLII
0.080954254	3.05891E-13	23233	EXOC6B
0.080927603	3.11407E-13	166614	DCLK2
0.080925278	3.11894E-13	5447	POR
0.080833897	3.31595E-13	10574	CCT7
0.080800936	3.38998E-13	157680	VPS13B
0.080800653	3.39061E-13	3621	ING1
0.080748186	3.51184E-13	51188	SS18L2
0.080745585	3.51794E-13	29882	ANAPC2
0.080653645	3.74108E-13	9706	ULK2
0.080636188	3.78497E-13	811	CALR
0.08063554	3.78662E-13	1656	DDX6
0.080632098	3.79534E-13	440061	TRIM64EP
0.080617711	3.83199E-13	145501	ISM2

region-wise, subcortex					
Spearman's corr coef	p-val	p-val, SA	p-val, SA (BH- correction)	GenID	Gene
0.058172457	0.735368133	0.754	1	375057	C1orf95
0.058172457	0.735368133	0.77	1	387978	C14orf23
0.057915058	0.73649925	0.764	1	4610	MYCL1
0.057915058	0.73649925	0.774	1	10841	FTCD
0.057915058	0.73649925	0.748	1	100130522	PARD6G-AS1
0.057657655	0.737630844	0.766	1	811	CALR
0.057657655	0.737630844	0.756	1	2251	FGF6
0.057657655	0.737630844	0.761	1	6576	SLC25A1
0.057657655	0.737630844	0.748	1	81606	LBH
0.057657655	0.737630844	0.768	1	140881	DEFB129
0.057657655	0.737630844	0.763	1	220965	FAM13C
0.057400256	0.738763094	0.739	1	3550	IK
0.057400256	0.738763094	0.763	1	5347	PLK1
0.057400256	0.738763094	0.755	1	55130	ARMC4
0.057400256	0.738763094	0.749	1	79571	GCC1
0.057142857	0.739896357	0.747	1	5116	PCNT
0.057142857	0.739896357	0.766	1	5871	MAP4K2
0.057142857	0.739896357	0.744	1	6097	RORC
0.057142857	0.739896357	0.759	1	10267	RAMP1
0.057142857	0.739896357	0.763	1	79663	HSPBAP1
0.057142857	0.739896357	0.787	1	114882	OSBPL8
0.057142857	0.739896357	0.771	1	202018	TAPT1
0.057142857	0.739896357	0.768	1	389856	USP27X
0.056885459	0.74102962	0.781	1	10159	ATP6AP2
0.056885459	0.74102962	0.753	1	11284	PNKP
0.056885459	0.74102962	0.77	1	22862	FNDC3A
0.056885459	0.74102962	0.746	1	23403	FBXO46
0.056885459	0.74102962	0.771	1	51763	INPP5K
0.056885459	0.74102962	0.767	1	56257	MEPCE
0.056885459	0.74102962	0.778	1	64978	MRPL38
0.056885459	0.74102962	0.778	1	80305	TRABD
0.056885459	0.74102962	0.757	1	84662	GLIS2
0.056885459	0.74102962	0.787	1	120376	AP002448.1
0.056885459	0.74102962	0.728	1	204962	SLC44A5
0.056885459	0.74102962	0.765	1	388722	C1orf53
0.056885459	0.74102962	0.766	1	390077	OR52N2
0.056628056	0.74216342	0.769	1	4863	NPAT
0.056628056	0.74216342	0.761	1	5026	P2RX5
0.056628056	0.74216342	0.728	1	5931	RBBP7
0.056628056	0.74216342	0.763	1	54935	DUSP23
0.056628056	0.74216342	0.783	1	114821	SCAND3
0.056370653	0.743297875	0.769	1	64221	ROBO3
0.056370653	0.743297875	0.754	1	153328	AC011427.1
0.056113254	0.744433284	0.763	1	55113	XKR8
0.056113254	0.744433284	0.756	1	55660	PRPF40A
0.056113254	0.744433284	0.756	1	91039	DPP9
0.056113254	0.744433284	0.764	1	342926	ZNF677
0.056113254	0.744433284	0.773	1	492307	C8orf22
0.055855855	0.745568752	0.746	1	415	ARSE
0.055855855	0.745568752	0.779	1	7486	WRN
0.055855855	0.745568752	0.756	1	8440	NCK2
0.055855855	0.745568752	0.764	1	55184	C20orf12
0.055855855	0.745568752	0.769	1	84132	USP42
0.055855855	0.745568752	0.791	1	92014	MCART1
0.055855855	0.745568752	0.778	1	114990	VASN
0.055855855	0.745568752	0.749	1	139431	FTH1P14
0.055855855	0.745568752	0.761	1	201562	PTPLB
0.055855855	0.745568752	0.754	1	219537	SMTNL1
0.055598456	0.746704698	0.766	1	5986	RFNG
0.055598456	0.746704698	0.798	1	10417	SPON2
0.055598456	0.746704698	0.752	1	25941	C18orf10
0.055598456	0.746704698	0.763	1	55607	PPP1R9A
0.055598456	0.746704698	0.783	1	90355	C5orf30
0.055341057	0.747841239	0.761	1	87	ACTN1
0.055341057	0.747841239	0.761	1	3628	INPP1
0.055341057	0.747841239	0.761	1	10279	PRSS16
0.055341057	0.747841239	0.766	1	10438	C1D
0.055341057	0.747841239	0.784	1	27094	KCNMB3
0.055341057	0.747841239	0.765	1	58498	MYL7
0.055341057	0.747841239	0.763	1	152330	CNTN4
0.055083655	0.748978257	0.784	1	11318	GPR182
0.055083655	0.748978257	0.772	1	23098	SARM1
0.055083655	0.748978257	0.779	1	84443	FRMPD3
0.054826252	0.750116348	0.765	1	829	CAPZA1
0.054826252	0.750116348	0.771	1	1014	CDH16
0.054826252	0.750116348	0.789	1	6123	RPL3L
0.054826252	0.750116348	0.786	1	9365	KL
0.054826252	0.750116348	0.783	1	23562	CLDN14
0.054826252	0.750116348	0.767	1	55432	YOD1

voxel-wise, subcortex			
Spearman's corr coef	p-val	GenID	Gene
0.080600619	3.876E-13	100270746	LOC100270746
0.080589108	3.90594E-13	7322	UBE2D2
0.080580093	3.92952E-13	5739	PTGIR
0.080570996	3.95348E-13	284422	AC005551.1
0.080532178	4.05727E-13	58500	ZNF250
0.080531344	4.05953E-13	8424	BBOX1
0.080516018	4.10127E-13	79369	B3GNT4
0.080452688	4.27824E-13	720	C4A
0.08038415	4.47824E-13	6556	SLC11A1
0.080361679	4.54579E-13	84146	ZNF644
0.080303632	4.72494E-13	124222	PAQR4
0.080276422	4.81127E-13	57596	BEGAIN
0.080267973	4.83839E-13	80863	PRRT1
0.080221541	4.99017E-13	79930	DOK3
0.080219403	4.99728E-13	81328	OR4A15
0.080190152	5.09541E-13	7932	OR2H2
0.080162853	5.18871E-13	653390	AC009093.1
0.080116048	5.35256E-13	762	CA4
0.080114655	5.35753E-13	90199	WFDC8
0.080065526	5.53514E-13	83640	FAM103A1
0.080062926	5.54472E-13	55898	UNC45A
0.079967648	5.90643E-13	54923	LIME1
0.079961054	5.9323E-13	10714	POLD3
0.079937279	6.0265E-13	8669	EIF3J
0.079858713	6.34845E-13	5321	PLA2G4A
0.079784796	6.66668E-13	10021	HCN4
0.079733625	6.89614E-13	220115	TPTE2P3
0.079644933	7.31228E-13	9130	FAM50A
0.079564236	7.71226E-13	81858	SHARPIN
0.079540372	7.83463E-13	126208	ZNF787
0.079459295	8.26468E-13	55628	ZNF407
0.079445742	8.3388E-13	642661	LOC642661
0.079410821	8.53282E-13	348262	AC174470.1
0.079383515	8.68757E-13	26205	GMEB2
0.079255179	9.45266E-13	4283	CXCL9
0.079235025	9.57864E-13	3612	IMPA1
0.079221278	9.66556E-13	548645	DNAJC25
0.079203732	9.7776E-13	253430	IPMK
0.079165377	1.0027E-12	474381	H2AFB2
0.079135105	1.02282E-12	137964	AGPAT6
0.07912758	1.02788E-12	100128977	MAPT-AS1
0.079124048	1.03026E-12	3932	LCK
0.079116903	1.03511E-12	339344	MYPOP
0.079022638	1.1011E-12	4696	NDUFA3
0.079008155	1.1116E-12	5317	PKP1
0.079002582	1.11566E-12	22827	PUF60
0.078928195	1.17135E-12	11138	TBC1D8
0.078897923	1.19479E-12	116931	MED12L
0.078876749	1.21145E-12	6653	SORL1
0.078860588	1.22432E-12	5230	PGK1
0.078824833	1.25328E-12	11103	KRR1
0.078786112	1.28541E-12	9804	TOMM20
0.078776643	1.29338E-12	170960	ZNF721
0.078686744	1.37156E-12	25801	GCA
0.078686744	1.37156E-12	140738	TMEM37
0.078655727	1.39961E-12	80208	SPG11
0.078637064	1.41674E-12	84852	C1orf203
0.078595646	1.45552E-12	9588	PRDX6
0.07857243	1.47771E-12	84256	FLYWCH1
0.078572333	1.47781E-12	55301	OLAH
0.07855209	1.49742E-12	9831	ZNF623
0.078417249	1.63479E-12	400954	EML6
0.078401923	1.65117E-12	64061	TSPYL2
0.078350477	1.70733E-12	23201	FAM168A
0.078336082	1.72336E-12	5202	PFDN2
0.078301907	1.76205E-12	84708	LNX1
0.078291789	1.77367E-12	163087	ZNF383
0.078118496	1.98468E-12	5427	POLE2
0.07807903	2.03607E-12	84910	TMEM87B
0.078078844	2.03632E-12	100131827	ZNF717
0.078072064	2.04528E-12	27306	PGDS
0.078070484	2.04737E-12	51015	ISOC1
0.078039937	2.08827E-12	989	SEPTIN7
0.078035474	2.09431E-12	7029	TFDP2
0.07800065	2.14204E-12	199834	LCE4A
0.077988207	2.15934E-12	10563	CXCL13
0.077974088	2.17916E-12	1951	CELSR3
0.077938803	2.22945E-12	54517	PUS7
0.077935271	2.23456E-12	5265	SERPINA1

region-wise, subcortex					
Spearman's corr coef	p-val	p-val, SA	p-val, SA (BH- correction)	GenID	Gene
0.054826252	0.750116348	0.775	1	124997	WDR81
0.054826252	0.750116348	0.776	1	147381	CBLN2
0.054826252	0.750116348	0.772	1	374650	GOLGA6L5
0.054568853	0.751254499	0.767	1	746	C11orf10
0.054568853	0.751254499	0.771	1	2564	GABRE
0.054568853	0.751254499	0.751	1	3042	HBM
0.054568853	0.751254499	0.772	1	9390	SLC22A13
0.054568853	0.751254499	0.766	1	166012	CHST13
0.054568853	0.751254499	0.79	1	207107	SFTA1P
0.054568853	0.751254499	0.775	1	286359	LOC286359
0.054311454	0.752393067	0.767	1	10594	PRPF8
0.054311454	0.752393067	0.766	1	11021	RAB35
0.054311454	0.752393067	0.785	1	29850	TRPM5
0.054311454	0.752393067	0.789	1	55906	ZC4H2
0.054311454	0.752393067	0.787	1	57647	DHX37
0.054311454	0.752393067	0.763	1	146664	MGAT5B
0.054311454	0.752393067	0.752	1	389137	ARMC10P1
0.054311454	0.752393067	0.767	1	100289060	LOC100289060
0.054054055	0.753532231	0.762	1	333	APLP1
0.054054055	0.753532231	0.793	1	4221	MEN1
0.054054055	0.753532231	0.774	1	11166	SOX21
0.054054055	0.753532231	0.777	1	54715	A2BP1
0.054054055	0.753532231	0.775	1	65005	MRPL9
0.054054055	0.753532231	0.776	1	130827	TMEM182
0.053796653	0.754672468	0.772	1	57478	USP31
0.053796653	0.754672468	0.771	1	92667	C20orf72
0.053539254	0.755812645	0.768	1	219	ALDH1B1
0.053539254	0.755812645	0.784	1	3033	HADH
0.053539254	0.755812645	0.794	1	3308	HSPA4
0.053539254	0.755812645	0.78	1	8648	NCOA1
0.053539254	0.755812645	0.788	1	9874	TLK1
0.053539254	0.755812645	0.788	1	11273	ATXN2L
0.053539254	0.755812645	0.779	1	22872	SEC31A
0.053539254	0.755812645	0.789	1	23251	KIAA1024
0.053539254	0.755812645	0.777	1	56916	SMARCAD1
0.053539254	0.755812645	0.752	1	80832	APOL4
0.053539254	0.755812645	0.771	1	340351	AGBL3
0.053539254	0.755812645	0.754	1	353497	POLN
0.053539254	0.755812645	0.761	1	100131785	LOC100131785
0.053281851	0.756953359	0.787	1	10328	COX4NB
0.053281851	0.756953359	0.763	1	55240	STEAP3
0.053281851	0.756953359	0.759	1	131583	FAM43A
0.053281851	0.756953359	0.76	1	339512	C1orf110
0.053024452	0.758094549	0.783	1	1141	CHRNB2
0.053024452	0.758094549	0.783	1	7538	ZFP36
0.053024452	0.758094549	0.774	1	50486	G0S2
0.053024452	0.758094549	0.768	1	116985	ARAP1
0.053024452	0.758094549	0.749	1	646892	SH2D7
0.05276705	0.759236217	0.781	1	120	ADD3
0.05276705	0.759236217	0.77	1	23443	SLC35A3
0.05276705	0.759236217	0.776	1	64374	SIL1
0.052509651	0.760379016	0.778	1	3191	HNRNPL
0.052509651	0.760379016	0.764	1	6223	RPS19
0.052509651	0.760379016	0.787	1	345895	RSHL1
0.052509651	0.760379016	0.766	1	441951	ZNFX1-AS1
0.052252252	0.761521697	0.793	1	7126	TNFAIP1
0.052252252	0.761521697	0.766	1	10999	SLC27A4
0.052252252	0.761521697	0.779	1	80006	C5orf44
0.052252252	0.761521697	0.76	1	129852	C2orf73
0.052252252	0.761521697	0.783	1	136371	ASB10
0.052252252	0.761521697	0.788	1	286046	XKR6
0.052252252	0.761521697	0.789	1	389075	RESP18
0.051994853	0.762664974	0.775	1	9368	SLC9A3R1
0.051994853	0.762664974	0.778	1	9530	BAG4
0.051994853	0.762664974	0.804	1	50804	MYEF2
0.051994853	0.762664974	0.804	1	133619	PRRC1
0.051737454	0.763808727	0.788	1	4835	NQO2
0.051737454	0.763808727	0.778	1	7266	DNAJC7
0.051737454	0.763808727	0.769	1	9200	PTPLA
0.051737454	0.763808727	0.802	1	140578	CHODL
0.051737454	0.763808727	0.771	1	222255	ATXN7L1
0.051737454	0.763808727	0.795	1	256979	SUNC1
0.051737454	0.763808727	0.773	1	341152	OR2AT4
0.051737454	0.763808727	0.781	1	441094	AC106818.2
0.051737454	0.763808727	0.799	1	643630	LOC643630
0.051480047	0.764953434	0.788	1	35	ACADS
0.051480047	0.764953434	0.767	1	4501	MT1X
0.051480047	0.764953434	0.804	1	8824	CES2
0.051480047	0.764953434	0.78	1	11116	FGFR1OP

voxel-wise, subcortex			
Spearman's corr coef	p-val	GenID	Gene
0.077934064	2.2363E-12	5990	RFX2
0.077925615	2.24854E-12	127540	HMGB4
0.077906951	2.27584E-12	93426	SYCE1
0.077886708	2.3058E-12	200035	NUDT17
0.077820957	2.40581E-12	339768	ESPNL
0.077804886	2.4309E-12	10814	CPLX2
0.07775604	2.50874E-12	9818	NUPL1
0.077750467	2.51778E-12	153768	PRELID2
0.07766448	2.66127E-12	7113	TMPRSS2
0.077587955	2.7957E-12	55343	SLC35C1
0.07757885	2.81213E-12	100128385	LINC00256B
0.077572167	2.82426E-12	643310	ST13P18
0.077529632	2.90261E-12	54674	LRRN3
0.077437699	3.0793E-12	65249	ZSWIM4
0.077271655	3.42556E-12	9721	GPRIN2
0.077270262	3.42863E-12	1476	CSTB
0.077256605	3.45877E-12	5167	ENPP1
0.077202559	3.58065E-12	346528	OR2A1
0.077178419	3.63645E-12	85359	DGCR6L
0.077160031	3.67952E-12	6910	TBX5
0.077033728	3.98913E-12	254048	UBN2
0.07701692	4.03222E-12	81553	FAM49A
0.07701423	4.03916E-12	84791	C1orf97
0.076955445	4.19371E-12	5252	PHF1
0.076943003	4.22715E-12	8687	KRT38
0.076902047	4.33913E-12	4688	NCF2
0.076852366	4.47882E-12	91056	AP5B1
0.076813452	4.59135E-12	8836	GGH
0.076748908	4.78411E-12	2358	FPR2
0.076706842	4.91397E-12	7319	UBE2A
0.076614901	5.20995E-12	8878	SQSTM1
0.076610357	5.22503E-12	441601	LOC441601
0.076558724	5.39932E-12	128853	DUSP15
0.076523341	5.52206E-12	8968	HIST1H3F
0.076514885	5.55179E-12	51249	TMEM69
0.076478951	5.67991E-12	90576	ZNF799
0.076424994	5.8777E-12	123207	C15orf40
0.076399922	5.97193E-12	171586	ABHD3
0.076358594	6.13042E-12	2002	ELK1
0.0763468	6.17642E-12	284018	C17orf58
0.07631699	6.29416E-12	54700	RRN3
0.076279379	6.44588E-12	6894	TARBP1
0.076233968	6.63383E-12	112724	RDH13
0.07623025	6.64946E-12	642987	TMEM232
0.076214284	6.71694E-12	51706	CYB5R1
0.076200627	6.77521E-12	11117	EMILIN1
0.076104976	7.19745E-12	55283	MCOLN3
0.076096155	7.23767E-12	340784	HMX3
0.076088816	7.2713E-12	2651	GCNT2
0.076066159	7.37608E-12	147947	ZNF542
0.076045267	7.474E-12	285267	ZNF619
0.076031424	7.5396E-12	440699	LRRC52
0.076026879	7.56125E-12	23302	WSCD1
0.07599967	7.69215E-12	196	AHR
0.07594952	7.9393E-12	7272	TTK
0.075927697	8.04929E-12	27141	CIDEB
0.075861298	8.39315E-12	132243	H1FOO
0.075817183	8.6295E-12	221443	C6orf130
0.075811617	8.65979E-12	219938	SPATA19
0.075793229	8.76058E-12	7922	SLC39A7
0.075636283	9.66883E-12	9136	RRP9
0.075626999	9.72532E-12	8484	GALR3
0.075529948	1.0336E-11	128240	APOA1BP
0.075519644	1.04029E-11	63874	ABHD4
0.075496987	1.05517E-11	2632	GBE1
0.07548742	1.06152E-11	4849	CNOT3
0.075419717	1.1075E-11	79874	RABEP2
0.075410709	1.11377E-11	84515	MCM8
0.075386383	1.13086E-11	23093	TTLL5
0.075345986	1.15981E-11	253260	RICTOR
0.075318493	1.17992E-11	387695	C10orf99
0.075269185	1.21686E-11	9131	AIFM1
0.075253122	1.22914E-11	4880	NPPC
0.075194888	1.27466E-11	468	ATF4
0.07518848	1.27977E-11	283933	ZNF843
0.075169355	1.29514E-11	7374	UNG
0.075153567	1.30797E-11	115827	RAB3C
0.075150967	1.31009E-11	10015	PDCD6IP
0.075137965	1.32076E-11	64881	PCDH20

region-wise, subcortex					
Spearman's corr coef	p-val	p-val, SA	p-val, SA (BH- correction)	GenID	Gene
0.051480047	0.764953434	0.79	1	79833	GEMIN6
0.051480047	0.764953434	0.784	1	130814	PQLC3
0.051480047	0.764953434	0.782	1	171023	ASXL1
0.051480047	0.764953434	0.772	1	644422	LOC644422
0.051222648	0.766098201	0.77	1	3489	IGFBP6
0.051222648	0.766098201	0.793	1	3816	KLK1
0.051222648	0.766098201	0.8	1	151254	ALS2CR11
0.05096525	0.767243445	0.793	1	1607	DGKB
0.05096525	0.767243445	0.802	1	6170	RPL39
0.05096525	0.767243445	0.771	1	7732	RNF112
0.05096525	0.767243445	0.769	1	10460	TACC3
0.05096525	0.767243445	0.798	1	81602	CDADC1
0.05096525	0.767243445	0.793	1	149371	EXOC8
0.05096525	0.767243445	0.771	1	388815	C21orf34
0.050707851	0.768389165	0.817	1	5045	FURIN
0.050707851	0.768389165	0.813	1	7102	TSPAN7
0.050707851	0.768389165	0.792	1	8293	SERF1A
0.050707851	0.768389165	0.788	1	10289	EIF1B
0.050707851	0.768389165	0.783	1	10634	GAS2L1
0.050707851	0.768389165	0.793	1	27249	MMADHC
0.050707851	0.768389165	0.783	1	54627	KIAA1383
0.050707851	0.768389165	0.799	1	90673	PPP1R3E
0.050707851	0.768389165	0.801	1	113157	RPLP0P2
0.050707851	0.768389165	0.799	1	220885	RPSAP15
0.050707851	0.768389165	0.789	1	728215	FAM155A
0.050707851	0.768389165	0.77	1	100129935	LOC100129935
0.050450452	0.769535899	0.794	1	655	BMP7
0.050450452	0.769535899	0.785	1	55796	MBNL3
0.050450452	0.769535899	0.762	1	79746	ECHDC3
0.050450452	0.769535899	0.788	1	148170	CDC42EP5
0.050450452	0.769535899	0.791	1	347468	OR13H1
0.050193049	0.770682633	0.787	1	6528	SLC5A5
0.050193049	0.770682633	0.775	1	55133	SRBD1
0.050193049	0.770682633	0.808	1	79716	NPEPL1
0.050193049	0.770682633	0.775	1	90441	ZNF622
0.050193049	0.770682633	0.77	1	201625	DNAH12
0.04993565	0.771829724	0.774	1	15	AANAT
0.04993565	0.771829724	0.809	1	754	PTTG1IP
0.04993565	0.771829724	0.809	1	9689	BZW1
0.04993565	0.771829724	0.798	1	22818	COPZ1
0.04993565	0.771829724	0.777	1	53916	RAB4B
0.04993565	0.771829724	0.797	1	55653	BCAS4
0.04993565	0.771829724	0.793	1	140733	MACROD2
0.04993565	0.771829724	0.798	1	147687	ZNF417
0.04993565	0.771829724	0.787	1	253190	SERHL2
0.049678247	0.772977412	0.802	1	6878	TAF6
0.049678247	0.772977412	0.793	1	51617	AC093310.1
0.049678247	0.772977412	0.772	1	57343	ZNF304
0.049678247	0.772977412	0.801	1	93953	ACRC
0.049678247	0.772977412	0.804	1	317649	EIF4E3
0.049420848	0.774125576	0.808	1	6156	RPL30
0.04916345	0.775274694	0.777	1	218	ALDH3A1
0.04916345	0.775274694	0.772	1	5170	PDPK1
0.04916345	0.775274694	0.801	1	8343	HIST1H2BF
0.04916345	0.775274694	0.79	1	9568	GABBR2
0.04916345	0.775274694	0.771	1	26515	FXC1
0.04916345	0.775274694	0.789	1	27043	PELP1
0.048906047	0.776423812	0.805	1	11285	B4GALT7
0.048906047	0.776423812	0.798	1	54788	DNAJB12
0.048648648	0.777573407	0.816	1	25843	MOBKL3
0.048648648	0.777573407	0.791	1	79657	RPAP3
0.048648648	0.777573407	0.801	1	89944	GLB1L2
0.048648648	0.777573407	0.802	1	387763	C11orf96
0.048391249	0.778723419	0.793	1	1111	CHEK1
0.048391249	0.778723419	0.778	1	3921	RPSA
0.048391249	0.778723419	0.799	1	5732	PTGER2
0.048391249	0.778723419	0.788	1	6202	RPS8
0.048391249	0.778723419	0.789	1	6319	SCD
0.048391249	0.778723419	0.783	1	9733	SART3
0.048391249	0.778723419	0.776	1	10748	RP11-904M10.1
0.04813385	0.779874504	0.801	1	5277	PIGA
0.04813385	0.779874504	0.798	1	6341	SCO1
0.04813385	0.779874504	0.804	1	7979	SHFM1
0.04813385	0.779874504	0.815	1	10884	MRPS30
0.04813385	0.779874504	0.778	1	23380	SRGAP2
0.04813385	0.779874504	0.789	1	374986	FAM73A
0.047876447	0.781025469	0.798	1	1536	CYBB
0.047876447	0.781025469	0.8	1	1821	DRP2
0.047876447	0.781025469	0.787	1	2178	FANCE

voxel-wise, subcortex			
Spearman's corr coef	p-val	GenID	Gene
0.075117625	1.33763E-11	10307	APBB3
0.07509283	1.35847E-11	283385	MORN3
0.075033672	1.4095E-11	51714	SELT
0.075012781	1.42795E-11	11161	C14orf1
0.075006835	1.43326E-11	145165	ST13P4
0.074979909	1.45749E-11	26156	RSL1D1
0.07489939	1.53237E-11	342898	SYCN
0.074786283	1.64397E-11	746	C11orf10
0.074680135	1.75591E-11	114984	FLYWCH2
0.074658126	1.78005E-11	2811	GP1BA
0.074645869	1.79362E-11	85365	ALG2
0.074591726	1.85482E-11	26251	KCNG2
0.074573897	1.87542E-11	3714	JAG2
0.074557923	1.89407E-11	5318	PKP2
0.074491523	1.97352E-11	2762	GMDS
0.074474901	1.99392E-11	93624	TADA2B
0.07446719	2.00345E-11	9351	SLC9A3R2
0.074417882	2.06546E-11	144195	SLC2A14
0.074416675	2.06701E-11	7009	TMBIM6
0.074342199	2.16433E-11	1128	CHRM1
0.074296415	2.22637E-11	375190	C2orf84
0.074278116	2.25165E-11	3798	KIF5A
0.074265584	2.26912E-11	27258	LSM3
0.074226208	2.3249E-11	149428	BNIPL
0.074205033	2.35544E-11	63977	PRDM15
0.074188873	2.37902E-11	168391	GALNTL5
0.074130923	2.46547E-11	51352	WT1-AS
0.074113466	2.49211E-11	401022	HOXD-AS1
0.074074373	2.55281E-11	10188	TNK2
0.074074	2.55339E-11	27430	MAT2B
0.073912688	2.81953E-11	9509	ADAMTS2
0.073885016	2.86783E-11	280664	WFDC10B
0.073817596	2.98894E-11	4609	MYC
0.073774785	3.06843E-11	728	C5AR1
0.073769957	3.07751E-11	8820	HESX1
0.073745348	3.12428E-11	28981	IFT81
0.073666133	3.27956E-11	1747	DLX3
0.073624715	3.36371E-11	22869	ZNF510
0.073543362	3.53524E-11	56097	PCDHGC5
0.073537044	3.54892E-11	51141	INSIG2
0.073536858	3.54932E-11	64747	MFSD1
0.073514111	3.59899E-11	57147	SCYL3
0.073511228	3.60534E-11	23534	TNPO3
0.073504634	3.61988E-11	64708	COPS7B
0.073501199	3.62748E-11	56833	SLAMF8
0.073489219	3.65411E-11	255928	SYT14
0.073472969	3.69055E-11	26259	FBXW8
0.073450401	3.74174E-11	23237	ARC
0.073429972	3.78866E-11	2322	FLT3
0.073428676	3.79166E-11	148304	C1orf74
0.073371097	3.92718E-11	9783	RIMS3
0.073365986	3.93943E-11	340595	ZCCHC16
0.073362365	3.94813E-11	730755	KRTAP2-1
0.073348157	3.98248E-11	7699	ZNF140
0.073336177	4.01165E-11	910	CD1B
0.073307946	4.08125E-11	6760	SS18
0.073201522	4.35438E-11	79711	IPO4
0.073129088	4.55043E-11	5297	PI4KA
0.073064171	4.73344E-11	57149	LYRM1
0.073022664	4.85422E-11	54793	KCTD9
0.073009938	4.89184E-11	84789	MGC2889
0.072975025	4.99654E-11	646808	LOC646808
0.072952174	5.06627E-11	10382	TUBB4
0.072844356	5.40821E-11	6498	SKIL
0.072812043	5.51503E-11	3481	IGF2
0.072741926	5.75395E-11	4246	SCGB2A1
0.072703764	5.88822E-11	80014	WWC2
0.072678313	5.97945E-11	5027	P2RX7
0.072657146	6.0564E-11	643965	TMEM88B
0.072643302	6.10722E-11	50814	NSDHL
0.072607182	6.24183E-11	23220	DTX4
0.072574116	6.36761E-11	257106	ARHGAP30
0.072558515	6.42782E-11	140625	ACTRT2
0.072539575	6.50167E-11	51619	UBE2D4
0.07253623	6.51481E-11	29889	GNL2
0.072487473	6.70907E-11	768096	HAR1A
0.072440855	6.90012E-11	10969	EBNA1BP2
0.072416343	7.00272E-11	619455	SPANXA2-OT1
0.072410487	7.02745E-11	412	STS

region-wise, subcortex					
Spearman's corr coef	p-val	p-val, SA	p-val, SA (BH- correction)	GenID	Gene
0.047876447	0.781025469	0.799	1	3642	INSM1
0.047876447	0.781025469	0.783	1	54808	DYM
0.047876447	0.781025469	0.795	1	55033	FKBP14
0.047876447	0.781025469	0.823	1	55168	MRPS18A
0.047876447	0.781025469	0.801	1	90957	DHX57
0.047876447	0.781025469	0.807	1	147949	ZNF583
0.047876447	0.781025469	0.787	1	148545	NBPF4
0.047876447	0.781025469	0.822	1	253558	LCLAT1
0.047619045	0.782176912	0.812	1	2045	EPHA7
0.047619045	0.782176912	0.807	1	6332	SCN7A
0.047619045	0.782176912	0.814	1	8165	AKAP1
0.047619045	0.782176912	0.793	1	9002	F2RL3
0.047619045	0.782176912	0.823	1	80781	COL18A1
0.047619045	0.782176912	0.799	1	116085	SLC22A12
0.047619045	0.782176912	0.807	1	100130691	AC074286.1
0.047361646	0.783328772	0.815	1	165	AEBP1
0.047361646	0.783328772	0.805	1	213	ALB
0.047361646	0.783328772	0.799	1	1591	CYP24A1
0.047361646	0.783328772	0.821	1	5740	PTGIS
0.047361646	0.783328772	0.805	1	8517	IKBKG
0.047361646	0.783328772	0.788	1	10421	CD2BP2
0.047361646	0.783328772	0.814	1	23179	RGL1
0.047361646	0.783328772	0.796	1	23275	POFUT2
0.047361646	0.783328772	0.794	1	54442	KCTD5
0.047361646	0.783328772	0.812	1	404266	RP11-357H14.1
0.047104247	0.784481704	0.799	1	4925	NUCB2
0.046846848	0.785634518	0.795	1	4360	MRC1
0.046846848	0.785634518	0.797	1	26973	CHORDC1
0.046846848	0.785634518	0.789	1	83987	CCDC8
0.046846848	0.785634518	0.791	1	96610	LL22NC03-80A10.2
0.046846848	0.785634518	0.81	1	150483	TEKT4
0.046846848	0.785634518	0.799	1	402778	AC068580.3
0.046589449	0.786787808	0.804	1	1345	COX6C
0.046589449	0.786787808	0.805	1	7125	TNNC2
0.046589449	0.786787808	0.81	1	8409	UXT
0.046589449	0.786787808	0.805	1	55954	ZMAT5
0.046589449	0.786787808	0.826	1	84969	C20orf100
0.046332043	0.787941575	0.817	1	4152	MBD1
0.046332043	0.787941575	0.79	1	10661	KLF1
0.046332043	0.787941575	0.814	1	51050	PI15
0.046332043	0.787941575	0.791	1	54842	MFSD6
0.046332043	0.787941575	0.793	1	58493	C9orf80
0.046332043	0.787941575	0.819	1	64328	XPO4
0.046332043	0.787941575	0.798	1	64651	CSRNP1
0.046332043	0.787941575	0.792	1	79165	LENG1
0.046332043	0.787941575	0.799	1	79825	CCDC48
0.046332043	0.787941575	0.814	1	84532	ACSS1
0.046332043	0.787941575	0.798	1	197358	NLRC3
0.046332043	0.787941575	0.811	1	440465	AC013356.3
0.046074644	0.7890957	0.823	1	153	ADRB1
0.046074644	0.7890957	0.807	1	10767	HBS1L
0.046074644	0.7890957	0.78	1	57121	LPAR5
0.046074644	0.7890957	0.806	1	400793	C1orf226
0.046074644	0.7890957	0.786	1	441869	ANKRD65
0.045817245	0.790250838	0.801	1	8678	BECN1
0.045817245	0.790250838	0.808	1	9245	GCNT3
0.045817245	0.790250838	0.817	1	25970	SH2B1
0.045817245	0.790250838	0.811	1	26190	FBXW2
0.045817245	0.790250838	0.824	1	29785	CYP2S1
0.045817245	0.790250838	0.816	1	29929	ALG6
0.045817245	0.790250838	0.799	1	64109	CRLF2
0.045817245	0.790250838	0.819	1	123207	C15orf40
0.045559846	0.791405916	0.823	1	6194	RPS6
0.045559846	0.791405916	0.812	1	7434	VIPR2
0.045559846	0.791405916	0.816	1	55718	POLR3E
0.045559846	0.791405916	0.801	1	55734	ZFP64
0.045559846	0.791405916	0.816	1	81704	DOCK8
0.045559846	0.791405916	0.807	1	259173	ALS2CL
0.045559846	0.791405916	0.807	1	342931	RFPL4A
0.045559846	0.791405916	0.807	1	387841	RP11-392P7.1
0.045302443	0.792561471	0.812	1	7155	TOP2B
0.045302443	0.792561471	0.798	1	7570	ZNF22
0.045302443	0.792561471	0.793	1	57291	SNHG13
0.045302443	0.792561471	0.801	1	64759	TNS3
0.045302443	0.792561471	0.801	1	90273	CEACAM21
0.045045044	0.793717384	0.814	1	2217	FCGRT
0.045045044	0.793717384	0.818	1	3169	FOXA1
0.045045044	0.793717384	0.802	1	4783	NFIL3
0.045045044	0.793717384	0.795	1	10520	ZNF211

voxel-wise, subcortex			
Spearman's corr coef	p-val	GenID	Gene
0.072368331	7.20796E-11	100288801	FRG2C
0.072330996	7.37163E-11	7940	LST1
0.072226338	7.85008E-11	10762	NUP50
0.072202288	7.96425E-11	4010	LMX1B
0.072200239	7.97403E-11	84798	C19orf48
0.072165601	8.14149E-11	2643	GCH1
0.072125763	8.33836E-11	256302	C17orf103
0.072082862	8.55561E-11	84912	SLC35B4
0.07201618	8.90424E-11	8674	VAMP4
0.072004944	8.96435E-11	64773	FAM113A
0.071959905	9.20923E-11	100128416	LOC100128416
0.071925454	9.40101E-11	79902	NUP85
0.071835093	9.92263E-11	84458	LCOR
0.071808718	1.00801E-10	66002	CYP4F12
0.071751423	1.04307E-10	9091	PIGQ
0.071712695	1.06745E-10	326	AIRE
0.071698301	1.07665E-10	4013	VWA5A
0.071684837	1.08533E-10	80194	TMEM134
0.071677312	1.09021E-10	81704	DOCK8
0.071531139	1.18934E-10	79441	HAUS3
0.071526594	1.19256E-10	79946	C10orf95
0.071523808	1.19454E-10	51123	ZNF706
0.071504489	1.20834E-10	400242	DICER1-AS
0.071458332	1.24195E-10	131578	LRRC15
0.071422115	1.26896E-10	2248	FGF3
0.071383581	1.29834E-10	10869	USP19
0.071348935	1.32531E-10	390882	OR7G2
0.071296655	1.36705E-10	642929	LOC642929
0.071272142	1.38706E-10	81545	FBXO38
0.071188003	1.45795E-10	147687	ZNF417
0.071142778	1.4975E-10	100286918	NDUFS5P2
0.071099497	1.53633E-10	158067	C9orf98
0.071073778	1.55988E-10	440356	AC120114.2
0.07105279	1.57935E-10	22861	AC055839.1
0.070896864	1.73163E-10	57132	CHMP1B
0.070818394	1.8136E-10	6538	SLC6A11
0.070793599	1.84028E-10	8294	HIST1H4I
0.070783108	1.85169E-10	11160	ERLIN2
0.070776328	1.85909E-10	5143	PDE4C
0.070776045	1.85941E-10	27201	GPR78
0.070754223	1.88344E-10	162417	NAGS
0.070673987	1.97447E-10	221424	C6orf154
0.070521407	2.15958E-10	221955	DAGLB
0.070513427	2.16971E-10	55161	TMEM33
0.070503205	2.18276E-10	29775	CARD10
0.070483148	2.20859E-10	8941	CDK5R2
0.070473395	2.22125E-10	4493	MT1E
0.070448048	2.25451E-10	3765	KCNJ9
0.070424922	2.28527E-10	80018	C12orf30
0.070414431	2.29936E-10	3138	HLA-K
0.070255905	2.52288E-10	55578	FAM48A
0.070255816	2.52301E-10	8817	FGF18
0.070153013	2.67916E-10	9638	FEZ1
0.070119299	2.7324E-10	100131897	AC008449.1
0.070071571	2.80955E-10	390259	BSX
0.070051879	2.84198E-10	2665	GDI2
0.070048168	2.84813E-10	245929	DEFB115
0.069937468	3.03783E-10	6370	CCL25
0.069908492	3.08948E-10	9761	MLEC
0.069856301	3.1847E-10	57553	MICAL3
0.069812655	3.26652E-10	219578	ZNF804B
0.069808759	3.27392E-10	64581	CLEC7A
0.069785818	3.31784E-10	259287	TAS2R41
0.069749601	3.38835E-10	128061	C1orf131
0.06967438	3.53951E-10	1482	NKX2-5
0.069645867	3.59851E-10	2928	GSC2
0.069630824	3.63001E-10	64403	CDH24
0.06962572	3.64077E-10	56033	BARX1
0.069448993	4.03291E-10	26220	DGCR5
0.069442309	4.04852E-10	2701	GJA4
0.069407299	4.13126E-10	58487	CREBZF
0.069363743	4.23652E-10	255738	PCSK9
0.069270879	4.46973E-10	9776	ATG13
0.069238745	4.5533E-10	23418	CRB1
0.069203824	4.64586E-10	54834	GDAP2
0.069188505	4.68704E-10	23729	SHPK
0.069179684	4.71093E-10	126119	JOSD2
0.069107339	4.91123E-10	26996	GPR160
0.069100469	4.9307E-10	113230	AC022098.1

region-wise, subcortex					
Spearman's corr coef	p-val	p-val, SA	p-val, SA (BH- correction)	GenID	Gene
0.045045044	0.793717384	0.82	1	92591	ASB16
0.045045044	0.793717384	0.778	1	338657	CCDC84
0.044787645	0.79487437	0.825	1	2980	GUCA2A
0.044787645	0.79487437	0.802	1	9363	RAB33A
0.044787645	0.79487437	0.828	1	148645	LINC00337
0.044530243	0.796031177	0.817	1	115426	UHRF2
0.044530243	0.796031177	0.807	1	157922	CAMSAP1
0.044272844	0.797188401	0.824	1	2628	GATM
0.044272844	0.797188401	0.832	1	3949	LDLR
0.044272844	0.797188401	0.823	1	22989	MYH15
0.044272844	0.797188401	0.805	1	54540	AC145098.2
0.044272844	0.797188401	0.823	1	56623	INPP5E
0.044272844	0.797188401	0.828	1	60592	SCOC
0.044272844	0.797188401	0.832	1	79740	ZBBX
0.044272844	0.797188401	0.816	1	284942	RPL23AP82
0.044272844	0.797188401	0.823	1	554203	JPX
0.044272844	0.797188401	0.815	1	647309	RP11-332P22.1
0.044015441	0.798346102	0.828	1	22982	DIP2C
0.044015441	0.798346102	0.807	1	23774	BRD1
0.044015441	0.798346102	0.804	1	54823	C1orf26
0.043758042	0.799504757	0.826	1	3434	IFIT1
0.043758042	0.799504757	0.813	1	5828	PXMP3
0.043758042	0.799504757	0.807	1	9628	RGS6
0.043758042	0.799504757	0.816	1	344805	TMPRSS7
0.043500643	0.800663352	0.819	1	3265	HRAS
0.043500643	0.800663352	0.82	1	5747	PTK2
0.043500643	0.800663352	0.814	1	11338	U2AF2
0.043500643	0.800663352	0.841	1	57733	GBA3
0.043243244	0.801822305	0.807	1	9667	SAFB2
0.043243244	0.801822305	0.802	1	9865	TRIL
0.043243244	0.801822305	0.814	1	23322	RPGRIP1L
0.043243244	0.801822305	0.808	1	23509	POFUT1
0.043243244	0.801822305	0.819	1	55220	KLHDC8A
0.043243244	0.801822305	0.82	1	122481	AK7
0.042985845	0.802981734	0.804	1	3801	KIFC3
0.042985845	0.802981734	0.815	1	6936	C2orf3
0.042985845	0.802981734	0.84	1	9184	BUB3
0.042985845	0.802981734	0.844	1	54892	NCAPG2
0.042985845	0.802981734	0.834	1	199786	FAM129C
0.042985845	0.802981734	0.817	1	219952	OR6Q1
0.042985845	0.802981734	0.826	1	339366	ADAMTSL5
0.042728439	0.804141581	0.829	1	940	CD28
0.042728439	0.804141581	0.82	1	3758	KCNJ1
0.042728439	0.804141581	0.819	1	5771	PTPN2
0.042728439	0.804141581	0.825	1	7179	TPTE
0.042728439	0.804141581	0.792	1	8756	ADAM7
0.042728439	0.804141581	0.81	1	23043	TNIK
0.042728439	0.804141581	0.819	1	64598	MOSPD3
0.042728439	0.804141581	0.83	1	84258	SYT3
0.042728439	0.804141581	0.823	1	196074	METT5D1
0.042728439	0.804141581	0.809	1	203228	C9orf72
0.04247104	0.805302262	0.839	1	3620	IDO1
0.04247104	0.805302262	0.81	1	9587	MAD2L1BP
0.04247104	0.805302262	0.823	1	9900	SV2A
0.04247104	0.805302262	0.813	1	54853	WDR55
0.04247104	0.805302262	0.809	1	55004	C11orf59
0.04247104	0.805302262	0.829	1	56108	PCDHGA7
0.04247104	0.805302262	0.823	1	151050	C2orf67
0.042213641	0.806462944	0.83	1	3201	HOXA4
0.042213641	0.806462944	0.8	1	23065	KIAA0090
0.042213641	0.806462944	0.808	1	23293	SMG6
0.042213641	0.806462944	0.807	1	25791	NGEF
0.042213641	0.806462944	0.805	1	57824	HMHB1
0.042213641	0.806462944	0.823	1	79752	ZFAND1
0.042213641	0.806462944	0.799	1	83452	RAB33B
0.042213641	0.806462944	0.833	1	342510	CD300E
0.042213641	0.806462944	0.798	1	645691	LOC645691
0.041956242	0.807623982	0.813	1	83729	INHBE
0.041698843	0.808785498	0.829	1	4236	MFAP1
0.041698843	0.808785498	0.811	1	8726	EED
0.041698843	0.808785498	0.823	1	55794	DDX28
0.041698843	0.808785498	0.837	1	64122	FN3K
0.041698843	0.808785498	0.808	1	93663	ARHGAP18
0.041698843	0.808785498	0.793	1	333929	SNAI3
0.041441441	0.809947908	0.822	1	3696	ITGB8
0.041441441	0.809947908	0.82	1	54858	PGPEP1
0.041441441	0.809947908	0.808	1	337969	KRTAP19-2
0.041184042	0.811110198	0.83	1	23090	ZNF423
0.041184042	0.811110198	0.813	1	51378	ANGPT4

voxel-wise, subcortex			
Spearman's corr coef	p-val	GenID	Gene
0.069067128	5.02615E-10	23394	ADNP
0.068996176	5.23538E-10	8521	GCM1
0.068968318	5.31979E-10	5073	PARN
0.068914831	5.48567E-10	128488	WFDC12
0.068853348	5.68257E-10	728229	TMEM191B
0.06884481	5.71047E-10	10269	ZMPSTE24
0.06882865	5.76359E-10	55011	PIH1D1
0.068796515	5.8707E-10	4808	NHLH2
0.068733923	6.08493E-10	64786	TBC1D15
0.068640128	6.42032E-10	414157	C10orf62
0.068480216	7.03409E-10	4159	MC3R
0.068473533	7.06096E-10	5742	PTGS1
0.068430349	7.23697E-10	6868	ADAM17
0.068415485	7.29851E-10	9436	NCR2
0.068410754	7.31821E-10	11105	PRDM7
0.068394035	7.38825E-10	6837	MED22
0.068360977	7.52867E-10	3622	ING2
0.068330146	7.66194E-10	5141	PDE4A
0.068310268	7.74913E-10	10529	NEBL
0.068309717	7.75155E-10	196374	KRT78
0.068282872	7.87082E-10	148046	CIRBP-AS1
0.068186946	8.3119E-10	254263	CNIH2
0.068181746	8.33647E-10	58485	TRAPPC1
0.068165399	8.41421E-10	7098	TLR3
0.068148315	8.49623E-10	1832	DSP
0.068143301	8.52042E-10	140876	C20orf175
0.068043187	9.01829E-10	57418	WDR18
0.067934908	9.58867E-10	8074	FGF23
0.067905284	9.75075E-10	27352	SGSM3
0.067866281	9.96821E-10	54960	GEMIN8
0.067821518	1.02237E-09	56884	FSTL5
0.067806289	1.0312E-09	129450	C2orf60
0.067784838	1.04377E-09	50964	SOST
0.067749828	1.06461E-09	4886	NPY1R
0.067691967	1.09994E-09	445577	C9orf129
0.067647211	1.12805E-09	64855	FAM129B
0.067635044	1.13581E-09	79774	GRTP1
0.067454234	1.25748E-09	6339	SCNN1D
0.0673583	1.3271E-09	360023	ZBTB41
0.067251973	1.40866E-09	84875	PARP10
0.06715966	1.48342E-09	9743	AP000751.3
0.06709633	1.53692E-09	51301	GCNT4
0.067088157	1.54396E-09	339456	TMEM52
0.067071348	1.55854E-09	9421	HAND1
0.067067631	1.56178E-09	4056	LTC4S
0.067064941	1.56413E-09	727982	AC022311.1
0.067048036	1.57898E-09	170680	PSORS1C2
0.067031875	1.5933E-09	22936	ELL2
0.067004852	1.61754E-09	6322	SCML1
0.06700179	1.62031E-09	283	ANG
0.066901311	1.71376E-09	85360	SYDE1
0.066815779	1.79743E-09	391358	TRMT112P6
0.066815317	1.79789E-09	1290	COL5A2
0.066812716	1.8005E-09	28978	TMEM14A
0.066803522	1.80975E-09	170384	FUT11
0.066739723	1.87517E-09	390197	OR4D10
0.066718176	1.89778E-09	440104	AC023055.2
0.066699885	1.91719E-09	414	ARSD
0.066637941	1.98434E-09	3420	IDH3B
0.066546746	2.08741E-09	124685	LOC124685
0.066520557	2.11795E-09	3753	KCNE1
0.066455737	2.19544E-09	169270	ZNF596
0.066385344	2.28272E-09	80263	TRIM45
0.06638284	2.28589E-09	2880	GPX5
0.066272788	2.42935E-09	7903	ST8SIA4
0.066204257	2.52308E-09	54072	LINC00158
0.066106372	2.66309E-09	1041	CDSN
0.066054739	2.73995E-09	54913	RPP25
0.066035517	2.76912E-09	84803	AGPAT9
0.06602855	2.77976E-09	374864	C18orf34
0.065985277	2.84677E-09	3848	KRT1
0.065963641	2.88087E-09	338094	FAM151A
0.065883778	3.01019E-09	5763	PTMS
0.06578283	3.18177E-09	1337	COX6A1
0.065773264	3.19853E-09	284697	BTBD8
0.06576398	3.21485E-09	55700	MAP7D1
0.06570185	3.32627E-09	776	CACNA1D
0.065650217	3.4217E-09	391356	PTRHD1
0.065629788	3.4602E-09	3983	ABLIM1

region-wise, subcortex					
Spearman's corr coef	p-val	p-val, SA	p-val, SA (BH- correction)	GenID	Gene
0.041184042	0.811110198	0.829	1	55298	RNF121
0.041184042	0.811110198	0.804	1	64782	AEN
0.041184042	0.811110198	0.831	1	400830	DEFB132
0.040926639	0.812272847	0.8	1	6234	RPS28
0.040926639	0.812272847	0.831	1	124976	SPNS2
0.040926639	0.812272847	0.828	1	128077	LIX1L
0.040926639	0.812272847	0.826	1	128229	C1orf182
0.04066924	0.813435972	0.82	1	823	CAPN1
0.04066924	0.813435972	0.833	1	6697	SPR
0.04066924	0.813435972	0.821	1	6767	ST13
0.04066924	0.813435972	0.827	1	9651	PLCH2
0.04066924	0.813435972	0.818	1	222166	C7orf41
0.040411841	0.814599931	0.835	1	1616	DAXX
0.040411841	0.814599931	0.841	1	5870	RAB6A
0.040411841	0.814599931	0.824	1	7204	TRIO
0.040411841	0.814599931	0.837	1	7453	WARS
0.040411841	0.814599931	0.835	1	51768	TM7SF3
0.040411841	0.814599931	0.835	1	79741	C10orf68
0.040411841	0.814599931	0.832	1	340120	ANKRD34B
0.040154438	0.815763891	0.846	1	4608	MYBPH
0.040154438	0.815763891	0.826	1	90060	CCDC120
0.040154438	0.815763891	0.83	1	246704	LINC00315
0.040154438	0.815763891	0.833	1	339453	TMEM240
0.03989704	0.816928148	0.823	1	2975	GTF3C1
0.03989704	0.816928148	0.827	1	4697	NDUFA4
0.03989704	0.816928148	0.812	1	7188	TRAF5
0.03989704	0.816928148	0.817	1	9985	REC8
0.03989704	0.816928148	0.837	1	10806	SDCCAG8
0.039639641	0.818092823	0.837	1	4688	NCF2
0.039639641	0.818092823	0.817	1	5684	PSMA3
0.039639641	0.818092823	0.844	1	11062	DUS4L
0.039639641	0.818092823	0.822	1	85320	ABCC11
0.039639641	0.818092823	0.827	1	284390	ZNF763
0.039639641	0.818092823	0.818	1	729475	RAD51AP2
0.039382242	0.819257915	0.832	1	1066	CES1
0.039382242	0.819257915	0.836	1	3215	HOXB5
0.039382242	0.819257915	0.832	1	8433	UTF1
0.039382242	0.819257915	0.825	1	8747	ADAM21
0.039382242	0.819257915	0.839	1	112483	SAT2
0.039382242	0.819257915	0.819	1	120534	C11orf46
0.039382242	0.819257915	0.817	1	146853	C17orf50
0.039124835	0.820423901	0.848	1	3237	HOXD11
0.039124835	0.820423901	0.811	1	6251	RSU1
0.039124835	0.820423901	0.819	1	8566	PDXK
0.039124835	0.820423901	0.809	1	26102	DKFZP434A062
0.039124835	0.820423901	0.83	1	55739	CARKD
0.039124835	0.820423901	0.807	1	64062	RBM26
0.039124835	0.820423901	0.819	1	79683	ZDHHC14
0.038867436	0.821589649	0.85	1	5290	PIK3CA
0.038867436	0.821589649	0.856	1	7003	TEAD1
0.038867436	0.821589649	0.81	1	8148	TAF15
0.038867436	0.821589649	0.852	1	26230	TIAM2
0.038867436	0.821589649	0.846	1	54982	CLN6
0.038867436	0.821589649	0.825	1	83549	UCK1
0.038867436	0.821589649	0.842	1	125206	SLC5A10
0.038610037	0.822755873	0.841	1	7135	TNNI1
0.038610037	0.822755873	0.827	1	9415	FADS2
0.038610037	0.822755873	0.827	1	79600	TCTN1
0.038352638	0.823922455	0.828	1	4799	NFX1
0.038352638	0.823922455	0.853	1	5992	RFX4
0.038352638	0.823922455	0.819	1	6770	STAR
0.038352638	0.823922455	0.845	1	29058	C20orf30
0.038352638	0.823922455	0.854	1	57724	KIAA1632
0.038352638	0.823922455	0.836	1	374569	ASPG
0.03809524	0.825089991	0.85	1	1750	DLX6
0.03809524	0.825089991	0.825	1	6204	RPS10
0.03809524	0.825089991	0.831	1	8526	DGKE
0.03809524	0.825089991	0.829	1	8550	MAPKAPK5
0.03809524	0.825089991	0.834	1	8778	SIGLEC5
0.03809524	0.825089991	0.843	1	23420	NOMO1
0.03809524	0.825089991	0.826	1	25806	VAX2
0.03809524	0.825089991	0.826	1	79745	CLIP4
0.03809524	0.825089991	0.835	1	94137	RP1L1
0.03809524	0.825089991	0.844	1	116143	WDR92
0.03809524	0.825089991	0.826	1	643749	TRAF3IP2-AS1
0.037837837	0.826257288	0.838	1	5306	PITPNA
0.037837837	0.826257288	0.851	1	9762	RP5-1187M17.1
0.037837837	0.826257288	0.837	1	55763	EXOC1
0.037837837	0.826257288	0.841	1	254439	C11orf86

voxel-wise, subcortex			
Spearman's corr coef	p-val	GenID	Gene
0.065590136	3.53612E-09	1588	CYP19A1
0.065525405	3.66355E-09	55643	BTBD2
0.06549903	3.71675E-09	55687	TRMU
0.065412112	3.89742E-09	376132	LRRC10
0.065383881	3.95792E-09	646625	PRHOXNB
0.065366887	3.99479E-09	350383	GPR142
0.065315902	4.1074E-09	285971	ZNF775
0.06530457	4.13284E-09	219557	C7orf62
0.06529928	4.14477E-09	112616	CMTM7
0.065189138	4.40091E-09	875	CBS
0.065149486	4.49685E-09	4249	MGAT5
0.065123759	4.56016E-09	80854	SETD7
0.065109551	4.59551E-09	146542	ZNF688
0.065092653	4.6379E-09	9098	USP6
0.065074265	4.68445E-09	79792	GSDMD
0.065039992	4.77243E-09	84826	SFT2D3
0.064961895	4.97895E-09	9400	RECQL5
0.06494648	5.02075E-09	59272	ACE2
0.064910077	5.12078E-09	245936	DEFB123
0.064909793	5.12157E-09	11016	ATF7
0.064900234	5.14815E-09	1500	CTNND1
0.064808294	5.41086E-09	158584	FAAH2
0.064805046	5.42037E-09	387590	AC005301.1
0.064770773	5.52177E-09	1979	EIF4EBP2
0.06471654	5.68601E-09	100128203	ENSAP3
0.064707629	5.71343E-09	56751	BARHL1
0.064690635	5.76611E-09	10648	SCGB1D1
0.064669549	5.83214E-09	5036	PA2G4
0.064636305	5.93771E-09	23676	SMPX
0.0646156	6.00441E-09	118425	RP11-610O8.1
0.064587459	6.09623E-09	203430	ZCCHC5
0.064536758	6.26513E-09	25823	TPSG1
0.064500257	6.38952E-09	80255	SLC35F5
0.064494498	6.40938E-09	4171	MCM2
0.064379625	6.81797E-09	23067	SETD1B
0.06416928	7.63276E-09	51626	DYNC2LI1
0.064048648	8.14195E-09	387680	FAM21A
0.064021908	8.25922E-09	51019	CCDC53
0.064007975	8.32097E-09	84187	TMEM164
0.063957453	8.54868E-09	57192	MCOLN1
0.063906565	8.78414E-09	107	ADCY1
0.063906007	8.78677E-09	6734	SRPR
0.063881867	8.90071E-09	5138	PDE2A
0.063806646	9.26496E-09	85481	PSKH2
0.063800327	9.29624E-09	5383	PMS2L5
0.063761696	9.48952E-09	92935	MARS2
0.063715167	9.72754E-09	151963	C3orf59
0.063689165	9.86309E-09	7064	THOP1
0.063688517	9.86648E-09	604	BCL6
0.063612923	1.02711E-08	51231	VRK3
0.063589059	1.04022E-08	170572	HTR3C
0.063508168	1.08586E-08	388743	CAPN8
0.063484028	1.09986E-08	8200	GDF5
0.063418001	1.13904E-08	55004	C11orf59
0.063386701	1.15808E-08	54020	SLC37A1
0.063352339	1.17934E-08	7141	TNP1
0.063307025	1.20796E-08	23218	NBEAL2
0.063290492	1.21857E-08	60506	NYX
0.063275076	1.22855E-08	80310	PDGFD
0.063269973	1.23187E-08	7278	TUBA3C
0.063265882	1.23453E-08	10452	TOMM40
0.063214712	1.26837E-08	9555	H2AFY
0.063150078	1.3124E-08	22875	ENPP4
0.063084051	1.35891E-08	6862	T
0.063072257	1.36739E-08	55702	CCDC94
0.063014492	1.40964E-08	53981	CPSF2
0.06300623	1.41578E-08	3653	IPW
0.063001677	1.41918E-08	10379	IRF9
0.062960632	1.45018E-08	30846	EHD2
0.062959239	1.45124E-08	5738	PTGFRN
0.062855691	1.53245E-08	80145	THOC7
0.062843807	1.54204E-08	57222	ERGIC1
0.062760413	1.61105E-08	128497	C20orf165
0.062752239	1.61797E-08	79192	IRX1
0.062734038	1.63349E-08	54797	MED18
0.062722899	1.64306E-08	5721	PSME2
0.06266588	1.6929E-08	146310	RNF151
0.062626779	1.72792E-08	100286979	ANAPC1P1
0.06260895	1.74413E-08	116179	TGM7

region-wise, subcortex					
Spearman's corr coef	p-val	p-val, SA	p-val, SA (BH- correction)	GenID	Gene
0.037837837	0.826257288	0.831	1	729409	LOC729409
0.037580434	0.827425003	0.822	1	1945	EFNA4
0.037580434	0.827425003	0.854	1	9278	ZBTB22
0.037580434	0.827425003	0.838	1	131034	CPNE4
0.037580434	0.827425003	0.831	1	163778	SPRR4
0.037580434	0.827425003	0.833	1	100170229	AC006276.1
0.037323035	0.828593135	0.834	1	55031	USP47
0.037323035	0.828593135	0.821	1	81847	RNF146
0.037323035	0.828593135	0.829	1	124995	MRPL10
0.037065636	0.829762101	0.84	1	1742	DLG4
0.037065636	0.829762101	0.857	1	10959	TMED2
0.037065636	0.829762101	0.824	1	23353	UNC84A
0.037065636	0.829762101	0.84	1	51384	WNT16
0.037065636	0.829762101	0.833	1	100132306	LOC100132306
0.036808237	0.830930889	0.845	1	1903	S1PR3
0.036808237	0.830930889	0.835	1	4210	MEFV
0.036808237	0.830930889	0.847	1	7423	VEGFB
0.036808237	0.830930889	0.827	1	8697	CDC23
0.036808237	0.830930889	0.875	1	9130	FAM50A
0.036808237	0.830930889	0.825	1	23623	RUSC1
0.036808237	0.830930889	0.838	1	54093	SETD4
0.036808237	0.830930889	0.858	1	54496	PRMT7
0.036808237	0.830930889	0.841	1	55776	C6orf64
0.036808237	0.830930889	0.848	1	642946	FLVCR1-AS1
0.036808237	0.830930889	0.829	1	728056	LOC728056
0.036550835	0.832100034	0.839	1	1365	CLDN3
0.036550835	0.832100034	0.85	1	64747	MFSD1
0.036293436	0.833269536	0.84	1	1417	CRYBB3
0.036293436	0.833269536	0.844	1	10644	IGF2BP2
0.036293436	0.833269536	0.845	1	22973	LAMB2P1
0.036293436	0.833269536	0.827	1	286333	LINC00256A
0.036293436	0.833269536	0.869	1	389199	LOC389199
0.036293436	0.833269536	0.849	1	100287430	LOC100287430
0.036036037	0.834439456	0.856	1	54469	ZFAND6
0.036036037	0.834439456	0.852	1	81300	OR4P4
0.036036037	0.834439456	0.85	1	387263	C6orf120
0.035778634	0.835610271	0.835	1	1937	EEF1G
0.035778634	0.835610271	0.866	1	1993	ELAVL2
0.035778634	0.835610271	0.813	1	9136	RRP9
0.035778634	0.835610271	0.861	1	54978	C2orf18
0.035778634	0.835610271	0.849	1	56984	PSMG2
0.035778634	0.835610271	0.855	1	57613	KIAA1467
0.035521235	0.836780846	0.843	1	10597	TRAPPC2P1
0.035521235	0.836780846	0.829	1	79155	TNIP2
0.035521235	0.836780846	0.834	1	200081	TXLNA
0.035521235	0.836780846	0.871	1	390282	LOC390282
0.035263833	0.837951779	0.854	1	4889	NPY5R
0.035263833	0.837951779	0.823	1	10069	RWDD2B
0.035263833	0.837951779	0.84	1	23201	FAM168A
0.035263833	0.837951779	0.846	1	63036	CELA2A
0.035006434	0.83912307	0.859	1	4670	HNRNPM
0.035006434	0.83912307	0.831	1	8533	COPS3
0.035006434	0.83912307	0.862	1	23513	SCRIB
0.035006434	0.83912307	0.843	1	25865	PRKD2
0.035006434	0.83912307	0.849	1	55205	ZNF532
0.035006434	0.83912307	0.865	1	57634	EP400
0.035006434	0.83912307	0.833	1	84060	C7orf64
0.035006434	0.83912307	0.854	1	152756	C4ORF39
0.034749035	0.840295255	0.839	1	9685	CLINT1
0.034749035	0.840295255	0.86	1	54989	ZNF770
0.034749035	0.840295255	0.839	1	55299	BRIX1
0.034749035	0.840295255	0.844	1	84105	PCBD2
0.034749035	0.840295255	0.863	1	144132	DNHD1
0.034491636	0.841467202	0.856	1	2054	STX2
0.034491636	0.841467202	0.864	1	4058	LTK
0.034491636	0.841467202	0.844	1	8325	FZD8
0.034491636	0.841467202	0.863	1	23548	TTC33
0.034491636	0.841467202	0.855	1	57699	CPNE5
0.034491636	0.841467202	0.863	1	96764	TGS1
0.034491636	0.841467202	0.847	1	163859	C1orf55
0.034491636	0.841467202	0.836	1	164091	PAQR7
0.034234237	0.842639565	0.848	1	1967	EIF2B1
0.034234237	0.842639565	0.847	1	3375	IAPP
0.034234237	0.842639565	0.844	1	55690	PACS1
0.034234237	0.842639565	0.87	1	57698	KIAA1598
0.034234237	0.842639565	0.854	1	79567	FAM65A
0.034234237	0.842639565	0.849	1	81832	NETO1
0.033976831	0.843812227	0.855	1	7555	CNBP
0.033976831	0.843812227	0.852	1	8798	DYRK4

voxel-wise, subcortex			
Spearman's corr coef	p-val	GenID	Gene
0.062587686	1.76364E-08	2558	GABRA5
0.06254524	1.80323E-08	54436	SH3TC1
0.062537909	1.81016E-08	63925	ZNF335
0.062465657	1.87981E-08	10902	BRD8
0.062456649	1.88867E-08	7044	LEFTY2
0.06241449	1.9307E-08	3680	ITGA9
0.062322456	2.02563E-08	399806	FLJ41350
0.062308993	2.03989E-08	154288	C6orf221
0.062294506	2.05534E-08	348013	FAM70B
0.062281225	2.06961E-08	6638	SNRPN
0.062203683	2.15484E-08	146845	WDR16
0.062201452	2.15734E-08	401466	C8orf59
0.06219653	2.16287E-08	577	BAI3
0.062143225	2.22365E-08	50509	COL5A3
0.062131062	2.23775E-08	63970	TP53AIP1
0.06210738	2.26545E-08	2628	GATM
0.062019527	2.37115E-08	147007	TMEM199
0.061944772	2.46484E-08	11214	AKAP13
0.061942358	2.46793E-08	219855	SLC37A2
0.061868805	2.56374E-08	4891	SLC11A2
0.061860356	2.57497E-08	84498	FAM120B
0.061844382	2.59634E-08	9857	CEP350
0.061810117	2.64276E-08	29114	TAGLN3
0.061792102	2.6675E-08	159686	CCDC147
0.0617752	2.6909E-08	51554	CCRL1
0.061740559	2.73948E-08	3070	HELLS
0.061725885	2.76032E-08	942	CD86
0.061690599	2.81107E-08	8533	COPS3
0.06165605	2.86163E-08	4656	MYOG
0.061616678	2.92032E-08	84260	TCHP
0.061546192	3.02832E-08	474354	LRRC18
0.061543684	3.03223E-08	5053	PAH
0.061535232	3.04546E-08	176	ACAN
0.061525296	3.06108E-08	9028	RHBDL1
0.061515361	3.07678E-08	9196	KCNAB3
0.061499573	3.10187E-08	55781	RIOK2
0.061417107	3.23624E-08	950	SCARB2
0.061407544	3.25218E-08	23357	ANGEL1
0.061265551	3.49807E-08	163227	ZNF100
0.061237689	3.54839E-08	337974	KRTAP19-7
0.061217073	3.58607E-08	2899	GRIK3
0.06121178	3.59581E-08	4917	NTN3
0.061169989	3.67359E-08	23362	PSD3
0.061168876	3.67569E-08	59344	ALOXE3
0.061156526	3.699E-08	171558	PTCRA
0.06114278	3.72512E-08	4683	NBN
0.061140925	3.72864E-08	221756	MGC39372
0.061139531	3.73131E-08	1789	DNMT3B
0.061119936	3.7689E-08	6415	SEPW1
0.061057903	3.89037E-08	51131	PHF11
0.061023168	3.96001E-08	2696	GIPR
0.060945068	4.12108E-08	2355	FOSL2
0.060929559	4.15382E-08	10068	IL18BP
0.060901422	4.21385E-08	91647	ATPAF2
0.060853317	4.31843E-08	23576	DDAH1
0.060835581	4.35763E-08	56270	WDR45L
0.060821094	4.38988E-08	3312	HSPA8
0.060820442	4.39135E-08	9816	URB2
0.06080772	4.41987E-08	200576	PIKFYVE
0.060762681	4.52234E-08	167359	AC114947.2
0.060753673	4.54312E-08	64772	ENGASE
0.060710955	4.64287E-08	148170	CDC42EP5
0.060643997	4.80353E-08	205147	FAM123C
0.060589578	4.93807E-08	51450	PRRX2
0.060552526	5.03174E-08	644192	RP11-327J17.3
0.060304016	5.70602E-08	26070	AP002380.3
0.060301322	5.7138E-08	90	ACVR1
0.060258046	5.84002E-08	148823	C1orf150
0.060248017	5.86966E-08	643866	CBLN3
0.060213007	5.97424E-08	1445	CSK
0.060193226	6.03413E-08	54828	BCAS3
0.060160536	6.13439E-08	115950	ZNF653
0.060132585	6.22138E-08	728039	SSR4P1
0.060111877	6.28661E-08	64689	GORASP1
0.060079742	6.38913E-08	6549	SLC9A2
0.060051885	6.47931E-08	92270	ATP6AP1L
0.060025882	6.56462E-08	9068	ANGPTL1
0.060010001	6.61725E-08	80321	CEP70
0.060007308	6.62622E-08	125965	COX6B2

region-wise, subcortex					
Spearman's corr coef	p-val	p-val, SA	p-val, SA (BH- correction)	GenID	Gene
0.033976831	0.843812227	0.871	1	10045	SH2D3A
0.033976831	0.843812227	0.838	1	27348	TOR1B
0.033976831	0.843812227	0.872	1	29915	HCFC2
0.033976831	0.843812227	0.865	1	58477	SRPRB
0.033719432	0.844985723	0.844	1	4791	NFKB2
0.033719432	0.844985723	0.85	1	7287	TULP1
0.033719432	0.844985723	0.848	1	55364	IMPACT
0.033719432	0.844985723	0.845	1	56341	PRMT8
0.033719432	0.844985723	0.857	1	113622	ADPRHL1
0.033719432	0.844985723	0.863	1	199223	TTC21A
0.033719432	0.844985723	0.859	1	256309	CCDC110
0.033719432	0.844985723	0.864	1	286151	FBXO43
0.033462033	0.846159041	0.856	1	966	CD59
0.033462033	0.846159041	0.865	1	3266	ERAS
0.033462033	0.846159041	0.869	1	5599	MAPK8
0.033462033	0.846159041	0.868	1	9728	SECISBP2L
0.033462033	0.846159041	0.87	1	284086	NEK8
0.033204634	0.847332716	0.83	1	9475	ROCK2
0.033204634	0.847332716	0.862	1	27091	CACNG5
0.033204634	0.847332716	0.855	1	126259	TMIGD2
0.033204634	0.847332716	0.868	1	392391	OR5C1
0.032947235	0.848506689	0.871	1	2195	FAT1
0.032947235	0.848506689	0.86	1	6092	ROBO2
0.032947235	0.848506689	0.868	1	9023	CH25H
0.032947235	0.848506689	0.853	1	9768	KIAA0101
0.032689832	0.84968096	0.859	1	6729	SRP54
0.032689832	0.84968096	0.856	1	23234	DNAJC9
0.032689832	0.84968096	0.849	1	84365	MKI67IP
0.032689832	0.84968096	0.85	1	158431	ZNF782
0.032689832	0.84968096	0.852	1	286343	LURAP1L
0.032432433	0.850856185	0.863	1	26271	FBXO5
0.032432433	0.850856185	0.858	1	285521	COX18
0.032175031	0.852031112	0.868	1	2060	EPS15
0.032175031	0.852031112	0.852	1	6188	RPS3P3
0.032175031	0.852031112	0.865	1	57184	C15orf17
0.032175031	0.852031112	0.879	1	81831	NETO2
0.032175031	0.852031112	0.871	1	124538	OR4D2
0.032175031	0.852031112	0.87	1	255349	TMEM211
0.032175031	0.852031112	0.869	1	283237	TTC9C
0.032175031	0.852031112	0.86	1	642757	FLJ32756
0.031917632	0.853206336	0.86	1	4104	MAGEA5
0.031917632	0.853206336	0.87	1	6517	SLC2A4
0.031917632	0.853206336	0.862	1	7644	ZNF91
0.031917632	0.853206336	0.87	1	8912	CACNA1H
0.031917632	0.853206336	0.837	1	10717	AP4B1
0.031917632	0.853206336	0.868	1	11019	LIAS
0.031917632	0.853206336	0.857	1	27199	OXGR1
0.031917632	0.853206336	0.86	1	91523	FAM113B
0.031917632	0.853206336	0.847	1	133308	NHEDC2
0.031917632	0.853206336	0.868	1	253769	WDR27
0.031917632	0.853206336	0.871	1	254187	TSGA10IP
0.031660229	0.854381919	0.856	1	3611	ILK
0.031660229	0.854381919	0.859	1	6205	RPS11P5
0.031660229	0.854381919	0.874	1	9780	FAM38A
0.031660229	0.854381919	0.878	1	51706	CYB5R1
0.031660229	0.854381919	0.866	1	54664	TMEM106B
0.031660229	0.854381919	0.868	1	57482	KIAA1211
0.031660229	0.854381919	0.863	1	120103	SLC36A4
0.031660229	0.854381919	0.865	1	125919	ZNF543
0.031660229	0.854381919	0.857	1	139818	DOCK11
0.031660229	0.854381919	0.862	1	644844	AC013356.1
0.031660229	0.854381919	0.87	1	692094	MSMP
0.03140283	0.855558395	0.865	1	1290	COL5A2
0.03140283	0.855558395	0.865	1	51131	PHF11
0.03140283	0.855558395	0.884	1	54931	RG9MTD1
0.03140283	0.855558395	0.863	1	642696	AC007601.1
0.031145431	0.856734633	0.854	1	287	ANK2
0.031145431	0.856734633	0.863	1	2557	GABRA4
0.031145431	0.856734633	0.873	1	55005	RMND1
0.031145431	0.856734633	0.856	1	126767	AADACL3
0.03088803	0.85791105	0.88	1	585	BBS4
0.03088803	0.85791105	0.863	1	6605	SMARCE1
0.03088803	0.85791105	0.87	1	8468	FKBP6
0.03088803	0.85791105	0.872	1	8498	RANBP3
0.03088803	0.85791105	0.866	1	83983	TSSK6
0.03088803	0.85791105	0.876	1	96626	LIMS3
0.03063063	0.859087884	0.879	1	6666	SOX12
0.03063063	0.859087884	0.885	1	8139	GAN
0.03063063	0.859087884	0.864	1	10309	CCNO

voxel-wise, subcortex			
Spearman's corr coef	p-val	GenID	Gene
0.059985951	6.69772E-08	57693	ZNF317
0.05992382	6.91004E-08	10838	ZNF275
0.059902929	6.98288E-08	143244	EIF5AL1
0.059893548	7.01583E-08	162083	C16orf82
0.059874974	7.08153E-08	51226	COPZ2
0.05986606	7.11325E-08	116135	LRRC3B
0.05957929	8.21099E-08	282809	WDR51B
0.059548087	8.33988E-08	57536	KIAA1328
0.05954558	8.35035E-08	164118	TTC24
0.059536848	8.3868E-08	51592	TRIM33
0.059536107	8.38989E-08	250	ALPP
0.059496358	8.55793E-08	115825	WDFY2
0.059489768	8.58606E-08	51271	UBAP1
0.059455685	8.73318E-08	131118	DNAJC19
0.059450019	8.75789E-08	29855	UBN1
0.059414268	8.91528E-08	6888	TALDO1
0.059399221	8.98231E-08	285141	AC007405.8
0.059308957	9.3949E-08	54926	UBE2R2
0.059279054	9.53555E-08	10016	PDCD6
0.059146997	1.01816E-07	26050	SLITRK5
0.059127402	1.0281E-07	3112	HLA-DOB
0.059025437	1.08136E-07	4690	NCK1
0.05901587	1.08649E-07	130355	C2orf76
0.059003334	1.09325E-07	8338	HIST2H2AC
0.058961358	1.11618E-07	56961	SHD
0.058955509	1.11942E-07	2564	GABRE
0.058950864	1.12199E-07	132660	LIN54
0.058944087	1.12575E-07	55293	UEVLD
0.058744237	1.24244E-07	11326	VSIG4
0.05874043	1.24477E-07	100289274	DNAJC3-AS1
0.058716565	1.25949E-07	441455	LOC441455
0.058666416	1.29097E-07	134145	FAM173B
0.058598902	1.33455E-07	9476	NAPSA
0.058505107	1.39747E-07	3340	NDST1
0.058466103	1.42447E-07	2702	GJA5
0.058329128	1.52335E-07	1499	CTNNB1
0.0582592	1.57635E-07	9973	CCS
0.058212951	1.61239E-07	537	ATP6AP1
0.058205988	1.61788E-07	401827	MSLNL
0.058172278	1.64473E-07	26146	TRAF3IP1
0.058148317	1.66407E-07	55106	SLFN12
0.058140796	1.67019E-07	51657	STYXL1
0.058117673	1.68914E-07	125111	GJD3
0.058081638	1.71908E-07	3267	AGFG1
0.058063809	1.73408E-07	246182	AKR7A2P1
0.058028519	1.76415E-07	55287	TMEM40
0.058013663	1.77696E-07	26270	FBXO6
0.057992674	1.79521E-07	646934	LOC646934
0.057989981	1.79756E-07	143570	XRRA1
0.057985336	1.80163E-07	6588	SLN
0.057978187	1.80791E-07	381	ARF5
0.057854861	1.91962E-07	245	ALOX12P2
0.057853375	1.921E-07	2533	FYB
0.05782143	1.95102E-07	7433	VIPR1
0.057801183	1.97029E-07	10203	CALCRL
0.057791062	1.97999E-07	81037	CLPTM1L
0.057788555	1.9824E-07	28998	MRPL13
0.057787441	1.98347E-07	2052	EPHX1
0.057774439	1.99602E-07	8357	HIST1H3H
0.057620373	2.15068E-07	4254	KITLG
0.05760533	2.16639E-07	11227	GALNT5
0.057584062	2.18878E-07	2857	GPR34
0.057521287	2.2562E-07	10726	NUDC
0.05749974	2.2798E-07	2010	EMD
0.057478011	2.30383E-07	124590	USH1G
0.057439379	2.34718E-07	23308	ICOSLG
0.057430647	2.35709E-07	51366	UBR5
0.057380222	2.41509E-07	2115	ETV1
0.057266925	2.55047E-07	253980	KCTD13
0.057255223	2.56487E-07	1464	CSPG4
0.057231452	2.59435E-07	219736	STOX1
0.057189662	2.64698E-07	221504	ZBTB9
0.057188828	2.64804E-07	9681	DEPDC5
0.057171367	2.67034E-07	10949	HNRNPA0
0.057168301	2.67427E-07	51014	TMED7
0.057160407	2.68443E-07	3396	ICT1
0.057108589	2.75203E-07	100506243	RP11-141M3.1
0.057084445	2.78408E-07	64755	C16orf58
0.057066709	2.80786E-07	9039	UBA3

region-wise, subcortex					
Spearman's corr coef	p-val	p-val, SA	p-val, SA (BH- correction)	GenID	Gene
0.03063063	0.859087884	0.862	1	29796	UCRC
0.03063063	0.859087884	0.857	1	55856	ACOT13
0.03063063	0.859087884	0.863	1	56899	ANKS1B
0.03063063	0.859087884	0.873	1	57144	PAK7
0.03063063	0.859087884	0.864	1	64693	CTAGE1
0.03063063	0.859087884	0.867	1	219409	GSX1
0.03063063	0.859087884	0.862	1	400986	ANKRD36C
0.030373231	0.860265017	0.87	1	6203	RPS9
0.030373231	0.860265017	0.864	1	146691	TOM1L2
0.030373231	0.860265017	0.881	1	150383	C22orf40
0.030115828	0.861442983	0.889	1	23483	TGDS
0.030115828	0.861442983	0.882	1	51290	ERGIC2
0.030115828	0.861442983	0.888	1	81567	TXNDC5
0.030115828	0.861442983	0.864	1	136647	C7orf11
0.030115828	0.861442983	0.877	1	266727	MDGA1
0.029858429	0.862620711	0.859	1	2019	EN1
0.029858429	0.862620711	0.887	1	8634	RTCD1
0.029858429	0.862620711	0.861	1	26001	RNF167
0.029858429	0.862620711	0.875	1	65003	MRPL11
0.029858429	0.862620711	0.891	1	79846	C7orf63
0.029858429	0.862620711	0.866	1	440026	TMEM41B
0.029858429	0.862620711	0.88	1	441191	RNF216L
0.02960103	0.863798738	0.853	1	650	BMP2
0.02960103	0.863798738	0.873	1	11120	BTN2A1
0.02960103	0.863798738	0.867	1	26492	OR8G2
0.02960103	0.863798738	0.872	1	80714	PBX4
0.02960103	0.863798738	0.903	1	124401	ANKS3
0.029343629	0.864977062	0.867	1	3795	KHK
0.029343629	0.864977062	0.869	1	4250	SCGB2A2
0.029343629	0.864977062	0.874	1	6741	SSB
0.029343629	0.864977062	0.878	1	8828	NRP2
0.029343629	0.864977062	0.873	1	10369	CACNG2
0.029343629	0.864977062	0.863	1	11337	GABARAP
0.029343629	0.864977062	0.868	1	85021	REPS1
0.029086228	0.866156161	0.881	1	6840	SVIL
0.029086228	0.866156161	0.872	1	22985	ACIN1
0.029086228	0.866156161	0.869	1	25871	C3orf17
0.029086228	0.866156161	0.884	1	26301	GBGT1
0.029086228	0.866156161	0.893	1	51659	GINS2
0.029086228	0.866156161	0.883	1	283518	KCNRG
0.029086228	0.866156161	0.873	1	731220	AC099797.1
0.028828828	0.867335081	0.869	1	1611	DAP
0.028828828	0.867335081	0.882	1	5533	PPP3CC
0.028828828	0.867335081	0.871	1	11007	CCDC85B
0.028828828	0.867335081	0.866	1	11148	HHLA2
0.028828828	0.867335081	0.874	1	57171	DOLPP1
0.028828828	0.867335081	0.879	1	114134	SLC2A13
0.028571429	0.86851424	0.876	1	23614	PPY2
0.028571429	0.86851424	0.892	1	57335	ZNF286
0.028571429	0.86851424	0.879	1	79012	CAMKV
0.028314028	0.869693696	0.885	1	10606	PAICS
0.028314028	0.869693696	0.872	1	284106	CISD3
0.028314028	0.869693696	0.868	1	541468	C1orf190
0.028056627	0.870873988	0.898	1	57511	COG6
0.028056627	0.870873988	0.879	1	57532	NUFIP2
0.028056627	0.870873988	0.901	1	90233	ZNF551
0.027799228	0.872053981	0.891	1	10922	FASTK
0.027799228	0.872053981	0.903	1	26330	GAPDHS
0.027799228	0.872053981	0.878	1	56254	RNF20
0.027799228	0.872053981	0.885	1	57053	CHRNA10
0.027799228	0.872053981	0.872	1	131870	NUDT16
0.027799228	0.872053981	0.894	1	197335	WDR90
0.027799228	0.872053981	0.87	1	201292	TRIM65
0.027799228	0.872053981	0.894	1	337976	KRTAP20-2
0.027541827	0.873234272	0.894	1	4773	NFATC2
0.027541827	0.873234272	0.884	1	5711	PSMD5
0.027541827	0.873234272	0.89	1	26469	PTPN18
0.027541827	0.873234272	0.872	1	345757	FAM174A
0.027541827	0.873234272	0.891	1	100506164	HSFX1
0.027284427	0.874414861	0.884	1	2077	ERF
0.027284427	0.874414861	0.876	1	10838	ZNF275
0.027284427	0.874414861	0.883	1	11031	RAB31
0.027284427	0.874414861	0.881	1	23040	MYT1L
0.027284427	0.874414861	0.882	1	27349	MCAT
0.027284427	0.874414861	0.895	1	84705	GTPBP3
0.027284427	0.874414861	0.882	1	100132062	LOC100132062
0.027027028	0.875595689	0.878	1	2186	BPTF
0.027027028	0.875595689	0.894	1	8031	NCOA4
0.027027028	0.875595689	0.888	1	29072	SETD2

voxel-wise, subcortex			
Spearman's corr coef	p-val	GenID	Gene
0.057029653	2.85816E-07	5984	RFC4
0.056950346	2.96879E-07	6293	VPS52
0.056898713	3.04302E-07	30001	ERO1L
0.056877073	3.07464E-07	55658	RNF126
0.056852188	3.11143E-07	2185	PTK2B
0.056837793	3.1329E-07	23768	FLRT2
0.056831572	3.14222E-07	643236	TMEM72
0.056808259	3.17739E-07	3364	HUS1
0.056797769	3.19334E-07	2042	EPHA3
0.056796003	3.19603E-07	57876	MUC3B
0.056794517	3.19829E-07	4059	BCAM
0.056791544	3.20283E-07	3476	IGBP1
0.056669332	3.39498E-07	2644	GCHFR
0.056666549	3.39949E-07	7343	UBTF
0.056617144	3.48039E-07	196527	ANO6
0.056588724	3.52776E-07	54957	TXNL4B
0.05658761	3.52963E-07	9101	USP8
0.056562722	3.57165E-07	100507135	AL139377.1
0.056499016	3.68142E-07	23582	CCNDBP1
0.056479886	3.71501E-07	10933	MORF4L1
0.056448776	3.77028E-07	84634	KISS1R
0.056437541	3.79043E-07	2195	FAT1
0.056404293	3.85067E-07	8085	MLL2
0.056394823	3.868E-07	1953	MEGF6
0.056377642	3.89963E-07	122042	RXFP2
0.056370396	3.91304E-07	1875	E2F5
0.0562324	4.17717E-07	147645	AC063977.1
0.056177143	4.28769E-07	4665	NAB2
0.056142598	4.35821E-07	55970	GNG12
0.056137305	4.36912E-07	55529	TMEM55A
0.056070071	4.50992E-07	28958	CCDC56
0.056052331	4.54781E-07	100507530	LOC100507530
0.056049917	4.55298E-07	54814	QPCTL
0.056016766	4.62466E-07	23042	PDXDC1
0.055988718	4.68616E-07	64940	STAG3L4
0.055986118	4.6919E-07	389336	C5orf46
0.055973675	4.71946E-07	3593	IL12B
0.055911548	4.85946E-07	8818	DPM2
0.055903003	4.87903E-07	57406	ABHD6
0.055858985	4.98102E-07	54849	DEF8
0.055839483	5.02686E-07	1230	CCR1
0.055807445	5.10305E-07	124221	TMPRSS8
0.055794813	5.13341E-07	153572	IRX2
0.055743832	5.25767E-07	84243	ZDHHC18
0.055737518	5.27326E-07	10193	RNF41
0.05568774	5.39772E-07	55374	TMCO6
0.055654123	5.48339E-07	343035	RD3
0.055608898	5.6007E-07	153339	TMEM167A
0.055574816	5.69071E-07	79596	RNF219
0.055567756	5.70952E-07	794	CALB2
0.055566177	5.71373E-07	1536	CYBB
0.055553827	5.74681E-07	79065	ATG9A
0.0555503	5.75629E-07	6543	SLC8A2
0.055535998	5.7949E-07	5662	PSD
0.055510458	5.86446E-07	53947	A4GALT
0.055428736	6.09249E-07	6349	CCL3L1
0.055368651	6.26557E-07	25864	ABHD14A
0.055249412	6.62318E-07	2952	GSTT1
0.055242542	6.64436E-07	821	CANX
0.055239104	6.655E-07	8241	RBM10
0.055188026	6.81487E-07	5430	POLR2A
0.055185243	6.82368E-07	2245	FGD1
0.055126086	7.01369E-07	8367	HIST1H4E
0.055068694	7.20292E-07	390260	OR6X1
0.054992266	7.46255E-07	9720	CCDC144A
0.054988179	7.47666E-07	6953	TCP10
0.054969884	7.54024E-07	253725	FAM21C
0.054903951	7.77376E-07	4345	CD200
0.054874513	7.88025E-07	114791	TUBGCP5
0.054856867	7.94478E-07	1053	CEBPE
0.054836903	8.01834E-07	84901	NFATC2IP
0.054833557	8.03075E-07	4299	AFF1
0.054816194	8.09539E-07	6850	SYK
0.054797526	8.16544E-07	197350	AC108134.5
0.054779138	8.23502E-07	2318	FLNC
0.05476521	8.28809E-07	5792	PTPRF
0.054741248	8.38017E-07	257000	PLAC2
0.054715898	8.47866E-07	844	CASQ1
0.054676242	8.63499E-07	1184	CLCN5

region-wise, subcortex					
Spearman's corr coef	p-val	p-val, SA	p-val, SA (BH- correction)	GenID	Gene
0.027027028	0.875595689	0.871	1	51307	FAM53C
0.026769627	0.876777291	0.871	1	8555	CDC14B
0.026769627	0.876777291	0.881	1	10642	IGF2BP1
0.026769627	0.876777291	0.893	1	57472	CNOT6
0.026769627	0.876777291	0.872	1	81609	SNX27
0.026769627	0.876777291	0.878	1	130355	C2orf76
0.026769627	0.876777291	0.877	1	339665	SLC35E4
0.026769627	0.876777291	0.905	1	388692	LOC388692
0.026512226	0.877958715	0.889	1	720	C4A
0.026512226	0.877958715	0.878	1	951	CD37
0.026512226	0.877958715	0.894	1	1474	CST6
0.026512226	0.877958715	0.884	1	5446	PON3
0.026512226	0.877958715	0.88	1	5536	PPP5C
0.026512226	0.877958715	0.875	1	7840	ALMS1
0.026512226	0.877958715	0.887	1	57515	SERINC1
0.026512226	0.877958715	0.89	1	80308	FLAD1
0.026512226	0.877958715	0.885	1	83444	INO80B
0.026512226	0.877958715	0.896	1	93621	MRFAP1
0.026512226	0.877958715	0.915	1	219431	OR4S2
0.026512226	0.877958715	0.877	1	648791	PPP1R3G
0.026254825	0.879140317	0.901	1	6433	SFRS8
0.026254825	0.879140317	0.882	1	94235	GNG8
0.026254825	0.879140317	0.906	1	116039	OSR2
0.026254825	0.879140317	0.888	1	283807	FBXL22
0.025997426	0.880322218	0.904	1	8915	BCL10
0.025997426	0.880322218	0.903	1	115265	DDIT4L
0.025997426	0.880322218	0.899	1	162387	MFSD6L
0.025997426	0.880322218	0.889	1	255809	C19orf38
0.025740024	0.881504893	0.898	1	509	ATP5C1
0.025740024	0.881504893	0.879	1	4261	CIITA
0.025740024	0.881504893	0.896	1	9527	GOSR1
0.025740024	0.881504893	0.88	1	10778	ZNF271
0.025740024	0.881504893	0.884	1	388512	CLEC17A
0.025740024	0.881504893	0.888	1	440556	RP1-163G9.1
0.025482625	0.88268733	0.888	1	5343	PLGLB1
0.025482625	0.88268733	0.893	1	5373	PMM2
0.025482625	0.88268733	0.893	1	84678	KDM2B
0.025482625	0.88268733	0.89	1	400406	LOC400406
0.025225226	0.883869946	0.883	1	2946	GSTM2
0.025225226	0.883869946	0.901	1	10379	IRF9
0.025225226	0.883869946	0.902	1	55277	FGGY
0.025225226	0.883869946	0.915	1	126917	IFFO2
0.025225226	0.883869946	0.892	1	100287049	RP11-574F21.2
0.024967825	0.88505286	0.892	1	4009	LMX1A
0.024967825	0.88505286	0.887	1	10456	HAX1
0.024967825	0.88505286	0.887	1	143678	AC068385.1
0.024967825	0.88505286	0.9	1	445582	POTEE
0.024710424	0.886236548	0.899	1	8737	RIPK1
0.024710424	0.886236548	0.896	1	10669	CGREF1
0.024710424	0.886236548	0.875	1	80124	VCPIP1
0.024710424	0.886236548	0.901	1	81034	SLC25A32
0.024710424	0.886236548	0.901	1	92960	PEX11G
0.024710424	0.886236548	0.889	1	221079	ARL5B
0.024710424	0.886236548	0.895	1	441052	LOC441052
0.024453023	0.887419939	0.91	1	955	ENTPD6
0.024453023	0.887419939	0.878	1	7031	TFF1
0.024453023	0.887419939	0.898	1	9815	GIT2
0.024453023	0.887419939	0.896	1	10260	DENND4A
0.024453023	0.887419939	0.898	1	84073	MYCBPAP
0.024453023	0.887419939	0.904	1	85294	KRTAP2-4
0.024195625	0.888603628	0.895	1	1894	ECT2
0.024195625	0.888603628	0.904	1	51696	HECA
0.024195625	0.888603628	0.922	1	112487	C14orf126
0.023938224	0.889787495	0.908	1	1761	DMRT1
0.023938224	0.889787495	0.891	1	26035	GLCE
0.023938224	0.889787495	0.9	1	26058	GIGYF2
0.023938224	0.889787495	0.905	1	64083	GOLPH3
0.023680823	0.890971541	0.895	1	814	CAMK4
0.023680823	0.890971541	0.895	1	4641	MYO1C
0.023680823	0.890971541	0.916	1	22888	UBOX5
0.023680823	0.890971541	0.893	1	100287932	TIMM23
0.023423424	0.892156482	0.896	1	4502	MT2A
0.023423424	0.892156482	0.897	1	10658	CUGBP1
0.023423424	0.892156482	0.91	1	11216	AKAP10
0.023423424	0.892156482	0.914	1	51174	TUBD1
0.023423424	0.892156482	0.909	1	51304	ZDHHC3
0.023423424	0.892156482	0.899	1	51375	SNX7
0.023423424	0.892156482	0.919	1	51807	TUBA8
0.023423424	0.892156482	0.902	1	79149	ZSCAN5A

voxel-wise, subcortex			
Spearman's corr coef	p-val	GenID	Gene
0.054663241	8.68682E-07	26001	RNF167
0.054653212	8.72704E-07	23127	GLT25D2
0.054474447	9.47437E-07	64928	MRPL14
0.054431912	9.66106E-07	23635	SSBP2
0.054396253	9.82029E-07	121601	AC079953.2
0.054391887	9.83996E-07	6692	SPINT1
0.054379631	9.89536E-07	5518	PPP2R1A
0.054337561	1.00879E-06	576	BAI2
0.054337282	1.00892E-06	285958	C7orf40
0.05430153	1.02557E-06	8482	SEMA7A
0.054198448	1.07505E-06	114569	MAL2
0.054183312	1.08251E-06	8623	ASMTL
0.054157864	1.09516E-06	9655	SOCS5
0.054154336	1.09692E-06	339398	LINGO4
0.054144681	1.10176E-06	5636	PRPSAP2
0.054143008	1.1026E-06	6452	SH3BP2
0.054137435	1.10541E-06	4594	MUT
0.054122206	1.11312E-06	7225	TRPC6
0.054118678	1.11491E-06	6001	RGS10
0.054097131	1.12592E-06	1965	EIF2S1
0.054003242	1.17513E-06	100131187	RP11-544M22.1
0.053984299	1.1853E-06	22978	NT5C2
0.053966563	1.1949E-06	653361	NCF1
0.05395003	1.20393E-06	51196	PLCE1
0.053949937	1.20398E-06	79366	NSBP1
0.053937122	1.21101E-06	945	CD33
0.053894125	1.23491E-06	2356	FPGS
0.053856794	1.25603E-06	653121	ZBTB8A
0.05382745	1.27287E-06	56000	NXF3
0.053751022	1.31776E-06	8336	HIST1H2AM
0.053730495	1.33008E-06	5783	PTPN13
0.053717125	1.33816E-06	60561	RINT1
0.053707838	1.3438E-06	1002	CDH4
0.053649981	1.37946E-06	6286	S100P
0.05362853	1.39291E-06	64236	PDLIM2
0.053601414	1.41009E-06	9148	NEURL
0.053555537	1.43963E-06	747	DAGLA
0.053513654	1.46711E-06	55213	RCBTB1
0.053378627	1.55919E-06	56900	TMEM167B
0.053357176	1.57432E-06	8269	TMEM187
0.053343616	1.58396E-06	440836	ODF3B
0.05330294	1.61321E-06	283902	HTA
0.053296257	1.61806E-06	9520	NPEPPS
0.053295325	1.61874E-06	56112	PCDHGA3
0.053261895	1.64325E-06	283635	FAM177A1
0.053256135	1.64751E-06	1432	MAPK14
0.053253815	1.64922E-06	26015	RPAP1
0.053133555	1.7407E-06	140947	C5ORF20
0.053102165	1.76536E-06	119358	SNRPD2P1
0.05309483	1.77117E-06	631	BFSP1
0.053079136	1.78367E-06	55279	ZNF654
0.053037532	1.81721E-06	84439	HHIPL1
0.053020723	1.83092E-06	257044	C1orf101
0.05291792	1.91704E-06	10876	FAM12A
0.052895073	1.93669E-06	3005	H1F0
0.052885417	1.94506E-06	388551	CEACAM16
0.052880865	1.94902E-06	127254	C1orf173
0.052842513	1.98266E-06	50808	AK3
0.052789394	2.03018E-06	284800	C20orf91
0.052783079	2.0359E-06	353332	KRTAP12-1
0.052780848	2.03793E-06	84815	MGC12916
0.052739058	2.07622E-06	160065	PATE1
0.052735344	2.07965E-06	353140	LCE2C
0.052726988	2.0874E-06	65062	ALS2CR4
0.052677583	2.1338E-06	51247	PAIP2
0.052654274	2.15603E-06	201305	SPNS3
0.052595861	2.21272E-06	2631	GBAS
0.052563079	2.24517E-06	284677	C1orf204
0.052545249	2.263E-06	64794	DDX31
0.05244096	2.37008E-06	9249	DHRS3
0.052402884	2.41038E-06	5277	PIGA
0.052384682	2.42988E-06	353088	ZNF429
0.052368987	2.44681E-06	7706	TRIM25
0.052353758	2.46334E-06	3846	KRTAP5-9
0.052350231	2.46718E-06	6729	SRP54
0.052313734	2.50731E-06	10916	MAGED2
0.052300639	2.52186E-06	64799	IQCH
0.052298967	2.52372E-06	80740	LY6G6C
0.052293394	2.52995E-06	51214	IGF2AS

region-wise, subcortex					
Spearman's corr coef	p-val	p-val, SA	p-val, SA (BH- correction)	GenID	Gene
0.023423424	0.892156482	0.907	1	541466	CT45A1
0.023166021	0.893341064	0.907	1	51645	PPIL1
0.023166021	0.893341064	0.894	1	140902	R3HDML
0.022908622	0.894525886	0.895	1	899	CCNF
0.022908622	0.894525886	0.909	1	22902	RUFY3
0.022908622	0.894525886	0.906	1	90025	UBE2CBP
0.022908622	0.894525886	0.904	1	284697	BTBD8
0.022651222	0.895710945	0.886	1	4921	DDR2
0.022651222	0.895710945	0.887	1	9716	AQR
0.022651222	0.895710945	0.902	1	23363	OBSL1
0.022651222	0.895710945	0.898	1	55740	ENAH
0.022393823	0.89689672	0.905	1	2787	GNG5
0.022393823	0.89689672	0.899	1	5499	PPP1CA
0.022393823	0.89689672	0.914	1	5551	PRF1
0.022393823	0.89689672	0.895	1	5994	RFXAP
0.022393823	0.89689672	0.902	1	23767	FLRT3
0.022136422	0.898082256	0.912	1	4287	ATXN3
0.022136422	0.898082256	0.928	1	4291	MLF1
0.022136422	0.898082256	0.916	1	9831	ZNF623
0.022136422	0.898082256	0.913	1	51438	MAGEC2
0.022136422	0.898082256	0.914	1	58508	MLL3
0.021879021	0.899267972	0.899	1	3139	HLA-L
0.021879021	0.899267972	0.925	1	3646	EIF3E
0.021879021	0.899267972	0.904	1	4853	NOTCH2
0.021879021	0.899267972	0.907	1	6240	RRM1
0.021879021	0.899267972	0.897	1	10347	ABCA7
0.021879021	0.899267972	0.919	1	55054	ATG16L1
0.021879021	0.899267972	0.915	1	64858	DCLRE1B
0.021879021	0.899267972	0.911	1	84572	GNPTG
0.021879021	0.899267972	0.889	1	84791	C1orf97
0.021879021	0.899267972	0.918	1	91289	LMF2
0.021879021	0.899267972	0.9	1	94241	TP53INP1
0.021621622	0.900453925	0.906	1	3956	LGALS1
0.021621622	0.900453925	0.902	1	7349	UCN
0.021621622	0.900453925	0.91	1	9638	FEZ1
0.021621622	0.900453925	0.913	1	79153	GDPD3
0.021364219	0.901640594	0.919	1	2177	FANCD2
0.021364219	0.901640594	0.904	1	3055	HCK
0.021364219	0.901640594	0.908	1	3663	IRF5
0.021364219	0.901640594	0.914	1	7620	ZNF69
0.021364219	0.901640594	0.918	1	8019	BRD3
0.021364219	0.901640594	0.921	1	54805	CNNM2
0.021364219	0.901640594	0.917	1	54962	TIPIN
0.021364219	0.901640594	0.916	1	55100	WDR70
0.021364219	0.901640594	0.908	1	55272	IMP3
0.021364219	0.901640594	0.917	1	81614	NIPA2
0.021106821	0.902826905	0.904	1	27333	GOLIM4
0.021106821	0.902826905	0.9	1	221830	TWISTNB
0.021106821	0.902826905	0.903	1	283796	GOLGA9P
0.021106821	0.902826905	0.915	1	651746	ANKRD33B
0.021106821	0.902826905	0.901	1	751071	METTL12
0.020849422	0.904013515	0.916	1	2937	GSS
0.020849422	0.904013515	0.904	1	3070	HELLS
0.020849422	0.904013515	0.935	1	10935	PRDX3
0.020849422	0.904013515	0.919	1	22824	HSPA4L
0.020849422	0.904013515	0.909	1	91942	NDUFAF2
0.020849422	0.904013515	0.91	1	126520	PLK5P
0.020849422	0.904013515	0.911	1	254028	LOC254028
0.020849422	0.904013515	0.912	1	283248	RCOR2
0.020592021	0.905200243	0.911	1	248	ALPI
0.020592021	0.905200243	0.909	1	482	ATP1B2
0.020592021	0.905200243	0.912	1	1787	TRDMT1
0.020592021	0.905200243	0.904	1	8411	EEA1
0.020592021	0.905200243	0.91	1	79412	KREMEN2
0.020592021	0.905200243	0.902	1	401149	FLJ14186
0.02033462	0.906387269	0.923	1	115	ADCY9
0.02033462	0.906387269	0.907	1	154	ADRB2
0.02033462	0.906387269	0.907	1	945	CD33
0.02033462	0.906387269	0.904	1	4035	LRP1
0.02033462	0.906387269	0.918	1	4086	SMAD1
0.02033462	0.906387269	0.912	1	54988	ACSM5
0.02033462	0.906387269	0.913	1	170622	COMMD6
0.02033462	0.906387269	0.911	1	286380	FOXD4L3
0.020077219	0.907574952	0.913	1	6143	RPL19P12
0.020077219	0.907574952	0.9	1	8078	USP5
0.020077219	0.907574952	0.919	1	8820	HESX1
0.020077219	0.907574952	0.905	1	58485	TRAPPC1
0.020077219	0.907574952	0.908	1	80161	ASMTL-AS1
0.020077219	0.907574952	0.924	1	113178	SCAMP4

voxel-wise, subcortex			
Spearman's corr coef	p-val	GenID	Gene
0.052287266	2.53681E-06	8615	USO1
0.052131716	2.71696E-06	4036	LRP2
0.052114349	2.73783E-06	653483	C6orf124
0.052098468	2.75703E-06	7913	DEK
0.052088812	2.76878E-06	284433	OR10H5
0.052087139	2.77082E-06	9064	MAP3K6
0.052068289	2.7939E-06	5130	PCYT1A
0.052040614	2.82812E-06	90246	LOC90246
0.052010711	2.86554E-06	10572	SIVA1
0.052010246	2.86613E-06	651430	FLJ38576
0.051986102	2.8967E-06	144402	CPNE8
0.051985081	2.898E-06	8313	AXIN2
0.051854417	3.06901E-06	9182	RASSF9
0.051837329	3.09208E-06	219858	OR8B12
0.051830273	3.10166E-06	619189	SERINC4
0.051775575	3.17684E-06	55520	ELAC1
0.051761087	3.19705E-06	4440	MSI1
0.051754121	3.20681E-06	100130794	NDUFS5P7
0.0517492	3.21373E-06	7443	VRK1
0.05174493	3.21973E-06	318	NUDT2
0.051737498	3.23021E-06	7705	ZNF146
0.051698033	3.28644E-06	388955	LOC388955
0.05166943	3.32777E-06	706	TSPO
0.051649556	3.35679E-06	24147	FJX1
0.051630333	3.38507E-06	26145	IRF2BP1
0.051605351	3.42219E-06	4604	MYBPC1
0.051591516	3.4429E-06	3745	KCNB1
0.051579721	3.46067E-06	374860	C21orf110
0.051574335	3.4688E-06	146562	C16orf71
0.051373929	3.78501E-06	10842	C7orf16
0.051312637	3.88711E-06	117166	WFIKKN1
0.051283754	3.93614E-06	8563	THOC5
0.051229987	4.02898E-06	4724	NDUFS4
0.051221073	4.04457E-06	7918	BAT4
0.05118281	4.11216E-06	4247	MGAT2
0.051171202	4.13289E-06	91612	CHURC1
0.051150121	4.17076E-06	144132	DNHD1
0.051144827	4.18034E-06	3958	LGALS3
0.051111396	4.24123E-06	29126	CD274
0.051035527	4.3826E-06	79893	GGNBP2
0.051008131	4.43474E-06	28970	C11orf54
0.050926778	4.59313E-06	140545	RNF32
0.050907835	4.63077E-06	64598	MOSPD3
0.050892696	4.66108E-06	2695	GIP
0.05089112	4.66424E-06	56181	FAM54B
0.050871152	4.70453E-06	5078	PAX4
0.050867993	4.71093E-06	51503	CWC15
0.05082212	4.80484E-06	51001	MTERFD1
0.050778564	4.8957E-06	9990	SLC12A6
0.050700929	5.06171E-06	7466	WFS1
0.050651245	5.17077E-06	6698	SPRR1A
0.050471921	5.5834E-06	135152	B3GAT2
0.050467744	5.59338E-06	1135	CHRNA2
0.050408587	5.73653E-06	23194	FBXL7
0.05039512	5.76961E-06	22994	AZI1
0.050285909	6.04466E-06	5571	PRKAG1
0.050268825	6.0888E-06	10170	DHRS9
0.050260838	6.10954E-06	407835	LOC407835
0.050220255	6.216E-06	478	ATP1A3
0.050219327	6.21845E-06	1635	DCTD
0.050140299	6.43094E-06	10498	CARM1
0.050078355	6.60232E-06	91404	SESTD1
0.050062105	6.648E-06	92799	SHKBP1
0.050056811	6.66294E-06	11037	STON1
0.050034616	6.72597E-06	60490	PPCDC
0.050028488	6.74346E-06	4189	DNAJB9
0.050001185	6.82196E-06	9751	SNPH
0.049993567	6.84401E-06	123803	NTAN1
0.049966082	6.92416E-06	202309	GAPT
0.049964037	6.93016E-06	23283	CSTF2T
0.049918719	7.06439E-06	10308	ZNF267
0.04982641	7.3455E-06	59352	LGR6
0.049757876	7.56112E-06	89849	ATG16L2
0.049676802	7.82398E-06	84326	C16orf13
0.049672533	7.83807E-06	57715	SEMA4G
0.04966566	7.8608E-06	8170	SLC14A2
0.049662687	7.87064E-06	388323	GLTPD2
0.049639005	7.94951E-06	51073	MRPL4
0.049622197	8.00593E-06	254427	C10orf47

region-wise, subcortex					
Spearman's corr coef	p-val	p-val, SA	p-val, SA (BH- correction)	GenID	Gene
0.01981982	0.908762395	0.913	1	286	ANK1
0.01981982	0.908762395	0.914	1	22954	TRIM32
0.01981982	0.908762395	0.907	1	25948	KBTBD2
0.01981982	0.908762395	0.917	1	55074	OXR1
0.01981982	0.908762395	0.923	1	130749	CPO
0.01981982	0.908762395	0.92	1	353144	LCE3C
0.01981982	0.908762395	0.907	1	440574	C1orf151
0.019562418	0.909949899	0.91	1	5586	PKN2
0.019562418	0.909949899	0.928	1	79897	RPP21
0.019562418	0.909949899	0.922	1	222696	ZSCAN23
0.019305019	0.91113776	0.924	1	905	CCNT2
0.019305019	0.91113776	0.909	1	9583	ENTPD4
0.019305019	0.91113776	0.937	1	22994	AZI1
0.019305019	0.91113776	0.923	1	57531	HACE1
0.019305019	0.91113776	0.928	1	285600	C5orf36
0.01904762	0.912326217	0.916	1	1677	DFFB
0.01904762	0.912326217	0.91	1	5089	PBX2
0.01904762	0.912326217	0.901	1	153478	PLEKHG4B
0.01904762	0.912326217	0.921	1	374897	SBSN
0.018790217	0.913514376	0.914	1	7019	TFAM
0.018790217	0.913514376	0.922	1	7994	MYST3
0.018790217	0.913514376	0.927	1	9730	VPRBP
0.018790217	0.913514376	0.924	1	200373	AC069154.2
0.018532818	0.914702773	0.915	1	3630	INS
0.018532818	0.914702773	0.921	1	5611	DNAJC3
0.018532818	0.914702773	0.924	1	51719	CAB39
0.018532818	0.914702773	0.931	1	100506060	LOC100506060
0.018275417	0.91589123	0.928	1	1185	CLCN6
0.018275417	0.91589123	0.924	1	29964	PRICKLE4
0.018275417	0.91589123	0.918	1	284367	SIGLECP3
0.018275417	0.91589123	0.913	1	387597	ILDR2
0.018018018	0.917080581	0.926	1	989	SEPTIN7
0.018018018	0.917080581	0.912	1	3232	HOXD3
0.018018018	0.917080581	0.924	1	4007	PRICKLE3
0.018018018	0.917080581	0.915	1	10575	CCT4
0.018018018	0.917080581	0.932	1	25909	AHCTF1
0.018018018	0.917080581	0.918	1	51008	ASCC1
0.018018018	0.917080581	0.928	1	93100	NAPRT1
0.018018018	0.917080581	0.924	1	132660	LIN54
0.018018018	0.917080581	0.929	1	253982	ASPHD1
0.018018018	0.917080581	0.921	1	388566	ZNF470
0.017760618	0.918269455	0.916	1	433	ASGR2
0.017760618	0.918269455	0.931	1	7009	TMBIM6
0.017760618	0.918269455	0.937	1	8192	CLPP
0.017760618	0.918269455	0.934	1	51185	CRBN
0.017760618	0.918269455	0.918	1	57677	ZFP14
0.017760618	0.918269455	0.94	1	83932	C1orf124
0.017760618	0.918269455	0.937	1	84553	FAXC
0.017503217	0.919458449	0.918	1	2623	GATA1
0.017503217	0.919458449	0.93	1	5479	PPIB
0.017503217	0.919458449	0.926	1	6615	SNAI1
0.017503217	0.919458449	0.942	1	26539	OR10H1
0.017503217	0.919458449	0.924	1	51639	AC008073.1
0.017503217	0.919458449	0.922	1	84964	ALKBH6
0.017503217	0.919458449	0.942	1	643310	ST13P18
0.017245818	0.92064774	0.925	1	2212	FCGR2A
0.017245818	0.92064774	0.938	1	50651	SLC45A1
0.017245818	0.92064774	0.927	1	78992	YIPF2
0.017245818	0.92064774	0.918	1	84515	MCM8
0.017245818	0.92064774	0.909	1	171024	SYNPO2
0.017245818	0.92064774	0.941	1	400684	LOC400684
0.016988415	0.921837151	0.928	1	762	CA4
0.016988415	0.921837151	0.937	1	7283	TUBG1
0.016988415	0.921837151	0.919	1	23484	LEPROTL1
0.016988415	0.921837151	0.933	1	81578	COL21A1
0.016988415	0.921837151	0.93	1	91319	DERL3
0.016988415	0.921837151	0.933	1	283208	P4HA3
0.016988415	0.921837151	0.928	1	341883	LRRC9
0.016988415	0.921837151	0.942	1	441228	LOC441228
0.016731016	0.923027277	0.924	1	4738	NEDD8
0.016731016	0.923027277	0.92	1	6233	RPS27A
0.016731016	0.923027277	0.926	1	25824	PRDX5
0.016731016	0.923027277	0.932	1	29092	LINC00339
0.016731016	0.923027277	0.917	1	55041	PLEKHB2
0.016731016	0.923027277	0.932	1	57560	IFT80
0.016731016	0.923027277	0.931	1	113746	ODF3
0.016731016	0.923027277	0.931	1	115950	ZNF653
0.016473617	0.924217045	0.945	1	27022	FOXD3
0.016473617	0.924217045	0.935	1	28969	BZW2

voxel-wise, subcortex			
Spearman's corr coef	p-val	GenID	Gene
0.049615882	8.02724E-06	56983	KTELC1
0.04961421	8.0329E-06	64943	NT5DC2
0.049611147	8.04323E-06	9824	ARHGAP11A
0.049603347	8.06968E-06	79632	FAM184A
0.049594801	8.09873E-06	81850	KRTAP1-3
0.049506485	8.40494E-06	56623	INPP5E
0.049486335	8.47635E-06	90288	C3orf25
0.049453273	8.59476E-06	23242	COBL
0.049435444	8.65928E-06	8574	AKR7A2
0.049384367	8.84665E-06	9040	UBE2M
0.049355023	8.95603E-06	8834	TMEM11
0.04933162	9.04421E-06	26033	ATRNL1
0.049254447	9.34086E-06	10207	INADL
0.049221296	9.47116E-06	23431	AP4E1
0.04920198	9.54784E-06	439916	C12orf37
0.049187679	9.60501E-06	373863	DND1
0.049130846	9.83545E-06	117177	RAB3IP
0.048979845	1.04737E-05	4015	LOX
0.048949011	1.06088E-05	79033	ERI3
0.048945669	1.06235E-05	54870	QRICH1
0.048900258	1.08258E-05	127435	PODN
0.048896916	1.08408E-05	5747	PTK2
0.048873883	1.09449E-05	896	CCND3
0.048843518	1.10837E-05	900	CCNG1
0.04871954	1.16677E-05	128866	CHMP4B
0.048718985	1.16704E-05	978	CDA
0.048704959	1.17383E-05	4544	MTNR1B
0.048689451	1.18138E-05	93183	PIGM
0.048662055	1.19484E-05	2710	GK
0.048645806	1.20289E-05	6594	SMARCA1
0.048644599	1.20349E-05	114049	WBSCR22
0.048441034	1.30887E-05	51548	SIRT6
0.048433233	1.31308E-05	26190	FBXW2
0.048408717	1.32639E-05	91433	RCCD1
0.048391353	1.33589E-05	3910	LAMA4
0.048208963	1.43974E-05	7100	TLR5
0.048178226	1.45798E-05	54069	C21orf45
0.04813569	1.48359E-05	4948	OCA2
0.048133835	1.48472E-05	2834	PRLHR
0.04800605	1.56431E-05	729873	TBC1D3E
0.047994256	1.57186E-05	5538	PPT1
0.047925815	1.61634E-05	8440	NCK2
0.047863316	1.65801E-05	2992	GYG1
0.047849387	1.66744E-05	7584	ZNF35
0.047836848	1.67597E-05	10043	TOM1
0.047720954	1.75679E-05	5794	PTPRH
0.04771176	1.76336E-05	55805	LRP2BP
0.047650933	1.80741E-05	79645	EFCAB1
0.047625486	1.82614E-05	342357	ZKSCAN2
0.047462042	1.95097E-05	112464	PRKCDBP
0.047315687	2.0696E-05	2868	GRK4
0.047306769	2.07705E-05	51699	VPS29
0.047253836	2.12178E-05	686	BTD
0.047148898	2.21317E-05	23070	FTSJD2
0.047044702	2.30762E-05	7274	TTPA
0.046992324	2.35653E-05	541465	CT45A4
0.046971153	2.37658E-05	124842	TMEM132E
0.046959173	2.38799E-05	56892	C8orf4
0.046953231	2.39368E-05	64147	KIF9
0.046946451	2.40018E-05	1266	CNN3
0.04691859	2.42707E-05	118812	MORN4
0.046876058	2.46865E-05	613212	CTXN3
0.04681031	2.53429E-05	820	CAMP
0.046758212	2.58747E-05	200150	PLD5
0.046751246	2.59466E-05	401264	FLJ37798
0.046672124	2.67768E-05	9016	SLC25A14
0.046572201	2.78613E-05	92345	NAF1
0.046554647	2.80562E-05	84179	MFSD7
0.04654694	2.81421E-05	84669	USP32
0.046538953	2.82314E-05	140462	ASB9
0.046497535	2.86989E-05	10199	MPHOSPH10
0.046478499	2.89163E-05	79074	C2orf49
0.0464238	2.95495E-05	6121	RPE65
0.046414327	2.96605E-05	10797	MTHFD2
0.046372537	3.01549E-05	113612	CYP2U1
0.046259243	3.15349E-05	127687	C1orf122
0.046254784	3.15905E-05	126661	RP11-291L19.1
0.046251163	3.16356E-05	81624	DIAPH3
0.04625079	3.16403E-05	56243	KIAA1217

region-wise, subcortex					
Spearman's corr coef	p-val	p-val, SA	p-val, SA (BH- correction)	GenID	Gene
0.016473617	0.924217045	0.937	1	51079	NDUFA13
0.016473617	0.924217045	0.913	1	56941	C3orf37
0.016473617	0.924217045	0.935	1	731157	LOC731157
0.016216217	0.925406933	0.918	1	389	RHOC
0.016216217	0.925406933	0.932	1	7258	TSPY1
0.016216217	0.925406933	0.922	1	8344	HIST1H2BE
0.016216217	0.925406933	0.94	1	9094	UNC119
0.016216217	0.925406933	0.938	1	284434	NWD1
0.016216217	0.925406933	0.929	1	339122	RAB43
0.016216217	0.925406933	0.919	1	442444	FAM47C
0.016216217	0.925406933	0.935	1	100128190	SLC9B1P1
0.016216217	0.925406933	0.929	1	100129726	LOC100129726
0.015958816	0.926596999	0.932	1	5744	PTHLH
0.015958816	0.926596999	0.946	1	6477	SIAH1
0.015958816	0.926596999	0.938	1	11335	CBX3
0.015958816	0.926596999	0.932	1	50839	TAS2R10
0.015958816	0.926596999	0.92	1	219541	MED19
0.015958816	0.926596999	0.935	1	220429	CTAGE10P
0.015701415	0.927787781	0.935	1	9880	ZBTB39
0.015701415	0.927787781	0.928	1	23462	HEY1
0.015701415	0.927787781	0.93	1	26689	AC005962.2
0.015701415	0.927787781	0.944	1	29104	N6AMT1
0.015701415	0.927787781	0.923	1	66002	CYP4F12
0.015701415	0.927787781	0.918	1	115939	C16orf42
0.015701415	0.927787781	0.932	1	134553	C5orf24
0.015701415	0.927787781	0.926	1	644511	RPL13AP6
0.015444015	0.928978145	0.934	1	5324	PLAG1
0.015444015	0.928978145	0.929	1	5723	PSPH
0.015444015	0.928978145	0.925	1	10257	ABCC4
0.015444015	0.928978145	0.925	1	11251	GPR44
0.015444015	0.928978145	0.923	1	51298	THEG
0.015444015	0.928978145	0.923	1	120787	OR52W1
0.015444015	0.928978145	0.922	1	137872	ADHFE1
0.015444015	0.928978145	0.939	1	147007	TMEM199
0.015444015	0.928978145	0.927	1	728470	LOC728470
0.015186615	0.930168748	0.938	1	8303	SNN
0.015186615	0.930168748	0.928	1	8720	MBTPS1
0.015186615	0.930168748	0.935	1	10209	EIF1
0.015186615	0.930168748	0.933	1	11317	RBPJL
0.014929214	0.93135941	0.938	1	5128	PCTK2
0.014929214	0.93135941	0.939	1	23392	KIAA0368
0.014929214	0.93135941	0.938	1	80724	ACAD10
0.014929214	0.93135941	0.932	1	85004	RERG
0.014929214	0.93135941	0.936	1	92170	MTG1
0.014929214	0.93135941	0.936	1	112970	KTI12
0.014929214	0.93135941	0.94	1	728643	RP11-314P12.3
0.014671815	0.932550788	0.948	1	4634	MYL3
0.014671815	0.932550788	0.935	1	5298	PI4KB
0.014671815	0.932550788	0.932	1	9455	HOMER2
0.014671815	0.932550788	0.944	1	9739	SETD1A
0.014671815	0.932550788	0.926	1	51495	PTPLAD1
0.014671815	0.932550788	0.934	1	90423	ATP6V1E2
0.014671815	0.932550788	0.931	1	91683	SYT12
0.014671815	0.932550788	0.942	1	144110	TMEM86A
0.014414414	0.933741808	0.954	1	2353	FOS
0.014414414	0.933741808	0.938	1	124975	GGT6
0.014414414	0.933741808	0.947	1	138804	OR13C4
0.014157014	0.934932947	0.932	1	11138	TBC1D8
0.014157014	0.934932947	0.929	1	27112	FAM155B
0.014157014	0.934932947	0.944	1	29126	CD274
0.014157014	0.934932947	0.948	1	51147	ING4
0.014157014	0.934932947	0.951	1	51265	CDKL3
0.014157014	0.934932947	0.935	1	54345	SOX18
0.014157014	0.934932947	0.936	1	80270	HSD3B7
0.014157014	0.934932947	0.94	1	161823	ADAL
0.014157014	0.934932947	0.945	1	442721	LMOD2
0.013899614	0.936124146	0.937	1	1602	DACH1
0.013899614	0.936124146	0.937	1	6133	RPL9
0.013899614	0.936124146	0.945	1	6702	SPRR2C
0.013899614	0.936124146	0.939	1	30844	EHD4
0.013642213	0.937315583	0.928	1	5434	POLR2E
0.013642213	0.937315583	0.943	1	8862	APLN
0.013642213	0.937315583	0.957	1	9180	OSMR
0.013642213	0.937315583	0.951	1	9315	C5orf13
0.013642213	0.937315583	0.934	1	23731	C9orf5
0.013642213	0.937315583	0.94	1	140730	RIMS4
0.013642213	0.937315583	0.95	1	146850	PIK3R6
0.013384813	0.938507736	0.933	1	783	CACNB2
0.013384813	0.938507736	0.943	1	2706	GJB2

voxel-wise, subcortex			
Spearman's corr coef	p-val	GenID	Gene
0.046215318	3.20859E-05	9889	ZBED4
0.046208724	3.21695E-05	196740	C10orf72
0.046120036	3.33128E-05	339291	LRRC30
0.046111584	3.34238E-05	291	SLC25A4
0.046105549	3.35032E-05	80148	PQLC1
0.046076573	3.38872E-05	201164	PLD6
0.046066917	3.4016E-05	6271	S100A1
0.046012033	3.47574E-05	10575	CCT4
0.045914058	3.61191E-05	134218	DNAJC21
0.04590895	3.61914E-05	50632	CALY
0.04585091	3.70234E-05	84548	TMEM185A
0.045816179	3.75298E-05	162073	ITPRIPL2
0.045784511	3.79974E-05	221079	ARL5B
0.045704924	3.9197E-05	6195	RPS6KA1
0.045692388	3.93893E-05	374928	ZNF773
0.045684494	3.95107E-05	440461	LOC440461
0.045621533	4.04924E-05	2250	FGF5
0.045597572	4.08721E-05	80028	FBXL18
0.045594692	4.09179E-05	7748	ZNF195
0.045388717	4.43274E-05	497661	C18orf32
0.045385186	4.43882E-05	126	ADH1C
0.04538101	4.446E-05	3359	HTR3A
0.045378409	4.45049E-05	134288	TMEM174
0.045360856	4.48088E-05	9750	FAM65B
0.04533764	4.52136E-05	282966	C10orf53
0.045324732	4.54402E-05	57407	NMRAL1
0.045323152	4.5468E-05	4628	MYH10
0.045307551	4.57435E-05	9943	OXSR1
0.045304764	4.57929E-05	2692	GHRHR
0.045286007	4.61266E-05	112840	WDR89
0.045273934	4.63426E-05	169981	SPIN3
0.045233257	4.70773E-05	1983	EIF5
0.045165095	4.83333E-05	4490	MT1B
0.045069166	5.01549E-05	286053	NSMCE2
0.045067959	5.01782E-05	283150	FOXR1
0.045053191	5.04646E-05	440017	DUX4L15
0.045003694	5.14354E-05	27044	SND1
0.044943515	5.26395E-05	283116	AC084851.1
0.044933114	5.28503E-05	347148	QRFP
0.044874981	5.40434E-05	494141	AC090099.4
0.044842664	5.47175E-05	11031	RAB31
0.044821955	5.51539E-05	58489	FAM108C1
0.044799201	5.5637E-05	55109	AGGF1
0.04479716	5.56804E-05	89857	KLHL6
0.044721104	5.73258E-05	341947	COX8C
0.044714604	5.74684E-05	79884	MAP9
0.044689715	5.80181E-05	219445	OR7E5P
0.044673	5.839E-05	4496	MT1H
0.044647366	5.89647E-05	4184	SMCP
0.044515871	6.19981E-05	54756	IL17RD
0.044503704	6.22862E-05	7353	UFD1L
0.044480022	6.28504E-05	163033	ZNF579
0.044474915	6.29727E-05	2125	EVPL
0.044453185	6.34957E-05	79646	PANK3
0.044435818	6.39166E-05	3081	HGD
0.044411767	6.45039E-05	400043	RP11-834C11.4
0.044394027	6.49403E-05	6774	STAT3
0.044375457	6.54002E-05	54499	TMCO1
0.044330228	6.65331E-05	6838	SURF6
0.044299584	6.73112E-05	389400	GFRAL
0.044170778	7.0677E-05	644591	PPIAL4G
0.044141527	7.14632E-05	1087	CEACAM7
0.044141248	7.14707E-05	64132	XYLT2
0.044106979	7.24024E-05	100129272	LOC100129272
0.043994054	7.55546E-05	26503	SLC17A5
0.043935362	7.72436E-05	85464	SSH2
0.04389859	7.83202E-05	81790	RNF170
0.043858193	7.95189E-05	133746	JMY
0.043856613	7.95662E-05	1070	CETN3
0.043774892	8.2046E-05	5594	MAPK1
0.043672737	8.52488E-05	54676	GTPBP2
0.043665774	8.54713E-05	387628	FAM88A
0.043650635	8.59569E-05	90488	C12orf23
0.04365008	8.59749E-05	27344	PCSK1N
0.043616462	8.7063E-05	283008	FAM22E
0.043579686	8.82685E-05	284306	ZNF547
0.043566965	8.86891E-05	202052	DNAJC18
0.043494437	9.11234E-05	23569	PADI4
0.043484129	9.14744E-05	125150	ZSWIM7

region-wise, subcortex					
Spearman's corr coef	p-val	p-val, SA	p-val, SA (BH- correction)	GenID	Gene
0.013384813	0.938507736	0.937	1	53981	CPSF2
0.013384813	0.938507736	0.931	1	83901	KRTAP9-8
0.013384813	0.938507736	0.956	1	128954	GAB4
0.013384813	0.938507736	0.944	1	267002	PGBD2
0.013127413	0.939699411	0.947	1	348	APOE
0.013127413	0.939699411	0.943	1	2939	GSTA2
0.013127413	0.939699411	0.948	1	4588	MUC6
0.013127413	0.939699411	0.939	1	5954	RCN1
0.013127413	0.939699411	0.932	1	6502	SKP2
0.013127413	0.939699411	0.93	1	6880	TAF9
0.013127413	0.939699411	0.954	1	29948	OSGIN1
0.013127413	0.939699411	0.948	1	83446	CCDC70
0.013127413	0.939699411	0.946	1	441601	LOC441601
0.012870012	0.940891147	0.947	1	2678	GGT1
0.012870012	0.940891147	0.946	1	3754	KCNF1
0.012870012	0.940891147	0.954	1	27120	DKKL1
0.012870012	0.940891147	0.952	1	55704	CCDC88A
0.012870012	0.940891147	0.955	1	57501	KIAA1257
0.012870012	0.940891147	0.948	1	79726	WDR59
0.012870012	0.940891147	0.939	1	80726	KIAA1683
0.012870012	0.940891147	0.942	1	100131897	AC008449.1
0.012612613	0.94208312	0.952	1	8724	SNX3
0.012612613	0.94208312	0.944	1	10660	LBX1
0.012612613	0.94208312	0.952	1	25833	POU2F3
0.012612613	0.94208312	0.948	1	29801	ZDHHC8
0.012612613	0.94208312	0.956	1	54463	FAM134B
0.012612613	0.94208312	0.955	1	55101	ATP5SL
0.012612613	0.94208312	0.949	1	128486	FITM2
0.012612613	0.94208312	0.953	1	284836	LINC00319
0.012355212	0.94327569	0.96	1	6850	SYK
0.012355212	0.94327569	0.941	1	10540	DCTN2
0.012355212	0.94327569	0.949	1	23440	OTP
0.012355212	0.94327569	0.943	1	57710	KIAA1614
0.012355212	0.94327569	0.939	1	150159	NHEDC1
0.012355212	0.94327569	0.935	1	204010	RPSAP52
0.012355212	0.94327569	0.951	1	391267	C21orf81
0.012097812	0.944467962	0.943	1	81552	VOPP1
0.012097812	0.944467962	0.954	1	81559	TRIM11
0.012097812	0.944467962	0.937	1	84616	KRTAP4-4
0.011840411	0.945660233	0.953	1	1471	CST3
0.011840411	0.945660233	0.958	1	27302	BMP10
0.011840411	0.945660233	0.938	1	84542	KIAA1841
0.011583011	0.946852624	0.943	1	710	SERPING1
0.011583011	0.946852624	0.945	1	11117	EMILIN1
0.011583011	0.946852624	0.958	1	23211	ZC3H4
0.011583011	0.946852624	0.948	1	54726	OTUD4
0.011583011	0.946852624	0.939	1	80148	PQLC1
0.011325611	0.948045731	0.935	1	5806	PTX3
0.011325611	0.948045731	0.95	1	7789	ZXDA
0.011325611	0.948045731	0.944	1	80758	PRR7
0.011325611	0.948045731	0.95	1	81689	ISCA1
0.011325611	0.948045731	0.959	1	128826	C20orf166
0.011325611	0.948045731	0.958	1	345222	MSANTD1
0.011068211	0.94923842	0.955	1	1163	CKS1B
0.011068211	0.94923842	0.949	1	6844	VAMP2
0.011068211	0.94923842	0.952	1	9812	KIAA0141
0.010810811	0.950431168	0.954	1	166	AES
0.010810811	0.950431168	0.96	1	9636	ISG15
0.010810811	0.950431168	0.954	1	26355	FAM162A
0.010810811	0.950431168	0.955	1	84302	TMEM246
0.010810811	0.950431168	0.94	1	90990	KIFC2
0.010810811	0.950431168	0.954	1	115704	EVI5L
0.010810811	0.950431168	0.957	1	145200	LINC00239
0.010810811	0.950431168	0.969	1	284383	OR2Z1
0.01055341	0.951624036	0.961	1	5708	PSMD2
0.01055341	0.951624036	0.951	1	54751	FBLIM1
0.01055341	0.951624036	0.968	1	57407	NMRAL1
0.01055341	0.951624036	0.95	1	84983	MGC14436
0.01055341	0.951624036	0.953	1	117178	SSX2IP
0.01055341	0.951624036	0.953	1	126075	AC024575.3
0.01055341	0.951624036	0.947	1	100270746	LOC100270746
0.01029601	0.952816963	0.951	1	5639	PRRG2
0.01029601	0.952816963	0.96	1	10247	HRSP12
0.01029601	0.952816963	0.961	1	10587	TXNRD2
0.01029601	0.952816963	0.954	1	11228	RASSF8
0.01029601	0.952816963	0.961	1	57486	NLN
0.01029601	0.952816963	0.964	1	285834	HCG22
0.01029601	0.952816963	0.944	1	341019	DCDC1
0.01029601	0.952816963	0.958	1	729830	FAM160A1

voxel-wise, subcortex			
Spearman's corr coef	p-val	GenID	Gene
0.043482922	9.15156E-05	94005	PIGS
0.043461002	9.22667E-05	26578	OSTF1
0.043419123	9.37181E-05	5062	PAK2
0.043406397	9.41633E-05	10085	EDIL3
0.043364052	9.56593E-05	55205	ZNF532
0.043266822	9.91796E-05	129138	ANKRD54
0.043263108	9.93165E-05	26057	ANKRD17
0.043246299	9.9938E-05	7414	VCL
0.043211196	0.000101248	641977	SEPTIN7P2
0.043195035	0.000101857	51647	FAM96B
0.04319058	0.000102025	3730	KAL1
0.043161232	0.000103141	246126	CYorf15B
0.043096691	0.000105635	65005	MRPL9
0.043068551	0.00010674	9683	N4BP1
0.043057874	0.000107163	55890	GPRC5C
0.042987201	0.000109997	51439	FAM8A1
0.042942163	0.00011184	6542	SLC7A2
0.042888485	0.000114075	197342	EME2
0.042811036	0.000117374	91894	C11orf52
0.04281085	0.000117382	343505	NBPF7
0.042781409	0.00011866	91151	TIGD7
0.042695973	0.000122442	10011	SRA1
0.042663377	0.000123914	64428	NARFL
0.042663191	0.000123923	3575	IL7R
0.042647313	0.000124646	8549	LGR5
0.042622611	0.000125779	37	ACADVL
0.042608958	0.00012641	26658	OR7C2
0.042607196	0.000126491	89883	OR6W1P
0.042544045	0.000129449	375307	C2orf62
0.042498078	0.000131642	554202	MIR31HG
0.042463716	0.000133305	51160	VPS28
0.042281605	0.000142451	11054	OGFR
0.042278357	0.000142619	136157	SSU72P8
0.042247616	0.000144223	399959	MIR100HG
0.042235728	0.000144847	81605	URM1
0.042224307	0.000145449	9413	C9orf61
0.042201184	0.000146676	91860	CALML4
0.042194217	0.000147047	763	CA5A
0.042169515	0.000148371	10228	STX6
0.042124383	0.00015082	2070	EYA4
0.042120021	0.000151058	4236	MFAP1
0.042109154	0.000151654	9675	KIAA0406
0.042033747	0.000155852	2837	UTS2R
0.042011552	0.000157108	85417	CCNB3
0.041979883	0.000158917	284346	ZNF575
0.041947287	0.000160799	145978	NCRNA00052
0.041946918	0.000160821	9227	LRAT
0.04186631	0.000165567	147744	TMEM190
0.041819967	0.000168354	915	CD3D
0.04178793	0.000170307	4481	MSR1
0.041753199	0.000172449	2475	MTOR
0.041746698	0.000172852	4255	MGMT
0.041738339	0.000173373	432	ASGR1
0.041736204	0.000173506	54971	BANP
0.04158093	0.000183449	392309	OR13J1
0.041564494	0.000184532	89832	CHRFAM7A
0.041525584	0.000187121	51411	BIN2
0.041462064	0.00019142	29781	NCAPH2
0.041442748	0.000192746	29959	NRBP1
0.04141758	0.000194486	5800	PTPRO
0.041373283	0.000197585	581	BAX
0.041370127	0.000197807	645027	EVPLL
0.041368548	0.000197918	440730	TRIM67
0.041288033	0.000203679	1043	CD52
0.041217361	0.000208865	4146	MATN1
0.041178357	0.00021178	85009	MGC16025
0.041155141	0.000213533	57574	MARCHF4
0.041127745	0.000215619	283600	C14orf68
0.041057259	0.000221075	131965	METTL6
0.041034043	0.000222901	26053	AUTS2
0.041022714	0.000223796	404281	YY2
0.041019835	0.000224024	23596	OPN3
0.040984176	0.000226869	124401	ANKS3
0.040888708	0.000234652	374768	SPEM1
0.040858619	0.000237156	9026	HIP1R
0.040857784	0.000237226	283491	OR7E156P
0.040835403	0.000239106	79077	DCTPP1
0.040830109	0.000239552	6573	SLC19A1
0.040769283	0.000244741	10671	DCTN6

region-wise, subcortex					
Spearman's corr coef	p-val	p-val, SA	p-val, SA (BH- correction)	GenID	Gene
0.01003861	0.954010606	0.952	1	2806	GOT2
0.01003861	0.954010606	0.958	1	3061	HCRTR1
0.01003861	0.954010606	0.959	1	4841	NONO
0.01003861	0.954010606	0.962	1	55229	PANK4
0.01003861	0.954010606	0.958	1	91947	ARRDC4
0.01003861	0.954010606	0.96	1	135398	C6ORF141
0.01003861	0.954010606	0.945	1	341405	ANKRD33
0.01003861	0.954010606	0.954	1	390916	NUDT19
0.009781209	0.955203831	0.965	1	47	ACLY
0.009781209	0.955203831	0.946	1	2671	GFER
0.009781209	0.955203831	0.96	1	3695	ITGB7
0.009781209	0.955203831	0.949	1	5571	PRKAG1
0.009781209	0.955203831	0.945	1	23184	MESDC2
0.009781209	0.955203831	0.962	1	64839	FBXL17
0.009781209	0.955203831	0.965	1	79979	TRMT2B
0.009781209	0.955203831	0.963	1	144165	PRICKLE1
0.00952381	0.956397116	0.959	1	224	ALDH3A2
0.00952381	0.956397116	0.964	1	6635	SNRPE
0.00952381	0.956397116	0.96	1	55844	PPP2R2D
0.00952381	0.956397116	0.96	1	56905	C15orf39
0.00952381	0.956397116	0.952	1	79778	MICALL2
0.00952381	0.956397116	0.961	1	100128108	RP11-89K11.1
0.009266409	0.957590461	0.971	1	3501	IGHG2
0.009266409	0.957590461	0.968	1	6916	TBXAS1
0.009266409	0.957590461	0.966	1	9601	PDIA4
0.009266409	0.957590461	0.965	1	56900	TMEM167B
0.009266409	0.957590461	0.96	1	79001	VKORC1
0.009266409	0.957590461	0.978	1	124245	ZC3H18
0.009266409	0.957590461	0.951	1	131118	DNAJC19
0.009266409	0.957590461	0.949	1	388955	LOC388955
0.009009009	0.958784461	0.963	1	9671	WSCD2
0.009009009	0.958784461	0.952	1	10802	SEC24A
0.009009009	0.958784461	0.969	1	23475	QPRT
0.009009009	0.958784461	0.963	1	83723	FAM57B
0.009009009	0.958784461	0.97	1	341880	SLC35F4
0.009009009	0.958784461	0.964	1	374977	C1orf175
0.008751608	0.959978044	0.96	1	744	MPPED2
0.008751608	0.959978044	0.96	1	6230	RPS25
0.008751608	0.959978044	0.967	1	8263	F8A1
0.008751608	0.959978044	0.966	1	84839	RAX2
0.008751608	0.959978044	0.966	1	283375	SLC39A5
0.008494208	0.961171627	0.957	1	2585	GALK2
0.008494208	0.961171627	0.958	1	51129	ANGPTL4
0.008494208	0.961171627	0.958	1	63917	GALNT11
0.008494208	0.961171627	0.962	1	79801	SHCBP1
0.008494208	0.961171627	0.967	1	85014	TMEM141
0.008494208	0.961171627	0.955	1	163175	LGI4
0.008494208	0.961171627	0.967	1	387338	NSUN4
0.008494208	0.961171627	0.973	1	400935	IL17REL
0.008236809	0.962365389	0.965	1	4049	LTA
0.008236809	0.962365389	0.956	1	6939	TCF15
0.008236809	0.962365389	0.971	1	9603	NFE2L3
0.008236809	0.962365389	0.96	1	10475	TRIM38
0.008236809	0.962365389	0.971	1	51399	TRAPPC4
0.008236809	0.962365389	0.965	1	126364	LRRC25
0.008236809	0.962365389	0.962	1	255919	TMEM188
0.007979408	0.963559151	0.962	1	26095	PTPN20B
0.007979408	0.963559151	0.957	1	27255	CNTN6
0.007979408	0.963559151	0.965	1	29066	ZC3H7A
0.007979408	0.963559151	0.963	1	54806	AHI1
0.007979408	0.963559151	0.968	1	57665	RDH14
0.007979408	0.963559151	0.966	1	64110	MAGEF1
0.007979408	0.963559151	0.97	1	84970	C1orf94
0.007979408	0.963559151	0.974	1	170082	TCEANC
0.007979408	0.963559151	0.969	1	100130890	LOC100130890
0.007722008	0.964753568	0.968	1	22837	COBLL1
0.007722008	0.964753568	0.97	1	22899	ARHGEF15
0.007722008	0.964753568	0.97	1	55780	C6orf70
0.007722008	0.964753568	0.965	1	79682	MLF1IP
0.007722008	0.964753568	0.971	1	85445	CNTNAP4
0.007722008	0.964753568	0.964	1	284252	KCTD1
0.007722008	0.964753568	0.961	1	347853	TBX10
0.007464607	0.965947568	0.967	1	3054	HCFC1
0.007464607	0.965947568	0.965	1	4113	MAGEB2
0.007464607	0.965947568	0.962	1	5411	PNN
0.007464607	0.965947568	0.968	1	6168	RPL37A
0.007464607	0.965947568	0.971	1	51306	FAM13B
0.007464607	0.965947568	0.975	1	100131998	RRN3P3
0.007207207	0.967141628	0.974	1	5977	DPF2

voxel-wise, subcortex			
Spearman's corr coef	p-val	GenID	Gene
0.04070669	0.00025019	9353	SLIT2
0.040663324	0.000254031	7709	ZBTB17
0.040640108	0.000256111	6861	SYT5
0.04060556	0.000259235	9528	TMEM59
0.040599618	0.000259775	23588	KLHDC2
0.040559683	0.000263438	2519	FUCA2
0.040522724	0.00026687	160777	CCDC60
0.040296689	0.000288793	84775	ZNF607
0.040267158	0.000291779	83639	TEX101
0.040224995	0.000296092	440570	LOC440570
0.040219329	0.000296675	56605	ERO1LB
0.040207814	0.000297866	93107	KCNG4
0.040134914	0.000305506	254028	LOC254028
0.040069353	0.000312534	26277	TINF2
0.040041216	0.000315597	55108	BSDC1
0.039942034	0.000326617	285989	ZNF789
0.039900895	0.000331292	56122	PCDHB14
0.039893929	0.00033209	163049	ZNF791
0.039877862	0.000333937	392196	RP11-1236K1.8
0.039795585	0.000343549	81502	HM13
0.03979196	0.000343978	2252	FGF7
0.039684795	0.000356899	114134	SLC2A13
0.039675694	0.000358017	28966	SNX24
0.039633438	0.000363251	83986	ITFG3
0.039544288	0.000374529	150726	FBXO41
0.039509371	0.000379035	1610	DAO
0.039490055	0.000381549	56034	PDGFC
0.039441019	0.000388002	388796	RP4-564F22.1
0.039423097	0.000390386	100129455	LOC100129455
0.039375365	0.000396801	285834	HCG22
0.039351869	0.000399995	5080	PAX6
0.039344534	0.000400998	57763	ANKRA2
0.039342396	0.00040129	84989	LOC84989
0.039215542	0.000419007	6570	SLC18A1
0.039213222	0.000419338	26531	OR11A1
0.03919762	0.000421569	8894	EIF2S2
0.039175797	0.000424708	7165	TPD52L2
0.039092403	0.000436902	55505	NOP10
0.039080795	0.000438626	100288911	LOC100288911
0.039067701	0.000440577	27069	GHITM
0.03906092	0.000441591	90332	EXOC3L2
0.039035011	0.000445484	84916	CIRH1A
0.03900845	0.000449509	55081	IFT57
0.038973164	0.000454908	79857	AC024940.3
0.038945116	0.000459243	374454	KRT77
0.038941961	0.000459733	93659	CGB5
0.038904626	0.000465569	60526	C2orf43
0.038849466	0.000474317	5464	PPA1
0.038804889	0.000481499	84067	FAM160A2
0.038755856	0.000489515	90135	BTBD6
0.038748518	0.000490726	83716	CRISPLD2
0.038716573	0.000496028	84983	MGC14436
0.038708773	0.000497331	91862	MARVELD3
0.038585726	0.000518307	159296	NKX2-3
0.03854505	0.000525419	85301	COL27A1
0.038510505	0.000531532	25878	MXRA5
0.038509391	0.00053173	114770	PGLYRP2
0.038501959	0.000533055	125919	ZNF543
0.03848571	0.00053596	1737	DLAT
0.038464628	0.000539753	2064	ERBB2
0.038437698	0.000544633	330	BIRC3
0.038427018	0.00054658	118460	EXOSC6
0.038374361	0.000556273	64137	ABCG4
0.038350496	0.00056072	84808	C1orf170
0.038324494	0.0005656	10208	USPL1
0.038236272	0.000582459	2280	FKBP1A
0.038208131	0.000587935	26952	SMR3A
0.038190953	0.000591301	126969	SLC44A3
0.038187701	0.00059194	122664	TPPP2
0.038166992	0.000596025	5890	RAD51L1
0.038101893	0.00060904	54620	FBXL19
0.038078398	0.000613802	25775	C22orf24
0.038070597	0.000615391	11174	ADAMTS6
0.037909105	0.00064915	8780	RIOK3
0.037871122	0.000657337	90441	ZNF622
0.037851155	0.000661679	56548	CHST7
0.03783416	0.000665396	2907	GRINA
0.037815031	0.000669602	55831	EMC3
0.037763953	0.000680956	9969	MED13

region-wise, subcortex					
Spearman's corr coef	p-val	p-val, SA	p-val, SA (BH- correction)	GenID	Gene
0.007207207	0.967141628	0.957	1	56919	DHX33
0.007207207	0.967141628	0.971	1	84541	KBTBD8
0.006949807	0.968335688	0.976	1	4111	MAGEA12
0.006949807	0.968335688	0.972	1	9491	PSMF1
0.006949807	0.968335688	0.98	1	64236	PDLIM2
0.006949807	0.968335688	0.967	1	84920	ALG10
0.006949807	0.968335688	0.967	1	158055	C9orf163
0.006949807	0.968335688	0.964	1	283726	FAM154B
0.006949807	0.968335688	0.964	1	346525	OR2A12
0.006949807	0.968335688	0.971	1	386674	KRTAP10-6
0.006692407	0.969530404	0.962	1	533	ATP6V0B
0.006692407	0.969530404	0.969	1	1964	EIF1AX
0.006692407	0.969530404	0.965	1	5530	PPP3CA
0.006692407	0.969530404	0.971	1	8408	ULK1
0.006692407	0.969530404	0.968	1	10654	PMVK
0.006692407	0.969530404	0.966	1	27090	ST6GALNAC4
0.006692407	0.969530404	0.972	1	50854	C6orf48
0.006692407	0.969530404	0.967	1	51738	GHRL
0.006692407	0.969530404	0.971	1	144124	OR10A5
0.006692407	0.969530404	0.972	1	201158	FAM18B2
0.006692407	0.969530404	0.969	1	222161	DKFZP586I1420
0.006692407	0.969530404	0.969	1	400027	LOC400027
0.006692407	0.969530404	0.965	1	431705	ASTL
0.006435006	0.970724702	0.983	1	4935	GPR143
0.006435006	0.970724702	0.963	1	6193	RPS5
0.006435006	0.970724702	0.969	1	9287	TAAR2
0.006435006	0.970724702	0.978	1	23316	CUX2
0.006435006	0.970724702	0.972	1	57092	PCNP
0.006435006	0.970724702	0.977	1	387509	GPR153
0.006177606	0.971918941	0.975	1	3910	LAMA4
0.006177606	0.971918941	0.965	1	23492	CBX7
0.006177606	0.971918941	0.976	1	54704	PDP1
0.006177606	0.971918941	0.966	1	151246	SGOL2
0.005920206	0.973113298	0.979	1	3305	HSPA1L
0.005920206	0.973113298	0.968	1	5261	PHKG2
0.005920206	0.973113298	0.979	1	9211	LGI1
0.005920206	0.973113298	0.977	1	116442	RAB39B
0.005920206	0.973113298	0.979	1	399473	SPRED3
0.005920206	0.973113298	0.976	1	641649	TMEM91
0.005920206	0.973113298	0.97	1	100130746	LOC100130746
0.005662805	0.974308312	0.982	1	1760	DMPK
0.005662805	0.974308312	0.966	1	4594	MUT
0.005662805	0.974308312	0.968	1	6777	STAT5B
0.005662805	0.974308312	0.982	1	8292	COLQ
0.005662805	0.974308312	0.981	1	10023	FRAT1
0.005662805	0.974308312	0.971	1	23596	OPN3
0.005662805	0.974308312	0.977	1	55614	KIF16B
0.005662805	0.974308312	0.977	1	59284	CACNG7
0.005662805	0.974308312	0.974	1	139324	HDX
0.005662805	0.974308312	0.976	1	353142	LCE3A
0.005662805	0.974308312	0.974	1	100130274	CCDC166
0.005405406	0.975502789	0.985	1	1519	CTSO
0.005405406	0.975502789	0.972	1	5148	PDE6G
0.005405406	0.975502789	0.975	1	7092	TLL1
0.005405406	0.975502789	0.979	1	23240	KIAA0922
0.005405406	0.975502789	0.971	1	64112	MOAP1
0.005405406	0.975502789	0.974	1	79784	MYH14
0.005405406	0.975502789	0.978	1	147948	ZNF582
0.005148005	0.976697326	0.979	1	6349	CCL3L1
0.005148005	0.976697326	0.975	1	64067	NPAS3
0.005148005	0.976697326	0.976	1	200316	APOBEC3F
0.005148005	0.976697326	0.985	1	344967	AC098591.2
0.005148005	0.976697326	0.981	1	348793	WDR53
0.004890604	0.977891862	0.98	1	2046	EPHA8
0.004890604	0.977891862	0.978	1	11232	POLG2
0.004890604	0.977891862	0.984	1	55334	SLC39A9
0.004890604	0.977891862	0.982	1	391190	OR2L8
0.004890604	0.977891862	0.98	1	643421	LOC643421
0.004633205	0.979086518	0.983	1	9825	SPATA2
0.004633205	0.979086518	0.985	1	84300	MNF1
0.004633205	0.979086518	0.975	1	145482	PTGR2
0.004633205	0.979086518	0.979	1	163154	PRR22
0.004633205	0.979086518	0.98	1	343472	BARHL2
0.004633205	0.979086518	0.979	1	399815	LOC399815
0.004633205	0.979086518	0.976	1	729992	ST13P1
0.004633205	0.979086518	0.982	1	100131138	AC002351.1
0.004375804	0.98028177	0.98	1	53944	CSNK1G1
0.004375804	0.98028177	0.972	1	55700	MAP7D1
0.004375804	0.98028177	0.986	1	145508	C14orf145

voxel-wise, subcortex			
Spearman's corr coef	p-val	GenID	Gene
0.037747145	0.000684731	160287	LDHAL6A
0.037683811	0.000699131	23060	ZNF609
0.037650656	0.000706782	221322	C6orf170
0.037537456	0.000733491	6331	SCN5A
0.037500959	0.000742301	10329	TMEM5
0.037474211	0.000748819	11183	MAP4K5
0.037472632	0.000749205	390072	OR52N4
0.037414405	0.000763587	57128	LYRM4
0.037359338	0.000777423	2569	GABRR1
0.037345685	0.000780889	284365	AC010325.3
0.037341323	0.000782	26272	FBXO4
0.037334263	0.0007838	92359	CRB3
0.037296839	0.000793408	10196	PRMT3
0.037269998	0.000800366	79754	ASB13
0.037250035	0.000805578	11132	CAPN10
0.037241768	0.000807746	646892	SH2D7
0.037221432	0.0008131	9920	KBTBD11
0.037165988	0.000827867	151313	FAHD2B
0.037154939	0.000830841	9331	B4GALT6
0.037106927	0.000843871	79725	THAP9
0.037031241	0.0008648	843	CASP10
0.036995117	0.000874958	51250	C6orf203
0.036965493	0.00088337	9265	CYTH3
0.036867611	0.000911702	144455	E2F7
0.036866032	0.000912165	338755	OR2AG2
0.036860276	0.000913859	56997	CABC1
0.036841054	0.000919532	9489	PGS1
0.036797125	0.000932623	6612	SUMO3
0.036777068	0.000938656	6608	SMO
0.036743727	0.000948766	2911	GRM1
0.036651976	0.000977111	6832	SUPV3L1
0.036622725	0.000986312	9910	RABGAP1L
0.036618639	0.000987604	10644	IGF2BP2
0.036586042	0.000997962	83461	CDCA3
0.036546018	0.00101082	345778	MTX3
0.036501907	0.001025169	2104	ESRRG
0.036467731	0.001036414	83854	ANGPTL6
0.036466524	0.001036813	9406	ZRANB2
0.036460765	0.001038721	354	KLK3
0.036453709	0.00104106	84162	KIAA1109
0.036453336	0.001041185	23533	PIK3R5
0.036338925	0.001079832	255394	TCP11L2
0.036243089	0.00111322	6749	SSRP1
0.036211047	0.001124595	57584	ARHGAP21
0.036204178	0.001127046	283143	LOC283143
0.036147993	0.001147293	3363	HTR7
0.036111776	0.001160521	84124	ZNF394
0.036110569	0.001160964	10410	IFITM3
0.036058005	0.001180419	219743	TYSND1
0.036044817	0.001185348	3937	LCP2
0.036040362	0.001187016	5896	RAG1
0.036015846	0.001196241	65993	MRPS34
0.036005352	0.001200209	54535	CCHCR1
0.035982691	0.001208822	57711	ZNF529
0.035956781	0.001218737	55672	NBPF1
0.035942759	0.001224134	51043	ZBTB7B
0.035864104	0.001254824	6932	TCF7
0.035837635	0.00126531	54467	ANKIB1
0.035829183	0.001268675	23385	NCSTN
0.035807826	0.001277215	10730	YME1L1
0.035677999	0.001330286	283208	P4HA3
0.035575196	0.001373744	124808	CCDC43
0.035574082	0.001374224	284615	ANKRD34A
0.035478152	0.001415967	30000	TNPO2
0.035465427	0.00142159	90326	THAP3
0.035455771	0.001425872	7403	KDM6A
0.03534656	0.001475132	79171	RBM42
0.035228621	0.001530084	267	AMFR
0.035218496	0.001534889	54581	SCAND2
0.035183951	0.001551387	347862	PDDC1
0.03512888	0.001578024	3166	HMX1
0.035118852	0.001582919	54487	DGCR8
0.035107151	0.00158865	9117	SEC22C
0.035107151	0.00158865	259292	TAS2R46
0.034995433	0.001644326	89932	PAPLN
0.034968689	0.001657917	144108	SPTY2D1
0.03492764	0.001678982	7580	ZNF32
0.034909163	0.001688541	94241	TP53INP1
0.034858271	0.001715135	728441	GGT2

region-wise, subcortex					
Spearman's corr coef	p-val	p-val, SA	p-val, SA (BH- correction)	GenID	Gene
0.004375804	0.98028177	0.984	1	145873	MESP2
0.004375804	0.98028177	0.984	1	146880	AC132812.1
0.004375804	0.98028177	0.987	1	153090	DAB2IP
0.004375804	0.98028177	0.977	1	283635	FAM177A1
0.004118404	0.981476426	0.979	1	6748	SSR4
0.004118404	0.981476426	0.985	1	55719	FAM178A
0.004118404	0.981476426	0.987	1	84905	ZNF341
0.003861004	0.982671201	0.978	1	5540	PPYR1
0.003861004	0.982671201	0.981	1	7850	IL1R2
0.003861004	0.982671201	0.986	1	64180	DPEP3
0.003861004	0.982671201	0.983	1	138199	C9orf41
0.003603603	0.983865976	0.983	1	9776	ATG13
0.003603603	0.983865976	0.987	1	387885	CCDC42B
0.003603603	0.983865976	0.993	1	389816	LRRC26
0.003603603	0.983865976	0.985	1	574407	C1orf145
0.003346203	0.985061347	0.981	1	537	ATP6AP1
0.003346203	0.985061347	0.981	1	2834	PRLHR
0.003346203	0.985061347	0.987	1	9411	ARHGAP29
0.003346203	0.985061347	0.983	1	22843	PPM1E
0.003346203	0.985061347	0.98	1	83881	MIXL1
0.003346203	0.985061347	0.982	1	728637	LOC728637
0.003088803	0.986256242	0.987	1	3014	H2AFX
0.003088803	0.986256242	0.982	1	23178	PASK
0.003088803	0.986256242	0.98	1	219986	OR4D11
0.003088803	0.986256242	0.988	1	729196	UNC93B5
0.002831403	0.987451136	0.993	1	473	RERE
0.002831403	0.987451136	0.984	1	2307	FOXS1
0.002831403	0.987451136	0.986	1	2330	FMO5
0.002831403	0.987451136	0.989	1	7479	WNT8B
0.002831403	0.987451136	0.99	1	8898	MTMR2
0.002831403	0.987451136	0.984	1	9308	CD83
0.002831403	0.987451136	0.984	1	57592	ZNF687
0.002831403	0.987451136	0.992	1	57657	HCN3
0.002831403	0.987451136	0.984	1	80139	ZNF703
0.002831403	0.987451136	0.989	1	390927	ZNF793
0.002831403	0.987451136	0.986	1	441554	DUSPP
0.002574003	0.98864603	0.992	1	1161	ERCC8
0.002574003	0.98864603	0.987	1	5071	PARK2
0.002574003	0.98864603	0.979	1	5520	PPP2R2A
0.002574003	0.98864603	0.989	1	8708	B3GALT1
0.002574003	0.98864603	0.991	1	26031	OSBPL3
0.002574003	0.98864603	0.995	1	83732	RIOK1
0.002574003	0.98864603	0.984	1	348013	FAM70B
0.002574003	0.98864603	0.992	1	642587	MIR205HG
0.002574003	0.98864603	0.992	1	100131816	RP1-215K18.2
0.002316602	0.989841521	0.992	1	5333	PLCD1
0.002316602	0.989841521	0.992	1	6652	SORD
0.002316602	0.989841521	0.998	1	11201	POLI
0.002316602	0.989841521	0.992	1	84519	ACRBP
0.002316602	0.989841521	0.982	1	284338	PRR19
0.002316602	0.989841521	0.992	1	442582	STAG3L2
0.002059202	0.991036534	0.993	1	1991	ELANE
0.002059202	0.991036534	0.989	1	7112	TMPO
0.002059202	0.991036534	0.988	1	118932	ANKRD22
0.002059202	0.991036534	0.993	1	138474	TAF1L
0.002059202	0.991036534	0.994	1	257101	ZNF683
0.001801802	0.992231488	0.991	1	613	BCR
0.001801802	0.992231488	0.992	1	4694	NDUFA1
0.001801802	0.992231488	0.991	1	8045	RASSF7
0.001801802	0.992231488	0.994	1	94234	FOXQ1
0.001801802	0.992231488	0.991	1	120526	DNAJC24
0.001801802	0.992231488	0.988	1	148430	LOC148430
0.001801802	0.992231488	0.991	1	253650	ANKRD18A
0.001801802	0.992231488	0.995	1	100289026	LOC100289026
0.001544402	0.993426502	0.994	1	5166	PDK4
0.001544402	0.993426502	0.987	1	57720	GPR107
0.001544402	0.993426502	0.995	1	64951	MRPS24
0.001544402	0.993426502	0.993	1	85352	KIAA1644
0.001544402	0.993426502	0.994	1	120776	OR2D2
0.001544402	0.993426502	0.99	1	128989	C22orf25
0.001544402	0.993426502	0.996	1	137994	LETM2
0.001287001	0.994621456	0.989	1	3750	KCND1
0.001287001	0.994621456	0.996	1	6320	CLEC11A
0.001287001	0.994621456	0.995	1	22879	MON1B
0.001287001	0.994621456	0.997	1	63933	CCDC90A
0.001287001	0.994621456	0.992	1	85441	RP4-697K14.1
0.001287001	0.994621456	0.991	1	286053	NSMCE2
0.001029601	0.995817065	0.991	1	1389	CREBL2
0.001029601	0.995817065	0.997	1	22845	DOLK

voxel-wise, subcortex			
Spearman's corr coef	p-val	GenID	Gene
0.034852233	0.001718316	170850	KCNG3
0.034845546	0.001721846	91687	CENPL
0.034577817	0.001868727	84306	PDCD2L
0.034535188	0.001893149	10670	RRAGA
0.034489777	0.001919486	79724	ZNF768
0.034471761	0.001930028	10519	CIB1
0.03446006	0.001936902	283450	C12orf51
0.034437403	0.001950281	8443	GNPAT
0.03432652	0.002016976	79036	C19orf50
0.034312867	0.002025332	51115	FAM82B
0.034275442	0.002048399	8312	AXIN1
0.03424396	0.002067989	79022	TMEM106C
0.034228083	0.002077932	284739	LINC00176
0.034207094	0.002091147	347733	TUBB2B
0.034197066	0.002097487	10586	MAB21L2
0.034153696	0.002125112	6911	TBX6
0.034140788	0.002133399	64599	GIGYF1
0.034109958	0.002153311	9659	PDE4DIP
0.034101039	0.002159103	255275	MYADML2
0.034075409	0.002175827	1973	EIF4A1
0.034050338	0.002192299	55036	CCDC40
0.033996474	0.00222808	93129	ORAI3
0.033961929	0.002251306	165918	RNF168
0.033731062	0.002412322	26019	UPF2
0.033721868	0.002418947	115548	FCHO2
0.033700418	0.002434468	55997	CFC1
0.033676736	0.002451712	8328	GFI1B
0.033637453	0.002480556	388815	C21orf34
0.033625845	0.002489143	54494	C11orf71
0.03361563	0.002496719	284186	TMEM105
0.033607457	0.002502794	85019	C18orf45
0.033600863	0.002507707	348110	C15orf38
0.033575978	0.002526326	55540	IL17RB
0.033532515	0.00255915	79004	CUEDC2
0.033509579	0.002576628	203076	C8orf74
0.033360157	0.002693192	51234	TMEM85
0.033323288	0.002722682	84057	MND1
0.033299699	0.002741703	2014	EMP3
0.03329413	0.002746215	139431	FTH1P14
0.033263762	0.002770921	10946	SF3A3
0.033236086	0.002793611	80726	KIAA1683
0.033195041	0.002827582	8161	COIL
0.033188354	0.00283315	84131	CEP78
0.033148516	0.002866541	51227	PIGP
0.033137556	0.002875787	3960	LGALS4
0.033130035	0.002882152	94059	LENG9
0.033075802	0.002928409	10944	C11orf58
0.033068929	0.002934321	353274	ZNF445
0.033061683	0.002940566	521	ATP5I
0.033061035	0.002941123	1504	CTRB1
0.033047382	0.00295292	338382	RAB7B
0.033001322	0.002993045	64081	PBLD
0.032909386	0.003074619	85285	KRTAP4-1
0.032881338	0.003099909	10273	STUB1
0.032875858	0.003104871	10036	CHAF1A
0.032743338	0.00322711	8476	CDC42BPA
0.032659482	0.003306712	136288	C7orf57
0.032620572	0.003344253	9397	NMT2
0.032568846	0.003394757	10250	SRRM1
0.03249418	0.003468883	9358	ITGBL1
0.032489166	0.003473915	112802	KRT71
0.032429546	0.003534237	4735	SEPTIN2
0.032331388	0.003635633	11184	MAP4K1
0.032322101	0.003645362	146713	AC021534.2
0.032286253	0.003683138	83903	GSG2
0.032240193	0.003732193	23384	CYTSA
0.032238986	0.003733491	7786	MAP3K12
0.032062449	0.003927137	59338	PLEKHA1
0.032054923	0.003935587	147808	ZNF784
0.032045916	0.003945728	642513	LOC642513
0.032024372	0.003970076	10239	AP3S2
0.032014064	0.003981777	26266	SLC13A4
0.032005798	0.003991181	22803	XRN2
0.031842634	0.004180981	84268	RPAIN
0.031838175	0.004186281	10475	TRIM38
0.03177122	0.004266601	55000	TUG1
0.031755246	0.004285968	225	ABCD2
0.031752553	0.004289239	63895	FAM38B
0.031739179	0.004305527	1232	CCR3

region-wise, subcortex					
Spearman's corr coef	p-val	p-val, SA	p-val, SA (BH- correction)	GenID	Gene
0.001029601	0.995817065	0.999	1	23327	NEDD4L
0.001029601	0.995817065	0.996	1	54734	RAB39
0.001029601	0.995817065	0.994	1	57643	ZSWIM5
0.001029601	0.995817065	0.994	1	165140	OXER1
0.001029601	0.995817065	0.988	1	767811	H2BFXP
0.000772201	0.997012079	0.998	1	5002	SLC22A18
0.000772201	0.997012079	0.997	1	440585	FAM183A
0.000514801	0.998207152	0.999	1	7166	TPH1
0.000514801	0.998207152	0.997	1	27242	TNFRSF21
0.000514801	0.998207152	0.997	1	51164	DCTN4
0.000514801	0.998207152	0.997	1	51690	LSM7
0.000514801	0.998207152	1	1	57717	PCDHB16
0.000514801	0.998207152	0.999	1	399671	HEATR4
0.0002574	0.999402225	0.998	1	1982	EIF4G2
0.0002574	0.999402225	0.999	1	4607	MYBPC3
0.0002574	0.999402225	0.998	1	7297	TYK2
0.0002574	0.999402225	0.997	1	7709	ZBTB17
0.0002574	0.999402225	0.996	1	8834	TMEM11
0.0002574	0.999402225	0.999	1	57162	PELI1
0.0002574	0.999402225	0.998	1	84250	ANKRD32
0.0002574	0.999402225	0.999	1	116092	DNTTIP1
0.0002574	0.999402225	0.997	1	287015	TRIM42
< 0.001	1	1	1	9746	CLSTN3
< 0.001	1	0.999	1	9962	SLC23A2
< 0.001	1	1	1	51063	CALHM2
< 0.001	1	1	1	55257	C20orf20
< 0.001	1	0.998	1	57614	KIAA1468
< 0.001	1	1	1	83550	GPR101
< 0.001	1	1	1	84064	HDHD2
< 0.001	1	0.998	1	84900	RNFT2
-0.0002574	0.999402225	1	1	7005	TEAD3
-0.0002574	0.999402225	0.998	1	9840	KIAA0748
-0.0002574	0.999402225	0.998	1	55149	MTPAP
-0.0002574	0.999402225	0.997	1	203414	CXorf24
-0.000514801	0.998207152	0.996	1	4129	MAOB
-0.000514801	0.998207152	0.992	1	4625	MYH7
-0.000514801	0.998207152	0.997	1	5965	RECQL
-0.000514801	0.998207152	1	1	54867	TMEM214
-0.000514801	0.998207152	0.998	1	147463	ANKRD29
-0.000514801	0.998207152	0.997	1	197407	ZNF48
-0.000514801	0.998207152	0.996	1	222662	LHFPL5
-0.000514801	0.998207152	0.995	1	387755	INSC
-0.000772201	0.997012079	0.996	1	142	PARP1
-0.000772201	0.997012079	0.996	1	3959	LGALS3BP
-0.000772201	0.997012079	0.998	1	4750	NEK1
-0.000772201	0.997012079	0.996	1	4919	ROR1
-0.000772201	0.997012079	0.997	1	6367	CCL22
-0.000772201	0.997012079	0.995	1	57722	IGDCC4
-0.000772201	0.997012079	0.998	1	79087	ALG12
-0.000772201	0.997012079	0.998	1	80339	PNPLA3
-0.000772201	0.997012079	0.998	1	164284	APCDD1L
-0.001029601	0.995817065	0.996	1	3560	IL2RB
-0.001029601	0.995817065	0.995	1	55750	AGK
-0.001287001	0.994621456	0.993	1	430	ASCL2
-0.001287001	0.994621456	0.995	1	1137	CHRNA4
-0.001287001	0.994621456	0.994	1	8568	RRP1
-0.001287001	0.994621456	0.992	1	9277	WDR46
-0.001287001	0.994621456	0.993	1	25978	CHMP2B
-0.001287001	0.994621456	0.994	1	116412	ZNF837
-0.001287001	0.994621456	0.993	1	285141	AC007405.8
-0.001287001	0.994621456	0.995	1	100131315	LOC100131315
-0.001544402	0.993426502	0.995	1	11095	ADAMTS8
-0.001544402	0.993426502	0.993	1	26470	SEZ6L2
-0.001544402	0.993426502	0.991	1	375759	C9orf50
-0.001801802	0.992231488	0.997	1	8934	RAB7L1
-0.001801802	0.992231488	0.992	1	9231	DLG5
-0.001801802	0.992231488	0.992	1	29800	ZDHHC1
-0.001801802	0.992231488	0.994	1	30012	TLX3
-0.001801802	0.992231488	0.992	1	55582	KIF27
-0.001801802	0.992231488	0.995	1	118430	MUCL1
-0.001801802	0.992231488	0.993	1	124222	PAQR4
-0.002059202	0.991036534	0.992	1	169436	C9orf96
-0.002059202	0.991036534	0.991	1	100128416	LOC100128416
-0.002316602	0.989841521	0.988	1	5049	PAFAH1B2
-0.002316602	0.989841521	0.986	1	7844	RNF103
-0.002316602	0.989841521	0.99	1	9782	MATR3
-0.002316602	0.989841521	0.987	1	23366	KIAA0895
-0.002316602	0.989841521	0.993	1	26003	GORASP2
-0.002316602	0.989841521	0.987	1	56945	MRPS22

voxel-wise, subcortex			
Spearman's corr coef	p-val	GenID	Gene
0.031726643	0.004320844	2192	FBLN1
0.031722743	0.004325623	2804	GOLGB1
0.03169442	0.004360449	161582	DYX1C1-CCPG1
0.031645201	0.004421572	220359	TIGD3
0.031616785	0.004457209	80308	FLAD1
0.031612232	0.004462945	143879	KBTBD3
0.031598952	0.004479713	144983	RP11-78J21.3
0.03154286	0.004551157	60509	AGBL5
0.031503394	0.004602043	6908	TBP
0.031450462	0.004671099	28972	SPCS1
0.031393811	0.004746043	8714	ABCC3
0.031388983	0.004752481	23373	CRTC1
0.031325184	0.004838292	51187	RSL24D1
0.031323884	0.004840053	403315	FAM92A3
0.031323697	0.004840305	123606	NIPA1
0.031322118	0.00484245	392264	LOC392264
0.031296488	0.00487734	10322	SMYD5
0.031281628	0.004897676	7342	UBP1
0.031270951	0.004912334	158219	TTC39B
0.03126993	0.004913734	26287	ANKRD2
0.031256836	0.004931768	54058	C21orf58
0.031239932	0.004955138	30010	NXPH1
0.031229904	0.004969054	79680	C22orf29
0.031221081	0.00498132	388574	AC139099.1
0.031037392	0.00524305	10238	DCAF7
0.030928368	0.005404206	23566	LPAR3
0.03091806	0.005419664	9477	MED20
0.030898929	0.005448478	1949	EFNB3
0.03087729	0.005481228	4072	EPCAM
0.030823801	0.005562957	1613	DAPK3
0.030804299	0.005593025	10501	SEMA6B
0.030777832	0.005634067	55329	MNS1
0.030662213	0.005816565	23424	TDRD7
0.030638626	0.005854443	27238	GPKOW
0.030633889	0.005862074	55246	CCDC25
0.030514464	0.006057525	2130	EWSR1
0.030506756	0.006070332	55131	RBM28
0.03044175	0.006179381	220064	ORAOV1
0.030428285	0.006202188	10226	M6PRBP1
0.030322881	0.006383344	100169750	PRINS
0.030307837	0.006409582	55739	CARKD
0.030269947	0.006476101	6524	SLC5A2
0.030246638	0.006517337	100128775	LOC100128775
0.030181725	0.006633402	132001	C3orf31
0.030175967	0.006643787	2023	ENO1
0.030082915	0.006813646	10713	USP39
0.030056635	0.00686232	84892	C3orf39
0.030042518	0.006888601	646817	SETP18
0.030026639	0.006918261	64087	MCCC2
0.029937394	0.007087114	5149	PDE6H
0.02990164	0.007155801	2300	FOXL1
0.029856229	0.00724389	492311	C5orf53
0.029797353	0.007359553	391257	SUMO1P1
0.029763363	0.007427079	7770	ZNF227
0.029759927	0.007433932	116986	AGAP2
0.029639481	0.007677911	10335	MRVI1
0.029638737	0.007679433	1180	CLCN1
0.029621279	0.007715402	23608	MKRN1
0.029513739	0.007940244	22853	LMTK2
0.029508818	0.007950672	10910	SUGT1
0.029491916	0.007986579	388272	C16orf87
0.029458856	0.00805724	54890	ALKBH5
0.029435081	0.008108404	79805	VASH2
0.029410938	0.008160651	115426	UHRF2
0.029407501	0.008168107	57636	ARHGAP23
0.029375184	0.008238574	84627	ZNF469
0.029347045	0.008300376	9753	ZSCAN12
0.029338965	0.008318204	57539	WDR35
0.029338688	0.008318815	157927	C9orf62
0.02932615	0.008346534	10389	SCML2
0.029248422	0.008520264	7179	TPTE
0.029187409	0.00865889	54840	APTX
0.029161777	0.008717728	143279	HECTD2
0.02908962	0.008885278	285381	DPH3
0.029016536	0.009057917	58509	C19orf29
0.02889256	0.009357614	100418883	LOC100418883
0.02882356	0.009528218	84328	LZIC
0.028804244	0.009576472	23196	FAM120A
0.028682403	0.009885903	4306	NR3C2

region-wise, subcortex					
Spearman's corr coef	p-val	p-val, SA	p-val, SA (BH- correction)	GenID	Gene
-0.002316602	0.989841521	0.992	1	137964	AGPAT6
-0.002316602	0.989841521	0.996	1	245973	ATP6V1C2
-0.002316602	0.989841521	0.983	1	340348	TSPAN33
-0.002316602	0.989841521	0.989	1	342897	NCCRP1
-0.002316602	0.989841521	0.984	1	100507135	AL139377.1
-0.002574003	0.98864603	0.99	1	123	ADFP
-0.002574003	0.98864603	0.996	1	3171	FOXA3
-0.002574003	0.98864603	0.994	1	3817	KLK2
-0.002574003	0.98864603	0.988	1	4762	NEUROG1
-0.002574003	0.98864603	0.99	1	6167	RPL37
-0.002574003	0.98864603	0.989	1	9038	TAAR5
-0.002574003	0.98864603	0.992	1	9118	INA
-0.002574003	0.98864603	0.991	1	10856	RUVBL2
-0.002574003	0.98864603	0.989	1	23236	PLCB1
-0.002574003	0.98864603	0.989	1	23400	ATP13A2
-0.002574003	0.98864603	0.989	1	57153	SLC44A2
-0.002574003	0.98864603	0.992	1	57541	ZNF398
-0.002574003	0.98864603	0.991	1	57575	PCDH10
-0.002574003	0.98864603	0.988	1	161635	CSNK1A1P1
-0.002831403	0.987451136	0.993	1	54947	LPCAT2
-0.002831403	0.987451136	0.982	1	55279	ZNF654
-0.002831403	0.987451136	0.991	1	65220	NADK
-0.002831403	0.987451136	0.987	1	129881	C2orf77
-0.002831403	0.987451136	0.986	1	138652	PRSS47
-0.002831403	0.987451136	0.987	1	246176	GAS2L2
-0.002831403	0.987451136	0.988	1	256006	ANKRD31
-0.002831403	0.987451136	0.99	1	285971	ZNF775
-0.003088803	0.986256242	0.988	1	1520	CTSS
-0.003088803	0.986256242	0.986	1	10277	UBE4B
-0.003088803	0.986256242	0.986	1	11079	RER1
-0.003088803	0.986256242	0.991	1	144245	ALG10B
-0.003088803	0.986256242	0.984	1	197370	NSMCE1
-0.003346203	0.985061347	0.982	1	4580	MTX1
-0.003346203	0.985061347	0.982	1	28959	TMEM176B
-0.003346203	0.985061347	0.986	1	55384	MEG3
-0.003346203	0.985061347	0.983	1	56951	C5orf15
-0.003346203	0.985061347	0.985	1	80321	CEP70
-0.003346203	0.985061347	0.98	1	85416	ZIC5
-0.003346203	0.985061347	0.99	1	85452	C1orf222
-0.003346203	0.985061347	0.986	1	201725	C4orf46
-0.003346203	0.985061347	0.985	1	644563	LOC644563
-0.003603603	0.983865976	0.986	1	10164	CHST4
-0.003603603	0.983865976	0.987	1	115825	WDFY2
-0.003603603	0.983865976	0.977	1	353134	LCE1D
-0.003861004	0.982671201	0.981	1	972	CD74
-0.003861004	0.982671201	0.982	1	9501	RPH3AL
-0.003861004	0.982671201	0.983	1	91419	XRCC6BP1
-0.003861004	0.982671201	0.981	1	117156	SCGB3A2
-0.003861004	0.982671201	0.982	1	118856	MMP21
-0.004118404	0.981476426	0.984	1	56	ACRV1
-0.004118404	0.981476426	0.982	1	6228	RPS23
-0.004118404	0.981476426	0.983	1	26000	TBC1D10B
-0.004118404	0.981476426	0.98	1	26275	HIBCH
-0.004118404	0.981476426	0.987	1	60468	BACH2
-0.004118404	0.981476426	0.983	1	122042	RXFP2
-0.004375804	0.98028177	0.98	1	3419	IDH3A
-0.004375804	0.98028177	0.98	1	27121	DKK4
-0.004375804	0.98028177	0.971	1	284366	KLK9
-0.004633205	0.979086518	0.976	1	7161	TP73
-0.004633205	0.979086518	0.972	1	55975	KLHL7
-0.004633205	0.979086518	0.989	1	63914	C6orf164
-0.004633205	0.979086518	0.97	1	79836	LONRF3
-0.004633205	0.979086518	0.978	1	149420	PDIK1L
-0.004633205	0.979086518	0.984	1	221527	ZBTB12
-0.004633205	0.979086518	0.987	1	100287825	LOC100287825
-0.004633205	0.979086518	0.984	1	100505929	C21orf37
-0.004890604	0.977891862	0.975	1	719	C3AR1
-0.004890604	0.977891862	0.977	1	2768	GNA12
-0.004890604	0.977891862	0.987	1	50832	TAS2R4
-0.004890604	0.977891862	0.981	1	261734	NPHP4
-0.005148005	0.976697326	0.974	1	1113	CHGA
-0.005148005	0.976697326	0.984	1	1350	COX7C
-0.005148005	0.976697326	0.972	1	4714	NDUFB8
-0.005148005	0.976697326	0.97	1	6508	SLC4A3
-0.005148005	0.976697326	0.97	1	23277	KIAA0664
-0.005148005	0.976697326	0.981	1	221937	FOXK1
-0.005148005	0.976697326	0.977	1	256051	ZNF549
-0.005148005	0.976697326	0.983	1	440097	DBX2
-0.005148005	0.976697326	0.982	1	442590	SPDYE5

voxel-wise, subcortex			
Spearman's corr coef	p-val	GenID	Gene
0.028673116	0.009909847	29902	C12orf24
0.028625291	0.010033984	81671	TMEM49
0.028600032	0.01010011	8913	CACNA1G
0.028597988	0.010105474	6041	RNASEL
0.028596224	0.010110107	199870	FAM76A
0.0285809	0.010150444	284309	ZNF776
0.028564092	0.01019485	10859	LILRB1
0.02852416	0.010301047	5156	PDGFRA
0.028521653	0.01030775	79663	HSPBAP1
0.028515244	0.010324904	22949	PTGR1
0.028486921	0.010400978	51522	TMEM14C
0.028423585	0.010572909	58	ACTA1
0.028404269	0.010625842	6867	TACC1
0.02837511	0.0107062	59277	NTN4
0.028336663	0.010812989	1468	SLC25A10
0.028232653	0.01110661	646450	C3orf71
0.028208323	0.011176314	1994	ELAVL1
0.028167089	0.011295313	10613	ERLIN1
0.028157895	0.011322012	7936	RDBP
0.028029185	0.011701559	5303	PIN4
0.028017947	0.011735225	221477	C6orf89
0.027927682	0.012008794	11014	KDELR2
0.027888305	0.012129887	440354	AC009086.2
0.027853761	0.012237013	8731	RNMT
0.027843544	0.012268861	7546	ZIC2
0.027830265	0.012310362	25911	RP11-529I10.1
0.027823672	0.012331013	128414	NKAIN4
0.027810764	0.012371533	84545	MRPL43
0.027803613	0.012394032	26092	TOR1AIP1
0.027779004	0.012471721	7799	PRDM2
0.027721891	0.012653712	139728	PNCK
0.027709074	0.012694882	22854	NTNG1
0.027698582	0.012728655	84868	HAVCR2
0.027629767	0.012952228	55156	ARMC1
0.027618809	0.01298814	83540	NUF2
0.027611565	0.013011931	84898	PLXDC2
0.027497156	0.013392848	1871	E2F3
0.02746948	0.013486453	286205	SCAI
0.027349962	0.013897398	5995	RGR
0.027331853	0.013960617	169693	C9orf71
0.027268983	0.014182076	9123	SLC16A3
0.027166367	0.014550177	51493	C22orf28
0.027152343	0.014601142	7417	VDAC2
0.027151972	0.014602492	115752	DIS3L
0.02712987	0.014683129	7101	NR2E1
0.027113248	0.014744022	25888	ZNF473
0.027084922	0.014848322	7473	WNT3
0.027059849	0.014941174	388591	RNF207
0.027004872	0.015146584	10261	IGSF6
0.026987229	0.015213025	84433	CARD11
0.026939774	0.015393003	7086	TKT
0.026914978	0.015487798	4060	LUM
0.026889903	0.015584169	79879	CCDC134
0.026843749	0.015762957	55676	SLC30A6
0.026825733	0.015833227	10464	PIBF1
0.026767785	0.016061122	441263	DTX2P1-UPK3BP1-PMS2P11
0.026746055	0.016147323	29999	FSCN3
0.026724417	0.016233573	90506	LRRC46
0.026706772	0.016304187	79166	LILRP2
0.026678542	0.016417736	100506164	HSFX1
0.02665226	0.016524082	151230	KLHL23
0.02652717	0.017038552	10598	AHSA1
0.026515376	0.017087778	9183	ZW10
0.0264578	0.017329866	9632	SEC24C
0.026434304	0.017429527	29904	EEF2K
0.026390564	0.017616389	57154	SMURF1
0.026356017	0.017765209	117854	TRIM6
0.02629129	0.018047003	8359	HIST1H4A
0.026210682	0.018403422	8061	FOSL1
0.026108623	0.018863508	6002	RGS12
0.026031358	0.019218476	5580	PRKCD
0.026028294	0.019232672	84320	ACBD6
0.026022071	0.019261539	8365	HIST1H4H
0.026019193	0.019274892	1738	DLD
0.026002012	0.01935483	201798	TIGD4
0.025960222	0.019550437	2495	FTHL16
0.025934592	0.019671265	8214	DGCR6
0.025911003	0.019783035	440068	CARD17
0.025712643	0.02074514	80315	CPEB4

region-wise, subcortex					
Spearman's corr coef	p-val	p-val, SA	p-val, SA (BH- correction)	GenID	Gene
-0.005148005	0.976697326	0.978	1	100129387	GABPB1-AS1
-0.005405406	0.975502789	0.975	1	2539	G6PD
-0.005405406	0.975502789	0.977	1	6451	SH3BGRL
-0.005405406	0.975502789	0.971	1	7317	UBA1
-0.005405406	0.975502789	0.983	1	9912	AC005277.1
-0.005662805	0.974308312	0.977	1	1445	CSK
-0.005662805	0.974308312	0.968	1	8911	CACNA1I
-0.005662805	0.974308312	0.971	1	60493	FASTKD5
-0.005662805	0.974308312	0.973	1	161436	EML5
-0.005920206	0.973113298	0.972	1	379	ARL4D
-0.005920206	0.973113298	0.98	1	950	SCARB2
-0.005920206	0.973113298	0.973	1	1158	CKM
-0.005920206	0.973113298	0.972	1	3773	KCNJ16
-0.005920206	0.973113298	0.973	1	10296	MAEA
-0.005920206	0.973113298	0.969	1	23765	IL17RA
-0.005920206	0.973113298	0.968	1	25879	DCAF13
-0.005920206	0.973113298	0.976	1	26958	COPG2
-0.005920206	0.973113298	0.968	1	29922	NME7
-0.005920206	0.973113298	0.974	1	55578	FAM48A
-0.005920206	0.973113298	0.97	1	57590	WDFY1
-0.005920206	0.973113298	0.974	1	80036	TRPM3
-0.005920206	0.973113298	0.971	1	153222	C5orf41
-0.005920206	0.973113298	0.98	1	646508	AC092070.5
-0.006177606	0.971918941	0.982	1	7805	LAPTM5
-0.006177606	0.971918941	0.976	1	140893	C20orf151
-0.006177606	0.971918941	0.98	1	162282	ANKFN1
-0.006177606	0.971918941	0.979	1	399967	PATE2
-0.006177606	0.971918941	0.975	1	100128203	ENSAP3
-0.006435006	0.970724702	0.966	1	7353	UFD1L
-0.006435006	0.970724702	0.964	1	645367	GGT8P
-0.006435006	0.970724702	0.963	1	731789	RP13-16H11.1
-0.006692407	0.969530404	0.972	1	2900	GRIK4
-0.006692407	0.969530404	0.971	1	4292	MLH1
-0.006692407	0.969530404	0.968	1	51100	SH3GLB1
-0.006692407	0.969530404	0.974	1	121981	PPIAP26
-0.006692407	0.969530404	0.978	1	122706	PSMB11
-0.006692407	0.969530404	0.972	1	255082	CASC2
-0.006692407	0.969530404	0.964	1	387066	SNHG5
-0.006949807	0.968335688	0.956	1	1038	CDR1
-0.006949807	0.968335688	0.972	1	4747	NEFL
-0.006949807	0.968335688	0.967	1	55055	ZWILCH
-0.007207207	0.967141628	0.971	1	2707	GJB3
-0.007207207	0.967141628	0.973	1	4585	MUC4
-0.007207207	0.967141628	0.968	1	7142	TNP2
-0.007207207	0.967141628	0.978	1	201255	LRRC45
-0.007207207	0.967141628	0.965	1	284131	AC120024.1
-0.007207207	0.967141628	0.981	1	387590	AC005301.1
-0.007464607	0.965947568	0.966	1	55966	AJAP1
-0.007464607	0.965947568	0.973	1	139081	MAGEC3
-0.007722008	0.964753568	0.979	1	80320	SP6
-0.007722008	0.964753568	0.964	1	150147	C21orf128
-0.007979408	0.963559151	0.962	1	1836	SLC26A2
-0.007979408	0.963559151	0.965	1	79999	AC007952.2
-0.007979408	0.963559151	0.966	1	89910	UBE3B
-0.008236809	0.962365389	0.958	1	1580	CYP4B1
-0.008236809	0.962365389	0.969	1	4784	NFIX
-0.008236809	0.962365389	0.963	1	51258	MRPL51
-0.008236809	0.962365389	0.958	1	79819	WDR78
-0.008236809	0.962365389	0.962	1	389692	MAFA
-0.008494208	0.961171627	0.954	1	4544	MTNR1B
-0.008494208	0.961171627	0.963	1	50624	CUZD1
-0.008494208	0.961171627	0.96	1	92799	SHKBP1
-0.008494208	0.961171627	0.961	1	94056	SYAP1
-0.008494208	0.961171627	0.976	1	374659	HDDC3
-0.008494208	0.961171627	0.965	1	729920	ISPD
-0.008751608	0.959978044	0.956	1	6665	SOX15
-0.008751608	0.959978044	0.96	1	6723	SRM
-0.008751608	0.959978044	0.954	1	8379	MAD1L1
-0.008751608	0.959978044	0.962	1	51239	ANKRD39
-0.008751608	0.959978044	0.956	1	57148	KIAA1219
-0.008751608	0.959978044	0.961	1	79171	RBM42
-0.008751608	0.959978044	0.953	1	645431	LOC645431
-0.009009009	0.958784461	0.958	1	492	ATP2B3
-0.009009009	0.958784461	0.96	1	4237	MFAP2
-0.009009009	0.958784461	0.961	1	9677	HISPPD2A
-0.009009009	0.958784461	0.961	1	23015	GOLGA8A
-0.009009009	0.958784461	0.948	1	23023	TMCC1
-0.009009009	0.958784461	0.958	1	54954	FAM120C
-0.009009009	0.958784461	0.96	1	55831	EMC3

voxel-wise, subcortex			
Spearman's corr coef	p-val	GenID	Gene
0.025662865	0.020992888	196403	DTX3
0.025616061	0.021228172	136319	MTPN
0.025615411	0.02123145	83451	ABHD11
0.025606589	0.021276066	166	AES
0.025603246	0.021292992	9156	EXO1
0.025600366	0.021307591	64777	RMND5B
0.025584394	0.021388667	124093	CCDC78
0.025538703	0.02162208	5335	PLCG1
0.025533132	0.021650705	26648	OR7E24
0.025465896	0.021998642	222161	DKFZP586I1420
0.025454754	0.022056766	283420	CLEC9A
0.025450388	0.022079578	652968	GATSL3
0.025430052	0.022186104	64800	EFCAB6
0.02540479	0.022319058	26696	OR2T1
0.025359195	0.022560783	991	CDC20
0.025300689	0.022874285	55845	BRK1
0.02523271	0.023243286	81848	SPRY4
0.02522946	0.023261052	100129138	RP11-364B6.1
0.025218131	0.02332308	3687	ITGAX
0.025216181	0.023333786	91661	ZNF765
0.025013082	0.024471005	80233	C17orf70
0.024993766	0.024581639	89891	WDR34
0.024873411	0.025280664	91227	GGTLC2
0.024824843	0.025567591	5313	PKLR
0.024738107	0.026087016	55328	RNLS
0.024701703	0.026307721	57019	CIAPIN1
0.024675144	0.026469762	23621	BACE1
0.0246666	0.026522074	57708	MIER1
0.024637347	0.02670184	5788	PTPRC
0.024616452	0.026830904	389740	GPR116P1
0.024603264	0.026912637	57338	JPH3
0.024578005	0.027069762	64121	RRAGC
0.024572155	0.02710627	55107	ANO1
0.024546709	0.027265567	116238	TLCD1
0.024544202	0.027281294	51361	HOOK1
0.024541788	0.027296472	9962	SLC23A2
0.024512906	0.027478414	140	ADORA3
0.024479661	0.027689146	115817	DHRS1
0.024471581	0.027740574	392352	LOC392352
0.02442942	0.028010255	84418	CYSTM1
0.024397288	0.028217312	3978	LIG1
0.024396174	0.0282245	9228	DLGAP2
0.024388187	0.028276181	147949	ZNF583
0.024384659	0.028299034	203328	SUSD3
0.024372401	0.028378567	246213	SLC17A8
0.024328381	0.028665749	22856	CHSY1
0.02432541	0.028685234	5523	PPP2R3A
0.024287056	0.028937632	2719	GPC3
0.024210906	0.029444458	5100	PCDH8
0.024205798	0.029478727	84295	PHF6
0.02420227	0.029502416	63901	FAM111A
0.024174968	0.029686285	730291	ZNF735
0.024120176	0.030058265	121364	OR10A7
0.024086744	0.030287195	27292	DIMT1
0.023952646	0.031220559	57228	AC139768.2
0.023949953	0.03123956	56904	SH3GLB2
0.023946146	0.03126644	4922	NTS
0.023933051	0.031358998	197370	NSMCE1
0.02392943	0.031384643	9015	TAF1A
0.023894977	0.03162951	118429	ANTXR2
0.023877146	0.031756863	26063	DECR2
0.023821611	0.032156345	54221	SNTG2
0.023798211	0.032325961	57642	COL20A1
0.023778336	0.032470636	9739	SETD1A
0.023774622	0.03249773	221711	SYCP2L
0.023768958	0.032539085	5169	ENPP3
0.023748526	0.032688644	84446	BRSK1
0.023741005	0.032743864	81543	LRRC3
0.02371528	0.032933276	4085	MAD2L1
0.023686677	0.033144981	26260	FBXO25
0.023640709	0.033487681	148156	ZNF558
0.023629194	0.033573985	4267	CD99
0.023622878	0.033621434	80228	ORAI2
0.023560101	0.034095921	6576	SLC25A1
0.023511253	0.03446909	28976	ACAD9
0.023503175	0.034531124	54716	SLC6A20
0.023496488	0.034582581	389558	FAM180A
0.023397865	0.035348766	6527	SLC5A4
0.023355611	0.035681441	100271715	AC019171.4

region-wise, subcortex					
Spearman's corr coef	p-val	p-val, SA	p-val, SA (BH- correction)	GenID	Gene
-0.009009009	0.958784461	0.95	1	116071	BATF2
-0.009266409	0.957590461	0.953	1	344	APOC2
-0.009266409	0.957590461	0.957	1	5293	PIK3CD
-0.009266409	0.957590461	0.965	1	6189	RPS3AP47
-0.009266409	0.957590461	0.956	1	10269	ZMPSTE24
-0.009266409	0.957590461	0.959	1	29125	C11orf21
-0.009266409	0.957590461	0.964	1	55172	C14orf104
-0.009266409	0.957590461	0.959	1	79816	TLE6
-0.009266409	0.957590461	0.96	1	84914	ZNF587
-0.009266409	0.957590461	0.963	1	374395	TMEM179B
-0.00952381	0.956397116	0.961	1	1133	CHRM5
-0.00952381	0.956397116	0.958	1	10427	SEC24B
-0.00952381	0.956397116	0.963	1	203859	ANO5
-0.00952381	0.956397116	0.959	1	100294362	LOC100294362
-0.009781209	0.955203831	0.958	1	3385	ICAM3
-0.009781209	0.955203831	0.954	1	4724	NDUFS4
-0.01003861	0.954010606	0.963	1	5866	RAB3IL1
-0.01003861	0.954010606	0.957	1	6271	S100A1
-0.01003861	0.954010606	0.959	1	7565	ZNF17
-0.01003861	0.954010606	0.962	1	10810	WASF3
-0.01003861	0.954010606	0.969	1	80775	TMEM177
-0.01003861	0.954010606	0.955	1	84883	AIFM2
-0.01029601	0.952816963	0.946	1	10693	CCT6B
-0.01029601	0.952816963	0.97	1	26036	ZNF451
-0.01029601	0.952816963	0.952	1	283902	HTA
-0.01029601	0.952816963	0.964	1	767846	PFN1P2
-0.01055341	0.951624036	0.947	1	27	ABL2
-0.01055341	0.951624036	0.952	1	2697	GJA1
-0.01055341	0.951624036	0.948	1	6835	SURF2
-0.01055341	0.951624036	0.948	1	23138	AC136632.1
-0.01055341	0.951624036	0.96	1	143162	FRMPD2
-0.01055341	0.951624036	0.954	1	144097	C11orf84
-0.010810811	0.950431168	0.958	1	961	CD47
-0.010810811	0.950431168	0.942	1	6293	VPS52
-0.010810811	0.950431168	0.961	1	26115	TANC2
-0.010810811	0.950431168	0.945	1	51752	ERAP1
-0.010810811	0.950431168	0.961	1	57546	PDP2
-0.010810811	0.950431168	0.945	1	91948	RP11-82I10.1
-0.010810811	0.950431168	0.964	1	145741	FAM148A
-0.010810811	0.950431168	0.956	1	387712	C10orf134
-0.011068211	0.94923842	0.96	1	4735	SEPTIN2
-0.011068211	0.94923842	0.957	1	5009	OTC
-0.011068211	0.94923842	0.949	1	10168	ZNF197
-0.011068211	0.94923842	0.957	1	23766	GABARAPL3
-0.011068211	0.94923842	0.951	1	26995	TRUB2
-0.011068211	0.94923842	0.953	1	728658	RPL13AP5
-0.011325611	0.948045731	0.96	1	23658	LSM5
-0.011325611	0.948045731	0.96	1	65987	KCTD14
-0.011325611	0.948045731	0.954	1	84777	MGC11082
-0.011325611	0.948045731	0.953	1	392376	OR13C2
-0.011325611	0.948045731	0.958	1	402665	IGLON5
-0.011583011	0.946852624	0.954	1	2737	GLI3
-0.011583011	0.946852624	0.951	1	6898	TAT
-0.011583011	0.946852624	0.949	1	7324	UBE2E1
-0.011583011	0.946852624	0.947	1	8355	HIST1H3G
-0.011583011	0.946852624	0.957	1	26056	RAB11FIP5
-0.011583011	0.946852624	0.943	1	151613	TTC14
-0.011840411	0.945660233	0.955	1	7617	ZNF66P
-0.011840411	0.945660233	0.941	1	51092	SIDT2
-0.011840411	0.945660233	0.951	1	57462	KIAA1161
-0.011840411	0.945660233	0.942	1	79639	TMEM53
-0.011840411	0.945660233	0.951	1	83896	KRTAP3-1
-0.011840411	0.945660233	0.943	1	91147	TMEM67
-0.011840411	0.945660233	0.947	1	149466	C1ORF210
-0.011840411	0.945660233	0.959	1	373856	USP41
-0.012097812	0.944467962	0.942	1	775	CACNA1C
-0.012097812	0.944467962	0.943	1	3937	LCP2
-0.012097812	0.944467962	0.942	1	79168	LILRA6
-0.012097812	0.944467962	0.944	1	83443	SF3B5
-0.012097812	0.944467962	0.951	1	160777	CCDC60
-0.012355212	0.94327569	0.949	1	3975	LHX1
-0.012355212	0.94327569	0.941	1	4909	AC008687.3
-0.012355212	0.94327569	0.934	1	5436	POLR2G
-0.012355212	0.94327569	0.948	1	85360	SYDE1
-0.012355212	0.94327569	0.942	1	161502	C15orf26
-0.012355212	0.94327569	0.933	1	390243	FOLR4
-0.012355212	0.94327569	0.944	1	653162	RPSAP9
-0.012612613	0.94208312	0.937	1	3958	LGALS3
-0.012612613	0.94208312	0.949	1	5789	PTPRD

voxel-wise, subcortex			
Spearman's corr coef	p-val	GenID	Gene
0.023307599	0.036062691	10856	RUVBL2
0.023274167	0.036330212	100129794	RP11-537P22.1
0.023249093	0.036531944	55002	TMCO3
0.02320916	0.036855232	983	CDC2
0.023091871	0.037818842	22982	DIP2C
0.022984982	0.038715601	170487	C20orf134
0.02297941	0.03876286	8458	TTF2
0.022974953	0.038800661	25796	PGLS
0.022968266	0.038857464	2538	G6PC
0.022966223	0.03887482	8224	SYN3
0.02293985	0.039099637	164127	C1orf65
0.022812624	0.040199663	4129	MAOB
0.022806494	0.040253326	257169	C9orf43
0.022783834	0.040452201	729852	LOC729852
0.022747617	0.040771779	54934	C12orf41
0.022723936	0.040981896	131177	FAM3D
0.022690319	0.041281741	57834	CYP4F11
0.022688739	0.041295905	293	SLC25A6
0.022678154	0.041390732	58492	ZNF77
0.022675924	0.041410714	54544	CRCT1
0.022633949	0.041788742	9112	MTA1
0.02263302	0.041797157	55743	CHFR
0.022624662	0.041872747	4796	NFKBIL2
0.022620205	0.041913155	91947	ARRDC4
0.022587979	0.042206075	126070	ZNF440
0.022489913	0.04310815	84148	MYST1
0.022465676	0.043333594	79031	PDCL3
0.022433173	0.043637495	64762	FAM59A
0.022368537	0.044247169	3155	HMGCL
0.022345228	0.044468779	255758	TCTEX1D2
0.022337614	0.044541378	10051	SMC4
0.02232099	0.044700246	1462	VCAN
0.022250969	0.045374561	57050	UTP3
0.022217909	0.045695912	643905	PRR21
0.022147888	0.046382848	23731	C9orf5
0.02209904	0.046867218	284323	ZNF780A
0.022009054	0.047770619	1783	DYNC1LI2
0.022007011	0.047791306	50511	SYCP3
0.021992246	0.047940992	3678	ITGA5
0.021978129	0.048084449	830	CAPZA2
0.021936154	0.048513174	29087	THYN1
0.02183233	0.049587447	1767	DNAH5
0.02165607	0.051456884	54872	PIGG
0.021634988	0.05168438	8861	LDB1
0.021593664	0.052132744	112936	VPS26B
0.021566268	0.052431788	8971	H1FX
0.021547139	0.05264144	7434	VIPR2
0.021544259	0.052673064	283297	OR10A4
0.021529214	0.052838523	149934	NCOR1P1
0.021440342	0.053824823	54539	NDUFB11
0.021428177	0.053961005	3605	IL17A
0.021370692	0.054608498	9236	CCPG1
0.021341996	0.054934122	442249	LOC442249
0.021340696	0.054948907	4148	MATN3
0.021302065	0.05538993	55835	CENPJ
0.02125749	0.055902485	3190	HNRNPK
0.021226471	0.05626148	837	CASP4
0.021195734	0.056619108	4974	OMG
0.021193318	0.056647282	9149	DYRK1B
0.021147722	0.057181504	9953	HS3ST3B1
0.021106396	0.057669301	168667	BMPER
0.021105746	0.057676986	55722	CEP72
0.02107445	0.058048744	9711	KIAA0226
0.021027552	0.058609534	3295	HSD17B4
0.020881381	0.060386352	55152	DALRD3
0.020867173	0.060561389	7479	WNT8B
0.020808017	0.061294809	5331	PLCB3
0.020764278	0.061841793	23247	KIAA0556
0.020684691	0.06284745	283951	C16orf91
0.020613741	0.063755326	286256	LCN12
0.020598419	0.063952811	54795	TRPM4
0.020562479	0.064417996	729220	FLJ45513
0.020562295	0.064420387	139322	APOOL
0.020541493	0.064690918	55716	LMBR1L
0.02047723	0.065532602	539	ATP5O
0.020454384	0.065834008	83937	RASSF4
0.020389749	0.066692866	7465	WEE1
0.020383898	0.066771053	337867	UBAC2
0.020261595	0.068423077	57561	ARRDC3

region-wise, subcortex					
Spearman's corr coef	p-val	p-val, SA	p-val, SA (BH- correction)	GenID	Gene
-0.012612613	0.94208312	0.955	1	7106	TSPAN4
-0.012612613	0.94208312	0.942	1	10914	PAPOLA
-0.012612613	0.94208312	0.947	1	54511	HMGCLL1
-0.012612613	0.94208312	0.947	1	122773	KLHDC1
-0.012612613	0.94208312	0.955	1	100507530	LOC100507530
-0.012870012	0.940891147	0.951	1	4907	NT5E
-0.012870012	0.940891147	0.94	1	7399	USH2A
-0.012870012	0.940891147	0.952	1	9581	PREPL
-0.012870012	0.940891147	0.934	1	26097	C1orf77
-0.012870012	0.940891147	0.95	1	64506	CPEB1
-0.013127413	0.939699411	0.95	1	23061	TBC1D9B
-0.013127413	0.939699411	0.937	1	54807	ZNF586
-0.013127413	0.939699411	0.957	1	55180	LINS
-0.013127413	0.939699411	0.947	1	57537	SORCS2
-0.013127413	0.939699411	0.94	1	79004	CUEDC2
-0.013127413	0.939699411	0.943	1	84075	FSCB
-0.013127413	0.939699411	0.946	1	283345	RPL13P5
-0.013384813	0.938507736	0.953	1	5027	P2RX7
-0.013384813	0.938507736	0.93	1	6830	SUPT6H
-0.013384813	0.938507736	0.957	1	8669	EIF3J
-0.013384813	0.938507736	0.942	1	26354	GNL3
-0.013384813	0.938507736	0.928	1	27141	CIDEB
-0.013384813	0.938507736	0.948	1	169792	GLIS3
-0.013642213	0.937315583	0.949	1	331	XIAP
-0.013642213	0.937315583	0.94	1	55200	PLEKHG6
-0.013642213	0.937315583	0.944	1	57175	CORO1B
-0.013642213	0.937315583	0.942	1	64423	INF2
-0.013642213	0.937315583	0.934	1	64714	PDIA2
-0.013642213	0.937315583	0.942	1	163590	TOR1AIP2
-0.013899614	0.936124146	0.934	1	891	CCNB1
-0.013899614	0.936124146	0.943	1	2357	FPR1
-0.013899614	0.936124146	0.937	1	5979	RET
-0.013899614	0.936124146	0.951	1	6993	DYNLT1
-0.013899614	0.936124146	0.92	1	10648	SCGB1D1
-0.014157014	0.934932947	0.928	1	1442	CSH1
-0.014157014	0.934932947	0.941	1	4284	MIP
-0.014157014	0.934932947	0.955	1	22978	NT5C2
-0.014157014	0.934932947	0.939	1	84133	ZNRF3
-0.014157014	0.934932947	0.94	1	84867	PTPN5
-0.014157014	0.934932947	0.944	1	128653	C20ORF141
-0.014157014	0.934932947	0.938	1	284618	RUSC1-AS1
-0.014414414	0.933741808	0.946	1	301	ANXA1
-0.014414414	0.933741808	0.925	1	1153	CIRBP
-0.014414414	0.933741808	0.947	1	5195	PEX14
-0.014414414	0.933741808	0.943	1	6942	TCF20
-0.014414414	0.933741808	0.931	1	9312	KCNB2
-0.014414414	0.933741808	0.932	1	10390	CEPT1
-0.014414414	0.933741808	0.945	1	10461	MERTK
-0.014414414	0.933741808	0.958	1	29980	DONSON
-0.014414414	0.933741808	0.939	1	54832	VPS13C
-0.014414414	0.933741808	0.941	1	56413	LTB4R2
-0.014671815	0.932550788	0.934	1	1553	CYP2A13
-0.014671815	0.932550788	0.923	1	6210	RPS15A
-0.014671815	0.932550788	0.927	1	11104	KATNA1
-0.014671815	0.932550788	0.936	1	146167	SLC38A8
-0.014671815	0.932550788	0.941	1	389705	LOC389705
-0.014671815	0.932550788	0.937	1	641977	SEPTIN7P2
-0.014929214	0.93135941	0.937	1	1892	ECHS1
-0.014929214	0.93135941	0.925	1	5422	POLA1
-0.014929214	0.93135941	0.929	1	8225	GTPBP6
-0.014929214	0.93135941	0.928	1	8888	MCM3AP
-0.014929214	0.93135941	0.948	1	63910	SLC17A9
-0.014929214	0.93135941	0.929	1	150368	FAM109B
-0.014929214	0.93135941	0.951	1	401264	FLJ37798
-0.014929214	0.93135941	0.927	1	442117	GALNTL6
-0.015186615	0.930168748	0.932	1	6548	SLC9A1
-0.015186615	0.930168748	0.932	1	10929	SRSF8
-0.015186615	0.930168748	0.938	1	79621	RNASEH2B
-0.015444015	0.928978145	0.94	1	1366	CLDN7
-0.015444015	0.928978145	0.933	1	2259	FGF14
-0.015444015	0.928978145	0.93	1	23633	KPNA6
-0.015444015	0.928978145	0.923	1	54600	UGT1A9
-0.015444015	0.928978145	0.943	1	139231	CXorf39
-0.015444015	0.928978145	0.945	1	157378	TMEM65
-0.015701415	0.927787781	0.925	1	84752	B3GNT9
-0.015701415	0.927787781	0.925	1	140832	WFDC10A
-0.015701415	0.927787781	0.947	1	285641	SLC36A3
-0.015701415	0.927787781	0.937	1	348840	AC073135.1
-0.015958816	0.926596999	0.929	1	9604	RNF14

voxel-wise, subcortex			
Spearman's corr coef	p-val	GenID	Gene
0.020222683	0.068955675	199	AIF1
0.020207824	0.069159947	54918	CMTM6
0.02016037	0.069815643	54881	TEX10
0.020038344	0.071525201	64374	SIL1
0.020027293	0.071681716	151354	FAM84A
0.020008627	0.071946688	55345	C4orf21
0.020006491	0.071977057	158	ADSL
0.02000222	0.072037823	54784	ALKBH4
0.019990982	0.072197907	100289410	MCF2L-AS1
0.019968603	0.072517566	27327	TNRC6A
0.019940741	0.072917171	10880	ACTL7B
0.019930063	0.073070817	374907	B3GNT8
0.019827724	0.074556567	92293	TMEM132C
0.019770427	0.075399131	284288	RSL24D1P11
0.019707741	0.07632982	27006	FGF22
0.019665487	0.076962434	7919	BAT1
0.01965685	0.07709226	596	BCL2
0.019631313	0.077477179	22876	INPP5F
0.019629177	0.077509485	22868	FASTKD2
0.019573644	0.078352205	25886	WDR51A
0.019544855	0.078792043	79100	RP1-10C16.2
0.019524053	0.079111099	8883	NAE1
0.019505851	0.079391122	11133	KPTN
0.019446231	0.080313995	28964	GIT1
0.019434344	0.080499023	283659	PRTG
0.019431464	0.080543905	26064	RAI14
0.0194271	0.080611952	389827	TMEM8C
0.019419579	0.080729365	220382	FAM181B
0.019406112	0.080939926	100132977	LOC100132977
0.019375375	0.081422128	1760	DMPK
0.019340642	0.081969827	23272	C3orf63
0.019333214	0.082087353	5378	PMS1
0.019329591	0.082144715	1508	CTSB
0.01924824	0.083441474	384	ARG2
0.019185556	0.084451936	8763	CD164
0.019168098	0.08473511	5657	PRTN3
0.019141445	0.08516892	113201	CASC4
0.01912055	0.085510269	6687	SPG7
0.019100027	0.085846588	55748	CNDP2
0.019058423	0.086531714	2121	EVC
0.019050807	0.086657621	7753	ZNF202
0.019040778	0.086823612	23423	TMED3
0.019032978	0.08695291	144608	C12orf60
0.019016169	0.087232031	3329	HSPD1
0.019005861	0.087403573	63924	CIDEC
0.019005675	0.087406673	23499	MACF1
0.018955247	0.088249788	23096	IQSEC2
0.018859411	0.0898702	55798	METTL2B
0.018853189	0.089976221	3881	KRT31
0.018797655	0.090927012	22977	AKR7A3
0.018777039	0.09128204	4552	MTRR
0.018726705	0.092153475	148229	ATP8B3
0.018715933	0.09234082	341567	H1FNT
0.018697731	0.092658088	79839	CCDC102B
0.018594092	0.094481401	9266	CYTH2
0.01850977	0.095985986	4714	NDUFB8
0.018475782	0.096597835	26166	RGS22
0.018471416	0.096676677	9771	RAPGEF5
0.018403346	0.097912237	116844	LRG1
0.018396288	0.09804105	219972	MPEG1
0.018373257	0.098462395	389	RHOC
0.018351713	0.098857857	91057	CCDC34
0.018332025	0.099220313	26589	MRPL46
0.018204242	0.101598777	9723	SEMA3E
0.018113604	0.103313252	5283	PIGH
0.018095959	0.103649683	399761	BMS1P5
0.017955082	0.10636694	9727	RAB11FIP3
0.017950717	0.106452025	57593	EBF4
0.017918121	0.10708914	2124	EVI2B
0.017912457	0.107200168	375287	RBM43
0.01790995	0.10724929	55788	LMBRD1
0.017898155	0.107480854	11118	BTN3A2
0.017880788	0.107822493	1200	TPP1
0.017821448	0.108996384	25996	REXO2
0.01781179	0.109188385	10079	ATP9A
0.017795539	0.109512083	10775	POP4
0.017781144	0.10979943	286103	C8ORF77
0.01778068	0.109808683	100506060	LOC100506060
0.017612221	0.113216214	83879	CDCA7

region-wise, subcortex					
Spearman's corr coef	p-val	p-val, SA	p-val, SA (BH- correction)	GenID	Gene
-0.015958816	0.926596999	0.927	1	283820	NOMO2
-0.015958816	0.926596999	0.943	1	728489	DNLZ
-0.016216217	0.925406933	0.927	1	4026	LPP
-0.016216217	0.925406933	0.922	1	6231	RPS26P20
-0.016216217	0.925406933	0.924	1	9113	LATS1
-0.016216217	0.925406933	0.928	1	56902	PNO1
-0.016216217	0.925406933	0.932	1	84146	ZNF644
-0.016216217	0.925406933	0.932	1	283687	AC015871.2
-0.016473617	0.924217045	0.923	1	3709	ITPR2
-0.016473617	0.924217045	0.93	1	22908	SACM1L
-0.016473617	0.924217045	0.923	1	89876	C3orf15
-0.016473617	0.924217045	0.923	1	221303	FAM162B
-0.016473617	0.924217045	0.907	1	441161	OOEP
-0.016731016	0.923027277	0.923	1	1815	DRD4
-0.016731016	0.923027277	0.919	1	7392	USF2
-0.016731016	0.923027277	0.93	1	22852	ANKRD26
-0.016731016	0.923027277	0.921	1	56243	KIAA1217
-0.016731016	0.923027277	0.933	1	79650	C16orf57
-0.016731016	0.923027277	0.933	1	165186	FAM179A
-0.016731016	0.923027277	0.919	1	333926	PPM1J
-0.016988415	0.921837151	0.93	1	5984	RFC4
-0.016988415	0.921837151	0.923	1	10814	CPLX2
-0.016988415	0.921837151	0.924	1	23135	KDM6B
-0.016988415	0.921837151	0.943	1	26018	LRIG1
-0.016988415	0.921837151	0.919	1	54943	DNAJC28
-0.016988415	0.921837151	0.929	1	164714	TTLL8
-0.016988415	0.921837151	0.927	1	653390	AC009093.1
-0.017245818	0.92064774	0.93	1	3108	HLA-DMA
-0.017245818	0.92064774	0.918	1	5250	SLC25A3
-0.017245818	0.92064774	0.925	1	64377	CHST8
-0.017245818	0.92064774	0.925	1	171586	ABHD3
-0.017503217	0.919458449	0.919	1	3281	HSBP1
-0.017503217	0.919458449	0.921	1	23382	AHCYL2
-0.017503217	0.919458449	0.916	1	116254	C6orf72
-0.017503217	0.919458449	0.924	1	283989	TSEN54
-0.017503217	0.919458449	0.918	1	338321	NLRP9
-0.017503217	0.919458449	0.931	1	441795	HMGB3P27
-0.017760618	0.918269455	0.938	1	970	CD70
-0.017760618	0.918269455	0.929	1	4076	CAPRIN1
-0.017760618	0.918269455	0.927	1	7508	XPC
-0.017760618	0.918269455	0.925	1	9607	CARTPT
-0.017760618	0.918269455	0.918	1	54886	PPAPR3
-0.017760618	0.918269455	0.923	1	79577	CDC73
-0.017760618	0.918269455	0.935	1	83882	TSPAN10
-0.017760618	0.918269455	0.919	1	84109	QRFPR
-0.018018018	0.917080581	0.917	1	326	AIRE
-0.018018018	0.917080581	0.91	1	2042	EPHA3
-0.018018018	0.917080581	0.927	1	79661	NEIL1
-0.018275417	0.91589123	0.913	1	26993	AKAP8L
-0.018275417	0.91589123	0.915	1	56154	TEX15
-0.018275417	0.91589123	0.915	1	79947	DHDDS
-0.018275417	0.91589123	0.918	1	388325	SCIMP
-0.018532818	0.914702773	0.928	1	1258	CNGB1
-0.018532818	0.914702773	0.921	1	4054	LTBP3
-0.018532818	0.914702773	0.93	1	5970	RELA
-0.018532818	0.914702773	0.92	1	9179	AP4M1
-0.018532818	0.914702773	0.928	1	9538	EI24
-0.018532818	0.914702773	0.933	1	10599	SLCO1B1
-0.018532818	0.914702773	0.923	1	55520	ELAC1
-0.018532818	0.914702773	0.947	1	340390	KIAA1875
-0.018790217	0.913514376	0.911	1	690	BTF3P11
-0.018790217	0.913514376	0.923	1	5264	PHYH
-0.018790217	0.913514376	0.911	1	10671	DCTN6
-0.018790217	0.913514376	0.919	1	28978	TMEM14A
-0.018790217	0.913514376	0.919	1	81551	STMN4
-0.018790217	0.913514376	0.921	1	119692	OR51S1
-0.01904762	0.912326217	0.913	1	10945	KDELR1
-0.01904762	0.912326217	0.923	1	27301	APEX2
-0.01904762	0.912326217	0.927	1	57396	CLK4
-0.01904762	0.912326217	0.924	1	127544	RNF19B
-0.01904762	0.912326217	0.915	1	100288252	LOC100288252
-0.019305019	0.91113776	0.925	1	4609	MYC
-0.019305019	0.91113776	0.925	1	10342	TFG
-0.019305019	0.91113776	0.931	1	57084	SLC17A6
-0.019305019	0.91113776	0.911	1	66000	TMEM108
-0.019305019	0.91113776	0.926	1	80045	GPR157
-0.019305019	0.91113776	0.921	1	162394	SLFN5
-0.019562418	0.909949899	0.929	1	5890	RAD51L1
-0.019562418	0.909949899	0.918	1	9476	NAPSA

voxel-wise, subcortex			
Spearman's corr coef	p-val	GenID	Gene
0.017492702	0.115683816	26750	RPS6KC1
0.017487872	0.115784451	50804	MYEF2
0.017441161	0.116760969	8518	IKBKAP
0.017361669	0.118437618	57393	TMEM27
0.017357768	0.118520372	91949	COG7
0.017345788	0.118774801	51163	DBR1
0.017331116	0.11908704	51265	CDKL3
0.017280782	0.120163001	23101	MCF2L2
0.017204538	0.121807307	55503	TRPV6
0.017186059	0.122208461	27333	GOLIM4
0.017160427	0.122766629	85478	CCDC65
0.017156526	0.122851714	23770	FKBP8
0.017121052	0.123627901	5438	POLR2I
0.017104615	0.123988844	340260	UNCX
0.017081955	0.12448775	9209	LRRFIP2
0.017067747	0.12480142	283726	FAM154B
0.01698621	0.12661323	253738	EBF3
0.016944699	0.127543435	79144	PPDPF
0.016940614	0.12763527	1748	DLX4
0.016916746	0.128172845	10576	CCT2
0.016877186	0.129067734	4946	OAZ1
0.016876722	0.129078254	661	POLR3D
0.016872915	0.129164636	283953	TMEM114
0.016829638	0.130149707	83549	UCK1
0.016742809	0.132143676	729317	LOC729317
0.016736308	0.13229391	337963	KRTAP23-1
0.016731201	0.132412031	79739	TTLL7
0.016703341	0.133057833	57659	ZBTB4
0.016627004	0.134839758	27347	STK39
0.016576115	0.136037871	121130	OR10P1
0.016567757	0.136235446	25758	C11orf41
0.016563484	0.13633655	55353	LAPTM4B
0.01652188	0.137323812	23089	PEG10
0.016477583	0.138381109	26267	FBXO10
0.016436815	0.139359608	90527	DUOXA1
0.01635333	0.141380057	780776	FAM18A
0.016314046	0.142338574	151188	ARL6IP6
0.016282657	0.14310801	79998	ANKRD53
0.016255263	0.143782139	51016	FAM158A
0.016255263	0.143782139	121256	TMEM132D
0.016224617	0.144539148	23336	SYNM
0.016223503	0.1445667	81442	OR6N2
0.016204372	0.145040914	8887	TAX1BP1
0.016057551	0.148719877	149685	ADIG
0.016054951	0.148785651	5825	ABCD3
0.016038699	0.14919737	731220	AC099797.1
0.016012697	0.149857923	55286	C4orf19
0.015942212	0.151659608	25904	CNOT10
0.015934411	0.151860014	580	BARD1
0.015857518	0.153846294	5625	PRODH
0.015841359	0.154266179	84904	C9orf100
0.015821207	0.154791102	147081	AC126544.1
0.015815914	0.154929176	401551	WDR38
0.015806349	0.155178934	1112	FOXN3
0.015758244	0.156439781	7323	UBE2D3
0.015725184	0.157310784	219771	CCNY
0.015696859	0.15805997	22796	COG2
0.015676707	0.158594623	23245	ASTN2
0.015665656	0.158888429	390010	NKX1-2
0.015641976	0.159519255	9941	EXOG
0.015603436	0.160550073	24150	TP53TG3
0.015523015	0.162717298	605	BCL7A
0.015518836	0.162830502	1674	DES
0.015401174	0.166042596	152078	C3orf55
0.015369878	0.166904971	79587	CARS2
0.015367093	0.166981846	4736	RPL10A
0.015355855	0.167292401	751071	METTL12
0.015311837	0.168513104	51564	HDAC7
0.015259832	0.169963881	23112	TNRC6B
0.015253982	0.17012766	53938	PPIL3
0.01525166	0.170192733	55785	FGD6
0.015250453	0.170226544	51399	TRAPPC4
0.015235965	0.170632854	8602	NOP14
0.01518721	0.172005475	8564	KMO
0.01518684	0.17201592	259296	TAS2R50
0.01516678	0.172583103	51481	VCX3A
0.015105767	0.174316764	84077	C3orf20
0.015017823	0.176838547	440905	AC018865.8
0.014932665	0.179306209	400013	TCP1P3

region-wise, subcortex					
Spearman's corr coef	p-val	p-val, SA	p-val, SA (BH- correction)	GenID	Gene
-0.019562418	0.909949899	0.924	1	10607	TBL3
-0.019562418	0.909949899	0.914	1	23217	ZFR2
-0.019562418	0.909949899	0.927	1	29802	VPREB3
-0.019562418	0.909949899	0.921	1	57555	NLGN2
-0.019562418	0.909949899	0.924	1	92737	DNER
-0.019562418	0.909949899	0.923	1	150372	NFAM1
-0.019562418	0.909949899	0.928	1	390151	OR8H2
-0.019562418	0.909949899	0.931	1	100131733	USP30-AS1
-0.01981982	0.908762395	0.908	1	5606	MAP2K3
-0.01981982	0.908762395	0.92	1	6518	SLC2A5
-0.01981982	0.908762395	0.905	1	9149	DYRK1B
-0.01981982	0.908762395	0.907	1	10926	DBF4
-0.01981982	0.908762395	0.912	1	22877	MLXIP
-0.01981982	0.908762395	0.92	1	79830	ZMYM1
-0.01981982	0.908762395	0.909	1	81876	RAB1B
-0.01981982	0.908762395	0.928	1	199699	DAND5
-0.01981982	0.908762395	0.913	1	282617	IL28B
-0.01981982	0.908762395	0.909	1	389813	AL355987.1
-0.020077219	0.907574952	0.909	1	6547	SLC8A3
-0.020077219	0.907574952	0.918	1	10637	LEFTY1
-0.020077219	0.907574952	0.899	1	10948	STARD3
-0.020077219	0.907574952	0.917	1	259282	BOD1L
-0.020077219	0.907574952	0.914	1	282809	WDR51B
-0.020077219	0.907574952	0.937	1	440193	CCDC88C
-0.02033462	0.906387269	0.919	1	4045	LSAMP
-0.02033462	0.906387269	0.904	1	5101	PCDH9
-0.02033462	0.906387269	0.926	1	8844	KSR1
-0.02033462	0.906387269	0.917	1	22849	CPEB3
-0.02033462	0.906387269	0.921	1	43847	KLK14
-0.02033462	0.906387269	0.935	1	55972	SLC25A40
-0.02033462	0.906387269	0.921	1	129531	MITD1
-0.02033462	0.906387269	0.904	1	135941	OR2A14
-0.02033462	0.906387269	0.923	1	497661	C18orf32
-0.020592021	0.905200243	0.895	1	2741	GLRA1
-0.020592021	0.905200243	0.922	1	27306	PGDS
-0.020849422	0.904013515	0.924	1	5923	RASGRF1
-0.020849422	0.904013515	0.921	1	94086	HSPB9
-0.020849422	0.904013515	0.902	1	284800	C20orf91
-0.021106821	0.902826905	0.911	1	3972	LHB
-0.021106821	0.902826905	0.913	1	11107	PRDM5
-0.021106821	0.902826905	0.924	1	23357	ANGEL1
-0.021106821	0.902826905	0.912	1	54718	BTN2A3
-0.021106821	0.902826905	0.902	1	89801	PPP1R3F
-0.021106821	0.902826905	0.911	1	112616	CMTM7
-0.021364219	0.901640594	0.912	1	5539	PPY
-0.021364219	0.901640594	0.906	1	9143	SYNGR3
-0.021364219	0.901640594	0.917	1	11244	ZHX1
-0.021364219	0.901640594	0.916	1	23590	PDSS1
-0.021364219	0.901640594	0.901	1	57035	C1orf63
-0.021364219	0.901640594	0.906	1	643376	AP001931.1
-0.021621622	0.900453925	0.916	1	2248	FGF3
-0.021621622	0.900453925	0.902	1	3704	ITPA
-0.021621622	0.900453925	0.898	1	23304	UBR2
-0.021621622	0.900453925	0.914	1	113179	ADAT3
-0.021621622	0.900453925	0.904	1	121340	SP7
-0.021621622	0.900453925	0.906	1	203069	R3HCC1
-0.021621622	0.900453925	0.902	1	441818	WBP11P1
-0.021621622	0.900453925	0.909	1	442075	AC034193.5
-0.021621622	0.900453925	0.873	1	728666	PRELID1P1
-0.021879021	0.899267972	0.902	1	6192	RPS4Y1
-0.021879021	0.899267972	0.921	1	10445	MCRS1
-0.021879021	0.899267972	0.896	1	25828	TXN2
-0.021879021	0.899267972	0.92	1	25862	USP49
-0.021879021	0.899267972	0.918	1	27102	EIF2AK1
-0.021879021	0.899267972	0.907	1	317754	POTED
-0.022136422	0.898082256	0.894	1	900	CCNG1
-0.022136422	0.898082256	0.917	1	3227	HOXC11
-0.022136422	0.898082256	0.905	1	4734	NEDD4
-0.022136422	0.898082256	0.913	1	6239	RREB1
-0.022136422	0.898082256	0.896	1	23194	FBXL7
-0.022136422	0.898082256	0.901	1	64319	FBRS
-0.022136422	0.898082256	0.906	1	161145	C14orf83
-0.022136422	0.898082256	0.906	1	196463	PLBD2
-0.022136422	0.898082256	0.904	1	203245	NAIF1
-0.022393823	0.89689672	0.904	1	777	CACNA1E
-0.022393823	0.89689672	0.9	1	6992	PPP1R11
-0.022393823	0.89689672	0.907	1	10107	TRIM10
-0.022393823	0.89689672	0.905	1	80256	KIAA1539
-0.022393823	0.89689672	0.89	1	116379	IL22RA2

voxel-wise, subcortex			
Spearman's corr coef	p-val	GenID	Gene
0.01486088	0.181406185	202151	RANBP3L
0.014800517	0.183186069	84891	ZSCAN10
0.014793366	0.18339777	137492	VPS37A
0.014765785	0.184216022	5875	RABGGTA
0.014716473	0.185685724	64645	HIAT1
0.014708951	0.185910702	55222	LRRC20
0.01470245	0.186105281	6730	SRP68
0.01467942	0.186795801	22919	MAPRE1
0.014659082	0.187407166	284802	FRG1B
0.014608656	0.188929319	81488	GRINL1A
0.014545135	0.19085972	9092	SART1
0.014509382	0.191952631	23095	KIF1B
0.014502789	0.192154661	30011	SH3KBP1
0.014437689	0.194157988	8892	EIF2B2
0.014434811	0.194246963	5212	VIT
0.014408065	0.195074692	643332	AL355922.1
0.014382434	0.195870399	9419	CRIPT
0.014312877	0.198041677	129446	XIRP2
0.014302198	0.198376611	83694	RPS6KL1
0.014276845	0.199173316	56704	JPH1
0.014205338	0.201433167	3184	HNRNPD
0.014125288	0.20398508	29928	TIMM22
0.014105415	0.204622254	92129	RIPPLY1
0.014103093	0.204696819	151835	CPNE9
0.014061489	0.206035748	26471	NUPR1
0.014058517	0.206131622	23625	FAM89B
0.014052667	0.20632048	645996	NAP1L6
0.014039852	0.206734583	440278	CATSPER2P1
0.013980696	0.208654061	27089	UQCRQ
0.013966209	0.209126055	115908	CTHRC1
0.013962494	0.209247202	8607	RUVBL1
0.013921262	0.210595474	84332	DYDC2
0.013917733	0.210711122	26683	OR4F3
0.013880401	0.211937815	79134	TMEM185B
0.0138752	0.212109044	57107	PDSS2
0.013855699	0.212752298	55143	CDCA8
0.013746395	0.216383293	10071	MUC12
0.013734694	0.216774598	23381	SMG5
0.013718628	0.217312753	10647	SCGB1D2
0.013625948	0.220435932	8794	TNFRSF10C
0.01361954	0.220653042	79794	C12orf49
0.013592609	0.221567228	2827	GPR3
0.013541626	0.223305255	64106	NPFFR1
0.013521845	0.2239822	5631	PRPS1
0.013508008	0.224456653	494470	RNF165
0.01336778	0.229305059	729359	KIAA1881
0.013327848	0.230699241	83478	ARHGAP24
0.013301474	0.231623396	3163	HMOX2
0.013285779	0.232174546	6120	RPE
0.013249841	0.233440146	26149	ZNF658
0.013232196	0.234063357	10254	STAM2
0.013212973	0.23474355	51528	JKAMP
0.013134966	0.237518385	64429	ZDHHC6
0.013077945	0.23956123	1524	CX3CR1
0.013056122	0.240346387	64145	ZFYVE20
0.013037549	0.241016045	6018	RLF
0.012990559	0.242716104	79987	SVEP1
0.012944312	0.244397432	55064	C9orf68
0.012816343	0.249092445	121551	BTBD11
0.012785882	0.250219285	64174	DPEP2
0.012664042	0.254761875	501	ALDH7A1
0.012613988	0.256644636	146	ADRA1D
0.012594114	0.257394791	9491	PSMF1
0.012574426	0.258139431	5589	PRKCSH
0.012563375	0.258558124	344657	LRRIQ4
0.012545545	0.259234518	57117	INTS12
0.012531336	0.259774446	63894	C14orf133
0.01243392	0.263497353	83460	TMEM93
0.012417668	0.264121979	10438	C1D
0.012416647	0.264161289	554226	NCRNA00164
0.012398631	0.264854968	219623	TMEM26
0.012395381	0.264980257	80011	C16orf94
0.01236975	0.265969634	10109	ARPC2
0.012360278	0.266335994	10114	HIPK3
0.012317837	0.26798138	282974	STK32C
0.012232865	0.271296769	90624	LYRM7
0.012217357	0.271904916	8751	ADAM15
0.012190518	0.272959441	6624	FSCN1
0.012183925	0.27321896	5352	PLOD2

region-wise, subcortex					
Spearman's corr coef	p-val	p-val, SA	p-val, SA (BH- correction)	GenID	Gene
-0.022651222	0.895710945	0.908	1	5457	POU4F1
-0.022651222	0.895710945	0.898	1	11316	COPE
-0.022651222	0.895710945	0.902	1	79094	CHAC1
-0.022651222	0.895710945	0.894	1	80210	ARMC9
-0.022651222	0.895710945	0.914	1	124460	SNX20
-0.022651222	0.895710945	0.885	1	284521	OR2L13
-0.022908622	0.894525886	0.909	1	957	ENTPD5
-0.022908622	0.894525886	0.901	1	9464	HAND2
-0.022908622	0.894525886	0.899	1	89866	SEC16B
-0.022908622	0.894525886	0.915	1	100272217	LOC100272217
-0.023166021	0.893341064	0.904	1	9700	ESPL1
-0.023166021	0.893341064	0.898	1	10330	CNPY2
-0.023166021	0.893341064	0.897	1	55958	KLHL9
-0.023166021	0.893341064	0.903	1	643418	LIPN
-0.023423424	0.892156482	0.897	1	23071	ERP44
-0.023423424	0.892156482	0.898	1	27030	MLH3
-0.023423424	0.892156482	0.896	1	51084	CRYL1
-0.023423424	0.892156482	0.902	1	51727	CMPK1
-0.023423424	0.892156482	0.905	1	64773	FAM113A
-0.023423424	0.892156482	0.905	1	148266	ZNF569
-0.023423424	0.892156482	0.909	1	163049	ZNF791
-0.023423424	0.892156482	0.897	1	284114	TMEM102
-0.023423424	0.892156482	0.89	1	677769	SCARNA17
-0.023680823	0.890971541	0.896	1	727	C5
-0.023680823	0.890971541	0.896	1	5701	PSMC2
-0.023680823	0.890971541	0.879	1	9421	HAND1
-0.023680823	0.890971541	0.899	1	65124	ANKRD57
-0.023680823	0.890971541	0.919	1	83540	NUF2
-0.023680823	0.890971541	0.901	1	92293	TMEM132C
-0.023680823	0.890971541	0.899	1	283643	C14orf80
-0.023938224	0.889787495	0.897	1	3017	HIST1H2BD
-0.023938224	0.889787495	0.917	1	28998	MRPL13
-0.023938224	0.889787495	0.886	1	284371	ZNF841
-0.023938224	0.889787495	0.887	1	100038246	TLX1NB
-0.023938224	0.889787495	0.896	1	100130958	AC009139.1
-0.023938224	0.889787495	0.894	1	100132733	ANKRD20A13P
-0.024195625	0.888603628	0.911	1	1454	CSNK1E
-0.024195625	0.888603628	0.905	1	6236	RRAD
-0.024453023	0.887419939	0.887	1	686	BTD
-0.024453023	0.887419939	0.891	1	29107	NXT1
-0.024453023	0.887419939	0.895	1	51406	NOL7
-0.024453023	0.887419939	0.9	1	113130	CDCA5
-0.024710424	0.886236548	0.904	1	5819	PVRL2
-0.024710424	0.886236548	0.894	1	58500	ZNF250
-0.024710424	0.886236548	0.882	1	81558	FAM117A
-0.024710424	0.886236548	0.897	1	652904	CKS1BP6
-0.024967825	0.88505286	0.891	1	51127	TRIM17
-0.024967825	0.88505286	0.909	1	55721	IQCC
-0.024967825	0.88505286	0.902	1	93650	ACPT
-0.024967825	0.88505286	0.905	1	151325	MYADML
-0.025225226	0.883869946	0.892	1	4849	CNOT3
-0.025225226	0.883869946	0.9	1	6978	TRGV5
-0.025225226	0.883869946	0.904	1	8665	EIF3F
-0.025225226	0.883869946	0.902	1	9152	SLC6A5
-0.025225226	0.883869946	0.878	1	25831	HECTD1
-0.025225226	0.883869946	0.881	1	50846	DHH
-0.025225226	0.883869946	0.882	1	54344	DPM3
-0.025225226	0.883869946	0.891	1	56971	CEACAM19
-0.025225226	0.883869946	0.899	1	64753	CCDC136
-0.025225226	0.883869946	0.896	1	83884	SLC25A2
-0.025225226	0.883869946	0.896	1	387856	C12orf68
-0.025482625	0.88268733	0.872	1	229	ALDOB
-0.025482625	0.88268733	0.883	1	2889	RAPGEF1
-0.025482625	0.88268733	0.889	1	4899	NRF1
-0.025482625	0.88268733	0.895	1	9726	ZNF646
-0.025482625	0.88268733	0.896	1	27327	TNRC6A
-0.025482625	0.88268733	0.889	1	54856	GON4L
-0.025482625	0.88268733	0.882	1	57130	ATP13A1
-0.025482625	0.88268733	0.877	1	79102	RNF26
-0.025482625	0.88268733	0.889	1	140290	TCP10L
-0.025740024	0.881504893	0.918	1	4131	MAP1B
-0.025740024	0.881504893	0.898	1	51363	CHST15
-0.025740024	0.881504893	0.884	1	123876	ACSM2A
-0.025997426	0.880322218	0.868	1	8174	MADCAM1
-0.025997426	0.880322218	0.886	1	11020	RABL4
-0.025997426	0.880322218	0.9	1	256355	RPS2P32
-0.025997426	0.880322218	0.892	1	389197	C4orf50
-0.025997426	0.880322218	0.9	1	100240734	LOC100240734
-0.026254825	0.879140317	0.895	1	27291	C10orf28

voxel-wise, subcortex			
Spearman's corr coef	p-val	GenID	Gene
0.012114832	0.275948524	171389	NLRP6
0.012073693	0.277582556	22993	HMGXB3
0.012009894	0.280129611	133482	SLCO6A1
0.011987791	0.281015664	4993	OR2C1
0.011974883	0.281534076	112476	PRRT2
0.011916564	0.283884168	3770	KCNJ14
0.01184385	0.286832899	131831	C3orf44
0.011837813	0.287078589	201158	FAM18B2
0.011836234	0.287142873	155060	RP4-800G7.1
0.011820911	0.287767321	5319	PLA2G1B
0.011795931	0.288787365	344328	ST13P2
0.011781351	0.289383799	80224	NUBPL
0.011748662	0.290724069	9880	ZBTB39
0.011712072	0.292229235	23358	USP24
0.011711515	0.292252183	11099	PTPN21
0.011701671	0.292658061	23633	KPNA6
0.01168802	0.293221503	200014	CC2D1B
0.011653567	0.2946468	340578	DCAF12L2
0.011575281	0.297902673	339559	ZNF642
0.011499874	0.301061302	23041	MON2
0.011477307	0.302010983	158801	NKAPP1
0.011462728	0.302625597	11107	PRDM5
0.011426509	0.304155886	118987	PDZD8
0.011347109	0.307528675	3799	KIF5B
0.011273652	0.310671031	83876	MRO
0.011243378	0.311972231	9897	KIAA0196
0.011241706	0.312044144	7356	SCGB1A1
0.011215147	0.313188821	154810	AMOTL1
0.011117823	0.317406893	388135	C15orf59
0.011116059	0.317483753	55311	ZNF444
0.01111578	0.317495853	2645	GCK
0.011101757	0.3181068	222194	RSBN1L
0.011018735	0.321739554	55308	DDX19A
0.010961623	0.324254215	11062	DUS4L
0.01091909	0.326135248	9512	PMPCB
0.010878229	0.327949077	84833	USMG5
0.010869221	0.328349799	286528	SSU72P1
0.010828453	0.330167323	673	BRAF
0.010788149	0.331970632	6628	SNRPB
0.010753231	0.333538085	4495	MT1G
0.010709213	0.335520774	57691	KIAA1586
0.010656651	0.337898284	55070	DET1
0.010638542	0.338719904	6507	SLC1A3
0.010629441	0.339133263	56006	C19orf61
0.01062229	0.339458346	1163	CKS1B
0.010562113	0.342201591	7472	WNT2
0.010557285	0.342422307	55114	ARHGAP17
0.010530353	0.343655139	80852	GRIP2
0.010512709	0.344464332	64149	C17orf75
0.010503329	0.344895005	23114	NFASC
0.010477048	0.346103489	7270	TTF1
0.010466276	0.346599638	116983	ACAP3
0.010449653	0.347366124	283349	RASSF3
0.01042755	0.348386943	6667	SP1
0.010417799	0.348837912	644157	DND1P1
0.010337285	0.352575719	85377	MICALL1
0.010318247	0.353463233	89958	C9orf140
0.010298374	0.354391158	113251	LARP4
0.010274972	0.355485857	11314	CD300A
0.010072524	0.365044683	147872	CCDC155
0.010064538	0.36542505	30	ACAA1
0.009996003	0.368699193	10922	FASTK
0.009976686	0.369625241	2670	GFAP
0.009928118	0.371960193	50613	UBQLN3
0.009898215	0.373402327	9692	KIAA0391
0.009803212	0.378006846	140290	TCP10L
0.009796712	0.378323138	64816	CYP3A43
0.009754272	0.380392402	23057	NMNAT2
0.009733749	0.381395549	29086	C19orf62
0.009684437	0.383812487	5298	PI4KB
0.009656763	0.385172993	5264	PHYH
0.009646826	0.385662228	64098	PARVG
0.009634382	0.38627544	10683	DLL3
0.009591663	0.388385028	120071	GYLTL1B
0.009578941	0.389014632	57567	ZNF319
0.009546902	0.390602976	5460	POU5F1
0.009533715	0.391257793	284111	SLC13A5
0.0095261	0.391636282	7503	XIST
0.009496476	0.393110782	54732	TMED9

region-wise, subcortex					
Spearman's corr coef	p-val	p-val, SA	p-val, SA (BH- correction)	GenID	Gene
-0.026254825	0.879140317	0.879	1	342574	KRT27
-0.026512226	0.877958715	0.87	1	794	CALB2
-0.026512226	0.877958715	0.894	1	4854	NOTCH3
-0.026512226	0.877958715	0.894	1	6472	SHMT2
-0.026512226	0.877958715	0.873	1	28977	MRPL42
-0.026512226	0.877958715	0.896	1	51362	CDC40
-0.026512226	0.877958715	0.88	1	79085	SLC25A23
-0.026512226	0.877958715	0.894	1	92579	G6PC3
-0.026512226	0.877958715	0.888	1	256714	MAP7D2
-0.026769627	0.876777291	0.882	1	89	ACTN3
-0.026769627	0.876777291	0.884	1	240	ALOX5
-0.026769627	0.876777291	0.877	1	3178	HNRNPA1P2
-0.026769627	0.876777291	0.886	1	6329	SCN4A
-0.026769627	0.876777291	0.875	1	9138	ARHGEF1
-0.026769627	0.876777291	0.89	1	29074	MRPL18
-0.026769627	0.876777291	0.884	1	54987	C1orf123
-0.027027028	0.875595689	0.889	1	6122	RPL3
-0.027027028	0.875595689	0.859	1	6339	SCNN1D
-0.027027028	0.875595689	0.884	1	10493	VAT1
-0.027027028	0.875595689	0.867	1	27244	SESN1
-0.027027028	0.875595689	0.896	1	51143	DYNC1LI1
-0.027027028	0.875595689	0.874	1	100134713	RP4-726N20.2
-0.027284427	0.874414861	0.875	1	10128	LRPPRC
-0.027284427	0.874414861	0.872	1	23576	DDAH1
-0.027284427	0.874414861	0.883	1	25790	CCDC19
-0.027284427	0.874414861	0.874	1	25898	RCHY1
-0.027284427	0.874414861	0.891	1	51337	C8orf55
-0.027284427	0.874414861	0.875	1	80705	TSGA10
-0.027284427	0.874414861	0.875	1	162515	SLC16A11
-0.027284427	0.874414861	0.886	1	100125288	AC011511.1
-0.027541827	0.873234272	0.872	1	750	C16orf3
-0.027541827	0.873234272	0.881	1	1604	CD55
-0.027541827	0.873234272	0.883	1	9104	RGN
-0.027541827	0.873234272	0.875	1	51244	C3orf19
-0.027541827	0.873234272	0.885	1	65062	ALS2CR4
-0.027541827	0.873234272	0.861	1	84188	FAR1
-0.027541827	0.873234272	0.895	1	157769	FAM91A1
-0.027541827	0.873234272	0.884	1	286101	ZNF252P
-0.027799228	0.872053981	0.884	1	140	ADORA3
-0.027799228	0.872053981	0.871	1	4316	MMP7
-0.027799228	0.872053981	0.876	1	4494	MT1F
-0.027799228	0.872053981	0.895	1	8140	SLC7A5
-0.027799228	0.872053981	0.856	1	55502	HES6
-0.027799228	0.872053981	0.88	1	57711	ZNF529
-0.027799228	0.872053981	0.887	1	91978	C19orf20
-0.027799228	0.872053981	0.882	1	139604	MAGEB16
-0.027799228	0.872053981	0.876	1	286411	RP1-177G6.1
-0.028056627	0.870873988	0.881	1	7079	TIMP4
-0.028314028	0.869693696	0.896	1	5032	P2RY11
-0.028314028	0.869693696	0.881	1	8357	HIST1H3H
-0.028314028	0.869693696	0.882	1	22984	PDCD11
-0.028314028	0.869693696	0.881	1	79759	ZNF668
-0.028314028	0.869693696	0.881	1	79831	JMJD5
-0.028571429	0.86851424	0.891	1	1673	DEFB4
-0.028571429	0.86851424	0.878	1	6768	ST14
-0.028571429	0.86851424	0.87	1	23334	KIAA0467
-0.028571429	0.86851424	0.898	1	51673	TPPP3
-0.028571429	0.86851424	0.886	1	81858	SHARPIN
-0.028571429	0.86851424	0.878	1	140947	C5ORF20
-0.028571429	0.86851424	0.888	1	400618	AC005152.2
-0.028571429	0.86851424	0.898	1	653657	AC010606.4
-0.028828828	0.867335081	0.88	1	5427	POLE2
-0.028828828	0.867335081	0.881	1	7780	SLC30A2
-0.028828828	0.867335081	0.868	1	10939	AFG3L2
-0.028828828	0.867335081	0.879	1	55258	THNSL2
-0.028828828	0.867335081	0.88	1	80755	AARSD1
-0.028828828	0.867335081	0.887	1	125931	CEACAM20
-0.028828828	0.867335081	0.893	1	169270	ZNF596
-0.029086228	0.866156161	0.891	1	678	ZFP36L2
-0.029086228	0.866156161	0.867	1	6003	RGS13
-0.029086228	0.866156161	0.845	1	8224	SYN3
-0.029086228	0.866156161	0.885	1	170589	GPHA2
-0.029343629	0.864977062	0.866	1	3268	AGFG2
-0.029343629	0.864977062	0.85	1	55891	LENEP
-0.029343629	0.864977062	0.883	1	386683	KRTAP12-3
-0.029343629	0.864977062	0.874	1	441864	TARM1
-0.02960103	0.863798738	0.871	1	10512	SEMA3C
-0.02960103	0.863798738	0.853	1	23589	CARHSP1
-0.02960103	0.863798738	0.872	1	64220	STRA6

voxel-wise, subcortex			
Spearman's corr coef	p-val	GenID	Gene
0.009490997	0.39338389	349565	NMNAT3
0.009483474	0.393758953	8568	RRP1
0.009470938	0.394384563	1315	COPB1
0.009415219	0.397172421	5333	PLCD1
0.009363399	0.399775714	5064	PALM
0.009346776	0.40061304	11169	WDHD1
0.009313622	0.402286083	8013	NR4A3
0.00929765	0.403093636	125981	ACER1
0.009234316	0.406305134	79695	GALNT12
0.00915445	0.410376698	677769	SCARNA17
0.009123155	0.411978722	6621	SNAPC4
0.009048211	0.415830106	10785	WDR4
0.009041432	0.416179568	26133	TRPC4AP
0.009032889	0.416620135	2110	ETFDH
0.008990264	0.418822646	135293	PM20D2
0.008876967	0.424709916	353219	KAAG1
0.008873995	0.424864948	3998	LMAN1
0.008845486	0.426354349	2153	F5
0.008759584	0.430860251	5744	PTHLH
0.00868362	0.434867799	9915	ARNT2
0.008637372	0.437318116	375719	AQP7P1
0.008612763	0.438625216	54553	DKFZP434I0714
0.008572738	0.440755934	401720	FIGNL2
0.008490366	0.445159525	6747	SSR3
0.008477272	0.445861787	56147	PCDHA1
0.008469471	0.446280509	26637	OR7E36P
0.008442911	0.447707772	1795	DOCK3
0.008425638	0.448637396	8431	NR0B2
0.008383012	0.450936049	2158	F9
0.008374376	0.451402634	341276	OR10A2
0.008313177	0.454716504	6039	RNASE6
0.008260523	0.45757857	123016	TTC8
0.008237399	0.458838731	2897	GRIK1
0.008234149	0.459016025	342933	ZSCAN5B
0.008231362	0.459167987	8456	FOXN1
0.008178615	0.462050885	9846	GAB2
0.008163942	0.462854624	199990	C1orf86
0.008156513	0.463261843	6847	SYCP1
0.008117694	0.465392977	59286	UBL5
0.00809262	0.466772437	84054	PCDHB19P
0.00808742	0.467058837	6830	SUPT6H
0.007990561	0.47241056	22795	NID2
0.007967809	0.473672599	91133	L3MBTL4
0.007881536	0.478474736	5096	PCCB
0.007878286	0.478656143	729956	AC135592.2
0.007787463	0.483741224	10420	TESK2
0.007724036	0.487309784	148898	C1ORF213
0.007533382	0.498121291	9789	SPCS2
0.007524931	0.498603463	3104	ZBTB48
0.007493821	0.500380516	27297	CRCP
0.007480912	0.501118958	731157	LOC731157
0.007440702	0.503422678	6799	SULT1A2
0.007429558	0.504062116	6604	SMARCD3
0.007429186	0.504083455	26534	OR10G2
0.007380246	0.506896794	54815	GATAD2A
0.007369566	0.507511795	285282	RABL3
0.007189035	0.517967165	55233	MOB1A
0.007170276	0.519059956	79927	GRRP1
0.007114463	0.522318244	5434	POLR2E
0.007106198	0.522801638	5034	P4HB
0.007081496	0.524247766	162282	ANKFN1
0.007038034	0.526797056	9873	FCHSD2
0.006997359	0.529188633	7574	ZNF26
0.006995316	0.529308975	56269	IRGC
0.006955013	0.531684339	3778	KCNMA1
0.006919816	0.53376323	22880	MORC2
0.006800855	0.540819824	387032	ZKSCAN4
0.006785254	0.541748762	91351	DDX60L
0.006760087	0.543248773	644762	LOC644762
0.006737056	0.544623375	1153	CIRBP
0.006573148	0.554455757	916	CD3E
0.00656934	0.554685235	5126	PCSK2
0.006516406	0.557879627	6693	SPN
0.006509535	0.558294952	140258	KRTAP13-1
0.006506377	0.558485925	126917	IFFO2
0.006481396	0.55999732	9903	KLHL21
0.006465237	0.560976088	7020	TFAP2A
0.006463566	0.561077356	3939	LDHA
0.006456694	0.561493874	27023	FOXB1

region-wise, subcortex					
Spearman's corr coef	p-val	p-val, SA	p-val, SA (BH- correction)	GenID	Gene
-0.02960103	0.863798738	0.865	1	89886	SLAMF9
-0.029858429	0.862620711	0.87	1	6626	SNRPA
-0.029858429	0.862620711	0.886	1	9894	TELO2
-0.029858429	0.862620711	0.868	1	26167	PCDHB5
-0.029858429	0.862620711	0.866	1	55244	SLC47A1
-0.029858429	0.862620711	0.874	1	81569	ACTL8
-0.029858429	0.862620711	0.855	1	89839	ARHGAP11B
-0.029858429	0.862620711	0.878	1	402117	VWC2L
-0.030115828	0.861442983	0.884	1	984	CDC2L1
-0.030115828	0.861442983	0.861	1	3762	KCNJ5
-0.030115828	0.861442983	0.861	1	7358	UGDH
-0.030115828	0.861442983	0.893	1	255057	C19orf26
-0.030373231	0.860265017	0.874	1	1794	DOCK2
-0.030373231	0.860265017	0.871	1	4063	LY9
-0.030373231	0.860265017	0.866	1	22944	KIN
-0.030373231	0.860265017	0.87	1	51352	WT1-AS
-0.030373231	0.860265017	0.888	1	92086	GGTLA3
-0.03063063	0.859087884	0.864	1	2850	GPR27
-0.03063063	0.859087884	0.871	1	54813	KLHL28
-0.03063063	0.859087884	0.864	1	147372	CCBE1
-0.03088803	0.85791105	0.871	1	3228	HOXC12
-0.03088803	0.85791105	0.857	1	23036	ZNF292
-0.03088803	0.85791105	0.87	1	84256	FLYWCH1
-0.03088803	0.85791105	0.875	1	124056	NOXO1
-0.03088803	0.85791105	0.876	1	392335	MYO5BP2
-0.03088803	0.85791105	0.871	1	654502	IQCJ
-0.031145431	0.856734633	0.864	1	11100	HNRNPUL1
-0.031145431	0.856734633	0.863	1	55830	GLT8D1
-0.031145431	0.856734633	0.868	1	124221	TMPRSS8
-0.031145431	0.856734633	0.875	1	147837	ZNF563
-0.031145431	0.856734633	0.864	1	390980	ZNF805
-0.03140283	0.855558395	0.877	1	10865	ARID5A
-0.03140283	0.855558395	0.875	1	57569	ARHGAP20
-0.031660229	0.854381919	0.86	1	4481	MSR1
-0.031660229	0.854381919	0.845	1	10057	ABCC5
-0.031660229	0.854381919	0.874	1	147172	LRRC37BP1
-0.031660229	0.854381919	0.849	1	285097	FLJ38379
-0.031660229	0.854381919	0.874	1	100131532	LOC100131532
-0.031917632	0.853206336	0.854	1	4522	MTHFD1
-0.031917632	0.853206336	0.874	1	5051	PAFAH2
-0.031917632	0.853206336	0.877	1	79664	NARG2
-0.031917632	0.853206336	0.873	1	81894	SLC25A28
-0.031917632	0.853206336	0.87	1	84167	C19orf44
-0.031917632	0.853206336	0.879	1	85007	AGXT2L2
-0.032175031	0.852031112	0.849	1	6464	SHC1
-0.032175031	0.852031112	0.85	1	7305	TYROBP
-0.032175031	0.852031112	0.848	1	8386	OR1D5
-0.032175031	0.852031112	0.858	1	127707	KLHDC7A
-0.032175031	0.852031112	0.857	1	285282	RABL3
-0.032175031	0.852031112	0.875	1	337974	KRTAP19-7
-0.032175031	0.852031112	0.869	1	375791	C9orf169
-0.032432433	0.850856185	0.871	1	2332	FMR1
-0.032432433	0.850856185	0.848	1	5073	PARN
-0.032432433	0.850856185	0.873	1	6348	CCL3
-0.032432433	0.850856185	0.865	1	7564	ZNF16
-0.032432433	0.850856185	0.856	1	53335	BCL11A
-0.032432433	0.850856185	0.853	1	55179	FAIM
-0.032432433	0.850856185	0.844	1	79807	GSTCD
-0.032432433	0.850856185	0.861	1	84458	LCOR
-0.032432433	0.850856185	0.867	1	148252	DIRAS1
-0.032432433	0.850856185	0.873	1	220929	ZNF438
-0.032432433	0.850856185	0.851	1	644604	EEF1A1P12
-0.032689832	0.84968096	0.866	1	9783	RIMS3
-0.032689832	0.84968096	0.851	1	152015	ROPN1B
-0.032947235	0.848506689	0.859	1	3110	MNX1
-0.032947235	0.848506689	0.849	1	7062	TCHH
-0.032947235	0.848506689	0.829	1	11102	RPP14
-0.032947235	0.848506689	0.859	1	27123	DKK2
-0.032947235	0.848506689	0.873	1	89958	C9orf140
-0.032947235	0.848506689	0.859	1	440461	LOC440461
-0.033204634	0.847332716	0.866	1	5153	PDE1B
-0.033204634	0.847332716	0.875	1	9092	SART1
-0.033204634	0.847332716	0.869	1	23348	DOCK9
-0.033204634	0.847332716	0.858	1	55799	CACNA2D3
-0.033204634	0.847332716	0.874	1	64582	GPR135
-0.033204634	0.847332716	0.865	1	122046	TEX26
-0.033462033	0.846159041	0.883	1	3860	KRT13
-0.033462033	0.846159041	0.847	1	8370	HIST2H4A
-0.033462033	0.846159041	0.873	1	9721	GPRIN2

voxel-wise, subcortex			
Spearman's corr coef	p-val	GenID	Gene
0.006448614	0.561983764	150142	ZNF295-AS1
0.00635742	0.567527592	7775	ZNF232
0.006347483	0.568133235	1794	DOCK2
0.006326124	0.569436073	594855	CPLX3
0.006317209	0.569980323	10458	BAIAP2
0.006219607	0.575954676	51175	TUBE1
0.006216357	0.576154172	11201	POLI
0.006207813	0.576678634	2547	XRCC6
0.006193511	0.577557087	1512	CTSH
0.006156365	0.579841554	553115	PEF1
0.006132777	0.581294417	51602	NOP58
0.006125162	0.581763804	7561	ZNF14
0.006124791	0.581786692	84749	USP30
0.006102317	0.583173096	339352	LOC339352
0.006090709	0.583889782	57158	JPH2
0.00603146	0.587554157	8633	UNC5C
0.006027188	0.587818742	220929	ZNF438
0.00601623	0.588497758	259289	TAS2R43
0.005978898	0.590813816	6198	RPS6KB1
0.005976484	0.590963781	5676	PSG7
0.005967104	0.591546416	54813	KLHL28
0.00596664	0.591575325	8639	AOC3
0.005960603	0.591950357	1654	DDX3X
0.005950017	0.592608511	374395	TMEM179B
0.005927172	0.594029844	54974	THG1L
0.005893554	0.596124232	152503	SH3D19
0.005873309	0.597387075	10892	MALT1
0.00585195	0.598720789	124491	TMEM170A
0.005844242	0.599202454	4069	LYZ
0.005840528	0.599434614	5928	RBBP4
0.005802546	0.601810932	150297	C22orf42
0.005743668	0.605502844	167410	LIX1
0.005684327	0.609234095	374819	LRRC37A3
0.005625172	0.612963796	148109	FAM187B
0.005581339	0.615733862	26175	C14orf109
0.005549764	0.617732763	7171	TPM4
0.00552014	0.619610667	6227	RPS21
0.005495995	0.621143103	8123	PAR5
0.005468228	0.62290746	387763	C11orf96
0.00545857	0.623521626	3652	IPP
0.005428296	0.625448585	373156	GSTK1
0.005415944	0.626235485	90313	TP53I13
0.005369047	0.629227161	55208	DCUN1D2
0.005360689	0.629760981	51232	CRIM1
0.0053202	0.63234973	10481	HOXB13
0.005304505	0.633354425	5816	PVALB
0.005197245	0.640238583	8115	TCL1A
0.005139575	0.643952847	64853	AIDA
0.005113109	0.6456604	54665	RSBN1
0.005051724	0.649627984	63931	MRPS14
0.00488633	0.660367608	2844	GPR21
0.004875186	0.661093771	23005	MAPKBP1
0.004802657	0.665827692	9836	LCMT2
0.004747123	0.66946137	80763	C12orf39
0.004705705	0.67217654	9994	CASP8AP2
0.00462454	0.677509606	11328	FKBP9
0.004536968	0.683281958	89944	GLB1L2
0.004461932	0.688242614	9256	BZRAP1
0.004448466	0.68913424	337876	CHSY3
0.00440937	0.691725612	64787	EPS8L2
0.004379653	0.693697691	105	ADARB2
0.004377331	0.693851829	9730	VPRBP
0.004337677	0.696486771	56135	PCDHAC1
0.004336377	0.696573198	25875	LETMD1
0.00431056	0.698290765	126295	ZNF57
0.004208129	0.705120444	6351	CCL4
0.004189742	0.706348956	4987	OPRL1
0.004167175	0.707857728	5229	PGGT1B
0.004161325	0.708249092	84861	KLHL22
0.004146002	0.709274471	23054	NCOA6
0.004139408	0.709715843	170371	C10orf128
0.004124086	0.710741937	64900	LPIN3
0.004123528	0.71077925	80723	TMEM22
0.004123157	0.710804164	121793	C13orf16
0.004119721	0.711034298	284467	FAM19A3
0.004031777	0.716934443	400798	RP5-990P15.1
0.004028527	0.717152774	55258	THNSL2
0.003921081	0.724385023	57794	SF4
0.003894893	0.726151526	54788	DNAJB12

region-wise, subcortex					
Spearman's corr coef	p-val	p-val, SA	p-val, SA (BH- correction)	GenID	Gene
-0.033462033	0.846159041	0.849	1	54777	C10orf92
-0.033462033	0.846159041	0.861	1	114049	WBSCR22
-0.033462033	0.846159041	0.872	1	140886	PABPC5
-0.033462033	0.846159041	0.865	1	348158	ACSM2
-0.033719432	0.844985723	0.859	1	573	BAG1
-0.033719432	0.844985723	0.849	1	4924	NUCB1
-0.033719432	0.844985723	0.872	1	9182	RASSF9
-0.033719432	0.844985723	0.855	1	57721	METTL14
-0.033719432	0.844985723	0.853	1	340286	FAM183B
-0.033976831	0.843812227	0.857	1	1394	CRHR1
-0.033976831	0.843812227	0.841	1	27122	DKK3
-0.033976831	0.843812227	0.861	1	64847	SPATA20
-0.033976831	0.843812227	0.859	1	79366	NSBP1
-0.033976831	0.843812227	0.848	1	84948	TIGD5
-0.033976831	0.843812227	0.851	1	119016	AGAP4
-0.033976831	0.843812227	0.851	1	140767	NRSN1
-0.033976831	0.843812227	0.845	1	200959	GABRR3
-0.033976831	0.843812227	0.853	1	729059	RP4-805N21.1
-0.034234237	0.842639565	0.859	1	6884	TAF13
-0.034234237	0.842639565	0.862	1	55841	WWC3
-0.034234237	0.842639565	0.856	1	137797	LYPD2
-0.034234237	0.842639565	0.862	1	158931	TCEAL6
-0.034234237	0.842639565	0.864	1	162083	C16orf82
-0.034234237	0.842639565	0.852	1	100130776	RP11-571M6.6
-0.034491636	0.841467202	0.857	1	5266	PI3
-0.034491636	0.841467202	0.87	1	22876	INPP5F
-0.034491636	0.841467202	0.841	1	25800	SLC39A6
-0.034491636	0.841467202	0.843	1	55296	TBC1D19
-0.034491636	0.841467202	0.837	1	81796	SLCO5A1
-0.034749035	0.840295255	0.838	1	2159	F10
-0.034749035	0.840295255	0.859	1	6629	SNRPB2
-0.034749035	0.840295255	0.862	1	9857	CEP350
-0.034749035	0.840295255	0.872	1	51720	UIMC1
-0.034749035	0.840295255	0.84	1	91750	LIN52
-0.034749035	0.840295255	0.864	1	220963	SLC16A9
-0.034749035	0.840295255	0.846	1	285220	EPHA6
-0.035006434	0.83912307	0.863	1	1850	DUSP8
-0.035006434	0.83912307	0.872	1	6336	SCN10A
-0.035006434	0.83912307	0.841	1	7918	BAT4
-0.035006434	0.83912307	0.826	1	10013	HDAC6
-0.035006434	0.83912307	0.858	1	64718	C16orf28
-0.035006434	0.83912307	0.857	1	79572	ATP13A3
-0.035006434	0.83912307	0.859	1	83715	ESPN
-0.035006434	0.83912307	0.868	1	284232	ANKRD20A9P
-0.035006434	0.83912307	0.831	1	390212	GPR152
-0.035263833	0.837951779	0.85	1	8295	TRRAP
-0.035263833	0.837951779	0.843	1	54467	ANKIB1
-0.035263833	0.837951779	0.841	1	79989	TTC26
-0.035263833	0.837951779	0.845	1	127579	DCST2
-0.035263833	0.837951779	0.853	1	139189	DGKK
-0.035263833	0.837951779	0.84	1	162962	ZNF836
-0.035521235	0.836780846	0.849	1	50	ACO2
-0.035521235	0.836780846	0.865	1	1524	CX3CR1
-0.035521235	0.836780846	0.86	1	2130	EWSR1
-0.035521235	0.836780846	0.874	1	6672	SP100
-0.035521235	0.836780846	0.864	1	29102	RNASEN
-0.035521235	0.836780846	0.844	1	55616	ASAP3
-0.035521235	0.836780846	0.857	1	56344	CABP5
-0.035521235	0.836780846	0.851	1	391192	OR2L3
-0.035778634	0.835610271	0.853	1	334	APLP2
-0.035778634	0.835610271	0.844	1	7156	TOP3A
-0.035778634	0.835610271	0.834	1	23423	TMED3
-0.035778634	0.835610271	0.847	1	57211	GPR126
-0.035778634	0.835610271	0.841	1	64397	ZFP106
-0.035778634	0.835610271	0.853	1	91355	LRP5L
-0.035778634	0.835610271	0.836	1	151174	AC016757.1
-0.035778634	0.835610271	0.843	1	219293	ATAD3C
-0.036036037	0.834439456	0.851	1	4000	LMNA
-0.036036037	0.834439456	0.845	1	7553	ZNF7
-0.036036037	0.834439456	0.844	1	10464	PIBF1
-0.036036037	0.834439456	0.847	1	51157	ZNF580
-0.036036037	0.834439456	0.818	1	79783	C7orf10
-0.036036037	0.834439456	0.841	1	122525	C14orf28
-0.036036037	0.834439456	0.839	1	133522	PPARGC1B
-0.036036037	0.834439456	0.855	1	151742	PPM1L
-0.036293436	0.833269536	0.844	1	10804	GJB6
-0.036293436	0.833269536	0.832	1	83850	ESYT3
-0.036293436	0.833269536	0.865	1	84450	ZNF512
-0.036293436	0.833269536	0.85	1	254272	TBC1D28

voxel-wise, subcortex			
Spearman's corr coef	p-val	GenID	Gene
0.003877434	0.727330029	124044	SPATA2L
0.003863411	0.728277028	84233	TMEM126A
0.003789582	0.733269751	6944	VPS72
0.003759122	0.735332906	25802	LMOD1
0.003747421	0.736126006	92667	C20orf72
0.003641461	0.743320465	113178	SCAMP4
0.003619545	0.744811356	64798	DEPDC6
0.003480524	0.754290521	162333	MARCHF10
0.00346418	0.755407512	388630	LOC388630
0.003414682	0.758793056	256051	ZNF549
0.003402146	0.759651303	90580	C19orf52
0.003286992	0.767548203	7779	SLC30A1
0.003260432	0.769373059	100129094	BTNL10
0.00321047	0.772809327	84314	TMEM107
0.003151407	0.776877165	54537	FAM35B
0.003131441	0.778253734	340393	AF205589.3
0.003126241	0.778612375	401	PHOX2A
0.003059656	0.783208609	10568	SLC34A2
0.003033932	0.784986317	122618	PLD4
0.002998457	0.787439704	181	AGRP
0.00283687	0.798641264	160298	C11orf42
0.002744097	0.805091441	83692	CD99L2
0.002724874	0.806429625	8694	DGAT1
0.002723388	0.806533098	79728	PALB2
0.002720138	0.806759417	728927	RP11-468B6.3
0.002701007	0.808091819	4179	CD46
0.002680855	0.809495986	201625	DNAH12
0.002664047	0.810667694	11096	ADAMTS5
0.002640923	0.812280297	6882	TAF11
0.002609349	0.814483523	23071	ERP44
0.002486487	0.823070407	22895	RPH3A
0.002479708	0.82354486	115286	SLC25A26
0.00243021	0.82701081	90529	C1orf201
0.002423338	0.827492297	7694	ZNF135
0.002341523	0.833229303	4829	NMBR
0.002340316	0.833314061	9662	CEP135
0.002302891	0.835941374	23645	PPP1R15A
0.00223157	0.84095341	9245	GCNT3
0.002229062	0.84112972	83590	TMUB1
0.002205289	0.842801929	84869	CBR4
0.002087999	0.851062119	23229	ARHGEF9
0.002073512	0.852083504	7486	WRN
0.002061625	0.852921784	5224	PGAM2
0.002027822	0.855306506	161823	ADAL
0.002017235	0.85605365	26229	B3GAT3
0.002007113	0.856768131	84335	AKT1S1
0.001992068	0.857830226	161145	C14orf83
0.00198919	0.858033538	254910	LCE5A
0.001866699	0.866691053	5914	RARA
0.001841997	0.868438959	84330	ZNF414
0.001824538	0.869674742	120863	DEPDC4
0.001676881	0.880138576	7070	THY1
0.001627291	0.883657575	5693	PSMB5
0.001586615	0.886545718	51130	ASB3
0.001586523	0.886552334	201134	CCDC46
0.001562099	0.888287246	50999	TMED5
0.001552998	0.888933837	9900	SV2A
0.001517895	0.891428649	868	CBLB
0.001509815	0.892003	7827	NPHS2
0.001495421	0.893026352	50837	TAS2R7
0.001457717	0.895707846	57118	CAMK1D
0.001400512	0.899778605	2773	GNAI3
0.001329377	0.904844224	129880	BBS5
0.001293902	0.907371879	23620	NTSR2
0.001281086	0.90828526	8398	PLA2G6
0.001227967	0.912072361	8548	BLZF1
0.001168254	0.916331947	79133	C20orf7
0.001159803	0.916934967	387733	IFITM5
0.001129715	0.919082463	115196	ZNF554
0.001120242	0.919758618	645121	CCNI2
0.001077803	0.922788799	338675	OR5AP2
0.001051336	0.924679101	5387	PMS2L3
0.000952434	0.931746602	51567	TTRAP
0.000911665	0.934661448	4821	NKX2-2
0.000830222	0.940487087	80757	TMEM121
0.000806727	0.942168295	328	APEX1
0.000777195	0.944281757	10634	GAS2L1
0.000724076	0.948084414	84725	PLEKHA8
0.000702438	0.949633718	474382	H2AFB1

region-wise, subcortex					
Spearman's corr coef	p-val	p-val, SA	p-val, SA (BH- correction)	GenID	Gene
-0.036550835	0.832100034	0.842	1	51	ACOX1
-0.036550835	0.832100034	0.848	1	22905	EPN2
-0.036550835	0.832100034	0.835	1	81872	KRTAP2-3
-0.036550835	0.832100034	0.833	1	150677	OTOS
-0.036550835	0.832100034	0.85	1	441212	RP9P
-0.036808237	0.830930889	0.853	1	741	ZNHIT2
-0.036808237	0.830930889	0.833	1	3309	HSPA5
-0.036808237	0.830930889	0.854	1	10808	HSPH1
-0.036808237	0.830930889	0.839	1	10973	ASCC3
-0.036808237	0.830930889	0.853	1	25782	RAB3GAP2
-0.036808237	0.830930889	0.852	1	153579	BTNL9
-0.037065636	0.829762101	0.853	1	598	BCL2L1
-0.037065636	0.829762101	0.825	1	113146	AHNAK2
-0.037065636	0.829762101	0.831	1	127687	C1orf122
-0.037065636	0.829762101	0.843	1	221443	C6orf130
-0.037065636	0.829762101	0.84	1	441531	PGAM4
-0.037323035	0.828593135	0.859	1	4249	MGAT5
-0.037323035	0.828593135	0.848	1	6715	SRD5A1
-0.037323035	0.828593135	0.847	1	84650	EBPL
-0.037323035	0.828593135	0.854	1	130617	ZFAND2B
-0.037323035	0.828593135	0.854	1	728373	AC116655.15
-0.037580434	0.827425003	0.833	1	6790	AURKA
-0.037580434	0.827425003	0.845	1	64925	CCDC71
-0.037580434	0.827425003	0.835	1	283514	SIAH3
-0.037580434	0.827425003	0.839	1	284358	MAMSTR
-0.037580434	0.827425003	0.837	1	386685	KRTAP10-12
-0.037580434	0.827425003	0.846	1	442229	SLC25A51P1
-0.037837837	0.826257288	0.839	1	2735	GLI1
-0.037837837	0.826257288	0.862	1	6733	SRPK2
-0.037837837	0.826257288	0.83	1	7791	ZYX
-0.03809524	0.825089991	0.847	1	3219	HOXB9
-0.03809524	0.825089991	0.835	1	4255	MGMT
-0.03809524	0.825089991	0.832	1	6000	RGS7
-0.03809524	0.825089991	0.863	1	6865	TACR2
-0.03809524	0.825089991	0.828	1	43849	KLK12
-0.03809524	0.825089991	0.849	1	54476	RNF216
-0.03809524	0.825089991	0.837	1	55154	MSTO1
-0.03809524	0.825089991	0.844	1	56912	C11orf60
-0.03809524	0.825089991	0.843	1	57658	CALCOCO1
-0.03809524	0.825089991	0.838	1	79071	ELOVL6
-0.03809524	0.825089991	0.819	1	116935	RPL3P2
-0.038352638	0.823922455	0.834	1	32	ACACB
-0.038352638	0.823922455	0.817	1	2249	FGF4
-0.038352638	0.823922455	0.836	1	27429	HTRA2
-0.038352638	0.823922455	0.829	1	139341	FUNDC1
-0.038610037	0.822755873	0.837	1	3280	HES1
-0.038610037	0.822755873	0.843	1	4931	NVL
-0.038610037	0.822755873	0.859	1	9732	DOCK4
-0.038610037	0.822755873	0.827	1	10015	PDCD6IP
-0.038610037	0.822755873	0.822	1	23288	IQCE
-0.038610037	0.822755873	0.839	1	55064	C9orf68
-0.038610037	0.822755873	0.841	1	84068	SLC10A7
-0.038610037	0.822755873	0.844	1	400710	AC010624.2
-0.038610037	0.822755873	0.825	1	643827	LOC643827
-0.038867436	0.821589649	0.842	1	1788	DNMT3A
-0.038867436	0.821589649	0.835	1	9520	NPEPPS
-0.038867436	0.821589649	0.822	1	10462	CLEC10A
-0.038867436	0.821589649	0.838	1	146057	TTBK2
-0.038867436	0.821589649	0.833	1	147991	DPY19L3
-0.039124835	0.820423901	0.846	1	517	ATP5G2
-0.039124835	0.820423901	0.833	1	5627	PROS1
-0.039124835	0.820423901	0.826	1	81693	AMN
-0.039124835	0.820423901	0.839	1	124491	TMEM170A
-0.039124835	0.820423901	0.827	1	221710	C6ORF228
-0.039124835	0.820423901	0.848	1	375775	PNPLA7
-0.039382242	0.819257915	0.833	1	6009	RHEB
-0.039382242	0.819257915	0.817	1	50636	ANO7
-0.039382242	0.819257915	0.851	1	84991	RBM17
-0.039382242	0.819257915	0.821	1	440348	NPIPL2
-0.039639641	0.818092823	0.836	1	25917	THUMPD3
-0.039639641	0.818092823	0.819	1	80255	SLC35F5
-0.039639641	0.818092823	0.813	1	81037	CLPTM1L
-0.03989704	0.816928148	0.853	1	1762	DMWD
-0.03989704	0.816928148	0.832	1	2643	GCH1
-0.03989704	0.816928148	0.846	1	4803	NGF
-0.03989704	0.816928148	0.832	1	11180	WDR6
-0.03989704	0.816928148	0.857	1	55157	DARS2
-0.03989704	0.816928148	0.831	1	55331	ACER3
-0.03989704	0.816928148	0.84	1	348110	C15orf38

voxel-wise, subcortex			
Spearman's corr coef	p-val	GenID	Gene
0.000651641	0.953271687	2904	GRIN2B
0.000620809	0.955480218	79581	GPR172A
0.000605672	0.956564665	51200	CPA4
0.000585241	0.958028436	8202	NCOA3
0.000576141	0.95868057	55150	C19orf73
0.000572426	0.958946705	9886	RHOBTB1
0.000568618	0.959219515	51244	C3orf19
0.000553296	0.960317552	10234	LRRC17
0.000507327	0.96361202	55937	APOM
0.000505284	0.963758469	54777	C10orf92
0.00042514	0.969503701	6645	SNTB2
0.000338311	0.975730002	285973	ATG9B
0.000305343	0.978094459	598	BCL2L1
0.000245445	0.982390821	57007	CXCR7
0.000229843	0.983509958	6742	SSBP1
0.000128527	0.990778446	144233	BCDIN3D
0.00010011	0.992817283	140609	NEK7
7.68002E-05	0.99448967	135932	TMEM139
-6.50062E-07	0.99995333	522	ATP5J
-3.3989E-05	0.997561276	517	ATP5G2
-6.14773E-05	0.995589018	79571	GCC1
-6.31489E-05	0.995469093	55515	ACCN4
-9.74164E-05	0.993010461	8859	STK19
-0.000153415	0.98899287	283820	NOMO2
-0.000156479	0.988773048	8360	HIST1H4D
-0.000163537	0.988266706	27341	RRP7A
-0.000171802	0.987673759	23082	PPRC1
-0.000242009	0.982637286	55745	MUDENG
-0.000248695	0.982157648	54623	PAF1
-0.000257146	0.981551468	83752	LONP2
-0.000309522	0.977794707	128439	SNHG11
-0.000329767	0.976342738	11338	U2AF2
-0.000343604	0.97535044	90522	YIF1B
-0.000357627	0.97434479	100288367	LOC100288367
-0.000577905	0.958554089	4000	LMNA
-0.000667521	0.952134311	159371	TMEM20
-0.000735684	0.947253287	83941	TM2D1
-0.00073912	0.947007298	23506	KIAA0240
-0.000823071	0.940998733	361	AQP4
-0.000884641	0.936594069	57718	PPP4R4
-0.000902843	0.935292304	7750	ZMYM2
-0.000914451	0.93446219	58490	RPRD1B
-0.000938782	0.932722509	126129	CPT1C
-0.000940175	0.93262291	59339	PLEKHA2
-0.00101874	0.927007735	114796	AC093734.11
-0.001066937	0.923564792	8531	CSDAP1
-0.001186177	0.915053189	9364	RAB28
-0.001334391	0.904486954	8453	CUL2
-0.001493006	0.893198073	25890	ABI3BP
-0.001493564	0.893158436	387923	SERP2
-0.001494864	0.893065989	57530	CGN
-0.001557363	0.888623774	55894	DEFB103B
-0.00160881	0.884969592	150223	YDJC
-0.001625341	0.883796036	10491	CRTAP
-0.00163797	0.882899523	57232	ZNF630
-0.001695361	0.878827751	122011	CSNK1A1L
-0.001697869	0.87864989	54765	TRIM44
-0.001723964	0.876799583	9679	FAM53B
-0.001885551	0.865357518	55352	C17orf79
-0.001900781	0.864280522	84992	PIGY
-0.001954457	0.860486686	85451	UNK
-0.002007484	0.856741905	7844	RNF103
-0.002008599	0.856663227	84450	ZNF512
-0.002018628	0.855955303	3162	HMOX1
-0.002019464	0.855896354	81567	TXNDC5
-0.002098028	0.850355089	79577	CDC73
-0.002113166	0.849288344	285636	C5orf51
-0.002171671	0.845167696	8797	TNFRSF10A
-0.002273731	0.837989807	79368	FCRL2
-0.002275495	0.837865829	284293	HMSD
-0.002295183	0.836482763	91603	ZNF830
-0.002297876	0.836293578	6596	HLTF
-0.002397707	0.829288602	9416	DDX23
-0.002417209	0.827921748	79651	RHBDF2
-0.002421574	0.827615917	1592	CYP26A1
-0.002511932	0.821290255	11157	LSM6
-0.0025501	0.818621755	7305	TYROBP
-0.002571831	0.817103386	26128	KIAA1279
-0.002620121	0.81373167	833	CARS

region-wise, subcortex					
Spearman's corr coef	p-val	p-val, SA	p-val, SA (BH- correction)	GenID	Gene
-0.040154438	0.815763891	0.838	1	8740	TNFSF14
-0.040154438	0.815763891	0.813	1	53841	MUPCDH
-0.040154438	0.815763891	0.829	1	81555	YIPF5
-0.040154438	0.815763891	0.84	1	84734	FAM167B
-0.040154438	0.815763891	0.83	1	84904	C9orf100
-0.040154438	0.815763891	0.825	1	116228	FAM36A
-0.040154438	0.815763891	0.821	1	100132594	PGAM1P5
-0.040411841	0.814599931	0.823	1	4343	MOV10
-0.040411841	0.814599931	0.835	1	5301	PIN1P1
-0.040411841	0.814599931	0.836	1	10565	ARFGEF1
-0.040411841	0.814599931	0.84	1	22869	ZNF510
-0.040411841	0.814599931	0.829	1	27236	ARFIP1
-0.040411841	0.814599931	0.833	1	27241	BBS9
-0.040411841	0.814599931	0.834	1	57147	SCYL3
-0.040411841	0.814599931	0.818	1	390892	OR7A10
-0.040411841	0.814599931	0.819	1	449520	GGNBP1
-0.04066924	0.813435972	0.816	1	9585	KIF20B
-0.04066924	0.813435972	0.823	1	54212	SNTG1
-0.04066924	0.813435972	0.816	1	55119	PRPF38B
-0.04066924	0.813435972	0.834	1	65082	VPS33A
-0.04066924	0.813435972	0.804	1	79848	CSPP1
-0.04066924	0.813435972	0.83	1	91373	UAP1L1
-0.04066924	0.813435972	0.843	1	126231	ZNF573
-0.040926639	0.812272847	0.813	1	1017	CDK2
-0.040926639	0.812272847	0.82	1	8351	HIST1H3D
-0.040926639	0.812272847	0.816	1	26503	SLC17A5
-0.040926639	0.812272847	0.844	1	54985	HCFC1R1
-0.040926639	0.812272847	0.828	1	64786	TBC1D15
-0.041184042	0.811110198	0.798	1	2108	ETFA
-0.041184042	0.811110198	0.806	1	3491	CYR61
-0.041184042	0.811110198	0.83	1	5313	PKLR
-0.041184042	0.811110198	0.827	1	7132	TNFRSF1A
-0.041184042	0.811110198	0.822	1	9159	PCSK7
-0.041184042	0.811110198	0.818	1	26223	FBXL21
-0.041184042	0.811110198	0.833	1	54433	GAR1
-0.041184042	0.811110198	0.838	1	55140	ELP3
-0.041184042	0.811110198	0.837	1	56135	PCDHAC1
-0.041184042	0.811110198	0.832	1	64403	CDH24
-0.041184042	0.811110198	0.827	1	83659	TEKT1
-0.041184042	0.811110198	0.858	1	339977	LRRC66
-0.041441441	0.809947908	0.809	1	3360	HTR4
-0.041441441	0.809947908	0.844	1	4891	SLC11A2
-0.041441441	0.809947908	0.812	1	6599	SMARCC1
-0.041441441	0.809947908	0.809	1	54453	RIN2
-0.041441441	0.809947908	0.82	1	57828	C19orf15
-0.041441441	0.809947908	0.826	1	79760	GEMIN7
-0.041698843	0.808785498	0.827	1	10266	RAMP2
-0.041698843	0.808785498	0.841	1	11235	PDCD10
-0.041698843	0.808785498	0.836	1	83699	SH3BGRL2
-0.041698843	0.808785498	0.837	1	122416	ANKRD9
-0.041698843	0.808785498	0.833	1	353137	LCE1F
-0.041698843	0.808785498	0.806	1	374900	ZNF568
-0.041956242	0.807623982	0.82	1	23394	ADNP
-0.041956242	0.807623982	0.817	1	26032	SUSD5
-0.041956242	0.807623982	0.836	1	55659	ZNF416
-0.041956242	0.807623982	0.818	1	78988	MRP63
-0.041956242	0.807623982	0.853	1	85443	DCLK3
-0.042213641	0.806462944	0.808	1	2568	GABRP
-0.042213641	0.806462944	0.807	1	4638	MYLK
-0.042213641	0.806462944	0.834	1	4689	NCF4
-0.042213641	0.806462944	0.816	1	10287	RGS19
-0.042213641	0.806462944	0.826	1	11247	NXPH4
-0.042213641	0.806462944	0.851	1	126119	JOSD2
-0.042213641	0.806462944	0.834	1	644139	PIRT
-0.04247104	0.805302262	0.809	1	120824	TRIM51GP
-0.04247104	0.805302262	0.806	1	168474	SEPHS1P1
-0.042728439	0.804141581	0.822	1	4015	LOX
-0.042728439	0.804141581	0.823	1	27124	INPP5J
-0.042728439	0.804141581	0.812	1	49861	CLDN20
-0.042728439	0.804141581	0.822	1	54953	C1orf27
-0.042728439	0.804141581	0.83	1	55633	TBC1D22B
-0.042728439	0.804141581	0.813	1	115653	KIR3DL3
-0.042728439	0.804141581	0.819	1	399670	RPL13AP17
-0.042728439	0.804141581	0.805	1	494119	SPANXN2
-0.042728439	0.804141581	0.827	1	728262	FAM157A
-0.042985845	0.802981734	0.822	1	1188	CLCNKB
-0.042985845	0.802981734	0.827	1	1958	EGR1
-0.042985845	0.802981734	0.822	1	23272	C3orf63
-0.042985845	0.802981734	0.815	1	337882	KRTAP19-1

voxel-wise, subcortex			
Spearman's corr coef	p-val	GenID	Gene
-0.002623186	0.813517809	54919	HEATR2
-0.002649095	0.811710298	25874	BRP44
-0.00265996	0.810952604	9741	LAPTM4A
-0.00268847	0.808965325	54982	CLN6
-0.002729053	0.806138635	142	PARP1
-0.00275747	0.804160833	161779	PGBD4
-0.002759327	0.80403161	2738	GLI4
-0.002768707	0.803379118	643253	CCT6P1
-0.0027714	0.803191781	196541	C13orf39
-0.002786258	0.802158415	9214	FAIM3
-0.002817833	0.799963772	23028	KDM1
-0.002871231	0.796255767	51601	LIPT1
-0.002911441	0.793466508	57510	XPO5
-0.00300115	0.78725338	56731	SLC2A4RG
-0.003012015	0.786501765	10089	KCNK7
-0.003083522	0.781560302	6648	SOD2
-0.003200905	0.77346772	3581	IL9R
-0.003382551	0.760993302	8678	BECN1
-0.003423319	0.758202016	51475	CABP2
-0.003441149	0.756982148	64151	NCAPG
-0.003486932	0.753852785	161291	TMEM30B
-0.003489068	0.753706932	387509	GPR153
-0.003500955	0.752895117	390407	EIF4A1P6
-0.003664028	0.741786361	3785	KCNQ2
-0.003683344	0.740473986	2969	GTF2I
-0.003699688	0.739364147	5948	RBP2
-0.003728941	0.737379134	168451	THAP5
-0.003741199	0.736547828	3012	HIST1H2AE
-0.003808156	0.73201263	3182	HNRNPAB
-0.003909751	0.725149095	51150	SDF4
-0.003922938	0.724259794	4327	MMP19
-0.003993887	0.719481647	65243	ZNF643
-0.004102634	0.712179303	6886	TAL1
-0.004176648	0.70722425	53940	FTHL17
-0.004183705	0.706752419	246778	IL27
-0.0042697	0.701012313	90627	STARD13
-0.004275086	0.700653315	152877	FAM53A
-0.004283072	0.700121164	79400	NOX5
-0.004294495	0.699360371	6936	C2orf3
-0.004333406	0.696770787	150568	AC013402.2
-0.004536039	0.683343232	646577	HMGB1P16
-0.004538639	0.683171511	129303	TMEM150A
-0.00454189	0.682956934	9719	ADAMTSL2
-0.004609867	0.678475499	901	CCNG2
-0.00468091	0.673803985	9377	COX5A
-0.004815937	0.664959848	10236	HNRNPR
-0.004837389	0.663559079	27153	ZNF777
-0.004870171	0.661420643	149499	C1orf92
-0.004895895	0.659744561	3885	KRT34
-0.004909175	0.658879995	5692	PSMB4
-0.004924219	0.657901049	7019	TFAM
-0.004924869	0.657858729	7978	MTERF
-0.004927934	0.657659411	2934	GSN
-0.004981517	0.654178023	3030	HADHA
-0.005049867	0.649748206	890	CCNA2
-0.005070576	0.648408473	2079	ERH
-0.005073362	0.648228288	644246	AC217773.2
-0.005109951	0.645864248	7013	TERF1
-0.005130103	0.644563735	57178	ZMIZ1
-0.00516047	0.64260608	8351	HIST1H3D
-0.005225476	0.638423562	5694	PSMB6
-0.005263551	0.635979235	22990	PCNX
-0.005296519	0.633865952	26261	FBXO24
-0.005305713	0.633277118	642521	C1DP3
-0.005312306	0.632854998	4258	MGST2
-0.005340909	0.631025136	113246	C12orf57
-0.005384927	0.628213465	6480	ST6GAL1
-0.005417709	0.626123071	6474	SHOX2
-0.005482343	0.622010291	56123	PCDHB13
-0.005487823	0.62166214	440577	LOC440577
-0.00550296	0.620700896	10804	GJB6
-0.00551076	0.62020576	23527	ACAP2
-0.005545771	0.617985666	678	ZFP36L2
-0.005595826	0.614817739	3232	HOXD3
-0.005679498	0.609538198	91869	RFT1
-0.005816568	0.600933075	27335	EIF3K
-0.005978805	0.590819597	10725	NFAT5
-0.005998864	0.589574635	23732	FRRS1L
-0.006087737	0.584073305	2352	FOLR3

region-wise, subcortex					
Spearman's corr coef	p-val	p-val, SA	p-val, SA (BH- correction)	GenID	Gene
-0.042985845	0.802981734	0.831	1	400745	SH2D5
-0.043243244	0.801822305	0.824	1	5509	PPP1R3D
-0.043243244	0.801822305	0.818	1	8729	GBF1
-0.043243244	0.801822305	0.825	1	10636	RGS14
-0.043243244	0.801822305	0.808	1	23753	SDF2L1
-0.043243244	0.801822305	0.825	1	90187	EMILIN3
-0.043243244	0.801822305	0.83	1	91646	TDRD12
-0.043243244	0.801822305	0.841	1	197135	PATL2
-0.043500643	0.800663352	0.803	1	1016	CDH18
-0.043500643	0.800663352	0.812	1	2632	GBE1
-0.043500643	0.800663352	0.809	1	5236	PGM1
-0.043500643	0.800663352	0.817	1	10002	NR2E3
-0.043500643	0.800663352	0.846	1	80324	PUS1
-0.043500643	0.800663352	0.801	1	90313	TP53I13
-0.043500643	0.800663352	0.834	1	92856	IMP4
-0.043758042	0.799504757	0.823	1	402	ARL2
-0.043758042	0.799504757	0.811	1	1070	CETN3
-0.043758042	0.799504757	0.804	1	6891	TAP2
-0.043758042	0.799504757	0.813	1	50939	IMPG2
-0.043758042	0.799504757	0.803	1	84569	LYZL1
-0.043758042	0.799504757	0.818	1	100128927	ZBTB42
-0.044015441	0.798346102	0.817	1	9785	DHX38
-0.044015441	0.798346102	0.82	1	23355	VPS8
-0.044015441	0.798346102	0.811	1	23649	POLA2
-0.044272844	0.797188401	0.803	1	1468	SLC25A10
-0.044272844	0.797188401	0.817	1	9110	MTMR4
-0.044272844	0.797188401	0.798	1	9550	ATP6V1G1
-0.044272844	0.797188401	0.811	1	79792	GSDMD
-0.044272844	0.797188401	0.806	1	83875	BCO2
-0.044272844	0.797188401	0.793	1	283229	EFCAB4A
-0.044272844	0.797188401	0.817	1	284359	IZUMO1
-0.044272844	0.797188401	0.83	1	644397	LOC644397
-0.044272844	0.797188401	0.818	1	645715	EEF1A1P24
-0.044530243	0.796031177	0.832	1	2810	SFN
-0.044530243	0.796031177	0.804	1	3931	LCAT
-0.044530243	0.796031177	0.835	1	4212	MEIS2
-0.044530243	0.796031177	0.802	1	25936	NSL1
-0.044530243	0.796031177	0.811	1	55684	C9orf86
-0.044530243	0.796031177	0.804	1	115861	NXNL1
-0.044530243	0.796031177	0.815	1	440145	C13orf37
-0.044787645	0.79487437	0.838	1	575	BAI1
-0.044787645	0.79487437	0.812	1	1736	DKC1
-0.044787645	0.79487437	0.797	1	7584	ZNF35
-0.044787645	0.79487437	0.802	1	9734	HDAC9
-0.044787645	0.79487437	0.798	1	85358	SHANK3
-0.044787645	0.79487437	0.798	1	259217	HSPA12A
-0.045045044	0.793717384	0.824	1	2530	FUT8
-0.045045044	0.793717384	0.832	1	3567	IL5
-0.045045044	0.793717384	0.806	1	54790	TET2
-0.045045044	0.793717384	0.827	1	57587	KIAA1430
-0.045045044	0.793717384	0.822	1	150684	COMMD1
-0.045045044	0.793717384	0.814	1	401466	C8orf59
-0.045302443	0.792561471	0.814	1	2049	EPHB3
-0.045302443	0.792561471	0.814	1	6183	MRPS12
-0.045302443	0.792561471	0.816	1	24148	PRPF6
-0.045302443	0.792561471	0.812	1	25996	REXO2
-0.045302443	0.792561471	0.818	1	125950	RAVER1
-0.045302443	0.792561471	0.795	1	100130932	LOC100130932
-0.045559846	0.791405916	0.801	1	8690	JRKL
-0.045559846	0.791405916	0.796	1	9439	MED23
-0.045559846	0.791405916	0.808	1	10024	TROAP
-0.045559846	0.791405916	0.797	1	27133	KCNH5
-0.045559846	0.791405916	0.788	1	54928	IMPAD1
-0.045559846	0.791405916	0.825	1	126868	C1orf161
-0.045559846	0.791405916	0.798	1	151306	GPBAR1
-0.045559846	0.791405916	0.812	1	388595	TMEM82
-0.045817245	0.790250838	0.796	1	429	ASCL1
-0.045817245	0.790250838	0.801	1	1382	CRABP2
-0.045817245	0.790250838	0.81	1	4582	MUC1
-0.045817245	0.790250838	0.826	1	6670	SP3
-0.045817245	0.790250838	0.798	1	51046	ST8SIA3
-0.045817245	0.790250838	0.808	1	51533	PHF7
-0.045817245	0.790250838	0.805	1	55311	ZNF444
-0.045817245	0.790250838	0.823	1	57666	FBRSL1
-0.045817245	0.790250838	0.81	1	90993	CREB3L1
-0.045817245	0.790250838	0.803	1	170958	ZNF525
-0.045817245	0.790250838	0.81	1	440352	SNX29P2
-0.045817245	0.790250838	0.807	1	100144596	LINC00029
-0.046074644	0.7890957	0.804	1	910	CD1B

voxel-wise, subcortex			
Spearman's corr coef	p-val	GenID	Gene
-0.006110582	0.582663	6388	SDF2
-0.006113647	0.582473934	147015	DHRS13
-0.006171781	0.578893006	79083	MLPH
-0.006212085	0.576416373	64693	CTAGE1
-0.006234373	0.575048983	9702	CEP57
-0.006272633	0.572705209	116071	BATF2
-0.006298079	0.571148932	51147	ING4
-0.006318509	0.56990093	54796	BNC2
-0.006361413	0.567284286	55364	IMPACT
-0.006365128	0.567057967	84186	ZCCHC7
-0.006392431	0.56539613	94101	ORMDL1
-0.006468766	0.560762227	6196	RPS6KA2
-0.006491054	0.559412777	10988	METAP2
-0.006519842	0.557672024	84142	FAM175A
-0.006536187	0.556684911	313	AOAH
-0.006541387	0.556370974	84680	ACCS
-0.00658002	0.554041803	55267	C22orf26
-0.006629332	0.551075697	100128239	AP000911.1
-0.006653384	0.549631774	5547	PRCP
-0.006654684	0.549553812	7174	TPP2
-0.006694802	0.547149718	64802	NMNAT1
-0.006809027	0.540333569	25913	POT1
-0.006879234	0.536165297	487	ATP2A1
-0.00692446	0.53348875	283948	NHLRC4
-0.006972193	0.53067112	64651	CSRNP1
-0.006998288	0.529133976	5558	PRIM2
-0.007023176	0.527670085	3631	INPP4A
-0.007055958	0.525744975	79573	TTC13
-0.007126536	0.521612585	2941	GSTA4
-0.007152724	0.520083487	3784	KCNQ1
-0.007169347	0.519114077	729769	UQCRHP2
-0.007221538	0.516076624	23389	MED13L
-0.007253112	0.514243484	100128190	SLC9B1P1
-0.007264071	0.513608038	4804	NGFR
-0.007298245	0.511629045	25884	CHRDL2
-0.007426586	0.504232705	340348	TSPAN33
-0.007481748	0.501071095	23174	ZCCHC14
-0.007552048	0.49705717	55929	DMAP1
-0.007580279	0.49545005	3936	LCP1
-0.007660608	0.49089241	151195	CCNYL1
-0.007681224	0.489726365	440419	TBC1D3P5
-0.007688747	0.489301324	355	FAS
-0.007694318	0.488986552	79925	SPEF2
-0.007701376	0.488588035	54961	SSH3
-0.00772645	0.487173617	93233	CCDC114
-0.00778412	0.483928978	10172	ZNF256
-0.007793964	0.483376265	26292	MYCBP
-0.007927691	0.475902379	157627	RP11-403C10.2
-0.007936606	0.475406319	51198	C9orf53
-0.007948679	0.474735111	8632	DNAH17
-0.007967901	0.473667473	6840	SVIL
-0.008002633	0.471741736	340286	FAM183B
-0.008043867	0.469461113	80235	PIGZ
-0.008098564	0.466445208	2679	GGT3P
-0.008159113	0.463119268	4090	SMAD5
-0.008277517	0.456653744	16	AARS
-0.00830677	0.455064207	64759	TNS3
-0.008335559	0.453502953	346	APOC4
-0.008341316	0.453191102	5226	PGD
-0.008382177	0.4509812	54955	C1orf109
-0.008396478	0.450209171	10157	AASS
-0.008412545	0.449342757	1047	CLGN
-0.008437711	0.447987556	120796	OR56A1
-0.008506339	0.444303572	442590	SPDYE5
-0.008584997	0.440102756	100129196	RP5-1166H10.2
-0.008652974	0.436490625	387266	KRTAP5-3
-0.008738875	0.431950659	80010	RMI1
-0.008739432	0.431921273	79767	ELMO3
-0.00880676	0.428382248	1727	CYB5R3
-0.008836849	0.426806152	8091	HMGA2
-0.008863408	0.425417691	9656	MDC1
-0.008930365	0.42192921	80013	C10orf97
-0.008937237	0.421572089	51091	SEPSECS
-0.008957017	0.42054525	5648	MASP1
-0.009008651	0.417871743	129787	TMEM18
-0.009085915	0.413889885	84804	MFSD9
-0.009095481	0.413398474	57817	HAMP
-0.009213421	0.407368064	1960	EGR3
-0.009264961	0.404749304	7326	AC087742.2

region-wise, subcortex					
Spearman's corr coef	p-val	p-val, SA	p-val, SA (BH- correction)	GenID	Gene
-0.046074644	0.7890957	0.798	1	2525	FUT3
-0.046074644	0.7890957	0.803	1	6731	SRP72
-0.046074644	0.7890957	0.814	1	9181	ARHGEF2
-0.046074644	0.7890957	0.823	1	22893	BAHD1
-0.046074644	0.7890957	0.809	1	57336	ZNF287
-0.046074644	0.7890957	0.803	1	79053	ALG8
-0.046074644	0.7890957	0.816	1	387486	LINC00320
-0.046332043	0.787941575	0.822	1	6343	SCT
-0.046332043	0.787941575	0.788	1	9091	PIGQ
-0.046332043	0.787941575	0.799	1	9881	LBA1
-0.046332043	0.787941575	0.819	1	26011	ODZ4
-0.046332043	0.787941575	0.81	1	339906	RP11-427P5.1
-0.046332043	0.787941575	0.797	1	647030	LOC647030
-0.046589449	0.786787808	0.809	1	56124	PCDHB12
-0.046589449	0.786787808	0.786	1	81890	QTRT1
-0.046589449	0.786787808	0.797	1	84232	MAF1
-0.046589449	0.786787808	0.811	1	149428	BNIPL
-0.046589449	0.786787808	0.813	1	285368	PRRT3
-0.046846848	0.785634518	0.818	1	1369	CPN1
-0.046846848	0.785634518	0.793	1	23287	AGTPBP1
-0.046846848	0.785634518	0.807	1	24138	IFIT5
-0.046846848	0.785634518	0.801	1	51764	GNG13
-0.046846848	0.785634518	0.785	1	80789	INTS5
-0.046846848	0.785634518	0.803	1	84647	PLA2G12B
-0.046846848	0.785634518	0.803	1	338773	TMEM119
-0.046846848	0.785634518	0.808	1	342371	ATXN1L
-0.046846848	0.785634518	0.79	1	374403	TBC1D10C
-0.047104247	0.784481704	0.775	1	1191	CLU
-0.047104247	0.784481704	0.804	1	5516	PPP2CB
-0.047104247	0.784481704	0.833	1	5626	PROP1
-0.047104247	0.784481704	0.798	1	5875	RABGGTA
-0.047104247	0.784481704	0.801	1	10942	PRSS21
-0.047104247	0.784481704	0.805	1	51368	TEX264
-0.047104247	0.784481704	0.786	1	79086	C19orf42
-0.047104247	0.784481704	0.799	1	201305	SPNS3
-0.047361646	0.783328772	0.808	1	1627	DBN1
-0.047361646	0.783328772	0.805	1	4637	MYL6
-0.047361646	0.783328772	0.794	1	90288	C3orf25
-0.047619045	0.782176912	0.794	1	1915	EEF1A1
-0.047619045	0.782176912	0.789	1	2215	FCGR3B
-0.047619045	0.782176912	0.785	1	6166	RPL36AL
-0.047619045	0.782176912	0.778	1	7384	UQCRC1
-0.047619045	0.782176912	0.786	1	23424	TDRD7
-0.047619045	0.782176912	0.801	1	219972	MPEG1
-0.047619045	0.782176912	0.799	1	100507436	MICA
-0.047876447	0.781025469	0.809	1	3857	KRT9
-0.047876447	0.781025469	0.794	1	5818	PVRL1
-0.047876447	0.781025469	0.803	1	6820	SULT2B1
-0.047876447	0.781025469	0.806	1	375307	C2orf62
-0.04813385	0.779874504	0.795	1	4134	MAP4
-0.04813385	0.779874504	0.808	1	5579	PRKCB
-0.04813385	0.779874504	0.804	1	22895	RPH3A
-0.04813385	0.779874504	0.815	1	29907	SNX15
-0.04813385	0.779874504	0.792	1	51068	NMD3
-0.04813385	0.779874504	0.788	1	125965	COX6B2
-0.04813385	0.779874504	0.808	1	219833	C11orf45
-0.048391249	0.778723419	0.811	1	183	AGT
-0.048391249	0.778723419	0.784	1	580	BARD1
-0.048391249	0.778723419	0.783	1	3295	HSD17B4
-0.048391249	0.778723419	0.804	1	8549	LGR5
-0.048391249	0.778723419	0.815	1	54551	MAGEL2
-0.048391249	0.778723419	0.809	1	55869	HDAC8
-0.048391249	0.778723419	0.795	1	56913	C1GALT1
-0.048391249	0.778723419	0.797	1	93109	TMEM44
-0.048391249	0.778723419	0.792	1	140596	DEFB104A
-0.048391249	0.778723419	0.792	1	152118	RP11-292E2.1
-0.048391249	0.778723419	0.808	1	342865	VSTM2B
-0.048648648	0.777573407	0.784	1	410	ARSA
-0.048648648	0.777573407	0.799	1	5202	PFDN2
-0.048648648	0.777573407	0.79	1	10874	NMU
-0.048648648	0.777573407	0.796	1	79728	PALB2
-0.048906047	0.776423812	0.809	1	50937	CDON
-0.048906047	0.776423812	0.793	1	58529	MYOZ1
-0.048906047	0.776423812	0.791	1	81888	HYI
-0.04916345	0.775274694	0.799	1	23471	TRAM1
-0.04916345	0.775274694	0.786	1	64943	NT5DC2
-0.04916345	0.775274694	0.793	1	154790	CLEC2L
-0.049420848	0.774125576	0.791	1	2533	FYB
-0.049420848	0.774125576	0.803	1	57117	INTS12

voxel-wise, subcortex			
Spearman's corr coef	p-val	GenID	Gene
-0.009297464	0.403103054	4016	LOXL1
-0.0093751	0.399186999	55762	ZNF701
-0.009427012	0.396581322	10953	TOMM34
-0.009444657	0.395698011	151647	FAM19A4
-0.009472516	0.394305736	83744	ZNF484
-0.009528236	0.391530097	3139	HLA-L
-0.009599279	0.388008416	57143	ADCK1
-0.009605593	0.387696326	146434	ZNF597
-0.00963011	0.386486113	81027	TUBB1
-0.009648312	0.385589033	27348	TOR1B
-0.009678865	0.384086221	375387	LRRC33
-0.009745171	0.380837023	10454	MAP3K7IP1
-0.009792254	0.378540188	57113	TRPC7
-0.009816585	0.377356559	8636	SSNA1
-0.009914095	0.37263605	1586	CYP17A1
-0.009927281	0.372000486	55277	FGGY
-0.009946319	0.371084064	81854	MGC3771
-0.009953841	0.370722413	7372	UMPS
-0.009961178	0.370369852	28962	OSTM1
-0.010056551	0.365805686	339175	METTL2A
-0.010092954	0.364072859	55900	ZNF302
-0.010230117	0.35758993	1349	COX7B
-0.010240797	0.357088298	144717	FAM109A
-0.010257605	0.356299579	1389	CREBL2
-0.010273857	0.35553804	60370	AVPI1
-0.01030636	0.354018062	155054	ZNF425
-0.010310539	0.353822917	10589	DRAP1
-0.0103501	0.351979107	7203	CCT3
-0.010364587	0.351305395	27177	IL1F8
-0.010365981	0.351240665	1132	CHRM4
-0.010380374	0.350572228	80267	EDEM3
-0.010385018	0.350356728	2657	GDF1
-0.0104256	0.348477095	643	CXCR5
-0.010487449	0.345624894	9371	KIF3B
-0.01052831	0.343748748	7813	EVI5
-0.010546884	0.342898101	2058	EPRS
-0.01067866	0.336901456	2961	GTF2E2
-0.010680982	0.336796433	2162	F13A1
-0.010716549	0.335189819	5146	PDE6C
-0.010734844	0.334365398	6389	SDHA
-0.010754718	0.333471239	440275	EIF2AK4
-0.010785549	0.332087159	2838	GPR15
-0.010830125	0.330092698	54627	KIAA1383
-0.010841826	0.329570383	4916	NTRK3
-0.010842568	0.329537272	54508	FLJ11235
-0.010844797	0.329437912	114803	MYSM1
-0.010845912	0.329388231	91452	ACBD5
-0.010963108	0.324188679	440307	TTLL13
-0.011026908	0.321380705	401397	AC073346.1
-0.011045481	0.320566267	285359	LOC285359
-0.011091728	0.318544239	202374	STK32A
-0.011120237	0.31730181	23143	LRCH1
-0.011225919	0.312724203	55744	C7orf44
-0.011226755	0.312688231	79713	TMEM149
-0.011234835	0.312340081	5822	PWP2H
-0.011248393	0.311756402	23191	CYFIP1
-0.011332158	0.308166564	51314	TXNDC3
-0.011334108	0.308083326	10569	SLU7
-0.011361132	0.306931227	8645	KCNK5
-0.011382027	0.306042403	2542	SLC37A4
-0.0113993	0.305308908	8697	CDC23
-0.011407287	0.304970205	10105	PPIF
-0.011443411	0.303441077	388403	YPEL2
-0.011450562	0.303139061	56111	PCDHGA4
-0.011464956	0.30253157	119587	CPXM2
-0.011529591	0.299813896	2956	MSH6
-0.011545379	0.299152553	91272	BOD1
-0.011560516	0.298519403	134187	POU5F2
-0.011576581	0.297848403	5413	SEPTIN5
-0.0116258	0.295798838	2149	F2R
-0.011631651	0.29555586	51075	TMX2
-0.011640473	0.295189708	4241	MFI2
-0.011653938	0.294631392	4726	NDUFS6
-0.011670933	0.293927759	84986	ARHGAP19
-0.011681984	0.293470889	4360	MRC1
-0.011718666	0.291957617	1490	CTGF
-0.011750612	0.29064396	338323	NLRP14
-0.011769	0.289889663	53342	IL17D
-0.011780979	0.289399028	494119	SPANXN2

region-wise, subcortex					
Spearman's corr coef	p-val	p-val, SA	p-val, SA (BH- correction)	GenID	Gene
-0.049420848	0.774125576	0.794	1	84981	MIR22HG
-0.049420848	0.774125576	0.784	1	124912	SPACA3
-0.049420848	0.774125576	0.781	1	373156	GSTK1
-0.049420848	0.774125576	0.782	1	375757	C9orf119
-0.049678247	0.772977412	0.793	1	2475	MTOR
-0.049678247	0.772977412	0.789	1	3594	IL12RB1
-0.049678247	0.772977412	0.813	1	6888	TALDO1
-0.049678247	0.772977412	0.792	1	144699	FBXL14
-0.04993565	0.771829724	0.796	1	6996	TDG
-0.04993565	0.771829724	0.789	1	54900	LAX1
-0.04993565	0.771829724	0.78	1	57105	CYSLTR2
-0.04993565	0.771829724	0.799	1	57192	MCOLN1
-0.04993565	0.771829724	0.804	1	391257	SUMO1P1
-0.04993565	0.771829724	0.82	1	401251	C6orf26
-0.04993565	0.771829724	0.765	1	401498	TMEM215
-0.050193049	0.770682633	0.796	1	6774	STAT3
-0.050450452	0.769535899	0.799	1	3822	KLRC2
-0.050450452	0.769535899	0.8	1	8733	GPAA1
-0.050450452	0.769535899	0.766	1	9132	KCNQ4
-0.050450452	0.769535899	0.786	1	81697	OR2B2
-0.050450452	0.769535899	0.784	1	126823	KLHDC9
-0.050450452	0.769535899	0.779	1	220296	HEPACAM
-0.050707851	0.768389165	0.81	1	841	CASP8
-0.050707851	0.768389165	0.803	1	6144	RPL21
-0.050707851	0.768389165	0.799	1	8653	DDX3Y
-0.050707851	0.768389165	0.813	1	9123	SLC16A3
-0.050707851	0.768389165	0.795	1	9760	TOX
-0.050707851	0.768389165	0.795	1	91272	BOD1
-0.050707851	0.768389165	0.797	1	116496	FAM129A
-0.050707851	0.768389165	0.788	1	128710	C20orf94
-0.050707851	0.768389165	0.793	1	100128998	C20orf181
-0.050707851	0.768389165	0.79	1	100133150	LOC100133150
-0.05096525	0.767243445	0.785	1	64105	CENPK
-0.05096525	0.767243445	0.772	1	84548	TMEM185A
-0.05096525	0.767243445	0.78	1	133418	EMB
-0.05096525	0.767243445	0.797	1	283129	C11orf85
-0.051222648	0.766098201	0.756	1	3480	IGF1R
-0.051222648	0.766098201	0.783	1	8968	HIST1H3F
-0.051222648	0.766098201	0.79	1	9150	CTDP1
-0.051222648	0.766098201	0.768	1	10249	GLYAT
-0.051222648	0.766098201	0.783	1	26259	FBXW8
-0.051480047	0.764953434	0.809	1	118	ADD1
-0.051480047	0.764953434	0.789	1	1778	DYNC1H1
-0.051480047	0.764953434	0.793	1	3700	ITIH4
-0.051480047	0.764953434	0.791	1	8674	VAMP4
-0.051480047	0.764953434	0.763	1	51762	RAB8B
-0.051480047	0.764953434	0.774	1	55686	MREG
-0.051480047	0.764953434	0.791	1	123970	C16orf78
-0.051480047	0.764953434	0.751	1	220004	C11orf66
-0.051737454	0.763808727	0.8	1	522	ATP5J
-0.051737454	0.763808727	0.767	1	3029	HAGH
-0.051737454	0.763808727	0.774	1	27031	NPHP3
-0.051737454	0.763808727	0.785	1	53940	FTHL17
-0.051737454	0.763808727	0.758	1	63970	TP53AIP1
-0.051737454	0.763808727	0.749	1	79447	C16orf53
-0.051737454	0.763808727	0.767	1	90506	LRRC46
-0.051737454	0.763808727	0.799	1	130502	TTC32
-0.051737454	0.763808727	0.762	1	149499	C1orf92
-0.051737454	0.763808727	0.776	1	256297	PTF1A
-0.051737454	0.763808727	0.786	1	259307	IL4I1
-0.051994853	0.762664974	0.749	1	23786	BCL2L13
-0.051994853	0.762664974	0.772	1	51614	ERGIC3
-0.051994853	0.762664974	0.78	1	56242	ZNF253
-0.051994853	0.762664974	0.769	1	84278	HIATL2
-0.051994853	0.762664974	0.759	1	84437	KIAA1826
-0.051994853	0.762664974	0.788	1	84517	AC078802.1
-0.052252252	0.761521697	0.767	1	1421	CRYGD
-0.052252252	0.761521697	0.762	1	2237	FEN1
-0.052252252	0.761521697	0.785	1	3196	TLX2
-0.052252252	0.761521697	0.789	1	3887	KRT81
-0.052252252	0.761521697	0.77	1	4543	MTNR1A
-0.052252252	0.761521697	0.793	1	26152	ZNF337
-0.052252252	0.761521697	0.767	1	79088	ZNF426
-0.052252252	0.761521697	0.793	1	83899	KRTAP9-2
-0.052509651	0.760379016	0.795	1	25825	BACE2
-0.052509651	0.760379016	0.772	1	51001	MTERFD1
-0.052509651	0.760379016	0.776	1	55723	ASF1B
-0.052509651	0.760379016	0.762	1	347404	LANCL3
-0.05276705	0.759236217	0.805	1	2968	GTF2H4

voxel-wise, subcortex			
Spearman's corr coef	p-val	GenID	Gene
-0.011791473	0.288969606	2581	GALC
-0.01183512	0.287188292	389458	LOC389458
-0.011898084	0.28463164	27113	BBC3
-0.011914985	0.283947974	1020	CDK5
-0.012171574	0.273705512	92196	DAPL1
-0.012222279	0.271711797	8999	CDKL2
-0.01223268	0.271303982	100506742	CASP12
-0.012271404	0.269789636	728492	SERF1B
-0.01233186	0.267436951	6518	SLC2A5
-0.012361578	0.266285688	23413	FREQ
-0.012396588	0.264933795	79086	C19orf42
-0.01241479	0.264232695	1579	CYP4A11
-0.012487875	0.261430889	5360	PLTP
-0.012550188	0.259058207	26122	EPC2
-0.012599593	0.257187843	10274	STAG1
-0.012600893	0.257138699	2618	GART
-0.012609808	0.256802231	114088	TRIM9
-0.012631168	0.2559973	7805	LAPTM5
-0.012669243	0.254566789	8658	TNKS
-0.012673422	0.254410148	6748	SSR4
-0.012688559	0.253843248	1773	DNASE1
-0.012751615	0.2514911	136	ADORA2B
-0.012810213	0.249318913	643037	LOC643037
-0.012832036	0.248513311	83642	RP3-402G11.1
-0.012876705	0.246870026	427	ASAH1
-0.012946262	0.244326338	79191	IRX3
-0.012985916	0.242884472	653562	LOC653562
-0.013040613	0.240905479	267004	PGBD3
-0.01305733	0.240302876	79145	CHCHD7
-0.013060765	0.240179226	28959	TMEM176B
-0.01309364	0.238997743	100132346	LOC100132346
-0.01316979	0.23627679	5803	PTPRZ1
-0.013173783	0.236134723	6423	SFRP2
-0.013229967	0.23414211	55608	ANKRD10
-0.013249841	0.233440146	9169	SCAF11
-0.013264977	0.23290655	50839	TAS2R10
-0.013281787	0.232314914	55806	HR
-0.013291259	0.231981993	84106	PRAM1
-0.01330974	0.231333479	1741	DLG3
-0.013318654	0.231021106	2963	GTF2F2
-0.013320233	0.230965793	1901	S1PR1
-0.013353572	0.229800433	10542	HBXIP
-0.013363973	0.229437724	100288560	LOC100288560
-0.013379203	0.228907317	220074	AP000812.2
-0.013398797	0.228226259	26519	TIMM10
-0.013497143	0.224829704	84624	FNDC1
-0.013551934	0.222953096	23457	ABCB9
-0.013585644	0.221804053	51629	SLC25A39
-0.013655201	0.219446674	10806	SDCCAG8
-0.013657336	0.219374612	387921	NHLRC3
-0.013659008	0.219318211	10664	CTCF
-0.01366718	0.219042495	57129	MRPL47
-0.013707113	0.217699081	23216	TBC1D1
-0.013737202	0.216690704	8329	HIST1H2AI
-0.013737944	0.216665864	55356	SLC22A15
-0.013840004	0.213270903	257364	SNX33
-0.013861828	0.212549999	4584	MUC3A
-0.013879751	0.211959183	5867	RAB4A
-0.013895074	0.211455062	6602	SMARCD1
-0.013953672	0.209535152	80164	AC010336.2
-0.013995183	0.208182812	56616	DIABLO
-0.014078669	0.205482066	26580	BSCL2
-0.014115444	0.204300568	728769	CTD-2037K23.2
-0.014195867	0.201733872	3816	KLK1
-0.014236728	0.200438917	163688	CALML6
-0.014240257	0.200327322	80774	LIMD2
-0.014240535	0.200318545	6627	SNRPA1
-0.014240628	0.200315595	348327	ZNF530
-0.014288268	0.198814094	81875	ISG20L2
-0.014317242	0.197904885	23231	AC092436.3
-0.014322628	0.197736219	9590	AKAP12
-0.014330615	0.197486296	610	HCN2
-0.014354946	0.196726412	55755	CDK5RAP2
-0.014355131	0.19672063	29095	ORMDL2
-0.014377048	0.196037903	1676	DFFA
-0.01439005	0.195633739	3746	KCNC1
-0.014445769	0.193908557	57535	KIAA1324
-0.014452456	0.193702266	343990	C2orf55
-0.014512818	0.191847399	5686	PSMA5

region-wise, subcortex					
Spearman's corr coef	p-val	p-val, SA	p-val, SA (BH- correction)	GenID	Gene
-0.05276705	0.759236217	0.776	1	4967	OGDH
-0.05276705	0.759236217	0.765	1	5167	ENPP1
-0.05276705	0.759236217	0.786	1	8527	DGKD
-0.05276705	0.759236217	0.77	1	8649	MAPKSP1
-0.05276705	0.759236217	0.795	1	9236	CCPG1
-0.05276705	0.759236217	0.782	1	9943	OXSR1
-0.05276705	0.759236217	0.752	1	10231	RCAN2
-0.05276705	0.759236217	0.786	1	57804	POLD4
-0.05276705	0.759236217	0.765	1	114131	UCN3
-0.05276705	0.759236217	0.782	1	120892	LRRK2
-0.05276705	0.759236217	0.776	1	152065	C3orf22
-0.053024452	0.758094549	0.779	1	4240	MFGE8
-0.053024452	0.758094549	0.783	1	5379	PMS2P1
-0.053024452	0.758094549	0.793	1	5930	RBBP6
-0.053024452	0.758094549	0.785	1	7703	PCGF2
-0.053281851	0.756953359	0.79	1	6667	SP1
-0.053281851	0.756953359	0.777	1	51175	TUBE1
-0.053281851	0.756953359	0.746	1	54960	GEMIN8
-0.053281851	0.756953359	0.782	1	219527	LRRC55
-0.053281851	0.756953359	0.786	1	284266	SIGLEC15
-0.053539254	0.755812645	0.768	1	3300	DNAJB2
-0.053539254	0.755812645	0.775	1	25813	SAMM50
-0.053539254	0.755812645	0.769	1	80759	KHDC1
-0.053539254	0.755812645	0.772	1	83590	TMUB1
-0.053539254	0.755812645	0.767	1	117285	DEFB118
-0.053539254	0.755812645	0.778	1	140771	AL135932.1
-0.053539254	0.755812645	0.789	1	340980	OR52B6
-0.053796653	0.754672468	0.772	1	6235	RPS29
-0.053796653	0.754672468	0.774	1	7095	SEC62
-0.053796653	0.754672468	0.786	1	29904	EEF2K
-0.053796653	0.754672468	0.768	1	56180	MOSPD1
-0.053796653	0.754672468	0.782	1	79727	LIN28
-0.053796653	0.754672468	0.767	1	255967	PAN3
-0.054054055	0.753532231	0.754	1	222	ALDH3B2
-0.054054055	0.753532231	0.776	1	4942	OAT
-0.054054055	0.753532231	0.778	1	11057	ABHD2
-0.054054055	0.753532231	0.784	1	161753	ODF3L1
-0.054054055	0.753532231	0.788	1	353139	LCE2A
-0.054311454	0.752393067	0.769	1	728	C5AR1
-0.054311454	0.752393067	0.77	1	2124	EVI2B
-0.054311454	0.752393067	0.773	1	5613	PRKX
-0.054311454	0.752393067	0.75	1	9294	S1PR2
-0.054311454	0.752393067	0.775	1	399940	AP004607.4
-0.054568853	0.751254499	0.775	1	9328	GTF3C5
-0.054568853	0.751254499	0.795	1	55110	MAGOHB
-0.054568853	0.751254499	0.758	1	64927	TTC23
-0.054568853	0.751254499	0.772	1	80739	C6orf25
-0.054568853	0.751254499	0.766	1	403282	OR6C65
-0.054826252	0.750116348	0.76	1	10692	RRH
-0.054826252	0.750116348	0.756	1	84435	GPR123
-0.054826252	0.750116348	0.775	1	643314	KIAA0754
-0.055083655	0.748978257	0.774	1	70	ACTC1
-0.055083655	0.748978257	0.751	1	27163	NAAA
-0.055083655	0.748978257	0.767	1	80217	C10orf79
-0.055083655	0.748978257	0.768	1	196527	ANO6
-0.055341057	0.747841239	0.78	1	9668	ZNF432
-0.055341057	0.747841239	0.765	1	11236	RNF139
-0.055341057	0.747841239	0.774	1	30968	STOML2
-0.055341057	0.747841239	0.788	1	51009	DERL2
-0.055341057	0.747841239	0.779	1	56122	PCDHB14
-0.055341057	0.747841239	0.763	1	56894	AGPAT3
-0.055341057	0.747841239	0.749	1	79692	ZNF322
-0.055341057	0.747841239	0.774	1	158801	NKAPP1
-0.055598456	0.746704698	0.769	1	664	BNIP3
-0.055598456	0.746704698	0.794	1	3177	SLC29A2
-0.055598456	0.746704698	0.761	1	23208	SYT11
-0.055598456	0.746704698	0.766	1	23438	HARS2
-0.055598456	0.746704698	0.781	1	56675	NRIP3
-0.055598456	0.746704698	0.764	1	64225	ATL2
-0.055598456	0.746704698	0.778	1	148304	C1orf74
-0.055855855	0.745568752	0.775	1	2017	CTTN
-0.055855855	0.745568752	0.774	1	10586	MAB21L2
-0.055855855	0.745568752	0.758	1	55150	C19orf73
-0.055855855	0.745568752	0.779	1	83737	ITCH
-0.055855855	0.745568752	0.799	1	112399	EGLN3
-0.055855855	0.745568752	0.79	1	119695	OR52R1
-0.055855855	0.745568752	0.763	1	256691	MAMDC2
-0.055855855	0.745568752	0.763	1	347088	GPR144
-0.056113254	0.744433284	0.768	1	10659	CUGBP2

voxel-wise, subcortex			
Spearman's corr coef	p-val	GenID	Gene
-0.014571974	0.190042332	3394	IRF8
-0.014590361	0.189483792	22934	RPIA
-0.014659268	0.187401533	386676	KRTAP10-9
-0.014714245	0.185752407	401805	LOC401805
-0.014733654	0.185172752	366	AQP9
-0.014761606	0.184340268	402644	LOC402644
-0.014869424	0.181155294	7088	TLE1
-0.014869609	0.18114984	5782	PTPN12
-0.014879731	0.180852935	8666	EIF3G
-0.014947245	0.178881899	347736	TXNDC6
-0.014988477	0.177686051	4190	MDH1
-0.015001665	0.177304804	9410	SNRNP40
-0.015048469	0.175956681	29988	SLC2A8
-0.015078929	0.175083458	648359	FAM108A4P
-0.01513372	0.173520893	55017	C14orf119
-0.015149321	0.173077852	84333	PCGF5
-0.015211634	0.171316788	80341	BPIL1
-0.015260389	0.169948295	240	ALOX5
-0.015280263	0.169392824	646498	RP11-694I15.6
-0.015283141	0.169312477	5522	PPP2R2C
-0.015334682	0.167878777	2720	GLB1
-0.015410275	0.165792465	3996	LLGL1
-0.015476024	0.163993806	1892	ECHS1
-0.015602879	0.160564989	84971	ATG4D
-0.015618573	0.160144612	22925	PLA2R1
-0.015660642	0.159021795	2020	EN2
-0.015777467	0.155935019	4999	ORC2
-0.015787311	0.155676991	5024	P2RX3
-0.01578889	0.155635655	93550	ANUBL1
-0.015859375	0.153798088	10594	PRPF8
-0.015862068	0.153728187	10721	POLQ
-0.015894478	0.152889088	26751	SH3YL1
-0.01591751	0.152294889	54554	WDR5B
-0.015983537	0.150601253	7802	DNALI1
-0.01601409	0.149822459	26167	PCDHB5
-0.016071852	0.148358405	55299	BRIX1
-0.016121071	0.147119582	122704	MRPL52
-0.016142245	0.146589041	25937	WWTR1
-0.016150231	0.14638941	55696	RBM22
-0.01617679	0.145726696	3592	IL12A
-0.01621338	0.144817546	80318	GKAP1
-0.016265107	0.143539637	90586	AOC4
-0.016285537	0.143037349	9188	DDX21
-0.016287023	0.143000841	6130	RPL7A
-0.016311632	0.142397657	163115	ZNF781
-0.016346829	0.141538367	51023	MRPS18C
-0.016353237	0.141382352	25813	SAMM50
-0.016390476	0.140478358	644096	SDHAF1
-0.016394468	0.140381709	390152	OR8H3
-0.016447773	0.139096081	283165	PRR10
-0.016467368	0.138625741	135924	OR9A2
-0.01652058	0.137354761	374900	ZNF568
-0.016527453	0.137191251	56098	PCDHGC4
-0.016594131	0.135612816	7109	TRAPPC10
-0.016640842	0.134515405	646127	LOC646127
-0.016669167	0.133853287	100133150	LOC100133150
-0.01671987	0.132674351	286257	C9orf142
-0.016764354	0.131646693	53918	PELO
-0.016768811	0.131544039	2253	FGF8
-0.016813572	0.130516812	8905	AP1S2
-0.016838368	0.129950508	11006	LILRB4
-0.016862884	0.129392415	214	ALCAM
-0.016866971	0.129299566	8403	SOX14
-0.016905138	0.128434926	122786	FRMD6
-0.016909132	0.12834473	84059	GPR98
-0.016986769	0.126600727	10328	COX4NB
-0.017000511	0.126293942	23140	ZZEF1
-0.017073134	0.124682434	53822	FXYD7
-0.017073411	0.124676324	55052	MRPL20
-0.017123096	0.123583093	3660	IRF2
-0.017186059	0.122208461	6284	S100A13
-0.017201103	0.12188179	6419	SETMAR
-0.017239735	0.121046074	57799	RAB40C
-0.017247442	0.120879859	10963	AP005668.1
-0.01726193	0.120567963	337967	KRTAP6-2
-0.01727521	0.120282546	55825	PECR
-0.017297776	0.119798861	7033	TFF3
-0.0173274	0.11916621	9379	NRXN2
-0.017401507	0.117595002	54498	SMOX

region-wise, subcortex					
Spearman's corr coef	p-val	p-val, SA	p-val, SA (BH- correction)	GenID	Gene
-0.056113254	0.744433284	0.754	1	51667	NUB1
-0.056113254	0.744433284	0.77	1	55512	SMPD3
-0.056113254	0.744433284	0.766	1	65258	MPPE1
-0.056113254	0.744433284	0.744	1	651302	ZNF192P1
-0.056370653	0.743297875	0.775	1	5467	PPARD
-0.056370653	0.743297875	0.767	1	7760	ZNF213
-0.056370653	0.743297875	0.771	1	29113	C6orf15
-0.056370653	0.743297875	0.774	1	266722	HS6ST3
-0.056628056	0.74216342	0.754	1	6369	CCL24
-0.056628056	0.74216342	0.763	1	7862	BRPF1
-0.056628056	0.74216342	0.763	1	29034	CPS1-IT1
-0.056628056	0.74216342	0.77	1	81619	TSPAN14
-0.056628056	0.74216342	0.749	1	116534	MRGPRE
-0.056628056	0.74216342	0.763	1	117608	ZNF354B
-0.056628056	0.74216342	0.783	1	153571	C5orf38
-0.056628056	0.74216342	0.767	1	401612	MCART6
-0.056885459	0.74102962	0.757	1	3747	KCNC2
-0.056885459	0.74102962	0.756	1	7086	TKT
-0.056885459	0.74102962	0.752	1	8969	HIST1H2AG
-0.056885459	0.74102962	0.759	1	10042	HMGXB4
-0.056885459	0.74102962	0.763	1	55138	FAM90A1
-0.056885459	0.74102962	0.783	1	60598	KCNK15
-0.056885459	0.74102962	0.774	1	115273	RAB42
-0.056885459	0.74102962	0.762	1	653604	HIST2H3D
-0.057142857	0.739896357	0.739	1	5822	PWP2H
-0.057142857	0.739896357	0.745	1	7292	TNFSF4
-0.057142857	0.739896357	0.774	1	8451	CUL4A
-0.057142857	0.739896357	0.784	1	83479	DDX59
-0.057142857	0.739896357	0.747	1	134218	DNAJC21
-0.057400256	0.738763094	0.758	1	2128	EVX1
-0.057400256	0.738763094	0.728	1	2804	GOLGB1
-0.057400256	0.738763094	0.761	1	10153	CEBPZ
-0.057400256	0.738763094	0.737	1	51227	PIGP
-0.057400256	0.738763094	0.725	1	55539	KCNQ1DN
-0.057400256	0.738763094	0.736	1	79934	ADCK4
-0.057400256	0.738763094	0.786	1	147694	ZNF548
-0.057400256	0.738763094	0.766	1	285367	RPUSD3
-0.057400256	0.738763094	0.74	1	285966	FAM115C
-0.057657655	0.737630844	0.738	1	3313	HSPA9
-0.057657655	0.737630844	0.774	1	10333	TLR6
-0.057657655	0.737630844	0.744	1	11183	MAP4K5
-0.057657655	0.737630844	0.749	1	55187	VPS13D
-0.057657655	0.737630844	0.768	1	63915	MUTED
-0.057657655	0.737630844	0.781	1	79906	MORN1
-0.057657655	0.737630844	0.755	1	90632	LINC00473
-0.057657655	0.737630844	0.763	1	219982	OR5A1
-0.057657655	0.737630844	0.761	1	255027	MPV17L
-0.057657655	0.737630844	0.782	1	286204	CRB2
-0.057657655	0.737630844	0.758	1	401176	RP11-19O2.2
-0.057657655	0.737630844	0.75	1	405753	DUOXA2
-0.057915058	0.73649925	0.756	1	266	AMELY
-0.057915058	0.73649925	0.749	1	1122	CHML
-0.057915058	0.73649925	0.748	1	9873	FCHSD2
-0.057915058	0.73649925	0.75	1	83988	NCALD
-0.057915058	0.73649925	0.754	1	440017	DUX4L15
-0.058172457	0.735368133	0.766	1	57701	KIAA1602
-0.058172457	0.735368133	0.746	1	114899	C1QTNF3
-0.058172457	0.735368133	0.775	1	136541	PRSS58
-0.058429856	0.734237075	0.74	1	6217	RPS16P1
-0.058429856	0.734237075	0.741	1	7288	TULP2
-0.058429856	0.734237075	0.755	1	9789	SPCS2
-0.058429856	0.734237075	0.754	1	10611	PDLIM5
-0.058429856	0.734237075	0.766	1	11164	NUDT5
-0.058429856	0.734237075	0.768	1	26257	NKX2-8
-0.058429856	0.734237075	0.787	1	51226	COPZ2
-0.058429856	0.734237075	0.751	1	51308	REEP2
-0.058429856	0.734237075	0.776	1	84952	CGNL1
-0.058429856	0.734237075	0.743	1	135927	C7orf34
-0.058429856	0.734237075	0.782	1	349152	DPY19L2P2
-0.058429856	0.734237075	0.756	1	388662	SLC6A17
-0.058429856	0.734237075	0.757	1	474354	LRRC18
-0.058687259	0.73310715	0.762	1	4254	KITLG
-0.058687259	0.73310715	0.728	1	10657	KHDRBS1
-0.058687259	0.73310715	0.747	1	51035	UBXN1
-0.058687259	0.73310715	0.737	1	79725	THAP9
-0.058687259	0.73310715	0.739	1	83941	TM2D1
-0.058687259	0.73310715	0.767	1	84669	USP32
-0.058687259	0.73310715	0.747	1	646405	TPTE2P1
-0.058944657	0.731977761	0.769	1	5355	PLP2

voxel-wise, subcortex			
Spearman's corr coef	p-val	GenID	Gene
-0.017421195	0.117180303	339883	C3orf35
-0.017584825	0.113778129	7873	ARMET
-0.017665153	0.112136647	642658	SCXB
-0.017675554	0.111925468	10938	EHD1
-0.017716881	0.111089543	120526	DNAJC24
-0.017764335	0.110135734	51759	C9orf78
-0.017787365	0.109675184	54949	SDHAF2
-0.01784225	0.108583741	23639	LRRC6
-0.01786788	0.108077005	79791	FBXO31
-0.017885897	0.107721895	9244	CRLF1
-0.017894719	0.107548386	10026	PIGK
-0.017911341	0.107222006	23061	TBC1D9B
-0.018091781	0.103729457	6247	RS1
-0.018120848	0.103175387	728642	CDC2L2
-0.018146386	0.102690533	172	AFG3L1P
-0.018151494	0.102593765	5439	POLR2J
-0.01817666	0.10211812	2151	F2RL2
-0.01820842	0.101520345	83869	TTTY14
-0.018267205	0.100421235	54993	ZSCAN2
-0.018410033	0.097790308	401562	LCNL1
-0.018538279	0.09547516	56913	C1GALT1
-0.018587591	0.094596714	4603	MYBL1
-0.018606909	0.094254367	80119	PIF1
-0.018696059	0.092687301	65983	GRAMD3
-0.018697917	0.092654832	368	ABCC6
-0.018700425	0.092611097	5330	PLCB2
-0.018788924	0.091077201	54819	ZCCHC10
-0.018875292	0.089600049	963	CD53
-0.018878076	0.089552753	123591	C15orf27
-0.018954691	0.088259153	55707	NECAP2
-0.019005861	0.087403573	5368	PNOC
-0.019011153	0.0873155	2693	GHSR
-0.019140238	0.085188627	2588	GALNS
-0.019176176	0.084604017	93081	C13orf27
-0.019227067	0.083781675	51562	MBIP
-0.019269042	0.083108343	6236	RRAD
-0.019387353	0.081233919	347918	EP400NL
-0.019475577	0.079858638	55589	BMP2K
-0.019510215	0.07932391	51428	DDX41
-0.019536775	0.078915842	1145	CHRNE
-0.019559713	0.078564808	51465	UBE2J1
-0.019579865	0.078257442	155435	RBM33
-0.019596023	0.078011684	6790	AURKA
-0.019602153	0.077918649	6336	SCN10A
-0.019688519	0.076617099	25821	MTO1
-0.019746002	0.07576064	23420	NOMO1
-0.019749809	0.075704217	25879	DCAF13
-0.019768568	0.075426623	191585	PLAC4
-0.019823732	0.074615017	11262	SP140
-0.019851126	0.074214652	1268	CNR1
-0.019857163	0.074126668	340602	CXorf67
-0.019873878	0.073883429	150275	CCDC117
-0.019889109	0.073662437	55661	DDX27
-0.019947428	0.072821096	8614	STC2
-0.019974917	0.072427273	400720	ZNF772
-0.020015035	0.071855627	254973	OR1L4
-0.020187858	0.069435194	51398	WDR83OS
-0.020189159	0.069417238	5494	PPM1A
-0.020190738	0.069395453	6555	SLC10A2
-0.020207454	0.069165051	6921	TCEB1
-0.020292705	0.067999683	54973	CPSF3L
-0.020318242	0.067653768	7525	YES1
-0.020350095	0.067224354	348378	FAM159A
-0.02046237	0.06572853	10009	ZBTB33
-0.020516604	0.065015838	9754	STARD8
-0.020632315	0.063516609	122773	KLHDC1
-0.02064253	0.063385651	343069	HNRNPCL1
-0.020696115	0.062702283	6450	SH3BGR
-0.020696765	0.062694028	100130302	FAM48B1
-0.020742919	0.062110346	4294	MAP3K10
-0.020745054	0.062083486	148345	C1orf127
-0.020749604	0.062026206	117583	PARD3B
-0.020766228	0.061817318	140700	SAMD10
-0.02085603	0.06069896	2752	GLUL
-0.020905342	0.060092047	1815	DRD4
-0.020921128	0.059898823	256227	AC099759.1
-0.020985113	0.059120864	55367	LRDD
-0.020995142	0.058999702	125113	KRT222
-0.021009073	0.058831722	389857	CENPVL1

region-wise, subcortex					
Spearman's corr coef	p-val	p-val, SA	p-val, SA (BH- correction)	GenID	Gene
-0.058944657	0.731977761	0.747	1	11000	SLC27A3
-0.058944657	0.731977761	0.774	1	23302	WSCD1
-0.058944657	0.731977761	0.761	1	55781	RIOK2
-0.058944657	0.731977761	0.771	1	283537	SLC46A3
-0.058944657	0.731977761	0.768	1	338398	TAS2R60
-0.05920206	0.730848849	0.77	1	29775	CARD10
-0.05920206	0.730848849	0.76	1	54516	MTRF1L
-0.05920206	0.730848849	0.769	1	127435	PODN
-0.05920206	0.730848849	0.748	1	283683	LOC283683
-0.05920206	0.730848849	0.773	1	100190986	LOC100190986
-0.059459459	0.729720533	0.723	1	4140	MARK3
-0.059459459	0.729720533	0.742	1	4194	MDM4
-0.059459459	0.729720533	0.749	1	4300	MLLT3
-0.059459459	0.729720533	0.767	1	10454	MAP3K7IP1
-0.059459459	0.729720533	0.739	1	144983	RP11-78J21.3
-0.059459459	0.729720533	0.777	1	643997	LOC643997
-0.059459459	0.729720533	0.738	1	100289361	LOC100289361
-0.059716858	0.728592277	0.775	1	1657	DMXL1
-0.059716858	0.728592277	0.732	1	2915	GRM5
-0.059716858	0.728592277	0.735	1	7290	HIRA
-0.059716858	0.728592277	0.766	1	7465	WEE1
-0.059716858	0.728592277	0.76	1	7761	ZNF214
-0.059716858	0.728592277	0.745	1	10412	NSA2
-0.059716858	0.728592277	0.745	1	22863	KIAA0831
-0.059716858	0.728592277	0.757	1	81442	OR6N2
-0.059716858	0.728592277	0.74	1	256472	TMEM151A
-0.059716858	0.728592277	0.746	1	400043	RP11-834C11.4
-0.059974257	0.727465153	0.758	1	1159	CKMT1B
-0.059974257	0.727465153	0.753	1	22919	MAPRE1
-0.059974257	0.727465153	0.734	1	91975	ZNF300
-0.059974257	0.727465153	0.761	1	222256	CTA-351J1.1
-0.060231656	0.726338565	0.734	1	843	CASP10
-0.060231656	0.726338565	0.734	1	2207	FCER1G
-0.060231656	0.726338565	0.744	1	3687	ITGAX
-0.060231656	0.726338565	0.752	1	8402	SLC25A11
-0.060231656	0.726338565	0.758	1	23769	FLRT1
-0.060231656	0.726338565	0.748	1	55237	C14orf115
-0.060231656	0.726338565	0.734	1	84152	PPP1R1B
-0.060231656	0.726338565	0.735	1	257106	ARHGAP30
-0.060489062	0.725212514	0.729	1	3777	KCNK3
-0.060489062	0.725212514	0.745	1	57010	CABP4
-0.060489062	0.725212514	0.753	1	65999	LRRC61
-0.060489062	0.725212514	0.727	1	79576	NKAP
-0.060489062	0.725212514	0.739	1	79719	AAGAB
-0.060746461	0.724086583	0.722	1	439	ASNA1
-0.060746461	0.724086583	0.751	1	868	CBLB
-0.060746461	0.724086583	0.714	1	4711	NDUFB5
-0.060746461	0.724086583	0.77	1	5021	OXTR
-0.060746461	0.724086583	0.756	1	7441	VPREB1
-0.060746461	0.724086583	0.748	1	23431	AP4E1
-0.060746461	0.724086583	0.726	1	25913	POT1
-0.060746461	0.724086583	0.746	1	26471	NUPR1
-0.060746461	0.724086583	0.724	1	51234	TMEM85
-0.060746461	0.724086583	0.753	1	57508	INTS2
-0.060746461	0.724086583	0.742	1	84124	ZNF394
-0.060746461	0.724086583	0.763	1	94134	ARHGAP12
-0.060746461	0.724086583	0.765	1	113026	PLCD3
-0.060746461	0.724086583	0.737	1	390197	OR4D10
-0.060746461	0.724086583	0.745	1	440084	LOC440084
-0.06100386	0.722961724	0.749	1	64794	DDX31
-0.06100386	0.722961724	0.727	1	142680	SLC34A3
-0.06100386	0.722961724	0.761	1	390271	OR8B3
-0.06100386	0.722961724	0.713	1	100132341	KIAA0664L3
-0.061261259	0.721837342	0.755	1	2597	GAPDH
-0.061261259	0.721837342	0.762	1	4189	DNAJB9
-0.061261259	0.721837342	0.747	1	5387	PMS2L3
-0.061261259	0.721837342	0.748	1	6232	RPS27
-0.061261259	0.721837342	0.75	1	6273	S100A2
-0.061261259	0.721837342	0.738	1	9325	TRIP4
-0.061261259	0.721837342	0.749	1	57468	SLC12A5
-0.061261259	0.721837342	0.748	1	79443	FYCO1
-0.061518662	0.720713735	0.753	1	2799	GNS
-0.061518662	0.720713735	0.752	1	5454	POU3F2
-0.061518662	0.720713735	0.743	1	23195	MDN1
-0.061518662	0.720713735	0.743	1	55120	FANCL
-0.061518662	0.720713735	0.767	1	148156	ZNF558
-0.061518662	0.720713735	0.761	1	282966	C10orf53
-0.061518662	0.720713735	0.746	1	338674	OR5F1
-0.061776061	0.719590068	0.748	1	10500	SEMA6C

voxel-wise, subcortex			
Spearman's corr coef	p-val	GenID	Gene
-0.021036562	0.058501441	147166	TRIM16L
-0.021036653	0.058500346	79089	TMUB2
-0.021067765	0.058128376	211	ALAS1
-0.021129241	0.057399206	51306	FAM13B
-0.021147443	0.057184789	160140	C11orf65
-0.021166945	0.056955781	124930	ANKRD13B
-0.021213656	0.056410335	3300	DNAJB2
-0.021221086	0.056323983	26073	POLDIP2
-0.021259997	0.055873547	339184	CCDC144NL
-0.021316737	0.055222087	79618	HMBOX1
-0.021322217	0.055159509	126820	WDR63
-0.021343112	0.054921433	64093	SMOC1
-0.021376079	0.054547533	26084	RP11-23D24.1
-0.02138193	0.054481413	8404	SPARCL1
-0.021437462	0.05385704	51397	COMMD10
-0.02147916	0.053392142	2000	ELF4
-0.021487517	0.053299379	9048	ARTN
-0.021656627	0.05145089	23213	SULF1
-0.021719033	0.050782427	84254	CAMKK1
-0.021724604	0.050723117	23376	KIAA0776
-0.02176073	0.050339852	79747	C6orf103
-0.021802055	0.049904432	440348	NPIPL2
-0.021813663	0.049782716	56995	TULP4
-0.021845331	0.04945185	10768	AHCYL1
-0.021849046	0.049413163	51172	NAGPA
-0.021925939	0.048618004	1479	CSTF3
-0.021943768	0.048435166	7783	ZP2
-0.021958906	0.048280388	6425	SFRP5
-0.021974694	0.048119418	8975	USP13
-0.021981101	0.048054222	7745	ZNF192
-0.021995403	0.047908951	972	CD74
-0.022061894	0.047238391	84166	NLRC5
-0.022071552	0.047141649	79159	NOL12
-0.022076102	0.047096137	552891	DNAJC25-GNG10
-0.022109164	0.046766486	728532	LOC728532
-0.022143058	0.046430551	7145	TNS1
-0.022191536	0.045953631	8575	PRKRA
-0.022306688	0.044837285	80742	PRR3
-0.022359159	0.044336218	5452	POU2F2
-0.022400763	0.043942317	84926	SPRYD3
-0.022462519	0.04336305	1509	CTSD
-0.02247422	0.043254025	549	AUH
-0.022527432	0.042761117	5939	RBMS2
-0.022577208	0.042304371	259234	DSCR10
-0.022603767	0.042062353	339059	AC138028.2
-0.022604696	0.042053904	10794	ZNF460
-0.022617418	0.04193842	10396	ATP8A1
-0.022646021	0.041679725	100288798	LOC100288798
-0.022649085	0.041652109	347732	CATSPER3
-0.022750217	0.040748779	641339	ZNF674
-0.022804173	0.040273629	51347	TAOK3
-0.022847354	0.03989679	26003	GORASP2
-0.022889331	0.039533339	256471	MFSD8
-0.022894345	0.0394901	55174	INTS10
-0.022942636	0.039075825	84034	EMILIN2
-0.022953594	0.038982362	259291	TAS2R45
-0.02301117	0.03849424	253943	YTHDF3
-0.023054538	0.038130041	9644	SH3PXD2A
-0.023069769	0.038002804	115948	CCDC151
-0.023104779	0.037711781	930	CD19
-0.023126509	0.037532073	56924	PAK6
-0.02314917	0.037345443	10816	SPINT3
-0.023197832	0.036947407	54816	ZNF280D
-0.023251694	0.036510989	54583	EGLN1
-0.023316327	0.035993136	4501	MT1X
-0.023342887	0.035782129	10245	TIMM17B
-0.02334688	0.035750512	23192	ATG4B
-0.023422102	0.035159163	7067	THRA
-0.023448383	0.034954499	79365	BHLHE41
-0.023451913	0.034927081	60626	RIC8A
-0.023490731	0.034626901	141	ADPRH
-0.023538278	0.034262195	24139	EML2
-0.023683241	0.033170514	7737	RNF113A
-0.023693642	0.033093333	1397	CRIP2
-0.023739332	0.03275615	115	ADCY9
-0.023825604	0.032127488	55719	FAM178A
-0.023832105	0.032080535	10268	RAMP3
-0.023839071	0.032030284	100289635	ZNF605
-0.023872688	0.031788763	166348	KLHDC6

region-wise, subcortex					
Spearman's corr coef	p-val	p-val, SA	p-val, SA (BH- correction)	GenID	Gene
-0.061776061	0.719590068	0.745	1	59272	ACE2
-0.061776061	0.719590068	0.753	1	162466	PHOSPHO1
-0.061776061	0.719590068	0.74	1	390535	GOLGA8E
-0.061776061	0.719590068	0.739	1	653639	LYPLA2P1
-0.06203346	0.718467534	0.752	1	55	ACPP
-0.06203346	0.718467534	0.738	1	10422	UBAC1
-0.062290862	0.717345595	0.74	1	716	C1S
-0.062290862	0.717345595	0.719	1	3123	HLA-DRB1
-0.062290862	0.717345595	0.744	1	6048	RNF5
-0.062290862	0.717345595	0.757	1	26280	KCTD9P2
-0.062290862	0.717345595	0.714	1	84269	CHCHD5
-0.062548265	0.716224253	0.745	1	23406	COTL1
-0.062548265	0.716224253	0.733	1	51714	SELT
-0.062548265	0.716224253	0.752	1	83464	APH1B
-0.062548265	0.716224253	0.717	1	149041	RC3H1
-0.062548265	0.716224253	0.746	1	388610	TRNP1
-0.062548265	0.716224253	0.72	1	401563	C9orf139
-0.06280566	0.715103447	0.738	1	27069	GHITM
-0.06280566	0.715103447	0.774	1	152815	THAP6
-0.06280566	0.715103447	0.757	1	440836	ODF3B
-0.06280566	0.715103447	0.735	1	100130794	NDUFS5P7
-0.063063063	0.713982701	0.737	1	274	BIN1
-0.063063063	0.713982701	0.737	1	8302	KLRC4
-0.063063063	0.713982701	0.754	1	26579	MYEOV
-0.063063063	0.713982701	0.723	1	149461	CLDN19
-0.063063063	0.713982701	0.732	1	388630	LOC388630
-0.063063063	0.713982701	0.729	1	643866	CBLN3
-0.063320458	0.712863147	0.733	1	55224	ETNK2
-0.063320458	0.712863147	0.749	1	201266	SLC39A11
-0.063577861	0.711744189	0.753	1	2535	FZD2
-0.063577861	0.711744189	0.714	1	8161	COIL
-0.063577861	0.711744189	0.743	1	29895	MYLPF
-0.063835263	0.710625768	0.743	1	346	APOC4
-0.063835263	0.710625768	0.743	1	1184	CLCN5
-0.063835263	0.710625768	0.731	1	1588	CYP19A1
-0.063835263	0.710625768	0.732	1	2303	FOXC2
-0.063835263	0.710625768	0.723	1	4552	MTRR
-0.063835263	0.710625768	0.74	1	9230	RAB11B
-0.063835263	0.710625768	0.729	1	23464	GCAT
-0.063835263	0.710625768	0.708	1	54890	ALKBH5
-0.063835263	0.710625768	0.722	1	339229	C17orf90
-0.064092666	0.709507525	0.76	1	208	AKT2
-0.064092666	0.709507525	0.71	1	2162	F13A1
-0.064092666	0.709507525	0.727	1	11272	PRR4
-0.064092666	0.709507525	0.73	1	51109	RDH11
-0.064092666	0.709507525	0.714	1	168448	CDC14C
-0.064350061	0.708390355	0.718	1	90	ACVR1
-0.064350061	0.708390355	0.738	1	1117	CHI3L2
-0.064350061	0.708390355	0.721	1	28511	NKIRAS2
-0.064350061	0.708390355	0.714	1	51780	KDM3B
-0.064607456	0.707273722	0.726	1	279	AMY2A
-0.064607456	0.707273722	0.736	1	2644	GCHFR
-0.064607456	0.707273722	0.749	1	6668	SP2
-0.064607456	0.707273722	0.751	1	51096	UTP18
-0.064864866	0.706157804	0.723	1	554	AVPR2
-0.064864866	0.706157804	0.744	1	713	C1QB
-0.064864866	0.706157804	0.709	1	5335	PLCG1
-0.064864866	0.706157804	0.738	1	9397	NMT2
-0.064864866	0.706157804	0.743	1	79009	DDX50
-0.065122269	0.705041945	0.716	1	1769	DNAH8
-0.065122269	0.705041945	0.734	1	4982	TNFRSF11B
-0.065122269	0.705041945	0.727	1	7105	TSPAN6
-0.065122269	0.705041945	0.733	1	11198	SUPT16H
-0.065122269	0.705041945	0.732	1	57697	FANCM
-0.065122269	0.705041945	0.72	1	148046	CIRBP-AS1
-0.065122269	0.705041945	0.741	1	163732	CITED4
-0.065379664	0.703927219	0.694	1	9169	SCAF11
-0.065379664	0.703927219	0.725	1	11143	MYST2
-0.065379664	0.703927219	0.709	1	25823	TPSG1
-0.065379664	0.703927219	0.715	1	284001	CCDC57
-0.065637067	0.702813089	0.737	1	2713	GK3P
-0.065637067	0.702813089	0.739	1	5453	POU3F1
-0.065637067	0.702813089	0.71	1	79872	CBLL1
-0.065637067	0.702813089	0.716	1	284412	LOC284412
-0.065637067	0.702813089	0.721	1	347475	AC004409.1
-0.06589447	0.701699555	0.714	1	9875	URB1
-0.06589447	0.701699555	0.729	1	26112	CCDC69
-0.06589447	0.701699555	0.702	1	28232	SLCO3A1
-0.06589447	0.701699555	0.729	1	81448	OR6K2

voxel-wise, subcortex			
Spearman's corr coef	p-val	GenID	Gene
-0.02388504	0.03170041	545	ATR
-0.02389284	0.031644728	128637	TBC1D20
-0.023947259	0.031258572	51024	FIS1
-0.023948932	0.031246753	115362	GBP5
-0.023976326	0.031053986	116254	C6orf72
-0.024005486	0.030849883	55568	GALNT10
-0.024100024	0.030196086	221154	EFHA1
-0.024177846	0.029666878	388699	LINC00302
-0.024180539	0.02964871	28965	SLC27A6
-0.024186296	0.029609881	554203	JPX
-0.024195861	0.029545499	10660	LBX1
-0.024213599	0.029426416	54470	ARMCX6
-0.024217779	0.029398413	56260	C8orf44
-0.024343891	0.028564284	23334	KIAA0467
-0.02442329	0.028049646	8813	DPM1
-0.024436848	0.027962578	7462	LAT2
-0.024469351	0.027754782	116092	DNTTIP1
-0.024683129	0.026420962	56965	PARP6
-0.024692602	0.026363151	7915	ALDH5A1
-0.024700588	0.026314519	3146	HMGB1
-0.024762252	0.025941508	26269	FBXO8
-0.02477256	0.02587962	390271	OR8B3
-0.024784168	0.025810068	342538	NACA2
-0.024803113	0.025696881	8886	DDX18
-0.024838217	0.025488308	140467	ZNF358
-0.024889199	0.025188012	7517	XRCC3
-0.024946218	0.02485577	23099	ZBTB43
-0.024975194	0.024688387	11000	SLC27A3
-0.025045864	0.024284258	54994	C20orf11
-0.025092576	0.024020283	23049	SMG1
-0.025099076	0.023983769	441376	AARD
-0.025123036	0.023849517	55048	VPS37C
-0.025195099	0.023449652	158800	RHOXF1
-0.025233639	0.02323821	9611	NCOR1
-0.02526252	0.023080846	55654	TMEM127
-0.025270229	0.023038983	10163	WASF2
-0.025288431	0.022940459	159013	CXorf38
-0.025472213	0.021965748	7570	ZNF22
-0.025472676	0.021963334	10316	NMUR1
-0.025636492	0.021125181	90121	TSR2
-0.025655344	0.021030532	54940	OCIAD1
-0.025687382	0.02087055	10490	VTI1B
-0.025687847	0.020868216	6609	SMPD1
-0.025897074	0.019849317	575	BAI1
-0.025934219	0.019673031	2145	EZH1
-0.025940906	0.019641435	91179	SCARF2
-0.025968023	0.019513797	3551	IKBKB
-0.025973132	0.019489828	221662	RBM24
-0.026023744	0.019253781	100128288	AC135178.1
-0.026104072	0.018884262	94121	SYTL4
-0.02611048	0.018855048	7311	RPS27AP11
-0.026141126	0.018715911	6520	SLC3A2
-0.026263338	0.018169917	51318	MRPL35
-0.02628191	0.018088169	4900	NRGN
-0.026295377	0.018029111	8577	TMEFF1
-0.026379513	0.017663872	440574	C1orf151
-0.026425481	0.017467082	9414	TJP2
-0.026431331	0.017442171	6789	STK4
-0.026437925	0.01741413	51202	DDX47
-0.02645687	0.017333804	51501	C11orf73
-0.0264799	0.017236592	26576	SRPK3
-0.026506182	0.017126232	100170229	AC006276.1
-0.026532371	0.01701688	414919	C8orf82
-0.02655215	0.016934702	80342	TRAF3IP3
-0.026575552	0.016837925	6716	SRD5A2
-0.026594033	0.016761843	93621	MRFAP1
-0.026602948	0.016725246	100129480	LOC100129480
-0.026604062	0.016720675	167681	PRSS23
-0.026612978	0.016684143	116540	MRPL53
-0.026617156	0.016667066	442229	SLC25A51P1
-0.026646502	0.016547462	9526	MPDU1
-0.026652073	0.016524844	10265	IRX5
-0.02671513	0.016270714	25972	UNC50
-0.026718289	0.016258072	133383	C5orf35
-0.026752833	0.016120395	79649	MAP7D3
-0.026756456	0.016106002	53919	SLCO1C1
-0.026757663	0.01610123	64760	FAM160B2
-0.026769642	0.016053783	115653	KIR3DL3
-0.026793787	0.015958512	51292	GMPR2

region-wise, subcortex					
Spearman's corr coef	p-val	p-val, SA	p-val, SA (BH- correction)	GenID	Gene
-0.06589447	0.701699555	0.732	1	84812	PLCD4
-0.066151865	0.700586677	0.722	1	27130	INVS
-0.066151865	0.700586677	0.751	1	28985	MCTS1
-0.066151865	0.700586677	0.716	1	53918	PELO
-0.066151865	0.700586677	0.719	1	55000	TUG1
-0.066151865	0.700586677	0.747	1	79608	RIC3
-0.066151865	0.700586677	0.723	1	284361	C19orf63
-0.066151865	0.700586677	0.743	1	646938	AC022748.1
-0.066409267	0.699473917	0.729	1	4542	MYO1F
-0.066409267	0.699473917	0.716	1	7389	UROD
-0.066409267	0.699473917	0.736	1	25840	METTL7A
-0.066409267	0.699473917	0.725	1	25880	TMEM186
-0.066409267	0.699473917	0.75	1	26073	POLDIP2
-0.066409267	0.699473917	0.724	1	80018	C12orf30
-0.066409267	0.699473917	0.745	1	163087	ZNF383
-0.066409267	0.699473917	0.704	1	200576	PIKFYVE
-0.066409267	0.699473917	0.716	1	100131471	LOC100131471
-0.066666663	0.698362231	0.715	1	1464	CSPG4
-0.066666663	0.698362231	0.728	1	7165	TPD52L2
-0.066666663	0.698362231	0.71	1	9896	FIG4
-0.066666663	0.698362231	0.705	1	167826	OLIG3
-0.066924065	0.69725126	0.749	1	2620	GAS2
-0.066924065	0.69725126	0.744	1	7512	XPNPEP2
-0.066924065	0.69725126	0.745	1	27136	MORC1
-0.066924065	0.69725126	0.703	1	27324	TOX3
-0.066924065	0.69725126	0.726	1	91949	COG7
-0.066924065	0.69725126	0.698	1	388228	SBK1
-0.067181468	0.696140885	0.726	1	3978	LIG1
-0.067181468	0.696140885	0.718	1	5683	PSMA2
-0.067181468	0.696140885	0.715	1	6427	SFRS2
-0.067181468	0.696140885	0.761	1	10933	MORF4L1
-0.067181468	0.696140885	0.722	1	26298	EHF
-0.067181468	0.696140885	0.691	1	80895	ILKAP
-0.067181468	0.696140885	0.741	1	84826	SFT2D3
-0.067181468	0.696140885	0.707	1	399664	MEX3D
-0.067438863	0.69503063	0.695	1	2358	FPR2
-0.067438863	0.69503063	0.716	1	6541	SLC7A1
-0.067438863	0.69503063	0.731	1	10797	MTHFD2
-0.067438863	0.69503063	0.729	1	27159	CHIA
-0.067438863	0.69503063	0.722	1	27336	HTATSF1
-0.067438863	0.69503063	0.698	1	55501	CHST12
-0.067438863	0.69503063	0.73	1	148753	FAM163A
-0.067438863	0.69503063	0.72	1	337975	KRTAP20-1
-0.067438863	0.69503063	0.73	1	399821	RP11-383C5.4
-0.067696266	0.693921447	0.737	1	283	ANG
-0.067696266	0.693921447	0.702	1	1327	COX4I1
-0.067696266	0.693921447	0.734	1	10282	BET1
-0.067696266	0.693921447	0.697	1	25915	NDUFAF3
-0.067696266	0.693921447	0.714	1	60313	GPBP1L1
-0.067696266	0.693921447	0.736	1	118427	OLFM3
-0.067696266	0.693921447	0.762	1	245937	DEFB124
-0.067953661	0.692812979	0.72	1	2068	ERCC2
-0.067953661	0.692812979	0.736	1	3418	IDH2
-0.067953661	0.692812979	0.738	1	4123	MAN2C1
-0.067953661	0.692812979	0.728	1	5230	PGK1
-0.067953661	0.692812979	0.719	1	10632	ATP5L
-0.067953661	0.692812979	0.715	1	114804	RNF157
-0.067953661	0.692812979	0.737	1	147929	ZNF565
-0.068211071	0.691705108	0.73	1	9655	SOCS5
-0.068211071	0.691705108	0.745	1	11024	LILRA1
-0.068211071	0.691705108	0.709	1	57460	PPM1H
-0.068211071	0.691705108	0.728	1	63951	DMRTA1
-0.068211071	0.691705108	0.743	1	127733	UBXN10
-0.068211071	0.691705108	0.725	1	150365	MEI1
-0.068211071	0.691705108	0.705	1	257000	PLAC2
-0.068468474	0.690597415	0.698	1	805	CALM2
-0.068468474	0.690597415	0.708	1	2995	GYPC
-0.068468474	0.690597415	0.712	1	220441	RNF152
-0.068725869	0.689490855	0.707	1	5037	PEBP1
-0.068725869	0.689490855	0.703	1	6407	SEMG2
-0.068725869	0.689490855	0.713	1	10157	AASS
-0.068725869	0.689490855	0.723	1	10916	MAGED2
-0.068725869	0.689490855	0.715	1	654466	KGFLP2
-0.068983272	0.688384891	0.704	1	262	AMD1
-0.068983272	0.688384891	0.736	1	3592	IL12A
-0.068983272	0.688384891	0.714	1	10370	CITED2
-0.068983272	0.688384891	0.724	1	50717	WDR42A
-0.068983272	0.688384891	0.718	1	56339	METTL3
-0.068983272	0.688384891	0.739	1	138802	OR13C8

voxel-wise, subcortex			
Spearman's corr coef	p-val	GenID	Gene
-0.026811989	0.015887013	4756	NEO1
-0.026828613	0.015821973	10881	ACTL7A
-0.026844865	0.015758613	3488	IGFBP5
-0.026996514	0.015178028	51101	ZC2HC1A
-0.027053719	0.014963967	2235	FECH
-0.027079908	0.014866848	8451	CUL4A
-0.02712114	0.01471508	23119	HIC2
-0.027152436	0.014600802	113444	C1orf212
-0.027170544	0.014535035	26873	OPLAH
-0.027178345	0.014506779	23293	SMG6
-0.027189676	0.014465836	113451	ADC
-0.02723063	0.01431868	331	XIAP
-0.027242145	0.014277536	387990	TOMM20L
-0.027272884	0.014168235	51599	LSR
-0.027295357	0.014088796	9463	PICK1
-0.02730808	0.014043993	3351	HTR1B
-0.027346155	0.013910671	775	CACNA1C
-0.027347084	0.013907424	4968	OGG1
-0.027356742	0.013873792	6642	SNX1
-0.027381444	0.013788093	284307	ZIK1
-0.027398624	0.013728771	51129	ANGPTL4
-0.027481925	0.013444302	100132831	LOC100132831
-0.027489262	0.013419494	6017	RLBP1
-0.027527986	0.013289242	23181	DIP2A
-0.027558818	0.013186347	126074	C19orf39
-0.027567547	0.013157342	5689	PSMB1
-0.027600423	0.013048603	143884	CWF19L2
-0.027644904	0.012902748	126668	TDRD10
-0.027654842	0.012870357	51334	PRR16
-0.02767323	0.012810623	2530	FUT8
-0.027686045	0.012769141	4298	MLLT1
-0.027692731	0.01274754	57508	INTS2
-0.027695332	0.012739143	28955	DEXI
-0.027700903	0.012721182	11340	EXOSC8
-0.027762566	0.01252387	83443	SF3B5
-0.027822929	0.012333337	391195	OR2T33
-0.027850231	0.01224801	23784	POTEH
-0.027876604	0.012166087	10952	SEC61B
-0.027893322	0.012114404	10573	MRPL28
-0.027962228	0.011903429	440567	UQCRHL
-0.027975136	0.011864278	115004	C6orf150
-0.028149445	0.011346591	55613	MTMR8
-0.028266085	0.011011463	64129	TINAGL1
-0.028330719	0.010829576	54455	FBXO42
-0.028347621	0.01078245	145226	RDH12
-0.028381983	0.010687216	10302	SNAPC5
-0.028398698	0.010641154	10004	NAALADL1
-0.028429344	0.010557172	10493	VAT1
-0.028450517	0.010499498	84706	GPT2
-0.028466862	0.010455157	53345	TM6SF2
-0.028471133	0.010443602	29107	NXT1
-0.028492585	0.010385728	79157	MFSD11
-0.028505586	0.010350788	29109	FHOD1
-0.028535211	0.010271561	162966	ZNF600
-0.028551091	0.010229321	7626	ZNF75D
-0.028553041	0.010224145	55830	GLT8D1
-0.028593902	0.010116209	10061	ABCF2
-0.028719177	0.009791578	9277	WDR46
-0.028776662	0.009645759	3691	ITGB4
-0.028804336	0.009576241	51735	RAPGEF6
-0.028842596	0.009480876	54502	RBM47
-0.028871015	0.009410593	23125	CAMTA2
-0.028894788	0.009352152	25970	SH2B1
-0.028921533	0.009286793	83394	PITPNM3
-0.028959701	0.009194228	23438	HARS2
-0.029008919	0.009076075	121642	ALKBH2
-0.029031115	0.009023243	2949	GSTM5
-0.029035294	0.009013325	6249	CLIP1
-0.029043188	0.008994614	138864	LOC138864
-0.029092221	0.008879191	1370	CPN2
-0.029140325	0.008767237	9213	XPR1
-0.029192053	0.00864827	644943	RASSF10
-0.029207468	0.008613095	121274	ZNF641
-0.029227806	0.008566883	51106	TFB1M
-0.02936274	0.00826586	100131479	LOC100131479
-0.029366268	0.008258114	91355	LRP5L
-0.029375741	0.008237357	11011	TLK2
-0.029471764	0.008029587	51011	FAHD2A
-0.029497767	0.007974139	9812	KIAA0141

region-wise, subcortex					
Spearman's corr coef	p-val	p-val, SA	p-val, SA (BH- correction)	GenID	Gene
-0.068983272	0.688384891	0.683	1	144363	LYRM5
-0.068983272	0.688384891	0.707	1	152137	CCDC50
-0.068983272	0.688384891	0.751	1	375387	LRRC33
-0.069240667	0.687279582	0.715	1	6296	ACSM3
-0.069240667	0.687279582	0.702	1	51490	C9orf114
-0.069240667	0.687279582	0.709	1	80835	TAS1R1
-0.069240667	0.687279582	0.725	1	114971	PTPMT1
-0.069240667	0.687279582	0.723	1	170394	PWWP2B
-0.069240667	0.687279582	0.714	1	100128496	C20orf78
-0.06949807	0.686174929	0.717	1	10139	ARFRP1
-0.06949807	0.686174929	0.692	1	10553	HTATIP2
-0.06949807	0.686174929	0.695	1	388574	AC139099.1
-0.06949807	0.686174929	0.719	1	390616	ANKRD34C
-0.069755472	0.685070455	0.707	1	5033	P4HA1
-0.069755472	0.685070455	0.734	1	5861	RAB1A
-0.069755472	0.685070455	0.713	1	51341	ZBTB7A
-0.069755472	0.685070455	0.722	1	124152	IQCK
-0.069755472	0.685070455	0.707	1	283899	INO80E
-0.070012867	0.683967054	0.693	1	566	AZU1
-0.070012867	0.683967054	0.71	1	1398	CRK
-0.070012867	0.683967054	0.709	1	2051	EPHB6
-0.070012867	0.683967054	0.679	1	3748	KCNC3
-0.070012867	0.683967054	0.696	1	4488	MSX2
-0.070012867	0.683967054	0.725	1	10162	U47924.1
-0.070012867	0.683967054	0.686	1	25972	UNC50
-0.070012867	0.683967054	0.712	1	84071	ARMC2
-0.070012867	0.683967054	0.714	1	84364	ARFGAP2
-0.07027027	0.682864368	0.703	1	6917	TCEA1P2
-0.07027027	0.682864368	0.711	1	9955	HS3ST3A1
-0.07027027	0.682864368	0.711	1	55301	OLAH
-0.07027027	0.682864368	0.725	1	79623	GALNT14
-0.07027027	0.682864368	0.706	1	147664	ERVV-1
-0.07027027	0.682864368	0.692	1	376132	LRRC10
-0.070527665	0.681762338	0.703	1	6428	SRSF3
-0.070527665	0.681762338	0.702	1	23157	SEPTIN6
-0.070527665	0.681762338	0.716	1	23235	SIK2
-0.070527665	0.681762338	0.693	1	25801	GCA
-0.070527665	0.681762338	0.722	1	27343	POLL
-0.070527665	0.681762338	0.713	1	55128	TRIM68
-0.070527665	0.681762338	0.697	1	79613	TMCO7
-0.070527665	0.681762338	0.72	1	114034	TOE1
-0.070785068	0.680660427	0.727	1	478	ATP1A3
-0.070785068	0.680660427	0.724	1	5229	PGGT1B
-0.070785068	0.680660427	0.706	1	7375	USP4
-0.070785068	0.680660427	0.71	1	11182	SLC2A6
-0.070785068	0.680660427	0.698	1	51091	SEPSECS
-0.070785068	0.680660427	0.724	1	55472	C8orf39
-0.070785068	0.680660427	0.705	1	83734	ATG10
-0.070785068	0.680660427	0.706	1	91464	ISX
-0.070785068	0.680660427	0.723	1	392360	CTSL3
-0.070785068	0.680660427	0.698	1	100127206	KIAA1024L
-0.071042471	0.679559708	0.677	1	3161	HMMR
-0.071042471	0.679559708	0.727	1	5435	POLR2F
-0.071042471	0.679559708	0.697	1	8226	HDHD1A
-0.071042471	0.679559708	0.739	1	10363	HMG20A
-0.071042471	0.679559708	0.698	1	80332	ADAM33
-0.071042471	0.679559708	0.722	1	84651	SPINK7
-0.071042471	0.679559708	0.7	1	137492	VPS37A
-0.071299866	0.678459704	0.699	1	6877	TAF5
-0.071299866	0.678459704	0.721	1	8712	PAGE1
-0.071299866	0.678459704	0.695	1	26608	TBL2
-0.071299866	0.678459704	0.715	1	29927	SEC61A1
-0.071299866	0.678459704	0.696	1	113835	ZNF257
-0.071299866	0.678459704	0.753	1	594855	CPLX3
-0.071557269	0.677360237	0.7	1	1475	CSTA
-0.071557269	0.677360237	0.683	1	9050	PSTPIP2
-0.071557269	0.677360237	0.685	1	9463	PICK1
-0.071557269	0.677360237	0.7	1	22809	ATF5
-0.071557269	0.677360237	0.713	1	54768	HYDIN
-0.071557269	0.677360237	0.692	1	64708	COPS7B
-0.071557269	0.677360237	0.711	1	220070	SHANK2-AS3
-0.071814671	0.676261008	0.705	1	2184	FAH
-0.071814671	0.676261008	0.706	1	3945	LDHB
-0.071814671	0.676261008	0.724	1	4646	MYO6
-0.071814671	0.676261008	0.724	1	55904	MLL5
-0.071814671	0.676261008	0.703	1	56952	PRTFDC1
-0.071814671	0.676261008	0.696	1	57727	NCOA5
-0.071814671	0.676261008	0.691	1	158158	RASEF
-0.071814671	0.676261008	0.695	1	100506742	CASP12

voxel-wise, subcortex			
Spearman's corr coef	p-val	GenID	Gene
-0.029549122	0.007865627	51433	ANAPC5
-0.029597227	0.007765181	84251	SGIP1
-0.029607905	0.007743049	7386	UQCRFS1
-0.029611435	0.007735737	158747	MOSPD2
-0.029643288	0.00767009	144363	LYRM5
-0.02966762	0.007620276	138162	C9orf116
-0.029675605	0.007603996	114926	C8orf40
-0.029727889	0.007498141	51611	DPH5
-0.029729653	0.007494591	5697	PYY
-0.02976197	0.007429851	54439	RBM27
-0.029764663	0.007424481	51384	WNT16
-0.029855208	0.007245885	6827	SUPT4H1
-0.029902941	0.007153292	8879	SGPL1
-0.029903034	0.007153114	11258	DCTN3
-0.029917428	0.007125398	132158	GLYCTK
-0.02994306	0.007076284	4070	TACSTD2
-0.029946402	0.007069905	4774	NFIA
-0.029956989	0.007049728	339521	FAM58BP
-0.029966461	0.007031715	7690	ZNF131
-0.029990328	0.006986522	259282	BOD1L
-0.029995436	0.006976883	5933	RBL1
-0.030022925	0.006925212	83943	IMMP2L
-0.030037226	0.00689847	126069	ZNF491
-0.030037411	0.006898123	8301	PICALM
-0.030039176	0.006894832	63971	KIF13A
-0.030050969	0.006872853	57624	KIAA1486
-0.030054592	0.006866119	7702	ZNF143
-0.030089509	0.006801485	26191	PTPN22
-0.030131763	0.006724	201232	SLC16A13
-0.030175781	0.006644121	9669	EIF5B
-0.030177174	0.006641608	433	ASGR2
-0.030235866	0.006536467	4055	LTBR
-0.030339783	0.006353982	7746	ZNF193
-0.030418904	0.006218115	200558	APLF
-0.030422062	0.006212751	2896	GRN
-0.030425962	0.006206128	6897	TARS
-0.030559411	0.005983285	8409	UXT
-0.030592658	0.005928902	161247	FITM1
-0.030629711	0.005868816	388588	RP1-286D6.2
-0.030647077	0.005840847	9043	SPAG9
-0.030678743	0.005790151	254295	PHYHD1
-0.030699082	0.005757799	55366	LGR4
-0.030727684	0.005712575	80306	MED28
-0.030754058	0.005671167	26472	PPP1R14B
-0.030783774	0.005624827	93611	FBXO44
-0.030784424	0.005623819	10101	NUBP2
-0.030886764	0.005466868	647219	ASCL5
-0.030899301	0.005447914	7105	TSPAN6
-0.030910352	0.005431259	23163	GGA3
-0.030938026	0.005389747	51654	CDK5RAP1
-0.03104965	0.005225201	1454	CSNK1E
-0.031075653	0.005187528	441478	NRARP
-0.0311232	0.005119275	79753	SNIP1
-0.031140288	0.005094949	246330	PELI3
-0.031202786	0.005006848	80150	ASRGL1
-0.031252097	0.004938314	254879	OR2T6
-0.031276893	0.004904165	79608	RIC3
-0.031348959	0.004806151	23378	RRP8
-0.031350166	0.004804525	140465	MYL6B
-0.031373754	0.004772837	259285	TAS2R39
-0.031390283	0.004750747	51013	EXOSC1
-0.03139279	0.004747405	6209	RPS15P5
-0.031403191	0.004733561	8512	MBL1P
-0.031455196	0.004664885	23173	METAP1
-0.03147544	0.004638397	10230	NBR2
-0.03152791	0.004570369	440163	RNASE13
-0.031528469	0.004569653	83733	SLC25A18
-0.031551592	0.004539971	63891	RNF123
-0.031552613	0.004538661	8302	KLRC4
-0.031605359	0.004471614	2987	GUK1
-0.031619847	0.004453359	728047	AC135983.8
-0.031640187	0.004427843	6919	TCEA2
-0.031763975	0.004275373	100132832	LOC100132832
-0.031776421	0.004260308	283748	PLA2G4D
-0.03179555	0.004237252	51100	SH3GLB1
-0.031811338	0.00421831	83448	PUS7L
-0.031833626	0.004191696	10020	GNE
-0.031876806	0.004140566	1725	DHPS
-0.031899188	0.004114288	728448	PPIEL

region-wise, subcortex					
Spearman's corr coef	p-val	p-val, SA	p-val, SA (BH- correction)	GenID	Gene
-0.072072074	0.675162971	0.717	1	325	APCS
-0.072072074	0.675162971	0.692	1	3799	KIF5B
-0.072072074	0.675162971	0.672	1	9260	PDLIM7
-0.072072074	0.675162971	0.697	1	9669	EIF5B
-0.072072074	0.675162971	0.696	1	10239	AP3S2
-0.072072074	0.675162971	0.687	1	92270	ATP6AP1L
-0.072072074	0.675162971	0.698	1	388021	TMEM179
-0.072072074	0.675162971	0.69	1	494141	AC090099.4
-0.072072074	0.675162971	0.714	1	728882	FAM182B
-0.072329476	0.67406553	0.721	1	1347	COX7A2
-0.072329476	0.67406553	0.703	1	7098	TLR3
-0.072329476	0.67406553	0.667	1	85012	TCEAL3
-0.072329476	0.67406553	0.683	1	144608	C12orf60
-0.072586872	0.672968805	0.697	1	1315	COPB1
-0.072586872	0.672968805	0.704	1	8459	TPST2
-0.072586872	0.672968805	0.693	1	9508	ADAMTS3
-0.072586872	0.672968805	0.708	1	10151	HNRNPA3P1
-0.072586872	0.672968805	0.702	1	23313	C22orf9
-0.072586872	0.672968805	0.682	1	23729	SHPK
-0.072586872	0.672968805	0.692	1	51249	TMEM69
-0.072586872	0.672968805	0.7	1	58488	PCTP
-0.072586872	0.672968805	0.714	1	94032	CAMK2N2
-0.072586872	0.672968805	0.693	1	114984	FLYWCH2
-0.072586872	0.672968805	0.696	1	134145	FAM173B
-0.072586872	0.672968805	0.714	1	150094	SIK1
-0.072586872	0.672968805	0.716	1	386653	IL31
-0.072844274	0.671872735	0.708	1	1412	CRYBA2
-0.072844274	0.671872735	0.713	1	5265	SERPINA1
-0.072844274	0.671872735	0.697	1	5620	PRM2
-0.072844274	0.671872735	0.709	1	7922	SLC39A7
-0.072844274	0.671872735	0.677	1	26580	BSCL2
-0.072844274	0.671872735	0.677	1	30009	TBX21
-0.072844274	0.671872735	0.706	1	57763	ANKRA2
-0.072844274	0.671872735	0.72	1	81620	CDT1
-0.072844274	0.671872735	0.682	1	196993	AC068181.1
-0.072844274	0.671872735	0.707	1	282616	IL28A
-0.072844274	0.671872735	0.72	1	338323	NLRP14
-0.072844274	0.671872735	0.699	1	389740	GPR116P1
-0.07310167	0.670776844	0.694	1	706	TSPO
-0.07310167	0.670776844	0.723	1	5782	PTPN12
-0.07310167	0.670776844	0.683	1	51078	THAP4
-0.07310167	0.670776844	0.68	1	55707	NECAP2
-0.07310167	0.670776844	0.695	1	92421	CHMP4C
-0.07310167	0.670776844	0.698	1	140691	TRIM69
-0.07310167	0.670776844	0.72	1	347736	TXNDC6
-0.073359072	0.669682086	0.696	1	4025	LPO
-0.073359072	0.669682086	0.706	1	6117	RPA1
-0.073359072	0.669682086	0.678	1	7707	ZNF148
-0.073359072	0.669682086	0.699	1	9117	SEC22C
-0.073359072	0.669682086	0.689	1	9525	VPS4B
-0.073359072	0.669682086	0.709	1	10290	SPEG
-0.073359072	0.669682086	0.712	1	51804	SIX4
-0.073359072	0.669682086	0.698	1	84527	ZNF559
-0.073359072	0.669682086	0.718	1	339855	KY
-0.073359072	0.669682086	0.688	1	441108	C5orf56
-0.073616475	0.668588102	0.677	1	1105	CHD1
-0.073616475	0.668588102	0.682	1	10026	PIGK
-0.073616475	0.668588102	0.687	1	23526	HMHA1
-0.073616475	0.668588102	0.698	1	23547	LILRA4
-0.073616475	0.668588102	0.686	1	23566	LPAR3
-0.07387387	0.667494774	0.7	1	3916	LAMP1
-0.07387387	0.667494774	0.687	1	4116	MAGOH
-0.07387387	0.667494774	0.686	1	51028	VPS36
-0.07387387	0.667494774	0.681	1	83755	KRTAP4-12
-0.07387387	0.667494774	0.727	1	199834	LCE4A
-0.07387387	0.667494774	0.696	1	692099	FAM86DP
-0.074131273	0.666401565	0.698	1	10411	RAPGEF3
-0.074131273	0.666401565	0.706	1	56922	MCCC1
-0.074131273	0.666401565	0.694	1	117145	THEM4
-0.074131273	0.666401565	0.68	1	147407	MCART2
-0.074388668	0.665309548	0.689	1	977	CD151
-0.074388668	0.665309548	0.687	1	5319	PLA2G1B
-0.074388668	0.665309548	0.726	1	10227	MFSD10
-0.074388668	0.665309548	0.693	1	10254	STAM2
-0.074388668	0.665309548	0.692	1	11215	AKAP11
-0.074388668	0.665309548	0.7	1	29085	PHPT1
-0.074388668	0.665309548	0.671	1	51629	SLC25A39
-0.074388668	0.665309548	0.706	1	54913	RPP25
-0.074388668	0.665309548	0.687	1	79007	DBNDD1

voxel-wise, subcortex			
Spearman's corr coef	p-val	GenID	Gene
-0.03198323	0.004016957	6566	SLC16A1
-0.031986762	0.004012919	51290	ERGIC2
-0.032017034	0.003978406	27095	TRAPPC3
-0.032214653	0.003759654	192666	KRT24
-0.032242049	0.003730208	5534	PPP3R1
-0.032246973	0.003724939	51367	POP5
-0.03230622	0.003662051	100129933	GLYATL1P2
-0.032436233	0.003527425	26493	OR8B8
-0.032481458	0.003481657	5077	PAX3
-0.032503933	0.003459118	284440	LOC284440
-0.032522876	0.00344022	198437	C20orf201
-0.032523621	0.003439481	10540	DCTN2
-0.032634132	0.003331123	9258	MFHAS1
-0.032672949	0.003293809	128178	EDARADD
-0.032719474	0.003249586	2326	FMO1
-0.032721516	0.003247658	4242	MFNG
-0.03274297	0.003227456	54991	C1orf159
-0.032851622	0.003126909	8529	CYP4F2
-0.032879669	0.00310142	90024	AP001816.1
-0.032892019	0.003090258	2962	GTF2F1
-0.032928422	0.003057566	57798	GATAD1
-0.03299417	0.002999319	651302	ZNF192P1
-0.032997236	0.002996629	92285	ZNF585B
-0.033004478	0.002990281	119395	CALHM3
-0.033011537	0.002984107	124961	ZFP3
-0.03306967	0.00293368	284348	LYPD5
-0.033080071	0.002924745	220296	HEPACAM
-0.033113781	0.002895944	8535	CBX4
-0.033114247	0.002895547	388585	HES5
-0.033163372	0.002854048	127550	A3GALT2P
-0.033192348	0.002829824	10915	TCERG1
-0.033218723	0.002807938	23405	DICER1
-0.033355236	0.002697108	4947	OAZ2
-0.033369627	0.00268566	7185	TRAF1
-0.033386994	0.002671905	27020	NPTN
-0.033406682	0.002656388	51119	SBDS
-0.033440951	0.002629576	860	RUNX2
-0.033448379	0.002623799	200420	ALMS1P
-0.033502705	0.002581887	4634	MYL3
-0.033517286	0.002570741	27094	KCNMB3
-0.033559445	0.002538767	79992	AGPAT4-IT1
-0.033571426	0.002529746	79544	OR4K1
-0.033614609	0.002497475	7756	ZNF207
-0.033691037	0.002441284	6100	RP9
-0.033696704	0.002437165	54900	LAX1
-0.033707939	0.002429014	84881	RPUSD4
-0.033741184	0.002405045	342908	ZNF404
-0.033770252	0.002384266	27106	ARRDC2
-0.033802569	0.002361355	51021	MRPS16
-0.033813156	0.002353896	3645	INSRR
-0.033901099	0.002292748	92856	IMP4
-0.033905093	0.002290005	2873	GPS1
-0.033944376	0.002263195	1183	CLCN4
-0.033983845	0.002236544	6778	STAT6
-0.034037244	0.002200948	144577	C12orf66
-0.034055628	0.002188815	7518	XRCC4
-0.034058414	0.002186979	100129503	PP13
-0.034060553	0.002185573	8320	EOMES
-0.034072809	0.002177529	55611	OTUB1
-0.034091383	0.00216539	160760	PPTC7
-0.034194466	0.002099136	212	ALAS2
-0.034303304	0.002031205	56655	POLE4
-0.034376111	0.001986893	163081	ZNF567
-0.034404714	0.00196973	23654	PLXNB2
-0.034409076	0.001967123	221545	C6orf136
-0.034411769	0.001965516	9513	FXR2
-0.034426164	0.001956946	84946	LTV1
-0.034559429	0.001879225	374659	HDDC3
-0.034571499	0.001872327	57655	GRAMD1A
-0.034614496	0.001847945	3601	IL15RA
-0.034636691	0.001835472	5082	PDCL
-0.034646999	0.001829707	10127	ZNF263
-0.034714419	0.001792403	79641	ROGDI
-0.034716837	0.001791079	56928	SPPL2B
-0.034724265	0.001787014	84101	USP44
-0.034739774	0.001778557	26065	LSM14A
-0.034754448	0.001770587	3772	KCNJ15
-0.034787506	0.001752755	79634	SCRN3
-0.034789085	0.001751908	146378	C16orf92

region-wise, subcortex					
Spearman's corr coef	p-val	p-val, SA	p-val, SA (BH- correction)	GenID	Gene
-0.074646071	0.664218247	0.679	1	2589	GALNT1
-0.074646071	0.664218247	0.681	1	9810	RNF40
-0.074646071	0.664218247	0.689	1	132158	GLYCTK
-0.074903473	0.663127661	0.726	1	126	ADH1C
-0.074903473	0.663127661	0.696	1	6046	BRD2
-0.074903473	0.663127661	0.691	1	54880	BCOR
-0.074903473	0.663127661	0.689	1	57659	ZBTB4
-0.074903473	0.663127661	0.717	1	253039	RP11-27I1.2
-0.074903473	0.663127661	0.7	1	285267	ZNF619
-0.074903473	0.663127661	0.673	1	339168	TMEM95
-0.075160868	0.662037253	0.687	1	3066	HDAC2
-0.075160868	0.662037253	0.694	1	7779	SLC30A1
-0.075160868	0.662037253	0.688	1	9413	C9orf61
-0.075160868	0.662037253	0.71	1	63928	AC130454.1
-0.075160868	0.662037253	0.691	1	114042	LINC00334
-0.075160868	0.662037253	0.704	1	148213	ZNF681
-0.075160868	0.662037253	0.688	1	157848	NKX6-3
-0.075160868	0.662037253	0.679	1	100131454	AC087392.3
-0.075418279	0.660948038	0.717	1	2618	GART
-0.075418279	0.660948038	0.671	1	3189	HNRNPH3
-0.075418279	0.660948038	0.673	1	5550	PREP
-0.075418279	0.660948038	0.683	1	7151	TOP1P1
-0.075418279	0.660948038	0.696	1	26251	KCNG2
-0.075418279	0.660948038	0.683	1	54929	TMEM161A
-0.075418279	0.660948038	0.698	1	64802	NMNAT1
-0.075418279	0.660948038	0.692	1	347862	PDDC1
-0.075675674	0.659859419	0.7	1	29889	GNL2
-0.075675674	0.659859419	0.691	1	125228	C18orf19
-0.075675674	0.659859419	0.67	1	164656	TMPRSS6
-0.075675674	0.659859419	0.675	1	203328	SUSD3
-0.075933076	0.658771634	0.684	1	23412	COMMD3
-0.075933076	0.658771634	0.698	1	284098	PIGW
-0.075933076	0.658771634	0.691	1	387601	SLC22A25
-0.075933076	0.658771634	0.668	1	387751	GVINP1
-0.075933076	0.658771634	0.686	1	389765	LOC389765
-0.076190479	0.657684445	0.686	1	3664	IRF6
-0.076190479	0.657684445	0.697	1	5339	PLEC1
-0.076190479	0.657684445	0.69	1	10987	COPS5
-0.076190479	0.657684445	0.663	1	51202	DDX47
-0.076190479	0.657684445	0.696	1	55687	TRMU
-0.076190479	0.657684445	0.685	1	441032	EEF1A1P9
-0.076447874	0.656597495	0.665	1	7184	HSP90B1
-0.076447874	0.656597495	0.69	1	10432	RBM14
-0.076447874	0.656597495	0.697	1	22795	NID2
-0.076447874	0.656597495	0.665	1	27113	BBC3
-0.076447874	0.656597495	0.681	1	27173	SLC39A1
-0.076447874	0.656597495	0.69	1	79170	ATAD4
-0.076447874	0.656597495	0.665	1	116285	ACSM1
-0.076447874	0.656597495	0.691	1	128439	SNHG11
-0.076447874	0.656597495	0.702	1	340578	DCAF12L2
-0.076447874	0.656597495	0.673	1	100130613	CXorf64
-0.076705277	0.655511796	0.704	1	6419	SETMAR
-0.076705277	0.655511796	0.693	1	8418	CMAH
-0.076705277	0.655511796	0.678	1	54106	TLR9
-0.076705277	0.655511796	0.672	1	130574	LYPD6
-0.076705277	0.655511796	0.712	1	197258	FUK
-0.076705277	0.655511796	0.691	1	100101467	ZNF397OS
-0.076962672	0.654426754	0.66	1	7299	TYR
-0.076962672	0.654426754	0.675	1	10283	SDCCAG10
-0.076962672	0.654426754	0.67	1	79634	SCRN3
-0.076962672	0.654426754	0.683	1	84498	FAM120B
-0.076962672	0.654426754	0.679	1	150142	ZNF295-AS1
-0.076962672	0.654426754	0.686	1	201163	FLCN
-0.076962672	0.654426754	0.676	1	440712	C1orf186
-0.076962672	0.654426754	0.694	1	729420	AL359392.2
-0.077220075	0.653342485	0.713	1	2864	FFAR1
-0.077220075	0.653342485	0.703	1	10163	WASF2
-0.077220075	0.653342485	0.672	1	63940	GPSM3
-0.077477477	0.652258277	0.683	1	103	ADAR
-0.077477477	0.652258277	0.656	1	2854	GPR32
-0.077477477	0.652258277	0.702	1	7770	ZNF227
-0.077477477	0.652258277	0.666	1	23533	PIK3R5
-0.077477477	0.652258277	0.663	1	348801	LNP1
-0.077734873	0.651175439	0.702	1	3667	IRS1
-0.077734873	0.651175439	0.668	1	23164	MPRIP
-0.077734873	0.651175439	0.674	1	27106	ARRDC2
-0.077734873	0.651175439	0.637	1	55328	RNLS
-0.077734873	0.651175439	0.666	1	56259	CTNNBL1
-0.077734873	0.651175439	0.722	1	56998	CTNNBIP1

voxel-wise, subcortex			
Spearman's corr coef	p-val	GenID	Gene
-0.034789179	0.001751858	57674	RNF213
-0.034806173	0.001742759	138649	ANKRD19P
-0.034952343	0.001666277	908	CCT6A
-0.034975465	0.001654464	79080	CCDC86
-0.035037689	0.001623058	4542	MYO1F
-0.035075393	0.001604295	594	BCKDHB
-0.035112817	0.001585872	8558	CDK10
-0.03513176	0.00157662	3117	HLA-DQA1
-0.035163522	0.001561219	26168	SENP3
-0.035175223	0.001555581	352909	C19orf51
-0.035197232	0.001545025	246175	CNOT6L
-0.03520596	0.001540857	9019	MPZL1
-0.035242084	0.001523717	10346	TRIM22
-0.035281461	0.001505236	55592	AC010203.2
-0.035319813	0.001487432	63922	CHTF18
-0.035392344	0.001454293	654817	NCF1C
-0.035523746	0.001395984	83868	TTTY13
-0.035559129	0.001380655	27165	GLS2
-0.035593212	0.001366037	2595	GANC
-0.035620328	0.001354507	342667	STAC2
-0.035630174	0.001350344	6731	SRP72
-0.035645775	0.001343771	286122	C8orf31
-0.035663791	0.001336217	51406	NOL7
-0.035718583	0.001313483	147179	WIPF2
-0.035733812	0.001307228	401634	GOLGA2P3Y
-0.035737246	0.00130582	8705	B3GALT4
-0.035749227	0.001300924	83787	ARMC10
-0.035774764	0.001290543	56127	PCDHB9
-0.035779685	0.001288551	23557	SNAPIN
-0.035879888	0.00124861	51258	MRPL51
-0.035937745	0.00122607	51665	ASB1
-0.036019001	0.001195052	85465	AC010896.1
-0.036047883	0.001184201	10985	GCN1L1
-0.036049556	0.001183575	130502	TTC32
-0.036150966	0.001146212	728489	DNLZ
-0.036186162	0.001133502	342371	ATXN1L
-0.036204457	0.001126947	284257	AC100775.1
-0.036219966	0.001121418	57156	TMEM63C
-0.036253303	0.001109617	4170	MCL1
-0.036255717	0.001108767	84296	GINS4
-0.036336232	0.001080757	51720	UIMC1
-0.036400959	0.001058715	26160	IFT172
-0.036442377	0.00104483	127002	ATXN7L2
-0.036486953	0.001030075	4084	MXD1
-0.036611672	0.000989808	4504	MT3
-0.036644455	0.000979469	84693	MCEE
-0.036650397	0.000977607	3756	KCNH1
-0.036674544	0.000970067	26061	HACL1
-0.036676679	0.000969403	866	SERPINA6
-0.036713734	0.000957947	1857	DVL3
-0.036716983	0.000956949	497258	BDNF-AS1
-0.036736205	0.000951061	55796	MBNL3
-0.03674512	0.000948341	770	CA11
-0.036766481	0.000941855	2196	FAT2
-0.036800656	0.000931564	653238	GTF2H2B
-0.036827959	0.000923416	11143	MYST2
-0.036857024	0.000914816	5355	PLP2
-0.036936238	0.000891751	5364	PLXNB1
-0.037004031	0.000872441	1355	COX15
-0.037004124	0.000872415	56945	MRPS22
-0.037012946	0.000869931	7408	VASP
-0.037070617	0.000853852	254013	C12orf72
-0.037076004	0.000852365	23788	MTCH2
-0.0371112	0.000842704	55841	WWC3
-0.037125871	0.000838707	85001	CTD-2514K5.2
-0.037164226	0.000828341	130132	RFTN2
-0.037209358	0.000816295	23109	DDN
-0.037219293	0.000813665	439	ASNA1
-0.037255049	0.000804266	7581	ZNF33A
-0.03729656	0.00079348	1933	EEF1B2
-0.037309002	0.000790273	1307	COL16A1
-0.037367325	0.000775401	64135	IFIH1
-0.037398156	0.000767646	145376	C14orf50
-0.037464462	0.000751208	27158	NDOR1
-0.037477743	0.000747955	26157	GIMAP2
-0.037499379	0.000742684	648771	LOC648771
-0.037536804	0.000733648	3703	STT3A
-0.037537176	0.000733558	27254	CSDC2
-0.037574694	0.000724604	3696	ITGB8

region-wise, subcortex					
Spearman's corr coef	p-val	p-val, SA	p-val, SA (BH- correction)	GenID	Gene
-0.077734873	0.651175439	0.666	1	130132	RFTN2
-0.077734873	0.651175439	0.689	1	729171	ANKRD20A8P
-0.077734873	0.651175439	0.68	1	100130109	SNRPEP4
-0.077992275	0.650093257	0.687	1	6941	TCF19
-0.077992275	0.650093257	0.671	1	7589	ZSCAN21
-0.077992275	0.650093257	0.688	1	27340	UTP20
-0.077992275	0.650093257	0.663	1	57502	NLGN4X
-0.077992275	0.650093257	0.669	1	57584	ARHGAP21
-0.077992275	0.650093257	0.675	1	91647	ATPAF2
-0.077992275	0.650093257	0.672	1	286238	LOC286238
-0.078249671	0.649011731	0.673	1	5464	PPA1
-0.078249671	0.649011731	0.689	1	25845	AC010442.2
-0.078249671	0.649011731	0.673	1	79594	MUL1
-0.078249671	0.649011731	0.676	1	401172	CTD-2324F15.2
-0.078507073	0.64793098	0.666	1	3682	ITGAE
-0.078507073	0.64793098	0.687	1	23522	MYST4
-0.078507073	0.64793098	0.682	1	57109	REXO4
-0.078764483	0.646850407	0.678	1	3182	HNRNPAB
-0.078764483	0.646850407	0.657	1	7638	ZNF221
-0.078764483	0.646850407	0.678	1	51473	DCDC2
-0.078764483	0.646850407	0.659	1	404037	HAPLN4
-0.078764483	0.646850407	0.675	1	548596	CKMT1A
-0.079021879	0.645771027	0.661	1	7360	UGP2
-0.079021879	0.645771027	0.655	1	8372	HYAL3
-0.079021879	0.645771027	0.67	1	26227	PHGDH
-0.079021879	0.645771027	0.66	1	55324	ABCF3
-0.079021879	0.645771027	0.66	1	140609	NEK7
-0.079021879	0.645771027	0.679	1	649946	RPL23AP64
-0.079279281	0.644692421	0.673	1	14	AAMP
-0.079279281	0.644692421	0.684	1	1050	CEBPA
-0.079279281	0.644692421	0.667	1	1431	CS
-0.079279281	0.644692421	0.679	1	4690	NCK1
-0.079279281	0.644692421	0.677	1	9826	ARHGEF11
-0.079279281	0.644692421	0.708	1	56935	C11orf75
-0.079279281	0.644692421	0.665	1	80263	TRIM45
-0.079279281	0.644692421	0.66	1	359845	FAM101B
-0.079536684	0.643614531	0.689	1	251	ALPPL2
-0.079536684	0.643614531	0.659	1	570	BAAT
-0.079536684	0.643614531	0.693	1	1946	EFNA5
-0.079536684	0.643614531	0.659	1	4759	NEU2
-0.079536684	0.643614531	0.677	1	6483	ST3GAL2
-0.079794079	0.642536819	0.692	1	3823	KLRC3
-0.079794079	0.642536819	0.656	1	10362	HMG20B
-0.079794079	0.642536819	0.662	1	51434	ANAPC7
-0.079794079	0.642536819	0.679	1	84989	LOC84989
-0.079794079	0.642536819	0.65	1	85280	KRTAP9-4
-0.079794079	0.642536819	0.652	1	222484	LNX2
-0.079794079	0.642536819	0.697	1	256815	C10orf67
-0.079794079	0.642536819	0.669	1	283927	NUDT7
-0.080051482	0.641460419	0.694	1	4947	OAZ2
-0.080051482	0.641460419	0.691	1	7080	NKX2-1
-0.080051482	0.641460419	0.65	1	26145	IRF2BP1
-0.080051482	0.641460419	0.656	1	51070	NOSIP
-0.080051482	0.641460419	0.652	1	64781	CERK
-0.080051482	0.641460419	0.633	1	337977	KRTAP21-1
-0.080308877	0.640384734	0.653	1	1351	COX8A
-0.080308877	0.640384734	0.686	1	5547	PRCP
-0.080308877	0.640384734	0.662	1	27004	TCL6
-0.080308877	0.640384734	0.672	1	93183	PIGM
-0.080308877	0.640384734	0.668	1	115948	CCDC151
-0.080308877	0.640384734	0.667	1	145165	ST13P4
-0.080308877	0.640384734	0.672	1	389151	AC130416.1
-0.080308877	0.640384734	0.651	1	643236	TMEM72
-0.080308877	0.640384734	0.66	1	100287015	RP11-115C21.2
-0.08056628	0.639309764	0.657	1	651	BMP3
-0.08056628	0.639309764	0.643	1	10516	FBLN5
-0.08056628	0.639309764	0.677	1	51231	VRK3
-0.08056628	0.639309764	0.652	1	79772	MCTP1
-0.08056628	0.639309764	0.627	1	153768	PRELID2
-0.080823682	0.638235033	0.663	1	2185	PTK2B
-0.080823682	0.638235033	0.683	1	26065	LSM14A
-0.080823682	0.638235033	0.653	1	57673	BEND3
-0.080823682	0.638235033	0.67	1	84950	PRPF38A
-0.080823682	0.638235033	0.669	1	353140	LCE2C
-0.081081077	0.637161493	0.683	1	481	ATP1B1
-0.081081077	0.637161493	0.704	1	3982	LIM2
-0.081081077	0.637161493	0.663	1	4686	NCBP1
-0.081081077	0.637161493	0.666	1	8233	ZRSR2
-0.081081077	0.637161493	0.649	1	9074	CLDN6

voxel-wise, subcortex			
Spearman's corr coef	p-val	GenID	Gene
-0.037769154	0.000679792	3212	HOXB2
-0.037777975	0.000677821	51105	PHF20L1
-0.037810944	0.000670504	57594	HOMEZ
-0.037845768	0.000662855	7812	CSDE1
-0.037869543	0.00065768	64114	TMBIM1
-0.03790576	0.000649868	4780	NFE2L2
-0.038009677	0.000627929	26184	OR1F2P
-0.03802333	0.000625099	93380	MMGT1
-0.038079698	0.000613538	222389	BEND7
-0.038093351	0.000610768	137209	ZNF572
-0.038130682	0.000603253	84292	AC010422.1
-0.038145263	0.000600341	4678	NASP
-0.038175721	0.0005943	168448	CDC14C
-0.038188074	0.000591867	2166	FAAH
-0.038231812	0.000583324	4499	MT1M
-0.038243793	0.000581005	29088	MRPL15
-0.038245	0.000580771	133619	PRRC1
-0.038317528	0.000566915	57830	KRTAP5-8
-0.038385507	0.000554208	56125	PCDHB11
-0.038399808	0.000551569	10175	CNIH
-0.038415316	0.00054872	115416	MALSU1
-0.03843816	0.000544549	26052	DNM3
-0.038509481	0.000531714	56882	CDC42SE1
-0.038521834	0.00052952	5394	EXOSC10
-0.038549691	0.000524603	150160	CESK1
-0.038589906	0.000517581	23255	KIAA0802
-0.038597334	0.000516293	571	BACH1
-0.038609408	0.000514206	10221	TRIB1
-0.038629651	0.000510726	64960	MRPS15
-0.038650826	0.000507108	132949	AASDH
-0.038659554	0.000505624	80700	UBXN6
-0.038681004	0.000501994	54859	C3orf75
-0.038701065	0.000498621	4149	MAX
-0.038704038	0.000498123	7693	ZNF134
-0.038722146	0.000495099	132321	C4orf33
-0.038732268	0.000493417	290	ANPEP
-0.03875697	0.000489333	5588	PRKCQ
-0.038765512	0.000487927	81309	OR4C15
-0.038769785	0.000487226	6811	STX5
-0.038842034	0.000475508	84985	FAM83A
-0.03884612	0.000474853	55130	ARMC4
-0.038856708	0.00047316	57533	TBC1D14
-0.038887631	0.000468248	90861	HN1L
-0.038906112	0.000465335	2167	FABP4
-0.038944468	0.000459343	4750	NEK1
-0.039021824	0.000447478	7021	TFAP2B
-0.03903947	0.000444812	79693	YRDC
-0.039062962	0.000441285	254958	REXO1L1
-0.039098531	0.000435995	55362	TMEM63B
-0.039100666	0.00043568	25764	HYPK
-0.039110512	0.000434227	10094	ARPC3
-0.039146822	0.000428908	3373	HYAL1
-0.039157778	0.000427315	81627	C1orf25
-0.0392018	0.00042097	8914	TIMELESS
-0.039207462	0.00042016	92340	C17orf72
-0.039217584	0.000418717	10435	CDC42EP2
-0.039265506	0.000411944	652904	CKS1BP6
-0.039290857	0.000408402	3707	ITPKB
-0.039368398	0.000397746	148413	RP4-758J18.2
-0.039566483	0.000371691	7150	TOP1
-0.039591372	0.000368532	2139	EYA2
-0.039596293	0.000367911	282775	OR5J2
-0.039619882	0.000364945	57473	ZNF512B
-0.039657492	0.000360263	219899	TBCEL
-0.039660465	0.000359895	23244	PDS5A
-0.039759364	0.000347861	112483	SAT2
-0.039804868	0.000342451	5635	PRPSAP1
-0.039829202	0.000339591	84856	RP11-178A10.1
-0.039905071	0.000330815	2950	GSTP1
-0.039915286	0.000329649	10587	TXNRD2
-0.039942034	0.000326617	55102	ATG2B
-0.040002953	0.000319806	60678	EEFSEC
-0.040026169	0.000317246	4500	MT1L
-0.040028676	0.000316971	10362	HMG20B
-0.040074088	0.000312022	492	ATP2B3
-0.040109284	0.000308236	4939	OAS2
-0.040149961	0.000303915	1428	CRYM
-0.040178936	0.000300871	57582	KCNT1
-0.040223137	0.000296282	51058	ZNF691

region-wise, subcortex					
Spearman's corr coef	p-val	p-val, SA	p-val, SA (BH- correction)	GenID	Gene
-0.081081077	0.637161493	0.666	1	54936	ADPRHL2
-0.081081077	0.637161493	0.686	1	165324	UBXN2A
-0.081081077	0.637161493	0.674	1	729614	RP11-169K16.9
-0.08133848	0.636088729	0.666	1	7032	TFF2
-0.08133848	0.636088729	0.664	1	23659	PLA2G15
-0.08133848	0.636088729	0.69	1	205860	TRIML2
-0.081595875	0.63501668	0.645	1	2131	EXT1
-0.081595875	0.63501668	0.677	1	5788	PTPRC
-0.081595875	0.63501668	0.662	1	8392	OR3A3
-0.081595875	0.63501668	0.695	1	22848	AAK1
-0.081595875	0.63501668	0.64	1	129684	CNTNAP5
-0.081595875	0.63501668	0.655	1	195814	SDR16C5
-0.081595875	0.63501668	0.674	1	283521	LINC00282
-0.081853278	0.633945346	0.686	1	9401	RECQL4
-0.081853278	0.633945346	0.666	1	57003	CCDC47
-0.081853278	0.633945346	0.662	1	83463	MXD3
-0.081853278	0.633945346	0.649	1	84290	CAPNS2
-0.081853278	0.633945346	0.648	1	112398	EGLN2
-0.081853278	0.633945346	0.66	1	114569	MAL2
-0.081853278	0.633945346	0.638	1	132949	AASDH
-0.081853278	0.633945346	0.622	1	149076	ZNF362
-0.082110681	0.63287431	0.686	1	4739	NEDD9
-0.082110681	0.63287431	0.648	1	6295	SAG
-0.082110681	0.63287431	0.659	1	7626	ZNF75D
-0.082110681	0.63287431	0.662	1	10308	ZNF267
-0.082110681	0.63287431	0.659	1	25850	ZNF345
-0.082110681	0.63287431	0.651	1	92104	TTC30A
-0.082110681	0.63287431	0.664	1	120237	DBX1
-0.082110681	0.63287431	0.668	1	441024	MTHFD2L
-0.082368083	0.631804466	0.645	1	5971	RELB
-0.082368083	0.631804466	0.667	1	6787	NEK4
-0.082368083	0.631804466	0.64	1	55293	UEVLD
-0.082368083	0.631804466	0.649	1	56946	C11orf30
-0.082368083	0.631804466	0.644	1	151194	FAM119A
-0.082625486	0.630735397	0.653	1	9533	POLR1C
-0.082625486	0.630735397	0.636	1	79590	MRPL24
-0.082625486	0.630735397	0.662	1	84217	ZMYND12
-0.082625486	0.630735397	0.647	1	202309	GAPT
-0.082625486	0.630735397	0.655	1	283685	AC100756.1
-0.082882881	0.629667103	0.677	1	7152	TOP1P2
-0.082882881	0.629667103	0.658	1	7275	TUB
-0.083140284	0.628598988	0.642	1	4917	NTN3
-0.083140284	0.628598988	0.694	1	9212	AURKB
-0.083140284	0.628598988	0.687	1	10112	KIF20A
-0.083140284	0.628598988	0.669	1	92482	BBIP1
-0.083140284	0.628598988	0.628	1	377047	PRSS45
-0.083397686	0.627532184	0.664	1	9443	MED7
-0.083397686	0.627532184	0.65	1	23788	MTCH2
-0.083397686	0.627532184	0.659	1	54994	C20orf11
-0.083397686	0.627532184	0.653	1	55768	NGLY1
-0.083397686	0.627532184	0.664	1	57019	CIAPIN1
-0.083397686	0.627532184	0.677	1	57678	GPAM
-0.083397686	0.627532184	0.667	1	57820	CCNB1IP1
-0.083397686	0.627532184	0.63	1	79812	MMRN2
-0.083397686	0.627532184	0.633	1	127623	OR2B11
-0.083397686	0.627532184	0.641	1	255313	CT47A11
-0.083397686	0.627532184	0.624	1	730051	ZNF814
-0.083655082	0.626466155	0.66	1	2145	EZH1
-0.083655082	0.626466155	0.655	1	5933	RBL1
-0.083655082	0.626466155	0.679	1	8428	STK24
-0.083655082	0.626466155	0.643	1	9129	PRPF3
-0.083655082	0.626466155	0.661	1	50484	RRM2B
-0.083655082	0.626466155	0.667	1	81926	FAM108A4
-0.083655082	0.626466155	0.65	1	144321	GLIPR1L2
-0.083655082	0.626466155	0.642	1	100131434	LOC100131434
-0.083912484	0.625400782	0.662	1	114	ADCY8
-0.083912484	0.625400782	0.64	1	4808	NHLH2
-0.083912484	0.625400782	0.655	1	4985	OPRD1
-0.083912484	0.625400782	0.669	1	6837	MED22
-0.083912484	0.625400782	0.645	1	57045	TWSG1
-0.083912484	0.625400782	0.647	1	79693	YRDC
-0.083912484	0.625400782	0.626	1	83706	FERMT3
-0.08416988	0.624335706	0.677	1	9459	ARHGEF6
-0.08416988	0.624335706	0.656	1	9863	MAGI2
-0.08416988	0.624335706	0.636	1	23499	MACF1
-0.08416988	0.624335706	0.635	1	54826	GIN1
-0.08416988	0.624335706	0.676	1	55744	C7orf44
-0.08416988	0.624335706	0.67	1	284654	RSPO1
-0.08416988	0.624335706	0.657	1	345651	ACTBL2

voxel-wise, subcortex			
Spearman's corr coef	p-val	GenID	Gene
-0.040231217	0.000295451	2119	ETV5
-0.040278394	0.000290639	1105	CHD1
-0.040300403	0.00028842	257160	RNF214
-0.040447503	0.000273986	1329	COX5B
-0.040457159	0.000273063	388939	C2orf71
-0.040479079	0.000270978	1069	CETN2
-0.040484462	0.000270468	262	AMD1
-0.040505078	0.000268524	284217	LAMA1
-0.040519197	0.0002672	389161	C3orf16
-0.040580858	0.00026149	9862	MED24
-0.040617634	0.000258139	203260	CCDC107
-0.040624507	0.000257517	9349	RPL23
-0.040642429	0.000255902	84461	NEURL4
-0.040651992	0.000255044	8435	SOAT2
-0.040663045	0.000254056	348021	LINC00442
-0.040676046	0.000252898	1776	DNASE1L3
-0.040681247	0.000252437	100287822	SETP10
-0.040834382	0.000239192	7455	ZAN
-0.040870693	0.000236148	6521	SLC4A1
-0.040880166	0.000235361	148545	NBPF4
-0.040893815	0.000234229	1629	DBT
-0.040920839	0.000232005	51074	APIP
-0.040925391	0.000231632	143630	UBQLNL
-0.040936813	0.000230699	5191	PEX7
-0.040949259	0.000229687	9801	MRPL19
-0.040975817	0.000227541	4927	NUP88
-0.041002657	0.000225391	55192	DNAJC17
-0.041083541	0.000219026	83758	RBP5
-0.041203059	0.000209929	26275	HIBCH
-0.041205753	0.000209729	1519	CTSO
-0.041347653	0.000199399	54520	CCDC93
-0.041378949	0.000197186	6199	RPS6KB2
-0.041413959	0.000194737	587	BCAT2
-0.041558739	0.000184913	10524	KAT5
-0.041582696	0.000183333	9785	DHX38
-0.041634142	0.000179982	10161	LPAR6
-0.041636743	0.000179815	2247	FGF2
-0.041645102	0.000179276	3567	IL5
-0.041713636	0.000174919	84777	MGC11082
-0.041753571	0.000172426	84705	GTPBP3
-0.041783843	0.000170558	729009	FTH1P20
-0.0419068	0.000163167	729732	LOC729732
-0.041968647	0.000159564	9403	RP4-604K5.1
-0.042049441	0.000154969	3643	INSR
-0.042053062	0.000154767	54929	TMEM161A
-0.042063743	0.000154169	1639	DCTN1
-0.042076278	0.000153471	83871	RAB34
-0.042107761	0.000151731	408263	C5orf40
-0.042148344	0.000149515	5199	CFP
-0.042168681	0.000148416	10125	RASGRP1
-0.042179175	0.000147852	1831	TSC22D3
-0.042183723	0.000147608	51192	CKLF
-0.042185675	0.000147504	11248	NXPH3
-0.042302035	0.000141397	25923	ATL3
-0.042340111	0.00013945	55243	KIRREL
-0.042359985	0.000138444	728763	LOC728763
-0.042459816	0.000133495	8726	EED
-0.042516187	0.000130774	8452	CUL3
-0.04254479	0.000129414	83464	APH1B
-0.042574041	0.000128036	4848	CNOT2
-0.042588715	0.00012735	392360	CTSL3
-0.042590849	0.000127251	10210	TOPORS
-0.042622704	0.000125775	5204	PFDN5
-0.042631153	0.000125386	390282	LOC390282
-0.042638212	0.000125062	152789	JAKMIP1
-0.042647406	0.000124642	378825	LINC00162
-0.042649169	0.000124561	343	AQP8
-0.042668577	0.000123678	1735	DIO3
-0.042671643	0.000123539	9581	PREPL
-0.042708233	0.000121892	55969	C20orf24
-0.042753831	0.000119868	26097	C1orf77
-0.042911887	0.000113096	84936	ZFYVE19
-0.042986736	0.000110016	2983	GUCY1B3
-0.042997696	0.000109572	129685	TAF8
-0.042999554	0.000109497	91373	UAP1L1
-0.043004476	0.000109298	10773	ZBTB6
-0.043037999	0.000107953	163255	ZNF540
-0.043041527	0.000107812	439933	MGC42157
-0.043053787	0.000107325	55065	GPR172B

region-wise, subcortex					
Spearman's corr coef	p-val	p-val, SA	p-val, SA (BH- correction)	GenID	Gene
-0.08416988	0.624335706	0.664	1	441150	C6orf226
-0.08416988	0.624335706	0.675	1	613209	DEFB136
-0.084427282	0.623271942	0.623	1	1558	CYP2C8
-0.084427282	0.623271942	0.641	1	5130	PCYT1A
-0.084427282	0.623271942	0.656	1	25807	RHBDD3
-0.084427282	0.623271942	0.622	1	84868	HAVCR2
-0.084427282	0.623271942	0.662	1	344593	LOC344593
-0.084684685	0.622208893	0.645	1	5774	PTPN3
-0.084684685	0.622208893	0.639	1	25904	CNOT10
-0.084684685	0.622208893	0.662	1	51433	ANAPC5
-0.084684685	0.622208893	0.653	1	161253	REM2
-0.084684685	0.622208893	0.648	1	646450	C3orf71
-0.08494208	0.621146619	0.649	1	5794	PTPRH
-0.08494208	0.621146619	0.673	1	54798	DCHS2
-0.08494208	0.621146619	0.633	1	57529	RGAG1
-0.08494208	0.621146619	0.659	1	64147	KIF9
-0.08494208	0.621146619	0.661	1	122618	PLD4
-0.08494208	0.621146619	0.658	1	134510	UBLCP1
-0.085199483	0.620085061	0.627	1	490	ATP2B1
-0.085199483	0.620085061	0.617	1	3713	IVL
-0.085199483	0.620085061	0.652	1	9058	SLC13A2
-0.085199483	0.620085061	0.63	1	22875	ENPP4
-0.085199483	0.620085061	0.634	1	116931	MED12L
-0.085199483	0.620085061	0.669	1	283507	SUGT1L1
-0.085199483	0.620085061	0.647	1	441549	CDNF
-0.085456878	0.619023919	0.641	1	373	TRIM23
-0.085456878	0.619023919	0.656	1	2868	GRK4
-0.085456878	0.619023919	0.661	1	4342	MOS
-0.085456878	0.619023919	0.651	1	9582	APOBEC3B
-0.085456878	0.619023919	0.651	1	27246	RNF115
-0.085456878	0.619023919	0.643	1	51305	KCNK9
-0.085456878	0.619023919	0.626	1	259287	TAS2R41
-0.085714288	0.61796385	0.656	1	1896	EDA
-0.085714288	0.61796385	0.647	1	4350	MPG
-0.085714288	0.61796385	0.662	1	10350	ABCA9
-0.085714288	0.61796385	0.65	1	23305	ACSL6
-0.085714288	0.61796385	0.645	1	115548	FCHO2
-0.085714288	0.61796385	0.662	1	132946	ARL9
-0.085714288	0.61796385	0.669	1	200014	CC2D1B
-0.085971691	0.616904616	0.618	1	8731	RNMT
-0.085971691	0.616904616	0.664	1	10017	BCL2L10
-0.085971691	0.616904616	0.656	1	10818	FRS2
-0.085971691	0.616904616	0.643	1	55848	C9orf46
-0.085971691	0.616904616	0.649	1	81492	AC011530.1
-0.085971691	0.616904616	0.634	1	83591	THAP2
-0.085971691	0.616904616	0.637	1	400242	DICER1-AS
-0.086229086	0.615846217	0.616	1	4926	NUMA1
-0.086229086	0.615846217	0.631	1	7745	ZNF192
-0.086229086	0.615846217	0.648	1	28904	IGKV1D-8
-0.086229086	0.615846217	0.643	1	54512	EXOSC4
-0.086229086	0.615846217	0.665	1	374354	NHLRC2
-0.086486489	0.614787996	0.651	1	54471	SMCR7L
-0.086486489	0.614787996	0.647	1	56659	KCNK13
-0.086486489	0.614787996	0.631	1	147166	TRIM16L
-0.086486489	0.614787996	0.626	1	646019	CBY3
-0.086743884	0.613731146	0.661	1	189	AGXT
-0.086743884	0.613731146	0.644	1	3364	HUS1
-0.086743884	0.613731146	0.633	1	8228	PNPLA4
-0.086743884	0.613731146	0.637	1	10399	GNB2L1
-0.086743884	0.613731146	0.654	1	51013	EXOSC1
-0.086743884	0.613731146	0.615	1	54101	RIPK4
-0.086743884	0.613731146	0.658	1	54939	COMMD4
-0.087001286	0.612675011	0.655	1	1767	DNAH5
-0.087001286	0.612675011	0.642	1	9400	RECQL5
-0.087001286	0.612675011	0.649	1	10730	YME1L1
-0.087001286	0.612675011	0.66	1	85476	GFM1
-0.087001286	0.612675011	0.664	1	90231	KIAA2013
-0.087001286	0.612675011	0.643	1	119559	SFXN4
-0.087258689	0.611619651	0.642	1	117	ADCYAP1R1
-0.087258689	0.611619651	0.648	1	3015	H2AFZ
-0.087258689	0.611619651	0.661	1	11257	TP53TG1
-0.087258689	0.611619651	0.663	1	83888	FGFBP2
-0.087258689	0.611619651	0.647	1	84814	PPAPDC3
-0.087258689	0.611619651	0.625	1	128061	C1orf131
-0.087258689	0.611619651	0.651	1	219557	C7orf62
-0.087258689	0.611619651	0.636	1	390431	OR4K2
-0.087516084	0.61056459	0.618	1	23534	TNPO3
-0.087516084	0.61056459	0.642	1	50674	NEUROG3
-0.087516084	0.61056459	0.631	1	51023	MRPS18C

voxel-wise, subcortex			
Spearman's corr coef	p-val	GenID	Gene
-0.043103933	0.000105352	8708	B3GALT1
-0.04311322	0.000104991	123920	CMTM3
-0.043123342	0.000104598	375323	LHFPL4
-0.043144424	0.000103785	11243	PMF1
-0.043186769	0.000102169	80059	LRRTM4
-0.043213144	0.000101175	2717	GLA
-0.043230973	0.000100508	55614	KIF16B
-0.043261062	9.93919E-05	221496	LEMD2
-0.043334708	9.67091E-05	219844	HYLS1
-0.043342508	9.64289E-05	1821	DRP2
-0.043431472	9.32879E-05	91749	KIAA1919
-0.043455802	9.24459E-05	2689	GH2
-0.043544397	8.94398E-05	728450	AL080274.2
-0.043545883	8.93903E-05	5162	PDHB
-0.043585259	8.80849E-05	3597	IL13RA1
-0.043669675	8.53465E-05	6883	TAF12
-0.043670047	8.53347E-05	51466	EVL
-0.043741737	8.30729E-05	22863	KIAA0831
-0.043814916	8.08225E-05	122525	C14orf28
-0.043818444	8.07155E-05	6505	SLC1A1
-0.043830238	8.03587E-05	10871	CD300C
-0.043854106	7.96412E-05	55751	TMEM184C
-0.043953471	7.67188E-05	718	C3
-0.043971673	7.61945E-05	84720	PIGO
-0.044018943	7.48489E-05	1719	DHFRP1
-0.044032685	7.44619E-05	55422	ZNF331
-0.044038352	7.43029E-05	6433	SFRS8
-0.04406872	7.34561E-05	1848	DUSP6
-0.044082649	7.30709E-05	51002	TPRKB
-0.044088777	7.29019E-05	158431	ZNF782
-0.044094723	7.27384E-05	48	ACO1
-0.044118587	7.20855E-05	90317	ZNF616
-0.044176165	7.05331E-05	6873	TAF2
-0.044224177	6.92628E-05	5992	RFX4
-0.044354375	6.5926E-05	64096	GFRA4
-0.044372205	6.54809E-05	31	ACACA
-0.044373877	6.54393E-05	5189	PEX1
-0.044445198	6.36889E-05	84932	RAB2B
-0.044538062	6.14761E-05	10450	PPIE
-0.044574004	6.06393E-05	55501	CHST12
-0.044662967	5.86143E-05	9639	ARHGEF10
-0.044714786	5.74645E-05	399947	C11orf87
-0.04477023	5.62578E-05	1129	CHRM2
-0.044776544	5.6122E-05	84100	ARL6
-0.044778865	5.60721E-05	63946	DMRTC2
-0.044806633	5.54787E-05	80155	NARG1
-0.044835236	5.48736E-05	1677	DFFB
-0.044842757	5.47157E-05	4168	MCF2
-0.044864953	5.42519E-05	166968	MIER3
-0.044933394	5.28447E-05	375189	PFN4
-0.044948623	5.25363E-05	51520	LARS
-0.044975091	5.20044E-05	22832	KIAA1009
-0.045006573	5.13784E-05	4855	NOTCH4
-0.045080587	4.99346E-05	9567	GTPBP1
-0.045117363	4.92318E-05	9540	TP53I3
-0.045157015	4.84843E-05	79847	TMEM180
-0.045170669	4.82295E-05	375748	C9orf102
-0.045256846	4.66499E-05	11278	KLF12
-0.045272447	4.63692E-05	6993	DYNLT1
-0.045325104	4.54337E-05	6158	RPL28
-0.045374043	4.45803E-05	23383	KIAA0892
-0.045389645	4.43114E-05	84269	CHCHD5
-0.045391593	4.4278E-05	220729	AC024560.3
-0.045424655	4.37138E-05	731275	LOC731275
-0.045473319	4.28957E-05	4137	MAPT
-0.045486875	4.26703E-05	54876	C4orf30
-0.045653105	3.99972E-05	9625	AATK
-0.045669358	3.97446E-05	10686	CLDN16
-0.045715418	3.90369E-05	729330	OC90
-0.045779496	3.80719E-05	10934	MORF4
-0.045780983	3.80499E-05	10975	UQCR11
-0.045799091	3.77815E-05	2004	ELK3
-0.045799185	3.77801E-05	26280	KCTD9P2
-0.045806147	3.76774E-05	5629	PROX1
-0.045833174	3.72812E-05	10935	PRDX3
-0.045855552	3.69562E-05	54906	C10orf18
-0.045867812	3.67792E-05	63906	GPATCH3
-0.045873292	3.67005E-05	2548	GAA
-0.045887686	3.64942E-05	3125	HLA-DRB3

region-wise, subcortex					
Spearman's corr coef	p-val	p-val, SA	p-val, SA (BH- correction)	GenID	Gene
-0.087516084	0.61056459	0.659	1	149281	METTL11B
-0.087516084	0.61056459	0.663	1	149837	LOC149837
-0.087773487	0.609510779	0.631	1	2063	NR2F6
-0.087773487	0.609510779	0.622	1	2665	GDI2
-0.087773487	0.609510779	0.645	1	3577	IL8RA
-0.087773487	0.609510779	0.644	1	10963	AP005668.1
-0.087773487	0.609510779	0.637	1	57655	GRAMD1A
-0.087773487	0.609510779	0.657	1	79933	SYNPO2L
-0.087773487	0.609510779	0.649	1	196968	C15orf51
-0.087773487	0.609510779	0.594	1	440957	C3orf78
-0.088030882	0.608457804	0.624	1	1796	DOK1
-0.088030882	0.608457804	0.654	1	3842	TNPO1
-0.088030882	0.608457804	0.638	1	5739	PTGIR
-0.088030882	0.608457804	0.661	1	7881	KCNAB1
-0.088030882	0.608457804	0.646	1	8890	EIF2B4
-0.088030882	0.608457804	0.635	1	9367	RAB9A
-0.088030882	0.608457804	0.652	1	54801	HAUS6
-0.088030882	0.608457804	0.652	1	143884	CWF19L2
-0.088030882	0.608457804	0.645	1	170572	HTR3C
-0.088030882	0.608457804	0.64	1	198437	C20orf201
-0.088288285	0.607405543	0.649	1	312	ANXA13
-0.088288285	0.607405543	0.622	1	4297	MLL
-0.088288285	0.607405543	0.654	1	4772	NFATC1
-0.088288285	0.607405543	0.649	1	23645	PPP1R15A
-0.088288285	0.607405543	0.641	1	27089	UQCRQ
-0.088288285	0.607405543	0.667	1	54549	SDK2
-0.088288285	0.607405543	0.619	1	80208	SPG11
-0.088288285	0.607405543	0.662	1	100128124	HGC6.3
-0.088545687	0.606354117	0.664	1	23761	PISD
-0.088803083	0.605302989	0.66	1	7525	YES1
-0.088803083	0.605302989	0.625	1	50805	IRX4
-0.088803083	0.605302989	0.619	1	51545	ZNF581
-0.088803083	0.605302989	0.637	1	79730	NSUN7
-0.088803083	0.605302989	0.639	1	80201	HKDC1
-0.088803083	0.605302989	0.651	1	80306	MED28
-0.088803083	0.605302989	0.659	1	81615	TMEM163
-0.088803083	0.605302989	0.639	1	114088	TRIM9
-0.088803083	0.605302989	0.632	1	160364	CLEC12A
-0.088803083	0.605302989	0.662	1	348807	CCDC37
-0.088803083	0.605302989	0.633	1	387804	VSTM5
-0.089060485	0.604253113	0.617	1	3396	ICT1
-0.089060485	0.604253113	0.655	1	8500	PPFIA1
-0.089060485	0.604253113	0.653	1	10482	NXF1
-0.089060485	0.604253113	0.653	1	10670	RRAGA
-0.089060485	0.604253113	0.597	1	54554	WDR5B
-0.089060485	0.604253113	0.648	1	84441	MAML2
-0.089060485	0.604253113	0.626	1	113444	C1orf212
-0.089060485	0.604253113	0.637	1	200159	C1orf100
-0.089060485	0.604253113	0.634	1	440888	ACTR3BP2
-0.089317888	0.603204131	0.623	1	6815	STYX
-0.089317888	0.603204131	0.641	1	7051	TGM1
-0.089317888	0.603204131	0.623	1	9738	AC012621.2
-0.089317888	0.603204131	0.606	1	24144	TFIP11
-0.089317888	0.603204131	0.644	1	147339	C18orf25
-0.089575291	0.602155805	0.647	1	406	ARNTL
-0.089575291	0.602155805	0.613	1	1373	CPS1
-0.089575291	0.602155805	0.627	1	1944	EFNA3
-0.089575291	0.602155805	0.636	1	2040	STOM
-0.089575291	0.602155805	0.638	1	23410	SIRT3
-0.089575291	0.602155805	0.648	1	51465	UBE2J1
-0.089575291	0.602155805	0.646	1	57568	SIPA1L2
-0.089575291	0.602155805	0.639	1	84771	DDX11L2
-0.089575291	0.602155805	0.63	1	133383	C5orf35
-0.089575291	0.602155805	0.626	1	100129094	BTNL10
-0.089832693	0.601107895	0.61	1	468	ATF4
-0.089832693	0.601107895	0.631	1	11162	NUDT6
-0.089832693	0.601107895	0.604	1	51585	PCF11
-0.089832693	0.601107895	0.612	1	64645	HIAT1
-0.089832693	0.601107895	0.646	1	165829	GPR156
-0.089832693	0.601107895	0.628	1	401105	AC022498.2
-0.089832693	0.601107895	0.62	1	401505	TOMM5
-0.090090089	0.600061238	0.63	1	6490	SILV
-0.090090089	0.600061238	0.611	1	23233	EXOC6B
-0.090090089	0.600061238	0.6	1	51665	ASB1
-0.090090089	0.600061238	0.64	1	134429	STARD4
-0.090090089	0.600061238	0.622	1	165100	C2orf57
-0.090090089	0.600061238	0.606	1	196872	RP11-477I4.3
-0.090347491	0.599015236	0.64	1	2187	FANCB
-0.090347491	0.599015236	0.623	1	8875	VNN2

voxel-wise, subcortex			
Spearman's corr coef	p-val	GenID	Gene
-0.045892701	3.64225E-05	202	AIM1
-0.045908395	3.61992E-05	56948	SDR39U1
-0.046013981	3.47308E-05	10084	PQBP1
-0.046081312	3.38242E-05	84300	MNF1
-0.046112791	3.34079E-05	340706	VWA2
-0.046136566	3.30968E-05	114814	GNRHR2
-0.046157088	3.28306E-05	391475	DYTN
-0.046179749	3.25388E-05	29957	SLC25A24
-0.046208259	3.21754E-05	9810	RNF40
-0.046216153	3.20754E-05	203414	CXorf24
-0.046222281	3.1998E-05	8470	SORBS2
-0.046259057	3.15372E-05	140732	SPAG4L
-0.0462832	3.12382E-05	1408	CRY2
-0.046287287	3.11878E-05	9104	RGN
-0.046298988	3.10441E-05	8890	EIF2B4
-0.046326477	3.07089E-05	252884	ZNF396
-0.046378948	3.00785E-05	23505	TMEM131
-0.046395477	2.98825E-05	10186	LHFP
-0.046472833	2.89812E-05	79852	EPHX3
-0.046475247	2.89536E-05	58157	NGB
-0.046490386	2.87803E-05	57455	REXO1
-0.04651453	2.85062E-05	8479	HIRIP3
-0.046535052	2.82751E-05	202018	TAPT1
-0.046552047	2.80851E-05	27131	SNX5
-0.046558641	2.80117E-05	6091	ROBO1
-0.046578608	2.77906E-05	11333	PDAP1
-0.046591889	2.76445E-05	50617	ATP6V0A4
-0.046617333	2.73664E-05	3371	TNC
-0.046690512	2.65816E-05	118487	CHCHD1
-0.046760533	2.58508E-05	10807	SDCCAG3
-0.046810493	2.5341E-05	713	C1QB
-0.046827767	2.5167E-05	10769	PLK2
-0.046941157	2.40526E-05	2188	FANCF
-0.047023252	2.32754E-05	81691	AC004381.2
-0.047029473	2.32174E-05	7024	TFCP2
-0.04704034	2.31165E-05	147111	NOTUM
-0.047073863	2.28081E-05	2810	SFN
-0.047155585	2.20724E-05	100128164	RP11-379K17.4
-0.04719951	2.16864E-05	3431	SP110
-0.047207959	2.16128E-05	81888	HYI
-0.047227833	2.14408E-05	9246	UBE2L6
-0.04729934	2.08327E-05	9093	DNAJA3
-0.047299527	2.08312E-05	6640	SNTA1
-0.047325157	2.06173E-05	5004	ORM1
-0.047458328	1.9539E-05	1234	CCR5
-0.047479592	1.93719E-05	27249	MMADHC
-0.047486931	1.93145E-05	90007	MIDN
-0.047525283	1.90174E-05	79730	NSUN7
-0.047539771	1.89063E-05	9559	VPS26A
-0.047561593	1.87401E-05	3895	KTN1
-0.047614437	1.83434E-05	7757	ZNF208
-0.047643408	1.81293E-05	60481	ELOVL5
-0.047671735	1.79223E-05	57724	KIAA1632
-0.047687057	1.78112E-05	5129	PCTK3
-0.047725409	1.75362E-05	81789	TIGD6
-0.047761351	1.72821E-05	23649	POLA2
-0.047791809	1.70695E-05	83608	C18orf21
-0.047803044	1.69917E-05	150248	C22orf15
-0.047902599	1.6317E-05	25926	NOL11
-0.048046634	1.5386E-05	5379	PMS2P1
-0.048053227	1.53446E-05	3779	KCNMB1
-0.048064001	1.52772E-05	283578	TMED8
-0.048080064	1.51772E-05	84874	ZNF514
-0.048143215	1.47902E-05	9023	CH25H
-0.048148602	1.47577E-05	171023	ASXL1
-0.048153058	1.47308E-05	51421	AMOTL2
-0.048165318	1.46571E-05	124454	EARS2
-0.048174694	1.46009E-05	7328	UBE2H
-0.048192155	1.44969E-05	387885	CCDC42B
-0.048218064	1.43438E-05	546	ATRX
-0.048246946	1.4175E-05	84311	MRPL45
-0.048318081	1.37672E-05	1836	SLC26A2
-0.048319194	1.37609E-05	203510	HMGB3P30
-0.048367113	1.34927E-05	55164	SHQ1
-0.04839126	1.33595E-05	55193	PBRM1
-0.048422463	1.31891E-05	4704	NDUFA9
-0.048473261	1.29162E-05	28952	CCDC22
-0.048493225	1.28104E-05	10825	NEU3
-0.04850474	1.27498E-05	3818	KLKB1

region-wise, subcortex					
Spearman's corr coef	p-val	p-val, SA	p-val, SA (BH- correction)	GenID	Gene
-0.090347491	0.599015236	0.622	1	10208	USPL1
-0.090347491	0.599015236	0.616	1	23041	MON2
-0.090347491	0.599015236	0.628	1	23244	PDS5A
-0.090604886	0.597970247	0.639	1	177	AGER
-0.090604886	0.597970247	0.611	1	6135	RPL11
-0.090604886	0.597970247	0.63	1	9856	KIAA0319
-0.090604886	0.597970247	0.621	1	51057	C2orf86
-0.090604886	0.597970247	0.661	1	84852	C1orf203
-0.090604886	0.597970247	0.6	1	390882	OR7G2
-0.090862289	0.596925437	0.627	1	2877	GPX2
-0.090862289	0.596925437	0.639	1	8326	FZD9
-0.090862289	0.596925437	0.632	1	9477	MED20
-0.090862289	0.596925437	0.636	1	23291	FBXW11
-0.090862289	0.596925437	0.624	1	55876	GSDMB
-0.090862289	0.596925437	0.635	1	90736	FAM104B
-0.090862289	0.596925437	0.643	1	93624	TADA2B
-0.090862289	0.596925437	0.627	1	169834	ZNF883
-0.091119692	0.595881999	0.618	1	9673	SLC25A44
-0.091119692	0.595881999	0.619	1	54811	ZNF562
-0.091119692	0.595881999	0.627	1	55282	LRRC36
-0.091119692	0.595881999	0.593	1	64975	MRPL41
-0.091119692	0.595881999	0.624	1	140699	C20orf132
-0.091119692	0.595881999	0.621	1	140801	RPL10L
-0.091119692	0.595881999	0.625	1	283596	SNHG10
-0.091377087	0.594839275	0.594	1	604	BCL6
-0.091377087	0.594839275	0.612	1	2912	GRM2
-0.091377087	0.594839275	0.617	1	3765	KCNJ9
-0.091377087	0.594839275	0.632	1	6096	RORB
-0.091377087	0.594839275	0.647	1	6704	SPRR2E
-0.091377087	0.594839275	0.623	1	7593	MZF1
-0.091377087	0.594839275	0.602	1	7913	DEK
-0.091377087	0.594839275	0.64	1	9632	SEC24C
-0.091377087	0.594839275	0.641	1	10672	GNA13
-0.091377087	0.594839275	0.612	1	56891	LGALS14
-0.091377087	0.594839275	0.636	1	57758	SCUBE2
-0.091377087	0.594839275	0.614	1	121441	NEDD1
-0.091377087	0.594839275	0.64	1	255275	MYADML2
-0.091377087	0.594839275	0.644	1	256302	C17orf103
-0.091377087	0.594839275	0.621	1	401491	RP11-125B21.2
-0.09163449	0.593797386	0.615	1	3420	IDH3B
-0.09163449	0.593797386	0.618	1	6657	SOX2
-0.09163449	0.593797386	0.622	1	9337	CNOT8
-0.09163449	0.593797386	0.625	1	9567	GTPBP1
-0.09163449	0.593797386	0.661	1	54793	KCTD9
-0.09163449	0.593797386	0.629	1	85446	ZFHX2
-0.09163449	0.593797386	0.621	1	135293	PM20D2
-0.09163449	0.593797386	0.619	1	160897	GPR180
-0.09163449	0.593797386	0.608	1	283377	SPRYD4
-0.091891885	0.592756331	0.631	1	683	BST1
-0.091891885	0.592756331	0.622	1	1473	CST5
-0.091891885	0.592756331	0.607	1	6596	HLTF
-0.091891885	0.592756331	0.627	1	6738	TROVE2
-0.091891885	0.592756331	0.629	1	7809	BSND
-0.091891885	0.592756331	0.63	1	26103	LRIT1
-0.091891885	0.592756331	0.628	1	347731	LRRTM3
-0.092149287	0.591715634	0.625	1	2906	GRIN2D
-0.092149287	0.591715634	0.63	1	3998	LMAN1
-0.092149287	0.591715634	0.622	1	50856	CLEC4A
-0.092149287	0.591715634	0.622	1	646817	SETP18
-0.09240669	0.590676188	0.614	1	6198	RPS6KB1
-0.09240669	0.590676188	0.617	1	9049	AIP
-0.09240669	0.590676188	0.636	1	9064	MAP3K6
-0.09240669	0.590676188	0.615	1	51214	IGF2AS
-0.092664085	0.589637578	0.621	1	9372	ZFYVE9
-0.092664085	0.589637578	0.624	1	22901	ARSG
-0.092664085	0.589637578	0.614	1	60482	SLC5A7
-0.092664085	0.589637578	0.613	1	60490	PPCDC
-0.092664085	0.589637578	0.612	1	65249	ZSWIM4
-0.092664085	0.589637578	0.622	1	79671	NLRX1
-0.092664085	0.589637578	0.61	1	148223	C19orf25
-0.092664085	0.589637578	0.64	1	167691	LCA5
-0.092921495	0.588599801	0.61	1	7381	UQCRB
-0.092921495	0.588599801	0.612	1	9209	LRRFIP2
-0.092921495	0.588599801	0.634	1	10184	LHFPL2
-0.092921495	0.588599801	0.593	1	54993	ZSCAN2
-0.092921495	0.588599801	0.629	1	60481	ELOVL5
-0.092921495	0.588599801	0.614	1	201627	FAM116A
-0.092921495	0.588599801	0.572	1	653562	LOC653562
-0.093178898	0.587562323	0.647	1	337	APOA4

voxel-wise, subcortex			
Spearman's corr coef	p-val	GenID	Gene
-0.048550151	1.25133E-05	55508	SLC35E3
-0.048570119	1.24107E-05	6629	SNRPB2
-0.04859928	1.22622E-05	2159	F10
-0.048624631	1.21346E-05	57209	ZNF248
-0.048625283	1.21313E-05	386684	KRTAP12-4
-0.048634939	1.2083E-05	51284	TLR7
-0.048682023	1.18502E-05	151903	CCDC12
-0.048698645	1.1769E-05	54414	SIAE
-0.048714619	1.16915E-05	6342	SCP2
-0.048766997	1.14408E-05	29015	SLC43A3
-0.048793275	1.13169E-05	50	ACO2
-0.048854753	1.10321E-05	79819	WDR78
-0.048855588	1.10283E-05	353149	TBC1D26
-0.048858374	1.10156E-05	55572	FOXRED1
-0.048881128	1.09121E-05	255812	SDHAP1
-0.048916973	1.07509E-05	8555	CDC14B
-0.048923753	1.07207E-05	81542	TMX1
-0.048956163	1.05773E-05	64850	AGXT2L1
-0.04899554	1.04055E-05	51103	NDUFAF1
-0.049078468	1.00525E-05	84514	GHDC
-0.049086083	1.00207E-05	1147	CHUK
-0.049167339	9.68689E-06	10715	LASS1
-0.049178392	9.64232E-06	92017	SNX29
-0.049217489	9.48622E-06	2908	NR3C1
-0.049260672	9.3166E-06	1187	CLCNKA
-0.049325306	9.06814E-06	64858	DCLRE1B
-0.04933738	9.02244E-06	56252	YLPM1
-0.049344249	8.99653E-06	2833	CXCR3
-0.049455598	8.58639E-06	57678	GPAM
-0.049484849	8.48164E-06	6169	RPL38
-0.049504165	8.41315E-06	55726	C12orf11
-0.049551155	8.24871E-06	729501	ZNF962P
-0.049639378	7.94825E-06	404220	C6orf201
-0.049666587	7.85772E-06	54936	ADPRHL2
-0.049701229	7.74389E-06	10311	DSCR3
-0.049708657	7.71967E-06	118432	RPL29P2
-0.049717572	7.69072E-06	1371	CPOX
-0.049741901	7.61223E-06	5950	RBP4
-0.049814247	7.38334E-06	3459	IFNGR1
-0.049842756	7.29496E-06	57462	KIAA1161
-0.04985724	7.25044E-06	645323	LINC00461
-0.049902003	7.11451E-06	30813	VSX1
-0.049962737	6.93396E-06	10137	RBM12
-0.049965523	6.92579E-06	1398	CRK
-0.049982611	6.87586E-06	29969	MDFIC
-0.050024029	6.75623E-06	56253	CRTAM
-0.050034616	6.72597E-06	9945	GFPT2
-0.050078262	6.60259E-06	92714	ARRDC1
-0.050087828	6.57582E-06	285195	SLC9A9
-0.050140481	6.43044E-06	57623	ZFAT
-0.050155435	6.38971E-06	391712	TRIM61
-0.050283682	6.0504E-06	8877	SPHK1
-0.050290648	6.03245E-06	1967	EIF2B1
-0.050331414	5.92856E-06	9261	MAPKAPK2
-0.050334107	5.92175E-06	83473	KATNAL2
-0.050397445	5.76389E-06	7096	TLR1
-0.050520398	5.46885E-06	728855	BX248398.1
-0.05052755	5.45214E-06	23592	LEMD3
-0.050547052	5.40683E-06	84950	PRPF38A
-0.050612614	5.25712E-06	51099	ABHD5
-0.050620601	5.23916E-06	100289143	LOC100289143
-0.050631743	5.21418E-06	6314	ATXN7
-0.050659418	5.15267E-06	1796	DOK1
-0.050738819	4.98002E-06	3549	IHH
-0.050745413	4.96595E-06	226	ALDOA
-0.050750054	4.95605E-06	4669	NAGLU
-0.050809119	4.8318E-06	23166	STAB1
-0.050856851	4.73358E-06	285590	SH3PXD2B
-0.050860845	4.72545E-06	10941	UGT2A1
-0.050864745	4.71752E-06	441212	RP9P
-0.050881274	4.68406E-06	81853	TMEM14B
-0.050898362	4.64971E-06	432369	ATP5EP2
-0.050987791	4.47384E-06	150684	COMMD1
-0.051028188	4.39651E-06	1373	CPS1
-0.051040728	4.37276E-06	10906	TRAFD1
-0.051072672	4.31283E-06	10654	PMVK
-0.051107034	4.24924E-06	79034	C7orf26
-0.051116131	4.23256E-06	2290	FOXG1B
-0.051179282	4.11845E-06	84310	C7orf50

region-wise, subcortex					
Spearman's corr coef	p-val	p-val, SA	p-val, SA (BH- correction)	GenID	Gene
-0.093178898	0.587562323	0.648	1	875	CBS
-0.093178898	0.587562323	0.632	1	8763	CD164
-0.093178898	0.587562323	0.62	1	10618	TGOLN2
-0.093178898	0.587562323	0.596	1	54883	CCDC49
-0.093178898	0.587562323	0.614	1	57002	C7orf36
-0.093178898	0.587562323	0.61	1	57693	ZNF317
-0.093178898	0.587562323	0.619	1	64241	ABCG8
-0.093436293	0.586526096	0.64	1	4974	OMG
-0.093436293	0.586526096	0.633	1	9489	PGS1
-0.093436293	0.586526096	0.611	1	10050	SLC17A4
-0.093436293	0.586526096	0.617	1	23093	TTLL5
-0.093436293	0.586526096	0.599	1	644768	SLC9B1P4
-0.093693696	0.585490823	0.621	1	1441	CSF3R
-0.093693696	0.585490823	0.615	1	10410	IFITM3
-0.093693696	0.585490823	0.589	1	22897	CEP164
-0.093693696	0.585490823	0.622	1	51463	GPR89B
-0.093693696	0.585490823	0.598	1	51806	CALML5
-0.093693696	0.585490823	0.624	1	127074	OR2T4
-0.093693696	0.585490823	0.638	1	146562	C16orf71
-0.093693696	0.585490823	0.612	1	153339	TMEM167A
-0.093693696	0.585490823	0.624	1	203430	ZCCHC5
-0.093693696	0.585490823	0.609	1	353174	ZACN
-0.093951091	0.584456265	0.628	1	2260	FGFR1
-0.093951091	0.584456265	0.613	1	2581	GALC
-0.093951091	0.584456265	0.604	1	5143	PDE4C
-0.093951091	0.584456265	0.607	1	8501	SLC43A1
-0.093951091	0.584456265	0.629	1	10175	CNIH
-0.093951091	0.584456265	0.607	1	54819	ZCCHC10
-0.093951091	0.584456265	0.599	1	55233	MOB1A
-0.093951091	0.584456265	0.599	1	153572	IRX2
-0.093951091	0.584456265	0.606	1	644096	SDHAF1
-0.094208494	0.583422065	0.608	1	581	BAX
-0.094208494	0.583422065	0.621	1	7163	TPD52
-0.094208494	0.583422065	0.59	1	26219	OR1J4
-0.094208494	0.583422065	0.612	1	55209	SETD5
-0.094208494	0.583422065	0.588	1	55702	CCDC94
-0.094208494	0.583422065	0.602	1	91603	ZNF830
-0.094208494	0.583422065	0.622	1	387640	C10orf140
-0.094208494	0.583422065	0.609	1	387680	FAM21A
-0.094465896	0.582389176	0.597	1	5549	PRELP
-0.094465896	0.582389176	0.596	1	56033	BARX1
-0.094465896	0.582389176	0.601	1	80207	OPA3
-0.094465896	0.582389176	0.635	1	81853	TMEM14B
-0.094465896	0.582389176	0.652	1	340393	AF205589.3
-0.094465896	0.582389176	0.596	1	388650	FAM69A
-0.094723292	0.581357121	0.579	1	242	ALOX12B
-0.094723292	0.581357121	0.623	1	2734	GLG1
-0.094723292	0.581357121	0.614	1	4507	MTAP
-0.094723292	0.581357121	0.615	1	4628	MYH10
-0.094723292	0.581357121	0.595	1	5813	PURA
-0.094723292	0.581357121	0.63	1	9970	NR1I3
-0.094723292	0.581357121	0.612	1	51367	POP5
-0.094723292	0.581357121	0.6	1	51574	LARP7
-0.094723292	0.581357121	0.604	1	79955	PDZD7
-0.094723292	0.581357121	0.619	1	83853	ROPN1L
-0.094980694	0.580325902	0.577	1	567	B2M
-0.094980694	0.580325902	0.612	1	594	BCKDHB
-0.094980694	0.580325902	0.633	1	3091	HIF1A
-0.094980694	0.580325902	0.627	1	3716	JAK1
-0.094980694	0.580325902	0.607	1	6720	SREBF1
-0.094980694	0.580325902	0.654	1	7903	ST8SIA4
-0.094980694	0.580325902	0.601	1	10435	CDC42EP2
-0.094980694	0.580325902	0.578	1	54971	BANP
-0.094980694	0.580325902	0.617	1	55111	PLEKHJ1
-0.094980694	0.580325902	0.602	1	147495	APCDD1
-0.094980694	0.580325902	0.648	1	255239	ANKK1
-0.094980694	0.580325902	0.622	1	653857	RP5-820A21.1
-0.09523809	0.579295456	0.601	1	101	ADAM8
-0.09523809	0.579295456	0.593	1	796	CALCA
-0.09523809	0.579295456	0.598	1	1349	COX7B
-0.09523809	0.579295456	0.579	1	3002	GZMB
-0.09523809	0.579295456	0.601	1	3609	ILF3
-0.09523809	0.579295456	0.584	1	4440	MSI1
-0.09523809	0.579295456	0.622	1	7543	ZFX
-0.09523809	0.579295456	0.599	1	23307	FKBP15
-0.09523809	0.579295456	0.596	1	27095	TRAPPC3
-0.09523809	0.579295456	0.6	1	51144	HSD17B12
-0.09523809	0.579295456	0.606	1	57191	VN1R1
-0.09523809	0.579295456	0.61	1	79711	IPO4

voxel-wise, subcortex			
Spearman's corr coef	p-val	GenID	Gene
-0.051198505	4.0843E-06	27440	CECR5
-0.051235836	4.01877E-06	29097	CNIH4
-0.051395103	3.75034E-06	255180	FLJ38723
-0.051429648	3.69442E-06	54863	C9orf167
-0.051470324	3.62961E-06	9044	BTAF1
-0.051476359	3.62008E-06	23593	HEBP2
-0.05151267	3.5633E-06	6259	RYK
-0.051522329	3.54833E-06	55786	ZNF415
-0.051545266	3.51304E-06	26575	RSG17
-0.05160201	3.42718E-06	100132644	LOC100132644
-0.051620394	3.39979E-06	10445	MCRS1
-0.051710755	3.26821E-06	114971	PTPMT1
-0.051714934	3.26224E-06	2824	GPM6B
-0.051814672	3.12294E-06	5910	RAP1GDS1
-0.051865377	3.05431E-06	12	SERPINA3
-0.051888313	3.02375E-06	10548	TM9SF1
-0.051910322	2.99469E-06	170626	XAGE3
-0.051950719	2.94207E-06	9905	SGSM2
-0.051968642	2.919E-06	100289060	LOC100289060
-0.051995017	2.88538E-06	56953	NT5M
-0.052070703	2.79093E-06	644186	C22orf41
-0.052082684	2.77626E-06	8332	HIST1H2AL
-0.052082684	2.77626E-06	441951	ZNFX1-AS1
-0.052105527	2.74848E-06	29915	HCFC2
-0.052117787	2.73369E-06	54858	PGPEP1
-0.052154098	2.69031E-06	51082	POLR1D
-0.052190315	2.6477E-06	23152	CIC
-0.052203778	2.63202E-06	442720	EIF3IP1
-0.052303053	2.51917E-06	7416	VDAC1
-0.052338529	2.47998E-06	57661	PHRF1
-0.052401584	2.41176E-06	644768	SLC9B1P4
-0.052490551	2.31857E-06	51741	WWOX
-0.052493706	2.31533E-06	7284	TUFM
-0.052530762	2.2776E-06	4715	NDUFB9
-0.052531224	2.27712E-06	168090	C6orf118
-0.052548219	2.26003E-06	79081	C11orf48
-0.052560851	2.24739E-06	51050	PI15
-0.052603755	2.20498E-06	163131	ZNF780B
-0.05269606	2.11633E-06	323	APBB2
-0.052707113	2.10594E-06	201931	TMEM192
-0.052708227	2.10491E-06	10960	LMAN2
-0.052762277	2.05485E-06	166647	GPR125
-0.052819297	2.0033E-06	54942	C9orf6
-0.052830905	1.99295E-06	646891	SDCCAG3P2
-0.05286805	1.9602E-06	55061	SUSD4
-0.052963238	1.8786E-06	90203	SNX21
-0.052975126	1.86865E-06	3127	HLA-DRB5
-0.052982088	1.86285E-06	29093	MRPL22
-0.053009577	1.84008E-06	388389	CCDC103
-0.053057309	1.80118E-06	117532	TMC2
-0.053140424	1.73534E-06	8653	DDX3Y
-0.053159278	1.72073E-06	140831	ZSWIM3
-0.053187508	1.69907E-06	6722	SRF
-0.053208403	1.68321E-06	4048	LTA4H
-0.053218156	1.67585E-06	54	ACP5
-0.053296439	1.61793E-06	11146	GLMN
-0.053310927	1.60743E-06	5731	PTGER1
-0.053380761	1.55769E-06	83734	ATG10
-0.053518206	1.4641E-06	54880	BCOR
-0.053573925	1.42772E-06	26150	RIBC2
-0.053575039	1.427E-06	4695	NDUFA2
-0.0535964	1.41329E-06	59343	SENP2
-0.053643018	1.38381E-06	5916	RARG
-0.053647473	1.38102E-06	3600	IL15
-0.053653046	1.37755E-06	26024	PTCD1
-0.053723346	1.33439E-06	6866	TAC3
-0.053738296	1.32539E-06	51444	RNF138
-0.053767085	1.30821E-06	26046	RNF160
-0.053771913	1.30534E-06	55298	RNF121
-0.053871468	1.24769E-06	83440	ADPGK
-0.053936567	1.21132E-06	29101	SSU72
-0.053940374	1.20922E-06	9610	RIN1
-0.054013088	1.16987E-06	94103	ORMDL3
-0.054034539	1.1585E-06	25828	TXN2
-0.054040391	1.15542E-06	9589	WTAP
-0.05405144	1.14961E-06	3101	HK3
-0.054128148	1.11011E-06	6727	SRP14
-0.054173745	1.08725E-06	1781	DYNC1I2
-0.054191854	1.07829E-06	57060	PCBP4

region-wise, subcortex					
Spearman's corr coef	p-val	p-val, SA	p-val, SA (BH- correction)	GenID	Gene
-0.09523809	0.579295456	0.623	1	90070	LACRT
-0.09523809	0.579295456	0.623	1	121642	ALKBH2
-0.095495492	0.578265488	0.59	1	9775	EIF4A3
-0.095495492	0.578265488	0.593	1	25894	PLEKHG4
-0.095495492	0.578265488	0.588	1	55526	DHTKD1
-0.095495492	0.578265488	0.597	1	259292	TAS2R46
-0.095495492	0.578265488	0.603	1	387778	SPDYC
-0.095752895	0.577236712	0.619	1	963	CD53
-0.095752895	0.577236712	0.627	1	8755	ADAM6
-0.095752895	0.577236712	0.639	1	9868	TOMM70A
-0.095752895	0.577236712	0.626	1	10077	TSPAN32
-0.095752895	0.577236712	0.606	1	51191	HERC5
-0.095752895	0.577236712	0.582	1	64960	MRPS15
-0.095752895	0.577236712	0.625	1	285679	AC136604.1
-0.09601029	0.57620877	0.598	1	4090	SMAD5
-0.09601029	0.57620877	0.585	1	23563	CHST5
-0.09601029	0.57620877	0.605	1	64965	MRPS9
-0.09601029	0.57620877	0.601	1	79958	DENND1C
-0.0962677	0.575181782	0.595	1	392	ARHGAP1
-0.0962677	0.575181782	0.567	1	1727	CYB5R3
-0.0962677	0.575181782	0.608	1	3162	HMOX1
-0.0962677	0.575181782	0.599	1	5830	PEX5
-0.0962677	0.575181782	0.635	1	51320	MEX3C
-0.0962677	0.575181782	0.613	1	100009676	LOC100009676
-0.096525095	0.574155033	0.601	1	414	ARSD
-0.096525095	0.574155033	0.615	1	9749	PHACTR2
-0.096525095	0.574155033	0.612	1	10063	COX17
-0.096525095	0.574155033	0.578	1	25900	IFFO1
-0.096525095	0.574155033	0.581	1	55527	FEM1A
-0.096525095	0.574155033	0.603	1	64763	ZNF574
-0.096525095	0.574155033	0.645	1	79074	C2orf49
-0.096525095	0.574155033	0.604	1	340252	ZNF680
-0.096782498	0.573129714	0.618	1	29988	SLC2A8
-0.096782498	0.573129714	0.6	1	150709	ANKAR
-0.096782498	0.573129714	0.576	1	171392	ZNF675
-0.097039901	0.572105169	0.613	1	3708	ITPR1
-0.097039901	0.572105169	0.611	1	4188	MDFI
-0.097039901	0.572105169	0.578	1	23493	HEY2
-0.097039901	0.572105169	0.604	1	23506	KIAA0240
-0.097039901	0.572105169	0.609	1	51027	BOLA1
-0.097039901	0.572105169	0.606	1	54625	PARP14
-0.097039901	0.572105169	0.586	1	283460	HNF1A-AS1
-0.097039901	0.572105169	0.609	1	100189589	AC005041.9
-0.097297296	0.5710814	0.616	1	9927	MFN2
-0.097297296	0.5710814	0.592	1	57524	CASKIN1
-0.097297296	0.5710814	0.614	1	80011	C16orf94
-0.097297296	0.5710814	0.597	1	83451	ABHD11
-0.097297296	0.5710814	0.612	1	92935	MARS2
-0.097297296	0.5710814	0.609	1	160140	C11orf65
-0.097554699	0.570058048	0.593	1	132	ADK
-0.097554699	0.570058048	0.61	1	2356	FPGS
-0.097554699	0.570058048	0.569	1	4780	NFE2L2
-0.097554699	0.570058048	0.613	1	53344	CHIC1
-0.097554699	0.570058048	0.588	1	54918	CMTM6
-0.097554699	0.570058048	0.598	1	94059	LENG9
-0.097554699	0.570058048	0.588	1	113612	CYP2U1
-0.097554699	0.570058048	0.61	1	135152	B3GAT2
-0.097554699	0.570058048	0.587	1	219855	SLC37A2
-0.097554699	0.570058048	0.594	1	285973	ATG9B
-0.097554699	0.570058048	0.58	1	728763	LOC728763
-0.097554699	0.570058048	0.61	1	100129196	RP5-1166H10.2
-0.097812094	0.569036067	0.599	1	1056	CEL
-0.097812094	0.569036067	0.651	1	3018	HIST1H2BB
-0.097812094	0.569036067	0.587	1	4213	MEIS3P1
-0.097812094	0.569036067	0.601	1	5589	PRKCSH
-0.097812094	0.569036067	0.601	1	11333	PDAP1
-0.097812094	0.569036067	0.586	1	54919	HEATR2
-0.097812094	0.569036067	0.577	1	57050	UTP3
-0.097812094	0.569036067	0.597	1	57715	SEMA4G
-0.097812094	0.569036067	0.61	1	79724	ZNF768
-0.098069496	0.56801492	0.595	1	8484	GALR3
-0.098069496	0.56801492	0.619	1	26175	C14orf109
-0.098069496	0.56801492	0.585	1	51119	SBDS
-0.098069496	0.56801492	0.614	1	55815	TSNAXIP1
-0.098069496	0.56801492	0.585	1	391109	OR10K1
-0.098326899	0.566994607	0.609	1	3053	SERPIND1
-0.098326899	0.566994607	0.596	1	5272	SERPINB9
-0.098326899	0.566994607	0.584	1	10207	INADL
-0.098326899	0.566994607	0.6	1	30819	KCNIP2

voxel-wise, subcortex			
Spearman's corr coef	p-val	GenID	Gene
-0.05419594	1.07628E-06	3338	DNAJC4
-0.054209501	1.06964E-06	55379	LRRC59
-0.05423541	1.05705E-06	152100	CMC1
-0.05440006	9.80318E-07	390535	GOLGA8E
-0.054422721	9.70186E-07	1374	CPT1A
-0.054433491	9.65408E-07	57091	CASS4
-0.054437298	9.63721E-07	79832	QSER1
-0.054471008	9.48932E-07	29942	PURG
-0.054529328	9.23862E-07	55665	URG4
-0.054543447	9.1789E-07	388759	C1orf229
-0.05465024	8.73899E-07	60675	PROK2
-0.054669186	8.6631E-07	6146	RPL22
-0.054708004	8.50955E-07	4292	MLH1
-0.054740228	8.38412E-07	127731	VWA5B1
-0.054792326	8.18507E-07	391123	VSIG8
-0.054863926	7.9189E-07	7294	TXK
-0.054874048	7.88197E-07	57105	CYSLTR2
-0.054888163	7.8307E-07	2193	FARSA
-0.054934781	7.66373E-07	120103	SLC36A4
-0.054961156	7.57077E-07	8611	PPAP2A
-0.055022635	7.35831E-07	83659	TEKT1
-0.055032477	7.32485E-07	57110	HRASLS
-0.055047337	7.27457E-07	38	ACAT1
-0.055053186	7.25488E-07	5565	PRKAB2
-0.055075567	7.17998E-07	8621	CDC2L5
-0.055139087	6.97152E-07	23658	LSM5
-0.055165648	6.88608E-07	4666	NACA
-0.055204652	6.76243E-07	2650	GCNT1
-0.0552678	6.56677E-07	10485	C1orf61
-0.055311725	6.43391E-07	2055	CLN8
-0.055369951	6.26176E-07	57577	KIAA1407
-0.055398371	6.17935E-07	24144	TFIP11
-0.055398464	6.1791E-07	65998	C11orf95
-0.055403665	6.16413E-07	58533	SNX6
-0.055486687	5.92994E-07	241	ALOX5AP
-0.055596918	5.63217E-07	81926	FAM108A4
-0.055647623	5.50011E-07	200205	C1orf69
-0.055672605	5.43613E-07	116093	DIRC1
-0.055764727	5.20638E-07	6590	SLPI
-0.055817194	5.07976E-07	347734	SLC35B2
-0.055834934	5.03762E-07	10083	USH1C
-0.055855457	4.98928E-07	122402	TDRD9
-0.055879228	4.93386E-07	92140	MTDH
-0.055921111	4.83765E-07	389895	LOC389895
-0.0559447	4.78426E-07	255082	CASC2
-0.055970237	4.72713E-07	2006	ELN
-0.055974323	4.71803E-07	150678	MYEOV2
-0.055974696	4.7172E-07	375260	WASH2P
-0.055984911	4.69458E-07	56895	AGPAT4
-0.056085393	4.47746E-07	51092	SIDT2
-0.056131918	4.38024E-07	4826	NNAT
-0.056136005	4.37179E-07	285521	COX18
-0.056145106	4.35305E-07	7365	UGT2B10
-0.056152999	4.33686E-07	6166	RPL36AL
-0.056167301	4.30767E-07	728666	PRELID1P1
-0.056249764	4.14301E-07	158358	KIAA2026
-0.056320623	4.0064E-07	5982	RFC2
-0.056399558	3.85933E-07	653319	KIAA0895L
-0.056421474	3.81942E-07	169436	C9orf96
-0.056433547	3.79762E-07	6635	SNRPE
-0.056503009	3.67444E-07	23386	NUDCD3
-0.056513134	3.65681E-07	55832	CAND1
-0.056583155	3.53712E-07	2281	FKBP1B
-0.056605626	3.49951E-07	55780	C6orf70
-0.056628194	3.46213E-07	92736	OTOP2
-0.056654289	3.41939E-07	55602	CDKN2AIP
-0.056667197	3.39843E-07	6262	RYR2
-0.056744371	3.27572E-07	79140	CCDC28B
-0.05681736	3.16361E-07	79153	GDPD3
-0.056932237	2.99461E-07	100130700	LOC100130700
-0.056939945	2.9836E-07	27342	RABGEF1
-0.056979969	2.927E-07	6182	MRPL12
-0.057010617	2.88435E-07	79701	C17orf101
-0.057025474	2.8639E-07	11122	PTPRT
-0.057031974	2.855E-07	7508	XPC
-0.057034574	2.85144E-07	23398	PPWD1
-0.057115741	2.7426E-07	9843	HEPH
-0.057130322	2.72348E-07	60313	GPBP1L1
-0.057164494	2.67917E-07	573	BAG1

region-wise, subcortex					
Spearman's corr coef	p-val	p-val, SA	p-val, SA (BH- correction)	GenID	Gene
-0.098326899	0.566994607	0.56	1	54014	BRWD1
-0.098326899	0.566994607	0.618	1	57107	PDSS2
-0.098326899	0.566994607	0.622	1	163227	ZNF100
-0.098584294	0.56597513	0.583	1	1783	DYNC1LI2
-0.098584294	0.56597513	0.622	1	3312	HSPA8
-0.098584294	0.56597513	0.569	1	6836	SURF4
-0.098584294	0.56597513	0.608	1	84818	IL17RC
-0.098584294	0.56597513	0.601	1	222487	GPR97
-0.098584294	0.56597513	0.588	1	445815	PALM2-AKAP2
-0.098584294	0.56597513	0.603	1	100128782	LINC00476
-0.098841697	0.564956129	0.583	1	86	ACTL6A
-0.098841697	0.564956129	0.613	1	1112	FOXN3
-0.098841697	0.564956129	0.586	1	7402	UTRN
-0.098841697	0.564956129	0.634	1	23432	GPR161
-0.098841697	0.564956129	0.61	1	26017	FAM32A
-0.098841697	0.564956129	0.623	1	84033	OBSCN
-0.099099092	0.56393832	0.608	1	54	ACP5
-0.099099092	0.56393832	0.576	1	5378	PMS1
-0.099099092	0.56393832	0.601	1	27131	SNX5
-0.099099092	0.56393832	0.567	1	57730	ANKRD36B
-0.099099092	0.56393832	0.626	1	79091	C16orf68
-0.099099092	0.56393832	0.565	1	94104	C21orf66
-0.099356495	0.562921405	0.574	1	111	ADCY5
-0.099356495	0.562921405	0.605	1	6195	RPS6KA1
-0.099356495	0.562921405	0.57	1	6608	SMO
-0.099356495	0.562921405	0.606	1	10887	PROKR1
-0.099356495	0.562921405	0.602	1	51316	PLAC8
-0.099356495	0.562921405	0.611	1	51742	ARID4B
-0.099356495	0.562921405	0.598	1	55905	RNF114
-0.099356495	0.562921405	0.585	1	64098	PARVG
-0.099356495	0.562921405	0.61	1	128338	DRAM2
-0.099356495	0.562921405	0.617	1	386679	KRTAP10-2
-0.099356495	0.562921405	0.607	1	100128569	C19orf71
-0.099613898	0.561905384	0.585	1	1293	COL6A3
-0.099613898	0.561905384	0.591	1	9849	ZNF518A
-0.099613898	0.561905384	0.593	1	51002	TPRKB
-0.099613898	0.561905384	0.599	1	84886	C1orf198
-0.099613898	0.561905384	0.59	1	90321	ZNF766
-0.099613898	0.561905384	0.581	1	93107	KCNG4
-0.099613898	0.561905384	0.609	1	163782	KANK4
-0.099613898	0.561905384	0.568	1	221336	BEND6
-0.0998713	0.560889661	0.583	1	7185	TRAF1
-0.0998713	0.560889661	0.596	1	7873	ARMET
-0.0998713	0.560889661	0.569	1	63971	KIF13A
-0.0998713	0.560889661	0.599	1	80329	ULBP1
-0.0998713	0.560889661	0.564	1	126299	ZNF428
-0.0998713	0.560889661	0.57	1	130589	GALM
-0.0998713	0.560889661	0.603	1	378938	MALAT1
-0.100128703	0.559875369	0.588	1	3570	IL6R
-0.100128703	0.559875369	0.568	1	4677	NARS
-0.100128703	0.559875369	0.602	1	9639	ARHGEF10
-0.100128703	0.559875369	0.598	1	57636	ARHGAP23
-0.100128703	0.559875369	0.587	1	64924	SLC30A5
-0.100128703	0.559875369	0.564	1	81544	GDPD5
-0.100128703	0.559875369	0.619	1	84206	MEX3B
-0.100386098	0.558861852	0.601	1	5990	RFX2
-0.100386098	0.558861852	0.592	1	6619	SNAPC3
-0.100386098	0.558861852	0.61	1	7029	TFDP2
-0.100386098	0.558861852	0.617	1	23200	ATP11B
-0.100386098	0.558861852	0.614	1	26959	HBP1
-0.100386098	0.558861852	0.585	1	90550	CCDC109A
-0.100386098	0.558861852	0.597	1	161424	C14orf21
-0.100386098	0.558861852	0.581	1	259294	TAS2R48
-0.100386098	0.558861852	0.587	1	349334	FOXD4L4
-0.100386098	0.558861852	0.602	1	402360	LOC402360
-0.100643501	0.557849288	0.587	1	6406	SEMG1
-0.100643501	0.557849288	0.595	1	23191	CYFIP1
-0.100643501	0.557849288	0.572	1	23529	CLCF1
-0.100643501	0.557849288	0.604	1	64398	MPP5
-0.100900903	0.556837559	0.607	1	949	SCARB1
-0.100900903	0.556837559	0.608	1	1407	CRY1
-0.100900903	0.556837559	0.604	1	2919	CXCL1
-0.100900903	0.556837559	0.595	1	6843	VAMP1
-0.100900903	0.556837559	0.581	1	8904	CPNE1
-0.100900903	0.556837559	0.579	1	9559	VPS26A
-0.100900903	0.556837559	0.608	1	25805	BAMBI
-0.100900903	0.556837559	0.604	1	26130	GAPVD1
-0.100900903	0.556837559	0.587	1	26249	KLHL3
-0.100900903	0.556837559	0.592	1	59343	SENP2

voxel-wise, subcortex			
Spearman's corr coef	p-val	GenID	Gene
-0.057212412	2.61821E-07	9738	AC012621.2
-0.057215851	2.61387E-07	2625	GATA3
-0.057347998	2.45287E-07	25898	RCHY1
-0.057379387	2.41606E-07	10732	TCFL5
-0.057400931	2.39111E-07	10042	HMGXB4
-0.057487391	2.29343E-07	84924	ZNF566
-0.057499554	2.28E-07	57153	SLC44A2
-0.05754469	2.23084E-07	191	AHCY
-0.057556204	2.21845E-07	51430	C1orf9
-0.057597157	2.17496E-07	127343	DMBX1
-0.05763644	2.13402E-07	9448	MAP4K4
-0.057643313	2.12694E-07	5859	QARS
-0.057676557	2.09298E-07	84888	AC012100.1
-0.057686958	2.08247E-07	4193	MDM2
-0.05782143	1.95102E-07	96610	LL22NC03-80A10.2
-0.057843626	1.93012E-07	4478	MSN
-0.057932962	1.84813E-07	133923	ZNF474
-0.057992674	1.79521E-07	56942	CMC2
-0.05808647	1.71504E-07	25915	NDUFAF3
-0.058101885	1.7022E-07	442582	STAG3L2
-0.05817144	1.6454E-07	5790	PTPRCAP
-0.05817822	1.63996E-07	2978	GUCA1A
-0.058182679	1.6364E-07	960	CD44
-0.058217596	1.60873E-07	8970	HIST1H2BJ
-0.058284458	1.557E-07	51285	RASL12
-0.058315106	1.53384E-07	22913	RALY
-0.058336556	1.51782E-07	23390	ZDHHC17
-0.058339622	1.51554E-07	4814	NINJ1
-0.058379088	1.48654E-07	377047	PRSS45
-0.05839042	1.47831E-07	3231	HOXD1
-0.058403049	1.46919E-07	60686	C14orf93
-0.058407784	1.46579E-07	534	ATP6V1G2
-0.058429517	1.45026E-07	22986	SORCS3
-0.058450226	1.43561E-07	4613	MYCN
-0.058492664	1.40603E-07	2621	GAS6
-0.058534175	1.37767E-07	2140	EYA3
-0.058579586	1.34728E-07	10494	STK25
-0.058579586	1.34728E-07	66035	SLC2A11
-0.058593702	1.33797E-07	79943	ZNF696
-0.058597047	1.33577E-07	9088	PKMYT1
-0.05868471	1.2794E-07	55040	EPN3
-0.058691956	1.27484E-07	3761	KCNJ4
-0.058697525	1.27135E-07	51209	RAB9B
-0.058713127	1.26162E-07	7369	UMOD
-0.058729842	1.25128E-07	54625	PARP14
-0.058784172	1.21823E-07	7710	ZNF154
-0.058806088	1.20513E-07	7357	UGCG
-0.058811288	1.20204E-07	8019	BRD3
-0.058825959	1.19338E-07	8605	PLA2G4C
-0.058855586	1.17605E-07	146556	C16orf89
-0.0588645	1.17089E-07	7568	ZNF20
-0.058881957	1.16084E-07	55644	OSGEP
-0.058956716	1.11875E-07	100129460	DPY19L1P1
-0.058970738	1.11102E-07	10903	MTMR11
-0.058979653	1.10613E-07	78987	CRELD1
-0.059112173	1.03589E-07	153830	RNF145
-0.059137061	1.02319E-07	1665	DHX15
-0.059190273	9.96536E-08	199746	U2AF1L4
-0.059253238	9.65868E-08	2793	GNGT2
-0.059256393	9.64353E-08	4524	MTHFR
-0.0593201	9.34298E-08	387486	LINC00320
-0.059377305	9.08086E-08	3336	HSPE1
-0.05939718	8.99147E-08	4729	NDUFV2
-0.059401821	8.97069E-08	768211	RELL1
-0.059417423	8.90126E-08	5465	PPARA
-0.059435811	8.82009E-08	112939	NACC1
-0.059446398	8.77372E-08	54878	DPP8
-0.059457172	8.72674E-08	780	DDR1
-0.059482243	8.61837E-08	8802	SUCLG1
-0.059547622	8.34184E-08	60489	APOBEC3G
-0.059566196	8.26485E-08	55839	CENPN
-0.059622657	8.03505E-08	9238	TBRG4
-0.05964049	7.96378E-08	79744	ZNF419
-0.059693143	7.75685E-08	9697	TRAM2
-0.059743289	7.56459E-08	93134	ZNF561
-0.059766691	7.4765E-08	729020	LOC729020
-0.059819903	7.2798E-08	9618	TRAF4
-0.059849251	7.17347E-08	26017	FAM32A
-0.059910543	6.95625E-08	54059	C21orf57

region-wise, subcortex					
Spearman's corr coef	p-val	p-val, SA	p-val, SA (BH- correction)	GenID	Gene
-0.100900903	0.556837559	0.572	1	221184	CPNE2
-0.101158299	0.555826128	0.564	1	1612	DAPK1
-0.101158299	0.555826128	0.611	1	2554	GABRA1
-0.101158299	0.555826128	0.588	1	3483	IGFALS
-0.101158299	0.555826128	0.609	1	10600	USP16
-0.101158299	0.555826128	0.576	1	51135	IRAK4
-0.101158299	0.555826128	0.565	1	55161	TMEM33
-0.101158299	0.555826128	0.615	1	64787	EPS8L2
-0.101158299	0.555826128	0.588	1	65980	BRD9
-0.101158299	0.555826128	0.597	1	84898	PLXDC2
-0.101158299	0.555826128	0.558	1	91612	CHURC1
-0.101158299	0.555826128	0.582	1	112724	RDH13
-0.101415701	0.554816127	0.573	1	6653	SORL1
-0.101415701	0.554816127	0.6	1	8870	IER3
-0.101415701	0.554816127	0.572	1	10150	MBNL2
-0.101415701	0.554816127	0.615	1	25855	BRMS1
-0.101415701	0.554816127	0.592	1	51115	FAM82B
-0.101415701	0.554816127	0.596	1	91304	C19orf6
-0.101415701	0.554816127	0.58	1	124685	LOC124685
-0.101415701	0.554816127	0.597	1	441394	SUGT1P1
-0.101673096	0.55380702	0.617	1	11103	KRR1
-0.101673096	0.55380702	0.578	1	51286	CEND1
-0.101673096	0.55380702	0.605	1	55166	CENPQ
-0.101673096	0.55380702	0.605	1	205147	FAM123C
-0.101673096	0.55380702	0.607	1	645191	LINGO3
-0.101673096	0.55380702	0.578	1	648262	LOC648262
-0.101930499	0.552798748	0.588	1	80017	C14orf159
-0.101930499	0.552798748	0.599	1	92949	ADAMTSL1
-0.102187902	0.551790893	0.59	1	8623	ASMTL
-0.102187902	0.551790893	0.575	1	9784	SNX17
-0.102187902	0.551790893	0.562	1	22936	ELL2
-0.102187902	0.551790893	0.596	1	26341	OR5H1
-0.102187902	0.551790893	0.568	1	130752	MDH1B
-0.102187902	0.551790893	0.575	1	345645	PSMC1P4
-0.102445297	0.550784349	0.594	1	4654	MYOD1
-0.102445297	0.550784349	0.611	1	54797	MED18
-0.102445297	0.550784349	0.595	1	79075	DSCC1
-0.102445297	0.550784349	0.575	1	79987	SVEP1
-0.102445297	0.550784349	0.572	1	80764	THAP7
-0.102445297	0.550784349	0.623	1	375704	ENHO
-0.1027027	0.549778759	0.57	1	3703	STT3A
-0.1027027	0.549778759	0.592	1	5593	PRKG2
-0.1027027	0.549778759	0.602	1	200765	TIGD1
-0.1027027	0.549778759	0.586	1	256227	AC099759.1
-0.102960095	0.548773944	0.578	1	1312	COMT
-0.102960095	0.548773944	0.577	1	7379	UPK2
-0.102960095	0.548773944	0.595	1	10584	COLEC10
-0.102960095	0.548773944	0.589	1	22870	SAPS1
-0.102960095	0.548773944	0.58	1	51522	TMEM14C
-0.102960095	0.548773944	0.581	1	57127	RHBG
-0.102960095	0.548773944	0.568	1	64135	IFIH1
-0.102960095	0.548773944	0.589	1	64174	DPEP2
-0.102960095	0.548773944	0.578	1	121364	OR10A7
-0.102960095	0.548773944	0.602	1	167153	PAPD4
-0.102960095	0.548773944	0.574	1	256148	OR4S1
-0.102960095	0.548773944	0.592	1	344167	FOXI3
-0.102960095	0.548773944	0.58	1	404734	ANKHD1-EIF4EBP3
-0.103217505	0.547769547	0.603	1	6624	FSCN1
-0.103217505	0.547769547	0.599	1	8175	SF3A2
-0.103217505	0.547769547	0.577	1	23112	TNRC6B
-0.103217505	0.547769547	0.581	1	91661	ZNF765
-0.103217505	0.547769547	0.584	1	643965	TMEM88B
-0.103474908	0.546766579	0.579	1	79089	TMUB2
-0.103474908	0.546766579	0.593	1	144455	E2F7
-0.103474908	0.546766579	0.591	1	646424	SPINK8
-0.103474908	0.546766579	0.588	1	730291	ZNF735
-0.103732303	0.545764446	0.593	1	583	BBS2
-0.103732303	0.545764446	0.574	1	2314	FLII
-0.103732303	0.545764446	0.578	1	5554	PRH1
-0.103732303	0.545764446	0.56	1	26287	ANKRD2
-0.103732303	0.545764446	0.573	1	51111	SUV420H1
-0.103732303	0.545764446	0.605	1	55081	IFT57
-0.103732303	0.545764446	0.584	1	55274	PHF10
-0.103732303	0.545764446	0.593	1	79913	ACTR5
-0.103732303	0.545764446	0.588	1	80741	LY6G5C
-0.103732303	0.545764446	0.6	1	80763	C12orf39
-0.103732303	0.545764446	0.582	1	81833	SPACA1
-0.103732303	0.545764446	0.575	1	84928	TMEM209
-0.103732303	0.545764446	0.582	1	92689	FAM114A1

voxel-wise, subcortex			
Spearman's corr coef	p-val	GenID	Gene
-0.059941188	6.85004E-08	4852	NPY
-0.060017709	6.59165E-08	6484	ST3GAL4
-0.060056899	6.46299E-08	10437	IFI30
-0.060186353	6.05507E-08	23179	RGL1
-0.060193971	6.03187E-08	2768	GNA12
-0.060194526	6.03019E-08	3823	KLRC3
-0.060243376	5.8834E-08	5962	RDX
-0.060317017	5.66868E-08	59307	SIGIRR
-0.060366329	5.52917E-08	54882	ANKHD1
-0.060399853	5.43623E-08	148203	ZNF738
-0.060461611	5.26895E-08	23252	OTUD3
-0.060487982	5.19906E-08	2354	FOSB
-0.060500983	5.16492E-08	55717	BRWD2
-0.06055364	5.02889E-08	55510	DDX43
-0.060573697	4.978E-08	258010	SVIP
-0.060580105	4.96184E-08	3916	LAMP1
-0.060755901	4.53797E-08	57644	MYH7B
-0.060760822	4.52663E-08	645317	CHCHD2P6
-0.060800198	4.43683E-08	80319	CXXC4
-0.060815986	4.40132E-08	89848	FCHSD1
-0.060887214	4.24448E-08	5473	PPBP
-0.060905509	4.20507E-08	10781	ZNF266
-0.060939405	4.13301E-08	9066	SYT7
-0.060946368	4.11836E-08	11315	PARK7
-0.060979057	4.05023E-08	3911	LAMA5
-0.061003666	3.99966E-08	81890	QTRT1
-0.06101732	3.97187E-08	8406	SRPX
-0.061019175	3.9681E-08	3007	HIST1H1D
-0.061028462	3.94932E-08	440051	KRTAP5-11
-0.061044157	3.91779E-08	27152	INTU
-0.061093841	3.81954E-08	55971	BAIAP2L1
-0.061103221	3.80126E-08	1716	DGUOK
-0.061112784	3.78271E-08	121536	AEBP2
-0.061189122	3.63777E-08	9811	KIAA0427
-0.061198037	3.62121E-08	5586	PKN2
-0.061226267	3.56922E-08	129531	MITD1
-0.061241683	3.54113E-08	5708	PSMD2
-0.061273813	3.48328E-08	79894	ZNF672
-0.061293688	3.44795E-08	386678	KRTAP10-11
-0.061315697	3.40923E-08	64398	MPP5
-0.06133882	3.36903E-08	6504	SLAMF1
-0.061428346	3.21761E-08	8089	YEATS4
-0.061449889	3.18217E-08	23331	TTC28
-0.061461497	3.16322E-08	91875	TTC5
-0.061472457	3.14545E-08	147807	ZNF524
-0.061482765	3.12881E-08	114904	C1QTNF6
-0.061565414	2.99849E-08	51752	ERAP1
-0.061592903	2.95633E-08	54621	AC131238.1
-0.06165605	2.86163E-08	6536	SLC6A9
-0.061662272	2.85245E-08	23456	ABCB10
-0.061703227	2.79281E-08	11156	PTP4A3
-0.061739258	2.74133E-08	29907	SNX15
-0.061745852	2.732E-08	55166	CENPQ
-0.061760619	2.71124E-08	55741	EDEM2
-0.061812438	2.63959E-08	5032	P2RY11
-0.061840761	2.60121E-08	5166	PDK4
-0.061881159	2.5474E-08	55791	C1orf103
-0.061920162	2.49647E-08	9121	SLC16A5
-0.061924897	2.49035E-08	60680	BRUNOL5
-0.061952479	2.45503E-08	401321	LOC401321
-0.062019158	2.37161E-08	163782	KANK4
-0.062123261	2.24684E-08	146705	C17orf56
-0.062165141	2.19846E-08	389033	AC079776.2
-0.062169414	2.19359E-08	340719	NANOS1
-0.062182602	2.1786E-08	10459	MAD2L2
-0.062212132	2.14539E-08	480	ATP1A4
-0.062240273	2.11421E-08	9456	HOMER1
-0.062416345	1.92884E-08	79957	PAQR6
-0.062450614	1.89464E-08	55063	ZCWPW1
-0.062463336	1.88209E-08	23526	HMHA1
-0.062465284	1.88018E-08	4772	NFATC1
-0.062584527	1.76656E-08	1196	CLK2
-0.062594742	1.75714E-08	7994	MYST3
-0.062594928	1.75697E-08	60674	GAS5
-0.062627152	1.72758E-08	3028	HSD17B10
-0.062677115	1.68296E-08	4739	NEDD9
-0.062723361	1.64266E-08	286499	FAM133A
-0.062724844	1.64139E-08	128869	PIGU
-0.062788554	1.58743E-08	57620	STIM2

region-wise, subcortex					
Spearman's corr coef	p-val	p-val, SA	p-val, SA (BH- correction)	GenID	Gene
-0.103732303	0.545764446	0.591	1	151300	LINC00608
-0.103732303	0.545764446	0.558	1	643155	C5orf43
-0.103989705	0.544763148	0.582	1	3626	INHBC
-0.103989705	0.544763148	0.577	1	5129	PCTK3
-0.103989705	0.544763148	0.586	1	79930	DOK3
-0.103989705	0.544763148	0.583	1	83667	SESN2
-0.103989705	0.544763148	0.589	1	115908	CTHRC1
-0.104247101	0.543762803	0.583	1	364	AQP7
-0.104247101	0.543762803	0.573	1	1152	CKB
-0.104247101	0.543762803	0.57	1	2115	ETV1
-0.104247101	0.543762803	0.548	1	2952	GSTT1
-0.104247101	0.543762803	0.564	1	5016	OVGP1
-0.104247101	0.543762803	0.555	1	51397	COMMD10
-0.104247101	0.543762803	0.576	1	51450	PRRX2
-0.104247101	0.543762803	0.556	1	79665	DHX40
-0.104247101	0.543762803	0.571	1	81605	URM1
-0.104247101	0.543762803	0.575	1	128853	DUSP15
-0.104247101	0.543762803	0.593	1	155061	ZNF746
-0.104247101	0.543762803	0.611	1	219477	OR8J1
-0.104247101	0.543762803	0.565	1	653483	C6orf124
-0.104504503	0.542762816	0.549	1	176	ACAN
-0.104504503	0.542762816	0.574	1	2107	ETF1
-0.104504503	0.542762816	0.572	1	25895	FAM119B
-0.104504503	0.542762816	0.575	1	51497	TH1L
-0.104504503	0.542762816	0.568	1	285093	CXXC11
-0.104761906	0.541764259	0.553	1	220	ALDH1A3
-0.104761906	0.541764259	0.555	1	7782	SLC30A4
-0.104761906	0.541764259	0.605	1	9513	FXR2
-0.104761906	0.541764259	0.551	1	23081	KDM4C
-0.104761906	0.541764259	0.563	1	51284	TLR7
-0.104761906	0.541764259	0.572	1	51294	PCDH12
-0.104761906	0.541764259	0.576	1	51366	UBR5
-0.104761906	0.541764259	0.562	1	55341	LSG1
-0.104761906	0.541764259	0.575	1	55663	ZNF446
-0.104761906	0.541764259	0.579	1	81025	GJA9
-0.104761906	0.541764259	0.617	1	90768	MGC45800
-0.104761906	0.541764259	0.589	1	123872	LRRC50
-0.104761906	0.541764259	0.567	1	339318	ZNF181
-0.105019301	0.540766478	0.578	1	1196	CLK2
-0.105019301	0.540766478	0.543	1	7678	ZNF124
-0.105019301	0.540766478	0.573	1	7690	ZNF131
-0.105019301	0.540766478	0.593	1	9213	XPR1
-0.105019301	0.540766478	0.567	1	23020	SNRNP200
-0.105019301	0.540766478	0.555	1	27335	EIF3K
-0.105019301	0.540766478	0.56	1	63874	ABHD4
-0.105019301	0.540766478	0.575	1	151449	GDF7
-0.105019301	0.540766478	0.573	1	100130302	FAM48B1
-0.105276704	0.53976965	0.533	1	6908	TBP
-0.105276704	0.53976965	0.584	1	114757	CYGB
-0.105276704	0.53976965	0.587	1	100049587	SIGLEC14
-0.105534099	0.538773239	0.552	1	19	ABCA1
-0.105534099	0.538773239	0.566	1	5756	TWF1
-0.105534099	0.538773239	0.558	1	5928	RBBP4
-0.105534099	0.538773239	0.574	1	23116	FAM179B
-0.105534099	0.538773239	0.585	1	55894	DEFB103B
-0.105534099	0.538773239	0.55	1	79134	TMEM185B
-0.105534099	0.538773239	0.558	1	80150	ASRGL1
-0.105534099	0.538773239	0.584	1	283234	CCDC88B
-0.105791502	0.537778258	0.58	1	816	CAMK2B
-0.105791502	0.537778258	0.566	1	2948	GSTM4
-0.105791502	0.537778258	0.566	1	4170	MCL1
-0.105791502	0.537778258	0.57	1	7020	TFAP2A
-0.105791502	0.537778258	0.568	1	9997	SCO2
-0.105791502	0.537778258	0.578	1	26048	ZNF500
-0.105791502	0.537778258	0.601	1	55716	LMBR1L
-0.105791502	0.537778258	0.576	1	100130717	CECR5-AS1
-0.106048904	0.536784053	0.561	1	2717	GLA
-0.106048904	0.536784053	0.592	1	4604	MYBPC1
-0.106048904	0.536784053	0.569	1	6785	ELOVL4
-0.106048904	0.536784053	0.557	1	7404	UTY
-0.106048904	0.536784053	0.576	1	26234	FBXL5
-0.106048904	0.536784053	0.588	1	54815	GATAD2A
-0.106048904	0.536784053	0.558	1	54949	SDHAF2
-0.106048904	0.536784053	0.553	1	83854	ANGPTL6
-0.1063063	0.535790801	0.565	1	231	AKR1B1
-0.1063063	0.535790801	0.554	1	308	ANXA5
-0.1063063	0.535790801	0.577	1	329	BIRC2
-0.1063063	0.535790801	0.583	1	4832	NME3
-0.1063063	0.535790801	0.577	1	6016	RIT1

voxel-wise, subcortex			
Spearman's corr coef	p-val	GenID	Gene
-0.062825233	1.55716E-08	4711	NDUFB5
-0.062845111	1.54099E-08	3769	KCNJ13
-0.062898137	1.49864E-08	1326	MAP3K8
-0.062898785	1.49813E-08	84865	CCDC142
-0.062928684	1.47476E-08	83987	CCDC8
-0.063039944	1.39087E-08	283149	BCL9L
-0.063049875	1.38361E-08	8370	HIST2H4A
-0.06307365	1.36638E-08	2532	DARC
-0.06309139	1.35366E-08	51293	CD320
-0.063094176	1.35168E-08	55870	ASH1L
-0.063121289	1.33248E-08	222611	GPR111
-0.063151471	1.31144E-08	5362	PLXNA2
-0.063255668	1.24122E-08	387254	SLC7A5P2
-0.063255854	1.24109E-08	646626	LOC646626
-0.063257523	1.24E-08	100132815	AC092329.2
-0.063341938	1.18586E-08	29967	LRP12
-0.063344911	1.18399E-08	3764	KCNJ8
-0.063437961	1.12705E-08	204	AK2
-0.063488297	1.09737E-08	126017	ZNF813
-0.063504919	1.08774E-08	53371	NUP54
-0.063605957	1.03092E-08	4194	MDM4
-0.063614875	1.02605E-08	22985	ACIN1
-0.063744701	9.57578E-09	26153	KIF26A
-0.063774049	9.42729E-09	145748	LYSMD4
-0.063849084	9.0577E-09	6117	RPA1
-0.063856043	9.02415E-09	3113	HLA-DPA1
-0.063861243	8.99914E-09	10357	HMGB1P1
-0.063873135	8.94226E-09	221120	ALKBH3
-0.063878708	8.91571E-09	23523	CABIN1
-0.063909911	8.76846E-09	657	BMPR1A
-0.063922539	8.70956E-09	64215	DNAJC1
-0.063993208	8.3869E-09	6128	RPL6P10
-0.064064719	8.07225E-09	84316	LSMD1
-0.064072609	8.03828E-09	2152	F3
-0.06408032	8.00516E-09	438	ASMT
-0.06408041	8.00477E-09	57719	ANO8
-0.06416975	7.63084E-09	3914	LAMB3
-0.064212933	7.45623E-09	84614	ZBTB37
-0.064273573	7.21759E-09	84230	LRRC8C
-0.064289451	7.15635E-09	64327	LMBR1
-0.064324372	7.02341E-09	9784	SNX17
-0.064428657	6.64055E-09	51728	POLR3K
-0.064431816	6.62928E-09	10885	WDR3
-0.064450108	6.56435E-09	23399	DULLARD
-0.064472586	6.48542E-09	8287	USP9Y
-0.064515859	6.33607E-09	8195	MKKS
-0.064549938	6.22077E-09	51104	FAM108B1
-0.064564057	6.17363E-09	23345	SYNE1
-0.064575575	6.13543E-09	4237	MFAP2
-0.064581797	6.11488E-09	84619	ZGPAT
-0.064594515	6.07309E-09	100130932	LOC100130932
-0.064599715	6.05608E-09	143888	KDELC2
-0.064604916	6.03913E-09	6155	RPL27
-0.064623863	5.97771E-09	65250	C5orf42
-0.064745985	5.59624E-09	644168	DRGX
-0.064798824	5.43864E-09	10327	AKR1A1
-0.064800866	5.43263E-09	246269	LACE1
-0.064801238	5.43153E-09	23443	SLC35A3
-0.064817578	5.38376E-09	54778	RNF111
-0.064839311	5.3208E-09	90321	ZNF766
-0.064937748	5.04455E-09	3588	IL10RB
-0.064956509	4.99352E-09	4213	MEIS3P1
-0.064995602	4.88878E-09	1653	DDX1
-0.065014459	4.83901E-09	646050	LOC646050
-0.065016128	4.83463E-09	4088	SMAD3
-0.065043986	4.76209E-09	1201	CLN3
-0.065047331	4.75345E-09	5315	PKM2
-0.06506674	4.70362E-09	3993	LLGL2
-0.065098129	4.62412E-09	5098	PCDHGC3
-0.065203346	4.36702E-09	22901	ARSG
-0.06526538	4.22202E-09	50717	WDR42A
-0.06526947	4.21262E-09	54812	AFTPH
-0.06529361	4.15758E-09	54826	GIN1
-0.065329455	4.07716E-09	25983	NGDN
-0.065433376	3.85245E-09	441168	FAM26F
-0.065441638	3.8351E-09	54108	CHRAC1
-0.065448329	3.82113E-09	51776	ZAK
-0.065499589	3.71561E-09	134429	STARD4
-0.065526888	3.66059E-09	23017	FAIM2

region-wise, subcortex					
Spearman's corr coef	p-val	p-val, SA	p-val, SA (BH- correction)	GenID	Gene
-0.1063063	0.535790801	0.608	1	6366	CCL21
-0.1063063	0.535790801	0.586	1	9589	WTAP
-0.1063063	0.535790801	0.602	1	84340	GFM2
-0.1063063	0.535790801	0.544	1	285175	C2orf21
-0.1063063	0.535790801	0.565	1	285440	CYP4V2
-0.106563702	0.534798026	0.579	1	3660	IRF2
-0.106563702	0.534798026	0.614	1	4499	MT1M
-0.106563702	0.534798026	0.575	1	6473	SHOX
-0.106563702	0.534798026	0.543	1	6818	SULT1A3
-0.106563702	0.534798026	0.57	1	10327	AKR1A1
-0.106563702	0.534798026	0.593	1	11219	TREX2
-0.106563702	0.534798026	0.569	1	29997	GLTSCR2
-0.106563702	0.534798026	0.568	1	57093	TRIM49
-0.106563702	0.534798026	0.537	1	83999	KREMEN1
-0.106563702	0.534798026	0.581	1	192134	B3GNT6
-0.106563702	0.534798026	0.573	1	285268	ZNF621
-0.106563702	0.534798026	0.562	1	340527	NHSL2
-0.106821112	0.533806503	0.577	1	5062	PAK2
-0.106821112	0.533806503	0.556	1	29880	ALG5
-0.106821112	0.533806503	0.605	1	55196	C12orf35
-0.106821112	0.533806503	0.567	1	56658	TRIM39
-0.106821112	0.533806503	0.585	1	64102	TNMD
-0.106821112	0.533806503	0.53	1	116540	MRPL53
-0.106821112	0.533806503	0.58	1	219578	ZNF804B
-0.107078508	0.532815993	0.567	1	427	ASAH1
-0.107078508	0.532815993	0.573	1	657	BMPR1A
-0.107078508	0.532815993	0.574	1	4640	MYO1A
-0.107078508	0.532815993	0.567	1	9824	ARHGAP11A
-0.107078508	0.532815993	0.579	1	151295	SLC23A3
-0.107078508	0.532815993	0.578	1	152877	FAM53A
-0.10733591	0.531826317	0.561	1	2950	GSTP1
-0.10733591	0.531826317	0.556	1	11118	BTN3A2
-0.10733591	0.531826317	0.541	1	51528	JKAMP
-0.10733591	0.531826317	0.569	1	79888	LPCAT1
-0.10733591	0.531826317	0.549	1	85444	LRRCC1
-0.10733591	0.531826317	0.566	1	197021	LCTL
-0.10733591	0.531826317	0.559	1	341346	C12orf70
-0.10733591	0.531826317	0.554	1	100286918	NDUFS5P2
-0.107593305	0.530837536	0.565	1	2047	EPHB1
-0.107593305	0.530837536	0.575	1	4060	LUM
-0.107593305	0.530837536	0.579	1	57514	CDGAP
-0.107593305	0.530837536	0.554	1	64137	ABCG4
-0.107593305	0.530837536	0.548	1	92070	CTBP1-AS1
-0.107850708	0.529849291	0.538	1	1738	DLD
-0.107850708	0.529849291	0.567	1	7436	VLDLR
-0.107850708	0.529849291	0.518	1	8034	SLC25A16
-0.107850708	0.529849291	0.563	1	11252	PACSIN2
-0.107850708	0.529849291	0.536	1	23301	EHBP1
-0.107850708	0.529849291	0.562	1	23544	SEZ6L
-0.107850708	0.529849291	0.574	1	54849	DEF8
-0.107850708	0.529849291	0.567	1	93979	CPA5
-0.107850708	0.529849291	0.553	1	118433	RPL23AP7
-0.107850708	0.529849291	0.588	1	150353	DNAJB7
-0.107850708	0.529849291	0.57	1	379025	RP11-349A22.5
-0.108108111	0.528862298	0.579	1	4717	NDUFC1
-0.108108111	0.528862298	0.549	1	8269	TMEM187
-0.108108111	0.528862298	0.563	1	54822	TRPM7
-0.108108111	0.528862298	0.581	1	83787	ARMC10
-0.108108111	0.528862298	0.554	1	91433	RCCD1
-0.108365506	0.527876258	0.565	1	1183	CLCN4
-0.108365506	0.527876258	0.553	1	4839	NOP2
-0.108365506	0.527876258	0.569	1	10085	EDIL3
-0.108365506	0.527876258	0.579	1	28996	HIPK2
-0.108365506	0.527876258	0.562	1	57664	PLEKHA4
-0.108365506	0.527876258	0.566	1	221357	GSTA5
-0.108622909	0.526891112	0.556	1	5608	MAP2K6
-0.108622909	0.526891112	0.554	1	5764	PTN
-0.108622909	0.526891112	0.546	1	9436	NCR2
-0.108622909	0.526891112	0.55	1	50808	AK3
-0.108622909	0.526891112	0.544	1	79832	QSER1
-0.108622909	0.526891112	0.584	1	160728	SLC5A8
-0.108622909	0.526891112	0.551	1	387921	NHLRC3
-0.108880304	0.525906384	0.549	1	26538	OR10H2
-0.108880304	0.525906384	0.55	1	28957	MRPS28
-0.108880304	0.525906384	0.573	1	219960	OR10Q1
-0.109137706	0.524923146	0.546	1	5226	PGD
-0.109137706	0.524923146	0.506	1	8636	SSNA1
-0.109137706	0.524923146	0.564	1	79796	ALG9
-0.109137706	0.524923146	0.571	1	401634	GOLGA2P3Y

voxel-wise, subcortex			
Spearman's corr coef	p-val	GenID	Gene
-0.065542676	3.62912E-09	171546	C14orf147
-0.065559953	3.595E-09	4297	MLL
-0.065584093	3.54784E-09	7388	UQCRH
-0.065601371	3.51444E-09	51755	CRKRS
-0.065614276	3.48971E-09	56001	NXF2
-0.065620035	3.47872E-09	5021	OXTR
-0.065650031	3.42205E-09	10981	RAB32
-0.06566935	3.38603E-09	119369	NUDT9P1
-0.065679565	3.36713E-09	414777	HCG18
-0.065680213	3.36594E-09	678655	AC005840.2
-0.065686248	3.35483E-09	8487	SIP1
-0.065763794	3.21519E-09	3135	HLA-G
-0.065807998	3.13813E-09	114792	KLHL32
-0.065862976	3.0448E-09	348825	TPRXL
-0.06588684	3.00514E-09	5269	SERPINB6
-0.06593699	2.92342E-09	11100	HNRNPUL1
-0.066044897	2.75484E-09	5756	TWF1
-0.066045828	2.75343E-09	84667	HES7
-0.066080466	2.70138E-09	22904	SBNO2
-0.066113621	2.65245E-09	5196	PF4
-0.066141665	2.61175E-09	51000	SLC35B3
-0.066163763	2.5801E-09	5015	OTX2
-0.066187911	2.54595E-09	442572	LOC442572
-0.066201843	2.52644E-09	9710	KIAA0355
-0.066256635	2.45113E-09	29780	PARVB
-0.066271216	2.43146E-09	112869	CCDC101
-0.066411257	2.25021E-09	6234	RPS28
-0.066412836	2.24823E-09	441024	MTHFD2L
-0.06647487	2.17228E-09	79954	NOL10
-0.066487402	2.15725E-09	341378	LOC341378
-0.066495948	2.14705E-09	4693	NDP
-0.066529661	2.10728E-09	653464	SRGAP2C
-0.066597916	2.02896E-09	10212	DDX39
-0.066603765	2.02237E-09	389549	FEZF1
-0.066678427	1.9402E-09	55179	FAIM
-0.066705175	1.91156E-09	2230	FDX1
-0.066799618	1.81369E-09	154141	MBOAT1
-0.066851348	1.76217E-09	6443	SGCB
-0.066889144	1.72543E-09	441394	SUGT1P1
-0.066893041	1.72168E-09	54969	C4orf27
-0.066901125	1.71394E-09	130617	ZFAND2B
-0.066920064	1.69593E-09	1292	COL6A2
-0.066948481	1.66925E-09	9025	RNF8
-0.066958606	1.65985E-09	142686	ASB14
-0.066974856	1.64486E-09	25855	BRMS1
-0.067004666	1.61771E-09	6676	SPAG4
-0.067035221	1.59033E-09	6599	SMARCC1
-0.067052305	1.57522E-09	2531	KDSR
-0.067084439	1.54717E-09	2782	GNB1
-0.067138769	1.50086E-09	6726	SRP9
-0.067150749	1.49083E-09	57827	C6orf47
-0.06718111	1.46571E-09	645644	FLJ42627
-0.067189097	1.45916E-09	388558	ZNF808
-0.067219377	1.43463E-09	340277	C7orf46
-0.067245282	1.41396E-09	57045	TWSG1
-0.067269892	1.39458E-09	3815	KIT
-0.067278899	1.38756E-09	150483	TEKT4
-0.067322455	1.35407E-09	7384	UQCRC1
-0.067323662	1.35315E-09	119774	OR52K2
-0.067376785	1.3134E-09	1802	DPH2
-0.067404181	1.29335E-09	114793	FMNL2
-0.067415416	1.28521E-09	80110	ZNF614
-0.067468718	1.24729E-09	64783	RBM15
-0.067472152	1.24488E-09	22877	MLXIP
-0.06748005	1.23937E-09	147686	ZNF418
-0.067485996	1.23523E-09	10611	PDLIM5
-0.06749101	1.23175E-09	54896	PQLC2
-0.067529641	1.20527E-09	54868	TMEM104
-0.067547008	1.19355E-09	5446	PON3
-0.067662068	1.11864E-09	11025	LILRB3
-0.067722149	1.08137E-09	222229	LRWD1
-0.06773933	1.07094E-09	6497	SKI
-0.067756049	1.06088E-09	285193	DUSP28
-0.067772761	1.05091E-09	286262	C9orf75
-0.067781769	1.04558E-09	153222	C5orf41
-0.06786219	9.99131E-10	10966	RAB40B
-0.067871384	9.9395E-10	81556	C15orf44
-0.067872129	9.93534E-10	9412	MED21
-0.067902125	9.76821E-10	64978	MRPL38

region-wise, subcortex					
Spearman's corr coef	p-val	p-val, SA	p-val, SA (BH- correction)	GenID	Gene
-0.109137706	0.524923146	0.556	1	100127983	RP11-122A3.2
-0.109395109	0.523940682	0.571	1	1942	EFNA1
-0.109395109	0.523940682	0.573	1	3481	IGF2
-0.109395109	0.523940682	0.56	1	6263	RYR3
-0.109395109	0.523940682	0.547	1	10021	HCN4
-0.109395109	0.523940682	0.538	1	27434	POLM
-0.109395109	0.523940682	0.57	1	29082	CHMP4A
-0.109395109	0.523940682	0.536	1	127795	C1orf87
-0.109395109	0.523940682	0.554	1	283551	C14orf182
-0.109395109	0.523940682	0.563	1	401152	C4orf3
-0.109652504	0.522959173	0.561	1	23621	BACE1
-0.109652504	0.522959173	0.54	1	55632	G2E3
-0.109652504	0.522959173	0.529	1	64215	DNAJC1
-0.109652504	0.522959173	0.552	1	121227	LRIG3
-0.109909907	0.52197814	0.554	1	5623	PSPN
-0.109909907	0.52197814	0.564	1	9846	GAB2
-0.109909907	0.52197814	0.562	1	79960	PHF17
-0.109909907	0.52197814	0.545	1	90326	THAP3
-0.11016731	0.520998478	0.543	1	667	DST
-0.11016731	0.520998478	0.526	1	3987	LIMS1
-0.11016731	0.520998478	0.528	1	8544	PIR
-0.11016731	0.520998478	0.565	1	114904	C1QTNF6
-0.11016731	0.520998478	0.574	1	387646	RP11-85G18.4
-0.11016731	0.520998478	0.578	1	728806	NSFP1
-0.11016731	0.520998478	0.539	1	100289635	ZNF605
-0.110424712	0.52001977	0.518	1	116	ADCYAP1
-0.110424712	0.52001977	0.535	1	4289	MKLN1
-0.110424712	0.52001977	0.587	1	5137	PDE1C
-0.110424712	0.52001977	0.558	1	54581	SCAND2
-0.110424712	0.52001977	0.543	1	54680	ZNHIT6
-0.110424712	0.52001977	0.551	1	63906	GPATCH3
-0.110682115	0.519041836	0.561	1	1238	CCBP2
-0.110682115	0.519041836	0.54	1	1326	MAP3K8
-0.110682115	0.519041836	0.559	1	3431	SP110
-0.110682115	0.519041836	0.542	1	25888	ZNF473
-0.110682115	0.519041836	0.58	1	25960	GPR124
-0.110682115	0.519041836	0.572	1	80143	SIKE1
-0.110682115	0.519041836	0.564	1	83752	LONP2
-0.110682115	0.519041836	0.533	1	90141	EFCAB11
-0.110682115	0.519041836	0.536	1	203260	CCDC107
-0.110682115	0.519041836	0.577	1	340485	ACER2
-0.11093951	0.518064916	0.553	1	712	C1QA
-0.11093951	0.518064916	0.583	1	22907	DHX30
-0.11093951	0.518064916	0.56	1	65989	DLK2
-0.11093951	0.518064916	0.549	1	134637	ADAT2
-0.11093951	0.518064916	0.568	1	338382	RAB7B
-0.111196913	0.517088532	0.553	1	4993	OR2C1
-0.111196913	0.517088532	0.563	1	9528	TMEM59
-0.111454308	0.51611346	0.536	1	1645	AKR1C1
-0.111454308	0.51611346	0.545	1	2559	GABRA6
-0.111454308	0.51611346	0.535	1	51124	IER3IP1
-0.111454308	0.51611346	0.542	1	54859	C3orf75
-0.111454308	0.51611346	0.574	1	115817	DHRS1
-0.111454308	0.51611346	0.562	1	129880	BBS5
-0.111711711	0.515139282	0.569	1	1197	CLK2P
-0.111711711	0.515139282	0.543	1	1371	CPOX
-0.111711711	0.515139282	0.528	1	646023	C22ORF45
-0.111969113	0.514166057	0.546	1	5636	PRPSAP2
-0.111969113	0.514166057	0.535	1	7775	ZNF232
-0.111969113	0.514166057	0.535	1	23421	ITGB3BP
-0.111969113	0.514166057	0.553	1	84879	MFSD2
-0.111969113	0.514166057	0.57	1	123103	KLHL33
-0.111969113	0.514166057	0.538	1	200172	SLFNL1
-0.111969113	0.514166057	0.556	1	642521	C1DP3
-0.111969113	0.514166057	0.566	1	728533	LOC728533
-0.112226509	0.513193309	0.528	1	3437	IFIT3
-0.112226509	0.513193309	0.566	1	4695	NDUFA2
-0.112226509	0.513193309	0.548	1	6049	RNF6
-0.112226509	0.513193309	0.556	1	9044	BTAF1
-0.112226509	0.513193309	0.541	1	50848	F11R
-0.112483911	0.512221932	0.508	1	4792	NFKBIA
-0.112483911	0.512221932	0.561	1	8546	AP3B1
-0.112483911	0.512221932	0.546	1	9843	HEPH
-0.112483911	0.512221932	0.547	1	22979	EFR3B
-0.112483911	0.512221932	0.519	1	64801	ARV1
-0.112483911	0.512221932	0.567	1	138311	FAM69B
-0.112741306	0.511251509	0.562	1	8802	SUCLG1
-0.112741306	0.511251509	0.492	1	9187	SLC24A1
-0.112741306	0.511251509	0.553	1	10592	SMC2

voxel-wise, subcortex			
Spearman's corr coef	p-val	GenID	Gene
-0.067931376	9.60784E-10	283652	SLC24A5
-0.068026654	9.10317E-10	10201	NME6
-0.068146549	8.50474E-10	8837	CFLAR
-0.068155743	8.46047E-10	222236	NAPEPLD
-0.068158247	8.44845E-10	5213	PFKM
-0.068164468	8.41865E-10	57053	CHRNA10
-0.068284824	7.8621E-10	56851	C15orf24
-0.068291515	7.83224E-10	5339	PLEC1
-0.06830024	7.79347E-10	51603	METTL13
-0.068355404	7.5526E-10	1395	CRHR2
-0.068389207	7.40861E-10	23328	SASH1
-0.068419293	7.28271E-10	55216	C11orf57
-0.06843508	7.21746E-10	3550	IK
-0.068440095	7.19686E-10	4650	MYO9B
-0.068478264	7.04191E-10	100093630	SNHG8
-0.068481982	7.02702E-10	2107	ETF1
-0.068599917	6.56954E-10	3872	KRT17
-0.068621837	6.4878E-10	10848	PPP1R13L
-0.068640597	6.41859E-10	9745	ZNF536
-0.068672635	6.30208E-10	3001	GZMA
-0.068675138	6.29306E-10	55829	AC023024.1
-0.068711825	6.16237E-10	84687	PPP1R9B
-0.068806179	5.83829E-10	55862	ECHDC1
-0.068811931	5.81909E-10	51726	DNAJB11
-0.068871923	5.62238E-10	10929	SRSF8
-0.068922073	5.46291E-10	8936	WASF1
-0.069061182	5.04338E-10	4548	MTR
-0.069077991	4.99487E-10	81892	C14orf156
-0.069092944	4.95208E-10	260341	TFAMP1
-0.069139659	4.82074E-10	91689	C22orf32
-0.069181725	4.70539E-10	7155	TOP2B
-0.069185533	4.69509E-10	10002	NR2E3
-0.069191009	4.6803E-10	151011	SEPTIN10
-0.069204383	4.64437E-10	643854	RP5-988G15.1
-0.06925147	4.52002E-10	219699	UNC5B
-0.069348231	4.27461E-10	9056	SLC7A7
-0.069381014	4.19448E-10	8804	CREG1
-0.069427915	4.08234E-10	152815	THAP6
-0.069458649	4.01045E-10	27068	PPA2
-0.069482796	3.95485E-10	391190	OR2L8
-0.069494404	3.92838E-10	1650	DDOST
-0.069568977	3.76244E-10	55225	RAVER2
-0.069577895	3.74306E-10	6777	STAT5B
-0.069595166	3.7058E-10	7114	TMSB4X
-0.069640204	3.61034E-10	4784	NFIX
-0.069663607	3.5617E-10	4326	MMP17
-0.06975285	3.38197E-10	4651	MYO10
-0.069832064	3.22988E-10	284406	ZFP82
-0.06983643	3.2217E-10	7739	ZNF185
-0.06994991	3.0159E-10	51573	GDE1
-0.069955297	3.00648E-10	401262	CRIP3
-0.069970526	2.97993E-10	4928	NUP98
-0.069974057	2.97381E-10	65125	WNK1
-0.069995604	2.93673E-10	8503	PIK3R3
-0.069998391	2.93197E-10	155370	SBDSP1
-0.070000336	2.92863E-10	7798	LUZP1
-0.070015289	2.90324E-10	80351	TNKS2
-0.070016965	2.9004E-10	116138	KLHDC3
-0.070026249	2.88475E-10	348235	FAM33A
-0.070104443	2.7562E-10	653240	KRTAP4-14
-0.070146695	2.68907E-10	115703	SNX26
-0.070203625	2.60114E-10	79726	WDR59
-0.070217736	2.57979E-10	57223	SMEK2
-0.070253305	2.52672E-10	4487	MSX1
-0.070373751	2.35481E-10	282808	RAB40AL
-0.070381179	2.34458E-10	388753	C1orf31
-0.070437461	2.26855E-10	50853	VILL
-0.070462346	2.23569E-10	55920	RCC2
-0.07047043	2.22512E-10	143903	LAYN
-0.070491232	2.19815E-10	1106	CHD2
-0.070568308	2.10094E-10	91695	RRP7B
-0.070588179	2.07657E-10	85013	TMEM128
-0.070686527	1.95997E-10	259290	TAS2R31
-0.07070408	1.93983E-10	51084	CRYL1
-0.070708908	1.93434E-10	79962	DNAJC22
-0.070729338	1.91123E-10	404672	GTF2H5
-0.070737787	1.90175E-10	57819	LSM2
-0.070744663	1.89407E-10	27129	HSPB7
-0.070769362	1.86673E-10	84079	ANKRD27

region-wise, subcortex					
Spearman's corr coef	p-val	p-val, SA	p-val, SA (BH- correction)	GenID	Gene
-0.112741306	0.511251509	0.573	1	11328	FKBP9
-0.112741306	0.511251509	0.558	1	146852	ODF4
-0.112998709	0.51028198	0.549	1	2287	FKBP3
-0.112998709	0.51028198	0.548	1	10423	CDIPT
-0.112998709	0.51028198	0.559	1	93436	ARMC6
-0.112998709	0.51028198	0.55	1	112574	SNX18
-0.112998709	0.51028198	0.516	1	124935	SLC43A2
-0.112998709	0.51028198	0.528	1	440104	AC023055.2
-0.112998709	0.51028198	0.554	1	100147773	RP11-466F5.8
-0.113256112	0.509312928	0.546	1	7748	ZNF195
-0.113256112	0.509312928	0.556	1	10694	CCT8
-0.113256112	0.509312928	0.554	1	55151	TMEM38B
-0.113256112	0.509312928	0.536	1	140831	ZSWIM3
-0.113256112	0.509312928	0.545	1	154661	RUNDC3B
-0.113513507	0.508345306	0.552	1	3653	IPW
-0.113513507	0.508345306	0.544	1	10103	TSPAN1
-0.113513507	0.508345306	0.564	1	10858	CYP46A1
-0.113513507	0.508345306	0.54	1	26658	OR7C2
-0.113513507	0.508345306	0.55	1	51324	SPG21
-0.113513507	0.508345306	0.528	1	90576	ZNF799
-0.113513507	0.508345306	0.532	1	152007	GLIPR2
-0.113513507	0.508345306	0.578	1	646024	RAET1K
-0.113770917	0.507378578	0.54	1	1822	ATN1
-0.113770917	0.507378578	0.52	1	4729	NDUFV2
-0.113770917	0.507378578	0.556	1	5523	PPP2R3A
-0.113770917	0.507378578	0.538	1	6734	SRPR
-0.113770917	0.507378578	0.571	1	9414	TJP2
-0.113770917	0.507378578	0.546	1	80072	HEXA-AS1
-0.113770917	0.507378578	0.535	1	158747	MOSPD2
-0.114028312	0.506412804	0.518	1	8658	TNKS
-0.114028312	0.506412804	0.544	1	9512	PMPCB
-0.114028312	0.506412804	0.5	1	51199	NIN
-0.114028312	0.506412804	0.552	1	142678	MIB2
-0.114028312	0.506412804	0.553	1	348932	SLC6A18
-0.114028312	0.506412804	0.538	1	391013	PLA2G2C
-0.114285715	0.505447924	0.543	1	368	ABCC6
-0.114285715	0.505447924	0.544	1	1053	CEBPE
-0.114285715	0.505447924	0.562	1	2147	F2
-0.114285715	0.505447924	0.572	1	5394	EXOSC10
-0.114285715	0.505447924	0.554	1	10733	PLK4
-0.114285715	0.505447924	0.532	1	54468	MIOS
-0.114285715	0.505447924	0.547	1	91754	NEK9
-0.114285715	0.505447924	0.525	1	114780	PKD1L2
-0.114285715	0.505447924	0.538	1	284339	TMEM145
-0.114285715	0.505447924	0.563	1	284349	ZNF283
-0.114543118	0.504483581	0.544	1	1515	CTSL2
-0.114543118	0.504483581	0.55	1	2899	GRIK3
-0.114543118	0.504483581	0.534	1	29071	C1GALT1C1
-0.114543118	0.504483581	0.527	1	55556	ENOSF1
-0.114800513	0.503520608	0.542	1	5978	REST
-0.114800513	0.503520608	0.523	1	6388	SDF2
-0.114800513	0.503520608	0.52	1	6556	SLC11A1
-0.114800513	0.503520608	0.51	1	27107	ZBTB11
-0.114800513	0.503520608	0.544	1	51314	TXNDC3
-0.114800513	0.503520608	0.526	1	163351	GBP6
-0.114800513	0.503520608	0.545	1	285601	GPR150
-0.114800513	0.503520608	0.51	1	728407	LOC728407
-0.115057915	0.502558589	0.54	1	291	SLC25A4
-0.115057915	0.502558589	0.528	1	5269	SERPINB6
-0.115057915	0.502558589	0.514	1	7988	ZNF212
-0.115057915	0.502558589	0.543	1	8443	GNPAT
-0.115057915	0.502558589	0.526	1	9219	MTA2
-0.115057915	0.502558589	0.549	1	27292	DIMT1
-0.115057915	0.502558589	0.537	1	115727	RASGRP4
-0.115057915	0.502558589	0.56	1	221756	MGC39372
-0.115315311	0.501597464	0.528	1	3683	ITGAL
-0.115315311	0.501597464	0.525	1	26024	PTCD1
-0.115315311	0.501597464	0.512	1	100129460	DPY19L1P1
-0.115572713	0.500636876	0.51	1	397	ARHGDIB
-0.115572713	0.500636876	0.563	1	476	ATP1A1
-0.115572713	0.500636876	0.534	1	54623	PAF1
-0.115572713	0.500636876	0.537	1	56751	BARHL1
-0.115572713	0.500636876	0.502	1	91746	YTHDC1
-0.115572713	0.500636876	0.551	1	128153	SPATA17
-0.115572713	0.500636876	0.508	1	162966	ZNF600
-0.115572713	0.500636876	0.524	1	222171	PRR15
-0.115830116	0.499677658	0.541	1	11064	CEP110
-0.115830116	0.499677658	0.515	1	11097	NUPL2
-0.115830116	0.499677658	0.545	1	23127	GLT25D2

voxel-wise, subcortex			
Spearman's corr coef	p-val	GenID	Gene
-0.070811711	1.82076E-10	64979	MRPL36
-0.070836693	1.79416E-10	29923	C7orf68
-0.070854612	1.77531E-10	56986	DTWD1
-0.070937261	1.69085E-10	253639	ZNF620
-0.070979983	1.64875E-10	10281	DSCR4
-0.070983514	1.64532E-10	94097	SFXN5
-0.071004219	1.62533E-10	11168	PSIP1
-0.071016759	1.61333E-10	6788	AP003355.2
-0.071050651	1.58134E-10	5160	PDHA1
-0.07105957	1.57303E-10	25790	CCDC19
-0.071081206	1.55305E-10	117584	RFFL
-0.071082324	1.55202E-10	147138	TMC8
-0.071085572	1.54904E-10	5535	PPP3R2
-0.071133487	1.50576E-10	8325	FZD8
-0.071134977	1.50443E-10	3123	HLA-DRB1
-0.071160048	1.48228E-10	389792	IER5L
-0.071233317	1.41934E-10	254559	LOC254559
-0.071266942	1.39135E-10	50626	CYHR1
-0.071288764	1.37346E-10	667	DST
-0.071327768	1.34206E-10	11275	KLHL2
-0.071415521	1.27395E-10	26234	FBXL5
-0.071428709	1.26401E-10	23591	FAM215A
-0.07144738	1.25006E-10	57188	ADAMTSL3
-0.071453601	1.24545E-10	81033	KCNH6
-0.071506158	1.20714E-10	283377	SPRYD4
-0.071512289	1.20275E-10	255027	MPV17L
-0.07157442	1.1591E-10	4738	NEDD8
-0.071580082	1.15521E-10	10948	STARD3
-0.071642026	1.11337E-10	1289	COL5A1
-0.071670718	1.0945E-10	6695	SPOCK1
-0.07167843	1.08948E-10	90693	CCDC126
-0.071684092	1.08581E-10	6874	TAF4
-0.071710929	1.06858E-10	646567	OSTCP2
-0.071835652	9.91929E-11	390154	OR5T3
-0.071854405	9.80879E-11	7476	WNT7A
-0.071855523	9.80224E-11	114783	LMTK3
-0.071959257	9.21284E-11	89978	ATPBD4
-0.071994454	9.02078E-11	645691	LOC645691
-0.072000489	8.98826E-11	51594	NBAS
-0.072027415	8.84454E-11	375743	PTAR1
-0.072038837	8.78425E-11	2342	FNTB
-0.07205797	8.68415E-11	6154	RPL26
-0.072117217	8.38119E-11	221937	FOXK1
-0.072127715	8.32862E-11	119559	SFXN4
-0.072143406	8.25062E-11	847	CAT
-0.072211199	7.92173E-11	8676	STX11
-0.072254106	7.72024E-11	23351	KIAA0323
-0.072370276	7.19953E-11	8263	F8A1
-0.072381049	7.15303E-11	116842	LEAP2
-0.072404079	7.05458E-11	712	C1QA
-0.072456457	6.83559E-11	10980	COPS6
-0.072473921	6.76406E-11	2309	FOXO3
-0.072540686	6.49733E-11	8928	FOXH1
-0.072592139	6.29876E-11	3111	HLA-DOA
-0.072620921	6.19031E-11	441272	SPDYE3
-0.072621949	6.18646E-11	399693	MGC50722
-0.072646745	6.09454E-11	259293	TAS2R30
-0.072683424	5.96102E-11	25839	COG4
-0.072733663	5.78276E-11	100272147	MTCP1NB
-0.072746761	5.73717E-11	50619	DEF6
-0.072755948	5.70538E-11	219988	PATL1
-0.072793655	5.57672E-11	117145	THEM4
-0.072848722	5.39393E-11	136259	KLF14
-0.072923854	5.15399E-11	113419	TEX261
-0.072938435	5.10863E-11	10782	ZNF274
-0.072952829	5.06426E-11	3822	KLRC2
-0.072952829	5.06426E-11	124446	TMEM219
-0.07300242	4.9142E-11	389765	LOC389765
-0.073068351	4.72144E-11	5436	POLR2G
-0.073102623	4.6242E-11	9752	PCDHA9
-0.073134288	4.53608E-11	163732	CITED4
-0.073144689	4.50749E-11	6429	SFRS4
-0.073236629	4.26237E-11	2018	EMX2
-0.073246002	4.23812E-11	27005	USP21
-0.073327452	4.03304E-11	25946	ZNF385A
-0.073411398	3.83185E-11	90196	SYS1
-0.073416322	3.82034E-11	83698	CALN1
-0.073419295	3.81343E-11	653479	MRPL45P2
-0.07348597	3.66137E-11	353376	TICAM2

region-wise, subcortex					
Spearman's corr coef	p-val	p-val, SA	p-val, SA (BH- correction)	GenID	Gene
-0.115830116	0.499677658	0.521	1	79794	C12orf49
-0.115830116	0.499677658	0.522	1	148709	RP11-480I12.7
-0.116087511	0.498719394	0.537	1	1314	COPA
-0.116087511	0.498719394	0.524	1	3267	AGFG1
-0.116087511	0.498719394	0.531	1	6990	DYNLT3
-0.116087511	0.498719394	0.533	1	22868	FASTKD2
-0.116087511	0.498719394	0.506	1	83551	TAAR8
-0.116087511	0.498719394	0.531	1	84614	ZBTB37
-0.116087511	0.498719394	0.556	1	84910	TMEM87B
-0.116087511	0.498719394	0.534	1	129303	TMEM150A
-0.116344914	0.497762054	0.522	1	1271	CNTFR
-0.116344914	0.497762054	0.509	1	54923	LIME1
-0.116344914	0.497762054	0.515	1	55063	ZCWPW1
-0.116344914	0.497762054	0.55	1	114881	OSBPL7
-0.116344914	0.497762054	0.574	1	124783	C17orf46
-0.116344914	0.497762054	0.539	1	399512	SLC25A35
-0.116602309	0.49680528	0.543	1	1263	PLK3
-0.116602309	0.49680528	0.554	1	3117	HLA-DQA1
-0.116602309	0.49680528	0.529	1	9351	SLC9A3R2
-0.116602309	0.49680528	0.53	1	10892	MALT1
-0.116602309	0.49680528	0.547	1	84236	RHBDD1
-0.116602309	0.49680528	0.509	1	114932	MRFAP1L1
-0.116602309	0.49680528	0.535	1	200403	VWA3B
-0.116602309	0.49680528	0.526	1	389362	PSMG4
-0.116859712	0.495849818	0.54	1	6746	SSR2
-0.116859712	0.495849818	0.55	1	23629	BRD7P3
-0.116859712	0.495849818	0.537	1	63920	C5orf54
-0.116859712	0.495849818	0.539	1	84159	ARID5B
-0.116859712	0.495849818	0.52	1	352954	GATS
-0.116859712	0.495849818	0.523	1	353145	LCE3E
-0.117117114	0.494895399	0.534	1	596	BCL2
-0.117117114	0.494895399	0.527	1	6197	RPS6KA3
-0.117117114	0.494895399	0.522	1	27132	CPNE7
-0.117117114	0.494895399	0.546	1	375033	PEAR1
-0.117117114	0.494895399	0.496	1	401397	AC073346.1
-0.117374517	0.493941814	0.529	1	8479	HIRIP3
-0.117374517	0.493941814	0.555	1	8725	URI1
-0.117374517	0.493941814	0.526	1	9792	SERTAD2
-0.117374517	0.493941814	0.536	1	51588	PIAS4
-0.117374517	0.493941814	0.535	1	84924	ZNF566
-0.117374517	0.493941814	0.559	1	115827	RAB3C
-0.117374517	0.493941814	0.482	1	282566	LINC00515
-0.117374517	0.493941814	0.527	1	375337	C3orf77
-0.117374517	0.493941814	0.516	1	645784	ANKRD36BP2
-0.117374517	0.493941814	0.531	1	653781	AC140481.7
-0.11763192	0.492989242	0.546	1	3208	HPCA
-0.11763192	0.492989242	0.493	1	23399	DULLARD
-0.11763192	0.492989242	0.5	1	51058	ZNF691
-0.11763192	0.492989242	0.563	1	56244	BTNL2
-0.11763192	0.492989242	0.536	1	116511	MAS1L
-0.11763192	0.492989242	0.531	1	100129933	GLYATL1P2
-0.117889315	0.492037207	0.514	1	7148	TNXB
-0.117889315	0.492037207	0.506	1	10623	POLR3C
-0.117889315	0.492037207	0.509	1	11006	LILRB4
-0.117889315	0.492037207	0.549	1	56098	PCDHGC4
-0.117889315	0.492037207	0.51	1	84634	KISS1R
-0.117889315	0.492037207	0.514	1	220416	LRRC63
-0.117889315	0.492037207	0.513	1	255101	CCDC108
-0.118146718	0.491086543	0.495	1	1195	CLK1
-0.118146718	0.491086543	0.534	1	9541	CIR1
-0.118146718	0.491086543	0.531	1	10897	YIF1A
-0.118146718	0.491086543	0.53	1	11260	XPOT
-0.118146718	0.491086543	0.503	1	23203	PMPCA
-0.118146718	0.491086543	0.517	1	59084	ENPP5
-0.118146718	0.491086543	0.511	1	134701	C6orf59
-0.11840412	0.490136832	0.54	1	946	SIGLEC6
-0.11840412	0.490136832	0.539	1	7718	ZNF165
-0.11840412	0.490136832	0.519	1	11330	CTRC
-0.11840412	0.490136832	0.522	1	55106	SLFN12
-0.11840412	0.490136832	0.532	1	84288	EFCAB2
-0.11840412	0.490136832	0.532	1	114791	TUBGCP5
-0.11840412	0.490136832	0.572	1	123720	WHAMM
-0.11840412	0.490136832	0.518	1	339456	TMEM52
-0.11840412	0.490136832	0.504	1	100132832	LOC100132832
-0.118661515	0.489188045	0.535	1	2923	PDIA3
-0.118661515	0.489188045	0.494	1	3784	KCNQ1
-0.118661515	0.489188045	0.539	1	8906	AP1G2
-0.118661515	0.489188045	0.518	1	57606	SLAIN2
-0.118661515	0.489188045	0.541	1	64853	AIDA

voxel-wise, subcortex			
Spearman's corr coef	p-val	GenID	Gene
-0.07356184	3.49555E-11	96626	LIMS3
-0.073562399	3.49437E-11	9310	ZNF235
-0.073572338	3.47319E-11	3987	LIMS1
-0.073589608	3.43672E-11	79176	FBXL15
-0.073610507	3.39309E-11	144486	LOC144486
-0.073643379	3.32554E-11	100049716	AC021054.1
-0.073662788	3.28628E-11	441549	CDNF
-0.073745348	3.12428E-11	6208	RPS14
-0.073746741	3.12163E-11	5699	PSMB10
-0.07387127	2.89213E-11	8344	HIST1H2BE
-0.073884368	2.86898E-11	3805	KIR2DL4
-0.073902659	2.83695E-11	25871	C3orf17
-0.073913246	2.81856E-11	283284	IGSF22
-0.0739998	2.6726E-11	7553	ZNF7
-0.074016511	2.64529E-11	4324	MMP15
-0.074022084	2.63625E-11	60343	FAM3A
-0.074089229	2.52958E-11	92126	DSEL
-0.074090712	2.52727E-11	29965	C16orf5
-0.074091643	2.52582E-11	135138	PACRG
-0.074192591	2.37357E-11	9372	ZFYVE9
-0.074209772	2.34857E-11	340351	AGBL3
-0.074223325	2.32903E-11	642413	CTSL1P6
-0.074264839	2.27017E-11	359821	MRPL42P5
-0.074274309	2.25694E-11	11232	POLG2
-0.074306257	2.21288E-11	22933	SIRT2
-0.074313499	2.20302E-11	25949	SYF2
-0.074340805	2.1662E-11	9189	ZBED1
-0.074419364	2.06358E-11	4248	MGAT3
-0.074562848	1.88829E-11	140862	C20orf82
-0.074616432	1.82665E-11	100286895	LOC100286895
-0.074618846	1.82392E-11	148741	ANKRD35
-0.074636862	1.80367E-11	4332	MNDA
-0.074664161	1.77339E-11	10550	ARL6IP5
-0.074665651	1.77176E-11	150946	FAM59B
-0.074694805	1.74001E-11	10507	SEMA4D
-0.074748114	1.6834E-11	57704	GBA2
-0.074760929	1.67006E-11	55103	RALGPS2
-0.074863918	1.56655E-11	8893	EIF2B5
-0.074937932	1.49607E-11	10421	CD2BP2
-0.07494536	1.48917E-11	55054	ATG16L1
-0.075005539	1.43441E-11	574	BAGE
-0.075024389	1.41767E-11	55559	HAUS7
-0.075041384	1.40274E-11	1841	DTYMK
-0.075049184	1.39594E-11	4610	MYCL1
-0.075089119	1.36161E-11	5891	RAGE
-0.075198695	1.27164E-11	116225	ZMYND19
-0.075210862	1.26201E-11	284161	GDPD1
-0.075219318	1.25536E-11	89853	FAM125B
-0.075294442	1.1978E-11	55006	TRMT61B
-0.075339667	1.1644E-11	2706	GJB2
-0.07534951	1.15725E-11	10371	SEMA3A
-0.075357869	1.15122E-11	153241	CEP120
-0.075430118	1.10032E-11	54551	MAGEL2
-0.075479157	1.06703E-11	8826	IQGAP1
-0.075494476	1.05684E-11	440515	ZNF506
-0.075514257	1.04381E-11	404734	ANKHD1-EIF4EBP3
-0.075537473	1.02873E-11	84747	UNC119B
-0.075545646	1.02347E-11	200726	LOC200726
-0.075570069	1.00791E-11	285489	DOK7
-0.075586602	9.9751E-12	3706	ITPKA
-0.075673148	9.44754E-12	79990	PLEKHH3
-0.075697854	9.30202E-12	64849	SLC13A3
-0.075769544	8.8921E-12	79745	CLIP4
-0.075809382	8.67199E-12	1062	CENPE
-0.075851455	8.44532E-12	84765	ZNF577
-0.075853311	8.43548E-12	1511	CTSG
-0.075854331	8.43007E-12	26716	OR2H1
-0.075891852	8.23318E-12	84304	NUDT22
-0.075906985	8.15504E-12	28989	NTMT1
-0.075945251	7.96073E-12	155066	ATP6V0E2
-0.075954258	7.91563E-12	5978	REST
-0.076054089	7.4325E-12	285525	YIPF7
-0.076062165	7.39472E-12	9580	SOX13
-0.076100335	7.21862E-12	55627	SMPD4
-0.076150298	6.99428E-12	284391	ZNF844
-0.076160327	6.95008E-12	63926	ANKRD5
-0.076211028	6.73079E-12	9495	AKAP5
-0.076255791	6.54282E-12	388677	NOTCH2NL
-0.076311417	6.31645E-12	84928	TMEM209

region-wise, subcortex					
Spearman's corr coef	p-val	p-val, SA	p-val, SA (BH- correction)	GenID	Gene
-0.118661515	0.489188045	0.532	1	81550	TDRD3
-0.118661515	0.489188045	0.538	1	284716	RIMKLA
-0.118918918	0.488239825	0.505	1	10155	TRIM28
-0.118918918	0.488239825	0.512	1	83593	RASSF5
-0.118918918	0.488239825	0.539	1	400668	PRSSL1
-0.118918918	0.488239825	0.524	1	644903	FLJ38668
-0.118918918	0.488239825	0.518	1	646890	LOC646890
-0.119176313	0.487293005	0.489	1	30	ACAA1
-0.119176313	0.487293005	0.536	1	7278	TUBA3C
-0.119176313	0.487293005	0.543	1	81669	CCNL2
-0.119176313	0.487293005	0.502	1	89891	WDR34
-0.119176313	0.487293005	0.515	1	159013	CXorf38
-0.119176313	0.487293005	0.547	1	391195	OR2T33
-0.119433716	0.486347079	0.512	1	763	CA5A
-0.119433716	0.486347079	0.528	1	7855	FZD5
-0.119433716	0.486347079	0.542	1	55655	NLRP2
-0.119433716	0.486347079	0.519	1	64976	MRPL40
-0.119433716	0.486347079	0.527	1	199857	ALG14
-0.119433716	0.486347079	0.511	1	284827	KRTAP13-4
-0.119691119	0.485402197	0.508	1	3107	HLA-C
-0.119691119	0.485402197	0.508	1	5279	PIGC
-0.119691119	0.485402197	0.533	1	7272	TTK
-0.119691119	0.485402197	0.532	1	27037	TRMT2A
-0.119691119	0.485402197	0.519	1	57332	CBX8
-0.119691119	0.485402197	0.507	1	91452	ACBD5
-0.119691119	0.485402197	0.523	1	145783	RP11-323F24.1
-0.119691119	0.485402197	0.498	1	400657	LOC400657
-0.119948514	0.484458208	0.5	1	6147	RPL23A
-0.119948514	0.484458208	0.538	1	9147	SDCCAG1
-0.119948514	0.484458208	0.521	1	10732	TCFL5
-0.119948514	0.484458208	0.489	1	23536	ADAT1
-0.119948514	0.484458208	0.506	1	55177	FAM82A2
-0.119948514	0.484458208	0.534	1	140686	WFDC3
-0.119948514	0.484458208	0.506	1	375341	C3orf62
-0.119948514	0.484458208	0.525	1	448835	LCE6A
-0.120205916	0.483514786	0.516	1	58472	SQRDL
-0.120205916	0.483514786	0.532	1	58506	SCAF1
-0.120205916	0.483514786	0.498	1	83450	LRRC48
-0.120205916	0.483514786	0.5	1	92659	MAFG-AS1
-0.120205916	0.483514786	0.533	1	118491	TTC18
-0.120205916	0.483514786	0.554	1	221545	C6orf136
-0.120463312	0.482572794	0.522	1	1339	COX6A2
-0.120463312	0.482572794	0.511	1	6129	RPL7
-0.120463312	0.482572794	0.534	1	23336	SYNM
-0.120463312	0.482572794	0.537	1	80227	PAAF1
-0.120463312	0.482572794	0.553	1	114336	CGB2
-0.120463312	0.482572794	0.544	1	148641	SLC35F3
-0.120463312	0.482572794	0.499	1	253832	ZDHHC20
-0.120720722	0.481631666	0.513	1	1300	COL10A1
-0.120720722	0.481631666	0.526	1	10101	NUBP2
-0.120720722	0.481631666	0.51	1	10557	RPP38
-0.120720722	0.481631666	0.535	1	23037	PDZD2
-0.120720722	0.481631666	0.526	1	51747	AC005921.3
-0.120720722	0.481631666	0.515	1	55238	SLC38A7
-0.120720722	0.481631666	0.534	1	440482	ANKRD20A5P
-0.120720722	0.481631666	0.491	1	100190939	XXyac-R12DG2.1
-0.120978124	0.480691612	0.507	1	2300	FOXL1
-0.120978124	0.480691612	0.512	1	2738	GLI4
-0.120978124	0.480691612	0.493	1	2956	MSH6
-0.120978124	0.480691612	0.494	1	3065	HDAC1
-0.120978124	0.480691612	0.526	1	5160	PDHA1
-0.120978124	0.480691612	0.532	1	8659	ALDH4A1
-0.120978124	0.480691612	0.526	1	9498	SLC4A8
-0.120978124	0.480691612	0.517	1	10912	GADD45G
-0.120978124	0.480691612	0.51	1	51206	GP6
-0.120978124	0.480691612	0.506	1	55892	MYNN
-0.120978124	0.480691612	0.536	1	79813	EHMT1
-0.120978124	0.480691612	0.523	1	113791	PIK3IP1
-0.120978124	0.480691612	0.519	1	171546	C14orf147
-0.12123552	0.479752034	0.538	1	5988	RFPL1
-0.12123552	0.479752034	0.514	1	6760	SS18
-0.12123552	0.479752034	0.523	1	285782	CAGE1
-0.12123552	0.479752034	0.517	1	374407	DNAJB13
-0.12123552	0.479752034	0.524	1	728819	LOC728819
-0.121492922	0.478813857	0.545	1	6429	SFRS4
-0.121492922	0.478813857	0.508	1	7464	CORO2A
-0.121492922	0.478813857	0.511	1	9957	HS3ST1
-0.121492922	0.478813857	0.522	1	158880	USP51
-0.121750325	0.477876663	0.534	1	1019	CDK4

voxel-wise, subcortex			
Spearman's corr coef	p-val	GenID	Gene
-0.076340854	6.19973E-12	2011	MARK2
-0.076342434	6.19354E-12	90120	C9ORF69
-0.076347545	6.1735E-12	51365	PLA1A
-0.076373361	6.07334E-12	6517	SLC2A4
-0.076379582	6.04942E-12	138151	NACC2
-0.076501332	5.59978E-12	552889	ATXN7L3B
-0.076517396	5.54295E-12	728255	KRTAP1-4
-0.076539963	5.46406E-12	80019	UBTD1
-0.076581471	5.32184E-12	79807	GSTCD
-0.07661175	5.22041E-12	183	AGT
-0.0766735	5.01935E-12	135458	HUS1B
-0.07672105	4.86972E-12	25849	AC110760.2
-0.0767738	4.70885E-12	28960	DCPS
-0.076780766	4.688E-12	11162	NUDT6
-0.076873071	4.4201E-12	4113	MAGEB2
-0.076891273	4.36906E-12	54920	DUS2L
-0.07689926	4.34685E-12	27439	CECR6
-0.076916717	4.2987E-12	5913	RAPSN
-0.076936312	4.24525E-12	779	CACNA1S
-0.076961756	4.17685E-12	1075	CTSC
-0.077044688	3.96128E-12	968	CD68
-0.077107467	3.8054E-12	9527	GOSR1
-0.077187516	3.61533E-12	23583	SMUG1
-0.077242866	3.48937E-12	283298	OLFML1
-0.077260137	3.45096E-12	359948	IRF2BP2
-0.077326812	3.30652E-12	8502	PK4P
-0.077356718	3.24367E-12	57529	RGAG1
-0.077377796	3.20007E-12	3035	HARS
-0.077403337	3.14803E-12	10067	SCAMP3
-0.077412903	3.12874E-12	51025	AC009171.1
-0.077435471	3.08371E-12	2918	GRM8
-0.077450886	3.05333E-12	5544	PRB3
-0.077459984	3.03554E-12	54344	DPM3
-0.077489518	2.97844E-12	51330	TNFRSF12A
-0.077625565	2.72882E-12	54507	ADAMTSL4
-0.077631883	2.71775E-12	29085	PHPT1
-0.07764414	2.69638E-12	196968	C15orf51
-0.077649243	2.68752E-12	402682	UFSP1
-0.077663265	2.66335E-12	729920	ISPD
-0.077732362	2.54735E-12	6641	SNTB1
-0.07778734	2.45859E-12	50856	CLEC4A
-0.077829592	2.39244E-12	3903	LAIR1
-0.077837303	2.38055E-12	84899	TMTC4
-0.077919297	2.25774E-12	22918	CD93
-0.077971123	2.18335E-12	5888	RAD51
-0.077981986	2.16806E-12	64949	MRPS26
-0.078090362	2.02119E-12	93436	ARMC6
-0.078180164	1.90691E-12	644006	LOC644006
-0.078185081	1.90084E-12	3508	IGHMBP2
-0.078228176	1.84843E-12	25963	TMEM87A
-0.078232072	1.84377E-12	2652	OPN1MW
-0.078281663	1.78537E-12	3385	ICAM3
-0.078304693	1.75886E-12	11144	DMC1
-0.078379355	1.67558E-12	55526	DHTKD1
-0.078408144	1.64451E-12	94104	C21orf66
-0.078426346	1.62515E-12	29843	SENP1
-0.078462288	1.58759E-12	100128782	LINC00476
-0.07848151	1.56785E-12	414152	C10orf105
-0.078498967	1.55014E-12	388507	ZNF788
-0.078508347	1.5407E-12	643837	RP11-206L10.11
-0.078523763	1.52531E-12	5886	RAD23A
-0.078558221	1.49146E-12	100128569	C19orf71
-0.078578278	1.47209E-12	7554	ZNF8
-0.078579575	1.47085E-12	147798	TMC4
-0.078621365	1.43133E-12	10300	KATNB1
-0.078677177	1.38016E-12	682	BSG
-0.078716181	1.34547E-12	10482	NXF1
-0.078755282	1.31155E-12	57717	PCDHB16
-0.078873403	1.21411E-12	220979	C10orf25
-0.07888028	1.20866E-12	2214	FCGR3A
-0.078919649	1.17792E-12	79576	NKAP
-0.078938223	1.16369E-12	9522	SCAMP1
-0.078947514	1.15663E-12	51631	LUC7L2
-0.078965902	1.14279E-12	147923	ZNF420
-0.078988649	1.12589E-12	64927	TTC23
-0.079005741	1.11336E-12	51206	GP6
-0.07903146	1.09475E-12	84939	MUM1
-0.079052083	1.08006E-12	4723	NDUFV1
-0.079059042	1.07514E-12	1942	EFNA1

region-wise, subcortex					
Spearman's corr coef	p-val	p-val, SA	p-val, SA (BH- correction)	GenID	Gene
-0.121750325	0.477876663	0.526	1	7737	RNF113A
-0.121750325	0.477876663	0.501	1	8050	PDHX
-0.121750325	0.477876663	0.531	1	10758	TRAF3IP2
-0.121750325	0.477876663	0.514	1	28972	SPCS1
-0.121750325	0.477876663	0.53	1	51142	CHCHD2
-0.121750325	0.477876663	0.508	1	79871	RPAP2
-0.12200772	0.476940513	0.545	1	1938	EEF2
-0.12200772	0.476940513	0.493	1	6730	SRP68
-0.12200772	0.476940513	0.54	1	9755	TBKBP1
-0.12200772	0.476940513	0.529	1	146540	ZNF785
-0.122265123	0.476004809	0.505	1	9946	CRYZL1
-0.122265123	0.476004809	0.55	1	30011	SH3KBP1
-0.122265123	0.476004809	0.518	1	54537	FAM35B
-0.122265123	0.476004809	0.518	1	56942	CMC2
-0.122265123	0.476004809	0.512	1	147183	KRT25
-0.122522518	0.475070477	0.523	1	3566	IL4R
-0.122522518	0.475070477	0.472	1	11331	PHB2
-0.122522518	0.475070477	0.497	1	55732	C1orf112
-0.122522518	0.475070477	0.505	1	64096	GFRA4
-0.122522518	0.475070477	0.513	1	83440	ADPGK
-0.122522518	0.475070477	0.484	1	126208	ZNF787
-0.122522518	0.475070477	0.511	1	128497	C20orf165
-0.122522518	0.475070477	0.52	1	148113	CILP2
-0.122522518	0.475070477	0.507	1	245929	DEFB115
-0.122522518	0.475070477	0.524	1	432369	ATP5EP2
-0.122779921	0.474137217	0.536	1	2181	ACSL3
-0.122779921	0.474137217	0.494	1	2797	GNRH2
-0.122779921	0.474137217	0.499	1	5005	ORM2
-0.122779921	0.474137217	0.513	1	51192	CKLF
-0.122779921	0.474137217	0.516	1	51200	CPA4
-0.122779921	0.474137217	0.514	1	53842	CLDN22
-0.122779921	0.474137217	0.51	1	54461	FBXW5
-0.122779921	0.474137217	0.5	1	147685	C19orf18
-0.123037323	0.473204881	0.516	1	2811	GP1BA
-0.123037323	0.473204881	0.485	1	4704	NDUFA9
-0.123037323	0.473204881	0.517	1	9757	AD000671.1
-0.123037323	0.473204881	0.5	1	9814	SFI1
-0.123037323	0.473204881	0.493	1	23205	ACSBG1
-0.123037323	0.473204881	0.503	1	60561	RINT1
-0.123037323	0.473204881	0.514	1	113230	AC022098.1
-0.123037323	0.473204881	0.464	1	115749	C12orf56
-0.123294719	0.472273529	0.503	1	1936	EEF1D
-0.123294719	0.472273529	0.51	1	4763	NF1
-0.123294719	0.472273529	0.513	1	10647	SCGB1D2
-0.123294719	0.472273529	0.494	1	51247	PAIP2
-0.123294719	0.472273529	0.517	1	55002	TMCO3
-0.123294719	0.472273529	0.499	1	56987	BBX
-0.123294719	0.472273529	0.487	1	84518	CNFN
-0.123294719	0.472273529	0.543	1	123169	LEO1
-0.123294719	0.472273529	0.492	1	164118	TTC24
-0.123294719	0.472273529	0.503	1	283131	NEAT1
-0.123294719	0.472273529	0.507	1	284433	OR10H5
-0.123552121	0.471342742	0.484	1	4851	NOTCH1
-0.123552121	0.471342742	0.526	1	23189	KANK1
-0.123552121	0.471342742	0.502	1	121551	BTBD11
-0.123552121	0.471342742	0.497	1	137075	CLDN23
-0.123552121	0.471342742	0.479	1	730144	LOC730144
-0.123809516	0.470413327	0.507	1	4327	MMP19
-0.123809516	0.470413327	0.504	1	22796	COG2
-0.123809516	0.470413327	0.472	1	55835	CENPJ
-0.123809516	0.470413327	0.498	1	344905	ATP13A5
-0.123809516	0.470413327	0.501	1	373861	HILS1
-0.123809516	0.470413327	0.531	1	389690	AC100803.1
-0.123809516	0.470413327	0.503	1	401250	MCCD1
-0.124066919	0.469484925	0.491	1	1495	CTNNA1
-0.124066919	0.469484925	0.502	1	5364	PLXNB1
-0.124066919	0.469484925	0.51	1	22794	CASC3
-0.124066919	0.469484925	0.506	1	79918	SETD6
-0.124066919	0.469484925	0.521	1	84154	RPF2
-0.124324329	0.468557507	0.524	1	642	BLMH
-0.124324329	0.468557507	0.536	1	23546	SYNGR4
-0.124324329	0.468557507	0.469	1	117289	TAGAP
-0.124324329	0.468557507	0.517	1	143425	SYT9
-0.124324329	0.468557507	0.498	1	144348	ZNF664
-0.124581724	0.467630595	0.514	1	8986	RPS6KA4
-0.124581724	0.467630595	0.504	1	54934	C12orf41
-0.124581724	0.467630595	0.496	1	63895	FAM38B
-0.124581724	0.467630595	0.496	1	79446	WDR25
-0.124581724	0.467630595	0.493	1	152503	SH3D19

voxel-wise, subcortex			
Spearman's corr coef	p-val	GenID	Gene
-0.079071023	1.06672E-12	348180	C16orf84
-0.079128511	1.02725E-12	56126	PCDHB10
-0.079141699	1.0184E-12	1855	DVL1
-0.07914383	1.01698E-12	774	CACNA1B
-0.079177819	9.94537E-13	56132	PCDHB3
-0.07919082	9.86084E-13	4238	MFAP3
-0.079198904	9.80865E-13	54865	GPATCH4
-0.079214685	9.70754E-13	728056	LOC728056
-0.079280809	9.29478E-13	5375	PMP2
-0.079314426	9.09156E-13	8349	HIST2H2BE
-0.079316378	9.07995E-13	54617	INO80
-0.07935594	8.84667E-13	5480	PPIC
-0.079373114	8.74726E-13	637	BID
-0.079377949	8.71944E-13	9817	KEAP1
-0.079402931	8.57727E-13	399512	SLC25A35
-0.079426885	8.44303E-13	3437	IFIT3
-0.0794423	8.35777E-13	8148	TAF15
-0.079491988	8.08857E-13	1844	DUSP2
-0.079501458	8.03824E-13	8106	PABPN1
-0.079571389	7.67596E-13	54541	DDIT4
-0.079572596	7.66985E-13	139562	OTUD6A
-0.079629429	7.3875E-13	57172	CAMK1G
-0.079662025	7.23023E-13	79714	CCDC51
-0.079706691	7.01997E-13	94233	OPN4
-0.07971774	6.96891E-13	643421	LOC643421
-0.079861596	6.33632E-13	5093	PCBP1
-0.079862982	6.33053E-13	1880	GPR183
-0.079864934	6.32234E-13	347689	SOX2-OT
-0.079893537	6.20373E-13	3309	HSPA5
-0.079902917	6.1653E-13	152687	ZNF595
-0.080024295	5.68869E-13	525	ATP6V1B1
-0.080027916	5.67502E-13	170591	S100Z
-0.080042399	5.62076E-13	162699	TCEB3C
-0.080060787	5.55259E-13	120364	LOC120364
-0.080125794	5.31803E-13	11004	KIF2C
-0.080201946	5.05561E-13	6506	SLC1A2
-0.080281347	4.79553E-13	11104	KATNA1
-0.08036641	4.53149E-13	79019	CENPM
-0.080378205	4.496E-13	51077	FCF1
-0.080421202	4.369E-13	643201	RP11-844P9.2
-0.080508865	4.1209E-13	90268	FAM105B
-0.080536261	4.04622E-13	3006	HIST1H1C
-0.080544248	4.02472E-13	10411	RAPGEF3
-0.080599971	3.87769E-13	9991	ROD1
-0.080666557	3.70891E-13	406	ARNTL
-0.08066795	3.70546E-13	84996	C21ORF119
-0.080708437	3.60648E-13	3766	KCNJ10
-0.080729984	3.55485E-13	80035	ANP32A-IT1
-0.080741964	3.52648E-13	9790	BMS1
-0.080744192	3.52123E-13	285987	DLX6-AS1
-0.080783844	3.42898E-13	83690	CRISPLD1
-0.080886371	3.20137E-13	340562	SATL1
-0.08095713	3.053E-13	158228	FAM201A
-0.080985084	2.99626E-13	5239	PGM5
-0.081018612	2.92958E-13	5238	PGM3
-0.081019633	2.92755E-13	5545	PRB4
-0.081054643	2.85954E-13	51252	FAM178B
-0.081072286	2.82585E-13	9129	PRPF3
-0.081141934	2.6966E-13	653	BMP5
-0.081152335	2.6778E-13	121441	NEDD1
-0.081383944	2.29107E-13	85302	FBF1
-0.081409484	2.25194E-13	83594	NUDT12
-0.081417374	2.23998E-13	5562	PRKAA1
-0.081423782	2.23032E-13	51588	PIAS4
-0.081430376	2.22042E-13	154796	AMOT
-0.081480712	2.14625E-13	134492	NUDCD2
-0.081602924	1.97613E-13	25900	IFFO1
-0.081607662	1.96981E-13	6917	TCEA1P2
-0.081638493	1.92916E-13	71	ACTG1
-0.081670158	1.88827E-13	4121	MAN1A1
-0.081704333	1.84509E-13	83548	COG3
-0.081732377	1.81039E-13	51300	TIMMDC1
-0.081753179	1.78505E-13	642	BLMH
-0.081753738	1.78438E-13	5813	PURA
-0.08177714	1.75631E-13	253039	RP11-27I1.2
-0.081803232	1.72553E-13	200316	APOBEC3F
-0.081832856	1.69121E-13	59351	PBOV1
-0.081881426	1.63642E-13	10541	ANP32B
-0.081896193	1.6201E-13	348093	RBPMS2

region-wise, subcortex					
Spearman's corr coef	p-val	p-val, SA	p-val, SA (BH- correction)	GenID	Gene
-0.124581724	0.467630595	0.527	1	375295	LOC375295
-0.124839127	0.466705143	0.503	1	2208	FCER2
-0.124839127	0.466705143	0.502	1	3030	HADHA
-0.124839127	0.466705143	0.513	1	4478	MSN
-0.124839127	0.466705143	0.515	1	8312	AXIN1
-0.124839127	0.466705143	0.506	1	29110	TBK1
-0.124839127	0.466705143	0.521	1	79673	ZNF329
-0.12509653	0.465780675	0.499	1	4678	NASP
-0.12509653	0.465780675	0.501	1	8821	INPP4B
-0.12509653	0.465780675	0.521	1	8836	GGH
-0.12509653	0.465780675	0.503	1	9258	MFHAS1
-0.12509653	0.465780675	0.491	1	11018	TMED1
-0.12509653	0.465780675	0.514	1	26146	TRAF3IP1
-0.12509653	0.465780675	0.49	1	29940	DSE
-0.12509653	0.465780675	0.501	1	55661	DDX27
-0.12509653	0.465780675	0.493	1	84912	SLC35B4
-0.12509653	0.465780675	0.496	1	123745	PLA2G4E
-0.125353917	0.464857221	0.501	1	335	APOA1
-0.125353917	0.464857221	0.501	1	9844	ELMO1
-0.125353917	0.464857221	0.493	1	51389	RWDD1
-0.125353917	0.464857221	0.506	1	79874	RABEP2
-0.125353917	0.464857221	0.517	1	116840	CNTROB
-0.125353917	0.464857221	0.525	1	353141	LCE2D
-0.125353917	0.464857221	0.528	1	389677	RBM12B
-0.12561132	0.463934183	0.494	1	5609	MAP2K7
-0.12561132	0.463934183	0.477	1	23347	AP001011.3
-0.12561132	0.463934183	0.474	1	54838	C10orf26
-0.12561132	0.463934183	0.471	1	55223	TRIM62
-0.12561132	0.463934183	0.518	1	83604	TMEM47
-0.12561132	0.463934183	0.49	1	84333	PCGF5
-0.125868723	0.463012695	0.481	1	3782	KCNN3
-0.125868723	0.463012695	0.525	1	4190	MDH1
-0.125868723	0.463012695	0.471	1	5193	PEX12
-0.125868723	0.463012695	0.52	1	6869	TACR1
-0.125868723	0.463012695	0.493	1	6991	TCTE3
-0.125868723	0.463012695	0.494	1	9994	CASP8AP2
-0.125868723	0.463012695	0.512	1	347344	ZNF81
-0.125868723	0.463012695	0.493	1	407835	LOC407835
-0.126126125	0.462092161	0.514	1	1814	DRD3
-0.126126125	0.462092161	0.495	1	6928	HNF1B
-0.126126125	0.462092161	0.508	1	9731	KIAA0562
-0.126126125	0.462092161	0.476	1	51014	TMED7
-0.126126125	0.462092161	0.518	1	55287	TMEM40
-0.126126125	0.462092161	0.527	1	64446	DNAI2
-0.126383528	0.461172581	0.478	1	1933	EEF1B2
-0.126383528	0.461172581	0.522	1	8780	RIOK3
-0.126383528	0.461172581	0.479	1	8872	CDC123
-0.126383528	0.461172581	0.496	1	10848	PPP1R13L
-0.126383528	0.461172581	0.527	1	162427	FAM134C
-0.126640916	0.460254073	0.46	1	747	DAGLA
-0.126640916	0.460254073	0.512	1	1041	CDSN
-0.126640916	0.460254073	0.486	1	22929	SEPHS1
-0.126640916	0.460254073	0.515	1	51021	MRPS16
-0.126640916	0.460254073	0.499	1	126661	RP11-291L19.1
-0.126898319	0.459336072	0.484	1	3181	HNRNPA2B1
-0.126898319	0.459336072	0.493	1	9350	CER1
-0.126898319	0.459336072	0.498	1	25803	SPDEF
-0.126898319	0.459336072	0.475	1	51082	POLR1D
-0.126898319	0.459336072	0.499	1	54629	FAM63B
-0.126898319	0.459336072	0.494	1	79651	RHBDF2
-0.126898319	0.459336072	0.512	1	152206	CCDC13
-0.127155721	0.458419472	0.509	1	241	ALOX5AP
-0.127155721	0.458419472	0.492	1	1610	DAO
-0.127155721	0.458419472	0.48	1	11012	KLK11
-0.127155721	0.458419472	0.489	1	25962	KIAA1429
-0.127155721	0.458419472	0.513	1	51230	PHF20
-0.127155721	0.458419472	0.496	1	51554	CCRL1
-0.127155721	0.458419472	0.477	1	56256	SERTAD4
-0.127155721	0.458419472	0.522	1	64114	TMBIM1
-0.127155721	0.458419472	0.467	1	642661	LOC642661
-0.127155721	0.458419472	0.485	1	100192378	RP11-65D13.1
-0.127413124	0.457503915	0.509	1	4744	NEFH
-0.127413124	0.457503915	0.53	1	83897	KRTAP3-2
-0.127413124	0.457503915	0.498	1	93550	ANUBL1
-0.127413124	0.457503915	0.483	1	114803	MYSM1
-0.127670527	0.456589252	0.508	1	7403	KDM6A
-0.127670527	0.456589252	0.49	1	8202	NCOA3
-0.127670527	0.456589252	0.489	1	51267	CLEC1A
-0.127670527	0.456589252	0.463	1	54541	DDIT4

voxel-wise, subcortex			
Spearman's corr coef	p-val	GenID	Gene
-0.081928045	1.58545E-13	3131	HLF
-0.081937335	1.57548E-13	5211	PFKL
-0.081974946	1.53576E-13	64768	IPPK
-0.082020544	1.48892E-13	283514	SIAH3
-0.082026951	1.48244E-13	728533	LOC728533
-0.082047194	1.46218E-13	1019	CDK4
-0.082091957	1.41835E-13	100133161	LOC100133161
-0.082110435	1.40063E-13	285093	CXXC11
-0.082156681	1.35724E-13	6136	RPL12
-0.082171172	1.34392E-13	100287596	DDX11L5
-0.0823166	1.21713E-13	220323	OAF
-0.082356997	1.18405E-13	23753	SDF2L1
-0.082402781	1.14763E-13	404203	SPINK6
-0.08242479	1.13051E-13	440434	AC183087.2
-0.082469918	1.09619E-13	5243	ABCB1
-0.082555912	1.03361E-13	9446	GSTO1
-0.082573839	1.02102E-13	80095	ZNF606
-0.08259882	1.00371E-13	152756	C4ORF39
-0.082626581	9.84826E-14	55695	NSUN5
-0.082666792	9.58084E-14	26353	HSPB8
-0.082679145	9.50013E-14	54407	SLC38A2
-0.082698368	9.3758E-14	149420	PDIK1L
-0.082716756	9.25844E-14	90634	N4BP2L1
-0.082726136	9.19913E-14	374946	C1orf187
-0.082808323	8.69507E-14	51181	DCXR
-0.082833394	8.54676E-14	60491	NIF3L1
-0.082852341	8.43641E-14	80054	LOC80054
-0.08288949	8.22397E-14	10195	ALG3
-0.082945578	7.91315E-14	2822	GPLD1
-0.083023958	7.4981E-14	5663	PSEN1
-0.08305683	7.33044E-14	5557	PRIM1
-0.083060637	7.3113E-14	54894	RNF43
-0.083100297	7.11445E-14	8675	STX16
-0.083174028	6.76224E-14	4670	HNRNPM
-0.083179228	6.73803E-14	90333	ZNF468
-0.083260395	6.37141E-14	122809	SOCS4
-0.083267733	6.33924E-14	151534	LBX2-AS1
-0.083291784	6.23494E-14	9907	AP5Z1
-0.083373688	5.89224E-14	10282	BET1
-0.08339709	5.79777E-14	6427	SFRS2
-0.083468415	5.51895E-14	254122	SNX32
-0.08351355	5.3493E-14	1544	CYP1A2
-0.083582081	5.10157E-14	9147	SDCCAG1
-0.083591744	5.06758E-14	9423	NTN1
-0.083630376	4.9338E-14	8991	SELENBP1
-0.083644956	4.88421E-14	28957	MRPS28
-0.083680332	4.76594E-14	27241	BBS9
-0.083688229	4.73995E-14	10655	DMRT2
-0.083717391	4.64507E-14	6282	S100A11
-0.083740883	4.57002E-14	8635	RNASET2
-0.083775617	4.46124E-14	1120	CHKB
-0.083832823	4.28754E-14	283899	INO80E
-0.083885476	4.13361E-14	7652	ZNF99
-0.083894759	4.10701E-14	51645	PPIL1
-0.08390303	4.08349E-14	255488	RNF144B
-0.083926059	4.01866E-14	10893	MMP24
-0.084019758	3.76515E-14	57335	ZNF286
-0.084056072	3.67118E-14	23370	ARHGEF18
-0.084096186	3.57007E-14	23291	FBXW11
-0.084112532	3.52964E-14	253558	LCLAT1
-0.084131941	3.48224E-14	23609	MKRN2
-0.084135562	3.47347E-14	10155	TRIM28
-0.084156834	3.42235E-14	441052	LOC441052
-0.084158964	3.41728E-14	145781	GCOM1
-0.084172711	3.3847E-14	6880	TAF9
-0.084192865	3.33749E-14	7673	ZNF222
-0.084253691	3.19884E-14	150776	AC093838.4
-0.084262051	3.18024E-14	89910	UBE3B
-0.084294461	3.10911E-14	79830	ZMYM1
-0.084357701	2.97481E-14	55317	C20orf29
-0.084360674	2.96863E-14	441161	OOEP
-0.084367447	2.95461E-14	7287	TULP1
-0.084370792	2.94772E-14	1908	EDN3
-0.084425122	2.83789E-14	51268	PIPOX
-0.084450938	2.78713E-14	338596	ST8SIA6
-0.084474713	2.74116E-14	92369	SPSB4
-0.084478147	2.73458E-14	55833	UBAP2
-0.084514923	2.66511E-14	10741	RBBP9
-0.084557921	2.58609E-14	92305	TMEM129

region-wise, subcortex					
Spearman's corr coef	p-val	p-val, SA	p-val, SA (BH- correction)	GenID	Gene
-0.127670527	0.456589252	0.501	1	55624	POMGNT1
-0.127670527	0.456589252	0.49	1	57224	NHSL1
-0.127670527	0.456589252	0.466	1	64772	ENGASE
-0.127670527	0.456589252	0.494	1	64799	IQCH
-0.127670527	0.456589252	0.493	1	92359	CRB3
-0.127670527	0.456589252	0.513	1	440730	TRIM67
-0.127927929	0.455675244	0.481	1	9790	BMS1
-0.127927929	0.455675244	0.486	1	51083	GAL
-0.127927929	0.455675244	0.5	1	55037	PTCD3
-0.127927929	0.455675244	0.482	1	55325	UFSP2
-0.127927929	0.455675244	0.52	1	81502	HM13
-0.127927929	0.455675244	0.514	1	83480	PUS3
-0.127927929	0.455675244	0.483	1	653361	NCF1
-0.128185332	0.454762697	0.511	1	8563	THOC5
-0.128185332	0.454762697	0.501	1	9816	URB2
-0.128185332	0.454762697	0.504	1	53371	NUP54
-0.128185332	0.454762697	0.479	1	83858	ATAD3B
-0.12844272	0.453851044	0.503	1	5573	PRKAR1A
-0.12844272	0.453851044	0.496	1	5738	PTGFRN
-0.12844272	0.453851044	0.525	1	10298	PAK4
-0.12844272	0.453851044	0.506	1	23181	DIP2A
-0.12844272	0.453851044	0.485	1	23478	SEC11B
-0.12844272	0.453851044	0.5	1	54795	TRPM4
-0.12844272	0.453851044	0.477	1	126755	LRRC38
-0.12844272	0.453851044	0.485	1	203076	C8orf74
-0.128700122	0.452940434	0.469	1	1051	CEBPB
-0.128700122	0.452940434	0.477	1	7145	TNS1
-0.128700122	0.452940434	0.464	1	8722	CTSF
-0.128700122	0.452940434	0.504	1	11277	TREX1
-0.128700122	0.452940434	0.472	1	170680	PSORS1C2
-0.128700122	0.452940434	0.508	1	374618	TEX9
-0.128957525	0.45203042	0.495	1	5519	PPP2R1B
-0.128957525	0.45203042	0.494	1	9440	MED17
-0.128957525	0.45203042	0.486	1	60680	BRUNOL5
-0.128957525	0.45203042	0.506	1	85455	DISP2
-0.128957525	0.45203042	0.491	1	158234	RG9MTD3
-0.128957525	0.45203042	0.498	1	284439	SLC25A42
-0.129214913	0.451121837	0.46	1	5357	PLS1
-0.129214913	0.451121837	0.491	1	84002	B3GNT5
-0.12947233	0.450214207	0.517	1	4166	CHST6
-0.12947233	0.450214207	0.495	1	5141	PDE4A
-0.12947233	0.450214207	0.477	1	5570	PKIB
-0.12947233	0.450214207	0.488	1	55260	TMEM143
-0.12947233	0.450214207	0.489	1	56964	WDR93
-0.12947233	0.450214207	0.528	1	60491	NIF3L1
-0.12947233	0.450214207	0.479	1	79701	C17orf101
-0.12947233	0.450214207	0.507	1	79777	ACBD4
-0.12947233	0.450214207	0.504	1	246330	PELI3
-0.12947233	0.450214207	0.498	1	283659	PRTG
-0.129729733	0.449307591	0.474	1	2318	FLNC
-0.129729733	0.449307591	0.458	1	6642	SNX1
-0.129729733	0.449307591	0.48	1	9706	ULK2
-0.129729733	0.449307591	0.483	1	54974	THG1L
-0.129729733	0.449307591	0.492	1	84279	C2orf7
-0.129729733	0.449307591	0.479	1	90390	MED30
-0.129729733	0.449307591	0.456	1	285195	SLC9A9
-0.129729733	0.449307591	0.5	1	342527	SMTNL2
-0.129987136	0.448401988	0.466	1	1603	DAD1
-0.129987136	0.448401988	0.481	1	5134	PDCD2
-0.129987136	0.448401988	0.507	1	220107	DLEU7
-0.129987136	0.448401988	0.501	1	253868	C20orf200
-0.129987136	0.448401988	0.478	1	283420	CLEC9A
-0.130244538	0.447496951	0.511	1	2246	FGF1
-0.130244538	0.447496951	0.48	1	27097	TAF5L
-0.130244538	0.447496951	0.48	1	55027	HEATR3
-0.130244538	0.447496951	0.487	1	63901	FAM111A
-0.130244538	0.447496951	0.503	1	79048	SECISBP2
-0.130244538	0.447496951	0.479	1	79870	BAALC
-0.130244538	0.447496951	0.458	1	84888	AC012100.1
-0.130244538	0.447496951	0.477	1	92249	LOC92249
-0.130244538	0.447496951	0.465	1	245936	DEFB123
-0.130244538	0.447496951	0.493	1	440307	TTLL13
-0.130501941	0.446593404	0.451	1	1500	CTNND1
-0.130501941	0.446593404	0.497	1	3060	HCRT
-0.130501941	0.446593404	0.485	1	9656	MDC1
-0.130501941	0.446593404	0.457	1	11016	ATF7
-0.130501941	0.446593404	0.466	1	55152	DALRD3
-0.130501941	0.446593404	0.487	1	55216	C11orf57
-0.130501941	0.446593404	0.494	1	57085	AGTRAP

voxel-wise, subcortex			
Spearman's corr coef	p-val	GenID	Gene
-0.084586337	2.53515E-14	8722	CTSF
-0.084603518	2.5048E-14	4602	MYB
-0.084661558	2.40496E-14	3036	HAS1
-0.084756188	2.25053E-14	869	CBLN1
-0.084798627	2.18446E-14	54939	COMMD4
-0.084855929	2.09828E-14	690	BTF3P11
-0.084966809	1.94093E-14	2045	EPHA7
-0.084979899	1.92314E-14	648791	PPP1R3G
-0.084996521	1.90078E-14	55096	EBLN2
-0.085006833	1.88703E-14	8358	HIST1H3B
-0.085025966	1.86178E-14	10738	RFPL3
-0.085055679	1.82324E-14	115939	C16orf42
-0.08510109	1.76584E-14	6301	SARS
-0.085158207	1.69616E-14	11276	AP1GBP1
-0.085202225	1.64431E-14	140578	CHODL
-0.085238256	1.60304E-14	60673	C12orf44
-0.085245594	1.59475E-14	387066	SNHG5
-0.085286267	1.54962E-14	84502	JPH4
-0.085300289	1.53435E-14	9635	CLCA2
-0.085307717	1.52632E-14	54861	SNRK
-0.085332975	1.49932E-14	1573	CYP2J2
-0.08539167	1.4384E-14	9758	FRMPD4
-0.085450731	1.37956E-14	54800	KLHL24
-0.085472278	1.35869E-14	2582	GALE
-0.085606374	1.2356E-14	9696	CROCC
-0.085755706	1.11141E-14	1774	DNASE1L1
-0.085757472	1.11002E-14	90736	FAM104B
-0.085817277	1.06386E-14	1514	CTSL1
-0.085844301	1.04362E-14	9798	IST1
-0.085870951	1.02404E-14	83985	SPNS1
-0.085905217	9.99393E-15	283985	FADS6
-0.085955828	9.64053E-15	26037	SIPA1L1
-0.085961126	9.60424E-15	23189	KANK1
-0.086021394	9.20094E-15	65109	UPF3B
-0.086035229	9.11074E-15	81532	MOB2
-0.086118534	8.58565E-15	1910	EDNRB
-0.086126611	8.53636E-15	403253	AC134982.1
-0.086156234	8.35791E-15	8671	SLC4A4
-0.086176574	8.23755E-15	55778	ZNF839
-0.086204432	8.07543E-15	11236	RNF139
-0.086206846	8.06148E-15	11224	RPL35
-0.086241394	7.86518E-15	10765	KDM5B
-0.086261362	7.7538E-15	64756	ATPAF1
-0.08627557	7.67553E-15	100129566	LOC100129566
-0.086314574	7.46464E-15	10240	MRPS31
-0.086352088	7.26719E-15	5332	PLCB4
-0.086363137	7.21002E-15	643630	LOC643630
-0.086498633	6.54389E-15	442421	PTGER4P2
-0.086537726	6.36322E-15	51287	CHCHD8
-0.08659029	6.12799E-15	29080	CCDC59
-0.086618803	6.00404E-15	5198	PFAS
-0.086639047	5.91749E-15	51389	RWDD1
-0.08667136	5.78195E-15	7867	MAPKAPK3
-0.086759679	5.42677E-15	121260	SLC15A4
-0.086825147	5.17746E-15	4125	MAN2B1
-0.086837403	5.13206E-15	54465	ETAA1
-0.086850226	5.08496E-15	152217	LOC152217
-0.087013111	4.52262E-15	6447	SCG5
-0.08702147	4.49546E-15	285220	EPHA6
-0.087050997	4.40084E-15	124540	MSI2
-0.087061308	4.36828E-15	79142	PHF23
-0.087087214	4.28748E-15	6546	SLC8A1
-0.087091208	4.27515E-15	2954	GSTZ1
-0.087106995	4.22676E-15	24148	PRPF6
-0.087118886	4.19068E-15	79092	CARD14
-0.087128267	4.16243E-15	81618	ITM2C
-0.087130398	4.15604E-15	25894	PLEKHG4
-0.087181754	4.00488E-15	119016	AGAP4
-0.087195128	3.96643E-15	9086	EIF1AY
-0.087216116	3.9068E-15	84920	ALG10
-0.087341301	3.56902E-15	102	ADAM10
-0.087404817	3.4088E-15	23149	FCHO1
-0.087419584	3.37257E-15	147407	MCART2
-0.087511055	3.1565E-15	11112	HIBADH
-0.08752387	3.12734E-15	115584	SLC5A11
-0.087546349	3.07683E-15	149840	C20orf196
-0.087560833	3.04471E-15	441294	CTAGE15P
-0.087567151	3.03079E-15	140699	C20orf132
-0.087716386	2.71986E-15	5019	OXCT1

region-wise, subcortex					
Spearman's corr coef	p-val	p-val, SA	p-val, SA (BH- correction)	GenID	Gene
-0.130501941	0.446593404	0.487	1	64093	SMOC1
-0.130501941	0.446593404	0.479	1	84296	GINS4
-0.130501941	0.446593404	0.496	1	125336	LOXHD1
-0.130501941	0.446593404	0.486	1	221458	KIF6
-0.130501941	0.446593404	0.469	1	727982	AC022311.1
-0.130759329	0.445690751	0.5	1	6385	SDC4
-0.130759329	0.445690751	0.472	1	51421	AMOTL2
-0.130759329	0.445690751	0.491	1	113655	MFSD3
-0.130759329	0.445690751	0.494	1	115362	GBP5
-0.130759329	0.445690751	0.498	1	441425	ANKRD20A1
-0.130759329	0.445690751	0.449	1	728642	CDC2L2
-0.131016731	0.444789141	0.479	1	4296	MAP3K11
-0.131016731	0.444789141	0.453	1	6658	SOX3
-0.131016731	0.444789141	0.492	1	10169	SERF2
-0.131016731	0.444789141	0.463	1	23137	SMC5
-0.131016731	0.444789141	0.486	1	54682	MANSC1
-0.131016731	0.444789141	0.511	1	79031	PDCL3
-0.131016731	0.444789141	0.501	1	80821	DDHD1
-0.131016731	0.444789141	0.491	1	90933	TRIM41
-0.131016731	0.444789141	0.48	1	116984	ARAP2
-0.131016731	0.444789141	0.485	1	728532	LOC728532
-0.131274134	0.443888187	0.478	1	663	BNIP2
-0.131274134	0.443888187	0.473	1	2329	FMO4
-0.131274134	0.443888187	0.492	1	5362	PLXNA2
-0.131274134	0.443888187	0.505	1	9255	AIMP1
-0.131274134	0.443888187	0.47	1	9719	ADAMTSL2
-0.131274134	0.443888187	0.505	1	54067	C21orf49
-0.131274134	0.443888187	0.487	1	93129	ORAI3
-0.131274134	0.443888187	0.441	1	168451	THAP5
-0.131274134	0.443888187	0.471	1	440311	LOC440311
-0.131531537	0.442988575	0.451	1	1232	CCR3
-0.131531537	0.442988575	0.47	1	8502	PK4P
-0.131531537	0.442988575	0.506	1	10043	TOM1
-0.131531537	0.442988575	0.503	1	23460	ABCA6
-0.131531537	0.442988575	0.464	1	51053	GMNN
-0.131531537	0.442988575	0.469	1	79166	LILRP2
-0.131788939	0.442090064	0.489	1	4928	NUP98
-0.131788939	0.442090064	0.454	1	7812	CSDE1
-0.131788939	0.442090064	0.493	1	23516	SLC39A14
-0.131788939	0.442090064	0.483	1	51692	CPSF3
-0.131788939	0.442090064	0.456	1	55898	UNC45A
-0.131788939	0.442090064	0.463	1	79713	TMEM149
-0.131788939	0.442090064	0.5	1	114793	FMNL2
-0.131788939	0.442090064	0.48	1	171558	PTCRA
-0.131788939	0.442090064	0.474	1	203427	SLC25A43
-0.131788939	0.442090064	0.496	1	222194	RSBN1L
-0.132046327	0.441192478	0.486	1	1805	DPT
-0.132046327	0.441192478	0.488	1	5074	PAWR
-0.132046327	0.441192478	0.482	1	9877	ZC3H11A
-0.132046327	0.441192478	0.461	1	90624	LYRM7
-0.132046327	0.441192478	0.481	1	389791	LOC389791
-0.132046327	0.441192478	0.498	1	100286946	LOC100286946
-0.13230373	0.440295547	0.454	1	9282	MED14
-0.13230373	0.440295547	0.499	1	9448	MAP4K4
-0.13230373	0.440295547	0.471	1	54108	CHRAC1
-0.13230373	0.440295547	0.477	1	55356	SLC22A15
-0.13230373	0.440295547	0.508	1	83786	FRMD8
-0.13230373	0.440295547	0.463	1	83933	HDAC10
-0.13230373	0.440295547	0.476	1	326624	RAB37
-0.132561132	0.439399987	0.493	1	5565	PRKAB2
-0.132561132	0.439399987	0.499	1	7056	THBD
-0.132561132	0.439399987	0.458	1	126017	ZNF813
-0.132561132	0.439399987	0.487	1	126147	NTN5
-0.132561132	0.439399987	0.457	1	401021	LOC401021
-0.132561132	0.439399987	0.451	1	441295	OR2A9P
-0.132818535	0.438505441	0.477	1	1781	DYNC1I2
-0.132818535	0.438505441	0.478	1	2875	GPT
-0.132818535	0.438505441	0.464	1	7462	LAT2
-0.132818535	0.438505441	0.48	1	9266	CYTH2
-0.132818535	0.438505441	0.469	1	23133	PHF8
-0.132818535	0.438505441	0.476	1	23247	KIAA0556
-0.132818535	0.438505441	0.472	1	51077	FCF1
-0.132818535	0.438505441	0.463	1	55343	SLC35C1
-0.132818535	0.438505441	0.497	1	79805	VASH2
-0.132818535	0.438505441	0.452	1	84366	PRAC
-0.132818535	0.438505441	0.48	1	145501	ISM2
-0.132818535	0.438505441	0.497	1	153241	CEP120
-0.132818535	0.438505441	0.467	1	199990	C1orf86
-0.133075938	0.437611967	0.472	1	2245	FGD1

voxel-wise, subcortex			
Spearman's corr coef	p-val	GenID	Gene
-0.087730221	2.69269E-15	60494	CCDC81
-0.087760404	2.63431E-15	54979	HRASLS2
-0.087815471	2.531E-15	80217	C10orf79
-0.087976128	2.25185E-15	64779	MTHFSD
-0.088014573	2.18969E-15	6904	TBCD
-0.088024236	2.17433E-15	5128	PCTK2
-0.088056736	2.12346E-15	9447	AIM2
-0.088133447	2.00796E-15	80067	C2orf37
-0.088147745	1.98713E-15	51527	C14orf129
-0.088158332	1.97184E-15	9454	HOMER3
-0.088176906	1.94529E-15	5365	PLXNB3
-0.088242099	1.85489E-15	54856	GON4L
-0.088254541	1.83812E-15	84232	MAF1
-0.088274047	1.81211E-15	144245	ALG10B
-0.088340349	1.72641E-15	83552	MFRP
-0.088348061	1.71671E-15	79840	NHEJ1
-0.088357441	1.70497E-15	205251	LINC00116
-0.088368863	1.69079E-15	54441	STAG3L1
-0.08841446	1.63533E-15	4489	MT1A
-0.088484012	1.55416E-15	7103	TSPAN8
-0.088514753	1.51957E-15	728698	EIF4A1P4
-0.088549115	1.48179E-15	25981	DNAH1
-0.088558771	1.47135E-15	283458	NME2P1
-0.088599078	1.42851E-15	55338	FLJ11292
-0.088603906	1.42347E-15	10884	MRPS30
-0.08861319	1.41381E-15	3344	FOXN2
-0.088756301	1.27287E-15	11060	WWP2
-0.088779606	1.25126E-15	9	NAT1
-0.088780813	1.25015E-15	11178	LZTS1
-0.088853896	1.18479E-15	2306	FOXD2
-0.088878602	1.16345E-15	164045	HFM1
-0.08896032	1.09556E-15	55652	SLC48A1
-0.089029416	1.04121E-15	5682	PSMA1
-0.089082628	1.00118E-15	2683	B4GALT1
-0.089087918	9.97279E-16	10669	CGREF1
-0.089098506	9.89528E-16	3985	LIMK2
-0.089165375	9.41912E-16	55998	NXF5
-0.089167044	9.40755E-16	9053	MAP7
-0.089232609	8.96317E-16	116461	TSEN15
-0.089233443	8.95772E-16	152007	GLIPR2
-0.089305878	8.49104E-16	197135	PATL2
-0.089351103	8.21191E-16	7643	ZNF90
-0.089411654	7.85229E-16	56129	PCDHB7
-0.089422517	7.78943E-16	284340	CXCL17
-0.089432828	7.73024E-16	112574	SNX18
-0.08946839	7.52945E-16	728643	RP11-314P12.3
-0.089493185	7.39246E-16	81539	SLC38A1
-0.089558847	7.04157E-16	3735	KARS
-0.089606389	6.7978E-16	201176	SH3D20
-0.089619949	6.72974E-16	79088	ZNF426
-0.089625798	6.70066E-16	51351	ZNF117
-0.089688115	6.39796E-16	55967	NDUFA12
-0.089696191	6.35972E-16	10617	STAMBP
-0.089716256	6.26572E-16	22862	FNDC3A
-0.089745596	6.13074E-16	440585	FAM183A
-0.089787759	5.94172E-16	515	ATP5F1
-0.089809678	5.84574E-16	654502	IQCJ
-0.089849979	5.67331E-16	84105	PCBD2
-0.08991573	5.40254E-16	645937	LOC645937
-0.089934304	5.3284E-16	55144	LRRC8D
-0.089938574	5.31151E-16	5683	PSMA2
-0.089947492	5.27639E-16	9946	CRYZL1
-0.089985751	5.12825E-16	7431	VIM
-0.090163127	4.49329E-16	2733	GLE1
-0.090195909	4.38473E-16	353144	LCE3C
-0.0902467	4.22166E-16	83596	BCL2L12
-0.090293229	4.07747E-16	14	AAMP
-0.09029945	4.05856E-16	7360	UGP2
-0.090369381	3.85191E-16	10224	ZNF443
-0.090373926	3.83884E-16	285605	DTWD2
-0.090376161	3.83243E-16	54776	LENG3
-0.09037783	3.82766E-16	4697	NDUFA4
-0.090390459	3.79168E-16	5521	PPP2R2B
-0.090491593	3.51535E-16	1345	COX6C
-0.090587333	3.27212E-16	123169	LEO1
-0.090590775	3.26369E-16	9367	RAB9A
-0.090639897	3.14569E-16	92689	FAM114A1
-0.090705924	2.99372E-16	8544	PIR
-0.090721995	2.95781E-16	23307	FKBP15

region-wise, subcortex					
Spearman's corr coef	p-val	p-val, SA	p-val, SA (BH- correction)	GenID	Gene
-0.133075938	0.437611967	0.472	1	7083	TK1
-0.133075938	0.437611967	0.442	1	8340	HIST1H2BL
-0.133075938	0.437611967	0.455	1	10550	ARL6IP5
-0.133075938	0.437611967	0.471	1	54885	TBC1D8B
-0.133075938	0.437611967	0.463	1	84218	TBC1D3
-0.133075938	0.437611967	0.478	1	113251	LARP4
-0.133075938	0.437611967	0.462	1	440567	UQCRHL
-0.133075938	0.437611967	0.484	1	100129794	RP11-537P22.1
-0.133333325	0.436719447	0.474	1	5989	RFX1
-0.133333325	0.436719447	0.479	1	7307	U2AF1
-0.133333325	0.436719447	0.49	1	9412	MED21
-0.133333325	0.436719447	0.46	1	9543	IGDCC3
-0.133333325	0.436719447	0.461	1	9694	TTC35
-0.133333325	0.436719447	0.48	1	9897	KIAA0196
-0.133333325	0.436719447	0.445	1	11086	ADAM29
-0.133333325	0.436719447	0.449	1	55839	CENPN
-0.133333325	0.436719447	0.448	1	84081	CCDC55
-0.133590728	0.435827583	0.464	1	2288	FKBP4
-0.133590728	0.435827583	0.457	1	7352	UCP3
-0.133590728	0.435827583	0.474	1	9487	PIGL
-0.133590728	0.435827583	0.459	1	10346	TRIM22
-0.133848131	0.434937119	0.448	1	2052	EPHX1
-0.133848131	0.434937119	0.498	1	10735	STAG2
-0.133848131	0.434937119	0.451	1	60489	APOBEC3G
-0.133848131	0.434937119	0.501	1	64866	CDCP1
-0.133848131	0.434937119	0.455	1	79901	CYBRD1
-0.133848131	0.434937119	0.5	1	245934	DEFB121
-0.133848131	0.434937119	0.476	1	339005	WHAMML1
-0.134105533	0.434047639	0.461	1	5817	PVR
-0.134105533	0.434047639	0.466	1	8074	FGF23
-0.134105533	0.434047639	0.467	1	8557	TCAP
-0.134105533	0.434047639	0.521	1	55672	NBPF1
-0.134105533	0.434047639	0.456	1	55762	ZNF701
-0.134105533	0.434047639	0.48	1	81556	C15orf44
-0.134105533	0.434047639	0.472	1	245939	DEFB128
-0.134105533	0.434047639	0.492	1	283576	ZDHHC22
-0.134105533	0.434047639	0.479	1	400750	LOC400750
-0.134362936	0.433159232	0.467	1	2519	FUCA2
-0.134362936	0.433159232	0.458	1	79892	C10orf119
-0.134362936	0.433159232	0.472	1	83596	BCL2L12
-0.134362936	0.433159232	0.501	1	171423	PDIA3P
-0.134362936	0.433159232	0.457	1	254783	OR6C74
-0.134362936	0.433159232	0.473	1	388558	ZNF808
-0.134362936	0.433159232	0.477	1	399761	BMS1P5
-0.134620324	0.432271421	0.465	1	4010	LMX1B
-0.134620324	0.432271421	0.461	1	10603	SH2B2
-0.134620324	0.432271421	0.48	1	10919	EHMT2
-0.134620324	0.432271421	0.506	1	51141	INSIG2
-0.134620324	0.432271421	0.45	1	55652	SLC48A1
-0.134620324	0.432271421	0.477	1	90317	ZNF616
-0.134877726	0.43138501	0.505	1	23076	RRP1B
-0.134877726	0.43138501	0.478	1	57578	KIAA1409
-0.134877726	0.43138501	0.463	1	80022	MYO15B
-0.134877726	0.43138501	0.472	1	84699	CREB3L3
-0.134877726	0.43138501	0.456	1	84996	C21ORF119
-0.134877726	0.43138501	0.492	1	222894	FERD3L
-0.134877726	0.43138501	0.477	1	442454	UQCRBP1
-0.135135129	0.430499643	0.438	1	3127	HLA-DRB5
-0.135135129	0.430499643	0.463	1	83759	RBM4B
-0.135135129	0.430499643	0.467	1	346528	OR2A1
-0.135135129	0.430499643	0.48	1	644186	C22orf41
-0.135392532	0.429615289	0.467	1	5698	PSMB9
-0.135392532	0.429615289	0.456	1	7113	TMPRSS2
-0.135392532	0.429615289	0.493	1	56893	UBQLN4
-0.135392532	0.429615289	0.48	1	57467	HHATL
-0.135649934	0.428731561	0.458	1	3329	HSPD1
-0.135649934	0.428731561	0.45	1	8330	HIST1H2AK
-0.135649934	0.428731561	0.478	1	10223	GPA33
-0.135649934	0.428731561	0.483	1	54553	DKFZP434I0714
-0.135649934	0.428731561	0.472	1	54841	BIVM
-0.135649934	0.428731561	0.459	1	150759	LINC00342
-0.135649934	0.428731561	0.449	1	219285	SAMD9L
-0.135907322	0.427849233	0.43	1	3077	HFE
-0.135907322	0.427849233	0.471	1	4149	MAX
-0.135907322	0.427849233	0.442	1	4258	MGST2
-0.135907322	0.427849233	0.431	1	4599	MX1
-0.135907322	0.427849233	0.483	1	163033	ZNF579
-0.135907322	0.427849233	0.456	1	221188	GPR114
-0.135907322	0.427849233	0.475	1	100128977	MAPT-AS1

voxel-wise, subcortex			
Spearman's corr coef	p-val	GenID	Gene
-0.09079396	2.80224E-16	5300	PIN1
-0.090810955	2.76671E-16	51267	CLEC1A
-0.09083315	2.72096E-16	30833	NT5C
-0.090840675	2.70561E-16	57085	AGTRAP
-0.090871967	2.64272E-16	56245	C21orf62
-0.090913199	2.56205E-16	2539	G6PD
-0.090924904	2.53959E-16	196047	EMX2OS
-0.090929642	2.53056E-16	4163	MCC
-0.090950347	2.49145E-16	84844	PHF5A
-0.090957686	2.47772E-16	145783	RP11-323F24.1
-0.09097477	2.44609E-16	9640	ZNF592
-0.091003843	2.39313E-16	54205	CYCS
-0.091041081	2.32698E-16	152273	FGD5
-0.091082871	2.25488E-16	399744	LOC399744
-0.091098562	2.22838E-16	8314	BAP1
-0.091114722	2.20138E-16	1616	DAXX
-0.091185302	2.0873E-16	2975	GTF3C1
-0.091222167	2.03005E-16	51447	IP6K2
-0.091233961	2.01207E-16	3107	HLA-C
-0.09144152	1.72004E-16	514	ATP5E
-0.091447741	1.71196E-16	64976	MRPL40
-0.091448948	1.7104E-16	165	AEBP1
-0.091490269	1.65776E-16	55795	PCID2
-0.091493711	1.65346E-16	23211	ZC3H4
-0.091554165	1.57949E-16	10983	CCNI
-0.091575153	1.55457E-16	157807	RLBP1L1
-0.091586478	1.54129E-16	4140	MARK3
-0.091621488	1.50094E-16	122769	PPIL5
-0.091681764	1.4339E-16	11209	RP5-1182A14.1
-0.091709159	1.40442E-16	127253	TYW3
-0.091716401	1.39673E-16	9987	HNRPDL
-0.091844	1.26778E-16	10297	APC2
-0.091847248	1.26466E-16	79706	PRKRIP1
-0.091864057	1.24862E-16	10577	NPC2
-0.091871671	1.24141E-16	57343	ZNF304
-0.091905385	1.21001E-16	27243	CHMP2A
-0.091936305	1.18189E-16	58528	RRAGD
-0.091978192	1.14482E-16	142689	ASB12
-0.092038549	1.09343E-16	27154	BRPF3
-0.092054062	1.0806E-16	25853	WDR40A
-0.092088513	1.05262E-16	27037	TRMT2A
-0.092097804	1.04519E-16	170622	COMMD6
-0.092106253	1.03849E-16	79850	FAM57A
-0.092181377	9.80701E-17	84816	RTN4IP1
-0.092193916	9.71368E-17	55035	NOL8
-0.092259482	9.23981E-17	4528	MTIF2
-0.092264496	9.20461E-17	83894	TTC29
-0.092274897	9.1318E-17	160622	GRASP
-0.092314459	8.86012E-17	8725	URI1
-0.092340834	8.68348E-17	4719	NDUFS1
-0.092348352	8.63379E-17	10169	SERF2
-0.092353649	8.59888E-17	80765	STARD5
-0.092462674	7.91143E-17	140730	RIMS4
-0.092487656	7.76171E-17	55544	RBM38
-0.092523031	7.55448E-17	3566	IL4R
-0.092527494	7.52874E-17	2197	FAU
-0.092552938	7.38348E-17	9849	ZNF518A
-0.092606612	7.08622E-17	147495	APCDD1
-0.09263958	6.90952E-17	483	ATP1B3
-0.092836455	5.94118E-17	2189	FANCG
-0.092851222	5.87418E-17	79868	ALG13
-0.092908524	5.62119E-17	51530	ZC3HC1
-0.092914186	5.59683E-17	91369	ANKRD40
-0.092987083	5.29182E-17	80227	PAAF1
-0.09308859	4.89424E-17	51608	C7orf20
-0.09308961	4.89038E-17	55604	LRRC16A
-0.093093231	4.8768E-17	11269	DDX19B
-0.093117006	4.78833E-17	399818	METTL10
-0.093138553	4.70949E-17	11341	SCRG1
-0.093173094	4.58588E-17	3105	HLA-A
-0.093234107	4.37519E-17	81490	PTDSS2
-0.09324117	4.35143E-17	5094	PCBP2
-0.093311653	4.12117E-17	56996	SLC12A9
-0.09333422	4.04998E-17	117155	CATSPER2
-0.0933709	3.93691E-17	401357	LOC401357
-0.093426339	3.77189E-17	8620	NPFF
-0.093434423	3.74841E-17	57609	DIP2B
-0.093438968	3.73527E-17	7059	THBS3
-0.09344241	3.72535E-17	284443	ZNF493

region-wise, subcortex					
Spearman's corr coef	p-val	p-val, SA	p-val, SA (BH- correction)	GenID	Gene
-0.136164725	0.426967978	0.475	1	2773	GNAI3
-0.136164725	0.426967978	0.456	1	8666	EIF3G
-0.136164725	0.426967978	0.461	1	10212	DDX39
-0.136164725	0.426967978	0.463	1	55015	PRPF39
-0.136164725	0.426967978	0.436	1	90586	AOC4
-0.136164725	0.426967978	0.469	1	150297	C22orf42
-0.136164725	0.426967978	0.458	1	151011	SEPTIN10
-0.136164725	0.426967978	0.444	1	100288165	HMGN2P15
-0.136422142	0.426087707	0.459	1	2055	CLN8
-0.136422142	0.426087707	0.451	1	8893	EIF2B5
-0.136422142	0.426087707	0.484	1	56848	SPHK2
-0.136422142	0.426087707	0.505	1	80228	ORAI2
-0.136422142	0.426087707	0.465	1	112752	C14orf179
-0.136679545	0.425208479	0.464	1	1109	AKR1C4
-0.136679545	0.425208479	0.473	1	5083	PAX9
-0.136679545	0.425208479	0.455	1	7091	TLE4
-0.136679545	0.425208479	0.453	1	55746	NUP133
-0.136679545	0.425208479	0.454	1	79365	BHLHE41
-0.136679545	0.425208479	0.466	1	81788	NUAK2
-0.136679545	0.425208479	0.483	1	132299	OCIAD2
-0.136936948	0.424329847	0.418	1	10577	NPC2
-0.136936948	0.424329847	0.476	1	51548	SIRT6
-0.136936948	0.424329847	0.454	1	53938	PPIL3
-0.136936948	0.424329847	0.496	1	57674	RNF213
-0.136936948	0.424329847	0.443	1	59344	ALOXE3
-0.136936948	0.424329847	0.467	1	85451	UNK
-0.136936948	0.424329847	0.463	1	90199	WFDC8
-0.136936948	0.424329847	0.467	1	151556	GPR155
-0.136936948	0.424329847	0.464	1	221687	RNF182
-0.137194335	0.423452675	0.456	1	8992	ATP6V0E1
-0.137194335	0.423452675	0.454	1	26873	OPLAH
-0.137194335	0.423452675	0.473	1	83744	ZNF484
-0.137194335	0.423452675	0.462	1	115004	C6orf150
-0.137194335	0.423452675	0.471	1	391769	H3.Y
-0.137451738	0.422576487	0.465	1	5588	PRKCQ
-0.137451738	0.422576487	0.441	1	51374	C2orf28
-0.137451738	0.422576487	0.441	1	64689	GORASP1
-0.137451738	0.422576487	0.463	1	65010	SLC26A6
-0.137709141	0.421701401	0.463	1	79157	MFSD11
-0.137709141	0.421701401	0.44	1	79763	ISOC2
-0.137709141	0.421701401	0.472	1	84465	MEGF11
-0.137709141	0.421701401	0.467	1	89792	GAL3ST3
-0.137709141	0.421701401	0.443	1	94239	H2AFV
-0.137709141	0.421701401	0.462	1	165918	RNF168
-0.137709141	0.421701401	0.449	1	170384	FUT11
-0.137709141	0.421701401	0.486	1	440419	TBC1D3P5
-0.137966543	0.420826912	0.461	1	1653	DDX1
-0.137966543	0.420826912	0.441	1	54831	BEST2
-0.137966543	0.420826912	0.463	1	55246	CCDC25
-0.137966543	0.420826912	0.481	1	79290	OR13A1
-0.138223946	0.419953793	0.48	1	372	ARCN1
-0.138223946	0.419953793	0.426	1	3593	IL12B
-0.138223946	0.419953793	0.486	1	6651	SON
-0.138223946	0.419953793	0.44	1	7932	OR2H2
-0.138223946	0.419953793	0.464	1	29095	ORMDL2
-0.138223946	0.419953793	0.419	1	29937	NENF
-0.138223946	0.419953793	0.46	1	64431	ACTR6
-0.138223946	0.419953793	0.458	1	84129	ACAD11
-0.138223946	0.419953793	0.473	1	144404	TMEM120B
-0.138223946	0.419953793	0.481	1	257068	PLCXD2
-0.138223946	0.419953793	0.45	1	646096	CHEK2P2
-0.138481334	0.419081777	0.442	1	23064	SETX
-0.138481334	0.419081777	0.457	1	27109	ATP5S
-0.138481334	0.419081777	0.452	1	55094	GPATCH1
-0.138481334	0.419081777	0.439	1	84875	PARP10
-0.138481334	0.419081777	0.445	1	96597	TBC1D27
-0.138481334	0.419081777	0.453	1	149483	CCDC17
-0.138481334	0.419081777	0.467	1	440077	ZNF705A
-0.138738737	0.418210804	0.434	1	1615	DARS
-0.138738737	0.418210804	0.473	1	5144	PDE4D
-0.138738737	0.418210804	0.446	1	7466	WFS1
-0.138738737	0.418210804	0.432	1	10188	TNK2
-0.138738737	0.418210804	0.439	1	22865	SLITRK3
-0.138738737	0.418210804	0.442	1	51125	GOLGA7
-0.138738737	0.418210804	0.457	1	64762	FAM59A
-0.138738737	0.418210804	0.437	1	80852	GRIP2
-0.138738737	0.418210804	0.461	1	81844	TRIM56
-0.138996139	0.417340338	0.432	1	11060	WWP2
-0.138996139	0.417340338	0.452	1	26005	C2CD3

voxel-wise, subcortex			
Spearman's corr coef	p-val	GenID	Gene
-0.093476117	3.62954E-17	8382	NME5
-0.093499981	3.56321E-17	10643	IGF2BP3
-0.093504816	3.54989E-17	404217	CTXN1
-0.09351512	3.52174E-17	84255	SLC37A3
-0.09353397	3.47075E-17	23081	KDM4C
-0.093545675	3.43947E-17	646890	LOC646890
-0.093548089	3.43304E-17	6387	CXCL12
-0.093592666	3.31665E-17	84695	LOXL3
-0.093601026	3.29526E-17	3032	HADHB
-0.093614116	3.26207E-17	25938	HEATR5A
-0.09375973	2.91404E-17	163071	ZNF114
-0.093766786	2.89814E-17	55148	UBR7
-0.093817957	2.7854E-17	114780	PKD1L2
-0.093895689	2.62228E-17	6736	SRY
-0.093905993	2.6014E-17	441191	RNF216L
-0.093909152	2.59502E-17	10904	BLCAP
-0.093915187	2.5829E-17	51773	RSF1
-0.093921505	2.57026E-17	57161	PELI2
-0.093941472	2.53069E-17	144347	FAM101A
-0.093988277	2.44031E-17	57458	TMCC3
-0.094008334	2.40254E-17	148362	BROX
-0.094057277	2.31283E-17	29920	PYCR2
-0.094066098	2.29702E-17	9731	KIAA0562
-0.094131105	2.18372E-17	164656	TMPRSS6
-0.094139554	2.16942E-17	57214	KIAA1199
-0.094180696	2.10101E-17	1874	E2F4
-0.094223782	2.03168E-17	26246	OR2L2
-0.094243281	2.00103E-17	6924	TCEB3
-0.094258606	1.97728E-17	11190	CEP250
-0.094301514	1.91223E-17	79885	HDAC11
-0.094306804	1.90435E-17	7398	USP1
-0.094321944	1.88199E-17	86	ACTL6A
-0.094331689	1.86775E-17	338328	GPIHBP1
-0.094353609	1.83608E-17	643376	AP001931.1
-0.094434403	1.72387E-17	202781	LOC202781
-0.094517328	1.61573E-17	284441	LOC284441
-0.094547607	1.57794E-17	333	APLP1
-0.094560139	1.56255E-17	4302	MLLT6
-0.0945841	1.53354E-17	55001	TTC22
-0.094609179	1.50376E-17	51124	IER3IP1
-0.094630346	1.47906E-17	85508	SCRT2
-0.094631091	1.47819E-17	63915	MUTED
-0.094633132	1.47583E-17	2686	GGT7
-0.094649762	1.45676E-17	84132	USP42
-0.094698049	1.40272E-17	10087	COL4A3BP
-0.094737701	1.35983E-17	29116	MYLIP
-0.094744578	1.35251E-17	8985	PLOD3
-0.094812833	1.28208E-17	27246	RNF115
-0.094848119	1.24708E-17	139189	DGKK
-0.094888143	1.20853E-17	340024	SLC6A19
-0.094892137	1.20476E-17	8546	AP3B1
-0.094933838	1.16597E-17	498	ATP5A1
-0.094951294	1.1501E-17	151636	DTX3L
-0.095041469	1.07145E-17	54801	HAUS6
-0.095062457	1.05392E-17	92291	CAPN13
-0.095068492	1.04893E-17	4296	MAP3K11
-0.095183648	9.58107E-18	27303	RBMS3
-0.095194697	9.49803E-18	414899	BLID
-0.095199987	9.45858E-18	55339	WDR33
-0.095212802	9.36371E-18	23274	CLEC16A
-0.095366403	8.29632E-18	163786	SASS6
-0.095421009	7.94662E-18	85450	ITPRIP
-0.095422499	7.93723E-18	5810	RAD1
-0.095474593	7.61756E-18	11135	CDC42EP1
-0.095480256	7.58361E-18	80349	WDR61
-0.09548685	7.54418E-18	3543	IGLL1
-0.095534772	7.26404E-18	352954	GATS
-0.095556967	7.13776E-18	283869	NPW
-0.095576838	7.02659E-18	729494	ARPC3P3
-0.095588543	6.96188E-18	3204	HOXA7
-0.095606372	6.86444E-18	9337	CNOT8
-0.095702119	6.36384E-18	7004	TEAD4
-0.095805943	5.86168E-18	81544	GDPD5
-0.095812164	5.83286E-18	157869	C8orf84
-0.095868349	5.57888E-18	4323	MMP14
-0.095878929	5.53225E-18	55113	XKR8
-0.095905773	5.41579E-18	2634	GBP2
-0.095924623	5.33545E-18	90233	ZNF551
-0.096044138	4.85262E-18	5976	UPF1

region-wise, subcortex					
Spearman's corr coef	p-val	p-val, SA	p-val, SA (BH- correction)	GenID	Gene
-0.138996139	0.417340338	0.476	1	158584	FAAH2
-0.138996139	0.417340338	0.458	1	727941	LOC727941
-0.139253542	0.416471422	0.463	1	6434	TRA2B
-0.139253542	0.416471422	0.439	1	6795	AURKC
-0.139253542	0.416471422	0.449	1	10062	NR1H3
-0.139253542	0.416471422	0.439	1	122786	FRMD6
-0.139253542	0.416471422	0.457	1	254295	PHYHD1
-0.139253542	0.416471422	0.428	1	286075	ZNF707
-0.139510944	0.415603518	0.464	1	22835	ZFP30
-0.139510944	0.415603518	0.464	1	23598	PATZ1
-0.139510944	0.415603518	0.454	1	54502	RBM47
-0.139510944	0.415603518	0.463	1	130576	LYPD6B
-0.139510944	0.415603518	0.459	1	353133	LCE1C
-0.139768332	0.414736629	0.446	1	202	AIM1
-0.139768332	0.414736629	0.421	1	6871	TADA2L
-0.139768332	0.414736629	0.436	1	57817	HAMP
-0.139768332	0.414736629	0.468	1	79646	PANK3
-0.139768332	0.414736629	0.445	1	441151	TMEM151B
-0.139768332	0.414736629	0.437	1	497258	BDNF-AS1
-0.139768332	0.414736629	0.446	1	728229	TMEM191B
-0.140025735	0.413870811	0.454	1	2595	GANC
-0.140025735	0.413870811	0.448	1	2992	GYG1
-0.140025735	0.413870811	0.44	1	4125	MAN2B1
-0.140025735	0.413870811	0.434	1	8520	HAT1
-0.140025735	0.413870811	0.436	1	8531	CSDAP1
-0.140025735	0.413870811	0.463	1	9047	SH2D2A
-0.140025735	0.413870811	0.461	1	10957	PNRC1
-0.140025735	0.413870811	0.441	1	55863	TMEM126B
-0.140025735	0.413870811	0.432	1	117248	GALNTL2
-0.140025735	0.413870811	0.442	1	651430	FLJ38576
-0.140025735	0.413870811	0.454	1	728431	RP11-422J8.1
-0.140283138	0.41300559	0.463	1	1523	CUX1
-0.140283138	0.41300559	0.499	1	1953	MEGF6
-0.140283138	0.41300559	0.449	1	8939	FUBP3
-0.140283138	0.41300559	0.442	1	25836	NIPBL
-0.140283138	0.41300559	0.43	1	51524	TMEM138
-0.140283138	0.41300559	0.433	1	646625	PRHOXNB
-0.14054054	0.41214186	0.431	1	2153	F5
-0.14054054	0.41214186	0.45	1	9745	ZNF536
-0.14054054	0.41214186	0.467	1	9798	IST1
-0.14054054	0.41214186	0.433	1	10130	PDIA6
-0.14054054	0.41214186	0.442	1	10272	FSTL3
-0.14054054	0.41214186	0.435	1	29894	CPSF1
-0.14054054	0.41214186	0.462	1	29999	FSCN3
-0.14054054	0.41214186	0.455	1	53354	PANK1
-0.14054054	0.41214186	0.438	1	83694	RPS6KL1
-0.14054054	0.41214186	0.457	1	283212	KLHL35
-0.14054054	0.41214186	0.476	1	541465	CT45A4
-0.140797943	0.411279082	0.41	1	37	ACADVL
-0.140797943	0.411279082	0.438	1	5213	PFKM
-0.140797943	0.411279082	0.45	1	8803	SUCLA2
-0.140797943	0.411279082	0.445	1	10910	SUGT1
-0.140797943	0.411279082	0.429	1	25834	MGAT4C
-0.140797943	0.411279082	0.464	1	55515	ACCN4
-0.140797943	0.411279082	0.423	1	55914	ERBB2IP
-0.140797943	0.411279082	0.43	1	146310	RNF151
-0.140797943	0.411279082	0.457	1	440093	RP11-428G5.1
-0.141055331	0.410417348	0.454	1	4798	NFRKB
-0.141055331	0.410417348	0.426	1	9697	TRAM2
-0.141055331	0.410417348	0.455	1	10864	SLC22A7
-0.141055331	0.410417348	0.421	1	64581	CLEC7A
-0.141055331	0.410417348	0.442	1	645181	LOC645181
-0.141312733	0.409556359	0.444	1	444	ASPH
-0.141312733	0.409556359	0.435	1	4621	MYH3
-0.141312733	0.409556359	0.45	1	6617	SNAPC1
-0.141312733	0.409556359	0.481	1	26092	TOR1AIP1
-0.141312733	0.409556359	0.435	1	54465	ETAA1
-0.141312733	0.409556359	0.467	1	55262	C7orf43
-0.141312733	0.409556359	0.439	1	79192	IRX1
-0.141312733	0.409556359	0.453	1	125893	ZNF816A
-0.141570136	0.408696711	0.446	1	3166	HMX1
-0.141570136	0.408696711	0.425	1	4586	MUC5AC
-0.141570136	0.408696711	0.459	1	6542	SLC7A2
-0.141570136	0.408696711	0.445	1	7541	ZFP161
-0.141570136	0.408696711	0.44	1	55333	SYNJ2BP
-0.141570136	0.408696711	0.439	1	57337	SENP7
-0.141570136	0.408696711	0.429	1	440570	LOC440570
-0.141827539	0.407838136	0.464	1	2794	GNL1
-0.141827539	0.407838136	0.499	1	8321	FZD1

voxel-wise, subcortex			
Spearman's corr coef	p-val	GenID	Gene
-0.096067637	4.76288E-18	1736	DKC1
-0.096068099	4.76114E-18	3852	KRT5
-0.096132644	4.52313E-18	10243	GPHN
-0.096160963	4.42243E-18	90338	ZNF160
-0.096208327	4.25898E-18	2353	FOS
-0.096214831	4.23698E-18	6281	S100A10
-0.096318372	3.90183E-18	10451	VAV3
-0.096344374	3.82187E-18	57453	DSCAML1
-0.096403629	3.64559E-18	260429	PRSS33
-0.096415423	3.61147E-18	55032	SLC35A5
-0.096429631	3.57079E-18	728819	LOC728819
-0.096512742	3.34178E-18	3553	IL1B
-0.096559733	3.21876E-18	92482	BBIP1
-0.096615084	3.07962E-18	55124	PIWIL2
-0.096620932	3.06523E-18	801	CALM1
-0.096750669	2.76328E-18	58505	OSTC
-0.096786141	2.68596E-18	10668	CGRRF1
-0.096837588	2.57761E-18	1822	ATN1
-0.096854307	2.54334E-18	7166	TPH1
-0.096889965	2.47171E-18	137868	SGCZ
-0.096901111	2.44975E-18	63917	GALNT11
-0.096907049	2.43813E-18	302	ANXA2
-0.096982554	2.29496E-18	64412	GZF1
-0.096986078	2.28848E-18	1353	COX11
-0.097080342	2.12182E-18	10950	BTG3
-0.09711238	2.06793E-18	63929	XPNPEP3
-0.097262911	1.83235E-18	541468	C1orf190
-0.097292908	1.78866E-18	207	AKT1
-0.097372495	1.67771E-18	57664	PLEKHA4
-0.097435087	1.59525E-18	7697	ZNF138
-0.097457744	1.5664E-18	1515	CTSL2
-0.09748143	1.53679E-18	10597	TRAPPC2P1
-0.097487465	1.52933E-18	165324	UBXN2A
-0.097490251	1.52589E-18	6453	ITSN1
-0.09752591	1.48266E-18	23184	MESDC2
-0.097567052	1.43428E-18	130749	CPO
-0.097631223	1.3619E-18	79665	DHX40
-0.097689636	1.29916E-18	83697	SLC4A9
-0.097700685	1.28762E-18	8218	CLTCL1
-0.097774327	1.21323E-18	7617	ZNF66P
-0.097809337	1.17938E-18	3018	HIST1H2BB
-0.097822987	1.16643E-18	1755	DMBT1
-0.097857349	1.13446E-18	440023	KRTAP5-6
-0.097863942	1.12842E-18	558	AXL
-0.097898118	1.09764E-18	23536	ADAT1
-0.097928017	1.07139E-18	4336	MOBP
-0.097936377	1.06417E-18	79956	ERMP1
-0.097947523	1.05461E-18	4245	MGAT1
-0.097957738	1.04592E-18	161357	MDGA2
-0.098007977	1.00422E-18	9792	SERTAD2
-0.098022372	9.92574E-19	51142	CHCHD2
-0.098047264	9.72748E-19	100287497	RP11-504P24.4
-0.09819492	8.62943E-19	23629	BRD7P3
-0.098205134	8.55814E-19	151056	PLB1
-0.098210707	8.51958E-19	51117	COQ4
-0.098235965	8.34659E-19	23512	SUZ12
-0.098266892	8.13954E-19	353497	POLN
-0.09826754	8.13525E-19	4122	MAN2A2
-0.098319732	7.7975E-19	29063	ZCCHC4
-0.098323628	7.77278E-19	8347	HIST1H2BC
-0.098440267	7.06931E-19	51614	ERGIC3
-0.098462649	6.94173E-19	7976	FZD3
-0.098480202	6.84324E-19	26823	RNU12-2P
-0.098517068	6.64084E-19	27316	RBMX
-0.098581426	6.30163E-19	27030	MLH3
-0.09858904	6.26262E-19	9517	SPTLC2
-0.098600924	6.20233E-19	2298	FOXD4
-0.098688036	5.77699E-19	23078	KIAA0564
-0.098699734	5.72212E-19	7110	TMF1
-0.098704197	5.70134E-19	84561	SLC12A8
-0.098734744	5.56092E-19	57184	C15orf17
-0.09873651	5.55297E-19	253461	ZBTB38
-0.098736696	5.55207E-19	5476	CTSA
-0.098771706	5.39564E-19	124995	MRPL10
-0.098802537	5.2615E-19	79763	ISOC2
-0.098804027	5.25512E-19	1351	COX8A
-0.098811358	5.22372E-19	51338	MS4A4A
-0.098850273	5.06024E-19	79613	TMCO7
-0.098887794	4.9074E-19	55599	RNPC3

region-wise, subcortex					
Spearman's corr coef	p-val	p-val, SA	p-val, SA (BH- correction)	GenID	Gene
-0.142084941	0.406980574	0.42	1	34	ACADM
-0.142084941	0.406980574	0.421	1	8892	EIF2B2
-0.142084941	0.406980574	0.44	1	83937	RASSF4
-0.142084941	0.406980574	0.435	1	85318	BAGE3
-0.142084941	0.406980574	0.423	1	154791	C7orf55
-0.142342329	0.406124055	0.455	1	6443	SGCB
-0.142342329	0.406124055	0.455	1	80318	GKAP1
-0.142342329	0.406124055	0.461	1	284443	ZNF493
-0.142342329	0.406124055	0.432	1	388531	RGS9BP
-0.142342329	0.406124055	0.47	1	100132249	LOC100132249
-0.142599732	0.405268222	0.429	1	9321	TRIP11
-0.142599732	0.405268222	0.421	1	9441	MED26
-0.142599732	0.405268222	0.435	1	11177	BAZ1A
-0.142599732	0.405268222	0.445	1	56996	SLC12A9
-0.142599732	0.405268222	0.462	1	253714	C6orf167
-0.142599732	0.405268222	0.452	1	284119	PTRF
-0.142599732	0.405268222	0.432	1	730092	RRN3P1
-0.142599732	0.405268222	0.444	1	100271715	AC019171.4
-0.142857134	0.40441379	0.444	1	2188	FANCF
-0.142857134	0.40441379	0.438	1	58496	LY6G5B
-0.142857134	0.40441379	0.407	1	89853	FAM125B
-0.142857134	0.40441379	0.432	1	146705	C17orf56
-0.143114537	0.40356046	0.44	1	3021	H3F3B
-0.143114537	0.40356046	0.446	1	7568	ZNF20
-0.143114537	0.40356046	0.449	1	7802	DNALI1
-0.143114537	0.40356046	0.445	1	55819	RNF130
-0.143114537	0.40356046	0.425	1	84286	TMEM175
-0.143114537	0.40356046	0.453	1	283651	HMGN2P46
-0.143114537	0.40356046	0.445	1	646498	RP11-694I15.6
-0.14337194	0.402708083	0.447	1	5093	PCBP1
-0.14337194	0.402708083	0.463	1	5878	RAB5C
-0.14337194	0.402708083	0.468	1	56101	PCDHGB5
-0.14337194	0.402708083	0.446	1	57798	GATAD1
-0.14337194	0.402708083	0.448	1	79739	TTLL7
-0.143629342	0.401856393	0.422	1	4691	NCL
-0.143629342	0.401856393	0.432	1	4720	NDUFS2
-0.143629342	0.401856393	0.445	1	7693	ZNF134
-0.143629342	0.401856393	0.446	1	50964	SOST
-0.143629342	0.401856393	0.441	1	51573	GDE1
-0.143629342	0.401856393	0.421	1	58512	DLGAP3
-0.143629342	0.401856393	0.411	1	115752	DIS3L
-0.143629342	0.401856393	0.456	1	441631	TSPAN11
-0.143629342	0.401856393	0.44	1	492311	C5orf53
-0.143886745	0.401006162	0.467	1	3712	IVD
-0.143886745	0.401006162	0.386	1	7518	XRCC4
-0.143886745	0.401006162	0.442	1	11267	SNF8
-0.143886745	0.401006162	0.443	1	22900	CARD8
-0.143886745	0.401006162	0.428	1	80267	EDEM3
-0.143886745	0.401006162	0.434	1	137814	NKX2-6
-0.143886745	0.401006162	0.44	1	284992	CCDC150
-0.143886745	0.401006162	0.433	1	399900	LOC399900
-0.143886745	0.401006162	0.44	1	399909	PCNXL3
-0.144144148	0.400156945	0.433	1	58	ACTA1
-0.144144148	0.400156945	0.443	1	3605	IL17A
-0.144144148	0.400156945	0.45	1	5450	POU2AF1
-0.144144148	0.400156945	0.419	1	10598	AHSA1
-0.144144148	0.400156945	0.43	1	23132	RAD54L2
-0.144144148	0.400156945	0.448	1	23155	CLCC1
-0.144144148	0.400156945	0.463	1	28984	C13orf15
-0.144144148	0.400156945	0.407	1	29105	C16orf80
-0.144144148	0.400156945	0.419	1	50814	NSDHL
-0.14440155	0.39930883	0.455	1	643	CXCR5
-0.14440155	0.39930883	0.466	1	7398	USP1
-0.14440155	0.39930883	0.442	1	9275	BCL7B
-0.14440155	0.39930883	0.44	1	10171	RCL1
-0.14440155	0.39930883	0.428	1	51024	FIS1
-0.14440155	0.39930883	0.432	1	54879	ST7L
-0.14440155	0.39930883	0.418	1	55212	BBS7
-0.14440155	0.39930883	0.474	1	84700	MYO18B
-0.14440155	0.39930883	0.442	1	100506658	OCLN
-0.144658953	0.398461342	0.429	1	3105	HLA-A
-0.144658953	0.398461342	0.434	1	8099	CDK2AP1
-0.144658953	0.398461342	0.438	1	23405	DICER1
-0.144658953	0.398461342	0.434	1	55353	LAPTM4B
-0.144658953	0.398461342	0.43	1	55529	TMEM55A
-0.144658953	0.398461342	0.413	1	150696	PROM2
-0.144658953	0.398461342	0.451	1	191585	PLAC4
-0.144916341	0.397615254	0.411	1	359	AQP2
-0.144916341	0.397615254	0.452	1	8481	OFD1

voxel-wise, subcortex			
Spearman's corr coef	p-val	GenID	Gene
-0.098918155	4.78711E-19	6720	SREBF1
-0.099131376	4.02041E-19	6493	SIM2
-0.099139921	3.99235E-19	29926	GMPPA
-0.09919332	3.82139E-19	55092	TMEM51
-0.099209942	3.76964E-19	91775	NXPE3
-0.099214397	3.75585E-19	6707	SPRR3
-0.09925656	3.62816E-19	79575	ABHD8
-0.099276617	3.56895E-19	84284	NTPCR
-0.099280521	3.55753E-19	25834	MGAT4C
-0.099300951	3.49837E-19	283796	GOLGA9P
-0.099321567	3.4397E-19	57165	GJC2
-0.09939976	3.22572E-19	64780	MICAL1
-0.099439412	3.12227E-19	10735	STAG2
-0.099448144	3.09993E-19	339390	CLEC4G
-0.099555962	2.83685E-19	9179	AP4M1
-0.099661827	2.60006E-19	221830	TWISTNB
-0.099666104	2.59089E-19	10712	C1orf2
-0.099670097	2.58239E-19	2901	GRIK5
-0.099678636	2.56426E-19	6782	HSPA13
-0.099723674	2.47085E-19	1763	DNA2
-0.099757113	2.40366E-19	152110	NEK10
-0.099813201	2.29498E-19	8355	HIST1H3G
-0.099827409	2.26824E-19	2329	FMO4
-0.099880159	2.1716E-19	389337	AC022100.1
-0.099881828	2.16861E-19	9060	PAPSS2
-0.099931322	2.08174E-19	649946	RPL23AP64
-0.099973209	2.01093E-19	27031	NPHP3
-0.099998377	1.96953E-19	344405	NCRNA00117
-0.100024566	1.92735E-19	57556	SEMA6A
-0.100052238	1.88375E-19	7458	EIF4H
-0.100064777	1.86432E-19	55041	PLEKHB2
-0.100070164	1.85601E-19	10591	C6orf108
-0.100162469	1.71951E-19	7597	ZBTB25
-0.100228496	1.628E-19	79627	OGFRL1
-0.100256912	1.59012E-19	57494	AC092490.2
-0.100257009	1.58998E-19	286380	FOXD4L3
-0.10027688	1.56402E-19	115019	SLC26A9
-0.100297034	1.5381E-19	6390	SDHB
-0.100315794	1.51437E-19	29119	CTNNA3
-0.100326009	1.50158E-19	3480	IGF1R
-0.100354798	1.46615E-19	5519	PPP2R1B
-0.100378379	1.43776E-19	9882	TBC1D4
-0.10041599	1.39358E-19	158248	TTC16
-0.100421846	1.38681E-19	286148	DPY19L4
-0.100657448	1.1402E-19	254228	FAM26E
-0.100715116	1.08678E-19	91975	ZNF300
-0.100843087	9.76872E-20	400579	FLJ35934
-0.100854695	9.6746E-20	4485	MST1
-0.100860916	9.62461E-20	63027	SLC22A23
-0.100925088	9.12304E-20	146456	TMED6
-0.100954987	8.89827E-20	57820	CCNB1IP1
-0.100963622	8.83431E-20	90987	ZNF251
-0.100979224	8.72005E-20	9470	EIF4E2
-0.1010186	8.43803E-20	84060	C7orf64
-0.10102696	8.37935E-20	116541	MRPL54
-0.101048321	8.23118E-20	644890	MEIG1
-0.101049244	8.22484E-20	27309	ZNF330
-0.101068839	8.09126E-20	29074	MRPL18
-0.101084538	7.98585E-20	5698	PSMB9
-0.10111212	7.80391E-20	5134	PDCD2
-0.101180561	7.36986E-20	51063	CALHM2
-0.101209067	7.19623E-20	9694	TTC35
-0.10121882	7.13775E-20	7957	EPM2A
-0.10123089	7.06602E-20	10285	SMNDC1
-0.101235814	7.03697E-20	51070	NOSIP
-0.101262189	6.88336E-20	90933	TRIM41
-0.101392105	6.17365E-20	84223	IQCG
-0.101425909	6.00111E-20	715	C1R
-0.101463802	5.81345E-20	9796	AC105206.1
-0.101497978	5.64919E-20	1281	COL3A1
-0.101499371	5.64256E-20	7290	HIRA
-0.10150215	5.62944E-20	3092	HIP1
-0.101605237	5.16275E-20	5836	PYGL
-0.101626128	5.07296E-20	10562	OLFM4
-0.101626225	5.07253E-20	55582	KIF27
-0.101641826	5.00652E-20	30827	CXXC1
-0.101688631	4.81339E-20	116228	FAM36A
-0.101696894	4.78005E-20	81894	SLC25A28
-0.101698101	4.77523E-20	390261	OR6M1

region-wise, subcortex					
Spearman's corr coef	p-val	p-val, SA	p-val, SA (BH- correction)	GenID	Gene
-0.144916341	0.397615254	0.438	1	55602	CDKN2AIP
-0.145173743	0.396770269	0.444	1	271	AMPD2
-0.145173743	0.396770269	0.465	1	3112	HLA-DOB
-0.145173743	0.396770269	0.422	1	6527	SLC5A4
-0.145173743	0.396770269	0.434	1	10226	M6PRBP1
-0.145173743	0.396770269	0.424	1	10903	MTMR11
-0.145173743	0.396770269	0.449	1	55644	OSGEP
-0.145173743	0.396770269	0.423	1	55727	BTBD7
-0.145173743	0.396770269	0.435	1	60370	AVPI1
-0.145173743	0.396770269	0.432	1	85395	C21orf70
-0.145173743	0.396770269	0.438	1	117584	RFFL
-0.145173743	0.396770269	0.456	1	201232	SLC16A13
-0.145173743	0.396770269	0.475	1	223075	CCDC129
-0.145173743	0.396770269	0.453	1	283600	C14orf68
-0.145173743	0.396770269	0.426	1	340706	VWA2
-0.145173743	0.396770269	0.445	1	441459	ANKRD18B
-0.145173743	0.396770269	0.409	1	100132288	TEKT4P2
-0.145431146	0.395926267	0.473	1	5238	PGM3
-0.145431146	0.395926267	0.429	1	5692	PSMB4
-0.145431146	0.395926267	0.42	1	10617	STAMBP
-0.145431146	0.395926267	0.447	1	10768	AHCYL1
-0.145431146	0.395926267	0.417	1	55254	TMEM39A
-0.145431146	0.395926267	0.452	1	139425	WDR42B
-0.145431146	0.395926267	0.457	1	441250	TYW1B
-0.145688549	0.395083398	0.41	1	2202	EFEMP1
-0.145688549	0.395083398	0.428	1	8795	TNFRSF10B
-0.145688549	0.395083398	0.444	1	84798	C19orf48
-0.145688549	0.395083398	0.445	1	360023	ZBTB41
-0.145945951	0.394241154	0.441	1	23381	SMG5
-0.145945951	0.394241154	0.438	1	55250	ELP2
-0.145945951	0.394241154	0.438	1	128272	ARHGEF19
-0.145945951	0.394241154	0.413	1	196441	ZFC3H1
-0.146203339	0.393400311	0.445	1	7100	TLR5
-0.146203339	0.393400311	0.42	1	64288	ZNF323
-0.146203339	0.393400311	0.43	1	283491	OR7E156P
-0.146203339	0.393400311	0.397	1	494115	RBMXL1
-0.146460742	0.392560571	0.419	1	10220	GDF11
-0.146460742	0.392560571	0.442	1	10793	ZNF273
-0.146460742	0.392560571	0.426	1	85365	ALG2
-0.146460742	0.392560571	0.421	1	89857	KLHL6
-0.146460742	0.392560571	0.442	1	169693	C9orf71
-0.146460742	0.392560571	0.395	1	619189	SERINC4
-0.146718144	0.391721845	0.412	1	3207	HOXA11
-0.146718144	0.391721845	0.431	1	5663	PSEN1
-0.146718144	0.391721845	0.42	1	7289	TULP3
-0.146718144	0.391721845	0.419	1	11145	PLA2G16
-0.146718144	0.391721845	0.453	1	23385	NCSTN
-0.146718144	0.391721845	0.42	1	29928	TIMM22
-0.146718144	0.391721845	0.428	1	100129827	AC009532.1
-0.146975547	0.390883863	0.415	1	4715	NDUFB9
-0.146975547	0.390883863	0.428	1	7554	ZNF8
-0.146975547	0.390883863	0.45	1	9410	SNRNP40
-0.146975547	0.390883863	0.436	1	10876	FAM12A
-0.146975547	0.390883863	0.434	1	54681	P4HTM
-0.146975547	0.390883863	0.436	1	123606	NIPA1
-0.14723295	0.390047222	0.405	1	5545	PRB4
-0.14723295	0.390047222	0.397	1	6573	SLC19A1
-0.14723295	0.390047222	0.409	1	7771	ZFP112
-0.14723295	0.390047222	0.461	1	8332	HIST1H2AL
-0.14723295	0.390047222	0.442	1	9526	MPDU1
-0.14723295	0.390047222	0.408	1	9590	AKAP12
-0.14723295	0.390047222	0.444	1	10161	LPAR6
-0.14723295	0.390047222	0.432	1	10514	MYBBP1A
-0.14723295	0.390047222	0.422	1	80258	EFHC2
-0.147490337	0.389211655	0.416	1	5198	PFAS
-0.147490337	0.389211655	0.438	1	55361	PI4K2A
-0.147490337	0.389211655	0.417	1	84273	NOA1
-0.147490337	0.389211655	0.442	1	84932	RAB2B
-0.147490337	0.389211655	0.41	1	91351	DDX60L
-0.14774774	0.38837716	0.414	1	22	ABCB7
-0.14774774	0.38837716	0.441	1	3190	HNRNPK
-0.14774774	0.38837716	0.421	1	5981	RFC1
-0.14774774	0.38837716	0.431	1	6637	SNRPG
-0.14774774	0.38837716	0.426	1	6862	T
-0.14774774	0.38837716	0.451	1	9019	MPZL1
-0.14774774	0.38837716	0.422	1	246744	STH
-0.14774774	0.38837716	0.406	1	339768	ESPNL
-0.148005143	0.38754335	0.443	1	1889	ECE1
-0.148005143	0.38754335	0.434	1	7054	TH

voxel-wise, subcortex			
Spearman's corr coef	p-val	GenID	Gene
-0.101726055	4.66429E-20	9878	TOX4
-0.101808704	4.35103E-20	3310	HSPA6
-0.101818457	4.31547E-20	401508	FAM74A4
-0.101850495	4.20066E-20	9045	RPL14
-0.10192813	3.93477E-20	80264	ZNF430
-0.101958588	3.83507E-20	54956	PARP16
-0.101960815	3.82786E-20	26230	TIAM2
-0.101991557	3.72995E-20	55732	C1orf112
-0.102002241	3.69652E-20	22915	MMRN1
-0.10201858	3.64595E-20	57180	ACTR3B
-0.102073468	3.48099E-20	2335	FN1
-0.102142647	3.28355E-20	100288570	AC108938.1
-0.102182768	3.17416E-20	382	ARF6
-0.102193817	3.14466E-20	220108	FAM124A
-0.102213971	3.09159E-20	652	BMP4
-0.102284268	2.91326E-20	51412	ACTL6B
-0.102387629	2.66934E-20	10897	YIF1A
-0.102425709	2.5847E-20	25816	TNFAIP8
-0.102478452	2.47177E-20	79590	MRPL24
-0.102509193	2.40823E-20	285150	AC062028.1
-0.102559432	2.30783E-20	22900	CARD8
-0.102578655	2.27054E-20	56104	PCDHGB1
-0.102624066	2.18475E-20	7409	VAV1
-0.102645986	2.1445E-20	25862	USP49
-0.102649324	2.13843E-20	160335	TMTC2
-0.102739222	1.98132E-20	10861	SLC26A1
-0.102741078	1.9782E-20	55118	CRTAC1
-0.102753244	1.95787E-20	7771	ZFP112
-0.102797359	1.88585E-20	27077	B9D1
-0.102892451	1.7394E-20	8099	CDK2AP1
-0.102919385	1.70001E-20	57677	ZFP14
-0.102921799	1.69653E-20	288	ANK3
-0.102927648	1.6881E-20	10608	MXD4
-0.102951422	1.65429E-20	23258	DENND5A
-0.102972127	1.6254E-20	202459	OSTCP1
-0.102978818	1.61616E-20	55342	STRBP
-0.102983363	1.60993E-20	6382	SDC1
-0.102991074	1.59939E-20	51191	HERC5
-0.103038251	1.53641E-20	9667	SAFB2
-0.103051811	1.51877E-20	57587	KIAA1430
-0.10305766	1.51123E-20	117178	SSX2IP
-0.103060633	1.50739E-20	128710	C20orf94
-0.103077441	1.48596E-20	55248	TMEM206
-0.103078462	1.48467E-20	27076	LYPD3
-0.103149131	1.39789E-20	58472	SQRDL
-0.103185818	1.35484E-20	5554	PRH1
-0.103207827	1.32963E-20	341883	LRRC9
-0.103213206	1.32354E-20	51678	MPP6
-0.103237078	1.29685E-20	10495	ENOX2
-0.103244044	1.28917E-20	57645	POGK
-0.10326577	1.26549E-20	399687	MYO18A
-0.103276916	1.25351E-20	55274	PHF10
-0.103360958	1.16669E-20	11331	PHB2
-0.103426613	1.10303E-20	1513	CTSK
-0.103483729	1.05045E-20	23064	SETX
-0.10352654	1.01267E-20	197258	FUK
-0.103553005	9.89991E-21	54067	C21orf49
-0.103555605	9.87791E-21	2272	FHIT
-0.103564613	9.80197E-21	4580	MTX1
-0.103608169	9.44315E-21	79156	PLEKHF1
-0.103629246	9.27421E-21	4008	LMO7
-0.103647448	9.13075E-21	1376	CPT2
-0.103651069	9.1024E-21	7543	ZFX
-0.103732333	8.48997E-21	282566	LINC00515
-0.103747189	8.38258E-21	654346	LGALS9C
-0.103761211	8.28239E-21	50863	OPCML
-0.103772447	8.20291E-21	4831	NME2
-0.10377951	8.15337E-21	7106	TSPAN4
-0.103806622	7.96594E-21	51360	MBTPS2
-0.103853986	7.64858E-21	9563	H6PD
-0.103863269	7.58783E-21	85415	RHPN2
-0.103877574	7.49529E-21	57511	COG6
-0.103943415	7.08313E-21	55916	NXT2
-0.103965424	6.95041E-21	7069	THRSP
-0.103977494	6.87869E-21	79603	LASS4
-0.103978612	6.87215E-21	643387	LOC643387
-0.104015015	6.66035E-21	6610	SMPD2
-0.104147069	5.94512E-21	23116	FAM179B
-0.104219131	5.58743E-21	84886	C1orf198

region-wise, subcortex					
Spearman's corr coef	p-val	p-val, SA	p-val, SA (BH- correction)	GenID	Gene
-0.148005143	0.38754335	0.448	1	29103	DNAJC15
-0.148005143	0.38754335	0.431	1	51312	SLC25A37
-0.148005143	0.38754335	0.414	1	55788	LMBRD1
-0.148005143	0.38754335	0.416	1	84214	RP13-254B10.1
-0.148005143	0.38754335	0.426	1	143903	LAYN
-0.148005143	0.38754335	0.401	1	253461	ZBTB38
-0.148262545	0.386711001	0.44	1	2670	GFAP
-0.148262545	0.386711001	0.423	1	3665	IRF7
-0.148262545	0.386711001	0.44	1	8942	KYNU
-0.148262545	0.386711001	0.395	1	23221	RHOBTB2
-0.148262545	0.386711001	0.436	1	26648	OR7E24
-0.148262545	0.386711001	0.401	1	51520	LARS
-0.148262545	0.386711001	0.441	1	57719	ANO8
-0.148262545	0.386711001	0.432	1	79169	C1orf35
-0.148262545	0.386711001	0.45	1	116512	MRGPRD
-0.148262545	0.386711001	0.433	1	134492	NUDCD2
-0.148262545	0.386711001	0.441	1	148254	ZNF555
-0.148262545	0.386711001	0.421	1	340277	C7orf46
-0.148262545	0.386711001	0.421	1	729046	LOC729046
-0.148519948	0.385879636	0.406	1	190	NR0B1
-0.148519948	0.385879636	0.471	1	2657	GDF1
-0.148519948	0.385879636	0.425	1	7284	TUFM
-0.148519948	0.385879636	0.433	1	23704	KCNE4
-0.148519948	0.385879636	0.386	1	58533	SNX6
-0.148519948	0.385879636	0.452	1	148345	C1orf127
-0.148777336	0.385049373	0.447	1	293	SLC25A6
-0.148777336	0.385049373	0.433	1	2000	ELF4
-0.148777336	0.385049373	0.396	1	3135	HLA-G
-0.148777336	0.385049373	0.428	1	6747	SSR3
-0.148777336	0.385049373	0.433	1	8358	HIST1H3B
-0.148777336	0.385049373	0.398	1	11099	PTPN21
-0.148777336	0.385049373	0.461	1	79750	ZNF385D
-0.148777336	0.385049373	0.429	1	81790	RNF170
-0.148777336	0.385049373	0.433	1	254050	LRRC43
-0.148777336	0.385049373	0.392	1	373863	DND1
-0.149034739	0.384220213	0.428	1	3930	LBR
-0.149034739	0.384220213	0.406	1	10715	LASS1
-0.149034739	0.384220213	0.446	1	11014	KDELR2
-0.149034739	0.384220213	0.444	1	64864	RFX7
-0.149292141	0.383391589	0.419	1	6505	SLC1A1
-0.149292141	0.383391589	0.419	1	51691	NAA38
-0.149292141	0.383391589	0.43	1	84675	TRIM55
-0.149292141	0.383391589	0.429	1	93349	SP140L
-0.149292141	0.383391589	0.406	1	200894	ARL13B
-0.149549544	0.382564515	0.405	1	221	ALDH3B1
-0.149549544	0.382564515	0.441	1	4331	MNAT1
-0.149549544	0.382564515	0.407	1	5494	PPM1A
-0.149549544	0.382564515	0.421	1	6120	RPE
-0.149549544	0.382564515	0.444	1	80031	SEMA6D
-0.149549544	0.382564515	0.417	1	84820	POLR2J4
-0.149549544	0.382564515	0.401	1	728448	PPIEL
-0.149806947	0.381738514	0.448	1	1503	CTPS
-0.149806947	0.381738514	0.384	1	6125	RPL5
-0.149806947	0.381738514	0.437	1	10549	PRDX4
-0.149806947	0.381738514	0.441	1	80303	EFHD1
-0.149806947	0.381738514	0.41	1	284532	OR14A16
-0.150064334	0.380913496	0.433	1	8886	DDX18
-0.150064334	0.380913496	0.402	1	9529	BAG5
-0.150064334	0.380913496	0.4	1	11157	LSM6
-0.150064334	0.380913496	0.43	1	25932	CLIC4
-0.150064334	0.380913496	0.442	1	113402	SFT2D1
-0.150064334	0.380913496	0.43	1	286042	AC068020.3
-0.150064334	0.380913496	0.436	1	100130449	AC110619.2
-0.150321737	0.380089194	0.397	1	7965	AIMP2
-0.150321737	0.380089194	0.441	1	8687	KRT38
-0.150321737	0.380089194	0.417	1	10058	ABCB6
-0.150321737	0.380089194	0.418	1	54964	C1orf56
-0.150321737	0.380089194	0.391	1	56851	C15orf24
-0.150321737	0.380089194	0.432	1	132228	C3orf45
-0.150321737	0.380089194	0.421	1	353131	LCE1A
-0.150321737	0.380089194	0.399	1	654346	LGALS9C
-0.150579154	0.379266351	0.419	1	2733	GLE1
-0.150579154	0.379266351	0.419	1	5775	PTPN4
-0.150579154	0.379266351	0.408	1	54969	C4orf27
-0.150579154	0.379266351	0.407	1	57570	TRMT5
-0.150579154	0.379266351	0.414	1	79811	SLTM
-0.150579154	0.379266351	0.407	1	84335	AKT1S1
-0.150579154	0.379266351	0.414	1	91316	AP000347.4
-0.150579154	0.379266351	0.401	1	137209	ZNF572

voxel-wise, subcortex			
Spearman's corr coef	p-val	GenID	Gene
-0.104227863	5.54554E-21	10023	FRAT1
-0.104230464	5.53313E-21	5450	POU2AF1
-0.104314603	5.14605E-21	6522	SLC4A2
-0.104341067	5.02992E-21	3636	INPPL1
-0.104354441	4.97227E-21	6929	TCF3
-0.104421489	4.69277E-21	55093	WDYHV1
-0.104439415	4.6207E-21	51239	ANKRD39
-0.104462907	4.52791E-21	23052	ENDOD1
-0.104484357	4.44484E-21	8343	HIST1H2BF
-0.104508415	4.35344E-21	83759	RBM4B
-0.104527265	4.2831E-21	54892	NCAPG2
-0.104537666	4.24477E-21	5324	PLAG1
-0.104587905	4.06442E-21	30815	ST6GALNAC6
-0.104625329	3.93496E-21	8303	SNN
-0.104626447	3.93116E-21	2760	GM2A
-0.104674645	3.77065E-21	745	C11orf9
-0.104684114	3.73986E-21	8673	VAMP8
-0.104703799	3.6767E-21	10520	ZNF211
-0.10477113	3.46851E-21	284325	C19orf54
-0.104785427	3.42582E-21	786	CACNG1
-0.104818955	3.32776E-21	27148	STK36
-0.104870871	3.18134E-21	283254	HARBI1
-0.104875043	3.16987E-21	23411	SIRT1
-0.104884051	3.14521E-21	221613	HIST1H2AA
-0.104974508	2.90787E-21	6139	RPL17P20
-0.105013512	2.8111E-21	84681	HINT2
-0.105047964	2.72826E-21	55905	RNF114
-0.105051123	2.72078E-21	6275	S100A4
-0.105064586	2.68915E-21	8225	GTPBP6
-0.105088733	2.63335E-21	55657	ZNF692
-0.105092444	2.62487E-21	114799	ESCO1
-0.105136462	2.52637E-21	56899	ANKS1B
-0.105145283	2.50709E-21	5814	PURB
-0.105174541	2.44411E-21	128346	C1orf162
-0.105182156	2.42802E-21	9879	DDX46
-0.105218932	2.35158E-21	518	ATP5G3
-0.105226919	2.3353E-21	5106	PCK2
-0.105244003	2.30087E-21	57468	SLC12A5
-0.105286904	2.21655E-21	23131	GPATCH8
-0.105305851	2.18028E-21	120379	PIH1D2
-0.105309471	2.17344E-21	146852	ODF4
-0.105333157	2.12908E-21	130507	UBR3
-0.105335757	2.12425E-21	56963	RGMA
-0.105347827	2.10206E-21	440900	AC010982.1
-0.105443105	1.93463E-21	25837	RAB26
-0.105452858	1.91825E-21	3704	ITPA
-0.105491772	1.85426E-21	64795	RMND5A
-0.105535232	1.78529E-21	55888	ZNF167
-0.105596334	1.69258E-21	4352	MPL
-0.105599776	1.68751E-21	286042	AC068020.3
-0.105602555	1.68341E-21	84866	TMEM25
-0.105625682	1.64978E-21	6159	RPL29
-0.105656795	1.60555E-21	200185	KRTCAP2
-0.105686232	1.56481E-21	5638	PRRG1
-0.105699793	1.54636E-21	5442	POLRMT
-0.105704807	1.53961E-21	54471	SMCR7L
-0.105746225	1.48488E-21	283234	CCDC88B
-0.105811134	1.40295E-21	139081	MAGEC3
-0.105818659	1.39375E-21	79443	FYCO1
-0.105877347	1.32398E-21	6257	RXRB
-0.105882086	1.3185E-21	7528	YY1
-0.105886266	1.31369E-21	27109	ATP5S
-0.105900563	1.29736E-21	22891	ZNF365
-0.105902426	1.29525E-21	113177	C19orf36
-0.105917096	1.27871E-21	3665	IRF7
-0.105957307	1.23448E-21	6352	CCL5
-0.105990648	1.19894E-21	54851	ANKRD49
-0.106019065	1.16945E-21	8649	MAPKSP1
-0.1060706	1.1178E-21	389741	MGC21881
-0.106077567	1.111E-21	10422	UBAC1
-0.106081374	1.10729E-21	64856	VWA1
-0.1060993	1.09002E-21	389362	PSMG4
-0.106103852	1.08568E-21	6671	SP4
-0.10610608	1.08355E-21	3060	HCRT
-0.106109239	1.08055E-21	2339	FNTA
-0.106131338	1.05981E-21	10217	CTDSPL
-0.10615743	1.03583E-21	6617	SNAPC1
-0.106238134	9.64982E-22	23450	SF3B3
-0.106297754	9.15748E-22	4682	NUBP1

region-wise, subcortex					
Spearman's corr coef	p-val	p-val, SA	p-val, SA (BH- correction)	GenID	Gene
-0.150836557	0.378444523	0.427	1	358	AQP1
-0.150836557	0.378444523	0.424	1	3212	HOXB2
-0.150836557	0.378444523	0.405	1	5096	PCCB
-0.150836557	0.378444523	0.409	1	7024	TFCP2
-0.150836557	0.378444523	0.426	1	8673	VAMP8
-0.150836557	0.378444523	0.413	1	283933	ZNF843
-0.150836557	0.378444523	0.399	1	387695	C10orf99
-0.15109396	0.377623826	0.407	1	6847	SYCP1
-0.15109396	0.377623826	0.411	1	84545	MRPL43
-0.15109396	0.377623826	0.392	1	115106	HAUS1
-0.15109396	0.377623826	0.431	1	255104	TMCO4
-0.15109396	0.377623826	0.417	1	100133161	LOC100133161
-0.151351348	0.376803726	0.432	1	7546	ZIC2
-0.151351348	0.376803726	0.417	1	51065	RPS27L
-0.151351348	0.376803726	0.407	1	51071	DERA
-0.151351348	0.376803726	0.415	1	115019	SLC26A9
-0.151351348	0.376803726	0.429	1	415116	PIM3
-0.151351348	0.376803726	0.426	1	643637	GLYATL1P4
-0.15160875	0.375985116	0.38	1	6627	SNRPA1
-0.15160875	0.375985116	0.409	1	8115	TCL1A
-0.15160875	0.375985116	0.402	1	23239	PHLPP1
-0.15160875	0.375985116	0.401	1	79962	DNAJC22
-0.15160875	0.375985116	0.416	1	84529	C15orf41
-0.15160875	0.375985116	0.432	1	348487	FAM131C
-0.15160875	0.375985116	0.399	1	401827	MSLNL
-0.15160875	0.375985116	0.443	1	729852	LOC729852
-0.151866153	0.375167578	0.426	1	81	ACTN4
-0.151866153	0.375167578	0.419	1	3006	HIST1H1C
-0.151866153	0.375167578	0.435	1	8941	CDK5R2
-0.151866153	0.375167578	0.412	1	11269	DDX19B
-0.151866153	0.375167578	0.445	1	55568	GALNT10
-0.151866153	0.375167578	0.404	1	79612	NARG1L
-0.151866153	0.375167578	0.405	1	253970	SFTA3
-0.151866153	0.375167578	0.428	1	339221	ENPP7
-0.151866153	0.375167578	0.408	1	400961	PAIP2B
-0.152123556	0.374351114	0.389	1	1031	CDKN2C
-0.152123556	0.374351114	0.36	1	7716	VEZF1
-0.152123556	0.374351114	0.406	1	8073	PTP4A2
-0.152123556	0.374351114	0.438	1	8100	IFT88
-0.152123556	0.374351114	0.404	1	8639	AOC3
-0.152123556	0.374351114	0.422	1	10054	UBA2
-0.152123556	0.374351114	0.396	1	55278	QRSL1
-0.152123556	0.374351114	0.407	1	79723	SUV39H2
-0.152123556	0.374351114	0.421	1	150274	HSCB
-0.152123556	0.374351114	0.428	1	728537	AC017099.3
-0.152380958	0.373535663	0.398	1	718	C3
-0.152380958	0.373535663	0.465	1	2037	EPB41L2
-0.152380958	0.373535663	0.415	1	4716	NDUFB10
-0.152380958	0.373535663	0.412	1	10452	TOMM40
-0.152380958	0.373535663	0.465	1	23130	ATG2A
-0.152380958	0.373535663	0.382	1	25776	CBY1
-0.152380958	0.373535663	0.402	1	25998	IBTK
-0.152380958	0.373535663	0.41	1	26577	PCOLCE2
-0.152380958	0.373535663	0.434	1	57787	MARK4
-0.152380958	0.373535663	0.428	1	60436	TGIF2
-0.152380958	0.373535663	0.419	1	647087	C7orf73
-0.152638346	0.372720897	0.417	1	9098	USP6
-0.152638346	0.372720897	0.382	1	29994	BAZ2B
-0.152638346	0.372720897	0.431	1	55486	PARL
-0.152638346	0.372720897	0.412	1	55916	NXT2
-0.152638346	0.372720897	0.436	1	58513	EPS15L1
-0.152638346	0.372720897	0.407	1	79710	MORC4
-0.152638346	0.372720897	0.389	1	125111	GJD3
-0.152638346	0.372720897	0.409	1	613212	CTXN3
-0.152895749	0.371907622	0.406	1	5260	PHKG1
-0.152895749	0.371907622	0.395	1	8850	KAT2B
-0.152895749	0.371907622	0.414	1	9469	CHST3
-0.152895749	0.371907622	0.414	1	9741	LAPTM4A
-0.152895749	0.371907622	0.397	1	80342	TRAF3IP3
-0.152895749	0.371907622	0.405	1	257364	SNX33
-0.153153151	0.371095359	0.421	1	23283	CSTF2T
-0.153153151	0.371095359	0.429	1	50999	TMED5
-0.153153151	0.371095359	0.43	1	54951	COMMD8
-0.153153151	0.371095359	0.435	1	56956	LHX9
-0.153153151	0.371095359	0.404	1	127253	TYW3
-0.153153151	0.371095359	0.412	1	140625	ACTRT2
-0.153410554	0.3702842	0.407	1	1512	CTSH
-0.153410554	0.3702842	0.391	1	6575	SLC20A2
-0.153410554	0.3702842	0.39	1	26277	TINF2

voxel-wise, subcortex			
Spearman's corr coef	p-val	GenID	Gene
-0.106320508	8.97618E-22	51110	LACTB2
-0.106365547	8.62773E-22	80221	ACSF2
-0.106426649	8.17632E-22	5605	MAP2K2
-0.106431477	8.14168E-22	79963	ABCA11P
-0.106576443	7.16615E-22	280636	C11orf31
-0.106604211	6.99293E-22	55713	ZNF334
-0.106605418	6.98556E-22	10840	ALDH1L1
-0.10661006	6.95703E-22	27111	SDCBP2
-0.106620088	6.89582E-22	166378	SPATA5
-0.106681101	6.53472E-22	338657	CCDC84
-0.106683984	6.51809E-22	874	CBR3
-0.106725305	6.28484E-22	914	CD2
-0.106822446	5.7684E-22	55347	ABHD10
-0.106841393	5.67273E-22	84331	C16orf14
-0.106868975	5.5362E-22	506	ATP5B
-0.106870554	5.52843E-22	643198	RBPJP6
-0.106888101	5.44342E-22	7478	WNT8A
-0.106920049	5.29189E-22	54629	FAM63B
-0.106925435	5.26674E-22	166379	BBS12
-0.107078664	4.59918E-22	80216	ALPK1
-0.107125007	4.41432E-22	9134	CCNE2
-0.107131965	4.38721E-22	29880	ALG5
-0.10713698	4.36779E-22	285613	RELL2
-0.107199758	4.13147E-22	3768	KCNJ12
-0.107210256	4.09323E-22	7594	ZNF43
-0.107229941	4.02241E-22	79691	QTRTD1
-0.107236534	3.99896E-22	644538	Z83826.4
-0.107240252	3.98584E-22	10579	TACC2
-0.107307948	3.75362E-22	8648	NCOA1
-0.107344635	3.63337E-22	400961	PAIP2B
-0.107392088	3.48353E-22	54209	TREM2
-0.107432857	3.35972E-22	80896	NPL
-0.107446693	3.31866E-22	6191	RPS4X
-0.10745997	3.27976E-22	55168	MRPS18A
-0.107529342	3.08368E-22	81788	NUAK2
-0.107582644	2.94096E-22	79960	PHF17
-0.107606329	2.87965E-22	1906	EDN1
-0.107693247	2.66532E-22	5010	CLDN11
-0.107698634	2.65258E-22	157574	FBXO16
-0.107706062	2.63507E-22	7942	TFEB
-0.107712291	2.62051E-22	80347	COASY
-0.107730396	2.57862E-22	26959	HBP1
-0.107791319	2.44239E-22	29950	SERTAD1
-0.107851863	2.31408E-22	83746	L3MBTL2
-0.107883252	2.25022E-22	644397	LOC644397
-0.107885577	2.24556E-22	1352	COX10
-0.107925229	2.16752E-22	8284	KDM5D
-0.107972778	2.07747E-22	55031	USP47
-0.108109474	1.83872E-22	6434	TRA2B
-0.10811802	1.82473E-22	10478	SLC25A17
-0.108142719	1.78489E-22	142679	DUSP19
-0.108143561	1.78354E-22	3185	HNRNPF
-0.108187765	1.71443E-22	4081	MAB21L1
-0.108226858	1.65552E-22	1974	EIF4A2
-0.108230576	1.65002E-22	60625	DHX35
-0.108246826	1.62619E-22	10595	ERN2
-0.108274035	1.58708E-22	10443	N4BP2L2
-0.108371176	1.45485E-22	374462	PTPRQ
-0.108375162	1.44967E-22	386724	AMIGO3
-0.108393274	1.42634E-22	78996	C7orf49
-0.108448438	1.35753E-22	57474	ZNF490
-0.108503416	1.29223E-22	113157	RPLP0P2
-0.108552076	1.23702E-22	169200	TMEM64
-0.10855449	1.23435E-22	6421	SFPQ
-0.108564146	1.22369E-22	55199	FAM86C
-0.108587734	1.19806E-22	100128361	LOC100128361
-0.108659796	1.123E-22	119392	C10orf78
-0.108668253	1.11449E-22	54443	ANLN
-0.108672522	1.11023E-22	8601	RGS20
-0.108694807	1.08823E-22	375611	SLC26A5
-0.108730845	1.05354E-22	54883	CCDC49
-0.108794361	9.95066E-23	148327	CREB3L4
-0.108801514	9.8869E-23	11167	FSTL1
-0.108807087	9.83735E-23	441250	TYW1B
-0.108819343	9.72946E-23	26769	SNORD81
-0.108824268	9.68652E-23	55069	C7orf42
-0.108828351	9.65101E-23	4173	MCM4
-0.108843304	9.52196E-23	57501	KIAA1257
-0.10890989	8.96809E-23	57035	C1orf63

region-wise, subcortex					
Spearman's corr coef	p-val	p-val, SA	p-val, SA (BH- correction)	GenID	Gene
-0.153410554	0.3702842	0.406	1	57530	CGN
-0.153410554	0.3702842	0.434	1	59283	CACNG8
-0.153410554	0.3702842	0.41	1	81671	TMEM49
-0.153410554	0.3702842	0.452	1	283481	LOC283481
-0.153410554	0.3702842	0.417	1	388115	C15orf52
-0.153667957	0.369473726	0.427	1	1757	SARDH
-0.153667957	0.369473726	0.45	1	2070	EYA4
-0.153667957	0.369473726	0.409	1	3881	KRT31
-0.153667957	0.369473726	0.426	1	80205	CHD9
-0.153667957	0.369473726	0.426	1	84295	PHF6
-0.153667957	0.369473726	0.396	1	89122	TRIM4
-0.153667957	0.369473726	0.413	1	148867	SLC30A7
-0.153925344	0.368664712	0.413	1	2146	EZH2
-0.153925344	0.368664712	0.391	1	2760	GM2A
-0.153925344	0.368664712	0.403	1	55786	ZNF415
-0.153925344	0.368664712	0.412	1	94101	ORMDL1
-0.153925344	0.368664712	0.412	1	114880	OSBPL6
-0.153925344	0.368664712	0.395	1	130872	AHSA2
-0.153925344	0.368664712	0.407	1	164045	HFM1
-0.153925344	0.368664712	0.384	1	100131943	LOC100131943
-0.154182747	0.367856711	0.425	1	8352	HIST1H3C
-0.154182747	0.367856711	0.423	1	11314	CD300A
-0.154182747	0.367856711	0.415	1	57708	MIER1
-0.154182747	0.367856711	0.412	1	113201	CASC4
-0.154182747	0.367856711	0.417	1	115294	PCMTD1
-0.15444015	0.367049783	0.404	1	3516	RBPSUH
-0.15444015	0.367049783	0.398	1	5094	PCBP2
-0.15444015	0.367049783	0.407	1	5420	PODXL
-0.15444015	0.367049783	0.388	1	5927	KDM5A
-0.15444015	0.367049783	0.415	1	8458	TTF2
-0.15444015	0.367049783	0.408	1	9274	BCL7C
-0.15444015	0.367049783	0.407	1	9802	DAZAP2
-0.15444015	0.367049783	0.405	1	51099	ABHD5
-0.15444015	0.367049783	0.418	1	64764	CREB3L2
-0.15444015	0.367049783	0.393	1	91227	GGTLC2
-0.15444015	0.367049783	0.406	1	285830	HLA-F-AS1
-0.154697552	0.366243631	0.41	1	2487	FRZB
-0.154697552	0.366243631	0.432	1	5196	PF4
-0.154697552	0.366243631	0.42	1	5825	ABCD3
-0.154697552	0.366243631	0.41	1	8859	STK19
-0.154697552	0.366243631	0.413	1	23097	CDC2L6
-0.154697552	0.366243631	0.386	1	79066	METTL16
-0.154697552	0.366243631	0.42	1	79133	C20orf7
-0.154697552	0.366243631	0.4	1	196051	PPAPDC1A
-0.154954955	0.365438849	0.4	1	100	ADA
-0.154954955	0.365438849	0.403	1	1979	EIF4EBP2
-0.154954955	0.365438849	0.423	1	6792	CDKL5
-0.154954955	0.365438849	0.366	1	7705	ZNF146
-0.154954955	0.365438849	0.432	1	8277	TKTL1
-0.154954955	0.365438849	0.392	1	8518	IKBKAP
-0.154954955	0.365438849	0.383	1	9988	DMTF1
-0.154954955	0.365438849	0.413	1	10039	PARP3
-0.154954955	0.365438849	0.39	1	57580	PREX1
-0.154954955	0.365438849	0.442	1	60681	FKBP10
-0.154954955	0.365438849	0.418	1	255031	FLJ35390
-0.154954955	0.365438849	0.391	1	338328	GPIHBP1
-0.155212343	0.36463517	0.432	1	2099	ESR1
-0.155212343	0.36463517	0.393	1	4669	NAGLU
-0.155212343	0.36463517	0.396	1	5317	PKP1
-0.155212343	0.36463517	0.403	1	5365	PLXNB3
-0.155212343	0.36463517	0.415	1	10297	APC2
-0.155212343	0.36463517	0.387	1	11276	AP1GBP1
-0.155212343	0.36463517	0.443	1	55676	SLC30A6
-0.155212343	0.36463517	0.408	1	57661	PHRF1
-0.155212343	0.36463517	0.41	1	79054	TRPM8
-0.155469745	0.363832504	0.406	1	3163	HMOX2
-0.155469745	0.363832504	0.417	1	3234	HOXD8
-0.155469745	0.363832504	0.382	1	6100	RP9
-0.155469745	0.363832504	0.415	1	9403	RP4-604K5.1
-0.155727148	0.36303091	0.387	1	8797	TNFRSF10A
-0.155727148	0.36303091	0.384	1	51128	SAR1B
-0.155727148	0.36303091	0.417	1	51778	MYOZ2
-0.155727148	0.36303091	0.404	1	115201	ATG4A
-0.155727148	0.36303091	0.379	1	118987	PDZD8
-0.155727148	0.36303091	0.409	1	255394	TCP11L2
-0.155727148	0.36303091	0.386	1	401022	HOXD-AS1
-0.155727148	0.36303091	0.441	1	497190	CLEC18B
-0.155984551	0.362230092	0.401	1	3306	HSPA2
-0.155984551	0.362230092	0.38	1	9531	BAG3

voxel-wise, subcortex			
Spearman's corr coef	p-val	GenID	Gene
-0.108920008	8.88681E-23	9877	ZC3H11A
-0.109052435	7.88732E-23	51264	MRPL27
-0.109109737	7.49006E-23	6201	RPS7
-0.109235384	6.68716E-23	389058	SP5
-0.109255068	6.56933E-23	11189	CELF3
-0.109256275	6.5621E-23	308	ANXA5
-0.109257951	6.55224E-23	122060	SLAIN1
-0.109287761	6.37818E-23	54807	ZNF586
-0.109296307	6.32907E-23	57480	PLEKHG1
-0.109311812	6.24105E-23	643382	C14orf176
-0.109316453	6.21491E-23	5428	POLG
-0.10932342	6.17594E-23	3292	HSD17B1
-0.10938035	5.8661E-23	285527	FRYL
-0.109538868	5.08223E-23	132241	RPL32P3
-0.10955596	5.00419E-23	7159	TP53BP2
-0.109576665	4.91121E-23	767	CA8
-0.109657645	4.56365E-23	9840	KIAA0748
-0.109677888	4.4807E-23	6161	RPL32
-0.109705195	4.37112E-23	3902	LAG3
-0.109717451	4.32274E-23	91526	ANKRD44
-0.109745957	4.21246E-23	10171	RCL1
-0.109762587	4.14937E-23	51506	UFC1
-0.109870955	3.76055E-23	2063	NR2F6
-0.109908663	3.63396E-23	9851	KIAA0753
-0.109909125	3.6324E-23	51107	APH1A
-0.109930389	3.56288E-23	152404	IGSF11
-0.109940425	3.53056E-23	1138	CHRNA5
-0.110046849	3.20489E-23	284131	AC120024.1
-0.11009226	3.07515E-23	84872	ZC3H10
-0.110108234	3.03076E-23	79037	PVRIG
-0.110156611	2.90017E-23	6813	STXBP2
-0.110217623	2.74337E-23	100130109	SNRPEP4
-0.110253751	2.65453E-23	3570	IL6R
-0.110272698	2.60908E-23	22909	MTMR15
-0.110278085	2.59628E-23	154091	SLC2A12
-0.110406049	2.31024E-23	4217	MAP3K5
-0.11043939	2.24099E-23	1852	DUSP9
-0.110454716	2.20982E-23	9554	SEC22B
-0.110505417	2.10984E-23	9084	VCY1B
-0.110516749	2.0881E-23	50649	ARHGEF4
-0.110542469	2.0396E-23	10561	IFI44
-0.110574514	1.98075E-23	83666	PARP9
-0.110596612	1.94111E-23	100287825	LOC100287825
-0.110616393	1.90634E-23	85444	LRRCC1
-0.110648714	1.85079E-23	112817	NPL2
-0.110722728	1.72958E-23	55321	C20orf46
-0.110724956	1.72606E-23	55486	PARL
-0.110747799	1.69034E-23	442194	OR10C1
-0.110760704	1.67048E-23	3115	HLA-DPB1
-0.110804357	1.60501E-23	6000	RGS7
-0.110860445	1.52461E-23	199731	CADM4
-0.11093279	1.42678E-23	23660	ZKSCAN5
-0.110971421	1.37709E-23	117289	TAGAP
-0.110972352	1.37592E-23	26140	TTLL3
-0.110979125	1.36739E-23	11194	ABCB8
-0.110997051	1.34508E-23	114883	OSBPL9
-0.111031972	1.30267E-23	344148	AC010974.2
-0.111041255	1.29161E-23	8292	COLQ
-0.111046918	1.28491E-23	846	CASR
-0.111078404	1.24829E-23	55746	NUP133
-0.111102544	1.22091E-23	9274	BCL7C
-0.111132175	1.18814E-23	8651	SOCS1
-0.111249648	1.06651E-23	4782	NFIC
-0.111259863	1.05654E-23	79670	ZCCHC6
-0.111302674	1.01574E-23	7266	DNAJC7
-0.111342326	9.79334E-24	2091	AC005393.1
-0.111366376	9.57886E-24	1028	CDKN1C
-0.111388482	9.38578E-24	100287482	RP11-305M3.3
-0.111388572	9.38506E-24	4303	FOXO4
-0.111467697	8.72524E-24	113263	GLCCI1
-0.111498155	8.48376E-24	9380	GRHPR
-0.111572079	7.92475E-24	219983	OR4D6
-0.111580804	7.86118E-24	57381	RHOJ
-0.111607738	7.66822E-24	2160	F11
-0.111607827	7.66766E-24	64077	LHPP
-0.11163383	7.48579E-24	85457	KIAA1737
-0.111661881	7.29441E-24	593	BCKDHA
-0.111679427	7.17724E-24	3850	KRT3
-0.111831449	6.2367E-24	10845	CLPX

region-wise, subcortex					
Spearman's corr coef	p-val	p-val, SA	p-val, SA (BH- correction)	GenID	Gene
-0.155984551	0.362230092	0.409	1	25938	HEATR5A
-0.155984551	0.362230092	0.382	1	53904	MYO3A
-0.155984551	0.362230092	0.403	1	55783	FTSJD1
-0.155984551	0.362230092	0.399	1	57412	AS3MT
-0.155984551	0.362230092	0.421	1	119392	C10orf78
-0.155984551	0.362230092	0.405	1	140467	ZNF358
-0.155984551	0.362230092	0.401	1	399665	FAM102A
-0.155984551	0.362230092	0.397	1	646050	LOC646050
-0.156241953	0.361430645	0.397	1	38	ACAT1
-0.156241953	0.361430645	0.406	1	5542	PRB1
-0.156241953	0.361430645	0.364	1	5757	PTMA
-0.156241953	0.361430645	0.405	1	8887	TAX1BP1
-0.156241953	0.361430645	0.403	1	8932	MBD2
-0.156241953	0.361430645	0.427	1	23030	KDM4B
-0.156241953	0.361430645	0.396	1	29119	CTNNA3
-0.156241953	0.361430645	0.42	1	151963	C3orf59
-0.156499341	0.36063233	0.391	1	873	CBR1
-0.156499341	0.36063233	0.425	1	930	CD19
-0.156499341	0.36063233	0.398	1	2588	GALNS
-0.156499341	0.36063233	0.419	1	3455	IFNAR2
-0.156499341	0.36063233	0.402	1	4099	MAG
-0.156499341	0.36063233	0.41	1	64285	RHBDF1
-0.156499341	0.36063233	0.41	1	80183	C13orf18
-0.156499341	0.36063233	0.386	1	93233	CCDC114
-0.156756744	0.359834999	0.386	1	4880	NPPC
-0.156756744	0.359834999	0.406	1	6356	CCL11
-0.156756744	0.359834999	0.407	1	55540	IL17RB
-0.156756744	0.359834999	0.414	1	84895	FAM73B
-0.157014146	0.359038472	0.394	1	725	C4BPB
-0.157014146	0.359038472	0.391	1	9026	HIP1R
-0.157014146	0.359038472	0.404	1	9139	CBFA2T2
-0.157014146	0.359038472	0.415	1	55731	C17orf63
-0.157014146	0.359038472	0.416	1	84851	TRIM52
-0.157014146	0.359038472	0.364	1	91664	ZNF845
-0.157014146	0.359038472	0.408	1	128346	C1orf162
-0.157014146	0.359038472	0.401	1	200185	KRTCAP2
-0.157271549	0.358243316	0.407	1	3996	LLGL1
-0.157271549	0.358243316	0.407	1	5896	RAG1
-0.157271549	0.358243316	0.383	1	6206	RPS12
-0.157271549	0.358243316	0.395	1	25926	NOL11
-0.157271549	0.358243316	0.402	1	85009	MGC16025
-0.157271549	0.358243316	0.414	1	219699	UNC5B
-0.157271549	0.358243316	0.397	1	254427	C10orf47
-0.157271549	0.358243316	0.399	1	728255	KRTAP1-4
-0.157528967	0.357449234	0.416	1	8359	HIST1H4A
-0.157528967	0.357449234	0.395	1	79736	TEFM
-0.157528967	0.357449234	0.377	1	90668	LRRC16B
-0.157528967	0.357449234	0.407	1	162540	AC217771.1
-0.157528967	0.357449234	0.385	1	339344	MYPOP
-0.157528967	0.357449234	0.412	1	729330	OC90
-0.157528967	0.357449234	0.404	1	729769	UQCRHP2
-0.157786369	0.356656253	0.401	1	3104	ZBTB48
-0.157786369	0.356656253	0.405	1	4283	CXCL9
-0.157786369	0.356656253	0.4	1	5463	POU6F1
-0.157786369	0.356656253	0.397	1	65125	WNK1
-0.158043757	0.355863959	0.395	1	2110	ETFDH
-0.158043757	0.355863959	0.383	1	3643	INSR
-0.158043757	0.355863959	0.38	1	9446	GSTO1
-0.158043757	0.355863959	0.403	1	11221	DUSP10
-0.158043757	0.355863959	0.377	1	51271	UBAP1
-0.158043757	0.355863959	0.425	1	84455	EFCAB7
-0.158043757	0.355863959	0.396	1	338755	OR2AG2
-0.15830116	0.355073154	0.386	1	8496	PPFIBP1
-0.15830116	0.355073154	0.393	1	25963	TMEM87A
-0.15830116	0.355073154	0.388	1	79923	NANOGP8
-0.158558562	0.354283333	0.373	1	3067	HDC
-0.158558562	0.354283333	0.369	1	6648	SOD2
-0.158558562	0.354283333	0.415	1	10307	APBB3
-0.158558562	0.354283333	0.393	1	57447	NDRG2
-0.158558562	0.354283333	0.375	1	84725	PLEKHA8
-0.158558562	0.354283333	0.386	1	283150	FOXR1
-0.158558562	0.354283333	0.427	1	414899	BLID
-0.158815965	0.353494585	0.384	1	43	ACHE
-0.158815965	0.353494585	0.424	1	3779	KCNMB1
-0.158815965	0.353494585	0.39	1	8503	PIK3R3
-0.158815965	0.353494585	0.404	1	23390	ZDHHC17
-0.158815965	0.353494585	0.386	1	23676	SMPX
-0.158815965	0.353494585	0.383	1	259289	TAS2R43
-0.158815965	0.353494585	0.383	1	440050	KRTAP5-7

voxel-wise, subcortex			
Spearman's corr coef	p-val	GenID	Gene
-0.111837767	6.20031E-24	57337	SENP7
-0.112039194	5.14582E-24	7175	TPR
-0.112058692	5.05369E-24	148254	ZNF555
-0.112144686	4.66654E-24	29896	TRA2A
-0.112149335	4.64646E-24	883	CCBL1
-0.112157598	4.61098E-24	65083	NOL6
-0.112222232	4.34271E-24	2872	MKNK2
-0.112226784	4.32441E-24	4940	OAS3
-0.112230308	4.31028E-24	8864	PER2
-0.112258449	4.19915E-24	1495	CTNNA1
-0.112283058	4.10434E-24	158880	USP51
-0.112299591	4.04181E-24	90592	ZNF700
-0.112308316	4.00921E-24	3290	HSD11B1
-0.112333111	3.91791E-24	51272	BET1L
-0.112421989	3.60736E-24	10418	SPON1
-0.1124309	3.57762E-24	1473	CST5
-0.112482719	3.40931E-24	57130	ATP13A1
-0.112582549	3.10691E-24	80776	B9D2
-0.112617008	3.00879E-24	6738	TROVE2
-0.112622857	2.99248E-24	54556	ING3
-0.112645425	2.93022E-24	5981	RFC1
-0.112665482	2.87601E-24	5925	RB1
-0.112669848	2.86434E-24	7752	ZNF200
-0.112694733	2.79868E-24	2815	GP9
-0.112723522	2.7246E-24	387071	FAM136BP
-0.112780541	2.58355E-24	646962	HRCT1
-0.11284174	2.4402E-24	9368	SLC9A3R1
-0.112850845	2.41956E-24	90113	VWA5B2
-0.112859569	2.39994E-24	222183	AC005077.3
-0.112895139	2.32157E-24	23545	ATP6V0A2
-0.113080591	1.95226E-24	10672	GNA13
-0.113124795	1.87321E-24	7855	FZD5
-0.113125354	1.87222E-24	55619	DOCK10
-0.113151357	1.82722E-24	54790	TET2
-0.113198623	1.74818E-24	23036	ZNF292
-0.11320132	1.74377E-24	4820	NKTR
-0.11322955	1.69828E-24	729614	RP11-169K16.9
-0.113237254	1.68607E-24	391192	OR2L3
-0.113297246	1.59394E-24	3033	HADH
-0.113333374	1.5409E-24	2304	FOXE1
-0.113355197	1.5097E-24	345895	RSHL1
-0.113360673	1.50199E-24	51161	C3orf18
-0.11339578	1.45334E-24	51135	IRAK4
-0.113530435	1.28086E-24	2261	FGFR3
-0.113566376	1.23837E-24	65989	DLK2
-0.11358235	1.21992E-24	6302	TSPAN31
-0.113590986	1.21008E-24	51742	ARID4B
-0.113624975	1.17204E-24	374291	NDUFS7
-0.113639086	1.15661E-24	54596	L1TD1
-0.113659151	1.135E-24	90141	EFCAB11
-0.113688774	1.10384E-24	55333	SYNJ2BP
-0.113715239	1.07671E-24	3684	ITGAM
-0.113718584	1.07332E-24	1523	CUX1
-0.113745421	1.04658E-24	153328	AC011427.1
-0.113768362	1.02424E-24	55775	TDP1
-0.113796778	9.97211E-25	83591	THAP2
-0.113812283	9.82775E-25	3065	HDAC1
-0.113908768	8.97437E-25	10793	ZNF273
-0.113984644	8.35516E-25	253152	EPHX4
-0.114025041	8.04302E-25	8665	EIF3F
-0.114030518	8.00147E-25	23404	EXOSC2
-0.114033028	7.98249E-25	8659	ALDH4A1
-0.114043057	7.9075E-25	3433	IFIT2
-0.114047974	7.8709E-25	27237	ARHGEF16
-0.114075743	7.66736E-25	646799	ZAR1L
-0.114078157	7.64996E-25	7923	HSD17B8
-0.114120319	7.35165E-25	25876	SPEF1
-0.11412283	7.3342E-25	26088	GGA1
-0.114135735	7.24544E-25	9975	NR1D2
-0.114158578	7.09088E-25	221035	REEP3
-0.114165172	7.04687E-25	4047	LSS
-0.114234358	6.60119E-25	64750	SMURF2
-0.114413217	5.57424E-25	3988	LIPA
-0.114482872	5.21859E-25	65988	ZNF747
-0.114485003	5.20807E-25	57634	EP400
-0.114546113	4.91528E-25	90827	ZNF479
-0.114554651	4.87568E-25	3811	KIR3DL1
-0.11458493	4.73785E-25	100272217	LOC100272217
-0.114586882	4.72908E-25	10428	CFDP1

region-wise, subcortex					
Spearman's corr coef	p-val	p-val, SA	p-val, SA (BH- correction)	GenID	Gene
-0.158815965	0.353494585	0.37	1	494513	DFNB59
-0.159073368	0.352707028	0.381	1	1548	CYP2A6
-0.159073368	0.352707028	0.403	1	10148	EBI3
-0.159073368	0.352707028	0.399	1	10668	CGRRF1
-0.159073368	0.352707028	0.383	1	55035	NOL8
-0.159073368	0.352707028	0.38	1	127665	ZNF648
-0.159330755	0.351920068	0.413	1	11039	SMA4
-0.159330755	0.351920068	0.395	1	51575	ESF1
-0.159330755	0.351920068	0.375	1	79109	MAPKAP1
-0.159330755	0.351920068	0.387	1	157927	C9orf62
-0.159330755	0.351920068	0.411	1	169200	TMEM64
-0.159588158	0.351134628	0.392	1	207	AKT1
-0.159588158	0.351134628	0.408	1	6094	ROM1
-0.159588158	0.351134628	0.384	1	8342	HIST1H2BM
-0.159588158	0.351134628	0.406	1	9690	UBE3C
-0.159588158	0.351134628	0.389	1	23378	RRP8
-0.159588158	0.351134628	0.395	1	84865	CCDC142
-0.159588158	0.351134628	0.367	1	90226	UCN2
-0.159588158	0.351134628	0.376	1	113675	SDSL
-0.159588158	0.351134628	0.415	1	728411	AC138951.4
-0.159845561	0.350350201	0.431	1	582	BBS1
-0.159845561	0.350350201	0.387	1	4077	NBR1
-0.159845561	0.350350201	0.396	1	7542	ZFPL1
-0.159845561	0.350350201	0.373	1	9377	COX5A
-0.159845561	0.350350201	0.382	1	84514	GHDC
-0.159845561	0.350350201	0.371	1	84938	ATG4C
-0.159845561	0.350350201	0.405	1	157777	C8orf45
-0.160102963	0.349566877	0.378	1	7093	TLL2
-0.160102963	0.349566877	0.393	1	23017	FAIM2
-0.160102963	0.349566877	0.366	1	23351	KIAA0323
-0.160102963	0.349566877	0.405	1	51088	KLHL5
-0.160102963	0.349566877	0.396	1	54874	FNBP1L
-0.160102963	0.349566877	0.388	1	55743	CHFR
-0.160102963	0.349566877	0.391	1	56246	MRAP
-0.160102963	0.349566877	0.402	1	353219	KAAG1
-0.160102963	0.349566877	0.405	1	441177	LINC00602
-0.160102963	0.349566877	0.411	1	644150	WIPF3
-0.160360366	0.348784268	0.393	1	55726	C12orf11
-0.160360366	0.348784268	0.382	1	57165	GJC2
-0.160360366	0.348784268	0.399	1	116179	TGM7
-0.160360366	0.348784268	0.39	1	129804	FBLN7
-0.160360366	0.348784268	0.4	1	359948	IRF2BP2
-0.160617754	0.348003089	0.396	1	4040	LRP6
-0.160617754	0.348003089	0.364	1	5158	PDE6B
-0.160617754	0.348003089	0.393	1	5998	RGS3
-0.160617754	0.348003089	0.38	1	9712	USP6NL
-0.160617754	0.348003089	0.387	1	9813	KIAA0494
-0.160617754	0.348003089	0.424	1	50807	ASAP1
-0.160617754	0.348003089	0.387	1	79603	LASS4
-0.160617754	0.348003089	0.395	1	339327	ZNF546
-0.160875157	0.347223014	0.384	1	58486	ZBED5
-0.160875157	0.347223014	0.383	1	84433	CARD11
-0.160875157	0.347223014	0.425	1	126070	ZNF440
-0.160875157	0.347223014	0.371	1	100132831	LOC100132831
-0.161132559	0.346443951	0.396	1	2720	GLB1
-0.161132559	0.346443951	0.41	1	3551	IKBKB
-0.161132559	0.346443951	0.394	1	23052	ENDOD1
-0.161132559	0.346443951	0.386	1	54454	ATAD2B
-0.161132559	0.346443951	0.407	1	55997	CFC1
-0.161132559	0.346443951	0.362	1	284293	HMSD
-0.161132559	0.346443951	0.392	1	730755	KRTAP2-1
-0.161389962	0.345665634	0.364	1	2253	FGF8
-0.161389962	0.345665634	0.393	1	2911	GRM1
-0.161389962	0.345665634	0.4	1	3001	GZMA
-0.161389962	0.345665634	0.4	1	5538	PPT1
-0.161389962	0.345665634	0.375	1	5831	PYCR1
-0.161389962	0.345665634	0.373	1	10011	SRA1
-0.161389962	0.345665634	0.407	1	10060	ABCC9
-0.161389962	0.345665634	0.414	1	10068	IL18BP
-0.161389962	0.345665634	0.398	1	23149	FCHO1
-0.161389962	0.345665634	0.368	1	24150	TP53TG3
-0.161389962	0.345665634	0.378	1	83939	EIF2A
-0.161389962	0.345665634	0.358	1	84992	PIGY
-0.161389962	0.345665634	0.408	1	390595	UBAP1L
-0.161389962	0.345665634	0.382	1	729967	MORN2
-0.161647364	0.344888806	0.385	1	2306	FOXD2
-0.161647364	0.344888806	0.377	1	23038	WDTC1
-0.161647364	0.344888806	0.395	1	160335	TMTC2
-0.161904752	0.344112962	0.393	1	1120	CHKB

voxel-wise, subcortex			
Spearman's corr coef	p-val	GenID	Gene
-0.11462681	4.55342E-25	57038	RARS2
-0.114637397	4.50798E-25	4862	NPAS2
-0.114658386	4.41916E-25	93164	AC007688.2
-0.114675842	4.34662E-25	6649	SOD3
-0.114679188	4.33285E-25	3587	IL10RA
-0.11469423	4.27146E-25	2783	GNB2
-0.114706121	4.22358E-25	286411	RP1-177G6.1
-0.114787653	3.90913E-25	11015	KDELR3
-0.114848763	3.68879E-25	286436	H2BFM
-0.114878945	3.58452E-25	29929	ALG6
-0.114885539	3.56211E-25	5137	PDE1C
-0.114898354	3.51899E-25	641311	RPL31P11
-0.114909306	3.4826E-25	78998	C8ORF51
-0.115035422	3.08906E-25	203429	FAM226A
-0.115035981	3.08742E-25	8664	EIF3D
-0.115144633	2.78411E-25	51479	ANKFY1
-0.1152023	2.6353E-25	91851	CHRDL1
-0.115212798	2.60909E-25	6348	CCL3
-0.115246043	2.52771E-25	4851	NOTCH1
-0.115316994	2.3624E-25	6907	TBL1X
-0.115356833	2.27432E-25	4638	MYLK
-0.115369461	2.24708E-25	414149	ACBD7
-0.115392581	2.19803E-25	58529	MYOZ1
-0.115408368	2.16516E-25	9775	EIF4A3
-0.115453042	2.07478E-25	1641	DCX
-0.115490556	2.00177E-25	54887	UHRF1BP1
-0.115519904	1.94645E-25	388561	ZNF761
-0.115549438	1.89227E-25	2059	EPS8
-0.115559183	1.87472E-25	4052	LTBP1
-0.115564667	1.86493E-25	9844	ELMO1
-0.115582399	1.83359E-25	255877	BCL6B
-0.115653165	1.71363E-25	53834	FGFRL1
-0.115674712	1.67868E-25	3454	IFNAR1
-0.115688458	1.65674E-25	10487	CAP1
-0.115691796	1.65146E-25	646090	RHPN2P1
-0.115764976	1.53974E-25	9863	MAGI2
-0.115777701	1.52109E-25	3695	ITGB7
-0.115802683	1.48514E-25	54811	ZNF562
-0.115943745	1.29736E-25	8242	KDM5C
-0.116151206	1.06313E-25	140803	TRPM6
-0.116226889	9.88565E-26	9911	TMCC2
-0.116234414	9.8143E-26	81844	TRIM56
-0.116258837	9.58661E-26	400618	AC005152.2
-0.116271935	9.46667E-26	7456	WIPF1
-0.116337404	8.88875E-26	219965	OR5B17
-0.116347522	8.80273E-26	22907	DHX30
-0.11635486	8.74084E-26	26747	NUFIP1
-0.116388947	8.45868E-26	3835	KIF22
-0.116411977	8.27326E-26	25842	ASF1A
-0.116419308	8.215E-26	6299	SALL1
-0.116431013	8.12294E-26	8801	SUCLG2
-0.116581082	7.02927E-26	858	CAV2
-0.116595201	6.93425E-26	51112	TTC15
-0.116595477	6.93238E-26	246176	GAS2L2
-0.116601698	6.89093E-26	10425	ARIH2
-0.116602816	6.88348E-26	79635	CCDC121
-0.116725765	6.11344E-26	5878	RAB5C
-0.116726793	6.10742E-26	84919	PPP1R15B
-0.116795696	5.71425E-26	64969	MRPS5
-0.116807491	5.6495E-26	8550	MAPKAPK5
-0.116831079	5.52223E-26	7559	ZNF12
-0.116866738	5.3351E-26	22938	SNW1
-0.116869986	5.31838E-26	51114	ZDHHC9
-0.116889209	5.22052E-26	390595	UBAP1L
-0.116966568	4.8442E-26	51524	TMEM138
-0.117050707	4.46534E-26	6203	RPS9
-0.117066771	4.39645E-26	3221	HOXC4
-0.117101692	4.25026E-26	9919	SEC16A
-0.117217585	3.79872E-26	1632	DCI
-0.11722938	3.75553E-26	2078	ERG
-0.117244519	3.70081E-26	632	BGLAP
-0.117305905	3.48689E-26	5396	PRRX1
-0.117307946	3.47998E-26	4919	ROR1
-0.117368124	3.28256E-26	79709	GLT25D1
-0.117393292	3.20331E-26	7592	ZNF41
-0.117403135	3.17285E-26	127428	C1orf83
-0.117405921	3.16429E-26	51324	SPG21
-0.117442325	3.05434E-26	2734	GLG1
-0.11751847	2.83654E-26	5028	P2RY1

region-wise, subcortex					
Spearman's corr coef	p-val	p-val, SA	p-val, SA (BH- correction)	GenID	Gene
-0.161904752	0.344112962	0.394	1	1482	NKX2-5
-0.161904752	0.344112962	0.384	1	3572	IL6ST
-0.161904752	0.344112962	0.389	1	4004	LMO1
-0.161904752	0.344112962	0.395	1	8242	KDM5C
-0.161904752	0.344112962	0.374	1	30833	NT5C
-0.161904752	0.344112962	0.384	1	51043	ZBTB7B
-0.161904752	0.344112962	0.362	1	51542	VPS54
-0.161904752	0.344112962	0.374	1	441208	ZNRF2P1
-0.162162155	0.343338281	0.365	1	534	ATP6V1G2
-0.162162155	0.343338281	0.392	1	1660	DHX9
-0.162162155	0.343338281	0.355	1	5934	RBL2
-0.162162155	0.343338281	0.416	1	6716	SRD5A2
-0.162162155	0.343338281	0.403	1	10194	TSHZ1
-0.162162155	0.343338281	0.396	1	64856	VWA1
-0.162162155	0.343338281	0.41	1	221749	C6orf145
-0.162162155	0.343338281	0.384	1	340784	HMX3
-0.162162155	0.343338281	0.382	1	348327	ZNF530
-0.162419558	0.342564672	0.372	1	629	CFB
-0.162419558	0.342564672	0.373	1	7702	ZNF143
-0.162419558	0.342564672	0.347	1	9878	TOX4
-0.162419558	0.342564672	0.396	1	83473	KATNAL2
-0.162419558	0.342564672	0.39	1	158314	LINC00475
-0.16267696	0.341791719	0.383	1	3766	KCNJ10
-0.16267696	0.341791719	0.372	1	10413	YAP1
-0.16267696	0.341791719	0.379	1	23060	ZNF609
-0.16267696	0.341791719	0.388	1	55156	ARMC1
-0.16267696	0.341791719	0.365	1	84223	IQCG
-0.16267696	0.341791719	0.417	1	159296	NKX2-3
-0.16267696	0.341791719	0.395	1	440335	LOC440335
-0.162934363	0.341020256	0.417	1	2521	FUS
-0.162934363	0.341020256	0.379	1	6342	SCP2
-0.162934363	0.341020256	0.396	1	6524	SLC5A2
-0.162934363	0.341020256	0.396	1	339500	ZNF678
-0.162934363	0.341020256	0.39	1	344148	AC010974.2
-0.162934363	0.341020256	0.387	1	387254	SLC7A5P2
-0.163191751	0.340249896	0.368	1	978	CDA
-0.163191751	0.340249896	0.359	1	3821	KLRK1
-0.163191751	0.340249896	0.387	1	5165	PDK3
-0.163191751	0.340249896	0.349	1	79957	PAQR6
-0.163191751	0.340249896	0.41	1	93659	CGB5
-0.163191751	0.340249896	0.398	1	127495	LRRC39
-0.163191751	0.340249896	0.382	1	284325	C19orf54
-0.163191751	0.340249896	0.382	1	374454	KRT77
-0.163449153	0.339480609	0.364	1	2200	FBN1
-0.163449153	0.339480609	0.357	1	7249	TSC2
-0.163449153	0.339480609	0.376	1	9557	CHD1L
-0.163449153	0.339480609	0.387	1	10105	PPIF
-0.163449153	0.339480609	0.383	1	10450	PPIE
-0.163449153	0.339480609	0.38	1	57232	ZNF630
-0.163449153	0.339480609	0.375	1	120224	TMEM45B
-0.163706556	0.338711947	0.397	1	307	ANXA4
-0.163706556	0.338711947	0.372	1	5781	PTPN11
-0.163706556	0.338711947	0.358	1	6536	SLC6A9
-0.163706556	0.338711947	0.374	1	6691	SPINK2
-0.163706556	0.338711947	0.35	1	26520	TIMM9
-0.163706556	0.338711947	0.393	1	51075	TMX2
-0.163706556	0.338711947	0.347	1	79023	NUP37
-0.163963959	0.337944806	0.362	1	9101	USP8
-0.163963959	0.337944806	0.378	1	83887	TTLL2
-0.164221361	0.337178737	0.405	1	5376	PMP22
-0.164221361	0.337178737	0.362	1	54942	C9orf6
-0.164221361	0.337178737	0.404	1	79660	PPP1R3B
-0.164221361	0.337178737	0.39	1	284186	TMEM105
-0.164478764	0.336413711	0.363	1	3936	LCP1
-0.164478764	0.336413711	0.366	1	9066	SYT7
-0.164478764	0.336413711	0.378	1	25925	ZNF521
-0.164478764	0.336413711	0.359	1	54739	XAF1
-0.164478764	0.336413711	0.394	1	79618	HMBOX1
-0.164478764	0.336413711	0.376	1	80010	RMI1
-0.164478764	0.336413711	0.372	1	124641	OVCA2
-0.164478764	0.336413711	0.354	1	643904	RNF222
-0.164478764	0.336413711	0.381	1	100131211	TMEM194B
-0.164736167	0.335649818	0.373	1	487	ATP2A1
-0.164736167	0.335649818	0.386	1	1298	COL9A2
-0.164736167	0.335649818	0.347	1	50862	RNF141
-0.164736167	0.335649818	0.367	1	51479	ANKFY1
-0.164736167	0.335649818	0.387	1	55717	BRWD2
-0.164736167	0.335649818	0.381	1	285636	C5orf51
-0.164993569	0.33488664	0.368	1	4987	OPRL1

voxel-wise, subcortex			
Spearman's corr coef	p-val	GenID	Gene
-0.117543451	2.76846E-26	4123	MAN2C1
-0.117547169	2.75849E-26	8909	AC004241.1
-0.117614865	2.58269E-26	7712	ZNF157
-0.117620535	2.56852E-26	838	CASP5
-0.117632233	2.53945E-26	23243	ANKRD28
-0.117659166	2.47374E-26	230	ALDOC
-0.117662229	2.46637E-26	4926	NUMA1
-0.117721573	2.32794E-26	23325	KIAA1033
-0.117762245	2.23751E-26	51315	KRCC1
-0.1177641	2.23348E-26	11238	CA5B
-0.11778193	2.19503E-26	400322	AC091565.3
-0.117894948	1.96606E-26	51460	SFMBT1
-0.117896155	1.96375E-26	84236	RHBDD1
-0.117928475	1.90282E-26	127066	OR14C36
-0.117934421	1.89181E-26	85007	AGXT2L2
-0.117973886	1.82034E-26	114879	OSBPL5
-0.117991716	1.78896E-26	84078	KBTBD7
-0.118078269	1.64397E-26	920	CD4
-0.118103899	1.60332E-26	163589	TDRD5
-0.118170299	1.5026E-26	84952	CGNL1
-0.118234187	1.4116E-26	27343	POLL
-0.118235491	1.40982E-26	1160	CKMT2
-0.118268922	1.36445E-26	4811	NID1
-0.118327521	1.2884E-26	2246	FGF1
-0.118379153	1.22489E-26	54921	CHTF8
-0.118388996	1.21316E-26	1312	COMT
-0.118498489	1.08973E-26	23341	DNAJC16
-0.118554763	1.03124E-26	80303	EFHD1
-0.118578628	1.00739E-26	633	BGN
-0.118598133	9.88288E-27	143458	LDLRAD3
-0.118612245	9.74708E-27	3576	IL8
-0.118618377	9.68857E-27	55858	TMEM165
-0.118672609	9.18635E-27	79848	CSPP1
-0.1186793	9.12628E-27	118611	C10orf90
-0.118713468	8.82519E-27	2186	BPTF
-0.118731767	8.66795E-27	3861	KRT14
-0.118769191	8.35503E-27	81786	TRIM7
-0.118799098	8.11307E-27	10057	ABCC5
-0.118813023	8.00284E-27	170958	ZNF525
-0.118995599	6.68728E-27	8243	SMC1A
-0.119010366	6.59071E-27	54663	WDR74
-0.119034231	6.43767E-27	10637	LEFTY1
-0.119055681	6.30307E-27	64852	TUT1
-0.119119108	5.92131E-27	91746	YTHDC1
-0.119185232	5.54768E-27	8073	PTP4A2
-0.119211607	5.40522E-27	10973	ASCC3
-0.119231388	5.3008E-27	56674	TMEM9B
-0.119266488	5.12036E-27	55749	CCAR1
-0.11930085	4.94965E-27	26986	PABPCP5
-0.119324155	4.8371E-27	255919	TMEM188
-0.119367525	4.63433E-27	8703	B4GALT3
-0.119393438	4.51731E-27	56302	TRPV5
-0.119409777	4.44492E-27	692094	MSMP
-0.119506545	4.03956E-27	122046	TEX26
-0.119531438	3.94134E-27	256356	GK5
-0.119537748	3.91682E-27	81533	ITFG1
-0.119544156	3.89211E-27	23236	PLCB1
-0.119579636	3.75785E-27	440181	LOC440181
-0.119590871	3.71633E-27	2920	CXCL2
-0.119616777	3.62228E-27	51081	MRPS7
-0.119655505	3.48603E-27	7920	BAT5
-0.119704634	3.32054E-27	113675	SDSL
-0.119711593	3.29771E-27	5971	RELB
-0.119719118	3.27322E-27	51009	DERL2
-0.119819596	2.96307E-27	5069	PAPPA
-0.119833432	2.92264E-27	1630	DCC
-0.119844764	2.89007E-27	51574	LARP7
-0.119863898	2.83568E-27	57419	SLC24A3
-0.119929552	2.65692E-27	339500	ZNF678
-0.120006815	2.46077E-27	84265	POLR3GL
-0.120032445	2.39893E-27	54852	PAQR5
-0.120034397	2.39428E-27	165545	DQX1
-0.120052785	2.35099E-27	54739	XAF1
-0.120113149	2.21411E-27	55086	CXorf57
-0.12014398	2.1473E-27	79939	SLC35E1
-0.120223843	1.98336E-27	11129	SFRS16
-0.120242693	1.94654E-27	90427	BMF
-0.120248362	1.93558E-27	134548	ANKRD43
-0.120256901	1.9192E-27	5304	PIP

region-wise, subcortex					
Spearman's corr coef	p-val	p-val, SA	p-val, SA (BH- correction)	GenID	Gene
-0.164993569	0.33488664	0.364	1	51081	MRPS7
-0.164993569	0.33488664	0.398	1	55217	TMLHE
-0.164993569	0.33488664	0.367	1	84106	PRAM1
-0.164993569	0.33488664	0.389	1	163050	ZNF564
-0.164993569	0.33488664	0.379	1	100130700	LOC100130700
-0.165250972	0.334124833	0.39	1	672	BRCA1
-0.165250972	0.334124833	0.355	1	883	CCBL1
-0.165250972	0.334124833	0.368	1	2059	EPS8
-0.165250972	0.334124833	0.352	1	2692	GHRHR
-0.165250972	0.334124833	0.355	1	6944	VPS72
-0.165250972	0.334124833	0.39	1	10899	JTB
-0.165250972	0.334124833	0.381	1	26637	OR7E36P
-0.165250972	0.334124833	0.386	1	51318	MRPL35
-0.165250972	0.334124833	0.375	1	55137	FIGN
-0.165250972	0.334124833	0.373	1	93058	COQ10A
-0.165250972	0.334124833	0.358	1	284739	LINC00176
-0.165508375	0.333364189	0.376	1	1121	CHM
-0.165508375	0.333364189	0.394	1	5283	PIGH
-0.165508375	0.333364189	0.384	1	8773	SNAP23
-0.165508375	0.333364189	0.368	1	10795	ZNF268
-0.165508375	0.333364189	0.367	1	51110	LACTB2
-0.165508375	0.333364189	0.364	1	54834	GDAP2
-0.165508375	0.333364189	0.401	1	114041	C21orf88
-0.165508375	0.333364189	0.355	1	259291	TAS2R45
-0.165765762	0.332604587	0.383	1	7103	TSPAN8
-0.165765762	0.332604587	0.372	1	8200	GDF5
-0.165765762	0.332604587	0.352	1	9126	SMC3
-0.165765762	0.332604587	0.352	1	10428	CFDP1
-0.165765762	0.332604587	0.395	1	29896	TRA2A
-0.165765762	0.332604587	0.359	1	768096	HAR1A
-0.166023165	0.331845701	0.395	1	2882	GPX7
-0.166023165	0.331845701	0.373	1	3336	HSPE1
-0.166023165	0.331845701	0.37	1	3770	KCNJ14
-0.166023165	0.331845701	0.391	1	54498	SMOX
-0.166023165	0.331845701	0.381	1	729317	LOC729317
-0.166280568	0.331088305	0.372	1	2538	G6PC
-0.166280568	0.331088305	0.381	1	7494	XBP1
-0.166280568	0.331088305	0.387	1	23469	PHF3
-0.166280568	0.331088305	0.377	1	26205	GMEB2
-0.166280568	0.331088305	0.337	1	89894	TMEM116
-0.166280568	0.331088305	0.357	1	134288	TMEM174
-0.16653797	0.330331951	0.349	1	1939	LGTN
-0.16653797	0.330331951	0.368	1	3920	LAMP2
-0.16653797	0.330331951	0.368	1	203429	FAM226A
-0.16653797	0.330331951	0.356	1	100128385	LINC00256B
-0.166795373	0.329576671	0.375	1	2701	GJA4
-0.166795373	0.329576671	0.356	1	3846	KRTAP5-9
-0.166795373	0.329576671	0.391	1	5291	PIK3CB
-0.166795373	0.329576671	0.382	1	6910	TBX5
-0.166795373	0.329576671	0.398	1	8837	CFLAR
-0.166795373	0.329576671	0.373	1	9048	ARTN
-0.166795373	0.329576671	0.381	1	10285	SMNDC1
-0.166795373	0.329576671	0.361	1	57217	TTC7A
-0.166795373	0.329576671	0.347	1	79096	C11orf49
-0.166795373	0.329576671	0.366	1	80854	SETD7
-0.167052761	0.328822136	0.385	1	5106	PCK2
-0.167052761	0.328822136	0.369	1	7673	ZNF222
-0.167052761	0.328822136	0.354	1	10178	ODZ1
-0.167052761	0.328822136	0.383	1	23500	DAAM2
-0.167052761	0.328822136	0.363	1	25956	SEC31B
-0.167052761	0.328822136	0.397	1	129685	TAF8
-0.167052761	0.328822136	0.359	1	389852	SPACA5
-0.167310163	0.328069061	0.348	1	27430	MAT2B
-0.167310163	0.328069061	0.367	1	79073	TMEM109
-0.167310163	0.328069061	0.377	1	221035	REEP3
-0.167567566	0.327316999	0.383	1	7388	UQCRH
-0.167567566	0.327316999	0.379	1	9295	SFRS11
-0.167567566	0.327316999	0.38	1	10274	STAG1
-0.167567566	0.327316999	0.366	1	81628	TSC22D4
-0.167567566	0.327316999	0.354	1	199692	ZNF627
-0.167824969	0.3265661	0.323	1	5430	POLR2A
-0.167824969	0.3265661	0.358	1	8085	MLL2
-0.167824969	0.3265661	0.377	1	9416	DDX23
-0.167824969	0.3265661	0.391	1	55729	ATF7IP
-0.167824969	0.3265661	0.378	1	83986	ITFG3
-0.167824969	0.3265661	0.383	1	84061	MAGT1
-0.167824969	0.3265661	0.374	1	252884	ZNF396
-0.167824969	0.3265661	0.334	1	375189	PFN4
-0.168082371	0.325816214	0.359	1	16	AARS

voxel-wise, subcortex			
Spearman's corr coef	p-val	GenID	Gene
-0.120304734	1.82999E-27	3655	ITGA6
-0.120319404	1.80347E-27	3315	HSPB1
-0.120390445	1.6803E-27	309	ANXA6
-0.120444678	1.59191E-27	85452	C1orf222
-0.120450713	1.58236E-27	60484	HAPLN2
-0.120453872	1.57738E-27	161742	SPRED1
-0.1204587	1.56982E-27	79802	HHIPL2
-0.120473377	1.547E-27	2108	ETFA
-0.120515443	1.48348E-27	6428	SRSF3
-0.120528817	1.46383E-27	7128	TNFAIP3
-0.1205329	1.45789E-27	55750	AGK
-0.12056578	1.41084E-27	374920	C19orf68
-0.120585464	1.3834E-27	10252	SPRY1
-0.120608307	1.35223E-27	25780	RASGRP3
-0.120743707	1.18124E-27	1485	CTAG1B
-0.12080472	1.11137E-27	5037	PEBP1
-0.120828405	1.08536E-27	24141	C20orf103
-0.120837964	1.07504E-27	64446	DNAI2
-0.120889418	1.02113E-27	50813	COPS7A
-0.120903812	1.00652E-27	162427	FAM134C
-0.12090715	1.00318E-27	8842	PROM1
-0.120924793	9.8562E-28	1027	CDKN1B
-0.120946251	9.64682E-28	6637	SNRPG
-0.12105184	8.67898E-28	169522	KCNV2
-0.121052206	8.67584E-28	7941	PLA2G7
-0.121187791	7.5735E-28	5660	PSAP
-0.121221691	7.32038E-28	10717	AP4B1
-0.12123014	7.25853E-28	51071	DERA
-0.121235341	7.22088E-28	55553	SOX6
-0.121279731	6.90632E-28	64770	CCDC14
-0.121293008	6.81489E-28	84938	ATG4C
-0.12130601	6.7265E-28	9770	RASSF2
-0.121309914	6.70022E-28	55633	TBC1D22B
-0.121329598	6.56908E-28	130940	CCDC148
-0.121369809	6.30909E-28	158471	PRUNE2
-0.1213817	6.23425E-28	55182	RNF220
-0.121390797	6.17751E-28	348995	NUP43
-0.121404544	6.0928E-28	5074	PAWR
-0.121469736	5.70644E-28	84447	SYVN1
-0.121472709	5.68943E-28	646278	LOC646278
-0.121649429	4.7629E-28	202299	C5ORF27
-0.121651381	4.7535E-28	3661	IRF3
-0.121652402	4.74861E-28	22836	RHOBTB3
-0.121653982	4.74109E-28	84337	ELOF1
-0.121677846	4.62853E-28	23205	ACSBG1
-0.121750005	4.30408E-28	1497	CTNS
-0.121772386	4.2081E-28	84890	ADO
-0.121774703	4.19823E-28	55196	C12orf35
-0.121778049	4.18418E-28	7494	XBP1
-0.121810742	4.04856E-28	284371	ZNF841
-0.121865623	3.83059E-28	10434	LYPLA1
-0.121874258	3.79737E-28	10184	LHFPL2
-0.121885031	3.75635E-28	653604	HIST2H3D
-0.121888928	3.74164E-28	22794	CASC3
-0.121902302	3.69153E-28	3758	KCNJ1
-0.121929325	3.59216E-28	168850	ZNF800
-0.121960342	3.4815E-28	100506658	OCLN
-0.121978179	3.41938E-28	9283	GPR37L1
-0.122036956	3.22238E-28	83604	TMEM47
-0.122045413	3.19497E-28	7586	ZKSCAN1
-0.122056276	3.16011E-28	6773	STAT2
-0.122059338	3.15035E-28	10174	SORBS3
-0.122061849	3.14237E-28	6323	SCN1A
-0.122152485	2.86733E-28	57580	PREX1
-0.122165114	2.83099E-28	11342	RNF13
-0.12216679	2.82619E-28	7297	TYK2
-0.122249991	2.59812E-28	6650	SOLH
-0.122324102	2.41037E-28	80346	REEP4
-0.122351035	2.34555E-28	57348	TTYH1
-0.122366823	2.30832E-28	4329	ALDH6A1
-0.122375362	2.28846E-28	284695	ZNF326
-0.122412972	2.20291E-28	4099	MAG
-0.122497387	2.02229E-28	150159	NHEDC1
-0.122553758	1.90995E-28	155400	NSUN5B
-0.122616909	1.79144E-28	85358	SHANK3
-0.122713581	1.62396E-28	11226	GALNT6
-0.122729279	1.59828E-28	26231	LRRC29
-0.122756116	1.5553E-28	10810	WASF3
-0.122802824	1.48321E-28	346329	LOC346329

region-wise, subcortex					
Spearman's corr coef	p-val	p-val, SA	p-val, SA (BH- correction)	GenID	Gene
-0.168082371	0.325816214	0.375	1	25821	MTO1
-0.168082371	0.325816214	0.372	1	84267	C9orf64
-0.168082371	0.325816214	0.356	1	122060	SLAIN1
-0.168339759	0.325067103	0.383	1	3138	HLA-K
-0.168339759	0.325067103	0.37	1	9358	ITGBL1
-0.168339759	0.325067103	0.399	1	10773	ZBTB6
-0.168597162	0.324319422	0.347	1	7544	ZFY
-0.168597162	0.324319422	0.35	1	8455	ATRN
-0.168597162	0.324319422	0.379	1	22880	MORC2
-0.168597162	0.324319422	0.338	1	65109	UPF3B
-0.168597162	0.324319422	0.355	1	84224	NBPF3
-0.168597162	0.324319422	0.349	1	90203	SNX21
-0.168597162	0.324319422	0.353	1	123096	SLC25A29
-0.168854564	0.323572785	0.362	1	23308	ICOSLG
-0.168854564	0.323572785	0.374	1	84693	MCEE
-0.169111967	0.322827309	0.358	1	2710	GK
-0.169111967	0.322827309	0.383	1	4719	NDUFS1
-0.169111967	0.322827309	0.351	1	55435	C4orf16
-0.169111967	0.322827309	0.349	1	388551	CEACAM16
-0.16936937	0.32208252	0.377	1	36	ACADSB
-0.16936937	0.32208252	0.345	1	3222	HOXC5
-0.16936937	0.32208252	0.36	1	3433	IFIT2
-0.16936937	0.32208252	0.357	1	9293	GPR52
-0.16936937	0.32208252	0.332	1	55252	ASXL2
-0.16936937	0.32208252	0.361	1	144233	BCDIN3D
-0.169626758	0.32133913	0.361	1	760	CA2
-0.169626758	0.32133913	0.368	1	3691	ITGB4
-0.169626758	0.32133913	0.361	1	10542	HBXIP
-0.169626758	0.32133913	0.358	1	80153	EDC3
-0.169626758	0.32133913	0.38	1	92999	ZBTB47
-0.169626758	0.32133913	0.362	1	285527	FRYL
-0.16988416	0.320596874	0.361	1	432	ASGR1
-0.16988416	0.320596874	0.363	1	4118	MAL
-0.16988416	0.320596874	0.373	1	6872	TAF1
-0.16988416	0.320596874	0.342	1	9953	HS3ST3B1
-0.16988416	0.320596874	0.348	1	30812	SOX8
-0.16988416	0.320596874	0.362	1	54407	SLC38A2
-0.16988416	0.320596874	0.367	1	54800	KLHL24
-0.16988416	0.320596874	0.386	1	79992	AGPAT4-IT1
-0.16988416	0.320596874	0.367	1	91369	ANKRD40
-0.170141563	0.31985572	0.362	1	2012	EMP1
-0.170141563	0.31985572	0.359	1	5338	PLD2
-0.170141563	0.31985572	0.389	1	7456	WIPF1
-0.170141563	0.31985572	0.378	1	64097	EPB41L4A
-0.170141563	0.31985572	0.351	1	246184	CDC26
-0.170398965	0.319115281	0.361	1	1613	DAPK3
-0.170398965	0.319115281	0.351	1	7756	ZNF207
-0.170398965	0.319115281	0.347	1	8021	NUP214
-0.170398965	0.319115281	0.388	1	27032	ATP2C1
-0.170398965	0.319115281	0.358	1	441722	LOC441722
-0.170656368	0.318376243	0.361	1	6152	RPL24
-0.170656368	0.318376243	0.339	1	7205	TRIP6
-0.170656368	0.318376243	0.369	1	23360	FNBP4
-0.170656368	0.318376243	0.336	1	51133	KCTD3
-0.170656368	0.318376243	0.356	1	55007	FAM118A
-0.170656368	0.318376243	0.363	1	79886	C9orf82
-0.170656368	0.318376243	0.359	1	80319	CXXC4
-0.170656368	0.318376243	0.347	1	112802	KRT71
-0.170656368	0.318376243	0.348	1	147744	TMEM190
-0.170656368	0.318376243	0.349	1	402644	LOC402644
-0.170913756	0.317638278	0.37	1	2172	FABP6
-0.170913756	0.317638278	0.35	1	3988	LIPA
-0.170913756	0.317638278	0.352	1	55227	LRRC1
-0.170913756	0.317638278	0.344	1	80146	UXS1
-0.170913756	0.317638278	0.364	1	162681	C18orf54
-0.170913756	0.317638278	0.364	1	353376	TICAM2
-0.171171159	0.316901445	0.354	1	2896	GRN
-0.171171159	0.316901445	0.358	1	3977	LIFR
-0.171171159	0.316901445	0.354	1	5654	HTRA1
-0.171171159	0.316901445	0.388	1	283303	C11ORF36
-0.171171159	0.316901445	0.372	1	441454	LOC441454
-0.171428576	0.316165745	0.37	1	3217	HOXB7
-0.171428576	0.316165745	0.35	1	4179	CD46
-0.171428576	0.316165745	0.357	1	8633	UNC5C
-0.171428576	0.316165745	0.358	1	10842	C7orf16
-0.171428576	0.316165745	0.353	1	50813	COPS7A
-0.171428576	0.316165745	0.361	1	56605	ERO1LB
-0.171428576	0.316165745	0.343	1	80196	RNF34
-0.171428576	0.316165745	0.352	1	114799	ESCO1

voxel-wise, subcortex			
Spearman's corr coef	p-val	GenID	Gene
-0.122808956	1.474E-28	6737	TRIM21
-0.122830778	1.44165E-28	81562	LMAN2L
-0.122843407	1.42324E-28	284942	RPL23AP82
-0.12288408	1.3656E-28	22835	ZFP30
-0.122939803	1.29035E-28	55892	MYNN
-0.122946486	1.28161E-28	5372	PMM1
-0.122950949	1.27579E-28	57484	RNF150
-0.122969151	1.25239E-28	4166	CHST6
-0.123015121	1.19513E-28	100130935	LOC100130935
-0.123094514	1.1023E-28	84281	C2orf88
-0.123177908	1.0125E-28	389332	LOC389332
-0.123223506	9.66491E-29	284402	SCGBL
-0.123237066	9.53214E-29	10549	PRDX4
-0.123245798	9.44765E-29	6391	SDHC
-0.123344511	8.54228E-29	100131176	RP4-555L14.5
-0.123349153	8.50191E-29	29899	GPSM2
-0.123372376	8.30264E-29	90589	ZNF625
-0.123459205	7.59797E-29	164395	TTLL9
-0.123486504	7.38895E-29	283481	LOC283481
-0.123530619	7.06312E-29	440184	LINC00238
-0.1235881	6.65986E-29	9762	RP5-1187M17.1
-0.123628497	6.39028E-29	10063	COX17
-0.123631097	6.37326E-29	4507	MTAP
-0.1236974	5.95513E-29	9552	SPAG7
-0.12370149	5.93023E-29	23324	MAN2B2
-0.123891398	4.88151E-29	10905	MAN1A2
-0.123944707	4.62181E-29	79058	ASPSCR1
-0.124008693	4.32813E-29	57461	ISY1
-0.124011755	4.31455E-29	96764	TGS1
-0.124044538	4.17172E-29	29103	DNAJC15
-0.124056235	4.12193E-29	7381	UQCRB
-0.124061435	4.09997E-29	23604	DAPK2
-0.124088831	3.98622E-29	1807	DPYS
-0.124119386	3.86307E-29	4717	NDUFC1
-0.124139078	3.78564E-29	8405	SPOP
-0.124150589	3.74118E-29	11197	WIF1
-0.124156252	3.71947E-29	51473	DCDC2
-0.124165356	3.68475E-29	6204	RPS10
-0.124197394	3.56539E-29	5830	PEX5
-0.124232128	3.44035E-29	9860	LRIG2
-0.12429212	3.23445E-29	79642	ARSJ
-0.124334276	3.09719E-29	5174	PDZK1
-0.124351554	3.04253E-29	7392	USF2
-0.124403559	2.88393E-29	3516	RBPSUH
-0.124470979	2.69046E-29	831	CAST
-0.124592632	2.37335E-29	11018	TMED1
-0.124613434	2.32296E-29	1946	EFNA5
-0.124627732	2.28895E-29	79064	TMEM223
-0.124664508	2.20372E-29	10060	ABCC9
-0.124680206	2.16832E-29	90102	PHLDB2
-0.124684572	2.15858E-29	5173	PDYN
-0.124772236	1.97175E-29	79993	ELOVL7
-0.124804273	1.90759E-29	3320	HSP90AA2
-0.124811612	1.89317E-29	1026	CDKN1A
-0.124819599	1.87759E-29	79872	CBLL1
-0.124899648	1.7285E-29	10227	MFSD10
-0.1249016	1.72504E-29	6939	TCF15
-0.124903359	1.72188E-29	6579	SLCO1A2
-0.125005141	1.54982E-29	64754	SMYD3
-0.125025764	1.51709E-29	161436	EML5
-0.125026688	1.51564E-29	5122	PCSK1
-0.125040427	1.49425E-29	6189	RPS3AP47
-0.125065416	1.45608E-29	645638	LOC645638
-0.125066623	1.45425E-29	100289361	LOC100289361
-0.125095963	1.41074E-29	4077	NBR1
-0.125110924	1.38904E-29	4800	NFYA
-0.12517722	1.29685E-29	4300	MLLT3
-0.125284016	1.16092E-29	57109	REXO4
-0.125292093	1.15124E-29	8625	RFXANK
-0.125387937	1.04226E-29	10152	ABI2
-0.125396669	1.03285E-29	118	ADD1
-0.125496224	9.31397E-30	28973	MRPS18B
-0.125704333	7.50152E-30	6004	RGS16
-0.125725597	7.3374E-30	666	BOK
-0.125762552	7.06045E-30	79005	SCNM1
-0.125789866	6.86239E-30	85025	TMEM60
-0.125801936	6.77673E-30	347	APOD
-0.125826076	6.60841E-30	7008	TEF
-0.125853285	6.42378E-30	3883	KRT33A

region-wise, subcortex					
Spearman's corr coef	p-val	p-val, SA	p-val, SA (BH- correction)	GenID	Gene
-0.171428576	0.316165745	0.333	1	253725	FAM21C
-0.171428576	0.316165745	0.39	1	284288	RSL24D1P11
-0.171685979	0.315430701	0.35	1	2582	GALE
-0.171685979	0.315430701	0.362	1	22854	NTNG1
-0.171685979	0.315430701	0.337	1	53838	C11orf24
-0.171685979	0.315430701	0.356	1	55677	IWS1
-0.171943381	0.314697087	0.338	1	3981	LIG4
-0.171943381	0.314697087	0.373	1	4036	LRP2
-0.171943381	0.314697087	0.353	1	4204	MECP2
-0.171943381	0.314697087	0.337	1	5962	RDX
-0.171943381	0.314697087	0.354	1	10078	TSSC4
-0.171943381	0.314697087	0.354	1	10850	CCL27
-0.171943381	0.314697087	0.357	1	55696	RBM22
-0.171943381	0.314697087	0.357	1	60488	MRPS35
-0.171943381	0.314697087	0.34	1	80035	ANP32A-IT1
-0.171943381	0.314697087	0.354	1	727936	GXYLT2
-0.172458172	0.313233137	0.367	1	2878	GPX3
-0.172458172	0.313233137	0.339	1	4741	NEFM
-0.172458172	0.313233137	0.348	1	5742	PTGS1
-0.172458172	0.313233137	0.34	1	6452	SH3BP2
-0.172458172	0.313233137	0.348	1	9028	RHBDL1
-0.172458172	0.313233137	0.362	1	63946	DMRTC2
-0.172458172	0.313233137	0.357	1	80194	TMEM134
-0.172458172	0.313233137	0.336	1	100130264	RP5-1027G4.3
-0.172715575	0.312502503	0.349	1	999	CDH1
-0.172715575	0.312502503	0.355	1	8829	NRP1
-0.172715575	0.312502503	0.355	1	9605	C16orf7
-0.172715575	0.312502503	0.352	1	23503	ZFYVE26
-0.172715575	0.312502503	0.36	1	54820	NDE1
-0.172715575	0.312502503	0.369	1	64779	MTHFSD
-0.172972977	0.311773241	0.356	1	3344	FOXN2
-0.172972977	0.311773241	0.351	1	4650	MYO9B
-0.172972977	0.311773241	0.36	1	6314	ATXN7
-0.172972977	0.311773241	0.312	1	57572	DOCK6
-0.172972977	0.311773241	0.359	1	84892	C3orf39
-0.172972977	0.311773241	0.33	1	100288083	LOC100288083
-0.17323038	0.311045051	0.359	1	2928	GSC2
-0.17323038	0.311045051	0.357	1	4211	MEIS1
-0.17323038	0.311045051	0.337	1	56992	KIF15
-0.17323038	0.311045051	0.368	1	197350	AC108134.5
-0.173487768	0.310318023	0.346	1	6834	SURF1
-0.173487768	0.310318023	0.319	1	11342	RNF13
-0.173487768	0.310318023	0.351	1	26064	RAI14
-0.173487768	0.310318023	0.333	1	113000	RPUSD1
-0.17374517	0.309591651	0.326	1	151903	CCDC12
-0.17374517	0.309591651	0.358	1	340205	TREML1
-0.174002573	0.308866799	0.326	1	23070	FTSJD2
-0.174002573	0.308866799	0.301	1	79894	ZNF672
-0.174002573	0.308866799	0.377	1	83666	PARP9
-0.174002573	0.308866799	0.35	1	337967	KRTAP6-2
-0.174002573	0.308866799	0.365	1	100131479	LOC100131479
-0.174259976	0.30814293	0.354	1	915	CD3D
-0.174259976	0.30814293	0.325	1	1292	COL6A2
-0.174259976	0.30814293	0.374	1	3142	HLX
-0.174259976	0.30814293	0.358	1	7942	TFEB
-0.174259976	0.30814293	0.357	1	22803	XRN2
-0.174259976	0.30814293	0.355	1	51074	APIP
-0.174259976	0.30814293	0.373	1	64855	FAM129B
-0.174517378	0.307420194	0.342	1	2339	FNTA
-0.174517378	0.307420194	0.367	1	2969	GTF2I
-0.174517378	0.307420194	0.364	1	3221	HOXC4
-0.174517378	0.307420194	0.354	1	9051	PSTPIP1
-0.174517378	0.307420194	0.364	1	10083	USH1C
-0.174517378	0.307420194	0.341	1	58494	JAM2
-0.174517378	0.307420194	0.35	1	80765	STARD5
-0.174517378	0.307420194	0.361	1	112939	NACC1
-0.174517378	0.307420194	0.346	1	219854	TMEM218
-0.174517378	0.307420194	0.329	1	440926	H3F3AP4
-0.174517378	0.307420194	0.331	1	642987	TMEM232
-0.174774766	0.306698561	0.362	1	1841	DTYMK
-0.174774766	0.306698561	0.347	1	8548	BLZF1
-0.174774766	0.306698561	0.361	1	9131	AIFM1
-0.174774766	0.306698561	0.337	1	28971	C11orf67
-0.174774766	0.306698561	0.357	1	55213	RCBTB1
-0.174774766	0.306698561	0.347	1	79056	PRRG4
-0.174774766	0.306698561	0.323	1	83746	L3MBTL2
-0.174774766	0.306698561	0.369	1	84299	MIEN1
-0.174774766	0.306698561	0.34	1	89781	HPS4
-0.174774766	0.306698561	0.348	1	161779	PGBD4

voxel-wise, subcortex			
Spearman's corr coef	p-val	GenID	Gene
-0.125866294	6.33729E-30	3145	HMBS
-0.125876963	6.26716E-30	122183	PRR20
-0.125891268	6.1744E-30	401236	RP3-486B10.1
-0.125899449	6.12197E-30	221785	ZNF498
-0.125942722	5.852E-30	4595	MUTYH
-0.125968158	5.69872E-30	56957	OTUD7B
-0.126003087	5.49483E-30	1798	DPAGT1
-0.12601617	5.42035E-30	90407	TMEM41A
-0.126042366	5.27423E-30	65009	NDRG4
-0.12606214	5.16651E-30	6942	TCF20
-0.12608248	5.05791E-30	200197	C1orf126
-0.126090184	5.0174E-30	115330	GPR146
-0.126113966	4.89435E-30	51617	AC093310.1
-0.126149163	4.71778E-30	79684	C11orf61
-0.126182586	4.55587E-30	51035	UBXN1
-0.12632969	3.90649E-30	84455	EFCAB7
-0.126351327	3.8191E-30	93349	SP140L
-0.126356527	3.79837E-30	10866	HCP5
-0.126366928	3.75723E-30	7347	UCHL3
-0.12637268	3.73473E-30	10456	HAX1
-0.126395345	3.64713E-30	170463	SSBP4
-0.126420796	3.55127E-30	285668	AC094086.1
-0.126437977	3.48795E-30	11221	DUSP10
-0.126467779	3.38083E-30	57463	AMIGO1
-0.126562044	3.06288E-30	57142	RTN4
-0.126636803	2.83206E-30	57540	PTCHD2
-0.126643762	2.81148E-30	55729	ATF7IP
-0.126649991	2.79313E-30	113510	HELQ
-0.126664191	2.75184E-30	22841	RAB11FIP2
-0.126670972	2.73235E-30	51128	SAR1B
-0.126711085	2.61977E-30	55313	CPPED1
-0.126724094	2.58425E-30	1785	DNM2
-0.126748323	2.5194E-30	28511	NKIRAS2
-0.126806095	2.37113E-30	6016	RIT1
-0.126807585	2.36742E-30	10218	ANGPTL7
-0.126807764	2.36698E-30	664701	ZNF826P
-0.126829028	2.31471E-30	477	ATP1A2
-0.126854852	2.25277E-30	2184	FAH
-0.126877606	2.19957E-30	10284	SAP18
-0.126879543	2.1951E-30	85315	PAQR8
-0.126898125	2.15265E-30	55435	C4orf16
-0.126940101	2.05974E-30	51768	TM7SF3
-0.126955971	2.02567E-30	79901	CYBRD1
-0.127026096	1.88168E-30	730144	LOC730144
-0.127165943	1.62414E-30	29072	SETD2
-0.127214238	1.54359E-30	79003	MIS12
-0.127250269	1.48609E-30	374666	WASH3P
-0.127334774	1.35943E-30	25870	SUMF2
-0.127346009	1.34343E-30	11234	HPS5
-0.127359942	1.32383E-30	84313	VPS25
-0.127379537	1.29674E-30	24	ABCA4
-0.127408981	1.25708E-30	50862	RNF141
-0.127423748	1.23768E-30	20	ABCA2
-0.12744306	1.21269E-30	389084	C2orf82
-0.127591461	1.0368E-30	9677	HISPPD2A
-0.127696022	9.28297E-31	27236	ARFIP1
-0.127696589	9.27739E-31	2947	GSTM3
-0.127761036	8.666E-31	55128	TRIM68
-0.127763167	8.64644E-31	54093	SETD4
-0.127781466	8.48073E-31	1601	DAB2
-0.127838761	7.98156E-31	23133	PHF8
-0.12786077	7.79774E-31	25793	FBXO7
-0.127901822	7.46609E-31	22882	ZHX2
-0.12794055	7.16593E-31	6218	RPS17
-0.127943605	7.14276E-31	100190986	LOC100190986
-0.12798892	6.8078E-31	79856	SNX22
-0.128001839	6.71529E-31	1889	ECE1
-0.12805301	6.36061E-31	7988	ZNF212
-0.12806201	6.30012E-31	4931	NVL
-0.128086254	6.14022E-31	11154	AP4S1
-0.128167048	5.63574E-31	11093	ADAMTS13
-0.128172427	5.60366E-31	5380	PMS2L2
-0.128201872	5.43123E-31	54758	KLHDC4
-0.128294826	4.92058E-31	6510	SLC1A5
-0.128307268	4.85588E-31	26118	WSB1
-0.12839587	4.41946E-31	100288083	LOC100288083
-0.128437281	4.22903E-31	79883	PODNL1
-0.128448889	4.17713E-31	342035	GLDN
-0.12848112	4.0363E-31	23500	DAAM2

region-wise, subcortex					
Spearman's corr coef	p-val	p-val, SA	p-val, SA (BH- correction)	GenID	Gene
-0.174774766	0.306698561	0.339	1	168850	ZNF800
-0.174774766	0.306698561	0.349	1	196374	KRT78
-0.175032169	0.305977613	0.363	1	2936	GSR
-0.175032169	0.305977613	0.35	1	3831	C14orf153
-0.175032169	0.305977613	0.343	1	4601	MXI1
-0.175032169	0.305977613	0.343	1	5194	PEX13
-0.175032169	0.305977613	0.353	1	5429	POLH
-0.175032169	0.305977613	0.338	1	11046	SLC35D2
-0.175032169	0.305977613	0.344	1	55061	SUSD4
-0.175032169	0.305977613	0.377	1	130507	UBR3
-0.175032169	0.305977613	0.348	1	439933	MGC42157
-0.175289571	0.305258185	0.327	1	3832	KIF11
-0.175289571	0.305258185	0.361	1	8675	STX16
-0.175289571	0.305258185	0.351	1	10507	SEMA4D
-0.175289571	0.305258185	0.345	1	30008	EFEMP2
-0.175289571	0.305258185	0.337	1	55572	FOXRED1
-0.175289571	0.305258185	0.362	1	64151	NCAPG
-0.175289571	0.305258185	0.34	1	388335	TMEM220
-0.175546974	0.30453977	0.346	1	8028	MLLT10
-0.175546974	0.30453977	0.34	1	9692	KIAA0391
-0.175546974	0.30453977	0.33	1	10541	ANP32B
-0.175546974	0.30453977	0.359	1	27129	HSPB7
-0.175546974	0.30453977	0.358	1	54187	NANS
-0.175546974	0.30453977	0.352	1	79641	ROGDI
-0.175804377	0.303822488	0.343	1	4089	SMAD4
-0.175804377	0.303822488	0.365	1	8913	CACNA1G
-0.175804377	0.303822488	0.367	1	55109	AGGF1
-0.175804377	0.303822488	0.359	1	56903	PAPOLB
-0.175804377	0.303822488	0.322	1	653238	GTF2H2B
-0.176061764	0.303105891	0.343	1	5763	PTMS
-0.176061764	0.303105891	0.339	1	26507	CNNM1
-0.176061764	0.303105891	0.347	1	57554	LRRC7
-0.176061764	0.303105891	0.355	1	84231	TRAF7
-0.176061764	0.303105891	0.325	1	114898	C1QTNF2
-0.176061764	0.303105891	0.328	1	140738	TMEM37
-0.176061764	0.303105891	0.35	1	150776	AC093838.4
-0.176061764	0.303105891	0.334	1	284988	RHOQP3
-0.176061764	0.303105891	0.363	1	286262	C9orf75
-0.176061764	0.303105891	0.344	1	400566	C17orf97
-0.176319167	0.302390754	0.36	1	3204	HOXA7
-0.176319167	0.302390754	0.354	1	5702	PSMC3
-0.176319167	0.302390754	0.345	1	6370	CCL25
-0.176319167	0.302390754	0.349	1	7252	TSHB
-0.176319167	0.302390754	0.343	1	51710	ZNF44
-0.176319167	0.302390754	0.35	1	81887	LAS1L
-0.176319167	0.302390754	0.338	1	85417	CCNB3
-0.176319167	0.302390754	0.331	1	286530	P2RY8
-0.176319167	0.302390754	0.363	1	653247	PRB2
-0.17657657	0.30167675	0.333	1	745	C11orf9
-0.17657657	0.30167675	0.335	1	9987	HNRPDL
-0.17657657	0.30167675	0.334	1	22861	AC055839.1
-0.17657657	0.30167675	0.312	1	54482	CCDC76
-0.17657657	0.30167675	0.331	1	57169	ZNFX1
-0.176833972	0.300963819	0.358	1	4528	MTIF2
-0.176833972	0.300963819	0.351	1	400617	KCNJ2-AS1
-0.176833972	0.300963819	0.351	1	728743	LOC728743
-0.177091375	0.300251573	0.318	1	2034	EPAS1
-0.177091375	0.300251573	0.336	1	4047	LSS
-0.177091375	0.300251573	0.324	1	22933	SIRT2
-0.177091375	0.300251573	0.354	1	64816	CYP3A43
-0.177091375	0.300251573	0.335	1	283254	HARBI1
-0.177348763	0.299540758	0.34	1	10224	ZNF443
-0.177348763	0.299540758	0.32	1	23099	ZBTB43
-0.177348763	0.299540758	0.339	1	23126	POGZ
-0.177348763	0.299540758	0.341	1	51338	MS4A4A
-0.177348763	0.299540758	0.335	1	54876	C4orf30
-0.177348763	0.299540758	0.34	1	55900	ZNF302
-0.177348763	0.299540758	0.335	1	84440	RAB11FIP4
-0.177606165	0.298831046	0.341	1	2263	FGFR2
-0.177606165	0.298831046	0.336	1	6134	RPL10
-0.177606165	0.298831046	0.358	1	7813	EVI5
-0.177606165	0.298831046	0.341	1	9444	QKI
-0.177606165	0.298831046	0.346	1	387036	GUSBP2
-0.177863568	0.298122495	0.326	1	2528	FUT6
-0.177863568	0.298122495	0.331	1	4124	MAN2A1
-0.177863568	0.298122495	0.34	1	84775	ZNF607
-0.177863568	0.298122495	0.337	1	146378	C16orf92
-0.178120971	0.297414929	0.332	1	1773	DNASE1
-0.178120971	0.297414929	0.363	1	3028	HSD17B10

voxel-wise, subcortex			
Spearman's corr coef	p-val	GenID	Gene
-0.128498942	3.96046E-31	2287	FKBP3
-0.128563955	3.69567E-31	5445	PON2
-0.128594235	3.57835E-31	261729	STEAP2
-0.128718391	3.13492E-31	160897	GPR180
-0.128720611	3.1275E-31	1687	DFNA5
-0.128736585	3.07471E-31	55167	MSL2
-0.128740683	3.06127E-31	340252	ZNF680
-0.128745779	3.04469E-31	7692	ZNF133
-0.128750235	3.03025E-31	2263	FGFR2
-0.128754228	3.0174E-31	56946	C11orf30
-0.128852487	2.71709E-31	728537	AC017099.3
-0.128867805	2.67302E-31	161176	C14orf49
-0.128881097	2.63535E-31	23076	RRP1B
-0.128888518	2.61457E-31	9533	POLR1C
-0.12894173	2.47015E-31	55151	TMEM38B
-0.128942847	2.4672E-31	2200	FBN1
-0.128992245	2.34037E-31	3400	ID4
-0.129049182	2.20222E-31	10165	SLC25A13
-0.129052609	2.19417E-31	390940	AC026369.2
-0.129059017	2.17921E-31	10957	PNRC1
-0.129068583	2.15701E-31	134285	TMEM171
-0.129074156	2.1442E-31	284207	METRNL
-0.129076391	2.1391E-31	388394	RPRML
-0.129118919	2.04398E-31	415116	PIM3
-0.129123569	2.03385E-31	224	ALDH3A2
-0.129141301	1.99565E-31	8293	SERF1A
-0.129194513	1.88519E-31	9927	MFN2
-0.129245028	1.78598E-31	8335	HIST1H2AB
-0.129400671	1.5117E-31	390284	SRP14P1
-0.129504785	1.35199E-31	6697	SPR
-0.129511833	1.3418E-31	56912	C11orf60
-0.129537746	1.30501E-31	399694	SHC4
-0.129567087	1.26457E-31	285268	ZNF621
-0.129574522	1.25452E-31	164153	UBL4B
-0.129590303	1.23343E-31	80205	CHD9
-0.129622713	1.19126E-31	3955	LFNG
-0.129648715	1.15846E-31	55701	AL161668.2
-0.129706115	1.08918E-31	4439	MSH5
-0.12971893	1.0743E-31	401152	C4orf3
-0.129779935	1.0061E-31	5236	PGM1
-0.129782632	1.00319E-31	84436	ZNF528
-0.129789695	9.9561E-32	5611	DNAJC3
-0.129843175	9.39954E-32	54910	SEMA4C
-0.129876435	9.06921E-32	64344	HIF3A
-0.129903361	8.81017E-32	7093	TLL2
-0.1299081	8.76549E-32	3354	HTR1E
-0.129909307	8.754E-32	8904	CPNE1
-0.129936323	8.50317E-32	797	CALCB
-0.129955083	8.33314E-32	83641	FAM107B
-0.129987314	8.04891E-32	116362	RBP7
-0.130040333	7.60212E-32	442454	UQCRBP1
-0.130055472	7.47913E-32	245802	MS4A6E
-0.130108684	7.06238E-32	5623	PSPN
-0.130142123	6.81234E-32	2013	EMP2
-0.130168125	6.62393E-32	92092	ZC3HAV1L
-0.130195603	6.43051E-32	757	TMEM50B
-0.130332589	5.54679E-32	1288	COL4A6
-0.130432978	4.97673E-32	84522	JAGN1
-0.130433902	4.97182E-32	79912	PYROXD1
-0.130451173	4.87984E-32	3170	FOXA2
-0.130497515	4.64146E-32	8654	PDE5A
-0.130553424	4.36907E-32	567	B2M
-0.130553693	4.36786E-32	57610	RANBP10
-0.130592987	4.18608E-32	222699	TOB2P1
-0.130596235	4.17138E-32	6285	S100B
-0.130633935	4.00463E-32	9321	TRIP11
-0.13066031	3.89194E-32	157773	C8orf48
-0.130684182	3.7926E-32	10220	GDF11
-0.130702093	3.71977E-32	4720	NDUFS2
-0.130731076	3.60485E-32	3293	HSD17B3
-0.130770355	3.45463E-32	6324	SCN1B
-0.130784571	3.40186E-32	64965	MRPS9
-0.130852818	3.15928E-32	84645	C22orf23
-0.130864993	3.11787E-32	9650	MTFR1
-0.130865082	3.11753E-32	8537	BCAS1
-0.130922556	2.92914E-32	1603	DAD1
-0.13094309	2.86465E-32	7080	NKX2-1
-0.13108851	2.4463E-32	103	ADAR
-0.131111264	2.3866E-32	127495	LRRC39

region-wise, subcortex					
Spearman's corr coef	p-val	p-val, SA	p-val, SA (BH- correction)	GenID	Gene
-0.178120971	0.297414929	0.331	1	26589	MRPL46
-0.178120971	0.297414929	0.314	1	55775	TDP1
-0.178120971	0.297414929	0.323	1	284161	GDPD1
-0.178378388	0.296708167	0.359	1	1129	CHRM2
-0.178378388	0.296708167	0.322	1	1514	CTSL1
-0.178378388	0.296708167	0.294	1	5525	PPP2R5A
-0.178378388	0.296708167	0.327	1	23192	ATG4B
-0.178378388	0.296708167	0.351	1	23259	DDHD2
-0.178378388	0.296708167	0.313	1	51741	WWOX
-0.178378388	0.296708167	0.341	1	55969	C20orf24
-0.178378388	0.296708167	0.318	1	64760	FAM160B2
-0.178635776	0.296002775	0.354	1	23136	EPB41L3
-0.178635776	0.296002775	0.303	1	25983	NGDN
-0.178635776	0.296002775	0.327	1	56112	PCDHGA3
-0.178635776	0.296002775	0.348	1	90139	TSPAN18
-0.178635776	0.296002775	0.334	1	144193	AMDHD1
-0.178893179	0.295298487	0.344	1	323	APBB2
-0.178893179	0.295298487	0.358	1	4352	MPL
-0.178893179	0.295298487	0.342	1	9801	MRPL19
-0.178893179	0.295298487	0.323	1	11141	IL1RAPL1
-0.178893179	0.295298487	0.323	1	11156	PTP4A3
-0.178893179	0.295298487	0.355	1	51596	CUTA
-0.178893179	0.295298487	0.323	1	55613	MTMR8
-0.178893179	0.295298487	0.332	1	79884	MAP9
-0.178893179	0.295298487	0.335	1	152485	ZNF827
-0.178893179	0.295298487	0.315	1	643641	ZNF862
-0.178893179	0.295298487	0.358	1	100131564	LOC100131564
-0.179150581	0.294595361	0.324	1	3231	HOXD1
-0.179150581	0.294595361	0.313	1	6924	TCEB3
-0.179150581	0.294595361	0.316	1	7221	TRPC2
-0.179150581	0.294595361	0.344	1	114883	OSBPL9
-0.179150581	0.294595361	0.362	1	202052	DNAJC18
-0.179150581	0.294595361	0.335	1	414919	C8orf82
-0.179150581	0.294595361	0.369	1	100132708	CYP4F30P
-0.179407984	0.29389286	0.34	1	1798	DPAGT1
-0.179407984	0.29389286	0.348	1	2494	NR5A2
-0.179407984	0.29389286	0.356	1	2934	GSN
-0.179407984	0.29389286	0.334	1	8714	ABCC3
-0.179407984	0.29389286	0.335	1	56833	SLAMF8
-0.179407984	0.29389286	0.326	1	84446	BRSK1
-0.179407984	0.29389286	0.352	1	90332	EXOC3L2
-0.179407984	0.29389286	0.345	1	197342	EME2
-0.179407984	0.29389286	0.336	1	200895	DHFRL1
-0.179665387	0.29319188	0.347	1	1687	DFNA5
-0.179665387	0.29319188	0.337	1	4595	MUTYH
-0.179665387	0.29319188	0.361	1	10240	MRPS31
-0.179665387	0.29319188	0.306	1	55818	KDM3A
-0.179665387	0.29319188	0.308	1	57459	GATAD2B
-0.179665387	0.29319188	0.346	1	80279	CDK5RAP3
-0.179665387	0.29319188	0.338	1	85016	C11orf70
-0.179665387	0.29319188	0.345	1	90827	ZNF479
-0.179665387	0.29319188	0.343	1	220979	C10orf25
-0.179922774	0.292491913	0.328	1	7175	TPR
-0.179922774	0.292491913	0.322	1	10628	TXNIP
-0.179922774	0.292491913	0.329	1	26140	TTLL3
-0.179922774	0.292491913	0.369	1	29956	LASS2
-0.179922774	0.292491913	0.323	1	29957	SLC25A24
-0.179922774	0.292491913	0.332	1	54621	AC131238.1
-0.179922774	0.292491913	0.311	1	124842	TMEM132E
-0.179922774	0.292491913	0.314	1	246126	CYorf15B
-0.180180177	0.291793138	0.349	1	587	BCAT2
-0.180180177	0.291793138	0.314	1	3688	ITGB1
-0.180180177	0.291793138	0.344	1	8187	ZNF239
-0.180180177	0.291793138	0.31	1	10755	GIPC1
-0.180180177	0.291793138	0.329	1	51447	IP6K2
-0.180180177	0.291793138	0.31	1	54558	SPATA6
-0.180180177	0.291793138	0.319	1	388561	ZNF761
-0.180180177	0.291793138	0.354	1	391322	LOC391322
-0.18043758	0.291094989	0.321	1	2829	XCR1
-0.18043758	0.291094989	0.355	1	9039	UBA3
-0.18043758	0.291094989	0.343	1	9517	SPTLC2
-0.18043758	0.291094989	0.34	1	9618	TRAF4
-0.18043758	0.291094989	0.346	1	51676	ASB2
-0.18043758	0.291094989	0.342	1	64429	ZDHHC6
-0.18043758	0.291094989	0.343	1	64599	GIGYF1
-0.18043758	0.291094989	0.344	1	127428	C1orf83
-0.18043758	0.291094989	0.342	1	159371	TMEM20
-0.18043758	0.291094989	0.313	1	163059	ZNF433
-0.180694982	0.290398359	0.335	1	1785	DNM2

voxel-wise, subcortex			
Spearman's corr coef	p-val	GenID	Gene
-0.131119341	2.36573E-32	140460	ASB7
-0.131126493	2.34744E-32	23329	TBC1D30
-0.131168559	2.24256E-32	55556	ENOSF1
-0.131186485	2.19927E-32	7378	UPP1
-0.131216303	2.12913E-32	51125	GOLGA7
-0.131278798	1.98921E-32	401431	RP4-751H13.1
-0.131295696	1.95299E-32	1298	COL9A2
-0.131302193	1.93922E-32	6010	RHO
-0.131324679	1.89234E-32	7916	BAT2
-0.131328106	1.8853E-32	199692	ZNF627
-0.131409273	1.72589E-32	10151	HNRNPA3P1
-0.131443352	1.663E-32	10111	RAD50
-0.131444663	1.66064E-32	8773	SNAP23
-0.131481707	1.59496E-32	83656	C8orf12
-0.131497681	1.56743E-32	646993	HMGB3P24
-0.131499544	1.56424E-32	7164	TPD52L1
-0.131542817	1.49221E-32	10630	PDPN
-0.131612375	1.3832E-32	54504	CPVL
-0.13161999	1.37179E-32	147525	LINC00526
-0.131636143	1.34782E-32	55653	BCAS4
-0.131708398	1.24564E-32	93953	ACRC
-0.131725475	1.22263E-32	1195	CLK1
-0.131762072	1.17475E-32	23306	TMEM194A
-0.131779164	1.15303E-32	91664	ZNF845
-0.131790489	1.13886E-32	50831	TAS2R3
-0.131822616	1.09958E-32	139324	HDX
-0.131856978	1.05908E-32	4066	LYL1
-0.131886512	1.02544E-32	81669	CCNL2
-0.131903604	1.00645E-32	162	AP1B1
-0.131904811	1.00512E-32	4054	LTBP3
-0.131923288	9.85027E-33	57459	GATAD2B
-0.131927833	9.80151E-33	6296	ACSM3
-0.132137805	7.7896E-33	120	ADD3
-0.132188052	7.37252E-33	51004	COQ6
-0.132213593	7.16911E-33	445329	SULT1A4
-0.132271901	6.72529E-33	642280	ZNF876P
-0.132380933	5.96739E-33	2187	FANCB
-0.132404611	5.81431E-33	163223	ZNF676
-0.132557377	4.91648E-33	51079	NDUFA13
-0.132656738	4.40789E-33	6872	TAF1
-0.132753327	3.96361E-33	8522	GAS7
-0.132788792	3.8119E-33	112495	GTF3C6
-0.132797256	3.77661E-33	6170	RPL39
-0.132816941	3.69558E-33	81846	SBF2
-0.132819444	3.68549E-33	79648	MCPH1
-0.132866621	3.49895E-33	100128822	LOC100128822
-0.132897541	3.38174E-33	26240	FAM50B
-0.132953733	3.17871E-33	1944	EFNA3
-0.133063585	2.81619E-33	1739	DLG1
-0.133075193	2.78038E-33	200634	KRTCAP3
-0.133088842	2.73884E-33	10623	POLR3C
-0.13316305	2.5235E-33	84275	SLC25A33
-0.133178651	2.4804E-33	966	CD59
-0.133187011	2.45762E-33	84061	MAGT1
-0.133203909	2.41219E-33	55177	FAM82A2
-0.133229166	2.34586E-33	11159	RABL2A
-0.133257672	2.27318E-33	404093	CUEDC1
-0.133288696	2.19659E-33	83695	C12orf32
-0.133288786	2.19637E-33	124512	C17orf95
-0.133296683	2.17728E-33	26958	COPG2
-0.133307368	2.15173E-33	3181	HNRNPA2B1
-0.133329093	2.10069E-33	151790	WDR49
-0.133354262	2.04305E-33	1378	CR1
-0.133382961	1.97926E-33	5627	PROS1
-0.133401528	1.93903E-33	10314	LANCL1
-0.133405611	1.93028E-33	83475	DOHH
-0.133425489	1.88833E-33	22929	SEPHS1
-0.133431986	1.87481E-33	1407	CRY1
-0.133489385	1.75945E-33	11176	BAZ2A
-0.133520588	1.69974E-33	7841	MOGS
-0.133531079	1.6801E-33	79411	GLB1L
-0.133531541	1.67923E-33	54948	MRPL16
-0.133582518	1.58709E-33	57611	ISLR2
-0.133593947	1.56716E-33	9328	GTF3C5
-0.133610293	1.53905E-33	219285	SAMD9L
-0.133649021	1.4744E-33	283248	RCOR2
-0.133665547	1.44764E-33	91316	AP000347.4
-0.133673251	1.43535E-33	2803	GOLGA4
-0.133703902	1.3874E-33	11180	WDR6

region-wise, subcortex					
Spearman's corr coef	p-val	p-val, SA	p-val, SA (BH- correction)	GenID	Gene
-0.180694982	0.290398359	0.327	1	25988	HINFP
-0.180694982	0.290398359	0.355	1	55326	AGPAT5
-0.180694982	0.290398359	0.322	1	56006	C19orf61
-0.180694982	0.290398359	0.314	1	79649	MAP7D3
-0.180952385	0.289702773	0.332	1	3421	IDH3G
-0.180952385	0.289702773	0.351	1	4796	NFKBIL2
-0.180952385	0.289702773	0.307	1	6302	TSPAN31
-0.180952385	0.289702773	0.341	1	23228	PLCL2
-0.180952385	0.289702773	0.335	1	56650	CLDND1
-0.180952385	0.289702773	0.326	1	148103	ZNF599
-0.180952385	0.289702773	0.356	1	645027	EVPLL
-0.180952385	0.289702773	0.336	1	100128174	LOC100128174
-0.180952385	0.289702773	0.329	1	100288560	LOC100288560
-0.181209773	0.28900823	0.333	1	3884	KRT33B
-0.181209773	0.28900823	0.338	1	26353	HSPB8
-0.181209773	0.28900823	0.325	1	54059	C21orf57
-0.181467175	0.288314849	0.335	1	98	ACYP2
-0.181467175	0.288314849	0.325	1	178	AGL
-0.181467175	0.288314849	0.318	1	3635	INPP5D
-0.181467175	0.288314849	0.324	1	6207	RPS13P2
-0.181467175	0.288314849	0.302	1	6520	SLC3A2
-0.181467175	0.288314849	0.314	1	9786	KIAA0586
-0.181467175	0.288314849	0.307	1	23433	RHOQ
-0.181467175	0.288314849	0.336	1	55421	C17orf85
-0.181467175	0.288314849	0.35	1	57122	NUP107
-0.181467175	0.288314849	0.334	1	57409	MIF4GD
-0.181724578	0.287622213	0.336	1	4684	NCAM1
-0.181724578	0.287622213	0.323	1	7681	MKRN3
-0.181724578	0.287622213	0.344	1	8751	ADAM15
-0.181724578	0.287622213	0.321	1	11161	C14orf1
-0.181724578	0.287622213	0.34	1	25878	MXRA5
-0.181724578	0.287622213	0.333	1	92815	HIST3H2A
-0.181724578	0.287622213	0.333	1	94274	PPP1R14A
-0.181724578	0.287622213	0.338	1	123920	CMTM3
-0.181724578	0.287622213	0.337	1	124512	C17orf95
-0.181724578	0.287622213	0.341	1	259293	TAS2R30
-0.181981981	0.286930919	0.324	1	9770	RASSF2
-0.181981981	0.286930919	0.328	1	10483	SEC23B
-0.181981981	0.286930919	0.327	1	23683	PRKD3
-0.181981981	0.286930919	0.337	1	83878	USHBP1
-0.181981981	0.286930919	0.332	1	115560	ZNF501
-0.182239383	0.286240727	0.33	1	250	ALPP
-0.182239383	0.286240727	0.323	1	2800	GOLGA1
-0.182239383	0.286240727	0.353	1	3818	KLKB1
-0.182239383	0.286240727	0.314	1	4643	MYO1E
-0.182239383	0.286240727	0.311	1	5191	PEX7
-0.182239383	0.286240727	0.327	1	55755	CDK5RAP2
-0.182239383	0.286240727	0.318	1	79944	L2HGDH
-0.182239383	0.286240727	0.301	1	114908	TMEM123
-0.182496771	0.285551667	0.331	1	1747	DLX3
-0.182496771	0.285551667	0.322	1	3125	HLA-DRB3
-0.182496771	0.285551667	0.341	1	3556	IL1RAP
-0.182496771	0.285551667	0.329	1	9806	SPOCK2
-0.182496771	0.285551667	0.335	1	26118	WSB1
-0.182496771	0.285551667	0.319	1	51493	C22orf28
-0.182496771	0.285551667	0.319	1	79905	TMC7
-0.182754174	0.284863412	0.351	1	1287	COL4A5
-0.182754174	0.284863412	0.31	1	29088	MRPL15
-0.182754174	0.284863412	0.3	1	54544	CRCT1
-0.182754174	0.284863412	0.324	1	56649	TMPRSS4
-0.182754174	0.284863412	0.351	1	90529	C1orf201
-0.182754174	0.284863412	0.321	1	389558	FAM180A
-0.183011577	0.284176469	0.338	1	5597	MAPK6
-0.183011577	0.284176469	0.327	1	6121	RPE65
-0.183011577	0.284176469	0.33	1	7917	BAT3
-0.183011577	0.284176469	0.352	1	9654	TTLL4
-0.183011577	0.284176469	0.321	1	65110	UPF3A
-0.183011577	0.284176469	0.327	1	119587	CPXM2
-0.183011577	0.284176469	0.332	1	222229	LRWD1
-0.183268979	0.283490628	0.334	1	10198	MPHOSPH9
-0.183268979	0.283490628	0.304	1	25793	FBXO7
-0.183268979	0.283490628	0.315	1	55379	LRRC59
-0.183268979	0.283490628	0.317	1	81328	OR4A15
-0.183268979	0.283490628	0.318	1	140679	SLC32A1
-0.183268979	0.283490628	0.314	1	219654	ZCCHC24
-0.183268979	0.283490628	0.343	1	254827	NAALADL2
-0.183526382	0.28280586	0.297	1	2013	EMP2
-0.183526382	0.28280586	0.329	1	2549	GAB1
-0.183526382	0.28280586	0.334	1	23048	FNBP1

voxel-wise, subcortex			
Spearman's corr coef	p-val	GenID	Gene
-0.133764818	1.29681E-33	4040	LRP6
-0.133949623	1.05637E-33	11068	CYB561D2
-0.133965597	1.03781E-33	29796	UCRC
-0.133965775	1.0376E-33	6217	RPS16P1
-0.134091705	9.02124E-34	84173	ELMOD3
-0.134108424	8.85521E-34	55783	FTSJD1
-0.134179562	8.18164E-34	10423	CDIPT
-0.134219021	7.83017E-34	64288	ZNF323
-0.134247348	7.58724E-34	56650	CLDND1
-0.134263143	7.45496E-34	56479	KCNQ5
-0.134281427	7.30483E-34	6764	ST5
-0.134296939	7.17975E-34	54149	C21orf91
-0.134304836	7.11677E-34	3795	KHK
-0.134331763	6.90668E-34	4694	NDUFA1
-0.134368822	6.62741E-34	23155	CLCC1
-0.13437058	6.61437E-34	6138	RPL15
-0.134388134	6.48637E-34	10998	SLC27A5
-0.134389535	6.47616E-34	23630	KCNE1L
-0.134441435	6.11231E-34	57685	CACHD1
-0.13445881	5.99499E-34	57578	KIAA1409
-0.134471908	5.9081E-34	84217	ZMYND12
-0.134475157	5.88679E-34	10808	HSPH1
-0.134502739	5.70846E-34	10645	CAMKK2
-0.134527162	5.5551E-34	5653	KLK6
-0.134532258	5.52358E-34	122953	JDP2
-0.134614259	5.0407E-34	202333	CMYA5
-0.134710014	4.52964E-34	27291	C10orf28
-0.134738982	4.38537E-34	143684	FAM76B
-0.13476564	4.25672E-34	6678	SPARC
-0.134798691	4.10238E-34	9986	RCE1
-0.134827852	3.97083E-34	317649	EIF4E3
-0.134829164	3.96498E-34	55819	RNF130
-0.134831846	3.95318E-34	4124	MAN2A1
-0.134832412	3.95061E-34	489	ATP2A3
-0.134896487	3.67749E-34	25925	ZNF521
-0.13490206	3.65467E-34	10513	APPBP2
-0.13491793	3.59028E-34	255043	TMEM86B
-0.135007739	3.24702E-34	87178	PNPT1
-0.135044053	3.11773E-34	5894	RAF1
-0.135067821	3.03579E-34	64919	BCL11B
-0.135113969	2.88288E-34	4058	LTK
-0.135132551	2.82344E-34	23451	SF3B1
-0.135165051	2.72251E-34	285367	RPUSD3
-0.135240555	2.50151E-34	26524	LATS2
-0.135324597	2.27648E-34	729535	FAM207BP
-0.135328874	2.26561E-34	57650	KIAA1524
-0.135358036	2.19263E-34	51096	UTP18
-0.135358676	2.19105E-34	25805	BAMBI
-0.135377064	2.14631E-34	10991	SLC38A3
-0.135468721	1.93639E-34	6233	RPS27A
-0.135517567	1.83299E-34	283598	C14orf177
-0.135564476	1.73885E-34	441722	LOC441722
-0.135596409	1.67753E-34	8226	HDHD1A
-0.135596886	1.67661E-34	10270	AKAP8
-0.13560088	1.66913E-34	648	BMI1
-0.135610163	1.65179E-34	64771	C6orf106
-0.135622144	1.62968E-34	54708	MARCHF5
-0.135655478	1.56972E-34	8604	SLC25A12
-0.135677025	1.53213E-34	5424	POLD1
-0.135724202	1.4529E-34	23382	AHCYL2
-0.135733202	1.43827E-34	79694	MANEA
-0.135749549	1.41203E-34	115399	LRRC56
-0.135763943	1.38935E-34	55191	NADSYN1
-0.135800168	1.33382E-34	7711	ZNF155
-0.135814458	1.31254E-34	79012	CAMKV
-0.135978281	1.09127E-34	153443	SRFBP1
-0.135982364	1.08625E-34	340947	LOC340947
-0.136051267	1.00502E-34	53407	STX18
-0.136070862	9.83037E-35	56521	DNAJC12
-0.136117294	9.32849E-35	390354	RPL18AP3
-0.136190474	8.58877E-35	9732	DOCK4
-0.136220574	8.3016E-35	4716	NDUFB10
-0.136242479	8.09869E-35	91807	MYLK3
-0.136305541	7.54162E-35	55020	TTC38
-0.136321053	7.41049E-35	9054	NFS1
-0.13634482	7.21412E-35	91768	CABLES1
-0.136352718	7.14994E-35	55765	C1orf106
-0.136355594	7.12673E-35	147991	DPY19L3
-0.136383086	6.90858E-35	100505741	SPATA1

region-wise, subcortex					
Spearman's corr coef	p-val	p-val, SA	p-val, SA (BH- correction)	GenID	Gene
-0.183526382	0.28280586	0.336	1	170487	C20orf134
-0.18378377	0.282121897	0.322	1	4336	MOBP
-0.18378377	0.282121897	0.333	1	4656	MYOG
-0.18378377	0.282121897	0.324	1	6579	SLCO1A2
-0.18378377	0.282121897	0.325	1	11167	FSTL1
-0.18378377	0.282121897	0.306	1	23119	HIC2
-0.18378377	0.282121897	0.336	1	64756	ATPAF1
-0.18378377	0.282121897	0.338	1	348995	NUP43
-0.184041172	0.281439394	0.31	1	4671	NAIP
-0.184041172	0.281439394	0.305	1	4804	NGFR
-0.184041172	0.281439394	0.293	1	54799	MBTD1
-0.184041172	0.281439394	0.33	1	55971	BAIAP2L1
-0.184041172	0.281439394	0.337	1	116362	RBP7
-0.184041172	0.281439394	0.313	1	120364	LOC120364
-0.184298575	0.280757874	0.324	1	51114	ZDHHC9
-0.184298575	0.280757874	0.316	1	57507	ZNF608
-0.184298575	0.280757874	0.341	1	80723	TMEM22
-0.184555978	0.280077457	0.322	1	140876	C20orf175
-0.18481338	0.279398113	0.291	1	1522	CTSZ
-0.18481338	0.279398113	0.343	1	6440	SFTPC
-0.18481338	0.279398113	0.312	1	8632	DNAH17
-0.18481338	0.279398113	0.276	1	29893	PSMC3IP
-0.18481338	0.279398113	0.337	1	55806	HR
-0.18481338	0.279398113	0.311	1	85001	CTD-2514K5.2
-0.18481338	0.279398113	0.314	1	401357	LOC401357
-0.185070768	0.278719574	0.339	1	538	ATP7A
-0.185070768	0.278719574	0.327	1	4050	LTB
-0.185070768	0.278719574	0.353	1	6426	SRSF1
-0.185070768	0.278719574	0.295	1	22807	IKZF2
-0.185070768	0.278719574	0.284	1	56127	PCDHB9
-0.185070768	0.278719574	0.302	1	79912	PYROXD1
-0.185070768	0.278719574	0.321	1	442720	EIF3IP1
-0.185328171	0.278042465	0.313	1	7326	AC087742.2
-0.185328171	0.278042465	0.337	1	27229	TUBGCP4
-0.185328171	0.278042465	0.311	1	55701	AL161668.2
-0.185328171	0.278042465	0.295	1	339829	CCDC39
-0.185328171	0.278042465	0.31	1	388523	ZNF728
-0.185585588	0.2773664	0.324	1	3111	HLA-DOA
-0.185585588	0.2773664	0.344	1	7417	VDAC2
-0.185585588	0.2773664	0.299	1	285381	DPH3
-0.185585588	0.2773664	0.324	1	374666	WASH3P
-0.185842991	0.276691377	0.317	1	5175	PECAM1
-0.185842991	0.276691377	0.314	1	8535	CBX4
-0.185842991	0.276691377	0.324	1	10548	TM9SF1
-0.185842991	0.276691377	0.326	1	10954	PDIA5
-0.185842991	0.276691377	0.318	1	23019	CNOT1
-0.185842991	0.276691377	0.289	1	23660	ZKSCAN5
-0.185842991	0.276691377	0.34	1	79029	SPATA5L1
-0.185842991	0.276691377	0.351	1	84327	ZBED3
-0.185842991	0.276691377	0.324	1	133482	SLCO6A1
-0.185842991	0.276691377	0.313	1	442113	LOC442113
-0.185842991	0.276691377	0.308	1	619455	SPANXA2-OT1
-0.186100394	0.276017159	0.317	1	63931	MRPS14
-0.186100394	0.276017159	0.316	1	84079	ANKRD27
-0.186100394	0.276017159	0.319	1	401265	KLHL31
-0.186357796	0.275344312	0.321	1	632	BGLAP
-0.186357796	0.275344312	0.336	1	3092	HIP1
-0.186357796	0.275344312	0.299	1	3612	IMPA1
-0.186357796	0.275344312	0.284	1	4778	NFE2
-0.186357796	0.275344312	0.306	1	6764	ST5
-0.186357796	0.275344312	0.295	1	8879	SGPL1
-0.186357796	0.275344312	0.289	1	10010	TANK
-0.186357796	0.275344312	0.315	1	55603	FAM46A
-0.186357796	0.275344312	0.32	1	80012	PHC3
-0.186357796	0.275344312	0.348	1	340526	RGAG4
-0.186357796	0.275344312	0.318	1	645996	NAP1L6
-0.186615184	0.274672627	0.307	1	6237	RRAS
-0.186615184	0.274672627	0.308	1	6330	SCN4B
-0.186615184	0.274672627	0.305	1	6856	SYPL1
-0.186615184	0.274672627	0.29	1	9936	CD302
-0.186615184	0.274672627	0.31	1	23545	ATP6V0A2
-0.186615184	0.274672627	0.315	1	26574	AATF
-0.186615184	0.274672627	0.336	1	29925	GMPPB
-0.186615184	0.274672627	0.311	1	143941	TTC36
-0.186615184	0.274672627	0.314	1	339291	LRRC30
-0.186872587	0.274001956	0.313	1	11083	DIDO1
-0.186872587	0.274001956	0.33	1	85317	BAGE4
-0.186872587	0.274001956	0.315	1	123283	TARSL2
-0.186872587	0.274001956	0.302	1	202374	STK32A

voxel-wise, subcortex			
Spearman's corr coef	p-val	GenID	Gene
-0.136435926	6.50757E-35	201627	FAM116A
-0.136475205	6.22469E-35	11145	PLA2G16
-0.136567146	5.60927E-35	7422	VEGFA
-0.136586547	5.48739E-35	8943	AP3D1
-0.136647016	5.12402E-35	203190	LGI3
-0.136692703	4.86536E-35	10875	FGL2
-0.136698648	4.83267E-35	79929	MAP6D1
-0.136711925	4.76049E-35	79811	SLTM
-0.136718601	4.72456E-35	148789	B3GALNT2
-0.136733189	4.64707E-35	8661	EIF3A
-0.136826888	4.17852E-35	1742	DLG4
-0.136995435	3.45058E-35	10657	KHDRBS1
-0.137003988	3.41724E-35	440077	ZNF705A
-0.137074649	3.1535E-35	440138	ALG11
-0.137089238	3.10163E-35	7728	ZNF175
-0.137090534	3.09703E-35	8693	GALNT4
-0.137105584	3.04447E-35	90871	C9orf123
-0.137119606	2.99631E-35	8348	HIST1H2BO
-0.13712424	2.98056E-35	60496	AASDHPPT
-0.137135759	2.94177E-35	1325	CORT
-0.137141332	2.92317E-35	6775	STAT4
-0.137208387	2.70848E-35	399821	RP11-383C5.4
-0.137245253	2.59715E-35	11057	ABHD2
-0.137249991	2.58317E-35	83878	USHBP1
-0.137264654	2.54044E-35	3083	HGFAC
-0.137278125	2.50174E-35	7385	UQCRC2
-0.137370348	2.25228E-35	5343	PLGLB1
-0.13738659	2.21096E-35	388799	C20orf107
-0.137404427	2.16645E-35	3306	HSPA2
-0.137445003	2.06854E-35	30061	SLC40A1
-0.137518749	1.90169E-35	208	AKT2
-0.137526825	1.88427E-35	5507	PPP1R3C
-0.137551337	1.83231E-35	10130	PDIA6
-0.137574375	1.78475E-35	100129034	LOC100129034
-0.137592107	1.74899E-35	9531	BAG3
-0.137592942	1.74734E-35	284312	ZSCAN1
-0.137602881	1.72762E-35	85440	DOCK7
-0.137623593	1.68726E-35	25836	NIPBL
-0.137671784	1.59692E-35	54933	RHBDL2
-0.137677908	1.58581E-35	7761	ZNF214
-0.137685061	1.5729E-35	84750	FUT10
-0.137709945	1.52882E-35	58496	LY6G5B
-0.137714788	1.52039E-35	23203	PMPCA
-0.137722209	1.50754E-35	10632	ATP5L
-0.137808487	1.36599E-35	303	ANXA2P1
-0.137862816	1.28373E-35	7404	UTY
-0.137863651	1.28249E-35	1317	SLC31A1
-0.137912869	1.21228E-35	114908	TMEM123
-0.137969047	1.13679E-35	79934	ADCK4
-0.137969792	1.13583E-35	51067	YARS2
-0.137987152	1.11349E-35	984	CDC2L1
-0.138035998	1.05293E-35	84883	AIFM2
-0.138095438	9.83625E-36	4992	OR1F1
-0.138215244	8.57424E-36	10129	FRY
-0.138225541	8.47354E-36	196385	DNAH10
-0.138263807	8.10971E-36	56658	TRIM39
-0.138288513	7.88316E-36	26020	LRP10
-0.138296023	7.81554E-36	143	PARP4
-0.138364479	7.22508E-36	7644	ZNF91
-0.13836503	7.22039E-36	79918	SETD6
-0.138369948	7.17978E-36	55256	ADI1
-0.13840124	6.92639E-36	83849	SYT15
-0.138404027	6.90427E-36	79659	DYNC2H1
-0.138449728	6.55128E-36	285368	PRRT3
-0.138481304	6.31778E-36	5455	POU3F3
-0.138487235	6.27497E-36	80243	PREX2
-0.13849625	6.21033E-36	84366	PRAC
-0.138520762	6.03777E-36	130872	AHSA2
-0.1385777	5.65531E-36	9187	SLC24A1
-0.13860333	5.49108E-36	57144	PAK7
-0.138616696	5.4072E-36	79740	ZBBX
-0.138661921	5.13316E-36	392	ARHGAP1
-0.138691083	4.96383E-36	65220	NADK
-0.138741687	4.68289E-36	4599	MX1
-0.13875246	4.62524E-36	84991	RBM17
-0.138753027	4.62224E-36	6871	TADA2L
-0.138784319	4.45862E-36	57467	HHATL
-0.138840318	4.18018E-36	7718	ZNF165
-0.138947487	3.69448E-36	29956	LASS2

region-wise, subcortex					
Spearman's corr coef	p-val	p-val, SA	p-val, SA (BH- correction)	GenID	Gene
-0.187129989	0.273332417	0.315	1	1103	CHAT
-0.187129989	0.273332417	0.305	1	3858	KRT10
-0.187129989	0.273332417	0.342	1	6299	SALL1
-0.187129989	0.273332417	0.306	1	9196	KCNAB3
-0.187129989	0.273332417	0.313	1	27006	FGF22
-0.187129989	0.273332417	0.333	1	51603	METTL13
-0.187129989	0.273332417	0.317	1	58476	TP53INP2
-0.187129989	0.273332417	0.333	1	79754	ASB13
-0.187129989	0.273332417	0.303	1	139760	GPR119
-0.187387392	0.272663563	0.307	1	102	ADAM10
-0.187387392	0.272663563	0.305	1	216	ALDH1A1
-0.187387392	0.272663563	0.316	1	546	ATRX
-0.187387392	0.272663563	0.311	1	7043	TGFB3
-0.187387392	0.272663563	0.328	1	28990	ASTE1
-0.187387392	0.272663563	0.291	1	54443	ANLN
-0.187387392	0.272663563	0.311	1	55023	PHIP
-0.187387392	0.272663563	0.298	1	91392	ZNF502
-0.187387392	0.272663563	0.324	1	113510	HELQ
-0.187387392	0.272663563	0.284	1	118738	ZNF488
-0.187387392	0.272663563	0.319	1	260429	PRSS33
-0.187387392	0.272663563	0.327	1	285800	PRR18
-0.187387392	0.272663563	0.321	1	399881	HNRNPKP3
-0.187387392	0.272663563	0.319	1	648359	FAM108A4P
-0.187644795	0.27199617	0.325	1	354	KLK3
-0.187644795	0.27199617	0.299	1	498	ATP5A1
-0.187644795	0.27199617	0.305	1	2827	GPR3
-0.187644795	0.27199617	0.308	1	84313	VPS25
-0.187644795	0.27199617	0.305	1	124751	KRBA2
-0.187644795	0.27199617	0.334	1	143244	EIF5AL1
-0.187644795	0.27199617	0.285	1	646891	SDCCAG3P2
-0.187902182	0.27132988	0.29	1	7082	TJP1
-0.187902182	0.27132988	0.35	1	10071	MUC12
-0.187902182	0.27132988	0.314	1	84765	ZNF577
-0.187902182	0.27132988	0.299	1	165679	SPTSSB
-0.188159585	0.270664603	0.316	1	6873	TAF2
-0.188159585	0.270664603	0.284	1	7323	UBE2D3
-0.188159585	0.270664603	0.313	1	10278	EFS
-0.188159585	0.270664603	0.293	1	64838	FNDC4
-0.188159585	0.270664603	0.303	1	353135	LCE1E
-0.188416988	0.27000013	0.329	1	9246	UBE2L6
-0.188416988	0.27000013	0.32	1	91614	DEPDC7
-0.188416988	0.27000013	0.296	1	139322	APOOL
-0.188416988	0.27000013	0.313	1	100310812	RP11-577H5.2
-0.18867439	0.269337058	0.315	1	23650	TRIM29
-0.18867439	0.269337058	0.305	1	26184	OR1F2P
-0.18867439	0.269337058	0.329	1	54508	FLJ11235
-0.18867439	0.269337058	0.301	1	55832	CAND1
-0.18867439	0.269337058	0.327	1	81850	KRTAP1-3
-0.18867439	0.269337058	0.323	1	131177	FAM3D
-0.188931793	0.268675059	0.305	1	10655	DMRT2
-0.188931793	0.268675059	0.294	1	29954	POMT2
-0.189189181	0.268014163	0.302	1	143	PARP4
-0.189189181	0.268014163	0.296	1	2843	GPR20
-0.189189181	0.268014163	0.323	1	8641	PCDHGB4
-0.189189181	0.268014163	0.307	1	29843	SENP1
-0.189189181	0.268014163	0.321	1	51259	TMEM216
-0.189189181	0.268014163	0.309	1	79885	HDAC11
-0.189189181	0.268014163	0.285	1	219858	OR8B12
-0.189446583	0.267354012	0.319	1	1636	ACE
-0.189446583	0.267354012	0.313	1	5476	CTSA
-0.189446583	0.267354012	0.276	1	54933	RHBDL2
-0.189446583	0.267354012	0.301	1	55367	LRDD
-0.189446583	0.267354012	0.266	1	80349	WDR61
-0.189446583	0.267354012	0.277	1	81576	CCDC130
-0.189446583	0.267354012	0.298	1	92285	ZNF585B
-0.189446583	0.267354012	0.293	1	388555	IGFL3
-0.189703986	0.266695231	0.276	1	10763	NES
-0.189703986	0.266695231	0.316	1	11093	ADAMTS13
-0.189703986	0.266695231	0.314	1	50835	TAS2R9
-0.189703986	0.266695231	0.304	1	56000	NXF3
-0.189703986	0.266695231	0.297	1	64979	MRPL36
-0.189703986	0.266695231	0.321	1	81856	ZNF611
-0.189961389	0.266037583	0.297	1	1236	CCR7
-0.189961389	0.266037583	0.312	1	10404	AP003117.1
-0.189961389	0.266037583	0.307	1	28987	NOB1
-0.189961389	0.266037583	0.283	1	150291	MORC2-AS1
-0.189961389	0.266037583	0.286	1	256987	SERINC5
-0.189961389	0.266037583	0.305	1	727764	MAFIP
-0.190218791	0.265380949	0.311	1	7033	TFF3

voxel-wise, subcortex			
Spearman's corr coef	p-val	GenID	Gene
-0.139017969	3.40605E-36	6494	SIPA1
-0.139021039	3.39402E-36	63920	C5orf54
-0.139049456	3.28461E-36	79809	TTC21B
-0.139091983	3.12728E-36	56915	EXOSC5
-0.139104053	3.08401E-36	84541	KBTBD8
-0.139112979	3.0524E-36	3572	IL6ST
-0.139144734	2.94252E-36	93166	PRDM6
-0.139155224	2.90713E-36	140901	STK35
-0.139180392	2.82381E-36	10776	ARPP19
-0.139197394	2.7689E-36	53838	C11orf24
-0.139233142	2.65692E-36	10181	RBM5
-0.139238432	2.64071E-36	9918	NCAPD2
-0.139276788	2.52625E-36	51692	CPSF3
-0.13929081	2.48565E-36	201456	FBXO15
-0.139320806	2.4009E-36	3563	IL3RA
-0.139349222	2.32331E-36	8500	PPFIA1
-0.139368355	2.27245E-36	147657	ZNF480
-0.139368534	2.272E-36	84002	B3GNT5
-0.139385536	2.22778E-36	92304	SCGB3A1
-0.139387116	2.2237E-36	285800	PRR18
-0.139392778	2.2092E-36	167691	LCA5
-0.139397144	2.19802E-36	50832	TAS2R4
-0.139475614	2.00728E-36	27233	SULT1C4
-0.139502451	1.9459E-36	2066	ERBB4
-0.139515087	1.91763E-36	57097	PARP11
-0.139523908	1.89814E-36	1466	CSRP2
-0.139538571	1.86617E-36	29980	DONSON
-0.139573395	1.79241E-36	84553	FAXC
-0.139710099	1.52981E-36	7544	ZFY
-0.139739811	1.47801E-36	646576	RP11-291L15.2
-0.139749661	1.46122E-36	84631	SLITRK2
-0.139765263	1.435E-36	57488	ESYT2
-0.13979359	1.38865E-36	9687	AC110754.1
-0.139838904	1.31751E-36	23048	FNBP1
-0.139899179	1.22849E-36	9444	QKI
-0.139927402	1.18889E-36	51133	KCTD3
-0.139945328	1.16438E-36	332	BIRC5
-0.140011176	1.07862E-36	374887	YJEFN3
-0.140053615	1.02673E-36	6657	SOX2
-0.140132919	9.36285E-37	6194	RPS6
-0.140179634	8.86775E-37	5132	PDC
-0.140196905	8.69131E-37	375757	C9orf119
-0.140219942	8.46143E-37	51663	ZFR
-0.140220031	8.46052E-37	1174	AP1S1
-0.140245944	8.20922E-37	2161	F12
-0.140276402	7.92332E-37	55270	NUDT15
-0.140389323	6.94676E-37	349334	FOXD4L4
-0.140469	6.33079E-37	283337	ZNF740
-0.140523791	5.93889E-37	55846	ITFG2
-0.140529454	5.89972E-37	9951	HS3ST4
-0.140652418	5.11109E-37	23469	PHF3
-0.140682593	4.93422E-37	256979	SUNC1
-0.14069958	4.83731E-37	9001	HAP1
-0.140741661	4.6052E-37	7389	UROD
-0.140760601	4.50454E-37	254552	NUDT8
-0.140796453	4.31964E-37	254042	METAP1D
-0.14081344	4.2348E-37	6431	SFRS6
-0.140820965	4.19768E-37	6529	SLC6A1
-0.140821055	4.19726E-37	2113	ETS1
-0.14084065	4.10218E-37	84942	WDR73
-0.140865356	3.98543E-37	221476	PI16
-0.140926272	3.71134E-37	94274	PPP1R14A
-0.140936583	3.66685E-37	125476	INO80C
-0.141020074	3.32554E-37	162681	C18orf54
-0.141024336	3.30898E-37	57828	C19orf15
-0.141028419	3.29323E-37	26152	ZNF337
-0.141031027	3.28322E-37	4646	MYO6
-0.141052753	3.20076E-37	8547	FCN3
-0.14113076	2.92125E-37	3122	HLA-DRA
-0.141154259	2.84193E-37	266623	SLC25A5P1
-0.141191497	2.72061E-37	91893	FDXACB1
-0.141299874	2.39592E-37	5934	RBL2
-0.141314641	2.3548E-37	59348	ZNF350
-0.141362458	2.2263E-37	10811	NOXA1
-0.141405001	2.11788E-37	4793	NFKBIB
-0.141417533	2.08696E-37	3137	HLA-J
-0.141484678	1.92872E-37	158572	LOC158572
-0.141523957	1.84176E-37	79797	ZNF408
-0.141541421	1.80434E-37	644037	HNRNPA1P12

region-wise, subcortex					
Spearman's corr coef	p-val	p-val, SA	p-val, SA (BH- correction)	GenID	Gene
-0.190218791	0.265380949	0.297	1	9860	LRIG2
-0.190218791	0.265380949	0.297	1	9940	DLEC1
-0.190218791	0.265380949	0.299	1	220729	AC024560.3
-0.190218791	0.265380949	0.299	1	339175	METTL2A
-0.190218791	0.265380949	0.327	1	653269	POTEI
-0.190476179	0.264725506	0.3	1	3597	IL13RA1
-0.190476179	0.264725506	0.292	1	9658	ZNF516
-0.190476179	0.264725506	0.306	1	23451	SF3B1
-0.190476179	0.264725506	0.303	1	25853	WDR40A
-0.190476179	0.264725506	0.298	1	26246	OR2L2
-0.190476179	0.264725506	0.302	1	30851	TAX1BP3
-0.190476179	0.264725506	0.308	1	54868	TMEM104
-0.190476179	0.264725506	0.319	1	56953	NT5M
-0.190733582	0.264070719	0.288	1	860	RUNX2
-0.190733582	0.264070719	0.303	1	4298	MLLT1
-0.190733582	0.264070719	0.282	1	9025	RNF8
-0.190733582	0.264070719	0.296	1	50619	DEF6
-0.190990984	0.263417393	0.323	1	1145	CHRNE
-0.190990984	0.263417393	0.311	1	6480	ST6GAL1
-0.190990984	0.263417393	0.301	1	6538	SLC6A11
-0.190990984	0.263417393	0.327	1	7458	EIF4H
-0.190990984	0.263417393	0.287	1	7869	SEMA3B
-0.190990984	0.263417393	0.303	1	10089	KCNK7
-0.190990984	0.263417393	0.308	1	23315	SLC9A8
-0.190990984	0.263417393	0.304	1	27238	GPKOW
-0.190990984	0.263417393	0.307	1	51161	C3orf18
-0.190990984	0.263417393	0.288	1	64172	OSGEPL1
-0.190990984	0.263417393	0.299	1	84645	C22orf23
-0.190990984	0.263417393	0.315	1	147184	TMEM99
-0.190990984	0.263417393	0.298	1	284391	ZNF844
-0.191248387	0.26276508	0.305	1	4702	NDUFA8
-0.191248387	0.26276508	0.276	1	7171	TPM4
-0.191248387	0.26276508	0.299	1	7200	TRH
-0.191248387	0.26276508	0.307	1	27201	GPR78
-0.191248387	0.26276508	0.296	1	51564	HDAC7
-0.191248387	0.26276508	0.326	1	57561	ARRDC3
-0.191248387	0.26276508	0.3	1	64771	C6orf106
-0.191248387	0.26276508	0.31	1	79591	C10orf76
-0.191248387	0.26276508	0.318	1	89797	NAV2
-0.191248387	0.26276508	0.314	1	114327	EFHC1
-0.191248387	0.26276508	0.299	1	390072	OR52N4
-0.19150579	0.262113869	0.308	1	5010	CLDN11
-0.19150579	0.262113869	0.276	1	8290	HIST3H3
-0.19150579	0.262113869	0.311	1	23213	SULF1
-0.19150579	0.262113869	0.3	1	65979	PHACTR4
-0.19150579	0.262113869	0.304	1	140803	TRPM6
-0.191763178	0.261463493	0.267	1	3137	HLA-J
-0.191763178	0.261463493	0.289	1	7576	ZNF28
-0.191763178	0.261463493	0.308	1	8738	CRADD
-0.191763178	0.261463493	0.304	1	10181	RBM5
-0.191763178	0.261463493	0.283	1	10425	ARIH2
-0.191763178	0.261463493	0.317	1	57222	ERGIC1
-0.191763178	0.261463493	0.313	1	91894	C11orf52
-0.191763178	0.261463493	0.287	1	93643	TJAP1
-0.191763178	0.261463493	0.31	1	283953	TMEM114
-0.19202058	0.260814458	0.29	1	5082	PDCL
-0.19202058	0.260814458	0.292	1	8642	DCHS1
-0.19202058	0.260814458	0.289	1	25764	HYPK
-0.19202058	0.260814458	0.324	1	29763	PACSIN3
-0.19202058	0.260814458	0.291	1	84971	ATG4D
-0.19202058	0.260814458	0.296	1	144347	FAM101A
-0.19202058	0.260814458	0.304	1	389400	GFRAL
-0.192277983	0.260166466	0.292	1	84142	FAM175A
-0.192277983	0.260166466	0.313	1	387644	LINC00202
-0.192277983	0.260166466	0.292	1	440061	TRIM64EP
-0.192277983	0.260166466	0.31	1	554202	MIR31HG
-0.1925354	0.259519547	0.3	1	7027	TFDP1
-0.1925354	0.259519547	0.296	1	7597	ZBTB25
-0.1925354	0.259519547	0.291	1	10667	FARS2
-0.1925354	0.259519547	0.295	1	25780	RASGRP3
-0.1925354	0.259519547	0.316	1	54973	CPSF3L
-0.1925354	0.259519547	0.288	1	93134	ZNF561
-0.192792803	0.258873492	0.288	1	105	ADARB2
-0.192792803	0.258873492	0.256	1	1477	CSTF1
-0.192792803	0.258873492	0.302	1	2631	GBAS
-0.192792803	0.258873492	0.326	1	9270	ITGB1BP1
-0.192792803	0.258873492	0.282	1	9381	OTOF
-0.192792803	0.258873492	0.271	1	56616	DIABLO
-0.192792803	0.258873492	0.276	1	64077	LHPP

voxel-wise, subcortex			
Spearman's corr coef	p-val	GenID	Gene
-0.141573176	1.73826E-37	28231	SLCO4A1
-0.141594172	1.6959E-37	5095	PCCA
-0.141605869	1.67273E-37	284648	LOC284648
-0.141631126	1.62377E-37	10113	PREB
-0.141836181	1.27567E-37	6572	SLC18A3
-0.141841844	1.26719E-37	100132356	LOC100132356
-0.141875833	1.21749E-37	284119	PTRF
-0.141882792	1.20753E-37	23193	GANAB
-0.141898215	1.18579E-37	3705	ITPK1
-0.141964704	1.09644E-37	3126	HLA-DRB4
-0.141976953	1.08071E-37	93653	ST7-AS1
-0.142004639	1.04601E-37	64764	CREB3L2
-0.142033234	1.01136E-37	55062	WIPI1
-0.142050698	9.90715E-38	1936	EEF1D
-0.142120525	9.12392E-38	6714	SRC
-0.142159164	8.71709E-38	23040	MYT1L
-0.142214507	8.16579E-38	728407	LOC728407
-0.142220646	8.1069E-38	256987	SERINC5
-0.142239958	7.92394E-38	51012	SLMO2
-0.142281935	7.54076E-38	55421	C17orf85
-0.142316759	7.23678E-38	6927	HNF1A
-0.142323717	7.17749E-38	23642	SNHG1
-0.142454013	6.15282E-38	11279	KLF8
-0.142505735	5.78757E-38	9365	KL
-0.142598599	5.18512E-38	137872	ADHFE1
-0.142599538	5.17943E-38	85366	MYLK2
-0.142606318	5.13798E-38	647030	LOC647030
-0.142645687	4.90385E-38	3382	ICA1
-0.142652377	4.86521E-38	89887	ZNF628
-0.142676234	4.72966E-38	5956	OPN1LW
-0.142883241	3.70033E-38	9778	KIAA0232
-0.142895028	3.64892E-38	51093	C1orf66
-0.142927438	3.51128E-38	6160	RPL31
-0.142944247	3.44189E-38	80323	CCDC68
-0.143066093	2.97822E-38	221458	KIF6
-0.143090889	2.89175E-38	29954	POMT2
-0.143112332	2.81899E-38	7965	AIMP2
-0.143150687	2.69335E-38	10052	GJC1
-0.143359542	2.10078E-38	80162	ATHL1
-0.143369108	2.07696E-38	10153	CEBPZ
-0.143383607	2.04146E-38	5597	MAPK6
-0.14353469	1.70519E-38	619279	ZNF704
-0.143557638	1.65914E-38	23113	CUL9
-0.143565714	1.64325E-38	50848	F11R
-0.1435671	1.64052E-38	977	CD151
-0.143599138	1.57903E-38	80829	ZFP91
-0.143619195	1.54171E-38	100130264	RP5-1027G4.3
-0.143635169	1.51262E-38	4041	LRP5
-0.143649474	1.48702E-38	8613	PPAP2B
-0.143664896	1.45991E-38	6092	ROBO2
-0.143680215	1.43347E-38	92399	MRRF
-0.143720701	1.36584E-38	9808	KIAA0087
-0.143786266	1.26303E-38	6137	RPL13
-0.143817008	1.21747E-38	57185	NIPAL3
-0.143872917	1.13881E-38	79692	ZNF322
-0.143904209	1.09701E-38	255967	PAN3
-0.143956497	1.03055E-38	2549	GAB1
-0.143966898	1.0178E-38	91353	IGLL3P
-0.143997163	9.81621E-39	9356	SLC22A6
-0.144018903	9.56422E-39	8079	MLF2
-0.144035801	9.37283E-39	83607	AMMECR1L
-0.144136086	8.31274E-39	57514	CDGAP
-0.14422366	7.48499E-39	5150	PDE7A
-0.144248083	7.26929E-39	10755	GIPC1
-0.144258022	7.1832E-39	124411	ZNF720
-0.144286066	6.94588E-39	441519	CT45A3
-0.144299358	6.83606E-39	55727	BTBD7
-0.144301489	6.81853E-39	23210	JMJD6
-0.144318104	6.6841E-39	5799	PTPRN2
-0.144353673	6.40512E-39	286451	YIPF6
-0.144371226	6.27161E-39	9475	ROCK2
-0.144385159	6.16778E-39	79602	ADIPOR2
-0.144404754	6.02441E-39	1463	NCAN
-0.144423693	5.88912E-39	54039	PCBP3
-0.144465297	5.60239E-39	22850	ADNP2
-0.144479886	5.5052E-39	11244	ZHX1
-0.144511089	5.30279E-39	79888	LPCAT1
-0.144567177	4.9574E-39	51652	VPS24
-0.14456746	4.95571E-39	79723	SUV39H2

region-wise, subcortex					
Spearman's corr coef	p-val	p-val, SA	p-val, SA (BH- correction)	GenID	Gene
-0.192792803	0.258873492	0.272	1	389084	C2orf82
-0.192792803	0.258873492	0.308	1	730094	C16ORF52
-0.193050191	0.258228749	0.302	1	2645	GCK
-0.193050191	0.258228749	0.297	1	5424	POLD1
-0.193050191	0.258228749	0.298	1	10509	SEMA4B
-0.193050191	0.258228749	0.317	1	27347	STK39
-0.193050191	0.258228749	0.284	1	27351	PPPDE2
-0.193050191	0.258228749	0.314	1	54765	TRIM44
-0.193050191	0.258228749	0.269	1	55051	C14orf102
-0.193050191	0.258228749	0.299	1	116844	LRG1
-0.193050191	0.258228749	0.3	1	118663	BTBD16
-0.193050191	0.258228749	0.291	1	283116	AC084851.1
-0.193307593	0.257585108	0.308	1	1180	CLCN1
-0.193307593	0.257585108	0.291	1	4241	MFI2
-0.193307593	0.257585108	0.309	1	5638	PRRG1
-0.193307593	0.257585108	0.312	1	25912	C1orf43
-0.193307593	0.257585108	0.306	1	55167	MSL2
-0.193307593	0.257585108	0.295	1	55503	TRPV6
-0.193307593	0.257585108	0.287	1	64940	STAG3L4
-0.193307593	0.257585108	0.318	1	150223	YDJC
-0.193307593	0.257585108	0.298	1	205251	LINC00116
-0.193307593	0.257585108	0.303	1	222389	BEND7
-0.193564996	0.256942511	0.269	1	6566	SLC16A1
-0.193564996	0.256942511	0.281	1	6698	SPRR1A
-0.193564996	0.256942511	0.304	1	8082	SSPN
-0.193564996	0.256942511	0.278	1	55749	CCAR1
-0.193564996	0.256942511	0.304	1	57704	GBA2
-0.193564996	0.256942511	0.293	1	100134229	LOC100134229
-0.193822399	0.256300986	0.31	1	1376	CPT2
-0.193822399	0.256300986	0.297	1	3148	HMGB2
-0.193822399	0.256300986	0.291	1	5305	PIP5K2A
-0.193822399	0.256300986	0.314	1	57732	ZFYVE28
-0.193822399	0.256300986	0.31	1	440934	AC010980.2
-0.194079801	0.255660295	0.276	1	4146	MATN1
-0.194079801	0.255660295	0.293	1	22981	NINL
-0.194079801	0.255660295	0.305	1	29916	SNX11
-0.194079801	0.255660295	0.323	1	388389	CCDC103
-0.194337189	0.255020976	0.283	1	4708	NDUFB2
-0.194337189	0.255020976	0.309	1	79100	RP1-10C16.2
-0.194337189	0.255020976	0.289	1	84946	LTV1
-0.194337189	0.255020976	0.289	1	123879	DCUN1D3
-0.194594592	0.25438267	0.307	1	8521	GCM1
-0.194594592	0.25438267	0.281	1	23140	ZZEF1
-0.194594592	0.25438267	0.311	1	26696	OR2T1
-0.194594592	0.25438267	0.297	1	55695	NSUN5
-0.194594592	0.25438267	0.303	1	80317	ZKSCAN3
-0.194594592	0.25438267	0.334	1	84872	ZC3H10
-0.194594592	0.25438267	0.298	1	94240	EPSTI1
-0.194594592	0.25438267	0.304	1	150356	CHADL
-0.194851995	0.253745437	0.299	1	20	ABCA2
-0.194851995	0.253745437	0.299	1	916	CD3E
-0.194851995	0.253745437	0.289	1	10281	DSCR4
-0.194851995	0.253745437	0.278	1	11011	TLK2
-0.194851995	0.253745437	0.3	1	26133	TRPC4AP
-0.194851995	0.253745437	0.267	1	85025	TMEM60
-0.194851995	0.253745437	0.288	1	284365	AC010325.3
-0.194851995	0.253745437	0.291	1	388323	GLTPD2
-0.194851995	0.253745437	0.262	1	100506144	RP11-244H3.1
-0.195109397	0.253109038	0.301	1	10284	SAP18
-0.195109397	0.253109038	0.291	1	204851	HIPK1
-0.195109397	0.253109038	0.306	1	399694	SHC4
-0.1953668	0.25247401	0.297	1	833	CARS
-0.1953668	0.25247401	0.291	1	952	CD38
-0.1953668	0.25247401	0.273	1	8341	HIST1H2BN
-0.1953668	0.25247401	0.273	1	56957	OTUD7B
-0.1953668	0.25247401	0.297	1	63035	BCORL1
-0.195624188	0.251840025	0.292	1	2920	CXCL2
-0.195624188	0.251840025	0.295	1	5253	PHF2
-0.195624188	0.251840025	0.289	1	9907	AP5Z1
-0.195624188	0.251840025	0.296	1	54814	QPCTL
-0.195624188	0.251840025	0.286	1	54882	ANKHD1
-0.195624188	0.251840025	0.275	1	84230	LRRC8C
-0.195624188	0.251840025	0.306	1	221476	PI16
-0.195624188	0.251840025	0.295	1	643253	CCT6P1
-0.19588159	0.251207113	0.283	1	6775	STAT4
-0.19588159	0.251207113	0.285	1	8537	BCAS1
-0.19588159	0.251207113	0.265	1	80095	ZNF606
-0.19588159	0.251207113	0.293	1	81488	GRINL1A
-0.196138993	0.250574976	0.307	1	3293	HSD17B3

voxel-wise, subcortex			
Spearman's corr coef	p-val	GenID	Gene
-0.144582868	4.865E-39	53944	CSNK1G1
-0.144619927	4.65307E-39	9445	ITM2B
-0.14469932	4.22968E-39	54558	SPATA6
-0.144699976	4.22628E-39	57531	HACE1
-0.144712701	4.16216E-39	863	CBFA2T3
-0.144729778	4.07764E-39	2921	CXCL3
-0.144756436	3.94898E-39	51542	VPS54
-0.144764975	3.90867E-39	6300	MAPK12
-0.144766554	3.90124E-39	9051	PSTPIP1
-0.144771114	3.87987E-39	29984	RHOD
-0.144812897	3.68969E-39	79971	GPR177
-0.144827753	3.62431E-39	25841	ABTB2
-0.144840568	3.56882E-39	163050	ZNF564
-0.144882828	3.3919E-39	397	ARHGDIB
-0.144883379	3.38961E-39	113791	PIK3IP1
-0.145013019	2.89974E-39	5349	FXYD3
-0.145024076	2.86136E-39	10293	TRAIP
-0.145039961	2.80713E-39	30968	STOML2
-0.145067543	2.71534E-39	84809	CROCCP2
-0.145081848	2.66895E-39	7482	WNT2B
-0.145104125	2.59821E-39	64834	ELOVL1
-0.145147964	2.46445E-39	80022	MYO15B
-0.145153716	2.44742E-39	58526	MID1IP1
-0.145160317	2.42799E-39	23	ABCF1
-0.145191237	2.33911E-39	64172	OSGEPL1
-0.145212412	2.28012E-39	56922	MCCC1
-0.145254388	2.16755E-39	57215	THAP11
-0.14535208	1.92641E-39	55914	ERBB2IP
-0.145360619	1.90661E-39	976	CD97
-0.145361185	1.90536E-39	2262	GPC5
-0.145366758	1.89254E-39	89797	NAV2
-0.145385042	1.85121E-39	8850	KAT2B
-0.145418659	1.77753E-39	5348	FXYD1
-0.145471692	1.6672E-39	348	APOE
-0.145502806	1.60564E-39	114932	MRFAP1L1
-0.145540878	1.53342E-39	115123	MARCHF3
-0.145540968	1.53324E-39	4643	MYO1E
-0.145545155	1.52552E-39	114899	C1QTNF3
-0.145567626	1.48462E-39	9765	ZFYVE16
-0.145567894	1.48412E-39	9757	AD000671.1
-0.14557673	1.46833E-39	4839	NOP2
-0.145590276	1.44448E-39	100287852	LOC100287852
-0.145632356	1.37277E-39	55037	PTCD3
-0.14565213	1.34033E-39	84859	LRCH3
-0.145758092	1.17896E-39	80312	TET1
-0.145823374	1.08932E-39	79068	FTO
-0.145864055	1.03691E-39	6376	CX3CL1
-0.145901382	9.9104E-40	57447	NDRG2
-0.145932123	9.54783E-40	55325	UFSP2
-0.146007523	8.7133E-40	84081	CCDC55
-0.146029726	8.48172E-40	55250	ELP2
-0.146045789	8.31794E-40	301	ANXA1
-0.146051273	8.26284E-40	643438	LOC643438
-0.146061212	8.16388E-40	51537	MTP18
-0.146092966	7.85506E-40	51201	ZDHHC2
-0.146105692	7.73478E-40	51604	PIGT
-0.146135494	7.45992E-40	23492	CBX7
-0.146137446	7.4421E-40	63982	ANO3
-0.146154627	7.28838E-40	158427	C9orf97
-0.14616558	7.19216E-40	91120	ZNF682
-0.146184072	7.03239E-40	23478	SEC11B
-0.146214902	6.77385E-40	11240	PADI2
-0.146229103	6.65799E-40	146712	B3GNTL1
-0.146246105	6.52181E-40	342574	KRT27
-0.146261513	6.40085E-40	23313	C22orf9
-0.146279529	6.26223E-40	2592	GALT
-0.14636144	5.6685E-40	23766	GABARAPL3
-0.146404251	5.38087E-40	84288	EFCAB2
-0.146423663	5.25599E-40	5617	PRL
-0.146435365	5.18099E-40	6631	SNRPC
-0.146449387	5.0933E-40	7541	ZFP161
-0.146475568	4.93358E-40	53828	FXYD4
-0.146488667	4.8555E-40	11046	SLC35D2
-0.146517172	4.68992E-40	11039	SMA4
-0.146576047	4.36538E-40	11066	SNRNP35
-0.146621555	4.12993E-40	27013	C2orf24
-0.146643102	4.02285E-40	57477	SHROOM4
-0.146654338	3.9682E-40	146429	SLC22A31
-0.146706536	3.72359E-40	388550	CEACAM22P

region-wise, subcortex					
Spearman's corr coef	p-val	p-val, SA	p-val, SA (BH- correction)	GenID	Gene
-0.196138993	0.250574976	0.269	1	6242	RTKN
-0.196138993	0.250574976	0.277	1	23604	DAPK2
-0.196138993	0.250574976	0.282	1	25870	SUMF2
-0.196138993	0.250574976	0.287	1	60678	EEFSEC
-0.196138993	0.250574976	0.296	1	91012	LASS5
-0.196138993	0.250574976	0.26	1	124790	HEXIM2
-0.196138993	0.250574976	0.303	1	196740	C10orf72
-0.196138993	0.250574976	0.286	1	642513	LOC642513
-0.196396396	0.24994421	0.269	1	1655	DDX5
-0.196396396	0.24994421	0.282	1	22834	ZNF652
-0.196396396	0.24994421	0.321	1	284312	ZSCAN1
-0.196396396	0.24994421	0.277	1	388336	AC007510.1
-0.196653798	0.249314591	0.286	1	1410	CRYAB
-0.196653798	0.249314591	0.26	1	5906	RAP1A
-0.196653798	0.249314591	0.303	1	51302	CYP39A1
-0.196653798	0.249314591	0.282	1	51317	PHF21A
-0.196653798	0.249314591	0.269	1	54205	CYCS
-0.196653798	0.249314591	0.277	1	55316	RSAD1
-0.196653798	0.249314591	0.27	1	64434	NOM1
-0.196653798	0.249314591	0.295	1	260341	TFAMP1
-0.196653798	0.249314591	0.29	1	389337	AC022100.1
-0.196911186	0.248685941	0.295	1	1499	CTNNB1
-0.196911186	0.248685941	0.296	1	4521	NUDT1
-0.196911186	0.248685941	0.292	1	6496	SIX3
-0.196911186	0.248685941	0.263	1	10826	C5orf4
-0.196911186	0.248685941	0.278	1	10881	ACTL7A
-0.196911186	0.248685941	0.294	1	23512	SUZ12
-0.196911186	0.248685941	0.297	1	56164	STK31
-0.196911186	0.248685941	0.284	1	100130933	C17ORF110
-0.197168589	0.248058408	0.277	1	2058	EPRS
-0.197168589	0.248058408	0.271	1	3915	LAMC1
-0.197168589	0.248058408	0.267	1	7174	TPP2
-0.197168589	0.248058408	0.296	1	55623	THUMPD1
-0.197168589	0.248058408	0.319	1	55861	DBNDD2
-0.197168589	0.248058408	0.296	1	79602	ADIPOR2
-0.197168589	0.248058408	0.288	1	399744	LOC399744
-0.197168589	0.248058408	0.294	1	644135	RP13-143G15.3
-0.197425991	0.247431636	0.283	1	4299	AFF1
-0.197425991	0.247431636	0.28	1	4520	MTF1
-0.197425991	0.247431636	0.285	1	4782	NFIC
-0.197425991	0.247431636	0.301	1	6373	CXCL11
-0.197425991	0.247431636	0.275	1	10749	KIF1C
-0.197425991	0.247431636	0.289	1	10780	ZNF234
-0.197425991	0.247431636	0.287	1	79844	ZDHHC11
-0.197683394	0.246806219	0.279	1	7013	TERF1
-0.197683394	0.246806219	0.257	1	9625	AATK
-0.197683394	0.246806219	0.284	1	22909	MTMR15
-0.197683394	0.246806219	0.273	1	54149	C21orf91
-0.197683394	0.246806219	0.295	1	87769	A2LD1
-0.197940797	0.24618195	0.307	1	2033	EP300
-0.197940797	0.24618195	0.296	1	4144	MAT2A
-0.197940797	0.24618195	0.282	1	5814	PURB
-0.197940797	0.24618195	0.269	1	79628	SH3TC2
-0.197940797	0.24618195	0.294	1	85235	HIST1H2AH
-0.197940797	0.24618195	0.299	1	374907	B3GNT8
-0.198198184	0.245558679	0.278	1	4303	FOXO4
-0.198198184	0.245558679	0.298	1	6591	SNAI2
-0.198198184	0.245558679	0.294	1	9121	SLC16A5
-0.198198184	0.245558679	0.276	1	23120	ATP10B
-0.198198184	0.245558679	0.284	1	51567	TTRAP
-0.198198184	0.245558679	0.28	1	94031	HTRA3
-0.198198184	0.245558679	0.285	1	122183	PRR20
-0.198198184	0.245558679	0.268	1	147015	DHRS13
-0.198198184	0.245558679	0.282	1	100132346	LOC100132346
-0.198455587	0.244936213	0.324	1	1902	LPAR1
-0.198455587	0.244936213	0.274	1	2524	FUT2
-0.198455587	0.244936213	0.288	1	5030	P2RY4
-0.198455587	0.244936213	0.28	1	7596	ZNF45
-0.198455587	0.244936213	0.289	1	22993	HMGXB3
-0.198455587	0.244936213	0.282	1	51228	GLTP
-0.198455587	0.244936213	0.272	1	51292	GMPR2
-0.198455587	0.244936213	0.268	1	57404	CYP20A1
-0.198455587	0.244936213	0.274	1	79364	ZXDC
-0.198455587	0.244936213	0.286	1	80155	NARG1
-0.198455587	0.244936213	0.312	1	92129	RIPPLY1
-0.198455587	0.244936213	0.262	1	113451	ADC
-0.198455587	0.244936213	0.284	1	128218	TMEM125
-0.198455587	0.244936213	0.283	1	138864	LOC138864
-0.198455587	0.244936213	0.281	1	257044	C1orf101

voxel-wise, subcortex			
Spearman's corr coef	p-val	GenID	Gene
-0.146710798	3.70425E-40	339290	LOC339290
-0.146715537	3.68295E-40	643394	SPINK9
-0.14677757	3.41454E-40	9913	SUPT7L
-0.146785006	3.38374E-40	154007	SNRNP48
-0.146811187	3.27736E-40	7542	ZFPL1
-0.146817967	3.2504E-40	10066	SCAMP2
-0.146859288	3.09054E-40	8295	TRRAP
-0.146887526	2.98583E-40	7639	ZNF85
-0.146934047	2.82093E-40	6222	RPS18
-0.146940276	2.79957E-40	3856	KRT8P9
-0.146951139	2.76269E-40	84701	COX4I2
-0.146977425	2.67536E-40	114907	FBXO32
-0.146997944	2.60913E-40	25976	TIPARP
-0.147022933	2.53066E-40	6374	CXCL5
-0.14703685	2.48801E-40	80336	PABPC1L
-0.147162601	2.13339E-40	125144	C17orf45
-0.147278026	1.8524E-40	23548	TTC33
-0.147295028	1.81423E-40	145389	SLC38A6
-0.147401541	1.59238E-40	2346	FOLH1
-0.147414446	1.56738E-40	84125	LRRIQ1
-0.147432283	1.53352E-40	8412	BCAR3
-0.14744927	1.50191E-40	4116	MAGOH
-0.147512332	1.39014E-40	6560	SLC12A4
-0.147531092	1.35859E-40	56648	EIF5A2
-0.147605568	1.23995E-40	729975	AC018755.8
-0.147606686	1.23824E-40	22839	DLGAP4
-0.147703812	1.09907E-40	54987	C1orf123
-0.14773047	1.0637E-40	1565	CYP2D6
-0.14775981	1.02606E-40	79818	AC010645.1
-0.147812471	9.61795E-41	54757	FAM20A
-0.147835404	9.35058E-41	202559	KHDRBS2
-0.147845432	9.23568E-41	10404	AP003117.1
-0.147849426	9.19056E-41	788	SLC25A20
-0.147855192	9.12582E-41	29925	GMPPB
-0.147912398	8.50588E-41	51228	GLTP
-0.147921771	8.40835E-41	6558	SLC12A2
-0.147955298	8.06896E-41	221481	C6orf81
-0.148006007	7.58102E-41	64397	ZFP106
-0.148012042	7.52497E-41	11020	RABL4
-0.148015752	7.4905E-41	27164	SALL3
-0.148035347	7.31198E-41	92342	C1orf156
-0.148105189	6.7097E-41	9255	AIMP1
-0.148122638	6.56705E-41	90990	KIFC2
-0.148130447	6.50427E-41	64418	TMEM168
-0.148146972	6.37311E-41	84329	HVCN1
-0.148177803	6.13573E-41	6242	RTKN
-0.148187935	6.05949E-41	54567	DLL4
-0.148215041	5.86051E-41	84532	ACSS1
-0.148217738	5.84117E-41	7102	TSPAN7
-0.148237616	5.69992E-41	55056	FLJ10038
-0.148256749	5.56708E-41	55690	PACS1
-0.14830029	5.27617E-41	79628	SH3TC2
-0.148339301	5.02842E-41	55183	RIF1
-0.148366049	4.86503E-41	6441	SFTPD
-0.148388058	4.73471E-41	10600	USP16
-0.148393437	4.70332E-41	147727	LOC147727
-0.148420095	4.55142E-41	84851	TRIM52
-0.148455754	4.35524E-41	55776	C6orf64
-0.148512408	4.06096E-41	91607	SLFN11
-0.148557439	3.84124E-41	390213	DOC2GP
-0.148575276	3.75772E-41	11177	BAZ1A
-0.148577601	3.74707E-41	5281	PIGF
-0.14857769	3.74651E-41	56246	MRAP
-0.148617625	3.56602E-41	388523	ZNF728
-0.148617804	3.56546E-41	84967	LSM10
-0.148618549	3.5621E-41	387521	TMEM189
-0.14869684	3.23364E-41	333932	HIST2H3A
-0.148714945	3.16189E-41	2902	GRIN1
-0.148820907	2.77345E-41	3337	DNAJB1
-0.148853868	2.66247E-41	2843	GPR20
-0.148871794	2.60417E-41	26027	ACOT11
-0.148877278	2.5868E-41	7572	ZNF24
-0.148930207	2.42256E-41	22802	CLCA4
-0.149016753	2.17622E-41	2905	GRIN2C
-0.149023905	2.15688E-41	9538	EI24
-0.149028465	2.14483E-41	200403	VWA3B
-0.149134785	1.87998E-41	128338	DRAM2
-0.149206489	1.72023E-41	84513	PPAPDC1B
-0.149226457	1.67791E-41	642517	BMS1P6

region-wise, subcortex					
Spearman's corr coef	p-val	p-val, SA	p-val, SA (BH- correction)	GenID	Gene
-0.19871299	0.244315073	0.312	1	9509	ADAMTS2
-0.19871299	0.244315073	0.295	1	10349	ABCA10
-0.19871299	0.244315073	0.288	1	50859	SPOCK3
-0.19871299	0.244315073	0.273	1	54776	LENG3
-0.19871299	0.244315073	0.296	1	54861	SNRK
-0.19871299	0.244315073	0.298	1	57691	KIAA1586
-0.198970392	0.243695021	0.271	1	8620	NPFF
-0.198970392	0.243695021	0.285	1	9144	SYNGR2
-0.198970392	0.243695021	0.31	1	10420	TESK2
-0.198970392	0.243695021	0.295	1	23245	ASTN2
-0.198970392	0.243695021	0.271	1	51660	BRP44L
-0.198970392	0.243695021	0.276	1	83692	CD99L2
-0.198970392	0.243695021	0.284	1	84266	ALKBH7
-0.198970392	0.243695021	0.278	1	255488	RNF144B
-0.199227795	0.243076101	0.303	1	2297	RP11-79P5.6
-0.199227795	0.243076101	0.291	1	3038	HAS3
-0.199227795	0.243076101	0.278	1	5859	QARS
-0.199227795	0.243076101	0.287	1	8819	SAP30
-0.199227795	0.243076101	0.267	1	11155	LDB3
-0.199227795	0.243076101	0.303	1	55658	RNF126
-0.199227795	0.243076101	0.288	1	280664	WFDC10B
-0.199227795	0.243076101	0.279	1	284427	SLC25A41
-0.199227795	0.243076101	0.295	1	353274	ZNF445
-0.199227795	0.243076101	0.276	1	644538	Z83826.4
-0.199485198	0.242457896	0.269	1	6285	S100B
-0.199485198	0.242457896	0.297	1	8736	MYOM1
-0.199485198	0.242457896	0.273	1	10196	PRMT3
-0.199485198	0.242457896	0.287	1	23114	NFASC
-0.199485198	0.242457896	0.284	1	28966	SNX24
-0.199485198	0.242457896	0.304	1	80162	ATHL1
-0.199485198	0.242457896	0.292	1	80264	ZNF430
-0.199485198	0.242457896	0.276	1	81610	FAM83D
-0.199485198	0.242457896	0.304	1	84893	FBXO18
-0.199485198	0.242457896	0.265	1	117196	MRGPRX4
-0.199485198	0.242457896	0.254	1	645644	FLJ42627
-0.1997426	0.241841048	0.268	1	4332	MNDA
-0.1997426	0.241841048	0.298	1	8661	EIF3A
-0.1997426	0.241841048	0.282	1	9670	IPO13
-0.1997426	0.241841048	0.294	1	10110	SGK2
-0.1997426	0.241841048	0.297	1	117283	IP6K3
-0.1997426	0.241841048	0.266	1	124599	CD300LB
-0.200000003	0.241225272	0.293	1	1132	CHRM4
-0.200000003	0.241225272	0.281	1	7507	XPA
-0.200000003	0.241225272	0.294	1	9341	VAMP3
-0.200000003	0.241225272	0.28	1	221496	LEMD2
-0.200257406	0.24061057	0.292	1	412	STS
-0.200257406	0.24061057	0.278	1	4642	MYO1D
-0.200257406	0.24061057	0.292	1	8625	RFXANK
-0.200257406	0.24061057	0.303	1	8701	DNAH11
-0.200257406	0.24061057	0.29	1	23384	CYTSA
-0.200257406	0.24061057	0.301	1	27086	FOXP1
-0.200257406	0.24061057	0.283	1	128312	HIST3H2BB
-0.200257406	0.24061057	0.266	1	219965	OR5B17
-0.200257406	0.24061057	0.267	1	220108	FAM124A
-0.200257406	0.24061057	0.276	1	347734	SLC35B2
-0.200514808	0.23999697	0.282	1	5162	PDHB
-0.200514808	0.23999697	0.262	1	7248	TSC1
-0.200514808	0.23999697	0.274	1	9238	TBRG4
-0.200514808	0.23999697	0.296	1	26153	KIF26A
-0.200514808	0.23999697	0.309	1	83461	CDCA3
-0.200514808	0.23999697	0.286	1	439949	LOC439949
-0.200514808	0.23999697	0.27	1	728957	ZNF705C
-0.200772196	0.239384115	0.279	1	3337	DNAJB1
-0.200772196	0.239384115	0.268	1	6386	SDCBP
-0.200772196	0.239384115	0.264	1	8398	PLA2G6
-0.200772196	0.239384115	0.291	1	9765	ZFYVE16
-0.200772196	0.239384115	0.268	1	126295	ZNF57
-0.200772196	0.239384115	0.291	1	148229	ATP8B3
-0.200772196	0.239384115	0.307	1	374768	SPEM1
-0.201029599	0.238772601	0.278	1	1234	CCR5
-0.201029599	0.238772601	0.288	1	25923	ATL3
-0.201029599	0.238772601	0.279	1	51700	CYB5R2
-0.201029599	0.238772601	0.276	1	64793	CCDC21
-0.201029599	0.238772601	0.267	1	83449	PMFBP1
-0.201287001	0.238162175	0.306	1	2634	GBP2
-0.201287001	0.238162175	0.278	1	3340	NDST1
-0.201287001	0.238162175	0.304	1	3769	KCNJ13
-0.201287001	0.238162175	0.287	1	8170	SLC14A2
-0.201287001	0.238162175	0.279	1	53407	STX18

voxel-wise, subcortex			
Spearman's corr coef	p-val	GenID	Gene
-0.149234429	1.66138E-41	55844	PPP2R2D
-0.149258211	1.61317E-41	1350	COX7C
-0.149332508	1.4708E-41	1130	LYST
-0.149338171	1.46071E-41	8552	INE1
-0.14934662	1.44558E-41	26528	DAZAP1
-0.149378479	1.38953E-41	8994	LIMD1
-0.149414033	1.32927E-41	148645	LINC00337
-0.149416268	1.32563E-41	586	BCAT1
-0.14941971	1.32002E-41	8028	MLLT10
-0.149420083	1.31946E-41	1327	COX4I1
-0.149422213	1.31582E-41	286140	RNF5P1
-0.149427593	1.30713E-41	113655	MFSD3
-0.149483874	1.21885E-41	9341	VAMP3
-0.149502724	1.19054E-41	3783	KCNN4
-0.149544328	1.13057E-41	1797	DOM3Z
-0.149559557	1.10927E-41	23511	NUP188
-0.149598092	1.05742E-41	25906	C11orf51
-0.149605528	1.04761E-41	51230	PHF20
-0.14961648	1.0336E-41	144321	GLIPR1L2
-0.149674058	9.61851E-42	6668	SP2
-0.149771765	8.51989E-42	100287430	LOC100287430
-0.149790153	8.32371E-42	3640	INSL3
-0.149888396	7.36522E-42	9557	CHD1L
-0.149944857	6.86636E-42	56903	PAPOLB
-0.14996019	6.73464E-42	550	AUP1
-0.149974942	6.61413E-42	11155	LDB3
-0.150009125	6.33667E-42	7205	TRIP6
-0.150022954	6.22737E-42	7766	ZNF223
-0.150063083	5.92469E-42	4089	SMAD4
-0.150123253	5.49309E-42	79027	ZNF655
-0.150124654	5.48748E-42	51302	CYP39A1
-0.150157988	5.26328E-42	400569	MED11
-0.150195971	5.01665E-42	375295	LOC375295
-0.150204048	4.9662E-42	120534	C11orf46
-0.150237575	4.76441E-42	255239	ANKK1
-0.150293857	4.43931E-42	471	ATIC
-0.150422752	3.7779E-42	100129543	ZNF730
-0.150428325	3.75268E-42	50865	HEBP1
-0.150473356	3.54809E-42	25936	NSL1
-0.150473922	3.54529E-42	3249	HPN
-0.150474191	3.54248E-42	5151	PDE8A
-0.150495738	3.44719E-42	54606	DDX56
-0.15056558	3.16133E-42	9670	IPO13
-0.15056929	3.14451E-42	11213	IRAK3
-0.150587305	3.07725E-42	9469	CHST3
-0.150628731	2.92031E-42	51204	TACO1
-0.150681287	2.73533E-42	92745	SLC38A5
-0.150721505	2.60081E-42	55230	USP40
-0.150800899	2.35418E-42	100127206	KIAA1024L
-0.150816873	2.30654E-42	641298	RP11-368J21.1
-0.150940195	1.97583E-42	10054	UBA2
-0.150944844	1.96462E-42	2915	GRM5
-0.151000842	1.8301E-42	6776	STAT5A
-0.151031956	1.76003E-42	26164	GTPBP5
-0.15108414	1.64793E-42	84273	NOA1
-0.151103079	1.60869E-42	79905	TMC7
-0.151243031	1.35085E-42	27230	SERP1
-0.151247963	1.34244E-42	7784	ZP3
-0.151263565	1.31722E-42	343099	CCDC18
-0.151263833	1.31722E-42	157739	TDH
-0.151322618	1.22193E-42	55254	TMEM39A
-0.151326329	1.21633E-42	58517	RBM25
-0.151378334	1.13785E-42	339453	TMEM240
-0.151380286	1.13785E-42	79781	IQCA1
-0.151388466	1.12384E-42	124857	WFIKKN2
-0.15139617	1.11543E-42	23007	PLCH1
-0.151471764	1.01174E-42	134	ADORA1
-0.151502594	9.75304E-43	221908	C7orf47
-0.151516899	9.55686E-43	2800	GOLGA1
-0.151659161	7.9874E-43	6168	RPL37A
-0.151712567	7.45491E-43	125061	AFMID
-0.151814446	6.55808E-43	1583	CYP11A1
-0.151820287	6.50202E-43	131405	TRIM71
-0.151850849	6.27782E-43	8227	SFRS17A
-0.15200825	5.15678E-43	55509	BATF3
-0.152021348	5.04467E-43	9361	LONP1
-0.152114496	4.48416E-43	51053	GMNN
-0.15215525	4.25995E-43	2526	FUT4
-0.152195841	4.03574E-43	1489	CTF1

region-wise, subcortex					
Spearman's corr coef	p-val	p-val, SA	p-val, SA (BH- correction)	GenID	Gene
-0.201287001	0.238162175	0.285	1	55308	DDX19A
-0.201287001	0.238162175	0.271	1	118611	C10orf90
-0.201287001	0.238162175	0.268	1	407975	MIR17HG
-0.201287001	0.238162175	0.295	1	653188	GUSBP3
-0.201544404	0.237552807	0.278	1	908	CCT6A
-0.201544404	0.237552807	0.279	1	1047	CLGN
-0.201544404	0.237552807	0.279	1	6275	S100A4
-0.201544404	0.237552807	0.276	1	6558	SLC12A2
-0.201544404	0.237552807	0.262	1	6655	SOS2
-0.201544404	0.237552807	0.252	1	9056	SLC7A7
-0.201544404	0.237552807	0.272	1	51300	TIMMDC1
-0.201544404	0.237552807	0.257	1	64149	C17orf75
-0.201544404	0.237552807	0.269	1	90592	ZNF700
-0.201544404	0.237552807	0.28	1	388591	RNF207
-0.201801807	0.236944243	0.284	1	2803	GOLGA4
-0.201801807	0.236944243	0.286	1	4973	OLR1
-0.201801807	0.236944243	0.306	1	6801	STRN
-0.201801807	0.236944243	0.295	1	221061	FAM171A1
-0.201801807	0.236944243	0.282	1	259290	TAS2R31
-0.202059194	0.236336991	0.267	1	2880	GPX5
-0.202059194	0.236336991	0.289	1	3676	ITGA4
-0.202059194	0.236336991	0.286	1	9644	SH3PXD2A
-0.202059194	0.236336991	0.287	1	50865	HEBP1
-0.202059194	0.236336991	0.274	1	54514	DDX4
-0.202059194	0.236336991	0.267	1	84747	UNC119B
-0.202059194	0.236336991	0.271	1	387628	FAM88A
-0.202316597	0.235730797	0.262	1	831	CAST
-0.202316597	0.235730797	0.261	1	1676	DFFA
-0.202316597	0.235730797	0.248	1	3292	HSD17B1
-0.202316597	0.235730797	0.277	1	5425	POLD2
-0.202316597	0.235730797	0.263	1	26022	TMEM98
-0.202316597	0.235730797	0.281	1	26266	SLC13A4
-0.202316597	0.235730797	0.289	1	51000	SLC35B3
-0.202316597	0.235730797	0.257	1	51104	FAM108B1
-0.202316597	0.235730797	0.266	1	51735	RAPGEF6
-0.202316597	0.235730797	0.269	1	121274	ZNF641
-0.202316597	0.235730797	0.282	1	221785	ZNF498
-0.202316597	0.235730797	0.284	1	402682	UFSP1
-0.202316597	0.235730797	0.287	1	729359	KIAA1881
-0.202574	0.235125706	0.277	1	1265	CNN2
-0.202574	0.235125706	0.271	1	6646	SOAT1
-0.202574	0.235125706	0.289	1	9188	DDX21
-0.202574	0.235125706	0.271	1	147808	ZNF784
-0.202574	0.235125706	0.278	1	254065	BRWD3
-0.202574	0.235125706	0.292	1	259266	ASPM
-0.202831402	0.234521344	0.265	1	4999	ORC2
-0.202831402	0.234521344	0.301	1	10845	CLPX
-0.202831402	0.234521344	0.289	1	60437	CDH26
-0.202831402	0.234521344	0.276	1	93377	OPALIN
-0.202831402	0.234521344	0.269	1	284459	AC008806.1
-0.202831402	0.234521344	0.285	1	440603	BCL2L15
-0.203088805	0.233918414	0.267	1	3155	HMGCL
-0.203088805	0.233918414	0.278	1	9331	B4GALT6
-0.203088805	0.233918414	0.297	1	23580	CDC42EP4
-0.203088805	0.233918414	0.252	1	26493	OR8B8
-0.203088805	0.233918414	0.262	1	54361	WNT4
-0.203346193	0.233316481	0.26	1	4041	LRP5
-0.203346193	0.233316481	0.263	1	27296	TP53TG5
-0.203346193	0.233316481	0.269	1	79035	OBFC2B
-0.203346193	0.233316481	0.256	1	121601	AC079953.2
-0.203346193	0.233316481	0.275	1	285512	FAM13A-AS1
-0.203603595	0.232715636	0.28	1	12	SERPINA3
-0.203603595	0.232715636	0.288	1	4286	MITF
-0.203603595	0.232715636	0.272	1	57060	PCBP4
-0.203603595	0.232715636	0.277	1	80740	LY6G6C
-0.203603595	0.232715636	0.245	1	136157	SSU72P8
-0.203603595	0.232715636	0.28	1	347689	SOX2-OT
-0.203860998	0.232115835	0.271	1	3012	HIST1H2AE
-0.203860998	0.232115835	0.262	1	3151	HMGN2
-0.203860998	0.232115835	0.274	1	3185	HNRNPF
-0.203860998	0.232115835	0.268	1	7439	BEST1
-0.203860998	0.232115835	0.258	1	28965	SLC27A6
-0.203860998	0.232115835	0.28	1	51726	DNAJB11
-0.203860998	0.232115835	0.284	1	84815	MGC12916
-0.203860998	0.232115835	0.262	1	389123	IQCF2
-0.203860998	0.232115835	0.269	1	646626	LOC646626
-0.203860998	0.232115835	0.259	1	100128361	LOC100128361
-0.204118401	0.231516823	0.249	1	2109	ETFB
-0.204118401	0.231516823	0.268	1	23383	KIAA0892

voxel-wise, subcortex			
Spearman's corr coef	p-val	GenID	Gene
-0.152213395	3.97969E-43	7289	TULP3
-0.152237535	3.86758E-43	283102	KRT8P41
-0.152312949	3.47522E-43	7376	NR1H2
-0.152331799	3.41917E-43	23098	SARM1
-0.152353063	3.30706E-43	25	ABL1
-0.15236105	3.30706E-43	121227	LRIG3
-0.152401254	3.13891E-43	203286	ANKS6
-0.152406275	3.11088E-43	6935	ZEB1
-0.152420104	3.05483E-43	375775	PNPLA7
-0.152452245	2.9147E-43	100287015	RP11-115C21.2
-0.152456611	2.9147E-43	6133	RPL9
-0.152527183	2.66247E-43	79970	ZNF767
-0.152571201	2.52234E-43	113189	CHST14
-0.152599245	2.43826E-43	79741	C10orf68
-0.152675867	2.18603E-43	2622	GAS8
-0.152681336	2.18603E-43	203427	SLC25A43
-0.152698711	2.12997E-43	55281	TMEM140
-0.152726203	2.07392E-43	11095	ADAMTS8
-0.152743742	2.01787E-43	22996	TTC39A
-0.152785718	1.90577E-43	7390	UROS
-0.152789623	1.90577E-43	8608	RDH16
-0.152860671	1.73761E-43	9275	BCL7B
-0.152883604	1.68156E-43	728411	AC138951.4
-0.152949825	1.56945E-43	51585	PCF11
-0.152989656	1.48538E-43	9487	PIGL
-0.153006837	1.45735E-43	11345	GABARAPL2
-0.153009534	1.45735E-43	84466	MEGF10
-0.153163224	1.17709E-43	4899	NRF1
-0.15331395	9.80909E-44	79845	RNF122
-0.153329819	9.52883E-44	1236	CCR7
-0.153352484	9.24857E-44	64801	ARV1
-0.153391674	8.96831E-44	8323	FZD6
-0.153423622	8.40779E-44	145853	C15orf61
-0.153431892	8.40779E-44	79658	ARHGAP10
-0.153487697	7.84727E-44	1660	DHX9
-0.15353775	7.28675E-44	51199	NIN
-0.153565988	7.28675E-44	57541	ZNF398
-0.153611585	6.72623E-44	401428	OR2A20P
-0.15392454	4.48416E-44	2648	KAT2A
-0.154029384	3.92364E-44	118663	BTBD16
-0.154032081	3.92364E-44	9375	TM9SF2
-0.154069036	3.92364E-44	5754	PTK7
-0.154077023	3.64338E-44	22893	BAHD1
-0.154081017	3.64338E-44	11083	DIDO1
-0.154111296	3.36312E-44	4691	NCL
-0.154258296	2.8026E-44	6141	RPL18
-0.154299259	2.8026E-44	10330	CNPY2
-0.154383674	2.52234E-44	1730	DIAPH2
-0.154388219	2.52234E-44	196513	DCP1B
-0.154469386	2.24208E-44	84164	ASCC2
-0.154478297	2.24208E-44	727936	GXYLT2
-0.15449743	2.24208E-44	25850	ZNF345
-0.154509321	2.24208E-44	8340	HIST1H2BL
-0.15453513	2.24208E-44	55255	WDR41
-0.154548779	1.96182E-44	55802	DCP1A
-0.154560387	1.96182E-44	84978	FRMD5
-0.154581934	1.96182E-44	1311	COMP
-0.154597625	1.68156E-44	10110	SGK2
-0.154655769	1.68156E-44	8449	DHX16
-0.154682696	1.68156E-44	219287	FAM123A
-0.154706568	1.68156E-44	246744	STH
-0.15470852	1.68156E-44	25912	C1orf43
-0.154767573	1.68156E-44	10319	LAMC3
-0.154867217	1.4013E-44	2879	GPX4
-0.15488255	1.12104E-44	8576	STK16
-0.154895738	1.12104E-44	10628	TXNIP
-0.155019805	1.12104E-44	55094	GPATCH1
-0.155065864	1.12104E-44	79258	MMEL1
-0.155072361	1.12104E-44	9440	MED17
-0.155130401	1.12104E-44	3965	LGALS9
-0.155171543	8.40779E-45	81832	NETO1
-0.155196428	8.40779E-45	252995	FNDC5
-0.155240446	8.40779E-45	80258	EFHC2
-0.155272394	8.40779E-45	27351	PPPDE2
-0.155302763	5.60519E-45	147650	NCRNA00085
-0.15531075	5.60519E-45	22890	ZBTB1
-0.155314744	5.60519E-45	6815	STYX
-0.155343354	5.60519E-45	572	BAD
-0.155349106	5.60519E-45	100129616	LOC100129616

region-wise, subcortex					
Spearman's corr coef	p-val	p-val, SA	p-val, SA (BH- correction)	GenID	Gene
-0.204118401	0.231516823	0.298	1	79797	ZNF408
-0.204118401	0.231516823	0.271	1	285961	SEPT7L
-0.204118401	0.231516823	0.252	1	442524	AC007276.5
-0.204375803	0.230919167	0.275	1	3636	INPPL1
-0.204375803	0.230919167	0.276	1	4056	LTC4S
-0.204375803	0.230919167	0.269	1	4616	GADD45B
-0.204375803	0.230919167	0.282	1	5375	PMP2
-0.204375803	0.230919167	0.253	1	6549	SLC9A2
-0.204375803	0.230919167	0.279	1	8313	AXIN2
-0.204375803	0.230919167	0.278	1	341378	LOC341378
-0.204375803	0.230919167	0.258	1	440434	AC183087.2
-0.204633191	0.23032257	0.269	1	1674	DES
-0.204633191	0.23032257	0.255	1	22828	RBM16
-0.204633191	0.23032257	0.274	1	55449	AL136419.1
-0.204633191	0.23032257	0.265	1	127396	ZNF684
-0.204633191	0.23032257	0.258	1	138639	PTPDC1
-0.204633191	0.23032257	0.261	1	139411	PTCHD1
-0.204890594	0.229727	0.248	1	889	KRIT1
-0.204890594	0.229727	0.262	1	933	CD22
-0.204890594	0.229727	0.278	1	3553	IL1B
-0.204890594	0.229727	0.291	1	23583	SMUG1
-0.204890594	0.229727	0.266	1	51661	FKBP7
-0.204890594	0.229727	0.266	1	84466	MEGF10
-0.205147997	0.229132235	0.265	1	1509	CTSD
-0.205147997	0.229132235	0.26	1	10581	IFITM2
-0.205147997	0.229132235	0.271	1	11262	SP140
-0.205147997	0.229132235	0.258	1	23303	KIF13B
-0.205147997	0.229132235	0.255	1	30817	EMR2
-0.205147997	0.229132235	0.258	1	55112	WDR60
-0.205147997	0.229132235	0.268	1	55374	TMCO6
-0.205147997	0.229132235	0.294	1	81033	KCNH6
-0.205147997	0.229132235	0.269	1	126074	C19orf39
-0.205147997	0.229132235	0.26	1	642394	ADARB2-AS1
-0.205405399	0.228538767	0.259	1	4034	LRCH4
-0.205405399	0.228538767	0.285	1	51643	TMBIM4
-0.205405399	0.228538767	0.277	1	94015	TTYH2
-0.205405399	0.228538767	0.249	1	119395	CALHM3
-0.205405399	0.228538767	0.262	1	122704	MRPL52
-0.205405399	0.228538767	0.276	1	192669	EIF2C3
-0.205662802	0.227946445	0.263	1	1160	CKMT2
-0.205662802	0.227946445	0.282	1	1267	CNP
-0.205662802	0.227946445	0.251	1	2214	FCGR3A
-0.205662802	0.227946445	0.28	1	2639	GCDH
-0.205662802	0.227946445	0.282	1	57594	HOMEZ
-0.205662802	0.227946445	0.243	1	83938	C10orf11
-0.205662802	0.227946445	0.29	1	130916	MTERFD2
-0.205662802	0.227946445	0.273	1	201176	SH3D20
-0.20592019	0.227355123	0.283	1	5287	PIK3C2B
-0.20592019	0.227355123	0.259	1	6050	RNH1
-0.20592019	0.227355123	0.285	1	9696	CROCC
-0.20592019	0.227355123	0.265	1	10186	LHFP
-0.20592019	0.227355123	0.256	1	23446	SLC44A1
-0.20592019	0.227355123	0.262	1	84445	LZTS2
-0.20592019	0.227355123	0.244	1	93081	C13orf27
-0.20592019	0.227355123	0.26	1	116064	LRRC58
-0.20592019	0.227355123	0.264	1	150678	MYEOV2
-0.20592019	0.227355123	0.266	1	253260	RICTOR
-0.206177592	0.226764575	0.254	1	1876	E2F6
-0.206177592	0.226764575	0.261	1	2252	FGF7
-0.206177592	0.226764575	0.249	1	2689	GH2
-0.206177592	0.226764575	0.262	1	3753	KCNE1
-0.206177592	0.226764575	0.256	1	4242	MFNG
-0.206177592	0.226764575	0.298	1	8705	B3GALT4
-0.206177592	0.226764575	0.285	1	9376	SLC22A8
-0.206177592	0.226764575	0.261	1	9553	MRPL33
-0.206177592	0.226764575	0.259	1	23556	PIGN
-0.206177592	0.226764575	0.253	1	79090	TRAPPC6A
-0.206177592	0.226764575	0.266	1	113419	TEX261
-0.206177592	0.226764575	0.268	1	122809	SOCS4
-0.206177592	0.226764575	0.261	1	122961	ISCA2
-0.206177592	0.226764575	0.255	1	219899	TBCEL
-0.206177592	0.226764575	0.259	1	494514	C18orf56
-0.20643501	0.226175368	0.254	1	4117	MAK
-0.20643501	0.226175368	0.271	1	9848	MFAP3L
-0.20643501	0.226175368	0.26	1	10144	FAM13A
-0.20643501	0.226175368	0.24	1	10664	CTCF
-0.20643501	0.226175368	0.26	1	23193	GANAB
-0.20643501	0.226175368	0.273	1	55230	USP40
-0.20643501	0.226175368	0.263	1	55714	ODZ3

voxel-wise, subcortex			
Spearman's corr coef	p-val	GenID	Gene
-0.155352354	5.60519E-45	55771	PRR11
-0.155378729	5.60519E-45	60684	C4orf41
-0.155416057	5.60519E-45	728637	LOC728637
-0.155424982	5.60519E-45	26022	TMEM98
-0.155427486	5.60519E-45	201973	CCDC111
-0.155458599	5.60519E-45	9235	IL32
-0.155464634	5.60519E-45	117246	FTSJ3
-0.155467883	5.60519E-45	5257	PHKB
-0.155544117	5.60519E-45	4641	MYO1C
-0.155591756	5.60519E-45	116143	WDR92
-0.155649334	5.60519E-45	9113	LATS1
-0.155653805	5.60519E-45	10237	SLC35B1
-0.155671999	5.60519E-45	2681	GGTA1
-0.15587306	5.60519E-45	131601	GPR175
-0.155925438	2.8026E-45	26574	AATF
-0.155930817	2.8026E-45	56919	DHX33
-0.155946419	2.8026E-45	730234	LOC730234
-0.155969083	2.8026E-45	55217	TMLHE
-0.155970931	2.8026E-45	1672	DEFB1
-0.156076521	2.8026E-45	51566	ARMCX3
-0.156102806	2.8026E-45	1191	CLU
-0.156123981	2.8026E-45	100133091	AC004980.7
-0.156131685	2.8026E-45	84674	CARD6
-0.1561407	2.8026E-45	688	KLF5
-0.156157404	2.8026E-45	56140	PCDHA8
-0.156183317	2.8026E-45	1073	CFL2
-0.156209141	2.8026E-45	51639	AC008073.1
-0.156213969	2.8026E-45	51386	EIF3L
-0.156255841	2.8026E-45	64434	NOM1
-0.15627265	2.8026E-45	29082	CHMP4A
-0.156276271	2.8026E-45	9124	PDLIM1
-0.156292528	2.8026E-45	79053	ALG8
-0.156344444	< 0.001	162540	AC217771.1
-0.156359851	< 0.001	7307	U2AF1
-0.156363115	< 0.001	5771	PTPN2
-0.156368673	< 0.001	339512	C1orf110
-0.156444266	< 0.001	419	ART3
-0.156468973	< 0.001	11267	SNF8
-0.156491354	< 0.001	389766	C9orf153
-0.156596854	< 0.001	26119	LDLRAP1
-0.156629547	< 0.001	1961	EGR4
-0.156646907	< 0.001	1663	DDX11
-0.156728357	< 0.001	54680	ZNHIT6
-0.156792432	< 0.001	125704	C18orf51
-0.1567927	< 0.001	53637	S1PR5
-0.156865135	< 0.001	387264	KRTAP5-1
-0.156865329	< 0.001	100127983	RP11-122A3.2
-0.156873226	< 0.001	11013	TMSB15A
-0.156877026	< 0.001	91107	TRIM47
-0.156905726	< 0.001	128218	TMEM125
-0.156951606	< 0.001	51056	LAP3
-0.157035172	< 0.001	6900	CNTN2
-0.157048181	< 0.001	79915	ATAD5
-0.157056347	< 0.001	6173	RPL36A
-0.157088861	< 0.001	441150	C6orf226
-0.157177821	< 0.001	5577	PRKAR2B
-0.157206982	< 0.001	4792	NFKBIA
-0.157208562	< 0.001	2861	GPR37
-0.157243013	< 0.001	2297	RP11-79P5.6
-0.157337084	< 0.001	57471	ERMN
-0.157372102	< 0.001	29799	YPEL1
-0.157414168	< 0.001	2990	GUSB
-0.157422334	< 0.001	440888	ACTR3BP2
-0.157482415	< 0.001	79710	MORC4
-0.157512695	< 0.001	5872	RAB13
-0.157545388	< 0.001	369	ARAF
-0.157553285	< 0.001	27296	TP53TG5
-0.157555044	< 0.001	216	ALDH1A1
-0.157593861	< 0.001	59283	CACNG8
-0.157617912	< 0.001	54820	NDE1
-0.157638341	< 0.001	79621	RNASEH2B
-0.157661378	< 0.001	2801	GOLGA2
-0.157668993	< 0.001	1474	CST6
-0.157679856	< 0.001	387036	GUSBP2
-0.157749876	< 0.001	51059	FAM135B
-0.15783374	< 0.001	10417	SPON2
-0.157887325	< 0.001	84817	TXNDC17
-0.157908678	< 0.001	3455	IFNAR2
-0.15791741	< 0.001	63935	PCIF1

region-wise, subcortex					
Spearman's corr coef	p-val	p-val, SA	p-val, SA (BH- correction)	GenID	Gene
-0.20643501	0.226175368	0.245	1	202020	FLJ39653
-0.20643501	0.226175368	0.267	1	202299	C5ORF27
-0.20643501	0.226175368	0.258	1	400506	C16orf88
-0.206692412	0.225587204	0.25	1	1462	VCAN
-0.206692412	0.225587204	0.26	1	6164	RPL34
-0.206692412	0.225587204	0.275	1	6534	SLC6A7
-0.206692412	0.225587204	0.266	1	10906	TRAFD1
-0.206692412	0.225587204	0.258	1	157657	C8orf37
-0.206692412	0.225587204	0.257	1	219595	FOLH1B
-0.206692412	0.225587204	0.247	1	246269	LACE1
-0.206692412	0.225587204	0.279	1	285671	RNF180
-0.206949815	0.225000173	0.259	1	5867	RAB4A
-0.206949815	0.225000173	0.265	1	11226	GALNT6
-0.206949815	0.225000173	0.277	1	84255	SLC37A3
-0.207207203	0.22441411	0.27	1	26472	PPP1R14B
-0.207207203	0.22441411	0.246	1	79943	ZNF696
-0.207464606	0.223828852	0.258	1	1654	DDX3X
-0.207464606	0.223828852	0.27	1	10129	FRY
-0.207464606	0.223828852	0.27	1	23166	STAB1
-0.207464606	0.223828852	0.259	1	55225	RAVER2
-0.207722008	0.22324492	0.265	1	8301	PICALM
-0.207722008	0.22324492	0.262	1	8804	CREG1
-0.207722008	0.22324492	0.252	1	23743	BHMT2
-0.207722008	0.22324492	0.277	1	28989	NTMT1
-0.207722008	0.22324492	0.284	1	50617	ATP6V0A4
-0.207722008	0.22324492	0.245	1	64327	LMBR1
-0.207979411	0.222662032	0.274	1	3726	JUNB
-0.207979411	0.222662032	0.249	1	5354	PLP1
-0.207979411	0.222662032	0.282	1	55193	PBRM1
-0.207979411	0.222662032	0.255	1	219988	PATL1
-0.207979411	0.222662032	0.25	1	389766	C9orf153
-0.208236814	0.222080275	0.261	1	8787	RGS9
-0.208236814	0.222080275	0.25	1	9261	MAPKAPK2
-0.208236814	0.222080275	0.257	1	81932	HDHD3
-0.208236814	0.222080275	0.293	1	399806	FLJ41350
-0.208236814	0.222080275	0.274	1	646799	ZAR1L
-0.208494201	0.221499234	0.244	1	976	CD97
-0.208494201	0.221499234	0.241	1	7096	TLR1
-0.208494201	0.221499234	0.246	1	11248	NXPH3
-0.208494201	0.221499234	0.249	1	22802	CLCA4
-0.208494201	0.221499234	0.244	1	26150	RIBC2
-0.208494201	0.221499234	0.253	1	79137	FAM134A
-0.208494201	0.221499234	0.278	1	84162	KIAA1109
-0.208494201	0.221499234	0.238	1	170692	ADAMTS18
-0.208494201	0.221499234	0.244	1	201798	TIGD4
-0.208751604	0.22091952	0.258	1	4723	NDUFV1
-0.208751604	0.22091952	0.251	1	6529	SLC6A1
-0.208751604	0.22091952	0.263	1	57593	EBF4
-0.208751604	0.22091952	0.255	1	83460	TMEM93
-0.208751604	0.22091952	0.263	1	90246	LOC90246
-0.208751604	0.22091952	0.264	1	135932	TMEM139
-0.208751604	0.22091952	0.288	1	150946	FAM59B
-0.208751604	0.22091952	0.253	1	399687	MYO18A
-0.208751604	0.22091952	0.23	1	646577	HMGB1P16
-0.209009007	0.220340848	0.233	1	3007	HIST1H1D
-0.209009007	0.220340848	0.284	1	5976	UPF1
-0.209009007	0.220340848	0.261	1	8863	PER3
-0.209009007	0.220340848	0.248	1	9366	RAB9BP1
-0.209009007	0.220340848	0.263	1	10468	FST
-0.209009007	0.220340848	0.254	1	29777	ABT1
-0.209009007	0.220340848	0.264	1	55748	CNDP2
-0.209009007	0.220340848	0.254	1	79963	ABCA11P
-0.209009007	0.220340848	0.271	1	89822	KCNK17
-0.209009007	0.220340848	0.266	1	140825	NEURL2
-0.209009007	0.220340848	0.246	1	284998	AC068057.2
-0.209009007	0.220340848	0.266	1	440181	LOC440181
-0.209009007	0.220340848	0.25	1	445347	TARP
-0.209266409	0.219763219	0.266	1	3895	KTN1
-0.209266409	0.219763219	0.256	1	340260	UNCX
-0.209523812	0.219186708	0.256	1	2887	GRB10
-0.209523812	0.219186708	0.25	1	4864	NPC1
-0.209523812	0.219186708	0.259	1	9710	KIAA0355
-0.209523812	0.219186708	0.249	1	337963	KRTAP23-1
-0.209523812	0.219186708	0.262	1	388969	C2orf68
-0.209523812	0.219186708	0.259	1	400960	PCBP1-AS1
-0.2097812	0.218610942	0.252	1	1106	CHD2
-0.2097812	0.218610942	0.253	1	8973	CHRNA6
-0.2097812	0.218610942	0.242	1	9635	CLCA2
-0.2097812	0.218610942	0.253	1	11105	PRDM7

voxel-wise, subcortex			
Spearman's corr coef	p-val	GenID	Gene
-0.158048525	< 0.001	4991	OR1D2
-0.158094406	< 0.001	9802	DAZAP2
-0.158114284	< 0.001	57169	ZNFX1
-0.158119574	< 0.001	84278	HIATL2
-0.158122823	< 0.001	81603	TRIM8
-0.158123478	< 0.001	23555	TSPAN15
-0.158132762	< 0.001	3930	LBR
-0.158151522	< 0.001	256435	ST6GALNAC3
-0.158241138	< 0.001	50624	CUZD1
-0.158243179	< 0.001	91683	SYT12
-0.158264726	< 0.001	23585	TMEM50A
-0.15827252	< 0.001	55793	FAM63A
-0.15827921	< 0.001	729566	ZBTB8OSP1
-0.158354625	< 0.001	115708	TRMT61A
-0.158378303	< 0.001	138639	PTPDC1
-0.158522338	< 0.001	22834	ZNF652
-0.158535331	< 0.001	7073	TIAL1
-0.158561066	< 0.001	4152	MBD1
-0.158561796	< 0.001	26469	PTPN18
-0.158573598	< 0.001	83875	BCO2
-0.158621699	< 0.001	10120	ACTR1B
-0.158637121	< 0.001	65975	STK33
-0.158639446	< 0.001	5893	RAD52
-0.158680573	< 0.001	440119	RP11-143E21.7
-0.158731103	< 0.001	441502	RPS26P11
-0.1587753	< 0.001	56133	PCDHB2
-0.15883632	< 0.001	166929	SGMS2
-0.158887014	< 0.001	283927	NUDT7
-0.158887386	< 0.001	254272	TBC1D28
-0.158894822	< 0.001	6048	RNF5
-0.15889816	< 0.001	10392	NOD1
-0.158903092	< 0.001	55623	THUMPD1
-0.158963636	< 0.001	54997	TESC
-0.158998922	< 0.001	11270	NRM
-0.159003749	< 0.001	55705	IPO9
-0.159030691	< 0.001	5606	MAP2K3
-0.159091234	< 0.001	1040	CDS1
-0.159094661	< 0.001	84129	ACAD11
-0.159103677	< 0.001	339665	SLC35E4
-0.159107208	< 0.001	79770	TXNDC15
-0.159113705	< 0.001	401399	RP11-155G14.1
-0.159205824	< 0.001	9968	MED12
-0.159228295	< 0.001	123720	WHAMM
-0.159242973	< 0.001	9988	DMTF1
-0.15933612	< 0.001	349075	ZNF713
-0.15939787	< 0.001	55622	TTC27
-0.159424439	< 0.001	51319	RSRC1
-0.159465954	< 0.001	8495	PPFIBP2
-0.15952231	< 0.001	64072	CDH23
-0.159598842	< 0.001	26778	SNORA70
-0.159633562	< 0.001	54715	A2BP1
-0.159702659	< 0.001	9881	LBA1
-0.159725606	< 0.001	284836	LINC00319
-0.15981847	< 0.001	55018	LINC00483
-0.159863964	< 0.001	4973	OLR1
-0.159887463	< 0.001	3061	HCRTR1
-0.159901768	< 0.001	10489	LRRC41
-0.159903988	< 0.001	9813	KIAA0494
-0.160047665	< 0.001	400966	RGPD1
-0.16006735	< 0.001	1285	COL4A3
-0.160108387	< 0.001	84154	RPF2
-0.160137087	< 0.001	1036	CDO1
-0.160165593	< 0.001	54453	RIN2
-0.16017814	< 0.001	3399	ID3
-0.160179436	< 0.001	1876	E2F6
-0.160199776	< 0.001	7094	TLN1
-0.160203025	< 0.001	3316	HSPB2
-0.160208881	< 0.001	2662	GDF10
-0.160259485	< 0.001	4799	NFX1
-0.160331175	< 0.001	6156	RPL30
-0.160387456	< 0.001	124975	GGT6
-0.160422653	< 0.001	9211	LGI1
-0.160551459	< 0.001	1410	CRYAB
-0.160573378	< 0.001	7840	ALMS1
-0.160585448	< 0.001	60436	TGIF2
-0.160645351	< 0.001	94240	EPSTI1
-0.160696045	< 0.001	3672	ITGA1
-0.16069828	< 0.001	92259	MRPS36
-0.160775259	< 0.001	7569	ZNF182

region-wise, subcortex					
Spearman's corr coef	p-val	p-val, SA	p-val, SA (BH- correction)	GenID	Gene
-0.2097812	0.218610942	0.246	1	51106	TFB1M
-0.2097812	0.218610942	0.26	1	51571	FAM49B
-0.2097812	0.218610942	0.253	1	83608	C18orf21
-0.2097812	0.218610942	0.258	1	127062	OR2M3
-0.2097812	0.218610942	0.275	1	340024	SLC6A19
-0.2097812	0.218610942	0.281	1	100132707	AC093726.2
-0.210038602	0.218036473	0.272	1	2158	F9
-0.210038602	0.218036473	0.275	1	5151	PDE8A
-0.210038602	0.218036473	0.245	1	6035	RNASE1
-0.210038602	0.218036473	0.277	1	7336	UBE2V2
-0.210038602	0.218036473	0.264	1	51274	KLF3
-0.210038602	0.218036473	0.279	1	79411	GLB1L
-0.210038602	0.218036473	0.247	1	85450	ITPRIP
-0.210038602	0.218036473	0.264	1	151188	ARL6IP6
-0.210038602	0.218036473	0.254	1	219995	MS4A15
-0.210296005	0.217463046	0.26	1	6609	SMPD1
-0.210296005	0.217463046	0.262	1	10335	MRVI1
-0.210296005	0.217463046	0.268	1	54896	PQLC2
-0.210296005	0.217463046	0.261	1	55766	H2AFJ
-0.210296005	0.217463046	0.262	1	57645	POGK
-0.210296005	0.217463046	0.244	1	84329	HVCN1
-0.210296005	0.217463046	0.273	1	133121	ENPP6
-0.210296005	0.217463046	0.254	1	155400	NSUN5B
-0.210296005	0.217463046	0.261	1	158219	TTC39B
-0.210553408	0.216890693	0.24	1	8883	NAE1
-0.210553408	0.216890693	0.233	1	10233	LRRC23
-0.210553408	0.216890693	0.269	1	113189	CHST14
-0.210553408	0.216890693	0.266	1	133022	TRAM1L1
-0.210553408	0.216890693	0.285	1	286528	SSU72P1
-0.21081081	0.216319159	0.238	1	837	CASP4
-0.21081081	0.216319159	0.248	1	54854	FAM83E
-0.21081081	0.216319159	0.257	1	58157	NGB
-0.21081081	0.216319159	0.254	1	90527	DUOXA1
-0.21081081	0.216319159	0.242	1	116448	OLIG1
-0.211068198	0.215748876	0.258	1	49860	CRNN
-0.211068198	0.215748876	0.254	1	55627	SMPD4
-0.211068198	0.215748876	0.24	1	93653	ST7-AS1
-0.211325601	0.215179637	0.246	1	141	ADPRH
-0.211325601	0.215179637	0.257	1	401	PHOX2A
-0.211325601	0.215179637	0.25	1	54930	HAUS4
-0.211325601	0.215179637	0.239	1	54979	HRASLS2
-0.211325601	0.215179637	0.24	1	79635	CCDC121
-0.211325601	0.215179637	0.262	1	84173	ELMOD3
-0.211325601	0.215179637	0.261	1	84859	LRCH3
-0.211325601	0.215179637	0.264	1	127550	A3GALT2P
-0.211325601	0.215179637	0.248	1	256643	CXorf23
-0.211583003	0.214611501	0.236	1	8412	BCAR3
-0.211583003	0.214611501	0.252	1	64231	MS4A6A
-0.211583003	0.214611501	0.268	1	164127	C1orf65
-0.211583003	0.214611501	0.253	1	219445	OR7E5P
-0.211583003	0.214611501	0.251	1	219793	C10orf27
-0.211583003	0.214611501	0.245	1	388284	C16orf86
-0.211840406	0.214044139	0.261	1	1839	HBEGF
-0.211840406	0.214044139	0.254	1	3134	HLA-F
-0.211840406	0.214044139	0.246	1	5660	PSAP
-0.211840406	0.214044139	0.25	1	7581	ZNF33A
-0.211840406	0.214044139	0.25	1	8291	DYSF
-0.211840406	0.214044139	0.262	1	11243	PMF1
-0.211840406	0.214044139	0.237	1	22847	ZNF507
-0.211840406	0.214044139	0.228	1	51604	PIGT
-0.211840406	0.214044139	0.24	1	55248	TMEM206
-0.211840406	0.214044139	0.266	1	115584	SLC5A11
-0.211840406	0.214044139	0.25	1	140862	C20orf82
-0.212097809	0.213478044	0.297	1	2796	GNRH1
-0.212097809	0.213478044	0.249	1	7923	HSD17B8
-0.212097809	0.213478044	0.254	1	22938	SNW1
-0.212097809	0.213478044	0.264	1	26020	LRP10
-0.212097809	0.213478044	0.286	1	50863	OPCML
-0.212097809	0.213478044	0.245	1	60506	NYX
-0.212097809	0.213478044	0.253	1	64418	TMEM168
-0.212355196	0.212913007	0.282	1	780	DDR1
-0.212355196	0.212913007	0.247	1	6692	SPINT1
-0.212355196	0.212913007	0.246	1	8382	NME5
-0.212355196	0.212913007	0.265	1	10437	IFI30
-0.212355196	0.212913007	0.254	1	146712	B3GNTL1
-0.212612599	0.212348998	0.264	1	5730	PTGDS
-0.212612599	0.212348998	0.257	1	5939	RBMS2
-0.212612599	0.212348998	0.268	1	9586	CREB5
-0.212612599	0.212348998	0.237	1	10061	ABCF2

voxel-wise, subcortex			
Spearman's corr coef	p-val	GenID	Gene
-0.160808131	< 0.001	153	ADRB1
-0.160829872	< 0.001	1605	DAG1
-0.16083777	< 0.001	494115	RBMXL1
-0.160902768	< 0.001	1287	COL4A5
-0.160954952	< 0.001	84807	NFKBID
-0.160959423	< 0.001	79029	SPATA5L1
-0.161005482	< 0.001	10536	LEPREL2
-0.161011055	< 0.001	58476	TP53INP2
-0.161025912	< 0.001	65110	UPF3A
-0.161123693	< 0.001	51027	BOLA1
-0.161136046	< 0.001	79801	SHCBP1
-0.161149696	< 0.001	221692	PHACTR1
-0.161151737	< 0.001	54512	EXOSC4
-0.161203012	< 0.001	63898	SH2D4A
-0.16128844	< 0.001	7368	UGT8
-0.161331624	< 0.001	79567	FAM65A
-0.161375836	< 0.001	8736	MYOM1
-0.161378056	< 0.001	871	SERPINH1
-0.161455601	< 0.001	1528	CYB5A
-0.16146563	< 0.001	2219	FCN1
-0.161473796	< 0.001	7058	THBS2
-0.16150789	< 0.001	117283	IP6K3
-0.161667049	< 0.001	340198	IFITM4P
-0.161682189	< 0.001	81609	SNX27
-0.161698252	< 0.001	57606	SLAIN2
-0.161718041	< 0.001	84504	NKX6-2
-0.161728159	< 0.001	6094	ROM1
-0.161733925	< 0.001	84309	NUDT16L1
-0.161751464	< 0.001	6303	SAT1
-0.161775798	< 0.001	5175	PECAM1
-0.161815181	< 0.001	2034	EPAS1
-0.161835521	< 0.001	79788	ZNF665
-0.16184248	< 0.001	348751	AC073043.2
-0.161945745	< 0.001	9725	TMEM63A
-0.161946401	< 0.001	100008589	RN28S1
-0.161956698	< 0.001	1839	HBEGF
-0.162081987	< 0.001	7078	TIMP3
-0.162105098	< 0.001	283860	LINC00304
-0.162112355	< 0.001	8418	CMAH
-0.162204653	< 0.001	65265	C8orf33
-0.162253499	< 0.001	79961	DENND2D
-0.162267894	< 0.001	404201	WDFY3-AS2
-0.162341177	< 0.001	57683	ZDBF2
-0.162360579	< 0.001	126755	LRRC38
-0.16243951	< 0.001	4430	MYO1B
-0.162465706	< 0.001	55314	TMEM144
-0.162600264	< 0.001	25844	YIPF3
-0.162604913	< 0.001	30817	EMR2
-0.162678927	< 0.001	4502	MT2A
-0.162691742	< 0.001	23209	MLC1
-0.162770584	< 0.001	1938	EEF2
-0.162783206	< 0.001	728806	NSFP1
-0.162860662	< 0.001	8992	ATP6V0E1
-0.162882492	< 0.001	100130933	C17ORF110
-0.162952602	< 0.001	5289	PIK3C3
-0.16296643	< 0.001	9181	ARHGEF2
-0.163022712	< 0.001	7726	TRIM26
-0.163037658	< 0.001	64793	CCDC21
-0.163096443	< 0.001	93643	TJAP1
-0.163128853	< 0.001	23187	PHLDB1
-0.1631556	< 0.001	64422	ATG3
-0.163181603	< 0.001	152816	C4orf26
-0.163242534	< 0.001	729096	RP11-464F9.1
-0.163341343	< 0.001	8520	HAT1
-0.163342267	< 0.001	57002	C7orf36
-0.163419068	< 0.001	84957	RELT
-0.163419619	< 0.001	9325	TRIP4
-0.163458258	< 0.001	9825	SPATA2
-0.16347535	< 0.001	84327	ZBED3
-0.163530782	< 0.001	873	CBR1
-0.163549364	< 0.001	51427	ZNF588
-0.163592815	< 0.001	79616	CCNJL
-0.163632661	< 0.001	9646	CTR9
-0.163634047	< 0.001	10399	GNB2L1
-0.163666829	< 0.001	259295	TAS2R20
-0.163790345	< 0.001	440465	AC013356.3
-0.163874105	< 0.001	7130	TNFAIP6
-0.164112404	< 0.001	3920	LAMP2
-0.164113984	< 0.001	342926	ZNF677

region-wise, subcortex					
Spearman's corr coef	p-val	p-val, SA	p-val, SA (BH- correction)	GenID	Gene
-0.212612599	0.212348998	0.224	1	10561	IFI44
-0.212612599	0.212348998	0.253	1	11068	CYB561D2
-0.212612599	0.212348998	0.252	1	57477	SHROOM4
-0.212612599	0.212348998	0.261	1	64087	MCCC2
-0.212612599	0.212348998	0.255	1	151790	WDR49
-0.212870002	0.211786076	0.265	1	2322	FLT3
-0.212870002	0.211786076	0.256	1	23555	TSPAN15
-0.212870002	0.211786076	0.261	1	51602	NOP58
-0.212870002	0.211786076	0.25	1	100131294	LOC100131294
-0.213127404	0.211223945	0.237	1	1119	CHKA
-0.213127404	0.211223945	0.256	1	5876	RABGGTB
-0.213127404	0.211223945	0.23	1	8910	SGCE
-0.213127404	0.211223945	0.239	1	27309	ZNF330
-0.213384822	0.210663065	0.284	1	5224	PGAM2
-0.213384822	0.210663065	0.252	1	11234	HPS5
-0.213384822	0.210663065	0.242	1	54470	ARMCX6
-0.213384822	0.210663065	0.261	1	79899	PRR5L
-0.213384822	0.210663065	0.257	1	81627	C1orf25
-0.213642225	0.210103244	0.249	1	51268	PIPOX
-0.213642225	0.210103244	0.261	1	90338	ZNF160
-0.213899612	0.209544495	0.242	1	830	CAPZA2
-0.213899612	0.209544495	0.249	1	2861	GPR37
-0.213899612	0.209544495	0.224	1	26508	HEYL
-0.213899612	0.209544495	0.266	1	55825	PECR
-0.213899612	0.209544495	0.27	1	285668	AC094086.1
-0.214157015	0.208986476	0.244	1	678655	AC005840.2
-0.214414418	0.208429813	0.232	1	4814	NINJ1
-0.214414418	0.208429813	0.248	1	4939	OAS2
-0.214414418	0.208429813	0.243	1	9662	CEP135
-0.214414418	0.208429813	0.254	1	23187	PHLDB1
-0.214414418	0.208429813	0.253	1	25842	ASF1A
-0.214414418	0.208429813	0.251	1	132241	RPL32P3
-0.214414418	0.208429813	0.255	1	254042	METAP1D
-0.21467182	0.207874149	0.25	1	5473	PPBP
-0.21467182	0.207874149	0.24	1	6753	SSTR3
-0.21467182	0.207874149	0.25	1	285498	RNF212
-0.214929223	0.207319558	0.245	1	9781	RNF144A
-0.214929223	0.207319558	0.208	1	51631	LUC7L2
-0.214929223	0.207319558	0.223	1	54620	FBXL19
-0.214929223	0.207319558	0.235	1	57158	JPH2
-0.214929223	0.207319558	0.259	1	58505	OSTC
-0.214929223	0.207319558	0.246	1	80135	RPF1
-0.214929223	0.207319558	0.255	1	80233	C17orf70
-0.214929223	0.207319558	0.256	1	653479	MRPL45P2
-0.215186611	0.206765696	0.229	1	825	CAPN3
-0.215186611	0.206765696	0.271	1	2166	FAAH
-0.215186611	0.206765696	0.246	1	51201	ZDHHC2
-0.215186611	0.206765696	0.29	1	56928	SPPL2B
-0.215186611	0.206765696	0.272	1	57471	ERMN
-0.215444013	0.206213161	0.247	1	1203	CLN5
-0.215444013	0.206213161	0.25	1	6882	TAF11
-0.215444013	0.206213161	0.241	1	8611	PPAP2A
-0.215444013	0.206213161	0.24	1	25946	ZNF385A
-0.215444013	0.206213161	0.246	1	54893	MTMR10
-0.215444013	0.206213161	0.248	1	83641	FAM107B
-0.215444013	0.206213161	0.25	1	85569	GALP
-0.215444013	0.206213161	0.247	1	116173	CMTM5
-0.215444013	0.206213161	0.264	1	343505	NBPF7
-0.215701416	0.205661684	0.226	1	2705	GJB1
-0.215701416	0.205661684	0.227	1	2908	NR3C1
-0.215701416	0.205661684	0.239	1	3122	HLA-DRA
-0.215701416	0.205661684	0.242	1	4815	NINJ2
-0.215701416	0.205661684	0.249	1	10291	SF3A1
-0.215701416	0.205661684	0.213	1	10985	GCN1L1
-0.215701416	0.205661684	0.259	1	90459	ERI1
-0.215701416	0.205661684	0.231	1	147807	ZNF524
-0.215701416	0.205661684	0.237	1	161176	C14orf49
-0.215701416	0.205661684	0.258	1	246778	IL27
-0.215958819	0.205111206	0.244	1	181	AGRP
-0.215958819	0.205111206	0.252	1	1992	SERPINB1
-0.215958819	0.205111206	0.25	1	3866	KRT15
-0.215958819	0.205111206	0.235	1	4752	NEK3
-0.215958819	0.205111206	0.244	1	9924	PAN2
-0.215958819	0.205111206	0.236	1	10311	DSCR3
-0.215958819	0.205111206	0.227	1	10352	WARS2
-0.215958819	0.205111206	0.255	1	23198	PSME4
-0.215958819	0.205111206	0.244	1	57545	CC2D2A
-0.215958819	0.205111206	0.243	1	83552	MFRP
-0.215958819	0.205111206	0.257	1	85315	PAQR8

voxel-wise, subcortex			
Spearman's corr coef	p-val	GenID	Gene
-0.164165795	< 0.001	729683	LOC729683
-0.164173514	< 0.001	4172	MCM3
-0.164227188	< 0.001	5008	OSM
-0.164259881	< 0.001	50651	SLC45A1
-0.164293587	< 0.001	8940	TOP3B
-0.164433539	< 0.001	5819	PVRL2
-0.164457768	< 0.001	162972	ZNF550
-0.164509222	< 0.001	10483	SEC23B
-0.164513588	< 0.001	55330	CNO
-0.164518416	< 0.001	25998	IBTK
-0.164565399	< 0.001	151258	SLC38A11
-0.164584622	< 0.001	1917	EEF1A2
-0.164673135	< 0.001	5193	PEX12
-0.164678797	< 0.001	8291	DYSF
-0.164681122	< 0.001	90665	TBL1Y
-0.164706096	< 0.001	285955	SPDYE1
-0.164711758	< 0.001	7276	TTR
-0.164726898	< 0.001	9507	ADAMTS4
-0.164729133	< 0.001	158234	RG9MTD3
-0.164760515	< 0.001	51362	CDC40
-0.164816231	< 0.001	55139	ANKZF1
-0.164825797	< 0.001	79899	PRR5L
-0.164843723	< 0.001	3142	HLX
-0.1648947	< 0.001	79736	TEFM
-0.165011629	< 0.001	116173	CMTM5
-0.16505982	< 0.001	6786	STIM1
-0.165090099	< 0.001	4864	NPC1
-0.165136993	< 0.001	121457	AC013283.1
-0.165156588	< 0.001	11065	UBE2C
-0.165222064	< 0.001	10419	PRMT5
-0.165289104	< 0.001	6124	RPL4
-0.165343255	< 0.001	79009	DDX50
-0.165349662	< 0.001	83606	C22orf13
-0.165360615	< 0.001	255403	ZNF718
-0.165365443	< 0.001	51006	SLC35C2
-0.165370926	< 0.001	55714	ODZ3
-0.165371299	< 0.001	442293	LOC442293
-0.165386245	< 0.001	157310	PEBP4
-0.165443912	< 0.001	85403	EAF1
-0.165495649	< 0.001	140432	RNF113B
-0.165521175	< 0.001	23239	PHLPP1
-0.165630668	< 0.001	9180	OSMR
-0.165655375	< 0.001	55848	C9orf46
-0.165693909	< 0.001	132228	C3orf45
-0.165891811	< 0.001	663	BNIP2
-0.16590853	< 0.001	91614	DEPDC7
-0.165959224	< 0.001	10750	GRAP
-0.166016161	< 0.001	9586	CREB5
-0.166081622	< 0.001	283229	EFCAB4A
-0.166109949	< 0.001	644619	INTS4L2
-0.166141346	< 0.001	5609	MAP2K7
-0.166143015	< 0.001	10078	TSSC4
-0.166185454	< 0.001	23015	GOLGA8A
-0.16620709	< 0.001	55721	IQCC
-0.166469246	< 0.001	8733	GPAA1
-0.166492462	< 0.001	58480	RHOU
-0.166571587	< 0.001	23519	ANP32D
-0.166577339	< 0.001	23493	HEY2
-0.166584775	< 0.001	253559	CADM2
-0.166591838	< 0.001	387273	KRTAP5-10
-0.166608736	< 0.001	283232	TMEM80
-0.166620061	< 0.001	123	ADFP
-0.166625455	< 0.001	84282	RNF135
-0.166634649	< 0.001	83860	TAF3
-0.166641235	< 0.001	55272	IMP3
-0.166797802	< 0.001	5414	SEPTIN4
-0.166822597	< 0.001	124245	ZC3H18
-0.166836157	< 0.001	8622	PDE8B
-0.166867465	< 0.001	9144	SYNGR2
-0.167006388	< 0.001	94081	SFXN1
-0.16700685	< 0.001	210	ALAD
-0.167075485	< 0.001	55033	FKBP14
-0.167156085	< 0.001	80031	SEMA6D
-0.167170942	< 0.001	246777	SPESP1
-0.167196199	< 0.001	283554	GPR137C
-0.167215526	< 0.001	391	RHOG
-0.167227507	< 0.001	54543	TOMM7
-0.167241707	< 0.001	23392	KIAA0368
-0.167246908	< 0.001	56259	CTNNBL1

region-wise, subcortex					
Spearman's corr coef	p-val	p-val, SA	p-val, SA (BH- correction)	GenID	Gene
-0.216216221	0.2045618	0.283	1	225	ABCD2
-0.216216221	0.2045618	0.234	1	403	ARL3
-0.216216221	0.2045618	0.251	1	871	SERPINH1
-0.216216221	0.2045618	0.227	1	9918	NCAPD2
-0.216216221	0.2045618	0.269	1	26534	OR10G2
-0.216216221	0.2045618	0.232	1	79064	TMEM223
-0.216216221	0.2045618	0.245	1	158586	ZXDB
-0.216216221	0.2045618	0.241	1	171017	ZNF384
-0.216216221	0.2045618	0.244	1	200916	RPL22L1
-0.216216221	0.2045618	0.224	1	284348	LYPD5
-0.216473609	0.204013109	0.253	1	230	ALDOC
-0.216473609	0.204013109	0.251	1	2192	FBLN1
-0.216473609	0.204013109	0.251	1	4793	NFKBIB
-0.216473609	0.204013109	0.225	1	10195	ALG3
-0.216473609	0.204013109	0.243	1	23371	TENC1
-0.216473609	0.204013109	0.253	1	29015	SLC43A3
-0.216473609	0.204013109	0.253	1	54961	SSH3
-0.216473609	0.204013109	0.23	1	79083	MLPH
-0.216473609	0.204013109	0.232	1	91404	SESTD1
-0.216473609	0.204013109	0.223	1	92140	MTDH
-0.216731012	0.203465775	0.24	1	2635	GBP3
-0.216731012	0.203465775	0.236	1	3772	KCNJ15
-0.216731012	0.203465775	0.237	1	7018	TF
-0.216731012	0.203465775	0.23	1	151516	ASPRV1
-0.216988415	0.202919468	0.269	1	7539	ZFP37
-0.216988415	0.202919468	0.259	1	9134	CCNE2
-0.216988415	0.202919468	0.233	1	157739	TDH
-0.217245817	0.202374175	0.236	1	991	CDC20
-0.217245817	0.202374175	0.271	1	6238	RRBP1
-0.217245817	0.202374175	0.232	1	10397	NDRG1
-0.217245817	0.202374175	0.247	1	23557	SNAPIN
-0.217245817	0.202374175	0.261	1	54414	SIAE
-0.217245817	0.202374175	0.245	1	54941	RNF125
-0.217245817	0.202374175	0.254	1	64421	DCLRE1C
-0.217245817	0.202374175	0.243	1	126668	TDRD10
-0.217245817	0.202374175	0.235	1	203190	LGI3
-0.217245817	0.202374175	0.224	1	388962	BOLA3
-0.21750322	0.201829627	0.239	1	1793	DOCK1
-0.21750322	0.201829627	0.227	1	5078	PAX4
-0.21750322	0.201829627	0.231	1	8309	ACOX2
-0.21750322	0.201829627	0.229	1	8605	PLA2G4C
-0.21750322	0.201829627	0.239	1	10677	AVIL
-0.21750322	0.201829627	0.234	1	10816	SPINT3
-0.21750322	0.201829627	0.256	1	24147	FJX1
-0.21750322	0.201829627	0.219	1	29923	C7orf68
-0.21750322	0.201829627	0.246	1	54796	BNC2
-0.21750322	0.201829627	0.233	1	55255	WDR41
-0.21750322	0.201829627	0.251	1	80820	EEPD1
-0.21750322	0.201829627	0.249	1	84844	PHF5A
-0.21750322	0.201829627	0.24	1	140766	ADAMTS14
-0.217760608	0.20128642	0.214	1	3113	HLA-DPA1
-0.217760608	0.20128642	0.23	1	5873	RAB27A
-0.217760608	0.20128642	0.247	1	51101	ZC2HC1A
-0.217760608	0.20128642	0.219	1	80829	ZFP91
-0.217760608	0.20128642	0.206	1	84245	MRI1
-0.217760608	0.20128642	0.249	1	333932	HIST2H3A
-0.217760608	0.20128642	0.246	1	729732	LOC729732
-0.21801801	0.200744227	0.244	1	26269	FBXO8
-0.21801801	0.200744227	0.257	1	51444	RNF138
-0.21801801	0.200744227	0.243	1	84681	HINT2
-0.21801801	0.200744227	0.262	1	113763	C7orf29
-0.21801801	0.200744227	0.228	1	114224	PRO2852
-0.218275413	0.200203106	0.258	1	6387	CXCL12
-0.218275413	0.200203106	0.217	1	27235	COQ2
-0.218275413	0.200203106	0.237	1	55159	RFWD3
-0.218275413	0.200203106	0.254	1	57209	ZNF248
-0.218275413	0.200203106	0.233	1	57609	DIP2B
-0.218275413	0.200203106	0.24	1	85285	KRTAP4-1
-0.218275413	0.200203106	0.242	1	126820	WDR63
-0.218275413	0.200203106	0.23	1	653464	SRGAP2C
-0.218532816	0.199662685	0.23	1	2592	GALT
-0.218532816	0.199662685	0.245	1	5676	PSG7
-0.218532816	0.199662685	0.238	1	8482	SEMA7A
-0.218532816	0.199662685	0.245	1	10630	PDPN
-0.218532816	0.199662685	0.247	1	29117	BRD7
-0.218532816	0.199662685	0.22	1	80005	DOCK5
-0.218532816	0.199662685	0.26	1	728724	RP11-1057N3.1
-0.218790218	0.199123591	0.246	1	5286	PIK3C2A
-0.218790218	0.199123591	0.226	1	6776	STAT5A

voxel-wise, subcortex			
Spearman's corr coef	p-val	GenID	Gene
-0.167257771	< 0.001	3732	CD82
-0.167292967	< 0.001	79973	ZNF442
-0.167362347	< 0.001	307	ANXA4
-0.167374134	< 0.001	51108	METTL9
-0.167412579	< 0.001	9940	DLEC1
-0.167449549	< 0.001	5526	PPP2R5B
-0.167544737	< 0.001	1111	CHEK1
-0.167551979	< 0.001	114805	GALNT13
-0.167559773	< 0.001	79228	THOC6
-0.167560145	< 0.001	23556	PIGN
-0.167634904	< 0.001	23091	ZC3H13
-0.167654037	< 0.001	117248	GALNTL2
-0.167744115	< 0.001	644135	RP13-143G15.3
-0.167794079	< 0.001	201161	CENPV
-0.167796671	< 0.001	94239	H2AFV
-0.167853042	< 0.001	57555	NLGN2
-0.16797182	< 0.001	2831	NPBWR1
-0.167975351	< 0.001	27107	ZBTB11
-0.168000609	< 0.001	285550	FAM200B
-0.168043137	< 0.001	64110	MAGEF1
-0.168059483	< 0.001	90649	ZNF486
-0.168070912	< 0.001	9815	GIT2
-0.168081954	< 0.001	284723	SLC25A34
-0.168086424	< 0.001	11098	AP000654.2
-0.168110937	< 0.001	10763	NES
-0.168176129	< 0.001	6856	SYPL1
-0.168184012	< 0.001	7249	TSC2
-0.168228403	< 0.001	255520	ELMOD2
-0.168315053	< 0.001	6210	RPS15A
-0.168419808	< 0.001	4700	NDUFA6
-0.168473482	< 0.001	51061	TXNDC11
-0.168475986	< 0.001	246184	CDC26
-0.168576106	< 0.001	56898	BDH2
-0.168631077	< 0.001	57146	TMEM159
-0.168640077	< 0.001	55624	POMGNT1
-0.168644443	< 0.001	399948	C11orf92
-0.168654382	< 0.001	1723	DHODH
-0.1686984	< 0.001	84336	TMEM101
-0.168719947	< 0.001	5338	PLD2
-0.168740377	< 0.001	54964	C1orf56
-0.168743446	< 0.001	26048	ZNF500
-0.168748453	< 0.001	51531	C9orf156
-0.168775767	< 0.001	51659	GINS2
-0.168822661	< 0.001	84976	DISP1
-0.168856829	< 0.001	283651	HMGN2P46
-0.168873265	< 0.001	51747	AC005921.3
-0.168878376	< 0.001	79903	NAT15
-0.168883294	< 0.001	10283	SDCCAG10
-0.168896392	< 0.001	2172	FABP6
-0.168897972	< 0.001	23347	AP001011.3
-0.168915436	< 0.001	54552	GNL3L
-0.168923691	< 0.001	54838	C10orf26
-0.168974221	< 0.001	5311	PKD2
-0.169099495	< 0.001	5147	PDE6D
-0.16921781	< 0.001	10278	EFS
-0.169405669	< 0.001	79685	SAP30L
-0.169419885	< 0.001	1793	DOCK1
-0.169436693	< 0.001	387082	SUMO4
-0.169519067	< 0.001	4126	MANBA
-0.169532806	< 0.001	10578	GNLY
-0.169573754	< 0.001	150356	CHADL
-0.169650093	< 0.001	100190939	XXyac-R12DG2.1
-0.169664204	< 0.001	729495	FAM108A5P
-0.169719741	< 0.001	81576	CCDC130
-0.16974844	< 0.001	85395	C21orf70
-0.169751406	< 0.001	79090	TRAPPC6A
-0.169799894	< 0.001	1850	DUSP8
-0.169816136	< 0.001	127003	C1orf194
-0.169885606	< 0.001	10514	MYBBP1A
-0.169919401	< 0.001	6238	RRBP1
-0.169938356	< 0.001	4642	MYO1D
-0.169939458	< 0.001	53335	BCL11A
-0.169983581	< 0.001	51547	SIRT7
-0.170098737	< 0.001	57572	DOCK6
-0.17009984	< 0.001	145200	LINC00239
-0.170107827	< 0.001	5829	PXN
-0.170170605	< 0.001	1937	EEF1G
-0.170211196	< 0.001	100131294	LOC100131294
-0.170237005	< 0.001	8021	NUP214

region-wise, subcortex					
Spearman's corr coef	p-val	p-val, SA	p-val, SA (BH- correction)	GenID	Gene
-0.218790218	0.199123591	0.256	1	54899	PXK
-0.218790218	0.199123591	0.229	1	340198	IFITM4P
-0.218790218	0.199123591	0.248	1	399947	C11orf87
-0.218790218	0.199123591	0.241	1	439916	C12orf37
-0.219047606	0.198585495	0.258	1	1545	CYP1B1
-0.219047606	0.198585495	0.237	1	3698	ITIH2
-0.219047606	0.198585495	0.251	1	6511	SLC1A6
-0.219047606	0.198585495	0.247	1	6887	TAL2
-0.219047606	0.198585495	0.253	1	7700	ZNF141
-0.219047606	0.198585495	0.219	1	10794	ZNF460
-0.219047606	0.198585495	0.256	1	29969	MDFIC
-0.219047606	0.198585495	0.209	1	84985	FAM83A
-0.219047606	0.198585495	0.242	1	643854	RP5-988G15.1
-0.219305009	0.198048443	0.248	1	332	BIRC5
-0.219305009	0.198048443	0.238	1	1737	DLAT
-0.219305009	0.198048443	0.253	1	3276	PRMT1
-0.219305009	0.198048443	0.239	1	8842	PROM1
-0.219305009	0.198048443	0.255	1	84166	NLRC5
-0.219305009	0.198048443	0.235	1	84817	TXNDC17
-0.219305009	0.198048443	0.231	1	85481	PSKH2
-0.219305009	0.198048443	0.291	1	341947	COX8C
-0.219562411	0.197512463	0.238	1	2335	FN1
-0.219562411	0.197512463	0.225	1	29902	C12orf24
-0.219562411	0.197512463	0.233	1	55846	ITFG2
-0.219562411	0.197512463	0.238	1	80312	TET1
-0.219562411	0.197512463	0.247	1	83543	AIF1L
-0.219562411	0.197512463	0.231	1	140258	KRTAP13-1
-0.219562411	0.197512463	0.223	1	145270	PRIMA1
-0.219562411	0.197512463	0.248	1	146050	ZSCAN29
-0.219562411	0.197512463	0.22	1	254958	REXO1L1
-0.219562411	0.197512463	0.231	1	574036	C1orf133
-0.219819814	0.196977213	0.234	1	1639	DCTN1
-0.219819814	0.196977213	0.231	1	5150	PDE7A
-0.219819814	0.196977213	0.232	1	7294	TXK
-0.219819814	0.196977213	0.221	1	8227	SFRS17A
-0.219819814	0.196977213	0.216	1	8322	FZD4
-0.219819814	0.196977213	0.204	1	10983	CCNI
-0.219819814	0.196977213	0.235	1	28973	MRPS18B
-0.219819814	0.196977213	0.229	1	28999	KLF15
-0.219819814	0.196977213	0.23	1	646791	ANP32BP1
-0.220077217	0.19644323	0.232	1	6911	TBX6
-0.220077217	0.19644323	0.248	1	25976	TIPARP
-0.220077217	0.19644323	0.249	1	245802	MS4A6E
-0.220077217	0.19644323	0.227	1	256435	ST6GALNAC3
-0.220334619	0.195910275	0.238	1	9869	SETDB1
-0.220334619	0.195910275	0.242	1	100132977	LOC100132977
-0.220592022	0.195378348	0.263	1	275	AMT
-0.220592022	0.195378348	0.23	1	6570	SLC18A1
-0.220592022	0.195378348	0.237	1	7536	SF1
-0.220592022	0.195378348	0.249	1	57571	ATPGD1
-0.220592022	0.195378348	0.228	1	100129138	RP11-364B6.1
-0.220849425	0.194847226	0.218	1	1856	DVL2
-0.220849425	0.194847226	0.228	1	3363	HTR7
-0.220849425	0.194847226	0.243	1	7852	CXCR4
-0.220849425	0.194847226	0.226	1	54462	KIAA1128
-0.220849425	0.194847226	0.246	1	79738	BBS10
-0.221106827	0.194317386	0.213	1	3106	HLA-B
-0.221106827	0.194317386	0.234	1	3746	KCNC1
-0.221106827	0.194317386	0.229	1	9725	TMEM63A
-0.221106827	0.194317386	0.246	1	23318	ZCCHC11
-0.221106827	0.194317386	0.212	1	55344	PLCXD1
-0.22136423	0.193788514	0.231	1	3115	HLA-DPB1
-0.22136423	0.193788514	0.229	1	4069	LYZ
-0.22136423	0.193788514	0.239	1	7007	TECTA
-0.22136423	0.193788514	0.235	1	7517	XRCC3
-0.22136423	0.193788514	0.226	1	8153	RND2
-0.22136423	0.193788514	0.249	1	23559	WBP1
-0.22136423	0.193788514	0.223	1	80347	COASY
-0.22136423	0.193788514	0.246	1	83903	GSG2
-0.221621618	0.193260685	0.236	1	3705	ITPK1
-0.221621618	0.193260685	0.225	1	5081	PAX7
-0.221621618	0.193260685	0.233	1	7728	ZNF175
-0.221621618	0.193260685	0.264	1	8349	HIST2H2BE
-0.221621618	0.193260685	0.215	1	10066	SCAMP2
-0.221621618	0.193260685	0.243	1	11123	RCAN3
-0.221621618	0.193260685	0.239	1	161357	MDGA2
-0.221621618	0.193260685	0.233	1	340745	LRIT2
-0.221621618	0.193260685	0.228	1	442421	PTGER4P2
-0.22187902	0.192733631	0.25	1	956	ENTPD3

voxel-wise, subcortex			
Spearman's corr coef	p-val	GenID	Gene
-0.170262173	< 0.001	92922	CCDC102A
-0.17028743	< 0.001	26005	C2CD3
-0.170301825	< 0.001	80975	TMPRSS5
-0.170353085	< 0.001	11141	IL1RAPL1
-0.170439079	< 0.001	57695	USP37
-0.170471311	< 0.001	43	ACHE
-0.170472607	< 0.001	100288165	HMGN2P15
-0.170530841	< 0.001	4118	MAL
-0.170566037	< 0.001	1306	COL15A1
-0.170576334	< 0.001	1607	DGKB
-0.17059584	< 0.001	10743	RAI1
-0.170642644	< 0.001	100192378	RP11-65D13.1
-0.170682028	< 0.001	2705	GJB1
-0.170694739	< 0.001	51710	ZNF44
-0.170757428	< 0.001	10849	CD3EAP
-0.170766905	< 0.001	23132	RAD54L2
-0.170798019	< 0.001	58486	ZBED5
-0.17091994	< 0.001	9516	LITAF
-0.171007425	< 0.001	3150	HMGN1
-0.171048284	< 0.001	5502	PPP1R1A
-0.171090081	< 0.001	25880	TMEM186
-0.171092212	< 0.001	729993	AC092324.1
-0.171248227	< 0.001	440556	RP1-163G9.1
-0.171249151	< 0.001	374354	NHLRC2
-0.171294108	< 0.001	1465	CSRP1
-0.171363562	< 0.001	6228	RPS23
-0.171394765	< 0.001	7444	VRK2
-0.171470448	< 0.001	55861	DBNDD2
-0.171489954	< 0.001	6193	RPS5
-0.171511501	< 0.001	645811	CCDC154
-0.171515584	< 0.001	729973	LOC729973
-0.171526268	< 0.001	643155	C5orf43
-0.171595544	< 0.001	2968	GTF2H4
-0.171622843	< 0.001	57062	DDX24
-0.171661019	< 0.001	347746	PAR-SN
-0.171670675	< 0.001	219995	MS4A15
-0.171689063	< 0.001	84657	GHRLOS2
-0.171702802	< 0.001	252983	STXBP4
-0.171706885	< 0.001	83743	GRWD1
-0.171720356	< 0.001	8912	CACNA1H
-0.171724066	< 0.001	100310812	RP11-577H5.2
-0.171735585	< 0.001	3700	ITIH4
-0.17174153	< 0.001	81857	MED25
-0.171747848	< 0.001	5876	RABGGTB
-0.17176187	< 0.001	6274	S100A3
-0.171789721	< 0.001	90390	MED30
-0.171867833	< 0.001	199857	ALG14
-0.171921417	< 0.001	84440	RAB11FIP4
-0.171930984	< 0.001	84945	ABHD13
-0.171953455	< 0.001	163059	ZNF433
-0.171971843	< 0.001	5909	RAP1GAP
-0.172002017	< 0.001	6125	RPL5
-0.172028676	< 0.001	51065	RPS27L
-0.172029972	< 0.001	563	AZGP1
-0.172068045	< 0.001	25962	KIAA1429
-0.172141314	< 0.001	57699	CPNE5
-0.172197595	< 0.001	5906	RAP1A
-0.172227502	< 0.001	1770	DNAH9
-0.172242537	< 0.001	389170	AC117392.3
-0.172328264	< 0.001	79673	ZNF329
-0.172342002	< 0.001	130557	ZNF513
-0.172524944	< 0.001	1477	CSTF1
-0.172531351	< 0.001	22847	ZNF507
-0.172547325	< 0.001	644563	LOC644563
-0.172584191	< 0.001	3077	HFE
-0.172594696	< 0.001	55677	IWS1
-0.172612891	< 0.001	7157	TP53
-0.172706127	< 0.001	10521	DDX17
-0.172741517	< 0.001	3623	INHA
-0.172743931	< 0.001	79073	TMEM109
-0.172749773	< 0.001	7043	TGFB3
-0.172802612	< 0.001	85407	NKD1
-0.172849327	< 0.001	641518	AC097067.1
-0.17287375	< 0.001	84219	WDR24
-0.172893628	< 0.001	79137	FAM134A
-0.172920555	< 0.001	51703	ACSL5
-0.172957703	< 0.001	255189	PLA2G4F
-0.172997549	< 0.001	4001	LMNB1
-0.173004419	< 0.001	3178	HNRNPA1P2

region-wise, subcortex					
Spearman's corr coef	p-val	p-val, SA	p-val, SA (BH- correction)	GenID	Gene
-0.22187902	0.192733631	0.227	1	6282	S100A11
-0.22187902	0.192733631	0.212	1	10215	OLIG2
-0.22187902	0.192733631	0.235	1	27295	PDLIM3
-0.22187902	0.192733631	0.22	1	60494	CCDC81
-0.22187902	0.192733631	0.236	1	84987	C12orf62
-0.22187902	0.192733631	0.219	1	123904	NRN1L
-0.222136423	0.192207888	0.239	1	4176	MCM7
-0.222136423	0.192207888	0.244	1	8403	SOX14
-0.222136423	0.192207888	0.215	1	8615	USO1
-0.222136423	0.192207888	0.232	1	10020	GNE
-0.222136423	0.192207888	0.204	1	10807	SDCCAG3
-0.222393826	0.191683114	0.209	1	3013	HIST1H2AD
-0.222393826	0.191683114	0.22	1	9088	PKMYT1
-0.222393826	0.191683114	0.226	1	23328	SASH1
-0.222393826	0.191683114	0.227	1	51012	SLMO2
-0.222393826	0.191683114	0.246	1	389170	AC117392.3
-0.222651228	0.191159353	0.218	1	7586	ZKSCAN1
-0.222651228	0.191159353	0.242	1	10210	TOPORS
-0.222651228	0.191159353	0.246	1	11132	CAPN10
-0.222651228	0.191159353	0.243	1	55032	SLC35A5
-0.222651228	0.191159353	0.238	1	55314	TMEM144
-0.222651228	0.191159353	0.222	1	57488	ESYT2
-0.222651228	0.191159353	0.223	1	79818	AC010645.1
-0.222651228	0.191159353	0.213	1	84504	NKX6-2
-0.222651228	0.191159353	0.24	1	389033	AC079776.2
-0.222908616	0.19063662	0.216	1	6389	SDHA
-0.222908616	0.19063662	0.205	1	9554	SEC22B
-0.222908616	0.19063662	0.241	1	11149	BVES
-0.222908616	0.19063662	0.225	1	29920	PYCR2
-0.222908616	0.19063662	0.203	1	64072	CDH23
-0.222908616	0.19063662	0.224	1	116461	TSEN15
-0.222908616	0.19063662	0.215	1	284695	ZNF326
-0.223166019	0.190114662	0.266	1	2326	FMO1
-0.223166019	0.190114662	0.227	1	2702	GJA5
-0.223166019	0.190114662	0.228	1	2954	GSTZ1
-0.223166019	0.190114662	0.213	1	3351	HTR1B
-0.223166019	0.190114662	0.239	1	4821	NKX2-2
-0.223166019	0.190114662	0.221	1	8476	CDC42BPA
-0.223166019	0.190114662	0.226	1	55266	TMEM19
-0.223166019	0.190114662	0.221	1	79152	FA2H
-0.223166019	0.190114662	0.21	1	155370	SBDSP1
-0.223166019	0.190114662	0.207	1	283446	MYO1H
-0.223166019	0.190114662	0.235	1	388796	RP4-564F22.1
-0.223166019	0.190114662	0.242	1	100133091	AC004980.7
-0.223423421	0.189594015	0.221	1	4055	LTBR
-0.223423421	0.189594015	0.249	1	6663	SOX10
-0.223423421	0.189594015	0.224	1	10049	DNAJB6
-0.223423421	0.189594015	0.227	1	54058	C21orf58
-0.223423421	0.189594015	0.219	1	79670	ZCCHC6
-0.223423421	0.189594015	0.233	1	219287	FAM123A
-0.223423421	0.189594015	0.215	1	339352	LOC339352
-0.223423421	0.189594015	0.216	1	642658	SCXB
-0.223423421	0.189594015	0.222	1	646567	OSTCP2
-0.223680824	0.189074323	0.223	1	2122	EVI1
-0.223680824	0.189074323	0.214	1	7351	UCP2
-0.223680824	0.189074323	0.228	1	8826	IQGAP1
-0.223680824	0.189074323	0.233	1	9175	MAP3K13
-0.223680824	0.189074323	0.242	1	9592	IER2
-0.223680824	0.189074323	0.256	1	54209	TREM2
-0.223680824	0.189074323	0.23	1	84282	RNF135
-0.223680824	0.189074323	0.236	1	90861	HN1L
-0.223938227	0.188555643	0.21	1	2346	FOLH1
-0.223938227	0.188555643	0.224	1	3459	IFNGR1
-0.223938227	0.188555643	0.226	1	22997	RP11-259P6.1
-0.223938227	0.188555643	0.224	1	25937	WWTR1
-0.223938227	0.188555643	0.228	1	55829	AC023024.1
-0.223938227	0.188555643	0.218	1	84229	CCDC135
-0.223938227	0.188555643	0.234	1	400013	TCP1P3
-0.224195614	0.188037768	0.209	1	824	CAPN2
-0.224195614	0.188037768	0.23	1	1633	DCK
-0.224195614	0.188037768	0.224	1	10392	NOD1
-0.224195614	0.188037768	0.204	1	151636	DTX3L
-0.224195614	0.188037768	0.219	1	386678	KRTAP10-11
-0.224453017	0.1875211	0.234	1	571	BACH1
-0.224453017	0.1875211	0.228	1	1149	CIDEA
-0.224453017	0.1875211	0.212	1	645317	CHCHD2P6
-0.22471042	0.187005535	0.212	1	6010	RHO
-0.22471042	0.187005535	0.242	1	8345	HIST1H2BH
-0.22471042	0.187005535	0.228	1	64108	RTP4

voxel-wise, subcortex			
Spearman's corr coef	p-val	GenID	Gene
-0.173010632	< 0.001	7700	ZNF141
-0.173018351	< 0.001	6165	RPL35A
-0.173058644	< 0.001	85449	KIAA1755
-0.173122257	< 0.001	28971	C11orf67
-0.173261926	< 0.001	29117	BRD7
-0.173265547	< 0.001	54951	COMMD8
-0.173274651	< 0.001	64425	POLR1E
-0.173288584	< 0.001	51616	TAF9B
-0.173431128	< 0.001	55904	MLL5
-0.173436806	< 0.001	23327	NEDD4L
-0.17349948	< 0.001	79892	C10orf119
-0.173562169	< 0.001	590	BCHE
-0.173588723	< 0.001	3026	HABP2
-0.173627362	< 0.001	760	CA2
-0.173645377	< 0.001	376693	RPS10P7
-0.173673972	< 0.001	5168	ENPP2
-0.173676014	< 0.001	11322	TMC6
-0.173712701	< 0.001	5454	POU3F2
-0.17372933	< 0.001	10588	MTHFS
-0.173750132	< 0.001	7732	RNF112
-0.173787653	< 0.001	253982	ASPHD1
-0.173792109	< 0.001	51435	SCARA3
-0.17381151	< 0.001	116369	SLC26A8
-0.173828781	< 0.001	26508	HEYL
-0.173831671	< 0.001	647087	C7orf73
-0.173885152	< 0.001	26585	GREM1
-0.173888311	< 0.001	1522	CTSZ
-0.173896953	< 0.001	348938	NIPAL4
-0.173932791	< 0.001	23126	POGZ
-0.173959628	< 0.001	5927	KDM5A
-0.174019441	< 0.001	8557	TCAP
-0.174101159	< 0.001	220963	SLC16A9
-0.174116209	< 0.001	8187	ZNF239
-0.174136639	< 0.001	51320	MEX3C
-0.174167007	< 0.001	197407	ZNF48
-0.174239442	< 0.001	157922	CAMSAP1
-0.174247891	< 0.001	11102	RPP14
-0.174336299	< 0.001	2109	ETFB
-0.174376503	< 0.001	220988	HNRNPA3
-0.174398422	< 0.001	2047	EPHB1
-0.174458697	< 0.001	115209	OMA1
-0.174485162	< 0.001	84294	UTP23
-0.174559727	< 0.001	51374	C2orf28
-0.174585924	< 0.001	57571	ATPGD1
-0.174603388	< 0.001	6050	RNH1
-0.174628451	< 0.001	7507	XPA
-0.174721047	< 0.001	2123	EVI2A
-0.174841866	< 0.001	6144	RPL21
-0.174849287	< 0.001	1992	SERPINB1
-0.174870834	< 0.001	2944	GSTM1
-0.174900457	< 0.001	54893	MTMR10
-0.174910769	< 0.001	4815	NINJ2
-0.174918935	< 0.001	283131	NEAT1
-0.175030008	< 0.001	57700	FAM160B1
-0.175184727	< 0.001	123283	TARSL2
-0.175201625	< 0.001	23020	SNRNP200
-0.175341383	< 0.001	100129917	RP11-440L14.1
-0.17535077	< 0.001	115361	GBP4
-0.175402582	< 0.001	24137	KIF4A
-0.17543602	< 0.001	4684	NCAM1
-0.175498709	< 0.001	285672	SREK1IP1
-0.175635591	< 0.001	4904	YBX1
-0.175653145	< 0.001	389197	C4orf50
-0.175661132	< 0.001	284988	RHOQP3
-0.175683409	< 0.001	57168	ASPHD2
-0.17570886	< 0.001	6192	RPS4Y1
-0.175728738	< 0.001	9854	C2CD2L
-0.175797075	< 0.001	55764	IFT122
-0.175800696	< 0.001	8888	MCM3AP
-0.175801441	< 0.001	23632	CA14
-0.175856143	< 0.001	4436	MSH2
-0.175916418	< 0.001	79054	TRPM8
-0.17606166	< 0.001	164781	WDR69
-0.176115334	< 0.001	140735	DYNLL2
-0.176144585	< 0.001	94031	HTRA3
-0.17617245	< 0.001	766	CA7
-0.176173002	< 0.001	113402	SFT2D1
-0.176174864	< 0.001	120425	AMICA1
-0.176175877	< 0.001	5425	POLD2

region-wise, subcortex					
Spearman's corr coef	p-val	p-val, SA	p-val, SA (BH- correction)	GenID	Gene
-0.22471042	0.187005535	0.226	1	124044	SPATA2L
-0.22471042	0.187005535	0.214	1	348235	FAM33A
-0.224967822	0.186490923	0.225	1	3739	KCNA4
-0.224967822	0.186490923	0.216	1	4904	YBX1
-0.224967822	0.186490923	0.234	1	5562	PRKAA1
-0.224967822	0.186490923	0.246	1	57178	ZMIZ1
-0.224967822	0.186490923	0.254	1	84641	HIATL1
-0.224967822	0.186490923	0.22	1	440163	RNASE13
-0.225225225	0.185977086	0.231	1	4337	MOCS1
-0.225225225	0.185977086	0.256	1	4603	MYBL1
-0.225225225	0.185977086	0.22	1	6932	TCF7
-0.225225225	0.185977086	0.235	1	8328	GFI1B
-0.225225225	0.185977086	0.218	1	84549	MAK16
-0.225225225	0.185977086	0.238	1	147011	PROCA1
-0.225225225	0.185977086	0.214	1	154141	MBOAT1
-0.225225225	0.185977086	0.228	1	641298	RP11-368J21.1
-0.225482613	0.185464486	0.225	1	55622	TTC27
-0.225482613	0.185464486	0.22	1	78996	C7orf49
-0.225740016	0.184952945	0.217	1	1368	CPM
-0.225740016	0.184952945	0.25	1	2838	GPR15
-0.225740016	0.184952945	0.236	1	5480	PPIC
-0.225740016	0.184952945	0.212	1	9204	ZMYM6
-0.225740016	0.184952945	0.235	1	9747	FAM115A
-0.225740016	0.184952945	0.251	1	23279	NUP160
-0.225740016	0.184952945	0.239	1	94107	TMEM203
-0.225740016	0.184952945	0.215	1	643905	PRR21
-0.225997418	0.184442386	0.209	1	2123	EVI2A
-0.225997418	0.184442386	0.221	1	3991	LIPE
-0.225997418	0.184442386	0.21	1	10230	NBR2
-0.225997418	0.184442386	0.22	1	57406	ABHD6
-0.225997418	0.184442386	0.233	1	79097	TRIM48
-0.225997418	0.184442386	0.222	1	83594	NUDT12
-0.226254821	0.183932826	0.198	1	3316	HSPB2
-0.226254821	0.183932826	0.224	1	5108	PCM1
-0.226254821	0.183932826	0.234	1	6555	SLC10A2
-0.226254821	0.183932826	0.246	1	9723	SEMA3E
-0.226254821	0.183932826	0.252	1	27156	RTDR1
-0.226254821	0.183932826	0.214	1	29098	RANGRF
-0.226254821	0.183932826	0.214	1	56948	SDR39U1
-0.226254821	0.183932826	0.246	1	58480	RHOU
-0.226254821	0.183932826	0.211	1	84062	DTNBP1
-0.226254821	0.183932826	0.232	1	115209	OMA1
-0.226254821	0.183932826	0.202	1	339398	LINGO4
-0.226512223	0.183424041	0.23	1	6726	SRP9
-0.226512223	0.183424041	0.209	1	9267	CYTH1
-0.226512223	0.183424041	0.221	1	22976	PAXIP1
-0.226769611	0.182916492	0.223	1	2149	F2R
-0.226769611	0.182916492	0.21	1	7368	UGT8
-0.226769611	0.182916492	0.218	1	10232	MSLN
-0.226769611	0.182916492	0.222	1	27250	PDCD4
-0.226769611	0.182916492	0.223	1	51204	TACO1
-0.226769611	0.182916492	0.228	1	90799	CEP95
-0.227027014	0.182409972	0.21	1	767	CA8
-0.227027014	0.182409972	0.222	1	2151	F2RL2
-0.227027014	0.182409972	0.22	1	9507	ADAMTS4
-0.227027014	0.182409972	0.232	1	85301	COL27A1
-0.227027014	0.182409972	0.207	1	122769	PPIL5
-0.227027014	0.182409972	0.228	1	347148	QRFP
-0.227027014	0.182409972	0.2	1	100144748	AC063965.2
-0.227284417	0.18190445	0.211	1	715	C1R
-0.227284417	0.18190445	0.215	1	928	CD9
-0.227284417	0.18190445	0.209	1	7627	ZNF75A
-0.227284417	0.18190445	0.209	1	57185	NIPAL3
-0.227284417	0.18190445	0.208	1	64983	MRPL32
-0.227284417	0.18190445	0.221	1	654817	NCF1C
-0.227541834	0.181399688	0.225	1	9043	SPAG9
-0.227541834	0.181399688	0.204	1	23148	NACAD
-0.227541834	0.181399688	0.209	1	80314	EPC1
-0.227541834	0.181399688	0.229	1	142689	ASB12
-0.227541834	0.181399688	0.207	1	348938	NIPAL4
-0.227799237	0.180896163	0.213	1	1317	SLC31A1
-0.227799237	0.180896163	0.234	1	1665	DHX15
-0.227799237	0.180896163	0.223	1	3126	HLA-DRB4
-0.227799237	0.180896163	0.194	1	100129616	LOC100129616
-0.228056625	0.180393681	0.237	1	1352	COX10
-0.228056625	0.180393681	0.212	1	2653	GCSH
-0.228056625	0.180393681	0.221	1	23232	TBC1D12
-0.228056625	0.180393681	0.201	1	30827	CXXC1
-0.228056625	0.180393681	0.201	1	55144	LRRC8D

voxel-wise, subcortex			
Spearman's corr coef	p-val	GenID	Gene
-0.176185071	< 0.001	4621	MYH3
-0.176240787	< 0.001	5973	RENBP
-0.176261872	< 0.001	79132	DHX58
-0.17629689	< 0.001	7535	ZAP70
-0.176359668	< 0.001	51233	C22orf43
-0.176381201	< 0.001	2528	FUT6
-0.176410645	< 0.001	81628	TSC22D4
-0.176485777	< 0.001	8352	HIST1H3C
-0.176504999	< 0.001	79066	METTL16
-0.176524028	< 0.001	10616	RBCK1
-0.176617637	< 0.001	50854	C6orf48
-0.176653951	< 0.001	84820	POLR2J4
-0.176724434	< 0.001	283807	FBXL22
-0.176734284	< 0.001	79932	KIAA0319L
-0.17683439	< 0.001	55506	H2AFY2
-0.176965699	< 0.001	9595	CYTIP
-0.176969334	< 0.001	26520	TIMM9
-0.176971927	< 0.001	51575	ESF1
-0.17700471	< 0.001	84274	COQ5
-0.177081794	< 0.001	729082	OIP5-AS1
-0.177114576	< 0.001	2999	GZMH
-0.177125245	< 0.001	388	RHOB
-0.177127481	< 0.001	10609	AC130686.2
-0.17714791	< 0.001	10215	OLIG2
-0.177152738	< 0.001	4293	MAP3K9
-0.177203074	< 0.001	126626	GABPB2
-0.177286461	< 0.001	6535	SLC6A8
-0.177289337	< 0.001	144348	ZNF664
-0.177348316	< 0.001	3280	HES1
-0.177372456	< 0.001	92106	OXNAD1
-0.177381933	< 0.001	4824	NKX3-1
-0.177391306	< 0.001	2521	FUS
-0.177428544	< 0.001	65991	FUNDC2
-0.177530512	< 0.001	22807	IKZF2
-0.177617714	< 0.001	613	BCR
-0.177630812	< 0.001	10400	PEMT
-0.177631095	< 0.001	2033	EP300
-0.177651063	< 0.001	5730	PTGDS
-0.177788779	< 0.001	35	ACADS
-0.177792028	< 0.001	348840	AC073135.1
-0.177877188	< 0.001	4601	MXI1
-0.177880257	< 0.001	1031	CDKN2C
-0.177894741	< 0.001	6167	RPL37
-0.1778965	< 0.001	2512	FTL
-0.177906439	< 0.001	50937	CDON
-0.177933559	< 0.001	147429	CHST9-AS1
-0.177968383	< 0.001	10557	RPP38
-0.177970514	< 0.001	11072	DUSP14
-0.17803748	< 0.001	80895	ILKAP
-0.178068027	< 0.001	9348	NDST3
-0.178073972	< 0.001	23446	SLC44A1
-0.178099602	< 0.001	2840	GPR17
-0.178102762	< 0.001	81565	NDEL1
-0.178164333	< 0.001	57513	CASKIN2
-0.178232774	< 0.001	7707	ZNF148
-0.178245485	< 0.001	163183	C19orf46
-0.178335756	< 0.001	94107	TMEM203
-0.178403452	< 0.001	149047	RP11-183M13.1
-0.178446636	< 0.001	30850	CDR2L
-0.178507462	< 0.001	219854	TMEM218
-0.178558633	< 0.001	11064	CEP110
-0.178573593	< 0.001	50809	HP1BP3
-0.178598478	< 0.001	5860	QDPR
-0.178633586	< 0.001	3158	HMGCS2
-0.178640172	< 0.001	81831	NETO2
-0.178642496	< 0.001	51333	ZNF771
-0.17878142	< 0.001	285830	HLA-F-AS1
-0.178911433	< 0.001	387751	GVINP1
-0.178933725	< 0.001	55119	PRPF38B
-0.178979412	< 0.001	2765	GML
-0.178992048	< 0.001	114034	TOE1
-0.179005042	< 0.001	1203	CLN5
-0.179010242	< 0.001	26227	PHGDH
-0.17902492	< 0.001	22917	ZP1
-0.179046929	< 0.001	3737	KCNA2
-0.179074049	< 0.001	146754	DNAH2
-0.17908825	< 0.001	5579	PRKCB
-0.179109707	< 0.001	6181	RPLP2
-0.179120004	< 0.001	100132341	KIAA0664L3

region-wise, subcortex					
Spearman's corr coef	p-val	p-val, SA	p-val, SA (BH- correction)	GenID	Gene
-0.228314027	0.179892153	0.211	1	1299	COL9A3
-0.228314027	0.179892153	0.215	1	3020	H3F3A
-0.228314027	0.179892153	0.191	1	54617	INO80
-0.228314027	0.179892153	0.206	1	57827	C6orf47
-0.228314027	0.179892153	0.209	1	84869	CBR4
-0.228314027	0.179892153	0.221	1	92736	OTOP2
-0.228314027	0.179892153	0.22	1	162963	ZNF610
-0.228314027	0.179892153	0.243	1	402573	C7orf61
-0.22857143	0.179391682	0.199	1	5414	SEPTIN4
-0.22857143	0.179391682	0.22	1	9650	MTFR1
-0.22857143	0.179391682	0.208	1	79027	ZNF655
-0.22857143	0.179391682	0.236	1	100129075	C14orf33
-0.228828833	0.178891912	0.22	1	2065	ERBB3
-0.228828833	0.178891912	0.216	1	4330	MN1
-0.228828833	0.178891912	0.233	1	8243	SMC1A
-0.228828833	0.178891912	0.209	1	55608	ANKRD10
-0.228828833	0.178891912	0.252	1	55771	PRR11
-0.228828833	0.178891912	0.237	1	60484	HAPLN2
-0.228828833	0.178891912	0.211	1	64129	TINAGL1
-0.228828833	0.178891912	0.212	1	79019	CENPM
-0.228828833	0.178891912	0.203	1	83742	MARVELD1
-0.228828833	0.178891912	0.194	1	284948	SH2D6
-0.229086235	0.178393424	0.205	1	9235	IL32
-0.229086235	0.178393424	0.197	1	10179	AP002373.1
-0.229086235	0.178393424	0.237	1	11066	SNRNP35
-0.229086235	0.178393424	0.231	1	81490	PTDSS2
-0.229086235	0.178393424	0.222	1	120379	PIH1D2
-0.229086235	0.178393424	0.221	1	286827	TRIM59
-0.229343623	0.177895904	0.218	1	7675	ZNF121
-0.229343623	0.177895904	0.215	1	10984	KCNQ1OT1
-0.229343623	0.177895904	0.215	1	30816	ERVWE1
-0.229343623	0.177895904	0.209	1	54778	RNF111
-0.229343623	0.177895904	0.214	1	79891	ZNF671
-0.229343623	0.177895904	0.231	1	81539	SLC38A1
-0.229343623	0.177895904	0.212	1	150082	LCA5L
-0.229343623	0.177895904	0.225	1	255180	FLJ38723
-0.229601026	0.177399367	0.202	1	2041	EPHA1
-0.229601026	0.177399367	0.214	1	2958	GTF2A2
-0.229601026	0.177399367	0.21	1	6883	TAF12
-0.229601026	0.177399367	0.23	1	9086	EIF1AY
-0.229601026	0.177399367	0.201	1	54464	XRN1
-0.229601026	0.177399367	0.211	1	64863	METTL4
-0.229601026	0.177399367	0.217	1	80209	C13orf23
-0.229601026	0.177399367	0.186	1	339290	LOC339290
-0.229601026	0.177399367	0.226	1	339487	ZBTB8OS
-0.229601026	0.177399367	0.219	1	642413	CTSL1P6
-0.229601026	0.177399367	0.231	1	100008589	RN28S1
-0.229858428	0.176903665	0.217	1	347	APOD
-0.229858428	0.176903665	0.221	1	7594	ZNF43
-0.229858428	0.176903665	0.213	1	10351	ABCA8
-0.229858428	0.176903665	0.227	1	54148	MRPL39
-0.229858428	0.176903665	0.198	1	80304	C2orf44
-0.229858428	0.176903665	0.21	1	113177	C19orf36
-0.230115831	0.176409125	0.231	1	4775	NFATC3
-0.230115831	0.176409125	0.227	1	7365	UGT2B10
-0.230115831	0.176409125	0.196	1	10544	PROCR
-0.230115831	0.176409125	0.207	1	26267	FBXO10
-0.230115831	0.176409125	0.227	1	56882	CDC42SE1
-0.230115831	0.176409125	0.226	1	79696	FAM164C
-0.230115831	0.176409125	0.202	1	80346	REEP4
-0.230115831	0.176409125	0.19	1	115361	GBP4
-0.230373234	0.175915629	0.216	1	3485	IGFBP2
-0.230373234	0.175915629	0.201	1	7608	ZNF56
-0.230373234	0.175915629	0.223	1	7984	ARHGEF5
-0.230373234	0.175915629	0.239	1	10825	NEU3
-0.230373234	0.175915629	0.204	1	26119	LDLRAP1
-0.230373234	0.175915629	0.193	1	58526	MID1IP1
-0.230373234	0.175915629	0.195	1	150160	CESK1
-0.230373234	0.175915629	0.21	1	252983	STXBP4
-0.230373234	0.175915629	0.218	1	344328	ST13P2
-0.230373234	0.175915629	0.224	1	347902	AMIGO2
-0.230630621	0.175423086	0.204	1	2235	FECH
-0.230630621	0.175423086	0.234	1	7227	TRPS1
-0.230630621	0.175423086	0.227	1	7444	VRK2
-0.230630621	0.175423086	0.205	1	11240	PADI2
-0.230630621	0.175423086	0.227	1	54552	GNL3L
-0.230630621	0.175423086	0.215	1	54587	MXRA8
-0.230888024	0.174931362	0.203	1	1755	DMBT1
-0.230888024	0.174931362	0.239	1	6707	SPRR3

voxel-wise, subcortex			
Spearman's corr coef	p-val	GenID	Gene
-0.179178521	< 0.001	53832	IL20RA
-0.179216966	< 0.001	1347	COX7A2
-0.179234415	< 0.001	1475	CSTA
-0.179236829	< 0.001	689	BTF3
-0.179283276	< 0.001	6102	RP2
-0.179294512	< 0.001	162967	ZNF320
-0.179381803	< 0.001	140801	RPL10L
-0.179427952	< 0.001	64062	RBM26
-0.179481357	< 0.001	10580	SORBS1
-0.179488316	< 0.001	57176	VARS2
-0.179527044	< 0.001	55659	ZNF416
-0.179545522	< 0.001	57653	LOC100499484-C9ORF174
-0.179557875	< 0.001	2122	EVI1
-0.179614887	< 0.001	79742	CXorf36
-0.179615259	< 0.001	26082	AC135731.1
-0.179644883	< 0.001	9510	ADAMTS1
-0.179661602	< 0.001	83861	RSPH3
-0.179706931	< 0.001	3659	IRF1
-0.179719552	< 0.001	1909	EDNRA
-0.179766446	< 0.001	11067	C10orf10
-0.179773048	< 0.001	100294362	LOC100294362
-0.179802671	< 0.001	79175	ZNF343
-0.17981112	< 0.001	4437	MSH3
-0.179880023	< 0.001	51691	NAA38
-0.179883555	< 0.001	6651	SON
-0.179955155	< 0.001	80005	DOCK5
-0.179960445	< 0.001	25861	DFNB31
-0.180006504	< 0.001	55027	HEATR3
-0.180011332	< 0.001	7039	TGFA
-0.180105969	< 0.001	374973	ATPAF1-AS1
-0.180168837	< 0.001	54476	RNF216
-0.18025957	< 0.001	3097	HIVEP2
-0.180269778	< 0.001	388419	BTBD17
-0.180298477	< 0.001	120376	AP002448.1
-0.180333495	< 0.001	10777	ARPP-21
-0.180356249	< 0.001	1295	COL8A1
-0.180375651	< 0.001	5376	PMP22
-0.180391535	< 0.001	4752	NEK3
-0.180398315	< 0.001	5745	PTH1R
-0.180473074	< 0.001	7716	VEZF1
-0.180487186	< 0.001	158586	ZXDB
-0.180510208	< 0.001	54899	PXK
-0.180544302	< 0.001	7608	ZNF56
-0.180604935	< 0.001	56339	METTL3
-0.180624813	< 0.001	81621	KAZALD1
-0.180667073	< 0.001	4710	NDUFB4
-0.180670321	< 0.001	4842	NOS1
-0.180782691	< 0.001	51684	SUFU
-0.180958197	< 0.001	3698	ITIH2
-0.180968881	< 0.001	4758	NEU1
-0.181016713	< 0.001	23503	ZFYVE26
-0.181055889	< 0.001	3821	KLRK1
-0.181108639	< 0.001	3882	KRT32
-0.181118116	< 0.001	51295	ECSIT
-0.181139007	< 0.001	9648	GCC2
-0.18115145	< 0.001	85378	TUBGCP6
-0.18116343	< 0.001	60468	BACH2
-0.181173086	< 0.001	26268	FBXO9
-0.181190833	< 0.001	5071	PARK2
-0.181232899	< 0.001	145482	PTGR2
-0.181288436	< 0.001	170959	ZNF431
-0.181290761	< 0.001	29911	HOOK2
-0.181291774	< 0.001	6440	SFTPC
-0.181293637	< 0.001	51422	PRKAG2
-0.181376651	< 0.001	1743	DLSTP
-0.181410462	< 0.001	115106	HAUS1
-0.181465521	< 0.001	151525	WDSUB1
-0.181495801	< 0.001	11123	RCAN3
-0.18149589	< 0.001	126520	PLK5P
-0.181506202	< 0.001	4034	LRCH4
-0.18151772	< 0.001	10072	DPP3
-0.181563869	< 0.001	79085	SLC25A23
-0.181575567	< 0.001	219595	FOLH1B
-0.181576133	< 0.001	286336	FAM78A
-0.181580767	< 0.001	94015	TTYH2
-0.181589872	< 0.001	100128302	LOC100128302
-0.181616813	< 0.001	400750	LOC400750
-0.181620523	< 0.001	202020	FLJ39653
-0.181646526	< 0.001	10809	STARD10

region-wise, subcortex					
Spearman's corr coef	p-val	p-val, SA	p-val, SA (BH- correction)	GenID	Gene
-0.230888024	0.174931362	0.22	1	8602	NOP14
-0.230888024	0.174931362	0.219	1	10312	TCIRG1
-0.230888024	0.174931362	0.219	1	83642	RP3-402G11.1
-0.230888024	0.174931362	0.191	1	148327	CREB3L4
-0.230888024	0.174931362	0.229	1	165055	CCDC138
-0.230888024	0.174931362	0.201	1	196528	ARID2
-0.230888024	0.174931362	0.209	1	386676	KRTAP10-9
-0.231145427	0.174440831	0.219	1	1544	CYP1A2
-0.231145427	0.174440831	0.205	1	55745	MUDENG
-0.231145427	0.174440831	0.22	1	84570	COL25A1
-0.231145427	0.174440831	0.213	1	151835	CPNE9
-0.231145427	0.174440831	0.224	1	152078	C3orf55
-0.231402829	0.173951298	0.185	1	3383	ICAM1
-0.231402829	0.173951298	0.204	1	7357	UGCG
-0.231402829	0.173951298	0.206	1	90333	ZNF468
-0.231402829	0.173951298	0.189	1	338675	OR5AP2
-0.231660232	0.173462734	0.21	1	1059	CENPB
-0.231660232	0.173462734	0.211	1	3655	ITGA6
-0.231660232	0.173462734	0.217	1	3669	ISG20
-0.231660232	0.173462734	0.217	1	26070	AP002380.3
-0.231660232	0.173462734	0.218	1	55192	DNAJC17
-0.231660232	0.173462734	0.185	1	81611	ANP32E
-0.231660232	0.173462734	0.203	1	284467	FAM19A3
-0.23191762	0.172975168	0.199	1	5681	PSKH1
-0.23191762	0.172975168	0.212	1	5893	RAD52
-0.23191762	0.172975168	0.217	1	6601	SMARCC2
-0.23191762	0.172975168	0.194	1	8125	ANP32A
-0.23191762	0.172975168	0.23	1	9717	SEC14L5
-0.23191762	0.172975168	0.214	1	51365	PLA1A
-0.23191762	0.172975168	0.232	1	140901	STK35
-0.23191762	0.172975168	0.205	1	155066	ATP6V0E2
-0.232175022	0.172488391	0.196	1	6938	TCF12
-0.232175022	0.172488391	0.218	1	25802	LMOD1
-0.232175022	0.172488391	0.222	1	91179	SCARF2
-0.232175022	0.172488391	0.179	1	286148	DPY19L4
-0.232432425	0.172002837	0.203	1	212	ALAS2
-0.232432425	0.172002837	0.199	1	5937	RBMS1
-0.232432425	0.172002837	0.215	1	55636	CHD7
-0.232432425	0.172002837	0.193	1	83478	ARHGAP24
-0.232432425	0.172002837	0.198	1	154810	AMOTL1
-0.232432425	0.172002837	0.228	1	161394	SAMD15
-0.232432425	0.172002837	0.191	1	340547	VSIG1
-0.232432425	0.172002837	0.209	1	414157	C10orf62
-0.232432425	0.172002837	0.218	1	730234	LOC730234
-0.232689828	0.171518236	0.205	1	11112	HIBADH
-0.232689828	0.171518236	0.218	1	56204	KIAA1370
-0.23294723	0.171034634	0.196	1	4340	MOG
-0.23294723	0.171034634	0.204	1	7692	ZNF133
-0.23294723	0.171034634	0.216	1	10419	PRMT5
-0.23294723	0.171034634	0.209	1	22882	ZHX2
-0.23294723	0.171034634	0.196	1	51056	LAP3
-0.23294723	0.171034634	0.202	1	51460	SFMBT1
-0.23294723	0.171034634	0.221	1	55139	ANKZF1
-0.23294723	0.171034634	0.202	1	55858	TMEM165
-0.23294723	0.171034634	0.199	1	723790	HIST2H2AA4
-0.23294723	0.171034634	0.221	1	100288801	FRG2C
-0.233204618	0.170551792	0.209	1	369	ARAF
-0.233204618	0.170551792	0.207	1	1380	CR2
-0.233204618	0.170551792	0.232	1	3678	ITGA5
-0.233204618	0.170551792	0.189	1	50809	HP1BP3
-0.233204618	0.170551792	0.213	1	84309	NUDT16L1
-0.233204618	0.170551792	0.204	1	127731	VWA5B1
-0.233204618	0.170551792	0.205	1	100132815	AC092329.2
-0.233462021	0.170070201	0.195	1	5829	PXN
-0.233462021	0.170070201	0.208	1	7328	UBE2H
-0.233462021	0.170070201	0.233	1	7978	MTERF
-0.233462021	0.170070201	0.222	1	26157	GIMAP2
-0.233462021	0.170070201	0.221	1	143879	KBTBD3
-0.233462021	0.170070201	0.182	1	202333	CMYA5
-0.233719423	0.169589564	0.195	1	391	RHOG
-0.233719423	0.169589564	0.206	1	3707	ITPKB
-0.233719423	0.169589564	0.195	1	11096	ADAMTS5
-0.233719423	0.169589564	0.181	1	54676	GTPBP2
-0.233976826	0.169109881	0.213	1	563	AZGP1
-0.233976826	0.169109881	0.189	1	8089	YEATS4
-0.233976826	0.169109881	0.191	1	9758	FRMPD4
-0.233976826	0.169109881	0.218	1	10009	ZBTB33
-0.233976826	0.169109881	0.213	1	282997	LOC282997
-0.234234229	0.168630987	0.187	1	1632	DCI

voxel-wise, subcortex			
Spearman's corr coef	p-val	GenID	Gene
-0.181664079	< 0.001	339005	WHAMML1
-0.181700572	< 0.001	5356	PLRG1
-0.181705028	< 0.001	57506	MAVS
-0.181730852	< 0.001	6418	SET
-0.181821764	< 0.001	5170	PDPK1
-0.181833372	< 0.001	80304	C2orf44
-0.181871071	< 0.001	25980	C20orf4
-0.181922421	< 0.001	10658	CUGBP1
-0.181947693	< 0.001	5354	PLP1
-0.181971923	< 0.001	349152	DPY19L2P2
-0.181980193	< 0.001	25932	CLIC4
-0.182011306	< 0.001	3189	HNRNPH3
-0.182019472	< 0.001	5260	PHKG1
-0.182046503	< 0.001	10039	PARP3
-0.182054758	< 0.001	80817	KIAA1712
-0.182103977	< 0.001	1729	DIAPH1
-0.182156265	< 0.001	145270	PRIMA1
-0.182176143	< 0.001	5118	PCOLCE
-0.182186082	< 0.001	83480	PUS3
-0.18224059	< 0.001	25961	NUDT13
-0.182366416	< 0.001	10813	UTP14A
-0.182381749	< 0.001	358	AQP1
-0.182392418	< 0.001	100129387	GABPB1-AS1
-0.182434484	< 0.001	9541	CIR1
-0.182483062	< 0.001	622	BDH1
-0.1825905	< 0.001	1634	DCN
-0.18267177	< 0.001	157777	C8orf45
-0.182680592	< 0.001	8566	PDXK
-0.182681426	< 0.001	9654	TTLL4
-0.182720423	< 0.001	54953	C1orf27
-0.182744294	< 0.001	3673	ITGA2
-0.182758033	< 0.001	57404	CYP20A1
-0.182772055	< 0.001	79364	ZXDC
-0.18279472	< 0.001	7018	TF
-0.182909966	< 0.001	1787	TRDMT1
-0.182955012	< 0.001	359845	FAM101B
-0.182957232	< 0.001	23264	ZC3H7B
-0.183066443	< 0.001	79039	DDX54
-0.183093369	< 0.001	55227	LRRC1
-0.183151603	< 0.001	4176	MCM7
-0.183172226	< 0.001	338799	AC084018.1
-0.183194876	< 0.001	9324	HMGN3
-0.183247343	< 0.001	85453	AP003115.1
-0.183286905	< 0.001	56257	MEPCE
-0.183346897	< 0.001	84935	C13orf33
-0.183367893	< 0.001	3834	KIF25
-0.183404848	< 0.001	84549	MAK16
-0.183528453	< 0.001	26233	FBXL6
-0.183573216	< 0.001	100131211	TMEM194B
-0.183629304	< 0.001	171017	ZNF384
-0.183662832	< 0.001	401251	C6orf26
-0.183699235	< 0.001	54148	MRPL39
-0.183718637	< 0.001	23250	ATP11A
-0.183784112	< 0.001	443	ASPA
-0.183792278	< 0.001	23279	NUP160
-0.183856547	< 0.001	57732	ZFYVE28
-0.183888122	< 0.001	643314	KIAA0754
-0.183924615	< 0.001	6385	SDC4
-0.183931023	< 0.001	1939	LGTN
-0.183981732	< 0.001	11198	SUPT16H
-0.184046641	< 0.001	8273	SLC10A3
-0.184151396	< 0.001	25791	NGEF
-0.184158638	< 0.001	10461	MERTK
-0.184170067	< 0.001	51661	FKBP7
-0.184182972	< 0.001	57409	MIF4GD
-0.184186876	< 0.001	10397	NDRG1
-0.184219465	< 0.001	3831	C14orf153
-0.184236556	< 0.001	11325	DDX42
-0.184238225	< 0.001	6819	SULT1C2
-0.18434836	< 0.001	8630	HSD17B6
-0.184358954	< 0.001	824	CAPN2
-0.184396192	< 0.001	51341	ZBTB7A
-0.184428602	< 0.001	400506	C16orf88
-0.184431851	< 0.001	8839	WISP2
-0.184432879	< 0.001	80174	DBF4B
-0.184590101	< 0.001	8819	SAP30
-0.184643582	< 0.001	196528	ARID2
-0.184650734	< 0.001	8545	CGGBP1
-0.184699208	< 0.001	494514	C18orf56

region-wise, subcortex					
Spearman's corr coef	p-val	p-val, SA	p-val, SA (BH- correction)	GenID	Gene
-0.234234229	0.168630987	0.201	1	3932	LCK
-0.234234229	0.168630987	0.21	1	5653	KLK6
-0.234234229	0.168630987	0.196	1	23250	ATP11A
-0.234234229	0.168630987	0.214	1	64969	MRPS5
-0.234234229	0.168630987	0.202	1	65975	STK33
-0.234234229	0.168630987	0.202	1	342035	GLDN
-0.234234229	0.168630987	0.194	1	392352	LOC392352
-0.234491631	0.168153301	0.195	1	54906	C10orf18
-0.234491631	0.168153301	0.209	1	64422	ATG3
-0.234491631	0.168153301	0.196	1	118442	GPR62
-0.234491631	0.168153301	0.195	1	149501	KRT8P45
-0.234491631	0.168153301	0.21	1	283948	NHLRC4
-0.234491631	0.168153301	0.219	1	390152	OR8H3
-0.234491631	0.168153301	0.174	1	404093	CUEDC1
-0.234749034	0.167676628	0.196	1	914	CD2
-0.234749034	0.167676628	0.197	1	1278	COL1A2
-0.234749034	0.167676628	0.208	1	4122	MAN2A2
-0.234749034	0.167676628	0.206	1	5465	PPARA
-0.234749034	0.167676628	0.21	1	5469	MED1
-0.234749034	0.167676628	0.208	1	56301	SLC7A10
-0.234749034	0.167676628	0.189	1	64798	DEPDC6
-0.235006437	0.167200893	0.207	1	2316	FLNA
-0.235006437	0.167200893	0.207	1	2531	KDSR
-0.235006437	0.167200893	0.211	1	5380	PMS2L2
-0.235006437	0.167200893	0.187	1	6414	SEPP1
-0.235006437	0.167200893	0.218	1	6431	SFRS6
-0.235006437	0.167200893	0.217	1	10400	PEMT
-0.235006437	0.167200893	0.228	1	27254	CSDC2
-0.235006437	0.167200893	0.202	1	29766	TMOD3
-0.235006437	0.167200893	0.218	1	58515	RP11-884K10.1
-0.235263839	0.166726142	0.202	1	7726	TRIM26
-0.235263839	0.166726142	0.204	1	9744	ACAP1
-0.235263839	0.166726142	0.203	1	55689	YEATS2
-0.235263839	0.166726142	0.202	1	83639	TEX101
-0.235263839	0.166726142	0.18	1	152273	FGD5
-0.235263839	0.166726142	0.215	1	200205	C1orf69
-0.235521242	0.166252166	0.179	1	3397	ID1
-0.235521242	0.166252166	0.203	1	23218	NBEAL2
-0.235521242	0.166252166	0.184	1	26030	PLEKHG3
-0.235521242	0.166252166	0.189	1	26610	ELP4
-0.235521242	0.166252166	0.187	1	51330	TNFRSF12A
-0.235521242	0.166252166	0.186	1	56961	SHD
-0.23577863	0.165779367	0.201	1	6461	SHB
-0.23577863	0.165779367	0.184	1	9623	TCL1B
-0.23577863	0.165779367	0.171	1	51642	MRPL48
-0.23577863	0.165779367	0.2	1	83695	C12orf32
-0.23577863	0.165779367	0.175	1	220359	TIGD3
-0.236036032	0.165307596	0.222	1	3690	ITGB3
-0.236036032	0.165307596	0.194	1	9454	HOMER3
-0.236036032	0.165307596	0.218	1	26261	FBXO24
-0.236036032	0.165307596	0.221	1	80742	PRR3
-0.236036032	0.165307596	0.22	1	124590	USH1G
-0.236293435	0.164836764	0.206	1	5289	PIK3C3
-0.236293435	0.164836764	0.188	1	5790	PTPRCAP
-0.236293435	0.164836764	0.205	1	81035	COLEC12
-0.236293435	0.164836764	0.192	1	85449	KIAA1755
-0.236293435	0.164836764	0.199	1	92399	MRRF
-0.236293435	0.164836764	0.203	1	220136	CCDC11
-0.236293435	0.164836764	0.18	1	221120	ALKBH3
-0.236293435	0.164836764	0.213	1	643332	AL355922.1
-0.236550838	0.164366677	0.206	1	7408	VASP
-0.236550838	0.164366677	0.221	1	27443	CECR2
-0.236550838	0.164366677	0.186	1	83607	AMMECR1L
-0.236550838	0.164366677	0.175	1	145781	GCOM1
-0.236550838	0.164366677	0.211	1	285908	LINC00174
-0.236550838	0.164366677	0.198	1	389741	MGC21881
-0.23680824	0.163897797	0.191	1	666	BOK
-0.23680824	0.163897797	0.187	1	1497	CTNS
-0.23680824	0.163897797	0.175	1	6391	SDHC
-0.23680824	0.163897797	0.192	1	6421	SFPQ
-0.23680824	0.163897797	0.193	1	10809	STARD10
-0.23680824	0.163897797	0.204	1	22918	CD93
-0.23680824	0.163897797	0.195	1	28513	CDH19
-0.23680824	0.163897797	0.209	1	51704	GPRC5B
-0.23680824	0.163897797	0.204	1	56985	C17orf48
-0.23680824	0.163897797	0.206	1	85457	KIAA1737
-0.23680824	0.163897797	0.202	1	389336	C5orf46
-0.237065628	0.163429871	0.217	1	2140	EYA3
-0.237065628	0.163429871	0.191	1	2837	UTS2R

voxel-wise, subcortex			
Spearman's corr coef	p-val	GenID	Gene
-0.184854761	< 0.001	116064	LRRC58
-0.184920415	< 0.001	439949	LOC439949
-0.1849325	< 0.001	55147	RBM23
-0.185000286	< 0.001	254428	SLC41A1
-0.185040593	< 0.001	4867	NPHP1
-0.185052574	< 0.001	57061	HYMAI
-0.185098916	< 0.001	5337	PLD1
-0.185120076	< 0.001	3029	HAGH
-0.185134843	< 0.001	6563	SLC14A1
-0.185155183	< 0.001	55603	FAM46A
-0.185192987	< 0.001	8321	FZD1
-0.185235232	< 0.001	4825	NKX6-1
-0.185235888	< 0.001	65979	PHACTR4
-0.185262069	< 0.001	272	AMPD3
-0.185267001	< 0.001	1768	DNAH6
-0.18528761	< 0.001	51642	MRPL48
-0.185338601	< 0.001	51704	GPRC5B
-0.185346961	< 0.001	64975	MRPL41
-0.185348898	< 0.001	79979	TRMT2B
-0.185356885	< 0.001	28984	C13orf15
-0.185360879	< 0.001	6781	STC1
-0.185364321	< 0.001	3635	INPP5D
-0.18536748	< 0.001	440295	AC135995.5
-0.18536897	< 0.001	56987	BBX
-0.185428396	< 0.001	3042	HBM
-0.185451433	< 0.001	6363	CCL19
-0.185462102	< 0.001	7060	THBS4
-0.185518205	< 0.001	63976	PRDM16
-0.185532689	< 0.001	4211	MEIS1
-0.185533702	< 0.001	7318	UBA7
-0.185540587	< 0.001	28990	ASTE1
-0.185558975	< 0.001	4343	MOV10
-0.185582742	< 0.001	341359	SYT10
-0.185605034	< 0.001	152	ADRA2C
-0.185623601	< 0.001	64789	DEM1
-0.185633451	< 0.001	10406	WFDC2
-0.185724452	< 0.001	2958	GTF2A2
-0.185729563	< 0.001	10351	ABCA8
-0.185743496	< 0.001	29903	CCDC106
-0.185745254	< 0.001	10290	SPEG
-0.185839146	< 0.001	84987	C12orf62
-0.185860783	< 0.001	1902	LPAR1
-0.185957089	< 0.001	26273	FBXO3
-0.185967296	< 0.001	22878	KIAA1012
-0.186000735	< 0.001	7696	ZNF137P
-0.186056167	< 0.001	2065	ERBB3
-0.186120436	< 0.001	2147	F2
-0.186138272	< 0.001	1786	DNMT1
-0.186202347	< 0.001	286827	TRIM59
-0.186249897	< 0.001	91754	NEK9
-0.18633458	< 0.001	122622	ADSSL1
-0.186339036	< 0.001	221037	JMJD1C
-0.186373129	< 0.001	57570	TRMT5
-0.186423734	< 0.001	83595	SOX7
-0.186493009	< 0.001	9486	CHST10
-0.186498404	< 0.001	55760	DHX32
-0.186521426	< 0.001	29105	C16orf80
-0.186577529	< 0.001	51078	THAP4
-0.186653763	< 0.001	25776	CBY1
-0.186697692	< 0.001	729046	LOC729046
-0.186789259	< 0.001	84527	ZNF559
-0.186805695	< 0.001	4790	NFKB1
-0.186882123	< 0.001	9374	PPT2
-0.186892986	< 0.001	10818	FRS2
-0.186896235	< 0.001	5510	PPP1R7
-0.186897069	< 0.001	54742	LY6K
-0.186924651	< 0.001	54482	CCDC76
-0.186959013	< 0.001	23361	ZNF629
-0.186991513	< 0.001	123879	DCUN1D3
-0.1870047	< 0.001	4037	LRP3
-0.187100545	< 0.001	58473	PLEKHB1
-0.187215507	< 0.001	253512	SLC25A30
-0.187249318	< 0.001	23743	BHMT2
-0.187252373	< 0.001	402176	RPL21P44
-0.187305406	< 0.001	84649	DGAT2
-0.1873198	< 0.001	4162	MCAM
-0.187444985	< 0.001	11343	MGLL
-0.187500328	< 0.001	9267	CYTH1
-0.187541097	< 0.001	6470	SHMT1

region-wise, subcortex					
Spearman's corr coef	p-val	p-val, SA	p-val, SA (BH- correction)	GenID	Gene
-0.237065628	0.163429871	0.193	1	6696	SPP1
-0.237065628	0.163429871	0.19	1	11200	CHEK2
-0.237065628	0.163429871	0.204	1	57513	CASKIN2
-0.237065628	0.163429871	0.205	1	283788	LOC283788
-0.237323031	0.162962988	0.189	1	9516	LITAF
-0.237323031	0.162962988	0.223	1	64132	XYLT2
-0.237323031	0.162962988	0.189	1	93010	B3GNT7
-0.237323031	0.162962988	0.19	1	664701	ZNF826P
-0.237580433	0.162496805	0.19	1	3371	TNC
-0.237580433	0.162496805	0.199	1	10474	TADA3L
-0.237580433	0.162496805	0.198	1	10875	FGL2
-0.237580433	0.162496805	0.191	1	10934	MORF4
-0.237580433	0.162496805	0.189	1	29093	MRPL22
-0.237580433	0.162496805	0.216	1	83869	TTTY14
-0.237580433	0.162496805	0.207	1	112817	NPL2
-0.237837836	0.162031814	0.211	1	1854	DUT
-0.237837836	0.162031814	0.214	1	1880	GPR183
-0.237837836	0.162031814	0.169	1	4238	MFAP3
-0.237837836	0.162031814	0.189	1	59271	C21orf63
-0.237837836	0.162031814	0.202	1	79856	SNX22
-0.237837836	0.162031814	0.199	1	441054	C4orf47
-0.237837836	0.162031814	0.205	1	643198	RBPJP6
-0.238095239	0.161567792	0.184	1	2913	GRM3
-0.238095239	0.161567792	0.195	1	51264	MRPL27
-0.238095239	0.161567792	0.183	1	58499	ZNF462
-0.238095239	0.161567792	0.21	1	93323	HAUS8
-0.238095239	0.161567792	0.213	1	260294	NSUN5C
-0.238352627	0.161104769	0.2	1	11135	CDC42EP1
-0.238352627	0.161104769	0.205	1	22927	HABP4
-0.238352627	0.161104769	0.199	1	79993	ELOVL7
-0.238352627	0.161104769	0.19	1	84196	USP48
-0.238352627	0.161104769	0.179	1	84939	MUM1
-0.238352627	0.161104769	0.21	1	388135	C15orf59
-0.238610029	0.160642698	0.194	1	3338	DNAJC4
-0.238610029	0.160642698	0.201	1	10588	MTHFS
-0.238610029	0.160642698	0.199	1	55191	NADSYN1
-0.238610029	0.160642698	0.208	1	80013	C10orf97
-0.238610029	0.160642698	0.2	1	87178	PNPT1
-0.238610029	0.160642698	0.213	1	147657	ZNF480
-0.238610029	0.160642698	0.21	1	286103	C8ORF77
-0.238867432	0.160181388	0.178	1	8801	SUCLG2
-0.238867432	0.160181388	0.186	1	51805	COQ3
-0.238867432	0.160181388	0.209	1	84809	CROCCP2
-0.238867432	0.160181388	0.193	1	147040	KCTD11
-0.238867432	0.160181388	0.187	1	388759	C1orf229
-0.238867432	0.160181388	0.189	1	400322	AC091565.3
-0.239124835	0.159721226	0.22	1	6432	SFRS7
-0.239124835	0.159721226	0.184	1	9646	CTR9
-0.239124835	0.159721226	0.219	1	84803	AGPAT9
-0.239124835	0.159721226	0.188	1	128637	TBC1D20
-0.239124835	0.159721226	0.23	1	100129455	LOC100129455
-0.239382237	0.159262061	0.198	1	5910	RAP1GDS1
-0.239382237	0.159262061	0.227	1	9111	NMI
-0.239382237	0.159262061	0.184	1	51277	DNAJC27
-0.239382237	0.159262061	0.174	1	55281	TMEM140
-0.239382237	0.159262061	0.204	1	143458	LDLRAD3
-0.239382237	0.159262061	0.175	1	387521	TMEM189
-0.239382237	0.159262061	0.195	1	100288797	RP5-860F19.1
-0.239639625	0.15880385	0.182	1	1511	CTSG
-0.239639625	0.15880385	0.217	1	5649	RELN
-0.239639625	0.15880385	0.201	1	6521	SLC4A1
-0.239639625	0.15880385	0.207	1	23258	DENND5A
-0.239639625	0.15880385	0.184	1	55657	ZNF692
-0.239639625	0.15880385	0.197	1	171389	NLRP6
-0.239639625	0.15880385	0.22	1	386757	SLC6A10P
-0.239639625	0.15880385	0.188	1	390260	OR6X1
-0.239639625	0.15880385	0.203	1	439931	AC002472.9
-0.239897028	0.158346385	0.199	1	204	AK2
-0.239897028	0.158346385	0.186	1	480	ATP1A4
-0.239897028	0.158346385	0.212	1	962	CD48
-0.239897028	0.158346385	0.2	1	1374	CPT1A
-0.239897028	0.158346385	0.196	1	1485	CTAG1B
-0.239897028	0.158346385	0.197	1	3603	IL16
-0.24015443	0.157890156	0.201	1	2683	B4GALT1
-0.24015443	0.157890156	0.219	1	3911	LAMA5
-0.24015443	0.157890156	0.165	1	7316	UBC
-0.24015443	0.157890156	0.203	1	51293	CD320
-0.24015443	0.157890156	0.197	1	121504	HIST4H4
-0.24015443	0.157890156	0.213	1	122011	CSNK1A1L

voxel-wise, subcortex			
Spearman's corr coef	p-val	GenID	Gene
-0.187645569	< 0.001	64838	FNDC4
-0.187678263	< 0.001	5089	PBX2
-0.187683374	< 0.001	1812	DRD1
-0.187747627	< 0.001	5111	PCNA
-0.187767133	< 0.001	7373	COL14A1
-0.187769085	< 0.001	7022	TFAP2C
-0.187849596	< 0.001	7776	ZNF236
-0.187885731	< 0.001	29901	SAC3D1
-0.187887773	< 0.001	6235	RPS29
-0.187889814	< 0.001	653073	AC120045.5
-0.187908947	< 0.001	261734	NPHP4
-0.187920928	< 0.001	100288252	LOC100288252
-0.187931135	< 0.001	415056	LOC415056
-0.187939867	< 0.001	9695	EDEM1
-0.187939957	< 0.001	23354	HAUS5
-0.187959373	< 0.001	11257	TP53TG1
-0.188077033	< 0.001	5184	PEPD
-0.188104883	< 0.001	23563	CHST5
-0.188110918	< 0.001	26502	NARF
-0.188186511	< 0.001	3688	ITGB1
-0.188196182	< 0.001	1265	CNN2
-0.18819803	< 0.001	9553	MRPL33
-0.188202679	< 0.001	25845	AC010442.2
-0.18820779	< 0.001	5287	PIK3C2B
-0.188253939	< 0.001	84740	AFAP1-AS1
-0.188254401	< 0.001	7248	TSC1
-0.188282266	< 0.001	53827	FXYD5
-0.188300923	< 0.001	10749	KIF1C
-0.188357294	< 0.001	79056	PRRG4
-0.18840085	< 0.001	5996	RGS1
-0.188433349	< 0.001	9600	PITPNM1
-0.188480154	< 0.001	93594	WDR67
-0.188533932	< 0.001	3017	HIST1H2BD
-0.188549444	< 0.001	26034	IPCEF1
-0.188567728	< 0.001	2327	FMO2
-0.188631907	< 0.001	729059	RP4-805N21.1
-0.188639894	< 0.001	55809	TRERF1
-0.188706189	< 0.001	64108	RTP4
-0.188736752	< 0.001	83955	NACAP1
-0.188743442	< 0.001	90342	FER1L5
-0.18881689	< 0.001	339541	C1orf228
-0.188996121	< 0.001	7789	ZXDA
-0.189100504	< 0.001	64324	NSD1
-0.189137191	< 0.001	54825	PCDH24
-0.18914099	< 0.001	4846	NOS3
-0.189153805	< 0.001	153527	ZMAT2
-0.189155951	< 0.001	100131564	LOC100131564
-0.189170897	< 0.001	201501	ZBTB7C
-0.189255968	< 0.001	8345	HIST1H2BH
-0.189328581	< 0.001	84224	NBPF3
-0.189340279	< 0.001	64167	ERAP2
-0.189345583	< 0.001	23241	PACS2
-0.189362571	< 0.001	84678	KDM2B
-0.189384207	< 0.001	11211	FZD10
-0.189414576	< 0.001	81	ACTN4
-0.189423487	< 0.001	92979	MARCHF9
-0.189516172	< 0.001	6696	SPP1
-0.189530656	< 0.001	898	CCNE1
-0.189553782	< 0.001	79871	RPAP2
-0.18959929	< 0.001	4671	NAIP
-0.18959938	< 0.001	100132288	TEKT4P2
-0.189646557	< 0.001	374650	GOLGA6L5
-0.189680457	< 0.001	285671	RNF180
-0.189759105	< 0.001	4337	MOCS1
-0.189928129	< 0.001	10544	PROCR
-0.189942434	< 0.001	79607	FAM118B
-0.190001771	< 0.001	55285	RBM41
-0.190005481	< 0.001	83543	AIF1L
-0.190012634	< 0.001	3133	HLA-E
-0.190013841	< 0.001	168474	SEPHS1P1
-0.190034837	< 0.001	120776	OR2D2
-0.190081909	< 0.001	149483	CCDC17
-0.190089524	< 0.001	80824	DUSP16
-0.190201715	< 0.001	5837	PYGM
-0.190231979	< 0.001	27250	PDCD4
-0.190237179	< 0.001	124152	IQCK
-0.190266997	< 0.001	8526	DGKE
-0.190320671	< 0.001	91445	RNF185
-0.190351218	< 0.001	201299	RDM1

region-wise, subcortex					
Spearman's corr coef	p-val	p-val, SA	p-val, SA (BH- correction)	GenID	Gene
-0.24015443	0.157890156	0.193	1	147645	AC063977.1
-0.24015443	0.157890156	0.189	1	403253	AC134982.1
-0.240411833	0.157434836	0.193	1	797	CALCB
-0.240411833	0.157434836	0.18	1	2104	ESRRG
-0.240411833	0.157434836	0.201	1	4649	MYO9A
-0.240411833	0.157434836	0.202	1	6901	TAZ
-0.240411833	0.157434836	0.199	1	23678	SGK3
-0.240411833	0.157434836	0.183	1	55143	CDCA8
-0.240411833	0.157434836	0.21	1	64834	ELOVL1
-0.240411833	0.157434836	0.184	1	192286	HIGD2A
-0.240669236	0.156980455	0.202	1	5142	PDE4B
-0.240669236	0.156980455	0.17	1	6546	SLC8A1
-0.240669236	0.156980455	0.191	1	9975	NR1D2
-0.240669236	0.156980455	0.211	1	10524	KAT5
-0.240669236	0.156980455	0.21	1	55596	ZCCHC8
-0.240669236	0.156980455	0.192	1	79685	SAP30L
-0.240669236	0.156980455	0.206	1	84899	TMTC4
-0.240669236	0.156980455	0.213	1	147660	ZNF578
-0.240926623	0.156526864	0.179	1	5168	ENPP2
-0.240926623	0.156526864	0.169	1	23325	KIAA1033
-0.240926623	0.156526864	0.205	1	55619	DOCK10
-0.240926623	0.156526864	0.197	1	57644	MYH7B
-0.240926623	0.156526864	0.184	1	80833	APOL3
-0.240926623	0.156526864	0.214	1	114987	WDR31
-0.240926623	0.156526864	0.191	1	200958	MUC20
-0.240926623	0.156526864	0.181	1	375260	WASH2P
-0.240926623	0.156526864	0.19	1	387071	FAM136BP
-0.240926623	0.156526864	0.173	1	389827	TMEM8C
-0.241184026	0.156074479	0.182	1	1486	CTBS
-0.241184026	0.156074479	0.176	1	2230	FDX1
-0.241184026	0.156074479	0.197	1	2286	FKBP2
-0.241184026	0.156074479	0.174	1	6422	SFRP1
-0.241184026	0.156074479	0.195	1	8817	FGF18
-0.241184026	0.156074479	0.197	1	9961	MVP
-0.241184026	0.156074479	0.195	1	10781	ZNF266
-0.241184026	0.156074479	0.168	1	23091	ZC3H13
-0.241184026	0.156074479	0.192	1	57602	USP36
-0.241441444	0.155622989	0.171	1	5973	RENBP
-0.241441444	0.155622989	0.181	1	9886	RHOBTB1
-0.241441444	0.155622989	0.206	1	645638	LOC645638
-0.241698846	0.155172467	0.199	1	800	CALD1
-0.241698846	0.155172467	0.188	1	1281	COL3A1
-0.241698846	0.155172467	0.183	1	10893	MMP24
-0.241698846	0.155172467	0.201	1	54221	SNTG2
-0.241698846	0.155172467	0.195	1	79998	ANKRD53
-0.241698846	0.155172467	0.207	1	150248	C22orf15
-0.241698846	0.155172467	0.167	1	283417	DPY19L2
-0.241698846	0.155172467	0.181	1	645937	LOC645937
-0.241956249	0.154722884	0.189	1	53637	S1PR5
-0.241956249	0.154722884	0.207	1	132321	C4orf33
-0.241956249	0.154722884	0.195	1	339761	CYP27C1
-0.242213652	0.154274076	0.183	1	3581	IL9R
-0.242213652	0.154274076	0.169	1	9633	MTL5
-0.242213652	0.154274076	0.198	1	11213	IRAK3
-0.242213652	0.154274076	0.171	1	29774	POM121L9P
-0.242213652	0.154274076	0.195	1	56302	TRPV5
-0.242213652	0.154274076	0.181	1	65986	ZBTB10
-0.242471039	0.153826475	0.173	1	1005	CDH7
-0.242471039	0.153826475	0.19	1	63935	PCIF1
-0.242471039	0.153826475	0.187	1	196513	DCP1B
-0.242728442	0.153379768	0.185	1	3903	LAIR1
-0.242728442	0.153379768	0.209	1	51326	ARL17P1
-0.242728442	0.153379768	0.187	1	79415	C17orf62
-0.242728442	0.153379768	0.191	1	148413	RP4-758J18.2
-0.242728442	0.153379768	0.186	1	644168	DRGX
-0.242985845	0.15293403	0.182	1	3601	IL15RA
-0.242985845	0.15293403	0.21	1	25798	BRI3
-0.242985845	0.15293403	0.18	1	56474	CTPS2
-0.242985845	0.15293403	0.209	1	92342	C1orf156
-0.242985845	0.15293403	0.181	1	100287596	DDX11L5
-0.243243247	0.152489036	0.185	1	7073	TIAL1
-0.243243247	0.152489036	0.193	1	10981	RAB32
-0.243243247	0.152489036	0.189	1	51090	PLLP
-0.243243247	0.152489036	0.193	1	55208	DCUN1D2
-0.243243247	0.152489036	0.174	1	92181	UBTD2
-0.24350065	0.15204519	0.188	1	994	CDC25B
-0.24350065	0.15204519	0.19	1	1027	CDKN1B
-0.24350065	0.15204519	0.191	1	1353	COX11
-0.24350065	0.15204519	0.194	1	3563	IL3RA

voxel-wise, subcortex			
Spearman's corr coef	p-val	GenID	Gene
-0.190371752	< 0.001	284338	PRR19
-0.190472409	< 0.001	23276	KLHL18
-0.190592393	< 0.001	645683	RPL13AP3
-0.190673932	< 0.001	402483	FLJ45340
-0.190744147	< 0.001	23130	ATG2A
-0.190760672	< 0.001	130916	MTERFD2
-0.190768942	< 0.001	51057	C2orf86
-0.190775529	< 0.001	22865	SLITRK3
-0.190794379	< 0.001	57396	CLK4
-0.190811187	< 0.001	2900	GRIK4
-0.190894678	< 0.001	9967	THRAP3
-0.190963298	< 0.001	148206	ZNF714
-0.19101578	< 0.001	5079	PAX5
-0.191028953	< 0.001	90864	SPSB3
-0.191055432	< 0.001	89941	RHOT2
-0.191064999	< 0.001	284184	C17orf89
-0.1910806	< 0.001	5931	RBBP7
-0.191143095	< 0.001	51780	KDM3B
-0.191212922	< 0.001	388969	C2orf68
-0.191221103	< 0.001	200132	TCTEX1D1
-0.191258624	< 0.001	23522	MYST4
-0.191278771	< 0.001	55015	PRPF39
-0.191320747	< 0.001	4889	NPY5R
-0.191348881	< 0.001	55090	MED9
-0.19135353	< 0.001	10260	DENND4A
-0.191368937	< 0.001	728621	CCDC30
-0.191411003	< 0.001	5046	PCSK6
-0.191447139	< 0.001	93100	NAPRT1
-0.191475093	< 0.001	730268	LOC730268
-0.191488743	< 0.001	2812	GP1BB
-0.191551611	< 0.001	100286946	LOC100286946
-0.191551983	< 0.001	84419	C15orf48
-0.191813126	< 0.001	29774	POM121L9P
-0.191892609	< 0.001	64332	NFKBIZ
-0.19191964	< 0.001	646791	ANP32BP1
-0.192074627	< 0.001	58503	PROL1
-0.192198604	< 0.001	26155	NOC2L
-0.192216352	< 0.001	672	BRCA1
-0.192332327	< 0.001	8612	PPAP2C
-0.192346916	< 0.001	5305	PIP5K2A
-0.192574903	< 0.001	23259	DDHD2
-0.192660436	< 0.001	54963	UCKL1
-0.192809567	< 0.001	55238	SLC38A7
-0.192849323	< 0.001	83700	JAM3
-0.192885354	< 0.001	144406	WDR66
-0.192900494	< 0.001	5757	PTMA
-0.19291079	< 0.001	7681	MKRN3
-0.192973569	< 0.001	23137	SMC5
-0.19303143	< 0.001	51205	ACP6
-0.193076104	< 0.001	255119	C4orf22
-0.193083152	< 0.001	10758	TRAF3IP2
-0.193157539	< 0.001	10841	FTCD
-0.193257749	< 0.001	1119	CHKA
-0.193298608	< 0.001	4174	MCM5
-0.19330743	< 0.001	222255	ATXN7L1
-0.193322659	< 0.001	55651	NHP2
-0.193376243	< 0.001	159963	SLC5A12
-0.193418592	< 0.001	5587	PRKD1
-0.193442181	< 0.001	53842	CLDN22
-0.193456009	< 0.001	98	ACYP2
-0.193487212	< 0.001	8969	HIST1H2AG
-0.193489358	< 0.001	5351	PLOD1
-0.193497807	< 0.001	167153	PAPD4
-0.193567365	< 0.001	26051	PPP1R16B
-0.193575352	< 0.001	391769	H3.Y
-0.19360061	< 0.001	51321	AMZ2
-0.193616301	< 0.001	144568	A2ML1
-0.193616584	< 0.001	439931	AC002472.9
-0.19362168	< 0.001	4708	NDUFB2
-0.193694308	< 0.001	2254	FGF9
-0.193716317	< 0.001	256815	C10orf67
-0.193765536	< 0.001	254956	MORN5
-0.193862572	< 0.001	1911	PHC1
-0.193867415	< 0.001	55051	C14orf102
-0.193921	< 0.001	441932	OR2W5
-0.193975508	< 0.001	153129	SLC38A9
-0.193976805	< 0.001	10994	ILVBL
-0.194019064	< 0.001	346288	SEPTIN14
-0.194041625	< 0.001	129807	NEU4

region-wise, subcortex					
Spearman's corr coef	p-val	p-val, SA	p-val, SA (BH- correction)	GenID	Gene
-0.24350065	0.15204519	0.159	1	3955	LFNG
-0.24350065	0.15204519	0.182	1	6900	CNTN2
-0.24350065	0.15204519	0.191	1	54441	STAG3L1
-0.24350065	0.15204519	0.191	1	158135	TTLL11
-0.24350065	0.15204519	0.198	1	163702	IL28RA
-0.243758038	0.151602343	0.197	1	4193	MDM2
-0.243758038	0.151602343	0.18	1	4756	NEO1
-0.243758038	0.151602343	0.188	1	7832	BTG2
-0.243758038	0.151602343	0.191	1	8348	HIST1H2BO
-0.243758038	0.151602343	0.198	1	9619	ABCG1
-0.243758038	0.151602343	0.198	1	23630	KCNE1L
-0.243758038	0.151602343	0.159	1	51181	DCXR
-0.243758038	0.151602343	0.18	1	90007	MIDN
-0.24401544	0.151160404	0.177	1	1647	GADD45A
-0.24401544	0.151160404	0.174	1	7746	ZNF193
-0.24401544	0.151160404	0.184	1	57819	LSM2
-0.24401544	0.151160404	0.194	1	81554	WBSCR16
-0.24401544	0.151160404	0.2	1	92196	DAPL1
-0.24401544	0.151160404	0.173	1	92305	TMEM129
-0.244272843	0.150719211	0.171	1	593	BCKDHA
-0.244272843	0.150719211	0.159	1	4855	NOTCH4
-0.244272843	0.150719211	0.195	1	4922	NTS
-0.244272843	0.150719211	0.179	1	56895	AGPAT4
-0.244272843	0.150719211	0.188	1	442293	LOC442293
-0.244530246	0.150279179	0.192	1	6813	STXBP2
-0.244530246	0.150279179	0.18	1	283932	FBXL19-AS1
-0.244530246	0.150279179	0.182	1	284837	AP001053.11
-0.244530246	0.150279179	0.173	1	548645	DNAJC25
-0.244530246	0.150279179	0.167	1	572558	RP11-561O23.6
-0.244787648	0.149840072	0.196	1	10268	RAMP3
-0.244787648	0.149840072	0.186	1	84808	C1orf170
-0.244787648	0.149840072	0.162	1	256364	EML3
-0.244787648	0.149840072	0.166	1	387266	KRTAP5-3
-0.245045036	0.149401963	0.182	1	1719	DHFRP1
-0.245045036	0.149401963	0.176	1	1954	MEGF8
-0.245045036	0.149401963	0.187	1	3908	LAMA2
-0.245045036	0.149401963	0.187	1	51593	SRRT
-0.245302439	0.148964763	0.176	1	788	SLC25A20
-0.245302439	0.148964763	0.2	1	4059	BCAM
-0.245302439	0.148964763	0.186	1	6425	SFRP5
-0.245302439	0.148964763	0.205	1	7130	TNFAIP6
-0.245302439	0.148964763	0.18	1	64857	PLEKHG2
-0.245302439	0.148964763	0.198	1	80164	AC010336.2
-0.245302439	0.148964763	0.163	1	85379	KIAA1671
-0.245302439	0.148964763	0.172	1	117246	FTSJ3
-0.245302439	0.148964763	0.176	1	201973	CCDC111
-0.245302439	0.148964763	0.18	1	221037	JMJD1C
-0.245302439	0.148964763	0.185	1	730183	LOC730183
-0.245559841	0.148528308	0.19	1	1776	DNASE1L3
-0.245559841	0.148528308	0.184	1	2073	ERCC5
-0.245559841	0.148528308	0.172	1	10140	TOB1
-0.245559841	0.148528308	0.183	1	11322	TMC6
-0.245559841	0.148528308	0.193	1	54920	DUS2L
-0.245559841	0.148528308	0.185	1	65991	FUNDC2
-0.245559841	0.148528308	0.173	1	125113	KRT222
-0.245817244	0.148092985	0.191	1	943	TNFRSF8
-0.245817244	0.148092985	0.183	1	4673	NAP1L1
-0.245817244	0.148092985	0.159	1	80127	C14orf45
-0.245817244	0.148092985	0.179	1	92106	OXNAD1
-0.245817244	0.148092985	0.186	1	253639	ZNF620
-0.245817244	0.148092985	0.172	1	256356	GK5
-0.246074647	0.147658616	0.182	1	9	NAT1
-0.246074647	0.147658616	0.173	1	5860	QDPR
-0.246074647	0.147658616	0.179	1	6199	RPS6KB2
-0.246074647	0.147658616	0.176	1	84034	EMILIN2
-0.246074647	0.147658616	0.161	1	116255	MOGAT1
-0.246074647	0.147658616	0.199	1	644619	INTS4L2
-0.246332034	0.147225231	0.198	1	1022	CDK7
-0.246332034	0.147225231	0.175	1	3382	ICA1
-0.246332034	0.147225231	0.186	1	6522	SLC4A2
-0.246332034	0.147225231	0.195	1	6736	SRY
-0.246332034	0.147225231	0.177	1	53827	FXYD5
-0.246332034	0.147225231	0.176	1	92922	CCDC102A
-0.246332034	0.147225231	0.192	1	644037	HNRNPA1P12
-0.246589437	0.146792531	0.189	1	54455	FBXO42
-0.246589437	0.146792531	0.187	1	79006	METRN
-0.24684684	0.146360993	0.187	1	3249	HPN
-0.24684684	0.146360993	0.181	1	22921	MSRB2
-0.24684684	0.146360993	0.175	1	26778	SNORA70

voxel-wise, subcortex			
Spearman's corr coef	p-val	GenID	Gene
-0.194051847	< 0.001	51126	NAT5
-0.194148794	< 0.001	1471	CST3
-0.194194764	< 0.001	10815	CPLX1
-0.19420284	< 0.001	257407	C2orf72
-0.194212779	< 0.001	55229	PANK4
-0.19428958	< 0.001	56902	PNO1
-0.194311872	< 0.001	9631	NUP155
-0.194339544	< 0.001	7869	SEMA3B
-0.19447884	< 0.001	64216	TFB2M
-0.194516823	< 0.001	5026	P2RX5
-0.194543943	< 0.001	55527	FEM1A
-0.194637358	< 0.001	81550	TDRD3
-0.194647849	< 0.001	889	KRIT1
-0.194684535	< 0.001	284992	CCDC150
-0.194704875	< 0.001	8125	ANP32A
-0.19472234	< 0.001	399940	AP004607.4
-0.194726512	< 0.001	6202	RPS8
-0.194771737	< 0.001	51	ACOX1
-0.194800615	< 0.001	5564	PRKAB1
-0.194811299	< 0.001	23473	CAPN7
-0.194823459	< 0.001	1959	EGR2
-0.194829032	< 0.001	100507436	MICA
-0.194901571	< 0.001	386757	SLC6A10P
-0.194930077	< 0.001	219654	ZCCHC24
-0.194932491	< 0.001	1305	COL13A1
-0.194940656	< 0.001	389787	LOC389787
-0.194957092	< 0.001	54941	RNF125
-0.195081905	< 0.001	1263	PLK3
-0.195087478	< 0.001	1267	CNP
-0.195104659	< 0.001	5531	PPP4C
-0.195117667	< 0.001	100130256	LOC100130256
-0.195131123	< 0.001	79955	PDZD7
-0.195257142	< 0.001	146923	RUNDC1
-0.195280835	< 0.001	80128	TRIM46
-0.195292816	< 0.001	5301	PIN1P1
-0.195293367	< 0.001	10926	DBF4
-0.19530116	< 0.001	253714	C6orf167
-0.195302933	< 0.001	51364	ZMYND10
-0.195313618	< 0.001	2635	GBP3
-0.195333675	< 0.001	4907	NT5E
-0.195369333	< 0.001	3208	HPCA
-0.195400909	< 0.001	100506282	LOC100506282
-0.19542338	< 0.001	27433	TOR2A
-0.195435166	< 0.001	56110	PCDHGA5
-0.195438325	< 0.001	23235	SIK2
-0.195492655	< 0.001	1975	EIF4B
-0.195544854	< 0.001	9909	DENND4B
-0.195576608	< 0.001	825	CAPN3
-0.195623785	< 0.001	79152	FA2H
-0.195659071	< 0.001	284451	ODF3L2
-0.195659354	< 0.001	1368	CPM
-0.195735961	< 0.001	57519	STARD9
-0.195744514	< 0.001	3257	HPS1
-0.195807472	< 0.001	572558	RP11-561O23.6
-0.195828453	< 0.001	3248	HPGD
-0.195850745	< 0.001	57684	ZBTB26
-0.195898756	< 0.001	7050	TGIF1
-0.196008712	< 0.001	643641	ZNF862
-0.196052551	< 0.001	5469	MED1
-0.196068883	< 0.001	962	CD48
-0.196088672	< 0.001	2653	GCSH
-0.196123675	< 0.001	119385	AGAP11
-0.196141973	< 0.001	9108	MTMR7
-0.19614318	< 0.001	220002	CYBASC3
-0.196149588	< 0.001	2766	GMPR
-0.196201965	< 0.001	118442	GPR62
-0.196228996	< 0.001	56341	PRMT8
-0.196238369	< 0.001	933	CD22
-0.196239859	< 0.001	64863	METTL4
-0.196292147	< 0.001	5334	PLCL1
-0.196301892	< 0.001	79169	C1orf35
-0.196303755	< 0.001	3977	LIFR
-0.196316004	< 0.001	85329	LGALS12
-0.196508795	< 0.001	11152	WDR45
-0.19651325	< 0.001	87	ACTN1
-0.196608722	< 0.001	54798	DCHS2
-0.19662042	< 0.001	6414	SEPP1
-0.196732968	< 0.001	85414	SLC45A3
-0.196773186	< 0.001	440926	H3F3AP4

region-wise, subcortex					
Spearman's corr coef	p-val	p-val, SA	p-val, SA (BH- correction)	GenID	Gene
-0.24684684	0.146360993	0.185	1	51531	C9orf156
-0.24684684	0.146360993	0.179	1	162417	NAGS
-0.247104242	0.145930365	0.173	1	288	ANK3
-0.247104242	0.145930365	0.184	1	3039	HBA1
-0.247104242	0.145930365	0.18	1	23569	PADI4
-0.247104242	0.145930365	0.166	1	57567	ZNF319
-0.247361645	0.14550069	0.163	1	3040	HBA2
-0.247361645	0.14550069	0.177	1	3394	IRF8
-0.247361645	0.14550069	0.181	1	5635	PRPSAP1
-0.247361645	0.14550069	0.18	1	23632	CA14
-0.247361645	0.14550069	0.184	1	57129	MRPL47
-0.247361645	0.14550069	0.185	1	199777	ZNF626
-0.247361645	0.14550069	0.178	1	256126	SYCE2
-0.247361645	0.14550069	0.168	1	339483	MTMR9LP
-0.247619033	0.145071805	0.184	1	1466	CSRP2
-0.247619033	0.145071805	0.152	1	54980	C2orf42
-0.247876436	0.144643992	0.172	1	1605	DAG1
-0.247876436	0.144643992	0.17	1	3035	HARS
-0.247876436	0.144643992	0.18	1	3315	HSPB1
-0.247876436	0.144643992	0.176	1	3684	ITGAM
-0.247876436	0.144643992	0.184	1	6279	S100A8
-0.247876436	0.144643992	0.194	1	51734	SEPX1
-0.247876436	0.144643992	0.16	1	54780	NSMCE4A
-0.247876436	0.144643992	0.174	1	55017	C14orf119
-0.247876436	0.144643992	0.171	1	57509	MTUS1
-0.247876436	0.144643992	0.176	1	65251	ZNF649
-0.247876436	0.144643992	0.198	1	253430	IPMK
-0.247876436	0.144643992	0.176	1	258010	SVIP
-0.247876436	0.144643992	0.162	1	729338	CETN4P
-0.247876436	0.144643992	0.162	1	768211	RELL1
-0.248133838	0.144217119	0.197	1	2352	FOLR3
-0.248133838	0.144217119	0.162	1	9817	KEAP1
-0.248133838	0.144217119	0.187	1	51530	ZC3HC1
-0.248133838	0.144217119	0.173	1	121260	SLC15A4
-0.248391256	0.143791184	0.174	1	438	ASMT
-0.248391256	0.143791184	0.176	1	6737	TRIM21
-0.248391256	0.143791184	0.175	1	9595	CYTIP
-0.248391256	0.143791184	0.178	1	130074	FAM168B
-0.248648658	0.143366188	0.182	1	80149	ZC3H12A
-0.248648658	0.143366188	0.185	1	83700	JAM3
-0.248648658	0.143366188	0.166	1	219623	TMEM26
-0.248648658	0.143366188	0.157	1	100288798	LOC100288798
-0.248906046	0.142941967	0.167	1	689	BTF3
-0.248906046	0.142941967	0.155	1	1378	CR1
-0.248906046	0.142941967	0.177	1	9488	PIGB
-0.248906046	0.142941967	0.2	1	26147	PHF19
-0.248906046	0.142941967	0.161	1	220988	HNRNPA3
-0.249163449	0.142518818	0.186	1	1289	COL5A1
-0.249163449	0.142518818	0.159	1	2840	GPR17
-0.249163449	0.142518818	0.183	1	11129	SFRS16
-0.249163449	0.142518818	0.171	1	27023	FOXB1
-0.249163449	0.142518818	0.175	1	55592	AC010203.2
-0.249163449	0.142518818	0.192	1	58509	C19orf29
-0.249163449	0.142518818	0.187	1	79809	TTC21B
-0.249163449	0.142518818	0.181	1	317762	CCDC85C
-0.249163449	0.142518818	0.167	1	386724	AMIGO3
-0.249163449	0.142518818	0.183	1	401428	OR2A20P
-0.249420851	0.142096594	0.178	1	3856	KRT8P9
-0.249678254	0.141675323	0.151	1	6693	SPN
-0.249678254	0.141675323	0.201	1	9360	PPIG
-0.249678254	0.141675323	0.174	1	114885	OSBPL11
-0.249678254	0.141675323	0.173	1	126626	GABPB2
-0.249935657	0.141254812	0.187	1	5396	PRRX1
-0.249935657	0.141254812	0.169	1	6676	SPAG4
-0.249935657	0.141254812	0.167	1	10750	GRAP
-0.249935657	0.141254812	0.179	1	56896	DPYSL5
-0.249935657	0.141254812	0.171	1	126859	C1orf125
-0.250193059	0.140835389	0.166	1	6102	RP2
-0.250193059	0.140835389	0.162	1	9423	NTN1
-0.250193059	0.140835389	0.189	1	26082	AC135731.1
-0.250193059	0.140835389	0.16	1	51287	CHCHD8
-0.250193059	0.140835389	0.165	1	84275	SLC25A33
-0.250193059	0.140835389	0.167	1	88455	ANKRD13A
-0.250193059	0.140835389	0.154	1	138151	NACC2
-0.250193059	0.140835389	0.178	1	140735	DYNLL2
-0.250193059	0.140835389	0.181	1	222236	NAPEPLD
-0.250450462	0.140416875	0.181	1	6525	SMTN
-0.250450462	0.140416875	0.152	1	7263	TST
-0.250707835	0.1399993	0.165	1	1743	DLSTP

voxel-wise, subcortex			
Spearman's corr coef	p-val	GenID	Gene
-0.196846828	< 0.001	57017	COQ9
-0.196910903	< 0.001	23598	PATZ1
-0.19691202	< 0.001	51283	BFAR
-0.196921125	< 0.001	143689	PIWIL4
-0.196938396	< 0.001	116835	HSPA12B
-0.196965888	< 0.001	285755	PPIL6
-0.196976468	< 0.001	440093	RP11-428G5.1
-0.19707036	< 0.001	79823	C2orf34
-0.197101563	< 0.001	10826	C5orf4
-0.197105736	< 0.001	112885	PHF21B
-0.197107032	< 0.001	23303	KIF13B
-0.19714056	< 0.001	6834	SURF1
-0.197142139	< 0.001	4340	MOG
-0.197232872	< 0.001	79612	NARG1L
-0.197308466	< 0.001	3038	HAS3
-0.197486401	< 0.001	9855	FARP2
-0.197560504	< 0.001	30812	SOX8
-0.197631732	< 0.001	79023	NUP37
-0.197706953	< 0.001	127396	ZNF684
-0.197814956	< 0.001	6187	RPS2
-0.197858691	< 0.001	6188	RPS3P3
-0.197910145	< 0.001	5194	PEX13
-0.197980911	< 0.001	9671	WSCD2
-0.197997808	< 0.001	22973	LAMB2P1
-0.198005706	< 0.001	26058	GIGYF2
-0.198030308	< 0.001	57509	MTUS1
-0.198074609	< 0.001	3832	KIF11
-0.198213816	< 0.001	9366	RAB9BP1
-0.198213816	< 0.001	54989	ZNF770
-0.198228031	< 0.001	146225	CMTM2
-0.198339552	< 0.001	3021	H3F3B
-0.198385715	< 0.001	55326	AGPAT5
-0.198448122	< 0.001	100133036	FAM95B1
-0.198460653	< 0.001	8800	PEX11A
-0.198511913	< 0.001	644511	RPL13AP6
-0.198630139	< 0.001	3066	HDAC2
-0.198688641	< 0.001	3276	PRMT1
-0.198697001	< 0.001	23417	MLYCD
-0.198714554	< 0.001	23310	NCAPD3
-0.198878929	< 0.001	85016	C11orf70
-0.198911428	< 0.001	9295	SFRS11
-0.198951915	< 0.001	730098	RP11-195F19.5
-0.19895786	< 0.001	196500	C12orf53
-0.199007258	< 0.001	54461	FBXW5
-0.199020267	< 0.001	54903	MKS1
-0.199023142	< 0.001	7075	TIE1
-0.199033171	< 0.001	256130	TMEM196
-0.199163929	< 0.001	9848	MFAP3L
-0.199227169	< 0.001	387978	C14orf23
-0.199230611	< 0.001	30816	ERVWE1
-0.199252144	< 0.001	6525	SMTN
-0.199258193	< 0.001	54360	CYTL1
-0.199265242	< 0.001	246329	STAC3
-0.199298114	< 0.001	6205	RPS11P5
-0.199320123	< 0.001	3357	HTR2B
-0.199357271	< 0.001	80704	SLC19A3
-0.199368134	< 0.001	2050	EPHB4
-0.199379474	< 0.001	116448	OLIG1
-0.199389592	< 0.001	7515	XRCC1
-0.199400738	< 0.001	953	ENTPD1
-0.199417263	< 0.001	6483	ST3GAL2
-0.199478835	< 0.001	200895	DHFRL1
-0.199490353	< 0.001	203562	TMEM31
-0.199503258	< 0.001	79844	ZDHHC11
-0.199565753	< 0.001	57475	PLEKHH1
-0.19962278	< 0.001	8165	AKAP1
-0.200007796	< 0.001	25865	PRKD2
-0.200049505	< 0.001	64839	FBXL17
-0.200097784	< 0.001	55893	ZNF395
-0.200124726	< 0.001	83650	AMAC1L2
-0.200152397	< 0.001	6035	RNASE1
-0.200193807	< 0.001	23429	RYBP
-0.200223342	< 0.001	7125	TNNC2
-0.200245544	< 0.001	85026	C9orf37
-0.200271264	< 0.001	23524	SRRM2
-0.200290859	< 0.001	7984	ARHGEF5
-0.200304791	< 0.001	115207	KCTD12
-0.200311661	< 0.001	55023	PHIP
-0.200339615	< 0.001	8341	HIST1H2BN

region-wise, subcortex					
Spearman's corr coef	p-val	p-val, SA	p-val, SA (BH- correction)	GenID	Gene
-0.250707835	0.1399993	0.165	1	7696	ZNF137P
-0.250707835	0.1399993	0.185	1	79684	C11orf61
-0.250707835	0.1399993	0.177	1	114907	FBXO32
-0.250707835	0.1399993	0.168	1	116444	GRIN3B
-0.250707835	0.1399993	0.171	1	153443	SRFBP1
-0.250707835	0.1399993	0.157	1	161247	FITM1
-0.250707835	0.1399993	0.162	1	646278	LOC646278
-0.250965238	0.139582649	0.158	1	7318	UBA7
-0.250965238	0.139582649	0.159	1	8985	PLOD3
-0.250965238	0.139582649	0.183	1	29914	UBIAD1
-0.250965238	0.139582649	0.171	1	54863	C9orf167
-0.250965238	0.139582649	0.16	1	55654	TMEM127
-0.250965238	0.139582649	0.16	1	57458	TMCC3
-0.25122264	0.139166772	0.187	1	1723	DHODH
-0.25122264	0.139166772	0.184	1	57830	KRTAP5-8
-0.25122264	0.139166772	0.172	1	92126	DSEL
-0.25122264	0.139166772	0.19	1	148362	BROX
-0.251480043	0.138751969	0.164	1	25874	BRP44
-0.251480043	0.138751969	0.165	1	54360	CYTL1
-0.251480043	0.138751969	0.181	1	57519	STARD9
-0.251480043	0.138751969	0.159	1	57688	ZSWIM6
-0.251480043	0.138751969	0.171	1	253559	CADM2
-0.251737446	0.138338059	0.167	1	471	ATIC
-0.251737446	0.138338059	0.176	1	2729	GCLC
-0.251737446	0.138338059	0.165	1	23241	PACS2
-0.251737446	0.138338059	0.181	1	25989	ULK3
-0.251737446	0.138338059	0.153	1	84816	RTN4IP1
-0.251737446	0.138338059	0.168	1	100286895	LOC100286895
-0.251994848	0.137925088	0.183	1	91807	MYLK3
-0.252252251	0.137512848	0.162	1	23	ABCF1
-0.252252251	0.137512848	0.186	1	8431	NR0B2
-0.252252251	0.137512848	0.155	1	8490	RP11-267N12.3
-0.252252251	0.137512848	0.181	1	8612	PPAP2C
-0.252252251	0.137512848	0.155	1	10430	TMEM147
-0.252252251	0.137512848	0.192	1	10538	BATF
-0.252252251	0.137512848	0.168	1	22913	RALY
-0.252252251	0.137512848	0.191	1	84447	SYVN1
-0.252252251	0.137512848	0.175	1	140680	C20orf96
-0.252509654	0.137101769	0.16	1	1584	CYP11B1
-0.252509654	0.137101769	0.172	1	1649	DDIT3
-0.252509654	0.137101769	0.177	1	3885	KRT34
-0.252509654	0.137101769	0.159	1	134187	POU5F2
-0.252509654	0.137101769	0.167	1	143684	FAM76B
-0.252509654	0.137101769	0.19	1	401551	WDR38
-0.252767056	0.13669154	0.174	1	51474	LIMA1
-0.252767056	0.13669154	0.165	1	53373	TPCN1
-0.252767056	0.13669154	0.164	1	84901	NFATC2IP
-0.252767056	0.13669154	0.179	1	200539	ANKRD23
-0.253024459	0.136282235	0.173	1	26531	OR11A1
-0.253281832	0.13587366	0.173	1	2072	ERCC4
-0.253281832	0.13587366	0.145	1	8638	OASL
-0.253281832	0.13587366	0.16	1	10992	SF3B2
-0.253281832	0.13587366	0.196	1	55693	KDM4D
-0.253281832	0.13587366	0.16	1	84190	C12orf26
-0.253281832	0.13587366	0.149	1	170371	C10orf128
-0.253281832	0.13587366	0.189	1	399693	MGC50722
-0.253539234	0.135466188	0.172	1	3834	KIF25
-0.253539234	0.135466188	0.171	1	54940	OCIAD1
-0.253539234	0.135466188	0.17	1	55506	H2AFY2
-0.253539234	0.135466188	0.168	1	84271	POLDIP3
-0.253539234	0.135466188	0.194	1	116093	DIRC1
-0.253539234	0.135466188	0.168	1	387328	ZNF322P1
-0.253539234	0.135466188	0.16	1	100128822	LOC100128822
-0.253796637	0.13505967	0.175	1	1586	CYP17A1
-0.253796637	0.13505967	0.156	1	10723	SLC12A7
-0.253796637	0.13505967	0.181	1	22995	CEP152
-0.253796637	0.13505967	0.18	1	27158	NDOR1
-0.253796637	0.13505967	0.151	1	148109	FAM187B
-0.253796637	0.13505967	0.164	1	152100	CMC1
-0.25405404	0.134654015	0.171	1	549	AUH
-0.25405404	0.134654015	0.158	1	890	CCNA2
-0.25405404	0.134654015	0.159	1	338799	AC084018.1
-0.254311442	0.13424927	0.179	1	443	ASPA
-0.254311442	0.13424927	0.176	1	4293	MAP3K9
-0.254311442	0.13424927	0.173	1	25839	COG4
-0.254311442	0.13424927	0.165	1	55741	EDEM2
-0.254311442	0.13424927	0.166	1	80817	KIAA1712
-0.254311442	0.13424927	0.142	1	285590	SH3PXD2B
-0.254568845	0.13384527	0.17	1	610	HCN2

voxel-wise, subcortex			
Spearman's corr coef	p-val	GenID	Gene
-0.200340167	< 0.001	6548	SLC9A1
-0.200400159	< 0.001	1318	SLC31A2
-0.200403601	< 0.001	132946	ARL9
-0.200469255	< 0.001	2737	GLI3
-0.200547636	< 0.001	200186	CRTC2
-0.200586349	< 0.001	29894	CPSF1
-0.200590998	< 0.001	7260	TSSC1
-0.200620532	< 0.001	81558	FAM117A
-0.200646341	< 0.001	89894	TMEM116
-0.200669006	< 0.001	23360	FNBP4
-0.200695932	< 0.001	7675	ZNF121
-0.200706154	< 0.001	2859	GPR35
-0.200771809	< 0.001	441204	LOC441204
-0.200846195	< 0.001	64419	MTMR14
-0.200850368	< 0.001	146330	FBXL16
-0.200901166	< 0.001	4520	MTF1
-0.200927734	< 0.001	730110	LOC730110
-0.200986609	< 0.001	7551	ZNF3
-0.200987816	< 0.001	3358	HTR2C
-0.201008707	< 0.001	7596	ZNF45
-0.201049849	< 0.001	388115	C15orf52
-0.201056629	< 0.001	400258	C14orf180
-0.201117545	< 0.001	25777	UNC84B
-0.201139927	< 0.001	162494	RHBDL3
-0.201146901	< 0.001	125893	ZNF816A
-0.201163888	< 0.001	6878	TAF6
-0.201190725	< 0.001	84445	LZTS2
-0.201209113	< 0.001	2882	GPX7
-0.201211438	< 0.001	8738	CRADD
-0.201215804	< 0.001	22937	SCAP
-0.201262146	< 0.001	6901	TAZ
-0.201311454	< 0.001	59271	C21orf63
-0.201452419	< 0.001	29940	DSE
-0.20145911	< 0.001	3918	LAMC2
-0.201463386	< 0.001	26226	FBXW4P1
-0.201492354	< 0.001	116935	RPL3P2
-0.201496348	< 0.001	51666	ASB4
-0.201545939	< 0.001	79738	BBS10
-0.201546222	< 0.001	54822	TRPM7
-0.201688021	< 0.001	8527	DGKD
-0.201703817	< 0.001	55567	DNAH3
-0.201729625	< 0.001	57217	TTC7A
-0.201762035	< 0.001	26135	SERBP1
-0.201762408	< 0.001	100	ADA
-0.201953158	< 0.001	140825	NEURL2
-0.201995879	< 0.001	292	SLC25A5
-0.202032834	< 0.001	26249	KLHL3
-0.202039897	< 0.001	4853	NOTCH2
-0.202073053	< 0.001	4686	NCBP1
-0.202109635	< 0.001	2027	ENO3
-0.202110663	< 0.001	392490	FLJ44635
-0.202193126	< 0.001	440311	LOC440311
-0.2022039	< 0.001	339483	MTMR9LP
-0.202307537	< 0.001	80759	KHDC1
-0.202311903	< 0.001	7045	TGFBI
-0.2023132	< 0.001	9330	GTF3C3
-0.202348575	< 0.001	3754	KCNF1
-0.202350348	< 0.001	151516	ASPRV1
-0.202363998	< 0.001	57205	ATP10D
-0.202385634	< 0.001	147685	C19orf18
-0.202395573	< 0.001	120824	TRIM51GP
-0.202593192	< 0.001	9788	MTSS1
-0.202608973	< 0.001	8438	RAD54L
-0.202654764	< 0.001	1521	CTSW
-0.20275858	< 0.001	23401	FRAT2
-0.202774644	< 0.001	26271	FBXO5
-0.202777907	< 0.001	27244	SESN1
-0.202827021	< 0.001	133121	ENPP6
-0.202830464	< 0.001	79937	CNTNAP3
-0.20289138	< 0.001	285025	CCDC141
-0.20293726	< 0.001	387267	KRTAP5-4
-0.202937439	< 0.001	50840	TAS2R14
-0.203020096	< 0.001	1060	CENPC1
-0.203024372	< 0.001	55075	UACA
-0.203037083	< 0.001	51127	TRIM17
-0.203044981	< 0.001	51571	FAM49B
-0.203107297	< 0.001	6122	RPL3
-0.203141838	< 0.001	644019	CBWD6
-0.203206107	< 0.001	1749	DLX5

region-wise, subcortex					
Spearman's corr coef	p-val	p-val, SA	p-val, SA (BH- correction)	GenID	Gene
-0.254568845	0.13384527	0.188	1	3811	KIR3DL1
-0.254568845	0.13384527	0.175	1	5605	MAP2K2
-0.254568845	0.13384527	0.188	1	6484	ST3GAL4
-0.254568845	0.13384527	0.188	1	27237	ARHGEF16
-0.254568845	0.13384527	0.161	1	129293	AC010087.3
-0.254568845	0.13384527	0.151	1	100130557	LOC100130557
-0.254826248	0.133442342	0.154	1	306	ANXA3
-0.254826248	0.133442342	0.163	1	6504	SLAMF1
-0.254826248	0.133442342	0.149	1	7157	TP53
-0.254826248	0.133442342	0.155	1	8449	DHX16
-0.254826248	0.133442342	0.175	1	79191	IRX3
-0.254826248	0.133442342	0.163	1	79858	NEK11
-0.254826248	0.133442342	0.17	1	84135	UTP15
-0.254826248	0.133442342	0.166	1	152573	SHISA3
-0.254826248	0.133442342	0.161	1	158471	PRUNE2
-0.25508365	0.133040369	0.146	1	8914	TIMELESS
-0.25508365	0.133040369	0.153	1	84836	ABHD14B
-0.25508365	0.133040369	0.169	1	100874392	ANKRD20A12P
-0.255341053	0.132639259	0.161	1	6948	TCN2
-0.255341053	0.132639259	0.155	1	23435	TARDBP
-0.255341053	0.132639259	0.179	1	58473	PLEKHB1
-0.255341053	0.132639259	0.176	1	222611	GPR111
-0.255341053	0.132639259	0.174	1	378825	LINC00162
-0.255341053	0.132639259	0.16	1	729494	ARPC3P3
-0.255598456	0.132238865	0.163	1	1355	COX15
-0.255598456	0.132238865	0.164	1	8975	USP13
-0.255598456	0.132238865	0.187	1	23524	SRRM2
-0.255598456	0.132238865	0.173	1	29063	ZCCHC4
-0.255598456	0.132238865	0.156	1	60675	PROK2
-0.255598456	0.132238865	0.173	1	100506243	RP11-141M3.1
-0.255855858	0.131839573	0.173	1	2736	GLI2
-0.255855858	0.131839573	0.168	1	7798	LUZP1
-0.255855858	0.131839573	0.174	1	26046	RNF160
-0.255855858	0.131839573	0.173	1	85376	RIMBP3
-0.255855858	0.131839573	0.166	1	91526	ANKRD44
-0.256113261	0.131441176	0.178	1	210	ALAD
-0.256113261	0.131441176	0.165	1	27115	PDE7B
-0.256113261	0.131441176	0.168	1	51455	REV1
-0.256113261	0.131441176	0.174	1	53828	FXYD4
-0.256113261	0.131441176	0.159	1	55199	FAM86C
-0.256113261	0.131441176	0.174	1	57326	PBXIP1
-0.256113261	0.131441176	0.154	1	79003	MIS12
-0.256113261	0.131441176	0.143	1	147111	NOTUM
-0.256113261	0.131441176	0.147	1	283337	ZNF740
-0.256370664	0.131043717	0.148	1	682	BSG
-0.256370664	0.131043717	0.16	1	9380	GRHPR
-0.256628066	0.130646944	0.149	1	1748	DLX4
-0.256628066	0.130646944	0.173	1	3170	FOXA2
-0.256628066	0.130646944	0.173	1	29780	PARVB
-0.256628066	0.130646944	0.166	1	125061	AFMID
-0.256628066	0.130646944	0.151	1	259296	TAS2R50
-0.256628066	0.130646944	0.162	1	445328	ARHGEF5L
-0.256885439	0.130251259	0.168	1	4174	MCM5
-0.256885439	0.130251259	0.163	1	7058	THBS2
-0.256885439	0.130251259	0.16	1	54910	SEMA4C
-0.256885439	0.130251259	0.198	1	55267	C22orf26
-0.256885439	0.130251259	0.153	1	148203	ZNF738
-0.257142872	0.129856452	0.144	1	1593	CYP27A1
-0.257142872	0.129856452	0.165	1	5888	RAD51
-0.257142872	0.129856452	0.166	1	26576	SRPK3
-0.257142872	0.129856452	0.162	1	56834	GPR137
-0.257142872	0.129856452	0.154	1	131096	KCNH8
-0.257142872	0.129856452	0.172	1	145376	C14orf50
-0.257400244	0.129462615	0.162	1	53832	IL20RA
-0.257657647	0.129069611	0.142	1	2686	GGT7
-0.257657647	0.129069611	0.16	1	55339	WDR33
-0.257657647	0.129069611	0.172	1	81309	OR4C15
-0.25791505	0.128677323	0.145	1	3032	HADHB
-0.25791505	0.128677323	0.168	1	3985	LIMK2
-0.25791505	0.128677323	0.16	1	5337	PLD1
-0.25791505	0.128677323	0.162	1	6396	SEC13
-0.25791505	0.128677323	0.184	1	7733	ZNF180
-0.25791505	0.128677323	0.151	1	26088	GGA1
-0.25791505	0.128677323	0.135	1	207063	DHRSX
-0.258172452	0.128286108	0.173	1	2078	ERG
-0.258172452	0.128286108	0.154	1	3815	KIT
-0.258172452	0.128286108	0.155	1	348378	FAM159A
-0.258429825	0.127895802	0.164	1	1318	SLC31A2
-0.258429825	0.127895802	0.155	1	7128	TNFAIP3

voxel-wise, subcortex			
Spearman's corr coef	p-val	GenID	Gene
-0.203214094	< 0.001	400960	PCBP1-AS1
-0.203222364	< 0.001	7852	CXCR4
-0.203243628	< 0.001	54454	ATAD2B
-0.203247055	< 0.001	9584	RBM39
-0.203639418	< 0.001	401494	PTPLAD2
-0.203668028	< 0.001	5817	PVR
-0.203704432	< 0.001	64231	MS4A6A
-0.203753918	< 0.001	10615	SPAG5
-0.203792274	< 0.001	629	CFB
-0.203803793	< 0.001	80149	ZC3H12A
-0.203813076	< 0.001	10413	YAP1
-0.203883931	< 0.001	30851	TAX1BP3
-0.203988597	< 0.001	10247	HRSP12
-0.204044968	< 0.001	79682	MLF1IP
-0.204064563	< 0.001	440934	AC010980.2
-0.204068556	< 0.001	51700	CYB5R2
-0.204099476	< 0.001	51363	CHST15
-0.204265237	< 0.001	26147	PHF19
-0.204302847	< 0.001	7107	GPR137B
-0.204328299	< 0.001	347240	KIF24
-0.204339817	< 0.001	26548	ITGB1BP2
-0.204368785	< 0.001	116151	C20orf108
-0.20437324	< 0.001	3712	IVD
-0.204393953	< 0.001	92086	GGTLA3
-0.204430908	< 0.001	23232	TBC1D12
-0.204481155	< 0.001	85317	BAGE4
-0.204489514	< 0.001	1387	CREBBP
-0.204489514	< 0.001	10517	FBXW10
-0.204498515	< 0.001	138241	C9orf85
-0.204524621	< 0.001	8481	OFD1
-0.204532608	< 0.001	6373	CXCL11
-0.204544112	< 0.001	91734	IDI2
-0.204602435	< 0.001	81559	TRIM11
-0.204676539	< 0.001	23371	TENC1
-0.204768211	< 0.001	7156	TOP3A
-0.204785109	< 0.001	29065	ASAP1-IT1
-0.204852059	< 0.001	25840	METTL7A
-0.204910845	< 0.001	23195	MDN1
-0.204917073	< 0.001	56301	SLC7A10
-0.204952732	< 0.001	126393	HSPB6
-0.20506306	< 0.001	8086	AAAS
-0.205064356	< 0.001	90806	ANGEL2
-0.205111533	< 0.001	9126	SMC3
-0.205223426	< 0.001	89	ACTN3
-0.205262527	< 0.001	79015	RP11-445H22.3
-0.205266982	< 0.001	158056	MAMDC4
-0.205353543	< 0.001	285908	LINC00174
-0.205381215	< 0.001	6787	NEK4
-0.205404237	< 0.001	93323	HAUS8
-0.20553565	< 0.001	114897	C1QTNF1
-0.205545589	< 0.001	9425	CDYL
-0.205622017	< 0.001	2286	FKBP2
-0.20567365	< 0.001	80004	ESRP2
-0.205795303	< 0.001	91544	UBXN11
-0.205828637	< 0.001	9961	MVP
-0.205830783	< 0.001	391322	LOC391322
-0.205902278	< 0.001	389677	RBM12B
-0.205947235	< 0.001	79582	SPAG16
-0.205972955	< 0.001	9546	APBA3
-0.205985397	< 0.001	84245	MRI1
-0.206014186	< 0.001	444	ASPH
-0.20601809	< 0.001	926	CD8B
-0.206056908	< 0.001	158405	KIAA1958
-0.206126183	< 0.001	6655	SOS2
-0.206154227	< 0.001	8896	BUD31
-0.206161559	< 0.001	729085	C3orf41
-0.206191927	< 0.001	9709	HERPUD1
-0.206302717	< 0.001	207107	SFTA1P
-0.20635204	< 0.001	415	ARSE
-0.206365407	< 0.001	51296	SLC15A3
-0.206405893	< 0.001	5900	RALGDS
-0.206508055	< 0.001	386681	KRTAP10-8
-0.206509352	< 0.001	57191	VN1R1
-0.206517518	< 0.001	2313	FLI1
-0.206609458	< 0.001	57608	KIAA1462
-0.206681713	< 0.001	4775	NFATC3
-0.206700101	< 0.001	6663	SOX10
-0.206759721	< 0.001	10049	DNAJB6
-0.206765845	< 0.001	83416	FCRL5

region-wise, subcortex					
Spearman's corr coef	p-val	p-val, SA	p-val, SA (BH- correction)	GenID	Gene
-0.258429825	0.127895802	0.139	1	54948	MRPL16
-0.258687258	0.12750642	0.134	1	26034	IPCEF1
-0.258687258	0.12750642	0.174	1	29899	GPSM2
-0.258687258	0.12750642	0.146	1	55313	CPPED1
-0.258687258	0.12750642	0.145	1	123099	DEGS2
-0.258687258	0.12750642	0.147	1	126393	HSPB6
-0.25894466	0.127117708	0.181	1	774	CACNA1B
-0.25894466	0.127117708	0.158	1	54916	C14orf101
-0.25894466	0.127117708	0.153	1	60343	FAM3A
-0.25894466	0.127117708	0.167	1	80019	UBTD1
-0.25894466	0.127117708	0.155	1	148523	C1orf51
-0.259202063	0.12673007	0.166	1	2308	FOXO1
-0.259202063	0.12673007	0.151	1	4618	MYF6
-0.259202063	0.12673007	0.159	1	57597	BAHCC1
-0.259202063	0.12673007	0.164	1	254428	SLC41A1
-0.259202063	0.12673007	0.159	1	389289	ANXA2R
-0.259202063	0.12673007	0.161	1	646962	HRCT1
-0.259459466	0.12634331	0.152	1	302	ANXA2
-0.259459466	0.12634331	0.139	1	9682	KDM4A
-0.259459466	0.12634331	0.184	1	51351	ZNF117
-0.259459466	0.12634331	0.181	1	128869	PIGU
-0.259459466	0.12634331	0.181	1	171019	ADAMTS19
-0.259716868	0.125957489	0.172	1	3187	HNRNPH1
-0.259716868	0.125957489	0.167	1	79879	CCDC134
-0.259716868	0.125957489	0.156	1	401507	FAM74A1
-0.259716868	0.125957489	0.154	1	653240	KRTAP4-14
-0.259974271	0.125572339	0.153	1	285359	LOC285359
-0.259974271	0.125572339	0.155	1	386684	KRTAP12-4
-0.260231644	0.125188261	0.164	1	9183	ZW10
-0.260231644	0.125188261	0.15	1	51150	SDF4
-0.260231644	0.125188261	0.14	1	58986	TMEM8A
-0.260231644	0.125188261	0.16	1	81848	SPRY4
-0.260231644	0.125188261	0.17	1	100128288	AC135178.1
-0.260489076	0.124805048	0.171	1	766	CA7
-0.260489076	0.124805048	0.176	1	7871	SLMAP
-0.260489076	0.124805048	0.156	1	8676	STX11
-0.260489076	0.124805048	0.168	1	9283	GPR37L1
-0.260489076	0.124805048	0.154	1	30061	SLC40A1
-0.260489076	0.124805048	0.157	1	55270	NUDT15
-0.260489076	0.124805048	0.17	1	83656	C8orf12
-0.260746449	0.124422774	0.15	1	2572	GAD2
-0.260746449	0.124422774	0.16	1	25876	SPEF1
-0.260746449	0.124422774	0.154	1	112703	FAM71E1
-0.260746449	0.124422774	0.158	1	161291	TMEM30B
-0.261003882	0.124041326	0.145	1	2342	FNTB
-0.261003882	0.124041326	0.163	1	23370	ARHGEF18
-0.261003882	0.124041326	0.15	1	55793	FAM63A
-0.261003882	0.124041326	0.163	1	92340	C17orf72
-0.261003882	0.124041326	0.136	1	117155	CATSPER2
-0.261261255	0.123660602	0.148	1	146	ADRA1D
-0.261261255	0.123660602	0.156	1	5159	PDGFRB
-0.261261255	0.123660602	0.16	1	5311	PKD2
-0.261261255	0.123660602	0.148	1	6584	SLC22A5
-0.261261255	0.123660602	0.171	1	7060	THBS4
-0.261261255	0.123660602	0.157	1	10314	LANCL1
-0.261261255	0.123660602	0.149	1	79544	OR4K1
-0.261518657	0.123280935	0.166	1	272	AMPD3
-0.261518657	0.123280935	0.138	1	5900	RALGDS
-0.261518657	0.123280935	0.171	1	9499	MYOT
-0.261518657	0.123280935	0.163	1	55769	ZNF83
-0.261518657	0.123280935	0.158	1	246777	SPESP1
-0.261518657	0.123280935	0.135	1	414152	C10orf105
-0.26177606	0.122902177	0.162	1	2030	SLC29A1
-0.26177606	0.122902177	0.16	1	8493	PPM1D
-0.26177606	0.122902177	0.152	1	58517	RBM25
-0.26177606	0.122902177	0.155	1	79400	NOX5
-0.26177606	0.122902177	0.144	1	84522	JAGN1
-0.262033463	0.122524261	0.171	1	10319	LAMC3
-0.262033463	0.122524261	0.152	1	29946	SERTAD3
-0.262033463	0.122524261	0.149	1	55062	WIPI1
-0.262033463	0.122524261	0.171	1	729993	AC092324.1
-0.262290865	0.122147053	0.145	1	8435	SOAT2
-0.262290865	0.122147053	0.135	1	114819	CROCCP3
-0.262290865	0.122147053	0.171	1	254013	C12orf72
-0.262290865	0.122147053	0.158	1	387733	IFITM5
-0.262548268	0.121770896	0.151	1	2976	GTF3C2
-0.262548268	0.121770896	0.148	1	5169	ENPP3
-0.262548268	0.121770896	0.146	1	5948	RBP2
-0.262548268	0.121770896	0.14	1	6788	AP003355.2

voxel-wise, subcortex			
Spearman's corr coef	p-val	GenID	Gene
-0.206930593	< 0.001	6237	RRAS
-0.206937373	< 0.001	7316	UBC
-0.206947491	< 0.001	374882	TMEM205
-0.206960678	< 0.001	54980	C2orf42
-0.206967831	< 0.001	144453	BEST3
-0.206975535	< 0.001	8354	HIST1H3I
-0.206980184	< 0.001	23135	KDM6B
-0.206986681	< 0.001	7430	VIL2
-0.207018718	< 0.001	283487	LINC00346
-0.207029298	< 0.001	3814	KISS1
-0.207036451	< 0.001	84893	FBXO18
-0.207070068	< 0.001	1593	CYP27A1
-0.207090318	< 0.001	399671	HEATR4
-0.207292587	< 0.001	8875	VNN2
-0.207462519	< 0.001	80177	MYCT1
-0.207484812	< 0.001	55260	TMEM143
-0.207509324	< 0.001	80196	RNF34
-0.207554087	< 0.001	219790	RTKN2
-0.207588077	< 0.001	2073	ERCC5
-0.207634509	< 0.001	29994	BAZ2B
-0.207698688	< 0.001	401242	XXBAC-BPG308K3.6
-0.207704723	< 0.001	971	CD72
-0.207809284	< 0.001	149501	KRT8P45
-0.207884699	< 0.001	64983	MRPL32
-0.20808278	< 0.001	9241	NOG
-0.208159387	< 0.001	6049	RNF6
-0.208177313	< 0.001	55601	AC068989.2
-0.20820564	< 0.001	56952	PRTFDC1
-0.208222166	< 0.001	10209	EIF1
-0.20824334	< 0.001	51805	COQ3
-0.208245113	< 0.001	140828	LINC00261
-0.208287552	< 0.001	1149	CIDEA
-0.208304629	< 0.001	84839	RAX2
-0.208329156	< 0.001	91748	C14orf43
-0.208373815	< 0.001	975	CD81
-0.208404005	< 0.001	138199	C9orf41
-0.208410591	< 0.001	645843	TMEM14E
-0.208414122	< 0.001	57615	ZNF492
-0.208461955	< 0.001	10636	RGS14
-0.208511263	< 0.001	26504	CNNM4
-0.208515167	< 0.001	1615	DARS
-0.208595216	< 0.001	925	CD8A
-0.208631516	< 0.001	10581	IFITM2
-0.208663374	< 0.001	6135	RPL11
-0.20870024	< 0.001	29941	PKN3
-0.208706737	< 0.001	1958	EGR1
-0.208718255	< 0.001	100240734	LOC100240734
-0.208765253	< 0.001	51540	SCLY
-0.208851054	< 0.001	387841	RP11-392P7.1
-0.208856538	< 0.001	348645	C22orf34
-0.20892489	< 0.001	85437	ZCRB1
-0.209091946	< 0.001	256309	CCDC110
-0.209133089	< 0.001	55266	TMEM19
-0.209187508	< 0.001	80210	ARMC9
-0.209193736	< 0.001	201514	ZNF584
-0.209243789	< 0.001	378708	APITD1
-0.20932886	< 0.001	57680	CHD8
-0.209357172	< 0.001	92312	MEX3A
-0.209408343	< 0.001	8872	CDC123
-0.209500372	< 0.001	6224	RPS20
-0.209506318	< 0.001	80758	PRR7
-0.209582657	< 0.001	10795	ZNF268
-0.209665492	< 0.001	54916	C14orf101
-0.209737927	< 0.001	163259	DENND2C
-0.209786221	< 0.001	23761	PISD
-0.209792808	< 0.001	150209	AIFM3
-0.20989728	< 0.001	284252	KCTD1
-0.209908888	< 0.001	85391	SNORD14E
-0.209932759	< 0.001	54985	HCFC1R1
-0.209954306	< 0.001	6143	RPL19P12
-0.21003826	< 0.001	51429	SNX9
-0.210097313	< 0.001	1535	CYBA
-0.210111707	< 0.001	790	CAD
-0.21019873	< 0.001	6129	RPL7
-0.210222036	< 0.001	23539	SLC16A8
-0.210246831	< 0.001	51660	BRP44L
-0.210257426	< 0.001	2687	GGT5
-0.210267633	< 0.001	56	ACRV1
-0.210311845	< 0.001	2729	GCLC

region-wise, subcortex					
Spearman's corr coef	p-val	p-val, SA	p-val, SA (BH- correction)	GenID	Gene
-0.262548268	0.121770896	0.17	1	8406	SRPX
-0.262548268	0.121770896	0.136	1	27013	C2orf24
-0.262548268	0.121770896	0.147	1	55751	TMEM184C
-0.262805641	0.121395603	0.155	1	10568	SLC34A2
-0.262805641	0.121395603	0.158	1	10994	ILVBL
-0.262805641	0.121395603	0.124	1	11174	ADAMTS6
-0.262805641	0.121395603	0.192	1	26052	DNM3
-0.262805641	0.121395603	0.146	1	118924	C10orf4
-0.262805641	0.121395603	0.158	1	200186	CRTC2
-0.263063073	0.121021233	0.164	1	477	ATP1A2
-0.263063073	0.121021233	0.145	1	4489	MT1A
-0.263063073	0.121021233	0.142	1	10627	MYL12A
-0.263063073	0.121021233	0.157	1	56111	PCDHGA4
-0.263063073	0.121021233	0.148	1	83868	TTTY13
-0.263320446	0.12064749	0.144	1	960	CD44
-0.263320446	0.12064749	0.135	1	4215	MAP3K3
-0.263320446	0.12064749	0.126	1	5894	RAF1
-0.263320446	0.12064749	0.14	1	8877	SPHK1
-0.263320446	0.12064749	0.154	1	493911	PHOSPHO2
-0.263577878	0.120274864	0.138	1	5046	PCSK6
-0.263577878	0.120274864	0.148	1	7422	VEGFA
-0.263577878	0.120274864	0.141	1	8645	KCNK5
-0.263577878	0.120274864	0.158	1	10174	SORBS3
-0.263577878	0.120274864	0.16	1	23609	MKRN2
-0.263577878	0.120274864	0.145	1	23642	SNHG1
-0.263577878	0.120274864	0.161	1	60625	DHX35
-0.263577878	0.120274864	0.155	1	85414	SLC45A3
-0.263835251	0.119903065	0.154	1	661	POLR3D
-0.263835251	0.119903065	0.164	1	10301	DLEU1
-0.263835251	0.119903065	0.172	1	22925	PLA2R1
-0.263835251	0.119903065	0.149	1	23473	CAPN7
-0.263835251	0.119903065	0.158	1	83475	DOHH
-0.263835251	0.119903065	0.148	1	339521	FAM58BP
-0.264092654	0.119532183	0.139	1	5008	OSM
-0.264092654	0.119532183	0.143	1	9304	SNORD22
-0.264092654	0.119532183	0.143	1	79691	QTRTD1
-0.264350057	0.11916212	0.16	1	8189	SYMPK
-0.264350057	0.11916212	0.138	1	9054	NFS1
-0.264350057	0.11916212	0.142	1	10762	NUP50
-0.264350057	0.11916212	0.131	1	27342	RABGEF1
-0.264350057	0.11916212	0.143	1	115416	MALSU1
-0.264607459	0.118792765	0.16	1	7957	EPM2A
-0.264607459	0.118792765	0.147	1	10941	UGT2A1
-0.264607459	0.118792765	0.135	1	56672	C11orf17
-0.264607459	0.118792765	0.149	1	138241	C9orf85
-0.264607459	0.118792765	0.142	1	286451	YIPF6
-0.264864862	0.118424445	0.144	1	6392	SDHD
-0.264864862	0.118424445	0.142	1	6678	SPARC
-0.264864862	0.118424445	0.147	1	55338	FLJ11292
-0.264864862	0.118424445	0.156	1	124093	CCDC78
-0.265122265	0.118057035	0.147	1	8869	ST3GAL5
-0.265122265	0.118057035	0.149	1	25980	C20orf4
-0.265122265	0.118057035	0.139	1	80174	DBF4B
-0.265122265	0.118057035	0.184	1	92283	ZNF461
-0.265122265	0.118057035	0.155	1	158787	RIBC1
-0.265379637	0.117690429	0.165	1	57475	PLEKHH1
-0.265379637	0.117690429	0.155	1	148741	ANKRD35
-0.265379637	0.117690429	0.156	1	221504	ZBTB9
-0.26563707	0.117324546	0.147	1	2327	FMO2
-0.26563707	0.117324546	0.157	1	7697	ZNF138
-0.26563707	0.117324546	0.147	1	9093	DNAJA3
-0.26563707	0.117324546	0.15	1	9356	SLC22A6
-0.26563707	0.117324546	0.151	1	115811	IQCD
-0.26563707	0.117324546	0.15	1	124411	ZNF720
-0.26563707	0.117324546	0.154	1	145226	RDH12
-0.26563707	0.117324546	0.132	1	283999	AC087645.1
-0.26563707	0.117324546	0.147	1	341567	H1FNT
-0.265894473	0.116959676	0.153	1	2532	DARC
-0.265894473	0.116959676	0.163	1	3805	KIR2DL4
-0.265894473	0.116959676	0.149	1	26278	SACS
-0.265894473	0.116959676	0.151	1	79970	ZNF767
-0.265894473	0.116959676	0.146	1	90196	SYS1
-0.266151875	0.116595671	0.121	1	6352	CCL5
-0.266151875	0.116595671	0.135	1	7551	ZNF3
-0.266151875	0.116595671	0.13	1	66035	SLC2A11
-0.266151875	0.116595671	0.15	1	79842	ZBTB3
-0.266151875	0.116595671	0.143	1	162967	ZNF320
-0.266151875	0.116595671	0.157	1	387990	TOMM20L
-0.266409278	0.116232559	0.144	1	27079	RPUSD2

voxel-wise, subcortex			
Spearman's corr coef	p-val	GenID	Gene
-0.210384458	< 0.001	2886	GRB7
-0.210403875	< 0.001	2053	EPHX2
-0.210438699	< 0.001	4043	LRPAP1
-0.210457638	< 0.001	79989	TTC26
-0.210470453	< 0.001	8863	PER3
-0.210522279	< 0.001	91942	NDUFAF2
-0.21054326	< 0.001	114757	CYGB
-0.210591465	< 0.001	54462	KIAA1128
-0.210601673	< 0.001	197259	MLKL
-0.21067141	< 0.001	200539	ANKRD23
-0.210676715	< 0.001	55349	CHDH
-0.21070309	< 0.001	100288788	LOC100288788
-0.210775793	< 0.001	1655	DDX5
-0.210784435	< 0.001	441454	LOC441454
-0.21080552	< 0.001	162387	MFSD6L
-0.210874423	< 0.001	3360	HTR4
-0.210884258	< 0.001	84876	ORAI1
-0.210905999	< 0.001	6230	RPS25
-0.210908785	< 0.001	22976	PAXIP1
-0.210920662	< 0.001	57730	ANKRD36B
-0.211016685	< 0.001	445582	POTEE
-0.211038709	< 0.001	6948	TCN2
-0.211090341	< 0.001	26235	FBXL4
-0.211108446	< 0.001	54875	CNTLN
-0.2112353	< 0.001	114790	STK11IP
-0.211272255	< 0.001	25921	ZDHHC5
-0.211284891	< 0.001	57721	METTL14
-0.211378679	< 0.001	89781	HPS4
-0.211420193	< 0.001	1029	CDKN2A
-0.211469978	< 0.001	442245	GSTM2P1
-0.211516783	< 0.001	51090	PLLP
-0.211528197	< 0.001	5286	PIK3C2A
-0.211606577	< 0.001	5443	POMC
-0.211776063	< 0.001	1271	CNTFR
-0.211887866	< 0.001	2830	GPR6
-0.21191369	< 0.001	56834	GPR137
-0.211937651	< 0.001	728841	NBPF8
-0.211963922	< 0.001	29914	UBIAD1
-0.21200636	< 0.001	2348	FOLR1
-0.212317094	< 0.001	284232	ANKRD20A9P
-0.21232833	< 0.001	221749	C6orf145
-0.212332144	< 0.001	51434	ANAPC7
-0.212380901	< 0.001	9937	DCLRE1A
-0.212450638	< 0.001	8631	SKAP1
-0.212454632	< 0.001	10150	MBNL2
-0.212482959	< 0.001	54879	ST7L
-0.212536722	< 0.001	161253	REM2
-0.212549254	< 0.001	127064	OR2T12
-0.212584183	< 0.001	91419	XRCC6BP1
-0.212613806	< 0.001	137835	TMEM71
-0.212662458	< 0.001	730183	LOC730183
-0.212788299	< 0.001	8153	RND2
-0.212788761	< 0.001	2323	FLT3LG
-0.212806225	< 0.001	83449	PMFBP1
-0.212814942	< 0.001	2736	GLI2
-0.21286045	< 0.001	3609	ILF3
-0.212881908	< 0.001	100132708	CYP4F30P
-0.212941155	< 0.001	51643	TMBIM4
-0.212994546	< 0.001	100125288	AC011511.1
-0.213004023	< 0.001	643749	TRAF3IP2-AS1
-0.21305947	< 0.001	56242	ZNF253
-0.213178515	< 0.001	389203	C4ORF52
-0.213354871	< 0.001	80199	FUZ
-0.213411793	< 0.001	26115	TANC2
-0.213412911	< 0.001	386617	KCTD8
-0.213430092	< 0.001	54885	TBC1D8B
-0.213442162	< 0.001	7755	ZNF205
-0.213524818	< 0.001	23294	ANKS1A
-0.213857561	< 0.001	190	NR0B1
-0.213908449	< 0.001	81887	LAS1L
-0.213943824	< 0.001	9668	ZNF432
-0.214112654	< 0.001	54681	P4HTM
-0.214133739	< 0.001	340888	LOC340888
-0.21419327	< 0.001	56918	C2orf83
-0.214220285	< 0.001	3106	HLA-B
-0.214229763	< 0.001	283643	C14orf80
-0.214237928	< 0.001	153090	DAB2IP
-0.214263752	< 0.001	22852	ANKRD26
-0.214287058	< 0.001	7991	TUSC3

region-wise, subcortex					
Spearman's corr coef	p-val	p-val, SA	p-val, SA (BH- correction)	GenID	Gene
-0.266409278	0.116232559	0.151	1	404203	SPINK6
-0.266666651	0.115870111	0.124	1	26099	C1orf144
-0.266666651	0.115870111	0.149	1	91977	MYOZ3
-0.266666651	0.115870111	0.159	1	164592	CCDC116
-0.266666651	0.115870111	0.152	1	257160	RNF214
-0.266924083	0.115508683	0.15	1	2272	FHIT
-0.266924083	0.115508683	0.139	1	4676	NAP1L4
-0.266924083	0.115508683	0.159	1	79058	ASPSCR1
-0.266924083	0.115508683	0.141	1	157310	PEBP4
-0.266924083	0.115508683	0.186	1	221613	HIST1H2AA
-0.267181456	0.115148112	0.146	1	11339	OIP5
-0.267181456	0.115148112	0.139	1	55667	DENND4C
-0.267181456	0.115148112	0.144	1	79585	CORO7
-0.267181456	0.115148112	0.152	1	80824	DUSP16
-0.267181456	0.115148112	0.131	1	387267	KRTAP5-4
-0.267438889	0.114788406	0.158	1	4829	NMBR
-0.267438889	0.114788406	0.149	1	5629	PROX1
-0.267438889	0.114788406	0.131	1	9388	LIPG
-0.267438889	0.114788406	0.15	1	9705	ST18
-0.267438889	0.114788406	0.149	1	23145	SSPO
-0.267438889	0.114788406	0.137	1	79258	MMEL1
-0.267438889	0.114788406	0.154	1	84735	CNDP1
-0.267438889	0.114788406	0.153	1	135924	OR9A2
-0.267438889	0.114788406	0.138	1	348093	RBPMS2
-0.267696261	0.114429578	0.161	1	5500	PPP1CB
-0.267696261	0.114429578	0.143	1	51093	C1orf66
-0.267696261	0.114429578	0.139	1	166968	MIER3
-0.267953664	0.114071406	0.148	1	1465	CSRP1
-0.267953664	0.114071406	0.152	1	1728	NQO1
-0.267953664	0.114071406	0.158	1	5190	PEX6
-0.268211067	0.113714248	0.148	1	953	ENTPD1
-0.268211067	0.113714248	0.136	1	7783	ZP2
-0.268468469	0.113357946	0.142	1	5241	PGR
-0.268468469	0.113357946	0.152	1	7298	TYMS
-0.268468469	0.113357946	0.127	1	200030	NBPF11
-0.268468469	0.113357946	0.154	1	646127	LOC646127
-0.268468469	0.113357946	0.151	1	729652	LOC729652
-0.268725872	0.113002494	0.132	1	2696	GIPR
-0.268725872	0.113002494	0.148	1	3290	HSD11B1
-0.268725872	0.113002494	0.122	1	10380	BPNT1
-0.268725872	0.113002494	0.146	1	80110	ZNF614
-0.268725872	0.113002494	0.127	1	89941	RHOT2
-0.268725872	0.113002494	0.162	1	164395	TTLL9
-0.268983275	0.112647764	0.135	1	7472	WNT2
-0.268983275	0.112647764	0.139	1	51616	TAF9B
-0.268983275	0.112647764	0.151	1	84219	WDR24
-0.268983275	0.112647764	0.134	1	163786	SASS6
-0.269240648	0.112294003	0.143	1	10365	KLF2
-0.269240648	0.112294003	0.145	1	85437	ZCRB1
-0.269240648	0.112294003	0.118	1	376940	ZC3H6
-0.269240648	0.112294003	0.159	1	390010	NKX1-2
-0.26949808	0.111941099	0.138	1	23150	FRMD4B
-0.26949808	0.111941099	0.143	1	58503	PROL1
-0.269755453	0.111589029	0.149	1	2313	FLI1
-0.269755453	0.111589029	0.155	1	54875	CNTLN
-0.269755453	0.111589029	0.162	1	57205	ATP10D
-0.269755453	0.111589029	0.143	1	80864	XXBAC-BPG300A18.12
-0.269755453	0.111589029	0.142	1	93164	AC007688.2
-0.270012885	0.111237653	0.129	1	6470	SHMT1
-0.270012885	0.111237653	0.155	1	10165	SLC25A13
-0.270012885	0.111237653	0.128	1	10513	APPBP2
-0.270012885	0.111237653	0.122	1	54878	DPP8
-0.270270258	0.110887319	0.12	1	6390	SDHB
-0.270270258	0.110887319	0.141	1	9572	NR1D1
-0.270270258	0.110887319	0.14	1	10690	FUT9
-0.270270258	0.110887319	0.12	1	89782	LMLN
-0.270270258	0.110887319	0.148	1	148823	C1orf150
-0.270270258	0.110887319	0.12	1	374291	NDUFS7
-0.270527661	0.110537775	0.147	1	2897	GRIK1
-0.270527661	0.110537775	0.145	1	55147	RBM23
-0.270527661	0.110537775	0.148	1	119504	C10orf104
-0.270527661	0.110537775	0.13	1	162699	TCEB3C
-0.270527661	0.110537775	0.146	1	353322	ANKRD37
-0.270527661	0.110537775	0.125	1	100130935	LOC100130935
-0.270785064	0.11018908	0.119	1	6618	SNAPC2
-0.270785064	0.11018908	0.121	1	11227	GALNT5
-0.271042466	0.10984122	0.139	1	1026	CDKN1A
-0.271042466	0.10984122	0.147	1	1535	CYBA
-0.271042466	0.10984122	0.132	1	10067	SCAMP3

voxel-wise, subcortex			
Spearman's corr coef	p-val	GenID	Gene
-0.214309067	< 0.001	192286	HIGD2A
-0.21432662	< 0.001	3485	IGFBP2
-0.214332193	< 0.001	10899	JTB
-0.214402303	< 0.001	54925	ZNF434
-0.214418843	< 0.001	339487	ZBTB8OS
-0.214514956	< 0.001	595	CCND1
-0.214566126	< 0.001	221938	MMD2
-0.214573458	< 0.001	969	CD69
-0.214665115	< 0.001	54496	PRMT7
-0.214731336	< 0.001	158787	RIBC1
-0.214829773	< 0.001	55072	RNF31
-0.214851037	< 0.001	442075	AC034193.5
-0.214911208	< 0.001	29128	UHRF1
-0.215051532	< 0.001	2887	GRB10
-0.215116352	< 0.001	55344	PLCXD1
-0.21513325	< 0.001	64127	NOD2
-0.215179503	< 0.001	3646	EIF3E
-0.215245619	< 0.001	122961	ISCA2
-0.215266421	< 0.001	83707	TRPT1
-0.215311095	< 0.001	1997	ELF1
-0.215321675	< 0.001	56927	GPR108
-0.215326041	< 0.001	100131998	RRN3P3
-0.215347871	< 0.001	122481	AK7
-0.215457723	< 0.001	4629	MYH11
-0.215538248	< 0.001	80153	EDC3
-0.215553939	< 0.001	644903	FLJ38668
-0.215587839	< 0.001	7562	ZNF708
-0.215611145	< 0.001	3991	LIPE
-0.215654984	< 0.001	7200	TRH
-0.215683028	< 0.001	84231	TRAF7
-0.21575211	< 0.001	26137	ZBTB20
-0.215821952	< 0.001	92715	WDR85
-0.215823993	< 0.001	55761	TTC17
-0.215907753	< 0.001	114826	SMYD4
-0.215979636	< 0.001	415117	STX19
-0.216068596	< 0.001	285741	LOC285741
-0.216108814	< 0.001	29997	GLTSCR2
-0.216142252	< 0.001	88	ACTN2
-0.216211155	< 0.001	642394	ADARB2-AS1
-0.216260284	< 0.001	192669	EIF2C3
-0.216354907	< 0.001	999	CDH1
-0.216410905	< 0.001	140733	MACROD2
-0.216446295	< 0.001	23288	IQCE
-0.21647805	< 0.001	8910	SGCE
-0.216504708	< 0.001	9623	TCL1B
-0.216682449	< 0.001	22997	RP11-259P6.1
-0.216684207	< 0.001	9841	ZBTB24
-0.216774017	< 0.001	23268	DNMBP
-0.21677494	< 0.001	541466	CT45A1
-0.216826111	< 0.001	653639	LYPLA2P1
-0.216980726	< 0.001	27	ABL2
-0.216981754	< 0.001	7439	BEST1
-0.217007577	< 0.001	162962	ZNF836
-0.217019916	< 0.001	55252	ASXL2
-0.217115387	< 0.001	5930	RBBP6
-0.217138603	< 0.001	4350	MPG
-0.217154026	< 0.001	60495	HPSE2
-0.217210665	< 0.001	57162	PELI1
-0.217277154	< 0.001	497190	CLEC18B
-0.217414051	< 0.001	23683	PRKD3
-0.21745342	< 0.001	113829	SLC35A4
-0.217500135	< 0.001	148430	LOC148430
-0.21753487	< 0.001	3921	RPSA
-0.217550188	< 0.001	284106	CISD3
-0.217602566	< 0.001	26993	AKAP8L
-0.217694223	< 0.001	63897	HEATR6
-0.217759416	< 0.001	219833	C11orf45
-0.217769533	< 0.001	79446	WDR25
-0.21780993	< 0.001	56649	TMPRSS4
-0.217916444	< 0.001	222235	FBXL13
-0.217977747	< 0.001	339324	ZNF260
-0.218027428	< 0.001	144811	C13orf31
-0.218160123	< 0.001	28513	CDH19
-0.218174994	< 0.001	151473	SLC16A14
-0.218249649	< 0.001	5696	PSMB8
-0.218259588	< 0.001	114825	PWWP2A
-0.218277231	< 0.001	7263	TST
-0.218318	< 0.001	154661	RUNDC3B
-0.218326539	< 0.001	51738	GHRL

region-wise, subcortex					
Spearman's corr coef	p-val	p-val, SA	p-val, SA (BH- correction)	GenID	Gene
-0.271042466	0.10984122	0.132	1	51252	FAM178B
-0.271299869	0.109494053	0.143	1	64216	TFB2M
-0.271299869	0.109494053	0.132	1	730091	RP11-392A22.2
-0.271557271	0.109147906	0.124	1	483	ATP1B3
-0.271557271	0.109147906	0.122	1	6829	SUPT5H
-0.271557271	0.109147906	0.135	1	29911	HOOK2
-0.271557271	0.109147906	0.176	1	284217	LAMA1
-0.271814644	0.108802564	0.141	1	2621	GAS6
-0.271814644	0.108802564	0.146	1	3320	HSP90AA2
-0.271814644	0.108802564	0.16	1	51426	POLK
-0.271814644	0.108802564	0.156	1	79140	CCDC28B
-0.271814644	0.108802564	0.137	1	442194	OR10C1
-0.271814644	0.108802564	0.147	1	442572	LOC442572
-0.272072077	0.108458057	0.128	1	5442	POLRMT
-0.272072077	0.108458057	0.134	1	10616	RBCK1
-0.272072077	0.108458057	0.121	1	11274	USP18
-0.272072077	0.108458057	0.126	1	55544	RBM38
-0.272072077	0.108458057	0.129	1	57188	ADAMTSL3
-0.27232945	0.108114235	0.163	1	6560	SLC12A4
-0.27232945	0.108114235	0.147	1	7633	ZNF79
-0.27232945	0.108114235	0.14	1	9584	RBM39
-0.27232945	0.108114235	0.127	1	9911	TMCC2
-0.27232945	0.108114235	0.14	1	151258	SLC38A11
-0.272586882	0.107771397	0.136	1	6714	SRC
-0.272586882	0.107771397	0.13	1	57380	MRS2
-0.272586882	0.107771397	0.134	1	150622	LOC150622
-0.272844285	0.10742943	0.143	1	3764	KCNJ8
-0.273101658	0.107088245	0.133	1	4879	NPPB
-0.273101658	0.107088245	0.127	1	5528	PPP2R5D
-0.273101658	0.107088245	0.125	1	51285	RASL12
-0.27335909	0.106747895	0.137	1	6363	CCL19
-0.27335909	0.106747895	0.12	1	54507	ADAMTSL4
-0.27335909	0.106747895	0.138	1	55065	GPR172B
-0.27335909	0.106747895	0.144	1	80298	MTERFD3
-0.27335909	0.106747895	0.115	1	84331	C16orf14
-0.27335909	0.106747895	0.109	1	441272	SPDYE3
-0.273616463	0.106408231	0.136	1	1573	CYP2J2
-0.273616463	0.106408231	0.132	1	55036	CCDC40
-0.273616463	0.106408231	0.144	1	84080	C16orf48
-0.273616463	0.106408231	0.133	1	730112	FAM166B
-0.273873895	0.106069535	0.132	1	8495	PPFIBP2
-0.273873895	0.106069535	0.136	1	23417	MLYCD
-0.273873895	0.106069535	0.118	1	79927	GRRP1
-0.273873895	0.106069535	0.128	1	168667	BMPER
-0.273873895	0.106069535	0.134	1	728441	GGT2
-0.274131268	0.105731703	0.14	1	1101	CHAD
-0.274131268	0.105731703	0.124	1	1997	ELF1
-0.274131268	0.105731703	0.143	1	2571	GAD1
-0.274131268	0.105731703	0.138	1	3993	LLGL2
-0.274131268	0.105731703	0.127	1	26191	PTPN22
-0.274131268	0.105731703	0.132	1	56155	TEX14
-0.274131268	0.105731703	0.126	1	79142	PHF23
-0.274131268	0.105731703	0.153	1	79956	ERMP1
-0.274131268	0.105731703	0.133	1	112869	CCDC101
-0.274388671	0.105394669	0.142	1	18	ABAT
-0.274388671	0.105394669	0.115	1	202915	TMEM184A
-0.274388671	0.105394669	0.134	1	285343	C3orf23
-0.274646074	0.105058305	0.152	1	5359	PLSCR1
-0.274646074	0.105058305	0.114	1	10993	SDS
-0.274903476	0.104722902	0.153	1	7503	XIST
-0.274903476	0.104722902	0.132	1	57556	SEMA6A
-0.274903476	0.104722902	0.135	1	128488	WFDC12
-0.274903476	0.104722902	0.137	1	160287	LDHAL6A
-0.275160879	0.104388334	0.116	1	7094	TLN1
-0.275160879	0.104388334	0.148	1	7473	WNT3
-0.275160879	0.104388334	0.136	1	10813	UTP14A
-0.275160879	0.104388334	0.115	1	11325	DDX42
-0.275160879	0.104388334	0.151	1	85366	MYLK2
-0.275418282	0.104054615	0.141	1	8320	EOMES
-0.275418282	0.104054615	0.129	1	51439	FAM8A1
-0.275418282	0.104054615	0.149	1	84674	CARD6
-0.275418282	0.104054615	0.132	1	92017	SNX29
-0.275418282	0.104054615	0.157	1	93166	PRDM6
-0.275675654	0.103721537	0.142	1	1288	COL4A6
-0.275675654	0.103721537	0.131	1	1528	CYB5A
-0.275675654	0.103721537	0.115	1	3912	LAMB1
-0.275675654	0.103721537	0.123	1	4163	MCC
-0.275675654	0.103721537	0.144	1	23269	MGA
-0.275675654	0.103721537	0.126	1	64150	DIO3OS

voxel-wise, subcortex			
Spearman's corr coef	p-val	GenID	Gene
-0.218491286	< 0.001	6581	SLC22A3
-0.21857515	< 0.001	22858	ICK
-0.218622327	< 0.001	80324	PUS1
-0.218679443	< 0.001	5525	PPP2R5A
-0.218703493	< 0.001	23198	PSME4
-0.218716577	< 0.001	441416	GPR116P2
-0.218729764	< 0.001	51116	MRPS2
-0.218752056	< 0.001	728658	RPL13AP5
-0.218815118	< 0.001	2850	GPR27
-0.21881707	< 0.001	84909	C9orf3
-0.218831927	< 0.001	2077	ERF
-0.218905479	< 0.001	22944	KIN
-0.218933702	< 0.001	27443	CECR2
-0.218956366	< 0.001	9688	NUP93
-0.219022483	< 0.001	22897	CEP164
-0.219026297	< 0.001	26030	PLEKHG3
-0.219046623	< 0.001	57124	CD248
-0.219057307	< 0.001	6446	SGK1
-0.219131872	< 0.001	57093	TRIM49
-0.219135866	< 0.001	9304	SNORD22
-0.219153419	< 0.001	34	ACADM
-0.219427377	< 0.001	205860	TRIML2
-0.219439074	< 0.001	3791	KDR
-0.219615713	< 0.001	574407	C1orf145
-0.219670683	< 0.001	55180	LINS
-0.21983172	< 0.001	127795	C1orf87
-0.219838127	< 0.001	6392	SDHD
-0.219969437	< 0.001	339451	KLHL17
-0.220032126	< 0.001	11149	BVES
-0.220072791	< 0.001	100289026	LOC100289026
-0.220170125	< 0.001	1181	CLCN2
-0.220192313	< 0.001	379	ARL4D
-0.220213115	< 0.001	81629	TSSK3
-0.220219523	< 0.001	30008	EFEMP2
-0.220256492	< 0.001	728378	A26C1B
-0.220282584	< 0.001	114885	OSBPL11
-0.22029902	< 0.001	100129827	AC009532.1
-0.220318526	< 0.001	7481	WNT11
-0.220358729	< 0.001	9705	ST18
-0.220378786	< 0.001	79718	TBL1XR1
-0.220453739	< 0.001	64222	TOR3A
-0.220497012	< 0.001	10430	TMEM147
-0.220751464	< 0.001	64115	C10orf54
-0.220761493	< 0.001	256006	ANKRD31
-0.22078675	< 0.001	4988	OPRM1
-0.220792323	< 0.001	124056	NOXO1
-0.220800221	< 0.001	79991	OBFC1
-0.220868006	< 0.001	8869	ST3GAL5
-0.220954195	< 0.001	204010	RPSAP52
-0.221055686	< 0.001	5654	HTRA1
-0.221106589	< 0.001	51174	TUBD1
-0.221180603	< 0.001	7082	TJP1
-0.221221268	< 0.001	112398	EGLN2
-0.221254885	< 0.001	131096	KCNH8
-0.221314698	< 0.001	131566	DCBLD2
-0.221326485	< 0.001	6938	TCF12
-0.221343026	< 0.001	8493	PPM1D
-0.221356019	< 0.001	25817	FAM19A5
-0.221360669	< 0.001	3304	HSPA1B
-0.221457615	< 0.001	388335	TMEM220
-0.221462354	< 0.001	260294	NSUN5C
-0.221470252	< 0.001	84735	CNDP1
-0.221590415	< 0.001	54532	USP53
-0.221595705	< 0.001	5858	PZP
-0.221600994	< 0.001	115024	NT5C3L
-0.22160992	< 0.001	23043	TNIK
-0.221611962	< 0.001	29777	ABT1
-0.2217208	< 0.001	84135	UTP15
-0.221747726	< 0.001	79230	ZNF557
-0.221773818	< 0.001	5530	PPP3CA
-0.221804842	< 0.001	10062	NR1H3
-0.221851826	< 0.001	54468	MIOS
-0.22193104	< 0.001	6207	RPS13P2
-0.221942753	< 0.001	49856	WDR8
-0.221981108	< 0.001	114881	OSBPL7
-0.22204797	< 0.001	133874	AC034199.2
-0.222054183	< 0.001	7565	ZNF17
-0.222109169	< 0.001	8356	HIST1H3J
-0.222178817	< 0.001	51111	SUV420H1

region-wise, subcortex					
Spearman's corr coef	p-val	p-val, SA	p-val, SA (BH- correction)	GenID	Gene
-0.275675654	0.103721537	0.129	1	166378	SPATA5
-0.275675654	0.103721537	0.134	1	542767	C1QTNF9B-AS1
-0.275933087	0.10338942	0.13	1	6909	TBX2
-0.275933087	0.10338942	0.144	1	65095	KRI1
-0.275933087	0.10338942	0.133	1	84890	ADO
-0.275933087	0.10338942	0.125	1	85415	RHPN2
-0.276447892	0.102727637	0.116	1	4825	NKX6-1
-0.276447892	0.102727637	0.129	1	54816	ZNF280D
-0.276447892	0.102727637	0.14	1	54887	UHRF1BP1
-0.276447892	0.102727637	0.147	1	84978	FRMD5
-0.276447892	0.102727637	0.142	1	199731	CADM4
-0.276705265	0.102398008	0.139	1	2990	GUSB
-0.276705265	0.102398008	0.136	1	23395	LARS2
-0.276705265	0.102398008	0.116	1	23644	EDC4
-0.276962668	0.102069005	0.124	1	1021	CDK6
-0.276962668	0.102069005	0.139	1	9514	GAL3ST1
-0.276962668	0.102069005	0.145	1	10686	CLDN16
-0.276962668	0.102069005	0.124	1	54606	DDX56
-0.276962668	0.102069005	0.138	1	57474	ZNF490
-0.276962668	0.102069005	0.146	1	644157	DND1P1
-0.27722007	0.101740964	0.126	1	2113	ETS1
-0.27722007	0.101740964	0.113	1	11065	UBE2C
-0.27722007	0.101740964	0.125	1	54970	TTC12
-0.27722007	0.101740964	0.147	1	84274	COQ5
-0.277477473	0.101413734	0.125	1	1395	CRHR2
-0.277477473	0.101413734	0.122	1	1504	CTRB1
-0.277477473	0.101413734	0.144	1	6535	SLC6A8
-0.277477473	0.101413734	0.127	1	7572	ZNF24
-0.277477473	0.101413734	0.122	1	8339	HIST1H2BG
-0.277477473	0.101413734	0.144	1	10578	GNLY
-0.277477473	0.101413734	0.111	1	23143	LRCH1
-0.277734876	0.101087317	0.128	1	3693	ITGB5
-0.277734876	0.101087317	0.124	1	9510	ADAMTS1
-0.277992278	0.100761592	0.129	1	10991	SLC38A3
-0.277992278	0.100761592	0.118	1	25841	ABTB2
-0.277992278	0.100761592	0.122	1	49856	WDR8
-0.277992278	0.100761592	0.127	1	55646	LYAR
-0.277992278	0.100761592	0.119	1	221927	C7orf27
-0.278249651	0.100436792	0.139	1	3303	HSPA1A
-0.278249651	0.100436792	0.111	1	3490	IGFBP7
-0.278249651	0.100436792	0.119	1	9968	MED12
-0.278249651	0.100436792	0.13	1	84077	C3orf20
-0.278249651	0.100436792	0.143	1	100132644	LOC100132644
-0.278507084	0.100112788	0.115	1	10815	CPLX1
-0.278507084	0.100112788	0.127	1	256236	NAPSB
-0.278507084	0.100112788	0.092	1	284565	NBPF15
-0.278507084	0.100112788	0.119	1	402483	FLJ45340
-0.278764457	0.099789605	0.121	1	7039	TGFA
-0.279021889	0.099467099	0.134	1	1955	MEGF9
-0.279021889	0.099467099	0.137	1	3133	HLA-E
-0.279021889	0.099467099	0.129	1	8350	HIST1H3A
-0.279021889	0.099467099	0.117	1	9754	STARD8
-0.279021889	0.099467099	0.11	1	51296	SLC15A3
-0.279021889	0.099467099	0.14	1	79034	C7orf26
-0.279279262	0.099145517	0.111	1	2774	GNAL
-0.279279262	0.099145517	0.136	1	22850	ADNP2
-0.279536664	0.098824732	0.114	1	2057	EPOR
-0.279536664	0.098824732	0.135	1	6927	HNF1A
-0.279536664	0.098824732	0.139	1	26137	ZBTB20
-0.279536664	0.098824732	0.117	1	55323	LARP6
-0.279536664	0.098824732	0.124	1	56126	PCDHB10
-0.279536664	0.098824732	0.117	1	84304	NUDT22
-0.279536664	0.098824732	0.121	1	389458	LOC389458
-0.279794097	0.098504752	0.129	1	3304	HSPA1B
-0.279794097	0.098504752	0.159	1	84807	NFKBID
-0.279794097	0.098504752	0.105	1	388407	C17orf82
-0.279794097	0.098504752	0.109	1	401082	FLJ25363
-0.279794097	0.098504752	0.12	1	401242	XXBAC-BPG308K3.6
-0.28005147	0.098185562	0.118	1	1024	CDK8
-0.28005147	0.098185562	0.112	1	85477	SCIN
-0.28005147	0.098185562	0.141	1	222699	TOB2P1
-0.280308902	0.097867072	0.143	1	2766	GMPR
-0.280308902	0.097867072	0.113	1	23131	GPATCH8
-0.280308902	0.097867072	0.13	1	219681	ARMC3
-0.280566275	0.097549483	0.136	1	9361	LONP1
-0.280566275	0.097549483	0.131	1	29901	SAC3D1
-0.280823678	0.097232692	0.136	1	2999	GZMH
-0.280823678	0.097232692	0.111	1	55125	CEP192
-0.280823678	0.097232692	0.134	1	79156	PLEKHF1

voxel-wise, subcortex			
Spearman's corr coef	p-val	GenID	Gene
-0.222210854	< 0.001	81932	HDHD3
-0.222236574	< 0.001	8350	HIST1H3A
-0.222249016	< 0.001	23590	PDSS1
-0.222283378	< 0.001	955	ENTPD6
-0.222305298	< 0.001	3908	LAMA2
-0.222310126	< 0.001	126823	KLHDC9
-0.222468466	< 0.001	80820	EEPD1
-0.222506627	< 0.001	150622	LOC150622
-0.222619548	< 0.001	89765	RSPH1
-0.222649649	< 0.001	203074	UNQ9391
-0.222687989	< 0.001	256236	NAPSB
-0.222691983	< 0.001	114822	RHPN1
-0.222706944	< 0.001	2180	ACSL1
-0.222736478	< 0.001	80833	APOL3
-0.222754031	< 0.001	55357	TBC1D2
-0.222831473	< 0.001	4287	ATXN3
-0.222837791	< 0.001	55245	UQCC
-0.223081291	< 0.001	8729	GBF1
-0.223144338	< 0.001	1198	CLK3
-0.223152146	< 0.001	55531	ELMOD1
-0.223189652	< 0.001	783	CACNB2
-0.223197371	< 0.001	9514	GAL3ST1
-0.223298773	< 0.001	147658	ZNF534
-0.223362297	< 0.001	2639	GCDH
-0.223466396	< 0.001	79149	ZSCAN5A
-0.223581269	< 0.001	112937	GLB1L3
-0.223621026	< 0.001	79716	NPEPL1
-0.223652691	< 0.001	58499	ZNF462
-0.223852813	< 0.001	22921	MSRB2
-0.223895535	< 0.001	341019	DCDC1
-0.223952278	< 0.001	345222	MSANTD1
-0.224009857	< 0.001	55384	MEG3
-0.224011064	< 0.001	727701	LINC00165
-0.224094823	< 0.001	128153	SPATA17
-0.224096492	< 0.001	1769	DNAH8
-0.224272013	< 0.001	146664	MGAT5B
-0.224282131	< 0.001	441124	LOC441124
-0.224341661	< 0.001	6863	TAC1
-0.22435011	< 0.001	4204	MECP2
-0.224351972	< 0.001	9617	MTRF1
-0.224363953	< 0.001	116841	SNAP47
-0.224376202	< 0.001	9619	ABCG1
-0.22443369	< 0.001	54212	SNTG1
-0.224492848	< 0.001	403277	OR5K3
-0.224560633	< 0.001	133022	TRAM1L1
-0.224606976	< 0.001	134466	ZNF300P1
-0.224622205	< 0.001	474344	GIMAP6
-0.224634379	< 0.001	11309	SLCO2B1
-0.224651366	< 0.001	147948	ZNF582
-0.224800229	< 0.001	5373	PMM2
-0.22484082	< 0.001	135228	CD109
-0.225001574	< 0.001	28514	DLL1
-0.225100562	< 0.001	84279	C2orf7
-0.225280628	< 0.001	6601	SMARCC2
-0.225387886	< 0.001	220004	C11orf66
-0.225392625	< 0.001	100240735	LOC100240735
-0.225432366	< 0.001	25907	TMEM158
-0.225534439	< 0.001	56121	PCDHB15
-0.225618005	< 0.001	442113	LOC442113
-0.225620061	< 0.001	64844	MARCHF7
-0.225629345	< 0.001	64857	PLEKHG2
-0.225700483	< 0.001	81626	C1orf14
-0.225723788	< 0.001	10432	RBM14
-0.225743011	< 0.001	9322	TRIP10
-0.225747466	< 0.001	729786	GOLGA8C
-0.225780159	< 0.001	220885	RPSAP15
-0.225819349	< 0.001	2794	GNL1
-0.225819528	< 0.001	84641	HIATL1
-0.225827798	< 0.001	6997	TDGF1
-0.225929111	< 0.001	56992	KIF15
-0.22594963	< 0.001	51046	ST8SIA3
-0.225974798	< 0.001	150274	HSCB
-0.226025134	< 0.001	256355	RPS2P32
-0.226145491	< 0.001	23097	CDC2L6
-0.22623232	< 0.001	10690	FUT9
-0.226245597	< 0.001	85235	HIST1H2AH
-0.226261199	< 0.001	57537	SORCS2
-0.226335406	< 0.001	55784	MCTP2
-0.226374865	< 0.001	147040	KCTD11

region-wise, subcortex					
Spearman's corr coef	p-val	p-val, SA	p-val, SA (BH- correction)	GenID	Gene
-0.28108108	0.096916698	0.128	1	7181	NR2C1
-0.28108108	0.096916698	0.11	1	9926	LPGAT1
-0.28108108	0.096916698	0.141	1	388677	NOTCH2NL
-0.281338483	0.096601352	0.14	1	4700	NDUFA6
-0.281338483	0.096601352	0.109	1	4940	OAS3
-0.281338483	0.096601352	0.14	1	9522	SCAMP1
-0.281338483	0.096601352	0.116	1	54520	CCDC93
-0.281338483	0.096601352	0.139	1	284273	ZADH2
-0.281595886	0.096286982	0.122	1	5132	PDC
-0.281595886	0.096286982	0.121	1	63892	THADA
-0.281595886	0.096286982	0.13	1	283487	LINC00346
-0.281853288	0.095973358	0.131	1	4162	MCAM
-0.281853288	0.095973358	0.105	1	10113	PREB
-0.281853288	0.095973358	0.133	1	54619	CCNJ
-0.281853288	0.095973358	0.122	1	84936	ZFYVE19
-0.282110661	0.09566053	0.125	1	6119	RPA3
-0.282110661	0.09566053	0.11	1	55251	PCMTD2
-0.282110661	0.09566053	0.146	1	55756	INTS9
-0.282110661	0.09566053	0.12	1	91775	NXPE3
-0.282110661	0.09566053	0.114	1	100128398	AC008969.2
-0.282110661	0.09566053	0.108	1	100506282	LOC100506282
-0.282368094	0.095348351	0.124	1	26505	CNNM3
-0.282368094	0.095348351	0.118	1	79598	CEP97
-0.282368094	0.095348351	0.125	1	84417	C2orf40
-0.282368094	0.095348351	0.14	1	728758	PIN4P1
-0.282625467	0.095037095	0.144	1	343	AQP8
-0.282625467	0.095037095	0.106	1	1998	ELF2
-0.282625467	0.095037095	0.11	1	55508	SLC35E3
-0.282625467	0.095037095	0.121	1	57020	C16orf62
-0.282625467	0.095037095	0.132	1	283102	KRT8P41
-0.282625467	0.095037095	0.132	1	347273	MURC
-0.282625467	0.095037095	0.114	1	388699	LINC00302
-0.282625467	0.095037095	0.111	1	401236	RP3-486B10.1
-0.282882899	0.094726659	0.125	1	23210	JMJD6
-0.282882899	0.094726659	0.111	1	643382	C14orf176
-0.283140272	0.094416983	0.114	1	684	BST2
-0.283140272	0.094416983	0.153	1	8399	PLA2G10
-0.283140272	0.094416983	0.118	1	9189	ZBED1
-0.283140272	0.094416983	0.129	1	10938	EHD1
-0.283140272	0.094416983	0.128	1	51148	CERCAM
-0.283140272	0.094416983	0.102	1	57038	RARS2
-0.283140272	0.094416983	0.11	1	201134	CCDC46
-0.283140272	0.094416983	0.099	1	404201	WDFY3-AS2
-0.283397675	0.094108082	0.133	1	969	CD69
-0.283397675	0.094108082	0.12	1	139285	FAM123B
-0.283655077	0.093799837	0.112	1	3441	IFNA4
-0.283655077	0.093799837	0.126	1	56125	PCDHB11
-0.283655077	0.093799837	0.113	1	79092	CARD14
-0.283655077	0.093799837	0.103	1	374920	C19orf68
-0.28391248	0.0934925	0.138	1	1201	CLN3
-0.28391248	0.0934925	0.103	1	2815	GP9
-0.28391248	0.0934925	0.128	1	7469	WHSC2
-0.28391248	0.0934925	0.098	1	7866	IFRD2
-0.28391248	0.0934925	0.114	1	10127	ZNF263
-0.28391248	0.0934925	0.129	1	54852	PAQR5
-0.28391248	0.0934925	0.112	1	64849	SLC13A3
-0.28391248	0.0934925	0.11	1	160298	C11orf42
-0.284169883	0.093185984	0.126	1	5648	MASP1
-0.284169883	0.093185984	0.116	1	57228	AC139768.2
-0.284169883	0.093185984	0.105	1	114792	KLHL32
-0.284169883	0.093185984	0.123	1	151827	LRRC34
-0.284169883	0.093185984	0.112	1	160760	PPTC7
-0.284427285	0.092880212	0.123	1	92259	MRPS36
-0.284684658	0.092575073	0.106	1	6625	SNRNP70
-0.284684658	0.092575073	0.11	1	26235	FBXL4
-0.284684658	0.092575073	0.13	1	27303	RBMS3
-0.284942091	0.092270859	0.143	1	6563	SLC14A1
-0.284942091	0.092270859	0.128	1	10478	SLC25A17
-0.284942091	0.092270859	0.105	1	84919	PPP1R15B
-0.284942091	0.092270859	0.12	1	126669	SHE
-0.285199463	0.091967456	0.12	1	3673	ITGA2
-0.285199463	0.091967456	0.12	1	23274	CLEC16A
-0.285199463	0.091967456	0.134	1	51477	ISYNA1
-0.285199463	0.091967456	0.128	1	192666	KRT24
-0.285456896	0.091664784	0.116	1	586	BCAT1
-0.285456896	0.091664784	0.121	1	1116	CHI3L1
-0.285456896	0.091664784	0.107	1	1786	DNMT1
-0.285456896	0.091664784	0.129	1	2309	FOXO3
-0.285456896	0.091664784	0.114	1	5455	POU3F3

voxel-wise, subcortex			
Spearman's corr coef	p-val	GenID	Gene
-0.226380527	< 0.001	54206	ERRFI1
-0.226451293	< 0.001	387119	C6orf204
-0.226456031	< 0.001	56969	RPL23AP32
-0.226480082	< 0.001	8986	RPS6KA4
-0.226495966	< 0.001	23520	ANP32C
-0.226498663	< 0.001	8290	HIST3H3
-0.226505071	< 0.001	57212	TP73-AS1
-0.226608142	< 0.001	64421	DCLRE1C
-0.226726085	< 0.001	5105	PCK1
-0.226900309	< 0.001	284009	LOC284009
-0.226942927	< 0.001	407975	MIR17HG
-0.227021396	< 0.001	814	CAMK4
-0.227077395	< 0.001	100132707	AC093726.2
-0.227090314	< 0.001	57472	CNOT6
-0.227199614	< 0.001	29091	STXBP6
-0.227221712	< 0.001	9332	CD163
-0.227230355	< 0.001	845	CASQ2
-0.227248833	< 0.001	89782	LMLN
-0.227273807	< 0.001	85443	DCLK3
-0.227318764	< 0.001	6176	RPLP1
-0.22753188	< 0.001	1297	COL9A1
-0.227535695	< 0.001	54514	DDX4
-0.227536812	< 0.001	100128398	AC008969.2
-0.227554262	< 0.001	1278	COL1A2
-0.227593079	< 0.001	375056	MIA3
-0.227638215	< 0.001	157695	C8orf42
-0.227640539	< 0.001	8233	ZRSR2
-0.227719381	< 0.001	6934	TCF7L2
-0.227791265	< 0.001	728978	RP11-790G19.2
-0.227819309	< 0.001	56241	SUSD2
-0.227885246	< 0.001	81571	C9orf45
-0.22803773	< 0.001	55184	C20orf12
-0.228108764	< 0.001	54891	INO80D
-0.228182048	< 0.001	23769	FLRT1
-0.228226244	< 0.001	401505	TOMM5
-0.228260979	< 0.001	84267	C9orf64
-0.228433713	< 0.001	27332	ZNF638
-0.22844699	< 0.001	23644	EDC4
-0.228500202	< 0.001	84191	FAM96A
-0.228552297	< 0.001	57412	AS3MT
-0.228701904	< 0.001	9786	KIAA0586
-0.228754833	< 0.001	388962	BOLA3
-0.228774801	< 0.001	23549	DNPEP
-0.22880201	< 0.001	55930	MYO5C
-0.228855133	< 0.001	79858	NEK11
-0.228913829	< 0.001	1537	CYC1
-0.228946701	< 0.001	648262	LOC648262
-0.228964627	< 0.001	100506144	RP11-244H3.1
-0.229022101	< 0.001	7917	BAT3
-0.229023784	< 0.001	23421	ITGB3BP
-0.229063705	< 0.001	84612	PARD6B
-0.22913745	< 0.001	3421	IDH3G
-0.229153231	< 0.001	140766	ADAMTS14
-0.229195207	< 0.001	131474	CHCHD4
-0.229219541	< 0.001	9842	PLEKHM1
-0.229251862	< 0.001	118856	MMP21
-0.229294017	< 0.001	2487	FRZB
-0.229518011	< 0.001	375341	C3orf62
-0.229525253	< 0.001	199221	DZIP1L
-0.229547083	< 0.001	26278	SACS
-0.229555815	< 0.001	79661	NEIL1
-0.229623511	< 0.001	928	CD9
-0.229710251	< 0.001	7046	TGFBR1
-0.229744703	< 0.001	10964	IFI44L
-0.229776919	< 0.001	51513	ETV7
-0.229844615	< 0.001	83450	LRRC48
-0.22994259	< 0.001	161003	STOML3
-0.23007381	< 0.001	130540	ALS2CR12
-0.230075017	< 0.001	154791	C7orf55
-0.230140865	< 0.001	57614	KIAA1468
-0.230158418	< 0.001	653436	ANKRD20A7P
-0.230176806	< 0.001	29102	RNASEN
-0.23019287	< 0.001	91304	C19orf6
-0.230196208	< 0.001	29075	RP5-1068E13.3
-0.230341181	< 0.001	7184	HSP90B1
-0.230354637	< 0.001	124790	HEXIM2
-0.230406746	< 0.001	9682	KDM4A
-0.230411842	< 0.001	55303	GIMAP4
-0.230547339	< 0.001	55824	PAG1

region-wise, subcortex					
Spearman's corr coef	p-val	p-val, SA	p-val, SA (BH- correction)	GenID	Gene
-0.285456896	0.091664784	0.123	1	7706	TRIM25
-0.285456896	0.091664784	0.111	1	11278	KLF12
-0.285456896	0.091664784	0.101	1	26952	SMR3A
-0.285456896	0.091664784	0.134	1	79823	C2orf34
-0.285456896	0.091664784	0.11	1	92291	CAPN13
-0.285456896	0.091664784	0.115	1	148932	MOBKL2C
-0.285456896	0.091664784	0.117	1	642597	C18orf42
-0.285714269	0.091362759	0.133	1	375743	PTAR1
-0.285971671	0.091061637	0.115	1	5925	RB1
-0.285971671	0.091061637	0.106	1	6510	SLC1A5
-0.285971671	0.091061637	0.118	1	126432	RINL
-0.285971671	0.091061637	0.116	1	338692	ANKRD13D
-0.286229074	0.090761289	0.113	1	622	BDH1
-0.286229074	0.090761289	0.132	1	5996	RGS1
-0.286229074	0.090761289	0.115	1	10521	DDX17
-0.286229074	0.090761289	0.126	1	64900	LPIN3
-0.286229074	0.090761289	0.117	1	257218	SHPRH
-0.286486477	0.090461738	0.118	1	5618	PRLR
-0.286486477	0.090461738	0.109	1	11013	TMSB15A
-0.286486477	0.090461738	0.127	1	55118	CRTAC1
-0.286743879	0.090162925	0.113	1	11025	LILRB3
-0.286743879	0.090162925	0.122	1	25777	UNC84B
-0.286743879	0.090162925	0.121	1	26027	ACOT11
-0.287001282	0.089864753	0.128	1	1028	CDKN1C
-0.287001282	0.089864753	0.112	1	7535	ZAP70
-0.287001282	0.089864753	0.106	1	84419	C15orf48
-0.287258685	0.089567475	0.096	1	2825	GPR1
-0.287258685	0.089567475	0.101	1	51116	MRPS2
-0.287258685	0.089567475	0.119	1	339324	ZNF260
-0.287516087	0.089270994	0.104	1	7841	MOGS
-0.287516087	0.089270994	0.115	1	11186	RASSF1
-0.28777349	0.088975251	0.14	1	1644	DDC
-0.28777349	0.088975251	0.109	1	2053	EPHX2
-0.28777349	0.088975251	0.116	1	9788	MTSS1
-0.28777349	0.088975251	0.112	1	54556	ING3
-0.28777349	0.088975251	0.123	1	57696	DDX55
-0.28777349	0.088975251	0.109	1	134285	TMEM171
-0.28777349	0.088975251	0.135	1	140700	SAMD10
-0.288030893	0.088680126	0.119	1	2569	GABRR1
-0.288030893	0.088680126	0.132	1	6897	TARS
-0.288030893	0.088680126	0.112	1	23111	SPG20
-0.288030893	0.088680126	0.106	1	60495	HPSE2
-0.288030893	0.088680126	0.124	1	90342	FER1L5
-0.288030893	0.088680126	0.116	1	342908	ZNF404
-0.288288295	0.088385902	0.087	1	2020	EN2
-0.288288295	0.088385902	0.092	1	9214	FAIM3
-0.288288295	0.088385902	0.116	1	56269	IRGC
-0.288288295	0.088385902	0.093	1	64784	CRTC3
-0.288288295	0.088385902	0.113	1	255738	PCSK9
-0.288545668	0.088092476	0.108	1	10766	TOB2
-0.288545668	0.088092476	0.113	1	115330	GPR146
-0.288545668	0.088092476	0.125	1	285525	YIPF7
-0.288545668	0.088092476	0.109	1	347732	CATSPER3
-0.288803101	0.087799765	0.128	1	3791	KDR
-0.288803101	0.087799765	0.11	1	9425	CDYL
-0.288803101	0.087799765	0.111	1	10244	RABEPK
-0.288803101	0.087799765	0.121	1	132204	SYNPR
-0.288803101	0.087799765	0.115	1	389058	SP5
-0.289060473	0.087507695	0.118	1	648	BMI1
-0.289060473	0.087507695	0.1	1	3732	CD82
-0.289060473	0.087507695	0.108	1	90649	ZNF486
-0.289060473	0.087507695	0.124	1	286122	C8orf31
-0.289317906	0.087216504	0.101	1	26053	AUTS2
-0.289832681	0.086636424	0.12	1	8086	AAAS
-0.289832681	0.086636424	0.121	1	8354	HIST1H3I
-0.289832681	0.086636424	0.112	1	8552	INE1
-0.289832681	0.086636424	0.11	1	84336	TMEM101
-0.289832681	0.086636424	0.109	1	129446	XIRP2
-0.289832681	0.086636424	0.113	1	149685	ADIG
-0.289832681	0.086636424	0.111	1	196385	DNAH10
-0.289832681	0.086636424	0.124	1	100128302	LOC100128302
-0.290090084	0.086347491	0.118	1	1306	COL15A1
-0.290090084	0.086347491	0.11	1	2852	GPER
-0.290090084	0.086347491	0.119	1	8839	WISP2
-0.290090084	0.086347491	0.102	1	51272	BET1L
-0.290090084	0.086347491	0.118	1	79174	CRELD2
-0.290090084	0.086347491	0.098	1	440905	AC018865.8
-0.290347487	0.08605919	0.115	1	83985	SPNS1
-0.290347487	0.08605919	0.124	1	91543	RSAD2

voxel-wise, subcortex			
Spearman's corr coef	p-val	GenID	Gene
-0.230556816	< 0.001	100130934	ZNF663
-0.230571941	< 0.001	9717	SEC14L5
-0.230572134	< 0.001	66008	TRAK2
-0.230601668	< 0.001	10766	TOB2
-0.230612338	< 0.001	84271	POLDIP3
-0.230624974	< 0.001	222256	CTA-351J1.1
-0.230667502	< 0.001	10733	PLK4
-0.230815902	< 0.001	83888	FGFBP2
-0.230852127	< 0.001	7733	ZNF180
-0.230879426	< 0.001	84914	ZNF587
-0.230934873	< 0.001	4286	MITF
-0.230984926	< 0.001	51171	HSD17B14
-0.231075004	< 0.001	84836	ABHD14B
-0.231108069	< 0.001	55112	WDR60
-0.231109366	< 0.001	56113	PCDHGA2
-0.231158584	< 0.001	55504	TNFRSF19
-0.231162578	< 0.001	7536	SF1
-0.231271416	< 0.001	80830	APOL6
-0.231333166	< 0.001	3669	ISG20
-0.231338933	< 0.001	6426	SRSF1
-0.231421947	< 0.001	6890	TAP1
-0.231431231	< 0.001	402573	C7orf61
-0.231468201	< 0.001	6461	SHB
-0.231520757	< 0.001	84988	PPP1R16A
-0.231528565	< 0.001	146050	ZSCAN29
-0.231535241	< 0.001	4798	NFRKB
-0.231553078	< 0.001	153562	MARVELD2
-0.231553257	< 0.001	401082	FLJ25363
-0.231608987	< 0.001	440957	C3orf78
-0.231681973	< 0.001	284001	CCDC57
-0.231687739	< 0.001	3172	HNF4A
-0.231744006	< 0.001	10267	RAMP1
-0.231792107	< 0.001	440145	C13orf37
-0.231853217	< 0.001	8641	PCDHGB4
-0.231913775	< 0.001	55472	C8orf39
-0.232004032	< 0.001	306	ANXA3
-0.232014433	< 0.001	55160	ARHGEF10L
-0.232040524	< 0.001	1299	COL9A3
-0.232186511	< 0.001	79025	C20orf195
-0.232241958	< 0.001	347902	AMIGO2
-0.232391849	< 0.001	55449	AL136419.1
-0.232452855	< 0.001	79789	CLMN
-0.232480809	< 0.001	7539	ZFP37
-0.232482105	< 0.001	94122	SYTL5
-0.232527792	< 0.001	4215	MAP3K3
-0.23259002	< 0.001	79269	WDR32
-0.232683808	< 0.001	1024	CDK8
-0.232725695	< 0.001	90423	ATP6V1E2
-0.23290205	< 0.001	10309	CCNO
-0.232943833	< 0.001	10850	CCL27
-0.232982382	< 0.001	1558	CYP2C8
-0.233014792	< 0.001	340596	LHFPL1
-0.233016461	< 0.001	24146	CLDN15
-0.233016461	< 0.001	222223	KIAA1324L
-0.233037069	< 0.001	129881	C2orf77
-0.23305741	< 0.001	79591	C10orf76
-0.233120278	< 0.001	5954	RCN1
-0.233163744	< 0.001	653188	GUSBP3
-0.23319681	< 0.001	196441	ZFC3H1
-0.233231068	< 0.001	3693	ITGB5
-0.233232558	< 0.001	128312	HIST3H2BB
-0.233241573	< 0.001	23087	TRIM35
-0.233282059	< 0.001	445347	TARP
-0.233333126	< 0.001	6152	RPL24
-0.233348921	< 0.001	80154	AGSK1
-0.233423218	< 0.001	644150	WIPF3
-0.233443648	< 0.001	28969	BZW2
-0.233506322	< 0.001	8473	OGT
-0.233534649	< 0.001	60437	CDH26
-0.233593524	< 0.001	391267	C21orf81
-0.233640701	< 0.001	9896	FIG4
-0.233661234	< 0.001	728724	RP11-1057N3.1
-0.233664662	< 0.001	9111	NMI
-0.233705252	< 0.001	4818	NKG7
-0.233717233	< 0.001	23435	TARDBP
-0.23373273	< 0.001	375337	C3orf77
-0.233739972	< 0.001	8772	FADD
-0.233750105	< 0.001	89122	TRIM4
-0.23378326	< 0.001	11339	OIP5

region-wise, subcortex					
Spearman's corr coef	p-val	p-val, SA	p-val, SA (BH- correction)	GenID	Gene
-0.290604889	0.085771762	0.111	1	1311	COMP
-0.290604889	0.085771762	0.124	1	3759	KCNJ2
-0.290604889	0.085771762	0.103	1	56674	TMEM9B
-0.290604889	0.085771762	0.108	1	285154	CYP1B1-AS1
-0.290862292	0.085485116	0.115	1	1634	DCN
-0.290862292	0.085485116	0.095	1	3661	IRF3
-0.290862292	0.085485116	0.129	1	5729	PTGDR
-0.290862292	0.085485116	0.115	1	64080	RBKS
-0.290862292	0.085485116	0.093	1	375061	FAM89A
-0.291119665	0.085199192	0.115	1	8994	LIMD1
-0.291119665	0.085199192	0.126	1	10449	ACAA2
-0.291119665	0.085199192	0.127	1	54976	C20orf27
-0.291119665	0.085199192	0.093	1	57061	HYMAI
-0.291119665	0.085199192	0.114	1	57687	VAT1L
-0.291119665	0.085199192	0.135	1	84281	C2orf88
-0.291119665	0.085199192	0.098	1	85377	MICALL1
-0.291377097	0.084913887	0.109	1	6182	MRPL12
-0.291377097	0.084913887	0.097	1	27148	STK36
-0.291377097	0.084913887	0.098	1	55342	STRBP
-0.291377097	0.084913887	0.117	1	55601	AC068989.2
-0.291377097	0.084913887	0.095	1	131474	CHCHD4
-0.291377097	0.084913887	0.109	1	145748	LYSMD4
-0.29163447	0.084629454	0.128	1	836	CASP3
-0.29163447	0.084629454	0.116	1	2981	GUCA2B
-0.29163447	0.084629454	0.096	1	8473	OGT
-0.291891903	0.084345795	0.107	1	7011	TEP1
-0.291891903	0.084345795	0.093	1	9311	ACCN3
-0.291891903	0.084345795	0.114	1	10234	LRRC17
-0.291891903	0.084345795	0.111	1	23085	ERC1
-0.291891903	0.084345795	0.118	1	29950	SERTAD1
-0.291891903	0.084345795	0.112	1	283652	SLC24A5
-0.292149276	0.084062852	0.112	1	1521	CTSW
-0.292149276	0.084062852	0.104	1	6374	CXCL5
-0.292149276	0.084062852	0.113	1	81857	MED25
-0.292149276	0.084062852	0.104	1	93343	FAM125A
-0.292149276	0.084062852	0.11	1	254552	NUDT8
-0.292149276	0.084062852	0.113	1	375719	AQP7P1
-0.292149276	0.084062852	0.092	1	400512	AC106739.1
-0.292406678	0.083780527	0.113	1	10455	PECI
-0.292406678	0.083780527	0.129	1	59352	LGR6
-0.292406678	0.083780527	0.109	1	84876	ORAI1
-0.292406678	0.083780527	0.111	1	376693	RPS10P7
-0.292406678	0.083780527	0.102	1	386681	KRTAP10-8
-0.292406678	0.083780527	0.1	1	100289574	AC133485.2
-0.292664081	0.083499067	0.121	1	10871	CD300C
-0.292664081	0.083499067	0.101	1	11023	VAX1
-0.292664081	0.083499067	0.117	1	54567	DLL4
-0.292664081	0.083499067	0.085	1	203562	TMEM31
-0.292921484	0.083218336	0.101	1	1073	CFL2
-0.292921484	0.083218336	0.102	1	2250	FGF5
-0.292921484	0.083218336	0.116	1	5187	PER1
-0.292921484	0.083218336	0.093	1	5412	UBL3
-0.292921484	0.083218336	0.114	1	7559	ZNF12
-0.292921484	0.083218336	0.104	1	81931	ZNF93
-0.292921484	0.083218336	0.105	1	148022	TICAM1
-0.293178886	0.082938388	0.088	1	506	ATP5B
-0.293178886	0.082938388	0.109	1	10263	CDK2AP2
-0.293178886	0.082938388	0.111	1	66008	TRAK2
-0.293178886	0.082938388	0.11	1	91833	WDR20
-0.293178886	0.082938388	0.103	1	114805	GALNT13
-0.293178886	0.082938388	0.084	1	729859	LOC729859
-0.293436289	0.08265914	0.094	1	11309	SLCO2B1
-0.293436289	0.08265914	0.113	1	79990	PLEKHH3
-0.293436289	0.08265914	0.116	1	84108	PCGF6
-0.293693662	0.082380503	0.113	1	93380	MMGT1
-0.293693662	0.082380503	0.117	1	136259	KLF14
-0.293951094	0.082102731	0.122	1	2793	GNGT2
-0.293951094	0.082102731	0.1	1	7827	NPHS2
-0.293951094	0.082102731	0.12	1	9855	FARP2
-0.293951094	0.082102731	0.1	1	9967	THRAP3
-0.293951094	0.082102731	0.12	1	27019	DNAI1
-0.293951094	0.082102731	0.123	1	431707	LHX8
-0.294208497	0.081825696	0.108	1	2006	ELN
-0.294208497	0.081825696	0.121	1	5914	RARA
-0.294208497	0.081825696	0.103	1	6494	SIPA1
-0.294208497	0.081825696	0.114	1	8346	HIST1H2BI
-0.294208497	0.081825696	0.1	1	79269	WDR32
-0.294465899	0.081549414	0.106	1	81575	APOLD1
-0.294465899	0.081549414	0.104	1	89848	FCHSD1

voxel-wise, subcortex			
Spearman's corr coef	p-val	GenID	Gene
-0.233840555	< 0.001	54799	MBTD1
-0.233903974	< 0.001	23353	UNC84A
-0.233917817	< 0.001	10147	SFRS14
-0.233955979	< 0.001	126231	ZNF573
-0.233980775	< 0.001	55224	ETNK2
-0.23404941	< 0.001	147837	ZNF563
-0.234185264	< 0.001	8603	C4orf8
-0.234218702	< 0.001	58494	JAM2
-0.234375	< 0.001	284434	NWD1
-0.234458476	< 0.001	113235	SLC46A1
-0.234459966	< 0.001	100132406	NBPF10
-0.234476864	< 0.001	285961	SEPT7L
-0.234532595	< 0.001	1627	DBN1
-0.23459053	< 0.001	23412	COMMD3
-0.234652668	< 0.001	57545	CC2D2A
-0.234757051	< 0.001	653583	PHLDB3
-0.234763727	< 0.001	26608	TBL2
-0.234775439	< 0.001	130074	FAM168B
-0.234813511	< 0.001	6279	S100A8
-0.234829575	< 0.001	115560	ZNF501
-0.234914824	< 0.001	401507	FAM74A1
-0.234962553	< 0.001	9924	PAN2
-0.234962657	< 0.001	116444	GRIN3B
-0.235007226	< 0.001	2012	EMP1
-0.235061646	< 0.001	284297	AC008735.3
-0.235067129	< 0.001	6223	RPS19
-0.235075757	< 0.001	153643	FAM81B
-0.235112265	< 0.001	642954	RBBP4P1
-0.235259816	< 0.001	51426	POLK
-0.235265955	< 0.001	30832	ZNF354C
-0.235271901	< 0.001	339122	RAB43
-0.235297248	< 0.001	23111	SPG20
-0.235365227	< 0.001	64285	RHBDF1
-0.235404968	< 0.001	81796	SLCO5A1
-0.235439152	< 0.001	55304	SPTLC3
-0.23548159	< 0.001	5463	POU6F1
-0.235523105	< 0.001	145508	C14orf145
-0.235601202	< 0.001	3891	KRT85
-0.235905334	< 0.001	2624	GATA2
-0.236070171	< 0.001	7027	TFDP1
-0.23610723	< 0.001	658	BMPR1B
-0.236117721	< 0.001	399815	LOC399815
-0.236255527	< 0.001	7181	NR2C1
-0.236311987	< 0.001	55689	YEATS2
-0.236363351	< 0.001	3148	HMGB2
-0.236411273	< 0.001	79897	RPP21
-0.236452967	< 0.001	100009676	LOC100009676
-0.236470327	< 0.001	728758	PIN4P1
-0.236568585	< 0.001	51596	CUTA
-0.236581206	< 0.001	54014	BRWD1
-0.236600339	< 0.001	23164	MPRIP
-0.236677602	< 0.001	55756	INTS9
-0.2366869	< 0.001	6147	RPL23A
-0.236708254	< 0.001	93377	OPALIN
-0.236742049	< 0.001	29763	PACSIN3
-0.23674652	< 0.001	84933	C8orf76
-0.236804366	< 0.001	79598	CEP97
-0.236884788	< 0.001	95	ACY1
-0.236951381	< 0.001	55876	GSDMB
-0.237024456	< 0.001	116159	CYYR1
-0.237080082	< 0.001	6206	RPS12
-0.237108782	< 0.001	5018	OXA1L
-0.237163946	< 0.001	4112	MAGEB1
-0.23717685	< 0.001	283507	SUGT1L1
-0.237187997	< 0.001	347273	MURC
-0.237190038	< 0.001	64745	METT11D1
-0.237267405	< 0.001	2137	EXTL3
-0.237442449	< 0.001	7768	ZNF225
-0.237541348	< 0.001	1059	CENPB
-0.237547755	< 0.001	80017	C14orf159
-0.23756057	< 0.001	85236	HIST1H2BK
-0.237755597	< 0.001	169834	ZNF883
-0.237768963	< 0.001	22838	RNF44
-0.23783806	< 0.001	213	ALB
-0.237911791	< 0.001	653162	RPSAP9
-0.237929255	< 0.001	90768	MGC45800
-0.237940207	< 0.001	305	ANXA2P3
-0.237949505	< 0.001	57727	NCOA5
-0.237967789	< 0.001	55512	SMPD3

region-wise, subcortex					
Spearman's corr coef	p-val	p-val, SA	p-val, SA (BH- correction)	GenID	Gene
-0.294465899	0.081549414	0.089	1	90268	FAM105B
-0.294723302	0.081273712	0.1	1	2323	FLT3LG
-0.294723302	0.081273712	0.096	1	3373	HYAL1
-0.294723302	0.081273712	0.124	1	6781	STC1
-0.294723302	0.081273712	0.105	1	9518	GDF15
-0.294723302	0.081273712	0.112	1	27233	SULT1C4
-0.294723302	0.081273712	0.122	1	125981	ACER1
-0.294723302	0.081273712	0.128	1	493869	GPX8
-0.294723302	0.081273712	0.106	1	644943	RASSF10
-0.294980675	0.080998868	0.116	1	3026	HABP2
-0.294980675	0.080998868	0.097	1	5334	PLCL1
-0.294980675	0.080998868	0.113	1	6345	SRL
-0.294980675	0.080998868	0.106	1	6468	FBXW4
-0.294980675	0.080998868	0.101	1	10712	C1orf2
-0.294980675	0.080998868	0.125	1	26509	MYOF
-0.294980675	0.080998868	0.113	1	140732	SPAG4L
-0.294980675	0.080998868	0.108	1	192670	EIF2C4
-0.295238107	0.080724746	0.086	1	6446	SGK1
-0.295238107	0.080724746	0.103	1	266623	SLC25A5P1
-0.295238107	0.080724746	0.108	1	728450	AL080274.2
-0.29549548	0.080451362	0.098	1	5315	PKM2
-0.29549548	0.080451362	0.113	1	10964	IFI44L
-0.29549548	0.080451362	0.118	1	259230	SGMS1
-0.295752913	0.08017873	0.091	1	2317	FLNB
-0.295752913	0.08017873	0.103	1	4323	MMP14
-0.295752913	0.08017873	0.104	1	127343	DMBX1
-0.296010286	0.079906672	0.112	1	51599	LSR
-0.296010286	0.079906672	0.099	1	55066	PDPR
-0.296010286	0.079906672	0.113	1	80709	AKNA
-0.296267688	0.079635456	0.083	1	971	CD72
-0.296267688	0.079635456	0.117	1	202459	OSTCP1
-0.296525091	0.07936497	0.1	1	4245	MGAT1
-0.296525091	0.07936497	0.099	1	9879	DDX46
-0.296525091	0.07936497	0.102	1	28955	DEXI
-0.296525091	0.07936497	0.11	1	80067	C2orf37
-0.296525091	0.07936497	0.08	1	115992	RNF166
-0.296525091	0.07936497	0.106	1	392309	OR13J1
-0.296782494	0.079095192	0.086	1	467	ATF3
-0.296782494	0.079095192	0.113	1	3164	NR4A1
-0.296782494	0.079095192	0.087	1	50840	TAS2R14
-0.296782494	0.079095192	0.098	1	54742	LY6K
-0.296782494	0.079095192	0.091	1	56898	BDH2
-0.297039896	0.078826062	0.09	1	126069	ZNF491
-0.297297299	0.07855773	0.089	1	975	CD81
-0.297297299	0.07855773	0.12	1	10418	SPON1
-0.297297299	0.07855773	0.113	1	387273	KRTAP5-10
-0.297554672	0.07829012	0.093	1	3257	HPS1
-0.297554672	0.07829012	0.114	1	27146	FAM184B
-0.297554672	0.07829012	0.12	1	57653	LOC100499484-C9ORF174
-0.297554672	0.07829012	0.104	1	112937	GLB1L3
-0.297812104	0.078023225	0.111	1	2833	CXCR3
-0.297812104	0.078023225	0.107	1	55075	UACA
-0.297812104	0.078023225	0.114	1	117286	CIB3
-0.297812104	0.078023225	0.107	1	389158	PLSCR5
-0.298069477	0.077756964	0.088	1	1763	DNA2
-0.298069477	0.077756964	0.1	1	4701	NDUFA7
-0.298069477	0.077756964	0.098	1	138716	C9orf23
-0.29832691	0.077491492	0.115	1	25	ABL1
-0.29832691	0.077491492	0.106	1	3357	HTR2B
-0.29832691	0.077491492	0.102	1	7078	TIMP3
-0.29832691	0.077491492	0.095	1	54903	MKS1
-0.298584282	0.077226743	0.099	1	1294	COL7A1
-0.298584282	0.077226743	0.108	1	28514	DLL1
-0.298584282	0.077226743	0.096	1	51198	C9orf53
-0.298584282	0.077226743	0.089	1	79714	CCDC51
-0.298584282	0.077226743	0.09	1	144811	C13orf31
-0.298841685	0.076962702	0.103	1	277	AMY1B
-0.298841685	0.076962702	0.099	1	5281	PIGF
-0.298841685	0.076962702	0.096	1	23310	NCAPD3
-0.298841685	0.076962702	0.115	1	283165	PRR10
-0.299099088	0.076699376	0.09	1	4487	MSX1
-0.299099088	0.076699376	0.11	1	83605	CCM2
-0.299099088	0.076699376	0.094	1	91748	C14orf43
-0.299099088	0.076699376	0.098	1	352909	C19orf51
-0.29935649	0.076436669	0.089	1	3192	HNRNPU
-0.299613893	0.076174766	0.1	1	3913	LAMB2
-0.299613893	0.076174766	0.097	1	4248	MGAT3
-0.299613893	0.076174766	0.1	1	9699	RIMS2
-0.299613893	0.076174766	0.117	1	11098	AP000654.2

voxel-wise, subcortex			
Spearman's corr coef	p-val	GenID	Gene
-0.2382541	< 0.001	3013	HIST1H2AD
-0.238306105	< 0.001	4625	MYH7
-0.238472521	< 0.001	56906	THAP10
-0.238577738	< 0.001	284998	AC068057.2
-0.238630578	< 0.001	219670	ENKUR
-0.238630861	< 0.001	10516	FBLN5
-0.238644227	< 0.001	400713	AC010332.1
-0.238796249	< 0.001	9197	SLC33A1
-0.238829687	< 0.001	93273	LEMD1
-0.238839895	< 0.001	6096	RORB
-0.23888281	< 0.001	27163	NAAA
-0.238901377	< 0.001	3642	INSM1
-0.238973349	< 0.001	3855	KRT7
-0.239051819	< 0.001	65095	KRI1
-0.239112929	< 0.001	4438	MSH4
-0.239295214	< 0.001	80142	PTGES2
-0.239304513	< 0.001	1390	CREM
-0.239322245	< 0.001	79002	C19orf43
-0.239324942	< 0.001	5764	PTN
-0.23944734	< 0.001	26355	FAM162A
-0.239574462	< 0.001	10858	CYP46A1
-0.239597127	< 0.001	23150	FRMD4B
-0.239752129	< 0.001	10803	CCR9
-0.239777282	< 0.001	10412	NSA2
-0.239790291	< 0.001	134701	C6orf59
-0.239846751	< 0.001	283446	MYO1H
-0.239939436	< 0.001	6258	RXRG
-0.239940271	< 0.001	115294	PCMTD1
-0.240020975	< 0.001	9429	ABCG2
-0.240021348	< 0.001	55200	PLEKHG6
-0.240096569	< 0.001	116966	WDR17
-0.240101859	< 0.001	81035	COLEC12
-0.240205958	< 0.001	57122	NUP107
-0.240226761	< 0.001	51326	ARL17P1
-0.240282938	< 0.001	6119	RPA3
-0.240390942	< 0.001	3187	HNRNPH1
-0.240392059	< 0.001	100132724	DCAF13P3
-0.240398377	< 0.001	26609	VCX
-0.240556061	< 0.001	84283	TMEM79
-0.240674004	< 0.001	9724	UTP14C
-0.240793064	< 0.001	283999	AC087645.1
-0.240819156	< 0.001	217	ALDH2
-0.240889639	< 0.001	10140	TOB1
-0.240922049	< 0.001	80178	C16orf59
-0.240965232	< 0.001	55794	DDX28
-0.240992814	< 0.001	3250	HPR
-0.241176784	< 0.001	57604	KIAA1456
-0.24127838	< 0.001	7579	ZSCAN20
-0.2413757	< 0.001	127700	C1orf102
-0.241399661	< 0.001	10747	MASP2
-0.241506457	< 0.001	56101	PCDHGB5
-0.24152112	< 0.001	163051	ZNF709
-0.241552427	< 0.001	56944	OLFML3
-0.241605818	< 0.001	254394	C6orf61
-0.241745308	< 0.001	79813	EHMT1
-0.241762206	< 0.001	92211	PCDH21
-0.241828695	< 0.001	150709	ANKAR
-0.241835013	< 0.001	8082	SSPN
-0.241876245	< 0.001	79768	C15orf29
-0.241962895	< 0.001	55332	DRAM1
-0.24203226	< 0.001	3613	IMPA2
-0.242173597	< 0.001	6618	SNAPC2
-0.242263317	< 0.001	729440	CCDC61
-0.242299348	< 0.001	9508	ADAMTS3
-0.242336214	< 0.001	9869	SETDB1
-0.242470592	< 0.001	55636	CHD7
-0.242522031	< 0.001	57291	SNHG13
-0.242812708	< 0.001	9120	SLC16A6
-0.242916346	< 0.001	6666	SOX12
-0.242944762	< 0.001	84759	PCGF1
-0.242989808	< 0.001	400986	ANKRD36C
-0.24306345	< 0.001	10233	LRRC23
-0.243111268	< 0.001	4354	MPP1
-0.243195131	< 0.001	8228	PNPLA4
-0.243195221	< 0.001	29122	TSP50
-0.243220195	< 0.001	642475	C8orf73
-0.243257165	< 0.001	178	AGL
-0.243286878	< 0.001	836	CASP3
-0.243532792	< 0.001	79154	DHRS11

region-wise, subcortex					
Spearman's corr coef	p-val	p-val, SA	p-val, SA (BH- correction)	GenID	Gene
-0.299613893	0.076174766	0.093	1	11176	BAZ2A
-0.299613893	0.076174766	0.104	1	64412	GZF1
-0.299613893	0.076174766	0.098	1	131566	DCBLD2
-0.299871296	0.075913556	0.093	1	1579	CYP4A11
-0.299871296	0.075913556	0.091	1	27068	PPA2
-0.299871296	0.075913556	0.106	1	79037	PVRIG
-0.299871296	0.075913556	0.105	1	79132	DHX58
-0.299871296	0.075913556	0.106	1	81542	TMX1
-0.300128669	0.075653061	0.097	1	4818	NKG7
-0.300128669	0.075653061	0.087	1	65065	NBEAL1
-0.300386101	0.075393155	0.109	1	1831	TSC22D3
-0.300386101	0.075393155	0.11	1	9540	TP53I3
-0.300386101	0.075393155	0.097	1	25816	TNFAIP8
-0.300386101	0.075393155	0.103	1	84054	PCDHB19P
-0.300643474	0.075134091	0.104	1	8335	HIST1H2AB
-0.300643474	0.075134091	0.1	1	9314	KLF4
-0.300643474	0.075134091	0.091	1	9976	CLEC2B
-0.300643474	0.075134091	0.099	1	23457	ABCB9
-0.300900906	0.074875712	0.095	1	1060	CENPC1
-0.300900906	0.074875712	0.1	1	1912	PHC2
-0.300900906	0.074875712	0.096	1	26716	OR2H1
-0.300900906	0.074875712	0.108	1	339059	AC138028.2
-0.300900906	0.074875712	0.109	1	391475	DYTN
-0.301158309	0.074618027	0.097	1	54505	DHX29
-0.301158309	0.074618027	0.098	1	58516	FAM60A
-0.301158309	0.074618027	0.099	1	90522	YIF1B
-0.301158309	0.074618027	0.106	1	93587	RG9MTD2
-0.301158309	0.074618027	0.085	1	147798	TMC4
-0.301158309	0.074618027	0.103	1	166793	ZNF509
-0.301415682	0.074360922	0.114	1	987	LRBA
-0.301415682	0.074360922	0.1	1	4758	NEU1
-0.301415682	0.074360922	0.102	1	201514	ZNF584
-0.301673114	0.074104637	0.096	1	341	APOC1
-0.301673114	0.074104637	0.091	1	4728	NDUFS8
-0.301673114	0.074104637	0.087	1	51537	MTP18
-0.301673114	0.074104637	0.091	1	56944	OLFML3
-0.301673114	0.074104637	0.091	1	80704	SLC19A3
-0.301930487	0.073849075	0.103	1	8123	PAR5
-0.301930487	0.073849075	0.119	1	11067	C10orf10
-0.301930487	0.073849075	0.105	1	11313	LYPLA2
-0.301930487	0.073849075	0.095	1	90427	BMF
-0.301930487	0.073849075	0.119	1	159963	SLC5A12
-0.30218792	0.073594183	0.103	1	1797	DOM3Z
-0.30218792	0.073594183	0.098	1	3250	HPR
-0.30218792	0.073594183	0.086	1	4790	NFKB1
-0.30218792	0.073594183	0.098	1	6487	ST3GAL3
-0.30218792	0.073594183	0.097	1	8603	C4orf8
-0.30218792	0.073594183	0.098	1	23619	ZIM2
-0.30218792	0.073594183	0.072	1	55802	DCP1A
-0.30218792	0.073594183	0.09	1	124404	SEPTIN12
-0.302445292	0.073339984	0.104	1	4584	MUC3A
-0.302445292	0.073339984	0.092	1	5836	PYGL
-0.302445292	0.073339984	0.095	1	55787	CXorf15
-0.302445292	0.073339984	0.09	1	124925	SEZ6
-0.302445292	0.073339984	0.089	1	100303728	RP3-404F18.2
-0.302702695	0.073086366	0.089	1	7162	TPBG
-0.302702695	0.073086366	0.109	1	132243	H1FOO
-0.302960098	0.072833553	0.094	1	595	CCND1
-0.302960098	0.072833553	0.085	1	6631	SNRPC
-0.302960098	0.072833553	0.099	1	10741	RBBP9
-0.302960098	0.072833553	0.11	1	441204	LOC441204
-0.3032175	0.072581463	0.101	1	5433	POLR2D
-0.3032175	0.072581463	0.094	1	5872	RAB13
-0.3032175	0.072581463	0.087	1	7069	THRSP
-0.3032175	0.072581463	0.088	1	55006	TRMT61B
-0.3032175	0.072581463	0.093	1	55020	TTC38
-0.3032175	0.072581463	0.108	1	80069	C6ORF208
-0.3032175	0.072581463	0.086	1	255812	SDHAP1
-0.3032175	0.072581463	0.084	1	390664	C1QTNF8
-0.303474903	0.072330035	0.123	1	5816	PVALB
-0.303474903	0.072330035	0.087	1	6886	TAL1
-0.303474903	0.072330035	0.116	1	11343	MGLL
-0.303474903	0.072330035	0.107	1	57506	MAVS
-0.303474903	0.072330035	0.099	1	140828	LINC00261
-0.303474903	0.072330035	0.102	1	283349	RASSF3
-0.303732306	0.072079182	0.114	1	753	C18orf1
-0.303732306	0.072079182	0.107	1	3672	ITGA1
-0.303732306	0.072079182	0.108	1	9617	MTRF1
-0.303732306	0.072079182	0.078	1	83657	DYNLRB2

voxel-wise, subcortex			
Spearman's corr coef	p-val	GenID	Gene
-0.243556559	< 0.001	51561	IL23A
-0.24358739	< 0.001	55607	PPP1R9A
-0.243623987	< 0.001	81856	ZNF611
-0.24368304	< 0.001	476	ATP1A1
-0.243753716	< 0.001	60681	FKBP10
-0.243828192	< 0.001	339983	NAT8L
-0.243907779	< 0.001	54726	OTUD4
-0.243945211	< 0.001	23433	RHOQ
-0.243961826	< 0.001	51259	TMEM216
-0.243991733	< 0.001	25798	BRI3
-0.244008914	< 0.001	3136	HLA-H
-0.244069561	< 0.001	400512	AC106739.1
-0.244097412	< 0.001	10332	CLEC4M
-0.244108096	< 0.001	440180	LOC440180
-0.244126573	< 0.001	5959	RDH5
-0.244392827	< 0.001	207063	DHRSX
-0.244436845	< 0.001	7881	KCNAB1
-0.244488567	< 0.001	389136	VGLL3
-0.244493678	< 0.001	80778	ZNF34
-0.244654059	< 0.001	80709	AKNA
-0.244660929	< 0.001	283011	FLJ37201
-0.24467653	< 0.001	22	ABCB7
-0.244702533	< 0.001	730087	ZNF726
-0.244840339	< 0.001	5253	PHF2
-0.244857803	< 0.001	55120	FANCL
-0.244864672	< 0.001	5350	PLN
-0.244902477	< 0.001	7298	TYMS
-0.244911388	< 0.001	10291	SF3A1
-0.24495931	< 0.001	84897	TBRG1
-0.244998395	< 0.001	91833	WDR20
-0.245000809	< 0.001	7979	SHFM1
-0.245110214	< 0.001	1006	CDH8
-0.245141223	< 0.001	10780	ZNF234
-0.245157301	< 0.001	28999	KLF15
-0.245205313	< 0.001	57189	KIAA1147
-0.245286196	< 0.001	54435	HCG4
-0.245421961	< 0.001	8330	HIST1H2AK
-0.245531172	< 0.001	3441	IFNA4
-0.245538414	< 0.001	79846	C7orf63
-0.245672047	< 0.001	22981	NINL
-0.24567382	< 0.001	27156	RTDR1
-0.245767146	< 0.001	150759	LINC00342
-0.245770112	< 0.001	57716	PRX
-0.245837167	< 0.001	339145	FAM92B
-0.245899484	< 0.001	253769	WDR27
-0.245965883	< 0.001	7074	TIAM1
-0.246028557	< 0.001	59274	MESDC1
-0.246035531	< 0.001	10537	UBD
-0.246044993	< 0.001	338692	ANKRD13D
-0.246084288	< 0.001	1943	EFNA2
-0.246118739	< 0.001	285512	FAM13A-AS1
-0.246161088	< 0.001	64866	CDCP1
-0.246180579	< 0.001	5966	REL
-0.24622646	< 0.001	199223	TTC21A
-0.246394634	< 0.001	57104	PNPLA2
-0.246395662	< 0.001	3020	H3F3A
-0.246399745	< 0.001	29078	NDUFAF4
-0.246451467	< 0.001	23589	CARHSP1
-0.246452957	< 0.001	727	C5
-0.246472552	< 0.001	1956	EGFR
-0.246497259	< 0.001	729652	LOC729652
-0.246556506	< 0.001	5208	PFKFB2
-0.246565327	< 0.001	54587	MXRA8
-0.246699527	< 0.001	2676	GFRA3
-0.246711686	< 0.001	403341	ZBTB34
-0.246711776	< 0.001	400684	LOC400684
-0.246765643	< 0.001	80314	EPC1
-0.246975422	< 0.001	8464	SUPT3H
-0.247258663	< 0.001	1649	DDIT3
-0.247461021	< 0.001	80198	MUS81
-0.247667834	< 0.001	730	C7
-0.247696534	< 0.001	8324	FZD7
-0.247778535	< 0.001	120892	LRRK2
-0.247833878	< 0.001	488	ATP2A2
-0.247858495	< 0.001	4117	MAK
-0.247932032	< 0.001	729859	LOC729859
-0.247934446	< 0.001	56164	STK31
-0.248011723	< 0.001	9049	AIP
-0.248017758	< 0.001	64225	ATL2

region-wise, subcortex					
Spearman's corr coef	p-val	p-val, SA	p-val, SA (BH- correction)	GenID	Gene
-0.303732306	0.072079182	0.091	1	93556	EGFEM1P
-0.303989679	0.071829125	0.088	1	8284	KDM5D
-0.303989679	0.071829125	0.105	1	23007	PLCH1
-0.303989679	0.071829125	0.09	1	84436	ZNF528
-0.303989679	0.071829125	0.095	1	148398	SAMD11
-0.304247111	0.071579754	0.105	1	1301	COL11A1
-0.304247111	0.071579754	0.106	1	3399	ID3
-0.304247111	0.071579754	0.087	1	10481	HOXB13
-0.304247111	0.071579754	0.093	1	26292	MYCBP
-0.304504484	0.071331106	0.108	1	23354	HAUS5
-0.304504484	0.071331106	0.094	1	80341	BPIL1
-0.304504484	0.071331106	0.113	1	84720	PIGO
-0.304761916	0.071083002	0.102	1	23264	ZC3H7B
-0.304761916	0.071083002	0.091	1	26585	GREM1
-0.304761916	0.071083002	0.103	1	79005	SCNM1
-0.304761916	0.071083002	0.087	1	284058	KIAA1267
-0.304761916	0.071083002	0.07	1	100129480	LOC100129480
-0.305019289	0.07083568	0.086	1	2619	GAS1
-0.305019289	0.07083568	0.095	1	153562	MARVELD2
-0.305276692	0.070589051	0.096	1	5725	PTBP1
-0.305276692	0.070589051	0.096	1	24139	EML2
-0.305276692	0.070589051	0.107	1	27065	AC110814.1
-0.305276692	0.070589051	0.116	1	285888	CNPY1
-0.305534095	0.0703431	0.082	1	4066	LYL1
-0.305534095	0.0703431	0.109	1	5585	PKN1
-0.305534095	0.0703431	0.092	1	8190	MIA
-0.305534095	0.0703431	0.088	1	10803	CCR9
-0.305534095	0.0703431	0.113	1	51809	GALNT7
-0.305534095	0.0703431	0.094	1	55275	VPS53
-0.305534095	0.0703431	0.091	1	79695	GALNT12
-0.305791497	0.070097849	0.09	1	162	AP1B1
-0.305791497	0.070097849	0.11	1	1874	E2F4
-0.305791497	0.070097849	0.098	1	6382	SDC1
-0.3060489	0.069853142	0.094	1	11061	LECT1
-0.3060489	0.069853142	0.101	1	30813	VSX1
-0.3060489	0.069853142	0.096	1	91801	ALKBH8
-0.3060489	0.069853142	0.087	1	259234	DSCR10
-0.306306303	0.069609225	0.086	1	518	ATP5G3
-0.306306303	0.069609225	0.09	1	1181	CLCN2
-0.306306303	0.069609225	0.091	1	9580	SOX13
-0.306563675	0.069365986	0.084	1	215	ABCD1
-0.306563675	0.069365986	0.097	1	1827	RCAN1
-0.306563675	0.069365986	0.078	1	5916	RARG
-0.306563675	0.069365986	0.096	1	9640	ZNF592
-0.306821108	0.069123417	0.094	1	8464	SUPT3H
-0.306821108	0.069123417	0.104	1	408263	C5orf40
-0.307078481	0.06888143	0.084	1	4682	NUBP1
-0.307078481	0.06888143	0.095	1	7867	MAPKAPK3
-0.307078481	0.06888143	0.1	1	10721	POLQ
-0.307078481	0.06888143	0.099	1	25896	INTS7
-0.307078481	0.06888143	0.093	1	64324	NSD1
-0.307335913	0.06864021	0.083	1	57140	RNPEPL1
-0.307335913	0.06864021	0.081	1	729085	C3orf41
-0.307593286	0.06839966	0.072	1	2167	FABP4
-0.307593286	0.06839966	0.123	1	5243	ABCB1
-0.307593286	0.06839966	0.097	1	10782	ZNF274
-0.307593286	0.06839966	0.077	1	100132724	DCAF13P3
-0.307850689	0.068159774	0.104	1	51171	HSD17B14
-0.307850689	0.068159774	0.086	1	158405	KIAA1958
-0.308108121	0.067920446	0.103	1	83860	TAF3
-0.308108121	0.067920446	0.1	1	284440	LOC284440
-0.308365494	0.067681931	0.089	1	5122	PCSK1
-0.308622926	0.067444049	0.084	1	1138	CHRNA5
-0.308622926	0.067444049	0.092	1	80307	FER1L4
-0.308622926	0.067444049	0.085	1	84701	COX4I2
-0.308880299	0.067206822	0.074	1	926	CD8B
-0.308880299	0.067206822	0.084	1	8091	HMGA2
-0.308880299	0.067206822	0.091	1	339883	C3orf35
-0.308880299	0.067206822	0.088	1	387032	ZKSCAN4
-0.308880299	0.067206822	0.088	1	728378	A26C1B
-0.308880299	0.067206822	0.087	1	100288788	LOC100288788
-0.309137702	0.066970266	0.09	1	2679	GGT3P
-0.309137702	0.066970266	0.095	1	26164	GTPBP5
-0.309137702	0.066970266	0.072	1	285605	DTWD2
-0.309395105	0.066734269	0.103	1	4613	MYCN
-0.309395105	0.066734269	0.092	1	57017	COQ9
-0.309652507	0.066499062	0.081	1	5079	PAX5
-0.309652507	0.066499062	0.09	1	5557	PRIM1
-0.309652507	0.066499062	0.09	1	7385	UQCRC2

voxel-wise, subcortex			
Spearman's corr coef	p-val	GenID	Gene
-0.248083785	< 0.001	6487	ST3GAL3
-0.248153344	< 0.001	199777	ZNF626
-0.248155475	< 0.001	55857	PLK1S1
-0.248188257	< 0.001	54538	ROBO4
-0.248285666	< 0.001	8353	HIST1H3E
-0.248396918	< 0.001	283579	C14orf178
-0.248434067	< 0.001	23086	EXPH5
-0.248591751	< 0.001	133522	PPARGC1B
-0.248634472	< 0.001	11080	DNAJB4
-0.248634756	< 0.001	3265	HRAS
-0.2487019	< 0.001	2619	GAS1
-0.24872233	< 0.001	10380	BPNT1
-0.248749807	< 0.001	10891	PPARGC1A
-0.248796612	< 0.001	29937	NENF
-0.248813704	< 0.001	2294	FOXF1
-0.248843238	< 0.001	379025	RP11-349A22.5
-0.248857722	< 0.001	3151	HMGN2
-0.248889491	< 0.001	3426	CFI
-0.248890698	< 0.001	374618	TEX9
-0.248950586	< 0.001	54768	HYDIN
-0.249015972	< 0.001	441295	OR2A9P
-0.249042526	< 0.001	80853	JHDM1D
-0.249117106	< 0.001	27436	EML4
-0.249165118	< 0.001	9441	MED26
-0.249297917	< 0.001	166785	MMAA
-0.249386415	< 0.001	10560	SLC19A2
-0.249411479	< 0.001	23240	KIAA0922
-0.249429598	< 0.001	57106	NAT14
-0.249456435	< 0.001	64105	CENPK
-0.249467105	< 0.001	157657	C8orf37
-0.249553844	< 0.001	8339	HIST1H2BG
-0.249640405	< 0.001	23452	ANGPTL2
-0.249657765	< 0.001	3134	HLA-F
-0.249668449	< 0.001	414328	IDNK
-0.24970299	< 0.001	25909	AHCTF1
-0.249718592	< 0.001	23559	WBP1
-0.249727041	< 0.001	389075	RESP18
-0.24986653	< 0.001	6253	RTN2
-0.249885753	< 0.001	400617	KCNJ2-AS1
-0.249886766	< 0.001	2557	GABRA4
-0.249930143	< 0.001	388849	LOC388849
-0.249930233	< 0.001	79035	OBFC2B
-0.250006288	< 0.001	4676	NAP1L4
-0.250101477	< 0.001	440084	LOC440084
-0.250179768	< 0.001	100130357	LOC100130357
-0.250393182	< 0.001	161198	CLEC14A
-0.250396043	< 0.001	114625	ERMAP
-0.250499219	< 0.001	1962	EHHADH
-0.250516593	< 0.001	79415	C17orf62
-0.250577986	< 0.001	6432	SFRS7
-0.250656545	< 0.001	2895	GRID2
-0.250660539	< 0.001	6792	CDKL5
-0.250719041	< 0.001	5585	PKN1
-0.250741333	< 0.001	124751	KRBA2
-0.25078097	< 0.001	338557	GPR120
-0.250843763	< 0.001	124641	OVCA2
-0.250873953	< 0.001	94120	SYTL3
-0.25097841	< 0.001	84842	HPDL
-0.251042485	< 0.001	116255	MOGAT1
-0.251078069	< 0.001	26509	MYOF
-0.251142591	< 0.001	389852	SPACA5
-0.251206219	< 0.001	284948	SH2D6
-0.251260549	< 0.001	10484	SEC23A
-0.251268625	< 0.001	3912	LAMB1
-0.251426131	< 0.001	23619	ZIM2
-0.25145036	< 0.001	1856	DVL2
-0.251474589	< 0.001	9976	CLEC2B
-0.251510441	< 0.001	80219	COQ10B
-0.251531065	< 0.001	23318	ZCCHC11
-0.251667559	< 0.001	142684	RAB40A
-0.251704901	< 0.001	132	ADK
-0.251728117	< 0.001	79109	MAPKAP1
-0.251785606	< 0.001	26035	GLCE
-0.25180158	< 0.001	51455	REV1
-0.251886189	< 0.001	7275	TUB
-0.251886636	< 0.001	5329	PLAUR
-0.251896501	< 0.001	201163	FLCN
-0.252043396	< 0.001	84913	ATOH8
-0.252132177	< 0.001	729171	ANKRD20A8P

region-wise, subcortex					
Spearman's corr coef	p-val	p-val, SA	p-val, SA (BH- correction)	GenID	Gene
-0.30990991	0.066264495	0.085	1	9679	FAM53B
-0.30990991	0.066264495	0.102	1	26683	OR4F3
-0.30990991	0.066264495	0.092	1	55510	DDX43
-0.310167313	0.066030577	0.077	1	793	CALB1
-0.310167313	0.066030577	0.072	1	2444	FRK
-0.310167313	0.066030577	0.082	1	2886	GRB7
-0.310167313	0.066030577	0.086	1	4126	MANBA
-0.310167313	0.066030577	0.102	1	170506	DHX36
-0.310424685	0.065797217	0.079	1	23452	ANGPTL2
-0.310424685	0.065797217	0.1	1	55285	RBM41
-0.310424685	0.065797217	0.106	1	90204	ZSWIM1
-0.310682118	0.065564618	0.068	1	4239	MFAP4
-0.310682118	0.065564618	0.112	1	8772	FADD
-0.310682118	0.065564618	0.085	1	131578	LRRC15
-0.310939491	0.065332688	0.101	1	958	CD40
-0.310939491	0.065332688	0.096	1	7980	TFPI2
-0.310939491	0.065332688	0.065	1	8519	IFITM1
-0.310939491	0.065332688	0.093	1	255403	ZNF718
-0.311196923	0.0651014	0.082	1	869	CBLN1
-0.311196923	0.0651014	0.077	1	5245	PHB
-0.311454296	0.064870656	0.097	1	3101	HK3
-0.311454296	0.064870656	0.117	1	55102	ATG2B
-0.311454296	0.064870656	0.097	1	57187	THOC2
-0.311454296	0.064870656	0.101	1	152816	C4orf26
-0.311711699	0.064640664	0.076	1	30834	ZNRD1
-0.311969101	0.06441135	0.081	1	838	CASP5
-0.311969101	0.06441135	0.087	1	4860	PNP
-0.312226504	0.064182661	0.079	1	9937	DCLRE1A
-0.312226504	0.064182661	0.091	1	51703	ACSL5
-0.312226504	0.064182661	0.088	1	203510	HMGB3P30
-0.312226504	0.064182661	0.089	1	221908	C7orf47
-0.312483907	0.063954644	0.088	1	2180	ACSL1
-0.312483907	0.063954644	0.075	1	195827	AAED1
-0.312483907	0.063954644	0.089	1	254394	C6orf61
-0.312483907	0.063954644	0.098	1	642938	FAM196A
-0.312483907	0.063954644	0.078	1	100128164	RP11-379K17.4
-0.312741309	0.063727126	0.087	1	382	ARF6
-0.312741309	0.063727126	0.088	1	79718	TBL1XR1
-0.312741309	0.063727126	0.079	1	84307	ZNF397
-0.312741309	0.063727126	0.077	1	84696	ABHD1
-0.312741309	0.063727126	0.092	1	125704	C18orf51
-0.312741309	0.063727126	0.08	1	286436	H2BFM
-0.312998682	0.063500389	0.09	1	5184	PEPD
-0.312998682	0.063500389	0.111	1	5443	POMC
-0.312998682	0.063500389	0.096	1	57189	KIAA1147
-0.312998682	0.063500389	0.087	1	122622	ADSSL1
-0.312998682	0.063500389	0.079	1	144717	FAM109A
-0.312998682	0.063500389	0.071	1	404220	C6orf201
-0.312998682	0.063500389	0.091	1	647024	C6orf132
-0.313256115	0.063274279	0.072	1	51059	FAM135B
-0.313513488	0.063048817	0.102	1	8529	CYP4F2
-0.313513488	0.063048817	0.091	1	83606	C22orf13
-0.313513488	0.063048817	0.088	1	114991	ZNF618
-0.31377092	0.062823892	0.086	1	8694	DGAT1
-0.31377092	0.062823892	0.085	1	92369	SPSB4
-0.314028293	0.062599711	0.082	1	1908	EDN3
-0.314028293	0.062599711	0.093	1	54532	USP53
-0.314028293	0.062599711	0.08	1	133923	ZNF474
-0.314285696	0.06237619	0.085	1	965	CD58
-0.314285696	0.06237619	0.092	1	5147	PDE6D
-0.314285696	0.06237619	0.079	1	9374	PPT2
-0.314285696	0.06237619	0.077	1	10201	NME6
-0.314285696	0.06237619	0.071	1	23224	SYNE2
-0.314285696	0.06237619	0.092	1	89883	OR6W1P
-0.314285696	0.06237619	0.088	1	389161	C3orf16
-0.314543098	0.062153284	0.081	1	7169	TPM2
-0.314543098	0.062153284	0.083	1	53345	TM6SF2
-0.314543098	0.062153284	0.088	1	124602	KIF19
-0.314800501	0.061930913	0.09	1	8545	CGGBP1
-0.314800501	0.061930913	0.081	1	10847	SRCAP
-0.315057933	0.061709274	0.088	1	95	ACY1
-0.315057933	0.061709274	0.075	1	5091	PC
-0.315057933	0.061709274	0.088	1	25861	DFNB31
-0.315057933	0.061709274	0.077	1	79776	ZFHX4
-0.315057933	0.061709274	0.084	1	84976	DISP1
-0.315057933	0.061709274	0.095	1	117532	TMC2
-0.315315306	0.061488297	0.08	1	24146	CLDN15
-0.315315306	0.061488297	0.089	1	80167	C4orf29
-0.315315306	0.061488297	0.064	1	84191	FAM96A

voxel-wise, subcortex			
Spearman's corr coef	p-val	GenID	Gene
-0.252162367	< 0.001	55799	CACNA2D3
-0.252245098	< 0.001	257218	SHPRH
-0.252373993	< 0.001	150291	MORC2-AS1
-0.25240317	< 0.001	919	CD247
-0.252418756	< 0.001	57140	RNPEPL1
-0.252472997	< 0.001	730130	AC004690.2
-0.252474934	< 0.001	23148	NACAD
-0.252474934	< 0.001	349114	LINC00265
-0.25250569	< 0.001	5116	PCNT
-0.252561957	< 0.001	54935	DUSP23
-0.252656132	< 0.001	26090	ABHD12
-0.25267145	< 0.001	79048	SECISBP2
-0.252699971	< 0.001	8379	MAD1L1
-0.252758294	< 0.001	7469	WHSC2
-0.25285235	< 0.001	3827	KNG1
-0.252901286	< 0.001	50838	TAS2R13
-0.252944857	< 0.001	1998	ELF2
-0.253015995	< 0.001	90799	CEP95
-0.253076166	< 0.001	51270	TFDP3
-0.253100961	< 0.001	89822	KCNK17
-0.25327155	< 0.001	7169	TPM2
-0.253350019	< 0.001	538	ATP7A
-0.253397018	< 0.001	54550	NECAB2
-0.253555626	< 0.001	285440	CYP4V2
-0.253595829	< 0.001	55698	RADIL
-0.253621757	< 0.001	6134	RPL10
-0.253657967	< 0.001	56893	UBQLN4
-0.25366345	< 0.001	146540	ZNF785
-0.253696322	< 0.001	8685	MARCO
-0.253739983	< 0.001	138429	PIP5KL1
-0.253756791	< 0.001	10919	EHMT2
-0.253767639	< 0.001	3040	HBA2
-0.25377813	< 0.001	55596	ZCCHC8
-0.253869504	< 0.001	56261	RP5-1022P6.1
-0.253880113	< 0.001	1912	PHC2
-0.253939062	< 0.001	5520	PPP2R2A
-0.253942609	< 0.001	1668	DEFA3
-0.254004002	< 0.001	9175	MAP3K13
-0.254005283	< 0.001	92840	REEP6
-0.254098982	< 0.001	81575	APOLD1
-0.254233748	< 0.001	6732	SRPK1
-0.254254729	< 0.001	4957	ODF2
-0.254377782	< 0.001	51474	LIMA1
-0.254420012	< 0.001	80279	CDK5RAP3
-0.254430622	< 0.001	10276	NET1
-0.254460245	< 0.001	6723	SRM
-0.254587382	< 0.001	10312	TCIRG1
-0.254691839	< 0.001	3352	HTR1D
-0.254700482	< 0.001	390664	C1QTNF8
-0.254767537	< 0.001	3039	HBA1
-0.254834205	< 0.001	59	ACTA2
-0.255063027	< 0.001	7352	UCP3
-0.255246997	< 0.001	83872	HMCN1
-0.255295008	< 0.001	5947	RBP1
-0.255314797	< 0.001	57835	SLC4A5
-0.255454928	< 0.001	494513	DFNB59
-0.255523562	< 0.001	6164	RPL34
-0.255541295	< 0.001	767811	H2BFXP
-0.25555855	< 0.001	10352	WARS2
-0.255650133	< 0.001	387496	RASL11A
-0.255723864	< 0.001	8342	HIST1H2BM
-0.255726844	< 0.001	857	CAV1
-0.256103873	< 0.001	10263	CDK2AP2
-0.256252378	< 0.001	9585	KIF20B
-0.256256372	< 0.001	27295	PDLIM3
-0.256379873	< 0.001	57326	PBXIP1
-0.256510258	< 0.001	124925	SEZ6
-0.256560862	< 0.001	254170	FBXO33
-0.256590784	< 0.001	80864	XXBAC-BPG300A18.12
-0.256700158	< 0.001	646096	CHEK2P2
-0.256783545	< 0.001	6605	SMARCE1
-0.256786168	< 0.001	9885	OSBPL2
-0.256817639	< 0.001	100289230	LOC100289230
-0.256881356	< 0.001	58512	DLGAP3
-0.25691089	< 0.001	375061	FAM89A
-0.256925374	< 0.001	10614	HEXIM1
-0.256933987	< 0.001	10166	SLC25A15
-0.256991863	< 0.001	7188	TRAF5
-0.25710535	< 0.001	388730	TMEM81

region-wise, subcortex					
Spearman's corr coef	p-val	p-val, SA	p-val, SA (BH- correction)	GenID	Gene
-0.315572739	0.061267931	0.088	1	3776	KCNK2
-0.315572739	0.061267931	0.08	1	5111	PCNA
-0.315572739	0.061267931	0.085	1	26823	RNU12-2P
-0.315830112	0.061048191	0.07	1	861	RUNX1
-0.315830112	0.061048191	0.081	1	6297	SALL2
-0.315830112	0.061048191	0.088	1	9254	CACNA2D2
-0.315830112	0.061048191	0.072	1	11152	WDR45
-0.315830112	0.061048191	0.076	1	23338	PHF15
-0.315830112	0.061048191	0.083	1	114790	STK11IP
-0.315830112	0.061048191	0.085	1	730130	AC004690.2
-0.316087514	0.060828988	0.097	1	23294	ANKS1A
-0.316087514	0.060828988	0.079	1	51561	IL23A
-0.316087514	0.060828988	0.086	1	55888	ZNF167
-0.316087514	0.060828988	0.08	1	55967	NDUFA12
-0.316087514	0.060828988	0.095	1	341276	OR10A2
-0.316344917	0.060610536	0.074	1	94103	ORMDL3
-0.316344917	0.060610536	0.08	1	619279	ZNF704
-0.316602319	0.060392685	0.094	1	3918	LAMC2
-0.316602319	0.060392685	0.097	1	51319	RSRC1
-0.316602319	0.060392685	0.08	1	63899	NSUN3
-0.316602319	0.060392685	0.087	1	85463	ZC3H12C
-0.316602319	0.060392685	0.088	1	114825	PWWP2A
-0.316859722	0.060175464	0.074	1	60672	MIIP
-0.316859722	0.060175464	0.081	1	201931	TMEM192
-0.316859722	0.060175464	0.082	1	388419	BTBD17
-0.317117125	0.059958767	0.075	1	7569	ZNF182
-0.317117125	0.059958767	0.067	1	55093	WDYHV1
-0.317117125	0.059958767	0.076	1	646960	PRSS56
-0.317374498	0.059742823	0.075	1	5959	RDH5
-0.317374498	0.059742823	0.087	1	7376	NR1H2
-0.317374498	0.059742823	0.088	1	79883	PODNL1
-0.317374498	0.059742823	0.077	1	643387	LOC643387
-0.31763193	0.059527472	0.095	1	6253	RTN2
-0.31763193	0.059527472	0.072	1	9674	KIAA0040
-0.31763193	0.059527472	0.091	1	55357	TBC1D2
-0.31763193	0.059527472	0.091	1	56129	PCDHB7
-0.31763193	0.059527472	0.071	1	79991	OBFC1
-0.31763193	0.059527472	0.085	1	92715	WDR85
-0.317889303	0.059312742	0.08	1	2842	GPR19
-0.317889303	0.059312742	0.074	1	9053	MAP7
-0.317889303	0.059312742	0.084	1	64342	HS1BP3
-0.317889303	0.059312742	0.074	1	65009	NDRG4
-0.317889303	0.059312742	0.097	1	90407	TMEM41A
-0.317889303	0.059312742	0.079	1	92312	MEX3A
-0.318146735	0.059098639	0.072	1	2551	GABPA
-0.318146735	0.059098639	0.072	1	6257	RXRB
-0.318404108	0.058885057	0.083	1	121130	OR10P1
-0.318404108	0.058885057	0.067	1	284402	SCGBL
-0.318661511	0.058672212	0.082	1	79630	C1orf54
-0.318661511	0.058672212	0.085	1	114897	C1QTNF1
-0.318661511	0.058672212	0.08	1	283748	PLA2G4D
-0.318661511	0.058672212	0.071	1	440138	ALG11
-0.318918914	0.05845996	0.074	1	8654	PDE5A
-0.318918914	0.05845996	0.073	1	80830	APOL6
-0.318918914	0.05845996	0.083	1	81565	NDEL1
-0.318918914	0.05845996	0.076	1	349114	LINC00265
-0.318918914	0.05845996	0.072	1	389203	C4ORF52
-0.319176316	0.058248326	0.08	1	7752	ZNF200
-0.319433719	0.05803721	0.081	1	6773	STAT2
-0.319433719	0.05803721	0.085	1	8218	CLTCL1
-0.319433719	0.05803721	0.075	1	285025	CCDC141
-0.319691122	0.057826828	0.078	1	5257	PHKB
-0.319691122	0.057826828	0.078	1	10265	IRX5
-0.319691122	0.057826828	0.074	1	11345	GABARAPL2
-0.319691122	0.057826828	0.085	1	56123	PCDHB13
-0.319691122	0.057826828	0.084	1	57091	CASS4
-0.319691122	0.057826828	0.081	1	283297	OR10A4
-0.319691122	0.057826828	0.091	1	415056	LOC415056
-0.319948494	0.057617031	0.068	1	1387	CREBBP
-0.319948494	0.057617031	0.079	1	57124	CD248
-0.319948494	0.057617031	0.085	1	142686	ASB14
-0.319948494	0.057617031	0.077	1	729566	ZBTB8OSP1
-0.320205927	0.057407849	0.081	1	4354	MPP1
-0.320205927	0.057407849	0.072	1	9960	USP3
-0.320205927	0.057407849	0.07	1	148789	B3GALNT2
-0.320205927	0.057407849	0.077	1	151647	FAM19A4
-0.3204633	0.05719918	0.073	1	5697	PYY
-0.3204633	0.05719918	0.083	1	64411	ARAP3
-0.3204633	0.05719918	0.085	1	79866	C13orf34

voxel-wise, subcortex			
Spearman's corr coef	p-val	GenID	Gene
-0.25722459	< 0.001	3786	KCNQ3
-0.257519811	< 0.001	8100	IFT88
-0.257521182	< 0.001	80012	PHC3
-0.257540524	< 0.001	8833	GMPS
-0.257614434	< 0.001	4331	MNAT1
-0.257634968	< 0.001	4649	MYO9A
-0.257705986	< 0.001	23338	PHF15
-0.25803864	< 0.001	54106	TLR9
-0.258122236	< 0.001	55278	QRSL1
-0.258209318	< 0.001	3068	HDGF
-0.258223087	< 0.001	401172	CTD-2324F15.2
-0.258224547	< 0.001	170692	ADAMTS18
-0.258300245	< 0.001	7412	VCAM1
-0.258333594	< 0.001	6396	SEC13
-0.258356422	< 0.001	644604	EEF1A1P12
-0.258693993	< 0.001	8629	JRK
-0.258791864	< 0.001	4616	GADD45B
-0.258813888	< 0.001	80209	C13orf23
-0.258815646	< 0.001	11030	RBPMS
-0.258840531	< 0.001	367	AR
-0.258877218	< 0.001	9960	USP3
-0.258952528	< 0.001	344	APOC2
-0.2589688	< 0.001	149134	LOC149134
-0.259049207	< 0.001	114991	ZNF618
-0.259055525	< 0.001	6468	FBXW4
-0.259062201	< 0.001	84133	ZNRF3
-0.259104848	< 0.001	286554	BCORP1
-0.259121448	< 0.001	104	ADARB1
-0.259132802	< 0.001	51480	VCX2
-0.25920096	< 0.001	64761	PARP12
-0.259262532	< 0.001	10449	ACAA2
-0.259263903	< 0.001	7782	SLC30A4
-0.259306639	< 0.001	85461	TANC1
-0.259469062	< 0.001	55297	CCDC91
-0.259626567	< 0.001	403313	PPAPDC2
-0.259688586	< 0.001	23012	STK38L
-0.259842575	< 0.001	116535	MRGPRF
-0.259889275	< 0.001	1827	RCAN1
-0.259900331	< 0.001	200894	ARL13B
-0.260002375	< 0.001	29893	PSMC3IP
-0.260233819	< 0.001	23462	HEY1
-0.260255069	< 0.001	79785	RERGL
-0.260295749	< 0.001	285175	C2orf21
-0.260410249	< 0.001	55679	LIMS2
-0.260429025	< 0.001	92999	ZBTB47
-0.260441273	< 0.001	2101	ESRRA
-0.260660797	< 0.001	5409	PNMT
-0.2608248	< 0.001	347730	LRRTM1
-0.26089251	< 0.001	124565	SLC38A10
-0.26097247	< 0.001	692099	FAM86DP
-0.261129767	< 0.001	2317	FLNB
-0.261187911	< 0.001	7832	BTG2
-0.261268348	< 0.001	1044	CDX1
-0.261321932	< 0.001	8932	MBD2
-0.261384428	< 0.001	6909	TBX2
-0.261437625	< 0.001	79190	IRX6
-0.261524737	< 0.001	283417	DPY19L2
-0.26154536	< 0.001	5153	PDE1B
-0.261595786	< 0.001	155038	GIMAP8
-0.261655957	< 0.001	57822	GRHL3
-0.261805385	< 0.001	2051	EPHB6
-0.261863321	< 0.001	162963	ZNF610
-0.261909097	< 0.001	403	ARL3
-0.261926562	< 0.001	2825	GPR1
-0.261942178	< 0.001	952	CD38
-0.261944771	< 0.001	3074	HEXB
-0.26194644	< 0.001	11332	ACOT7
-0.261954337	< 0.001	5327	PLAT
-0.262092441	< 0.001	148867	SLC30A7
-0.262131989	< 0.001	64065	PERP
-0.262141466	< 0.001	164592	CCDC116
-0.262145162	< 0.001	645191	LINGO3
-0.262148619	< 0.001	4935	GPR143
-0.26218605	< 0.001	89876	C3orf15
-0.262230426	< 0.001	26338	OR5L2
-0.262267023	< 0.001	5495	PPM1B
-0.262277424	< 0.001	973	CD79A
-0.262528449	< 0.001	64321	SOX17
-0.262529552	< 0.001	134265	AFAP1L1

region-wise, subcortex					
Spearman's corr coef	p-val	p-val, SA	p-val, SA (BH- correction)	GenID	Gene
-0.3204633	0.05719918	0.061	1	83416	FCRL5
-0.3204633	0.05719918	0.077	1	201780	SLC10A4
-0.3204633	0.05719918	0.086	1	254879	OR2T6
-0.3204633	0.05719918	0.074	1	400569	MED11
-0.3204633	0.05719918	0.074	1	401562	LCNL1
-0.3204633	0.05719918	0.08	1	441124	LOC441124
-0.320720732	0.056991216	0.078	1	4710	NDUFB4
-0.320720732	0.056991216	0.077	1	10866	HCP5
-0.320720732	0.056991216	0.062	1	57716	PRX
-0.320720732	0.056991216	0.069	1	134466	ZNF300P1
-0.321235508	0.056577131	0.068	1	51295	ECSIT
-0.321235508	0.056577131	0.084	1	84733	CBX2
-0.321235508	0.056577131	0.084	1	84795	PYROXD2
-0.321235508	0.056577131	0.069	1	84909	C9orf3
-0.32149291	0.056370981	0.063	1	5042	PABPC3
-0.32149291	0.056370981	0.07	1	5239	PGM5
-0.32149291	0.056370981	0.07	1	440689	HIST2H2BF
-0.321750313	0.056165349	0.079	1	5276	SERPINI2
-0.321750313	0.056165349	0.081	1	5341	PLEK
-0.321750313	0.056165349	0.085	1	26226	FBXW4P1
-0.321750313	0.056165349	0.055	1	149047	RP11-183M13.1
-0.322007746	0.055960406	0.078	1	54955	C1orf109
-0.322007746	0.055960406	0.082	1	79852	EPHX3
-0.322265118	0.055756092	0.075	1	9724	UTP14C
-0.322265118	0.055756092	0.076	1	23429	RYBP
-0.322265118	0.055756092	0.079	1	79742	CXorf36
-0.322522521	0.055552356	0.068	1	4357	MPST
-0.322522521	0.055552356	0.077	1	92241	RCSD1
-0.322522521	0.055552356	0.078	1	221806	VWDE
-0.322779924	0.055349123	0.066	1	5077	PAX3
-0.322779924	0.055349123	0.077	1	51205	ACP6
-0.323037326	0.055146586	0.09	1	1583	CYP11A1
-0.323037326	0.055146586	0.075	1	2004	ELK3
-0.323037326	0.055146586	0.077	1	5947	RBP1
-0.323037326	0.055146586	0.09	1	23519	ANP32D
-0.323037326	0.055146586	0.08	1	100170765	C19orf69
-0.323294729	0.054944638	0.079	1	1003	CDH5
-0.323294729	0.054944638	0.07	1	1962	EHHADH
-0.323294729	0.054944638	0.09	1	9420	CYP7B1
-0.323552132	0.054743312	0.077	1	4143	MAT1A
-0.323552132	0.054743312	0.076	1	84957	RELT
-0.323809505	0.054542467	0.074	1	23474	ETHE1
-0.323809505	0.054542467	0.08	1	57539	WDR35
-0.324066937	0.054342303	0.088	1	5956	OPN1LW
-0.324066937	0.054342303	0.069	1	9600	PITPNM1
-0.324066937	0.054342303	0.081	1	197259	MLKL
-0.32432431	0.054142732	0.077	1	6424	SFRP4
-0.32432431	0.054142732	0.078	1	10385	BTN2A2
-0.32432431	0.054142732	0.074	1	116159	CYYR1
-0.324581742	0.053943753	0.067	1	2624	GATA2
-0.324581742	0.053943753	0.073	1	26123	TCTN3
-0.324839115	0.053745385	0.075	1	1135	CHRNA2
-0.324839115	0.053745385	0.061	1	8334	HIST1H2AC
-0.324839115	0.053745385	0.078	1	51429	SNX9
-0.324839115	0.053745385	0.084	1	57624	KIAA1486
-0.324839115	0.053745385	0.066	1	157638	FAM84B
-0.324839115	0.053745385	0.081	1	169714	QSOX2
-0.325096518	0.053547494	0.083	1	4958	OMD
-0.325096518	0.053547494	0.07	1	391712	TRIM61
-0.32535392	0.053350281	0.069	1	8353	HIST1H3E
-0.32535392	0.053350281	0.071	1	92840	REEP6
-0.325611323	0.053153649	0.078	1	2298	FOXD4
-0.325611323	0.053153649	0.07	1	57615	ZNF492
-0.325868726	0.052957606	0.081	1	1198	CLK3
-0.325868726	0.052957606	0.076	1	132430	PABPC4L
-0.325868726	0.052957606	0.071	1	257236	CCDC96
-0.326126128	0.05276208	0.083	1	84679	SLC9A7
-0.326383501	0.052567199	0.076	1	1506	CTRL
-0.326383501	0.052567199	0.063	1	9923	ZBTB40
-0.326383501	0.052567199	0.068	1	9980	DOPEY2
-0.326898307	0.052179184	0.069	1	5837	PYGM
-0.326898307	0.052179184	0.084	1	84293	C10orf57
-0.326898307	0.052179184	0.069	1	259285	TAS2R39
-0.326898307	0.052179184	0.074	1	284207	METRNL
-0.326898307	0.052179184	0.071	1	326340	ZAR1
-0.327155739	0.05198596	0.063	1	7757	ZNF208
-0.327155739	0.05198596	0.074	1	23341	DNAJC16
-0.327155739	0.05198596	0.064	1	643669	LOC643669
-0.327155739	0.05198596	0.067	1	727701	LINC00165

voxel-wise, subcortex			
Spearman's corr coef	p-val	GenID	Gene
-0.262620568	< 0.001	5158	PDE6B
-0.262634754	< 0.001	9350	CER1
-0.262637824	< 0.001	8277	TKTL1
-0.262683898	< 0.001	114882	OSBPL8
-0.262687236	< 0.001	7593	MZF1
-0.262731254	< 0.001	440459	LOC440459
-0.263263851	< 0.001	55693	KDM4D
-0.263300985	< 0.001	23178	PASK
-0.263307959	< 0.001	84196	USP48
-0.263466835	< 0.001	29766	TMOD3
-0.263473988	< 0.001	116984	ARAP2
-0.263505101	< 0.001	27079	RPUSD2
-0.2636002	< 0.001	84073	MYCBPAP
-0.263632417	< 0.001	84266	ALKBH7
-0.26364997	< 0.001	64123	ELTD1
-0.263714701	< 0.001	81611	ANP32E
-0.263736427	< 0.001	23261	CAMTA1
-0.263813525	< 0.001	387328	ZNF322P1
-0.263818532	< 0.001	57552	AADACL1
-0.263878256	< 0.001	171425	CLYBL
-0.263887614	< 0.001	1775	DNASE1L2
-0.263928682	< 0.001	441208	ZNRF2P1
-0.263972223	< 0.001	7633	ZNF79
-0.264214218	< 0.001	56941	C3orf37
-0.264220923	< 0.001	2916	GRM6
-0.264358819	< 0.001	8490	RP11-267N12.3
-0.264380097	< 0.001	6625	SNRNP70
-0.26438418	< 0.001	9576	SPAG6
-0.26439476	< 0.001	3782	KCNN3
-0.264452249	< 0.001	4522	MTHFD1
-0.264519483	< 0.001	440603	BCL2L15
-0.264621258	< 0.001	83550	GPR101
-0.264751732	< 0.001	54886	PPAPR3
-0.264868468	< 0.001	63899	NSUN3
-0.264873207	< 0.001	9499	MYOT
-0.26489234	< 0.001	6913	TBX15
-0.264938772	< 0.001	5781	PTPN11
-0.264973402	< 0.001	3418	IDH2
-0.264977217	< 0.001	90204	ZSWIM1
-0.264982432	< 0.001	55552	ZNF823
-0.265013069	< 0.001	84218	TBC1D3
-0.26508233	< 0.001	4144	MAT2A
-0.265235752	< 0.001	1294	COL7A1
-0.265342474	< 0.001	197322	ACSF3
-0.265416473	< 0.001	9926	LPGAT1
-0.265448511	< 0.001	9401	RECQL4
-0.265598863	< 0.001	643955	RP11-196D18.6
-0.265753776	< 0.001	201562	PTPLB
-0.265791655	< 0.001	2946	GSTM2
-0.265997827	< 0.001	134553	C5orf24
-0.266022503	< 0.001	79944	L2HGDH
-0.266023636	< 0.001	364	AQP7
-0.266049087	< 0.001	23580	CDC42EP4
-0.266114742	< 0.001	7048	TGFBR2
-0.266180843	< 0.001	2037	EPB41L2
-0.266224325	< 0.001	10198	MPHOSPH9
-0.26644358	< 0.001	440482	ANKRD20A5P
-0.26644823	< 0.001	154043	CNKSR3
-0.266552955	< 0.001	55125	CEP192
-0.266640812	< 0.001	9923	ZBTB40
-0.266657174	< 0.001	816	CAMK2B
-0.266710848	< 0.001	57194	ATP10A
-0.266841143	< 0.001	3397	ID1
-0.266919613	< 0.001	10954	PDIA5
-0.267000943	< 0.001	7773	ZNF230
-0.267050356	< 0.001	94	ACVRL1
-0.267052948	< 0.001	51148	CERCAM
-0.267053872	< 0.001	9031	BAZ1B
-0.267077655	< 0.001	83538	TTC25
-0.267160952	< 0.001	90025	UBE2CBP
-0.267258197	< 0.001	79842	ZBTB3
-0.26737985	< 0.001	115265	DDIT4L
-0.267438352	< 0.001	5681	PSKH1
-0.267512828	< 0.001	30819	KCNIP2
-0.267599016	< 0.001	30820	KCNIP1
-0.267613113	< 0.001	93343	FAM125A
-0.267632633	< 0.001	6424	SFRP4
-0.267689824	< 0.001	84107	ZIC4
-0.267733306	< 0.001	5919	RARRES2

region-wise, subcortex					
Spearman's corr coef	p-val	p-val, SA	p-val, SA (BH- correction)	GenID	Gene
-0.327413112	0.051793419	0.082	1	3068	HDGF
-0.327413112	0.051793419	0.07	1	10451	VAV3
-0.327413112	0.051793419	0.068	1	85508	SCRT2
-0.327413112	0.051793419	0.076	1	387264	KRTAP5-1
-0.327670515	0.051601432	0.098	1	7412	VCAM1
-0.327927917	0.051410023	0.065	1	1187	CLCNKA
-0.327927917	0.051410023	0.068	1	2801	GOLGA2
-0.327927917	0.051410023	0.081	1	25961	NUDT13
-0.327927917	0.051410023	0.066	1	147525	LINC00526
-0.327927917	0.051410023	0.083	1	163183	C19orf46
-0.327927917	0.051410023	0.058	1	203286	ANKS6
-0.32818532	0.051219184	0.05	1	4160	MC4R
-0.32818532	0.051219184	0.069	1	7050	TGIF1
-0.32818532	0.051219184	0.074	1	7920	BAT5
-0.32818532	0.051219184	0.067	1	79642	ARSJ
-0.32818532	0.051219184	0.08	1	100287822	SETP10
-0.328442723	0.051028855	0.065	1	1108	CHD4
-0.328700125	0.050839163	0.064	1	83758	RBP5
-0.328700125	0.050839163	0.07	1	196549	EEF1DP3
-0.328957528	0.050650042	0.056	1	2879	GPX4
-0.328957528	0.050650042	0.054	1	5826	ABCD4
-0.328957528	0.050650042	0.082	1	9794	MAML1
-0.328957528	0.050650042	0.077	1	64167	ERAP2
-0.328957528	0.050650042	0.061	1	80199	FUZ
-0.328957528	0.050650042	0.081	1	81532	MOB2
-0.329214931	0.05046149	0.075	1	27252	KLHL20
-0.329214931	0.05046149	0.072	1	29128	UHRF1
-0.329214931	0.05046149	0.076	1	64224	HERPUD2
-0.329214931	0.05046149	0.071	1	158511	CSAG1
-0.329214931	0.05046149	0.071	1	400931	RP4-695O20__B.1
-0.329472333	0.050273418	0.064	1	4722	NDUFS3
-0.329472333	0.050273418	0.066	1	7021	TFAP2B
-0.329729736	0.050086025	0.077	1	1910	EDNRB
-0.329729736	0.050086025	0.071	1	79230	ZNF557
-0.329987139	0.049899168	0.077	1	51107	APH1A
-0.329987139	0.049899168	0.067	1	152687	ZNF595
-0.329987139	0.049899168	0.079	1	254228	FAM26E
-0.329987139	0.049899168	0.067	1	389125	MUSTN1
-0.329987139	0.049899168	0.077	1	647219	ASCL5
-0.330244511	0.049712881	0.073	1	5351	PLOD1
-0.330244511	0.049712881	0.068	1	7356	SCGB1A1
-0.330244511	0.049712881	0.069	1	55761	TTC17
-0.330244511	0.049712881	0.051	1	64106	NPFFR1
-0.330501944	0.049527071	0.065	1	3549	IHH
-0.330501944	0.049527071	0.072	1	5024	P2RX3
-0.330501944	0.049527071	0.076	1	11059	WWP1
-0.330501944	0.049527071	0.079	1	55080	TAPBPL
-0.330501944	0.049527071	0.055	1	55871	CBWD1
-0.330501944	0.049527071	0.07	1	221395	GPR116
-0.330759317	0.04934191	0.059	1	11004	KIF2C
-0.330759317	0.04934191	0.074	1	129563	DIS3L2
-0.331016749	0.049157325	0.07	1	151534	LBX2-AS1
-0.331016749	0.049157325	0.085	1	350383	GPR142
-0.331016749	0.049157325	0.075	1	729501	ZNF962P
-0.331274122	0.048973285	0.067	1	3965	LGALS9
-0.331274122	0.048973285	0.064	1	26504	CNNM4
-0.331531525	0.048789795	0.054	1	10147	SFRS14
-0.331531525	0.048789795	0.083	1	57552	AADACL1
-0.331531525	0.048789795	0.066	1	441519	CT45A3
-0.331788927	0.048606791	0.075	1	1192	CLIC1
-0.331788927	0.048606791	0.055	1	7327	UBE2G2
-0.331788927	0.048606791	0.067	1	10615	SPAG5
-0.331788927	0.048606791	0.058	1	147658	ZNF534
-0.33204633	0.048424415	0.071	1	5174	PDZK1
-0.33204633	0.048424415	0.062	1	84171	LOXL4
-0.33204633	0.048424415	0.066	1	91607	SLFN11
-0.332303733	0.048242621	0.059	1	23261	CAMTA1
-0.332561135	0.04806136	0.062	1	8651	SOCS1
-0.332561135	0.04806136	0.07	1	340596	LHFPL1
-0.332818508	0.047880568	0.071	1	23059	CLUAP1
-0.332818508	0.047880568	0.078	1	80746	TSEN2
-0.332818508	0.047880568	0.059	1	285555	C4orf37
-0.332818508	0.047880568	0.062	1	346288	SEPTIN14
-0.332818508	0.047880568	0.065	1	386680	KRTAP10-5
-0.333075941	0.047700409	0.077	1	2176	FANCC
-0.333075941	0.047700409	0.078	1	9546	APBA3
-0.333075941	0.047700409	0.081	1	100133036	FAM95B1
-0.333333313	0.047520798	0.084	1	6397	SEC14L1
-0.333333313	0.047520798	0.076	1	55245	UQCC

voxel-wise, subcortex			
Spearman's corr coef	p-val	GenID	Gene
-0.267742038	< 0.001	5142	PDE4B
-0.267753631	< 0.001	56052	ALG1
-0.267773688	< 0.001	389151	AC130416.1
-0.267807215	< 0.001	2259	FGF14
-0.2678518	< 0.001	58515	RP11-884K10.1
-0.267943919	< 0.001	1612	DAPK1
-0.268102348	< 0.001	50618	ITSN2
-0.268188536	< 0.001	147184	TMEM99
-0.268414199	< 0.001	389289	ANXA2R
-0.268495262	< 0.001	9270	ITGB1BP1
-0.268502772	< 0.001	93058	COQ10A
-0.268601954	< 0.001	9744	ACAP1
-0.26876086	< 0.001	100189589	AC005041.9
-0.268778592	< 0.001	64423	INF2
-0.268924296	< 0.001	1633	DCK
-0.268951595	< 0.001	723790	HIST2H2AA4
-0.268961459	< 0.001	1788	DNMT3A
-0.268967867	< 0.001	8701	DNAH11
-0.268989503	< 0.001	442524	AC007276.5
-0.269091547	< 0.001	55080	TAPBPL
-0.269331157	< 0.001	27146	FAM184B
-0.269392818	< 0.001	19	ABCA1
-0.269413412	< 0.001	668	FOXL2
-0.269502193	< 0.001	2072	ERCC4
-0.269526362	< 0.001	113763	C7orf29
-0.269547909	< 0.001	401131	LOC401131
-0.269633323	< 0.001	200916	RPL22L1
-0.269858062	< 0.001	727764	MAFIP
-0.269911736	< 0.001	81839	VANGL1
-0.270047694	< 0.001	253827	MSRB3
-0.270246536	< 0.001	84688	C9orf24
-0.270287097	< 0.001	10008	KCNE3
-0.270482421	< 0.001	163747	C1orf177
-0.270498484	< 0.001	23474	ETHE1
-0.270502746	< 0.001	30835	CD209
-0.270621061	< 0.001	9616	RNF7
-0.270629495	< 0.001	23765	IL17RA
-0.270636111	< 0.001	2268	FGR
-0.270649016	< 0.001	7062	TCHH
-0.270655125	< 0.001	231	AKR1B1
-0.270685136	< 0.001	54830	NUP62CL
-0.27073139	< 0.001	929	CD14
-0.270933837	< 0.001	50859	SPOCK3
-0.270955563	< 0.001	645784	ANKRD36BP2
-0.271004766	< 0.001	1915	EEF1A1
-0.271009147	< 0.001	56204	KIAA1370
-0.271025777	< 0.001	284058	KIAA1267
-0.271113902	< 0.001	114327	EFHC1
-0.271190047	< 0.001	23322	RPGRIP1L
-0.271199793	< 0.001	1152	CKB
-0.271353573	< 0.001	399967	PATE2
-0.271460474	< 0.001	8408	ULK1
-0.271532267	< 0.001	158511	CSAG1
-0.271533281	< 0.001	55159	RFWD3
-0.271567553	< 0.001	2817	GPC1
-0.271572918	< 0.001	286676	ILDR1
-0.271746218	< 0.001	728957	ZNF705C
-0.27175504	< 0.001	57018	CCNL1
-0.271825612	< 0.001	8942	KYNU
-0.271968365	< 0.001	56155	TEX14
-0.271979392	< 0.001	5241	PGR
-0.272065312	< 0.001	139411	PTCHD1
-0.27217108	< 0.001	9022	CLIC3
-0.272215366	< 0.001	9204	ZMYM6
-0.272223085	< 0.001	1728	NQO1
-0.272288084	< 0.001	57569	ARHGAP20
-0.272300452	< 0.001	3268	AGFG2
-0.272344649	< 0.001	55275	VPS53
-0.272496194	< 0.001	8638	OASL
-0.27252081	< 0.001	128602	C20orf85
-0.272551745	< 0.001	26136	TES
-0.272561967	< 0.001	647589	AC226150.1
-0.272715271	< 0.001	125336	LOXHD1
-0.272808313	< 0.001	2878	GPX3
-0.272812307	< 0.001	133	ADM
-0.272900909	< 0.001	9021	SOCS3
-0.272909075	< 0.001	55808	ST6GALNAC1
-0.272919029	< 0.001	50507	NOX4
-0.27308926	< 0.001	285888	CNPY1

region-wise, subcortex					
Spearman's corr coef	p-val	p-val, SA	p-val, SA (BH- correction)	GenID	Gene
-0.333333313	0.047520798	0.07	1	63929	XPNPEP3
-0.333333313	0.047520798	0.054	1	144100	PLEKHA7
-0.333590746	0.047341757	0.07	1	54708	MARCHF5
-0.333590746	0.047341757	0.059	1	55857	PLK1S1
-0.333848119	0.04716317	0.065	1	8899	PRPF4B
-0.333848119	0.04716317	0.063	1	23607	CD2AP
-0.334105521	0.046985202	0.061	1	133	ADM
-0.334362924	0.046807781	0.054	1	51755	CRKRS
-0.334362924	0.046807781	0.07	1	158800	RHOXF1
-0.334620327	0.046630908	0.071	1	257	ALX3
-0.334620327	0.046630908	0.05	1	22937	SCAP
-0.334620327	0.046630908	0.056	1	57161	PELI2
-0.334620327	0.046630908	0.054	1	644006	LOC644006
-0.334877729	0.046454594	0.061	1	7579	ZSCAN20
-0.334877729	0.046454594	0.068	1	10873	ME3
-0.334877729	0.046454594	0.064	1	57018	CCNL1
-0.335135132	0.046278726	0.061	1	5913	RAPSN
-0.335135132	0.046278726	0.068	1	7562	ZNF708
-0.335135132	0.046278726	0.063	1	22915	MMRN1
-0.335135132	0.046278726	0.062	1	53632	PRKAG3
-0.335135132	0.046278726	0.067	1	128344	C1orf88
-0.335392505	0.046103474	0.053	1	7110	TMF1
-0.335392505	0.046103474	0.082	1	114826	SMYD4
-0.335392505	0.046103474	0.064	1	285613	RELL2
-0.335649937	0.045928765	0.078	1	2687	GGT5
-0.335649937	0.045928765	0.062	1	4991	OR1D2
-0.335649937	0.045928765	0.071	1	5966	REL
-0.335649937	0.045928765	0.068	1	7768	ZNF225
-0.335649937	0.045928765	0.072	1	55362	TMEM63B
-0.335649937	0.045928765	0.069	1	84284	NTPCR
-0.335649937	0.045928765	0.054	1	84444	DOT1L
-0.335649937	0.045928765	0.061	1	283869	NPW
-0.33590734	0.045754611	0.064	1	29091	STXBP6
-0.33590734	0.045754611	0.068	1	730110	LOC730110
-0.336164743	0.045580897	0.05	1	55303	GIMAP4
-0.336164743	0.045580897	0.055	1	57589	KIAA1432
-0.336164743	0.045580897	0.057	1	80778	ZNF34
-0.336164743	0.045580897	0.063	1	116966	WDR17
-0.336164743	0.045580897	0.058	1	161198	CLEC14A
-0.336164743	0.045580897	0.063	1	388550	CEACAM22P
-0.336164743	0.045580897	0.068	1	388799	C20orf107
-0.336422145	0.045407802	0.065	1	28960	DCPS
-0.336422145	0.045407802	0.055	1	79770	TXNDC15
-0.336679518	0.045235235	0.061	1	920	CD4
-0.336679518	0.045235235	0.073	1	1601	DAB2
-0.336679518	0.045235235	0.072	1	9021	SOCS3
-0.336679518	0.045235235	0.058	1	55182	RNF220
-0.336936951	0.045063205	0.071	1	835	CASP2
-0.337194324	0.044891726	0.06	1	5004	ORM1
-0.337194324	0.044891726	0.074	1	8853	ASAP2
-0.337451756	0.044720683	0.066	1	367	AR
-0.337451756	0.044720683	0.066	1	26155	NOC2L
-0.337709129	0.044550251	0.051	1	828	CAPS
-0.337709129	0.044550251	0.069	1	7455	ZAN
-0.337709129	0.044550251	0.045	1	23511	NUP188
-0.337709129	0.044550251	0.061	1	79694	MANEA
-0.337966532	0.044380344	0.057	1	1302	COL11A2
-0.337966532	0.044380344	0.061	1	284004	HEXDC
-0.337966532	0.044380344	0.069	1	340602	CXorf67
-0.338223934	0.044210982	0.056	1	3543	IGLL1
-0.338223934	0.044210982	0.063	1	5797	PTPRM
-0.338223934	0.044210982	0.051	1	7764	ZNF217
-0.338223934	0.044210982	0.059	1	10610	ST6GALNAC2
-0.338223934	0.044210982	0.063	1	23456	ABCB10
-0.338223934	0.044210982	0.064	1	55567	DNAH3
-0.338223934	0.044210982	0.063	1	79864	C11orf63
-0.338223934	0.044210982	0.052	1	80219	COQ10B
-0.338223934	0.044210982	0.057	1	91107	TRIM47
-0.338481337	0.044042055	0.045	1	5208	PFKFB2
-0.338481337	0.044042055	0.043	1	5598	MAPK7
-0.338481337	0.044042055	0.069	1	8874	ARHGEF7
-0.338481337	0.044042055	0.05	1	23505	TMEM131
-0.338481337	0.044042055	0.075	1	113829	SLC35A4
-0.338481337	0.044042055	0.058	1	149840	C20orf196
-0.338738739	0.043873727	0.06	1	3145	HMBS
-0.338738739	0.043873727	0.058	1	10891	PPARGC1A
-0.338738739	0.043873727	0.069	1	27436	EML4
-0.338738739	0.043873727	0.065	1	83707	TRPT1
-0.338738739	0.043873727	0.057	1	140432	RNF113B

voxel-wise, subcortex			
Spearman's corr coef	p-val	GenID	Gene
-0.273094714	< 0.001	285555	C4orf37
-0.273117304	< 0.001	55323	LARP6
-0.273385108	< 0.001	10474	TADA3L
-0.273603618	< 0.001	64342	HS1BP3
-0.273752868	< 0.001	8209	C21orf33
-0.27378872	< 0.001	26099	C1orf144
-0.273846477	< 0.001	29946	SERTAD3
-0.273855299	< 0.001	81554	WBSCR16
-0.273984939	< 0.001	30834	ZNRD1
-0.274008632	< 0.001	800	CALD1
-0.274025708	< 0.001	92610	TIFA
-0.274082899	< 0.001	8906	AP1G2
-0.2742199	< 0.001	641649	TMEM91
-0.274248391	< 0.001	3439	IFNA1
-0.274319351	< 0.001	6829	SUPT5H
-0.274331808	< 0.001	2787	GNG5
-0.274633884	< 0.001	3075	CFH
-0.274687737	< 0.001	1968	EIF2S3
-0.274693787	< 0.001	26610	ELP4
-0.274695635	< 0.001	7122	CLDN5
-0.27470836	< 0.001	123096	SLC25A29
-0.274717182	< 0.001	6887	TAL2
-0.274810433	< 0.001	57554	LRRC7
-0.274842173	< 0.001	87769	A2LD1
-0.27499783	< 0.001	9936	CD302
-0.275053561	< 0.001	317754	POTED
-0.275107145	< 0.001	729648	ZNF812
-0.275231123	< 0.001	126859	C1orf125
-0.27523315	< 0.001	152206	CCDC13
-0.275474429	< 0.001	23640	HSPBP1
-0.27559644	< 0.001	4086	SMAD1
-0.275638133	< 0.001	8553	BHLHE40
-0.275703698	< 0.001	22828	RBM16
-0.275753945	< 0.001	10681	GNB5
-0.275856376	< 0.001	84858	ZNF503
-0.275915354	< 0.001	5366	PMAIP1
-0.275989443	< 0.001	55055	ZWILCH
-0.276131719	< 0.001	79671	NLRX1
-0.276146859	< 0.001	7402	UTRN
-0.276245475	< 0.001	22889	KIAA0907
-0.276255786	< 0.001	256364	EML3
-0.276456952	< 0.001	54433	GAR1
-0.276602089	< 0.001	2784	GNB3
-0.276666075	< 0.001	51690	LSM7
-0.276700348	< 0.001	22984	PDCD11
-0.276792467	< 0.001	147660	ZNF578
-0.276797116	< 0.001	79696	FAM164C
-0.276884228	< 0.001	23315	SLC9A8
-0.276964456	< 0.001	7866	IFRD2
-0.277047932	< 0.001	84867	PTPN5
-0.277083963	< 0.001	3321	IGSF3
-0.277106255	< 0.001	283212	KLHL35
-0.277156413	< 0.001	92815	HIST3H2A
-0.277300626	< 0.001	2176	FANCC
-0.277316779	< 0.001	5433	POLR2D
-0.277359784	< 0.001	10847	SRCAP
-0.277440578	< 0.001	1192	CLIC1
-0.277443171	< 0.001	100132733	ANKRD20A13P
-0.277522385	< 0.001	388152	AC103965.1
-0.277609974	< 0.001	83714	NRIP2
-0.277634561	< 0.001	196294	IMMP1L
-0.27764219	< 0.001	57658	CALCOCO1
-0.277700692	< 0.001	283596	SNHG10
-0.277728468	< 0.001	399900	LOC399900
-0.277909189	< 0.001	650623	LOC650623
-0.277935088	< 0.001	643827	LOC643827
-0.27805841	< 0.001	6095	RORA
-0.278128058	< 0.001	53405	CLIC5
-0.278198093	< 0.001	22979	EFR3B
-0.278401554	< 0.001	2316	FLNA
-0.278443813	< 0.001	51277	DNAJC27
-0.278465271	< 0.001	4807	NHLH1
-0.278550684	< 0.001	56165	TDRD1
-0.278723806	< 0.001	341880	SLC35F4
-0.278724909	< 0.001	10179	AP002373.1
-0.278779238	< 0.001	60482	SLC5A7
-0.278871357	< 0.001	645715	EEF1A1P24
-0.278892726	< 0.001	344593	LOC344593
-0.278909057	< 0.001	1392	CRH

region-wise, subcortex					
Spearman's corr coef	p-val	p-val, SA	p-val, SA (BH- correction)	GenID	Gene
-0.338996142	0.043705929	0.069	1	790	CAD
-0.338996142	0.043705929	0.055	1	9772	KIAA0195
-0.338996142	0.043705929	0.067	1	84283	TMEM79
-0.339253515	0.043538649	0.062	1	2	A2M
-0.339253515	0.043538649	0.059	1	3850	KRT3
-0.339253515	0.043538649	0.056	1	7004	TEAD4
-0.339253515	0.043538649	0.069	1	283860	LINC00304
-0.339510947	0.043371815	0.055	1	8209	C21orf33
-0.33976832	0.043205597	0.072	1	1690	COCH
-0.33976832	0.043205597	0.054	1	54538	ROBO4
-0.33976832	0.043205597	0.074	1	79887	PLBD1
-0.33976832	0.043205597	0.054	1	84287	ZDHHC16
-0.340283126	0.042874705	0.065	1	1513	CTSK
-0.340283126	0.042874705	0.058	1	54925	ZNF434
-0.340283126	0.042874705	0.057	1	64170	CARD9
-0.340540528	0.042710006	0.058	1	4038	LRP4
-0.340797931	0.042545784	0.051	1	929	CD14
-0.340797931	0.042545784	0.069	1	63897	HEATR6
-0.340797931	0.042545784	0.072	1	93099	DMKN
-0.341055334	0.042382136	0.065	1	7122	CLDN5
-0.341055334	0.042382136	0.064	1	7639	ZNF85
-0.341055334	0.042382136	0.062	1	8692	HYAL2
-0.341055334	0.042382136	0.073	1	10625	IVNS1ABP
-0.341055334	0.042382136	0.052	1	56165	TDRD1
-0.341055334	0.042382136	0.056	1	401321	LOC401321
-0.341312736	0.042219002	0.066	1	9861	PSMD6
-0.341312736	0.042219002	0.047	1	11319	ECD
-0.341312736	0.042219002	0.057	1	26025	PCDHGA12
-0.341570139	0.042056397	0.059	1	5531	PPP4C
-0.341570139	0.042056397	0.062	1	8702	B4GALT4
-0.342084944	0.041732617	0.062	1	7072	TIA1
-0.342084944	0.041732617	0.053	1	221091	LRRN4CL
-0.342084944	0.041732617	0.063	1	283579	C14orf178
-0.342084944	0.041732617	0.046	1	723961	INS-IGF2
-0.342084944	0.041732617	0.069	1	100289143	LOC100289143
-0.342342317	0.041571528	0.07	1	6695	SPOCK1
-0.342342317	0.041571528	0.055	1	55332	DRAM1
-0.342342317	0.041571528	0.053	1	55703	POLR3B
-0.342342317	0.041571528	0.05	1	389799	C9orf171
-0.34259975	0.041410945	0.042	1	51233	C22orf43
-0.34259975	0.041410945	0.067	1	53822	FXYD7
-0.342857152	0.041250799	0.062	1	6091	ROBO1
-0.342857152	0.041250799	0.061	1	6513	SLC2A1
-0.343114525	0.041091245	0.059	1	27332	ZNF638
-0.343371958	0.040932182	0.067	1	6418	SET
-0.343371958	0.040932182	0.049	1	390205	AP003108.2
-0.343371958	0.040932182	0.049	1	402176	RPL21P44
-0.34362933	0.040773641	0.068	1	7857	SCG2
-0.34362933	0.040773641	0.078	1	56113	PCDHGA2
-0.34362933	0.040773641	0.046	1	65988	ZNF747
-0.34362933	0.040773641	0.047	1	85391	SNORD14E
-0.34362933	0.040773641	0.069	1	196541	C13orf39
-0.343886763	0.040615566	0.051	1	842	CASP9
-0.343886763	0.040615566	0.053	1	129607	CMPK2
-0.344144136	0.040457953	0.055	1	2817	GPC1
-0.344144136	0.040457953	0.059	1	3429	IFI27
-0.344144136	0.040457953	0.058	1	79767	ELMO3
-0.344401538	0.040300898	0.06	1	266743	NPAS4
-0.344658941	0.040144343	0.055	1	9718	ECE2
-0.344658941	0.040144343	0.057	1	30850	CDR2L
-0.344916344	0.039988298	0.054	1	2009	EML1
-0.344916344	0.039988298	0.072	1	51112	TTC15
-0.344916344	0.039988298	0.054	1	646999	LOC646999
-0.345173746	0.039832648	0.069	1	22858	ICK
-0.345173746	0.039832648	0.053	1	23528	ZNF281
-0.345173746	0.039832648	0.055	1	55268	ECHDC2
-0.345431149	0.039677598	0.051	1	7273	TTN
-0.345431149	0.039677598	0.064	1	8439	NSMAF
-0.345431149	0.039677598	0.06	1	54661	PCDHB17
-0.345431149	0.039677598	0.065	1	84842	HPDL
-0.345688522	0.039523024	0.053	1	7592	ZNF41
-0.345688522	0.039523024	0.057	1	54206	ERRFI1
-0.345688522	0.039523024	0.059	1	92745	SLC38A5
-0.345688522	0.039523024	0.054	1	116842	LEAP2
-0.345688522	0.039523024	0.043	1	100289137	LOC100289137
-0.345945954	0.039368946	0.048	1	9563	H6PD
-0.345945954	0.039368946	0.04	1	64115	C10orf54
-0.345945954	0.039368946	0.045	1	84942	WDR73
-0.346203327	0.039215293	0.06	1	4992	OR1F1

voxel-wise, subcortex			
Spearman's corr coef	p-val	GenID	Gene
-0.278949648	< 0.001	196883	ADCY4
-0.278998971	< 0.001	6261	RYR1
-0.27906099	< 0.001	5873	RAB27A
-0.27906248	< 0.001	3491	CYR61
-0.279063791	< 0.001	8692	HYAL2
-0.279232323	< 0.001	64399	HHIP
-0.279261202	< 0.001	54704	PDP1
-0.279270411	< 0.001	3682	ITGAE
-0.279289454	< 0.001	84915	C12orf34
-0.279432744	< 0.001	9904	RBM19
-0.279543906	< 0.001	11200	CHEK2
-0.27958262	< 0.001	5542	PRB1
-0.279656738	< 0.001	3547	IGSF1
-0.279687762	< 0.001	51734	SEPX1
-0.27971822	< 0.001	5179	PENK
-0.27979511	< 0.001	94086	HSPB9
-0.279806525	< 0.001	744	MPPED2
-0.279858053	< 0.001	2321	FLT1
-0.279872	< 0.001	6442	SGCA
-0.27991879	< 0.001	118430	MUCL1
-0.280149281	< 0.001	10010	TANK
-0.28017205	< 0.001	6297	SALL2
-0.280240774	< 0.001	100131532	LOC100131532
-0.280246347	< 0.001	91252	SLC39A13
-0.280347466	< 0.001	2828	GPR4
-0.280364007	< 0.001	8644	AKR1C3
-0.28038007	< 0.001	170575	GIMAP1
-0.280422956	< 0.001	27065	AC110814.1
-0.280437738	< 0.001	64776	C11orf1
-0.280509055	< 0.001	148022	TICAM1
-0.280526131	< 0.001	1486	CTBS
-0.280742437	< 0.001	30849	PIK3R4
-0.280750871	< 0.001	2146	EZH2
-0.280800849	< 0.001	7273	TTN
-0.280876607	< 0.001	1209	CLPTM1
-0.280890167	< 0.001	441032	EEF1A1P9
-0.280901402	< 0.001	653509	SFTPA1
-0.281178802	< 0.001	7862	BRPF1
-0.281284302	< 0.001	11319	ECD
-0.281341225	< 0.001	79600	TCTN1
-0.281411439	< 0.001	64080	RBKS
-0.281563997	< 0.001	51477	ISYNA1
-0.281582952	< 0.001	8519	IFITM1
-0.281662345	< 0.001	221395	GPR116
-0.281753451	< 0.001	6996	TDG
-0.281801105	< 0.001	29916	SNX11
-0.281931311	< 0.001	56896	DPYSL5
-0.281966388	< 0.001	126669	SHE
-0.282193184	< 0.001	93587	RG9MTD2
-0.282214254	< 0.001	10365	KLF2
-0.282292813	< 0.001	55282	LRRC36
-0.282378078	< 0.001	148641	SLC35F3
-0.282452077	< 0.001	4057	LTF
-0.282495916	< 0.001	1954	MEGF8
-0.282798111	< 0.001	10058	ABCB6
-0.282838881	< 0.001	441459	ANKRD18B
-0.282877237	< 0.001	115992	RNF166
-0.282916784	< 0.001	79913	ACTR5
-0.28302896	< 0.001	363	AQP6
-0.28324005	< 0.001	9870	KIAA0317
-0.283306003	< 0.001	112703	FAM71E1
-0.28333357	< 0.001	83999	KREMEN1
-0.283373028	< 0.001	9080	CLDN9
-0.283406943	< 0.001	6895	TARBP2
-0.283503234	< 0.001	59347	FKSG2
-0.283566952	< 0.001	84071	ARMC2
-0.283572137	< 0.001	57380	MRS2
-0.283573717	< 0.001	25989	ULK3
-0.283593506	< 0.001	2202	EFEMP1
-0.28367877	< 0.001	64784	CRTC3
-0.283715725	< 0.001	125962	OR7G1
-0.283808112	< 0.001	643911	CRNDE
-0.28389141	< 0.001	5702	PSMC3
-0.283903599	< 0.001	4071	TM4SF1
-0.284016222	< 0.001	147011	PROCA1
-0.284090698	< 0.001	842	CASP9
-0.28415069	< 0.001	5195	PEX14
-0.284223139	< 0.001	114815	SORCS1
-0.284242451	< 0.001	7299	TYR

region-wise, subcortex					
Spearman's corr coef	p-val	p-val, SA	p-val, SA (BH- correction)	GenID	Gene
-0.346203327	0.039215293	0.06	1	8745	ADAM23
-0.346203327	0.039215293	0.056	1	56261	RP5-1022P6.1
-0.346203327	0.039215293	0.055	1	56288	PARD3
-0.346203327	0.039215293	0.059	1	64785	GINS3
-0.34646076	0.039062198	0.05	1	9429	ABCG2
-0.34646076	0.039062198	0.06	1	10008	KCNE3
-0.34646076	0.039062198	0.033	1	83697	SLC4A9
-0.346718132	0.038909607	0.061	1	1104	RCC1
-0.346718132	0.038909607	0.067	1	5332	PLCB4
-0.346718132	0.038909607	0.056	1	9001	HAP1
-0.346975535	0.038757507	0.06	1	5696	PSMB8
-0.346975535	0.038757507	0.051	1	11030	RBPMS
-0.346975535	0.038757507	0.072	1	55164	SHQ1
-0.346975535	0.038757507	0.06	1	64094	SMOC2
-0.346975535	0.038757507	0.053	1	79709	GLT25D1
-0.346975535	0.038757507	0.06	1	221092	HNRNPUL2
-0.347232938	0.038605861	0.058	1	115207	KCTD12
-0.34749034	0.038454667	0.06	1	1392	CRH
-0.34749034	0.038454667	0.06	1	1843	DUSP1
-0.34749034	0.038454667	0.045	1	2788	AC005512.1
-0.34749034	0.038454667	0.06	1	55096	EBLN2
-0.34749034	0.038454667	0.052	1	124565	SLC38A10
-0.347747743	0.038303997	0.057	1	10391	CORO2B
-0.347747743	0.038303997	0.051	1	27161	EIF2C2
-0.347747743	0.038303997	0.054	1	55243	KIRREL
-0.347747743	0.038303997	0.057	1	79903	NAT15
-0.347747743	0.038303997	0.063	1	284233	CYP4F35P
-0.348005146	0.038153838	0.05	1	2563	GABRD
-0.348005146	0.038153838	0.066	1	64745	METT11D1
-0.348005146	0.038153838	0.057	1	116835	HSPA12B
-0.348262519	0.038004167	0.056	1	10276	NET1
-0.348262519	0.038004167	0.062	1	56052	ALG1
-0.348262519	0.038004167	0.048	1	196294	IMMP1L
-0.348519951	0.037854876	0.044	1	3827	KNG1
-0.348519951	0.037854876	0.051	1	9435	CHST2
-0.348777324	0.037706167	0.045	1	2872	MKNK2
-0.348777324	0.037706167	0.036	1	7369	UMOD
-0.348777324	0.037706167	0.051	1	27040	LAT
-0.348777324	0.037706167	0.055	1	162972	ZNF550
-0.349034756	0.037557904	0.055	1	8771	TNFRSF6B
-0.349034756	0.037557904	0.047	1	51566	ARMCX3
-0.349292129	0.037410151	0.059	1	3590	IL11RA
-0.349292129	0.037410151	0.056	1	29127	RACGAP1P
-0.349292129	0.037410151	0.046	1	440180	LOC440180
-0.349292129	0.037410151	0.053	1	100130357	LOC100130357
-0.349549532	0.037262797	0.051	1	81603	TRIM8
-0.349549532	0.037262797	0.057	1	283598	C14orf177
-0.349806964	0.03711598	0.05	1	10388	SYCP2
-0.349806964	0.03711598	0.054	1	100132406	NBPF10
-0.35032177	0.036823779	0.057	1	4168	MCF2
-0.350579143	0.036678404	0.044	1	134	ADORA1
-0.350579143	0.036678404	0.054	1	84913	ATOH8
-0.350836545	0.036533434	0.058	1	2859	GPR35
-0.350836545	0.036533434	0.052	1	51427	ZNF588
-0.350836545	0.036533434	0.051	1	80177	MYCT1
-0.350836545	0.036533434	0.058	1	94120	SYTL3
-0.351093948	0.036388971	0.063	1	1241	LTB4R
-0.351093948	0.036388971	0.041	1	348645	C22orf34
-0.351351351	0.036245011	0.059	1	5118	PCOLCE
-0.351351351	0.036245011	0.05	1	11270	NRM
-0.351351351	0.036245011	0.055	1	115708	TRMT61A
-0.351608753	0.036101483	0.049	1	2674	GFRA1
-0.351608753	0.036101483	0.06	1	3883	KRT33A
-0.351608753	0.036101483	0.056	1	440051	KRTAP5-11
-0.351866156	0.035958394	0.056	1	4071	TM4SF1
-0.351866156	0.035958394	0.048	1	84709	C4orf49
-0.352123529	0.035815831	0.047	1	9124	PDLIM1
-0.352123529	0.035815831	0.048	1	55340	GIMAP5
-0.352123529	0.035815831	0.057	1	56132	PCDHB3
-0.352123529	0.035815831	0.052	1	64321	SOX17
-0.352123529	0.035815831	0.056	1	253943	YTHDF3
-0.352380961	0.035673708	0.065	1	1039	CDR2
-0.352380961	0.035673708	0.052	1	51684	SUFU
-0.352380961	0.035673708	0.058	1	80896	NPL
-0.352380961	0.035673708	0.066	1	642954	RBBP4P1
-0.352638334	0.035532076	0.043	1	7629	ZNF76
-0.352638334	0.035532076	0.041	1	8916	HERC3
-0.352638334	0.035532076	0.045	1	28231	SLCO4A1
-0.352638334	0.035532076	0.051	1	79706	PRKRIP1

voxel-wise, subcortex			
Spearman's corr coef	p-val	GenID	Gene
-0.284368008	< 0.001	79660	PPP1R3B
-0.284401447	< 0.001	259230	SGMS1
-0.28446579	< 0.001	6674	SPAG1
-0.284765005	< 0.001	389125	MUSTN1
-0.284771591	< 0.001	84168	ANTXR1
-0.285311997	< 0.001	6014	RIT2
-0.285351843	< 0.001	3625	INHBB
-0.285362422	< 0.001	57125	PLXDC1
-0.285378933	< 0.001	339263	C17orf51
-0.285427511	< 0.001	684	BST2
-0.285452038	< 0.001	2535	FZD2
-0.285587072	< 0.001	118924	C10orf4
-0.285697281	< 0.001	84229	CCDC135
-0.285796851	< 0.001	6875	TAF4B
-0.28586632	< 0.001	653781	AC140481.7
-0.285975337	< 0.001	2040	STOM
-0.286004484	< 0.001	10627	MYL12A
-0.28607285	< 0.001	8309	ACOX2
-0.286073685	< 0.001	55709	KBTBD4
-0.286087781	< 0.001	402	ARL2
-0.286118239	< 0.001	79974	C7orf58
-0.286237389	< 0.001	128272	ARHGEF19
-0.286255419	< 0.001	84940	CORO6
-0.286307603	< 0.001	6626	SNRPA
-0.286390245	< 0.001	5159	PDGFRB
-0.286414593	< 0.001	1382	CRABP2
-0.286438823	< 0.001	2796	GNRH1
-0.286482573	< 0.001	100874392	ANKRD20A12P
-0.286578506	< 0.001	57575	PCDH10
-0.286640614	< 0.001	65986	ZBTB10
-0.286745936	< 0.001	79866	C13orf34
-0.286833704	< 0.001	339501	MPN2
-0.286838233	< 0.001	6345	SRL
-0.286876887	< 0.001	57696	DDX55
-0.286952466	< 0.001	51317	PHF21A
-0.287052304	< 0.001	170506	DHX36
-0.287072092	< 0.001	730091	RP11-392A22.2
-0.287189364	< 0.001	158135	TTLL11
-0.287384003	< 0.001	84448	ABLIM2
-0.287391543	< 0.001	26108	PYGO1
-0.287411213	< 0.001	147172	LRRC37BP1
-0.287467122	< 0.001	2120	ETV6
-0.287622124	< 0.001	8189	SYMPK
-0.287700236	< 0.001	9306	SOCS6
-0.28771916	< 0.001	64063	PRSS22
-0.28788504	< 0.001	55110	MAGOHB
-0.287926733	< 0.001	54619	CCNJ
-0.287930638	< 0.001	9170	LPAR2
-0.288174957	< 0.001	2976	GTF3C2
-0.288234383	< 0.001	285203	C3orf64
-0.288302362	< 0.001	1843	DUSP1
-0.288535178	< 0.001	79922	MRM1
-0.28854093	< 0.001	6197	RPS6KA3
-0.28855142	< 0.001	399949	C11orf88
-0.288555533	< 0.001	10677	AVIL
-0.288567215	< 0.001	2	A2M
-0.288575202	< 0.001	7016	TESK1
-0.288582832	< 0.001	4722	NDUFS3
-0.288605124	< 0.001	4291	MLF1
-0.288636208	< 0.001	2177	FANCD2
-0.288786113	< 0.001	29803	REPIN1
-0.288790107	< 0.001	57336	ZNF287
-0.288857043	< 0.001	958	CD40
-0.288858086	< 0.001	5042	PABPC3
-0.288951486	< 0.001	344838	PAQR9
-0.288974255	< 0.001	6603	SMARCD2
-0.289179772	< 0.001	284439	SLC25A42
-0.289243758	< 0.001	112609	MRAP2
-0.289266795	< 0.001	80298	MTERFD3
-0.289379627	< 0.001	646262	RP1-74M1.1
-0.289454281	< 0.001	56672	C11orf17
-0.289485306	< 0.001	84518	CNFN
-0.28956601	< 0.001	25812	POM121L1P
-0.289585769	< 0.001	124404	SEPTIN12
-0.28976658	< 0.001	196549	EEF1DP3
-0.289785892	< 0.001	57493	HEG1
-0.289876342	< 0.001	2913	GRM3
-0.28993383	< 0.001	8190	MIA
-0.290000796	< 0.001	753	C18orf1

region-wise, subcortex					
Spearman's corr coef	p-val	p-val, SA	p-val, SA (BH- correction)	GenID	Gene
-0.352638334	0.035532076	0.06	1	83849	SYT15
-0.352638334	0.035532076	0.053	1	283985	FADS6
-0.352638334	0.035532076	0.059	1	403341	ZBTB34
-0.352895766	0.035390813	0.05	1	388	RHOB
-0.353153139	0.035250109	0.044	1	1770	DNAH9
-0.353153139	0.035250109	0.047	1	5152	PDE9A
-0.353153139	0.035250109	0.054	1	10434	LYPLA1
-0.353153139	0.035250109	0.051	1	347746	PAR-SN
-0.353153139	0.035250109	0.039	1	643036	SLED1
-0.353153139	0.035250109	0.042	1	729975	AC018755.8
-0.353410542	0.035109863	0.059	1	22891	ZNF365
-0.353410542	0.035109863	0.038	1	79854	LINC00115
-0.353667945	0.034970045	0.054	1	303	ANXA2P1
-0.353667945	0.034970045	0.053	1	3902	LAG3
-0.353667945	0.034970045	0.052	1	55679	LIMS2
-0.353667945	0.034970045	0.048	1	255877	BCL6B
-0.353925347	0.034830723	0.043	1	5810	RAD1
-0.353925347	0.034830723	0.048	1	56937	PMEPA1
-0.353925347	0.034830723	0.054	1	129530	LYG1
-0.353925347	0.034830723	0.055	1	100287852	LOC100287852
-0.35418275	0.034691758	0.039	1	22987	SV2C
-0.35418275	0.034691758	0.057	1	23051	ZHX3
-0.35418275	0.034691758	0.052	1	79973	ZNF442
-0.354440153	0.034553345	0.052	1	84612	PARD6B
-0.354440153	0.034553345	0.058	1	723972	ANP32AP1
-0.354697526	0.03441539	0.037	1	2622	GAS8
-0.354697526	0.03441539	0.052	1	2693	GHSR
-0.354697526	0.03441539	0.059	1	4023	LPL
-0.354697526	0.03441539	0.053	1	9013	TAF1C
-0.354697526	0.03441539	0.059	1	23105	FSTL4
-0.354697526	0.03441539	0.045	1	23256	SCFD1
-0.354697526	0.03441539	0.066	1	79785	RERGL
-0.354954958	0.034277856	0.043	1	353323	KRTAP12-2
-0.355212331	0.034140743	0.058	1	147872	CCDC155
-0.355469763	0.034004118	0.055	1	6890	TAP1
-0.355469763	0.034004118	0.046	1	7022	TFAP2C
-0.355469763	0.034004118	0.064	1	23102	TBC1D2B
-0.355469763	0.034004118	0.032	1	135458	HUS1B
-0.355984539	0.033732273	0.045	1	2550	GABBR1
-0.355984539	0.033732273	0.053	1	8940	TOP3B
-0.355984539	0.033732273	0.045	1	10237	SLC35B1
-0.355984539	0.033732273	0.041	1	80176	SPSB1
-0.356241941	0.033596937	0.038	1	292	SLC25A5
-0.356241941	0.033596937	0.043	1	4057	LTF
-0.356241941	0.033596937	0.057	1	90594	ZNF439
-0.356499344	0.033462133	0.044	1	9631	NUP155
-0.356499344	0.033462133	0.049	1	9811	KIAA0427
-0.356499344	0.033462133	0.051	1	60485	SAV1
-0.356499344	0.033462133	0.043	1	400713	AC010332.1
-0.357014149	0.033193842	0.043	1	785	CACNB4
-0.357014149	0.033193842	0.042	1	728841	NBPF8
-0.357528955	0.032927267	0.048	1	3136	HLA-H
-0.357528955	0.032927267	0.049	1	79841	AGBL2
-0.357528955	0.032927267	0.057	1	79929	MAP6D1
-0.357786357	0.032794688	0.042	1	309	ANXA6
-0.35804376	0.032662522	0.044	1	132320	SCLT1
-0.358301163	0.032530826	0.057	1	6935	ZEB1
-0.358558536	0.032399505	0.044	1	1735	DIO3
-0.358558536	0.032399505	0.046	1	3689	ITGB2
-0.358815968	0.032268658	0.047	1	57104	PNPLA2
-0.358815968	0.032268658	0.05	1	83690	CRISPLD1
-0.358815968	0.032268658	0.052	1	195828	ZNF367
-0.359073341	0.032138273	0.045	1	653	BMP5
-0.359073341	0.032138273	0.042	1	5255	PHKA1
-0.359073341	0.032138273	0.043	1	10537	UBD
-0.359073341	0.032138273	0.043	1	441376	AARD
-0.359330773	0.032008313	0.046	1	5731	PTGER1
-0.359330773	0.032008313	0.046	1	6274	S100A3
-0.359330773	0.032008313	0.051	1	131873	AC128683.1
-0.359588146	0.031878781	0.04	1	5919	RARRES2
-0.359588146	0.031878781	0.042	1	9885	OSBPL2
-0.359845549	0.031749647	0.053	1	200162	SPAG17
-0.360102952	0.031620972	0.046	1	3882	KRT32
-0.360102952	0.031620972	0.055	1	54039	PCBP3
-0.360102952	0.031620972	0.037	1	55698	RADIL
-0.360102952	0.031620972	0.031	1	79582	SPAG16
-0.360102952	0.031620972	0.045	1	80198	MUS81
-0.360102952	0.031620972	0.046	1	92304	SCGB3A1
-0.360360354	0.031492755	0.03	1	23520	ANP32C

voxel-wise, subcortex			
Spearman's corr coef	p-val	GenID	Gene
-0.290133119	< 0.001	23228	PLCL2
-0.290133327	< 0.001	2852	GPER
-0.290136009	< 0.001	79923	NANOGP8
-0.290225804	< 0.001	65251	ZNF649
-0.290274739	< 0.001	64224	HERPUD2
-0.29041329	< 0.001	3015	H2AFZ
-0.290433556	< 0.001	5245	PHB
-0.290527821	< 0.001	79664	NARG2
-0.290723383	< 0.001	23403	FBXO46
-0.290725052	< 0.001	10723	SLC12A7
-0.29080382	< 0.001	63928	AC130454.1
-0.291019261	< 0.001	100129128	RP11-257K9.7
-0.291023701	< 0.001	5190	PEX6
-0.291047126	< 0.001	124923	SGK494
-0.291247785	< 0.001	79776	ZFHX4
-0.291423589	< 0.001	169611	OLFML2A
-0.291491657	< 0.001	200162	SPAG17
-0.291579038	< 0.001	152015	ROPN1B
-0.291590661	< 0.001	55871	CBWD1
-0.291777223	< 0.001	4212	MEIS2
-0.29185003	< 0.001	1750	DLX6
-0.29200381	< 0.001	3603	IL16
-0.292014688	< 0.001	730112	FAM166B
-0.292055279	< 0.001	84152	PPP1R1B
-0.292061776	< 0.001	5187	PER1
-0.292124182	< 0.001	780529	LOC780529
-0.292269498	< 0.001	6876	TAGLN
-0.292282045	< 0.001	5322	PLA2G5
-0.292398512	< 0.001	6386	SDCBP
-0.292771816	< 0.001	23395	LARS2
-0.292829484	< 0.001	5528	PPP2R5D
-0.292933404	< 0.001	200232	FAM209A
-0.292951137	< 0.001	54505	DHX29
-0.293043256	< 0.001	2589	GALNT1
-0.293111712	< 0.001	1021	CDK6
-0.293119431	< 0.001	6511	SLC1A6
-0.293149799	< 0.001	56154	TEX15
-0.293177545	< 0.001	646960	PRSS56
-0.293188602	< 0.001	26206	SPAG8
-0.293275058	< 0.001	85379	KIAA1671
-0.293367565	< 0.001	84074	QRICH2
-0.293384463	< 0.001	6926	TBX3
-0.293427259	< 0.001	18	ABAT
-0.293597221	< 0.001	79841	AGBL2
-0.293643177	< 0.001	728299	KRTAP19-8
-0.293758631	< 0.001	83938	C10orf11
-0.2937693	< 0.001	1301	COL11A1
-0.293789357	< 0.001	6513	SLC2A1
-0.293790013	< 0.001	284837	AP001053.11
-0.293846011	< 0.001	2875	GPT
-0.293923259	< 0.001	26505	CNNM3
-0.294081032	< 0.001	645332	AP003716.1
-0.294092178	< 0.001	1545	CYP1B1
-0.294187576	< 0.001	3192	HNRNPU
-0.294208735	< 0.001	7227	TRPS1
-0.294248015	< 0.001	441425	ANKRD20A1
-0.294319808	< 0.001	4050	LTB
-0.294440717	< 0.001	253970	SFTA3
-0.294476569	< 0.001	10391	CORO2B
-0.294629991	< 0.001	115677	NOSTRIN
-0.294655502	< 0.001	84190	C12orf26
-0.294685692	< 0.001	144404	TMEM120B
-0.294878304	< 0.001	8745	ADAM23
-0.294962168	< 0.001	4925	NUCB2
-0.295170546	< 0.001	57697	FANCM
-0.295299917	< 0.001	490	ATP2B1
-0.295555383	< 0.001	54329	GPR85
-0.295565784	< 0.001	5255	PHKA1
-0.295683354	< 0.001	727941	LOC727941
-0.295712709	< 0.001	3141	HLCS
-0.295731932	< 0.001	9569	GTF2IRD1
-0.295732766	< 0.001	5549	PRELP
-0.295740932	< 0.001	51029	PPPDE1
-0.295838445	< 0.001	3726	JUNB
-0.295889884	< 0.001	91147	TMEM67
-0.295977175	< 0.001	192670	EIF2C4
-0.296030402	< 0.001	5429	POLH
-0.296151221	< 0.001	4701	NDUFA7
-0.296181679	< 0.001	254065	BRWD3

region-wise, subcortex					
Spearman's corr coef	p-val	p-val, SA	p-val, SA (BH- correction)	GenID	Gene
-0.360360354	0.031492755	0.058	1	57835	SLC4A5
-0.360360354	0.031492755	0.036	1	79845	RNF122
-0.360360354	0.031492755	0.04	1	253827	MSRB3
-0.360360354	0.031492755	0.051	1	344758	GPR149
-0.360617757	0.031364955	0.052	1	55531	ELMOD1
-0.360617757	0.031364955	0.047	1	79583	AC009163.1
-0.360617757	0.031364955	0.046	1	129401	NUP35
-0.36087516	0.031237528	0.042	1	6324	SCN1B
-0.361132532	0.0311106	0.045	1	898	CCNE1
-0.361389965	0.030984072	0.042	1	5526	PPP2R5B
-0.361389965	0.030984072	0.024	1	79081	C11orf48
-0.361389965	0.030984072	0.054	1	285150	AC062028.1
-0.36190477	0.030732265	0.04	1	2822	GPLD1
-0.36190477	0.030732265	0.044	1	26108	PYGO1
-0.36190477	0.030732265	0.042	1	64761	PARP12
-0.36190477	0.030732265	0.041	1	203111	C8orf47
-0.362162143	0.030607035	0.052	1	1295	COL8A1
-0.362419546	0.030482236	0.043	1	9252	RPS6KA5
-0.362676948	0.03035784	0.049	1	8614	STC2
-0.362676948	0.03035784	0.044	1	11080	DNAJB4
-0.362676948	0.03035784	0.039	1	79675	FASTKD1
-0.362676948	0.03035784	0.05	1	149478	RP11-269F19.1
-0.362934351	0.030233864	0.045	1	56648	EIF5A2
-0.363191754	0.030110257	0.053	1	489	ATP2A3
-0.363191754	0.030110257	0.043	1	7450	VWF
-0.363191754	0.030110257	0.049	1	30832	ZNF354C
-0.363449156	0.029987121	0.042	1	2160	F11
-0.363449156	0.029987121	0.042	1	11320	MGAT4A
-0.363449156	0.029987121	0.043	1	29065	ASAP1-IT1
-0.363449156	0.029987121	0.042	1	64065	PERP
-0.363449156	0.029987121	0.035	1	79932	KIAA0319L
-0.363449156	0.029987121	0.042	1	83872	HMCN1
-0.363449156	0.029987121	0.059	1	163486	DENND1B
-0.363706559	0.029864416	0.035	1	94	ACVRL1
-0.363706559	0.029864416	0.048	1	3439	IFNA1
-0.363706559	0.029864416	0.035	1	10072	DPP3
-0.363706559	0.029864416	0.041	1	375611	SLC26A5
-0.363963962	0.029742114	0.04	1	9902	MRC2
-0.364221364	0.029620174	0.051	1	155038	GIMAP8
-0.364478767	0.029498704	0.054	1	362	AQP5
-0.364478767	0.029498704	0.04	1	3141	HLCS
-0.364478767	0.029498704	0.035	1	7075	TIE1
-0.364478767	0.029498704	0.045	1	9709	HERPUD1
-0.364478767	0.029498704	0.043	1	645843	TMEM14E
-0.36473617	0.029377645	0.04	1	285	ANGPT2
-0.36473617	0.029377645	0.051	1	3075	CFH
-0.36473617	0.029377645	0.053	1	56927	GPR108
-0.365250975	0.029136727	0.045	1	3587	IL10RA
-0.365250975	0.029136727	0.039	1	7124	TNF
-0.365250975	0.029136727	0.054	1	51360	MBTPS2
-0.365250975	0.029136727	0.042	1	170575	GIMAP1
-0.365508348	0.029016903	0.036	1	147276	C18orf15
-0.36576578	0.028897488	0.038	1	6793	STK10
-0.36576578	0.028897488	0.046	1	147650	NCRNA00085
-0.366280556	0.028659895	0.055	1	6913	TBX15
-0.366280556	0.028659895	0.044	1	7123	CLEC3B
-0.366280556	0.028659895	0.039	1	51540	SCLY
-0.366280556	0.028659895	0.051	1	51547	SIRT7
-0.366280556	0.028659895	0.043	1	440295	AC135995.5
-0.366537958	0.028541651	0.043	1	448834	KPRP
-0.366795361	0.028423861	0.046	1	8788	DLK1
-0.366795361	0.028423861	0.039	1	646090	RHPN2P1
-0.366795361	0.028423861	0.044	1	100418883	LOC100418883
-0.367052764	0.028306477	0.045	1	3720	JARID2
-0.367052764	0.028306477	0.039	1	161003	STOML3
-0.367052764	0.028306477	0.045	1	220869	CBWD5
-0.367310166	0.028189508	0.044	1	63027	SLC22A23
-0.367567539	0.028072879	0.04	1	80168	MOGAT2
-0.367824972	0.0279567	0.055	1	56906	THAP10
-0.367824972	0.0279567	0.045	1	64410	KLHL25
-0.368082345	0.027840935	0.035	1	56105	PCDHGA11
-0.368339777	0.027725542	0.049	1	8604	SLC25A12
-0.368339777	0.027725542	0.044	1	56104	PCDHGB1
-0.36859715	0.027610522	0.039	1	4988	OPRM1
-0.36859715	0.027610522	0.036	1	64399	HHIP
-0.368854553	0.027495936	0.038	1	56652	C10orf2
-0.368854553	0.027495936	0.04	1	135892	TRIM50
-0.368854553	0.027495936	0.043	1	158056	MAMDC4
-0.368854553	0.027495936	0.047	1	342909	ZNF284

voxel-wise, subcortex			
Spearman's corr coef	p-val	GenID	Gene
-0.296219379	< 0.001	6232	RPS27
-0.296322733	< 0.001	10992	SF3B2
-0.296360075	< 0.001	56288	PARD3
-0.296361089	< 0.001	11274	USP18
-0.29636842	< 0.001	4773	NFATC2
-0.296377987	< 0.001	8334	HIST1H2AC
-0.296380877	< 0.001	3697	ITIH1
-0.29641968	< 0.001	54839	LRRC49
-0.296505392	< 0.001	3720	JARID2
-0.296586394	< 0.001	9457	FHL5
-0.296613038	< 0.001	5634	PRPS2
-0.296710998	< 0.001	79070	KDELC1
-0.296716124	< 0.001	84249	PSD2
-0.296735257	< 0.001	23428	SLC7A8
-0.296875	< 0.001	5029	P2RY2
-0.296958953	< 0.001	80167	C4orf29
-0.297040313	< 0.001	3699	ITIH3
-0.297055721	< 0.001	57787	MARK4
-0.297173679	< 0.001	8788	DLK1
-0.297221869	< 0.001	100147773	RP11-466F5.8
-0.297363222	< 0.001	148932	MOBKL2C
-0.297554702	< 0.001	9420	CYP7B1
-0.297664374	< 0.001	2668	GDNF
-0.297694474	< 0.001	100130557	LOC100130557
-0.29775241	< 0.001	25956	SEC31B
-0.297822058	< 0.001	92241	RCSD1
-0.297976047	< 0.001	84287	ZDHHC16
-0.298047721	< 0.001	3429	IFI27
-0.298099905	< 0.001	11097	NUPL2
-0.298148304	< 0.001	3767	KCNJ11
-0.298158705	< 0.001	135398	C6ORF141
-0.298207074	< 0.001	286046	XKR6
-0.298336923	< 0.001	728264	MIR143HG
-0.298433661	< 0.001	11215	AKAP11
-0.29858914	< 0.001	341350	OVCH1
-0.298754811	< 0.001	730232	EEF1A1P32
-0.29886958	< 0.001	10301	DLEU1
-0.298912674	< 0.001	463	ATBF1
-0.298917681	< 0.001	100272228	RP13-507I23.1
-0.29893133	< 0.001	5552	SRGN
-0.299060881	< 0.001	445328	ARHGEF5L
-0.29908058	< 0.001	285533	RNF175
-0.299309224	< 0.001	27229	TUBGCP4
-0.29932037	< 0.001	90459	ERI1
-0.299423635	< 0.001	7375	USP4
-0.299443781	< 0.001	83657	DYNLRB2
-0.299585491	< 0.001	161394	SAMD15
-0.299647063	< 0.001	23224	SYNE2
-0.299683273	< 0.001	23281	MTUS2
-0.299847096	< 0.001	88455	ANKRD13A
-0.299928635	< 0.001	7327	UBE2G2
-0.299964666	< 0.001	3708	ITPR1
-0.300079346	< 0.001	1136	CHRNA3
-0.300114453	< 0.001	64427	TTC31
-0.300261647	< 0.001	79676	OGFOD2
-0.300300837	< 0.001	152573	SHISA3
-0.30030632	< 0.001	26586	CKAP2
-0.300311327	< 0.001	84062	DTNBP1
-0.300394624	< 0.001	166793	ZNF509
-0.300431401	< 0.001	148398	SAMD11
-0.300473094	< 0.001	282997	LOC282997
-0.300485641	< 0.001	340120	ANKRD34B
-0.300531417	< 0.001	26123	TCTN3
-0.300535053	< 0.001	151556	GPR155
-0.300592333	< 0.001	9814	SFI1
-0.300620109	< 0.001	144100	PLEKHA7
-0.300665259	< 0.001	55818	KDM3A
-0.301008195	< 0.001	79630	C1orf54
-0.301011443	< 0.001	362	AQP5
-0.301028997	< 0.001	64097	EPB41L4A
-0.301053971	< 0.001	84696	ABHD1
-0.301140338	< 0.001	146956	EME1
-0.301364541	< 0.001	5740	PTGIS
-0.301535398	< 0.001	150082	LCA5L
-0.301747054	< 0.001	9572	NR1D1
-0.301750571	< 0.001	9718	ECE2
-0.30176422	< 0.001	8346	HIST1H2BI
-0.301940292	< 0.001	9674	KIAA0040
-0.30196926	< 0.001	132430	PABPC4L

region-wise, subcortex					
Spearman's corr coef	p-val	p-val, SA	p-val, SA (BH- correction)	GenID	Gene
-0.368854553	0.027495936	0.033	1	347442	DCAF8L2
-0.369111955	0.027381748	0.035	1	574	BAGE
-0.369111955	0.027381748	0.045	1	5029	P2RY2
-0.369369358	0.027267966	0.037	1	4913	NTHL1
-0.369369358	0.027267966	0.041	1	5327	PLAT
-0.36962676	0.027154554	0.041	1	1909	EDNRA
-0.36962676	0.027154554	0.04	1	56969	RPL23AP32
-0.36962676	0.027154554	0.044	1	84915	C12orf34
-0.36962676	0.027154554	0.03	1	134265	AFAP1L1
-0.369884163	0.027041508	0.044	1	2321	FLT1
-0.369884163	0.027041508	0.039	1	23591	FAM215A
-0.369884163	0.027041508	0.038	1	79922	MRM1
-0.370141536	0.02692889	0.049	1	26528	DAZAP1
-0.370398968	0.026816666	0.041	1	550	AUP1
-0.370398968	0.026816666	0.042	1	5256	PHKA2
-0.370398968	0.026816666	0.038	1	9427	ECEL1
-0.371171176	0.026482284	0.031	1	633	BGN
-0.371171176	0.026482284	0.051	1	4629	MYH11
-0.371171176	0.026482284	0.039	1	6895	TARBP2
-0.371171176	0.026482284	0.044	1	83992	CTTNBP2
-0.371685982	0.026261322	0.032	1	339390	CLEC4G
-0.371685982	0.026261322	0.045	1	641518	AC097067.1
-0.371943355	0.026151383	0.051	1	100272228	RP13-507I23.1
-0.372200787	0.026041863	0.044	1	7035	TFPI
-0.372200787	0.026041863	0.039	1	54829	ASPN
-0.372200787	0.026041863	0.034	1	90589	ZNF625
-0.37245816	0.025932726	0.027	1	2648	KAT2A
-0.37245816	0.025932726	0.029	1	55297	CCDC91
-0.37245816	0.025932726	0.049	1	222223	KIAA1324L
-0.37245816	0.025932726	0.035	1	645879	FAM90A12P
-0.372715592	0.025823982	0.036	1	2791	GNG11
-0.372715592	0.025823982	0.041	1	9486	CHST10
-0.372715592	0.025823982	0.037	1	79627	OGFRL1
-0.372972965	0.025715595	0.041	1	1009	CDH11
-0.372972965	0.025715595	0.044	1	780529	LOC780529
-0.373230368	0.025607564	0.051	1	3547	IGSF1
-0.373230368	0.025607564	0.047	1	3685	ITGAV
-0.373230368	0.025607564	0.045	1	339403	RXFP4
-0.373487771	0.025499946	0.034	1	1956	EGFR
-0.373745173	0.025392706	0.04	1	6474	SHOX2
-0.373745173	0.025392706	0.032	1	8323	FZD6
-0.373745173	0.025392706	0.039	1	10395	DLC1
-0.374002546	0.025285855	0.029	1	649	BMP1
-0.374002546	0.025285855	0.035	1	259295	TAS2R20
-0.374002546	0.025285855	0.04	1	401131	LOC401131
-0.374259979	0.025179308	0.041	1	80178	C16orf59
-0.374517351	0.025073206	0.036	1	84182	FAM188B
-0.374774784	0.024967477	0.035	1	3449	IFNA16
-0.374774784	0.024967477	0.026	1	9375	TM9SF2
-0.374774784	0.024967477	0.034	1	55626	AMBRA1
-0.374774784	0.024967477	0.035	1	84804	MFSD9
-0.375032157	0.024862099	0.038	1	54963	UCKL1
-0.375032157	0.024862099	0.038	1	643911	CRNDE
-0.375289589	0.024757067	0.03	1	2294	FOXF1
-0.375289589	0.024757067	0.032	1	5501	PPP1CC
-0.375289589	0.024757067	0.031	1	8896	BUD31
-0.375289589	0.024757067	0.037	1	27198	HCAR1
-0.375546962	0.024652431	0.044	1	3617	IMPG1
-0.375546962	0.024652431	0.053	1	10678	B3GNT1
-0.375546962	0.024652431	0.044	1	154043	CNKSR3
-0.375546962	0.024652431	0.062	1	729839	LOC729839
-0.375804365	0.024548186	0.04	1	2027	ENO3
-0.375804365	0.024548186	0.032	1	9842	PLEKHM1
-0.375804365	0.024548186	0.04	1	23268	DNMBP
-0.376061767	0.024444308	0.046	1	79939	SLC35E1
-0.376061767	0.024444308	0.033	1	729082	OIP5-AS1
-0.37631917	0.024340777	0.044	1	4148	MATN3
-0.37631917	0.024340777	0.037	1	5552	SRGN
-0.37631917	0.024340777	0.028	1	9330	GTF3C3
-0.37631917	0.024340777	0.047	1	162494	RHBDL3
-0.376576573	0.02423759	0.034	1	5745	PTH1R
-0.376576573	0.02423759	0.029	1	9034	CCRL2
-0.376576573	0.02423759	0.05	1	284340	CXCL17
-0.376833975	0.024134791	0.028	1	9108	MTMR7
-0.376833975	0.024134791	0.032	1	22889	KIAA0907
-0.376833975	0.024134791	0.039	1	196883	ADCY4
-0.377091348	0.024032379	0.046	1	2905	GRIN2C
-0.377091348	0.024032379	0.033	1	6323	SCN1A
-0.377091348	0.024032379	0.036	1	9866	TRIM66

voxel-wise, subcortex			
Spearman's corr coef	p-val	GenID	Gene
-0.30197233	< 0.001	3490	IGFBP7
-0.302321881	< 0.001	285877	POM121L12
-0.30237481	< 0.001	5326	PLAGL2
-0.302716106	< 0.001	339201	C17orf65
-0.302997202	< 0.001	27252	KLHL20
-0.3030155	< 0.001	221092	HNRNPUL2
-0.303069919	< 0.001	4673	NAP1L1
-0.303158134	< 0.001	9747	FAM115A
-0.30316788	< 0.001	57597	BAHCC1
-0.30323419	< 0.001	118491	TTC18
-0.303293824	< 0.001	2981	GUCA2B
-0.303294182	< 0.001	51176	LEF1
-0.303325951	< 0.001	5139	PDE3A
-0.303473055	< 0.001	389799	C9orf171
-0.303680688	< 0.001	100128174	LOC100128174
-0.30368489	< 0.001	653269	POTEI
-0.30370307	< 0.001	23145	SSPO
-0.303744137	< 0.001	5016	OVGP1
-0.303747922	< 0.001	126147	NTN5
-0.304200292	< 0.001	4958	OMD
-0.304311544	< 0.001	729305	RP11-469N6.1
-0.304568768	< 0.001	129563	DIS3L2
-0.304669708	< 0.001	26151	NAT9
-0.304683566	< 0.001	408186	OVOS
-0.304781079	< 0.001	8870	IER3
-0.304842532	< 0.001	6793	STK10
-0.305049628	< 0.001	2277	FIGF
-0.305072844	< 0.001	729839	LOC729839
-0.305176109	< 0.001	9980	DOPEY2
-0.30525431	< 0.001	51167	CYB5R4
-0.30536446	< 0.001	285154	CYP1B1-AS1
-0.305396289	< 0.001	220869	CBWD5
-0.305399269	< 0.001	653247	PRB2
-0.305590034	< 0.001	55766	H2AFJ
-0.305753201	< 0.001	9488	PIGB
-0.305775285	< 0.001	9699	RIMS2
-0.305915982	< 0.001	197021	LCTL
-0.305949509	< 0.001	2919	CXCL1
-0.30601126	< 0.001	4618	MYF6
-0.30623731	< 0.001	114987	WDR31
-0.30664748	< 0.001	222698	NKAPL
-0.3067424	< 0.001	6559	SLC12A3
-0.306905389	< 0.001	4005	LMO2
-0.306957483	< 0.001	83742	MARVELD1
-0.306959063	< 0.001	27122	DKK3
-0.306985527	< 0.001	152485	ZNF827
-0.307014495	< 0.001	83547	RILP
-0.307108015	< 0.001	342909	ZNF284
-0.30711478	< 0.001	7764	ZNF217
-0.307143569	< 0.001	8045	RASSF7
-0.307175159	< 0.001	5453	POU3F1
-0.307214051	< 0.001	137075	CLDN23
-0.307251304	< 0.001	139818	DOCK11
-0.307272553	< 0.001	28985	MCTS1
-0.307365149	< 0.001	8743	TNFSF10
-0.307450593	< 0.001	5797	PTPRM
-0.307739496	< 0.001	10978	CLP1
-0.307752311	< 0.001	642938	FAM196A
-0.307785481	< 0.001	80760	ITIH5
-0.307791024	< 0.001	3045	HBD
-0.307801157	< 0.001	83482	SCRT1
-0.307902098	< 0.001	55684	C9orf86
-0.308072239	< 0.001	271	AMPD2
-0.308150232	< 0.001	256949	KANK3
-0.308486432	< 0.001	72	ACTG2
-0.308545858	< 0.001	54930	HAUS4
-0.308620423	< 0.001	387644	LINC00202
-0.308656931	< 0.001	4702	NDUFA8
-0.308780164	< 0.001	26025	PCDHGA12
-0.308898181	< 0.001	542767	C1QTNF9B-AS1
-0.308956325	< 0.001	339488	TFAP2E
-0.309008509	< 0.001	283788	LOC283788
-0.309179932	< 0.001	57649	PHF12
-0.309209466	< 0.001	493911	PHOSPHO2
-0.309237599	< 0.001	23678	SGK3
-0.309250534	< 0.001	55145	THAP1
-0.30940932	< 0.001	1116	CHI3L1
-0.30946058	< 0.001	56920	SEMA3G
-0.309505731	< 0.001	85004	RERG

region-wise, subcortex					
Spearman's corr coef	p-val	p-val, SA	p-val, SA (BH- correction)	GenID	Gene
-0.377091348	0.024032379	0.037	1	79015	RP11-445H22.3
-0.377091348	0.024032379	0.029	1	151393	FAM82A1
-0.377348781	0.02393033	0.031	1	6495	SIX1
-0.377348781	0.02393033	0.034	1	64844	MARCHF7
-0.377348781	0.02393033	0.033	1	146956	EME1
-0.377348781	0.02393033	0.03	1	390284	SRP14P1
-0.377606153	0.023828575	0.04	1	3291	HSD11B2
-0.377606153	0.023828575	0.037	1	3786	KCNQ3
-0.377606153	0.023828575	0.038	1	51006	SLC35C2
-0.377606153	0.023828575	0.043	1	55018	LINC00483
-0.377606153	0.023828575	0.033	1	153129	SLC38A9
-0.377606153	0.023828575	0.015	1	256949	KANK3
-0.377863586	0.023727253	0.038	1	55145	THAP1
-0.378120989	0.023626266	0.047	1	51435	SCARA3
-0.378120989	0.023626266	0.034	1	729096	RP11-464F9.1
-0.378378361	0.023525663	0.033	1	5326	PLAGL2
-0.378378361	0.023525663	0.039	1	9445	ITM2B
-0.378378361	0.023525663	0.036	1	57639	CCDC146
-0.378378361	0.023525663	0.041	1	80760	ITIH5
-0.378378361	0.023525663	0.048	1	138429	PIP5KL1
-0.378378361	0.023525663	0.037	1	148206	ZNF714
-0.378893167	0.023325462	0.035	1	6876	TAGLN
-0.378893167	0.023325462	0.028	1	59351	PBOV1
-0.378893167	0.023325462	0.037	1	124274	GPR139
-0.379150599	0.02322593	0.027	1	23078	KIAA0564
-0.379150599	0.02322593	0.033	1	79002	C19orf43
-0.379665375	0.023027912	0.032	1	6926	TBX3
-0.379665375	0.023027912	0.026	1	57684	ZBTB26
-0.379665375	0.023027912	0.032	1	127845	GOLT1A
-0.379922777	0.0229294	0.026	1	51751	HIGD1B
-0.379922777	0.0229294	0.039	1	643438	LOC643438
-0.38018018	0.022831265	0.026	1	79840	NHEJ1
-0.380437583	0.022733504	0.033	1	4430	MYO1B
-0.380437583	0.022733504	0.042	1	57822	GRHL3
-0.380437583	0.022733504	0.036	1	81626	C1orf14
-0.380437583	0.022733504	0.031	1	91353	IGLL3P
-0.380437583	0.022733504	0.025	1	388931	AC104665.1
-0.380694985	0.022636071	0.04	1	10489	LRRC41
-0.380694985	0.022636071	0.037	1	130557	ZNF513
-0.380952358	0.022538966	0.027	1	973	CD79A
-0.380952358	0.022538966	0.037	1	23154	NCDN
-0.380952358	0.022538966	0.032	1	57786	RBAK
-0.380952358	0.022538966	0.029	1	151473	SLC16A14
-0.380952358	0.022538966	0.033	1	474344	GIMAP6
-0.380952358	0.022538966	0.032	1	646993	HMGB3P24
-0.381209791	0.022442251	0.036	1	786	CACNG1
-0.381209791	0.022442251	0.04	1	136895	C7orf31
-0.381467164	0.022345865	0.029	1	55040	EPN3
-0.381467164	0.022345865	0.025	1	57685	CACHD1
-0.381467164	0.022345865	0.031	1	85291	KRTAP4-2
-0.381981969	0.022154156	0.034	1	1080	CFTR
-0.381981969	0.022154156	0.035	1	1489	CTF1
-0.381981969	0.022154156	0.034	1	339451	KLHL17
-0.382239372	0.022058787	0.029	1	6892	TAPBP
-0.382239372	0.022058787	0.036	1	163071	ZNF114
-0.382496774	0.021963805	0.043	1	283663	AC016525.1
-0.382754177	0.021869145	0.033	1	3588	IL10RB
-0.38301158	0.021774847	0.039	1	7074	TIAM1
-0.38301158	0.021774847	0.018	1	154007	SNRNP48
-0.383268982	0.021680828	0.029	1	5544	PRB3
-0.383268982	0.021680828	0.027	1	23329	TBC1D30
-0.383526355	0.021587214	0.028	1	6559	SLC12A3
-0.383526355	0.021587214	0.021	1	403313	PPAPDC2
-0.383783787	0.021493938	0.029	1	5090	PBX3
-0.38404116	0.021400981	0.018	1	3976	LIF
-0.38404116	0.021400981	0.035	1	7481	WNT11
-0.38404116	0.021400981	0.031	1	79971	GPR177
-0.38404116	0.021400981	0.032	1	284648	LOC284648
-0.384298593	0.02130834	0.036	1	9904	RBM19
-0.384298593	0.02130834	0.031	1	255520	ELMOD2
-0.384298593	0.02130834	0.035	1	493754	LOC493754
-0.384813368	0.021124128	0.033	1	2625	GATA3
-0.384813368	0.021124128	0.027	1	4957	ODF2
-0.384813368	0.021124128	0.034	1	128602	C20orf85
-0.384813368	0.021124128	0.037	1	160857	CCDC122
-0.385070801	0.021032535	0.04	1	515	ATP5F1
-0.385070801	0.021032535	0.04	1	26206	SPAG8
-0.385328174	0.020941256	0.024	1	115677	NOSTRIN
-0.385328174	0.020941256	0.029	1	127066	OR14C36

voxel-wise, subcortex			
Spearman's corr coef	p-val	GenID	Gene
-0.309562176	< 0.001	3303	HSPA1A
-0.309653103	< 0.001	3053	SERPIND1
-0.309875309	< 0.001	378938	MALAT1
-0.31012094	< 0.001	9360	PPIG
-0.310293138	< 0.001	387646	RP11-85G18.4
-0.310532808	< 0.001	85027	C5ORF62
-0.310664862	< 0.001	285343	C3orf23
-0.310803413	< 0.001	9452	ITM2A
-0.310866654	< 0.001	195827	AAED1
-0.310966492	< 0.001	3090	HIC1
-0.31120646	< 0.001	168537	GIMAP7
-0.311485529	< 0.001	729967	MORN2
-0.311513186	< 0.001	5826	ABCD4
-0.31166476	< 0.001	3759	KCNJ2
-0.311720192	< 0.001	3449	IFNA16
-0.3117356	< 0.001	284565	NBPF15
-0.311784357	< 0.001	25966	C2CD2
-0.311843067	< 0.001	79796	ALG9
-0.31189248	< 0.001	139285	FAM123B
-0.311915308	< 0.001	79174	CRELD2
-0.31202507	< 0.001	4240	MFGE8
-0.312093347	< 0.001	2324	FLT4
-0.312136799	< 0.001	2302	FOXJ1
-0.312266618	< 0.001	7450	VWF
-0.312337011	< 0.001	119504	C10orf104
-0.312753618	< 0.001	4913	NTHL1
-0.312888443	< 0.001	1302	COL11A2
-0.313044846	< 0.001	949	SCARB1
-0.313280344	< 0.001	5003	SLC22A18AS
-0.313307375	< 0.001	84570	COL25A1
-0.313381582	< 0.001	9592	IER2
-0.313424379	< 0.001	132014	IL17RE
-0.313481033	< 0.001	23704	KCNE4
-0.313529968	< 0.001	5831	PYCR1
-0.313566655	< 0.001	160857	CCDC122
-0.313624322	< 0.001	1842	ECM2
-0.31379807	< 0.001	80144	FRAS1
-0.31383419	< 0.001	83439	TCF7L1
-0.313892156	< 0.001	80201	HKDC1
-0.313928932	< 0.001	493869	GPX8
-0.314036548	< 0.001	9781	RNF144A
-0.314163327	< 0.001	85015	USP45
-0.314188659	< 0.001	170394	PWWP2B
-0.314191282	< 0.001	23528	ZNF281
-0.314225912	< 0.001	29057	FAM156A
-0.314317465	< 0.001	255104	TMCO4
-0.314373851	< 0.001	448834	KPRP
-0.314403921	< 0.001	4661	MYT1
-0.314476281	< 0.001	54780	NSMCE4A
-0.314780682	< 0.001	54660	PCDHB18
-0.314785063	< 0.001	85289	KRTAP4-5
-0.314952493	< 0.001	392862	GRID2IP
-0.315288305	< 0.001	150165	XKR3
-0.315331578	< 0.001	8436	SDPR
-0.315375686	< 0.001	150135	LINC00479
-0.315530121	< 0.001	84733	CBX2
-0.315672398	< 0.001	400710	AC010624.2
-0.315740645	< 0.001	347731	LRRTM3
-0.315868348	< 0.001	401491	RP11-125B21.2
-0.315891653	< 0.001	4038	LRP4
-0.315923959	< 0.001	25769	SLC24A2
-0.315949321	< 0.001	6422	SFRP1
-0.316004306	< 0.001	23059	CLUAP1
-0.316029191	< 0.001	649	BMP1
-0.316160679	< 0.001	2328	FMO3
-0.316175073	< 0.001	221294	NT5DC1
-0.316212416	< 0.001	6646	SOAT1
-0.316223651	< 0.001	400745	SH2D5
-0.316368788	< 0.001	257177	C1orf192
-0.316671073	< 0.001	79097	TRIM48
-0.316801071	< 0.001	162137	MGC34800
-0.316870451	< 0.001	1638	DCT
-0.316900343	< 0.001	79624	C6orf211
-0.317149967	< 0.001	11313	LYPLA2
-0.317256778	< 0.001	140679	SLC32A1
-0.317261875	< 0.001	84299	MIEN1
-0.317312598	< 0.001	2894	GRID1
-0.317673653	< 0.001	80176	SPSB1
-0.317923635	< 0.001	57589	KIAA1432

region-wise, subcortex					
Spearman's corr coef	p-val	p-val, SA	p-val, SA (BH- correction)	GenID	Gene
-0.385585606	0.02085029	0.026	1	5754	PTK7
-0.385842979	0.020759676	0.034	1	3768	KCNJ12
-0.385842979	0.020759676	0.03	1	9197	SLC33A1
-0.385842979	0.020759676	0.036	1	51475	CABP2
-0.386100382	0.020669412	0.03	1	8576	STK16
-0.386100382	0.020669412	0.039	1	51283	BFAR
-0.386100382	0.020669412	0.037	1	54112	GPR88
-0.386100382	0.020669412	0.026	1	55711	FAR2
-0.386357784	0.020579478	0.033	1	4615	MYD88
-0.386357784	0.020579478	0.034	1	7045	TGFBI
-0.386615187	0.020489816	0.039	1	55076	TMEM45A
-0.386615187	0.020489816	0.035	1	55160	ARHGEF10L
-0.38687259	0.020400539	0.027	1	83650	AMAC1L2
-0.38687259	0.020400539	0.023	1	166929	SGMS2
-0.387129992	0.020311579	0.03	1	3362	HTR6
-0.387129992	0.020311579	0.03	1	6478	SIAH2
-0.387129992	0.020311579	0.028	1	6819	SULT1C2
-0.387129992	0.020311579	0.039	1	25966	C2CD2
-0.387129992	0.020311579	0.031	1	55552	ZNF823
-0.387129992	0.020311579	0.043	1	64419	MTMR14
-0.387129992	0.020311579	0.026	1	255189	PLA2G4F
-0.387129992	0.020311579	0.043	1	338596	ST8SIA6
-0.387387365	0.020222949	0.03	1	11211	FZD10
-0.387387365	0.020222949	0.035	1	84891	ZSCAN10
-0.387387365	0.020222949	0.03	1	114785	MBD6
-0.387644798	0.020134611	0.03	1	25973	PARS2
-0.387644798	0.020134611	0.034	1	257407	C2orf72
-0.387644798	0.020134611	0.038	1	415117	STX19
-0.38790217	0.020046618	0.037	1	25844	YIPF3
-0.38790217	0.020046618	0.024	1	650623	LOC650623
-0.388159603	0.019958969	0.029	1	163747	C1orf177
-0.388416976	0.019871632	0.032	1	9882	TBC1D4
-0.388416976	0.019871632	0.033	1	10166	SLC25A15
-0.388416976	0.019871632	0.022	1	442523	DPY19L2P4
-0.388674378	0.019784618	0.027	1	81786	TRIM7
-0.388674378	0.019784618	0.029	1	729020	LOC729020
-0.388931781	0.019697895	0.035	1	129807	NEU4
-0.388931781	0.019697895	0.039	1	222698	NKAPL
-0.389189184	0.019611511	0.034	1	83548	COG3
-0.389189184	0.019611511	0.026	1	85329	LGALS12
-0.389189184	0.019611511	0.032	1	284723	SLC25A34
-0.389446586	0.019525468	0.027	1	55656	INTS8
-0.389446586	0.019525468	0.033	1	83857	TMTC1
-0.389703989	0.019439729	0.034	1	7378	UPP1
-0.389961362	0.019354273	0.033	1	3659	IRF1
-0.389961362	0.019354273	0.031	1	10106	CTDSP2
-0.389961362	0.019354273	0.033	1	64123	ELTD1
-0.389961362	0.019354273	0.031	1	84759	PCGF1
-0.389961362	0.019354273	0.026	1	728464	C6orf186
-0.390218794	0.01926918	0.028	1	7015	TERT
-0.390218794	0.01926918	0.016	1	23063	WAPAL
-0.390218794	0.01926918	0.025	1	51117	COQ4
-0.390218794	0.01926918	0.033	1	169522	KCNV2
-0.390476167	0.01918439	0.029	1	388533	KRTDAP
-0.3907336	0.019099917	0.028	1	6664	SOX11
-0.3907336	0.019099917	0.027	1	152559	PAQR3
-0.3907336	0.019099917	0.03	1	286676	ILDR1
-0.390990973	0.019015718	0.032	1	9031	BAZ1B
-0.390990973	0.019015718	0.034	1	57480	PLEKHG1
-0.390990973	0.019015718	0.031	1	84933	C8orf76
-0.390990973	0.019015718	0.036	1	124923	SGK494
-0.390990973	0.019015718	0.02	1	148418	SAMD13
-0.391248375	0.018931873	0.038	1	90507	SCRN2
-0.391248375	0.018931873	0.036	1	124857	WFIKKN2
-0.391505778	0.018848347	0.03	1	29909	GPR171
-0.39176318	0.018765122	0.035	1	5069	PAPPA
-0.39176318	0.018765122	0.03	1	84264	HAGHL
-0.392020613	0.018682206	0.023	1	10517	FBXW10
-0.392020613	0.018682206	0.025	1	57493	HEG1
-0.392020613	0.018682206	0.023	1	114789	SLC25A25
-0.392277986	0.018599564	0.028	1	22890	ZBTB1
-0.392277986	0.018599564	0.021	1	100132356	LOC100132356
-0.392535388	0.018517267	0.036	1	23086	EXPH5
-0.392792791	0.018435284	0.033	1	919	CD247
-0.393050194	0.0183536	0.033	1	79652	TMEM204
-0.393050194	0.0183536	0.025	1	130340	AP1S3
-0.393050194	0.0183536	0.037	1	165904	XIRP1
-0.393307596	0.018272186	0.027	1	25921	ZDHHC5
-0.393564999	0.018191112	0.025	1	3861	KRT14

voxel-wise, subcortex			
Spearman's corr coef	p-val	GenID	Gene
-0.318076134	< 0.001	256126	SYCE2
-0.318122923	< 0.001	400891	AC002472.1
-0.31818682	< 0.001	23410	SIRT3
-0.318308681	< 0.001	653857	RP5-820A21.1
-0.318549365	< 0.001	3858	KRT10
-0.318693608	< 0.001	64388	GREM2
-0.31872797	< 0.001	5499	PPP1CA
-0.318743378	< 0.001	55815	TSNAXIP1
-0.31885156	< 0.001	55769	ZNF83
-0.31887126	< 0.001	7545	ZIC1
-0.31892547	< 0.001	7124	TNF
-0.319322407	< 0.001	401190	RGS7BP
-0.319399387	< 0.001	26045	LRRTM2
-0.319411814	< 0.001	55626	AMBRA1
-0.319428831	< 0.001	440854	CAPN14
-0.319711119	< 0.001	7436	VLDLR
-0.319711417	< 0.001	6746	SSR2
-0.319743633	< 0.001	11186	RASSF1
-0.319830358	< 0.001	121504	HIST4H4
-0.319877177	< 0.001	26507	CNNM1
-0.319919348	< 0.001	23607	CD2AP
-0.319946557	< 0.001	51809	GALNT7
-0.32008639	< 0.001	90594	ZNF439
-0.320226729	< 0.001	1051	CEBPB
-0.320386559	< 0.001	6584	SLC22A5
-0.320471883	< 0.001	10993	SDS
-0.320513874	< 0.001	6263	RYR3
-0.320527226	< 0.001	1814	DRD3
-0.320549518	< 0.001	9518	GDF15
-0.320555478	< 0.001	717	C2
-0.320817351	< 0.001	27161	EIF2C2
-0.321078956	< 0.001	100303728	RP3-404F18.2
-0.321105599	< 0.001	79958	DENND1C
-0.321295232	< 0.001	132299	OCIAD2
-0.321426928	< 0.001	374403	TBC1D10C
-0.321451068	< 0.001	2030	SLC29A1
-0.321485251	< 0.001	9970	NR1I3
-0.321501136	< 0.001	129530	LYG1
-0.321562886	< 0.001	26223	FBXL21
-0.32161358	< 0.001	9293	GPR52
-0.321681738	< 0.001	57602	USP36
-0.321737945	< 0.001	90952	ESAM
-0.321766526	< 0.001	2948	GSTM4
-0.321771652	< 0.001	84108	PCGF6
-0.321870178	< 0.001	5725	PTBP1
-0.321981788	< 0.001	57003	CCDC47
-0.322038263	< 0.001	23102	TBC1D2B
-0.322054058	< 0.001	9861	PSMD6
-0.322206825	< 0.001	467	ATF3
-0.322548926	< 0.001	26207	PITPNC1
-0.322562665	< 0.001	554235	ASPDH
-0.322609663	< 0.001	386680	KRTAP10-5
-0.322977215	< 0.001	84417	C2orf40
-0.323024035	< 0.001	828	CAPS
-0.323114008	< 0.001	353333	KRTAP10-10
-0.323147088	< 0.001	23085	ERC1
-0.323174298	< 0.001	3915	LAMC1
-0.323230952	< 0.001	1286	COL4A4
-0.323243022	< 0.001	11043	MID2
-0.32340312	< 0.001	201780	SLC10A4
-0.323556811	< 0.001	1636	ACE
-0.323591262	< 0.001	574036	C1orf133
-0.323671967	< 0.001	4860	PNP
-0.323788315	< 0.001	159195	USP54
-0.323810965	< 0.001	9122	SLC16A4
-0.323846817	< 0.001	256374	LOC256374
-0.323909968	< 0.001	60672	MIIP
-0.324428618	< 0.001	835	CASP2
-0.324576557	< 0.001	53373	TPCN1
-0.3246153	< 0.001	10398	MYL9
-0.324718833	< 0.001	3690	ITGB3
-0.324748546	< 0.001	80755	AARSD1
-0.325145364	< 0.001	54874	FNBP1L
-0.32520926	< 0.001	6330	SCN4B
-0.325256795	< 0.001	63916	ELMO2
-0.325309277	< 0.001	5359	PLSCR1
-0.325582117	< 0.001	55073	LRRC37A4P
-0.325740278	< 0.001	84171	LOXL4
-0.325741023	< 0.001	200958	MUC20

region-wise, subcortex					
Spearman's corr coef	p-val	p-val, SA	p-val, SA (BH- correction)	GenID	Gene
-0.393564999	0.018191112	0.032	1	5617	PRL
-0.393822372	0.018110352	0.027	1	81698	LINC00597
-0.394079804	0.018029882	0.028	1	285513	GPRIN3
-0.394337177	0.017949682	0.019	1	3640	INSL3
-0.39459461	0.017869817	0.023	1	84164	ASCC2
-0.394851983	0.017790254	0.023	1	222235	FBXL13
-0.395109385	0.017710999	0.039	1	1807	DPYS
-0.395109385	0.017710999	0.028	1	8528	DDO
-0.395109385	0.017710999	0.025	1	29062	WDR91
-0.395109385	0.017710999	0.032	1	29803	REPIN1
-0.395109385	0.017710999	0.022	1	135228	CD109
-0.395109385	0.017710999	0.026	1	654254	ZNF732
-0.395366788	0.017632039	0.036	1	1396	CRIP1
-0.395366788	0.017632039	0.032	1	3090	HIC1
-0.395366788	0.017632039	0.026	1	10398	MYL9
-0.395366788	0.017632039	0.027	1	25812	POM121L1P
-0.395366788	0.017632039	0.022	1	130399	ACVR1C
-0.395624191	0.017553328	0.025	1	2268	FGR
-0.395624191	0.017553328	0.02	1	147923	ZNF420
-0.395624191	0.017553328	0.028	1	389136	VGLL3
-0.395881593	0.017474962	0.028	1	7712	ZNF157
-0.395881593	0.017474962	0.03	1	90693	CCDC126
-0.395881593	0.017474962	0.031	1	122953	JDP2
-0.396138996	0.017396878	0.018	1	5366	PMAIP1
-0.396138996	0.017396878	0.024	1	441416	GPR116P2
-0.396653801	0.017241584	0.019	1	668	FOXL2
-0.396653801	0.017241584	0.027	1	9022	CLIC3
-0.396911174	0.017164389	0.025	1	2784	GNB3
-0.396911174	0.017164389	0.024	1	3625	INHBB
-0.396911174	0.017164389	0.025	1	8631	SKAP1
-0.396911174	0.017164389	0.025	1	9828	ARHGEF17
-0.397168607	0.017087493	0.039	1	9748	SLK
-0.397168607	0.017087493	0.028	1	400891	AC002472.1
-0.397425979	0.017010875	0.035	1	2977	GUCY1A2
-0.397425979	0.017010875	0.019	1	50834	TAS2R1
-0.397683382	0.016934531	0.023	1	1040	CDS1
-0.397683382	0.016934531	0.022	1	1071	CETP
-0.397683382	0.016934531	0.035	1	1663	DDX11
-0.397683382	0.016934531	0.022	1	9778	KIAA0232
-0.397940785	0.016858498	0.027	1	64766	S100PBP
-0.398198187	0.016782759	0.023	1	55759	WDR12
-0.39845559	0.016707312	0.026	1	9934	P2RY14
-0.39845559	0.016707312	0.018	1	55753	OGDHL
-0.39845559	0.016707312	0.028	1	168537	GIMAP7
-0.39845559	0.016707312	0.021	1	169611	OLFML2A
-0.39845559	0.016707312	0.031	1	390429	OR4N2
-0.398712993	0.016632138	0.023	1	3710	ITPR3
-0.398712993	0.016632138	0.027	1	400579	FLJ35934
-0.399227798	0.016482646	0.022	1	23401	FRAT2
-0.399227798	0.016482646	0.032	1	84101	USP44
-0.399227798	0.016482646	0.022	1	92797	HELB
-0.399742603	0.01633431	0.024	1	56102	PCDHGB3
-0.399742603	0.01633431	0.016	1	64852	TUT1
-0.399742603	0.01633431	0.026	1	83714	NRIP2
-0.399742603	0.01633431	0.021	1	641700	ECSCR
-0.400000006	0.016260525	0.024	1	4324	MMP15
-0.400000006	0.016260525	0.027	1	5211	PFKL
-0.400000006	0.016260525	0.023	1	29984	RHOD
-0.400000006	0.016260525	0.031	1	158228	FAM201A
-0.400257379	0.01618707	0.027	1	22878	KIAA1012
-0.400257379	0.01618707	0.025	1	152831	KLB
-0.400514811	0.0161139	0.025	1	7652	ZNF99
-0.400514811	0.0161139	0.024	1	8436	SDPR
-0.400514811	0.0161139	0.022	1	9120	SLC16A6
-0.400514811	0.0161139	0.026	1	10052	GJC1
-0.400772184	0.016040992	0.025	1	1209	CLPTM1
-0.401029617	0.015968347	0.035	1	23415	KCNH4
-0.401029617	0.015968347	0.022	1	55920	RCC2
-0.401029617	0.015968347	0.029	1	90102	PHLDB2
-0.401286989	0.015895996	0.026	1	857	CAV1
-0.401286989	0.015895996	0.02	1	80144	FRAS1
-0.401544392	0.01582394	0.024	1	6569	SLC34A1
-0.401544392	0.01582394	0.028	1	23361	ZNF629
-0.401801795	0.015752161	0.019	1	55808	ST6GALNAC1
-0.401801795	0.015752161	0.023	1	64118	DUS1L
-0.402059197	0.015680641	0.028	1	90113	VWA5B2
-0.4023166	0.015609383	0.019	1	9122	SLC16A4
-0.4023166	0.015609383	0.025	1	400916	CHCHD10
-0.402574003	0.015538413	0.033	1	757	TMEM50B

voxel-wise, subcortex			
Spearman's corr coef	p-val	GenID	Gene
-0.325921744	< 0.001	114224	PRO2852
-0.325993329	< 0.001	80185	C8orf41
-0.326028258	< 0.001	1602	DACH1
-0.326046824	< 0.001	583	BBS2
-0.326085716	< 0.001	63892	THADA
-0.326190382	< 0.001	2550	GABBR1
-0.32621035	< 0.001	7424	VEGFC
-0.32638225	< 0.001	8884	SLC5A6
-0.326401949	< 0.001	55753	OGDHL
-0.326518565	< 0.001	79834	AC107883.1
-0.326565295	< 0.001	1104	RCC1
-0.326573849	< 0.001	284273	ZADH2
-0.326588243	< 0.001	29090	C18orf55
-0.326902777	< 0.001	4239	MFAP4
-0.326907784	< 0.001	80168	MOGAT2
-0.326911598	< 0.001	93190	C1orf158
-0.327053666	< 0.001	441054	C4orf47
-0.327187479	< 0.001	5108	PCM1
-0.327258438	< 0.001	7083	TK1
-0.327341557	< 0.001	79609	C14orf138
-0.327379644	< 0.001	440193	CCDC88C
-0.327572614	< 0.001	387640	C10orf140
-0.327617079	< 0.001	416	ARSF
-0.327629805	< 0.001	55632	G2E3
-0.327682555	< 0.001	4969	OGN
-0.327859938	< 0.001	9794	MAML1
-0.327924371	< 0.001	6664	SOX11
-0.328027666	< 0.001	8846	ALKBH1
-0.328220814	< 0.001	400931	RP4-695O20__B.1
-0.328255922	< 0.001	7052	TGM2
-0.32840088	< 0.001	653641	GOLGA6C
-0.328435719	< 0.001	79177	ZNF576
-0.32851845	< 0.001	4357	MPST
-0.328521132	< 0.001	8483	CILP
-0.328529507	< 0.001	79652	TMEM204
-0.328598976	< 0.001	84922	FIZ1
-0.328614295	< 0.001	219681	ARMC3
-0.32866323	< 0.001	171019	ADAMTS19
-0.32872358	< 0.001	4615	MYD88
-0.328795284	< 0.001	92283	ZNF461
-0.328818679	< 0.001	723972	ANP32AP1
-0.328837067	< 0.001	4778	NFE2
-0.328910619	< 0.001	92797	HELB
-0.329013705	< 0.001	167465	ZNF366
-0.329563379	< 0.001	3685	ITGAV
-0.329869002	< 0.001	100289574	AC133485.2
-0.3300291	< 0.001	93556	EGFEM1P
-0.330039501	< 0.001	4053	LTBP2
-0.330213517	< 0.001	9435	CHST2
-0.33074072	< 0.001	56287	GKN1
-0.330813438	< 0.001	55268	ECHDC2
-0.330847621	< 0.001	56102	PCDHGB3
-0.330972522	< 0.001	341	APOC1
-0.33127296	< 0.001	84838	ZNF496
-0.331330895	< 0.001	115811	IQCD
-0.331397653	< 0.001	647024	C6orf132
-0.331418186	< 0.001	317762	CCDC85C
-0.331641257	< 0.001	9254	CACNA2D2
-0.331679612	< 0.001	56985	C17orf48
-0.332050592	< 0.001	4635	MYL4
-0.332127035	< 0.001	28987	NOB1
-0.332321018	< 0.001	79891	ZNF671
-0.332331419	< 0.001	9314	KLF4
-0.332393646	< 0.001	23169	SLC35D1
-0.33250761	< 0.001	25896	INTS7
-0.332670778	< 0.001	54429	TAS2R5
-0.332675785	< 0.001	3235	HOXD9
-0.332758248	< 0.001	400566	C17orf97
-0.332826793	< 0.001	25988	HINFP
-0.333127111	< 0.001	84709	C4orf49
-0.333131015	< 0.001	3739	KCNA4
-0.333135098	< 0.001	2633	GBP1
-0.333412588	< 0.001	7072	TIA1
-0.333527356	< 0.001	2889	RAPGEF1
-0.333706886	< 0.001	440689	HIST2H2BF
-0.333726466	< 0.001	138716	C9orf23
-0.333796859	< 0.001	149478	RP11-269F19.1
-0.333918601	< 0.001	7691	ZNF132
-0.333942205	< 0.001	10324	KBTBD10

region-wise, subcortex					
Spearman's corr coef	p-val	p-val, SA	p-val, SA (BH- correction)	GenID	Gene
-0.402574003	0.015538413	0.02	1	401508	FAM74A4
-0.402831376	0.015467734	0.027	1	79041	TMEM38A
-0.403088808	0.015397327	0.02	1	1297	COL9A1
-0.403088808	0.015397327	0.02	1	26090	ABHD12
-0.403088808	0.015397327	0.022	1	79177	ZNF576
-0.403088808	0.015397327	0.019	1	131601	GPR175
-0.403088808	0.015397327	0.022	1	162137	MGC34800
-0.403346181	0.015327144	0.025	1	8553	BHLHE40
-0.403346181	0.015327144	0.027	1	50838	TAS2R13
-0.403603613	0.01525728	0.025	1	3737	KCNA2
-0.403603613	0.01525728	0.026	1	6493	SIM2
-0.403603613	0.01525728	0.021	1	83743	GRWD1
-0.403860986	0.015187671	0.021	1	866	SERPINA6
-0.403860986	0.015187671	0.025	1	285203	C3orf64
-0.404118389	0.015118347	0.025	1	6875	TAF4B
-0.404118389	0.015118347	0.022	1	131544	CRYBG3
-0.404375792	0.015049292	0.026	1	8833	GMPS
-0.404375792	0.015049292	0.012	1	84838	ZNF496
-0.404633194	0.014980458	0.025	1	4969	OGN
-0.404890597	0.014911937	0.035	1	22894	DIS3
-0.405148	0.014843669	0.02	1	1638	DCT
-0.405148	0.014843669	0.023	1	3814	KISS1
-0.405148	0.014843669	0.022	1	246329	STAC3
-0.405148	0.014843669	0.024	1	439921	MXRA7
-0.405405372	0.014775679	0.015	1	440119	RP11-143E21.7
-0.405662805	0.014707925	0.027	1	222183	AC005077.3
-0.405662805	0.014707925	0.02	1	341350	OVCH1
-0.405662805	0.014707925	0.019	1	389834	LOC389834
-0.405920208	0.014640447	0.018	1	2277	FIGF
-0.405920208	0.014640447	0.032	1	4914	NTRK1
-0.405920208	0.014640447	0.031	1	81621	KAZALD1
-0.405920208	0.014640447	0.023	1	257415	FAM133B
-0.406435013	0.0145063	0.025	1	26045	LRRTM2
-0.406692386	0.014439595	0.028	1	57106	NAT14
-0.406692386	0.014439595	0.023	1	84864	MINA
-0.406692386	0.014439595	0.025	1	728264	MIR143HG
-0.406949818	0.014373181	0.024	1	114044	MCM3AP-AS1
-0.407464623	0.014241118	0.026	1	7048	TGFBR2
-0.407721996	0.014175464	0.022	1	1164	CKS2
-0.407721996	0.014175464	0.022	1	5596	MAPK4
-0.407721996	0.014175464	0.016	1	153643	FAM81B
-0.407721996	0.014175464	0.021	1	285955	SPDYE1
-0.407979399	0.014110055	0.016	1	3248	HPGD
-0.407979399	0.014110055	0.021	1	9688	NUP93
-0.407979399	0.014110055	0.025	1	729648	ZNF812
-0.408236802	0.014044932	0.02	1	6581	SLC22A3
-0.408236802	0.014044932	0.022	1	55612	FERMT1
-0.408494204	0.013980049	0.028	1	26524	LATS2
-0.408494204	0.013980049	0.024	1	56253	CRTAM
-0.408751607	0.013915436	0.018	1	284185	LINC00482
-0.40900901	0.013851049	0.016	1	10595	ERN2
-0.409266382	0.013786931	0.017	1	305	ANXA2P3
-0.409523815	0.013723077	0.031	1	144568	A2ML1
-0.409781188	0.013659461	0.025	1	283554	GPR137C
-0.410295993	0.013532983	0.014	1	7186	TRAF2
-0.410295993	0.013532983	0.022	1	25817	FAM19A5
-0.410295993	0.013532983	0.024	1	54830	NUP62CL
-0.410553396	0.013470119	0.019	1	7046	TGFBR1
-0.410810798	0.013407516	0.017	1	1029	CDKN2A
-0.410810798	0.013407516	0.027	1	9480	ONECUT2
-0.410810798	0.013407516	0.022	1	252995	FNDC5
-0.410810798	0.013407516	0.021	1	286144	C8orf83
-0.411068201	0.013345155	0.021	1	57419	SLC24A3
-0.411325604	0.013283013	0.024	1	9080	CLDN9
-0.411325604	0.013283013	0.026	1	9569	GTF2IRD1
-0.411325604	0.013283013	0.024	1	283578	TMED8
-0.411583006	0.013221152	0.02	1	79940	LINC00472
-0.411840379	0.013159524	0.023	1	142684	RAB40A
-0.412097812	0.013098152	0.02	1	9663	LPIN2
-0.412355185	0.013036985	0.025	1	5015	OTX2
-0.41287002	0.012915459	0.02	1	220002	CYBASC3
-0.413127393	0.012855047	0.02	1	22996	TTC39A
-0.413127393	0.012855047	0.026	1	79190	IRX6
-0.413127393	0.012855047	0.023	1	219983	OR4D6
-0.413384825	0.012794862	0.025	1	5634	PRPS2
-0.413384825	0.012794862	0.018	1	257240	KLHL34
-0.413642198	0.012734929	0.016	1	157869	C8orf84
-0.41389963	0.01267525	0.016	1	286410	ATP11C
-0.414157003	0.012615808	0.027	1	2652	OPN1MW

voxel-wise, subcortex			
Spearman's corr coef	p-val	GenID	Gene
-0.334201932	< 0.001	114785	MBD6
-0.334436059	< 0.001	23554	TSPAN12
-0.334525585	< 0.001	5325	PLAGL1
-0.334686339	< 0.001	83886	PRSS27
-0.334823489	< 0.001	85376	RIMBP3
-0.334831744	< 0.001	55316	RSAD1
-0.334974855	< 0.001	643036	SLED1
-0.334982485	< 0.001	6295	SAG
-0.335039675	< 0.001	203062	TSNARE1
-0.335152239	< 0.001	57688	ZSWIM6
-0.335437149	< 0.001	10194	TSHZ1
-0.336012453	< 0.001	91646	TDRD12
-0.336017668	< 0.001	23212	RRS1
-0.336052388	< 0.001	57096	RPGRIP1
-0.336159378	< 0.001	55646	LYAR
-0.336363852	< 0.001	22995	CEP152
-0.336625755	< 0.001	1804	DPP6
-0.336671621	< 0.001	7980	TFPI2
-0.336732924	< 0.001	55066	PDPR
-0.336929053	< 0.001	388284	C16orf86
-0.337075949	< 0.001	1396	CRIP1
-0.337150812	< 0.001	83857	TMTC1
-0.337164551	< 0.001	84787	SUV420H2
-0.337212473	< 0.001	7351	UCP2
-0.337277472	< 0.001	79572	ATP13A3
-0.33733356	< 0.001	51593	SRRT
-0.337441474	< 0.001	1647	GADD45A
-0.337545037	< 0.001	10667	FARS2
-0.337558568	< 0.001	113878	DTX2
-0.337904781	< 0.001	23415	KCNH4
-0.338062018	< 0.001	374897	SBSN
-0.338142604	< 0.001	9427	ECEL1
-0.338647813	< 0.001	1506	CTRL
-0.33875525	< 0.001	5501	PPP1CC
-0.338860482	< 0.001	91977	MYOZ3
-0.338948131	< 0.001	23256	SCFD1
-0.339609981	< 0.001	284427	SLC25A41
-0.339745939	< 0.001	23037	PDZD2
-0.339796454	< 0.001	8874	ARHGEF7
-0.340001702	< 0.001	9868	TOMM70A
-0.340017587	< 0.001	8720	MBTPS1
-0.340039134	< 0.001	27086	FOXP1
-0.340125769	< 0.001	2135	EXTL2
-0.340595394	< 0.001	91392	ZNF502
-0.340608209	< 0.001	8565	YARS
-0.340813637	< 0.001	119391	GSTO2
-0.340991378	< 0.001	64170	CARD9
-0.341031015	< 0.001	23105	FSTL4
-0.341044664	< 0.001	112752	C14orf179
-0.341124177	< 0.001	340547	VSIG1
-0.341353834	< 0.001	7857	SCG2
-0.341444641	< 0.001	55703	POLR3B
-0.341473818	< 0.001	348807	CCDC37
-0.341525078	< 0.001	199704	ZNF585A
-0.34167859	< 0.001	339403	RXFP4
-0.341884941	< 0.001	1005	CDH7
-0.342031002	< 0.001	219931	TPCN2
-0.342110395	< 0.001	57643	ZSWIM5
-0.342121542	< 0.001	79854	LINC00115
-0.342163533	< 0.001	3263	HPX
-0.342360973	< 0.001	284233	CYP4F35P
-0.342523277	< 0.001	283521	LINC00282
-0.34258616	< 0.001	151887	CCDC80
-0.342936456	< 0.001	56474	CTPS2
-0.343009919	< 0.001	399665	FAM102A
-0.343136579	< 0.001	376940	ZC3H6
-0.343257487	< 0.001	339829	CCDC39
-0.343319237	< 0.001	29990	PILRB
-0.34341535	< 0.001	339327	ZNF546
-0.343443215	< 0.001	284076	TTLL6
-0.343519926	< 0.001	2444	FRK
-0.343562633	< 0.001	3976	LIF
-0.343837172	< 0.001	23584	VSIG2
-0.344134688	< 0.001	79006	METRN
-0.344153285	< 0.001	8702	B4GALT4
-0.344320714	< 0.001	165904	XIRP1
-0.344337702	< 0.001	84795	PYROXD2
-0.344418675	< 0.001	9663	LPIN2
-0.344610184	< 0.001	284029	LINC00324

region-wise, subcortex					
Spearman's corr coef	p-val	p-val, SA	p-val, SA (BH- correction)	GenID	Gene
-0.414414406	0.012556599	0.024	1	858	CAV2
-0.414414406	0.012556599	0.014	1	51126	NAT5
-0.414414406	0.012556599	0.018	1	408186	OVOS
-0.414929211	0.012438864	0.023	1	283870	C16orf79
-0.415186614	0.012380364	0.017	1	54763	ROPN1
-0.415186614	0.012380364	0.022	1	150209	AIFM3
-0.415186614	0.012380364	0.027	1	728978	RP11-790G19.2
-0.415701389	0.012264041	0.022	1	9322	TRIP10
-0.415958822	0.012206248	0.019	1	258	AMBN
-0.415958822	0.012206248	0.02	1	3045	HBD
-0.415958822	0.012206248	0.015	1	146395	GSG1L
-0.416216195	0.012148682	0.015	1	2302	FOXJ1
-0.416473627	0.012091348	0.018	1	4318	MMP9
-0.416731	0.012034226	0.016	1	7052	TGM2
-0.416731	0.012034226	0.021	1	9452	ITM2A
-0.416731	0.012034226	0.019	1	79175	ZNF343
-0.416731	0.012034226	0.023	1	79788	ZNF665
-0.416988403	0.011977348	0.016	1	2304	FOXE1
-0.416988403	0.011977348	0.017	1	3736	KCNA1
-0.416988403	0.011977348	0.022	1	56140	PCDHA8
-0.416988403	0.011977348	0.018	1	403277	OR5K3
-0.417245805	0.01192071	0.009	1	57194	ATP10A
-0.417245805	0.01192071	0.022	1	114822	RHPN1
-0.417245805	0.01192071	0.016	1	100130256	LOC100130256
-0.417503208	0.011864296	0.025	1	6442	SGCA
-0.417503208	0.011864296	0.02	1	10747	MASP2
-0.417503208	0.011864296	0.017	1	132014	IL17RE
-0.417503208	0.011864296	0.019	1	388730	TMEM81
-0.417760611	0.011808117	0.018	1	55824	PAG1
-0.418018013	0.011752132	0.024	1	7367	UGT2B17
-0.418018013	0.011752132	0.017	1	163051	ZNF709
-0.418018013	0.011752132	0.014	1	386677	KRTAP10-1
-0.418275386	0.011696401	0.024	1	6014	RIT2
-0.418275386	0.011696401	0.017	1	9457	FHL5
-0.418275386	0.011696401	0.036	1	26268	FBXO9
-0.418275386	0.011696401	0.024	1	83955	NACAP1
-0.418790191	0.011585613	0.016	1	127833	SYT2
-0.419047624	0.011530533	0.018	1	29952	DPP7
-0.419047624	0.011530533	0.016	1	64427	TTC31
-0.419047624	0.011530533	0.017	1	84945	ABHD13
-0.419304997	0.011475705	0.028	1	10003	NAALAD2
-0.419304997	0.011475705	0.025	1	80323	CCDC68
-0.419304997	0.011475705	0.024	1	728485	LOC728485
-0.419562399	0.011421096	0.019	1	2676	GFRA3
-0.419562399	0.011421096	0.02	1	5590	PRKCZ
-0.419819832	0.011366708	0.019	1	49	ACR
-0.419819832	0.011366708	0.024	1	50831	TAS2R3
-0.419819832	0.011366708	0.02	1	221178	SPATA13
-0.420077205	0.011312517	0.021	1	201501	ZBTB7C
-0.420334637	0.011258571	0.014	1	4824	NKX3-1
-0.42059201	0.011204853	0.019	1	165545	DQX1
-0.42059201	0.011204853	0.016	1	221294	NT5DC1
-0.420849413	0.011151346	0.02	1	64332	NFKBIZ
-0.421106815	0.011098064	0.02	1	4081	MAB21L1
-0.421106815	0.011098064	0.019	1	9909	DENND4B
-0.421106815	0.011098064	0.014	1	56918	C2orf83
-0.421106815	0.011098064	0.018	1	339983	NAT8L
-0.421364218	0.011044967	0.018	1	57096	RPGRIP1
-0.421364218	0.011044967	0.021	1	84740	AFAP1-AS1
-0.421879023	0.010939483	0.019	1	151	ADRA2B
-0.421879023	0.010939483	0.017	1	23281	MTUS2
-0.421879023	0.010939483	0.014	1	171425	CLYBL
-0.422651201	0.010782844	0.018	1	26225	ARL5A
-0.422651201	0.010782844	0.018	1	26548	ITGB1BP2
-0.422651201	0.010782844	0.011	1	79070	KDELC1
-0.422908634	0.010731074	0.016	1	1136	CHRNA3
-0.422908634	0.010731074	0.019	1	6603	SMARCD2
-0.422908634	0.010731074	0.016	1	8743	TNFSF10
-0.423166007	0.010679515	0.013	1	2035	EPB41
-0.423166007	0.010679515	0.016	1	5456	POU3F4
-0.423423409	0.010628135	0.018	1	199704	ZNF585A
-0.424195617	0.010475351	0.013	1	55599	RNPC3
-0.42445302	0.010424848	0.021	1	554235	ASPDH
-0.424710393	0.010374518	0.022	1	1537	CYC1
-0.424967825	0.010324431	0.018	1	6539	SLC6A12
-0.425482631	0.010224866	0.016	1	64768	IPPK
-0.425482631	0.010224866	0.02	1	100129917	RP11-440L14.1
-0.425740004	0.010175371	0.012	1	10484	SEC23A
-0.425997406	0.010126103	0.014	1	26136	TES

voxel-wise, subcortex			
Spearman's corr coef	p-val	GenID	Gene
-0.344739735	< 0.001	7007	TECTA
-0.344810128	< 0.001	83939	EIF2A
-0.345002353	< 0.001	51454	GULP1
-0.345072657	< 0.001	2572	GAD2
-0.345171362	< 0.001	1854	DUT
-0.345374078	< 0.001	5649	RELN
-0.345404536	< 0.001	79867	TCTN2
-0.345553607	< 0.001	654254	ZNF732
-0.34567219	< 0.001	373861	HILS1
-0.345692068	< 0.001	200030	NBPF11
-0.345721602	< 0.001	348801	LNP1
-0.345726341	< 0.001	79447	C16orf53
-0.345789105	< 0.001	1108	CHD4
-0.345962852	< 0.001	2842	GPR19
-0.34605822	< 0.001	7057	THBS1
-0.346220106	< 0.001	111	ADCY5
-0.346287996	< 0.001	64118	DUS1L
-0.346410483	< 0.001	257	ALX3
-0.346562207	< 0.001	22844	FRMPD1
-0.346731126	< 0.001	84293	C10orf57
-0.346762061	< 0.001	10878	CFHR3
-0.346798569	< 0.001	56652	C10orf2
-0.346866459	< 0.001	643669	LOC643669
-0.34703064	< 0.001	3043	HBB
-0.34728387	< 0.001	266743	NPAS4
-0.34751603	< 0.001	729338	CETN4P
-0.34758839	< 0.001	442523	DPY19L2P4
-0.347754061	< 0.001	93109	TMEM44
-0.347796023	< 0.001	10090	UST
-0.347914636	< 0.001	151126	ZNF385B
-0.348035991	< 0.001	3913	LAMB2
-0.348119766	< 0.001	4256	MGP
-0.348146141	< 0.001	85002	FAM86B1
-0.348179847	< 0.001	2571	GAD1
-0.348488271	< 0.001	221927	C7orf27
-0.348517597	< 0.001	202915	TMEM184A
-0.348900765	< 0.001	9673	SLC25A44
-0.34902826	< 0.001	79940	LINC00472
-0.349106848	< 0.001	54464	XRN1
-0.349228024	< 0.001	84033	OBSCN
-0.34960106	< 0.001	29992	PILRA
-0.349644065	< 0.001	7123	CLEC3B
-0.349759042	< 0.001	9658	ZNF516
-0.349767298	< 0.001	60485	SAV1
-0.349852175	< 0.001	7011	TEP1
-0.349978	< 0.001	493754	LOC493754
-0.349985719	< 0.001	994	CDC25B
-0.350205064	< 0.001	9767	PHF16
-0.350401014	< 0.001	10244	RABEPK
-0.350437969	< 0.001	389432	SAMD5
-0.350512743	< 0.001	347442	DCAF8L2
-0.350558043	< 0.001	285498	RNF212
-0.350618035	< 0.001	79585	CORO7
-0.350656033	< 0.001	64411	ARAP3
-0.350737005	< 0.001	55138	FAM90A1
-0.350818276	< 0.001	56975	FAM20C
-0.350870818	< 0.001	3383	ICAM1
-0.35115099	< 0.001	100129842	ZNF737
-0.351174682	< 0.001	279	AMY2A
-0.351673573	< 0.001	121512	FGD4
-0.351698995	< 0.001	283663	AC016525.1
-0.351789564	< 0.001	220136	CCDC11
-0.351798177	< 0.001	215	ABCD1
-0.351817876	< 0.001	286144	C8orf83
-0.351833194	< 0.001	5152	PDE9A
-0.351933867	< 0.001	130399	ACVR1C
-0.351973236	< 0.001	5500	PPP1CB
-0.35208106	< 0.001	283870	C16orf79
-0.352281362	< 0.001	1164	CKS2
-0.352381378	< 0.001	578	BAK1
-0.352647066	< 0.001	55667	DENND4C
-0.352688223	< 0.001	7629	ZNF76
-0.352688402	< 0.001	57187	THOC2
-0.353022903	< 0.001	90853	SPOCD1
-0.353069156	< 0.001	3167	HMX2
-0.353132755	< 0.001	388407	C17orf82
-0.353137314	< 0.001	353323	KRTAP12-2
-0.353180498	< 0.001	7091	TLE4
-0.353287011	< 0.001	285382	C3orf70

region-wise, subcortex					
Spearman's corr coef	p-val	p-val, SA	p-val, SA (BH- correction)	GenID	Gene
-0.425997406	0.010126103	0.016	1	440900	AC010982.1
-0.426254809	0.010077045	0.014	1	6004	RGS16
-0.426254809	0.010077045	0.021	1	26586	CKAP2
-0.426254809	0.010077045	0.013	1	79676	OGFOD2
-0.426254809	0.010077045	0.012	1	90864	SPSB3
-0.426254809	0.010077045	0.011	1	167465	ZNF366
-0.426254809	0.010077045	0.02	1	440459	LOC440459
-0.426512212	0.010028181	0.018	1	5564	PRKAB1
-0.426769644	0.00997953	0.009	1	93589	CACNA2D4
-0.427027017	0.00993106	0.015	1	284076	TTLL6
-0.427284449	0.009882802	0.014	1	3043	HBB
-0.427284449	0.009882802	0.028	1	284451	ODF3L2
-0.427541822	0.009834757	0.022	1	57212	TP73-AS1
-0.427799225	0.009786902	0.009	1	3172	HNF4A
-0.427799225	0.009786902	0.018	1	23593	HEBP2
-0.427799225	0.009786902	0.016	1	80853	JHDM1D
-0.42831403	0.00969179	0.017	1	163223	ZNF676
-0.42831403	0.00969179	0.014	1	644256	DND1P2
-0.428828835	0.009597483	0.02	1	3321	IGSF3
-0.428828835	0.009597483	0.017	1	85026	C9orf37
-0.429086208	0.009550599	0.009	1	148281	SYT6
-0.429343641	0.009503943	0.015	1	4846	NOS3
-0.429343641	0.009503943	0.014	1	8356	HIST1H3J
-0.429343641	0.009503943	0.01	1	10901	DHRS4
-0.429601014	0.009457483	0.014	1	116441	TM4SF18
-0.429601014	0.009457483	0.014	1	221400	TDRD6
-0.429858446	0.009411207	0.013	1	79834	AC107883.1
-0.430115819	0.009365137	0.025	1	79828	METTL8
-0.430373222	0.00931923	0.021	1	363	AQP6
-0.430373222	0.00931923	0.01	1	8644	AKR1C3
-0.430373222	0.00931923	0.013	1	27076	LYPD3
-0.430373222	0.00931923	0.007	1	388507	ZNF788
-0.430630624	0.009273546	0.012	1	2101	ESRRA
-0.430630624	0.009273546	0.017	1	85462	FHDC1
-0.4311454	0.009182745	0.015	1	4807	NHLH1
-0.431402832	0.009137617	0.009	1	7766	ZNF223
-0.431402832	0.009137617	0.015	1	149563	C1orf64
-0.431660205	0.009092689	0.019	1	30849	PIK3R4
-0.431917638	0.009047961	0.016	1	578	BAK1
-0.431917638	0.009047961	0.018	1	3426	CFI
-0.431917638	0.009047961	0.01	1	6732	SRPK1
-0.431917638	0.009047961	0.019	1	55709	KBTBD4
-0.431917638	0.009047961	0.017	1	64116	SLC39A8
-0.43217501	0.009003422	0.011	1	488	ATP2A2
-0.43217501	0.009003422	0.013	1	29990	PILRB
-0.432432443	0.008959042	0.018	1	2921	CXCL3
-0.432432443	0.008959042	0.023	1	3310	HSPA6
-0.432689816	0.00891488	0.011	1	219790	RTKN2
-0.432947218	0.008870896	0.009	1	91252	SLC39A13
-0.432947218	0.008870896	0.018	1	339145	FAM92B
-0.432947218	0.008870896	0.011	1	339416	ANKRD45
-0.433204621	0.008827107	0.015	1	25957	SFRS18
-0.433204621	0.008827107	0.011	1	84787	SUV420H2
-0.433462024	0.008783505	0.007	1	653436	ANKRD20A7P
-0.433719456	0.008740058	0.011	1	54891	INO80D
-0.433976829	0.008696826	0.011	1	51270	TFDP3
-0.434234232	0.008653768	0.012	1	342132	ZNF774
-0.434234232	0.008653768	0.014	1	378108	TRIM74
-0.434491634	0.008610904	0.012	1	5018	OXA1L
-0.434491634	0.008610904	0.015	1	50618	ITSN2
-0.434491634	0.008610904	0.008	1	51167	CYB5R4
-0.434749037	0.008568203	0.01	1	4438	MSH4
-0.434749037	0.008568203	0.011	1	353333	KRTAP10-10
-0.43500644	0.008525694	0.018	1	23428	SLC7A8
-0.43500644	0.008525694	0.007	1	119391	GSTO2
-0.435263842	0.008483375	0.012	1	8547	FCN3
-0.435263842	0.008483375	0.016	1	10332	CLEC4M
-0.435263842	0.008483375	0.017	1	257177	C1orf192
-0.435521215	0.008441228	0.018	1	4091	SMAD6
-0.435778648	0.008399253	0.013	1	7515	XRCC1
-0.436293453	0.008315867	0.019	1	643955	RP11-196D18.6
-0.436550826	0.008274446	0.015	1	2137	EXTL3
-0.436808228	0.008233199	0.012	1	150763	GPAT2
-0.436808228	0.008233199	0.018	1	387119	C6orf204
-0.437065631	0.008192111	0.008	1	79658	ARHGAP10
-0.437580436	0.0081105	0.013	1	2916	GRM6
-0.437580436	0.0081105	0.018	1	3235	HOXD9
-0.437580436	0.0081105	0.017	1	85407	NKD1
-0.437837839	0.008069962	0.016	1	285489	DOK7

voxel-wise, subcortex			
Spearman's corr coef	p-val	GenID	Gene
-0.35335955	< 0.001	6869	TACR1
-0.353402734	< 0.001	116441	TM4SF18
-0.353449345	< 0.001	149563	C1orf64
-0.353871316	< 0.001	641700	ECSCR
-0.353880972	< 0.001	6508	SLC4A3
-0.353881717	< 0.001	56172	ANKH
-0.353886366	< 0.001	23063	WAPAL
-0.354314029	< 0.001	80135	RPF1
-0.354471236	< 0.001	644256	DND1P2
-0.354533732	< 0.001	83605	CCM2
-0.354997993	< 0.001	114819	CROCCP3
-0.355035394	< 0.001	340526	RGAG4
-0.355091214	< 0.001	582	BBS1
-0.355352551	< 0.001	2301	FOXE3
-0.355367959	< 0.001	22927	HABP4
-0.355579317	< 0.001	51751	HIGD1B
-0.355663627	< 0.001	1241	LTB4R
-0.355847418	< 0.001	5341	PLEK
-0.35588941	< 0.001	390429	OR4N2
-0.356127053	< 0.001	151393	FAM82A1
-0.35614869	< 0.001	131873	AC128683.1
-0.356179595	< 0.001	10388	SYCP2
-0.356414557	< 0.001	390205	AP003108.2
-0.356687129	< 0.001	79919	C2orf54
-0.356692404	< 0.001	7186	TRAF2
-0.35673058	< 0.001	22894	DIS3
-0.357030988	< 0.001	29062	WDR91
-0.357116342	< 0.001	5412	UBL3
-0.357171029	< 0.001	375612	LHFPL3
-0.35747844	< 0.001	55711	FAR2
-0.357603878	< 0.001	157638	FAM84B
-0.357849061	< 0.001	196264	MPZL3
-0.3578915	< 0.001	2296	FOXC1
-0.357928932	< 0.001	9633	MTL5
-0.358184755	< 0.001	79041	TMEM38A
-0.358401716	< 0.001	11082	ESM1
-0.358477205	< 0.001	8399	PLA2G10
-0.358726919	< 0.001	23051	ZHX3
-0.35872969	< 0.001	132320	SCLT1
-0.358802408	< 0.001	169714	QSOX2
-0.358819604	< 0.001	64518	TEKT3
-0.358939201	< 0.001	723961	INS-IGF2
-0.358966231	< 0.001	84529	C15orf41
-0.358995855	< 0.001	55007	FAM118A
-0.359698385	< 0.001	58190	CTDSP1
-0.359734893	< 0.001	80745	THUMPD2
-0.359768957	< 0.001	55787	CXorf15
-0.359810114	< 0.001	55759	WDR12
-0.35983333	< 0.001	5133	PDCD1
-0.359864891	< 0.001	1847	DUSP
-0.360013962	< 0.001	55656	INTS8
-0.360527396	< 0.001	93589	CACNA2D4
-0.360998601	< 0.001	9311	ACCN3
-0.36103797	< 0.001	6998	TDGF1P3
-0.361098349	< 0.001	81610	FAM83D
-0.36128509	< 0.001	92565	FANK1
-0.361348331	< 0.001	54661	PCDHB17
-0.361574918	< 0.001	6496	SIX3
-0.361598045	< 0.001	114044	MCM3AP-AS1
-0.362178743	< 0.001	275	AMT
-0.362206787	< 0.001	124602	KIF19
-0.362419277	< 0.001	284004	HEXDC
-0.36243096	< 0.001	84264	HAGHL
-0.362487346	< 0.001	6327	SCN2B
-0.36254251	< 0.001	57639	CCDC146
-0.362592548	< 0.001	6801	STRN
-0.362617344	< 0.001	2957	GTF2A1
-0.362641603	< 0.001	55251	PCMTD2
-0.362679392	< 0.001	2563	GABRD
-0.362956673	< 0.001	2791	GNG11
-0.363114268	< 0.001	7030	TFE3
-0.363185495	< 0.001	53632	PRKAG3
-0.36341694	< 0.001	51312	SLC25A37
-0.363801688	< 0.001	146395	GSG1L
-0.363885254	< 0.001	773	CACNA1A
-0.363974601	< 0.001	92162	TMEM88
-0.364378452	< 0.001	57020	C16orf62
-0.364774913	< 0.001	57786	RBAK
-0.364912897	< 0.001	401265	KLHL31

region-wise, subcortex					
Spearman's corr coef	p-val	p-val, SA	p-val, SA (BH- correction)	GenID	Gene
-0.438095212	0.008029581	0.011	1	2242	FES
-0.438095212	0.008029581	0.013	1	54757	FAM20A
-0.438610017	0.007949368	0.015	1	9808	KIAA0087
-0.439124823	0.007869828	0.012	1	1842	ECM2
-0.439124823	0.007869828	0.014	1	24137	KIF4A
-0.439124823	0.007869828	0.013	1	219931	TPCN2
-0.439382225	0.007830329	0.015	1	5915	RARB
-0.439382225	0.007830329	0.015	1	26151	NAT9
-0.439382225	0.007830329	0.022	1	144453	BEST3
-0.439639628	0.007790993	0.008	1	8608	RDH16
-0.439897031	0.007751835	0.013	1	2301	FOXE3
-0.440154433	0.007712843	0.021	1	9170	LPAR2
-0.440154433	0.007712843	0.01	1	158572	LOC158572
-0.440411836	0.007674003	0.012	1	463	ATBF1
-0.440411836	0.007674003	0.015	1	83547	RILP
-0.440669239	0.007635354	0.021	1	5095	PCCA
-0.440926641	0.007596863	0.014	1	730	C7
-0.440926641	0.007596863	0.008	1	9870	KIAA0317
-0.440926641	0.007596863	0.007	1	81629	TSSK3
-0.440926641	0.007596863	0.015	1	91734	IDI2
-0.441184044	0.007558549	0.009	1	7475	WNT6
-0.441184044	0.007558549	0.01	1	23169	SLC35D1
-0.441184044	0.007558549	0.011	1	63926	ANKRD5
-0.441184044	0.007558549	0.012	1	91445	RNF185
-0.442213655	0.007406923	0.008	1	590	BCHE
-0.442213655	0.007406923	0.016	1	5451	POU2F1
-0.443243235	0.007257929	0.008	1	56133	PCDHB2
-0.443500638	0.007221091	0.012	1	347252	IGFBPL1
-0.443758041	0.007184397	0.018	1	642517	BMS1P6
-0.443758041	0.007184397	0.012	1	729495	FAM108A5P
-0.444015443	0.00714788	0.007	1	41	ACCN2
-0.444015443	0.00714788	0.007	1	10849	CD3EAP
-0.444272846	0.007111525	0.008	1	5495	PPM1B
-0.444530219	0.007075333	0.013	1	2324	FLT4
-0.444530219	0.007075333	0.014	1	29941	PKN3
-0.444787651	0.007039277	0.014	1	10878	CFHR3
-0.444787651	0.007039277	0.011	1	139562	OTUD6A
-0.444787651	0.007039277	0.013	1	146429	SLC22A31
-0.445045024	0.007003402	0.012	1	24	ABCA4
-0.445045024	0.007003402	0.006	1	6103	RPGR
-0.445045024	0.007003402	0.006	1	79867	TCTN2
-0.445302457	0.006967682	0.004	1	6998	TDGF1P3
-0.445559829	0.006932124	0.016	1	646576	RP11-291L15.2
-0.445817232	0.006896718	0.01	1	5105	PCK1
-0.446074635	0.006861453	0.006	1	3384	ICAM2
-0.446332037	0.00682636	0.015	1	5997	RGS2
-0.44658944	0.006791422	0.01	1	51705	EMCN
-0.446846843	0.006756642	0.014	1	5178	PEG3AS
-0.446846843	0.006756642	0.012	1	5329	PLAUR
-0.446846843	0.006756642	0.011	1	10082	GPC6
-0.446846843	0.006756642	0.008	1	51136	RNFT1
-0.446846843	0.006756642	0.014	1	152519	NIPAL1
-0.446846843	0.006756642	0.02	1	153769	SH3RF2
-0.447104216	0.006721998	0.009	1	90952	ESAM
-0.447361648	0.006687521	0.008	1	286464	CXorf59
-0.447619021	0.006653198	0.019	1	268	AMH
-0.447619021	0.006653198	0.005	1	1813	DRD2
-0.448133856	0.006584994	0.014	1	2615	LRRC32
-0.448133856	0.006584994	0.011	1	79609	C14orf138
-0.448133856	0.006584994	0.006	1	116337	PANX3
-0.448648661	0.006517414	0.015	1	121512	FGD4
-0.448906034	0.006483852	0.01	1	115123	MARCHF3
-0.448906034	0.006483852	0.015	1	168002	DACT2
-0.449163467	0.006450431	0.011	1	26135	SERBP1
-0.449163467	0.006450431	0.016	1	54825	PCDH24
-0.44942084	0.006417152	0.017	1	654790	PCP4L1
-0.449678242	0.006384034	0.008	1	79768	C15orf29
-0.449935645	0.006351063	0.008	1	1305	COL13A1
-0.449935645	0.006351063	0.009	1	54429	TAS2R5
-0.449935645	0.006351063	0.015	1	54477	PLEKHA5
-0.449935645	0.006351063	0.014	1	440021	KRTAP5-2
-0.450193048	0.006318243	0.009	1	26338	OR5L2
-0.450193048	0.006318243	0.009	1	84249	PSD2
-0.450707853	0.006253023	0.011	1	81571	C9orf45
-0.450965226	0.006220642	0.011	1	1375	CPT1B
-0.450965226	0.006220642	0.004	1	1968	EIF2S3
-0.450965226	0.006220642	0.016	1	114879	OSBPL5
-0.451222658	0.006188399	0.007	1	4053	LTBP2
-0.451222658	0.006188399	0.01	1	10560	SLC19A2

voxel-wise, subcortex			
Spearman's corr coef	p-val	GenID	Gene
-0.365025729	< 0.001	27019	DNAI1
-0.365151018	< 0.001	204851	HIPK1
-0.365197808	< 0.001	6540	SLC6A13
-0.365496576	< 0.001	51705	EMCN
-0.365504384	< 0.001	652995	UCA1
-0.3655352	< 0.001	195828	ZNF367
-0.365641534	< 0.001	5451	POU2F1
-0.365665108	< 0.001	3689	ITGB2
-0.365911216	< 0.001	10082	GPC6
-0.366491824	< 0.001	78989	COLEC11
-0.366560727	< 0.001	2615	LRRC32
-0.366682202	< 0.001	9772	KIAA0195
-0.36674124	< 0.001	285	ANGPT2
-0.366821766	< 0.001	7127	TNFAIP2
-0.366982609	< 0.001	4143	MAT1A
-0.367022187	< 0.001	84690	SPATA22
-0.367077142	< 0.001	221061	FAM171A1
-0.367121905	< 0.001	342132	ZNF774
-0.367178202	< 0.001	730092	RRN3P1
-0.36723724	< 0.001	10251	SPRY3
-0.367290735	< 0.001	5863	RGL2
-0.367319077	< 0.001	2774	GNAL
-0.367320746	< 0.001	956	ENTPD3
-0.367486507	< 0.001	91543	RSAD2
-0.367512584	< 0.001	1388	CREBL1
-0.367620409	< 0.001	10144	FAM13A
-0.367750049	< 0.001	8916	HERC3
-0.367822766	< 0.001	3274	HRH2
-0.368543863	< 0.001	100289350	LOC100289350
-0.36859104	< 0.001	10455	PECI
-0.368624568	< 0.001	148103	ZNF599
-0.369143784	< 0.001	100129075	C14orf33
-0.369208694	< 0.001	84444	DOT1L
-0.369270176	< 0.001	84630	TTBK1
-0.369319201	< 0.001	84307	ZNF397
-0.369712144	< 0.001	8322	FZD4
-0.369761348	< 0.001	10538	BATF
-0.370360613	< 0.001	11023	VAX1
-0.370384306	< 0.001	257240	KLHL34
-0.370558143	< 0.001	2637	GBX2
-0.371402949	< 0.001	3710	ITPR3
-0.371545941	< 0.001	29028	ATAD2
-0.371690542	< 0.001	54738	FEV
-0.371734858	< 0.001	258	AMBN
-0.371806353	< 0.001	257415	FAM133B
-0.371917605	< 0.001	130752	MDH1B
-0.372099996	< 0.001	57687	VAT1L
-0.372363001	< 0.001	3617	IMPG1
-0.372665077	< 0.001	23762	OSBP2
-0.372870237	< 0.001	9034	CCRL2
-0.372933179	< 0.001	1003	CDH5
-0.373013616	< 0.001	6550	SLC9A3
-0.373746216	< 0.001	221400	TDRD6
-0.373828143	< 0.001	221981	THSD7A
-0.374195337	< 0.001	93986	FOXP2
-0.374287546	< 0.001	79875	THSD4
-0.37438488	< 0.001	284185	LINC00482
-0.374438167	< 0.001	8787	RGS9
-0.374547929	< 0.001	29909	GPR171
-0.374584258	< 0.001	81491	GPR63
-0.374587029	< 0.001	151827	LRRC34
-0.374800354	< 0.001	4728	NDUFS8
-0.375006139	< 0.001	56105	PCDHGA11
-0.375070214	< 0.001	388125	FAM148B
-0.375282973	< 0.001	6495	SIX1
-0.376235873	< 0.001	100144748	AC063965.2
-0.376400232	< 0.001	27198	HCAR1
-0.37656945	< 0.001	353322	ANKRD37
-0.37659061	< 0.001	3776	KCNK2
-0.376809418	< 0.001	11059	WWP1
-0.377314329	< 0.001	123904	NRN1L
-0.377426594	< 0.001	50834	TAS2R1
-0.377605826	< 0.001	9481	SLC25A27
-0.377648264	< 0.001	6383	SDC2
-0.377849966	< 0.001	9934	P2RY14
-0.378087431	< 0.001	2057	EPOR
-0.378406256	< 0.001	3291	HSD11B2
-0.378445804	< 0.001	6103	RPGR
-0.378879964	< 0.001	6539	SLC6A12

region-wise, subcortex					
Spearman's corr coef	p-val	p-val, SA	p-val, SA (BH- correction)	GenID	Gene
-0.451480031	0.006156294	0.011	1	1006	CDH8
-0.451480031	0.006156294	0.013	1	120425	AMICA1
-0.451994836	0.006092538	0.01	1	7711	ZNF155
-0.451994836	0.006092538	0.014	1	9306	SOCS6
-0.452252239	0.006060879	0.009	1	1388	CREBL1
-0.452252239	0.006060879	0.011	1	91608	RASL10B
-0.452252239	0.006060879	0.01	1	113878	DTX2
-0.452509642	0.006029354	0.014	1	79817	MOBKL2B
-0.452767044	0.005997966	0.011	1	2219	FCN1
-0.453024447	0.005966726	0.005	1	1044	CDX1
-0.453024447	0.005966726	0.008	1	2765	GML
-0.453024447	0.005966726	0.008	1	63898	SH2D4A
-0.45328185	0.005935633	0.011	1	4017	LOXL2
-0.45328185	0.005935633	0.015	1	64063	PRSS22
-0.453539222	0.005904682	0.01	1	78989	COLEC11
-0.453539222	0.005904682	0.013	1	283011	FLJ37201
-0.453796655	0.005873849	0.005	1	390261	OR6M1
-0.45431146	0.005812637	0.011	1	6310	ATXN1
-0.454568833	0.005782242	0.006	1	1285	COL4A3
-0.454568833	0.005782242	0.01	1	51733	UPB1
-0.454568833	0.005782242	0.016	1	93273	LEMD1
-0.454826236	0.005751972	0.011	1	55504	TNFRSF19
-0.455341041	0.005691862	0.01	1	3767	KCNJ11
-0.455341041	0.005691862	0.012	1	92162	TMEM88
-0.455341041	0.005691862	0.008	1	285877	POM121L12
-0.455598474	0.005662009	0.012	1	846	CASR
-0.455855846	0.005632298	0.011	1	23387	AP006216.1
-0.456370652	0.005573262	0.013	1	7016	TESK1
-0.456370652	0.005573262	0.008	1	53820	DSCR6
-0.456370652	0.005573262	0.007	1	166815	TIGD2
-0.45714286	0.005485726	0.013	1	5350	PLN
-0.45714286	0.005485726	0.01	1	51454	GULP1
-0.457400233	0.00545682	0.008	1	6095	RORA
-0.457915038	0.005399398	0.014	1	200232	FAM209A
-0.458687246	0.005314238	0.005	1	56241	SUSD2
-0.458944649	0.005286111	0.011	1	5139	PDE3A
-0.458944649	0.005286111	0.014	1	57125	PLXDC1
-0.459202051	0.005258121	0.011	1	26207	PITPNC1
-0.459202051	0.005258121	0.011	1	84922	FIZ1
-0.459202051	0.005258121	0.004	1	347730	LRRTM1
-0.459459454	0.005230241	0.009	1	53405	CLIC5
-0.459459454	0.005230241	0.006	1	92565	FANK1
-0.459459454	0.005230241	0.007	1	170959	ZNF431
-0.459716856	0.005202507	0.01	1	219670	ENKUR
-0.459716856	0.005202507	0.011	1	255119	C4orf22
-0.460231662	0.005147417	0.005	1	9241	NOG
-0.460489035	0.005120053	0.01	1	3614	IMPDH1
-0.461261243	0.005038749	0.009	1	5133	PDCD1
-0.461776048	0.004985158	0.007	1	4635	MYL4
-0.461776048	0.004985158	0.008	1	8884	SLC5A6
-0.461776048	0.004985158	0.004	1	56920	SEMA3G
-0.46203348	0.004958551	0.009	1	284009	LOC284009
-0.462548256	0.00490572	0.008	1	3697	ITIH1
-0.462548256	0.00490572	0.005	1	338557	GPR120
-0.462805659	0.004879472	0.014	1	30835	CD209
-0.463063061	0.004853362	0.005	1	79624	C6orf211
-0.463063061	0.004853362	0.011	1	100129842	ZNF737
-0.463835239	0.004775744	0.007	1	390213	DOC2GP
-0.464350045	0.004724606	0.009	1	9084	VCY1B
-0.464607477	0.004699217	0.012	1	80154	AGSK1
-0.46486485	0.004673935	0.011	1	6997	TDGF1
-0.465122253	0.004648785	0.009	1	729305	RP11-469N6.1
-0.465379655	0.004623747	0.008	1	23012	STK38L
-0.465894461	0.004574033	0.007	1	6540	SLC6A13
-0.466409236	0.004524772	0.006	1	10681	GNB5
-0.466409236	0.004524772	0.007	1	26233	FBXL6
-0.466409236	0.004524772	0.007	1	79154	DHRS11
-0.466409236	0.004524772	0.009	1	159195	USP54
-0.466666669	0.00450032	0.004	1	5858	PZP
-0.466666669	0.00450032	0.01	1	390154	OR5T3
-0.467181474	0.004451749	0.008	1	79789	CLMN
-0.467181474	0.004451749	0.004	1	399949	C11orf88
-0.467438847	0.004427641	0.004	1	2633	GBP1
-0.467438847	0.004427641	0.005	1	651250	LRRC37A16P
-0.467696249	0.004403649	0.008	1	717	C2
-0.467696249	0.004403649	0.005	1	168544	ZNF467
-0.467953652	0.004379768	0.007	1	653509	SFTPA1
-0.46872586	0.00430879	0.011	1	2869	GRK5
-0.468983263	0.00428536	0.002	1	10218	ANGPTL7

voxel-wise, subcortex			
Spearman's corr coef	p-val	GenID	Gene
-0.379201353	< 0.001	27299	ADAMDEC1
-0.379508853	< 0.001	793	CALB1
-0.379839808	< 0.001	23286	WWC1
-0.380070031	< 0.001	5937	RBMS1
-0.38029626	< 0.001	79864	C11orf63
-0.380379736	< 0.001	100170765	C19orf69
-0.38052851	< 0.001	10003	NAALAD2
-0.380776554	< 0.001	5590	PRKCZ
-0.380821973	< 0.001	9381	OTOF
-0.381030738	< 0.001	389421	LIN28B
-0.381155819	< 0.001	80069	C6ORF208
-0.381171137	< 0.001	326340	ZAR1
-0.381206721	< 0.001	2035	EPB41
-0.381318599	< 0.001	1009	CDH11
-0.38133496	< 0.001	7871	SLMAP
-0.381452888	< 0.001	5729	PTGDR
-0.381681174	< 0.001	3362	HTR6
-0.381758064	< 0.001	152559	PAQR3
-0.382018745	< 0.001	163933	FAM43B
-0.382811815	< 0.001	2022	ENG
-0.382941365	< 0.001	23225	NUP210
-0.383080572	< 0.001	83992	CTTNBP2
-0.38317129	< 0.001	5915	RARB
-0.383367598	< 0.001	400916	CHCHD10
-0.383474857	< 0.001	221091	LRRN4CL
-0.383587152	< 0.001	6533	SLC6A6
-0.383646846	< 0.001	4160	MC4R
-0.383786798	< 0.001	11035	RIPK3
-0.383917004	< 0.001	64150	DIO3OS
-0.384004295	< 0.001	5091	PC
-0.384300172	< 0.001	148523	C1orf51
-0.385393381	< 0.001	126432	RINL
-0.385561675	< 0.001	55340	GIMAP5
-0.386435717	< 0.001	389158	PLSCR5
-0.386626005	< 0.001	56929	FEM1C
-0.387109369	< 0.001	388931	AC104665.1
-0.387544543	< 0.001	54112	GPR88
-0.387732774	< 0.001	5598	MAPK7
-0.387785792	< 0.001	57703	CWC22
-0.38778618	< 0.001	7547	ZIC3
-0.388318866	< 0.001	129521	NMS
-0.388937145	< 0.001	3590	IL11RA
-0.389336109	< 0.001	135892	TRIM50
-0.389366001	< 0.001	7035	TFPI
-0.389608681	< 0.001	79413	ZBED2
-0.389740735	< 0.001	3614	IMPDH1
-0.389820486	< 0.001	651250	LRRC37A16P
-0.390057951	< 0.001	81931	ZNF93
-0.390072614	< 0.001	10142	AKAP9
-0.390120536	< 0.001	27115	PDE7B
-0.390165955	< 0.001	100125556	RP11-666A20.1
-0.39036423	< 0.001	144193	AMDHD1
-0.390756041	< 0.001	114041	C21orf88
-0.390969068	< 0.001	10106	CTDSP2
-0.391057193	< 0.001	8853	ASAP2
-0.391787022	< 0.001	4330	MN1
-0.39182511	< 0.001	431707	LHX8
-0.392017066	< 0.001	965	CD58
-0.392319322	< 0.001	79675	FASTKD1
-0.393059671	< 0.001	386682	KRTAP10-3
-0.393203974	< 0.001	3384	ICAM2
-0.393293947	< 0.001	378108	TRIM74
-0.39349547	< 0.001	3164	NR4A1
-0.393647134	< 0.001	10317	B3GALT5
-0.394148707	< 0.001	2674	GFRA1
-0.394221783	< 0.001	840	CASP7
-0.39428845	< 0.001	491	ATP2B2
-0.394320607	< 0.001	10100	TSPAN2
-0.394755751	< 0.001	9748	SLK
-0.394995928	< 0.001	10873	ME3
-0.395844519	< 0.001	85291	KRTAP4-2
-0.396083564	< 0.001	1666	DECR1
-0.396094322	< 0.001	80307	FER1L4
-0.396423638	< 0.001	23742	C15orf2
-0.396616787	< 0.001	54477	PLEKHA5
-0.396702051	< 0.001	100293090	MTND4P12
-0.396719873	< 0.001	54829	ASPN
-0.396989018	< 0.001	2308	FOXO1
-0.397031724	< 0.001	160518	DENND5B

region-wise, subcortex					
Spearman's corr coef	p-val	p-val, SA	p-val, SA (BH- correction)	GenID	Gene
-0.468983263	0.00428536	0.008	1	400258	C14orf180
-0.469240665	0.004262039	0.014	1	154197	PNLDC1
-0.469498068	0.004238818	0.007	1	2254	FGF9
-0.470012873	0.00419273	0.01	1	7547	ZIC3
-0.470785052	0.004124403	0.013	1	317671	RFESD
-0.470785052	0.004124403	0.004	1	647589	AC226150.1
-0.471299857	0.004079402	0.008	1	151887	CCDC80
-0.47155726	0.004057061	0.004	1	23225	NUP210
-0.471814662	0.004034818	0.006	1	84690	SPATA22
-0.472072065	0.00401269	0.006	1	72	ACTG2
-0.472072065	0.00401269	0.004	1	1666	DECR1
-0.472329468	0.003990668	0.007	1	55304	SPTLC3
-0.472329468	0.003990668	0.003	1	85289	KRTAP4-5
-0.472844243	0.003946933	0.007	1	8711	TNK1
-0.472844243	0.003946933	0.01	1	56975	FAM20C
-0.473101676	0.003925227	0.003	1	23584	VSIG2
-0.473616481	0.003882126	0.006	1	11043	MID2
-0.473616481	0.003882126	0.006	1	56963	RGMA
-0.474646062	0.003797158	0.01	1	5778	PTPN7
-0.474903464	0.003776169	0.003	1	7276	TTR
-0.474903464	0.003776169	0.009	1	284029	LINC00324
-0.475160867	0.003755278	0.005	1	83595	SOX7
-0.475160867	0.003755278	0.005	1	112609	MRAP2
-0.475675672	0.003713812	0.007	1	10317	B3GALT5
-0.475675672	0.003713812	0.004	1	133874	AC034199.2
-0.475933075	0.003693231	0.005	1	7057	THBS1
-0.475933075	0.003693231	0.005	1	8629	JRK
-0.475933075	0.003693231	0.01	1	386682	KRTAP10-3
-0.476190478	0.003672739	0.012	1	7545	ZIC1
-0.476190478	0.003672739	0.006	1	51409	HEMK1
-0.476190478	0.003672739	0.011	1	55124	PIWIL2
-0.476190478	0.003672739	0.006	1	55349	CHDH
-0.47644788	0.003652356	0.009	1	654	BMP6
-0.476962686	0.003611884	0.001	1	3167	HMX2
-0.476962686	0.003611884	0.004	1	100293090	MTND4P12
-0.477477491	0.003571798	0.011	1	104	ADARB1
-0.477477491	0.003571798	0.009	1	7773	ZNF230
-0.477734864	0.003551905	0.006	1	64388	GREM2
-0.477734864	0.003551905	0.004	1	85002	FAM86B1
-0.478507072	0.003492798	0.005	1	58190	CTDSP1
-0.47927925	0.003434559	0.004	1	79961	DENND2D
-0.47927925	0.003434559	0.004	1	84107	ZIC4
-0.479794055	0.003396197	0.004	1	4005	LMO2
-0.479794055	0.003396197	0.005	1	23087	TRIM35
-0.479794055	0.003396197	0.007	1	388125	FAM148B
-0.480566263	0.003339367	0.006	1	84168	ANTXR1
-0.481338471	0.003283373	0.007	1	29078	NDUFAF4
-0.482110679	0.003228194	0.007	1	845	CASQ2
-0.482110679	0.003228194	0.006	1	119385	AGAP11
-0.482625484	0.00319187	0.006	1	5304	PIP
-0.482625484	0.00319187	0.004	1	163882	C1orf71
-0.482882887	0.003173829	0.005	1	11082	ESM1
-0.482882887	0.003173829	0.01	1	85027	C5ORF62
-0.48314026	0.003155892	0.012	1	340888	LOC340888
-0.483655065	0.003120278	0.007	1	84623	KIRREL3
-0.483655065	0.003120278	0.004	1	157285	AC068353.1
-0.483655065	0.003120278	0.005	1	401190	RGS7BP
-0.483912498	0.003102595	0.005	1	375612	LHFPL3
-0.484169871	0.003085007	0.009	1	125962	OR7G1
-0.484169871	0.003085007	0.007	1	200132	TCTEX1D1
-0.484427303	0.003067508	0.003	1	441932	OR2W5
-0.484684676	0.003050095	0.003	1	2135	EXTL2
-0.484942079	0.003032772	0.002	1	26609	VCX
-0.485199481	0.003015525	0.003	1	8324	FZD7
-0.485199481	0.003015525	0.007	1	164153	UBL4B
-0.485456884	0.002998371	0.002	1	56110	PCDHGA5
-0.485456884	0.002998371	0.009	1	83886	PRSS27
-0.485714257	0.002981302	0.003	1	947	CD34
-0.485714257	0.002981302	0.009	1	1879	EBF1
-0.485714257	0.002981302	0.004	1	57680	CHD8
-0.485971689	0.002964319	0.01	1	9576	SPAG6
-0.486486495	0.002930598	0.003	1	85015	USP45
-0.486743867	0.002913869	0.01	1	65083	NOL6
-0.4870013	0.002897226	0.005	1	157506	RDH10
-0.487516075	0.002864176	0.006	1	2348	FOLR1
-0.487516075	0.002864176	0.002	1	55073	LRRC37A4P
-0.487773478	0.002847779	0.004	1	1911	PHC1
-0.488030881	0.002831464	0.002	1	54660	PCDHB18
-0.488545686	0.002799073	0.005	1	23212	RRS1

voxel-wise, subcortex			
Spearman's corr coef	p-val	GenID	Gene
-0.397046864	< 0.001	5336	PLCG2
-0.397217453	< 0.001	130340	AP1S3
-0.397849679	< 0.001	41	ACCN2
-0.39800486	< 0.001	4521	NUDT1
-0.398056418	< 0.001	4157	MC1R
-0.39839536	< 0.001	3880	KRT19
-0.398666352	< 0.001	163882	C1orf71
-0.398702949	< 0.001	163702	IL28RA
-0.39878875	< 0.001	9260	PDLIM7
-0.398835093	< 0.001	91801	ALKBH8
-0.39900291	< 0.001	646999	LOC646999
-0.399067521	< 0.001	2982	GUCY1A3
-0.399131596	< 0.001	1022	CDK7
-0.399281681	< 0.001	3736	KCNA1
-0.399516076	< 0.001	11061	LECT1
-0.400043726	< 0.001	5778	PTPN7
-0.400336087	< 0.001	947	CD34
-0.40046832	< 0.001	79734	KCTD17
-0.400606126	< 0.001	254050	LRRC43
-0.40103963	< 0.001	11113	CIT
-0.40126121	< 0.001	10625	IVNS1ABP
-0.401359469	< 0.001	54976	C20orf27
-0.401466459	< 0.001	785	CACNB4
-0.401542783	< 0.001	11320	MGAT4A
-0.401621908	< 0.001	1644	DDC
-0.401673079	< 0.001	128344	C1orf88
-0.401753962	< 0.001	91608	RASL10B
-0.401852131	< 0.001	100289137	LOC100289137
-0.401934505	< 0.001	136895	C7orf31
-0.402356833	< 0.001	64116	SLC39A8
-0.402922392	< 0.001	166815	TIGD2
-0.403055847	< 0.001	116337	PANX3
-0.40320164	< 0.001	4995	OR3A2
-0.403241009	< 0.001	2551	GABPA
-0.403702289	< 0.001	10678	B3GNT1
-0.403754771	< 0.001	5256	PHKA2
-0.404135495	< 0.001	81698	LINC00597
-0.404157341	< 0.001	286826	LIN9
-0.404321432	< 0.001	6478	SIAH2
-0.404609948	< 0.001	51409	HEMK1
-0.404955417	< 0.001	283383	GPR133
-0.404979557	< 0.001	133584	EGFLAM
-0.405046433	< 0.001	148418	SAMD13
-0.40507412	< 0.001	84679	SLC9A7
-0.405098617	< 0.001	317671	RFESD
-0.405114323	< 0.001	8387	OR1E1
-0.405550331	< 0.001	943	TNFRSF8
-0.405829281	< 0.001	654429	LRTM2
-0.405886114	< 0.001	6397	SEC14L1
-0.406246066	< 0.001	58986	TMEM8A
-0.406322122	< 0.001	92181	UBTD2
-0.406359941	< 0.001	84182	FAM188B
-0.40645501	< 0.001	25957	SFRS18
-0.407045931	< 0.001	10610	ST6GALNAC2
-0.407092929	< 0.001	5618	PRLR
-0.407386571	< 0.001	84623	KIRREL3
-0.407402426	< 0.001	4318	MMP9
-0.407937825	< 0.001	83891	SNX25
-0.408401579	< 0.001	9013	TAF1C
-0.408408076	< 0.001	64094	SMOC2
-0.408689469	< 0.001	221806	VWDE
-0.408827573	< 0.001	1039	CDR2
-0.409553975	< 0.001	987	LRBA
-0.409632802	< 0.001	1101	CHAD
-0.409934253	< 0.001	728464	C6orf186
-0.410337001	< 0.001	386677	KRTAP10-1
-0.410560817	< 0.001	8439	NSMAF
-0.411396325	< 0.001	145173	B3GALTL
-0.411785632	< 0.001	196996	GRAMD2
-0.411956489	< 0.001	85477	SCIN
-0.412080854	< 0.001	388533	KRTDAP
-0.412464559	< 0.001	146802	SLC47A2
-0.412525296	< 0.001	5276	SERPINI2
-0.412765086	< 0.001	138065	RNF183
-0.412942171	< 0.001	64785	GINS3
-0.413151205	< 0.001	132204	SYNPR
-0.413338333	< 0.001	134728	IRAK1BP1
-0.413494736	< 0.001	4091	SMAD6
-0.414412796	< 0.001	56130	PCDHB6

region-wise, subcortex					
Spearman's corr coef	p-val	p-val, SA	p-val, SA (BH- correction)	GenID	Gene
-0.488803059	0.002783002	0.003	1	29075	RP5-1068E13.3
-0.489060491	0.002767013	0.002	1	2828	GPR4
-0.489317864	0.002751105	0.003	1	374987	NEXN-AS1
-0.489575297	0.00273527	0.004	1	2982	GUCY1A3
-0.489832699	0.002719522	0.006	1	5322	PLA2G5
-0.489832699	0.002719522	0.005	1	9099	USP2
-0.489832699	0.002719522	0.001	1	57522	SRGAP1
-0.490090072	0.002703857	0.008	1	79413	ZBED2
-0.490347505	0.002688267	0.002	1	1075	CTSC
-0.490347505	0.002688267	0.004	1	57101	ANO2
-0.490604877	0.002672757	0.005	1	2296	FOXC1
-0.49086231	0.002657318	0.006	1	51666	ASB4
-0.49086231	0.002657318	0.002	1	134728	IRAK1BP1
-0.491634488	0.002611494	0.003	1	4256	MGP
-0.491891891	0.002596363	0.005	1	2022	ENG
-0.492406696	0.002566352	0.006	1	652	BMP4
-0.492921501	0.002536641	0.002	1	22822	PHLDA1
-0.492921501	0.002536641	0.006	1	646262	RP1-74M1.1
-0.493436307	0.002507237	0.007	1	203062	TSNARE1
-0.493951082	0.002478132	0.004	1	491	ATP2B2
-0.493951082	0.002478132	0.005	1	3554	IL1R1
-0.494208485	0.002463688	0.003	1	59	ACTA2
-0.494208485	0.002463688	0.001	1	6383	SDC2
-0.494465888	0.002449318	0.009	1	11113	CIT
-0.494465888	0.002449318	0.005	1	83482	SCRT1
-0.494465888	0.002449318	0.004	1	94081	SFXN1
-0.49472329	0.002435028	0.004	1	10978	CLP1
-0.495238066	0.002406655	0.007	1	79616	CCNJL
-0.495495498	0.002392581	0.003	1	25953	PNKD
-0.495495498	0.002392581	0.002	1	51776	ZAK
-0.495752871	0.002378579	0.004	1	151126	ZNF385B
-0.496525079	0.002336998	0.007	1	5863	RGL2
-0.496782511	0.002323285	0.008	1	7226	TRPM2
-0.496782511	0.002323285	0.002	1	339201	C17orf65
-0.497297317	0.002296066	0.005	1	3741	KCNA5
-0.49755469	0.002282553	0.008	1	23762	OSBP2
-0.497812092	0.002269122	0.005	1	29028	ATAD2
-0.498069495	0.002255757	0.004	1	116535	MRGPRF
-0.4985843	0.002229229	0.004	1	80185	C8orf41
-0.498841703	0.002216071	0.002	1	1668	DEFA3
-0.498841703	0.002216071	0.004	1	56121	PCDHB15
-0.498841703	0.002216071	0.003	1	284370	ZNF615
-0.499099076	0.002202981	0.004	1	1308	COL17A1
-0.499613881	0.002177	0.005	1	79025	C20orf195
-0.500128686	0.002151295	0.006	1	2328	FMO3
-0.500128686	0.002151295	0.002	1	7222	TRPC3
-0.501158297	0.002100681	0.003	1	83439	TCF7L1
-0.501158297	0.002100681	0.005	1	93986	FOXP2
-0.50141567	0.002088192	0.005	1	23223	RRP12
-0.501673102	0.00207577	0.005	1	54839	LRRC49
-0.502187908	0.002051114	0.001	1	100129543	ZNF730
-0.502702653	0.002026725	0.001	1	93190	C1orf158
-0.502960086	0.002014619	0.002	1	59347	FKSG2
-0.503217459	0.002002583	0.008	1	2831	NPBWR1
-0.503732324	0.001978699	0.007	1	222537	HS3ST5
-0.503989697	0.001966852	0.003	1	64518	TEKT3
-0.503989697	0.001966852	0.006	1	440854	CAPN14
-0.504247129	0.001955062	0.002	1	23286	WWC1
-0.504247129	0.001955062	0.001	1	55188	RIC8B
-0.504504502	0.001943343	0.004	1	4661	MYT1
-0.504761934	0.001931679	0.001	1	339501	MPN2
-0.505019307	0.001920083	0.003	1	56172	ANKH
-0.50527668	0.001908538	0.005	1	2668	GDNF
-0.50527668	0.001908538	0.004	1	5325	PLAGL1
-0.505534112	0.001897064	0.005	1	56311	ANKRD7
-0.505791485	0.001885648	0.005	1	11035	RIPK3
-0.506306291	0.001862994	0.004	1	642475	C8orf73
-0.506563663	0.001851759	0.004	1	5003	SLC22A18AS
-0.507593274	0.001807413	0.004	1	49854	ZNF295
-0.508108079	0.001785594	0.004	1	6674	SPAG1
-0.508622885	0.001764002	0.003	1	7373	COL14A1
-0.508622885	0.001764002	0.008	1	8685	MARCO
-0.508880317	0.001753296	0.003	1	90075	ZNF30
-0.508880317	0.001753296	0.003	1	197322	ACSF3
-0.50913769	0.001742649	0.005	1	729786	GOLGA8C
-0.509395123	0.001732056	0.002	1	57604	KIAA1456
-0.509395123	0.001732056	0.002	1	64981	MRPL34
-0.509652495	0.001721516	0.004	1	9162	DGKI
-0.510424674	0.001690248	0.002	1	29122	TSP50

voxel-wise, subcortex			
Spearman's corr coef	p-val	GenID	Gene
-0.415181458	< 0.001	123099	DEGS2
-0.415565372	< 0.001	56256	SERTAD4
-0.415628612	< 0.001	50515	CHST11
-0.415876657	< 0.001	153769	SH3RF2
-0.416158408	< 0.001	10867	TSPAN9
-0.416417867	< 0.001	9902	MRC2
-0.416505814	< 0.001	130367	SGPP2
-0.416767061	< 0.001	7015	TERT
-0.416866034	< 0.001	53820	DSCR6
-0.416902274	< 0.001	347252	IGFBPL1
-0.417130232	< 0.001	1690	COCH
-0.417442173	< 0.001	5225	PGC
-0.418821514	< 0.001	9099	USP2
-0.419027507	< 0.001	2977	GUCY1A2
-0.419572264	< 0.001	150763	GPAT2
-0.419685721	< 0.001	2788	AC005512.1
-0.419879436	< 0.001	65065	NBEAL1
-0.421010852	< 0.001	152519	NIPAL1
-0.421085507	< 0.001	4879	NPPB
-0.421241879	< 0.001	339416	ANKRD45
-0.421453446	< 0.001	7367	UGT2B17
-0.421875507	< 0.001	10385	BTN2A2
-0.421941161	< 0.001	25973	PARS2
-0.422336787	< 0.001	23223	RRP12
-0.422387749	< 0.001	80127	C14orf45
-0.422448575	< 0.001	56311	ANKRD7
-0.423809618	< 0.001	7222	TRPC3
-0.424165666	< 0.001	687	KLF9
-0.424276292	< 0.001	29098	RANGRF
-0.424564272	< 0.001	168544	ZNF467
-0.424639851	< 0.001	54970	TTC12
-0.424711913	< 0.001	79828	METTL8
-0.424773961	< 0.001	277	AMY1B
-0.425274968	< 0.001	8899	PRPF4B
-0.425343305	< 0.001	54586	C9orf11
-0.425706416	< 0.001	80746	TSEN2
-0.425741702	< 0.001	203111	C8orf47
-0.425750911	< 0.001	8741	TNFSF13
-0.425962269	< 0.001	7162	TPBG
-0.426032394	< 0.001	196483	FAM86A
-0.426443696	< 0.001	129401	NUP35
-0.426679462	< 0.001	4703	NEB
-0.427386463	< 0.001	84080	C16orf48
-0.427481264	< 0.001	1080	CFTR
-0.427507728	< 0.001	64981	MRPL34
-0.427622527	< 0.001	25953	PNKD
-0.427672863	< 0.001	1375	CPT1B
-0.428216666	< 0.001	5090	PBX3
-0.428216964	< 0.001	79583	AC009163.1
-0.42842266	< 0.001	8528	DDO
-0.428490371	< 0.001	1540	CYLD
-0.428782523	< 0.001	22822	PHLDA1
-0.428813994	< 0.001	6310	ATXN1
-0.429058224	< 0.001	8771	TNFRSF6B
-0.429384112	< 0.001	254531	LPCAT4
-0.429689616	< 0.001	3554	IL1R1
-0.429741442	< 0.001	7226	TRPM2
-0.429914176	< 0.001	2242	FES
-0.429995239	< 0.001	90075	ZNF30
-0.431020021	< 0.001	4023	LPL
-0.431446105	< 0.001	26074	C20orf26
-0.431738347	< 0.001	25914	RTTN
-0.431922883	< 0.001	79887	PLBD1
-0.432079345	< 0.001	2009	EML1
-0.432239354	< 0.001	93099	DMKN
-0.432475984	< 0.001	2869	GRK5
-0.43323794	< 0.001	55612	FERMT1
-0.433387816	< 0.001	1308	COL17A1
-0.433410853	< 0.001	286464	CXorf59
-0.434181452	< 0.001	118980	SFXN2
-0.43420729	< 0.001	3741	KCNA5
-0.434228629	< 0.001	6710	SPTB
-0.434440553	< 0.001	861	RUNX1
-0.434715271	< 0.001	127845	GOLT1A
-0.434743673	< 0.001	23154	NCDN
-0.434839338	< 0.001	29127	RACGAP1P
-0.435402095	< 0.001	10395	DLC1
-0.435796112	< 0.001	284370	ZNF615
-0.436090976	< 0.001	55188	RIC8B

region-wise, subcortex					
Spearman's corr coef	p-val	p-val, SA	p-val, SA (BH- correction)	GenID	Gene
-0.510682106	0.001679937	0.002	1	25769	SLC24A2
-0.510682106	0.001679937	< 0.001	< 0.001	51765	STK3
-0.511196911	0.001659477	0.003	1	54586	C9orf11
-0.513256133	0.00157983	0.003	1	2138	EYA1
-0.513256133	0.00157983	0.002	1	392862	GRID2IP
-0.513770878	0.001560454	0.001	1	3263	HPX
-0.514543116	0.001531783	0.004	1	64127	NOD2
-0.514543116	0.001531783	0.003	1	652995	UCA1
-0.514800489	0.00152233	0.001	1	7424	VEGFC
-0.515315294	0.001503575	0.004	1	100130934	ZNF663
-0.516602337	0.001457586	0.003	1	57649	PHF12
-0.517117083	0.001439542	0.002	1	9020	MAP3K14
-0.517631948	0.001421691	0.001	1	160851	DGKH
-0.517889321	0.001412841	0.003	1	79228	THOC6
-0.518146694	0.001404043	0.001	1	143686	SESN3
-0.518918931	0.001377923	0.003	1	730087	ZNF726
-0.519176304	0.001369317	0.001	1	7030	TFE3
-0.519176304	0.001369317	0.002	1	730232	EEF1A1P32
-0.51969111	0.001352242	0.003	1	57703	CWC22
-0.519948542	0.001343772	0.005	1	25788	RAD54B
-0.519948542	0.001343772	0.004	1	79974	C7orf58
-0.520205915	0.001335353	< 0.001	< 0.001	339488	TFAP2E
-0.520463288	0.00132698	0.003	1	389432	SAMD5
-0.520978153	0.00131037	0.004	1	2120	ETV6
-0.521492898	0.001293947	< 0.001	< 0.001	51176	LEF1
-0.521750331	0.001285807	0.003	1	3699	ITIH3
-0.521750331	0.001285807	0.001	1	10846	PDE10A
-0.522007763	0.001277705	0.003	1	1381	CRABP1
-0.522007763	0.001277705	0.003	1	57801	HES4
-0.522779942	0.001253683	0.001	1	1672	DEFB1
-0.522779942	0.001253683	0.002	1	1943	EFNA2
-0.523037314	0.001245765	0.001	1	29992	PILRA
-0.524066925	0.00121453	0.002	1	728299	KRTAP19-8
-0.524324298	0.001206829	0.005	1	6241	RRM2
-0.52458173	0.001199174	0.004	1	339263	C17orf51
-0.524839103	0.001191561	0.004	1	286826	LIN9
-0.525096536	0.001183991	0.001	1	80868	HCG4B
-0.525611281	0.001168976	< 0.001	< 0.001	645249	RP5-1121A15.1
-0.525868714	0.001161535	0.004	1	8483	CILP
-0.525868714	0.001161535	0.002	1	84940	CORO6
-0.526126146	0.001154133	0.004	1	687	KLF9
-0.526126146	0.001154133	0.001	1	56929	FEM1C
-0.526126146	0.001154133	0.004	1	57464	FAM40B
-0.526640892	0.001139454	0.001	1	7127	TNFAIP2
-0.526898324	0.00113218	< 0.001	< 0.001	100289350	LOC100289350
-0.52741313	0.001117751	0.002	1	126375	ZNF792
-0.527670503	0.001110595	0.002	1	8273	SLC10A3
-0.527927935	0.001103482	0.001	1	2662	GDF10
-0.529214919	0.001068519	< 0.001	< 0.001	840	CASP7
-0.529214919	0.001068519	0.002	1	11332	ACOT7
-0.529214919	0.001068519	0.001	1	85461	TANC1
-0.529472351	0.001061648	0.001	1	120114	FAT3
-0.530244529	0.00104126	0.001	1	54738	FEV
-0.530501962	0.001034543	0.001	1	8178	ELL
-0.530501962	0.001034543	0.004	1	51480	VCX2
-0.530759275	0.001027864	0.002	1	10867	TSPAN9
-0.530759275	0.001027864	0.002	1	22844	FRMPD1
-0.53127414	0.001014617	0.001	1	3891	KRT85
-0.531531513	0.001008054	0.001	1	81029	WNT5B
-0.531788945	0.001001528	0.001	1	135	ADORA2A
-0.532046318	0.000995035	0.002	1	3880	KRT19
-0.532046318	0.000995035	0.001	1	84074	QRICH2
-0.532561123	0.000982168	0.005	1	129521	NMS
-0.532818556	0.000975789	0.001	1	23062	GGA2
-0.532818556	0.000975789	< 0.001	< 0.001	56287	GKN1
-0.533075929	0.000969448	0.003	1	10614	HEXIM1
-0.533333302	0.00096314	0.001	1	6934	TCF7L2
-0.533848166	0.000950641	0.001	1	23554	TSPAN12
-0.534877777	0.000926068	0.002	1	10142	AKAP9
-0.53513509	0.000920014	< 0.001	< 0.001	7448	VTN
-0.535392523	0.000913997	0.001	1	344838	PAQR9
-0.535649955	0.000908009	0.004	1	256374	LOC256374
-0.536936939	0.000878607	0.003	1	1847	DUSP
-0.536936939	0.000878607	0.001	1	50507	NOX4
-0.536936939	0.000878607	0.003	1	84727	SPSB2
-0.536936939	0.000878607	0.003	1	138065	RNF183
-0.537194312	0.000872829	0.006	1	9481	SLC25A27
-0.537451744	0.000867085	0.001	1	29842	TFCP2L1
-0.537709117	0.000861373	0.001	1	118980	SFXN2

voxel-wise, subcortex			
Spearman's corr coef	p-val	GenID	Gene
-0.436233521	< 0.001	147276	C18orf15
-0.437116593	< 0.001	6899	TBX1
-0.438080519	< 0.001	344758	GPR149
-0.438568652	< 0.001	6569	SLC34A1
-0.439374715	< 0.001	389834	LOC389834
-0.439950287	< 0.001	127833	SYT2
-0.440026909	< 0.001	4881	NPR1
-0.440029055	< 0.001	81029	WNT5B
-0.440546304	< 0.001	85463	ZC3H12C
-0.440678269	< 0.001	257236	CCDC96
-0.44228217	< 0.001	54763	ROPN1
-0.443277687	< 0.001	221178	SPATA13
-0.44347316	< 0.001	152831	KLB
-0.443479955	< 0.001	79817	MOBKL2B
-0.444622934	< 0.001	27040	LAT
-0.444679767	< 0.001	400831	RP4-545L17.1
-0.444717467	< 0.001	49	ACR
-0.444922358	< 0.001	10901	DHRS4
-0.445227236	< 0.001	645249	RP5-1121A15.1
-0.446857303	< 0.001	49854	ZNF295
-0.44685933	< 0.001	55076	TMEM45A
-0.447626591	< 0.001	80325	ABTB1
-0.448203206	< 0.001	6892	TAPBP
-0.448472053	< 0.001	4914	NTRK1
-0.448548764	< 0.001	58516	FAM60A
-0.448552489	< 0.001	5456	POU3F4
-0.449864209	< 0.001	157285	AC068353.1
-0.450083941	< 0.001	9866	TRIM66
-0.450588286	< 0.001	79698	ZMAT4
-0.45157209	< 0.001	340061	TMEM173
-0.453066677	< 0.001	161497	STRC
-0.453347981	< 0.001	4017	LOXL2
-0.453352332	< 0.001	374987	NEXN-AS1
-0.455180138	< 0.001	29842	TFCP2L1
-0.455831856	< 0.001	57801	HES4
-0.45606032	< 0.001	56128	PCDHB8
-0.456292391	< 0.001	645879	FAM90A12P
-0.45645982	< 0.001	84684	INSM2
-0.456465036	< 0.001	11255	HRH3
-0.456940234	< 0.001	151	ADRA2B
-0.457051665	< 0.001	7448	VTN
-0.457879484	< 0.001	9252	RPS6KA5
-0.457990915	< 0.001	84864	MINA
-0.458005011	< 0.001	25788	RAD54B
-0.458663166	< 0.001	3757	KCNH2
-0.45902589	< 0.001	5596	MAPK4
-0.45922482	< 0.001	10666	CD226
-0.459234953	< 0.001	1071	CETP
-0.46026352	< 0.001	126375	ZNF792
-0.460572869	< 0.001	728485	LOC728485
-0.460766196	< 0.001	100129781	LOC100129781
-0.461150199	< 0.001	1813	DRD2
-0.46124652	< 0.001	23387	AP006216.1
-0.461571455	< 0.001	80326	WNT10A
-0.46247828	< 0.001	5178	PEG3AS
-0.462731808	< 0.001	90507	SCRN2
-0.462807119	< 0.001	56937	PMEPA1
-0.462856054	< 0.001	285513	GPRIN3
-0.463536948	< 0.001	163486	DENND1B
-0.463711351	< 0.001	701	BUB1B
-0.464141995	< 0.001	51136	RNFT1
-0.464725286	< 0.001	64766	S100PBP
-0.465016514	< 0.001	1628	DBP
-0.466324806	< 0.001	1004	CDH6
-0.467568189	< 0.001	439921	MXRA7
-0.468197346	< 0.001	222537	HS3ST5
-0.468431175	< 0.001	9162	DGKI
-0.469656646	< 0.001	286410	ATP11C
-0.47002393	< 0.001	114789	SLC25A25
-0.47003442	< 0.001	343637	RSPO4
-0.470149398	< 0.001	285622	NBPF22P
-0.471016675	< 0.001	131544	CRYBG3
-0.471215129	< 0.001	6241	RRM2
-0.472091764	< 0.001	56099	PCDHGB7
-0.473032981	< 0.001	168002	DACT2
-0.473189086	< 0.001	9828	ARHGEF17
-0.475019276	< 0.001	5066	PAM
-0.478243321	< 0.001	1879	EBF1
-0.478407681	< 0.001	390874	ONECUT3

region-wise, subcortex					
Spearman's corr coef	p-val	p-val, SA	p-val, SA (BH- correction)	GenID	Gene
-0.538223922	0.000850052	< 0.001	< 0.001	1286	COL4A4
-0.539253533	0.000827806	0.001	1	8565	YARS
-0.539253533	0.000827806	< 0.001	< 0.001	10100	TSPAN2
-0.540283144	0.000806076	0.001	1	283383	GPR133
-0.540797949	0.000795404	0.002	1	133584	EGFLAM
-0.541055322	0.000790114	0.002	1	2894	GRID1
-0.541055322	0.000790114	0.003	1	27299	ADAMDEC1
-0.541312754	0.000784856	0.001	1	84630	TTBK1
-0.542084932	0.000769269	< 0.001	< 0.001	150084	IGSF5
-0.542342365	0.000764133	0.001	1	57512	GPR158
-0.542599738	0.000759031	< 0.001	< 0.001	127064	OR2T12
-0.542599738	0.000759031	0.004	1	100125556	RP11-666A20.1
-0.543114543	0.000748914	0.001	1	10324	KBTBD10
-0.543114543	0.000748914	0.002	1	65265	C8orf33
-0.543371975	0.000743898	0.002	1	1628	DBP
-0.543371975	0.000743898	0.004	1	6327	SCN2B
-0.543629289	0.000738915	0.002	1	6533	SLC6A6
-0.543629289	0.000738915	< 0.001	< 0.001	80325	ABTB1
-0.544401526	0.00072414	0.002	1	2637	GBX2
-0.544658899	0.000719275	0.002	1	84657	GHRLOS2
-0.545431137	0.000704852	0.001	1	80004	ESRP2
-0.545945942	0.00069538	0.001	1	4157	MC1R
-0.546203315	0.000690687	< 0.001	< 0.001	56099	PCDHGB7
-0.546203315	0.000690687	0.002	1	150135	LINC00479
-0.546203315	0.000690687	0.002	1	163933	FAM43B
-0.54671818	0.000681383	< 0.001	< 0.001	389421	LIN28B
-0.546975493	0.000676774	0.001	1	400831	RP4-545L17.1
-0.546975493	0.000676774	0.002	1	729440	CCDC61
-0.548519969	0.000649696	0.001	1	23551	RASD2
-0.548777342	0.000645278	0.001	1	83891	SNX25
-0.549034715	0.000640885	0.001	1	1775	DNASE1L2
-0.549034715	0.000640885	< 0.001	< 0.001	196996	GRAMD2
-0.54954952	0.000632183	< 0.001	< 0.001	653641	GOLGA6C
-0.549806952	0.000627873	0.002	1	113235	SLC46A1
-0.550064325	0.000623584	0.002	1	654429	LRTM2
-0.550321758	0.000619326	0.001	1	4112	MAGEB1
-0.551093936	0.000606701	< 0.001	< 0.001	145173	B3GALTL
-0.552123487	0.000590227	< 0.001	< 0.001	85403	EAF1
-0.552380919	0.00058617	0.002	1	9641	IKBKE
-0.552380919	0.00058617	< 0.001	< 0.001	286554	BCORP1
-0.553667963	0.000566259	0.001	1	6710	SPTB
-0.553667963	0.000566259	0.003	1	23742	C15orf2
-0.554440141	0.000554604	0.001	1	146802	SLC47A2
-0.554954946	0.000546955	0.001	1	196483	FAM86A
-0.555212379	0.000543164	0.001	1	254531	LPCAT4
-0.555212379	0.000543164	0.002	1	388152	AC103965.1
-0.555469751	0.000539398	0.001	1	221981	THSD7A
-0.555984557	0.000531934	0.002	1	84189	SLITRK6
-0.556499302	0.000524563	< 0.001	< 0.001	8741	TNFSF13
-0.556499302	0.000524563	0.001	1	282996	RBM20
-0.557014167	0.000517282	0.001	1	728635	DHRS4L1
-0.559845507	0.000478829	< 0.001	< 0.001	158248	TTC16
-0.560875118	0.000465492	0.001	1	6550	SLC9A3
-0.561647356	0.000455706	0.001	1	80745	THUMPD2
-0.562676966	0.000442946	0.001	1	26074	C20orf26
-0.562934339	0.000439806	< 0.001	< 0.001	3158	HMGCS2
-0.562934339	0.000439806	0.002	1	196264	MPZL3
-0.563449144	0.000433585	< 0.001	< 0.001	645332	AP003716.1
-0.563706577	0.000430505	< 0.001	< 0.001	116151	C20orf108
-0.564221323	0.000424403	< 0.001	< 0.001	285622	NBPF22P
-0.564478755	0.000421382	0.001	1	1540	CYLD
-0.566537976	0.000397887	0.001	1	644019	CBWD6
-0.566795349	0.000395032	< 0.001	< 0.001	285533	RNF175
-0.567567527	0.000386584	< 0.001	< 0.001	92610	TIFA
-0.568082392	0.00038104	< 0.001	< 0.001	5336	PLCG2
-0.569369316	0.000367491	0.001	1	4995	OR3A2
-0.570398927	0.000356965	< 0.001	< 0.001	773	CACNA1A
-0.570656359	0.000354376	< 0.001	< 0.001	416	ARSF
-0.571171165	0.000349249	< 0.001	< 0.001	29090	C18orf55
-0.572200775	0.000339191	0.001	1	81491	GPR63
-0.572458148	0.000336716	0.002	1	3757	KCNH2
-0.575289607	0.000310554	< 0.001	< 0.001	54329	GPR85
-0.576061785	0.000303741	0.001	1	4902	NRTN
-0.576319158	0.0003015	< 0.001	< 0.001	56130	PCDHB6
-0.576576591	0.000299274	< 0.001	< 0.001	90853	SPOCD1
-0.576833963	0.000297064	< 0.001	< 0.001	3751	KCND2
-0.578120947	0.000286224	< 0.001	< 0.001	54836	BSPRY
-0.578378379	0.0002841	< 0.001	< 0.001	771	CA12
-0.579665363	0.000273686	0.001	1	9627	SNCAIP

voxel-wise, subcortex			
Spearman's corr coef	p-val	GenID	Gene
-0.478413075	< 0.001	4902	NRTN
-0.47883153	< 0.001	91624	NEXN
-0.480184406	< 0.001	143686	SESN3
-0.480657548	< 0.001	29952	DPP7
-0.481593162	< 0.001	57101	ANO2
-0.4820849	< 0.001	124274	GPR139
-0.482460171	< 0.001	10846	PDE10A
-0.482517749	< 0.001	654	BMP6
-0.482553869	< 0.001	83541	FAM110A
-0.482578754	< 0.001	440021	KRTAP5-2
-0.48410669	< 0.001	8178	ELL
-0.484799176	< 0.001	7475	WNT6
-0.48593086	< 0.001	150379	PNPLA5
-0.485996604	< 0.001	1381	CRABP1
-0.486345947	< 0.001	8711	TNK1
-0.486970752	< 0.001	27092	CACNG4
-0.48710978	< 0.001	6553	SLC9A5
-0.488486528	< 0.001	268	AMH
-0.488657296	< 0.001	256076	COL29A1
-0.488704294	< 0.001	728635	DHRS4L1
-0.489157379	< 0.001	654790	PCP4L1
-0.489293158	< 0.001	85397	RGS8
-0.489867717	< 0.001	3751	KCND2
-0.490109622	< 0.001	160851	DGKH
-0.492380202	< 0.001	125058	TBC1D16
-0.492624074	< 0.001	22987	SV2C
-0.493019313	< 0.001	23062	GGA2
-0.493172824	< 0.001	135	ADORA2A
-0.49320811	< 0.001	57512	GPR158
-0.495251238	< 0.001	57464	FAM40B
-0.495402157	< 0.001	22874	PLEKHA6
-0.495595127	< 0.001	157506	RDH10
-0.496398598	< 0.001	5997	RGS2
-0.497042358	< 0.001	154197	PNLDC1
-0.497309148	< 0.001	57522	SRGAP1
-0.499095351	< 0.001	3738	KCNA3
-0.49993524	< 0.001	5583	PRKCH
-0.500123739	< 0.001	4092	SMAD7
-0.50042814	< 0.001	148281	SYT6
-0.500478745	< 0.001	64410	KLHL25
-0.500561535	< 0.001	136306	SVOPL
-0.500615776	< 0.001	5157	PDGFRL
-0.501000941	< 0.001	84189	SLITRK6
-0.501803339	< 0.001	80868	HCG4B
-0.502064466	< 0.001	145447	ABHD12B
-0.502158046	< 0.001	85462	FHDC1
-0.503231943	< 0.001	129607	CMPK2
-0.507031679	< 0.001	9480	ONECUT2
-0.507416308	< 0.001	26225	ARL5A
-0.508548558	< 0.001	57562	KIAA1377
-0.510961771	< 0.001	51733	UPB1
-0.515823662	< 0.001	23463	ICMT
-0.51741761	< 0.001	80215	RUNX1-IT1
-0.518377841	< 0.001	9069	CLDN12
-0.518522441	< 0.001	9641	IKBKE
-0.520616412	< 0.001	387914	TMEM46
-0.521389008	< 0.001	2138	EYA1
-0.521693707	< 0.001	153020	RASGEF1B
-0.522237659	< 0.001	57186	C20orf74
-0.525138497	< 0.001	771	CA12
-0.525558352	< 0.001	23551	RASD2
-0.526489675	< 0.001	3624	INHBA
-0.528961778	< 0.001	1013	CDH15
-0.530067623	< 0.001	90293	KLHL13
-0.532631755	< 0.001	646658	TMEM90A
-0.532928765	< 0.001	51765	STK3
-0.533897519	< 0.001	55024	BANK1
-0.534932911	< 0.001	54836	BSPRY
-0.536795795	< 0.001	150084	IGSF5
-0.536915839	< 0.001	5121	PCP4
-0.542470634	< 0.001	57633	LRRN1
-0.542942762	< 0.001	80311	KLHL15
-0.544966578	< 0.001	374655	ZNF710
-0.544983864	< 0.001	9627	SNCAIP
-0.545810282	< 0.001	3632	INPP5A
-0.545933902	< 0.001	10235	RASGRP2
-0.546801805	< 0.001	84727	SPSB2
-0.547943592	< 0.001	120114	FAT3
-0.548263431	< 0.001	9020	MAP3K14

region-wise, subcortex					
Spearman's corr coef	p-val	p-val, SA	p-val, SA (BH- correction)	GenID	Gene
-0.579665363	0.000273686	0.001	1	22874	PLEKHA6
-0.581209779	0.000261643	< 0.001	< 0.001	27092	CACNG4
-0.582496762	0.000251971	< 0.001	< 0.001	160518	DENND5B
-0.58326894	0.000246324	0.001	1	9069	CLDN12
-0.58326894	0.000246324	< 0.001	< 0.001	81839	VANGL1
-0.583526373	0.000244467	< 0.001	< 0.001	150379	PNPLA5
-0.585585594	0.000230055	< 0.001	< 0.001	199221	DZIP1L
-0.586872578	0.00022144	< 0.001	< 0.001	79734	KCTD17
-0.589446604	0.000205071	< 0.001	< 0.001	8846	ALKBH1
-0.590733588	0.000197301	< 0.001	< 0.001	10090	UST
-0.594079792	0.000178323	< 0.001	< 0.001	390874	ONECUT3
-0.595624208	0.000170131	< 0.001	< 0.001	29057	FAM156A
-0.596138954	0.000167476	< 0.001	< 0.001	388849	LOC388849
-0.596396387	0.000166162	< 0.001	< 0.001	7691	ZNF132
-0.599742591	0.000149913	< 0.001	< 0.001	254170	FBXO33
-0.600257337	0.000147544	< 0.001	< 0.001	1004	CDH6
-0.600772202	0.000145209	< 0.001	< 0.001	79875	THSD4
-0.602573991	0.000137296	< 0.001	< 0.001	85397	RGS8
-0.603088796	0.000135107	0.001	1	79698	ZMAT4
-0.603603601	0.00013295	0.001	1	1804	DPP6
-0.603860974	0.000131884	< 0.001	< 0.001	10251	SPRY3
-0.604633152	0.00012873	0.001	1	340061	TMEM173
-0.606435001	0.000121633	0.001	1	136306	SVOPL
-0.606692374	0.000120648	< 0.001	< 0.001	84858	ZNF503
-0.607464612	0.000117737	0.001	1	2957	GTF2A1
-0.607464612	0.000117737	< 0.001	< 0.001	25914	RTTN
-0.607721984	0.000116781	< 0.001	< 0.001	7755	ZNF205
-0.60823679	0.00011489	< 0.001	< 0.001	50515	CHST11
-0.610296011	0.000107599	< 0.001	< 0.001	83541	FAM110A
-0.611068189	0.000104975	< 0.001	< 0.001	91624	NEXN
-0.612612605	9.98983E-05	< 0.001	< 0.001	1013	CDH15
-0.614156961	9.50445E-05	< 0.001	< 0.001	22838	RNF44
-0.614414394	9.42566E-05	< 0.001	< 0.001	125058	TBC1D16
-0.614671826	9.34748E-05	< 0.001	< 0.001	161497	STRC
-0.620077193	7.83513E-05	< 0.001	< 0.001	203074	UNQ9391
-0.621364236	7.50941E-05	< 0.001	< 0.001	5157	PDGFRL
-0.622393847	7.25773E-05	< 0.001	< 0.001	6899	TBX1
-0.625225186	6.60458E-05	< 0.001	< 0.001	80311	KLHL15
-0.626254797	6.38061E-05	< 0.001	< 0.001	54084	C21orf29
-0.626254797	6.38061E-05	< 0.001	< 0.001	153020	RASGEF1B
-0.62754184	6.11037E-05	< 0.001	< 0.001	10235	RASGRP2
-0.632689834	5.13034E-05	< 0.001	< 0.001	80326	WNT10A
-0.640411854	3.92667E-05	< 0.001	< 0.001	4311	MME
-0.642213643	3.68587E-05	< 0.001	< 0.001	374655	ZNF710
-0.643758059	3.49035E-05	< 0.001	< 0.001	9767	PHF16
-0.644015491	3.45872E-05	< 0.001	< 0.001	5066	PAM
-0.645302415	3.30438E-05	< 0.001	< 0.001	387914	TMEM46
-0.645559847	3.27429E-05	< 0.001	< 0.001	109	ADCY3
-0.64864862	2.93227E-05	< 0.001	< 0.001	3274	HRH2
-0.649420798	2.85208E-05	< 0.001	< 0.001	57186	C20orf74
-0.651737452	2.62348E-05	< 0.001	< 0.001	701	BUB1B
-0.653796613	2.43459E-05	< 0.001	< 0.001	63916	ELMO2
-0.654054046	2.41188E-05	< 0.001	< 0.001	343637	RSPO4
-0.654826224	2.34494E-05	< 0.001	< 0.001	150165	XKR3
-0.655083656	2.32301E-05	< 0.001	< 0.001	4881	NPR1
-0.656628072	2.19537E-05	< 0.001	< 0.001	11255	HRH3
-0.658172429	2.07428E-05	< 0.001	< 0.001	1277	COL1A1
-0.659459472	1.97816E-05	< 0.001	< 0.001	4703	NEB
-0.66023165	1.92248E-05	< 0.001	< 0.001	646658	TMEM90A
-0.661518633	1.83293E-05	< 0.001	< 0.001	90293	KLHL13
-0.664350033	1.64944E-05	< 0.001	< 0.001	10666	CD226
-0.664607465	1.63365E-05	< 0.001	< 0.001	4092	SMAD7
-0.665122271	1.60249E-05	< 0.001	< 0.001	285382	C3orf70
-0.665637016	1.57187E-05	< 0.001	< 0.001	3632	INPP5A
-0.666151881	1.54181E-05	< 0.001	< 0.001	79919	C2orf54
-0.666409254	1.52699E-05	< 0.001	< 0.001	80215	RUNX1-IT1
-0.667696238	1.45483E-05	< 0.001	< 0.001	3738	KCNA3
-0.66795367	1.44079E-05	< 0.001	< 0.001	56128	PCDHB8
-0.668983221	1.38591E-05	< 0.001	< 0.001	23463	ICMT
-0.675160825	1.09593E-05	< 0.001	< 0.001	8038	ADAM12
-0.676447868	1.04327E-05	< 0.001	< 0.001	55024	BANK1
-0.67850703	9.64002E-06	< 0.001	< 0.001	57562	KIAA1377
-0.679021895	9.45111E-06	< 0.001	< 0.001	100129781	LOC100129781
-0.681338489	8.64424E-06	< 0.001	< 0.001	57633	LRRN1
-0.681853235	8.47423E-06	< 0.001	< 0.001	340146	SLC35D3
-0.683140278	8.06315E-06	< 0.001	< 0.001	387496	RASL11A
-0.688288271	6.60462E-06	< 0.001	< 0.001	5121	PCP4
-0.68906045	6.4095E-06	< 0.001	< 0.001	145447	ABHD12B
-0.691634476	5.79932E-06	< 0.001	< 0.001	6553	SLC9A5

voxel-wise, subcortex			
Spearman's corr coef	p-val	GenID	Gene
-0.549173594	< 0.001	340146	SLC35D3
-0.557477057	< 0.001	4311	MME
-0.558562458	< 0.001	54084	C21orf29
-0.562510192	< 0.001	2914	GRM4
-0.564779282	< 0.001	1277	COL1A1
-0.569896579	< 0.001	51279	C1RL
-0.575270355	< 0.001	8038	ADAM12
-0.575566947	< 0.001	109	ADCY3
-0.58736223	< 0.001	9542	NRG2
-0.591522276	< 0.001	282996	RBM20
-0.625812292	< 0.001	56114	PCDHGA1

region-wise, subcortex					
Spearman's corr coef	p-val	p-val, SA	p-val, SA (BH- correction)	GenID	Gene
-0.695238113	5.04115E-06	< 0.001	< 0.001	256076	COL29A1
-0.697554648	4.60726E-06	< 0.001	< 0.001	3624	INHBA
-0.699099064	4.33921E-06	< 0.001	< 0.001	51279	C1RL
-0.700900853	4.0467E-06	< 0.001	< 0.001	5583	PRKCH
-0.705534101	3.38449E-06	< 0.001	< 0.001	2914	GRM4
-0.711196899	2.72705E-06	< 0.001	< 0.001	84684	INSM2
-0.715572715	2.31393E-06	< 0.001	< 0.001	8387	OR1E1
-0.718404114	2.08391E-06	< 0.001	< 0.001	130367	SGPP2
-0.719691098	1.98799E-06	< 0.001	< 0.001	5225	PGC
-0.731016695	1.3339E-06	< 0.001	< 0.001	9542	NRG2
-0.740283072	9.8743E-07	< 0.001	< 0.001	56114	PCDHGA1

Emotion (single-site) Cortex

voxel-wise, cortex			
Spearman's corr coef	p-val	GenID	Gene
0.328194857	< 0.001	245711	SPDYA
0.298659474	< 0.001	84660	CCDC62
0.295964718	< 0.001	90780	PYGO2
0.289521992	< 0.001	8532	CPZ
0.285584927	< 0.001	121643	FOXN4
0.282777727	< 0.001	257019	FRMD3
0.282284588	< 0.001	8929	PHOX2B
0.280825406	< 0.001	25814	ATXN10
0.276376724	< 0.001	170626	XAGE3
0.273480684	< 0.001	57536	KIAA1328
0.273387939	< 0.001	25975	EGFL6
0.272640556	< 0.001	200312	RNF215
0.272379637	< 0.001	51382	ATP6V1D
0.271965027	< 0.001	54072	LINC00158
0.271777332	< 0.001	711	ERC2-IT1
0.269577354	< 0.001	5511	PPP1R8
0.268109292	< 0.001	79139	DERL1
0.266408086	< 0.001	57156	TMEM63C
0.265974164	< 0.001	221806	VWDE
0.26574561	< 0.001	4884	NPTX1
0.264867723	< 0.001	83989	FAM172A
0.264651954	< 0.001	7567	ZNF19
0.264044911	< 0.001	51340	CRNKL1
0.257401705	< 0.001	541466	CT45A1
0.257302225	< 0.001	162979	ZNF296
0.256674945	< 0.001	55164	SHQ1
0.25574559	< 0.001	642938	FAM196A
0.253203988	< 0.001	23327	NEDD4L
0.250779063	< 0.001	220115	TPTE2P3
0.250141859	< 0.001	541465	CT45A4
0.249326184	< 0.001	6781	STC1
0.249097854	< 0.001	5199	CFP
0.2489831	< 0.001	80072	HEXA-AS1
0.248951569	< 0.001	8237	USP11
0.248571321	< 0.001	282890	ZNF311
0.247913346	< 0.001	140886	PABPC5
0.247424513	< 0.001	150572	SMYD1
0.245078415	< 0.001	7436	VLDLR
0.244748935	< 0.001	728255	KRTAP1-4
0.244653568	< 0.001	123207	C15orf40
0.244578257	< 0.001	781	CACNA2D1
0.244312838	< 0.001	51123	ZNF706
0.244150564	< 0.001	1741	DLG3
0.24255155	< 0.001	54903	MKS1
0.242420062	< 0.001	80320	SP6
0.241374895	< 0.001	5865	RAB3B
0.239813104	< 0.001	84789	MGC2889
0.23689197	< 0.001	26260	FBXO25
0.236658216	< 0.001	4288	MKI67
0.235888138	< 0.001	339896	GADL1
0.235468775	< 0.001	6602	SMARCD1
0.234405607	< 0.001	23784	POTEH
0.23319678	< 0.001	150383	C22orf40
0.231458351	< 0.001	8999	CDKL2
0.230697438	< 0.001	57456	KIAA1143
0.230324194	< 0.001	10772	FUSIP1
0.229996532	< 0.001	8974	P4HA2
0.229921445	< 0.001	54521	WDR44
0.229592249	< 0.001	685	BTC
0.229484305	< 0.001	9991	ROD1
0.228211984	< 0.001	5787	PTPRB
0.228075758	< 0.001	643226	GRXCR2
0.228064165	< 0.001	81537	SGPP1
0.227801651	< 0.001	375484	C5orf25
0.227779001	< 0.001	51032	CELA2B
0.227097839	< 0.001	89890	KBTBD6
0.227088243	< 0.001	83592	AKR1CL2
0.22597611	< 0.001	5009	OTC
0.225939155	< 0.001	8880	FUBP1
0.225770712	< 0.001	152519	NIPAL1
0.22574757	< 0.001	10124	ARL4A
0.225063488	< 0.001	440895	AC013271.3
0.224465117	< 0.001	8761	PABPC4
0.224100217	< 0.001	4633	MYL2
0.223023862	< 0.001	54545	MTMR12
0.222654998	< 0.001	56649	TMPRSS4

region-wise, cortex					
Spearman's corr coef	p-val	p-val, SA	p-val, SA (BH- correction)	GenID	Gene
0.428802162	8.40016E-11	< 0.001	< 0.001	121643	FOXN4
0.393436819	3.47579E-09	< 0.001	< 0.001	146850	PIK3R6
0.389991969	4.88259E-09	0.001	1	23429	RYBP
0.388352782	5.73178E-09	< 0.001	< 0.001	6781	STC1
0.384260684	8.52078E-09	< 0.001	< 0.001	84660	CCDC62
0.378879279	1.42347E-08	< 0.001	< 0.001	8880	FUBP1
0.378321439	1.50045E-08	< 0.001	< 0.001	150572	SMYD1
0.354859203	1.25909E-07	< 0.001	< 0.001	56649	TMPRSS4
0.348708093	2.13905E-07	< 0.001	< 0.001	1741	DLG3
0.346211106	2.64402E-07	< 0.001	< 0.001	3303	HSPA1A
0.345657796	2.77048E-07	< 0.001	< 0.001	1475	CSTA
0.343362957	3.35956E-07	0.001	1	4929	NR4A2
0.340946317	4.10897E-07	< 0.001	< 0.001	25814	ATXN10
0.338863343	4.88111E-07	< 0.001	< 0.001	146779	AC008026.2
0.334177107	7.15785E-07	0.001	1	730	C7
0.329671651	1.02817E-06	0.002	1	245711	SPDYA
0.32865575	1.11476E-06	< 0.001	< 0.001	84172	POLR1B
0.327525169	1.21931E-06	< 0.001	< 0.001	83592	AKR1CL2
0.326845527	1.28661E-06	0.001	1	1576	CYP3A4
0.323719442	1.64448E-06	0.001	1	51382	ATP6V1D
0.322719097	1.7778E-06	0.002	1	200312	RNF215
0.320719689	2.07582E-06	0.002	1	7136	TNNI2
0.320603073	2.0946E-06	< 0.001	< 0.001	3698	ITIH2
0.318226576	2.51443E-06	0.001	1	940	CD28
0.315798283	3.02559E-06	< 0.001	< 0.001	51340	CRNKL1
0.315448403	3.10694E-06	< 0.001	< 0.001	643226	GRXCR2
0.314751923	3.27511E-06	0.001	1	8999	CDKL2
0.313251406	3.66735E-06	0.004	1	541466	CT45A1
0.312641084	3.83934E-06	0.003	1	7436	VLDLR
0.311513752	4.17741E-06	0.002	1	9021	SOCS3
0.309019983	5.02864E-06	0.003	1	83989	FAM172A
0.307897836	5.46331E-06	< 0.001	< 0.001	541465	CT45A4
0.306253493	6.16521E-06	0.002	1	7412	VCAM1
0.305472136	6.528E-06	0.001	1	6887	TAL2
0.303926885	7.30604E-06	0.006	1	149018	LELP1
0.30389902	7.32085E-06	0.001	1	57091	CASS4
0.303538144	7.5152E-06	0.002	1	123207	C15orf40
0.303287417	7.65309E-06	< 0.001	< 0.001	139081	MAGEC3
0.303008825	7.80914E-06	0.003	1	64768	IPPK
0.302839071	7.90571E-06	0.004	1	219771	CCNY
0.302730232	7.96821E-06	0.004	1	131034	CPNE4
0.300240368	9.53295E-06	0.005	1	8532	CPZ
0.300204098	9.55778E-06	0.008	1	4288	MKI67
0.299174577	1.02882E-05	0.003	1	84929	FIBCD1
0.298010319	1.1178E-05	0.001	1	170626	XAGE3
0.297375381	1.16934E-05	0.005	1	89780	WNT3A
0.295902073	1.29777E-05	0.006	1	89822	KCNK17
0.29531765	1.35232E-05	0.006	1	158158	RASEF
0.295061082	1.37695E-05	0.001	1	3726	JUNB
0.294546664	1.42761E-05	0.001	1	201243	C17orf74
0.294209123	1.46182E-05	0.002	1	5552	SRGN
0.292899728	1.60196E-05	0.005	1	8237	USP11
0.290463626	1.89717E-05	0.005	1	6792	CDKL5
0.29017663	1.93515E-05	0.004	1	51752	ERAP1
0.288895726	2.11362E-05	0.005	1	90780	PYGO2
0.288676083	2.14575E-05	0.002	1	114042	LINC00334
0.288466811	2.17678E-05	0.002	1	5009	OTC
0.288081974	2.23497E-05	0.004	1	154881	KCTD7
0.287599295	2.31002E-05	0.005	1	79414	LRFN3
0.287462592	2.33171E-05	0.004	1	9107	MTMR6
0.287020057	2.40323E-05	0.006	1	54903	MKS1
0.286875606	2.42702E-05	0.003	1	3054	HCFC1
0.286230922	2.53595E-05	0.006	1	403253	AC134982.1
0.286113679	2.55625E-05	0.007	1	92345	NAF1
0.285732716	2.62327E-05	0.006	1	389332	LOC389332
0.285415888	2.68027E-05	0.003	1	284233	CYP4F35P
0.284632593	2.82625E-05	0.005	1	337968	KRTAP6-3
0.283844084	2.98075E-05	0.004	1	4881	NPR1
0.283671111	3.0157E-05	0.007	1	337975	KRTAP20-1
0.281987876	3.37652E-05	0.008	1	8685	MARCO
0.281976223	3.37915E-05	0.009	1	6594	SMARCA1
0.281974256	3.37959E-05	0.005	1	260429	PRSS33
0.281224012	3.55336E-05	0.006	1	55164	SHQ1
0.281003058	3.60612E-05	0.006	1	29956	LASS2
0.279936641	3.87131E-05	0.004	1	1545	CYP1B1
0.27915138	4.0782E-05	0.006	1	3430	IFI35

0.222480983	< 0.001	84083	ZRANB3
0.222159579	< 0.001	131377	KBTBD5
0.222026482	< 0.001	374	AREG
0.221249163	< 0.001	540	ATP7B
0.220946714	< 0.001	84898	PLXDC2
0.220471784	< 0.001	126306	JSRP1
0.22027047	< 0.001	79054	TRPM8
0.219470441	< 0.001	123904	NRN1L
0.219207734	< 0.001	10677	AVIL
0.219056293	< 0.001	59269	HIVEP3
0.218717024	< 0.001	5985	RFC5
0.218657702	< 0.001	114783	LMTK3
0.218468487	< 0.001	4074	M6PR
0.217964649	< 0.001	4624	MYH6
0.21768707	< 0.001	6376	CX3CL1
0.217241392	< 0.001	55714	ODZ3
0.217014939	< 0.001	90768	MGC45800
0.216972068	< 0.001	169166	SNX31
0.216728106	< 0.001	23429	RYBP
0.216726378	< 0.001	6176	RPLP1
0.215528935	< 0.001	7178	TPT1
0.214966968	< 0.001	22871	NLGN1
0.21469453	< 0.001	56986	DTWD1
0.214519814	< 0.001	6594	SMARCA1
0.21424669	< 0.001	6769	STAC
0.213986546	< 0.001	79153	GDPD3
0.213792309	< 0.001	3054	HCFC1
0.213320434	< 0.001	6401	SELE
0.213260695	< 0.001	653	BMP5
0.213193595	< 0.001	2019	EN1
0.212916225	< 0.001	3698	ITIH2
0.212732345	< 0.001	1577	CYP3A5
0.212687761	< 0.001	4018	LPA
0.212684631	< 0.001	646934	LOC646934
0.212446496	< 0.001	80024	SLC24A6
0.212408915	< 0.001	1000	CDH2
0.212399751	< 0.001	23627	PRND
0.211437106	< 0.001	27315	PGAP2
0.21121107	< 0.001	400798	RP5-990P15.1
0.211081028	< 0.001	22992	KDM2A
0.210846514	< 0.001	126402	CCDC105
0.210664108	< 0.001	146850	PIK3R6
0.208034292	< 0.001	148645	LINC00337
0.207378343	< 0.001	135293	PM20D2
0.206786156	< 0.001	84560	MT4
0.205662414	< 0.001	7025	NR2F1
0.205527797	< 0.001	4951	OCM2
0.205170602	< 0.001	9722	NOS1AP
0.205004096	< 0.001	5316	PKNOX1
0.204973817	< 0.001	1475	CSTA
0.204905733	< 0.001	9097	USP14
0.204629481	< 0.001	4762	NEUROG1
0.20446068	< 0.001	25850	ZNF345
0.203886405	< 0.001	4929	NR4A2
0.203779221	< 0.001	440917	LOC440917
0.203110486	< 0.001	26577	PCOLCE2
0.202483565	< 0.001	9203	ZMYM3
0.202452451	< 0.001	26037	SIPA1L1
0.202098176	< 0.001	54851	ANKRD49
0.202062264	< 0.001	23080	AVL9
0.201651379	< 0.001	100129794	RP11-537P22.1
0.201607332	< 0.001	347730	LRRTM1
0.201551139	< 0.001	92345	NAF1
0.201328292	< 0.001	100287825	LOC100287825
0.201142475	< 0.001	85458	DIXDC1
0.201027438	< 0.001	389158	PLSCR5
0.200962633	< 0.001	5139	PDE3A
0.200868368	< 0.001	3623	INHA
0.200176015	< 0.001	79414	LRFN3
0.200044438	< 0.001	80714	PBX4
0.19991295	< 0.001	56164	STK31
0.19978179	< 0.001	60385	TSKS
0.199227542	< 0.001	60592	SCOC
0.198973149	< 0.001	118856	MMP21
0.198870212	< 0.001	54431	DNAJC10
0.198825479	< 0.001	131034	CPNE4
0.198729262	< 0.001	122740	OR4K14
0.198245093	< 0.001	728673	LOC728673
0.198187098	< 0.001	654433	AC016683.6
0.198126584	< 0.001	80790	AC099524.1
0.197969809	< 0.001	387893	SETD8

0.278594196	4.23127E-05	0.006	1	54716	SLC6A20
0.278094679	4.3731E-05	0.005	1	401190	RGS7BP
0.275989026	5.02158E-05	0.003	1	27286	SRPX2
0.275865912	5.06218E-05	0.007	1	23405	DICER1
0.275185615	5.29208E-05	0.004	1	286151	FBXO43
0.275067717	5.3329E-05	0.006	1	84894	LINGO1
0.274568826	5.50896E-05	0.006	1	169166	SNX31
0.273812085	5.7865E-05	0.007	1	414777	HCG18
0.273795873	5.79258E-05	0.008	1	6928	HNF1B
0.272853196	6.15705E-05	0.006	1	781	CACNA2D1
0.271861285	6.56377E-05	0.01	1	659	BMPR2
0.271716803	6.62509E-05	0.007	1	8974	P4HA2
0.27156195	6.69138E-05	0.01	1	65977	PLEKHA3
0.271353334	6.78169E-05	0.005	1	51481	VCX3A
0.270799369	7.02709E-05	0.005	1	23327	NEDD4L
0.270743012	7.05252E-05	0.008	1	90768	MGC45800
0.27022016	7.29258E-05	0.005	1	84560	MT4
0.269794494	7.49367E-05	0.009	1	22926	ATF6
0.269620866	7.57718E-05	0.009	1	81501	TM7SF4
0.269260615	7.75323E-05	0.007	1	58531	PRM3
0.268639296	8.06593E-05	0.003	1	56062	KLHL4
0.26786375	8.47286E-05	0.006	1	654434	LINC00338
0.267836541	8.48747E-05	0.007	1	301	ANXA1
0.267827481	8.49234E-05	0.007	1	5175	PECAM1
0.26718089	8.84702E-05	0.015	1	11074	TRIM31
0.266992986	8.95266E-05	0.005	1	10523	CHERP
0.266432554	9.27483E-05	0.006	1	284040	CDRT4
0.26624015	9.38791E-05	0.007	1	140462	ASB9
0.266089171	9.47756E-05	0.012	1	474382	H2AFB1
0.266073614	9.48683E-05	0.008	1	26091	HERC4
0.265862405	9.61371E-05	0.006	1	29915	HCFC2
0.265436113	9.87463E-05	0.01	1	26260	FBXO25
0.264843285	0.000102485	0.009	1	711	ERC2-IT1
0.264634639	0.000103832	0.01	1	646993	HMGB3P24
0.264604211	0.00010403	0.007	1	162979	ZNF296
0.264577627	0.000104204	0.007	1	54600	UGT1A9
0.264481097	0.000104835	0.008	1	150290	DUSP18
0.263622642	0.000110606	0.011	1	55619	DOCK10
0.26328963	0.000112923	0.005	1	123904	NRN1L
0.263129592	0.000114053	0.011	1	1232	CCR3
0.26259768	0.000117885	0.011	1	157848	NKX6-3
0.262377381	0.000119508	0.007	1	4283	CXCL9
0.262083888	0.000121701	0.011	1	6373	CXCL11
0.26141268	0.00012686	0.009	1	23704	KCNE4
0.260797828	0.000131765	0.014	1	2779	GNAT1
0.260534137	0.000133923	0.006	1	7188	TRAF5
0.260238051	0.000136385	0.01	1	342979	AC022098.3
0.26008901	0.00013764	0.009	1	6401	SELE
0.25876084	0.000149313	0.015	1	257019	FRMD3
0.258395433	0.000152682	0.012	1	339896	GADL1
0.258131742	0.000155158	0.013	1	9332	CD163
0.258061767	0.000155822	0.013	1	79802	HHIPL2
0.257310212	0.000163115	0.017	1	54467	ANKIB1
0.257126182	0.000164948	0.011	1	6521	SLC4A1
0.256261259	0.000173828	0.005	1	1947	EFNB1
0.25615111	0.00017499	0.01	1	55539	KCNQ1DN
0.256013751	0.00017645	0.005	1	130814	PQLC3
0.255599111	0.000180925	0.012	1	2891	GRIA2
0.254754245	0.000190374	0.01	1	224	ALDH3A2
0.254597455	0.000192177	0.016	1	390535	GOLGA8E
0.254044801	0.000198661	0.01	1	79883	PODNL1
0.253813505	0.000201435	0.008	1	10537	UBD
0.253497988	0.000205277	0.018	1	126792	B3GALT6
0.253387839	0.000206635	0.02	1	4312	MMP1
0.253187001	0.000209131	0.006	1	1490	CTGF
0.253012061	0.000211328	0.016	1	467	ATF3
0.252610356	0.000216456	0.014	1	6617	SNAPC1
0.25237906	0.000219461	0.009	1	64231	MS4A6A
0.252186	0.000221998	0.017	1	5956	OPN1LW
0.251915812	0.000225596	0.015	1	1769	DNAH8
0.251892507	0.000225909	0.017	1	128239	IQGAP3
0.2517519	0.000227805	0.013	1	4074	M6PR
0.251689076	0.000228657	0.017	1	26037	SIPA1L1
0.251421481	0.000232319	0.01	1	26289	AK5
0.25081116	0.000240877	0.013	1	910	CD1B
0.250547469	0.000244664	0.013	1	5754	PTK7
0.250521541	0.00024504	0.013	1	3337	DNAJB1
0.250504076	0.000245293	0.009	1	728255	KRTAP1-4
0.250219643	0.000249451	0.016	1	3383	ICAM1
0.250001281	0.000252686	0.009	1	375686	SPATC1
0.249350816	0.00026256	0.02	1	2294	FOXF1

0.197925493	< 0.001	84203	TXNDC2
0.19787693	< 0.001	10665	C6orf10
0.19738929	< 0.001	93210	PGAP3
0.19735615	< 0.001	11063	SOX30
0.197261885	< 0.001	140462	ASB9
0.197261468	< 0.001	90293	KLHL13
0.197216734	< 0.001	10523	CHERP
0.197125256	< 0.001	89780	WNT3A
0.197037235	< 0.001	26575	RSG17
0.196915537	< 0.001	4015	LOX
0.196817592	< 0.001	90060	CCDC120
0.196660951	< 0.001	55634	ZNF673
0.196576819	< 0.001	26689	AC005962.2
0.196365029	< 0.001	10554	AGPAT1
0.195996448	< 0.001	150290	DUSP18
0.195712611	< 0.001	574	BAGE
0.195623904	< 0.001	26751	SH3YL1
0.195258588	< 0.001	6223	RPS19
0.195230737	< 0.001	79647	AKIRIN1
0.195118472	< 0.001	84894	LINGO1
0.195033938	< 0.001	25890	ABI3BP
0.194751918	< 0.001	10046	MAMLD1
0.194688275	< 0.001	9324	HMGN3
0.194688007	< 0.001	119678	OR52E2
0.194497317	< 0.001	645121	CCNI2
0.193907142	< 0.001	5460	POU5F1
0.193809345	< 0.001	128372	OR6N1
0.19349508	< 0.001	54926	UBE2R2
0.193391308	< 0.001	132200	C3orf49
0.193128094	< 0.001	84620	ST6GAL2
0.193092182	< 0.001	55752	SEPTIN11
0.193053707	< 0.001	219431	OR4S2
0.192871213	< 0.001	163351	GBP6
0.192647129	< 0.001	149628	PYHIN1
0.192607671	< 0.001	10486	CAP2
0.192601979	< 0.001	375686	SPATC1
0.19218491	< 0.001	126731	C1orf96
0.192106828	< 0.001	1769	DNAH8
0.191948995	< 0.001	23317	DNAJC13
0.191794574	< 0.001	128061	C1orf131
0.191579655	< 0.001	22941	SHANK2
0.191500187	< 0.001	10472	ZNF238
0.191027343	< 0.001	414777	HCG18
0.190843329	< 0.001	158158	RASEF
0.190628961	< 0.001	83659	TEKT1
0.190614507	< 0.001	23635	SSBP2
0.190578252	< 0.001	5173	PDYN
0.190516606	< 0.001	116447	TOP1MT
0.190367013	< 0.001	8831	SYNGAP1
0.190013081	< 0.001	164045	HFM1
0.189992666	< 0.001	9698	PUM1
0.189837411	< 0.001	10951	CBX1
0.189751759	< 0.001	623	BDKRB1
0.189674795	< 0.001	6093	ROCK1
0.189639017	< 0.001	404281	YY2
0.189526558	< 0.001	91869	RFT1
0.189361915	< 0.001	1525	CXADR
0.189339906	< 0.001	11062	DUS4L
0.188804328	< 0.001	100288870	ERLEC1P1
0.188546821	< 0.001	57554	LRRC7
0.188170105	< 0.001	2890	GRIA1
0.188099191	< 0.001	10950	BTG3
0.187942684	< 0.001	65977	PLEKHA3
0.187887594	< 0.001	2664	GDI1
0.187712476	< 0.001	220965	FAM13C
0.187341392	< 0.001	10293	TRAIP
0.187333271	< 0.001	115350	FCRL1
0.187286034	< 0.001	10973	ASCC3
0.18704471	< 0.001	54467	ANKIB1
0.187041312	< 0.001	653781	AC140481.7
0.186761498	< 0.001	4151	MB
0.186490104	< 0.001	124221	TMPRSS8
0.186239123	< 0.001	9464	HAND2
0.186226487	< 0.001	201229	C17orf108
0.186196536	< 0.001	57695	USP37
0.186182365	< 0.001	27032	ATP2C1
0.185903534	< 0.001	55122	AKIRIN2
0.185887843	< 0.001	3303	HSPA1A
0.185840741	< 0.001	51669	TMEM66
0.18573384	< 0.001	7412	VCAM1
0.18562144	< 0.001	100272228	RP13-507I23.1

0.248954952	0.000268743	0.014	1	1510	CTSE
0.248642653	0.000273717	0.013	1	6363	CCL19
0.248638779	0.000273779	0.011	1	84628	NTNG2
0.248500764	0.000276004	0.022	1	1001	CDH3
0.248494938	0.000276099	0.014	1	11063	SOX30
0.247189432	0.000298005	0.021	1	128646	SIRPD
0.246854469	0.000303881	0.021	1	245934	DEFB121
0.246540889	0.000309479	0.015	1	120400	FAM55A
0.246202037	0.000315636	0.021	1	54072	LINC00158
0.246042654	0.000318571	0.02	1	221223	CES7
0.245452419	0.000329663	0.012	1	8407	TAGLN2
0.245340988	0.000331797	0.014	1	645121	CCNI2
0.245162174	0.000335248	0.016	1	219621	C10orf107
0.245055258	0.000337328	0.014	1	1555	CYP2B7P1
0.244700223	0.000344319	0.022	1	64105	CENPK
0.244075641	0.000356946	0.021	1	80714	PBX4
0.243530765	0.000368311	0.022	1	6175	RPLP0P3
0.243514568	0.000368654	0.016	1	64121	RRAGC
0.243116111	0.000377184	0.013	1	26751	SH3YL1
0.243071407	0.000378152	0.02	1	10572	SIVA1
0.242997557	0.000379757	0.016	1	3586	IL10
0.242970988	0.000380336	0.013	1	161198	CLEC14A
0.242782444	0.000384468	0.021	1	4151	MB
0.242781147	0.000384496	0.018	1	388611	CD164L2
0.242076248	0.000400315	0.012	1	1525	CXADR
0.241966754	0.000402826	0.025	1	81575	APOLD1
0.241390765	0.000416275	0.019	1	170463	SSBP4
0.241064236	0.000424084	0.016	1	685	BTC
0.241019532	0.000425163	0.027	1	9722	NOS1AP
0.240960568	0.000426591	0.023	1	139886	SPIN4
0.240774617	0.000431123	0.014	1	310	ANXA7
0.240669012	0.000433717	0.019	1	51233	C22orf43
0.240650222	0.00043418	0.02	1	51365	PLA1A
0.240200594	0.000445396	0.02	1	23392	KIAA0368
0.239650533	0.000459482	0.015	1	6431	SFRS6
0.239375174	0.000466688	0.02	1	9620	CELSR1
0.239361569	0.000467046	0.017	1	8840	WISP1
0.239062235	0.000475004	0.02	1	25903	OLFML2B
0.238857508	0.000480518	0.012	1	7567	ZNF19
0.238836765	0.00048108	0.024	1	3280	HES1
0.238816038	0.000481642	0.017	1	246213	SLC17A8
0.238330767	0.00049498	0.016	1	79139	DERL1
0.238162965	0.000499671	0.017	1	51032	CELA2B
0.238074198	0.000502169	0.026	1	5935	RBM3
0.238036618	0.00050323	0.015	1	87	ACTN1
0.23772563	0.000512091	0.02	1	7128	TNFAIP3
0.23755458	0.000517026	0.021	1	201229	C17orf108
0.23718594	0.000527811	0.02	1	9464	HAND2
0.236978605	0.000533969	0.018	1	79583	AC009163.1
0.23623094	0.000556727	0.026	1	119678	OR52E2
0.236212149	0.000557311	0.024	1	153657	TTC23L
0.23551695	0.000579296	0.032	1	10124	ARL4A
0.235474199	0.000580674	0.015	1	340554	ZC3H12B
0.235343963	0.000584889	0.023	1	202	AIM1
0.235127568	0.000591957	0.033	1	645104	CLRN2
0.235081568	0.00059347	0.02	1	51602	NOP58
0.234937742	0.000598222	0.023	1	11238	CA5B
0.234928668	0.000598523	0.017	1	100131532	LOC100131532
0.234543175	0.00061144	0.02	1	5158	PDE6B
0.2344881	0.000613307	0.021	1	9053	MAP7
0.234315753	0.000619181	0.029	1	388125	FAM148B
0.234251618	0.00062138	0.014	1	29940	DSE
0.234171927	0.000624123	0.019	1	7050	TGIF1
0.23412852	0.000625622	0.014	1	2077	ERF
0.234047532	0.000628427	0.023	1	79931	TNIP3
0.233961359	0.000631424	0.026	1	645181	LOC645181
0.233960062	0.000631469	0.026	1	774	CACNA1B
0.233817533	0.000636457	0.027	1	25890	ABI3BP
0.233805224	0.000636889	0.019	1	6442	SGCA
0.233685359	0.000641114	0.03	1	55001	TTC22
0.233379543	0.00065201	0.027	1	84898	PLXDC2
0.233159259	0.000659965	0.022	1	100129794	RP11-537P22.1
0.232898816	0.000669485	0.024	1	22974	TPX2
0.232872248	0.000670464	0.029	1	1382	CRABP2
0.232664928	0.000678142	0.028	1	8655	DYNLL1
0.232489988	0.000684685	0.023	1	10950	BTG3
0.232306629	0.000691606	0.022	1	401027	C2orf66
0.232097372	0.000699582	0.025	1	9032	TM4SF5
0.231921136	0.000706365	0.038	1	89890	KBTBD6
0.231756568	0.000712754	0.021	1	4610	MYCL1
0.231503889	0.000722668	0.021	1	132884	EVC2

0.185601503	< 0.001	10903	MTMR11
0.185073435	< 0.001	222967	RSPH10B
0.185022131	< 0.001	26289	AK5
0.185004458	< 0.001	7903	ST8SIA4
0.184841558	< 0.001	57577	KIAA1407
0.184735283	< 0.001	10682	EBP
0.184634209	< 0.001	55706	TMEM48
0.184541479	< 0.001	157376	FER1L6-AS2
0.184385732	< 0.001	145978	NCRNA00052
0.184365034	< 0.001	55863	TMEM126B
0.184358999	< 0.001	3739	KCNA4
0.184323415	< 0.001	100101267	POM121C
0.184099182	< 0.001	8600	TNFSF11
0.184011936	< 0.001	1540	CYLD
0.18400909	< 0.001	168455	C7ORF74
0.183734849	< 0.001	2779	GNAT1
0.183714002	< 0.001	337975	KRTAP20-1
0.183692753	< 0.001	257101	ZNF683
0.183217257	< 0.001	147699	PPM1N
0.183213025	< 0.001	91300	C19orf22
0.183059722	< 0.001	100506164	HSFX1
0.182960168	< 0.001	7531	YWHAE
0.182753861	< 0.001	146771	TCAM1
0.182741776	< 0.001	2790	GNG10
0.182669878	< 0.001	55472	C8orf39
0.18253693	< 0.001	3980	LIG3
0.182399169	< 0.001	644054	FAM25C
0.182376117	< 0.001	64768	IPPK
0.181992874	< 0.001	64577	ALDH8A1
0.181878254	< 0.001	80381	CD276
0.181814	< 0.001	6887	TAL2
0.181753293	< 0.001	63934	ZNF667
0.181615397	< 0.001	126299	ZNF428
0.181406319	< 0.001	2312	FLG
0.181047767	< 0.001	55512	SMPD3
0.180974379	< 0.001	26091	HERC4
0.180250138	< 0.001	222029	DKFZp434L192
0.1799936	< 0.001	23704	KCNE4
0.179935873	< 0.001	5128	PCTK2
0.1796128	< 0.001	25	ABL1
0.179444134	< 0.001	6181	RPLP2
0.179414898	< 0.001	474382	H2AFB1
0.17935884	< 0.001	2825	GPR1
0.178687662	< 0.001	56853	BRUNOL4
0.178644463	< 0.001	5451	POU2F1
0.17855908	< 0.001	23177	CEP68
0.1784385	< 0.001	150590	C2orf15
0.178336039	< 0.001	3283	HSD3B1
0.178204194	< 0.001	1947	EFNB1
0.178184181	< 0.001	26273	FBXO3
0.178109646	< 0.001	84171	LOXL4
0.177887425	< 0.001	9107	MTMR6
0.177707866	< 0.001	56704	JPH1
0.177406043	< 0.001	4858	NOVA2
0.177345455	< 0.001	401546	C9orf152
0.177253693	< 0.001	3985	LIMK2
0.177186802	< 0.001	158038	LINGO2
0.177101299	< 0.001	645104	CLRN2
0.177041143	< 0.001	64766	S100PBP
0.176637962	< 0.001	55218	EXD2
0.176488549	< 0.001	26045	LRRTM2
0.176148444	< 0.001	139081	MAGEC3
0.176103294	< 0.001	22926	ATF6
0.17596145	< 0.001	64711	HS3ST6
0.175947413	< 0.001	8735	MYH13
0.175823763	< 0.001	79697	C14orf169
0.175757557	< 0.001	5862	RAB2A
0.175596192	< 0.001	4887	NPY2R
0.175518945	< 0.001	6398	SECTM1
0.175267622	< 0.001	256356	GK5
0.175153777	< 0.001	401498	TMEM215
0.175086677	< 0.001	6196	RPS6KA2
0.175063744	< 0.001	1576	CYP3A4
0.174982399	< 0.001	22905	EPN2
0.174974412	< 0.001	4610	MYCL1
0.174910516	< 0.001	55602	CDKN2AIP
0.174880564	< 0.001	6659	SOX4
0.17476213	< 0.001	245940	AC144830.1
0.174535677	< 0.001	64105	CENPK
0.174492121	< 0.001	202181	LOC202181
0.174158067	< 0.001	8512	MBL1P

0.231448829	0.000724845	0.023	1	23096	IQSEC2
0.231419668	0.000726	0.029	1	26696	OR2T1
0.230530754	0.000762048	0.027	1	643827	LOC643827
0.230517805	0.000762585	0.019	1	220965	FAM13C
0.23049058	0.000763715	0.019	1	64693	CTAGE1
0.230343521	0.000769847	0.018	1	10022	INSL5
0.230152383	0.000777887	0.031	1	442459	LOC442459
0.22992757	0.00078744	0.034	1	375	ARF1
0.229892582	0.000788937	0.029	1	2	A2M
0.229888037	0.000789131	0.029	1	440895	AC013271.3
0.229872495	0.000789797	0.022	1	374	AREG
0.229828432	0.000791687	0.026	1	23562	CLDN14
0.229696915	0.000797355	0.02	1	6728	SRP19
0.229657397	0.000799065	0.029	1	1277	COL1A1
0.229299113	0.000814724	0.019	1	6814	STXBP3
0.229216173	0.00081839	0.032	1	4018	LPA
0.228974506	0.000829156	0.027	1	1540	CYLD
0.228769124	0.000838407	0.026	1	641700	ECSCR
0.228638902	0.000844322	0.026	1	6713	SQLE
0.228430927	0.000853849	0.031	1	100130932	LOC100130932
0.228173718	0.000865769	0.024	1	57093	TRIM49
0.228160113	0.000866403	0.024	1	100288929	LOC100288929
0.227727965	0.000886787	0.026	1	22953	P2RX2
0.22763142	0.000891402	0.024	1	57574	MARCHF4
0.22755821	0.000894915	0.034	1	151556	GPR155
0.227462322	0.000899537	0.02	1	255743	NPNT
0.227445483	0.00090035	0.018	1	3739	KCNA4
0.227219358	0.000911345	0.025	1	9518	GDF15
0.227079421	0.00091821	0.021	1	3488	IGFBP5
0.226971224	0.000923551	0.031	1	441054	C4orf47
0.22695826	0.000924192	0.037	1	2885	GRB2
0.226944655	0.000924866	0.025	1	338675	OR5AP2
0.226746395	0.00093474	0.02	1	5616	PRKY
0.226733446	0.000935388	0.037	1	25975	EGFL6
0.226730198	0.000935551	0.025	1	684	BST2
0.226585075	0.000942845	0.028	1	80270	HSD3B7
0.226476222	0.000948351	0.038	1	151507	MSL3P1
0.226145148	0.000965279	0.038	1	219844	HYLS1
0.226143211	0.000965378	0.021	1	126299	ZNF428
0.2259482	0.000975479	0.017	1	574	BAGE
0.225793347	0.000983569	0.031	1	1043	CD52
0.225129902	0.001018933	0.025	1	7258	TSPY1
0.225057989	0.001022837	0.034	1	22893	BAHD1
0.225032717	0.001024211	0.019	1	7135	TNNI1
0.224696457	0.001042666	0.027	1	969	CD69
0.224675074	0.001043849	0.018	1	2775	GNAO1
0.224621952	0.001046795	0.033	1	168544	ZNF467
0.22445415	0.001056153	0.024	1	54	ACP5
0.224352434	0.00106186	0.036	1	3623	INHA
0.224330395	0.001063102	0.032	1	55076	TMEM45A
0.224264309	0.00106683	0.037	1	284417	TMEM150B
0.224240348	0.001068184	0.022	1	79153	GDPD3
0.224170372	0.001072149	0.028	1	4295	MLN
0.224100396	0.001076129	0.035	1	729515	TMEM242
0.223755717	0.001095928	0.03	1	55504	TNFRSF19
0.223702595	0.001099008	0.035	1	83700	JAM3
0.22358337	0.001105953	0.023	1	6347	CCL2
0.22326526	0.001124676	0.031	1	8073	PTP4A2
0.223240644	0.001126138	0.023	1	4685	NCAM2
0.223068297	0.001136416	0.029	1	23641	LDOC1
0.223003507	0.001140301	0.043	1	57536	KIAA1328
0.222967222	0.001142484	0.031	1	3139	HLA-L
0.222951025	0.001143458	0.027	1	9184	BUB3
0.2226381	0.001162447	0.032	1	7273	TTN
0.222585618	0.001165659	0.035	1	3486	IGFBP3
0.22246252	0.001173228	0.029	1	56896	DPYSL5
0.22241652	0.001176067	0.03	1	652904	CKS1BP6
0.222413927	0.001176227	0.022	1	2920	CXCL2
0.222228631	0.00118773	0.03	1	127829	ARL8A
0.221785471	0.001215663	0.031	1	5996	RGS1
0.221749842	0.001217934	0.022	1	84171	LOXL4
0.2217291	0.001219259	0.038	1	441124	LOC441124
0.221551582	0.001230646	0.034	1	140886	PABPC5
0.22148484	0.001234953	0.033	1	9097	USP14
0.221380532	0.001241711	0.023	1	27071	DAPP1
0.221335828	0.001244618	0.023	1	84083	ZRANB3
0.221328706	0.001245082	0.019	1	147699	PPM1N
0.221271038	0.001248843	0.033	1	150280	HORMAD2
0.221126556	0.001258309	0.029	1	57456	KIAA1143
0.22106047	0.00126266	0.029	1	27042	C1orf107
0.221035212	0.001264327	0.035	1	221806	VWDE

0.174144521	< 0.001	5584	PRKCI
0.174103811	< 0.001	56107	PCDHGA9
0.174099013	< 0.001	10129	FRY
0.174075052	< 0.001	3326	HSP90AB1
0.174043238	< 0.001	151556	GPR155
0.174017951	< 0.001	55660	PRPF40A
0.173677087	< 0.001	26994	RNF11
0.173503712	< 0.001	3116	HLA-DPB2
0.173422784	< 0.001	7465	WEE1
0.173421949	< 0.001	84524	ZC3H8
0.173387289	< 0.001	6874	TAF4
0.173296973	< 0.001	6157	RPL27A
0.173121646	< 0.001	6328	SCN3A
0.172893032	< 0.001	9856	KIAA0319
0.172776133	< 0.001	5754	PTK7
0.172635809	< 0.001	4660	PPP1R12B
0.172535926	< 0.001	4072	EPCAM
0.172495276	< 0.001	6792	CDKL5
0.172430605	< 0.001	4312	MMP1
0.172388509	< 0.001	4683	NBN
0.172355726	< 0.001	340393	AF205589.3
0.171495885	< 0.001	55859	BEX1
0.171419129	< 0.001	23129	PLXND1
0.171340436	< 0.001	80173	IFT74
0.171325982	< 0.001	10966	RAB40B
0.171270609	< 0.001	3188	HNRNPH2
0.171257481	< 0.001	26086	GPSM1
0.171092093	< 0.001	80262	C16orf70
0.171041593	< 0.001	6500	SKP1
0.171004489	< 0.001	2885	GRB2
0.170949206	< 0.001	127262	TPRG1L
0.170943022	< 0.001	126326	GIPC3
0.17053172	< 0.001	541471	AC068491.1
0.17024608	< 0.001	400579	FLJ35934
0.170195371	< 0.001	25822	DNAJB5
0.170189261	< 0.001	79574	EPS8L3
0.170185581	< 0.001	89796	NAV1
0.170136943	< 0.001	114770	PGLYRP2
0.169863611	< 0.001	6234	RPS28
0.16980657	< 0.001	84960	KIAA1984
0.169541359	< 0.001	84186	ZCCHC7
0.169118106	< 0.001	4685	NCAM2
0.169024542	< 0.001	92552	ATXN3L
0.168994531	< 0.001	10563	CXCL13
0.16882211	< 0.001	201243	C17orf74
0.168801144	< 0.001	7587	ZNF37A
0.168536752	< 0.001	2116	ETV2
0.168530226	< 0.001	4927	NUP88
0.168488964	< 0.001	85443	DCLK3
0.168306828	< 0.001	729683	LOC729683
0.168289945	< 0.001	81871	KRTAP4-6
0.168243676	< 0.001	6175	RPLP0P3
0.168088153	< 0.001	50624	CUZD1
0.168023065	< 0.001	219844	HYLS1
0.168018967	< 0.001	79677	SMC6
0.168012857	< 0.001	286234	C9orf79
0.168005764	< 0.001	153733	CCDC112
0.167927265	< 0.001	54716	SLC6A20
0.167918518	< 0.001	128646	SIRPD
0.167851135	< 0.001	114134	SLC2A13
0.167824745	< 0.001	10808	HSPH1
0.167718947	< 0.001	6814	STXBP3
0.167570084	< 0.001	55762	ZNF701
0.167524725	< 0.001	283298	OLFML1
0.167423785	< 0.001	127829	ARL8A
0.167208314	< 0.001	10537	UBD
0.16716066	< 0.001	201562	PTPLB
0.167082652	< 0.001	624	BDKRB2
0.1668116	< 0.001	4082	MARCKS
0.166702747	< 0.001	4361	MRE11A
0.166472465	< 0.001	4314	MMP3
0.166349515	< 0.001	146198	ZFP90
0.166121811	< 0.001	55719	FAM178A
0.166024208	< 0.001	729515	TMEM242
0.165877908	< 0.001	57335	ZNF286
0.165845618	< 0.001	8854	ALDH1A2
0.165832624	< 0.001	2690	GHR
0.165734336	< 0.001	144195	SLC2A14
0.16550225	< 0.001	4291	MLF1
0.165479675	< 0.001	3142	HLX
0.165476263	< 0.001	221002	RASGEF1A

0.220888138	0.001274074	0.031	1	78998	C8ORF51
0.220594645	0.00129373	0.033	1	79054	TRPM8
0.220358163	0.001309771	0.033	1	2168	FABP1
0.220338717	0.001311096	0.029	1	221613	HIST1H2AA
0.220337421	0.001311185	0.03	1	1773	DNASE1
0.220128149	0.001325546	0.035	1	254272	TBC1D28
0.220087335	0.001328362	0.034	1	4015	LOX
0.219828829	0.001346335	0.031	1	124739	USP43
0.219753668	0.001351602	0.038	1	6731	SRP72
0.219631225	0.001360223	0.034	1	6423	SFRP2
0.219284594	0.0013849	0.034	1	64081	PBLD
0.21927163	0.001385832	0.033	1	3118	HLA-DQA2
0.219020903	0.001403949	0.046	1	257000	PLAC2
0.218972951	0.001407438	0.038	1	143941	TTC36
0.218620509	0.001433331	0.038	1	395	ARHGAP6
0.218513593	0.001441271	0.039	1	4633	MYL2
0.218315989	0.001456051	0.033	1	155185	AMZ1
0.21814236	0.001469154	0.036	1	93210	PGAP3
0.21792984	0.001485338	0.038	1	6631	SNRPC
0.217842385	0.001492045	0.05	1	403315	FAM92A3
0.217687532	0.001503988	0.03	1	131408	FAM131A
0.217649952	0.001506899	0.032	1	143630	UBQLNL
0.217314988	0.001533083	0.041	1	80790	AC099524.1
0.217226237	0.001540088	0.035	1	164592	CCDC116
0.21721068	0.001541319	0.036	1	23179	RGL1
0.217165336	0.001544912	0.047	1	80731	THSD7B
0.217119977	0.001548516	0.033	1	23253	ANKRD12
0.217113495	0.00154903	0.025	1	55683	KIAA1310
0.217027321	0.001555898	0.046	1	79647	AKIRIN1
0.217013717	0.001556986	0.037	1	865	CBFB
0.216969669	0.001560509	0.034	1	6677	SPAM1
0.216969013	0.00156056	0.038	1	6332	SCN7A
0.216943756	0.001562584	0.026	1	2308	FOXO1
0.216705322	0.001581805	0.045	1	80072	HEXA-AS1
0.216695607	0.001582593	0.035	1	7127	TNFAIP2
0.216543347	0.001594986	0.044	1	54521	WDR44
0.216534927	0.001595674	0.029	1	939	CD27
0.216157198	0.001626815	0.037	1	83986	ITFG3
0.216123521	0.001629618	0.037	1	84970	C1orf94
0.216093704	0.001632104	0.046	1	50940	PDE11A
0.216072977	0.001633833	0.035	1	4009	LMX1A
0.215625927	0.001671553	0.032	1	389649	C8orf86
0.215594828	0.001674206	0.039	1	55762	ZNF701
0.2155132	0.00168119	0.026	1	257101	ZNF683
0.215370014	0.001693504	0.048	1	7093	TLL2
0.215290964	0.001700336	0.042	1	27189	IL17C
0.21519962	0.001708262	0.038	1	10463	SLC30A9
0.215183422	0.001709672	0.04	1	728858	C12orf71
0.215163335	0.001711422	0.029	1	828	CAPS
0.215151668	0.001712438	0.032	1	84789	MGC2889
0.21497868	0.001727576	0.032	1	23677	SH3BP4
0.214941099	0.001730882	0.032	1	6720	SREBF1
0.214801162	0.001743238	0.035	1	2103	ESRRB
0.214643717	0.001757237	0.03	1	4239	MFAP4
0.214576349	0.00176326	0.04	1	132864	CPEB2
0.214488879	0.001771105	0.036	1	221044	UCMA
0.214428619	0.001776528	0.033	1	23193	GANAB
0.214423433	0.001776995	0.028	1	7903	ST8SIA4
0.214348927	0.001783725	0.028	1	100289143	LOC100289143
0.214168817	0.001800086	0.04	1	54829	ASPN
0.214089766	0.001807311	0.043	1	59	ACTA2
0.213986754	0.001816766	0.035	1	145814	AC069029.1
0.213798225	0.001834184	0.037	1	5737	PTGFR
0.213791743	0.001834787	0.036	1	80024	SLC24A6
0.213789791	0.001834967	0.045	1	126306	JSRP1
0.21374768	0.001838881	0.044	1	152519	NIPAL1
0.213261113	0.00188465	0.039	1	81833	SPACA1
0.212879509	0.001921271	0.054	1	27198	HCAR1
0.212849051	0.001924222	0.032	1	57156	TMEM63C
0.212812766	0.001927741	0.029	1	93663	ARHGAP18
0.212606743	0.001947845	0.038	1	135228	CD109
0.212597668	0.001948734	0.029	1	22829	NLGN4Y
0.212513447	0.001957009	0.051	1	54905	CYP2W1
0.212361842	0.001971988	0.039	1	8854	ALDH1A2
0.212197274	0.001988363	0.047	1	56913	C1GALT1
0.212069631	0.002001149	0.039	1	114783	LMTK3
0.211950421	0.002013159	0.047	1	7141	TNP1
0.211886927	0.002019582	0.033	1	92291	CAPN13
0.211747631	0.00203374	0.04	1	5858	PZP
0.211689964	0.002039627	0.028	1	6643	SNX2
0.211316139	0.00207817	0.039	1	84203	TXNDC2

0.16545786	< 0.001	9620	CELSR1
0.165212169	< 0.001	145837	LOC145837
0.16519396	< 0.001	79883	PODNL1
0.165173128	< 0.001	128239	IQGAP3
0.165160969	< 0.001	219771	CCNY
0.165154159	< 0.001	57501	KIAA1257
0.165026829	< 0.001	4218	RAB8A
0.165025443	< 0.001	9061	PAPSS1
0.164914504	< 0.001	10463	SLC30A9
0.164892763	< 0.001	337978	KRTAP21-2
0.164797947	< 0.001	100129935	LOC100129935
0.164794892	< 0.001	63922	CHTF18
0.164693058	< 0.001	135228	CD109
0.164441168	< 0.001	4109	MAGEA10
0.164401159	< 0.001	54065	C21orf51
0.164301276	< 0.001	114335	CGB1
0.164259017	< 0.001	10658	CUGBP1
0.164018273	< 0.001	32	ACACB
0.163991943	< 0.001	149499	C1orf92
0.16395165	< 0.001	407738	FAM19A1
0.163846552	< 0.001	3868	KRT16
0.163760692	< 0.001	6583	SLC22A4
0.163754717	< 0.001	23774	BRD1
0.16365914	< 0.001	1136	CHRNA3
0.16358842	< 0.001	266811	NPSA
0.163496375	< 0.001	9184	BUB3
0.163449287	< 0.001	2923	PDIA3
0.163384616	< 0.001	348378	FAM159A
0.163368285	< 0.001	5194	PEX13
0.163214773	< 0.001	389332	LOC389332
0.163198024	< 0.001	23295	MGRN1
0.16314961	< 0.001	22990	PCNX
0.163074583	< 0.001	100129933	GLYATL1P2
0.162982136	< 0.001	7638	ZNF221
0.162970811	< 0.001	286436	H2BFM
0.162771091	< 0.001	1756	DMD
0.162645638	< 0.001	6643	SNX2
0.162581176	< 0.001	8573	CASK
0.162515119	< 0.001	6476	SI
0.162435025	< 0.001	25929	GEMIN5
0.162203848	< 0.001	81025	GJA9
0.162022814	< 0.001	57621	ZBTB2
0.161969543	< 0.001	2741	GLRA1
0.161950305	< 0.001	23593	HEBP2
0.161946684	< 0.001	25950	RWDD3
0.161866799	< 0.001	4283	CXCL9
0.161801502	< 0.001	152756	C4ORF39
0.161758021	< 0.001	100506282	LOC100506282
0.161749616	< 0.001	619279	ZNF704
0.161703423	< 0.001	57535	KIAA1324
0.161315739	< 0.001	2775	GNAO1
0.161279127	< 0.001	7204	TRIO
0.161114708	< 0.001	2702	GJA5
0.161053643	< 0.001	11251	GPR44
0.160986751	< 0.001	23033	DOPEY1
0.160829067	< 0.001	84140	FAM161A
0.16062443	< 0.001	100271715	AC019171.4
0.160599485	< 0.001	100130386	LINC00552
0.160316065	< 0.001	10438	C1D
0.160172418	< 0.001	400236	RP11-33N16.1
0.160059541	< 0.001	55027	HEATR3
0.159883365	< 0.001	100506144	RP11-244H3.1
0.159877747	< 0.001	949	SCARB1
0.159506038	< 0.001	56244	BTNL2
0.159337997	< 0.001	150864	FAM117B
0.159321472	< 0.001	84929	FIBCD1
0.159303054	< 0.001	143686	SESN3
0.15909633	< 0.001	611	OPN1SW
0.158926561	< 0.001	153020	RASGEF1B
0.158892453	< 0.001	23148	NACAD
0.158751786	< 0.001	80014	WWC2
0.15862675	< 0.001	120775	OR2D3
0.1585702	< 0.001	10572	SIVA1
0.158565685	< 0.001	768206	PRCD
0.158507213	< 0.001	4761	NEUROD2
0.158198014	< 0.001	8840	WISP1
0.15815106	< 0.001	22829	NLGN4Y
0.158034697	< 0.001	5926	ARID4A
0.157843605	< 0.001	57654	KIAA1530
0.1578352	< 0.001	158046	NXNL2
0.157796368	< 0.001	90410	IFT20

0.211177483	0.002092634	0.039	1	5067	CNTN3
0.211095199	0.002101262	0.042	1	567	B2M
0.211016804	0.002109511	0.042	1	389289	ANXA2R
0.210938409	0.002117789	0.036	1	79730	NSUN7
0.210821137	0.002130227	0.048	1	2831	NPBWR1
0.210584655	0.002155514	0.053	1	167826	OLIG3
0.210492015	0.002165496	0.04	1	26223	FBXL21
0.210469976	0.002167876	0.037	1	23129	PLXND1
0.210369557	0.00217875	0.038	1	57580	PREX1
0.210122064	0.002205771	0.053	1	1565	CYP2D6
0.210119471	0.002206055	0.031	1	80262	C16orf70
0.21010004	0.002208189	0.035	1	4072	EPCAM
0.209605694	0.002263126	0.037	1	126402	CCDC105
0.209434003	0.002282497	0.045	1	7832	BTG2
0.209434003	0.002282497	0.047	1	10058	ABCB6
0.209418446	0.002284258	0.031	1	100287825	LOC100287825
0.209332272	0.002294041	0.046	1	729501	ZNF962P
0.209277853	0.002300244	0.035	1	11251	GPR44
0.208756953	0.002360353	0.044	1	286234	C9orf79
0.208754361	0.002360655	0.062	1	54892	NCAPG2
0.208628669	0.002375377	0.046	1	8600	TNFSF11
0.208611816	0.002377355	0.039	1	10163	WASF2
0.208451137	0.002396307	0.055	1	53827	FXYD5
0.208385706	0.002404062	0.045	1	340371	NRBP2
0.208284631	0.002416091	0.045	1	127254	C1orf173
0.208017707	0.002448116	0.052	1	27315	PGAP2
0.207838878	0.002469788	0.043	1	387885	CCDC42B
0.207726151	0.002483535	0.061	1	100132644	LOC100132644
0.207497448	0.002511646	0.054	1	10410	IFITM3
0.207326397	0.002532856	0.047	1	347365	ITIH5L
0.207316682	0.002534066	0.046	1	440224	CXADRP3
0.207246706	0.002542795	0.062	1	55510	DDX43
0.207202643	0.002548305	0.042	1	5199	CFP
0.207077608	0.002564	0.042	1	79992	AGPAT4-IT1
0.206933767	0.002582166	0.041	1	10808	HSPH1
0.206883878	0.002588491	0.038	1	6275	S100A4
0.206820384	0.002596565	0.049	1	440917	LOC440917
0.206726447	0.00260855	0.041	1	23769	FLRT1
0.206700534	0.002611866	0.049	1	123722	FSD2
0.206575483	0.002627917	0.048	1	6504	SLAMF1
0.206464693	0.002642212	0.031	1	84189	SLITRK6
0.20645757	0.002643134	0.049	1	3990	LIPC
0.206404448	0.002650015	0.038	1	2564	GABRE
0.206375286	0.002653799	0.04	1	27145	FILIP1
0.206370756	0.002654389	0.039	1	222487	GPR97
0.206258669	0.002668985	0.046	1	4016	LOXL1
0.205861509	0.002721289	0.042	1	7482	WNT2B
0.205788299	0.002731035	0.046	1	81551	STMN4
0.205717668	0.002740462	0.035	1	4082	MARCKS
0.205712497	0.002741153	0.046	1	100272228	RP13-507I23.1
0.205582917	0.002758533	0.048	1	63914	C6orf164
0.20554857	0.002763157	0.05	1	85417	CCNB3
0.205326989	0.002793153	0.048	1	122706	PSMB11
0.205306903	0.002795887	0.048	1	83449	PMFBP1
0.205089867	0.002825579	0.045	1	51700	CYB5R2
0.205081433	0.00282674	0.051	1	999	CDH1
0.204924002	0.002848465	0.061	1	100287822	SETP10
0.204597458	0.002894013	0.054	1	10516	FBLN5
0.20453915	0.002902215	0.049	1	51191	HERC5
0.204498976	0.002907878	0.051	1	10507	SEMA4D
0.204346076	0.002929522	0.06	1	51123	ZNF706
0.20419383	0.002951222	0.049	1	84140	FAM161A
[truncated: 873,372 more chars]
