# Supplementary material for: Regional gene expression patterns are associated with task‐specific brain activation during reward and emotion processing measured with functional MRI
Source: Hum Brain Mapp. 2022 Jul 7;43(17):5266–80. doi: 10.1002/hbm.26001 (PMC9812247; doi:10.1002/hbm.26001)
Supplement: Supplementary file 4 — TABLE S6: Master regulators associated with Major Depressive Disorder and task‐specific functional brain activation. Transcription factor binding motifs identified with RcisTarget (Aibar et al., 2017), corresponding target genes, normalized enrichment scores and AUC values are provided for single‐site and meta‐analytical imaging data. (excel‐file) [file HBM-43-5266-s001.pdf]

fMRI Reward Pos Cortex

"motif"	"NES"	"AUC"	"TF_highConf"	"nEnrGenes"	"enrichedGenes"
"cisbp__M5741"	4.51	0.0765	"POU4F2 (directAnnotation). "	16	"BAIAP2L2;C12orf64;CA10;CCDC68;DGCR5;GAP43;HDAC9;KLHL14;NEUROD6;OLFM3;OPTN;OR14I1;RIMS1;SERPINI2;TUBB2C;VSTM2L"
"cisbp__M6545"	4.03	0.0715	"HIVEP2 (directAnnotation). "	56	"ASS1;B4GALT2;BAIAP2L2;C2orf40;C6orf105;CA10;CCK;CCNYL1;CDC34;CLTA;COL11A1;DCP2;DGCR5;DLEU7;FAM83H;FBXO2;FREM3;FRMPD2;FUT9;GNB1;GPA33;GPR116;GRASP;HDAC9;HDC;KCNJ12;KCNN2;KRT83;LASS6;LOC646627;MEPE;MYBPC2;NAT14;NEK2;NEUROD6;NT5DC2;OPTN;PARP8;PCDH10;PGAM5;PPEF1;PPP1R14C;PRELP;PVRL3;RIMS1;RMND5B;SH2D5;SHC1;SNX10;SYP;TPST1;TTPAL;TUBB2C;VSTM2L;WDR86;WSB2"
"cisbp__M5743"	3.55	0.0666	"POU4F3 (directAnnotation). "	10	"BAIAP2L2;C12orf64;CA10;CCDC68;GAP43;HDAC9;OPTN;RIMS1;SERPINI2;VSTM2L"
"cisbp__M5742"	3.41	0.0652	"POU4F2 (directAnnotation). "	10	"BAIAP2L2;C12orf64;CA10;CCDC68;GAP43;KLHL14;OLFM3;OPTN;OR14I1;SERPINI2"
"cisbp__M5740"	3.39	0.0649	"POU4F1 (directAnnotation). "	10	"BAIAP2L2;C12orf64;CA10;CCDC68;GAP43;HDAC9;OPTN;RIMS1;SERPINI2;VSTM2L"
"cisbp__M6344"	3.19	0.0629	"MEIS2 (directAnnotation). "	12	"AP1M1;CCNYL1;CLIC5;COL11A1;COL23A1;COL5A3;GNG3;GOLPH3L;KIAA1715;PHF20L1;RIMS1;SERPINB2"
"cisbp__M5226"	3.19	0.0628	"TCF12; TCF3; TCF4 (inferredBy_Orthology). "	12	"CLIC5;COL5A3;GOLPH3L;GPR116;KIAA1715;LRFN3;MYBPC2;PARP8;RIMS1;SERPINB2;SH2D5;WFDC1"
"cisbp__M0720"	3.19	0.0628	""	14	"CA10;DCP2;HDAC9;HDC;KCNA5;KCNJ12;NEUROD6;PCDH10;PIK3CD;PPEF1;PRELP;RIMS1;VIT;YBX2"
"cisbp__M4795"	3.03	0.0612	"IRX1; IRX2; IRX3; IRX4; IRX5; IRX6 (inferredBy_Orthology). "	12	"B4GALT2;GPR116;HDC;KCNN2;MEPE;MYBPC2;NBAS;NEUROD6;PIK3CD;PPEF1;PRELP;RIMS1"

fMRI Reward Neg Cortex

"motif"	"NES"	"AUC"	"TF_highConf"	"nEnrGenes"	"enrichedGenes"
"cisbp__M4710"	5.39	0.128	""	11	"BHLHE22;CD209;CUX1;EDIL3;FBXO21;FEZF1;ITGA11;MAML3;TAP2;USH2A;VAV3"
"cisbp__M2444"	4.77	0.115	"HSF1; HSF2; HSF4 (inferredBy_Orthology). "	5	"CUX1;EDIL3;ITGA11;UNC13C;ZFP37"
"cisbp__M0667"	4.54	0.111	""	4	"BHLHE22;FEZF1;KIAA1530;MAML3"
"cisbp__M2053"	4.47	0.11	"SIX1; SIX2 (inferredBy_Orthology). "	4	"MAML3;MMP3;UNC13C;VAV3"
"cisbp__M2568"	4.33	0.107	"HSF1; HSF2; HSF4 (inferredBy_Orthology). "	6	"CUX1;EDIL3;FEZF1;ITGA11;MMP3;UNC13C"
"cisbp__M0674"	4.24	0.105	""	4	"BHLHE22;FEZF1;KIAA1530;MAML3"
"cisbp__M5835"	4.23	0.105	"SOX7 (directAnnotation). "	5	"FEZF1;SLC35F4;USH2A;VAV3;ZFP37"
"cisbp__M3727"	4.19	0.104	"PAX6 (directAnnotation). "	5	"BHLHE22;EDIL3;FEZF1;PPIL5;PRR16"
"cisbp__M5021"	4.09	0.102	""	5	"CUX1;EDIL3;FEZF1;KIAA1530;POLR3G"
"cisbp__M5095"	4.01	0.1	""	7	"BHLHE22;CNTN5;MAML3;POLR3G;UNC13C;USH2A;VAV3"
"cisbp__M0629"	4	0.1	"DMRT3 (directAnnotation). "	6	"MAML3;MMP3;SLC35F4;UNC13C;USH2A;VAV3"
"cisbp__M0938"	3.97	0.0994	"SIX6 (inferredBy_Orthology). "	4	"MAML3;MMP3;UNC13C;VAV3"
"cisbp__M6404"	3.8	0.0961	"TP63 (directAnnotation). "	5	"BHLHE22;CNTN5;PRR16;SLC35F4;VAV3"
"cisbp__M5610"	3.78	0.0957	"MAFK (directAnnotation). "	5	"BHLHE22;CNTN5;FEZF1;INADL;VAV3"
"cisbp__M4567"	3.68	0.0936	"FOXA2 (directAnnotation). "	8	"BHLHE22;EDIL3;FEZF1;MAML3;MMP3;PPIL5;VAV3;ZNF30"
"cisbp__M1897"	3.63	0.0927	"PAX6 (directAnnotation). "	3	"BHLHE22;EDIL3;VAV3"
"cisbp__M1569"	3.63	0.0926	""	6	"CNTN5;EDIL3;USH2A;VAV3;ZFP37;ZNF30"
"cisbp__M0678"	3.59	0.0919	""	3	"BHLHE22;FEZF1;MAML3"
"cisbp__M3296"	3.55	0.091	"FOXD1 (inferredBy_Orthology). "	10	"BHLHE22;FEZF1;MAML3;MMP3;PPIL5;PRR16;SLC35F4;UNC13C;VAV3;ZNF30"
"cisbp__M0498"	3.52	0.0904	""	5	"BHLHE22;CNTN5;FEZF1;POLR3G;VAV3"
"cisbp__M1175"	3.47	0.0894	""	6	"BHLHE22;CNTN5;FEZF1;KIAA1530;USH2A;VAV3"
"cisbp__M4924"	3.47	0.0893	""	5	"CUX1;FEZF1;INADL;MAML3;ZFP37"
"cisbp__M4799"	3.46	0.0892	"ZNF853 (inferredBy_Orthology). "	4	"BHLHE22;EDIL3;FEZF1;VAV3"
"cisbp__M1726"	3.46	0.0892	""	3	"HDAC8;KIAA1530;MAML3"
"cisbp__M5096"	3.45	0.0891	""	7	"BHLHE22;CNTN5;ITGA11;POLR3G;UNC13C;USH2A;VAV3"
"cisbp__M0671"	3.45	0.0891	""	3	"BHLHE22;FEZF1;MAML3"
"cisbp__M5608"	3.41	0.0882	"MAFG (directAnnotation). "	5	"BHLHE22;CNTN5;FEZF1;INADL;VAV3"
"cisbp__M4237"	3.35	0.087	""	4	"BHLHE22;HDAC8;KIAA1530;MAML3"
"cisbp__M2176"	3.34	0.0869	""	3	"BHLHE22;EDIL3;VAV3"
"cisbp__M1720"	3.29	0.0859	""	6	"BHLHE22;CNTN5;FEZF1;HDAC8;KIAA1530;MAML3"
"cisbp__M4664"	3.29	0.0859	"FOXA1 (directAnnotation). "	10	"BHLHE22;EDIL3;FEZF1;HDAC8;MAML3;MMP3;PPIL5;UNC13C;VAV3;ZNF30"
"cisbp__M4545"	3.27	0.0855	"PRDM1 (directAnnotation). "	11	"BHLHE22;CD209;EDIL3;FBXO21;FEZF1;INADL;ITGA11;MMP3;SLC35F4;TAP2;VAV3"
"cisbp__M0672"	3.27	0.0854	""	4	"BHLHE22;FEZF1;HDAC8;KIAA1530"
"cisbp__M0564"	3.24	0.0849	""	4	"CNTN5;ITGA11;UNC13C;ZFP37"
"cisbp__M5708"	3.24	0.0848	"PAX6 (directAnnotation). "	4	"BHLHE22;CD209;EDIL3;PPIL5"
"cisbp__M6501"	3.23	0.0847	"TAL1 (directAnnotation). "	2	"BHLHE22;SLC35F4"
"cisbp__M1566"	3.23	0.0845	""	13	"CNTN5;EDIL3;INADL;INSR;KIAA1530;MAML3;POLR3G;PPIL5;SPAG5;USH2A;VAV3;ZFP37;ZNF30"
"cisbp__M6293"	3.21	0.0843	"HOXA7 (directAnnotation). "	4	"FEZF1;MAML3;MMP3;SLC35F4"
"cisbp__M4768"	3.21	0.0842	"PRDM1 (inferredBy_Orthology). "	6	"BHLHE22;CD209;EDIL3;MMP3;TAP2;VAV3"
"cisbp__M3988"	3.18	0.0836	"STAT5A (directAnnotation). "	6	"EDIL3;FEZF1;ITGA11;MMP3;UNC13C;ZFP37"
"cisbp__M0679"	3.16	0.0833	""	3	"BHLHE22;FEZF1;MAML3"
"cisbp__M0821"	3.16	0.0832	""	12	"CNTN5;CUX1;EDIL3;INADL;INSR;KIAA1530;MAML3;PRR16;UNC13C;VAV3;ZFP37;ZNF30"
"cisbp__M5335"	3.14	0.0829	"CUX2 (directAnnotation). "	14	"CD209;CPN2;FBXO21;GK5;IGFL4;INADL;ITGA11;MAML3;OR1N2;PPIL5;SLC35F4;TAP2;USH2A;VAV3"
"cisbp__M4814"	3.13	0.0827	""	4	"EDIL3;FEZF1;MMP3;UNC13C"
"cisbp__M0677"	3.1	0.0821	""	3	"BHLHE22;FEZF1;MAML3"
"cisbp__M1746"	3.09	0.0818	""	3	"BHLHE22;FEZF1;MAML3"
"cisbp__M0737"	3.09	0.0817	"FOXB1 (inferredBy_Orthology). "	4	"BHLHE22;EDIL3;FEZF1;MAML3"
"cisbp__M5749"	3.05	0.081	"PRDM1 (directAnnotation). "	3	"BHLHE22;EDIL3;TAP2"
"cisbp__M1736"	3.05	0.081	""	2	"BHLHE22;MAML3"
"cisbp__M4453"	3.03	0.0805	"BCL11A (directAnnotation). "	3	"EDIL3;ITGA11;UNC13C"
"cisbp__M4566"	3.02	0.0803	"FOXA1 (directAnnotation). "	6	"BHLHE22;EDIL3;FEZF1;MAML3;MMP3;VAV3"
"cisbp__M0725"	3.01	0.0801	"FOXP1 (inferredBy_Orthology). "	4	"BHLHE22;FEZF1;INSR;MAML3"
"cisbp__M3401"	3	0.08	"HMX3 (inferredBy_Orthology). "	4	"HCG4;ITGA11;MYO9B;PRR16"

fMRI Reward Pos Subc

"motif"	"NES"	"AUC"	"TF_highConf"	"nEnrGenes"	"enrichedGenes"
"cisbp__M4454"	4.3	0.0458	"BRCA1 (directAnnotation). "	117	"ANAPC10;ANKHD1-EIF4EBP3;ASPH;B3GNT5;BOD1;C10orf26;C12orf48;C14orf135;C1orf9;C20orf72;C20orf94;C4orf29;C9orf5;CAPN7;CBLB;CCDC90B;CDC25A;CFLAR;CHUK;CIRBP;CRY1;CUL4A;DDX3Y;DERL2;DGUOK;DHFRL1;DRAM2;EIF4A3;ERLIN2;FAM76A;FNDC3A;FOS;GGNBP2;GKAP1;GPN3;HELLS;HELQ;HMGB2;HSPA13;IKZF5;INTS2;KIF27;LARP1B;LARP7;LIFR;LIMS1;LOC728407;LSM14A;MAK16;MBNL3;ME2;MED28;MPP5;MRPS31;MSH5;MTAP;MUTYH;MX1;NID2;NINL;NKX2-1;NOC3L;NOL11;NXT1;OFD1;PAPOLA;PARG;PDIK1L;PFDN5;PLRG1;PNPLA4;PPAPDC1B;PPP1R3B;PRDM13;PSME2;RPL10;RPL24;RPL7;RPP38;RPS28;RPS6;SEC11C;SERPINB6;SH3GLB1;SIM1;SLU7;SMARCAD1;SNRPF;SRI;SRRM1;SSBP1;SSR1;STXBP4;SYTL4;TAF7;TDRD3;TEX9;TGFB1;THAP2;THG1L;TIGD6;TMCO1;TPH1;TRIM39;TRIP4;TRPS1;TSG101;TTC14;TTC32;UAP1L1;UBXN2A;UPF3A;USP51;VAT1L;WRN;ZKSCAN2;ZZZ3"
"cisbp__M4528"	4.07	0.0445	"BRCA1 (directAnnotation). "	277	"ACHE;ADAL;ADH5;ANAPC10;ANKHD1-EIF4EBP3;ANKRA2;APPL2;ARFGAP3;ARFIP1;ARIH1;AS3MT;ASB1;ASPH;ATF1;ATF7;ATP10D;AVP;B3GNT5;BCAR3;BMPR1A;BOD1;BPTF;C10orf26;C12orf48;C14orf135;C17orf101;C19orf42;C1orf112;C1orf212;C1orf56;C1orf9;C1orf97;C20orf72;C20orf94;C21orf34;C2orf40;C3orf38;C4orf29;C5orf41;C6orf145;C9orf5;CAPN7;CBLB;CCDC127;CCDC15;CCDC90B;CDC25A;CFLAR;CHUK;CIRBP;CLNS1A;CNIH;COMMD6;COPG2;COPS4;CRY1;CRYL1;CST6;CUL4A;DDX3Y;DDX50;DERL2;DGUOK;DHFRL1;DRAM2;DUSP18;EFC4A;EIF4A3;ERLIN2;FAM46A;FAM76A;FIP1L1;FNDC3A;FOS;FYTTD1;FZD5;GALNT10;GGNBP2;GKAP1;GLG1;GMNN;GPN3;GPX3;HAS3;HELLS;HELQ;HIST1H2AD;HIST1H3G;HIST4H4;HLA-A;HMGB2;HMGN4;HSP90B1;HSPA13;HSPBAP1;IKZF5;IMPACT;INTS2;ITGB3BP;KCTD3;KIAA1430;KIAA1530;KIF27;KLHDC1;KLHDC9;KLHL21;LARP1B;LARP7;LCMT2;LIFR;LIG1;LIMS1;LOC389765;LOC728407;LPP;LSM14A;MAK16;MBNL3;MDM1;ME2;MED28;METAP2;MPP5;MRPS26;MRPS31;MSH5;MTAP;MTL5;MUTYH;MX1;MYST2;N6AMT2;NDUFC1;NEDD1;NEK1;NHLRC2;NID2;NINL;NKX2-1;NOC3L;NOL11;NT5C2;NUP54;NXT1;OFD1;PAPOLA;PARG;PBXIP1;PCGF5;PDIK1L;PFDN5;PHF11;PHLDA2;PIF1;PIN4;PITX2;PLCD1;PLRG1;PNPLA4;PNPO;POLA1;PPAPDC1A;PPAPDC1B;PPP1R3B;PPP4R1;PRDM13;PRDX6;PRMT2;PRRC1;PSMB1;PSME1;PSME2;PTCH1;PTGDR;PTOV1;RAD21;RAN;RAP1B;RASAL3;RBBP8;RCBTB2;RCL1;REST;RNF135;RPL10;RPL13A;RPL24;RPL27A;RPL7;RPP38;RPS28;RPS6;RRM1;RSPRY1;SEC11C;SERPINB6;SH3GLB1;SIM1;SLAIN2;SLC16A8;SLC22A5;SLC25A1;SLC26A2;SLU7;SMARCAD1;SMC5;SMEK1;SNRPF;SOX2;SRI;SRRM1;SRSF3;SSBP1;SSR1;STAG1;STAT5B;STX2;STXBP4;SUMF2;SUPT16H;SYF2;SYTL4;TAF7;TARSL2;TDRD3;TEX9;TGFB1;THAP2;THG1L;THNSL2;TIGD6;TMCO1;TMED10;TMEM138;TMEM62;TNPO1;TOB1;TRAF3IP1;TRIM39;TRIM4;TRIM52;TRIP4;TRMT112;TRPS1;TSEN34;TSG101;TSPAN4;TTC14;TTC32;TTLL3;TUBB2B;TXNDC5;TYMP;UAP1L1;UBXN2A;UPF3A;USP24;USP51;UTP18;VAT1;VAT1L;WRB;WRN;ZCCHC11;ZCCHC3;ZFC3H1;ZFX;ZKSCAN2;ZMYND12;ZNF25;ZNF33A;ZNF345;ZNF471;ZNF512;ZNF616;ZNF841;ZZZ3"
"cisbp__M4688"	3.95	0.0438	"ELF1 (directAnnotation). "	252	"ACE2;ACTL6A;ADAL;ADH5;AEBP1;ALOX5;ANKHD1-EIF4EBP3;ANKS3;ANP32E;APPL2;ARF4;ARFIP1;ARID4B;ARIH1;ASB1;ASPH;ATP7A;B3GNT5;BBS9;BCL7C;BMP2K;BOD1;BRIX1;BTBD8;C10orf26;C11orf54;C11orf71;C12orf48;C12orf57;C14orf109;C15orf24;C15orf38;C1orf112;C1orf212;C1orf97;C2orf84;C5orf41;C5orf43;C6orf226;C6orf70;C6orf72;C8orf45;C9orf64;CARD8;CBFB;CCDC15;CCDC152;CCDC88B;CCDC90B;CCR7;CD274;CDC16;CDKL3;CDNF;CHUK;CIRBP;CITED4;COL7A1;COMMD6;COX16;CREBL2;CRY1;CSGALNACT2;CSTF1;CWC15;CYB5D2;DBR1;DCLRE1B;DDX50;DERL2;DHRS12;DUSP18;DYNLT1;EFC4A;EFHC1;EIF5B;ELAC1;ERAP1;ERLIN2;EXOSC3;FAM114A1;FAM173B;FAM46A;FBXO15;FBXO36;FCGBP;FNDC3A;GKAP1;GLIPR1L2;GMNN;GNA13;GTF2A2;GTF2F2;H2AFY;HAUS6;HEATR5A;HELLS;HEPH;HFM1;HIST1H3B;HLA-A;HMGN4;HSPA13;HSPA5;HSPBAP1;IFT88;IL28RA;IL7R;IMPACT;INTS4;IRF7;IRF9;KIAA1530;KLHDC9;LARP7;LCA5;LEPROT;LIFR;LIG1;LIMS1;LOC100131564;LOC100272217;LOC389705;LOC728407;LOC729852;LPP;LSM5;LSM6;MAEA;MAK16;MDM1;MLH3;MPP5;MRPL20;MRPL51;MRPS26;MYST2;NAA38;NEDD1;NFATC1;NKX2-1;NOC3L;NOL11;NOL7;NR1H3;NSUN5;NUP54;NXT1;PARG;PARP9;PBLD;PCNP;PDCD10;PDLIM5;PFDN5;PIGV;PITX2;PLK3;PLRG1;PNPLA4;PNPO;POLA1;POP5;PPP1R3B;PRKY;PROCA1;PTCH1;PTGR2;RAD17;RAP2C;RBBP8;RBM34;RBM8A;RDBP;REST;RFX4;RHBDD1;RPIA;RPL10;RPL13A;RPL27A;RPL7;RPN1;RPS6;RTDR1;SALL4;SCAMP2;SETD4;SH3GLB1;SLC25A1;SLC25A20;SLC26A2;SMARCA5;SMEK1;SP140L;SPIN4;SPR;SRP72;SSBP1;SSR1;STAG1;STAT5B;STK32B;STT3A;SYF2;TAF1A;TAF7;TDRD3;TFAM;TGDS;THNSL2;TIGD6;TMCO1;TMED10;TMEM138;TMEM20;TMEM222;TPRKB;TRAF3IP1;TRAPPC4;TRIM42;TRIM52;TRIP4;TRMT112;TSG101;TSPAN4;TTC14;TTC30B;TTLL3;TXNDC5;TYMP;UBR1;UMPS;USP24;UTP18;VMO1;WDR61;WRB;XIAP;YES1;ZNF197;ZNF273;ZNF331;ZNF394;ZNF512;ZNF529;ZNF610;ZNF660;ZNF671;ZNF691;ZNF701;ZNF92"
"cisbp__M5580"	3.92	0.0437	"IRF9 (directAnnotation). "	77	"ACADM;APPL2;ARID4B;ASPH;BET3L;BTN3A1;C10orf26;C17orf101;C1orf212;C2orf3;CARD8;CASP10;CCDC102A;CCDC88B;CD274;CLEC1A;CRNKL1;DYNLT1;FAM123B;FAM76A;FSTL5;GNA13;GTF2F2;HERC5;HEY2;HLA-A;HLA-DRB1;HSPA13;IFI44;IFIT3;IGFBP6;IRF7;KLRK1;LRP10;LST1;MDK;ME2;METAP2;MS4A6E;MX1;MYOZ1;NEDD1;PARP14;PDIK1L;PMFBP1;POLI;POP5;PPAPDC1A;PRICKLE4;PRKY;PTCH1;RL3;RARRES3;RPL24;RTP4;SAMD4A;SAMD9L;SLC35F5;SLU7;SMARCAD1;SP140L;SRRM1;SSBP1;TFAM;TMCO1;TMED10;TNFSF11;TPH1;TRAF3IP1;TRIM34;TRIM38;TRMT11;TSEN34;UBR1;ZMYND12;ZNF273;ZNF518A"

"motif"	"NES"	"AUC"	"TF_highConf"	"nEnrGenes"	"enrichedGenes"
"cisbp__M4739"	3.89	0.0435	"ETV6; ETV7 (inferredBy_Orthology). "	262	"ADAL;ADH5;AEBP1;ALG14;ALOX5;ANKHD1-EIF4EBP3;ANKS3;ANP32E;APPL2;ARF4;ARFIP1;ARIH1;ASB1;ATAD2B;ATP7A;B3GNT5;BCL7C;BMP2K;BMPR1A;BOD1;BRIX1;C10orf26;C11orf71;C12orf48;C12orf57;C14orf109;C14orf135;C15orf24;C15orf38;C1orf112;C1orf212;C1orf97;C20orf94;C2orf84;C5orf43;C6orf226;C6orf70;C7orf11;C9orf64;CARD8;CBFB;CBLB;CCDC15;CCDC152;CCDC88B;CD274;CDC16;CDC26;CDK4;CDKL3;CETN3;CFLAR;CGA;CHUK;CIRBP;CITED4;CLEC11A;CNIH;CNIH4;COMMD6;COX16;CREBL2;CRY1;CSGALNACT2;CSTF1;CWC15;CWF19L2;CXXC4;CYB5D2;DCLRE1B;DDX50;DDX6;DERL2;DGUOK;DLEC1;DNAJC10;DUSP18;EFC4A;EFHC1;EIF5B;ERCC4;ERLIN2;EXOSC3;FAM173B;FAM177A1;FAM46A;FBXO15;FIP1L1;FNDC3A;FOS;FTSJD1;GALNT10;GCNT2;GMNN;GTF2A2;GTF2F2;H2AFY;HAUS6;HEPH;HFM1;HIST1H3B;HMGB2;HMGN4;HSPA13;HSPA5;HSPBAP1;IFT20;IGF2BP2;IGFBP6;IL7R;IMPACT;INTS2;INTS4;ITGB3BP;KIAA1530;KLRK1;LARP7;LAX1;LEPROT;LIMS1;LOC100131564;LOC100272217;LOC389705;LOC728407;LOC729852;LRP10;LRRC70;LSM5;LST1;MAEA;MAGEH1;MAK16;MED28;METTL12;MGAT2;MRPL20;MRPL51;MRPS26;MRPS31;MSH5;MSN;MUTYH;MYST2;NAA38;NCF2;NEDD1;NEK9;NFATC1;NHLRC2;NHP2L1;NOC3L;NOL11;NOL7;NR1H3;NSUN5;NUP54;NXT1;OFD1;OGFOD1;PAPOLA;PARG;PARP3;PATZ1;PBXIP1;PCNP;PDCD10;PFDN5;PHIP;PLCD1;PLRG1;PMFBP1;PNPLA4;POLA1;POLI;POP5;PPAPDC1B;PSME2;PTCH1;RAD17;RAP1B;RAP2C;RBBP8;RBM34;RBM8A;RCBTB2;RDBP;REST;RFX4;RPIA;RPL10;RPL13A;RPL24;RPL7;RPS6;RRP15;S100A13;SALL4;SAMD4A;SCAMP2;SCARNA17;SETD4;SKIL;SLAIN2;SLC25A1;SLC7A7;SMARCA5;SNAPC1;SOX2;SP140L;SPR;SRP72;SRRM1;SSBP1;STAG1;STAT5B;STT3A;SYF2;TAF1A;TAF7;TDRD3;TFAM;TGDS;TGIF2;THAP2;THAP6;TIGD6;TMCO1;TMED10;TMEM138;TMEM176A;TMEM199;TMEM20;TMEM222;TOR1B;TPRKB;TRAPPC2P1;TRAPPC4;TRIM39;TRMT112;TSEN34;TSG101;TTC14;TTC30B;TTF1;TXNDC5;TYMP;UBR1;UFD1L;USP16;USP24;UTP18;WDR61;XIAP;YES1;ZKSCAN2;ZNF197;ZNF394;ZNF512;ZNF518A;ZNF529;ZNF671;ZZZ3"
"cisbp__M6276"	3.88	0.0435	"HINFP (directAnnotation). "	80	"ANAPC10;ANP32E;APPL2;ARF4;ATF1;ATPBD4;BTF3;C11orf71;C11orf96;C1orf212;C1orf9;C9orf64;CALCR;CBFB;CBLB;CCDC15;CDC73;CDK4;CHUK;CKLF;CLEC1A;COMMD6;CSTF1;CTXN2;DERL2;DRAM2;DYNLT1;EFC2;EFC4A;EFHC1;EIF5B;ELAC1;EXOSC3;FAM46A;FSTL5;FTSJD1;GCNT2;GGNBP2;HAUS6;HSPA5;LCMT2;METAP2;MPL;MRPS26;MXRA8;MYST2;NDUFC1;NHLRC2;NRARP;NT5C2;NUDT6;PATZ1;PIF1;POP5;PRDM13;PRRX1;PTCH1;RAN;REST;RFX4;RNF135;RPL24;RPS6;SCAMP2;SH3GLB1;SLAIN2;SLC35F5;SMARCA5;TARSL2;TDRD3;TEX9;TFAM;TGIF2;TMED4;TP53INP1;TRIM39;TRPS1;TSEN34;TUBB2B;ZFP36L2"
"cisbp__M2323"	3.86	0.0434	"ZBTB33 (directAnnotation). "	184	"ANAPC10;ANKHD1-EIF4EBP3;ANKRA2;ANKS3;APPL2;ARFIP1;ARIH1;ASPH;ATF1;ATP10D;BCAR3;BOD1;BPTF;C10orf26;C12orf48;C14orf135;C17orf101;C1orf9;C20orf72;C20orf94;C21orf34;C4orf29;C5orf41;C6orf145;C9orf5;CAPN7;CBLB;CCDC15;CCDC90B;CDC16;CDC25A;CFLAR;CHUK;CIRBP;CNIH;COMMD6;COPG2;COPS4;CRY1;CRYL1;CSTF1;CUL4A;DDX3Y;DERL2;DGUOK;DHFRL1;DRAM2;EFC4A;EIF4A3;ERLIN2;FAM76A;FNDC3A;FOS;FZD5;GGNBP2;GKAP1;GPN3;HELLS;HELQ;HIST1H3G;HMGB2;HSPA13;HSPBAP1;IKZF5;ITGB3;KIAA1530;KIF27;LARP1B;LARP7;LIFR;LIG1;LIMS1;LOC389765;LOC728407;LPP;LSM14A;MAK16;MDM1;ME2;MED28;METAP2;MPP5;MRPS26;MSH5;MTAP;MUTYH;MX1;MYST2;NEDD1;NEK9;NINL;NKX2-1;NOC3L;NOL11;NT5C2;NUP54;NXT1;OFD1;PAPOLA;PARG;PDIK1L;PFDN5;PHIP;PLCD1;PLRG1;PNPLA4;POLA1;PPAPDC1A;PPAPDC1B;PPP1R3B;PRDM13;PRMT2;PSMB1;PSME2;PTCH1;PUS3;RAP1B;RBBP8;RCL1;RPL10;RPL23;RPL24;RPL27A;RPL7;RPP38;RPS28;RPS6;RSPRY1;SALL4;SEC11C;SERPINB6;SH3GLB1;SIM1;SLAIN2;SLC16A8;SLU7;SMARCAD1;SMC5;SMEK1;SNAPC1;SNRPF;SRI;SRP72;SRRM1;SRSF3;SSBP1;SSR1;STXBP4;SUPT16H;SYTL4;TAF7;TDRD3;TEX9;TGFB1;THAP2;THG1L;TIGD6;TMCO1;TMEM216;TNPO1;TOB1;TRIM39;TRIP4;TRMT112;TRPS1;TSEN34;TSG101;TSPAN4;TTC14;TTC32;UAP1L1;UBXN2A;UPF3A;USP24;USP51;UTP18;VAT1L;WBP5;WRN;ZCCHC11;ZFX;ZKSCAN2;ZNF345;ZZZ3"
"cisbp__M5426"	3.77	0.0428	"ETV6 (directAnnotation). "	165	"ADAL;ANKHD1-EIF4EBP3;ANKS3;APPL2;ARF4;ARFIP1;ASB1;ATP7A;B3GNT5;BCL7C;BMP2K;BTF3;C10orf26;C12orf48;C12orf57;C15orf24;C1orf26;C1orf97;C20orf94;C21orf34;C5orf43;C6orf226;CBFB;CBLB;CCDC15;CCDC88B;CD74;CD86;CDC26;CETN3;CFLAR;CHUK;CIRBP;CLEC1A;COMMD6;CSGALNACT2;CSTF1;CWF19L2;CYBA;DDX50;DDX6;DERL2;DGUOK;DIRC2;DLEC1;EFHC1;EXOSC3;FAM173B;FAM46A;FBXO15;FCGBP;FIP1L1;G3BP1;GALNT10;GCNT2;GJB6;GTF2A2;HAUS6;HSPA5;IL7R;INPP5D;INTS2;INTS4;ISYNA1;KIAA1530;KLRK1;LAX1;LEPROT;LIMS1;LOC100131564;LOC100272217;LOC389705;LOC728407;LOC729852;LPP;LRP10;LRRC70;LSM5;LST1;MAEA;MAGEH1;MAK16;METTL12;MGAT2;MRPL51;MSH5;MUTYH;MYO1F;MYST2;NAA38;NCF2;NFE2;NHP2L1;NOC3L;NOL11;NOL7;NR1H3;NSUN5;NUP54;OGFOD1;PARG;PATZ1;PCNP;PDCD10;PLCD1;PLRG1;PMFBP1;PNPLA4;POLA1;PRPF40A;RAP1B;RAP2C;RBBP8;RBM34;RBM8A;RDBP;REST;RND2;RPIA;RPL13A;RPS6;SAMD4A;SAMD9L;SCAMP2;SKIL;SLC7A7;SOX2;SPR;SRP72;SSBP1;STAG1;STT3A;SYF2;TAF1A;TAF7;TDRD3;TFAM;TGDS;TGIF2;THAP2;TIGD6;TMCO1;TMED10;TMEM138;TMEM176A;TMEM199;TMEM222;TOR1B;TPRKB;TRAPPC4;TRIM38;TRIM39;TRMT112;TSG101;TTC14;TTC30B;TTF1;TXNDC5;TYMP;UBR1;UFD1L;UTP18;ZNF394;ZNF512;ZNF518A"

"motif"	"NES"	"AUC"	"TF_highConf"	"nEnrGenes"	"enrichedGenes"
"cisbp__M5424"	3.71	0.0425	"ETV5 (directAnnotation). "	278	"ADAL;ADH5;AEBP1;AKR7L;ANKHD1-EIF4EBP3;ANKS3;ANP32E;APPL2;ARF4;ARFIP1;ARID4B;ARIH1;ASB1;ASCC1;ATF7;ATP7A;B3GNT5;BAG3;BCL7C;BMP2K;BTF3;C10orf26;C11orf71;C11orf96;C12orf48;C12orf57;C14orf109;C14orf135;C15orf24;C1orf112;C1orf212;C1orf26;C1orf9;C1orf97;C20orf94;C21orf34;C2orf84;C5orf43;C6orf226;C6orf70;C7orf11;C9orf5;C9orf64;CALCR;CARD8;CBFB;CBLB;CCDC15;CCDC152;CCDC88B;CD274;CDC16;CDC25A;CDC26;CDKL3;CETN3;CFLAR;CGA;CHUK;CLEC11A;CNIH;COMMD6;COMT;COX16;CREBL2;CRNKL1;CRY1;CRYL1;CSGALNACT2;CSTF1;CUL4A;CWF19L2;CXXC4;CYB5D2;DCLRE1B;DDX50;DDX6;DERL2;DKFZP434I0714;DLEC1;DNAJC10;DUSP18;EFC4A;EFHC1;EMP3;ERLIN2;EXOSC3;FAM123B;FAM164C;FAM173B;FAM177A1;FAM46A;FBXO15;FCGBP;FNDC3A;FOS;FSTL5;FTSJD1;G3BP1;GALNT10;GJB6;GMNN;GTF2A2;GTF2F2;HAUS6;HIST4H4;HMGN4;HSP90B1;HSPA5;HSPBAP1;IFT20;IL11;IL7R;INTS2;INTS4;ISYNA1;KIAA1530;KLHL1;KLRK1;LARP7;LAX1;LEPROT;LIFR;LIMS1;LOC100131564;LOC100272217;LOC389705;LOC728407;LOC729852;LPP;LRP10;LRRC37A3;LRRC70;LSM14A;LSM5;LST1;MAEA;MAGEH1;MAK16;METTL12;MGAT2;MPL;MRPL20;MRPL50;MRPL51;MRPS31;MSH5;MUTYH;MXRA8;MYO1F;MYST2;NAA38;NCF2;NEDD1;NFATC1;NFE2;NHP2L1;NID2;NOC3L;NOL11;NOL7;NR1H3;NSUN5;NUDT6;NUP54;NXT1;OFD1;OGFOD1;PAPOLA;PARG;PARN;PARP3;PATZ1;PBXIP1;PCNP;PDCD10;PIGV;PLCD1;PLRG1;PMFBP1;PNPLA4;PODNL1;POLA1;POP5;PRPF40A;PTCH1;RAD17;RAD21;RAP1B;RAP2C;RBBP8;RBM34;RBM8A;RDBP;REST;RFX4;RND2;RPIA;RPL10;RPL13A;RPL24;RPS6;RRP15;RSPRY1;S100A13;SALL4;SAMD4A;SCAMP2;SCARNA17;SKIL;SLAIN2;SLC7A7;SMAD5;SMARCAD1;SMEK1;SNRPF;SOAT1;SPR;SSBP1;STAG1;STT3A;SUGT1P1;SYF2;TAF1A;TAF7;TDRD3;TFAM;TGDS;TGIF2;THAP2;THAP6;THAP9;TIGD6;TMCO1;TMED10;TMEM138;TMEM199;TMEM222;TNPO1;TOR1B;TPRKB;TRAF3IP1;TRAF3IP2;TRAPPC2P1;TRAPPC4;TRIM38;TRIM39;TRIP4;TRMT112;TSEN34;TSG101;TSPAN4;TTC14;TTC30B;TTF1;TXNDC5;TYMP;UBR1;UFD1L;USP16;USP24;UTP18;WDR61;XIAP;YES1;ZFX;ZKSCAN2;ZNF197;ZNF285;ZNF394;ZNF512;ZNF518A;ZNF529;ZNF583;ZNF660;ZNF671;ZNF701;ZZZ3"
"cisbp__M4617"	3.67	0.0423	"ELF1 (directAnnotation). "	177	"ADAL;ANKHD1-EIF4EBP3;ANKS3;APPL2;ARF4;ARFIP1;ARIH1;ASB1;ATF7;ATP7A;B3GNT5;BCL7C;BMP2K;BTF3;C10orf26;C12orf48;C12orf57;C14orf109;C15orf24;C15orf38;C1orf97;C5orf41;C5orf43;C6orf226;C6orf70;C9orf64;CBFB;CBLB;CCDC15;CCDC152;CCDC88B;CDC16;CDC26;CDKL3;CETN3;CHUK;CIRBP;CLEC11A;COMMD6;CSGALNACT2;CSTF1;CWF19L2;CYB5D2;DDX50;DLEC1;DNAJC10;EFC4A;EFHC1;ERLIN2;EXOSC3;FAM173B;FAM177A1;FAM46A;FBXO15;FBXO36;FIP1L1;GALNT10;GJB6;GTF2A2;HAUS6;HMGN4;IL7R;IMPACT;INTS2;INTS4;ISYNA1;KIAA1530;KLRK1;LAX1;LEPROT;LIMS1;LOC100131564;LOC100272217;LOC389705;LOC728407;LOC729852;LSM5;LST1;MAEA;MAGEH1;MAK16;MGAT2;MRPL51;MRPS26;MUTYH;MYO1F;MYST2;NAA38;NCF2;NEDD1;NFATC1;NHP2L1;NOC3L;NOL11;NOL7;NR1H3;NUP54;NXT1;OGFOD1;PARG;PARP3;PATZ1;PCNP;PDCD10;PFDN5;PITX2;PLRG1;PMFBP1;PNPLA4;POLA1;PPP4R1;R18;RAP1B;RAP2C;RBBP8;RBM34;RBM8A;RDBP;REST;RFX4;RND2;RPIA;RPL10;RPL13A;RPS6;SAMD4A;SCAMP2;SETD4;SLC25A1;SLC7A7;SP140L;SPR;SRP72;SSBP1;SSR4;STAG1;STAT5B;SYF2;TAF1A;TAF7;TDRD3;TGDS;TGFB1;TGIF2;THAP2;TIGD6;TMCO1;TMED10;TMEM138;TMEM199;TMEM222;TOR1B;TPRKB;TRAPPC4;TRIM39;TRMT112;TSG101;TTC14;TTC30B;TTF1;TTLL3;TXNDC5;TYMP;UBR1;UFD1L;USP24;UTP18;WDR61;XIAP;YES1;ZNF197;ZNF285;ZNF394;ZNF512;ZNF518A;ZNF671;ZNF701"
"cisbp__M4549"	3.63	0.0421	"NR2C2 (directAnnotation). "	116	"ADAL;ANKS3;APPL2;ARFIP1;ARIH1;ASB1;ATF7;ATP7A;BBS9;BMP2K;C10orf26;C12orf48;C15orf24;C1orf9;C2orf84;C5orf41;C6orf226;C7orf11;C9orf64;CCDC15;CCDC152;CDC16;CDKL3;CLNS1A;COMMD6;COX16;CRY1;CSGALNACT2;CSTF1;DDX50;DNAJC10;EFC4A;EFHC1;FAM46A;FBXO36;FIP1L1;GMNN;GTF2A2;HEATR5A;IFT20;IMPACT;INTS4;IRF9;KIAA1530;KLRK1;LAX1;LEPROT;LIFR;LIMS1;LOC100131564;LOC100272217;LOC389705;LOC728407;LOC729852;LST1;MAK16;MRPL51;MRPS26;MYST2;NAA38;NEDD1;NFATC1;NOC3L;NUP54;PAPOLA;PARG;PATZ1;PDCD10;PFDN5;PLRG1;PNPLA4;PROCA1;PTCH1;RAP2C;RBBP8;RBM34;RDBP;REST;RND2;RPL5;RPS6;S100A13;SCAMP2;SETD4;SPR;SRP72;SSBP1;STAG1;STAT5B;SUGT1P1;TAF1A;TAF7;TGFB1;TGIF2;TIGD6;TMCO1;TMED10;TMEM138;TMEM199;TMEM222;TPRKB;TRAPPC4;TRMT112;TSG101;TTC14;TTC30B;TTF1;TTLL3;UBR1;USP24;UTP18;ZFP90;ZNF394;ZNF518A;ZNF660;ZNF701"
"cisbp__M4563"	3.6	0.0419	"ELF1 (directAnnotation). "	185	"ADAL;ANKHD1-EIF4EBP3;ANKS3;APPL2;ARF4;ARFIP1;ARIH1;ASB1;ATF7;ATP7A;B3GNT5;BCL7C;BMP2K;BTF3;C10orf26;C12orf48;C12orf57;C15orf24;C15orf38;C1orf9;C1orf97;C5orf41;C5orf43;C6orf226;C6orf70;C7orf11;C9orf64;CALCR;CBFB;CBLB;CCDC15;CCDC88B;CDC16;CDC26;CDKL3;CETN3;CHUK;CIRBP;CLEC11A;COMMD6;CSGALNACT2;CSTF1;CWF19L2;CYB5D2;DDX50;DLEC1;DNAJC10;DUSP18;EFC4A;EFHC1;ERLIN2;EXOSC3;FAM173B;FAM177A1;FAM46A;FAM76A;FBXO15;FBXO36;FCGBP;FIP1L1;FNDC3A;GALNT10;GJB6;GTF2A2;GTF2F2;HAUS6;HEATR5A;HSPA5;IFT20;IL7R;IMPACT;INPP5D;INTS2;INTS4;IRF9;ISYNA1;KIAA1530;KLRK1;LEPROT;LIMS1;LOC100131564;LOC100272217;LOC389705;LOC728407;LOC729852;LRRC70;LSM5;LST1;MAEA;MAGEH1;MAK16;MGAT2;MRPL51;MRPS26;MUTYH;MYO1F;MYST2;NAA38;NCF2;NEDD1;NFATC1;NHP2L1;NOC3L;NR1H3;NUP54;OGFOD1;PARG;PARP3;PATZ1;PCNP;PDCD10;PFDN5;PITX2;PLRG1;PNPLA4;POLA1;POLI;PRPF40A;PTCH1;R18;RAP1B;RAP2C;RBBP8;RBM34;RBM8A;RDBP;REST;RFX4;RND2;RPL10;RPL13A;RPS6;S100A13;SAMD4A;SAMD9L;SCAMP2;SETD4;SNRPF;SOX2;SP140L;SPR;SRP72;SSBP1;SSR4;STAG1;STAT5B;SYF2;TAF1A;TAF7;TDRD3;TGFB1;TGIF2;THAP2;TIGD6;TMCO1;TMED10;TMEM138;TMEM199;TMEM222;TOR1B;TPRKB;TRAPPC4;TRIM38;TRIM39;TRIP4;TRMT112;TSG101;TTC14;TTC30B;TTF1;TTLL3;UBR1;UFD1L;USP24;UTP18;WDR61;XIAP;ZFX;ZNF197;ZNF394;ZNF512;ZNF518A;ZNF660;ZNF671;ZNF701"

"motif"	"NES"	"AUC"	"TF_highConf"	"nEnrGenes"	"enrichedGenes"
"cisbp__M4540"	3.58	0.0418	"GPA (directAnnotation). "	215	"ACO1;ADAL;ADH5;ANKHD1-EIF4EBP3;ANKS3;APPL2;ARF4;ARFIP1;ARIH1;ASB1;ASCC1;ATAD2B;ATF7;ATP7A;B3GNT5;BCL7C;BMP2K;BTF3;C10orf26;C12orf48;C12orf57;C14orf109;C15orf24;C15orf38;C1orf9;C1orf97;C2orf84;C5orf41;C5orf43;C6orf226;C7orf11;C9orf64;CALCR;CBFB;CBLB;CCDC15;CCDC152;CCDC88B;CDC16;CDC26;CDKL3;CETN3;CFLAR;CHUK;CIRBP;CLEC18B;COMMD6;COX16;CSGALNACT2;CSTF1;CWF19L2;CXXC4;CYB5D2;DDX50;DDX6;DERL2;DLEC1;DNAJC10;DUSP18;EFC4A;EFHC1;ERLIN2;EXOSC3;FAM173B;FAM177A1;FAM46A;FBXO15;FBXO36;FCGBP;FIP1L1;FTSJD1;GALNT10;GJB6;GMNN;GTF2A2;H2AFY;HAUS6;HMGN4;HSPA5;IFT20;IL7R;IMPACT;INTS2;INTS4;IRF9;ISYNA1;KIAA1530;KLRK1;LAX1;LEPROT;LIFR;LIMS1;LOC100131564;LOC100272217;LOC389705;LOC728407;LOC729852;LRP10;LRRC70;LSM5;LST1;MAEA;MAGEH1;MAK16;METTL12;MGAT2;MRPL20;MRPL51;MRPS26;MRPS31;MUTYH;MYO1F;MYST2;NAA38;NCF2;NEDD1;NFATC1;NHP2L1;NOC3L;NOL11;NR1H3;NSUN5;NUDT6;NUP54;NXT1;OGFOD1;PAPOLA;PARG;PARP3;PATZ1;PBXIP1;PCNP;PDCD10;PFDN5;PITX2;PLCD1;PLRG1;PNPLA4;POLA1;POLI;PRPF40A;R18;RAD17;RAP1B;RAP2C;RBBP8;RBM34;RBM8A;RDBP;REST;RND2;RPIA;RPL10;RPL13A;RPS6;RRP15;S100A13;SAMD4A;SCAMP2;SETD4;SKIL;SLAIN2;SMARCAD1;SOX2;SP140L;SPR;SRP72;SRRM1;SSBP1;STAT5B;STT3A;SYF2;TAF1A;TAF7;TDRD3;TFAM;TGFB1;TGIF2;THAP2;TIGD6;TMCO1;TMED10;TMEM138;TMEM199;TMEM222;TOR1B;TPRKB;TRAPPC2P1;TRAPPC4;TRIM38;TRIM39;TRMT112;TSEN34;TSG101;TTC14;TTC30B;TTF1;TXNDC5;TYMP;UBR1;UFD1L;USP24;UTP18;WDR61;XIAP;YES1;ZFX;ZNF197;ZNF394;ZNF512;ZNF518A;ZNF529;ZNF660;ZNF671;ZNF701"
"cisbp__M4936"	3.57	0.0418	"ELF1; ELF2; ELF4 (inferredBy_Orthology). "	233	"AASDH;ACE2;ACO1;ACTL6A;ADAL;ADAP2;AEBP1;ANKHD1-EIF4EBP3;ANKS3;APOM;APPL2;ARFIP1;ARIH1;ASB1;ATF7;ATP7A;AVP;BBS9;BCL7C;BMP2K;BRIX1;C10orf26;C12orf48;C15orf24;C15orf38;C1orf63;C2orf84;C5orf41;C5orf43;C6orf145;C6orf226;C6orf72;C9orf64;CBFB;CBLB;CCDC15;CCDC152;CCDC88B;CD274;CDC16;CDKL3;CFLAR;CHUK;CIRBP;CITED4;CLK1;COMMD6;COX16;CRY1;CSGALNACT2;CSTF1;CWC15;CWF19L2;CYB5D2;DCLRE1B;DDX50;DHRS12;DUSP18;DUT;EFC4A;EFHC1;EID1;ERAP1;ERLIN2;FAM123B;FAM173B;FAM46A;FAM76A;FBXO15;FBXO36;FMO2;GKAP1;GLG1;GMNN;GTF2A2;GTF2F2;H2AFY;HAUS6;HEATR5A;HEPH;HFM1;HIST1H3B;HMGN4;HSPA13;HSPA5;IFT20;IL18;IL7R;IMPACT;INTS4;KIAA1530;KIF27;KLHDC9;KLHL1;KLHL21;LAX1;LEPROT;LIMS1;LOC100131564;LOC100272217;LOC389705;LOC728407;LOC729852;LPP;LSM5;LSM6;MAEA;MAK16;MDK;MESDC2;MLH3;MPP5;MRPL20;MRPL51;MRPS26;MX1;MYST2;NAA38;NEDD1;NFATC1;NHP2L1;NKX2-1;NOC3L;NOL7;NR1H3;NSUN5;NUP54;NXT1;PARG;PARN;PCBD2;PCNP;PDCD10;PDLIM5;PIN4;PITX2;PLK3;PLRG1;PNPLA4;PNPO;POLD3;POP5;PPP1R3B;PQLC3;PRKY;PSME2;PTCH1;PTOV1;RAD17;RAP2C;RBBP8;RBM34;RBM8A;RCBTB2;RDBP;REST;RHBDD1;RPIA;RPL10;RPL13A;RPL7;RPS6;RRM1;RRP15;RTDR1;S100A13;SAMD4A;SCAMP2;SETD4;SLC25A1;SP140L;SPR;SRP72;SSBP1;SSR1;STAG1;STAT5B;STK32B;SUGT1P1;SYF2;TAF1A;TAF7;TDRD3;TFAM;TGDS;TGFB1;TGIF2;THEM4;TIGD6;TMCO1;TMED10;TMEM138;TMEM199;TMEM20;TMEM222;TOR1B;TPRKB;TRAF3IP1;TRAPPC4;TRIM38;TRIM4;TRIM52;TRIP4;TRMT112;TRNAU1AP;TSG101;TSPAN4;TTC14;TTC30B;TTLL3;TWF1;TXNDC5;TYMP;UBR1;UFD1L;UPF3A;USP16;USP24;UTP18;XIAP;YES1;ZFP90;ZNF197;ZNF331;ZNF394;ZNF501;ZNF518A;ZNF529;ZNF610;ZNF660;ZNF671;ZNF701;ZNHIT6"
"cisbp__M4460"	3.56	0.0417	"ELF1 (directAnnotation). "	258	"ADAL;ADH5;ANKHD1-EIF4EBP3;ANKS3;ANP32E;APPL2;AQP1;ARF4;ARFIP1;ARIH1;ASB1;ASPH;ATAD2B;ATF7;ATP7A;B3GNT5;BBS9;BCL7C;BMP2K;BTF3;C10orf26;C12orf48;C12orf57;C14orf109;C15orf24;C15orf38;C1orf9;C1orf97;C20orf94;C2orf84;C5orf41;C5orf43;C6orf226;C6orf70;C7orf11;C8orf59;C9orf64;CALCR;CAPN7;CARD8;CBFB;CBLB;CCDC15;CCDC152;CCDC88B;CDC16;CDC26;CDKL3;CENPE;CETN3;CHUK;CIRBP;CLEC11A;CLEC18B;COMMD6;COX16;CREBL2;CRY1;CSGALNACT2;CST6;CSTF1;CWC15;CWF19L2;CYB5D2;DDX50;DDX6;DERL2;DHRS12;DLEC1;DNAJC10;DUSP18;EFC4A;EFHC1;ERLIN2;EXOSC3;FAM123B;FAM164C;FAM173B;FAM177A1;FAM46A;FAM76A;FBXO15;FBXO36;FCGBP;FIP1L1;FNDC3A;FTSJD1;GALNT10;GJB6;GPX3;GTF2A2;GTF2F2;HAUS6;HDAC1;HEATR5A;HIST4H4;HMGB2;HMGN4;HSPA5;IFT20;IL7R;IMPACT;INPP5D;INTS2;INTS4;IRF9;ISYNA1;KCTD3;KIAA1530;KLHDC9;KLHL21;KLRK1;LAX1;LEPROT;LIFR;LIMS1;LOC100131564;LOC100272217;LOC389705;LOC728407;LOC729852;LRP10;LRRC70;LSM5;LST1;MAEA;MAGEH1;MAK16;ME1;ME2;MED28;MGAT2;MRPL51;MRPS26;MRPS31;MUTYH;MYO1F;MYST2;NAA38;NCF2;NEDD1;NFATC1;NHP2L1;NOC3L;NOL11;NR1H3;NSUN5;NUDT6;NUDT7;NUP54;NXT1;OGFOD1;PAPOLA;PARG;PARP3;PATZ1;PCNP;PDCD10;PIGV;PITX2;PLCD1;PLRG1;PNPLA4;POLA1;POLD3;POLI;PPP1R3B;PPP4R1;PRKY;PRPF40A;PTCH1;R18;RAD21;RAP1B;RAP2C;RBBP8;RBM34;RBM8A;RDBP;REST;RFX4;RND2;RPIA;RPL10;RPL13A;RPL23;RPS6;RSPRY1;S100A13;SAMD4A;SAMD9L;SCAMP2;SETD4;SLAIN2;SLC25A1;SLC26A2;SMARCAD1;SNRPF;SOX2;SP140L;SPR;SRP72;SRRM1;SSBP1;SSR4;STAG1;STAT5B;STT3A;SUGT1P1;SYF2;TAF1A;TAF7;TAGLN2;TDRD3;TGFB1;TGIF2;THAP2;TIGD6;TMCO1;TMED10;TMEM138;TMEM199;TMEM222;TOR1B;TPRKB;TRAPPC2P1;TRAPPC4;TRIM38;TRIP4;TRMT112;TSG101;TTC14;TTC30B;TTF1;TTLL3;TXNDC5;TYMP;UBR1;UFD1L;USP24;UTP18;WDR61;XIAP;YES1;ZFP90;ZFX;ZNF197;ZNF394;ZNF493;ZNF512;ZNF518A;ZNF529;ZNF660;ZNF671;ZNF691;ZNF701;ZNF92;ZZZ3"

"motif"	"NES"	"AUC"	"TF_highConf"	"nEnrGenes"	"enrichedGenes"
"cisbp__M0713"	3.55	0.0416	"ERF; ETV3; ETV3L (inferredBy_Orthology). "	187	"ADAL;AEBP1;ANKHD1-EIF4EBP3;ANKS3;APPL2;ARF4;ARFIP1;ARID4B;ARIH1;ASB1;ATAD2B;ATF7;ATP7A;B3GNT5;BCAR3;BCL7C;BMP2K;BOD1;C10orf26;C12orf48;C12orf57;C14orf109;C15orf24;C15orf38;C1orf112;C1orf212;C1orf9;C5orf43;C6orf226;C6orf70;C7orf11;C9orf64;CARD8;CBLB;CCDC15;CCDC152;CD74;CDC16;CDC26;CDKL3;CETN3;CHUK;CLEC11A;CNIH;COX16;CSGALNACT2;CSTF1;CWF19L2;CXXC4;CYB5D2;DCLRE1B;DDX50;DLEC1;DUSP18;EFC4A;ERCC4;EXOSC3;FAM173B;FAM177A1;FAM46A;FBXO15;FCGBP;FNDC3A;GCNT2;GJB6;GTF2A2;GTF2F2;HAUS6;HDAC1;HEPH;HFM1;HMGN4;HSPA13;HSPBAP1;IFT20;IGFBP6;IL7R;IMPACT;KIAA1530;LAX1;LEPROT;LOC100131564;LOC100272217;LOC389705;LOC728407;LOC729852;LPP;LRRC70;LSM5;MAGEH1;MAK16;MRPL20;MRPL51;MRPS26;MRPS31;MSH5;MUTYH;MYST2;NAA38;NEDD1;NEK9;NFATC1;NOC3L;NOL11;NOL7;NR1H3;NSUN5;NUP54;NXT1;OFD1;PAPOLA;PARG;PARP3;PATZ1;PDCD10;PHIP;PLCD1;PLRG1;PNPLA4;POLA1;POP5;PTOV1;RAP2C;RBBP8;RBM34;RBM8A;RDBP;REST;RND2;RPIA;RPL10;RPS6;RRP15;S100A13;SALL4;SAMD4A;SCAMP2;SCARNA17;SETD4;SKIL;SLAIN2;SLC25A1;SLC7A7;SOX2;SP140L;SRRM1;SSBP1;SSPN;SYF2;TAF1A;TAF7;TFAM;TGDS;TGIF2;THAP6;TIGD6;TMCO1;TMED10;TMEM138;TMEM199;TMEM222;TOR1B;TPRKB;TRAPPC2P1;TRAPPC4;TRIM39;TRIM52;TRMT112;TSEN34;TSG101;TTC14;TTC30B;TTF1;TXNDC5;TYMP;UBR1;USP16;USP24;UTP18;WDR61;XIAP;YES1;ZNF197;ZNF394;ZNF518A;ZNF660;ZNF671"
"cisbp__M0576"	3.52	0.0415	""	96	"ALG10;ANAPC10;ANP32E;BAG3;BCL7C;BOD1;BPTF;C10orf26;C11orf54;C1orf9;C20orf72;C20orf94;C5orf41;CDC25A;CDNF;CHUK;CIRBP;CRNKL1;CSTF1;CTXN2;DDX3Y;DDX50;DERL2;DGUOK;ERLIN2;FAM46A;FAM76A;FNDC3A;GGNBP2;HELLS;HEY2;HIST1H3B;HIST1H3G;HIST4H4;HMGN4;HSP90B1;HSPA13;KIF27;KLHL21;LARP7;LEPROT;LIFR;LIG1;LMAN1;LOC728407;LOC729852;LSM14A;MAK16;MBNL3;METAP2;METTL12;MPP5;NAA38;NHLRC2;NOL11;NR1H3;NUDT6;PARG;PHLDA2;POLA1;POU3F2;PPP1R3B;PRDX6;PRRX1;RAD21;RBBP8;RBM8A;REST;RPS6;SBDS;SH3GLB1;SLU7;SMARCAD1;SMEK1;SRRM1;SRSF3;SSBP1;STAG1;TAF7;TDRD3;TEX9;THAP2;TIGD6;TMCO1;TMED10;TSPAN4;TTC14;TTC32;VAT1L;WBP5;YES1;ZCCHC11;ZFP36L2;ZFP90;ZKSCAN2;ZZZ3"
"cisbp__M0670"	3.5	0.0414	"E2F2 (inferredBy_Orthology). "	60	"ANAPC10;ANKHD1-EIF4EBP3;ANP32E;C10orf26;C20orf72;C20orf94;CBX5;CDC25A;CDNF;CTXN2;DDX3Y;DERL2;DKFZP434I0714;FAM76A;FBXO15;FGGY;HELLS;HES1;HHEX;HIST1H2AG;HIST1H2AI;HIST1H2BJ;HIST1H3G;HIST4H4;HMGB2;LARP7;LIG1;LOC728407;MSH5;MYST2;NHLRC2;NHP2L1;NKAP;NKX2-1;NPM1;PARG;PLRG1;PNPO;POLA1;POU3F2;PPIL1;PRDM13;PRRX1;PSMB1;RAD21;RBBP8;RPL10;RPL13A;RPL7;SMAD5OS;SMARCAD1;SRSF3;STAG1;TDRD3;TMED10;TTLL3;UBXN2A;USP51;ZFP36L2;ZNF518A"
"cisbp__M4310"	3.48	0.0413	""	204	"ACTL6A;ADAL;ADH5;ADPRH;AEBP1;ALK;ANKHD1-EIF4EBP3;ANKS3;ANP32E;APPL2;ARF4;ARFIP1;ARID4B;ARIH1;ASB1;ATP7A;BCAR3;BCL7C;BMP2K;BOD1;BPTF;BRIX1;C10orf26;C11orf96;C12orf48;C12orf57;C14orf109;C15orf24;C1orf112;C20orf94;C2orf40;C5orf43;C6orf145;C6orf226;C7orf11;C9orf64;CCDC15;CCDC152;CCDC88B;CD274;CDC16;CDKL3;CETN3;CLEC11A;CLNS1A;CNIH4;COX16;CRY1;CSGALNACT2;CSTF1;CUL4A;CWF19L2;CYB5D2;DCLRE1B;DDX50;DDX6;DKK1;DLEC1;DNAJC10;DUSP18;EFC2;EFC4A;ERLIN2;EXOSC3;FAM123B;FAM173B;FAM46A;FAM76A;FNDC3A;G3BP1;GPN3;GTF2A2;HAUS6;HDAC1;HEPH;HSPBAP1;IFT88;IGFBP6;IMPACT;INTS4;KIAA1530;LARP7;LEPROT;LIG1;LOC100131564;LOC100272217;LOC389705;LOC728407;LOC729852;LRP10;LRRC37A3;LSM5;MAK16;MDM1;MRPL50;MRPL51;MRPS26;MRPS31;MSH5;MTHFD2L;MYST2;NAA38;NEDD1;NFATC1;NHLRC2;NID2;NKX2-1;NOC3L;NOL7;NR1H3;NSUN5;NUDT6;NUP54;NXT1;PARG;PARP3;PATZ1;PDCD10;PDIK1L;PIGV;PITX2;PLCD1;PLRG1;PNPLA4;PNPO;POLA1;POLD3;PPP4R1;PROCA1;PTCH1;PTOV1;R18;RAP2C;RBBP8;RBM34;RBM8A;RDBP;REST;RPIA;RPL10;RPL13A;RPL7;RPS27L;RPS6;RRP15;RSPRY1;S100A13;SALL4;SAMD4A;SCAMP2;SFXN4;SIM1;SLAIN2;SLC25A1;SMARCAD1;SMEK1;SNAPC1;SSBP1;STAG1;STAT5B;STT3A;SUGT1P1;SYF2;TAF1A;TAF7;TFAM;TGDS;TGIF2;THAP2;THAP6;THAP9;THEM4;TIGD6;TMCO1;TMED10;TMEM138;TMEM199;TMEM20;TMEM222;TPRKB;TRAF3IP1;TRAPPC4;TRIM39;TRIM52;TRIP4;TRMT112;TSEN34;TSG101;TTC14;TTC30B;TTF1;TYMP;UBR1;USP16;USP24;UTP18;WDR61;YES1;ZKSCAN2;ZNF197;ZNF394;ZNF518A;ZNF691;ZNHIT6"
"cisbp__M5372"	3.48	0.0412	"EHF (directAnnotation). "	249	"ACE2;ADAL;ADAP2;ADH5;ANKHD1-EIF4EBP3;ANKS3;APPL2;ARF4;ARFIP1;ARIH1;AS3MT;ASB1;ASPH;ATF7;ATP7A;B3GNT5;BMP2K;C10orf26;C12orf48;C12orf57;C14orf135;C15orf24;C15orf38;C1orf26;C1orf9;C20orf94;C21orf34;C2orf84;C5orf41;C5orf43;C6orf226;C7orf11;C8orf59;CALCR;CBFB;CBLB;CCDC15;CCDC88B;CD74;CDC16;CDC26;CDKL3;CFLAR;CHUK;CIRBP;CITED4;CNIH;COMMD6;COX16;CREBL2;CRYL1;CSGALNACT2;CSTF1;CWF19L2;CYB5D2;DDX50;DERL2;DLEC1;DNAJC10;EFC4A;EFHC1;EIF5B;ERLIN2;EXOSC3;FP6;FAM123B;FAM173B;FBXO15;FBXO36;FCGBP;FMO2;FSTL5;G3BP1;GJB6;GTF2A2;GTF2F2;HAS3;HAUS6;HEATR5A;HEPH;HIST1H3B;HIST4H4;HLA-A;HSPA13;HSPA5;HSPBAP1;IFT20;IL18;IL7R;IMPACT;INPP5D;INTS2;INTS4;IRF9;ISYNA1;KCTD3;KLRK1;LARP7;LEPROT;LIMS1;LIX1;LOC100131564;LOC100272217;LOC389705;LOC728407;LOC729852;LPP;LRP10;LRRC70;LSM5;LST1;MAEA;MAGEH1;MAK16;METAP2;METTL12;MFSD1;MGAT2;MRPL20;MRPL51;MRPS26;MSH5;MUTYH;MX1;MYO1F;MYOZ1;MYST2;NCF2;NEDD1;NFATC1;NHP2L1;NOC3L;NR1H3;NSUN5;NUP54;NXT1;PARG;PARN;PATZ1;PCNP;PDCD10;PDLIM5;PFDN5;PITX2;PLRG1;PMFBP1;PNPLA4;PNPO;POLA1;POLD3;PPAPDC1A;PPP1R3B;PQLC3;PRDM13;PRKY;PRPF40A;PRRC1;R18;RAD17;RAD21;RAP2C;RBBP8;RBM34;RBM8A;RDBP;REST;RFX4;RND2;RPIA;RPL10;RPL13A;RPL7;RPS6;RRP15;S100A13;SAMD4A;SAMD9L;SCAMP2;SETD4;SKIL;SLC25A1;SLC7A7;SOX2;SP140L;SPR;SRP72;SRRM1;SRSF3;SSBP1;SSPN;SSR1;SSR4;STAG1;STAT5B;STT3A;SUGT1P1;SYF2;SYTL4;TAF1A;TAF7;TAS2R46;TDRD3;TGDS;TGFB1;THNSL2;TIGD6;TMCO1;TMED10;TMEM138;TMEM199;TMEM222;TOR1B;TPRKB;TRAF3IP1;TRAPPC4;TRIM38;TRIM39;TRIP4;TRMT112;TSG101;TTC14;TTC30B;TTF1;TTLL3;TXNDC5;TYMP;UBR1;UFD1L;USP16;USP24;UTP18;VMO1;WDR61;XIAP;YES1;ZFP90;ZFX;ZNF197;ZNF285;ZNF331;ZNF394;ZNF512;ZNF518A;ZNF529;ZNF583;ZNF610;ZNF671;ZNF691;ZNF701"
"cisbp__M4602"	3.47	0.0412	"NR2C2 (directAnnotation). "	61	"ANKS3;ARFIP1;ATP7A;BBS9;C12orf48;C15orf24;C6orf226;CDC16;CDKL3;CLNS1A;CRY1;CSTF1;DDX50;EFC4A;FAM46A;FBXO36;IFT20;IMPACT;IRF9;LEPROT;LIFR;LIMS1;LOC389705;LOC728407;LOC729852;LST1;MAK16;MRPL51;MRPS26;MYST2;NFATC1;PARG;PFDN5;PNPLA4;RAP2C;RBBP8;RBM34;RPS6;SCAMP2;SETD4;SPR;SSBP1;STAT5B;SUGT1P1;TAF1A;TAF7;TGFB1;TGIF2;TIGD6;TMCO1;TMED10;TPRKB;TRAPPC4;TRMT112;TSG101;TTC14;TTC30B;TTLL3;UTP18;ZNF394;ZNF701"

"motif"	"NES"	"AUC"	"TF_highConf"	"nEnrGenes"	"enrichedGenes"
"cisbp__M5422"	3.45	0.0411	"ETV3 (directAnnotation). "	237	"ADAL;ADH5;AEBP1;ANKHD1-EIF4EBP3;ANKS3;APPL2;ARF4;ARFIP1;ARIH1;ASB1;ASPH;ATP7A;B3GNT5;BAG3;BCL7C;BMP2K;BRIX1;BTF3;C10orf26;C12orf48;C12orf57;C14orf109;C14orf135;C15orf24;C1orf112;C1orf212;C1orf63;C1orf9;C1orf97;C21orf34;C2orf84;C5orf41;C5orf43;C6orf226;C6orf70;C7orf11;C9orf64;CARD8;CBLB;CCDC147;CCDC15;CCKAR;CD74;CDC16;CDC26;CDK4;CDKL3;CETN3;CFLAR;CGA;CHUK;CIRBP;CLEC11A;CNIH;COMMD6;COX16;CSGALNACT2;CSTF1;CWC15;CWF19L2;CXXC4;CYB5D2;DCLRE1B;DDX50;DDX6;DLEC1;DNAJC10;DUSP18;EFC4A;EFHC1;EMP3;ERCC4;EXOSC3;FAM123B;FAM173B;FAM177A1;FAM46A;FBXO15;FCGBP;FGGY;FNDC3A;FOS;FTSJD1;GCNT2;GJB6;GLT8D1;GTF2A2;GTF2F2;HAS3;HAUS6;HEATR5A;HFM1;HSP90B1;HSPA13;IFT20;IL11;IL18;IL7R;INTS2;INTS4;KIAA1530;KLRK1;LARP7;LAX1;LEPROT;LIMS1;LOC100131564;LOC100272217;LOC389705;LOC728407;LOC729852;LPP;LRP10;LRRC70;LSM5;LST1;MAGEH1;MAK16;MBNL3;METTL12;MGAT2;MRPL20;MRPL50;MRPL51;MRPS26;MUTYH;MYO1F;MYST2;NAA38;NCF2;NEDD1;NHP2L1;NOC3L;NOL11;NOL7;NR1H3;NSUN5;NUDT6;NUP54;PAPOLA;PARG;PARN;PARP3;PATZ1;PCNP;PDCD10;PHIP;PITX2;PLCD1;PLRG1;PMFBP1;PNPLA4;POLA1;POLD3;POP5;PPAPDC1A;PSMB1;PSME2;PTCH1;PTOV1;RAD17;RAP2C;RBBP8;RBM34;RBM8A;RDBP;REST;RFX4;RND2;RPIA;RPL10;RPL24;RPS6;RRP15;S100A13;SAMD4A;SCAMP2;SCARNA17;SKIL;SLAIN2;SLC7A7;SMAD5;SMAD5OS;SMARCAD1;SMEK1;SNRPF;SP140L;SRP72;SRRM1;SSBP1;STAG1;STT3A;SYF2;TAF1A;TAF7;TFAM;TGDS;TGIF2;THAP6;THAP9;TIGD6;TMCO1;TMED10;TMEM138;TMEM176A;TMEM199;TMEM222;TOR1B;TPRKB;TRAPPC4;TRIM38;TRIM39;TRMT112;TSEN34;TSG101;TTC14;TTC30B;TTF1;TXNDC5;TYMP;UBR1;UFD1L;USP16;USP24;UTP18;WDR61;XIAP;YES1;ZFP90;ZNF197;ZNF285;ZNF394;ZNF512;ZNF518A;ZNF654;ZNF671;ZNHIT6"
"cisbp__M4952"	3.43	0.041	"ETV1; ETV4; ETV5 (inferredBy_Orthology). "	155	"ADAL;ADH5;AEBP1;ANKHD1-EIF4EBP3;ANKS3;APPL2;ARF4;ARFIP1;ARIH1;ASB1;ATAD2B;ATOX1;ATP7A;B3GNT5;BCL7C;BMP2K;C10orf26;C12orf48;C12orf57;C14orf109;C15orf24;C15orf38;C1orf112;C1orf97;C20orf94;C5orf43;C6orf226;C6orf70;C7orf11;C9orf64;CARD8;CBLB;CCDC15;CCDC152;CDC16;CDC26;CHUK;CNIH;COMMD6;CSGALNACT2;CSTF1;CWF19L2;CXXC4;CYB5D2;DCLRE1B;DDX50;DLEC1;DNAJC10;DUSP18;EFC4A;EIF5B;EXOSC3;FAM173B;FAM177A1;FAM46A;GMNN;GTF2A2;HAUS6;HFM1;HMGN4;HSP90B1;IFT20;IMPACT;INTS4;KLRK1;LAX1;LEPROT;LIMS1;LOC100131564;LOC100272217;LOC389705;LOC728407;LOC729852;LRP10;LRRC70;LSM5;LST1;MAK16;METTL12;MRPL51;MRPS26;MRPS31;MUTYH;MYST2;NAA38;NEDD1;NEK9;NFATC1;NOC3L;NOL7;NR1H3;NSUN5;NUP54;OFD1;PAPOLA;PARG;PARP3;PCNP;PDCD10;PHIP;PLCD1;PLRG1;PNPLA4;POLA1;PTCH1;R18;RAP2C;RBBP8;RBM34;RBM8A;RDBP;REST;RND2;RPIA;RPL10;RPS6;SAMD4A;SCAMP2;SSBP1;SYF2;TAF1A;TAF7;TFAM;TGIF2;THAP6;TIGD6;TMCO1;TMED10;TMEM138;TMEM199;TMEM20;TOR1B;TPRKB;TRAPPC2P1;TRAPPC4;TRIM39;TRMT112;TSEN34;TSG101;TTC14;TTC30B;TTF1;TYMP;UBR1;USP16;USP24;UTP18;WDR61;XIAP;YES1;ZNF197;ZNF394;ZNF518A;ZNF529;ZNF671"
"cisbp__M5373"	3.41	0.0409	"ELF1 (directAnnotation). "	276	"ACE2;ACO1;ADAL;ADAP2;AEBP1;ANKHD1-EIF4EBP3;ANKS3;APPL2;ARF4;ARFIP1;ARIH1;AS3MT;ASB1;ASPH;ATF7;ATP7A;ATPBD4;B3GNT5;BCL7C;BMP2K;BRIX1;BTF3;C10orf26;C12orf48;C12orf57;C14orf135;C15orf24;C15orf38;C1orf9;C20orf94;C21orf34;C2orf84;C5orf41;C5orf43;C6orf226;C6orf70;C7orf11;C8orf59;C9orf64;CARD8;CBFB;CBLB;CCDC102B;CCDC147;CCDC15;CCDC152;CCDC88B;CD274;CDC16;CDC26;CDKL3;CFLAR;CHUK;CIRBP;CITED4;CLEC11A;CLK1;CNIH;COMMD6;COX16;CRY1;CSGALNACT2;CSTF1;CWC15;CWF19L2;CYB5D2;DCLRE1B;DDX50;DLEC1;DNAJC10;DUSP18;EFC4A;EFHC1;EIF5B;ELAC1;EPS8;ERAP1;EXOSC3;FAM123B;FAM173B;FAM46A;FBXO15;FBXO36;FCGBP;FNDC3A;FOS;GJB6;GMNN;GTF2A2;GTF2F2;H2AFY;HAUS6;HEATR5A;HEPH;HFM1;HIST1H3B;HIST4H4;HMGN4;HSPA13;HSPA5;HSPBAP1;IFT20;IL11;IL18;IL7R;IMPACT;INPP5D;INTS2;INTS4;ISYNA1;ITGB3BP;KLHDC9;KLRK1;LARP7;LAX1;LEPROT;LIG1;LIMS1;LOC100131564;LOC100272217;LOC389705;LOC728407;LOC729852;LPP;LRRC70;LSM5;LSM6;LST1;MAEA;MAGEH1;MAK16;ME2;METTL12;MFSD1;MGAT2;MLH3;MPP5;MRPL20;MRPL51;MRPS26;MSH5;MUTYH;MX1;MYO1F;MYST2;NAA38;NCF2;NEDD1;NFATC1;NHP2L1;NOC3L;NOL11;NOL7;NR1H3;NSUN5;NUP54;NXT1;PARG;PARN;PARP3;PATZ1;PCNP;PDCD10;PDLIM5;PFDN5;PITX2;PLRG1;PMFBP1;PNPLA4;PNPO;POLA1;POLD3;PQLC3;PRKY;PTGR2;RAD17;RAP2C;RBBP8;RBM34;RBM8A;RDBP;REST;RIMBP3;RND2;RPH3AL;RPIA;RPL10;RPL13A;RPS6;RRP15;RTDR1;S100A13;SALL4;SAMD4A;SCAMP2;SETD4;SKIL;SLAIN2;SLC25A1;SLC25A20;SLC7A7;SMARCAD1;SP140L;SPR;SRP72;SRRM1;SRSF3;SSBP1;SSPN;SSR1;SSR4;STAG1;STAT5B;STK32B;STT3A;SYF2;TAF1A;TAF7;TAS2R46;TDRD3;TFAM;TGDS;TGFB1;TGIF2;THAP2;THEM4;THNSL2;TIGD6;TMCO1;TMED10;TMEM138;TMEM199;TMEM222;TOR1B;TPRKB;TRAF3IP1;TRAPPC2P1;TRAPPC4;TRIM38;TRIM39;TRIM42;TRIP4;TRMT112;TRNAU1AP;TSG101;TSPAN4;TTC14;TTC30B;TTF1;TTLL3;TWF1;TXNDC5;TYMP;UBR1;UFD1L;USP16;USP24;UTP18;WDR61;XIAP;YES1;ZFP90;ZFX;ZNF197;ZNF285;ZNF331;ZNF394;ZNF512;ZNF518A;ZNF529;ZNF610;ZNF660;ZNF671;ZNF701;ZNF765;ZNF92"
"cisbp__M6213"	3.4	0.0408	"ERG (directAnnotation). "	74	"ANKHD1-EIF4EBP3;ANKS3;APPL2;ARFIP1;ATP7A;BCL7C;BMP2K;C12orf57;C5orf43;CBLB;CCDC15;CDC26;CHUK;COMMD6;CSGALNACT2;CXXC4;DDX50;FAM173B;FOS;GJB6;HAUS6;IL7R;LAX1;LIMS1;LOC100131564;LOC100272217;LOC728407;LOC729852;LRRC70;LSM5;LST1;MAGEH1;MRPL51;MUTYH;MYO1F;MYST2;NCF2;NEDD1;NOC3L;NR1H3;NUP54;PARG;PATZ1;PNPLA4;POLA1;RAP2C;RBM34;RDBP;REST;RPIA;RPS6;RSPRY1;SAMD4A;SKIL;SLAIN2;SSBP1;TAF1A;TFAM;TGIF2;THAP6;TMEM138;TMEM199;TOR1B;TPRKB;TRAPPC4;TRMT112;TSG101;TTC30B;TTF1;UBR1;YES1;ZNF197;ZNF394;ZNF671"

"motif"	"NES"	"AUC"	"TF_highConf"	"nEnrGenes"	"enrichedGenes"
"cisbp__M5374"	3.4	0.0408	"ELF1 (directAnnotation). "	273	"ACE2;ACO1;ACTL6A;ADAL;ADH5;ALG14;ANKHD1-EIF4EBP3;ANKS3;APOM;APPL2;ARF4;ARFIP1;ARID4B;ARIH1;AS3MT;ASB1;ASPH;ATP7A;ATPBD4;AVP;B3GNT5;BOD1;BRIX1;BTBD8;C11orf83;C12orf48;C14orf135;C15orf24;C1orf63;C1QTNF3;C20orf94;C2orf84;C5orf41;C5orf43;C6orf145;C6orf226;C6orf70;C8orf59;C9orf64;CALCR;CBFB;CCDC147;CCDC15;CCDC152;CCDC88B;CCNDBP1;CD274;CD74;CDC16;CDC26;CDK4;CDKL3;CDNF;CENPE;CGA;CHUK;CIRBP;CLK1;CNIH;COMMD6;COX16;CRISPLD2;CRNKL1;CRY1;CSTF1;CWC15;CWF19L2;CYB5D2;DDX50;DNAJC10;DPH5;DYNLT1;EFHC1;EIF5B;ELAC1;EMP3;EPS8;ERAP1;EXOSC3;FAM136A;FAM173B;FBXO36;FIP1L1;FMO2;FNDC3A;FRAT1;FSD1L;G3BP1;GCNT2;GLIPR1L2;GLT8D1;GTF2A2;GTF2F2;HAS3;HAUS6;HEATR5A;HEPH;HFM1;HIST1H3B;HSPA13;HSPA5;HSPBAP1;IFT20;IL7R;IMPACT;INPP5D;INTS2;INTS4;ITGB3BP;KIAA1530;KIAA1683;KLHDC9;KLHL21;KLRK1;LARP7;LEPROT;LOC100131564;LOC389705;LOC728407;LOC729852;LPP;LRP10;LRRC37A3;LRRCC1;LSM5;LSM6;LST1;MAEA;MAGEH1;MAK16;MBNL3;MED28;METAP2;METTL12;MLH3;MPP5;MRPL20;MRPL50;MRPL51;MRPS26;MS4A6E;MUTYH;MYOZ1;MYST2;NCF2;NFATC1;NHLRC2;NHP2L1;NOC3L;NOL11;NOL7;NR1H3;NTSR2;NUP54;PAPOLA;PARG;PATZ1;PCNP;PDCD10;PFDN5;PHF17;PIGV;PITX2;PLRG1;PMFBP1;PNPLA4;POLA1;POLD3;POP5;POT1;PQLC3;PRKD3;PRKY;PRRC1;PSME2;R18;RAD17;RAP1B;RAP2C;RBBP8;RBM34;RBM8A;RCBTB2;RDBP;REST;RIMBP3;RPH3AL;RPIA;RPL13A;RPN1;RPS6;RRP15;RTDR1;S100A13;SALL4;SAMD4A;SCAMP2;SETD4;SKIL;SLC25A1;SLC25A20;SLC7A10;SLC7A7;SMARCA5;SNRPF;SPR;SRRM1;SRSF3;SSBP1;SSR1;SSR4;STAG1;STAT5B;STT3A;SUGT1P1;SUMF1;SYF2;SYTL4;TAF1A;TAF7;TDRD3;TEX9;TFAM;TGDS;THAP2;THEM4;THG1L;THNSL2;THUMPD1;TIGD6;TMCO1;TMED10;TMEM138;TMEM222;TOR1B;TPRKB;TRAF3IP1;TRAPPC4;TRIM38;TRIM42;TRIP4;TRMT112;TSG101;TTC14;TTC30B;TTF1;TTLL3;TWF1;TYMP;UBR1;UFD1L;USP24;UTP18;WRB;XIAP;YES1;ZFX;ZNF197;ZNF273;ZNF285;ZNF331;ZNF394;ZNF501;ZNF512;ZNF518A;ZNF583;ZNF660;ZNF671;ZNF691;ZNF701;ZNF765;ZNHIT6;ZZZ3"
"cisbp__M5377"	3.4	0.0408	"ELF4 (directAnnotation). "	228	"ACO1;ADAL;ADAP2;ADH5;ANKHD1-EIF4EBP3;ANKS3;APPL2;ARF4;ARFIP1;ARIH1;ASB1;ASPH;ATF7;ATP7A;B3GNT5;BCL7C;BMP2K;BRIX1;C10orf26;C11orf83;C12orf48;C12orf57;C14orf135;C15orf24;C15orf38;C1orf9;C20orf94;C21orf34;C2orf84;C5orf41;C5orf43;C6orf226;C6orf70;C7orf11;C9orf64;CALCR;CBLB;CCDC102B;CCDC15;CCDC88B;CD274;CDC16;CDC26;CDKL3;CFLAR;CHUK;CIRBP;COMMD6;COX16;CREBL2;CSGALNACT2;CSTF1;CWF19L2;CYB5D2;DDX50;DERL2;DLEC1;EFC4A;EFHC1;EIF5B;EPS8;ERAP1;EXOSC3;FAM123B;FAM173B;FAM46A;FBXO15;FBXO36;FCGBP;FNDC3A;GJB6;GTF2A2;GTF2F2;HAUS6;HEATR5A;HEPH;HIST4H4;HMGN4;HSPA13;HSPA5;IFT20;IL18;IL7R;IMPACT;INPP5D;INTS2;INTS4;IRF9;ISYNA1;KIAA1530;KLRK1;LARP7;LEPROT;LIG1;LIMS1;LOC100131564;LOC100272217;LOC389705;LOC728407;LOC729852;LPP;LRP10;LRRC70;LSM5;LST1;MAEA;MAGEH1;MAK16;MED28;METAP2;METTL12;MGAT2;MPP5;MRPL20;MRPL51;MRPS26;MTHFD2L;MUTYH;MYO1F;MYST2;NCF2;NEDD1;NFATC1;NHP2L1;NOC3L;NOL11;NR1H3;NSUN5;NUP54;NXT1;PARG;PARN;PATZ1;PCNP;PDCD10;PDLIM5;PFDN5;PITX2;PLRG1;PMFBP1;PNPLA4;POLD3;PRPF40A;R18;RAD17;RAP2C;RBBP8;RBM34;RBM8A;RDBP;REST;RFX4;RND2;RPIA;RPL10;RPS6;RRP15;S100A13;SAMD4A;SAMD9L;SCAMP2;SETD4;SKIL;SLC25A1;SLC7A7;SMARCAD1;SP140L;SPR;SRP72;SRRM1;SSBP1;SSPN;SSR1;SSR4;STAT5B;STT3A;SUGT1P1;SYF2;TAF1A;TAF7;TDRD3;TFAM;TGDS;TGFB1;THBS4;THEM4;THNSL2;TIGD6;TMCO1;TMED10;TMEM138;TMEM199;TMEM222;TOR1B;TPRKB;TRAF3IP1;TRAPPC4;TRIM38;TRIM39;TRIM42;TRIP4;TRMT112;TSG101;TTC14;TTC30B;TTF1;TTLL3;TWF1;TXNDC5;TYMP;UBR1;USP16;USP24;UTP18;WDR61;XIAP;YES1;ZFP90;ZFX;ZNF197;ZNF394;ZNF512;ZNF518A;ZNF660;ZNF671;ZNF701;ZNF83;ZNF92"
"cisbp__M5431"	3.39	0.0408	"FLI1 (directAnnotation). "	261	"ACO1;ADAL;ADH5;ADPRH;AEBP1;ANKHD1-EIF4EBP3;ANKS3;APPL2;AQP1;ARF4;ARFIP1;ARIH1;ASB1;ASCC1;ATF7;ATP7A;B3GNT5;BAG3;BCL7C;BMP2K;BTBD8;BTF3;C10orf26;C11orf71;C12orf48;C12orf57;C14orf109;C14orf135;C15orf24;C1orf112;C1orf212;C1orf9;C1orf97;C21orf34;C2orf84;C5orf43;C6orf226;C6orf70;C7orf11;C9orf5;C9orf64;CALCR;CARD8;CBFB;CBLB;CBX5;CCDC15;CCDC152;CCDC88B;CD274;CDC16;CDC26;CDKL3;CDNF;CETN3;CFLAR;CGA;CHUK;CLEC11A;CNIH;COMMD6;COX16;CRNKL1;CSGALNACT2;CSTF1;CTXN2;CWF19L2;CXXC4;CYB5D2;DCLRE1B;DDX50;DDX6;DGUOK;DKFZP434I0714;DLEC1;DNAJC10;DUSP18;EFC4A;EFHC1;EMP3;ERLIN2;EXOSC3;FAM123B;FAM173B;FAM177A1;FAM46A;FBXO15;FCGBP;FNDC3A;FOS;FTSJD1;GALNT10;GJB6;GMNN;GTF2A2;GTF2F2;HAUS6;HEPH;HFM1;HIST4H4;HMGB2;HMGN4;HSP90B1;HSPA13;HSPA5;HSPBAP1;IFT20;IL11;IL18;IL7R;INTS2;INTS4;ISYNA1;KIAA1530;KLRK1;LARP7;LAX1;LCA5;LEPROT;LIFR;LIMS1;LOC100131564;LOC100272217;LOC389705;LOC728407;LOC729852;LPP;LRP10;LRRC37A3;LRRC70;LSM5;LST1;MAEA;MAGEH1;MAK16;METTL12;MGAT2;MPL;MRPL20;MRPL50;MRPL51;MRPS31;MSH5;MUTYH;MYO1F;MYST2;NAA38;NCF2;NEDD1;NEK9;NFATC1;NFE2;NHP2L1;NKAP;NOC3L;NOL11;NOL7;NR1H3;NSUN5;NUDT6;NUP54;NXT1;OGFOD1;PAPOLA;PARG;PARN;PARP3;PATZ1;PBXIP1;PCNP;PDCD10;PDIK1L;PLCD1;PLRG1;PMCH;PMFBP1;PNPLA4;PODNL1;POLA1;POP5;PPAPDC1B;PRPF18;PRRX1;PSMB1;PSME2;PTCH1;RAD21;RAP1B;RAP2C;RBBP8;RBM34;RBM8A;RDBP;REST;RFX4;RND2;RPIA;RPL10;RPL13A;RPL24;RPS6;RRP15;RSPRY1;S100A13;SAMD4A;SCAMP2;SCARNA17;SKIL;SLAIN2;SLC7A7;SMAD5OS;SNRPF;SOX2;SP140L;SPR;SSBP1;STAG1;STT3A;SYF2;TAF1A;TAF7;TFAM;TGDS;TGIF2;THAP6;TIGD6;TMCO1;TMED10;TMEM138;TMEM199;TOR1B;TPRKB;TRAF3IP1;TRAF3IP2;TRAPPC2P1;TRAPPC4;TRIM38;TRIM39;TRMT112;TSEN34;TSG101;TSPAN4;TTC14;TTC30B;TTF1;TXNDC5;TYMP;UBR1;UFD1L;USP16;USP24;UTP18;WDR61;XIAP;YES1;ZKSCAN2;ZNF197;ZNF394;ZNF518A;ZNF529;ZNF671"

"motif"	"NES"	"AUC"	"TF_highConf"	"nEnrGenes"	"enrichedGenes"
"cisbp__M5420"	3.37	0.0407	"ETV1 (directAnnotation). "	137	"ADAL;ANKHD1-EIF4EBP3;ANKS3;APPL2;ARF4;ARFIP1;ASB1;ATP7A;BCL7C;BMP2K;C10orf26;C12orf48;C12orf57;C14orf109;C15orf24;C1orf112;C1orf97;C5orf43;C6orf226;C7orf11;C9orf64;CBLB;CCDC15;CDC26;CDKL3;CETN3;CHUK;CLEC11A;COMMD6;CSGALNACT2;CSTF1;CXXC4;CYB5D2;DCLRE1B;DDX50;DLEC1;DNAJC10;EFHC1;EXOSC3;FAM173B;FBXO15;FOS;GJB6;GTF2A2;HAUS6;IFT20;IL7R;INTS2;INTS4;KIAA1530;KLRK1;LAX1;LEPROT;LIMS1;LOC100131564;LOC100272217;LOC389705;LOC728407;LOC729852;LRP10;LRRC70;LSM5;LST1;MAGEH1;MAK16;METTL12;MPL;MRPL51;MUTYH;MYO1F;MYST2;NAA38;NCF2;NEDD1;NOC3L;NR1H3;NSUN5;NUP54;OGFOD1;PARG;PARP3;PATZ1;PCNP;PDCD10;PLRG1;PNPLA4;POLA1;POP5;RAP1B;RAP2C;RBBP8;RBM34;RBM8A;RDBP;REST;RPIA;RPL10;RPS6;RRP15;SAMD4A;SCAMP2;SKIL;SLAIN2;SLC7A7;SSBP1;STT3A;SYF2;TAF1A;TAF7;TFAM;TGDS;TGIF2;THAP6;TIGD6;TMCO1;TMED10;TMEM138;TMEM199;TOR1B;TPRKB;TRAPPC4;TRMT112;TSEN34;TSG101;TTC30B;TTF1;TXNDC5;UBR1;UFD1L;USP16;UTP18;WDR61;XIAP;YES1;ZNF197;ZNF394;ZNF518A"
"cisbp__M4522"	3.36	0.0406	"ELK4 (directAnnotation). "	249	"ACO1;ADAL;ADAP2;ADH5;AEBP1;ANKHD1-EIF4EBP3;ANKS3;APPL2;AQP1;ARF4;ARFIP1;ARIH1;ASB1;ATAD2B;ATF7;ATP7A;B3GNT5;BCL7C;BMP2K;BTBD8;BTF3;C10orf26;C11orf71;C12orf48;C12orf57;C14orf109;C15orf24;C15orf38;C1orf112;C1orf9;C1orf97;C5orf41;C5orf43;C6orf226;C6orf70;C7orf11;C9orf5;C9orf64;CALCR;CBFB;CBLB;CCDC15;CCDC152;CCDC88B;CDC16;CDC26;CDKL3;CDNF;CETN3;CFLAR;CGA;CHUK;CIRBP;CLEC11A;CNIH;COMMD6;COX16;CSGALNACT2;CSTF1;CWF19L2;CXXC4;CYB5D2;CYBA;DCLRE1B;DDX50;DDX6;DGUOK;DLEC1;DNAJC10;DUSP18;EFC4A;EFHC1;ERLIN2;EXOSC3;FAM123B;FAM173B;FAM177A1;FAM46A;FAM76A;FBXO15;FBXO36;FCGBP;FIP1L1;GALNT10;GJB6;GTF2A2;GTF2F2;HAUS6;HHEX;HIST4H4;HMGB2;HMGN4;HSPA5;IFT20;IL7R;IMPACT;INPP5D;INTS4;IRF9;ISYNA1;KIAA1530;KLRK1;LAX1;LEPROT;LIFR;LIMS1;LOC100131564;LOC100272217;LOC389705;LOC728407;LOC729852;LPP;LRRC70;LSM5;LST1;MAGEH1;MAK16;MDK;ME2;METTL12;MGAT2;MRPL20;MRPL51;MRPS26;MRPS31;MSH5;MUTYH;MYO1F;MYST2;NAA38;NCF2;NEDD1;NFATC1;NOC3L;NOL11;NOL7;NR1H3;NSUN5;NUP54;NXT1;OFD1;OGFOD1;PAPOLA;PARG;PARN;PARP3;PATZ1;PBXIP1;PCNP;PDCD10;PFDN5;PITX2;PLCD1;PLRG1;PNPLA4;POLA1;POLI;POP5;PPAPDC1B;PPP4R1;PRPF40A;PRRX1;PTCH1;RAP1B;RAP2C;RBBP8;RBM34;RBM8A;RDBP;REST;RFX4;RND2;RPIA;RPL10;RPL13A;RPL5;RPS6;RSPRY1;S100A13;SAMD4A;SCAMP2;SH3GLB1;SKIL;SLAIN2;SLC7A7;SMAD5OS;SMARCAD1;SNRPF;SOX2;SP140L;SPR;SRP72;SRRM1;SSBP1;STAG1;STAT5B;STT3A;SYF2;TAF1A;TAF7;TDRD3;TFAM;TGDS;TGFB1;TGIF2;THAP6;TIGD6;TMCO1;TMED10;TMEM138;TMEM199;TMEM20;TMEM222;TOR1B;TPRKB;TRAF3IP1;TRAF3IP2;TRAPPC2P1;TRAPPC4;TRIM38;TRIM39;TRMT112;TSEN34;TSG101;TSPAN4;TTC14;TTC30B;TTF1;TTLL3;TXNDC5;TYMP;UBR1;UFD1L;USP16;USP24;UTP18;WDR61;XIAP;YES1;ZFX;ZKSCAN2;ZNF197;ZNF394;ZNF512;ZNF518A;ZNF529;ZNF660;ZNF671;ZNF701"
"cisbp__M5423"	3.35	0.0405	"ETV4 (directAnnotation). "	269	"ADAL;ADH5;AEBP1;ANKHD1-EIF4EBP3;ANKS3;APPL2;AQP1;ARF4;ARFIP1;ARIH1;ASB1;ASCC1;ATF7;ATP7A;B3GNT5;BAG3;BCL7C;BMP2K;BTF3;C10orf26;C12orf48;C12orf57;C14orf109;C14orf135;C15orf24;C1orf112;C1orf212;C1orf26;C1orf9;C1orf97;C20orf94;C21orf34;C5orf43;C6orf226;C7orf11;C9orf5;C9orf64;CALCR;CARD8;CBFB;CBLB;CCDC15;CCDC152;CCDC88B;CD274;CD74;CDC16;CDC26;CDKL3;CDNF;CETN3;CFLAR;CGA;CHUK;CLEC11A;CLEC1A;CNIH;COMMD6;COMT;COX16;CREBL2;CRYL1;CSGALNACT2;CSTF1;CUL4A;CWF19L2;CXXC4;CYB5D2;DCLRE1B;DDX50;DDX6;DERL2;DGUOK;DKFZP434I0714;DLEC1;DNAJC10;DUSP18;EFC4A;EFHC1;ERLIN2;EXOSC3;FAM123B;FAM173B;FAM177A1;FAM46A;FBXO15;FBXO36;FCGBP;FOS;FSTL5;FTSJD1;G3BP1;GALNT10;GJB6;GMNN;GTF2A2;GTF2F2;HAUS6;HEPH;HFM1;HIST4H4;HMGN4;HSP90B1;HSPA13;HSPA5;HSPBAP1;IFT20;IL11;IL7R;INTS2;INTS4;ISYNA1;KIAA1530;KLRK1;LARP7;LAX1;LCA5;LEPROT;LIFR;LIMS1;LOC100131564;LOC100272217;LOC389705;LOC728407;LOC729852;LPP;LRP10;LRRC70;LSM5;LST1;MAEA;MAGEH1;MAK16;MBNL3;METTL12;MGAT2;MPL;MRPL20;MRPL50;MRPL51;MRPS31;MSH5;MUTYH;MYO1F;MYOZ1;MYST2;NAA38;NCF2;NEDD1;NEK9;NFE2;NHP2L1;NOC3L;NOL11;NOL7;NR1H3;NSUN5;NUDT6;NUP54;NXT1;OGFOD1;PAPOLA;PARG;PARN;PARP3;PATZ1;PBXIP1;PCNP;PDCD10;PIGV;PIN4;PLCD1;PLRG1;PMFBP1;PNPLA4;PODNL1;POLA1;POLD3;POP5;PPAPDC1B;PRPF18;PRPF40A;PSME2;PTCH1;R18;RAD17;RAD21;RAP1B;RAP2C;RBBP8;RBM34;RBM8A;RDBP;REST;RFX4;RND2;RPIA;RPL10;RPL13A;RPL24;RPS6;RRP15;RSPRY1;S100A13;SAMD4A;SCAMP2;SCARNA17;SKIL;SLAIN2;SLC7A7;SMAD5OS;SNRPF;SPR;SSBP1;STAG1;STT3A;SUGT1P1;SYF2;TAF1A;TAF7;TFAM;TGDS;TGIF2;THAP6;TIGD6;TMCO1;TMED10;TMEM138;TMEM199;TMEM222;TNFSF11;TNPO1;TOR1B;TPRKB;TRAF3IP1;TRAF3IP2;TRAPPC2P1;TRAPPC4;TRIM38;TRIM39;TRIP4;TRMT112;TSEN34;TSG101;TSPAN4;TTC14;TTC30B;TTF1;TTLL3;TXNDC5;TYMP;UBR1;UFD1L;USP16;USP24;UTP18;VAT1L;WDR61;XIAP;YES1;ZFX;ZKSCAN2;ZNF197;ZNF394;ZNF512;ZNF518A;ZNF529;ZNF583;ZNF671"

"motif"	"NES"	"AUC"	"TF_highConf"	"nEnrGenes"	"enrichedGenes"
"cisbp__M0711"	3.34	0.0405	""	278	"ACO1;ADAL;ADH5;AEBP1;ALOX5;ANKHD1-EIF4EBP3;ANKS3;APPL2;ARF4;ARFIP1;ARID4B;ARIH1;ASB1;ATAD2B;ATF7;ATOX1;ATP7A;B3GNT5;BBS9;BCAR3;BCL7C;BMP2K;BMPR1A;BOD1;BTF3;C10orf26;C11orf71;C11orf83;C12orf48;C12orf57;C14orf109;C14orf135;C15orf24;C15orf38;C1orf112;C1orf212;C1orf63;C1orf9;C1orf97;C2orf84;C5orf43;C6orf226;C6orf70;C7orf11;C9orf64;CARD8;CBLB;CCDC147;CCDC15;CCDC152;CCDC88B;CD274;CD74;CDC16;CDC26;CDK4;CDKL3;CETN3;CFLAR;CGA;CHD1;CHUK;CIRBP;CLEC11A;CNIH;CNIH4;COMMD6;COX16;CREBL2;CRY1;CSGALNACT2;CSTF1;CWF19L2;CXXC4;CYB5D2;DCLRE1B;DDX50;DDX6;DERL2;DGUOK;DLEC1;DNAJC10;DUSP18;EFC2;EFC4A;EMP3;ERCC4;EXOSC3;FAM173B;FAM177A1;FAM46A;FBXO15;FCGBP;FNDC3A;FOS;FPGS;FRAT1;GALNT10;GCNT2;GJB6;GMNN;GNG12;GTF2A2;GTF2F2;HAUS6;HDAC1;HEATR5A;HELQ;HEPH;HFM1;HIST4H4;HMGN4;HSPA13;HSPBAP1;IFT20;IGFBP6;IL28RA;IL7R;IMPACT;INTS4;IRF7;ITGB3BP;KIAA1530;KLRK1;LAX1;LEPROT;LIMS1;LOC100131564;LOC100272217;LOC389705;LOC389765;LOC728407;LOC729852;LPP;LRP10;LRRC70;LSM5;LST1;MAGEH1;MAK16;MESDC2;MGAT2;MRPL20;MRPL23;MRPL51;MRPS26;MRPS31;MSH5;MUTYH;MXRA8;MYST2;NAA38;NEDD1;NEK9;NFATC1;NHP2L1;NKX2-1;NOC3L;NOL11;NOL7;NR1H3;NSUN5;NUP54;NXT1;OFD1;OGFOD1;PAPOLA;PARG;PARP3;PATZ1;PCNP;PDCD10;PFDN5;PHIP;PIN4;PLCD1;PLRG1;PNPLA4;PNPO;POLA1;POLI;POP5;POT1;PTGR2;PTOV1;R18;RAP2C;RBBP8;RBM34;RBM8A;RCBTB2;RDBP;REST;RFX4;RND2;RPIA;RPL10;RPL13A;RPL24;RPS6;RRP15;S100A13;SALL4;SAMD4A;SCAMP2;SCARNA17;SETD4;SH3GLB1;SKIL;SLAIN2;SLC25A1;SLC7A7;SMAD5;SOX2;SP140L;SPR;SRP72;SRRM1;SSBP1;SSPN;SSR1;STAG1;STAT5B;SYF2;TAF1A;TAF7;TFAM;TGDS;TGIF2;THAP6;THEM4;TIGD6;TMCO1;TMED10;TMEM138;TMEM199;TMEM20;TMEM222;TOR1B;TPRKB;TRAF3IP1;TRAPPC2P1;TRAPPC4;TRIM38;TRIM39;TRIM52;TRIP4;TRMT112;TSEN34;TSG101;TSPAN4;TTC14;TTC30B;TTF1;TXNDC5;TYMP;UBR1;UFD1L;USP16;USP24;UTP18;VAT1L;WDR61;XIAP;YES1;ZFP90;ZKSCAN2;ZNF197;ZNF285;ZNF394;ZNF493;ZNF512;ZNF518A;ZNF529;ZNF660;ZNF671;ZNF701;ZNF92"
"cisbp__M0669"	3.33	0.0405	"E2F3 (inferredBy_Orthology). "	235	"ACTL6A;AEBP1;ALG10;ALG14;ANAPC10;ANKHD1-EIF4EBP3;ANP32E;APH1B;ARFGAP3;ARIH1;ATP10D;ATP7A;BCAR3;BCL7C;BMP2K;C10orf26;C14orf135;C1orf112;C1orf63;C1orf9;C20orf72;C20orf94;C21orf34;C3orf38;C5orf43;C9orf5;CAPN7;CBLB;CBX5;CCDC102A;CDC25A;CDC73;CDNF;CETN3;CFLAR;CKLF;CLK1;CNIH;COMMD6;CREBL2;CRISPLD2;CRNKL1;CRY1;CSGALNACT2;CSTF1;CTXN2;CXCL12;CXXC4;DDX3Y;DDX50;DERL2;DGUOK;DKFZP434I0714;EFC2;EFHC1;ERCC4;FAM177A1;FAM18B2;FAM46A;FAM76A;FBXO15;FGGY;FIP1L1;FITM2;FNDC3A;FOS;FRAT1;GAA;GCNT2;GLIPR1L2;GMNN;GTF2F2;HAS3;HAUS6;HELLS;HES1;HEY2;HHEX;HIST1H2AG;HIST1H2AI;HIST1H2BJ;HIST1H3B;HIST1H3G;HIST4H4;HMGB2;HMGN4;HSP90B1;HSPA13;HSPA5;IL11;IL18;IL28RA;INTS2;IQCG;ISYNA1;KIAA1530;KIF6;KLHDC10;KLHL21;LARP7;LIG1;LOC389705;LOC728407;LOC729852;LPIN1;LRRC37A3;LSM5;LSM6;MAEA;MAOB;MBNL3;MED28;METAP2;MGAT2;MORC4;MPP5;MRPL51;MSH5;MUTED;MUTYH;MYST2;NAA38;NDUFC1;NHLRC2;NHP2L1;NKAP;NKAPP1;NKX2-1;NOC3L;NOL7;NPM1;NUP205;OXTR;PARG;PARP3;PATZ1;PCSK1N;PDCD10;PDIK1L;PDLIM5;PHIP;PIN4;PITX2;PLRG1;PMFBP1;PNPO;POLA1;POP5;POU3F2;PPA1;PPIL1;PPP4R1;PRDM13;PRDX6;PRPF18;PRRX1;PSENEN;PSMB1;PTCH1;PTOV1;PUS3;RAD17;RAD21;RAN;RBBP8;RCL1;REST;RPL10;RPL13A;RPL24;RPL7;RPN1;RPS27L;RPS6;RRM1;RTDR1;S100A13;SAMD4A;SBDS;SEC11C;SENP8;SIM1;SLAIN2;SLC35F5;SLU7;SMAD5;SMAD5OS;SMARCA5;SMARCAD1;SMC5;SNRPF;SRRM1;SRSF3;SSPN;STAG1;STT3A;SUMF1;SYF2;TAF7;TAGLN2;TDRD3;THAP9;THG1L;THNSL2;TIGD6;TMCO1;TMED1;TMED10;TMED4;TMEM138;TMEM164;TMEM220;TMEM62;TMEM9;TRAF3IP1;TRIM39;TRMT112;TRPS1;TTLL3;TWISTNB;UBXN2A;USP51;VAT1;WBP5;YES1;ZCCHC11;ZCCHC3;ZFC3H1;ZFP36L2;ZFX;ZNF394;ZNF518A;ZNF583;ZNF654;ZNF691"
"cisbp__M4951"	3.3	0.0402	"FEV (inferredBy_Orthology). "	174	"ADAL;AEBP1;ANKHD1-EIF4EBP3;ANKS3;APPL2;ARFIP1;ARIH1;ASB1;ATAD2B;ATP7A;BCAR3;BCL7C;BMP2K;BOD1;BTF3;C10orf26;C11orf71;C12orf48;C12orf57;C14orf109;C15orf24;C1orf112;C1orf131;C2orf84;C5orf43;C6orf226;C6orf70;C7orf11;C9orf64;CBLB;CCDC15;CCDC152;CD74;CDC16;CDC26;CDKL3;CETN3;CHUK;CLEC11A;CNIH;COX16;CREBL2;CSGALNACT2;CSTF1;CWF19L2;CXXC4;CYB5D2;DCLRE1B;DDX50;DLEC1;DNAJC10;DUSP18;EFC2;EFC4A;EXOSC3;FAM114A1;FAM173B;FAM177A1;FAM46A;FBXO15;FOS;GTF2A2;GTF2F2;HAUS6;HDAC1;HEPH;HMGN4;HSPA5;HSPBAP1;IFT20;IGFBP6;IMPACT;INTS4;KIAA1530;LARP7;LAX1;LEPROT;LOC100131564;LOC100272217;LOC389705;LOC728407;LOC729852;LPP;LRRC70;LSM5;LST1;MAGEH1;MAK16;MRPL20;MRPL51;MRPS26;MRPS31;MSH5;MUTYH;MYST2;NAA38;NEDD1;NEK9;NFATC1;NOC3L;NOL7;NR1H3;NSUN5;NUP205;NUP54;NXT1;OFD1;PAPOLA;PARG;PARP3;PCNP;PDCD10;PHIP;PLCD1;PLRG1;PNPLA4;POLA1;PTOV1;RAP2C;RBBP8;RBM34;RBM8A;RDBP;REST;RPIA;RPL10;RPL13A;RPL24;RPS6;RRP15;S100A13;SAMD4A;SCAMP2;SLAIN2;SOX2;SRP72;SSBP1;STAG1;SYF2;TAF1A;TAF7;TFAM;TGDS;TGIF2;THAP6;TIGD6;TMCO1;TMED10;TMEM138;TMEM199;TMEM222;TOR1B;TPRKB;TRAPPC2P1;TRAPPC4;TRMT112;TSEN34;TSG101;TTC14;TTC30B;TTF1;TYMP;UBR1;USP16;USP24;UTP18;WDR61;XIAP;YES1;ZNF197;ZNF285;ZNF394;ZNF529;ZNF671"
"cisbp__M6339"	3.29	0.0402	"MECP2 (directAnnotation). "	214	"ACHE;ADAL;ADPRH;AEBP1;ANAPC10;ANKS3;ANP32E;APPL2;ARFIP1;ARIH1;ASB1;ATP7A;BAG3;BCAR3;BCL7C;BMP2K;BOD1;C10orf26;C12orf48;C12orf57;C14orf109;C14orf135;C15orf24;C15orf51;C1orf112;C2orf84;C5orf43;C6orf226;C6orf72;C7orf11;CALCR;CCDC15;CCDC88B;CDC16;CDC73;CDKL3;CDNF;CETN3;CIB1;CLEC11A;CNIH;CNIH4;COPG2;COX16;CSGALNACT2;CSTF1;CUL4A;CXXC4;CYB5D2;DDX50;DDX6;DHFRL1;DKFZP434I0714;DKK1;DNAJC10;DUT;EFC2;EFC4A;EIF1B;EIF5B;EPS8;ERLIN2;FAM164C;FAM173B;FAM46A;FBXO36;FNDC3A;FPGS;FZD5;GNA13;GNG8;GTF2A2;GTF2F2;H2AFY;HAUS6;HDAC1;HEPH;HHEX;HIPK3;HMGB2;HMGN4;HSPA13;HSPA5;HSPBAP1;IGF2BP2;IGFBP6;IL28RA;IMPACT;INTS4;IRF7;ITGB3;KIAA1530;KLHDC10;KLHL21;LEPROT;LIG1;LMAN1;LOC100131564;LOC100272217;LOC389705;LOC728407;LOC729852;MAK16;MDK;MDM1;MED28;METTL12;MRPL50;MRPL51;MRPS31;MTAP;MTHFD2L;MX1;MYST2;NAA38;NDUFC1;NEDD1;NFATC1;NKX2-1;NOC3L;NOL7;NR1H3;NSUN5;NXT1;OXTR;PAPOLA;PARG;PARP14;PARP3;PATZ1;PBLD;PDCD10;PDIK1L;PLCD1;PLRG1;PNPLA4;PNPO;POLA1;POLD3;POU3F2;PPAPDC1B;PPP1R3B;PPP4R1;PQLC3;PRDM5;PRKY;PRRX1;PTCH1;PTGR2;R18;RAD17;RBBP8;RBM34;RCBTB2;RCL1;RDBP;REST;RPIA;RPL10;RPL7;RPS27L;RPS6;RRM1;RRP15;S100A13;SAMD4A;SCAMP2;SDF2L1;SIM1;SLC25A1;SMARCAD1;SMO;SOAT1;SSBP1;SSR1;STAG1;STAT5B;SYF2;TAF1A;TAF7;TGDS;THEM4;TIGD6;TMED10;TMEM138;TMEM164;TMEM222;TNPO1;TP53INP1;TRAF3IP1;TRAPPC4;TRIM52;TRMT112;TSG101;TTC14;TTC30B;TTLL3;TXNDC5;TYMP;UBR1;UFD1L;USP24;UTP18;VMO1;WDR61;YES1;ZCCHC11;ZCCHC3;ZFP36L2;ZFP90;ZFX;ZNF394;ZNF660;ZNF691"

"motif"	"NES"	"AUC"	"TF_highConf"	"nEnrGenes"	"enrichedGenes"
"cisbp__M0700"	3.28	0.0401	"ETV4 (inferredBy_Orthology). "	197	"ADAL;AEBP1;ANKHD1-EIF4EBP3;ANKS3;APPL2;ARF4;ARFIP1;ARIH1;ASB1;ASCC1;ATF7;ATP7A;BCL7C;BMP2K;BTF3;C10orf26;C12orf48;C12orf57;C14orf109;C14orf135;C15orf24;C1orf112;C1orf212;C1orf9;C1orf97;C21orf34;C5orf43;C6orf226;C6orf70;C7orf11;C9orf64;CBLB;CCDC15;CCDC88B;CD274;CDC16;CDC26;CDKL3;CETN3;CFLAR;CHUK;CLEC11A;CNIH;COMMD6;COX16;CRY1;CSGALNACT2;CSTF1;CWF19L2;CXXC4;CYB5D2;DCLRE1B;DDX50;DKFZP434I0714;DLEC1;DNAJC10;DUSP18;EFC4A;EFHC1;EXOSC3;FAM173B;FAM46A;FBXO15;FBXO36;FOS;GJB6;GTF2A2;HAUS6;HMGB2;HMGN4;IL11;IL7R;INPP5D;INTS2;INTS4;ISYNA1;KIAA1530;KLRK1;LARP7;LAX1;LEPROT;LIMS1;LOC100131564;LOC100272217;LOC389705;LOC728407;LOC729852;LPP;LRP10;LRRC70;LSM5;LST1;MAEA;MAGEH1;MAK16;METTL12;MPL;MRPL50;MRPL51;MRPS31;MSH5;MUTYH;MYO1F;MYST2;NAA38;NCF2;NEDD1;NFATC1;NFE2;NOC3L;NOL11;NOL7;NR1H3;NSUN5;NUDT6;NUP54;NXT1;OGFOD1;PARG;PARN;PARP3;PATZ1;PCNP;PDCD10;PLCD1;PLRG1;PNPLA4;POLA1;POP5;PSME2;PTCH1;RAP1B;RAP2C;RBBP8;RBM34;RBM8A;RDBP;REST;RFX4;RND2;RPIA;RPL10;RPL13A;RPS6;RRP15;RSPRY1;SAMD4A;SCAMP2;SKIL;SLAIN2;SLC7A7;SPR;SSBP1;STAG1;STT3A;SYF2;TAF1A;TAF7;TDRD3;TFAM;TGDS;TGIF2;THAP2;TIGD6;TMCO1;TMED10;TMEM138;TMEM199;TMEM222;TOR1B;TPRKB;TRAPPC4;TRIM39;TRMT112;TSEN34;TSG101;TTC14;TTC30B;TTF1;TXNDC5;UBR1;UFD1L;USP16;USP24;UTP18;WDR61;XIAP;YES1;ZKSCAN2;ZNF197;ZNF394;ZNF512;ZNF518A;ZNF529;ZNF671;ZNF701;ZZZ3"
"cisbp__M0698"	3.26	0.0401	"ETV5 (inferredBy_Orthology). "	132	"ADAL;AEBP1;ANKHD1-EIF4EBP3;ANKS3;APPL2;ARF4;ARFIP1;ARIH1;ASB1;ATP7A;BCL7C;BMP2K;BTF3;C10orf26;C12orf57;C14orf109;C15orf24;C1orf97;C21orf34;C5orf43;C7orf11;CBLB;CCDC15;CDC26;CDKL3;CETN3;CFLAR;CHUK;CLEC11A;COMMD6;COX16;CSGALNACT2;CSTF1;CXXC4;CYB5D2;DCLRE1B;DDX50;DKFZP434I0714;DLEC1;DNAJC10;EXOSC3;FAM173B;FOS;GJB6;HAUS6;IL7R;INTS4;KIAA1530;KLRK1;LAX1;LEPROT;LIMS1;LOC100131564;LOC100272217;LOC728407;LOC729852;LRRC70;LSM5;LST1;MAGEH1;MAK16;MPL;MRPL51;MUTYH;MYO1F;MYST2;NAA38;NCF2;NEDD1;NFE2;NOC3L;NR1H3;NUP54;PARG;PARP3;PATZ1;PCNP;PDCD10;PLRG1;PNPLA4;POLA1;POP5;PSME2;RAP2C;RBBP8;RBM34;RBM8A;RDBP;REST;RPIA;RPL10;RPL13A;RPS6;RRP15;SAMD4A;SCAMP2;SKIL;SLAIN2;SSBP1;STAG1;STT3A;TAF1A;TAF7;TDRD3;TFAM;TGDS;TGIF2;THAP6;TIGD6;TMCO1;TMED10;TMEM138;TMEM199;TOR1B;TPRKB;TRAPPC4;TRMT112;TSEN34;TSG101;TTC30B;TTF1;TXNDC5;UBR1;UFD1L;USP16;UTP18;WDR61;XIAP;YES1;ZNF197;ZNF394;ZZZ3"
"cisbp__M0676"	3.26	0.04	""	253	"ACTL6A;AEBP1;ANAPC10;ANKHD1-EIF4EBP3;ANP32E;APH1B;ARID4B;ARIH1;ASB1;ATF1;ATP10D;BAG3;BBS9;BCAR3;BCAS2;BCL7C;BCO2;BMP2K;BOD1;BTBD8;BTF3;C10orf26;C14orf135;C15orf24;C15orf38;C1orf112;C1orf9;C20orf72;C20orf94;C2orf84;C4orf29;C6orf145;C6orf226;C6orf72;CBLB;CBX5;CCDC102A;CDC25A;CDC73;CDNF;CETN3;CIRBP;CLCC1;CLK1;CNIH;COL7A1;COMMD6;CREBL2;CRISPLD2;CSTF1;CTXN2;CXXC4;DDX3Y;DDX50;DERL2;DKFZP434I0714;DKK1;DNAJC10;EFC2;EFHC1;EIF4A3;ERCC4;FAM123B;FAM164C;FAM173B;FAM18B2;FAM46A;FAM76A;FBXO15;FBXO22;FGGY;FIP1L1;FNDC3A;FOS;GAA;GJB6;GLG1;GLIPR1L2;GMNN;GSTCD;GTF2F2;H2AFY;HAUS6;HELLS;HES1;HEY2;HHEX;HIST1H2AG;HIST1H2AI;HIST1H2BJ;HIST1H3B;HIST1H3G;HIST4H4;HMGB2;HSP90B1;HSPA13;HSPA5;HSPBAP1;IL18;IL28RA;INTS2;ITGB3BP;KIAA1407;KIAA1530;KIF6;KLHDC10;KLHL1;KLHL21;LARP7;LCA5;LCMT2;LEPROT;LIFR;LIG1;LOC389705;LOC728407;LOC729852;LPIN1;LSM5;LSM6;MAEA;MAK16;MBNL3;MED28;METAP2;METTL12;MORC4;MSH5;MTAP;MUTED;MUTYH;MYST2;NDUFC1;NFATC1;NHLRC2;NHP2L1;NID2;NKAP;NOC3L;NOL7;NPM1;NRARP;NUDT6;NXT1;OXTR;PARG;PAWR;PCNP;PCSK1N;PDIK1L;PHLDA2;PIN4;PITX2;PLRG1;PMFBP1;PNPO;POLA1;POLD3;POP5;POT1;POU3F2;PPAPDC1A;PPIL1;PRDM13;PRDX6;PRKY;PRPF18;PRRX1;PSMB1;PSME2;PTCH1;PTOV1;RAD17;RAD21;RAP1B;RBBP8;RBM8A;RCL1;REST;RNF135;RPL10;RPL13A;RPL23;RPL7;RPS27L;RRM1;RTDR1;S100A13;SAMD4A;SARNP;SEC11C;SH3GLB1;SIM1;SLC24A1;SLC25A1;SLC35F5;SLU7;SMAD5OS;SMARCA5;SMARCAD1;SMC5;SMO;SNRPF;SPIN4;SPRYD4;SRP72;SRRM1;SRSF3;SSR1;STAG1;STT3A;SUMF1;SUPT16H;TAF7;TAGLN2;TDRD3;TEX9;TFAM;TFPI2;TGDS;THAP2;THG1L;TIGD6;TMCO1;TMED1;TMED10;TMEM138;TMEM62;TMEM9;TRAF3IP1;TRH;TRIM39;TRIP4;TRMT112;TSEN34;TSPAN4;TWF1;TWISTNB;TXNDC5;UBXN2A;USP51;VAT1;WBP5;YES1;ZCCHC11;ZCCHC3;ZFC3H1;ZFP36L2;ZNF394;ZNF518A;ZNF583;ZNF691;ZZZ3"
"cisbp__M1787"	3.26	0.04	""	235	"ADAL;ADPRH;AEBP1;ANKHD1-EIF4EBP3;ANKS3;ANP32E;APPL2;ARF4;ARFIP1;ARHGAP19;ARID4B;ARIH1;AS3MT;ASB1;ASPH;ATF7;ATP7A;BBS9;BCAR3;BMP2K;BOD1;C11orf71;C11orf96;C12orf48;C12orf57;C15orf24;C15orf38;C17orf101;C1orf112;C1orf131;C1orf63;C1orf9;C1orf97;C5orf43;C6orf145;C6orf226;C6orf72;C7orf11;C9orf64;CALCR;CCDC15;CCDC152;CCDC88B;CD274;CD38;CDC16;CDC25A;CDKL3;CDNF;CETN3;CHUK;CLEC11A;CLNS1A;COMMD6;COPS4;COX16;CREBL2;CSTF1;CUL4A;CWF19L2;DBR1;DDX50;DDX6;DERL2;DKFZP434I0714;DNAJC10;EFC2;EFC4A;EFHC1;ERLIN2;EXOSC3;FAM123B;FAM164C;FAM46A;FAM76A;FBXO15;FBXO36;FIP1L1;FNDC3A;FOS;FTSJD1;FZD5;GGNBP2;GKAP1;GLG1;GTF2A2;HAS3;HAUS6;HDAC1;HIST1H3B;HIST1H3G;HMGN4;HSPA13;HSPBAP1;IFT20;IFT88;IGFBP6;IKZF5;IL11;INTS4;IRF7;KIAA1530;KLHDC10;KLHL21;LARP7;LAX1;LEPROT;LIG1;LOC100131564;LOC100272217;LOC389705;LOC389765;LOC728407;LOC729852;LSM5;MAGEH1;MAK16;MAP7D3;MDM1;MED28;METAP2;METTL12;MRPL50;MRPL51;MRPS26;MRPS31;MSN;MTHFD2L;MUTYH;MYST2;NAA38;NEDD1;NFATC1;NHLRC2;NHP2L1;NKAPP1;NKX2-1;NOC3L;NOL7;NPHP3;NR1H3;NSUN5;NT5C2;NUDT6;NUP205;NXT1;OSTM1;PAPOLA;PARG;PARN;PATZ1;PBLD;PDCD10;PDIK1L;PDLIM5;PFDN5;PLRG1;PNPLA4;POLA1;PPAPDC1B;PPIL1;PRRX1;PTCH1;PTGR2;PTOV1;R18;RAD17;RAP1B;RAP2C;RBBP8;RBM34;RBM8A;RDBP;REST;RPIA;RPL13A;RPL24;RPL7;RPS27L;RPS6;RRP15;S100A13;SAMD4A;SBDS;SCAMP2;SIM1;SLAIN2;SMARCAD1;SPR;SSBP1;STAG1;STAT5B;STT3A;SYF2;TAF1A;TAF7;TDRD3;TGDS;TGIF2;THEM4;TIGD6;TMCO1;TMED10;TMEM138;TMEM222;TMEM38B;TNPO1;TPRKB;TRAF3IP1;TRAPPC4;TRIM52;TRIP4;TRMT112;TSEN34;TSG101;TTC14;TTC30B;TYMP;UBR1;UFD1L;USP24;UTP18;VMO1;WDR61;WRB;YES1;ZFX;ZKSCAN2;ZNF285;ZNF394;ZNF518A;ZNF529;ZNF660;ZNF691;ZZZ3"

"motif"	"NES"	"AUC"	"TF_highConf"	"nEnrGenes"	"enrichedGenes"
"cisbp__M0092"	3.23	0.0399	""	70	"ANAPC10;ANKHD1-EIF4EBP3;ARF4;ARIH1;ATF1;ATF7;ATP7A;C11orf54;C12orf57;C14orf135;C1orf9;C20orf72;C4orf29;C5orf41;CBX5;CDK4;CDNF;CIRBP;CSTF1;CTXN2;DDX3Y;DERL2;ERLIN2;FAM46A;FAM76A;FNDC3A;GAA;GGNBP2;GPN3;HELLS;HES1;HIST1H2AG;HIST1H2BJ;HMGN4;HSPBAP1;IQCG;LARP7;LIG1;LOC728407;LRP10;LSM1;NAA38;NEURL2;PARG;POU3F2;PPP1R3B;PRDX6;PRRX1;RND2;RPL5;RSPRY1;SAMD4A;SENP6;SLU7;SMARCA5;SMARCAD1;SRRM1;SSR4;STAG1;STT3A;TDRD3;THAP2;TIGD6;TMED1;TRMT112;TSPAN4;ZFP36L2;ZKSCAN2;ZNF331;ZZZ3"
"cisbp__M5378"	3.23	0.0399	"ELF5 (directAnnotation). "	77	"ADAL;ANKS3;APPL2;ARFIP1;ATP7A;B3GNT5;C10orf26;C12orf48;C15orf24;C5orf41;CBLB;CCDC15;CCDC88B;CFLAR;CHUK;COMMD6;CSGALNACT2;DDX50;FBXO36;FCGBP;GJB6;IL7R;INPP5D;INTS4;LEPROT;LIMS1;LOC100131564;LOC389705;LOC728407;LOC729852;LST1;MAGEH1;MAK16;MRPL51;MUTYH;MYO1F;MYST2;NAA38;NCF2;NFATC1;NOC3L;NOL7;PARG;PATZ1;PITX2;PNPLA4;RAD21;RBM34;RDBP;RPS6;SCAMP2;SKIL;SLC7A7;SP140L;SSBP1;SSR4;STAT5B;TAF1A;TAF7;TDRD3;TIGD6;TMED10;TMEM222;TPRKB;TRAPPC4;TRIP4;TRMT112;TSG101;TTC14;TTC30B;TTF1;TTLL3;UBR1;UTP18;ZNF394;ZNF512;ZNF701"
"cisbp__M5401"	3.22	0.0398	"ERG (directAnnotation). "	144	"ADAL;AEBP1;ANKHD1-EIF4EBP3;ANKS3;APPL2;ARF4;ARFIP1;ASB1;ATP7A;BCL7C;BMP2K;BTF3;C10orf26;C12orf48;C12orf57;C14orf109;C15orf24;C1orf112;C1orf97;C5orf43;C6orf226;C9orf64;CBLB;CCDC15;CDC26;CDKL3;CETN3;CHUK;CLEC11A;CNIH;COMMD6;COX16;CSGALNACT2;CSTF1;CWF19L2;CXXC4;CYB5D2;DCLRE1B;DDX50;DDX6;DLEC1;DNAJC10;EXOSC3;FAM173B;FAM46A;FBXO15;FOS;GJB6;GTF2A2;HAUS6;IL7R;INTS4;KIAA1530;KLRK1;LAX1;LEPROT;LIMS1;LOC100131564;LOC100272217;LOC389705;LOC728407;LOC729852;LRP10;LRRC70;LSM5;LST1;MAGEH1;MAK16;METTL12;MPL;MRPL51;MUTYH;MYO1F;MYST2;NAA38;NCF2;NEDD1;NFE2;NOC3L;NOL7;NR1H3;NSUN5;NUP54;OGFOD1;PARG;PARP3;PATZ1;PCNP;PDCD10;PLCD1;PLRG1;PNPLA4;POLA1;POP5;RAP2C;RBBP8;RBM34;RBM8A;RDBP;REST;RPIA;RPL10;RPS6;SAMD4A;SCAMP2;SKIL;SLAIN2;SLC7A7;SMAD5OS;SSBP1;STAG1;STT3A;SYF2;TAF1A;TAF7;TFAM;TGDS;TGIF2;THAP6;TIGD6;TMCO1;TMED10;TMEM138;TMEM199;TOR1B;TPRKB;TRAPPC4;TSEN34;TSG101;TTC14;TTC30B;TTF1;TXNDC5;UBR1;USP16;UTP18;WDR61;XIAP;YES1;ZNF197;ZNF394;ZNF518A;ZNF671"
"cisbp__M5475"	3.22	0.0398	"GPA (directAnnotation). "	192	"ADAL;ADH5;ANKHD1-EIF4EBP3;ANKS3;APPL2;ARF4;ARFIP1;ARIH1;ASB1;ASCC1;ATP7A;B3GNT5;BCAR3;BCL7C;BMP2K;C10orf26;C12orf48;C12orf57;C14orf109;C15orf24;C1orf112;C1orf212;C1orf97;C5orf43;C6orf226;C6orf70;C7orf11;C9orf64;CBLB;CCDC15;CCDC152;CCDC88B;CDC16;CDC26;CDKL3;CETN3;CFLAR;CHUK;CLEC11A;CNIH;COMMD6;COX16;CSGALNACT2;CSTF1;CWF19L2;CXXC4;CYB5D2;DCLRE1B;DDX50;DDX6;DLEC1;DNAJC10;DUSP18;EFC4A;EFHC1;ERLIN2;EXOSC3;FAM123B;FAM173B;FAM177A1;FAM46A;FBXO15;FCGBP;FOS;GALNT10;GJB6;GMNN;GTF2A2;GTF2F2;HAUS6;HEPH;IFT20;IL11;IL7R;IMPACT;INTS2;INTS4;ISYNA1;KIAA1530;KLRK1;LAX1;LEPROT;LIMS1;LOC100131564;LOC100272217;LOC389705;LOC728407;LOC729852;LRP10;LRRC70;LSM5;LST1;MAGEH1;MAK16;METTL12;MGAT2;MRPL20;MRPL51;MRPS26;MUTYH;MYO1F;MYST2;NAA38;NCF2;NEDD1;NEK9;NFATC1;NHP2L1;NOC3L;NOL11;NOL7;NR1H3;NSUN5;NUP54;NXT1;OFD1;OGFOD1;PARG;PARP3;PATZ1;PCNP;PDCD10;PHIP;PIGV;PLCD1;PLRG1;PNPLA4;POLA1;POP5;RAD17;RAP2C;RBBP8;RBM34;RBM8A;RDBP;REST;RND2;RPIA;RPL10;RPL13A;RPS6;RRP15;S100A13;SAMD4A;SCAMP2;SKIL;SLAIN2;SLC7A7;SNRPF;SSBP1;STAT5B;STT3A;SYF2;TAF1A;TAF7;TFAM;TGDS;TGIF2;THAP6;TIGD6;TMCO1;TMED10;TMEM138;TMEM199;TMEM222;TOR1B;TPRKB;TRAPPC4;TRIM38;TRIM39;TRMT112;TSEN34;TSG101;TSPAN4;TTC14;TTC30B;TTF1;TXNDC5;TYMP;UBR1;UFD1L;USP16;USP24;UTP18;WDR61;XIAP;YES1;ZNF197;ZNF394;ZNF512;ZNF518A;ZNF671"
"cisbp__M4700"	3.22	0.0398	"NR2C2 (directAnnotation). "	155	"ACE2;ACO1;ALG14;ALK;ANKS3;ANP32E;APPL2;ARFIP1;ARIH1;ATF7;BBS9;BTBD8;C12orf48;C15orf24;C15orf38;C1orf112;C2orf84;C6orf145;C6orf226;C9orf64;CCDC102B;CCDC15;CCDC152;CD274;CDC16;CDKL3;CLNS1A;COMMD6;COMT;COX16;CRY1;CSTF1;CWC15;DUSP18;DYNLT1;EFC4A;EFHC1;EIF5B;ERAP1;EXOSC3;FP6;FAM123B;FAM173B;FAM177A1;FAM46A;FAM76A;FBXO36;FIP1L1;FPGS;GMNN;GTF2A2;HEATR5A;HIST4H4;HSPA5;IFT20;IL4R;IMPACT;INTS4;IRF9;KLRK1;LARP7;LAX1;LCA5;LEPROT;LIFR;LIMS1;LOC100131564;LOC389705;LOC389765;LOC728407;LOC729852;LRP10;LRRCC1;LSM5;LSM6;LST1;MAEA;MAK16;MDK;MPP5;MRPL20;MRPL51;MRPS26;MYO1F;MYST2;NFATC1;NUP54;PARG;PCNP;PDCD10;PFDN5;PIGV;PITX2;PLCD1;PLRG1;PNPLA4;PROCA1;PSMB1;PTCH1;RAP2C;RBBP8;RBM34;RBM8A;REST;RND2;RPL13A;RPL27A;RPS6;S100A13;SCAMP2;SETD4;SLC26A2;SLC7A10;SMC5;SPR;SRP72;SSBP1;STAG1;STAT5B;STT3A;SUGT1P1;SYTL4;TAF1A;TAF7;TDRD3;TGFB1;TGIF2;TIGD6;TMCO1;TMED10;TMEM138;TPRKB;TRAF3IP1;TRAPPC4;TRMT112;TRNAU1AP;TSG101;TTC14;TTC30B;TTF1;TTLL3;TXNDC5;UAP1L1;USP24;UTP18;WDR61;WRB;ZFP90;ZNF394;ZNF501;ZNF518A;ZNF654;ZNF701;ZNF765;ZNF92"

"motif"	"NES"	"AUC"	"TF_highConf"	"nEnrGenes"	"enrichedGenes"
"cisbp__M5376"	3.21	0.0398	"ELF3 (directAnnotation). "	273	"AASDH;ADAL;ADAP2;ADH5;AIRE;ANKS3;APPL2;ARF4;ARFIP1;ARIH1;AS3MT;ASB1;ASPH;ATP7A;B3GNT5;BBS9;BMP2K;BRIX1;BTF3;C10orf26;C12orf48;C12orf57;C15orf24;C17orf97;C1orf26;C1orf63;C1orf97;C20orf94;C2orf84;C5orf41;C5orf43;C6orf145;C6orf226;C7orf11;C7orf55;C8orf59;C9orf64;CBFB;CBLB;CCDC102B;CCDC15;CCDC152;CCDC88B;CCDC90B;CD274;CD38;CD74;CDC16;CDKL3;CFLAR;CHUK;CIRBP;CLEC18B;CLK1;CLNS1A;CNIH;COMMD6;CREBL2;CRYL1;CSGALNACT2;CSTF1;CWC15;CWF19L2;CYB5D2;DDX50;DERL2;DGUOK;DKFZP434I0714;DLEC1;DNAJC10;DPH5;EFC4A;EFHC1;EIF5B;EMP1;ERAP1;ERLIN2;EXOSC3;FP6;FAM173B;FBXO15;FBXO36;FCGBP;FMO2;FSTL5;GCNT2;GSTCD;GTF2A2;GTF2F2;H2AFY;HAUS6;HEATR5A;HEPH;HIST1H2AI;HIST4H4;HLA-A;HSPA13;HSPA5;IL18;IL28RA;IL7R;IMPACT;INPP5D;INTS2;INTS4;IQCK;IRF9;ISYNA1;ITGB3BP;KCTD3;KLHL1;KLRK1;LARP7;LCN12;LEPROT;LIG1;LILRB1;LIMS1;LOC100131564;LOC100272217;LOC389705;LOC728407;LOC729852;LPP;LRRCC1;LSM5;LST1;MAEA;MAGEH1;MAK16;MAP7D3;MBNL3;MDK;MFSD1;MGAT2;MLH3;MRPL20;MRPL51;MRPS26;MS4A6E;MUTYH;MX1;MYO1F;MYST2;NAA38;NCF2;NFATC1;NHLRC2;NHP2L1;NID2;NOC3L;NOL11;NR1H3;NUP54;NXT1;PARG;PARN;PATZ1;PCNP;PDLIM5;PFDN5;PITX2;PLRG1;PMFBP1;PNPLA4;PNPO;POLA1;POLD3;PPP1R3B;PQLC3;PRDM13;PRKY;R18;RAD17;RAD21;RAP1B;RAP2C;RBBP8;RBM34;RBM8A;RCL1;RDBP;REST;RFX4;RIMBP3;RND2;RPAP3;RPL10;RPL13A;RPL23;RPL7;RPS6;RTDR1;S100A13;SAMD4A;SAMD9L;SCAMP2;SETD4;SLC22A5;SLC25A1;SLC7A7;SMARCAD1;SOX2;SP140L;SPR;SRP72;SRRM1;SRSF3;SSBP1;SSPN;SSR1;STAT5B;STT3A;SUGT1P1;SYF2;SYTL4;TAF1A;TAF7;TAS2R46;TDRD3;TFAM;TGDS;THEM4;THNSL2;TIGD6;TLR3;TMCO1;TMED10;TMEM138;TMEM199;TMEM222;TOR1B;TPRKB;TRAF3IP1;TRAPPC4;TRIM38;TRIM39;TRIM42;TRIP4;TRMT112;TSG101;TTC14;TTC30B;TTF1;TTLL3;TWF1;TYMP;UBR1;UFD1L;UMPS;USP16;USP24;UTP18;VMO1;WDR61;XIAP;ZFP90;ZFX;ZNF197;ZNF285;ZNF331;ZNF394;ZNF512;ZNF518A;ZNF529;ZNF583;ZNF610;ZNF660;ZNF671;ZNF691;ZNF701;ZNF765;ZNF92"
"cisbp__M5384"	3.2	0.0397	"ELK3 (directAnnotation). "	197	"ADAL;ADH5;ANKHD1-EIF4EBP3;ANKS3;APPL2;ARF4;ARFIP1;ARIH1;ASB1;ASCC1;ATP7A;B3GNT5;BCL7C;BMP2K;C10orf26;C12orf48;C12orf57;C14orf109;C14orf135;C15orf24;C1orf112;C1orf212;C1orf97;C21orf34;C5orf43;C6orf226;C7orf11;C9orf64;CARD8;CBFB;CBLB;CCDC15;CCDC152;CDC16;CDC26;CDKL3;CETN3;CFLAR;CHUK;CLEC11A;CNIH;COMMD6;COX16;CSGALNACT2;CSTF1;CUL4A;CWF19L2;CXXC4;CYB5D2;DCLRE1B;DDX50;DDX6;DKFZP434I0714;DLEC1;DNAJC10;DUSP18;EFC4A;EFHC1;ERLIN2;EXOSC3;FAM173B;FAM177A1;FAM46A;FBXO15;FBXO36;FCGBP;FOS;FTSJD1;GALNT10;GJB6;GMNN;GTF2A2;HAUS6;IFT20;IL7R;INTS2;INTS4;KIAA1530;KLRK1;LARP7;LAX1;LEPROT;LIMS1;LOC100131564;LOC100272217;LOC389705;LOC728407;LOC729852;LRP10;LRRC70;LSM5;LST1;MAGEH1;MAK16;METTL12;MGAT2;MPL;MRPL20;MRPL51;MRPS31;MSH5;MUTYH;MYO1F;MYST2;NAA38;NCF2;NEDD1;NHP2L1;NOC3L;NOL11;NOL7;NR1H3;NSUN5;NUDT6;NUP54;NXT1;OGFOD1;PAPOLA;PARG;PARP3;PATZ1;PCNP;PDCD10;PLCD1;PLRG1;PNPLA4;POLA1;POP5;PRPF40A;PTCH1;R18;RAD17;RAP1B;RAP2C;RBBP8;RBM34;RBM8A;RDBP;REST;RND2;RPIA;RPL10;RPL13A;RPS6;RRP15;S100A13;SAMD4A;SCAMP2;SCARNA17;SKIL;SLAIN2;SLC7A7;SNRPF;SPR;SSBP1;STT3A;SYF2;TAF1A;TAF7;TFAM;TGDS;TGIF2;THAP6;TIGD6;TMCO1;TMED10;TMEM138;TMEM199;TMEM222;TOR1B;TPRKB;TRAPPC2P1;TRAPPC4;TRIM38;TRIM39;TRMT112;TSEN34;TSG101;TTC14;TTC30B;TTF1;TXNDC5;TYMP;UBR1;UFD1L;USP16;USP24;UTP18;WDR61;XIAP;YES1;ZNF197;ZNF394;ZNF512;ZNF518A;ZNF529;ZNF671"
"cisbp__M5418"	3.2	0.0397	"ETS1 (directAnnotation). "	173	"ADAL;AEBP1;ANKHD1-EIF4EBP3;ANKS3;APPL2;ARF4;ARFIP1;ARIH1;ASB1;ATP7A;B3GNT5;BCL7C;BMP2K;BTF3;C10orf26;C12orf48;C12orf57;C14orf109;C15orf24;C1orf112;C1orf212;C1orf97;C5orf43;C6orf226;C7orf11;C9orf64;CBLB;CCDC15;CDC16;CDC26;CDKL3;CETN3;CFLAR;CGA;CHUK;CLEC11A;CNIH;COMMD6;COX16;CSGALNACT2;CSTF1;CWF19L2;CXXC4;CYB5D2;DCLRE1B;DDX50;DDX6;DLEC1;DNAJC10;DUSP18;EFC4A;EFHC1;EXOSC3;FAM173B;FAM46A;FBXO15;FOS;FTSJD1;GJB6;GTF2A2;HAUS6;HSP90B1;IFT20;IL11;IL7R;INTS4;KIAA1530;KLRK1;LAX1;LEPROT;LIMS1;LOC100131564;LOC100272217;LOC389705;LOC728407;LOC729852;LPP;LRP10;LRRC70;LSM5;LST1;MAGEH1;MAK16;METTL12;MPL;MRPL50;MRPL51;MSH5;MUTYH;MYO1F;MYST2;NAA38;NCF2;NEDD1;NFATC1;NFE2;NOC3L;NOL11;NOL7;NR1H3;NSUN5;NUP54;PARG;PARN;PARP3;PATZ1;PCNP;PDCD10;PLCD1;PLRG1;PNPLA4;POLA1;POP5;PTCH1;RAD21;RAP1B;RAP2C;RBBP8;RBM34;RBM8A;RDBP;REST;RND2;RPIA;RPL10;RPS6;RRP15;S100A13;SAMD4A;SCAMP2;SKIL;SLAIN2;SLC7A7;SMAD5OS;SSBP1;STAG1;STT3A;SYF2;TAF1A;TAF7;TFAM;TGDS;TGIF2;THAP6;TIGD6;TMCO1;TMED10;TMEM138;TMEM199;TOR1B;TPRKB;TRAF3IP1;TRAPPC4;TRMT112;TSEN34;TSG101;TTC14;TTC30B;TTF1;TXNDC5;UBR1;UFD1L;USP16;USP24;UTP18;WDR61;XIAP;YES1;ZNF197;ZNF394;ZNF518A;ZNF529;ZNF671"
"cisbp__M5429"	3.19	0.0397	"FEV (directAnnotation). "	212	"ADAL;ADH5;AEBP1;ANKHD1-EIF4EBP3;ANKS3;APPL2;ARF4;ARFIP1;ARIH1;ASB1;ASCC1;ATF7;ATP7A;B3GNT5;BCL7C;BMP2K;BTF3;C10orf26;C12orf48;C12orf57;C14orf109;C14orf135;C15orf24;C1orf112;C1orf212;C1orf9;C1orf97;C21orf34;C5orf43;C6orf226;C7orf11;C9orf64;CARD8;CBFB;CBLB;CCDC15;CCDC152;CDC16;CDC26;CDKL3;CETN3;CFLAR;CGA;CHUK;CLEC11A;CNIH;COMMD6;COX16;CSGALNACT2;CSTF1;CWF19L2;CXXC4;CYB5D2;DCLRE1B;DDX50;DDX6;DGUOK;DKFZP434I0714;DLEC1;DNAJC10;DUSP18;EFHC1;ERLIN2;EXOSC3;FAM173B;FAM46A;FBXO15;FCGBP;FOS;FTSJD1;GJB6;GMNN;GTF2A2;GTF2F2;HAUS6;HEPH;HFM1;IFT20;IL11;IL7R;INTS2;INTS4;KIAA1530;KLRK1;LARP7;LAX1;LCA5;LEPROT;LIMS1;LOC100131564;LOC100272217;LOC389705;LOC728407;LOC729852;LRP10;LRRC70;LSM5;LST1;MAGEH1;MAK16;METTL12;MGAT2;MPL;MRPL20;MRPL50;MRPL51;MRPS31;MSH5;MUTYH;MYO1F;MYOZ1;MYST2;NAA38;NCF2;NEDD1;NFE2;NHP2L1;NOC3L;NOL11;NOL7;NR1H3;NSUN5;NUDT6;NUP54;OGFOD1;PAPOLA;PARG;PARN;PARP3;PATZ1;PCNP;PDCD10;PLCD1;PLRG1;PMFBP1;PNPLA4;PODNL1;POLA1;POP5;PSME2;PTCH1;RAD17;RAD21;RAP1B;RAP2C;RBBP8;RBM34;RBM8A;RDBP;REST;RFX4;RND2;RPIA;RPL10;RPL13A;RPS6;RRP15;RSPRY1;S100A13;SAMD4A;SCAMP2;SCARNA17;SKIL;SLAIN2;SLC7A7;SMAD5OS;SNRPF;SPR;SSBP1;STAG1;STT3A;SYF2;TAF1A;TAF7;TFAM;TGDS;TGIF2;THAP6;TIGD6;TMCO1;TMED10;TMEM138;TMEM199;TOR1B;TPRKB;TRAF3IP1;TRAPPC4;TRIM38;TRIM39;TRMT112;TSEN34;TSG101;TSPAN4;TTC14;TTC30B;TTF1;TXNDC5;TYMP;UBR1;UFD1L;USP16;USP24;UTP18;WDR61;XIAP;YES1;ZKSCAN2;ZNF197;ZNF394;ZNF518A;ZNF529;ZNF671"

"motif"	"NES"	"AUC"	"TF_highConf"	"nEnrGenes"	"enrichedGenes"
"cisbp__M5385"	3.19	0.0396	"ELK4 (directAnnotation). "	128	"ADAL;ANKHD1-EIF4EBP3;ANKS3;APPL2;ARFIP1;ASB1;ATP7A;BCL7C;BMP2K;C10orf26;C12orf48;C12orf57;C14orf109;C15orf24;C1orf112;C1orf97;C5orf43;C6orf226;C9orf64;CBLB;CCDC15;CDC26;CDKL3;CETN3;CHUK;CLEC11A;CNIH;COMMD6;COX16;CSGALNACT2;CSTF1;CXXC4;CYB5D2;DCLRE1B;DDX50;DLEC1;DNAJC10;EXOSC3;FAM173B;FAM46A;FBXO15;FOS;GJB6;GTF2A2;HAUS6;IL7R;INTS4;KIAA1530;KLRK1;LAX1;LEPROT;LIMS1;LOC100131564;LOC100272217;LOC389705;LOC728407;LOC729852;LRRC70;LSM5;LST1;MAGEH1;MAK16;METTL12;MRPL51;MUTYH;MYO1F;MYST2;NAA38;NCF2;NEDD1;NOC3L;NR1H3;NSUN5;NUP54;PARG;PARP3;PATZ1;PCNP;PDCD10;PLRG1;PNPLA4;POLA1;POP5;RAP2C;RBBP8;RBM34;RBM8A;RDBP;REST;RPIA;RPL10;RPS6;SAMD4A;SCAMP2;SKIL;SLAIN2;SLC7A7;SSBP1;STT3A;SYF2;TAF1A;TAF7;TFAM;TGDS;TGIF2;THAP6;TIGD6;TMED10;TMEM138;TMEM199;TOR1B;TPRKB;TRAPPC4;TRMT112;TSEN34;TSG101;TTC14;TTC30B;TTF1;TXNDC5;UBR1;USP16;UTP18;WDR61;XIAP;YES1;ZNF197;ZNF394"
"cisbp__M4620"	3.18	0.0396	"GPA (directAnnotation). "	276	"ACO1;ADAL;ADH5;ALOX5;ANKHD1-EIF4EBP3;ANKS3;APOM;APPL2;ARF4;ARFIP1;ARIH1;ASB1;ASCC1;ATAD2B;ATF7;ATP7A;B3GNT5;BCL7C;BMP2K;BTBD8;BTF3;C10orf26;C12orf48;C12orf57;C14orf109;C14orf135;C15orf24;C15orf38;C1orf26;C1orf9;C1orf97;C20orf94;C21orf34;C2orf84;C5orf41;C5orf43;C6orf226;C6orf70;C7orf11;C9orf5;C9orf64;CALCR;CARD8;CBFB;CBLB;CCDC15;CCDC152;CCDC88B;CD274;CDC16;CDC26;CDK4;CDKL3;CETN3;CFLAR;CHUK;CIRBP;CLEC11A;CNIH;COMMD6;COX16;CRNKL1;CSGALNACT2;CSTF1;CWF19L2;CXXC4;CYB5D2;CYBA;DDX50;DDX6;DERL2;DGUOK;DLEC1;DNAJC10;DUSP18;EFC4A;EFHC1;ERCC4;ERLIN2;EXOSC3;FAM123B;FAM173B;FAM177A1;FAM46A;FBXO15;FBXO36;FCGBP;FIP1L1;FNDC3A;FTSJD1;GALNT10;GJB6;GMNN;GTF2A2;GTF2F2;H2AFY;HAUS6;HEPH;HHEX;HMGB2;HMGN4;HSPA5;IFT20;IL7R;IMPACT;INPP5D;INTS2;INTS4;IRF9;ISYNA1;KIAA1530;KLRK1;LARP7;LAX1;LEPROT;LIFR;LIMS1;LOC100131564;LOC100272217;LOC389705;LOC728407;LOC729852;LRP10;LRRC70;LSM5;LST1;MAEA;MAGEH1;MAK16;MDK;ME2;METTL12;MGAT2;MPL;MRPL20;MRPL23;MRPL51;MRPS26;MRPS31;MSH5;MTHFD2L;MUTYH;MYO1F;MYST2;NAA38;NCF2;NEDD1;NEK9;NFATC1;NFE2;NHP2L1;NKAP;NOC3L;NOL11;NR1H3;NSUN5;NUDT6;NUP54;NXT1;OFD1;OGFOD1;PAPOLA;PARG;PARP3;PATZ1;PBXIP1;PCNP;PDCD10;PFDN5;PITX2;PLCD1;PLRG1;PMFBP1;PNPLA4;POLA1;POLD3;POLI;PPAPDC1B;PPP4R1;PRDM13;PRPF40A;PSME2;PTCH1;R18;RAD17;RAP1B;RAP2C;RBBP8;RBM34;RBM8A;RCBTB2;RDBP;REST;RFX4;RND2;RPIA;RPL10;RPL13A;RPL24;RPL5;RPS6;RRP15;S100A13;SAMD4A;SCAMP2;SCARNA17;SETD4;SKIL;SLAIN2;SLC25A1;SLC35F5;SLC7A7;SMARCAD1;SNRPF;SOX2;SP140L;SPR;SRP72;SRRM1;SSBP1;STAG1;STAT5B;STT3A;SYF2;TAF1A;TAF7;TAGLN2;TDRD3;TFAM;TGDS;TGFB1;TGIF2;THAP2;TIGD6;TMCO1;TMED10;TMEM138;TMEM176A;TMEM199;TMEM20;TMEM222;TOR1B;TPRKB;TRAPPC2P1;TRAPPC4;TRIM38;TRIM39;TRIM52;TRMT112;TSEN34;TSG101;TTC14;TTC30B;TTF1;TTLL3;TXNDC5;TYMP;UBR1;UFD1L;USP16;USP24;UTP18;WDR61;XIAP;YES1;ZFP90;ZFX;ZNF197;ZNF394;ZNF512;ZNF518A;ZNF529;ZNF562;ZNF660;ZNF671;ZNF701"
"cisbp__M1720"	3.17	0.0396	""	103	"ACTL6A;ALG10;ANAPC10;ANKHD1-EIF4EBP3;ANP32E;APH1B;BOD1;C10orf26;C11orf71;C17orf97;C1orf9;C20orf72;C21orf34;CBX5;CDC73;CDNF;CIB1;CKLF;CNIH;COMMD6;CRISPLD2;CSTF1;CTXN2;CWF19L2;DDX3Y;DERL2;DGUOK;DKFZP434I0714;FAM46A;FAM76A;FBXO15;FIP1L1;FNDC3A;GTF2F2;HAUS6;HELLS;HIST1H2AG;HIST1H2AI;HIST1H2BJ;HIST1H3G;HIST4H4;HMGN4;HSPA13;INTS2;IQCG;ITGB3BP;KIAA1530;KLHDC10;LARP7;LIG1;LOC728407;LOC729852;LSM5;MBNL3;NAA38;NDUFC1;NHLRC2;NKAP;PARG;PHIP;PITX2;POLA1;POU3F2;PPIL1;PPP1R3B;PRDM13;PRRX1;PSENEN;PSMB1;PUS3;R18;RAD21;RAN;RBBP8;REST;RPIA;RPL13A;RPL7;RPS27L;RPS6;SBDS;SENP8;SLU7;SMAD5OS;SMARCA5;SMARCAD1;SNAPC1;STAG1;TAF7;TDRD3;THAP2;THAP9;TIGD6;TMED10;TMED4;TTLL3;TWISTNB;UBXN2A;UFD1L;ZCCHC11;ZKSCAN2;ZNF518A;ZZZ3"
"cisbp__M5398"	3.16	0.0395	"ERF (directAnnotation). "	252	"ACO1;ADAL;ADH5;ALOX5;ANKHD1-EIF4EBP3;ANKS3;APPL2;AQP1;ARF4;ARFIP1;ASB1;ASCC1;ATP7A;B3GNT5;BAG3;BCAR3;BCAS2;BCL7C;BMP2K;BRIX1;C10orf26;C12orf48;C12orf57;C12orf60;C14orf109;C14orf135;C15orf24;C1orf112;C1orf212;C1orf26;C1orf9;C1orf97;C20orf94;C21orf34;C2orf84;C5orf43;C6orf226;C6orf70;C7orf11;C7orf55;C9orf64;CALCR;CARD8;CBFB;CBLB;CBX5;CCDC15;CCDC152;CCKAR;CD274;CD74;CDC16;CDC26;CDK4;CDKL3;CETN3;CFLAR;CGA;CHD1;CHUK;CLEC11A;CLEC18B;CLEC1A;CNIH;COMMD6;COX16;CREBL2;CRNKL1;CSGALNACT2;CSTF1;CWF19L2;CXorf57;CXXC4;CYB5D2;DCLRE1B;DDX50;DDX6;DGUOK;DLEC1;DNAJC10;DUSP18;EFC4A;EMP3;ERCC4;ERLIN2;EXOSC3;FAM173B;FAM177A1;FAM46A;FBXO15;FBXO36;FCGBP;FOS;FTSJD1;GALNT10;GCNT2;GJB6;GMNN;GTF2A2;HAUS6;HFM1;HSP90B1;HSPA13;IFT20;IL18;IL1A;IL7R;INTS4;ISYNA1;KIAA1530;KLRK1;LAX1;LEPROT;LIMS1;LOC100131564;LOC100272217;LOC389705;LOC728407;LOC729852;LPP;LRP10;LRRC70;LSM5;LST1;MAGEH1;MAK16;METTL12;MGAT2;MPL;MRPL50;MRPL51;MRPS31;MSH5;MUTYH;MYO1F;MYOZ1;MYST2;NAA38;NCF2;NEDD1;NFE2;NKAP;NOC3L;NOL7;NR1H3;NSUN5;NUDT6;NUP54;OGFOD1;PAPOLA;PARG;PARN;PARP3;PATZ1;PCNP;PDCD10;PHIP;PLCD1;PLRG1;PMFBP1;PNPLA4;POLA1;POLD3;POP5;PPAPDC1B;PRDM13;PRPF18;PSMB1;PSME2;PUS3;R18;RAD17;RAD21;RAP1B;RAP2C;RBBP8;RBM34;RBM8A;RDBP;REST;RFX4;RND2;RPIA;RPL10;RPL13A;RPL24;RPL5;RPS6;SAMD4A;SCAMP2;SCARNA17;SH3GLB1;SKIL;SLAIN2;SLC7A7;SMAD5;SMAD5OS;SMEK1;SOX2;SP140L;SPR;SRP72;SRRM1;SSBP1;STAG1;STAT5B;STT3A;TAF1A;TAF7;TFAM;TGDS;TGIF2;THAP6;THAP9;TIGD6;TMCO1;TMED10;TMEM138;TMEM199;TMEM222;TNFSF11;TOR1B;TPRKB;TRAF3IP2;TRAPPC2P1;TRAPPC4;TRIM38;TRIM39;TRMT112;TSEN34;TSG101;TSPAN4;TTC14;TTC30B;TTF1;TTLL3;TXNDC5;UBR1;UFD1L;USP16;USP24;UTP18;WDR61;XIAP;YES1;ZNF136;ZNF197;ZNF394;ZNF518A;ZNF529;ZNF583;ZNF671"
"cisbp__M5579"	3.16	0.0395	"IRF8 (directAnnotation). "	63	"ACADM;ARF4;ARID4B;ASPH;ATF7;C17orf101;C1orf212;C1orf26;C2orf3;C6orf145;CARD8;CASP10;CBX5;CCDC88B;CD274;CD59;CD74;CFLAR;CLEC1A;CWF19L2;DYNLT1;EXOSC3;FAM123B;FAM76A;GNA13;GTF2F2;HERC5;HEY2;HIBCH;IFI44;IFI6;IFIT3;IRF7;KLHL21;LIX1;LRP10;MDK;ME2;MGAT2;MX1;NEDD1;PARP14;PATZ1;PGRMC2;PMFBP1;PPAPDC1A;PTCH1;RARRES3;RTP4;SAMD4A;SAMD9L;SLC35F5;SMARCAD1;SRRM1;TMED10;TNFSF11;TPH1;TRAF3IP1;TRIM34;TRMT11;ZMYND12;ZNF273;ZNF518A"

"motif"	"NES"	"AUC"	"TF_highConf"	"nEnrGenes"	"enrichedGenes"
"cisbp__M6209"	3.16	0.0395	"ELK4 (directAnnotation). "	100	"ANKHD1-EIF4EBP3;ANKS3;APPL2;ARFIP1;ASB1;ATP7A;BCL7C;BMP2K;C12orf48;C12orf57;C15orf24;C5orf43;C6orf226;C9orf64;CBLB;CCDC15;CDC26;CHUK;CLEC11A;CSGALNACT2;CSTF1;CWF19L2;CYB5D2;DDX50;DNAJC10;DUSP18;FAM173B;FAM46A;FOS;GJB6;HAUS6;IFT20;IL7R;INTS4;LAX1;LEPROT;LIMS1;LOC100131564;LOC100272217;LOC389705;LOC728407;LOC729852;LRP10;LRRC70;LSM5;LST1;MAK16;MRPL51;MUTYH;MYO1F;MYST2;NAA38;NEDD1;NOC3L;NOL7;NR1H3;NUP54;PARG;PATZ1;PLCD1;PNPLA4;POP5;RAP2C;RBBP8;RBM34;RDBP;REST;RPIA;RPS6;SAMD4A;SCAMP2;SKIL;SLAIN2;SSBP1;TAF1A;TAF7;TFAM;TGDS;TGIF2;TIGD6;TMED10;TMEM138;TMEM199;TOR1B;TPRKB;TRAPPC4;TRMT112;TSEN34;TSG101;TTC14;TTC30B;TTF1;TXNDC5;UBR1;USP16;XIAP;YES1;ZNF197;ZNF394;ZNF671"
"cisbp__M1891"	3.15	0.0394	"GPA (inferredBy_Orthology). "	262	"ACO1;ADAL;AEBP1;ALOX5;ANKHD1-EIF4EBP3;ANKS3;APOM;APPL2;AQP1;ARF4;ARFIP1;ARIH1;ASB1;ATAD2B;ATF7;ATP7A;B3GNT5;BCL7C;BMP2K;BTF3;C10orf26;C12orf48;C12orf57;C14orf109;C15orf24;C15orf38;C1orf97;C20orf94;C2orf84;C5orf41;C5orf43;C6orf226;C6orf70;C7orf11;C9orf5;C9orf64;CALCR;CBFB;CBLB;CCDC15;CCDC152;CCDC88B;CD274;CDC16;CDC26;CDKL3;CDNF;CETN3;CFLAR;CHUK;CIRBP;CITED4;CLEC11A;CLEC18B;CNIH;COMMD6;COX16;CSGALNACT2;CSTF1;CWC15;CWF19L2;CXXC4;CYB5D2;CYBA;DDX50;DDX6;DERL2;DGUOK;DLEC1;DNAJC10;DUSP18;EFC4A;EFHC1;ERLIN2;EXOSC3;FAM123B;FAM173B;FAM177A1;FAM46A;FBXO15;FBXO36;FCGBP;FIP1L1;FTSJD1;GALNT10;GJB6;GMNN;GTF2A2;GTF2F2;HAUS6;HDAC1;HFM1;HHEX;HMGB2;HMGN4;HSPA5;HSPBAP1;IFT20;IGF2BP2;IL7R;IMPACT;INPP5D;INTS2;INTS4;IRF9;ISYNA1;KIAA1530;KLRK1;LAX1;LEPROT;LIFR;LIMS1;LOC100131564;LOC100272217;LOC389705;LOC728407;LOC729852;LPP;LRP10;LRRC70;LSM1;LSM14A;LSM5;LST1;MAEA;MAGEH1;MAK16;MBNL3;MDK;MED28;METTL12;MGAT2;MRPL20;MRPL51;MRPS26;MRPS31;MUTYH;MYO1F;MYST2;NAA38;NCF2;NEDD1;NEK9;NFATC1;NHP2L1;NOC3L;NOL11;NOL7;NR1H3;NSUN5;NUDT6;NUP54;NXT1;OFD1;OGFOD1;PAPOLA;PARG;PARP3;PATZ1;PBXIP1;PCNP;PDCD10;PFDN5;PITX2;PLCD1;PLRG1;PMFBP1;PNPLA4;POLA1;POLI;POP5;PPAPDC1B;PPP4R1;PRPF40A;PRRX1;PTCH1;R18;RAD17;RAP1B;RAP2C;RBBP8;RBM34;RBM8A;RDBP;REST;RFX4;RND2;RPIA;RPL10;RPL13A;RPS6;RRP15;S100A13;SAMD4A;SCAMP2;SETD4;SH3GLB1;SLAIN2;SLC7A7;SMARCAD1;SNRPF;SOX2;SP140L;SPR;SRP72;SRRM1;SSBP1;STAG1;STAT5B;STT3A;SYF2;TAF1A;TAF7;TDRD3;TFAM;TGDS;TGFB1;TGIF2;THAP2;THAP6;TIGD6;TMCO1;TMED10;TMEM138;TMEM199;TMEM20;TMEM216;TMEM222;TOR1B;TPRKB;TRAF3IP2;TRAPPC2P1;TRAPPC4;TRIM38;TRIM39;TRMT112;TSEN34;TSG101;TTC14;TTC30B;TTF1;TXNDC5;TYMP;UBR1;UFD1L;USP16;USP24;UTP18;WDR61;XIAP;YES1;ZFX;ZKSCAN2;ZNF197;ZNF285;ZNF394;ZNF512;ZNF518A;ZNF562;ZNF660;ZNF671;ZNF701"
"cisbp__M5416"	3.15	0.0394	"ETS1 (directAnnotation). "	92	"ANKHD1-EIF4EBP3;ANKS3;APPL2;ARFIP1;ATP7A;BCL7C;BMP2K;C10orf26;C12orf57;C15orf24;C5orf43;CBLB;CCDC15;CDC26;CHUK;CLEC11A;COMMD6;CSGALNACT2;CSTF1;CXXC4;DDX50;EXOSC3;FAM173B;FOS;GJB6;HAUS6;IL7R;INTS4;KIAA1530;KLRK1;LAX1;LEPROT;LIMS1;LOC100131564;LOC100272217;LOC728407;LOC729852;LRRC70;LSM5;LST1;MAGEH1;MRPL51;MUTYH;MYO1F;MYST2;NAA38;NCF2;NEDD1;NOC3L;NR1H3;NUP54;PARG;PATZ1;PCNP;PLRG1;PNPLA4;POP5;RAP2C;RBBP8;RBM34;RDBP;REST;RPIA;RPS6;SAMD4A;SCAMP2;SKIL;SLAIN2;SLC7A7;SSBP1;STT3A;TAF1A;TAF7;TFAM;TGIF2;THAP6;TIGD6;TMED10;TMEM138;TMEM199;TOR1B;TPRKB;TRAPPC4;TRMT112;TSG101;TTC30B;TTF1;TXNDC5;UBR1;YES1;ZNF197;ZNF394"
"cisbp__M5380"	3.14	0.0394	"ELK1 (directAnnotation). "	204	"ADAL;ADH5;AEBP1;ANKHD1-EIF4EBP3;ANKS3;APPL2;ARF4;ARFIP1;ARIH1;ASB1;ASCC1;ATF7;ATP7A;B3GNT5;BCL7C;BMP2K;BTF3;C10orf26;C12orf48;C12orf57;C14orf109;C14orf135;C15orf24;C1orf112;C1orf212;C1orf97;C21orf34;C5orf43;C6orf226;C6orf70;C7orf11;C9orf64;CARD8;CBFB;CBLB;CCDC15;CCDC152;CDC16;CDC26;CDK4;CDKL3;CETN3;CFLAR;CHUK;CLEC11A;CNIH;COMMD6;COX16;CSGALNACT2;CSTF1;CUL4A;CWF19L2;CXXC4;CYB5D2;DCLRE1B;DDX50;DDX6;DLEC1;DNAJC10;DUSP18;EFC4A;EFHC1;ERLIN2;EXOSC3;FAM123B;FAM173B;FAM177A1;FAM46A;FBXO15;FBXO36;FCGBP;FOS;FTSJD1;GALNT10;GJB6;GMNN;GTF2A2;HAUS6;IFT20;IL7R;INTS2;INTS4;KIAA1530;KLRK1;LARP7;LAX1;LEPROT;LIMS1;LOC100131564;LOC100272217;LOC389705;LOC728407;LOC729852;LRP10;LRRC70;LSM5;LST1;MAGEH1;MAK16;METTL12;MGAT2;MPL;MRPL20;MRPL51;MRPS31;MSH5;MUTYH;MYO1F;MYST2;NAA38;NCF2;NEDD1;NHP2L1;NOC3L;NOL11;NOL7;NR1H3;NSUN5;NUDT6;NUP54;NXT1;OGFOD1;PARG;PARP3;PATZ1;PCNP;PDCD10;PLCD1;PLRG1;PNPLA4;PODNL1;POLA1;POP5;PRPF40A;R18;RAD21;RAP1B;RAP2C;RBBP8;RBM34;RBM8A;RDBP;REST;RFX4;RND2;RPIA;RPL10;RPL13A;RPS6;RRP15;S100A13;SAMD4A;SCAMP2;SCARNA17;SKIL;SLAIN2;SLC7A7;SNRPF;SPR;SSBP1;STAG1;STT3A;SYF2;TAF1A;TAF7;TFAM;TGDS;TGIF2;THAP6;TIGD6;TMCO1;TMED10;TMEM138;TMEM199;TMEM222;TOR1B;TPRKB;TRAPPC2P1;TRAPPC4;TRIM38;TRIM39;TRMT112;TSEN34;TSG101;TTC14;TTC30B;TTF1;TXNDC5;TYMP;UBR1;UFD1L;USP16;USP24;UTP18;WDR61;XIAP;YES1;ZKSCAN2;ZNF197;ZNF394;ZNF512;ZNF518A;ZNF529;ZNF671"
"cisbp__M1817"	3.14	0.0394	""	129	"ADAL;ADH5;ADPRH;AEBP1;ANP32E;APPL2;ARF4;ARFIP1;ARID4B;ARIH1;ATP7A;BAG3;BCAR3;BMP2K;BOD1;BPTF;C12orf57;C14orf109;C15orf24;C1orf112;C6orf145;C6orf226;C7orf11;CCDC15;CDC16;CDC73;CDKL3;CDNF;CETN3;CHUK;CLEC11A;COX16;CRY1;CYB5D2;DDX50;DNAJC10;DYNLT1;EIF5B;ERLIN2;FAM173B;FAM46A;FNDC3A;GPN3;HAUS6;HEPH;IGFBP6;INTS4;KIAA1530;LARP7;LEPROT;LOC100131564;LOC389705;LOC728407;LOC729852;LRP10;LRRC37A3;LSM5;MAK16;MRPL50;MRPL51;MRPS31;MYST2;NAA38;NEDD1;NFATC1;NKX2-1;NOC3L;NR1H3;NXT1;PARG;PBLD;PDCD10;PITX2;PLRG1;PNPLA4;PRDM13;PRRX1;PTCH1;R18;RBBP8;RBM34;REST;RPIA;RPL13A;RPS27L;RPS6;RRP15;RSPRY1;SAMD4A;SCAMP2;SIM1;SLAIN2;SLC25A1;SMARCAD1;SMEK1;SNAPC1;SSBP1;STAG1;STAT5B;STT3A;SUGT1P1;SYF2;TAF1A;TAF7;TFAM;TGDS;TGIF2;THAP2;THEM4;TMED10;TMEM138;TMEM222;TPRKB;TRAF3IP1;TRAPPC4;TRIM52;TRIP4;TRMT112;TSEN34;TTC14;TTC30B;TXNDC5;TYMP;UBR1;USP24;UTP18;WDR61;YES1;ZFP90"

"motif"	"NES"	"AUC"	"TF_highConf"	"nEnrGenes"	"enrichedGenes"
"cisbp__M1760"	3.14	0.0394	""	201	"ACO1;ADH5;ALG10;ALG14;ANAPC10;ANP32E;APH1B;ARF4;ATAD2B;ATF1;ATP10D;BAG3;BAT1;BBS9;BCAR3;BCAS2;BMP2K;BMPR1A;BOD1;BTF3;C11orf71;C12orf48;C12orf57;C14orf109;C14orf135;C1orf112;C1orf9;C20orf132;C20orf94;C7orf11;CALCB;CBX5;CCDC15;CD274;CDK4;CDNF;CETN3;CIRBP;CLEC11A;CLK1;CNIH;COX16;CREBL2;CSTF1;CTXN2;CWC15;CXCL12;CXXC4;CYB5D2;DBR1;DDX50;DERL2;DKFZP434I0714;DRAM2;DUSP18;DUT;EFC2;EFHC1;EIF5B;ERCC4;ERLIN2;FAIM;FAM123B;FAM46A;FBXO15;FSD1L;FZD5;G3BP1;GKAP1;GLG1;GPN3;HAS3;HAUS6;HHEX;HMGB2;HSPA5;HSPBAP1;IL28RA;INTS2;INTS4;ISCU;ISYNA1;KIF27;KLHDC10;KLHDC9;LARP7;LCA5;LIFR;LMAN1;LOC100272217;LOC389765;LOC728407;LRP10;LSM14A;LSM5;LSM6;MAEA;MBNL3;METTL12;MRPL51;MUTYH;MYST2;NDUFC1;NEDD1;NEURL2;NHLRC2;NOC3L;NOVA1;NR1H3;NT5C2;NUDT6;PARG;PARP3;PAWR;PBLD;PCGF5;PDCD10;PDIK1L;PHIP;PIF1;PIGV;PLCD1;PLK3;PLRG1;PNPLA4;POP5;POU3F2;PPA1;PPAPDC1B;PPP4R1;PRDM13;PRDM5;PRPF40A;PRRX1;PTCH1;PTOV1;R18;RAD17;RAN;RAP1B;RBBP8;RBM8A;RCL1;REST;ROCK1;RPIA;RPL10;RPL13A;RPL7;RRM1;RRP15;S100A13;SAMD4A;SDF2L1;SEC11C;SENP6;SIM1;SLAIN2;SLC25A1;SLC26A2;SMAD5;SMARCA5;SMARCAD1;SMEK1;SOAT1;SOX2;SRP72;SRRM1;SRSF3;SSBP1;STAG1;STT3A;TDRD3;TGDS;TGIF2;THAP2;THAP9;TIGD6;TMCO1;TMED10;TMEM138;TMEM222;TP53INP1;TRIM39;TRIP4;TRMT112;TSEN34;TSPAN4;TWF1;TYMP;UFD1L;USP16;USP24;UTP18;VAT1L;WRB;YES1;ZFC3H1;ZFX;ZKSCAN2;ZZZ3"
"cisbp__M5399"	3.14	0.0394	"ERG (directAnnotation). "	85	"ANKHD1-EIF4EBP3;ANKS3;APPL2;ARFIP1;ATP7A;BCL7C;BMP2K;C12orf57;C5orf43;CBLB;CCDC15;CDC26;CHUK;CLEC11A;COMMD6;CSGALNACT2;CXXC4;DCLRE1B;DDX50;FAM173B;FOS;GJB6;HAUS6;IL7R;LAX1;LEPROT;LIMS1;LOC100131564;LOC100272217;LOC728407;LOC729852;LRRC70;LSM5;LST1;MAGEH1;MAK16;MRPL51;MUTYH;MYO1F;MYST2;NAA38;NEDD1;NOC3L;NR1H3;NUP54;PARG;PATZ1;PCNP;PLRG1;PNPLA4;POP5;RAP2C;RBBP8;RBM34;RDBP;REST;RPIA;RPS6;SAMD4A;SKIL;SLAIN2;SSBP1;STT3A;TAF1A;TAF7;TFAM;TGIF2;THAP6;TIGD6;TMED10;TMEM138;TMEM199;TOR1B;TPRKB;TRAPPC4;TRMT112;TSG101;TTC30B;TTF1;TXNDC5;UBR1;XIAP;YES1;ZNF197;ZNF394"
"cisbp__M5572"	3.14	0.0394	"IRF3 (directAnnotation). "	26	"ARID4B;BTN3A1;C6orf145;CD274;CDKL3;GNA13;HERC5;IFI44;IFI6;IFIT3;IGFBP6;IL15;MDK;MX1;PARP14;PRICKLE4;RARRES3;RTP4;SAMD9L;SP140L;SSBP1;TFAM;TRIM34;TTC30B;XIAP;ZNF583"
"cisbp__M6252"	3.13	0.0393	"GPA (directAnnotation). "	159	"ADAL;ANKHD1-EIF4EBP3;ANKS3;APPL2;ARFIP1;ASB1;ATF7;ATP7A;BCL7C;BMP2K;BTF3;C10orf26;C12orf48;C12orf57;C14orf109;C15orf24;C1orf97;C5orf41;C5orf43;C6orf226;C9orf64;CALCR;CBFB;CBLB;CCDC15;CCDC152;CCDC88B;CDC16;CDC26;CDKL3;CETN3;CHUK;COMMD6;CSGALNACT2;CSTF1;CWF19L2;CYB5D2;DDX50;DGUOK;DLEC1;DNAJC10;DUSP18;EFC4A;EFHC1;ERLIN2;EXOSC3;FAM173B;FAM177A1;FAM46A;FBXO15;FBXO36;FIP1L1;GALNT10;GJB6;GTF2A2;HAUS6;IL7R;INTS2;INTS4;ISYNA1;KIAA1530;KLRK1;LAX1;LEPROT;LIMS1;LOC100131564;LOC100272217;LOC389705;LOC728407;LOC729852;LRP10;LRRC70;LSM5;LST1;MAEA;MAGEH1;MAK16;METTL12;MGAT2;MRPL51;MRPS26;MUTYH;MYO1F;MYST2;NCF2;NEDD1;NHP2L1;NOC3L;NR1H3;NSUN5;NUP54;OGFOD1;PARG;PARP3;PATZ1;PCNP;PDCD10;PFDN5;PITX2;PLRG1;PMFBP1;PNPLA4;POLA1;PRPF40A;RAP1B;RAP2C;RBBP8;RBM34;RDBP;REST;RND2;RPIA;RPL10;RPL13A;RPS6;SAMD4A;SCAMP2;SKIL;SOX2;SP140L;SPR;SRP72;SRRM1;SSBP1;STAT5B;STT3A;SYF2;TAF1A;TAF7;TDRD3;TFAM;TGFB1;TGIF2;TIGD6;TMED10;TMEM138;TMEM199;TMEM222;TOR1B;TPRKB;TRAPPC4;TRMT112;TSEN34;TSG101;TTC14;TTC30B;TTF1;TXNDC5;UBR1;UFD1L;UTP18;XIAP;YES1;ZNF197;ZNF394;ZNF512;ZNF518A;ZNF671;ZNF701"
"cisbp__M2096"	3.12	0.0393	""	248	"ACTL6A;ALG14;ALOX5;ANAPC10;ANP32E;ARFIP1;ARIH1;ASB1;ATAD2B;BCAR3;BOD1;BPTF;BTBD7;BTF3P11;C10orf26;C11orf71;C14orf135;C17orf97;C19orf42;C1orf112;C1orf63;C1orf9;C1orf97;C20orf72;C20orf94;C21orf34;C3orf38;C4orf29;C6orf145;C8orf45;CALCR;CBX5;CCDC66;CCDC90B;CD274;CDC26;CDC73;CDNF;CENPE;CFLAR;CKLF;CLK1;CNIH;COMMD6;COPG2;CREBL2;CRISPLD2;CRYL1;CSTF1;CTXN2;CWF19L2;CXCL12;CXXC4;DDAH2;DDX3Y;DERL2;DKK1;DPH5;DYNLT1;EFC2;ERCC4;ERLIN2;FAIM;FAM164C;FAM46A;FAM70A;FAM76A;FBXO15;FGGY;FIP1L1;FITM2;FOS;FSTL5;FZD5;GGNBP2;GLB1;GPN3;GSTCD;GTF2F2;H2AFY;HAUS6;HELLS;HERC5;HEY2;HIST1H2AG;HIST1H2AI;HIST1H2BJ;HIST1H3G;HIST4H4;HMGN4;HSPA13;HSPA5;HYI;IL12A;INTS2;INTS4;ISYNA1;ITGB3BP;KIAA1407;KIAA1530;KLHDC1;KLHL1;KLHL21;LARP1B;LARP7;LEPREL1;LEPROT;LIFR;LIG1;LOC728407;LOC729852;LSM5;LSM6;MAK16;MAOB;MBNL3;ME2;MED28;METAP2;METTL12;MFSD1;MORC4;MPP5;MRPL13;MRPS31;MUTYH;MYST2;NAA38;NDUFC1;NEK1;NHLRC2;NID2;NKAP;NKAPP1;NOC3L;NOL11;NOL7;NOVA1;NPHP3;NPM1;NR1H3;NT5C2;PAPOLA;PARG;PATZ1;PAWR;PCSK1N;PDIK1L;PHKG1;PIGV;PITX2;PLK3;POLA1;POLD3;POP5;PPA1;PRDX6;PRMT2;PRPF40A;PTCH1;PTGR2;PUS3;R18;RAD17;RAD21;RAP1B;RBBP8;RBM8A;RCL1;REST;RFX4;RHBDD1;RND2;ROCK1;RPAP3;RPIA;RPL10;RPL13A;RPL27A;RPL5;RPL7;RPP38;RPS27L;SALL4;SBDS;SEC11C;SENP8;SFXN4;SLAIN2;SLU7;SMAD5;SMARCA5;SMARCAD1;SMEK1;SNAPC1;SNRPF;SRRM1;SRSF3;SSBP1;SSPN;STAG1;STAT5B;STYX;SUPT16H;TAF7;TDRD3;TFPI2;TGDS;TGIF2;THAP9;TIGD6;TMCO1;TMED4;TMEM138;TMEM164;TMEM62;TMEM9;TNPO1;TRAF3IP1;TRIM39;TRIM52;TRIP4;TSEN34;TSPAN4;TTF1;TTLL3;TUBB2B;TWF1;TWISTNB;TYMP;UBXN2A;USP16;USP51;VAT1;WBP5;WRB;XIAP;ZCCHC11;ZCCHC3;ZFP90;ZFX;ZMYND12;ZNF345;ZNF394;ZNF471;ZNF583;ZNF610;ZNF701"

"motif"	"NES"	"AUC"	"TF_highConf"	"nEnrGenes"	"enrichedGenes"
"cisbp__M0710"	3.12	0.0393	""	236	"ADAL;ADH5;AEBP1;ANKHD1-EIF4EBP3;ANKS3;APPL2;ARF4;ARFIP1;ARID4B;ARIH1;ASB1;ASCC1;ATF7;ATP7A;BCL7C;BMP2K;BOD1;BTF3;C10orf26;C12orf48;C12orf57;C14orf109;C14orf135;C15orf24;C1orf112;C1orf212;C1orf9;C1orf97;C21orf34;C5orf43;C6orf226;C6orf70;C7orf11;C9orf64;CARD8;CBFB;CBLB;CCDC15;CCDC152;CCDC88B;CD274;CDC16;CDC25A;CDC26;CDKL3;CDNF;CETN3;CFLAR;CGA;CHUK;CLEC11A;CNIH;COMMD6;COX16;CREBL2;CRY1;CSGALNACT2;CSTF1;CWF19L2;CXXC4;CYB5D2;DCLRE1B;DDX50;DDX6;DERL2;DKFZP434I0714;DLEC1;DNAJC10;DUSP18;EFC4A;EFHC1;EXOSC3;FAM173B;FAM177A1;FAM46A;FBXO15;FBXO36;FCGBP;FOS;FTSJD1;GALNT10;GJB6;GMNN;GTF2A2;GTF2F2;HAUS6;HMGB2;HMGN4;HSPA5;IFT20;IL11;IL7R;INPP5D;INTS2;INTS4;ISYNA1;KIAA1530;KLRK1;LARP7;LAX1;LEPROT;LIFR;LIMS1;LOC100131564;LOC100272217;LOC389705;LOC728407;LOC729852;LPP;LRP10;LRRC70;LSM5;LST1;MAEA;MAGEH1;MAK16;METTL12;MGAT2;MPL;MRPL50;MRPL51;MRPS31;MSH5;MUTYH;MYO1F;MYST2;NAA38;NCF2;NEDD1;NEK9;NFATC1;NFE2;NHP2L1;NOC3L;NOL11;NOL7;NR1H3;NSUN5;NUDT6;NUP54;NXT1;OGFOD1;PARG;PARP3;PATZ1;PBXIP1;PCNP;PDCD10;PLCD1;PLRG1;PNPLA4;POLA1;POP5;PPAPDC1B;PRPF18;PSME2;PTCH1;R18;RAD21;RAP1B;RAP2C;RBBP8;RBM34;RBM8A;RDBP;REST;RFX4;RND2;RPIA;RPL10;RPL13A;RPS6;RRP15;RSPRY1;S100A13;SALL4;SAMD4A;SCAMP2;SKIL;SLAIN2;SLC7A7;SPR;SSBP1;STAG1;STAT5B;STT3A;SYF2;TAF1A;TAF7;TDRD3;TFAM;TGDS;TGIF2;TIGD6;TMCO1;TMED10;TMEM138;TMEM199;TMEM222;TOR1B;TPRKB;TRAF3IP2;TRAPPC2P1;TRAPPC4;TRIM39;TRIP4;TRMT112;TSEN34;TSG101;TSPAN4;TTC14;TTC30B;TTF1;TXNDC5;TYMP;UBR1;UFD1L;USP16;USP24;UTP18;WDR61;XIAP;YES1;ZFX;ZKSCAN2;ZNF197;ZNF285;ZNF394;ZNF512;ZNF518A;ZNF529;ZNF583;ZNF671;ZNF701;ZZZ3"
"cisbp__M5433"	3.11	0.0392	"FLI1 (directAnnotation). "	126	"ADAL;ADH5;AEBP1;ANKHD1-EIF4EBP3;ANKS3;APPL2;ARFIP1;ASB1;ATP7A;BCL7C;BMP2K;C10orf26;C12orf57;C14orf109;C15orf24;C1orf112;C1orf97;C5orf43;C6orf226;C9orf64;CBLB;CCDC15;CDC26;CDKL3;CHUK;CLEC11A;CNIH;COMMD6;CSGALNACT2;CSTF1;CXXC4;CYB5D2;DCLRE1B;DDX50;DNAJC10;EXOSC3;FAM173B;FAM46A;FOS;GJB6;GTF2A2;HAUS6;IL7R;INTS4;KIAA1530;KLRK1;LAX1;LEPROT;LIMS1;LOC100131564;LOC100272217;LOC728407;LOC729852;LRP10;LRRC70;LSM5;LST1;MAGEH1;MAK16;METTL12;MRPL51;MUTYH;MYO1F;MYST2;NAA38;NCF2;NEDD1;NOC3L;NR1H3;NSUN5;NUP54;PARG;PARP3;PATZ1;PCNP;PDCD10;PLCD1;PLRG1;PNPLA4;POP5;RAP2C;RBBP8;RBM34;RBM8A;RDBP;REST;RPIA;RPL10;RPS6;SAMD4A;SCAMP2;SKIL;SLAIN2;SLC7A7;SMAD5OS;SSBP1;STT3A;SYF2;TAF1A;TAF7;TFAM;TGDS;TGIF2;THAP6;TIGD6;TMCO1;TMED10;TMEM138;TMEM199;TOR1B;TPRKB;TRAPPC4;TRMT112;TSEN34;TSG101;TTC14;TTC30B;TTF1;TXNDC5;UBR1;USP16;WDR61;XIAP;YES1;ZNF197;ZNF394"
"cisbp__M0703"	3.08	0.039	"ELK4 (inferredBy_Orthology). "	183	"ADAL;AEBP1;ANKHD1-EIF4EBP3;ANKS3;APPL2;ARF4;ARFIP1;ARIH1;ASB1;ATF7;ATP7A;BCL7C;BMP2K;BOD1;BTF3;C10orf26;C12orf57;C14orf109;C14orf135;C15orf24;C1orf112;C1orf9;C1orf97;C21orf34;C5orf43;C6orf226;C6orf70;C7orf11;C9orf64;CBLB;CCDC15;CCDC88B;CD274;CDC16;CDC25A;CDC26;CDKL3;CDNF;CETN3;CFLAR;CHUK;CLEC11A;CNIH;COMMD6;COX16;CRY1;CSGALNACT2;CSTF1;CWF19L2;CXXC4;CYB5D2;DCLRE1B;DDX50;DKFZP434I0714;DLEC1;DNAJC10;EFC4A;EFHC1;EXOSC3;FAM173B;FBXO36;FOS;GJB6;GTF2A2;HAUS6;HMGB2;HMGN4;HSPA5;IL11;INTS2;INTS4;KIAA1530;KLRK1;LARP7;LAX1;LEPROT;LIMS1;LOC100131564;LOC100272217;LOC389705;LOC728407;LOC729852;LRP10;LRRC70;LSM5;LST1;MAEA;MAGEH1;MAK16;METTL12;MRPL50;MRPL51;MRPS31;MSH5;MUTYH;MYO1F;MYST2;NAA38;NCF2;NEDD1;NFATC1;NFE2;NOC3L;NOL7;NR1H3;NUDT6;NUP54;NXT1;PARG;PARP3;PATZ1;PCNP;PDCD10;PLRG1;PNPLA4;POLA1;POP5;PRRX1;PSME2;PTCH1;R18;RAP1B;RAP2C;RBBP8;RBM34;RBM8A;RDBP;REST;RPIA;RPL10;RPL13A;RPS6;RRP15;RSPRY1;S100A13;SAMD4A;SCAMP2;SKIL;SLAIN2;SPR;SSBP1;STAG1;STT3A;SYF2;TAF1A;TAF7;TDRD3;TFAM;TGDS;TGIF2;THAP2;TIGD6;TMCO1;TMED10;TMEM138;TMEM199;TMEM222;TOR1B;TPRKB;TRAPPC4;TRIM39;TRMT112;TSEN34;TSG101;TTC14;TTC30B;TTF1;TXNDC5;UBR1;UFD1L;USP16;USP24;UTP18;WDR61;XIAP;YES1;ZNF197;ZNF394;ZNF512;ZNF518A;ZNF529;ZNF671;ZZZ3"
"cisbp__M4505"	3.08	0.039	"GPA (directAnnotation). "	216	"ACO1;ADAL;ADH5;ANKHD1-EIF4EBP3;ANKS3;APPL2;ARF4;ARFIP1;ARIH1;ASB1;ASCC1;ATP7A;B3GNT5;BCL7C;BMP2K;BTF3;C10orf26;C12orf48;C12orf57;C14orf109;C14orf135;C15orf24;C15orf38;C1orf26;C1orf97;C21orf34;C2orf84;C5orf41;C5orf43;C6orf226;C7orf11;C9orf64;CALCR;CBFB;CBLB;CCDC15;CCDC152;CCDC88B;CD274;CDC16;CDC26;CDKL3;CETN3;CFLAR;CHUK;CIRBP;CLEC11A;CLEC18B;CNIH;COMMD6;COX16;CSGALNACT2;CSTF1;CWF19L2;CXXC4;CYB5D2;DDX50;DGUOK;DLEC1;DNAJC10;DUSP18;EFC4A;EFHC1;ERLIN2;EXOSC3;FAM173B;FAM177A1;FAM46A;FBXO15;FBXO36;FCGBP;FIP1L1;FOS;FTSJD1;GALNT10;GJB6;GMNN;GTF2A2;GTF2F2;HAUS6;HMGN4;HSPA5;IFT20;IL7R;IMPACT;INTS2;INTS4;ISYNA1;KIAA1530;KLRK1;LAX1;LEPROT;LIFR;LIMS1;LOC100131564;LOC100272217;LOC389705;LOC728407;LOC729852;LPP;LRP10;LRRC70;LSM5;LST1;MAGEH1;MAK16;METTL12;MGAT2;MRPL20;MRPL51;MRPS26;MUTYH;MYO1F;MYST2;NAA38;NCF2;NEDD1;NFATC1;NHP2L1;NOC3L;NOL7;NR1H3;NSUN5;NUDT6;NUP54;NXT1;OGFOD1;PAPOLA;PARG;PARP3;PATZ1;PCNP;PDCD10;PFDN5;PITX2;PLCD1;PLRG1;PMFBP1;PNPLA4;POLA1;POLI;PPAPDC1B;PPP4R1;PRPF40A;R18;RAP1B;RAP2C;RBBP8;RBM34;RBM8A;RDBP;REST;RND2;RPIA;RPL10;RPL13A;RPS6;RRP15;S100A13;SAMD4A;SCAMP2;SETD4;SKIL;SLAIN2;SLC7A7;SOX2;SP140L;SPR;SRP72;SRRM1;SSBP1;STAG1;STAT5B;STT3A;SYF2;TAF1A;TAF7;TDRD3;TFAM;TGDS;TGFB1;TGIF2;THAP2;TIGD6;TMCO1;TMED10;TMEM138;TMEM199;TMEM222;TOR1B;TPRKB;TRAPPC4;TRMT112;TSEN34;TSG101;TTC14;TTC30B;TTF1;TXNDC5;TYMP;UBR1;UFD1L;USP16;USP24;UTP18;WDR61;XIAP;YES1;ZNF197;ZNF285;ZNF394;ZNF512;ZNF518A;ZNF529;ZNF660;ZNF671"

"motif"	"NES"	"AUC"	"TF_highConf"	"nEnrGenes"	"enrichedGenes"
"cisbp__M5375"	3.08	0.039	"ELF3 (directAnnotation). "	252	"ADAL;ADAP2;ADH5;AIRE;ANKHD1-EIF4EBP3;ANKS3;APPL2;ARF4;ARFIP1;ARIH1;AS3MT;ASB1;ASPH;ATP7A;B3GNT5;BMP2K;BTF3;C10orf26;C12orf48;C12orf57;C15orf24;C17orf97;C1orf63;C1orf9;C20orf94;C2orf84;C5orf41;C5orf43;C6orf226;C6orf70;C8orf59;C9orf64;CBFB;CBLB;CCDC15;CCDC152;CCDC88B;CCDC90B;CD274;CD74;CDC26;CDKL3;CFLAR;CHUK;CIRBP;CITED4;CLEC18B;CLK1;CNIH;COMMD6;CREBL2;CRYL1;CSTF1;CWC15;CWF19L2;CYB5D2;DDX50;DERL2;DLEC1;DNAJC10;EFC4A;EFHC1;EIF5B;ERAP1;ERLIN2;EXOSC3;FAM173B;FBXO15;FBXO36;FCGBP;FSTL5;GJB6;GTF2A2;GTF2F2;HAS3;HAUS6;HEATR5A;HEPH;HIST1H3B;HIST4H4;HLA-A;HSPA13;HSPA5;HSPBAP1;IL18;IL28RA;IL7R;IMPACT;INPP5D;INTS2;INTS4;IQCK;IRF9;ISYNA1;ITGB3BP;KCTD3;KLRK1;LARP7;LCN12;LEPROT;LIG1;LILRB1;LIMS1;LOC100131564;LOC100272217;LOC389705;LOC728407;LOC729852;LPP;LSM5;LST1;MAEA;MAGEH1;MAK16;MAP7D3;METAP2;METTL12;MGAT2;MLH3;MPP5;MRPL20;MRPL50;MRPL51;MRPS26;MS4A6E;MSH5;MUTYH;MX1;MYST2;NCF2;NEDD1;NFATC1;NHP2L1;NOC3L;NOL11;NR1H3;NSUN5;NUP54;NXT1;PARG;PARN;PARP14;PATZ1;PCNP;PDCD10;PDLIM5;PFDN5;PIGV;PITX2;PLRG1;PMFBP1;PNPLA4;PNPO;POLA1;POLD3;POT1;PQLC3;PRDM13;PRKY;R18;RAD17;RAD21;RAP2C;RBBP8;RBM34;RBM8A;RDBP;REST;RFX4;RIMBP3;RND2;RPL10;RPL13A;RPL7;RPS6;RTDR1;S100A13;SAMD4A;SAMD9L;SCAMP2;SETD4;SKIL;SLC25A1;SLC25A20;SLC7A7;SMARCAD1;SP140L;SPR;SRP72;SRRM1;SRSF3;SSBP1;SSPN;SSR1;SSR4;STAT5B;STT3A;SUGT1P1;SYF2;SYTL4;TAF1A;TAF7;TDRD3;TGDS;THBS4;THNSL2;TIGD6;TLR3;TMCO1;TMED10;TMEM138;TMEM176A;TMEM222;TPRKB;TRAF3IP1;TRAPPC4;TRIM38;TRIM39;TRIM42;TRIP4;TRMT112;TSG101;TTC14;TTC30B;TTF1;TTLL3;TWF1;TYMP;UBR1;UFD1L;UMPS;USP16;USP24;UTP18;VMO1;XIAP;ZFP90;ZFX;ZNF197;ZNF285;ZNF331;ZNF394;ZNF512;ZNF518A;ZNF529;ZNF610;ZNF660;ZNF671;ZNF691;ZNF701;ZNF765;ZNF92"
"cisbp__M3049"	3.07	0.039	"ETS1 (directAnnotation). "	60	"ANKHD1-EIF4EBP3;ANKS3;APPL2;ARFIP1;ATP7A;B3GNT5;BCL7C;BMP2K;CBLB;CDC26;CHUK;CSGALNACT2;CXXC4;DDX50;FOS;GJB6;IL11;IL7R;INTS4;LAX1;LEPROT;LIMS1;LOC100131564;LOC100272217;LOC729852;LRP10;LRRC70;LST1;MAK16;MPL;MRPL51;MYO1F;MYST2;NAA38;NEDD1;NOC3L;NR1H3;PARG;RAP2C;RPS6;SKIL;SLAIN2;SSBP1;TAF1A;TAF7;TFAM;TGIF2;TIGD6;TMED10;TMEM138;TMEM199;TPRKB;TRAPPC4;TRMT112;TTC30B;TXNDC5;UBR1;WDR61;YES1;ZNF197"
"cisbp__M5154"	3.07	0.039	"YY1; YY2 (inferredBy_Orthology). "	107	"ALG10;ANAPC10;ANKHD1-EIF4EBP3;ARFGAP3;ARID4B;ASPH;ATF1;BAG3;BPTF;BTF3P11;C10orf26;C11orf54;C11orf71;C14orf135;C1orf131;C20orf94;C21orf34;C5orf43;C9orf64;CARD8;CASP10;CBLB;CBX5;CFLAR;CKLF;CLK1;CLNS1A;CREBL2;CST6;CSTF1;CTXN2;DDX3Y;DNAJC10;DUT;DYRK4;EFC2;FAM76A;FBXO22;FPGS;GLG1;GNG8;HMGB2;HSPA5;IL7R;KDELC2;KIAA1430;KIF11;KLHL1;LCA5;LIMS1;LIX1;MDM1;MESDC2;MRPL51;MUTED;MYST2;NDUFC1;NHP2L1;NKX2-1;NPM1;NR1H3;NRARP;PATZ1;PCSK1N;PHIP;PHLDA2;PIGV;PMFBP1;PNPLA4;PRMT2;PSMB1;RBM8A;RCL1;RPL10;RPL13A;RPN1;RPS6;RRM1;RTDR1;SAMD4A;SERPINB6;SETD4;SLAIN2;SLC16A8;SPRYD4;SRP72;STAG1;THAP9;TMCO1;TMED1;TMED10;TOR1B;TRIP6;UBR1;UBXN2A;UFD1L;UNC119B;USP51;VAT1L;XIAP;YES1;ZFC3H1;ZFP36L2;ZFP90;ZNF187;ZNF518A;ZZZ3"
"cisbp__M5860"	3.07	0.039	"SPDEF (directAnnotation). "	172	"ADAL;ANAPC10;ANKHD1-EIF4EBP3;ANKS3;APPL2;ARF4;ARFIP1;ARID4B;ARIH1;ATP7A;B3GNT5;BCAR3;BMP2K;C10orf26;C12orf48;C12orf57;C14orf109;C15orf24;C15orf38;C1orf212;C1orf63;C20orf94;C2orf84;C6orf226;C6orf70;C6orf72;C7orf11;C9orf64;CARD8;CBLB;CCDC15;CCDC90B;CFLAR;CGA;CHUK;CIRBP;CNIH;COMMD6;CRY1;CSGALNACT2;CSTF1;CWF19L2;CYB5D2;DDX50;DERL2;DNAJC10;EFC4A;EFHC1;ERCC4;FAIM;FAM173B;FAM46A;FBXO36;FCGBP;FOS;FPGS;GNG8;GTF2A2;GTF2F2;HAUS6;HEPH;HES1;HIBCH;HSPA13;IL11;IL18;IL28RA;IL7R;IMPACT;INTS4;ITGB3;ITGB3BP;KIAA1683;KLRK1;LEPROT;LIMS1;LOC100131564;LOC100272217;LOC389705;LOC728407;LOC729852;LPP;LRP10;LST1;MPP5;MRPL51;MRPS26;MSH5;MYF6;MYST2;NEDD1;NFATC1;NOC3L;NOL11;NOL7;NR1H3;NUP205;NUP54;NXT1;PARG;PARN;PATZ1;PCNP;PDCD10;PIGV;PITX2;PLRG1;PNPLA4;POP5;PPAPDC1B;PROCA1;PTCH1;RAP2C;RBBP8;RBM34;RBM8A;RDBP;REST;RFX4;RIMBP3;RPIA;RPL10;RPS6;RSPRY1;RTDR1;SALL4;SAMD4A;SCAMP2;SETD4;SKIL;SLAIN2;SLC25A1;SMEK1;SNAPC1;SP140L;SRP72;SSBP1;STAT5B;STK32B;TAF1A;TAF7;TFAM;TGDS;TMCO1;TMED10;TMEM138;TMEM199;TMEM222;TNFSF11;TPRKB;TRAPPC4;TRIM42;TRIP4;TRMT112;TSG101;TSPAN4;TTC14;TTC30B;TTF1;TXNDC5;UBR1;USP24;UTP18;VMO1;XIAP;YES1;ZNF197;ZNF394;ZNF512;ZNF518A;ZNF529;ZNF701"
"cisbp__M0692"	3.05	0.0389	"ELK3 (inferredBy_Orthology). "	163	"ADAL;AEBP1;ANKHD1-EIF4EBP3;ANKS3;APPL2;ARF4;ARFIP1;ARIH1;ASB1;ATP7A;BCL7C;BMP2K;BTF3;C10orf26;C12orf48;C12orf57;C14orf109;C14orf135;C15orf24;C1orf97;C21orf34;C5orf43;C6orf226;C7orf11;C9orf64;CBLB;CCDC15;CCDC88B;CDC16;CDC26;CDKL3;CETN3;CFLAR;CHUK;CLEC11A;COMMD6;COX16;CSGALNACT2;CSTF1;CWF19L2;CXXC4;CYB5D2;DCLRE1B;DDX50;DKFZP434I0714;DLEC1;DNAJC10;DUSP18;EFC4A;EFHC1;EXOSC3;FAM173B;FAM46A;FBXO15;FBXO36;FOS;GJB6;GTF2A2;HAUS6;INTS2;INTS4;KIAA1530;KLRK1;LARP7;LAX1;LEPROT;LIMS1;LOC100131564;LOC100272217;LOC389705;LOC728407;LOC729852;LRRC70;LSM5;LST1;MAGEH1;MAK16;MRPL51;MRPS31;MUTYH;MYO1F;MYST2;NAA38;NCF2;NEDD1;NFATC1;NFE2;NOC3L;NOL7;NR1H3;NUDT6;NUP54;NXT1;PARG;PARP3;PATZ1;PCNP;PDCD10;PLRG1;PNPLA4;POLA1;POP5;PSME2;PTCH1;RAP2C;RBBP8;RBM34;RBM8A;RDBP;REST;RPIA;RPL10;RPL13A;RPS6;RRP15;S100A13;SAMD4A;SCAMP2;SKIL;SLAIN2;SPR;SSBP1;STAG1;STT3A;SYF2;TAF1A;TAF7;TDRD3;TFAM;TGDS;TGIF2;TIGD6;TMCO1;TMED10;TMEM138;TMEM199;TMEM222;TOR1B;TPRKB;TRAPPC4;TRIM39;TRMT112;TSEN34;TSG101;TTC14;TTC30B;TTF1;TXNDC5;UBR1;UFD1L;USP16;USP24;UTP18;WDR61;XIAP;YES1;ZNF197;ZNF394;ZNF512;ZNF518A;ZNF529;ZNF671;ZZZ3"

"motif"	"NES"	"AUC"	"TF_highConf"	"nEnrGenes"	"enrichedGenes"
"cisbp__M6005"	3.03	0.0388	"ELK3 (inferredBy_Orthology). "	283	"ACO1;ADAL;ADH5;AEBP1;AKR7L;ALOX5;ANKHD1-EIF4EBP3;ANKS3;APPL2;ARF4;ARFIP1;ARIH1;ASB1;ASCC1;ATAD2B;ATOX1;ATP7A;B3GNT5;BAG3;BCAR3;BCL7C;BMP2K;BOD1;BRIX1;BTF3;C10orf26;C11orf83;C12orf48;C12orf57;C14orf109;C14orf135;C15orf24;C1orf112;C1orf212;C1orf26;C1orf9;C1orf97;C20orf94;C21orf34;C2orf84;C5orf43;C6orf226;C6orf70;C7orf11;C9orf64;CALCR;CARD8;CBFB;CBLB;CCDC147;CCDC15;CCDC152;CCDC88B;CD274;CDC16;CDC26;CDK4;CDKL3;CETN3;CFLAR;CGA;CHUK;CITED4;CLEC11A;CNIH;COMMD6;COX16;CREBL2;CSGALNACT2;CSTF1;CUL4A;CWF19L2;CXXC4;CYB5D2;DCLRE1B;DDX50;DDX6;DGUOK;DLEC1;DNAJC10;DUSP18;EFC4A;EFHC1;EMP3;ERCC4;ERLIN2;EXOSC3;FAM123B;FAM173B;FAM177A1;FAM46A;FBXO15;FBXO36;FCGBP;FOS;FTSJD1;GALNT10;GCNT2;GJB6;GMNN;GTF2A2;GTF2F2;HAUS6;HEPH;HFM1;HSP90B1;HSPA13;HSPA5;IFT20;IGFBP6;IL11;IL7R;IMPACT;INTS2;INTS4;IRF7;ISYNA1;KIAA1530;KLRK1;LARP7;LAX1;LEPROT;LIMS1;LOC100131564;LOC100272217;LOC389705;LOC728407;LOC729852;LRP10;LRRC37A3;LRRC70;LSM5;LST1;MAGEH1;MAK16;MESDC2;METAP2;METTL12;MGAT2;MPL;MRPL20;MRPL50;MRPL51;MRPS26;MRPS31;MSH5;MUTYH;MXRA8;MYO1F;MYOZ1;MYST2;NAA38;NCF2;NEDD1;NEK9;NFATC1;NHP2L1;NOC3L;NOL11;NOL7;NR1H3;NSUN5;NUDT6;NUP54;NXT1;OFD1;OGFOD1;PAPOLA;PARG;PARP3;PATZ1;PCNP;PDCD10;PFDN5;PHIP;PIN4;PLCD1;PLRG1;PMFBP1;PNPLA4;PNPO;POLA1;POLD3;POLI;POP5;PRDM13;PRPF40A;PTCH1;PTOV1;R18;RAD17;RAP1B;RAP2C;RBBP8;RBM34;RBM8A;RCBTB2;RDBP;REST;RFX4;RND2;RPIA;RPL10;RPL13A;RPL24;RPS6;RRP15;RSPRY1;S100A13;SAMD4A;SCAMP2;SCARNA17;SERHL2;SETD4;SH3GLB1;SKIL;SLAIN2;SLC7A7;SMAD5;SMARCAD1;SNRPF;SP140L;SPR;SRP72;SRRM1;SSBP1;STAG1;STT3A;SUGT1P1;SYF2;TAF1A;TAF7;TFAM;TGDS;TGIF2;THAP6;THEM4;TIGD6;TMCO1;TMED10;TMEM138;TMEM199;TMEM20;TMEM222;TOR1B;TPRKB;TRAF3IP1;TRAPPC2P1;TRAPPC4;TRIM38;TRIM39;TRMT112;TSEN34;TSG101;TSPAN4;TTC14;TTC30B;TTF1;TWF1;TXNDC5;TYMP;UBR1;UFD1L;USP16;USP24;UTP18;WDR61;XIAP;YES1;ZFC3H1;ZFP90;ZKSCAN2;ZNF136;ZNF197;ZNF394;ZNF512;ZNF518A;ZNF529;ZNF562;ZNF583;ZNF660;ZNF671;ZNF701"
"cisbp__M5383"	3.02	0.0387	"ELK1 (directAnnotation). "	259	"ACO1;ADAL;ADH5;AEBP1;ALOX5;ANKHD1-EIF4EBP3;ANKS3;APPL2;ARF4;ARFIP1;ARIH1;ASB1;ASCC1;ATAD2B;ATF7;ATOX1;ATP7A;B3GNT5;BCL7C;BMP2K;BOD1;BRIX1;BTF3;C10orf26;C12orf48;C12orf57;C14orf109;C14orf135;C15orf24;C15orf38;C1orf112;C1orf212;C1orf26;C1orf9;C1orf97;C20orf94;C21orf34;C2orf84;C5orf43;C6orf226;C7orf11;C9orf64;CARD8;CBFB;CBLB;CCDC147;CCDC15;CCDC152;CCDC88B;CD274;CDC16;CDC26;CDK4;CDKL3;CETN3;CFLAR;CHUK;CIRBP;CITED4;CLEC11A;CNIH;CNIH4;COMMD6;COX16;CREBL2;CSGALNACT2;CSTF1;CUL4A;CWF19L2;CXXC4;CYB5D2;DCLRE1B;DDX50;DDX6;DGUOK;DLEC1;DNAJC10;DUSP18;EFC4A;EFHC1;EIF5B;EMP3;EPS8;ERCC4;ERLIN2;EXOSC3;FAM123B;FAM164C;FAM173B;FAM177A1;FAM46A;FBXO15;FBXO36;FCGBP;FOS;FTSJD1;GALNT10;GCNT2;GJB6;GMNN;GTF2A2;GTF2F2;HAUS6;HDAC1;HFM1;HMGN4;HSPA13;IFT20;IL7R;IMPACT;INTS2;INTS4;IRF7;ISYNA1;KIAA1530;KLRK1;LARP7;LAX1;LEPROT;LIMS1;LOC100131564;LOC100272217;LOC389705;LOC728407;LOC729852;LRP10;LRRC37A3;LRRC70;LSM5;LST1;MAGEH1;MAK16;MESDC2;METTL12;MGAT2;MPL;MRPL20;MRPL51;MRPS26;MRPS31;MSH5;MUTYH;MYO1F;MYOZ1;MYST2;NAA38;NCF2;NEDD1;NEK9;NHP2L1;NOC3L;NOL11;NOL7;NR1H3;NSUN5;NUDT6;NUP54;NXT1;OFD1;OGFOD1;OSTM1;PAPOLA;PARG;PARP3;PATZ1;PCNP;PDCD10;PFDN5;PHIP;PIN4;PLCD1;PLRG1;PMFBP1;PNPLA4;POLA1;POLD3;POLI;POP5;PSMD10;R18;RAD17;RAP1B;RAP2C;RBBP8;RBM34;RBM8A;RCBTB2;RDBP;REST;RFX4;RND2;RPIA;RPL10;RPL13A;RPS6;RRP15;S100A13;SAMD4A;SCAMP2;SCARNA17;SETD4;SH3GLB1;SKIL;SLAIN2;SLC7A7;SMARCAD1;SNRPF;SP140L;SPR;SRRM1;SSBP1;STT3A;SYF2;TAF1A;TAF7;TFAM;TGDS;TGIF2;THAP6;THEM4;TIGD6;TMCO1;TMED10;TMEM138;TMEM199;TMEM20;TMEM222;TOR1B;TPRKB;TRAPPC2P1;TRAPPC4;TRIM38;TRIM39;TRMT112;TSEN34;TSG101;TTC14;TTC30B;TTF1;TXNDC5;TYMP;UBR1;UFD1L;USP16;USP24;UTP18;WDR61;XIAP;YES1;ZFP90;ZKSCAN2;ZNF197;ZNF394;ZNF512;ZNF518A;ZNF529;ZNF562;ZNF671;ZNF701"
"cisbp__M4554"	3.01	0.0387	"NR2C2 (directAnnotation). "	87	"ANKS3;ARFIP1;ARIH1;BBS9;C12orf48;C15orf24;C6orf226;CCDC15;CDC16;CDKL3;CLNS1A;CNIH;COX16;CRY1;CSGALNACT2;CSTF1;CYB5D2;DDX50;DLEC1;DNAJC10;DUSP18;EFC4A;FAM46A;FBXO36;GMNN;GTF2A2;HEATR5A;IFT20;IMPACT;IRF9;LEPROT;LIMS1;LOC100131564;LOC389705;LOC728407;LOC729852;LST1;MAEA;MAK16;MRPL51;MRPS26;MYST2;NEDD1;NFATC1;PARG;PCNP;PDCD10;PFDN5;PLRG1;PNPLA4;PROCA1;PTCH1;RAP2C;RBBP8;RBM34;RBM8A;RND2;RPS6;S100A13;SALL4;SCAMP2;SETD4;SPR;SRP72;SSBP1;STAT5B;SUGT1P1;TAF1A;TAF7;TGIF2;TIGD6;TMCO1;TMED10;TMEM138;TMEM199;TPRKB;TRAPPC4;TRMT112;TSG101;TTC14;TTC30B;USP24;UTP18;ZFP90;ZNF394;ZNF518A;ZNF701"
"cisbp__M1400"	3.01	0.0387	""	249	"AASDH;ADAL;AIRE;ALG14;ALK;AMIGO2;ANAPC10;ANKRA2;ANP32E;APPL2;ARF4;ARFIP1;ARIH1;AS3MT;ATF1;ATF7;ATP10D;ATP7A;B3GNT5;BCAR3;BCAS2;BCO2;BET3L;BRAP;C11orf71;C12orf48;C12orf60;C14orf135;C15orf38;C17orf101;C17orf97;C19orf42;C1orf212;C1orf63;C1orf9;C20orf132;C20orf72;C21orf34;C2orf3;C4orf29;C5orf41;C7orf55;CALCR;CBLB;CBX5;CCDC102A;CCDC15;CCDC152;CCDC66;CCDC90B;CD38;CD59;CDC26;CDNF;CETN3;CFLAR;CGA;CHUK;CIB1;CITED4;CLEC18B;CLK1;CNIH;CNIH4;COMMD6;COX16;CREBL2;CXorf57;DDX3Y;DERL2;DGUOK;DHRS12;DIRC2;DKFZP434I0714;DNAJC10;EID1;FAM123B;FAM177A1;FAM46A;FAM76A;FBXO15;FITM2;FNDC3A;FOS;FSD1L;FSTL5;FTSJD1;FZD5;G3BP1;GKAP1;GLG1;GLIPR1L2;GMNN;GNA13;HADH;HAS3;HAUS6;HEATR5A;HELLS;HELQ;HEYL;HIST1H2AA;HIST1H2AG;HIST1H2BJ;HIST1H3G;HMGN4;HSPA13;HSPA5;HSPBAP1;IFIT3;IFT20;IKZF5;IL18;ITGB3BP;KIAA1407;KIAA1530;KIF27;KLHDC10;KLHL1;KLHL21;LARP1B;LARP7;LEPREL1;LEPROT;LIFR;LIMS1;LOC728407;LPP;LRRCC1;LSM5;MAEA;MAP7D3;MBNL3;MED28;METAP2;MLH1;MPP5;MSN;MUTED;MX1;MYST2;NAA38;NDUFC1;NEK1;NEURL2;NFATC1;NHLRC2;NKAP;NOC3L;NOL7;NPM1;NUP205;OFD1;PARG;PARN;PBLD;PDIK1L;PITX2;PLK3;PODNL1;POLA1;POU3F2;PPAPDC1B;PRDM13;PRDX6;PRPF18;PRPF38A;PRPF40A;PSENEN;PSMB1;PTOV1;R18;RAD17;RAN;RBBP8;RBM8A;RCBTB2;RPIA;RPL13A;RPL23;RPL23AP7;RPL24;RPL5;RPS27L;RSPRY1;S100A13;SALL4;SAMD4A;SARNP;SEC11C;SENP8;SKIL;SLU7;SMAD5OS;SMARCA5;SMARCAD1;SNAPC1;SOAT1;SOX2;SPRYD4;SRRM1;SRSF3;SSR1;SSR4;STAG1;STT3A;STYX;SUGT1P1;SUMF1;SYF2;TAF7;TEX9;TFAM;THAP9;TIGD6;TMED10;TMED4;TMEM38B;TMEM62;TNPO1;TP53INP1;TRAF3IP1;TRAF3IP2;TRAPPC2P1;TRIM39;TRIM52;TRMT112;TSG101;TTC14;TTC30B;TTF1;TUBB2B;TWISTNB;TXNDC5;TXNDC6;USP16;UTP18;VAT1L;WRB;ZCCHC9;ZFX;ZKSCAN2;ZNF267;ZNF394;ZNF518A;ZNF583;ZNF92;ZNHIT6;ZZZ3"

"motif"	"NES"	"AUC"	"TF_highConf"	"nEnrGenes"	"enrichedGenes"
"cisbp__M4568"	3.01	0.0387	"GPA (directAnnotation). "	281	"ACO1;ADAL;ADH5;ALOX5;ANKHD1-EIF4EBP3;ANKS3;ANP32E;APPL2;ARF4;ARFIP1;ARIH1;ASB1;ATAD2B;ATF7;ATP7A;B3GNT5;BCL7C;BMP2K;BRIX1;BTBD8;BTF3;C10orf26;C11orf96;C12orf48;C12orf57;C14orf109;C15orf24;C15orf38;C1orf112;C1orf26;C1orf56;C1orf9;C1orf97;C20orf94;C2orf84;C5orf41;C5orf43;C6orf226;C7orf11;C9orf64;CALCR;CAPN7;CARD8;CBFB;CBLB;CCDC15;CCDC152;CCDC88B;CDC16;CDC26;CDKL3;CETN3;CFLAR;CHUK;CIRBP;CLEC11A;CLEC1A;CNIH;COMMD6;COX16;CREBL2;CSGALNACT2;CSTF1;CTXN2;CWC15;CWF19L2;CXXC4;CYB5D2;CYBA;DCLRE1B;DDX50;DDX6;DERL2;DGUOK;DLEC1;DNAJC10;DUSP18;EFC4A;EFHC1;ERCC4;ERLIN2;EXOSC3;FP6;FAM123B;FAM164C;FAM173B;FAM177A1;FAM46A;FBXO15;FBXO36;FCGBP;FIP1L1;FNDC3A;FTSJD1;GALNT10;GJB6;GMNN;GTF2A2;GTF2F2;H2AFY;HAUS6;HEATR5A;HFM1;HMGB2;HMGN4;HSPA5;HSPBAP1;IFT20;IGF2BP2;IL7R;IMPACT;INPP5D;INTS2;INTS4;IRF9;ISYNA1;KIAA1530;KLRK1;LARP7;LAX1;LEPROT;LIFR;LIMS1;LOC100131564;LOC100272217;LOC389705;LOC728407;LOC729852;LRP10;LRRC70;LSM5;LST1;MAEA;MAGEH1;MAK16;MDK;ME2;MED28;METTL12;MGAT2;MPL;MRPL20;MRPL23;MRPL51;MRPS26;MRPS31;MUTYH;MYO1F;MYST2;NAA38;NCF2;NEDD1;NEK9;NFATC1;NHP2L1;NOC3L;NOL11;NR1H3;NSUN5;NUDT6;NUP54;NXT1;OFD1;OGFOD1;PAPOLA;PARG;PARP3;PATZ1;PBXIP1;PCNP;PDCD10;PITX2;PLCD1;PLRG1;PMFBP1;PNPLA4;POLA1;POLD3;POLI;POP5;PPP4R1;PRPF40A;PRRX1;PSME2;PTCH1;R18;RAD21;RAP1B;RAP2C;RBBP8;RBM34;RBM8A;RCBTB2;RDBP;REST;RFX4;RND2;RPIA;RPL10;RPL13A;RPL23;RPL24;RPL5;RPS6;RRP15;S100A13;SAMD4A;SCAMP2;SCARNA17;SETD4;SH3GLB1;SKIL;SLAIN2;SLC25A1;SLC35F5;SMARCAD1;SNRPF;SOX2;SP140L;SPIN4;SPR;SRP72;SRRM1;SSBP1;STAG1;STAT5B;STT3A;SUGT1P1;SYF2;TAF1A;TAF7;TAGLN2;TDRD3;TFAM;TGFB1;TGIF2;THAP2;TIGD6;TMCO1;TMED10;TMEM138;TMEM199;TMEM20;TMEM222;TOR1B;TPRKB;TRAPPC2P1;TRAPPC4;TRIM38;TRMT112;TSEN34;TSG101;TSPAN4;TTC14;TTC30B;TTF1;TTLL3;TXNDC5;TYMP;UBR1;UFD1L;USP16;USP24;UTP18;WDR61;XIAP;YES1;ZCCHC3;ZFP90;ZFX;ZKSCAN2;ZNF197;ZNF394;ZNF512;ZNF518A;ZNF529;ZNF562;ZNF583;ZNF671;ZNF691;ZNF701"
"cisbp__M4462"	3	0.0386	"GPA (directAnnotation). "	218	"ACO1;ADAL;ADH5;ANKHD1-EIF4EBP3;ANKS3;APPL2;ARF4;ARFIP1;ARIH1;ASB1;ATF7;ATP7A;B3GNT5;BCL7C;BMP2K;BTF3;C10orf26;C12orf48;C12orf57;C14orf109;C14orf135;C15orf24;C15orf38;C1orf112;C1orf97;C21orf34;C2orf84;C5orf41;C5orf43;C6orf226;C7orf11;C9orf64;CALCR;CBFB;CBLB;CCDC15;CCDC152;CCDC88B;CD274;CDC16;CDC26;CDKL3;CETN3;CFLAR;CHUK;CIRBP;CLEC11A;CLEC18B;CNIH;COMMD6;COX16;CSGALNACT2;CSTF1;CWF19L2;CXXC4;CYB5D2;DDX50;DGUOK;DLEC1;DNAJC10;DUSP18;EFC4A;EFHC1;ERLIN2;EXOSC3;FAM173B;FAM177A1;FAM46A;FBXO15;FBXO36;FCGBP;FIP1L1;FOS;FTSJD1;GALNT10;GJB6;GMNN;GTF2A2;HAUS6;HMGN4;HSPA5;IFT20;IL7R;IMPACT;INTS2;INTS4;ISYNA1;KIAA1530;KLRK1;LAX1;LEPROT;LIFR;LIMS1;LOC100131564;LOC100272217;LOC389705;LOC728407;LOC729852;LPP;LRP10;LRRC70;LSM5;LST1;MAGEH1;MAK16;METTL12;MGAT2;MRPL20;MRPL51;MRPS26;MUTYH;MYO1F;MYST2;NAA38;NCF2;NEDD1;NFATC1;NHP2L1;NOC3L;NOL7;NR1H3;NSUN5;NUDT6;NUP54;NXT1;OFD1;OGFOD1;PAPOLA;PARG;PARP3;PATZ1;PCNP;PDCD10;PFDN5;PITX2;PLCD1;PLRG1;PMFBP1;PNPLA4;POLA1;POLI;PPAPDC1B;PRPF40A;R18;RAP1B;RAP2C;RBBP8;RBM34;RBM8A;RDBP;REST;RND2;RPIA;RPL10;RPL13A;RPS6;RRP15;S100A13;SAMD4A;SCAMP2;SETD4;SKIL;SLAIN2;SLC25A1;SLC7A7;SMARCAD1;SOX2;SP140L;SPR;SRP72;SRRM1;SSBP1;STAT5B;STT3A;SYF2;TAF1A;TAF7;TDRD3;TFAM;TGDS;TGFB1;TGIF2;THAP2;TIGD6;TMCO1;TMED10;TMEM138;TMEM199;TMEM222;TOR1B;TPRKB;TRAF3IP2;TRAPPC2P1;TRAPPC4;TRMT112;TSEN34;TSG101;TTC14;TTC30B;TTF1;TXNDC5;TYMP;UBR1;UFD1L;USP16;USP24;UTP18;WDR61;XIAP;YES1;ZNF197;ZNF285;ZNF394;ZNF512;ZNF518A;ZNF529;ZNF671;ZNF701"
"cisbp__M1903"	3	0.0386	"ELK4 (directAnnotation). "	246	"ACO1;ADAL;ADH5;ALOX5;ANKHD1-EIF4EBP3;ANKS3;APPL2;ARF4;ARFIP1;ARIH1;ASB1;ATAD2B;ATF7;ATP7A;B3GNT5;BCL7C;BMP2K;BTBD8;BTF3;C10orf26;C12orf48;C12orf57;C14orf109;C15orf24;C15orf38;C1orf112;C1orf26;C1orf97;C20orf94;C5orf41;C5orf43;C6orf226;C7orf11;C9orf5;C9orf64;CALCR;CBFB;CBLB;CCDC15;CCDC152;CCDC88B;CD274;CDC16;CDC26;CDKL3;CDNF;CETN3;CFLAR;CHUK;CIRBP;CLEC11A;CLEC18B;CNIH;COMMD6;COX16;CSGALNACT2;CSTF1;CTXN2;CWF19L2;CXXC4;CYB5D2;CYBA;DDX50;DGUOK;DLEC1;DNAJC10;DUSP18;EFC4A;EFHC1;ERLIN2;EXOSC3;FAM123B;FAM173B;FAM177A1;FAM46A;FBXO15;FBXO30;FBXO36;FCGBP;FIP1L1;FTSJD1;GALNT10;GJB6;GMNN;GTF2A2;GTF2F2;HAUS6;HHEX;HIST4H4;HMGB2;HMGN4;HSPA5;IFT20;IL7R;IMPACT;INTS2;INTS4;IRF9;ISYNA1;KIAA1530;KLRK1;LARP7;LAX1;LEPROT;LIFR;LIMS1;LOC100131564;LOC100272217;LOC389705;LOC728407;LOC729852;LPP;LRP10;LRRC70;LSM5;LST1;MAEA;MAGEH1;MAK16;MDK;ME2;METTL12;MGAT2;MPL;MRPL20;MRPL50;MRPL51;MRPS26;MRPS31;MSH5;MUTYH;MYO1F;MYST2;NAA38;NCF2;NEDD1;NEK9;NFATC1;NHP2L1;NOC3L;NOL11;NOL7;NR1H3;NSUN5;NUP54;NXT1;OFD1;OGFOD1;PAPOLA;PARG;PARP3;PARP9;PATZ1;PBXIP1;PCNP;PDCD10;PFDN5;PITX2;PLCD1;PLRG1;PMFBP1;PNPLA4;POLA1;POLI;PPAPDC1B;PPP4R1;PRPF40A;PTCH1;RAP1B;RAP2C;RBBP8;RBM34;RBM8A;RDBP;REST;RFX4;RND2;RPIA;RPL10;RPL13A;RPS6;SAMD4A;SCAMP2;SH3GLB1;SLAIN2;SLC7A7;SMAD5OS;SMARCAD1;SNRPF;SOX2;SP140L;SPR;SRP72;SRRM1;SSBP1;STAG1;STAT5B;STT3A;SYF2;TAF1A;TAF7;TDRD3;TFAM;TGDS;TGFB1;TGIF2;THAP6;TIGD6;TMCO1;TMED10;TMEM138;TMEM199;TMEM20;TMEM222;TOR1B;TPRKB;TRAF3IP2;TRAPPC2P1;TRAPPC4;TRIM38;TRMT112;TSEN34;TSG101;TTC14;TTC30B;TTF1;TTLL3;TXNDC5;TYMP;UBR1;UFD1L;USP24;UTP18;WDR61;XIAP;YES1;ZFX;ZKSCAN2;ZNF197;ZNF394;ZNF512;ZNF518A;ZNF529;ZNF660;ZNF671;ZNF701"

fMRI Reward Neg Subc

"motif"	"NES"	"AUC"	"TF_highConf"	"nEnrGenes"	"enrichedGenes"
"cisbp__M1943"	5.08	0.112	""	22	"C1orf95;CACNA1C;CACNA1D;DUSP6;FMNL1;FRMPD4;KCND3;NEUROG2;NPAS2;PCDHB12;PCDHB18;PCDHB2;PCSK2;RGS7BP;RIMS1;RNF6;RTN4R;SHANK2;SIRPA;SLC12A5;SYT7;XKR4"
"cisbp__M1794"	4.07	0.0977	""	17	"ATP8B1;CDKL5;DBC1;DLGAP4;DUSP6;FMNL1;GRIN2B;GRM5;HS6ST3;LRRTM1;MGLL;NPAS2;PCSK2;RASAL2;RNF6;SIRPA;SLITRK3"
"cisbp__M0021"	3.91	0.0955	""	16	"ATP8B1;C7orf65;CACNA1C;CACNA1D;CDKL5;DUSP6;FMNL1;GRM5;KCNQ3;NEUROG2;NPAS2;PCDHB18;PSD3;SIRPA;SLITRK3;XKR4"
"cisbp__M0039"	3.88	0.0951	""	20	"C7orf65;CACNA1C;CACNA1D;CDKL5;DUSP6;FMNL1;GRM5;KCNQ3;NEUROG2;NPAS2;PCDHB12;PCDHB18;PCDHB2;PSD3;RIMS1;RTN2;SCN10A;SIRPA;SLITRK3;TPM1"
"cisbp__M0029"	3.75	0.0932	""	44	"ARNTL;ATP8B1;CACNA1C;CACNA1D;CALHM2;CYB561D1;DLGAP4;DUSP6;ELOVL3;EPHA6;FMNL1;FZD8;GALNTL4;GRIN2A;HS2ST1;HS6ST3;KCND3;KCNQ3;LRRTM1;MEF2A;MGAT5B;MGLL;MYH10;NCK2;NEUROG2;NPAS2;PCDHAC2;PCDHB18;PCSK2;PSD3;R15;RGS7BP;RIMS1;RNF6;RTN4R;SHANK2;SIRPA;SLITRK3;SOCS7;STK32C;SYT7;TPM1;WBSCR17;XKR4"
"cisbp__M0033"	3.63	0.0915	""	25	"CACNA1C;CACNA1D;CYB561D1;DLGAP4;DUSP6;FMNL1;FZD8;HS2ST1;HS6ST3;KCNQ3;LRRTM1;MGLL;NCK2;NEUROG2;NPAS2;PCDHAC2;PSD3;RGS7BP;RIMS1;RNF6;SHANK2;SIRPA;SOCS7;SYT7;XKR4"
"cisbp__M0605"	3.62	0.0913	""	40	"CACNA1C;CACNA1D;CDKL5;DBC1;DLGAP4;DUSP6;ELOVL3;FMNL1;GRIN2B;GRM5;HECW1;HLF;HOOK1;HS6ST3;KCNQ3;LAMB1;LRRTM1;MEF2A;MGLL;MYH10;NCK2;NNT;NPAS2;PCDHAC2;PCDHB12;PCDHB18;PCSK2;PSD3;PTPRN2;R15;RNF6;RTN2;SEMA6B;SIRPA;SLC12A5;SLITRK3;STK32C;TMSB10;TPM1;VTA1"
"cisbp__M0012"	3.48	0.0894	""	62	"ACVR1B;ARNTL;ATP8B1;C1orf95;C7orf65;CACNA1C;CACNA1D;CDKL5;CYB561D1;CYFIP2;DBC1;DIRAS2;DUSP6;ELOVL3;FMNL1;FZD8;GALNTL4;GRIN2A;GRM5;HIST1H4K;HS2ST1;HS6ST3;KCND3;KCNQ3;LRRTM1;MEF2A;MGAT5B;MGLL;MSC;MTUS2;NCK2;NEUROG2;NPAS2;OTUB2;PCDHAC2;PCDHB12;PCDHB18;PCDHB2;PCSK2;PREPL;PSD3;PTK2B;R15;R3D;RGS7BP;RIMS1;RNF6;RTN2;RTN4R;SCN10A;SEMA6B;SHANK2;SHANK3;SIRPA;SLC12A5;SLITRK3;SNPH;STK32C;SYNPO;TMSB10;TPM1;XKR4"
"cisbp__M1275"	3.44	0.0888	""	28	"ACVR1B;CACNA1C;CACNA1D;CDKL5;DBC1;DLGAP4;DUSP6;ELOVL3;FMNL1;GRIN2B;GRM5;HS6ST3;LRRTM1;MGLL;MSC;NCK2;NEUROG2;NPAS2;PCSK2;PREPL;RASAL2;RTN2;SEMA6B;SIRPA;SLC12A5;SLITRK3;TMSB10;TPM1"
"cisbp__M0007"	3.38	0.0879	""	63	"ACVR1B;AIM1;ARNTL;ATP8B1;BRSK2;C11orf87;C7orf65;CACNA1C;CACNA1D;CCKBR;CDKL5;CPNE6;CYFIP2;DDX10;DIRAS2;DUSP6;ELOVL3;EPHA6;FMNL1;FZD8;GPR21;GRIN2A;GRIN2B;GRM5;HLF;HS2ST1;HS6ST3;KCND3;KCNQ3;LRRTM1;LRRTM2;MEF2A;MGAT5B;MGLL;MTUS2;NEUROG2;NNT;NPAS2;PCDHAC2;PCDHB12;PCDHB18;PCDHB2;PCSK2;PREPL;PSD3;R15;R3D;RGS7BP;RIMS1;RNF6;RTN2;RTN4R;SEMA6B;SHANK2;SHANK3;SIRPA;SLITRK3;SNPH;SOCS7;STK32C;TMSB10;TPM1;XKR4"
"cisbp__M0615"	3.36	0.0876	""	47	"ACVR1B;ATP8B1;BRSK2;CACNA1C;CACNA1D;CDKL5;CPNE6;DBC1;DLGAP4;DUSP6;ELOVL3;FMNL1;GPC1;GRIN2B;GRM5;HOOK1;HS6ST3;KCND3;KCNQ3;LAMB1;LRRTM1;MEF2A;MGLL;MSC;MYH10;NCK2;NEUROG2;NNT;NPAS2;PCDHB18;PCSK2;PSD3;PTPRN2;R15;RASAL2;RIMS1;RNF6;RTN2;SCN10A;SEMA6B;SIRPA;SLC12A5;SLITRK3;SOCS7;STK32C;TMSB10;TPM1"
"cisbp__M0006"	3.26	0.0863	""	25	"ACVR1B;ARNTL;CACNA1C;CACNA1D;CPNE6;DIRAS2;DUSP6;FMNL1;FZD8;HIST1H4K;HS2ST1;LRRTM1;MGAT5B;MGLL;NEUROG2;NPAS2;PCDHB12;PCDHB18;PCDHB2;RASAL2;RIMS1;SHANK2;SIRPA;SLITRK3;SOCS7"
"cisbp__M2529"	3.26	0.0863	""	15	"ATP8B1;CACNA1C;DUSP6;GRIN2A;HS2ST1;HS6ST3;KCNQ3;NEUROG2;PCDHAC2;R15;RIMS1;SHANK2;SOCS7;SYT7;XKR4"
"cisbp__M0580"	3.25	0.086	""	21	"CACNA1C;CACNA1D;CDKL5;DBC1;DDX10;DUSP6;FMNL1;GRM5;KCNQ3;LAMB1;LRRTM1;NEUROG2;NPAS2;PCDHB12;PCDHB18;PSD3;RTN2;SEMA6B;SIRPA;SLITRK3;SOCS7"
"cisbp__M0720"	3.21	0.0855	""	26	"ACVR1B;ATL1;CYB561D1;DBC1;DLGAP4;DUSP6;FRMPD4;HIST1H4J;HIST1H4K;HYMAI;KCNQ3;KIAA1486;MEF2A;NEUROG2;NNT;PCSK2;PSD3;RIMS1;RTN2;SHANK2;SIRPA;SLC12A5;SLITRK3;TBC1D30;TMSB10;VTA1"
"cisbp__M0607"	3.19	0.0853	"KDM2B (directAnnotation). "	28	"ACVR1B;CACNA1C;CACNA1D;CDKL5;DBC1;DLGAP4;DUSP6;FMNL1;HS6ST3;KCND3;KCNQ3;LRRTM1;MEF2A;MGLL;NCK2;NEUROG2;NNT;NPAS2;PCSK2;PSD3;RIMS1;RTN2;SEMA6B;SIRPA;SLC12A5;SLITRK3;TMSB10;TPM1"
"cisbp__M0431"	3.14	0.0846	"OSR1 (inferredBy_Orthology). "	29	"ARNTL;CACNA1C;CALHM2;FMNL1;FRMPD4;GPR21;GRIN2A;GRM5;GTPBP1;HS6ST3;IQCJ;KCND3;KCNQ3;KIAA1486;MGAT4B;MYH10;NCK2;NNT;PPFIA4;PSD3;RASAL2;RIMS1;RNF6;RTN2;SCN10A;SLITRK3;SYNPO;TIAM2;ZC3H12D"
"cisbp__M0741"	3.13	0.0844	""	8	"DUSP6;KCNQ3;NEUROG2;PCSK2;PSD3;RIMS1;SIRPA;SLC12A5"
"cisbp__M0614"	3.13	0.0844	""	34	"ACVR1B;ATP8B1;CACNA1C;CACNA1D;CDKL5;DBC1;DLGAP4;DUSP6;FMNL1;GRM5;KCND3;KCNQ3;LRRTM1;MEF2A;MGLL;MYH10;NCK2;NEUROG2;NNT;NPAS2;PCDHB18;PCSK2;PSD3;R15;RIMS1;RTN2;RTN4R;SCN10A;SEMA6B;SIRPA;SLITRK3;SOCS7;TMSB10;TPM1"
"cisbp__M0022"	3.13	0.0844	""	22	"CACNA1C;CACNA1D;CYB561D1;DUSP6;FMNL1;FZD8;HS6ST3;KCNQ3;LRRTM1;MEF2A;NCK2;NEUROG2;NPAS2;PCDHAC2;RGS7BP;RIMS1;RNF6;SHANK2;SOCS7;STK32C;SYT7;XKR4"
"cisbp__M2376"	3.09	0.0838	""	28	"ACVR1B;BAI1;CA7;CACNA1C;CALHM2;CDKL5;CYFIP2;EXTL1;FAM89B;FMNL1;GPR21;GRM5;HLF;LYVE1;MAL2;MEF2A;MGAT4B;MGAT5B;OR2T5;PAK7;PCLO;RASAL2;RIMS1;RS1;SLC12A5;SLITRK3;SYN3;ZC3H12D"
"cisbp__M0041"	3.05	0.0833	""	39	"ARNTL;BRSK2;C7orf65;CACNA1C;CACNA1D;CDKL5;CYFIP2;DIRAS2;DUSP6;EXTL1;FMNL1;FZD8;GRIN2A;GRM5;KCND3;KCNQ3;LRRTM1;MGLL;NEUROG2;NPAS2;PCDHAC2;PCDHB12;PCDHB18;PCDHB2;PSD3;RIMS1;RNF6;RTN2;RTN4R;SCN10A;SEMA6B;SHANK2;SIRPA;SLITRK3;SNPH;SOCS7;STK32C;TMSB10;XKR4"
"cisbp__M4195"	3.04	0.0831	""	65	"HD8;ACVR1B;ATL1;C11orf87;C7orf65;CACNA1C;CACNA1D;CALHM2;CDH3;CDKL5;CPNE6;CYFIP2;DUSP6;ELOVL3;EPHA3;EPHA6;EXTL1;FMNL1;FRMPD4;GRB2;GPC1;GRIN2A;GRIN2B;GRM5;HECW1;HOOK1;HS6ST3;KCND3;KCNQ3;KRT222;LRRTM1;MEF2A;MGLL;MSC;MTUS2;NEUROG2;NNT;NPAS2;PAK7;PCDHAC2;PCDHB2;PCLO;PCNXL2;PCSK2;PREPL;PRRT1;PSD3;R15;RASAL2;RIMS1;RNF6;RTN2;SCN10A;SEMA6B;SHANK2;SIRPA;SLITRK3;STK32C;TBC1D30;TMSB10;TPM1;UNC13A;VTA1;WBSCR17;ZC3H12D"
"cisbp__M1385"	3.03	0.083	""	31	"ATL1;CACNA1C;CYFIP2;DBC1;DUSP6;ELOVL3;FOXD4;GRIN2A;GRM5;HECW1;KCNQ3;KIAA1486;MEF2A;MGAT4B;MGLL;MTUS2;NEUROG2;OTUB2;PI15;PREPL;PTK2B;RGS7BP;RTN2;SCN10A;SIRPA;SLC12A5;SLITRK3;TMSB10;TPM1;UPP2;XKR4"

"motif"	"NES"	"AUC"	"TF_highConf"	"nEnrGenes"	"enrichedGenes"
"cisbp__M1419"	3.03	0.083	"MYRF (inferredBy_Orthology). "	67	"ATL1;C11orf87;C1orf95;C7orf65;CACNA1C;CACNA1D;CACNA1F;CALHM2;CDH22;CDKL5;CPNE6;CYB561D1;CYFIP2;DBC1;DIRAS2;DLGAP4;DUSP6;ELOVL3;EXTL1;FAM89B;FRMPD4;GPR21;GRIN2B;GRM5;GTPBP1;HECW1;HIST1H4J;HOOK1;HS6ST3;HYMAI;IQCJ;KCND3;KCNQ3;KIAA1486;LRRTM1;LRRTM2;MEF2A;MGAT5B;MGLL;MTUS2;NNT;NPAS2;OR2T5;PCDHB12;PCDHB18;PCDHB2;PCLO;PCNXL2;PREPL;PSD3;RASAL2;RIMS1;RTKN2;RTN2;SCN10A;SEMA6B;SHANK2;SIRPA;SLC12A5;SLITRK3;STK32C;SYN3;TIAM2;TMSB10;TPM1;UPP2;ZC3H12D"
"cisbp__M0023"	3.02	0.0829	""	67	"ACVR1B;ARNTL;ATP8B1;C11orf87;C7orf65;CACNA1C;CACNA1D;CDH22;CDKL5;CYFIP2;DBC1;DDX10;DIRAS2;DUSP6;ELOVL3;EPHA6;FMNL1;FZD8;GALNTL4;GPR21;GRIN2A;GRM5;HIST1H4K;HLF;HS2ST1;HS6ST3;HTT;KCND3;KCNQ3;LRRTM1;MEF2A;MGAT5B;MGLL;MTUS2;NCK2;NEUROG2;NPAS2;NR1D1;OTUB2;PCDHAC2;PCDHB12;PCDHB18;PCDHB2;PCSK2;PPP4R4;PREPL;PSD3;PTK2B;R3D;RGS7BP;RIMS1;RNF6;RTN2;RTN4R;SEMA6B;SHANK2;SHANK3;SIRPA;SLC12A5;SLITRK3;SNPH;SOCS7;STK32C;SYNPO;TMSB10;TPM1;XKR4"

fMRI Emotion Pos Subc

"motif"	"NES"	"AUC"	"TF_highConf"	"nEnrGenes"	"enrichedGenes"
"cisbp__M3447"	4.18	0.0469	"XBP1 (directAnnotation). "	46	"ANKRD16;ARHGAP6;ARMCX1;ARMCX2;ARMCX5;BEX5;BHLHB9;CALM3;CCDC104;CCDC149;CCDC99;CDC5L;CHCHD6;DCP2;ETNK1;G3BP2;GOPC;GPSM1;GRPR;GTF2B;LOC145837;MIPOL1;MON1A;NUAK1;OR5M9;OSR1;PAK1IP1;PPFIA2;PRKCI;PRNP;PSME3;RFTN1;RPTOR;RTN1;SCARA5;SEC24D;SLC1A4;SLITRK1;SMYD2;SOX5;SRP19;SULF2;UBXN8;VAC14;WDR5;YWHAH"
"cisbp__M0673"	4.09	0.0465	""	203	"ACOT2;ACOX3;ACTR8;AGAP3;ANKLE2;ANKRD16;AP3S1;APAF1;ARHGAP6;ARHGDIA;ARMC7;ARRB2;ASXL3;ATF2;ATP6V1D;AZIN1;B4GALNT3;B4GALT2;B4GALT5;BRE;C10orf46;C11orf80;C12orf65;C1orf93;C1QL4;C6orf1;C9orf40;C39L;CALM3;CAMLG;CBX1;CCDC157;CCDC87;CCRN4L;CD276;CDC14A;CDC5L;CDKN2AIPNL;CHCHD6;CHD5;CHERP;CHIC2;CREG2;CXorf26;CYS1;DCP2;DRD5;DUSP3;EBAG9;EFHD2;EFNB1;FAM58A;FAT4;FBXW9;FDX1L;FMN2;FMNL3;FOXP4;FRMD3;G3BP2;GADD45GIP1;GALNT2;GATA4;GDI1;GFRA2;GLOD4;GMFB;GNAI1;GNB4;GPC4;GPR176;GPRIN1;GPSM1;GTDC1;HCN1;HDAC4;HERC4;HHAT;HK1;HPRT1;HSF2BP;HSPB11;INTS3;JAK2;JUND;KCNN2;KIAA1239;KIAA1549;KLHL12;KPNA1;KPNA3;KPNA4;LOC728024;LPHN2;LRRC24;MADD;MAP4K3;MAPK9;MAST2;MBOAT2;MCM6;MEAF6;METTL5;MMP16;MNT;MPHOSPH6;MRPL49;MSL3;MTFMT;MYL12B;NAA10;NGFRAP1;NHS;NOV;NPAS1;NPNT;NUDCD1;NUDT4;OPHN1;PCYOX1L;PCYT1B;PDE1A;PDK1;PGRMC1;PHF13;PIK3AP1;PITPNB;PLAA;PLAU;POLB;PPFIA2;PPP1R14C;PRDM8;PRNP;PSMD1;PSME3;PTGER4;PTPRK;PUM1;PUSL1;PVRL3;PXDN;R12;RALYL;RASA1;RASL11B;RFTN1;RHOF;RNF38;RPRD2;RPTOR;SATB1;SCML4;SCUBE1;SEC24D;SEC63;SERGEF;SETDB2;SHISA5;SKAP2;SLC24A4;SLC25A14;SLC4A11;SMYD2;SNAP29;SOX4;SOX5;SP8;SPIN1;SRP19;SSBP3;SSSCA1;ST3GAL1;STT3B;STX12;SULF2;TCP1;TECPR1;TLE2;TM2D3;TMEM117;TOPBP1;TP53BP1;TP53I11;TSR1;TWIST2;TXNL4A;UAP1;UBE2S;UBE2T;USP10;VAPA;VAV2;VDR;VEZT;VGLL4;VMA21;WDR5;WDR82;YTHDC2;ZCCHC17;ZDHHC21;ZNF697"
"cisbp__M5780"	4.03	0.0462	"RFX5 (directAnnotation). "	51	"ARHGAP25;BAIAP3;C10orf107;C10orf46;C21orf59;C9orf117;CCDC105;CDC14A;CHD5;DNAJA1;DYNLL1;EFHD2;FGA;FYN;GALNT2;GPATCH2;GPR179;HMGCR;KIAA1967;LDOC1L;LOC145837;LOC642852;LRRC27;MYADM;NHS;NT5C3;NUAK1;NUDCD1;OSTN;PCYT1B;PNMA1;PNMA5;PNMAL1;PPM1G;PRPF40B;PUM1;RALYL;RPTOR;RTN1;SCML4;SLC25A14;SPATA7;SSSCA1;SULF2;THTPA;TMIE;TP53BP1;TPM3;TTLL1;UFM1;ZNF483"
"cisbp__M5779"	3.82	0.0452	"RFX5 (directAnnotation). "	54	"ARHGAP25;ATF2;BAIAP3;BHLHB9;C10orf107;C10orf46;C21orf59;C9orf117;CCDC105;CCDC28A;CHD5;DNAJA1;DYNLL1;EFHD2;FGA;FYN;GALNT2;GPATCH2;GPR179;HMGCR;KIAA0947;KIAA1967;LDOC1L;LOC642852;LRRC27;MYADM;NHS;NT5C3;NUAK1;NUDCD1;OSTN;PCYT1B;PNMA1;PNMA5;PNMAL1;PPM1G;PRNP;PRPF40B;PUM1;RALYL;RPTOR;RTN1;SCML4;SLC25A14;SSSCA1;SULF2;TGFB2;THTPA;TMIE;TP53BP1;TPM3;TTLL1;UFM1;ZNF483"
"cisbp__M3830"	3.75	0.0449	"RFX1 (directAnnotation). "	43	"ANKRD16;BAIAP3;C10orf107;C10orf46;C21orf59;C9orf117;CCDC105;CDC14A;CDH10;CHD5;DNAJA1;DNAJA4;EFHD2;FYN;GPATCH2;GPR179;KIAA1967;KIF3C;LDOC1L;LOC642852;MYADM;NANOS3;NHS;NUDCD1;PCYT1B;PNMA1;PNMA5;PNMAL1;PPM1G;PRNP;PUM1;RALYL;RPTOR;RTN1;SCML4;SLC25A14;THTPA;TMIE;TP53BP1;TPM3;TTLL1;UFM1;YTHDC2"
"cisbp__M2199"	3.73	0.0447	""	98	"ACTR10;ACVR2A;AGAP3;APAF1;ATP6V0C;AZIN1;C11orf80;C12orf65;C6orf1;CALM3;CBX1;CDC42SE2;CDC5L;CDKN2AIPNL;CHD5;CHERP;CMAS;CREG2;DCP2;DNAJA4;EBAG9;EFHD2;EFNB1;FAM58A;FMN1;G3BP2;GALNT2;GFRA2;GLUD2;GMFB;GOPC;HCN1;HDAC4;KCNN2;KIAA1239;KIAA1549;KPNA3;LOC145837;LPHN2;MADD;MAMLD1;MAP4K3;MEAF6;MMP16;MNT;MSL3;MYADM;MYL12B;N1;NHS;NOBOX;NR4A2;NT5C3;NTNG2;OPTN;OSR1;PDZRN4;PEX11B;PHF13;PPM1F;PPP1R2P3;PRNP;PSME3;PTPRK;RALYL;RAPH1;RASA1;RFK;RFTN1;RTN1;SLC39A10;SLC4A11;SLC6A15;SLCO2A1;SMYD2;SOX5;SP8;SPATS2L;SPIN1;SSBP3;SSSCA1;STT3B;STXBP5;TLE2;TM2D3;TMEM117;TMEM170B;TP53BP1;TPM3;TUBB2A;TYRO3;UBE2K;VAPA;VAV2;VMA21;YWHAH;ZDHHC21;ZNF697"
"cisbp__M2158"	3.64	0.0443	""	64	"ACVR2A;ANKRD16;APAF1;ATP6V0C;BAIAP3;C3orf14;CALM3;CCDC149;CCDC157;CDC14A;CHIC2;CTBP2;DENND3;DNAJA4;FAT4;G3BP2;GATA4;GDI1;GLDC;GLRA2;GOLGA7B;HK1;HSPB11;INTS3;KIAA1239;KIAA1549;KIF3C;MAPK1IP1L;MEAF6;MYL12B;N1;NHS;NT5C3;NUDT4;OPHN1;OPTN;PAPOLG;PDZRN4;PHF13;PPP1R2P3;PRKCI;PUM1;RASL11B;RNASEH2A;SAE1;SCARA5;SCUBE1;SLC39A10;SMYD2;SNUPN;SOX5;SP8;SPATA7;SSBP3;TDRKH;TM2D3;TMEM117;TMEM132A;TRAPPC2L;TYRO3;VAPA;VDR;VMA21;ZC3HAV1"
"cisbp__M0396"	3.59	0.0441	"OSR2 (inferredBy_Orthology). "	34	"ANKRD16;ARHGAP25;ARMCX1;ARMCX2;ATP8B4;BEX2;C10orf32;CTBP2;F8;FGA;FOXP4;GALNT2;GLRA2;GPATCH2;KIAA1967;LIPC;LOR;METTL5;MMP16;MYCBP2;NDUFA11;PCDH17;PCYT1B;PDE1A;RASA1;RNF38;RPRD2;SLC25A14;SLC36A1;SLITRK1;SMYD1;SOX5;SPATS2L;UCMA"
"cisbp__M5777"	3.57	0.044	"RFX4 (directAnnotation). "	39	"ARHGAP25;BAIAP3;C10orf107;C10orf46;C21orf59;CCDC105;CDC14A;CHD5;DNAJA1;DYNLL1;EFHD2;FYN;GALNT2;GPATCH2;GPR179;HMGCR;KIAA1967;LDOC1L;LOC642852;LRRC27;MYADM;OSTN;PCYT1B;PNMA1;PNMA5;PNMAL1;PPM1G;PUM1;RALYL;RPTOR;RTN1;SCML4;SLC25A14;SSSCA1;THTPA;TMIE;TP53BP1;TPM3;UFM1"
"cisbp__M6077"	3.55	0.0439	"RFX3 (inferredBy_Orthology). "	37	"ARHGAP25;BAIAP3;C10orf107;C10orf46;C21orf59;CCDC105;CHD5;DNAJA1;EFHD2;GALNT2;GPATCH2;GPR179;HMGCR;KIAA1967;LDOC1L;LOC642852;LRRC27;MYADM;NHS;NUDCD1;OSTN;PCYT1B;PNMA1;PNMA5;PNMAL1;PPM1G;PUM1;RALYL;RPTOR;SCML4;SLC25A14;SULF2;THTPA;TMIE;TP53BP1;TPM3;UFM1"
"cisbp__M5775"	3.52	0.0437	"RFX3 (directAnnotation). "	40	"ARHGAP25;BAIAP3;C10orf107;C10orf46;C21orf59;C9orf117;CCDC105;CDC14A;CHD5;DNAJA1;DYNLL1;EFHD2;FYN;GALNT2;GPATCH2;GPR179;HMGCR;KIAA1967;LDOC1L;LOC642852;LRRC27;MYADM;NHS;OSTN;PCYT1B;PNMA1;PNMA5;PNMAL1;PPM1G;PUM1;RALYL;RPTOR;RTN1;SCML4;SLC25A14;THTPA;TMIE;TP53BP1;TPM3;UFM1"
"cisbp__M6139"	3.5	0.0437	"AHR (directAnnotation). "	41	"AGAP3;ANKRD16;AP2A2;APAF1;ARRB2;ATP6V1D;C12orf65;C12orf73;CALM3;CCDC87;CDC14A;CXorf26;DCP2;EBAG9;G3BP2;GPR176;GPSM1;GRK6;JUND;LRRC24;M6PR;MMP16;NPAS1;NR4A2;POLB;PPFIA2;PRDM8;PTPRK;RALYL;RPRD2;SEL1L;SLC1A4;SLITRK1;SOX4;SP8;SPATS2L;TNIP1;TP53BP1;UBE2S;VAV2;VEZT"
"cisbp__M6075"	3.5	0.0436	"RFX2 (inferredBy_Orthology). "	41	"ARHGAP25;BAIAP3;C10orf107;C10orf46;C21orf59;CCDC105;CDC14A;CHD5;DNAJA1;DYNLL1;EFHD2;FYN;GALNT2;GPATCH2;GPR179;HMGCR;KIAA1967;LDOC1L;LOC642852;LRRC27;MYADM;NHS;NUDCD1;OSTN;PCYT1B;PNMA1;PNMA5;PNMAL1;PPM1G;PUM1;RALYL;RPTOR;RTN1;SCML4;SLC25A14;SSSCA1;THTPA;TMIE;TP53BP1;TPM3;UFM1"

"motif"	"NES"	"AUC"	"TF_highConf"	"nEnrGenes"	"enrichedGenes"
"cisbp__M4678"	3.43	0.0433	"MXI1 (directAnnotation). "	53	"ARHGAP25;BAIAP3;C10orf107;C10orf46;C21orf59;C9orf117;CCDC105;CCDC28A;CDC14A;CHCHD6;CHD5;DNAJA1;DYNLL1;EFHD2;FYN;GALNT2;GPATCH2;GPR179;HMGCR;JUND;KIAA1967;LDOC1L;LOC642852;LRRC27;MYADM;NGFRAP1;NHS;NT5C3;NUAK1;OSTN;PCYT1B;PNMA1;PNMA3;PNMA5;PNMAL1;PPM1G;PPP1R2P3;PUM1;RALYL;RFK;RSPO2;RTN1;SCML4;SETDB2;SLC25A14;SSSCA1;THTPA;TMEM170B;TMIE;TP53BP1;TPM3;TTLL1;UFM1"
"cisbp__M1529"	3.43	0.0433	"RFX7 (inferredBy_Orthology). "	44	"ATP8B4;AZIN1;BAIAP3;C4orf45;C9orf117;CDC14A;CHD5;EFHD2;GPATCH2;GPR179;HDAC4;JUND;KCNN2;KIAA1967;KIF3C;LDOC1L;MNT;MYADM;NANOS3;NGFRAP1;PDE1A;PDZRN4;PNMA1;PNMA5;PNMAL1;PPM1G;PRPF40B;PUM1;RTN1;SCML4;SLC25A14;SLC36A1;SLITRK1;SP8;SULF2;THTPA;TMEM117;TMIE;TP53BP1;TPM3;TTLL1;TTLL10;TWIST2;UFM1"
"cisbp__M6453"	3.36	0.043	"RFX3 (directAnnotation). "	61	"ARHGAP25;BAIAP3;BHLHB9;C10orf107;C10orf46;C21orf59;C9orf117;CCDC105;CDC14A;CDH10;CHD5;DNAJA1;EFHD2;FGA;FOXP4;FYN;GALNT2;GPATCH2;GPR179;HMGCR;KCNN2;KIAA1967;LDOC1L;LOC642852;LRRC27;MMP1;MYADM;NGFRAP1;NHS;NT5C3;NUAK1;NUDCD1;OSTN;PCYT1B;PDE1A;PDZRN4;PIK3CG;PNMA1;PNMA5;PNMAL1;PPM1G;PRNP;PRPF40B;PRSS2;PUM1;RALYL;RPTOR;RTN1;SCML4;SLC25A14;SSSCA1;SULF2;SYTL2;THTPA;TMEM117;TMIE;TP53BP1;TPM3;TTLL1;TTLL10;UFM1"
"cisbp__M5773"	3.33	0.0428	"RFX2 (directAnnotation). "	38	"ARHGAP25;BAIAP3;C10orf107;C10orf46;C21orf59;CCDC105;CHD5;DNAJA1;EFHD2;FYN;GALNT2;GPATCH2;GPR179;HMGCR;KIAA1967;LDOC1L;LOC642852;LRRC27;MYADM;NHS;NUDCD1;OSTN;PCYT1B;PNMA1;PNMA5;PNMAL1;PPM1G;PUM1;RALYL;RPTOR;RTN1;SCML4;SLC25A14;THTPA;TMIE;TP53BP1;TPM3;UFM1"
"cisbp__M2392"	3.23	0.0424	"RFX2 (directAnnotation). "	80	"ARHGAP25;BAIAP3;BHLHB9;C10orf107;C10orf46;C12orf65;C21orf59;C4orf45;C9orf117;CADM3;CCDC105;CCDC28A;CDC14A;CHCHD6;CHD5;CNPY3;DNAJA1;DNAJA4;DYNLL1;EFHD2;FGA;FMN1;FYN;GALNT2;GPATCH2;GPR179;HMGCR;JAK3;JUND;KCNN2;KIAA0947;KIAA1967;LDOC1L;LOC642852;LRRC27;MCFD2;MYADM;NGFRAP1;NHS;NLK;NT5C3;NUAK1;NUDCD1;OPHN1;OSTN;P2RY6;PCYT1B;PDE1A;PIK3CG;PNMA1;PNMA5;PNMAL1;PPM1G;PPP1R2P3;PRPF40B;PRSS2;PTGER4;PUM1;RALYL;RASA1;RFK;RPTOR;RSPO2;RTN1;SCML4;SLC25A14;SPATA7;SSSCA1;SULF2;SYTL2;TGFB2;THTPA;TMIE;TP53BP1;TPM3;TTLL1;TTLL10;UFM1;VGLL4;ZNF483"
"cisbp__M1524"	3.21	0.0422	""	36	"ATP8B4;AZIN1;BAIAP3;C4orf45;C9orf117;CDC14A;CHD5;EFHD2;GPATCH2;GPR179;JUND;KCNN2;KIAA1967;MYADM;NANOS3;NGFRAP1;PDE1A;PDZRN4;PNMA1;PNMA5;PNMAL1;PPM1G;PRPF40B;PUM1;RALYL;RTN1;SCML4;SLC25A14;SLC36A1;SLITRK1;SULF2;TMEM117;TP53BP1;TPM3;TTLL10;TWIST2"
"cisbp__M0384"	3.19	0.0422	""	31	"ANKRD16;ARHGAP25;ARMCX2;ATF2;ATP8B4;CPZ;EFTUD1;ETNK1;FGA;GLRA2;GPATCH2;GPR173;KIAA1967;LIPC;LRRC28;MRPL49;MYCBP2;NDUFA11;PCDH17;PCYT1B;PDE1A;RASA1;RNF38;SCAND1;SLC36A1;SLITRK1;SMYD1;SSSCA1;TNIP3;TRMT6;UCMA"
"cisbp__M2308"	3.13	0.0419	"RFX1 (inferredBy_Orthology). "	46	"ARHGAP25;BAIAP3;C10orf107;C10orf46;C21orf59;C9orf117;CCDC105;CCDC28A;CDC14A;CHCHD6;CHD5;DNAJA1;DYNLL1;EFHD2;GALNT2;GPATCH2;GPR179;HMGCR;KIAA1967;LDOC1L;LOC642852;LRRC27;MYADM;NHS;OSTN;PCYT1B;PDE1A;PNMA1;PNMA5;PNMAL1;PPM1G;PUM1;RALYL;RTN1;SCML4;SLC25A14;SLITRK1;SSSCA1;SULF2;THTPA;TMIE;TP53BP1;TPM3;TTLL1;TTLL10;UFM1"
"cisbp__M1533"	3.11	0.0418	""	43	"ARHGAP25;ATP8B4;AZIN1;BAIAP3;C4orf45;C9orf117;CDC14A;CHD5;EFHD2;GPATCH2;GPR179;JUND;KCNN2;KIAA1967;KIF3C;LDOC1L;MYADM;NANOS3;NGFRAP1;NHS;PDE1A;PDZRN4;PIK3CG;PNMA1;PNMA5;PNMAL1;PPM1G;PRPF40B;PUM1;RALYL;RTN1;SCML4;SLC25A14;SLC36A1;SLITRK1;SULF2;TMEM117;TMIE;TP53BP1;TPM3;TTLL1;TTLL10;TWIST2"
"cisbp__M2110"	3.1	0.0417	""	40	"ANKRD16;ARHGAP25;ATP8B4;BEX2;CALHM1;CCDC105;CCIN;CHIC2;EGFL7;F8;FARSB;GDA;GPC4;INTS3;ITGA2B;JAK3;KIAA1967;LIPC;M6PR;MADD;MYADM;MYCBP2;NLK;NOV;NPM2;OPHN1;PIK3CG;PPFIA2;PTGER4;RASA1;SLC25A14;SLC4A11;SOX4;SPATS2L;SSTR1;TAC4;TFAP2D;TMEM150C;TMIE;VGLL4"
"cisbp__M2529"	3.08	0.0416	""	96	"LIM3;ACVR2A;AGAP3;ANKRD16;APAF1;ARHGAP25;ARMCX1;ASXL3;B4GALT5;C12orf65;C21orf59;CADM3;CBX1;CHCHD6;CHD5;CHERP;CHIC2;DAK;DENND3;DNAJA1;EBAG9;EFHD2;FAM174B;FRMD3;FYN;G3BP2;GALNT2;GDI1;GFRA2;GMFB;GOLGA7B;GTDC1;HCN1;HDAC4;HIVEP1;JAK3;KCNN2;KIAA1239;KIAA1549;KIAA1967;LASS6;MAMLD1;MAP4K3;MCFD2;MMP16;MRPL49;MTFMT;MYADM;MYCBP2;MYL12B;N1;NGFRAP1;NHS;NT5C3;OPHN1;OPTN;PCMT1;PDZRN4;PHF13;PIK3AP1;PIK3CG;PNMA2;POLR2L;PPFIA2;PTPRK;PUM1;PVRL3;RALYL;RASA1;RASL11B;RNASEH2A;RTN1;SATB1;SLC24A4;SLC25A14;SLC6A15;SLCO2A1;SMYD2;SP8;SPATS2L;SPIN1;SSBP3;STT3B;STXBP5;TMEM117;TMEM170B;TOPBP1;TP53BP1;TTC9B;TUBB8;TWIST2;TYRO3;USP15;VAV2;YWHAH;ZNF697"
"cisbp__M5010"	3.08	0.0416	"TCF12; TCF3; TCF4 (inferredBy_Orthology). "	24	"ATF2;B4GALNT3;DACH2;DNAJA4;EBAG9;GLDC;GOLGA7B;KCNN2;KRTAP5-5;MYO7A;PCYOX1L;PIK3AP1;PNMA3;RALYL;REP15;RNF38;RTN1;SATB2;SKAP2;SLC25A14;TAC4;UCMA;WNT10B;ZNF483"
"cisbp__M5371"	3.08	0.0416	"EGR4 (directAnnotation). "	188	"LIM3;ACTR10;AKAP7;ANKRD16;ANKRD50;APAF1;ARHGAP25;ARRB2;ASXL3;ATF2;ATP1B4;AZIN1;B4GALNT3;B4GALT2;BAIAP3;BEX5;BHMT;BTNL8;C10orf46;C15orf23;C16orf73;C19orf12;C1orf91;C1QL4;C3orf14;C6orf1;C9orf40;CALM3;CCDC28A;CCNH;CHD5;CHIC2;CMAS;CPSF6;CREG2;CSGALNACT1;CSNK1G3;CTBP2;CTNNAL1;CXorf26;DCP2;DDA1;DNAJA1;EBP;EPS8L1;FP3;FAM126A;FAM173A;FARSB;FAT4;FBXO21;FDX1L;G3BP2;GALNT2;GATA4;GDAP1L1;GFRA2;GLDC;GLRA2;GMIP;GNAI1;GOPC;GPSM1;GRK6;GRPR;GTDC1;HCN1;HERC4;KIAA0947;KIF3C;KLK7;KPNA1;KPNA3;KPNA4;LCMT1;LEPREL1;LIPH;LOC642852;LRP11;LRRC27;M6PR;MADD;MAMLD1;MBP;MCFD2;MED31;METTL5;MMP16;MNT;MPHOSPH6;MPP3;MRPL49;MSL3;MUM1L1;MYL12B;NAA11;N1;NANOS3;NDUFA5;NGFRAP1;NHS;NLK;NOV;NPDC1;NR4A2;NUDCD1;NUDT14;NUDT4;OAS1;OSBPL10;P2RY6;PEX11B;PHF13;PLAA;PLEKHM3;POLB;PPFIA2;PPM1F;PPM1G;PPP1R14C;PPP1R2;PRDM8;PROSC;PRSS1;PRSS2;PTGER4;PTTG1;PUSL1;R12;RALYL;RAPH1;RASA1;RHCG;RNASEH2A;RPRD2;RRAGB;RSPO2;SATB2;SCAND1;SCML4;SETDB2;SKAP2;SLC17A7;SLC1A4;SLC25A14;SLC39A10;SLC9A6;SLITRK1;SOX4;SOX5;SP8;STEAP1;STX7;SUB1;SULF2;SYTL2;TDRKH;TFAP2D;THEMIS;TM2D3;TMEM54;TP53BP1;TRIM15;TTC9B;TUBB2A;TUBB8;TWIST2;UAP1;UBE2S;UBE2T;UBXN8;UFM1;USP15;VAC14;VAPA;VAV2;VDR;VEZT;VGLL4;VPS18;WIPI2;XYLT1;YWHAH;ZC3HAV1;ZDHHC21;ZNF280C;ZNF697;ZNF821"
"cisbp__M0431"	3.07	0.0416	"OSR1 (inferredBy_Orthology). "	30	"ANKRD16;ARHGAP6;ARMCX1;ARMCX2;ATP8B4;BEX2;C15orf23;CALM3;F8;FGA;FMN1;FOXP4;GPATCH2;KIAA1967;MYCBP2;NANOS3;OPHN1;PCDH17;PDE1A;PIK3CG;RASA1;RPRD2;SLC24A4;SLC25A14;SLC36A1;SMYD1;SPATS2L;SULF2;TWIST2;UCMA"

"motif"	"NES"	"AUC"	"TF_highConf"	"nEnrGenes"	"enrichedGenes"
"cisbp__M2258"	3.05	0.0415	"OSR1; OSR2 (inferredBy_Orthology). "	42	"ANKRD16;ARHGAP25;ARMCX2;ATP8B4;B4GALT2;C12orf73;CDC42SE2;CPZ;CTBP2;ETNK1;FOXP4;FYN;G3BP2;GPR173;KCNN2;KIAA1967;LOC642852;LOR;LRRC28;MADD;MAGEB18;MMP1;MMP16;NANOS3;NDUFA11;NUDT10;PCDH17;PCYT1B;PDE1A;PDZRN4;PPP1R2P3;RNF38;SLC36A1;SLC9A6;SLITRK1;SMYD1;SPATS2L;SYTL1;TNIP3;UCMA;VGLL4;VIP"
"cisbp__M1789"	3.01	0.0413	""	194	"ACOT2;ACVR2A;ADCK2;AGAP3;ANKRD16;AP2S1;APAF1;ARHGAP6;ARHGDIA;ARRB2;ASXL3;ATF2;ATP6V0C;AZIN1;B4GALT2;B4GALT5;BAIAP3;BEX2;BEX5;BRE;C10orf46;C11orf80;C12orf65;C14orf169;C16orf73;C1QL4;C6orf1;C9orf40;CALM3;CAMLG;CBX1;CCDC149;CCRN4L;CDC14A;CDC5L;CELSR1;CHCHD6;CHD5;CHERP;CHIC2;CMAS;CREG2;CTBP2;CXorf26;DENND3;DNAJA1;DRD5;EBAG9;EFHD2;EIF4EBP1;FAM132A;FAM175B;FAM58A;FBXW9;FMN2;FMNL3;FOXP4;G3BP2;GADD45GIP1;GALNT2;GATA4;GDI1;GLOD4;GMFB;GNAI1;GNB4;GPC4;GPR176;GPRIN1;GPSM1;GRK6;HCN1;HDAC4;HERC4;HHAT;HPRT1;HSF2BP;HSPB11;INTS3;JAK2;JUND;KCMF1;KCNN2;KIAA0947;KIAA1239;KIAA1549;KIAA1967;KIF3C;KLHL12;KPNA1;KPNA3;KPNA4;LCMT1;LDOC1L;LPHN2;LRP11;LRRC24;M6PR;MADD;MAP4K3;MAPK9;MAST2;MEAF6;MFSD4;MMP16;MMP28;MNT;MPHOSPH6;MSL3;N1;NGFRAP1;NHS;NOLC1;NOV;NPAS1;NUDCD1;NUDT4;PDK1;PDZRN4;PHF13;PIK3AP1;PITPNB;PLAA;POLB;PPM1G;PPP1R14C;PRDM8;PRKCI;PRNP;PROSC;PRPF40B;PTGER4;PTPRK;PTTG1;PUM1;PUSL1;PVRL3;R12;R3GAP1;RALYL;RAPH1;RASA1;RASL11B;RFTN1;RHCG;RHOF;RNF38;RPRD2;SATB1;SATB2;SCUBE1;SEC24D;SEC63;SEL1L;SKAP2;SLC1A4;SLC24A4;SLC39A10;SLC4A11;SLCO2A1;SMYD2;SOX4;SOX5;SP8;SPIN1;SRP19;SSBP3;STT3B;STX12;SYTL2;TAF1B;TECPR1;TLE2;TMEM170B;TP53BP1;TP53I11;TPM3;TUBB8;TWIST2;UBE2S;UBE2T;USP15;VAV2;VEZT;VGLL4;VMA21;WDR5;WDR82;WIPI2;XYLT1;ZCCHC17;ZNF277;ZNF697;ZNF821"

fMRI Emotion Neg Subc

"motif"	"NES"	"AUC"	"TF_highConf"	"nEnrGenes"	"enrichedGenes"
"cisbp__M2710"	3.97	0.0916	""	10	"ELMO2;KCND2;LEF1;LRRN1;NPR1;PHF16;RNF44;TFAP2E;VTN;ZNF503"
"cisbp__M1669"	3.93	0.0911	""	16	"ADCY3;CA12;CACNA1A;DZIP1L;FAM110A;GPR85;HRH2;INHBA;NRG2;PCP4;PNPLA5;SMAD7;TNFSF13;UST;VANGL1;ZNF710"
"cisbp__M0512"	3.89	0.0905	"EGR1; EGR2; EGR3; EGR4; WT1 (inferredBy_Orthology). "	9	"CACNA1A;COL1A1;GPR85;KCTD17;NPR1;SMAD7;TMEM90A;UST;ZNF710"
"cisbp__M0511"	3.82	0.0895	"EGR1; EGR2; EGR3; EGR4; WT1 (inferredBy_Orthology). "	11	"CACNA1A;ELMO2;GPR85;KCND2;KCTD17;LEF1;RASGRP2;SLC35D3;SLC9A5;SMAD7;TMEM90A"
"cisbp__M2382"	3.74	0.0885	""	11	"GPR85;HMGCS2;HRH3;KLHL13;LRRN1;MME;SMAD7;TFAP2E;THSD4;UST;ZNF503"
"cisbp__M0568"	3.72	0.0881	""	11	"CACNA1A;CDH6;COL1A1;KLHL13;LEF1;NPR1;SMAD7;TNFSF13;UST;WNT10A;ZNF710"
"cisbp__M0397"	3.62	0.0867	"ZNF263 (inferredBy_Orthology). "	15	"CDH6;COL1A1;DZIP1L;GRM4;INSM2;LEF1;LRRN1;NRG2;RNF175;TMEM90A;TNFSF13;TSPAN2;UST;ZNF205;ZNF503"
"cisbp__M6483"	3.57	0.086	"SP4 (directAnnotation). "	9	"CACNA1A;CDH6;CHST11;LEF1;MME;SMAD7;TMEM90A;UST;ZNF503"
"cisbp__M5371"	3.55	0.0858	"EGR4 (directAnnotation). "	13	"HD12B;ADCY3;C3orf70;CACNA1A;CASP7;CDH6;GRAMD2;LEF1;NPR1;NRG2;PCP4;RASGRP2;SGPP2"
"cisbp__M1381"	3.42	0.0839	""	9	"CACNA1A;CHST11;GPR85;KLHL13;LEF1;LRRN1;PCDHGA1;RASGRP2;ZNF710"
"cisbp__M0654"	3.39	0.0836	""	11	"CACNA1A;CHST11;COL1A1;ELMO2;KCND2;LEF1;LRRN1;SMAD7;TFAP2E;UST;ZNF710"
"cisbp__M0550"	3.31	0.0823	""	14	"ADCY3;COL1A1;FAM110A;GKN1;GPR85;KIAA1377;NPR1;RASGRP2;SMAD7;TBC1D16;TFAP2E;TMEM90A;TNFSF13;ZNF710"
"cisbp__M4959"	3.24	0.0815	"PBX4 (inferredBy_Orthology). "	17	"HD12B;BSPRY;CACNA1A;CD226;CHST11;COL1A1;HMGCS2;KCND2;LEF1;LIN28B;LRRN1;PCP4;PGC;RNF44;SMAD7;UST;ZNF503"
"cisbp__M1740"	3.21	0.0811	""	11	"ALKBH1;CACNA1A;GPR85;NPR1;PRKCH;RTTN;SLC35D3;SLC9A5;SMAD7;TMEM90A;ZNF710"
"cisbp__M5430"	3.21	0.081	"FIGLA (directAnnotation). "	9	"CHST11;FAM110A;LRRN1;NEB;PHF16;PNPLA5;SGPP2;TNFSF13;WNT10A"
"cisbp__M5065"	3.19	0.0807	""	16	"C1RL;C2orf54;CACNA1A;CDH6;COL1A1;FAM110A;GOLGA6C;LEF1;MME;NEXN;SMAD7;TNFSF13;UST;VTN;WNT10A;ZNF503"
"cisbp__M6408"	3.14	0.08	"PAX5 (directAnnotation). "	16	"CA12;CACNA1A;COL1A1;GPR85;KCND2;LIN28B;NEXN;PNPLA5;PRKCH;RASGEF1B;SMAD7;TFAP2E;TMEM90A;UST;VTN;ZNF710"
"cisbp__M0542"	3.13	0.0799	""	6	"COL1A1;KCND2;LEF1;SMAD7;UST;ZNF710"
"cisbp__M5297"	3.09	0.0793	"BARHL2 (directAnnotation). "	20	"C18orf55;C2orf54;CHST11;DZIP1L;FAM110A;HRH2;KCNA3;KCND2;LIN28B;LRRN1;NRG2;RASGRP2;SLC35D3;TFAP2E;TSPAN2;UST;WNT10A;ZNF205;ZNF503;ZNF710"
"cisbp__M0499"	3.08	0.0792	""	25	"ADCY3;C2orf54;CACNA1A;CDH6;COL1A1;ELMO2;FAM110A;KCND2;KLHL13;LEF1;LIN28B;MME;NPR1;NRG2;PNPLA5;RASGRP2;SMAD7;TBX1;TMEM90A;TNFSF13;UST;VTN;WNT10A;ZNF503;ZNF710"
"cisbp__M6512"	3.05	0.0787	"TCF7L2 (directAnnotation). "	12	"C2orf54;CD226;COL1A1;KCND2;LEF1;LRRN1;NRG2;PGC;SMAD7;UST;ZNF503;ZNF710"
"cisbp__M0439"	3.03	0.0784	"ZIC2 (inferredBy_Orthology). "	12	"CACNA1A;COL1A1;DZIP1L;ELMO2;GPR85;KLHL13;LIN28B;SMAD7;TNFSF13;UST;WNT10A;ZNF710"
"cisbp__M4291"	3	0.0781	"EGR1; EGR2; EGR3; EGR4; WT1 (inferredBy_Orthology). "	14	"CACNA1A;CDH6;GPR85;KCND2;KCTD17;LEF1;NPR1;PAM;RASGRP2;RNF44;SLC35D3;SLC9A5;SMAD7;UST"

Neurosynth Reward Pos Cortex

"motif"	"NES"	"AUC"	"TF_highConf"	"nEnrGenes"	"enrichedGenes"
"cisbp__M3717"	4.49	0.16	"PAX4 (directAnnotation). "	11	"ADCYAP1;ASB2;BAIAP2L2;CBLN2;HAPLN3;HYAL2;KRT31;NANOS3;PABPC4L;SULF1;TRPC5"
"cisbp__M0015"	4.27	0.154	""	10	"ADCYAP1;CBLN2;COL23A1;DACH1;GALNTL1;KCNT2;PABPC4L;TGM2;TLE3;TRPC5"
"cisbp__M1428"	4.08	0.149	""	16	"ADCYAP1;ASB2;BAIAP2L2;DACH1;HYAL2;KRT31;LHX6;MAFF;MYCT1;MYLK3;NANOS3;PLK2;S1PR2;TGM2;TLE3;TRPC5"
"cisbp__M0431"	3.94	0.146	"OSR1 (inferredBy_Orthology). "	7	"DACH1;HYAL2;KCNT2;LHX6;NANOS3;TLE3;TRPC5"
"cisbp__M5236"	3.88	0.144	"AHR; AHRR (inferredBy_Orthology). "	9	"ADCYAP1;ASB2;BAIAP2L2;DACH1;HYAL2;KLF4;LHX6;MYLK3;S1PR2"
"cisbp__M2258"	3.73	0.14	"OSR1; OSR2 (inferredBy_Orthology). "	5	"DACH1;FMO2;KCNT2;SULF1;TLE3"
"cisbp__M0039"	3.66	0.138	""	8	"ADCYAP1;CBLN2;DACH1;KCNT2;LHX6;SULF1;TLE3;TRPC5"
"cisbp__M0396"	3.65	0.138	"OSR2 (inferredBy_Orthology). "	9	"ADCYAP1;DACH1;FMO2;HYAL2;KCNT2;LHX6;NANOS3;SULF1;TLE3"
"cisbp__M0394"	3.61	0.137	"PLAGL1 (inferredBy_Orthology). "	10	"BAIAP2L2;FHL3;HAPLN3;HYAL2;MYLK3;NANOS3;PTP4A3;SULF1;TLE3;TRPC5"
"cisbp__M5608"	3.61	0.137	"MAFG (directAnnotation). "	8	"ADCYAP1;HYAL2;KLF4;KRT31;MYLK3;S1PR2;SULF1;TLE3"
"cisbp__M0035"	3.51	0.135	""	6	"ADCYAP1;CBLN2;DACH1;LHX6;TLE3;TRPC5"
"cisbp__M1631"	3.46	0.133	"TBX20 (inferredBy_Orthology). "	9	"ASB2;HYAL2;KCNT2;KRT31;MAFF;NANOS3;S1PR2;SULF1;TRPC5"
"cisbp__M1635"	3.46	0.133	""	7	"HYAL2;KRT31;MAFF;NANOS3;S1PR2;SULF1;TRPC5"
"cisbp__M0003"	3.44	0.133	""	20	"ADCYAP1;ASB2;BAIAP2L2;CBLN2;COLEC12;FHL3;FMO2;GALNTL1;GRIN2D;HAPLN3;HYAL2;KCNT2;KRT31;MAFF;MYCT1;NANOS3;PABPC4L;SULF1;TGM2;TRPC5"
"cisbp__M4210"	3.39	0.132	""	9	"ADCYAP1;DACH1;GRIN2D;KLF4;KRT31;LHX6;PLK2;TLE3;TRPC5"
"cisbp__M0400"	3.37	0.131	"ZSCAN10 (inferredBy_Orthology). "	8	"FHL3;HYAL2;KCNT2;LHX6;NANOS3;SULF1;TLE3;TRPC5"
"cisbp__M5124"	3.29	0.129	"OSR1; OSR2 (inferredBy_Orthology). "	6	"DACH1;FMO2;HYAL2;MYCT1;SULF1;TLE3"
"cisbp__M5610"	3.26	0.128	"MAFK (directAnnotation). "	6	"ADCYAP1;KLF4;KRT31;MYLK3;SULF1;TLE3"
"cisbp__M1626"	3.23	0.127	"TBX2 (inferredBy_Orthology). "	19	"ASB2;COL23A1;DACH1;FHL3;FMO2;GALNTL1;GRIN2D;HAPLN3;HYAL2;KCNT2;KRT31;LHX6;MAFF;NANOS3;PLK2;S1PR2;SULF1;TGM2;TRPC5"
"cisbp__M5204"	3.23	0.127	"OSR1; OSR2 (inferredBy_Orthology). "	5	"DACH1;FMO2;HYAL2;SULF1;TLE3"
"cisbp__M0439"	3.21	0.127	"ZIC2 (inferredBy_Orthology). "	11	"BAIAP2L2;FHL3;FMO2;GRIN2D;HYAL2;KRT31;LHX6;NANOS3;SULF1;TLE3;TRPC5"
"cisbp__M4926"	3.18	0.126	""	8	"ADCYAP1;KCNT2;KLF4;MYLK3;NANOS3;PABPC4L;SULF1;TLE3"
"cisbp__M0577"	3.16	0.126	""	15	"ADCYAP1;ASB2;BAIAP2L2;DACH1;HYAL2;KCNT2;KRT31;MAFF;MYLK3;NANOS3;PLK2;PTP4A3;S1PR2;SULF1;TLE3"
"cisbp__M2081"	3.16	0.125	""	7	"ADCYAP1;BAIAP2L2;LHX6;PTP4A3;S1PR2;TGM2;TLE3"
"cisbp__M0021"	3.14	0.125	""	14	"ADCYAP1;CBLN2;COL23A1;DACH1;GRIN2D;KCNT2;KLF4;LHX6;PTP4A3;S1PR2;SULF1;TGM2;TLE3;TRPC5"
"cisbp__M1624"	3.05	0.123	""	8	"ASB2;COL23A1;HYAL2;KCNT2;KRT31;NANOS3;SULF1;TRPC5"
"cisbp__M2786"	3.05	0.123	""	8	"ASB2;HYAL2;KRT31;LHX6;MYCT1;SULF1;TLE3;TRPC5"
"cisbp__M0026"	3.03	0.122	""	8	"ADCYAP1;CBLN2;DACH1;GALNTL1;KCNT2;LHX6;PABPC4L;TLE3"

Neurosynth Reward Neg Cortex

"motif"	"NES"	"AUC"	"TF_highConf"	"nEnrGenes"	"enrichedGenes"
"cisbp__M5848"	5.21	0.114	"SOX9 (directAnnotation). "	5	"KRTAP10-4;LGR6;POM121C;PRKAB2;SPDYC"
"cisbp__M2189"	4.42	0.1	""	4	"C21orf81;HOXB9;LGR6;NOX5"
"cisbp__M5204"	4.4	0.0995	"OSR1; OSR2 (inferredBy_Orthology). "	5	"C20orf103;GJA9;KRTAP10-4;PAX8;TOPORS"
"cisbp__M1547"	4.2	0.096	""	4	"C1orf135;HOXB9;KIAA1530;TOPORS"
"cisbp__M2485"	4.09	0.0941	""	4	"HOXB9;INSRR;LGR6;TOPORS"
"cisbp__M6186"	3.97	0.0918	"DBP (directAnnotation). "	3	"AKNA;HOXB9;PAX8"
"cisbp__M5109"	3.92	0.091	"IRX1; IRX2; IRX3; IRX4; IRX5; IRX6 (inferredBy_Orthology). "	4	"HOXB9;IL22RA2;KRTAP10-4;PAX8"
"cisbp__M1872"	3.84	0.0895	""	5	"C8orf39;IL22RA2;KRTAP10-4;LGR6;PAX8"
"cisbp__M5123"	3.83	0.0893	"OSR1; OSR2 (inferredBy_Orthology). "	4	"GJA9;KRTAP10-4;PAX8;TOPORS"
"cisbp__M4105"	3.77	0.0884	""	4	"IL22RA2;INSRR;OR6C76;SH2D4A"
"cisbp__M0620"	3.71	0.0873	""	9	"AKNA;C14orf182;C20orf103;HOXB9;IL22RA2;INSRR;KRTAP10-4;LGR6;PAX8"
"cisbp__M1303"	3.7	0.0871	""	4	"C8orf39;HOXB9;INSRR;OR6C76"
"cisbp__M2281"	3.65	0.0861	"FOXH1 (directAnnotation). "	6	"GPR77;IL22RA2;KRTAP10-4;LGR6;PAX8;SH2D4A"
"cisbp__M1548"	3.63	0.0858	""	4	"C1orf135;HOXB9;KIAA1530;TOPORS"
"cisbp__M4795"	3.58	0.0848	"IRX1; IRX2; IRX3; IRX4; IRX5; IRX6 (inferredBy_Orthology). "	5	"AKNA;HOXB9;IL22RA2;KRTAP10-4;PAX8"
"cisbp__M5124"	3.56	0.0846	"OSR1; OSR2 (inferredBy_Orthology). "	3	"KRTAP10-4;PAX8;TOPORS"
"cisbp__M6247"	3.55	0.0844	"FOXO4 (directAnnotation). "	5	"C8orf39;HOXB9;IL22RA2;KRTAP10-4;LGR6"
"cisbp__M0384"	3.55	0.0843	""	4	"GJA9;KRTAP10-4;PAX8;TOPORS"
"cisbp__M0568"	3.49	0.0832	""	4	"HOXB9;INSRR;KRTAP10-4;LGR6"
"cisbp__M4808"	3.42	0.082	""	4	"AKNA;C20orf103;HOXB9;PAX8"
"cisbp__M0765"	3.35	0.0808	""	4	"C14orf182;C1orf135;C8orf39;SH2D4A"
"cisbp__M5493"	3.31	0.0801	"GMEB2 (directAnnotation). "	4	"C1orf135;HOXB9;KIAA1530;TOPORS"
"cisbp__M1248"	3.28	0.0795	""	5	"HOXB9;LGR6;NOX5;PAX8;ULBP2"
"cisbp__M1894"	3.23	0.0786	"PPARG (directAnnotation). "	7	"INSRR;KRTAP10-4;PAX8;POM121C;SERPINA5;SH2D4A;TOPORS"
"cisbp__M2377"	3.22	0.0785	""	3	"C8orf39;HOXB9;IL22RA2"
"cisbp__M4886"	3.22	0.0784	""	3	"C1orf135;HOXB9;TOPORS"
"cisbp__M6250"	3.19	0.078	"FOXQ1 (directAnnotation). "	4	"C14orf182;C8orf39;IL22RA2;KRTAP10-4"
"cisbp__M0081"	3.19	0.0779	""	4	"AKNA;C20orf103;INSRR;PAX8"
"cisbp__M5815"	3.15	0.0772	"SOX14 (directAnnotation). "	5	"C20orf103;GJA9;GPR77;LGR6;SPANXN2"
"cisbp__M1949"	3.15	0.0771	""	3	"C1orf135;HOXB9;KIAA1530"
"cisbp__M1192"	3.12	0.0765	""	3	"HOXB9;INSRR;OR6C76"
"cisbp__M4270"	3.11	0.0765	"GATA1; GATA2; GATA3; GATA4; GATA5; GATA6; TRPS1 (inferredBy_Orthology). "	3	"C8orf39;HOXB9;SH2D4A"
"cisbp__M4339"	3.1	0.0762	""	2	"C14orf182;C1orf135"
"cisbp__M4988"	3.09	0.0761	"GRHL1; GRHL2; GRHL3 (inferredBy_Orthology). "	3	"HOXB9;KRTAP10-4;PAX8"
"cisbp__M0606"	3.07	0.0758	""	3	"C1orf135;PAX8;TOPORS"
"cisbp__M0080"	3.06	0.0756	""	2	"INSRR;PAX8"
"cisbp__M0564"	3.06	0.0755	""	6	"CCDC88B;GPR77;HOXB9;POM121C;SECTM1;ULBP2"
"cisbp__M6225"	3.04	0.0752	"MECOM (directAnnotation). "	5	"HOXB9;LGR6;OR6C76;REXO1;SERPINA5"
"cisbp__M0431"	3.02	0.0748	"OSR1 (inferredBy_Orthology). "	4	"KRTAP10-4;LGR6;PAX8;TOPORS"
"cisbp__M0008"	3	0.0745	""	4	"C20orf103;HOXB9;INSRR;PAX8"

Neurosynth Reward Pos Subc

"motif"	"NES"	"AUC"	"TF_highConf"	"nEnrGenes"	"enrichedGenes"
"cisbp__M3830"	3.42	0.0545	"RFX1 (directAnnotation). "	22	"C16orf82;C9orf9;CCDC37;EFNA5;FAM133A;KIAA1009;KIAA1407;KLHDC9;LCLAT1;LMAN2L;LRRC36;LYST;MZF1;NUCB2;PCSK1N;RSPH1;SIX3;SSBP2;TEX9;TRIM66;TTC26;ZSCAN18"
"cisbp__M2309"	3.41	0.0545	"RFX5 (directAnnotation). "	26	"ADK;C16orf48;C16orf82;C1orf87;C9orf9;CCDC30;CST6;CXorf57;DDC;GHRL;INSRR;ITM2C;KIAA1009;KIAA1407;KLHDC9;LRRC36;MEPCE;MZF1;NUCB2;NWD1;PCSK1N;ROPN1B;SIX3;TEX9;TRIM66;TTC26"
"cisbp__M1129"	3.41	0.0544	""	41	"ADAMTS9;AMOT;BET3L;BLCAP;C16orf82;C9orf68;C9orf9;CCDC88C;CLU;CYR61;DDC;FAM196A;FNDC3A;GRID2;GUCA1A;HIST1H4H;HMGN1;HSPB9;ID4;KCNN3;LOC100289230;LRRC36;MYH7;PDLIM3;PRDM13;PTCH1;RXRG;SALL3;SERPINI2;SLC12A1;SMAD1;SSBP2;TMEM27;TRAF3IP2;TUBB2B;VAX1;WRN;YAP1;ZNF292;ZNF658;ZXDA"
"cisbp__M1271"	3.39	0.0543	""	21	"AMOT;ANP32E;C1orf9;CLDN23;CYR61;EFNA5;F3;FAM122C;HERC5;HIST1H4H;ID4;PHF21B;PTCH1;RBM43;S100A13;SIX3;SMAD1;SSBP2;VAX1;YAP1;ZNF395"
"cisbp__M1531"	3.21	0.0529	"RFX3 (inferredBy_Orthology). "	37	"ADK;C1orf87;C9orf9;CCDC11;CCDC30;CXorf57;CYR61;DGKK;FSTL5;GEM;GPC3;HKDC1;HMGN1;ID4;ITM2C;KIAA1407;KLHDC9;LCLAT1;LRRC36;MMP21;MZF1;PCSK1N;PDLIM3;RDH10;ROPN1B;RSPH1;S100A13;SIX3;SLC12A1;SSBP2;TRIM66;TTC26;WDR48;XRN1;YAP1;ZNF292;ZSCAN18"
"cisbp__M1595"	3.2	0.0528	"SOX6 (inferredBy_Orthology). "	17	"AMOT;BET3L;C9orf9;CCDC88C;CYR61;FEZF1;HMGN1;ID4;LOC100289230;MYH7;PHF21B;PRDM13;SALL3;SIX3;SSBP2;WRN;ZNF292"
"cisbp__M1539"	3.15	0.0524	""	33	"ABI3BP;ADK;AMOT;C9orf68;C9orf9;CCDC11;CCDC30;CCDC37;CXorf57;CYR61;DGKK;FNDC3B;HMGN1;ID4;ITM2C;KIAA1407;KLHDC9;LCLAT1;LRRC36;MMP21;MZF1;PCSK1N;PHF21B;RDH10;RSPH1;S100A13;SIX3;SSBP2;TRIM66;TTC26;XRN1;YAP1;ZNF292"
"cisbp__M1591"	3.13	0.0523	"SOX5 (inferredBy_Orthology). "	20	"AMOT;BET3L;C16orf82;C9orf9;CCDC88C;CYR61;FEZF1;HMGN1;HSPB9;ID4;LOC100289230;MYH7;PRDM13;SALL3;SSBP2;TRAF3IP2;TUBB2B;WRN;ZNF292;ZXDA"
"cisbp__M2555"	3.12	0.0521	"GRHL1; GRHL2; GRHL3 (inferredBy_Orthology). "	77	"ABI3BP;AMOT;ANP32E;BET3L;C4orf47;C5;CCDC30;CLU;DIAPH3;ERMAP;FAM122C;FAM133A;FAM167A;FNBP1L;FNDC3A;FSTL5;FUT10;GABRG1;GCNT2;GEM;GHRL;GRAMD1C;GRID2;GSTM1;GSTM2P1;GUCA1A;HAUS1;HERC5;HMGN1;IRS4;KCNN4;LIX1;LOC100129034;LOC282997;LRRC34;LRRC36;MEPCE;MNAT1;MRAS;MSH5;MYOZ1;NPHP1;PBXIP1;PHF21B;PLCB2;PPAPDC1B;PPIL4;PRDM13;PRLR;PTCH1;RAB34;RBM12;RBM43;RGS9;RYK;S100A13;SERP1;SFTA3;SFTPD;SIX3;SLC10A4;SLC22A5;SLC35F4;SRI;SSBP2;TNFSF11;TPH1;TTC26;UBE2CBP;VAX1;WDR48;WRN;XRN1;YAP1;ZNF292;ZNF334;ZNF395"
"cisbp__M1602"	3.09	0.0519	"SRY (inferredBy_Orthology). "	26	"AMOT;BET3L;C16orf82;C9orf9;CCDC88C;CYR61;FEZF1;GUCA1A;HMGN1;HSPB9;ID4;LOC100289230;LRRC36;MYH7;PHF21B;PNMT;PRDM13;PTCH1;RXRG;SALL3;SIX3;SSBP2;TRAF3IP2;WRN;ZNF292;ZXDA"
"cisbp__M1530"	3.03	0.0515	"RFX7 (inferredBy_Orthology). "	46	"ADAMTS9;ADK;AMOT;ANP32E;C1orf87;C9orf9;CCDC11;CCDC30;CCDC37;CHST7;CXorf57;CYR61;DGKK;EFNA5;FAM167A;FNDC3B;FSTL5;GPC3;HKDC1;HMGN1;ID4;ITM2C;KIAA1407;KLHDC9;LCLAT1;LMAN2L;LRRC36;MZF1;PCSK1N;PDLIM3;PRLR;RDH10;ROPN1B;RSPH1;S100A13;SIX3;SLC12A1;SSBP2;TEX9;TRIM66;TTC26;VAX1;XRN1;YAP1;ZNF292;ZSCAN18"
"cisbp__M1528"	3.02	0.0513	"RFX4 (inferredBy_Orthology). "	32	"ABI3BP;ADK;C1orf87;C9orf9;CCDC11;CCDC30;CCDC37;CXorf57;CYR61;DGKK;EFNA5;GPC3;HKDC1;ID4;KIAA1407;KLHDC9;LCLAT1;LRRC36;MMP21;MZF1;NWD1;PCSK1N;RDH10;RSPH1;S100A13;SIX3;SSBP2;TRIM66;TTC26;XRN1;YAP1;ZSCAN18"

Neurosynth Reward Neg Subc

"motif"	"NES"	"AUC"	"TF_highConf"	"nEnrGenes"	"enrichedGenes"
"cisbp__M2384"	4.32	0.0515	"ESRRA (directAnnotation). "	56	"ACSL6;ADRBK1;AES;ARPC1A;ATP1A4;ATP2A2;BHLHA15;C15orf59;CAPN1;CKMT1A;CKMT1B;DAND5;DEPDC5;DIRAS1;DNASE1;DNM1;DYSFIP1;FEM1B;GAS2L1;GPR61;HOMER2;HS6ST3;HSD3B7;IL18BP;INPP5J;JDP2;KHDRBS3;LCK;LYNX1;MAP1A;MAPK10;MAST1;MGAT4B;MYL2;NDRG3;NLGN3;PANK1;PPP5C;RHOT1;SCN8A;SERTAD1;SH3BP1;SLC25A19;SLC47A1;SLC6A7;STOX2;SYP;TCL1A;TFR2;TMEM211;TTC7B;UCK2;UPK3A;WNK2;ZCCHC5;ZKSCAN4"
"cisbp__M1459"	4.03	0.0498	""	65	"ABHD2;AKT3;ATP1B1;ATP6V0A1;BAG5;BTBD1;C3orf22;CAPN1;CKMT1A;CKMT1B;CRTAM;DEPDC5;DNM1;DYSFIP1;EHD3;EIF4G3;ELOVL4;GABRA1;GPR61;GRM1;HOMER2;HSD3B7;ICA1;IL18BP;INPP5J;JDP2;LRRC59;LYNX1;LYSMD2;MAP1A;MAPK10;MAST1;MGAT4B;NCALD;NDRG3;NFYC;NLGN3;NRBF2;PANK1;PCNXL3;PPEF1;PPFIA4;PPP2CA;PRR3;PRSSL1;PTPRS;RNF208;RNF26;RPS6KL1;RUSC2;SCN10A;SCN8A;SETD5;SH3BP1;SLC6A7;SMS;STOX2;SYP;SYT1;TGM1;TMEM119;TMEM211;UCK2;ULK2;WNK4"
"cisbp__M6215"	3.89	0.049	"ESRRB (directAnnotation). "	80	"ABHD2;ADRBK1;AES;AKT3;ARPC1A;ATP2A2;C15orf59;C1orf100;C3orf22;CA4;CAPN1;CCL11;CKMT1A;CKMT1B;DAND5;DEPDC5;DHCR24;DIRAS1;DNASE1;DNM1;DYSFIP1;EFHA2;FAM162B;FEM1B;GABRA1;GAS2L1;GPR61;GRM1;HMG20A;HOMER2;HSD3B7;IBSP;ICA1;IL12B;IL18BP;INPP5J;JDP2;KCNC1;LCK;LYNX1;MANSC1;MAP1A;MAPK10;MAST1;MGAT4B;MOSPD1;MYL2;NDRG3;NFAM1;NFYC;NLGN3;OR2T5;PANK1;PANK2;PPP5C;PRR3;RHOT1;RNF26;RPS6KL1;SCN8A;SERTAD1;SH3BP1;SLC25A19;SLC47A1;SLC6A7;STOX2;SUMO1;SYP;TCL1A;TFR2;TGM1;TMEM119;TMEM211;TTC7B;UCK2;UPK3A;WNK2;ZBTB22;ZCCHC5;ZKSCAN4"
"cisbp__M1958"	3.82	0.0486	"ESRRB (inferredBy_Orthology). "	92	"ACSL6;ACTBL2;ADRBK1;AES;AKT3;ALPP;ARHGEF3;ARPC1A;ATP1A4;ATP1B1;ATP2A2;BHLHA15;BRD2;C15orf59;C1orf100;C1orf172;C20orf151;C20orf200;C3orf22;C5orf13;CA4;CAPN1;CKMT1A;CKMT1B;DAND5;DEPDC5;DIRAS1;DNASE1;DNM1;DYSFIP1;EFHA2;EIF4G3;ELP3;FEM1B;GABRA1;GAS2L1;GPR61;GRM1;HMG20A;HOMER2;HSD3B7;IBSP;IL12B;IL18BP;INPP5J;JDP2;KCNJ11;KHDRBS3;LCE3B;LCK;LDLRAD2;LOC100134229;LYNX1;MANSC1;MAP1A;MAPK10;MAST1;MGAT4B;MOSPD1;MYL2;NDRG3;NFAM1;NFYC;NLGN3;OR2T5;PANK1;PPP5C;REEP2;RHOT1;RNF26;RPS6KL1;RSPO3;SCN8A;SERTAD1;SH3BP1;SLC25A19;SLC6A7;SNAP91;STOX2;SYP;TCL1A;TFR2;TMEM119;TMEM211;TMEM8B;UCK2;UPK3A;WNK2;ZBTB22;ZCCHC5;ZFYVE27;ZKSCAN4"
"cisbp__M6443"	3.79	0.0485	"RARA (directAnnotation). "	32	"ABHD2;AKT3;ATP1B1;BAG5;C3orf22;CAPN1;CRTAM;DIRAS1;DNM1;EHD3;EIF4G3;GABRA1;GPR61;GRM1;HOMER2;HSD3B7;ICA1;INPP5J;JDP2;LYNX1;MAP1A;MAPK10;PANK1;PCNXL3;PPP2CA;SH3BP1;STOX2;SYT1;TGM1;TMEM119;TMEM211;UCK2"
"cisbp__M6214"	3.74	0.0482	"ESRRA (directAnnotation). "	33	"ADRBK1;C15orf59;C3orf22;CAPN1;CKMT1A;CKMT1B;DEPDC5;DIRAS1;DNM1;DYSFIP1;GPR61;HOMER2;HSD3B7;IL18BP;INPP5J;JDP2;LYNX1;MAP1A;MAST1;MGAT4B;MYL2;NDRG3;NLGN3;PANK1;PPP5C;SCN8A;SH3BP1;SLC6A7;STOX2;SYP;TFR2;TMEM211;UCK2"
"cisbp__M5034"	3.66	0.0477	""	37	"ABHD2;ADRBK1;AES;C3orf22;CAPN1;CKMT1A;CKMT1B;DEPDC5;DIRAS1;DNM1;DYSFIP1;GABRA1;GLRB;GPR61;HOMER2;HSD3B7;IL18BP;INPP5J;JDP2;KCNC1;LYNX1;MAP1A;MAST1;MGAT4B;NFAM1;NLGN3;PANK1;PPP2CA;PPP5C;PRR3;SLC22A8;SLC6A7;SYP;TMEM211;TNR;TTC7B;UCK2"
"cisbp__M6430"	3.51	0.0468	"PPARA (directAnnotation). "	42	"ABHD2;ADRBK1;ATP1B1;BAG5;BHLHA15;C3orf22;CAPN1;CKMT1A;CKMT1B;CRTAM;DEPDC5;DIRAS1;DNM1;DYSFIP1;EHD3;GABRA1;GLRB;GPR61;HOMER2;HSD3B7;IL18BP;INPP5J;JDP2;KCNC1;LYNX1;MAP1A;MAPK10;MGAT4B;NLGN3;PANK1;PPP2CA;PRSSL1;RPS6KL1;SH3BP1;SLC6A7;STOX2;SYP;TMEM119;TMEM211;TMEM8B;TTC7B;UCK2"
"cisbp__M1494"	3.45	0.0465	""	50	"ABHD2;AES;ATP1B1;ATP6V0A1;BAG5;BTBD1;C3orf22;CAPN1;CKMT1A;CKMT1B;CRTAM;DEPDC5;DIRAS1;DNM1;DYSFIP1;ELOVL4;GABRA1;GPR61;GRM1;HOMER2;HSD3B7;ICA1;IL18BP;INPP5J;JDP2;KCNC1;LYNX1;MAP1A;MAPK10;MAST1;MGAT4B;NCALD;NLGN3;NRBF2;PANK1;PPP2CA;PPP5C;PRR3;PRSSL1;PTPRS;RNF26;SCN8A;SH3BP1;SLC6A7;STOX2;SYP;TMEM119;TMEM211;UCK2;ULK2"
"cisbp__M1458"	3.41	0.0462	""	107	"ABHD2;ACP2;ADCY10;ADRBK1;AES;AFF3;AKT3;ARFGEF2;ATP1A4;ATP1B1;ATP6V0A1;BAG5;BHLHA15;BRD2;BTBD1;C12orf5;C16orf57;C3orf22;C9orf169;CACNG7;CAPN1;CCL11;CHPF;CKMT1A;CKMT1B;CNTNAP1;CRTAM;DAND5;DEPDC5;DES;DNM1;DYSFIP1;EHD3;EIF4G3;ELOVL4;GABRA1;GLRB;GPR61;GRM1;HIF1AN;HOMER2;HSD3B7;ICA1;IGF1;IL18BP;INPP5J;ITGA4;JDP2;KCNC1;KLC2;LCE3B;LCK;LCN15;LRFN1;LRRC59;LYNX1;LYSMD2;MAP1A;MAPK10;MAST1;MGAT4B;MPZ;NCALD;NDRG3;NEUROG1;NFYC;NLGN3;NNT;NRBF2;PANK1;PANK2;PCNXL3;PPEF1;PPFIA4;PPP2CA;PPP5C;PQLC1;PRR3;PRSSL1;PTPRS;RAB6B;RAD51C;RNF208;RNF26;RPS6KL1;RUSC2;SCN10A;SCN8A;SETD5;SH3BP1;SLC16A7;SLC6A7;SMS;STOX2;SULT4A1;SYP;SYT1;TGM1;TMEM119;TMEM211;TMEM8B;TTC7B;TXNL1;UCK2;ULK2;WNK4;ZSCAN10"
"cisbp__M4938"	3.38	0.0461	""	39	"ABHD2;ADCY10;ADRBK1;ATP1B1;BAG5;C3orf22;CACNG7;CAPN1;CKMT1A;CKMT1B;DIRAS1;DNM1;DYSFIP1;EHD3;EIF4G3;GABRA1;GLRB;GPR61;HSD3B7;ICA1;INPP5J;JDP2;LYNX1;MAP1A;NEUROG1;NLGN3;PANK1;PCNXL3;PPP2CA;RPS6KL1;SH3BP1;SLC6A7;STOX2;SULT4A1;SYT1;TMEM119;TMEM211;UCK2;ULK2"
"cisbp__M1449"	3.35	0.0459	"ESRRG (inferredBy_Orthology). "	50	"ABHD2;ADRBK1;AES;ATP1B1;C15orf59;C1orf100;C3orf22;CAPN1;CKMT1A;CKMT1B;DEPDC5;DIRAS1;DNM1;DYSFIP1;GABRA1;GAS2L1;GPR61;HOMER2;HSD3B7;IL18BP;INPP5J;JDP2;KCNC1;LYNX1;MANSC1;MAP1A;MGAT4B;MYL2;NDRG3;NLGN3;PANK1;PPP5C;PRR3;RHOT1;RPS6KL1;RSPO3;SCN8A;SH3BP1;SLC25A19;SLC6A7;STOX2;SYP;TFR2;TGM1;TMEM119;TMEM211;TTC7B;UCK2;WNK2;ZCCHC5"
"cisbp__M1432"	3.31	0.0457	"NR2E1 (directAnnotation). "	36	"ABHD2;AKT3;ATP1B1;ATP6V0A1;BAG5;C3orf22;CHPF;CKMT1A;CKMT1B;CRTAM;DNM1;DYSFIP1;EHD3;GABRA1;GRM1;HSD3B7;ICA1;INPP5J;JDP2;KIAA1217;LYNX1;MAPK10;NLGN3;NRBF2;PANK1;PARD6A;PPP2CA;PRR3;PTPRS;RAD51C;SETD5;SH3BP1;SULT4A1;TMEM119;UCK2;WNK4"
"cisbp__M1448"	3.29	0.0456	"NR5A2 (inferredBy_Orthology). "	71	"ABHD2;ADRBK1;AES;ARPC1A;ATP1A4;ATP1B1;ATP2A2;BAG5;BHLHA15;C15orf59;C1orf100;C20orf151;C3orf22;CA4;CAPN1;CKMT1A;CKMT1B;DAND5;DEPDC5;DHCR24;DIRAS1;DNASE1;DNM1;DYSFIP1;EFHA2;FGFR4;GABRA1;GAS2L1;GPR61;HOMER2;HSD3B7;IL18BP;INPP5J;JDP2;KCNC1;LYNX1;MAP1A;MAPK10;MAST1;MGAT4B;MYL2;NCALD;NDRG3;NFAM1;NLGN3;OR2T5;PANK1;PPP5C;PRR3;RHOT1;RPS6KL1;RSPO3;SCN8A;SH3BP1;SLC25A19;SLC47A1;SLC6A7;SNAP91;STOX2;SUMO1;SYP;TCL1A;TDO2;TFR2;TGM1;TMEM119;TMEM211;TTC7B;UCK2;WNK2;ZBTB22"
"cisbp__M4564"	3.24	0.0453	"ESR1 (directAnnotation). "	30	"ADRBK1;ATP2A2;C15orf59;C1orf100;CA9;CAPN1;CKMT1A;CKMT1B;DAND5;DEPDC5;DIRAS1;DYSFIP1;GPR61;HOMER2;HSD3B7;IL18BP;INPP5J;JDP2;MAP1A;MGAT4B;NDRG3;PPP5C;RHOT1;RPS6KL1;SH3BP1;SLC6A7;SYP;TFR2;TMEM211;UCK2"

"motif"	"NES"	"AUC"	"TF_highConf"	"nEnrGenes"	"enrichedGenes"
"cisbp__M1457"	3.2	0.0451	"NR2F1 (inferredBy_Orthology). "	70	"ABHD2;ACP2;ACVR1;ADCY10;ADRBK1;AKT3;ARFGEF2;ATP1A4;ATP6V0A1;BAG5;BHLHA15;C15orf59;C20orf200;C3orf22;C9orf169;CA9;CACNG7;CAPN1;CHPF;CKMT1A;CKMT1B;CRTAM;DAND5;DIRAS1;DNM1;DYSFIP1;EHD3;GABRA1;GALNT5;GLRB;GPR61;GRM1;GUCY2GP;HOMER2;HSD3B7;ICA1;IGF1;IKBKG;IL18BP;INPP5J;JDP2;KLC2;LYNX1;MANSC1;MAP1A;MAPK10;MGAT4B;MLL2;MYL2;NCALD;NFYC;NLGN3;NRBF2;PANK1;PPP2CA;PRSSL1;RAB6B;RPS6KL1;RSPO3;SH3BP1;SLC16A7;SLC25A19;SLC6A7;SMS;STOX2;SYP;TMEM211;TMEM8B;TTC7B;UCK2"
"cisbp__M1422"	3.18	0.0449	""	84	"ABHD2;ADCY10;ADRBK1;AES;AKT3;ARFGEF2;ATP1A4;ATP1B1;BAG5;BHLHA15;C16orf57;C20orf200;C3orf22;CACNG7;CAPN1;CKMT1A;CKMT1B;CLASP1;CRTAM;CRYBB2;DAND5;DES;DHCR24;DIRAS1;DLEU7;DNM1;DYSFIP1;EHD3;EIF4G3;EPHA4;GABRA1;GLRB;GPR61;GRM1;HOMER2;HSD3B7;ICA1;IGF1;IL18BP;INPP5J;ITGA4;JDP2;KCNC1;KLC2;LCK;LDLRAD2;LYNX1;MAP1A;MAPK10;MEX3B;MGAT4B;MLL2;NCALD;NEUROG1;NLGN3;PANK1;PANK2;PCNXL3;PGP;PPP2CA;PQLC1;PRR3;PRSSL1;RAB6B;RAD51C;RPS6KL1;RSPO1;RSPO3;SH3BP1;SLC16A7;SLC25A19;SLC6A7;SMS;STOX2;SYT1;SYT3;TGM1;TMEM119;TMEM211;TMEM8B;TPD52;TTC7B;UCK2;ULK2"
"cisbp__M6521"	3.17	0.0449	"THRA (directAnnotation). "	63	"ABHD2;ADRBK1;AKT3;ASNA1;ATP1B1;BAG5;BHLHA15;BRSK1;C3orf22;C5orf13;CACNG7;CAPN1;CRTAM;CRYBB2;DEPDC5;DES;DLEU7;DNM1;EHD3;EIF4G3;EPS15;FOXI1;GABRA1;GPR61;GRHL2;GRM1;HOMER2;HSD3B7;ICA1;IGF1;INPP5J;JDP2;LCK;LDLRAD2;LYNX1;LYSMD2;MAP1A;MGAT4B;MLL2;MPZ;NCALD;NPEPPS;PANK2;PCNXL3;POLDIP2;PPEF1;PPP2CA;PQLC1;PRR3;RAB6B;RPS6KL1;RUSC2;SH3BP1;SLC16A7;SLC6A7;SULT4A1;SYT1;TGM1;TMEM119;TMEM211;TUBA8;UCK2;ULK2"
"cisbp__M1442"	3.14	0.0447	"RARB (inferredBy_Orthology). "	27	"ATP1B1;C3orf22;CAPN1;CKMT1A;CKMT1B;CRTAM;DEPDC5;DNM1;DYSFIP1;GPR61;GRM1;HOMER2;HSD3B7;ICA1;IGF1;IL18BP;INPP5J;LYNX1;MAP1A;MAPK10;NLGN3;PANK1;PPP5C;PRSSL1;SLC6A7;TTC7B;UCK2"
"cisbp__M5413"	3.11	0.0446	"ESRRG (directAnnotation). "	46	"ADRBK1;AES;C15orf59;C5orf13;CA9;CAPN1;CCL11;CKMT1A;CKMT1B;DAND5;DEPDC5;DIRAS1;DNM1;DYSFIP1;FEM1B;GABRA1;GAS2L1;GPR61;HOMER2;HSD3B7;IBSP;IL18BP;INPP5J;JDP2;LCK;LYNX1;MANSC1;MAP1A;MAST1;MGAT4B;MYL2;NDRG3;NLGN3;PANK1;PPP5C;PRR3;RHOT1;SCN8A;SH3BP1;SLC6A7;STOX2;SYP;TFR2;TMEM211;ZBTB22;ZCCHC5"
"cisbp__M1439"	3.09	0.0444	"NR2C1 (inferredBy_Orthology). "	44	"ABHD2;ADCY10;ATP1B1;ATP6V0A1;BAG5;C3orf22;CACNG7;CAPN1;CCL11;CKMT1A;CKMT1B;CRTAM;DIRAS1;DNM1;DYSFIP1;EHD3;EIF4G3;GABRA1;GLRB;GPR61;GRM1;HOMER2;HSD3B7;ICA1;IGF1;IL18BP;INPP5J;JDP2;LYNX1;MAP1A;MAPK10;MGAT4B;NLGN3;PANK1;PPP2CA;PRR3;PRSSL1;RPS6KL1;SH3BP1;SLC6A7;STOX2;TMEM211;TTC7B;UCK2"
"cisbp__M4612"	3.08	0.0444	"CTCFL (directAnnotation). "	133	"ACP2;ACSL6;ADRBK1;AP2A1;ARPC1A;ASNS;ATP1A3;ATP1B1;ATXN7L3;BCL9;BEND5;BTBD1;BTBD3;C15orf59;C17orf63;C20orf177;C3orf33;C5orf13;C7orf70;CA4;CAPN1;CCBE1;CCNJL;CHFR;CLSTN3;CLTC;CPNE4;CRTAM;CYB561D1;DEPDC5;DES;DNM1;EHD3;ELAVL3;EPHA4;ETS2;EXTL3;FAM135B;FRMPD4;FXYD7;GALNTL4;GPR124;GRAMD1B;GRHL2;HCFC1;HEATR7A;HIF1AN;HSD3B7;ICA1;IGF1;IL1RAP;IRF2BP1;ITGA8;JDP2;KCNC1;KCNC4;KCNJ11;KCNJ9;KHK;KIAA1217;LCK;LOC100134229;LRRC59;MAPK8IP3;MAST1;MATN4;MLL2;MTOR;MYO5B;NAPA;NCK2;NCKIPSD;NEUROG1;NFYC;NNT;NPEPPS;PAK4;PCDHA3;PCDHA5;PCDHAC1;PCNXL2;PCNXL3;PELI3;PGM2;PIP4K2C;PKN3;PLK1;POU5F2;PPARD;PREP;PRICKLE1;PRKACA;PRND;PRR3;PSMD8;RAB11FIP5;RAD51C;RELA;REPS1;RERE;RNF208;RNF26;RSPO1;SCN8A;SEMA7A;SIRT4;SLC22A8;SMTNL2;SNAI2;SNX20;SPDEF;SPOCK1;STC2;SV2B;SYNJ1;SYT1;TADA2B;TCL1A;THBD;TNR;TRIM3;TRIM37;TRIO;TSFM;TSPAN5;TTC7B;TUBA8;UBE2O;ULK2;VPS36;WNK2;ZDHHC14;ZNF554"
"cisbp__M5221"	3.08	0.0444	"NR2F1; NR2F2 (inferredBy_Orthology). "	30	"ABHD2;ADRBK1;C3orf22;CAPN1;CKMT1A;CKMT1B;DEPDC5;DIRAS1;DNM1;DYSFIP1;EIF4G3;GABRA1;GPR61;GRM1;HOMER2;HSD3B7;IL18BP;INPP5J;JDP2;KCNC1;LYNX1;MAP1A;MGAT4B;NLGN3;PANK1;SH3BP1;SLC6A7;SYP;TMEM211;TTC7B"
"cisbp__M4979"	3.08	0.0443	"NR5A1 (inferredBy_Orthology). "	32	"ABHD2;ADRBK1;AES;C15orf59;CAPN1;CKMT1A;CKMT1B;DEPDC5;DIRAS1;DYSFIP1;GABRA1;GPR61;HOMER2;HSD3B7;IL18BP;INPP5J;JDP2;KCNC1;LYNX1;MAP1A;MGAT4B;NLGN3;PANK1;PPP2CA;PPP5C;PRR3;SH3BP1;SLC6A7;SNAP91;SYP;TMEM211;TTC7B"
"cisbp__M1447"	3.08	0.0443	"ESRRA (inferredBy_Orthology). "	35	"ATP1B1;C15orf59;C3orf22;CA9;CAPN1;CKMT1A;CKMT1B;DEPDC5;DIRAS1;DNM1;DYSFIP1;GABRA1;GPR61;HOMER2;HSD3B7;IL18BP;INPP5J;LYNX1;MAP1A;MGAT4B;MYL2;NLGN3;PANK1;PPP5C;PRR3;RHOT1;RPS6KL1;RSPO3;SCN8A;SH3BP1;SLC6A7;STOX2;SYP;TMEM211;TTC7B"
"cisbp__M3154"	3.04	0.0441	"TCF3 (directAnnotation). "	31	"ATP1A3;ATP6AP2;CA9;CCBE1;CCNJL;CYFIP2;DHRS7C;DYSFIP1;ELAVL3;FAM135B;FOXI1;FRMPD4;GALNTL4;ICA1;IGF1;INPP5J;KCNC4;LARP1;MAP1A;MAST1;MYH10;PF4;PF4V1;SLC30A2;STC2;STOX2;SV2B;TSFM;ZDHHC14;ZNF668;ZSCAN10"
"cisbp__M5407"	3.03	0.0441	"ESRRA (directAnnotation). "	33	"ADRBK1;C15orf59;C3orf22;CAPN1;CKMT1A;CKMT1B;DEPDC5;DYSFIP1;EFHA2;GABRA1;GAS2L1;GPR61;HOMER2;IL18BP;INPP5J;JDP2;LYNX1;MAP1A;MGAT4B;MYL2;NDRG3;NLGN3;PANK1;PPP5C;RHOT1;RSPO3;SCN8A;SLC6A7;STOX2;SYP;TFR2;TMEM211;ZBTB22"
"cisbp__M1430"	3.01	0.044	""	32	"ABHD2;ATP1B1;ATP6V0A1;BAG5;C3orf22;CAPN1;CKMT1A;CKMT1B;CRTAM;DEPDC5;DIRAS1;DNM1;DYSFIP1;GABRA1;HOMER2;HSD3B7;ICA1;INPP5J;JDP2;LYNX1;MAP1A;MAPK10;MGAT4B;NLGN3;PANK1;PRR3;PRSSL1;RPS6KL1;SLC6A7;STOX2;TTC7B;UCK2"

Neurosynth Emotion Pos Subc

"motif"	"NES"	"AUC"	"TF_highConf"	"nEnrGenes"	"enrichedGenes"
"cisbp__M6212"	3.64	0.0555	"EPAS1 (directAnnotation). "	82	"ABCC12;ACCN1;AK5;ARHGAP6;ATP2B4;BCL9L;C15orf23;C1orf173;C2orf80;C2orf81;CCDC6;CCNA1;CDC14A;CDH10;CLPS;COL24A1;CSRNP3;DACH2;DOC2A;EBAG9;EPB41L4B;ERC2;ETV4;GPM6A;GPR179;GRIA1;HOPX;HOXB4;HPCAL4;IGFBP5;IL18R1;IP6K1;IQSEC3;KCNV1;LMO3;MAMLD1;MAP2;MCOLN3;MMP16;NEGR1;NEK6;NEUROD2;NHS;NLRP10;NOL4;NPAS1;NRXN3;PAFAH1B3;PIK3AP1;PIP5K1B;PKIA;PORCN;PPP2R5C;PRKG1;PRSS3;PSD;RALYL;RRAS2;SAMD14;SCML4;SCN3B;SEMA4F;SETBP1;SLIT1;SLITRK5;SLN;SMAP2;SOX4;SOX5;SPC24;SST;SULF2;TBR1;TGFB2;TNFAIP8L1;TSC22D1;TSKU;TXLNB;WDR54;WNT10B;ZMYM3;ZNF804A"
"cisbp__M1901"	3.57	0.055	"RREB1 (directAnnotation). "	159	"ACCN1;ACPL2;ADCK5;AGBL4;AHDC1;AKAP14;ANKRD50;ANO1;AQP9;ARHGAP6;ARMCX2;ATP2B4;BAI3;BAIAP3;BEGAIN;BIRC3;C15orf23;C15orf57;C16orf45;C17orf108;C17orf76;C1orf157;C21orf56;C2orf61;C2orf80;C2orf81;C5orf33;C7orf52;CAB39L;CADM3;CCDC34;CD36;CD5;CDC14A;CDC42SE2;CHD3;CHL1;CLPS;CMTM8;CNN1;CPNE8;CSRNP3;DACH2;DCLK1;DGKA;DYNLL1;EBAG9;EFNB1;EFNB3;ELL3;ENC1;EPB41L4B;ETV4;FAM126A;FAM149A;FAM5C;FAM71F1;FBXO11;FEZF2;FREM2;GABRA2;GABRB1;GLT8D2;GPM6A;GPR176;GRIA1;GSG1;HMGA1;HN1;HOPX;HOXB4;HOXC6;HPCAL4;ICAM5;IGFBP5;IQSEC3;KAL1;KCNA6;KCNV1;KIAA1522;KIAA1984;KLF10;LANCL2;LDB2;LMO3;LRRTM4;LY6H;MAMLD1;MAP2;MEST;MORN4;MSL3;MUM1L1;MYCNOS;NBEA;NEGR1;NHS;NNAT;NOL4;NPR3;NUDT4;NUDT4P1;PAFAH1B3;PCDH1;PDGFC;PDGFD;PDZRN4;PIM2;PKIA;PKNOX2;PPP1R2P3;PRKG1;PROC;PROSC;PTPRR;PTTG1;PURG;RALYL;RFC5;RFTN1;RFWD2;RPA2;RSPH9;RUNX1T1;SAMD14;SCML4;SCN3A;SEMA4A;SETBP1;SLIT1;SLITRK5;SMAP2;SNAPC4;SOX5;SPDYA;ST6GALNAC5;SYNGAP1;SYT14;SYT5;TAF1B;TBR1;TC2N;TDRKH;TMEM74;TP63;TSKU;TXLNB;TYRO3;UNC5A;VGLL4;WDR5;WNT7B;XKR7;ZC3H12B;ZMYM3;ZMYND8;ZNF483;ZNF804A;ZNF831"
"cisbp__M2332"	3.39	0.0539	""	64	"ABCC12;AK5;ARHGAP6;ASTN1;ATP2B4;C11orf20;C16orf45;C1orf173;C2orf81;C3orf26;CCDC6;CDC14A;CHD3;CLPS;COL24A1;DGKA;DOC2A;EBAG9;ENC1;ETV4;FAM149A;FAM190A;GPM6A;HOPX;HOXC6;IL18R1;JAK2;L1CAM;LMO3;MAMLD1;MAP2;MMP16;NHS;NOL4;NRXN3;PAFAH1B3;PIM2;PIP5K1B;PKIA;PORCN;PRDM8;PRKG1;RALYL;RRAS2;SAMD14;SCML4;SCN3B;SCN5A;SETBP1;SLIT1;SLITRK5;SMAP2;SMARCA2;SOX4;SOX5;SST;SULF2;TARBP1;TGFB2;TSKU;TXLNB;WNT10B;ZC3H12B;ZNF804A"
"cisbp__M0059"	3.17	0.0524	""	45	"ABCC12;ARHGAP6;ASTN1;C1orf173;CDC14A;CLPS;COL24A1;DGKA;DOC2A;EBAG9;EPB41L4B;ETV4;FAM149A;HOXC6;L1CAM;LMO3;MAP2;MMP16;NHS;NOL4;NRXN3;PAFAH1B3;PCDH1;PIP5K1B;PKIA;PORCN;PPP2R5C;PRDM8;PRKG1;RALYL;RRAS2;SAMD14;SCML4;SCN5A;SETBP1;SLIT1;SMAP2;SMARCA2;SOX4;SOX5;SST;SULF2;TGFB2;TSKU;TXLNB"
"cisbp__M6112"	3.1	0.052	"ZNF740 (inferredBy_Orthology). "	65	"ACCN1;AHDC1;ARHGAP6;ASTN1;ATOH7;BAI3;BEGAIN;BHLHB9;C2orf80;C2orf81;CACNB1;CADM3;CCT5;CHD3;CNN1;DBH;DGKA;EFCAB5;ETV4;FAM110C;FAM5C;FBXO11;FGF13;GPR179;GRIA1;HN1;HOXB4;HOXC6;HPCAL4;ICAM5;IGFBP5;KCNA6;KCNV1;KIAA1522;KLHL14;LANCL2;MAMLD1;MEF2C;MEST;NBEA;NEGR1;NEUROD2;NNAT;NOL4;NPR3;NTSR1;PAFAH1B3;PCDH11Y;PCDH7;PDGFC;PIK3AP1;PRDM8;PRKG1;SATB1;SEMA4A;SETBP1;TBR1;TNNT3;TP63;TSKU;VGLL4;XKR7;ZC3H12B;ZMYM3;ZNF804A"
"cisbp__M5780"	3.1	0.052	"RFX5 (directAnnotation). "	66	"ARID4A;ARL6;BAIAP3;C10orf107;C10orf46;C1orf114;C1orf230;C2orf66;CCDC113;CCDC34;CCDC74A;CCDC74B;CCNA1;CDC14A;CENPH;CLRN3;DCAF15;DOC2A;DYNC2LI1;DYNLL1;EFCAB1;FAM81A;FAM92A1;GOT1L1;GPR179;HMGCR;HOXC6;HPCAL4;HSD11B1L;ICA1L;IP6K1;KLF10;LOC145837;LOC643037;LOC728392;LPAR4;LRCH2;LRRC27;MAPRE3;NEGR1;NEK2;NEURL;NHS;NOL4;NPR3;OSTN;PNMAL1;PRPF40B;PSD;PUM1;RAB36;RALYL;RFC5;RILPL2;RSBN1;RSPH9;SCML4;SCN2A;SULF2;TTC23L;ULK4;VWA3A;WDR52;WDR54;ZNF454;ZNF497"
"cisbp__M0054"	3.05	0.0517	""	69	"ABCC12;ACCN1;AK5;ARHGAP6;ASTN1;ATP2B4;C16orf45;C1orf173;C2orf81;CD200R1;CDC14A;CLPS;COL24A1;DCLK1;DGKA;DOC2A;EBAG9;EPB41L4B;ETV4;FAM149A;GPM6A;GRIA1;GRPR;HOPX;HOXC6;IL18R1;IP6K1;IQSEC3;KLF10;L1CAM;LMO3;MAP2;MMP16;MNT;NHS;NOL4;NRXN3;PAFAH1B3;PCDH1;PDZRN4;PIM2;PIP5K1B;PKIA;PORCN;PPP2R5C;PRDM8;PRKG1;RALYL;RRAS2;SAMD14;SATB1;SCML4;SCN2A;SCN3B;SCN5A;SEMA4F;SETBP1;SLIT1;SMAP2;SOX4;SOX5;SST;SULF2;TGFB2;TSKU;TXLNB;WNT10B;ZC3H12B;ZMYM3"
"cisbp__M6535"	3	0.0514	"WT1 (directAnnotation). "	152	"ABR;ADCY2;AHDC1;ANO1;ARF1;ARG1;ARHGAP6;ASTN1;ATOH7;ATP2B4;BAI3;BAIAP3;BCL9L;BEGAIN;BHLHB9;BRS3;C15orf23;C16orf73;C16orf87;C1orf157;C2orf80;C2orf81;C5orf33;CADM1;CADM3;CANX;CCDC149;CCNA1;CDC14A;CDC42SE2;CDH10;CHL1;CNN1;COL24A1;CSRNP3;DCP2;DNAL4;E2F5;EBAG9;EFCAB5;ETV4;FAM110C;FAM126A;FAM190A;FBXO11;FEZF2;FMN2;GABRA2;GABRB3;GDAP1L1;GLDC;GRIA1;GSG1;HMGA1;HN1;HOXC6;HPCAL4;HRH1;HS3ST6;HTR1A;ICAM5;IGFBP5;IP6K1;IQSEC3;JAK2;KCNA6;KCNV1;KIAA1239;KIAA1522;KLF10;KLHL14;KLK7;KRT16P2;LANCL2;LTBP4;MAMLD1;MAP2;MAPK13;MCOLN3;MEF2C;MNT;MORN3;MUM1L1;MYCNOS;NBEA;NEGR1;NEURL;NEUROD2;NHS;NNAT;NOL4;NPDC1;NPTX1;NRXN3;NTSR1;NUDT11;NUDT4P1;NUMBL;PAFAH1B3;PCDH1;PCDH7;PDGFC;PHF13;PIK3AP1;PKIA;PKNOX2;PORCN;PPP1R13B;PRKG1;PSD;PTGER4;PTTG1;RAB9B;RALYL;RFX3;RRAS2;RTN4RL2;SAMD14;SCML4;SCN3B;SEC24D;SEMA4A;SEMA4F;SETBP1;SLIT1;SMAP2;SMARCA2;SOX4;SOX5;SSTR2;ST6GAL2;TBR1;TGFB2;TNFAIP8L1;TNNT3;TP63;TRPC4;TSC22D1;TSKU;TSPYL2;TXLNB;VGLL4;VSTM2L;VWCE;WNT7B;XKR7;XPNPEP1;ZC3H12B;ZCCHC12;ZMYM3;ZMYND8;ZNF804A"

Neurosynth Emotion Neg Subc

"motif"	"NES"	"AUC"	"TF_highConf"	"nEnrGenes"	"enrichedGenes"
"cisbp__M0689"	3.93	0.0443	"ETV3 (inferredBy_Orthology). "	217	"ACAA1;ACOT7;ACVRL1;AGPAT4;ALKBH1;AMICA1;ANK3;ANKRD44;AP1B1;APBA3;APOLD1;ARMCX3;ARMCX6;ATG2B;ATP10A;ATP1A2;B3GALNT2;BMP1;BRCA1;BTBD16;C11orf61;C14orf119;C1orf66;C20orf4;C2orf24;C4orf37;C5orf51;C7orf47;C9orf167;CAMTA1;CASKIN2;CCDC51;CCDC76;CCL5;CCNI;CCT6P1;CD40;CDK6;CFLAR;CHD7;CLDN5;COG3;CPSF3;CRADD;CSRP1;CSRP2;CTDSP1;CWC22;CXorf36;CYBASC3;CYLD;CYTL1;DCUN1D3;DDX18;DFNB31;DPP8;DSCR3;EBF1;ECSCR;EEF1B2;ELP2;EMCN;ERBB2IP;ESRRA;FAM110A;FAM119A;FAM96A;FBXO32;FKBP2;FLI1;FLT1;FRYL;GCN1L1;GGA1;GNG11;GPR108;GPR116;GREM1;HAGHL;HYAL2;ICAM1;IL17RE;IL23A;INTS9;IPPK;IRF1;ITGA5;ITGB1;ITPR3;KDELC1;KIAA0240;KIAA1267;KIAA1712;KLHL20;KRTCAP2;LAG3;LEF1;LLGL2;LMO2;LRRC32;LRRC41;LRRC49;LRRC58;LYRM7;MAN2A1;MAP3K3;MAP7;METT11D1;MFSD11;MGAT1;MIDN;MKRN2;MORC2;MRGPRF;MTIF2;MYO1E;NBR1;NCAM1;NDST1;NDUFS1;NDUFS7;NFKBIZ;NGDN;NKAPL;NOL6;NOSTRIN;NR1H2;NUP155;OLFML3;OLIG2;ORC2;OSM;OTX2;PAPPA;PAQR5;PELI2;PHF23;PICALM;PITPNC1;PLEKHB1;PLEKHG3;PLEKHH1;PLOD3;PLP1;PPM1A;PPP1CC;PPP4C;PRKAB1;PRUNE2;PSMB4;PSTPIP1;PTPRH;QARS;RAB32;RABGEF1;RAI14;RASGRP3;RB1;RBMS3;REEP4;RELT;RGL2;RNF144B;RNPEPL1;RRS1;RXRB;SASS6;SCGB3A1;SENP7;SLC16A6;SLC39A13;SLC39A8;SLC44A1;SLCO6A1;SMAD7;SMNDC1;SMUG1;SOX13;SOX8;SPN;SRFBP1;SYVN1;TAL1;TENC1;TFB1M;TFE3;THAP1;THAP10;TIE1;TJP2;TM9SF2;TMEM101;TMEM206;TMX2;TPM2;TRPT1;UACA;UBE2D3;UBL4B;USHBP1;USP13;USP3;WDR45;XIRP2;ZBTB20;ZBTB26;ZC3H10;ZDHHC5;ZKSCAN1;ZNF167;ZNF284;ZNF366;ZNF528;ZNF585A;ZNF641;ZNF655;ZNF768"
"cisbp__M0704"	3.79	0.0438	"ELF5 (inferredBy_Orthology). "	202	"ACAA1;ACOT7;ACVRL1;AGPAT4;ALKBH1;AMICA1;AMPD3;ANKRD44;ANKS1A;APOLD1;ARMCX6;ATG2B;B3GALNT2;BDH2;BMP1;BTBD16;C11orf61;C14orf119;C2orf24;C2orf49;C4orf37;C9orf167;CAMTA1;CARD14;CASKIN2;CCDC51;CCDC76;CCL5;CCNI;CCRL2;CCT6P1;CD40;CDK6;CFLAR;CLCA2;CLDN5;COG3;CPSF3;CRADD;CRB3;CSRP1;CTDSP1;CWC22;CXorf36;CYBASC3;CYLD;CYTL1;DCUN1D3;DDX18;DISP1;DMBT1;DPP8;DSCR3;EBF1;ECSCR;EEF1B2;ELP2;EMCN;EPN3;ERBB2IP;ESM1;ESRRA;ETF1;ETV6;EXTL2;FAM110A;FAM96A;FGFR2;FKBP2;FLI1;FLT1;FRYL;GCN1L1;GKN1;GNG11;GPR108;GPR116;GREM1;HAGHL;HPS1;HYAL2;ICAM1;IL23A;INTS9;IPPK;IRF1;ITGA5;ITGAV;ITGB1;ITPR3;KIAA0040;KIAA0240;KIAA1267;KIAA1712;KLHL20;LEF1;LLGL2;LMO2;LRRC49;MAN2A1;MAP3K3;MAP7;METT11D1;MFSD11;MGAT1;MIDN;MKS1;MORC2;MTIF2;MYO1E;NBR1;NDST1;NDUFS1;NFKBIZ;NKAPL;NOL6;NOSTRIN;NOTCH4;NR1H2;NUP155;ORC2;OSM;OTX2;PC;PELI2;PHF23;PICALM;PITPNC1;PLA2R1;PLEKHB1;PLOD3;PPM1A;PPP1CC;PPP1R15B;PPP4C;PRKAB1;PRUNE2;PSMB4;PTPRH;QARS;RABGEF1;RAI14;RASGRP3;RB1;RBMS3;REEP4;RELT;RGL2;RNF144B;RNPEPL1;RRS1;SASS6;SCGB3A1;SCNM1;SCRT2;SENP7;SGK1;SLC39A13;SLC39A8;SMAD7;SMUG1;SOX13;SOX8;SPN;SRFBP1;SYVN1;TAF1;TAL1;TDGF1;TENC1;THAP1;THAP10;TIE1;TJP2;TM9SF2;TMEM101;TMEM206;TMX2;TRPT1;UACA;UBE2D3;UBL4B;USHBP1;USP8;VCAN;WDR73;WWP2;XIRP2;ZBTB26;ZBTB7B;ZC3H10;ZDHHC5;ZKSCAN1;ZNF167;ZNF223;ZNF284;ZNF3;ZNF366;ZNF641;ZNF655;ZNF768;ZNF823"
"cisbp__M0714"	3.58	0.043	""	195	"ACAA1;ACOT7;ACVRL1;ADIPOR2;AGPAT4;ALKBH1;AMICA1;ANKRD44;AP1B1;APOLD1;ARL6IP5;ARMCX3;ARMCX6;ATG2B;B3GALNT2;BRCA1;BTBD16;C11orf61;C14orf119;C1orf66;C20orf4;C2orf24;C4orf37;C5orf51;C7orf47;C9orf167;CAMTA1;CASKIN2;CCDC51;CCDC76;CCL5;CCT6P1;CD40;CDK6;CFLAR;CLDN5;COG3;CPSF3;CRADD;CREB3L2;CSRP1;CTDSP1;CWC22;CXorf36;CYBASC3;CYLD;CYTL1;DCUN1D3;DDX18;DFNB31;DPP8;DSCR3;EBF1;ECSCR;EEF1B2;ELP2;EMCN;ERBB2IP;ESRRA;FAM110A;FAM119A;FAM96A;FBXO32;FKBP2;FLI1;FLT1;FNTA;FRYL;GCN1L1;GGA1;GNG11;GPR108;GPR116;GREM1;HYAL2;ICAM1;IL17RE;INTS9;IPPK;IRF1;ITGA5;ITGB1;ITPR3;KDELC1;KIAA1267;KIAA1712;KIAA1958;KLHL20;KRTCAP2;LEF1;LLGL2;LMO2;LRRC32;LRRC41;LRRC49;LRRC58;LYRM7;MAN2A1;MAP3K3;MAP7;METT11D1;MFSD11;MGAT1;MIDN;MORC2;MRPL47;MTIF2;MYO1E;NBR1;NDST1;NDUFS1;NDUFS7;NFKBIZ;NGDN;NKAPL;NOL6;NOSTRIN;NR1H2;NUP155;OLIG2;ORC2;OSM;OTUD7B;OTX2;PAQR5;PELI2;PHF23;PICALM;PITPNC1;PLEKHB1;PLEKHH1;PLOD3;PPM1A;PPP1CC;PPP4C;PRKAB1;PRUNE2;PSMB4;PSTPIP1;PTPRH;QARS;RABGEF1;RAI14;RASGRP3;RBMS3;REEP4;RELT;RGL2;RNF144B;RNPEPL1;RRS1;RXRB;SASS6;SCGB3A1;SENP7;SLC39A13;SLC39A8;SMAD7;SMNDC1;SMUG1;SOX8;SPN;SRFBP1;SYVN1;TAL1;TENC1;TFB1M;TFE3;THAP1;THAP10;TIE1;TJP2;TM9SF2;TMEM101;TMEM206;TMX2;TRPT1;UACA;UBE2D3;UBL4B;USHBP1;XIRP2;ZBTB20;ZBTB26;ZC3H10;ZDHHC5;ZKSCAN1;ZNF167;ZNF284;ZNF366;ZNF528;ZNF585A;ZNF641;ZNF655;ZNF768"
"cisbp__M0707"	3.45	0.0425	"ETS1 (inferredBy_Orthology). "	238	"ACAA1;ACOT7;ACVRL1;ADIPOR2;ADO;AGPAT4;ALKBH1;ALKBH3;AMICA1;ANKRD44;ANKS1A;AP1B1;APBA3;APOLD1;ARMCX6;ATG2B;B3GALNT2;BET1L;BRCA1;BTBD16;C11orf61;C14orf119;C16orf7;C19orf48;C1orf66;C20orf4;C2orf24;C2orf49;C4orf37;C5orf51;C7orf47;C9orf167;CAMTA1;CASKIN2;CCDC51;CCDC76;CCL5;CCT6P1;CD40;CDK6;CFLAR;CHD8;CLDN5;CNDP2;COG3;CPSF3;CRADD;CRB3;CRTC2;CSRP1;CSRP2;CTDSP1;CWC22;CXorf36;CYBASC3;CYLD;CYTL1;DCUN1D3;DDX18;DFNB31;DISP1;DMBT1;DPP8;DSCR3;EBF1;ECSCR;EEF1B2;EIF5A2;ELP2;EMCN;EPN3;ERBB2IP;ESRRA;ETF1;EXTL2;FAM110A;FAM119A;FAM49B;FAM76B;FAM96A;FBXO32;FGFR2;FKBP2;FLI1;FLT1;FNTA;FRYL;GCN1L1;GGA1;GNG11;GPR108;GPR116;GREM1;HAVCR2;HEATR6;HYAL2;ICAM1;IL17RE;INTS9;IPPK;IRF1;ITGA1;ITGA5;ITGB1;ITPR3;KDELC1;KIAA0556;KIAA0564;KIAA1267;KIAA1712;KIAA1958;KLHL20;KRTCAP2;LLGL2;LMO2;LRRC32;LRRC41;LRRC49;LYRM7;MAN2A1;MAP3K3;MAP7;METT11D1;MFSD11;MGAT1;MIDN;MKRN2;MLKL;MORC2;MPZL3;MRPL47;MTIF2;MYO1E;NBR1;NDST1;NDUFS1;NDUFS7;NFASC;NFKBIZ;NGDN;NKAPL;NOL6;NOSTRIN;NR1H2;NUDT12;NUP155;OLIG2;ORC2;OSM;OTUD7B;OTX2;PAQR5;PELI2;PHC2;PHF23;PICALM;PITPNC1;PLEKHH1;PLOD3;PPM1A;PPP1CC;PPP4C;PRKAB1;PRUNE2;PSMB4;PSMD6;PSTPIP1;PTPRH;QARS;RAB32;RABGEF1;RAI14;RASGRP3;RB1;RBMS3;RELT;RGL2;RNF144B;RNF214;RNPEPL1;RRP12;RRS1;RXRB;SASS6;SCGB3A1;SCNM1;SCRT2;SENP7;SGK1;SLC35C2;SLC39A13;SLC39A8;SLC44A1;SMAD7;SMNDC1;SMUG1;SOX13;SPN;SRFBP1;SYVN1;TAF1;TAF12;TAL1;TENC1;TFB1M;TFE3;TFPI;THAP1;THAP10;TIE1;TJP2;TM9SF2;TMEM101;TMEM206;TMX2;TRIM35;TRPT1;UACA;UBE2D3;UBL4B;USHBP1;VWF;WDR45;WDR73;WWP2;XIRP2;ZC3H10;ZDHHC5;ZFYVE16;ZKSCAN1;ZNF167;ZNF175;ZNF284;ZNF366;ZNF585A;ZNF641;ZNF655;ZNF768"

"motif"	"NES"	"AUC"	"TF_highConf"	"nEnrGenes"	"enrichedGenes"
"cisbp__M0701"	3.43	0.0424	"SPDEF (inferredBy_Orthology). "	134	"ACAA1;ACVRL1;ALKBH1;ANKRD44;ANKS1A;ARMCX3;ATG2B;C11orf61;C14orf119;C1orf66;C2orf24;C4orf37;C9orf167;CAMTA1;CCDC51;CCDC76;CCT6P1;CDK6;CHD8;CLDN5;CLP1;COG3;CPSF3;CRB3;CXorf36;CYBASC3;DCUN1D3;DDIT3;DDX18;DPP8;DSCR3;EBF1;ECSCR;EEF1B2;ELP2;EMCN;ERBB2IP;ESM1;ESRRA;ETFB;FAM110A;FAM119A;FAM49B;FAM96A;FBXO32;FGFR2;FKBP2;FLI1;FLT1;FOXP2;FRYL;GATA3;GCN1L1;GGA1;GNG11;GPR108;GPR116;GREM1;HOMER3;IPPK;IRF1;ITGB1;ITPR3;KIAA1267;KIAA1712;KLHL20;LLGL2;LRRC41;LRRC49;LRRFIP2;MTIF2;NCAM1;NDUFS1;NDUFS7;NFKBIZ;NKAPL;NOL6;NOSTRIN;NR1H2;NUP155;ORC2;PCBP4;PELI2;PICALM;PITPNC1;PLA2R1;PLP1;PPM1A;PPP4C;PRDM6;PRKAB1;PRUNE2;RABGEF1;RAI14;RBMS3;RGL2;RNPEPL1;RRP12;RRS1;SASS6;SENP7;SGK1;SLC16A6;SLC39A13;SLC39A8;SMAD7;SOX13;SRFBP1;SYVN1;TAF12;TAL1;TENC1;TFB1M;TIE1;TJP2;TM9SF2;TMEM101;TMX2;TRPT1;UACA;UBE2D3;UBL4B;USHBP1;USP3;WTAP;XBP1;ZBTB7B;ZKSCAN1;ZNF167;ZNF528;ZNF641;ZNF655;ZNF768;ZNF823"
"cisbp__M0706"	3.38	0.0423	"ELF4 (inferredBy_Orthology). "	112	"ACAA1;ACOT7;AGPAT4;ALKBH1;ANKRD44;ARMCX3;ARMCX6;ATG2B;BRCA1;C14orf119;C20orf4;C2orf24;C4orf37;C9orf167;CAMTA1;CASKIN2;CCDC76;CCNI;CCT6P1;CD40;CHD7;CLDN5;COG3;CPSF3;CRADD;CXorf36;CYBASC3;CYTL1;DDX18;DPP8;DSCR3;EBF1;EMCN;ERBB2IP;EXTL2;FAM110A;FAM96A;FKBP2;FLI1;FLT1;FRYL;GCN1L1;GNG11;GPR116;GREM1;HPS1;HYAL2;INTS9;IPPK;IRF1;ITGA5;ITGB1;ITPKB;ITPR3;KIAA1267;KIAA1712;KLHL20;LMO2;LRRC49;MAP7;MIDN;MTIF2;NBR1;NFKBIZ;NKAPL;NOL6;NR1H2;ORC2;PELI2;PHF23;PICALM;PITPNC1;PLOD3;PPP1CC;PPP4C;PRUNE2;PSMB4;QARS;RABGEF1;RAI14;RASGRP3;RBMS3;RRS1;SASS6;SENP7;SLC39A13;SLC39A8;SMUG1;SOX8;SPN;SRFBP1;SYVN1;TAL1;THAP10;TJP2;TMEM101;TMX2;TRPT1;UACA;UBE2D3;USHBP1;USP13;USP3;WTAP;ZC3H10;ZDHHC5;ZKSCAN1;ZNF167;ZNF284;ZNF366;ZNF655;ZNF768"
"cisbp__M0699"	3.34	0.0421	"FLI1 (inferredBy_Orthology). "	217	"ACAA1;ACOT7;ACVRL1;ADIPOR2;ADO;AGPAT4;ALKBH1;ALKBH3;AMICA1;ANKRD44;ANKS1A;AP1B1;APBA3;APOLD1;ATG2B;B3GALNT2;BRCA1;BTBD16;C11orf61;C14orf119;C19orf48;C1orf66;C20orf4;C2orf24;C3orf64;C4orf37;C5orf51;C7orf47;C9orf167;CAMTA1;CASKIN2;CCDC51;CCDC76;CCL5;CCT6P1;CDK6;CFLAR;CHD8;CLDN5;COG3;CPSF3;CRADD;CRB3;CRTC2;CSRP2;CTDSP1;CWC22;CXorf36;CYBASC3;CYLD;CYTL1;DCUN1D3;DDX18;DFNB31;DPP8;DSCR3;EBF1;ECSCR;EEF1B2;EIF5A2;ELP2;EMCN;EPN3;ERBB2IP;ESRRA;ETF1;EXTL2;FAM110A;FAM119A;FAM96A;FBXO32;FKBP2;FLI1;FLT1;FNTA;FRYL;GCN1L1;GGA1;GNG11;GPR108;GPR116;GREM1;HEATR6;HYAL2;ICAM1;IL17RE;INTS9;IPPK;IRF1;ITGA1;ITGA5;ITGB1;ITPR3;KDELC1;KIAA0556;KIAA0564;KIAA1267;KIAA1712;KIAA1958;KLHL20;KRTCAP2;LLGL2;LMO2;LRRC41;LRRC49;LRRC58;LYRM7;MAP3K3;MAP7;METT11D1;MFSD11;MGAT1;MIDN;MKRN2;MORC2;MPZL3;MRPL47;MTIF2;MYO1E;NBR1;NDUFS1;NDUFS7;NFKBIZ;NGDN;NKAPL;NOL6;NOSTRIN;NR1H2;NUDT12;NUP155;OLIG2;ORC2;OSM;OTUD7B;OTX2;PELI2;PHC2;PHF23;PICALM;PITPNC1;PLEKHH1;PLOD3;PPM1A;PPP1CC;PPP4C;PRKAB1;PRUNE2;PSMB4;PSMD6;PSTPIP1;PTPRH;QARS;RABGEF1;RAI14;RASGRP3;RBMS3;RELT;RGL2;RNF144B;RNF214;RNPEPL1;RRS1;RXRB;SASS6;SCGB3A1;SCNM1;SCRT2;SENP7;SGK1;SLC39A13;SLC39A8;SLC44A1;SMAD7;SMNDC1;SMUG1;SOX13;SPN;SRFBP1;STC2;SYVN1;TAF1;TAF12;TAL1;TENC1;TFB1M;TFE3;THAP1;THAP10;TIE1;TJP2;TM9SF2;TMEM101;TMEM206;TMX2;TRIM35;TRPT1;UACA;UBE2D3;UBL4B;USHBP1;WDR45;WDR73;WWP2;XIRP2;ZBTB26;ZC3H10;ZDHHC5;ZFYVE16;ZKSCAN1;ZNF167;ZNF175;ZNF284;ZNF366;ZNF585A;ZNF641;ZNF655;ZNF768"
"cisbp__M0705"	3.3	0.042	"ETV6 (inferredBy_Orthology). "	330	"AAAS;ACAA1;ACOT7;ACP6;ACVRL1;ADIPOR2;ADO;AGPAT4;ALG11;ALKBH1;ALKBH3;AMICA1;AMPD3;ANKRD44;ANKS1A;AP1B1;APBA3;APOLD1;ARF6;ARHGEF5;ARL6IP5;ARMCX3;ARMCX6;ASPDH;ATG2B;ATP10A;B3GALNT2;B4GALT1;BDH2;BET1L;BMP1;BRCA1;BTBD16;C11orf61;C14orf119;C16orf7;C19orf48;C1orf66;C2;C20orf4;C2orf24;C2orf49;C4orf37;C5orf51;C7orf47;C9orf167;CAMTA1;CAPN13;CARD14;CARD9;CASKIN2;CCDC51;CCDC76;CCL5;CCNI;CCT6P1;CD40;CDK6;CFLAR;CLCA2;CLDN5;CLN3;CLP1;COG3;COX17;CPSF3;CRADD;CRB3;CRTC2;CSRP1;CSRP2;CTDSP1;CWC22;CXorf36;CYBASC3;CYLD;CYTL1;DCUN1D3;DDIT3;DDX18;DFNB31;DISP1;DMBT1;DPP8;DSCR3;EBF1;ECSCR;EEF1B2;EIF5A2;ELP2;EMCN;EPN3;ERBB2IP;ERRFI1;ESM1;ESRRA;ETF1;ETV6;EXTL2;FAM110A;FAM119A;FAM96A;FBXO32;FCRL5;FGFR2;FKBP2;FLI1;FLT1;FNTA;FOXO3;FRYL;GCN1L1;GGA1;GIMAP1;GNG11;GPR108;GPR116;GPR85;GREM1;GTF2A1;GTF2A2;HAGHL;HEATR6;HIC1;HIGD2A;HK3;HPS1;HYAL2;ICAM1;IFITM1;IL17RE;IL23A;INTS9;IPPK;IRF1;ITGA5;ITGAV;ITGB1;ITPR3;KDELC1;KIAA0040;KIAA0240;KIAA0556;KIAA1267;KIAA1712;KIAA1958;KLHL20;KRTCAP2;L3MBTL2;LAG3;LEF1;LLGL2;LMO2;LOC284648;LRRC32;LRRC41;LRRC49;LRRC58;LSR;LTC4S;LYRM7;MAN2A1;MAP3K3;MAP7;MAT2B;MAX;METT11D1;MFSD11;MGAT1;MIDN;MKRN2;MKS1;MOGAT2;MORC2;MPZL3;MRGPRF;MRPL47;MRRF;MTIF2;MYBL1;MYO1E;NBR1;NCAM1;NDE1;NDST1;NDUFS1;NDUFS7;NFASC;NFKBIZ;NGDN;NKAPL;NOL6;NOSTRIN;NOTCH4;NPR1;NR1H2;NUDT12;NUP155;OLFML3;ORC2;OSBPL2;OSM;OTUD7B;OTX2;OXA1L;PAPPA;PC;PELI2;PHF23;PICALM;PIGT;PITPNC1;PKN1;PLA2G4F;PLA2G6;PLA2R1;PLEKHB1;PLEKHH1;PLOD3;PLP1;PPM1A;PPP1CC;PPP1R15B;PPP4C;PRKAB1;PRUNE2;PSMB4;PSMD6;PSTPIP1;PTAR1;PTPRH;PXN;QARS;RAB32;RABGEF1;RAI14;RASGRP3;RB1;RBMS3;RCAN2;REEP4;RELT;RG9MTD2;RGL2;RNF144B;RNPEPL1;RPA3;RRS1;RXRB;SASS6;SCGB3A1;SCNM1;SCRT2;SENP7;SGK1;SLC12A4;SLC16A4;SLC26A9;SLC35A4;SLC35C2;SLC39A13;SLC39A8;SLCO6A1;SMAD7;SMNDC1;SMUG1;SOX13;SOX8;SPN;SRFBP1;SRGN;SSH3;SYVN1;TAF1;TAF11;TAF12;TAL1;TDGF1;TENC1;TFB1M;TFE3;TFPI;THAP1;THAP10;TIE1;TJP2;TLN1;TM9SF2;TMEM101;TMEM206;TMX2;TRIM35;TRPT1;TSPAN8;UACA;UBE2D3;UBL4B;UNC5B;USHBP1;USP8;VCAN;VWF;WDR45;WDR73;WIPF1;WTAP;WWP2;XIRP2;YME1L1;ZAP70;ZBTB20;ZBTB26;ZBTB7B;ZC3H10;ZDHHC5;ZFYVE16;ZKSCAN1;ZMAT4;ZNF167;ZNF175;ZNF223;ZNF274;ZNF284;ZNF3;ZNF330;ZNF366;ZNF585A;ZNF641;ZNF655;ZNF768"
"cisbp__M6365"	3.3	0.042	"NFATC4 (directAnnotation). "	100	"AAAS;ABL1;APBB2;APOLD1;APPBP2;ASPDH;ATP10A;ATP1A2;C14orf178;C17orf75;CAMTA1;CAV1;CCDC91;CCL5;CCNI;CD97;CDH5;CDK6;CHKA;CNKSR3;COL3A1;CTDSP1;CXCR4;DAAM2;DOCK1;DYNLL2;EBF1;ENPP2;FBXO32;FLI1;GNG11;GPR116;GREM1;GSN;HMX2;HYAL2;ICK;INO80D;ITGA5;ITPKB;JARID2;KIAA1267;KRT14;LAG3;MAP7;MAX;MGP;MIDN;MRGPRF;MUSTN1;MYO1E;NDE1;NFKBIZ;NKG7;NPR1;NR3C1;NTN1;NUDT12;OLFML3;ORC2;OTX2;OXA1L;P2RY14;PAPPA;PELI2;PICALM;PITPNC1;PLEKHG3;PRUNE2;PTGER1;RASGRP3;RB1;RBMS3;REEP4;RGMA;RHOBTB1;SDC1;SEPP1;SGK1;SLC14A1;SLC25A12;SLC43A3;SLC6A6;SLCO6A1;SORBS3;SOX17;STX16;SUZ12;TAF11;TAS2R20;TBC1D4;TM4SF1;TM4SF18;TPM2;TTC39B;UACA;VEGFA;WWP2;ZNF3;ZNF366"

"motif"	"NES"	"AUC"	"TF_highConf"	"nEnrGenes"	"enrichedGenes"
"cisbp__M0709"	3.29	0.0419	"ERG (inferredBy_Orthology). "	224	"ACAA1;ACOT7;ACVRL1;ADIPOR2;ADO;AGPAT4;ALKBH1;ALKBH3;AMICA1;ANKRD44;ANKS1A;AP1B1;APBA3;APOLD1;ASCC2;ATG2B;B3GALNT2;BRCA1;BTBD16;C11orf61;C14orf119;C16orf7;C19orf48;C1orf66;C20orf4;C2orf24;C3orf64;C4orf37;C7orf47;C9orf167;CAMTA1;CASKIN2;CCDC51;CCDC76;CCL5;CCT6P1;CDK6;CFLAR;CHD8;CLDN5;COG3;CPSF3;CRADD;CRB3;CRTC2;CSRP2;CTDSP1;CWC22;CXorf36;CYBASC3;CYLD;CYTL1;DCUN1D3;DDX18;DFNB31;DPP8;DSCR3;EBF1;ECSCR;EEF1B2;EIF5A2;ELP2;EMCN;EPN3;ERBB2IP;ESRRA;ETF1;EXTL2;FAM110A;FAM119A;FAM96A;FBXO32;FGFR2;FKBP2;FLI1;FLT1;FNTA;FRYL;GCN1L1;GGA1;GNG11;GPR108;GPR116;GREM1;HEATR6;HYAL2;ICAM1;IL17RE;INTS9;IPPK;IRF1;ITGA1;ITGA5;ITGB1;ITPR3;KDELC1;KIAA0556;KIAA0564;KIAA1267;KIAA1712;KIAA1958;KLHL20;KRTCAP2;LLGL2;LMO2;LRRC41;LRRC49;LRRC58;LYRM7;MAP3K3;MAP7;MAT2B;METT11D1;MFSD11;MGAT1;MIDN;MKRN2;MORC2;MPZL3;MRPL47;MTIF2;MYO1E;NBR1;NDUFS1;NDUFS7;NFASC;NFKBIZ;NGDN;NKAPL;NOL6;NOSTRIN;NR1H2;NUDT12;NUP155;OCIAD1;OLIG2;ORC2;OSM;OTUD7B;OTX2;PELI2;PHC2;PHF23;PICALM;PITPNC1;PLEKHH1;PLOD3;PPM1A;PPP1CC;PPP4C;PRKAB1;PRUNE2;PSMB4;PSMD6;PSTPIP1;PTPRH;QARS;RABGEF1;RAI14;RASGRP3;RBMS3;RELT;RGL2;RNF144B;RNF214;RNPEPL1;RRP12;RRS1;RXRB;SASS6;SCGB3A1;SCNM1;SCRT2;SENP7;SGK1;SLC39A13;SLC39A8;SLC44A1;SMAD7;SMNDC1;SMUG1;SOX13;SPN;SRFBP1;STC2;SYVN1;TAF1;TAF12;TAL1;TENC1;TFB1M;TFE3;THAP1;THAP10;TIE1;TJP2;TM9SF2;TMEM101;TMEM206;TMX2;TRIM35;TRPT1;UACA;UBE2D3;UBL4B;USHBP1;VWF;WDR45;WDR73;WWP2;XIRP2;ZBTB26;ZC3H10;ZDHHC5;ZFYVE16;ZKSCAN1;ZNF167;ZNF175;ZNF284;ZNF366;ZNF585A;ZNF641;ZNF655;ZNF768"
"cisbp__M1265"	3.28	0.0419	"IRF7 (inferredBy_Orthology). "	144	"ABTB2;ACSL5;AGPAT4;ANK3;APBB2;APOLD1;AR;ARL6IP5;ARMCX3;ARMCX6;ATP10A;ATP1A2;BACE1;BRCA1;C14orf178;C5orf4;C6orf47;C9orf167;CCND1;CCNI;CD14;CDK6;CDKN1C;CDKN2A;CDX1;CFL2;CNKSR3;COL3A1;COLEC11;CSRP2;CXCR4;CYTIP;CYTL1;DCLRE1A;DCUN1D3;DENND4B;DLL4;DPP8;DPYS;DUSP1;EBF1;EMCN;ENPP2;ERG;ESRRG;ETS1;ETV6;EVI5;FAM63A;FAM96A;FBN1;FLI1;FOXO3;FOXP2;HAUS5;HPS1;ICAM1;ITPKB;JARID2;JPH2;KIAA1267;KIAA1712;LAG3;LANCL1;LEF1;LLGL2;LMO2;MAP7;METT11D1;MGAT1;MMP14;MXD3;MYCN;MYO1E;NBR1;NCAM1;NCF1C;NDE1;NDUFS7;NKAPL;NR3C1;NTN1;NUP160;ORC2;OTX2;PAPPA;PAX3;PDPN;PELI2;PICALM;PITPNC1;PLEKHA5;PLEKHG1;PLEKHG4;PLOD3;PLP1;PNPT1;PPP1CC;PROX1;PRUNE2;PTH1R;RBMS3;REEP4;RHOBTB1;RNF111;SDC1;SEPP1;SGK1;SGMS1;SLC15A3;SLC26A9;SLC39A8;SMAD6;SMAD7;SOX8;STAT5A;STX16;SYS1;TAL1;TBC1D4;TIE1;TJP2;TM4SF18;TMEM101;TRAF4;TRPC3;TTC17;TTC35;UACA;UBE2D3;UNC5B;USP13;VAMP8;VEGFA;WNT11;WTAP;ZBTB20;ZHX2;ZNF24;ZNF34;ZNF366;ZNF462;ZNF503;ZNF692"
"cisbp__M0696"	3.26	0.0418	"EHF (inferredBy_Orthology). "	157	"ACAA1;ACOT7;ACVRL1;AGPAT4;ALKBH1;AMICA1;ANKRD44;ANKS1A;ATG2B;BET1L;BTBD16;C11orf61;C14orf119;C1orf66;C2orf24;C2orf49;C4orf37;C9orf167;CAMTA1;CCDC51;CCDC76;CD40;CDK6;CFLAR;CLDN5;CLN3;COG3;CPSF3;CRADD;CRB3;CSRP1;CWC22;CXorf36;CYBASC3;CYLD;DCUN1D3;DDIT3;DDX18;DISP1;DMBT1;DPP8;DSCR3;EBF1;ECSCR;EEF1B2;ELP2;EPN3;ERBB2IP;ESRRA;ETF1;EXTL2;FAM110A;FAM96A;FGFR2;FKBP2;FLI1;FLT1;FRYL;GCN1L1;GGA1;GNG11;GPR116;GREM1;INTS9;IPPK;IRF1;ITGA5;ITGAV;ITGB1;ITPR3;KIAA0040;KIAA1267;KIAA1712;KLHL20;LLGL2;LMO2;LRRC49;MAN2A1;MAP3K3;MGAT1;MIDN;MKS1;MORC2;MTIF2;MYO1E;NBR1;NDUFS1;NGDN;NKAPL;NOL6;NOSTRIN;NR1H2;NUP155;ORC2;OSM;PC;PELI2;PHF23;PICALM;PITPNC1;PLEKHB1;PLOD3;PPM1A;PPP1CC;PPP4C;PRKAB1;PRUNE2;PSMB4;PTAR1;PTPRH;QARS;RABGEF1;RAI14;RASGRP3;RBMS3;RGL2;RNF144B;RRS1;SASS6;SCGB3A1;SCNM1;SENP7;SLC39A13;SLC39A8;SMAD7;SMNDC1;SMUG1;SOX13;SPN;SRFBP1;SYVN1;TAF1;TFB1M;THAP1;THAP10;TIE1;TJP2;TM9SF2;TMEM101;TMEM206;TMX2;TRPT1;UACA;UBE2D3;USHBP1;WDR73;WWP2;ZBTB7B;ZC3H10;ZDHHC5;ZKSCAN1;ZNF167;ZNF284;ZNF366;ZNF641;ZNF655;ZNF768"
"cisbp__M1521"	3.26	0.0418	""	184	"AAAS;ABL1;ALG11;AMICA1;ANK3;APBB2;APOLD1;AR;ARL6IP5;ARMCX3;ARMCX6;ASF1A;ASPDH;ATP10A;ATP1A2;BDH2;BGN;BMF;BRCA1;C14orf178;C5orf4;CASKIN2;CCDC91;CCL5;CCND1;CCNE1;CCNI;CD40;CDK6;CDKN1C;CDX1;CHD7;COG3;COL13A1;CSRP2;CTDSP1;CXCR4;CXorf36;CYLD;CYTL1;DAAM2;DEXI;DYNLL2;EBF1;EEF1B2;EMCN;EMILIN2;ERMP1;ERRFI1;ESCO1;EVI5;FAM26E;FAM96A;FBXO32;FLI1;FRYL;GDNF;GNG11;GPR116;GRB7;GREM1;HIGD1B;HMCN1;HMX2;HNF1A;HPS1;HYAL2;INO80D;INTS9;IRF1;ITGA5;ITGA6;ITPKB;JARID2;KCNJ15;KDELC1;KIAA0240;KIAA1267;KRT14;KRTCAP2;L3MBTL2;LAG3;LLGL2;LMO2;LOC284648;LRRC32;MAB21L1;MAX;METT11D1;MFSD11;MGAT1;MRGPRF;MTUS1;MUSTN1;MXD3;MYCN;MYO1E;NAT8L;NBR1;NCAM1;NDE1;NDUFS1;NFKB1;NFKBIZ;NKAPL;NPBWR1;NR1D2;NR3C1;NTN1;OLIG2;OR10C1;ORC2;OTUD7B;OTX2;OXA1L;PAPPA;PELI2;PHF23;PHOX2A;PICALM;PITPNC1;PKD2;PLEKHB1;PLEKHG3;PLP1;PPP1CC;PRDM6;PRUNE2;PTGER1;QARS;RAI14;RASGRP3;RBMS3;REEP4;RELT;RHOBTB1;SASH1;SDC1;SGK1;SGMS1;SLC14A1;SLC26A5;SLC35B1;SLC44A1;SLC7A8;SLCO6A1;SMAD7;SMTN;SORBS3;SOX13;SOX8;TAF11;TAL1;TBC1D4;TBX2;TFDP2;TFPI;TIE1;TJP2;TM9SF2;TMEM206;TNFAIP3;TPM2;TTC35;UACA;UBE2D3;UBL4B;UNC5B;USP13;VEGFA;VWA5B2;WNT11;WTAP;WWP2;XAF1;XBP1;YIPF6;ZAP70;ZBTB20;ZMAT4;ZNF366;ZNF462;ZNF768;ZSCAN20"
"cisbp__M3050"	3.24	0.0418	"ETS1 (directAnnotation). "	125	"ABL1;ADORA1;AGPAT4;ALG11;ALKBH1;ANK3;ANKRD44;APOLD1;ASPDH;C14orf119;C19orf48;C1orf156;C4orf37;C9orf167;CASKIN2;CCDC51;CCDC76;CCL5;CCT6P1;CD14;CDK6;CNDP2;COG3;CPSF3;CRADD;CWC22;CXorf36;CYBASC3;CYTL1;DCUN1D3;DDX18;DFNB31;DISP1;DPP8;EBF1;ECSCR;EEF1B2;EIF5A2;EMCN;FAM110A;FAM96A;FLI1;FOXP2;FRYL;GNG11;GPR116;HIGD1B;HK3;HYAL2;ICAM1;INTS9;IPPK;ITGA1;ITPR3;KCNJ15;KDELC1;KIAA0040;KIAA1958;KLHL20;KRTCAP2;LRRC49;LYRM7;MGAT1;MLKL;MORC2;MRGPRF;MYL4;MYO1E;NDUFS1;NFKBIZ;NGDN;NKAPL;NOL6;NR1H2;NUP155;ORC2;OTX2;PELI2;PHF23;PICALM;PLAT;PLEKHB1;PLEKHG3;PLEKHH1;PLOD3;PPP4C;PRUNE2;PSMB4;PSTPIP1;PTGER1;RBMS3;RELT;RNF144B;RRP12;RRS1;SASS6;SCRT2;SENP7;SLC16A4;SMUG1;SPN;SRFBP1;SYVN1;TBC1D4;TFB1M;TFE3;TIE1;TJP2;TM4SF18;TMEM101;TMEM206;TMX2;TPM2;TRPT1;UBL4B;UNC5B;USHBP1;USP13;XIRP2;ZKSCAN1;ZMAT4;ZNF167;ZNF200;ZNF641;ZNF768"

"motif"	"NES"	"AUC"	"TF_highConf"	"nEnrGenes"	"enrichedGenes"
"cisbp__M6222"	3.23	0.0417	"ETV4 (directAnnotation). "	154	"ABCA2;ACOT7;ACP6;ADAMTS1;AGPAT4;AMICA1;AMPD3;ANK3;ANKRD44;APOLD1;ARHGEF5;B4GALT1;C11orf61;C14orf119;C2;C2orf49;C4orf37;C9orf167;CAMTA1;CAPN13;CASKIN2;CCDC51;CCNI;CD40;CLDN5;COG3;CPSF3;CRADD;CRTC2;CSRP1;CTDSP1;CXorf36;CYBASC3;CYTL1;DDX18;DFNB31;DMBT1;DPP8;DSCR3;ECSCR;EMCN;EPN3;ESAM;ESRRA;ETF1;EXTL2;FAM110A;FAM96A;FCN1;FERMT1;FGR;FKBP2;FLI1;FLT1;GNG11;GPR116;GRB7;GREM1;HK3;HYAL2;IFITM1;IL17RE;IL23A;INTS9;IPPK;ITGA5;ITGB1;ITPR3;KIAA1267;KLHL20;KRTAP5-4;KRTCAP2;LMO2;LRRC32;LRRC49;LSM2;LTC4S;MAN2A1;MAP7;MIDN;MKS1;MORC2;MYO1E;NDST1;NGDN;NKAPL;NKG7;NOL6;NOSTRIN;NOTCH4;NR1H2;ORC2;OTX2;PAQR5;PAQR8;PELI2;PHF23;PICALM;PITPNC1;PLEKHB1;PLOD3;PPP1CC;PPP4C;PSTPIP1;PTPRH;QARS;RAB32;RAI14;RASGRP3;RBMS3;RBP5;RCAN2;RELT;REPIN1;RG9MTD2;RGL2;RNF144B;ROBO4;SCGB3A1;SENP7;SH3BP2;SLC16A4;SLC18A1;SLC39A13;SLC39A8;SMUG1;SOX13;SPN;SRGN;TCF12;TDGF1;TENC1;TFB1M;TFE3;TFPI;TIE1;TMEM101;TMEM206;TMX2;TRIM44;UACA;UBE2D3;UBL4B;USHBP1;VWF;WDR45;WIPF1;ZC3H10;ZNF167;ZNF284;ZNF366;ZNF641;ZNF655;ZNF768"
"cisbp__M0697"	3.23	0.0417	"EHF (inferredBy_Orthology). "	158	"ACAA1;ACOT7;ACVRL1;AGPAT4;ALKBH1;AMICA1;ANKRD44;ANKS1A;ATG2B;BET1L;BTBD16;C11orf61;C14orf119;C1orf66;C2orf24;C2orf49;C4orf37;C9orf167;CAMTA1;CCDC51;CCDC76;CD40;CDK6;CFLAR;CLDN5;CLN3;COG3;CPSF3;CRADD;CRB3;CSRP1;CWC22;CXorf36;CYBASC3;CYLD;DCUN1D3;DDIT3;DDX18;DISP1;DMBT1;DPP8;DSCR3;EBF1;ECSCR;EEF1B2;ELP2;EPN3;ERBB2IP;ESRRA;ETF1;EXTL2;FAM110A;FAM96A;FGFR2;FKBP2;FLI1;FLT1;FRYL;GCN1L1;GGA1;GNG11;GPR116;GREM1;INTS9;IPPK;IRF1;ITGA5;ITGAV;ITGB1;ITPR3;KIAA0040;KIAA1267;KIAA1712;KLHL20;LLGL2;LMO2;LRRC49;MAN2A1;MAP3K3;MGAT1;MIDN;MKS1;MORC2;MTIF2;MYO1E;NBR1;NDUFS1;NGDN;NKAPL;NOL6;NOSTRIN;NR1H2;NUP155;ORC2;OSM;OTX2;PC;PELI2;PHF23;PICALM;PITPNC1;PLEKHB1;PLOD3;PPM1A;PPP1CC;PPP4C;PRKAB1;PRUNE2;PSMB4;PTAR1;PTPRH;QARS;RABGEF1;RAI14;RASGRP3;RBMS3;RGL2;RNF144B;RRS1;SASS6;SCGB3A1;SCNM1;SENP7;SLC39A13;SLC39A8;SMAD7;SMNDC1;SMUG1;SOX13;SPN;SRFBP1;SYVN1;TAF1;TFB1M;THAP1;THAP10;TIE1;TJP2;TM9SF2;TMEM101;TMEM206;TMX2;TRPT1;UACA;UBE2D3;USHBP1;WDR73;WWP2;ZBTB7B;ZC3H10;ZDHHC5;ZKSCAN1;ZNF167;ZNF284;ZNF366;ZNF641;ZNF655;ZNF768"
"cisbp__M6220"	3.23	0.0417	"ETS1 (directAnnotation). "	112	"ACOT7;AGPAT4;ALKBH3;AMICA1;ANKRD44;AP1B1;APOLD1;B4GALT1;BTBD16;C11orf61;C14orf119;C2orf24;C4orf37;C9orf167;CASKIN2;CCDC51;CCL5;CLDN5;COG3;CPSF3;CRADD;CWC22;CXorf36;CYBASC3;CYTL1;DCUN1D3;DDX18;DFNB31;DPP8;DSCR3;ECSCR;ELP2;EMCN;EPN3;ERBB2IP;EXTL2;FAM110A;FAM96A;FKBP2;FLI1;FLT1;GNG11;GPR116;GREM1;HAVCR2;HK3;HYAL2;IL17RE;INTS9;IPPK;ITGB1;ITPR3;KIAA1712;KIAA1958;KLHL20;LRRC41;LRRC49;MGAT1;MTIF2;NFKBIZ;NGDN;NKAPL;NOL6;NOSTRIN;NR1H2;NUP155;ORC2;PELI2;PHF23;PICALM;PKN1;PLEKHH1;PLOD3;PPP4C;PSMB4;PSTPIP1;RAB32;RAI14;RASGRP3;RBMS3;RELT;RGL2;RRS1;SCGB3A1;SCRT2;SENP7;SLC39A13;SLC39A8;SMNDC1;SMUG1;SPN;SRFBP1;SYVN1;TENC1;TFB1M;TFE3;TFPI;THAP1;THAP10;TIE1;TMEM206;TMX2;TRPT1;USHBP1;WIPF1;ZC3H10;ZNF167;ZNF284;ZNF366;ZNF641;ZNF655;ZNF768"
"cisbp__M0648"	3.16	0.0415	""	184	"ABCB10;ABL1;ABTB2;ADAMTS1;ANK3;APOLD1;ARMCX3;ATP10A;ATP1A2;ATP5G3;BAZ1B;BMP1;C14orf178;C5orf4;C6orf47;CAV1;CCND1;CCNE1;CCT6P1;CD9;CDA;CDK6;CDKN1C;CDX1;CENPN;CFL2;CHD7;CLDN11;COL6A2;CREB5;CRTAC1;CSRP2;CTDSP1;CXCR4;CYTIP;CYTL1;CYYR1;DAAM2;DCLRE1A;DOCK1;DYNLL2;EBF1;EMCN;ENO3;ENPP2;ERG;ESRRG;FAM119A;FAM123B;FBN1;FGFR2;FGL2;FHIT;FLI1;FOXE1;FOXO3;FRYL;GATA3;GBX2;GLT25D1;GNG11;GPR85;GRB10;GRINL1A;GTF2A1;HBEGF;HEG1;HMX2;HPSE2;ICAM1;ID1;INO80D;ITGA1;ITGA5;ITGA6;ITGB1;ITPKB;JARID2;KCND2;KCNK5;KEAP1;KIAA1267;KIAA1712;KLF2;KLHL24;LEF1;LHFP;LHPP;LITAF;LLGL2;LMO2;LRRC32;MAT2B;MFSD11;MGAT1;MIER3;MMP14;MRPS14;MXD3;MYCN;MYO1E;NAT8L;NDE1;NFE2L2;NFKBIZ;NR3C1;NTN1;NUP160;OLIG1;OSM;OTX2;PABPC4L;PDPN;PELI2;PFKFB2;PHOX2A;PICALM;PITPNC1;PLEKHA5;PLEKHG1;PLEKHG3;PLOD3;PLP1;PPARGC1A;PPP1CC;PRDM6;PRUNE2;PTGER1;PTH1R;RAI14;RB1;RBMS3;REEP4;RGMA;RHOB;RHOBTB1;RNF44;SASH1;SDC1;SEMA3B;SEPP1;SFMBT1;SGK1;SH3PXD2B;SHC4;SLC12A2;SLC26A9;SLC35D2;SLC46A1;SLC9A3;SMAD6;SMAD7;SNRNP48;SOX8;SPSB1;SRSF1;STX16;SURF4;TAGLN;TAL1;TBC1D2;TBC1D4;TBX2;TDRD1;TFAP2E;TFDP2;THBS1;TJP2;TPM2;TRIM44;TRIP10;UACA;UGT8;USP13;VAMP8;VCAM1;VEGFA;WNT11;WTAP;ZBTB7B;ZHX2;ZNF366;ZNF462;ZNF692"
"cisbp__M0687"	3.15	0.0414	"ELF3 (inferredBy_Orthology). "	423	"AAAS;ABL1;ACAA1;ACOT7;ACP6;ACVRL1;ADAMTS1;ADIPOR2;ADM;ADO;AGPAT4;ALG11;ALKBH1;AMICA1;AMPD3;ANKRD44;ANKS1A;APBA3;APBB2;APOD;APOLD1;AR;ARHGEF16;ARL6IP5;ARMCX3;ARMCX6;ASPDH;ATG2B;ATOH8;ATP10A;ATP1A2;AUH;B3GALNT2;B4GALT1;BAZ1B;BDH2;BGN;BMP1;BRCA1;BTBD16;C10orf62;C11orf61;C14orf119;C14orf178;C19orf48;C1orf14;C1orf144;C2;C20orf4;C2orf24;C2orf49;C4orf26;C4orf37;C5orf4;C5orf51;C6orf204;C6orf47;C9orf167;CAMTA1;CAPN13;CARD14;CARD9;CASKIN2;CASP5;CASS4;CCDC76;CCL5;CCND1;CCNI;CCRL2;CCT6P1;CD14;CD226;CD40;CDC42EP1;CDK6;CDKN1C;CDKN2A;CFLAR;CHD7;CLCA2;CLDN5;CLIC1;CLP1;CNDP2;COG3;COL13A1;COX17;CPSF3;CRADD;CRB3;CRTC2;CSRP1;CSRP2;CTDSP1;CTF1;CWC22;CXCR4;CXorf36;CYBASC3;CYLD;CYTL1;CYYR1;DCPS;DCUN1D3;DDIT3;DDX18;DDX23;DENND4B;DFNB31;DHRS11;DISP1;DMBT1;DPP8;DPYS;DSCR3;DUSP1;EBF1;ECSCR;EDNRA;EEF1B2;EMCN;EMILIN2;EPN3;ERBB2IP;ESAM;ESM1;ESRRA;ESRRG;ETF1;ETV6;EXTL2;FAIM3;FAM110A;FAM119A;FAM49B;FAM63A;FAM96A;FBXO32;FCN1;FCRL5;FERMT1;FGFR2;FIZ1;FKBP2;FLI1;FLT1;FOXO3;FRYL;GALNT13;GCN1L1;GDNF;GGA1;GIMAP1;GKN1;GLDN;GNE;GNG11;GPR108;GPR116;GRB7;GREM1;GTF2A1;GZF1;HAGHL;HCP5;HIC1;HIGD1B;HLA-B;HLA-H;HN1L;HNF1A;HPGD;HPS1;HSD11B1;HSPB1;HYAL2;ICAM1;ID1;IL23A;INO80D;INTS9;IPPK;IQGAP1;IRF1;ITGA5;ITGAV;ITGB1;ITIH2;ITPKB;ITPR3;KDELC1;KIAA0040;KIAA0240;KIAA1267;KIAA1324L;KIAA1712;KIRREL;KLHL20;KRI1;KRTCAP2;L3MBTL2;LAG3;LANCL1;LCP1;LEF1;LHFP;LHPP;LLGL2;LMO2;LOC284648;LRRC32;LRRC49;MAN2A1;MAP3K3;MAP7;MAT2B;MAX;METT11D1;MFSD11;MGAT1;MID1IP1;MIDN;MKS1;MLKL;MMP14;MOGAT2;MORC2;MRGPRF;MRPS14;MTIF2;MYCN;MYL12A;MYO1E;NBR1;NCAM1;NDE1;NDST1;NDUFS1;NDUFS7;NET1;NFASC;NFKBIZ;NKAPL;NOL6;NOSTRIN;NOTCH4;NPBWR1;NPR1;NR1H2;NR3C1;NTN1;NUP155;ODF2;OLFML3;OLIG2;OR10C1;ORC2;OSM;OTX2;OXA1L;P2RY14;PABPC4L;PAPPA;PAQR5;PAX3;PC;PCDHGA12;PELI2;PHF23;PHOX2A;PICALM;PIGT;PIP;PITPNC1;PLA2G4F;PLA2R1;PLEKHB1;PLEKHG1;PLEKHG3;PLEKHG4;PLEKHH1;PLOD3;PLP1;POMC;PPM1A;PPP1CC;PPP1R15B;PPP4C;PRDM6;PRKAB1;PROCR;PRR5L;PRUNE2;PSMB4;PTPRH;PVALB;PXN;QARS;RAB32;RABGEF1;RAI14;RASGRP3;RB1;RBMS3;RCAN2;REEP4;RELT;REPIN1;RG9MTD2;RGMA;RHOB;RHOBTB1;RNPEPL1;RPA3;RRS1;SAP30L;SASS6;SCGB3A1;SCNM1;SCRT2;SDC1;SDPR;SEMA7A;SENP7;SEPP1;S

"motif"	"NES"	"AUC"	"TF_highConf"	"nEnrGenes"	"enrichedGenes"
"cisbp__M6307"	3.09	0.0412	"IRF1 (directAnnotation). "	166	"ABTB2;ACSL5;AGPAT4;APOD;APOL6;APOLD1;ARMCX3;ARMCX6;ATP10A;BACE1;BAZ1B;BCHE;BRCA1;BTN3A2;C11orf52;C2;C4orf26;C5orf4;CAPN3;CCDC28B;CCL19;CCL5;CCND1;CD40;CDK6;CDKN1B;CDKN1C;CDKN2A;CDX1;CNDP2;CNKSR3;CNP;COLEC11;CRYAB;CUEDC1;CYLD;CYTH1;CYTL1;DENND4B;DLL4;DPP8;DTX3L;EBF1;ENPP2;ERG;ESRRG;ETS1;ETV6;EVI5;FAM123B;FAM63A;FBN1;FGD4;FGF9;FHIT;FIZ1;FLI1;FLT3LG;FNTB;GATA3;GNG11;GPR116;GRB7;HCP5;HLA-B;HLA-H;IFI27;IFI44L;IFNA1;IRF7;ITPKB;JARID2;JPH2;KIAA1109;KIAA1267;KIAA1712;LAG3;LANCL1;LBR;LEF1;LMO2;LOC284648;METT11D1;MGAT1;MITF;MMP14;MORC2;MXD3;MYCN;MYO1E;NAALADL2;NBR1;NCF1C;NDUFA12;NFASC;NKAPL;NPBWR1;NR3C1;NTN1;NUP160;OAS2;OAS3;OLFML3;OLR1;OR10P1;ORC2;OSM;OTX2;PAPPA;PARP4;PAX3;PCDHB19P;PELI2;PGM5;PHKA1;PITPNC1;PLEKHG1;PLP1;PNPT1;PRKAB1;PRKRIP1;PRR5L;PRUNE2;PSMB4;PSMB8;PSMD6;QARS;RASGRP3;RBM41;RBMS3;RNF111;RTP4;SCRT2;SDPR;SENP7;SGK1;SGMS1;SIX1;SLC15A3;SLC26A6;SLC37A2;SLC39A13;SLC6A12;SMAD7;SRGN;STAT2;STAT5A;TAL1;TAS2R13;TCF12;TJP2;TNFSF10;TRIM44;TRPC3;UACA;UBA7;USO1;VCAM1;WDR45;ZBTB20;ZHX2;ZNF34;ZNF366;ZNF385A;ZNF503;ZNF692"
"cisbp__M6494"	3.09	0.0412	"STAT2 (directAnnotation). "	106	"ACSL5;AGPAT4;AMPD3;APOLD1;ARMCX3;ARMCX6;ATP10A;ATP1A2;BRCA1;C11orf52;C5orf4;CCL5;CD14;CD40;CDK6;CDKN1C;CNDP2;CNKSR3;CWC22;CYTIP;DENND4B;DLL4;DPP8;EBF1;EGFLAM;EMCN;ENPP2;ERG;ESRRG;ETV6;EVI5;FAM123B;FAM63A;FBN1;FGF9;FIZ1;FLI1;FRYL;GPR116;GRB7;HLA-B;HLA-H;HPS1;ICAM1;IFI27;IFI44L;ITGB1;ITPKB;JARID2;KCNJ15;LANCL1;LEF1;LMO2;LOC284648;METT11D1;MGAT1;MLKL;MMP14;MYCN;MYO1E;NCF1C;NKAPL;NR3C1;NTN1;NUP160;OAS2;ORC2;OTX2;PARP4;PAX3;PELI2;PITPNC1;PLEKHG1;PLEKHG4;PNPT1;PRKAB1;PRUNE2;PSMB4;PVALB;RASGRP3;RBMS3;RNF111;RTP4;SDPR;SGK1;SGMS1;SIX1;SLC15A3;SLC39A13;SLC39A8;SPG20;TAL1;TBC1D4;TDRD7;TMEM206;USP13;VEGFA;VSIG2;XIRP2;ZBTB20;ZC3H13;ZHX2;ZNF34;ZNF366;ZNF503;ZNF692"
"cisbp__M6308"	3.08	0.0412	"IRF2 (directAnnotation). "	73	"ABTB2;ACSL5;APOL6;ATP10A;BRCA1;C11orf52;CCL5;CCND1;CD40;CDK6;CDKN1C;CNKSR3;CUEDC1;DLL4;EBF1;ENPP2;ERG;ETS1;ETV6;EVI5;FAM123B;FAM63A;FBN1;FLI1;FLT3LG;GPR116;GRB7;HLA-B;HLA-H;IFI27;IFI44L;LEF1;LMO2;LOC284648;MGAT1;MXD3;MYO1E;NAALADL2;NCF1C;NR3C1;NUP160;OAS2;OAS3;ORC2;OTX2;PARP4;PAX3;PELI2;PLP1;PNPT1;PRUNE2;PSMB4;PSMB8;RASGRP3;RBMS3;RNF111;RTP4;SCRT2;SGK1;SGMS1;SLC15A3;SLC37A2;SLC39A13;TAL1;TCF12;TRIM44;UBA7;USO1;ZBTB20;ZHX2;ZNF366;ZNF385A;ZNF692"
"cisbp__M0660"	3.03	0.041	""	132	"ABCB10;ABL1;ABTB2;ADORA1;AMPD3;APOLD1;AR;ARMCX3;ATP10A;ATP1A2;ATP5G3;C14orf178;C5orf4;C6orf47;CAV1;CD9;CDA;CDKN1C;CDX1;CFL2;CLDN11;CNKSR3;COL3A1;CREB5;CSRP2;CTDSP1;CXCR4;CYTL1;CYYR1;DCLRE1A;DYNLL2;EBF1;EMCN;ENO3;FHIT;FLI1;FOXO3;FOXP2;FRYL;GATA3;GBX2;GLT25D1;GNG11;GRB10;GRINL1A;GTF2A1;HEG1;HPSE2;ICAM1;ICK;INO80D;ITGA1;ITGA5;ITGA6;ITGB1;ITPKB;JARID2;KCND2;KIAA1267;KIAA1712;KLHL24;LEF1;LHFP;LHPP;LLGL2;LMO2;MFSD11;MGAT1;MIER3;MORF4;MRPS14;MYCN;MYL4;NDE1;NFKBIZ;NKX6-1;NR3C1;NTN1;OLIG1;OSM;OTX2;PDPN;PELI2;PHOX2A;PITPNC1;PLEKHG1;PLOD3;PLP1;PPARGC1A;PPP1CC;PRDM6;PRUNE2;PTGER1;RBMS3;REEP4;RGMA;RGS8;RHOB;RHOBTB1;SASH1;SEPP1;SFMBT1;SHC4;SLC12A2;SLC26A9;SLC35D2;SLC45A3;SLC46A1;SMAD6;SNRNP48;SOX8;SRSF1;STX16;SURF4;TAL1;TBC1D4;TDRD1;TFAP2E;TFDP2;THBS1;TJP2;TPM2;TRIP10;UACA;USP13;VCAM1;VEGFA;WNT11;WTAP;ZHX2;ZNF366;ZNF462"
