# Supplementary material for: Regional gene expression patterns are associated with task‐specific brain activation during reward and emotion processing measured with functional MRI
Source: Hum Brain Mapp. 2022 Jul 7;43(17):5266–80. doi: 10.1002/hbm.26001 (PMC9812247; doi:10.1002/hbm.26001)
Supplement: Supplementary file 3 — TABLE S4: Enriched biological programs for emotion and reward processing based on ontological structure. Specific gene categories listed within the gene ontology (GO) knowledgebase were significantly enriched for genes showing expression patterns strongly correlated with single‐site imaging data in the subcortex. Resulting GO categories and corresponding p values are provided for both paradigms (excel‐file) [file HBM-43-5266-s004.pdf]

Emotion COR positive

GOIDlabel	GOName	size	categoryScores	pValPerm	pValPermCorr
GO:0015012	heparan sulfate proteoglycan biosynthetic process	9	0.099673677	< 0.001	< 0.001

Emotion SUBC positive

GOIDlabel	GOName	size	categoryScores	pValPerm	pValPermCorr
GO:0006607	NLS-bearing protein import into nucleus	5	0.554800515	< 0.001	< 0.001
GO:0070757	interleukin-35-mediated signaling pathway	5	0.519433719	< 0.001	< 0.001
GO:0098792	xenophagy	7	0.48758963	< 0.001	< 0.001
GO:1905098	negative regulation of guanyl-nucleotide exchange factor activity	5	0.486589447	< 0.001	< 0.001
GO:0098703	calcium ion import across plasma membrane	5	0.466306306	< 0.001	< 0.001
GO:1990035	calcium ion import into cell	5	0.466306306	< 0.001	< 0.001
GO:0006054	N-acetylneuraminate metabolic process	5	0.462445302	< 0.001	< 0.001
GO:0051024	positive regulation of immunoglobulin secretion	5	0.461621622	0.0002	0.000356582
GO:0031646	positive regulation of neurological system process	6	0.459244959	< 0.001	< 0.001
GO:0021877	forebrain neuron fate commitment	7	0.459238831	0.0072	0.008495371
GO:1900246	positive regulation of RIG-I signaling pathway	5	0.457760618	< 0.001	< 0.001
GO:0002692	negative regulation of cellular extravasation	5	0.451480051	0.0048	0.005840847
GO:0035493	SNARE complex assembly	6	0.451051051	0.0005	0.000791911
GO:0072160	nephron tubule epithelial cell differentiation	5	0.44967825	< 0.001	< 0.001
GO:0019433	triglyceride catabolic process	7	0.448097077	< 0.001	< 0.001
GO:0046461	neutral lipid catabolic process	7	0.448097077	< 0.001	< 0.001
GO:0046464	acylglycerol catabolic process	7	0.448097077	< 0.001	< 0.001
GO:0006525	arginine metabolic process	6	0.448005148	< 0.001	< 0.001
GO:0072087	renal vesicle development	6	0.447275847	< 0.001	< 0.001
GO:0010919	regulation of inositol phosphate biosynthetic process	6	0.443200343	< 0.001	< 0.001
GO:0060732	positive regulation of inositol phosphate biosynthetic process	6	0.443200343	< 0.001	< 0.001
GO:0018410	C-terminal protein amino acid modification	6	0.442900043	0.0001	0.000192793
GO:0051000	positive regulation of nitric-oxide synthase activity	7	0.442801986	< 0.001	< 0.001
GO:0018065	protein-cofactor linkage	6	0.442170742	< 0.001	< 0.001
GO:2000696	regulation of epithelial cell differentiation involved in kidney development	5	0.4402574	< 0.001	< 0.001
GO:0048566	embryonic digestive tract development	7	0.438058467	< 0.001	< 0.001
GO:0009415	response to water	6	0.436979837	0.0001	0.000192793
GO:0032226	positive regulation of synaptic transmission, dopaminergic	5	0.436138996	< 0.001	< 0.001
GO:0060439	trachea morphogenesis	6	0.434963535	0.0001	0.000192793
GO:0031547	brain-derived neurotrophic factor receptor signaling pathway	5	0.434954955	0.0005	0.000791911
GO:0098659	inorganic cation import into cell	6	0.434706135	< 0.001	< 0.001
GO:0099587	inorganic ion import into cell	6	0.434706135	< 0.001	< 0.001
GO:0021902	commitment of neuronal cell to specific neuron type in forebrain	5	0.434697555	0.0084	0.009757868
GO:1990709	presynaptic active zone organization	6	0.431746032	0.0025	0.003237479
GO:0007202	activation of phospholipase C activity	11	0.427963028	< 0.001	< 0.001
GO:0090036	regulation of protein kinase C signaling	6	0.427027027	< 0.001	< 0.001
GO:0006474	N-terminal protein amino acid acetylation	8	0.426898327	< 0.001	< 0.001
GO:0045176	apical protein localization	7	0.426291598	< 0.001	< 0.001
GO:1903044	protein localization to membrane raft	5	0.425431145	0.0002	0.000356582
GO:1905097	regulation of guanyl-nucleotide exchange factor activity	6	0.424281424	< 0.001	< 0.001
GO:1902932	positive regulation of alcohol biosynthetic process	7	0.423607281	0.0004	0.000648934
GO:0014049	positive regulation of glutamate secretion	7	0.423570509	0.0006	0.000927725
GO:0033540	fatty acid beta-oxidation using acyl-CoA oxidase	6	0.422565423	< 0.001	< 0.001
GO:0032211	negative regulation of telomere maintenance via telomerase	8	0.421235521	< 0.001	< 0.001
GO:0051957	positive regulation of amino acid transport	8	0.41994852	0.0002	0.000356582
GO:0001958	endochondral ossification	10	0.419459459	< 0.001	< 0.001
GO:0036075	replacement ossification	10	0.419459459	< 0.001	< 0.001
GO:0050718	positive regulation of interleukin-1 beta secretion	10	0.417734878	< 0.001	< 0.001
GO:0002076	osteoblast development	5	0.417657658	< 0.001	< 0.001
GO:0072077	renal vesicle morphogenesis	5	0.417451737	< 0.001	< 0.001
GO:0072283	metanephric renal vesicle morphogenesis	5	0.417451737	< 0.001	< 0.001
GO:0071107	response to parathyroid hormone	5	0.416628057	0.0001	0.000192793
GO:0060767	epithelial cell proliferation involved in prostate gland development	5	0.416370656	< 0.001	< 0.001
GO:0071498	cellular response to fluid shear stress	6	0.414886315	0.0007	0.001063182
GO:0060330	regulation of response to interferon-gamma	9	0.414414414	0.0001	0.000192793
GO:0060334	regulation of interferon-gamma-mediated signaling pathway	9	0.414414414	0.0001	0.000192793
GO:0050995	negative regulation of lipid catabolic process	5	0.411068211	0.0002	0.000356582
GO:1904321	response to forskolin	5	0.410604891	< 0.001	< 0.001
GO:1904322	cellular response to forskolin	5	0.410604891	< 0.001	< 0.001
GO:0006195	purine nucleotide catabolic process	12	0.409202059	< 0.001	< 0.001
GO:0097094	craniofacial suture morphogenesis	6	0.408365508	< 0.001	< 0.001
GO:0051152	positive regulation of smooth muscle cell differentiation	6	0.408065208	0.0002	0.000356582
GO:0002689	negative regulation of leukocyte chemotaxis	6	0.407893608	< 0.001	< 0.001
GO:0071318	cellular response to ATP	9	0.407693408	< 0.001	< 0.001
GO:0035589	G-protein coupled purinergic nucleotide receptor signaling pathway	6	0.407507508	0.0004	0.000648934
GO:0060534	trachea cartilage development	5	0.407104247	< 0.001	< 0.001
GO:0003351	epithelial cilium movement	7	0.406618864	0.0006	0.000927725
GO:0050951	sensory perception of temperature stimulus	8	0.406467181	< 0.001	< 0.001
GO:0006048	UDP-N-acetylglucosamine biosynthetic process	6	0.406349206	< 0.001	< 0.001
GO:1902414	protein localization to cell junction	5	0.405817246	< 0.001	< 0.001
GO:0071569	protein ufmylation	5	0.405611326	< 0.001	< 0.001
GO:1990564	protein polyufmylation	5	0.405611326	< 0.001	< 0.001
GO:1990592	protein K69-linked ufmylation	5	0.405611326	< 0.001	< 0.001
GO:1903997	positive regulation of non-membrane spanning protein tyrosine kinase activity	5	0.405508366	0.0003	0.000505993
GO:0002833	positive regulation of response to biotic stimulus	20	0.405495495	< 0.001	< 0.001
GO:0045063	T-helper 1 cell differentiation	5	0.404890605	< 0.001	< 0.001
GO:0060438	trachea development	8	0.404150579	0.0011	0.001576346
GO:1903995	regulation of non-membrane spanning protein tyrosine kinase activity	6	0.403732304	< 0.001	< 0.001
GO:0034114	regulation of heterotypic cell-cell adhesion	6	0.403088803	< 0.001	< 0.001
GO:0032516	positive regulation of phosphoprotein phosphatase activity	8	0.402574003	0.0001	0.000192793
GO:0035162	embryonic hemopoiesis	5	0.401750322	< 0.001	< 0.001

GOIDlabel	GOName	size	categoryScores	pValPerm	pValPermCorr
GO:0021960	anterior commissure morphogenesis	5	0.401750322	0.0059	0.007066401
GO:0032700	negative regulation of interleukin-17 production	6	0.401501502	< 0.001	< 0.001
GO:0006359	regulation of transcription from RNA polymerase III promoter	9	0.401401401	< 0.001	< 0.001
GO:0010992	ubiquitin homeostasis	5	0.401132561	0.0001	0.000192793
GO:0033561	regulation of water loss via skin	7	0.400551572	0.0001	0.000192793
GO:0050961	detection of temperature stimulus involved in sensory perception	6	0.4001287	< 0.001	< 0.001
GO:0050965	detection of temperature stimulus involved in sensory perception of pain	6	0.4001287	< 0.001	< 0.001
GO:0021895	cerebral cortex neuron differentiation	15	0.39955384	0.0004	0.000648934
GO:0031115	negative regulation of microtubule polymerization	8	0.398938224	< 0.001	< 0.001
GO:0070885	negative regulation of calcineurin-NFAT signaling cascade	7	0.39823497	0.0004	0.000648934
GO:1904357	negative regulation of telomere maintenance via telomere lengthening	10	0.397400257	< 0.001	< 0.001
GO:0008105	asymmetric protein localization	8	0.396750322	< 0.001	< 0.001
GO:1904429	regulation of t-circle formation	5	0.396499356	0.0014	0.00194839
GO:0033004	negative regulation of mast cell activation	6	0.395967396	< 0.001	< 0.001
GO:0099558	maintenance of synapse structure	11	0.395928396	0.0048	0.005840847
GO:0070102	interleukin-6-mediated signaling pathway	7	0.395918367	0.0001	0.000192793
GO:0003407	neural retina development	22	0.395858196	0.0002	0.000356582
GO:0046325	negative regulation of glucose import	6	0.395795796	< 0.001	< 0.001
GO:0072574	hepatocyte proliferation	5	0.393770914	< 0.001	< 0.001
GO:0072575	epithelial cell proliferation involved in liver morphogenesis	5	0.393770914	< 0.001	< 0.001
GO:0016081	synaptic vesicle docking	8	0.392664093	0.0012	0.001702047
GO:0016191	synaptic vesicle uncoating	5	0.392123552	0.0011	0.001576346
GO:0072318	clathrin coat disassembly	5	0.392123552	0.0011	0.001576346
GO:0050999	regulation of nitric-oxide synthase activity	16	0.391924067	< 0.001	< 0.001
GO:1990504	dense core granule exocytosis	5	0.391248391	0.0018	0.002428764
GO:0032620	interleukin-17 production	9	0.390847991	< 0.001	< 0.001
GO:0040019	positive regulation of embryonic development	11	0.390803791	< 0.001	< 0.001
GO:0010807	regulation of synaptic vesicle priming	5	0.39047619	0.0063	0.007507211
GO:0034616	response to laminar fluid shear stress	5	0.39032175	< 0.001	< 0.001
GO:0051954	positive regulation of amine transport	17	0.38958286	0.0001	0.000192793
GO:0099640	axo-dendritic protein transport	6	0.389146289	0.0016	0.002186748
GO:0099641	anterograde axonal protein transport	6	0.389146289	0.0016	0.002186748
GO:0031915	positive regulation of synaptic plasticity	6	0.389060489	0.0006	0.000927725
GO:0036037	CD8-positive, alpha-beta T cell activation	5	0.388880309	< 0.001	< 0.001
GO:0072321	chaperone-mediated protein transport	7	0.388564074	0.0003	0.000505993
GO:0006040	amino sugar metabolic process	16	0.387612613	< 0.001	< 0.001
GO:1901071	glucosamine-containing compound metabolic process	7	0.386615187	< 0.001	< 0.001
GO:0039531	regulation of viral-induced cytoplasmic pattern recognition receptor signaling pathway	9	0.386414986	< 0.001	< 0.001
GO:0043162	ubiquitin-dependent protein catabolic process via the multivesicular body sorting pathway	6	0.385971686	< 0.001	< 0.001
GO:0010454	negative regulation of cell fate commitment	5	0.385379665	0.0024	0.003116943
GO:0045161	neuronal ion channel clustering	5	0.384658945	0.0018	0.002428764
GO:0010226	response to lithium ion	6	0.384255684	< 0.001	< 0.001
GO:0070106	interleukin-27-mediated signaling pathway	6	0.383998284	0.0011	0.001576346
GO:0050716	positive regulation of interleukin-1 secretion	13	0.383704584	< 0.001	< 0.001
GO:2001169	regulation of ATP biosynthetic process	6	0.383354783	0.0001	0.000192793
GO:0042416	dopamine biosynthetic process	6	0.383097383	< 0.001	< 0.001
GO:0032958	inositol phosphate biosynthetic process	10	0.383011583	0.0016	0.002186748
GO:0033006	regulation of mast cell activation involved in immune response	12	0.382990133	< 0.001	< 0.001
GO:1905050	positive regulation of metallopeptidase activity	5	0.382960103	0.0019	0.00254969
GO:0009249	protein lipoylation	5	0.382548263	0.0005	0.000791911
GO:0006904	vesicle docking involved in exocytosis	21	0.381638782	< 0.001	< 0.001
GO:1904376	negative regulation of protein localization to cell periphery	9	0.381467181	< 0.001	< 0.001
GO:0090148	membrane fission	6	0.381295581	0.001	0.001450349
GO:0001682	tRNA 5'-leader removal	6	0.381166881	< 0.001	< 0.001
GO:0009084	glutamine family amino acid biosynthetic process	8	0.381048906	< 0.001	< 0.001
GO:0007288	sperm axoneme assembly	5	0.380643501	0.0113	0.012793882
GO:0060004	reflex	8	0.380212355	0.0002	0.000356582
GO:0032594	protein transport within lipid bilayer	6	0.38005148	0.009	0.010394529
GO:0031111	negative regulation of microtubule polymerization or depolymerization	19	0.379800853	< 0.001	< 0.001
GO:0010453	regulation of cell fate commitment	7	0.379738923	0.0035	0.004390436
GO:0060425	lung morphogenesis	15	0.37971686	0.0011	0.001576346
GO:0030857	negative regulation of epithelial cell differentiation	7	0.379628608	< 0.001	< 0.001
GO:1901985	positive regulation of protein acetylation	16	0.379472329	0.0001	0.000192793
GO:1901841	regulation of high voltage-gated calcium channel activity	8	0.379343629	0.0022	0.002896881
GO:0002820	negative regulation of adaptive immune response	12	0.379279279	< 0.001	< 0.001
GO:0010092	specification of animal organ identity	7	0.378709322	0.0015	0.002069969
GO:0021892	cerebral cortex GABAergic interneuron differentiation	7	0.37841515	0.0073	0.008600218
GO:0097154	GABAergic neuron differentiation	7	0.37841515	0.0073	0.008600218
GO:0048278	vesicle docking	24	0.378389103	< 0.001	< 0.001
GO:0042462	eye photoreceptor cell development	13	0.378239778	< 0.001	< 0.001
GO:0061436	establishment of skin barrier	5	0.378172458	0.0011	0.001576346
GO:0072576	liver morphogenesis	6	0.378078078	< 0.001	< 0.001
GO:0002701	negative regulation of production of molecular mediator of immune response	9	0.376662377	< 0.001	< 0.001
GO:1905048	regulation of metallopeptidase activity	8	0.376190476	0.0001	0.000192793
GO:0099560	synaptic membrane adhesion	17	0.376167764	0.0014	0.00194839
GO:0032225	regulation of synaptic transmission, dopaminergic	10	0.376113256	< 0.001	< 0.001
GO:0046349	amino sugar biosynthetic process	7	0.376025005	< 0.001	< 0.001
GO:0032715	negative regulation of interleukin-6 production	12	0.375439725	< 0.001	< 0.001
GO:0048643	positive regulation of skeletal muscle tissue development	6	0.375375375	0.0001	0.000192793
GO:0070528	protein kinase C signaling	12	0.375010725	< 0.001	< 0.001
GO:0007435	salivary gland morphogenesis	12	0.374817675	< 0.001	< 0.001
GO:0051445	regulation of meiotic cell cycle	10	0.374594595	< 0.001	< 0.001
GO:0031342	negative regulation of cell killing	5	0.374568855	< 0.001	< 0.001

GOIDlabel	GOName	size	categoryScores	pValPerm	pValPermCorr
GO:2000104	negative regulation of DNA-dependent DNA replication	8	0.374259974	0.0013	0.001826625
GO:0070493	thrombin-activated receptor signaling pathway	5	0.373899614	< 0.001	< 0.001
GO:0060413	atrial septum morphogenesis	7	0.373230373	< 0.001	< 0.001
GO:0042762	regulation of sulfur metabolic process	7	0.372936202	0.0109	0.012398148
GO:1903421	regulation of synaptic vesicle recycling	16	0.372747748	0.0011	0.001576346
GO:0010470	regulation of gastrulation	10	0.372689833	< 0.001	< 0.001
GO:0031579	membrane raft organization	7	0.37238463	< 0.001	< 0.001
GO:1903959	regulation of anion transmembrane transport	6	0.372114972	< 0.001	< 0.001
GO:0072202	cell differentiation involved in metanephros development	6	0.372072072	< 0.001	< 0.001
GO:0014048	regulation of glutamate secretion	10	0.372046332	0.0029	0.003693519
GO:0032768	regulation of monooxygenase activity	20	0.371917632	< 0.001	< 0.001
GO:0098501	polynucleotide dephosphorylation	5	0.371891892	< 0.001	< 0.001
GO:0072673	lamellipodium morphogenesis	5	0.371891892	0.0036	0.004505419
GO:0016082	synaptic vesicle priming	11	0.371802972	0.0062	0.00739947
GO:0048715	negative regulation of oligodendrocyte differentiation	5	0.370965251	0.0107	0.012191187
GO:0015874	norepinephrine transport	10	0.370553411	0.0002	0.000356582
GO:1905476	negative regulation of protein localization to membrane	12	0.37035607	< 0.001	< 0.001
GO:1904263	positive regulation of TORC1 signaling	5	0.36983269	0.0049	0.005958506
GO:0009154	purine ribonucleotide catabolic process	8	0.368918919	< 0.001	< 0.001
GO:0002704	negative regulation of leukocyte mediated immunity	11	0.368831169	< 0.001	< 0.001
GO:0042403	thyroid hormone metabolic process	5	0.368803089	0.0004	0.000648934
GO:0045671	negative regulation of osteoclast differentiation	7	0.368707483	< 0.001	< 0.001
GO:0090185	negative regulation of kidney development	5	0.368545689	< 0.001	< 0.001
GO:0010310	regulation of hydrogen peroxide metabolic process	8	0.368532819	< 0.001	< 0.001
GO:0072319	vesicle uncoating	6	0.368425568	0.0011	0.001576346
GO:0010829	negative regulation of glucose transport	9	0.368396968	< 0.001	< 0.001
GO:0072523	purine-containing compound catabolic process	15	0.368168168	< 0.001	< 0.001
GO:0031365	N-terminal protein amino acid modification	11	0.367871768	< 0.001	< 0.001
GO:0033235	positive regulation of protein sumoylation	7	0.367089539	< 0.001	< 0.001
GO:0002686	negative regulation of leukocyte migration	18	0.367052767	< 0.001	< 0.001
GO:0002828	regulation of type 2 immune response	7	0.367015996	0.0004	0.000648934
GO:0061437	renal system vasculature development	7	0.366685052	< 0.001	< 0.001
GO:0061440	kidney vasculature development	7	0.366685052	< 0.001	< 0.001
GO:0072012	glomerulus vasculature development	7	0.366685052	< 0.001	< 0.001
GO:0060080	inhibitory postsynaptic potential	6	0.366580867	0.0112	0.012688637
GO:0031666	positive regulation of lipopolysaccharide-mediated signaling pathway	5	0.366486486	< 0.001	< 0.001
GO:0032770	positive regulation of monooxygenase activity	10	0.366229086	0.0001	0.000192793
GO:0018146	keratan sulfate biosynthetic process	9	0.365851566	0.0001	0.000192793
GO:0043951	negative regulation of cAMP-mediated signaling	7	0.365839309	0.0008	0.001193265
GO:0060536	cartilage morphogenesis	5	0.364942085	< 0.001	< 0.001
GO:1901983	regulation of protein acetylation	28	0.364892443	< 0.001	< 0.001
GO:0061014	positive regulation of mRNA catabolic process	9	0.364764765	< 0.001	< 0.001
GO:0033623	regulation of integrin activation	5	0.364375804	< 0.001	< 0.001
GO:0046039	GTP metabolic process	12	0.364242814	< 0.001	< 0.001
GO:0043304	regulation of mast cell degranulation	11	0.364151164	< 0.001	< 0.001
GO:0071435	potassium ion export	5	0.363912484	0.0004	0.000648934
GO:0097623	potassium ion export across plasma membrane	5	0.363912484	0.0004	0.000648934
GO:0090197	positive regulation of chemokine secretion	8	0.363352638	< 0.001	< 0.001
GO:0039528	cytoplasmic pattern recognition receptor signaling pathway in response to virus	10	0.363140283	< 0.001	< 0.001
GO:0098779	mitophagy in response to mitochondrial depolarization	9	0.362877163	0.0001	0.000192793
GO:0021680	cerebellar Purkinje cell layer development	6	0.362419562	0.0027	0.003470695
GO:0034405	response to fluid shear stress	10	0.362316602	< 0.001	< 0.001
GO:0003171	atrioventricular valve development	9	0.362219362	< 0.001	< 0.001
GO:0003181	atrioventricular valve morphogenesis	9	0.362219362	< 0.001	< 0.001
GO:0006290	pyrimidine dimer repair	6	0.361861862	0.0012	0.001702047
GO:2000756	regulation of peptidyl-lysine acetylation	21	0.361720905	< 0.001	< 0.001
GO:0032201	telomere maintenance via semi-conservative replication	10	0.361647362	0.0023	0.003013139
GO:0023019	signal transduction involved in regulation of gene expression	8	0.361615187	0.0003	0.000505993
GO:0035590	purinergic nucleotide receptor signaling pathway	10	0.361338481	< 0.001	< 0.001
GO:0010922	positive regulation of phosphatase activity	13	0.361290961	< 0.001	< 0.001
GO:0006596	polyamine biosynthetic process	7	0.360801618	0.0001	0.000192793
GO:0045932	negative regulation of muscle contraction	11	0.360734761	< 0.001	< 0.001
GO:0043696	dedifferentiation	5	0.360669241	< 0.001	< 0.001
GO:0043697	cell dedifferentiation	5	0.360669241	< 0.001	< 0.001
GO:0060441	epithelial tube branching involved in lung morphogenesis	8	0.360456885	< 0.001	< 0.001
GO:0016048	detection of temperature stimulus	7	0.360397132	< 0.001	< 0.001
GO:0001754	eye photoreceptor cell differentiation	15	0.36006864	< 0.001	< 0.001
GO:0010592	positive regulation of lamellipodium assembly	10	0.36002574	< 0.001	< 0.001
GO:0015813	L-glutamate transport	11	0.35984556	0.0008	0.001193265
GO:0060219	camera-type eye photoreceptor cell differentiation	5	0.35984556	0.0023	0.003013139
GO:0003128	heart field specification	5	0.35958816	0.0153	0.01697069
GO:0003139	secondary heart field specification	5	0.35958816	0.0153	0.01697069
GO:0032660	regulation of interleukin-17 production	7	0.359367531	< 0.001	< 0.001
GO:0002279	mast cell activation involved in immune response	18	0.359244959	< 0.001	< 0.001
GO:0002823	negative regulation of adaptive immune response based on somatic recombination of immune receptors built from immunoglobulin superfamily domains	10	0.358944659	0.0001	0.000192793
GO:1902745	positive regulation of lamellipodium organization	12	0.358880309	< 0.001	< 0.001
GO:0035850	epithelial cell differentiation involved in kidney development	11	0.358722359	0.0001	0.000192793
GO:1903077	negative regulation of protein localization to plasma membrane	8	0.358365508	< 0.001	< 0.001
GO:1904888	cranial skeletal system development	19	0.358301158	< 0.001	< 0.001
GO:0051955	regulation of amino acid transport	15	0.358266838	0.0017	0.00231289
GO:0003215	cardiac right ventricle morphogenesis	7	0.358264387	0.0015	0.002069969
GO:0050849	negative regulation of calcium-mediated signaling	13	0.358202158	0.0002	0.000356582

GOIDlabel	GOName	size	categoryScores	pValPerm	pValPermCorr
GO:0032731	positive regulation of interleukin-1 beta production	15	0.358112398	< 0.001	< 0.001
GO:0044331	cell-cell adhesion mediated by cadherin	11	0.358043758	< 0.001	< 0.001
GO:0009268	response to pH	13	0.357984358	< 0.001	< 0.001
GO:0001829	trophectodermal cell differentiation	5	0.357889318	0.0131	0.01468722
GO:0015804	neutral amino acid transport	7	0.3576025	< 0.001	< 0.001
GO:0034393	positive regulation of smooth muscle cell apoptotic process	7	0.357234786	0.0018	0.002428764
GO:0015936	coenzyme A metabolic process	6	0.356499356	< 0.001	< 0.001
GO:0009445	putrescine metabolic process	5	0.356447876	0.0003	0.000505993
GO:0021762	substantia nigra development	18	0.356413556	< 0.001	< 0.001
GO:2001171	positive regulation of ATP biosynthetic process	5	0.356241956	0.0001	0.000192793
GO:0048701	embryonic cranial skeleton morphogenesis	13	0.355925156	< 0.001	< 0.001
GO:0006047	UDP-N-acetylglucosamine metabolic process	7	0.35583747	< 0.001	< 0.001
GO:0034390	smooth muscle cell apoptotic process	12	0.355619906	0.0014	0.00194839
GO:0034391	regulation of smooth muscle cell apoptotic process	12	0.355619906	0.0014	0.00194839
GO:0034058	endosomal vesicle fusion	6	0.355126555	0.0002	0.000356582
GO:0008272	sulfate transport	6	0.354826255	0.01	0.011478335
GO:1902358	sulfate transmembrane transport	6	0.354826255	0.01	0.011478335
GO:1901385	regulation of voltage-gated calcium channel activity	13	0.354459954	< 0.001	< 0.001
GO:0060180	female mating behavior	6	0.354397254	0.0007	0.001063182
GO:0019228	neuronal action potential	8	0.354054054	< 0.001	< 0.001
GO:0045746	negative regulation of Notch signaling pathway	14	0.354035668	< 0.001	< 0.001
GO:0045622	regulation of T-helper cell differentiation	7	0.353925354	< 0.001	< 0.001
GO:2000758	positive regulation of peptidyl-lysine acetylation	11	0.353340353	< 0.001	< 0.001
GO:0035137	hindlimb morphogenesis	11	0.353340353	< 0.001	< 0.001
GO:0039535	regulation of RIG-I signaling pathway	8	0.352863578	< 0.001	< 0.001
GO:0032892	positive regulation of organic acid transport	14	0.352748667	< 0.001	< 0.001
GO:0072215	regulation of metanephros development	6	0.352509653	0.0037	0.004617737
GO:0051546	keratinocyte migration	5	0.352432432	< 0.001	< 0.001
GO:0051547	regulation of keratinocyte migration	5	0.352432432	< 0.001	< 0.001
GO:1902743	regulation of lamellipodium organization	17	0.352396094	< 0.001	< 0.001
GO:0010591	regulation of lamellipodium assembly	15	0.352295152	< 0.001	< 0.001
GO:0090342	regulation of cell aging	22	0.352275652	< 0.001	< 0.001
GO:2000279	negative regulation of DNA biosynthetic process	13	0.352004752	< 0.001	< 0.001
GO:0043923	positive regulation by host of viral transcription	7	0.351939695	0.0005	0.000791911
GO:0003333	amino acid transmembrane transport	12	0.351887602	< 0.001	< 0.001
GO:0061179	negative regulation of insulin secretion involved in cellular response to glucose stimulus	6	0.351265551	< 0.001	< 0.001
GO:0033206	meiotic cytokinesis	5	0.351145431	< 0.001	< 0.001
GO:2000341	regulation of chemokine (C-X-C motif) ligand 2 production	6	0.351136851	< 0.001	< 0.001
GO:0048747	muscle fiber development	18	0.351065351	< 0.001	< 0.001
GO:0001956	positive regulation of neurotransmitter secretion	16	0.351061776	0.0121	0.013633945
GO:0006312	mitotic recombination	8	0.350740026	< 0.001	< 0.001
GO:0060350	endochondral bone morphogenesis	17	0.350624574	0.0011	0.001576346
GO:0048172	regulation of short-term neuronal synaptic plasticity	11	0.35048555	0.0077	0.00902229
GO:0009166	nucleotide catabolic process	19	0.350457224	< 0.001	< 0.001
GO:1905383	protein localization to presynapse	8	0.3502574	0.0054	0.006510843
GO:0061013	regulation of mRNA catabolic process	12	0.3498284	< 0.001	< 0.001
GO:2000772	regulation of cellular senescence	19	0.349359886	0.0002	0.000356582
GO:0010863	positive regulation of phospholipase C activity	16	0.349308237	0.0001	0.000192793
GO:0048488	synaptic vesicle endocytosis	31	0.349209117	0.0005	0.000791911
GO:0016577	histone demethylation	8	0.348938224	0.0078	0.009125612
GO:0070076	histone lysine demethylation	8	0.348938224	0.0078	0.009125612
GO:0046530	photoreceptor cell differentiation	22	0.348894349	< 0.001	< 0.001
GO:0050812	regulation of acyl-CoA biosynthetic process	5	0.348880309	0.0093	0.010727257
GO:0009954	proximal/distal pattern formation	8	0.348777349	0.0043	0.005276385
GO:1900120	regulation of receptor binding	5	0.348571429	< 0.001	< 0.001
GO:0034214	protein hexamerization	6	0.348562849	< 0.001	< 0.001
GO:0040020	regulation of meiotic nuclear division	5	0.348365508	< 0.001	< 0.001
GO:1901032	negative regulation of response to reactive oxygen species	7	0.348078691	< 0.001	< 0.001
GO:1903206	negative regulation of hydrogen peroxide-induced cell death	7	0.348078691	< 0.001	< 0.001
GO:0051127	positive regulation of actin nucleation	8	0.348069498	0.0003	0.000505993
GO:1902894	negative regulation of pri-miRNA transcription from RNA polymerase II promoter	7	0.348005148	0.0006	0.000927725
GO:0051590	positive regulation of neurotransmitter transport	18	0.347890748	0.0109	0.012398148
GO:0043330	response to exogenous dsRNA	10	0.347850708	< 0.001	< 0.001
GO:0007026	negative regulation of microtubule depolymerization	12	0.347833548	0.0002	0.000356582
GO:0051023	regulation of immunoglobulin secretion	7	0.34756389	< 0.001	< 0.001
GO:1902992	negative regulation of amyloid precursor protein catabolic process	8	0.347522523	< 0.001	< 0.001
GO:0050706	regulation of interleukin-1 beta secretion	15	0.347387387	< 0.001	< 0.001
GO:0001660	fever generation	5	0.347335907	< 0.001	< 0.001
GO:0036119	response to platelet-derived growth factor	6	0.347275847	< 0.001	< 0.001
GO:0045668	negative regulation of osteoblast differentiation	13	0.347252747	0.0011	0.001576346
GO:0007193	adenylate cyclase-inhibiting G-protein coupled receptor signaling pathway	32	0.346927284	0.0008	0.001193265
GO:0007431	salivary gland development	13	0.346896347	< 0.001	< 0.001
GO:0010839	negative regulation of keratinocyte proliferation	6	0.346803947	< 0.001	< 0.001
GO:0070200	establishment of protein localization to telomere	7	0.346387204	0.0005	0.000791911
GO:1900151	regulation of nuclear-transcribed mRNA catabolic process, deadenylation-dependent decay	7	0.346129803	< 0.001	< 0.001
GO:1900153	positive regulation of nuclear-transcribed mRNA catabolic process, deadenylation-dependent decay	7	0.346129803	< 0.001	< 0.001
GO:0009595	detection of biotic stimulus	5	0.345842986	< 0.001	< 0.001
GO:1900242	regulation of synaptic vesicle endocytosis	12	0.345817246	0.001	0.001450349
GO:0043303	mast cell degranulation	17	0.345658263	< 0.001	< 0.001
GO:2001258	negative regulation of cation channel activity	13	0.345648946	< 0.001	< 0.001
GO:0045822	negative regulation of heart contraction	9	0.345602746	< 0.001	< 0.001
GO:0051150	regulation of smooth muscle cell differentiation	9	0.345431145	0.0001	0.000192793
GO:0046503	glycerolipid catabolic process	12	0.345280995	< 0.001	< 0.001

GOIDlabel	GOName	size	categoryScores	pValPerm	pValPermCorr
GO:0014047	glutamate secretion	30	0.345208065	0.0019	0.00254969
GO:0010971	positive regulation of G2/M transition of mitotic cell cycle	12	0.345023595	0.0005	0.000791911
GO:2001212	regulation of vasculogenesis	5	0.344916345	0.0002	0.000356582
GO:0001963	synaptic transmission, dopaminergic	14	0.344787645	< 0.001	< 0.001
GO:0000083	regulation of transcription involved in G1/S transition of mitotic cell cycle	10	0.344607465	< 0.001	< 0.001
GO:0007019	microtubule depolymerization	17	0.344552956	< 0.001	< 0.001
GO:0032695	negative regulation of interleukin-12 production	5	0.344401544	0.0003	0.000505993
GO:0006482	protein demethylation	10	0.344375804	0.0066	0.007843941
GO:0008214	protein dealkylation	10	0.344375804	0.0066	0.007843941
GO:0051145	smooth muscle cell differentiation	16	0.344288932	< 0.001	< 0.001
GO:0044068	modulation by symbiont of host cellular process	17	0.344234991	< 0.001	< 0.001
GO:0043508	negative regulation of JUN kinase activity	5	0.344195624	0.0001	0.000192793
GO:2000343	positive regulation of chemokine (C-X-C motif) ligand 2 production	5	0.343680824	< 0.001	< 0.001
GO:0032732	positive regulation of interleukin-1 production	18	0.343472043	< 0.001	< 0.001
GO:0090382	phagosome maturation	19	0.342979069	< 0.001	< 0.001
GO:0043084	penile erection	5	0.342805663	0.0019	0.00254969
GO:0051353	positive regulation of oxidoreductase activity	17	0.34256946	< 0.001	< 0.001
GO:0051123	RNA polymerase II transcriptional preinitiation complex assembly	5	0.342548263	0.0016	0.002186748
GO:0070527	platelet aggregation	27	0.342523476	< 0.001	< 0.001
GO:0033028	myeloid cell apoptotic process	8	0.342213642	0.0008	0.001193265
GO:0033032	regulation of myeloid cell apoptotic process	8	0.342213642	0.0008	0.001193265
GO:0006865	amino acid transport	56	0.342195256	0.0001	0.000192793
GO:0071157	negative regulation of cell cycle arrest	8	0.341859717	< 0.001	< 0.001
GO:0015837	amine transport	36	0.341727442	< 0.001	< 0.001
GO:0042461	photoreceptor cell development	18	0.341698842	< 0.001	< 0.001
GO:0006144	purine nucleobase metabolic process	7	0.341680456	< 0.001	< 0.001
GO:0032927	positive regulation of activin receptor signaling pathway	6	0.341527242	< 0.001	< 0.001
GO:0002739	regulation of cytokine secretion involved in immune response	8	0.341505792	< 0.001	< 0.001
GO:0090239	regulation of histone H4 acetylation	7	0.341165655	< 0.001	< 0.001
GO:0070741	response to interleukin-6	14	0.341110498	< 0.001	< 0.001
GO:0048490	anterograde synaptic vesicle transport	13	0.340995941	< 0.001	< 0.001
GO:0099514	synaptic vesicle cytoskeletal transport	13	0.340995941	< 0.001	< 0.001
GO:0099517	synaptic vesicle transport along microtubule	13	0.340995941	< 0.001	< 0.001
GO:0033198	response to ATP	15	0.340849421	< 0.001	< 0.001
GO:0033137	negative regulation of peptidyl-serine phosphorylation	11	0.340587341	< 0.001	< 0.001
GO:0071354	cellular response to interleukin-6	13	0.340580141	< 0.001	< 0.001
GO:0035518	histone H2A monoubiquitination	8	0.340540541	< 0.001	< 0.001
GO:0043247	telomere maintenance in response to DNA damage	7	0.34028314	0.006	0.007178216
GO:0051764	actin crosslink formation	6	0.34019734	0.0002	0.000356582
GO:0031114	regulation of microtubule depolymerization	13	0.34016434	0.0001	0.000192793
GO:0036465	synaptic vesicle recycling	36	0.34016159	0.0004	0.000648934
GO:0051953	negative regulation of amine transport	11	0.34011934	0.0037	0.004617737
GO:0001710	mesodermal cell fate commitment	5	0.34007722	0.0071	0.008390203
GO:0043653	mitochondrial fragmentation involved in apoptotic process	5	0.34002574	< 0.001	< 0.001
GO:0014046	dopamine secretion	9	0.33996854	< 0.001	< 0.001
GO:0014059	regulation of dopamine secretion	9	0.33996854	< 0.001	< 0.001
GO:0060795	cell fate commitment involved in formation of primary germ layer	7	0.339915426	0.0021	0.002780108
GO:0097320	membrane tubulation	5	0.33976834	0.0038	0.004725081
GO:0003338	metanephros morphogenesis	11	0.33955774	< 0.001	< 0.001
GO:0070229	negative regulation of lymphocyte apoptotic process	9	0.339339339	< 0.001	< 0.001
GO:0032205	negative regulation of telomere maintenance	17	0.339086986	0.0001	0.000192793
GO:0002377	immunoglobulin production	32	0.338972008	< 0.001	< 0.001
GO:0051382	kinetochore assembly	7	0.338849053	< 0.001	< 0.001
GO:0090281	negative regulation of calcium ion import	10	0.338532819	0.0003	0.000505993
GO:0014068	positive regulation of phosphatidylinositol 3-kinase signaling	34	0.338451056	< 0.001	< 0.001
GO:2000010	positive regulation of protein localization to cell surface	8	0.338256113	0.0004	0.000648934
GO:0007379	segment specification	5	0.338172458	0.0081	0.00945
GO:0007157	heterophilic cell-cell adhesion via plasma membrane cell adhesion molecules	11	0.337755938	0.0007	0.001063182
GO:0048305	immunoglobulin secretion	9	0.337709138	< 0.001	< 0.001
GO:2000369	regulation of clathrin-dependent endocytosis	20	0.337696268	0.001	0.001450349
GO:1905523	positive regulation of macrophage migration	9	0.337680538	< 0.001	< 0.001
GO:0042092	type 2 immune response	8	0.337483912	< 0.001	< 0.001
GO:0019933	cAMP-mediated signaling	25	0.337431145	0.0001	0.000192793
GO:0010447	response to acidic pH	8	0.337226512	< 0.001	< 0.001
GO:0033233	regulation of protein sumoylation	8	0.337226512	< 0.001	< 0.001
GO:0071542	dopaminergic neuron differentiation	9	0.337108537	< 0.001	< 0.001
GO:0090383	phagosome acidification	14	0.337065637	0.0018	0.002428764
GO:0035067	negative regulation of histone acetylation	9	0.337022737	< 0.001	< 0.001
GO:2000757	negative regulation of peptidyl-lysine acetylation	9	0.337022737	< 0.001	< 0.001
GO:0043301	negative regulation of leukocyte degranulation	5	0.336782497	0.0004	0.000648934
GO:1901299	negative regulation of hydrogen peroxide-mediated programmed cell death	5	0.336731017	< 0.001	< 0.001
GO:0071260	cellular response to mechanical stimulus	20	0.336679537	< 0.001	< 0.001
GO:0072583	clathrin-dependent endocytosis	45	0.336668097	0.0003	0.000505993
GO:1900274	regulation of phospholipase C activity	17	0.336573548	< 0.001	< 0.001
GO:0010452	histone H3-K36 methylation	7	0.336532451	0.0022	0.002896881
GO:0035735	intraciliary transport involved in cilium assembly	22	0.336410436	< 0.001	< 0.001
GO:0051125	regulation of actin nucleation	11	0.336398736	0.0016	0.002186748
GO:0060338	regulation of type I interferon-mediated signaling pathway	11	0.336258336	< 0.001	< 0.001
GO:1902042	negative regulation of extrinsic apoptotic signaling pathway via death domain receptors	8	0.336164736	< 0.001	< 0.001
GO:0090557	establishment of endothelial intestinal barrier	5	0.336164736	0.0015	0.002069969
GO:0042088	T-helper 1 type immune response	11	0.336047736	< 0.001	< 0.001
GO:0051058	negative regulation of small GTPase mediated signal transduction	29	0.336013846	< 0.001	< 0.001
GO:0098840	protein transport along microtubule	31	0.335923942	< 0.001	< 0.001

GOIDlabel	GOName	size	categoryScores	pValPerm	pValPermCorr
GO:0099118	microtubule-based protein transport	31	0.335923942	< 0.001	< 0.001
GO:0051770	positive regulation of nitric-oxide synthase biosynthetic process	7	0.335907336	< 0.001	< 0.001
GO:0007076	mitotic chromosome condensation	6	0.335649936	0.0007	0.001063182
GO:1901298	regulation of hydrogen peroxide-mediated programmed cell death	6	0.335564136	0.0001	0.000192793
GO:0046173	polyol biosynthetic process	17	0.335543947	< 0.001	< 0.001
GO:0035307	positive regulation of protein dephosphorylation	20	0.335469755	0.0001	0.000192793
GO:0015012	heparan sulfate proteoglycan biosynthetic process	9	0.335220935	0.0013	0.001826625
GO:0048857	neural nucleus development	23	0.335202283	< 0.001	< 0.001
GO:0003188	heart valve formation	5	0.335083655	< 0.001	< 0.001
GO:0042475	odontogenesis of dentin-containing tooth	19	0.335080946	< 0.001	< 0.001
GO:0035065	regulation of histone acetylation	19	0.334945472	< 0.001	< 0.001
GO:0001768	establishment of T cell polarity	5	0.334929215	< 0.001	< 0.001
GO:0070199	establishment of protein localization to chromosome	10	0.334903475	0.0003	0.000505993
GO:0014812	muscle cell migration	31	0.33476149	< 0.001	< 0.001
GO:0035588	G-protein coupled purinergic receptor signaling pathway	8	0.33458816	0.0001	0.000192793
GO:0035025	positive regulation of Rho protein signal transduction	6	0.334534535	< 0.001	< 0.001
GO:0050704	regulation of interleukin-1 secretion	18	0.334534535	< 0.001	< 0.001
GO:0006293	nucleotide-excision repair, preincision complex stabilization	10	0.334388674	0.0002	0.000356582
GO:0006295	nucleotide-excision repair, DNA incision, 3'-to lesion	10	0.334388674	0.0002	0.000356582
GO:0038179	neurotrophin signaling pathway	18	0.334205634	0.0012	0.001702047
GO:0051952	regulation of amine transport	34	0.334060111	0.0002	0.000356582
GO:0048308	organelle inheritance	7	0.334031991	0.0229	0.024839743
GO:0048313	Golgi inheritance	7	0.334031991	0.0229	0.024839743
GO:0098787	mRNA cleavage involved in mRNA processing	5	0.333951094	0.0001	0.000192793
GO:0042744	hydrogen peroxide catabolic process	9	0.333905334	< 0.001	< 0.001
GO:0046854	phosphatidylinositol phosphorylation	15	0.333830974	0.0011	0.001576346
GO:1903975	regulation of glial cell migration	6	0.333719434	0.0009	0.001319479
GO:1904948	midbrain dopaminergic neuron differentiation	5	0.333539254	< 0.001	< 0.001
GO:0043984	histone H4-K16 acetylation	10	0.333487773	< 0.001	< 0.001
GO:0046640	regulation of alpha-beta T cell proliferation	6	0.333462033	0.0002	0.000356582
GO:0008090	retrograde axonal transport	7	0.333370105	0.0022	0.002896881
GO:0090329	regulation of DNA-dependent DNA replication	18	0.333290433	0.0019	0.00254969
GO:0071295	cellular response to vitamin	8	0.333236808	< 0.001	< 0.001
GO:0070202	regulation of establishment of protein localization to chromosome	7	0.333112705	0.0004	0.000648934
GO:0072210	metanephric nephron development	12	0.333097383	< 0.001	< 0.001
GO:2000651	positive regulation of sodium ion transmembrane transporter activity	6	0.333033033	0.0005	0.000791911
GO:0033598	mammary gland epithelial cell proliferation	13	0.333016533	< 0.001	< 0.001
GO:0016445	somatic diversification of immunoglobulins	19	0.332940459	0.0002	0.000356582
GO:0072170	metanephric tubule development	8	0.332915058	< 0.001	< 0.001
GO:0072207	metanephric epithelium development	8	0.332915058	< 0.001	< 0.001
GO:0072234	metanephric nephron tubule development	8	0.332915058	< 0.001	< 0.001
GO:0072243	metanephric nephron epithelium development	8	0.332915058	< 0.001	< 0.001
GO:0090195	chemokine secretion	9	0.332875733	< 0.001	< 0.001
GO:0090196	regulation of chemokine secretion	9	0.332875733	< 0.001	< 0.001
GO:0015800	acidic amino acid transport	13	0.332858133	0.0001	0.000192793
GO:0060397	JAK-STAT cascade involved in growth hormone signaling pathway	7	0.332855304	< 0.001	< 0.001
GO:1903393	positive regulation of adherens junction organization	14	0.332726604	< 0.001	< 0.001
GO:0043949	regulation of cAMP-mediated signaling	15	0.332715573	0.0001	0.000192793
GO:0055093	response to hyperoxia	7	0.332450818	0.0007	0.001063182
GO:0010421	hydrogen peroxide-mediated programmed cell death	7	0.332340504	< 0.001	< 0.001
GO:0097468	programmed cell death in response to reactive oxygen species	7	0.332340504	< 0.001	< 0.001
GO:0019935	cyclic-nucleotide-mediated signaling	34	0.332243168	0.0001	0.000192793
GO:0042308	negative regulation of protein import into nucleus	17	0.332243168	< 0.001	< 0.001
GO:1904590	negative regulation of protein import	17	0.332243168	< 0.001	< 0.001
GO:0018200	peptidyl-glutamic acid modification	9	0.332017732	< 0.001	< 0.001
GO:0046337	phosphatidylethanolamine metabolic process	10	0.331969112	0.0001	0.000192793
GO:0033003	regulation of mast cell activation	17	0.331955485	< 0.001	< 0.001
GO:0002576	platelet degranulation	49	0.331883487	< 0.001	< 0.001
GO:0043244	regulation of protein complex disassembly	43	0.331824848	< 0.001	< 0.001
GO:0046823	negative regulation of nucleocytoplasmic transport	22	0.331718732	< 0.001	< 0.001
GO:0045686	negative regulation of glial cell differentiation	8	0.331402831	0.0137	0.015318601
GO:0051261	protein depolymerization	52	0.331402831	< 0.001	< 0.001
GO:0046580	negative regulation of Ras protein signal transduction	26	0.331323631	< 0.001	< 0.001
GO:2001267	regulation of cysteine-type endopeptidase activity involved in apoptotic signaling pathway	7	0.331274131	0.0083	0.009660392
GO:0050702	interleukin-1 beta secretion	16	0.331241956	< 0.001	< 0.001
GO:0060349	bone morphogenesis	28	0.33123736	< 0.001	< 0.001
GO:1902188	positive regulation of viral release from host cell	7	0.331163817	0.001	0.001450349
GO:0070884	regulation of calcineurin-NFAT signaling cascade	16	0.331145431	< 0.001	< 0.001
GO:0048243	norepinephrine secretion	8	0.331081081	0.0004	0.000648934
GO:0016578	histone deubiquitination	12	0.330973831	0.0012	0.001702047
GO:0090343	positive regulation of cell aging	5	0.330965251	0.0001	0.000192793
GO:2000774	positive regulation of cellular senescence	5	0.330965251	0.0001	0.000192793
GO:0042743	hydrogen peroxide metabolic process	17	0.33074419	< 0.001	< 0.001
GO:0039529	RIG-I signaling pathway	9	0.330730731	< 0.001	< 0.001
GO:1901292	nucleoside phosphate catabolic process	21	0.330710302	< 0.001	< 0.001
GO:0048706	embryonic skeletal system development	31	0.330634782	< 0.001	< 0.001
GO:1902259	regulation of delayed rectifier potassium channel activity	9	0.330616331	0.0031	0.003925117
GO:0006919	activation of cysteine-type endopeptidase activity involved in apoptotic process	23	0.330501931	< 0.001	< 0.001
GO:0003283	atrial septum development	8	0.330469755	< 0.001	< 0.001
GO:0001881	receptor recycling	15	0.33041613	< 0.001	< 0.001
GO:0045010	actin nucleation	22	0.33036153	0.0009	0.001319479
GO:0051220	cytoplasmic sequestering of protein	20	0.33029601	< 0.001	< 0.001
GO:0043650	dicarboxylic acid biosynthetic process	8	0.330276705	0.0001	0.000192793

GOIDlabel	GOName	size	categoryScores	pValPerm	pValPermCorr
GO:0002374	cytokine secretion involved in immune response	10	0.33019305	< 0.001	< 0.001
GO:0075733	intracellular transport of virus	28	0.330161794	< 0.001	< 0.001
GO:0009261	ribonucleotide catabolic process	9	0.33007293	0.0001	0.000192793
GO:0002711	positive regulation of T cell mediated immunity	6	0.32990133	0.0006	0.000927725
GO:0051938	L-glutamate import	6	0.32990133	0.0322	0.03424022
GO:0060914	heart formation	6	0.32968683	0.0019	0.00254969
GO:0019054	modulation by virus of host process	16	0.329649292	< 0.001	< 0.001
GO:2000144	positive regulation of DNA-templated transcription, initiation	8	0.329633205	0.0003	0.000505993
GO:0042073	intraciliary transport	23	0.329550669	< 0.001	< 0.001
GO:0002448	mast cell mediated immunity	18	0.32954383	< 0.001	< 0.001
GO:0002691	regulation of cellular extravasation	12	0.32953668	0.0003	0.000505993
GO:0015807	L-amino acid transport	18	0.329386529	< 0.001	< 0.001
GO:1905521	regulation of macrophage migration	14	0.329343629	< 0.001	< 0.001
GO:1904037	positive regulation of epithelial cell apoptotic process	8	0.329182754	< 0.001	< 0.001
GO:0030011	maintenance of cell polarity	6	0.329172029	< 0.001	< 0.001
GO:0030201	heparan sulfate proteoglycan metabolic process	12	0.329129129	0.0016	0.002186748
GO:0031110	regulation of microtubule polymerization or depolymerization	33	0.329121329	< 0.001	< 0.001
GO:0006361	transcription initiation from RNA polymerase I promoter	22	0.329097929	< 0.001	< 0.001
GO:0014911	positive regulation of smooth muscle cell migration	14	0.329031072	< 0.001	< 0.001
GO:0051930	regulation of sensory perception of pain	11	0.328957529	0.0001	0.000192793
GO:0051931	regulation of sensory perception	11	0.328957529	0.0001	0.000192793
GO:0033127	regulation of histone phosphorylation	6	0.328871729	< 0.001	< 0.001
GO:0009994	oocyte differentiation	14	0.3288656	< 0.001	< 0.001
GO:0032024	positive regulation of insulin secretion	27	0.328862196	0.0004	0.000648934
GO:0006835	dicarboxylic acid transport	46	0.328851211	0.0007	0.001063182
GO:0045978	negative regulation of nucleoside metabolic process	8	0.328700129	0.0001	0.000192793
GO:1903579	negative regulation of ATP metabolic process	8	0.328700129	0.0001	0.000192793
GO:0015988	energy coupled proton transmembrane transport, against electrochemical gradient	13	0.328581329	0.0023	0.003013139
GO:0015991	ATP hydrolysis coupled proton transport	13	0.328581329	0.0023	0.003013139
GO:0090662	ATP hydrolysis coupled transmembrane transport	13	0.328581329	0.0023	0.003013139
GO:0014909	smooth muscle cell migration	27	0.328547595	< 0.001	< 0.001
GO:0010464	regulation of mesenchymal cell proliferation	8	0.328539254	< 0.001	< 0.001
GO:1900181	negative regulation of protein localization to nucleus	23	0.328465111	< 0.001	< 0.001
GO:0090183	regulation of kidney development	15	0.328219648	< 0.001	< 0.001
GO:0000726	non-recombinational repair	28	0.328203714	0.0001	0.000192793
GO:0045576	mast cell activation	21	0.328148557	< 0.001	< 0.001
GO:0003209	cardiac atrium morphogenesis	12	0.328142428	< 0.001	< 0.001
GO:0031650	regulation of heat generation	5	0.328082368	< 0.001	< 0.001
GO:0031652	positive regulation of heat generation	5	0.328082368	< 0.001	< 0.001
GO:0016559	peroxisome fission	7	0.328001471	0.0002	0.000356582
GO:0051341	regulation of oxidoreductase activity	28	0.327817614	< 0.001	< 0.001
GO:0090241	negative regulation of histone H4 acetylation	5	0.327722008	< 0.001	< 0.001
GO:0070207	protein homotrimerization	11	0.327670528	0.0002	0.000356582
GO:0018198	peptidyl-cysteine modification	19	0.327670528	< 0.001	< 0.001
GO:0033605	positive regulation of catecholamine secretion	8	0.327606178	0.0001	0.000192793
GO:2000107	negative regulation of leukocyte apoptotic process	11	0.327459927	< 0.001	< 0.001
GO:0034113	heterotypic cell-cell adhesion	16	0.327445302	< 0.001	< 0.001
GO:0035306	positive regulation of dephosphorylation	25	0.327423423	< 0.001	< 0.001
GO:0098930	axonal transport	34	0.327375274	0.0001	0.000192793
GO:1903727	positive regulation of phospholipid metabolic process	19	0.327264106	< 0.001	< 0.001
GO:0021695	cerebellar cortex development	15	0.327241527	0.0002	0.000356582
GO:0060042	retina morphogenesis in camera-type eye	12	0.327220077	0.0003	0.000505993
GO:1902581	multi-organism cellular localization	29	0.32708472	< 0.001	< 0.001
GO:1902583	multi-organism intracellular transport	29	0.32708472	< 0.001	< 0.001
GO:0072425	signal transduction involved in G2 DNA damage checkpoint	6	0.327069927	0.0005	0.000791911
GO:0002639	positive regulation of immunoglobulin production	16	0.326978764	0.0006	0.000927725
GO:0008089	anterograde axonal transport	30	0.326924067	< 0.001	< 0.001
GO:0033599	regulation of mammary gland epithelial cell proliferation	8	0.326833977	< 0.001	< 0.001
GO:1901880	negative regulation of protein depolymerization	29	0.326809568	< 0.001	< 0.001
GO:0010518	positive regulation of phospholipase activity	21	0.326677698	< 0.001	< 0.001
GO:0043552	positive regulation of phosphatidylinositol 3-kinase activity	13	0.326660727	< 0.001	< 0.001
GO:0060231	mesenchymal to epithelial transition	5	0.326589447	< 0.001	< 0.001
GO:2000380	regulation of mesoderm development	6	0.326426426	< 0.001	< 0.001
GO:0042635	positive regulation of hair cycle	5	0.326383526	< 0.001	< 0.001
GO:0051798	positive regulation of hair follicle development	5	0.326383526	< 0.001	< 0.001
GO:1903423	positive regulation of synaptic vesicle recycling	7	0.326346755	0.0088	0.010185307
GO:0050777	negative regulation of immune response	40	0.326274131	< 0.001	< 0.001
GO:0051897	positive regulation of protein kinase B signaling	64	0.326242761	< 0.001	< 0.001
GO:0030168	platelet activation	64	0.326234717	0.0002	0.000356582
GO:0016188	synaptic vesicle maturation	5	0.326229086	0.0193	0.021134184
GO:0043114	regulation of vascular permeability	14	0.326126126	< 0.001	< 0.001
GO:2000047	regulation of cell-cell adhesion mediated by cadherin	9	0.326126126	< 0.001	< 0.001
GO:0002700	regulation of production of molecular mediator of immune response	43	0.326078238	< 0.001	< 0.001
GO:0044766	multi-organism transport	32	0.326029601	< 0.001	< 0.001
GO:1902579	multi-organism localization	32	0.326029601	< 0.001	< 0.001
GO:0016925	protein sumoylation	29	0.325824347	< 0.001	< 0.001
GO:0035456	response to interferon-beta	6	0.325782926	< 0.001	< 0.001
GO:0035458	cellular response to interferon-beta	6	0.325782926	< 0.001	< 0.001
GO:1901068	guanosine-containing compound metabolic process	21	0.325684869	< 0.001	< 0.001
GO:0072395	signal transduction involved in cell cycle checkpoint	25	0.325436293	< 0.001	< 0.001
GO:0072401	signal transduction involved in DNA integrity checkpoint	25	0.325436293	< 0.001	< 0.001
GO:0072422	signal transduction involved in DNA damage checkpoint	25	0.325436293	< 0.001	< 0.001
GO:0019377	glycolipid catabolic process	5	0.325302445	< 0.001	< 0.001

GOIDlabel	GOName	size	categoryScores	pValPerm	pValPermCorr
GO:0043242	negative regulation of protein complex disassembly	30	0.325199485	< 0.001	< 0.001
GO:0002707	negative regulation of lymphocyte mediated immunity	6	0.325096525	< 0.001	< 0.001
GO:0098739	import across plasma membrane	14	0.325059754	< 0.001	< 0.001
GO:0042339	keratan sulfate metabolic process	11	0.325026325	0.0001	0.000192793
GO:0070897	DNA-templated transcriptional preinitiation complex assembly	12	0.325010725	0.0003	0.000505993
GO:0030219	megakaryocyte differentiation	25	0.324983269	< 0.001	< 0.001
GO:0035690	cellular response to drug	27	0.324982125	< 0.001	< 0.001
GO:0001953	negative regulation of cell-matrix adhesion	15	0.324976405	< 0.001	< 0.001
GO:0035272	exocrine system development	16	0.324887387	< 0.001	< 0.001
GO:0045292	mRNA cis splicing, via spliceosome	6	0.324753325	0.0003	0.000505993
GO:0002762	negative regulation of myeloid leukocyte differentiation	15	0.324736165	< 0.001	< 0.001
GO:0042501	serine phosphorylation of STAT protein	5	0.324581725	0.0002	0.000356582
GO:0006953	acute-phase response	14	0.324563339	< 0.001	< 0.001
GO:0000460	maturation of 5.8S rRNA	5	0.324478764	0.0001	0.000192793
GO:0071379	cellular response to prostaglandin stimulus	10	0.324324324	< 0.001	< 0.001
GO:0071380	cellular response to prostaglandin E stimulus	10	0.324324324	< 0.001	< 0.001
GO:0016239	positive regulation of macroautophagy	34	0.324082065	< 0.001	< 0.001
GO:0031503	protein complex localization	38	0.32398564	< 0.001	< 0.001
GO:1901879	regulation of protein depolymerization	39	0.323928324	< 0.001	< 0.001
GO:0014014	negative regulation of gliogenesis	12	0.323788074	0.0034	0.00427293
GO:0002287	alpha-beta T cell activation involved in immune response	14	0.323754367	< 0.001	< 0.001
GO:0002292	T cell differentiation involved in immune response	14	0.323754367	< 0.001	< 0.001
GO:0002293	alpha-beta T cell differentiation involved in immune response	14	0.323754367	< 0.001	< 0.001
GO:0002294	CD4-positive, alpha-beta T cell differentiation involved in immune response	14	0.323754367	< 0.001	< 0.001
GO:0042093	T-helper cell differentiation	14	0.323754367	< 0.001	< 0.001
GO:0006270	DNA replication initiation	13	0.323690724	0.0008	0.001193265
GO:0042769	DNA damage response, detection of DNA damage	18	0.323666524	0.0001	0.000192793
GO:0045429	positive regulation of nitric oxide biosynthetic process	13	0.323552124	< 0.001	< 0.001
GO:1904407	positive regulation of nitric oxide metabolic process	13	0.323552124	< 0.001	< 0.001
GO:0042430	indole-containing compound metabolic process	7	0.323515352	0.001	0.001450349
GO:0032835	glomerulus development	18	0.323437723	< 0.001	< 0.001
GO:0002440	production of molecular mediator of immune response	58	0.323263658	< 0.001	< 0.001
GO:0002118	aggressive behavior	6	0.323208923	0.0051	0.006185009
GO:0050850	positive regulation of calcium-mediated signaling	19	0.323186344	< 0.001	< 0.001
GO:0002328	pro-B cell differentiation	6	0.323080223	< 0.001	< 0.001
GO:0072148	epithelial cell fate commitment	5	0.323037323	0.0005	0.000791911
GO:0006012	galactose metabolic process	5	0.322985843	0.0112	0.012688637
GO:0021542	dentate gyrus development	9	0.322980123	0.0021	0.002780108
GO:0007260	tyrosine phosphorylation of STAT protein	26	0.322859123	0.0001	0.000192793
GO:0009065	glutamine family amino acid catabolic process	13	0.322760123	< 0.001	< 0.001
GO:1904896	ESCRT complex disassembly	7	0.32270638	0.0002	0.000356582
GO:1904903	ESCRT III complex disassembly	7	0.32270638	0.0002	0.000356582
GO:0010573	vascular endothelial growth factor production	10	0.322651223	< 0.001	< 0.001
GO:0008088	axo-dendritic transport	38	0.322651223	0.0001	0.000192793
GO:0021955	central nervous system neuron axonogenesis	22	0.322639523	< 0.001	< 0.001
GO:0051280	negative regulation of release of sequestered calcium ion into cytosol	8	0.322522523	0.002	0.002664694
GO:0015682	ferric iron transport	19	0.322522523	0.0026	0.003356522
GO:0033572	transferrin transport	19	0.322522523	0.0026	0.003356522
GO:0072512	trivalent inorganic cation transport	19	0.322522523	0.0026	0.003356522
GO:2000786	positive regulation of autophagosome assembly	8	0.322490347	0.0002	0.000356582
GO:1902774	late endosome to lysosome transport	5	0.322419562	< 0.001	< 0.001
GO:0003382	epithelial cell morphogenesis	13	0.322185922	< 0.001	< 0.001
GO:0048025	negative regulation of mRNA splicing, via spliceosome	11	0.322124722	0.0004	0.000648934
GO:0043388	positive regulation of DNA binding	23	0.322097253	< 0.001	< 0.001
GO:0042401	cellular biogenic amine biosynthetic process	8	0.321943372	0.0001	0.000192793
GO:0044786	cell cycle DNA replication	20	0.321917632	0.0005	0.000791911
GO:0044803	multi-organism membrane organization	17	0.321780604	0.0001	0.000192793
GO:1901655	cellular response to ketone	30	0.321604462	< 0.001	< 0.001
GO:0021894	cerebral cortex GABAergic interneuron development	5	0.321544402	0.0083	0.009660392
GO:0001960	negative regulation of cytokine-mediated signaling pathway	21	0.321505179	< 0.001	< 0.001
GO:0070671	response to interleukin-12	20	0.321454311	< 0.001	< 0.001
GO:0021952	central nervous system projection neuron axonogenesis	16	0.321364221	0.0008	0.001193265
GO:0070233	negative regulation of T cell apoptotic process	5	0.321338481	< 0.001	< 0.001
GO:1904589	regulation of protein import	40	0.321287001	< 0.001	< 0.001
GO:0042573	retinoic acid metabolic process	6	0.321278421	0.0001	0.000192793
GO:0098962	regulation of postsynaptic neurotransmitter receptor activity	6	0.321278421	0.0227	0.024637638
GO:0050432	catecholamine secretion	19	0.321276163	< 0.001	< 0.001
GO:0008156	negative regulation of DNA replication	15	0.321252681	0.0012	0.001702047
GO:0014832	urinary bladder smooth muscle contraction	5	0.321184041	0.0004	0.000648934
GO:0014848	urinary tract smooth muscle contraction	5	0.321184041	0.0004	0.000648934
GO:0051293	establishment of spindle localization	17	0.321129533	< 0.001	< 0.001
GO:0030206	chondroitin sulfate biosynthetic process	14	0.32107005	< 0.001	< 0.001
GO:0072171	mesonephric tubule morphogenesis	18	0.321063921	< 0.001	< 0.001
GO:0098880	maintenance of postsynaptic specialization structure	5	0.320926641	0.0182	0.020002603
GO:0031113	regulation of microtubule polymerization	23	0.32079906	0.0006	0.000927725
GO:0006362	transcription elongation from RNA polymerase I promoter	18	0.320692121	< 0.001	< 0.001
GO:0000132	establishment of mitotic spindle orientation	11	0.320650521	0.0047	0.005730776
GO:0061025	membrane fusion	57	0.32060331	< 0.001	< 0.001
GO:1902106	negative regulation of leukocyte differentiation	29	0.320569831	< 0.001	< 0.001
GO:1900027	regulation of ruffle assembly	10	0.32048906	0.0001	0.000192793
GO:0035587	purinergic receptor signaling pathway	12	0.32042042	< 0.001	< 0.001
GO:0098586	cellular response to virus	20	0.32034749	< 0.001	< 0.001
GO:0033260	nuclear DNA replication	16	0.320286358	0.0013	0.001826625

GOIDlabel	GOName	size	categoryScores	pValPerm	pValPermCorr
GO:0048199	vesicle targeting, to, from or within Golgi	35	0.3202574	< 0.001	< 0.001
GO:0032786	positive regulation of DNA-templated transcription, elongation	10	0.32007722	0.0003	0.000505993
GO:0046794	transport of virus	29	0.319886389	< 0.001	< 0.001
GO:2000738	positive regulation of stem cell differentiation	8	0.319851995	< 0.001	< 0.001
GO:0048569	post-embryonic animal organ development	5	0.31984556	< 0.001	< 0.001
GO:0007213	G-protein coupled acetylcholine receptor signaling pathway	11	0.31980812	0.0016	0.002186748
GO:0035082	axoneme assembly	17	0.319797108	0.0128	0.014368758
GO:0033238	regulation of cellular amine metabolic process	52	0.31974557	0.0001	0.000192793
GO:0002320	lymphoid progenitor cell differentiation	7	0.319544034	< 0.001	< 0.001
GO:1902667	regulation of axon guidance	14	0.319415334	0.0001	0.000192793
GO:0090218	positive regulation of lipid kinase activity	15	0.319399399	< 0.001	< 0.001
GO:0061098	positive regulation of protein tyrosine kinase activity	28	0.319378562	< 0.001	< 0.001
GO:0007000	nucleolus organization	6	0.319347919	< 0.001	< 0.001
GO:0030574	collagen catabolic process	6	0.319305019	0.0002	0.000356582
GO:0044243	multicellular organism catabolic process	6	0.319305019	0.0002	0.000356582
GO:0061005	cell differentiation involved in kidney development	14	0.319286634	< 0.001	< 0.001
GO:0022408	negative regulation of cell-cell adhesion	55	0.319166959	< 0.001	< 0.001
GO:0045989	positive regulation of striated muscle contraction	5	0.319124839	< 0.001	< 0.001
GO:0060452	positive regulation of cardiac muscle contraction	5	0.319124839	< 0.001	< 0.001
GO:0051969	regulation of transmission of nerve impulse	6	0.319004719	< 0.001	< 0.001
GO:0016447	somatic recombination of immunoglobulin gene segments	18	0.318961819	0.0006	0.000927725
GO:0007270	neuron-neuron synaptic transmission	65	0.318910999	0.0001	0.000192793
GO:0000729	DNA double-strand break processing	9	0.318833119	0.0003	0.000505993
GO:0050688	regulation of defense response to virus	37	0.318703259	< 0.001	< 0.001
GO:0010758	regulation of macrophage chemotaxis	10	0.318687259	0.0001	0.000192793
GO:0043154	negative regulation of cysteine-type endopeptidase activity involved in apoptotic process	33	0.318427518	< 0.001	< 0.001
GO:0035116	embryonic hindlimb morphogenesis	9	0.318346918	< 0.001	< 0.001
GO:0051894	positive regulation of focal adhesion assembly	11	0.318310518	0.0002	0.000356582
GO:0090161	Golgi ribbon formation	5	0.318249678	0.0001	0.000192793
GO:0021796	cerebral cortex regionalization	5	0.318249678	0.0004	0.000648934
GO:0002031	G-protein coupled receptor internalization	6	0.318232518	< 0.001	< 0.001
GO:0060850	regulation of transcription involved in cell fate commitment	8	0.318018018	< 0.001	< 0.001
GO:0050974	detection of mechanical stimulus involved in sensory perception	9	0.317975118	0.0016	0.002186748
GO:0071377	cellular response to glucagon stimulus	12	0.317975118	0.0001	0.000192793
GO:0034587	piRNA metabolic process	6	0.317932218	0.0041	0.005057384
GO:0035864	response to potassium ion	7	0.317926089	0.0013	0.001826625
GO:0035865	cellular response to potassium ion	7	0.317926089	0.0013	0.001826625
GO:0021819	layer formation in cerebral cortex	9	0.317917918	0.0047	0.005730776
GO:0033604	negative regulation of catecholamine secretion	6	0.317889318	0.0015	0.002069969
GO:1903428	positive regulation of reactive oxygen species biosynthetic process	16	0.31787323	< 0.001	< 0.001
GO:0042306	regulation of protein import into nucleus	39	0.317770518	< 0.001	< 0.001
GO:0006646	phosphatidylethanolamine biosynthetic process	6	0.317760618	< 0.001	< 0.001
GO:0071514	genetic imprinting	10	0.317657658	< 0.001	< 0.001
GO:0032272	negative regulation of protein polymerization	32	0.317438867	< 0.001	< 0.001
GO:0048207	vesicle targeting, rough ER to cis-Golgi	33	0.317436917	< 0.001	< 0.001
GO:0048208	COPII vesicle coating	33	0.317436917	< 0.001	< 0.001
GO:0010517	regulation of phospholipase activity	24	0.317428142	< 0.001	< 0.001
GO:0030042	actin filament depolymerization	28	0.317374517	< 0.001	< 0.001
GO:0030834	regulation of actin filament depolymerization	25	0.317302445	< 0.001	< 0.001
GO:1902430	negative regulation of beta-amyloid formation	7	0.317264203	< 0.001	< 0.001
GO:0050860	negative regulation of T cell receptor signaling pathway	8	0.317245817	< 0.001	< 0.001
GO:0048820	hair follicle maturation	6	0.317245817	0.0006	0.000927725
GO:0043367	CD4-positive, alpha-beta T cell differentiation	20	0.317232947	< 0.001	< 0.001
GO:0002718	regulation of cytokine production involved in immune response	21	0.317166146	< 0.001	< 0.001
GO:0002053	positive regulation of mesenchymal cell proliferation	6	0.317160017	< 0.001	< 0.001
GO:0071711	basement membrane organization	6	0.317074217	0.0002	0.000356582
GO:0034629	cellular protein complex localization	10	0.316705277	< 0.001	< 0.001
GO:0071257	cellular response to electrical stimulus	5	0.316550837	0.0012	0.001702047
GO:0097091	synaptic vesicle clustering	11	0.316461916	0.0033	0.004153051
GO:0090331	negative regulation of platelet aggregation	5	0.316396396	< 0.001	< 0.001
GO:0070986	left/right axis specification	6	0.316302016	< 0.001	< 0.001
GO:0014910	regulation of smooth muscle cell migration	25	0.316272844	< 0.001	< 0.001
GO:0031954	positive regulation of protein autophosphorylation	15	0.316190476	0.0013	0.001826625
GO:0032418	lysosome localization	35	0.316124288	< 0.001	< 0.001
GO:0035774	positive regulation of insulin secretion involved in cellular response to glucose stimulus	14	0.316124288	0.0001	0.000192793
GO:0001702	gastrulation with mouth forming second	10	0.316113256	< 0.001	< 0.001
GO:0051386	regulation of neurotrophin TRK receptor signaling pathway	7	0.316050745	0.0112	0.012688637
GO:0042417	dopamine metabolic process	14	0.315977202	< 0.001	< 0.001
GO:0042551	neuron maturation	18	0.315858716	< 0.001	< 0.001
GO:0032434	regulation of proteasomal ubiquitin-dependent protein catabolic process	55	0.315708436	< 0.001	< 0.001
GO:0006521	regulation of cellular amino acid metabolic process	42	0.315689159	0.0003	0.000505993
GO:0030641	regulation of cellular pH	27	0.315677582	< 0.001	< 0.001
GO:0030279	negative regulation of ossification	23	0.315662246	< 0.001	< 0.001
GO:0030858	positive regulation of epithelial cell differentiation	18	0.315644216	< 0.001	< 0.001
GO:0071391	cellular response to estrogen stimulus	5	0.315624196	0.0005	0.000791911
GO:0006473	protein acetylation	84	0.315609487	< 0.001	< 0.001
GO:0098926	postsynaptic signal transduction	5	0.315521236	0.0001	0.000192793
GO:0099527	postsynapse to nucleus signaling pathway	5	0.315521236	0.0001	0.000192793
GO:0014066	regulation of phosphatidylinositol 3-kinase signaling	53	0.315441587	< 0.001	< 0.001
GO:0006349	regulation of gene expression by genetic imprinting	6	0.315401115	0.0003	0.000505993
GO:1901984	negative regulation of protein acetylation	11	0.315385515	< 0.001	< 0.001
GO:0051409	response to nitrosative stress	7	0.315352087	< 0.001	< 0.001
GO:0050650	chondroitin sulfate proteoglycan biosynthetic process	16	0.315315315	0.0002	0.000356582

GOIDlabel	GOName	size	categoryScores	pValPerm	pValPermCorr
GO:0006303	double-strand break repair via nonhomologous end joining	26	0.315285615	0.0001	0.000192793
GO:0006901	vesicle coating	34	0.31512605	< 0.001	< 0.001
GO:0031952	regulation of protein autophosphorylation	21	0.315070172	0.0002	0.000356582
GO:0035635	entry of bacterium into host cell	5	0.315057915	0.0014	0.00194839
GO:0071670	smooth muscle cell chemotaxis	5	0.315057915	0.0006	0.000927725
GO:0043921	modulation by host of viral transcription	8	0.315057915	0.0023	0.003013139
GO:0052472	modulation by host of symbiont transcription	8	0.315057915	0.0023	0.003013139
GO:0071599	otic vesicle development	5	0.314954955	0.0001	0.000192793
GO:0043116	negative regulation of vascular permeability	6	0.314843415	0.0001	0.000192793
GO:0002699	positive regulation of immune effector process	68	0.314819441	< 0.001	< 0.001
GO:0031958	corticosteroid receptor signaling pathway	9	0.314800515	0.0003	0.000505993
GO:0042220	response to cocaine	22	0.314765415	< 0.001	< 0.001
GO:0010466	negative regulation of peptidase activity	62	0.314746544	< 0.001	< 0.001
GO:2000049	positive regulation of cell-cell adhesion mediated by cadherin	5	0.314697555	0.0171	0.018858857
GO:0060117	auditory receptor cell development	8	0.31463964	0.0001	0.000192793
GO:0018196	peptidyl-asparagine modification	14	0.314451186	< 0.001	< 0.001
GO:0060428	lung epithelium development	8	0.314414414	< 0.001	< 0.001
GO:0051294	establishment of spindle orientation	12	0.314392964	0.0033	0.004153051
GO:1905522	negative regulation of macrophage migration	5	0.314337194	0.0001	0.000192793
GO:0046834	lipid phosphorylation	21	0.314236686	0.0029	0.003693519
GO:0010649	regulation of cell communication by electrical coupling	5	0.314182754	< 0.001	< 0.001
GO:1901844	regulation of cell communication by electrical coupling involved in cardiac conduction	5	0.314182754	< 0.001	< 0.001
GO:0098742	cell-cell adhesion via plasma-membrane adhesion molecules	88	0.314133614	0.0001	0.000192793
GO:0051193	regulation of cofactor metabolic process	36	0.314071214	< 0.001	< 0.001
GO:1904385	cellular response to angiotensin	7	0.314065085	0.021	0.022870617
GO:0097581	lamellipodium organization	38	0.314028314	< 0.001	< 0.001
GO:0045986	negative regulation of smooth muscle contraction	7	0.313918	0.0041	0.005057384
GO:0010951	negative regulation of endopeptidase activity	60	0.313916774	< 0.001	< 0.001
GO:0048011	neurotrophin TRK receptor signaling pathway	15	0.313908194	0.0013	0.001826625
GO:0044241	lipid digestion	5	0.313873874	< 0.001	< 0.001
GO:0045910	negative regulation of DNA recombination	13	0.313850114	0.0041	0.005057384
GO:0045652	regulation of megakaryocyte differentiation	21	0.313770914	< 0.001	< 0.001
GO:0072567	chemokine (C-X-C motif) ligand 2 production	7	0.313697371	< 0.001	< 0.001
GO:0046068	cGMP metabolic process	8	0.313642214	0.0001	0.000192793
GO:0042246	tissue regeneration	14	0.313495128	0.0009	0.001319479
GO:0071526	semaphorin-plexin signaling pathway	10	0.313436293	< 0.001	< 0.001
GO:0030890	positive regulation of B cell proliferation	13	0.313295713	< 0.001	< 0.001
GO:0032332	positive regulation of chondrocyte differentiation	9	0.313284713	0.0007	0.001063182
GO:1901800	positive regulation of proteasomal protein catabolic process	44	0.313174213	< 0.001	< 0.001
GO:0046755	viral budding	15	0.313101673	0.0003	0.000505993
GO:1902590	multi-organism organelle organization	15	0.313101673	0.0003	0.000505993
GO:1902592	multi-organism membrane budding	15	0.313101673	0.0003	0.000505993
GO:0007162	negative regulation of cell adhesion	85	0.3129866	< 0.001	< 0.001
GO:0097479	synaptic vesicle localization	111	0.312952335	0.001	0.001450349
GO:1902083	negative regulation of peptidyl-cysteine S-nitrosylation	5	0.312947233	< 0.001	< 0.001
GO:0007253	cytoplasmic sequestering of NF-kappaB	5	0.312947233	0.0002	0.000356582
GO:0042345	regulation of NF-kappaB import into nucleus	5	0.312947233	0.0002	0.000356582
GO:0042347	negative regulation of NF-kappaB import into nucleus	5	0.312947233	0.0002	0.000356582
GO:0042348	NF-kappaB import into nucleus	5	0.312947233	0.0002	0.000356582
GO:0072273	metanephric nephron morphogenesis	9	0.312941513	< 0.001	< 0.001
GO:2000370	positive regulation of clathrin-dependent endocytosis	8	0.312934363	0.0114	0.012893596
GO:0032536	regulation of cell projection size	5	0.312895753	0.0193	0.021134184
GO:0019233	sensory perception of pain	41	0.312791537	< 0.001	< 0.001
GO:0051968	positive regulation of synaptic transmission, glutamatergic	12	0.312741313	0.0018	0.002428764
GO:0060856	establishment of blood-brain barrier	5	0.312741313	0.0021	0.002780108
GO:0051882	mitochondrial depolarization	7	0.31266777	< 0.001	< 0.001
GO:0070206	protein trimerization	17	0.312605042	< 0.001	< 0.001
GO:0046822	regulation of nucleocytoplasmic transport	59	0.312584255	< 0.001	< 0.001
GO:1904591	positive regulation of protein import	18	0.312512513	< 0.001	< 0.001
GO:0001188	RNA polymerase I transcriptional preinitiation complex assembly	7	0.312483912	0.0001	0.000192793
GO:0070268	cornification	19	0.312416176	< 0.001	< 0.001
GO:0021871	forebrain regionalization	11	0.312390312	0.0017	0.00231289
GO:1903170	negative regulation of calcium ion transmembrane transport	14	0.312373598	0.0001	0.000192793
GO:0060193	positive regulation of lipase activity	25	0.312339768	< 0.001	< 0.001
GO:0033119	negative regulation of RNA splicing	14	0.312226512	0.0002	0.000356582
GO:0034350	regulation of glial cell apoptotic process	5	0.312226512	0.0045	0.005509289
GO:2000807	regulation of synaptic vesicle clustering	6	0.312097812	0.0006	0.000927725
GO:0050433	regulation of catecholamine secretion	18	0.312040612	< 0.001	< 0.001
GO:0030835	negative regulation of actin filament depolymerization	17	0.311969112	< 0.001	< 0.001
GO:0034219	carbohydrate transmembrane transport	10	0.311943372	< 0.001	< 0.001
GO:0021549	cerebellum development	31	0.311877776	< 0.001	< 0.001
GO:1901661	quinone metabolic process	9	0.311854712	< 0.001	< 0.001
GO:0072111	cell proliferation involved in kidney development	7	0.311822026	< 0.001	< 0.001
GO:0033622	integrin activation	8	0.311808237	< 0.001	< 0.001
GO:0030901	midbrain development	29	0.31169396	< 0.001	< 0.001
GO:0043090	amino acid import	8	0.311647362	0.0187	0.020510412
GO:0043092	L-amino acid import	8	0.311647362	0.0187	0.020510412
GO:0021826	substrate-independent telencephalic tangential migration	6	0.311583012	0.0016	0.002186748
GO:0021830	interneuron migration from the subpallium to the cortex	6	0.311583012	0.0016	0.002186748
GO:0021843	substrate-independent telencephalic tangential interneuron migration	6	0.311583012	0.0016	0.002186748
GO:0060315	negative regulation of ryanodine-sensitive calcium-release channel activity	6	0.311497211	0.0132	0.014787065
GO:0010882	regulation of cardiac muscle contraction by calcium ion signaling	13	0.311434511	< 0.001	< 0.001
GO:0006363	termination of RNA polymerase I transcription	18	0.311425711	< 0.001	< 0.001

GOIDlabel	GOName	size	categoryScores	pValPerm	pValPermCorr
GO:0032890	regulation of organic acid transport	24	0.311336336	< 0.001	< 0.001
GO:0006261	DNA-dependent DNA replication	53	0.31131347	0.0005	0.000791911
GO:0060021	palate development	27	0.311273178	< 0.001	< 0.001
GO:0099550	trans-synaptic signalling, modulating synaptic transmission	5	0.311248391	0.0411	0.043118695
GO:2000169	regulation of peptidyl-cysteine S-nitrosylation	6	0.311239811	0.0004	0.000648934
GO:0002702	positive regulation of production of molecular mediator of immune response	33	0.311220311	< 0.001	< 0.001
GO:0006541	glutamine metabolic process	10	0.311171171	< 0.001	< 0.001
GO:0090174	organelle membrane fusion	30	0.311136851	< 0.001	< 0.001
GO:0030517	negative regulation of axon extension	15	0.311093951	< 0.001	< 0.001
GO:0090493	catecholamine uptake	8	0.311036036	0.0001	0.000192793
GO:0032435	negative regulation of proteasomal ubiquitin-dependent protein catabolic process	14	0.310994668	0.0002	0.000356582
GO:1900016	negative regulation of cytokine production involved in inflammatory response	8	0.310971686	< 0.001	< 0.001
GO:0060761	negative regulation of response to cytokine stimulus	23	0.310905937	0.0002	0.000356582
GO:0090231	regulation of spindle checkpoint	5	0.310888031	0.0459	0.047857148
GO:0090266	regulation of mitotic cell cycle spindle assembly checkpoint	5	0.310888031	0.0459	0.047857148
GO:1903504	regulation of mitotic spindle checkpoint	5	0.310888031	0.0459	0.047857148
GO:0045907	positive regulation of vasoconstriction	9	0.310882311	< 0.001	< 0.001
GO:0001836	release of cytochrome c from mitochondria	29	0.310770869	< 0.001	< 0.001
GO:0008045	motor neuron axon guidance	14	0.310700496	0.0045	0.005509289
GO:0006383	transcription from RNA polymerase III promoter	25	0.310692407	0.0002	0.000356582
GO:2001251	negative regulation of chromosome organization	33	0.310635311	0.0005	0.000791911
GO:0045744	negative regulation of G-protein coupled receptor protein signaling pathway	19	0.310600826	< 0.001	< 0.001
GO:0048489	synaptic vesicle transport	104	0.310570736	0.0014	0.00194839
GO:0097480	establishment of synaptic vesicle localization	104	0.310570736	0.0014	0.00194839
GO:2001234	negative regulation of apoptotic signaling pathway	80	0.310472973	< 0.001	< 0.001
GO:0046825	regulation of protein export from nucleus	13	0.31042471	< 0.001	< 0.001
GO:1903313	positive regulation of mRNA metabolic process	17	0.310364146	< 0.001	< 0.001
GO:0048705	skeletal system morphogenesis	61	0.310331878	< 0.001	< 0.001
GO:0071709	membrane assembly	16	0.310312098	< 0.001	< 0.001
GO:0051583	dopamine uptake involved in synaptic transmission	6	0.31025311	0.0031	0.003925117
GO:0051934	catecholamine uptake involved in synaptic transmission	6	0.31025311	0.0031	0.003925117
GO:0050701	interleukin-1 secretion	20	0.31019305	< 0.001	< 0.001
GO:0043124	negative regulation of I-kappaB kinase/NF-kappaB signaling	22	0.31016731	< 0.001	< 0.001
GO:1903205	regulation of hydrogen peroxide-induced cell death	9	0.31011011	< 0.001	< 0.001
GO:0016572	histone phosphorylation	15	0.30994423	0.0055	0.006618125
GO:0006296	nucleotide-excision repair, DNA incision, 5'-to lesion	16	0.30990991	0.0002	0.000356582
GO:0044106	cellular amine metabolic process	65	0.30985843	< 0.001	< 0.001
GO:0019043	establishment of viral latency	6	0.30978121	< 0.001	< 0.001
GO:0075713	establishment of integrated proviral latency	6	0.30978121	< 0.001	< 0.001
GO:1903825	organic acid transmembrane transport	21	0.309762824	0.0003	0.000505993
GO:1905039	carboxylic acid transmembrane transport	21	0.309762824	0.0003	0.000505993
GO:0071467	cellular response to pH	5	0.30970399	< 0.001	< 0.001
GO:0061178	regulation of insulin secretion involved in cellular response to glucose stimulus	26	0.30963271	< 0.001	< 0.001
GO:0010574	regulation of vascular endothelial growth factor production	9	0.30962391	< 0.001	< 0.001
GO:0046425	regulation of JAK-STAT cascade	38	0.309503488	< 0.001	< 0.001
GO:0051937	catecholamine transport	26	0.309434709	< 0.001	< 0.001
GO:0061136	regulation of proteasomal protein catabolic process	77	0.309401795	< 0.001	< 0.001
GO:0043543	protein acylation	99	0.309392509	< 0.001	< 0.001
GO:0060041	retina development in camera-type eye	49	0.309337326	< 0.001	< 0.001
GO:0071349	cellular response to interleukin-12	19	0.309327372	< 0.001	< 0.001
GO:0048741	skeletal muscle fiber development	12	0.309287859	0.002	0.002664694
GO:0050715	positive regulation of cytokine secretion	43	0.309275388	< 0.001	< 0.001
GO:0050909	sensory perception of taste	10	0.309240669	0.0009	0.001319479
GO:0090656	t-circle formation	9	0.309194909	0.0001	0.000192793
GO:0001889	liver development	37	0.309123796	< 0.001	< 0.001
GO:0061008	hepaticobiliary system development	37	0.309123796	< 0.001	< 0.001
GO:0002793	positive regulation of peptide secretion	33	0.309090909	< 0.001	< 0.001
GO:0051653	spindle localization	20	0.309021879	0.0003	0.000505993
GO:1902043	positive regulation of extrinsic apoptotic signaling pathway via death domain receptors	6	0.309009009	0.0001	0.000192793
GO:0099504	synaptic vesicle cycle	99	0.308997309	0.001	0.001450349
GO:0051693	actin filament capping	15	0.308863149	0.0006	0.000927725
GO:0051383	kinetochore organization	8	0.308848134	< 0.001	< 0.001
GO:1903955	positive regulation of protein targeting to mitochondrion	21	0.30883128	< 0.001	< 0.001
GO:0070198	protein localization to chromosome, telomeric region	14	0.308825152	0.0003	0.000505993
GO:0009649	entrainment of circadian clock	12	0.308815959	0.0123	0.013827585
GO:0090184	positive regulation of kidney development	11	0.308810109	< 0.001	< 0.001
GO:0099003	vesicle-mediated transport in synapse	110	0.308807769	0.001	0.001450349
GO:0014061	regulation of norepinephrine secretion	7	0.308733223	0.0001	0.000192793
GO:0019226	transmission of nerve impulse	22	0.308716509	< 0.001	< 0.001
GO:0008542	visual learning	22	0.308622909	0.0037	0.004617737
GO:2000106	regulation of leukocyte apoptotic process	24	0.308526384	< 0.001	< 0.001
GO:2000074	regulation of type B pancreatic cell development	5	0.308519949	0.0101	0.011583277
GO:0006885	regulation of pH	34	0.308501779	< 0.001	< 0.001
GO:0008593	regulation of Notch signaling pathway	31	0.308481754	< 0.001	< 0.001
GO:1900034	regulation of cellular response to heat	35	0.308416988	< 0.001	< 0.001
GO:0001706	endoderm formation	12	0.308344058	< 0.001	< 0.001
GO:0001709	cell fate determination	17	0.308274661	0.0047	0.005730776
GO:0095500	acetylcholine receptor signaling pathway	15	0.308211068	< 0.001	< 0.001
GO:1903831	signal transduction involved in cellular response to ammonium ion	15	0.308211068	< 0.001	< 0.001
GO:0010954	positive regulation of protein processing	7	0.30814488	< 0.001	< 0.001
GO:0035249	synaptic transmission, glutamatergic	35	0.308071337	0.0033	0.004153051
GO:0090114	COPII-coated vesicle budding	38	0.308067466	< 0.001	< 0.001
GO:0018279	protein N-linked glycosylation via asparagine	13	0.308048708	< 0.001	< 0.001

GOIDlabel	GOName	size	categoryScores	pValPerm	pValPermCorr
GO:0051966	regulation of synaptic transmission, glutamatergic	26	0.308048708	0.0014	0.00194839
GO:0034394	protein localization to cell surface	24	0.307947233	< 0.001	< 0.001
GO:0031570	DNA integrity checkpoint	58	0.307886211	< 0.001	< 0.001
GO:0050848	regulation of calcium-mediated signaling	47	0.307724746	< 0.001	< 0.001
GO:1902751	positive regulation of cell cycle G2/M phase transition	15	0.307696268	0.0004	0.000648934
GO:0050731	positive regulation of peptidyl-tyrosine phosphorylation	60	0.307687688	< 0.001	< 0.001
GO:0034332	adherens junction organization	60	0.307666238	< 0.001	< 0.001
GO:0002831	regulation of response to biotic stimulus	67	0.307631726	< 0.001	< 0.001
GO:2000117	negative regulation of cysteine-type endopeptidase activity	35	0.307600662	< 0.001	< 0.001
GO:0006534	cysteine metabolic process	7	0.307593308	< 0.001	< 0.001
GO:0007188	adenylate cyclase-modulating G-protein coupled receptor signaling pathway	55	0.307588628	< 0.001	< 0.001
GO:0046427	positive regulation of JAK-STAT cascade	20	0.307554698	< 0.001	< 0.001
GO:0030166	proteoglycan biosynthetic process	25	0.307490347	< 0.001	< 0.001
GO:0032228	regulation of synaptic transmission, GABAergic	15	0.307456027	0.0002	0.000356582
GO:0021879	forebrain neuron differentiation	24	0.307410982	0.0045	0.005509289
GO:0007259	JAK-STAT cascade	46	0.307341503	< 0.001	< 0.001
GO:0071305	cellular response to vitamin D	5	0.307284427	0.0004	0.000648934
GO:0090201	negative regulation of release of cytochrome c from mitochondria	12	0.307228657	< 0.001	< 0.001
GO:0006152	purine nucleoside catabolic process	5	0.307129987	0.0003	0.000505993
GO:0046130	purine ribonucleoside catabolic process	5	0.307129987	0.0003	0.000505993
GO:0006903	vesicle targeting	45	0.307101387	< 0.001	< 0.001
GO:0032891	negative regulation of organic acid transport	5	0.307078507	0.0053	0.006400268
GO:0033262	regulation of nuclear cell cycle DNA replication	5	0.307027027	< 0.001	< 0.001
GO:1901136	carbohydrate derivative catabolic process	64	0.306986004	< 0.001	< 0.001
GO:1901976	regulation of cell cycle checkpoint	11	0.306984907	0.0068	0.008063902
GO:2000042	negative regulation of double-strand break repair via homologous recombination	5	0.306975547	0.0001	0.000192793
GO:0002819	regulation of adaptive immune response	41	0.306883887	< 0.001	< 0.001
GO:0009301	snRNA transcription	36	0.306799657	< 0.001	< 0.001
GO:0042795	snRNA transcription from RNA polymerase II promoter	36	0.306799657	< 0.001	< 0.001
GO:0032728	positive regulation of interferon-beta production	9	0.306792507	0.0014	0.00194839
GO:0021697	cerebellar cortex formation	8	0.306756757	0.0072	0.008495371
GO:0044801	single-organism membrane fusion	41	0.306752048	< 0.001	< 0.001
GO:0035722	interleukin-12-mediated signaling pathway	18	0.306692407	< 0.001	< 0.001
GO:0010569	regulation of double-strand break repair via homologous recombination	17	0.306639413	< 0.001	< 0.001
GO:0006294	nucleotide-excision repair, preincision complex assembly	14	0.306637249	0.0001	0.000192793
GO:0002637	regulation of immunoglobulin production	22	0.306587107	0.0004	0.000648934
GO:0060993	kidney morphogenesis	29	0.306572582	< 0.001	< 0.001
GO:0021761	limbic system development	49	0.306474404	< 0.001	< 0.001
GO:0006497	protein lipidation	30	0.306366366	< 0.001	< 0.001
GO:0070203	regulation of establishment of protein localization to telomere	6	0.306306306	0.002	0.002664694
GO:1903405	protein localization to nuclear body	6	0.306306306	0.002	0.002664694
GO:1904816	positive regulation of protein localization to chromosome, telomeric region	6	0.306306306	0.002	0.002664694
GO:1904851	positive regulation of establishment of protein localization to telomere	6	0.306306306	0.002	0.002664694
GO:1904867	protein localization to Cajal body	6	0.306306306	0.002	0.002664694
GO:1904869	regulation of protein localization to Cajal body	6	0.306306306	0.002	0.002664694
GO:1904871	positive regulation of protein localization to Cajal body	6	0.306306306	0.002	0.002664694
GO:1900117	regulation of execution phase of apoptosis	9	0.306220506	0.0024	0.003116943
GO:0006370	7-methylguanosine mRNA capping	17	0.306200318	0.0003	0.000505993
GO:1902600	hydrogen ion transmembrane transport	38	0.306157285	< 0.001	< 0.001
GO:1903523	negative regulation of blood circulation	12	0.306134706	< 0.001	< 0.001
GO:0009308	amine metabolic process	67	0.306129584	0.0001	0.000192793
GO:0030318	melanocyte differentiation	7	0.306085678	0.0001	0.000192793
GO:0006611	protein export from nucleus	28	0.306076485	0.0002	0.000356582
GO:0050655	dermatan sulfate proteoglycan metabolic process	7	0.306048906	0.019	0.020831
GO:0021800	cerebral cortex tangential migration	6	0.306006006	0.0051	0.006185009
GO:0045793	positive regulation of cell size	6	0.305963106	0.0007	0.001063182
GO:1905517	macrophage migration	19	0.305954074	< 0.001	< 0.001
GO:0072331	signal transduction by p53 class mediator	106	0.305937204	0.0004	0.000648934
GO:0051057	positive regulation of small GTPase mediated signal transduction	24	0.305920206	< 0.001	< 0.001
GO:0051648	vesicle localization	180	0.305918776	0.0001	0.000192793
GO:0006906	vesicle fusion	28	0.305763927	< 0.001	< 0.001
GO:0035136	forelimb morphogenesis	12	0.305705706	0.0001	0.000192793
GO:0051196	regulation of coenzyme metabolic process	35	0.3056959	< 0.001	< 0.001
GO:0050798	activated T cell proliferation	12	0.305598456	< 0.001	< 0.001
GO:0043627	response to estrogen	21	0.30555862	< 0.001	< 0.001
GO:0031297	replication fork processing	12	0.305491205	0.0005	0.000791911
GO:0045005	DNA-dependent DNA replication maintenance of fidelity	12	0.305491205	0.0005	0.000791911
GO:0050714	positive regulation of protein secretion	93	0.305404022	< 0.001	< 0.001
GO:0071392	cellular response to estradiol stimulus	7	0.305313477	0.0001	0.000192793
GO:0006367	transcription initiation from RNA polymerase II promoter	67	0.305299756	< 0.001	< 0.001
GO:0003230	cardiac atrium development	13	0.305296505	< 0.001	< 0.001
GO:0018394	peptidyl-lysine acetylation	70	0.305210517	< 0.001	< 0.001
GO:0035909	aorta morphogenesis	10	0.305199485	0.0039	0.004841626
GO:0006026	aminoglycan catabolic process	22	0.305183105	0.0001	0.000192793
GO:0006027	glycosaminoglycan catabolic process	22	0.305183105	0.0001	0.000192793
GO:0017145	stem cell division	18	0.305176605	< 0.001	< 0.001
GO:1901020	negative regulation of calcium ion transmembrane transporter activity	12	0.305169455	0.0009	0.001319479
GO:2000402	negative regulation of lymphocyte migration	7	0.305166391	0.0006	0.000927725
GO:0033762	response to glucagon	14	0.305074462	< 0.001	< 0.001
GO:0072413	signal transduction involved in mitotic cell cycle checkpoint	17	0.305004164	0.0008	0.001193265
GO:0072431	signal transduction involved in mitotic G1 DNA damage checkpoint	17	0.305004164	0.0008	0.001193265
GO:1902400	intracellular signal transduction involved in G1 DNA damage checkpoint	17	0.305004164	0.0008	0.001193265
GO:1902402	signal transduction involved in mitotic DNA damage checkpoint	17	0.305004164	0.0008	0.001193265

GOIDlabel	GOName	size	categoryScores	pValPerm	pValPermCorr
GO:1902403	signal transduction involved in mitotic DNA integrity checkpoint	17	0.305004164	0.0008	0.001193265
GO:0001959	regulation of cytokine-mediated signaling pathway	60	0.304869155	< 0.001	< 0.001
GO:0033522	histone H2A ubiquitination	12	0.304869155	< 0.001	< 0.001
GO:0060675	ureteric bud morphogenesis	17	0.304867893	< 0.001	< 0.001
GO:1900180	regulation of protein localization to nucleus	62	0.304770208	< 0.001	< 0.001
GO:0016574	histone ubiquitination	20	0.304749035	< 0.001	< 0.001
GO:0043403	skeletal muscle tissue regeneration	11	0.304668305	0.001	0.001450349
GO:0021533	cell differentiation in hindbrain	6	0.304633205	0.0028	0.003584743
GO:0021872	forebrain generation of neurons	28	0.304633205	0.0041	0.005057384
GO:0098751	bone cell development	10	0.304607465	< 0.001	< 0.001
GO:0051168	nuclear export	74	0.304598421	0.0001	0.000192793
GO:0006278	RNA-dependent DNA biosynthetic process	35	0.304585402	0.0001	0.000192793
GO:0009064	glutamine family amino acid metabolic process	30	0.304564565	< 0.001	< 0.001
GO:0006406	mRNA export from nucleus	39	0.304550705	0.0009	0.001319479
GO:0071427	mRNA-containing ribonucleoprotein complex export from nucleus	39	0.304550705	0.0009	0.001319479
GO:0006635	fatty acid beta-oxidation	20	0.304504505	< 0.001	< 0.001
GO:0086011	membrane repolarization during action potential	9	0.304504505	0.0024	0.003116943
GO:0007200	phospholipase C-activating G-protein coupled receptor signaling pathway	25	0.30436036	0.0009	0.001319479
GO:0007221	positive regulation of transcription of Notch receptor target	5	0.304350064	0.0001	0.000192793
GO:0031338	regulation of vesicle fusion	9	0.304304304	0.0083	0.009660392
GO:0048546	digestive tract morphogenesis	12	0.304290004	< 0.001	< 0.001
GO:0000077	DNA damage checkpoint	57	0.304247104	< 0.001	< 0.001
GO:0001825	blastocyst formation	14	0.304173561	0.0152	0.016877091
GO:0007187	G-protein coupled receptor signaling pathway, coupled to cyclic nucleotide second messenger	62	0.304122556	< 0.001	< 0.001
GO:0021954	central nervous system neuron development	45	0.304069784	< 0.001	< 0.001
GO:0042752	regulation of circadian rhythm	52	0.304034254	0.0007	0.001063182
GO:0030705	cytoskeleton-dependent intracellular transport	85	0.304032099	< 0.001	< 0.001
GO:1905144	response to acetylcholine	17	0.304004845	< 0.001	< 0.001
GO:1905145	cellular response to acetylcholine	17	0.304004845	< 0.001	< 0.001
GO:0061213	positive regulation of mesonephros development	7	0.303989704	< 0.001	< 0.001
GO:0061217	regulation of mesonephros development	7	0.303989704	< 0.001	< 0.001
GO:0097530	granulocyte migration	29	0.303936449	< 0.001	< 0.001
GO:0001656	metanephros development	23	0.303933747	< 0.001	< 0.001
GO:0042307	positive regulation of protein import into nucleus	17	0.303929139	< 0.001	< 0.001
GO:0000018	regulation of DNA recombination	40	0.303880309	0.0002	0.000356582
GO:0006388	tRNA splicing, via endonucleolytic cleavage and ligation	5	0.303835264	0.0038	0.004725081
GO:0032436	positive regulation of proteasomal ubiquitin-dependent protein catabolic process	36	0.303782354	0.0001	0.000192793
GO:0050686	negative regulation of mRNA processing	15	0.303749464	0.0001	0.000192793
GO:2000001	regulation of DNA damage checkpoint	6	0.303732304	0.0002	0.000356582
GO:0034109	homotypic cell-cell adhesion	36	0.303710854	< 0.001	< 0.001
GO:0009310	amine catabolic process	10	0.303680824	< 0.001	< 0.001
GO:1901890	positive regulation of cell junction assembly	13	0.303653104	< 0.001	< 0.001
GO:0019217	regulation of fatty acid metabolic process	34	0.303641457	< 0.001	< 0.001
GO:0046717	acid secretion	50	0.303624196	0.0001	0.000192793
GO:0007004	telomere maintenance via telomerase	34	0.303580892	0.0003	0.000505993
GO:0006297	nucleotide-excision repair, DNA gap filling	8	0.303507079	0.0135	0.015116867
GO:0002204	somatic recombination of immunoglobulin genes involved in immune response	16	0.303458816	0.0008	0.001193265
GO:0002208	somatic diversification of immunoglobulins involved in immune response	16	0.303458816	0.0008	0.001193265
GO:0045190	isotype switching	16	0.303458816	0.0008	0.001193265
GO:0060122	inner ear receptor stereocilium organization	10	0.303423423	< 0.001	< 0.001
GO:2000178	negative regulation of neural precursor cell proliferation	9	0.303417703	0.0001	0.000192793
GO:0006638	neutral lipid metabolic process	31	0.303391871	< 0.001	< 0.001
GO:0090199	regulation of release of cytochrome c from mitochondria	24	0.303335478	< 0.001	< 0.001
GO:0003299	muscle hypertrophy in response to stress	9	0.303274703	0.0029	0.003693519
GO:0014887	cardiac muscle adaptation	9	0.303274703	0.0029	0.003693519
GO:0014898	cardiac muscle hypertrophy in response to stress	9	0.303274703	0.0029	0.003693519
GO:0015872	dopamine transport	15	0.303251823	< 0.001	< 0.001
GO:0051895	negative regulation of focal adhesion assembly	9	0.303217503	< 0.001	< 0.001
GO:1903392	negative regulation of adherens junction organization	9	0.303217503	< 0.001	< 0.001
GO:0043486	histone exchange	15	0.303200343	0.0068	0.008063902
GO:1990266	neutrophil migration	20	0.303166023	< 0.001	< 0.001
GO:0048709	oligodendrocyte differentiation	27	0.30315077	< 0.001	< 0.001
GO:1901524	regulation of macromitophagy	6	0.303088803	0.0004	0.000648934
GO:0045123	cellular extravasation	25	0.303063063	< 0.001	< 0.001
GO:0030534	adult behavior	63	0.303062246	< 0.001	< 0.001
GO:0033135	regulation of peptidyl-serine phosphorylation	60	0.303007293	< 0.001	< 0.001
GO:0050690	regulation of defense response to virus by virus	17	0.303005527	< 0.001	< 0.001
GO:0042273	ribosomal large subunit biogenesis	13	0.302979903	0.003	0.003811001
GO:0006977	DNA damage response, signal transduction by p53 class mediator resulting in cell cycle arrest	16	0.302960103	0.0008	0.001193265
GO:0043491	protein kinase B signaling	91	0.30290636	< 0.001	< 0.001
GO:0071300	cellular response to retinoic acid	17	0.302869256	0.0028	0.003584743
GO:0015849	organic acid transport	106	0.302865399	< 0.001	< 0.001
GO:0046942	carboxylic acid transport	106	0.302865399	< 0.001	< 0.001
GO:1902805	positive regulation of synaptic vesicle transport	14	0.302813017	0.0111	0.012593827
GO:0048713	regulation of oligodendrocyte differentiation	12	0.302745603	< 0.001	< 0.001
GO:0051004	regulation of lipoprotein lipase activity	6	0.302745603	0.0013	0.001826625
GO:1903146	regulation of mitophagy	19	0.302743345	0.0004	0.000648934
GO:0010833	telomere maintenance via telomere lengthening	37	0.302695746	0.0001	0.000192793
GO:0001824	blastocyst development	35	0.302592388	0.0004	0.000648934
GO:1903792	negative regulation of anion transport	7	0.302555617	0.0001	0.000192793
GO:0009070	serine family amino acid biosynthetic process	7	0.302518845	0.0018	0.002428764
GO:0046578	regulation of Ras protein signal transduction	100	0.30236036	< 0.001	< 0.001
GO:0002200	somatic diversification of immune receptors	23	0.302333389	0.0014	0.00194839

GOIDlabel	GOName	size	categoryScores	pValPerm	pValPermCorr
GO:0002092	positive regulation of receptor internalization	10	0.302290862	0.0004	0.000648934
GO:0034661	ncRNA catabolic process	6	0.302230802	0.0013	0.001826625
GO:0031146	SCF-dependent proteasomal ubiquitin-dependent protein catabolic process	49	0.302172143	0.0003	0.000505993
GO:0051767	nitric-oxide synthase biosynthetic process	8	0.302155727	< 0.001	< 0.001
GO:0051769	regulation of nitric-oxide synthase biosynthetic process	8	0.302155727	< 0.001	< 0.001
GO:0045026	plasma membrane fusion	6	0.302145002	0.025	0.026960686
GO:0006353	DNA-templated transcription, termination	34	0.302134908	< 0.001	< 0.001
GO:0006405	RNA export from nucleus	44	0.302100152	0.0002	0.000356582
GO:0050482	arachidonic acid secretion	9	0.301959102	< 0.001	< 0.001
GO:1903963	arachidonate transport	9	0.301959102	< 0.001	< 0.001
GO:0043525	positive regulation of neuron apoptotic process	21	0.301795673	< 0.001	< 0.001
GO:2000679	positive regulation of transcription regulatory region DNA binding	10	0.301776062	< 0.001	< 0.001
GO:0043624	cellular protein complex disassembly	101	0.301741912	< 0.001	< 0.001
GO:1902041	regulation of extrinsic apoptotic signaling pathway via death domain receptors	17	0.301733666	< 0.001	< 0.001
GO:0019048	modulation by virus of host morphology or physiology	21	0.301697616	< 0.001	< 0.001
GO:0046579	positive regulation of Ras protein signal transduction	23	0.301684293	< 0.001	< 0.001
GO:0090277	positive regulation of peptide hormone secretion	32	0.301681145	< 0.001	< 0.001
GO:0050796	regulation of insulin secretion	64	0.301661036	0.0007	0.001063182
GO:0044030	regulation of DNA methylation	7	0.30163633	0.0011	0.001576346
GO:0010970	transport along microtubule	76	0.301629073	< 0.001	< 0.001
GO:0099111	microtubule-based transport	76	0.301629073	< 0.001	< 0.001
GO:0001845	phagolysosome assembly	5	0.301621622	< 0.001	< 0.001
GO:0006968	cellular defense response	9	0.301615902	< 0.001	< 0.001
GO:0021766	hippocampus development	42	0.301611816	< 0.001	< 0.001
GO:1904356	regulation of telomere maintenance via telomere lengthening	28	0.301599559	< 0.001	< 0.001
GO:0036445	neuronal stem cell division	6	0.301587302	0.0029	0.003693519
GO:0055057	neuroblast division	6	0.301587302	0.0029	0.003693519
GO:0014029	neural crest formation	7	0.301562787	0.0002	0.000356582
GO:0030204	chondroitin sulfate metabolic process	20	0.301557272	0.0003	0.000505993
GO:0042531	positive regulation of tyrosine phosphorylation of STAT protein	17	0.301445984	0.0002	0.000356582
GO:0060478	acrosomal vesicle exocytosis	5	0.301415701	0.0117	0.013205263
GO:0047496	vesicle transport along microtubule	25	0.301405405	0.0001	0.000192793
GO:0006368	transcription elongation from RNA polymerase II promoter	36	0.301379951	0.0003	0.000505993
GO:0010831	positive regulation of myotube differentiation	6	0.301372801	0.0046	0.0056228
GO:0030032	lamellipodium assembly	32	0.301359395	< 0.001	< 0.001
GO:1902803	regulation of synaptic vesicle transport	58	0.301340257	0.0027	0.003470695
GO:0070534	protein K63-linked ubiquitination	18	0.301315601	0.0001	0.000192793
GO:0072006	nephron development	42	0.30129313	< 0.001	< 0.001
GO:0006940	regulation of smooth muscle contraction	22	0.301287001	< 0.001	< 0.001
GO:0034314	Arp2/3 complex-mediated actin nucleation	17	0.301264289	0.0047	0.005730776
GO:0000086	G2/M transition of mitotic cell cycle	121	0.301254029	< 0.001	< 0.001
GO:0097202	activation of cysteine-type endopeptidase activity	8	0.301190476	0.0006	0.000927725
GO:0001822	kidney development	86	0.30110742	< 0.001	< 0.001
GO:0006516	glycoprotein catabolic process	9	0.301072501	0.001	0.001450349
GO:0044003	modification by symbiont of host morphology or physiology	23	0.301068771	< 0.001	< 0.001
GO:0046633	alpha-beta T cell proliferation	7	0.301047987	0.0007	0.001063182
GO:0002286	T cell activation involved in immune response	21	0.30103573	< 0.001	< 0.001
GO:1903426	regulation of reactive oxygen species biosynthetic process	29	0.300892025	< 0.001	< 0.001
GO:0030207	chondroitin sulfate catabolic process	8	0.300772201	0.0282	0.030230669
GO:0015732	prostaglandin transport	6	0.300772201	< 0.001	< 0.001
GO:0032310	prostaglandin secretion	6	0.300772201	< 0.001	< 0.001
GO:2000008	regulation of protein localization to cell surface	16	0.300659588	0.0001	0.000192793
GO:0051650	establishment of vesicle localization	171	0.300623932	0.0001	0.000192793
GO:1902410	mitotic cytokinetic process	5	0.300540541	< 0.001	< 0.001
GO:0048148	behavioral response to cocaine	8	0.300514801	0.0075	0.008822377
GO:0043241	protein complex disassembly	115	0.300491299	< 0.001	< 0.001
GO:0033683	nucleotide-excision repair, DNA incision	17	0.300461806	0.0002	0.000356582
GO:0051099	positive regulation of binding	76	0.300433516	< 0.001	< 0.001
GO:0010463	mesenchymal cell proliferation	11	0.3004329	< 0.001	< 0.001
GO:0043371	negative regulation of CD4-positive, alpha-beta T cell differentiation	5	0.3003861	0.0022	0.002896881
GO:0046639	negative regulation of alpha-beta T cell differentiation	5	0.3003861	0.0022	0.002896881
GO:0051453	regulation of intracellular pH	24	0.30036465	< 0.001	< 0.001
GO:0006826	iron ion transport	26	0.3003267	< 0.001	< 0.001
GO:0009408	response to heat	72	0.300239525	< 0.001	< 0.001
GO:0009118	regulation of nucleoside metabolic process	42	0.300239015	< 0.001	< 0.001
GO:1903578	regulation of ATP metabolic process	42	0.300239015	< 0.001	< 0.001
GO:0001662	behavioral fear response	26	0.3001584	0.0002	0.000356582
GO:0002209	behavioral defense response	26	0.3001584	0.0002	0.000356582
GO:0016073	snRNA metabolic process	37	0.300045219	< 0.001	< 0.001
GO:0090344	negative regulation of cell aging	12	0.3000429	< 0.001	< 0.001
GO:0043029	T cell homeostasis	12	0.30002145	< 0.001	< 0.001
GO:0055007	cardiac muscle cell differentiation	38	0.299878074	< 0.001	< 0.001
GO:0035107	appendage morphogenesis	42	0.299852914	< 0.001	< 0.001
GO:0035108	limb morphogenesis	42	0.299852914	< 0.001	< 0.001
GO:0031109	microtubule polymerization or depolymerization	43	0.299811439	< 0.001	< 0.001
GO:1901673	regulation of mitotic spindle assembly	8	0.2997426	0.0013	0.001826625
GO:0051354	negative regulation of oxidoreductase activity	5	0.2996139	0.0024	0.003116943
GO:0072080	nephron tubule development	27	0.299604366	< 0.001	< 0.001
GO:1901796	regulation of signal transduction by p53 class mediator	81	0.299537633	0.0005	0.000791911
GO:0014065	phosphatidylinositol 3-kinase signaling	64	0.299521396	< 0.001	< 0.001
GO:0097049	motor neuron apoptotic process	5	0.29951094	0.0048	0.005840847
GO:2000671	regulation of motor neuron apoptotic process	5	0.29951094	0.0048	0.005840847
GO:0090200	positive regulation of release of cytochrome c from mitochondria	12	0.299442299	< 0.001	< 0.001

GOIDlabel	GOName	size	categoryScores	pValPerm	pValPermCorr
GO:0072009	nephron epithelium development	30	0.299407979	< 0.001	< 0.001
GO:0006022	aminoglycan metabolic process	62	0.299377257	< 0.001	< 0.001
GO:0030203	glycosaminoglycan metabolic process	62	0.299377257	< 0.001	< 0.001
GO:0051284	positive regulation of sequestering of calcium ion	9	0.299356499	0.0054	0.006510843
GO:1902950	regulation of dendritic spine maintenance	7	0.299282956	0.0048	0.005840847
GO:0009219	pyrimidine deoxyribonucleotide metabolic process	5	0.299253539	0.0009	0.001319479
GO:0006582	melanin metabolic process	13	0.299198099	< 0.001	< 0.001
GO:0042438	melanin biosynthetic process	13	0.299198099	< 0.001	< 0.001
GO:0044550	secondary metabolite biosynthetic process	13	0.299198099	< 0.001	< 0.001
GO:0098657	import into cell	29	0.299187858	< 0.001	< 0.001
GO:0032410	negative regulation of transporter activity	30	0.299167739	< 0.001	< 0.001
GO:0050654	chondroitin sulfate proteoglycan metabolic process	22	0.299145899	0.0004	0.000648934
GO:0050651	dermatan sulfate proteoglycan biosynthetic process	6	0.299141999	0.0193	0.021134184
GO:0014904	myotube cell development	14	0.299135871	0.0026	0.003356522
GO:0046683	response to organophosphorus	54	0.299132466	< 0.001	< 0.001
GO:0099623	regulation of cardiac muscle cell membrane repolarization	9	0.299127699	0.0001	0.000192793
GO:0042310	vasoconstriction	21	0.299086842	< 0.001	< 0.001
GO:0051896	regulation of protein kinase B signaling	83	0.299068087	< 0.001	< 0.001
GO:0021953	central nervous system neuron differentiation	82	0.29899865	0.0001	0.000192793
GO:0019229	regulation of vasoconstriction	19	0.298868794	< 0.001	< 0.001
GO:0048736	appendage development	51	0.298781134	< 0.001	< 0.001
GO:0060173	limb development	51	0.298781134	< 0.001	< 0.001
GO:0042098	T cell proliferation	52	0.298722899	< 0.001	< 0.001
GO:1903050	regulation of proteolysis involved in cellular protein catabolic process	90	0.298715859	0.0001	0.000192793
GO:0071426	ribonucleoprotein complex export from nucleus	44	0.298707149	0.0004	0.000648934
GO:0035023	regulation of Rho protein signal transduction	54	0.298693932	< 0.001	< 0.001
GO:1903312	negative regulation of mRNA metabolic process	19	0.298692678	< 0.001	< 0.001
GO:0036120	cellular response to platelet-derived growth factor stimulus	5	0.298687259	0.0002	0.000356582
GO:0035710	CD4-positive, alpha-beta T cell activation	22	0.298677899	< 0.001	< 0.001
GO:1901658	glycosyl compound catabolic process	14	0.298676227	0.0002	0.000356582
GO:0007212	dopamine receptor signaling pathway	15	0.298635779	< 0.001	< 0.001
GO:0042053	regulation of dopamine metabolic process	10	0.298635779	< 0.001	< 0.001
GO:0042069	regulation of catecholamine metabolic process	10	0.298635779	< 0.001	< 0.001
GO:0015844	monoamine transport	31	0.298617512	< 0.001	< 0.001
GO:0048012	hepatocyte growth factor receptor signaling pathway	10	0.298584299	0.0004	0.000648934
GO:0021801	cerebral cortex radial glia guided migration	14	0.298529141	0.0017	0.00231289
GO:0022030	telencephalon glial cell migration	14	0.298529141	0.0017	0.00231289
GO:0046824	positive regulation of nucleocytoplasmic transport	26	0.298495198	< 0.001	< 0.001
GO:0021544	subpallium development	11	0.298490698	0.0046	0.0056228
GO:0099622	cardiac muscle cell membrane repolarization	13	0.298485298	0.0005	0.000791911
GO:0008333	endosome to lysosome transport	31	0.298468053	< 0.001	< 0.001
GO:0071242	cellular response to ammonium ion	37	0.298340812	0.0001	0.000192793
GO:0048704	embryonic skeletal system morphogenesis	21	0.298326898	< 0.001	< 0.001
GO:0071786	endoplasmic reticulum tubular network organization	10	0.298301158	< 0.001	< 0.001
GO:0017014	protein nitrosylation	9	0.298269698	< 0.001	< 0.001
GO:0018119	peptidyl-cysteine S-nitrosylation	9	0.298269698	< 0.001	< 0.001
GO:0061326	renal tubule development	28	0.298198198	< 0.001	< 0.001
GO:0071166	ribonucleoprotein complex localization	45	0.298143858	0.0006	0.000927725
GO:0044346	fibroblast apoptotic process	10	0.298120978	< 0.001	< 0.001
GO:0040001	establishment of mitotic spindle localization	13	0.298109098	0.0015	0.002069969
GO:0008217	regulation of blood pressure	47	0.298053068	< 0.001	< 0.001
GO:0000244	spliceosomal tri-snRNP complex assembly	8	0.298037323	0.0003	0.000505993
GO:0006120	mitochondrial electron transport, NADH to ubiquinone	22	0.298034398	0.0018	0.002428764
GO:0060191	regulation of lipase activity	34	0.297978651	< 0.001	< 0.001
GO:2000269	regulation of fibroblast apoptotic process	9	0.297955098	< 0.001	< 0.001
GO:0021799	cerebral cortex radially oriented cell migration	18	0.297912198	0.0007	0.001063182
GO:0006900	membrane budding	63	0.297861126	< 0.001	< 0.001
GO:0045428	regulation of nitric oxide biosynthetic process	19	0.29785274	< 0.001	< 0.001
GO:0006023	aminoglycan biosynthetic process	46	0.297840076	< 0.001	< 0.001
GO:0006024	glycosaminoglycan biosynthetic process	46	0.297840076	< 0.001	< 0.001
GO:0006595	polyamine metabolic process	9	0.297783498	0.0006	0.000927725
GO:0045995	regulation of embryonic development	37	0.297714703	< 0.001	< 0.001
GO:0021575	hindbrain morphogenesis	11	0.297695098	0.0024	0.003116943
GO:0021587	cerebellum morphogenesis	11	0.297695098	0.0024	0.003116943
GO:0007632	visual behavior	25	0.29762677	0.0046	0.0056228
GO:0010565	regulation of cellular ketone metabolic process	83	0.29758571	< 0.001	< 0.001
GO:1903052	positive regulation of proteolysis involved in cellular protein catabolic process	50	0.297580438	0.0001	0.000192793
GO:0050872	white fat cell differentiation	7	0.297517926	0.0009	0.001319479
GO:0009264	deoxyribonucleotide catabolic process	7	0.297297297	0.0002	0.000356582
GO:0060307	regulation of ventricular cardiac muscle cell membrane repolarization	7	0.297223754	< 0.001	< 0.001
GO:0009309	amine biosynthetic process	9	0.297211497	0.0003	0.000505993
GO:0035773	insulin secretion involved in cellular response to glucose stimulus	29	0.297199663	< 0.001	< 0.001
GO:2000272	negative regulation of receptor activity	20	0.297155727	0.0004	0.000648934
GO:1900408	negative regulation of cellular response to oxidative stress	17	0.297145885	< 0.001	< 0.001
GO:1902883	negative regulation of response to oxidative stress	17	0.297145885	< 0.001	< 0.001
GO:1903202	negative regulation of oxidative stress-induced cell death	17	0.297145885	< 0.001	< 0.001
GO:0030330	DNA damage response, signal transduction by p53 class mediator	35	0.297120794	< 0.001	< 0.001
GO:0002697	regulation of immune effector process	129	0.297069827	< 0.001	< 0.001
GO:0034776	response to histamine	6	0.297039897	0.0243	0.026242446
GO:0071420	cellular response to histamine	6	0.297039897	0.0243	0.026242446
GO:1904837	beta-catenin-TCF complex assembly	12	0.296996997	0.0001	0.000192793
GO:0008306	associative learning	39	0.296947497	0.0024	0.003116943
GO:0042158	lipoprotein biosynthetic process	31	0.296907045	< 0.001	< 0.001

GOIDlabel	GOName	size	categoryScores	pValPerm	pValPermCorr
GO:0035024	negative regulation of Rho protein signal transduction	13	0.296901297	< 0.001	< 0.001
GO:0036005	response to macrophage colony-stimulating factor	8	0.296879022	< 0.001	< 0.001
GO:0036006	cellular response to macrophage colony-stimulating factor stimulus	8	0.296879022	< 0.001	< 0.001
GO:0032252	secretory granule localization	9	0.296839697	0.0094	0.010831045
GO:0043476	pigment accumulation	8	0.296718147	< 0.001	< 0.001
GO:0043482	cellular pigment accumulation	8	0.296718147	< 0.001	< 0.001
GO:0010661	positive regulation of muscle cell apoptotic process	14	0.296672182	0.0022	0.002896881
GO:0042770	signal transduction in response to DNA damage	50	0.296658945	< 0.001	< 0.001
GO:0050657	nucleic acid transport	70	0.296646442	< 0.001	< 0.001
GO:0050658	RNA transport	70	0.296646442	< 0.001	< 0.001
GO:0034122	negative regulation of toll-like receptor signaling pathway	17	0.296631085	0.0006	0.000927725
GO:0002534	cytokine production involved in inflammatory response	17	0.296585661	< 0.001	< 0.001
GO:0097696	STAT cascade	49	0.296577627	< 0.001	< 0.001
GO:0032608	interferon-beta production	17	0.296509955	< 0.001	< 0.001
GO:0032648	regulation of interferon-beta production	17	0.296509955	< 0.001	< 0.001
GO:0010923	negative regulation of phosphatase activity	57	0.296493486	0.0007	0.001063182
GO:1904892	regulation of STAT cascade	41	0.29648115	< 0.001	< 0.001
GO:0002791	regulation of peptide secretion	75	0.29648048	0.0001	0.000192793
GO:0090087	regulation of peptide transport	75	0.29648048	0.0001	0.000192793
GO:0099072	regulation of postsynaptic specialization membrane neurotransmitter receptor levels	16	0.296428571	0.0078	0.009125612
GO:0006000	fructose metabolic process	5	0.296422136	0.0007	0.001063182
GO:0044839	cell cycle G2/M phase transition	132	0.296351546	< 0.001	< 0.001
GO:1902884	positive regulation of response to oxidative stress	7	0.296304468	0.0003	0.000505993
GO:2000027	regulation of organ morphogenesis	102	0.296207132	< 0.001	< 0.001
GO:0072088	nephron epithelium morphogenesis	22	0.296138996	< 0.001	< 0.001
GO:0032486	Rap protein signal transduction	9	0.296096096	< 0.001	< 0.001
GO:0030220	platelet formation	7	0.296083839	0.0002	0.000356582
GO:0036344	platelet morphogenesis	7	0.296083839	0.0002	0.000356582
GO:0051056	regulation of small GTPase mediated signal transduction	149	0.296070759	< 0.001	< 0.001
GO:0006475	internal protein amino acid acetylation	70	0.29606913	< 0.001	< 0.001
GO:0033138	positive regulation of peptidyl-serine phosphorylation	47	0.296010296	< 0.001	< 0.001
GO:0044843	cell cycle G1/S phase transition	105	0.295975976	< 0.001	< 0.001
GO:0042304	regulation of fatty acid biosynthetic process	16	0.295833333	< 0.001	< 0.001
GO:0032509	endosome transport via multivesicular body sorting pathway	7	0.295789667	0.0011	0.001576346
GO:0032984	macromolecular complex disassembly	129	0.295754891	< 0.001	< 0.001
GO:2000050	regulation of non-canonical Wnt signaling pathway	6	0.295709996	0.0088	0.010185307
GO:0043300	regulation of leukocyte degranulation	19	0.295698706	< 0.001	< 0.001
GO:0061180	mammary gland epithelium development	26	0.295663796	< 0.001	< 0.001
GO:0009226	nucleotide-sugar biosynthetic process	11	0.295659296	< 0.001	< 0.001
GO:0030521	androgen receptor signaling pathway	20	0.295649936	0.0012	0.001702047
GO:0048013	ephrin receptor signaling pathway	53	0.295616911	0.0001	0.000192793
GO:0032305	positive regulation of icosanoid secretion	7	0.29560581	< 0.001	< 0.001
GO:2000193	positive regulation of fatty acid transport	7	0.29560581	< 0.001	< 0.001
GO:0001570	vasculogenesis	28	0.295596617	< 0.001	< 0.001
GO:0003298	physiological muscle hypertrophy	14	0.295587424	0.0011	0.001576346
GO:0003301	physiological cardiac muscle hypertrophy	14	0.295587424	0.0011	0.001576346
GO:0061049	cell growth involved in cardiac muscle cell development	14	0.295587424	0.0011	0.001576346
GO:0017156	calcium ion regulated exocytosis	78	0.295558196	0.0027	0.003470695
GO:0002260	lymphocyte homeostasis	21	0.295507753	< 0.001	< 0.001
GO:0098661	inorganic anion transmembrane transport	34	0.295503066	< 0.001	< 0.001
GO:0007265	Ras protein signal transduction	156	0.295488895	< 0.001	< 0.001
GO:1903533	regulation of protein targeting	85	0.295480354	< 0.001	< 0.001
GO:0006654	phosphatidic acid biosynthetic process	14	0.29534841	< 0.001	< 0.001
GO:0046473	phosphatidic acid metabolic process	14	0.29534841	< 0.001	< 0.001
GO:1904645	response to beta-amyloid	18	0.295323895	0.0003	0.000505993
GO:0035567	non-canonical Wnt signaling pathway	79	0.295316293	< 0.001	< 0.001
GO:0032204	regulation of telomere maintenance	38	0.295305832	0.0002	0.000356582
GO:1903311	regulation of mRNA metabolic process	50	0.295299871	< 0.001	< 0.001
GO:0060037	pharyngeal system development	5	0.295289575	< 0.001	< 0.001
GO:0045651	positive regulation of macrophage differentiation	8	0.29520592	< 0.001	< 0.001
GO:0048103	somatic stem cell division	10	0.295109395	0.0001	0.000192793
GO:0051236	establishment of RNA localization	71	0.295096706	< 0.001	< 0.001
GO:0042634	regulation of hair cycle	8	0.29501287	< 0.001	< 0.001
GO:0035066	positive regulation of histone acetylation	9	0.294952095	0.0002	0.000356582
GO:0060445	branching involved in salivary gland morphogenesis	7	0.294943924	< 0.001	< 0.001
GO:0032271	regulation of protein polymerization	88	0.294939745	< 0.001	< 0.001
GO:0022037	metencephalon development	34	0.294859565	< 0.001	< 0.001
GO:0060765	regulation of androgen receptor signaling pathway	9	0.294837695	0.0038	0.004725081
GO:0006734	NADH metabolic process	18	0.294823395	0.0001	0.000192793
GO:0048538	thymus development	16	0.294787645	0.0002	0.000356582
GO:0002228	natural killer cell mediated immunity	17	0.294783859	< 0.001	< 0.001
GO:0042730	fibrinolysis	7	0.294723295	< 0.001	< 0.001
GO:0055002	striated muscle cell development	46	0.294661742	< 0.001	< 0.001
GO:0002753	cytoplasmic pattern recognition receptor signaling pathway	22	0.294653095	0.0001	0.000192793
GO:0034383	low-density lipoprotein particle clearance	11	0.294606295	0.0013	0.001826625
GO:0090169	regulation of spindle assembly	9	0.294580295	0.0009	0.001319479
GO:0001736	establishment of planar polarity	73	0.294518785	< 0.001	< 0.001
GO:0007164	establishment of tissue polarity	73	0.294518785	< 0.001	< 0.001
GO:0030324	lung development	51	0.294516365	< 0.001	< 0.001
GO:0098781	ncRNA transcription	52	0.294510445	< 0.001	< 0.001
GO:1904380	endoplasmic reticulum mannose trimming	9	0.294465894	0.001	0.001450349
GO:0005513	detection of calcium ion	7	0.294465894	0.0133	0.014896
GO:1905209	positive regulation of cardiocyte differentiation	12	0.294444444	< 0.001	< 0.001

GOIDlabel	GOName	size	categoryScores	pValPerm	pValPermCorr
GO:0000737	DNA catabolic process, endonucleolytic	9	0.294437294	0.0013	0.001826625
GO:0006308	DNA catabolic process	9	0.294437294	0.0013	0.001826625
GO:0006309	apoptotic DNA fragmentation	9	0.294437294	0.0013	0.001826625
GO:0018393	internal peptidyl-lysine acetylation	67	0.294415951	< 0.001	< 0.001
GO:0031348	negative regulation of defense response	58	0.294399325	< 0.001	< 0.001
GO:0060964	regulation of gene silencing by miRNA	36	0.294372944	< 0.001	< 0.001
GO:1904526	regulation of microtubule binding	5	0.294362934	0.009	0.010394529
GO:0042509	regulation of tyrosine phosphorylation of STAT protein	24	0.294294294	0.0004	0.000648934
GO:0032495	response to muramyl dipeptide	7	0.294282037	< 0.001	< 0.001
GO:0030004	cellular monovalent inorganic cation homeostasis	31	0.294266617	< 0.001	< 0.001
GO:0046324	regulation of glucose import	23	0.294264451	0.0001	0.000192793
GO:0006310	DNA recombination	97	0.294216455	< 0.001	< 0.001
GO:0043981	histone H4-K5 acetylation	7	0.294208494	< 0.001	< 0.001
GO:0043982	histone H4-K8 acetylation	7	0.294208494	< 0.001	< 0.001
GO:0031987	locomotion involved in locomotory behavior	5	0.294208494	< 0.001	< 0.001
GO:0090263	positive regulation of canonical Wnt signaling pathway	66	0.294110994	0.0001	0.000192793
GO:0003018	vascular process in circulatory system	47	0.294098962	< 0.001	< 0.001
GO:0048385	regulation of retinoic acid receptor signaling pathway	7	0.29409818	< 0.001	< 0.001
GO:0030323	respiratory tube development	53	0.294087079	< 0.001	< 0.001
GO:0039702	viral budding via host ESCRT complex	12	0.294079794	0.0005	0.000791911
GO:0060147	regulation of posttranscriptional gene silencing	37	0.294055445	< 0.001	< 0.001
GO:0060966	regulation of gene silencing by RNA	37	0.294055445	< 0.001	< 0.001
GO:0021591	ventricular system development	13	0.293990694	0.0002	0.000356582
GO:0050708	regulation of protein secretion	159	0.293983471	< 0.001	< 0.001
GO:0009755	hormone-mediated signaling pathway	78	0.293927994	< 0.001	< 0.001
GO:0051642	centrosome localization	10	0.293925354	0.0019	0.00254969
GO:0050891	multicellular organismal water homeostasis	24	0.293918919	< 0.001	< 0.001
GO:0006735	NADH regeneration	13	0.293911494	0.0006	0.000927725
GO:0061621	canonical glycolysis	13	0.293911494	0.0006	0.000927725
GO:0061718	glucose catabolic process to pyruvate	13	0.293911494	0.0006	0.000927725
GO:1903391	regulation of adherens junction organization	32	0.293910875	< 0.001	< 0.001
GO:0061318	renal filtration cell differentiation	5	0.293899614	0.0076	0.008916717
GO:0072010	glomerular epithelium development	5	0.293899614	0.0076	0.008916717
GO:0072112	glomerular visceral epithelial cell differentiation	5	0.293899614	0.0076	0.008916717
GO:0072311	glomerular epithelial cell differentiation	5	0.293899614	0.0076	0.008916717
GO:0060968	regulation of gene silencing	43	0.293891233	< 0.001	< 0.001
GO:0000082	G1/S transition of mitotic cell cycle	100	0.293868726	< 0.001	< 0.001
GO:1903793	positive regulation of anion transport	21	0.293853037	< 0.001	< 0.001
GO:0018023	peptidyl-lysine trimethylation	15	0.293745174	0.0005	0.000791911
GO:0008286	insulin receptor signaling pathway	52	0.293743194	< 0.001	< 0.001
GO:0002093	auditory receptor cell morphogenesis	6	0.293693694	0.0003	0.000505993
GO:0060088	auditory receptor cell stereocilium organization	6	0.293693694	0.0003	0.000505993
GO:0098877	neurotransmitter receptor transport to plasma membrane	8	0.293693694	0.0006	0.000927725
GO:0098969	neurotransmitter receptor transport to postsynaptic membrane	8	0.293693694	0.0006	0.000927725
GO:0099637	neurotransmitter receptor transport	8	0.293693694	0.0006	0.000927725
GO:1903540	establishment of protein localization to postsynaptic membrane	8	0.293693694	0.0006	0.000927725
GO:0061517	macrophage proliferation	5	0.293693694	< 0.001	< 0.001
GO:0061518	microglial cell proliferation	5	0.293693694	< 0.001	< 0.001
GO:0060831	smoothened signaling pathway involved in dorsal/ventral neural tube patterning	7	0.293693694	0.0016	0.002186748
GO:1903729	regulation of plasma membrane organization	50	0.293688546	< 0.001	< 0.001
GO:0008154	actin polymerization or depolymerization	88	0.293681994	< 0.001	< 0.001
GO:1903169	regulation of calcium ion transmembrane transport	60	0.293603604	0.0013	0.001826625
GO:0051258	protein polymerization	113	0.293586633	< 0.001	< 0.001
GO:0070979	protein K11-linked ubiquitination	19	0.293571767	0.0002	0.000356582
GO:0045861	negative regulation of proteolysis	92	0.293509037	< 0.001	< 0.001
GO:0090330	regulation of platelet aggregation	9	0.293464893	0.0007	0.001063182
GO:0051797	regulation of hair follicle development	6	0.293393393	< 0.001	< 0.001
GO:0006639	acylglycerol metabolic process	30	0.293341913	< 0.001	< 0.001
GO:2001237	negative regulation of extrinsic apoptotic signaling pathway	24	0.293339768	< 0.001	< 0.001
GO:0002741	positive regulation of cytokine secretion involved in immune response	6	0.293264693	< 0.001	< 0.001
GO:0045923	positive regulation of fatty acid metabolic process	9	0.293236093	0.0002	0.000356582
GO:0051155	positive regulation of striated muscle cell differentiation	20	0.293217503	< 0.001	< 0.001
GO:0006352	DNA-templated transcription, initiation	91	0.293210007	< 0.001	< 0.001
GO:0033363	secretory granule organization	14	0.293197279	< 0.001	< 0.001
GO:0006409	tRNA export from nucleus	12	0.293178893	0.0001	0.000192793
GO:0051031	tRNA transport	12	0.293178893	0.0001	0.000192793
GO:0071431	tRNA-containing ribonucleoprotein complex export from nucleus	12	0.293178893	0.0001	0.000192793
GO:0042771	intrinsic apoptotic signaling pathway in response to DNA damage by p53 class mediator	18	0.293178893	0.0009	0.001319479
GO:1902750	negative regulation of cell cycle G2/M phase transition	55	0.293141453	0.0004	0.000648934
GO:0002934	desmosome organization	5	0.293127413	0.0004	0.000648934
GO:0090276	regulation of peptide hormone secretion	74	0.293105847	0.0002	0.000356582
GO:0060359	response to ammonium ion	71	0.293091885	< 0.001	< 0.001
GO:0014074	response to purine-containing compound	61	0.293073401	< 0.001	< 0.001
GO:0072001	renal system development	92	0.293069778	< 0.001	< 0.001
GO:0060713	labyrinthine layer morphogenesis	7	0.293068579	0.0001	0.000192793
GO:0045071	negative regulation of viral genome replication	16	0.293066281	< 0.001	< 0.001
GO:0032720	negative regulation of tumor necrosis factor production	20	0.293024453	< 0.001	< 0.001
GO:0006489	dolichyl diphosphate biosynthetic process	5	0.293024453	0.0179	0.019692915
GO:0046465	dolichyl diphosphate metabolic process	5	0.293024453	0.0179	0.019692915
GO:0008064	regulation of actin polymerization or depolymerization	74	0.293004974	< 0.001	< 0.001
GO:0030832	regulation of actin filament length	74	0.293004974	< 0.001	< 0.001
GO:0000724	double-strand break repair via homologous recombination	45	0.292955813	< 0.001	< 0.001
GO:0000725	recombinational repair	45	0.292955813	< 0.001	< 0.001

GOIDlabel	GOName	size	categoryScores	pValPerm	pValPermCorr
GO:0046189	phenol-containing compound biosynthetic process	22	0.292909793	< 0.001	< 0.001
GO:0051494	negative regulation of cytoskeleton organization	63	0.292835693	< 0.001	< 0.001
GO:0032526	response to retinoic acid	29	0.292761727	0.0007	0.001063182
GO:0009225	nucleotide-sugar metabolic process	17	0.292739799	< 0.001	< 0.001
GO:0048246	macrophage chemotaxis	15	0.292612613	< 0.001	< 0.001
GO:0010972	negative regulation of G2/M transition of mitotic cell cycle	48	0.292535393	0.0009	0.001319479
GO:0055013	cardiac muscle cell development	25	0.292530245	0.0002	0.000356582
GO:0043370	regulation of CD4-positive, alpha-beta T cell differentiation	11	0.292476892	< 0.001	< 0.001
GO:0007338	single fertilization	42	0.292449592	< 0.001	< 0.001
GO:0006577	amino-acid betaine metabolic process	9	0.292406692	0.0005	0.000791911
GO:0035150	regulation of tube size	35	0.29238463	< 0.001	< 0.001
GO:0050880	regulation of blood vessel size	35	0.29238463	< 0.001	< 0.001
GO:0007156	homophilic cell adhesion via plasma membrane adhesion molecules	46	0.292350736	0.0001	0.000192793
GO:0006165	nucleoside diphosphate phosphorylation	48	0.292347705	< 0.001	< 0.001
GO:0002381	immunoglobulin production involved in immunoglobulin mediated immune response	17	0.292346128	0.0012	0.001702047
GO:0052547	regulation of peptidase activity	127	0.292345889	< 0.001	< 0.001
GO:0035305	negative regulation of dephosphorylation	62	0.29232366	0.0003	0.000505993
GO:0006354	DNA-templated transcription, elongation	52	0.292287892	< 0.001	< 0.001
GO:1900424	regulation of defense response to bacterium	8	0.292245817	< 0.001	< 0.001
GO:0009306	protein secretion	199	0.292221726	< 0.001	< 0.001
GO:0048641	regulation of skeletal muscle tissue development	15	0.292200772	< 0.001	< 0.001
GO:0045023	G0 to G1 transition	13	0.292149292	0.0001	0.000192793
GO:0070316	regulation of G0 to G1 transition	13	0.292149292	0.0001	0.000192793
GO:0007626	locomotory behavior	84	0.292094135	< 0.001	< 0.001
GO:0050922	negative regulation of chemotaxis	16	0.292084942	< 0.001	< 0.001
GO:0060260	regulation of transcription initiation from RNA polymerase II promoter	7	0.292002206	0.0025	0.003237479
GO:0019932	second-messenger-mediated signaling	114	0.291991239	< 0.001	< 0.001
GO:0018205	peptidyl-lysine modification	152	0.291981643	< 0.001	< 0.001
GO:0072538	T-helper 17 type immune response	5	0.291943372	< 0.001	< 0.001
GO:0098976	excitatory chemical synaptic transmission	5	0.291943372	0.0067	0.007954042
GO:0031334	positive regulation of protein complex assembly	102	0.291934792	< 0.001	< 0.001
GO:0032210	regulation of telomere maintenance via telomerase	26	0.291872092	0.0002	0.000356582
GO:1905606	regulation of presynapse assembly	17	0.291816186	0.001	0.001450349
GO:0052312	modulation of transcription in other organism involved in symbiotic interaction	9	0.291806092	0.0017	0.00231289
GO:0001655	urogenital system development	104	0.291778042	< 0.001	< 0.001
GO:0032103	positive regulation of response to external stimulus	108	0.291698842	< 0.001	< 0.001
GO:0021696	cerebellar cortex morphogenesis	10	0.291685972	0.0028	0.003584743
GO:0006369	termination of RNA polymerase II transcription	16	0.291682754	0.0002	0.000356582
GO:0002888	positive regulation of myeloid leukocyte mediated immunity	8	0.291666667	< 0.001	< 0.001
GO:0060071	Wnt signaling pathway, planar cell polarity pathway	64	0.291646557	0.0001	0.000192793
GO:0090175	regulation of establishment of planar polarity	64	0.291646557	0.0001	0.000192793
GO:0045324	late endosome to vacuole transport	6	0.291591592	< 0.001	< 0.001
GO:0035296	regulation of tube diameter	33	0.291509692	< 0.001	< 0.001
GO:0097746	regulation of blood vessel diameter	33	0.291509692	< 0.001	< 0.001
GO:0051817	modification of morphology or physiology of other organism involved in symbiotic interaction	44	0.291505792	< 0.001	< 0.001
GO:0007292	female gamete generation	34	0.291407374	< 0.001	< 0.001
GO:0002367	cytokine production involved in immune response	25	0.291366795	< 0.001	< 0.001
GO:0002822	regulation of adaptive immune response based on somatic recombination of immune receptors built from immunoglobulin superfamily domains	35	0.291362383	< 0.001	< 0.001
GO:0046339	diacylglycerol metabolic process	5	0.291325611	0.0004	0.000648934
GO:0047484	regulation of response to osmotic stress	7	0.291303548	0.0036	0.004505419
GO:0043243	positive regulation of protein complex disassembly	16	0.291296654	< 0.001	< 0.001
GO:0097485	neuron projection guidance	109	0.291207066	0.0001	0.000192793
GO:0015740	C4-dicarboxylate transport	8	0.291184041	0.0112	0.012688637
GO:0090311	regulation of protein deacetylation	19	0.291146786	0.0003	0.000505993
GO:0002715	regulation of natural killer cell mediated immunity	8	0.291119691	< 0.001	< 0.001
GO:0070507	regulation of microtubule cytoskeleton organization	80	0.291100386	< 0.001	< 0.001
GO:0046785	microtubule polymerization	34	0.291074268	0.0001	0.000192793
GO:0046883	regulation of hormone secretion	90	0.291065351	0.0001	0.000192793
GO:0043302	positive regulation of leukocyte degranulation	8	0.291055341	< 0.001	< 0.001
GO:0002931	response to ischemia	25	0.291037323	< 0.001	< 0.001
GO:0019098	reproductive behavior	19	0.290997765	0.0001	0.000192793
GO:0097306	cellular response to alcohol	20	0.290926641	< 0.001	< 0.001
GO:1903038	negative regulation of leukocyte cell-cell adhesion	32	0.29090251	< 0.001	< 0.001
GO:0090494	dopamine uptake	7	0.290899062	< 0.001	< 0.001
GO:0070669	response to interleukin-2	6	0.290819391	< 0.001	< 0.001
GO:0000075	cell cycle checkpoint	83	0.290806469	< 0.001	< 0.001
GO:0000394	RNA splicing, via endonucleolytic cleavage and ligation	6	0.290776491	0.0029	0.003693519
GO:0061418	regulation of transcription from RNA polymerase II promoter in response to hypoxia	45	0.290747891	0.0012	0.001702047
GO:0042596	fear response	27	0.290747891	0.0006	0.000927725
GO:0007411	axon guidance	107	0.290691493	< 0.001	< 0.001
GO:0008637	apoptotic mitochondrial changes	58	0.29064927	< 0.001	< 0.001
GO:0050707	regulation of cytokine secretion	63	0.290633491	< 0.001	< 0.001
GO:0046031	ADP metabolic process	47	0.290582984	< 0.001	< 0.001
GO:0006739	NADP metabolic process	10	0.290579151	< 0.001	< 0.001
GO:0007018	microtubule-based movement	108	0.290564374	< 0.001	< 0.001
GO:1904063	negative regulation of cation transmembrane transport	28	0.290522155	< 0.001	< 0.001
GO:0048675	axon extension	65	0.290517771	< 0.001	< 0.001
GO:0033627	cell adhesion mediated by integrin	23	0.290504169	< 0.001	< 0.001
GO:0050858	negative regulation of antigen receptor-mediated signaling pathway	10	0.290501931	< 0.001	< 0.001
GO:1904894	positive regulation of STAT cascade	22	0.29047619	< 0.001	< 0.001
GO:1903510	mucopolysaccharide metabolic process	44	0.29047034	< 0.001	< 0.001
GO:0033173	calcineurin-NFAT signaling cascade	21	0.290470062	0.0001	0.000192793

GOIDlabel	GOName	size	categoryScores	pValPerm	pValPermCorr
GO:0097720	calcineurin-mediated signaling	21	0.290470062	0.0001	0.000192793
GO:0006096	glycolytic process	41	0.290447939	< 0.001	< 0.001
GO:0010614	negative regulation of cardiac muscle hypertrophy	10	0.29042471	0.0003	0.000505993
GO:0043523	regulation of neuron apoptotic process	93	0.290413916	< 0.001	< 0.001
GO:0006836	neurotransmitter transport	104	0.29039699	0.0018	0.002428764
GO:0052548	regulation of endopeptidase activity	115	0.290363158	< 0.001	< 0.001
GO:0015711	organic anion transport	148	0.290352708	< 0.001	< 0.001
GO:0001501	skeletal system development	169	0.290350537	< 0.001	< 0.001
GO:0090670	RNA localization to Cajal body	11	0.29034749	0.0024	0.003116943
GO:0090671	telomerase RNA localization to Cajal body	11	0.29034749	0.0024	0.003116943
GO:0090672	telomerase RNA localization	11	0.29034749	0.0024	0.003116943
GO:1904872	regulation of telomerase RNA localization to Cajal body	11	0.29034749	0.0024	0.003116943
GO:0010950	positive regulation of endopeptidase activity	56	0.290315315	< 0.001	< 0.001
GO:0030593	neutrophil chemotaxis	19	0.290279753	< 0.001	< 0.001
GO:0030837	negative regulation of actin filament polymerization	24	0.290272415	0.0001	0.000192793
GO:0099518	vesicle cytoskeletal trafficking	28	0.290246369	0.0005	0.000791911
GO:0010771	negative regulation of cell morphogenesis involved in differentiation	44	0.29024219	0.0013	0.001826625
GO:0097529	myeloid leukocyte migration	55	0.29021645	< 0.001	< 0.001
GO:0043280	positive regulation of cysteine-type endopeptidase activity involved in apoptotic process	40	0.290199485	< 0.001	< 0.001
GO:0007140	male meiosis	12	0.29011154	< 0.001	< 0.001
GO:0018108	peptidyl-tyrosine phosphorylation	127	0.290057662	< 0.001	< 0.001
GO:0038003	opioid receptor signaling pathway	5	0.29003861	< 0.001	< 0.001
GO:0006888	ER to Golgi vesicle-mediated transport	84	0.290037997	0.0002	0.000356582
GO:0071277	cellular response to calcium ion	28	0.289988969	< 0.001	< 0.001
GO:0042698	ovulation cycle	33	0.28997309	0.0023	0.003013139
GO:0008033	tRNA processing	48	0.289950665	0.0002	0.000356582
GO:0014075	response to amine	21	0.289943004	< 0.001	< 0.001
GO:0010801	negative regulation of peptidyl-threonine phosphorylation	9	0.28991849	0.0064	0.007617974
GO:0010921	regulation of phosphatase activity	94	0.28984912	0.0003	0.000505993
GO:0006576	cellular biogenic amine metabolic process	17	0.28983269	< 0.001	< 0.001
GO:0015672	monovalent inorganic cation transport	164	0.289812286	< 0.001	< 0.001
GO:0003148	outflow tract septum morphogenesis	7	0.289795918	0.0068	0.008063902
GO:0016573	histone acetylation	65	0.28977725	< 0.001	< 0.001
GO:0035987	endodermal cell differentiation	11	0.28973909	< 0.001	< 0.001
GO:0045117	azole transport	5	0.28972973	0.0001	0.000192793
GO:0051932	synaptic transmission, GABAergic	18	0.28971829	0.0002	0.000356582
GO:0033555	multicellular organismal response to stress	38	0.289690442	0.0004	0.000648934
GO:0048477	oogenesis	26	0.28965449	< 0.001	< 0.001
GO:0031670	cellular response to nutrient	12	0.28963964	0.0038	0.004725081
GO:0055117	regulation of cardiac muscle contraction	31	0.289633412	< 0.001	< 0.001
GO:0060759	regulation of response to cytokine stimulus	67	0.289632916	< 0.001	< 0.001
GO:0018209	peptidyl-serine modification	128	0.289617519	< 0.001	< 0.001
GO:0006818	hydrogen transport	44	0.28961624	0.0001	0.000192793
GO:0015992	proton transport	44	0.28961624	0.0001	0.000192793
GO:0045980	negative regulation of nucleotide metabolic process	15	0.28959245	< 0.001	< 0.001
GO:1900543	negative regulation of purine nucleotide metabolic process	15	0.28959245	< 0.001	< 0.001
GO:0044091	membrane biogenesis	21	0.28957529	< 0.001	< 0.001
GO:0007224	smoothened signaling pathway	44	0.28956944	0.0017	0.00231289
GO:0002562	somatic diversification of immune receptors via germline recombination within a single locus	22	0.28950509	0.0039	0.004841626
GO:0016444	somatic cell DNA recombination	22	0.28950509	0.0039	0.004841626
GO:2000727	positive regulation of cardiac muscle cell differentiation	10	0.289472329	< 0.001	< 0.001
GO:1903358	regulation of Golgi organization	10	0.289472329	0.0021	0.002780108
GO:0010952	positive regulation of peptidase activity	67	0.289398567	< 0.001	< 0.001
GO:0006403	RNA localization	86	0.289392715	< 0.001	< 0.001
GO:0000422	mitophagy	31	0.289392618	0.0002	0.000356582
GO:0061726	mitochondrion disassembly	31	0.289392618	0.0002	0.000356582
GO:0031572	G2 DNA damage checkpoint	13	0.289377289	0.0004	0.000648934
GO:0042102	positive regulation of T cell proliferation	23	0.289373846	< 0.001	< 0.001
GO:0032651	regulation of interleukin-1 beta production	27	0.289365556	< 0.001	< 0.001
GO:0060026	convergent extension	6	0.289360789	0.0001	0.000192793
GO:0008344	adult locomotory behavior	31	0.289276373	< 0.001	< 0.001
GO:0030177	positive regulation of Wnt signaling pathway	82	0.289223718	0.0001	0.000192793
GO:0048599	oocyte development	12	0.289189189	< 0.001	< 0.001
GO:0035725	sodium ion transmembrane transport	43	0.289162252	< 0.001	< 0.001
GO:0006821	chloride transport	35	0.289111969	< 0.001	< 0.001
GO:0021543	pallium development	82	0.289110714	< 0.001	< 0.001
GO:0010827	regulation of glucose transport	32	0.289108752	0.0001	0.000192793
GO:0001578	microtubule bundle formation	30	0.289094809	0.0002	0.000356582
GO:2001057	reactive nitrogen species metabolic process	28	0.289023718	< 0.001	< 0.001
GO:0018212	peptidyl-tyrosine modification	129	0.289016591	< 0.001	< 0.001
GO:0002495	antigen processing and presentation of peptide antigen via MHC class II	47	0.2890112	< 0.001	< 0.001
GO:0002504	antigen processing and presentation of peptide or polysaccharide antigen via MHC class II	47	0.2890112	< 0.001	< 0.001
GO:0022612	gland morphogenesis	38	0.2890063	< 0.001	< 0.001
GO:0000723	telomere maintenance	63	0.289003289	0.0003	0.000505993
GO:0032200	telomere organization	63	0.289003289	0.0003	0.000505993
GO:2000278	regulation of DNA biosynthetic process	46	0.288998937	0.0001	0.000192793
GO:0043524	negative regulation of neuron apoptotic process	71	0.288911849	< 0.001	< 0.001
GO:0070988	demethylation	20	0.288893179	0.0031	0.003925117
GO:0070672	response to interleukin-15	5	0.288854569	< 0.001	< 0.001
GO:0046037	GMP metabolic process	7	0.28883986	< 0.001	< 0.001
GO:0035303	regulation of dephosphorylation	113	0.288821312	< 0.001	< 0.001
GO:0003281	ventricular septum development	25	0.288813385	< 0.001	< 0.001
GO:0045446	endothelial cell differentiation	33	0.288756289	< 0.001	< 0.001

GOIDlabel	GOName	size	categoryScores	pValPerm	pValPermCorr
GO:0051169	nuclear transport	149	0.288733988	< 0.001	< 0.001
GO:0018105	peptidyl-serine phosphorylation	121	0.288692471	< 0.001	< 0.001
GO:0016233	telomere capping	20	0.288661519	0.0026	0.003356522
GO:0050434	positive regulation of viral transcription	22	0.288650989	0.001	0.001450349
GO:0061333	renal tubule morphogenesis	23	0.28864641	< 0.001	< 0.001
GO:0048469	cell maturation	48	0.288583226	< 0.001	< 0.001
GO:0045738	negative regulation of DNA repair	13	0.288565489	< 0.001	< 0.001
GO:0031333	negative regulation of protein complex assembly	55	0.288508249	< 0.001	< 0.001
GO:0051047	positive regulation of secretion	162	0.288499611	< 0.001	< 0.001
GO:0031397	negative regulation of protein ubiquitination	41	0.288495464	0.0001	0.000192793
GO:0099531	presynaptic process involved in chemical synaptic transmission	90	0.288482768	0.0042	0.005168936
GO:0032652	regulation of interleukin-1 production	31	0.288454353	< 0.001	< 0.001
GO:0050728	negative regulation of inflammatory response	41	0.288413849	< 0.001	< 0.001
GO:0006641	triglyceride metabolic process	26	0.288347688	< 0.001	< 0.001
GO:0002675	positive regulation of acute inflammatory response	10	0.288339768	0.0105	0.011988591
GO:1903409	reactive oxygen species biosynthetic process	34	0.288333712	< 0.001	< 0.001
GO:0045601	regulation of endothelial cell differentiation	6	0.288331188	0.0001	0.000192793
GO:0036152	phosphatidylethanolamine acyl-chain remodeling	6	0.288288288	0.0053	0.006400268
GO:0002698	negative regulation of immune effector process	31	0.288279985	< 0.001	< 0.001
GO:0006110	regulation of glycolytic process	28	0.288269903	< 0.001	< 0.001
GO:0048146	positive regulation of fibroblast proliferation	20	0.288262548	< 0.001	< 0.001
GO:0017158	regulation of calcium ion-dependent exocytosis	53	0.288244579	0.002	0.002664694
GO:0042095	interferon-gamma biosynthetic process	5	0.288185328	< 0.001	< 0.001
GO:0045072	regulation of interferon-gamma biosynthetic process	5	0.288185328	< 0.001	< 0.001
GO:0044351	macropinocytosis	5	0.288185328	0.0002	0.000356582
GO:0030900	forebrain development	168	0.288151927	< 0.001	< 0.001
GO:0051028	mRNA transport	57	0.288143783	0.0002	0.000356582
GO:0086013	membrane repolarization during cardiac muscle cell action potential	7	0.288104431	0.0033	0.004153051
GO:1990126	retrograde transport, endosome to plasma membrane	9	0.288088088	0.0003	0.000505993
GO:0051410	detoxification of nitrogen compound	5	0.288082368	0.0378	0.039873355
GO:0070317	negative regulation of G0 to G1 transition	12	0.288073788	0.0001	0.000192793
GO:0051402	neuron apoptotic process	102	0.288071265	< 0.001	< 0.001
GO:2000785	regulation of autophagosome assembly	20	0.288069498	< 0.001	< 0.001
GO:0000186	activation of MAPKK activity	20	0.288056628	< 0.001	< 0.001
GO:2000573	positive regulation of DNA biosynthetic process	32	0.288022844	0.0003	0.000505993
GO:0060969	negative regulation of gene silencing	7	0.287957345	0.0057	0.006849633
GO:0042129	regulation of T cell proliferation	39	0.287918688	< 0.001	< 0.001
GO:2000271	positive regulation of fibroblast apoptotic process	6	0.287902188	< 0.001	< 0.001
GO:1904375	regulation of protein localization to cell periphery	45	0.287870728	< 0.001	< 0.001
GO:0042711	maternal behavior	8	0.287870013	0.0007	0.001063182
GO:0060746	parental behavior	8	0.287870013	0.0007	0.001063182
GO:0042476	odontogenesis	32	0.287845882	< 0.001	< 0.001
GO:0007041	lysosomal transport	52	0.287837838	< 0.001	< 0.001
GO:0051222	positive regulation of protein transport	171	0.287826172	< 0.001	< 0.001
GO:0031571	mitotic G1 DNA damage checkpoint	20	0.287824968	0.0023	0.003013139
GO:0044819	mitotic G1/S transition checkpoint	20	0.287824968	0.0023	0.003013139
GO:0019934	cGMP-mediated signaling	9	0.287802088	0.0338	0.03585693
GO:0016079	synaptic vesicle exocytosis	65	0.287761608	0.0055	0.006618125
GO:0045620	negative regulation of lymphocyte differentiation	15	0.287756328	0.0001	0.000192793
GO:0042311	vasodilation	10	0.287722008	0.0054	0.006510843
GO:0032413	negative regulation of ion transmembrane transporter activity	25	0.287701416	< 0.001	< 0.001
GO:1904951	positive regulation of establishment of protein localization	191	0.287667024	< 0.001	< 0.001
GO:0043087	regulation of GTPase activity	187	0.287650982	< 0.001	< 0.001
GO:0043161	proteasome-mediated ubiquitin-dependent protein catabolic process	182	0.287634888	0.0001	0.000192793
GO:0034134	toll-like receptor 2 signaling pathway	9	0.287630488	0.0001	0.000192793
GO:0007623	circadian rhythm	87	0.287622598	< 0.001	< 0.001
GO:0050853	B cell receptor signaling pathway	10	0.287567568	< 0.001	< 0.001
GO:0006606	protein import into nucleus	60	0.287533248	< 0.001	< 0.001
GO:0044744	protein targeting to nucleus	60	0.287533248	< 0.001	< 0.001
GO:1902593	single-organism nuclear import	60	0.287533248	< 0.001	< 0.001
GO:0030041	actin filament polymerization	72	0.287523238	< 0.001	< 0.001
GO:0051149	positive regulation of muscle cell differentiation	42	0.287430287	< 0.001	< 0.001
GO:0002230	positive regulation of defense response to virus by host	7	0.287405773	0.0023	0.003013139
GO:0050670	regulation of lymphocyte proliferation	52	0.287387387	< 0.001	< 0.001
GO:0070661	leukocyte proliferation	79	0.287314077	< 0.001	< 0.001
GO:0016601	Rac protein signal transduction	14	0.287313844	0.0006	0.000927725
GO:0051924	regulation of calcium ion transport	95	0.287302039	< 0.001	< 0.001
GO:0030856	regulation of epithelial cell differentiation	36	0.287087087	< 0.001	< 0.001
GO:1901216	positive regulation of neuron death	39	0.287054087	< 0.001	< 0.001
GO:0010390	histone monoubiquitination	11	0.287001287	0.0002	0.000356582
GO:1903201	regulation of oxidative stress-induced cell death	24	0.287001287	< 0.001	< 0.001
GO:0045445	myoblast differentiation	33	0.286985687	< 0.001	< 0.001
GO:2000780	negative regulation of double-strand break repair	11	0.286977887	< 0.001	< 0.001
GO:0003085	negative regulation of systemic arterial blood pressure	7	0.286964516	< 0.001	< 0.001
GO:0050671	positive regulation of lymphocyte proliferation	35	0.286957161	< 0.001	< 0.001
GO:0048678	response to axon injury	33	0.286876487	< 0.001	< 0.001
GO:0070232	regulation of T cell apoptotic process	8	0.286872587	< 0.001	< 0.001
GO:0001913	T cell mediated cytotoxicity	8	0.286872587	0.0003	0.000505993
GO:1903076	regulation of protein localization to plasma membrane	41	0.286806667	< 0.001	< 0.001
GO:0070663	regulation of leukocyte proliferation	55	0.286795367	< 0.001	< 0.001
GO:0060541	respiratory system development	57	0.286784529	< 0.001	< 0.001
GO:0003341	cilium movement	19	0.286784529	0.0023	0.003013139
GO:0010765	positive regulation of sodium ion transport	16	0.286776062	0.0004	0.000648934

GOIDlabel	GOName	size	categoryScores	pValPerm	pValPermCorr
GO:0006813	potassium ion transport	74	0.286733452	0.0003	0.000505993
GO:0072348	sulfur compound transport	14	0.286707115	< 0.001	< 0.001
GO:0051346	negative regulation of hydrolase activity	156	0.286704287	< 0.001	< 0.001
GO:0002703	regulation of leukocyte mediated immunity	54	0.28666762	< 0.001	< 0.001
GO:0010389	regulation of G2/M transition of mitotic cell cycle	102	0.286665657	0.0001	0.000192793
GO:1901215	negative regulation of neuron death	97	0.286653664	< 0.001	< 0.001
GO:0035428	hexose transmembrane transport	9	0.286629487	0.0002	0.000356582
GO:1904659	glucose transmembrane transport	9	0.286629487	0.0002	0.000356582
GO:0043281	regulation of cysteine-type endopeptidase activity involved in apoptotic process	71	0.286627875	< 0.001	< 0.001
GO:0009452	7-methylguanosine RNA capping	19	0.286581318	0.0003	0.000505993
GO:0036260	RNA capping	19	0.286581318	0.0003	0.000505993
GO:0045471	response to ethanol	46	0.28655923	< 0.001	< 0.001
GO:0006939	smooth muscle contraction	39	0.286559087	< 0.001	< 0.001
GO:0007096	regulation of exit from mitosis	6	0.286529387	< 0.001	< 0.001
GO:0006913	nucleocytoplasmic transport	146	0.286504117	< 0.001	< 0.001
GO:0009914	hormone transport	114	0.286500034	< 0.001	< 0.001
GO:0048021	regulation of melanin biosynthetic process	8	0.286486486	0.0001	0.000192793
GO:1900376	regulation of secondary metabolite biosynthetic process	8	0.286486486	0.0001	0.000192793
GO:0055001	muscle cell development	51	0.286476392	< 0.001	< 0.001
GO:0045979	positive regulation of nucleoside metabolic process	17	0.286456204	< 0.001	< 0.001
GO:1903580	positive regulation of ATP metabolic process	17	0.286456204	< 0.001	< 0.001
GO:0006656	phosphatidylcholine biosynthetic process	12	0.286443586	< 0.001	< 0.001
GO:1903305	regulation of regulated secretory pathway	73	0.286405388	0.0001	0.000192793
GO:0048017	inositol lipid-mediated signaling	76	0.286391655	< 0.001	< 0.001
GO:0001946	lymphangiogenesis	6	0.286357786	0.0012	0.001702047
GO:0007214	gamma-aminobutyric acid signaling pathway	6	0.286357786	0.0226	0.024538959
GO:0002824	positive regulation of adaptive immune response based on somatic recombination of immune receptors built from immunoglobulin superfamily domains	23	0.286307425	0.0001	0.000192793
GO:0001832	blastocyst growth	7	0.286302629	0.0001	0.000192793
GO:0014003	oligodendrocyte development	11	0.286275886	< 0.001	< 0.001
GO:0033182	regulation of histone ubiquitination	6	0.286229086	0.0001	0.000192793
GO:0050730	regulation of peptidyl-tyrosine phosphorylation	85	0.286229086	< 0.001	< 0.001
GO:0035304	regulation of protein dephosphorylation	74	0.28615604	0.0003	0.000505993
GO:0007584	response to nutrient	51	0.286143286	< 0.001	< 0.001
GO:0031329	regulation of cellular catabolic process	157	0.286140554	< 0.001	< 0.001
GO:0015931	nucleobase-containing compound transport	82	0.286128637	< 0.001	< 0.001
GO:0001658	branching involved in ureteric bud morphogenesis	15	0.286091806	0.0001	0.000192793
GO:1903532	positive regulation of secretion by cell	154	0.286061943	< 0.001	< 0.001
GO:0014741	negative regulation of muscle hypertrophy	11	0.286041886	0.0001	0.000192793
GO:0034389	lipid particle organization	8	0.286036036	0.0009	0.001319479
GO:0098974	postsynaptic actin cytoskeleton organization	5	0.286023166	0.0035	0.004390436
GO:0034766	negative regulation of ion transmembrane transport	32	0.286019949	< 0.001	< 0.001
GO:0032655	regulation of interleukin-12 production	17	0.28601711	< 0.001	< 0.001
GO:0051452	intracellular pH reduction	22	0.285971686	< 0.001	< 0.001
GO:1903321	negative regulation of protein modification by small protein conjugation or removal	42	0.285965557	0.0001	0.000192793
GO:0007413	axonal fasciculation	16	0.285955598	0.0001	0.000192793
GO:0010948	negative regulation of cell cycle process	135	0.285937366	0.0001	0.000192793
GO:2000241	regulation of reproductive process	42	0.2858675	< 0.001	< 0.001
GO:0022412	cellular process involved in reproduction in multicellular organism	86	0.285848972	< 0.001	< 0.001
GO:0071375	cellular response to peptide hormone stimulus	123	0.285816827	< 0.001	< 0.001
GO:0006757	ATP generation from ADP	42	0.2857817	< 0.001	< 0.001
GO:0031349	positive regulation of defense response	180	0.285727156	< 0.001	< 0.001
GO:0001738	morphogenesis of a polarized epithelium	80	0.285698198	< 0.001	< 0.001
GO:0043254	regulation of protein complex assembly	176	0.285680648	< 0.001	< 0.001
GO:0046651	lymphocyte proliferation	70	0.285666483	< 0.001	< 0.001
GO:0071361	cellular response to ethanol	7	0.285603971	0.0003	0.000505993
GO:0060065	uterus development	7	0.285603971	0.0166	0.018341116
GO:0015698	inorganic anion transport	49	0.285588212	< 0.001	< 0.001
GO:0048821	erythrocyte development	11	0.285573886	0.0005	0.000791911
GO:0030049	muscle filament sliding	16	0.285505148	< 0.001	< 0.001
GO:0033275	actin-myosin filament sliding	16	0.285505148	< 0.001	< 0.001
GO:0072028	nephron morphogenesis	23	0.285456885	< 0.001	< 0.001
GO:0023061	signal release	189	0.285429647	0.0001	0.000192793
GO:0046879	hormone secretion	110	0.285410085	< 0.001	< 0.001
GO:0071280	cellular response to copper ion	9	0.285399685	0.0013	0.001826625
GO:0070071	proton-transporting two-sector ATPase complex assembly	6	0.285242385	0.0062	0.00739947
GO:0006688	glycosphingolipid biosynthetic process	12	0.285242385	0.0073	0.008600218
GO:0031644	regulation of neurological system process	26	0.285209385	< 0.001	< 0.001
GO:0050663	cytokine secretion	67	0.285191802	< 0.001	< 0.001
GO:0030888	regulation of B cell proliferation	15	0.285182325	< 0.001	< 0.001
GO:0046148	pigment biosynthetic process	27	0.285170885	< 0.001	< 0.001
GO:0022407	regulation of cell-cell adhesion	140	0.285164552	< 0.001	< 0.001
GO:0046466	membrane lipid catabolic process	7	0.285162714	< 0.001	< 0.001
GO:0055012	ventricular cardiac muscle cell differentiation	5	0.285148005	0.0037	0.004617737
GO:0010742	macrophage derived foam cell differentiation	7	0.285089171	0.0083	0.009660392
GO:0090077	foam cell differentiation	7	0.285089171	0.0083	0.009660392
GO:0007269	neurotransmitter secretion	87	0.285019009	0.006	0.007178216
GO:0099643	signal release from synapse	87	0.285019009	0.006	0.007178216
GO:1904029	regulation of cyclin-dependent protein kinase activity	39	0.284961885	< 0.001	< 0.001
GO:0051289	protein homotetramerization	33	0.284934285	< 0.001	< 0.001
GO:0072078	nephron tubule morphogenesis	21	0.284929828	< 0.001	< 0.001
GO:1903214	regulation of protein targeting to mitochondrion	27	0.284923018	0.0001	0.000192793
GO:0051281	positive regulation of release of sequestered calcium ion into cytosol	17	0.284896661	0.0023	0.003013139

GOIDlabel	GOName	size	categoryScores	pValPerm	pValPermCorr
GO:0046939	nucleotide phosphorylation	50	0.284859717	< 0.001	< 0.001
GO:0002089	lens morphogenesis in camera-type eye	6	0.284770485	< 0.001	< 0.001
GO:0007160	cell-matrix adhesion	82	0.284766299	< 0.001	< 0.001
GO:1905330	regulation of morphogenesis of an epithelium	85	0.284748278	< 0.001	< 0.001
GO:0018242	protein O-linked glycosylation via serine	5	0.284736165	0.006	0.007178216
GO:0070228	regulation of lymphocyte apoptotic process	14	0.28470307	< 0.001	< 0.001
GO:0001952	regulation of cell-matrix adhesion	47	0.284701114	< 0.001	< 0.001
GO:0030073	insulin secretion	77	0.28469137	< 0.001	< 0.001
GO:0046474	glycerophospholipid biosynthetic process	90	0.284681825	< 0.001	< 0.001
GO:0001649	osteoblast differentiation	74	0.284681206	< 0.001	< 0.001
GO:0043966	histone H3 acetylation	26	0.284674785	< 0.001	< 0.001
GO:0001818	negative regulation of cytokine production	85	0.284669543	< 0.001	< 0.001
GO:1901988	negative regulation of cell cycle phase transition	101	0.284664297	0.0001	0.000192793
GO:0050673	epithelial cell proliferation	136	0.284631691	< 0.001	< 0.001
GO:0030878	thyroid gland development	9	0.284627485	0.0013	0.001826625
GO:0008053	mitochondrial fusion	11	0.284567685	< 0.001	< 0.001
GO:0032611	interleukin-1 beta production	29	0.284560422	< 0.001	< 0.001
GO:0030833	regulation of actin filament polymerization	63	0.284500827	< 0.001	< 0.001
GO:0051973	positive regulation of telomerase activity	16	0.284459459	< 0.001	< 0.001
GO:0045589	regulation of regulatory T cell differentiation	5	0.284427284	0.0004	0.000648934
GO:0051568	histone H3-K4 methylation	23	0.284427284	0.0001	0.000192793
GO:0051893	regulation of focal adhesion assembly	29	0.284427284	< 0.001	< 0.001
GO:0090109	regulation of cell-substrate junction assembly	29	0.284427284	< 0.001	< 0.001
GO:0032330	regulation of chondrocyte differentiation	15	0.284410124	< 0.001	< 0.001
GO:0043044	ATP-dependent chromatin remodeling	25	0.284406692	0.0035	0.004390436
GO:1902514	regulation of calcium ion transmembrane transport via high voltage-gated calcium channel	7	0.284390513	0.0121	0.013633945
GO:0051965	positive regulation of synapse assembly	33	0.284349284	0.002	0.002664694
GO:0032309	icosanoid secretion	14	0.284335356	< 0.001	< 0.001
GO:0071715	icosanoid transport	14	0.284335356	< 0.001	< 0.001
GO:1901571	fatty acid derivative transport	14	0.284335356	< 0.001	< 0.001
GO:0006029	proteoglycan metabolic process	36	0.284291434	< 0.001	< 0.001
GO:0010575	positive regulation of vascular endothelial growth factor production	7	0.284243427	< 0.001	< 0.001
GO:0006289	nucleotide-excision repair	53	0.28423302	0.0001	0.000192793
GO:0010001	glial cell differentiation	62	0.284228007	< 0.001	< 0.001
GO:0070665	positive regulation of leukocyte proliferation	37	0.284225538	< 0.001	< 0.001
GO:0034199	activation of protein kinase A activity	11	0.284216684	< 0.001	< 0.001
GO:0006929	substrate-dependent cell migration	10	0.284195624	< 0.001	< 0.001
GO:0050680	negative regulation of epithelial cell proliferation	50	0.284175032	< 0.001	< 0.001
GO:2001044	regulation of integrin-mediated signaling pathway	5	0.284169884	0.0248	0.026750339
GO:1901031	regulation of response to reactive oxygen species	10	0.284144144	< 0.001	< 0.001
GO:1902749	regulation of cell cycle G2/M phase transition	112	0.28404578	< 0.001	< 0.001
GO:1902036	regulation of hematopoietic stem cell differentiation	42	0.284004413	0.0008	0.001193265
GO:0050691	regulation of defense response to virus by host	9	0.283941084	0.0007	0.001063182
GO:1901992	positive regulation of mitotic cell cycle phase transition	31	0.283937394	0.0002	0.000356582
GO:0060325	face morphogenesis	7	0.283912484	0.0004	0.000648934
GO:0070571	negative regulation of neuron projection regeneration	8	0.283912484	0.0334	0.035446504
GO:1903828	negative regulation of cellular protein localization	60	0.283895324	< 0.001	< 0.001
GO:0036296	response to increased oxygen levels	12	0.283891034	0.0001	0.000192793
GO:0099625	ventricular cardiac muscle cell membrane repolarization	9	0.283855284	0.0002	0.000356582
GO:0007569	cell aging	48	0.283853496	< 0.001	< 0.001
GO:0032615	interleukin-12 production	18	0.283840984	< 0.001	< 0.001
GO:0009713	catechol-containing compound biosynthetic process	9	0.283826684	< 0.001	< 0.001
GO:0042423	catecholamine biosynthetic process	9	0.283826684	< 0.001	< 0.001
GO:0015696	ammonium transport	28	0.28380217	< 0.001	< 0.001
GO:1901991	negative regulation of mitotic cell cycle phase transition	94	0.283759139	0.0001	0.000192793
GO:0019722	calcium-mediated signaling	75	0.283754612	< 0.001	< 0.001
GO:0097421	liver regeneration	7	0.283728627	0.0042	0.005168936
GO:0006820	anion transport	186	0.283709055	< 0.001	< 0.001
GO:0042267	natural killer cell mediated cytotoxicity	16	0.283687259	< 0.001	< 0.001
GO:0009205	purine ribonucleoside triphosphate metabolic process	115	0.283641654	0.0001	0.000192793
GO:1902570	protein localization to nucleolus	6	0.283612184	0.0002	0.000356582
GO:0006779	porphyrin-containing compound biosynthetic process	9	0.283597884	0.0015	0.002069969
GO:0006783	heme biosynthetic process	9	0.283597884	0.0015	0.002069969
GO:0001767	establishment of lymphocyte polarity	6	0.283569284	0.0004	0.000648934
GO:0001541	ovarian follicle development	13	0.283556084	0.0017	0.00231289
GO:0050806	positive regulation of synaptic transmission	89	0.28354229	0.0031	0.003925117
GO:0032944	regulation of mononuclear cell proliferation	53	0.283528812	< 0.001	< 0.001
GO:0002062	chondrocyte differentiation	35	0.283522706	< 0.001	< 0.001
GO:0097194	execution phase of apoptosis	24	0.283494208	0.0002	0.000356582
GO:1902882	regulation of response to oxidative stress	28	0.283480419	< 0.001	< 0.001
GO:2000779	regulation of double-strand break repair	32	0.283437902	< 0.001	< 0.001
GO:0071548	response to dexamethasone	13	0.283437283	< 0.001	< 0.001
GO:1900182	positive regulation of protein localization to nucleus	30	0.283414843	0.0001	0.000192793
GO:0019372	lipoxygenase pathway	5	0.283397683	0.0001	0.000192793
GO:0009048	dosage compensation by inactivation of X chromosome	6	0.283397683	0.0037	0.004617737
GO:0031345	negative regulation of cell projection organization	82	0.283360015	0.0008	0.001193265
GO:0060712	spongiotrophoblast layer development	5	0.283346203	< 0.001	< 0.001
GO:0007044	cell-substrate junction assembly	39	0.283344883	< 0.001	< 0.001
GO:0043568	positive regulation of insulin-like growth factor receptor signaling pathway	5	0.283294723	0.0208	0.022657367
GO:0060749	mammary gland alveolus development	5	0.283294723	< 0.001	< 0.001
GO:0061377	mammary gland lobule development	5	0.283294723	< 0.001	< 0.001
GO:2000479	regulation of cAMP-dependent protein kinase activity	11	0.283280683	< 0.001	< 0.001
GO:0042180	cellular ketone metabolic process	108	0.283264217	< 0.001	< 0.001

GOIDlabel	GOName	size	categoryScores	pValPerm	pValPermCorr
GO:1900015	regulation of cytokine production involved in inflammatory response	16	0.283252896	< 0.001	< 0.001
GO:0046887	positive regulation of hormone secretion	41	0.283221898	< 0.001	< 0.001
GO:0002886	regulation of myeloid leukocyte mediated immunity	19	0.283221567	< 0.001	< 0.001
GO:0090189	regulation of branching involved in ureteric bud morphogenesis	6	0.283183183	0.0008	0.001193265
GO:0090190	positive regulation of branching involved in ureteric bud morphogenesis	6	0.283183183	0.0008	0.001193265
GO:0070987	error-free translesion synthesis	9	0.283168883	0.0003	0.000505993
GO:0048284	organelle fusion	59	0.283166459	< 0.001	< 0.001
GO:0043455	regulation of secondary metabolic process	10	0.283166023	0.0003	0.000505993
GO:0007266	Rho protein signal transduction	72	0.283147433	< 0.001	< 0.001
GO:0071549	cellular response to dexamethasone stimulus	9	0.283083083	0.0002	0.000356582
GO:0050954	sensory perception of mechanical stimulus	59	0.283061754	< 0.001	< 0.001
GO:0007283	spermatogenesis	167	0.283004647	< 0.001	< 0.001
GO:0098656	anion transmembrane transport	65	0.283001683	< 0.001	< 0.001
GO:0010676	positive regulation of cellular carbohydrate metabolic process	25	0.282996139	< 0.001	< 0.001
GO:0036159	inner dynein arm assembly	7	0.282993197	0.0477	0.049647689
GO:1901565	organonitrogen compound catabolic process	115	0.282985843	< 0.001	< 0.001
GO:0043967	histone H4 acetylation	27	0.282968683	< 0.001	< 0.001
GO:0048259	regulation of receptor-mediated endocytosis	44	0.282947233	< 0.001	< 0.001
GO:0030202	heparin metabolic process	6	0.282925783	0.0021	0.002780108
GO:0009144	purine nucleoside triphosphate metabolic process	118	0.282922147	0.0001	0.000192793
GO:0043401	steroid hormone mediated signaling pathway	64	0.282858752	< 0.001	< 0.001
GO:0032943	mononuclear cell proliferation	71	0.282810376	< 0.001	< 0.001
GO:0048015	phosphatidylinositol-mediated signaling	75	0.282745603	< 0.001	< 0.001
GO:2000142	regulation of DNA-templated transcription, initiation	12	0.282732733	0.0005	0.000791911
GO:0043516	regulation of DNA damage response, signal transduction by p53 class mediator	15	0.282728443	< 0.001	< 0.001
GO:0048659	smooth muscle cell proliferation	54	0.282682683	< 0.001	< 0.001
GO:0017157	regulation of exocytosis	99	0.282659283	0.0001	0.000192793
GO:0015833	peptide transport	98	0.282643868	< 0.001	< 0.001
GO:0035195	gene silencing by miRNA	50	0.282620335	< 0.001	< 0.001
GO:0016042	lipid catabolic process	83	0.282594471	< 0.001	< 0.001
GO:1903977	positive regulation of glial cell migration	5	0.282574003	0.0203	0.022152908
GO:0030518	intracellular steroid hormone receptor signaling pathway	47	0.282521427	< 0.001	< 0.001
GO:0016241	regulation of macroautophagy	91	0.282520825	< 0.001	< 0.001
GO:0035019	somatic stem cell population maintenance	28	0.282496782	< 0.001	< 0.001
GO:0090066	regulation of anatomical structure size	194	0.282464939	< 0.001	< 0.001
GO:0048002	antigen processing and presentation of peptide antigen	89	0.282457739	0.0002	0.000356582
GO:0031998	regulation of fatty acid beta-oxidation	6	0.282410982	0.0002	0.000356582
GO:1901653	cellular response to peptide	148	0.282376778	< 0.001	< 0.001
GO:0019369	arachidonic acid metabolic process	11	0.282368082	< 0.001	< 0.001
GO:0006140	regulation of nucleotide metabolic process	70	0.282334988	< 0.001	< 0.001
GO:1905037	autophagosome organization	39	0.282295482	0.0001	0.000192793
GO:0035308	negative regulation of protein dephosphorylation	35	0.282243059	0.0022	0.002896881
GO:1900244	positive regulation of synaptic vesicle endocytosis	6	0.282239382	0.0187	0.020510412
GO:0070588	calcium ion transmembrane transport	110	0.282157482	< 0.001	< 0.001
GO:0050729	positive regulation of inflammatory response	38	0.282110682	< 0.001	< 0.001
GO:0019896	axonal transport of mitochondrion	5	0.282110682	0.0003	0.000505993
GO:0060142	regulation of syncytium formation by plasma membrane fusion	6	0.282067782	0.0006	0.000927725
GO:0060143	positive regulation of syncytium formation by plasma membrane fusion	6	0.282067782	0.0006	0.000927725
GO:0002821	positive regulation of adaptive immune response	26	0.282021582	0.0001	0.000192793
GO:0048661	positive regulation of smooth muscle cell proliferation	36	0.281989132	< 0.001	< 0.001
GO:0006163	purine nucleotide metabolic process	182	0.281936725	< 0.001	< 0.001
GO:0099054	presynapse assembly	25	0.281904762	0.0009	0.001319479
GO:0009266	response to temperature stimulus	93	0.281886495	< 0.001	< 0.001
GO:2000379	positive regulation of reactive oxygen species metabolic process	32	0.281861326	< 0.001	< 0.001
GO:0097553	calcium ion transmembrane import into cytosol	56	0.281857878	0.0011	0.001576346
GO:1902656	calcium ion import into cytosol	56	0.281857878	0.0011	0.001576346
GO:0003214	cardiac left ventricle morphogenesis	6	0.281853282	0.0395	0.041520716
GO:2000134	negative regulation of G1/S transition of mitotic cell cycle	32	0.281845238	< 0.001	< 0.001
GO:0002902	regulation of B cell apoptotic process	6	0.281810382	< 0.001	< 0.001
GO:0006376	mRNA splice site selection	10	0.281801802	0.001	0.001450349
GO:0035115	embryonic forelimb morphogenesis	9	0.281738882	0.0001	0.000192793
GO:0045665	negative regulation of neuron differentiation	97	0.281683451	< 0.001	< 0.001
GO:0034605	cellular response to heat	56	0.281674021	< 0.001	< 0.001
GO:0036473	cell death in response to oxidative stress	35	0.28166207	< 0.001	< 0.001
GO:0048663	neuron fate commitment	19	0.281609429	0.0009	0.001319479
GO:0048016	inositol phosphate-mediated signaling	25	0.281606178	< 0.001	< 0.001
GO:0071156	regulation of cell cycle arrest	38	0.281595882	< 0.001	< 0.001
GO:0019886	antigen processing and presentation of exogenous peptide antigen via MHC class II	45	0.281584442	< 0.001	< 0.001
GO:0001516	prostaglandin biosynthetic process	12	0.281552982	0.0002	0.000356582
GO:0046457	prostanoid biosynthetic process	12	0.281552982	0.0002	0.000356582
GO:0008608	attachment of spindle microtubules to kinetochore	11	0.281549082	< 0.001	< 0.001
GO:2001056	positive regulation of cysteine-type endopeptidase activity	51	0.281520176	< 0.001	< 0.001
GO:0032802	low-density lipoprotein particle receptor catabolic process	10	0.281441441	0.0056	0.006732458
GO:0007628	adult walking behavior	11	0.281432081	0.0014	0.00194839
GO:0090659	walking behavior	11	0.281432081	0.0014	0.00194839
GO:0001764	neuron migration	77	0.281428739	0.0001	0.000192793
GO:0090312	positive regulation of protein deacetylation	9	0.281424281	0.0016	0.002186748
GO:0048232	male gamete generation	168	0.281422749	< 0.001	< 0.001
GO:2000177	regulation of neural precursor cell proliferation	31	0.281404907	0.0001	0.000192793
GO:0002478	antigen processing and presentation of exogenous peptide antigen	84	0.281390574	0.0002	0.000356582
GO:0035194	posttranscriptional gene silencing by RNA	53	0.281377334	< 0.001	< 0.001
GO:0010977	negative regulation of neuron projection development	69	0.281372055	0.0006	0.000927725
GO:0051053	negative regulation of DNA metabolic process	54	0.281371848	0.0004	0.000648934

GOIDlabel	GOName	size	categoryScores	pValPerm	pValPermCorr
GO:1902166	negative regulation of intrinsic apoptotic signaling pathway in response to DNA damage by p53 class mediator	8	0.281370656	0.0409	0.042933877
GO:0006360	transcription from RNA polymerase I promoter	33	0.281307281	0.0001	0.000192793
GO:0016236	macroautophagy	152	0.281294452	0.0002	0.000356582
GO:0032946	positive regulation of mononuclear cell proliferation	36	0.281288431	< 0.001	< 0.001
GO:0006970	response to osmotic stress	30	0.281261261	< 0.001	< 0.001
GO:0045061	thymic T cell selection	5	0.281235521	0.0108	0.012299937
GO:1903362	regulation of cellular protein catabolic process	103	0.281231023	< 0.001	< 0.001
GO:0006809	nitric oxide biosynthetic process	23	0.281215377	0.0002	0.000356582
GO:0009952	anterior/posterior pattern specification	54	0.281176415	< 0.001	< 0.001
GO:0002479	antigen processing and presentation of exogenous peptide antigen via MHC class I, TAP-dependent	38	0.281175913	0.0014	0.00194839
GO:0042590	antigen processing and presentation of exogenous peptide antigen via MHC class I	39	0.281166881	0.0015	0.002069969
GO:0034763	negative regulation of transmembrane transport	36	0.281109681	< 0.001	< 0.001
GO:0046627	negative regulation of insulin receptor signaling pathway	11	0.281104481	0.0048	0.005840847
GO:0016180	snRNA processing	5	0.281081081	0.0002	0.000356582
GO:0061615	glycolytic process through fructose-6-phosphate	14	0.281081081	0.001	0.001450349
GO:0061620	glycolytic process through glucose-6-phosphate	14	0.281081081	0.001	0.001450349
GO:0051701	interaction with host	75	0.281067353	< 0.001	< 0.001
GO:0044070	regulation of anion transport	36	0.281066781	< 0.001	< 0.001
GO:0002790	peptide secretion	95	0.281040439	< 0.001	< 0.001
GO:0045684	positive regulation of epidermis development	11	0.281034281	< 0.001	< 0.001
GO:0032515	negative regulation of phosphoprotein phosphatase activity	31	0.281014655	0.0038	0.004725081
GO:0097066	response to thyroid hormone	6	0.280995281	0.0042	0.005168936
GO:0045862	positive regulation of proteolysis	131	0.280986766	< 0.001	< 0.001
GO:0070498	interleukin-1-mediated signaling pathway	54	0.280985748	0.0006	0.000927725
GO:0045661	regulation of myoblast differentiation	21	0.280983024	< 0.001	< 0.001
GO:0097305	response to alcohol	63	0.280974852	< 0.001	< 0.001
GO:0006650	glycerophospholipid metabolic process	125	0.280963707	< 0.001	< 0.001
GO:0071621	granulocyte chemotaxis	27	0.280947614	< 0.001	< 0.001
GO:0007281	germ cell development	71	0.280943318	< 0.001	< 0.001
GO:0051580	regulation of neurotransmitter uptake	9	0.280938081	0.0076	0.008916717
GO:0051310	metaphase plate congression	25	0.280936937	0.0002	0.000356582
GO:0009798	axis specification	25	0.280916345	< 0.001	< 0.001
GO:0051926	negative regulation of calcium ion transport	22	0.280870481	< 0.001	< 0.001
GO:1904706	negative regulation of vascular smooth muscle cell proliferation	7	0.280860452	0.0043	0.005276385
GO:0042493	response to drug	107	0.280859765	< 0.001	< 0.001
GO:0002709	regulation of T cell mediated immunity	12	0.280845131	0.0002	0.000356582
GO:0050920	regulation of chemotaxis	73	0.280844837	< 0.001	< 0.001
GO:0090398	cellular senescence	34	0.280831251	0.0004	0.000648934
GO:0071322	cellular response to carbohydrate stimulus	62	0.280827832	< 0.001	< 0.001
GO:0006733	oxidoreduction coenzyme metabolic process	71	0.280827306	< 0.001	< 0.001
GO:0035372	protein localization to microtubule	6	0.280823681	0.0206	0.022453086
GO:0032675	regulation of interleukin-6 production	37	0.280802811	< 0.001	< 0.001
GO:0003013	circulatory system process	174	0.280800012	< 0.001	< 0.001
GO:0031498	chromatin disassembly	8	0.280791506	0.0233	0.025253349
GO:0070555	response to interleukin-1	82	0.280767178	0.0002	0.000356582
GO:0042472	inner ear morphogenesis	27	0.280718814	< 0.001	< 0.001
GO:0010639	negative regulation of organelle organization	139	0.280705166	< 0.001	< 0.001
GO:0035051	cardiocyte differentiation	49	0.280697607	< 0.001	< 0.001
GO:0035767	endothelial cell chemotaxis	10	0.280694981	0.0025	0.003237479
GO:1902186	regulation of viral release from host cell	14	0.280694981	0.0003	0.000505993
GO:0060828	regulation of canonical Wnt signaling pathway	116	0.280690543	< 0.001	< 0.001
GO:0051881	regulation of mitochondrial membrane potential	25	0.280689833	0.0001	0.000192793
GO:0045191	regulation of isotype switching	13	0.280665281	0.0056	0.006732458
GO:0071229	cellular response to acid chemical	74	0.280614978	< 0.001	< 0.001
GO:1904950	negative regulation of establishment of protein localization	87	0.280586991	< 0.001	< 0.001
GO:0006090	pyruvate metabolic process	52	0.280586081	< 0.001	< 0.001
GO:0048660	regulation of smooth muscle cell proliferation	53	0.280585707	< 0.001	< 0.001
GO:0042402	cellular biogenic amine catabolic process	9	0.280566281	0.0002	0.000356582
GO:0044342	type B pancreatic cell proliferation	8	0.280534106	0.0101	0.011583277
GO:0016050	vesicle organization	139	0.280523689	< 0.001	< 0.001
GO:0006283	transcription-coupled nucleotide-excision repair	39	0.280506881	0.0006	0.000927725
GO:0006302	double-strand break repair	80	0.280501931	< 0.001	< 0.001
GO:0006007	glucose catabolic process	15	0.28049764	0.0021	0.002780108
GO:0032844	regulation of homeostatic process	181	0.28044398	< 0.001	< 0.001
GO:0051131	chaperone-mediated protein complex assembly	8	0.28043758	0.0003	0.000505993
GO:0034381	plasma lipoprotein particle clearance	17	0.280414869	0.0002	0.000356582
GO:0033046	negative regulation of sister chromatid segregation	16	0.280405405	0.0012	0.001702047
GO:0033048	negative regulation of mitotic sister chromatid segregation	16	0.280405405	0.0012	0.001702047
GO:0051985	negative regulation of chromosome segregation	16	0.280405405	0.0012	0.001702047
GO:0050852	T cell receptor signaling pathway	80	0.280379665	0.0002	0.000356582
GO:0007599	hemostasis	126	0.280368123	< 0.001	< 0.001
GO:0034968	histone lysine methylation	46	0.280342454	0.0003	0.000505993
GO:0046386	deoxyribose phosphate catabolic process	8	0.280341055	0.0002	0.000356582
GO:0043551	regulation of phosphatidylinositol 3-kinase activity	21	0.280333395	< 0.001	< 0.001
GO:0001505	regulation of neurotransmitter levels	109	0.280320688	0.0009	0.001319479
GO:0007612	learning	79	0.28030888	0.0035	0.004390436
GO:1901214	regulation of neuron death	142	0.280303442	< 0.001	< 0.001
GO:0042471	ear morphogenesis	36	0.28030173	< 0.001	< 0.001
GO:2001233	regulation of apoptotic signaling pathway	154	0.280298852	< 0.001	< 0.001
GO:0006949	syncytium formation	16	0.280292793	0.0098	0.011263122
GO:0048732	gland development	141	0.280286974	< 0.001	< 0.001
GO:0002638	negative regulation of immunoglobulin production	5	0.2802574	0.0036	0.004505419

GOIDlabel	GOName	size	categoryScores	pValPerm	pValPermCorr
GO:0050000	chromosome localization	31	0.280209241	0.0001	0.000192793
GO:0051303	establishment of chromosome localization	31	0.280209241	0.0001	0.000192793
GO:0061577	calcium ion transmembrane transport via high voltage-gated calcium channel	8	0.28018018	0.0195	0.021348865
GO:0007015	actin filament organization	154	0.28015678	< 0.001	< 0.001
GO:0051660	establishment of centrosome localization	6	0.28013728	0.0136	0.015222535
GO:1900029	positive regulation of ruffle assembly	7	0.280125023	0.0003	0.000505993
GO:0032387	negative regulation of intracellular transport	42	0.280118894	< 0.001	< 0.001
GO:0009199	ribonucleoside triphosphate metabolic process	117	0.28009768	0.0001	0.000192793
GO:0007045	cell-substrate adherens junction assembly	37	0.280086264	< 0.001	< 0.001
GO:0048041	focal adhesion assembly	37	0.280086264	< 0.001	< 0.001
GO:2000778	positive regulation of interleukin-6 secretion	8	0.280083655	0.0016	0.002186748
GO:0045216	cell-cell junction organization	94	0.280056957	< 0.001	< 0.001
GO:0042276	error-prone translesion synthesis	9	0.28005148	0.0073	0.008600218
GO:0032507	maintenance of protein location in cell	36	0.28004433	< 0.001	< 0.001
GO:0030072	peptide hormone secretion	93	0.280029338	< 0.001	< 0.001
GO:0090279	regulation of calcium ion import	51	0.279945492	0.0009	0.001319479
GO:1904705	regulation of vascular smooth muscle cell proliferation	23	0.279928376	< 0.001	< 0.001
GO:1990874	vascular smooth muscle cell proliferation	23	0.279928376	< 0.001	< 0.001
GO:0015909	long-chain fatty acid transport	21	0.279904394	< 0.001	< 0.001
GO:0070252	actin-mediated cell contraction	44	0.27989353	< 0.001	< 0.001
GO:0016579	protein deubiquitination	148	0.279870604	0.0001	0.000192793
GO:0034080	CENP-A containing nucleosome assembly	13	0.27983368	0.0141	0.015736555
GO:0061641	CENP-A containing chromatin organization	13	0.27983368	0.0141	0.015736555
GO:0002532	production of molecular mediator involved in inflammatory response	23	0.279749315	< 0.001	< 0.001
GO:1903556	negative regulation of tumor necrosis factor superfamily cytokine production	21	0.279745051	< 0.001	< 0.001
GO:0030178	negative regulation of Wnt signaling pathway	86	0.279737212	< 0.001	< 0.001
GO:0009886	post-embryonic animal morphogenesis	5	0.27969112	0.0002	0.000356582
GO:0032703	negative regulation of interleukin-2 production	8	0.27966538	< 0.001	< 0.001
GO:1905332	positive regulation of morphogenesis of an epithelium	9	0.27965108	0.0004	0.000648934
GO:0090317	negative regulation of intracellular protein transport	32	0.279641248	< 0.001	< 0.001
GO:0000041	transition metal ion transport	35	0.27963964	< 0.001	< 0.001
GO:0070997	neuron death	154	0.279636965	< 0.001	< 0.001
GO:0018958	phenol-containing compound metabolic process	42	0.279628608	< 0.001	< 0.001
GO:0019884	antigen processing and presentation of exogenous antigen	85	0.279615414	0.0002	0.000356582
GO:0033628	regulation of cell adhesion mediated by integrin	19	0.27953668	< 0.001	< 0.001
GO:1904031	positive regulation of cyclin-dependent protein kinase activity	17	0.27953668	0.0003	0.000505993
GO:1904707	positive regulation of vascular smooth muscle cell proliferation	16	0.279520592	< 0.001	< 0.001
GO:0010800	positive regulation of peptidyl-threonine phosphorylation	18	0.27950808	< 0.001	< 0.001
GO:1904814	regulation of protein localization to chromosome, telomeric region	7	0.279499908	0.0026	0.003356522
GO:0031331	positive regulation of cellular catabolic process	100	0.279472329	< 0.001	< 0.001
GO:0060402	calcium ion transport into cytosol	63	0.279434537	0.0008	0.001193265
GO:2000300	regulation of synaptic vesicle exocytosis	43	0.279387028	0.0055	0.006618125
GO:0071352	cellular response to interleukin-2	5	0.279382239	< 0.001	< 0.001
GO:0038095	Fc-epsilon receptor signaling pathway	69	0.279353888	0.0002	0.000356582
GO:0045667	regulation of osteoblast differentiation	42	0.279328308	< 0.001	< 0.001
GO:0046128	purine ribonucleoside metabolic process	143	0.279309879	0.0001	0.000192793
GO:0021537	telencephalon development	116	0.27929925	< 0.001	< 0.001
GO:0043550	regulation of lipid kinase activity	24	0.279290004	0.0001	0.000192793
GO:0072332	intrinsic apoptotic signaling pathway by p53 class mediator	29	0.279288155	0.0014	0.00194839
GO:1900077	negative regulation of cellular response to insulin stimulus	12	0.279257829	0.002	0.002664694
GO:0060412	ventricular septum morphogenesis	17	0.279248997	< 0.001	< 0.001
GO:0090090	negative regulation of canonical Wnt signaling pathway	77	0.279182336	0.0001	0.000192793
GO:0036507	protein demannosylation	12	0.279150579	< 0.001	< 0.001
GO:0036508	protein alpha-1,2-demannosylation	12	0.279150579	< 0.001	< 0.001
GO:1903749	positive regulation of establishment of protein localization to mitochondrion	36	0.279150579	< 0.001	< 0.001
GO:0071383	cellular response to steroid hormone stimulus	80	0.279140927	< 0.001	< 0.001
GO:1904062	regulation of cation transmembrane transport	125	0.279106306	0.0012	0.001702047
GO:2000116	regulation of cysteine-type endopeptidase activity	84	0.279034136	< 0.001	< 0.001
GO:0044773	mitotic DNA damage checkpoint	30	0.278996139	0.0006	0.000927725
GO:0044774	mitotic DNA integrity checkpoint	30	0.278996139	0.0006	0.000927725
GO:0060218	hematopoietic stem cell differentiation	44	0.278992629	0.0008	0.001193265
GO:0033005	positive regulation of mast cell activation	6	0.278978979	0.0003	0.000505993
GO:0007141	male meiosis I	5	0.278918919	0.0001	0.000192793
GO:0022038	corpus callosum development	12	0.278893179	0.0005	0.000791911
GO:0009150	purine ribonucleotide metabolic process	176	0.278828829	< 0.001	< 0.001
GO:0006513	protein monoubiquitination	35	0.278815959	< 0.001	< 0.001
GO:0044783	G1 DNA damage checkpoint	21	0.27880125	0.0027	0.003470695
GO:0030522	intracellular receptor signaling pathway	98	0.27874084	< 0.001	< 0.001
GO:0060259	regulation of feeding behavior	10	0.278738739	0.0089	0.010294435
GO:0030433	ER-associated ubiquitin-dependent protein catabolic process	31	0.278714659	0.0001	0.000192793
GO:0014888	striated muscle adaptation	15	0.278712999	< 0.001	< 0.001
GO:0043666	regulation of phosphoprotein phosphatase activity	62	0.278702205	0.0009	0.001319479
GO:1990776	response to angiotensin	11	0.278694279	0.0107	0.012191187
GO:0032612	interleukin-1 production	34	0.278666061	< 0.001	< 0.001
GO:0007034	vacuolar transport	58	0.278649092	< 0.001	< 0.001
GO:0006266	DNA ligation	6	0.278635779	0.0281	0.030129444
GO:0030336	negative regulation of cell migration	111	0.278634619	< 0.001	< 0.001
GO:0003254	regulation of membrane depolarization	13	0.278625879	< 0.001	< 0.001
GO:0070886	positive regulation of calcineurin-NFAT signaling cascade	7	0.278617393	0.0001	0.000192793
GO:0021795	cerebral cortex cell migration	24	0.278603604	0.0009	0.001319479
GO:0007405	neuroblast proliferation	18	0.278592879	0.004	0.004954389
GO:0007596	blood coagulation	124	0.278567277	< 0.001	< 0.001
GO:0050817	coagulation	124	0.278567277	< 0.001	< 0.001

GOIDlabel	GOName	size	categoryScores	pValPerm	pValPermCorr
GO:0008015	blood circulation	172	0.278560953	< 0.001	< 0.001
GO:0018904	ether metabolic process	5	0.278558559	0.0005	0.000791911
GO:0002695	negative regulation of leukocyte activation	47	0.278556368	< 0.001	< 0.001
GO:1901889	negative regulation of cell junction assembly	11	0.278530479	< 0.001	< 0.001
GO:0045581	negative regulation of T cell differentiation	12	0.278528529	0.0005	0.000791911
GO:0051154	negative regulation of striated muscle cell differentiation	10	0.278507079	0.0002	0.000356582
GO:0032306	regulation of prostaglandin secretion	5	0.278507079	< 0.001	< 0.001
GO:0032308	positive regulation of prostaglandin secretion	5	0.278507079	< 0.001	< 0.001
GO:0051101	regulation of DNA binding	45	0.278501359	< 0.001	< 0.001
GO:0006338	chromatin remodeling	53	0.278443943	0.0009	0.001319479
GO:0051224	negative regulation of protein transport	85	0.278422288	< 0.001	< 0.001
GO:0006775	fat-soluble vitamin metabolic process	12	0.278399828	< 0.001	< 0.001
GO:0009141	nucleoside triphosphate metabolic process	124	0.27838253	0.0001	0.000192793
GO:0007223	Wnt signaling pathway, calcium modulating pathway	16	0.278378378	0.0094	0.010831045
GO:0009566	fertilization	54	0.278368845	< 0.001	< 0.001
GO:0045663	positive regulation of myoblast differentiation	11	0.278366678	0.002	0.002664694
GO:0071347	cellular response to interleukin-1	69	0.278331748	0.0003	0.000505993
GO:0043462	regulation of ATPase activity	27	0.278325945	< 0.001	< 0.001
GO:0033280	response to vitamin D	10	0.278275418	< 0.001	< 0.001
GO:0001556	oocyte maturation	7	0.278212907	< 0.001	< 0.001
GO:0090002	establishment of protein localization to plasma membrane	25	0.278198198	< 0.001	< 0.001
GO:0008209	androgen metabolic process	5	0.278198198	0.0081	0.00945
GO:0072659	protein localization to plasma membrane	118	0.278162424	< 0.001	< 0.001
GO:1904035	regulation of epithelial cell apoptotic process	19	0.278141299	< 0.001	< 0.001
GO:0002250	adaptive immune response	96	0.278096847	< 0.001	< 0.001
GO:0060078	regulation of postsynaptic membrane potential	63	0.27808625	0.0036	0.004505419
GO:0050865	regulation of cell activation	170	0.278055871	< 0.001	< 0.001
GO:0051588	regulation of neurotransmitter transport	63	0.278012707	0.0047	0.005730776
GO:0098780	response to mitochondrial depolarisation	13	0.277992278	0.0021	0.002780108
GO:0055123	digestive system development	35	0.277984924	< 0.001	< 0.001
GO:0009063	cellular amino acid catabolic process	39	0.277959278	0.0007	0.001063182
GO:0045930	negative regulation of mitotic cell cycle	130	0.277940798	0.0001	0.000192793
GO:0009135	purine nucleoside diphosphate metabolic process	55	0.277931438	< 0.001	< 0.001
GO:0009179	purine ribonucleoside diphosphate metabolic process	55	0.277931438	< 0.001	< 0.001
GO:0042278	purine nucleoside metabolic process	145	0.277770381	0.0001	0.000192793
GO:0003205	cardiac chamber development	69	0.277749799	< 0.001	< 0.001
GO:0050770	regulation of axonogenesis	87	0.277743754	0.0002	0.000356582
GO:0070646	protein modification by small protein removal	156	0.277687028	0.0001	0.000192793
GO:0051147	regulation of muscle cell differentiation	69	0.277663999	< 0.001	< 0.001
GO:1900542	regulation of purine nucleotide metabolic process	68	0.277647816	< 0.001	< 0.001
GO:0015908	fatty acid transport	31	0.277626936	< 0.001	< 0.001
GO:0036303	lymph vessel morphogenesis	8	0.277606178	0.009	0.010394529
GO:0043547	positive regulation of GTPase activity	146	0.277593836	< 0.001	< 0.001
GO:0031047	gene silencing by RNA	63	0.277567363	< 0.001	< 0.001
GO:0090075	relaxation of muscle	10	0.277554698	0.0002	0.000356582
GO:2000146	negative regulation of cell motility	115	0.277546864	< 0.001	< 0.001
GO:0048841	regulation of axon extension involved in axon guidance	8	0.277541828	< 0.001	< 0.001
GO:1900407	regulation of cellular response to oxidative stress	25	0.277539254	< 0.001	< 0.001
GO:0042100	B cell proliferation	17	0.277538042	< 0.001	< 0.001
GO:0038128	ERBB2 signaling pathway	17	0.277492619	< 0.001	< 0.001
GO:0055006	cardiac cell development	28	0.277459092	0.0001	0.000192793
GO:0001776	leukocyte homeostasis	28	0.277440706	0.0001	0.000192793
GO:0097178	ruffle assembly	19	0.277396193	< 0.001	< 0.001
GO:0046486	glycerolipid metabolic process	147	0.277395179	< 0.001	< 0.001
GO:1905475	regulation of protein localization to membrane	88	0.277389727	< 0.001	< 0.001
GO:0050878	regulation of body fluid levels	183	0.277352294	< 0.001	< 0.001
GO:0048260	positive regulation of receptor-mediated endocytosis	23	0.27733199	0.0006	0.000927725
GO:0009437	carnitine metabolic process	7	0.277330392	0.0016	0.002186748
GO:0090502	RNA phosphodiester bond hydrolysis, endonucleolytic	27	0.277315411	0.001	0.001450349
GO:0061097	regulation of protein tyrosine kinase activity	40	0.277297297	0.0001	0.000192793
GO:0003231	cardiac ventricle development	54	0.277291577	< 0.001	< 0.001
GO:0050851	antigen receptor-mediated signaling pathway	87	0.277225994	0.0001	0.000192793
GO:0036257	multivesicular body organization	17	0.277204936	0.0008	0.001193265
GO:0036258	multivesicular body assembly	17	0.277204936	0.0008	0.001193265
GO:0042110	T cell activation	142	0.277185636	< 0.001	< 0.001
GO:0070489	T cell aggregation	142	0.277185636	< 0.001	< 0.001
GO:0071593	lymphocyte aggregation	142	0.277185636	< 0.001	< 0.001
GO:0050863	regulation of T cell activation	93	0.277175793	< 0.001	< 0.001
GO:1902930	regulation of alcohol biosynthetic process	36	0.277170027	< 0.001	< 0.001
GO:0010959	regulation of metal ion transport	142	0.277158446	< 0.001	< 0.001
GO:0042692	muscle cell differentiation	135	0.277151437	< 0.001	< 0.001
GO:0071897	DNA biosynthetic process	80	0.277129987	0.0002	0.000356582
GO:0016570	histone modification	177	0.277129914	< 0.001	< 0.001
GO:0060560	developmental growth involved in morphogenesis	113	0.277119851	0.0001	0.000192793
GO:0046323	glucose import	25	0.277096525	0.0002	0.000356582
GO:0048568	embryonic organ development	126	0.277083206	< 0.001	< 0.001
GO:0006487	protein N-linked glycosylation	22	0.277079677	< 0.001	< 0.001
GO:0003015	heart process	105	0.27707054	< 0.001	< 0.001
GO:0097164	ammonium ion metabolic process	69	0.277067129	< 0.001	< 0.001
GO:0044273	sulfur compound catabolic process	16	0.277043115	0.0015	0.002069969
GO:2001026	regulation of endothelial cell chemotaxis	8	0.277027027	0.0015	0.002069969
GO:2001028	positive regulation of endothelial cell chemotaxis	8	0.277027027	0.0015	0.002069969
GO:1901989	positive regulation of cell cycle phase transition	35	0.276999448	0.0003	0.000505993

GOIDlabel	GOName	size	categoryScores	pValPerm	pValPermCorr
GO:0043616	keratinocyte proliferation	17	0.276947536	< 0.001	< 0.001
GO:1903319	positive regulation of protein maturation	8	0.276930502	0.0001	0.000192793
GO:0016569	covalent chromatin modification	180	0.276894037	< 0.001	< 0.001
GO:1903035	negative regulation of response to wounding	32	0.276874196	< 0.001	< 0.001
GO:0060119	inner ear receptor cell development	16	0.276850064	< 0.001	< 0.001
GO:0001835	blastocyst hatching	10	0.276833977	< 0.001	< 0.001
GO:0035188	hatching	10	0.276833977	< 0.001	< 0.001
GO:0071684	organism emergence from protective structure	10	0.276833977	< 0.001	< 0.001
GO:0099565	chemical synaptic transmission, postsynaptic	56	0.276815591	0.0028	0.003584743
GO:0043407	negative regulation of MAP kinase activity	34	0.276796124	0.0005	0.000791911
GO:0090313	regulation of protein targeting to membrane	20	0.276782497	< 0.001	< 0.001
GO:0050684	regulation of mRNA processing	39	0.276744877	< 0.001	< 0.001
GO:0034330	cell junction organization	107	0.27673655	< 0.001	< 0.001
GO:0060396	growth hormone receptor signaling pathway	13	0.276725077	0.0002	0.000356582
GO:0002792	negative regulation of peptide secretion	19	0.276718824	0.0019	0.00254969
GO:0090278	negative regulation of peptide hormone secretion	19	0.276718824	0.0019	0.00254969
GO:0034142	toll-like receptor 4 signaling pathway	16	0.276673102	< 0.001	< 0.001
GO:0046033	AMP metabolic process	6	0.276662377	0.0021	0.002780108
GO:0001659	temperature homeostasis	14	0.276631734	< 0.001	< 0.001
GO:1903599	positive regulation of mitophagy	7	0.276631734	0.0003	0.000505993
GO:0016441	posttranscriptional gene silencing	54	0.276609943	< 0.001	< 0.001
GO:0010524	positive regulation of calcium ion transport into cytosol	21	0.276545934	0.0021	0.002780108
GO:0030104	water homeostasis	28	0.276539805	< 0.001	< 0.001
GO:0009303	rRNA transcription	15	0.276533677	0.0002	0.000356582
GO:0002694	regulation of leukocyte activation	156	0.276528727	< 0.001	< 0.001
GO:1903725	regulation of phospholipid metabolic process	36	0.276512227	< 0.001	< 0.001
GO:0006004	fucose metabolic process	8	0.276512227	< 0.001	< 0.001
GO:0045088	regulation of innate immune response	159	0.276483492	< 0.001	< 0.001
GO:2000773	negative regulation of cellular senescence	9	0.276476476	0.0002	0.000356582
GO:0032011	ARF protein signal transduction	10	0.276473616	0.0053	0.006400268
GO:0032012	regulation of ARF protein signal transduction	10	0.276473616	0.0053	0.006400268
GO:0001101	response to acid chemical	121	0.276458513	< 0.001	< 0.001
GO:0032886	regulation of microtubule-based process	93	0.276398057	< 0.001	< 0.001
GO:0043434	response to peptide hormone	162	0.276370021	< 0.001	< 0.001
GO:0035265	organ growth	60	0.276336336	< 0.001	< 0.001
GO:0001508	action potential	48	0.276303089	0.0002	0.000356582
GO:0060348	bone development	67	0.27626347	< 0.001	< 0.001
GO:0048745	smooth muscle tissue development	5	0.276241956	0.0023	0.003013139
GO:0071248	cellular response to metal ion	70	0.276205185	< 0.001	< 0.001
GO:0045851	pH reduction	24	0.276179751	0.0002	0.000356582
GO:0001654	eye development	121	0.276147931	< 0.001	< 0.001
GO:0006937	regulation of muscle contraction	69	0.276119598	< 0.001	< 0.001
GO:0034762	regulation of transmembrane transport	183	0.276117335	0.0003	0.000505993
GO:0042921	glucocorticoid receptor signaling pathway	8	0.276093951	0.0008	0.001193265
GO:0042326	negative regulation of phosphorylation	192	0.276072501	< 0.001	< 0.001
GO:0046329	negative regulation of JNK cascade	19	0.276041455	0.0002	0.000356582
GO:0006260	DNA replication	107	0.276041328	0.0001	0.000192793
GO:0001954	positive regulation of cell-matrix adhesion	20	0.275997426	< 0.001	< 0.001
GO:0002285	lymphocyte activation involved in immune response	43	0.27595702	< 0.001	< 0.001
GO:1903037	regulation of leukocyte cell-cell adhesion	102	0.275940647	< 0.001	< 0.001
GO:0050771	negative regulation of axonogenesis	32	0.275868726	0.0013	0.001826625
GO:0006658	phosphatidylserine metabolic process	9	0.275847276	0.0003	0.000505993
GO:0002244	hematopoietic progenitor cell differentiation	68	0.27579302	0.0001	0.000192793
GO:1901990	regulation of mitotic cell cycle phase transition	190	0.275724446	0.0001	0.000192793
GO:0071326	cellular response to monosaccharide stimulus	58	0.275720055	< 0.001	< 0.001
GO:0071331	cellular response to hexose stimulus	58	0.275720055	< 0.001	< 0.001
GO:0071333	cellular response to glucose stimulus	58	0.275720055	< 0.001	< 0.001
GO:0007263	nitric oxide mediated signal transduction	10	0.275701416	< 0.001	< 0.001
GO:0070374	positive regulation of ERK1 and ERK2 cascade	75	0.275644788	< 0.001	< 0.001
GO:0030838	positive regulation of actin filament polymerization	39	0.275570076	0.0005	0.000791911
GO:0000423	macromitophagy	14	0.27552859	0.0007	0.001063182
GO:0003334	keratinocyte development	5	0.275521236	0.0036	0.004505419
GO:0060271	cilium assembly	151	0.275517144	< 0.001	< 0.001
GO:0072384	organelle transport along microtubule	42	0.275479561	0.0001	0.000192793
GO:0032535	regulation of cellular component size	164	0.275473208	< 0.001	< 0.001
GO:0071872	cellular response to epinephrine stimulus	5	0.275469755	< 0.001	< 0.001
GO:0042990	regulation of transcription factor import into nucleus	9	0.275446875	0.0003	0.000505993
GO:0042991	transcription factor import into nucleus	9	0.275446875	0.0003	0.000505993
GO:0042992	negative regulation of transcription factor import into nucleus	9	0.275446875	0.0003	0.000505993
GO:0042994	cytoplasmic sequestering of transcription factor	9	0.275446875	0.0003	0.000505993
GO:0042063	gliogenesis	100	0.275446589	< 0.001	< 0.001
GO:0061003	positive regulation of dendritic spine morphogenesis	11	0.275441675	0.0015	0.002069969
GO:0032784	regulation of DNA-templated transcription, elongation	17	0.275433417	0.0003	0.000505993
GO:0031099	regeneration	55	0.275408915	< 0.001	< 0.001
GO:1990778	protein localization to cell periphery	129	0.275388345	< 0.001	< 0.001
GO:0010721	negative regulation of cell development	132	0.275379275	< 0.001	< 0.001
GO:0042249	establishment of planar polarity of embryonic epithelium	5	0.275366795	0.0018	0.002428764
GO:0009126	purine nucleoside monophosphate metabolic process	117	0.275363275	0.0001	0.000192793
GO:0009167	purine ribonucleoside monophosphate metabolic process	117	0.275363275	0.0001	0.000192793
GO:0007605	sensory perception of sound	51	0.275362758	< 0.001	< 0.001
GO:1901652	response to peptide	192	0.275348563	< 0.001	< 0.001
GO:0032206	positive regulation of telomere maintenance	27	0.275294342	0.0001	0.000192793
GO:0051250	negative regulation of lymphocyte activation	35	0.275293252	0.0001	0.000192793

GOIDlabel	GOName	size	categoryScores	pValPerm	pValPermCorr
GO:0007286	spermatid development	39	0.275292875	< 0.001	< 0.001
GO:0051170	nuclear import	73	0.275273708	< 0.001	< 0.001
GO:0032845	negative regulation of homeostatic process	83	0.275272519	< 0.001	< 0.001
GO:0051782	negative regulation of cell division	5	0.275263835	0.0003	0.000505993
GO:0060766	negative regulation of androgen receptor signaling pathway	6	0.275246675	0.031	0.033048728
GO:0030111	regulation of Wnt signaling pathway	147	0.275239671	< 0.001	< 0.001
GO:0008299	isoprenoid biosynthetic process	8	0.275225225	0.0057	0.006849633
GO:0050890	cognition	143	0.275166275	0.0014	0.00194839
GO:0034333	adherens junction assembly	41	0.275160875	< 0.001	< 0.001
GO:0009259	ribonucleotide metabolic process	183	0.275124305	< 0.001	< 0.001
GO:0046928	regulation of neurotransmitter secretion	57	0.275106686	0.0075	0.008822377
GO:0002683	negative regulation of immune system process	138	0.275026579	< 0.001	< 0.001
GO:0002685	regulation of leukocyte migration	63	0.275013789	< 0.001	< 0.001
GO:0045475	locomotor rhythm	5	0.275006435	0.0155	0.017160828
GO:0046632	alpha-beta T cell differentiation	27	0.274998808	< 0.001	< 0.001
GO:0009651	response to salt stress	12	0.274989275	0.0002	0.000356582
GO:0006284	base-excision repair	16	0.274967825	0.0143	0.015940017
GO:0060563	neuroepithelial cell differentiation	19	0.274944117	< 0.001	< 0.001
GO:1902807	negative regulation of cell cycle G1/S phase transition	33	0.274942475	< 0.001	< 0.001
GO:0071241	cellular response to inorganic substance	78	0.274933175	< 0.001	< 0.001
GO:0038111	interleukin-7-mediated signaling pathway	8	0.274903475	< 0.001	< 0.001
GO:1902806	regulation of cell cycle G1/S phase transition	57	0.274903475	0.0001	0.000192793
GO:1900409	positive regulation of cellular response to oxidative stress	6	0.274903475	0.0009	0.001319479
GO:1903209	positive regulation of oxidative stress-induced cell death	6	0.274903475	0.0009	0.001319479
GO:0019362	pyridine nucleotide metabolic process	67	0.274895791	< 0.001	< 0.001
GO:0046496	nicotinamide nucleotide metabolic process	67	0.274895791	< 0.001	< 0.001
GO:0044782	cilium organization	152	0.274879767	< 0.001	< 0.001
GO:0072521	purine-containing compound metabolic process	192	0.2748713	< 0.001	< 0.001
GO:1902307	positive regulation of sodium ion transmembrane transport	10	0.274851995	< 0.001	< 0.001
GO:0051146	striated muscle cell differentiation	85	0.274848967	< 0.001	< 0.001
GO:0048515	spermatid differentiation	42	0.274829932	< 0.001	< 0.001
GO:0071396	cellular response to lipid	195	0.274822955	< 0.001	< 0.001
GO:0001933	negative regulation of protein phosphorylation	177	0.274820583	< 0.001	< 0.001
GO:0060070	canonical Wnt signaling pathway	132	0.274804025	< 0.001	< 0.001
GO:0007568	aging	127	0.274796056	< 0.001	< 0.001
GO:0007620	copulation	7	0.274793161	0.0012	0.001702047
GO:0045844	positive regulation of striated muscle tissue development	24	0.2747855	< 0.001	< 0.001
GO:0048636	positive regulation of muscle organ development	24	0.2747855	< 0.001	< 0.001
GO:1901863	positive regulation of muscle tissue development	24	0.2747855	< 0.001	< 0.001
GO:0007094	mitotic spindle assembly checkpoint	13	0.274764875	0.0018	0.002428764
GO:0031577	spindle checkpoint	13	0.274764875	0.0018	0.002428764
GO:0071173	spindle assembly checkpoint	13	0.274764875	0.0018	0.002428764
GO:0071174	mitotic spindle checkpoint	13	0.274764875	0.0018	0.002428764
GO:0043372	positive regulation of CD4-positive, alpha-beta T cell differentiation	5	0.274749035	< 0.001	< 0.001
GO:2000516	positive regulation of CD4-positive, alpha-beta T cell activation	5	0.274749035	< 0.001	< 0.001
GO:0045956	positive regulation of calcium ion-dependent exocytosis	16	0.2747426	0.018	0.019794872
GO:0010799	regulation of peptidyl-threonine phosphorylation	25	0.274728443	0.0003	0.000505993
GO:0031623	receptor internalization	41	0.274683743	< 0.001	< 0.001
GO:0002758	innate immune response-activating signal transduction	117	0.274650475	0.0001	0.000192793
GO:0019076	viral release from host cell	17	0.274646075	0.0006	0.000927725
GO:0035890	exit from host	17	0.274646075	0.0006	0.000927725
GO:0035891	exit from host cell	17	0.274646075	0.0006	0.000927725
GO:0052126	movement in host environment	17	0.274646075	0.0006	0.000927725
GO:0052192	movement in environment of other organism involved in symbiotic interaction	17	0.274646075	0.0006	0.000927725
GO:0009612	response to mechanical stimulus	64	0.274646075	< 0.001	< 0.001
GO:0070486	leukocyte aggregation	144	0.2746282	< 0.001	< 0.001
GO:0006816	calcium ion transport	154	0.27460596	< 0.001	< 0.001
GO:0042759	long-chain fatty acid biosynthetic process	6	0.274603175	0.0006	0.000927725
GO:1990138	neuron projection extension	91	0.274564046	0.0001	0.000192793
GO:0001937	negative regulation of endothelial cell proliferation	9	0.274560275	< 0.001	< 0.001
GO:0010522	regulation of calcium ion transport into cytosol	46	0.274550948	0.0011	0.001576346
GO:0002474	antigen processing and presentation of peptide antigen via MHC class I	46	0.274545353	0.0007	0.001063182
GO:0015758	glucose transport	44	0.274540775	0.0001	0.000192793
GO:0022029	telencephalon cell migration	30	0.274508795	0.0009	0.001319479
GO:0021978	telencephalon regionalization	7	0.274498989	0.0001	0.000192793
GO:0042440	pigment metabolic process	29	0.274477433	< 0.001	< 0.001
GO:0006282	regulation of DNA repair	49	0.27446747	< 0.001	< 0.001
GO:0018022	peptidyl-lysine methylation	49	0.274462217	0.0002	0.000356582
GO:0030488	tRNA methylation	9	0.274445874	0.0004	0.000648934
GO:0032729	positive regulation of interferon-gamma production	17	0.274434098	< 0.001	< 0.001
GO:0007009	plasma membrane organization	143	0.274428274	< 0.001	< 0.001
GO:0045948	positive regulation of translational initiation	7	0.274425446	0.0002	0.000356582
GO:0034612	response to tumor necrosis factor	117	0.274395274	0.0001	0.000192793
GO:0050868	negative regulation of T cell activation	28	0.274351903	0.0001	0.000192793
GO:0021885	forebrain cell migration	33	0.274349674	0.0016	0.002186748
GO:0045913	positive regulation of carbohydrate metabolic process	34	0.274343251	< 0.001	< 0.001
GO:0032102	negative regulation of response to external stimulus	111	0.274337658	< 0.001	< 0.001
GO:0046112	nucleobase biosynthetic process	5	0.274337194	< 0.001	< 0.001
GO:0021772	olfactory bulb development	14	0.274315131	0.0449	0.046859714
GO:0021988	olfactory lobe development	14	0.274315131	0.0449	0.046859714
GO:0048520	positive regulation of behavior	13	0.274309474	0.0028	0.003584743
GO:0097193	intrinsic apoptotic signaling pathway	114	0.274300616	< 0.001	< 0.001
GO:0033209	tumor necrosis factor-mediated signaling pathway	81	0.274296519	0.0001	0.000192793

GOIDlabel	GOName	size	categoryScores	pValPerm	pValPermCorr
GO:0006399	tRNA metabolic process	71	0.274287164	0.0002	0.000356582
GO:0045165	cell fate commitment	71	0.274269038	< 0.001	< 0.001
GO:0019882	antigen processing and presentation	102	0.274267545	0.0002	0.000356582
GO:2000649	regulation of sodium ion transmembrane transporter activity	18	0.274259974	0.0024	0.003116943
GO:0051702	interaction with symbiont	28	0.274241589	< 0.001	< 0.001
GO:0030809	negative regulation of nucleotide biosynthetic process	8	0.274227799	0.0001	0.000192793
GO:1900372	negative regulation of purine nucleotide biosynthetic process	8	0.274227799	0.0001	0.000192793
GO:0009132	nucleoside diphosphate metabolic process	59	0.274196715	< 0.001	< 0.001
GO:0060491	regulation of cell projection assembly	75	0.274189618	< 0.001	< 0.001
GO:0031124	mRNA 3'-end processing	35	0.274182754	0.0014	0.00194839
GO:0010523	negative regulation of calcium ion transport into cytosol	10	0.274182754	0.0021	0.002780108
GO:0060416	response to growth hormone	18	0.274159874	< 0.001	< 0.001
GO:0031102	neuron projection regeneration	23	0.274153657	0.0011	0.001576346
GO:0007618	mating	19	0.274131274	0.0009	0.001319479
GO:0008630	intrinsic apoptotic signaling pathway in response to DNA damage	40	0.274099099	< 0.001	< 0.001
GO:0071320	cellular response to cAMP	24	0.274088374	< 0.001	< 0.001
GO:1902476	chloride transmembrane transport	27	0.274074074	< 0.001	< 0.001
GO:0031589	cell-substrate adhesion	119	0.274072872	< 0.001	< 0.001
GO:0043010	camera-type eye development	105	0.274062634	< 0.001	< 0.001
GO:0051249	regulation of lymphocyte activation	127	0.27405831	< 0.001	< 0.001
GO:0048863	stem cell differentiation	98	0.274041972	< 0.001	< 0.001
GO:0045815	positive regulation of gene expression, epigenetic	22	0.274037674	0.0001	0.000192793
GO:0070838	divalent metal ion transport	160	0.273978443	< 0.001	< 0.001
GO:0072511	divalent inorganic cation transport	160	0.273978443	< 0.001	< 0.001
GO:0051052	regulation of DNA metabolic process	173	0.273972073	< 0.001	< 0.001
GO:2000736	regulation of stem cell differentiation	58	0.273936005	0.0004	0.000648934
GO:0050994	regulation of lipid catabolic process	17	0.273889015	< 0.001	< 0.001
GO:0071214	cellular response to abiotic stimulus	101	0.273871325	< 0.001	< 0.001
GO:0002706	regulation of lymphocyte mediated immunity	33	0.273866074	< 0.001	< 0.001
GO:0006814	sodium ion transport	67	0.273858507	< 0.001	< 0.001
GO:0030195	negative regulation of blood coagulation	18	0.273816674	< 0.001	< 0.001
GO:0050819	negative regulation of coagulation	18	0.273816674	< 0.001	< 0.001
GO:1900047	negative regulation of hemostasis	18	0.273816674	< 0.001	< 0.001
GO:0034765	regulation of ion transmembrane transport	175	0.273809156	0.0004	0.000648934
GO:0031647	regulation of protein stability	124	0.273801221	< 0.001	< 0.001
GO:0046209	nitric oxide metabolic process	25	0.27378121	< 0.001	< 0.001
GO:1990416	cellular response to brain-derived neurotrophic factor stimulus	5	0.273770914	0.0302	0.032240379
GO:0060419	heart growth	34	0.273730033	< 0.001	< 0.001
GO:0000266	mitochondrial fission	12	0.273702274	0.0008	0.001193265
GO:1904862	inhibitory synapse assembly	7	0.273616474	0.018	0.019794872
GO:0097106	postsynaptic density organization	13	0.273596674	0.0055	0.006618125
GO:0099084	postsynaptic specialization organization	13	0.273596674	0.0055	0.006618125
GO:0002082	regulation of oxidative phosphorylation	9	0.273559274	0.0006	0.000927725
GO:0001657	ureteric bud development	30	0.273539254	< 0.001	< 0.001
GO:0048545	response to steroid hormone	120	0.273522094	< 0.001	< 0.001
GO:0007059	chromosome segregation	108	0.27349254	< 0.001	< 0.001
GO:0030149	sphingolipid catabolic process	5	0.273462033	0.0025	0.003237479
GO:0002768	immune response-regulating cell surface receptor signaling pathway	145	0.273407003	< 0.001	< 0.001
GO:0033157	regulation of intracellular protein transport	122	0.27340549	< 0.001	< 0.001
GO:0042255	ribosome assembly	13	0.273398673	0.0022	0.002896881
GO:0007611	learning or memory	130	0.273370953	0.0017	0.00231289
GO:0090022	regulation of neutrophil chemotaxis	10	0.273359073	< 0.001	< 0.001
GO:1902622	regulation of neutrophil migration	10	0.273359073	< 0.001	< 0.001
GO:0009185	ribonucleoside diphosphate metabolic process	56	0.273313109	< 0.001	< 0.001
GO:0002757	immune response-activating signal transduction	184	0.273301718	< 0.001	< 0.001
GO:0046034	ATP metabolic process	105	0.273292885	0.0003	0.000505993
GO:0060047	heart contraction	103	0.273259112	< 0.001	< 0.001
GO:0051051	negative regulation of transport	192	0.273242439	< 0.001	< 0.001
GO:0001885	endothelial cell development	21	0.273236502	< 0.001	< 0.001
GO:1901657	glycosyl compound metabolic process	166	0.27322262	0.0001	0.000192793
GO:0045017	glycerolipid biosynthetic process	99	0.273164073	< 0.001	< 0.001
GO:1904646	cellular response to beta-amyloid	16	0.273149936	0.0001	0.000192793
GO:0002446	neutrophil mediated immunity	190	0.273149089	< 0.001	< 0.001
GO:0030278	regulation of ossification	73	0.273133407	< 0.001	< 0.001
GO:0009615	response to virus	113	0.273117618	< 0.001	< 0.001
GO:0010657	muscle cell apoptotic process	34	0.273116814	< 0.001	< 0.001
GO:0051271	negative regulation of cellular component movement	123	0.273116322	< 0.001	< 0.001
GO:1903531	negative regulation of secretion by cell	74	0.273115587	< 0.001	< 0.001
GO:0048565	digestive tract development	33	0.273093873	< 0.001	< 0.001
GO:0051591	response to cAMP	38	0.273081352	< 0.001	< 0.001
GO:0044057	regulation of system process	194	0.273080444	< 0.001	< 0.001
GO:0007189	adenylate cyclase-activating G-protein coupled receptor signaling pathway	25	0.273029601	< 0.001	< 0.001
GO:0002720	positive regulation of cytokine production involved in immune response	15	0.272981553	< 0.001	< 0.001
GO:0032846	positive regulation of homeostatic process	93	0.272974357	< 0.001	< 0.001
GO:0002283	neutrophil activation involved in immune response	185	0.272969495	< 0.001	< 0.001
GO:0043312	neutrophil degranulation	185	0.272969495	< 0.001	< 0.001
GO:0044827	modulation by host of viral genome replication	8	0.272940798	0.0001	0.000192793
GO:0071804	cellular potassium ion transport	56	0.272917816	0.0004	0.000648934
GO:0071805	potassium ion transmembrane transport	56	0.272917816	0.0004	0.000648934
GO:0002764	immune response-regulating signaling pathway	199	0.272905066	< 0.001	< 0.001
GO:0022406	membrane docking	82	0.272850551	< 0.001	< 0.001
GO:0010460	positive regulation of heart rate	8	0.272844273	0.0114	0.012893596
GO:0072350	tricarboxylic acid metabolic process	11	0.272820873	0.0005	0.000791911

GOIDlabel	GOName	size	categoryScores	pValPerm	pValPermCorr
GO:0051016	barbed-end actin filament capping	7	0.272807501	< 0.001	< 0.001
GO:0006336	DNA replication-independent nucleosome assembly	15	0.272792793	0.0203	0.022152908
GO:0034724	DNA replication-independent nucleosome organization	15	0.272792793	0.0203	0.022152908
GO:1903747	regulation of establishment of protein localization to mitochondrion	44	0.272774073	< 0.001	< 0.001
GO:0051607	defense response to virus	84	0.27277073	< 0.001	< 0.001
GO:0032722	positive regulation of chemokine production	20	0.272767053	< 0.001	< 0.001
GO:0046607	positive regulation of centrosome cycle	6	0.272758473	0.0083	0.009660392
GO:0030048	actin filament-based movement	51	0.272743332	< 0.001	< 0.001
GO:0098722	asymmetric stem cell division	7	0.272733958	0.0024	0.003116943
GO:0006644	phospholipid metabolic process	166	0.272678358	< 0.001	< 0.001
GO:0008654	phospholipid biosynthetic process	110	0.272675793	< 0.001	< 0.001
GO:0042491	auditory receptor cell differentiation	12	0.272672673	< 0.001	< 0.001
GO:0032925	regulation of activin receptor signaling pathway	10	0.272664093	< 0.001	< 0.001
GO:0060048	cardiac muscle contraction	53	0.272645152	< 0.001	< 0.001
GO:0031532	actin cytoskeleton reorganization	38	0.272634288	0.0001	0.000192793
GO:1901019	regulation of calcium ion transmembrane transporter activity	40	0.272631918	0.0008	0.001193265
GO:0002063	chondrocyte development	10	0.272612613	< 0.001	< 0.001
GO:0036474	cell death in response to hydrogen peroxide	12	0.272608323	< 0.001	< 0.001
GO:0060333	interferon-gamma-mediated signaling pathway	23	0.272598064	< 0.001	< 0.001
GO:0007159	leukocyte cell-cell adhesion	162	0.272597995	< 0.001	< 0.001
GO:0002090	regulation of receptor internalization	20	0.272561133	< 0.001	< 0.001
GO:0045648	positive regulation of erythrocyte differentiation	10	0.272561133	< 0.001	< 0.001
GO:0046165	alcohol biosynthetic process	60	0.272561133	< 0.001	< 0.001
GO:0050900	leukocyte migration	124	0.272539129	< 0.001	< 0.001
GO:0046434	organophosphate catabolic process	41	0.272517814	< 0.001	< 0.001
GO:0043046	DNA methylation involved in gamete generation	7	0.27251333	0.0211	0.022970266
GO:0048588	developmental cell growth	113	0.272498035	0.0001	0.000192793
GO:0015893	drug transport	7	0.272476558	< 0.001	< 0.001
GO:0033865	nucleoside bisphosphate metabolic process	13	0.272468072	< 0.001	< 0.001
GO:0033875	ribonucleoside bisphosphate metabolic process	13	0.272468072	< 0.001	< 0.001
GO:0034032	purine nucleoside bisphosphate metabolic process	13	0.272468072	< 0.001	< 0.001
GO:0003002	regionalization	112	0.272458172	0.0001	0.000192793
GO:0001678	cellular glucose homeostasis	64	0.272454151	< 0.001	< 0.001
GO:0009127	purine nucleoside monophosphate biosynthetic process	29	0.272409355	0.0001	0.000192793
GO:0009168	purine ribonucleoside monophosphate biosynthetic process	29	0.272409355	0.0001	0.000192793
GO:0002705	positive regulation of leukocyte mediated immunity	32	0.272401866	< 0.001	< 0.001
GO:1901685	glutathione derivative metabolic process	8	0.272393822	0.0097	0.01115531
GO:1901687	glutathione derivative biosynthetic process	8	0.272393822	0.0097	0.01115531
GO:0071679	commissural neuron axon guidance	6	0.272372372	0.0047	0.005730776
GO:0051091	positive regulation of sequence-specific DNA binding transcription factor activity	98	0.272360991	< 0.001	< 0.001
GO:0042886	amide transport	108	0.272355689	< 0.001	< 0.001
GO:0048562	embryonic organ morphogenesis	77	0.272322787	< 0.001	< 0.001
GO:0001503	ossification	140	0.272290862	< 0.001	< 0.001
GO:0019079	viral genome replication	48	0.272270485	< 0.001	< 0.001
GO:0048333	mesodermal cell differentiation	7	0.272255929	0.0001	0.000192793
GO:1903429	regulation of cell maturation	5	0.272226512	0.0026	0.003356522
GO:0061515	myeloid cell development	22	0.272200772	< 0.001	< 0.001
GO:0030325	adrenal gland development	6	0.272200772	0.0001	0.000192793
GO:0060323	head morphogenesis	10	0.272123552	< 0.001	< 0.001
GO:1900119	positive regulation of execution phase of apoptosis	7	0.272108844	0.0024	0.003116943
GO:0050931	pigment cell differentiation	13	0.272091872	< 0.001	< 0.001
GO:0002429	immune response-activating cell surface receptor signaling pathway	131	0.272089756	< 0.001	< 0.001
GO:0060602	branch elongation of an epithelium	5	0.272072072	< 0.001	< 0.001
GO:0000045	autophagosome assembly	38	0.272058525	0.0001	0.000192793
GO:0007423	sensory organ development	177	0.27205753	< 0.001	< 0.001
GO:0000079	regulation of cyclin-dependent protein serine/threonine kinase activity	36	0.272050622	< 0.001	< 0.001
GO:0032680	regulation of tumor necrosis factor production	45	0.272032032	< 0.001	< 0.001
GO:0071941	nitrogen cycle metabolic process	5	0.272020592	0.002	0.002664694
GO:0045089	positive regulation of innate immune response	138	0.272017981	< 0.001	< 0.001
GO:0051592	response to calcium ion	54	0.271914772	< 0.001	< 0.001
GO:0019693	ribose phosphate metabolic process	188	0.271891344	< 0.001	< 0.001
GO:0008645	hexose transport	45	0.271889032	0.0001	0.000192793
GO:0015749	monosaccharide transport	45	0.271889032	0.0001	0.000192793
GO:0097503	sialylation	9	0.271871872	0.0001	0.000192793
GO:0014745	negative regulation of muscle adaptation	5	0.271866152	0.0002	0.000356582
GO:1990173	protein localization to nucleoplasm	7	0.271851443	0.0029	0.003693519
GO:0032388	positive regulation of intracellular transport	107	0.271833917	< 0.001	< 0.001
GO:0033143	regulation of intracellular steroid hormone receptor signaling pathway	31	0.271756549	< 0.001	< 0.001
GO:0008585	female gonad development	26	0.271755272	0.0009	0.001319479
GO:0051491	positive regulation of filopodium assembly	17	0.271754107	0.0056	0.006732458
GO:0043470	regulation of carbohydrate catabolic process	31	0.271748246	< 0.001	< 0.001
GO:0003401	axis elongation	7	0.271704357	0.0002	0.000356582
GO:2000243	positive regulation of reproductive process	21	0.271679843	0.0009	0.001319479
GO:0007204	positive regulation of cytosolic calcium ion concentration	109	0.271677707	< 0.001	< 0.001
GO:0060046	regulation of acrosome reaction	5	0.271660232	0.0406	0.042635523
GO:1903320	regulation of protein modification by small protein conjugation or removal	121	0.271638108	< 0.001	< 0.001
GO:0048511	rhythmic process	131	0.271612288	0.0004	0.000648934
GO:0035904	aorta development	19	0.271611461	0.0007	0.001063182
GO:0006891	intra-Golgi vesicle-mediated transport	10	0.271608752	0.0004	0.000648934
GO:0002218	activation of innate immune response	127	0.271607941	0.0001	0.000192793
GO:0008406	gonad development	64	0.271529118	< 0.001	< 0.001
GO:0048525	negative regulation of viral process	30	0.271488631	< 0.001	< 0.001
GO:0001910	regulation of leukocyte mediated cytotoxicity	13	0.271458271	< 0.001	< 0.001

GOIDlabel	GOName	size	categoryScores	pValPerm	pValPermCorr
GO:0031145	anaphase-promoting complex-dependent catabolic process	54	0.271457171	0.0014	0.00194839
GO:0042176	regulation of protein catabolic process	169	0.271455225	< 0.001	< 0.001
GO:1903008	organelle disassembly	40	0.271435006	0.0001	0.000192793
GO:0050678	regulation of epithelial cell proliferation	118	0.271417665	< 0.001	< 0.001
GO:0009066	aspartate family amino acid metabolic process	18	0.271399971	0.0003	0.000505993
GO:0045879	negative regulation of smoothened signaling pathway	12	0.271342771	< 0.001	< 0.001
GO:0032688	negative regulation of interferon-beta production	6	0.271342771	0.0001	0.000192793
GO:0034695	response to prostaglandin E	13	0.271339471	< 0.001	< 0.001
GO:0051048	negative regulation of secretion	79	0.271280322	< 0.001	< 0.001
GO:0070911	global genome nucleotide-excision repair	13	0.271280071	0.0004	0.000648934
GO:0072337	modified amino acid transport	9	0.271271271	0.0048	0.005840847
GO:0003279	cardiac septum development	36	0.271264121	< 0.001	< 0.001
GO:0061138	morphogenesis of a branching epithelium	54	0.271261738	< 0.001	< 0.001
GO:0018345	protein palmitoylation	13	0.271240471	< 0.001	< 0.001
GO:0008334	histone mRNA metabolic process	12	0.271235521	0.0002	0.000356582
GO:1903364	positive regulation of cellular protein catabolic process	61	0.271228137	< 0.001	< 0.001
GO:0006479	protein methylation	75	0.271217503	< 0.001	< 0.001
GO:0008213	protein alkylation	75	0.271217503	< 0.001	< 0.001
GO:0045069	regulation of viral genome replication	34	0.271178742	< 0.001	< 0.001
GO:0070936	protein K48-linked ubiquitination	24	0.271128271	0.0004	0.000648934
GO:1904358	positive regulation of telomere maintenance via telomere lengthening	19	0.271123755	0.0005	0.000791911
GO:0009896	positive regulation of catabolic process	126	0.271099671	< 0.001	< 0.001
GO:0043620	regulation of DNA-templated transcription in response to stress	55	0.271065871	0.0016	0.002186748
GO:0010660	regulation of muscle cell apoptotic process	31	0.271050774	< 0.001	< 0.001
GO:0016322	neuron remodeling	7	0.270932157	0.0003	0.000505993
GO:0009636	response to toxic substance	65	0.270931591	< 0.001	< 0.001
GO:0070391	response to lipoteichoic acid	5	0.270888031	0.0001	0.000192793
GO:0071223	cellular response to lipoteichoic acid	5	0.270888031	0.0001	0.000192793
GO:1904019	epithelial cell apoptotic process	30	0.270862291	< 0.001	< 0.001
GO:0009116	nucleoside metabolic process	161	0.270833034	0.0001	0.000192793
GO:0050866	negative regulation of cell activation	56	0.270821842	< 0.001	< 0.001
GO:0002115	store-operated calcium entry	8	0.270785071	0.0005	0.000791911
GO:2001256	regulation of store-operated calcium entry	8	0.270785071	0.0005	0.000791911
GO:0007190	activation of adenylate cyclase activity	13	0.270785071	0.0053	0.006400268
GO:0003208	cardiac ventricle morphogenesis	33	0.270769471	< 0.001	< 0.001
GO:0032273	positive regulation of protein polymerization	52	0.270755371	0.0004	0.000648934
GO:0098727	maintenance of cell number	53	0.270751075	< 0.001	< 0.001
GO:0001779	natural killer cell differentiation	8	0.270720721	0.001	0.001450349
GO:0022602	ovulation cycle process	25	0.270702703	0.0055	0.006618125
GO:0043954	cellular component maintenance	32	0.270688546	< 0.001	< 0.001
GO:0045947	negative regulation of translational initiation	8	0.270688546	0.0002	0.000356582
GO:0086003	cardiac muscle cell contraction	24	0.270656371	0.0004	0.000648934
GO:2000179	positive regulation of neural precursor cell proliferation	17	0.2706488	0.0111	0.012593827
GO:0045137	development of primary sexual characteristics	67	0.270616032	< 0.001	< 0.001
GO:0001909	leukocyte mediated cytotoxicity	28	0.270582828	< 0.001	< 0.001
GO:0046322	negative regulation of fatty acid oxidation	6	0.270570571	0.0032	0.004042263
GO:0051260	protein homooligomerization	148	0.270546802	< 0.001	< 0.001
GO:1903522	regulation of blood circulation	111	0.270525352	< 0.001	< 0.001
GO:0048193	Golgi vesicle transport	152	0.270502269	0.0002	0.000356582
GO:1902513	regulation of organelle transport along microtubule	5	0.27047619	0.0088	0.010185307
GO:0042060	wound healing	180	0.27044902	< 0.001	< 0.001
GO:0009119	ribonucleoside metabolic process	150	0.270410982	0.0001	0.000192793
GO:0010508	positive regulation of autophagy	50	0.270409266	< 0.001	< 0.001
GO:0035601	protein deacylation	34	0.270406541	< 0.001	< 0.001
GO:0098732	macromolecule deacylation	34	0.270406541	< 0.001	< 0.001
GO:0071158	positive regulation of cell cycle arrest	28	0.270371392	0.0002	0.000356582
GO:0030516	regulation of axon extension	47	0.270363372	< 0.001	< 0.001
GO:0003073	regulation of systemic arterial blood pressure	24	0.270280995	< 0.001	< 0.001
GO:0045185	maintenance of protein location	38	0.270229628	< 0.001	< 0.001
GO:0046888	negative regulation of hormone secretion	26	0.27013167	0.0024	0.003116943
GO:0050727	regulation of inflammatory response	104	0.27013167	< 0.001	< 0.001
GO:0050805	negative regulation of synaptic transmission	32	0.270085264	0.0022	0.002896881
GO:0042119	neutrophil activation	188	0.27006079	< 0.001	< 0.001
GO:0003158	endothelium development	39	0.27004587	< 0.001	< 0.001
GO:0030210	heparin biosynthetic process	5	0.27001287	0.0007	0.001063182
GO:0046631	alpha-beta T cell activation	32	0.269964607	< 0.001	< 0.001
GO:0050768	negative regulation of neurogenesis	119	0.26996312	0.0001	0.000192793
GO:0043405	regulation of MAP kinase activity	142	0.269949426	< 0.001	< 0.001
GO:0000209	protein polyubiquitination	159	0.26992707	0.0002	0.000356582
GO:2001236	regulation of extrinsic apoptotic signaling pathway	43	0.269893149	< 0.001	< 0.001
GO:0010762	regulation of fibroblast migration	13	0.26987427	< 0.001	< 0.001
GO:0010675	regulation of cellular carbohydrate metabolic process	54	0.269860337	< 0.001	< 0.001
GO:0009161	ribonucleoside monophosphate metabolic process	125	0.269837838	0.0003	0.000505993
GO:0008544	epidermis development	111	0.269834313	< 0.001	< 0.001
GO:0042987	amyloid precursor protein catabolic process	21	0.269829013	< 0.001	< 0.001
GO:0021700	developmental maturation	100	0.269801802	< 0.001	< 0.001
GO:0033002	muscle cell proliferation	70	0.269795918	< 0.001	< 0.001
GO:0070371	ERK1 and ERK2 cascade	113	0.269730413	< 0.001	< 0.001
GO:0032869	cellular response to insulin stimulus	80	0.269697555	< 0.001	< 0.001
GO:0032635	interleukin-6 production	41	0.269655021	< 0.001	< 0.001
GO:0046545	development of primary female sexual characteristics	29	0.269622332	0.0011	0.001576346
GO:0007638	mechanosensory behavior	5	0.26960103	0.0123	0.013827585
GO:0031223	auditory behavior	5	0.26960103	0.0123	0.013827585

GOIDlabel	GOName	size	categoryScores	pValPerm	pValPermCorr
GO:0072524	pyridine-containing compound metabolic process	71	0.269559701	< 0.001	< 0.001
GO:0008356	asymmetric cell division	10	0.26954955	0.0037	0.004617737
GO:0002064	epithelial cell development	66	0.26950977	< 0.001	< 0.001
GO:0018026	peptidyl-lysine monomethylation	5	0.269446589	0.0016	0.002186748
GO:1901838	positive regulation of transcription of nuclear large rRNA transcript from RNA polymerase I promoter	5	0.269446589	0.032	0.034034245
GO:0043618	regulation of transcription from RNA polymerase II promoter in response to stress	53	0.269405794	0.0021	0.002780108
GO:0060149	negative regulation of posttranscriptional gene silencing	6	0.269369369	0.0024	0.003116943
GO:0060965	negative regulation of gene silencing by miRNA	6	0.269369369	0.0024	0.003116943
GO:0060967	negative regulation of gene silencing by RNA	6	0.269369369	0.0024	0.003116943
GO:0010817	regulation of hormone levels	163	0.269322785	< 0.001	< 0.001
GO:0030501	positive regulation of bone mineralization	18	0.269312169	< 0.001	< 0.001
GO:0045931	positive regulation of mitotic cell cycle	58	0.269307238	< 0.001	< 0.001
GO:0006921	cellular component disassembly involved in execution phase of apoptosis	12	0.269283569	0.0011	0.001576346
GO:0030262	apoptotic nuclear changes	12	0.269283569	0.0011	0.001576346
GO:0072176	nephric duct development	7	0.269277441	0.0001	0.000192793
GO:0071356	cellular response to tumor necrosis factor	108	0.269259736	0.0001	0.000192793
GO:0038093	Fc receptor signaling pathway	102	0.26925581	0.0001	0.000192793
GO:0043112	receptor metabolic process	77	0.269223955	< 0.001	< 0.001
GO:0051090	regulation of sequence-specific DNA binding transcription factor activity	162	0.269202536	< 0.001	< 0.001
GO:0007219	Notch signaling pathway	62	0.269186698	< 0.001	< 0.001
GO:0042407	cristae formation	14	0.269185512	0.0071	0.008390203
GO:0060079	excitatory postsynaptic potential	53	0.269133824	0.0026	0.003356522
GO:0019827	stem cell population maintenance	52	0.269131769	< 0.001	< 0.001
GO:0018158	protein oxidation	6	0.269111969	0.0002	0.000356582
GO:1904350	regulation of protein catabolic process in the vacuole	5	0.269086229	0.0093	0.010727257
GO:0001975	response to amphetamine	16	0.269079794	< 0.001	< 0.001
GO:0043687	post-translational protein modification	182	0.269061055	< 0.001	< 0.001
GO:0048645	animal organ formation	18	0.269054769	< 0.001	< 0.001
GO:0032259	methylation	126	0.269040469	< 0.001	< 0.001
GO:0002221	pattern recognition receptor signaling pathway	69	0.269028034	< 0.001	< 0.001
GO:0046599	regulation of centriole replication	9	0.268954669	< 0.001	< 0.001
GO:0051209	release of sequestered calcium ion into cytosol	49	0.268951751	0.0007	0.001063182
GO:0051282	regulation of sequestering of calcium ion	49	0.268951751	0.0007	0.001063182
GO:0051283	negative regulation of sequestering of calcium ion	49	0.268951751	0.0007	0.001063182
GO:0042391	regulation of membrane potential	160	0.268933398	0.0002	0.000356582
GO:0030308	negative regulation of cell growth	70	0.268924435	< 0.001	< 0.001
GO:0045933	positive regulation of muscle contraction	17	0.268892422	< 0.001	< 0.001
GO:2000045	regulation of G1/S transition of mitotic cell cycle	52	0.268824869	0.0003	0.000505993
GO:0008016	regulation of heart contraction	92	0.268795814	< 0.001	< 0.001
GO:0036230	granulocyte activation	191	0.268685439	< 0.001	< 0.001
GO:0009948	anterior/posterior axis specification	12	0.268682969	0.0001	0.000192793
GO:0035637	multicellular organismal signaling	72	0.268665094	0.0001	0.000192793
GO:0051443	positive regulation of ubiquitin-protein transferase activity	15	0.268657229	0.0004	0.000648934
GO:0031638	zymogen activation	16	0.268613256	< 0.001	< 0.001
GO:0000381	regulation of alternative mRNA splicing, via spliceosome	20	0.268584299	0.0001	0.000192793
GO:0010543	regulation of platelet activation	14	0.268578783	0.0001	0.000192793
GO:0001919	regulation of receptor recycling	9	0.268554269	0.0009	0.001319479
GO:0006400	tRNA modification	27	0.268535202	0.0001	0.000192793
GO:0043950	positive regulation of cAMP-mediated signaling	6	0.268511369	0.0074	0.008712331
GO:0048024	regulation of mRNA splicing, via spliceosome	29	0.268503972	< 0.001	< 0.001
GO:0002360	T cell lineage commitment	5	0.268468468	0.0025	0.003237479
GO:0061045	negative regulation of wound healing	26	0.268458568	< 0.001	< 0.001
GO:0000768	syncytium formation by plasma membrane fusion	15	0.268434148	0.0173	0.01905998
GO:0040029	regulation of gene expression, epigenetic	113	0.26840241	0.0002	0.000356582
GO:0008347	glial cell migration	26	0.268399168	< 0.001	< 0.001
GO:0045821	positive regulation of glycolytic process	10	0.268365508	0.0103	0.011785137
GO:0051194	positive regulation of cofactor metabolic process	10	0.268365508	0.0103	0.011785137
GO:0051197	positive regulation of coenzyme metabolic process	10	0.268365508	0.0103	0.011785137
GO:0098876	vesicle-mediated transport to the plasma membrane	33	0.268351468	0.0002	0.000356582
GO:0051279	regulation of release of sequestered calcium ion into cytosol	38	0.268346542	0.003	0.003811001
GO:0021670	lateral ventricle development	6	0.268339768	0.039	0.041027059
GO:0033146	regulation of intracellular estrogen receptor signaling pathway	17	0.268317057	< 0.001	< 0.001
GO:0008643	carbohydrate transport	54	0.268311168	< 0.001	< 0.001
GO:0009164	nucleoside catabolic process	11	0.268304668	0.0005	0.000791911
GO:0045777	positive regulation of blood pressure	10	0.268288288	< 0.001	< 0.001
GO:0006520	cellular amino acid metabolic process	145	0.268282075	0.0001	0.000192793
GO:0003206	cardiac chamber morphogenesis	55	0.268271908	< 0.001	< 0.001
GO:0060561	apoptotic process involved in morphogenesis	7	0.26824784	0.0007	0.001063182
GO:0000226	microtubule cytoskeleton organization	200	0.268227799	< 0.001	< 0.001
GO:0045785	positive regulation of cell adhesion	138	0.268151381	< 0.001	< 0.001
GO:0034205	beta-amyloid formation	15	0.268142428	0.0001	0.000192793
GO:0048738	cardiac muscle tissue development	75	0.268121836	< 0.001	< 0.001
GO:1902808	positive regulation of cell cycle G1/S phase transition	17	0.26810508	0.0021	0.002780108
GO:0032956	regulation of actin cytoskeleton organization	139	0.268098108	< 0.001	< 0.001
GO:1990823	response to leukemia inhibitory factor	38	0.268089142	< 0.001	< 0.001
GO:1990830	cellular response to leukemia inhibitory factor	38	0.268089142	< 0.001	< 0.001
GO:0031396	regulation of protein ubiquitination	109	0.268074103	0.0001	0.000192793
GO:1901607	alpha-amino acid biosynthetic process	26	0.268052668	< 0.001	< 0.001
GO:0043368	positive T cell selection	8	0.268050193	0.0123	0.013827585
GO:0038065	collagen-activated signaling pathway	5	0.268005148	< 0.001	< 0.001
GO:0009743	response to carbohydrate	89	0.267930531	< 0.001	< 0.001
GO:0002220	innate immune response activating cell surface receptor signaling pathway	56	0.267861739	0.0004	0.000648934
GO:0002223	stimulatory C-type lectin receptor signaling pathway	56	0.267861739	0.0004	0.000648934

GOIDlabel	GOName	size	categoryScores	pValPerm	pValPermCorr
GO:0051013	microtubule severing	5	0.267850708	< 0.001	< 0.001
GO:0044242	cellular lipid catabolic process	55	0.267836668	< 0.001	< 0.001
GO:0009746	response to hexose	76	0.267818194	< 0.001	< 0.001
GO:0030808	regulation of nucleotide biosynthetic process	32	0.267816924	0.0001	0.000192793
GO:1900371	regulation of purine nucleotide biosynthetic process	32	0.267816924	0.0001	0.000192793
GO:0016571	histone methylation	56	0.267815775	0.0003	0.000505993
GO:0006476	protein deacetylation	33	0.267805468	< 0.001	< 0.001
GO:0002065	columnar/cuboidal epithelial cell differentiation	36	0.267803518	< 0.001	< 0.001
GO:0071887	leukocyte apoptotic process	29	0.267793902	< 0.001	< 0.001
GO:0048598	embryonic morphogenesis	184	0.267756421	< 0.001	< 0.001
GO:0051216	cartilage development	65	0.267739828	< 0.001	< 0.001
GO:0031663	lipopolysaccharide-mediated signaling pathway	27	0.267724868	< 0.001	< 0.001
GO:0046660	female sex differentiation	33	0.267719668	0.0012	0.001702047
GO:0051983	regulation of chromosome segregation	39	0.267709468	0.0001	0.000192793
GO:0051054	positive regulation of DNA metabolic process	106	0.267708409	< 0.001	< 0.001
GO:0051153	regulation of striated muscle cell differentiation	34	0.267696268	< 0.001	< 0.001
GO:0045920	negative regulation of exocytosis	15	0.267679108	< 0.001	< 0.001
GO:2001020	regulation of response to DNA damage stimulus	85	0.267665985	< 0.001	< 0.001
GO:0060326	cell chemotaxis	79	0.267653911	< 0.001	< 0.001
GO:0007422	peripheral nervous system development	23	0.267651502	0.0009	0.001319479
GO:0042594	response to starvation	72	0.267546118	< 0.001	< 0.001
GO:1904668	positive regulation of ubiquitin protein ligase activity	6	0.267524668	0.0003	0.000505993
GO:0031103	axon regeneration	19	0.267520152	0.0015	0.002069969
GO:0001945	lymph vessel development	11	0.267509068	0.0028	0.003584743
GO:0050808	synapse organization	179	0.267497825	0.0003	0.000505993
GO:0030850	prostate gland development	15	0.267438867	< 0.001	< 0.001
GO:0045717	negative regulation of fatty acid biosynthetic process	7	0.267438867	< 0.001	< 0.001
GO:0006099	tricarboxylic acid cycle	6	0.267353067	0.015	0.016665296
GO:1905269	positive regulation of chromatin organization	37	0.267313646	0.0007	0.001063182
GO:0071397	cellular response to cholesterol	8	0.267310167	< 0.001	< 0.001
GO:0043433	negative regulation of sequence-specific DNA binding transcription factor activity	60	0.267267267	< 0.001	< 0.001
GO:2000273	positive regulation of receptor activity	24	0.267235092	0.0003	0.000505993
GO:0031294	lymphocyte costimulation	21	0.267156953	< 0.001	< 0.001
GO:0031295	T cell costimulation	21	0.267156953	< 0.001	< 0.001
GO:0050774	negative regulation of dendrite morphogenesis	10	0.267155727	0.0019	0.00254969
GO:0006991	response to sterol depletion	6	0.267138567	< 0.001	< 0.001
GO:0035329	hippo signaling	11	0.267111267	< 0.001	< 0.001
GO:0006469	negative regulation of protein kinase activity	99	0.267095667	< 0.001	< 0.001
GO:1902224	ketone body metabolic process	5	0.267078507	0.0043	0.005276385
GO:0051900	regulation of mitochondrial depolarization	6	0.267052767	0.0001	0.000192793
GO:0051384	response to glucocorticoid	46	0.267047171	< 0.001	< 0.001
GO:0050709	negative regulation of protein secretion	49	0.266960838	< 0.001	< 0.001
GO:0072163	mesonephric epithelium development	31	0.266924067	< 0.001	< 0.001
GO:0072164	mesonephric tubule development	31	0.266924067	< 0.001	< 0.001
GO:0040013	negative regulation of locomotion	131	0.266843507	< 0.001	< 0.001
GO:0090316	positive regulation of intracellular protein transport	78	0.266841567	< 0.001	< 0.001
GO:0035315	hair cell differentiation	13	0.266825067	< 0.001	< 0.001
GO:0038202	TORC1 signaling	21	0.266813753	< 0.001	< 0.001
GO:0007616	long-term memory	16	0.266811454	0.0113	0.012793882
GO:2000108	positive regulation of leukocyte apoptotic process	9	0.266809667	< 0.001	< 0.001
GO:0016525	negative regulation of angiogenesis	36	0.266788217	< 0.001	< 0.001
GO:2000181	negative regulation of blood vessel morphogenesis	36	0.266788217	< 0.001	< 0.001
GO:0060236	regulation of mitotic spindle organization	17	0.266787796	0.0017	0.00231289
GO:1903322	positive regulation of protein modification by small protein conjugation or removal	73	0.266772448	< 0.001	< 0.001
GO:0043331	response to dsRNA	32	0.266771236	< 0.001	< 0.001
GO:0033120	positive regulation of RNA splicing	12	0.266752467	0.0003	0.000505993
GO:1901532	regulation of hematopoietic progenitor cell differentiation	48	0.266725654	0.0011	0.001576346
GO:0060401	cytosolic calcium ion transport	70	0.266699761	0.0004	0.000648934
GO:0010469	regulation of receptor activity	197	0.266698025	< 0.001	< 0.001
GO:0006625	protein targeting to peroxisome	32	0.266690798	0.0004	0.000648934
GO:0043574	peroxisomal transport	32	0.266690798	0.0004	0.000648934
GO:0072662	protein localization to peroxisome	32	0.266690798	0.0004	0.000648934
GO:0072663	establishment of protein localization to peroxisome	32	0.266690798	0.0004	0.000648934
GO:2001032	regulation of double-strand break repair via nonhomologous end joining	7	0.266666667	0.0024	0.003116943
GO:0043414	macromolecule methylation	108	0.266638067	< 0.001	< 0.001
GO:0030851	granulocyte differentiation	12	0.266623767	< 0.001	< 0.001
GO:0032042	mitochondrial DNA metabolic process	5	0.266615187	0.0168	0.018543137
GO:0007131	reciprocal meiotic recombination	9	0.266609467	0.0004	0.000648934
GO:0035825	reciprocal DNA recombination	9	0.266609467	0.0004	0.000648934
GO:0070266	necroptotic process	9	0.266609467	0.0018	0.002428764
GO:0032970	regulation of actin filament-based process	153	0.266584231	< 0.001	< 0.001
GO:0009069	serine family amino acid metabolic process	16	0.266554054	0.0002	0.000356582
GO:0006954	inflammatory response	191	0.266541336	< 0.001	< 0.001
GO:0070286	axonemal dynein complex assembly	10	0.266435006	0.0408	0.042837226
GO:0030217	T cell differentiation	66	0.266381966	< 0.001	< 0.001
GO:0030500	regulation of bone mineralization	25	0.266357786	< 0.001	< 0.001
GO:0071243	cellular response to arsenic-containing substance	8	0.266312741	< 0.001	< 0.001
GO:0034284	response to monosaccharide	77	0.266278895	< 0.001	< 0.001
GO:0007566	embryo implantation	17	0.266272996	0.0001	0.000192793
GO:0006337	nucleosome disassembly	7	0.266262181	0.0316	0.033622052
GO:0072089	stem cell proliferation	38	0.266260245	0.0008	0.001193265
GO:0007416	synapse assembly	92	0.266252588	0.001	0.001450349
GO:0051262	protein tetramerization	54	0.2662472	< 0.001	< 0.001

GOIDlabel	GOName	size	categoryScores	pValPerm	pValPermCorr
GO:1903900	regulation of viral life cycle	74	0.266190128	0.0003	0.000505993
GO:0090314	positive regulation of protein targeting to membrane	17	0.266151866	< 0.001	< 0.001
GO:0060113	inner ear receptor cell differentiation	19	0.266138319	< 0.001	< 0.001
GO:0006109	regulation of carbohydrate metabolic process	79	0.266129059	< 0.001	< 0.001
GO:0043279	response to alkaloid	59	0.2660777	< 0.001	< 0.001
GO:0007218	neuropeptide signaling pathway	33	0.266050466	0.0004	0.000648934
GO:0090224	regulation of spindle organization	18	0.266037466	0.001	0.001450349
GO:0009123	nucleoside monophosphate metabolic process	131	0.266030043	0.0002	0.000356582
GO:0043270	positive regulation of ion transport	105	0.266024392	< 0.001	< 0.001
GO:0043271	negative regulation of ion transport	49	0.266010033	< 0.001	< 0.001
GO:0071901	negative regulation of protein serine/threonine kinase activity	58	0.2659921	< 0.001	< 0.001
GO:0002708	positive regulation of lymphocyte mediated immunity	24	0.265980266	0.0001	0.000192793
GO:0043457	regulation of cellular respiration	12	0.265958816	0.0001	0.000192793
GO:0031629	synaptic vesicle fusion to presynaptic active zone membrane	5	0.265945946	0.0388	0.040832561
GO:0099500	vesicle fusion to plasma membrane	5	0.265945946	0.0388	0.040832561
GO:0098754	detoxification	36	0.265930216	< 0.001	< 0.001
GO:0051480	regulation of cytosolic calcium ion concentration	118	0.265916279	< 0.001	< 0.001
GO:0006415	translational termination	48	0.265830116	0.0009	0.001319479
GO:0071230	cellular response to amino acid stimulus	33	0.265816466	< 0.001	< 0.001
GO:0051098	regulation of binding	157	0.265789538	< 0.001	< 0.001
GO:0010043	response to zinc ion	14	0.265784151	0.0013	0.001826625
GO:0045830	positive regulation of isotype switching	11	0.265777466	0.0059	0.007066401
GO:1902305	regulation of sodium ion transmembrane transport	24	0.265776491	0.001	0.001450349
GO:0032506	cytokinetic process	14	0.265765766	0.0005	0.000791911
GO:0034315	regulation of Arp2/3 complex-mediated actin nucleation	8	0.265765766	0.014	0.015631405
GO:0045066	regulatory T cell differentiation	7	0.26574738	0.001	0.001450349
GO:1901030	positive regulation of mitochondrial outer membrane permeabilization involved in apoptotic signaling pathway	18	0.265694266	0.0023	0.003013139
GO:0019068	virion assembly	20	0.265649936	0.0016	0.002186748
GO:0014013	regulation of gliogenesis	43	0.26563108	< 0.001	< 0.001
GO:0006898	receptor-mediated endocytosis	111	0.265588368	< 0.001	< 0.001
GO:0055067	monovalent inorganic cation homeostasis	45	0.265545546	< 0.001	< 0.001
GO:0032481	positive regulation of type I interferon production	35	0.265526751	0.0002	0.000356582
GO:0071398	cellular response to fatty acid	23	0.265525152	< 0.001	< 0.001
GO:0051208	sequestering of calcium ion	51	0.265510889	0.0005	0.000791911
GO:0033044	regulation of chromosome organization	73	0.265404348	0.0003	0.000505993
GO:0009749	response to glucose	74	0.265390101	< 0.001	< 0.001
GO:0010035	response to inorganic substance	189	0.26536877	< 0.001	< 0.001
GO:1901654	response to ketone	68	0.265353168	< 0.001	< 0.001
GO:0046475	glycerophospholipid catabolic process	6	0.265336765	0.0002	0.000356582
GO:0046321	positive regulation of fatty acid oxidation	6	0.265336765	0.0072	0.008495371
GO:0007031	peroxisome organization	38	0.265318702	0.0004	0.000648934
GO:0042269	regulation of natural killer cell mediated cytotoxicity	7	0.26523258	< 0.001	< 0.001
GO:0042726	flavin-containing compound metabolic process	5	0.265173745	0.0287	0.030748374
GO:0010212	response to ionizing radiation	63	0.265167208	0.0001	0.000192793
GO:0098900	regulation of action potential	22	0.265157365	0.0002	0.000356582
GO:0016458	gene silencing	85	0.265140435	0.0002	0.000356582
GO:0010506	regulation of autophagy	150	0.265110253	< 0.001	< 0.001
GO:2000725	regulation of cardiac muscle cell differentiation	16	0.265057915	0.0001	0.000192793
GO:0042451	purine nucleoside biosynthetic process	36	0.265057915	0.0002	0.000356582
GO:0046129	purine ribonucleoside biosynthetic process	36	0.265057915	0.0002	0.000356582
GO:0042490	mechanoreceptor differentiation	21	0.265024208	< 0.001	< 0.001
GO:0048167	regulation of synaptic plasticity	100	0.265006435	0.0072	0.008495371
GO:0030902	hindbrain development	47	0.26498535	< 0.001	< 0.001
GO:0001701	in utero embryonic development	130	0.264940105	< 0.001	< 0.001
GO:0006470	protein dephosphorylation	166	0.264936193	< 0.001	< 0.001
GO:0031529	ruffle organization	24	0.264929215	< 0.001	< 0.001
GO:0071902	positive regulation of protein serine/threonine kinase activity	144	0.26492564	< 0.001	< 0.001
GO:0032640	tumor necrosis factor production	47	0.264903201	< 0.001	< 0.001
GO:2000021	regulation of ion homeostasis	75	0.264895753	< 0.001	< 0.001
GO:2000514	regulation of CD4-positive, alpha-beta T cell activation	13	0.264884665	0.0001	0.000192793
GO:0016266	O-glycan processing	14	0.264846479	< 0.001	< 0.001
GO:0051225	spindle assembly	35	0.264813385	< 0.001	< 0.001
GO:0036297	interstrand cross-link repair	24	0.26474689	0.0016	0.002186748
GO:0010769	regulation of cell morphogenesis involved in differentiation	144	0.264707565	0.0002	0.000356582
GO:0010631	epithelial cell migration	116	0.264698442	< 0.001	< 0.001
GO:0090130	tissue migration	116	0.264698442	< 0.001	< 0.001
GO:0090132	epithelium migration	116	0.264698442	< 0.001	< 0.001
GO:0019395	fatty acid oxidation	33	0.264677665	< 0.001	< 0.001
GO:0032355	response to estradiol	41	0.264663967	< 0.001	< 0.001
GO:0070125	mitochondrial translational elongation	41	0.264638855	0.0007	0.001063182
GO:0070126	mitochondrial translational termination	41	0.264638855	0.0007	0.001063182
GO:0032212	positive regulation of telomere maintenance via telomerase	18	0.264636065	0.0005	0.000791911
GO:2000648	positive regulation of stem cell proliferation	11	0.264607465	0.0272	0.029199205
GO:0048846	axon extension involved in axon guidance	13	0.264607465	0.0001	0.000192793
GO:1902284	neuron projection extension involved in neuron projection guidance	13	0.264607465	0.0001	0.000192793
GO:0001755	neural crest cell migration	11	0.264584065	< 0.001	< 0.001
GO:1902165	regulation of intrinsic apoptotic signaling pathway in response to DNA damage by p53 class mediator	9	0.264578865	0.0475	0.049449046
GO:0031123	RNA 3'-end processing	41	0.264576074	0.0006	0.000927725
GO:0043583	ear development	71	0.26456396	< 0.001	< 0.001
GO:0036294	cellular response to decreased oxygen levels	94	0.26454996	0.0003	0.000505993
GO:0017038	protein import	75	0.264545689	0.0001	0.000192793
GO:0010544	negative regulation of platelet activation	9	0.264521665	< 0.001	< 0.001

GOIDlabel	GOName	size	categoryScores	pValPerm	pValPermCorr
GO:0006997	nucleus organization	55	0.264518545	< 0.001	< 0.001
GO:2001259	positive regulation of cation channel activity	25	0.264442728	0.0004	0.000648934
GO:0007548	sex differentiation	80	0.264440154	< 0.001	< 0.001
GO:0051238	sequestering of metal ion	54	0.264435864	0.0005	0.000791911
GO:0021681	cerebellar granular layer development	6	0.264392964	< 0.001	< 0.001
GO:0033081	regulation of T cell differentiation in thymus	9	0.264378664	0.0076	0.008916717
GO:2000398	regulation of thymocyte aggregation	9	0.264378664	0.0076	0.008916717
GO:0045839	negative regulation of mitotic nuclear division	21	0.264350064	0.0001	0.000192793
GO:0051784	negative regulation of nuclear division	21	0.264350064	0.0001	0.000192793
GO:1904064	positive regulation of cation transmembrane transport	48	0.264317889	0.0004	0.000648934
GO:0030514	negative regulation of BMP signaling pathway	12	0.264264264	0.0041	0.005057384
GO:0061448	connective tissue development	81	0.26421342	< 0.001	< 0.001
GO:0043248	proteasome assembly	9	0.264207064	0.0046	0.0056228
GO:0050711	negative regulation of interleukin-1 secretion	5	0.264144144	0.0104	0.011884458
GO:0050713	negative regulation of interleukin-1 beta secretion	5	0.264144144	0.0104	0.011884458
GO:0031664	regulation of lipopolysaccharide-mediated signaling pathway	12	0.264135564	< 0.001	< 0.001
GO:0071453	cellular response to oxygen levels	103	0.264095163	0.0003	0.000505993
GO:0010165	response to X-ray	11	0.264092664	0.006	0.007178216
GO:1901606	alpha-amino acid catabolic process	34	0.264085093	0.0008	0.001193265
GO:1901888	regulation of cell junction assembly	37	0.264030053	< 0.001	< 0.001
GO:0007389	pattern specification process	141	0.264021468	< 0.001	< 0.001
GO:0048387	negative regulation of retinoic acid receptor signaling pathway	5	0.263989704	< 0.001	< 0.001
GO:0050732	negative regulation of peptidyl-tyrosine phosphorylation	17	0.263971535	0.0012	0.001702047
GO:0051851	modification by host of symbiont morphology or physiology	26	0.263963964	< 0.001	< 0.001
GO:0048839	inner ear development	59	0.263944332	< 0.001	< 0.001
GO:0036475	neuron death in response to oxidative stress	12	0.263942514	< 0.001	< 0.001
GO:0048485	sympathetic nervous system development	6	0.263921064	0.0265	0.028498706
GO:0030282	bone mineralization	40	0.263873874	< 0.001	< 0.001
GO:1903902	positive regulation of viral life cycle	49	0.263866782	0.0009	0.001319479
GO:0034504	protein localization to nucleus	107	0.263866537	< 0.001	< 0.001
GO:0030258	lipid modification	74	0.26382135	< 0.001	< 0.001
GO:0001504	neurotransmitter uptake	17	0.263774699	0.0005	0.000791911
GO:0072073	kidney epithelium development	44	0.263753364	< 0.001	< 0.001
GO:0007173	epidermal growth factor receptor signaling pathway	49	0.263751215	< 0.001	< 0.001
GO:0010823	negative regulation of mitochondrion organization	24	0.263738739	0.0001	0.000192793
GO:0010812	negative regulation of cell-substrate adhesion	22	0.263729964	< 0.001	< 0.001
GO:0001895	retina homeostasis	22	0.263718264	< 0.001	< 0.001
GO:0038127	ERBB signaling pathway	64	0.263690476	< 0.001	< 0.001
GO:0061387	regulation of extent of cell growth	51	0.263628334	< 0.001	< 0.001
GO:0007158	neuron cell-cell adhesion	11	0.263601264	0.0212	0.023069835
GO:0060148	positive regulation of posttranscriptional gene silencing	11	0.263577864	< 0.001	< 0.001
GO:1904036	negative regulation of epithelial cell apoptotic process	9	0.263577864	0.0004	0.000648934
GO:0002456	T cell mediated immunity	20	0.263564994	0.0001	0.000192793
GO:0007126	meiotic nuclear division	48	0.263556414	< 0.001	< 0.001
GO:0007050	cell cycle arrest	99	0.263546664	< 0.001	< 0.001
GO:1903046	meiotic cell cycle process	52	0.263538264	< 0.001	< 0.001
GO:0008631	intrinsic apoptotic signaling pathway in response to oxidative stress	21	0.263528835	0.0001	0.000192793
GO:0050792	regulation of viral process	79	0.263483375	0.0003	0.000505993
GO:0071456	cellular response to hypoxia	89	0.263433257	0.0004	0.000648934
GO:1901798	positive regulation of signal transduction by p53 class mediator	7	0.263394006	0.0088	0.010185307
GO:0034644	cellular response to UV	29	0.263355967	< 0.001	< 0.001
GO:0009895	negative regulation of catabolic process	70	0.263346203	< 0.001	< 0.001
GO:0045921	positive regulation of exocytosis	44	0.263338013	0.0001	0.000192793
GO:0071353	cellular response to interleukin-4	13	0.263300663	< 0.001	< 0.001
GO:0010761	fibroblast migration	17	0.263290181	< 0.001	< 0.001
GO:0007250	activation of NF-kappaB-inducing kinase activity	5	0.263268983	0.0128	0.014368758
GO:0032147	activation of protein kinase activity	128	0.263233993	< 0.001	< 0.001
GO:0046488	phosphatidylinositol metabolic process	72	0.263209638	< 0.001	< 0.001
GO:0031016	pancreas development	20	0.263191763	< 0.001	< 0.001
GO:0030198	extracellular matrix organization	105	0.263188086	< 0.001	< 0.001
GO:0043062	extracellular structure organization	105	0.263188086	< 0.001	< 0.001
GO:0046782	regulation of viral transcription	29	0.263169574	0.0005	0.000791911
GO:0090280	positive regulation of calcium ion import	24	0.263148863	< 0.001	< 0.001
GO:1902275	regulation of chromatin organization	61	0.263113699	< 0.001	< 0.001
GO:0045600	positive regulation of fat cell differentiation	26	0.263063063	< 0.001	< 0.001
GO:0001667	ameboidal-type cell migration	149	0.263045788	< 0.001	< 0.001
GO:0002526	acute inflammatory response	36	0.263041613	< 0.001	< 0.001
GO:0043487	regulation of RNA stability	90	0.263040183	0.0003	0.000505993
GO:1903555	regulation of tumor necrosis factor superfamily cytokine production	47	0.26303568	< 0.001	< 0.001
GO:0010810	regulation of cell-substrate adhesion	76	0.263032581	< 0.001	< 0.001
GO:0030836	positive regulation of actin filament depolymerization	9	0.262948663	< 0.001	< 0.001
GO:0071294	cellular response to zinc ion	9	0.262891463	0.0148	0.01645324
GO:0050772	positive regulation of axonogenesis	38	0.262880173	< 0.001	< 0.001
GO:0051148	negative regulation of muscle cell differentiation	20	0.262857143	< 0.001	< 0.001
GO:0042753	positive regulation of circadian rhythm	6	0.262848563	0.0013	0.001826625
GO:0060986	endocrine hormone secretion	13	0.262785863	< 0.001	< 0.001
GO:2000677	regulation of transcription regulatory region DNA binding	19	0.262765021	< 0.001	< 0.001
GO:0032963	collagen metabolic process	23	0.262749706	< 0.001	< 0.001
GO:0044259	multicellular organismal macromolecule metabolic process	23	0.262749706	< 0.001	< 0.001
GO:0050821	protein stabilization	79	0.262747015	0.0001	0.000192793
GO:0043488	regulation of mRNA stability	89	0.262721791	0.0003	0.000505993
GO:0033673	negative regulation of kinase activity	107	0.262704627	< 0.001	< 0.001
GO:0008360	regulation of cell shape	48	0.262666238	< 0.001	< 0.001

GOIDlabel	GOName	size	categoryScores	pValPerm	pValPermCorr
GO:0031960	response to corticosteroid	50	0.262646075	< 0.001	< 0.001
GO:0009394	2'-deoxyribonucleotide metabolic process	12	0.262634063	0.0001	0.000192793
GO:0006692	prostanoid metabolic process	17	0.262623969	< 0.001	< 0.001
GO:0006693	prostaglandin metabolic process	17	0.262623969	< 0.001	< 0.001
GO:0007586	digestion	41	0.262541984	< 0.001	< 0.001
GO:1903901	negative regulation of viral life cycle	29	0.262521635	< 0.001	< 0.001
GO:0031018	endocrine pancreas development	15	0.262496782	< 0.001	< 0.001
GO:0031069	hair follicle morphogenesis	8	0.262483912	< 0.001	< 0.001
GO:0048730	epidermis morphogenesis	8	0.262483912	< 0.001	< 0.001
GO:0032922	circadian regulation of gene expression	25	0.262455598	0.0016	0.002186748
GO:0097119	postsynaptic density protein 95 clustering	7	0.262437948	0.0067	0.007954042
GO:0035418	protein localization to synapse	33	0.262423462	0.0091	0.010505533
GO:0065004	protein-DNA complex assembly	73	0.262403695	0.0002	0.000356582
GO:0060999	positive regulation of dendritic spine development	21	0.262376662	0.004	0.004954389
GO:0007614	short-term memory	5	0.262342342	0.0148	0.01645324
GO:0032760	positive regulation of tumor necrosis factor production	24	0.262280137	< 0.001	< 0.001
GO:0033273	response to vitamin	24	0.262183612	< 0.001	< 0.001
GO:0030326	embryonic limb morphogenesis	33	0.262150462	< 0.001	< 0.001
GO:0035113	embryonic appendage morphogenesis	33	0.262150462	< 0.001	< 0.001
GO:0060998	regulation of dendritic spine development	39	0.262066462	0.0083	0.009660392
GO:0070536	protein K63-linked deubiquitination	19	0.262060557	< 0.001	< 0.001
GO:0050870	positive regulation of T cell activation	59	0.262042187	< 0.001	< 0.001
GO:0045747	positive regulation of Notch signaling pathway	14	0.262033462	< 0.001	< 0.001
GO:0060972	left/right pattern formation	9	0.262004862	< 0.001	< 0.001
GO:0010935	regulation of macrophage cytokine production	5	0.261981982	0.0002	0.000356582
GO:0061684	chaperone-mediated autophagy	5	0.261981982	0.0003	0.000505993
GO:0071774	response to fibroblast growth factor	56	0.261890973	< 0.001	< 0.001
GO:0046777	protein autophosphorylation	94	0.261863687	0.0005	0.000791911
GO:1904377	positive regulation of protein localization to cell periphery	27	0.261861862	0.0001	0.000192793
GO:0045103	intermediate filament-based process	12	0.261861862	< 0.001	< 0.001
GO:0045104	intermediate filament cytoskeleton organization	12	0.261861862	< 0.001	< 0.001
GO:0003091	renal water homeostasis	16	0.261856499	< 0.001	< 0.001
GO:0007084	mitotic nuclear envelope reassembly	7	0.261849605	0.0038	0.004725081
GO:0031440	regulation of mRNA 3'-end processing	8	0.261840412	0.0058	0.006954338
GO:0030260	entry into host cell	38	0.261837025	< 0.001	< 0.001
GO:0044409	entry into host	38	0.261837025	< 0.001	< 0.001
GO:0051806	entry into cell of other organism involved in symbiotic interaction	38	0.261837025	< 0.001	< 0.001
GO:0051828	entry into other organism involved in symbiotic interaction	38	0.261837025	< 0.001	< 0.001
GO:0051961	negative regulation of nervous system development	131	0.261823219	< 0.001	< 0.001
GO:0070841	inclusion body assembly	8	0.261808237	0.0009	0.001319479
GO:0060324	face development	14	0.261794448	0.0001	0.000192793
GO:0007617	mating behavior	15	0.261793222	< 0.001	< 0.001
GO:0016054	organic acid catabolic process	80	0.261782497	< 0.001	< 0.001
GO:0046395	carboxylic acid catabolic process	80	0.261782497	< 0.001	< 0.001
GO:0051865	protein autoubiquitination	31	0.261767759	< 0.001	< 0.001
GO:0033630	positive regulation of cell adhesion mediated by integrin	9	0.261747462	0.0003	0.000505993
GO:0010759	positive regulation of macrophage chemotaxis	7	0.26173929	0.0001	0.000192793
GO:0060294	cilium movement involved in cell motility	5	0.261724582	< 0.001	< 0.001
GO:0045669	positive regulation of osteoblast differentiation	27	0.261690262	< 0.001	< 0.001
GO:1901343	negative regulation of vasculature development	38	0.26168123	< 0.001	< 0.001
GO:0090257	regulation of muscle system process	96	0.261676855	< 0.001	< 0.001
GO:2000637	positive regulation of gene silencing by miRNA	10	0.261673102	< 0.001	< 0.001
GO:0036150	phosphatidylserine acyl-chain remodeling	5	0.261673102	0.0001	0.000192793
GO:0010560	positive regulation of glycoprotein biosynthetic process	6	0.261561562	< 0.001	< 0.001
GO:1903020	positive regulation of glycoprotein metabolic process	6	0.261561562	< 0.001	< 0.001
GO:0045824	negative regulation of innate immune response	13	0.261558262	< 0.001	< 0.001
GO:1903306	negative regulation of regulated secretory pathway	10	0.261518662	< 0.001	< 0.001
GO:0097061	dendritic spine organization	47	0.261513185	0.0041	0.005057384
GO:0071824	protein-DNA complex subunit organization	81	0.261483706	0.0004	0.000648934
GO:0044344	cellular response to fibroblast growth factor stimulus	55	0.261439101	< 0.001	< 0.001
GO:0002449	lymphocyte mediated immunity	63	0.261420604	< 0.001	< 0.001
GO:0006661	phosphatidylinositol biosynthetic process	56	0.261371576	< 0.001	< 0.001
GO:1904874	positive regulation of telomerase RNA localization to Cajal body	10	0.261338481	0.0052	0.006299215
GO:0001676	long-chain fatty acid metabolic process	30	0.261329901	< 0.001	< 0.001
GO:0008361	regulation of cell size	81	0.261321639	< 0.001	< 0.001
GO:0060840	artery development	25	0.261271557	< 0.001	< 0.001
GO:0061028	establishment of endothelial barrier	16	0.261245174	< 0.001	< 0.001
GO:0002712	regulation of B cell mediated immunity	14	0.261206104	0.0016	0.002186748
GO:0002889	regulation of immunoglobulin mediated immune response	14	0.261206104	0.0016	0.002186748
GO:0036503	ERAD pathway	42	0.261187718	0.0001	0.000192793
GO:1902991	regulation of amyloid precursor protein catabolic process	16	0.261164736	0.0004	0.000648934
GO:0048813	dendrite morphogenesis	82	0.261123144	0.002	0.002664694
GO:0003300	cardiac muscle hypertrophy	35	0.261040632	0.0004	0.000648934
GO:0016358	dendrite development	129	0.261037782	0.0004	0.000648934
GO:0007600	sensory perception	186	0.261024619	< 0.001	< 0.001
GO:0045732	positive regulation of protein catabolic process	90	0.261012441	0.0001	0.000192793
GO:0044745	amino acid transmembrane import	5	0.260952381	0.0417	0.043705741
GO:0089718	amino acid import across plasma membrane	5	0.260952381	0.0417	0.043705741
GO:0010822	positive regulation of mitochondrion organization	68	0.260901658	0.0001	0.000192793
GO:0043392	negative regulation of DNA binding	19	0.260895482	0.0006	0.000927725
GO:0042773	ATP synthesis coupled electron transport	33	0.260879061	0.0069	0.008171729
GO:0043122	regulation of I-kappaB kinase/NF-kappaB signaling	92	0.260872363	< 0.001	< 0.001
GO:0009152	purine ribonucleotide biosynthetic process	67	0.260838664	< 0.001	< 0.001

GOIDlabel	GOName	size	categoryScores	pValPerm	pValPermCorr
GO:0048844	artery morphogenesis	16	0.260810811	< 0.001	< 0.001
GO:0014031	mesenchymal cell development	26	0.260805861	< 0.001	< 0.001
GO:0014032	neural crest cell development	26	0.260805861	< 0.001	< 0.001
GO:0045787	positive regulation of cell cycle	136	0.260769173	< 0.001	< 0.001
GO:0009314	response to radiation	172	0.260768908	< 0.001	< 0.001
GO:0031100	animal organ regeneration	19	0.260760008	< 0.001	< 0.001
GO:0031056	regulation of histone modification	54	0.260717861	< 0.001	< 0.001
GO:0072393	microtubule anchoring at microtubule organizing center	7	0.260672918	0.014	0.015631405
GO:0061050	regulation of cell growth involved in cardiac muscle cell development	9	0.260660661	0.0074	0.008712331
GO:0008625	extrinsic apoptotic signaling pathway via death domain receptors	28	0.260636146	0.0001	0.000192793
GO:1901799	negative regulation of proteasomal protein catabolic process	18	0.260632061	0.0006	0.000927725
GO:0032868	response to insulin	100	0.260617761	< 0.001	< 0.001
GO:0060996	dendritic spine development	54	0.260603461	0.0043	0.005276385
GO:0050867	positive regulation of cell activation	100	0.260576577	< 0.001	< 0.001
GO:0009799	specification of symmetry	42	0.260574861	< 0.001	< 0.001
GO:0009855	determination of bilateral symmetry	42	0.260574861	< 0.001	< 0.001
GO:0042775	mitochondrial ATP synthesis coupled electron transport	32	0.260553411	0.0077	0.00902229
GO:0098915	membrane repolarization during ventricular cardiac muscle cell action potential	5	0.260540541	0.0042	0.005168936
GO:0043410	positive regulation of MAPK cascade	199	0.260539506	< 0.001	< 0.001
GO:0001912	positive regulation of leukocyte mediated cytotoxicity	8	0.260521236	< 0.001	< 0.001
GO:0014896	muscle hypertrophy	36	0.260517661	0.0006	0.000927725
GO:0014897	striated muscle hypertrophy	36	0.260517661	0.0006	0.000927725
GO:0038061	NIK/NF-kappaB signaling	70	0.260496415	0.0005	0.000791911
GO:0060216	definitive hemopoiesis	6	0.26048906	< 0.001	< 0.001
GO:0046470	phosphatidylcholine metabolic process	26	0.26047916	0.0001	0.000192793
GO:0010811	positive regulation of cell-substrate adhesion	43	0.26045913	< 0.001	< 0.001
GO:0043001	Golgi to plasma membrane protein transport	13	0.26044946	0.0002	0.000356582
GO:0071496	cellular response to external stimulus	112	0.260443096	< 0.001	< 0.001
GO:0090596	sensory organ morphogenesis	74	0.260440363	< 0.001	< 0.001
GO:0043542	endothelial cell migration	85	0.260428496	< 0.001	< 0.001
GO:0021987	cerebral cortex development	56	0.260383343	< 0.001	< 0.001
GO:0009187	cyclic nucleotide metabolic process	33	0.26037206	0.0009	0.001319479
GO:0046541	saliva secretion	5	0.26033462	0.0242	0.026144902
GO:0042542	response to hydrogen peroxide	50	0.26030888	< 0.001	< 0.001
GO:0070509	calcium ion import	78	0.26029106	0.0008	0.001193265
GO:0034349	glial cell apoptotic process	6	0.26027456	0.0007	0.001063182
GO:2001021	negative regulation of response to DNA damage stimulus	38	0.260245208	< 0.001	< 0.001
GO:0050982	detection of mechanical stimulus	12	0.26023166	0.0009	0.001319479
GO:0022617	extracellular matrix disassembly	24	0.26023166	0.0016	0.002186748
GO:0000578	embryonic axis specification	11	0.26023166	< 0.001	< 0.001
GO:0070570	regulation of neuron projection regeneration	15	0.2602145	0.0077	0.00902229
GO:0070372	regulation of ERK1 and ERK2 cascade	106	0.260207377	< 0.001	< 0.001
GO:0032303	regulation of icosanoid secretion	8	0.260199485	< 0.001	< 0.001
GO:0014850	response to muscle activity	7	0.260194889	0.0054	0.006510843
GO:0001819	positive regulation of cytokine production	151	0.26016177	< 0.001	< 0.001
GO:0048514	blood vessel morphogenesis	182	0.260100132	< 0.001	< 0.001
GO:0072595	maintenance of protein localization in organelle	16	0.260054698	< 0.001	< 0.001
GO:0006091	generation of precursor metabolites and energy	183	0.26002349	< 0.001	< 0.001
GO:0046513	ceramide biosynthetic process	14	0.25997426	0.0002	0.000356582
GO:0001539	cilium or flagellum-dependent cell motility	10	0.25997426	0.0062	0.00739947
GO:0060285	cilium-dependent cell motility	10	0.25997426	0.0062	0.00739947
GO:1903078	positive regulation of protein localization to plasma membrane	25	0.259953668	0.0008	0.001193265
GO:0000289	nuclear-transcribed mRNA poly(A) tail shortening	11	0.25995086	0.0018	0.002428764
GO:0035278	miRNA mediated inhibition of translation	7	0.259937489	0.0001	0.000192793
GO:0040033	negative regulation of translation, ncRNA-mediated	7	0.259937489	0.0001	0.000192793
GO:0045974	regulation of translation, ncRNA-mediated	7	0.259937489	0.0001	0.000192793
GO:0007549	dosage compensation	7	0.259863946	0.0001	0.000192793
GO:0007093	mitotic cell cycle checkpoint	56	0.25984556	0.0002	0.000356582
GO:0002027	regulation of heart rate	35	0.259841883	0.0003	0.000505993
GO:0043409	negative regulation of MAPK cascade	74	0.259835125	0.0006	0.000927725
GO:0060669	embryonic placenta morphogenesis	8	0.259813385	0.0001	0.000192793
GO:0001823	mesonephros development	33	0.25977146	< 0.001	< 0.001
GO:0007143	female meiotic division	14	0.259735245	0.0005	0.000791911
GO:0071378	cellular response to growth hormone stimulus	14	0.259698474	< 0.001	< 0.001
GO:0048384	retinoic acid receptor signaling pathway	11	0.25964666	< 0.001	< 0.001
GO:0045454	cell redox homeostasis	30	0.25963964	< 0.001	< 0.001
GO:0070341	fat cell proliferation	6	0.25963106	0.0349	0.036944094
GO:0070344	regulation of fat cell proliferation	6	0.25963106	0.0349	0.036944094
GO:1901797	negative regulation of signal transduction by p53 class mediator	17	0.259626013	< 0.001	< 0.001
GO:0048872	homeostasis of number of cells	98	0.259619678	< 0.001	< 0.001
GO:0034501	protein localization to kinetochore	5	0.2596139	0.0109	0.012398148
GO:0071459	protein localization to chromosome, centromeric region	5	0.2596139	0.0109	0.012398148
GO:0032253	dense core granule localization	6	0.25958816	0.0265	0.028498706
GO:0099519	dense core granule cytoskeletal transport	6	0.25958816	0.0265	0.028498706
GO:1901950	dense core granule transport	6	0.25958816	0.0265	0.028498706
GO:0034446	substrate adhesion-dependent cell spreading	32	0.259547941	< 0.001	< 0.001
GO:2000059	negative regulation of protein ubiquitination involved in ubiquitin-dependent protein catabolic process	6	0.25950236	0.0116	0.013103344
GO:0051321	meiotic cell cycle	70	0.259481522	< 0.001	< 0.001
GO:2001243	negative regulation of intrinsic apoptotic signaling pathway	40	0.259388674	< 0.001	< 0.001
GO:0008584	male gonad development	39	0.259360459	< 0.001	< 0.001
GO:0046546	development of primary male sexual characteristics	39	0.259360459	< 0.001	< 0.001
GO:0036295	cellular response to increased oxygen levels	9	0.259345059	0.0002	0.000356582
GO:0006458	'de novo' protein folding	15	0.259287859	< 0.001	< 0.001

GOIDlabel	GOName	size	categoryScores	pValPerm	pValPermCorr
GO:0033500	carbohydrate homeostasis	93	0.259282324	< 0.001	< 0.001
GO:0042593	glucose homeostasis	93	0.259282324	< 0.001	< 0.001
GO:0009101	glycoprotein biosynthetic process	121	0.259280768	< 0.001	< 0.001
GO:0007368	determination of left/right symmetry	41	0.259271118	< 0.001	< 0.001
GO:0071384	cellular response to corticosteroid stimulus	18	0.259259259	< 0.001	< 0.001
GO:1904666	regulation of ubiquitin protein ligase activity	13	0.259241659	0.0001	0.000192793
GO:0042149	cellular response to glucose starvation	10	0.259227799	0.0022	0.002896881
GO:0021884	forebrain neuron development	15	0.259219219	0.0026	0.003356522
GO:0051963	regulation of synapse assembly	56	0.259202059	0.0011	0.001576346
GO:0032941	secretion by tissue	30	0.259193479	< 0.001	< 0.001
GO:0097502	mannosylation	14	0.259165288	< 0.001	< 0.001
GO:0000187	activation of MAPK activity	68	0.259156636	< 0.001	< 0.001
GO:0046629	gamma-delta T cell activation	6	0.259116259	0.0002	0.000356582
GO:0055098	response to low-density lipoprotein particle	6	0.259116259	0.0302	0.032240379
GO:0071404	cellular response to low-density lipoprotein particle stimulus	6	0.259116259	0.0302	0.032240379
GO:0034440	lipid oxidation	34	0.2590885	< 0.001	< 0.001
GO:0051348	negative regulation of transferase activity	121	0.259074423	< 0.001	< 0.001
GO:1902254	negative regulation of intrinsic apoptotic signaling pathway by p53 class mediator	12	0.259073359	0.0206	0.022453086
GO:0030101	natural killer cell activation	17	0.259050647	< 0.001	< 0.001
GO:0009100	glycoprotein metabolic process	139	0.259005768	< 0.001	< 0.001
GO:0071474	cellular hyperosmotic response	5	0.258944659	0.0179	0.019692915
GO:0007369	gastrulation	58	0.258935783	< 0.001	< 0.001
GO:0009109	coenzyme catabolic process	5	0.258893179	0.0002	0.000356582
GO:0043648	dicarboxylic acid metabolic process	40	0.258886744	< 0.001	< 0.001
GO:0006164	purine nucleotide biosynthetic process	68	0.258853812	< 0.001	< 0.001
GO:2000191	regulation of fatty acid transport	10	0.258841699	< 0.001	< 0.001
GO:0042303	molting cycle	33	0.258804259	< 0.001	< 0.001
GO:0042633	hair cycle	33	0.258804259	< 0.001	< 0.001
GO:1900087	positive regulation of G1/S transition of mitotic cell cycle	16	0.258751609	0.002	0.002664694
GO:0060314	regulation of ryanodine-sensitive calcium-release channel activity	17	0.258747823	0.0015	0.002069969
GO:2000601	positive regulation of Arp2/3 complex-mediated actin nucleation	6	0.258730159	0.0157	0.01736447
GO:0098542	defense response to other organism	134	0.258700705	< 0.001	< 0.001
GO:0001763	morphogenesis of a branching structure	60	0.258682969	< 0.001	< 0.001
GO:0002460	adaptive immune response based on somatic recombination of immune receptors built from immunoglobulin superfamily domains	62	0.258629136	< 0.001	< 0.001
GO:0032479	regulation of type I interferon production	49	0.258597957	0.0001	0.000192793
GO:0051899	membrane depolarization	26	0.258588259	< 0.001	< 0.001
GO:0007340	acrosome reaction	12	0.258537109	0.0006	0.000927725
GO:0015718	monocarboxylic acid transport	45	0.258509939	< 0.001	< 0.001
GO:0043255	regulation of carbohydrate biosynthetic process	40	0.258507079	< 0.001	< 0.001
GO:0055081	anion homeostasis	14	0.258485016	0.0003	0.000505993
GO:0002433	immune response-regulating cell surface receptor signaling pathway involved in phagocytosis	44	0.258435708	0.0001	0.000192793
GO:0038094	Fc-gamma receptor signaling pathway	44	0.258435708	0.0001	0.000192793
GO:0038096	Fc-gamma receptor signaling pathway involved in phagocytosis	44	0.258435708	0.0001	0.000192793
GO:0048285	organelle fission	163	0.258425121	< 0.001	< 0.001
GO:0006677	glycosylceramide metabolic process	7	0.258393087	0.0274	0.029402224
GO:0007520	myoblast fusion	11	0.258383058	0.0312	0.033235719
GO:1903054	negative regulation of extracellular matrix organization	5	0.258378378	0.0001	0.000192793
GO:0061640	cytoskeleton-dependent cytokinesis	32	0.258365508	< 0.001	< 0.001
GO:0070302	regulation of stress-activated protein kinase signaling cascade	94	0.258224486	< 0.001	< 0.001
GO:0045494	photoreceptor cell maintenance	13	0.258172458	< 0.001	< 0.001
GO:0030810	positive regulation of nucleotide biosynthetic process	23	0.258116502	0.0006	0.000927725
GO:1900373	positive regulation of purine nucleotide biosynthetic process	23	0.258116502	0.0006	0.000927725
GO:1900745	positive regulation of p38MAPK cascade	7	0.258098915	0.001	0.001450349
GO:0009880	embryonic pattern specification	21	0.258000858	< 0.001	< 0.001
GO:0014706	striated muscle tissue development	131	0.257962215	< 0.001	< 0.001
GO:1903203	regulation of oxidative stress-induced neuron death	11	0.257961858	< 0.001	< 0.001
GO:0010830	regulation of myotube differentiation	13	0.257875458	< 0.001	< 0.001
GO:0032409	regulation of transporter activity	114	0.257874416	0.0012	0.001702047
GO:0045981	positive regulation of nucleotide metabolic process	35	0.257863578	< 0.001	< 0.001
GO:1900544	positive regulation of purine nucleotide metabolic process	35	0.257863578	< 0.001	< 0.001
GO:0060411	cardiac septum morphogenesis	24	0.257797083	< 0.001	< 0.001
GO:1903307	positive regulation of regulated secretory pathway	27	0.257791124	0.0011	0.001576346
GO:0035728	response to hepatocyte growth factor	7	0.257767972	0.0001	0.000192793
GO:0035729	cellular response to hepatocyte growth factor stimulus	7	0.257767972	0.0001	0.000192793
GO:0060706	cell differentiation involved in embryonic placenta development	5	0.257760618	< 0.001	< 0.001
GO:0051403	stress-activated MAPK cascade	124	0.257751069	< 0.001	< 0.001
GO:0031098	stress-activated protein kinase signaling cascade	126	0.257725072	< 0.001	< 0.001
GO:0002052	positive regulation of neuroblast proliferation	7	0.257694429	0.0381	0.040181967
GO:0070542	response to fatty acid	36	0.257693408	< 0.001	< 0.001
GO:2001222	regulation of neuron migration	20	0.257619048	< 0.001	< 0.001
GO:0060537	muscle tissue development	137	0.257580625	< 0.001	< 0.001
GO:0051092	positive regulation of NF-kappaB transcription factor activity	60	0.257546118	< 0.001	< 0.001
GO:0032873	negative regulation of stress-activated MAPK cascade	22	0.257528958	0.0004	0.000648934
GO:0070303	negative regulation of stress-activated protein kinase signaling cascade	22	0.257528958	0.0004	0.000648934
GO:0006732	coenzyme metabolic process	132	0.257513358	< 0.001	< 0.001
GO:0032602	chemokine production	30	0.257365937	< 0.001	< 0.001
GO:1905207	regulation of cardiocyte differentiation	19	0.25733252	< 0.001	< 0.001
GO:0044272	sulfur compound biosynthetic process	75	0.257273273	< 0.001	< 0.001
GO:0097191	extrinsic apoptotic signaling pathway	75	0.257273273	< 0.001	< 0.001
GO:0007051	spindle organization	67	0.257265795	0.0001	0.000192793
GO:0019674	NAD metabolic process	34	0.257248845	< 0.001	< 0.001
GO:0045841	negative regulation of mitotic metaphase/anaphase transition	15	0.257211497	0.0027	0.003470695

GOIDlabel	GOName	size	categoryScores	pValPerm	pValPermCorr
GO:1902100	negative regulation of metaphase/anaphase transition of cell cycle	15	0.257211497	0.0027	0.003470695
GO:2000816	negative regulation of mitotic sister chromatid separation	15	0.257211497	0.0027	0.003470695
GO:0016202	regulation of striated muscle tissue development	45	0.257205777	< 0.001	< 0.001
GO:0048634	regulation of muscle organ development	45	0.257205777	< 0.001	< 0.001
GO:0000387	spliceosomal snRNP assembly	24	0.257175032	0.0004	0.000648934
GO:0034764	positive regulation of transmembrane transport	57	0.257151889	< 0.001	< 0.001
GO:2000377	regulation of reactive oxygen species metabolic process	65	0.257075537	< 0.001	< 0.001
GO:0007095	mitotic G2 DNA damage checkpoint	7	0.257069314	0.0106	0.012082345
GO:0044818	mitotic G2/M transition checkpoint	7	0.257069314	0.0106	0.012082345
GO:0009247	glycolipid biosynthetic process	23	0.257064518	0.0004	0.000648934
GO:0051251	positive regulation of lymphocyte activation	84	0.25702335	< 0.001	< 0.001
GO:0050803	regulation of synapse structure or activity	95	0.257007383	0.0003	0.000505993
GO:0009913	epidermal cell differentiation	74	0.2570072	< 0.001	< 0.001
GO:0048754	branching morphogenesis of an epithelial tube	44	0.256973207	< 0.001	< 0.001
GO:0090129	positive regulation of synapse maturation	6	0.256885457	0.0132	0.014787065
GO:1901861	regulation of muscle tissue development	46	0.256879861	< 0.001	< 0.001
GO:0002088	lens development in camera-type eye	29	0.256876581	0.0004	0.000648934
GO:0070169	positive regulation of biomineral tissue development	19	0.256844815	< 0.001	< 0.001
GO:0032872	regulation of stress-activated MAPK cascade	93	0.25680796	< 0.001	< 0.001
GO:0061351	neural precursor cell proliferation	55	0.256801217	< 0.001	< 0.001
GO:0030879	mammary gland development	55	0.256796537	< 0.001	< 0.001
GO:0072593	reactive oxygen species metabolic process	89	0.256784232	< 0.001	< 0.001
GO:0050884	neuromuscular process controlling posture	8	0.256756757	< 0.001	< 0.001
GO:0060291	long-term synaptic potentiation	44	0.256745057	0.0036	0.004505419
GO:0035994	response to muscle stretch	7	0.256738371	0.0005	0.000791911
GO:0051651	maintenance of location in cell	44	0.256709957	< 0.001	< 0.001
GO:0032986	protein-DNA complex disassembly	8	0.256692407	0.0341	0.036153894
GO:0045662	negative regulation of myoblast differentiation	8	0.256660232	< 0.001	< 0.001
GO:0014033	neural crest cell differentiation	28	0.256637249	< 0.001	< 0.001
GO:0031669	cellular response to nutrient levels	80	0.256618404	< 0.001	< 0.001
GO:0009260	ribonucleotide biosynthetic process	73	0.256585744	< 0.001	< 0.001
GO:0022898	regulation of transmembrane transporter activity	108	0.256556557	0.0015	0.002069969
GO:0070482	response to oxygen levels	157	0.256551001	0.0001	0.000192793
GO:0002190	cap-independent translational initiation	7	0.256517742	0.0001	0.000192793
GO:0015810	aspartate transport	6	0.256499356	0.013	0.014581154
GO:0060337	type I interferon signaling pathway	23	0.25648257	0.0002	0.000356582
GO:0071357	cellular response to type I interferon	23	0.25648257	0.0002	0.000356582
GO:1902115	regulation of organelle assembly	74	0.256481965	< 0.001	< 0.001
GO:0043647	inositol phosphate metabolic process	31	0.256461992	< 0.001	< 0.001
GO:0090305	nucleic acid phosphodiester bond hydrolysis	104	0.256390456	0.0001	0.000192793
GO:0008543	fibroblast growth factor receptor signaling pathway	45	0.256387816	< 0.001	< 0.001
GO:0048169	regulation of long-term neuronal synaptic plasticity	17	0.256370656	0.0147	0.016362266
GO:1902017	regulation of cilium assembly	23	0.256370656	< 0.001	< 0.001
GO:0045646	regulation of erythrocyte differentiation	21	0.256358399	< 0.001	< 0.001
GO:2000463	positive regulation of excitatory postsynaptic potential	19	0.256357109	0.0234	0.025351564
GO:0044264	cellular polysaccharide metabolic process	37	0.256328916	< 0.001	< 0.001
GO:0002755	MyD88-dependent toll-like receptor signaling pathway	12	0.256284856	0.0023	0.003013139
GO:0031330	negative regulation of cellular catabolic process	41	0.256263929	0.0002	0.000356582
GO:0002312	B cell activation involved in immune response	21	0.256260342	0.0014	0.00194839
GO:0031346	positive regulation of cell projection organization	171	0.256227656	< 0.001	< 0.001
GO:0032239	regulation of nucleobase-containing compound transport	5	0.256216216	0.0048	0.005840847
GO:0042982	amyloid precursor protein metabolic process	23	0.256202787	< 0.001	< 0.001
GO:0045685	regulation of glial cell differentiation	21	0.256199056	< 0.001	< 0.001
GO:0050864	regulation of B cell activation	36	0.256184756	< 0.001	< 0.001
GO:2001257	regulation of cation channel activity	70	0.256131642	0.0028	0.003584743
GO:1905268	negative regulation of chromatin organization	23	0.256113256	< 0.001	< 0.001
GO:1903557	positive regulation of tumor necrosis factor superfamily cytokine production	25	0.256072072	< 0.001	< 0.001
GO:0009581	detection of external stimulus	37	0.256057602	< 0.001	< 0.001
GO:0097484	dendrite extension	21	0.256027456	0.0081	0.00945
GO:0010459	negative regulation of heart rate	6	0.256027456	0.0003	0.000505993
GO:0009142	nucleoside triphosphate biosynthetic process	28	0.256012135	0.0001	0.000192793
GO:0031058	positive regulation of histone modification	34	0.256007268	0.0005	0.000791911
GO:0007016	cytoskeletal anchoring at plasma membrane	5	0.255958816	< 0.001	< 0.001
GO:0031648	protein destabilization	21	0.255941656	< 0.001	< 0.001
GO:0009190	cyclic nucleotide biosynthetic process	29	0.255926863	0.0008	0.001193265
GO:0052652	cyclic purine nucleotide metabolic process	29	0.255926863	0.0008	0.001193265
GO:0050769	positive regulation of neurogenesis	194	0.255886372	< 0.001	< 0.001
GO:1904427	positive regulation of calcium ion transmembrane transport	28	0.255874242	0.0043	0.005276385
GO:0030317	flagellated sperm motility	23	0.255867047	0.001	0.001450349
GO:0097722	sperm motility	23	0.255867047	0.001	0.001450349
GO:0006171	cAMP biosynthetic process	24	0.255845131	0.0018	0.002428764
GO:0030814	regulation of cAMP metabolic process	24	0.255845131	0.0018	0.002428764
GO:0030817	regulation of cAMP biosynthetic process	24	0.255845131	0.0018	0.002428764
GO:0045761	regulation of adenylate cyclase activity	24	0.255845131	0.0018	0.002428764
GO:0023058	adaptation of signaling pathway	10	0.255830116	0.0001	0.000192793
GO:0071385	cellular response to glucocorticoid stimulus	17	0.255810432	< 0.001	< 0.001
GO:1900038	negative regulation of cellular response to hypoxia	5	0.255804376	0.0006	0.000927725
GO:1903297	regulation of hypoxia-induced intrinsic apoptotic signaling pathway	5	0.255804376	0.0006	0.000927725
GO:1903298	negative regulation of hypoxia-induced intrinsic apoptotic signaling pathway	5	0.255804376	0.0006	0.000927725
GO:1990144	intrinsic apoptotic signaling pathway in response to hypoxia	5	0.255804376	0.0006	0.000927725
GO:0035360	positive regulation of peroxisome proliferator activated receptor signaling pathway	5	0.255752896	< 0.001	< 0.001
GO:0002066	columnar/cuboidal epithelial cell development	21	0.255733284	< 0.001	< 0.001
GO:0055074	calcium ion homeostasis	150	0.255730588	< 0.001	< 0.001

GOIDlabel	GOName	size	categoryScores	pValPerm	pValPermCorr
GO:0051186	cofactor metabolic process	153	0.255721267	< 0.001	< 0.001
GO:0002431	Fc receptor mediated stimulatory signaling pathway	45	0.255707136	0.0001	0.000192793
GO:0000717	nucleotide-excision repair, DNA duplex unwinding	12	0.255705706	0.0005	0.000791911
GO:0006778	porphyrin-containing compound metabolic process	11	0.255692056	0.0084	0.009757868
GO:0042168	heme metabolic process	11	0.255692056	0.0084	0.009757868
GO:0009648	photoperiodism	10	0.255598456	0.0111	0.012593827
GO:0048592	eye morphogenesis	44	0.255580906	< 0.001	< 0.001
GO:0051972	regulation of telomerase activity	21	0.255549427	< 0.001	< 0.001
GO:0001942	hair follicle development	27	0.255541256	< 0.001	< 0.001
GO:0022404	molting cycle process	27	0.255541256	< 0.001	< 0.001
GO:0022405	hair cycle process	27	0.255541256	< 0.001	< 0.001
GO:0090307	mitotic spindle assembly	21	0.255475884	0.0001	0.000192793
GO:0045058	T cell selection	13	0.255459855	0.0024	0.003116943
GO:0031398	positive regulation of protein ubiquitination	62	0.255448997	0.0001	0.000192793
GO:0010656	negative regulation of muscle cell apoptotic process	16	0.255405405	< 0.001	< 0.001
GO:0046622	positive regulation of organ growth	17	0.255356197	< 0.001	< 0.001
GO:0006517	protein deglycosylation	14	0.255304284	0.0001	0.000192793
GO:0002696	positive regulation of leukocyte activation	97	0.25528533	< 0.001	< 0.001
GO:0055017	cardiac muscle tissue growth	32	0.255252574	< 0.001	< 0.001
GO:1903706	regulation of hemopoiesis	156	0.255192555	< 0.001	< 0.001
GO:0008652	cellular amino acid biosynthetic process	33	0.255114855	< 0.001	< 0.001
GO:0022618	ribonucleoprotein complex assembly	87	0.255110283	0.0008	0.001193265
GO:0032799	low-density lipoprotein receptor particle metabolic process	12	0.255062205	0.0079	0.009236608
GO:0050710	negative regulation of cytokine secretion	21	0.255059141	< 0.001	< 0.001
GO:0032753	positive regulation of interleukin-4 production	5	0.255032175	0.0014	0.00194839
GO:0002028	regulation of sodium ion transport	33	0.255029055	< 0.001	< 0.001
GO:0051569	regulation of histone H3-K4 methylation	10	0.255006435	< 0.001	< 0.001
GO:0048524	positive regulation of viral process	53	0.255001093	0.0009	0.001319479
GO:0019751	polyol metabolic process	42	0.254985598	< 0.001	< 0.001
GO:0007517	muscle organ development	138	0.254964281	< 0.001	< 0.001
GO:0098869	cellular oxidant detoxification	32	0.254946911	0.0001	0.000192793
GO:0006275	regulation of DNA replication	44	0.254931555	0.0005	0.000791911
GO:0009582	detection of abiotic stimulus	39	0.254885655	0.0001	0.000192793
GO:0000280	nuclear division	146	0.254882671	< 0.001	< 0.001
GO:0050807	regulation of synapse organization	92	0.254882211	0.0002	0.000356582
GO:0019692	deoxyribose phosphate metabolic process	13	0.254865855	0.0001	0.000192793
GO:0051928	positive regulation of calcium ion transport	46	0.254854233	0.0007	0.001063182
GO:0007492	endoderm development	20	0.254851995	< 0.001	< 0.001
GO:0035239	tube morphogenesis	122	0.254811486	< 0.001	< 0.001
GO:0032496	response to lipopolysaccharide	103	0.254808762	< 0.001	< 0.001
GO:0000963	mitochondrial RNA processing	6	0.254783355	0.0027	0.003470695
GO:0071560	cellular response to transforming growth factor beta stimulus	88	0.25477068	< 0.001	< 0.001
GO:0044723	single-organism carbohydrate metabolic process	179	0.254762983	< 0.001	< 0.001
GO:0007215	glutamate receptor signaling pathway	49	0.254736953	0.0147	0.016362266
GO:0045823	positive regulation of heart contraction	15	0.254688975	0.0011	0.001576346
GO:0046320	regulation of fatty acid oxidation	15	0.254671815	< 0.001	< 0.001
GO:0034502	protein localization to chromosome	33	0.254639055	0.0005	0.000791911
GO:0006536	glutamate metabolic process	17	0.254614278	< 0.001	< 0.001
GO:0034063	stress granule assembly	10	0.254594595	0.0006	0.000927725
GO:0045638	negative regulation of myeloid cell differentiation	26	0.254509455	< 0.001	< 0.001
GO:0034121	regulation of toll-like receptor signaling pathway	27	0.254492588	0.0004	0.000648934
GO:0007033	vacuole organization	82	0.254490379	0.0002	0.000356582
GO:0033189	response to vitamin A	5	0.254465894	0.0102	0.011688041
GO:0001782	B cell homeostasis	11	0.254451854	0.0003	0.000505993
GO:0031055	chromatin remodeling at centromere	15	0.254448734	0.0153	0.01697069
GO:1901998	toxin transport	15	0.254448734	0.0001	0.000192793
GO:0003151	outflow tract morphogenesis	27	0.254406788	< 0.001	< 0.001
GO:0006584	catecholamine metabolic process	22	0.254381654	< 0.001	< 0.001
GO:0009712	catechol-containing compound metabolic process	22	0.254381654	< 0.001	< 0.001
GO:0009156	ribonucleoside monophosphate biosynthetic process	37	0.254381022	0.0002	0.000356582
GO:0045682	regulation of epidermis development	23	0.254345028	< 0.001	< 0.001
GO:0045666	positive regulation of neuron differentiation	165	0.254342654	0.0001	0.000192793
GO:1901881	positive regulation of protein depolymerization	13	0.254331254	0.0001	0.000192793
GO:0060074	synapse maturation	18	0.254325754	0.0011	0.001576346
GO:0002040	sprouting angiogenesis	37	0.254269714	0.0007	0.001063182
GO:0032653	regulation of interleukin-10 production	12	0.254268554	0.0015	0.002069969
GO:0035270	endocrine system development	36	0.254254254	< 0.001	< 0.001
GO:1903829	positive regulation of cellular protein localization	152	0.254216623	< 0.001	< 0.001
GO:0044088	regulation of vacuole organization	25	0.254198198	< 0.001	< 0.001
GO:0009062	fatty acid catabolic process	27	0.254177988	< 0.001	< 0.001
GO:0071826	ribonucleoprotein complex subunit organization	92	0.254177159	0.0003	0.000505993
GO:0031929	TOR signaling	48	0.254166667	< 0.001	< 0.001
GO:0098813	nuclear chromosome segregation	91	0.254130426	< 0.001	< 0.001
GO:0060688	regulation of morphogenesis of a branching structure	16	0.254118404	< 0.001	< 0.001
GO:1905477	positive regulation of protein localization to membrane	64	0.25411036	< 0.001	< 0.001
GO:0046661	male sex differentiation	43	0.254107929	< 0.001	< 0.001
GO:0009416	response to light stimulus	119	0.254023772	< 0.001	< 0.001
GO:0034249	negative regulation of cellular amide metabolic process	97	0.253916066	< 0.001	< 0.001
GO:0010824	regulation of centrosome duplication	20	0.253912484	< 0.001	< 0.001
GO:0007339	binding of sperm to zona pellucida	14	0.253906968	< 0.001	< 0.001
GO:0035036	sperm-egg recognition	14	0.253906968	< 0.001	< 0.001
GO:0071622	regulation of granulocyte chemotaxis	17	0.253902642	< 0.001	< 0.001
GO:0072522	purine-containing compound biosynthetic process	70	0.253855488	< 0.001	< 0.001

GOIDlabel	GOName	size	categoryScores	pValPerm	pValPermCorr
GO:0002224	toll-like receptor signaling pathway	57	0.253846328	< 0.001	< 0.001
GO:0008277	regulation of G-protein coupled receptor protein signaling pathway	56	0.253838022	< 0.001	< 0.001
GO:0043588	skin development	101	0.253783911	< 0.001	< 0.001
GO:0046718	viral entry into host cell	33	0.253773254	< 0.001	< 0.001
GO:0006890	retrograde vesicle-mediated transport, Golgi to ER	38	0.253749238	0.0003	0.000505993
GO:1901342	regulation of vasculature development	95	0.253688275	< 0.001	< 0.001
GO:0043153	entrainment of circadian clock by photoperiod	8	0.253667954	0.041	0.043030491
GO:0019371	cyclooxygenase pathway	6	0.253667954	< 0.001	< 0.001
GO:1903051	negative regulation of proteolysis involved in cellular protein catabolic process	24	0.253657229	0.0005	0.000791911
GO:0009112	nucleobase metabolic process	12	0.253603604	< 0.001	< 0.001
GO:1901028	regulation of mitochondrial outer membrane permeabilization involved in apoptotic signaling pathway	19	0.253593443	0.002	0.002664694
GO:0046605	regulation of centrosome cycle	24	0.253592879	< 0.001	< 0.001
GO:0090646	mitochondrial tRNA processing	5	0.253590734	0.0009	0.001319479
GO:0070723	response to cholesterol	12	0.253582154	< 0.001	< 0.001
GO:0034508	centromere complex assembly	18	0.253567854	0.0088	0.010185307
GO:2000826	regulation of heart morphogenesis	11	0.253492453	< 0.001	< 0.001
GO:1901605	alpha-amino acid metabolic process	76	0.253457969	< 0.001	< 0.001
GO:0060249	anatomical structure homeostasis	161	0.253454519	< 0.001	< 0.001
GO:0030316	osteoclast differentiation	31	0.253364886	< 0.001	< 0.001
GO:0016331	morphogenesis of embryonic epithelium	59	0.253356019	< 0.001	< 0.001
GO:0006874	cellular calcium ion homeostasis	146	0.253340033	< 0.001	< 0.001
GO:0036293	response to decreased oxygen levels	147	0.253329131	0.0002	0.000356582
GO:0072503	cellular divalent inorganic cation homeostasis	149	0.253316404	< 0.001	< 0.001
GO:0050871	positive regulation of B cell activation	28	0.253309432	< 0.001	< 0.001
GO:1903707	negative regulation of hemopoiesis	44	0.253305253	< 0.001	< 0.001
GO:0006942	regulation of striated muscle contraction	40	0.253236808	< 0.001	< 0.001
GO:0061307	cardiac neural crest cell differentiation involved in heart development	5	0.253230373	0.0032	0.004042263
GO:0061308	cardiac neural crest cell development involved in heart development	5	0.253230373	0.0032	0.004042263
GO:0070585	protein localization to mitochondrion	71	0.253223848	0.0002	0.000356582
GO:0001502	cartilage condensation	7	0.25320831	0.0358	0.037852319
GO:0072132	mesenchyme morphogenesis	15	0.253196053	< 0.001	< 0.001
GO:0046390	ribose phosphate biosynthetic process	75	0.253178893	< 0.001	< 0.001
GO:0032606	type I interferon production	51	0.253175865	0.0002	0.000356582
GO:1903039	positive regulation of leukocyte cell-cell adhesion	64	0.253157175	< 0.001	< 0.001
GO:0006790	sulfur compound metabolic process	133	0.253107673	< 0.001	< 0.001
GO:0006457	protein folding	90	0.253095953	0.0001	0.000192793
GO:1902692	regulation of neuroblast proliferation	8	0.253088803	0.0148	0.01645324
GO:0002688	regulation of leukocyte chemotaxis	36	0.253010153	< 0.001	< 0.001
GO:0051606	detection of stimulus	88	0.253006903	< 0.001	< 0.001
GO:0007249	I-kappaB kinase/NF-kappaB signaling	103	0.253001962	< 0.001	< 0.001
GO:0098773	skin epidermis development	28	0.252996874	< 0.001	< 0.001
GO:0015985	energy coupled proton transport, down electrochemical gradient	8	0.252960103	0.0196	0.021436632
GO:0015986	ATP synthesis coupled proton transport	8	0.252960103	0.0196	0.021436632
GO:0042776	mitochondrial ATP synthesis coupled proton transport	8	0.252960103	0.0196	0.021436632
GO:0042157	lipoprotein metabolic process	41	0.252942838	0.0001	0.000192793
GO:0086001	cardiac muscle cell action potential	24	0.252895753	0.0033	0.004153051
GO:0071604	transforming growth factor beta production	15	0.252887173	< 0.001	< 0.001
GO:0071634	regulation of transforming growth factor beta production	15	0.252887173	< 0.001	< 0.001
GO:0071559	response to transforming growth factor beta	90	0.252829973	< 0.001	< 0.001
GO:0000491	small nucleolar ribonucleoprotein complex assembly	5	0.252818533	0.0143	0.015940017
GO:0045737	positive regulation of cyclin-dependent protein serine/threonine kinase activity	15	0.252801373	0.0005	0.000791911
GO:0032609	interferon-gamma production	30	0.252767053	< 0.001	< 0.001
GO:0021782	glial cell development	28	0.252711896	< 0.001	< 0.001
GO:0042632	cholesterol homeostasis	21	0.25269351	0.0001	0.000192793
GO:0055092	sterol homeostasis	21	0.25269351	0.0001	0.000192793
GO:0009124	nucleoside monophosphate biosynthetic process	39	0.252681253	0.0002	0.000356582
GO:0002237	response to molecule of bacterial origin	106	0.252655351	< 0.001	< 0.001
GO:0005976	polysaccharide metabolic process	43	0.252647332	< 0.001	< 0.001
GO:0070373	negative regulation of ERK1 and ERK2 cascade	30	0.252646933	0.0024	0.003116943
GO:0010976	positive regulation of neuron projection development	133	0.252616096	0.0001	0.000192793
GO:2001235	positive regulation of apoptotic signaling pathway	73	0.252615433	< 0.001	< 0.001
GO:0042058	regulation of epidermal growth factor receptor signaling pathway	36	0.252609753	0.0001	0.000192793
GO:1902110	positive regulation of mitochondrial membrane permeability involved in apoptotic process	26	0.252588853	0.0007	0.001063182
GO:0050687	negative regulation of defense response to virus	6	0.252552553	0.0214	0.023282786
GO:0048708	astrocyte differentiation	23	0.252532035	0.0011	0.001576346
GO:0035335	peptidyl-tyrosine dephosphorylation	59	0.252527103	< 0.001	< 0.001
GO:0010821	regulation of mitochondrion organization	99	0.252525253	0.0002	0.000356582
GO:1902230	negative regulation of intrinsic apoptotic signaling pathway in response to DNA damage	17	0.252524794	0.0021	0.002780108
GO:0006979	response to oxidative stress	160	0.252524131	< 0.001	< 0.001
GO:0018126	protein hydroxylation	16	0.252493565	0.0002	0.000356582
GO:0045727	positive regulation of translation	60	0.252475332	0.0001	0.000192793
GO:0010038	response to metal ion	126	0.252411595	< 0.001	< 0.001
GO:0030511	positive regulation of transforming growth factor beta receptor signaling pathway	10	0.252406692	0.0002	0.000356582
GO:1903846	positive regulation of cellular response to transforming growth factor beta stimulus	10	0.252406692	0.0002	0.000356582
GO:0050921	positive regulation of chemotaxis	51	0.252378429	< 0.001	< 0.001
GO:0001553	luteinization	5	0.252355212	0.0002	0.000356582
GO:0032412	regulation of ion transmembrane transporter activity	104	0.252319077	0.0018	0.002428764
GO:0033151	V(D)J recombination	6	0.252295152	0.0386	0.040653752
GO:0010632	regulation of epithelial cell migration	93	0.252291001	< 0.001	< 0.001
GO:0001666	response to hypoxia	142	0.25228488	0.0002	0.000356582
GO:0043467	regulation of generation of precursor metabolites and energy	55	0.252275652	0.0002	0.000356582
GO:0019320	hexose catabolic process	23	0.252263444	0.0017	0.00231289
GO:0045924	regulation of female receptivity	5	0.252252252	0.0169	0.018649704

GOIDlabel	GOName	size	categoryScores	pValPerm	pValPermCorr
GO:0044794	positive regulation by host of viral process	6	0.252252252	0.0015	0.002069969
GO:2001224	positive regulation of neuron migration	7	0.252215481	0.0003	0.000505993
GO:0031667	response to nutrient levels	156	0.252211002	< 0.001	< 0.001
GO:0045922	negative regulation of fatty acid metabolic process	12	0.252187902	< 0.001	< 0.001
GO:0033692	cellular polysaccharide biosynthetic process	26	0.252133452	< 0.001	< 0.001
GO:0030098	lymphocyte differentiation	94	0.252115337	< 0.001	< 0.001
GO:0098815	modulation of excitatory postsynaptic potential	26	0.252054252	0.0145	0.016156289
GO:0050679	positive regulation of epithelial cell proliferation	59	0.252003577	< 0.001	< 0.001
GO:0021545	cranial nerve development	14	0.251994852	0.0014	0.00194839
GO:0071706	tumor necrosis factor superfamily cytokine production	50	0.251953668	< 0.001	< 0.001
GO:0060562	epithelial tube morphogenesis	109	0.251949984	< 0.001	< 0.001
GO:0061337	cardiac conduction	52	0.251940402	0.0017	0.00231289
GO:2000310	regulation of N-methyl-D-aspartate selective glutamate receptor activity	23	0.251927704	0.0128	0.014368758
GO:0002029	desensitization of G-protein coupled receptor protein signaling pathway	9	0.251909052	0.0001	0.000192793
GO:0022401	negative adaptation of signaling pathway	9	0.251909052	0.0001	0.000192793
GO:0031290	retinal ganglion cell axon guidance	11	0.251901252	0.0366	0.038652804
GO:0072655	establishment of protein localization to mitochondrion	70	0.251866152	0.0002	0.000356582
GO:0032649	regulation of interferon-gamma production	26	0.251856252	< 0.001	< 0.001
GO:0050996	positive regulation of lipid catabolic process	9	0.251851852	0.0007	0.001063182
GO:0040017	positive regulation of locomotion	192	0.251836658	< 0.001	< 0.001
GO:1902668	negative regulation of axon guidance	6	0.251823252	0.003	0.003811001
GO:0071470	cellular response to osmotic stress	12	0.251780352	0.0013	0.001826625
GO:0007498	mesoderm development	46	0.251776621	< 0.001	< 0.001
GO:0070670	response to interleukin-4	15	0.251771772	< 0.001	< 0.001
GO:0010042	response to manganese ion	8	0.251769627	0.001	0.001450349
GO:0006936	muscle contraction	128	0.251767616	< 0.001	< 0.001
GO:0043406	positive regulation of MAP kinase activity	101	0.251719612	< 0.001	< 0.001
GO:0001558	regulation of cell growth	180	0.251608752	< 0.001	< 0.001
GO:0030816	positive regulation of cAMP metabolic process	16	0.251608752	0.0058	0.006954338
GO:0030819	positive regulation of cAMP biosynthetic process	16	0.251608752	0.0058	0.006954338
GO:0045762	positive regulation of adenylate cyclase activity	16	0.251608752	0.0058	0.006954338
GO:0072507	divalent inorganic cation homeostasis	156	0.251574102	< 0.001	< 0.001
GO:0044262	cellular carbohydrate metabolic process	107	0.251501702	< 0.001	< 0.001
GO:0034694	response to prostaglandin	15	0.251497211	< 0.001	< 0.001
GO:0042113	B cell activation	67	0.251483893	< 0.001	< 0.001
GO:0010837	regulation of keratinocyte proliferation	14	0.251480051	< 0.001	< 0.001
GO:0071360	cellular response to exogenous dsRNA	6	0.251437151	0.0148	0.01645324
GO:0034976	response to endoplasmic reticulum stress	118	0.251408067	0.0002	0.000356582
GO:1990748	cellular detoxification	34	0.25132864	< 0.001	< 0.001
GO:0055094	response to lipoprotein particle	7	0.251296194	0.0053	0.006400268
GO:0071402	cellular response to lipoprotein particle stimulus	7	0.251296194	0.0053	0.006400268
GO:0030071	regulation of mitotic metaphase/anaphase transition	22	0.251269451	0.0058	0.006954338
GO:1902099	regulation of metaphase/anaphase transition of cell cycle	22	0.251269451	0.0058	0.006954338
GO:0014009	glial cell proliferation	22	0.251222651	0.0004	0.000648934
GO:2000249	regulation of actin cytoskeleton reorganization	16	0.251206564	0.0013	0.001826625
GO:0046685	response to arsenic-containing substance	11	0.251175851	< 0.001	< 0.001
GO:0034243	regulation of transcription elongation from RNA polymerase II promoter	8	0.251158301	0.0022	0.002896881
GO:0035794	positive regulation of mitochondrial membrane permeability	27	0.251098718	0.0007	0.001063182
GO:1902686	mitochondrial outer membrane permeabilization involved in programmed cell death	27	0.251098718	0.0007	0.001063182
GO:1905710	positive regulation of membrane permeability	27	0.251098718	0.0007	0.001063182
GO:0046710	GDP metabolic process	6	0.251093951	< 0.001	< 0.001
GO:0006720	isoprenoid metabolic process	36	0.251051051	< 0.001	< 0.001
GO:0032414	positive regulation of ion transmembrane transporter activity	40	0.251029601	0.0003	0.000505993
GO:0086009	membrane repolarization	17	0.250919827	0.0008	0.001193265
GO:1902893	regulation of pri-miRNA transcription from RNA polymerase II promoter	20	0.250875161	< 0.001	< 0.001
GO:0006909	phagocytosis	100	0.250870013	< 0.001	< 0.001
GO:0007589	body fluid secretion	34	0.250866833	< 0.001	< 0.001
GO:0044724	single-organism carbohydrate catabolic process	63	0.250842679	< 0.001	< 0.001
GO:1901099	negative regulation of signal transduction in absence of ligand	7	0.250744622	0.0003	0.000505993
GO:2001240	negative regulation of extrinsic apoptotic signaling pathway in absence of ligand	7	0.250744622	0.0003	0.000505993
GO:0071478	cellular response to radiation	65	0.250739531	< 0.001	< 0.001
GO:0031214	biomineral tissue development	49	0.250734116	< 0.001	< 0.001
GO:0032148	activation of protein kinase B activity	15	0.250725011	< 0.001	< 0.001
GO:0051235	maintenance of location	115	0.250685468	< 0.001	< 0.001
GO:0060538	skeletal muscle organ development	59	0.250668586	< 0.001	< 0.001
GO:0001523	retinoid metabolic process	24	0.250654226	< 0.001	< 0.001
GO:0009267	cellular response to starvation	58	0.250650158	< 0.001	< 0.001
GO:0016052	carbohydrate catabolic process	65	0.250604891	< 0.001	< 0.001
GO:0048662	negative regulation of smooth muscle cell proliferation	15	0.250604891	< 0.001	< 0.001
GO:0006855	drug transmembrane transport	5	0.250604891	0.0254	0.027370209
GO:0045834	positive regulation of lipid metabolic process	46	0.250590342	< 0.001	< 0.001
GO:0008038	neuron recognition	24	0.250589876	0.0017	0.00231289
GO:0021602	cranial nerve morphogenesis	10	0.250579151	0.0002	0.000356582
GO:0014902	myotube differentiation	36	0.25047905	0.0016	0.002186748
GO:1902742	apoptotic process involved in development	11	0.25047385	< 0.001	< 0.001
GO:0007099	centriole replication	14	0.25045045	< 0.001	< 0.001
GO:0098534	centriole assembly	14	0.25045045	< 0.001	< 0.001
GO:1901021	positive regulation of calcium ion transmembrane transporter activity	18	0.25043615	0.0042	0.005168936
GO:1901379	regulation of potassium ion transmembrane transport	33	0.25043485	0.0003	0.000505993
GO:0043030	regulation of macrophage activation	14	0.250413679	< 0.001	< 0.001
GO:0090181	regulation of cholesterol metabolic process	35	0.250406325	0.0005	0.000791911
GO:0071071	regulation of phospholipid biosynthetic process	8	0.250353925	< 0.001	< 0.001
GO:0033077	T cell differentiation in thymus	22	0.25033345	0.002	0.002664694

GOIDlabel	GOName	size	categoryScores	pValPerm	pValPermCorr
GO:0071594	thymocyte aggregation	22	0.25033345	0.002	0.002664694
GO:0048762	mesenchymal cell differentiation	69	0.250327346	< 0.001	< 0.001
GO:0097300	programmed necrotic cell death	10	0.25029601	0.0066	0.007843941
GO:0043266	regulation of potassium ion transport	37	0.250269575	0.0002	0.000356582
GO:0097104	postsynaptic membrane assembly	6	0.25023595	0.0198	0.021642233
GO:0006941	striated muscle contraction	69	0.250234085	< 0.001	< 0.001
GO:0006379	mRNA cleavage	8	0.250225225	0.0031	0.003925117
GO:0021536	diencephalon development	20	0.25020592	< 0.001	< 0.001
GO:0045333	cellular respiration	65	0.25020097	0.0005	0.000791911
GO:2000351	regulation of endothelial cell apoptotic process	10	0.25019305	0.0008	0.001193265
GO:0006999	nuclear pore organization	6	0.25015015	0.0004	0.000648934
GO:0010635	regulation of mitochondrial fusion	5	0.25014157	0.0102	0.011688041
GO:0006301	postreplication repair	21	0.250119507	< 0.001	< 0.001
GO:0035821	modification of morphology or physiology of other organism	57	0.250053061	< 0.001	< 0.001
GO:0010739	positive regulation of protein kinase A signaling	5	0.25003861	0.0002	0.000356582
GO:0007254	JNK cascade	97	0.250028526	< 0.001	< 0.001
GO:0046634	regulation of alpha-beta T cell activation	20	0.25	0.0002	0.000356582
GO:0045637	regulation of myeloid cell differentiation	76	0.249972905	< 0.001	< 0.001
GO:0035561	regulation of chromatin binding	7	0.249972421	0.0063	0.007507211
GO:0019216	regulation of lipid metabolic process	164	0.249954484	< 0.001	< 0.001
GO:0010559	regulation of glycoprotein biosynthetic process	14	0.24993565	< 0.001	< 0.001
GO:0060485	mesenchyme development	83	0.249882929	< 0.001	< 0.001
GO:0099601	regulation of neurotransmitter receptor activity	38	0.249861139	0.0112	0.012688637
GO:0042254	ribosome biogenesis	105	0.249842496	0.0011	0.001576346
GO:0035282	segmentation	26	0.24983665	0.0037	0.004617737
GO:1903367	positive regulation of fear response	5	0.24978121	0.0071	0.008390203
GO:2000987	positive regulation of behavioral fear response	5	0.24978121	0.0071	0.008390203
GO:0009145	purine nucleoside triphosphate biosynthetic process	21	0.24976405	0.0013	0.001826625
GO:0009206	purine ribonucleoside triphosphate biosynthetic process	21	0.24976405	0.0013	0.001826625
GO:0000819	sister chromatid segregation	70	0.249737084	0.0001	0.000192793
GO:1901626	regulation of postsynaptic membrane organization	9	0.24967825	0.0001	0.000192793
GO:0032642	regulation of chemokine production	29	0.249651622	< 0.001	< 0.001
GO:0006766	vitamin metabolic process	42	0.249647607	< 0.001	< 0.001
GO:0031057	negative regulation of histone modification	22	0.24959635	< 0.001	< 0.001
GO:0017148	negative regulation of translation	91	0.249567935	0.0001	0.000192793
GO:1903779	regulation of cardiac conduction	24	0.249517375	0.0001	0.000192793
GO:0010634	positive regulation of epithelial cell migration	54	0.249516183	< 0.001	< 0.001
GO:0043502	regulation of muscle adaptation	36	0.2495138	< 0.001	< 0.001
GO:0032411	positive regulation of transporter activity	43	0.249438808	0.0001	0.000192793
GO:0006837	serotonin transport	6	0.249377949	0.0001	0.000192793
GO:0090128	regulation of synapse maturation	10	0.249369369	0.0053	0.006400268
GO:0055025	positive regulation of cardiac muscle tissue development	19	0.249353113	< 0.001	< 0.001
GO:0034767	positive regulation of ion transmembrane transport	54	0.249335049	0.0003	0.000505993
GO:0030216	keratinocyte differentiation	59	0.249303056	< 0.001	< 0.001
GO:0043518	negative regulation of DNA damage response, signal transduction by p53 class mediator	9	0.249249249	< 0.001	< 0.001
GO:0019042	viral latency	8	0.249227799	0.0001	0.000192793
GO:0060135	maternal process involved in female pregnancy	24	0.249184899	< 0.001	< 0.001
GO:0046328	regulation of JNK cascade	76	0.249163449	< 0.001	< 0.001
GO:0050685	positive regulation of mRNA processing	8	0.249163449	0.0002	0.000356582
GO:0034470	ncRNA processing	141	0.249145194	0.0007	0.001063182
GO:1900449	regulation of glutamate receptor signaling pathway	38	0.249136354	0.0144	0.016048175
GO:0021756	striatum development	9	0.249134849	0.0041	0.005057384
GO:0006626	protein targeting to mitochondrion	47	0.249125113	0.0003	0.000505993
GO:0001936	regulation of endothelial cell proliferation	42	0.249120549	< 0.001	< 0.001
GO:0051272	positive regulation of cellular component movement	186	0.24910671	< 0.001	< 0.001
GO:1903427	negative regulation of reactive oxygen species biosynthetic process	11	0.249046449	< 0.001	< 0.001
GO:0022409	positive regulation of cell-cell adhesion	75	0.249043329	< 0.001	< 0.001
GO:0046365	monosaccharide catabolic process	24	0.249002574	0.0026	0.003356522
GO:0090501	RNA phosphodiester bond hydrolysis	50	0.248957529	0.002	0.002664694
GO:0051438	regulation of ubiquitin-protein transferase activity	25	0.248957529	0.0002	0.000356582
GO:1901184	regulation of ERBB signaling pathway	40	0.248835264	< 0.001	< 0.001
GO:0051495	positive regulation of cytoskeleton organization	91	0.24880422	0.0002	0.000356582
GO:0032092	positive regulation of protein binding	41	0.248761654	< 0.001	< 0.001
GO:0001947	heart looping	19	0.248757028	< 0.001	< 0.001
GO:0061371	determination of heart left/right asymmetry	19	0.248757028	< 0.001	< 0.001
GO:0006356	regulation of transcription from RNA polymerase I promoter	15	0.248751609	0.0006	0.000927725
GO:0043489	RNA stabilization	17	0.248739496	0.001	0.001450349
GO:0031639	plasminogen activation	5	0.248648649	0.0002	0.000356582
GO:0010743	regulation of macrophage derived foam cell differentiation	5	0.248648649	0.0444	0.046382679
GO:0007052	mitotic spindle organization	41	0.248617258	0.0002	0.000356582
GO:0072678	T cell migration	16	0.248616474	0.0002	0.000356582
GO:0000245	spliceosomal complex assembly	20	0.248610039	0.0037	0.004617737
GO:0018210	peptidyl-threonine modification	59	0.248591933	0.0027	0.003470695
GO:0048864	stem cell development	29	0.248577642	< 0.001	< 0.001
GO:0050854	regulation of antigen receptor-mediated signaling pathway	17	0.248572943	< 0.001	< 0.001
GO:2000147	positive regulation of cell motility	179	0.248558055	< 0.001	< 0.001
GO:0002832	negative regulation of response to biotic stimulus	15	0.248545689	0.0005	0.000791911
GO:0090068	positive regulation of cell cycle process	98	0.248477924	< 0.001	< 0.001
GO:0090102	cochlea development	15	0.248477048	0.0029	0.003693519
GO:0022613	ribonucleoprotein complex biogenesis	176	0.248459986	0.0014	0.00194839
GO:0019318	hexose metabolic process	98	0.248378116	< 0.001	< 0.001
GO:0070050	neuron cellular homeostasis	16	0.248342986	0.0208	0.022657367
GO:0045778	positive regulation of ossification	41	0.2482908	< 0.001	< 0.001

GOIDlabel	GOName	size	categoryScores	pValPerm	pValPermCorr
GO:0045019	negative regulation of nitric oxide biosynthetic process	5	0.248288288	0.0298	0.031844809
GO:1904406	negative regulation of nitric oxide metabolic process	5	0.248288288	0.0298	0.031844809
GO:0086010	membrane depolarization during action potential	6	0.248262548	0.0299	0.031945354
GO:0000271	polysaccharide biosynthetic process	30	0.248228228	< 0.001	< 0.001
GO:0060997	dendritic spine morphogenesis	35	0.248214745	0.0098	0.011263122
GO:0007067	mitotic nuclear division	106	0.248187271	0.0001	0.000192793
GO:0048286	lung alveolus development	16	0.248149936	0.0003	0.000505993
GO:0010659	cardiac muscle cell apoptotic process	20	0.248120978	< 0.001	< 0.001
GO:0010828	positive regulation of glucose transport	18	0.248105248	0.0025	0.003237479
GO:0010257	NADH dehydrogenase complex assembly	26	0.248104148	0.0064	0.007617974
GO:0032981	mitochondrial respiratory chain complex I assembly	26	0.248104148	0.0064	0.007617974
GO:0097031	mitochondrial respiratory chain complex I biogenesis	26	0.248104148	0.0064	0.007617974
GO:0098810	neurotransmitter reuptake	12	0.248005148	0.0058	0.006954338
GO:0050918	positive chemotaxis	26	0.247995248	< 0.001	< 0.001
GO:0050905	neuromuscular process	54	0.247981315	0.0004	0.000648934
GO:0060306	regulation of membrane repolarization	13	0.247935848	0.0007	0.001063182
GO:0097352	autophagosome maturation	18	0.247919348	0.0078	0.009125612
GO:0097576	vacuole fusion	18	0.247919348	0.0078	0.009125612
GO:0071312	cellular response to alkaloid	13	0.247916048	0.0033	0.004153051
GO:0042044	fluid transport	7	0.247913219	0.0001	0.000192793
GO:0007567	parturition	5	0.247876448	< 0.001	< 0.001
GO:0032793	positive regulation of CREB transcription factor activity	8	0.247812098	0.0097	0.01115531
GO:0034599	cellular response to oxidative stress	113	0.247794444	< 0.001	< 0.001
GO:0015701	bicarbonate transport	12	0.247747748	0.0025	0.003237479
GO:0002521	leukocyte differentiation	151	0.24770087	< 0.001	< 0.001
GO:0003170	heart valve development	24	0.247694123	< 0.001	< 0.001
GO:0001525	angiogenesis	157	0.247681348	< 0.001	< 0.001
GO:1902229	regulation of intrinsic apoptotic signaling pathway in response to DNA damage	21	0.247655819	0.0056	0.006732458
GO:0045742	positive regulation of epidermal growth factor receptor signaling pathway	12	0.247597598	0.0001	0.000192793
GO:0071479	cellular response to ionizing radiation	29	0.24759242	0.0022	0.002896881
GO:0032633	interleukin-4 production	7	0.247582276	0.001	0.001450349
GO:0032673	regulation of interleukin-4 production	7	0.247582276	0.001	0.001450349
GO:0007163	establishment or maintenance of cell polarity	87	0.247580586	< 0.001	< 0.001
GO:0061647	histone H3-K9 modification	15	0.247567568	0.0039	0.004841626
GO:0043903	regulation of symbiosis, encompassing mutualism through parasitism	91	0.247556819	0.0003	0.000505993
GO:0043473	pigmentation	33	0.247556648	< 0.001	< 0.001
GO:0098760	response to interleukin-7	11	0.247548848	< 0.001	< 0.001
GO:0098761	cellular response to interleukin-7	11	0.247548848	< 0.001	< 0.001
GO:0022904	respiratory electron transport chain	39	0.247533248	0.0068	0.008063902
GO:0034250	positive regulation of cellular amide metabolic process	68	0.2475282	0.0001	0.000192793
GO:0033059	cellular pigmentation	24	0.247522523	< 0.001	< 0.001
GO:0048871	multicellular organismal homeostasis	122	0.247509336	< 0.001	< 0.001
GO:0007272	ensheathment of neurons	41	0.247499765	< 0.001	< 0.001
GO:0008366	axon ensheathment	41	0.247499765	< 0.001	< 0.001
GO:0030335	positive regulation of cell migration	175	0.247495495	< 0.001	< 0.001
GO:1900363	regulation of mRNA polyadenylation	6	0.247447447	0.0043	0.005276385
GO:0071216	cellular response to biotic stimulus	73	0.247396908	< 0.001	< 0.001
GO:0043484	regulation of RNA splicing	44	0.247390897	< 0.001	< 0.001
GO:0043649	dicarboxylic acid catabolic process	11	0.247361647	0.0003	0.000505993
GO:1901659	glycosyl compound biosynthetic process	44	0.247361647	0.0003	0.000505993
GO:1901264	carbohydrate derivative transport	14	0.247269719	0.0002	0.000356582
GO:0061053	somite development	20	0.247245817	0.0032	0.004042263
GO:0031424	keratinization	36	0.247240097	0.0001	0.000192793
GO:0034329	cell junction assembly	82	0.247239225	< 0.001	< 0.001
GO:0022900	electron transport chain	70	0.247214562	0.0003	0.000505993
GO:0030100	regulation of endocytosis	103	0.247159226	< 0.001	< 0.001
GO:0042177	negative regulation of protein catabolic process	54	0.247137614	< 0.001	< 0.001
GO:0071222	cellular response to lipopolysaccharide	64	0.24708816	< 0.001	< 0.001
GO:0006721	terpenoid metabolic process	30	0.246924067	< 0.001	< 0.001
GO:0034340	response to type I interferon	24	0.246921922	0.0004	0.000648934
GO:0072606	interleukin-8 secretion	9	0.246904047	< 0.001	< 0.001
GO:0046636	negative regulation of alpha-beta T cell activation	7	0.246883618	0.0062	0.00739947
GO:2000515	negative regulation of CD4-positive, alpha-beta T cell activation	7	0.246883618	0.0062	0.00739947
GO:0051156	glucose 6-phosphate metabolic process	8	0.246846847	0.0064	0.007617974
GO:0048168	regulation of neuronal synaptic plasticity	31	0.24683024	0.0122	0.013738029
GO:0010881	regulation of cardiac muscle contraction by regulation of the release of sequestered calcium ion	11	0.246823447	0.0001	0.000192793
GO:0043500	muscle adaptation	41	0.246777788	< 0.001	< 0.001
GO:0008037	cell recognition	47	0.246770174	< 0.001	< 0.001
GO:0045987	positive regulation of smooth muscle contraction	11	0.246706447	< 0.001	< 0.001
GO:1903069	regulation of ER-associated ubiquitin-dependent protein catabolic process	5	0.246640927	0.0099	0.011373214
GO:0019985	translesion synthesis	17	0.24663487	< 0.001	< 0.001
GO:0048608	reproductive structure development	145	0.246600098	< 0.001	< 0.001
GO:0002011	morphogenesis of an epithelial sheet	21	0.246589447	0.0004	0.000648934
GO:2000171	negative regulation of dendrite development	14	0.246571061	0.0112	0.012688637
GO:0003179	heart valve morphogenesis	22	0.246566047	< 0.001	< 0.001
GO:0032528	microvillus organization	9	0.246560847	0.0356	0.037648219
GO:0048008	platelet-derived growth factor receptor signaling pathway	24	0.246514372	< 0.001	< 0.001
GO:0007274	neuromuscular synaptic transmission	10	0.246486486	0.0002	0.000356582
GO:0002042	cell migration involved in sprouting angiogenesis	22	0.246484146	0.0103	0.011785137
GO:0045725	positive regulation of glycogen biosynthetic process	7	0.246442361	0.0001	0.000192793
GO:0021683	cerebellar granular layer morphogenesis	5	0.246435006	< 0.001	< 0.001
GO:0006907	pinocytosis	8	0.246396396	0.0003	0.000505993
GO:0009791	post-embryonic development	36	0.246389246	< 0.001	< 0.001

GOIDlabel	GOName	size	categoryScores	pValPerm	pValPermCorr
GO:0042026	protein refolding	6	0.246332046	< 0.001	< 0.001
GO:0090207	regulation of triglyceride metabolic process	11	0.246332046	< 0.001	< 0.001
GO:2001022	positive regulation of response to DNA damage stimulus	38	0.246311725	< 0.001	< 0.001
GO:0045649	regulation of macrophage differentiation	11	0.246308646	< 0.001	< 0.001
GO:2000242	negative regulation of reproductive process	16	0.246299871	0.0002	0.000356582
GO:0003007	heart morphogenesis	91	0.246281132	< 0.001	< 0.001
GO:0009410	response to xenobiotic stimulus	49	0.24625325	< 0.001	< 0.001
GO:1903539	protein localization to postsynaptic membrane	13	0.246153846	0.0068	0.008063902
GO:0042455	ribonucleoside biosynthetic process	41	0.246149983	0.0003	0.000505993
GO:0048255	mRNA stabilization	16	0.246074646	0.0004	0.000648934
GO:0005996	monosaccharide metabolic process	103	0.246049656	< 0.001	< 0.001
GO:0021904	dorsal/ventral neural tube patterning	9	0.246046046	0.0149	0.016557598
GO:0010171	body morphogenesis	14	0.246001103	< 0.001	< 0.001
GO:0045580	regulation of T cell differentiation	37	0.245977251	< 0.001	< 0.001
GO:0048714	positive regulation of oligodendrocyte differentiation	5	0.245920206	0.003	0.003811001
GO:0071868	cellular response to monoamine stimulus	17	0.245892952	0.0005	0.000791911
GO:0071870	cellular response to catecholamine stimulus	17	0.245892952	0.0005	0.000791911
GO:0071346	cellular response to interferon-gamma	53	0.245890095	< 0.001	< 0.001
GO:0009163	nucleoside biosynthetic process	43	0.245889078	0.0003	0.000505993
GO:0070189	kynurenine metabolic process	5	0.245868726	0.0024	0.003116943
GO:0097052	L-kynurenine metabolic process	5	0.245868726	0.0024	0.003116943
GO:0061458	reproductive system development	147	0.245855768	< 0.001	< 0.001
GO:0072178	nephric duct morphogenesis	5	0.245817246	0.0022	0.002896881
GO:0017185	peptidyl-lysine hydroxylation	5	0.245817246	0.0152	0.016877091
GO:0015850	organic hydroxy compound transport	77	0.24581056	< 0.001	< 0.001
GO:0030595	leukocyte chemotaxis	59	0.24580852	< 0.001	< 0.001
GO:0007127	meiosis I	25	0.245776062	0.0015	0.002069969
GO:0030218	erythrocyte differentiation	48	0.245699271	< 0.001	< 0.001
GO:0007030	Golgi organization	50	0.245683398	< 0.001	< 0.001
GO:0061614	pri-miRNA transcription from RNA polymerase II promoter	22	0.245676846	< 0.001	< 0.001
GO:0036151	phosphatidylcholine acyl-chain remodeling	8	0.245624196	0.0027	0.003470695
GO:0071772	response to BMP	43	0.245619706	< 0.001	< 0.001
GO:0071773	cellular response to BMP stimulus	43	0.245619706	< 0.001	< 0.001
GO:0060421	positive regulation of heart growth	15	0.245611326	< 0.001	< 0.001
GO:0007080	mitotic metaphase plate congression	21	0.245608874	0.0039	0.004841626
GO:0010458	exit from mitosis	14	0.245596617	0.0007	0.001063182
GO:0033108	mitochondrial respiratory chain complex assembly	36	0.245588446	0.0041	0.005057384
GO:0006754	ATP biosynthetic process	19	0.245546298	0.0015	0.002069969
GO:0010880	regulation of release of sequestered calcium ion into cytosol by sarcoplasmic reticulum	14	0.245394374	< 0.001	< 0.001
GO:0002717	positive regulation of natural killer cell mediated immunity	6	0.245388245	< 0.001	< 0.001
GO:0032543	mitochondrial translation	61	0.245378399	0.004	0.004954389
GO:0006875	cellular metal ion homeostasis	181	0.245377817	< 0.001	< 0.001
GO:0032088	negative regulation of NF-kappaB transcription factor activity	36	0.245323895	< 0.001	< 0.001
GO:0031668	cellular response to extracellular stimulus	95	0.245299736	< 0.001	< 0.001
GO:0046676	negative regulation of insulin secretion	17	0.245287304	0.0021	0.002780108
GO:0015980	energy derivation by oxidation of organic compounds	102	0.24527721	0.0002	0.000356582
GO:1900451	positive regulation of glutamate receptor signaling pathway	9	0.245273845	0.0003	0.000505993
GO:0045776	negative regulation of blood pressure	14	0.245265674	0.0002	0.000356582
GO:0043576	regulation of respiratory gaseous exchange	12	0.245238095	0.0004	0.000648934
GO:0009991	response to extracellular stimulus	171	0.245237719	< 0.001	< 0.001
GO:2000401	regulation of lymphocyte migration	18	0.245216645	< 0.001	< 0.001
GO:0048640	negative regulation of developmental growth	39	0.245203445	0.0013	0.001826625
GO:0030224	monocyte differentiation	12	0.245195195	0.0004	0.000648934
GO:1903131	mononuclear cell differentiation	12	0.245195195	0.0004	0.000648934
GO:0033014	tetrapyrrole biosynthetic process	12	0.245195195	0.0006	0.000927725
GO:0034341	response to interferon-gamma	58	0.245160431	< 0.001	< 0.001
GO:0003416	endochondral bone growth	8	0.24514157	0.0111	0.012593827
GO:0044282	small molecule catabolic process	131	0.245119711	< 0.001	< 0.001
GO:0001510	RNA methylation	20	0.245006435	0.0002	0.000356582
GO:0035973	aggrephagy	6	0.244959245	0.0028	0.003584743
GO:0007194	negative regulation of adenylate cyclase activity	7	0.244897959	< 0.001	< 0.001
GO:0030800	negative regulation of cyclic nucleotide metabolic process	7	0.244897959	< 0.001	< 0.001
GO:0030803	negative regulation of cyclic nucleotide biosynthetic process	7	0.244897959	< 0.001	< 0.001
GO:0030815	negative regulation of cAMP metabolic process	7	0.244897959	< 0.001	< 0.001
GO:0030818	negative regulation of cAMP biosynthetic process	7	0.244897959	< 0.001	< 0.001
GO:0031280	negative regulation of cyclase activity	7	0.244897959	< 0.001	< 0.001
GO:0051350	negative regulation of lyase activity	7	0.244897959	< 0.001	< 0.001
GO:0003012	muscle system process	160	0.244850386	< 0.001	< 0.001
GO:1902108	regulation of mitochondrial membrane permeability involved in apoptotic process	28	0.244833609	0.0007	0.001063182
GO:0000070	mitotic sister chromatid segregation	64	0.244783623	0.0001	0.000192793
GO:0001935	endothelial cell proliferation	44	0.244764245	< 0.001	< 0.001
GO:0016485	protein processing	63	0.244673245	< 0.001	< 0.001
GO:0015949	nucleobase-containing small molecule interconversion	12	0.244637495	< 0.001	< 0.001
GO:0071219	cellular response to molecule of bacterial origin	65	0.244633205	< 0.001	< 0.001
GO:0007029	endoplasmic reticulum organization	24	0.24462677	0.0001	0.000192793
GO:0035872	nucleotide-binding domain, leucine rich repeat containing receptor signaling pathway	12	0.244573145	0.0006	0.000927725
GO:0070423	nucleotide-binding oligomerization domain containing signaling pathway	12	0.244573145	0.0006	0.000927725
GO:0051783	regulation of nuclear division	68	0.244484821	< 0.001	< 0.001
GO:0034260	negative regulation of GTPase activity	21	0.244456702	< 0.001	< 0.001
GO:0046677	response to antibiotic	12	0.244444444	< 0.001	< 0.001
GO:0044319	wound healing, spreading of cells	12	0.244401544	0.0014	0.00194839
GO:0090504	epiboly	12	0.244401544	0.0014	0.00194839
GO:0090505	epiboly involved in wound healing	12	0.244401544	0.0014	0.00194839

GOIDlabel	GOName	size	categoryScores	pValPerm	pValPermCorr
GO:0043393	regulation of protein binding	91	0.244397302	< 0.001	< 0.001
GO:1902475	L-alpha-amino acid transmembrane transport	6	0.244358644	0.0128	0.014368758
GO:0031050	dsRNA fragmentation	19	0.244340581	0.0003	0.000505993
GO:0070918	production of small RNA involved in gene silencing by RNA	19	0.244340581	0.0003	0.000505993
GO:0002714	positive regulation of B cell mediated immunity	12	0.244315744	0.0015	0.002069969
GO:0002891	positive regulation of immunoglobulin mediated immune response	12	0.244315744	0.0015	0.002069969
GO:0042790	transcription of nuclear large rRNA transcript from RNA polymerase I promoter	6	0.244315744	0.033	0.035035756
GO:1901836	regulation of transcription of nuclear large rRNA transcript from RNA polymerase I promoter	6	0.244315744	0.033	0.035035756
GO:2001223	negative regulation of neuron migration	7	0.244309616	< 0.001	< 0.001
GO:0030193	regulation of blood coagulation	29	0.244290596	< 0.001	< 0.001
GO:0050818	regulation of coagulation	29	0.244290596	< 0.001	< 0.001
GO:1900046	regulation of hemostasis	29	0.244290596	< 0.001	< 0.001
GO:0045444	fat cell differentiation	82	0.244247732	< 0.001	< 0.001
GO:0030099	myeloid cell differentiation	136	0.244231206	< 0.001	< 0.001
GO:0007091	metaphase/anaphase transition of mitotic cell cycle	24	0.244176319	0.0012	0.001702047
GO:0010965	regulation of mitotic sister chromatid separation	24	0.244176319	0.0012	0.001702047
GO:0044784	metaphase/anaphase transition of cell cycle	24	0.244176319	0.0012	0.001702047
GO:0006414	translational elongation	55	0.244151164	0.0024	0.003116943
GO:0007565	female pregnancy	66	0.244140244	< 0.001	< 0.001
GO:0009262	deoxyribonucleotide metabolic process	13	0.244114444	0.0001	0.000192793
GO:1903671	negative regulation of sprouting angiogenesis	8	0.244079794	0.0016	0.002186748
GO:0031128	developmental induction	6	0.244058344	0.0102	0.011688041
GO:0090049	regulation of cell migration involved in sprouting angiogenesis	15	0.244049764	0.0018	0.002428764
GO:0010718	positive regulation of epithelial to mesenchymal transition	13	0.243975844	< 0.001	< 0.001
GO:0045540	regulation of cholesterol biosynthetic process	30	0.243963964	0.0011	0.001576346
GO:0042364	water-soluble vitamin biosynthetic process	6	0.243886744	0.0038	0.004725081
GO:1901617	organic hydroxy compound biosynthetic process	105	0.243863455	< 0.001	< 0.001
GO:0043900	regulation of multi-organism process	116	0.243846802	0.0001	0.000192793
GO:0031341	regulation of cell killing	18	0.243843844	< 0.001	< 0.001
GO:0010507	negative regulation of autophagy	35	0.243831587	0.0004	0.000648934
GO:0045740	positive regulation of DNA replication	15	0.243826684	0.0002	0.000356582
GO:0007088	regulation of mitotic nuclear division	64	0.243798263	0.0001	0.000192793
GO:0090559	regulation of membrane permeability	31	0.243749741	0.0004	0.000648934
GO:0010804	negative regulation of tumor necrosis factor-mediated signaling pathway	9	0.243729444	0.0007	0.001063182
GO:0009110	vitamin biosynthetic process	9	0.243700844	0.0018	0.002428764
GO:0018107	peptidyl-threonine phosphorylation	55	0.243687844	0.0038	0.004725081
GO:0001838	embryonic epithelial tube formation	52	0.243663994	< 0.001	< 0.001
GO:0072175	epithelial tube formation	52	0.243663994	< 0.001	< 0.001
GO:2000480	negative regulation of cAMP-dependent protein kinase activity	5	0.243655084	0.0002	0.000356582
GO:0072329	monocarboxylic acid catabolic process	35	0.243647729	< 0.001	< 0.001
GO:0015914	phospholipid transport	27	0.243643644	< 0.001	< 0.001
GO:0045070	positive regulation of viral genome replication	19	0.243636117	0.0014	0.00194839
GO:0048536	spleen development	13	0.243599644	< 0.001	< 0.001
GO:0031116	positive regulation of microtubule polymerization	11	0.243570844	0.0046	0.0056228
GO:0070085	glycosylation	99	0.243557844	< 0.001	< 0.001
GO:0014015	positive regulation of gliogenesis	24	0.243543544	< 0.001	< 0.001
GO:1900271	regulation of long-term synaptic potentiation	23	0.243489452	0.0123	0.013827585
GO:0061029	eyelid development in camera-type eye	5	0.243397683	0.0078	0.009125612
GO:0006869	lipid transport	107	0.243377957	< 0.001	< 0.001
GO:0031065	positive regulation of histone deacetylation	8	0.243307593	0.0117	0.013205263
GO:0046637	regulation of alpha-beta T cell differentiation	16	0.243291506	0.0002	0.000356582
GO:0045606	positive regulation of epidermal cell differentiation	6	0.243243243	0.0003	0.000505993
GO:0021915	neural tube development	65	0.243211563	< 0.001	< 0.001
GO:0000302	response to reactive oxygen species	82	0.243208714	0.0001	0.000192793
GO:0030397	membrane disassembly	7	0.243206472	0.0094	0.010831045
GO:0051081	nuclear envelope disassembly	7	0.243206472	0.0094	0.010831045
GO:0071359	cellular response to dsRNA	27	0.24320511	< 0.001	< 0.001
GO:0007006	mitochondrial membrane organization	57	0.243202601	0.0011	0.001576346
GO:0009072	aromatic amino acid family metabolic process	6	0.243071643	0.0005	0.000791911
GO:0009074	aromatic amino acid family catabolic process	6	0.243071643	0.0005	0.000791911
GO:1903524	positive regulation of blood circulation	26	0.243015543	< 0.001	< 0.001
GO:0070306	lens fiber cell differentiation	14	0.242967457	0.0123	0.013827585
GO:0006006	glucose metabolic process	81	0.242944532	< 0.001	< 0.001
GO:0007256	activation of JNKK activity	6	0.242857143	0.0058	0.006954338
GO:0070167	regulation of biomineral tissue development	29	0.242826077	< 0.001	< 0.001
GO:0034454	microtubule anchoring at centrosome	5	0.242779923	0.0103	0.011785137
GO:0002573	myeloid leukocyte differentiation	67	0.242774544	< 0.001	< 0.001
GO:0071425	hematopoietic stem cell proliferation	8	0.242728443	< 0.001	< 0.001
GO:0046716	muscle cell cellular homeostasis	10	0.242728443	< 0.001	< 0.001
GO:0003229	ventricular cardiac muscle tissue development	24	0.242717718	< 0.001	< 0.001
GO:0007205	protein kinase C-activating G-protein coupled receptor signaling pathway	11	0.242705043	0.0073	0.008600218
GO:0048339	paraxial mesoderm development	8	0.242696268	0.0018	0.002428764
GO:0072376	protein activation cascade	14	0.242691671	< 0.001	< 0.001
GO:0060420	regulation of heart growth	23	0.242683677	< 0.001	< 0.001
GO:0071466	cellular response to xenobiotic stimulus	45	0.242676963	< 0.001	< 0.001
GO:0044236	multicellular organism metabolic process	29	0.242675188	< 0.001	< 0.001
GO:0070734	histone H3-K27 methylation	7	0.2426549	0.0001	0.000192793
GO:0060711	labyrinthine layer development	19	0.242633611	< 0.001	< 0.001
GO:1902652	secondary alcohol metabolic process	63	0.242618128	0.0031	0.003925117
GO:0033013	tetrapyrrole metabolic process	20	0.242612613	0.0002	0.000356582
GO:0071501	cellular response to sterol depletion	5	0.242574003	< 0.001	< 0.001
GO:0006878	cellular copper ion homeostasis	8	0.242535393	0.0041	0.005057384
GO:0046326	positive regulation of glucose import	14	0.2425262	0.0053	0.006400268

GOIDlabel	GOName	size	categoryScores	pValPerm	pValPermCorr
GO:0007032	endosome organization	41	0.242508711	0.0016	0.002186748
GO:1904353	regulation of telomere capping	14	0.242507814	0.0079	0.009236608
GO:1902105	regulation of leukocyte differentiation	76	0.242477816	< 0.001	< 0.001
GO:0042446	hormone biosynthetic process	19	0.242443948	< 0.001	< 0.001
GO:0002693	positive regulation of cellular extravasation	7	0.242434271	< 0.001	< 0.001
GO:0072529	pyrimidine-containing compound catabolic process	11	0.242424242	0.0007	0.001063182
GO:0043123	positive regulation of I-kappaB kinase/NF-kappaB signaling	67	0.24240189	< 0.001	< 0.001
GO:0045687	positive regulation of glial cell differentiation	11	0.242354042	< 0.001	< 0.001
GO:0031279	regulation of cyclase activity	30	0.242256542	0.0011	0.001576346
GO:0035235	ionotropic glutamate receptor signaling pathway	14	0.242250414	0.0222	0.02411918
GO:0050886	endocrine process	20	0.242226512	< 0.001	< 0.001
GO:0010770	positive regulation of cell morphogenesis involved in differentiation	71	0.242217268	< 0.001	< 0.001
GO:0009060	aerobic respiration	17	0.242213642	0.0018	0.002428764
GO:0007613	memory	60	0.242192192	0.0023	0.003013139
GO:0009617	response to bacterium	162	0.242142142	< 0.001	< 0.001
GO:0003143	embryonic heart tube morphogenesis	20	0.242020592	< 0.001	< 0.001
GO:0048854	brain morphogenesis	16	0.241956242	0.0007	0.001063182
GO:0048532	anatomical structure arrangement	6	0.241956242	0.0153	0.01697069
GO:0050856	regulation of T cell receptor signaling pathway	13	0.241936442	0.0018	0.002428764
GO:1905515	non-motile cilium assembly	22	0.241932842	< 0.001	< 0.001
GO:0050773	regulation of dendrite development	78	0.241824242	0.0017	0.00231289
GO:0006323	DNA packaging	57	0.241789158	0.0021	0.002780108
GO:0070920	regulation of production of small RNA involved in gene silencing by RNA	6	0.241784642	0.0003	0.000505993
GO:1903798	regulation of production of miRNAs involved in gene silencing by miRNA	6	0.241784642	0.0003	0.000505993
GO:0006972	hyperosmotic response	10	0.241776062	< 0.001	< 0.001
GO:0000377	RNA splicing, via transesterification reactions with bulged adenosine as nucleophile	145	0.241737896	0.0012	0.001702047
GO:0000398	mRNA splicing, via spliceosome	145	0.241737896	0.0012	0.001702047
GO:0097035	regulation of membrane lipid distribution	16	0.241731017	0.0006	0.000927725
GO:0014072	response to isoquinoline alkaloid	20	0.241711712	0.0014	0.00194839
GO:0043278	response to morphine	20	0.241711712	0.0014	0.00194839
GO:0006111	regulation of gluconeogenesis	18	0.241670242	< 0.001	< 0.001
GO:0055065	metal ion homeostasis	198	0.241649442	< 0.001	< 0.001
GO:0001921	positive regulation of receptor recycling	5	0.241647362	0.0003	0.000505993
GO:0010633	negative regulation of epithelial cell migration	35	0.241640007	< 0.001	< 0.001
GO:0045765	regulation of angiogenesis	86	0.241632995	< 0.001	< 0.001
GO:0030520	intracellular estrogen receptor signaling pathway	22	0.241616942	< 0.001	< 0.001
GO:0016051	carbohydrate biosynthetic process	77	0.241611927	< 0.001	< 0.001
GO:0010906	regulation of glucose metabolic process	44	0.241593542	< 0.001	< 0.001
GO:1903115	regulation of actin filament-based movement	14	0.241570142	0.0001	0.000192793
GO:0000281	mitotic cytokinesis	24	0.241516517	0.001	0.001450349
GO:0009411	response to UV	54	0.241493875	0.0001	0.000192793
GO:0048593	camera-type eye morphogenesis	33	0.241449241	< 0.001	< 0.001
GO:0044090	positive regulation of vacuole organization	12	0.241419991	0.0003	0.000505993
GO:0006805	xenobiotic metabolic process	44	0.241400491	< 0.001	< 0.001
GO:1901186	positive regulation of ERBB signaling pathway	13	0.241382041	< 0.001	< 0.001
GO:0034162	toll-like receptor 9 signaling pathway	10	0.241364221	< 0.001	< 0.001
GO:0014037	Schwann cell differentiation	13	0.241342441	0.0002	0.000356582
GO:0002067	glandular epithelial cell differentiation	15	0.241184041	0.0012	0.001702047
GO:0031167	rRNA methylation	5	0.241184041	0.0028	0.003584743
GO:0007519	skeletal muscle tissue development	55	0.241179361	< 0.001	< 0.001
GO:0030799	regulation of cyclic nucleotide metabolic process	26	0.241154341	0.0007	0.001063182
GO:0030802	regulation of cyclic nucleotide biosynthetic process	26	0.241154341	0.0007	0.001063182
GO:0046626	regulation of insulin receptor signaling pathway	21	0.24114727	0.0077	0.00902229
GO:0006695	cholesterol biosynthetic process	41	0.241001978	0.0029	0.003693519
GO:1902653	secondary alcohol biosynthetic process	41	0.241001978	0.0029	0.003693519
GO:0007183	SMAD protein complex assembly	6	0.240883741	< 0.001	< 0.001
GO:0071871	response to epinephrine	6	0.240883741	< 0.001	< 0.001
GO:0043038	amino acid activation	20	0.240836551	0.0027	0.003470695
GO:0043039	tRNA aminoacylation	20	0.240836551	0.0027	0.003470695
GO:0036124	histone H3-K9 trimethylation	6	0.240755041	0.0007	0.001063182
GO:0003016	respiratory system process	15	0.240720721	0.0001	0.000192793
GO:0006486	protein glycosylation	92	0.240652454	< 0.001	< 0.001
GO:0043413	macromolecule glycosylation	92	0.240652454	< 0.001	< 0.001
GO:0061001	regulation of dendritic spine morphogenesis	27	0.240650174	0.0114	0.012893596
GO:0032727	positive regulation of interferon-alpha production	7	0.240632469	0.0084	0.009757868
GO:1901387	positive regulation of voltage-gated calcium channel activity	6	0.240626341	0.0126	0.014156008
GO:0014743	regulation of muscle hypertrophy	28	0.240577312	0.0001	0.000192793
GO:0010664	negative regulation of striated muscle cell apoptotic process	13	0.240570241	< 0.001	< 0.001
GO:0070059	intrinsic apoptotic signaling pathway in response to endoplasmic reticulum stress	26	0.240550441	0.0002	0.000356582
GO:0010611	regulation of cardiac muscle hypertrophy	27	0.240516707	0.0001	0.000192793
GO:0045773	positive regulation of axon extension	23	0.240512562	0.0015	0.002069969
GO:0046688	response to copper ion	16	0.240444015	0.002	0.002664694
GO:0048679	regulation of axon regeneration	13	0.24043164	0.0168	0.018543137
GO:0000375	RNA splicing, via transesterification reactions	149	0.240287459	0.0014	0.00194839
GO:0006182	cGMP biosynthetic process	6	0.24028314	0.0005	0.000791911
GO:0001707	mesoderm formation	23	0.240255162	< 0.001	< 0.001
GO:0000731	DNA synthesis involved in DNA repair	21	0.24024024	< 0.001	< 0.001
GO:0032091	negative regulation of protein binding	40	0.240160875	< 0.001	< 0.001
GO:0006687	glycosphingolipid metabolic process	25	0.24015444	0.0004	0.000648934
GO:0046058	cAMP metabolic process	26	0.24012474	0.0024	0.003116943
GO:1902253	regulation of intrinsic apoptotic signaling pathway by p53 class mediator	14	0.240117669	0.0304	0.032428267
GO:0048023	positive regulation of melanin biosynthetic process	5	0.24010296	0.0073	0.008600218
GO:1900378	positive regulation of secondary metabolite biosynthetic process	5	0.24010296	0.0073	0.008600218

GOIDlabel	GOName	size	categoryScores	pValPerm	pValPermCorr
GO:0001837	epithelial to mesenchymal transition	49	0.240101909	< 0.001	< 0.001
GO:1902117	positive regulation of organelle assembly	28	0.240080897	0.0001	0.000192793
GO:0072527	pyrimidine-containing compound metabolic process	28	0.240007354	0.0004	0.000648934
GO:1990048	anterograde neuronal dense core vesicle transport	5	0.24	0.0288	0.030849395
GO:0002756	MyD88-independent toll-like receptor signaling pathway	15	0.23998284	0.0004	0.000648934
GO:0086019	cell-cell signaling involved in cardiac conduction	10	0.23994852	0.0348	0.036852675
GO:0031281	positive regulation of cyclase activity	21	0.23989704	0.0025	0.003237479
GO:2001252	positive regulation of chromosome organization	34	0.23989704	0.0001	0.000192793
GO:0002068	glandular epithelial cell development	9	0.23981124	0.01	0.011478335
GO:0045833	negative regulation of lipid metabolic process	26	0.23977824	< 0.001	< 0.001
GO:0030148	sphingolipid biosynthetic process	31	0.239755885	0.0008	0.001193265
GO:1904738	vascular associated smooth muscle cell migration	10	0.23969112	0.0071	0.008390203
GO:1904752	regulation of vascular associated smooth muscle cell migration	10	0.23969112	0.0071	0.008390203
GO:0045619	regulation of lymphocyte differentiation	44	0.23964549	< 0.001	< 0.001
GO:0060395	SMAD protein signal transduction	14	0.239621254	< 0.001	< 0.001
GO:0055003	cardiac myofibril assembly	6	0.23959674	< 0.001	< 0.001
GO:0019227	neuronal action potential propagation	5	0.23958816	0.0315	0.033528856
GO:0098870	action potential propagation	5	0.23958816	0.0315	0.033528856
GO:0045807	positive regulation of endocytosis	59	0.239556748	< 0.001	< 0.001
GO:0090050	positive regulation of cell migration involved in sprouting angiogenesis	7	0.239529325	0.0229	0.024839743
GO:0033045	regulation of sister chromatid segregation	30	0.23950236	0.0001	0.000192793
GO:1904779	regulation of protein localization to centrosome	9	0.239496639	0.0019	0.00254969
GO:0061512	protein localization to cilium	21	0.239455782	< 0.001	< 0.001
GO:2000311	regulation of alpha-amino-3-hydroxy-5-methyl-4-isoxazole propionate selective glutamate receptor activity	12	0.239446589	0.0248	0.026750339
GO:0042552	myelination	40	0.239446589	< 0.001	< 0.001
GO:0008203	cholesterol metabolic process	62	0.239432059	0.0032	0.004042263
GO:0051298	centrosome duplication	26	0.239372339	< 0.001	< 0.001
GO:0043902	positive regulation of multi-organism process	72	0.239285714	0.0004	0.000648934
GO:0051497	negative regulation of stress fiber assembly	12	0.239253539	0.0051	0.006185009
GO:0050435	beta-amyloid metabolic process	19	0.239165481	0.0004	0.000648934
GO:0018027	peptidyl-lysine dimethylation	9	0.239153439	0.0021	0.002780108
GO:0032508	DNA duplex unwinding	33	0.239140439	0.0015	0.002069969
GO:0043200	response to amino acid	52	0.239075339	< 0.001	< 0.001
GO:0021532	neural tube patterning	16	0.239044402	0.0082	0.009562538
GO:1903034	regulation of response to wounding	56	0.239028314	< 0.001	< 0.001
GO:0099574	regulation of protein catabolic process at synapse, modulating synaptic transmission	5	0.239021879	0.0002	0.000356582
GO:0006244	pyrimidine nucleotide catabolic process	5	0.239021879	0.01	0.011478335
GO:0060251	regulation of glial cell proliferation	12	0.239017589	0.0122	0.013738029
GO:0046940	nucleoside monophosphate phosphorylation	7	0.239014525	< 0.001	< 0.001
GO:0002761	regulation of myeloid leukocyte differentiation	37	0.239013531	< 0.001	< 0.001
GO:0034728	nucleosome organization	48	0.238969326	0.0035	0.004390436
GO:2001242	regulation of intrinsic apoptotic signaling pathway	65	0.238938719	0.0001	0.000192793
GO:0097205	renal filtration	5	0.238918919	< 0.001	< 0.001
GO:0034248	regulation of cellular amide metabolic process	200	0.238906049	0.0002	0.000356582
GO:0010876	lipid localization	118	0.238893615	< 0.001	< 0.001
GO:0046902	regulation of mitochondrial membrane permeability	30	0.238850279	0.0003	0.000505993
GO:0010658	striated muscle cell apoptotic process	21	0.238708096	< 0.001	< 0.001
GO:0032006	regulation of TOR signaling	41	0.238697931	< 0.001	< 0.001
GO:0035268	protein mannosylation	11	0.238680239	0.0005	0.000791911
GO:0032008	positive regulation of TOR signaling	16	0.238642214	0.0008	0.001193265
GO:0051017	actin filament bundle assembly	65	0.238625879	< 0.001	< 0.001
GO:0010511	regulation of phosphatidylinositol biosynthetic process	5	0.238610039	< 0.001	< 0.001
GO:0031112	positive regulation of microtubule polymerization or depolymerization	14	0.238610039	0.0019	0.00254969
GO:0010934	macrophage cytokine production	6	0.238610039	0.0029	0.003693519
GO:1904030	negative regulation of cyclin-dependent protein kinase activity	12	0.238588589	0.0046	0.0056228
GO:0061051	positive regulation of cell growth involved in cardiac muscle cell development	5	0.238558559	0.005	0.006070546
GO:1904355	positive regulation of telomere capping	9	0.238552839	0.0022	0.002896881
GO:0001974	blood vessel remodeling	14	0.238462953	< 0.001	< 0.001
GO:0046835	carbohydrate phosphorylation	14	0.238462953	0.0006	0.000927725
GO:0019682	glyceraldehyde-3-phosphate metabolic process	8	0.238449163	0.0177	0.019488753
GO:0097062	dendritic spine maintenance	10	0.238352638	0.0011	0.001576346
GO:0003071	renal system process involved in regulation of systemic arterial blood pressure	5	0.238352638	< 0.001	< 0.001
GO:0051306	mitotic sister chromatid separation	25	0.23832175	0.0009	0.001319479
GO:0048512	circadian behavior	14	0.238279095	0.0017	0.00231289
GO:0097345	mitochondrial outer membrane permeabilization	25	0.23827027	0.0013	0.001826625
GO:0043267	negative regulation of potassium ion transport	9	0.238266838	0.0001	0.000192793
GO:0030852	regulation of granulocyte differentiation	6	0.238223938	0.0007	0.001063182
GO:0021675	nerve development	24	0.238191763	0.0049	0.005958506
GO:0071482	cellular response to light stimulus	39	0.238141438	< 0.001	< 0.001
GO:0006418	tRNA aminoacylation for protein translation	18	0.238109538	0.0019	0.00254969
GO:0000154	rRNA modification	10	0.238095238	0.0008	0.001193265
GO:0002262	myeloid cell homeostasis	59	0.238064699	< 0.001	< 0.001
GO:0016197	endosomal transport	138	0.238022495	0.0002	0.000356582
GO:1990089	response to nerve growth factor	24	0.238020163	0.0007	0.001063182
GO:1990090	cellular response to nerve growth factor stimulus	24	0.238020163	0.0007	0.001063182
GO:0060317	cardiac epithelial to mesenchymal transition	10	0.237940798	< 0.001	< 0.001
GO:0016246	RNA interference	5	0.237940798	< 0.001	< 0.001
GO:0036315	cellular response to sterol	10	0.237915058	< 0.001	< 0.001
GO:0006112	energy reserve metabolic process	35	0.237867255	< 0.001	< 0.001
GO:0061099	negative regulation of protein tyrosine kinase activity	9	0.237837838	0.0027	0.003470695
GO:1901615	organic hydroxy compound metabolic process	186	0.237832302	< 0.001	< 0.001
GO:0042059	negative regulation of epidermal growth factor receptor signaling pathway	20	0.237824968	0.0005	0.000791911

GOIDlabel	GOName	size	categoryScores	pValPerm	pValPermCorr
GO:1904018	positive regulation of vasculature development	53	0.237823268	< 0.001	< 0.001
GO:0008535	respiratory chain complex IV assembly	6	0.237666238	0.0201	0.021947949
GO:1903432	regulation of TORC1 signaling	16	0.237660875	< 0.001	< 0.001
GO:0006305	DNA alkylation	18	0.237609038	0.0001	0.000192793
GO:0006306	DNA methylation	18	0.237609038	0.0001	0.000192793
GO:1900739	regulation of protein insertion into mitochondrial membrane involved in apoptotic signaling pathway	15	0.237597598	0.0031	0.003925117
GO:1900740	positive regulation of protein insertion into mitochondrial membrane involved in apoptotic signaling pathway	15	0.237597598	0.0031	0.003925117
GO:1900024	regulation of substrate adhesion-dependent cell spreading	20	0.237528958	< 0.001	< 0.001
GO:0044706	multi-multicellular organism process	76	0.237488993	< 0.001	< 0.001
GO:2000352	negative regulation of endothelial cell apoptotic process	7	0.237470123	0.002	0.002664694
GO:0006664	glycolipid metabolic process	41	0.237454876	< 0.001	< 0.001
GO:0032924	activin receptor signaling pathway	14	0.237378195	< 0.001	< 0.001
GO:0045911	positive regulation of DNA recombination	17	0.237338179	0.0009	0.001319479
GO:0035148	tube formation	57	0.237323037	< 0.001	< 0.001
GO:0070168	negative regulation of biomineral tissue development	8	0.237323037	0.0002	0.000356582
GO:0051705	multi-organism behavior	35	0.237264203	0.0009	0.001319479
GO:0051567	histone H3-K9 methylation	14	0.237249494	0.0099	0.011373214
GO:0070304	positive regulation of stress-activated protein kinase signaling cascade	66	0.237248937	< 0.001	< 0.001
GO:0005977	glycogen metabolic process	30	0.237237237	< 0.001	< 0.001
GO:0006073	cellular glucan metabolic process	30	0.237237237	< 0.001	< 0.001
GO:0044042	glucan metabolic process	30	0.237237237	< 0.001	< 0.001
GO:0006631	fatty acid metabolic process	127	0.237221699	< 0.001	< 0.001
GO:0000188	inactivation of MAPK activity	15	0.237220077	0.0081	0.00945
GO:0055022	negative regulation of cardiac muscle tissue growth	8	0.237194337	0.0015	0.002069969
GO:0061117	negative regulation of heart growth	8	0.237194337	0.0015	0.002069969
GO:0006333	chromatin assembly or disassembly	52	0.237120087	0.0033	0.004153051
GO:0043901	negative regulation of multi-organism process	41	0.237103305	0.0001	0.000192793
GO:0034111	negative regulation of homotypic cell-cell adhesion	7	0.237102409	< 0.001	< 0.001
GO:0001773	myeloid dendritic cell activation	8	0.237033462	< 0.001	< 0.001
GO:0072091	regulation of stem cell proliferation	19	0.2369979	< 0.001	< 0.001
GO:0032480	negative regulation of type I interferon production	11	0.236995437	< 0.001	< 0.001
GO:0032438	melanosome organization	13	0.236986437	0.0003	0.000505993
GO:0048753	pigment granule organization	13	0.236986437	0.0003	0.000505993
GO:1901185	negative regulation of ERBB signaling pathway	23	0.23692015	0.0003	0.000505993
GO:0033866	nucleoside bisphosphate biosynthetic process	6	0.236894037	0.0186	0.020421455
GO:0034030	ribonucleoside bisphosphate biosynthetic process	6	0.236894037	0.0186	0.020421455
GO:0034033	purine nucleoside bisphosphate biosynthetic process	6	0.236894037	0.0186	0.020421455
GO:0060603	mammary gland duct morphogenesis	11	0.236878437	< 0.001	< 0.001
GO:0006304	DNA modification	32	0.236832368	0.0008	0.001193265
GO:0006417	regulation of translation	184	0.236806838	0.0002	0.000356582
GO:0055072	iron ion homeostasis	28	0.236780658	0.0003	0.000505993
GO:0007178	transmembrane receptor protein serine/threonine kinase signaling pathway	104	0.236763687	< 0.001	< 0.001
GO:0016064	immunoglobulin mediated immune response	28	0.236743887	< 0.001	< 0.001
GO:0019724	B cell mediated immunity	28	0.236743887	< 0.001	< 0.001
GO:0051875	pigment granule localization	14	0.236716308	< 0.001	< 0.001
GO:0009081	branched-chain amino acid metabolic process	5	0.236705277	0.0019	0.00254969
GO:0009083	branched-chain amino acid catabolic process	5	0.236705277	0.0019	0.00254969
GO:0010714	positive regulation of collagen metabolic process	7	0.236697922	0.0006	0.000927725
GO:0032967	positive regulation of collagen biosynthetic process	7	0.236697922	0.0006	0.000927725
GO:0044253	positive regulation of multicellular organismal metabolic process	7	0.236697922	0.0006	0.000927725
GO:2000404	regulation of T cell migration	12	0.236679537	0.0001	0.000192793
GO:0055088	lipid homeostasis	37	0.236655188	0.0001	0.000192793
GO:0070208	protein heterotrimerization	5	0.236653797	0.0042	0.005168936
GO:1902850	microtubule cytoskeleton organization involved in mitosis	23	0.236651558	0.0004	0.000648934
GO:0009165	nucleotide biosynthetic process	102	0.23663159	< 0.001	< 0.001
GO:0042538	hyperosmotic salinity response	6	0.236593737	0.0003	0.000505993
GO:0034101	erythrocyte homeostasis	53	0.236579976	< 0.001	< 0.001
GO:1903363	negative regulation of cellular protein catabolic process	28	0.236578415	0.0004	0.000648934
GO:0071985	multivesicular body sorting pathway	6	0.236550837	0.0001	0.000192793
GO:1902003	regulation of beta-amyloid formation	13	0.236511237	0.0007	0.001063182
GO:0009151	purine deoxyribonucleotide metabolic process	7	0.236477294	< 0.001	< 0.001
GO:0001961	positive regulation of cytokine-mediated signaling pathway	15	0.236465036	0.0001	0.000192793
GO:0010594	regulation of endothelial cell migration	64	0.236410071	< 0.001	< 0.001
GO:0036035	osteoclast development	6	0.236336336	0.0156	0.0172574
GO:1903859	regulation of dendrite extension	15	0.236327756	0.0187	0.020510412
GO:0006298	mismatch repair	9	0.236322036	0.0212	0.023069835
GO:0046467	membrane lipid biosynthetic process	42	0.236238279	0.0003	0.000505993
GO:1904667	negative regulation of ubiquitin protein ligase activity	6	0.236121836	0.0432	0.045198993
GO:0010663	positive regulation of striated muscle cell apoptotic process	7	0.236109579	0.0095	0.010941603
GO:0010666	positive regulation of cardiac muscle cell apoptotic process	7	0.236109579	0.0095	0.010941603
GO:0006066	alcohol metabolic process	121	0.236070072	< 0.001	< 0.001
GO:0014912	negative regulation of smooth muscle cell migration	11	0.236036036	0.0006	0.000927725
GO:0071867	response to monoamine	18	0.236007436	0.0012	0.001702047
GO:0071869	response to catecholamine	18	0.236007436	0.0012	0.001702047
GO:0060038	cardiac muscle cell proliferation	16	0.235955598	< 0.001	< 0.001
GO:0046135	pyrimidine nucleoside catabolic process	6	0.235950236	0.0045	0.005509289
GO:0046620	regulation of organ growth	35	0.235933076	< 0.001	< 0.001
GO:0098743	cell aggregation	8	0.235907336	0.0182	0.020002603
GO:0045926	negative regulation of growth	100	0.235899614	< 0.001	< 0.001
GO:0051489	regulation of filopodium assembly	24	0.235853711	0.0111	0.012593827
GO:0061041	regulation of wound healing	44	0.235796186	< 0.001	< 0.001
GO:0060976	coronary vasculature development	17	0.235718071	< 0.001	< 0.001

GOIDlabel	GOName	size	categoryScores	pValPerm	pValPermCorr
GO:0051100	negative regulation of binding	66	0.235657736	< 0.001	< 0.001
GO:1900744	regulation of p38MAPK cascade	12	0.235649936	0.0076	0.008916717
GO:1900273	positive regulation of long-term synaptic potentiation	15	0.235624196	0.0173	0.01905998
GO:1901293	nucleoside phosphate biosynthetic process	103	0.2356137	< 0.001	< 0.001
GO:0060081	membrane hyperpolarization	6	0.235607036	0.0012	0.001702047
GO:0001704	formation of primary germ layer	36	0.235542686	< 0.001	< 0.001
GO:0061572	actin filament bundle organization	66	0.235529036	< 0.001	< 0.001
GO:2001238	positive regulation of extrinsic apoptotic signaling pathway	17	0.235521236	< 0.001	< 0.001
GO:0055024	regulation of cardiac muscle tissue development	29	0.235459104	< 0.001	< 0.001
GO:0035094	response to nicotine	15	0.235452595	< 0.001	< 0.001
GO:0071103	DNA conformation change	95	0.235450789	0.0009	0.001319479
GO:0009953	dorsal/ventral pattern formation	27	0.235435435	0.0039	0.004841626
GO:0046697	decidualization	9	0.235435435	0.0163	0.018013333
GO:0051560	mitochondrial calcium ion homeostasis	11	0.235404235	0.0023	0.003013139
GO:0032365	intracellular lipid transport	12	0.235263835	0.0003	0.000505993
GO:0001938	positive regulation of endothelial cell proliferation	34	0.235256265	< 0.001	< 0.001
GO:0022010	central nervous system myelination	6	0.235220935	0.0004	0.000648934
GO:0032291	axon ensheathment in central nervous system	6	0.235220935	0.0004	0.000648934
GO:0090023	positive regulation of neutrophil chemotaxis	8	0.235199485	< 0.001	< 0.001
GO:1902624	positive regulation of neutrophil migration	8	0.235199485	< 0.001	< 0.001
GO:0006839	mitochondrial transport	103	0.235158876	0.0006	0.000927725
GO:0090136	epithelial cell-cell adhesion	6	0.235049335	< 0.001	< 0.001
GO:1903509	liposaccharide metabolic process	42	0.235049335	< 0.001	< 0.001
GO:0000338	protein deneddylation	7	0.235006435	0.0104	0.011884458
GO:0048483	autonomic nervous system development	9	0.234920635	0.0114	0.012893596
GO:0032733	positive regulation of interleukin-10 production	9	0.234920635	0.0032	0.004042263
GO:0031497	chromatin assembly	43	0.234904672	0.0024	0.003116943
GO:0032874	positive regulation of stress-activated MAPK cascade	65	0.234899515	< 0.001	< 0.001
GO:0070301	cellular response to hydrogen peroxide	34	0.234877735	< 0.001	< 0.001
GO:0035357	peroxisome proliferator activated receptor signaling pathway	11	0.234819235	< 0.001	< 0.001
GO:0009451	RNA modification	47	0.234814754	0.0003	0.000505993
GO:0034754	cellular hormone metabolic process	29	0.234811166	< 0.001	< 0.001
GO:0071218	cellular response to misfolded protein	8	0.234749035	< 0.001	< 0.001
GO:1903543	positive regulation of exosomal secretion	6	0.234706135	0.0391	0.041116248
GO:0009201	ribonucleoside triphosphate biosynthetic process	23	0.234681887	0.0033	0.004153051
GO:0051305	chromosome movement towards spindle pole	6	0.234620335	0.0002	0.000356582
GO:0046916	cellular transition metal ion homeostasis	35	0.234601949	0.0006	0.000927725
GO:0070613	regulation of protein processing	27	0.234558368	< 0.001	< 0.001
GO:0008380	RNA splicing	182	0.234555277	0.0014	0.00194839
GO:0036314	response to sterol	14	0.234546792	0.0005	0.000791911
GO:0032007	negative regulation of TOR signaling	18	0.234534535	< 0.001	< 0.001
GO:0042787	protein ubiquitination involved in ubiquitin-dependent protein catabolic process	13	0.234491634	0.0009	0.001319479
GO:2000058	regulation of protein ubiquitination involved in ubiquitin-dependent protein catabolic process	13	0.234491634	0.0009	0.001319479
GO:0000096	sulfur amino acid metabolic process	15	0.234440154	< 0.001	< 0.001
GO:0045598	regulation of fat cell differentiation	50	0.234419562	< 0.001	< 0.001
GO:0002673	regulation of acute inflammatory response	22	0.234339534	0.0003	0.000505993
GO:0001841	neural tube formation	48	0.234336122	< 0.001	< 0.001
GO:0050953	sensory perception of light stimulus	59	0.2343084	< 0.001	< 0.001
GO:0003014	renal system process	38	0.234288424	< 0.001	< 0.001
GO:0006119	oxidative phosphorylation	42	0.234271006	0.0084	0.009757868
GO:0016125	sterol metabolic process	64	0.234262387	0.0029	0.003693519
GO:0055021	regulation of cardiac muscle tissue growth	22	0.234222534	< 0.001	< 0.001
GO:0061036	positive regulation of cartilage development	16	0.234185972	< 0.001	< 0.001
GO:0008202	steroid metabolic process	100	0.234177606	0.0003	0.000505993
GO:0009068	aspartate family amino acid catabolic process	7	0.234160691	0.0104	0.011884458
GO:0071675	regulation of mononuclear cell migration	18	0.234076934	0.0004	0.000648934
GO:0031665	negative regulation of lipopolysaccharide-mediated signaling pathway	5	0.234028314	0.0033	0.004153051
GO:0051304	chromosome separation	30	0.233959674	0.001	0.001450349
GO:0006767	water-soluble vitamin metabolic process	29	0.233950206	0.0001	0.000192793
GO:0030252	growth hormone secretion	6	0.233933934	0.011	0.012498738
GO:0016024	CDP-diacylglycerol biosynthetic process	5	0.233873874	< 0.001	< 0.001
GO:0046341	CDP-diacylglycerol metabolic process	5	0.233873874	< 0.001	< 0.001
GO:0021511	spinal cord patterning	6	0.233805234	0.0063	0.007507211
GO:0021513	spinal cord dorsal/ventral patterning	6	0.233805234	0.0063	0.007507211
GO:0042147	retrograde transport, endosome to Golgi	32	0.233799871	0.0004	0.000648934
GO:1903204	negative regulation of oxidative stress-induced neuron death	9	0.233748034	0.0001	0.000192793
GO:0032232	negative regulation of actin filament bundle assembly	14	0.233701048	0.0019	0.00254969
GO:0001756	somitogenesis	17	0.233643728	0.0044	0.005395416
GO:0051604	protein maturation	75	0.233616474	< 0.001	< 0.001
GO:0000288	nuclear-transcribed mRNA catabolic process, deadenylation-dependent decay	32	0.233574646	0.0027	0.003470695
GO:1901725	regulation of histone deacetylase activity	5	0.233513514	0.0038	0.004725081
GO:0035883	enteroendocrine cell differentiation	11	0.233462033	0.0076	0.008916717
GO:0035050	embryonic heart tube development	21	0.233449776	< 0.001	< 0.001
GO:0060343	trabecula formation	11	0.233438633	0.0188	0.020615909
GO:0051187	cofactor catabolic process	8	0.233429858	0.0003	0.000505993
GO:0035924	cellular response to vascular endothelial growth factor stimulus	14	0.233370105	0.0069	0.008171729
GO:0019748	secondary metabolic process	23	0.233305355	< 0.001	< 0.001
GO:0006622	protein targeting to lysosome	9	0.233261833	0.0004	0.000648934
GO:0016126	sterol biosynthetic process	43	0.233234563	0.0022	0.002896881
GO:0016101	diterpenoid metabolic process	26	0.233214533	< 0.001	< 0.001
GO:0050795	regulation of behavior	26	0.233194733	0.0001	0.000192793
GO:0014855	striated muscle cell proliferation	19	0.233028517	< 0.001	< 0.001
GO:0034643	establishment of mitochondrion localization, microtubule-mediated	10	0.232972973	0.0044	0.005395416

GOIDlabel	GOName	size	categoryScores	pValPerm	pValPermCorr
GO:0047497	mitochondrion transport along microtubule	10	0.232972973	0.0044	0.005395416
GO:0048247	lymphocyte chemotaxis	9	0.232890033	0.0008	0.001193265
GO:0060612	adipose tissue development	14	0.232763376	< 0.001	< 0.001
GO:0051988	regulation of attachment of spindle microtubules to kinetochore	6	0.232732733	0.0001	0.000192793
GO:0007007	inner mitochondrial membrane organization	18	0.232718433	0.0131	0.01468722
GO:0007601	visual perception	58	0.232623264	< 0.001	< 0.001
GO:0030225	macrophage differentiation	19	0.232622096	< 0.001	< 0.001
GO:0010155	regulation of proton transport	7	0.232579518	0.0031	0.003925117
GO:0051339	regulation of lyase activity	30	0.232561133	< 0.001	< 0.001
GO:0055023	positive regulation of cardiac muscle tissue growth	14	0.232524361	< 0.001	< 0.001
GO:0033194	response to hydroperoxide	12	0.232518233	0.0008	0.001193265
GO:0048265	response to pain	7	0.232469204	< 0.001	< 0.001
GO:1900076	regulation of cellular response to insulin stimulus	23	0.232466006	0.0085	0.009867669
GO:0035646	endosome to melanosome transport	6	0.232432432	0.0326	0.034638301
GO:0043485	endosome to pigment granule transport	6	0.232432432	0.0326	0.034638301
GO:0048757	pigment granule maturation	6	0.232432432	0.0326	0.034638301
GO:0050869	negative regulation of B cell activation	5	0.232380952	< 0.001	< 0.001
GO:0002687	positive regulation of leukocyte migration	43	0.232372572	0.0001	0.000192793
GO:0046635	positive regulation of alpha-beta T cell activation	11	0.232362232	0.0029	0.003693519
GO:0042445	hormone metabolic process	52	0.232333432	< 0.001	< 0.001
GO:0070932	histone H3 deacetylation	7	0.232322118	0.0082	0.009562538
GO:0000959	mitochondrial RNA metabolic process	12	0.232303732	0.013	0.014581154
GO:0007100	mitotic centrosome separation	5	0.232175032	0.0032	0.004042263
GO:0051299	centrosome separation	5	0.232175032	0.0032	0.004042263
GO:0007631	feeding behavior	43	0.232151088	0.0005	0.000791911
GO:0006623	protein targeting to vacuole	10	0.232149292	0.0003	0.000505993
GO:0072666	establishment of protein localization to vacuole	10	0.232149292	0.0003	0.000505993
GO:0055070	copper ion homeostasis	9	0.232117832	0.0053	0.006400268
GO:0086002	cardiac muscle cell action potential involved in contraction	19	0.231985369	0.0061	0.007291396
GO:0032988	ribonucleoprotein complex disassembly	5	0.231969112	0.0462	0.04815136
GO:0030509	BMP signaling pathway	39	0.231944032	< 0.001	< 0.001
GO:1903909	regulation of receptor clustering	6	0.231917632	< 0.001	< 0.001
GO:0009395	phospholipid catabolic process	9	0.231917632	< 0.001	< 0.001
GO:1901380	negative regulation of potassium ion transmembrane transport	7	0.23188086	0.0001	0.000192793
GO:0000380	alternative mRNA splicing, via spliceosome	26	0.231788932	0.0012	0.001702047
GO:0051043	regulation of membrane protein ectodomain proteolysis	7	0.231697003	0.0014	0.00194839
GO:0042481	regulation of odontogenesis	5	0.231557272	0.0011	0.001576346
GO:0034637	cellular carbohydrate biosynthetic process	30	0.231514372	< 0.001	< 0.001
GO:0061339	establishment or maintenance of monopolar cell polarity	11	0.231402831	0.0001	0.000192793
GO:0048332	mesoderm morphogenesis	24	0.231370656	< 0.001	< 0.001
GO:0043534	blood vessel endothelial cell migration	47	0.231359019	0.0005	0.000791911
GO:0015748	organophosphate ester transport	34	0.231349837	< 0.001	< 0.001
GO:0006334	nucleosome assembly	39	0.231343431	0.0019	0.00254969
GO:0086065	cell communication involved in cardiac conduction	20	0.231299871	0.0008	0.001193265
GO:0010595	positive regulation of endothelial cell migration	37	0.231256739	0.0001	0.000192793
GO:0046890	regulation of lipid biosynthetic process	73	0.23122653	< 0.001	< 0.001
GO:0034067	protein localization to Golgi apparatus	14	0.231163817	< 0.001	< 0.001
GO:0007097	nuclear migration	6	0.231145431	0.0304	0.032428267
GO:0040023	establishment of nucleus localization	6	0.231145431	0.0304	0.032428267
GO:0032755	positive regulation of interleukin-6 production	25	0.231114543	< 0.001	< 0.001
GO:0031468	nuclear envelope reassembly	12	0.231081081	0.0018	0.002428764
GO:0046006	regulation of activated T cell proliferation	9	0.231059631	< 0.001	< 0.001
GO:0033617	mitochondrial respiratory chain complex IV assembly	5	0.230939511	0.0251	0.02705773
GO:0097034	mitochondrial respiratory chain complex IV biogenesis	5	0.230939511	0.0251	0.02705773
GO:0030801	positive regulation of cyclic nucleotide metabolic process	18	0.230859431	0.0028	0.003584743
GO:0030804	positive regulation of cyclic nucleotide biosynthetic process	18	0.230859431	0.0028	0.003584743
GO:0061462	protein localization to lysosome	16	0.230711068	0.0001	0.000192793
GO:0051297	centrosome organization	47	0.230575865	0.0006	0.000927725
GO:0000715	nucleotide-excision repair, DNA damage recognition	14	0.230557088	0.0063	0.007507211
GO:0007179	transforming growth factor beta receptor signaling pathway	67	0.230434699	< 0.001	< 0.001
GO:0048814	regulation of dendrite morphogenesis	51	0.230433795	0.0025	0.003237479
GO:0051788	response to misfolded protein	9	0.23043043	0.0005	0.000791911
GO:0035176	social behavior	28	0.230281302	0.0021	0.002780108
GO:0051703	intraspecies interaction between organisms	28	0.230281302	0.0021	0.002780108
GO:0007040	lysosome organization	31	0.230240379	< 0.001	< 0.001
GO:0080171	lytic vacuole organization	31	0.230240379	< 0.001	< 0.001
GO:1902187	negative regulation of viral release from host cell	7	0.230226145	0.0008	0.001193265
GO:0072665	protein localization to vacuole	17	0.230206677	0.0002	0.000356582
GO:0006364	rRNA processing	83	0.230072413	0.0036	0.004505419
GO:0006515	misfolded or incompletely synthesized protein catabolic process	8	0.22985843	0.0003	0.000505993
GO:0007252	I-kappaB phosphorylation	8	0.22979408	0.0038	0.004725081
GO:0097006	regulation of plasma lipoprotein particle levels	28	0.229766501	< 0.001	< 0.001
GO:0006879	cellular iron ion homeostasis	24	0.229761905	0.005	0.006070546
GO:0044728	DNA methylation or demethylation	23	0.229757708	0.001	0.001450349
GO:0016053	organic acid biosynthetic process	112	0.229738923	< 0.001	< 0.001
GO:0046394	carboxylic acid biosynthetic process	112	0.229738923	< 0.001	< 0.001
GO:0051668	localization within membrane	50	0.229683398	0.0009	0.001319479
GO:0033047	regulation of mitotic sister chromatid segregation	29	0.229565526	0.0002	0.000356582
GO:0045722	positive regulation of gluconeogenesis	7	0.229564258	0.048	0.049940701
GO:0002467	germinal center formation	6	0.22955813	0.0002	0.000356582
GO:0061035	regulation of cartilage development	23	0.229545073	< 0.001	< 0.001
GO:0001893	maternal placenta development	17	0.229510182	< 0.001	< 0.001
GO:0044060	regulation of endocrine process	13	0.229462429	< 0.001	< 0.001

GOIDlabel	GOName	size	categoryScores	pValPerm	pValPermCorr
GO:0032689	negative regulation of interferon-gamma production	8	0.229440154	0.0081	0.00945
GO:0042454	ribonucleoside catabolic process	7	0.229417172	0.022	0.023906696
GO:0009583	detection of light stimulus	21	0.229343629	0.0001	0.000192793
GO:0001708	cell fate specification	19	0.229330082	0.0038	0.004725081
GO:0046459	short-chain fatty acid metabolic process	7	0.229270086	0.003	0.003811001
GO:0031076	embryonic camera-type eye development	9	0.229229229	< 0.001	< 0.001
GO:0010640	regulation of platelet-derived growth factor receptor signaling pathway	11	0.229179829	0.0003	0.000505993
GO:0060045	positive regulation of cardiac muscle cell proliferation	9	0.229172029	< 0.001	< 0.001
GO:0046426	negative regulation of JAK-STAT cascade	13	0.228987229	0.0009	0.001319479
GO:0048710	regulation of astrocyte differentiation	10	0.228906049	< 0.001	< 0.001
GO:0070227	lymphocyte apoptotic process	19	0.228801734	0.0001	0.000192793
GO:0038066	p38MAPK cascade	15	0.228743029	0.0005	0.000791911
GO:0090085	regulation of protein deubiquitination	5	0.228674389	0.0014	0.00194839
GO:0002183	cytoplasmic translational initiation	13	0.228670429	0.001	0.001450349
GO:0036480	neuron intrinsic apoptotic signaling pathway in response to oxidative stress	5	0.228622909	0.0006	0.000927725
GO:1903376	regulation of oxidative stress-induced neuron intrinsic apoptotic signaling pathway	5	0.228622909	0.0006	0.000927725
GO:1902306	negative regulation of sodium ion transmembrane transport	5	0.228571429	0.0116	0.013103344
GO:2000650	negative regulation of sodium ion transmembrane transporter activity	5	0.228571429	0.0116	0.013103344
GO:0035196	production of miRNAs involved in gene silencing by miRNA	17	0.22846544	0.0043	0.005276385
GO:0051188	cofactor biosynthetic process	68	0.22840109	0.0001	0.000192793
GO:0045730	respiratory burst	13	0.228393228	< 0.001	< 0.001
GO:0034110	regulation of homotypic cell-cell adhesion	12	0.228378378	< 0.001	< 0.001
GO:0007184	SMAD protein import into nucleus	5	0.228365508	0.0005	0.000791911
GO:0060390	regulation of SMAD protein import into nucleus	5	0.228365508	0.0005	0.000791911
GO:0032392	DNA geometric change	38	0.228347897	0.0003	0.000505993
GO:0034614	cellular response to reactive oxygen species	62	0.228268361	< 0.001	< 0.001
GO:1903317	regulation of protein maturation	28	0.228268064	0.0001	0.000192793
GO:0032647	regulation of interferon-alpha production	9	0.228228228	0.0016	0.002186748
GO:0031023	microtubule organizing center organization	52	0.228210078	0.0003	0.000505993
GO:0055076	transition metal ion homeostasis	42	0.2281792	0.0002	0.000356582
GO:0030575	nuclear body organization	7	0.227983085	< 0.001	< 0.001
GO:0003272	endocardial cushion formation	5	0.227953668	< 0.001	< 0.001
GO:0030301	cholesterol transport	23	0.22789995	0.0012	0.001702047
GO:0032461	positive regulation of protein oligomerization	8	0.227863578	< 0.001	< 0.001
GO:0072386	plus-end-directed organelle transport along microtubule	5	0.227799228	0.0129	0.014478006
GO:0030212	hyaluronan metabolic process	13	0.227759628	< 0.001	< 0.001
GO:0007176	regulation of epidermal growth factor-activated receptor activity	9	0.227742028	0.0028	0.003584743
GO:0098868	bone growth	9	0.227713428	0.0042	0.005168936
GO:1902175	regulation of oxidative stress-induced intrinsic apoptotic signaling pathway	12	0.227606178	0.0007	0.001063182
GO:0045736	negative regulation of cyclin-dependent protein serine/threonine kinase activity	11	0.227588628	0.0113	0.012793882
GO:0071875	adrenergic receptor signaling pathway	8	0.227574003	0.0053	0.006400268
GO:0071674	mononuclear cell migration	24	0.227509653	< 0.001	< 0.001
GO:0043536	positive regulation of blood vessel endothelial cell migration	21	0.227505056	0.0037	0.004617737
GO:0048026	positive regulation of mRNA splicing, via spliceosome	5	0.227490347	0.0089	0.010294435
GO:0097711	ciliary basal body docking	51	0.227466122	< 0.001	< 0.001
GO:0070296	sarcoplasmic reticulum calcium ion transport	17	0.227329851	< 0.001	< 0.001
GO:0030194	positive regulation of blood coagulation	9	0.227227227	< 0.001	< 0.001
GO:0050820	positive regulation of coagulation	9	0.227227227	< 0.001	< 0.001
GO:1900048	positive regulation of hemostasis	9	0.227227227	< 0.001	< 0.001
GO:0006538	glutamate catabolic process	6	0.227198627	0.0079	0.009236608
GO:0006342	chromatin silencing	18	0.227127127	0.0038	0.004725081
GO:0002437	inflammatory response to antigenic stimulus	14	0.22710057	0.0002	0.000356582
GO:0031649	heat generation	8	0.227091377	< 0.001	< 0.001
GO:0048010	vascular endothelial growth factor receptor signaling pathway	35	0.226975547	< 0.001	< 0.001
GO:0010665	regulation of cardiac muscle cell apoptotic process	18	0.226926927	< 0.001	< 0.001
GO:0016072	rRNA metabolic process	86	0.226892341	0.0041	0.005057384
GO:0061298	retina vasculature development in camera-type eye	7	0.226806398	0.0004	0.000648934
GO:0045880	positive regulation of smoothened signaling pathway	8	0.226705277	0.0426	0.044579861
GO:0060443	mammary gland morphogenesis	15	0.226683827	< 0.001	< 0.001
GO:0009235	cobalamin metabolic process	9	0.226626627	< 0.001	< 0.001
GO:0050901	leukocyte tethering or rolling	10	0.226589447	0.0004	0.000648934
GO:0032613	interleukin-10 production	15	0.226529387	0.0016	0.002186748
GO:0007622	rhythmic behavior	15	0.226529387	0.0058	0.006954338
GO:0021781	glial cell fate commitment	6	0.226512227	0.0029	0.003693519
GO:0051654	establishment of mitochondrion localization	13	0.226334026	0.0008	0.001193265
GO:0035264	multicellular organism growth	63	0.226332455	< 0.001	< 0.001
GO:0033144	negative regulation of intracellular steroid hormone receptor signaling pathway	15	0.226306306	0.0027	0.003470695
GO:2000482	regulation of interleukin-8 secretion	8	0.226254826	< 0.001	< 0.001
GO:0048638	regulation of developmental growth	139	0.226147422	< 0.001	< 0.001
GO:0072577	endothelial cell apoptotic process	13	0.226076626	< 0.001	< 0.001
GO:0000038	very long-chain fatty acid metabolic process	8	0.225836551	0.0008	0.001193265
GO:0032735	positive regulation of interleukin-12 production	11	0.225833626	< 0.001	< 0.001
GO:0048144	fibroblast proliferation	37	0.225788723	< 0.001	< 0.001
GO:0072676	lymphocyte migration	25	0.225770914	< 0.001	< 0.001
GO:0002438	acute inflammatory response to antigenic stimulus	5	0.225637066	0.0311	0.033148797
GO:0001890	placenta development	61	0.225621875	< 0.001	< 0.001
GO:0006893	Golgi to plasma membrane transport	17	0.22554319	0.0008	0.001193265
GO:0043631	RNA polyadenylation	16	0.225530888	0.0069	0.008171729
GO:0055010	ventricular cardiac muscle tissue morphogenesis	22	0.225529426	< 0.001	< 0.001
GO:2000781	positive regulation of double-strand break repair	14	0.225501011	0.0003	0.000505993
GO:0045814	negative regulation of gene expression, epigenetic	29	0.225376115	0.0009	0.001319479
GO:0086004	regulation of cardiac muscle cell contraction	11	0.225318825	0.0002	0.000356582
GO:0048066	developmental pigmentation	17	0.225255508	< 0.001	< 0.001

GOIDlabel	GOName	size	categoryScores	pValPerm	pValPermCorr
GO:0001894	tissue homeostasis	75	0.225235521	< 0.001	< 0.001
GO:0014733	regulation of skeletal muscle adaptation	6	0.225225225	< 0.001	< 0.001
GO:1900006	positive regulation of dendrite development	36	0.225203775	0.0008	0.001193265
GO:0032048	cardiolipin metabolic process	5	0.225173745	0.0012	0.001702047
GO:0043535	regulation of blood vessel endothelial cell migration	36	0.225117975	0.0003	0.000505993
GO:0033574	response to testosterone	15	0.225019305	< 0.001	< 0.001
GO:1900272	negative regulation of long-term synaptic potentiation	6	0.225010725	0.0204	0.022253048
GO:0000733	DNA strand renaturation	5	0.224916345	0.0038	0.004725081
GO:0031060	regulation of histone methylation	23	0.224878294	0.0022	0.002896881
GO:0019511	peptidyl-proline hydroxylation	7	0.224783968	0.0123	0.013827585
GO:0018149	peptide cross-linking	5	0.224710425	0.0005	0.000791911
GO:0007098	centrosome cycle	46	0.224654468	0.0008	0.001193265
GO:0032623	interleukin-2 production	18	0.224596025	< 0.001	< 0.001
GO:0032663	regulation of interleukin-2 production	18	0.224596025	< 0.001	< 0.001
GO:0045197	establishment or maintenance of epithelial cell apical/basal polarity	18	0.224453024	< 0.001	< 0.001
GO:1900017	positive regulation of cytokine production involved in inflammatory response	5	0.224401544	0.0185	0.020328182
GO:0030010	establishment of cell polarity	63	0.224379482	< 0.001	< 0.001
GO:0046827	positive regulation of protein export from nucleus	7	0.224379482	0.0024	0.003116943
GO:2000726	negative regulation of cardiac muscle cell differentiation	6	0.224367224	0.0029	0.003693519
GO:0034198	cellular response to amino acid starvation	25	0.224185328	0.0001	0.000192793
GO:0032692	negative regulation of interleukin-1 production	12	0.224174174	0.0016	0.002186748
GO:0014808	release of sequestered calcium ion into cytosol by sarcoplasmic reticulum	16	0.224131274	< 0.001	< 0.001
GO:1903514	calcium ion transport from endoplasmic reticulum to cytosol	16	0.224131274	< 0.001	< 0.001
GO:0071636	positive regulation of transforming growth factor beta production	9	0.224109824	0.0004	0.000648934
GO:0006637	acyl-CoA metabolic process	30	0.224066924	0.0004	0.000648934
GO:0035383	thioester metabolic process	30	0.224066924	0.0004	0.000648934
GO:0032897	negative regulation of viral transcription	9	0.223995424	0.0012	0.001702047
GO:0030307	positive regulation of cell growth	77	0.22399171	< 0.001	< 0.001
GO:0035809	regulation of urine volume	8	0.223970399	0.0001	0.000192793
GO:2000378	negative regulation of reactive oxygen species metabolic process	22	0.223879724	0.0002	0.000356582
GO:1904893	negative regulation of STAT cascade	14	0.223717595	0.0015	0.002069969
GO:0045927	positive regulation of growth	113	0.223623877	< 0.001	< 0.001
GO:0035666	TRIF-dependent toll-like receptor signaling pathway	11	0.223540424	0.0011	0.001576346
GO:0046638	positive regulation of alpha-beta T cell differentiation	10	0.223397683	0.0025	0.003237479
GO:0006101	citrate metabolic process	8	0.223391248	0.0105	0.011988591
GO:0032366	intracellular sterol transport	6	0.223380523	0.0265	0.028498706
GO:0032367	intracellular cholesterol transport	6	0.223380523	0.0265	0.028498706
GO:0070849	response to epidermal growth factor	19	0.223247307	< 0.001	< 0.001
GO:0015918	sterol transport	27	0.22312789	0.0004	0.000648934
GO:0010002	cardioblast differentiation	5	0.223114543	0.0011	0.001576346
GO:1903055	positive regulation of extracellular matrix organization	9	0.223108823	0.0004	0.000648934
GO:0043968	histone H2A acetylation	6	0.223080223	0.0052	0.006299215
GO:0045670	regulation of osteoclast differentiation	18	0.222994423	< 0.001	< 0.001
GO:0044270	cellular nitrogen compound catabolic process	185	0.22293645	0.0042	0.005168936
GO:0010644	cell communication by electrical coupling	10	0.222882883	< 0.001	< 0.001
GO:0086064	cell communication by electrical coupling involved in cardiac conduction	10	0.222882883	< 0.001	< 0.001
GO:0007229	integrin-mediated signaling pathway	30	0.222874303	0.0001	0.000192793
GO:0019730	antimicrobial humoral response	17	0.222863199	< 0.001	< 0.001
GO:0034655	nucleobase-containing compound catabolic process	166	0.222862105	0.0087	0.010084685
GO:0048806	genitalia development	11	0.222861823	0.0002	0.000356582
GO:1904994	regulation of leukocyte adhesion to vascular endothelial cell	8	0.222844273	0.0042	0.005168936
GO:0042733	embryonic digit morphogenesis	17	0.222726929	< 0.001	< 0.001
GO:0010907	positive regulation of glucose metabolic process	15	0.222531103	< 0.001	< 0.001
GO:0046330	positive regulation of JNK cascade	56	0.222274315	< 0.001	< 0.001
GO:0044380	protein localization to cytoskeleton	28	0.222265122	0.0001	0.000192793
GO:0040036	regulation of fibroblast growth factor receptor signaling pathway	7	0.222173194	< 0.001	< 0.001
GO:0060415	muscle tissue morphogenesis	28	0.222164001	< 0.001	< 0.001
GO:2000641	regulation of early endosome to late endosome transport	11	0.222136422	0.0005	0.000791911
GO:0051898	negative regulation of protein kinase B signaling	18	0.221993422	0.0003	0.000505993
GO:0043174	nucleoside salvage	7	0.221989336	0.0024	0.003116943
GO:1902902	negative regulation of autophagosome assembly	7	0.221952565	0.0109	0.012398148
GO:0001843	neural tube closure	40	0.221930502	< 0.001	< 0.001
GO:0060606	tube closure	40	0.221930502	< 0.001	< 0.001
GO:0033619	membrane protein proteolysis	17	0.221894163	0.0017	0.00231289
GO:0038084	vascular endothelial growth factor signaling pathway	10	0.221879022	0.0192	0.02103747
GO:0006998	nuclear envelope organization	27	0.221859955	0.0009	0.001319479
GO:0007064	mitotic sister chromatid cohesion	8	0.221846847	< 0.001	< 0.001
GO:0045724	positive regulation of cilium assembly	11	0.221785422	0.0002	0.000356582
GO:1901862	negative regulation of muscle tissue development	16	0.221750322	< 0.001	< 0.001
GO:0032930	positive regulation of superoxide anion generation	6	0.221750322	0.011	0.012498738
GO:0070664	negative regulation of leukocyte proliferation	14	0.221695165	0.0022	0.002896881
GO:0030510	regulation of BMP signaling pathway	21	0.221682907	< 0.001	< 0.001
GO:0009311	oligosaccharide metabolic process	17	0.221667045	0.0007	0.001063182
GO:0006633	fatty acid biosynthetic process	56	0.2216492	< 0.001	< 0.001
GO:0007585	respiratory gaseous exchange	30	0.221613042	< 0.001	< 0.001
GO:0043567	regulation of insulin-like growth factor receptor signaling pathway	7	0.221400993	0.0331	0.035135023
GO:0021517	ventral spinal cord development	15	0.221381381	0.0003	0.000505993
GO:0034453	microtubule anchoring	15	0.221329901	0.0005	0.000791911
GO:0050906	detection of stimulus involved in sensory perception	37	0.22125987	< 0.001	< 0.001
GO:0071695	anatomical structure maturation	19	0.221255842	0.0008	0.001193265
GO:0003309	type B pancreatic cell differentiation	10	0.221235521	0.0115	0.013001255
GO:0000910	cytokinesis	60	0.221179751	0.0001	0.000192793
GO:0006643	membrane lipid metabolic process	65	0.221158301	0.0002	0.000356582

GOIDlabel	GOName	size	categoryScores	pValPerm	pValPermCorr
GO:0006094	gluconeogenesis	34	0.221152245	0.0001	0.000192793
GO:0019319	hexose biosynthetic process	34	0.221152245	0.0001	0.000192793
GO:0046364	monosaccharide biosynthetic process	34	0.221152245	0.0001	0.000192793
GO:0051646	mitochondrion localization	22	0.221130221	0.0001	0.000192793
GO:0014020	primary neural tube formation	43	0.221112807	< 0.001	< 0.001
GO:0010667	negative regulation of cardiac muscle cell apoptotic process	11	0.221083421	0.0006	0.000927725
GO:0032801	receptor catabolic process	22	0.221024921	0.0008	0.001193265
GO:0010738	regulation of protein kinase A signaling	9	0.221021021	0.0001	0.000192793
GO:0001844	protein insertion into mitochondrial membrane involved in apoptotic signaling pathway	17	0.221015974	0.0024	0.003116943
GO:0051349	positive regulation of lyase activity	22	0.221013221	< 0.001	< 0.001
GO:0006892	post-Golgi vesicle-mediated transport	30	0.221003861	0.0011	0.001576346
GO:0021522	spinal cord motor neuron differentiation	13	0.220908821	0.0022	0.002896881
GO:0061756	leukocyte adhesion to vascular endothelial cell	15	0.220866581	0.001	0.001450349
GO:0060252	positive regulation of glial cell proliferation	8	0.220785071	0.0085	0.009867669
GO:0045739	positive regulation of DNA repair	29	0.220769538	0.0002	0.000356582
GO:0034204	lipid translocation	9	0.220763621	0.0008	0.001193265
GO:0045332	phospholipid translocation	9	0.220763621	0.0008	0.001193265
GO:0071624	positive regulation of granulocyte chemotaxis	9	0.220706421	< 0.001	< 0.001
GO:0010944	negative regulation of transcription by competitive promoter binding	5	0.220694981	0.0267	0.028702367
GO:0036148	phosphatidylglycerol acyl-chain remodeling	5	0.220694981	0.0154	0.017060599
GO:0009108	coenzyme biosynthetic process	53	0.220689153	0.0001	0.000192793
GO:0006446	regulation of translational initiation	29	0.220680779	0.0013	0.001826625
GO:0035358	regulation of peroxisome proliferator activated receptor signaling pathway	9	0.220649221	0.0003	0.000505993
GO:0001892	embryonic placenta development	32	0.220608108	< 0.001	< 0.001
GO:0010996	response to auditory stimulus	9	0.220592021	0.0012	0.001702047
GO:0003323	type B pancreatic cell development	8	0.220592021	0.0124	0.013937105
GO:0007602	phototransduction	16	0.22046332	0.0001	0.000192793
GO:0060644	mammary gland epithelial cell differentiation	6	0.22042042	0.0002	0.000356582
GO:0045843	negative regulation of striated muscle tissue development	15	0.2203861	< 0.001	< 0.001
GO:0048635	negative regulation of muscle organ development	15	0.2203861	< 0.001	< 0.001
GO:0006505	GPI anchor metabolic process	11	0.22033462	0.0043	0.005276385
GO:0001783	B cell apoptotic process	9	0.22022022	0.0012	0.001702047
GO:0061383	trabecula morphogenesis	20	0.22018018	0.0004	0.000648934
GO:1903018	regulation of glycoprotein metabolic process	16	0.22014157	0.0003	0.000505993
GO:0045582	positive regulation of T cell differentiation	18	0.22013442	0.0002	0.000356582
GO:0006690	icosanoid metabolic process	34	0.22007722	< 0.001	< 0.001
GO:1901568	fatty acid derivative metabolic process	34	0.22007722	< 0.001	< 0.001
GO:0044788	modulation by host of viral process	11	0.22005382	< 0.001	< 0.001
GO:0008589	regulation of smoothened signaling pathway	26	0.22004752	0.0026	0.003356522
GO:0009988	cell-cell recognition	20	0.21997426	< 0.001	< 0.001
GO:0006509	membrane protein ectodomain proteolysis	13	0.21993862	0.0033	0.004153051
GO:0006851	mitochondrial calcium ion transport	12	0.21988417	0.0083	0.009660392
GO:0010842	retina layer formation	6	0.21986272	0.0137	0.015318601
GO:0000097	sulfur amino acid biosynthetic process	6	0.21981982	0.0019	0.00254969
GO:1903019	negative regulation of glycoprotein metabolic process	6	0.21964822	0.02	0.021843169
GO:0006220	pyrimidine nucleotide metabolic process	17	0.219638126	0.0009	0.001319479
GO:0072378	blood coagulation, fibrin clot formation	6	0.21960532	< 0.001	< 0.001
GO:1904262	negative regulation of TORC1 signaling	6	0.21956242	0.0001	0.000192793
GO:0006986	response to unfolded protein	67	0.219493267	< 0.001	< 0.001
GO:0008210	estrogen metabolic process	6	0.219476619	0.0098	0.011263122
GO:2000678	negative regulation of transcription regulatory region DNA binding	9	0.219419419	0.0179	0.019692915
GO:0031032	actomyosin structure organization	68	0.219388296	< 0.001	< 0.001
GO:0010596	negative regulation of endothelial cell migration	24	0.219305019	0.0002	0.000356582
GO:0035966	response to topologically incorrect protein	73	0.219255655	< 0.001	< 0.001
GO:0019439	aromatic compound catabolic process	186	0.219206764	0.0042	0.005168936
GO:1901623	regulation of lymphocyte chemotaxis	5	0.219202059	0.0005	0.000791911
GO:0032607	interferon-alpha production	10	0.219124839	0.0052	0.006299215
GO:0061323	cell proliferation involved in heart morphogenesis	5	0.219099099	0.0002	0.000356582
GO:2000136	regulation of cell proliferation involved in heart morphogenesis	5	0.219099099	0.0002	0.000356582
GO:0055008	cardiac muscle tissue morphogenesis	27	0.219066686	< 0.001	< 0.001
GO:1904467	regulation of tumor necrosis factor secretion	7	0.219010848	0.0004	0.000648934
GO:0090630	activation of GTPase activity	17	0.218987054	< 0.001	< 0.001
GO:0006493	protein O-linked glycosylation	34	0.218941631	< 0.001	< 0.001
GO:0000291	nuclear-transcribed mRNA catabolic process, exonucleolytic	19	0.218844408	0.0137	0.015318601
GO:0043928	exonucleolytic nuclear-transcribed mRNA catabolic process involved in deadenylation-dependent decay	19	0.218844408	0.0137	0.015318601
GO:0099024	plasma membrane invagination	17	0.218835642	0.0043	0.005276385
GO:0021561	facial nerve development	5	0.218790219	0.0293	0.031335286
GO:0021604	cranial nerve structural organization	5	0.218790219	0.0293	0.031335286
GO:0021610	facial nerve morphogenesis	5	0.218790219	0.0293	0.031335286
GO:0021612	facial nerve structural organization	5	0.218790219	0.0293	0.031335286
GO:0021783	preganglionic parasympathetic fiber development	5	0.218790219	0.0293	0.031335286
GO:0048486	parasympathetic nervous system development	5	0.218790219	0.0293	0.031335286
GO:0031343	positive regulation of cell killing	11	0.218766819	< 0.001	< 0.001
GO:1901361	organic cyclic compound catabolic process	192	0.218715144	0.0038	0.004725081
GO:0010712	regulation of collagen metabolic process	12	0.218682969	< 0.001	< 0.001
GO:0044246	regulation of multicellular organismal metabolic process	12	0.218682969	< 0.001	< 0.001
GO:0030261	chromosome condensation	11	0.218603019	0.0186	0.020421455
GO:0048145	regulation of fibroblast proliferation	36	0.218454168	< 0.001	< 0.001
GO:0050775	positive regulation of dendrite morphogenesis	19	0.218437987	0.0007	0.001063182
GO:0010717	regulation of epithelial to mesenchymal transition	30	0.218395538	< 0.001	< 0.001
GO:0007625	grooming behavior	9	0.218361218	< 0.001	< 0.001
GO:0046686	response to cadmium ion	23	0.218253036	0.0009	0.001319479
GO:0007350	blastoderm segmentation	5	0.218172458	0.024	0.025944389

GOIDlabel	GOName	size	categoryScores	pValPerm	pValPermCorr
GO:0050885	neuromuscular process controlling balance	25	0.21814157	0.0009	0.001319479
GO:0006694	steroid biosynthetic process	68	0.218108865	0.0011	0.001576346
GO:0006833	water transport	5	0.218069498	< 0.001	< 0.001
GO:0046700	heterocycle catabolic process	183	0.21798848	0.0061	0.007291396
GO:0033962	cytoplasmic mRNA processing body assembly	8	0.217985843	0.0083	0.009660392
GO:0072330	monocarboxylic acid biosynthetic process	80	0.217905405	< 0.001	< 0.001
GO:0048311	mitochondrion distribution	6	0.217803518	0.0062	0.00739947
GO:0090287	regulation of cellular response to growth factor stimulus	80	0.21770592	< 0.001	< 0.001
GO:0035089	establishment of apical/basal cell polarity	10	0.217683398	< 0.001	< 0.001
GO:0061162	establishment of monopolar cell polarity	10	0.217683398	< 0.001	< 0.001
GO:0010662	regulation of striated muscle cell apoptotic process	19	0.217638691	< 0.001	< 0.001
GO:0097435	fibril organization	11	0.217526618	0.0003	0.000505993
GO:0016482	cytosolic transport	72	0.217517518	0.0003	0.000505993
GO:0032368	regulation of lipid transport	32	0.217471042	< 0.001	< 0.001
GO:0010332	response to gamma radiation	23	0.217324156	0.005	0.006070546
GO:0006665	sphingolipid metabolic process	49	0.217214299	0.0001	0.000192793
GO:0090151	establishment of protein localization to mitochondrial membrane	7	0.217209046	0.0153	0.01697069
GO:0043011	myeloid dendritic cell differentiation	6	0.217160017	< 0.001	< 0.001
GO:0071276	cellular response to cadmium ion	18	0.217160017	0.0005	0.000791911
GO:0090140	regulation of mitochondrial fission	9	0.217131417	0.0086	0.009975188
GO:0033559	unsaturated fatty acid metabolic process	40	0.217091377	< 0.001	< 0.001
GO:1901224	positive regulation of NIK/NF-kappaB signaling	23	0.217021991	0.0019	0.00254969
GO:0072698	protein localization to microtubule cytoskeleton	25	0.216936937	0.0002	0.000356582
GO:0048639	positive regulation of developmental growth	75	0.216768769	< 0.001	< 0.001
GO:1903861	positive regulation of dendrite extension	14	0.216749402	0.0269	0.028900119
GO:0099010	modification of postsynaptic structure	9	0.216731017	0.0024	0.003116943
GO:0046847	filopodium assembly	33	0.216731017	0.0072	0.008495371
GO:0071806	protein transmembrane transport	20	0.216589447	0.0149	0.016557598
GO:0006575	cellular modified amino acid metabolic process	59	0.216578322	< 0.001	< 0.001
GO:0051573	negative regulation of histone H3-K9 methylation	5	0.216576577	0.009	0.010394529
GO:0006956	complement activation	10	0.216550837	0.0001	0.000192793
GO:0001759	organ induction	5	0.216525097	0.0106	0.012082345
GO:0070265	necrotic cell death	12	0.216430716	0.0003	0.000505993
GO:0001906	cell killing	42	0.216424588	< 0.001	< 0.001
GO:0051984	positive regulation of chromosome segregation	10	0.216422136	0.0004	0.000648934
GO:0070875	positive regulation of glycogen metabolic process	8	0.216377091	0.0016	0.002186748
GO:0050810	regulation of steroid biosynthetic process	39	0.216374616	0.0047	0.005730776
GO:0035929	steroid hormone secretion	5	0.216370656	0.024	0.025944389
GO:0005978	glycogen biosynthetic process	20	0.216241956	< 0.001	< 0.001
GO:0009250	glucan biosynthetic process	20	0.216241956	< 0.001	< 0.001
GO:0016575	histone deacetylation	22	0.216216216	0.0001	0.000192793
GO:0086091	regulation of heart rate by cardiac conduction	13	0.216196416	0.014	0.015631405
GO:0044546	NLRP3 inflammasome complex assembly	8	0.216055341	0.0059	0.007066401
GO:0006084	acetyl-CoA metabolic process	12	0.216044616	0.008	0.009347459
GO:0090303	positive regulation of wound healing	13	0.215998416	< 0.001	< 0.001
GO:0009200	deoxyribonucleoside triphosphate metabolic process	7	0.215958816	0.0012	0.001702047
GO:0060043	regulation of cardiac muscle cell proliferation	13	0.215919216	< 0.001	< 0.001
GO:0042130	negative regulation of T cell proliferation	12	0.215873016	0.0034	0.00427293
GO:0048009	insulin-like growth factor receptor signaling pathway	13	0.215840016	0.0018	0.002428764
GO:0043506	regulation of JUN kinase activity	40	0.215682111	< 0.001	< 0.001
GO:0032881	regulation of polysaccharide metabolic process	18	0.215644216	0.0013	0.001826625
GO:0002690	positive regulation of leukocyte chemotaxis	28	0.215637066	< 0.001	< 0.001
GO:0032691	negative regulation of interleukin-1 beta production	11	0.215607816	0.0011	0.001576346
GO:0042755	eating behavior	12	0.215572716	< 0.001	< 0.001
GO:0030490	maturation of SSU-rRNA	6	0.215529816	0.0244	0.026339912
GO:0051905	establishment of pigment granule localization	13	0.215483615	< 0.001	< 0.001
GO:0071472	cellular response to salt stress	6	0.215186615	0.0238	0.025753945
GO:0007595	lactation	19	0.21515952	< 0.001	< 0.001
GO:0060389	pathway-restricted SMAD protein phosphorylation	15	0.215083655	0.0037	0.004617737
GO:0048535	lymph node development	8	0.21502574	0.0014	0.00194839
GO:0070231	T cell apoptotic process	12	0.214929215	0.0021	0.002780108
GO:0006884	cell volume homeostasis	10	0.214877735	< 0.001	< 0.001
GO:0061384	heart trabecula morphogenesis	16	0.21483269	< 0.001	< 0.001
GO:0042537	benzene-containing compound metabolic process	12	0.214779065	< 0.001	< 0.001
GO:0031062	positive regulation of histone methylation	14	0.214726972	0.0122	0.013738029
GO:1900026	positive regulation of substrate adhesion-dependent cell spreading	17	0.214717238	< 0.001	< 0.001
GO:0099068	postsynapse assembly	9	0.214614615	0.0173	0.01905998
GO:0030214	hyaluronan catabolic process	7	0.214598272	0.0002	0.000356582
GO:0045943	positive regulation of transcription from RNA polymerase I promoter	12	0.214543115	0.0031	0.003925117
GO:0035088	establishment or maintenance of apical/basal cell polarity	19	0.214482151	< 0.001	< 0.001
GO:0061245	establishment or maintenance of bipolar cell polarity	19	0.214482151	< 0.001	< 0.001
GO:0010766	negative regulation of sodium ion transport	6	0.214371514	0.0004	0.000648934
GO:0060347	heart trabecula formation	9	0.214300014	0.0105	0.011988591
GO:0007603	phototransduction, visible light	11	0.214180414	0.0022	0.002896881
GO:1902004	positive regulation of beta-amyloid formation	6	0.214157014	0.0372	0.039263438
GO:1902993	positive regulation of amyloid precursor protein catabolic process	6	0.214157014	0.0372	0.039263438
GO:0019218	regulation of steroid metabolic process	48	0.214130202	0.0035	0.004390436
GO:0051055	negative regulation of lipid biosynthetic process	16	0.213963964	0.0024	0.003116943
GO:0060571	morphogenesis of an epithelial fold	5	0.213951094	0.0312	0.033235719
GO:0051205	protein insertion into membrane	25	0.213920206	< 0.001	< 0.001
GO:0030038	contractile actin filament bundle assembly	46	0.213843657	< 0.001	< 0.001
GO:0043149	stress fiber assembly	46	0.213843657	< 0.001	< 0.001
GO:0010719	negative regulation of epithelial to mesenchymal transition	13	0.213780814	< 0.001	< 0.001

GOIDlabel	GOName	size	categoryScores	pValPerm	pValPermCorr
GO:0045745	positive regulation of G-protein coupled receptor protein signaling pathway	11	0.213759214	0.0025	0.003237479
GO:0007171	activation of transmembrane receptor protein tyrosine kinase activity	6	0.213728014	0.0304	0.032428267
GO:0045214	sarcomere organization	10	0.213719434	0.0091	0.010505533
GO:0021854	hypothalamus development	5	0.213693694	0.0029	0.003693519
GO:0050919	negative chemotaxis	18	0.213685114	0.0004	0.000648934
GO:0070873	regulation of glycogen metabolic process	14	0.213587056	0.0002	0.000356582
GO:1903670	regulation of sprouting angiogenesis	24	0.213513514	0.0072	0.008495371
GO:0048644	muscle organ morphogenesis	30	0.213359073	< 0.001	< 0.001
GO:0072528	pyrimidine-containing compound biosynthetic process	14	0.21331127	0.0007	0.001063182
GO:0051444	negative regulation of ubiquitin-protein transferase activity	8	0.213223938	0.0236	0.025557996
GO:0042742	defense response to bacterium	51	0.213137507	< 0.001	< 0.001
GO:0046719	regulation by virus of viral protein levels in host cell	5	0.213127413	0.0473	0.049269314
GO:0060760	positive regulation of response to cytokine stimulus	20	0.213050193	0.0007	0.001063182
GO:0032371	regulation of sterol transport	11	0.212987013	0.0006	0.000927725
GO:0032374	regulation of cholesterol transport	11	0.212987013	0.0006	0.000927725
GO:0042116	macrophage activation	23	0.212914778	< 0.001	< 0.001
GO:1903350	response to dopamine	6	0.212870013	0.0346	0.036655244
GO:1903351	cellular response to dopamine	6	0.212870013	0.0346	0.036655244
GO:1900037	regulation of cellular response to hypoxia	8	0.212870013	0.0009	0.001319479
GO:1903649	regulation of cytoplasmic transport	15	0.212801373	0.0001	0.000192793
GO:0086005	ventricular cardiac muscle cell action potential	14	0.212741313	0.0033	0.004153051
GO:1902065	response to L-glutamate	7	0.212722927	0.0052	0.006299215
GO:0006959	humoral immune response	39	0.212711613	< 0.001	< 0.001
GO:1902116	negative regulation of organelle assembly	15	0.212612613	0.0018	0.002428764
GO:0010612	regulation of cardiac muscle adaptation	5	0.212612613	0.0024	0.003116943
GO:1903242	regulation of cardiac muscle hypertrophy in response to stress	5	0.212612613	0.0024	0.003116943
GO:0090150	establishment of protein localization to membrane	153	0.212498212	0.0047	0.005730776
GO:0032231	regulation of actin filament bundle assembly	44	0.212402012	< 0.001	< 0.001
GO:1903844	regulation of cellular response to transforming growth factor beta stimulus	41	0.212399159	< 0.001	< 0.001
GO:0001569	branching involved in blood vessel morphogenesis	10	0.212329472	0.0003	0.000505993
GO:0090100	positive regulation of transmembrane receptor protein serine/threonine kinase signaling pathway	27	0.212298012	0.0032	0.004042263
GO:0007062	sister chromatid cohesion	17	0.212294648	< 0.001	< 0.001
GO:0048070	regulation of developmental pigmentation	6	0.212226512	0.0093	0.010727257
GO:0032456	endocytic recycling	22	0.212214812	0.0013	0.001826625
GO:0032370	positive regulation of lipid transport	23	0.212176151	< 0.001	< 0.001
GO:0070935	3'-UTR-mediated mRNA stabilization	8	0.212033462	0.0007	0.001063182
GO:0031641	regulation of myelination	14	0.211987498	< 0.001	< 0.001
GO:0043931	ossification involved in bone maturation	6	0.211969112	0.0004	0.000648934
GO:0039694	viral RNA genome replication	11	0.211934012	0.0005	0.000791911
GO:0039703	RNA replication	11	0.211934012	0.0005	0.000791911
GO:0061000	negative regulation of dendritic spine development	7	0.211913955	0.0335	0.035545651
GO:0060632	regulation of microtubule-based movement	11	0.211887212	0.0008	0.001193265
GO:0060444	branching involved in mammary gland duct morphogenesis	7	0.211730097	< 0.001	< 0.001
GO:0018208	peptidyl-proline modification	27	0.211697412	0.0059	0.007066401
GO:0019359	nicotinamide nucleotide biosynthetic process	16	0.211615187	0.0003	0.000505993
GO:0019363	pyridine nucleotide biosynthetic process	16	0.211615187	0.0003	0.000505993
GO:0001573	ganglioside metabolic process	7	0.21154624	0.0028	0.003584743
GO:0016226	iron-sulfur cluster assembly	6	0.211540112	0.0376	0.039670129
GO:0031163	metallo-sulfur cluster assembly	6	0.211540112	0.0376	0.039670129
GO:0051085	chaperone mediated protein folding requiring cofactor	9	0.211325611	0.0003	0.000505993
GO:0010225	response to UV-C	9	0.211239811	0.0002	0.000356582
GO:0044275	cellular carbohydrate catabolic process	16	0.211196911	0.0001	0.000192793
GO:1904754	positive regulation of vascular associated smooth muscle cell migration	7	0.211068211	0.0013	0.001826625
GO:0006636	unsaturated fatty acid biosynthetic process	27	0.211039611	< 0.001	< 0.001
GO:0006488	dolichol-linked oligosaccharide biosynthetic process	8	0.210939511	0.0196	0.021436632
GO:0006490	oligosaccharide-lipid intermediate biosynthetic process	8	0.210939511	0.0196	0.021436632
GO:0016558	protein import into peroxisome matrix	5	0.210913771	0.0314	0.033435586
GO:0051385	response to mineralocorticoid	13	0.210890011	< 0.001	< 0.001
GO:1905331	negative regulation of morphogenesis of an epithelium	7	0.210884354	0.0448	0.046773416
GO:0017144	drug metabolic process	9	0.210782211	< 0.001	< 0.001
GO:0009148	pyrimidine nucleoside triphosphate biosynthetic process	5	0.210553411	0.0471	0.049070445
GO:0044065	regulation of respiratory system process	6	0.210553411	0.0106	0.012082345
GO:0045639	positive regulation of myeloid cell differentiation	32	0.210513192	0.0002	0.000356582
GO:0046456	icosanoid biosynthetic process	20	0.210501931	0.0012	0.001702047
GO:1901570	fatty acid derivative biosynthetic process	20	0.210501931	0.0012	0.001702047
GO:0090092	regulation of transmembrane receptor protein serine/threonine kinase signaling pathway	66	0.21037011	< 0.001	< 0.001
GO:0010803	regulation of tumor necrosis factor-mediated signaling pathway	24	0.210349635	0.0003	0.000505993
GO:1903365	regulation of fear response	6	0.21033891	0.0154	0.017060599
GO:2000822	regulation of behavioral fear response	6	0.21033891	0.0154	0.017060599
GO:0006825	copper ion transport	5	0.21029601	0.0208	0.022657367
GO:0045472	response to ether	5	0.21024453	0.0014	0.00194839
GO:0032570	response to progesterone	15	0.21010725	< 0.001	< 0.001
GO:0090288	negative regulation of cellular response to growth factor stimulus	48	0.210054698	< 0.001	< 0.001
GO:0006098	pentose-phosphate shunt	5	0.20998713	0.0273	0.029300735
GO:0050779	RNA destabilization	15	0.20990133	0.003	0.003811001
GO:0061157	mRNA destabilization	15	0.20990133	0.003	0.003811001
GO:0051492	regulation of stress fiber assembly	39	0.20966901	< 0.001	< 0.001
GO:0045143	homologous chromosome segregation	13	0.209484209	0.0014	0.00194839
GO:0090394	negative regulation of excitatory postsynaptic potential	5	0.209420849	0.0008	0.001193265
GO:0009584	detection of visible light	18	0.209337909	0.0012	0.001702047
GO:0048771	tissue remodeling	48	0.209239597	0.0001	0.000192793
GO:0045806	negative regulation of endocytosis	25	0.209194337	< 0.001	< 0.001
GO:0051084	'de novo' posttranslational protein folding	11	0.209126009	0.0003	0.000505993

GOIDlabel	GOName	size	categoryScores	pValPerm	pValPermCorr
GO:0042074	cell migration involved in gastrulation	5	0.209111969	0.0004	0.000648934
GO:0072173	metanephric tubule morphogenesis	5	0.209111969	0.003	0.003811001
GO:0072282	metanephric nephron tubule morphogenesis	5	0.209111969	0.003	0.003811001
GO:0055026	negative regulation of cardiac muscle tissue development	10	0.209060489	0.0001	0.000192793
GO:0046889	positive regulation of lipid biosynthetic process	22	0.208880309	0.0004	0.000648934
GO:2001014	regulation of skeletal muscle cell differentiation	6	0.208794509	< 0.001	< 0.001
GO:0018216	peptidyl-arginine methylation	9	0.208780209	0.0024	0.003116943
GO:0002192	IRES-dependent translational initiation	6	0.208751609	0.0015	0.002069969
GO:0001774	microglial cell activation	11	0.208751609	0.0003	0.000505993
GO:0032945	negative regulation of mononuclear cell proliferation	13	0.208751609	0.0021	0.002780108
GO:0050672	negative regulation of lymphocyte proliferation	13	0.208751609	0.0021	0.002780108
GO:0051781	positive regulation of cell division	25	0.208720721	< 0.001	< 0.001
GO:2001239	regulation of extrinsic apoptotic signaling pathway in absence of ligand	9	0.208379808	0.0008	0.001193265
GO:0000002	mitochondrial genome maintenance	12	0.208344058	0.0015	0.002069969
GO:0043501	skeletal muscle adaptation	7	0.20827358	0.0001	0.000192793
GO:0007077	mitotic nuclear envelope disassembly	6	0.208193908	0.0399	0.041924869
GO:0032885	regulation of polysaccharide biosynthetic process	16	0.208188546	0.0015	0.002069969
GO:0045217	cell-cell junction maintenance	5	0.208185328	0.0256	0.027580223
GO:0014823	response to activity	23	0.208113704	0.0002	0.000356582
GO:0060575	intestinal epithelial cell differentiation	5	0.208082368	0.0003	0.000505993
GO:0016242	negative regulation of macroautophagy	15	0.208013728	0.0023	0.003013139
GO:0070584	mitochondrion morphogenesis	14	0.207997794	0.007	0.008288344
GO:0006085	acetyl-CoA biosynthetic process	7	0.207979408	0.0138	0.015427224
GO:0007257	activation of JUN kinase activity	19	0.207843934	0.0001	0.000192793
GO:0006213	pyrimidine nucleoside metabolic process	14	0.207758779	0.0117	0.013205263
GO:0007129	synapsis	10	0.207670528	0.0006	0.000927725
GO:0010669	epithelial structure maintenance	14	0.20753815	< 0.001	< 0.001
GO:1902895	positive regulation of pri-miRNA transcription from RNA polymerase II promoter	15	0.207481767	< 0.001	< 0.001
GO:0065002	intracellular protein transmembrane transport	18	0.207478907	0.0148	0.01645324
GO:0085029	extracellular matrix assembly	8	0.207464607	0.0048	0.005840847
GO:0045453	bone resorption	17	0.207358619	0.0069	0.008171729
GO:0042532	negative regulation of tyrosine phosphorylation of STAT protein	6	0.207335907	0.0347	0.036753979
GO:0000413	protein peptidyl-prolyl isomerization	20	0.207117117	0.0063	0.007507211
GO:1903708	positive regulation of hemopoiesis	54	0.206911674	0.0001	0.000192793
GO:0030183	B cell differentiation	33	0.206895207	0.0003	0.000505993
GO:0051775	response to redox state	9	0.206492206	0.005	0.006070546
GO:0051204	protein insertion into mitochondrial membrane	19	0.206394364	0.0041	0.005057384
GO:0070601	centromeric sister chromatid cohesion	5	0.206383526	0.0027	0.003470695
GO:0090051	negative regulation of cell migration involved in sprouting angiogenesis	6	0.206263406	0.0109	0.012398148
GO:0048873	homeostasis of number of cells within a tissue	9	0.206120406	0.0016	0.002186748
GO:0010613	positive regulation of cardiac muscle hypertrophy	17	0.206086759	0.0001	0.000192793
GO:0014742	positive regulation of muscle hypertrophy	17	0.206086759	0.0001	0.000192793
GO:0048799	animal organ maturation	8	0.206081081	0.0003	0.000505993
GO:0070977	bone maturation	8	0.206081081	0.0003	0.000505993
GO:0046621	negative regulation of organ growth	12	0.206027456	0.0002	0.000356582
GO:0010677	negative regulation of cellular carbohydrate metabolic process	13	0.205979606	0.0027	0.003470695
GO:0043276	anoikis	13	0.205979606	0.0036	0.004505419
GO:0090101	negative regulation of transmembrane receptor protein serine/threonine kinase signaling pathway	37	0.205913249	0.0001	0.000192793
GO:0050879	multicellular organismal movement	19	0.205879564	0.0001	0.000192793
GO:0050881	musculoskeletal movement	19	0.205879564	0.0001	0.000192793
GO:0070098	chemokine-mediated signaling pathway	11	0.205639406	0.0003	0.000505993
GO:0036499	PERK-mediated unfolded protein response	6	0.205577006	0.0045	0.005509289
GO:0051180	vitamin transport	8	0.205148005	0.026	0.028
GO:0002407	dendritic cell chemotaxis	5	0.205148005	0.0324	0.034446114
GO:0045132	meiotic chromosome segregation	23	0.205069666	0.0001	0.000192793
GO:0009147	pyrimidine nucleoside triphosphate metabolic process	6	0.205019305	0.0411	0.043118695
GO:0045912	negative regulation of carbohydrate metabolic process	16	0.205003218	0.0034	0.00427293
GO:0098901	regulation of cardiac muscle cell action potential	10	0.204942085	0.0043	0.005276385
GO:0003197	endocardial cushion development	14	0.204927376	0.0001	0.000192793
GO:0007039	protein catabolic process in the vacuole	7	0.204706748	0.0413	0.043311702
GO:0051785	positive regulation of nuclear division	18	0.204561705	0.0001	0.000192793
GO:0007043	cell-cell junction assembly	33	0.204469404	< 0.001	< 0.001
GO:0071364	cellular response to epidermal growth factor stimulus	17	0.204436369	< 0.001	< 0.001
GO:0032400	melanosome localization	13	0.204395604	0.0003	0.000505993
GO:0010862	positive regulation of pathway-restricted SMAD protein phosphorylation	8	0.204375804	0.0206	0.022453086
GO:0048671	negative regulation of collateral sprouting	5	0.204272844	0.042	0.043994573
GO:0003203	endocardial cushion morphogenesis	10	0.204118404	< 0.001	< 0.001
GO:0042104	positive regulation of activated T cell proliferation	7	0.204044861	0.0002	0.000356582
GO:0046856	phosphatidylinositol dephosphorylation	12	0.204011154	0.0272	0.029199205
GO:0016254	preassembly of GPI anchor in ER membrane	6	0.203989704	0.0132	0.014787065
GO:0006506	GPI anchor biosynthetic process	10	0.203963964	0.0059	0.007066401
GO:0017015	regulation of transforming growth factor beta receptor signaling pathway	40	0.203925354	< 0.001	< 0.001
GO:0030239	myofibril assembly	14	0.203897775	0.0006	0.000927725
GO:0097009	energy homeostasis	10	0.203758044	0.0047	0.005730776
GO:0044458	motile cilium assembly	5	0.203758044	0.0022	0.002896881
GO:0002763	positive regulation of myeloid leukocyte differentiation	21	0.203701661	0.0005	0.000791911
GO:0032604	granulocyte macrophage colony-stimulating factor production	6	0.203603604	0.0008	0.001193265
GO:0032645	regulation of granulocyte macrophage colony-stimulating factor production	6	0.203603604	0.0008	0.001193265
GO:0010324	membrane invagination	21	0.203591346	0.0087	0.010084685
GO:0021846	cell proliferation in forebrain	12	0.203582154	0.0006	0.000927725
GO:0010869	regulation of receptor biosynthetic process	6	0.203517804	0.0029	0.003693519
GO:0032800	receptor biosynthetic process	6	0.203517804	0.0029	0.003693519
GO:0035970	peptidyl-threonine dephosphorylation	9	0.203517804	0.0018	0.002428764

GOIDlabel	GOName	size	categoryScores	pValPerm	pValPermCorr
GO:0099563	modification of synaptic structure	11	0.203439803	0.0112	0.012688637
GO:0051290	protein heterotetramerization	13	0.203425403	0.0011	0.001576346
GO:0005979	regulation of glycogen biosynthetic process	12	0.203303303	0.0004	0.000648934
GO:0010962	regulation of glucan biosynthetic process	12	0.203303303	0.0004	0.000648934
GO:0032459	regulation of protein oligomerization	16	0.203265766	0.0004	0.000648934
GO:0022011	myelination in peripheral nervous system	9	0.203060203	0.0009	0.001319479
GO:0032292	peripheral nervous system axon ensheathment	9	0.203060203	0.0009	0.001319479
GO:0045109	intermediate filament organization	5	0.203037323	0.0096	0.011052066
GO:0045577	regulation of B cell differentiation	9	0.203003003	0.0002	0.000356582
GO:0010259	multicellular organism aging	13	0.202910603	0.017	0.018752398
GO:0072584	caveolin-mediated endocytosis	6	0.202831403	0.0237	0.02566115
GO:0009215	purine deoxyribonucleoside triphosphate metabolic process	5	0.202779923	0.002	0.002664694
GO:0045992	negative regulation of embryonic development	11	0.202550603	< 0.001	< 0.001
GO:0071677	positive regulation of mononuclear cell migration	9	0.202545403	0.0035	0.004390436
GO:0045604	regulation of epidermal cell differentiation	15	0.202436722	< 0.001	< 0.001
GO:0006672	ceramide metabolic process	23	0.202428515	0.0001	0.000192793
GO:0032964	collagen biosynthetic process	13	0.202356202	< 0.001	< 0.001
GO:0030968	endoplasmic reticulum unfolded protein response	48	0.202295152	0.0003	0.000505993
GO:0014044	Schwann cell development	10	0.202239382	0.0009	0.001319479
GO:0045621	positive regulation of lymphocyte differentiation	21	0.202059202	0.0015	0.002069969
GO:0045780	positive regulation of bone resorption	5	0.201750322	0.0342	0.036245695
GO:0046852	positive regulation of bone remodeling	5	0.201750322	0.0342	0.036245695
GO:0008206	bile acid metabolic process	14	0.201654716	0.0006	0.000927725
GO:0008207	C21-steroid hormone metabolic process	10	0.201647362	0.0022	0.002896881
GO:0050926	regulation of positive chemotaxis	7	0.201617944	0.0015	0.002069969
GO:0050927	positive regulation of positive chemotaxis	7	0.201617944	0.0015	0.002069969
GO:0035967	cellular response to topologically incorrect protein	60	0.201613042	0.0002	0.000356582
GO:0070131	positive regulation of mitochondrial translation	12	0.201587302	0.0154	0.017060599
GO:1903541	regulation of exosomal secretion	7	0.201544402	0.0252	0.027160112
GO:0003009	skeletal muscle contraction	15	0.201389961	< 0.001	< 0.001
GO:0035246	peptidyl-arginine N-methylation	6	0.201244101	0.0055	0.006618125
GO:0042398	cellular modified amino acid biosynthetic process	15	0.201201201	0.004	0.004954389
GO:0051645	Golgi localization	11	0.201170001	0.0006	0.000927725
GO:0002861	regulation of inflammatory response to antigenic stimulus	7	0.201139915	0.0029	0.003693519
GO:0043113	receptor clustering	28	0.200726236	0.0088	0.010185307
GO:0018195	peptidyl-arginine modification	10	0.200720721	0.003	0.003811001
GO:0045766	positive regulation of angiogenesis	45	0.200720721	0.0011	0.001576346
GO:1990774	tumor necrosis factor secretion	8	0.200611326	0.0053	0.006400268
GO:0022600	digestive system process	30	0.200600601	< 0.001	< 0.001
GO:0032637	interleukin-8 production	23	0.200503609	0.0011	0.001576346
GO:0034035	purine ribonucleoside bisphosphate metabolic process	7	0.200441258	0.004	0.004954389
GO:0050427	3'-phosphoadenosine 5'-phosphosulfate metabolic process	7	0.200441258	0.004	0.004954389
GO:0051302	regulation of cell division	58	0.200412728	< 0.001	< 0.001
GO:0006071	glycerol metabolic process	5	0.2002574	0.0009	0.001319479
GO:1901222	regulation of NIK/NF-kappaB signaling	31	0.200166065	0.0039	0.004841626
GO:0036498	IRE1-mediated unfolded protein response	29	0.200062131	0.0003	0.000505993
GO:0045954	positive regulation of natural killer cell mediated cytotoxicity	5	0.2	< 0.001	< 0.001
GO:0034620	cellular response to unfolded protein	55	0.19987364	0.0003	0.000505993
GO:0072525	pyridine-containing compound biosynthetic process	19	0.199674863	0.0001	0.000192793
GO:0042036	negative regulation of cytokine biosynthetic process	11	0.199649	0.0001	0.000192793
GO:0044058	regulation of digestive system process	9	0.1996282	0.0001	0.000192793
GO:0035584	calcium-mediated signaling using intracellular calcium source	10	0.19956242	0.0063	0.007507211
GO:0048670	regulation of collateral sprouting	7	0.199558742	0.0086	0.009975188
GO:0035810	positive regulation of urine volume	5	0.19953668	0.0011	0.001576346
GO:0030970	retrograde protein transport, ER to cytosol	14	0.199466814	0.0084	0.009757868
GO:1903513	endoplasmic reticulum to cytosol transport	14	0.199466814	0.0084	0.009757868
GO:0051647	nucleus localization	10	0.199433719	0.0314	0.033435586
GO:0060312	regulation of blood vessel remodeling	5	0.199382239	0.0033	0.004153051
GO:0031063	regulation of histone deacetylation	12	0.199377949	< 0.001	< 0.001
GO:1904886	beta-catenin destruction complex disassembly	7	0.199374885	0.0017	0.00231289
GO:1902107	positive regulation of leukocyte differentiation	42	0.199331985	0.0008	0.001193265
GO:0006401	RNA catabolic process	128	0.199288127	0.0387	0.04075113
GO:0080154	regulation of fertilization	7	0.199264571	0.0382	0.040279571
GO:0033197	response to vitamin E	5	0.199124839	0.0009	0.001319479
GO:0090025	regulation of monocyte chemotaxis	9	0.198970399	0.0041	0.005057384
GO:0045599	negative regulation of fat cell differentiation	21	0.198933628	< 0.001	< 0.001
GO:0045198	establishment of epithelial cell apical/basal polarity	8	0.198809524	0.0026	0.003356522
GO:0010737	protein kinase A signaling	11	0.198806599	< 0.001	< 0.001
GO:0035269	protein O-linked mannosylation	10	0.198532819	0.0028	0.003584743
GO:0006471	protein ADP-ribosylation	11	0.198525799	0.0012	0.001702047
GO:0098885	modification of postsynaptic actin cytoskeleton	7	0.198418827	0.0032	0.004042263
GO:0061002	negative regulation of dendritic spine morphogenesis	5	0.198404118	0.0316	0.033622052
GO:0032965	regulation of collagen biosynthetic process	10	0.198378378	< 0.001	< 0.001
GO:0051292	nuclear pore complex assembly	5	0.198198198	0.0054	0.006510843
GO:0032527	protein exit from endoplasmic reticulum	19	0.198184651	0.0052	0.006299215
GO:0001941	postsynaptic membrane organization	24	0.198166023	0.0041	0.005057384
GO:0001964	startle response	18	0.198098098	0.0103	0.011785137
GO:0042780	tRNA 3'-end processing	5	0.198095238	0.0009	0.001319479
GO:0043628	ncRNA 3'-end processing	5	0.198095238	0.0009	0.001319479
GO:0046471	phosphatidylglycerol metabolic process	9	0.197997998	0.0024	0.003116943
GO:0032781	positive regulation of ATPase activity	19	0.197696945	0.0005	0.000791911
GO:0006221	pyrimidine nucleotide biosynthetic process	11	0.197683398	0.0009	0.001319479
GO:0010039	response to iron ion	9	0.197683398	0.0277	0.029712346

GOIDlabel	GOName	size	categoryScores	pValPerm	pValPermCorr
GO:0021510	spinal cord development	32	0.197627091	0.0023	0.003013139
GO:0014002	astrocyte development	7	0.197536312	0.0086	0.009975188
GO:1903053	regulation of extracellular matrix organization	17	0.197425997	< 0.001	< 0.001
GO:0010955	negative regulation of protein processing	9	0.197397397	0.0215	0.023377465
GO:1903318	negative regulation of protein maturation	9	0.197397397	0.0215	0.023377465
GO:0032878	regulation of establishment or maintenance of cell polarity	11	0.197379197	< 0.001	< 0.001
GO:0006749	glutathione metabolic process	22	0.197098397	0.0001	0.000192793
GO:0050829	defense response to Gram-negative bacterium	20	0.197001287	0.0002	0.000356582
GO:1901616	organic hydroxy compound catabolic process	20	0.196936937	< 0.001	< 0.001
GO:0050892	intestinal absorption	7	0.196800883	0.016	0.017689034
GO:0048596	embryonic camera-type eye morphogenesis	7	0.196764111	0.0004	0.000648934
GO:0071539	protein localization to centrosome	19	0.196762176	0.0006	0.000927725
GO:1905508	protein localization to microtubule organizing center	19	0.196762176	0.0006	0.000927725
GO:0032464	positive regulation of protein homooligomerization	5	0.196550837	0.001	0.001450349
GO:0001522	pseudouridine synthesis	7	0.196286082	0.0444	0.046382679
GO:1902176	negative regulation of oxidative stress-induced intrinsic apoptotic signaling pathway	8	0.196171171	0.0028	0.003584743
GO:1905214	regulation of RNA binding	6	0.195967396	0.0041	0.005057384
GO:0006378	mRNA polyadenylation	15	0.195933076	0.0181	0.019900793
GO:0007020	microtubule nucleation	7	0.195808053	0.0367	0.03875084
GO:0090141	positive regulation of mitochondrial fission	5	0.195778636	0.0105	0.011988591
GO:0072531	pyrimidine-containing compound transmembrane transport	5	0.195572716	0.0116	0.013103344
GO:0007606	sensory perception of chemical stimulus	35	0.195418275	< 0.001	< 0.001
GO:0051044	positive regulation of membrane protein ectodomain proteolysis	5	0.195366795	0.0238	0.025753945
GO:1904781	positive regulation of protein localization to centrosome	6	0.195323895	0.0205	0.022357619
GO:0032104	regulation of response to extracellular stimulus	9	0.195252395	0.0021	0.002780108
GO:0032107	regulation of response to nutrient levels	9	0.195252395	0.0021	0.002780108
GO:0055069	zinc ion homeostasis	6	0.194894895	0.0148	0.01645324
GO:0005980	glycogen catabolic process	8	0.19488417	0.0018	0.002428764
GO:0009251	glucan catabolic process	8	0.19488417	0.0018	0.002428764
GO:0052173	response to defenses of other organism involved in symbiotic interaction	5	0.194851995	0.02	0.021843169
GO:0052200	response to host defenses	5	0.194851995	0.02	0.021843169
GO:0075136	response to host	5	0.194851995	0.02	0.021843169
GO:0046839	phospholipid dephosphorylation	13	0.194574795	0.0104	0.011884458
GO:0043032	positive regulation of macrophage activation	6	0.194551695	0.004	0.004954389
GO:0051683	establishment of Golgi localization	6	0.194251394	0.0037	0.004617737
GO:0046655	folic acid metabolic process	6	0.194122694	0.001	0.001450349
GO:0015721	bile acid and bile salt transport	8	0.194111969	0.0065	0.007730194
GO:0061082	myeloid leukocyte cytokine production	9	0.194108394	0.002	0.002664694
GO:0097028	dendritic cell differentiation	12	0.194036894	0.0031	0.003925117
GO:1902914	regulation of protein polyubiquitination	9	0.194022594	0.014	0.015631405
GO:0016486	peptide hormone processing	7	0.194006251	0.0004	0.000648934
GO:0031061	negative regulation of histone methylation	10	0.193976834	0.0005	0.000791911
GO:0061081	positive regulation of myeloid leukocyte cytokine production involved in immune response	7	0.19396948	0.0001	0.000192793
GO:1905208	negative regulation of cardiocyte differentiation	7	0.193712079	0.0096	0.011052066
GO:0033687	osteoblast proliferation	8	0.193564994	0.0101	0.011583277
GO:0007528	neuromuscular junction development	16	0.193436293	0.0005	0.000791911
GO:0070572	positive regulation of neuron projection regeneration	5	0.193256113	0.0053	0.006400268
GO:0046849	bone remodeling	24	0.193114543	0.002	0.002664694
GO:0036514	dopaminergic neuron axon guidance	5	0.193101673	0.009	0.010394529
GO:1904938	planar cell polarity pathway involved in axon guidance	5	0.193101673	0.009	0.010394529
GO:0035384	thioester biosynthetic process	17	0.192717087	0.004	0.004954389
GO:0071616	acyl-CoA biosynthetic process	17	0.192717087	0.004	0.004954389
GO:0015074	DNA integration	6	0.192664093	< 0.001	< 0.001
GO:0045838	positive regulation of membrane potential	6	0.192621193	0.0365	0.038569808
GO:1903036	positive regulation of response to wounding	17	0.192520251	0.0002	0.000356582
GO:0035914	skeletal muscle cell differentiation	20	0.192496782	< 0.001	< 0.001
GO:0034103	regulation of tissue remodeling	22	0.192453492	0.0003	0.000505993
GO:1900221	regulation of beta-amyloid clearance	8	0.192374517	< 0.001	< 0.001
GO:0044793	negative regulation by host of viral process	5	0.192277992	0.0043	0.005276385
GO:1905244	regulation of modification of synaptic structure	5	0.192226512	0.0101	0.011583277
GO:1903845	negative regulation of cellular response to transforming growth factor beta stimulus	29	0.192135978	0.0014	0.00194839
GO:2000403	positive regulation of lymphocyte migration	8	0.191988417	0.0047	0.005730776
GO:0101023	vascular endothelial cell proliferation	11	0.191903592	0.0002	0.000356582
GO:1905562	regulation of vascular endothelial cell proliferation	11	0.191903592	0.0002	0.000356582
GO:0060134	prepulse inhibition	10	0.191763192	0.0111	0.012593827
GO:2001244	positive regulation of intrinsic apoptotic signaling pathway	23	0.191740809	0.0162	0.017906484
GO:0030213	hyaluronan biosynthetic process	5	0.191711712	0.0239	0.025846628
GO:0002548	monocyte chemotaxis	14	0.191487406	< 0.001	< 0.001
GO:0051904	pigment granule transport	12	0.191462891	0.0003	0.000505993
GO:1990542	mitochondrial transmembrane transport	27	0.191410458	0.0244	0.026339912
GO:0048194	Golgi vesicle budding	5	0.191402831	0.0087	0.010084685
GO:0043297	apical junction assembly	25	0.191258687	0.0002	0.000356582
GO:0032462	regulation of protein homooligomerization	10	0.191093951	0.0147	0.016362266
GO:0030865	cortical cytoskeleton organization	16	0.191071429	0.0001	0.000192793
GO:0061077	chaperone-mediated protein folding	24	0.191001716	0.0038	0.004725081
GO:0010883	regulation of lipid storage	10	0.190913771	0.0123	0.013827585
GO:0032677	regulation of interleukin-8 production	22	0.190885691	0.0033	0.004153051
GO:0021515	cell differentiation in spinal cord	17	0.19085472	0.0001	0.000192793
GO:0060512	prostate gland morphogenesis	6	0.190776491	0.0106	0.012082345
GO:0060740	prostate gland epithelium morphogenesis	6	0.190776491	0.0106	0.012082345
GO:0042474	middle ear morphogenesis	6	0.190519091	0.0031	0.003925117
GO:0086014	atrial cardiac muscle cell action potential	6	0.190519091	0.0423	0.044274492
GO:0086026	atrial cardiac muscle cell to AV node cell signaling	6	0.190519091	0.0423	0.044274492

GOIDlabel	GOName	size	categoryScores	pValPerm	pValPermCorr
GO:0086066	atrial cardiac muscle cell to AV node cell communication	6	0.190519091	0.0423	0.044274492
GO:1904861	excitatory synapse assembly	11	0.19049959	0.0238	0.025753945
GO:1905314	semi-lunar valve development	15	0.19032175	0.0003	0.000505993
GO:1901017	negative regulation of potassium ion transmembrane transporter activity	6	0.19017589	0.0002	0.000356582
GO:0090162	establishment of epithelial cell polarity	15	0.18996139	< 0.001	< 0.001
GO:0042089	cytokine biosynthetic process	30	0.18961819	0.0005	0.000791911
GO:0042107	cytokine metabolic process	30	0.18961819	0.0005	0.000791911
GO:0050766	positive regulation of phagocytosis	19	0.189446589	0.0003	0.000505993
GO:0033344	cholesterol efflux	12	0.189253539	0.0071	0.008390203
GO:0034356	NAD biosynthesis via nicotinamide riboside salvage pathway	8	0.189189189	0.0413	0.043311702
GO:0034375	high-density lipoprotein particle remodeling	6	0.189189189	0.0081	0.00945
GO:0043507	positive regulation of JUN kinase activity	34	0.189181619	0.0003	0.000505993
GO:0006081	cellular aldehyde metabolic process	24	0.189167739	0.0022	0.002896881
GO:0017004	cytochrome complex assembly	9	0.189160589	0.0177	0.019488753
GO:0051570	regulation of histone H3-K9 methylation	9	0.188960389	0.0478	0.049742191
GO:0019915	lipid storage	16	0.188674389	0.0289	0.030950377
GO:0035640	exploration behavior	13	0.188397188	0.0156	0.0172574
GO:0000272	polysaccharide catabolic process	9	0.188388388	0.0008	0.001193265
GO:0044247	cellular polysaccharide catabolic process	9	0.188388388	0.0008	0.001193265
GO:0051657	maintenance of organelle location	5	0.188314028	0.0227	0.024637638
GO:0000212	meiotic spindle organization	5	0.188005148	0.0142	0.01584489
GO:0060179	male mating behavior	6	0.187945088	0.0065	0.007730194
GO:0032233	positive regulation of actin filament bundle assembly	24	0.187934363	0.0003	0.000505993
GO:0051412	response to corticosterone	7	0.187828645	< 0.001	< 0.001
GO:0002181	cytoplasmic translation	30	0.187782068	0.0309	0.032948619
GO:0006555	methionine metabolic process	6	0.187430287	0.0241	0.02604728
GO:0090045	positive regulation of deacetylase activity	5	0.187284427	0.0136	0.015222535
GO:0071825	protein-lipid complex subunit organization	15	0.187250107	0.0002	0.000356582
GO:0070262	peptidyl-serine dephosphorylation	10	0.187155727	0.0114	0.012893596
GO:0045124	regulation of bone resorption	9	0.186958387	0.0072	0.008495371
GO:1901223	negative regulation of NIK/NF-kappaB signaling	5	0.186924067	0.0074	0.008712331
GO:0048668	collateral sprouting	11	0.186825787	0.0061	0.007291396
GO:0051291	protein heterooligomerization	46	0.1867271	0.0002	0.000356582
GO:0097734	extracellular exosome biogenesis	8	0.186647362	0.0383	0.040353519
GO:1990182	exosomal secretion	8	0.186647362	0.0383	0.040353519
GO:0009409	response to cold	14	0.186486486	0.0008	0.001193265
GO:0070830	bicellular tight junction assembly	19	0.186452618	< 0.001	< 0.001
GO:0002026	regulation of the force of heart contraction	11	0.186357786	< 0.001	< 0.001
GO:0045955	negative regulation of calcium ion-dependent exocytosis	5	0.186254826	0.0168	0.018543137
GO:0072643	interferon-gamma secretion	6	0.186229086	0.0287	0.030748374
GO:0010642	negative regulation of platelet-derived growth factor receptor signaling pathway	7	0.186173929	0.0025	0.003237479
GO:0051457	maintenance of protein location in nucleus	9	0.186157586	0.0009	0.001319479
GO:0019400	alditol metabolic process	6	0.185928786	0.0026	0.003356522
GO:0003176	aortic valve development	12	0.185757186	< 0.001	< 0.001
GO:0036010	protein localization to endosome	7	0.1854385	< 0.001	< 0.001
GO:0060716	labyrinthine layer blood vessel development	8	0.18523166	0.0009	0.001319479
GO:1902237	positive regulation of endoplasmic reticulum stress-induced intrinsic apoptotic signaling pathway	5	0.185070785	0.0104	0.011884458
GO:0009299	mRNA transcription	5	0.185019305	0.0041	0.005057384
GO:0090110	cargo loading into COPII-coated vesicle	6	0.184984985	0.0437	0.045686748
GO:0061548	ganglion development	6	0.184899185	0.0192	0.02103747
GO:0010715	regulation of extracellular matrix disassembly	9	0.184841985	0.0132	0.014787065
GO:0009220	pyrimidine ribonucleotide biosynthetic process	8	0.18478121	0.0008	0.001193265
GO:0006103	2-oxoglutarate metabolic process	8	0.18465251	0.0086	0.009975188
GO:0014829	vascular smooth muscle contraction	6	0.184384384	0.0001	0.000192793
GO:0045616	regulation of keratinocyte differentiation	9	0.184212784	0.0065	0.007730194
GO:0006700	C21-steroid hormone biosynthetic process	7	0.18418827	0.0017	0.00231289
GO:0043537	negative regulation of blood vessel endothelial cell migration	16	0.184185972	0.0043	0.005276385
GO:2000036	regulation of stem cell population maintenance	8	0.184169884	0.0016	0.002186748
GO:0009593	detection of chemical stimulus	31	0.184157429	0.0007	0.001063182
GO:0044743	intracellular protein transmembrane import	14	0.183986027	0.0393	0.041318521
GO:0045721	negative regulation of gluconeogenesis	5	0.183835264	0.0051	0.006185009
GO:0002920	regulation of humoral immune response	15	0.183766624	0.0014	0.00194839
GO:0050765	negative regulation of phagocytosis	8	0.183719434	0.0137	0.015318601
GO:0006750	glutathione biosynthetic process	7	0.183710241	0.0118	0.013309789
GO:0019184	nonribosomal peptide biosynthetic process	7	0.183710241	0.0118	0.013309789
GO:2000251	positive regulation of actin cytoskeleton reorganization	9	0.183669384	0.008	0.009347459
GO:0051602	response to electrical stimulus	16	0.183478121	< 0.001	< 0.001
GO:0046850	regulation of bone remodeling	12	0.183440583	0.0046	0.0056228
GO:0044033	multi-organism metabolic process	114	0.183399941	0.0403	0.042328707
GO:0046174	polyol catabolic process	9	0.183268983	0.0021	0.002780108
GO:2000209	regulation of anoikis	9	0.183240383	0.004	0.004954389
GO:1904294	positive regulation of ERAD pathway	6	0.183183183	0.0155	0.017160828
GO:0060674	placenta blood vessel development	15	0.182891463	0.0024	0.003116943
GO:0030199	collagen fibril organization	11	0.182566983	< 0.001	< 0.001
GO:0006699	bile acid biosynthetic process	12	0.182561133	0.002	0.002664694
GO:0031122	cytoplasmic microtubule organization	18	0.182282282	0.0007	0.001063182
GO:2001138	regulation of phospholipid transport	6	0.182024882	0.0055	0.006618125
GO:2001140	positive regulation of phospholipid transport	6	0.182024882	0.0055	0.006618125
GO:0060263	regulation of respiratory burst	6	0.181981982	0.0275	0.029503673
GO:0006984	ER-nucleus signaling pathway	16	0.181965894	0.001	0.001450349
GO:0043173	nucleotide salvage	6	0.181810382	0.002	0.002664694
GO:0035902	response to immobilization stress	11	0.181537382	0.0242	0.026144902
GO:0043268	positive regulation of potassium ion transport	13	0.181486981	0.0008	0.001193265

GOIDlabel	GOName	size	categoryScores	pValPerm	pValPermCorr
GO:0003156	regulation of animal organ formation	9	0.181209781	< 0.001	< 0.001
GO:0000303	response to superoxide	6	0.181166881	0.0328	0.034837107
GO:0097709	connective tissue replacement	5	0.181158301	0.0271	0.029103418
GO:0006911	phagocytosis, engulfment	12	0.181145431	0.0239	0.025846628
GO:0032350	regulation of hormone metabolic process	7	0.181136238	0.0067	0.007954042
GO:0030277	maintenance of gastrointestinal epithelium	9	0.181009581	0.0041	0.005057384
GO:0009435	NAD biosynthetic process	14	0.180933995	0.0029	0.003693519
GO:0050830	defense response to Gram-positive bacterium	17	0.180922099	0.0002	0.000356582
GO:0032743	positive regulation of interleukin-2 production	10	0.180540541	0.0103	0.011785137
GO:0045655	regulation of monocyte differentiation	5	0.18043758	0.0154	0.017060599
GO:0045657	positive regulation of monocyte differentiation	5	0.18043758	0.0154	0.017060599
GO:0046931	pore complex assembly	7	0.180069866	0.013	0.014581154
GO:0070129	regulation of mitochondrial translation	14	0.179720537	0.034	0.036054945
GO:1900103	positive regulation of endoplasmic reticulum unfolded protein response	7	0.17966538	0.0139	0.015532589
GO:0038034	signal transduction in absence of ligand	17	0.179453403	0.0019	0.00254969
GO:0097192	extrinsic apoptotic signaling pathway in absence of ligand	17	0.179453403	0.0019	0.00254969
GO:0030512	negative regulation of transforming growth factor beta receptor signaling pathway	28	0.179306858	0.0031	0.003925117
GO:0042572	retinol metabolic process	7	0.179297665	0.0111	0.012593827
GO:0033688	regulation of osteoblast proliferation	6	0.179279279	0.0016	0.002186748
GO:0098927	vesicle-mediated transport between endosomal compartments	25	0.179202059	0.0038	0.004725081
GO:0021983	pituitary gland development	9	0.179064779	0.0268	0.028804137
GO:0032682	negative regulation of chemokine production	7	0.179003493	0.0033	0.004153051
GO:0070192	chromosome organization involved in meiotic cell cycle	16	0.178989704	< 0.001	< 0.001
GO:0046134	pyrimidine nucleoside biosynthetic process	9	0.178978979	0.0204	0.022253048
GO:0032401	establishment of melanosome localization	12	0.178700129	0.0004	0.000648934
GO:0010927	cellular component assembly involved in morphogenesis	24	0.178657229	0.0016	0.002186748
GO:0040015	negative regulation of multicellular organism growth	7	0.178451921	0.0085	0.009867669
GO:0051496	positive regulation of stress fiber assembly	21	0.178219035	0.0009	0.001319479
GO:0019731	antibacterial humoral response	11	0.178191178	0.0027	0.003470695
GO:0032460	negative regulation of protein oligomerization	6	0.177992278	0.0445	0.046469179
GO:2000406	positive regulation of T cell migration	7	0.177606178	0.0434	0.045399458
GO:0042035	regulation of cytokine biosynthetic process	29	0.177464164	0.0019	0.00254969
GO:0042135	neurotransmitter catabolic process	5	0.177451737	0.0028	0.003584743
GO:0046164	alcohol catabolic process	16	0.177059202	0.0006	0.000927725
GO:0006448	regulation of translational elongation	6	0.177005577	0.0234	0.025351564
GO:0048048	embryonic eye morphogenesis	8	0.176930502	0.0021	0.002780108
GO:0030431	sleep	10	0.176499356	< 0.001	< 0.001
GO:0001732	formation of cytoplasmic translation initiation complex	7	0.176355948	0.0239	0.025846628
GO:1901381	positive regulation of potassium ion transmembrane transport	9	0.176233376	0.0167	0.018447833
GO:0090026	positive regulation of monocyte chemotaxis	7	0.176061776	0.0076	0.008916717
GO:0032717	negative regulation of interleukin-8 production	8	0.175675676	0.0069	0.008171729
GO:0055089	fatty acid homeostasis	6	0.175632776	0.0215	0.023377465
GO:1900225	regulation of NLRP3 inflammasome complex assembly	7	0.175583747	0.0172	0.018961404
GO:0098911	regulation of ventricular cardiac muscle cell action potential	6	0.175203775	0.0115	0.013001255
GO:0006760	folic acid-containing compound metabolic process	7	0.17466446	0.0012	0.001702047
GO:0018202	peptidyl-histidine modification	6	0.174603175	0.044	0.045991489
GO:1901201	regulation of extracellular matrix assembly	6	0.174517375	0.0076	0.008916717
GO:1901203	positive regulation of extracellular matrix assembly	6	0.174517375	0.0076	0.008916717
GO:0071168	protein localization to chromatin	11	0.174330174	0.0034	0.00427293
GO:0050764	regulation of phagocytosis	30	0.174294294	0.0011	0.001576346
GO:0033147	negative regulation of intracellular estrogen receptor signaling pathway	6	0.174259974	0.0041	0.005057384
GO:1904292	regulation of ERAD pathway	15	0.173968254	0.0065	0.007730194
GO:0044062	regulation of excretion	7	0.173929031	0.0475	0.049449046
GO:0003044	regulation of systemic arterial blood pressure mediated by a chemical signal	9	0.173888174	0.001	0.001450349
GO:0042228	interleukin-8 biosynthetic process	5	0.173487773	0.0146	0.016261006
GO:0045414	regulation of interleukin-8 biosynthetic process	5	0.173487773	0.0146	0.016261006
GO:1901016	regulation of potassium ion transmembrane transporter activity	23	0.172883442	0.0088	0.010185307
GO:0034138	toll-like receptor 3 signaling pathway	9	0.172858573	0.0327	0.034737724
GO:0034367	macromolecular complex remodeling	7	0.172825887	0.011	0.012498738
GO:0034368	protein-lipid complex remodeling	7	0.172825887	0.011	0.012498738
GO:0034369	plasma lipoprotein particle remodeling	7	0.172825887	0.011	0.012498738
GO:0003180	aortic valve morphogenesis	11	0.172715573	< 0.001	< 0.001
GO:0014819	regulation of skeletal muscle contraction	5	0.172509653	0.0003	0.000505993
GO:0045022	early endosome to late endosome transport	24	0.172415272	0.0106	0.012082345
GO:0045116	protein neddylation	6	0.172372372	0.0475	0.049449046
GO:0050802	circadian sleep/wake cycle, sleep	6	0.172029172	< 0.001	< 0.001
GO:0045647	negative regulation of erythrocyte differentiation	8	0.171428571	0.0009	0.001319479
GO:0030948	negative regulation of vascular endothelial growth factor receptor signaling pathway	6	0.171299871	0.0143	0.015940017
GO:0032928	regulation of superoxide anion generation	8	0.171171171	0.039	0.041027059
GO:0030859	polarized epithelial cell differentiation	10	0.170888031	0.0262	0.028209763
GO:1902235	regulation of endoplasmic reticulum stress-induced intrinsic apoptotic signaling pathway	16	0.170865508	0.0077	0.00902229
GO:0007398	ectoderm development	7	0.170325427	0.0054	0.006510843
GO:0010763	positive regulation of fibroblast migration	6	0.17022737	0.032	0.034034245
GO:0035813	regulation of renal sodium excretion	5	0.17014157	0.0388	0.040832561
GO:0071108	protein K48-linked deubiquitination	21	0.169982227	0.0078	0.009125612
GO:0007588	excretion	20	0.16993565	0.0126	0.014156008
GO:0030513	positive regulation of BMP signaling pathway	8	0.16988417	0.0075	0.008822377
GO:0006206	pyrimidine nucleobase metabolic process	6	0.16979837	0.0366	0.038652804
GO:0061158	3'-UTR-mediated mRNA destabilization	7	0.16962677	< 0.001	< 0.001
GO:0006107	oxaloacetate metabolic process	5	0.16957529	0.0421	0.044090775
GO:0046885	regulation of hormone biosynthetic process	5	0.16952381	0.0035	0.004390436
GO:0071236	cellular response to antibiotic	5	0.169420849	0.0028	0.003584743
GO:0043651	linoleic acid metabolic process	6	0.169412269	0.0218	0.023694127

GOIDlabel	GOName	size	categoryScores	pValPerm	pValPermCorr
GO:1900101	regulation of endoplasmic reticulum unfolded protein response	9	0.169083369	0.0117	0.013205263
GO:0070193	synaptonemal complex organization	6	0.168768769	0.008	0.009347459
GO:1902236	negative regulation of endoplasmic reticulum stress-induced intrinsic apoptotic signaling pathway	9	0.168625769	0.0211	0.022970266
GO:0003376	sphingosine-1-phosphate signaling pathway	5	0.167876448	0.0097	0.01115531
GO:0090520	sphingolipid mediated signaling pathway	5	0.167876448	0.0097	0.01115531
GO:0008211	glucocorticoid metabolic process	6	0.167782068	0.0168	0.018543137
GO:0042558	pteridine-containing compound metabolic process	12	0.167589018	0.0033	0.004153051
GO:0048261	negative regulation of receptor-mediated endocytosis	10	0.167490347	0.0081	0.00945
GO:0065005	protein-lipid complex assembly	10	0.167052767	0.0014	0.00194839
GO:0009218	pyrimidine ribonucleotide metabolic process	9	0.166394966	0.0108	0.012299937
GO:0035791	platelet-derived growth factor receptor-beta signaling pathway	5	0.166280566	0.0312	0.033235719
GO:0071827	plasma lipoprotein particle organization	14	0.165949623	0.001	0.001450349
GO:0002024	diet induced thermogenesis	6	0.165722866	0.0036	0.004505419
GO:1990845	adaptive thermogenesis	6	0.165722866	0.0036	0.004505419
GO:0097242	beta-amyloid clearance	10	0.165585586	0.0033	0.004153051
GO:0097150	neuronal stem cell population maintenance	7	0.165545137	0.0067	0.007954042
GO:1902074	response to salt	8	0.16541184	0.0456	0.047571892
GO:0034308	primary alcohol metabolic process	17	0.1651904	0.0084	0.009757868
GO:0060049	regulation of protein glycosylation	8	0.16489704	0.0061	0.007291396
GO:0044320	cellular response to leptin stimulus	8	0.164800515	0.0005	0.000791911
GO:0051712	positive regulation of killing of cells of other organism	5	0.164684685	0.0245	0.02644258
GO:0016056	rhodopsin mediated signaling pathway	9	0.164507365	0.003	0.003811001
GO:2000114	regulation of establishment of cell polarity	10	0.164375804	< 0.001	< 0.001
GO:0034123	positive regulation of toll-like receptor signaling pathway	8	0.164285714	0.0054	0.006510843
GO:0022410	circadian sleep/wake cycle process	7	0.163706564	0.0013	0.001826625
GO:0042133	neurotransmitter metabolic process	9	0.163706564	0.0018	0.002428764
GO:0045840	positive regulation of mitotic nuclear division	15	0.163569284	0.005	0.006070546
GO:0034969	histone arginine methylation	5	0.163500644	0.0047	0.005730776
GO:0016338	calcium-independent cell-cell adhesion via plasma membrane cell-adhesion molecules	6	0.163234663	0.0191	0.020936389
GO:0051181	cofactor transport	6	0.163105963	0.0354	0.03746604
GO:0035313	wound healing, spreading of epidermal cells	5	0.163088803	0.0006	0.000927725
GO:0043094	cellular metabolic compound salvage	12	0.162977263	0.0155	0.017160828
GO:0010874	regulation of cholesterol efflux	9	0.162819963	0.0093	0.010727257
GO:0021516	dorsal spinal cord development	6	0.162419562	0.0018	0.002428764
GO:0006801	superoxide metabolic process	17	0.162419562	0.0058	0.006954338
GO:0071447	cellular response to hydroperoxide	6	0.162333762	0.0089	0.010294435
GO:0061311	cell surface receptor signaling pathway involved in heart development	10	0.162239382	0.0172	0.018961404
GO:0032098	regulation of appetite	9	0.162047762	0.0009	0.001319479
GO:0034105	positive regulation of tissue remodeling	9	0.161876162	0.0004	0.000648934
GO:0008655	pyrimidine-containing compound salvage	5	0.161853282	0.0143	0.015940017
GO:0043097	pyrimidine nucleoside salvage	5	0.161853282	0.0143	0.015940017
GO:0002544	chronic inflammatory response	5	0.161801802	0.0156	0.0172574
GO:0046132	pyrimidine ribonucleoside biosynthetic process	7	0.161389961	0.0309	0.032948619
GO:0042745	circadian sleep/wake cycle	8	0.161325611	0.0019	0.00254969
GO:1903651	positive regulation of cytoplasmic transport	9	0.161161161	0.0017	0.00231289
GO:0046653	tetrahydrofolate metabolic process	5	0.161029601	0.012	0.013526909
GO:0048665	neuron fate specification	8	0.160971686	0.0094	0.010831045
GO:0030449	regulation of complement activation	9	0.160503361	0.0034	0.00427293
GO:2000257	regulation of protein activation cascade	9	0.160503361	0.0034	0.00427293
GO:0030947	regulation of vascular endothelial growth factor receptor signaling pathway	8	0.16042471	0.017	0.018752398
GO:0038007	netrin-activated signaling pathway	8	0.159877735	0.002	0.002664694
GO:0016339	calcium-dependent cell-cell adhesion via plasma membrane cell adhesion molecules	8	0.159813385	0.005	0.006070546
GO:0035247	peptidyl-arginine omega-N-methylation	5	0.15979408	0.0075	0.008822377
GO:0090070	positive regulation of ribosome biogenesis	5	0.15953668	0.0418	0.043793563
GO:2000234	positive regulation of rRNA processing	5	0.15953668	0.0418	0.043793563
GO:0098801	regulation of renal system process	9	0.159445159	0.0004	0.000648934
GO:0032467	positive regulation of cytokinesis	13	0.159429759	0.0007	0.001063182
GO:0040014	regulation of multicellular organism growth	33	0.158714559	0.0007	0.001063182
GO:1903726	negative regulation of phospholipid metabolic process	5	0.158661519	0.0069	0.008171729
GO:0061450	trophoblast cell migration	5	0.158404118	0.0045	0.005509289
GO:1901163	regulation of trophoblast cell migration	5	0.158404118	0.0045	0.005509289
GO:0032095	regulation of response to food	8	0.157657658	0.0016	0.002186748
GO:0055090	acylglycerol homeostasis	8	0.156853282	0.0297	0.031750504
GO:0070328	triglyceride homeostasis	8	0.156853282	0.0297	0.031750504
GO:0000305	response to oxygen radical	7	0.156756757	0.0384	0.040450994
GO:0033145	positive regulation of intracellular steroid hormone receptor signaling pathway	6	0.156713857	0.0437	0.045686748
GO:0032096	negative regulation of response to food	5	0.156653797	0.0013	0.001826625
GO:0032099	negative regulation of appetite	5	0.156653797	0.0013	0.001826625
GO:0032105	negative regulation of response to extracellular stimulus	5	0.156653797	0.0013	0.001826625
GO:0032108	negative regulation of response to nutrient levels	5	0.156653797	0.0013	0.001826625
GO:0032465	regulation of cytokinesis	36	0.156249106	0.0023	0.003013139
GO:2000737	negative regulation of stem cell differentiation	8	0.156241956	0.0049	0.005958506
GO:0010288	response to lead ion	10	0.155984556	0.0226	0.024538959
GO:1905564	positive regulation of vascular endothelial cell proliferation	8	0.15472973	0.0366	0.038652804
GO:2000060	positive regulation of protein ubiquitination involved in ubiquitin-dependent protein catabolic process	6	0.154440154	0.0177	0.019488753
GO:0060192	negative regulation of lipase activity	5	0.154285714	0.0217	0.023590183
GO:0035627	ceramide transport	6	0.153925354	0.0271	0.029103418
GO:0070861	regulation of protein exit from endoplasmic reticulum	12	0.153839554	0.0269	0.028900119
GO:0001990	regulation of systemic arterial blood pressure by hormone	6	0.152595453	0.0236	0.025557996
GO:0035641	locomotory exploration behavior	7	0.152380952	0.029	0.031051318
GO:0001661	conditioned taste aversion	5	0.152226512	0.0037	0.004617737
GO:0070828	heterochromatin organization	6	0.151737452	0.0398	0.041827927
GO:0002021	response to dietary excess	11	0.151655552	0.0021	0.002780108

GOIDlabel	GOName	size	categoryScores	pValPerm	pValPermCorr
GO:0007608	sensory perception of smell	24	0.151555127	0.0152	0.016877091
GO:0006067	ethanol metabolic process	7	0.151498437	0.0257	0.02768244
GO:1903573	negative regulation of response to endoplasmic reticulum stress	19	0.15125652	0.012	0.013526909
GO:0021527	spinal cord association neuron differentiation	5	0.150733591	0.0465	0.048454686
GO:0042181	ketone biosynthetic process	10	0.14993565	0.036	0.038048895
GO:0009067	aspartate family amino acid biosynthetic process	6	0.14976405	0.0456	0.047571892
GO:0030866	cortical actin cytoskeleton organization	13	0.149391149	0.001	0.001450349
GO:0046460	neutral lipid biosynthetic process	11	0.149268749	0.0071	0.008390203
GO:0046463	acylglycerol biosynthetic process	11	0.149268749	0.0071	0.008390203
GO:0032402	melanosome transport	11	0.149151749	0.0053	0.006400268
GO:0060039	pericardium development	9	0.149006149	0.0041	0.005057384
GO:0071545	inositol phosphate catabolic process	7	0.148850892	0.0198	0.021642233
GO:0019471	4-hydroxyproline metabolic process	5	0.148622909	0.0441	0.046087101
GO:0042554	superoxide anion generation	10	0.148314028	0.0435	0.045495258
GO:0006730	one-carbon metabolic process	7	0.147527119	0.0139	0.015532589
GO:0034498	early endosome to Golgi transport	5	0.147284427	0.046	0.04795216
GO:0007135	meiosis II	5	0.146924067	0.0383	0.040353519
GO:0007635	chemosensory behavior	5	0.146718147	0.0414	0.043408149
GO:0009620	response to fungus	9	0.146575147	0.0106	0.012082345
GO:0010866	regulation of triglyceride biosynthetic process	7	0.146497518	0.0015	0.002069969
GO:0003159	morphogenesis of an endothelium	6	0.145602746	0.0187	0.020510412
GO:0003352	regulation of cilium movement	5	0.145585586	0.009	0.010394529
GO:0019432	triglyceride biosynthetic process	10	0.143552124	0.0043	0.005276385
GO:0044321	response to leptin	10	0.142265122	0.0002	0.000356582
GO:0032094	response to food	11	0.141944542	0.0008	0.001193265
GO:0043555	regulation of translation in response to stress	10	0.141879022	0.0339	0.035955957
GO:0022400	regulation of rhodopsin mediated signaling pathway	7	0.14127597	0.0233	0.025253349
GO:0035336	long-chain fatty-acyl-CoA metabolic process	5	0.140797941	0.0077	0.00902229
GO:0010875	positive regulation of cholesterol efflux	7	0.140209597	0.0355	0.037549814
GO:0032373	positive regulation of sterol transport	7	0.140209597	0.0355	0.037549814
GO:0032376	positive regulation of cholesterol transport	7	0.140209597	0.0355	0.037549814
GO:0036342	post-anal tail morphogenesis	7	0.139841883	0.0051	0.006185009
GO:0051964	negative regulation of synapse assembly	6	0.138567139	0.002	0.002664694
GO:0043496	regulation of protein homodimerization activity	7	0.137672366	0.0118	0.013309789
GO:2000811	negative regulation of anoikis	7	0.134840963	0.0084	0.009757868
GO:0009312	oligosaccharide biosynthetic process	5	0.133899614	0.0383	0.040353519
GO:0035385	Roundabout signaling pathway	5	0.132509653	0.028	0.03002818
GO:1903010	regulation of bone development	8	0.132496782	0.0197	0.021541643
GO:1901522	positive regulation of transcription from RNA polymerase II promoter involved in cellular response to chemical stimulus	8	0.132432432	0.0158	0.017471496
GO:0034377	plasma lipoprotein particle assembly	9	0.131674532	0.012	0.013526909
GO:0061085	regulation of histone H3-K27 methylation	5	0.130965251	0.0359	0.037950626
GO:0007271	synaptic transmission, cholinergic	9	0.12987273	0.0016	0.002186748
GO:0048147	negative regulation of fibroblast proliferation	15	0.128236808	0.005	0.006070546
GO:2000810	regulation of bicellular tight junction assembly	7	0.127964699	0.0067	0.007954042
GO:0042219	cellular modified amino acid catabolic process	6	0.125697126	0.0102	0.011688041
GO:0032725	positive regulation of granulocyte macrophage colony-stimulating factor production	5	0.125508366	0.0284	0.030439032
GO:0035337	fatty-acyl-CoA metabolic process	11	0.125330525	0.0267	0.028702367
GO:0046949	fatty-acyl-CoA biosynthetic process	10	0.122522523	0.0402	0.042231882
GO:0030953	astral microtubule organization	5	0.12020592	0.0226	0.024538959
GO:0055078	sodium ion homeostasis	11	0.11997192	0.0006	0.000927725
GO:0010867	positive regulation of triglyceride biosynthetic process	5	0.11984556	0.023	0.024938202
GO:0090208	positive regulation of triglyceride metabolic process	5	0.11984556	0.023	0.024938202
GO:0006691	leukotriene metabolic process	10	0.116628057	0.0302	0.032240379
GO:1904396	regulation of neuromuscular junction development	5	0.116550837	0.0248	0.026750339
GO:0019081	viral translation	6	0.115658516	0.0156	0.0172574
GO:0014067	negative regulation of phosphatidylinositol 3-kinase signaling	6	0.107164307	0.0391	0.041116248
GO:0071340	skeletal muscle acetylcholine-gated channel clustering	6	0.105448305	0.0016	0.002186748
GO:1903265	positive regulation of tumor necrosis factor-mediated signaling pathway	5	0.102393822	0.0445	0.046469179

Reward COR positive

GOIDlabel	GOName	size	categoryScores	pValPerm	pValPermCorr
GO:0019673	GDP-mannose metabolic process	5	0.427965778	0.0046	0.014132412
GO:0036295	cellular response to increased oxygen levels	7	0.401269407	0.001	0.010787313
GO:1902047	polyamine transmembrane transport	6	0.393502498	0.0021	0.012314604
GO:0045161	neuronal ion channel clustering	5	0.385680142	0.0003	0.007956881
GO:1902267	regulation of polyamine transmembrane transport	5	0.385507543	0.0031	0.013277185
GO:1902414	protein localization to cell junction	5	0.380598847	0.0002	0.006319126
GO:0033182	regulation of histone ubiquitination	5	0.379305912	0.0092	0.017033109
GO:1903365	regulation of fear response	5	0.377964515	0.0028	0.012972436
GO:2000822	regulation of behavioral fear response	5	0.377964515	0.0028	0.012972436
GO:0070669	response to interleukin-2	6	0.376501573	< 0.001	< 0.001
GO:0035815	positive regulation of renal sodium excretion	5	0.37134279	0.0054	0.014720792
GO:0015893	drug transport	8	0.368887536	0.0007	0.010220707
GO:1901725	regulation of histone deacetylase activity	5	0.365805639	0.0007	0.010220707
GO:0002692	negative regulation of cellular extravasation	5	0.364732728	0.0027	0.012827773
GO:0098914	membrane repolarization during atrial cardiac muscle cell action potential	5	0.364561425	0.0046	0.014132412
GO:0097237	cellular response to toxic substance	5	0.361999138	0.0027	0.012827773
GO:2000765	regulation of cytoplasmic translation	6	0.35945983	0.004	0.013824268
GO:0060712	spongiotrophoblast layer development	5	0.359129232	0.0001	0.004220438
GO:1905383	protein localization to presynapse	5	0.356184948	0.0053	0.014634479
GO:0051004	regulation of lipoprotein lipase activity	8	0.354073465	0.0001	0.004220438
GO:0038110	interleukin-2-mediated signaling pathway	5	0.352811758	0.0001	0.004220438
GO:0071352	cellular response to interleukin-2	5	0.352811758	0.0001	0.004220438
GO:0018410	C-terminal protein amino acid modification	6	0.352285712	0.0004	0.008964341
GO:0000097	sulfur amino acid biosynthetic process	6	0.350812188	0.0158	0.021871104
GO:2000848	positive regulation of corticosteroid hormone secretion	5	0.349922933	0.0015	0.011704453
GO:2000108	positive regulation of leukocyte apoptotic process	5	0.345713443	0.0001	0.004220438
GO:0098792	xenophagy	6	0.344722642	0.0039	0.013791927
GO:0001780	neutrophil homeostasis	6	0.344703422	0.0023	0.012498684
GO:0006558	L-phenylalanine metabolic process	5	0.344246355	0.0035	0.01351837
GO:0006559	L-phenylalanine catabolic process	5	0.344246355	0.0035	0.01351837
GO:1902221	erythrose 4-phosphate/phosphoenolpyruvate family amino acid metabolic process	5	0.344246355	0.0035	0.01351837
GO:1902222	erythrose 4-phosphate/phosphoenolpyruvate family amino acid catabolic process	5	0.344246355	0.0035	0.01351837
GO:0042780	tRNA 3'-end processing	5	0.343693832	0.0108	0.018152791
GO:0043628	ncRNA 3'-end processing	5	0.343693832	0.0108	0.018152791
GO:0015846	polyamine transport	7	0.342630473	0.0036	0.01353394
GO:0015695	organic cation transport	6	0.340051767	0.0001	0.004220438
GO:0033523	histone H2B ubiquitination	6	0.339067833	0.0027	0.012827773
GO:0048268	clathrin coat assembly	7	0.338209992	0.0054	0.014720792
GO:0061051	positive regulation of cell growth involved in cardiac muscle cell development	5	0.337659876	0.0005	0.009604651
GO:2001044	regulation of integrin-mediated signaling pathway	8	0.337377913	0.0056	0.014771533
GO:0090050	positive regulation of cell migration involved in sprouting angiogenesis	8	0.337113896	0.0002	0.006319126
GO:0021542	dentate gyrus development	9	0.336348339	0.0065	0.015491756
GO:0007494	midgut development	5	0.335971467	0.0088	0.016916437
GO:0032594	protein transport within lipid bilayer	5	0.335133871	0.0057	0.01480566
GO:0036123	histone H3-K9 dimethylation	5	0.334400456	0.0043	0.014102439
GO:0043252	sodium-independent organic anion transport	5	0.33340607	0.0017	0.012001709
GO:0006489	dolichyl diphosphate biosynthetic process	6	0.333331174	0.0126	0.019370699
GO:0046465	dolichyl diphosphate metabolic process	6	0.333331174	0.0126	0.019370699
GO:0015671	oxygen transport	7	0.333090158	0.0051	0.014547706
GO:0070672	response to interleukin-15	6	0.333065538	0.0003	0.007956881
GO:0099624	atrial cardiac muscle cell membrane repolarization	6	0.332305559	0.0079	0.01629604
GO:0048875	chemical homeostasis within a tissue	6	0.332008392	0.0002	0.006319126
GO:0015721	bile acid and bile salt transport	8	0.331787731	0.0045	0.014132412
GO:0035723	interleukin-15-mediated signaling pathway	5	0.331675159	0.0001	0.004220438
GO:0071350	cellular response to interleukin-15	5	0.331675159	0.0001	0.004220438
GO:0099072	regulation of postsynaptic specialization membrane neurotransmitter receptor levels	11	0.331401395	0.0038	0.01366393
GO:2000251	positive regulation of actin cytoskeleton reorganization	9	0.330098332	0.0077	0.016177834
GO:0043117	positive regulation of vascular permeability	5	0.329010765	0.0132	0.019860109
GO:0001502	cartilage condensation	6	0.328712993	0.0083	0.016559903
GO:0098743	cell aggregation	6	0.328712993	0.0083	0.016559903
GO:0005513	detection of calcium ion	6	0.328692693	0.0125	0.019314538
GO:0018065	protein-cofactor linkage	5	0.328245733	0.0178	0.023348367
GO:0006527	arginine catabolic process	5	0.328207637	0.0049	0.014447629
GO:0006677	glycosylceramide metabolic process	8	0.327160804	0.0038	0.01366393
GO:0090178	regulation of establishment of planar polarity involved in neural tube closure	6	0.32693172	0.0164	0.022306469
GO:0090179	planar cell polarity pathway involved in neural tube closure	6	0.32693172	0.0164	0.022306469
GO:1900087	positive regulation of G1/S transition of mitotic cell cycle	14	0.326385947	0.0008	0.010465158
GO:0070213	protein auto-ADP-ribosylation	5	0.326212386	0.004	0.013824268
GO:0048243	norepinephrine secretion	8	0.326176654	0.0084	0.016633151
GO:0015874	norepinephrine transport	9	0.325787973	0.0114	0.018551872
GO:0033604	negative regulation of catecholamine secretion	6	0.325259076	0.0113	0.018488002
GO:2000727	positive regulation of cardiac muscle cell differentiation	9	0.325036129	0.001	0.010787313
GO:2000311	regulation of alpha-amino-3-hydroxy-5-methyl-4-isoxazole propionate selective glutamate receptor activity	10	0.324753072	0.0132	0.019860109
GO:0032042	mitochondrial DNA metabolic process	6	0.324745729	0.0019	0.012288367
GO:0032228	regulation of synaptic transmission, GABAergic	14	0.324092314	0.0001	0.004220438
GO:0031649	heat generation	7	0.323408602	0.0293	0.033401538
GO:0051932	synaptic transmission, GABAergic	16	0.323292721	0.0001	0.004220438
GO:0035307	positive regulation of protein dephosphorylation	25	0.323171021	0.0036	0.01353394
GO:0043476	pigment accumulation	8	0.322682892	< 0.001	< 0.001
GO:0043482	cellular pigment accumulation	8	0.322682892	< 0.001	< 0.001

GOIDlabel	GOName	size	categoryScores	pValPerm	pValPermCorr
GO:0014829	vascular smooth muscle contraction	10	0.322662969	0.0011	0.010890753
GO:0015988	energy coupled proton transmembrane transport, against electrochemical gradient	12	0.322266351	0.0057	0.01480566
GO:0015991	ATP hydrolysis coupled proton transport	12	0.322266351	0.0057	0.01480566
GO:0090662	ATP hydrolysis coupled transmembrane transport	12	0.322266351	0.0057	0.01480566
GO:0035646	endosome to melanosome transport	7	0.322067093	< 0.001	< 0.001
GO:0043485	endosome to pigment granule transport	7	0.322067093	< 0.001	< 0.001
GO:0048757	pigment granule maturation	7	0.322067093	< 0.001	< 0.001
GO:0010447	response to acidic pH	7	0.321878464	0.004	0.013824268
GO:0071447	cellular response to hydroperoxide	7	0.321753143	0.0004	0.008964341
GO:0016556	mRNA modification	5	0.321718082	0.0016	0.011845327
GO:0001696	gastric acid secretion	6	0.321589411	0.0011	0.010890753
GO:0031650	regulation of heat generation	6	0.32132723	0.0283	0.032434212
GO:1903613	regulation of protein tyrosine phosphatase activity	5	0.321124094	0.0239	0.028592965
GO:0045713	low-density lipoprotein particle receptor biosynthetic process	5	0.320622366	0.0004	0.008964341
GO:0071340	skeletal muscle acetylcholine-gated channel clustering	7	0.318416864	0.008	0.016379603
GO:0036296	response to increased oxygen levels	13	0.318300457	0.0027	0.012827773
GO:0021819	layer formation in cerebral cortex	9	0.318084526	0.0031	0.013277185
GO:0044062	regulation of excretion	11	0.317914739	0.0024	0.012603815
GO:0060027	convergent extension involved in gastrulation	5	0.31722663	0.0083	0.016559903
GO:1905146	lysosomal protein catabolic process	6	0.317186763	0.0051	0.014547706
GO:0014049	positive regulation of glutamate secretion	5	0.316917455	0.0022	0.012422266
GO:0032802	low-density lipoprotein particle receptor catabolic process	13	0.316789372	0.0026	0.012805111
GO:0014048	regulation of glutamate secretion	8	0.316504585	0.0013	0.011218806
GO:0038084	vascular endothelial growth factor signaling pathway	14	0.316448103	0.0047	0.014168613
GO:0060368	regulation of Fc receptor mediated stimulatory signaling pathway	5	0.316324244	0.0136	0.020157703
GO:0060369	positive regulation of Fc receptor mediated stimulatory signaling pathway	5	0.316324244	0.0136	0.020157703
GO:0002674	negative regulation of acute inflammatory response	5	0.316200627	0.0019	0.012288367
GO:1905165	regulation of lysosomal protein catabolic process	5	0.315412011	0.0085	0.016693954
GO:0034505	tooth mineralization	6	0.314974208	0.0033	0.013333753
GO:0035970	peptidyl-threonine dephosphorylation	7	0.314837164	0.0073	0.015844069
GO:0035458	cellular response to interferon-beta	8	0.314536935	0.0058	0.014878261
GO:0014061	regulation of norepinephrine secretion	7	0.314508034	0.0091	0.016994897
GO:0000338	protein deneddylation	7	0.31448397	0.0083	0.016559903
GO:0060600	dichotomous subdivision of an epithelial terminal unit	6	0.314009496	0.0028	0.012972436
GO:0007205	protein kinase C-activating G-protein coupled receptor signaling pathway	12	0.313897195	< 0.001	< 0.001
GO:0050432	catecholamine secretion	22	0.313810102	0.0012	0.010961137
GO:0070307	lens fiber cell development	6	0.313805626	0.0051	0.014547706
GO:0021801	cerebral cortex radial glia guided migration	12	0.313727015	0.0087	0.016840777
GO:0022030	telencephalon glial cell migration	12	0.313727015	0.0087	0.016840777
GO:0032799	low-density lipoprotein receptor particle metabolic process	17	0.313631419	0.0021	0.012314604
GO:0031652	positive regulation of heat generation	5	0.313412096	0.0325	0.036312077
GO:0035493	SNARE complex assembly	5	0.313361042	0.0037	0.013609033
GO:0006525	arginine metabolic process	8	0.313024429	0.0003	0.007956881
GO:0002691	regulation of cellular extravasation	10	0.312750467	0.008	0.016379603
GO:0034695	response to prostaglandin E	12	0.311802341	< 0.001	< 0.001
GO:0071380	cellular response to prostaglandin E stimulus	12	0.311802341	< 0.001	< 0.001
GO:0042026	protein refolding	6	0.311221613	0.0125	0.019314538
GO:1904396	regulation of neuromuscular junction development	6	0.311122053	0.0078	0.01620539
GO:0007214	gamma-aminobutyric acid signaling pathway	7	0.311048841	0.0012	0.010961137
GO:0051386	regulation of neurotrophin TRK receptor signaling pathway	9	0.310721727	0.0001	0.004220438
GO:1900746	regulation of vascular endothelial growth factor signaling pathway	7	0.310718601	0.0048	0.014269203
GO:1902547	regulation of cellular response to vascular endothelial growth factor stimulus	7	0.310718601	0.0048	0.014269203
GO:0010888	negative regulation of lipid storage	5	0.310626156	0.0092	0.017033109
GO:0051410	detoxification of nitrogen compound	5	0.310610607	0.0231	0.027930615
GO:0034755	iron ion transmembrane transport	6	0.310584518	0.0035	0.01351837
GO:0030638	polyketide metabolic process	6	0.310220834	0.023	0.027838811
GO:0030647	aminoglycoside antibiotic metabolic process	6	0.310220834	0.023	0.027838811
GO:0044597	daunorubicin metabolic process	6	0.310220834	0.023	0.027838811
GO:0044598	doxorubicin metabolic process	6	0.310220834	0.023	0.027838811
GO:1904886	beta-catenin destruction complex disassembly	8	0.309911303	0.0013	0.011218806
GO:0010043	response to zinc ion	23	0.309827479	0.0076	0.016084627
GO:0086014	atrial cardiac muscle cell action potential	9	0.309793081	0.0022	0.012422266
GO:0086026	atrial cardiac muscle cell to AV node cell signaling	9	0.309793081	0.0022	0.012422266
GO:0086066	atrial cardiac muscle cell to AV node cell communication	9	0.309793081	0.0022	0.012422266
GO:2000402	negative regulation of lymphocyte migration	6	0.309477269	< 0.001	< 0.001
GO:0050433	regulation of catecholamine secretion	21	0.309331678	0.001	0.010787313
GO:0050860	negative regulation of T cell receptor signaling pathway	10	0.309042317	0.0023	0.012498684
GO:0030949	positive regulation of vascular endothelial growth factor receptor signaling pathway	5	0.308942671	< 0.001	< 0.001
GO:0048147	negative regulation of fibroblast proliferation	11	0.308865796	0.0011	0.010890753
GO:1902808	positive regulation of cell cycle G1/S phase transition	19	0.308721747	0.0015	0.011704453
GO:0007213	G-protein coupled acetylcholine receptor signaling pathway	12	0.308465045	< 0.001	< 0.001
GO:0007039	protein catabolic process in the vacuole	8	0.30842277	0.0063	0.015311728
GO:0034694	response to prostaglandin	15	0.308385829	< 0.001	< 0.001
GO:0009268	response to pH	14	0.308310383	0.002	0.012314604
GO:0007191	adenylate cyclase-activating dopamine receptor signaling pathway	6	0.308229643	0.0015	0.011704453
GO:0033227	dsRNA transport	5	0.308156129	0.0007	0.010220707
GO:0034392	negative regulation of smooth muscle cell apoptotic process	5	0.308093932	0.0043	0.014102439
GO:0042249	establishment of planar polarity of embryonic epithelium	9	0.308093874	0.0056	0.014771533
GO:2000833	positive regulation of steroid hormone secretion	6	0.308048018	0.0009	0.010729485
GO:0046951	ketone body biosynthetic process	5	0.307627449	0.0204	0.025564109
GO:0071435	potassium ion export	6	0.307621488	0.0095	0.01725157
GO:0097623	potassium ion export across plasma membrane	6	0.307621488	0.0095	0.01725157
GO:0070106	interleukin-27-mediated signaling pathway	5	0.307022058	0.0001	0.004220438

GOIDlabel	GOName	size	categoryScores	pValPerm	pValPermCorr
GO:2001032	regulation of double-strand break repair via nonhomologous end joining	9	0.30693141	0.0008	0.010465158
GO:0016578	histone deubiquitination	11	0.306588747	< 0.001	< 0.001
GO:0022417	protein maturation by protein folding	5	0.306345139	0.0116	0.018682786
GO:0021978	telencephalon regionalization	5	0.306342288	0.0108	0.018152791
GO:0046950	cellular ketone body metabolic process	6	0.306217072	0.0188	0.02420971
GO:0009226	nucleotide-sugar biosynthetic process	10	0.306176557	0.0229	0.027770092
GO:0000002	mitochondrial genome maintenance	9	0.305955827	0.0037	0.013609033
GO:1904350	regulation of protein catabolic process in the vacuole	7	0.305903091	0.007	0.015816335
GO:0051957	positive regulation of amino acid transport	6	0.305694655	0.0012	0.010961137
GO:2000052	positive regulation of non-canonical Wnt signaling pathway	5	0.305609651	0.0178	0.023348367
GO:0051443	positive regulation of ubiquitin-protein transferase activity	12	0.305553198	0.0018	0.01210186
GO:0014047	glutamate secretion	24	0.305512165	< 0.001	< 0.001
GO:2001046	positive regulation of integrin-mediated signaling pathway	5	0.305475408	0.013	0.019676963
GO:0006555	methionine metabolic process	6	0.305312614	0.0094	0.017156187
GO:0060192	negative regulation of lipase activity	6	0.305154528	0.0057	0.01480566
GO:0035360	positive regulation of peroxisome proliferator activated receptor signaling pathway	5	0.30502577	0.0189	0.024289798
GO:0071294	cellular response to zinc ion	16	0.30469779	0.0177	0.02329117
GO:0060602	branch elongation of an epithelium	7	0.304449219	0.0219	0.026855949
GO:0042415	norepinephrine metabolic process	5	0.304220828	0.0006	0.009940401
GO:0045117	azole transport	8	0.303801997	0.0053	0.014634479
GO:0060439	trachea morphogenesis	5	0.30350063	0.0244	0.029011063
GO:0051937	catecholamine transport	25	0.303351356	0.0018	0.01210186
GO:0033194	response to hydroperoxide	12	0.303307601	0.0011	0.010890753
GO:0046184	aldehyde biosynthetic process	7	0.302847813	0.0051	0.014547706
GO:1901890	positive regulation of cell junction assembly	16	0.302735971	0.0046	0.014132412
GO:0010225	response to UV-C	6	0.302423227	0.0072	0.015844069
GO:0051901	positive regulation of mitochondrial depolarization	6	0.302241385	0.0141	0.020504577
GO:0045880	positive regulation of smoothened signaling pathway	6	0.302207263	0.0229	0.027770092
GO:1900221	regulation of beta-amyloid clearance	6	0.30204313	0.022	0.026944291
GO:1903727	positive regulation of phospholipid metabolic process	24	0.301087596	0.0041	0.013887639
GO:0035306	positive regulation of dephosphorylation	31	0.300924198	0.0057	0.01480566
GO:0051894	positive regulation of focal adhesion assembly	14	0.300731147	0.0075	0.015990044
GO:0060081	membrane hyperpolarization	7	0.300651827	0.0013	0.011218806
GO:0032509	endosome transport via multivesicular body sorting pathway	6	0.300604808	0.0157	0.021779607
GO:0016188	synaptic vesicle maturation	5	0.300304445	0.0004	0.008964341
GO:0060044	negative regulation of cardiac muscle cell proliferation	6	0.300166616	0.0133	0.019917275
GO:0099560	synaptic membrane adhesion	11	0.29993312	0.009	0.016956012
GO:0010954	positive regulation of protein processing	11	0.299891183	0.0162	0.022196303
GO:1903319	positive regulation of protein maturation	11	0.299891183	0.0162	0.022196303
GO:0022038	corpus callosum development	9	0.299841302	0.0034	0.01347416
GO:0001821	histamine secretion	6	0.299703157	0.0065	0.015491756
GO:0018216	peptidyl-arginine methylation	6	0.299479202	0.0015	0.011704453
GO:0046710	GDP metabolic process	5	0.299210024	0.0273	0.031601321
GO:0031665	negative regulation of lipopolysaccharide-mediated signaling pathway	5	0.299162339	0.003	0.013210967
GO:0071379	cellular response to prostaglandin stimulus	14	0.299040702	< 0.001	< 0.001
GO:0015682	ferric iron transport	18	0.298880189	0.0016	0.011845327
GO:0033572	transferrin transport	18	0.298880189	0.0016	0.011845327
GO:0072512	trivalent inorganic cation transport	18	0.298880189	0.0016	0.011845327
GO:0006703	estrogen biosynthetic process	5	0.298868714	0.0005	0.009604651
GO:0032211	negative regulation of telomere maintenance via telomerase	8	0.2987277	0.0002	0.006319126
GO:0060441	epithelial tube branching involved in lung morphogenesis	11	0.298533436	0.0112	0.018392048
GO:2000345	regulation of hepatocyte proliferation	7	0.298410487	0.0124	0.019242297
GO:0090177	establishment of planar polarity involved in neural tube closure	7	0.29825018	0.0073	0.015844069
GO:0030850	prostate gland development	18	0.298209836	0.0052	0.014616626
GO:0051608	histamine transport	7	0.298196127	0.0085	0.016693954
GO:0043031	negative regulation of macrophage activation	8	0.298036885	0.0086	0.016782045
GO:0008299	isoprenoid biosynthetic process	12	0.297813361	0.0141	0.020504577
GO:1904923	regulation of mitophagy in response to mitochondrial depolarization	5	0.297791139	0.0148	0.020979063
GO:1903012	positive regulation of bone development	5	0.297679183	0.0007	0.010220707
GO:0042574	retinal metabolic process	6	0.297540059	0.0006	0.009940401
GO:0080111	DNA demethylation	5	0.297450865	0.0056	0.014771533
GO:1902224	ketone body metabolic process	7	0.296961799	0.0191	0.02440579
GO:0009309	amine biosynthetic process	11	0.296935614	0.0094	0.017156187
GO:0042401	cellular biogenic amine biosynthetic process	11	0.296935614	0.0094	0.017156187
GO:0009437	carnitine metabolic process	8	0.296816254	0.0167	0.02255007
GO:0072337	modified amino acid transport	11	0.296788601	0.0207	0.025805821
GO:0061469	regulation of type B pancreatic cell proliferation	6	0.296780728	0.0085	0.016693954
GO:0070886	positive regulation of calcineurin-NFAT signaling cascade	6	0.29675978	0.0038	0.01366393
GO:0090218	positive regulation of lipid kinase activity	18	0.296712412	0.0053	0.014634479
GO:0030206	chondroitin sulfate biosynthetic process	17	0.296608071	0.0071	0.01583804
GO:0035864	response to potassium ion	6	0.296568003	0.012	0.018931514
GO:0035865	cellular response to potassium ion	6	0.296568003	0.012	0.018931514
GO:0071549	cellular response to dexamethasone stimulus	8	0.296514822	0.0054	0.014720792
GO:0001835	blastocyst hatching	13	0.296467962	0.012	0.018931514
GO:0035188	hatching	13	0.296467962	0.012	0.018931514
GO:0071684	organism emergence from protective structure	13	0.296467962	0.012	0.018931514
GO:0071548	response to dexamethasone	12	0.296456026	0.0118	0.018836996
GO:2000849	regulation of glucocorticoid secretion	5	0.296430045	0.0002	0.006319126
GO:0009756	carbohydrate mediated signaling	5	0.296331566	0.0056	0.014771533
GO:2000773	negative regulation of cellular senescence	5	0.296329233	< 0.001	< 0.001
GO:1902570	protein localization to nucleolus	5	0.296224793	0.0031	0.013277185
GO:0006826	iron ion transport	30	0.296206522	0.0025	0.012769435
GO:1902473	regulation of protein localization to synapse	6	0.29618769	0.0022	0.012422266

GOIDlabel	GOName	size	categoryScores	pValPerm	pValPermCorr
GO:1902474	positive regulation of protein localization to synapse	6	0.29618769	0.0022	0.012422266
GO:0043555	regulation of translation in response to stress	7	0.296143232	0.0067	0.015576759
GO:0035812	renal sodium excretion	9	0.295734525	0.0037	0.013609033
GO:0035813	regulation of renal sodium excretion	9	0.295734525	0.0037	0.013609033
GO:0007194	negative regulation of adenylate cyclase activity	8	0.295634498	0.0059	0.014942532
GO:0030815	negative regulation of cAMP metabolic process	8	0.295634498	0.0059	0.014942532
GO:0030818	negative regulation of cAMP biosynthetic process	8	0.295634498	0.0059	0.014942532
GO:0009950	dorsal/ventral axis specification	7	0.295587896	0.0023	0.012498684
GO:0043558	regulation of translational initiation in response to stress	6	0.29545298	0.0049	0.014447629
GO:0055093	response to hyperoxia	9	0.295374297	0.0021	0.012314604
GO:0033173	calcineurin-NFAT signaling cascade	19	0.295307431	0.0001	0.004220438
GO:0097720	calcineurin-mediated signaling	19	0.295307431	0.0001	0.004220438
GO:0051450	myoblast proliferation	6	0.295290359	0.0027	0.012827773
GO:0016048	detection of temperature stimulus	8	0.295219198	0.0013	0.011218806
GO:0035510	DNA dealkylation	8	0.295067915	0.0105	0.017924712
GO:0006577	amino-acid betaine metabolic process	11	0.295061436	0.0195	0.02481272
GO:0097242	beta-amyloid clearance	10	0.294807074	0.0179	0.02344231
GO:0030809	negative regulation of nucleotide biosynthetic process	11	0.29467918	0.0107	0.018084595
GO:1900372	negative regulation of purine nucleotide biosynthetic process	11	0.29467918	0.0107	0.018084595
GO:0051561	positive regulation of mitochondrial calcium ion concentration	7	0.294632902	0.0013	0.011218806
GO:0042255	ribosome assembly	11	0.294607676	0.0072	0.015844069
GO:1900451	positive regulation of glutamate receptor signaling pathway	9	0.294592362	0.011	0.018323826
GO:0061045	negative regulation of wound healing	29	0.294543435	0.0138	0.020339434
GO:0021799	cerebral cortex radially oriented cell migration	17	0.294436715	0.0037	0.013609033
GO:0019226	transmission of nerve impulse	21	0.294416937	0.0003	0.007956881
GO:0043586	tongue development	7	0.294183264	0.0002	0.006319126
GO:0055069	zinc ion homeostasis	8	0.293851173	0.003	0.013210967
GO:0048741	skeletal muscle fiber development	12	0.293733472	0.0042	0.013948535
GO:0071467	cellular response to pH	7	0.2937066	< 0.001	< 0.001
GO:0071242	cellular response to ammonium ion	35	0.293403867	0.0016	0.011845327
GO:0048172	regulation of short-term neuronal synaptic plasticity	11	0.293282794	0.0006	0.009940401
GO:0070572	positive regulation of neuron projection regeneration	6	0.2931305	0.0133	0.019917275
GO:0060343	trabecula formation	14	0.292887478	0.0071	0.01583804
GO:0016137	glycoside metabolic process	11	0.292828915	0.0161	0.02211694
GO:1901992	positive regulation of mitotic cell cycle phase transition	29	0.292766466	0.0006	0.009940401
GO:0010869	regulation of receptor biosynthetic process	10	0.292704532	< 0.001	< 0.001
GO:0009642	response to light intensity	6	0.292623631	0.0055	0.01475
GO:0035933	glucocorticoid secretion	6	0.292459931	0.0007	0.010220707
GO:0051969	regulation of transmission of nerve impulse	7	0.292316592	0.0027	0.012827773
GO:0032354	response to follicle-stimulating hormone	5	0.292218223	0.0163	0.022248961
GO:0071372	cellular response to follicle-stimulating hormone stimulus	5	0.292218223	0.0163	0.022248961
GO:1904398	positive regulation of neuromuscular junction development	5	0.292009083	0.0078	0.01620539
GO:0031630	regulation of synaptic vesicle fusion to presynaptic membrane	5	0.291917083	0.0074	0.015911789
GO:0043552	positive regulation of phosphatidylinositol 3-kinase activity	16	0.291898261	0.006	0.015037711
GO:0071371	cellular response to gonadotropin stimulus	6	0.291547697	0.0105	0.017924712
GO:0051481	negative regulation of cytosolic calcium ion concentration	5	0.291534826	0.0071	0.01583804
GO:2000647	negative regulation of stem cell proliferation	5	0.291402397	0.0296	0.033690394
GO:0009071	serine family amino acid catabolic process	5	0.291392808	0.0062	0.015209334
GO:0006188	IMP biosynthetic process	5	0.291307027	0.019	0.024347961
GO:0046040	IMP metabolic process	5	0.291307027	0.019	0.024347961
GO:0032516	positive regulation of phosphoprotein phosphatase activity	12	0.291257333	0.0061	0.015143924
GO:0045780	positive regulation of bone resorption	5	0.291182631	0.0114	0.018551872
GO:0046852	positive regulation of bone remodeling	5	0.291182631	0.0114	0.018551872
GO:0071071	regulation of phospholipid biosynthetic process	6	0.290871729	0.0099	0.017505138
GO:2000846	regulation of corticosteroid hormone secretion	7	0.290857352	0.0001	0.004220438
GO:0032536	regulation of cell projection size	6	0.290819898	0.0024	0.012603815
GO:0060180	female mating behavior	5	0.290779642	0.014	0.020462083
GO:0043046	DNA methylation involved in gamete generation	6	0.290767418	0.0163	0.022248961
GO:0060433	bronchus development	6	0.290597454	0.0067	0.015576759
GO:0006953	acute-phase response	13	0.290406476	0.006	0.015037711
GO:0039694	viral RNA genome replication	9	0.290312094	0.0022	0.012422266
GO:0039703	RNA replication	9	0.290312094	0.0022	0.012422266
GO:0051953	negative regulation of amine transport	12	0.290241977	0.0026	0.012805111
GO:0023019	signal transduction involved in regulation of gene expression	9	0.290134859	0.0175	0.023154462
GO:0015837	amine transport	36	0.290028676	0.0004	0.008964341
GO:0001829	trophectodermal cell differentiation	5	0.289961483	0.0044	0.014132412
GO:0002371	dendritic cell cytokine production	8	0.289950533	0.0006	0.009940401
GO:0002730	regulation of dendritic cell cytokine production	8	0.289950533	0.0006	0.009940401
GO:0001963	synaptic transmission, dopaminergic	12	0.289616329	0.002	0.012314604
GO:0051354	negative regulation of oxidoreductase activity	6	0.289483295	0.0278	0.032045375
GO:0072531	pyrimidine-containing compound transmembrane transport	7	0.289478451	0.0007	0.010220707
GO:0002190	cap-independent translational initiation	6	0.289476816	0.025	0.029554283
GO:0018146	keratan sulfate biosynthetic process	14	0.289471509	0.0013	0.011218806
GO:0070262	peptidyl-serine dephosphorylation	8	0.288741889	0.0121	0.018970228
GO:0035930	corticosteroid hormone secretion	8	0.288576353	0.0004	0.008964341
GO:0032332	positive regulation of chondrocyte differentiation	12	0.288469558	0.0016	0.011845327
GO:0030195	negative regulation of blood coagulation	17	0.288427699	0.0121	0.018970228
GO:1900047	negative regulation of hemostasis	17	0.288427699	0.0121	0.018970228
GO:0051900	regulation of mitochondrial depolarization	11	0.288405692	0.0071	0.01583804
GO:0090313	regulation of protein targeting to membrane	17	0.288343473	0.0037	0.013609033
GO:0018200	peptidyl-glutamic acid modification	10	0.288340455	0.0029	0.013089617
GO:0019359	nicotinamide nucleotide biosynthetic process	14	0.288159248	0.0086	0.016782045
GO:0019363	pyridine nucleotide biosynthetic process	14	0.288159248	0.0086	0.016782045

GOIDlabel	GOName	size	categoryScores	pValPerm	pValPermCorr
GO:0043029	T cell homeostasis	8	0.288048644	0.0005	0.009604651
GO:0045738	negative regulation of DNA repair	13	0.287964019	0.0046	0.014132412
GO:0031063	regulation of histone deacetylation	12	0.287837863	0.0009	0.010729485
GO:0010764	negative regulation of fibroblast migration	6	0.287810219	0.0059	0.014942532
GO:0060312	regulation of blood vessel remodeling	5	0.287378723	0.0173	0.023005658
GO:0090314	positive regulation of protein targeting to membrane	16	0.287317578	0.0037	0.013609033
GO:0021533	cell differentiation in hindbrain	7	0.286951483	0.0024	0.012603815
GO:0060039	pericardium development	7	0.286861704	0.0083	0.016559903
GO:0034067	protein localization to Golgi apparatus	8	0.286805985	0.001	0.010787313
GO:0010752	regulation of cGMP-mediated signaling	6	0.286781365	0.001	0.010787313
GO:1901989	positive regulation of cell cycle phase transition	35	0.286748341	0.0022	0.012422266
GO:0006882	cellular zinc ion homeostasis	6	0.286554603	0.0023	0.012498684
GO:0018027	peptidyl-lysine dimethylation	7	0.286514248	0.0021	0.012314604
GO:0006705	mineralocorticoid biosynthetic process	5	0.286265902	0.0245	0.029070183
GO:0008212	mineralocorticoid metabolic process	5	0.286265902	0.0245	0.029070183
GO:0032341	aldosterone metabolic process	5	0.286265902	0.0245	0.029070183
GO:0032342	aldosterone biosynthetic process	5	0.286265902	0.0245	0.029070183
GO:0002833	positive regulation of response to biotic stimulus	19	0.286100669	0.0169	0.022703485
GO:0050650	chondroitin sulfate proteoglycan biosynthetic process	19	0.286029537	0.0096	0.017302743
GO:0002228	natural killer cell mediated immunity	18	0.285969124	0.0119	0.018881943
GO:0070131	positive regulation of mitochondrial translation	10	0.285911375	0.0032	0.013333753
GO:0090344	negative regulation of cell aging	9	0.285853007	0.0013	0.011218806
GO:0071609	chemokine (C-C motif) ligand 5 production	5	0.285832591	0.0029	0.013089617
GO:0014904	myotube cell development	14	0.28582091	0.0048	0.014269203
GO:0072321	chaperone-mediated protein transport	10	0.285794754	0.014	0.020462083
GO:0090502	RNA phosphodiester bond hydrolysis, endonucleolytic	21	0.285739122	0.0019	0.012288367
GO:1905276	regulation of epithelial tube formation	7	0.285715859	0.0133	0.019917275
GO:0090312	positive regulation of protein deacetylation	11	0.285619399	0.0014	0.011465722
GO:0042267	natural killer cell mediated cytotoxicity	16	0.285530365	0.0134	0.01999969
GO:0021871	forebrain regionalization	9	0.285381197	0.0207	0.025805821
GO:0030201	heparan sulfate proteoglycan metabolic process	13	0.284853834	0.0041	0.013887639
GO:0003401	axis elongation	11	0.284839571	0.0157	0.021779607
GO:0070873	regulation of glycogen metabolic process	12	0.284699685	0.0001	0.004220438
GO:0035900	response to isolation stress	5	0.284444027	0.0015	0.011704453
GO:0035690	cellular response to drug	28	0.284408411	0.0068	0.015633241
GO:0007176	regulation of epidermal growth factor-activated receptor activity	10	0.28434464	< 0.001	< 0.001
GO:0099054	presynapse assembly	30	0.284079566	0.0017	0.012001709
GO:0051590	positive regulation of neurotransmitter transport	17	0.284047486	0.0012	0.010961137
GO:2000249	regulation of actin cytoskeleton reorganization	22	0.284045608	0.01	0.017574468
GO:0046033	AMP metabolic process	9	0.283984916	0.033	0.036785425
GO:0072672	neutrophil extravasation	5	0.283705947	0.0218	0.026744664
GO:0032225	regulation of synaptic transmission, dopaminergic	7	0.283675367	0.0009	0.010729485
GO:0051560	mitochondrial calcium ion homeostasis	13	0.283628619	0.0011	0.010890753
GO:0097711	ciliary basal body docking	38	0.283589947	0.0038	0.01366393
GO:0060347	heart trabecula formation	9	0.283409731	0.0071	0.01583804
GO:0060263	regulation of respiratory burst	6	0.283362003	0.0046	0.014132412
GO:0061179	negative regulation of insulin secretion involved in cellular response to glucose stimulus	5	0.283288445	0.0166	0.02247277
GO:0007614	short-term memory	6	0.283244518	0.0062	0.015209334
GO:0030800	negative regulation of cyclic nucleotide metabolic process	9	0.283238543	0.0102	0.0177
GO:0030803	negative regulation of cyclic nucleotide biosynthetic process	9	0.283238543	0.0102	0.0177
GO:0031280	negative regulation of cyclase activity	9	0.283238543	0.0102	0.0177
GO:0009067	aspartate family amino acid biosynthetic process	6	0.283233936	0.0076	0.016084627
GO:0042762	regulation of sulfur metabolic process	7	0.283197037	0.0132	0.019860109
GO:0051350	negative regulation of lyase activity	10	0.283131655	0.0119	0.018881943
GO:0039532	negative regulation of viral-induced cytoplasmic pattern recognition receptor signaling pathway	6	0.283114076	0.0139	0.020388077
GO:0090383	phagosome acidification	13	0.283002954	0.0046	0.014132412
GO:1905097	regulation of guanyl-nucleotide exchange factor activity	5	0.282908002	0.0065	0.015491756
GO:0039702	viral budding via host ESCRT complex	13	0.282867893	0.0109	0.018225506
GO:0007183	SMAD protein complex assembly	8	0.282846874	0.0143	0.020629391
GO:0031338	regulation of vesicle fusion	10	0.282747973	0.0052	0.014616626
GO:0002922	positive regulation of humoral immune response	6	0.282743049	0.0222	0.027148985
GO:0061299	retina vasculature morphogenesis in camera-type eye	7	0.28266632	0.0088	0.016916437
GO:0003376	sphingosine-1-phosphate signaling pathway	5	0.282587166	0.0032	0.013333753
GO:0008210	estrogen metabolic process	9	0.282494618	0.0038	0.01366393
GO:0002820	negative regulation of adaptive immune response	13	0.282477762	0.0007	0.010220707
GO:0051952	regulation of amine transport	34	0.282351607	0.0005	0.009604651
GO:0048680	positive regulation of axon regeneration	5	0.282312459	0.0121	0.018970228
GO:0071360	cellular response to exogenous dsRNA	10	0.282302352	0.0117	0.018739446
GO:0071377	cellular response to glucagon stimulus	9	0.282268287	< 0.001	< 0.001
GO:1905606	regulation of presynapse assembly	21	0.282260616	0.0022	0.012422266
GO:1900449	regulation of glutamate receptor signaling pathway	35	0.282147154	0.0017	0.012001709
GO:0051954	positive regulation of amine transport	15	0.282079823	0.0001	0.004220438
GO:0014029	neural crest formation	8	0.281941605	0.0008	0.010465158
GO:0010591	regulation of lamellipodium assembly	11	0.281936776	0.0009	0.010729485
GO:0010592	positive regulation of lamellipodium assembly	11	0.281936776	0.0009	0.010729485
GO:1905476	negative regulation of protein localization to membrane	16	0.281784005	0.0023	0.012498684
GO:0016574	histone ubiquitination	18	0.281778336	0.0019	0.012288367
GO:0051489	regulation of filopodium assembly	21	0.281707994	0.002	0.012314604
GO:1900739	regulation of protein insertion into mitochondrial membrane involved in apoptotic signaling pathway	10	0.281629451	0.0033	0.013333753
GO:1900740	positive regulation of protein insertion into mitochondrial membrane involved in apoptotic signaling pathway	10	0.281629451	0.0033	0.013333753
GO:0030397	membrane disassembly	8	0.281401749	0.0001	0.004220438
GO:0051081	nuclear envelope disassembly	8	0.281401749	0.0001	0.004220438

GOIDlabel	GOName	size	categoryScores	pValPerm	pValPermCorr
GO:1903035	negative regulation of response to wounding	35	0.281396524	0.0116	0.018682786
GO:0015844	monoamine transport	32	0.281237751	0.0051	0.014547706
GO:0043550	regulation of lipid kinase activity	24	0.281074239	0.0042	0.013948535
GO:0048557	embryonic digestive tract morphogenesis	6	0.281026349	0.0045	0.014132412
GO:0007616	long-term memory	15	0.281016587	0.0059	0.014942532
GO:0089711	L-glutamate transmembrane transport	6	0.280849474	0.0127	0.019457181
GO:0098712	L-glutamate import across plasma membrane	6	0.280849474	0.0127	0.019457181
GO:1903998	regulation of eating behavior	5	0.280807534	< 0.001	< 0.001
GO:0055091	phospholipid homeostasis	5	0.280615757	0.0161	0.02211694
GO:0045540	regulation of cholesterol biosynthetic process	29	0.280569681	0.0102	0.0177
GO:2000780	negative regulation of double-strand break repair	12	0.280393249	0.0104	0.017832977
GO:0001956	positive regulation of neurotransmitter secretion	16	0.280369979	0.0006	0.009940401
GO:0032104	regulation of response to extracellular stimulus	14	0.28026236	0.0177	0.02329117
GO:0032107	regulation of response to nutrient levels	14	0.28026236	0.0177	0.02329117
GO:0035304	regulation of protein dephosphorylation	68	0.280220345	0.0025	0.012769435
GO:0090311	regulation of protein deacetylation	17	0.280175068	0.0011	0.010890753
GO:0021697	cerebellar cortex formation	10	0.279979915	0.0012	0.010961137
GO:0032352	positive regulation of hormone metabolic process	5	0.279972529	0.0133	0.019917275
GO:0042448	progesterone metabolic process	7	0.279937395	0.012	0.018931514
GO:0009072	aromatic amino acid family metabolic process	10	0.279906185	0.0118	0.018836996
GO:2001256	regulation of store-operated calcium entry	6	0.279869212	0.0133	0.019917275
GO:0048845	venous blood vessel morphogenesis	5	0.279863424	0.006	0.015037711
GO:0032881	regulation of polysaccharide metabolic process	17	0.279813163	0.0002	0.006319126
GO:0030239	myofibril assembly	17	0.27973755	0.0083	0.016559903
GO:0048016	inositol phosphate-mediated signaling	25	0.279615359	< 0.001	< 0.001
GO:0042059	negative regulation of epidermal growth factor receptor signaling pathway	20	0.279463674	0.0005	0.009604651
GO:0042753	positive regulation of circadian rhythm	6	0.279297123	0.0001	0.004220438
GO:2000969	positive regulation of alpha-amino-3-hydroxy-5-methyl-4-isoxazole propionate selective glutamate receptor activity	5	0.27926218	0.0459	0.048535808
GO:0022010	central nervous system myelination	7	0.278813949	0.0082	0.016514246
GO:0032291	axon ensheathment in central nervous system	7	0.278813949	0.0082	0.016514246
GO:0035924	cellular response to vascular endothelial growth factor stimulus	21	0.278726946	0.0047	0.014168613
GO:2001028	positive regulation of endothelial cell chemotaxis	8	0.278710564	0.0241	0.028760826
GO:0034122	negative regulation of toll-like receptor signaling pathway	15	0.278706374	0.007	0.015816335
GO:0050961	detection of temperature stimulus involved in sensory perception	7	0.278679927	0.0011	0.010890753
GO:0050965	detection of temperature stimulus involved in sensory perception of pain	7	0.278679927	0.0011	0.010890753
GO:0035338	long-chain fatty-acyl-CoA biosynthetic process	5	0.278678558	0.0159	0.021956962
GO:0010753	positive regulation of cGMP-mediated signaling	5	0.278598478	0.0081	0.016514175
GO:0051965	positive regulation of synapse assembly	35	0.278594665	0.0005	0.009604651
GO:1900363	regulation of mRNA polyadenylation	7	0.278542019	0.0093	0.017114131
GO:0034260	negative regulation of GTPase activity	17	0.278493901	0.0038	0.01366393
GO:2001169	regulation of ATP biosynthetic process	9	0.278439384	0.0197	0.024979254
GO:0001662	behavioral fear response	21	0.278413242	0.0105	0.017924712
GO:0002209	behavioral defense response	21	0.278413242	0.0105	0.017924712
GO:0042596	fear response	21	0.278413242	0.0105	0.017924712
GO:0044346	fibroblast apoptotic process	7	0.278348947	0.0071	0.01583804
GO:2000269	regulation of fibroblast apoptotic process	7	0.278348947	0.0071	0.01583804
GO:0031579	membrane raft organization	9	0.278314413	0.013	0.019676963
GO:0046928	regulation of neurotransmitter secretion	52	0.278298877	0.0008	0.010465158
GO:0002063	chondrocyte development	12	0.278298504	0.0071	0.01583804
GO:0072215	regulation of metanephros development	7	0.278292117	0.0076	0.016084627
GO:0010923	negative regulation of phosphatase activity	44	0.27818848	0.0019	0.012288367
GO:0007270	neuron-neuron synaptic transmission	58	0.27815809	0.0001	0.004220438
GO:0051882	mitochondrial depolarization	12	0.278020558	0.0098	0.017418875
GO:2000178	negative regulation of neural precursor cell proliferation	10	0.27795512	0.0083	0.016559903
GO:0032892	positive regulation of organic acid transport	11	0.27788933	0.0016	0.011845327
GO:0032878	regulation of establishment or maintenance of cell polarity	11	0.277877903	0.0055	0.01475
GO:0072497	mesenchymal stem cell differentiation	7	0.277818785	0.0207	0.025805821
GO:0044827	modulation by host of viral genome replication	8	0.277758809	0.0091	0.016994897
GO:2000637	positive regulation of gene silencing by miRNA	8	0.277683167	0.007	0.015816335
GO:0042310	vasoconstriction	25	0.277661832	0.005	0.014547706
GO:0060766	negative regulation of androgen receptor signaling pathway	5	0.27747866	0.0163	0.022248961
GO:0032960	regulation of inositol trisphosphate biosynthetic process	5	0.277428902	0.0012	0.010961137
GO:0045429	positive regulation of nitric oxide biosynthetic process	14	0.277367389	0.013	0.019676963
GO:1904407	positive regulation of nitric oxide metabolic process	14	0.277367389	0.013	0.019676963
GO:0032095	regulation of response to food	12	0.277301612	0.0129	0.019597425
GO:0014046	dopamine secretion	11	0.277257104	0.0033	0.013333753
GO:0014059	regulation of dopamine secretion	11	0.277257104	0.0033	0.013333753
GO:0043011	myeloid dendritic cell differentiation	7	0.277206064	0.0169	0.022703485
GO:0034969	histone arginine methylation	5	0.27716508	0.0026	0.012805111
GO:0043985	histone H4-R3 methylation	5	0.27716508	0.0026	0.012805111
GO:0045663	positive regulation of myoblast differentiation	6	0.277001206	0.0066	0.015525305
GO:0050819	negative regulation of coagulation	18	0.276900567	0.013	0.019676963
GO:0097067	cellular response to thyroid hormone stimulus	5	0.276894001	0.0166	0.02247277
GO:0086064	cell communication by electrical coupling involved in cardiac conduction	7	0.276878045	0.0301	0.034151923
GO:0001990	regulation of systemic arterial blood pressure by hormone	5	0.276855905	0.0012	0.010961137
GO:1905209	positive regulation of cardiocyte differentiation	13	0.276836209	0.0011	0.010890753
GO:1905144	response to acetylcholine	18	0.276829155	0.0008	0.010465158
GO:1905145	cellular response to acetylcholine	18	0.276829155	0.0008	0.010465158
GO:0044546	NLRP3 inflammasome complex assembly	5	0.276814699	0.0139	0.020388077
GO:1900225	regulation of NLRP3 inflammasome complex assembly	5	0.276814699	0.0139	0.020388077
GO:0051764	actin crosslink formation	8	0.276808188	0.0203	0.025471918
GO:0010390	histone monoubiquitination	11	0.276662456	0.0005	0.009604651

GOIDlabel	GOName	size	categoryScores	pValPerm	pValPermCorr
GO:0031065	positive regulation of histone deacetylation	10	0.276635751	0.0011	0.010890753
GO:0033145	positive regulation of intracellular steroid hormone receptor signaling pathway	7	0.276601672	0.0087	0.016840777
GO:0010907	positive regulation of glucose metabolic process	10	0.276391107	0.0129	0.019597425
GO:0044745	amino acid transmembrane import	7	0.276382499	0.0115	0.018599441
GO:0089718	amino acid import across plasma membrane	7	0.276382499	0.0115	0.018599441
GO:0060765	regulation of androgen receptor signaling pathway	11	0.276348405	0.0128	0.019522448
GO:0046755	viral budding	16	0.27625837	0.0082	0.016514246
GO:1902590	multi-organism organelle organization	16	0.27625837	0.0082	0.016514246
GO:1902592	multi-organism membrane budding	16	0.27625837	0.0082	0.016514246
GO:0098962	regulation of postsynaptic neurotransmitter receptor activity	8	0.276192204	0.0052	0.014616626
GO:0032793	positive regulation of CREB transcription factor activity	8	0.276115915	0.0089	0.016916437
GO:0010459	negative regulation of heart rate	5	0.276085431	0.0135	0.020081554
GO:0002115	store-operated calcium entry	7	0.276064291	0.0142	0.020567234
GO:1901524	regulation of macromitophagy	9	0.2758995	0.0055	0.01475
GO:0042481	regulation of odontogenesis	7	0.275862889	0.0033	0.013333753
GO:0035305	negative regulation of dephosphorylation	47	0.275646954	0.0025	0.012769435
GO:0007340	acrosome reaction	12	0.2755861	0.0001	0.004220438
GO:0008631	intrinsic apoptotic signaling pathway in response to oxidative stress	19	0.275583935	0.0061	0.015143924
GO:0030049	muscle filament sliding	24	0.275537076	0.0119	0.018881943
GO:0033275	actin-myosin filament sliding	24	0.275537076	0.0119	0.018881943
GO:0070208	protein heterotrimerization	5	0.275380524	0.0001	0.004220438
GO:0097066	response to thyroid hormone	8	0.275324189	0.0125	0.019314538
GO:0032800	receptor biosynthetic process	11	0.275313543	< 0.001	< 0.001
GO:0051963	regulation of synapse assembly	56	0.275276685	0.0014	0.011465722
GO:0043113	receptor clustering	28	0.275219763	0.0037	0.013609033
GO:1905288	vascular associated smooth muscle cell apoptotic process	6	0.275215613	0.0026	0.012805111
GO:1905459	regulation of vascular associated smooth muscle cell apoptotic process	6	0.275215613	0.0026	0.012805111
GO:0031954	positive regulation of protein autophosphorylation	11	0.275177015	0.0193	0.024606968
GO:0070129	regulation of mitochondrial translation	12	0.275074697	0.0026	0.012805111
GO:0050858	negative regulation of antigen receptor-mediated signaling pathway	14	0.275008133	0.0009	0.010729485
GO:0007223	Wnt signaling pathway, calcium modulating pathway	16	0.274950598	0.002	0.012314604
GO:0046627	negative regulation of insulin receptor signaling pathway	14	0.274931775	0.0004	0.008964341
GO:0061314	Notch signaling involved in heart development	7	0.274914189	0.024	0.028671074
GO:0042058	regulation of epidermal growth factor receptor signaling pathway	36	0.274904589	0.0001	0.004220438
GO:0051668	localization within membrane	50	0.274794543	0.0046	0.014132412
GO:0010829	negative regulation of glucose transport	10	0.274759713	0.004	0.013824268
GO:2000463	positive regulation of excitatory postsynaptic potential	18	0.274625743	< 0.001	< 0.001
GO:0006534	cysteine metabolic process	7	0.274543039	0.0087	0.016840777
GO:1904037	positive regulation of epithelial cell apoptotic process	10	0.274539559	0.0023	0.012498684
GO:0033692	cellular polysaccharide biosynthetic process	23	0.274470386	0.0006	0.009940401
GO:0090181	regulation of cholesterol metabolic process	36	0.274458946	0.0092	0.017033109
GO:0007289	spermatid nucleus differentiation	7	0.274393283	0.0039	0.013791927
GO:0048712	negative regulation of astrocyte differentiation	6	0.274370545	0.0205	0.025639412
GO:0060512	prostate gland morphogenesis	9	0.274350892	0.0101	0.017653628
GO:0042274	ribosomal small subunit biogenesis	9	0.274239455	0.011	0.018323826
GO:1902950	regulation of dendritic spine maintenance	5	0.274191511	0.0047	0.014168613
GO:0032461	positive regulation of protein oligomerization	7	0.274086367	0.0128	0.019522448
GO:0033280	response to vitamin D	11	0.274010925	0.0157	0.021779607
GO:0016056	rhodopsin mediated signaling pathway	13	0.273923582	0.0007	0.010220707
GO:0071498	cellular response to fluid shear stress	6	0.273922419	0.0006	0.009940401
GO:0015732	prostaglandin transport	5	0.273916545	0.0104	0.017832977
GO:0032310	prostaglandin secretion	5	0.273916545	0.0104	0.017832977
GO:0095500	acetylcholine receptor signaling pathway	17	0.273899959	0.0004	0.008964341
GO:1903831	signal transduction involved in cellular response to ammonium ion	17	0.273899959	0.0004	0.008964341
GO:0000188	inactivation of MAPK activity	13	0.27389657	0.0011	0.010890753
GO:0070884	regulation of calcineurin-NFAT signaling cascade	15	0.273886655	< 0.001	< 0.001
GO:0007520	myoblast fusion	13	0.273875638	0.0032	0.013333753
GO:0033540	fatty acid beta-oxidation using acyl-CoA oxidase	10	0.273820527	0.0143	0.020629391
GO:1904357	negative regulation of telomere maintenance via telomere lengthening	11	0.273714544	0.0007	0.010220707
GO:0051491	positive regulation of filopodium assembly	16	0.2736029	0.0069	0.015750415
GO:0000154	rRNA modification	11	0.273574952	0.0029	0.013089617
GO:0033574	response to testosterone	16	0.273476804	0.005	0.014547706
GO:0042940	D-amino acid transport	5	0.273421036	0.0103	0.017772187
GO:0032515	negative regulation of phosphoprotein phosphatase activity	28	0.273392659	0.003	0.013210967
GO:0031629	synaptic vesicle fusion to presynaptic active zone membrane	6	0.273374302	0.0044	0.014132412
GO:0099500	vesicle fusion to plasma membrane	6	0.273374302	0.0044	0.014132412
GO:0000271	polysaccharide biosynthetic process	28	0.27333023	0.0004	0.008964341
GO:0035456	response to interferon-beta	12	0.273236517	0.0011	0.010890753
GO:0006688	glycosphingolipid biosynthetic process	12	0.273227122	0.0005	0.009604651
GO:1903725	regulation of phospholipid metabolic process	35	0.273184241	0.0019	0.012288367
GO:0001967	suckling behavior	7	0.273107308	0.0029	0.013089617
GO:0005976	polysaccharide metabolic process	41	0.273035701	0.0011	0.010890753
GO:0007260	tyrosine phosphorylation of STAT protein	24	0.273032808	0.0017	0.012001709
GO:2000344	positive regulation of acrosome reaction	5	0.27296803	0.0022	0.012422266
GO:0009225	nucleotide-sugar metabolic process	17	0.272810737	0.0094	0.017156187
GO:0021892	cerebral cortex GABAergic interneuron differentiation	6	0.272713451	0.0099	0.017505138
GO:0097154	GABAergic neuron differentiation	6	0.272713451	0.0099	0.017505138
GO:0090151	establishment of protein localization to mitochondrial membrane	9	0.272552054	0.0079	0.01629604
GO:0072525	pyridine-containing compound biosynthetic process	15	0.272466129	0.0127	0.019457181
GO:0021795	cerebral cortex cell migration	23	0.272431665	0.0053	0.014634479
GO:0045737	positive regulation of cyclin-dependent protein serine/threonine kinase activity	9	0.272408653	0.002	0.012314604
GO:0046459	short-chain fatty acid metabolic process	7	0.272402401	0.0172	0.022941269
GO:0036005	response to macrophage colony-stimulating factor	6	0.272396847	0.0359	0.039290895

GOIDlabel	GOName	size	categoryScores	pValPerm	pValPermCorr
GO:0036006	cellular response to macrophage colony-stimulating factor stimulus	6	0.272396847	0.0359	0.039290895
GO:0007635	chemosensory behavior	5	0.272349422	0.0004	0.008964341
GO:0043407	negative regulation of MAP kinase activity	37	0.272260965	0.0016	0.011845327
GO:0034314	Arp2/3 complex-mediated actin nucleation	19	0.272168994	0.0039	0.013791927
GO:1903393	positive regulation of adherens junction organization	17	0.272128391	0.0032	0.013333753
GO:0001844	protein insertion into mitochondrial membrane involved in apoptotic signaling pathway	11	0.272051462	0.0038	0.01366393
GO:0017062	respiratory chain complex III assembly	6	0.271974205	0.02	0.025188412
GO:0034551	mitochondrial respiratory chain complex III assembly	6	0.271974205	0.02	0.025188412
GO:0097033	mitochondrial respiratory chain complex III biogenesis	6	0.271974205	0.02	0.025188412
GO:0016139	glycoside catabolic process	5	0.271958613	0.0139	0.020388077
GO:0001833	inner cell mass cell proliferation	5	0.271815817	0.0106	0.018010344
GO:0090281	negative regulation of calcium ion import	12	0.271779297	0.0178	0.023348367
GO:0009074	aromatic amino acid family catabolic process	8	0.271747821	0.0123	0.019143634
GO:0050856	regulation of T cell receptor signaling pathway	20	0.271726926	0.0012	0.010961137
GO:0006595	polyamine metabolic process	11	0.271662137	0.0138	0.020339434
GO:0035269	protein O-linked mannosylation	11	0.271578618	0.0116	0.018682786
GO:0042730	fibrinolysis	5	0.271473211	0.0031	0.013277185
GO:0035249	synaptic transmission, glutamatergic	34	0.271441548	0.0001	0.004220438
GO:0035774	positive regulation of insulin secretion involved in cellular response to glucose stimulus	17	0.271405038	0.0016	0.011845327
GO:0048521	negative regulation of behavior	8	0.271402818	< 0.001	< 0.001
GO:1901655	cellular response to ketone	31	0.271326486	0.0012	0.010961137
GO:0051683	establishment of Golgi localization	6	0.271127841	0.0106	0.018010344
GO:0061178	regulation of insulin secretion involved in cellular response to glucose stimulus	29	0.270982028	0.0021	0.012314604
GO:0098883	synapse disassembly	7	0.270925205	0.0333	0.037012803
GO:0006596	polyamine biosynthetic process	10	0.27088337	0.0169	0.022703485
GO:1900037	regulation of cellular response to hypoxia	6	0.270832186	0.0124	0.019242297
GO:0032927	positive regulation of activin receptor signaling pathway	5	0.270802253	0.0373	0.040638515
GO:0006398	mRNA 3'-end processing by stem-loop binding and cleavage	5	0.270716732	0.0022	0.012422266
GO:0032785	negative regulation of DNA-templated transcription, elongation	6	0.27068425	0.0078	0.01620539
GO:0008207	C21-steroid hormone metabolic process	19	0.27058793	0.0206	0.025725529
GO:0046717	acid secretion	47	0.27045157	0.0002	0.006319126
GO:1900077	negative regulation of cellular response to insulin stimulus	15	0.270441075	0.0002	0.006319126
GO:0060359	response to ammonium ion	72	0.270409742	0.002	0.012314604
GO:0032925	regulation of activin receptor signaling pathway	7	0.270370238	0.0156	0.021692929
GO:0061041	regulation of wound healing	49	0.270349453	0.0119	0.018881943
GO:0030204	chondroitin sulfate metabolic process	24	0.270268033	0.0088	0.016916437
GO:0035303	regulation of dephosphorylation	96	0.270255251	0.0023	0.012498684
GO:0016081	synaptic vesicle docking	5	0.270240919	< 0.001	< 0.001
GO:1901623	regulation of lymphocyte chemotaxis	5	0.270082833	0.0156	0.021692929
GO:0038179	neurotrophin signaling pathway	20	0.270072013	0.0001	0.004220438
GO:0050728	negative regulation of inflammatory response	53	0.269972065	0.0034	0.01347416
GO:0005979	regulation of glycogen biosynthetic process	10	0.269949238	0.0001	0.004220438
GO:0010962	regulation of glucan biosynthetic process	10	0.269949238	0.0001	0.004220438
GO:0007193	adenylate cyclase-inhibiting G-protein coupled receptor signaling pathway	36	0.26980632	0.0053	0.014634479
GO:0042509	regulation of tyrosine phosphorylation of STAT protein	23	0.269710345	0.0042	0.013948535
GO:0033605	positive regulation of catecholamine secretion	8	0.269687521	< 0.001	< 0.001
GO:0051588	regulation of neurotransmitter transport	57	0.269660693	0.0006	0.009940401
GO:0098801	regulation of renal system process	15	0.269657902	0.0094	0.017156187
GO:0060046	regulation of acrosome reaction	6	0.269622354	0.0015	0.011704453
GO:0071636	positive regulation of transforming growth factor beta production	6	0.269612852	0.0216	0.026572596
GO:0014072	response to isoquinoline alkaloid	23	0.269568034	0.0018	0.01210186
GO:0043278	response to morphine	23	0.269568034	0.0018	0.01210186
GO:0006835	dicarboxylic acid transport	44	0.269529509	0.001	0.010787313
GO:0035336	long-chain fatty-acyl-CoA metabolic process	6	0.269517611	0.021	0.026005997
GO:0045010	actin nucleation	22	0.26951449	0.004	0.013824268
GO:0060438	trachea development	8	0.269482301	0.0166	0.02247277
GO:0032885	regulation of polysaccharide biosynthetic process	15	0.269327995	0.0002	0.006319126
GO:0043551	regulation of phosphatidylinositol 3-kinase activity	20	0.269324604	0.0073	0.015844069
GO:0035810	positive regulation of urine volume	7	0.269322503	0.0014	0.011465722
GO:0035268	protein mannosylation	12	0.269247224	0.0146	0.020879842
GO:0006470	protein dephosphorylation	148	0.269232941	0.0014	0.011465722
GO:0048066	developmental pigmentation	19	0.269218762	0.0017	0.012001709
GO:1902914	regulation of protein polyubiquitination	12	0.269174768	0.004	0.013824268
GO:0010927	cellular component assembly involved in morphogenesis	28	0.269167333	0.0044	0.014132412
GO:0016126	sterol biosynthetic process	44	0.26916289	0.0075	0.015990044
GO:0019317	fucose catabolic process	5	0.269063568	0.0024	0.012603815
GO:0042354	L-fucose metabolic process	5	0.269063568	0.0024	0.012603815
GO:0042355	L-fucose catabolic process	5	0.269063568	0.0024	0.012603815
GO:0006516	glycoprotein catabolic process	9	0.269056312	0.0007	0.010220707
GO:1901071	glucosamine-containing compound metabolic process	5	0.269033247	0.0264	0.030750363
GO:0070542	response to fatty acid	40	0.268990259	0.001	0.010787313
GO:0043666	regulation of phosphoprotein phosphatase activity	56	0.268961812	0.0025	0.012769435
GO:0015669	gas transport	9	0.26885892	0.0152	0.0213213
GO:0032700	negative regulation of interleukin-17 production	8	0.268855303	0.0021	0.012314604
GO:0035308	negative regulation of protein dephosphorylation	30	0.268845012	0.0042	0.013948535
GO:1900006	positive regulation of dendrite development	27	0.268839675	0.0011	0.010890753
GO:0030193	regulation of blood coagulation	25	0.268836546	0.0159	0.021956962
GO:1900046	regulation of hemostasis	25	0.268836546	0.0159	0.021956962
GO:0031214	biomineral tissue development	59	0.268823685	0.0036	0.01353394
GO:0061684	chaperone-mediated autophagy	11	0.268796094	0.0065	0.015491756
GO:0019229	regulation of vasoconstriction	19	0.268686194	0.0028	0.012972436
GO:0090036	regulation of protein kinase C signaling	6	0.268650083	0.0022	0.012422266
GO:0010878	cholesterol storage	7	0.268645733	0.0002	0.006319126

GOIDlabel	GOName	size	categoryScores	pValPerm	pValPermCorr
GO:0050812	regulation of acyl-CoA biosynthetic process	5	0.268641919	0.0091	0.016994897
GO:0009312	oligosaccharide biosynthetic process	6	0.268607538	0.0067	0.015576759
GO:1904385	cellular response to angiotensin	10	0.26860175	0.0008	0.010465158
GO:0043950	positive regulation of cAMP-mediated signaling	8	0.268375538	0.0023	0.012498684
GO:0014866	skeletal myofibril assembly	5	0.26836203	0.0414	0.044386204
GO:0099601	regulation of neurotransmitter receptor activity	38	0.268245423	0.0008	0.010465158
GO:0045453	bone resorption	23	0.26822053	0.0005	0.009604651
GO:0097028	dendritic cell differentiation	14	0.268205443	0.0074	0.015911789
GO:1901184	regulation of ERBB signaling pathway	39	0.268193086	0.0002	0.006319126
GO:2000114	regulation of establishment of cell polarity	10	0.268160405	0.0063	0.015311728
GO:0032024	positive regulation of insulin secretion	34	0.268093543	0.0012	0.010961137
GO:0071312	cellular response to alkaloid	16	0.268088927	0.0045	0.014132412
GO:0030213	hyaluronan biosynthetic process	5	0.268085509	0.0069	0.015750415
GO:1900125	regulation of hyaluronan biosynthetic process	5	0.268085509	0.0069	0.015750415
GO:0033539	fatty acid beta-oxidation using acyl-CoA dehydrogenase	7	0.267988954	0.0266	0.030933467
GO:0015740	C4-dicarboxylate transport	11	0.267948297	0.0022	0.012422266
GO:0046835	carbohydrate phosphorylation	12	0.267928546	0.031	0.034967226
GO:0046173	polyol biosynthetic process	17	0.267888809	0.01	0.017574468
GO:1903429	regulation of cell maturation	7	0.267868446	0.0015	0.011704453
GO:0003351	epithelial cilium movement	7	0.267745347	0.0171	0.022860624
GO:0010921	regulation of phosphatase activity	78	0.267708595	0.0027	0.012827773
GO:0003094	glomerular filtration	9	0.267699912	0.0091	0.016994897
GO:0010893	positive regulation of steroid biosynthetic process	5	0.267675263	0.0213	0.026276211
GO:0006029	proteoglycan metabolic process	46	0.267615905	0.0073	0.015844069
GO:0015012	heparan sulfate proteoglycan biosynthetic process	9	0.267581419	0.0011	0.010890753
GO:0070570	regulation of neuron projection regeneration	18	0.267575084	0.0034	0.01347416
GO:0032692	negative regulation of interleukin-1 production	15	0.267557433	0.0305	0.034510959
GO:1903034	regulation of response to wounding	64	0.267531597	0.0094	0.017156187
GO:0039531	regulation of viral-induced cytoplasmic pattern recognition receptor signaling pathway	9	0.267501513	0.011	0.018323826
GO:0007099	centriole replication	9	0.267500937	0.0141	0.020504577
GO:0098534	centriole assembly	9	0.267500937	0.0141	0.020504577
GO:1901185	negative regulation of ERBB signaling pathway	23	0.267488636	0.001	0.010787313
GO:0060068	vagina development	6	0.267487979	0.0092	0.017033109
GO:0035357	peroxisome proliferator activated receptor signaling pathway	12	0.267477829	0.0016	0.011845327
GO:0035929	steroid hormone secretion	9	0.267476029	0.0003	0.007956881
GO:0016114	terpenoid biosynthetic process	7	0.267472306	0.0275	0.031743861
GO:0010870	positive regulation of receptor biosynthetic process	5	0.267438653	0.0015	0.011704453
GO:0051383	kinetochore organization	9	0.26729721	0.0008	0.010465158
GO:1902930	regulation of alcohol biosynthetic process	39	0.267248558	0.0123	0.019143634
GO:0032703	negative regulation of interleukin-2 production	11	0.267199686	0.0233	0.028125386
GO:0098586	cellular response to virus	21	0.267110412	0.0094	0.017156187
GO:0010676	positive regulation of cellular carbohydrate metabolic process	21	0.266996938	0.0089	0.016916437
GO:0060997	dendritic spine morphogenesis	28	0.266910537	0.0016	0.011845327
GO:0048820	hair follicle maturation	6	0.266862762	0.0315	0.035393121
GO:0035641	locomotory exploration behavior	7	0.266857918	0.0015	0.011704453
GO:2000831	regulation of steroid hormone secretion	8	0.266834363	0.0001	0.004220438
GO:0006851	mitochondrial calcium ion transport	15	0.266813134	0.0074	0.015911789
GO:0015802	basic amino acid transport	5	0.266757588	0.021	0.026005997
GO:0006695	cholesterol biosynthetic process	43	0.266742189	0.0073	0.015844069
GO:1902653	secondary alcohol biosynthetic process	43	0.266742189	0.0073	0.015844069
GO:0015872	dopamine transport	14	0.266413557	0.0051	0.014547706
GO:0001765	membrane raft assembly	6	0.266398223	0.0027	0.012827773
GO:0005980	glycogen catabolic process	10	0.266383753	0.007	0.015816335
GO:0009251	glucan catabolic process	10	0.266383753	0.007	0.015816335
GO:0002715	regulation of natural killer cell mediated immunity	12	0.266373819	0.0164	0.022306469
GO:0036257	multivesicular body organization	19	0.266352143	0.0064	0.015399417
GO:0036258	multivesicular body assembly	19	0.266352143	0.0064	0.015399417
GO:0002717	positive regulation of natural killer cell mediated immunity	8	0.266234738	0.0085	0.016693954
GO:0050803	regulation of synapse structure or activity	88	0.266211119	0.0023	0.012498684
GO:0050807	regulation of synapse organization	88	0.266211119	0.0023	0.012498684
GO:0002089	lens morphogenesis in camera-type eye	9	0.26620803	0.0003	0.007956881
GO:0060999	positive regulation of dendritic spine development	14	0.266152548	0.0027	0.012827773
GO:2001026	regulation of endothelial cell chemotaxis	9	0.266074565	0.0325	0.036312077
GO:0060445	branching involved in salivary gland morphogenesis	12	0.265975689	0.0031	0.013277185
GO:0001961	positive regulation of cytokine-mediated signaling pathway	18	0.265852841	0.0072	0.015844069
GO:0072583	clathrin-dependent endocytosis	38	0.265754718	0.0008	0.010465158
GO:0007215	glutamate receptor signaling pathway	53	0.265712182	0.0047	0.014168613
GO:0009065	glutamine family amino acid catabolic process	15	0.265654615	0.0036	0.01353394
GO:0044264	cellular polysaccharide metabolic process	34	0.265604796	0.0008	0.010465158
GO:0045986	negative regulation of smooth muscle contraction	9	0.265574823	0.004	0.013824268
GO:0042573	retinoic acid metabolic process	8	0.265569028	0.0145	0.020788247
GO:0031290	retinal ganglion cell axon guidance	11	0.26555557	0.003	0.013210967
GO:0006890	retrograde vesicle-mediated transport, Golgi to ER	47	0.265455516	0.0019	0.012288367
GO:0033032	regulation of myeloid cell apoptotic process	11	0.265432588	0.0074	0.015911789
GO:0007076	mitotic chromosome condensation	8	0.265372231	0.0012	0.010961137
GO:0046579	positive regulation of Ras protein signal transduction	27	0.265282791	0.0055	0.01475
GO:0007288	sperm axoneme assembly	6	0.265277368	0.0124	0.019242297
GO:0034244	negative regulation of transcription elongation from RNA polymerase II promoter	5	0.265242814	0.0087	0.016840777
GO:0051412	response to corticosterone	5	0.265193574	0.0263	0.030677143
GO:0032691	negative regulation of interleukin-1 beta production	14	0.265156089	0.0326	0.036409735
GO:0021885	forebrain cell migration	29	0.265020028	0.008	0.016379603
GO:2000242	negative regulation of reproductive process	17	0.265008505	0.0091	0.016994897
GO:0039535	regulation of RIG-I signaling pathway	7	0.264994393	0.0062	0.015209334

GOIDlabel	GOName	size	categoryScores	pValPerm	pValPermCorr
GO:0016075	rRNA catabolic process	5	0.264975364	0.0147	0.02091937
GO:0060231	mesenchymal to epithelial transition	7	0.264867221	0.0006	0.009940401
GO:1990776	response to angiotensin	11	0.264814969	0.0034	0.01347416
GO:0042490	mechanoreceptor differentiation	24	0.264785834	0.0141	0.020504577
GO:0071243	cellular response to arsenic-containing substance	12	0.26473519	0.0042	0.013948535
GO:0090279	regulation of calcium ion import	45	0.264734808	0.0028	0.012972436
GO:0086013	membrane repolarization during cardiac muscle cell action potential	10	0.264714393	0.0036	0.01353394
GO:0097090	presynaptic membrane organization	5	0.264676815	0.0138	0.020339434
GO:0035150	regulation of tube size	38	0.264668713	0.0032	0.013333753
GO:0050880	regulation of blood vessel size	38	0.264668713	0.0032	0.013333753
GO:0072087	renal vesicle development	7	0.264627871	0.004	0.013824268
GO:0050654	chondroitin sulfate proteoglycan metabolic process	26	0.264563723	0.0111	0.018389742
GO:0016233	telomere capping	22	0.264537259	0.0011	0.010890753
GO:0051458	corticotropin secretion	5	0.264532723	0.0279	0.032115827
GO:0051459	regulation of corticotropin secretion	5	0.264532723	0.0279	0.032115827
GO:0044803	multi-organism membrane organization	24	0.264473819	0.0085	0.016693954
GO:0035567	non-canonical Wnt signaling pathway	85	0.264432902	0.0063	0.015311728
GO:0010644	cell communication by electrical coupling	8	0.264411244	0.0311	0.035052671
GO:0009435	NAD biosynthetic process	10	0.264314513	0.0102	0.0177
GO:2001171	positive regulation of ATP biosynthetic process	6	0.264302937	0.0254	0.029880529
GO:0042559	pteridine-containing compound biosynthetic process	9	0.264286812	0.0187	0.024129301
GO:0000291	nuclear-transcribed mRNA catabolic process, exonucleolytic	19	0.264230328	0.0079	0.01629604
GO:0043928	exonucleolytic nuclear-transcribed mRNA catabolic process involved in deadenylation-dependent decay	19	0.264230328	0.0079	0.01629604
GO:0032958	inositol phosphate biosynthetic process	12	0.26411548	0.0127	0.019457181
GO:0051893	regulation of focal adhesion assembly	27	0.264007498	0.0024	0.012603815
GO:0090109	regulation of cell-substrate junction assembly	27	0.264007498	0.0024	0.012603815
GO:0032350	regulation of hormone metabolic process	12	0.263965385	0.0067	0.015576759
GO:0021670	lateral ventricle development	6	0.263937526	0.0466	0.049159132
GO:0035296	regulation of tube diameter	35	0.263896863	0.0045	0.014132412
GO:0097746	regulation of blood vessel diameter	35	0.263896863	0.0045	0.014132412
GO:0090231	regulation of spindle checkpoint	7	0.263895752	0.0002	0.006319126
GO:0090266	regulation of mitotic cell cycle spindle assembly checkpoint	7	0.263895752	0.0002	0.006319126
GO:1903504	regulation of mitotic spindle checkpoint	7	0.263895752	0.0002	0.006319126
GO:0060113	inner ear receptor cell differentiation	22	0.263776927	0.02	0.025188412
GO:0036315	cellular response to sterol	11	0.263754427	0.0055	0.01475
GO:0098815	modulation of excitatory postsynaptic potential	25	0.263753231	< 0.001	< 0.001
GO:0006023	aminoglycan biosynthetic process	60	0.263710699	0.0046	0.014132412
GO:0006024	glycosaminoglycan biosynthetic process	60	0.263710699	0.0046	0.014132412
GO:0050974	detection of mechanical stimulus involved in sensory perception	10	0.263704068	0.0147	0.02091937
GO:0034698	response to gonadotropin	7	0.263668249	0.0057	0.01480566
GO:0019233	sensory perception of pain	51	0.263610937	0.0014	0.011465722
GO:1904181	positive regulation of membrane depolarization	7	0.263599573	0.0101	0.017653628
GO:0021680	cerebellar Purkinje cell layer development	8	0.263597166	0.0021	0.012314604
GO:0010922	positive regulation of phosphatase activity	18	0.263581257	0.0099	0.017505138
GO:0030490	maturation of SSU-rRNA	5	0.263576174	0.0045	0.014132412
GO:0000394	RNA splicing, via endonucleolytic cleavage and ligation	6	0.263545551	0.0021	0.012314604
GO:0006388	tRNA splicing, via endonucleolytic cleavage and ligation	6	0.263545551	0.0021	0.012314604
GO:0000096	sulfur amino acid metabolic process	17	0.263504174	0.0164	0.022306469
GO:0072210	metanephric nephron development	16	0.263473743	0.0116	0.018682786
GO:0021987	cerebral cortex development	49	0.263395896	0.005	0.014547706
GO:0036314	response to sterol	17	0.263364687	0.011	0.018323826
GO:0022406	membrane docking	65	0.26329148	0.0029	0.013089617
GO:0006026	aminoglycan catabolic process	34	0.263258394	0.0091	0.016994897
GO:0006027	glycosaminoglycan catabolic process	34	0.263258394	0.0091	0.016994897
GO:0051044	positive regulation of membrane protein ectodomain proteolysis	5	0.263185625	0.0022	0.012422266
GO:0071499	cellular response to laminar fluid shear stress	5	0.263167743	0.0015	0.011704453
GO:0030730	sequestering of triglyceride	9	0.263145945	0.0021	0.012314604
GO:0050732	negative regulation of peptidyl-tyrosine phosphorylation	20	0.263137162	0.0018	0.01210186
GO:0050931	pigment cell differentiation	18	0.263093033	0.0015	0.011704453
GO:0072224	metanephric glomerulus development	5	0.26303635	0.0378	0.041067193
GO:0008286	insulin receptor signaling pathway	53	0.26296338	0.0029	0.013089617
GO:0050909	sensory perception of taste	15	0.262953506	0.0001	0.004220438
GO:0032330	regulation of chondrocyte differentiation	25	0.26293927	0.0074	0.015911789
GO:0050857	positive regulation of antigen receptor-mediated signaling pathway	12	0.262931132	0.0056	0.014771533
GO:0007175	negative regulation of epidermal growth factor-activated receptor activity	5	0.262865306	0.007	0.015816335
GO:0072173	metanephric tubule morphogenesis	6	0.262844315	0.0322	0.036060507
GO:0031365	N-terminal protein amino acid modification	11	0.262635929	0.004	0.013824268
GO:0032836	glomerular basement membrane development	6	0.262562049	0.013	0.019676963
GO:0003018	vascular process in circulatory system	54	0.262548587	0.0056	0.014771533
GO:2000758	positive regulation of peptidyl-lysine acetylation	13	0.262523857	0.0196	0.0248852
GO:0046165	alcohol biosynthetic process	62	0.262503439	0.0082	0.016514246
GO:0046164	alcohol catabolic process	12	0.262429447	0.0041	0.013887639
GO:0050951	sensory perception of temperature stimulus	9	0.262387046	0.0018	0.01210186
GO:0061517	macrophage proliferation	6	0.262380423	0.0389	0.042009675
GO:0061518	microglial cell proliferation	6	0.262380423	0.0389	0.042009675
GO:1904467	regulation of tumor necrosis factor secretion	8	0.262379775	0.0053	0.014634479
GO:1990774	tumor necrosis factor secretion	8	0.262379775	0.0053	0.014634479
GO:0034105	positive regulation of tissue remodeling	6	0.262359907	0.0208	0.025885837
GO:0007286	spermatid development	42	0.262306378	0.0077	0.016177834
GO:0035329	hippo signaling	14	0.262298326	0.007	0.015816335
GO:0090277	positive regulation of peptide hormone secretion	43	0.262235958	0.0009	0.010729485
GO:0060586	multicellular organismal iron ion homeostasis	5	0.262234777	0.0044	0.014132412
GO:1903077	negative regulation of protein localization to plasma membrane	12	0.262188863	0.0056	0.014771533

GOIDlabel	GOName	size	categoryScores	pValPerm	pValPermCorr
GO:0002097	tRNA wobble base modification	8	0.262161597	0.001	0.010787313
GO:0002098	tRNA wobble uridine modification	8	0.262161597	0.001	0.010787313
GO:0035773	insulin secretion involved in cellular response to glucose stimulus	32	0.262135236	0.0023	0.012498684
GO:0006750	glutathione biosynthetic process	9	0.26209967	0.0072	0.015844069
GO:0060079	excitatory postsynaptic potential	50	0.26208558	0.0006	0.009940401
GO:0051881	regulation of mitochondrial membrane potential	34	0.262072941	0.0073	0.015844069
GO:0060740	prostate gland epithelium morphogenesis	8	0.262060526	0.0134	0.01999969
GO:0010635	regulation of mitochondrial fusion	8	0.26204028	0.0085	0.016693954
GO:0060071	Wnt signaling pathway, planar cell polarity pathway	71	0.261975262	0.0088	0.016916437
GO:0097062	dendritic spine maintenance	9	0.26190559	< 0.001	< 0.001
GO:0007632	visual behavior	23	0.261889997	0.0025	0.012769435
GO:0070670	response to interleukin-4	13	0.261798615	0.0015	0.011704453
GO:0031952	regulation of protein autophosphorylation	19	0.261794728	0.0127	0.019457181
GO:0002793	positive regulation of peptide secretion	44	0.261793315	0.0007	0.010220707
GO:0072574	hepatocyte proliferation	9	0.261752831	0.009	0.016956012
GO:0072575	epithelial cell proliferation involved in liver morphogenesis	9	0.261752831	0.009	0.016956012
GO:0072576	liver morphogenesis	9	0.261752831	0.009	0.016956012
GO:0019218	regulation of steroid metabolic process	56	0.261715986	0.0073	0.015844069
GO:0046949	fatty-acyl-CoA biosynthetic process	10	0.261692879	0.0303	0.034331687
GO:0046425	regulation of JAK-STAT cascade	38	0.261673402	0.0019	0.012288367
GO:0090110	cargo loading into COPII-coated vesicle	8	0.261668227	0.0012	0.010961137
GO:0050818	regulation of coagulation	26	0.26160973	0.0164	0.022306469
GO:0010885	regulation of cholesterol storage	6	0.261535355	0.0027	0.012827773
GO:2000302	positive regulation of synaptic vesicle exocytosis	7	0.261523724	0.0002	0.006319126
GO:0090175	regulation of establishment of planar polarity	72	0.261498533	0.008	0.016379603
GO:0051589	negative regulation of neurotransmitter transport	5	0.261491514	0.0386	0.041834152
GO:0035358	regulation of peroxisome proliferator activated receptor signaling pathway	8	0.26148374	0.0097	0.017363901
GO:0035767	endothelial cell chemotaxis	14	0.261458842	0.0151	0.02121706
GO:0048011	neurotrophin TRK receptor signaling pathway	17	0.261442655	0.0002	0.006319126
GO:0033522	histone H2A ubiquitination	11	0.261309044	0.0042	0.013948535
GO:0002433	immune response-regulating cell surface receptor signaling pathway involved in phagocytosis	43	0.261269453	0.0117	0.018739446
GO:0038094	Fc-gamma receptor signaling pathway	43	0.261269453	0.0117	0.018739446
GO:0038096	Fc-gamma receptor signaling pathway involved in phagocytosis	43	0.261269453	0.0117	0.018739446
GO:0008343	adult feeding behavior	5	0.261258014	0.0112	0.018392048
GO:0030317	flagellated sperm motility	27	0.261141988	0.0068	0.015633241
GO:0097722	sperm motility	27	0.261141988	0.0068	0.015633241
GO:0002702	positive regulation of production of molecular mediator of immune response	35	0.261137802	0.0072	0.015844069
GO:0042462	eye photoreceptor cell development	12	0.261049435	0.0001	0.004220438
GO:0070050	neuron cellular homeostasis	17	0.260967331	0.0039	0.013791927
GO:0048641	regulation of skeletal muscle tissue development	19	0.260934258	0.0016	0.011845327
GO:0042269	regulation of natural killer cell mediated cytotoxicity	11	0.260915479	0.0228	0.027701114
GO:0000050	urea cycle	6	0.26087688	0.0016	0.011845327
GO:0019627	urea metabolic process	6	0.26087688	0.0016	0.011845327
GO:0039529	RIG-I signaling pathway	8	0.260822403	0.0097	0.017363901
GO:0002693	positive regulation of cellular extravasation	5	0.260768207	0.0221	0.027038129
GO:0050706	regulation of interleukin-1 beta secretion	16	0.260743036	0.0177	0.02329117
GO:0050746	regulation of lipoprotein metabolic process	5	0.260738663	0.0329	0.036702257
GO:0014902	myotube differentiation	36	0.260681468	0.002	0.012314604
GO:0045026	plasma membrane fusion	7	0.260671467	0.0005	0.009604651
GO:0050718	positive regulation of interleukin-1 beta secretion	9	0.260663795	0.0205	0.025639412
GO:0032205	negative regulation of telomere maintenance	13	0.260663507	0.0009	0.010729485
GO:1901387	positive regulation of voltage-gated calcium channel activity	6	0.260588352	0.0037	0.013609033
GO:2000601	positive regulation of Arp2/3 complex-mediated actin nucleation	6	0.260520755	0.0018	0.01210186
GO:0048193	Golgi vesicle transport	159	0.260490442	0.0023	0.012498684
GO:0050810	regulation of steroid biosynthetic process	43	0.26047375	0.0107	0.018084595
GO:2000300	regulation of synaptic vesicle exocytosis	40	0.260394016	0.0008	0.010465158
GO:0043114	regulation of vascular permeability	18	0.26037685	0.0114	0.018551872
GO:0046325	negative regulation of glucose import	7	0.260345669	0.0039	0.013791927
GO:1902992	negative regulation of amyloid precursor protein catabolic process	9	0.260311485	0.0006	0.009940401
GO:0046685	response to arsenic-containing substance	17	0.260304117	0.0021	0.012314604
GO:0098779	mitophagy in response to mitochondrial depolarization	11	0.260264876	0.0078	0.01620539
GO:0045123	cellular extravasation	23	0.260244743	0.0137	0.020264364
GO:0046475	glycerophospholipid catabolic process	9	0.260232442	0.0006	0.009940401
GO:0002431	Fc receptor mediated stimulatory signaling pathway	45	0.26018709	0.0126	0.019370699
GO:0071398	cellular response to fatty acid	31	0.260181182	0.0003	0.007956881
GO:0006576	cellular biogenic amine metabolic process	24	0.260172566	0.0119	0.018881943
GO:0060996	dendritic spine development	43	0.260132627	0.0005	0.009604651
GO:0060425	lung morphogenesis	20	0.260026175	0.0135	0.020081554
GO:0032930	positive regulation of superoxide anion generation	9	0.259977605	0.0315	0.035393121
GO:0060148	positive regulation of posttranscriptional gene silencing	9	0.259941179	0.0031	0.013277185
GO:1901661	quinone metabolic process	18	0.259939739	0.0089	0.016916437
GO:0097106	postsynaptic density organization	10	0.259910785	0.0029	0.013089617
GO:0099084	postsynaptic specialization organization	10	0.259910785	0.0029	0.013089617
GO:0021859	pyramidal neuron differentiation	5	0.259845866	0.0075	0.015990044
GO:0021860	pyramidal neuron development	5	0.259845866	0.0075	0.015990044
GO:0071391	cellular response to estrogen stimulus	7	0.259841238	0.0005	0.009604651
GO:0003338	metanephros morphogenesis	15	0.259827812	0.0066	0.015525305
GO:0010573	vascular endothelial growth factor production	15	0.2597803	0.0168	0.022626974
GO:0051955	regulation of amino acid transport	13	0.259769315	0.0006	0.009940401
GO:0010743	regulation of macrophage derived foam cell differentiation	9	0.259706497	0.0014	0.011465722
GO:0008090	retrograde axonal transport	8	0.259673527	0.0133	0.019917275
GO:0051043	regulation of membrane protein ectodomain proteolysis	8	0.259660731	0.0024	0.012603815
GO:0045745	positive regulation of G-protein coupled receptor protein signaling pathway	13	0.259652994	0.0029	0.013089617

GOIDlabel	GOName	size	categoryScores	pValPerm	pValPermCorr
GO:2001267	regulation of cysteine-type endopeptidase activity involved in apoptotic signaling pathway	8	0.259649069	0.0045	0.014132412
GO:0008542	visual learning	22	0.259645785	0.0026	0.012805111
GO:0034637	cellular carbohydrate biosynthetic process	28	0.259624981	0.0007	0.010220707
GO:0051149	positive regulation of muscle cell differentiation	36	0.25960611	0.0008	0.010465158
GO:1904376	negative regulation of protein localization to cell periphery	14	0.25947231	0.0009	0.010729485
GO:0021543	pallium development	73	0.259377484	0.004	0.013824268
GO:0001832	blastocyst growth	9	0.259363258	0.0055	0.01475
GO:0032105	negative regulation of response to extracellular stimulus	9	0.259307971	0.0109	0.018225506
GO:0032108	negative regulation of response to nutrient levels	9	0.259307971	0.0109	0.018225506
GO:0008033	tRNA processing	47	0.259285646	0.0045	0.014132412
GO:0010637	negative regulation of mitochondrial fusion	6	0.25926773	0.0077	0.016177834
GO:0007263	nitric oxide mediated signal transduction	15	0.259183288	0.0118	0.018836996
GO:0070358	actin polymerization-dependent cell motility	5	0.259132147	0.018	0.023514686
GO:0086011	membrane repolarization during action potential	12	0.259119038	0.0049	0.014447629
GO:0022029	telencephalon cell migration	26	0.259111853	0.0092	0.017033109
GO:0019934	cGMP-mediated signaling	15	0.259009048	0.0092	0.017033109
GO:0006885	regulation of pH	37	0.259006092	0.0082	0.016514246
GO:0042531	positive regulation of tyrosine phosphorylation of STAT protein	19	0.258996445	0.0051	0.014547706
GO:0043266	regulation of potassium ion transport	37	0.258974328	0.0004	0.008964341
GO:0034661	ncRNA catabolic process	7	0.258943444	0.0083	0.016559903
GO:0035459	cargo loading into vesicle	9	0.258852431	0.0024	0.012603815
GO:0006112	energy reserve metabolic process	31	0.258784701	0.0006	0.009940401
GO:0045722	positive regulation of gluconeogenesis	5	0.258775547	0.0286	0.032739101
GO:1902803	regulation of synaptic vesicle transport	53	0.258739241	0.0009	0.010729485
GO:0034121	regulation of toll-like receptor signaling pathway	25	0.258645761	0.0115	0.018599441
GO:0070885	negative regulation of calcineurin-NFAT signaling cascade	9	0.258637906	< 0.001	< 0.001
GO:0071361	cellular response to ethanol	9	0.258609975	0.0113	0.018488002
GO:0032102	negative regulation of response to external stimulus	138	0.25855624	0.0064	0.015399417
GO:0071295	cellular response to vitamin	9	0.258518262	0.0287	0.032827577
GO:0060191	regulation of lipase activity	37	0.258517301	0.0059	0.014942532
GO:0061036	positive regulation of cartilage development	18	0.25843094	0.0042	0.013948535
GO:0002686	negative regulation of leukocyte migration	21	0.258407297	0.0058	0.014878261
GO:1905330	regulation of morphogenesis of an epithelium	98	0.258373893	0.0071	0.01583804
GO:2000177	regulation of neural precursor cell proliferation	39	0.258321823	0.0112	0.018392048
GO:0044380	protein localization to cytoskeleton	17	0.258289475	0.0027	0.012827773
GO:0071385	cellular response to glucocorticoid stimulus	16	0.258253346	0.0121	0.018970228
GO:0035337	fatty-acyl-CoA metabolic process	11	0.258240152	0.0314	0.035301342
GO:0051125	regulation of actin nucleation	13	0.258175598	0.0036	0.01353394
GO:0043330	response to exogenous dsRNA	20	0.258166657	0.0046	0.014132412
GO:0002548	monocyte chemotaxis	12	0.258130138	0.0208	0.025885837
GO:0055003	cardiac myofibril assembly	7	0.258100258	0.0302	0.034245225
GO:0010889	regulation of sequestering of triglyceride	8	0.258098013	0.0027	0.012827773
GO:0031943	regulation of glucocorticoid metabolic process	6	0.258084677	0.0107	0.018084595
GO:0001736	establishment of planar polarity	80	0.258041582	0.0073	0.015844069
GO:0007164	establishment of tissue polarity	80	0.258041582	0.0073	0.015844069
GO:0035902	response to immobilization stress	11	0.258031177	0.0063	0.015311728
GO:0006022	aminoglycan metabolic process	82	0.258018097	0.0044	0.014132412
GO:0030203	glycosaminoglycan metabolic process	82	0.258018097	0.0044	0.014132412
GO:0032096	negative regulation of response to food	8	0.257965033	0.0103	0.017772187
GO:0032099	negative regulation of appetite	8	0.257965033	0.0103	0.017772187
GO:0048488	synaptic vesicle endocytosis	25	0.257943601	0.0005	0.009604651
GO:0072170	metanephric tubule development	13	0.257933286	0.0209	0.025971158
GO:0032148	activation of protein kinase B activity	15	0.25791886	0.0062	0.015209334
GO:1905475	regulation of protein localization to membrane	81	0.257875936	0.0045	0.014132412
GO:0034138	toll-like receptor 3 signaling pathway	11	0.257816195	0.0063	0.015311728
GO:0046427	positive regulation of JAK-STAT cascade	26	0.257784171	0.0034	0.01347416
GO:0031348	negative regulation of defense response	82	0.257766588	0.0045	0.014132412
GO:0099565	chemical synaptic transmission, postsynaptic	53	0.257757084	0.0008	0.010465158
GO:0044272	sulfur compound biosynthetic process	98	0.25773162	0.0072	0.015844069
GO:0048169	regulation of long-term neuronal synaptic plasticity	14	0.257717908	0.009	0.016956012
GO:0032232	negative regulation of actin filament bundle assembly	8	0.257709116	0.0188	0.02420971
GO:0060856	establishment of blood-brain barrier	5	0.257698231	0.0065	0.015491756
GO:0001779	natural killer cell differentiation	11	0.25769604	0.0001	0.004220438
GO:0045954	positive regulation of natural killer cell mediated cytotoxicity	7	0.257637477	0.0166	0.02247277
GO:0060078	regulation of postsynaptic membrane potential	68	0.257589332	0.0012	0.010961137
GO:1905477	positive regulation of protein localization to membrane	59	0.257548284	0.0035	0.01351837
GO:0040009	regulation of growth rate	5	0.257546365	0.0054	0.014720792
GO:0048701	embryonic cranial skeleton morphogenesis	18	0.257521515	0.021	0.026005997
GO:0051938	L-glutamate import	8	0.25747474	0.0064	0.015399417
GO:0006891	intra-Golgi vesicle-mediated transport	10	0.257466544	0.0279	0.032115827
GO:0061318	renal filtration cell differentiation	6	0.257451254	0.0095	0.01725157
GO:0072112	glomerular visceral epithelial cell differentiation	6	0.257451254	0.0095	0.01725157
GO:0072311	glomerular epithelial cell differentiation	6	0.257451254	0.0095	0.01725157
GO:0050854	regulation of antigen receptor-mediated signaling pathway	30	0.257442702	0.0022	0.012422266
GO:0015813	L-glutamate transport	11	0.257429108	0.0113	0.018488002
GO:0050806	positive regulation of synaptic transmission	93	0.257373869	0.0005	0.009604651
GO:0010882	regulation of cardiac muscle contraction by calcium ion signaling	10	0.25736936	0.0169	0.022703485
GO:0008211	glucocorticoid metabolic process	11	0.257357015	0.0027	0.012827773
GO:0000041	transition metal ion transport	44	0.257343881	0.0034	0.01347416
GO:0001659	temperature homeostasis	14	0.257342408	0.0032	0.013333753
GO:0034383	low-density lipoprotein particle clearance	17	0.257335777	0.0124	0.019242297
GO:0015780	nucleotide-sugar transport	5	0.257293687	0.0125	0.019314538
GO:0015781	pyrimidine nucleotide-sugar transport	5	0.257293687	0.0125	0.019314538

GOIDlabel	GOName	size	categoryScores	pValPerm	pValPermCorr
GO:0097009	energy homeostasis	13	0.257273233	0.0018	0.01210186
GO:0044848	biological phase	5	0.257168255	0.0401	0.043160499
GO:0044851	hair cycle phase	5	0.257168255	0.0401	0.043160499
GO:0048819	regulation of hair follicle maturation	5	0.257168255	0.0401	0.043160499
GO:0060330	regulation of response to interferon-gamma	7	0.257166922	0.0049	0.014447629
GO:0060334	regulation of interferon-gamma-mediated signaling pathway	7	0.257166922	0.0049	0.014447629
GO:1901096	regulation of autophagosome maturation	5	0.257147263	0.0151	0.02121706
GO:1902751	positive regulation of cell cycle G2/M phase transition	12	0.257046969	0.0147	0.02091937
GO:0045834	positive regulation of lipid metabolic process	61	0.257029062	0.0018	0.01210186
GO:0070527	platelet aggregation	27	0.256978292	0.0187	0.024129301
GO:1901203	positive regulation of extracellular matrix assembly	5	0.256967667	0.0244	0.029011063
GO:0071384	cellular response to corticosteroid stimulus	19	0.256954764	0.0083	0.016559903
GO:0098780	response to mitochondrial depolarisation	14	0.256889994	0.0077	0.016177834
GO:1903510	mucopolysaccharide metabolic process	59	0.256861013	0.0035	0.01351837
GO:0000715	nucleotide-excision repair, DNA damage recognition	15	0.256855106	0.0143	0.020629391
GO:0045191	regulation of isotype switching	13	0.256808146	0.0008	0.010465158
GO:0005978	glycogen biosynthetic process	17	0.256790099	0.0005	0.009604651
GO:0009250	glucan biosynthetic process	17	0.256790099	0.0005	0.009604651
GO:0031663	lipopolysaccharide-mediated signaling pathway	22	0.256736982	0.0191	0.02440579
GO:0060711	labyrinthine layer development	22	0.256653698	0.0088	0.016916437
GO:0072677	eosinophil migration	6	0.256643548	0.0246	0.029170878
GO:0007567	parturition	8	0.256636043	0.0088	0.016916437
GO:1902915	negative regulation of protein polyubiquitination	5	0.256625839	0.0062	0.015209334
GO:0002704	negative regulation of leukocyte mediated immunity	15	0.256612794	0.0198	0.025062084
GO:0051127	positive regulation of actin nucleation	8	0.256585183	0.0113	0.018488002
GO:2000241	regulation of reproductive process	49	0.256579613	0.0061	0.015143924
GO:0044406	adhesion of symbiont to host	7	0.25657808	0.019	0.024347961
GO:0044650	adhesion of symbiont to host cell	7	0.25657808	0.019	0.024347961
GO:0071941	nitrogen cycle metabolic process	7	0.256570675	0.0032	0.013333753
GO:0005977	glycogen metabolic process	27	0.256527023	0.0005	0.009604651
GO:0006073	cellular glucan metabolic process	27	0.256527023	0.0005	0.009604651
GO:0044042	glucan metabolic process	27	0.256527023	0.0005	0.009604651
GO:0007218	neuropeptide signaling pathway	30	0.256524681	0.0053	0.014634479
GO:0030501	positive regulation of bone mineralization	19	0.256517675	0.02	0.025188412
GO:0000272	polysaccharide catabolic process	11	0.256434887	0.0089	0.016916437
GO:0044247	cellular polysaccharide catabolic process	11	0.256434887	0.0089	0.016916437
GO:0060281	regulation of oocyte development	6	0.256423048	0.0036	0.01353394
GO:2000779	regulation of double-strand break repair	27	0.256379399	0.007	0.015816335
GO:0000491	small nucleolar ribonucleoprotein complex assembly	5	0.25637342	0.0275	0.031743861
GO:0048246	macrophage chemotaxis	16	0.256364981	0.0177	0.02329117
GO:0060760	positive regulation of response to cytokine stimulus	21	0.256360326	0.012	0.018931514
GO:0032098	regulation of appetite	13	0.256331596	0.0196	0.0248852
GO:1900272	negative regulation of long-term synaptic potentiation	9	0.256331119	0.0088	0.016916437
GO:0023058	adaptation of signaling pathway	10	0.256319644	0.0202	0.025368462
GO:0031167	rRNA methylation	6	0.256304916	0.0051	0.014547706
GO:0060479	lung cell differentiation	6	0.256269498	0.0165	0.022405589
GO:0060487	lung epithelial cell differentiation	6	0.256269498	0.0165	0.022405589
GO:0009954	proximal/distal pattern formation	11	0.256216842	0.0093	0.017114131
GO:0006998	nuclear envelope organization	23	0.256216145	0.0015	0.011704453
GO:1904668	positive regulation of ubiquitin protein ligase activity	5	0.256178533	0.0067	0.015576759
GO:0071709	membrane assembly	17	0.256136565	0.0013	0.011218806
GO:2001057	reactive nitrogen species metabolic process	32	0.256107038	0.0122	0.019049527
GO:0002192	IRES-dependent translational initiation	5	0.255963692	0.0374	0.040724444
GO:0009133	nucleoside diphosphate biosynthetic process	5	0.255963174	0.0457	0.0483597
GO:0009136	purine nucleoside diphosphate biosynthetic process	5	0.255963174	0.0457	0.0483597
GO:0009180	purine ribonucleoside diphosphate biosynthetic process	5	0.255963174	0.0457	0.0483597
GO:0009188	ribonucleoside diphosphate biosynthetic process	5	0.255963174	0.0457	0.0483597
GO:0006911	phagocytosis, engulfment	18	0.25594188	0.0119	0.018881943
GO:1901616	organic hydroxy compound catabolic process	18	0.255900774	0.0098	0.017418875
GO:0045671	negative regulation of osteoclast differentiation	9	0.255875579	0.0056	0.014771533
GO:1904892	regulation of STAT cascade	39	0.255872622	0.0028	0.012972436
GO:0001539	cilium or flagellum-dependent cell motility	10	0.255806643	0.0281	0.032268957
GO:0060285	cilium-dependent cell motility	10	0.255806643	0.0281	0.032268957
GO:0032400	melanosome localization	11	0.255768971	0.0088	0.016916437
GO:0010523	negative regulation of calcium ion transport into cytosol	9	0.255750463	0.0185	0.023962142
GO:0051280	negative regulation of release of sequestered calcium ion into cytosol	9	0.255750463	0.0185	0.023962142
GO:0051284	positive regulation of sequestering of calcium ion	9	0.255750463	0.0185	0.023962142
GO:0086019	cell-cell signaling involved in cardiac conduction	13	0.255741415	0.01	0.017574468
GO:0048515	spermatid differentiation	44	0.255711573	0.0074	0.015911789
GO:0051057	positive regulation of small GTPase mediated signal transduction	29	0.255703865	0.009	0.016956012
GO:0010971	positive regulation of G2/M transition of mitotic cell cycle	10	0.255701684	0.0094	0.017156187
GO:0086065	cell communication involved in cardiac conduction	20	0.255682831	0.0191	0.02440579
GO:0036017	response to erythropoietin	5	0.255656332	0.0059	0.014942532
GO:0007379	segment specification	5	0.255602168	0.0102	0.0177
GO:0046599	regulation of centriole replication	5	0.255594393	0.0191	0.02440579
GO:0046058	cAMP metabolic process	31	0.255530732	0.0009	0.010729485
GO:1903421	regulation of synaptic vesicle recycling	12	0.255509303	0.0017	0.012001709
GO:0015696	ammonium transport	36	0.255476297	0.0084	0.016633151
GO:0007130	synaptonemal complex assembly	7	0.25544075	0.0049	0.014447629
GO:0006285	base-excision repair, AP site formation	6	0.255377349	0.0013	0.011218806
GO:0070723	response to cholesterol	16	0.255350219	0.0156	0.021692929
GO:1905331	negative regulation of morphogenesis of an epithelium	5	0.255335754	0.004	0.013824268
GO:0048308	organelle inheritance	11	0.255283405	0.0017	0.012001709

GOIDlabel	GOName	size	categoryScores	pValPerm	pValPermCorr
GO:0048313	Golgi inheritance	11	0.255283405	0.0017	0.012001709
GO:0007200	phospholipase C-activating G-protein coupled receptor signaling pathway	35	0.255211359	0.0006	0.009940401
GO:0032025	response to cobalt ion	5	0.255209026	0.0001	0.004220438
GO:0007416	synapse assembly	90	0.255190986	0.0008	0.010465158
GO:0030166	proteoglycan biosynthetic process	33	0.255167451	0.0076	0.016084627
GO:1902259	regulation of delayed rectifier potassium channel activity	7	0.255165673	0.0018	0.01210186
GO:0002823	negative regulation of adaptive immune response based on somatic recombination of immune receptors built from immunoglobulin superfamily domains	11	0.255108945	0.0004	0.008964341
GO:0008206	bile acid metabolic process	14	0.255074228	0.0103	0.017772187
GO:0002862	negative regulation of inflammatory response to antigenic stimulus	6	0.255030294	0.017	0.022784886
GO:0060029	convergent extension involved in organogenesis	5	0.254944945	0.0037	0.013609033
GO:0007141	male meiosis I	7	0.25492114	0.0173	0.023005658
GO:0030148	sphingolipid biosynthetic process	41	0.254910471	0.0002	0.006319126
GO:0035809	regulation of urine volume	10	0.254864347	0.0005	0.009604651
GO:0046626	regulation of insulin receptor signaling pathway	25	0.25484885	0.0005	0.009604651
GO:0001909	leukocyte mediated cytotoxicity	30	0.254840548	0.0121	0.018970228
GO:0055081	anion homeostasis	16	0.254828422	0.0037	0.013609033
GO:0017158	regulation of calcium ion-dependent exocytosis	52	0.254808175	0.0007	0.010220707
GO:0031146	SCF-dependent proteasomal ubiquitin-dependent protein catabolic process	51	0.254805133	0.0099	0.017505138
GO:0010663	positive regulation of striated muscle cell apoptotic process	8	0.254779344	0.0002	0.006319126
GO:0010666	positive regulation of cardiac muscle cell apoptotic process	8	0.254779344	0.0002	0.006319126
GO:0051279	regulation of release of sequestered calcium ion into cytosol	32	0.254741159	0.0037	0.013609033
GO:0060122	inner ear receptor stereocilium organization	12	0.254732912	0.0162	0.022196303
GO:0006171	cAMP biosynthetic process	28	0.2547314	0.0019	0.012288367
GO:0030814	regulation of cAMP metabolic process	28	0.2547314	0.0019	0.012288367
GO:0030817	regulation of cAMP biosynthetic process	28	0.2547314	0.0019	0.012288367
GO:0045761	regulation of adenylate cyclase activity	28	0.2547314	0.0019	0.012288367
GO:0045740	positive regulation of DNA replication	18	0.254679784	0.0017	0.012001709
GO:0046322	negative regulation of fatty acid oxidation	6	0.254635728	0.0277	0.03194284
GO:0032370	positive regulation of lipid transport	23	0.254612751	0.0127	0.019457181
GO:0045838	positive regulation of membrane potential	5	0.25461141	0.0161	0.02211694
GO:0030282	bone mineralization	45	0.254600842	0.0059	0.014942532
GO:0006694	steroid biosynthetic process	81	0.254599646	0.0097	0.017363901
GO:0045956	positive regulation of calcium ion-dependent exocytosis	15	0.254454015	0.0002	0.006319126
GO:0050711	negative regulation of interleukin-1 secretion	6	0.254445463	0.0266	0.030933467
GO:0050713	negative regulation of interleukin-1 beta secretion	6	0.254445463	0.0266	0.030933467
GO:0031664	regulation of lipopolysaccharide-mediated signaling pathway	11	0.254437492	0.0147	0.02091937
GO:0070200	establishment of protein localization to telomere	8	0.254412798	0.0249	0.029484292
GO:1904888	cranial skeletal system development	25	0.254411704	0.019	0.024347961
GO:0060119	inner ear receptor cell development	19	0.254380242	0.0231	0.027930615
GO:0006303	double-strand break repair via nonhomologous end joining	28	0.254311889	0.0013	0.011218806
GO:0002448	mast cell mediated immunity	23	0.254293931	0.0158	0.021871104
GO:0031468	nuclear envelope reassembly	9	0.254285649	0.0074	0.015911789
GO:0006997	nucleus organization	54	0.25425731	0.0011	0.010890753
GO:0042551	neuron maturation	21	0.25418717	0.0019	0.012288367
GO:1902116	negative regulation of organelle assembly	16	0.254179233	0.0079	0.01629604
GO:0046890	regulation of lipid biosynthetic process	84	0.254169106	0.0062	0.015209334
GO:0097061	dendritic spine organization	39	0.254120254	0.0008	0.010465158
GO:0032401	establishment of melanosome localization	10	0.254004205	0.0069	0.015750415
GO:0032402	melanosome transport	10	0.254004205	0.0069	0.015750415
GO:0007269	neurotransmitter secretion	75	0.253972778	0.0007	0.010220707
GO:0099643	signal release from synapse	75	0.253972778	0.0007	0.010220707
GO:0006904	vesicle docking involved in exocytosis	16	0.253965914	0.0011	0.010890753
GO:0002138	retinoic acid biosynthetic process	5	0.253935528	0.0356	0.03903645
GO:0016102	diterpenoid biosynthetic process	5	0.253935528	0.0356	0.03903645
GO:0042391	regulation of membrane potential	177	0.253930238	0.0017	0.012001709
GO:0060405	regulation of penile erection	5	0.253921534	0.0351	0.038590645
GO:0090049	regulation of cell migration involved in sprouting angiogenesis	20	0.25390417	0.0029	0.013089617
GO:0072077	renal vesicle morphogenesis	6	0.253903393	0.0025	0.012769435
GO:0035563	positive regulation of chromatin binding	6	0.253842275	0.0003	0.007956881
GO:0030207	chondroitin sulfate catabolic process	10	0.253791566	0.0097	0.017363901
GO:0050996	positive regulation of lipid catabolic process	10	0.253749194	0.003	0.013210967
GO:0002042	cell migration involved in sprouting angiogenesis	28	0.253743085	0.0007	0.010220707
GO:0016601	Rac protein signal transduction	14	0.253735403	0.0152	0.0213213
GO:0010800	positive regulation of peptidyl-threonine phosphorylation	14	0.253688477	0.0032	0.013333753
GO:0090183	regulation of kidney development	21	0.253683418	0.0108	0.018152791
GO:0009415	response to water	6	0.25366756	0.0027	0.012827773
GO:0061437	renal system vasculature development	9	0.253659929	0.0402	0.043252028
GO:0061440	kidney vasculature development	9	0.253659929	0.0402	0.043252028
GO:0045931	positive regulation of mitotic cell cycle	58	0.253616488	0.0003	0.007956881
GO:0007173	epidermal growth factor receptor signaling pathway	50	0.253614588	0.0003	0.007956881
GO:0006415	translational termination	58	0.253611662	0.0205	0.025639412
GO:0006901	vesicle coating	40	0.253582653	0.0048	0.014269203
GO:0048199	vesicle targeting, to, from or within Golgi	40	0.253582653	0.0048	0.014269203
GO:0001911	negative regulation of leukocyte mediated cytotoxicity	7	0.25354483	0.0377	0.040981651
GO:0002707	negative regulation of lymphocyte mediated immunity	7	0.25354483	0.0377	0.040981651
GO:0046889	positive regulation of lipid biosynthetic process	27	0.25354302	0.0009	0.010729485
GO:0021537	telencephalon development	110	0.253497659	0.0069	0.015750415
GO:0061001	regulation of dendritic spine morphogenesis	19	0.253468622	0.0064	0.015399417
GO:0003044	regulation of systemic arterial blood pressure mediated by a chemical signal	7	0.253452644	0.0055	0.01475
GO:1901525	negative regulation of macromitophagy	5	0.253444166	0.0121	0.018970228
GO:0006949	syncytium formation	19	0.253440047	0.0128	0.019522448
GO:0007603	phototransduction, visible light	15	0.253427321	0.0001	0.004220438

GOIDlabel	GOName	size	categoryScores	pValPerm	pValPermCorr
GO:0008652	cellular amino acid biosynthetic process	35	0.253414659	0.0066	0.015525305
GO:0006304	DNA modification	31	0.2533865	0.0035	0.01351837
GO:0090394	negative regulation of excitatory postsynaptic potential	6	0.253379463	0.0031	0.013277185
GO:2000739	regulation of mesenchymal stem cell differentiation	6	0.25332612	0.0275	0.031743861
GO:0034754	cellular hormone metabolic process	48	0.253303363	0.0124	0.019242297
GO:0019886	antigen processing and presentation of exogenous peptide antigen via MHC class II	57	0.253264707	0.0088	0.016916437
GO:0009084	glutamine family amino acid biosynthetic process	10	0.253233471	0.0044	0.014132412
GO:0001824	blastocyst development	43	0.253149758	0.0047	0.014168613
GO:0007009	plasma membrane organization	143	0.253149254	0.0014	0.011465722
GO:0043931	ossification involved in bone maturation	5	0.253112445	0.0117	0.018739446
GO:0070296	sarcoplasmic reticulum calcium ion transport	16	0.253069798	0.0093	0.017114131
GO:0090239	regulation of histone H4 acetylation	7	0.253069647	0.0098	0.017418875
GO:1900076	regulation of cellular response to insulin stimulus	26	0.253030482	0.0003	0.007956881
GO:0009713	catechol-containing compound biosynthetic process	9	0.253006421	0.0097	0.017363901
GO:0042423	catecholamine biosynthetic process	9	0.253006421	0.0097	0.017363901
GO:0048643	positive regulation of skeletal muscle tissue development	7	0.252995047	0.0017	0.012001709
GO:1990823	response to leukemia inhibitory factor	35	0.252956692	0.0052	0.014616626
GO:1990830	cellular response to leukemia inhibitory factor	35	0.252956692	0.0052	0.014616626
GO:0099509	regulation of presynaptic cytosolic calcium ion concentration	8	0.252949532	0.0007	0.010220707
GO:0071322	cellular response to carbohydrate stimulus	64	0.252944673	0.0005	0.009604651
GO:0070571	negative regulation of neuron projection regeneration	8	0.252926856	0.0035	0.01351837
GO:1901160	primary amino compound metabolic process	7	0.25291767	0.0013	0.011218806
GO:1900543	negative regulation of purine nucleotide metabolic process	22	0.252891266	0.0057	0.01480566
GO:2000179	positive regulation of neural precursor cell proliferation	26	0.252872147	0.0146	0.020879842
GO:0032091	negative regulation of protein binding	45	0.252848278	0.0038	0.01366393
GO:0021591	ventricular system development	13	0.252832874	0.0357	0.03911643
GO:2000696	regulation of epithelial cell differentiation involved in kidney development	8	0.252799383	0.0037	0.013609033
GO:0048207	vesicle targeting, rough ER to cis-Golgi	38	0.252736469	0.0046	0.014132412
GO:0048208	COPII vesicle coating	38	0.252736469	0.0046	0.014132412
GO:0002495	antigen processing and presentation of peptide antigen via MHC class II	60	0.252729756	0.011	0.018323826
GO:0002504	antigen processing and presentation of peptide or polysaccharide antigen via MHC class II	60	0.252729756	0.011	0.018323826
GO:0031532	actin cytoskeleton reorganization	43	0.252698191	0.0126	0.019370699
GO:1901021	positive regulation of calcium ion transmembrane transporter activity	17	0.252697092	0.0046	0.014132412
GO:0090382	phagosome maturation	21	0.252675543	0.0045	0.014132412
GO:0010613	positive regulation of cardiac muscle hypertrophy	13	0.252632526	0.0025	0.012769435
GO:0014742	positive regulation of muscle hypertrophy	13	0.252632526	0.0025	0.012769435
GO:0021766	hippocampus development	40	0.252622832	0.0061	0.015143924
GO:0006888	ER to Golgi vesicle-mediated transport	97	0.252587168	0.0044	0.014132412
GO:0045428	regulation of nitric oxide biosynthetic process	21	0.25257188	0.018	0.023514686
GO:1902916	positive regulation of protein polyubiquitination	5	0.252520271	0.0102	0.0177
GO:0000082	G1/S transition of mitotic cell cycle	89	0.252479513	0.0008	0.010465158
GO:1902745	positive regulation of lamellipodium organization	15	0.252479497	0.0023	0.012498684
GO:0001574	ganglioside biosynthetic process	6	0.252469606	0.0016	0.011845327
GO:0045851	pH reduction	24	0.252468148	0.0094	0.017156187
GO:0030325	adrenal gland development	13	0.252455801	0.0176	0.023223003
GO:0071305	cellular response to vitamin D	6	0.252420582	0.029	0.033098697
GO:0045907	positive regulation of vasoconstriction	7	0.252370293	0.0105	0.017924712
GO:0048599	oocyte development	18	0.252363063	0.0012	0.010961137
GO:0001738	morphogenesis of a polarized epithelium	86	0.252344472	0.0084	0.016633151
GO:0050853	B cell receptor signaling pathway	22	0.252312403	0.013	0.019676963
GO:0034219	carbohydrate transmembrane transport	9	0.252300362	0.012	0.018931514
GO:0007045	cell-substrate adherens junction assembly	36	0.25228474	0.0009	0.010729485
GO:0048041	focal adhesion assembly	36	0.25228474	0.0009	0.010729485
GO:0097484	dendrite extension	21	0.252274096	0.0021	0.012314604
GO:0001522	pseudouridine synthesis	11	0.252263163	0.0101	0.017653628
GO:0034381	plasma lipoprotein particle clearance	25	0.252245357	0.0145	0.020788247
GO:0051865	protein autoubiquitination	31	0.252239646	0.0028	0.012972436
GO:0035637	multicellular organismal signaling	79	0.252221692	0.0012	0.010961137
GO:0046677	response to antibiotic	13	0.252153384	0.0018	0.01210186
GO:0032784	regulation of DNA-templated transcription, elongation	18	0.252139108	0.0043	0.014102439
GO:0002712	regulation of B cell mediated immunity	18	0.252135365	0.0025	0.012769435
GO:0002889	regulation of immunoglobulin mediated immune response	18	0.252135365	0.0025	0.012769435
GO:0006282	regulation of DNA repair	44	0.252110585	0.0024	0.012603815
GO:0030212	hyaluronan metabolic process	14	0.252097808	0.0073	0.015844069
GO:0046887	positive regulation of hormone secretion	60	0.252093115	0.0012	0.010961137
GO:0009108	coenzyme biosynthetic process	65	0.25202147	0.0061	0.015143924
GO:0006862	nucleotide transport	8	0.252014299	0.0008	0.010465158
GO:1900242	regulation of synaptic vesicle endocytosis	9	0.25200521	0.0006	0.009940401
GO:0060706	cell differentiation involved in embryonic placenta development	7	0.252000068	0.0032	0.013333753
GO:0031648	protein destabilization	23	0.251994024	0.0004	0.008964341
GO:0048679	regulation of axon regeneration	15	0.251965415	0.007	0.015816335
GO:0071276	cellular response to cadmium ion	20	0.251939435	0.0162	0.022196303
GO:0050775	positive regulation of dendrite morphogenesis	17	0.251825867	< 0.001	< 0.001
GO:0032305	positive regulation of icosanoid secretion	6	0.251784999	0.01	0.017574468
GO:2000193	positive regulation of fatty acid transport	6	0.251784999	0.01	0.017574468
GO:0060444	branching involved in mammary gland duct morphogenesis	8	0.251760649	0.0183	0.023783007
GO:0048714	positive regulation of oligodendrocyte differentiation	9	0.251759443	0.0304	0.034411276
GO:0072659	protein localization to plasma membrane	113	0.251735921	0.0009	0.010729485
GO:0032481	positive regulation of type I interferon production	36	0.251640159	0.0088	0.016916437
GO:0071482	cellular response to light stimulus	47	0.251627441	0.0035	0.01351837
GO:0045844	positive regulation of striated muscle tissue development	28	0.251606659	0.0062	0.015209334
GO:0048636	positive regulation of muscle organ development	28	0.251606659	0.0062	0.015209334
GO:1901863	positive regulation of muscle tissue development	28	0.251606659	0.0062	0.015209334

GOIDlabel	GOName	size	categoryScores	pValPerm	pValPermCorr
GO:0090195	chemokine secretion	9	0.251605677	0.0235	0.028248857
GO:0090196	regulation of chemokine secretion	9	0.251605677	0.0235	0.028248857
GO:0090197	positive regulation of chemokine secretion	9	0.251605677	0.0235	0.028248857
GO:0006865	amino acid transport	57	0.251605146	0.0005	0.009604651
GO:1903306	negative regulation of regulated secretory pathway	11	0.251574159	0.0397	0.042801678
GO:0043650	dicarboxylic acid biosynthetic process	8	0.251548626	0.0075	0.015990044
GO:0046209	nitric oxide metabolic process	29	0.25154122	0.0125	0.019314538
GO:1901617	organic hydroxy compound biosynthetic process	105	0.251532808	0.009	0.016956012
GO:0032196	transposition	7	0.251515074	0.0071	0.01583804
GO:1901888	regulation of cell junction assembly	36	0.251487148	0.003	0.013210967
GO:1903391	regulation of adherens junction organization	30	0.251471635	0.002	0.012314604
GO:1904263	positive regulation of TORC1 signaling	8	0.251463428	0.0099	0.017505138
GO:0060538	skeletal muscle organ development	66	0.251457558	0.0017	0.012001709
GO:0072234	metanephric nephron tubule development	12	0.25145371	0.0217	0.026655917
GO:0045913	positive regulation of carbohydrate metabolic process	29	0.251447119	0.0094	0.017156187
GO:2000279	negative regulation of DNA biosynthetic process	20	0.251424425	0.0012	0.010961137
GO:1990126	retrograde transport, endosome to plasma membrane	8	0.251423907	0.0025	0.012769435
GO:2000045	regulation of G1/S transition of mitotic cell cycle	52	0.251412394	0.0016	0.011845327
GO:0030168	platelet activation	71	0.251410374	0.0033	0.013333753
GO:0043627	response to estrogen	36	0.251394176	0.0018	0.01210186
GO:0060020	Bergmann glial cell differentiation	5	0.251373241	0.0182	0.023684988
GO:1901607	alpha-amino acid biosynthetic process	28	0.251368188	0.0075	0.015990044
GO:0048144	fibroblast proliferation	39	0.251365447	0.0093	0.017114131
GO:0045070	positive regulation of viral genome replication	15	0.251360024	0.0065	0.015491756
GO:0016051	carbohydrate biosynthetic process	91	0.251347676	0.0033	0.013333753
GO:0072273	metanephric nephron morphogenesis	11	0.251345276	0.0105	0.017924712
GO:0006270	DNA replication initiation	10	0.25133981	0.0003	0.007956881
GO:1905037	autophagosome organization	40	0.251315352	0.0026	0.012805111
GO:0048278	vesicle docking	20	0.251302297	0.001	0.010787313
GO:0071359	cellular response to dsRNA	25	0.251284143	0.0089	0.016916437
GO:0050704	regulation of interleukin-1 secretion	17	0.251280265	0.0121	0.018970228
GO:0050766	positive regulation of phagocytosis	20	0.251277807	0.0102	0.0177
GO:0061311	cell surface receptor signaling pathway involved in heart development	13	0.251276436	0.0228	0.027701114
GO:0001954	positive regulation of cell-matrix adhesion	29	0.251269909	0.0038	0.01366393
GO:0032615	interleukin-12 production	15	0.251265864	0.0014	0.011465722
GO:0048247	lymphocyte chemotaxis	9	0.251259414	0.0154	0.021487162
GO:0010867	positive regulation of triglyceride biosynthetic process	7	0.251054145	0.0074	0.015911789
GO:0090208	positive regulation of triglyceride metabolic process	7	0.251054145	0.0074	0.015911789
GO:0032651	regulation of interleukin-1 beta production	30	0.251048172	0.028	0.032186083
GO:0033059	cellular pigmentation	26	0.251002427	0.0062	0.015209334
GO:0009566	fertilization	50	0.250995857	0.0022	0.012422266
GO:0071453	cellular response to oxygen levels	112	0.2509871	0.0066	0.015525305
GO:0071397	cellular response to cholesterol	10	0.250970252	0.0099	0.017505138
GO:1902991	regulation of amyloid precursor protein catabolic process	15	0.25083359	0.0001	0.004220438
GO:0051204	protein insertion into mitochondrial membrane	12	0.250813591	0.006	0.015037711
GO:0044788	modulation by host of viral process	10	0.250810352	0.0158	0.021871104
GO:0016093	polyprenol metabolic process	5	0.250804651	0.0109	0.018225506
GO:0035335	peptidyl-tyrosine dephosphorylation	48	0.250804467	0.0019	0.012288367
GO:0010863	positive regulation of phospholipase C activity	19	0.250735196	0.0089	0.016916437
GO:1900274	regulation of phospholipase C activity	19	0.250735196	0.0089	0.016916437
GO:0060998	regulation of dendritic spine development	29	0.250730505	0.0007	0.010220707
GO:0006925	inflammatory cell apoptotic process	8	0.250711224	0.0038	0.01366393
GO:0048148	behavioral response to cocaine	7	0.250709651	0.0002	0.006319126
GO:0055025	positive regulation of cardiac muscle tissue development	20	0.250671962	0.0102	0.0177
GO:0044843	cell cycle G1/S phase transition	99	0.250663012	0.001	0.010787313
GO:0016241	regulation of macroautophagy	86	0.250608918	0.0039	0.013791927
GO:0016079	synaptic vesicle exocytosis	55	0.250602956	0.0005	0.009604651
GO:0021544	subpallium development	13	0.250590866	0.0046	0.014132412
GO:0097205	renal filtration	10	0.250569077	0.0062	0.015209334
GO:0035272	exocrine system development	22	0.250542478	0.0056	0.014771533
GO:0060294	cilium movement involved in cell motility	5	0.250530462	0.0227	0.027637692
GO:0001763	morphogenesis of a branching structure	69	0.250521411	0.0068	0.015633241
GO:0017156	calcium ion regulated exocytosis	71	0.250502795	0.0007	0.010220707
GO:0071229	cellular response to acid chemical	102	0.250493405	0.001	0.010787313
GO:0001820	serotonin secretion	5	0.250470338	0.0245	0.029070183
GO:0050912	detection of chemical stimulus involved in sensory perception of taste	5	0.250449346	0.0005	0.009604651
GO:0051875	pigment granule localization	12	0.250361794	0.0057	0.01480566
GO:0051966	regulation of synaptic transmission, glutamatergic	25	0.250311423	< 0.001	< 0.001
GO:1901685	glutathione derivative metabolic process	13	0.250295825	0.0194	0.024690909
GO:1901687	glutathione derivative biosynthetic process	13	0.250295825	0.0194	0.024690909
GO:0006399	tRNA metabolic process	71	0.250248072	0.0057	0.01480566
GO:0003382	epithelial cell morphogenesis	12	0.25022023	0.0112	0.018392048
GO:0060134	prepulse inhibition	10	0.25020509	0.0003	0.007956881
GO:0035115	embryonic forelimb morphogenesis	10	0.250194724	0.0167	0.02255007
GO:0060315	negative regulation of ryanodine-sensitive calcium-release channel activity	7	0.25017986	0.012	0.018931514
GO:0050710	negative regulation of cytokine secretion	29	0.250134399	0.009	0.016956012
GO:1903909	regulation of receptor clustering	9	0.250132512	0.0359	0.039290895
GO:0006521	regulation of cellular amino acid metabolic process	46	0.25013167	0.0155	0.021605834
GO:0061081	positive regulation of myeloid leukocyte cytokine production involved in immune response	11	0.250123703	0.0165	0.022405589
GO:0035066	positive regulation of histone acetylation	11	0.250091898	0.0184	0.023875404
GO:0019184	nonribosomal peptide biosynthetic process	10	0.250077974	0.0128	0.019522448
GO:1902074	response to salt	8	0.250033366	0.0032	0.013333753
GO:0006086	acetyl-CoA biosynthetic process from pyruvate	7	0.250031955	0.0174	0.023064374

GOIDlabel	GOName	size	categoryScores	pValPerm	pValPermCorr
GO:0000423	macromitophagy	17	0.250028155	0.0057	0.01480566
GO:0072207	metanephric epithelium development	14	0.25002742	0.022	0.026944291
GO:0007004	telomere maintenance via telomerase	31	0.250025942	0.0067	0.015576759
GO:0030808	regulation of nucleotide biosynthetic process	39	0.250005005	0.0044	0.014132412
GO:1900371	regulation of purine nucleotide biosynthetic process	39	0.250005005	0.0044	0.014132412
GO:0030574	collagen catabolic process	9	0.249967227	0.0023	0.012498684
GO:0044243	multicellular organism catabolic process	9	0.249967227	0.0023	0.012498684
GO:0032801	receptor catabolic process	28	0.249934123	0.0018	0.01210186
GO:0043486	histone exchange	16	0.249891964	0.0042	0.013948535
GO:0090114	COPII-coated vesicle budding	43	0.249886818	0.0041	0.013887639
GO:0007084	mitotic nuclear envelope reassembly	7	0.249864614	0.0094	0.017156187
GO:0006900	membrane budding	70	0.24986354	0.006	0.015037711
GO:0006378	mRNA polyadenylation	13	0.249825256	0.0089	0.016916437
GO:0043631	RNA polyadenylation	13	0.249825256	0.0089	0.016916437
GO:0050709	negative regulation of protein secretion	60	0.249783809	0.0089	0.016916437
GO:0014819	regulation of skeletal muscle contraction	7	0.249723373	0.0185	0.023962142
GO:0032924	activin receptor signaling pathway	11	0.24972201	0.0062	0.015209334
GO:0099531	presynaptic process involved in chemical synaptic transmission	78	0.249707522	0.0008	0.010465158
GO:0032543	mitochondrial translation	71	0.249633176	0.0172	0.022941269
GO:0030534	adult behavior	69	0.249614755	0.001	0.010787313
GO:0031058	positive regulation of histone modification	36	0.249614486	0.0055	0.01475
GO:0036473	cell death in response to oxidative stress	36	0.24961301	0.0066	0.015525305
GO:0060993	kidney morphogenesis	35	0.249601051	0.0092	0.017033109
GO:0006809	nitric oxide biosynthetic process	27	0.249584059	0.0119	0.018881943
GO:1904894	positive regulation of STAT cascade	27	0.249549313	0.005	0.014547706
GO:0007259	JAK-STAT cascade	47	0.249544204	0.0026	0.012805111
GO:0070528	protein kinase C signaling	12	0.249484979	0.0148	0.020979063
GO:0010453	regulation of cell fate commitment	9	0.249453376	0.0041	0.013887639
GO:0001964	startle response	14	0.249404702	< 0.001	< 0.001
GO:1903010	regulation of bone development	12	0.249386175	0.0024	0.012603815
GO:1903307	positive regulation of regulated secretory pathway	28	0.249355185	0.0011	0.010890753
GO:1903861	positive regulation of dendrite extension	15	0.249340672	0.004	0.013824268
GO:0060314	regulation of ryanodine-sensitive calcium-release channel activity	14	0.24933945	0.0111	0.018389742
GO:0035384	thioester biosynthetic process	21	0.249336858	0.0119	0.018881943
GO:0071616	acyl-CoA biosynthetic process	21	0.249336858	0.0119	0.018881943
GO:0035850	epithelial cell differentiation involved in kidney development	17	0.249310747	0.0066	0.015525305
GO:0006563	L-serine metabolic process	6	0.249246123	0.0068	0.015633241
GO:0010675	regulation of cellular carbohydrate metabolic process	55	0.249230436	0.0011	0.010890753
GO:0031032	actomyosin structure organization	64	0.249211514	0.003	0.013210967
GO:0043403	skeletal muscle tissue regeneration	13	0.249196102	0.0058	0.014878261
GO:0061138	morphogenesis of a branching epithelium	68	0.24915632	0.0066	0.015525305
GO:0031639	plasminogen activation	5	0.249140343	0.0323	0.03615849
GO:0048520	positive regulation of behavior	11	0.249117938	0.0013	0.011218806
GO:0001101	response to acid chemical	158	0.249076394	0.0032	0.013333753
GO:0051307	meiotic chromosome separation	5	0.249046788	0.0153	0.021378589
GO:0030091	protein repair	5	0.249042123	0.0205	0.025639412
GO:0046503	glycerolipid catabolic process	22	0.249036834	0.0075	0.015990044
GO:0010640	regulation of platelet-derived growth factor receptor signaling pathway	8	0.249021844	0.008	0.016379603
GO:0070306	lens fiber cell differentiation	16	0.248990502	0.0082	0.016514246
GO:0051438	regulation of ubiquitin-protein transferase activity	21	0.248956021	0.0045	0.014132412
GO:0070374	positive regulation of ERK1 and ERK2 cascade	81	0.248955299	0.014	0.020462083
GO:0034453	microtubule anchoring	8	0.248918829	0.0032	0.013333753
GO:0001505	regulation of neurotransmitter levels	98	0.248877595	0.0012	0.010961137
GO:0045830	positive regulation of isotype switching	11	0.248864789	0.0035	0.01351837
GO:1903076	regulation of protein localization to plasma membrane	43	0.248801432	0.0033	0.013333753
GO:0045069	regulation of viral genome replication	30	0.24879277	0.0044	0.014132412
GO:0007026	negative regulation of microtubule depolymerization	8	0.24878925	0.0062	0.015209334
GO:0031114	regulation of microtubule depolymerization	8	0.24878925	0.0062	0.015209334
GO:0048715	negative regulation of oligodendrocyte differentiation	8	0.248745517	0.0215	0.026489026
GO:0031440	regulation of mRNA 3'-end processing	12	0.248735583	0.0119	0.018881943
GO:1900151	regulation of nuclear-transcribed mRNA catabolic process, deadenylation-dependent decay	8	0.248729482	0.0074	0.015911789
GO:1900153	positive regulation of nuclear-transcribed mRNA catabolic process, deadenylation-dependent decay	8	0.248729482	0.0074	0.015911789
GO:0051341	regulation of oxidoreductase activity	37	0.248718604	0.0061	0.015143924
GO:0014808	release of sequestered calcium ion into cytosol by sarcoplasmic reticulum	14	0.248718306	0.0127	0.019457181
GO:1903514	calcium ion transport from endoplasmic reticulum to cytosol	14	0.248718306	0.0127	0.019457181
GO:0009069	serine family amino acid metabolic process	20	0.248701007	0.0092	0.017033109
GO:1902806	regulation of cell cycle G1/S phase transition	62	0.248683968	0.0019	0.012288367
GO:0007519	skeletal muscle tissue development	61	0.248673644	0.0018	0.01210186
GO:0071478	cellular response to radiation	78	0.24864119	0.0026	0.012805111
GO:0002183	cytoplasmic translational initiation	11	0.248623419	0.0253	0.029811412
GO:1901800	positive regulation of proteasomal protein catabolic process	48	0.248608596	0.0025	0.012769435
GO:0030858	positive regulation of epithelial cell differentiation	19	0.248504331	0.007	0.015816335
GO:0046580	negative regulation of Ras protein signal transduction	17	0.248484112	0.0046	0.014132412
GO:0046532	regulation of photoreceptor cell differentiation	6	0.2484639	0.0112	0.018392048
GO:0046533	negative regulation of photoreceptor cell differentiation	6	0.2484639	0.0112	0.018392048
GO:2000027	regulation of organ morphogenesis	127	0.248457552	0.0073	0.015844069
GO:0016236	macroautophagy	153	0.248430112	0.0039	0.013791927
GO:0002088	lens development in camera-type eye	29	0.248422442	0.0096	0.017302743
GO:0048168	regulation of neuronal synaptic plasticity	29	0.248415025	0.0031	0.013277185
GO:0046513	ceramide biosynthetic process	22	0.248413973	0.0004	0.008964341
GO:0032486	Rap protein signal transduction	9	0.248401054	0.0029	0.013089617
GO:0002011	morphogenesis of an epithelial sheet	21	0.248390256	0.0101	0.017653628
GO:0040036	regulation of fibroblast growth factor receptor signaling pathway	11	0.248370623	0.0109	0.018225506

GOIDlabel	GOName	size	categoryScores	pValPerm	pValPermCorr
GO:0043954	cellular component maintenance	26	0.24834811	0.0004	0.008964341
GO:0031279	regulation of cyclase activity	31	0.248335903	0.0018	0.01210186
GO:0001510	RNA methylation	20	0.248285578	0.0014	0.011465722
GO:0072282	metanephric nephron tubule morphogenesis	5	0.248275536	0.0337	0.03733539
GO:0051904	pigment granule transport	11	0.2482659	0.0037	0.013609033
GO:0051905	establishment of pigment granule localization	11	0.2482659	0.0037	0.013609033
GO:2000095	regulation of Wnt signaling pathway, planar cell polarity pathway	7	0.248256358	0.0473	0.049788567
GO:0014911	positive regulation of smooth muscle cell migration	15	0.248250138	0.0097	0.017363901
GO:0009636	response to toxic substance	82	0.248211369	0.0094	0.017156187
GO:1903531	negative regulation of secretion by cell	92	0.24818616	0.0103	0.017772187
GO:0048145	regulation of fibroblast proliferation	38	0.248175338	0.0091	0.016994897
GO:0035561	regulation of chromatin binding	9	0.248137146	0.0006	0.009940401
GO:0030032	lamellipodium assembly	27	0.248074564	0.0056	0.014771533
GO:0010742	macrophage derived foam cell differentiation	11	0.248060106	0.0058	0.014878261
GO:0090077	foam cell differentiation	11	0.248060106	0.0058	0.014878261
GO:0032957	inositol trisphosphate metabolic process	6	0.248058319	0.0051	0.014547706
GO:0032959	inositol trisphosphate biosynthetic process	6	0.248058319	0.0051	0.014547706
GO:0015800	acidic amino acid transport	13	0.248028898	0.0054	0.014720792
GO:0051385	response to mineralocorticoid	14	0.24802756	0.0301	0.034151923
GO:0002831	regulation of response to biotic stimulus	77	0.247990487	0.0094	0.017156187
GO:0099068	postsynapse assembly	8	0.247987807	0.0011	0.010890753
GO:0042491	auditory receptor cell differentiation	12	0.247930036	0.0227	0.027637692
GO:0071277	cellular response to calcium ion	31	0.247920833	0.0053	0.014634479
GO:0046461	neutral lipid catabolic process	14	0.247910013	0.0168	0.022626974
GO:0046464	acylglycerol catabolic process	14	0.247910013	0.0168	0.022626974
GO:0031053	primary miRNA processing	5	0.24789302	0.0421	0.045028154
GO:0043200	response to amino acid	53	0.24785288	0.0053	0.014634479
GO:0090075	relaxation of muscle	12	0.247802833	0.0189	0.024289798
GO:0048754	branching morphogenesis of an epithelial tube	51	0.24769635	0.0085	0.016693954
GO:0018197	peptidyl-aspartic acid modification	5	0.247678438	0.0029	0.013089617
GO:0009451	RNA modification	51	0.247677498	0.0021	0.012314604
GO:0019054	modulation by virus of host process	15	0.247612871	0.0024	0.012603815
GO:0044068	modulation by symbiont of host cellular process	15	0.247612871	0.0024	0.012603815
GO:2000021	regulation of ion homeostasis	85	0.24759537	0.0026	0.012805111
GO:0033014	tetrapyrrole biosynthetic process	18	0.247566928	0.015	0.021138192
GO:0061136	regulation of proteasomal protein catabolic process	82	0.247542222	0.0034	0.01347416
GO:0002026	regulation of the force of heart contraction	8	0.24750545	0.0168	0.022626974
GO:0001773	myeloid dendritic cell activation	11	0.247498795	0.0377	0.040981651
GO:0050862	positive regulation of T cell receptor signaling pathway	8	0.247489253	0.0045	0.014132412
GO:0043301	negative regulation of leukocyte degranulation	7	0.247479812	0.0427	0.045577146
GO:0040037	negative regulation of fibroblast growth factor receptor signaling pathway	8	0.247448112	0.0166	0.02247277
GO:0032094	response to food	15	0.247446233	0.0149	0.021043429
GO:0050691	regulation of defense response to virus by host	13	0.247368147	0.0206	0.025725529
GO:0006813	potassium ion transport	75	0.247360936	0.0002	0.006319126
GO:0035987	endodermal cell differentiation	18	0.247343765	0.0044	0.014132412
GO:0010827	regulation of glucose transport	31	0.247339736	0.0091	0.016994897
GO:0045980	negative regulation of nucleotide metabolic process	23	0.247274603	0.0044	0.014132412
GO:0006704	glucocorticoid biosynthetic process	7	0.247241758	0.0083	0.016559903
GO:0060159	regulation of dopamine receptor signaling pathway	6	0.247125353	0.0094	0.017156187
GO:0006754	ATP biosynthetic process	23	0.247108292	0.0236	0.028327839
GO:0010976	positive regulation of neuron projection development	119	0.2471079	0.0012	0.010961137
GO:0046329	negative regulation of JNK cascade	18	0.247104909	0.0002	0.006319126
GO:0002526	acute inflammatory response	57	0.247073533	0.0074	0.015911789
GO:0050999	regulation of nitric-oxide synthase activity	20	0.247063717	0.0061	0.015143924
GO:0051193	regulation of cofactor metabolic process	35	0.247024547	0.0046	0.014132412
GO:0042572	retinol metabolic process	15	0.246999964	0.0098	0.017418875
GO:0044839	cell cycle G2/M phase transition	121	0.246988855	0.0077	0.016177834
GO:0006383	transcription from RNA polymerase III promoter	28	0.246987784	0.0033	0.013333753
GO:0051181	cofactor transport	11	0.246985899	0.0123	0.019143634
GO:0051382	kinetochore assembly	8	0.246966565	0.0023	0.012498684
GO:0050777	negative regulation of immune response	56	0.24694618	0.007	0.015816335
GO:0045824	negative regulation of innate immune response	22	0.246919402	0.01	0.017574468
GO:0000768	syncytium formation by plasma membrane fusion	18	0.246899023	0.0143	0.020629391
GO:0000726	non-recombinational repair	29	0.246875855	0.0009	0.010729485
GO:0046849	bone remodeling	35	0.246875458	0.0018	0.01210186
GO:0001952	regulation of cell-matrix adhesion	44	0.246863447	0.002	0.012314604
GO:0001678	cellular glucose homeostasis	60	0.246851295	0.0007	0.010220707
GO:1905517	macrophage migration	23	0.246833585	0.0312	0.035137982
GO:0047484	regulation of response to osmotic stress	7	0.2468247	0.0046	0.014132412
GO:0001754	eye photoreceptor cell differentiation	17	0.246788995	0.0011	0.010890753
GO:0032011	ARF protein signal transduction	10	0.246784346	0.0015	0.011704453
GO:0032012	regulation of ARF protein signal transduction	10	0.246784346	0.0015	0.011704453
GO:0046847	filopodium assembly	33	0.246767147	0.007	0.015816335
GO:0003341	cilium movement	21	0.246718939	0.0139	0.020388077
GO:1903672	positive regulation of sprouting angiogenesis	15	0.246708067	0.0041	0.013887639
GO:2000310	regulation of N-methyl-D-aspartate selective glutamate receptor activity	23	0.246698148	0.0014	0.011465722
GO:0010866	regulation of triglyceride biosynthetic process	10	0.24668768	0.0076	0.016084627
GO:0036465	synaptic vesicle recycling	29	0.246679744	0.0004	0.008964341
GO:0051897	positive regulation of protein kinase B signaling	58	0.246653158	0.0127	0.019457181
GO:0015810	aspartate transport	8	0.246635006	0.0047	0.014168613
GO:0070371	ERK1 and ERK2 cascade	129	0.246619731	0.0114	0.018551872
GO:0034390	smooth muscle cell apoptotic process	14	0.24658609	0.0039	0.013791927
GO:0034391	regulation of smooth muscle cell apoptotic process	14	0.24658609	0.0039	0.013791927

GOIDlabel	GOName	size	categoryScores	pValPerm	pValPermCorr
GO:0033555	multicellular organismal response to stress	37	0.24657772	0.0187	0.024129301
GO:0006700	C21-steroid hormone biosynthetic process	13	0.246563265	0.0283	0.032434212
GO:0048706	embryonic skeletal system development	41	0.246537451	0.0132	0.019860109
GO:0032890	regulation of organic acid transport	20	0.24653387	0.0007	0.010220707
GO:0050808	synapse organization	174	0.246530848	0.0021	0.012314604
GO:0006699	bile acid biosynthetic process	10	0.246496163	0.0168	0.022626974
GO:0010092	specification of animal organ identity	5	0.246462472	0.0291	0.033199724
GO:0060026	convergent extension	9	0.246428868	0.0053	0.014634479
GO:0001706	endoderm formation	20	0.246408956	0.0026	0.012805111
GO:0060384	innervation	10	0.246359069	0.006	0.015037711
GO:1901215	negative regulation of neuron death	95	0.246347829	0.0012	0.010961137
GO:0071333	cellular response to glucose stimulus	57	0.246335685	0.0008	0.010465158
GO:1903729	regulation of plasma membrane organization	51	0.24632579	0.004	0.013824268
GO:0030521	androgen receptor signaling pathway	20	0.246307043	0.0042	0.013948535
GO:0007030	Golgi organization	47	0.246270249	0.0015	0.011704453
GO:0051775	response to redox state	11	0.246249422	0.001	0.010787313
GO:0070169	positive regulation of biomineral tissue development	21	0.246218828	0.016	0.022026667
GO:0043255	regulation of carbohydrate biosynthetic process	38	0.246205845	0.0031	0.013277185
GO:0007528	neuromuscular junction development	18	0.2461903	0.0038	0.01366393
GO:0048678	response to axon injury	36	0.2461885	0.0118	0.018836996
GO:0034143	regulation of toll-like receptor 4 signaling pathway	6	0.246135589	0.0068	0.015633241
GO:0055089	fatty acid homeostasis	6	0.246116368	0.0014	0.011465722
GO:0001776	leukocyte homeostasis	29	0.246107499	0.0031	0.013277185
GO:0070304	positive regulation of stress-activated protein kinase signaling cascade	51	0.246103169	0.0056	0.014771533
GO:0002714	positive regulation of B cell mediated immunity	16	0.246090209	0.0059	0.014942532
GO:0002891	positive regulation of immunoglobulin mediated immune response	16	0.246090209	0.0059	0.014942532
GO:0039528	cytoplasmic pattern recognition receptor signaling pathway in response to virus	11	0.246066951	0.0132	0.019860109
GO:0003091	renal water homeostasis	15	0.245985537	0.0042	0.013948535
GO:0045820	negative regulation of glycolytic process	6	0.245948996	0.0021	0.012314604
GO:0051195	negative regulation of cofactor metabolic process	6	0.245948996	0.0021	0.012314604
GO:0051198	negative regulation of coenzyme metabolic process	6	0.245948996	0.0021	0.012314604
GO:0030199	collagen fibril organization	19	0.245937652	0.0009	0.010729485
GO:0032261	purine nucleotide salvage	5	0.245921352	0.0169	0.022703485
GO:0010880	regulation of release of sequestered calcium ion into cytosol by sarcoplasmic reticulum	12	0.245904075	0.0073	0.015844069
GO:0050920	regulation of chemotaxis	72	0.245853593	0.0179	0.02344231
GO:0002062	chondrocyte differentiation	47	0.245851793	0.0121	0.018970228
GO:0090670	RNA localization to Cajal body	12	0.245842093	0.0228	0.027701114
GO:0090671	telomerase RNA localization to Cajal body	12	0.245842093	0.0228	0.027701114
GO:0090672	telomerase RNA localization	12	0.245842093	0.0228	0.027701114
GO:1904872	regulation of telomerase RNA localization to Cajal body	12	0.245842093	0.0228	0.027701114
GO:0050796	regulation of insulin secretion	75	0.245821179	0.0018	0.01210186
GO:0060563	neuroepithelial cell differentiation	19	0.245803504	0.0309	0.034874839
GO:0002260	lymphocyte homeostasis	17	0.245791697	0.0043	0.014102439
GO:1903859	regulation of dendrite extension	16	0.245759865	0.0038	0.01366393
GO:0010324	membrane invagination	28	0.245728743	0.0084	0.016633151
GO:0007605	sensory perception of sound	46	0.245718167	0.0184	0.023875404
GO:0071326	cellular response to monosaccharide stimulus	58	0.245680939	0.0008	0.010465158
GO:0071331	cellular response to hexose stimulus	58	0.245680939	0.0008	0.010465158
GO:0044321	response to leptin	8	0.245680822	0.0204	0.025564109
GO:0007628	adult walking behavior	11	0.245676449	0.0007	0.010220707
GO:0046467	membrane lipid biosynthetic process	57	0.245675449	0.0027	0.012827773
GO:0045576	mast cell activation	27	0.245673857	0.0179	0.02344231
GO:0007599	hemostasis	125	0.245673111	0.0033	0.013333753
GO:0071287	cellular response to manganese ion	6	0.245645782	0.0095	0.01725157
GO:0010522	regulation of calcium ion transport into cytosol	40	0.245625006	0.0049	0.014447629
GO:0009110	vitamin biosynthetic process	8	0.245525976	0.0344	0.037979912
GO:0016322	neuron remodeling	7	0.245478934	0.0216	0.026572596
GO:0031398	positive regulation of protein ubiquitination	56	0.245456559	0.0058	0.014878261
GO:0060028	convergent extension involved in axis elongation	5	0.24545176	0.0132	0.019860109
GO:0019228	neuronal action potential	8	0.245424256	0.0011	0.010890753
GO:0048873	homeostasis of number of cells within a tissue	9	0.245411245	0.0066	0.015525305
GO:0044728	DNA methylation or demethylation	21	0.245382984	0.0056	0.014771533
GO:2000050	regulation of non-canonical Wnt signaling pathway	8	0.245370319	0.0362	0.03958177
GO:1904375	regulation of protein localization to cell periphery	46	0.245359207	0.002	0.012314604
GO:0048813	dendrite morphogenesis	67	0.245310264	0.0006	0.009940401
GO:0061025	membrane fusion	66	0.245298354	0.0027	0.012827773
GO:0071539	protein localization to centrosome	12	0.245285121	0.0019	0.012288367
GO:1905508	protein localization to microtubule organizing center	12	0.245285121	0.0019	0.012288367
GO:0034308	primary alcohol metabolic process	24	0.245250891	0.0068	0.015633241
GO:1902902	negative regulation of autophagosome assembly	10	0.245243397	0.0153	0.021378589
GO:0022612	gland morphogenesis	51	0.245205215	0.0088	0.016916437
GO:1902749	regulation of cell cycle G2/M phase transition	96	0.245175452	0.0105	0.017924712
GO:0014068	positive regulation of phosphatidylinositol 3-kinase signaling	35	0.245159578	0.0098	0.017418875
GO:0072028	nephron morphogenesis	25	0.245146732	0.0108	0.018152791
GO:0072078	nephron tubule morphogenesis	25	0.245146732	0.0108	0.018152791
GO:0072088	nephron epithelium morphogenesis	25	0.245146732	0.0108	0.018152791
GO:0006560	proline metabolic process	6	0.245125308	0.014	0.020462083
GO:0010565	regulation of cellular ketone metabolic process	97	0.245121786	0.0099	0.017505138
GO:0042554	superoxide anion generation	15	0.245117965	0.0329	0.036702257
GO:0008209	androgen metabolic process	8	0.245094965	0.0211	0.026113057
GO:0097696	STAT cascade	48	0.245083762	0.0035	0.01351837
GO:0051058	negative regulation of small GTPase mediated signal transduction	20	0.245078314	0.0056	0.014771533
GO:0007596	blood coagulation	122	0.245059963	0.0034	0.01347416

GOIDlabel	GOName	size	categoryScores	pValPerm	pValPermCorr
GO:0070509	calcium ion import	76	0.244998889	0.0035	0.01351837
GO:0007190	activation of adenylate cyclase activity	15	0.244997198	0.0001	0.004220438
GO:0060713	labyrinthine layer morphogenesis	8	0.244933802	0.0126	0.019370699
GO:0060009	Sertoli cell development	6	0.2449331	0.0135	0.020081554
GO:0043536	positive regulation of blood vessel endothelial cell migration	24	0.244922787	0.0015	0.011704453
GO:0048490	anterograde synaptic vesicle transport	15	0.244911676	0.0104	0.017832977
GO:0099514	synaptic vesicle cytoskeletal transport	15	0.244911676	0.0104	0.017832977
GO:0099517	synaptic vesicle transport along microtubule	15	0.244911676	0.0104	0.017832977
GO:0090280	positive regulation of calcium ion import	24	0.244902541	0.0047	0.014168613
GO:0031111	negative regulation of microtubule polymerization or depolymerization	16	0.24489096	0.0052	0.014616626
GO:0051592	response to calcium ion	56	0.244863227	0.0073	0.015844069
GO:0002699	positive regulation of immune effector process	71	0.244856965	0.0109	0.018225506
GO:0006729	tetrahydrobiopterin biosynthetic process	5	0.244842999	0.0272	0.031504487
GO:0046146	tetrahydrobiopterin metabolic process	5	0.244842999	0.0272	0.031504487
GO:0032652	regulation of interleukin-1 production	33	0.244842159	0.0264	0.030750363
GO:1903036	positive regulation of response to wounding	23	0.244833512	0.009	0.016956012
GO:0010906	regulation of glucose metabolic process	40	0.244830916	0.0004	0.008964341
GO:0007281	germ cell development	77	0.24479762	0.0029	0.013089617
GO:0051091	positive regulation of sequence-specific DNA binding transcription factor activity	96	0.244797407	0.0053	0.014634479
GO:0007283	spermatogenesis	168	0.244786406	0.0045	0.014132412
GO:0048232	male gamete generation	168	0.244786406	0.0045	0.014132412
GO:0021696	cerebellar cortex morphogenesis	15	0.244764907	0.0036	0.01353394
GO:0023035	CD40 signaling pathway	5	0.244755404	0.0302	0.034245225
GO:0000086	G2/M transition of mitotic cell cycle	113	0.244654911	0.0073	0.015844069
GO:0097479	synaptic vesicle localization	94	0.24458945	0.0011	0.010890753
GO:0032731	positive regulation of interleukin-1 beta production	14	0.244585027	0.0291	0.033199724
GO:0050716	positive regulation of interleukin-1 secretion	10	0.244585008	0.0089	0.016916437
GO:0002639	positive regulation of immunoglobulin production	18	0.244562145	0.0049	0.014447629
GO:0042339	keratan sulfate metabolic process	19	0.244560914	0.0025	0.012769435
GO:0006958	complement activation, classical pathway	13	0.244543932	0.0329	0.036702257
GO:1901379	regulation of potassium ion transmembrane transport	33	0.244521904	0.0007	0.010220707
GO:0009395	phospholipid catabolic process	16	0.244521661	0.0011	0.010890753
GO:0016125	sterol metabolic process	79	0.244508306	0.0062	0.015209334
GO:0042180	cellular ketone metabolic process	134	0.244496075	0.0103	0.017772187
GO:0048489	synaptic vesicle transport	89	0.244459124	0.0011	0.010890753
GO:0097480	establishment of synaptic vesicle localization	89	0.244459124	0.0011	0.010890753
GO:0006491	N-glycan processing	6	0.244450851	0.0122	0.019049527
GO:0019216	regulation of lipid metabolic process	180	0.244435273	0.0049	0.014447629
GO:0043473	pigmentation	44	0.244428342	0.0044	0.014132412
GO:0070302	regulation of stress-activated protein kinase signaling cascade	84	0.244400948	0.0035	0.01351837
GO:0009127	purine nucleoside monophosphate biosynthetic process	37	0.2444001	0.0229	0.027770092
GO:0009168	purine ribonucleoside monophosphate biosynthetic process	37	0.2444001	0.0229	0.027770092
GO:0034332	adherens junction organization	62	0.244382272	0.0017	0.012001709
GO:1901264	carbohydrate derivative transport	24	0.244366193	0.0031	0.013277185
GO:0010818	T cell chemotaxis	6	0.244356259	0.0191	0.02440579
GO:0010833	telomere maintenance via telomere lengthening	37	0.244337097	0.0072	0.015844069
GO:0070125	mitochondrial translational elongation	53	0.244310935	0.0243	0.028921902
GO:0045471	response to ethanol	62	0.244306385	0.0052	0.014616626
GO:0031098	stress-activated protein kinase signaling cascade	116	0.244302972	0.0029	0.013089617
GO:0072171	mesonephric tubule morphogenesis	23	0.24429655	0.0112	0.018392048
GO:0070372	regulation of ERK1 and ERK2 cascade	118	0.244250788	0.0115	0.018599441
GO:0031345	negative regulation of cell projection organization	75	0.244250618	0.0007	0.010220707
GO:0060124	positive regulation of growth hormone secretion	6	0.244244174	< 0.001	< 0.001
GO:1902475	L-alpha-amino acid transmembrane transport	12	0.244199361	0.0211	0.026113057
GO:0055023	positive regulation of cardiac muscle tissue growth	15	0.244189837	0.0119	0.018881943
GO:0060457	negative regulation of digestive system process	7	0.24418611	0.001	0.010787313
GO:0061013	regulation of mRNA catabolic process	16	0.244182651	0.0025	0.012769435
GO:0010812	negative regulation of cell-substrate adhesion	18	0.244171178	0.0021	0.012314604
GO:0031055	chromatin remodeling at centromere	11	0.244168979	0.0139	0.020388077
GO:0034080	CENP-A containing nucleosome assembly	11	0.244168979	0.0139	0.020388077
GO:0061641	CENP-A containing chromatin organization	11	0.244168979	0.0139	0.020388077
GO:0007613	memory	62	0.244140608	0.0023	0.012498684
GO:0008202	steroid metabolic process	130	0.244025219	0.0068	0.015633241
GO:0043405	regulation of MAP kinase activity	153	0.244013778	0.0064	0.015399417
GO:0051238	sequestering of metal ion	52	0.244006704	0.0044	0.014132412
GO:0031343	positive regulation of cell killing	14	0.243997573	0.0172	0.022941269
GO:0001657	ureteric bud development	40	0.243995588	0.0083	0.016559903
GO:0072163	mesonephric epithelium development	40	0.243995588	0.0083	0.016559903
GO:0072164	mesonephric tubule development	40	0.243995588	0.0083	0.016559903
GO:0035024	negative regulation of Rho protein signal transduction	8	0.243994681	0.0001	0.004220438
GO:0060716	labyrinthine layer blood vessel development	10	0.243982079	0.0148	0.020979063
GO:0006940	regulation of smooth muscle contraction	25	0.243975652	0.0069	0.015750415
GO:0042493	response to drug	122	0.243968391	0.0077	0.016177834
GO:0033239	negative regulation of cellular amine metabolic process	5	0.243961865	0.0234	0.02817551
GO:0009247	glycolipid biosynthetic process	28	0.243960597	0.0126	0.019370699
GO:0006012	galactose metabolic process	9	0.243950894	0.0069	0.015750415
GO:0051092	positive regulation of NF-kappaB transcription factor activity	55	0.243897783	0.0061	0.015143924
GO:1903350	response to dopamine	5	0.243861053	0.0204	0.025564109
GO:0050921	positive regulation of chemotaxis	52	0.243801746	0.0199	0.025133639
GO:0045599	negative regulation of fat cell differentiation	18	0.243747816	0.0033	0.013333753
GO:0090501	RNA phosphodiester bond hydrolysis	47	0.243742447	0.0043	0.014102439
GO:1990778	protein localization to cell periphery	126	0.24373637	0.0008	0.010465158
GO:0050817	coagulation	123	0.24367919	0.0035	0.01351837

GOIDlabel	GOName	size	categoryScores	pValPerm	pValPermCorr
GO:0071300	cellular response to retinoic acid	29	0.243673968	0.0031	0.013277185
GO:0006054	N-acetylneuraminate metabolic process	6	0.243658262	0.0427	0.045577146
GO:0010881	regulation of cardiac muscle contraction by regulation of the release of sequestered calcium ion	9	0.243637458	0.0161	0.02211694
GO:1903792	negative regulation of anion transport	8	0.243628783	0.0032	0.013333753
GO:0007143	female meiotic division	7	0.243617075	0.0016	0.011845327
GO:2000781	positive regulation of double-strand break repair	11	0.243616267	0.0058	0.014878261
GO:0009743	response to carbohydrate	104	0.243615028	0.0009	0.010729485
GO:0071248	cellular response to metal ion	89	0.243607501	0.0072	0.015844069
GO:0032526	response to retinoic acid	49	0.24358029	0.0048	0.014269203
GO:0034384	high-density lipoprotein particle clearance	5	0.243563281	0.0057	0.01480566
GO:0061333	renal tubule morphogenesis	26	0.243546576	0.013	0.019676963
GO:1900016	negative regulation of cytokine production involved in inflammatory response	7	0.243526925	0.0051	0.014547706
GO:0032869	cellular response to insulin stimulus	84	0.243503771	0.001	0.010787313
GO:0010842	retina layer formation	9	0.243495353	0.0119	0.018881943
GO:0090207	regulation of triglyceride metabolic process	15	0.243491495	0.0089	0.016916437
GO:0060478	acrosomal vesicle exocytosis	5	0.243470762	< 0.001	< 0.001
GO:0007618	mating	20	0.243441801	0.0088	0.016916437
GO:0072243	metanephric nephron epithelium development	13	0.243438128	0.0232	0.028016374
GO:1901020	negative regulation of calcium ion transmembrane transporter activity	13	0.243428161	0.0037	0.013609033
GO:0010661	positive regulation of muscle cell apoptotic process	16	0.243405989	0.0003	0.007956881
GO:0050690	regulation of defense response to virus by virus	19	0.243405755	0.0087	0.016840777
GO:0051196	regulation of coenzyme metabolic process	34	0.243405013	0.0046	0.014132412
GO:0048013	ephrin receptor signaling pathway	53	0.243382932	0.0011	0.010890753
GO:0060218	hematopoietic stem cell differentiation	42	0.243376984	0.0198	0.025062084
GO:0042445	hormone metabolic process	70	0.243373856	0.0099	0.017505138
GO:0045742	positive regulation of epidermal growth factor receptor signaling pathway	11	0.243326954	< 0.001	< 0.001
GO:1901186	positive regulation of ERBB signaling pathway	11	0.243326954	< 0.001	< 0.001
GO:0043331	response to dsRNA	36	0.243300662	0.007	0.015816335
GO:1904353	regulation of telomere capping	15	0.243297559	0.0023	0.012498684
GO:0006631	fatty acid metabolic process	161	0.243296951	0.0106	0.018010344
GO:0002562	somatic diversification of immune receptors via germline recombination within a single locus	20	0.243280735	0.0003	0.007956881
GO:0016444	somatic cell DNA recombination	20	0.243280735	0.0003	0.007956881
GO:1902837	amino acid import into cell	8	0.243270175	0.0055	0.01475
GO:0045739	positive regulation of DNA repair	25	0.243264007	0.0022	0.012422266
GO:0032210	regulation of telomere maintenance via telomerase	26	0.243230603	0.0051	0.014547706
GO:0046622	positive regulation of organ growth	21	0.243223835	0.0115	0.018599441
GO:1990138	neuron projection extension	80	0.243208398	0.0013	0.011218806
GO:0008203	cholesterol metabolic process	75	0.243180178	0.0064	0.015399417
GO:0010389	regulation of G2/M transition of mitotic cell cycle	90	0.24317839	0.0095	0.01725157
GO:0021587	cerebellum morphogenesis	17	0.243141587	0.0053	0.014634479
GO:0003014	renal system process	48	0.243132389	0.0024	0.012603815
GO:0010544	negative regulation of platelet activation	10	0.243126213	0.0398	0.042885501
GO:0051155	positive regulation of striated muscle cell differentiation	23	0.243113497	0.003	0.013210967
GO:0099558	maintenance of synapse structure	11	0.243090649	0.004	0.013824268
GO:0051926	negative regulation of calcium ion transport	21	0.243085494	0.0087	0.016840777
GO:0060837	blood vessel endothelial cell differentiation	6	0.243078182	0.0193	0.024606968
GO:0006244	pyrimidine nucleotide catabolic process	6	0.243055074	0.0034	0.01347416
GO:0009223	pyrimidine deoxyribonucleotide catabolic process	6	0.243055074	0.0034	0.01347416
GO:0006004	fucose metabolic process	9	0.243042404	0.014	0.020462083
GO:0006771	riboflavin metabolic process	7	0.24299917	0.0126	0.019370699
GO:0048704	embryonic skeletal system morphogenesis	28	0.242992321	0.0133	0.019917275
GO:0070584	mitochondrion morphogenesis	11	0.242939396	0.0175	0.023154462
GO:0001676	long-chain fatty acid metabolic process	48	0.242926926	0.0081	0.016514175
GO:1903305	regulation of regulated secretory pathway	77	0.242925226	0.0024	0.012603815
GO:0008306	associative learning	38	0.242913936	0.0052	0.014616626
GO:0007202	activation of phospholipase C activity	13	0.2428935	0.0037	0.013609033
GO:0072073	kidney epithelium development	57	0.242888418	0.0061	0.015143924
GO:0033238	regulation of cellular amine metabolic process	57	0.242865276	0.0177	0.02329117
GO:0051896	regulation of protein kinase B signaling	82	0.242865194	0.0073	0.015844069
GO:0072507	divalent inorganic cation homeostasis	177	0.242853488	0.0027	0.012827773
GO:0070257	positive regulation of mucus secretion	5	0.242844379	0.0008	0.010465158
GO:0010839	negative regulation of keratinocyte proliferation	7	0.242827941	0.0148	0.020979063
GO:2000379	positive regulation of reactive oxygen species metabolic process	38	0.242801987	0.0181	0.023607986
GO:0019433	triglyceride catabolic process	11	0.2428011	0.0228	0.027701114
GO:0009396	folic acid-containing compound biosynthetic process	5	0.242745381	0.0163	0.022248961
GO:0006909	phagocytosis	118	0.242734169	0.0048	0.014269203
GO:0050954	sensory perception of mechanical stimulus	53	0.24271817	0.0167	0.02255007
GO:0090141	positive regulation of mitochondrial fission	10	0.242713635	0.0135	0.020081554
GO:0050702	interleukin-1 beta secretion	18	0.24270658	0.0278	0.032045375
GO:0071867	response to monoamine	14	0.242692347	0.0078	0.01620539
GO:0071869	response to catecholamine	14	0.242692347	0.0078	0.01620539
GO:0036294	cellular response to decreased oxygen levels	104	0.242657255	0.0078	0.01620539
GO:0061003	positive regulation of dendritic spine morphogenesis	9	0.242629912	0.0022	0.012422266
GO:0072080	nephron tubule development	34	0.242575481	0.0111	0.018389742
GO:0034644	cellular response to UV	33	0.242567859	0.0079	0.01629604
GO:0034315	regulation of Arp2/3 complex-mediated actin nucleation	10	0.242559177	0.0007	0.010220707
GO:0007492	endoderm development	27	0.242531673	0.0045	0.014132412
GO:0001973	adenosine receptor signaling pathway	8	0.242516675	0.01	0.017574468
GO:0032611	interleukin-1 beta production	33	0.242498436	0.0339	0.037513837
GO:0016050	vesicle organization	150	0.242485732	0.0033	0.013333753
GO:0090276	regulation of peptide hormone secretion	87	0.242478554	0.0015	0.011704453
GO:0060326	cell chemotaxis	91	0.24245457	0.0115	0.018599441
GO:0042220	response to cocaine	24	0.24243963	0.0034	0.01347416

GOIDlabel	GOName	size	categoryScores	pValPerm	pValPermCorr
GO:0032872	regulation of stress-activated MAPK cascade	82	0.242438817	0.0035	0.01351837
GO:0071868	cellular response to monoamine stimulus	13	0.242423628	0.0062	0.015209334
GO:0071870	cellular response to catecholamine stimulus	13	0.242423628	0.0062	0.015209334
GO:0002689	negative regulation of leukocyte chemotaxis	5	0.242408477	0.0373	0.040638515
GO:0034470	ncRNA processing	132	0.242394066	0.0062	0.015209334
GO:0050890	cognition	142	0.242387389	0.0043	0.014102439
GO:0071353	cellular response to interleukin-4	12	0.24236928	0.0033	0.013333753
GO:1903802	L-glutamate(1-) import into cell	7	0.242368678	0.0055	0.01475
GO:1990123	L-glutamate import into cell	7	0.242368678	0.0055	0.01475
GO:0006720	isoprenoid metabolic process	58	0.24234228	0.0062	0.015209334
GO:0090201	negative regulation of release of cytochrome c from mitochondria	11	0.242307524	0.0226	0.027544941
GO:0010595	positive regulation of endothelial cell migration	48	0.242302897	0.0042	0.013948535
GO:0070444	oligodendrocyte progenitor proliferation	5	0.242283045	0.0072	0.015844069
GO:0070445	regulation of oligodendrocyte progenitor proliferation	5	0.242283045	0.0072	0.015844069
GO:0050708	regulation of protein secretion	196	0.242268578	0.005	0.014547706
GO:0072503	cellular divalent inorganic cation homeostasis	170	0.242215832	0.0027	0.012827773
GO:0034104	negative regulation of tissue remodeling	12	0.242206766	< 0.001	< 0.001
GO:0051289	protein homotetramerization	44	0.242204744	0.0015	0.011704453
GO:0044106	cellular amine metabolic process	76	0.242164494	0.0153	0.021378589
GO:0051567	histone H3-K9 methylation	12	0.242098568	0.0042	0.013948535
GO:0006836	neurotransmitter transport	92	0.242089263	0.001	0.010787313
GO:0070126	mitochondrial translational termination	54	0.242058231	0.0241	0.028760826
GO:0044262	cellular carbohydrate metabolic process	107	0.242034927	0.0028	0.012972436
GO:0009311	oligosaccharide metabolic process	19	0.242033722	0.004	0.013824268
GO:0099024	plasma membrane invagination	22	0.242030814	0.0085	0.016693954
GO:0042723	thiamine-containing compound metabolic process	5	0.242014299	0.0032	0.013333753
GO:0008088	axo-dendritic transport	36	0.241979968	0.004	0.013824268
GO:0023061	signal release	195	0.241971239	0.001	0.010787313
GO:0051282	regulation of sequestering of calcium ion	45	0.241940295	0.0047	0.014168613
GO:0055074	calcium ion homeostasis	169	0.241934526	0.0027	0.012827773
GO:0007431	salivary gland development	17	0.241928274	0.0098	0.017418875
GO:0051452	intracellular pH reduction	22	0.241918788	0.0104	0.017832977
GO:0031346	positive regulation of cell projection organization	162	0.241897018	0.0013	0.011218806
GO:0008053	mitochondrial fusion	14	0.241896086	0.0084	0.016633151
GO:0051403	stress-activated MAPK cascade	111	0.241866126	0.0033	0.013333753
GO:0006261	DNA-dependent DNA replication	47	0.241861435	0.0018	0.01210186
GO:0045651	positive regulation of macrophage differentiation	5	0.241829002	0.0175	0.023154462
GO:0001913	T cell mediated cytotoxicity	11	0.241797337	0.0112	0.018392048
GO:0061337	cardiac conduction	61	0.241792193	0.0021	0.012314604
GO:0001508	action potential	49	0.241790546	0.0032	0.013333753
GO:0003149	membranous septum morphogenesis	7	0.241784649	0.0053	0.014634479
GO:0045773	positive regulation of axon extension	20	0.241784621	0.0014	0.011465722
GO:0006874	cellular calcium ion homeostasis	165	0.241762673	0.0027	0.012827773
GO:0061154	endothelial tube morphogenesis	7	0.241753735	0.0106	0.018010344
GO:0045920	negative regulation of exocytosis	16	0.241748355	0.0365	0.039857035
GO:0072698	protein localization to microtubule cytoskeleton	16	0.241745683	0.0042	0.013948535
GO:0090102	cochlea development	24	0.241743199	0.0184	0.023875404
GO:1905269	positive regulation of chromatin organization	39	0.241739752	0.0051	0.014547706
GO:0032874	positive regulation of stress-activated MAPK cascade	50	0.241735601	0.006	0.015037711
GO:0051956	negative regulation of amino acid transport	5	0.24173415	0.0117	0.018739446
GO:0072593	reactive oxygen species metabolic process	107	0.241717726	0.0103	0.017772187
GO:0002279	mast cell activation involved in immune response	22	0.241671528	0.0185	0.023962142
GO:0043303	mast cell degranulation	22	0.241671528	0.0185	0.023962142
GO:0060688	regulation of morphogenesis of a branching structure	22	0.241642609	0.01	0.017574468
GO:1901990	regulation of mitotic cell cycle phase transition	181	0.241642009	0.0044	0.014132412
GO:0032436	positive regulation of proteasomal ubiquitin-dependent protein catabolic process	36	0.241638636	0.0014	0.011465722
GO:2000047	regulation of cell-cell adhesion mediated by cadherin	6	0.2416377	0.0175	0.023154462
GO:0042178	xenobiotic catabolic process	7	0.241567882	0.0269	0.031225818
GO:0042116	macrophage activation	35	0.241558368	0.0267	0.031037274
GO:0006779	porphyrin-containing compound biosynthetic process	16	0.241520459	0.0126	0.019370699
GO:0045730	respiratory burst	16	0.241508797	0.0226	0.027544941
GO:0098814	spontaneous synaptic transmission	5	0.241492356	0.0027	0.012827773
GO:0010883	regulation of lipid storage	15	0.241479831	0.0024	0.012603815
GO:0030799	regulation of cyclic nucleotide metabolic process	30	0.241474691	0.0019	0.012288367
GO:0030802	regulation of cyclic nucleotide biosynthetic process	30	0.241474691	0.0019	0.012288367
GO:0071456	cellular response to hypoxia	100	0.241464368	0.0092	0.017033109
GO:0061326	renal tubule development	35	0.241460258	0.0122	0.019049527
GO:0003159	morphogenesis of an endothelium	9	0.241431886	0.018	0.023514686
GO:0071230	cellular response to amino acid stimulus	36	0.241430267	0.0011	0.010890753
GO:0001914	regulation of T cell mediated cytotoxicity	7	0.241426086	0.0147	0.02091937
GO:1901214	regulation of neuron death	145	0.241415941	0.0021	0.012314604
GO:0042987	amyloid precursor protein catabolic process	23	0.241414497	0.0007	0.010220707
GO:0006109	regulation of carbohydrate metabolic process	79	0.241388841	0.0017	0.012001709
GO:0042304	regulation of fatty acid biosynthetic process	18	0.24138675	0.0082	0.016514246
GO:0051607	defense response to virus	105	0.241370417	0.0046	0.014132412
GO:0060539	diaphragm development	5	0.241362	0.0045	0.014132412
GO:2001022	positive regulation of response to DNA damage stimulus	31	0.241344888	0.0028	0.012972436
GO:0007435	salivary gland morphogenesis	16	0.241320665	0.0098	0.017418875
GO:0021549	cerebellum development	42	0.241317179	0.005	0.014547706
GO:1903322	positive regulation of protein modification by small protein conjugation or removal	62	0.241315461	0.005	0.014547706
GO:0035418	protein localization to synapse	30	0.241298637	0.0001	0.004220438
GO:0007188	adenylate cyclase-modulating G-protein coupled receptor signaling pathway	66	0.241295633	0.0026	0.012805111
GO:0009308	amine metabolic process	77	0.241260175	0.0154	0.021487162

GOIDlabel	GOName	size	categoryScores	pValPerm	pValPermCorr
GO:0038127	ERBB signaling pathway	60	0.241248554	0.0005	0.009604651
GO:0002832	negative regulation of response to biotic stimulus	22	0.24124386	0.0072	0.015844069
GO:0000963	mitochondrial RNA processing	6	0.24123903	0.0206	0.025725529
GO:0034333	adherens junction assembly	41	0.241226531	0.0008	0.010465158
GO:0006687	glycosphingolipid metabolic process	37	0.241221607	0.0023	0.012498684
GO:0051048	negative regulation of secretion	102	0.241209583	0.0103	0.017772187
GO:0032844	regulation of homeostatic process	193	0.241205927	0.0036	0.01353394
GO:0043491	protein kinase B signaling	95	0.241149873	0.0054	0.014720792
GO:0046578	regulation of Ras protein signal transduction	101	0.241142405	0.0029	0.013089617
GO:1904837	beta-catenin-TCF complex assembly	11	0.24111328	0.0036	0.01353394
GO:0060193	positive regulation of lipase activity	28	0.241059795	0.01	0.017574468
GO:0000731	DNA synthesis involved in DNA repair	18	0.241019539	0.0213	0.026276211
GO:0031115	negative regulation of microtubule polymerization	8	0.240992669	0.0071	0.01583804
GO:0050688	regulation of defense response to virus	46	0.240990099	0.0097	0.017363901
GO:0007204	positive regulation of cytosolic calcium ion concentration	115	0.240954955	0.0024	0.012603815
GO:0042594	response to starvation	83	0.240948845	0.0016	0.011845327
GO:1904874	positive regulation of telomerase RNA localization to Cajal body	10	0.240936594	0.0362	0.03958177
GO:0071241	cellular response to inorganic substance	99	0.240925404	0.0066	0.015525305
GO:0002637	regulation of immunoglobulin production	27	0.240903302	0.0019	0.012288367
GO:1901987	regulation of cell cycle phase transition	197	0.240895782	0.0047	0.014168613
GO:0030641	regulation of cellular pH	28	0.240884078	0.0084	0.016633151
GO:0031145	anaphase-promoting complex-dependent catabolic process	51	0.240883388	0.0097	0.017363901
GO:0061383	trabecula morphogenesis	20	0.24082872	0.0086	0.016782045
GO:0051346	negative regulation of hydrolase activity	149	0.240826627	0.0047	0.014168613
GO:0032201	telomere maintenance via semi-conservative replication	9	0.240821715	0.0131	0.019786886
GO:1903052	positive regulation of proteolysis involved in cellular protein catabolic process	57	0.240819616	0.0031	0.013277185
GO:0042475	odontogenesis of dentin-containing tooth	28	0.240798	0.0031	0.013277185
GO:1901216	positive regulation of neuron death	46	0.240794829	0.0067	0.015576759
GO:0070202	regulation of establishment of protein localization to chromosome	7	0.240768938	0.0356	0.03903645
GO:0070203	regulation of establishment of protein localization to telomere	7	0.240768938	0.0356	0.03903645
GO:0072009	nephron epithelium development	40	0.240712293	0.0101	0.017653628
GO:0021681	cerebellar granular layer development	7	0.240691746	0.0261	0.030517735
GO:0051100	negative regulation of binding	76	0.240679132	0.0037	0.013609033
GO:0050982	detection of mechanical stimulus	14	0.240641395	0.0281	0.032268957
GO:0008347	glial cell migration	27	0.240592025	0.0173	0.023005658
GO:0010977	negative regulation of neuron projection development	66	0.240588153	0.0006	0.009940401
GO:0042446	hormone biosynthetic process	30	0.24055762	0.0143	0.020629391
GO:0060316	positive regulation of ryanodine-sensitive calcium-release channel activity	5	0.240538399	0.0187	0.024129301
GO:0051650	establishment of vesicle localization	158	0.240528743	0.0019	0.012288367
GO:0051260	protein homooligomerization	160	0.240491718	0.0012	0.010961137
GO:0046697	decidualization	10	0.240489159	0.0007	0.010220707
GO:0050727	regulation of inflammatory response	141	0.240480329	0.0036	0.01353394
GO:0060675	ureteric bud morphogenesis	22	0.240447541	0.0096	0.017302743
GO:0051648	vesicle localization	166	0.240443894	0.0025	0.012769435
GO:0001836	release of cytochrome c from mitochondria	33	0.240443555	0.0088	0.016916437
GO:0009062	fatty acid catabolic process	46	0.240442573	0.017	0.022784886
GO:0043243	positive regulation of protein complex disassembly	14	0.240435828	0.0131	0.019786886
GO:0098930	axonal transport	31	0.240401221	0.0067	0.015576759
GO:0010817	regulation of hormone levels	195	0.240381018	0.0039	0.013791927
GO:0019395	fatty acid oxidation	49	0.24032882	0.0166	0.02247277
GO:0021575	hindbrain morphogenesis	18	0.240328597	0.0083	0.016559903
GO:0030177	positive regulation of Wnt signaling pathway	80	0.240327235	0.0133	0.019917275
GO:1903358	regulation of Golgi organization	10	0.240324724	0.0029	0.013089617
GO:0045746	negative regulation of Notch signaling pathway	20	0.240283518	0.0006	0.009940401
GO:0003071	renal system process involved in regulation of systemic arterial blood pressure	8	0.24025893	0.0103	0.017772187
GO:0000045	autophagosome assembly	38	0.240258768	0.0026	0.012805111
GO:0090263	positive regulation of canonical Wnt signaling pathway	69	0.240245792	0.0117	0.018739446
GO:0033500	carbohydrate homeostasis	87	0.240235474	0.0006	0.009940401
GO:0042593	glucose homeostasis	87	0.240235474	0.0006	0.009940401
GO:0001823	mesonephros development	41	0.240231399	0.0096	0.017302743
GO:0070646	protein modification by small protein removal	128	0.240135061	0.0053	0.014634479
GO:0090184	positive regulation of kidney development	15	0.240125734	0.014	0.020462083
GO:0007216	G-protein coupled glutamate receptor signaling pathway	6	0.240118608	0.0129	0.019597425
GO:0060560	developmental growth involved in morphogenesis	108	0.240111121	0.0016	0.011845327
GO:0032026	response to magnesium ion	8	0.240085781	0.0046	0.014132412
GO:0031669	cellular response to nutrient levels	86	0.240076711	0.0034	0.01347416
GO:1990089	response to nerve growth factor	26	0.240075714	0.0009	0.010729485
GO:0003254	regulation of membrane depolarization	22	0.240072411	0.0025	0.012769435
GO:0046479	glycosphingolipid catabolic process	6	0.240069152	0.0036	0.01353394
GO:0046514	ceramide catabolic process	6	0.240069152	0.0036	0.01353394
GO:0009410	response to xenobiotic stimulus	45	0.240062788	0.009	0.016956012
GO:0051645	Golgi localization	8	0.240058893	0.0147	0.02091937
GO:0030316	osteoclast differentiation	40	0.240058246	0.0066	0.015525305
GO:1902652	secondary alcohol metabolic process	77	0.240057215	0.0073	0.015844069
GO:0071214	cellular response to abiotic stimulus	140	0.240054395	0.0025	0.012769435
GO:0021695	cerebellar cortex development	21	0.240034906	0.0038	0.01366393
GO:0035640	exploration behavior	13	0.240014363	0.0043	0.014102439
GO:0016053	organic acid biosynthetic process	137	0.240007291	0.0074	0.015911789
GO:0046394	carboxylic acid biosynthetic process	137	0.240007291	0.0074	0.015911789
GO:0098876	vesicle-mediated transport to the plasma membrane	30	0.239994212	0.0003	0.007956881
GO:0051388	positive regulation of neurotrophin TRK receptor signaling pathway	5	0.23994778	0.0007	0.010220707
GO:0014066	regulation of phosphatidylinositol 3-kinase signaling	52	0.239919223	0.0053	0.014634479
GO:1904950	negative regulation of establishment of protein localization	98	0.239915517	0.0079	0.01629604

GOIDlabel	GOName	size	categoryScores	pValPerm	pValPermCorr
GO:0015850	organic hydroxy compound transport	86	0.239912221	0.0097	0.017363901
GO:0070920	regulation of production of small RNA involved in gene silencing by RNA	5	0.23990165	0.0073	0.015844069
GO:1903798	regulation of production of miRNAs involved in gene silencing by miRNA	5	0.23990165	0.0073	0.015844069
GO:0090520	sphingolipid mediated signaling pathway	6	0.239901348	0.005	0.014547706
GO:0002791	regulation of peptide secretion	89	0.239880981	0.0014	0.011465722
GO:0090087	regulation of peptide transport	89	0.239880981	0.0014	0.011465722
GO:0002706	regulation of lymphocyte mediated immunity	34	0.239870681	0.0042	0.013948535
GO:0019882	antigen processing and presentation	129	0.239844639	0.0145	0.020788247
GO:0045214	sarcomere organization	12	0.23983591	0.0105	0.017924712
GO:0051928	positive regulation of calcium ion transport	48	0.239828028	0.0044	0.014132412
GO:0030073	insulin secretion	89	0.239815566	0.0015	0.011704453
GO:0001818	negative regulation of cytokine production	122	0.239811818	0.0081	0.016514175
GO:0060216	definitive hemopoiesis	6	0.239756004	0.0311	0.035052671
GO:0032846	positive regulation of homeostatic process	96	0.239747891	0.0049	0.014447629
GO:0070988	demethylation	16	0.239736648	0.0027	0.012827773
GO:0048008	platelet-derived growth factor receptor signaling pathway	25	0.239733353	0.0096	0.017302743
GO:0010656	negative regulation of muscle cell apoptotic process	18	0.23970554	0.0028	0.012972436
GO:0018107	peptidyl-threonine phosphorylation	45	0.239664723	< 0.001	< 0.001
GO:0051054	positive regulation of DNA metabolic process	101	0.239640101	0.0024	0.012603815
GO:0006458	'de novo' protein folding	14	0.239631238	0.0052	0.014616626
GO:0032655	regulation of interleukin-12 production	14	0.239614115	0.0014	0.011465722
GO:0044091	membrane biogenesis	20	0.239610552	0.0031	0.013277185
GO:0061005	cell differentiation involved in kidney development	23	0.239579889	0.0143	0.020629391
GO:0099003	vesicle-mediated transport in synapse	95	0.239577431	0.0008	0.010465158
GO:0003333	amino acid transmembrane transport	20	0.239570771	0.0109	0.018225506
GO:1904031	positive regulation of cyclin-dependent protein kinase activity	12	0.239562931	0.0092	0.017033109
GO:0006939	smooth muscle contraction	47	0.2395406	0.0026	0.012805111
GO:0006782	protoporphyrinogen IX biosynthetic process	5	0.239534683	0.0331	0.03684717
GO:0006085	acetyl-CoA biosynthetic process	9	0.239509948	0.0118	0.018836996
GO:0072595	maintenance of protein localization in organelle	13	0.239478008	0.0023	0.012498684
GO:0007617	mating behavior	12	0.239429574	0.0029	0.013089617
GO:1905516	positive regulation of fertilization	6	0.239426442	0.0033	0.013333753
GO:1904356	regulation of telomere maintenance via telomere lengthening	30	0.23941979	0.0041	0.013887639
GO:0007611	learning or memory	129	0.239405709	0.0036	0.01353394
GO:1902036	regulation of hematopoietic stem cell differentiation	41	0.239397008	0.021	0.026005997
GO:0034199	activation of protein kinase A activity	7	0.239394664	0.0001	0.004220438
GO:0042492	gamma-delta T cell differentiation	5	0.23935742	0.0289	0.033004108
GO:0051208	sequestering of calcium ion	47	0.239330214	0.0058	0.014878261
GO:1901985	positive regulation of protein acetylation	16	0.239323697	0.0141	0.020504577
GO:0045778	positive regulation of ossification	45	0.23930435	0.0111	0.018389742
GO:0009615	response to virus	141	0.239296744	0.0065	0.015491756
GO:0001894	tissue homeostasis	74	0.239259105	0.0035	0.01351837
GO:0051188	cofactor biosynthetic process	88	0.239253857	0.0076	0.016084627
GO:1903170	negative regulation of calcium ion transmembrane transport	16	0.239251295	0.0091	0.016994897
GO:0006278	RNA-dependent DNA biosynthetic process	33	0.239244914	0.0079	0.01629604
GO:0099504	synaptic vesicle cycle	81	0.239234762	0.0009	0.010729485
GO:0006400	tRNA modification	27	0.239229819	0.0082	0.016514246
GO:0097306	cellular response to alcohol	25	0.239218408	0.0147	0.02091937
GO:0019884	antigen processing and presentation of exogenous antigen	106	0.239200869	0.0153	0.021378589
GO:0048546	digestive tract morphogenesis	20	0.239191365	0.0084	0.016633151
GO:0048265	response to pain	14	0.239183729	0.0218	0.026744664
GO:0007140	male meiosis	11	0.239153699	0.0138	0.020339434
GO:0071804	cellular potassium ion transport	59	0.239152167	0.0006	0.009940401
GO:0071805	potassium ion transmembrane transport	59	0.239152167	0.0006	0.009940401
GO:0051224	negative regulation of protein transport	95	0.239141351	0.0081	0.016514175
GO:0051788	response to misfolded protein	11	0.239118006	0.0116	0.018682786
GO:0055094	response to lipoprotein particle	10	0.239076753	0.0066	0.015525305
GO:0055098	response to low-density lipoprotein particle	10	0.239076753	0.0066	0.015525305
GO:0071402	cellular response to lipoprotein particle stimulus	10	0.239076753	0.0066	0.015525305
GO:0071404	cellular response to low-density lipoprotein particle stimulus	10	0.239076753	0.0066	0.015525305
GO:0006739	NADP metabolic process	14	0.239058871	0.0124	0.019242297
GO:0051209	release of sequestered calcium ion into cytosol	44	0.239050419	0.0052	0.014616626
GO:0051283	negative regulation of sequestering of calcium ion	44	0.239050419	0.0052	0.014616626
GO:0051353	positive regulation of oxidoreductase activity	24	0.239021617	0.0043	0.014102439
GO:0070507	regulation of microtubule cytoskeleton organization	62	0.239016507	0.0024	0.012603815
GO:0045666	positive regulation of neuron differentiation	152	0.239003433	0.0013	0.011218806
GO:0032720	negative regulation of tumor necrosis factor production	28	0.23897844	0.0096	0.017302743
GO:1903556	negative regulation of tumor necrosis factor superfamily cytokine production	28	0.23897844	0.0096	0.017302743
GO:1903147	negative regulation of mitophagy	7	0.238960576	0.0163	0.022248961
GO:0014009	glial cell proliferation	27	0.238954584	0.0144	0.02072711
GO:0043279	response to alkaloid	68	0.238948958	0.0055	0.01475
GO:0036124	histone H3-K9 trimethylation	5	0.238937067	0.0316	0.035498582
GO:0090030	regulation of steroid hormone biosynthetic process	6	0.238920654	0.0357	0.03911643
GO:0001701	in utero embryonic development	156	0.238919366	0.0031	0.013277185
GO:0030111	regulation of Wnt signaling pathway	168	0.238895903	0.0063	0.015311728
GO:0060491	regulation of cell projection assembly	68	0.238886912	0.0015	0.011704453
GO:0010719	negative regulation of epithelial to mesenchymal transition	13	0.238885634	0.0091	0.016994897
GO:0042254	ribosome biogenesis	97	0.23887865	0.0052	0.014616626
GO:0042246	tissue regeneration	22	0.238877166	0.0169	0.022703485
GO:0006635	fatty acid beta-oxidation	37	0.238875114	0.0224	0.027324219
GO:0009267	cellular response to starvation	66	0.238870334	0.0014	0.011465722
GO:0045454	cell redox homeostasis	29	0.238860571	0.0089	0.016916437
GO:0032103	positive regulation of response to external stimulus	121	0.238845773	0.01	0.017574468

GOIDlabel	GOName	size	categoryScores	pValPerm	pValPermCorr
GO:0030336	negative regulation of cell migration	123	0.238818097	0.0037	0.013609033
GO:0090659	walking behavior	12	0.238811052	0.0007	0.010220707
GO:0001711	endodermal cell fate commitment	5	0.238797122	0.027	0.031323034
GO:0070987	error-free translesion synthesis	9	0.238731181	0.0369	0.040233038
GO:0014812	muscle cell migration	31	0.238720086	0.007	0.015816335
GO:0015711	organic anion transport	172	0.238684848	0.0031	0.013277185
GO:0030816	positive regulation of cAMP metabolic process	19	0.238683775	0.0007	0.010220707
GO:0030819	positive regulation of cAMP biosynthetic process	19	0.238683775	0.0007	0.010220707
GO:0045762	positive regulation of adenylate cyclase activity	19	0.238683775	0.0007	0.010220707
GO:0048871	multicellular organismal homeostasis	124	0.23866863	0.0018	0.01210186
GO:0044331	cell-cell adhesion mediated by cadherin	7	0.238662545	0.0164	0.022306469
GO:0002093	auditory receptor cell morphogenesis	5	0.238660805	0.0163	0.022248961
GO:0060088	auditory receptor cell stereocilium organization	5	0.238660805	0.0163	0.022248961
GO:0048644	muscle organ morphogenesis	29	0.23859985	0.0164	0.022306469
GO:0002688	regulation of leukocyte chemotaxis	34	0.238597914	0.0139	0.020388077
GO:0071260	cellular response to mechanical stimulus	35	0.238552552	0.0104	0.017832977
GO:0060628	regulation of ER to Golgi vesicle-mediated transport	8	0.23850557	0.0089	0.016916437
GO:0050707	regulation of cytokine secretion	74	0.238493033	0.0162	0.022196303
GO:0032612	interleukin-1 production	37	0.238481931	0.0314	0.035301342
GO:0007032	endosome organization	40	0.238470325	0.0025	0.012769435
GO:2000146	negative regulation of cell motility	129	0.238465732	0.0038	0.01366393
GO:0034103	regulation of tissue remodeling	29	0.238464597	0.0057	0.01480566
GO:2001257	regulation of cation channel activity	73	0.238453224	0.0012	0.010961137
GO:0002478	antigen processing and presentation of exogenous peptide antigen	105	0.238451801	0.0153	0.021378589
GO:0097305	response to alcohol	85	0.238446467	0.0087	0.016840777
GO:0045124	regulation of bone resorption	15	0.238413397	0.0021	0.012314604
GO:0048167	regulation of synaptic plasticity	101	0.238404999	0.0009	0.010729485
GO:0043123	positive regulation of I-kappaB kinase/NF-kappaB signaling	82	0.238402034	0.0041	0.013887639
GO:0019319	hexose biosynthetic process	41	0.238393722	0.0066	0.015525305
GO:0050773	regulation of dendrite development	62	0.23834738	0.0004	0.008964341
GO:0006081	cellular aldehyde metabolic process	39	0.238336188	0.0091	0.016994897
GO:0015849	organic acid transport	122	0.238320719	0.0035	0.01351837
GO:0046942	carboxylic acid transport	122	0.238320719	0.0035	0.01351837
GO:0002673	regulation of acute inflammatory response	38	0.238316079	0.0169	0.022703485
GO:0070979	protein K11-linked ubiquitination	17	0.238301979	0.0025	0.012769435
GO:0031668	cellular response to extracellular stimulus	103	0.238205413	0.0046	0.014132412
GO:0038093	Fc receptor signaling pathway	105	0.238186955	0.0102	0.0177
GO:0051899	membrane depolarization	36	0.238178492	0.0046	0.014132412
GO:0014065	phosphatidylinositol 3-kinase signaling	61	0.238167753	0.0042	0.013948535
GO:0070613	regulation of protein processing	37	0.238131158	0.0253	0.029811412
GO:1903317	regulation of protein maturation	37	0.238131158	0.0253	0.029811412
GO:0045932	negative regulation of muscle contraction	15	0.238107332	0.0078	0.01620539
GO:1903409	reactive oxygen species biosynthetic process	40	0.238071741	0.0141	0.020504577
GO:1902743	regulation of lamellipodium organization	17	0.238069805	0.004	0.013824268
GO:0031047	gene silencing by RNA	53	0.238024369	0.0046	0.014132412
GO:0051216	cartilage development	81	0.238009732	0.0061	0.015143924
GO:0008643	carbohydrate transport	52	0.237986384	0.0082	0.016514246
GO:0021761	limbic system development	52	0.23798449	0.0042	0.013948535
GO:0016239	positive regulation of macroautophagy	34	0.237954175	0.0044	0.014132412
GO:0006937	regulation of muscle contraction	66	0.237951504	0.0107	0.018084595
GO:0055067	monovalent inorganic cation homeostasis	53	0.237941512	0.0083	0.016559903
GO:0002230	positive regulation of defense response to virus by host	12	0.237930781	0.025	0.029554283
GO:0070252	actin-mediated cell contraction	56	0.23791652	0.0056	0.014771533
GO:0007612	learning	76	0.237912134	0.0062	0.015209334
GO:0008089	anterograde axonal transport	25	0.23791153	0.0115	0.018599441
GO:0001658	branching involved in ureteric bud morphogenesis	20	0.237908537	0.0071	0.01583804
GO:0021692	cerebellar Purkinje cell layer morphogenesis	5	0.237877892	0.0046	0.014132412
GO:0032835	glomerulus development	24	0.237874145	0.0245	0.029070183
GO:0014909	smooth muscle cell migration	28	0.237863555	0.0066	0.015525305
GO:0002700	regulation of production of molecular mediator of immune response	54	0.23785439	0.006	0.015037711
GO:0008645	hexose transport	41	0.237803823	0.009	0.016956012
GO:0015749	monosaccharide transport	41	0.237803823	0.009	0.016956012
GO:0015758	glucose transport	41	0.237803823	0.009	0.016956012
GO:2000353	positive regulation of endothelial cell apoptotic process	6	0.237800447	0.0178	0.023348367
GO:0006732	coenzyme metabolic process	162	0.237788553	0.0073	0.015844069
GO:0046324	regulation of glucose import	19	0.237763221	0.0069	0.015750415
GO:0032222	regulation of synaptic transmission, cholinergic	5	0.237752719	0.0095	0.01725157
GO:0035315	hair cell differentiation	13	0.237745343	0.0201	0.025275816
GO:0046328	regulation of JNK cascade	67	0.237744074	0.0023	0.012498684
GO:0000387	spliceosomal snRNP assembly	23	0.237712606	0.0111	0.018389742
GO:0014874	response to stimulus involved in regulation of muscle adaptation	5	0.237677563	0.0005	0.009604651
GO:0046330	positive regulation of JNK cascade	44	0.237643195	0.0057	0.01480566
GO:0048839	inner ear development	74	0.237641316	0.0141	0.020504577
GO:0034123	positive regulation of toll-like receptor signaling pathway	10	0.237639208	0.0228	0.027701114
GO:0030072	peptide hormone secretion	107	0.237619568	0.0016	0.011845327
GO:0009313	oligosaccharide catabolic process	5	0.237613551	0.0013	0.011218806
GO:0072283	metanephric renal vesicle morphogenesis	5	0.23754643	0.0006	0.009940401
GO:0021554	optic nerve development	6	0.237545609	0.0119	0.018881943
GO:0031123	RNA 3'-end processing	46	0.237538999	0.0047	0.014168613
GO:0014075	response to amine	20	0.237482029	0.0051	0.014547706
GO:0046883	regulation of hormone secretion	108	0.23747794	0.002	0.012314604
GO:0072584	caveolin-mediated endocytosis	5	0.237465573	0.0159	0.021956962
GO:0070199	establishment of protein localization to chromosome	12	0.237463111	0.0262	0.030579007

GOIDlabel	GOName	size	categoryScores	pValPerm	pValPermCorr
GO:0010642	negative regulation of platelet-derived growth factor receptor signaling pathway	6	0.237461815	0.0097	0.017363901
GO:0030277	maintenance of gastrointestinal epithelium	7	0.237450986	0.0347	0.038238117
GO:0018212	peptidyl-tyrosine modification	155	0.237445826	0.0006	0.009940401
GO:0034440	lipid oxidation	50	0.237431908	0.0173	0.023005658
GO:0070586	cell-cell adhesion involved in gastrulation	6	0.237422941	0.0143	0.020629391
GO:0060421	positive regulation of heart growth	17	0.237417339	0.0115	0.018599441
GO:0017157	regulation of exocytosis	103	0.237413173	0.0022	0.012422266
GO:0009749	response to glucose	84	0.237395128	0.0011	0.010890753
GO:0010959	regulation of metal ion transport	146	0.237383819	0.0036	0.01353394
GO:0001656	metanephros development	40	0.237369004	0.009	0.016956012
GO:0051271	negative regulation of cellular component movement	138	0.237353758	0.0042	0.013948535
GO:0034762	regulation of transmembrane transport	192	0.237347192	0.0017	0.012001709
GO:0070207	protein homotrimerization	15	0.237309214	0.0046	0.014132412
GO:0060291	long-term synaptic potentiation	49	0.237296867	0.0021	0.012314604
GO:0032768	regulation of monooxygenase activity	24	0.237282834	0.0074	0.015911789
GO:0043583	ear development	84	0.237275037	0.0146	0.020879842
GO:0007584	response to nutrient	74	0.237273856	0.0134	0.01999969
GO:0006783	heme biosynthetic process	15	0.237264121	0.0138	0.020339434
GO:0051480	regulation of cytosolic calcium ion concentration	132	0.237201745	0.0033	0.013333753
GO:1900408	negative regulation of cellular response to oxidative stress	20	0.237196049	0.0084	0.016633151
GO:1902883	negative regulation of response to oxidative stress	20	0.237196049	0.0084	0.016633151
GO:1903202	negative regulation of oxidative stress-induced cell death	20	0.237196049	0.0084	0.016633151
GO:0019318	hexose metabolic process	119	0.237167764	0.0042	0.013948535
GO:0021772	olfactory bulb development	11	0.237144171	0.0283	0.032434212
GO:0021988	olfactory lobe development	11	0.237144171	0.0283	0.032434212
GO:0030500	regulation of bone mineralization	33	0.237126658	0.0077	0.016177834
GO:0090103	cochlea morphogenesis	9	0.237106841	0.0053	0.014634479
GO:1905332	positive regulation of morphogenesis of an epithelium	13	0.237104826	0.0147	0.02091937
GO:0001906	cell killing	40	0.23710191	0.0153	0.021378589
GO:0050701	interleukin-1 secretion	20	0.237081566	0.0201	0.025275816
GO:0019079	viral genome replication	41	0.23707635	0.0043	0.014102439
GO:0009746	response to hexose	85	0.237053543	0.0012	0.010961137
GO:0006471	protein ADP-ribosylation	16	0.237046515	0.0073	0.015844069
GO:0006633	fatty acid biosynthetic process	66	0.237042883	0.0076	0.016084627
GO:0001556	oocyte maturation	10	0.237027079	0.002	0.012314604
GO:0002920	regulation of humoral immune response	25	0.237010441	0.0318	0.035688587
GO:2000738	positive regulation of stem cell differentiation	11	0.237007995	0.0166	0.02247277
GO:0030048	actin filament-based movement	60	0.236983346	0.0032	0.013333753
GO:0097035	regulation of membrane lipid distribution	13	0.236959897	0.0109	0.018225506
GO:0006721	terpenoid metabolic process	49	0.236914425	0.0042	0.013948535
GO:0043116	negative regulation of vascular permeability	6	0.236901603	0.0283	0.032434212
GO:0019730	antimicrobial humoral response	25	0.236874953	0.0071	0.01583804
GO:1902175	regulation of oxidative stress-induced intrinsic apoptotic signaling pathway	12	0.23685258	0.0102	0.0177
GO:0044273	sulfur compound catabolic process	25	0.236847638	0.0112	0.018392048
GO:0032418	lysosome localization	38	0.236773446	0.0173	0.023005658
GO:0006837	serotonin transport	7	0.236723495	0.0357	0.03911643
GO:0046326	positive regulation of glucose import	9	0.23670414	0.0115	0.018599441
GO:0010574	regulation of vascular endothelial growth factor production	13	0.236692766	0.023	0.027838811
GO:0002675	positive regulation of acute inflammatory response	14	0.236668701	0.018	0.023514686
GO:0071280	cellular response to copper ion	11	0.236665083	0.005	0.014547706
GO:0007265	Ras protein signal transduction	152	0.236664813	0.0036	0.01353394
GO:0033363	secretory granule organization	18	0.236662027	0.0212	0.026203164
GO:0015833	peptide transport	113	0.23665794	0.0021	0.012314604
GO:0006805	xenobiotic metabolic process	41	0.23664735	0.0146	0.020879842
GO:0010657	muscle cell apoptotic process	36	0.236582372	0.0008	0.010465158
GO:0042311	vasodilation	12	0.236540403	0.0062	0.015209334
GO:0006903	vesicle targeting	49	0.236533252	0.0048	0.014269203
GO:0043030	regulation of macrophage activation	24	0.236503635	0.0344	0.037979912
GO:0071375	cellular response to peptide hormone stimulus	125	0.23649901	0.001	0.010787313
GO:0009914	hormone transport	132	0.236493912	0.0021	0.012314604
GO:0000098	sulfur amino acid catabolic process	6	0.23648436	0.0324	0.036242368
GO:0002703	regulation of leukocyte mediated immunity	63	0.236481265	0.0072	0.015844069
GO:0001910	regulation of leukocyte mediated cytotoxicity	19	0.236459866	0.0234	0.02817551
GO:0002204	somatic recombination of immunoglobulin genes involved in immune response	18	0.236428138	0.0003	0.007956881
GO:0002208	somatic diversification of immunoglobulins involved in immune response	18	0.236428138	0.0003	0.007956881
GO:0016447	somatic recombination of immunoglobulin gene segments	18	0.236428138	0.0003	0.007956881
GO:0045190	isotype switching	18	0.236428138	0.0003	0.007956881
GO:0043547	positive regulation of GTPase activity	150	0.236406786	0.0022	0.012422266
GO:0007254	JNK cascade	84	0.236395522	0.0028	0.012972436
GO:1901654	response to ketone	79	0.236380924	0.0088	0.016916437
GO:0030866	cortical actin cytoskeleton organization	11	0.236359985	0.0055	0.01475
GO:0060249	anatomical structure homeostasis	163	0.236340861	0.0046	0.014132412
GO:0072089	stem cell proliferation	50	0.236328572	0.0125	0.019314538
GO:0001774	microglial cell activation	17	0.236291453	0.0417	0.044666432
GO:0007229	integrin-mediated signaling pathway	46	0.236280115	0.0101	0.017653628
GO:0048675	axon extension	55	0.23624572	0.0012	0.010961137
GO:0033144	negative regulation of intracellular steroid hormone receptor signaling pathway	12	0.236244964	0.0006	0.009940401
GO:0072386	plus-end-directed organelle transport along microtubule	5	0.236213844	0.0073	0.015844069
GO:0006936	muscle contraction	152	0.236201663	0.0055	0.01475
GO:0042036	negative regulation of cytokine biosynthetic process	16	0.236177205	0.0133	0.019917275
GO:0031341	regulation of cell killing	21	0.236175562	0.0224	0.027324219
GO:0006040	amino sugar metabolic process	16	0.236127965	0.0288	0.032902905
GO:0071466	cellular response to xenobiotic stimulus	42	0.236113704	0.0126	0.019370699

GOIDlabel	GOName	size	categoryScores	pValPerm	pValPermCorr
GO:0019217	regulation of fatty acid metabolic process	41	0.236107166	0.0074	0.015911789
GO:0099566	regulation of postsynaptic cytosolic calcium ion concentration	6	0.236101888	0.0313	0.035223161
GO:1903428	positive regulation of reactive oxygen species biosynthetic process	19	0.236080473	0.0112	0.018392048
GO:0015748	organophosphate ester transport	35	0.236070826	0.0021	0.012314604
GO:0050922	negative regulation of chemotaxis	18	0.236052648	0.0237	0.028436066
GO:0046888	negative regulation of hormone secretion	29	0.236038081	0.0059	0.014942532
GO:0002762	negative regulation of myeloid leukocyte differentiation	17	0.23603001	0.0032	0.013333753
GO:0001974	blood vessel remodeling	17	0.236022311	< 0.001	< 0.001
GO:0042476	odontogenesis	44	0.23601669	0.0071	0.01583804
GO:0000288	nuclear-transcribed mRNA catabolic process, deadenylation-dependent decay	33	0.236009691	0.0031	0.013277185
GO:0007187	G-protein coupled receptor signaling pathway, coupled to cyclic nucleotide second messenger	73	0.23598745	0.0033	0.013333753
GO:0038202	TORC1 signaling	21	0.235949664	0.0027	0.012827773
GO:0010659	cardiac muscle cell apoptotic process	18	0.235936099	0.0004	0.008964341
GO:0002931	response to ischemia	24	0.235934893	0.0048	0.014269203
GO:0016202	regulation of striated muscle tissue development	57	0.235931253	0.0024	0.012603815
GO:1903050	regulation of proteolysis involved in cellular protein catabolic process	99	0.235908423	0.003	0.013210967
GO:0060137	maternal process involved in parturition	5	0.235897672	0.0294	0.033482529
GO:0019748	secondary metabolic process	27	0.235888035	0.0131	0.019786886
GO:0016358	dendrite development	104	0.235879536	0.0009	0.010729485
GO:0010586	miRNA metabolic process	9	0.235877429	0.0034	0.01347416
GO:0008277	regulation of G-protein coupled receptor protein signaling pathway	70	0.235849246	0.0032	0.013333753
GO:0070997	neuron death	156	0.235783282	0.0025	0.012769435
GO:1901626	regulation of postsynaptic membrane organization	9	0.235777077	0.019	0.024347961
GO:1903532	positive regulation of secretion by cell	183	0.235772206	0.0039	0.013791927
GO:1903078	positive regulation of protein localization to plasma membrane	25	0.235770426	0.001	0.010787313
GO:0006665	sphingolipid metabolic process	67	0.235767672	0.0012	0.010961137
GO:2000736	regulation of stem cell differentiation	62	0.235724291	0.017	0.022784886
GO:0009070	serine family amino acid biosynthetic process	8	0.235718173	0.0166	0.02247277
GO:0016559	peroxisome fission	7	0.235704822	0.0158	0.021871104
GO:0060041	retina development in camera-type eye	66	0.235675005	0.0039	0.013791927
GO:0097104	postsynaptic membrane assembly	5	0.235671946	0.0083	0.016559903
GO:0031667	response to nutrient levels	183	0.235670497	0.0054	0.014720792
GO:2000737	negative regulation of stem cell differentiation	8	0.235624876	0.0374	0.040724444
GO:1904862	inhibitory synapse assembly	5	0.235613117	0.0385	0.041733596
GO:0030595	leukocyte chemotaxis	65	0.23560295	0.0151	0.02121706
GO:0002790	peptide secretion	109	0.23558777	0.0017	0.012001709
GO:0051262	protein tetramerization	71	0.235580398	0.0047	0.014168613
GO:0007338	single fertilization	37	0.235554471	0.0021	0.012314604
GO:0043507	positive regulation of JUN kinase activity	27	0.235536781	0.002	0.012314604
GO:0006520	cellular amino acid metabolic process	179	0.23553437	0.0103	0.017772187
GO:0010517	regulation of phospholipase activity	25	0.235507588	0.012	0.018931514
GO:0048002	antigen processing and presentation of peptide antigen	112	0.23545676	0.0179	0.02344231
GO:0014910	regulation of smooth muscle cell migration	25	0.23543461	0.0051	0.014547706
GO:0016486	peptide hormone processing	5	0.235434299	0.0057	0.01480566
GO:0071496	cellular response to external stimulus	136	0.235396933	0.0058	0.014878261
GO:0061448	connective tissue development	103	0.235364611	0.0066	0.015525305
GO:1901606	alpha-amino acid catabolic process	48	0.235357027	0.0072	0.015844069
GO:0033028	myeloid cell apoptotic process	14	0.235345519	0.005	0.014547706
GO:0001504	neurotransmitter uptake	13	0.235322223	0.0071	0.01583804
GO:0046777	protein autophosphorylation	97	0.235290766	0.0003	0.007956881
GO:0048771	tissue remodeling	60	0.235279388	0.0011	0.010890753
GO:0035588	G-protein coupled purinergic receptor signaling pathway	14	0.235267402	0.008	0.016379603
GO:1902170	cellular response to reactive nitrogen species	7	0.235266106	0.0052	0.014616626
GO:0034765	regulation of ion transmembrane transport	184	0.235259676	0.0013	0.011218806
GO:0044242	cellular lipid catabolic process	87	0.23524372	0.0082	0.016514246
GO:0032479	regulation of type I interferon production	60	0.23522585	0.0068	0.015633241
GO:0032606	type I interferon production	60	0.23522585	0.0068	0.015633241
GO:0019731	antibacterial humoral response	14	0.235217699	0.0274	0.03166636
GO:0032735	positive regulation of interleukin-12 production	10	0.235201964	0.0068	0.015633241
GO:0007189	adenylate cyclase-activating G-protein coupled receptor signaling pathway	29	0.235150066	0.0003	0.007956881
GO:0002697	regulation of immune effector process	160	0.235131611	0.01	0.017574468
GO:0034284	response to monosaccharide	87	0.235127487	0.0014	0.011465722
GO:0072330	monocarboxylic acid biosynthetic process	98	0.235124363	0.0081	0.016514175
GO:0008593	regulation of Notch signaling pathway	41	0.235122337	0.0047	0.014168613
GO:0031396	regulation of protein ubiquitination	101	0.235119554	0.0048	0.014269203
GO:0043388	positive regulation of DNA binding	20	0.235114693	0.0129	0.019597425
GO:1903426	regulation of reactive oxygen species biosynthetic process	31	0.235109692	0.0156	0.021692929
GO:0009066	aspartate family amino acid metabolic process	23	0.235109259	0.0131	0.019786886
GO:0030178	negative regulation of Wnt signaling pathway	111	0.235084801	0.0048	0.014269203
GO:0018095	protein polyglutamylation	5	0.235081327	0.0044	0.014132412
GO:0033197	response to vitamin E	7	0.235028237	0.0079	0.01629604
GO:0016579	protein deubiquitination	120	0.235027854	0.0052	0.014616626
GO:0060415	muscle tissue morphogenesis	28	0.235024072	0.0174	0.023064374
GO:0051055	negative regulation of lipid biosynthetic process	22	0.234988042	0.0161	0.02211694
GO:0043087	regulation of GTPase activity	189	0.234952039	0.0023	0.012498684
GO:0006790	sulfur compound metabolic process	180	0.234933564	0.0087	0.016840777
GO:0047496	vesicle transport along microtubule	27	0.234930737	0.0099	0.017505138
GO:0001942	hair follicle development	27	0.23492805	0.0112	0.018392048
GO:0022404	molting cycle process	27	0.23492805	0.0112	0.018392048
GO:0022405	hair cycle process	27	0.23492805	0.0112	0.018392048
GO:0098773	skin epidermis development	27	0.23492805	0.0112	0.018392048
GO:1900182	positive regulation of protein localization to nucleus	36	0.234881077	0.0142	0.020567234
GO:0051298	centrosome duplication	21	0.234850097	0.0026	0.012805111

GOIDlabel	GOName	size	categoryScores	pValPerm	pValPermCorr
GO:0035589	G-protein coupled purinergic nucleotide receptor signaling pathway	8	0.23483218	0.0202	0.025368462
GO:0021762	substantia nigra development	23	0.234828975	0.0061	0.015143924
GO:0018108	peptidyl-tyrosine phosphorylation	152	0.234817602	0.0006	0.009940401
GO:0032868	response to insulin	109	0.234801525	0.0007	0.010220707
GO:0090140	regulation of mitochondrial fission	14	0.234792774	0.0078	0.01620539
GO:0015858	nucleoside transport	9	0.234767948	0.0001	0.004220438
GO:0030539	male genitalia development	6	0.234761181	0.0337	0.03733539
GO:0033762	response to glucagon	13	0.23475148	< 0.001	< 0.001
GO:0016072	rRNA metabolic process	79	0.234747513	0.0056	0.014771533
GO:0050714	positive regulation of protein secretion	111	0.234747056	0.0079	0.01629604
GO:0090342	regulation of cell aging	22	0.234742667	0.0029	0.013089617
GO:2001259	positive regulation of cation channel activity	30	0.234725504	0.003	0.013210967
GO:1901532	regulation of hematopoietic progenitor cell differentiation	48	0.234704815	0.0197	0.024979254
GO:0043508	negative regulation of JUN kinase activity	9	0.234648592	0.002	0.012314604
GO:0051924	regulation of calcium ion transport	92	0.234559719	0.0067	0.015576759
GO:0061000	negative regulation of dendritic spine development	6	0.234535283	< 0.001	< 0.001
GO:1903038	negative regulation of leukocyte cell-cell adhesion	41	0.234528108	0.0093	0.017114131
GO:0010038	response to metal ion	158	0.234454222	0.0059	0.014942532
GO:2001021	negative regulation of response to DNA damage stimulus	30	0.23444704	0.0259	0.0303391
GO:0010744	positive regulation of macrophage derived foam cell differentiation	5	0.234435507	0.0006	0.009940401
GO:0034114	regulation of heterotypic cell-cell adhesion	9	0.234430612	0.0157	0.021779607
GO:0008344	adult locomotory behavior	31	0.234410093	0.0007	0.010220707
GO:0097320	membrane tubulation	7	0.234409591	0.0053	0.014634479
GO:0051898	negative regulation of protein kinase B signaling	23	0.234392915	0.0018	0.01210186
GO:0046426	negative regulation of JAK-STAT cascade	7	0.234378863	0.0114	0.018551872
GO:1904893	negative regulation of STAT cascade	7	0.234378863	0.0114	0.018551872
GO:0071480	cellular response to gamma radiation	14	0.234373957	0.005	0.014547706
GO:0034605	cellular response to heat	47	0.234356988	0.0041	0.013887639
GO:0036297	interstrand cross-link repair	20	0.234352188	0.0098	0.017418875
GO:0009994	oocyte differentiation	21	0.234348258	< 0.001	< 0.001
GO:0032048	cardiolipin metabolic process	6	0.234347394	0.0054	0.014720792
GO:0009190	cyclic nucleotide biosynthetic process	36	0.234343254	0.0023	0.012498684
GO:0052652	cyclic purine nucleotide metabolic process	36	0.234343254	0.0023	0.012498684
GO:1901861	regulation of muscle tissue development	58	0.234333967	0.0028	0.012972436
GO:0009187	cyclic nucleotide metabolic process	42	0.234277545	0.0022	0.012422266
GO:0007126	meiotic nuclear division	45	0.234263542	0.0018	0.01210186
GO:0006066	alcohol metabolic process	144	0.234249949	0.0071	0.01583804
GO:0002705	positive regulation of leukocyte mediated immunity	38	0.234249323	0.0073	0.015844069
GO:0001953	negative regulation of cell-matrix adhesion	9	0.234243731	0.003	0.013210967
GO:2000772	regulation of cellular senescence	17	0.234230468	0.0011	0.010890753
GO:0072010	glomerular epithelium development	7	0.234204857	0.0112	0.018392048
GO:0072329	monocarboxylic acid catabolic process	54	0.234193579	0.0147	0.02091937
GO:0010506	regulation of autophagy	149	0.234186047	0.0028	0.012972436
GO:0071677	positive regulation of mononuclear cell migration	8	0.234158047	0.0384	0.04164081
GO:0019048	modulation by virus of host morphology or physiology	24	0.234136289	0.0017	0.012001709
GO:0044003	modification by symbiont of host morphology or physiology	24	0.234136289	0.0017	0.012001709
GO:0021884	forebrain neuron development	15	0.234131084	0.0065	0.015491756
GO:1903046	meiotic cell cycle process	48	0.234113423	0.0018	0.01210186
GO:0060292	long term synaptic depression	12	0.234094176	< 0.001	< 0.001
GO:0009314	response to radiation	181	0.234079452	0.0018	0.01210186
GO:0009156	ribonucleoside monophosphate biosynthetic process	47	0.234072163	0.0171	0.022860624
GO:0007409	axonogenesis	199	0.234066202	0.001	0.010787313
GO:0051705	multi-organism behavior	36	0.234055842	0.0026	0.012805111
GO:0051047	positive regulation of secretion	196	0.234044801	0.0038	0.01366393
GO:0010799	regulation of peptidyl-threonine phosphorylation	21	0.234026779	0.005	0.014547706
GO:0002449	lymphocyte mediated immunity	73	0.234015779	0.0064	0.015399417
GO:0032607	interferon-alpha production	11	0.234010725	0.0415	0.044476923
GO:0071806	protein transmembrane transport	29	0.233971169	0.0054	0.014720792
GO:0042471	ear morphogenesis	40	0.233956714	0.009	0.016956012
GO:0032873	negative regulation of stress-activated MAPK cascade	21	0.233951747	0.0008	0.010465158
GO:0070303	negative regulation of stress-activated protein kinase signaling cascade	21	0.233951747	0.0008	0.010465158
GO:0000244	spliceosomal tri-snRNP complex assembly	7	0.233929966	0.0087	0.016840777
GO:0022412	cellular process involved in reproduction in multicellular organism	90	0.233909447	0.005	0.014547706
GO:0050730	regulation of peptidyl-tyrosine phosphorylation	103	0.233900775	0.001	0.010787313
GO:0042886	amide transport	127	0.233893858	0.0017	0.012001709
GO:0048634	regulation of muscle organ development	58	0.233891188	0.0021	0.012314604
GO:0002683	negative regulation of immune system process	169	0.233883541	0.0071	0.01583804
GO:0016572	histone phosphorylation	18	0.233832175	0.0006	0.009940401
GO:0050849	negative regulation of calcium-mediated signaling	18	0.233824616	0.0011	0.010890753
GO:0002438	acute inflammatory response to antigenic stimulus	11	0.233808818	0.0086	0.016782045
GO:0090305	nucleic acid phosphodiester bond hydrolysis	97	0.233797112	0.0039	0.013791927
GO:0030214	hyaluronan catabolic process	9	0.233791214	0.0167	0.02255007
GO:0097553	calcium ion transmembrane import into cytosol	54	0.233789222	0.0047	0.014168613
GO:1902656	calcium ion import into cytosol	54	0.233789222	0.0047	0.014168613
GO:0060252	positive regulation of glial cell proliferation	5	0.233783727	0.033	0.036785425
GO:0010837	regulation of keratinocyte proliferation	17	0.233763452	0.0141	0.020504577
GO:1903405	protein localization to nuclear body	6	0.2337509	0.0388	0.041925173
GO:1904816	positive regulation of protein localization to chromosome, telomeric region	6	0.2337509	0.0388	0.041925173
GO:1904851	positive regulation of establishment of protein localization to telomere	6	0.2337509	0.0388	0.041925173
GO:1904867	protein localization to Cajal body	6	0.2337509	0.0388	0.041925173
GO:1904869	regulation of protein localization to Cajal body	6	0.2337509	0.0388	0.041925173
GO:1904871	positive regulation of protein localization to Cajal body	6	0.2337509	0.0388	0.041925173
GO:1990173	protein localization to nucleoplasm	6	0.2337509	0.0388	0.041925173

GOIDlabel	GOName	size	categoryScores	pValPerm	pValPermCorr
GO:0043687	post-translational protein modification	187	0.233748463	0.0048	0.014269203
GO:0010875	positive regulation of cholesterol efflux	7	0.233745038	0.025	0.029554283
GO:0035587	purinergic receptor signaling pathway	17	0.233740737	0.0076	0.016084627
GO:0060443	mammary gland morphogenesis	16	0.23374048	0.0123	0.019143634
GO:0006743	ubiquinone metabolic process	9	0.23374039	0.0072	0.015844069
GO:2000369	regulation of clathrin-dependent endocytosis	14	0.233721437	0.001	0.010787313
GO:0009798	axis specification	29	0.233714291	0.0058	0.014878261
GO:0051495	positive regulation of cytoskeleton organization	93	0.233694067	0.0045	0.014132412
GO:0021794	thalamus development	6	0.233683088	0.0176	0.023223003
GO:1903312	negative regulation of mRNA metabolic process	15	0.233675313	0.0061	0.015143924
GO:0071383	cellular response to steroid hormone stimulus	84	0.233670022	0.0041	0.013887639
GO:0061351	neural precursor cell proliferation	64	0.233658461	0.0111	0.018389742
GO:0006414	translational elongation	67	0.233653703	0.0253	0.029811412
GO:0042752	regulation of circadian rhythm	54	0.233652709	0.0001	0.004220438
GO:0014821	phasic smooth muscle contraction	8	0.233642162	0.0123	0.019143634
GO:0016101	diterpenoid metabolic process	44	0.233634682	0.006	0.015037711
GO:1900119	positive regulation of execution phase of apoptosis	5	0.233607241	0.001	0.010787313
GO:0072006	nephron development	56	0.233591974	0.0122	0.019049527
GO:0016558	protein import into peroxisome matrix	6	0.233578345	0.0059	0.014942532
GO:0043409	negative regulation of MAPK cascade	78	0.233558327	0.0053	0.014634479
GO:0007080	mitotic metaphase plate congression	21	0.233531789	0.0024	0.012603815
GO:0070266	necroptotic process	15	0.23349684	0.0179	0.02344231
GO:0005996	monosaccharide metabolic process	130	0.233494318	0.0046	0.014132412
GO:0051310	metaphase plate congression	26	0.233442437	0.0055	0.01475
GO:0007100	mitotic centrosome separation	5	0.233437234	< 0.001	< 0.001
GO:0070206	protein trimerization	25	0.233436093	0.0069	0.015750415
GO:0060271	cilium assembly	140	0.233379247	0.0036	0.01353394
GO:0055088	lipid homeostasis	48	0.233351285	0.0015	0.011704453
GO:1900180	regulation of protein localization to nucleus	65	0.233341166	0.0107	0.018084595
GO:0099623	regulation of cardiac muscle cell membrane repolarization	12	0.233340028	0.0176	0.023223003
GO:0030705	cytoskeleton-dependent intracellular transport	80	0.233339078	0.0045	0.014132412
GO:0046605	regulation of centrosome cycle	22	0.233290386	0.0015	0.011704453
GO:0048588	developmental cell growth	109	0.233282924	0.0012	0.010961137
GO:0018210	peptidyl-threonine modification	50	0.233272384	0.0001	0.004220438
GO:0010810	regulation of cell-substrate adhesion	80	0.233216626	0.0046	0.014132412
GO:0001569	branching involved in blood vessel morphogenesis	12	0.233214337	0.0109	0.018225506
GO:0042558	pteridine-containing compound metabolic process	18	0.233210558	0.01	0.017574468
GO:0050663	cytokine secretion	84	0.23319652	0.0131	0.019786886
GO:0032204	regulation of telomere maintenance	37	0.23319509	0.0049	0.014447629
GO:0006094	gluconeogenesis	39	0.233194178	0.0067	0.015576759
GO:0033143	regulation of intracellular steroid hormone receptor signaling pathway	32	0.233190136	0.0088	0.016916437
GO:0050878	regulation of body fluid levels	191	0.233186173	0.0028	0.012972436
GO:0032331	negative regulation of chondrocyte differentiation	6	0.23318421	0.0068	0.015633241
GO:0097006	regulation of plasma lipoprotein particle levels	34	0.233175432	0.0129	0.019597425
GO:0007530	sex determination	6	0.233161966	0.009	0.016956012
GO:0046879	hormone secretion	129	0.233151183	0.0021	0.012314604
GO:0007411	axon guidance	111	0.233148733	0.002	0.012314604
GO:0006284	base-excision repair	14	0.233113713	0.0009	0.010729485
GO:0051056	regulation of small GTPase mediated signal transduction	152	0.233105895	0.0036	0.01353394
GO:0060021	palate development	31	0.233104735	0.0033	0.013333753
GO:0035036	sperm-egg recognition	11	0.233101908	0.0128	0.019522448
GO:0003073	regulation of systemic arterial blood pressure	28	0.233078495	0.0028	0.012972436
GO:1904355	positive regulation of telomere capping	12	0.233060031	0.0055	0.01475
GO:0007156	homophilic cell adhesion via plasma membrane adhesion molecules	47	0.233011246	0.0005	0.009604651
GO:0043406	positive regulation of MAP kinase activity	103	0.233001513	0.0094	0.017156187
GO:0009145	purine nucleoside triphosphate biosynthetic process	29	0.232987221	0.0189	0.024289798
GO:0098542	defense response to other organism	174	0.232951527	0.0046	0.014132412
GO:0001946	lymphangiogenesis	6	0.232938659	0.0057	0.01480566
GO:0006301	postreplication repair	18	0.232906264	0.0338	0.0374175
GO:0042398	cellular modified amino acid biosynthetic process	22	0.232895584	0.0135	0.020081554
GO:0006140	regulation of nucleotide metabolic process	82	0.232886474	0.0043	0.014102439
GO:0044557	relaxation of smooth muscle	5	0.232883156	0.0176	0.023223003
GO:0060087	relaxation of vascular smooth muscle	5	0.232883156	0.0176	0.023223003
GO:0097091	synaptic vesicle clustering	8	0.232865663	0.0174	0.023064374
GO:0019372	lipoxygenase pathway	10	0.232863201	0.0135	0.020081554
GO:0051817	modification of morphology or physiology of other organism involved in symbiotic interaction	46	0.232844648	0.0075	0.015990044
GO:0032465	regulation of cytokinesis	27	0.232823358	0.0014	0.011465722
GO:0032891	negative regulation of organic acid transport	6	0.23281923	0.0163	0.022248961
GO:0001889	liver development	58	0.232812826	0.003	0.013210967
GO:0061008	hepaticobiliary system development	58	0.232812826	0.003	0.013210967
GO:0050769	positive regulation of neurogenesis	192	0.232790723	0.0027	0.012827773
GO:0070862	negative regulation of protein exit from endoplasmic reticulum	5	0.232782084	0.0254	0.029880529
GO:0006979	response to oxidative stress	197	0.232737962	0.0048	0.014269203
GO:0098661	inorganic anion transmembrane transport	36	0.232725898	0.0159	0.021956962
GO:2000272	negative regulation of receptor activity	26	0.23272183	0.0024	0.012603815
GO:0071695	anatomical structure maturation	22	0.2327213	0.0045	0.014132412
GO:0010212	response to ionizing radiation	63	0.232700121	0.0023	0.012498684
GO:1901028	regulation of mitochondrial outer membrane permeabilization involved in apoptotic signaling pathway	19	0.232648414	0.0032	0.013333753
GO:0060541	respiratory system development	84	0.232631187	0.0054	0.014720792
GO:0043535	regulation of blood vessel endothelial cell migration	42	0.232620605	0.0036	0.01353394
GO:0032412	regulation of ion transmembrane transporter activity	109	0.232611136	0.0012	0.010961137
GO:0006685	sphingomyelin catabolic process	6	0.23260089	0.0169	0.022703485
GO:0035590	purinergic nucleotide receptor signaling pathway	11	0.232591486	0.0152	0.0213213

GOIDlabel	GOName	size	categoryScores	pValPerm	pValPermCorr
GO:0097300	programmed necrotic cell death	17	0.2325711	0.0148	0.020979063
GO:0006898	receptor-mediated endocytosis	126	0.232564485	0.0067	0.015576759
GO:0048747	muscle fiber development	22	0.232552966	0.0121	0.018970228
GO:0050680	negative regulation of epithelial cell proliferation	56	0.232534451	0.0112	0.018392048
GO:0010634	positive regulation of epithelial cell migration	68	0.2325259	0.0033	0.013333753
GO:0098722	asymmetric stem cell division	5	0.232509192	0.0236	0.028327839
GO:0042307	positive regulation of protein import into nucleus	25	0.23250857	0.0147	0.02091937
GO:0021700	developmental maturation	121	0.23250363	0.0014	0.011465722
GO:0032727	positive regulation of interferon-alpha production	7	0.232479796	0.045	0.047741284
GO:0043001	Golgi to plasma membrane protein transport	12	0.232456842	0.0004	0.008964341
GO:0006361	transcription initiation from RNA polymerase I promoter	20	0.232440645	0.0065	0.015491756
GO:0097485	neuron projection guidance	112	0.23241901	0.0021	0.012314604
GO:0006497	protein lipidation	36	0.23241077	0.0098	0.017418875
GO:0071902	positive regulation of protein serine/threonine kinase activity	137	0.232405994	0.0078	0.01620539
GO:0045862	positive regulation of proteolysis	146	0.232400014	0.0042	0.013948535
GO:0044782	cilium organization	144	0.232394042	0.0035	0.01351837
GO:0045833	negative regulation of lipid metabolic process	32	0.232361076	0.0102	0.0177
GO:0070167	regulation of biomineral tissue development	38	0.232342302	0.008	0.016379603
GO:0034650	cortisol metabolic process	5	0.232333483	0.044	0.046783376
GO:0034651	cortisol biosynthetic process	5	0.232333483	0.044	0.046783376
GO:0010569	regulation of double-strand break repair via homologous recombination	11	0.232318547	0.0257	0.030153693
GO:0070193	synaptonemal complex organization	8	0.232280129	0.0091	0.016994897
GO:0031113	regulation of microtubule polymerization	18	0.232277016	0.0051	0.014547706
GO:0032956	regulation of actin cytoskeleton organization	137	0.232274467	0.0095	0.01725157
GO:0032480	negative regulation of type I interferon production	22	0.232268305	0.0116	0.018682786
GO:0001655	urogenital system development	146	0.232259066	0.0082	0.016514246
GO:0060964	regulation of gene silencing by miRNA	30	0.232254138	0.0063	0.015311728
GO:0000187	activation of MAPK activity	70	0.232243056	0.0074	0.015911789
GO:2000725	regulation of cardiac muscle cell differentiation	17	0.232236406	0.0005	0.009604651
GO:0032620	interleukin-17 production	14	0.232228414	0.004	0.013824268
GO:0099622	cardiac muscle cell membrane repolarization	16	0.232223763	0.0076	0.016084627
GO:0043524	negative regulation of neuron apoptotic process	63	0.232209759	0.0035	0.01351837
GO:0000132	establishment of mitotic spindle orientation	10	0.232207533	0.0013	0.011218806
GO:1904591	positive regulation of protein import	26	0.232200227	0.0151	0.02121706
GO:0032496	response to lipopolysaccharide	121	0.232196678	0.0126	0.019370699
GO:0060259	regulation of feeding behavior	11	0.23218779	0.0007	0.010220707
GO:0032409	regulation of transporter activity	118	0.23218532	0.0016	0.011845327
GO:0010762	regulation of fibroblast migration	14	0.232182784	0.007	0.015816335
GO:0007517	muscle organ development	158	0.232180832	0.0023	0.012498684
GO:0031110	regulation of microtubule polymerization or depolymerization	25	0.23216187	0.0041	0.013887639
GO:0035023	regulation of Rho protein signal transduction	55	0.232152144	0.0012	0.010961137
GO:0050872	white fat cell differentiation	8	0.232148121	0.0003	0.007956881
GO:0071347	cellular response to interleukin-1	73	0.232131689	0.0071	0.01583804
GO:0090025	regulation of monocyte chemotaxis	8	0.232129818	0.0256	0.03006687
GO:0030324	lung development	76	0.232121592	0.0078	0.01620539
GO:0043648	dicarboxylic acid metabolic process	48	0.2321015	0.0061	0.015143924
GO:0009266	response to temperature stimulus	91	0.232098137	0.0027	0.012827773
GO:0034446	substrate adhesion-dependent cell spreading	42	0.232071161	0.0072	0.015844069
GO:0010543	regulation of platelet activation	15	0.232046165	0.0412	0.04417997
GO:0060828	regulation of canonical Wnt signaling pathway	131	0.232033239	0.0064	0.015399417
GO:0034393	positive regulation of smooth muscle cell apoptotic process	8	0.232032634	0.0052	0.014616626
GO:0002768	immune response-regulating cell surface receptor signaling pathway	182	0.232030806	0.0073	0.015844069
GO:0019068	virion assembly	23	0.232029289	0.0147	0.02091937
GO:0007413	axonal fasciculation	11	0.232014508	0.0106	0.018010344
GO:0030513	positive regulation of BMP signaling pathway	15	0.232007809	0.0122	0.019049527
GO:0010758	regulation of macrophage chemotaxis	11	0.231993187	0.0208	0.025885837
GO:0003085	negative regulation of systemic arterial blood pressure	8	0.231991169	0.0033	0.013333753
GO:0072524	pyridine-containing compound metabolic process	79	0.23195452	0.0081	0.016514175
GO:0006364	rRNA processing	75	0.231942208	0.0057	0.01480566
GO:0032715	negative regulation of interleukin-6 production	16	0.231937475	0.0142	0.020567234
GO:0030540	female genitalia development	7	0.231916129	0.0097	0.017363901
GO:0006733	oxidoreduction coenzyme metabolic process	86	0.231886335	0.0073	0.015844069
GO:0045978	negative regulation of nucleoside metabolic process	13	0.231881613	0.0061	0.015143924
GO:1903579	negative regulation of ATP metabolic process	13	0.231881613	0.0061	0.015143924
GO:0010518	positive regulation of phospholipase activity	24	0.231875466	0.0107	0.018084595
GO:0031281	positive regulation of cyclase activity	22	0.231860251	0.001	0.010787313
GO:0009063	cellular amino acid catabolic process	59	0.231847999	0.0123	0.019143634
GO:0043038	amino acid activation	22	0.231808243	0.0104	0.017832977
GO:0043039	tRNA aminoacylation	22	0.231808243	0.0104	0.017832977
GO:0097421	liver regeneration	15	0.231796078	0.0085	0.016693954
GO:0035195	gene silencing by miRNA	45	0.2317824	0.0065	0.015491756
GO:0097581	lamellipodium organization	36	0.231768888	0.0078	0.01620539
GO:0060732	positive regulation of inositol phosphate biosynthetic process	8	0.23175809	0.0347	0.038238117
GO:0030323	respiratory tube development	79	0.231756314	0.0083	0.016559903
GO:0001675	acrosome assembly	7	0.231725834	0.0171	0.022860624
GO:0019362	pyridine nucleotide metabolic process	77	0.231669627	0.0075	0.015990044
GO:0046496	nicotinamide nucleotide metabolic process	77	0.231669627	0.0075	0.015990044
GO:0090646	mitochondrial tRNA processing	5	0.23166123	0.0262	0.030579007
GO:0030210	heparin biosynthetic process	5	0.2316519	0.0239	0.028592965
GO:0015914	phospholipid transport	24	0.231646922	0.0051	0.014547706
GO:0040013	negative regulation of locomotion	146	0.231643497	0.0041	0.013887639
GO:0070373	negative regulation of ERK1 and ERK2 cascade	37	0.231618224	0.008	0.016379603
GO:0046323	glucose import	20	0.231612443	0.0044	0.014132412

GOIDlabel	GOName	size	categoryScores	pValPerm	pValPermCorr
GO:0032928	regulation of superoxide anion generation	13	0.231584978	0.0356	0.03903645
GO:0022400	regulation of rhodopsin mediated signaling pathway	10	0.231563787	0.003	0.013210967
GO:0044342	type B pancreatic cell proliferation	12	0.231560482	0.0148	0.020979063
GO:0098900	regulation of action potential	18	0.231549	0.0213	0.026276211
GO:0006000	fructose metabolic process	7	0.231541647	0.0256	0.03006687
GO:0050772	positive regulation of axonogenesis	34	0.231541636	0.0012	0.010961137
GO:0045995	regulation of embryonic development	47	0.231541494	0.0047	0.014168613
GO:0071479	cellular response to ionizing radiation	33	0.231540408	0.0054	0.014720792
GO:0002381	immunoglobulin production involved in immunoglobulin mediated immune response	20	0.231540268	0.0006	0.009940401
GO:0032803	regulation of low-density lipoprotein particle receptor catabolic process	5	0.231540203	0.0164	0.022306469
GO:0014850	response to muscle activity	10	0.231465048	0.0071	0.01583804
GO:0008584	male gonad development	49	0.231417648	0.0069	0.015750415
GO:0035584	calcium-mediated signaling using intracellular calcium source	11	0.231417386	0.0012	0.010961137
GO:0006547	histidine metabolic process	5	0.231407774	0.0058	0.014878261
GO:0052803	imidazole-containing compound metabolic process	5	0.231407774	0.0058	0.014878261
GO:2000679	positive regulation of transcription regulatory region DNA binding	9	0.23140596	0.0253	0.029811412
GO:0042074	cell migration involved in gastrulation	5	0.231393261	0.0353	0.038781037
GO:0050764	regulation of phagocytosis	34	0.231368954	0.0128	0.019522448
GO:0002819	regulation of adaptive immune response	47	0.231334774	0.002	0.012314604
GO:1902476	chloride transmembrane transport	27	0.231313143	0.0136	0.020157703
GO:0043647	inositol phosphate metabolic process	33	0.231310975	0.0125	0.019314538
GO:0000578	embryonic axis specification	14	0.231277955	0.0087	0.016840777
GO:0045089	positive regulation of innate immune response	151	0.231262694	0.0114	0.018551872
GO:0001960	negative regulation of cytokine-mediated signaling pathway	22	0.231249582	0.0173	0.023005658
GO:0061548	ganglion development	7	0.231219922	0.002	0.012314604
GO:0015807	L-amino acid transport	23	0.231210522	0.0093	0.017114131
GO:1902108	regulation of mitochondrial membrane permeability involved in apoptotic process	26	0.231207406	0.0025	0.012769435
GO:0036109	alpha-linolenic acid metabolic process	6	0.231205761	0.0431	0.045953199
GO:0007569	cell aging	46	0.231199029	0.0019	0.012288367
GO:0046189	phenol-containing compound biosynthetic process	20	0.23118464	0.0085	0.016693954
GO:1903169	regulation of calcium ion transmembrane transport	55	0.231173095	0.0052	0.014616626
GO:0036119	response to platelet-derived growth factor	7	0.231163278	0.0041	0.013887639
GO:0036120	cellular response to platelet-derived growth factor stimulus	7	0.231163278	0.0041	0.013887639
GO:0045132	meiotic chromosome segregation	20	0.231149913	0.0068	0.015633241
GO:0007625	grooming behavior	7	0.231144026	0.0013	0.011218806
GO:0007219	Notch signaling pathway	76	0.231117835	0.0069	0.015750415
GO:0007212	dopamine receptor signaling pathway	23	0.231107817	0.0023	0.012498684
GO:0035278	miRNA mediated inhibition of translation	6	0.231085037	0.0111	0.018389742
GO:0040033	negative regulation of translation, ncRNA-mediated	6	0.231085037	0.0111	0.018389742
GO:0045974	regulation of translation, ncRNA-mediated	6	0.231085037	0.0111	0.018389742
GO:1903825	organic acid transmembrane transport	35	0.231080718	0.0088	0.016916437
GO:1905039	carboxylic acid transmembrane transport	35	0.231080718	0.0088	0.016916437
GO:0072091	regulation of stem cell proliferation	26	0.231079522	0.0188	0.02420971
GO:0015718	monocarboxylic acid transport	54	0.231068696	0.0087	0.016840777
GO:0035082	axoneme assembly	24	0.231058257	0.0098	0.017418875
GO:0032530	regulation of microvillus organization	6	0.231047243	0.001	0.010787313
GO:0042181	ketone biosynthetic process	19	0.231034683	0.0116	0.018682786
GO:0002695	negative regulation of leukocyte activation	65	0.231033571	0.0122	0.019049527
GO:0008217	regulation of blood pressure	64	0.231031903	0.0077	0.016177834
GO:0070588	calcium ion transmembrane transport	108	0.231023331	0.0046	0.014132412
GO:0061035	regulation of cartilage development	34	0.23098422	0.0051	0.014547706
GO:0032438	melanosome organization	15	0.230972995	0.012	0.018931514
GO:0048753	pigment granule organization	15	0.230972995	0.012	0.018931514
GO:0042726	flavin-containing compound metabolic process	8	0.23096766	0.0139	0.020388077
GO:0045216	cell-cell junction organization	93	0.230966354	0.0037	0.013609033
GO:1900542	regulation of purine nucleotide metabolic process	79	0.230927569	0.0045	0.014132412
GO:0050832	defense response to fungus	7	0.230920225	0.008	0.016379603
GO:0051090	regulation of sequence-specific DNA binding transcription factor activity	162	0.230891861	0.0064	0.015399417
GO:1901343	negative regulation of vasculature development	52	0.230886325	0.0073	0.015844069
GO:0002708	positive regulation of lymphocyte mediated immunity	26	0.230877778	0.0055	0.01475
GO:0042219	cellular modified amino acid catabolic process	7	0.230865062	0.002	0.012314604
GO:2000377	regulation of reactive oxygen species metabolic process	79	0.230852725	0.0102	0.0177
GO:0046320	regulation of fatty acid oxidation	18	0.230842941	0.0168	0.022626974
GO:0030810	positive regulation of nucleotide biosynthetic process	27	0.230835958	0.0031	0.013277185
GO:1900373	positive regulation of purine nucleotide biosynthetic process	27	0.230835958	0.0031	0.013277185
GO:1904062	regulation of cation transmembrane transport	133	0.230831976	0.0015	0.011704453
GO:0040019	positive regulation of embryonic development	15	0.23081871	0.0057	0.01480566
GO:0010042	response to manganese ion	10	0.230816248	0.0142	0.020567234
GO:0090129	positive regulation of synapse maturation	6	0.230811194	< 0.001	< 0.001
GO:2000181	negative regulation of blood vessel morphogenesis	48	0.230803393	0.007	0.015816335
GO:0035455	response to interferon-alpha	6	0.230788734	0.011	0.018323826
GO:0021872	forebrain generation of neurons	29	0.230778844	0.0129	0.019597425
GO:0016054	organic acid catabolic process	119	0.23076472	0.0125	0.019314538
GO:0046395	carboxylic acid catabolic process	119	0.23076472	0.0125	0.019314538
GO:0019985	translesion synthesis	15	0.230761609	0.0331	0.03684717
GO:0001708	cell fate specification	21	0.230747454	0.0178	0.023348367
GO:0048010	vascular endothelial growth factor receptor signaling pathway	45	0.230743525	0.0077	0.016177834
GO:0043470	regulation of carbohydrate catabolic process	30	0.230730035	0.0021	0.012314604
GO:0022898	regulation of transmembrane transporter activity	113	0.230696684	0.0015	0.011704453
GO:0000959	mitochondrial RNA metabolic process	12	0.230692846	0.0082	0.016514246
GO:0009612	response to mechanical stimulus	84	0.230669676	0.006	0.015037711
GO:0090493	catecholamine uptake	6	0.230658076	0.0242	0.028862294
GO:0090494	dopamine uptake	6	0.230658076	0.0242	0.028862294

GOIDlabel	GOName	size	categoryScores	pValPerm	pValPermCorr
GO:1903522	regulation of blood circulation	117	0.230645433	0.0035	0.01351837
GO:0042659	regulation of cell fate specification	6	0.230641014	0.009	0.016956012
GO:1904377	positive regulation of protein localization to cell periphery	26	0.230614484	0.001	0.010787313
GO:0050850	positive regulation of calcium-mediated signaling	24	0.230613911	0.0114	0.018551872
GO:2000106	regulation of leukocyte apoptotic process	26	0.23060965	0.0022	0.012422266
GO:0032886	regulation of microtubule-based process	71	0.230600875	0.0031	0.013277185
GO:0009582	detection of abiotic stimulus	46	0.230600657	0.0035	0.01351837
GO:0046655	folic acid metabolic process	10	0.230593114	0.0129	0.019597425
GO:0000209	protein polyubiquitination	154	0.230525524	0.0044	0.014132412
GO:0060339	negative regulation of type I interferon-mediated signaling pathway	5	0.230506425	0.0104	0.017832977
GO:0051146	striated muscle cell differentiation	94	0.230498552	0.0044	0.014132412
GO:0009896	positive regulation of catabolic process	141	0.230492982	0.0031	0.013277185
GO:0032728	positive regulation of interferon-beta production	11	0.23046946	0.0062	0.015209334
GO:0050918	positive chemotaxis	24	0.230439412	0.0295	0.033583186
GO:0060043	regulation of cardiac muscle cell proliferation	17	0.230434883	0.022	0.026944291
GO:0032434	regulation of proteasomal ubiquitin-dependent protein catabolic process	57	0.23041873	0.0031	0.013277185
GO:0090278	negative regulation of peptide hormone secretion	20	0.230372636	0.0115	0.018599441
GO:0002429	immune response-activating cell surface receptor signaling pathway	167	0.230351733	0.0077	0.016177834
GO:0043122	regulation of I-kappaB kinase/NF-kappaB signaling	99	0.230333023	0.0046	0.014132412
GO:0016441	posttranscriptional gene silencing	47	0.230329686	0.0055	0.01475
GO:0035194	posttranscriptional gene silencing by RNA	47	0.230329686	0.0055	0.01475
GO:0050848	regulation of calcium-mediated signaling	53	0.2303153	0.0049	0.014447629
GO:0070482	response to oxygen levels	186	0.230300996	0.0068	0.015633241
GO:1900024	regulation of substrate adhesion-dependent cell spreading	25	0.230299307	0.012	0.018931514
GO:0043241	protein complex disassembly	116	0.230291439	0.0102	0.0177
GO:1901019	regulation of calcium ion transmembrane transporter activity	35	0.230272629	0.0066	0.015525305
GO:0097529	myeloid leukocyte migration	64	0.23023133	0.0213	0.026276211
GO:0032303	regulation of icosanoid secretion	8	0.23022145	0.018	0.023514686
GO:2000191	regulation of fatty acid transport	8	0.23022145	0.018	0.023514686
GO:0022410	circadian sleep/wake cycle process	10	0.230203471	0.0003	0.007956881
GO:0050802	circadian sleep/wake cycle, sleep	10	0.230203471	0.0003	0.007956881
GO:0002281	macrophage activation involved in immune response	8	0.230184358	0.0183	0.023783007
GO:0022037	metencephalon development	46	0.230182344	0.0042	0.013948535
GO:0006816	calcium ion transport	167	0.230164085	0.0045	0.014132412
GO:0014044	Schwann cell development	10	0.230144124	0.0041	0.013887639
GO:0045911	positive regulation of DNA recombination	16	0.230097783	0.0035	0.01351837
GO:0060074	synapse maturation	17	0.230093939	0.0002	0.006319126
GO:0031050	dsRNA fragmentation	14	0.230067691	0.0082	0.016514246
GO:0070918	production of small RNA involved in gene silencing by RNA	14	0.230067691	0.0082	0.016514246
GO:0010660	regulation of muscle cell apoptotic process	34	0.230034752	0.0009	0.010729485
GO:0061099	negative regulation of protein tyrosine kinase activity	12	0.230033291	0.0042	0.013948535
GO:0002573	myeloid leukocyte differentiation	80	0.230022611	0.0064	0.015399417
GO:0002031	G-protein coupled receptor internalization	5	0.230021024	0.0375	0.040825645
GO:0097151	positive regulation of inhibitory postsynaptic potential	5	0.22998837	0.0006	0.009940401
GO:0098828	modulation of inhibitory postsynaptic potential	5	0.22998837	0.0006	0.009940401
GO:0006879	cellular iron ion homeostasis	30	0.229986729	0.0103	0.017772187
GO:1904948	midbrain dopaminergic neuron differentiation	7	0.229974487	0.0166	0.02247277
GO:0035136	forelimb morphogenesis	11	0.229955858	0.0206	0.025725529
GO:0045922	negative regulation of fatty acid metabolic process	12	0.22995306	0.0284	0.032523034
GO:0006942	regulation of striated muscle contraction	35	0.2299502	0.0139	0.020388077
GO:0008637	apoptotic mitochondrial changes	63	0.229944252	0.0049	0.014447629
GO:0035627	ceramide transport	6	0.229909543	0.0006	0.009940401
GO:0043488	regulation of mRNA stability	86	0.229876978	0.0126	0.019370699
GO:0002690	positive regulation of leukocyte chemotaxis	28	0.229850184	0.0135	0.020081554
GO:0031929	TOR signaling	50	0.229842672	0.0016	0.011845327
GO:0000723	telomere maintenance	63	0.229817121	0.0084	0.016633151
GO:0032200	telomere organization	63	0.229817121	0.0084	0.016633151
GO:0099518	vesicle cytoskeletal trafficking	29	0.229811026	0.0096	0.017302743
GO:0051225	spindle assembly	26	0.229802624	0.0062	0.015209334
GO:0006302	double-strand break repair	75	0.229802071	0.0026	0.012805111
GO:0061450	trophoblast cell migration	5	0.229785191	0.0188	0.02420971
GO:1901163	regulation of trophoblast cell migration	5	0.229785191	0.0188	0.02420971
GO:0010508	positive regulation of autophagy	53	0.229754469	0.0014	0.011465722
GO:0022616	DNA strand elongation	5	0.229751242	0.0451	0.047829824
GO:0001822	kidney development	127	0.229738161	0.0071	0.01583804
GO:0006110	regulation of glycolytic process	27	0.229735404	0.0041	0.013887639
GO:1903829	positive regulation of cellular protein localization	149	0.229734405	0.004	0.013824268
GO:0021514	ventral spinal cord interneuron differentiation	6	0.22969833	0.0229	0.027770092
GO:0036010	protein localization to endosome	10	0.229693449	0.0051	0.014547706
GO:0072395	signal transduction involved in cell cycle checkpoint	24	0.22967641	0.0018	0.01210186
GO:0034243	regulation of transcription elongation from RNA polymerase II promoter	12	0.22967587	0.009	0.016956012
GO:0048814	regulation of dendrite morphogenesis	42	0.229672445	0.0008	0.010465158
GO:2000042	negative regulation of double-strand break repair via homologous recombination	5	0.229666756	0.0397	0.042801678
GO:0050866	negative regulation of cell activation	76	0.229657894	0.016	0.022026667
GO:0006644	phospholipid metabolic process	186	0.229639658	0.0058	0.014878261
GO:0002029	desensitization of G-protein coupled receptor protein signaling pathway	8	0.229621501	0.0317	0.035590175
GO:0022401	negative adaptation of signaling pathway	8	0.229621501	0.0317	0.035590175
GO:0045686	negative regulation of glial cell differentiation	13	0.229614985	0.0183	0.023783007
GO:0032147	activation of protein kinase activity	136	0.229582208	0.0043	0.014102439
GO:0098742	cell-cell adhesion via plasma-membrane adhesion molecules	86	0.229571881	0.0041	0.013887639
GO:0002534	cytokine production involved in inflammatory response	16	0.229548856	0.0011	0.010890753
GO:0043467	regulation of generation of precursor metabolites and energy	54	0.229548667	0.0017	0.012001709
GO:0048477	oogenesis	31	0.229522347	0.0008	0.010465158

GOIDlabel	GOName	size	categoryScores	pValPerm	pValPermCorr
GO:0019722	calcium-mediated signaling	90	0.229506496	0.0027	0.012827773
GO:0021756	striatum development	10	0.229489234	0.0051	0.014547706
GO:0045806	negative regulation of endocytosis	30	0.229478695	0.002	0.012314604
GO:0032732	positive regulation of interleukin-1 production	16	0.229460014	0.0264	0.030750363
GO:0071222	cellular response to lipopolysaccharide	74	0.229453754	0.0078	0.01620539
GO:0019674	NAD metabolic process	37	0.229449674	0.0097	0.017363901
GO:0055090	acylglycerol homeostasis	10	0.229446214	0.0004	0.008964341
GO:0070328	triglyceride homeostasis	10	0.229446214	0.0004	0.008964341
GO:0010575	positive regulation of vascular endothelial growth factor production	10	0.229445436	0.0308	0.034775552
GO:1903320	regulation of protein modification by small protein conjugation or removal	110	0.229408577	0.0039	0.013791927
GO:0060603	mammary gland duct morphogenesis	11	0.229390188	0.0253	0.029811412
GO:0048146	positive regulation of fibroblast proliferation	25	0.229374323	0.0245	0.029070183
GO:0043506	regulation of JUN kinase activity	39	0.229361974	0.003	0.013210967
GO:0007252	I-kappaB phosphorylation	9	0.22933515	0.033	0.036785425
GO:0031329	regulation of cellular catabolic process	178	0.229333467	0.003	0.013210967
GO:0032970	regulation of actin filament-based process	151	0.229328618	0.0086	0.016782045
GO:0030208	dermatan sulfate biosynthetic process	9	0.229301604	0.0142	0.020567234
GO:0006664	glycolipid metabolic process	56	0.229298711	0.0095	0.01725157
GO:1905521	regulation of macrophage migration	18	0.229292101	0.0377	0.040981651
GO:0090090	negative regulation of canonical Wnt signaling pathway	94	0.229269416	0.0079	0.01629604
GO:0009100	glycoprotein metabolic process	184	0.229262822	0.0032	0.013333753
GO:0042755	eating behavior	16	0.229243213	0.0001	0.004220438
GO:0010770	positive regulation of cell morphogenesis involved in differentiation	68	0.229239044	0.0015	0.011704453
GO:0003413	chondrocyte differentiation involved in endochondral bone morphogenesis	6	0.229237462	0.0093	0.017114131
GO:0006293	nucleotide-excision repair, preincision complex stabilization	11	0.229229864	0.0145	0.020788247
GO:0006295	nucleotide-excision repair, DNA incision, 3'-to lesion	11	0.229229864	0.0145	0.020788247
GO:0031647	regulation of protein stability	134	0.229215879	0.0045	0.014132412
GO:0010421	hydrogen peroxide-mediated programmed cell death	5	0.229199237	0.0143	0.020629391
GO:0097468	programmed cell death in response to reactive oxygen species	5	0.229199237	0.0143	0.020629391
GO:0010594	regulation of endothelial cell migration	80	0.229194507	0.0041	0.013887639
GO:0000018	regulation of DNA recombination	31	0.229194346	0.0033	0.013333753
GO:0042769	DNA damage response, detection of DNA damage	18	0.229194198	0.0248	0.029383934
GO:0042754	negative regulation of circadian rhythm	9	0.229170586	< 0.001	< 0.001
GO:0048639	positive regulation of developmental growth	78	0.229147688	0.0027	0.012827773
GO:0051930	regulation of sensory perception of pain	22	0.229143106	0.0029	0.013089617
GO:0051931	regulation of sensory perception	22	0.229143106	0.0029	0.013089617
GO:0097345	mitochondrial outer membrane permeabilization	25	0.229139371	0.0029	0.013089617
GO:1902110	positive regulation of mitochondrial membrane permeability involved in apoptotic process	25	0.229139371	0.0029	0.013089617
GO:0046785	microtubule polymerization	25	0.229127709	0.0033	0.013333753
GO:0031589	cell-substrate adhesion	127	0.229126591	0.0047	0.014168613
GO:0006643	membrane lipid metabolic process	87	0.229121549	0.0045	0.014132412
GO:0001892	embryonic placenta development	36	0.229107956	0.0022	0.012422266
GO:0044801	single-organism membrane fusion	45	0.229095948	0.0032	0.013333753
GO:0007588	excretion	30	0.22907618	0.0077	0.016177834
GO:0051222	positive regulation of protein transport	193	0.229024923	0.006	0.015037711
GO:0010639	negative regulation of organelle organization	140	0.229022936	0.0033	0.013333753
GO:0070838	divalent metal ion transport	176	0.228952566	0.0047	0.014168613
GO:0072511	divalent inorganic cation transport	176	0.228952566	0.0047	0.014168613
GO:0006260	DNA replication	103	0.228950484	0.0017	0.012001709
GO:1990090	cellular response to nerve growth factor stimulus	24	0.228948502	0.0012	0.010961137
GO:0032663	regulation of interleukin-2 production	20	0.228947271	0.0112	0.018392048
GO:0002237	response to molecule of bacterial origin	127	0.228946222	0.0139	0.020388077
GO:1903670	regulation of sprouting angiogenesis	32	0.22893765	0.0048	0.014269203
GO:0051220	cytoplasmic sequestering of protein	21	0.228921075	0.0051	0.014547706
GO:0007565	female pregnancy	78	0.228900326	0.0031	0.013277185
GO:0051152	positive regulation of smooth muscle cell differentiation	6	0.228899694	0.0164	0.022306469
GO:2001014	regulation of skeletal muscle cell differentiation	8	0.228884523	0.0014	0.011465722
GO:0060070	canonical Wnt signaling pathway	148	0.228883249	0.0068	0.015633241
GO:0015672	monovalent inorganic cation transport	183	0.228878555	0.0016	0.011845327
GO:0009064	glutamine family amino acid metabolic process	35	0.228873143	0.0021	0.012314604
GO:0000226	microtubule cytoskeleton organization	173	0.228868886	0.0023	0.012498684
GO:0007626	locomotory behavior	87	0.228858743	0.0021	0.012314604
GO:0051052	regulation of DNA metabolic process	177	0.228850502	0.0022	0.012422266
GO:0051453	regulation of intracellular pH	25	0.228842896	0.0087	0.016840777
GO:0030900	forebrain development	177	0.228824103	0.0076	0.016084627
GO:0051409	response to nitrosative stress	8	0.228803212	0.0089	0.016916437
GO:1904427	positive regulation of calcium ion transmembrane transport	27	0.228800135	0.0058	0.014878261
GO:0055002	striated muscle cell development	56	0.228797034	0.0042	0.013948535
GO:0032613	interleukin-10 production	16	0.228749761	0.0117	0.018739446
GO:0007623	circadian rhythm	93	0.228743636	0.0004	0.008964341
GO:0031023	microtubule organizing center organization	42	0.228726028	0.002	0.012314604
GO:0001941	postsynaptic membrane organization	19	0.22870145	0.0056	0.014771533
GO:0072189	ureter development	5	0.228678072	0.0332	0.036915846
GO:0032780	negative regulation of ATPase activity	7	0.228669446	0.0126	0.019370699
GO:0006111	regulation of gluconeogenesis	14	0.228630572	0.0059	0.014942532
GO:0006457	protein folding	92	0.228624443	0.0147	0.02091937
GO:0006006	glucose metabolic process	95	0.228622148	0.0036	0.01353394
GO:0010919	regulation of inositol phosphate biosynthetic process	10	0.228617299	0.0247	0.029271449
GO:0038003	opioid receptor signaling pathway	6	0.228609222	0.0002	0.006319126
GO:0045732	positive regulation of protein catabolic process	103	0.228608421	0.0036	0.01353394
GO:0090398	cellular senescence	29	0.22860179	0.0022	0.012422266
GO:2001225	regulation of chloride transport	5	0.228585293	0.0475	0.049962707
GO:1901223	negative regulation of NIK/NF-kappaB signaling	7	0.228577074	0.0249	0.029484292

GOIDlabel	GOName	size	categoryScores	pValPerm	pValPermCorr
GO:0048538	thymus development	16	0.228573048	0.0032	0.013333753
GO:0016525	negative regulation of angiogenesis	47	0.228571801	0.0066	0.015525305
GO:0045088	regulation of innate immune response	173	0.228568953	0.0115	0.018599441
GO:0060147	regulation of posttranscriptional gene silencing	31	0.228568691	0.0054	0.014720792
GO:0060966	regulation of gene silencing by RNA	31	0.228568691	0.0054	0.014720792
GO:0006513	protein monoubiquitination	33	0.22853175	0.0016	0.011845327
GO:2000278	regulation of DNA biosynthetic process	53	0.228518729	0.005	0.014547706
GO:0043534	blood vessel endothelial cell migration	55	0.228469615	0.0019	0.012288367
GO:0050805	negative regulation of synaptic transmission	31	0.228467578	0.0036	0.01353394
GO:0034508	centromere complex assembly	14	0.228466192	0.0134	0.01999969
GO:0009235	cobalamin metabolic process	7	0.228464156	0.015	0.021138192
GO:0007162	negative regulation of cell adhesion	96	0.228435101	0.0122	0.019049527
GO:0098754	detoxification	48	0.228410103	0.0145	0.020788247
GO:0003158	endothelium development	46	0.228405174	0.0059	0.014942532
GO:0050434	positive regulation of viral transcription	20	0.22839734	0.0157	0.021779607
GO:1902882	regulation of response to oxidative stress	39	0.228394155	0.0067	0.015576759
GO:0050821	protein stabilization	82	0.228392326	0.0051	0.014547706
GO:0051851	modification by host of symbiont morphology or physiology	25	0.228351637	0.0243	0.028921902
GO:0016082	synaptic vesicle priming	5	0.2283435	0.013	0.019676963
GO:0002218	activation of innate immune response	136	0.228339645	0.0143	0.020629391
GO:0046364	monosaccharide biosynthetic process	44	0.228289625	0.0085	0.016693954
GO:0060251	regulation of glial cell proliferation	17	0.228267948	0.0139	0.020388077
GO:1901605	alpha-amino acid metabolic process	96	0.228257295	0.007	0.015816335
GO:0051604	protein maturation	96	0.228246362	0.0113	0.018488002
GO:0050651	dermatan sulfate proteoglycan biosynthetic process	11	0.228231756	0.0138	0.020339434
GO:0031331	positive regulation of cellular catabolic process	110	0.228228823	0.0035	0.01351837
GO:0046546	development of primary male sexual characteristics	50	0.228223666	0.0071	0.01583804
GO:0031102	neuron projection regeneration	27	0.228212568	0.0072	0.015844069
GO:0048285	organelle fission	164	0.228165397	0.0016	0.011845327
GO:0017145	stem cell division	17	0.228148583	0.022	0.026944291
GO:0007006	mitochondrial membrane organization	59	0.22812626	0.008	0.016379603
GO:1904321	response to forskolin	5	0.228111813	0.0076	0.016084627
GO:1904322	cellular response to forskolin	5	0.228111813	0.0076	0.016084627
GO:0000212	meiotic spindle organization	6	0.228098899	0.0092	0.017033109
GO:0060401	cytosolic calcium ion transport	66	0.228095051	0.0054	0.014720792
GO:0050000	chromosome localization	32	0.228080285	0.0046	0.014132412
GO:0051303	establishment of chromosome localization	32	0.228080285	0.0046	0.014132412
GO:0090002	establishment of protein localization to plasma membrane	22	0.228061242	< 0.001	< 0.001
GO:0002455	humoral immune response mediated by circulating immunoglobulin	16	0.228056597	0.0365	0.039857035
GO:1903509	liposaccharide metabolic process	57	0.22802937	0.011	0.018323826
GO:0002720	positive regulation of cytokine production involved in immune response	17	0.228013059	0.0096	0.017302743
GO:1903362	regulation of cellular protein catabolic process	117	0.227979969	0.0036	0.01353394
GO:0007015	actin filament organization	158	0.227962746	0.0082	0.016514246
GO:0032275	luteinizing hormone secretion	6	0.227958738	0.0018	0.01210186
GO:0072202	cell differentiation involved in metanephros development	12	0.22790939	0.0149	0.021043429
GO:0014706	striated muscle tissue development	151	0.227887198	0.0038	0.01366393
GO:0051258	protein polymerization	106	0.227880934	0.0051	0.014547706
GO:0098656	anion transmembrane transport	79	0.227858771	0.0089	0.016916437
GO:0060402	calcium ion transport into cytosol	62	0.227857179	0.0053	0.014634479
GO:1901016	regulation of potassium ion transmembrane transporter activity	21	0.227853347	0.0002	0.006319126
GO:0001523	retinoid metabolic process	42	0.22784946	0.0086	0.016782045
GO:0031295	T cell costimulation	24	0.227846274	0.0056	0.014771533
GO:0042474	middle ear morphogenesis	10	0.227838532	0.0301	0.034151923
GO:1903523	negative regulation of blood circulation	14	0.227818855	0.0063	0.015311728
GO:0060135	maternal process involved in female pregnancy	26	0.227807812	0.0033	0.013333753
GO:0090303	positive regulation of wound healing	17	0.227804362	0.0108	0.018152791
GO:1903779	regulation of cardiac conduction	31	0.227761667	0.0056	0.014771533
GO:0051496	positive regulation of stress fiber assembly	26	0.227756678	0.0041	0.013887639
GO:0032469	endoplasmic reticulum calcium ion homeostasis	10	0.227752751	0.005	0.014547706
GO:0010039	response to iron ion	14	0.227673912	0.0125	0.019314538
GO:0043487	regulation of RNA stability	87	0.227654049	0.0132	0.019860109
GO:0022408	negative regulation of cell-cell adhesion	60	0.227651464	0.0134	0.01999969
GO:0010155	regulation of proton transport	10	0.227649477	0.0089	0.016916437
GO:1903902	positive regulation of viral life cycle	48	0.227642917	0.0101	0.017653628
GO:0019432	triglyceride biosynthetic process	17	0.227638577	0.0011	0.010890753
GO:0043434	response to peptide hormone	172	0.2276345	0.0015	0.011704453
GO:0006825	copper ion transport	7	0.227634298	0.0174	0.023064374
GO:0006637	acyl-CoA metabolic process	42	0.227629793	0.0181	0.023607986
GO:0035383	thioester metabolic process	42	0.227629793	0.0181	0.023607986
GO:0018105	peptidyl-serine phosphorylation	94	0.227587886	0.001	0.010787313
GO:0021879	forebrain neuron differentiation	25	0.227547265	0.0112	0.018392048
GO:2000479	regulation of cAMP-dependent protein kinase activity	8	0.227544194	0.0015	0.011704453
GO:0070268	cornification	21	0.227536127	0.0042	0.013948535
GO:0090066	regulation of anatomical structure size	182	0.227522134	0.0022	0.012422266
GO:1900044	regulation of protein K63-linked ubiquitination	6	0.227509748	0.0091	0.016994897
GO:0007339	binding of sperm to zona pellucida	10	0.227502017	0.011	0.018323826
GO:0042542	response to hydrogen peroxide	57	0.227484321	0.0055	0.01475
GO:0055072	iron ion homeostasis	39	0.227455907	0.0084	0.016633151
GO:0060537	muscle tissue development	157	0.227429515	0.0042	0.013948535
GO:0043902	positive regulation of multi-organism process	69	0.227425325	0.0067	0.015576759
GO:1902692	regulation of neuroblast proliferation	13	0.227410654	0.0216	0.026572596
GO:0042088	T-helper 1 type immune response	15	0.227395157	0.0031	0.013277185

GOIDlabel	GOName	size	categoryScores	pValPerm	pValPermCorr
GO:1901030	positive regulation of mitochondrial outer membrane permeabilization involved in apoptotic signaling pathway	14	0.227386803	0.0008	0.010465158
GO:0048863	stem cell differentiation	109	0.227383781	0.0112	0.018392048
GO:0000186	activation of MAPKK activity	19	0.227363449	0.0009	0.010729485
GO:0045840	positive regulation of mitotic nuclear division	16	0.227355982	0.001	0.010787313
GO:0060253	negative regulation of glial cell proliferation	11	0.227348619	0.0202	0.025368462
GO:0070664	negative regulation of leukocyte proliferation	27	0.227340792	0.0161	0.02211694
GO:0045669	positive regulation of osteoblast differentiation	31	0.227339492	0.0077	0.016177834
GO:1903201	regulation of oxidative stress-induced cell death	29	0.227317398	0.0084	0.016633151
GO:0046916	cellular transition metal ion homeostasis	43	0.227311307	0.005	0.014547706
GO:0010658	striated muscle cell apoptotic process	19	0.227307458	0.001	0.010787313
GO:0015908	fatty acid transport	37	0.227290824	0.0076	0.016084627
GO:0042472	inner ear morphogenesis	30	0.227287694	0.0068	0.015633241
GO:0043523	regulation of neuron apoptotic process	88	0.227266396	0.0028	0.012972436
GO:0006749	glutathione metabolic process	23	0.227252121	0.015	0.021138192
GO:0000038	very long-chain fatty acid metabolic process	9	0.227245768	0.0411	0.044080913
GO:0001501	skeletal system development	199	0.227196323	0.0047	0.014168613
GO:0045926	negative regulation of growth	109	0.227178013	0.0052	0.014616626
GO:0001912	positive regulation of leukocyte mediated cytotoxicity	13	0.227166747	0.0202	0.025368462
GO:0007249	I-kappaB kinase/NF-kappaB signaling	106	0.227144426	0.0061	0.015143924
GO:1905048	regulation of metallopeptidase activity	7	0.227144121	0.0133	0.019917275
GO:0034599	cellular response to oxidative stress	134	0.227143676	0.0059	0.014942532
GO:0000266	mitochondrial fission	18	0.227137642	0.0077	0.016177834
GO:0021895	cerebral cortex neuron differentiation	14	0.227128571	0.0057	0.01480566
GO:0008038	neuron recognition	20	0.227128506	0.0123	0.019143634
GO:0019935	cyclic-nucleotide-mediated signaling	47	0.227116302	0.003	0.013210967
GO:0002250	adaptive immune response	129	0.227098868	0.0064	0.015399417
GO:0043471	regulation of cellular carbohydrate catabolic process	5	0.227092548	0.0021	0.012314604
GO:0002437	inflammatory response to antigenic stimulus	21	0.227007384	0.0243	0.028921902
GO:2000644	regulation of receptor catabolic process	8	0.226996077	0.002	0.012314604
GO:0045921	positive regulation of exocytosis	46	0.226977387	0.0008	0.010465158
GO:0030258	lipid modification	89	0.226909685	0.0045	0.014132412
GO:2000573	positive regulation of DNA biosynthetic process	30	0.226905696	0.0131	0.019786886
GO:0042632	cholesterol homeostasis	31	0.226905403	0.0016	0.011845327
GO:0055092	sterol homeostasis	31	0.226905403	0.0016	0.011845327
GO:0060850	regulation of transcription involved in cell fate commitment	8	0.226901809	0.0035	0.01351837
GO:0097503	sialylation	8	0.226873949	0.0005	0.009604651
GO:0050891	multicellular organismal water homeostasis	21	0.226866082	0.0001	0.004220438
GO:0051000	positive regulation of nitric-oxide synthase activity	10	0.226851662	0.0025	0.012769435
GO:0008654	phospholipid biosynthetic process	122	0.226844709	0.0045	0.014132412
GO:0072348	sulfur compound transport	16	0.226843417	0.0058	0.014878261
GO:0010288	response to lead ion	12	0.226833024	0.0102	0.0177
GO:0000083	regulation of transcription involved in G1/S transition of mitotic cell cycle	12	0.22682255	0.0002	0.006319126
GO:0000280	nuclear division	142	0.226813914	0.0019	0.012288367
GO:0046620	regulation of organ growth	43	0.226808572	0.0071	0.01583804
GO:0038095	Fc-epsilon receptor signaling pathway	71	0.226801102	0.0119	0.018881943
GO:0046718	viral entry into host cell	48	0.226797714	0.0049	0.014447629
GO:0044743	intracellular protein transmembrane import	19	0.226782392	0.0074	0.015911789
GO:0090503	RNA phosphodiester bond hydrolysis, exonucleolytic	12	0.226779681	0.0032	0.013333753
GO:0070555	response to interleukin-1	84	0.226777182	0.0074	0.015911789
GO:0034109	homotypic cell-cell adhesion	35	0.226761864	0.0283	0.032434212
GO:0006310	DNA recombination	83	0.226756431	0.0025	0.012769435
GO:0007568	aging	134	0.226724238	0.0045	0.014132412
GO:0010985	negative regulation of lipoprotein particle clearance	5	0.226717289	0.0263	0.030677143
GO:0001654	eye development	149	0.226708806	0.0068	0.015633241
GO:0030202	heparin metabolic process	6	0.22669729	0.0243	0.028921902
GO:0051304	chromosome separation	27	0.226680997	0.0026	0.012805111
GO:0051702	interaction with symbiont	27	0.226671159	0.023	0.027838811
GO:0031128	developmental induction	10	0.22666779	0.0256	0.03006687
GO:0009152	purine ribonucleotide biosynthetic process	87	0.226652928	0.0079	0.01629604
GO:0050852	T cell receptor signaling pathway	101	0.226639226	0.0087	0.016840777
GO:1903055	positive regulation of extracellular matrix organization	11	0.226637822	0.0016	0.011845327
GO:0046834	lipid phosphorylation	14	0.22663608	< 0.001	< 0.001
GO:0043112	receptor metabolic process	84	0.226634522	0.0026	0.012805111
GO:0003012	muscle system process	188	0.226613999	0.0063	0.015311728
GO:0043101	purine-containing compound salvage	8	0.226611391	0.0308	0.034775552
GO:0042176	regulation of protein catabolic process	188	0.226609471	0.0024	0.012603815
GO:0050851	antigen receptor-mediated signaling pathway	115	0.22660104	0.0093	0.017114131
GO:0002888	positive regulation of myeloid leukocyte mediated immunity	15	0.22658892	0.0166	0.02247277
GO:0071415	cellular response to purine-containing compound	6	0.226586501	0.0283	0.032434212
GO:0033209	tumor necrosis factor-mediated signaling pathway	82	0.226577262	0.008	0.016379603
GO:0045198	establishment of epithelial cell apical/basal polarity	7	0.226576566	0.0466	0.049159132
GO:0016458	gene silencing	68	0.22657649	0.0036	0.01353394
GO:0030205	dermatan sulfate metabolic process	10	0.226563868	0.0136	0.020157703
GO:0045727	positive regulation of translation	57	0.226551787	0.0107	0.018084595
GO:2000785	regulation of autophagosome assembly	19	0.226537693	0.0064	0.015399417
GO:0072676	lymphocyte migration	28	0.226518765	0.012	0.018931514
GO:0048709	oligodendrocyte differentiation	49	0.226489669	0.0135	0.020081554
GO:0009206	purine ribonucleoside triphosphate biosynthetic process	28	0.226479845	0.021	0.026005997
GO:0006584	catecholamine metabolic process	24	0.226474577	0.0073	0.015844069
GO:0009712	catechol-containing compound metabolic process	24	0.226474577	0.0073	0.015844069
GO:0031000	response to caffeine	5	0.226413038	0.0172	0.022941269
GO:1901841	regulation of high voltage-gated calcium channel activity	9	0.226382774	0.013	0.019676963

GOIDlabel	GOName	size	categoryScores	pValPerm	pValPermCorr
GO:0072350	tricarboxylic acid metabolic process	17	0.226382427	0.0238	0.028526451
GO:1903749	positive regulation of establishment of protein localization to mitochondrion	30	0.226378182	0.0034	0.01347416
GO:0006090	pyruvate metabolic process	66	0.226345072	0.0068	0.015633241
GO:0051024	positive regulation of immunoglobulin secretion	7	0.226332033	0.0053	0.014634479
GO:0008016	regulation of heart contraction	103	0.226330367	0.004	0.013824268
GO:0006369	termination of RNA polymerase II transcription	18	0.226328063	0.0025	0.012769435
GO:1904018	positive regulation of vasculature development	83	0.226277849	0.0042	0.013948535
GO:0072001	renal system development	135	0.226271654	0.0086	0.016782045
GO:0033044	regulation of chromosome organization	69	0.226239418	0.0028	0.012972436
GO:0060428	lung epithelium development	11	0.226227764	0.019	0.024347961
GO:0042113	B cell activation	90	0.226210507	0.0028	0.012972436
GO:0030335	positive regulation of cell migration	199	0.226209703	0.0068	0.015633241
GO:0015697	quaternary ammonium group transport	5	0.2262096	0.0069	0.015750415
GO:0061647	histone H3-K9 modification	13	0.22620956	0.0051	0.014547706
GO:0051895	negative regulation of focal adhesion assembly	8	0.226197387	0.0026	0.012805111
GO:1901889	negative regulation of cell junction assembly	8	0.226197387	0.0026	0.012805111
GO:1903392	negative regulation of adherens junction organization	8	0.226197387	0.0026	0.012805111
GO:0051101	regulation of DNA binding	50	0.226192677	0.005	0.014547706
GO:0051783	regulation of nuclear division	75	0.226179088	0.0017	0.012001709
GO:0044088	regulation of vacuole organization	25	0.226165906	0.0099	0.017505138
GO:0003208	cardiac ventricle morphogenesis	31	0.226161622	0.0115	0.018599441
GO:0009409	response to cold	16	0.226155193	0.0011	0.010890753
GO:0031099	regeneration	76	0.226136289	0.009	0.016956012
GO:0045672	positive regulation of osteoclast differentiation	10	0.226131205	0.0272	0.031504487
GO:1903533	regulation of protein targeting	90	0.226113582	0.0078	0.01620539
GO:0016540	protein autoprocessing	8	0.22610814	0.0053	0.014634479
GO:0050655	dermatan sulfate proteoglycan metabolic process	12	0.226039463	0.0136	0.020157703
GO:1901293	nucleoside phosphate biosynthetic process	126	0.226028778	0.0089	0.016916437
GO:0045912	negative regulation of carbohydrate metabolic process	22	0.226022971	0.0012	0.010961137
GO:0045822	negative regulation of heart contraction	10	0.226014714	0.0132	0.019860109
GO:0009581	detection of external stimulus	44	0.226013489	0.0033	0.013333753
GO:0030100	regulation of endocytosis	105	0.226001953	0.0079	0.01629604
GO:0018023	peptidyl-lysine trimethylation	15	0.22599113	0.0055	0.01475
GO:0009408	response to heat	67	0.225968437	0.0052	0.014616626
GO:0007620	copulation	9	0.225960204	0.0338	0.0374175
GO:0010761	fibroblast migration	15	0.225947765	0.0091	0.016994897
GO:0051017	actin filament bundle assembly	67	0.225947259	0.0072	0.015844069
GO:0061572	actin filament bundle organization	67	0.225947259	0.0072	0.015844069
GO:0045638	negative regulation of myeloid cell differentiation	28	0.225920806	0.0052	0.014616626
GO:0071320	cellular response to cAMP	25	0.225895501	0.0173	0.023005658
GO:0045116	protein neddylation	6	0.225893471	0.0189	0.024289798
GO:0007160	cell-matrix adhesion	77	0.225889447	0.0048	0.014269203
GO:2000243	positive regulation of reproductive process	24	0.22588791	0.0075	0.015990044
GO:0010811	positive regulation of cell-substrate adhesion	56	0.225880822	0.0082	0.016514246
GO:0010771	negative regulation of cell morphogenesis involved in differentiation	37	0.225857826	0.0011	0.010890753
GO:0030038	contractile actin filament bundle assembly	41	0.225838253	0.0052	0.014616626
GO:0043149	stress fiber assembly	41	0.225838253	0.0052	0.014616626
GO:0007088	regulation of mitotic nuclear division	66	0.225824206	0.0017	0.012001709
GO:0048732	gland development	178	0.225819609	0.0037	0.013609033
GO:0006919	activation of cysteine-type endopeptidase activity involved in apoptotic process	31	0.225803526	0.01	0.017574468
GO:0051147	regulation of muscle cell differentiation	73	0.225799219	0.001	0.010787313
GO:0009124	nucleoside monophosphate biosynthetic process	51	0.225789923	0.0182	0.023684988
GO:0018195	peptidyl-arginine modification	8	0.2257782	0.0033	0.013333753
GO:0007566	embryo implantation	20	0.225762619	0.0044	0.014132412
GO:0070741	response to interleukin-6	12	0.225761085	0.001	0.010787313
GO:0048806	genitalia development	16	0.225759411	0.0135	0.020081554
GO:0097178	ruffle assembly	20	0.2257253	0.0016	0.011845327
GO:0018904	ether metabolic process	7	0.225723569	0.032	0.035892144
GO:0006766	vitamin metabolic process	61	0.225722898	0.01	0.017574468
GO:0060395	SMAD protein signal transduction	13	0.225698422	0.0332	0.036915846
GO:0090257	regulation of muscle system process	95	0.22567976	0.0077	0.016177834
GO:0033003	regulation of mast cell activation	20	0.225630513	0.0307	0.034703304
GO:0044706	multi-multicellular organism process	93	0.225620917	0.0023	0.012498684
GO:0051281	positive regulation of release of sequestered calcium ion into cytosol	17	0.225617761	0.0056	0.014771533
GO:0030307	positive regulation of cell growth	75	0.225611102	0.0033	0.013333753
GO:0019915	lipid storage	26	0.225602734	0.0028	0.012972436
GO:0048017	inositol lipid-mediated signaling	75	0.225597194	0.0075	0.015990044
GO:0045777	positive regulation of blood pressure	14	0.225586216	0.0016	0.011845327
GO:0030318	melanocyte differentiation	11	0.225564086	0.0117	0.018739446
GO:0032459	regulation of protein oligomerization	17	0.225551905	0.0046	0.014132412
GO:0071219	cellular response to molecule of bacterial origin	79	0.22554207	0.0093	0.017114131
GO:0036293	response to decreased oxygen levels	175	0.225526469	0.0075	0.015990044
GO:0022600	digestive system process	35	0.225522389	0.006	0.015037711
GO:0048535	lymph node development	6	0.225520285	0.0007	0.010220707
GO:0051497	negative regulation of stress fiber assembly	6	0.225515534	0.0168	0.022626974
GO:0044282	small molecule catabolic process	177	0.225508854	0.0101	0.017653628
GO:0061458	reproductive system development	175	0.225507573	0.0034	0.01347416
GO:0003214	cardiac left ventricle morphogenesis	7	0.225489402	0.0187	0.024129301
GO:0034587	piRNA metabolic process	6	0.225481843	0.0102	0.0177
GO:0009165	nucleotide biosynthetic process	124	0.225477427	0.0096	0.017302743
GO:0055076	transition metal ion homeostasis	57	0.225450915	0.0065	0.015491756
GO:0030260	entry into host cell	54	0.225426029	0.0054	0.014720792
GO:0044409	entry into host	54	0.225426029	0.0054	0.014720792

GOIDlabel	GOName	size	categoryScores	pValPerm	pValPermCorr
GO:0051806	entry into cell of other organism involved in symbiotic interaction	54	0.225426029	0.0054	0.014720792
GO:0051828	entry into other organism involved in symbiotic interaction	54	0.225426029	0.0054	0.014720792
GO:0032259	methylation	126	0.225423595	0.0024	0.012603815
GO:0045661	regulation of myoblast differentiation	19	0.225417452	0.0133	0.019917275
GO:0045940	positive regulation of steroid metabolic process	9	0.225406472	0.0207	0.025805821
GO:0019377	glycolipid catabolic process	8	0.225335204	0.0054	0.014720792
GO:0034198	cellular response to amino acid starvation	29	0.225322514	0.0029	0.013089617
GO:0040001	establishment of mitotic spindle localization	11	0.225303987	0.0015	0.011704453
GO:0048608	reproductive structure development	172	0.225275447	0.0038	0.01366393
GO:0022613	ribonucleoprotein complex biogenesis	155	0.22526952	0.0066	0.015525305
GO:0009101	glycoprotein biosynthetic process	164	0.225267475	0.0034	0.01347416
GO:0048284	organelle fusion	63	0.22524203	0.0067	0.015576759
GO:1902600	hydrogen ion transmembrane transport	52	0.225241957	0.0091	0.016994897
GO:0061418	regulation of transcription from RNA polymerase II promoter in response to hypoxia	51	0.225236267	0.0191	0.02440579
GO:0030252	growth hormone secretion	11	0.225220114	< 0.001	< 0.001
GO:0009416	response to light stimulus	123	0.225211893	0.0012	0.010961137
GO:0072678	T cell migration	21	0.225210129	0.0208	0.025885837
GO:0048661	positive regulation of smooth muscle cell proliferation	37	0.22518836	0.0127	0.019457181
GO:0044030	regulation of DNA methylation	6	0.225150122	0.0141	0.020504577
GO:0044829	positive regulation by host of viral genome replication	5	0.225148092	0.0301	0.034151923
GO:0001945	lymph vessel development	10	0.225138762	0.0065	0.015491756
GO:1903432	regulation of TORC1 signaling	18	0.225136516	0.0038	0.01366393
GO:0035601	protein deacylation	34	0.225111299	0.0005	0.009604651
GO:0098732	macromolecule deacylation	34	0.225111299	0.0005	0.009604651
GO:0031646	positive regulation of neurological system process	17	0.225110042	0.0017	0.012001709
GO:0006801	superoxide metabolic process	27	0.225091356	0.016	0.022026667
GO:0007051	spindle organization	56	0.225082872	0.0058	0.014878261
GO:0048305	immunoglobulin secretion	12	0.225037821	0.0006	0.009940401
GO:0007044	cell-substrate junction assembly	45	0.225032105	0.0031	0.013277185
GO:0071625	vocalization behavior	9	0.225021623	0.0052	0.014616626
GO:0055078	sodium ion homeostasis	14	0.225003482	0.0092	0.017033109
GO:1903719	regulation of I-kappaB phosphorylation	5	0.224987156	0.0047	0.014168613
GO:0006164	purine nucleotide biosynthetic process	90	0.224975666	0.0083	0.016559903
GO:0048569	post-embryonic animal organ development	5	0.224959944	0.0156	0.021692929
GO:0016042	lipid catabolic process	127	0.224951937	0.0115	0.018599441
GO:0009135	purine nucleoside diphosphate metabolic process	61	0.224940371	0.0071	0.01583804
GO:0009179	purine ribonucleoside diphosphate metabolic process	61	0.224940371	0.0071	0.01583804
GO:0072401	signal transduction involved in DNA integrity checkpoint	23	0.224934073	0.0016	0.011845327
GO:0072422	signal transduction involved in DNA damage checkpoint	23	0.224934073	0.0016	0.011845327
GO:2001020	regulation of response to DNA damage stimulus	73	0.22492309	0.0058	0.014878261
GO:0006564	L-serine biosynthetic process	5	0.224905521	0.0188	0.02420971
GO:0061028	establishment of endothelial barrier	14	0.224862797	0.006	0.015037711
GO:0060562	epithelial tube morphogenesis	120	0.224857966	0.0067	0.015576759
GO:0042158	lipoprotein biosynthetic process	39	0.224855995	0.0111	0.018389742
GO:0021683	cerebellar granular layer morphogenesis	5	0.224843064	0.0307	0.034703304
GO:0050994	regulation of lipid catabolic process	24	0.224840753	0.0027	0.012827773
GO:0006869	lipid transport	123	0.224775635	0.0052	0.014616626
GO:1903828	negative regulation of cellular protein localization	68	0.224774014	0.0041	0.013887639
GO:0048015	phosphatidylinositol-mediated signaling	74	0.224751189	0.0073	0.015844069
GO:0070254	mucus secretion	8	0.224732501	0.0013	0.011218806
GO:2000134	negative regulation of G1/S transition of mitotic cell cycle	36	0.224727912	0.0035	0.01351837
GO:0034504	protein localization to nucleus	104	0.224721138	0.0086	0.016782045
GO:0001885	endothelial cell development	20	0.224715364	0.0048	0.014269203
GO:0051098	regulation of binding	173	0.224701759	0.0032	0.013333753
GO:0035176	social behavior	28	0.224663755	0.0024	0.012603815
GO:0051703	intraspecies interaction between organisms	28	0.224663755	0.0024	0.012603815
GO:0019932	second-messenger-mediated signaling	145	0.224651476	0.0036	0.01353394
GO:0006418	tRNA aminoacylation for protein translation	20	0.224648502	0.0105	0.017924712
GO:0016197	endosomal transport	132	0.224634429	0.0013	0.011218806
GO:0048864	stem cell development	32	0.224630539	0.0093	0.017114131
GO:0006906	vesicle fusion	30	0.2246106	0.0046	0.014132412
GO:0090174	organelle membrane fusion	30	0.2246106	0.0046	0.014132412
GO:0097502	mannosylation	19	0.224597629	0.0169	0.022703485
GO:0031124	mRNA 3'-end processing	41	0.224593287	0.0043	0.014102439
GO:1900026	positive regulation of substrate adhesion-dependent cell spreading	20	0.224591487	0.0161	0.02211694
GO:0051297	centrosome organization	40	0.224585041	0.0026	0.012805111
GO:0050678	regulation of epithelial cell proliferation	134	0.224576376	0.0116	0.018682786
GO:0045670	regulation of osteoclast differentiation	24	0.22457177	0.0067	0.015576759
GO:0034330	cell junction organization	111	0.22455444	0.0041	0.013887639
GO:0002474	antigen processing and presentation of peptide antigen via MHC class I	57	0.224528475	0.023	0.027838811
GO:0002758	innate immune response-activating signal transduction	127	0.224527868	0.0136	0.020157703
GO:0042692	muscle cell differentiation	147	0.224522358	0.0032	0.013333753
GO:0010876	lipid localization	139	0.224521991	0.0038	0.01366393
GO:0006506	GPI anchor biosynthetic process	14	0.224489055	0.0325	0.036312077
GO:0002360	T cell lineage commitment	6	0.224481281	0.0106	0.018010344
GO:0009118	regulation of nucleoside metabolic process	49	0.224470148	0.0091	0.016994897
GO:1903578	regulation of ATP metabolic process	49	0.224470148	0.0091	0.016994897
GO:0072522	purine-containing compound biosynthetic process	94	0.224466476	0.0095	0.01725157
GO:0045717	negative regulation of fatty acid biosynthetic process	8	0.224420217	0.0269	0.031225818
GO:0051205	protein insertion into membrane	20	0.224413965	0.0051	0.014547706
GO:0051445	regulation of meiotic cell cycle	15	0.22439239	0.0034	0.01347416
GO:0045776	negative regulation of blood pressure	15	0.224389107	0.0089	0.016916437
GO:0001666	response to hypoxia	170	0.224385332	0.008	0.016379603

GOIDlabel	GOName	size	categoryScores	pValPerm	pValPermCorr
GO:0003016	respiratory system process	12	0.224358613	0.001	0.010787313
GO:0007007	inner mitochondrial membrane organization	20	0.224299028	0.0126	0.019370699
GO:0090169	regulation of spindle assembly	7	0.224292281	0.021	0.026005997
GO:1901673	regulation of mitotic spindle assembly	7	0.224292281	0.021	0.026005997
GO:0051348	negative regulation of transferase activity	130	0.224239447	0.0039	0.013791927
GO:0006575	cellular modified amino acid metabolic process	72	0.224211649	0.0058	0.014878261
GO:0051988	regulation of attachment of spindle microtubules to kinetochore	7	0.224201946	0.0056	0.014771533
GO:0019471	4-hydroxyproline metabolic process	7	0.224195838	0.015	0.021138192
GO:0055024	regulation of cardiac muscle tissue development	36	0.224175691	0.0055	0.01475
GO:0002532	production of molecular mediator involved in inflammatory response	27	0.224159302	0.0064	0.015399417
GO:0046688	response to copper ion	15	0.224146709	0.0124	0.019242297
GO:0031670	cellular response to nutrient	14	0.224117998	0.0199	0.025133639
GO:0033673	negative regulation of kinase activity	118	0.22411767	0.0038	0.01366393
GO:1902932	positive regulation of alcohol biosynthetic process	10	0.224117424	0.0461	0.04873838
GO:0051235	maintenance of location	125	0.224110292	0.0036	0.01353394
GO:0014003	oligodendrocyte development	18	0.224106871	0.0046	0.014132412
GO:0032368	regulation of lipid transport	39	0.224104141	0.0121	0.018970228
GO:0032945	negative regulation of mononuclear cell proliferation	25	0.224098039	0.0192	0.02450649
GO:0050672	negative regulation of lymphocyte proliferation	25	0.224098039	0.0192	0.02450649
GO:0071526	semaphorin-plexin signaling pathway	9	0.224094849	0.0141	0.020504577
GO:1901653	cellular response to peptide	158	0.224089773	0.0015	0.011704453
GO:1901342	regulation of vasculature development	141	0.224078209	0.0048	0.014269203
GO:0006625	protein targeting to peroxisome	40	0.224076574	0.0096	0.017302743
GO:0043574	peroxisomal transport	40	0.224076574	0.0096	0.017302743
GO:0072662	protein localization to peroxisome	40	0.224076574	0.0096	0.017302743
GO:0072663	establishment of protein localization to peroxisome	40	0.224076574	0.0096	0.017302743
GO:0006818	hydrogen transport	60	0.224055529	0.0083	0.016559903
GO:0015992	proton transport	60	0.224055529	0.0083	0.016559903
GO:1903364	positive regulation of cellular protein catabolic process	71	0.224035914	0.0048	0.014269203
GO:1904589	regulation of protein import	48	0.224011018	0.0128	0.019522448
GO:0009185	ribonucleoside diphosphate metabolic process	62	0.223984744	0.0076	0.016084627
GO:0046850	regulation of bone remodeling	21	0.223950996	0.0035	0.01351837
GO:0051339	regulation of lyase activity	35	0.223942937	0.0037	0.013609033
GO:0045744	negative regulation of G-protein coupled receptor protein signaling pathway	20	0.223919234	0.0176	0.023223003
GO:0051250	negative regulation of lymphocyte activation	50	0.223917666	0.0112	0.018392048
GO:0048713	regulation of oligodendrocyte differentiation	20	0.223913273	0.0269	0.031225818
GO:0010632	regulation of epithelial cell migration	109	0.2239004	0.0032	0.013333753
GO:2000322	regulation of glucocorticoid receptor signaling pathway	6	0.223889107	0.0002	0.006319126
GO:0051291	protein heterooligomerization	45	0.223884643	0.0096	0.017302743
GO:1904707	positive regulation of vascular smooth muscle cell proliferation	18	0.223879532	0.0115	0.018599441
GO:0002064	epithelial cell development	86	0.223869263	0.0019	0.012288367
GO:0007018	microtubule-based movement	109	0.223858495	0.0081	0.016514175
GO:0032984	macromolecular complex disassembly	132	0.22385417	0.0099	0.017505138
GO:0007033	vacuole organization	89	0.223835397	0.0065	0.015491756
GO:0043401	steroid hormone mediated signaling pathway	63	0.223827937	0.0034	0.01347416
GO:0031397	negative regulation of protein ubiquitination	39	0.223796574	0.0068	0.015633241
GO:0030198	extracellular matrix organization	152	0.223781846	0.0086	0.016782045
GO:0043062	extracellular structure organization	152	0.223781846	0.0086	0.016782045
GO:1990748	cellular detoxification	46	0.223775838	0.0148	0.020979063
GO:0001893	maternal placenta development	15	0.223759096	0.0028	0.012972436
GO:0060080	inhibitory postsynaptic potential	9	0.223754057	0.0103	0.017772187
GO:0098813	nuclear chromosome segregation	91	0.223746827	0.0027	0.012827773
GO:0043280	positive regulation of cysteine-type endopeptidase activity involved in apoptotic process	50	0.223690722	0.0084	0.016633151
GO:0050687	negative regulation of defense response to virus	11	0.223679896	0.0075	0.015990044
GO:0010824	regulation of centrosome duplication	17	0.223665622	0.0022	0.012422266
GO:0006476	protein deacetylation	32	0.223664855	0.0004	0.008964341
GO:0043949	regulation of cAMP-mediated signaling	17	0.223657618	0.0007	0.010220707
GO:0050829	defense response to Gram-negative bacterium	21	0.223614647	0.0196	0.0248852
GO:0046686	response to cadmium ion	28	0.223614585	0.021	0.026005997
GO:0006892	post-Golgi vesicle-mediated transport	33	0.223613065	0.0009	0.010729485
GO:0032212	positive regulation of telomere maintenance via telomerase	17	0.223612723	0.018	0.023514686
GO:0044275	cellular carbohydrate catabolic process	18	0.223605114	0.0036	0.01353394
GO:0035239	tube morphogenesis	138	0.223597837	0.0059	0.014942532
GO:0007292	female gamete generation	40	0.22358071	0.0008	0.010465158
GO:1904262	negative regulation of TORC1 signaling	6	0.223580494	0.0037	0.013609033
GO:0010739	positive regulation of protein kinase A signaling	5	0.223565419	0.0098	0.017418875
GO:1901565	organonitrogen compound catabolic process	167	0.223561482	0.0091	0.016994897
GO:0046390	ribose phosphate biosynthetic process	99	0.223556168	0.0073	0.015844069
GO:0014823	response to activity	28	0.22354341	0.0041	0.013887639
GO:0007019	microtubule depolymerization	12	0.22353201	0.0053	0.014634479
GO:2001251	negative regulation of chromosome organization	30	0.22352931	0.0016	0.011845327
GO:0051580	regulation of neurotransmitter uptake	7	0.223526287	0.0011	0.010890753
GO:1904358	positive regulation of telomere maintenance via telomere lengthening	19	0.223523968	0.0175	0.023154462
GO:0065002	intracellular protein transmembrane transport	25	0.223474144	0.0053	0.014634479
GO:0070098	chemokine-mediated signaling pathway	20	0.223442255	0.0144	0.02072711
GO:2001135	regulation of endocytic recycling	5	0.223424956	0.0035	0.01351837
GO:0048857	neural nucleus development	31	0.223421286	0.0057	0.01480566
GO:0032623	interleukin-2 production	24	0.223411934	0.0138	0.020339434
GO:0032007	negative regulation of TOR signaling	17	0.223359054	0.0001	0.004220438
GO:0001819	positive regulation of cytokine production	162	0.223350996	0.0128	0.019522448
GO:0051384	response to glucocorticoid	47	0.223343195	0.018	0.023514686
GO:0021782	glial cell development	41	0.223332266	0.0071	0.01583804
GO:0002440	production of molecular mediator of immune response	70	0.223305207	0.0066	0.015525305

GOIDlabel	GOName	size	categoryScores	pValPerm	pValPermCorr
GO:1903203	regulation of oxidative stress-induced neuron death	16	0.223298309	0.0086	0.016782045
GO:0045665	negative regulation of neuron differentiation	101	0.223292545	0.001	0.010787313
GO:0050886	endocrine process	28	0.223283466	0.007	0.015816335
GO:0046031	ADP metabolic process	54	0.223273009	0.0071	0.01583804
GO:0035821	modification of morphology or physiology of other organism	56	0.223269096	0.0082	0.016514246
GO:0045927	positive regulation of growth	113	0.223251339	0.0046	0.014132412
GO:0018209	peptidyl-serine modification	106	0.223247399	0.0009	0.010729485
GO:0090316	positive regulation of intracellular protein transport	82	0.223235241	0.004	0.013824268
GO:0046661	male sex differentiation	56	0.223206968	0.0082	0.016514246
GO:0018126	protein hydroxylation	14	0.223197435	0.0116	0.018682786
GO:0016266	O-glycan processing	25	0.223192389	0.0046	0.014132412
GO:0010970	transport along microtubule	73	0.223060993	0.0047	0.014168613
GO:0099111	microtubule-based transport	73	0.223060993	0.0047	0.014168613
GO:0001188	RNA polymerase I transcriptional preinitiation complex assembly	5	0.223022485	0.0196	0.0248852
GO:0048524	positive regulation of viral process	51	0.223021819	0.0082	0.016514246
GO:0045598	regulation of fat cell differentiation	56	0.222986638	0.0019	0.012288367
GO:0032387	negative regulation of intracellular transport	43	0.222954785	0.016	0.022026667
GO:0046851	negative regulation of bone remodeling	8	0.22294788	0.0001	0.004220438
GO:0030838	positive regulation of actin filament polymerization	40	0.222931165	0.0026	0.012805111
GO:0051571	positive regulation of histone H3-K4 methylation	6	0.222920507	0.0198	0.025062084
GO:0031294	lymphocyte costimulation	25	0.222901614	0.0033	0.013333753
GO:0032845	negative regulation of homeostatic process	80	0.222894105	0.0033	0.013333753
GO:0035428	hexose transmembrane transport	8	0.222871591	0.0174	0.023064374
GO:1904659	glucose transmembrane transport	8	0.222871591	0.0174	0.023064374
GO:0061162	establishment of monopolar cell polarity	10	0.222846777	0.0239	0.028592965
GO:0033077	T cell differentiation in thymus	26	0.222820382	0.0037	0.013609033
GO:0071594	thymocyte aggregation	26	0.222820382	0.0037	0.013609033
GO:0050771	negative regulation of axonogenesis	26	0.222816894	0.0011	0.010890753
GO:0007067	mitotic nuclear division	103	0.222808736	0.002	0.012314604
GO:0050953	sensory perception of light stimulus	73	0.222792464	0.0021	0.012314604
GO:0044786	cell cycle DNA replication	23	0.222740308	0.0023	0.012498684
GO:0048799	animal organ maturation	7	0.222735117	0.0089	0.016916437
GO:0070977	bone maturation	7	0.222735117	0.0089	0.016916437
GO:0009584	detection of visible light	19	0.222713693	0.0005	0.009604651
GO:0031547	brain-derived neurotrophic factor receptor signaling pathway	6	0.222697632	< 0.001	< 0.001
GO:0046341	CDP-diacylglycerol metabolic process	10	0.222696336	0.0038	0.01366393
GO:1904754	positive regulation of vascular associated smooth muscle cell migration	9	0.222695688	0.0041	0.013887639
GO:0009260	ribonucleotide biosynthetic process	94	0.222690629	0.0072	0.015844069
GO:0015698	inorganic anion transport	61	0.222685683	0.0073	0.015844069
GO:0048681	negative regulation of axon regeneration	7	0.222662183	0.0114	0.018551872
GO:0043501	skeletal muscle adaptation	7	0.222661998	0.0426	0.045512417
GO:0038128	ERBB2 signaling pathway	11	0.222649982	0.0053	0.014634479
GO:0034405	response to fluid shear stress	11	0.222627011	0.0012	0.010961137
GO:0042306	regulation of protein import into nucleus	46	0.222608532	0.0129	0.019597425
GO:0031644	regulation of neurological system process	43	0.222596334	0.002	0.012314604
GO:0090287	regulation of cellular response to growth factor stimulus	115	0.222544904	0.0052	0.014616626
GO:0060968	regulation of gene silencing	34	0.222544691	0.0055	0.01475
GO:0001503	ossification	160	0.222514181	0.0053	0.014634479
GO:0002221	pattern recognition receptor signaling pathway	70	0.222502653	0.0128	0.019522448
GO:0006839	mitochondrial transport	127	0.222470466	0.0075	0.015990044
GO:0010823	negative regulation of mitochondrion organization	33	0.222458755	0.0087	0.016840777
GO:0035794	positive regulation of mitochondrial membrane permeability	27	0.222455296	0.004	0.013824268
GO:1902686	mitochondrial outer membrane permeabilization involved in programmed cell death	27	0.222455296	0.004	0.013824268
GO:1905710	positive regulation of membrane permeability	27	0.222455296	0.004	0.013824268
GO:0051402	neuron apoptotic process	97	0.222453095	0.0032	0.013333753
GO:0008334	histone mRNA metabolic process	16	0.222448355	0.0027	0.012827773
GO:1901201	regulation of extracellular matrix assembly	8	0.222445601	0.0293	0.033401538
GO:0033559	unsaturated fatty acid metabolic process	46	0.222436108	0.0172	0.022941269
GO:0006636	unsaturated fatty acid biosynthetic process	29	0.22243166	0.026	0.030450071
GO:0009755	hormone-mediated signaling pathway	78	0.222413389	0.0037	0.013609033
GO:0006352	DNA-templated transcription, initiation	97	0.222404283	0.0041	0.013887639
GO:0070534	protein K63-linked ubiquitination	26	0.22240284	0.0067	0.015576759
GO:0070498	interleukin-1-mediated signaling pathway	55	0.222396762	0.0186	0.024075487
GO:0007601	visual perception	72	0.222382414	0.0021	0.012314604
GO:0042110	T cell activation	171	0.222382221	0.0121	0.018970228
GO:0070489	T cell aggregation	171	0.222382221	0.0121	0.018970228
GO:0043542	endothelial cell migration	107	0.222374696	0.003	0.013210967
GO:0031342	negative regulation of cell killing	8	0.222370608	0.0427	0.045577146
GO:0010874	regulation of cholesterol efflux	9	0.222341938	0.0205	0.025639412
GO:0002576	platelet degranulation	61	0.222337881	0.0204	0.025564109
GO:1902807	negative regulation of cell cycle G1/S phase transition	38	0.222336721	0.003	0.013210967
GO:0051302	regulation of cell division	59	0.222328783	0.0046	0.014132412
GO:0006757	ATP generation from ADP	48	0.222322502	0.0064	0.015399417
GO:0070168	negative regulation of biomineral tissue development	12	0.222304253	0.0033	0.013333753
GO:0002224	toll-like receptor signaling pathway	55	0.222304196	0.0126	0.019370699
GO:0033273	response to vitamin	36	0.222296262	0.0197	0.024979254
GO:0021781	glial cell fate commitment	9	0.222272398	0.0189	0.024289798
GO:0006959	humoral immune response	60	0.222253112	0.0202	0.025368462
GO:0071901	negative regulation of protein serine/threonine kinase activity	64	0.22225174	0.0031	0.013277185
GO:0048251	elastic fiber assembly	6	0.222251233	0.0331	0.03684717
GO:0016485	protein processing	81	0.222251009	0.0166	0.02247277
GO:0051153	regulation of striated muscle cell differentiation	40	0.222249808	0.0005	0.009604651
GO:0032786	positive regulation of DNA-templated transcription, elongation	13	0.222219836	0.0056	0.014771533

GOIDlabel	GOName	size	categoryScores	pValPerm	pValPermCorr
GO:0043393	regulation of protein binding	102	0.22221485	0.0036	0.01353394
GO:0043616	keratinocyte proliferation	22	0.222183499	0.0138	0.020339434
GO:0050863	regulation of T cell activation	115	0.222181081	0.0107	0.018084595
GO:0060038	cardiac muscle cell proliferation	21	0.222178793	0.0227	0.027637692
GO:0008543	fibroblast growth factor receptor signaling pathway	45	0.222166403	0.0082	0.016514246
GO:0046794	transport of virus	28	0.222165388	0.0093	0.017114131
GO:0075733	intracellular transport of virus	28	0.222165388	0.0093	0.017114131
GO:0009201	ribonucleoside triphosphate biosynthetic process	30	0.222141524	0.0186	0.024075487
GO:0034135	regulation of toll-like receptor 2 signaling pathway	5	0.222136686	0.0424	0.045323868
GO:0030449	regulation of complement activation	20	0.222130725	0.0379	0.041160368
GO:2000257	regulation of protein activation cascade	20	0.222130725	0.0379	0.041160368
GO:0031334	positive regulation of protein complex assembly	100	0.222122873	0.0038	0.01366393
GO:0003231	cardiac ventricle development	55	0.222104951	0.0077	0.016177834
GO:0030104	water homeostasis	24	0.222083807	0.0001	0.004220438
GO:0006469	negative regulation of protein kinase activity	109	0.222075649	0.0042	0.013948535
GO:0009620	response to fungus	11	0.222039431	0.0116	0.018682786
GO:0061014	positive regulation of mRNA catabolic process	13	0.222039123	0.0046	0.014132412
GO:0097164	ammonium ion metabolic process	91	0.221977666	0.0132	0.019860109
GO:2000641	regulation of early endosome to late endosome transport	10	0.22196927	0.0098	0.017418875
GO:0007159	leukocyte cell-cell adhesion	195	0.221944697	0.0118	0.018836996
GO:0010665	regulation of cardiac muscle cell apoptotic process	16	0.221941622	0.0006	0.009940401
GO:0071593	lymphocyte aggregation	172	0.221935845	0.012	0.018931514
GO:0022407	regulation of cell-cell adhesion	158	0.221928173	0.0114	0.018551872
GO:0045165	cell fate commitment	76	0.221922871	0.0054	0.014720792
GO:0034982	mitochondrial protein processing	5	0.221913033	0.0245	0.029070183
GO:1903793	positive regulation of anion transport	18	0.221902955	0.0065	0.015491756
GO:0046337	phosphatidylethanolamine metabolic process	10	0.221900723	0.0028	0.012972436
GO:0045821	positive regulation of glycolytic process	8	0.221891167	0.0133	0.019917275
GO:0090288	negative regulation of cellular response to growth factor stimulus	62	0.221860909	0.0141	0.020504577
GO:0046902	regulation of mitochondrial membrane permeability	32	0.221782807	0.0055	0.01475
GO:0090130	tissue migration	145	0.221779085	0.0031	0.013277185
GO:1903037	regulation of leukocyte cell-cell adhesion	128	0.221771114	0.0093	0.017114131
GO:2000060	positive regulation of protein ubiquitination involved in ubiquitin-dependent protein catabolic process	9	0.221750916	0.006	0.015037711
GO:0070486	leukocyte aggregation	173	0.221716973	0.0122	0.019049527
GO:0009202	deoxyribonucleoside triphosphate biosynthetic process	5	0.221685234	0.0227	0.027637692
GO:0043094	cellular metabolic compound salvage	18	0.221663523	0.0212	0.026203164
GO:0051249	regulation of lymphocyte activation	166	0.221661565	0.0082	0.016514246
GO:0003015	heart process	121	0.221650888	0.0042	0.013948535
GO:0010507	negative regulation of autophagy	42	0.221634902	0.0051	0.014547706
GO:0002821	positive regulation of adaptive immune response	31	0.221620813	0.0037	0.013609033
GO:0051306	mitotic sister chromatid separation	22	0.221597863	0.0018	0.01210186
GO:0034113	heterotypic cell-cell adhesion	21	0.221585261	0.0274	0.03166636
GO:0002181	cytoplasmic translation	25	0.221573589	0.0287	0.032827577
GO:0042428	serotonin metabolic process	5	0.221571205	0.0007	0.010220707
GO:0032231	regulation of actin filament bundle assembly	43	0.221560037	0.0107	0.018084595
GO:0030516	regulation of axon extension	39	0.221559251	0.0003	0.007956881
GO:0007224	smoothened signaling pathway	55	0.221536266	0.0142	0.020567234
GO:2001234	negative regulation of apoptotic signaling pathway	92	0.221532537	0.01	0.017574468
GO:0008037	cell recognition	51	0.221461617	0.0064	0.015399417
GO:1902430	negative regulation of beta-amyloid formation	8	0.221446388	0.0027	0.012827773
GO:0045446	endothelial cell differentiation	37	0.221433523	0.0075	0.015990044
GO:0031529	ruffle organization	27	0.221421019	0.0038	0.01366393
GO:0007127	meiosis I	26	0.22140967	0.0065	0.015491756
GO:1903845	negative regulation of cellular response to transforming growth factor beta stimulus	32	0.22134799	0.013	0.019676963
GO:0015865	purine nucleotide transport	6	0.221340944	< 0.001	< 0.001
GO:0015867	ATP transport	6	0.221340944	< 0.001	< 0.001
GO:0015868	purine ribonucleotide transport	6	0.221340944	< 0.001	< 0.001
GO:0051503	adenine nucleotide transport	6	0.221340944	< 0.001	< 0.001
GO:0001933	negative regulation of protein phosphorylation	189	0.221305303	0.0056	0.014771533
GO:0043624	cellular protein complex disassembly	100	0.221294594	0.0136	0.020157703
GO:0060306	regulation of membrane repolarization	15	0.221274211	0.0104	0.017832977
GO:0030801	positive regulation of cyclic nucleotide metabolic process	21	0.221273964	0.0008	0.010465158
GO:0030804	positive regulation of cyclic nucleotide biosynthetic process	21	0.221273964	0.0008	0.010465158
GO:0051602	response to electrical stimulus	18	0.221268884	0.0245	0.029070183
GO:0002040	sprouting angiogenesis	50	0.221240624	0.0042	0.013948535
GO:0035116	embryonic hindlimb morphogenesis	7	0.221181099	0.0171	0.022860624
GO:0010769	regulation of cell morphogenesis involved in differentiation	132	0.221178058	0.0009	0.010729485
GO:0019751	polyol metabolic process	43	0.22116434	0.0105	0.017924712
GO:0060236	regulation of mitotic spindle organization	14	0.221160274	0.0114	0.018551872
GO:0090224	regulation of spindle organization	14	0.221160274	0.0114	0.018551872
GO:0033013	tetrapyrrole metabolic process	27	0.221155191	0.0199	0.025133639
GO:0002824	positive regulation of adaptive immune response based on somatic recombination of immune receptors built from immunoglobulin superfamily domains	28	0.221141995	0.0038	0.01366393
GO:0043900	regulation of multi-organism process	123	0.221141535	0.0099	0.017505138
GO:0071472	cellular response to salt stress	6	0.221113318	0.0292	0.033300671
GO:0002716	negative regulation of natural killer cell mediated immunity	5	0.221092283	0.0435	0.046336957
GO:0045953	negative regulation of natural killer cell mediated cytotoxicity	5	0.221092283	0.0435	0.046336957
GO:0014074	response to purine-containing compound	66	0.221078685	0.0065	0.015491756
GO:0043537	negative regulation of blood vessel endothelial cell migration	19	0.221077183	0.0094	0.017156187
GO:1901976	regulation of cell cycle checkpoint	12	0.221071205	0.0005	0.009604651
GO:0045766	positive regulation of angiogenesis	75	0.221068319	0.0053	0.014634479
GO:0090330	regulation of platelet aggregation	8	0.221063808	0.0356	0.03903645
GO:0072384	organelle transport along microtubule	41	0.22106107	0.0046	0.014132412

GOIDlabel	GOName	size	categoryScores	pValPerm	pValPermCorr
GO:0044058	regulation of digestive system process	16	0.221048906	0.0011	0.010890753
GO:0071356	cellular response to tumor necrosis factor	117	0.221047327	0.0068	0.015633241
GO:0006305	DNA alkylation	17	0.221011243	0.0058	0.014878261
GO:0006306	DNA methylation	17	0.221011243	0.0058	0.014878261
GO:0009988	cell-cell recognition	20	0.221006178	0.0139	0.020388077
GO:0033860	regulation of NAD(P)H oxidase activity	5	0.220993803	0.0208	0.025885837
GO:0033864	positive regulation of NAD(P)H oxidase activity	5	0.220993803	0.0208	0.025885837
GO:0032682	negative regulation of chemokine production	8	0.22098687	0.0152	0.0213213
GO:0009265	2'-deoxyribonucleotide biosynthetic process	6	0.220944865	0.0259	0.0303391
GO:0046385	deoxyribose phosphate biosynthetic process	6	0.220944865	0.0259	0.0303391
GO:0051492	regulation of stress fiber assembly	36	0.220937703	0.0104	0.017832977
GO:0006767	water-soluble vitamin metabolic process	41	0.220936264	0.0077	0.016177834
GO:0043010	camera-type eye development	128	0.220906147	0.0087	0.016840777
GO:0045472	response to ether	5	0.220895064	0.0121	0.018970228
GO:0044724	single-organism carbohydrate catabolic process	79	0.220820965	0.0062	0.015209334
GO:0016242	negative regulation of macroautophagy	18	0.220816151	0.0161	0.02211694
GO:0070936	protein K48-linked ubiquitination	25	0.220811408	0.0016	0.011845327
GO:0002685	regulation of leukocyte migration	72	0.220794284	0.0126	0.019370699
GO:0060042	retina morphogenesis in camera-type eye	24	0.220787805	0.009	0.016956012
GO:0007405	neuroblast proliferation	26	0.220787195	0.0191	0.02440579
GO:0072655	establishment of protein localization to mitochondrion	74	0.22078668	0.0075	0.015990044
GO:0030488	tRNA methylation	8	0.220766263	0.0281	0.032268957
GO:0006359	regulation of transcription from RNA polymerase III promoter	10	0.220754342	0.0061	0.015143924
GO:0072378	blood coagulation, fibrin clot formation	9	0.220751721	0.0078	0.01620539
GO:1903204	negative regulation of oxidative stress-induced neuron death	12	0.220709248	0.0049	0.014447629
GO:0030512	negative regulation of transforming growth factor beta receptor signaling pathway	31	0.220670291	0.0141	0.020504577
GO:0071674	mononuclear cell migration	21	0.220665315	0.0261	0.030517735
GO:1904896	ESCRT complex disassembly	6	0.220632365	0.0145	0.020788247
GO:1904903	ESCRT III complex disassembly	6	0.220632365	0.0145	0.020788247
GO:0043414	macromolecule methylation	100	0.220627808	0.0012	0.010961137
GO:0071887	leukocyte apoptotic process	34	0.220624641	0.0024	0.012603815
GO:0006893	Golgi to plasma membrane transport	17	0.220596286	0.0007	0.010220707
GO:1900407	regulation of cellular response to oxidative stress	34	0.220580966	0.0074	0.015911789
GO:0006907	pinocytosis	8	0.220567198	0.0098	0.017418875
GO:0030902	hindbrain development	57	0.220537545	0.006	0.015037711
GO:0002223	stimulatory C-type lectin receptor signaling pathway	62	0.220536512	0.0129	0.019597425
GO:0090101	negative regulation of transmembrane receptor protein serine/threonine kinase signaling pathway	44	0.220493147	0.0108	0.018152791
GO:0010310	regulation of hydrogen peroxide metabolic process	8	0.220465479	0.0106	0.018010344
GO:0006067	ethanol metabolic process	5	0.220464345	0.0313	0.035223161
GO:0043270	positive regulation of ion transport	112	0.220464345	0.0057	0.01480566
GO:0007059	chromosome segregation	107	0.220448032	0.0026	0.012805111
GO:0030865	cortical cytoskeleton organization	13	0.220438429	0.0067	0.015576759
GO:0002479	antigen processing and presentation of exogenous peptide antigen via MHC class I, TAP-dependent	47	0.220415877	0.0225	0.027440413
GO:0034142	toll-like receptor 4 signaling pathway	14	0.22041057	0.0229	0.027770092
GO:0035019	somatic stem cell population maintenance	36	0.220400347	0.0114	0.018551872
GO:0045445	myoblast differentiation	32	0.22036955	0.0078	0.01620539
GO:0003205	cardiac chamber development	73	0.220346801	0.0045	0.014132412
GO:0098869	cellular oxidant detoxification	44	0.22034098	0.0158	0.021871104
GO:0046148	pigment biosynthetic process	32	0.220337317	0.009	0.016956012
GO:0035196	production of miRNAs involved in gene silencing by miRNA	13	0.220332175	0.0115	0.018599441
GO:0010801	negative regulation of peptidyl-threonine phosphorylation	9	0.220308131	0.024	0.028671074
GO:0072376	protein activation cascade	33	0.220282621	0.0175	0.023154462
GO:1901998	toxin transport	20	0.220273152	0.0108	0.018152791
GO:0048638	regulation of developmental growth	143	0.220252217	0.0017	0.012001709
GO:0045765	regulation of angiogenesis	129	0.220240445	0.0061	0.015143924
GO:0006684	sphingomyelin metabolic process	9	0.220238878	0.0095	0.01725157
GO:0050868	negative regulation of T cell activation	35	0.220223588	0.0136	0.020157703
GO:0090026	positive regulation of monocyte chemotaxis	6	0.22021469	0.0444	0.04718265
GO:0001977	renal system process involved in regulation of blood volume	6	0.220209939	0.0108	0.018152791
GO:0003093	regulation of glomerular filtration	6	0.220209939	0.0108	0.018152791
GO:0032413	negative regulation of ion transmembrane transporter activity	28	0.22018552	0.0009	0.010729485
GO:0042745	circadian sleep/wake cycle	11	0.22016867	0.0007	0.010220707
GO:0030149	sphingolipid catabolic process	11	0.220148173	0.0014	0.011465722
GO:0046716	muscle cell cellular homeostasis	10	0.220105153	0.0183	0.023783007
GO:0021800	cerebral cortex tangential migration	7	0.220085975	0.0123	0.019143634
GO:0070585	protein localization to mitochondrion	77	0.220076619	0.0079	0.01629604
GO:0032273	positive regulation of protein polymerization	49	0.220076323	0.0032	0.013333753
GO:0006817	phosphate ion transport	6	0.220055308	0.0249	0.029484292
GO:0044319	wound healing, spreading of cells	13	0.219998958	0.0084	0.016633151
GO:0090504	epiboly	13	0.219998958	0.0084	0.016633151
GO:0090505	epiboly involved in wound healing	13	0.219998958	0.0084	0.016633151
GO:0060571	morphogenesis of an epithelial fold	5	0.219998121	0.0327	0.036514368
GO:0070255	regulation of mucus secretion	7	0.219968799	0.0026	0.012805111
GO:0000413	protein peptidyl-prolyl isomerization	20	0.219953676	0.0112	0.018392048
GO:2000811	negative regulation of anoikis	8	0.219953319	0.0023	0.012498684
GO:0072529	pyrimidine-containing compound catabolic process	14	0.219933535	0.0147	0.02091937
GO:0002792	negative regulation of peptide secretion	21	0.219926964	0.0166	0.02247277
GO:0016064	immunoglobulin mediated immune response	45	0.219923282	0.0091	0.016994897
GO:0019724	B cell mediated immunity	45	0.219923282	0.0091	0.016994897
GO:0055021	regulation of cardiac muscle tissue growth	29	0.219911178	0.0054	0.014720792
GO:0000422	mitophagy	38	0.219883628	0.0038	0.01366393
GO:0061726	mitochondrion disassembly	38	0.219883628	0.0038	0.01366393
GO:0001764	neuron migration	78	0.219879514	0.0029	0.013089617

GOIDlabel	GOName	size	categoryScores	pValPerm	pValPermCorr
GO:0052312	modulation of transcription in other organism involved in symbiotic interaction	8	0.219870227	0.0211	0.026113057
GO:0060047	heart contraction	118	0.21985974	0.0044	0.014132412
GO:0006941	striated muscle contraction	70	0.219844756	0.0073	0.015844069
GO:0033081	regulation of T cell differentiation in thymus	9	0.21981602	0.0004	0.008964341
GO:2000398	regulation of thymocyte aggregation	9	0.21981602	0.0004	0.008964341
GO:0050855	regulation of B cell receptor signaling pathway	12	0.219813968	0.0106	0.018010344
GO:0006821	chloride transport	41	0.219809025	0.0088	0.016916437
GO:0042590	antigen processing and presentation of exogenous peptide antigen via MHC class I	49	0.219798972	0.0264	0.030750363
GO:1905207	regulation of cardiocyte differentiation	22	0.219781584	0.0011	0.010890753
GO:0030278	regulation of ossification	89	0.219763383	0.0054	0.014720792
GO:0021532	neural tube patterning	12	0.219735141	0.0324	0.036242368
GO:0030518	intracellular steroid hormone receptor signaling pathway	48	0.219724748	0.005	0.014547706
GO:0071500	cellular response to nitrosative stress	5	0.219717972	0.0174	0.023064374
GO:0001890	placenta development	65	0.219705812	0.003	0.013210967
GO:2000678	negative regulation of transcription regulatory region DNA binding	12	0.21970577	0.0026	0.012805111
GO:0045606	positive regulation of epidermal cell differentiation	6	0.21970523	0.0337	0.03733539
GO:0031638	zymogen activation	20	0.219704043	0.0163	0.022248961
GO:0014033	neural crest cell differentiation	33	0.219683654	0.0055	0.01475
GO:0007131	reciprocal meiotic recombination	9	0.219677947	0.0241	0.028760826
GO:0031642	negative regulation of myelination	5	0.219661994	0.0005	0.009604651
GO:0031645	negative regulation of neurological system process	5	0.219661994	0.0005	0.009604651
GO:1903900	regulation of viral life cycle	79	0.219648754	0.008	0.016379603
GO:0010332	response to gamma radiation	26	0.219639976	0.0003	0.007956881
GO:0044344	cellular response to fibroblast growth factor stimulus	56	0.219638675	0.0083	0.016559903
GO:0050870	positive regulation of T cell activation	70	0.219604961	0.0144	0.02072711
GO:0030575	nuclear body organization	8	0.219565231	0.0044	0.014132412
GO:0006672	ceramide metabolic process	34	0.219546052	0.0029	0.013089617
GO:1902275	regulation of chromatin organization	63	0.219534012	0.0029	0.013089617
GO:0007631	feeding behavior	44	0.219518568	0.0006	0.009940401
GO:0022618	ribonucleoprotein complex assembly	72	0.219475786	0.0102	0.0177
GO:0032463	negative regulation of protein homooligomerization	7	0.219473809	0.0205	0.025639412
GO:0070198	protein localization to chromosome, telomeric region	13	0.219456423	0.0146	0.020879842
GO:0045930	negative regulation of mitotic cell cycle	129	0.219416034	0.0097	0.017363901
GO:0031960	response to corticosteroid	56	0.219407632	0.0199	0.025133639
GO:0034329	cell junction assembly	82	0.219401769	0.0034	0.01347416
GO:0033688	regulation of osteoblast proliferation	5	0.219374071	0.0038	0.01366393
GO:1902959	regulation of aspartic-type endopeptidase activity involved in amyloid precursor protein catabolic process	5	0.21937122	0.0002	0.006319126
GO:0090083	regulation of inclusion body assembly	9	0.219367102	0.0105	0.017924712
GO:0010894	negative regulation of steroid biosynthetic process	11	0.219334066	0.0262	0.030579007
GO:0045939	negative regulation of steroid metabolic process	11	0.219334066	0.0262	0.030579007
GO:0050715	positive regulation of cytokine secretion	45	0.219326962	0.0301	0.034151923
GO:0097193	intrinsic apoptotic signaling pathway	117	0.219298547	0.0075	0.015990044
GO:0010828	positive regulation of glucose transport	13	0.219263151	0.0201	0.025275816
GO:2000316	regulation of T-helper 17 type immune response	5	0.219239309	0.0068	0.015633241
GO:0051701	interaction with host	93	0.219235188	0.0047	0.014168613
GO:0045981	positive regulation of nucleotide metabolic process	39	0.219223248	0.0047	0.014168613
GO:1900544	positive regulation of purine nucleotide metabolic process	39	0.219223248	0.0047	0.014168613
GO:0050673	epithelial cell proliferation	161	0.219215191	0.0115	0.018599441
GO:0051494	negative regulation of cytoskeleton organization	57	0.219179676	0.0096	0.017302743
GO:0010631	epithelial cell migration	140	0.219158868	0.0028	0.012972436
GO:0090132	epithelium migration	140	0.219158868	0.0028	0.012972436
GO:0050913	sensory perception of bitter taste	6	0.219153873	0.0091	0.016994897
GO:0033005	positive regulation of mast cell activation	10	0.219126446	0.0324	0.036242368
GO:0033344	cholesterol efflux	15	0.219122084	0.0224	0.027324219
GO:0050768	negative regulation of neurogenesis	131	0.219121753	0.0027	0.012827773
GO:0014031	mesenchymal cell development	32	0.219104782	0.0119	0.018881943
GO:0048368	lateral mesoderm development	6	0.219101826	0.0428	0.045650175
GO:0042147	retrograde transport, endosome to Golgi	38	0.219090676	0.0027	0.012827773
GO:2001239	regulation of extrinsic apoptotic signaling pathway in absence of ligand	14	0.219052123	0.0153	0.021378589
GO:0015936	coenzyme A metabolic process	10	0.219049088	0.0083	0.016559903
GO:0002200	somatic diversification of immune receptors	24	0.219048699	0.0019	0.012288367
GO:0021954	central nervous system neuron development	45	0.219023892	0.002	0.012314604
GO:0101023	vascular endothelial cell proliferation	7	0.21900492	0.0251	0.029642198
GO:1905562	regulation of vascular endothelial cell proliferation	7	0.21900492	0.0251	0.029642198
GO:0010714	positive regulation of collagen metabolic process	9	0.218982974	0.0201	0.025275816
GO:0032967	positive regulation of collagen biosynthetic process	9	0.218982974	0.0201	0.025275816
GO:0044253	positive regulation of multicellular organismal metabolic process	9	0.218982974	0.0201	0.025275816
GO:0055008	cardiac muscle tissue morphogenesis	25	0.218966831	0.0175	0.023154462
GO:0051583	dopamine uptake involved in synaptic transmission	5	0.218958901	0.0235	0.028248857
GO:0051934	catecholamine uptake involved in synaptic transmission	5	0.218958901	0.0235	0.028248857
GO:2000401	regulation of lymphocyte migration	21	0.218947473	0.0066	0.015525305
GO:0006096	glycolytic process	47	0.218939949	0.0067	0.015576759
GO:0014037	Schwann cell differentiation	15	0.218927888	0.0011	0.010890753
GO:0032233	positive regulation of actin filament bundle assembly	31	0.218916717	0.0053	0.014634479
GO:0002244	hematopoietic progenitor cell differentiation	72	0.218906702	0.0153	0.021378589
GO:0033865	nucleoside bisphosphate metabolic process	21	0.218883733	0.0017	0.012001709
GO:0033875	ribonucleoside bisphosphate metabolic process	21	0.218883733	0.0017	0.012001709
GO:0034032	purine nucleoside bisphosphate metabolic process	21	0.218883733	0.0017	0.012001709
GO:0031109	microtubule polymerization or depolymerization	32	0.218871824	0.0037	0.013609033
GO:0009953	dorsal/ventral pattern formation	32	0.21887065	0.0072	0.015844069
GO:0010677	negative regulation of cellular carbohydrate metabolic process	18	0.21886081	0.0026	0.012805111
GO:0043161	proteasome-mediated ubiquitin-dependent protein catabolic process	190	0.218850403	0.0038	0.01366393

GOIDlabel	GOName	size	categoryScores	pValPerm	pValPermCorr
GO:1904814	regulation of protein localization to chromosome, telomeric region	8	0.218832789	0.0441	0.046872463
GO:0071897	DNA biosynthetic process	88	0.218827502	0.0066	0.015525305
GO:0018345	protein palmitoylation	12	0.218821828	0.0105	0.017924712
GO:0019371	cyclooxygenase pathway	7	0.218820918	0.0192	0.02450649
GO:0019062	virion attachment to host cell	5	0.218795891	0.025	0.029554283
GO:0034250	positive regulation of cellular amide metabolic process	68	0.218776393	0.0058	0.014878261
GO:0050731	positive regulation of peptidyl-tyrosine phosphorylation	78	0.218754855	0.0022	0.012422266
GO:0043525	positive regulation of neuron apoptotic process	25	0.218733849	0.006	0.015037711
GO:0034035	purine ribonucleoside bisphosphate metabolic process	11	0.218733411	0.0008	0.010465158
GO:0050427	3'-phosphoadenosine 5'-phosphosulfate metabolic process	11	0.218733411	0.0008	0.010465158
GO:0032535	regulation of cellular component size	148	0.218696193	0.0023	0.012498684
GO:1900452	regulation of long term synaptic depression	5	0.218674346	0.0004	0.008964341
GO:0006778	porphyrin-containing compound metabolic process	20	0.218597053	0.0236	0.028327839
GO:0008406	gonad development	84	0.218585517	0.0055	0.01475
GO:0002220	innate immune response activating cell surface receptor signaling pathway	63	0.2185726	0.0124	0.019242297
GO:0090068	positive regulation of cell cycle process	101	0.21853551	0.001	0.010787313
GO:0042742	defense response to bacterium	67	0.218492971	0.0082	0.016514246
GO:0060761	negative regulation of response to cytokine stimulus	26	0.21848274	0.0147	0.02091937
GO:0043090	amino acid import	11	0.218442212	0.0055	0.01475
GO:0043092	L-amino acid import	11	0.218442212	0.0055	0.01475
GO:0051785	positive regulation of nuclear division	22	0.218430727	0.0006	0.009940401
GO:0006363	termination of RNA polymerase I transcription	17	0.218418147	0.0042	0.013948535
GO:0043173	nucleotide salvage	9	0.218392959	0.0223	0.027242468
GO:0051983	regulation of chromosome segregation	39	0.218388363	0.0027	0.012827773
GO:1904058	positive regulation of sensory perception of pain	6	0.21837597	0.0051	0.014547706
GO:0045787	positive regulation of cell cycle	138	0.218358702	0.0012	0.010961137
GO:0018958	phenol-containing compound metabolic process	41	0.218344882	0.0103	0.017772187
GO:0043254	regulation of protein complex assembly	168	0.218321107	0.004	0.013824268
GO:0033157	regulation of intracellular protein transport	125	0.218306996	0.0066	0.015525305
GO:0034620	cellular response to unfolded protein	59	0.218291598	0.0005	0.009604651
GO:0071675	regulation of mononuclear cell migration	16	0.218279407	0.0294	0.033482529
GO:0009411	response to UV	55	0.218279061	0.0068	0.015633241
GO:0030041	actin filament polymerization	72	0.218260015	0.0086	0.016782045
GO:0031056	regulation of histone modification	57	0.218236798	0.0036	0.01353394
GO:0032006	regulation of TOR signaling	44	0.218228466	0.0021	0.012314604
GO:0060841	venous blood vessel development	9	0.218195424	0.0018	0.01210186
GO:0032271	regulation of protein polymerization	79	0.218155304	0.0061	0.015143924
GO:0007098	centrosome cycle	38	0.218127941	0.0027	0.012827773
GO:0090596	sensory organ morphogenesis	96	0.218127571	0.0034	0.01347416
GO:0033260	nuclear DNA replication	19	0.218085623	0.0017	0.012001709
GO:0070265	necrotic cell death	21	0.218064673	0.0182	0.023684988
GO:0045785	positive regulation of cell adhesion	158	0.218060129	0.0124	0.019242297
GO:0045137	development of primary sexual characteristics	89	0.218052655	0.0055	0.01475
GO:0044320	cellular response to leptin stimulus	7	0.21803271	0.0279	0.032115827
GO:0014014	negative regulation of gliogenesis	24	0.218012286	0.0119	0.018881943
GO:0006853	carnitine shuttle	7	0.217982545	0.0171	0.022860624
GO:1902001	fatty acid transmembrane transport	7	0.217982545	0.0171	0.022860624
GO:0010596	negative regulation of endothelial cell migration	28	0.217972688	0.0053	0.014634479
GO:0072148	epithelial cell fate commitment	8	0.217971577	0.0129	0.019597425
GO:1902750	negative regulation of cell cycle G2/M phase transition	50	0.217918929	0.0215	0.026489026
GO:0001578	microtubule bundle formation	33	0.217909016	0.0086	0.016782045
GO:0050770	regulation of axonogenesis	76	0.217880002	0.0001	0.004220438
GO:0042749	regulation of circadian sleep/wake cycle	8	0.217873259	< 0.001	< 0.001
GO:0045187	regulation of circadian sleep/wake cycle, sleep	8	0.217873259	< 0.001	< 0.001
GO:1900181	negative regulation of protein localization to nucleus	22	0.21786117	0.0064	0.015399417
GO:0061077	chaperone-mediated protein folding	21	0.217858581	0.0133	0.019917275
GO:0007548	sex differentiation	105	0.217852571	0.005	0.014547706
GO:0033138	positive regulation of peptidyl-serine phosphorylation	38	0.217825545	0.0116	0.018682786
GO:0035967	cellular response to topologically incorrect protein	65	0.217820563	0.001	0.010787313
GO:0061384	heart trabecula morphogenesis	15	0.217789152	0.0099	0.017505138
GO:0045947	negative regulation of translational initiation	7	0.21776837	0.0072	0.015844069
GO:0060420	regulation of heart growth	31	0.217763593	0.0054	0.014720792
GO:0048705	skeletal system morphogenesis	80	0.217734011	0.0072	0.015844069
GO:0051321	meiotic cell cycle	60	0.217725205	0.0033	0.013333753
GO:0030879	mammary gland development	56	0.217698328	0.0053	0.014634479
GO:0051961	negative regulation of nervous system development	139	0.217640539	0.0026	0.012805111
GO:0044070	regulation of anion transport	33	0.217634702	0.0037	0.013609033
GO:0045779	negative regulation of bone resorption	6	0.217626358	0.0009	0.010729485
GO:0034612	response to tumor necrosis factor	124	0.217586979	0.0076	0.016084627
GO:0006047	UDP-N-acetylglucosamine metabolic process	7	0.217540867	0.0451	0.047829824
GO:0030099	myeloid cell differentiation	147	0.217485527	0.0057	0.01480566
GO:2000010	positive regulation of protein localization to cell surface	8	0.217476263	0.0194	0.024690909
GO:0071108	protein K48-linked deubiquitination	16	0.217474562	0.0012	0.010961137
GO:0002521	leukocyte differentiation	189	0.217461296	0.0037	0.013609033
GO:0032277	negative regulation of gonadotropin secretion	5	0.217453716	0.0128	0.019522448
GO:0002377	immunoglobulin production	40	0.217450671	0.0025	0.012769435
GO:0060261	positive regulation of transcription initiation from RNA polymerase II promoter	6	0.217443004	0.0012	0.010961137
GO:0052547	regulation of peptidase activity	150	0.217420052	0.0083	0.016559903
GO:0000910	cytokinesis	52	0.217415261	0.0015	0.011704453
GO:0045987	positive regulation of smooth muscle contraction	11	0.21740523	0.0194	0.024690909
GO:0061298	retina vasculature development in camera-type eye	13	0.217393532	0.0062	0.015209334
GO:0060674	placenta blood vessel development	14	0.217361123	0.0275	0.031743861
GO:0070301	cellular response to hydrogen peroxide	41	0.217326046	0.0023	0.012498684

GOIDlabel	GOName	size	categoryScores	pValPerm	pValPermCorr
GO:0043300	regulation of leukocyte degranulation	23	0.217322797	0.0307	0.034703304
GO:0000070	mitotic sister chromatid segregation	60	0.217291916	0.003	0.013210967
GO:0014032	neural crest cell development	29	0.217216793	0.0082	0.016514246
GO:0016570	histone modification	166	0.217143472	0.0008	0.010465158
GO:0048545	response to steroid hormone	137	0.217107418	0.0115	0.018599441
GO:0071774	response to fibroblast growth factor	57	0.217105623	0.0084	0.016633151
GO:0001837	epithelial to mesenchymal transition	61	0.2170939	0.007	0.015816335
GO:2001233	regulation of apoptotic signaling pathway	173	0.217093639	0.009	0.016956012
GO:0060117	auditory receptor cell development	8	0.217085421	0.0196	0.0248852
GO:0033137	negative regulation of peptidyl-serine phosphorylation	11	0.217074098	0.0074	0.015911789
GO:0030510	regulation of BMP signaling pathway	31	0.217044014	0.006	0.015037711
GO:0055001	muscle cell development	63	0.217039748	0.0054	0.014720792
GO:0042157	lipoprotein metabolic process	53	0.217034204	0.0078	0.01620539
GO:1903054	negative regulation of extracellular matrix organization	8	0.217022252	0.024	0.028671074
GO:1901988	negative regulation of cell cycle phase transition	105	0.216993243	0.0096	0.017302743
GO:0039530	MDA-5 signaling pathway	5	0.216988011	0.0094	0.017156187
GO:0033619	membrane protein proteolysis	25	0.216971528	0.0104	0.017832977
GO:0042982	amyloid precursor protein metabolic process	27	0.21695934	0.0019	0.012288367
GO:1902581	multi-organism cellular localization	29	0.216950084	0.0096	0.017302743
GO:1902583	multi-organism intracellular transport	29	0.216950084	0.0096	0.017302743
GO:0010821	regulation of mitochondrion organization	111	0.216948586	0.0046	0.014132412
GO:0009126	purine nucleoside monophosphate metabolic process	157	0.216930919	0.0182	0.023684988
GO:0009167	purine ribonucleoside monophosphate metabolic process	157	0.216930919	0.0182	0.023684988
GO:0034162	toll-like receptor 9 signaling pathway	8	0.216928469	0.0217	0.026655917
GO:0016569	covalent chromatin modification	168	0.216923618	0.0008	0.010465158
GO:0071218	cellular response to misfolded protein	9	0.216923538	0.013	0.019676963
GO:0010952	positive regulation of peptidase activity	75	0.216901331	0.006	0.015037711
GO:0010830	regulation of myotube differentiation	20	0.216867049	0.0011	0.010890753
GO:0045979	positive regulation of nucleoside metabolic process	18	0.216830746	0.0151	0.02121706
GO:1903580	positive regulation of ATP metabolic process	18	0.216830746	0.0151	0.02121706
GO:0030101	natural killer cell activation	24	0.216796137	0.0063	0.015311728
GO:0072520	seminiferous tubule development	6	0.216779562	0.038	0.041253474
GO:0043618	regulation of transcription from RNA polymerase II promoter in response to stress	66	0.216761323	0.019	0.024347961
GO:2001204	regulation of osteoclast development	6	0.216754726	0.003	0.013210967
GO:0086009	membrane repolarization	20	0.216700994	0.0063	0.015311728
GO:0097435	fibril organization	11	0.21667264	0.0179	0.02344231
GO:1904779	regulation of protein localization to centrosome	7	0.216662139	0.0024	0.012603815
GO:0044387	negative regulation of protein kinase activity by regulation of protein phosphorylation	5	0.216650329	0.0346	0.038164288
GO:0033683	nucleotide-excision repair, DNA incision	18	0.216649767	0.021	0.026005997
GO:0051968	positive regulation of synaptic transmission, glutamatergic	12	0.216646096	0.0021	0.012314604
GO:0006760	folic acid-containing compound metabolic process	14	0.21663826	0.006	0.015037711
GO:0000245	spliceosomal complex assembly	21	0.216635298	0.0117	0.018739446
GO:0048511	rhythmic process	153	0.216625203	0.0008	0.010465158
GO:0035265	organ growth	70	0.216607753	0.0067	0.015576759
GO:0016575	histone deacetylation	27	0.216605687	0.0004	0.008964341
GO:0003206	cardiac chamber morphogenesis	58	0.216594793	0.0064	0.015399417
GO:0019098	reproductive behavior	15	0.216567399	0.001	0.010787313
GO:0070099	regulation of chemokine-mediated signaling pathway	6	0.216537466	0.0376	0.040911404
GO:2001244	positive regulation of intrinsic apoptotic signaling pathway	21	0.216536201	0.0117	0.018739446
GO:0000819	sister chromatid segregation	67	0.216483981	0.0029	0.013089617
GO:0046530	photoreceptor cell differentiation	25	0.216459693	0.0021	0.012314604
GO:0001570	vasculogenesis	37	0.216446603	0.0022	0.012422266
GO:0003009	skeletal muscle contraction	17	0.216426753	0.0202	0.025368462
GO:2000273	positive regulation of receptor activity	30	0.216408838	0.0055	0.01475
GO:2000036	regulation of stem cell population maintenance	9	0.216407384	0.0054	0.014720792
GO:0007266	Rho protein signal transduction	66	0.216406945	0.0033	0.013333753
GO:0001975	response to amphetamine	13	0.216378433	0.0095	0.01725157
GO:0006493	protein O-linked glycosylation	45	0.21637807	0.0039	0.013791927
GO:1990314	cellular response to insulin-like growth factor stimulus	6	0.216375709	0.0128	0.019522448
GO:0009648	photoperiodism	12	0.216365666	0.0025	0.012769435
GO:0060693	regulation of branching involved in salivary gland morphogenesis	6	0.216350441	0.0223	0.027242468
GO:0034341	response to interferon-gamma	71	0.216347652	0.0149	0.021043429
GO:0019369	arachidonic acid metabolic process	19	0.216332766	0.0013	0.011218806
GO:0072079	nephron tubule formation	8	0.216297422	0.0042	0.013948535
GO:0006991	response to sterol depletion	7	0.216260446	0.0007	0.010220707
GO:0016331	morphogenesis of embryonic epithelium	64	0.216236662	0.0068	0.015633241
GO:0007050	cell cycle arrest	100	0.21619959	0.005	0.014547706
GO:0051194	positive regulation of cofactor metabolic process	9	0.216158161	0.0109	0.018225506
GO:0051197	positive regulation of coenzyme metabolic process	9	0.216158161	0.0109	0.018225506
GO:0033684	regulation of luteinizing hormone secretion	5	0.216146787	< 0.001	< 0.001
GO:0007157	heterophilic cell-cell adhesion via plasma membrane cell adhesion molecules	15	0.216123635	0.0136	0.020157703
GO:0007031	peroxisome organization	46	0.216107857	0.0093	0.017114131
GO:0055010	ventricular cardiac muscle tissue morphogenesis	19	0.216090113	0.0144	0.02072711
GO:0061097	regulation of protein tyrosine kinase activity	44	0.216072426	0.0001	0.004220438
GO:0006367	transcription initiation from RNA polymerase II promoter	81	0.216029398	0.0045	0.014132412
GO:0014745	negative regulation of muscle adaptation	6	0.216001443	0.0475	0.049962707
GO:0048102	autophagic cell death	6	0.216000795	0.0287	0.032827577
GO:0050830	defense response to Gram-positive bacterium	20	0.215990968	0.0237	0.028436066
GO:0010633	negative regulation of epithelial cell migration	37	0.215989086	0.004	0.013824268
GO:1901032	negative regulation of response to reactive oxygen species	9	0.215987117	0.0215	0.026489026
GO:1903206	negative regulation of hydrogen peroxide-induced cell death	9	0.215987117	0.0215	0.026489026
GO:1900015	regulation of cytokine production involved in inflammatory response	15	0.215984209	0.0019	0.012288367
GO:0002696	positive regulation of leukocyte activation	121	0.215978495	0.0149	0.021043429

GOIDlabel	GOName	size	categoryScores	pValPerm	pValPermCorr
GO:0098703	calcium ion import across plasma membrane	6	0.215961057	0.0031	0.013277185
GO:1990035	calcium ion import into cell	6	0.215961057	0.0031	0.013277185
GO:0010524	positive regulation of calcium ion transport into cytosol	23	0.215933264	0.0063	0.015311728
GO:0010721	negative regulation of cell development	148	0.215931441	0.0017	0.012001709
GO:1990440	positive regulation of transcription from RNA polymerase II promoter in response to endoplasmic reticulum stress	6	0.215911601	0.002	0.012314604
GO:0032722	positive regulation of chemokine production	23	0.21590301	0.0275	0.031743861
GO:0035728	response to hepatocyte growth factor	9	0.215897996	0.0064	0.015399417
GO:0060242	contact inhibition	7	0.215892998	0.0101	0.017653628
GO:0071826	ribonucleoprotein complex subunit organization	79	0.215882233	0.0108	0.018152791
GO:0043921	modulation by host of viral transcription	7	0.215876708	0.0238	0.028526451
GO:0052472	modulation by host of symbiont transcription	7	0.215876708	0.0238	0.028526451
GO:0006690	icosanoid metabolic process	40	0.215830874	0.0137	0.020264364
GO:1901568	fatty acid derivative metabolic process	40	0.215830874	0.0137	0.020264364
GO:0017038	protein import	90	0.215804382	0.012	0.018931514
GO:0061082	myeloid leukocyte cytokine production	14	0.21579424	0.0304	0.034411276
GO:0007271	synaptic transmission, cholinergic	13	0.21575536	0.004	0.013824268
GO:0070231	T cell apoptotic process	12	0.215737318	0.002	0.012314604
GO:1901991	negative regulation of mitotic cell cycle phase transition	98	0.215712738	0.0093	0.017114131
GO:0052548	regulation of endopeptidase activity	136	0.215683715	0.0087	0.016840777
GO:0009161	ribonucleoside monophosphate metabolic process	167	0.215669115	0.0165	0.022405589
GO:0046782	regulation of viral transcription	30	0.215653179	0.0149	0.021043429
GO:0046676	negative regulation of insulin secretion	17	0.215615972	0.0235	0.028248857
GO:0050729	positive regulation of inflammatory response	50	0.21561015	0.0035	0.01351837
GO:0006289	nucleotide-excision repair	52	0.215606917	0.0066	0.015525305
GO:0003407	neural retina development	32	0.215592757	0.0204	0.025564109
GO:0006735	NADH regeneration	17	0.215584949	0.0149	0.021043429
GO:0061621	canonical glycolysis	17	0.215584949	0.0149	0.021043429
GO:0061718	glucose catabolic process to pyruvate	17	0.215584949	0.0149	0.021043429
GO:0007091	metaphase/anaphase transition of mitotic cell cycle	21	0.215578492	0.0017	0.012001709
GO:0010965	regulation of mitotic sister chromatid separation	21	0.215578492	0.0017	0.012001709
GO:0006084	acetyl-CoA metabolic process	17	0.215573287	0.0214	0.026388313
GO:0035729	cellular response to hepatocyte growth factor stimulus	8	0.215568056	0.0047	0.014168613
GO:0006568	tryptophan metabolic process	5	0.215566015	0.0469	0.0494125
GO:0006586	indolalkylamine metabolic process	5	0.215566015	0.0469	0.0494125
GO:0070085	glycosylation	136	0.215557747	0.0034	0.01347416
GO:0040020	regulation of meiotic nuclear division	11	0.215556733	0.0022	0.012422266
GO:0032660	regulation of interleukin-17 production	12	0.215509908	0.0102	0.0177
GO:0043271	negative regulation of ion transport	56	0.215507501	0.0019	0.012288367
GO:0051053	negative regulation of DNA metabolic process	60	0.215502738	0.0026	0.012805111
GO:0071732	cellular response to nitric oxide	6	0.215488527	0.016	0.022026667
GO:0009142	nucleoside triphosphate biosynthetic process	38	0.21548181	0.0231	0.027930615
GO:0000012	single strand break repair	5	0.215480753	< 0.001	< 0.001
GO:0007257	activation of JUN kinase activity	14	0.215476125	0.0051	0.014547706
GO:0007422	peripheral nervous system development	28	0.215465481	0.0002	0.006319126
GO:0030431	sleep	12	0.215452893	0.0001	0.004220438
GO:0051251	positive regulation of lymphocyte activation	101	0.215387161	0.0112	0.018392048
GO:0006294	nucleotide-excision repair, preincision complex assembly	17	0.21537709	0.0163	0.022248961
GO:0001825	blastocyst formation	17	0.21536558	0.0045	0.014132412
GO:0042303	molting cycle	33	0.215350507	0.0048	0.014269203
GO:0042633	hair cycle	33	0.215350507	0.0048	0.014269203
GO:0010951	negative regulation of endopeptidase activity	70	0.215342011	0.0101	0.017653628
GO:0006360	transcription from RNA polymerase I promoter	29	0.215280621	0.0076	0.016084627
GO:0043649	dicarboxylic acid catabolic process	13	0.215258775	0.0101	0.017653628
GO:0071800	podosome assembly	7	0.215255286	0.0397	0.042801678
GO:0035966	response to topologically incorrect protein	87	0.215254824	0.0017	0.012001709
GO:0045807	positive regulation of endocytosis	57	0.215235895	0.0112	0.018392048
GO:2000104	negative regulation of DNA-dependent DNA replication	8	0.215230666	0.0007	0.010220707
GO:0006353	DNA-templated transcription, termination	38	0.215229063	0.004	0.013824268
GO:0043620	regulation of DNA-templated transcription in response to stress	69	0.215199455	0.0181	0.023607986
GO:0006486	protein glycosylation	126	0.215193644	0.0034	0.01347416
GO:0043413	macromolecule glycosylation	126	0.215193644	0.0034	0.01347416
GO:0080154	regulation of fertilization	8	0.215188391	0.0043	0.014102439
GO:0006956	complement activation	25	0.215175673	0.0289	0.033004108
GO:0021675	nerve development	24	0.215169008	0.0052	0.014616626
GO:0043903	regulation of symbiosis, encompassing mutualism through parasitism	93	0.215151358	0.0103	0.017772187
GO:0060048	cardiac muscle contraction	57	0.215145622	0.0082	0.016514246
GO:0060348	bone development	85	0.215142431	0.0031	0.013277185
GO:0016052	carbohydrate catabolic process	82	0.215120666	0.0063	0.015311728
GO:0038061	NIK/NF-kappaB signaling	80	0.215117495	0.0156	0.021692929
GO:0032755	positive regulation of interleukin-6 production	29	0.215097513	0.011	0.018323826
GO:0010826	negative regulation of centrosome duplication	5	0.215063251	0.0234	0.02817551
GO:0046034	ATP metabolic process	141	0.215059677	0.0199	0.025133639
GO:0050774	negative regulation of dendrite morphogenesis	8	0.215050066	0.0001	0.004220438
GO:1903747	regulation of establishment of protein localization to mitochondrion	40	0.215044397	0.0068	0.015633241
GO:0046501	protoporphyrinogen IX metabolic process	6	0.215042777	0.0321	0.035976391
GO:1903321	negative regulation of protein modification by small protein conjugation or removal	43	0.214972172	0.0058	0.014878261
GO:0008154	actin polymerization or depolymerization	83	0.214927405	0.01	0.017574468
GO:0006650	glycerophospholipid metabolic process	137	0.214909009	0.0064	0.015399417
GO:0070536	protein K63-linked deubiquitination	11	0.214899416	0.0014	0.011465722
GO:0046006	regulation of activated T cell proliferation	8	0.21488048	0.0104	0.017832977
GO:0030593	neutrophil chemotaxis	24	0.214788695	0.0182	0.023684988
GO:0021877	forebrain neuron fate commitment	6	0.214750146	0.0198	0.025062084

GOIDlabel	GOName	size	categoryScores	pValPerm	pValPermCorr
GO:2000677	regulation of transcription regulatory region DNA binding	23	0.21470638	0.0036	0.01353394
GO:0046466	membrane lipid catabolic process	14	0.214679217	0.0044	0.014132412
GO:0060419	heart growth	43	0.214671761	0.0097	0.017363901
GO:0042451	purine nucleoside biosynthetic process	51	0.214626322	0.0172	0.022941269
GO:0046129	purine ribonucleoside biosynthetic process	51	0.214626322	0.0172	0.022941269
GO:0033135	regulation of peptidyl-serine phosphorylation	50	0.21459923	0.0083	0.016559903
GO:0006921	cellular component disassembly involved in execution phase of apoptosis	12	0.214597351	0.0002	0.006319126
GO:0030262	apoptotic nuclear changes	12	0.214597351	0.0002	0.006319126
GO:0001667	ameboidal-type cell migration	173	0.214594233	0.0039	0.013791927
GO:0008589	regulation of smoothened signaling pathway	27	0.2145893	0.0107	0.018084595
GO:1904861	excitatory synapse assembly	9	0.214573271	0.0038	0.01366393
GO:0061213	positive regulation of mesonephros development	10	0.214540271	0.0138	0.020339434
GO:0061217	regulation of mesonephros development	10	0.214540271	0.0138	0.020339434
GO:1990542	mitochondrial transmembrane transport	35	0.214534422	0.0121	0.018970228
GO:0031953	negative regulation of protein autophosphorylation	7	0.214532608	0.0088	0.016916437
GO:0046487	glyoxylate metabolic process	5	0.214529905	0.0157	0.021779607
GO:0001558	regulation of cell growth	185	0.214521997	0.0015	0.011704453
GO:0033006	regulation of mast cell activation involved in immune response	14	0.214475963	0.0244	0.029011063
GO:0043304	regulation of mast cell degranulation	14	0.214475963	0.0244	0.029011063
GO:0035635	entry of bacterium into host cell	6	0.214452547	0.024	0.028671074
GO:0048536	spleen development	12	0.214409786	0.0046	0.014132412
GO:0002886	regulation of myeloid leukocyte mediated immunity	28	0.214372624	0.0218	0.026744664
GO:0006283	transcription-coupled nucleotide-excision repair	38	0.214349054	0.0153	0.021378589
GO:0002067	glandular epithelial cell differentiation	23	0.214335898	0.0001	0.004220438
GO:0030968	endoplasmic reticulum unfolded protein response	51	0.214330959	0.0002	0.006319126
GO:1900271	regulation of long-term synaptic potentiation	25	0.214321232	0.0043	0.014102439
GO:0050792	regulation of viral process	84	0.214319096	0.0087	0.016840777
GO:0051342	regulation of cyclic-nucleotide phosphodiesterase activity	6	0.214269841	0.0295	0.033583186
GO:1903025	regulation of RNA polymerase II regulatory region sequence-specific DNA binding	7	0.214257346	0.0046	0.014132412
GO:1905063	regulation of vascular smooth muscle cell differentiation	7	0.214240316	0.0076	0.016084627
GO:0000302	response to reactive oxygen species	96	0.214223341	0.0033	0.013333753
GO:0055017	cardiac muscle tissue growth	40	0.214210494	0.0097	0.017363901
GO:0051131	chaperone-mediated protein complex assembly	9	0.214201236	0.0041	0.013887639
GO:0007220	Notch receptor processing	6	0.214198789	0.0301	0.034151923
GO:0050867	positive regulation of cell activation	123	0.214187896	0.0169	0.022703485
GO:0070911	global genome nucleotide-excision repair	16	0.214182537	0.0111	0.018389742
GO:0048469	cell maturation	69	0.214173324	0.0041	0.013887639
GO:0032653	regulation of interleukin-10 production	14	0.214147204	0.0158	0.021871104
GO:0045143	homologous chromosome segregation	14	0.214073344	0.0092	0.017033109
GO:0046683	response to organophosphorus	60	0.2140504	0.0076	0.016084627
GO:0016254	preassembly of GPI anchor in ER membrane	9	0.214040703	0.0367	0.040037623
GO:0042168	heme metabolic process	19	0.214030291	0.0261	0.030517735
GO:0030901	midbrain development	40	0.214019268	0.013	0.019676963
GO:0046939	nucleotide phosphorylation	61	0.213981953	0.0085	0.016693954
GO:0021953	central nervous system neuron differentiation	88	0.213975315	0.0026	0.012805111
GO:2000648	positive regulation of stem cell proliferation	18	0.213964179	0.0136	0.020157703
GO:0002027	regulation of heart rate	39	0.213953553	0.0112	0.018392048
GO:0071257	cellular response to electrical stimulus	8	0.213947514	0.0338	0.0374175
GO:0045923	positive regulation of fatty acid metabolic process	13	0.213905476	0.0085	0.016693954
GO:0061180	mammary gland epithelium development	30	0.213901319	0.0101	0.017653628
GO:0033235	positive regulation of protein sumoylation	5	0.21384884	0.0006	0.009940401
GO:0022011	myelination in peripheral nervous system	8	0.213846767	0.0036	0.01353394
GO:0032292	peripheral nervous system axon ensheathment	8	0.213846767	0.0036	0.01353394
GO:0030004	cellular monovalent inorganic cation homeostasis	34	0.213843146	0.0111	0.018389742
GO:0002460	adaptive immune response based on somatic recombination of immune receptors built from immunoglobulin superfamily domains	78	0.213831799	0.0054	0.014720792
GO:0048562	embryonic organ morphogenesis	96	0.213809338	0.0097	0.017363901
GO:0002822	regulation of adaptive immune response based on somatic recombination of immune receptors built from immunoglobulin superfamily domains	40	0.213788716	0.0026	0.012805111
GO:0098727	maintenance of cell number	62	0.213699946	0.0092	0.017033109
GO:0048762	mesenchymal cell differentiation	86	0.213682097	0.0084	0.016633151
GO:0071706	tumor necrosis factor superfamily cytokine production	66	0.213665023	0.0196	0.0248852
GO:1903555	regulation of tumor necrosis factor superfamily cytokine production	66	0.213665023	0.0196	0.0248852
GO:0051180	vitamin transport	13	0.213637747	0.0207	0.025805821
GO:1901017	negative regulation of potassium ion transmembrane transporter activity	7	0.213592608	< 0.001	< 0.001
GO:1903008	organelle disassembly	52	0.213558778	0.0039	0.013791927
GO:0002312	B cell activation involved in immune response	30	0.213547915	0.0016	0.011845327
GO:0031103	axon regeneration	24	0.21353621	0.009	0.016956012
GO:0071621	granulocyte chemotaxis	31	0.213528798	0.0171	0.022860624
GO:0022409	positive regulation of cell-cell adhesion	86	0.213517336	0.0151	0.02121706
GO:0090317	negative regulation of intracellular protein transport	32	0.213517111	0.0141	0.020504577
GO:0032675	regulation of interleukin-6 production	48	0.213512967	0.0102	0.0177
GO:0006409	tRNA export from nucleus	10	0.213502477	0.0056	0.014771533
GO:0051031	tRNA transport	10	0.213502477	0.0056	0.014771533
GO:0071431	tRNA-containing ribonucleoprotein complex export from nucleus	10	0.213502477	0.0056	0.014771533
GO:0051591	response to cAMP	42	0.21341308	0.0081	0.016514175
GO:0043153	entrainment of circadian clock by photoperiod	10	0.213376008	0.0036	0.01353394
GO:0030514	negative regulation of BMP signaling pathway	15	0.213344693	0.0104	0.017832977
GO:0045667	regulation of osteoblast differentiation	56	0.213330674	0.0036	0.01353394
GO:0006582	melanin metabolic process	11	0.213330455	0.0108	0.018152791
GO:0042438	melanin biosynthetic process	11	0.213330455	0.0108	0.018152791
GO:0032642	regulation of chemokine production	34	0.213287712	0.0258	0.030258742
GO:0060391	positive regulation of SMAD protein import into nucleus	6	0.213256968	0.0322	0.036060507

GOIDlabel	GOName	size	categoryScores	pValPerm	pValPermCorr
GO:0090559	regulation of membrane permeability	34	0.213194415	0.006	0.015037711
GO:0042407	cristae formation	16	0.21318049	0.0239	0.028592965
GO:0060155	platelet dense granule organization	5	0.213180215	0.0469	0.0494125
GO:0009123	nucleoside monophosphate metabolic process	175	0.213171248	0.0167	0.02255007
GO:0044784	metaphase/anaphase transition of cell cycle	22	0.213170838	0.0018	0.01210186
GO:0050435	beta-amyloid metabolic process	19	0.213136676	0.0005	0.009604651
GO:0045684	positive regulation of epidermis development	11	0.213123365	0.028	0.032186083
GO:0010662	regulation of striated muscle cell apoptotic process	17	0.213121051	0.0012	0.010961137
GO:2001242	regulation of intrinsic apoptotic signaling pathway	63	0.21310712	0.0141	0.020504577
GO:0090042	tubulin deacetylation	5	0.213105318	0.0055	0.01475
GO:0071346	cellular response to interferon-gamma	62	0.213079453	0.0158	0.021871104
GO:0046823	negative regulation of nucleocytoplasmic transport	22	0.213058988	0.0145	0.020788247
GO:0055117	regulation of cardiac muscle contraction	27	0.213049331	0.0221	0.027038129
GO:0008361	regulation of cell size	70	0.213031495	0.0006	0.009940401
GO:0045747	positive regulation of Notch signaling pathway	20	0.21302336	0.0165	0.022405589
GO:2001056	positive regulation of cysteine-type endopeptidase activity	59	0.213012127	0.008	0.016379603
GO:0050765	negative regulation of phagocytosis	10	0.212983385	0.0101	0.017653628
GO:1903019	negative regulation of glycoprotein metabolic process	9	0.212983054	0.0139	0.020388077
GO:0009303	rRNA transcription	13	0.21296334	0.0221	0.027038129
GO:0042063	gliogenesis	132	0.212960665	0.0111	0.018389742
GO:0032373	positive regulation of sterol transport	8	0.212959478	0.0404	0.043394538
GO:0032376	positive regulation of cholesterol transport	8	0.212959478	0.0404	0.043394538
GO:0034614	cellular response to reactive oxygen species	73	0.212943356	0.0047	0.014168613
GO:1903706	regulation of hemopoiesis	179	0.212940607	0.0107	0.018084595
GO:0036475	neuron death in response to oxidative stress	17	0.212925082	0.0072	0.015844069
GO:0060412	ventricular septum morphogenesis	22	0.212919573	0.0258	0.030258742
GO:0009214	cyclic nucleotide catabolic process	5	0.212864043	0.0013	0.011218806
GO:0086003	cardiac muscle cell contraction	29	0.212853435	0.0031	0.013277185
GO:2001258	negative regulation of cation channel activity	15	0.212805992	0.0018	0.01210186
GO:0060219	camera-type eye photoreceptor cell differentiation	9	0.212792285	0.0127	0.019457181
GO:0010566	regulation of ketone biosynthetic process	7	0.212785889	0.0263	0.030677143
GO:0045649	regulation of macrophage differentiation	7	0.212785703	0.0362	0.03958177
GO:0032640	tumor necrosis factor production	65	0.212780156	0.0193	0.024606968
GO:0032680	regulation of tumor necrosis factor production	65	0.212780156	0.0193	0.024606968
GO:0032456	endocytic recycling	18	0.212773856	0.0028	0.012972436
GO:0046486	glycerolipid metabolic process	172	0.212706174	0.0057	0.01480566
GO:0050879	multicellular organismal movement	20	0.212703884	0.0163	0.022248961
GO:0050881	musculoskeletal movement	20	0.212703884	0.0163	0.022248961
GO:0001959	regulation of cytokine-mediated signaling pathway	62	0.212700435	0.0132	0.019860109
GO:0006775	fat-soluble vitamin metabolic process	17	0.212656168	0.017	0.022784886
GO:0090128	regulation of synapse maturation	9	0.212631896	< 0.001	< 0.001
GO:0006354	DNA-templated transcription, elongation	53	0.212573425	0.0073	0.015844069
GO:0090189	regulation of branching involved in ureteric bud morphogenesis	9	0.212559188	0.0143	0.020629391
GO:0090190	positive regulation of branching involved in ureteric bud morphogenesis	9	0.212559188	0.0143	0.020629391
GO:0030308	negative regulation of cell growth	75	0.212550791	0.002	0.012314604
GO:0030833	regulation of actin filament polymerization	61	0.21255011	0.0073	0.015844069
GO:2001238	positive regulation of extrinsic apoptotic signaling pathway	23	0.212549347	0.0069	0.015750415
GO:0048738	cardiac muscle tissue development	87	0.212504512	0.0058	0.014878261
GO:0001845	phagolysosome assembly	7	0.212469347	0.0081	0.016514175
GO:0010972	negative regulation of G2/M transition of mitotic cell cycle	46	0.212450107	0.0217	0.026655917
GO:0006403	RNA localization	85	0.212424543	0.0086	0.016782045
GO:0032410	negative regulation of transporter activity	32	0.212422293	0.0014	0.011465722
GO:0000281	mitotic cytokinesis	22	0.212318388	0.0043	0.014102439
GO:1903053	regulation of extracellular matrix organization	20	0.212304847	0.0029	0.013089617
GO:0097352	autophagosome maturation	20	0.212302255	0.0196	0.0248852
GO:0097576	vacuole fusion	20	0.212302255	0.0196	0.0248852
GO:0051482	positive regulation of cytosolic calcium ion concentration involved in phospholipase C-activating G-protein coupled signaling pathway	10	0.212297396	0.0001	0.004220438
GO:0006986	response to unfolded protein	79	0.212279128	0.0012	0.010961137
GO:0030502	negative regulation of bone mineralization	8	0.212272873	0.0002	0.006319126
GO:0046474	glycerophospholipid biosynthetic process	100	0.212236675	0.0055	0.01475
GO:0007602	phototransduction	22	0.212222617	0.001	0.010787313
GO:0043281	regulation of cysteine-type endopeptidase activity involved in apoptotic process	82	0.212198591	0.0103	0.017772187
GO:1903978	regulation of microglial cell activation	8	0.212181682	0.0407	0.043676206
GO:0042278	purine nucleoside metabolic process	195	0.212117531	0.0146	0.020879842
GO:0031442	positive regulation of mRNA 3'-end processing	6	0.212095512	0.0114	0.018551872
GO:0060350	endochondral bone morphogenesis	23	0.212095484	0.0016	0.011845327
GO:0071875	adrenergic receptor signaling pathway	5	0.212093828	0.0032	0.013333753
GO:0006275	regulation of DNA replication	48	0.212081232	0.0014	0.011465722
GO:1900034	regulation of cellular response to heat	32	0.212072593	0.0037	0.013609033
GO:0043502	regulation of muscle adaptation	36	0.212035186	0.0056	0.014771533
GO:1901136	carbohydrate derivative catabolic process	88	0.212000773	0.0119	0.018881943
GO:0033008	positive regulation of mast cell activation involved in immune response	6	0.212000272	0.0272	0.031504487
GO:0043306	positive regulation of mast cell degranulation	6	0.212000272	0.0272	0.031504487
GO:0045017	glycerolipid biosynthetic process	115	0.211975325	0.0047	0.014168613
GO:0006626	protein targeting to mitochondrion	56	0.211962023	0.0085	0.016693954
GO:0033622	integrin activation	10	0.211932373	0.0434	0.046247475
GO:0046128	purine ribonucleoside metabolic process	193	0.211911205	0.0145	0.020788247
GO:0006266	DNA ligation	8	0.211907785	0.0085	0.016693954
GO:0006336	DNA replication-independent nucleosome assembly	13	0.211889632	0.0177	0.02329117
GO:0034724	DNA replication-independent nucleosome organization	13	0.211889632	0.0177	0.02329117
GO:0032635	interleukin-6 production	49	0.211884656	0.0087	0.016840777
GO:0010948	negative regulation of cell cycle process	142	0.211874289	0.0085	0.016693954

GOIDlabel	GOName	size	categoryScores	pValPerm	pValPermCorr
GO:0045008	depyrimidination	5	0.211874063	0.0038	0.01366393
GO:0071168	protein localization to chromatin	7	0.211849776	0.0161	0.02211694
GO:0042130	negative regulation of T cell proliferation	17	0.211826789	0.017	0.022784886
GO:0070633	transepithelial transport	8	0.211817566	0.0356	0.03903645
GO:0002052	positive regulation of neuroblast proliferation	10	0.21177338	0.0188	0.02420971
GO:0061615	glycolytic process through fructose-6-phosphate	18	0.211750833	0.0126	0.019370699
GO:0061620	glycolytic process through glucose-6-phosphate	18	0.211750833	0.0126	0.019370699
GO:0070232	regulation of T cell apoptotic process	9	0.211730317	0.004	0.013824268
GO:0061462	protein localization to lysosome	17	0.211714056	0.0108	0.018152791
GO:1903039	positive regulation of leukocyte cell-cell adhesion	77	0.211693491	0.0146	0.020879842
GO:0051570	regulation of histone H3-K9 methylation	8	0.211692847	0.0147	0.02091937
GO:0042744	hydrogen peroxide catabolic process	7	0.211679287	0.0266	0.030933467
GO:0032648	regulation of interferon-beta production	17	0.211660166	0.0076	0.016084627
GO:0090100	positive regulation of transmembrane receptor protein serine/threonine kinase signaling pathway	37	0.211608384	0.0114	0.018551872
GO:0055007	cardiac muscle cell differentiation	46	0.211606748	0.004	0.013824268
GO:0050900	leukocyte migration	158	0.211574771	0.0186	0.024075487
GO:0046456	icosanoid biosynthetic process	21	0.211534591	0.0388	0.041925173
GO:1901570	fatty acid derivative biosynthetic process	21	0.211534591	0.0388	0.041925173
GO:0010001	glial cell differentiation	96	0.211492864	0.0118	0.018836996
GO:0019081	viral translation	5	0.211491806	0.0387	0.041895413
GO:0006488	dolichol-linked oligosaccharide biosynthetic process	13	0.211476974	0.0079	0.01629604
GO:0006490	oligosaccharide-lipid intermediate biosynthetic process	13	0.211476974	0.0079	0.01629604
GO:0015909	long-chain fatty acid transport	30	0.21147604	0.0117	0.018739446
GO:0002828	regulation of type 2 immune response	10	0.211473017	0.0038	0.01366393
GO:0042092	type 2 immune response	10	0.211473017	0.0038	0.01366393
GO:0098840	protein transport along microtubule	27	0.21145657	0.0082	0.016514246
GO:0099118	microtubule-based protein transport	27	0.21145657	0.0082	0.016514246
GO:0051023	regulation of immunoglobulin secretion	10	0.211455524	0.001	0.010787313
GO:0006479	protein methylation	68	0.211451202	0.0014	0.011465722
GO:0008213	protein alkylation	68	0.211451202	0.0014	0.011465722
GO:0050679	positive regulation of epithelial cell proliferation	72	0.211450931	0.0101	0.017653628
GO:0061053	somite development	31	0.211443335	0.0149	0.021043429
GO:0032388	positive regulation of intracellular transport	114	0.211388252	0.0051	0.014547706
GO:0044766	multi-organism transport	32	0.211380765	0.0119	0.018881943
GO:1902579	multi-organism localization	32	0.211380765	0.0119	0.018881943
GO:0010528	regulation of transposition	5	0.211320762	0.0332	0.036915846
GO:0010529	negative regulation of transposition	5	0.211320762	0.0332	0.036915846
GO:0043302	positive regulation of leukocyte degranulation	10	0.211317911	0.0305	0.034510959
GO:0034764	positive regulation of transmembrane transport	69	0.211284792	0.0073	0.015844069
GO:0032411	positive regulation of transporter activity	51	0.211284135	0.0093	0.017114131
GO:0016445	somatic diversification of immunoglobulins	22	0.211239116	0.0017	0.012001709
GO:0014854	response to inactivity	5	0.211220727	0.0057	0.01480566
GO:0034763	negative regulation of transmembrane transport	42	0.211203642	0.0033	0.013333753
GO:0009151	purine deoxyribonucleotide metabolic process	10	0.211184445	0.0342	0.037780741
GO:0010466	negative regulation of peptidase activity	74	0.211168255	0.0117	0.018739446
GO:0032414	positive regulation of ion transmembrane transporter activity	47	0.211159996	0.0071	0.01583804
GO:0090162	establishment of epithelial cell polarity	13	0.211020957	0.0387	0.041895413
GO:0061387	regulation of extent of cell growth	45	0.210994195	0.0001	0.004220438
GO:0022617	extracellular matrix disassembly	29	0.21097955	0.0045	0.014132412
GO:0043276	anoikis	15	0.210973405	0.0003	0.007956881
GO:0033628	regulation of cell adhesion mediated by integrin	22	0.210936432	0.0096	0.017302743
GO:0040015	negative regulation of multicellular organism growth	5	0.210922956	0.0376	0.040911404
GO:0010950	positive regulation of endopeptidase activity	64	0.210922077	0.0078	0.01620539
GO:0045444	fat cell differentiation	99	0.210910346	0.0012	0.010961137
GO:0048853	forebrain morphogenesis	5	0.210903001	0.0328	0.036618961
GO:1902305	regulation of sodium ion transmembrane transport	26	0.210834214	0.0023	0.012498684
GO:0097191	extrinsic apoptotic signaling pathway	88	0.210821357	0.0057	0.01480566
GO:0015931	nucleobase-containing compound transport	84	0.210790101	0.0043	0.014102439
GO:0008625	extrinsic apoptotic signaling pathway via death domain receptors	37	0.210789203	0.0058	0.014878261
GO:0006656	phosphatidylcholine biosynthetic process	15	0.210784911	0.0159	0.021956962
GO:0006474	N-terminal protein amino acid acetylation	6	0.210764956	0.0384	0.04164081
GO:2000649	regulation of sodium ion transmembrane transporter activity	21	0.210760575	0.0006	0.009940401
GO:0072132	mesenchyme morphogenesis	28	0.210751135	0.0162	0.022196303
GO:0036303	lymph vessel morphogenesis	7	0.210746137	0.0025	0.012769435
GO:0071985	multivesicular body sorting pathway	6	0.210745736	0.006	0.015037711
GO:0003229	ventricular cardiac muscle tissue development	22	0.210737293	0.0116	0.018682786
GO:0035094	response to nicotine	19	0.210693233	0.0109	0.018225506
GO:0097094	craniofacial suture morphogenesis	8	0.210689423	0.0345	0.038068511
GO:0019827	stem cell population maintenance	61	0.210686579	0.0091	0.016994897
GO:1904646	cellular response to beta-amyloid	17	0.210678418	0.0145	0.020788247
GO:0006509	membrane protein ectodomain proteolysis	18	0.210662157	0.0075	0.015990044
GO:0090092	regulation of transmembrane receptor protein serine/threonine kinase signaling pathway	89	0.21064041	0.0079	0.01629604
GO:0071493	cellular response to UV-B	5	0.210630627	0.0318	0.035688587
GO:1902510	regulation of apoptotic DNA fragmentation	6	0.210628251	0.0009	0.010729485
GO:0009263	deoxyribonucleotide biosynthetic process	7	0.210623592	0.0285	0.032631089
GO:1901739	regulation of myoblast fusion	8	0.210607464	0.0035	0.01351837
GO:1901741	positive regulation of myoblast fusion	8	0.210607464	0.0035	0.01351837
GO:0035282	segmentation	29	0.210578616	0.0153	0.021378589
GO:0097194	execution phase of apoptosis	25	0.210565526	0.0014	0.011465722
GO:0009132	nucleoside diphosphate metabolic process	71	0.210541126	0.0102	0.0177
GO:0006362	transcription elongation from RNA polymerase I promoter	16	0.210508013	0.0034	0.01347416
GO:0035025	positive regulation of Rho protein signal transduction	7	0.210503454	0.0109	0.018225506
GO:0045197	establishment or maintenance of epithelial cell apical/basal polarity	15	0.210477119	0.0284	0.032523034

GOIDlabel	GOName	size	categoryScores	pValPerm	pValPermCorr
GO:0046822	regulation of nucleocytoplasmic transport	65	0.210462852	0.0094	0.017156187
GO:2000008	regulation of protein localization to cell surface	17	0.210453256	0.0111	0.018389742
GO:0022900	electron transport chain	95	0.210436736	0.0308	0.034775552
GO:0007163	establishment or maintenance of cell polarity	82	0.210422005	0.0053	0.014634479
GO:0060485	mesenchyme development	109	0.210404577	0.0088	0.016916437
GO:0009205	purine ribonucleoside triphosphate metabolic process	155	0.210352568	0.017	0.022784886
GO:1902946	protein localization to early endosome	5	0.210303311	0.028	0.032186083
GO:0043268	positive regulation of potassium ion transport	18	0.210210144	0.0013	0.011218806
GO:0090329	regulation of DNA-dependent DNA replication	21	0.210172103	0.0013	0.011218806
GO:0030728	ovulation	9	0.210118215	0.0038	0.01366393
GO:0048592	eye morphogenesis	61	0.210111781	0.0013	0.011218806
GO:0002698	negative regulation of immune effector process	43	0.21009387	0.0143	0.020629391
GO:0070192	chromosome organization involved in meiotic cell cycle	15	0.210068169	0.0155	0.021605834
GO:0031112	positive regulation of microtubule polymerization or depolymerization	9	0.21006552	0.0128	0.019522448
GO:0071216	cellular response to biotic stimulus	90	0.21004473	0.0083	0.016559903
GO:0086002	cardiac muscle cell action potential involved in contraction	22	0.210035861	0.0031	0.013277185
GO:0046660	female sex differentiation	50	0.210024406	0.003	0.013210967
GO:0030903	notochord development	9	0.210020311	0.02	0.025188412
GO:0033630	positive regulation of cell adhesion mediated by integrin	10	0.209996728	0.0151	0.02121706
GO:0055123	digestive system development	59	0.209986072	0.01	0.017574468
GO:1902805	positive regulation of synaptic vesicle transport	12	0.209980142	< 0.001	< 0.001
GO:0046321	positive regulation of fatty acid oxidation	9	0.209955954	0.0224	0.027324219
GO:0042440	pigment metabolic process	36	0.209955774	0.0153	0.021378589
GO:0060840	artery development	37	0.20994386	0.0118	0.018836996
GO:0030890	positive regulation of B cell proliferation	19	0.209919513	0.0233	0.028125386
GO:0001958	endochondral ossification	12	0.209904879	0.0062	0.015209334
GO:0036075	replacement ossification	12	0.209904879	0.0062	0.015209334
GO:0042273	ribosomal large subunit biogenesis	11	0.209895916	0.0118	0.018836996
GO:0070471	uterine smooth muscle contraction	5	0.209847454	0.0184	0.023875404
GO:0048565	digestive tract development	57	0.209766519	0.0095	0.01725157
GO:0009199	ribonucleoside triphosphate metabolic process	157	0.209729032	0.0167	0.02255007
GO:0003207	cardiac chamber formation	7	0.2097208	0.0331	0.03684717
GO:0032507	maintenance of protein location in cell	39	0.209712579	0.0063	0.015311728
GO:0032608	interferon-beta production	19	0.209669358	0.0087	0.016840777
GO:0046460	neutral lipid biosynthetic process	19	0.209638941	0.0024	0.012603815
GO:0046463	acylglycerol biosynthetic process	19	0.209638941	0.0024	0.012603815
GO:0008272	sulfate transport	7	0.209600477	0.0457	0.0483597
GO:1902358	sulfate transmembrane transport	7	0.209600477	0.0457	0.0483597
GO:0006505	GPI anchor metabolic process	15	0.209595812	0.0363	0.039683607
GO:0031100	animal organ regeneration	33	0.209585948	0.0098	0.017418875
GO:0045071	negative regulation of viral genome replication	16	0.2095433	0.0107	0.018084595
GO:0010822	positive regulation of mitochondrion organization	73	0.209538048	0.004	0.013824268
GO:0031122	cytoplasmic microtubule organization	17	0.209534392	0.0032	0.013333753
GO:0042308	negative regulation of protein import into nucleus	18	0.209503941	0.0142	0.020567234
GO:1904590	negative regulation of protein import	18	0.209503941	0.0142	0.020567234
GO:0071545	inositol phosphate catabolic process	5	0.209465197	0.0032	0.013333753
GO:2000144	positive regulation of DNA-templated transcription, initiation	8	0.209376144	0.0121	0.018970228
GO:0050905	neuromuscular process	49	0.209375936	0.0005	0.009604651
GO:0070661	leukocyte proliferation	111	0.209343865	0.0183	0.023783007
GO:0009119	ribonucleoside metabolic process	200	0.20934371	0.0141	0.020504577
GO:1901381	positive regulation of potassium ion transmembrane transport	16	0.209297344	0.0027	0.012827773
GO:0010996	response to auditory stimulus	9	0.209272355	0.0002	0.006319126
GO:0055006	cardiac cell development	37	0.209272153	0.0051	0.014547706
GO:0030183	B cell differentiation	41	0.209263952	0.0026	0.012805111
GO:1902115	regulation of organelle assembly	63	0.209261454	0.0079	0.01629604
GO:0035089	establishment of apical/basal cell polarity	9	0.209244856	0.0333	0.037012803
GO:0014855	striated muscle cell proliferation	24	0.209205856	0.024	0.028671074
GO:0007586	digestion	46	0.209197009	0.0115	0.018599441
GO:0051099	positive regulation of binding	80	0.209154937	0.0027	0.012827773
GO:2000209	regulation of anoikis	12	0.209144144	0.0001	0.004220438
GO:0042552	myelination	52	0.209133969	0.0066	0.015525305
GO:0071622	regulation of granulocyte chemotaxis	15	0.209125442	0.0259	0.0303391
GO:0000075	cell cycle checkpoint	76	0.209113943	0.0026	0.012805111
GO:0006606	protein import into nucleus	67	0.209065723	0.017	0.022784886
GO:0044744	protein targeting to nucleus	67	0.209065723	0.017	0.022784886
GO:1902593	single-organism nuclear import	67	0.209065723	0.017	0.022784886
GO:0010944	negative regulation of transcription by competitive promoter binding	5	0.209054951	0.03	0.034118804
GO:0042461	photoreceptor cell development	18	0.209053799	0.0006	0.009940401
GO:0006814	sodium ion transport	64	0.209027659	0.0029	0.013089617
GO:1903707	negative regulation of hemopoiesis	48	0.208985815	0.0118	0.018836996
GO:0046628	positive regulation of insulin receptor signaling pathway	6	0.208932931	0.0125	0.019314538
GO:1900078	positive regulation of cellular response to insulin stimulus	6	0.208932931	0.0125	0.019314538
GO:0048568	embryonic organ development	159	0.208883378	0.0057	0.01480566
GO:0006641	triglyceride metabolic process	40	0.20883172	0.0041	0.013887639
GO:0008356	asymmetric cell division	10	0.208800459	0.028	0.032186083
GO:0010460	positive regulation of heart rate	12	0.208776789	0.004	0.013824268
GO:0048103	somatic stem cell division	11	0.208764224	0.0319	0.035793868
GO:2000404	regulation of T cell migration	14	0.208703053	0.0133	0.019917275
GO:0032941	secretion by tissue	38	0.208685857	0.0014	0.011465722
GO:0071624	positive regulation of granulocyte chemotaxis	11	0.20864725	0.0301	0.034151923
GO:0090022	regulation of neutrophil chemotaxis	11	0.20864725	0.0301	0.034151923
GO:0090023	positive regulation of neutrophil chemotaxis	11	0.20864725	0.0301	0.034151923
GO:1902622	regulation of neutrophil migration	11	0.20864725	0.0301	0.034151923

GOIDlabel	GOName	size	categoryScores	pValPerm	pValPermCorr
GO:1902624	positive regulation of neutrophil migration	11	0.20864725	0.0301	0.034151923
GO:1903311	regulation of mRNA metabolic process	52	0.208609001	0.0035	0.01351837
GO:0033108	mitochondrial respiratory chain complex assembly	51	0.208595125	0.0315	0.035393121
GO:0034767	positive regulation of ion transmembrane transport	65	0.208586315	0.0073	0.015844069
GO:0030194	positive regulation of blood coagulation	8	0.208545707	0.0243	0.028921902
GO:0050820	positive regulation of coagulation	8	0.208545707	0.0243	0.028921902
GO:1900048	positive regulation of hemostasis	8	0.208545707	0.0243	0.028921902
GO:1902106	negative regulation of leukocyte differentiation	35	0.208512868	0.0081	0.016514175
GO:0031570	DNA integrity checkpoint	51	0.208485517	0.0014	0.011465722
GO:0007016	cytoskeletal anchoring at plasma membrane	6	0.208475303	0.0252	0.029736
GO:0009880	embryonic pattern specification	22	0.208455002	0.0164	0.022306469
GO:1903146	regulation of mitophagy	27	0.208451163	0.0036	0.01353394
GO:0046824	positive regulation of nucleocytoplasmic transport	33	0.20842834	0.0142	0.020567234
GO:0032757	positive regulation of interleukin-8 production	19	0.208426086	0.0257	0.030153693
GO:0033262	regulation of nuclear cell cycle DNA replication	11	0.208418367	0.0021	0.012314604
GO:0050995	negative regulation of lipid catabolic process	9	0.208390789	0.0028	0.012972436
GO:0060759	regulation of response to cytokine stimulus	69	0.208387002	0.0142	0.020567234
GO:0019933	cAMP-mediated signaling	33	0.208363001	0.0027	0.012827773
GO:0045793	positive regulation of cell size	5	0.208352376	0.0174	0.023064374
GO:0051145	smooth muscle cell differentiation	27	0.208332287	0.0142	0.020567234
GO:1903115	regulation of actin filament-based movement	14	0.208326627	0.0079	0.01629604
GO:0006296	nucleotide-excision repair, DNA incision, 5'-to lesion	17	0.208324814	0.025	0.029554283
GO:0001649	osteoblast differentiation	91	0.208288487	0.0063	0.015311728
GO:0097186	amelogenesis	7	0.208275999	0.0157	0.021779607
GO:0060008	Sertoli cell differentiation	9	0.208272872	0.0127	0.019457181
GO:0070897	DNA-templated transcriptional preinitiation complex assembly	12	0.208194981	0.0068	0.015633241
GO:0048659	smooth muscle cell proliferation	59	0.208181811	0.0106	0.018010344
GO:0015980	energy derivation by oxidation of organic compounds	127	0.208163899	0.0162	0.022196303
GO:0010561	negative regulation of glycoprotein biosynthetic process	8	0.208149683	0.0129	0.019597425
GO:1904063	negative regulation of cation transmembrane transport	36	0.20814297	0.0021	0.012314604
GO:0003298	physiological muscle hypertrophy	17	0.208132504	0.001	0.010787313
GO:0003301	physiological cardiac muscle hypertrophy	17	0.208132504	0.001	0.010787313
GO:0061049	cell growth involved in cardiac muscle cell development	17	0.208132504	0.001	0.010787313
GO:1900017	positive regulation of cytokine production involved in inflammatory response	5	0.20809581	0.0404	0.043394538
GO:0071318	cellular response to ATP	10	0.20809283	0.0173	0.023005658
GO:0032964	collagen biosynthetic process	17	0.208083874	0.0107	0.018084595
GO:0043277	apoptotic cell clearance	18	0.208051509	0.0005	0.009604651
GO:0007052	mitotic spindle organization	38	0.208042226	0.006	0.015037711
GO:0070849	response to epidermal growth factor	24	0.208020374	0.0018	0.01210186
GO:0002687	positive regulation of leukocyte migration	45	0.208004242	0.0154	0.021487162
GO:0032693	negative regulation of interleukin-10 production	6	0.20795958	0.0203	0.025471918
GO:0046037	GMP metabolic process	6	0.207929561	0.013	0.019676963
GO:0034248	regulation of cellular amide metabolic process	194	0.207898102	0.0048	0.014269203
GO:0051151	negative regulation of smooth muscle cell differentiation	8	0.207897814	0.021	0.026005997
GO:0010611	regulation of cardiac muscle hypertrophy	26	0.207896369	0.0023	0.012498684
GO:0019320	hexose catabolic process	33	0.20789377	0.0094	0.017156187
GO:0030522	intracellular receptor signaling pathway	105	0.207850549	0.0056	0.014771533
GO:0019064	fusion of virus membrane with host plasma membrane	6	0.207849438	0.0336	0.037253154
GO:0039663	membrane fusion involved in viral entry into host cell	6	0.207849438	0.0336	0.037253154
GO:0044800	multi-organism membrane fusion	6	0.207849438	0.0336	0.037253154
GO:0035725	sodium ion transmembrane transport	45	0.207845925	0.0012	0.010961137
GO:0042976	activation of Janus kinase activity	6	0.207844039	0.0024	0.012603815
GO:0086001	cardiac muscle cell action potential	27	0.20784128	0.0101	0.017653628
GO:0006446	regulation of translational initiation	33	0.207789557	0.0059	0.014942532
GO:0070286	axonemal dynein complex assembly	11	0.207750919	0.0134	0.01999969
GO:0002082	regulation of oxidative phosphorylation	15	0.207734113	0.0057	0.01480566
GO:0048660	regulation of smooth muscle cell proliferation	58	0.207709255	0.0099	0.017505138
GO:0098927	vesicle-mediated transport between endosomal compartments	24	0.207707064	0.0018	0.01210186
GO:0036035	osteoclast development	9	0.207685161	0.0021	0.012314604
GO:0032770	positive regulation of monooxygenase activity	13	0.207642445	0.0032	0.013333753
GO:0006165	nucleoside diphosphate phosphorylation	59	0.20763253	0.0087	0.016840777
GO:0010560	positive regulation of glycoprotein biosynthetic process	6	0.207625699	0.0053	0.014634479
GO:0032733	positive regulation of interleukin-10 production	9	0.207616629	0.0353	0.038781037
GO:0070242	thymocyte apoptotic process	5	0.207559873	0.0045	0.014132412
GO:0006744	ubiquinone biosynthetic process	8	0.207521874	0.0133	0.019917275
GO:1901663	quinone biosynthetic process	8	0.207521874	0.0133	0.019917275
GO:0009144	purine nucleoside triphosphate metabolic process	161	0.207516942	0.019	0.024347961
GO:0051170	nuclear import	79	0.207516788	0.0178	0.023348367
GO:0030261	chromosome condensation	12	0.207505623	0.0033	0.013333753
GO:0008064	regulation of actin polymerization or depolymerization	69	0.207496309	0.0106	0.018010344
GO:0030832	regulation of actin filament length	69	0.207496309	0.0106	0.018010344
GO:0009895	negative regulation of catabolic process	87	0.207471039	0.0021	0.012314604
GO:0033002	muscle cell proliferation	78	0.207459041	0.0154	0.021487162
GO:0030851	granulocyte differentiation	14	0.207456322	0.0059	0.014942532
GO:0051606	detection of stimulus	107	0.207426596	0.0085	0.016693954
GO:0044550	secondary metabolite biosynthetic process	12	0.207376477	0.0147	0.02091937
GO:0002679	respiratory burst involved in defense response	7	0.207337665	0.0418	0.044765253
GO:0006417	regulation of translation	174	0.207330201	0.0064	0.015399417
GO:0048645	animal organ formation	22	0.207328022	0.0287	0.032827577
GO:0007585	respiratory gaseous exchange	30	0.207303092	0.0032	0.013333753
GO:0002065	columnar/cuboidal epithelial cell differentiation	47	0.207274293	0.0029	0.013089617
GO:0003002	regionalization	102	0.207273614	0.0089	0.016916437
GO:0072331	signal transduction by p53 class mediator	109	0.207263486	0.0062	0.015209334

GOIDlabel	GOName	size	categoryScores	pValPerm	pValPermCorr
GO:0042733	embryonic digit morphogenesis	25	0.207239503	0.0144	0.02072711
GO:0001756	somitogenesis	21	0.207210924	0.0145	0.020788247
GO:0033045	regulation of sister chromatid segregation	31	0.207207119	0.0035	0.01351837
GO:0007272	ensheathment of neurons	53	0.207188677	0.0056	0.014771533
GO:0008366	axon ensheathment	53	0.207188677	0.0056	0.014771533
GO:0009952	anterior/posterior pattern specification	55	0.207181457	0.0146	0.020879842
GO:0044060	regulation of endocrine process	20	0.20714114	0.0097	0.017363901
GO:0007595	lactation	21	0.207085603	0.0024	0.012603815
GO:0070278	extracellular matrix constituent secretion	7	0.207085357	0.0057	0.01480566
GO:0042033	chemokine biosynthetic process	6	0.207070455	0.0454	0.048095053
GO:0045073	regulation of chemokine biosynthetic process	6	0.207070455	0.0454	0.048095053
GO:0050755	chemokine metabolic process	6	0.207070455	0.0454	0.048095053
GO:0050686	negative regulation of mRNA processing	12	0.207059657	0.0152	0.0213213
GO:0002825	regulation of T-helper 1 type immune response	9	0.207031222	0.0071	0.01583804
GO:2001243	negative regulation of intrinsic apoptotic signaling pathway	38	0.20702084	0.0198	0.025062084
GO:0009791	post-embryonic development	24	0.206999781	0.0078	0.01620539
GO:2001235	positive regulation of apoptotic signaling pathway	79	0.206988616	0.0069	0.015750415
GO:0031623	receptor internalization	43	0.206965873	0.0092	0.017033109
GO:0034389	lipid particle organization	13	0.206905541	0.0134	0.01999969
GO:0016482	cytosolic transport	70	0.20688786	0.0021	0.012314604
GO:0042098	T cell proliferation	68	0.206885863	0.0206	0.025725529
GO:0098781	ncRNA transcription	49	0.20687975	0.0085	0.016693954
GO:0042455	ribonucleoside biosynthetic process	57	0.20687919	0.0164	0.022306469
GO:0006661	phosphatidylinositol biosynthetic process	57	0.206843181	0.0039	0.013791927
GO:0070227	lymphocyte apoptotic process	20	0.206838056	0.0039	0.013791927
GO:0008630	intrinsic apoptotic signaling pathway in response to DNA damage	40	0.206790501	0.0181	0.023607986
GO:0051150	regulation of smooth muscle cell differentiation	15	0.206785425	0.0114	0.018551872
GO:0097530	granulocyte migration	36	0.206775088	0.0192	0.02450649
GO:0043967	histone H4 acetylation	22	0.206769597	0.0096	0.017302743
GO:0042149	cellular response to glucose starvation	14	0.206745213	0.0158	0.021871104
GO:0043455	regulation of secondary metabolic process	8	0.206738248	0.0019	0.012288367
GO:0034616	response to laminar fluid shear stress	9	0.206735494	0.002	0.012314604
GO:0032460	negative regulation of protein oligomerization	9	0.206706411	0.0121	0.018970228
GO:0017004	cytochrome complex assembly	19	0.206666068	0.0188	0.02420971
GO:0060260	regulation of transcription initiation from RNA polymerase II promoter	10	0.206643882	0.0059	0.014942532
GO:0040014	regulation of multicellular organism growth	31	0.206628571	0.0129	0.019597425
GO:0060544	regulation of necroptotic process	6	0.206564451	0.0326	0.036409735
GO:0002763	positive regulation of myeloid leukocyte differentiation	22	0.206559916	0.0266	0.030933467
GO:0030010	establishment of cell polarity	59	0.206538184	0.0035	0.01351837
GO:0035148	tube formation	62	0.206508293	0.0067	0.015576759
GO:0042743	hydrogen peroxide metabolic process	18	0.20650758	0.0268	0.031128485
GO:0060986	endocrine hormone secretion	23	0.206487323	0.0092	0.017033109
GO:0043392	negative regulation of DNA binding	25	0.206469755	0.0064	0.015399417
GO:0046545	development of primary female sexual characteristics	43	0.206460637	0.003	0.013210967
GO:0015918	sterol transport	33	0.20645266	0.0156	0.021692929
GO:0000717	nucleotide-excision repair, DNA duplex unwinding	14	0.206449218	0.0167	0.02255007
GO:0071474	cellular hyperosmotic response	8	0.206442647	0.0067	0.015576759
GO:0032943	mononuclear cell proliferation	101	0.206406878	0.0191	0.02440579
GO:0006536	glutamate metabolic process	18	0.206380017	0.0063	0.015311728
GO:0055013	cardiac muscle cell development	34	0.206375304	0.0048	0.014269203
GO:0046488	phosphatidylinositol metabolic process	71	0.206318416	0.0056	0.014771533
GO:0006368	transcription elongation from RNA polymerase II promoter	45	0.206292595	0.0107	0.018084595
GO:0046651	lymphocyte proliferation	100	0.206285157	0.0187	0.024129301
GO:1904064	positive regulation of cation transmembrane transport	56	0.206258645	0.0055	0.01475
GO:0048021	regulation of melanin biosynthetic process	6	0.206248063	0.0022	0.012422266
GO:1900376	regulation of secondary metabolite biosynthetic process	6	0.206248063	0.0022	0.012422266
GO:0061640	cytoskeleton-dependent cytokinesis	28	0.20624342	0.0027	0.012827773
GO:0036474	cell death in response to hydrogen peroxide	12	0.206182518	0.0092	0.017033109
GO:0006968	cellular defense response	17	0.206177087	0.0106	0.018010344
GO:0042133	neurotransmitter metabolic process	10	0.206151743	0.0308	0.034775552
GO:0016073	snRNA metabolic process	36	0.206149151	0.0056	0.014771533
GO:0048340	paraxial mesoderm morphogenesis	5	0.20614358	0.0473	0.049788567
GO:1901881	positive regulation of protein depolymerization	11	0.206088426	0.0208	0.025885837
GO:0050864	regulation of B cell activation	51	0.206079598	0.0109	0.018225506
GO:0031062	positive regulation of histone methylation	14	0.206053726	0.0116	0.018682786
GO:0051651	maintenance of location in cell	46	0.206047601	0.0068	0.015633241
GO:0098751	bone cell development	14	0.206033179	0.0008	0.010465158
GO:0003281	ventricular septum development	31	0.206005716	0.0132	0.019860109
GO:0018208	peptidyl-proline modification	25	0.205989226	0.0144	0.02072711
GO:0002761	regulation of myeloid leukocyte differentiation	44	0.205981583	0.009	0.016956012
GO:0006638	neutral lipid metabolic process	51	0.205941381	0.0048	0.014269203
GO:0046174	polyol catabolic process	6	0.205909215	0.0024	0.012603815
GO:0019682	glyceraldehyde-3-phosphate metabolic process	10	0.205865893	0.0232	0.028016374
GO:1990868	response to chemokine	5	0.205792681	0.0121	0.018970228
GO:1990869	cellular response to chemokine	5	0.205792681	0.0121	0.018970228
GO:0015812	gamma-aminobutyric acid transport	5	0.205680207	0.0075	0.015990044
GO:2001224	positive regulation of neuron migration	8	0.205672788	0.0002	0.006319126
GO:1903524	positive regulation of blood circulation	25	0.205661444	0.012	0.018931514
GO:0090656	t-circle formation	9	0.205654377	0.0065	0.015491756
GO:0030071	regulation of mitotic metaphase/anaphase transition	20	0.20565293	0.002	0.012314604
GO:0071425	hematopoietic stem cell proliferation	8	0.205612048	0.0012	0.010961137
GO:1901385	regulation of voltage-gated calcium channel activity	16	0.205592854	0.0164	0.022306469
GO:0046470	phosphatidylcholine metabolic process	32	0.205582772	0.0135	0.020081554

GOIDlabel	GOName	size	categoryScores	pValPerm	pValPermCorr
GO:0048872	homeostasis of number of cells	83	0.205558203	0.0071	0.01583804
GO:0032602	chemokine production	37	0.205554018	0.0229	0.027770092
GO:2000169	regulation of peptidyl-cysteine S-nitrosylation	5	0.205552442	0.012	0.018931514
GO:0048259	regulation of receptor-mediated endocytosis	43	0.205515105	0.0065	0.015491756
GO:0014743	regulation of muscle hypertrophy	27	0.20550349	0.0039	0.013791927
GO:0048745	smooth muscle tissue development	6	0.205502986	0.022	0.026944291
GO:2001236	regulation of extrinsic apoptotic signaling pathway	62	0.205498103	0.008	0.016379603
GO:0048566	embryonic digestive tract development	15	0.205496551	0.0051	0.014547706
GO:0050871	positive regulation of B cell activation	33	0.205424081	0.0149	0.021043429
GO:0032570	response to progesterone	21	0.205418112	0.0324	0.036242368
GO:0000377	RNA splicing, via transesterification reactions with bulged adenosine as nucleophile	138	0.205410694	0.0098	0.017418875
GO:0000398	mRNA splicing, via spliceosome	138	0.205410694	0.0098	0.017418875
GO:0033146	regulation of intracellular estrogen receptor signaling pathway	13	0.205404124	0.0239	0.028592965
GO:0032508	DNA duplex unwinding	33	0.205379906	0.0057	0.01480566
GO:0045136	development of secondary sexual characteristics	5	0.205318164	0.0393	0.042425803
GO:0008360	regulation of cell shape	55	0.20530808	0.0145	0.020788247
GO:0019058	viral life cycle	199	0.205287034	0.0135	0.020081554
GO:0046885	regulation of hormone biosynthetic process	7	0.20526015	0.0255	0.029992067
GO:0008585	female gonad development	39	0.205232815	0.0021	0.012314604
GO:0070228	regulation of lymphocyte apoptotic process	14	0.205226274	0.0033	0.013333753
GO:0045685	regulation of glial cell differentiation	31	0.205214451	0.0196	0.0248852
GO:0046838	phosphorylated carbohydrate dephosphorylation	5	0.205154377	0.0113	0.018488002
GO:0010717	regulation of epithelial to mesenchymal transition	36	0.205134299	0.0113	0.018488002
GO:0009141	nucleoside triphosphate metabolic process	169	0.205090606	0.0184	0.023875404
GO:0006929	substrate-dependent cell migration	13	0.205086159	0.0209	0.025971158
GO:0061050	regulation of cell growth involved in cardiac muscle cell development	12	0.205002597	0.0004	0.008964341
GO:0021517	ventral spinal cord development	18	0.204961924	0.0063	0.015311728
GO:0045637	regulation of myeloid cell differentiation	80	0.204961402	0.0067	0.015576759
GO:0035825	reciprocal DNA recombination	10	0.204956769	0.0141	0.020504577
GO:0045022	early endosome to late endosome transport	22	0.204926754	0.0027	0.012827773
GO:0086091	regulation of heart rate by cardiac conduction	15	0.204906622	0.014	0.020462083
GO:0071559	response to transforming growth factor beta	105	0.204897268	0.0085	0.016693954
GO:0007158	neuron cell-cell adhesion	9	0.204826802	0.0308	0.034775552
GO:0031333	negative regulation of protein complex assembly	58	0.204825328	0.0055	0.01475
GO:2000116	regulation of cysteine-type endopeptidase activity	93	0.204791788	0.0097	0.017363901
GO:0071560	cellular response to transforming growth factor beta stimulus	104	0.204735125	0.0078	0.01620539
GO:0048009	insulin-like growth factor receptor signaling pathway	11	0.204734448	0.0044	0.014132412
GO:0030033	microvillus assembly	9	0.204687865	0.0024	0.012603815
GO:1901796	regulation of signal transduction by p53 class mediator	79	0.204682527	0.0089	0.016916437
GO:1902176	negative regulation of oxidative stress-induced intrinsic apoptotic signaling pathway	7	0.204627806	0.016	0.022026667
GO:0006622	protein targeting to lysosome	10	0.20459693	0.0136	0.020157703
GO:0097120	receptor localization to synapse	7	0.204545802	0.0317	0.035590175
GO:0006007	glucose catabolic process	22	0.204532364	0.0162	0.022196303
GO:0008380	RNA splicing	164	0.204518958	0.0101	0.017653628
GO:0046365	monosaccharide catabolic process	36	0.204508831	0.0129	0.019597425
GO:0006298	mismatch repair	9	0.20449105	0.0168	0.022626974
GO:0000375	RNA splicing, via transesterification reactions	139	0.204483577	0.01	0.017574468
GO:0061158	3'-UTR-mediated mRNA destabilization	9	0.204471613	0.025	0.029554283
GO:0045861	negative regulation of proteolysis	109	0.204462651	0.0092	0.017033109
GO:2000403	positive regulation of lymphocyte migration	11	0.204434061	0.0131	0.019786886
GO:0001516	prostaglandin biosynthetic process	12	0.204394118	0.0274	0.03166636
GO:0046457	prostanoid biosynthetic process	12	0.204394118	0.0274	0.03166636
GO:0035051	cardiocyte differentiation	60	0.204326931	0.007	0.015816335
GO:0009394	2'-deoxyribonucleotide metabolic process	19	0.204321309	0.0208	0.025885837
GO:0042430	indole-containing compound metabolic process	9	0.204305752	0.0091	0.016994897
GO:0070663	regulation of leukocyte proliferation	87	0.204297555	0.019	0.024347961
GO:0030301	cholesterol transport	31	0.204253689	0.0194	0.024690909
GO:0048663	neuron fate commitment	19	0.204252292	0.0205	0.025639412
GO:0060351	cartilage development involved in endochondral bone morphogenesis	10	0.204225169	0.0003	0.007956881
GO:1903624	regulation of DNA catabolic process	7	0.2042213	0.0041	0.013887639
GO:0051315	attachment of mitotic spindle microtubules to kinetochore	7	0.204203159	0.0145	0.020788247
GO:0001573	ganglioside metabolic process	9	0.204194027	0.0044	0.014132412
GO:0042698	ovulation cycle	51	0.204190139	0.002	0.012314604
GO:0010257	NADH dehydrogenase complex assembly	37	0.204156741	0.0403	0.043327371
GO:0032981	mitochondrial respiratory chain complex I assembly	37	0.204156741	0.0403	0.043327371
GO:0097031	mitochondrial respiratory chain complex I biogenesis	37	0.204156741	0.0403	0.043327371
GO:0040029	regulation of gene expression, epigenetic	83	0.204155585	0.0034	0.01347416
GO:1902105	regulation of leukocyte differentiation	98	0.204150693	0.0111	0.018389742
GO:0032944	regulation of mononuclear cell proliferation	82	0.204140446	0.0212	0.026203164
GO:0002285	lymphocyte activation involved in immune response	58	0.20413874	0.0066	0.015525305
GO:0045652	regulation of megakaryocyte differentiation	16	0.204029246	0.0187	0.024129301
GO:0061339	establishment or maintenance of monopolar cell polarity	11	0.204016583	0.0152	0.0213213
GO:0050670	regulation of lymphocyte proliferation	81	0.203962193	0.0209	0.025971158
GO:0010737	protein kinase A signaling	14	0.203917901	0.0182	0.023684988
GO:0030219	megakaryocyte differentiation	22	0.203861736	0.0085	0.016693954
GO:0032922	circadian regulation of gene expression	23	0.203816709	0.0001	0.004220438
GO:0034139	regulation of toll-like receptor 3 signaling pathway	5	0.20381583	0.0104	0.017832977
GO:0034141	positive regulation of toll-like receptor 3 signaling pathway	5	0.20381583	0.0104	0.017832977
GO:0034976	response to endoplasmic reticulum stress	127	0.20381565	0.0018	0.01210186
GO:0016024	CDP-diacylglycerol biosynthetic process	9	0.203753891	0.005	0.014547706
GO:0060416	response to growth hormone	14	0.203680494	0.0008	0.010465158
GO:0033198	response to ATP	16	0.203651281	0.0074	0.015911789
GO:0043966	histone H3 acetylation	19	0.203627791	0.0087	0.016840777

GOIDlabel	GOName	size	categoryScores	pValPerm	pValPermCorr
GO:0006639	acylglycerol metabolic process	50	0.203606638	0.0047	0.014168613
GO:1902099	regulation of metaphase/anaphase transition of cell cycle	21	0.203603272	0.0022	0.012422266
GO:1903313	positive regulation of mRNA metabolic process	23	0.203595242	0.0026	0.012805111
GO:0002456	T cell mediated immunity	21	0.203585501	0.003	0.013210967
GO:0042073	intraciliary transport	22	0.203569713	0.007	0.015816335
GO:0060123	regulation of growth hormone secretion	8	0.203565679	< 0.001	< 0.001
GO:0009650	UV protection	5	0.203476334	0.0166	0.02247277
GO:0033627	cell adhesion mediated by integrin	25	0.203455602	0.0143	0.020629391
GO:0051639	actin filament network formation	5	0.203427353	0.0018	0.01210186
GO:0051294	establishment of spindle orientation	13	0.20341609	0.0027	0.012827773
GO:0035735	intraciliary transport involved in cilium assembly	19	0.203415009	0.007	0.015816335
GO:0014013	regulation of gliogenesis	54	0.203390399	0.0191	0.02440579
GO:0071772	response to BMP	53	0.203389556	0.0059	0.014942532
GO:0071773	cellular response to BMP stimulus	53	0.203389556	0.0059	0.014942532
GO:0072413	signal transduction involved in mitotic cell cycle checkpoint	18	0.20331354	0.002	0.012314604
GO:0072431	signal transduction involved in mitotic G1 DNA damage checkpoint	18	0.20331354	0.002	0.012314604
GO:1902400	intracellular signal transduction involved in G1 DNA damage checkpoint	18	0.20331354	0.002	0.012314604
GO:1902402	signal transduction involved in mitotic DNA damage checkpoint	18	0.20331354	0.002	0.012314604
GO:1902403	signal transduction involved in mitotic DNA integrity checkpoint	18	0.20331354	0.002	0.012314604
GO:0070841	inclusion body assembly	13	0.203266676	0.0054	0.014720792
GO:0072665	protein localization to vacuole	19	0.203250717	0.0148	0.020979063
GO:0035088	establishment or maintenance of apical/basal cell polarity	17	0.20319556	0.0234	0.02817551
GO:0061245	establishment or maintenance of bipolar cell polarity	17	0.20319556	0.0234	0.02817551
GO:0043044	ATP-dependent chromatin remodeling	27	0.203177843	0.0059	0.014942532
GO:0090630	activation of GTPase activity	20	0.203174157	0.0066	0.015525305
GO:0050685	positive regulation of mRNA processing	11	0.203123904	0.0022	0.012422266
GO:0010259	multicellular organism aging	14	0.203112385	0.0004	0.008964341
GO:0002718	regulation of cytokine production involved in immune response	29	0.203111807	0.0143	0.020629391
GO:0045185	maintenance of protein location	42	0.203104024	0.0075	0.015990044
GO:0035855	megakaryocyte development	5	0.203059609	0.0009	0.010729485
GO:0007589	body fluid secretion	43	0.20305474	0.002	0.012314604
GO:0032933	SREBP signaling pathway	5	0.203040432	0.0013	0.011218806
GO:0045724	positive regulation of cilium assembly	9	0.203012414	0.0136	0.020157703
GO:0051236	establishment of RNA localization	68	0.203003955	0.0067	0.015576759
GO:1904019	epithelial cell apoptotic process	37	0.202990113	0.014	0.020462083
GO:0009081	branched-chain amino acid metabolic process	13	0.20295798	0.034	0.037588528
GO:0009083	branched-chain amino acid catabolic process	13	0.20295798	0.034	0.037588528
GO:0002028	regulation of sodium ion transport	32	0.202918676	0.0049	0.014447629
GO:0002367	cytokine production involved in immune response	31	0.20291673	0.0134	0.01999969
GO:0048012	hepatocyte growth factor receptor signaling pathway	8	0.202871623	0.0086	0.016782045
GO:0034766	negative regulation of ion transmembrane transport	37	0.202839907	0.0026	0.012805111
GO:0010664	negative regulation of striated muscle cell apoptotic process	10	0.202831162	0.0148	0.020979063
GO:0006356	regulation of transcription from RNA polymerase I promoter	13	0.202786238	0.0206	0.025725529
GO:0046621	negative regulation of organ growth	17	0.202698131	0.0026	0.012805111
GO:0034249	negative regulation of cellular amide metabolic process	94	0.202695593	0.0077	0.016177834
GO:0035235	ionotropic glutamate receptor signaling pathway	19	0.202670069	0.0053	0.014634479
GO:0006397	mRNA processing	191	0.202669237	0.0084	0.016633151
GO:0097734	extracellular exosome biogenesis	12	0.202631525	0.0073	0.015844069
GO:1990182	exosomal secretion	12	0.202631525	0.0073	0.015844069
GO:0001895	retina homeostasis	18	0.202590852	0.0074	0.015911789
GO:0001838	embryonic epithelial tube formation	57	0.202542468	0.0078	0.01620539
GO:0072175	epithelial tube formation	57	0.202542468	0.0078	0.01620539
GO:0060612	adipose tissue development	18	0.202539524	0.0139	0.020388077
GO:0006734	NADH metabolic process	23	0.202537599	0.0141	0.020504577
GO:1901018	positive regulation of potassium ion transmembrane transporter activity	11	0.202526193	0.0029	0.013089617
GO:0002864	regulation of acute inflammatory response to antigenic stimulus	9	0.202518288	0.0094	0.017156187
GO:0043433	negative regulation of sequence-specific DNA binding transcription factor activity	62	0.202516792	0.0099	0.017505138
GO:1902236	negative regulation of endoplasmic reticulum stress-induced intrinsic apoptotic signaling pathway	9	0.202506194	0.0128	0.019522448
GO:0034145	positive regulation of toll-like receptor 4 signaling pathway	5	0.202466399	0.0116	0.018682786
GO:0021846	cell proliferation in forebrain	13	0.202464983	0.0302	0.034245225
GO:0032206	positive regulation of telomere maintenance	26	0.202457856	0.0134	0.01999969
GO:0050795	regulation of behavior	29	0.202425934	0.0013	0.011218806
GO:1902306	negative regulation of sodium ion transmembrane transport	9	0.202370856	0.0098	0.017418875
GO:0009301	snRNA transcription	35	0.202368104	0.0067	0.015576759
GO:0042795	snRNA transcription from RNA polymerase II promoter	35	0.202368104	0.0067	0.015576759
GO:0009583	detection of light stimulus	25	0.202314273	0.0024	0.012603815
GO:1902235	regulation of endoplasmic reticulum stress-induced intrinsic apoptotic signaling pathway	12	0.202303475	0.0092	0.017033109
GO:0031330	negative regulation of cellular catabolic process	49	0.202291134	0.0036	0.01353394
GO:0043462	regulation of ATPase activity	36	0.202124765	0.0106	0.018010344
GO:0030098	lymphocyte differentiation	124	0.202078764	0.0033	0.013333753
GO:0043543	protein acylation	87	0.202070412	0.0048	0.014269203
GO:0043267	negative regulation of potassium ion transport	12	0.202066346	0.0006	0.009940401
GO:1904738	vascular associated smooth muscle cell migration	11	0.20204263	0.0128	0.019522448
GO:1904752	regulation of vascular associated smooth muscle cell migration	11	0.20204263	0.0128	0.019522448
GO:0048485	sympathetic nervous system development	9	0.20203208	0.0072	0.015844069
GO:0006323	DNA packaging	46	0.202006524	0.0174	0.023064374
GO:0044849	estrous cycle	9	0.202006308	0.003	0.013210967
GO:0006487	protein N-linked glycosylation	35	0.201964744	0.0111	0.018389742
GO:0030433	ER-associated ubiquitin-dependent protein catabolic process	35	0.201950972	0.0038	0.01366393
GO:0036480	neuron intrinsic apoptotic signaling pathway in response to oxidative stress	6	0.201920139	0.0066	0.015525305
GO:1903376	regulation of oxidative stress-induced neuron intrinsic apoptotic signaling pathway	6	0.201920139	0.0066	0.015525305
GO:0002320	lymphoid progenitor cell differentiation	9	0.201919059	0.024	0.028671074
GO:0038034	signal transduction in absence of ligand	22	0.20182853	0.0051	0.014547706

GOIDlabel	GOName	size	categoryScores	pValPerm	pValPermCorr
GO:0097192	extrinsic apoptotic signaling pathway in absence of ligand	22	0.20182853	0.0051	0.014547706
GO:0050657	nucleic acid transport	67	0.201811818	0.0065	0.015491756
GO:0050658	RNA transport	67	0.201811818	0.0065	0.015491756
GO:0006913	nucleocytoplasmic transport	152	0.20180988	0.0099	0.017505138
GO:0032988	ribonucleoprotein complex disassembly	7	0.201795589	0.0187	0.024129301
GO:0048593	camera-type eye morphogenesis	50	0.201781757	0.0042	0.013948535
GO:0030279	negative regulation of ossification	32	0.201758138	0.0004	0.008964341
GO:0010712	regulation of collagen metabolic process	16	0.201744452	0.0142	0.020567234
GO:0044246	regulation of multicellular organismal metabolic process	16	0.201744452	0.0142	0.020567234
GO:0035313	wound healing, spreading of epidermal cells	5	0.201730911	0.0159	0.021956962
GO:1902229	regulation of intrinsic apoptotic signaling pathway in response to DNA damage	15	0.201729356	0.0466	0.049159132
GO:0021915	neural tube development	64	0.201720399	0.0137	0.020264364
GO:0044794	positive regulation by host of viral process	7	0.20168193	0.04	0.043076923
GO:0035137	hindlimb morphogenesis	9	0.201668972	0.0123	0.019143634
GO:2000650	negative regulation of sodium ion transmembrane transporter activity	8	0.20160645	0.007	0.015816335
GO:0000077	DNA damage checkpoint	49	0.201581017	0.002	0.012314604
GO:0007043	cell-cell junction assembly	25	0.20157609	0.0063	0.015311728
GO:0090343	positive regulation of cell aging	5	0.201569197	0.0124	0.019242297
GO:0071103	DNA conformation change	85	0.201562169	0.0121	0.018970228
GO:1902043	positive regulation of extrinsic apoptotic signaling pathway via death domain receptors	8	0.201543119	0.0167	0.02255007
GO:0009262	deoxyribonucleotide metabolic process	20	0.201540041	0.0224	0.027324219
GO:0033033	negative regulation of myeloid cell apoptotic process	6	0.201495553	0.0353	0.038781037
GO:0051293	establishment of spindle localization	15	0.201483588	0.0021	0.012314604
GO:0042129	regulation of T cell proliferation	53	0.201477049	0.0224	0.027324219
GO:1902017	regulation of cilium assembly	21	0.201473432	0.0035	0.01351837
GO:0016571	histone methylation	53	0.20142935	0.0017	0.012001709
GO:0032008	positive regulation of TOR signaling	20	0.201379947	0.0082	0.016514246
GO:1903649	regulation of cytoplasmic transport	16	0.2013626	0.0093	0.017114131
GO:0002753	cytoplasmic pattern recognition receptor signaling pathway	21	0.201330093	0.0235	0.028248857
GO:0021955	central nervous system neuron axonogenesis	20	0.201291898	0.001	0.010787313
GO:2000756	regulation of peptidyl-lysine acetylation	25	0.201264635	0.0104	0.017832977
GO:0007094	mitotic spindle assembly checkpoint	14	0.201223037	0.0009	0.010729485
GO:0031577	spindle checkpoint	14	0.201223037	0.0009	0.010729485
GO:0071173	spindle assembly checkpoint	14	0.201223037	0.0009	0.010729485
GO:0071174	mitotic spindle checkpoint	14	0.201223037	0.0009	0.010729485
GO:0006896	Golgi to vacuole transport	5	0.201194196	0.0089	0.016916437
GO:0090160	Golgi to lysosome transport	5	0.201194196	0.0089	0.016916437
GO:0006475	internal protein amino acid acetylation	60	0.201148714	0.0033	0.013333753
GO:1904035	regulation of epithelial cell apoptotic process	27	0.201148306	0.0163	0.022248961
GO:0032392	DNA geometric change	38	0.201146532	0.0071	0.01583804
GO:0086004	regulation of cardiac muscle cell contraction	11	0.201083159	0.0159	0.021956962
GO:0007369	gastrulation	69	0.201067558	0.0112	0.018392048
GO:1901838	positive regulation of transcription of nuclear large rRNA transcript from RNA polymerase I promoter	6	0.201000995	0.0388	0.041925173
GO:0046434	organophosphate catabolic process	55	0.200985575	0.0076	0.016084627
GO:0065004	protein-DNA complex assembly	66	0.200970799	0.0165	0.022405589
GO:0071392	cellular response to estradiol stimulus	10	0.200945535	0.0116	0.018682786
GO:0051784	negative regulation of nuclear division	23	0.200937755	0.002	0.012314604
GO:1903557	positive regulation of tumor necrosis factor superfamily cytokine production	35	0.200936446	0.0364	0.039777887
GO:0030852	regulation of granulocyte differentiation	8	0.200925353	0.012	0.018931514
GO:0010763	positive regulation of fibroblast migration	5	0.200925191	0.0078	0.01620539
GO:2000105	positive regulation of DNA-dependent DNA replication	6	0.200920656	0.0002	0.006319126
GO:0010803	regulation of tumor necrosis factor-mediated signaling pathway	27	0.200901675	0.0127	0.019457181
GO:0045948	positive regulation of translational initiation	11	0.200865938	0.0047	0.014168613
GO:0008608	attachment of spindle microtubules to kinetochore	15	0.200852713	0.0047	0.014168613
GO:0035518	histone H2A monoubiquitination	5	0.200798463	0.0043	0.014102439
GO:0010715	regulation of extracellular matrix disassembly	7	0.200797834	0.0009	0.010729485
GO:0043500	muscle adaptation	45	0.200779861	0.0125	0.019314538
GO:0009649	entrainment of circadian clock	15	0.200770215	0.0017	0.012001709
GO:0036003	positive regulation of transcription from RNA polymerase II promoter in response to stress	10	0.200706462	0.0052	0.014616626
GO:0014912	negative regulation of smooth muscle cell migration	9	0.200686047	0.0325	0.036312077
GO:0017148	negative regulation of translation	85	0.200679403	0.0082	0.016514246
GO:0032355	response to estradiol	48	0.200678101	0.004	0.013824268
GO:0072643	interferon-gamma secretion	6	0.200672513	0.0422	0.045118417
GO:0034502	protein localization to chromosome	27	0.200589774	0.0089	0.016916437
GO:0061512	protein localization to cilium	17	0.200502384	0.0042	0.013948535
GO:0036498	IRE1-mediated unfolded protein response	31	0.200500048	< 0.001	< 0.001
GO:0000380	alternative mRNA splicing, via spliceosome	17	0.200490264	0.0142	0.020567234
GO:0030888	regulation of B cell proliferation	28	0.200395446	0.0174	0.023064374
GO:0071711	basement membrane organization	8	0.200380313	0.0148	0.020979063
GO:0002861	regulation of inflammatory response to antigenic stimulus	13	0.200366707	0.0271	0.031420132
GO:0007129	synapsis	11	0.200349744	0.0164	0.022306469
GO:0010873	positive regulation of cholesterol esterification	5	0.200317986	0.0072	0.015844069
GO:0032088	negative regulation of NF-kappaB transcription factor activity	33	0.200289942	0.027	0.031323034
GO:0051972	regulation of telomerase activity	21	0.200289269	0.0091	0.016994897
GO:0070293	renal absorption	7	0.200288405	0.0085	0.016693954
GO:0032309	icosanoid secretion	14	0.200275725	0.0039	0.013791927
GO:0071715	icosanoid transport	14	0.200275725	0.0039	0.013791927
GO:1901571	fatty acid derivative transport	14	0.200275725	0.0039	0.013791927
GO:0061515	myeloid cell development	27	0.200240104	0.0047	0.014168613
GO:0009163	nucleoside biosynthetic process	62	0.20023205	0.0176	0.023223003
GO:0071731	response to nitric oxide	7	0.200228058	0.0113	0.018488002
GO:1901361	organic cyclic compound catabolic process	199	0.200185349	0.0152	0.0213213
GO:0032968	positive regulation of transcription elongation from RNA polymerase II promoter	8	0.200179952	0.0226	0.027544941

GOIDlabel	GOName	size	categoryScores	pValPerm	pValPermCorr
GO:0098885	modification of postsynaptic actin cytoskeleton	5	0.200168452	0.0012	0.010961137
GO:1904645	response to beta-amyloid	21	0.200114153	0.0087	0.016840777
GO:0048710	regulation of astrocyte differentiation	13	0.200112135	0.019	0.024347961
GO:0008544	epidermis development	126	0.200022892	0.0082	0.016514246
GO:0051169	nuclear transport	154	0.199982633	0.0105	0.017924712
GO:0045061	thymic T cell selection	8	0.199923225	0.0256	0.03006687
GO:0007178	transmembrane receptor protein serine/threonine kinase signaling pathway	128	0.199883096	0.0056	0.014771533
GO:1990416	cellular response to brain-derived neurotrophic factor stimulus	7	0.199847282	0.0336	0.037253154
GO:0042537	benzene-containing compound metabolic process	11	0.199781601	0.0117	0.018739446
GO:1905666	regulation of protein localization to endosome	5	0.19973877	0.0132	0.019860109
GO:1905668	positive regulation of protein localization to endosome	5	0.19973877	0.0132	0.019860109
GO:0042100	B cell proliferation	32	0.199737968	0.0123	0.019143634
GO:0061098	positive regulation of protein tyrosine kinase activity	30	0.199725121	0.0001	0.004220438
GO:0018158	protein oxidation	8	0.199720434	0.014	0.020462083
GO:0060142	regulation of syncytium formation by plasma membrane fusion	11	0.199712571	0.0148	0.020979063
GO:0060143	positive regulation of syncytium formation by plasma membrane fusion	11	0.199712571	0.0148	0.020979063
GO:0090150	establishment of protein localization to membrane	133	0.199697907	0.0119	0.018881943
GO:0033598	mammary gland epithelial cell proliferation	14	0.199667724	0.0023	0.012498684
GO:0006654	phosphatidic acid biosynthetic process	16	0.1996388	0.0059	0.014942532
GO:0046473	phosphatidic acid metabolic process	16	0.1996388	0.0059	0.014942532
GO:0003300	cardiac muscle hypertrophy	35	0.199583016	0.011	0.018323826
GO:0046854	phosphatidylinositol phosphorylation	9	0.199567064	0.001	0.010787313
GO:0048665	neuron fate specification	7	0.199547956	0.0348	0.038319101
GO:0007035	vacuolar acidification	8	0.199520235	0.0342	0.037780741
GO:0032092	positive regulation of protein binding	47	0.199453178	0.0025	0.012769435
GO:0042533	tumor necrosis factor biosynthetic process	11	0.19942738	0.0465	0.049080504
GO:0042534	regulation of tumor necrosis factor biosynthetic process	11	0.19942738	0.0465	0.049080504
GO:0043951	negative regulation of cAMP-mediated signaling	7	0.199413194	0.0084	0.016633151
GO:0046469	platelet activating factor metabolic process	5	0.199359104	0.0383	0.041555752
GO:0070665	positive regulation of leukocyte proliferation	52	0.199357589	0.0212	0.026203164
GO:0035914	skeletal muscle cell differentiation	25	0.199353403	0.0009	0.010729485
GO:0045103	intermediate filament-based process	14	0.199341278	0.0236	0.028327839
GO:0045104	intermediate filament cytoskeleton organization	14	0.199341278	0.0236	0.028327839
GO:0051156	glucose 6-phosphate metabolic process	11	0.19928343	0.0216	0.026572596
GO:0071156	regulation of cell cycle arrest	40	0.199259426	0.0019	0.012288367
GO:0033047	regulation of mitotic sister chromatid segregation	26	0.199205384	0.0043	0.014102439
GO:0003184	pulmonary valve morphogenesis	11	0.199204505	0.0366	0.039958686
GO:0042759	long-chain fatty acid biosynthetic process	13	0.199199802	0.002	0.012314604
GO:1904666	regulation of ubiquitin protein ligase activity	10	0.199095801	0.0234	0.02817551
GO:0071364	cellular response to epidermal growth factor stimulus	22	0.19908276	0.0015	0.011704453
GO:0048708	astrocyte differentiation	35	0.199025384	0.0167	0.02255007
GO:0007389	pattern specification process	145	0.198987509	0.007	0.015816335
GO:0003007	heart morphogenesis	104	0.198977775	0.0047	0.014168613
GO:0007597	blood coagulation, intrinsic pathway	5	0.198943935	0.0051	0.014547706
GO:0032760	positive regulation of tumor necrosis factor production	34	0.198870418	0.0356	0.03903645
GO:0046541	saliva secretion	6	0.19882861	0.0239	0.028592965
GO:1901659	glycosyl compound biosynthetic process	63	0.198826388	0.0197	0.024979254
GO:1902041	regulation of extrinsic apoptotic signaling pathway via death domain receptors	27	0.198820307	0.0046	0.014132412
GO:1904705	regulation of vascular smooth muscle cell proliferation	28	0.198770114	0.0109	0.018225506
GO:1990874	vascular smooth muscle cell proliferation	28	0.198770114	0.0109	0.018225506
GO:1900029	positive regulation of ruffle assembly	8	0.198759933	0.0021	0.012314604
GO:1903955	positive regulation of protein targeting to mitochondrion	20	0.198752547	0.006	0.015037711
GO:0051781	positive regulation of cell division	29	0.198733794	0.0017	0.012001709
GO:0090199	regulation of release of cytochrome c from mitochondria	26	0.198713584	0.0189	0.024289798
GO:0032677	regulation of interleukin-8 production	29	0.198671105	0.0351	0.038590645
GO:0042053	regulation of dopamine metabolic process	12	0.198667284	0.0463	0.048905115
GO:0042069	regulation of catecholamine metabolic process	12	0.198667284	0.0463	0.048905115
GO:0006878	cellular copper ion homeostasis	8	0.198616749	0.003	0.013210967
GO:0019692	deoxyribose phosphate metabolic process	20	0.19860865	0.0243	0.028921902
GO:0071514	genetic imprinting	8	0.198517297	0.0197	0.024979254
GO:0051299	centrosome separation	6	0.198475724	0.0002	0.006319126
GO:0006972	hyperosmotic response	13	0.198431102	0.0153	0.021378589
GO:0035999	tetrahydrofolate interconversion	5	0.198420437	0.0001	0.004220438
GO:0048854	brain morphogenesis	13	0.198364718	0.0162	0.022196303
GO:0051187	cofactor catabolic process	9	0.198347153	0.0321	0.035976391
GO:0042177	negative regulation of protein catabolic process	63	0.198344109	0.0031	0.013277185
GO:0032965	regulation of collagen biosynthetic process	14	0.198339636	0.014	0.020462083
GO:0051349	positive regulation of lyase activity	25	0.198333827	0.004	0.013824268
GO:0071354	cellular response to interleukin-6	10	0.198320921	0.0101	0.017653628
GO:0048640	negative regulation of developmental growth	45	0.198297136	0.0052	0.014616626
GO:0003176	aortic valve development	15	0.198253972	0.0465	0.049080504
GO:0044783	G1 DNA damage checkpoint	22	0.19822548	0.0022	0.012422266
GO:1990266	neutrophil migration	27	0.198216903	0.0205	0.025639412
GO:2000142	regulation of DNA-templated transcription, initiation	13	0.19821341	0.0121	0.018970228
GO:0021545	cranial nerve development	14	0.19819784	0.0045	0.014132412
GO:0018205	peptidyl-lysine modification	148	0.198156701	0.0012	0.010961137
GO:0034367	macromolecular complex remodeling	9	0.198146882	0.0232	0.028016374
GO:0034368	protein-lipid complex remodeling	9	0.198146882	0.0232	0.028016374
GO:0034369	plasma lipoprotein particle remodeling	9	0.198146882	0.0232	0.028016374
GO:0034629	cellular protein complex localization	9	0.198120247	0.0037	0.013609033
GO:0007093	mitotic cell cycle checkpoint	56	0.198052249	0.0067	0.015576759
GO:0014897	striated muscle hypertrophy	36	0.198019283	0.0132	0.019860109
GO:0046636	negative regulation of alpha-beta T cell activation	10	0.197974946	0.0064	0.015399417

GOIDlabel	GOName	size	categoryScores	pValPerm	pValPermCorr
GO:0032528	microvillus organization	12	0.197962701	0.0002	0.006319126
GO:0055022	negative regulation of cardiac muscle tissue growth	13	0.197902422	0.0013	0.011218806
GO:0061117	negative regulation of heart growth	13	0.197902422	0.0013	0.011218806
GO:0045600	positive regulation of fat cell differentiation	34	0.197901208	0.0009	0.010729485
GO:0050779	RNA destabilization	12	0.197870268	0.0276	0.031846578
GO:0061157	mRNA destabilization	12	0.197870268	0.0276	0.031846578
GO:0030217	T cell differentiation	84	0.197858051	0.0067	0.015576759
GO:0002374	cytokine secretion involved in immune response	6	0.197834742	0.0336	0.037253154
GO:0002739	regulation of cytokine secretion involved in immune response	6	0.197834742	0.0336	0.037253154
GO:0006119	oxidative phosphorylation	61	0.197769974	0.035	0.03849534
GO:0043651	linoleic acid metabolic process	9	0.197755843	0.0061	0.015143924
GO:0033687	osteoblast proliferation	6	0.197755699	0.0194	0.024690909
GO:0097113	AMPA glutamate receptor clustering	5	0.197740927	0.0387	0.041895413
GO:0097688	glutamate receptor clustering	5	0.197740927	0.0387	0.041895413
GO:0060976	coronary vasculature development	18	0.197668521	0.0288	0.032902905
GO:0030878	thyroid gland development	8	0.197644099	0.0174	0.023064374
GO:0001841	neural tube formation	48	0.197476106	0.0085	0.016693954
GO:0060045	positive regulation of cardiac muscle cell proliferation	10	0.197454818	0.0447	0.047449128
GO:0031016	pancreas development	29	0.197392522	0.0011	0.010890753
GO:0032462	regulation of protein homooligomerization	11	0.197375091	0.0189	0.024289798
GO:0021522	spinal cord motor neuron differentiation	14	0.197367519	0.0069	0.015750415
GO:0050798	activated T cell proliferation	9	0.19735127	0.0009	0.010729485
GO:0050684	regulation of mRNA processing	38	0.197287784	0.0057	0.01480566
GO:0018394	peptidyl-lysine acetylation	62	0.197276342	0.005	0.014547706
GO:0035107	appendage morphogenesis	46	0.197264653	0.0114	0.018551872
GO:0035108	limb morphogenesis	46	0.197264653	0.0114	0.018551872
GO:0009109	coenzyme catabolic process	5	0.197262005	0.0312	0.035137982
GO:0010884	positive regulation of lipid storage	8	0.197120278	0.0015	0.011704453
GO:0036503	ERAD pathway	46	0.19707851	0.0041	0.013887639
GO:0018393	internal peptidyl-lysine acetylation	57	0.1970497	0.0034	0.01347416
GO:0046839	phospholipid dephosphorylation	15	0.197045695	0.0003	0.007956881
GO:0032276	regulation of gonadotropin secretion	7	0.19698156	0.0003	0.007956881
GO:1903844	regulation of cellular response to transforming growth factor beta stimulus	52	0.196961058	0.0102	0.0177
GO:0001843	neural tube closure	41	0.196957463	0.009	0.016956012
GO:0060606	tube closure	41	0.196957463	0.009	0.016956012
GO:0032609	interferon-gamma production	31	0.196909509	0.0144	0.02072711
GO:0071712	ER-associated misfolded protein catabolic process	7	0.196889374	0.0359	0.039290895
GO:0070830	bicellular tight junction assembly	13	0.19688832	0.0075	0.015990044
GO:1903959	regulation of anion transmembrane transport	7	0.196845502	0.0111	0.018389742
GO:0015985	energy coupled proton transport, down electrochemical gradient	11	0.196829566	0.0428	0.045650175
GO:0015986	ATP synthesis coupled proton transport	11	0.196829566	0.0428	0.045650175
GO:0042776	mitochondrial ATP synthesis coupled proton transport	11	0.196829566	0.0428	0.045650175
GO:0045933	positive regulation of muscle contraction	18	0.196774645	0.0229	0.027770092
GO:0098657	import into cell	35	0.196720144	0.0047	0.014168613
GO:0035065	regulation of histone acetylation	21	0.19670784	0.0063	0.015311728
GO:0006692	prostanoid metabolic process	18	0.19669625	0.0209	0.025971158
GO:0006693	prostaglandin metabolic process	18	0.19669625	0.0209	0.025971158
GO:0009219	pyrimidine deoxyribonucleotide metabolic process	9	0.196695602	0.0125	0.019314538
GO:0031116	positive regulation of microtubule polymerization	8	0.19669526	0.0222	0.027148985
GO:0048483	autonomic nervous system development	17	0.196623563	0.005	0.014547706
GO:0019439	aromatic compound catabolic process	192	0.196616028	0.0156	0.021692929
GO:0031571	mitotic G1 DNA damage checkpoint	21	0.196615346	0.0035	0.01351837
GO:0044819	mitotic G1/S transition checkpoint	21	0.196615346	0.0035	0.01351837
GO:0006401	RNA catabolic process	111	0.196582663	0.0234	0.02817551
GO:0052173	response to defenses of other organism involved in symbiotic interaction	7	0.19655321	0.0071	0.01583804
GO:0052200	response to host defenses	7	0.19655321	0.0071	0.01583804
GO:0075136	response to host	7	0.19655321	0.0071	0.01583804
GO:0009215	purine deoxyribonucleoside triphosphate metabolic process	8	0.19639399	0.0419	0.044864037
GO:1901799	negative regulation of proteasomal protein catabolic process	20	0.196345755	0.0091	0.016994897
GO:0002262	myeloid cell homeostasis	49	0.196319704	0.0093	0.017114131
GO:2001237	negative regulation of extrinsic apoptotic signaling pathway	36	0.196319213	0.0048	0.014269203
GO:0051123	RNA polymerase II transcriptional preinitiation complex assembly	6	0.196288654	0.0111	0.018389742
GO:0031641	regulation of myelination	23	0.196265433	0.0078	0.01620539
GO:1901068	guanosine-containing compound metabolic process	22	0.196214597	0.0059	0.014942532
GO:0031503	protein complex localization	35	0.196195277	0.0061	0.015143924
GO:0017015	regulation of transforming growth factor beta receptor signaling pathway	51	0.196070949	0.01	0.017574468
GO:0003279	cardiac septum development	46	0.196038642	0.0052	0.014616626
GO:0006406	mRNA export from nucleus	41	0.196025003	0.0063	0.015311728
GO:0071427	mRNA-containing ribonucleoprotein complex export from nucleus	41	0.196025003	0.0063	0.015311728
GO:0010165	response to X-ray	12	0.195990083	0.0115	0.018599441
GO:0010890	positive regulation of sequestering of triglyceride	5	0.195953002	0.0191	0.02440579
GO:0002295	T-helper cell lineage commitment	5	0.195943931	0.0274	0.03166636
GO:0002363	alpha-beta T cell lineage commitment	5	0.195943931	0.0274	0.03166636
GO:0043369	CD4-positive or CD8-positive, alpha-beta T cell lineage commitment	5	0.195943931	0.0274	0.03166636
GO:0043373	CD4-positive, alpha-beta T cell lineage commitment	5	0.195943931	0.0274	0.03166636
GO:0051290	protein heterotetramerization	15	0.195941599	0.0403	0.043327371
GO:0072332	intrinsic apoptotic signaling pathway by p53 class mediator	32	0.195940303	0.0262	0.030579007
GO:0032946	positive regulation of mononuclear cell proliferation	49	0.195926711	0.0252	0.029736
GO:0060669	embryonic placenta morphogenesis	11	0.195923576	0.0059	0.014942532
GO:0031018	endocrine pancreas development	21	0.195900874	0.001	0.010787313
GO:0007034	vacuolar transport	51	0.195849624	0.0012	0.010961137
GO:0031958	corticosteroid receptor signaling pathway	11	0.195811903	0.0092	0.017033109
GO:0042921	glucocorticoid receptor signaling pathway	11	0.195811903	0.0092	0.017033109

GOIDlabel	GOName	size	categoryScores	pValPerm	pValPermCorr
GO:1902884	positive regulation of response to oxidative stress	11	0.195799651	0.0275	0.031743861
GO:0048844	artery morphogenesis	25	0.195733133	0.0123	0.019143634
GO:0035264	multicellular organism growth	63	0.19569361	0.0045	0.014132412
GO:0010955	negative regulation of protein processing	8	0.195684871	0.0264	0.030750363
GO:1903318	negative regulation of protein maturation	8	0.195684871	0.0264	0.030750363
GO:0090322	regulation of superoxide metabolic process	17	0.195676268	0.0378	0.041067193
GO:0015701	bicarbonate transport	13	0.195609678	0.0449	0.047643935
GO:0071166	ribonucleoprotein complex localization	46	0.195579624	0.0083	0.016559903
GO:0006658	phosphatidylserine metabolic process	10	0.195565691	0.0125	0.019314538
GO:0003334	keratinocyte development	5	0.195555714	0.0018	0.01210186
GO:0021983	pituitary gland development	19	0.195551567	0.0178	0.023348367
GO:0031998	regulation of fatty acid beta-oxidation	10	0.195542886	0.014	0.020462083
GO:2001212	regulation of vasculogenesis	8	0.195541201	0.0008	0.010465158
GO:0001935	endothelial cell proliferation	51	0.195472956	0.0129	0.019597425
GO:0014896	muscle hypertrophy	37	0.195463909	0.0155	0.021605834
GO:0050671	positive regulation of lymphocyte proliferation	48	0.195454788	0.025	0.029554283
GO:0035904	aorta development	24	0.195422799	0.0113	0.018488002
GO:0036015	response to interleukin-3	6	0.195394345	0.0006	0.009940401
GO:0036016	cellular response to interleukin-3	6	0.195394345	0.0006	0.009940401
GO:0051797	regulation of hair follicle development	9	0.195368286	0.042	0.044946141
GO:0090307	mitotic spindle assembly	20	0.195235137	0.0092	0.017033109
GO:1902850	microtubule cytoskeleton organization involved in mitosis	20	0.195235137	0.0092	0.017033109
GO:0014020	primary neural tube formation	43	0.195213428	0.0081	0.016514175
GO:0051340	regulation of ligase activity	6	0.195205809	0.0228	0.027701114
GO:0071470	cellular response to osmotic stress	18	0.195191843	0.0037	0.013609033
GO:0032729	positive regulation of interferon-gamma production	20	0.195175466	0.0209	0.025971158
GO:0033046	negative regulation of sister chromatid segregation	17	0.195132571	0.0023	0.012498684
GO:0033048	negative regulation of mitotic sister chromatid segregation	17	0.195132571	0.0023	0.012498684
GO:0021536	diencephalon development	36	0.195110568	0.0149	0.021043429
GO:0010872	regulation of cholesterol esterification	6	0.194998699	0.0043	0.014102439
GO:0045058	T cell selection	16	0.194989925	0.0213	0.026276211
GO:0043588	skin development	109	0.194979757	0.006	0.015037711
GO:0071824	protein-DNA complex subunit organization	76	0.194967407	0.0149	0.021043429
GO:0072070	loop of Henle development	5	0.194945399	0.0252	0.029736
GO:0032637	interleukin-8 production	32	0.194940564	0.0243	0.028921902
GO:0003416	endochondral bone growth	10	0.194913911	0.0028	0.012972436
GO:0030856	regulation of epithelial cell differentiation	48	0.194858322	0.0036	0.01353394
GO:0006473	protein acetylation	73	0.194725477	0.0048	0.014269203
GO:0035270	endocrine system development	54	0.194722241	0.0073	0.015844069
GO:0045836	positive regulation of meiotic nuclear division	6	0.194630048	0.0046	0.014132412
GO:0050873	brown fat cell differentiation	14	0.194618602	0.0092	0.017033109
GO:0043244	regulation of protein complex disassembly	37	0.194564809	0.0168	0.022626974
GO:0097201	negative regulation of transcription from RNA polymerase II promoter in response to stress	6	0.194451229	0.0371	0.040443477
GO:0034115	negative regulation of heterotypic cell-cell adhesion	5	0.194438228	0.0261	0.030517735
GO:0070587	regulation of cell-cell adhesion involved in gastrulation	5	0.194438228	0.0261	0.030517735
GO:0021510	spinal cord development	42	0.194399552	0.0117	0.018739446
GO:0009060	aerobic respiration	31	0.19439326	0.0271	0.031420132
GO:0055026	negative regulation of cardiac muscle tissue development	15	0.194336034	0.0014	0.011465722
GO:1905245	regulation of aspartic-type peptidase activity	6	0.194246279	0.0002	0.006319126
GO:0043154	negative regulation of cysteine-type endopeptidase activity involved in apoptotic process	32	0.194071011	0.0149	0.021043429
GO:0046640	regulation of alpha-beta T cell proliferation	14	0.19402578	0.0254	0.029880529
GO:0006910	phagocytosis, recognition	6	0.193993385	0.0021	0.012314604
GO:0030225	macrophage differentiation	18	0.193977332	0.0259	0.0303391
GO:0001759	organ induction	6	0.193945873	0.0415	0.044476923
GO:0048821	erythrocyte development	12	0.193906999	0.0101	0.017653628
GO:0040034	regulation of development, heterochronic	5	0.19386886	0.0437	0.046498601
GO:0048505	regulation of timing of cell differentiation	5	0.19386886	0.0437	0.046498601
GO:0044270	cellular nitrogen compound catabolic process	186	0.193836706	0.0167	0.02255007
GO:0090153	regulation of sphingolipid biosynthetic process	7	0.193825396	0.0056	0.014771533
GO:1905038	regulation of membrane lipid metabolic process	7	0.193825396	0.0056	0.014771533
GO:2000303	regulation of ceramide biosynthetic process	7	0.193825396	0.0056	0.014771533
GO:0071426	ribonucleoprotein complex export from nucleus	45	0.193775333	0.0073	0.015844069
GO:0050885	neuromuscular process controlling balance	26	0.193749129	0.0053	0.014634479
GO:0098760	response to interleukin-7	10	0.19369963	0.0174	0.023064374
GO:0098761	cellular response to interleukin-7	10	0.19369963	0.0174	0.023064374
GO:0048596	embryonic camera-type eye morphogenesis	10	0.193679546	0.0079	0.01629604
GO:0006977	DNA damage response, signal transduction by p53 class mediator resulting in cell cycle arrest	17	0.193675041	0.0026	0.012805111
GO:0007368	determination of left/right symmetry	49	0.19359382	0.0092	0.017033109
GO:0045333	cellular respiration	94	0.193591967	0.0339	0.037513837
GO:0014002	astrocyte development	11	0.193582562	0.02	0.025188412
GO:0014015	positive regulation of gliogenesis	29	0.193577147	0.0436	0.046426372
GO:0042990	regulation of transcription factor import into nucleus	8	0.193564966	0.0094	0.017156187
GO:0042991	transcription factor import into nucleus	8	0.193564966	0.0094	0.017156187
GO:0042992	negative regulation of transcription factor import into nucleus	8	0.193564966	0.0094	0.017156187
GO:0042994	cytoplasmic sequestering of transcription factor	8	0.193564966	0.0094	0.017156187
GO:1902742	apoptotic process involved in development	11	0.193561947	0.0355	0.038985945
GO:0045839	negative regulation of mitotic nuclear division	21	0.193545691	0.0026	0.012805111
GO:0002328	pro-B cell differentiation	7	0.193496451	0.0314	0.035301342
GO:0046700	heterocycle catabolic process	184	0.193356049	0.0156	0.021692929
GO:0086010	membrane depolarization during action potential	10	0.193335644	0.0003	0.007956881
GO:0051985	negative regulation of chromosome segregation	18	0.193325767	0.0024	0.012603815
GO:0001936	regulation of endothelial cell proliferation	47	0.193234647	0.0126	0.019370699
GO:0051305	chromosome movement towards spindle pole	5	0.19315566	0.0155	0.021605834

GOIDlabel	GOName	size	categoryScores	pValPerm	pValPermCorr
GO:0072578	neurotransmitter-gated ion channel clustering	6	0.193140758	0.0462	0.048826247
GO:0030509	BMP signaling pathway	50	0.193018228	0.0058	0.014878261
GO:0036445	neuronal stem cell division	6	0.192998006	0.0446	0.047360367
GO:0055057	neuroblast division	6	0.192998006	0.0446	0.047360367
GO:0030520	intracellular estrogen receptor signaling pathway	18	0.192988719	0.0187	0.024129301
GO:0006405	RNA export from nucleus	45	0.192983666	0.0081	0.016514175
GO:1901099	negative regulation of signal transduction in absence of ligand	10	0.192853612	0.0147	0.02091937
GO:2001240	negative regulation of extrinsic apoptotic signaling pathway in absence of ligand	10	0.192853612	0.0147	0.02091937
GO:0080182	histone H3-K4 trimethylation	5	0.192819014	0.0082	0.016514246
GO:0042102	positive regulation of T cell proliferation	30	0.19272196	0.0313	0.035223161
GO:0046471	phosphatidylglycerol metabolic process	8	0.19269436	0.0089	0.016916437
GO:0007501	mesodermal cell fate specification	6	0.192650951	0.0422	0.045118417
GO:1902107	positive regulation of leukocyte differentiation	53	0.19260341	0.0217	0.026655917
GO:0003156	regulation of animal organ formation	10	0.192585514	0.0459	0.048535808
GO:0003309	type B pancreatic cell differentiation	15	0.192567027	0.0002	0.006319126
GO:0035883	enteroendocrine cell differentiation	16	0.192563323	0.0005	0.009604651
GO:1900120	regulation of receptor binding	12	0.192546208	0.0015	0.011704453
GO:0007184	SMAD protein import into nucleus	12	0.192537462	0.023	0.027838811
GO:0060390	regulation of SMAD protein import into nucleus	12	0.192537462	0.023	0.027838811
GO:0071827	plasma lipoprotein particle organization	12	0.192532495	0.0279	0.032115827
GO:0045841	negative regulation of mitotic metaphase/anaphase transition	15	0.192511395	0.0014	0.011465722
GO:2000816	negative regulation of mitotic sister chromatid separation	15	0.192511395	0.0014	0.011465722
GO:0032371	regulation of sterol transport	17	0.19247339	0.0254	0.029880529
GO:0032374	regulation of cholesterol transport	17	0.19247339	0.0254	0.029880529
GO:1902003	regulation of beta-amyloid formation	12	0.192403456	0.0008	0.010465158
GO:0034110	regulation of homotypic cell-cell adhesion	11	0.192392324	0.0303	0.034331687
GO:0007041	lysosomal transport	46	0.192322244	0.0012	0.010961137
GO:0046633	alpha-beta T cell proliferation	15	0.192320569	0.0311	0.035052671
GO:0060397	JAK-STAT cascade involved in growth hormone signaling pathway	7	0.192291185	< 0.001	< 0.001
GO:0031223	auditory behavior	6	0.192265887	0.0001	0.004220438
GO:0070391	response to lipoteichoic acid	6	0.192225933	0.0367	0.040037623
GO:0071223	cellular response to lipoteichoic acid	6	0.192225933	0.0367	0.040037623
GO:0007179	transforming growth factor beta receptor signaling pathway	79	0.192207062	0.0045	0.014132412
GO:0032488	Cdc42 protein signal transduction	5	0.192173713	0.0257	0.030153693
GO:0032489	regulation of Cdc42 protein signal transduction	5	0.192173713	0.0257	0.030153693
GO:0045687	positive regulation of glial cell differentiation	18	0.192120111	0.0372	0.040544844
GO:0060349	bone morphogenesis	40	0.191977628	0.005	0.014547706
GO:0010458	exit from mitosis	16	0.191977304	0.001	0.010787313
GO:1900424	regulation of defense response to bacterium	10	0.191932826	0.0369	0.040233038
GO:0032649	regulation of interferon-gamma production	28	0.191830024	0.0164	0.022306469
GO:0006309	apoptotic DNA fragmentation	9	0.191788318	0.0003	0.007956881
GO:0048512	circadian behavior	22	0.191770406	0.0004	0.008964341
GO:0099563	modification of synaptic structure	9	0.191765281	0.0014	0.011465722
GO:0036158	outer dynein arm assembly	8	0.191718957	0.0045	0.014132412
GO:1905314	semi-lunar valve development	19	0.191714609	0.0417	0.044666432
GO:0051028	mRNA transport	52	0.191694586	0.0065	0.015491756
GO:0009310	amine catabolic process	12	0.191660431	0.0427	0.045577146
GO:0006970	response to osmotic stress	34	0.191540539	0.0013	0.011218806
GO:0000724	double-strand break repair via homologous recombination	34	0.191511345	0.0138	0.020339434
GO:0000725	recombinational repair	34	0.191511345	0.0138	0.020339434
GO:0016925	protein sumoylation	28	0.191431107	0.0013	0.011218806
GO:0050869	negative regulation of B cell activation	11	0.191428259	0.004	0.013824268
GO:0000381	regulation of alternative mRNA splicing, via spliceosome	14	0.191370529	0.0176	0.023223003
GO:0009162	deoxyribonucleoside monophosphate metabolic process	6	0.191313916	0.038	0.041253474
GO:0034655	nucleobase-containing compound catabolic process	164	0.191200256	0.0171	0.022860624
GO:0030517	negative regulation of axon extension	13	0.191145198	0.0136	0.020157703
GO:0001938	positive regulation of endothelial cell proliferation	37	0.190932476	0.0089	0.016916437
GO:2000171	negative regulation of dendrite development	14	0.19083972	< 0.001	< 0.001
GO:0042748	circadian sleep/wake cycle, non-REM sleep	6	0.190817846	0.0018	0.01210186
GO:1900117	regulation of execution phase of apoptosis	12	0.190754568	0.0051	0.014547706
GO:0045582	positive regulation of T cell differentiation	28	0.190692741	0.0314	0.035301342
GO:1902100	negative regulation of metaphase/anaphase transition of cell cycle	16	0.190642564	0.0019	0.012288367
GO:0051168	nuclear export	71	0.190619515	0.0051	0.014547706
GO:0048260	positive regulation of receptor-mediated endocytosis	22	0.190614682	0.0151	0.02121706
GO:1902117	positive regulation of organelle assembly	24	0.190549834	0.0107	0.018084595
GO:0010831	positive regulation of myotube differentiation	12	0.19052759	0.0037	0.013609033
GO:0051654	establishment of mitochondrion localization	10	0.190460036	0.0005	0.009604651
GO:0060393	regulation of pathway-restricted SMAD protein phosphorylation	18	0.190435229	0.0058	0.014878261
GO:0048736	appendage development	61	0.190428158	0.0103	0.017772187
GO:0060173	limb development	61	0.190428158	0.0103	0.017772187
GO:0060317	cardiac epithelial to mesenchymal transition	14	0.190411185	0.0078	0.01620539
GO:0006308	DNA catabolic process	13	0.190405603	0.0007	0.010220707
GO:0040018	positive regulation of multicellular organism growth	14	0.190368054	0.0178	0.023348367
GO:0098739	import across plasma membrane	24	0.190326419	0.004	0.013824268
GO:0072538	T-helper 17 type immune response	6	0.190318968	0.0122	0.019049527
GO:0032274	gonadotropin secretion	9	0.190294133	0.0013	0.011218806
GO:0045580	regulation of T cell differentiation	48	0.190233555	0.017	0.022784886
GO:0031268	pseudopodium organization	5	0.190194012	0.02	0.025188412
GO:0031269	pseudopodium assembly	5	0.190194012	0.02	0.025188412
GO:0031272	regulation of pseudopodium assembly	5	0.190194012	0.02	0.025188412
GO:0031274	positive regulation of pseudopodium assembly	5	0.190194012	0.02	0.025188412
GO:0007040	lysosome organization	33	0.190155366	0.0117	0.018739446
GO:0080171	lytic vacuole organization	33	0.190155366	0.0117	0.018739446

GOIDlabel	GOName	size	categoryScores	pValPerm	pValPermCorr
GO:0046135	pyrimidine nucleoside catabolic process	7	0.190150732	0.0425	0.045422366
GO:0051084	'de novo' posttranslational protein folding	11	0.190123992	0.0082	0.016514246
GO:0046415	urate metabolic process	7	0.19007243	0.0407	0.043676206
GO:1902188	positive regulation of viral release from host cell	7	0.190052808	0.0452	0.047918298
GO:0042787	protein ubiquitination involved in ubiquitin-dependent protein catabolic process	17	0.189908268	0.0028	0.012972436
GO:2000058	regulation of protein ubiquitination involved in ubiquitin-dependent protein catabolic process	17	0.189908268	0.0028	0.012972436
GO:2000351	regulation of endothelial cell apoptotic process	17	0.189853464	0.0177	0.02329117
GO:0045494	photoreceptor cell maintenance	11	0.189852584	0.0053	0.014634479
GO:0022602	ovulation cycle process	37	0.189805073	0.003	0.013210967
GO:0007096	regulation of exit from mitosis	10	0.189753704	0.0019	0.012288367
GO:0003417	growth plate cartilage development	7	0.189498952	0.0012	0.010961137
GO:0034205	beta-amyloid formation	15	0.189496792	0.0013	0.011218806
GO:0006370	7-methylguanosine mRNA capping	22	0.18949424	0.0182	0.023684988
GO:0034433	steroid esterification	7	0.189481551	0.0078	0.01620539
GO:0034434	sterol esterification	7	0.189481551	0.0078	0.01620539
GO:0034435	cholesterol esterification	7	0.189481551	0.0078	0.01620539
GO:0009651	response to salt stress	9	0.189457919	0.0173	0.023005658
GO:0035333	Notch receptor processing, ligand-dependent	5	0.189448676	0.0462	0.048826247
GO:0051457	maintenance of protein location in nucleus	5	0.18942872	0.0069	0.015750415
GO:0002313	mature B cell differentiation involved in immune response	9	0.189398601	0.0138	0.020339434
GO:0002021	response to dietary excess	8	0.189386381	0.0009	0.010729485
GO:0006611	protein export from nucleus	25	0.189385182	0.0051	0.014547706
GO:0042771	intrinsic apoptotic signaling pathway in response to DNA damage by p53 class mediator	20	0.189369438	0.04	0.043076923
GO:0000737	DNA catabolic process, endonucleolytic	10	0.189249254	0.0007	0.010220707
GO:0046598	positive regulation of viral entry into host cell	6	0.189216903	0.0075	0.015990044
GO:1901798	positive regulation of signal transduction by p53 class mediator	5	0.189151941	0.0221	0.027038129
GO:0044236	multicellular organism metabolic process	36	0.189133252	0.0057	0.01480566
GO:0070059	intrinsic apoptotic signaling pathway in response to endoplasmic reticulum stress	24	0.189125712	0.0074	0.015911789
GO:0009452	7-methylguanosine RNA capping	24	0.189110648	0.0187	0.024129301
GO:0036260	RNA capping	24	0.189110648	0.0187	0.024129301
GO:0010667	negative regulation of cardiac muscle cell apoptotic process	8	0.189103899	0.008	0.016379603
GO:1903541	regulation of exosomal secretion	11	0.189075819	0.0073	0.015844069
GO:0006623	protein targeting to vacuole	11	0.189064157	0.0213	0.026276211
GO:0072666	establishment of protein localization to vacuole	11	0.189064157	0.0213	0.026276211
GO:0032272	negative regulation of protein polymerization	30	0.188968069	0.0187	0.024129301
GO:0034968	histone lysine methylation	42	0.188967186	0.0016	0.011845327
GO:0006541	glutamine metabolic process	8	0.188938039	0.0126	0.019370699
GO:0042089	cytokine biosynthetic process	43	0.188908134	0.0238	0.028526451
GO:0042107	cytokine metabolic process	43	0.188908134	0.0238	0.028526451
GO:0001709	cell fate determination	15	0.18888043	0.0187	0.024129301
GO:2000378	negative regulation of reactive oxygen species metabolic process	24	0.188861425	0.0177	0.02329117
GO:0071786	endoplasmic reticulum tubular network organization	6	0.188842852	0.0367	0.040037623
GO:0051646	mitochondrion localization	17	0.188820875	0.0014	0.011465722
GO:0090154	positive regulation of sphingolipid biosynthetic process	6	0.188785406	0.002	0.012314604
GO:2000304	positive regulation of ceramide biosynthetic process	6	0.188785406	0.002	0.012314604
GO:0015937	coenzyme A biosynthetic process	6	0.188698372	0.0066	0.015525305
GO:0032963	collagen metabolic process	34	0.188611834	0.0051	0.014547706
GO:0044259	multicellular organismal macromolecule metabolic process	34	0.188611834	0.0051	0.014547706
GO:0060333	interferon-gamma-mediated signaling pathway	35	0.188540478	0.0381	0.041354271
GO:0032781	positive regulation of ATPase activity	25	0.188478236	0.0142	0.020567234
GO:0016573	histone acetylation	54	0.18844447	0.0033	0.013333753
GO:0043901	negative regulation of multi-organism process	45	0.188442311	0.014	0.020462083
GO:0043489	RNA stabilization	19	0.188420926	0.0028	0.012972436
GO:1903214	regulation of protein targeting to mitochondrion	28	0.18839522	0.0134	0.01999969
GO:0002826	negative regulation of T-helper 1 type immune response	5	0.188384835	0.0134	0.01999969
GO:0051569	regulation of histone H3-K4 methylation	12	0.188249167	0.015	0.021138192
GO:0042340	keratan sulfate catabolic process	6	0.188240096	0.0182	0.023684988
GO:0070102	interleukin-6-mediated signaling pathway	5	0.188194355	0.0112	0.018392048
GO:0045668	negative regulation of osteoblast differentiation	21	0.18811388	0.0002	0.006319126
GO:0045943	positive regulation of transcription from RNA polymerase I promoter	8	0.188092215	0.0391	0.042217778
GO:0014067	negative regulation of phosphatidylinositol 3-kinase signaling	6	0.188003183	0.0191	0.02440579
GO:0097202	activation of cysteine-type endopeptidase activity	9	0.187989362	0.0266	0.030933467
GO:0043171	peptide catabolic process	5	0.187976922	0.0227	0.027637692
GO:0099010	modification of postsynaptic structure	6	0.187943577	0.003	0.013210967
GO:0009799	specification of symmetry	52	0.187822729	0.008	0.016379603
GO:0009855	determination of bilateral symmetry	52	0.187822729	0.008	0.016379603
GO:0042770	signal transduction in response to DNA damage	45	0.187818059	0.0098	0.017418875
GO:0001955	blood vessel maturation	5	0.187772447	0.0217	0.026655917
GO:2000117	negative regulation of cysteine-type endopeptidase activity	34	0.187754253	0.0157	0.021779607
GO:2001252	positive regulation of chromosome organization	33	0.187705781	0.0089	0.016916437
GO:2000778	positive regulation of interleukin-6 secretion	7	0.187603773	0.0329	0.036702257
GO:0006338	chromatin remodeling	52	0.187587568	0.0022	0.012422266
GO:1904177	regulation of adipose tissue development	5	0.187583781	0.0168	0.022626974
GO:0043383	negative T cell selection	6	0.187566504	0.0304	0.034411276
GO:0045060	negative thymic T cell selection	6	0.187566504	0.0304	0.034411276
GO:0018196	peptidyl-asparagine modification	24	0.18756456	0.0133	0.019917275
GO:0006402	mRNA catabolic process	98	0.187550447	0.0234	0.02817551
GO:0055070	copper ion homeostasis	11	0.187508154	0.0137	0.020264364
GO:0051653	spindle localization	17	0.187495134	0.0045	0.014132412
GO:0098810	neurotransmitter reuptake	8	0.187446913	0.015	0.021138192
GO:0046634	regulation of alpha-beta T cell activation	31	0.187333652	0.0213	0.026276211
GO:0046631	alpha-beta T cell activation	47	0.18733204	0.0187	0.024129301
GO:0006334	nucleosome assembly	30	0.187318533	0.0395	0.042617839

GOIDlabel	GOName	size	categoryScores	pValPerm	pValPermCorr
GO:0006515	misfolded or incompletely synthesized protein catabolic process	12	0.187191374	0.0176	0.023223003
GO:1901222	regulation of NIK/NF-kappaB signaling	38	0.187140315	0.0158	0.021871104
GO:0032365	intracellular lipid transport	19	0.187118457	0.0097	0.017363901
GO:0001916	positive regulation of T cell mediated cytotoxicity	5	0.187075055	0.0335	0.037227945
GO:1903708	positive regulation of hemopoiesis	67	0.187037752	0.0181	0.023607986
GO:0030947	regulation of vascular endothelial growth factor receptor signaling pathway	14	0.187026685	0.0176	0.023223003
GO:0018206	peptidyl-methionine modification	5	0.187001195	0.003	0.013210967
GO:1903818	positive regulation of voltage-gated potassium channel activity	8	0.186992578	0.0041	0.013887639
GO:1903573	negative regulation of response to endoplasmic reticulum stress	20	0.186984415	0.0154	0.021487162
GO:0010470	regulation of gastrulation	15	0.186927681	0.0136	0.020157703
GO:0003177	pulmonary valve development	13	0.186927515	0.0309	0.034874839
GO:0000460	maturation of 5.8S rRNA	6	0.186900254	0.0055	0.01475
GO:0007029	endoplasmic reticulum organization	22	0.186854666	0.0036	0.01353394
GO:1903671	negative regulation of sprouting angiogenesis	14	0.186831207	0.0148	0.020979063
GO:0000956	nuclear-transcribed mRNA catabolic process	91	0.186750804	0.0246	0.029170878
GO:0045992	negative regulation of embryonic development	12	0.186708585	0.0108	0.018152791
GO:0010559	regulation of glycoprotein biosynthetic process	18	0.18669408	0.0069	0.015750415
GO:0098877	neurotransmitter receptor transport to plasma membrane	6	0.18668105	0.0043	0.014102439
GO:0098969	neurotransmitter receptor transport to postsynaptic membrane	6	0.18668105	0.0043	0.014102439
GO:0099637	neurotransmitter receptor transport	6	0.18668105	0.0043	0.014102439
GO:0048026	positive regulation of mRNA splicing, via spliceosome	6	0.186659669	0.0041	0.013887639
GO:0042044	fluid transport	13	0.186629086	0.0185	0.023962142
GO:0060795	cell fate commitment involved in formation of primary germ layer	13	0.186585029	0.0244	0.029011063
GO:0010862	positive regulation of pathway-restricted SMAD protein phosphorylation	12	0.186567777	0.011	0.018323826
GO:0061577	calcium ion transmembrane transport via high voltage-gated calcium channel	9	0.186541105	0.0194	0.024690909
GO:0044241	lipid digestion	8	0.186533708	0.0281	0.032268957
GO:0043484	regulation of RNA splicing	36	0.186500684	0.0097	0.017363901
GO:0090148	membrane fission	6	0.186405911	0.0239	0.028592965
GO:0006884	cell volume homeostasis	8	0.186402348	0.0194	0.024690909
GO:0046856	phosphatidylinositol dephosphorylation	11	0.186364328	0.0003	0.007956881
GO:0051546	keratinocyte migration	7	0.18634575	0.0032	0.013333753
GO:0007062	sister chromatid cohesion	16	0.186307027	0.0134	0.01999969
GO:0006376	mRNA splice site selection	8	0.18626953	0.0277	0.03194284
GO:0010738	regulation of protein kinase A signaling	8	0.186145458	0.0027	0.012827773
GO:0050906	detection of stimulus involved in sensory perception	49	0.186082414	0.0153	0.021378589
GO:0045619	regulation of lymphocyte differentiation	63	0.186029002	0.0145	0.020788247
GO:0035886	vascular smooth muscle cell differentiation	12	0.185996227	0.0246	0.029170878
GO:0007622	rhythmic behavior	23	0.185969457	0.0004	0.008964341
GO:1904714	regulation of chaperone-mediated autophagy	5	0.18595446	0.0396	0.042717761
GO:0031497	chromatin assembly	32	0.185941737	0.0395	0.042617839
GO:0010766	negative regulation of sodium ion transport	10	0.18592984	0.018	0.023514686
GO:0060632	regulation of microtubule-based movement	8	0.185919668	0.031	0.034967226
GO:0030326	embryonic limb morphogenesis	37	0.185741523	0.0148	0.020979063
GO:0035113	embryonic appendage morphogenesis	37	0.185741523	0.0148	0.020979063
GO:0072577	endothelial cell apoptotic process	18	0.185698556	0.0153	0.021378589
GO:0046637	regulation of alpha-beta T cell differentiation	21	0.185689033	0.0177	0.02329117
GO:0002286	T cell activation involved in immune response	29	0.18563617	0.0223	0.027242468
GO:0060307	regulation of ventricular cardiac muscle cell membrane repolarization	9	0.185611595	0.0298	0.033898013
GO:0051642	centrosome localization	7	0.185605856	0.0115	0.018599441
GO:0018149	peptide cross-linking	10	0.18558244	0.0229	0.027770092
GO:0032743	positive regulation of interleukin-2 production	10	0.18556702	0.0117	0.018739446
GO:0045719	negative regulation of glycogen biosynthetic process	5	0.185484349	0.0018	0.01210186
GO:0070874	negative regulation of glycogen metabolic process	5	0.185484349	0.0018	0.01210186
GO:0045639	positive regulation of myeloid cell differentiation	33	0.185437213	0.0185	0.023962142
GO:0008045	motor neuron axon guidance	11	0.18542724	0.0031	0.013277185
GO:0010804	negative regulation of tumor necrosis factor-mediated signaling pathway	8	0.185416741	0.0331	0.03684717
GO:0051647	nucleus localization	10	0.185399863	0.025	0.029554283
GO:0043174	nucleoside salvage	10	0.18534259	0.0178	0.023348367
GO:0043297	apical junction assembly	17	0.18529992	0.0115	0.018599441
GO:0031297	replication fork processing	10	0.185258234	0.021	0.026005997
GO:0045005	DNA-dependent DNA replication maintenance of fidelity	10	0.185258234	0.021	0.026005997
GO:0006379	mRNA cleavage	8	0.185257359	0.0294	0.033482529
GO:0034728	nucleosome organization	41	0.185245899	0.0262	0.030579007
GO:1901658	glycosyl compound catabolic process	20	0.185157292	0.0406	0.043585072
GO:1900027	regulation of ruffle assembly	11	0.185111068	0.0018	0.01210186
GO:0042035	regulation of cytokine biosynthetic process	42	0.1850433	0.0242	0.028862294
GO:0045815	positive regulation of gene expression, epigenetic	14	0.185037469	0.0039	0.013791927
GO:0032506	cytokinetic process	9	0.184977667	0.0082	0.016514246
GO:0007250	activation of NF-kappaB-inducing kinase activity	10	0.18492729	0.0182	0.023684988
GO:0071158	positive regulation of cell cycle arrest	27	0.18483182	0.0004	0.008964341
GO:1903363	negative regulation of cellular protein catabolic process	32	0.18482238	0.0045	0.014132412
GO:0008333	endosome to lysosome transport	24	0.184789689	0.0001	0.004220438
GO:0060389	pathway-restricted SMAD protein phosphorylation	20	0.184755663	0.0098	0.017418875
GO:0018022	peptidyl-lysine methylation	48	0.184750734	0.0016	0.011845327
GO:0070292	N-acylphosphatidylethanolamine metabolic process	5	0.184743159	0.016	0.022026667
GO:0030889	negative regulation of B cell proliferation	7	0.184720094	0.0124	0.019242297
GO:0001541	ovarian follicle development	27	0.184691827	0.0039	0.013791927
GO:0042094	interleukin-2 biosynthetic process	7	0.184618467	0.0234	0.02817551
GO:0045076	regulation of interleukin-2 biosynthetic process	7	0.184618467	0.0234	0.02817551
GO:0031640	killing of cells of other organism	12	0.184365049	0.0092	0.017033109
GO:0044364	disruption of cells of other organism	12	0.184365049	0.0092	0.017033109
GO:0045577	regulation of B cell differentiation	12	0.184302635	0.0201	0.025275816
GO:0010226	response to lithium ion	11	0.184175628	< 0.001	< 0.001

GOIDlabel	GOName	size	categoryScores	pValPerm	pValPermCorr
GO:2000786	positive regulation of autophagosome assembly	6	0.184100277	0.0128	0.019522448
GO:1901983	regulation of protein acetylation	30	0.184089133	0.0077	0.016177834
GO:0061635	regulation of protein complex stability	6	0.18401713	0.0145	0.020788247
GO:0001937	negative regulation of endothelial cell proliferation	13	0.183985683	0.0279	0.032115827
GO:1903205	regulation of hydrogen peroxide-induced cell death	11	0.183898801	0.0138	0.020339434
GO:0072527	pyrimidine-containing compound metabolic process	42	0.183898597	0.0133	0.019917275
GO:0021602	cranial nerve morphogenesis	9	0.183878121	0.0002	0.006319126
GO:0060055	angiogenesis involved in wound healing	7	0.183867097	0.0033	0.013333753
GO:0009948	anterior/posterior axis specification	13	0.183725828	0.0173	0.023005658
GO:0035020	regulation of Rac protein signal transduction	5	0.183612197	0.0337	0.03733539
GO:0045910	negative regulation of DNA recombination	10	0.183534839	0.0368	0.040139144
GO:1902895	positive regulation of pri-miRNA transcription from RNA polymerase II promoter	16	0.183431921	0.0271	0.031420132
GO:0007350	blastoderm segmentation	6	0.183384355	0.0421	0.045028154
GO:0003323	type B pancreatic cell development	13	0.183378756	0.0001	0.004220438
GO:0021952	central nervous system projection neuron axonogenesis	14	0.183289084	0.0018	0.01210186
GO:0002066	columnar/cuboidal epithelial cell development	33	0.18322142	0.0004	0.008964341
GO:0043567	regulation of insulin-like growth factor receptor signaling pathway	7	0.183189586	0.0048	0.014269203
GO:0046606	negative regulation of centrosome cycle	6	0.183100794	0.0134	0.01999969
GO:2001214	positive regulation of vasculogenesis	5	0.1830703	0.0039	0.013791927
GO:0003283	atrial septum development	8	0.183021481	0.02	0.025188412
GO:0060413	atrial septum morphogenesis	8	0.183021481	0.02	0.025188412
GO:0046597	negative regulation of viral entry into host cell	9	0.18297755	0.0032	0.013333753
GO:0033189	response to vitamin A	8	0.182915712	0.002	0.012314604
GO:0007606	sensory perception of chemical stimulus	48	0.182909287	0.0146	0.020879842
GO:0046653	tetrahydrofolate metabolic process	10	0.18290405	0.0058	0.014878261
GO:0015949	nucleobase-containing small molecule interconversion	14	0.182837595	0.0436	0.046426372
GO:0010224	response to UV-B	8	0.182689922	0.0066	0.015525305
GO:0048255	mRNA stabilization	17	0.182666731	0.0028	0.012972436
GO:0048048	embryonic eye morphogenesis	13	0.182643248	0.0017	0.012001709
GO:0046039	GTP metabolic process	14	0.182504115	0.0021	0.012314604
GO:0045292	mRNA cis splicing, via spliceosome	5	0.182449618	0.0346	0.038164288
GO:0072553	terminal button organization	5	0.182408931	0.0207	0.025805821
GO:0008156	negative regulation of DNA replication	17	0.182252842	0.0041	0.013887639
GO:0018198	peptidyl-cysteine modification	15	0.182140271	0.0004	0.008964341
GO:0021515	cell differentiation in spinal cord	18	0.182130826	0.0112	0.018392048
GO:0006220	pyrimidine nucleotide metabolic process	26	0.18200632	0.0147	0.02091937
GO:1902893	regulation of pri-miRNA transcription from RNA polymerase II promoter	21	0.181952357	0.0398	0.042885501
GO:0061614	pri-miRNA transcription from RNA polymerase II promoter	24	0.18193923	0.0239	0.028592965
GO:0046632	alpha-beta T cell differentiation	34	0.181928057	0.0139	0.020388077
GO:0035909	aorta morphogenesis	13	0.18184654	0.009	0.016956012
GO:0006538	glutamate catabolic process	7	0.181829381	0.0241	0.028760826
GO:0031060	regulation of histone methylation	24	0.181792968	0.0108	0.018152791
GO:0046825	regulation of protein export from nucleus	11	0.18177277	0.0091	0.016994897
GO:0051446	positive regulation of meiotic cell cycle	7	0.181736085	0.0132	0.019860109
GO:0006333	chromatin assembly or disassembly	42	0.181695576	0.032	0.035892144
GO:0006730	one-carbon metabolic process	12	0.181677697	0.0034	0.01347416
GO:0032467	positive regulation of cytokinesis	10	0.181553842	0.0022	0.012422266
GO:0002701	negative regulation of production of molecular mediator of immune response	12	0.181544123	0.024	0.028671074
GO:0086012	membrane depolarization during cardiac muscle cell action potential	7	0.181519318	0.0002	0.006319126
GO:0060411	cardiac septum morphogenesis	35	0.181457046	0.0104	0.017832977
GO:0071825	protein-lipid complex subunit organization	14	0.181448698	0.0344	0.037979912
GO:0001704	formation of primary germ layer	47	0.181447242	0.0102	0.0177
GO:0035067	negative regulation of histone acetylation	8	0.18138134	0.0154	0.021487162
GO:0045682	regulation of epidermis development	28	0.181363592	0.0236	0.028327839
GO:0085029	extracellular matrix assembly	19	0.181320634	0.0307	0.034703304
GO:0001783	B cell apoptotic process	9	0.181190374	0.0193	0.024606968
GO:0009913	epidermal cell differentiation	86	0.181183156	0.0123	0.019143634
GO:0045843	negative regulation of striated muscle tissue development	24	0.181048053	0.0042	0.013948535
GO:0030836	positive regulation of actin filament depolymerization	8	0.181015119	0.034	0.037588528
GO:0043457	regulation of cellular respiration	16	0.180990742	0.0072	0.015844069
GO:0090324	negative regulation of oxidative phosphorylation	5	0.180832996	0.021	0.026005997
GO:0090051	negative regulation of cell migration involved in sprouting angiogenesis	10	0.180791272	0.0283	0.032434212
GO:0035973	aggrephagy	5	0.180693569	0.0115	0.018599441
GO:2001222	regulation of neuron migration	19	0.180686722	0.0018	0.01210186
GO:0009264	deoxyribonucleotide catabolic process	11	0.180678538	0.021	0.026005997
GO:0033599	regulation of mammary gland epithelial cell proliferation	8	0.18063189	0.0119	0.018881943
GO:0010759	positive regulation of macrophage chemotaxis	6	0.180575955	0.0452	0.047918298
GO:0046639	negative regulation of alpha-beta T cell differentiation	7	0.180470842	0.0065	0.015491756
GO:0071157	negative regulation of cell cycle arrest	12	0.180434175	0.021	0.026005997
GO:0017014	protein nitrosylation	7	0.18041716	0.0025	0.012769435
GO:0018119	peptidyl-cysteine S-nitrosylation	7	0.18041716	0.0025	0.012769435
GO:0061756	leukocyte adhesion to vascular endothelial cell	13	0.180254318	0.0387	0.041895413
GO:0048261	negative regulation of receptor-mediated endocytosis	14	0.180246023	0.0026	0.012805111
GO:0046596	regulation of viral entry into host cell	16	0.180181199	0.0064	0.015399417
GO:0045589	regulation of regulatory T cell differentiation	10	0.180111891	0.044	0.046783376
GO:0051568	histone H3-K4 methylation	18	0.180095363	0.0122	0.019049527
GO:0033127	regulation of histone phosphorylation	8	0.180082315	0.0139	0.020388077
GO:0018279	protein N-linked glycosylation via asparagine	23	0.180074533	0.0184	0.023875404
GO:0044773	mitotic DNA damage checkpoint	29	0.180034577	0.0077	0.016177834
GO:0044774	mitotic DNA integrity checkpoint	29	0.180034577	0.0077	0.016177834
GO:0044033	multi-organism metabolic process	92	0.179953025	0.0338	0.0374175
GO:0003198	epithelial to mesenchymal transition involved in endocardial cushion formation	9	0.179801003	0.0383	0.041555752
GO:0032816	positive regulation of natural killer cell activation	9	0.179600013	0.0151	0.02121706

GOIDlabel	GOName	size	categoryScores	pValPerm	pValPermCorr
GO:0070932	histone H3 deacetylation	7	0.179588782	0.0146	0.020879842
GO:2000826	regulation of heart morphogenesis	14	0.179569068	0.0268	0.031128485
GO:1901862	negative regulation of muscle tissue development	25	0.179537677	0.0057	0.01480566
GO:1903599	positive regulation of mitophagy	11	0.179472279	0.0033	0.013333753
GO:0003170	heart valve development	28	0.179274323	0.027	0.031323034
GO:0071634	regulation of transforming growth factor beta production	14	0.179252711	0.0234	0.02817551
GO:0035710	CD4-positive, alpha-beta T cell activation	31	0.179251502	0.0108	0.018152791
GO:0035722	interleukin-12-mediated signaling pathway	25	0.17924711	0.0361	0.039494834
GO:0010939	regulation of necrotic cell death	9	0.179156566	0.0325	0.036312077
GO:0008535	respiratory chain complex IV assembly	13	0.179135855	0.0256	0.03006687
GO:1903018	regulation of glycoprotein metabolic process	21	0.179091509	0.0107	0.018084595
GO:0033561	regulation of water loss via skin	6	0.179067445	0.0023	0.012498684
GO:0043576	regulation of respiratory gaseous exchange	9	0.179047432	0.0051	0.014547706
GO:0009166	nucleotide catabolic process	27	0.17892678	0.0189	0.024289798
GO:0003151	outflow tract morphogenesis	36	0.178918069	0.0168	0.022626974
GO:0002068	glandular epithelial cell development	17	0.178836007	< 0.001	< 0.001
GO:1901224	positive regulation of NIK/NF-kappaB signaling	28	0.178817235	0.0129	0.019597425
GO:0009154	purine ribonucleotide catabolic process	13	0.178792971	0.0121	0.018970228
GO:0009261	ribonucleotide catabolic process	13	0.178792971	0.0121	0.018970228
GO:0072606	interleukin-8 secretion	16	0.178631116	0.0282	0.032370935
GO:0000079	regulation of cyclin-dependent protein serine/threonine kinase activity	36	0.178625258	0.0058	0.014878261
GO:0061037	negative regulation of cartilage development	8	0.178559766	0.0009	0.010729485
GO:0007638	mechanosensory behavior	7	0.178554745	< 0.001	< 0.001
GO:0048635	negative regulation of muscle organ development	25	0.178510429	0.0029	0.013089617
GO:2000370	positive regulation of clathrin-dependent endocytosis	6	0.17850421	0.0012	0.010961137
GO:0043242	negative regulation of protein complex disassembly	24	0.178199214	0.0197	0.024979254
GO:0045621	positive regulation of lymphocyte differentiation	34	0.178097714	0.0256	0.03006687
GO:1901292	nucleoside phosphate catabolic process	31	0.178066401	0.0236	0.028327839
GO:0048286	lung alveolus development	18	0.17801865	< 0.001	< 0.001
GO:0001702	gastrulation with mouth forming second	9	0.178004036	0.0147	0.02091937
GO:1904029	regulation of cyclin-dependent protein kinase activity	39	0.177897948	0.007	0.015816335
GO:0048525	negative regulation of viral process	32	0.177884014	0.0113	0.018488002
GO:1903901	negative regulation of viral life cycle	32	0.177884014	0.0113	0.018488002
GO:0060004	reflex	6	0.177876833	0.0267	0.031037274
GO:0090084	negative regulation of inclusion body assembly	6	0.177875754	0.0184	0.023875404
GO:1900273	positive regulation of long-term synaptic potentiation	12	0.17787435	0.0068	0.015633241
GO:0002709	regulation of T cell mediated immunity	12	0.177802542	0.0053	0.014634479
GO:0070671	response to interleukin-12	27	0.177797718	0.0365	0.039857035
GO:0043248	proteasome assembly	7	0.177720074	0.0205	0.025639412
GO:1904380	endoplasmic reticulum mannose trimming	8	0.177606392	0.0031	0.013277185
GO:0045604	regulation of epidermal cell differentiation	18	0.177544032	0.0336	0.037253154
GO:0030299	intestinal cholesterol absorption	6	0.177524596	0.0322	0.036060507
GO:0098856	intestinal lipid absorption	6	0.177524596	0.0322	0.036060507
GO:1903051	negative regulation of proteolysis involved in cellular protein catabolic process	27	0.177440801	0.0072	0.015844069
GO:0006482	protein demethylation	8	0.177363432	0.0038	0.01366393
GO:0008214	protein dealkylation	8	0.177363432	0.0038	0.01366393
GO:0009200	deoxyribonucleoside triphosphate metabolic process	13	0.177280689	0.0321	0.035976391
GO:0034394	protein localization to cell surface	28	0.177150391	0.0137	0.020264364
GO:0021854	hypothalamus development	11	0.177055992	0.003	0.013210967
GO:0032897	negative regulation of viral transcription	10	0.177054485	0.0301	0.034151923
GO:0000289	nuclear-transcribed mRNA poly(A) tail shortening	10	0.176983216	0.0007	0.010220707
GO:0070317	negative regulation of G0 to G1 transition	13	0.17678221	0.004	0.013824268
GO:0033120	positive regulation of RNA splicing	10	0.176631799	0.0106	0.018010344
GO:0090237	regulation of arachidonic acid secretion	6	0.176526409	0.0331	0.03684717
GO:0031293	membrane protein intracellular domain proteolysis	10	0.176517252	0.0472	0.049710455
GO:0030218	erythrocyte differentiation	37	0.176466149	0.0113	0.018488002
GO:0060749	mammary gland alveolus development	5	0.176391301	0.0044	0.014132412
GO:0061377	mammary gland lobule development	5	0.176391301	0.0044	0.014132412
GO:0002287	alpha-beta T cell activation involved in immune response	19	0.176374415	0.0089	0.016916437
GO:0002292	T cell differentiation involved in immune response	19	0.176374415	0.0089	0.016916437
GO:0002293	alpha-beta T cell differentiation involved in immune response	19	0.176374415	0.0089	0.016916437
GO:0002294	CD4-positive, alpha-beta T cell differentiation involved in immune response	19	0.176374415	0.0089	0.016916437
GO:0042093	T-helper cell differentiation	19	0.176374415	0.0089	0.016916437
GO:0045879	negative regulation of smoothened signaling pathway	14	0.176314239	0.0176	0.023223003
GO:0006612	protein targeting to membrane	79	0.176291483	0.0331	0.03684717
GO:0034101	erythrocyte homeostasis	40	0.176286958	0.0162	0.022196303
GO:0071450	cellular response to oxygen radical	9	0.176273011	0.0458	0.048447786
GO:0071451	cellular response to superoxide	9	0.176273011	0.0458	0.048447786
GO:0010713	negative regulation of collagen metabolic process	6	0.176259693	0.0109	0.018225506
GO:0044252	negative regulation of multicellular organismal metabolic process	6	0.176259693	0.0109	0.018225506
GO:0031057	negative regulation of histone modification	18	0.176144872	0.0045	0.014132412
GO:1905268	negative regulation of chromatin organization	18	0.176144872	0.0045	0.014132412
GO:0042417	dopamine metabolic process	17	0.176061653	0.0203	0.025471918
GO:0071349	cellular response to interleukin-12	26	0.176038678	0.0349	0.038392656
GO:0018243	protein O-linked glycosylation via threonine	5	0.175741335	0.0341	0.037691875
GO:1902042	negative regulation of extrinsic apoptotic signaling pathway via death domain receptors	14	0.175667827	0.0052	0.014616626
GO:0098868	bone growth	12	0.175662767	0.0002	0.006319126
GO:0045662	negative regulation of myoblast differentiation	11	0.175607539	0.0308	0.034775552
GO:0007498	mesoderm development	45	0.175573113	0.0294	0.033482529
GO:0019835	cytolysis	10	0.175463648	0.0429	0.045748395
GO:0021548	pons development	5	0.175382403	0.0163	0.022248961
GO:0048668	collateral sprouting	9	0.175288645	0.0004	0.008964341
GO:0046339	diacylglycerol metabolic process	8	0.175192343	0.0177	0.02329117

GOIDlabel	GOName	size	categoryScores	pValPerm	pValPermCorr
GO:0003197	endocardial cushion development	22	0.17515531	0.0113	0.018488002
GO:0006829	zinc II ion transport	6	0.175098453	0.0131	0.019786886
GO:0002902	regulation of B cell apoptotic process	6	0.175065842	0.018	0.023514686
GO:0043367	CD4-positive, alpha-beta T cell differentiation	25	0.17502404	0.0094	0.017156187
GO:0043568	positive regulation of insulin-like growth factor receptor signaling pathway	5	0.174974489	0.022	0.026944291
GO:0003143	embryonic heart tube morphogenesis	24	0.17496828	0.0183	0.023783007
GO:0010835	regulation of protein ADP-ribosylation	7	0.17491007	0.0262	0.030579007
GO:0071501	cellular response to sterol depletion	6	0.174890263	0.0006	0.009940401
GO:1901879	regulation of protein depolymerization	32	0.174725415	0.0273	0.031601321
GO:0048024	regulation of mRNA splicing, via spliceosome	24	0.174723107	0.0102	0.0177
GO:0007063	regulation of sister chromatid cohesion	8	0.174597092	0.0216	0.026572596
GO:2000107	negative regulation of leukocyte apoptotic process	16	0.174578465	0.0082	0.016514246
GO:0031076	embryonic camera-type eye development	15	0.174506711	0.0117	0.018739446
GO:1903020	positive regulation of glycoprotein metabolic process	8	0.174316554	0.0047	0.014168613
GO:0021783	preganglionic parasympathetic fiber development	6	0.174133308	0.0264	0.030750363
GO:0035404	histone-serine phosphorylation	5	0.174038154	0.0041	0.013887639
GO:0003203	endocardial cushion morphogenesis	18	0.17395557	0.0142	0.020567234
GO:0045823	positive regulation of heart contraction	18	0.173952546	0.012	0.018931514
GO:0046638	positive regulation of alpha-beta T cell differentiation	16	0.173885138	0.0439	0.046702815
GO:0003179	heart valve morphogenesis	26	0.173874666	0.0303	0.034331687
GO:1903543	positive regulation of exosomal secretion	10	0.173694642	0.0128	0.019522448
GO:1900744	regulation of p38MAPK cascade	10	0.173441186	0.015	0.021138192
GO:0051261	protein depolymerization	41	0.173404155	0.011	0.018323826
GO:0045066	regulatory T cell differentiation	11	0.173399756	0.0468	0.049334111
GO:0061626	pharyngeal arch artery morphogenesis	6	0.1733878	0.0384	0.04164081
GO:0060561	apoptotic process involved in morphogenesis	7	0.17338419	0.0357	0.03911643
GO:0006413	translational initiation	83	0.173358925	0.033	0.036785425
GO:0043368	positive T cell selection	10	0.173315365	0.0468	0.049334111
GO:0036037	CD8-positive, alpha-beta T cell activation	7	0.173197597	0.0261	0.030517735
GO:0046386	deoxyribose phosphate catabolic process	12	0.173127671	0.029	0.033098697
GO:0006544	glycine metabolic process	8	0.173092846	0.0218	0.026744664
GO:0009593	detection of chemical stimulus	42	0.172938069	0.0297	0.03379756
GO:0045814	negative regulation of gene expression, epigenetic	17	0.172670506	0.0164	0.022306469
GO:0060972	left/right pattern formation	7	0.172517864	0.0051	0.014547706
GO:0002090	regulation of receptor internalization	22	0.172408127	0.0169	0.022703485
GO:0001782	B cell homeostasis	9	0.172370968	0.0151	0.02121706
GO:0042297	vocal learning	5	0.17236996	0.001	0.010787313
GO:0098596	imitative learning	5	0.17236996	0.001	0.010787313
GO:0098597	observational learning	5	0.17236996	0.001	0.010787313
GO:0098598	learned vocalization behavior or vocal learning	5	0.17236996	0.001	0.010787313
GO:1905515	non-motile cilium assembly	21	0.172343365	0.015	0.021138192
GO:0060338	regulation of type I interferon-mediated signaling pathway	11	0.172226834	0.0298	0.033898013
GO:0010463	mesenchymal cell proliferation	18	0.172157523	0.034	0.037588528
GO:0030853	negative regulation of granulocyte differentiation	6	0.172137366	0.0067	0.015576759
GO:0051148	negative regulation of muscle cell differentiation	31	0.172030422	0.0104	0.017832977
GO:0030216	keratinocyte differentiation	66	0.171868353	0.0157	0.021779607
GO:0045023	G0 to G1 transition	14	0.171819251	0.0078	0.01620539
GO:0070316	regulation of G0 to G1 transition	14	0.171819251	0.0078	0.01620539
GO:0015804	neutral amino acid transport	11	0.171770011	0.0097	0.017363901
GO:0019080	viral gene expression	84	0.171766787	0.042	0.044946141
GO:1903540	establishment of protein localization to postsynaptic membrane	7	0.171658573	0.0063	0.015311728
GO:0050482	arachidonic acid secretion	11	0.171592842	0.0112	0.018392048
GO:1903963	arachidonate transport	11	0.171592842	0.0112	0.018392048
GO:1990709	presynaptic active zone organization	5	0.171549727	0.0332	0.036915846
GO:0030220	platelet formation	6	0.171508478	0.0103	0.017772187
GO:0036344	platelet morphogenesis	6	0.171508478	0.0103	0.017772187
GO:0045063	T-helper 1 cell differentiation	9	0.171491849	0.0172	0.022941269
GO:0021984	adenohypophysis development	5	0.171470425	0.0475	0.049962707
GO:1901380	negative regulation of potassium ion transmembrane transport	10	0.171448527	0.004	0.013824268
GO:0050892	intestinal absorption	11	0.171421327	0.0254	0.029880529
GO:0003209	cardiac atrium morphogenesis	13	0.171361739	0.0198	0.025062084
GO:0003230	cardiac atrium development	13	0.171361739	0.0198	0.025062084
GO:2000514	regulation of CD4-positive, alpha-beta T cell activation	19	0.171063535	0.0199	0.025133639
GO:0090385	phagosome-lysosome fusion	5	0.171053701	0.0257	0.030153693
GO:0010718	positive regulation of epithelial to mesenchymal transition	19	0.171001542	0.0245	0.029070183
GO:0051547	regulation of keratinocyte migration	6	0.170984981	0.0009	0.010729485
GO:0030857	negative regulation of epithelial cell differentiation	14	0.170892024	< 0.001	< 0.001
GO:0051973	positive regulation of telomerase activity	17	0.170754267	0.0179	0.02344231
GO:0033629	negative regulation of cell adhesion mediated by integrin	5	0.170500918	0.0034	0.01347416
GO:1902947	regulation of tau-protein kinase activity	5	0.170456603	0.0342	0.037780741
GO:0099625	ventricular cardiac muscle cell membrane repolarization	12	0.170442155	0.0216	0.026572596
GO:0070861	regulation of protein exit from endoplasmic reticulum	10	0.170419154	0.0118	0.018836996
GO:0032689	negative regulation of interferon-gamma production	9	0.170397342	0.0232	0.028016374
GO:0048642	negative regulation of skeletal muscle tissue development	8	0.170391781	0.0279	0.032115827
GO:1904526	regulation of microtubule binding	5	0.170299294	0.0251	0.029642198
GO:0032369	negative regulation of lipid transport	7	0.170106222	0.0086	0.016782045
GO:0051085	chaperone mediated protein folding requiring cofactor	9	0.170090529	0.008	0.016379603
GO:0030837	negative regulation of actin filament polymerization	22	0.170050032	0.0304	0.034411276
GO:0098659	inorganic cation import into cell	9	0.170046616	0.008	0.016379603
GO:0099587	inorganic ion import into cell	9	0.170046616	0.008	0.016379603
GO:1900101	regulation of endoplasmic reticulum unfolded protein response	5	0.169960835	0.0086	0.016782045
GO:0031069	hair follicle morphogenesis	7	0.169917037	0.0471	0.049614174
GO:0071604	transforming growth factor beta production	15	0.169685956	0.0265	0.03085441

GOIDlabel	GOName	size	categoryScores	pValPerm	pValPermCorr
GO:0006984	ER-nucleus signaling pathway	17	0.169505435	0.0098	0.017418875
GO:0021527	spinal cord association neuron differentiation	5	0.169462994	0.0389	0.042009675
GO:0035050	embryonic heart tube development	26	0.169404753	0.0148	0.020979063
GO:1901880	negative regulation of protein depolymerization	23	0.169345032	0.0235	0.028248857
GO:0043370	regulation of CD4-positive, alpha-beta T cell differentiation	14	0.169281269	0.0157	0.021779607
GO:0071542	dopaminergic neuron differentiation	11	0.169232038	0.0016	0.011845327
GO:0021511	spinal cord patterning	11	0.169102106	0.0365	0.039857035
GO:2000757	negative regulation of peptidyl-lysine acetylation	10	0.169000269	0.0259	0.0303391
GO:0021513	spinal cord dorsal/ventral patterning	9	0.168868315	0.0345	0.038068511
GO:0048532	anatomical structure arrangement	6	0.168551279	0.0447	0.047449128
GO:0072673	lamellipodium morphogenesis	8	0.168463166	0.0358	0.039211138
GO:0006103	2-oxoglutarate metabolic process	11	0.168215553	0.0284	0.032523034
GO:0070935	3'-UTR-mediated mRNA stabilization	8	0.16814667	0.0173	0.023005658
GO:0090166	Golgi disassembly	5	0.168114858	0.0311	0.035052671
GO:0045616	regulation of keratinocyte differentiation	10	0.168089202	0.029	0.033098697
GO:0033233	regulation of protein sumoylation	8	0.168067141	0.0028	0.012972436
GO:0051984	positive regulation of chromosome segregation	10	0.168050847	0.0023	0.012498684
GO:0097011	cellular response to granulocyte macrophage colony-stimulating factor stimulus	5	0.167992536	0.0437	0.046498601
GO:0097012	response to granulocyte macrophage colony-stimulating factor	5	0.167992536	0.0437	0.046498601
GO:1904152	regulation of retrograde protein transport, ER to cytosol	6	0.167869264	0.0314	0.035301342
GO:0031061	negative regulation of histone methylation	8	0.167669173	0.0217	0.026655917
GO:0045188	regulation of circadian sleep/wake cycle, non-REM sleep	5	0.167501693	0.0033	0.013333753
GO:0045988	negative regulation of striated muscle contraction	5	0.167376779	0.0426	0.045512417
GO:0060037	pharyngeal system development	10	0.167369393	0.0273	0.031601321
GO:0000729	DNA double-strand break processing	5	0.167343866	0.0313	0.035223161
GO:0002091	negative regulation of receptor internalization	8	0.167308297	0.0056	0.014771533
GO:0033866	nucleoside bisphosphate biosynthetic process	10	0.167065531	0.0036	0.01353394
GO:0034030	ribonucleoside bisphosphate biosynthetic process	10	0.167065531	0.0036	0.01353394
GO:0034033	purine nucleoside bisphosphate biosynthetic process	10	0.167065531	0.0036	0.01353394
GO:0001947	heart looping	22	0.16685539	0.016	0.022026667
GO:0060396	growth hormone receptor signaling pathway	11	0.166780176	0.0007	0.010220707
GO:0071378	cellular response to growth hormone stimulus	11	0.166780176	0.0007	0.010220707
GO:0003188	heart valve formation	8	0.166758721	0.0213	0.026276211
GO:0031498	chromatin disassembly	8	0.166758721	0.0078	0.01620539
GO:0006182	cGMP biosynthetic process	9	0.16668218	0.0159	0.021956962
GO:0032435	negative regulation of proteasomal ubiquitin-dependent protein catabolic process	14	0.166465897	0.0093	0.017114131
GO:0046635	positive regulation of alpha-beta T cell activation	22	0.166453991	0.042	0.044946141
GO:0019083	viral transcription	78	0.166427071	0.0463	0.048905115
GO:0072567	chemokine (C-X-C motif) ligand 2 production	6	0.166387533	0.0178	0.023348367
GO:2000341	regulation of chemokine (C-X-C motif) ligand 2 production	6	0.166387533	0.0178	0.023348367
GO:0048662	negative regulation of smooth muscle cell proliferation	20	0.166387382	0.0394	0.042525817
GO:0045601	regulation of endothelial cell differentiation	8	0.166379542	0.0053	0.014634479
GO:0030330	DNA damage response, signal transduction by p53 class mediator	37	0.166192494	0.0137	0.020264364
GO:0045475	locomotor rhythm	8	0.166190681	0.0031	0.013277185
GO:0045579	positive regulation of B cell differentiation	5	0.166165997	0.0374	0.040724444
GO:1902004	positive regulation of beta-amyloid formation	5	0.166126086	0.0011	0.010890753
GO:1902993	positive regulation of amyloid precursor protein catabolic process	5	0.166126086	0.0011	0.010890753
GO:0043124	negative regulation of I-kappaB kinase/NF-kappaB signaling	11	0.165871594	0.0105	0.017924712
GO:1901031	regulation of response to reactive oxygen species	15	0.165797043	0.0156	0.021692929
GO:1903539	protein localization to postsynaptic membrane	11	0.165709974	0.0004	0.008964341
GO:0045620	negative regulation of lymphocyte differentiation	18	0.165498696	0.0172	0.022941269
GO:0046068	cGMP metabolic process	13	0.165175857	0.0112	0.018392048
GO:0043249	erythrocyte maturation	7	0.16489957	0.0188	0.02420971
GO:0048384	retinoic acid receptor signaling pathway	13	0.16478443	0.0016	0.011845327
GO:0032814	regulation of natural killer cell activation	11	0.164693725	0.0239	0.028592965
GO:0046689	response to mercury ion	6	0.164493958	0.0144	0.02072711
GO:1902514	regulation of calcium ion transmembrane transport via high voltage-gated calcium channel	8	0.164385328	0.0282	0.032370935
GO:0006206	pyrimidine nucleobase metabolic process	11	0.16433491	0.015	0.021138192
GO:1902186	regulation of viral release from host cell	16	0.164209911	0.018	0.023514686
GO:0001302	replicative cell aging	5	0.164102847	0.0044	0.014132412
GO:0032527	protein exit from endoplasmic reticulum	24	0.164012196	0.0135	0.020081554
GO:0098915	membrane repolarization during ventricular cardiac muscle cell action potential	7	0.163985115	0.0354	0.038883511
GO:0051016	barbed-end actin filament capping	7	0.163936431	0.028	0.032186083
GO:0060324	face development	20	0.16387116	0.0292	0.033300671
GO:0043162	ubiquitin-dependent protein catabolic process via the multivesicular body sorting pathway	6	0.163819717	0.0445	0.047271541
GO:2000352	negative regulation of endothelial cell apoptotic process	11	0.163700564	0.0473	0.049788567
GO:0033119	negative regulation of RNA splicing	11	0.163656625	0.0352	0.038693232
GO:2000074	regulation of type B pancreatic cell development	6	0.163521687	< 0.001	< 0.001
GO:0000303	response to superoxide	11	0.163488055	0.0268	0.031128485
GO:0000305	response to oxygen radical	11	0.163488055	0.0268	0.031128485
GO:0002335	mature B cell differentiation	12	0.16347493	0.0162	0.022196303
GO:0046641	positive regulation of alpha-beta T cell proliferation	10	0.163333279	0.0399	0.042985243
GO:0032634	interleukin-5 production	5	0.163270693	0.0349	0.038392656
GO:0032674	regulation of interleukin-5 production	5	0.163270693	0.0349	0.038392656
GO:0010614	negative regulation of cardiac muscle hypertrophy	13	0.163160212	0.0059	0.014942532
GO:0048387	negative regulation of retinoic acid receptor signaling pathway	5	0.163087469	0.0029	0.013089617
GO:0007274	neuromuscular synaptic transmission	9	0.162805333	0.002	0.012314604
GO:0090200	positive regulation of release of cytochrome c from mitochondria	16	0.162769159	0.0377	0.040981651
GO:2000406	positive regulation of T cell migration	9	0.162700662	0.0358	0.039211138
GO:0002507	tolerance induction	8	0.162626299	0.0167	0.02255007
GO:1903862	positive regulation of oxidative phosphorylation	5	0.162278121	0.019	0.024347961
GO:0019388	galactose catabolic process	5	0.16221074	0.0231	0.027930615
GO:0086005	ventricular cardiac muscle cell action potential	13	0.162088996	0.0294	0.033482529

GOIDlabel	GOName	size	categoryScores	pValPerm	pValPermCorr
GO:1904994	regulation of leukocyte adhesion to vascular endothelial cell	7	0.162055875	0.0262	0.030579007
GO:0070229	negative regulation of lymphocyte apoptotic process	10	0.162039956	0.0085	0.016693954
GO:0006144	purine nucleobase metabolic process	9	0.161985964	0.0218	0.026744664
GO:0038066	p38MAPK cascade	13	0.161838012	0.0231	0.027930615
GO:0014741	negative regulation of muscle hypertrophy	14	0.161740814	0.0096	0.017302743
GO:1902187	negative regulation of viral release from host cell	8	0.161676326	0.0065	0.015491756
GO:1904292	regulation of ERAD pathway	12	0.161586754	0.0051	0.014547706
GO:0002467	germinal center formation	5	0.161509461	0.0073	0.015844069
GO:0002634	regulation of germinal center formation	5	0.161509461	0.0073	0.015844069
GO:0060337	type I interferon signaling pathway	31	0.161421657	0.0072	0.015844069
GO:0071357	cellular response to type I interferon	31	0.161421657	0.0072	0.015844069
GO:0044090	positive regulation of vacuole organization	11	0.161404785	0.0454	0.048095053
GO:0048385	regulation of retinoic acid receptor signaling pathway	9	0.161269827	< 0.001	< 0.001
GO:0032966	negative regulation of collagen biosynthetic process	5	0.161181627	0.0218	0.026744664
GO:2000001	regulation of DNA damage checkpoint	5	0.161116838	0.0159	0.021956962
GO:0032616	interleukin-13 production	7	0.161059231	0.0141	0.020504577
GO:0032656	regulation of interleukin-13 production	7	0.161059231	0.0141	0.020504577
GO:0006195	purine nucleotide catabolic process	19	0.161000599	0.0312	0.035137982
GO:0070734	histone H3-K27 methylation	6	0.160935514	0.0243	0.028921902
GO:0007064	mitotic sister chromatid cohesion	8	0.160457963	0.0203	0.025471918
GO:0061371	determination of heart left/right asymmetry	23	0.160338138	0.0178	0.023348367
GO:0060049	regulation of protein glycosylation	12	0.160213114	0.0149	0.021043429
GO:0031424	keratinization	42	0.159927146	0.0277	0.03194284
GO:0003272	endocardial cushion formation	13	0.15978788	0.0449	0.047643935
GO:0002903	negative regulation of B cell apoptotic process	5	0.159775699	0.0312	0.035137982
GO:0010749	regulation of nitric oxide mediated signal transduction	5	0.159700285	0.0467	0.049255637
GO:0009299	mRNA transcription	6	0.159692424	0.0041	0.013887639
GO:0060575	intestinal epithelial cell differentiation	9	0.15930196	0.0083	0.016559903
GO:0017144	drug metabolic process	7	0.159283819	0.0106	0.018010344
GO:0006833	water transport	10	0.159187024	0.0385	0.041733596
GO:2000515	negative regulation of CD4-positive, alpha-beta T cell activation	8	0.159162663	0.0165	0.022405589
GO:0045623	negative regulation of T-helper cell differentiation	5	0.159078048	0.0406	0.043585072
GO:0001707	mesoderm formation	26	0.159056409	0.0349	0.038392656
GO:0048332	mesoderm morphogenesis	26	0.159056409	0.0349	0.038392656
GO:0045581	negative regulation of T cell differentiation	13	0.159026058	0.0348	0.038319101
GO:0010832	negative regulation of myotube differentiation	5	0.158912188	0.0214	0.026388313
GO:0072523	purine-containing compound catabolic process	22	0.15868334	0.0348	0.038319101
GO:0060576	intestinal epithelial cell development	8	0.15867642	0.0033	0.013333753
GO:0001881	receptor recycling	14	0.158218776	0.0174	0.023064374
GO:0048333	mesodermal cell differentiation	13	0.158104654	0.0323	0.03615849
GO:0031643	positive regulation of myelination	8	0.157955315	0.0387	0.041895413
GO:1900118	negative regulation of execution phase of apoptosis	6	0.157396507	0.0364	0.039777887
GO:0019076	viral release from host cell	20	0.15732187	0.0122	0.019049527
GO:0035890	exit from host	20	0.15732187	0.0122	0.019049527
GO:0035891	exit from host cell	20	0.15732187	0.0122	0.019049527
GO:0052126	movement in host environment	20	0.15732187	0.0122	0.019049527
GO:0052192	movement in environment of other organism involved in symbiotic interaction	20	0.15732187	0.0122	0.019049527
GO:0071285	cellular response to lithium ion	8	0.157134597	0.0018	0.01210186
GO:0036499	PERK-mediated unfolded protein response	5	0.156924193	0.0204	0.025564109
GO:0030511	positive regulation of transforming growth factor beta receptor signaling pathway	12	0.156838887	0.0199	0.025133639
GO:1903846	positive regulation of cellular response to transforming growth factor beta stimulus	12	0.156838887	0.0199	0.025133639
GO:0009147	pyrimidine nucleoside triphosphate metabolic process	12	0.156798502	0.019	0.024347961
GO:0008655	pyrimidine-containing compound salvage	7	0.156720755	0.0207	0.025805821
GO:0043097	pyrimidine nucleoside salvage	7	0.156720755	0.0207	0.025805821
GO:0009112	nucleobase metabolic process	19	0.156626367	0.0144	0.02072711
GO:0060065	uterus development	8	0.156443619	0.0217	0.026655917
GO:0036507	protein demannosylation	12	0.156212591	0.0011	0.010890753
GO:0036508	protein alpha-1,2-demannosylation	12	0.156212591	0.0011	0.010890753
GO:0003190	atrioventricular valve formation	5	0.156165122	0.0234	0.02817551
GO:0060973	cell migration involved in heart development	8	0.156114651	0.0433	0.046157928
GO:1903651	positive regulation of cytoplasmic transport	9	0.15568643	0.021	0.026005997
GO:0042772	DNA damage response, signal transduction resulting in transcription	9	0.155417626	0.0321	0.035976391
GO:0034340	response to type I interferon	33	0.155279566	0.0086	0.016782045
GO:0030970	retrograde protein transport, ER to cytosol	17	0.155190965	0.0212	0.026203164
GO:1903513	endoplasmic reticulum to cytosol transport	17	0.155190965	0.0212	0.026203164
GO:0006517	protein deglycosylation	14	0.155155816	0.002	0.012314604
GO:0051549	positive regulation of keratinocyte migration	5	0.154878666	0.0027	0.012827773
GO:0071888	macrophage apoptotic process	5	0.154875815	0.0411	0.044080913
GO:0048486	parasympathetic nervous system development	7	0.154745792	0.0135	0.020081554
GO:0045622	regulation of T-helper cell differentiation	11	0.154612747	0.0111	0.018389742
GO:0055012	ventricular cardiac muscle cell differentiation	8	0.15415105	0.0085	0.016693954
GO:0045086	positive regulation of interleukin-2 biosynthetic process	5	0.154108969	0.0445	0.047271541
GO:0021516	dorsal spinal cord development	7	0.154027187	0.012	0.018931514
GO:0044458	motile cilium assembly	5	0.153939739	0.0398	0.042885501
GO:0035907	dorsal aorta development	5	0.153690171	0.0057	0.01480566
GO:0035912	dorsal aorta morphogenesis	5	0.153690171	0.0057	0.01480566
GO:1904706	negative regulation of vascular smooth muscle cell proliferation	10	0.153573162	0.0475	0.049962707
GO:0009113	purine nucleobase biosynthetic process	5	0.15314309	0.0198	0.025062084
GO:0071816	tail-anchored membrane protein insertion into ER membrane	6	0.152859529	0.0175	0.023154462
GO:0003084	positive regulation of systemic arterial blood pressure	5	0.15283573	0.028	0.032186083
GO:0019400	alditol metabolic process	7	0.152768423	0.0306	0.034617335
GO:0001867	complement activation, lectin pathway	5	0.152701227	0.0151	0.02121706
GO:0072528	pyrimidine-containing compound biosynthetic process	22	0.152609992	0.0143	0.020629391

GOIDlabel	GOName	size	categoryScores	pValPerm	pValPermCorr
GO:1902667	regulation of axon guidance	10	0.152563745	0.0405	0.043493871
GO:0003171	atrioventricular valve development	14	0.152258531	0.0191	0.02440579
GO:0003181	atrioventricular valve morphogenesis	14	0.152258531	0.0191	0.02440579
GO:1903423	positive regulation of synaptic vesicle recycling	5	0.152096614	0.0059	0.014942532
GO:1901070	guanosine-containing compound biosynthetic process	7	0.151924867	0.0172	0.022941269
GO:0046112	nucleobase biosynthetic process	7	0.15168274	0.0137	0.020264364
GO:0001919	regulation of receptor recycling	11	0.151565412	0.0251	0.029642198
GO:0042634	regulation of hair cycle	12	0.151196604	0.0224	0.027324219
GO:0006342	chromatin silencing	10	0.15104923	0.014	0.020462083
GO:0009048	dosage compensation by inactivation of X chromosome	5	0.150964097	0.007	0.015816335
GO:0045048	protein insertion into ER membrane	7	0.150787537	0.0117	0.018739446
GO:0070233	negative regulation of T cell apoptotic process	6	0.150720187	0.0112	0.018392048
GO:0016339	calcium-dependent cell-cell adhesion via plasma membrane cell adhesion molecules	13	0.150502578	0.0004	0.008964341
GO:0060644	mammary gland epithelial cell differentiation	9	0.149942356	0.0107	0.018084595
GO:0002711	positive regulation of T cell mediated immunity	9	0.149697164	0.0163	0.022248961
GO:0008298	intracellular mRNA localization	5	0.149685675	0.0376	0.040911404
GO:0052564	response to immune response of other organism involved in symbiotic interaction	5	0.149274392	0.0288	0.032902905
GO:0052572	response to host immune response	5	0.149274392	0.0288	0.032902905
GO:0016577	histone demethylation	7	0.149113937	0.0096	0.017302743
GO:0070076	histone lysine demethylation	7	0.149113937	0.0096	0.017302743
GO:1903715	regulation of aerobic respiration	6	0.148541107	0.0061	0.015143924
GO:0006312	mitotic recombination	7	0.148519542	0.01	0.017574468
GO:0033483	gas homeostasis	5	0.148423839	0.0262	0.030579007
GO:0006978	DNA damage response, signal transduction by p53 class mediator resulting in transcription of p21 class mediator	8	0.148341232	0.0345	0.038068511
GO:0006337	nucleosome disassembly	7	0.148119329	0.0209	0.025971158
GO:0002866	positive regulation of acute inflammatory response to antigenic stimulus	6	0.147823242	0.0434	0.046247475
GO:0010765	positive regulation of sodium ion transport	12	0.147801321	0.0094	0.017156187
GO:1901388	regulation of transforming growth factor beta activation	5	0.147734999	0.034	0.037588528
GO:0043371	negative regulation of CD4-positive, alpha-beta T cell differentiation	6	0.14770187	0.0265	0.03085441
GO:1901984	negative regulation of protein acetylation	12	0.147057648	0.0254	0.029880529
GO:0060325	face morphogenesis	11	0.146987401	0.0336	0.037253154
GO:0009218	pyrimidine ribonucleotide metabolic process	13	0.146794537	0.0189	0.024289798
GO:2000009	negative regulation of protein localization to cell surface	7	0.146350766	0.0228	0.027701114
GO:0009208	pyrimidine ribonucleoside triphosphate metabolic process	8	0.14633649	0.0176	0.023223003
GO:0007256	activation of JNKK activity	6	0.14624044	0.003	0.013210967
GO:0051154	negative regulation of striated muscle cell differentiation	14	0.145852352	0.0102	0.0177
GO:0006089	lactate metabolic process	7	0.145744524	0.0347	0.038238117
GO:0006213	pyrimidine nucleoside metabolic process	23	0.145334066	0.0284	0.032523034
GO:0060323	head morphogenesis	12	0.144597059	0.0427	0.045577146
GO:0098976	excitatory chemical synaptic transmission	7	0.144374878	0.0025	0.012769435
GO:0046827	positive regulation of protein export from nucleus	5	0.143518512	0.0216	0.026572596
GO:0030042	actin filament depolymerization	22	0.143323331	0.0411	0.044080913
GO:0009220	pyrimidine ribonucleotide biosynthetic process	12	0.143199338	0.0153	0.021378589
GO:0006221	pyrimidine nucleotide biosynthetic process	17	0.142896321	0.0288	0.032902905
GO:0048670	regulation of collateral sprouting	6	0.142321335	0.0001	0.004220438
GO:0045646	regulation of erythrocyte differentiation	16	0.142080076	0.0139	0.020388077
GO:0036150	phosphatidylserine acyl-chain remodeling	6	0.141673874	0.0347	0.038238117
GO:0071679	commissural neuron axon guidance	8	0.141605576	0.0421	0.045028154
GO:0046051	UTP metabolic process	6	0.141313646	0.0254	0.029880529
GO:0099022	vesicle tethering	6	0.141307599	0.0153	0.021378589
GO:0006222	UMP biosynthetic process	6	0.141028573	0.0336	0.037253154
GO:0009173	pyrimidine ribonucleoside monophosphate metabolic process	6	0.141028573	0.0336	0.037253154
GO:0009174	pyrimidine ribonucleoside monophosphate biosynthetic process	6	0.141028573	0.0336	0.037253154
GO:0046049	UMP metabolic process	6	0.141028573	0.0336	0.037253154
GO:0045625	regulation of T-helper 1 cell differentiation	5	0.140951042	0.0468	0.049334111
GO:0035162	embryonic hemopoiesis	8	0.140238036	0.0178	0.023348367
GO:0006241	CTP biosynthetic process	7	0.140107856	0.016	0.022026667
GO:0009209	pyrimidine ribonucleoside triphosphate biosynthetic process	7	0.140107856	0.016	0.022026667
GO:0046036	CTP metabolic process	7	0.140107856	0.016	0.022026667
GO:0046131	pyrimidine ribonucleoside metabolic process	13	0.139827795	0.014	0.020462083
GO:0060394	negative regulation of pathway-restricted SMAD protein phosphorylation	5	0.139610163	0.0441	0.046872463
GO:0055075	potassium ion homeostasis	6	0.138862235	0.0348	0.038319101
GO:0002070	epithelial cell maturation	9	0.138830705	0.0052	0.014616626
GO:0001755	neural crest cell migration	15	0.138152145	0.0178	0.023348367
GO:1905208	negative regulation of cardiocyte differentiation	9	0.137369346	0.0118	0.018836996
GO:0010107	potassium ion import	8	0.136822668	0.0261	0.030517735
GO:1990573	potassium ion import across plasma membrane	8	0.136822668	0.0261	0.030517735
GO:0046132	pyrimidine ribonucleoside biosynthetic process	11	0.136737853	0.0173	0.023005658
GO:1904996	positive regulation of leukocyte adhesion to vascular endothelial cell	5	0.136684019	0.0332	0.036915846
GO:0007549	dosage compensation	6	0.136683372	0.0181	0.023607986
GO:0036152	phosphatidylethanolamine acyl-chain remodeling	9	0.135673594	0.0288	0.032902905
GO:0014832	urinary bladder smooth muscle contraction	5	0.135516257	0.0199	0.025133639
GO:0014848	urinary tract smooth muscle contraction	5	0.135516257	0.0199	0.025133639
GO:0072396	response to cell cycle checkpoint signaling	5	0.135061436	0.0013	0.011218806
GO:0072402	response to DNA integrity checkpoint signaling	5	0.135061436	0.0013	0.011218806
GO:0072423	response to DNA damage checkpoint signaling	5	0.135061436	0.0013	0.011218806
GO:0032736	positive regulation of interleukin-13 production	5	0.134840635	0.0192	0.02450649
GO:1904294	positive regulation of ERAD pathway	5	0.134491032	0.0171	0.022860624
GO:0036151	phosphatidylcholine acyl-chain remodeling	10	0.134485201	0.0325	0.036312077
GO:0034643	establishment of mitochondrion localization, microtubule-mediated	8	0.13439631	0.0095	0.01725157
GO:0047497	mitochondrion transport along microtubule	8	0.13439631	0.0095	0.01725157
GO:0033631	cell-cell adhesion mediated by integrin	6	0.133246948	0.0378	0.041067193

GOIDlabel	GOName	size	categoryScores	pValPerm	pValPermCorr
GO:0046134	pyrimidine nucleoside biosynthetic process	16	0.132899353	0.0219	0.026855949
GO:0006071	glycerol metabolic process	5	0.132289751	0.0404	0.043394538
GO:0018242	protein O-linked glycosylation via serine	5	0.132140994	0.0173	0.023005658
GO:0006183	GTP biosynthetic process	5	0.13158899	0.0223	0.027242468
GO:0006228	UTP biosynthetic process	5	0.13158899	0.0223	0.027242468
GO:0042789	mRNA transcription from RNA polymerase II promoter	5	0.13131843	0.0303	0.034331687
GO:0019042	viral latency	5	0.131111363	0.0098	0.017418875
GO:0019043	establishment of viral latency	5	0.131111363	0.0098	0.017418875
GO:0075713	establishment of integrated proviral latency	5	0.131111363	0.0098	0.017418875
GO:1900122	positive regulation of receptor binding	6	0.130389308	0.0032	0.013333753
GO:0032986	protein-DNA complex disassembly	8	0.129834334	0.0404	0.043394538
GO:0009148	pyrimidine nucleoside triphosphate biosynthetic process	10	0.129268497	0.0252	0.029736
GO:0036363	transforming growth factor beta activation	6	0.129071062	0.0361	0.039494834
GO:0045648	positive regulation of erythrocyte differentiation	10	0.128230831	0.0087	0.016840777
GO:0008340	determination of adult lifespan	6	0.128190576	0.0347	0.038238117
GO:2000726	negative regulation of cardiac muscle cell differentiation	8	0.127836718	0.0256	0.03006687
GO:0010171	body morphogenesis	18	0.127740794	0.0251	0.029642198
GO:0009129	pyrimidine nucleoside monophosphate metabolic process	7	0.126184267	0.0247	0.029271449
GO:0009130	pyrimidine nucleoside monophosphate biosynthetic process	7	0.126184267	0.0247	0.029271449
GO:0003148	outflow tract septum morphogenesis	16	0.123506121	0.0106	0.018010344
GO:0030011	maintenance of cell polarity	7	0.12241242	0.0206	0.025725529
GO:1902307	positive regulation of sodium ion transmembrane transport	8	0.122267431	0.028	0.032186083
GO:0032633	interleukin-4 production	13	0.121902194	0.0171	0.022860624
GO:0032673	regulation of interleukin-4 production	13	0.121902194	0.0171	0.022860624
GO:0032239	regulation of nucleobase-containing compound transport	6	0.121841441	0.0417	0.044666432
GO:2000059	negative regulation of protein ubiquitination involved in ubiquitin-dependent protein catabolic process	7	0.119100391	0.031	0.034967226
GO:0003174	mitral valve development	7	0.119074661	0.016	0.022026667
GO:0003183	mitral valve morphogenesis	7	0.119074661	0.016	0.022026667
GO:0045736	negative regulation of cyclin-dependent protein serine/threonine kinase activity	16	0.116446194	0.0253	0.029811412
GO:0032753	positive regulation of interleukin-4 production	12	0.115891301	0.0149	0.021043429
GO:1904030	negative regulation of cyclin-dependent protein kinase activity	17	0.115847555	0.0241	0.028760826
GO:0048070	regulation of developmental pigmentation	5	0.111384839	0.0221	0.027038129
GO:0061052	negative regulation of cell growth involved in cardiac muscle cell development	7	0.110247398	0.0465	0.049080504
GO:0002069	columnar/cuboidal epithelial cell maturation	6	0.101164372	0.0074	0.015911789

Reward SUBC positive

GOIDlabel	GOName	size	categoryScores	pValPerm	pValPermCorr
GO:0006729	tetrahydrobiopterin biosynthetic process	5	0.535186615	< 0.001	< 0.001
GO:0046146	tetrahydrobiopterin metabolic process	5	0.535186615	< 0.001	< 0.001
GO:0000054	ribosomal subunit export from nucleus	5	0.516808237	0.0001	0.000877824
GO:0033750	ribosome localization	5	0.516808237	0.0001	0.000877824
GO:0033753	establishment of ribosome localization	5	0.516808237	0.0001	0.000877824
GO:0001514	selenocysteine incorporation	5	0.514028314	0.0002	0.001341684
GO:0006451	translational readthrough	5	0.514028314	0.0002	0.001341684
GO:0034975	protein folding in endoplasmic reticulum	6	0.497039897	< 0.001	< 0.001
GO:0060456	positive regulation of digestive system process	5	0.495444015	< 0.001	< 0.001
GO:0032817	regulation of natural killer cell proliferation	5	0.48993565	0.0005	0.002177563
GO:0032819	positive regulation of natural killer cell proliferation	5	0.48993565	0.0005	0.002177563
GO:0032525	somite rostral/caudal axis specification	5	0.488597169	< 0.001	< 0.001
GO:0002579	positive regulation of antigen processing and presentation	5	0.487052767	< 0.001	< 0.001
GO:0071428	rRNA-containing ribonucleoprotein complex export from nucleus	6	0.485971686	0.0001	0.000877824
GO:0051096	positive regulation of helicase activity	5	0.483294723	< 0.001	< 0.001
GO:0009263	deoxyribonucleotide biosynthetic process	6	0.47979408	0.0008	0.002313249
GO:0043508	negative regulation of JUN kinase activity	8	0.475933076	0.0001	0.000877824
GO:0045329	carnitine biosynthetic process	5	0.475881596	< 0.001	< 0.001
GO:0046689	response to mercury ion	5	0.475881596	0.0001	0.000877824
GO:0001787	natural killer cell proliferation	6	0.475203775	0.0009	0.002399874
GO:0010421	hydrogen peroxide-mediated programmed cell death	5	0.474337194	0.0001	0.000877824
GO:0097468	programmed cell death in response to reactive oxygen species	5	0.474337194	0.0001	0.000877824
GO:0008105	asymmetric protein localization	7	0.472145615	0.0001	0.000877824
GO:0051095	regulation of helicase activity	7	0.468284611	< 0.001	< 0.001
GO:0070424	regulation of nucleotide-binding oligomerization domain containing signaling pathway	5	0.467644788	0.0001	0.000877824
GO:0035434	copper ion transmembrane transport	5	0.465637066	0.0006	0.002177563
GO:0051764	actin crosslink formation	5	0.464247104	< 0.001	< 0.001
GO:0042559	pteridine-containing compound biosynthetic process	11	0.45971686	< 0.001	< 0.001
GO:0051280	negative regulation of release of sequestered calcium ion into cytosol	5	0.458584299	< 0.001	< 0.001
GO:0051284	positive regulation of sequestering of calcium ion	5	0.458584299	< 0.001	< 0.001
GO:0051974	negative regulation of telomerase activity	5	0.458275418	0.0002	0.001341684
GO:0045955	negative regulation of calcium ion-dependent exocytosis	6	0.457014157	0.0009	0.002399874
GO:0071236	cellular response to antibiotic	9	0.455998856	0.0002	0.001341684
GO:0031573	intra-S DNA damage checkpoint	5	0.454723295	0.0004	0.002007244
GO:0042711	maternal behavior	5	0.454517375	< 0.001	< 0.001
GO:0060746	parental behavior	5	0.454517375	< 0.001	< 0.001
GO:0034063	stress granule assembly	9	0.452595453	0.0001	0.000877824
GO:0009265	2'-deoxyribonucleotide biosynthetic process	5	0.45045045	0.0009	0.002399874
GO:0046385	deoxyribose phosphate biosynthetic process	5	0.45045045	0.0009	0.002399874
GO:1904667	negative regulation of ubiquitin protein ligase activity	7	0.447104247	0.0002	0.001341684
GO:0001866	NK T cell proliferation	5	0.446229086	0.0005	0.002177563
GO:0036480	neuron intrinsic apoptotic signaling pathway in response to oxidative stress	5	0.444015444	0.0008	0.002313249
GO:1903376	regulation of oxidative stress-induced neuron intrinsic apoptotic signaling pathway	5	0.444015444	0.0008	0.002313249
GO:0048642	negative regulation of skeletal muscle tissue development	5	0.443912484	0.0018	0.003459409
GO:0033194	response to hydroperoxide	13	0.443758044	< 0.001	< 0.001
GO:2000669	negative regulation of dendritic cell apoptotic process	5	0.443140283	0.0005	0.002177563
GO:0009597	detection of virus	6	0.442642643	0.0001	0.000877824
GO:0002480	antigen processing and presentation of exogenous peptide antigen via MHC class I, TAP-independent	9	0.441841842	0.0012	0.002741075
GO:0051315	attachment of mitotic spindle microtubules to kinetochore	8	0.441537967	0.0001	0.000877824
GO:0071671	regulation of smooth muscle cell chemotaxis	6	0.441441441	< 0.001	< 0.001
GO:1903862	positive regulation of oxidative phosphorylation	5	0.441235521	0.0001	0.000877824
GO:0045351	type I interferon biosynthetic process	5	0.441235521	< 0.001	< 0.001
GO:0032928	regulation of superoxide anion generation	16	0.441071429	0.0005	0.002177563
GO:0035520	monoubiquitinated protein deubiquitination	5	0.440772201	0.0025	0.004279479
GO:0071638	negative regulation of monocyte chemotactic protein-1 production	5	0.44020592	0.0014	0.002975058
GO:0048532	anatomical structure arrangement	5	0.439073359	< 0.001	< 0.001
GO:0033864	positive regulation of NAD(P)H oxidase activity	6	0.438009438	< 0.001	< 0.001
GO:0045579	positive regulation of B cell differentiation	8	0.437612613	< 0.001	< 0.001
GO:0019511	peptidyl-proline hydroxylation	7	0.437065637	< 0.001	< 0.001
GO:0072641	type I interferon secretion	6	0.437022737	0.0005	0.002177563
GO:0072642	interferon-alpha secretion	6	0.437022737	0.0005	0.002177563
GO:1902739	regulation of interferon-alpha secretion	6	0.437022737	0.0005	0.002177563
GO:1902741	positive regulation of interferon-alpha secretion	6	0.437022737	0.0005	0.002177563
GO:0097201	negative regulation of transcription from RNA polymerase II promoter in response to stress	8	0.436840412	0.0015	0.003088691
GO:0010869	regulation of receptor biosynthetic process	11	0.436784837	0.0007	0.002248538
GO:1901739	regulation of myoblast fusion	8	0.436454311	0.0019	0.003579279
GO:0045176	apical protein localization	5	0.436241956	0.0001	0.000877824
GO:0010944	negative regulation of transcription by competitive promoter binding	7	0.435999265	< 0.001	< 0.001
GO:0032056	positive regulation of translation in response to stress	5	0.434954955	< 0.001	< 0.001
GO:0010870	positive regulation of receptor biosynthetic process	8	0.434073359	0.0007	0.002248538
GO:0006825	copper ion transport	12	0.433998284	0.0006	0.002177563
GO:0061469	regulation of type B pancreatic cell proliferation	5	0.433925354	0.0013	0.002859821
GO:1903897	regulation of PERK-mediated unfolded protein response	6	0.432947233	0.0021	0.003804235
GO:0032930	positive regulation of superoxide anion generation	12	0.432882883	0.0011	0.002610912
GO:0032800	receptor biosynthetic process	13	0.432531433	0.0008	0.002313249
GO:0051132	NK T cell activation	6	0.432303732	0.0013	0.002859821
GO:0032875	regulation of DNA endoreduplication	5	0.43021879	0.0008	0.002313249
GO:0043102	amino acid salvage	6	0.429343629	0.0002	0.001341684
GO:0071265	L-methionine biosynthetic process	6	0.429343629	0.0002	0.001341684
GO:0071267	L-methionine salvage	6	0.429343629	0.0002	0.001341684
GO:1903209	positive regulation of oxidative stress-induced cell death	11	0.429203229	0.0001	0.000877824

GOIDlabel	GOName	size	categoryScores	pValPerm	pValPermCorr
GO:0003174	mitral valve development	6	0.428528529	< 0.001	< 0.001
GO:0003183	mitral valve morphogenesis	6	0.428528529	< 0.001	< 0.001
GO:0071763	nuclear membrane organization	5	0.428365508	0.0004	0.002007244
GO:0070525	tRNA threonylcarbamoyladenosine metabolic process	8	0.427767053	0.0034	0.005263104
GO:0042554	superoxide anion generation	17	0.426572791	0.0005	0.002177563
GO:0070208	protein heterotrimerization	7	0.426034197	0.0006	0.002177563
GO:0021542	dentate gyrus development	6	0.425053625	< 0.001	< 0.001
GO:1901033	positive regulation of response to reactive oxygen species	7	0.424416253	0.0001	0.000877824
GO:0033860	regulation of NAD(P)H oxidase activity	7	0.424195624	0.0001	0.000877824
GO:0035795	negative regulation of mitochondrial membrane permeability	5	0.424092664	0.0009	0.002399874
GO:1901741	positive regulation of myoblast fusion	7	0.424048538	0.0028	0.004605006
GO:0042023	DNA endoreduplication	6	0.423509224	< 0.001	< 0.001
GO:0009086	methionine biosynthetic process	9	0.423223223	0.0002	0.001341684
GO:0070933	histone H4 deacetylation	6	0.422779923	0.0008	0.002313249
GO:0044342	type B pancreatic cell proliferation	7	0.421879022	0.0017	0.003334595
GO:1901678	iron coordination entity transport	6	0.420592021	< 0.001	< 0.001
GO:0090343	positive regulation of cell aging	7	0.419599191	0.0006	0.002177563
GO:0051451	myoblast migration	7	0.419047619	0.0006	0.002177563
GO:0030889	negative regulation of B cell proliferation	9	0.418761619	0.0062	0.008281828
GO:1902884	positive regulation of response to oxidative stress	14	0.418293804	0.0001	0.000877824
GO:0060700	regulation of ribonuclease activity	6	0.417932218	0.0006	0.002177563
GO:0090169	regulation of spindle assembly	15	0.417915058	0.0003	0.001720882
GO:0045876	positive regulation of sister chromatid cohesion	7	0.417613532	0.0007	0.002248538
GO:0002082	regulation of oxidative phosphorylation	13	0.417285417	0.0002	0.001341684
GO:0072321	chaperone-mediated protein transport	9	0.417217217	0.0001	0.000877824
GO:0090646	mitochondrial tRNA processing	6	0.417117117	0.0012	0.002741075
GO:0036336	dendritic cell migration	16	0.416618404	0.0008	0.002313249
GO:1902931	negative regulation of alcohol biosynthetic process	5	0.416370656	0.0002	0.001341684
GO:1905063	regulation of vascular smooth muscle cell differentiation	7	0.415922044	0.0003	0.001720882
GO:2001034	positive regulation of double-strand break repair via nonhomologous end joining	7	0.415333701	0.0013	0.002859821
GO:0031340	positive regulation of vesicle fusion	5	0.414826255	< 0.001	< 0.001
GO:0017062	respiratory chain complex III assembly	6	0.414714715	0.0006	0.002177563
GO:0034551	mitochondrial respiratory chain complex III assembly	6	0.414714715	0.0006	0.002177563
GO:0097033	mitochondrial respiratory chain complex III biogenesis	6	0.414714715	0.0006	0.002177563
GO:2000774	positive regulation of cellular senescence	6	0.414586015	0.0005	0.002177563
GO:0002385	mucosal immune response	6	0.414500215	0.0005	0.002177563
GO:0042532	negative regulation of tyrosine phosphorylation of STAT protein	6	0.414242814	0.0003	0.001720882
GO:0006999	nuclear pore organization	8	0.414157014	0.0005	0.002177563
GO:0060287	epithelial cilium movement involved in determination of left/right asymmetry	5	0.413899614	0.0007	0.002248538
GO:0045820	negative regulation of glycolytic process	5	0.413230373	0.0042	0.006097175
GO:0051195	negative regulation of cofactor metabolic process	5	0.413230373	0.0042	0.006097175
GO:0051198	negative regulation of coenzyme metabolic process	5	0.413230373	0.0042	0.006097175
GO:1900409	positive regulation of cellular response to oxidative stress	13	0.413186813	0.0001	0.000877824
GO:0090322	regulation of superoxide metabolic process	24	0.412172887	0.0006	0.002177563
GO:0002604	regulation of dendritic cell antigen processing and presentation	6	0.411840412	0.0022	0.003910907
GO:0048102	autophagic cell death	7	0.411619783	0.0021	0.003804235
GO:0002921	negative regulation of humoral immune response	5	0.411531532	0.002	0.003693422
GO:0045916	negative regulation of complement activation	5	0.411531532	0.002	0.003693422
GO:2000258	negative regulation of protein activation cascade	5	0.411531532	0.002	0.003693422
GO:0009120	deoxyribonucleoside metabolic process	7	0.411104983	0.0009	0.002399874
GO:0002475	antigen processing and presentation via MHC class Ib	6	0.411068211	0.0036	0.005475609
GO:0009130	pyrimidine nucleoside monophosphate biosynthetic process	10	0.411016731	0.0015	0.003088691
GO:0002834	regulation of response to tumor cell	7	0.410884354	< 0.001	< 0.001
GO:0002837	regulation of immune response to tumor cell	7	0.410884354	< 0.001	< 0.001
GO:0046498	S-adenosylhomocysteine metabolic process	6	0.41042471	0.0014	0.002975058
GO:0035994	response to muscle stretch	5	0.41024453	0.0018	0.003459409
GO:0051292	nuclear pore complex assembly	6	0.40956671	0.0002	0.001341684
GO:0080182	histone H3-K4 trimethylation	8	0.409427284	0.0006	0.002177563
GO:0000076	DNA replication checkpoint	6	0.409395109	0.0037	0.005561344
GO:0017004	cytochrome complex assembly	19	0.409225767	0.0009	0.002399874
GO:0046007	negative regulation of activated T cell proliferation	5	0.408906049	0.0028	0.004605006
GO:0072050	S-shaped body morphogenesis	5	0.408442728	0.0002	0.001341684
GO:0071712	ER-associated misfolded protein catabolic process	5	0.407824968	< 0.001	< 0.001
GO:0002251	organ or tissue specific immune response	7	0.40728075	0.001	0.002498236
GO:0003180	aortic valve morphogenesis	16	0.40719112	0.0001	0.000877824
GO:0051151	negative regulation of smooth muscle cell differentiation	10	0.406924067	0.0012	0.002741075
GO:0036363	transforming growth factor beta activation	6	0.406778207	0.0006	0.002177563
GO:0032486	Rap protein signal transduction	7	0.40676595	0.0009	0.002399874
GO:0001911	negative regulation of leukocyte mediated cytotoxicity	8	0.406306306	0.0009	0.002399874
GO:0031342	negative regulation of cell killing	8	0.406306306	0.0009	0.002399874
GO:0051409	response to nitrosative stress	7	0.406251149	0.0001	0.000877824
GO:0003084	positive regulation of systemic arterial blood pressure	6	0.405619906	0.0001	0.000877824
GO:1904026	regulation of collagen fibril organization	5	0.405559846	0.002	0.003693422
GO:0032823	regulation of natural killer cell differentiation	7	0.405552491	0.0004	0.002007244
GO:0070900	mitochondrial tRNA modification	5	0.405199485	0.0057	0.007745437
GO:0070901	mitochondrial tRNA methylation	5	0.405199485	0.0057	0.007745437
GO:1900864	mitochondrial RNA modification	5	0.405199485	0.0057	0.007745437
GO:0045047	protein targeting to ER	95	0.404077762	0.0009	0.002399874
GO:0036302	atrioventricular canal development	5	0.404066924	0.0048	0.006748379
GO:0003176	aortic valve development	18	0.403918204	0.0005	0.002177563
GO:1900122	positive regulation of receptor binding	5	0.403861004	0.0021	0.003804235
GO:2000508	regulation of dendritic cell chemotaxis	5	0.403861004	0.0045	0.006417207
GO:1901673	regulation of mitotic spindle assembly	14	0.403842618	0.0005	0.002177563

GOIDlabel	GOName	size	categoryScores	pValPerm	pValPermCorr
GO:2000973	regulation of pro-B cell differentiation	5	0.403655084	0.0002	0.001341684
GO:0034091	regulation of maintenance of sister chromatid cohesion	6	0.403389103	0.0001	0.000877824
GO:0034182	regulation of maintenance of mitotic sister chromatid cohesion	6	0.403389103	0.0001	0.000877824
GO:0060405	regulation of penile erection	5	0.403294723	< 0.001	< 0.001
GO:0002115	store-operated calcium entry	5	0.402831403	< 0.001	< 0.001
GO:0061419	positive regulation of transcription from RNA polymerase II promoter in response to hypoxia	5	0.402676963	0.0073	0.009463568
GO:0032060	bleb assembly	10	0.402548263	0.0012	0.002741075
GO:0071731	response to nitric oxide	10	0.402419562	0.0006	0.002177563
GO:0006614	SRP-dependent cotranslational protein targeting to membrane	90	0.402379522	0.001	0.002498236
GO:0071394	cellular response to testosterone stimulus	5	0.402007722	0.0021	0.003804235
GO:0010560	positive regulation of glycoprotein biosynthetic process	12	0.401951952	0.0002	0.001341684
GO:0006222	UMP biosynthetic process	7	0.401581173	0.0017	0.003334595
GO:0009173	pyrimidine ribonucleoside monophosphate metabolic process	7	0.401581173	0.0017	0.003334595
GO:0009174	pyrimidine ribonucleoside monophosphate biosynthetic process	7	0.401581173	0.0017	0.003334595
GO:0046049	UMP metabolic process	7	0.401581173	0.0017	0.003334595
GO:0022417	protein maturation by protein folding	6	0.401415701	0.0005	0.002177563
GO:1904732	regulation of electron carrier activity	6	0.401287001	0.0025	0.004279479
GO:0002327	immature B cell differentiation	5	0.401184041	0.0015	0.003088691
GO:0042276	error-prone translesion synthesis	15	0.401029601	0.0002	0.001341684
GO:0021783	preganglionic parasympathetic fiber development	5	0.400823681	< 0.001	< 0.001
GO:0035456	response to interferon-beta	17	0.400166553	0.0007	0.002248538
GO:1901533	negative regulation of hematopoietic progenitor cell differentiation	7	0.399705828	0.0007	0.002248538
GO:0051444	negative regulation of ubiquitin-protein transferase activity	12	0.39963535	0.0001	0.000877824
GO:0032825	positive regulation of natural killer cell differentiation	6	0.3996139	0.0008	0.002313249
GO:0006613	cotranslational protein targeting to membrane	93	0.399316367	0.0009	0.002399874
GO:0072599	establishment of protein localization to endoplasmic reticulum	97	0.399307408	0.0006	0.002177563
GO:0043101	purine-containing compound salvage	13	0.399267399	0.0002	0.001341684
GO:0000578	embryonic axis specification	17	0.399212658	< 0.001	< 0.001
GO:0006369	termination of RNA polymerase II transcription	26	0.398920899	< 0.001	< 0.001
GO:0060019	radial glial cell differentiation	6	0.398670099	0.0004	0.002007244
GO:2001198	regulation of dendritic cell differentiation	6	0.398627199	0.0001	0.000877824
GO:0044829	positive regulation by host of viral genome replication	6	0.398627199	0.0002	0.001341684
GO:0019755	one-carbon compound transport	5	0.398558559	0.0009	0.002399874
GO:0018023	peptidyl-lysine trimethylation	25	0.398496782	0.0008	0.002313249
GO:0060009	Sertoli cell development	8	0.398487773	0.0003	0.001720882
GO:0045777	positive regulation of blood pressure	15	0.398438438	< 0.001	< 0.001
GO:0032740	positive regulation of interleukin-17 production	6	0.398326898	0.0022	0.003910907
GO:0071360	cellular response to exogenous dsRNA	14	0.397756941	0.0021	0.003804235
GO:0001768	establishment of T cell polarity	5	0.397683398	0.001	0.002498236
GO:0008334	histone mRNA metabolic process	18	0.397154297	< 0.001	< 0.001
GO:1902459	positive regulation of stem cell population maintenance	7	0.397131826	0.0002	0.001341684
GO:0032196	transposition	6	0.396954097	0.0006	0.002177563
GO:0071732	cellular response to nitric oxide	7	0.39672734	0.0012	0.002741075
GO:0070474	positive regulation of uterine smooth muscle contraction	5	0.396705277	0.0001	0.000877824
GO:0009396	folic acid-containing compound biosynthetic process	7	0.396396396	0.0005	0.002177563
GO:0070431	nucleotide-binding oligomerization domain containing 2 signaling pathway	6	0.396353496	0.0007	0.002248538
GO:0034661	ncRNA catabolic process	13	0.395921196	< 0.001	< 0.001
GO:0043616	keratinocyte proliferation	15	0.395555556	< 0.001	< 0.001
GO:0031392	regulation of prostaglandin biosynthetic process	5	0.395469755	< 0.001	< 0.001
GO:2001279	regulation of unsaturated fatty acid biosynthetic process	5	0.395469755	< 0.001	< 0.001
GO:1902358	sulfate transmembrane transport	6	0.395066495	< 0.001	< 0.001
GO:0009067	aspartate family amino acid biosynthetic process	14	0.394962309	0.0001	0.000877824
GO:0043628	ncRNA 3'-end processing	9	0.394737595	< 0.001	< 0.001
GO:0014889	muscle atrophy	6	0.394551695	0.0035	0.005358035
GO:0035356	cellular triglyceride homeostasis	5	0.394543115	0.0001	0.000877824
GO:0006616	SRP-dependent cotranslational protein targeting to membrane, translocation	5	0.393925354	0.002	0.003693422
GO:0043094	cellular metabolic compound salvage	27	0.393393393	0.0001	0.000877824
GO:0048539	bone marrow development	5	0.392998713	0.0013	0.002859821
GO:0002407	dendritic cell chemotaxis	14	0.39297665	0.0019	0.003579279
GO:0000183	chromatin silencing at rDNA	10	0.392818533	0.0026	0.004377754
GO:0006206	pyrimidine nucleobase metabolic process	11	0.392675793	0.0004	0.002007244
GO:2000059	negative regulation of protein ubiquitination involved in ubiquitin-dependent protein catabolic process	12	0.392492492	0.0008	0.002313249
GO:1904385	cellular response to angiotensin	10	0.392406692	0.001	0.002498236
GO:0033617	mitochondrial respiratory chain complex IV assembly	12	0.392342342	0.0009	0.002399874
GO:0097034	mitochondrial respiratory chain complex IV biogenesis	12	0.392342342	0.0009	0.002399874
GO:0090070	positive regulation of ribosome biogenesis	9	0.392106392	< 0.001	< 0.001
GO:2000234	positive regulation of rRNA processing	9	0.392106392	< 0.001	< 0.001
GO:0070972	protein localization to endoplasmic reticulum	104	0.392065142	0.0004	0.002007244
GO:0032735	positive regulation of interleukin-12 production	19	0.392061234	0.0001	0.000877824
GO:0008340	determination of adult lifespan	7	0.391873506	0.0012	0.002741075
GO:0002396	MHC protein complex assembly	6	0.391720292	0.0025	0.004279479
GO:1990776	response to angiotensin	13	0.391584992	0.0007	0.002248538
GO:0010523	negative regulation of calcium ion transport into cytosol	8	0.390830116	< 0.001	< 0.001
GO:0072257	metanephric nephron tubule epithelial cell differentiation	5	0.390785071	0.0001	0.000877824
GO:0072307	regulation of metanephric nephron tubule epithelial cell differentiation	5	0.390785071	0.0001	0.000877824
GO:0050798	activated T cell proliferation	16	0.390749678	0.0007	0.002248538
GO:0005984	disaccharide metabolic process	5	0.390733591	0.0024	0.004156304
GO:0002176	male germ cell proliferation	5	0.390579151	0.0008	0.002313249
GO:0036093	germ cell proliferation	5	0.390579151	0.0008	0.002313249
GO:0008535	respiratory chain complex IV assembly	13	0.390555391	0.0014	0.002975058
GO:2000371	regulation of DNA topoisomerase (ATP-hydrolyzing) activity	6	0.39021879	0.0012	0.002741075
GO:2000373	positive regulation of DNA topoisomerase (ATP-hydrolyzing) activity	6	0.39021879	0.0012	0.002741075
GO:0009129	pyrimidine nucleoside monophosphate metabolic process	11	0.38970399	0.0028	0.004605006

GOIDlabel	GOName	size	categoryScores	pValPerm	pValPermCorr
GO:1903799	negative regulation of production of miRNAs involved in gene silencing by miRNA	5	0.389292149	0.0108	0.013015961
GO:2000271	positive regulation of fibroblast apoptotic process	5	0.389240669	0.0001	0.000877824
GO:0035458	cellular response to interferon-beta	10	0.389034749	0.0002	0.001341684
GO:2000347	positive regulation of hepatocyte proliferation	6	0.388888889	0.0007	0.002248538
GO:0019614	catechol-containing compound catabolic process	5	0.388777349	0.0001	0.000877824
GO:0042424	catecholamine catabolic process	5	0.388777349	0.0001	0.000877824
GO:0032968	positive regulation of transcription elongation from RNA polymerase II promoter	7	0.388674389	0.0022	0.003910907
GO:0002428	antigen processing and presentation of peptide antigen via MHC class Ib	5	0.388622909	0.0056	0.007655255
GO:0019509	L-methionine biosynthetic process from methylthioadenosine	5	0.388571429	0.0014	0.002975058
GO:0060576	intestinal epithelial cell development	7	0.388564074	0.0031	0.004916949
GO:0032816	positive regulation of natural killer cell activation	13	0.388278388	0.0015	0.003088691
GO:0071157	negative regulation of cell cycle arrest	15	0.388211068	0.0007	0.002248538
GO:0033240	positive regulation of cellular amine metabolic process	7	0.388122817	0.0004	0.002007244
GO:0097048	dendritic cell apoptotic process	6	0.388030888	< 0.001	< 0.001
GO:2000668	regulation of dendritic cell apoptotic process	6	0.388030888	< 0.001	< 0.001
GO:0097435	fibril organization	10	0.387696268	0.001	0.002498236
GO:0048308	organelle inheritance	5	0.387593308	0.0001	0.000877824
GO:0048313	Golgi inheritance	5	0.387593308	0.0001	0.000877824
GO:0034433	steroid esterification	8	0.387580438	0.0009	0.002399874
GO:0034434	sterol esterification	8	0.387580438	0.0009	0.002399874
GO:0034435	cholesterol esterification	8	0.387580438	0.0009	0.002399874
GO:0010745	negative regulation of macrophage derived foam cell differentiation	8	0.387580438	0.0011	0.002610912
GO:1902916	positive regulation of protein polyubiquitination	7	0.387313844	0.0011	0.002610912
GO:0060142	regulation of syncytium formation by plasma membrane fusion	12	0.387087087	0.003	0.004825593
GO:0034471	ncRNA 5'-end processing	6	0.387001287	0.0066	0.008731949
GO:0035965	cardiolipin acyl-chain remodeling	5	0.386975547	0.0019	0.003579279
GO:0060100	positive regulation of phagocytosis, engulfment	7	0.386762272	0.0048	0.006748379
GO:1905155	positive regulation of membrane invagination	7	0.386762272	0.0048	0.006748379
GO:0060368	regulation of Fc receptor mediated stimulatory signaling pathway	6	0.386615187	0.0001	0.000877824
GO:1903306	negative regulation of regulated secretory pathway	13	0.386179586	0.0003	0.001720882
GO:0033962	cytoplasmic mRNA processing body assembly	12	0.386014586	< 0.001	< 0.001
GO:0090023	positive regulation of neutrophil chemotaxis	14	0.385806214	0.0005	0.002177563
GO:0045292	mRNA cis splicing, via spliceosome	10	0.385740026	0.0004	0.002007244
GO:1900102	negative regulation of endoplasmic reticulum unfolded protein response	9	0.385585586	0.0011	0.002610912
GO:0051085	chaperone mediated protein folding requiring cofactor	8	0.385553411	0.0001	0.000877824
GO:0000184	nuclear-transcribed mRNA catabolic process, nonsense-mediated decay	111	0.385437175	0.0015	0.003088691
GO:0045915	positive regulation of catecholamine metabolic process	5	0.385276705	0.0004	0.002007244
GO:0045964	positive regulation of dopamine metabolic process	5	0.385276705	0.0004	0.002007244
GO:0035812	renal sodium excretion	12	0.385135135	< 0.001	< 0.001
GO:0035813	regulation of renal sodium excretion	12	0.385135135	< 0.001	< 0.001
GO:0055070	copper ion homeostasis	9	0.384841985	0.0001	0.000877824
GO:0031167	rRNA methylation	12	0.384620335	0.002	0.003693422
GO:2001032	regulation of double-strand break repair via nonhomologous end joining	12	0.384598885	< 0.001	< 0.001
GO:0072173	metanephric tubule morphogenesis	5	0.384350064	0.0001	0.000877824
GO:0072282	metanephric nephron tubule morphogenesis	5	0.384350064	0.0001	0.000877824
GO:0060099	regulation of phagocytosis, engulfment	8	0.383751609	0.0062	0.008281828
GO:0006515	misfolded or incompletely synthesized protein catabolic process	12	0.383569284	0.0004	0.002007244
GO:0060333	interferon-gamma-mediated signaling pathway	52	0.383209583	0.0022	0.003910907
GO:0046543	development of secondary female sexual characteristics	5	0.382857143	0.0001	0.000877824
GO:0006983	ER overload response	11	0.382754183	0.0002	0.001341684
GO:0048490	anterograde synaptic vesicle transport	8	0.382625483	0.0002	0.001341684
GO:0099514	synaptic vesicle cytoskeletal transport	8	0.382625483	0.0002	0.001341684
GO:0099517	synaptic vesicle transport along microtubule	8	0.382625483	0.0002	0.001341684
GO:0007131	reciprocal meiotic recombination	16	0.382593308	< 0.001	< 0.001
GO:0035825	reciprocal DNA recombination	16	0.382593308	< 0.001	< 0.001
GO:0061085	regulation of histone H3-K27 methylation	6	0.382453882	< 0.001	< 0.001
GO:0035666	TRIF-dependent toll-like receptor signaling pathway	19	0.382361309	0.0004	0.002007244
GO:0014824	artery smooth muscle contraction	5	0.382187902	0.0008	0.002313249
GO:0006555	methionine metabolic process	10	0.381981982	0.0006	0.002177563
GO:0090162	establishment of epithelial cell polarity	17	0.381966841	0.0024	0.004156304
GO:0006409	tRNA export from nucleus	24	0.381724582	0.0006	0.002177563
GO:0051031	tRNA transport	24	0.381724582	0.0006	0.002177563
GO:0071431	tRNA-containing ribonucleoprotein complex export from nucleus	24	0.381724582	0.0006	0.002177563
GO:0043518	negative regulation of DNA damage response, signal transduction by p53 class mediator	9	0.381695982	0.0001	0.000877824
GO:0006568	tryptophan metabolic process	6	0.381681682	0.0023	0.004032435
GO:0017182	peptidyl-diphthamide metabolic process	6	0.381467181	0.0008	0.002313249
GO:0017183	peptidyl-diphthamide biosynthetic process from peptidyl-histidine	6	0.381467181	0.0008	0.002313249
GO:0018202	peptidyl-histidine modification	6	0.381467181	0.0008	0.002313249
GO:0043084	penile erection	7	0.38143041	< 0.001	< 0.001
GO:0008272	sulfate transport	7	0.381393639	< 0.001	< 0.001
GO:0070986	left/right axis specification	8	0.381048906	< 0.001	< 0.001
GO:0060340	positive regulation of type I interferon-mediated signaling pathway	5	0.380694981	0.008	0.010168329
GO:0045621	positive regulation of lymphocyte differentiation	49	0.380679221	0.0014	0.002975058
GO:0036474	cell death in response to hydrogen peroxide	16	0.38043758	0.0005	0.002177563
GO:1903020	positive regulation of glycoprotein metabolic process	13	0.38037818	0.0003	0.001720882
GO:0007183	SMAD protein complex assembly	10	0.38030888	0.0001	0.000877824
GO:0006349	regulation of gene expression by genetic imprinting	9	0.37995138	< 0.001	< 0.001
GO:0034086	maintenance of sister chromatid cohesion	10	0.37981982	0.0008	0.002313249
GO:0034088	maintenance of mitotic sister chromatid cohesion	10	0.37981982	0.0008	0.002313249
GO:0045683	negative regulation of epidermis development	7	0.379812466	0.0009	0.002399874
GO:0044794	positive regulation by host of viral process	11	0.37964198	0.0006	0.002177563
GO:0090026	positive regulation of monocyte chemotaxis	9	0.37960818	0.0002	0.001341684
GO:0032069	regulation of nuclease activity	14	0.379591837	0.0008	0.002313249

GOIDlabel	GOName	size	categoryScores	pValPerm	pValPermCorr
GO:0035933	glucocorticoid secretion	7	0.379555065	< 0.001	< 0.001
GO:1903054	negative regulation of extracellular matrix organization	8	0.379472329	0.0001	0.000877824
GO:0044849	estrous cycle	8	0.379472329	0.0003	0.001720882
GO:0032727	positive regulation of interferon-alpha production	16	0.379198842	0.0001	0.000877824
GO:0097340	inhibition of cysteine-type endopeptidase activity	5	0.378790219	0.0063	0.008390784
GO:0097341	zymogen inhibition	5	0.378790219	0.0063	0.008390784
GO:0038155	interleukin-23-mediated signaling pathway	5	0.378481338	< 0.001	< 0.001
GO:1904959	regulation of cytochrome-c oxidase activity	5	0.378275418	0.0031	0.004916949
GO:0000966	RNA 5'-end processing	7	0.378231293	0.008	0.010168329
GO:0014842	regulation of skeletal muscle satellite cell proliferation	6	0.378206778	< 0.001	< 0.001
GO:0014857	regulation of skeletal muscle cell proliferation	6	0.378206778	< 0.001	< 0.001
GO:0010887	negative regulation of cholesterol storage	6	0.378035178	0.002	0.003693422
GO:0071494	cellular response to UV-C	6	0.377863578	< 0.001	< 0.001
GO:0006612	protein targeting to membrane	127	0.377853444	0.0008	0.002313249
GO:0034508	centromere complex assembly	23	0.377751665	< 0.001	< 0.001
GO:0006586	indolalkylamine metabolic process	8	0.377702703	0.0002	0.001341684
GO:0042430	indole-containing compound metabolic process	8	0.377702703	0.0002	0.001341684
GO:0032071	regulation of endodeoxyribonuclease activity	6	0.377691978	0.001	0.002498236
GO:0002716	negative regulation of natural killer cell mediated immunity	6	0.377691978	0.0038	0.005666214
GO:0045953	negative regulation of natural killer cell mediated cytotoxicity	6	0.377691978	0.0038	0.005666214
GO:0045582	positive regulation of T cell differentiation	41	0.377668958	0.0028	0.004605006
GO:0035886	vascular smooth muscle cell differentiation	16	0.37733269	0.0006	0.002177563
GO:0016254	preassembly of GPI anchor in ER membrane	13	0.377269577	0.0002	0.001341684
GO:0051571	positive regulation of histone H3-K4 methylation	11	0.377231777	0.0005	0.002177563
GO:0001835	blastocyst hatching	13	0.377170577	0.0003	0.001720882
GO:0035188	hatching	13	0.377170577	0.0003	0.001720882
GO:0071684	organism emergence from protective structure	13	0.377170577	0.0003	0.001720882
GO:0007035	vacuolar acidification	11	0.376974377	0.0077	0.009887588
GO:0051006	positive regulation of lipoprotein lipase activity	5	0.376885457	0.003	0.004825593
GO:0061365	positive regulation of triglyceride lipase activity	5	0.376885457	0.003	0.004825593
GO:0000185	activation of MAPKKK activity	6	0.376662377	0.0031	0.004916949
GO:0006458	'de novo' protein folding	14	0.376558191	0.0002	0.001341684
GO:1901841	regulation of high voltage-gated calcium channel activity	6	0.376447876	< 0.001	< 0.001
GO:0048641	regulation of skeletal muscle tissue development	19	0.376278534	0.0033	0.005149584
GO:0060575	intestinal epithelial cell differentiation	8	0.376254826	0.0033	0.005149584
GO:0045662	negative regulation of myoblast differentiation	14	0.376080162	0.0005	0.002177563
GO:2001014	regulation of skeletal muscle cell differentiation	7	0.375988233	0.0068	0.008946408
GO:0045672	positive regulation of osteoclast differentiation	13	0.375487575	0.0021	0.003804235
GO:0002483	antigen processing and presentation of endogenous peptide antigen	9	0.375403975	0.0079	0.010077402
GO:0036089	cleavage furrow formation	5	0.375341055	0.0068	0.008946408
GO:1905456	regulation of lymphoid progenitor cell differentiation	6	0.375289575	0.0012	0.002741075
GO:0001302	replicative cell aging	5	0.375186615	0.0001	0.000877824
GO:0006578	amino-acid betaine biosynthetic process	7	0.375179261	0.0003	0.001720882
GO:0090281	negative regulation of calcium ion import	8	0.375160875	< 0.001	< 0.001
GO:0010911	regulation of isomerase activity	8	0.375	0.0031	0.004916949
GO:0010912	positive regulation of isomerase activity	8	0.375	0.0031	0.004916949
GO:0045472	response to ether	7	0.374958632	0.0013	0.002859821
GO:0035739	CD4-positive, alpha-beta T cell proliferation	7	0.374848318	0.0051	0.007095023
GO:2000561	regulation of CD4-positive, alpha-beta T cell proliferation	7	0.374848318	0.0051	0.007095023
GO:0060143	positive regulation of syncytium formation by plasma membrane fusion	11	0.374704575	0.004	0.005880507
GO:0060602	branch elongation of an epithelium	9	0.374660375	0.0004	0.002007244
GO:0002864	regulation of acute inflammatory response to antigenic stimulus	7	0.374480603	0.0004	0.002007244
GO:0010837	regulation of keratinocyte proliferation	13	0.374457974	< 0.001	< 0.001
GO:0031293	membrane protein intracellular domain proteolysis	15	0.374414414	0.001	0.002498236
GO:0097186	amelogenesis	7	0.374370289	0.0007	0.002248538
GO:0032814	regulation of natural killer cell activation	17	0.374214551	0.0011	0.002610912
GO:0031062	positive regulation of histone methylation	26	0.374200574	0.0007	0.002248538
GO:0060842	arterial endothelial cell differentiation	6	0.374088374	0.0002	0.001341684
GO:2000737	negative regulation of stem cell differentiation	16	0.374034749	0.0002	0.001341684
GO:0008156	negative regulation of DNA replication	21	0.374014831	0.0001	0.000877824
GO:0010878	cholesterol storage	10	0.374002574	0.0009	0.002399874
GO:0001955	blood vessel maturation	5	0.374002574	0.0023	0.004032435
GO:0002830	positive regulation of type 2 immune response	8	0.373552124	0.0003	0.001720882
GO:0060122	inner ear receptor stereocilium organization	18	0.373516374	0.0001	0.000877824
GO:0045628	regulation of T-helper 2 cell differentiation	7	0.373377459	0.0014	0.002975058
GO:0042492	gamma-delta T cell differentiation	6	0.373359073	0.0003	0.001720882
GO:0036124	histone H3-K9 trimethylation	9	0.373316173	0.0016	0.003223775
GO:0000245	spliceosomal complex assembly	37	0.373160806	< 0.001	< 0.001
GO:0090022	regulation of neutrophil chemotaxis	16	0.373133848	0.0014	0.002975058
GO:0010934	macrophage cytokine production	10	0.373101673	0.0002	0.001341684
GO:0036499	PERK-mediated unfolded protein response	13	0.372992773	0.0072	0.009362497
GO:0033539	fatty acid beta-oxidation using acyl-CoA dehydrogenase	10	0.372895753	0.0006	0.002177563
GO:0001921	positive regulation of receptor recycling	11	0.372809173	0.01	0.012232246
GO:0051412	response to corticosterone	9	0.372744173	0.0005	0.002177563
GO:0071426	ribonucleoprotein complex export from nucleus	88	0.372698023	0.0006	0.002177563
GO:0000956	nuclear-transcribed mRNA catabolic process	172	0.372682649	0.0004	0.002007244
GO:0003190	atrioventricular valve formation	5	0.372664093	0.0009	0.002399874
GO:0000338	protein deneddylation	6	0.372586873	0.0007	0.002248538
GO:0097084	vascular smooth muscle cell development	9	0.372400972	0.0069	0.009046225
GO:0050869	negative regulation of B cell activation	15	0.372389532	0.0011	0.002610912
GO:0032075	positive regulation of nuclease activity	7	0.372347858	0.0033	0.005149584
GO:0050862	positive regulation of T cell receptor signaling pathway	8	0.372297297	0.0003	0.001720882
GO:0071166	ribonucleoprotein complex localization	89	0.372249939	0.0006	0.002177563

GOIDlabel	GOName	size	categoryScores	pValPerm	pValPermCorr
GO:0070098	chemokine-mediated signaling pathway	26	0.372210672	0.0038	0.005666214
GO:0032025	response to cobalt ion	8	0.372039897	0.0034	0.005263104
GO:1903332	regulation of protein folding	7	0.371833058	0.0043	0.006204188
GO:0043174	nucleoside salvage	12	0.371707422	0.0003	0.001720882
GO:0006801	superoxide metabolic process	38	0.371672424	0.0007	0.002248538
GO:1902074	response to salt	10	0.371660232	0.0001	0.000877824
GO:1900246	positive regulation of RIG-I signaling pathway	6	0.371643072	< 0.001	< 0.001
GO:0014841	skeletal muscle satellite cell proliferation	7	0.371538886	0.0001	0.000877824
GO:0014856	skeletal muscle cell proliferation	7	0.371538886	0.0001	0.000877824
GO:0050691	regulation of defense response to virus by host	24	0.371503647	0.0001	0.000877824
GO:0006044	N-acetylglucosamine metabolic process	8	0.371428571	0.0003	0.001720882
GO:0014820	tonic smooth muscle contraction	6	0.371128271	0.0018	0.003459409
GO:0007567	parturition	5	0.370604891	0.0002	0.001341684
GO:0071624	positive regulation of granulocyte chemotaxis	15	0.370570571	0.0011	0.002610912
GO:0060330	regulation of response to interferon-gamma	10	0.37047619	0.0007	0.002248538
GO:0060334	regulation of interferon-gamma-mediated signaling pathway	10	0.37047619	0.0007	0.002248538
GO:0002707	negative regulation of lymphocyte mediated immunity	14	0.370435742	0.0002	0.001341684
GO:0010742	macrophage derived foam cell differentiation	18	0.37039897	0.0005	0.002177563
GO:0090077	foam cell differentiation	18	0.37039897	0.0005	0.002177563
GO:0097421	liver regeneration	21	0.370325427	0.0005	0.002177563
GO:0051574	positive regulation of histone H3-K9 methylation	7	0.370251885	0.0037	0.005561344
GO:0060453	regulation of gastric acid secretion	8	0.370173745	0.0031	0.004916949
GO:0042538	hyperosmotic salinity response	7	0.370031256	< 0.001	< 0.001
GO:1902667	regulation of axon guidance	7	0.369994484	< 0.001	< 0.001
GO:0051084	'de novo' posttranslational protein folding	13	0.36996337	0.0001	0.000877824
GO:0045048	protein insertion into ER membrane	5	0.36993565	0.0015	0.003088691
GO:2000781	positive regulation of double-strand break repair	18	0.36992707	0.0007	0.002248538
GO:0001767	establishment of lymphocyte polarity	6	0.36988417	0.0007	0.002248538
GO:0033033	negative regulation of myeloid cell apoptotic process	8	0.369851995	0.0218	0.023945433
GO:0002866	positive regulation of acute inflammatory response to antigenic stimulus	6	0.36984127	0.0002	0.001341684
GO:0010888	negative regulation of lipid storage	11	0.36974377	0.0051	0.007095023
GO:0060281	regulation of oocyte development	5	0.36962677	0.0006	0.002177563
GO:0045005	DNA-dependent DNA replication maintenance of fidelity	22	0.369392769	0.0001	0.000877824
GO:0071670	smooth muscle cell chemotaxis	8	0.369369369	< 0.001	< 0.001
GO:1904177	regulation of adipose tissue development	6	0.369197769	0.0008	0.002313249
GO:0010224	response to UV-B	9	0.369197769	0.0004	0.002007244
GO:1903204	negative regulation of oxidative stress-induced neuron death	9	0.369169169	0.0004	0.002007244
GO:0006376	mRNA splice site selection	16	0.369160232	0.0003	0.001720882
GO:0006406	mRNA export from nucleus	80	0.369137709	0.0008	0.002313249
GO:0071427	mRNA-containing ribonucleoprotein complex export from nucleus	80	0.369137709	0.0008	0.002313249
GO:0000097	sulfur amino acid biosynthetic process	12	0.369047619	< 0.001	< 0.001
GO:2000678	negative regulation of transcription regulatory region DNA binding	12	0.369047619	< 0.001	< 0.001
GO:0039528	cytoplasmic pattern recognition receptor signaling pathway in response to virus	22	0.369041769	0.0001	0.000877824
GO:0046006	regulation of activated T cell proliferation	14	0.368946498	0.0009	0.002399874
GO:0097709	connective tissue replacement	6	0.368897469	0.001	0.002498236
GO:0035735	intraciliary transport involved in cilium assembly	29	0.368854569	< 0.001	< 0.001
GO:2000104	negative regulation of DNA-dependent DNA replication	13	0.368795169	0.0001	0.000877824
GO:0032607	interferon-alpha production	21	0.36863394	0.0002	0.001341684
GO:0016075	rRNA catabolic process	7	0.368523626	0.001	0.002498236
GO:0010259	multicellular organism aging	19	0.36848879	0.0004	0.002007244
GO:0006402	mRNA catabolic process	190	0.368437309	0.0001	0.000877824
GO:0010528	regulation of transposition	5	0.368391248	0.0017	0.003334595
GO:0010529	negative regulation of transposition	5	0.368391248	0.0017	0.003334595
GO:1902176	negative regulation of oxidative stress-induced intrinsic apoptotic signaling pathway	12	0.368382668	0.0006	0.002177563
GO:0001696	gastric acid secretion	9	0.368339768	0.0044	0.006304227
GO:2000810	regulation of bicellular tight junction assembly	13	0.368220968	0.0003	0.001720882
GO:0035584	calcium-mediated signaling using intracellular calcium source	5	0.368133848	0.0007	0.002248538
GO:0045542	positive regulation of cholesterol biosynthetic process	5	0.368030888	0.0003	0.001720882
GO:0042149	cellular response to glucose starvation	25	0.367907336	0.0016	0.003223775
GO:0046600	negative regulation of centriole replication	7	0.367677882	0.0015	0.003088691
GO:0010225	response to UV-C	11	0.367614368	< 0.001	< 0.001
GO:1990314	cellular response to insulin-like growth factor stimulus	6	0.367567568	0.0115	0.013693853
GO:0097150	neuronal stem cell population maintenance	17	0.367537285	0.0005	0.002177563
GO:0010955	negative regulation of protein processing	19	0.367391452	0.0045	0.006417207
GO:1903318	negative regulation of protein maturation	19	0.367391452	0.0045	0.006417207
GO:0071027	nuclear RNA surveillance	5	0.367361647	0.0022	0.003910907
GO:0071028	nuclear mRNA surveillance	5	0.367361647	0.0022	0.003910907
GO:0038065	collagen-activated signaling pathway	5	0.367361647	0.0129	0.015093024
GO:0018158	protein oxidation	8	0.367342342	0.0001	0.000877824
GO:2000615	regulation of histone H3-K9 acetylation	6	0.367310167	0.0026	0.004377754
GO:0015949	nucleobase-containing small molecule interconversion	16	0.367181467	0.0003	0.001720882
GO:1901213	regulation of transcription from RNA polymerase II promoter involved in heart development	6	0.367181467	0.0033	0.005149584
GO:0032647	regulation of interferon-alpha production	20	0.367129987	0.0002	0.001341684
GO:0006620	posttranslational protein targeting to membrane	6	0.367095667	0.0004	0.002007244
GO:0009950	dorsal/ventral axis specification	8	0.367052767	< 0.001	< 0.001
GO:0051969	regulation of transmission of nerve impulse	9	0.367024167	0.0003	0.001720882
GO:0031297	replication fork processing	18	0.366966967	< 0.001	< 0.001
GO:0071346	cellular response to interferon-gamma	87	0.366922588	0.0006	0.002177563
GO:0090713	immunological memory process	6	0.366881167	0.0117	0.013882722
GO:1904714	regulation of chaperone-mediated autophagy	6	0.366838267	0.0004	0.002007244
GO:0042274	ribosomal small subunit biogenesis	23	0.366828941	0.0003	0.001720882
GO:0046599	regulation of centriole replication	13	0.366815167	0.0004	0.002007244
GO:0046716	muscle cell cellular homeostasis	12	0.366709567	0.0007	0.002248538

GOIDlabel	GOName	size	categoryScores	pValPerm	pValPermCorr
GO:0070071	proton-transporting two-sector ATPase complex assembly	7	0.366648281	0.0006	0.002177563
GO:0060969	negative regulation of gene silencing	12	0.366645217	0.0044	0.006304227
GO:2001185	regulation of CD8-positive, alpha-beta T cell activation	6	0.366623767	0.0099	0.012121556
GO:0002468	dendritic cell antigen processing and presentation	7	0.366611509	0.0038	0.005666214
GO:0010044	response to aluminum ion	5	0.366537967	0.0036	0.005475609
GO:0051602	response to electrical stimulus	20	0.366396396	0.0002	0.001341684
GO:0060337	type I interferon signaling pathway	53	0.366387412	0.0012	0.002741075
GO:0071357	cellular response to type I interferon	53	0.366387412	0.0012	0.002741075
GO:0045059	positive thymic T cell selection	5	0.366383526	0.0154	0.017651835
GO:0006266	DNA ligation	13	0.366359766	< 0.001	< 0.001
GO:0050847	progesterone receptor signaling pathway	5	0.366332046	< 0.001	< 0.001
GO:0031643	positive regulation of myelination	9	0.366251966	0.0033	0.005149584
GO:0071801	regulation of podosome assembly	7	0.366059937	0.0117	0.013882722
GO:0032007	negative regulation of TOR signaling	21	0.365814794	0.0004	0.002007244
GO:0019083	viral transcription	149	0.365553281	0.0006	0.002177563
GO:0071481	cellular response to X-ray	6	0.365551266	0.0002	0.001341684
GO:0045687	positive regulation of glial cell differentiation	25	0.365456885	0.0014	0.002975058
GO:0039529	RIG-I signaling pathway	17	0.365387236	< 0.001	< 0.001
GO:0044062	regulation of excretion	13	0.365310365	< 0.001	< 0.001
GO:0042756	drinking behavior	5	0.365302445	0.0007	0.002248538
GO:1905564	positive regulation of vascular endothelial cell proliferation	11	0.365297765	0.0001	0.000877824
GO:0006405	RNA export from nucleus	89	0.365262534	0.0008	0.002313249
GO:0060027	convergent extension involved in gastrulation	5	0.365199485	0.0002	0.001341684
GO:0033540	fatty acid beta-oxidation using acyl-CoA oxidase	10	0.365173745	< 0.001	< 0.001
GO:0051385	response to mineralocorticoid	16	0.365074003	0.0003	0.001720882
GO:0001731	formation of translation preinitiation complex	5	0.364993565	0.0001	0.000877824
GO:0002418	immune response to tumor cell	9	0.364879165	0.0002	0.001341684
GO:0051123	RNA polymerase II transcriptional preinitiation complex assembly	11	0.364665965	0.0004	0.002007244
GO:1902415	regulation of mRNA binding	5	0.364633205	0.004	0.005880507
GO:0002756	MyD88-independent toll-like receptor signaling pathway	20	0.364620335	0.0002	0.001341684
GO:0098586	cellular response to virus	32	0.364422458	0.0001	0.000877824
GO:0001561	fatty acid alpha-oxidation	5	0.364324324	0.0011	0.002610912
GO:0043558	regulation of translational initiation in response to stress	9	0.364307164	0.0002	0.001341684
GO:0048670	regulation of collateral sprouting	9	0.364164164	0.0008	0.002313249
GO:0090502	RNA phosphodiester bond hydrolysis, endonucleolytic	44	0.364110214	< 0.001	< 0.001
GO:0006298	mismatch repair	20	0.363899614	0.0007	0.002248538
GO:0033483	gas homeostasis	7	0.363816878	0.0076	0.009790742
GO:1902170	cellular response to reactive nitrogen species	8	0.363803089	0.0028	0.004605006
GO:0061684	chaperone-mediated autophagy	12	0.363728014	0.0009	0.002399874
GO:0010998	regulation of translational initiation by eIF2 alpha phosphorylation	6	0.363706564	0.0001	0.000877824
GO:0046654	tetrahydrofolate biosynthetic process	6	0.363577864	0.0006	0.002177563
GO:0036500	ATF6-mediated unfolded protein response	10	0.363552124	0.0006	0.002177563
GO:0019388	galactose catabolic process	5	0.363397683	0.003	0.004825593
GO:0098659	inorganic cation import into cell	8	0.363320463	0.0098	0.012008345
GO:0099587	inorganic ion import into cell	8	0.363320463	0.0098	0.012008345
GO:0051028	mRNA transport	105	0.363311883	0.0007	0.002248538
GO:1903203	regulation of oxidative stress-induced neuron death	14	0.363283692	0.001	0.002498236
GO:0002828	regulation of type 2 immune response	13	0.363251163	0.0003	0.001720882
GO:0071514	genetic imprinting	16	0.363111326	< 0.001	< 0.001
GO:0071479	cellular response to ionizing radiation	46	0.363012702	0.0004	0.002007244
GO:0002753	cytoplasmic pattern recognition receptor signaling pathway	40	0.362985843	0.0001	0.000877824
GO:0019883	antigen processing and presentation of endogenous antigen	11	0.362864163	0.0027	0.004491543
GO:0046485	ether lipid metabolic process	5	0.362728443	0.0124	0.014577606
GO:0098801	regulation of renal system process	19	0.36269051	< 0.001	< 0.001
GO:0010883	regulation of lipid storage	24	0.362666238	0.0013	0.002859821
GO:0090136	epithelial cell-cell adhesion	9	0.362648363	0.0015	0.003088691
GO:0010935	regulation of macrophage cytokine production	8	0.362580438	0.0003	0.001720882
GO:0098869	cellular oxidant detoxification	57	0.36251891	0.0003	0.001720882
GO:0009157	deoxyribonucleoside monophosphate biosynthetic process	7	0.362456334	0.0023	0.004032435
GO:0001916	positive regulation of T cell mediated cytotoxicity	10	0.362445302	0.0024	0.004156304
GO:0071218	cellular response to misfolded protein	10	0.362342342	0.0002	0.001341684
GO:0009880	embryonic pattern specification	26	0.362320562	< 0.001	< 0.001
GO:0033605	positive regulation of catecholamine secretion	6	0.362290862	< 0.001	< 0.001
GO:0021591	ventricular system development	15	0.362265122	0.0001	0.000877824
GO:0060008	Sertoli cell differentiation	12	0.362226512	0.0007	0.002248538
GO:0061311	cell surface receptor signaling pathway involved in heart development	14	0.362180548	< 0.001	< 0.001
GO:0051569	regulation of histone H3-K4 methylation	15	0.362076362	< 0.001	< 0.001
GO:0032781	positive regulation of ATPase activity	36	0.362004862	0.0011	0.002610912
GO:0051290	protein heterotetramerization	22	0.361939862	0.0002	0.001341684
GO:1903051	negative regulation of proteolysis involved in cellular protein catabolic process	37	0.361821281	0.0008	0.002313249
GO:0051103	DNA ligation involved in DNA repair	8	0.361808237	0.0001	0.000877824
GO:0045723	positive regulation of fatty acid biosynthetic process	9	0.361790362	0.0023	0.004032435
GO:0048711	positive regulation of astrocyte differentiation	9	0.361733162	0.0017	0.003334595
GO:1904706	negative regulation of vascular smooth muscle cell proliferation	9	0.361733162	0.0081	0.010256567
GO:0060674	placenta blood vessel development	16	0.361727799	0.0006	0.002177563
GO:0014807	regulation of somitogenesis	5	0.361544402	0.0157	0.017969846
GO:0046500	S-adenosylmethionine metabolic process	11	0.361460161	0.0016	0.003223775
GO:1905522	negative regulation of macrophage migration	6	0.361432861	< 0.001	< 0.001
GO:0070987	error-free translesion synthesis	16	0.361422136	0.0005	0.002177563
GO:0032070	regulation of deoxyribonuclease activity	8	0.361293436	0.0022	0.003910907
GO:0043970	histone H3-K9 acetylation	8	0.361164736	0.0035	0.005358035
GO:0043330	response to exogenous dsRNA	23	0.361143752	0.0045	0.006417207
GO:0030279	negative regulation of ossification	35	0.36099283	0.0005	0.002177563

GOIDlabel	GOName	size	categoryScores	pValPerm	pValPermCorr
GO:0070269	pyroptosis	5	0.360978121	0.0001	0.000877824
GO:0071472	cellular response to salt stress	9	0.360960961	0.0005	0.002177563
GO:0044789	modulation by host of viral release from host cell	5	0.360926641	0.001	0.002498236
GO:0044791	positive regulation by host of viral release from host cell	5	0.360926641	0.001	0.002498236
GO:0045197	establishment or maintenance of epithelial cell apical/basal polarity	18	0.360918061	0.0032	0.005044175
GO:0021904	dorsal/ventral neural tube patterning	12	0.360918061	< 0.001	< 0.001
GO:0006353	DNA-templated transcription, termination	53	0.360850878	< 0.001	< 0.001
GO:0042090	interleukin-12 biosynthetic process	5	0.360823681	0.0002	0.001341684
GO:0045075	regulation of interleukin-12 biosynthetic process	5	0.360823681	0.0002	0.001341684
GO:1903205	regulation of hydrogen peroxide-induced cell death	14	0.360654532	0.0012	0.002741075
GO:0098840	protein transport along microtubule	37	0.360541236	< 0.001	< 0.001
GO:0099118	microtubule-based protein transport	37	0.360541236	< 0.001	< 0.001
GO:1905214	regulation of RNA binding	8	0.360456885	0.0012	0.002741075
GO:0060379	cardiac muscle cell myoblast differentiation	7	0.360397132	0.0088	0.010992238
GO:0022616	DNA strand elongation	12	0.36033891	0.0005	0.002177563
GO:0043171	peptide catabolic process	10	0.3602574	0.0008	0.002313249
GO:0008210	estrogen metabolic process	14	0.360250046	0.0028	0.004605006
GO:0072710	response to hydroxyurea	6	0.36023166	0.0034	0.005263104
GO:0060253	negative regulation of glial cell proliferation	10	0.36020592	0.0025	0.004279479
GO:0051150	regulation of smooth muscle cell differentiation	20	0.36019305	0.0006	0.002177563
GO:0034340	response to type I interferon	56	0.360093767	0.0012	0.002741075
GO:0031053	primary miRNA processing	6	0.36001716	0.0028	0.004605006
GO:0035457	cellular response to interferon-alpha	8	0.35997426	0.001	0.002498236
GO:0044827	modulation by host of viral genome replication	10	0.3598713	0.0003	0.001720882
GO:1903613	regulation of protein tyrosine phosphatase activity	5	0.35953668	0.008	0.010168329
GO:0046184	aldehyde biosynthetic process	7	0.359514617	0.0013	0.002859821
GO:0002230	positive regulation of defense response to virus by host	17	0.359497312	< 0.001	< 0.001
GO:0050966	detection of mechanical stimulus involved in sensory perception of pain	6	0.359073359	0.0065	0.008621124
GO:0033574	response to testosterone	28	0.359018202	0.0009	0.002399874
GO:0001840	neural plate development	5	0.358970399	0.0001	0.000877824
GO:0034341	response to interferon-gamma	99	0.358914759	0.0009	0.002399874
GO:0034123	positive regulation of toll-like receptor signaling pathway	14	0.35885273	0.0002	0.001341684
GO:0051148	negative regulation of muscle cell differentiation	41	0.358841071	0.0007	0.002248538
GO:0046134	pyrimidine nucleoside biosynthetic process	22	0.358734059	0.0005	0.002177563
GO:0048668	collateral sprouting	12	0.358494208	0.0014	0.002975058
GO:0048368	lateral mesoderm development	7	0.358411473	< 0.001	< 0.001
GO:0003081	regulation of systemic arterial blood pressure by renin-angiotensin	8	0.358365508	0.0017	0.003334595
GO:0071604	transforming growth factor beta production	18	0.358229658	0.0006	0.002177563
GO:0030223	neutrophil differentiation	7	0.358227615	0.002	0.003693422
GO:0035455	response to interferon-alpha	17	0.358180029	0.0015	0.003088691
GO:0071459	protein localization to chromosome, centromeric region	13	0.358142758	0.0007	0.002248538
GO:0042073	intraciliary transport	33	0.358137358	< 0.001	< 0.001
GO:0048087	positive regulation of developmental pigmentation	6	0.358129558	0.0002	0.001341684
GO:0045779	negative regulation of bone resorption	6	0.357957958	0.0047	0.006644432
GO:0010826	negative regulation of centrosome duplication	10	0.357940798	0.0005	0.002177563
GO:0007549	dosage compensation	12	0.357936508	0.0006	0.002177563
GO:1903363	negative regulation of cellular protein catabolic process	43	0.357852204	0.0008	0.002313249
GO:0061756	leukocyte adhesion to vascular endothelial cell	20	0.357773488	0.0016	0.003223775
GO:0043379	memory T cell differentiation	5	0.357683398	0.0109	0.013101792
GO:0090715	immunological memory formation process	5	0.357683398	0.0109	0.013101792
GO:0019080	viral gene expression	161	0.357679241	0.0007	0.002248538
GO:0019915	lipid storage	37	0.357556785	0.0008	0.002313249
GO:0070168	negative regulation of biomineral tissue development	16	0.35751287	0.0006	0.002177563
GO:0071025	RNA surveillance	7	0.357418643	0.0029	0.004714719
GO:0019062	virion attachment to host cell	5	0.357323037	0.0067	0.008845888
GO:0032905	transforming growth factor beta1 production	5	0.357323037	0.0148	0.017062301
GO:0090201	negative regulation of release of cytochrome c from mitochondria	11	0.357294957	0.0001	0.000877824
GO:0045978	negative regulation of nucleoside metabolic process	12	0.357293007	0.0029	0.004714719
GO:1903579	negative regulation of ATP metabolic process	12	0.357293007	0.0029	0.004714719
GO:0007140	male meiosis	13	0.357212157	0.0008	0.002313249
GO:0090205	positive regulation of cholesterol metabolic process	6	0.357142857	0.0009	0.002399874
GO:0035914	skeletal muscle cell differentiation	33	0.357099957	0.0021	0.003804235
GO:0003093	regulation of glomerular filtration	6	0.357099957	0.0176	0.019838132
GO:0061314	Notch signaling involved in heart development	7	0.3570877	< 0.001	< 0.001
GO:0042053	regulation of dopamine metabolic process	10	0.356988417	0.002	0.003693422
GO:0042069	regulation of catecholamine metabolic process	10	0.356988417	0.002	0.003693422
GO:0031646	positive regulation of neurological system process	15	0.356911197	0.0022	0.003910907
GO:0036462	TRAIL-activated apoptotic signaling pathway	6	0.356670957	0.0041	0.005981983
GO:1900034	regulation of cellular response to heat	50	0.356607465	0.0008	0.002313249
GO:0045136	development of secondary sexual characteristics	7	0.356499356	0.0014	0.002975058
GO:0035872	nucleotide-binding domain, leucine rich repeat containing receptor signaling pathway	20	0.356486486	0.0007	0.002248538
GO:0045589	regulation of regulatory T cell differentiation	16	0.356483269	0.0027	0.004491543
GO:0072528	pyrimidine-containing compound biosynthetic process	28	0.356379849	0.0005	0.002177563
GO:0030970	retrograde protein transport, ER to cytosol	23	0.356230765	0.001	0.002498236
GO:1903513	endoplasmic reticulum to cytosol transport	23	0.356230765	0.001	0.002498236
GO:2000849	regulation of glucocorticoid secretion	6	0.356156156	< 0.001	< 0.001
GO:0002861	regulation of inflammatory response to antigenic stimulus	12	0.356070356	0.001	0.002498236
GO:0009048	dosage compensation by inactivation of X chromosome	10	0.355984556	0.0023	0.004032435
GO:0040015	negative regulation of multicellular organism growth	6	0.355984556	< 0.001	< 0.001
GO:0034501	protein localization to kinetochore	10	0.355958816	0.0006	0.002177563
GO:0006346	methylation-dependent chromatin silencing	12	0.355920206	0.0009	0.002399874
GO:1903427	negative regulation of reactive oxygen species biosynthetic process	16	0.355904118	0.0006	0.002177563
GO:0019372	lipoxygenase pathway	9	0.355870156	0.0008	0.002313249

GOIDlabel	GOName	size	categoryScores	pValPerm	pValPermCorr
GO:0051788	response to misfolded protein	11	0.355820756	0.0002	0.001341684
GO:0002755	MyD88-dependent toll-like receptor signaling pathway	22	0.355785656	0.0003	0.001720882
GO:0039530	MDA-5 signaling pathway	7	0.355763927	0.0091	0.011322589
GO:0045019	negative regulation of nitric oxide biosynthetic process	8	0.355759331	0.0021	0.003804235
GO:1904406	negative regulation of nitric oxide metabolic process	8	0.355759331	0.0021	0.003804235
GO:0060765	regulation of androgen receptor signaling pathway	19	0.35575425	0.0029	0.004714719
GO:0006891	intra-Golgi vesicle-mediated transport	13	0.355746956	0.0015	0.003088691
GO:0009651	response to salt stress	15	0.355744316	0.0001	0.000877824
GO:0009218	pyrimidine ribonucleotide metabolic process	15	0.355675676	0.0033	0.005149584
GO:0101023	vascular endothelial cell proliferation	13	0.355667756	0.0001	0.000877824
GO:1905562	regulation of vascular endothelial cell proliferation	13	0.355667756	0.0001	0.000877824
GO:0000731	DNA synthesis involved in DNA repair	36	0.355648506	0.0005	0.002177563
GO:0042558	pteridine-containing compound metabolic process	25	0.355583012	0.0008	0.002313249
GO:0007221	positive regulation of transcription of Notch receptor target	14	0.355469755	0.0022	0.003910907
GO:1902107	positive regulation of leukocyte differentiation	78	0.355436755	0.0019	0.003579279
GO:2000001	regulation of DNA damage checkpoint	12	0.355426855	0.0002	0.001341684
GO:0018208	peptidyl-proline modification	28	0.355414598	< 0.001	< 0.001
GO:0042769	DNA damage response, detection of DNA damage	28	0.355405405	< 0.001	< 0.001
GO:1990748	cellular detoxification	60	0.355272415	0.0002	0.001341684
GO:0060628	regulation of ER to Golgi vesicle-mediated transport	10	0.355238095	0.0058	0.007856196
GO:0070206	protein trimerization	31	0.355220658	0.0006	0.002177563
GO:0042953	lipoprotein transport	5	0.355109395	< 0.001	< 0.001
GO:0044872	lipoprotein localization	5	0.355109395	< 0.001	< 0.001
GO:0001914	regulation of T cell mediated cytotoxicity	14	0.355028498	0.0012	0.002741075
GO:0050665	hydrogen peroxide biosynthetic process	8	0.355019305	0.0004	0.002007244
GO:0035088	establishment or maintenance of apical/basal cell polarity	20	0.354980695	0.0042	0.006097175
GO:0061245	establishment or maintenance of bipolar cell polarity	20	0.354980695	0.0042	0.006097175
GO:1905314	semi-lunar valve development	21	0.354893669	< 0.001	< 0.001
GO:1902894	negative regulation of pri-miRNA transcription from RNA polymerase II promoter	5	0.354851995	0.0009	0.002399874
GO:0051894	positive regulation of focal adhesion assembly	15	0.354800515	0.0019	0.003579279
GO:0045661	regulation of myoblast differentiation	23	0.354787085	0.0005	0.002177563
GO:0071634	regulation of transforming growth factor beta production	16	0.354777992	0.0007	0.002248538
GO:0046426	negative regulation of JAK-STAT cascade	10	0.354671815	0.0006	0.002177563
GO:1904893	negative regulation of STAT cascade	10	0.354671815	0.0006	0.002177563
GO:1905709	negative regulation of membrane permeability	7	0.354660783	0.0034	0.005263104
GO:0035722	interleukin-12-mediated signaling pathway	30	0.354628915	0.0002	0.001341684
GO:0071349	cellular response to interleukin-12	30	0.354628915	0.0002	0.001341684
GO:0045604	regulation of epidermal cell differentiation	27	0.354602221	0.0008	0.002313249
GO:1903800	positive regulation of production of miRNAs involved in gene silencing by miRNA	5	0.354440154	0.0109	0.013101792
GO:0000154	rRNA modification	20	0.354427284	0.0007	0.002248538
GO:0090084	negative regulation of inclusion body assembly	8	0.354375804	0.0012	0.002741075
GO:2000121	regulation of removal of superoxide radicals	8	0.354375804	0.0014	0.002975058
GO:0000387	spliceosomal snRNP assembly	27	0.354325754	0.0001	0.000877824
GO:0007099	centriole replication	18	0.354225654	0.0005	0.002177563
GO:0090069	regulation of ribosome biogenesis	12	0.354225654	0.0003	0.001720882
GO:2000232	regulation of rRNA processing	12	0.354225654	0.0003	0.001720882
GO:0003188	heart valve formation	8	0.354214929	0.0001	0.000877824
GO:0045624	positive regulation of T-helper cell differentiation	10	0.354157014	0.0019	0.003579279
GO:0043615	astrocyte cell migration	5	0.354131274	0.0016	0.003223775
GO:0090239	regulation of histone H4 acetylation	9	0.353982554	0.0001	0.000877824
GO:0000715	nucleotide-excision repair, DNA damage recognition	17	0.353940495	0.0002	0.001341684
GO:0044806	G-quadruplex DNA unwinding	7	0.353851811	0.0022	0.003910907
GO:0006413	translational initiation	157	0.353815508	0.0008	0.002313249
GO:0034755	iron ion transmembrane transport	8	0.353796654	0.0001	0.000877824
GO:0046642	negative regulation of alpha-beta T cell proliferation	6	0.353796654	0.005	0.006974174
GO:0002507	tolerance induction	14	0.353778268	0.0013	0.002859821
GO:0043313	regulation of neutrophil degranulation	5	0.353770914	0.0111	0.013284563
GO:0032988	ribonucleoprotein complex disassembly	11	0.353667954	0.0006	0.002177563
GO:0003085	negative regulation of systemic arterial blood pressure	6	0.353667954	0.0014	0.002975058
GO:0071605	monocyte chemotactic protein-1 production	8	0.353635779	0.0049	0.006860215
GO:0071637	regulation of monocyte chemotactic protein-1 production	8	0.353635779	0.0049	0.006860215
GO:0070423	nucleotide-binding oligomerization domain containing signaling pathway	19	0.353559575	0.0005	0.002177563
GO:0090557	establishment of endothelial intestinal barrier	5	0.353513514	0.0006	0.002177563
GO:0050657	nucleic acid transport	121	0.353485008	0.0007	0.002248538
GO:0050658	RNA transport	121	0.353485008	0.0007	0.002248538
GO:0033077	T cell differentiation in thymus	32	0.353410553	0.0007	0.002248538
GO:0071594	thymocyte aggregation	32	0.353410553	0.0007	0.002248538
GO:0046606	negative regulation of centrosome cycle	11	0.353363753	0.0006	0.002177563
GO:0036109	alpha-linolenic acid metabolic process	10	0.353333333	0.0049	0.006860215
GO:0060603	mammary gland duct morphogenesis	15	0.353324753	0.0005	0.002177563
GO:0002717	positive regulation of natural killer cell mediated immunity	10	0.353178893	0.0003	0.001720882
GO:0045652	regulation of megakaryocyte differentiation	33	0.353168753	0.0001	0.000877824
GO:0060457	negative regulation of digestive system process	9	0.353124553	< 0.001	< 0.001
GO:0070670	response to interleukin-4	19	0.353085416	0.0002	0.001341684
GO:1904380	endoplasmic reticulum mannose trimming	13	0.352875953	0.0004	0.002007244
GO:0010592	positive regulation of lamellipodium assembly	7	0.352858981	0.0012	0.002741075
GO:0043383	negative T cell selection	6	0.352767053	0.0005	0.002177563
GO:0045060	negative thymic T cell selection	6	0.352767053	0.0005	0.002177563
GO:0050849	negative regulation of calcium-mediated signaling	10	0.352741313	0.001	0.002498236
GO:0098754	detoxification	62	0.35273384	< 0.001	< 0.001
GO:0072643	interferon-gamma secretion	9	0.352724153	0.009	0.011215682
GO:2000483	negative regulation of interleukin-8 secretion	5	0.352689833	0.0018	0.003459409
GO:0071478	cellular response to radiation	105	0.352648158	0.0005	0.002177563

GOIDlabel	GOName	size	categoryScores	pValPerm	pValPermCorr
GO:1902624	positive regulation of neutrophil migration	16	0.352606178	0.003	0.004825593
GO:1904886	beta-catenin destruction complex disassembly	10	0.352561133	0.0018	0.003459409
GO:0099022	vesicle tethering	7	0.352491267	0.0001	0.000877824
GO:0034036	purine ribonucleoside bisphosphate biosynthetic process	5	0.352483912	0.0015	0.003088691
GO:0050428	3'-phosphoadenosine 5'-phosphosulfate biosynthetic process	5	0.352483912	0.0015	0.003088691
GO:0006297	nucleotide-excision repair, DNA gap filling	18	0.352180752	0.0011	0.002610912
GO:0045064	T-helper 2 cell differentiation	8	0.352123552	0.0024	0.004156304
GO:0060837	blood vessel endothelial cell differentiation	7	0.352086781	0.0006	0.002177563
GO:0006221	pyrimidine nucleotide biosynthetic process	20	0.352007722	0.0016	0.003223775
GO:0042092	type 2 immune response	15	0.351986272	0.0004	0.002007244
GO:0009948	anterior/posterior axis specification	19	0.351960984	< 0.001	< 0.001
GO:1904036	negative regulation of epithelial cell apoptotic process	23	0.351944491	0.0006	0.002177563
GO:0007143	female meiotic division	13	0.351866152	0.0009	0.002399874
GO:0016973	poly(A)+ mRNA export from nucleus	6	0.351823252	0.001	0.002498236
GO:0071359	cellular response to dsRNA	50	0.351619048	0.001	0.002498236
GO:0007492	endoderm development	40	0.351544402	0.0002	0.001341684
GO:0030214	hyaluronan catabolic process	8	0.351480051	< 0.001	< 0.001
GO:0006957	complement activation, alternative pathway	6	0.351480051	0.0003	0.001720882
GO:0002710	negative regulation of T cell mediated immunity	7	0.351461666	0.0002	0.001341684
GO:1901798	positive regulation of signal transduction by p53 class mediator	9	0.351437151	0.0022	0.003910907
GO:0045591	positive regulation of regulatory T cell differentiation	9	0.351408551	0.0107	0.012912536
GO:0051168	nuclear export	131	0.351365106	0.0007	0.002248538
GO:2000403	positive regulation of lymphocyte migration	15	0.351334191	0.0031	0.004916949
GO:2000406	positive regulation of T cell migration	15	0.351334191	0.0031	0.004916949
GO:1904996	positive regulation of leukocyte adhesion to vascular endothelial cell	9	0.351236951	0.0019	0.003579279
GO:0035437	maintenance of protein localization in endoplasmic reticulum	5	0.351145431	0.0059	0.007964563
GO:0035728	response to hepatocyte growth factor	9	0.351093951	0.0039	0.005778819
GO:0048662	negative regulation of smooth muscle cell proliferation	22	0.351093951	0.0026	0.004377754
GO:0060766	negative regulation of androgen receptor signaling pathway	8	0.351061776	0.0018	0.003459409
GO:0051236	establishment of RNA localization	122	0.350948372	0.0007	0.002248538
GO:0051665	membrane raft localization	5	0.350888031	0.0043	0.006204188
GO:0045793	positive regulation of cell size	6	0.350879451	< 0.001	< 0.001
GO:0007042	lysosomal lumen acidification	8	0.350868726	0.0136	0.015813319
GO:0060716	labyrinthine layer blood vessel development	13	0.350836551	0.0001	0.000877824
GO:0015074	DNA integration	9	0.350836551	0.0001	0.000877824
GO:0031055	chromatin remodeling at centromere	17	0.350730562	< 0.001	< 0.001
GO:1900037	regulation of cellular response to hypoxia	6	0.350707851	0.0003	0.001720882
GO:0010830	regulation of myotube differentiation	23	0.350512003	0.0118	0.013977955
GO:0000027	ribosomal large subunit assembly	7	0.350432065	0.0068	0.008946408
GO:0002479	antigen processing and presentation of exogenous peptide antigen via MHC class I, TAP-dependent	59	0.350426456	0.0005	0.002177563
GO:0043922	negative regulation by host of viral transcription	6	0.35040755	0.0031	0.004916949
GO:0060242	contact inhibition	7	0.350284979	< 0.001	< 0.001
GO:0034105	positive regulation of tissue remodeling	12	0.35027885	0.0072	0.009362497
GO:1903573	negative regulation of response to endoplasmic reticulum stress	32	0.350176963	0.0013	0.002859821
GO:0071826	ribonucleoprotein complex subunit organization	139	0.350042128	0.0001	0.000877824
GO:0072182	regulation of nephron tubule epithelial cell differentiation	7	0.349843721	< 0.001	< 0.001
GO:0036342	post-anal tail morphogenesis	6	0.34980695	0.0002	0.001341684
GO:1901031	regulation of response to reactive oxygen species	20	0.34970399	0.001	0.002498236
GO:0015669	gas transport	9	0.34952095	0.0033	0.005149584
GO:0070671	response to interleukin-12	31	0.349516337	0.0003	0.001720882
GO:0043368	positive T cell selection	11	0.34950275	0.0042	0.006097175
GO:0009650	UV protection	8	0.349420849	0.0004	0.002007244
GO:0044033	multi-organism metabolic process	174	0.349388305	0.0006	0.002177563
GO:0046719	regulation by virus of viral protein levels in host cell	7	0.349292149	0.0012	0.002741075
GO:0046951	ketone body biosynthetic process	5	0.349292149	0.0118	0.013977955
GO:0051984	positive regulation of chromosome segregation	17	0.349125596	0.0023	0.004032435
GO:0030853	negative regulation of granulocyte differentiation	5	0.349086229	< 0.001	< 0.001
GO:0061512	protein localization to cilium	25	0.349045045	< 0.001	< 0.001
GO:0022618	ribonucleoprotein complex assembly	130	0.349044649	< 0.001	< 0.001
GO:0032790	ribosome disassembly	6	0.348991849	0.0006	0.002177563
GO:2000114	regulation of establishment of cell polarity	13	0.348658549	0.0018	0.003459409
GO:0043928	exonucleolytic nuclear-transcribed mRNA catabolic process involved in deadenylation-dependent decay	31	0.3486528	< 0.001	< 0.001
GO:0071353	cellular response to interleukin-4	17	0.348565372	0.0007	0.002248538
GO:0030219	megakaryocyte differentiation	43	0.348424172	0.0007	0.002248538
GO:0000288	nuclear-transcribed mRNA catabolic process, deadenylation-dependent decay	56	0.348400441	< 0.001	< 0.001
GO:0002833	positive regulation of response to biotic stimulus	21	0.348397377	0.0001	0.000877824
GO:0072160	nephron tubule epithelial cell differentiation	8	0.348391248	0.0001	0.000877824
GO:0048681	negative regulation of axon regeneration	5	0.348365508	0.0208	0.022969745
GO:0046638	positive regulation of alpha-beta T cell differentiation	23	0.348307314	0.0058	0.007856196
GO:0009219	pyrimidine deoxyribonucleotide metabolic process	11	0.348262548	0.0016	0.003223775
GO:0019430	removal of superoxide radicals	15	0.348228228	0.0012	0.002741075
GO:0034505	tooth mineralization	5	0.348211068	0.0091	0.011322589
GO:0045580	regulation of T cell differentiation	66	0.348204048	0.0007	0.002248538
GO:0007253	cytoplasmic sequestering of NF-kappaB	5	0.348108108	0.0008	0.002313249
GO:0042345	regulation of NF-kappaB import into nucleus	5	0.348108108	0.0008	0.002313249
GO:0042347	negative regulation of NF-kappaB import into nucleus	5	0.348108108	0.0008	0.002313249
GO:0042348	NF-kappaB import into nucleus	5	0.348108108	0.0008	0.002313249
GO:0031100	animal organ regeneration	46	0.348049913	0.0011	0.002610912
GO:0039531	regulation of viral-induced cytoplasmic pattern recognition receptor signaling pathway	17	0.347990007	< 0.001	< 0.001
GO:0030157	pancreatic juice secretion	9	0.347919348	0.0007	0.002248538
GO:0000291	nuclear-transcribed mRNA catabolic process, exonucleolytic	32	0.347779923	< 0.001	< 0.001
GO:0072074	kidney mesenchyme development	9	0.347776348	0.0001	0.000877824
GO:0006972	hyperosmotic response	18	0.347747748	0.0002	0.001341684

GOIDlabel	GOName	size	categoryScores	pValPerm	pValPermCorr
GO:0033234	negative regulation of protein sumoylation	5	0.347644788	0.0021	0.003804235
GO:0045577	regulation of B cell differentiation	16	0.347619048	0.0002	0.001341684
GO:1902563	regulation of neutrophil activation	7	0.347600662	0.0148	0.017062301
GO:0060831	smoothened signaling pathway involved in dorsal/ventral neural tube patterning	9	0.347518948	< 0.001	< 0.001
GO:0048535	lymph node development	11	0.347513748	0.0002	0.001341684
GO:0001771	immunological synapse formation	10	0.347464607	0.0041	0.005981983
GO:1903798	regulation of production of miRNAs involved in gene silencing by miRNA	10	0.347413127	0.0144	0.016652368
GO:0051383	kinetochore organization	14	0.347398419	0.0001	0.000877824
GO:1902175	regulation of oxidative stress-induced intrinsic apoptotic signaling pathway	18	0.347361647	0.0008	0.002313249
GO:0031123	RNA 3'-end processing	85	0.347281399	0.0001	0.000877824
GO:0035729	cellular response to hepatocyte growth factor stimulus	8	0.347265122	0.0033	0.005149584
GO:1904994	regulation of leukocyte adhesion to vascular endothelial cell	12	0.347211497	0.002	0.003693422
GO:1902475	L-alpha-amino acid transmembrane transport	16	0.347136422	0.001	0.002498236
GO:0035563	positive regulation of chromatin binding	11	0.347045747	0.0001	0.000877824
GO:0034770	histone H4-K20 methylation	7	0.347012318	0.0042	0.006097175
GO:0060760	positive regulation of response to cytokine stimulus	35	0.34694613	0.001	0.002498236
GO:0007220	Notch receptor processing	9	0.346861147	0.0088	0.010992238
GO:0072678	T cell migration	30	0.346761047	0.0013	0.002859821
GO:0010889	regulation of sequestering of triglyceride	8	0.346750322	0.0011	0.002610912
GO:0042104	positive regulation of activated T cell proliferation	9	0.346746747	0.0004	0.002007244
GO:0003401	axis elongation	16	0.346685972	0.0008	0.002313249
GO:0009220	pyrimidine ribonucleotide biosynthetic process	14	0.346644604	0.0031	0.004916949
GO:0046132	pyrimidine ribonucleoside biosynthetic process	14	0.346644604	0.0031	0.004916949
GO:0010885	regulation of cholesterol storage	9	0.346603747	0.0013	0.002859821
GO:0045910	negative regulation of DNA recombination	19	0.346582673	< 0.001	< 0.001
GO:0008089	anterograde axonal transport	20	0.346550837	0.0009	0.002399874
GO:0046835	carbohydrate phosphorylation	14	0.346534289	0.0002	0.001341684
GO:0007100	mitotic centrosome separation	7	0.346534289	0.0019	0.003579279
GO:0051299	centrosome separation	7	0.346534289	0.0019	0.003579279
GO:0019934	cGMP-mediated signaling	10	0.346486486	0.0005	0.002177563
GO:0048714	positive regulation of oligodendrocyte differentiation	12	0.346460746	0.0016	0.003223775
GO:0002501	peptide antigen assembly with MHC protein complex	5	0.346409266	0.008	0.010168329
GO:0098543	detection of other organism	5	0.346306306	0.0001	0.000877824
GO:1905153	regulation of membrane invagination	9	0.346289146	0.0101	0.012328539
GO:0048194	Golgi vesicle budding	5	0.346254826	0.0014	0.002975058
GO:0036475	neuron death in response to oxidative stress	15	0.346151866	0.0008	0.002313249
GO:0009642	response to light intensity	5	0.346100386	0.0006	0.002177563
GO:1901522	positive regulation of transcription from RNA polymerase II promoter involved in cellular response to chemical stimulus	20	0.346036036	0.0008	0.002313249
GO:0048486	parasympathetic nervous system development	6	0.346031746	0.0001	0.000877824
GO:0042990	regulation of transcription factor import into nucleus	9	0.346031746	0.0006	0.002177563
GO:0042991	transcription factor import into nucleus	9	0.346031746	0.0006	0.002177563
GO:0042992	negative regulation of transcription factor import into nucleus	9	0.346031746	0.0006	0.002177563
GO:0042994	cytoplasmic sequestering of transcription factor	9	0.346031746	0.0006	0.002177563
GO:1903894	regulation of IRE1-mediated unfolded protein response	9	0.345974546	0.0086	0.010769857
GO:0006342	chromatin silencing	38	0.345973041	0.0021	0.003804235
GO:0033523	histone H2B ubiquitination	9	0.345917346	0.0078	0.009981807
GO:0033344	cholesterol efflux	22	0.345899146	< 0.001	< 0.001
GO:0002360	T cell lineage commitment	7	0.345872403	0.0007	0.002248538
GO:0002363	alpha-beta T cell lineage commitment	7	0.345872403	0.0007	0.002248538
GO:0035333	Notch receptor processing, ligand-dependent	7	0.345762089	0.0167	0.018954426
GO:0042590	antigen processing and presentation of exogenous peptide antigen via MHC class I	62	0.345613817	0.0008	0.002313249
GO:0044058	regulation of digestive system process	20	0.345611326	< 0.001	< 0.001
GO:0050766	positive regulation of phagocytosis	30	0.345602746	0.0007	0.002248538
GO:0009404	toxin metabolic process	11	0.345594946	0.004	0.005880507
GO:0071364	cellular response to epidermal growth factor stimulus	32	0.345551802	0.001	0.002498236
GO:0046688	response to copper ion	18	0.345545546	0.0002	0.001341684
GO:0038113	interleukin-9-mediated signaling pathway	5	0.345534106	0.0001	0.000877824
GO:0071355	cellular response to interleukin-9	5	0.345534106	0.0001	0.000877824
GO:0008655	pyrimidine-containing compound salvage	8	0.345495495	0.0005	0.002177563
GO:0043097	pyrimidine nucleoside salvage	8	0.345495495	0.0005	0.002177563
GO:0045721	negative regulation of gluconeogenesis	10	0.345353925	0.0005	0.002177563
GO:0006635	fatty acid beta-oxidation	46	0.345336019	0.0007	0.002248538
GO:0035809	regulation of urine volume	11	0.345243945	0.0003	0.001720882
GO:1901620	regulation of smoothened signaling pathway involved in dorsal/ventral neural tube patterning	6	0.345216645	< 0.001	< 0.001
GO:0032239	regulation of nucleobase-containing compound transport	10	0.345173745	0.0009	0.002399874
GO:0060947	cardiac vascular smooth muscle cell differentiation	5	0.345173745	0.0008	0.002313249
GO:2000107	negative regulation of leukocyte apoptotic process	28	0.345136974	0.0009	0.002399874
GO:0032728	positive regulation of interferon-beta production	23	0.345106597	0.0012	0.002741075
GO:0090224	regulation of spindle organization	25	0.345019305	0.0015	0.003088691
GO:0031122	cytoplasmic microtubule organization	16	0.34494852	0.0001	0.000877824
GO:0002524	hypersensitivity	5	0.344916345	0.0006	0.002177563
GO:0002883	regulation of hypersensitivity	5	0.344916345	0.0006	0.002177563
GO:0002885	positive regulation of hypersensitivity	5	0.344916345	0.0006	0.002177563
GO:0031124	mRNA 3'-end processing	75	0.344902617	0.0003	0.001720882
GO:0003348	cardiac endothelial cell differentiation	5	0.344864865	0.0036	0.005475609
GO:0060956	endocardial cell differentiation	5	0.344864865	0.0036	0.005475609
GO:1902622	regulation of neutrophil migration	19	0.344835061	0.0049	0.006860215
GO:0006370	7-methylguanosine mRNA capping	21	0.344830545	0.0001	0.000877824
GO:0014829	vascular smooth muscle contraction	14	0.344732488	0.0039	0.005778819
GO:0006054	N-acetylneuraminate metabolic process	6	0.344530245	0.0127	0.014886353
GO:0034644	cellular response to UV	51	0.344522674	0.0006	0.002177563
GO:0006853	carnitine shuttle	8	0.344465894	0.0012	0.002741075

GOIDlabel	GOName	size	categoryScores	pValPerm	pValPermCorr
GO:1902882	regulation of response to oxidative stress	47	0.34445631	0.0006	0.002177563
GO:0007064	mitotic sister chromatid cohesion	14	0.34441993	0.001	0.002498236
GO:1904469	positive regulation of tumor necrosis factor secretion	10	0.344401544	0.0014	0.002975058
GO:0009798	axis specification	42	0.344364773	< 0.001	< 0.001
GO:0010664	negative regulation of striated muscle cell apoptotic process	19	0.344279618	0.001	0.002498236
GO:0014061	regulation of norepinephrine secretion	7	0.344254459	< 0.001	< 0.001
GO:1902065	response to L-glutamate	5	0.344247104	0.0086	0.010769857
GO:0090150	establishment of protein localization to membrane	191	0.344214222	0.0002	0.001341684
GO:0055078	sodium ion homeostasis	18	0.344172744	0.0003	0.001720882
GO:0010743	regulation of macrophage derived foam cell differentiation	13	0.344104544	0.0016	0.003223775
GO:0046635	positive regulation of alpha-beta T cell activation	31	0.344102628	0.0102	0.012424427
GO:0006457	protein folding	126	0.344017487	0.0002	0.001341684
GO:0007184	SMAD protein import into nucleus	13	0.343965944	0.0037	0.005561344
GO:0060390	regulation of SMAD protein import into nucleus	13	0.343965944	0.0037	0.005561344
GO:2000249	regulation of actin cytoskeleton reorganization	20	0.343951094	< 0.001	< 0.001
GO:0015791	polyol transport	5	0.343886744	< 0.001	< 0.001
GO:0051225	spindle assembly	57	0.343864165	0.0009	0.002399874
GO:0042983	amyloid precursor protein biosynthetic process	9	0.343858144	0.0103	0.012519912
GO:0042984	regulation of amyloid precursor protein biosynthetic process	9	0.343858144	0.0103	0.012519912
GO:0015802	basic amino acid transport	8	0.343822394	0.0058	0.007856196
GO:0002437	inflammatory response to antigenic stimulus	22	0.343804844	0.0004	0.002007244
GO:0072676	lymphocyte migration	42	0.343794815	0.0008	0.002313249
GO:0045814	negative regulation of gene expression, epigenetic	54	0.343786644	0.0006	0.002177563
GO:0050961	detection of temperature stimulus involved in sensory perception	6	0.343758044	< 0.001	< 0.001
GO:0050965	detection of temperature stimulus involved in sensory perception of pain	6	0.343758044	< 0.001	< 0.001
GO:0075733	intracellular transport of virus	51	0.343715144	0.0009	0.002399874
GO:0019985	translesion synthesis	32	0.343709781	0.0005	0.002177563
GO:1901890	positive regulation of cell junction assembly	18	0.343700844	0.0015	0.003088691
GO:0015936	coenzyme A metabolic process	8	0.343661519	0.0001	0.000877824
GO:0051193	regulation of cofactor metabolic process	52	0.343634294	0.001	0.002498236
GO:0039535	regulation of RIG-I signaling pathway	15	0.343595024	< 0.001	< 0.001
GO:0031077	post-embryonic camera-type eye development	6	0.343586444	0.0064	0.008500875
GO:0043691	reverse cholesterol transport	8	0.343500644	< 0.001	< 0.001
GO:0001780	neutrophil homeostasis	8	0.343500644	0.0028	0.004605006
GO:0010501	RNA secondary structure unwinding	7	0.343482258	0.0044	0.006304227
GO:0070571	negative regulation of neuron projection regeneration	6	0.343457743	0.0217	0.023852035
GO:0048672	positive regulation of collateral sprouting	6	0.343457743	0.0013	0.002859821
GO:0060260	regulation of transcription initiation from RNA polymerase II promoter	15	0.343440583	0.0006	0.002177563
GO:0010155	regulation of proton transport	14	0.343390329	0.0013	0.002859821
GO:0009452	7-methylguanosine RNA capping	24	0.343382668	0.0001	0.000877824
GO:0036260	RNA capping	24	0.343382668	0.0001	0.000877824
GO:1901998	toxin transport	26	0.343282843	0.0002	0.001341684
GO:0032373	positive regulation of sterol transport	15	0.343148863	0.0002	0.001341684
GO:0032376	positive regulation of cholesterol transport	15	0.343148863	0.0002	0.001341684
GO:0035815	positive regulation of renal sodium excretion	9	0.343143143	0.0001	0.000877824
GO:0098534	centriole assembly	19	0.343141638	0.0006	0.002177563
GO:0046931	pore complex assembly	10	0.343140283	0.0002	0.001341684
GO:0048548	regulation of pinocytosis	9	0.343114543	0.0021	0.003804235
GO:0009262	deoxyribonucleotide metabolic process	20	0.343037323	0.0004	0.002007244
GO:2000345	regulation of hepatocyte proliferation	9	0.343028743	0.0003	0.001720882
GO:0000038	very long-chain fatty acid metabolic process	16	0.343018018	0.0007	0.002248538
GO:2001224	positive regulation of neuron migration	6	0.342942943	0.0012	0.002741075
GO:0010603	regulation of cytoplasmic mRNA processing body assembly	6	0.342857143	0.0006	0.002177563
GO:0046598	positive regulation of viral entry into host cell	6	0.342814243	0.0079	0.010077402
GO:1902946	protein localization to early endosome	5	0.342805663	0.002	0.003693422
GO:1902965	regulation of protein localization to early endosome	5	0.342805663	0.002	0.003693422
GO:1902966	positive regulation of protein localization to early endosome	5	0.342805663	0.002	0.003693422
GO:0006403	RNA localization	145	0.342784361	0.0007	0.002248538
GO:1903707	negative regulation of hemopoiesis	76	0.342755537	0.0002	0.001341684
GO:0090307	mitotic spindle assembly	35	0.342636514	0.0007	0.002248538
GO:0051350	negative regulation of lyase activity	11	0.342623143	0.001	0.002498236
GO:0031047	gene silencing by RNA	95	0.342621418	0.0002	0.001341684
GO:1990403	embryonic brain development	8	0.342599743	0.0008	0.002313249
GO:0042509	regulation of tyrosine phosphorylation of STAT protein	33	0.342521743	< 0.001	< 0.001
GO:0042135	neurotransmitter catabolic process	11	0.342506143	< 0.001	< 0.001
GO:0006984	ER-nucleus signaling pathway	32	0.342495174	0.0034	0.005263104
GO:2000404	regulation of T cell migration	21	0.342464914	0.0013	0.002859821
GO:2000811	negative regulation of anoikis	9	0.342399542	0.008	0.010168329
GO:0045454	cell redox homeostasis	46	0.342370321	0.0002	0.001341684
GO:0001833	inner cell mass cell proliferation	8	0.342342342	0.0023	0.004032435
GO:0018126	protein hydroxylation	17	0.342296919	0.0001	0.000877824
GO:0001919	regulation of receptor recycling	19	0.342288153	0.0038	0.005666214
GO:0045039	protein import into mitochondrial inner membrane	5	0.342239382	0.004	0.005880507
GO:1901799	negative regulation of proteasomal protein catabolic process	26	0.342193842	0.0013	0.002859821
GO:0015727	lactate transport	5	0.342136422	0.0018	0.003459409
GO:0035873	lactate transmembrane transport	5	0.342136422	0.0018	0.003459409
GO:0071482	cellular response to light stimulus	64	0.342105051	0.0006	0.002177563
GO:0042177	negative regulation of protein catabolic process	69	0.342099864	0.0011	0.002610912
GO:0048266	behavioral response to pain	9	0.342027742	0.0026	0.004377754
GO:0017148	negative regulation of translation	147	0.341808281	0.0005	0.002177563
GO:0055094	response to lipoprotein particle	13	0.341807742	0.0016	0.003223775
GO:0071402	cellular response to lipoprotein particle stimulus	13	0.341807742	0.0016	0.003223775
GO:0061014	positive regulation of mRNA catabolic process	21	0.341778513	0.0001	0.000877824

GOIDlabel	GOName	size	categoryScores	pValPerm	pValPermCorr
GO:0032786	positive regulation of DNA-templated transcription, elongation	15	0.341776062	0.0009	0.002399874
GO:0044818	mitotic G2/M transition checkpoint	16	0.341763192	0.0011	0.002610912
GO:0006220	pyrimidine nucleotide metabolic process	30	0.341758902	0.0007	0.002248538
GO:0035195	gene silencing by miRNA	80	0.341753539	0.0005	0.002177563
GO:0033689	negative regulation of osteoblast proliferation	6	0.341741742	0.0057	0.007745437
GO:0035930	corticosteroid hormone secretion	9	0.341741742	< 0.001	< 0.001
GO:0061099	negative regulation of protein tyrosine kinase activity	18	0.341727442	0.0043	0.006204188
GO:0050821	protein stabilization	105	0.341719679	0.0005	0.002177563
GO:0000289	nuclear-transcribed mRNA poly(A) tail shortening	22	0.341698842	0.0001	0.000877824
GO:0060479	lung cell differentiation	10	0.341698842	0.0008	0.002313249
GO:0060487	lung epithelial cell differentiation	10	0.341698842	0.0008	0.002313249
GO:0071385	cellular response to glucocorticoid stimulus	31	0.341678084	0.0001	0.000877824
GO:0010831	positive regulation of myotube differentiation	12	0.341677392	0.0125	0.014684332
GO:0030490	maturation of SSU-rRNA	12	0.341634492	0.0002	0.001341684
GO:0071450	cellular response to oxygen radical	17	0.341615565	0.0016	0.003223775
GO:0071451	cellular response to superoxide	17	0.341615565	0.0016	0.003223775
GO:0045671	negative regulation of osteoclast differentiation	13	0.341589942	0.0028	0.004605006
GO:1902902	negative regulation of autophagosome assembly	11	0.341570142	0.0009	0.002399874
GO:0032570	response to progesterone	23	0.34155895	0.0001	0.000877824
GO:0045581	negative regulation of T cell differentiation	20	0.341544402	0.0001	0.000877824
GO:0021575	hindbrain morphogenesis	22	0.341511642	0.0017	0.003334595
GO:0006301	postreplication repair	37	0.341500574	0.0005	0.002177563
GO:0072665	protein localization to vacuole	25	0.341498069	0.0012	0.002741075
GO:0051170	nuclear import	101	0.341399391	0.0005	0.002177563
GO:0016925	protein sumoylation	46	0.341357507	0.0018	0.003459409
GO:0051196	regulation of coenzyme metabolic process	51	0.341348071	0.001	0.002498236
GO:0006833	water transport	10	0.341338481	< 0.001	< 0.001
GO:0006662	glycerol ether metabolic process	7	0.341312741	0.0053	0.007330056
GO:0000281	mitotic cytokinesis	30	0.341252681	0.0022	0.003910907
GO:2000378	negative regulation of reactive oxygen species metabolic process	31	0.341238012	0.0006	0.002177563
GO:2000279	negative regulation of DNA biosynthetic process	21	0.341165655	0.0007	0.002248538
GO:0014911	positive regulation of smooth muscle cell migration	20	0.341158301	0.0006	0.002177563
GO:1903867	extraembryonic membrane development	5	0.341158301	0.0056	0.007655255
GO:1905208	negative regulation of cardiocyte differentiation	12	0.341119691	0.0008	0.002313249
GO:0006471	protein ADP-ribosylation	20	0.341093951	0.0011	0.002610912
GO:0031503	protein complex localization	48	0.341092879	< 0.001	< 0.001
GO:0035194	posttranscriptional gene silencing by RNA	83	0.341021228	0.0005	0.002177563
GO:0008631	intrinsic apoptotic signaling pathway in response to oxidative stress	26	0.341015741	0.0002	0.001341684
GO:1904357	negative regulation of telomere maintenance via telomere lengthening	17	0.340994776	0.0005	0.002177563
GO:0045619	regulation of lymphocyte differentiation	82	0.340989421	0.0007	0.002248538
GO:0032435	negative regulation of proteasomal ubiquitin-dependent protein catabolic process	20	0.340978121	0.0012	0.002741075
GO:0010872	regulation of cholesterol esterification	5	0.340900901	0.004	0.005880507
GO:0003071	renal system process involved in regulation of systemic arterial blood pressure	9	0.340855141	0.0203	0.022456501
GO:0033866	nucleoside bisphosphate biosynthetic process	9	0.340740741	0.0017	0.003334595
GO:0034030	ribonucleoside bisphosphate biosynthetic process	9	0.340740741	0.0017	0.003334595
GO:0034033	purine nucleoside bisphosphate biosynthetic process	9	0.340740741	0.0017	0.003334595
GO:0060972	left/right pattern formation	11	0.340727741	< 0.001	< 0.001
GO:0030422	production of siRNA involved in RNA interference	6	0.340626341	0.0031	0.004916949
GO:0050870	positive regulation of T cell activation	98	0.340558926	0.0014	0.002975058
GO:0070471	uterine smooth muscle contraction	7	0.340540541	< 0.001	< 0.001
GO:0007340	acrosome reaction	7	0.340503769	0.0003	0.001720882
GO:0042026	protein refolding	13	0.34046134	0.0049	0.006860215
GO:0016458	gene silencing	135	0.34045474	0.0007	0.002248538
GO:0036120	cellular response to platelet-derived growth factor stimulus	12	0.34041184	0.0007	0.002248538
GO:1903053	regulation of extracellular matrix organization	27	0.34036894	0.0022	0.003910907
GO:0002577	regulation of antigen processing and presentation	12	0.34036894	0.0089	0.011106266
GO:0033211	adiponectin-activated signaling pathway	6	0.34032604	0.0028	0.004605006
GO:0032287	peripheral nervous system myelin maintenance	7	0.34028314	0.0047	0.006644432
GO:0043462	regulation of ATPase activity	49	0.340193838	0.0008	0.002313249
GO:0043470	regulation of carbohydrate catabolic process	48	0.340149078	0.0019	0.003579279
GO:0030513	positive regulation of BMP signaling pathway	19	0.340147666	0.0021	0.003804235
GO:0097202	activation of cysteine-type endopeptidase activity	8	0.34009009	0.0012	0.002741075
GO:0032211	negative regulation of telomere maintenance via telomerase	13	0.34006534	0.0004	0.002007244
GO:0048569	post-embryonic animal organ development	12	0.34004719	0.0008	0.002313249
GO:2001046	positive regulation of integrin-mediated signaling pathway	5	0.33997426	0.0007	0.002248538
GO:0006517	protein deglycosylation	17	0.339965175	0.0005	0.002177563
GO:0045668	negative regulation of osteoblast differentiation	21	0.339890911	0.0019	0.003579279
GO:0010667	negative regulation of cardiac muscle cell apoptotic process	17	0.339844046	0.0006	0.002177563
GO:0009112	nucleobase metabolic process	20	0.33972973	0.0025	0.004279479
GO:1902581	multi-organism cellular localization	52	0.33972874	0.0009	0.002399874
GO:1902583	multi-organism intracellular transport	52	0.33972874	0.0009	0.002399874
GO:0036503	ERAD pathway	62	0.339726824	0.0001	0.000877824
GO:0006930	substrate-dependent cell migration, cell extension	7	0.339694797	0.001	0.002498236
GO:0042178	xenobiotic catabolic process	8	0.339607465	0.0005	0.002177563
GO:0090025	regulation of monocyte chemotaxis	13	0.33959014	0.0003	0.001720882
GO:0006188	IMP biosynthetic process	5	0.33956242	0.0031	0.004916949
GO:0046040	IMP metabolic process	5	0.33956242	0.0031	0.004916949
GO:0071622	regulation of granulocyte chemotaxis	23	0.339555705	0.0022	0.003910907
GO:0044319	wound healing, spreading of cells	16	0.339430502	0.0024	0.004156304
GO:0090504	epiboly	16	0.339430502	0.0024	0.004156304
GO:0090505	epiboly involved in wound healing	16	0.339430502	0.0024	0.004156304
GO:0071480	cellular response to gamma radiation	22	0.339429039	0.0004	0.002007244
GO:0007635	chemosensory behavior	5	0.339407979	0.0004	0.002007244

GOIDlabel	GOName	size	categoryScores	pValPerm	pValPermCorr
GO:0016246	RNA interference	9	0.339396539	0.0043	0.006204188
GO:0016441	posttranscriptional gene silencing	84	0.339388368	0.0005	0.002177563
GO:0070841	inclusion body assembly	14	0.339290311	0.0007	0.002248538
GO:0060612	adipose tissue development	26	0.339263439	0.0011	0.002610912
GO:0051382	kinetochore assembly	11	0.339253539	0.0001	0.000877824
GO:0006084	acetyl-CoA metabolic process	25	0.339222651	0.0013	0.002859821
GO:0032096	negative regulation of response to food	10	0.339202059	0.0008	0.002313249
GO:0032099	negative regulation of appetite	10	0.339202059	0.0008	0.002313249
GO:0032105	negative regulation of response to extracellular stimulus	10	0.339202059	0.0008	0.002313249
GO:0032108	negative regulation of response to nutrient levels	10	0.339202059	0.0008	0.002313249
GO:0036473	cell death in response to oxidative stress	47	0.339160437	0.0005	0.002177563
GO:0002328	pro-B cell differentiation	7	0.339106453	0.0001	0.000877824
GO:0048710	regulation of astrocyte differentiation	13	0.339015939	0.0039	0.005778819
GO:1903708	positive regulation of hemopoiesis	105	0.338978979	0.0023	0.004032435
GO:0018344	protein geranylgeranylation	5	0.338944659	0.0083	0.010468217
GO:0016072	rRNA metabolic process	137	0.338941653	0.0001	0.000877824
GO:0060965	negative regulation of gene silencing by miRNA	6	0.338867439	0.0209	0.023072181
GO:0021680	cerebellar Purkinje cell layer development	15	0.338807379	0.0017	0.003334595
GO:0031935	regulation of chromatin silencing	13	0.338778339	0.001	0.002498236
GO:0009595	detection of biotic stimulus	8	0.338706564	< 0.001	< 0.001
GO:0060964	regulation of gene silencing by miRNA	54	0.338629105	0.0006	0.002177563
GO:0070920	regulation of production of small RNA involved in gene silencing by RNA	11	0.338621739	0.0132	0.015415723
GO:0060050	positive regulation of protein glycosylation	6	0.338610039	0.0001	0.000877824
GO:0021681	cerebellar granular layer development	7	0.338554881	0.0014	0.002975058
GO:0009081	branched-chain amino acid metabolic process	19	0.338549075	0.0022	0.003910907
GO:0009083	branched-chain amino acid catabolic process	19	0.338549075	0.0022	0.003910907
GO:0006506	GPI anchor biosynthetic process	23	0.338537295	0.0011	0.002610912
GO:0045898	regulation of RNA polymerase II transcriptional preinitiation complex assembly	6	0.338524239	0.0003	0.001720882
GO:0035307	positive regulation of protein dephosphorylation	17	0.338420774	0.001	0.002498236
GO:1905521	regulation of macrophage migration	21	0.338407796	0.0004	0.002007244
GO:0046950	cellular ketone body metabolic process	6	0.338395538	0.0033	0.005149584
GO:1902224	ketone body metabolic process	6	0.338395538	0.0033	0.005149584
GO:0045954	positive regulation of natural killer cell mediated cytotoxicity	9	0.338395538	0.0001	0.000877824
GO:0006364	rRNA processing	132	0.338317538	0.0001	0.000877824
GO:1900543	negative regulation of purine nucleotide metabolic process	21	0.338272967	0.0027	0.004491543
GO:0001975	response to amphetamine	14	0.338242324	0.0001	0.000877824
GO:0090501	RNA phosphodiester bond hydrolysis	95	0.338194134	< 0.001	< 0.001
GO:0061614	pri-miRNA transcription from RNA polymerase II promoter	27	0.338166738	0.0004	0.002007244
GO:0048563	post-embryonic animal organ morphogenesis	7	0.338113624	0.0003	0.001720882
GO:0030903	notochord development	9	0.338109538	0.0031	0.004916949
GO:0043162	ubiquitin-dependent protein catabolic process via the multivesicular body sorting pathway	12	0.338095238	0.0042	0.006097175
GO:0046839	phospholipid dephosphorylation	20	0.338082368	0.0037	0.005561344
GO:0042531	positive regulation of tyrosine phosphorylation of STAT protein	28	0.338076852	< 0.001	< 0.001
GO:0009411	response to UV	86	0.338059322	0.0006	0.002177563
GO:0034249	negative regulation of cellular amide metabolic process	158	0.33801867	0.0005	0.002177563
GO:0007595	lactation	30	0.338009438	0.001	0.002498236
GO:1903025	regulation of RNA polymerase II regulatory region sequence-specific DNA binding	8	0.337934363	0.0061	0.008182551
GO:0010758	regulation of macrophage chemotaxis	12	0.337837838	0.0001	0.000877824
GO:0002098	tRNA wobble uridine modification	8	0.337773488	0.0031	0.004916949
GO:0000272	polysaccharide catabolic process	15	0.337726298	0.002	0.003693422
GO:0044247	cellular polysaccharide catabolic process	15	0.337726298	0.002	0.003693422
GO:0046794	transport of virus	53	0.337694568	0.001	0.002498236
GO:0001954	positive regulation of cell-matrix adhesion	31	0.337675925	0.0012	0.002741075
GO:0009062	fatty acid catabolic process	63	0.337631509	0.0011	0.002610912
GO:0043331	response to dsRNA	61	0.337586767	0.0012	0.002741075
GO:0002792	negative regulation of peptide secretion	23	0.337563651	0.0008	0.002313249
GO:0050856	regulation of T cell receptor signaling pathway	21	0.33752528	0.0001	0.000877824
GO:1901797	negative regulation of signal transduction by p53 class mediator	21	0.337500766	< 0.001	< 0.001
GO:0070849	response to epidermal growth factor	34	0.337459308	0.0012	0.002741075
GO:1903201	regulation of oxidative stress-induced cell death	38	0.337458511	0.0009	0.002399874
GO:1900112	regulation of histone H3-K9 trimethylation	7	0.337451737	0.0043	0.006204188
GO:0045824	negative regulation of innate immune response	28	0.337433352	0.0014	0.002975058
GO:0046633	alpha-beta T cell proliferation	20	0.337413127	0.0119	0.014078095
GO:1902993	positive regulation of amyloid precursor protein catabolic process	6	0.337408837	0.0038	0.005666214
GO:0060119	inner ear receptor cell development	24	0.337387387	0.0001	0.000877824
GO:0006379	mRNA cleavage	16	0.337387387	< 0.001	< 0.001
GO:1902188	positive regulation of viral release from host cell	12	0.337323037	0.001	0.002498236
GO:0035912	dorsal aorta morphogenesis	6	0.337237237	0.0021	0.003804235
GO:0000717	nucleotide-excision repair, DNA duplex unwinding	15	0.337211497	0.0004	0.002007244
GO:0046676	negative regulation of insulin secretion	21	0.337206594	0.0013	0.002859821
GO:0002315	marginal zone B cell differentiation	6	0.337194337	0.0048	0.006748379
GO:0061462	protein localization to lysosome	23	0.337104807	0.0017	0.003334595
GO:0002704	negative regulation of leukocyte mediated immunity	21	0.33709628	0.0001	0.000877824
GO:1901385	regulation of voltage-gated calcium channel activity	11	0.337030537	0.0001	0.000877824
GO:0006400	tRNA modification	47	0.337024563	0.0006	0.002177563
GO:0071384	cellular response to corticosteroid stimulus	35	0.33701048	0.0003	0.001720882
GO:0060968	regulation of gene silencing	69	0.336992894	0.0007	0.002248538
GO:0006110	regulation of glycolytic process	43	0.336960881	0.0019	0.003579279
GO:0070472	regulation of uterine smooth muscle contraction	6	0.336936937	< 0.001	< 0.001
GO:0002763	positive regulation of myeloid leukocyte differentiation	30	0.336936937	0.0018	0.003459409
GO:0048147	negative regulation of fibroblast proliferation	22	0.336878437	0.0002	0.001341684
GO:0035690	cellular response to drug	34	0.336868802	0.0009	0.002399874
GO:0021695	cerebellar cortex development	26	0.336847837	0.0011	0.002610912

GOIDlabel	GOName	size	categoryScores	pValPerm	pValPermCorr
GO:0030449	regulation of complement activation	26	0.336837937	0.0048	0.006748379
GO:2000257	regulation of protein activation cascade	26	0.336837937	0.0048	0.006748379
GO:0031647	regulation of protein stability	170	0.336723446	0.0008	0.002313249
GO:0001895	retina homeostasis	34	0.336634113	0.0001	0.000877824
GO:0060147	regulation of posttranscriptional gene silencing	56	0.336578415	0.0006	0.002177563
GO:0060966	regulation of gene silencing by RNA	56	0.336578415	0.0006	0.002177563
GO:0006336	DNA replication-independent nucleosome assembly	20	0.336576577	< 0.001	< 0.001
GO:0034724	DNA replication-independent nucleosome organization	20	0.336576577	< 0.001	< 0.001
GO:1901977	negative regulation of cell cycle checkpoint	5	0.336576577	0.0007	0.002248538
GO:0051145	smooth muscle cell differentiation	34	0.336558407	0.0003	0.001720882
GO:2000401	regulation of lymphocyte migration	28	0.336541644	0.001	0.002498236
GO:1901550	regulation of endothelial cell development	8	0.336486486	0.0041	0.005981983
GO:1903140	regulation of establishment of endothelial barrier	8	0.336486486	0.0041	0.005981983
GO:0070120	ciliary neurotrophic factor-mediated signaling pathway	5	0.336473616	0.0044	0.006304227
GO:0006004	fucose metabolic process	10	0.336396396	0.0001	0.000877824
GO:0097306	cellular response to alcohol	28	0.336376172	< 0.001	< 0.001
GO:1900115	extracellular regulation of signal transduction	5	0.336267696	0.0003	0.001720882
GO:1900116	extracellular negative regulation of signal transduction	5	0.336267696	0.0003	0.001720882
GO:0009118	regulation of nucleoside metabolic process	65	0.336208296	0.0009	0.002399874
GO:1903578	regulation of ATP metabolic process	65	0.336208296	0.0009	0.002399874
GO:0030433	ER-associated ubiquitin-dependent protein catabolic process	48	0.336159374	0.0002	0.001341684
GO:1903039	positive regulation of leukocyte cell-cell adhesion	106	0.336096744	0.0014	0.002975058
GO:1900407	regulation of cellular response to oxidative stress	45	0.336096096	0.0011	0.002610912
GO:0090503	RNA phosphodiester bond hydrolysis, exonucleolytic	29	0.336084853	0.0015	0.003088691
GO:0010824	regulation of centrosome duplication	24	0.336078936	0.0001	0.000877824
GO:0046677	response to antibiotic	21	0.336042165	0.0007	0.002248538
GO:0014044	Schwann cell development	20	0.336036036	0.0026	0.004377754
GO:0010656	negative regulation of muscle cell apoptotic process	22	0.335942436	0.0017	0.003334595
GO:0007569	cell aging	59	0.335929149	0.0007	0.002248538
GO:1902004	positive regulation of beta-amyloid formation	5	0.335907336	0.0015	0.003088691
GO:0031581	hemidesmosome assembly	6	0.335864436	0.002	0.003693422
GO:0051568	histone H3-K4 methylation	27	0.335821536	< 0.001	< 0.001
GO:0019852	L-ascorbic acid metabolic process	6	0.335692836	0.0177	0.019919142
GO:0033865	nucleoside bisphosphate metabolic process	20	0.335688546	0.0001	0.000877824
GO:0033875	ribonucleoside bisphosphate metabolic process	20	0.335688546	0.0001	0.000877824
GO:0034032	purine nucleoside bisphosphate metabolic process	20	0.335688546	0.0001	0.000877824
GO:0002715	regulation of natural killer cell mediated immunity	13	0.335649936	0.0005	0.002177563
GO:0042269	regulation of natural killer cell mediated cytotoxicity	13	0.335649936	0.0005	0.002177563
GO:0014037	Schwann cell differentiation	23	0.335627553	0.0024	0.004156304
GO:0046940	nucleoside monophosphate phosphorylation	10	0.335624196	0.0004	0.002007244
GO:0007062	sister chromatid cohesion	28	0.335613164	0.0006	0.002177563
GO:0070932	histone H3 deacetylation	14	0.335558007	0.0015	0.003088691
GO:0045920	negative regulation of exocytosis	19	0.335487367	0.0013	0.002859821
GO:0043369	CD4-positive or CD8-positive, alpha-beta T cell lineage commitment	6	0.335435435	0.0004	0.002007244
GO:0060216	definitive hemopoiesis	12	0.335371085	0.001	0.002498236
GO:0010606	positive regulation of cytoplasmic mRNA processing body assembly	5	0.335341055	0.0051	0.007095023
GO:0009147	pyrimidine nucleoside triphosphate metabolic process	12	0.335328185	0.0037	0.005561344
GO:0070102	interleukin-6-mediated signaling pathway	13	0.335313335	0.0003	0.001720882
GO:0090394	negative regulation of excitatory postsynaptic potential	5	0.335289575	0.0001	0.000877824
GO:0045616	regulation of keratinocyte differentiation	18	0.335235235	0.0047	0.006644432
GO:0009451	RNA modification	90	0.335132275	0.0009	0.002399874
GO:0007141	male meiosis I	7	0.335098364	0.0026	0.004377754
GO:0001967	suckling behavior	6	0.335092235	0.001	0.002498236
GO:0040029	regulation of gene expression, epigenetic	182	0.334944206	0.0006	0.002177563
GO:0010832	negative regulation of myotube differentiation	8	0.334942085	0.0115	0.013693853
GO:0043201	response to leucine	5	0.334929215	0.0035	0.005358035
GO:0051972	regulation of telomerase activity	32	0.33490991	0.0001	0.000877824
GO:0014075	response to amine	20	0.334787645	< 0.001	< 0.001
GO:0010332	response to gamma radiation	37	0.334773383	0.0004	0.002007244
GO:0042306	regulation of protein import into nucleus	54	0.334768101	0.0006	0.002177563
GO:0045066	regulatory T cell differentiation	18	0.334720435	0.0039	0.005778819
GO:2000515	negative regulation of CD4-positive, alpha-beta T cell activation	17	0.334711182	0.0001	0.000877824
GO:1902236	negative regulation of endoplasmic reticulum stress-induced intrinsic apoptotic signaling pathway	12	0.334620335	0.0036	0.005475609
GO:0043486	histone exchange	23	0.334575569	0.0001	0.000877824
GO:0051053	negative regulation of DNA metabolic process	83	0.334564513	0.0004	0.002007244
GO:0000726	non-recombinational repair	48	0.334539897	0.0002	0.001341684
GO:0002069	columnar/cuboidal epithelial cell maturation	6	0.334534535	0.0167	0.018954426
GO:0001682	tRNA 5'-leader removal	6	0.334491634	0.0002	0.001341684
GO:1902893	regulation of pri-miRNA transcription from RNA polymerase II promoter	23	0.334486039	0.0003	0.001720882
GO:0002863	positive regulation of inflammatory response to antigenic stimulus	9	0.334477334	0.0012	0.002741075
GO:0071549	cellular response to dexamethasone stimulus	18	0.334477334	< 0.001	< 0.001
GO:0032527	protein exit from endoplasmic reticulum	32	0.334467503	0.002	0.003693422
GO:1903393	positive regulation of adherens junction organization	18	0.334463034	0.0016	0.003223775
GO:0061042	vascular wound healing	8	0.334459459	0.0106	0.012825859
GO:0032469	endoplasmic reticulum calcium ion homeostasis	14	0.334436477	0.0136	0.015813319
GO:0090085	regulation of protein deubiquitination	9	0.334248534	0.0011	0.002610912
GO:0036037	CD8-positive, alpha-beta T cell activation	8	0.334202059	0.0134	0.015626386
GO:0007519	skeletal muscle tissue development	74	0.334161188	0.0035	0.005358035
GO:1903557	positive regulation of tumor necrosis factor superfamily cytokine production	48	0.334148434	0.001	0.002498236
GO:0050732	negative regulation of peptidyl-tyrosine phosphorylation	27	0.334076934	0.0039	0.005778819
GO:0050974	detection of mechanical stimulus involved in sensory perception	13	0.334065934	0.004	0.005880507
GO:0048024	regulation of mRNA splicing, via spliceosome	52	0.334031284	0.0015	0.003088691
GO:0051865	protein autoubiquitination	42	0.334001348	0.0041	0.005981983

GOIDlabel	GOName	size	categoryScores	pValPerm	pValPermCorr
GO:1902237	positive regulation of endoplasmic reticulum stress-induced intrinsic apoptotic signaling pathway	7	0.333884905	0.0026	0.004377754
GO:0060236	regulation of mitotic spindle organization	24	0.333773059	0.0023	0.004032435
GO:1904262	negative regulation of TORC1 signaling	5	0.333745174	0.0019	0.003579279
GO:0010662	regulation of striated muscle cell apoptotic process	22	0.333742834	0.0018	0.003459409
GO:0071280	cellular response to copper ion	11	0.333684334	0.0009	0.002399874
GO:0002291	T cell activation via T cell receptor contact with antigen bound to MHC molecule on antigen presenting cell	6	0.333633634	0.01	0.012232246
GO:0031060	regulation of histone methylation	40	0.333603604	0.001	0.002498236
GO:0006368	transcription elongation from RNA polymerase II promoter	56	0.333549366	0.0003	0.001720882
GO:0061028	establishment of endothelial barrier	22	0.333497133	0.0022	0.003910907
GO:0043516	regulation of DNA damage response, signal transduction by p53 class mediator	16	0.333381596	0.0009	0.002399874
GO:0009886	post-embryonic animal morphogenesis	8	0.333365508	0.0001	0.000877824
GO:0051298	centrosome duplication	34	0.333348475	0.0005	0.002177563
GO:1904589	regulation of protein import	56	0.33327358	0.0006	0.002177563
GO:0008033	tRNA processing	80	0.333272201	0.0006	0.002177563
GO:0018195	peptidyl-arginine modification	9	0.333247533	0.0014	0.002975058
GO:0051291	protein heterooligomerization	66	0.333247533	0.0007	0.002248538
GO:0042490	mechanoreceptor differentiation	30	0.333221793	0.0001	0.000877824
GO:0060113	inner ear receptor cell differentiation	30	0.333221793	0.0001	0.000877824
GO:0036119	response to platelet-derived growth factor	13	0.333194733	0.0005	0.002177563
GO:0035019	somatic stem cell population maintenance	37	0.333131587	0.0001	0.000877824
GO:0046607	positive regulation of centrosome cycle	5	0.333127413	0.0102	0.012424427
GO:1902850	microtubule cytoskeleton organization involved in mitosis	37	0.333117674	0.0008	0.002313249
GO:0043589	skin morphogenesis	5	0.333075933	0.012	0.014185865
GO:0070922	small RNA loading onto RISC	9	0.333047333	0.0062	0.008281828
GO:1903313	positive regulation of mRNA metabolic process	41	0.333038265	0.0005	0.002177563
GO:0042254	ribosome biogenesis	169	0.333019579	0.0001	0.000877824
GO:0006703	estrogen biosynthetic process	8	0.332979408	0.0272	0.02926158
GO:0042273	ribosomal large subunit biogenesis	20	0.332960103	0.0014	0.002975058
GO:0006152	purine nucleoside catabolic process	9	0.332932933	0.0006	0.002177563
GO:0046130	purine ribonucleoside catabolic process	9	0.332932933	0.0006	0.002177563
GO:0003073	regulation of systemic arterial blood pressure	30	0.332921493	0.0024	0.004156304
GO:0007289	spermatid nucleus differentiation	10	0.332921493	0.0054	0.007432873
GO:0051251	positive regulation of lymphocyte activation	137	0.332856109	0.0006	0.002177563
GO:0015931	nucleobase-containing compound transport	150	0.332844273	0.0009	0.002399874
GO:0001779	natural killer cell differentiation	11	0.332818533	0.0035	0.005358035
GO:0071824	protein-DNA complex subunit organization	134	0.332808928	0.0001	0.000877824
GO:1903845	negative regulation of cellular response to transforming growth factor beta stimulus	46	0.332784959	0.0009	0.002399874
GO:0032660	regulation of interleukin-17 production	11	0.332748333	0.0019	0.003579279
GO:0046637	regulation of alpha-beta T cell differentiation	31	0.332727197	0.0013	0.002859821
GO:0032760	positive regulation of tumor necrosis factor production	46	0.332717811	0.001	0.002498236
GO:0061640	cytoskeleton-dependent cytokinesis	37	0.332658527	0.0017	0.003334595
GO:0002689	negative regulation of leukocyte chemotaxis	8	0.332561133	0.0006	0.002177563
GO:0070613	regulation of protein processing	59	0.332521868	0.0026	0.004377754
GO:0010875	positive regulation of cholesterol efflux	12	0.332475332	< 0.001	< 0.001
GO:0045980	negative regulation of nucleotide metabolic process	22	0.332444132	0.0034	0.005263104
GO:0031330	negative regulation of cellular catabolic process	66	0.332409032	0.0008	0.002313249
GO:0042787	protein ubiquitination involved in ubiquitin-dependent protein catabolic process	20	0.332406692	0.0009	0.002399874
GO:2000058	regulation of protein ubiquitination involved in ubiquitin-dependent protein catabolic process	20	0.332406692	0.0009	0.002399874
GO:0010586	miRNA metabolic process	18	0.332403832	0.0029	0.004714719
GO:0032516	positive regulation of phosphoprotein phosphatase activity	9	0.332389532	0.0005	0.002177563
GO:0032945	negative regulation of mononuclear cell proliferation	37	0.332387213	0.0008	0.002313249
GO:0050672	negative regulation of lymphocyte proliferation	37	0.332387213	0.0008	0.002313249
GO:2000767	positive regulation of cytoplasmic translation	7	0.332377275	0.0011	0.002610912
GO:1901068	guanosine-containing compound metabolic process	26	0.332283932	0.001	0.002498236
GO:1990868	response to chemokine	10	0.332277992	0.0203	0.022456501
GO:1990869	cellular response to chemokine	10	0.332277992	0.0203	0.022456501
GO:0001510	RNA methylation	46	0.332230989	0.0012	0.002741075
GO:0034103	regulation of tissue remodeling	36	0.332210782	0.0022	0.003910907
GO:0006971	hypotonic response	8	0.332207207	0.0098	0.012008345
GO:0018216	peptidyl-arginine methylation	5	0.332200772	0.0008	0.002313249
GO:0030852	regulation of granulocyte differentiation	9	0.332160732	0.0001	0.000877824
GO:0032464	positive regulation of protein homooligomerization	6	0.332132132	0.0006	0.002177563
GO:0050764	regulation of phagocytosis	47	0.332112051	0.0011	0.002610912
GO:1901623	regulation of lymphocyte chemotaxis	8	0.332110682	0.0079	0.010077402
GO:0000462	maturation of SSU-rRNA from tricistronic rRNA transcript (SSU-rRNA, 5.8S rRNA, LSU-rRNA)	7	0.332046332	0.001	0.002498236
GO:0019692	deoxyribose phosphate metabolic process	20	0.332033462	0.0005	0.002177563
GO:0006855	drug transmembrane transport	7	0.332009561	0.0048	0.006748379
GO:0002474	antigen processing and presentation of peptide antigen via MHC class I	76	0.331917632	0.0009	0.002399874
GO:0070314	G1 to G0 transition	6	0.331917632	0.0088	0.010992238
GO:1905666	regulation of protein localization to endosome	7	0.331899246	0.0025	0.004279479
GO:1900101	regulation of endoplasmic reticulum unfolded protein response	19	0.331856669	0.0082	0.010366713
GO:1901796	regulation of signal transduction by p53 class mediator	109	0.331722811	0.0003	0.001720882
GO:0043983	histone H4-K12 acetylation	6	0.331703132	0.0001	0.000877824
GO:0051125	regulation of actin nucleation	12	0.331638782	0.0015	0.003088691
GO:0006692	prostanoid metabolic process	21	0.331556046	< 0.001	< 0.001
GO:0006693	prostaglandin metabolic process	21	0.331556046	< 0.001	< 0.001
GO:0060338	regulation of type I interferon-mediated signaling pathway	15	0.331531532	0.0002	0.001341684
GO:0042780	tRNA 3'-end processing	6	0.331531532	0.0003	0.001720882
GO:0034135	regulation of toll-like receptor 2 signaling pathway	6	0.331488631	0.0023	0.004032435
GO:0006606	protein import into nucleus	84	0.331482503	0.0006	0.002177563
GO:0044744	protein targeting to nucleus	84	0.331482503	0.0006	0.002177563
GO:1902593	single-organism nuclear import	84	0.331482503	0.0006	0.002177563
GO:0031440	regulation of mRNA 3'-end processing	21	0.331482503	0.0006	0.002177563

GOIDlabel	GOName	size	categoryScores	pValPerm	pValPermCorr
GO:0031050	dsRNA fragmentation	31	0.331456802	0.0009	0.002399874
GO:0070918	production of small RNA involved in gene silencing by RNA	31	0.331456802	0.0009	0.002399874
GO:0006516	glycoprotein catabolic process	10	0.331402831	0.0001	0.000877824
GO:0050684	regulation of mRNA processing	79	0.331388169	0.0008	0.002313249
GO:0007129	synapsis	15	0.331359931	0.0004	0.002007244
GO:0006968	cellular defense response	23	0.331285323	0.0084	0.010571327
GO:0048144	fibroblast proliferation	50	0.331284427	0.0007	0.002248538
GO:0048145	regulation of fibroblast proliferation	50	0.331284427	0.0007	0.002248538
GO:0007260	tyrosine phosphorylation of STAT protein	35	0.331266777	< 0.001	< 0.001
GO:0006213	pyrimidine nucleoside metabolic process	32	0.331258044	0.0007	0.002248538
GO:0045682	regulation of epidermis development	39	0.331221331	0.0004	0.002007244
GO:0046634	regulation of alpha-beta T cell activation	47	0.331175552	0.0039	0.005778819
GO:0019395	fatty acid oxidation	61	0.33116442	0.0006	0.002177563
GO:0061081	positive regulation of myeloid leukocyte cytokine production involved in immune response	11	0.331157131	0.0041	0.005981983
GO:0031099	regeneration	103	0.331134186	0.0028	0.004605006
GO:1905332	positive regulation of morphogenesis of an epithelium	18	0.331131131	0.001	0.002498236
GO:0021587	cerebellum morphogenesis	20	0.331119691	0.0029	0.004714719
GO:0006851	mitochondrial calcium ion transport	15	0.331119691	0.0007	0.002248538
GO:0002645	positive regulation of tolerance induction	5	0.331119691	0.0265	0.028585731
GO:0072711	cellular response to hydroxyurea	5	0.331068211	0.0073	0.009463568
GO:0019336	phenol-containing compound catabolic process	6	0.331059631	0.0008	0.002313249
GO:1905668	positive regulation of protein localization to endosome	6	0.330973831	0.0037	0.005561344
GO:0042102	positive regulation of T cell proliferation	48	0.330941656	0.0013	0.002859821
GO:0071276	cellular response to cadmium ion	23	0.330893627	0.001	0.002498236
GO:0045639	positive regulation of myeloid cell differentiation	53	0.330890459	0.0019	0.003579279
GO:0043489	RNA stabilization	23	0.330882435	0.0007	0.002248538
GO:0042733	embryonic digit morphogenesis	33	0.330868531	0.0001	0.000877824
GO:0050919	negative chemotaxis	12	0.330866581	0.0003	0.001720882
GO:0042743	hydrogen peroxide metabolic process	23	0.330860053	0.0001	0.000877824
GO:0051127	positive regulation of actin nucleation	8	0.330855856	0.0007	0.002248538
GO:0019827	stem cell population maintenance	99	0.330754131	0.0003	0.001720882
GO:0021532	neural tube patterning	20	0.330746461	< 0.001	< 0.001
GO:0006657	CDP-choline pathway	5	0.330707851	0.0025	0.004279479
GO:0051782	negative regulation of cell division	8	0.330630631	0.0002	0.001341684
GO:0061179	negative regulation of insulin secretion involved in cellular response to glucose stimulus	7	0.330612245	0.0026	0.004377754
GO:0002246	wound healing involved in inflammatory response	6	0.330587731	0.0072	0.009362497
GO:0090594	inflammatory response to wounding	6	0.330587731	0.0072	0.009362497
GO:0044766	multi-organism transport	56	0.330575473	0.0009	0.002399874
GO:1902579	multi-organism localization	56	0.330575473	0.0009	0.002399874
GO:0046850	regulation of bone remodeling	24	0.330566281	0.0022	0.003910907
GO:0061626	pharyngeal arch artery morphogenesis	5	0.330553411	0.0004	0.002007244
GO:0050688	regulation of defense response to virus	69	0.330550426	0.0005	0.002177563
GO:0007252	I-kappaB phosphorylation	12	0.330544831	0.0001	0.000877824
GO:0065004	protein-DNA complex assembly	116	0.330513025	< 0.001	< 0.001
GO:1902003	regulation of beta-amyloid formation	13	0.33048213	0.0027	0.004491543
GO:1902991	regulation of amyloid precursor protein catabolic process	13	0.33048213	0.0027	0.004491543
GO:0000460	maturation of 5.8S rRNA	9	0.33041613	0.0008	0.002313249
GO:0001910	regulation of leukocyte mediated cytotoxicity	26	0.33040293	0.0022	0.003910907
GO:0009208	pyrimidine ribonucleoside triphosphate metabolic process	9	0.33038753	0.0055	0.007544447
GO:0055090	acylglycerol homeostasis	13	0.33038313	0.0018	0.003459409
GO:0070328	triglyceride homeostasis	13	0.33038313	0.0018	0.003459409
GO:0034035	purine ribonucleoside bisphosphate metabolic process	12	0.33037323	0.0008	0.002313249
GO:0050427	3'-phosphoadenosine 5'-phosphosulfate metabolic process	12	0.33037323	0.0008	0.002313249
GO:0072203	cell proliferation involved in metanephros development	6	0.33028743	0.0001	0.000877824
GO:0016558	protein import into peroxisome matrix	10	0.33016731	0.0027	0.004491543
GO:1900151	regulation of nuclear-transcribed mRNA catabolic process, deadenylation-dependent decay	14	0.330152602	< 0.001	< 0.001
GO:1900153	positive regulation of nuclear-transcribed mRNA catabolic process, deadenylation-dependent decay	14	0.330152602	< 0.001	< 0.001
GO:0006896	Golgi to vacuole transport	6	0.33011583	0.0047	0.006644432
GO:0090160	Golgi to lysosome transport	6	0.33011583	0.0047	0.006644432
GO:0072329	monocarboxylic acid catabolic process	74	0.330035827	0.0012	0.002741075
GO:0043302	positive regulation of leukocyte degranulation	15	0.33000429	0.0098	0.012008345
GO:0000466	maturation of 5.8S rRNA from tricistronic rRNA transcript (SSU-rRNA, 5.8S rRNA, LSU-rRNA)	5	0.32993565	0.0143	0.016557758
GO:0018196	peptidyl-asparagine modification	27	0.32992993	0.0002	0.001341684
GO:0018279	protein N-linked glycosylation via asparagine	27	0.32992993	0.0002	0.001341684
GO:0010884	positive regulation of lipid storage	10	0.32990991	0.0009	0.002399874
GO:1904293	negative regulation of ERAD pathway	10	0.32990991	0.0026	0.004377754
GO:0043696	dedifferentiation	5	0.32988417	0.0001	0.000877824
GO:0043697	cell dedifferentiation	5	0.32988417	0.0001	0.000877824
GO:0033047	regulation of mitotic sister chromatid segregation	37	0.329868865	0.0001	0.000877824
GO:0050864	regulation of B cell activation	63	0.329868644	0.0002	0.001341684
GO:0030517	negative regulation of axon extension	8	0.32985843	0.0001	0.000877824
GO:0072525	pyridine-containing compound biosynthetic process	22	0.32979993	0.0003	0.001720882
GO:0045494	photoreceptor cell maintenance	20	0.32979408	0.0001	0.000877824
GO:0006479	protein methylation	105	0.329788564	0.0003	0.001720882
GO:0008213	protein alkylation	105	0.329788564	0.0003	0.001720882
GO:0046831	regulation of RNA export from nucleus	9	0.32978693	0.0022	0.003910907
GO:0050901	leukocyte tethering or rolling	13	0.32974953	0.0038	0.005666214
GO:0098727	maintenance of cell number	100	0.329740026	0.0003	0.001720882
GO:0006699	bile acid biosynthetic process	17	0.329638883	0.0012	0.002741075
GO:0002690	positive regulation of leukocyte chemotaxis	43	0.329550148	0.0021	0.003804235
GO:0045727	positive regulation of translation	89	0.32943184	0.0005	0.002177563
GO:0006303	double-strand break repair via nonhomologous end joining	44	0.329413829	0.0002	0.001341684
GO:0032878	regulation of establishment or maintenance of cell polarity	15	0.329403689	0.0019	0.003579279

GOIDlabel	GOName	size	categoryScores	pValPerm	pValPermCorr
GO:0065002	intracellular protein transmembrane transport	35	0.329376724	0.001	0.002498236
GO:1900408	negative regulation of cellular response to oxidative stress	24	0.329365079	0.0014	0.002975058
GO:1902883	negative regulation of response to oxidative stress	24	0.329365079	0.0014	0.002975058
GO:1903202	negative regulation of oxidative stress-induced cell death	24	0.329365079	0.0014	0.002975058
GO:1901607	alpha-amino acid biosynthetic process	39	0.329327129	0.0001	0.000877824
GO:0001912	positive regulation of leukocyte mediated cytotoxicity	19	0.329269119	0.0017	0.003334595
GO:0003351	epithelial cilium movement	10	0.329240669	< 0.001	< 0.001
GO:0006294	nucleotide-excision repair, preincision complex assembly	21	0.329202672	0.0001	0.000877824
GO:0010874	regulation of cholesterol efflux	14	0.329086229	< 0.001	< 0.001
GO:0032941	secretion by tissue	47	0.329078014	0.0002	0.001341684
GO:0022011	myelination in peripheral nervous system	18	0.329043329	0.0027	0.004491543
GO:0032292	peripheral nervous system axon ensheathment	18	0.329043329	0.0027	0.004491543
GO:0006260	DNA replication	162	0.329009962	0.0005	0.002177563
GO:0042771	intrinsic apoptotic signaling pathway in response to DNA damage by p53 class mediator	33	0.329004329	< 0.001	< 0.001
GO:0045342	MHC class II biosynthetic process	6	0.329000429	0.0001	0.000877824
GO:0045346	regulation of MHC class II biosynthetic process	6	0.329000429	0.0001	0.000877824
GO:2000035	regulation of stem cell division	6	0.329000429	0.0192	0.021362011
GO:0070664	negative regulation of leukocyte proliferation	40	0.328944659	0.0006	0.002177563
GO:0010665	regulation of cardiac muscle cell apoptotic process	20	0.328918919	0.0016	0.003223775
GO:0043568	positive regulation of insulin-like growth factor receptor signaling pathway	5	0.328906049	0.0141	0.016361854
GO:0071806	protein transmembrane transport	43	0.328891682	0.0013	0.002859821
GO:1902895	positive regulation of pri-miRNA transcription from RNA polymerase II promoter	18	0.328828829	0.0002	0.001341684
GO:1902106	negative regulation of leukocyte differentiation	49	0.328794684	0.0001	0.000877824
GO:0007520	myoblast fusion	16	0.328780566	0.0036	0.005475609
GO:0051930	regulation of sensory perception of pain	14	0.328773672	< 0.001	< 0.001
GO:0051931	regulation of sensory perception	14	0.328773672	< 0.001	< 0.001
GO:0019064	fusion of virus membrane with host plasma membrane	7	0.328773672	0.0086	0.010769857
GO:0039663	membrane fusion involved in viral entry into host cell	7	0.328773672	0.0086	0.010769857
GO:0044800	multi-organism membrane fusion	7	0.328773672	0.0086	0.010769857
GO:0021522	spinal cord motor neuron differentiation	16	0.328764479	< 0.001	< 0.001
GO:0009409	response to cold	29	0.328744508	0.0002	0.001341684
GO:0001516	prostaglandin biosynthetic process	15	0.328717289	< 0.001	< 0.001
GO:0046457	prostanoid biosynthetic process	15	0.328717289	< 0.001	< 0.001
GO:1901032	negative regulation of response to reactive oxygen species	9	0.328700129	0.0051	0.007095023
GO:1903206	negative regulation of hydrogen peroxide-induced cell death	9	0.328700129	0.0051	0.007095023
GO:0036005	response to macrophage colony-stimulating factor	6	0.328700129	0.0011	0.002610912
GO:0036006	cellular response to macrophage colony-stimulating factor stimulus	6	0.328700129	0.0011	0.002610912
GO:0006958	complement activation, classical pathway	17	0.328669846	0.0078	0.009981807
GO:0032615	interleukin-12 production	29	0.328655749	0.0001	0.000877824
GO:0032655	regulation of interleukin-12 production	29	0.328655749	0.0001	0.000877824
GO:0043414	macromolecule methylation	173	0.32857366	0.0007	0.002248538
GO:0030512	negative regulation of transforming growth factor beta receptor signaling pathway	45	0.328551409	0.0009	0.002399874
GO:0010212	response to ionizing radiation	95	0.328548398	0.0006	0.002177563
GO:0048549	positive regulation of pinocytosis	6	0.328485628	0.0022	0.003910907
GO:0072527	pyrimidine-containing compound metabolic process	50	0.328468468	0.0004	0.002007244
GO:0046131	pyrimidine ribonucleoside metabolic process	17	0.328412446	0.0076	0.009790742
GO:0055098	response to low-density lipoprotein particle	11	0.328395928	0.004	0.005880507
GO:0071404	cellular response to low-density lipoprotein particle stimulus	11	0.328395928	0.004	0.005880507
GO:0043044	ATP-dependent chromatin remodeling	39	0.328389928	0.0006	0.002177563
GO:0070126	mitochondrial translational termination	76	0.328385152	0.003	0.004825593
GO:0035280	miRNA loading onto RISC involved in gene silencing by miRNA	7	0.328332414	0.0079	0.010077402
GO:0060759	regulation of response to cytokine stimulus	99	0.328317928	0.0005	0.002177563
GO:0009108	coenzyme biosynthetic process	91	0.328312614	0.001	0.002498236
GO:0006730	one-carbon metabolic process	17	0.328261034	0.0006	0.002177563
GO:0009148	pyrimidine nucleoside triphosphate biosynthetic process	10	0.328236808	0.0042	0.006097175
GO:0072189	ureter development	8	0.328217503	0.0001	0.000877824
GO:0000413	protein peptidyl-prolyl isomerization	21	0.328197585	0.0001	0.000877824
GO:0048247	lymphocyte chemotaxis	17	0.328185328	0.0045	0.006417207
GO:0050871	positive regulation of B cell activation	42	0.328160814	< 0.001	< 0.001
GO:0050746	regulation of lipoprotein metabolic process	9	0.328156728	0.0007	0.002248538
GO:0001977	renal system process involved in regulation of blood volume	7	0.328148557	0.0272	0.02926158
GO:0009394	2'-deoxyribonucleotide metabolic process	19	0.328117591	0.0005	0.002177563
GO:0006625	protein targeting to peroxisome	52	0.328116028	0.0004	0.002007244
GO:0043574	peroxisomal transport	52	0.328116028	0.0004	0.002007244
GO:0072662	protein localization to peroxisome	52	0.328116028	0.0004	0.002007244
GO:0072663	establishment of protein localization to peroxisome	52	0.328116028	0.0004	0.002007244
GO:0048291	isotype switching to IgG isotypes	7	0.328111785	0.0006	0.002177563
GO:0007589	body fluid secretion	51	0.328104575	0.0003	0.001720882
GO:0045116	protein neddylation	10	0.328082368	0.001	0.002498236
GO:1902235	regulation of endoplasmic reticulum stress-induced intrinsic apoptotic signaling pathway	21	0.328050499	0.0045	0.006417207
GO:1901201	regulation of extracellular matrix assembly	11	0.328021528	0.0047	0.006644432
GO:0001961	positive regulation of cytokine-mediated signaling pathway	28	0.327927928	0.002	0.003693422
GO:0060351	cartilage development involved in endochondral bone morphogenesis	8	0.327895753	0.0006	0.002177563
GO:0031648	protein destabilization	27	0.327889795	0.0009	0.002399874
GO:0009162	deoxyribonucleoside monophosphate metabolic process	9	0.327813528	0.0019	0.003579279
GO:0006289	nucleotide-excision repair	72	0.327788503	0.0001	0.000877824
GO:1901685	glutathione derivative metabolic process	17	0.327746234	< 0.001	< 0.001
GO:1901687	glutathione derivative biosynthetic process	17	0.327746234	< 0.001	< 0.001
GO:0006783	heme biosynthetic process	17	0.327731092	0.0064	0.008500875
GO:0003094	glomerular filtration	12	0.327670528	0.0222	0.024321919
GO:0045776	negative regulation of blood pressure	20	0.327657658	0.0004	0.002007244
GO:0002011	morphogenesis of an epithelial sheet	29	0.327581769	0.0014	0.002975058
GO:0043001	Golgi to plasma membrane protein transport	21	0.32757247	0.0017	0.003334595

GOIDlabel	GOName	size	categoryScores	pValPerm	pValPermCorr
GO:1903055	positive regulation of extracellular matrix organization	15	0.327550408	0.006	0.00807902
GO:0006307	DNA dealkylation involved in DNA repair	10	0.327516088	0.0035	0.005358035
GO:0042340	keratan sulfate catabolic process	8	0.327509653	0.0008	0.002313249
GO:0048243	norepinephrine secretion	8	0.327413127	< 0.001	< 0.001
GO:0002688	regulation of leukocyte chemotaxis	55	0.327394407	0.0013	0.002859821
GO:0035196	production of miRNAs involved in gene silencing by miRNA	29	0.327324369	0.0009	0.002399874
GO:1902570	protein localization to nucleolus	13	0.327314127	0.0004	0.002007244
GO:0032392	DNA geometric change	58	0.327306617	0.0008	0.002313249
GO:0001832	blastocyst growth	11	0.327272727	0.0006	0.002177563
GO:2000377	regulation of reactive oxygen species metabolic process	97	0.32721676	0.0006	0.002177563
GO:0035864	response to potassium ion	6	0.327155727	0.0004	0.002007244
GO:0021545	cranial nerve development	18	0.327141427	0.0004	0.002007244
GO:0003171	atrioventricular valve development	17	0.327080021	0.0001	0.000877824
GO:0003181	atrioventricular valve morphogenesis	17	0.327080021	0.0001	0.000877824
GO:0034080	CENP-A containing nucleosome assembly	16	0.327043115	0.0001	0.000877824
GO:0061641	CENP-A containing chromatin organization	16	0.327043115	0.0001	0.000877824
GO:0070125	mitochondrial translational elongation	75	0.327035607	0.003	0.004825593
GO:1903317	regulation of protein maturation	60	0.327031317	0.0028	0.004605006
GO:0055076	transition metal ion homeostasis	69	0.32700651	0.0008	0.002313249
GO:0002097	tRNA wobble base modification	10	0.326975547	0.0044	0.006304227
GO:0002281	macrophage activation involved in immune response	6	0.326898327	0.0014	0.002975058
GO:0007098	centrosome cycle	64	0.326874196	0.0007	0.002248538
GO:0051147	regulation of muscle cell differentiation	96	0.326836658	0.0009	0.002399874
GO:0033144	negative regulation of intracellular steroid hormone receptor signaling pathway	22	0.326816427	0.0009	0.002399874
GO:1903311	regulation of mRNA metabolic process	102	0.326812527	0.0008	0.002313249
GO:0002295	T-helper cell lineage commitment	5	0.326795367	0.0003	0.001720882
GO:0043373	CD4-positive, alpha-beta T cell lineage commitment	5	0.326795367	0.0003	0.001720882
GO:0000303	response to superoxide	20	0.326769627	0.0007	0.002248538
GO:0000305	response to oxygen radical	20	0.326769627	0.0007	0.002248538
GO:0017038	protein import	114	0.326742532	0.0006	0.002177563
GO:0046851	negative regulation of bone remodeling	8	0.326673102	0.0066	0.008731949
GO:0001990	regulation of systemic arterial blood pressure by hormone	10	0.326666667	0.0058	0.007856196
GO:0006553	lysine metabolic process	9	0.326640927	0.0013	0.002859821
GO:0006554	lysine catabolic process	9	0.326640927	0.0013	0.002859821
GO:0046949	fatty-acyl-CoA biosynthetic process	20	0.326615187	0.0039	0.005778819
GO:0061077	chaperone-mediated protein folding	27	0.326612327	0.0005	0.002177563
GO:0046112	nucleobase biosynthetic process	8	0.326576577	0.0071	0.009268394
GO:0014896	muscle hypertrophy	40	0.326544402	0.0007	0.002248538
GO:0014823	response to activity	37	0.326508748	0.0014	0.002975058
GO:0070193	synaptonemal complex organization	10	0.326486486	0.0005	0.002177563
GO:2000482	regulation of interleukin-8 secretion	15	0.326435006	0.0019	0.003579279
GO:0009299	mRNA transcription	17	0.32642895	0.0068	0.008946408
GO:0001885	endothelial cell development	35	0.326412944	0.0016	0.003223775
GO:0061154	endothelial tube morphogenesis	11	0.326336726	0.0035	0.005358035
GO:0035561	regulation of chromatin binding	17	0.326277538	< 0.001	< 0.001
GO:2000142	regulation of DNA-templated transcription, initiation	20	0.326241956	0.0003	0.001720882
GO:0034243	regulation of transcription elongation from RNA polymerase II promoter	16	0.326190476	0.0047	0.006644432
GO:0050860	negative regulation of T cell receptor signaling pathway	10	0.326151866	0.0004	0.002007244
GO:0007063	regulation of sister chromatid cohesion	14	0.326144512	0.0043	0.006204188
GO:0008298	intracellular mRNA localization	6	0.326126126	0.0135	0.015731487
GO:0015697	quaternary ammonium group transport	6	0.326126126	0.0102	0.012424427
GO:0072606	interleukin-8 secretion	16	0.326093951	0.0019	0.003579279
GO:0007320	insemination	6	0.326083226	< 0.001	< 0.001
GO:0006505	GPI anchor metabolic process	24	0.326061776	0.0012	0.002741075
GO:0007084	mitotic nuclear envelope reassembly	7	0.326052583	0.0003	0.001720882
GO:2001243	negative regulation of intrinsic apoptotic signaling pathway	59	0.326030146	0.0006	0.002177563
GO:0002438	acute inflammatory response to antigenic stimulus	10	0.326023166	0.0009	0.002399874
GO:0061052	negative regulation of cell growth involved in cardiac muscle cell development	5	0.326023166	0.0026	0.004377754
GO:0046477	glycosylceramide catabolic process	5	0.325971686	0.0083	0.010468217
GO:0036010	protein localization to endosome	14	0.325960655	0.0085	0.010671887
GO:0071281	cellular response to iron ion	8	0.325933076	0.0041	0.005981983
GO:0060443	mammary gland morphogenesis	19	0.325922915	0.0031	0.004916949
GO:0030217	T cell differentiation	116	0.325908667	0.0007	0.002248538
GO:0003256	regulation of transcription from RNA polymerase II promoter involved in myocardial precursor cell differentiation	5	0.325868726	0.0153	0.017559319
GO:0009895	negative regulation of catabolic process	99	0.325853126	0.0011	0.002610912
GO:2000042	negative regulation of double-strand break repair via homologous recombination	11	0.325845326	< 0.001	< 0.001
GO:0035418	protein localization to synapse	8	0.325836551	0.0007	0.002248538
GO:0003170	heart valve development	37	0.325833942	< 0.001	< 0.001
GO:0006414	translational elongation	102	0.325777879	0.0024	0.004156304
GO:0006296	nucleotide-excision repair, DNA incision, 5'-to lesion	28	0.325776797	0.0016	0.003223775
GO:0033683	nucleotide-excision repair, DNA incision	29	0.325717836	0.0019	0.003579279
GO:0008608	attachment of spindle microtubules to kinetochore	20	0.325701416	0.0027	0.004491543
GO:0034440	lipid oxidation	63	0.325701211	0.0006	0.002177563
GO:0090278	negative regulation of peptide hormone secretion	22	0.325681526	0.0009	0.002399874
GO:0021766	hippocampus development	36	0.325675676	0.0002	0.001341684
GO:0090305	nucleic acid phosphodiester bond hydrolysis	183	0.325647896	0.0005	0.002177563
GO:0043618	regulation of transcription from RNA polymerase II promoter in response to stress	85	0.325626467	0.0011	0.002610912
GO:0098930	axonal transport	25	0.325621622	0.001	0.002498236
GO:0051013	microtubule severing	7	0.325611326	0.0097	0.011922488
GO:0051898	negative regulation of protein kinase B signaling	28	0.325602133	0.0037	0.005561344
GO:2000379	positive regulation of reactive oxygen species metabolic process	46	0.325588943	0.0008	0.002313249
GO:0044786	cell cycle DNA replication	37	0.325576542	0.0008	0.002313249

GOIDlabel	GOName	size	categoryScores	pValPerm	pValPermCorr
GO:0046628	positive regulation of insulin receptor signaling pathway	11	0.325564526	0.0006	0.002177563
GO:0030953	astral microtubule organization	5	0.325559846	0.0005	0.002177563
GO:0045739	positive regulation of DNA repair	39	0.325545326	0.0008	0.002313249
GO:0006261	DNA-dependent DNA replication	86	0.325542486	0.0008	0.002313249
GO:0046039	GTP metabolic process	18	0.325539826	0.0013	0.002859821
GO:0071677	positive regulation of mononuclear cell migration	14	0.325537783	0.0002	0.001341684
GO:0060538	skeletal muscle organ development	79	0.325520095	0.003	0.004825593
GO:0032733	positive regulation of interleukin-10 production	18	0.325496925	0.0006	0.002177563
GO:0043301	negative regulation of leukocyte degranulation	7	0.32546424	0.0003	0.001720882
GO:0051342	regulation of cyclic-nucleotide phosphodiesterase activity	5	0.325456885	0.017	0.019243517
GO:0030316	osteoclast differentiation	50	0.325410553	0.0037	0.005561344
GO:0032006	regulation of TOR signaling	51	0.325404396	0.0011	0.002610912
GO:2000773	negative regulation of cellular senescence	12	0.325396825	0.0007	0.002248538
GO:0010970	transport along microtubule	83	0.32539114	0.0001	0.000877824
GO:0099111	microtubule-based transport	83	0.32539114	0.0001	0.000877824
GO:0002696	positive regulation of leukocyte activation	160	0.325379665	0.0006	0.002177563
GO:0001711	endodermal cell fate commitment	8	0.32532175	0.0007	0.002248538
GO:2000209	regulation of anoikis	12	0.325311025	0.0078	0.009981807
GO:0030888	regulation of B cell proliferation	32	0.325265444	0.0006	0.002177563
GO:0046653	tetrahydrofolate metabolic process	14	0.325243611	0.0015	0.003088691
GO:0070339	response to bacterial lipopeptide	5	0.325199485	0.042	0.043800687
GO:0071220	cellular response to bacterial lipoprotein	5	0.325199485	0.042	0.043800687
GO:0071221	cellular response to bacterial lipopeptide	5	0.325199485	0.042	0.043800687
GO:0007031	peroxisome organization	60	0.325186615	0.0005	0.002177563
GO:0019377	glycolipid catabolic process	11	0.325143325	0.0035	0.005358035
GO:0000380	alternative mRNA splicing, via spliceosome	38	0.325110072	0.0017	0.003334595
GO:0050779	RNA destabilization	21	0.325084268	< 0.001	< 0.001
GO:0034599	cellular response to oxidative stress	167	0.325074947	0.0006	0.002177563
GO:0032481	positive regulation of type I interferon production	52	0.325056925	0.0002	0.001341684
GO:0042304	regulation of fatty acid biosynthetic process	26	0.325056925	0.0001	0.000877824
GO:0051923	sulfation	5	0.325045045	0.0036	0.005475609
GO:0000381	regulation of alternative mRNA splicing, via spliceosome	27	0.325029792	0.0014	0.002975058
GO:0060688	regulation of morphogenesis of a branching structure	27	0.325010725	0.0028	0.004605006
GO:0006878	cellular copper ion homeostasis	6	0.324924925	0.0002	0.001341684
GO:1901071	glucosamine-containing compound metabolic process	11	0.324909325	0.0002	0.001341684
GO:0050922	negative regulation of chemotaxis	19	0.324852672	0.0003	0.001720882
GO:1903350	response to dopamine	9	0.324839125	0.002	0.003693422
GO:0003044	regulation of systemic arterial blood pressure mediated by a chemical signal	13	0.324839125	0.0043	0.006204188
GO:0022409	positive regulation of cell-cell adhesion	121	0.324828488	0.0021	0.003804235
GO:0061157	mRNA destabilization	20	0.324800515	< 0.001	< 0.001
GO:0010890	positive regulation of sequestering of triglyceride	5	0.324736165	0.0004	0.002007244
GO:0051154	negative regulation of striated muscle cell differentiation	20	0.324723295	0.0014	0.002975058
GO:0043217	myelin maintenance	11	0.324698725	0.0041	0.005981983
GO:0070911	global genome nucleotide-excision repair	19	0.324690104	0.0011	0.002610912
GO:0045620	negative regulation of lymphocyte differentiation	24	0.324688975	0.0001	0.000877824
GO:0002221	pattern recognition receptor signaling pathway	110	0.324675325	0.0003	0.001720882
GO:0030850	prostate gland development	24	0.324667525	0.0006	0.002177563
GO:0006686	sphingomyelin biosynthetic process	5	0.324633205	0.0102	0.012424427
GO:0016024	CDP-diacylglycerol biosynthetic process	10	0.324607465	0.0002	0.001341684
GO:0010042	response to manganese ion	12	0.324560275	0.0078	0.009981807
GO:0046629	gamma-delta T cell activation	7	0.324544953	0.0029	0.004714719
GO:0034205	beta-amyloid formation	18	0.324538825	0.0063	0.008390784
GO:0050765	negative regulation of phagocytosis	11	0.324534925	0.0014	0.002975058
GO:0010803	regulation of tumor necrosis factor-mediated signaling pathway	38	0.324473346	0.001	0.002498236
GO:0060149	negative regulation of posttranscriptional gene silencing	8	0.324453024	0.0101	0.012328539
GO:0060967	negative regulation of gene silencing by RNA	8	0.324453024	0.0101	0.012328539
GO:0045685	regulation of glial cell differentiation	37	0.324449546	0.0028	0.004605006
GO:0008088	axo-dendritic transport	30	0.324444444	0.0017	0.003334595
GO:0006283	transcription-coupled nucleotide-excision repair	54	0.324419658	0.0004	0.002007244
GO:0045880	positive regulation of smoothened signaling pathway	17	0.32440003	< 0.001	< 0.001
GO:0070723	response to cholesterol	17	0.324339466	0.0001	0.000877824
GO:0045670	regulation of osteoclast differentiation	32	0.324332368	0.0058	0.007856196
GO:1901654	response to ketone	109	0.324319601	0.0004	0.002007244
GO:1904666	regulation of ubiquitin protein ligase activity	14	0.324305939	0.0008	0.002313249
GO:0046686	response to cadmium ion	39	0.324198924	0.0024	0.004156304
GO:0010677	negative regulation of cellular carbohydrate metabolic process	23	0.324111689	0.004	0.005880507
GO:0045667	regulation of osteoblast differentiation	64	0.324066924	0.0003	0.001720882
GO:0019885	antigen processing and presentation of endogenous peptide antigen via MHC class I	6	0.324024024	0.0197	0.021861066
GO:0046037	GMP metabolic process	7	0.323993381	0.0071	0.009268394
GO:0001959	regulation of cytokine-mediated signaling pathway	89	0.323983052	0.0005	0.002177563
GO:0043471	regulation of cellular carbohydrate catabolic process	6	0.323981124	0.0043	0.006204188
GO:0050731	positive regulation of peptidyl-tyrosine phosphorylation	78	0.323974524	0.0005	0.002177563
GO:0045638	negative regulation of myeloid cell differentiation	45	0.323969684	0.0004	0.002007244
GO:0032715	negative regulation of interleukin-6 production	19	0.32393145	0.0006	0.002177563
GO:0030488	tRNA methylation	20	0.323912484	0.0006	0.002177563
GO:0018022	peptidyl-lysine methylation	76	0.323870487	0.0006	0.002177563
GO:0006415	translational termination	87	0.323850945	0.003	0.004825593
GO:0042982	amyloid precursor protein metabolic process	35	0.323728627	0.005	0.006974174
GO:0003299	muscle hypertrophy in response to stress	9	0.323580724	0.0006	0.002177563
GO:0014887	cardiac muscle adaptation	9	0.323580724	0.0006	0.002177563
GO:0014898	cardiac muscle hypertrophy in response to stress	9	0.323580724	0.0006	0.002177563
GO:0043619	regulation of transcription from RNA polymerase II promoter in response to oxidative stress	9	0.323580724	0.0087	0.010877987
GO:0006390	transcription from mitochondrial promoter	10	0.323552124	0.0187	0.020874952

GOIDlabel	GOName	size	categoryScores	pValPerm	pValPermCorr
GO:0001881	receptor recycling	21	0.323527609	0.0041	0.005981983
GO:0051297	centrosome organization	67	0.323486813	0.0007	0.002248538
GO:0006335	DNA replication-dependent nucleosome assembly	8	0.323455598	0.0009	0.002399874
GO:0034723	DNA replication-dependent nucleosome organization	8	0.323455598	0.0009	0.002399874
GO:0032095	regulation of response to food	12	0.323337623	0.0007	0.002248538
GO:0034377	plasma lipoprotein particle assembly	13	0.323274923	< 0.001	< 0.001
GO:0060444	branching involved in mammary gland duct morphogenesis	10	0.323268983	0.0022	0.003910907
GO:0071214	cellular response to abiotic stimulus	188	0.323257756	0.0015	0.003088691
GO:0035020	regulation of Rac protein signal transduction	5	0.323243243	0.0259	0.02799537
GO:0045625	regulation of T-helper 1 cell differentiation	8	0.323198198	0.0004	0.002007244
GO:0032986	protein-DNA complex disassembly	13	0.323195723	0.0006	0.002177563
GO:0045058	T cell selection	19	0.323186344	0.0003	0.001720882
GO:0051188	cofactor biosynthetic process	121	0.323167087	0.001	0.002498236
GO:0045648	positive regulation of erythrocyte differentiation	18	0.323151723	0.0031	0.004916949
GO:0002347	response to tumor cell	14	0.323147637	0.0002	0.001341684
GO:0060601	lateral sprouting from an epithelium	5	0.323140283	0.014	0.01625173
GO:0042256	mature ribosome assembly	6	0.323123123	0.0015	0.003088691
GO:0042044	fluid transport	14	0.323110866	< 0.001	< 0.001
GO:0071243	cellular response to arsenic-containing substance	15	0.323071643	0.0019	0.003579279
GO:0010658	striated muscle cell apoptotic process	23	0.323026132	0.0014	0.002975058
GO:0060512	prostate gland morphogenesis	15	0.323003003	0.0055	0.007544447
GO:0010559	regulation of glycoprotein biosynthetic process	31	0.322995807	0.0018	0.003459409
GO:0007130	synaptonemal complex assembly	9	0.322980123	0.0002	0.001341684
GO:0000244	spliceosomal tri-snRNP complex assembly	8	0.322940798	0.0035	0.005358035
GO:0034250	positive regulation of cellular amide metabolic process	101	0.322930285	0.0005	0.002177563
GO:0006487	protein N-linked glycosylation	43	0.322881686	0.001	0.002498236
GO:0014912	negative regulation of smooth muscle cell migration	12	0.322865723	0.001	0.002498236
GO:0006354	DNA-templated transcription, elongation	79	0.322864637	< 0.001	< 0.001
GO:0070317	negative regulation of G0 to G1 transition	28	0.322816694	0.0006	0.002177563
GO:0051607	defense response to virus	165	0.322769003	0.0006	0.002177563
GO:0040014	regulation of multicellular organism growth	45	0.322734163	0.0002	0.001341684
GO:0050982	detection of mechanical stimulus	17	0.322719358	0.0036	0.005475609
GO:0045879	negative regulation of smoothened signaling pathway	15	0.322676963	0.0037	0.005561344
GO:0034605	cellular response to heat	75	0.322639211	0.0008	0.002313249
GO:0000768	syncytium formation by plasma membrane fusion	23	0.32258967	0.0037	0.005561344
GO:0071168	protein localization to chromatin	19	0.32253607	0.0023	0.004032435
GO:0019884	antigen processing and presentation of exogenous antigen	124	0.322530826	0.0013	0.002859821
GO:0030521	androgen receptor signaling pathway	39	0.322456522	0.0034	0.005263104
GO:0043467	regulation of generation of precursor metabolites and energy	82	0.322440908	0.0014	0.002975058
GO:0051220	cytoplasmic sequestering of protein	21	0.322412208	0.0002	0.001341684
GO:0071156	regulation of cell cycle arrest	68	0.322393822	0.0008	0.002313249
GO:0006566	threonine metabolic process	5	0.322368082	0.0022	0.003910907
GO:0014067	negative regulation of phosphatidylinositol 3-kinase signaling	9	0.322350922	0.0038	0.005666214
GO:0034504	protein localization to nucleus	160	0.322321429	0.0006	0.002177563
GO:0048050	post-embryonic eye morphogenesis	5	0.322316602	0.0055	0.007544447
GO:1903426	regulation of reactive oxygen species biosynthetic process	44	0.322306072	0.0007	0.002248538
GO:1902253	regulation of intrinsic apoptotic signaling pathway by p53 class mediator	22	0.322218322	0.0001	0.000877824
GO:1990173	protein localization to nucleoplasm	10	0.322213642	< 0.001	< 0.001
GO:0031069	hair follicle morphogenesis	13	0.322205722	0.0001	0.000877824
GO:0002827	positive regulation of T-helper 1 type immune response	6	0.322179322	0.0021	0.003804235
GO:0046651	lymphocyte proliferation	134	0.322142185	0.0009	0.002399874
GO:0043496	regulation of protein homodimerization activity	18	0.322079222	0.0107	0.012912536
GO:1902116	negative regulation of organelle assembly	27	0.322074455	0.0008	0.002313249
GO:0000083	regulation of transcription involved in G1/S transition of mitotic cell cycle	18	0.322064922	0.0012	0.002741075
GO:0003016	respiratory system process	14	0.322062879	< 0.001	< 0.001
GO:0007127	meiosis I	37	0.322049463	0.0006	0.002177563
GO:0030502	negative regulation of bone mineralization	9	0.321979122	0.0024	0.004156304
GO:0046033	AMP metabolic process	10	0.321930502	0.0031	0.004916949
GO:0097066	response to thyroid hormone	15	0.321870442	0.0041	0.005981983
GO:0097205	renal filtration	13	0.321869122	0.0197	0.021861066
GO:0006547	histidine metabolic process	5	0.321853282	< 0.001	< 0.001
GO:0052803	imidazole-containing compound metabolic process	5	0.321853282	< 0.001	< 0.001
GO:1904152	regulation of retrograde protein transport, ER to cytosol	9	0.321836122	0.0041	0.005981983
GO:0006690	icosanoid metabolic process	53	0.321803744	0.0005	0.002177563
GO:1901568	fatty acid derivative metabolic process	53	0.321803744	0.0005	0.002177563
GO:0050671	positive regulation of lymphocyte proliferation	68	0.321773034	0.0005	0.002177563
GO:0000963	mitochondrial RNA processing	10	0.321750322	0.0072	0.009362497
GO:2000134	negative regulation of G1/S transition of mitotic cell cycle	70	0.32173929	0.0024	0.004156304
GO:0045912	negative regulation of carbohydrate metabolic process	25	0.32172973	0.0044	0.006304227
GO:0032461	positive regulation of protein oligomerization	14	0.321695165	0.0019	0.003579279
GO:0071548	response to dexamethasone	23	0.321683174	0.0001	0.000877824
GO:0051131	chaperone-mediated protein complex assembly	11	0.321680122	0.0005	0.002177563
GO:0090398	cellular senescence	41	0.321668707	0.0006	0.002177563
GO:0006334	nucleosome assembly	62	0.321638228	0.0001	0.000877824
GO:0050730	regulation of peptidyl-tyrosine phosphorylation	109	0.321481114	0.0006	0.002177563
GO:0007398	ectoderm development	11	0.321446121	0.0029	0.004714719
GO:0097305	response to alcohol	98	0.321435138	0.0002	0.001341684
GO:0050867	positive regulation of cell activation	167	0.32143281	0.0006	0.002177563
GO:0061097	regulation of protein tyrosine kinase activity	40	0.321428571	0.0007	0.002248538
GO:0071476	cellular hypotonic response	7	0.321382607	0.0097	0.011922488
GO:0031054	pre-miRNA processing	9	0.321321321	0.0057	0.007745437
GO:0008217	regulation of blood pressure	79	0.321313719	0.0027	0.004491543
GO:1904153	negative regulation of retrograde protein transport, ER to cytosol	8	0.321299871	0.0054	0.007432873

GOIDlabel	GOName	size	categoryScores	pValPerm	pValPermCorr
GO:0043173	nucleotide salvage	9	0.321292721	0.0018	0.003459409
GO:0032943	mononuclear cell proliferation	135	0.321290815	0.0009	0.002399874
GO:1902105	regulation of leukocyte differentiation	139	0.321261446	0.0013	0.002859821
GO:0061734	parkin-mediated mitophagy in response to mitochondrial depolarization	5	0.321235521	0.002	0.003693422
GO:0061418	regulation of transcription from RNA polymerase II promoter in response to hypoxia	57	0.321212942	0.0017	0.003334595
GO:0030318	melanocyte differentiation	13	0.321195921	< 0.001	< 0.001
GO:0072673	lamellipodium morphogenesis	10	0.321184041	0.0195	0.021665533
GO:0061158	3'-UTR-mediated mRNA destabilization	10	0.321132561	0.0028	0.004605006
GO:0044650	adhesion of symbiont to host cell	7	0.321125207	0.0032	0.005044175
GO:0035723	interleukin-15-mediated signaling pathway	6	0.321106821	0.0006	0.002177563
GO:0070672	response to interleukin-15	6	0.321106821	0.0006	0.002177563
GO:0071350	cellular response to interleukin-15	6	0.321106821	0.0006	0.002177563
GO:0008354	germ cell migration	6	0.321106821	0.0094	0.011616482
GO:0070661	leukocyte proliferation	145	0.32107398	0.0005	0.002177563
GO:2000726	negative regulation of cardiac muscle cell differentiation	10	0.321055341	0.0008	0.002313249
GO:0010759	positive regulation of macrophage chemotaxis	8	0.321042471	0.0011	0.002610912
GO:0060669	embryonic placenta morphogenesis	13	0.320978121	0.0007	0.002248538
GO:0048002	antigen processing and presentation of peptide antigen	134	0.320966596	0.0013	0.002859821
GO:0033688	regulation of osteoblast proliferation	14	0.32094135	0.0069	0.009046225
GO:0007224	smoothened signaling pathway	77	0.32094135	< 0.001	< 0.001
GO:2000138	positive regulation of cell proliferation involved in heart morphogenesis	5	0.320926641	0.0001	0.000877824
GO:2000778	positive regulation of interleukin-6 secretion	18	0.320920921	0.0007	0.002248538
GO:0031532	actin cytoskeleton reorganization	48	0.320913771	0.0002	0.001341684
GO:0002478	antigen processing and presentation of exogenous peptide antigen	123	0.320898599	0.0015	0.003088691
GO:0002888	positive regulation of myeloid leukocyte mediated immunity	19	0.320856195	0.0146	0.016871405
GO:0008589	regulation of smoothened signaling pathway	44	0.320855271	0.0001	0.000877824
GO:0050857	positive regulation of antigen receptor-mediated signaling pathway	12	0.320849421	0.0002	0.001341684
GO:0031570	DNA integrity checkpoint	94	0.32080287	0.0007	0.002248538
GO:0002920	regulation of humoral immune response	33	0.320751921	0.0041	0.005981983
GO:0021675	nerve development	29	0.320711845	0.0007	0.002248538
GO:0048538	thymus development	18	0.320706421	0.0034	0.005263104
GO:0019098	reproductive behavior	12	0.320699271	< 0.001	< 0.001
GO:0034375	high-density lipoprotein particle remodeling	9	0.320692121	0.0003	0.001720882
GO:0042100	B cell proliferation	39	0.320667921	0.0019	0.003579279
GO:0090329	regulation of DNA-dependent DNA replication	26	0.320661321	0.0005	0.002177563
GO:0045069	regulation of viral genome replication	60	0.320660661	0.0006	0.002177563
GO:2001252	positive regulation of chromosome organization	50	0.320653797	0.0001	0.000877824
GO:0030101	natural killer cell activation	32	0.320632239	0.0024	0.004156304
GO:0042772	DNA damage response, signal transduction resulting in transcription	12	0.320613471	0.0005	0.002177563
GO:0001706	endoderm formation	27	0.320577721	0.0006	0.002177563
GO:0039532	negative regulation of viral-induced cytoplasmic pattern recognition receptor signaling pathway	9	0.320549121	0.003	0.004825593
GO:0031341	regulation of cell killing	33	0.320502321	0.005	0.006974174
GO:0010972	negative regulation of G2/M transition of mitotic cell cycle	61	0.320484419	0.0009	0.002399874
GO:0009066	aspartate family amino acid metabolic process	36	0.32046332	0.0005	0.002177563
GO:0001824	blastocyst development	55	0.32040716	0.0005	0.002177563
GO:0051900	regulation of mitochondrial depolarization	13	0.32038412	0.0097	0.011922488
GO:0010823	negative regulation of mitochondrion organization	35	0.320382423	0.0021	0.003804235
GO:0006551	leucine metabolic process	5	0.32036036	0.0103	0.012519912
GO:0006552	leucine catabolic process	5	0.32036036	0.0103	0.012519912
GO:0061178	regulation of insulin secretion involved in cellular response to glucose stimulus	26	0.32031482	0.0002	0.001341684
GO:0035773	insulin secretion involved in cellular response to glucose stimulus	28	0.320297849	0.0003	0.001720882
GO:0044774	mitotic DNA integrity checkpoint	65	0.32027324	0.0011	0.002610912
GO:0000722	telomere maintenance via recombination	5	0.3202574	0.0007	0.002248538
GO:0043488	regulation of mRNA stability	116	0.320252519	0.0003	0.001720882
GO:0097696	STAT cascade	72	0.320180895	0.0002	0.001341684
GO:0071397	cellular response to cholesterol	10	0.32018018	< 0.001	< 0.001
GO:0048255	mRNA stabilization	19	0.320178825	0.0017	0.003334595
GO:0033137	negative regulation of peptidyl-serine phosphorylation	13	0.32014652	0.0101	0.012328539
GO:0034284	response to monosaccharide	100	0.320133848	0.0012	0.002741075
GO:0032946	positive regulation of mononuclear cell proliferation	69	0.320112659	0.0005	0.002177563
GO:0032627	interleukin-23 production	5	0.32010296	0.0018	0.003459409
GO:0032667	regulation of interleukin-23 production	5	0.32010296	0.0018	0.003459409
GO:0050711	negative regulation of interleukin-1 secretion	6	0.32007722	0.01	0.012232246
GO:0060441	epithelial tube branching involved in lung morphogenesis	15	0.32006864	0.0005	0.002177563
GO:0030071	regulation of mitotic metaphase/anaphase transition	27	0.32003432	< 0.001	< 0.001
GO:1905523	positive regulation of macrophage migration	12	0.32003432	0.001	0.002498236
GO:0014745	negative regulation of muscle adaptation	6	0.31999142	0.0124	0.014577606
GO:0036507	protein demannosylation	15	0.31998284	0.0006	0.002177563
GO:0036508	protein alpha-1,2-demannosylation	15	0.31998284	0.0006	0.002177563
GO:0019724	B cell mediated immunity	63	0.319973034	0.0003	0.001720882
GO:0019433	triglyceride catabolic process	12	0.31996997	< 0.001	< 0.001
GO:0048265	response to pain	14	0.319966906	0.0018	0.003459409
GO:0070233	negative regulation of T cell apoptotic process	12	0.31992707	0.0006	0.002177563
GO:0014897	striated muscle hypertrophy	39	0.31990892	0.0009	0.002399874
GO:0006356	regulation of transcription from RNA polymerase I promoter	25	0.319907336	0.0005	0.002177563
GO:0003413	chondrocyte differentiation involved in endochondral bone morphogenesis	5	0.31984556	0.0049	0.006860215
GO:0051250	negative regulation of lymphocyte activation	69	0.319795572	0.0003	0.001720882
GO:0009267	cellular response to starvation	81	0.319729253	0.0014	0.002975058
GO:0000077	DNA damage checkpoint	89	0.319717149	0.0006	0.002177563
GO:0045669	positive regulation of osteoblast differentiation	35	0.31969112	< 0.001	< 0.001
GO:0034085	establishment of sister chromatid cohesion	5	0.31969112	0.0147	0.016962357
GO:0071921	cohesin loading	5	0.31969112	0.0147	0.016962357
GO:1901991	negative regulation of mitotic cell cycle phase transition	150	0.319684256	0.001	0.002498236

GOIDlabel	GOName	size	categoryScores	pValPerm	pValPermCorr
GO:0035384	thioester biosynthetic process	35	0.319669057	0.0047	0.006644432
GO:0071616	acyl-CoA biosynthetic process	35	0.319669057	0.0047	0.006644432
GO:0031644	regulation of neurological system process	40	0.319658945	0.0002	0.001341684
GO:1902807	negative regulation of cell cycle G1/S phase transition	71	0.319658491	0.0024	0.004156304
GO:0043487	regulation of RNA stability	119	0.319595946	0.0003	0.001720882
GO:0001732	formation of cytoplasmic translation initiation complex	12	0.31954097	0.0009	0.002399874
GO:0034728	nucleosome organization	80	0.319520592	0.0004	0.002007244
GO:2000047	regulation of cell-cell adhesion mediated by cadherin	6	0.319476619	0.0011	0.002610912
GO:2000846	regulation of corticosteroid hormone secretion	8	0.319465894	0.0002	0.001341684
GO:0048715	negative regulation of oligodendrocyte differentiation	9	0.319462319	0.0009	0.002399874
GO:0006521	regulation of cellular amino acid metabolic process	43	0.319457664	0.0007	0.002248538
GO:0009437	carnitine metabolic process	11	0.319363519	< 0.001	< 0.001
GO:1990090	cellular response to nerve growth factor stimulus	26	0.319344619	0.0012	0.002741075
GO:0046602	regulation of mitotic centrosome separation	5	0.319330759	0.0029	0.004714719
GO:0001553	luteinization	7	0.319323405	< 0.001	< 0.001
GO:0009743	response to carbohydrate	115	0.319194225	0.0009	0.002399874
GO:0048713	regulation of oligodendrocyte differentiation	24	0.319176319	0.0011	0.002610912
GO:0034614	cellular response to reactive oxygen species	88	0.319123669	0.0014	0.002975058
GO:2000677	regulation of transcription regulatory region DNA binding	29	0.31908756	0.0007	0.002248538
GO:1904894	positive regulation of STAT cascade	42	0.319072133	0.0002	0.001341684
GO:0021535	cell migration in hindbrain	5	0.319021879	0.0044	0.006304227
GO:0009953	dorsal/ventral pattern formation	47	0.318990115	< 0.001	< 0.001
GO:0071425	hematopoietic stem cell proliferation	10	0.318970399	0.0061	0.008182551
GO:0060428	lung epithelium development	18	0.318961819	0.0046	0.006532041
GO:0070734	histone H3-K27 methylation	14	0.31895569	0.0014	0.002975058
GO:0006890	retrograde vesicle-mediated transport, Golgi to ER	51	0.318944154	0.0013	0.002859821
GO:0019229	regulation of vasoconstriction	21	0.318918919	0.0023	0.004032435
GO:1901532	regulation of hematopoietic progenitor cell differentiation	64	0.318910875	0.001	0.002498236
GO:0010591	regulation of lamellipodium assembly	10	0.318893179	0.0027	0.004491543
GO:0048070	regulation of developmental pigmentation	9	0.318890319	0.0031	0.004916949
GO:0031929	TOR signaling	60	0.318880309	0.0016	0.003223775
GO:0046605	regulation of centrosome cycle	32	0.318822394	0.0001	0.000877824
GO:0045023	G0 to G1 transition	31	0.31881928	0.0008	0.002313249
GO:1903321	negative regulation of protein modification by small protein conjugation or removal	56	0.318794815	0.0008	0.002313249
GO:0048846	axon extension involved in axon guidance	7	0.318771833	< 0.001	< 0.001
GO:1902284	neuron projection extension involved in neuron projection guidance	7	0.318771833	< 0.001	< 0.001
GO:0071871	response to epinephrine	7	0.318771833	0.004	0.005880507
GO:0014812	muscle cell migration	43	0.318727365	0.0006	0.002177563
GO:0033143	regulation of intracellular steroid hormone receptor signaling pathway	51	0.318717036	0.0012	0.002741075
GO:2000785	regulation of autophagosome assembly	24	0.318715144	0.0006	0.002177563
GO:0090263	positive regulation of canonical Wnt signaling pathway	83	0.31866462	0.0006	0.002177563
GO:0051348	negative regulation of transferase activity	145	0.318654418	0.0006	0.002177563
GO:0050910	detection of mechanical stimulus involved in sensory perception of sound	5	0.318610039	0.0057	0.007745437
GO:0010614	negative regulation of cardiac muscle hypertrophy	15	0.318610039	0.0028	0.004605006
GO:0014741	negative regulation of muscle hypertrophy	15	0.318610039	0.0028	0.004605006
GO:0045943	positive regulation of transcription from RNA polymerase I promoter	16	0.318581081	0.0007	0.002248538
GO:0006892	post-Golgi vesicle-mediated transport	53	0.318578956	0.0037	0.005561344
GO:0031145	anaphase-promoting complex-dependent catabolic process	60	0.318567139	0.0009	0.002399874
GO:0031960	response to corticosteroid	89	0.318557402	0.0005	0.002177563
GO:0044788	modulation by host of viral process	17	0.318525248	0.0006	0.002177563
GO:0003300	cardiac muscle hypertrophy	38	0.318512497	0.0009	0.002399874
GO:0016559	peroxisome fission	5	0.318507079	0.0009	0.002399874
GO:0016485	protein processing	125	0.318492664	0.0028	0.004605006
GO:0010833	telomere maintenance via telomere lengthening	52	0.318453618	< 0.001	< 0.001
GO:0035966	response to topologically incorrect protein	130	0.318439758	0.0019	0.003579279
GO:0006310	DNA recombination	142	0.318424058	< 0.001	< 0.001
GO:0051604	protein maturation	144	0.318420206	0.0027	0.004491543
GO:0000050	urea cycle	6	0.318404118	0.0001	0.000877824
GO:0019627	urea metabolic process	6	0.318404118	0.0001	0.000877824
GO:0071941	nitrogen cycle metabolic process	6	0.318404118	0.0001	0.000877824
GO:0075525	viral translational termination-reinitiation	5	0.318404118	0.002	0.003693422
GO:0046325	negative regulation of glucose import	8	0.318371943	0.0001	0.000877824
GO:0034067	protein localization to Golgi apparatus	12	0.318318318	0.0013	0.002859821
GO:0031497	chromatin assembly	71	0.318306234	0.0002	0.001341684
GO:0032330	regulation of chondrocyte differentiation	20	0.318223938	0.0001	0.000877824
GO:0045198	establishment of epithelial cell apical/basal polarity	7	0.318220261	0.0184	0.020586938
GO:0042094	interleukin-2 biosynthetic process	12	0.318211068	0.0047	0.006644432
GO:0007223	Wnt signaling pathway, calcium modulating pathway	19	0.318200908	0.0004	0.002007244
GO:0043620	regulation of DNA-templated transcription in response to stress	89	0.318158287	0.0011	0.002610912
GO:0043372	positive regulation of CD4-positive, alpha-beta T cell differentiation	13	0.318126918	0.0091	0.011322589
GO:0043407	negative regulation of MAP kinase activity	32	0.318122587	0.0005	0.002177563
GO:0022038	corpus callosum development	7	0.318109947	0.0017	0.003334595
GO:0072331	signal transduction by p53 class mediator	165	0.318098358	0.0007	0.002248538
GO:0032508	DNA duplex unwinding	51	0.318096248	0.0008	0.002313249
GO:0031023	microtubule organizing center organization	71	0.318085087	0.0006	0.002177563
GO:0046640	regulation of alpha-beta T cell proliferation	18	0.318075218	0.0183	0.020489441
GO:0046636	negative regulation of alpha-beta T cell activation	20	0.318056628	0.0004	0.002007244
GO:0045637	regulation of myeloid cell differentiation	131	0.31803472	0.0006	0.002177563
GO:0072273	metanephric nephron morphogenesis	16	0.318034106	0.0001	0.000877824
GO:0051384	response to glucocorticoid	79	0.318029422	0.0004	0.002007244
GO:0045861	negative regulation of proteolysis	159	0.317983212	0.0006	0.002177563
GO:2001238	positive regulation of extrinsic apoptotic signaling pathway	39	0.317975118	0.0021	0.003804235
GO:0045851	pH reduction	27	0.317975118	0.0034	0.005263104

GOIDlabel	GOName	size	categoryScores	pValPerm	pValPermCorr
GO:0051452	intracellular pH reduction	27	0.317975118	0.0034	0.005263104
GO:0045071	negative regulation of viral genome replication	35	0.317962861	0.0007	0.002248538
GO:0042795	snRNA transcription from RNA polymerase II promoter	51	0.317934741	0.0002	0.001341684
GO:0035774	positive regulation of insulin secretion involved in cellular response to glucose stimulus	13	0.317928918	0.0001	0.000877824
GO:0045923	positive regulation of fatty acid metabolic process	18	0.317903618	0.0029	0.004714719
GO:0071044	histone mRNA catabolic process	8	0.317889318	< 0.001	< 0.001
GO:0002931	response to ischemia	30	0.317889318	0.0024	0.004156304
GO:0002070	epithelial cell maturation	11	0.317865918	0.0151	0.01736105
GO:1903409	reactive oxygen species biosynthetic process	54	0.317855951	0.0006	0.002177563
GO:0042255	ribosome assembly	19	0.317848676	0.0011	0.002610912
GO:1902036	regulation of hematopoietic stem cell differentiation	54	0.317813051	0.0011	0.002610912
GO:0009636	response to toxic substance	117	0.317792518	0.0002	0.001341684
GO:0042398	cellular modified amino acid biosynthetic process	32	0.317784749	0.0025	0.004279479
GO:0001826	inner cell mass cell differentiation	5	0.317734878	0.0001	0.000877824
GO:0048841	regulation of axon extension involved in axon guidance	6	0.317717718	< 0.001	< 0.001
GO:0086069	bundle of His cell to Purkinje myocyte communication	5	0.317683398	0.0075	0.00968541
GO:0048026	positive regulation of mRNA splicing, via spliceosome	12	0.317674818	0.0043	0.006204188
GO:0006978	DNA damage response, signal transduction by p53 class mediator resulting in transcription of p21 class mediator	11	0.317655318	0.0007	0.002248538
GO:0070417	cellular response to cold	7	0.317631918	0.0023	0.004032435
GO:1902806	regulation of cell cycle G1/S phase transition	102	0.317591541	0.0023	0.004032435
GO:0002053	positive regulation of mesenchymal cell proliferation	16	0.317583655	0.0062	0.008281828
GO:0090344	negative regulation of cell aging	13	0.317552718	0.0011	0.002610912
GO:0007617	mating behavior	9	0.317517518	< 0.001	< 0.001
GO:0000018	regulation of DNA recombination	49	0.317505844	< 0.001	< 0.001
GO:0050920	regulation of chemotaxis	93	0.317499066	0.0004	0.002007244
GO:0090183	regulation of kidney development	30	0.317494637	0.0001	0.000877824
GO:0035306	positive regulation of dephosphorylation	24	0.317492492	0.0008	0.002313249
GO:0000729	DNA double-strand break processing	16	0.317471042	0.0005	0.002177563
GO:0010660	regulation of muscle cell apoptotic process	33	0.317468117	0.0021	0.003804235
GO:0010659	cardiac muscle cell apoptotic process	21	0.317411289	0.0011	0.002610912
GO:0003179	heart valve morphogenesis	32	0.317398649	< 0.001	< 0.001
GO:0046461	neutral lipid catabolic process	15	0.317374517	0.0005	0.002177563
GO:0046464	acylglycerol catabolic process	15	0.317374517	0.0005	0.002177563
GO:0006348	chromatin silencing at telomere	5	0.317374517	0.0123	0.014492124
GO:0001836	release of cytochrome c from mitochondria	35	0.317337746	0.0015	0.003088691
GO:0044773	mitotic DNA damage checkpoint	62	0.317337153	0.001	0.002498236
GO:0045191	regulation of isotype switching	19	0.31730678	< 0.001	< 0.001
GO:0090184	positive regulation of kidney development	22	0.317304317	0.0004	0.002007244
GO:0016064	immunoglobulin mediated immune response	62	0.317291485	0.0003	0.001720882
GO:0043484	regulation of RNA splicing	83	0.317284582	0.0015	0.003088691
GO:0002021	response to dietary excess	14	0.317227432	0.0011	0.002610912
GO:0042116	macrophage activation	43	0.317200922	0.0035	0.005358035
GO:0015693	magnesium ion transport	5	0.317168597	0.0176	0.019838132
GO:0043353	enucleate erythrocyte differentiation	5	0.317168597	0.0257	0.027793331
GO:1902231	positive regulation of intrinsic apoptotic signaling pathway in response to DNA damage	5	0.317168597	0.0084	0.010571327
GO:0048146	positive regulation of fibroblast proliferation	27	0.317164784	0.0016	0.003223775
GO:0048570	notochord morphogenesis	6	0.317117117	0.0251	0.027199847
GO:0007052	mitotic spindle organization	61	0.317104458	0.0009	0.002399874
GO:0001649	osteoblast differentiation	120	0.317042042	0.0006	0.002177563
GO:0070862	negative regulation of protein exit from endoplasmic reticulum	9	0.317031317	0.0078	0.009981807
GO:0005980	glycogen catabolic process	14	0.317025188	0.0037	0.005561344
GO:0009251	glucan catabolic process	14	0.317025188	0.0037	0.005561344
GO:1901679	nucleotide transmembrane transport	5	0.317014157	0.0298	0.031859654
GO:0031397	negative regulation of protein ubiquitination	52	0.317013167	0.0008	0.002313249
GO:0071378	cellular response to growth hormone stimulus	14	0.316988417	0.0001	0.000877824
GO:0007259	JAK-STAT cascade	69	0.316964169	0.0002	0.001341684
GO:0006338	chromatin remodeling	80	0.316936937	0.0009	0.002399874
GO:1902750	negative regulation of cell cycle G2/M phase transition	67	0.316936553	0.0008	0.002313249
GO:0006399	tRNA metabolic process	117	0.316923517	0.0014	0.002975058
GO:0072593	reactive oxygen species metabolic process	143	0.316920917	0.0005	0.002177563
GO:0032722	positive regulation of chemokine production	30	0.316911197	0.0001	0.000877824
GO:0061136	regulation of proteasomal protein catabolic process	91	0.316890831	0.0006	0.002177563
GO:0016054	organic acid catabolic process	149	0.316842442	0.0009	0.002399874
GO:0046395	carboxylic acid catabolic process	149	0.316842442	0.0009	0.002399874
GO:0090002	establishment of protein localization to plasma membrane	27	0.31684065	0.001	0.002498236
GO:0045103	intermediate filament-based process	25	0.316839125	0.0022	0.003910907
GO:0045104	intermediate filament cytoskeleton organization	25	0.316839125	0.0022	0.003910907
GO:0031442	positive regulation of mRNA 3'-end processing	11	0.316789517	0.0032	0.005044175
GO:0045686	negative regulation of glial cell differentiation	12	0.316773917	0.0024	0.004156304
GO:0070391	response to lipoteichoic acid	6	0.316731017	0.0038	0.005666214
GO:0071223	cellular response to lipoteichoic acid	6	0.316731017	0.0038	0.005666214
GO:0043031	negative regulation of macrophage activation	6	0.316731017	0.0048	0.006748379
GO:0033687	osteoblast proliferation	16	0.316682754	0.0055	0.007544447
GO:0055072	iron ion homeostasis	53	0.316670309	0.0012	0.002741075
GO:2000117	negative regulation of cysteine-type endopeptidase activity	54	0.316664283	0.0006	0.002177563
GO:0045738	negative regulation of DNA repair	17	0.31664774	< 0.001	< 0.001
GO:0002371	dendritic cell cytokine production	9	0.316630917	0.0004	0.002007244
GO:0002730	regulation of dendritic cell cytokine production	9	0.316630917	0.0004	0.002007244
GO:0050701	interleukin-1 secretion	29	0.316620068	0.0001	0.000877824
GO:1902042	negative regulation of extrinsic apoptotic signaling pathway via death domain receptors	22	0.316614017	0.0061	0.008182551
GO:0045766	positive regulation of angiogenesis	95	0.316607736	0.0028	0.004605006
GO:0021554	optic nerve development	8	0.316570142	0.0049	0.006860215

GOIDlabel	GOName	size	categoryScores	pValPerm	pValPermCorr
GO:0044843	cell cycle G1/S phase transition	163	0.31655968	0.0008	0.002313249
GO:0009301	snRNA transcription	52	0.316513217	0.0003	0.001720882
GO:0044275	cellular carbohydrate catabolic process	24	0.316505792	0.0013	0.002859821
GO:0045086	positive regulation of interleukin-2 biosynthetic process	7	0.316492002	0.0199	0.022056122
GO:0032371	regulation of sterol transport	24	0.316462891	0.001	0.002498236
GO:0032374	regulation of cholesterol transport	24	0.316462891	0.001	0.002498236
GO:0070665	positive regulation of leukocyte proliferation	71	0.316439176	0.0005	0.002177563
GO:0016073	snRNA metabolic process	55	0.316438516	0.0003	0.001720882
GO:0070192	chromosome organization involved in meiotic cell cycle	20	0.316435006	0.0002	0.001341684
GO:0035510	DNA dealkylation	17	0.316405481	0.0023	0.004032435
GO:0009408	response to heat	102	0.316387816	0.0008	0.002313249
GO:0033238	regulation of cellular amine metabolic process	52	0.316384516	0.0004	0.002007244
GO:0050852	T cell receptor signaling pathway	116	0.316338259	0.0007	0.002248538
GO:0002887	negative regulation of myeloid leukocyte mediated immunity	5	0.316293436	0.0002	0.001341684
GO:0043305	negative regulation of mast cell degranulation	5	0.316293436	0.0002	0.001341684
GO:0010459	negative regulation of heart rate	8	0.316248391	0.0002	0.001341684
GO:0010390	histone monoubiquitination	21	0.316246859	0.0009	0.002399874
GO:0048753	pigment granule organization	19	0.316195895	0.0002	0.001341684
GO:1903362	regulation of cellular protein catabolic process	134	0.316189324	0.0004	0.002007244
GO:1903307	positive regulation of regulated secretory pathway	18	0.316187616	0.002	0.003693422
GO:1902166	negative regulation of intrinsic apoptotic signaling pathway in response to DNA damage by p53 class mediator	13	0.316127116	0.0001	0.000877824
GO:0032488	Cdc42 protein signal transduction	7	0.316087516	0.0007	0.002248538
GO:0021696	cerebellar cortex morphogenesis	16	0.316055341	0.0018	0.003459409
GO:0060740	prostate gland epithelium morphogenesis	14	0.316032359	0.0057	0.007745437
GO:0042417	dopamine metabolic process	18	0.316016016	0.0003	0.001720882
GO:0002320	lymphoid progenitor cell differentiation	10	0.316010296	0.0009	0.002399874
GO:0072332	intrinsic apoptotic signaling pathway by p53 class mediator	52	0.315998416	0.0006	0.002177563
GO:0046916	cellular transition metal ion homeostasis	55	0.315947116	0.0004	0.002007244
GO:0006879	cellular iron ion homeostasis	43	0.315943851	0.0005	0.002177563
GO:2001268	negative regulation of cysteine-type endopeptidase activity involved in apoptotic signaling pathway	5	0.315933076	0.0003	0.001720882
GO:0035337	fatty-acyl-CoA metabolic process	23	0.315919646	0.0032	0.005044175
GO:0002523	leukocyte migration involved in inflammatory response	8	0.315894466	0.0007	0.002248538
GO:0097320	membrane tubulation	12	0.315851566	0.0055	0.007544447
GO:1902165	regulation of intrinsic apoptotic signaling pathway in response to DNA damage by p53 class mediator	15	0.315830116	0.0002	0.001341684
GO:0071294	cellular response to zinc ion	17	0.315814975	0.0016	0.003223775
GO:0050851	antigen receptor-mediated signaling pathway	136	0.315799833	0.0007	0.002248538
GO:0002643	regulation of tolerance induction	10	0.315778636	0.0144	0.016652368
GO:0006622	protein targeting to lysosome	16	0.315765766	0.0073	0.009463568
GO:1903428	positive regulation of reactive oxygen species biosynthetic process	24	0.315744316	0.0006	0.002177563
GO:0090197	positive regulation of chemokine secretion	12	0.315744316	< 0.001	< 0.001
GO:0044803	multi-organism membrane organization	28	0.315710609	0.0016	0.003223775
GO:0061082	myeloid leukocyte cytokine production	20	0.315688546	0.0019	0.003579279
GO:0002724	regulation of T cell cytokine production	12	0.315679966	0.0049	0.006860215
GO:2000045	regulation of G1/S transition of mitotic cell cycle	94	0.315622005	0.0027	0.004491543
GO:0051775	response to redox state	7	0.315609487	0.0003	0.001720882
GO:0032642	regulation of chemokine production	46	0.315583907	0.0002	0.001341684
GO:0050858	negative regulation of antigen receptor-mediated signaling pathway	14	0.315572716	0.0014	0.002975058
GO:0070584	mitochondrion morphogenesis	15	0.315572716	0.0011	0.002610912
GO:0051262	protein tetramerization	94	0.315515211	0.0005	0.002177563
GO:0002695	negative regulation of leukocyte activation	83	0.315495185	0.0003	0.001720882
GO:0060675	ureteric bud morphogenesis	36	0.315486915	0.0001	0.000877824
GO:0014015	positive regulation of gliogenesis	44	0.315484965	0.0033	0.005149584
GO:0060632	regulation of microtubule-based movement	14	0.315480787	< 0.001	< 0.001
GO:0070229	negative regulation of lymphocyte apoptotic process	16	0.31547619	0.0005	0.002177563
GO:0006275	regulation of DNA replication	58	0.315475081	0.0005	0.002177563
GO:0050667	homocysteine metabolic process	5	0.315469755	0.001	0.002498236
GO:0099024	plasma membrane invagination	32	0.315444015	0.0048	0.006748379
GO:1904356	regulation of telomere maintenance via telomere lengthening	41	0.315434598	0.0001	0.000877824
GO:1903050	regulation of proteolysis involved in cellular protein catabolic process	110	0.315420615	0.0006	0.002177563
GO:0006302	double-strand break repair	124	0.315365135	0.0003	0.001720882
GO:1901988	negative regulation of cell cycle phase transition	158	0.31534301	0.0009	0.002399874
GO:0002548	monocyte chemotaxis	20	0.315341055	0.0005	0.002177563
GO:0048298	positive regulation of isotype switching to IgA isotypes	5	0.315315315	0.0014	0.002975058
GO:0000082	G1/S transition of mitotic cell cycle	155	0.315311994	0.0008	0.002313249
GO:0006970	response to osmotic stress	49	0.315278544	0.0028	0.004605006
GO:0048290	isotype switching to IgA isotypes	6	0.315272415	0.0011	0.002610912
GO:0048296	regulation of isotype switching to IgA isotypes	6	0.315272415	0.0011	0.002610912
GO:0051965	positive regulation of synapse assembly	23	0.315248167	< 0.001	< 0.001
GO:1903672	positive regulation of sprouting angiogenesis	22	0.315233415	0.0011	0.002610912
GO:0010839	negative regulation of keratinocyte proliferation	5	0.315212355	0.0002	0.001341684
GO:0071897	DNA biosynthetic process	122	0.315199274	0.0004	0.002007244
GO:0006760	folic acid-containing compound metabolic process	20	0.315173745	0.0038	0.005666214
GO:2000514	regulation of CD4-positive, alpha-beta T cell activation	31	0.315149251	0.0024	0.004156304
GO:1903555	regulation of tumor necrosis factor superfamily cytokine production	81	0.315140537	0.0014	0.002975058
GO:0007406	negative regulation of neuroblast proliferation	5	0.315109395	0.0041	0.005981983
GO:0072111	cell proliferation involved in kidney development	10	0.315006435	0.0001	0.000877824
GO:0030278	regulation of ossification	102	0.314992303	0.0002	0.001341684
GO:0042976	activation of Janus kinase activity	7	0.314984372	< 0.001	< 0.001
GO:1902115	regulation of organelle assembly	106	0.314972924	0.0005	0.002177563
GO:0006893	Golgi to plasma membrane transport	28	0.314947601	0.0032	0.005044175
GO:0070498	interleukin-1-mediated signaling pathway	67	0.314927294	0.0011	0.002610912
GO:0061213	positive regulation of mesonephros development	13	0.314919315	0.0009	0.002399874

GOIDlabel	GOName	size	categoryScores	pValPerm	pValPermCorr
GO:0061217	regulation of mesonephros development	13	0.314919315	0.0009	0.002399874
GO:0090189	regulation of branching involved in ureteric bud morphogenesis	13	0.314919315	0.0009	0.002399874
GO:0090190	positive regulation of branching involved in ureteric bud morphogenesis	13	0.314919315	0.0009	0.002399874
GO:0090083	regulation of inclusion body assembly	11	0.314917515	0.0013	0.002859821
GO:0071467	cellular response to pH	9	0.314914915	0.0135	0.015731487
GO:0007077	mitotic nuclear envelope disassembly	7	0.314910829	< 0.001	< 0.001
GO:0009164	nucleoside catabolic process	19	0.314868252	0.0033	0.005149584
GO:0042113	B cell activation	121	0.314849442	0.0008	0.002313249
GO:0050777	negative regulation of immune response	71	0.314822267	0.0001	0.000877824
GO:0009749	response to glucose	93	0.314808818	0.001	0.002498236
GO:0006596	polyamine biosynthetic process	6	0.314800515	0.0023	0.004032435
GO:0036490	regulation of translation in response to endoplasmic reticulum stress	5	0.314800515	0.0002	0.001341684
GO:0001913	T cell mediated cytotoxicity	18	0.314771915	0.0069	0.009046225
GO:0045840	positive regulation of mitotic nuclear division	27	0.314667048	0.0023	0.004032435
GO:0030857	negative regulation of epithelial cell differentiation	22	0.314636715	0.0004	0.002007244
GO:0009266	response to temperature stimulus	134	0.314625713	0.0005	0.002177563
GO:0043153	entrainment of circadian clock by photoperiod	9	0.314600315	0.0015	0.003088691
GO:0036065	fucosylation	5	0.314594595	0.0382	0.040087041
GO:0036066	protein O-linked fucosylation	5	0.314594595	0.0382	0.040087041
GO:0046822	regulation of nucleocytoplasmic transport	89	0.314557575	0.0008	0.002313249
GO:0051785	positive regulation of nuclear division	28	0.314524729	0.0029	0.004714719
GO:1905517	macrophage migration	27	0.314514515	0.0004	0.002007244
GO:2000516	positive regulation of CD4-positive, alpha-beta T cell activation	17	0.314512832	0.0153	0.017559319
GO:1903909	regulation of receptor clustering	7	0.314506343	0.0003	0.001720882
GO:0014888	striated muscle adaptation	18	0.314485914	0.003	0.004825593
GO:0030705	cytoskeleton-dependent intracellular transport	89	0.314467919	0.0001	0.000877824
GO:0008630	intrinsic apoptotic signaling pathway in response to DNA damage	63	0.314465486	0.0004	0.002007244
GO:1900180	regulation of protein localization to nucleus	92	0.314431201	0.0006	0.002177563
GO:0042308	negative regulation of protein import into nucleus	18	0.314428714	0.0003	0.001720882
GO:1904590	negative regulation of protein import	18	0.314428714	0.0003	0.001720882
GO:0045807	positive regulation of endocytosis	73	0.314416177	0.0015	0.003088691
GO:0000096	sulfur amino acid metabolic process	26	0.314404514	0.0001	0.000877824
GO:0032543	mitochondrial translation	104	0.314369864	0.0045	0.006417207
GO:1905268	negative regulation of chromatin organization	30	0.314362934	0.0001	0.000877824
GO:0042790	transcription of nuclear large rRNA transcript from RNA polymerase I promoter	10	0.314362934	0.0011	0.002610912
GO:1901836	regulation of transcription of nuclear large rRNA transcript from RNA polymerase I promoter	10	0.314362934	0.0011	0.002610912
GO:0050921	positive regulation of chemotaxis	69	0.314326749	0.0005	0.002177563
GO:0048025	negative regulation of mRNA splicing, via spliceosome	13	0.314325314	0.003	0.004825593
GO:0032490	detection of molecule of bacterial origin	7	0.314285714	0.0002	0.001341684
GO:0098581	detection of external biotic stimulus	7	0.314285714	0.0002	0.001341684
GO:0071887	leukocyte apoptotic process	58	0.3142724	0.0017	0.003334595
GO:0006493	protein O-linked glycosylation	60	0.314199914	0.0006	0.002177563
GO:2001242	regulation of intrinsic apoptotic signaling pathway	106	0.314132731	0.0011	0.002610912
GO:0071470	cellular response to osmotic stress	27	0.314123647	0.01	0.012232246
GO:0030183	B cell differentiation	64	0.314108752	0.0027	0.004491543
GO:0051310	metaphase plate congression	37	0.314042228	0.0001	0.000877824
GO:0045117	azole transport	11	0.314004914	0.0042	0.006097175
GO:0051004	regulation of lipoprotein lipase activity	10	0.314002574	0.0039	0.005778819
GO:0034198	cellular response to amino acid starvation	25	0.313966538	0.0006	0.002177563
GO:2001044	regulation of integrin-mediated signaling pathway	10	0.313951094	0.0041	0.005981983
GO:0051016	barbed-end actin filament capping	9	0.313942514	0.0009	0.002399874
GO:0045446	endothelial cell differentiation	59	0.313927972	0.0008	0.002313249
GO:0071435	potassium ion export	7	0.313918	0.0028	0.004605006
GO:0097623	potassium ion export across plasma membrane	7	0.313918	0.0028	0.004605006
GO:0006986	response to unfolded protein	121	0.313904932	0.0025	0.004279479
GO:0070663	regulation of leukocyte proliferation	115	0.313902971	0.0008	0.002313249
GO:0051973	positive regulation of telomerase activity	26	0.313899614	0.0001	0.000877824
GO:0002720	positive regulation of cytokine production involved in immune response	26	0.313830314	0.0021	0.003804235
GO:0002224	toll-like receptor signaling pathway	82	0.31381486	0.0006	0.002177563
GO:0042246	tissue regeneration	36	0.313799514	0.0076	0.009790742
GO:0032480	negative regulation of type I interferon production	31	0.313795823	0.0029	0.004714719
GO:0060788	ectodermal placode formation	8	0.313738739	0.0121	0.014285532
GO:0071696	ectodermal placode development	8	0.313738739	0.0121	0.014285532
GO:0071697	ectodermal placode morphogenesis	8	0.313738739	0.0121	0.014285532
GO:0032104	regulation of response to extracellular stimulus	15	0.313736594	0.0008	0.002313249
GO:0032107	regulation of response to nutrient levels	15	0.313736594	0.0008	0.002313249
GO:1901658	glycosyl compound catabolic process	24	0.313728014	0.0023	0.004032435
GO:1903830	magnesium ion transmembrane transport	8	0.313706564	0.022	0.024152627
GO:0032648	regulation of interferon-beta production	35	0.313645891	0.0006	0.002177563
GO:0032230	positive regulation of synaptic transmission, GABAergic	6	0.313642214	< 0.001	< 0.001
GO:0016556	mRNA modification	13	0.313632314	0.0027	0.004491543
GO:0080009	mRNA methylation	13	0.313632314	0.0027	0.004491543
GO:0016126	sterol biosynthetic process	48	0.313626126	0.0016	0.003223775
GO:0045601	regulation of endothelial cell differentiation	19	0.313567703	0.0012	0.002741075
GO:0019079	viral genome replication	78	0.313563014	0.0007	0.002248538
GO:0042307	positive regulation of protein import into nucleus	31	0.313538423	0.0012	0.002741075
GO:0035751	regulation of lysosomal lumen pH	10	0.313487773	0.025	0.027100697
GO:0016571	histone methylation	80	0.313481338	0.0011	0.002610912
GO:0031668	cellular response to extracellular stimulus	122	0.313454438	0.0011	0.002610912
GO:0043371	negative regulation of CD4-positive, alpha-beta T cell differentiation	12	0.313449163	0.0001	0.000877824
GO:0009072	aromatic amino acid family metabolic process	15	0.313427713	0.0015	0.003088691
GO:0043968	histone H2A acetylation	12	0.313427713	0.0001	0.000877824
GO:0045471	response to ethanol	70	0.313417908	0.0002	0.001341684

GOIDlabel	GOName	size	categoryScores	pValPerm	pValPermCorr
GO:0050854	regulation of antigen receptor-mediated signaling pathway	32	0.313416988	0.0004	0.002007244
GO:0071706	tumor necrosis factor superfamily cytokine production	85	0.313410553	0.0014	0.002975058
GO:0006623	protein targeting to vacuole	17	0.313407525	0.0045	0.006417207
GO:0072666	establishment of protein localization to vacuole	17	0.313407525	0.0045	0.006417207
GO:0009746	response to hexose	96	0.313392857	0.0014	0.002975058
GO:1902254	negative regulation of intrinsic apoptotic signaling pathway by p53 class mediator	17	0.313362102	< 0.001	< 0.001
GO:0034138	toll-like receptor 3 signaling pathway	14	0.313348042	0.0124	0.014577606
GO:0006388	tRNA splicing, via endonucleolytic cleavage and ligation	15	0.313324753	0.0026	0.004377754
GO:1903902	positive regulation of viral life cycle	75	0.313317889	0.0008	0.002313249
GO:2000484	positive regulation of interleukin-8 secretion	10	0.313307593	0.0187	0.020874952
GO:0046427	positive regulation of JAK-STAT cascade	39	0.313295713	0.0002	0.001341684
GO:1903533	regulation of protein targeting	103	0.3132911	0.0005	0.002177563
GO:0006085	acetyl-CoA biosynthetic process	15	0.313290433	0.0058	0.007856196
GO:0032984	macromolecular complex disassembly	184	0.31326031	0.0018	0.003459409
GO:0070316	regulation of G0 to G1 transition	30	0.313256113	0.0008	0.002313249
GO:2000106	regulation of leukocyte apoptotic process	42	0.313243856	0.0007	0.002248538
GO:0032606	type I interferon production	86	0.313241148	0.0006	0.002177563
GO:0072524	pyridine-containing compound metabolic process	111	0.313230605	0.0018	0.003459409
GO:0000188	inactivation of MAPK activity	9	0.313198913	0.004	0.005880507
GO:0032691	negative regulation of interleukin-1 beta production	14	0.313164185	0.011	0.013192134
GO:0042594	response to starvation	100	0.313142857	0.0014	0.002975058
GO:0030851	granulocyte differentiation	18	0.313141713	0.0013	0.002859821
GO:0060148	positive regulation of posttranscriptional gene silencing	15	0.313135993	0.0014	0.002975058
GO:2000637	positive regulation of gene silencing by miRNA	15	0.313135993	0.0014	0.002975058
GO:0002637	regulation of immunoglobulin production	34	0.313134984	< 0.001	< 0.001
GO:0002312	B cell activation involved in immune response	42	0.313133542	0.0006	0.002177563
GO:0032602	chemokine production	51	0.313109748	0.0001	0.000877824
GO:0050670	regulation of lymphocyte proliferation	109	0.313083726	0.0009	0.002399874
GO:2000780	negative regulation of double-strand break repair	16	0.313079151	< 0.001	< 0.001
GO:2001020	regulation of response to DNA damage stimulus	123	0.31305103	0.0006	0.002177563
GO:0045428	regulation of nitric oxide biosynthetic process	32	0.313014801	0.0007	0.002248538
GO:0046349	amino sugar biosynthetic process	9	0.312998713	0.0001	0.000877824
GO:2000696	regulation of epithelial cell differentiation involved in kidney development	11	0.31292851	0.0001	0.000877824
GO:0002831	regulation of response to biotic stimulus	102	0.312925531	0.0005	0.002177563
GO:0007194	negative regulation of adenylate cyclase activity	9	0.312912913	0.0037	0.005561344
GO:0030800	negative regulation of cyclic nucleotide metabolic process	9	0.312912913	0.0037	0.005561344
GO:0030803	negative regulation of cyclic nucleotide biosynthetic process	9	0.312912913	0.0037	0.005561344
GO:0030815	negative regulation of cAMP metabolic process	9	0.312912913	0.0037	0.005561344
GO:0030818	negative regulation of cAMP biosynthetic process	9	0.312912913	0.0037	0.005561344
GO:0031280	negative regulation of cyclase activity	9	0.312912913	0.0037	0.005561344
GO:0035726	common myeloid progenitor cell proliferation	5	0.312895753	0.0011	0.002610912
GO:0050435	beta-amyloid metabolic process	22	0.312893413	0.0083	0.010468217
GO:0071901	negative regulation of protein serine/threonine kinase activity	64	0.312890122	0.0008	0.002313249
GO:2000779	regulation of double-strand break repair	41	0.312860596	0.0001	0.000877824
GO:0022400	regulation of rhodopsin mediated signaling pathway	10	0.312844273	0.0071	0.009268394
GO:0090196	regulation of chemokine secretion	13	0.312820513	< 0.001	< 0.001
GO:0050866	negative regulation of cell activation	93	0.3127939	0.0004	0.002007244
GO:0002063	chondrocyte development	15	0.312792793	0.0002	0.001341684
GO:0006739	NADP metabolic process	22	0.312764713	0.0022	0.003910907
GO:0007093	mitotic cell cycle checkpoint	100	0.312759331	0.0007	0.002248538
GO:0072170	metanephric tubule development	14	0.312741313	0.0001	0.000877824
GO:0072234	metanephric nephron tubule development	14	0.312741313	0.0001	0.000877824
GO:0032438	melanosome organization	18	0.312727013	0.0002	0.001341684
GO:1901888	regulation of cell junction assembly	54	0.312717479	0.0016	0.003223775
GO:0009435	NAD biosynthetic process	17	0.31271103	0.0009	0.002399874
GO:0048730	epidermis morphogenesis	16	0.31269305	0.0001	0.000877824
GO:0032675	regulation of interleukin-6 production	67	0.312614534	0.0012	0.002741075
GO:0070373	negative regulation of ERK1 and ERK2 cascade	33	0.312561913	0.0014	0.002975058
GO:0006695	cholesterol biosynthetic process	45	0.312558273	0.0022	0.003910907
GO:1902653	secondary alcohol biosynthetic process	45	0.312558273	0.0022	0.003910907
GO:0070131	positive regulation of mitochondrial translation	11	0.312554113	0.0044	0.006304227
GO:0044344	cellular response to fibroblast growth factor stimulus	62	0.312533732	0.0003	0.001720882
GO:0006140	regulation of nucleotide metabolic process	92	0.312517486	0.0018	0.003459409
GO:0071675	regulation of mononuclear cell migration	26	0.312464112	0.0003	0.001720882
GO:0010001	glial cell differentiation	125	0.312424196	0.0015	0.003088691
GO:0010640	regulation of platelet-derived growth factor receptor signaling pathway	13	0.312404712	0.004	0.005880507
GO:0003159	morphogenesis of an endothelium	15	0.312398112	0.0044	0.006304227
GO:2000425	regulation of apoptotic cell clearance	5	0.312380952	0.0121	0.014285532
GO:0030098	lymphocyte differentiation	168	0.312376662	0.0014	0.002975058
GO:0048339	paraxial mesoderm development	11	0.312366912	< 0.001	< 0.001
GO:0070301	cellular response to hydrogen peroxide	54	0.312340912	0.0014	0.002975058
GO:0046661	male sex differentiation	91	0.312319855	0.0006	0.002177563
GO:0002369	T cell cytokine production	16	0.31230695	0.0037	0.005561344
GO:0061326	renal tubule development	52	0.312305712	0.0002	0.001341684
GO:0032434	regulation of proteasomal ubiquitin-dependent protein catabolic process	64	0.312298906	0.0013	0.002859821
GO:1900026	positive regulation of substrate adhesion-dependent cell spreading	22	0.312261612	0.0015	0.003088691
GO:0000302	response to reactive oxygen species	127	0.3122407	0.0014	0.002975058
GO:0050931	pigment cell differentiation	20	0.312175032	0.0004	0.002007244
GO:0045602	negative regulation of endothelial cell differentiation	5	0.312175032	0.0201	0.022258436
GO:0006271	DNA strand elongation involved in DNA replication	5	0.312123552	0.0045	0.006417207
GO:0032944	regulation of mononuclear cell proliferation	110	0.312121212	0.001	0.002498236
GO:0014819	regulation of skeletal muscle contraction	6	0.312097812	0.0003	0.001720882
GO:0043129	surfactant homeostasis	6	0.312097812	0.0008	0.002313249

GOIDlabel	GOName	size	categoryScores	pValPerm	pValPermCorr
GO:0048875	chemical homeostasis within a tissue	6	0.312097812	0.0008	0.002313249
GO:0051156	glucose 6-phosphate metabolic process	17	0.3120751	0.0014	0.002975058
GO:0042133	neurotransmitter metabolic process	18	0.312054912	< 0.001	< 0.001
GO:0016052	carbohydrate catabolic process	114	0.31204588	0.0018	0.003459409
GO:0002181	cytoplasmic translation	52	0.312003762	0.0006	0.002177563
GO:1904892	regulation of STAT cascade	56	0.312001287	0.0002	0.001341684
GO:0070189	kynurenine metabolic process	5	0.311969112	0.0029	0.004714719
GO:0001765	membrane raft assembly	6	0.311969112	0.0181	0.020286898
GO:0006883	cellular sodium ion homeostasis	5	0.311917632	0.0391	0.040970783
GO:1904018	positive regulation of vasculature development	105	0.311885763	0.0021	0.003804235
GO:0032732	positive regulation of interleukin-1 production	24	0.311883312	0.0001	0.000877824
GO:0007051	spindle organization	91	0.311870112	0.0009	0.002399874
GO:0001562	response to protozoan	7	0.311822026	< 0.001	< 0.001
GO:0042832	defense response to protozoan	7	0.311822026	< 0.001	< 0.001
GO:0071774	response to fibroblast growth factor	65	0.311806752	0.0004	0.002007244
GO:0034154	toll-like receptor 7 signaling pathway	5	0.311763192	0.0003	0.001720882
GO:0048515	spermatid differentiation	55	0.311758512	0.0001	0.000877824
GO:0072425	signal transduction involved in G2 DNA damage checkpoint	6	0.311754612	0.0005	0.002177563
GO:0014910	regulation of smooth muscle cell migration	32	0.311743887	0.0008	0.002313249
GO:0019682	glyceraldehyde-3-phosphate metabolic process	13	0.311711712	0.0078	0.009981807
GO:0034134	toll-like receptor 2 signaling pathway	11	0.311711712	0.0014	0.002975058
GO:0002517	T cell tolerance induction	6	0.311668812	0.0185	0.020677043
GO:0032526	response to retinoic acid	47	0.311640516	0.0002	0.001341684
GO:0006656	phosphatidylcholine biosynthetic process	27	0.311625912	0.0019	0.003579279
GO:0070757	interleukin-35-mediated signaling pathway	6	0.311625912	< 0.001	< 0.001
GO:0006333	chromatin assembly or disassembly	86	0.311621921	0.0004	0.002007244
GO:0002313	mature B cell differentiation involved in immune response	13	0.311592912	0.016	0.018267288
GO:0010464	regulation of mesenchymal cell proliferation	20	0.311570142	0.0061	0.008182551
GO:0002248	connective tissue replacement involved in inflammatory response wound healing	5	0.311557272	0.0091	0.011322589
GO:0045979	positive regulation of nucleoside metabolic process	23	0.311543842	0.0013	0.002859821
GO:1903580	positive regulation of ATP metabolic process	23	0.311543842	0.0013	0.002859821
GO:0046888	negative regulation of hormone secretion	29	0.311534194	0.0008	0.002313249
GO:0018212	peptidyl-tyrosine modification	157	0.311514973	0.0006	0.002177563
GO:0035608	protein deglutamylation	5	0.311505792	0.0031	0.004916949
GO:0009416	response to light stimulus	144	0.311500787	0.0007	0.002248538
GO:0043500	muscle adaptation	56	0.311463504	0.0035	0.005358035
GO:0031572	G2 DNA damage checkpoint	18	0.311454311	0.0011	0.002610912
GO:0006241	CTP biosynthetic process	8	0.311422136	0.0057	0.007745437
GO:0009209	pyrimidine ribonucleoside triphosphate biosynthetic process	8	0.311422136	0.0057	0.007745437
GO:0046036	CTP metabolic process	8	0.311422136	0.0057	0.007745437
GO:2001021	negative regulation of response to DNA damage stimulus	46	0.311415142	< 0.001	< 0.001
GO:0034502	protein localization to chromosome	56	0.311376172	0.0003	0.001720882
GO:0060033	anatomical structure regression	8	0.311357786	0.0064	0.008500875
GO:0045717	negative regulation of fatty acid biosynthetic process	10	0.311351351	< 0.001	< 0.001
GO:0009109	coenzyme catabolic process	7	0.311343997	< 0.001	< 0.001
GO:0060218	hematopoietic stem cell differentiation	58	0.311338925	0.001	0.002498236
GO:0050863	regulation of T cell activation	153	0.311321405	0.0013	0.002859821
GO:0048524	positive regulation of viral process	77	0.311290511	0.0008	0.002313249
GO:0002576	platelet degranulation	75	0.311282711	0.0007	0.002248538
GO:0030277	maintenance of gastrointestinal epithelium	12	0.311239811	0.0001	0.000877824
GO:0035810	positive regulation of urine volume	6	0.311239811	0.0035	0.005358035
GO:0071158	positive regulation of cell cycle arrest	53	0.311172628	0.0013	0.002859821
GO:1902914	regulation of protein polyubiquitination	14	0.31116014	0.0027	0.004491543
GO:0044743	intracellular protein transmembrane import	24	0.311132561	0.003	0.004825593
GO:1990542	mitochondrial transmembrane transport	39	0.311130911	0.0044	0.006304227
GO:1902389	ceramide 1-phosphate transport	6	0.311111111	0.0043	0.006204188
GO:0043555	regulation of translation in response to stress	13	0.311097911	0.0003	0.001720882
GO:0003014	renal system process	58	0.311072649	0.002	0.003693422
GO:0051153	regulation of striated muscle cell differentiation	48	0.311062849	0.0056	0.007655255
GO:0071356	cellular response to tumor necrosis factor	157	0.31103788	0.0003	0.001720882
GO:1904292	regulation of ERAD pathway	20	0.311029601	0.0035	0.005358035
GO:0032088	negative regulation of NF-kappaB transcription factor activity	47	0.31102166	0.0001	0.000877824
GO:1903779	regulation of cardiac conduction	21	0.311013054	0.0009	0.002399874
GO:0045070	positive regulation of viral genome replication	24	0.311003861	0.0007	0.002248538
GO:0035067	negative regulation of histone acetylation	7	0.310976282	0.0001	0.000877824
GO:0070278	extracellular matrix constituent secretion	7	0.310976282	0.0143	0.016557758
GO:0046856	phosphatidylinositol dephosphorylation	15	0.310905191	0.0071	0.009268394
GO:0033209	tumor necrosis factor-mediated signaling pathway	107	0.31089621	0.0003	0.001720882
GO:0007179	transforming growth factor beta receptor signaling pathway	105	0.310875774	0.0012	0.002741075
GO:0033673	negative regulation of kinase activity	127	0.310830065	0.0007	0.002248538
GO:0019882	antigen processing and presentation	152	0.310805731	0.0015	0.003088691
GO:0072401	signal transduction involved in DNA integrity checkpoint	45	0.310762191	0.0011	0.002610912
GO:0072422	signal transduction involved in DNA damage checkpoint	45	0.310762191	0.0011	0.002610912
GO:0030111	regulation of Wnt signaling pathway	191	0.310733321	0.0005	0.002177563
GO:0007097	nuclear migration	7	0.310718882	0.002	0.003693422
GO:0040023	establishment of nucleus localization	7	0.310718882	0.002	0.003693422
GO:0033262	regulation of nuclear cell cycle DNA replication	10	0.310707851	0.0064	0.008500875
GO:0010657	muscle cell apoptotic process	34	0.310697252	0.0018	0.003459409
GO:2001234	negative regulation of apoptotic signaling pathway	133	0.310691787	0.0019	0.003579279
GO:0007080	mitotic metaphase plate congression	29	0.310682111	0.0008	0.002313249
GO:0006949	syncytium formation	24	0.310628486	0.0037	0.005561344
GO:0046031	ADP metabolic process	71	0.310613229	0.003	0.004825593
GO:0001709	cell fate determination	18	0.310610611	0.0001	0.000877824

GOIDlabel	GOName	size	categoryScores	pValPerm	pValPermCorr
GO:0018108	peptidyl-tyrosine phosphorylation	156	0.310560011	0.0006	0.002177563
GO:0021782	glial cell development	70	0.310557088	0.0026	0.004377754
GO:0032210	regulation of telomere maintenance via telomerase	37	0.310549932	0.0001	0.000877824
GO:0071496	cellular response to external stimulus	172	0.310508515	0.0013	0.002859821
GO:0009163	nucleoside biosynthetic process	75	0.310489918	0.002	0.003693422
GO:0014012	peripheral nervous system axon regeneration	5	0.31047619	0.0352	0.037152965
GO:1902808	positive regulation of cell cycle G1/S phase transition	29	0.31046909	0.0018	0.003459409
GO:0007040	lysosome organization	40	0.31045045	0.0005	0.002177563
GO:0080171	lytic vacuole organization	40	0.31045045	0.0005	0.002177563
GO:0042168	heme metabolic process	22	0.31043641	0.0136	0.015813319
GO:0007625	grooming behavior	9	0.31042471	0.0055	0.007544447
GO:0060856	establishment of blood-brain barrier	6	0.31042471	0.0031	0.004916949
GO:0003158	endothelium development	72	0.31042471	0.0009	0.002399874
GO:0002285	lymphocyte activation involved in immune response	88	0.31041886	0.0006	0.002177563
GO:0006611	protein export from nucleus	37	0.310396883	0.0015	0.003088691
GO:0007095	mitotic G2 DNA damage checkpoint	13	0.31036531	0.0033	0.005149584
GO:0072080	nephron tubule development	51	0.310354052	0.0002	0.001341684
GO:0043241	protein complex disassembly	158	0.310344884	0.0022	0.003910907
GO:0046456	icosanoid biosynthetic process	33	0.31029991	0.0005	0.002177563
GO:1901570	fatty acid derivative biosynthetic process	33	0.31029991	0.0005	0.002177563
GO:0006977	DNA damage response, signal transduction by p53 class mediator resulting in cell cycle arrest	38	0.310275689	0.0015	0.003088691
GO:0072431	signal transduction involved in mitotic G1 DNA damage checkpoint	38	0.310275689	0.0015	0.003088691
GO:1902400	intracellular signal transduction involved in G1 DNA damage checkpoint	38	0.310275689	0.0015	0.003088691
GO:0006446	regulation of translational initiation	55	0.31026091	0.0016	0.003223775
GO:0055003	cardiac myofibril assembly	9	0.31025311	0.0003	0.001720882
GO:0043370	regulation of CD4-positive, alpha-beta T cell differentiation	21	0.31025311	0.0006	0.002177563
GO:1903037	regulation of leukocyte cell-cell adhesion	168	0.310233192	0.0012	0.002741075
GO:0006360	transcription from RNA polymerase I promoter	47	0.310222076	< 0.001	< 0.001
GO:0006367	transcription initiation from RNA polymerase II promoter	109	0.310155503	0.0012	0.002741075
GO:0032731	positive regulation of interleukin-1 beta production	19	0.310140215	0.0001	0.000877824
GO:0061008	hepaticobiliary system development	81	0.310100577	0.0015	0.003088691
GO:0006119	oxidative phosphorylation	80	0.309990347	0.0065	0.008621124
GO:0072413	signal transduction involved in mitotic cell cycle checkpoint	39	0.30998911	0.0017	0.003334595
GO:1902402	signal transduction involved in mitotic DNA damage checkpoint	39	0.30998911	0.0017	0.003334595
GO:1902403	signal transduction involved in mitotic DNA integrity checkpoint	39	0.30998911	0.0017	0.003334595
GO:0002712	regulation of B cell mediated immunity	27	0.30996711	0.0002	0.001341684
GO:0002889	regulation of immunoglobulin mediated immune response	27	0.30996711	0.0002	0.001341684
GO:0071474	cellular hyperosmotic response	10	0.30996139	0.0028	0.004605006
GO:0045445	myoblast differentiation	45	0.30994423	0.0022	0.003910907
GO:0070232	regulation of T cell apoptotic process	17	0.30990991	0.0029	0.004714719
GO:0045603	positive regulation of endothelial cell differentiation	9	0.30988131	0.0014	0.002975058
GO:0006363	termination of RNA polymerase I transcription	23	0.309876336	< 0.001	< 0.001
GO:0007286	spermatid development	50	0.309863578	0.0001	0.000877824
GO:0072395	signal transduction involved in cell cycle checkpoint	46	0.309853953	0.0009	0.002399874
GO:0050796	regulation of insulin secretion	76	0.309838786	0.0008	0.002313249
GO:0060993	kidney morphogenesis	49	0.309820608	0.0001	0.000877824
GO:0032755	positive regulation of interleukin-6 production	46	0.309814784	0.0014	0.002975058
GO:0070507	regulation of microtubule cytoskeleton organization	90	0.30980981	0.0009	0.002399874
GO:0071800	podosome assembly	10	0.30980695	0.0211	0.023264758
GO:2001022	positive regulation of response to DNA damage stimulus	54	0.30978121	0.0017	0.003334595
GO:0042136	neurotransmitter biosynthetic process	6	0.30978121	< 0.001	< 0.001
GO:0002244	hematopoietic progenitor cell differentiation	101	0.309746805	0.0007	0.002248538
GO:2000036	regulation of stem cell population maintenance	18	0.30972401	0.0015	0.003088691
GO:0000394	RNA splicing, via endonucleolytic cleavage and ligation	16	0.30971686	0.0018	0.003459409
GO:0032769	negative regulation of monooxygenase activity	5	0.30970399	0.0156	0.017858595
GO:0043248	proteasome assembly	9	0.30968111	0.0044	0.006304227
GO:0071559	response to transforming growth factor beta	133	0.309658316	0.0011	0.002610912
GO:0048251	elastic fiber assembly	7	0.309615738	0.0025	0.004279479
GO:0060644	mammary gland epithelial cell differentiation	13	0.30961291	< 0.001	< 0.001
GO:0060055	angiogenesis involved in wound healing	13	0.30961291	0.016	0.018267288
GO:0050685	positive regulation of mRNA processing	22	0.30952381	0.0035	0.005358035
GO:0006282	regulation of DNA repair	66	0.30952381	0.0005	0.002177563
GO:0018230	peptidyl-L-cysteine S-palmitoylation	5	0.309498069	0.0117	0.013882722
GO:0018231	peptidyl-S-diacylglycerol-L-cysteine biosynthetic process from peptidyl-cysteine	5	0.309498069	0.0117	0.013882722
GO:0071456	cellular response to hypoxia	122	0.309496382	0.0011	0.002610912
GO:0048305	immunoglobulin secretion	14	0.309450267	0.0001	0.000877824
GO:0010827	regulation of glucose transport	38	0.309422204	0.0006	0.002177563
GO:0001658	branching involved in ureteric bud morphogenesis	32	0.309395109	< 0.001	< 0.001
GO:0033197	response to vitamin E	6	0.309395109	0.0164	0.018653793
GO:0016139	glycoside catabolic process	5	0.309395109	0.0017	0.003334595
GO:0035967	cellular response to topologically incorrect protein	99	0.309392509	0.0019	0.003579279
GO:0071322	cellular response to carbohydrate stimulus	63	0.309391024	0.0005	0.002177563
GO:0032493	response to bacterial lipoprotein	6	0.309352209	0.0101	0.012328539
GO:0002335	mature B cell differentiation	16	0.309330759	0.0192	0.021362011
GO:0045576	mast cell activation	31	0.309303774	0.0004	0.002007244
GO:1902804	negative regulation of synaptic vesicle transport	5	0.309292149	0.0223	0.024414688
GO:0030879	mammary gland development	70	0.309277441	0.0006	0.002177563
GO:0044724	single-organism carbohydrate catabolic process	110	0.309273429	0.002	0.003693422
GO:0048708	astrocyte differentiation	44	0.309266409	0.0036	0.005475609
GO:0002711	positive regulation of T cell mediated immunity	23	0.30922724	0.0028	0.004605006
GO:0060828	regulation of canonical Wnt signaling pathway	146	0.309213519	0.0006	0.002177563
GO:0000959	mitochondrial RNA metabolic process	24	0.309212784	0.0113	0.013488462
GO:0015804	neutral amino acid transport	18	0.309209209	0.0078	0.009981807

GOIDlabel	GOName	size	categoryScores	pValPerm	pValPermCorr
GO:0031953	negative regulation of protein autophosphorylation	9	0.309194909	0.0133	0.015521132
GO:0070227	lymphocyte apoptotic process	36	0.309187759	0.0025	0.004279479
GO:0071498	cellular response to fluid shear stress	10	0.309163449	0.0039	0.005778819
GO:0000075	cell cycle checkpoint	127	0.309137709	0.0006	0.002177563
GO:0006779	porphyrin-containing compound biosynthetic process	20	0.309099099	0.0035	0.005358035
GO:0030866	cortical actin cytoskeleton organization	21	0.309076423	0.0042	0.006097175
GO:1990089	response to nerve growth factor	27	0.309051909	0.0012	0.002741075
GO:1902099	regulation of metaphase/anaphase transition of cell cycle	28	0.30904578	< 0.001	< 0.001
GO:0061333	renal tubule morphogenesis	41	0.309043538	0.0002	0.001341684
GO:0046631	alpha-beta T cell activation	73	0.309031928	0.0042	0.006097175
GO:0006352	DNA-templated transcription, initiation	139	0.309022898	0.0008	0.002313249
GO:0045143	homologous chromosome segregation	17	0.30901658	0.0001	0.000877824
GO:0034104	negative regulation of tissue remodeling	10	0.309009009	0.0119	0.014078095
GO:0090101	negative regulation of transmembrane receptor protein serine/threonine kinase signaling pathway	61	0.308947824	0.0016	0.003223775
GO:0035987	endodermal cell differentiation	25	0.308942085	0.0008	0.002313249
GO:0007088	regulation of mitotic nuclear division	91	0.30893688	0.0009	0.002399874
GO:0033559	unsaturated fatty acid metabolic process	65	0.308919909	0.002	0.003693422
GO:0050951	sensory perception of temperature stimulus	7	0.30891708	0.0001	0.000877824
GO:0051152	positive regulation of smooth muscle cell differentiation	7	0.30891708	0.0001	0.000877824
GO:0032640	tumor necrosis factor production	81	0.308912087	0.0019	0.003579279
GO:0033005	positive regulation of mast cell activation	9	0.308908909	0.0129	0.015093024
GO:0050868	negative regulation of T cell activation	52	0.308885259	0.0004	0.002007244
GO:0032608	interferon-beta production	37	0.308852482	0.0006	0.002177563
GO:0070813	hydrogen sulfide metabolic process	5	0.308828829	0.0071	0.009268394
GO:0035907	dorsal aorta development	7	0.308806766	0.0054	0.007432873
GO:0032886	regulation of microtubule-based process	106	0.308805031	0.0005	0.002177563
GO:0016601	Rac protein signal transduction	17	0.308774321	0.0053	0.007330056
GO:0031669	cellular response to nutrient levels	106	0.308758894	0.0014	0.002975058
GO:0060028	convergent extension involved in axis elongation	5	0.308725869	0.003	0.004825593
GO:0019068	virion assembly	30	0.308691549	0.0017	0.003334595
GO:0001676	long-chain fatty acid metabolic process	54	0.308689642	0.0017	0.003334595
GO:0034612	response to tumor necrosis factor	166	0.308680281	0.0003	0.001720882
GO:0019362	pyridine nucleotide metabolic process	109	0.308670138	0.0019	0.003579279
GO:0046496	nicotinamide nucleotide metabolic process	109	0.308670138	0.0019	0.003579279
GO:0043032	positive regulation of macrophage activation	17	0.308653191	0.0049	0.006860215
GO:0042130	negative regulation of T cell proliferation	29	0.308649536	0.0007	0.002248538
GO:0010453	regulation of cell fate commitment	11	0.308622909	< 0.001	< 0.001
GO:0050884	neuromuscular process controlling posture	6	0.308580009	0.0009	0.002399874
GO:0010324	membrane invagination	39	0.308523909	0.0024	0.004156304
GO:0007004	telomere maintenance via telomerase	47	0.308518853	< 0.001	< 0.001
GO:0048643	positive regulation of skeletal muscle tissue development	7	0.308512594	0.0071	0.009268394
GO:0051321	meiotic cell cycle	88	0.308497133	0.0008	0.002313249
GO:0044106	cellular amine metabolic process	78	0.308494208	0.0004	0.002007244
GO:0042098	T cell proliferation	96	0.308472758	0.0008	0.002313249
GO:1990823	response to leukemia inhibitory factor	65	0.308468468	< 0.001	< 0.001
GO:1990830	cellular response to leukemia inhibitory factor	65	0.308468468	< 0.001	< 0.001
GO:0051882	mitochondrial depolarization	15	0.308468468	0.008	0.010168329
GO:0002381	immunoglobulin production involved in immunoglobulin mediated immune response	27	0.308460842	0.0001	0.000877824
GO:0014014	negative regulation of gliogenesis	22	0.308459108	0.0041	0.005981983
GO:0009132	nucleoside diphosphate metabolic process	91	0.308458851	0.0023	0.004032435
GO:0032479	regulation of type I interferon production	84	0.308451308	0.0006	0.002177563
GO:0060396	growth hormone receptor signaling pathway	13	0.308444708	< 0.001	< 0.001
GO:0097028	dendritic cell differentiation	26	0.308424908	0.0005	0.002177563
GO:0006837	serotonin transport	6	0.308408408	0.0003	0.001720882
GO:0046341	CDP-diacylglycerol metabolic process	11	0.308388908	0.0004	0.002007244
GO:0002687	positive regulation of leukocyte migration	69	0.308354317	0.0016	0.003223775
GO:1903146	regulation of mitophagy	23	0.308331934	0.0034	0.005263104
GO:2000756	regulation of peptidyl-lysine acetylation	34	0.308327655	0.0006	0.002177563
GO:0001678	cellular glucose homeostasis	64	0.308309202	0.0006	0.002177563
GO:1900221	regulation of beta-amyloid clearance	9	0.308308308	< 0.001	< 0.001
GO:0006469	negative regulation of protein kinase activity	118	0.308300068	0.0006	0.002177563
GO:0030730	sequestering of triglyceride	11	0.308295308	0.0053	0.007330056
GO:0033233	regulation of protein sumoylation	10	0.308262548	0.0044	0.006304227
GO:0030178	negative regulation of Wnt signaling pathway	115	0.30826031	0.0004	0.002007244
GO:1903509	liposaccharide metabolic process	74	0.308257679	0.0023	0.004032435
GO:0043154	negative regulation of cysteine-type endopeptidase activity involved in apoptotic process	50	0.30823166	0.0006	0.002177563
GO:0007173	epidermal growth factor receptor signaling pathway	69	0.308231213	0.0027	0.004491543
GO:0032635	interleukin-6 production	70	0.308214745	0.0014	0.002975058
GO:0033135	regulation of peptidyl-serine phosphorylation	59	0.308186637	0.0004	0.002007244
GO:0032680	regulation of tumor necrosis factor production	78	0.308180708	0.0018	0.003459409
GO:0030258	lipid modification	115	0.308179733	0.0009	0.002399874
GO:0071888	macrophage apoptotic process	9	0.308165308	0.0121	0.014285532
GO:0043624	cellular protein complex disassembly	134	0.308152289	0.0023	0.004032435
GO:0090050	positive regulation of cell migration involved in sprouting angiogenesis	13	0.308127908	0.0014	0.002975058
GO:0042552	myelination	78	0.308124608	0.0044	0.006304227
GO:0007603	phototransduction, visible light	11	0.308061308	0.0112	0.013379119
GO:0016056	rhodopsin mediated signaling pathway	11	0.308061308	0.0112	0.013379119
GO:0009309	amine biosynthetic process	9	0.308050908	0.0001	0.000877824
GO:0042401	cellular biogenic amine biosynthetic process	9	0.308050908	0.0001	0.000877824
GO:0009135	purine nucleoside diphosphate metabolic process	77	0.308044594	0.0033	0.005149584
GO:0009179	purine ribonucleoside diphosphate metabolic process	77	0.308044594	0.0033	0.005149584
GO:1904035	regulation of epithelial cell apoptotic process	46	0.30804096	0.0044	0.006304227
GO:0035024	negative regulation of Rho protein signal transduction	11	0.308037908	0.0014	0.002975058

GOIDlabel	GOName	size	categoryScores	pValPerm	pValPermCorr
GO:0009649	entrainment of circadian clock	10	0.308005148	0.0026	0.004377754
GO:0002820	negative regulation of adaptive immune response	21	0.307997794	0.0001	0.000877824
GO:0071560	cellular response to transforming growth factor beta stimulus	131	0.307970566	0.0011	0.002610912
GO:1901659	glycosyl compound biosynthetic process	76	0.307959087	0.0022	0.003910907
GO:0034162	toll-like receptor 9 signaling pathway	16	0.307947233	0.0008	0.002313249
GO:0044088	regulation of vacuole organization	34	0.307941555	0.0025	0.004279479
GO:0034968	histone lysine methylation	67	0.307935227	0.0011	0.002610912
GO:0002886	regulation of myeloid leukocyte mediated immunity	31	0.307925437	0.007	0.009166016
GO:0071103	DNA conformation change	151	0.307860936	0.0008	0.002313249
GO:2000109	regulation of macrophage apoptotic process	7	0.307850708	0.0094	0.011616482
GO:0010039	response to iron ion	28	0.307841515	0.0056	0.007655255
GO:0071326	cellular response to monosaccharide stimulus	60	0.307790648	0.0005	0.002177563
GO:0071331	cellular response to hexose stimulus	60	0.307790648	0.0005	0.002177563
GO:1903351	cellular response to dopamine	8	0.307786358	0.0038	0.005666214
GO:0000724	double-strand break repair via homologous recombination	71	0.30777095	< 0.001	< 0.001
GO:0000725	recombinational repair	71	0.30777095	< 0.001	< 0.001
GO:0008625	extrinsic apoptotic signaling pathway via death domain receptors	54	0.307764908	0.0028	0.004605006
GO:0034367	macromolecular complex remodeling	13	0.307751708	0.0006	0.002177563
GO:0034368	protein-lipid complex remodeling	13	0.307751708	0.0006	0.002177563
GO:0034369	plasma lipoprotein particle remodeling	13	0.307751708	0.0006	0.002177563
GO:0030510	regulation of BMP signaling pathway	38	0.307749102	0.0046	0.006532041
GO:1900182	positive regulation of protein localization to nucleus	55	0.307738388	0.0009	0.002399874
GO:0090199	regulation of release of cytochrome c from mitochondria	28	0.307712815	0.0019	0.003579279
GO:0097193	intrinsic apoptotic signaling pathway	175	0.307697739	0.0006	0.002177563
GO:0046824	positive regulation of nucleocytoplasmic transport	49	0.307656344	0.0011	0.002610912
GO:0071347	cellular response to interleukin-1	91	0.30762725	0.0011	0.002610912
GO:0032692	negative regulation of interleukin-1 production	16	0.307625483	0.0116	0.013792313
GO:0050434	positive regulation of viral transcription	34	0.307616019	0.0012	0.002741075
GO:0033619	membrane protein proteolysis	43	0.307593308	0.0043	0.006204188
GO:0016575	histone deacetylation	41	0.307593308	0.002	0.003693422
GO:0070555	response to interleukin-1	100	0.307570142	0.0007	0.002248538
GO:0051306	mitotic sister chromatid separation	30	0.307533248	< 0.001	< 0.001
GO:0098739	import across plasma membrane	22	0.307523108	0.0063	0.008390784
GO:0036314	response to sterol	20	0.307516088	0.0005	0.002177563
GO:0034389	lipid particle organization	12	0.307507508	0.002	0.003693422
GO:0006488	dolichol-linked oligosaccharide biosynthetic process	17	0.307487319	0.0018	0.003459409
GO:0006490	oligosaccharide-lipid intermediate biosynthetic process	17	0.307487319	0.0018	0.003459409
GO:0010389	regulation of G2/M transition of mitotic cell cycle	123	0.307467746	0.0005	0.002177563
GO:0055006	cardiac cell development	38	0.307464607	0.0006	0.002177563
GO:0097067	cellular response to thyroid hormone stimulus	10	0.307464607	0.0152	0.017453982
GO:0035264	multicellular organism growth	92	0.307450618	0.0007	0.002248538
GO:0090317	negative regulation of intracellular protein transport	36	0.307428857	0.0007	0.002248538
GO:0072088	nephron epithelium morphogenesis	41	0.307404966	0.0001	0.000877824
GO:0051702	interaction with symbiont	52	0.307355707	0.001	0.002498236
GO:0046425	regulation of JAK-STAT cascade	53	0.307350477	0.0002	0.001341684
GO:0070970	interleukin-2 secretion	5	0.307335907	0.0013	0.002859821
GO:0030520	intracellular estrogen receptor signaling pathway	34	0.307313196	0.001	0.002498236
GO:0015918	sterol transport	48	0.307303732	0.001	0.002498236
GO:0000910	cytokinesis	68	0.30730184	0.002	0.003693422
GO:0072171	mesonephric tubule morphogenesis	37	0.30728721	0.0001	0.000877824
GO:0006012	galactose metabolic process	8	0.307239382	0.0094	0.011616482
GO:0030593	neutrophil chemotaxis	31	0.307227965	0.0029	0.004714719
GO:0043506	regulation of JUN kinase activity	46	0.307212803	0.0006	0.002177563
GO:0006637	acyl-CoA metabolic process	62	0.307211359	0.0033	0.005149584
GO:0035383	thioester metabolic process	62	0.307211359	0.0033	0.005149584
GO:1903214	regulation of protein targeting to mitochondrion	31	0.307211359	0.0002	0.001341684
GO:0031579	membrane raft organization	15	0.307181467	0.018	0.020203241
GO:0060231	mesenchymal to epithelial transition	9	0.307164307	0.0011	0.002610912
GO:0035304	regulation of protein dephosphorylation	67	0.307147659	0.0005	0.002177563
GO:0001783	B cell apoptotic process	13	0.307137907	0.0024	0.004156304
GO:1901342	regulation of vasculature development	176	0.307131157	0.0018	0.003459409
GO:0007018	microtubule-based movement	130	0.307120087	< 0.001	< 0.001
GO:0060394	negative regulation of pathway-restricted SMAD protein phosphorylation	8	0.307110682	0.0103	0.012519912
GO:1904591	positive regulation of protein import	32	0.307102638	0.0012	0.002741075
GO:0055007	cardiac muscle cell differentiation	52	0.307088407	0.0006	0.002177563
GO:0007091	metaphase/anaphase transition of mitotic cell cycle	29	0.307078507	< 0.001	< 0.001
GO:0010965	regulation of mitotic sister chromatid separation	29	0.307078507	< 0.001	< 0.001
GO:0060736	prostate gland growth	5	0.307078507	0.0032	0.005044175
GO:0035065	regulation of histone acetylation	29	0.307051879	0.0007	0.002248538
GO:0033500	carbohydrate homeostasis	104	0.307048807	0.0005	0.002177563
GO:0042593	glucose homeostasis	104	0.307048807	0.0005	0.002177563
GO:0045815	positive regulation of gene expression, epigenetic	37	0.307022853	0.0003	0.001720882
GO:0034121	regulation of toll-like receptor signaling pathway	32	0.306973938	0.0002	0.001341684
GO:0007351	tripartite regional subdivision	7	0.306968193	0.0015	0.003088691
GO:0008595	anterior/posterior axis specification, embryo	7	0.306968193	0.0015	0.003088691
GO:0045930	negative regulation of mitotic cell cycle	200	0.306931789	0.001	0.002498236
GO:2000009	negative regulation of protein localization to cell surface	7	0.306931421	0.0081	0.010256567
GO:0014003	oligodendrocyte development	30	0.306898327	0.0036	0.005475609
GO:0002758	innate immune response-activating signal transduction	173	0.306891036	0.0007	0.002248538
GO:0032024	positive regulation of insulin secretion	29	0.306883238	< 0.001	< 0.001
GO:0032933	SREBP signaling pathway	5	0.306872587	0.0342	0.036175369
GO:0071501	cellular response to sterol depletion	5	0.306872587	0.0342	0.036175369
GO:0031116	positive regulation of microtubule polymerization	15	0.306855427	0.0061	0.008182551

GOIDlabel	GOName	size	categoryScores	pValPerm	pValPermCorr
GO:0014743	regulation of muscle hypertrophy	30	0.306838267	0.0038	0.005666214
GO:0051101	regulation of DNA binding	70	0.306810075	0.0008	0.002313249
GO:0001889	liver development	78	0.306781507	0.0012	0.002741075
GO:0036353	histone H2A-K119 monoubiquitination	5	0.306769627	0.0004	0.002007244
GO:0002702	positive regulation of production of molecular mediator of immune response	46	0.306748363	0.0003	0.001720882
GO:0055089	fatty acid homeostasis	7	0.306747564	0.0013	0.002859821
GO:0002218	activation of innate immune response	187	0.306738519	0.0006	0.002177563
GO:0002823	negative regulation of adaptive immune response based on somatic recombination of immune receptors built from immunoglobulin superfamily domains	20	0.306731017	0.0001	0.000877824
GO:1904044	response to aldosterone	5	0.306718147	0.0028	0.004605006
GO:0050713	negative regulation of interleukin-1 beta secretion	5	0.306718147	0.0114	0.01358747
GO:0006700	C21-steroid hormone biosynthetic process	15	0.306683827	0.0095	0.011730963
GO:0045651	positive regulation of macrophage differentiation	9	0.306678107	0.0023	0.004032435
GO:0033260	nuclear DNA replication	31	0.306621829	0.0017	0.003334595
GO:0046329	negative regulation of JNK cascade	18	0.306620907	0.0008	0.002313249
GO:0030073	insulin secretion	90	0.306603747	0.0008	0.002313249
GO:0033145	positive regulation of intracellular steroid hormone receptor signaling pathway	13	0.306603307	0.0064	0.008500875
GO:0019359	nicotinamide nucleotide biosynthetic process	20	0.306602317	0.0004	0.002007244
GO:0019363	pyridine nucleotide biosynthetic process	20	0.306602317	0.0004	0.002007244
GO:0010818	T cell chemotaxis	9	0.306592307	0.0069	0.009046225
GO:0085029	extracellular matrix assembly	25	0.306574003	0.0029	0.004714719
GO:0002286	T cell activation involved in immune response	46	0.306541324	0.0009	0.002399874
GO:0051092	positive regulation of NF-kappaB transcription factor activity	84	0.306490164	0.0007	0.002248538
GO:0045922	negative regulation of fatty acid metabolic process	16	0.306467181	< 0.001	< 0.001
GO:0010819	regulation of T cell chemotaxis	5	0.306460746	0.0192	0.021362011
GO:0010820	positive regulation of T cell chemotaxis	5	0.306460746	0.0192	0.021362011
GO:0090342	regulation of cell aging	29	0.306421693	0.0003	0.001720882
GO:0048678	response to axon injury	39	0.306418506	0.0013	0.002859821
GO:0097191	extrinsic apoptotic signaling pathway	134	0.306410035	0.0017	0.003334595
GO:2000278	regulation of DNA biosynthetic process	66	0.306376506	0.0004	0.002007244
GO:0002573	myeloid leukocyte differentiation	112	0.306354569	0.003	0.004825593
GO:0071333	cellular response to glucose stimulus	58	0.306350686	0.0005	0.002177563
GO:0006907	pinocytosis	17	0.306336589	0.0023	0.004032435
GO:0044242	cellular lipid catabolic process	121	0.306331834	0.003	0.004825593
GO:0045408	regulation of interleukin-6 biosynthetic process	15	0.306306306	0.0048	0.006748379
GO:1900542	regulation of purine nucleotide metabolic process	89	0.30629763	0.0022	0.003910907
GO:0072207	metanephric epithelium development	16	0.306290219	0.0001	0.000877824
GO:0072243	metanephric nephron epithelium development	16	0.306290219	0.0001	0.000877824
GO:1901722	regulation of cell proliferation involved in kidney development	5	0.306254826	0.0002	0.001341684
GO:0009312	oligosaccharide biosynthetic process	9	0.306249106	0.0218	0.023945433
GO:0050830	defense response to Gram-positive bacterium	27	0.306210973	0.0023	0.004032435
GO:0036294	cellular response to decreased oxygen levels	128	0.306177606	0.0012	0.002741075
GO:0006925	inflammatory cell apoptotic process	12	0.306156156	0.0041	0.005981983
GO:0000012	single strand break repair	5	0.306151866	0.0075	0.00968541
GO:0046546	development of primary male sexual characteristics	78	0.306105006	0.0006	0.002177563
GO:0016226	iron-sulfur cluster assembly	11	0.306095706	0.0056	0.007655255
GO:0031163	metallo-sulfur cluster assembly	11	0.306095706	0.0056	0.007655255
GO:0000469	cleavage involved in rRNA processing	6	0.306091806	0.0092	0.011424708
GO:0038095	Fc-epsilon receptor signaling pathway	70	0.306085678	0.0009	0.002399874
GO:0002228	natural killer cell mediated immunity	22	0.306084006	0.0004	0.002007244
GO:2000243	positive regulation of reproductive process	25	0.306079794	< 0.001	< 0.001
GO:1904705	regulation of vascular smooth muscle cell proliferation	30	0.306074646	0.0026	0.004377754
GO:1990874	vascular smooth muscle cell proliferation	30	0.306074646	0.0026	0.004377754
GO:0002685	regulation of leukocyte migration	102	0.306071618	0.001	0.002498236
GO:0001886	endothelial cell morphogenesis	8	0.305952381	0.0118	0.013977955
GO:0032094	response to food	21	0.305950849	0.0008	0.002313249
GO:0043576	regulation of respiratory gaseous exchange	10	0.305945946	0.0007	0.002248538
GO:1902749	regulation of cell cycle G2/M phase transition	132	0.305920206	0.0003	0.001720882
GO:0043537	negative regulation of blood vessel endothelial cell migration	24	0.305877306	0.0013	0.002859821
GO:0009185	ribonucleoside diphosphate metabolic process	78	0.305864106	0.0037	0.005561344
GO:0030177	positive regulation of Wnt signaling pathway	102	0.305829359	0.0007	0.002248538
GO:0050853	B cell receptor signaling pathway	26	0.305821206	0.0058	0.007856196
GO:0055088	lipid homeostasis	55	0.305814906	0.0016	0.003223775
GO:0009308	amine metabolic process	79	0.305814313	0.0004	0.002007244
GO:0034976	response to endoplasmic reticulum stress	174	0.30580482	0.0006	0.002177563
GO:0031061	negative regulation of histone methylation	14	0.305791506	0.0003	0.001720882
GO:0002204	somatic recombination of immunoglobulin genes involved in immune response	26	0.305761806	0.0001	0.000877824
GO:0002208	somatic diversification of immunoglobulins involved in immune response	26	0.305761806	0.0001	0.000877824
GO:0045190	isotype switching	26	0.305761806	0.0001	0.000877824
GO:0051304	chromosome separation	39	0.305751906	< 0.001	< 0.001
GO:0048302	regulation of isotype switching to IgG isotypes	6	0.305748606	0.0026	0.004377754
GO:0048304	positive regulation of isotype switching to IgG isotypes	6	0.305748606	0.0026	0.004377754
GO:0045911	positive regulation of DNA recombination	22	0.305744706	< 0.001	< 0.001
GO:0033146	regulation of intracellular estrogen receptor signaling pathway	25	0.305709138	0.0008	0.002313249
GO:0070085	glycosylation	170	0.305705201	0.0009	0.002399874
GO:0036003	positive regulation of transcription from RNA polymerase II promoter in response to stress	21	0.305693449	0.0091	0.011322589
GO:0002718	regulation of cytokine production involved in immune response	40	0.305682111	0.0007	0.002248538
GO:0006486	protein glycosylation	158	0.305667693	0.001	0.002498236
GO:0043413	macromolecule glycosylation	158	0.305667693	0.001	0.002498236
GO:0072078	nephron tubule morphogenesis	39	0.305659506	0.0001	0.000877824
GO:0021936	regulation of cerebellar granule cell precursor proliferation	5	0.305637066	0.0394	0.041258002
GO:0006165	nucleoside diphosphate phosphorylation	78	0.305583606	0.0027	0.004491543
GO:0060546	negative regulation of necroptotic process	9	0.305562706	0.0107	0.012912536

GOIDlabel	GOName	size	categoryScores	pValPerm	pValPermCorr
GO:0045124	regulation of bone resorption	18	0.305519806	0.0108	0.013015961
GO:0034244	negative regulation of transcription elongation from RNA polymerase II promoter	9	0.305476905	0.0083	0.010468217
GO:0045948	positive regulation of translational initiation	18	0.305462605	0.0027	0.004491543
GO:0050792	regulation of viral process	141	0.305428225	0.0007	0.002248538
GO:0044458	motile cilium assembly	12	0.305426855	< 0.001	< 0.001
GO:1903038	negative regulation of leukocyte cell-cell adhesion	60	0.305413985	0.0004	0.002007244
GO:0070059	intrinsic apoptotic signaling pathway in response to endoplasmic reticulum stress	37	0.305408884	0.002	0.003693422
GO:0007272	ensheathment of neurons	81	0.305394283	0.0043	0.006204188
GO:0008366	axon ensheathment	81	0.305394283	0.0043	0.006204188
GO:0043651	linoleic acid metabolic process	11	0.305393705	0.0021	0.003804235
GO:0046685	response to arsenic-containing substance	21	0.30538702	0.0035	0.005358035
GO:0033120	positive regulation of RNA splicing	20	0.305379665	0.0038	0.005666214
GO:0006098	pentose-phosphate shunt	12	0.305341055	0.0089	0.011106266
GO:0097284	hepatocyte apoptotic process	10	0.305328185	0.0013	0.002859821
GO:0000070	mitotic sister chromatid segregation	82	0.305304957	0.0008	0.002313249
GO:0043300	regulation of leukocyte degranulation	27	0.305286239	0.0037	0.005561344
GO:1901203	positive regulation of extracellular matrix assembly	8	0.305276705	0.0062	0.008281828
GO:0071867	response to monoamine	18	0.305248105	0.0016	0.003223775
GO:0071869	response to catecholamine	18	0.305248105	0.0016	0.003223775
GO:0030301	cholesterol transport	45	0.305242385	0.0009	0.002399874
GO:0071104	response to interleukin-9	6	0.305190905	0.0004	0.002007244
GO:0032682	negative regulation of chemokine production	13	0.305138105	0.0016	0.003223775
GO:0000209	protein polyubiquitination	195	0.305113025	0.0007	0.002248538
GO:0032609	interferon-gamma production	50	0.305111969	0.0022	0.003910907
GO:0043030	regulation of macrophage activation	29	0.305108064	0.0069	0.009046225
GO:0032700	negative regulation of interleukin-17 production	6	0.305105105	0.0012	0.002741075
GO:0045765	regulation of angiogenesis	161	0.305100842	0.0021	0.003804235
GO:1901800	positive regulation of proteasomal protein catabolic process	53	0.305063014	0.0006	0.002177563
GO:1901984	negative regulation of protein acetylation	12	0.305062205	0.0001	0.000877824
GO:0045655	regulation of monocyte differentiation	13	0.305039105	0.0031	0.004916949
GO:0032784	regulation of DNA-templated transcription, elongation	28	0.305028498	0.0022	0.003910907
GO:2000757	negative regulation of peptidyl-lysine acetylation	10	0.305019305	0.0002	0.001341684
GO:0002583	regulation of antigen processing and presentation of peptide antigen	5	0.305019305	0.0249	0.02700148
GO:0046755	viral budding	20	0.305006435	0.0015	0.003088691
GO:1902590	multi-organism organelle organization	20	0.305006435	0.0015	0.003088691
GO:1902592	multi-organism membrane budding	20	0.305006435	0.0015	0.003088691
GO:0003177	pulmonary valve development	13	0.304999505	0.0006	0.002177563
GO:0042789	mRNA transcription from RNA polymerase II promoter	14	0.304982534	0.0211	0.023264758
GO:0042089	cytokine biosynthetic process	65	0.304975745	0.001	0.002498236
GO:0042107	cytokine metabolic process	65	0.304975745	0.001	0.002498236
GO:0006096	glycolytic process	65	0.304951985	0.0034	0.005263104
GO:0060544	regulation of necroptotic process	11	0.304925705	0.0122	0.014387602
GO:0002698	negative regulation of immune effector process	71	0.30492142	0.0007	0.002248538
GO:0032873	negative regulation of stress-activated MAPK cascade	25	0.304916345	0.0008	0.002313249
GO:0070303	negative regulation of stress-activated protein kinase signaling cascade	25	0.304916345	0.0008	0.002313249
GO:0002761	regulation of myeloid leukocyte differentiation	62	0.304890605	0.0029	0.004714719
GO:0002699	positive regulation of immune effector process	103	0.304884357	0.0002	0.001341684
GO:0030522	intracellular receptor signaling pathway	165	0.304836785	0.0008	0.002313249
GO:0003091	renal water homeostasis	14	0.304835448	< 0.001	< 0.001
GO:0044272	sulfur compound biosynthetic process	128	0.304830277	0.0007	0.002248538
GO:0045986	negative regulation of smooth muscle contraction	6	0.304804805	0.0001	0.000877824
GO:0002700	regulation of production of molecular mediator of immune response	67	0.304800323	0.0002	0.001341684
GO:0060307	regulation of ventricular cardiac muscle cell membrane repolarization	9	0.304790505	0.0003	0.001720882
GO:1902275	regulation of chromatin organization	95	0.304775452	0.001	0.002498236
GO:0071453	cellular response to oxygen levels	137	0.304771299	0.0012	0.002741075
GO:0001894	tissue homeostasis	114	0.304750615	0.001	0.002498236
GO:0043902	positive regulation of multi-organism process	102	0.304749287	0.0004	0.002007244
GO:0032637	interleukin-8 production	43	0.304725988	0.0011	0.002610912
GO:0006081	cellular aldehyde metabolic process	41	0.304724236	0.0024	0.004156304
GO:0050686	negative regulation of mRNA processing	19	0.304721263	0.0029	0.004714719
GO:0006111	regulation of gluconeogenesis	25	0.304720721	0.0002	0.001341684
GO:0006278	RNA-dependent DNA biosynthetic process	49	0.304714627	< 0.001	< 0.001
GO:0038127	ERBB signaling pathway	82	0.304705402	0.0022	0.003910907
GO:0048754	branching morphogenesis of an epithelial tube	74	0.304699294	0.002	0.003693422
GO:0033007	negative regulation of mast cell activation involved in immune response	6	0.304676105	0.0003	0.001720882
GO:1904896	ESCRT complex disassembly	8	0.30466538	0.002	0.003693422
GO:1904903	ESCRT III complex disassembly	8	0.30466538	0.002	0.003693422
GO:0006884	cell volume homeostasis	16	0.304633205	0.0021	0.003804235
GO:0010611	regulation of cardiac muscle hypertrophy	29	0.30455776	0.0038	0.005666214
GO:0072497	mesenchymal stem cell differentiation	5	0.304555985	0.0034	0.005263104
GO:2000739	regulation of mesenchymal stem cell differentiation	5	0.304555985	0.0034	0.005263104
GO:0003223	ventricular compact myocardium morphogenesis	5	0.304555985	0.0119	0.014078095
GO:0003254	regulation of membrane depolarization	19	0.304531599	0.0094	0.011616482
GO:0002562	somatic diversification of immune receptors via germline recombination within a single locus	34	0.304489363	0.0001	0.000877824
GO:0016444	somatic cell DNA recombination	34	0.304489363	0.0001	0.000877824
GO:0001892	embryonic placenta development	49	0.304472986	0.0008	0.002313249
GO:0019217	regulation of fatty acid metabolic process	49	0.30446248	0.0004	0.002007244
GO:0042744	hydrogen peroxide catabolic process	10	0.304453024	0.0001	0.000877824
GO:0051353	positive regulation of oxidoreductase activity	27	0.304447304	0.0007	0.002248538
GO:0006378	mRNA polyadenylation	28	0.304412576	0.0007	0.002248538
GO:0072028	nephron morphogenesis	43	0.304384783	0.0001	0.000877824
GO:0042310	vasoconstriction	29	0.304380242	0.0037	0.005561344
GO:1904467	regulation of tumor necrosis factor secretion	14	0.304375804	0.0021	0.003804235

GOIDlabel	GOName	size	categoryScores	pValPerm	pValPermCorr
GO:1903844	regulation of cellular response to transforming growth factor beta stimulus	65	0.304373824	0.0012	0.002741075
GO:0090630	activation of GTPase activity	22	0.304352404	0.0039	0.005778819
GO:0006911	phagocytosis, engulfment	27	0.304351971	0.0048	0.006748379
GO:0060502	epithelial cell proliferation involved in lung morphogenesis	5	0.304350064	0.0066	0.008731949
GO:2000794	regulation of epithelial cell proliferation involved in lung morphogenesis	5	0.304350064	0.0066	0.008731949
GO:0032677	regulation of interleukin-8 production	42	0.304339033	0.001	0.002498236
GO:0044273	sulfur compound catabolic process	31	0.30433844	0.0011	0.002610912
GO:0009313	oligosaccharide catabolic process	9	0.304332904	0.0027	0.004491543
GO:0006109	regulation of carbohydrate metabolic process	112	0.30430456	0.0009	0.002399874
GO:1903405	protein localization to nuclear body	9	0.304247104	< 0.001	< 0.001
GO:1904851	positive regulation of establishment of protein localization to telomere	9	0.304247104	< 0.001	< 0.001
GO:1904867	protein localization to Cajal body	9	0.304247104	< 0.001	< 0.001
GO:1904869	regulation of protein localization to Cajal body	9	0.304247104	< 0.001	< 0.001
GO:1904871	positive regulation of protein localization to Cajal body	9	0.304247104	< 0.001	< 0.001
GO:0050848	regulation of calcium-mediated signaling	39	0.304240504	0.0003	0.001720882
GO:2000273	positive regulation of receptor activity	25	0.304236808	0.001	0.002498236
GO:0042129	regulation of T cell proliferation	80	0.304231017	0.0013	0.002859821
GO:0031112	positive regulation of microtubule polymerization or depolymerization	16	0.304198842	0.005	0.006974174
GO:2000772	regulation of cellular senescence	27	0.304189904	< 0.001	< 0.001
GO:0060292	long term synaptic depression	6	0.304161304	0.0001	0.000877824
GO:0000723	telomere maintenance	97	0.304159535	0.0004	0.002007244
GO:0044030	regulation of DNA methylation	13	0.304148104	0.0007	0.002248538
GO:0032703	negative regulation of interleukin-2 production	11	0.304130104	0.0017	0.003334595
GO:0032200	telomere organization	99	0.304124904	0.0005	0.002177563
GO:0002027	regulation of heart rate	40	0.304124839	0.001	0.002498236
GO:0042493	response to drug	158	0.304111888	0.0007	0.002248538
GO:0043900	regulation of multi-organism process	192	0.304099635	0.0007	0.002248538
GO:0045540	regulation of cholesterol biosynthetic process	33	0.304091104	0.0014	0.002975058
GO:0030856	regulation of epithelial cell differentiation	76	0.304087923	0.0015	0.003088691
GO:0060560	developmental growth involved in morphogenesis	99	0.304057304	0.0006	0.002177563
GO:0031056	regulation of histone modification	79	0.304045094	0.0008	0.002313249
GO:0008652	cellular amino acid biosynthetic process	54	0.304027837	0.0002	0.001341684
GO:0010988	regulation of low-density lipoprotein particle clearance	7	0.304026475	0.0087	0.010877987
GO:0042921	glucocorticoid receptor signaling pathway	9	0.304018304	0.001	0.002498236
GO:0002089	lens morphogenesis in camera-type eye	12	0.304011154	0.0015	0.003088691
GO:1900078	positive regulation of cellular response to insulin stimulus	13	0.304009504	0.0008	0.002313249
GO:0002377	immunoglobulin production	53	0.303975134	< 0.001	< 0.001
GO:0044783	G1 DNA damage checkpoint	45	0.303926784	0.0017	0.003334595
GO:0098760	response to interleukin-7	20	0.303925354	0.0008	0.002313249
GO:0098761	cellular response to interleukin-7	20	0.303925354	0.0008	0.002313249
GO:0021697	cerebellar cortex formation	12	0.303925354	0.0028	0.004605006
GO:0018904	ether metabolic process	10	0.303912484	0.0152	0.017453982
GO:0051341	regulation of oxidoreductase activity	51	0.303888763	0.0011	0.002610912
GO:0061448	connective tissue development	126	0.303887561	0.0007	0.002248538
GO:0006664	glycolipid metabolic process	73	0.303883923	0.0024	0.004156304
GO:1901605	alpha-amino acid metabolic process	130	0.303868924	0.0005	0.002177563
GO:2000535	regulation of entry of bacterium into host cell	5	0.303835264	0.0104	0.012619802
GO:0043092	L-amino acid import	9	0.303818104	0.001	0.002498236
GO:0007602	phototransduction	14	0.303787461	0.0147	0.016962357
GO:0071260	cellular response to mechanical stimulus	52	0.303776854	0.0048	0.006748379
GO:0006733	oxidoreduction coenzyme metabolic process	121	0.303753576	0.002	0.003693422
GO:0031058	positive regulation of histone modification	53	0.30375173	0.001	0.002498236
GO:0098781	ncRNA transcription	74	0.303746217	0.0004	0.002007244
GO:0055013	cardiac muscle cell development	34	0.303709592	0.0006	0.002177563
GO:0031571	mitotic G1 DNA damage checkpoint	44	0.303708904	0.0017	0.003334595
GO:0044819	mitotic G1/S transition checkpoint	44	0.303708904	0.0017	0.003334595
GO:0072523	purine-containing compound catabolic process	29	0.3036968	0.0009	0.002399874
GO:0032418	lysosome localization	41	0.303694635	0.0006	0.002177563
GO:0034142	toll-like receptor 4 signaling pathway	19	0.303691662	0.0001	0.000877824
GO:0043303	mast cell degranulation	24	0.303657229	0.0005	0.002177563
GO:0043666	regulation of phosphoprotein phosphatase activity	59	0.303649412	0.0004	0.002007244
GO:0061005	cell differentiation involved in kidney development	32	0.303643822	0.0021	0.003804235
GO:0010922	positive regulation of phosphatase activity	16	0.303635779	0.0003	0.001720882
GO:0032506	cytokinetic process	12	0.303603604	0.0079	0.010077402
GO:1901989	positive regulation of cell cycle phase transition	51	0.303580892	0.0013	0.002859821
GO:0070228	regulation of lymphocyte apoptotic process	24	0.303549979	0.0018	0.003459409
GO:0051904	pigment granule transport	12	0.303539254	0.0001	0.000877824
GO:0051905	establishment of pigment granule localization	12	0.303539254	0.0001	0.000877824
GO:0006304	DNA modification	62	0.303524723	0.0008	0.002313249
GO:0033138	positive regulation of peptidyl-serine phosphorylation	42	0.303523932	< 0.001	< 0.001
GO:0033157	regulation of intracellular protein transport	155	0.303519741	0.0007	0.002248538
GO:0006000	fructose metabolic process	6	0.303517804	0.0002	0.001341684
GO:0007176	regulation of epidermal growth factor-activated receptor activity	10	0.303500644	0.0077	0.009887588
GO:0060976	coronary vasculature development	28	0.303493289	0.0015	0.003088691
GO:0030539	male genitalia development	10	0.303474903	0.0006	0.002177563
GO:0008584	male gonad development	77	0.303458189	0.0006	0.002177563
GO:0017144	drug metabolic process	15	0.303457743	< 0.001	< 0.001
GO:0048546	digestive tract morphogenesis	20	0.303449163	0.0125	0.014684332
GO:0061013	regulation of mRNA catabolic process	27	0.303446303	0.0001	0.000877824
GO:0051048	negative regulation of secretion	120	0.303438438	0.0006	0.002177563
GO:0031076	embryonic camera-type eye development	25	0.303423423	0.002	0.003693422
GO:0043903	regulation of symbiosis, encompassing mutualism through parasitism	157	0.303406045	0.0008	0.002313249
GO:0017185	peptidyl-lysine hydroxylation	6	0.303389103	0.001	0.002498236

GOIDlabel	GOName	size	categoryScores	pValPerm	pValPermCorr
GO:0003160	endocardium morphogenesis	5	0.303371943	0.0315	0.033525301
GO:0072176	nephric duct development	6	0.303346203	0.0002	0.001341684
GO:2000050	regulation of non-canonical Wnt signaling pathway	7	0.303327818	0.0342	0.036175369
GO:0000819	sister chromatid segregation	95	0.303325882	0.0009	0.002399874
GO:0045841	negative regulation of mitotic metaphase/anaphase transition	19	0.303325882	< 0.001	< 0.001
GO:2000816	negative regulation of mitotic sister chromatid separation	19	0.303325882	< 0.001	< 0.001
GO:0039536	negative regulation of RIG-I signaling pathway	7	0.303291046	0.0065	0.008621124
GO:0030330	DNA damage response, signal transduction by p53 class mediator	65	0.303276903	0.0008	0.002313249
GO:0060049	regulation of protein glycosylation	16	0.303265766	0.0011	0.002610912
GO:0016447	somatic recombination of immunoglobulin gene segments	28	0.303263468	0.0001	0.000877824
GO:0022010	central nervous system myelination	13	0.303197703	0.0059	0.007964563
GO:0032291	axon ensheathment in central nervous system	13	0.303197703	0.0059	0.007964563
GO:0070646	protein modification by small protein removal	179	0.303190181	0.0006	0.002177563
GO:0071868	cellular response to monoamine stimulus	14	0.303180732	0.003	0.004825593
GO:0071870	cellular response to catecholamine stimulus	14	0.303180732	0.003	0.004825593
GO:0009791	post-embryonic development	61	0.303154208	0.0003	0.001720882
GO:1902041	regulation of extrinsic apoptotic signaling pathway via death domain receptors	36	0.303153153	0.0023	0.004032435
GO:0006090	pyruvate metabolic process	84	0.30314396	0.003	0.004825593
GO:0006809	nitric oxide biosynthetic process	39	0.303092103	0.0008	0.002313249
GO:0016180	snRNA processing	13	0.303059103	0.001	0.002498236
GO:0010828	positive regulation of glucose transport	19	0.30302784	0.0022	0.003910907
GO:0097300	programmed necrotic cell death	24	0.303024453	0.0085	0.010671887
GO:0032623	interleukin-2 production	33	0.303022503	0.0014	0.002975058
GO:0072719	cellular response to cisplatin	5	0.303011583	0.0142	0.016456919
GO:0006284	base-excision repair	22	0.303006903	0.0012	0.002741075
GO:0046939	nucleotide phosphorylation	79	0.302995944	0.0031	0.004916949
GO:0060070	canonical Wnt signaling pathway	168	0.302989214	0.0007	0.002248538
GO:2001258	negative regulation of cation channel activity	15	0.302942943	0.0002	0.001341684
GO:0050718	positive regulation of interleukin-1 beta secretion	14	0.302941717	< 0.001	< 0.001
GO:1990774	tumor necrosis factor secretion	16	0.302895753	0.0023	0.004032435
GO:0006195	purine nucleotide catabolic process	24	0.302874303	0.0007	0.002248538
GO:0006654	phosphatidic acid biosynthetic process	21	0.302874303	0.0033	0.005149584
GO:0046473	phosphatidic acid metabolic process	21	0.302874303	0.0033	0.005149584
GO:0030224	monocyte differentiation	22	0.302831403	0.0031	0.004916949
GO:1903131	mononuclear cell differentiation	22	0.302831403	0.0031	0.004916949
GO:0008543	fibroblast growth factor receptor signaling pathway	48	0.302809953	0.0002	0.001341684
GO:0035567	non-canonical Wnt signaling pathway	81	0.302807569	0.0004	0.002007244
GO:2000831	regulation of steroid hormone secretion	10	0.302805663	< 0.001	< 0.001
GO:0090090	negative regulation of canonical Wnt signaling pathway	97	0.302798233	0.0006	0.002177563
GO:0060547	negative regulation of necrotic cell death	11	0.302796303	0.0132	0.015415723
GO:0007620	copulation	12	0.302745603	< 0.001	< 0.001
GO:0006757	ATP generation from ADP	66	0.302726103	0.0035	0.005358035
GO:0045649	regulation of macrophage differentiation	11	0.302726103	0.0019	0.003579279
GO:0097502	mannosylation	26	0.302702703	< 0.001	< 0.001
GO:0007517	muscle organ development	197	0.302650439	0.002	0.003693422
GO:0046324	regulation of glucose import	30	0.302642643	0.0006	0.002177563
GO:0035089	establishment of apical/basal cell polarity	8	0.302638353	0.0224	0.024519959
GO:2000725	regulation of cardiac muscle cell differentiation	17	0.302596714	0.0042	0.006097175
GO:0042692	muscle cell differentiation	191	0.302596239	0.0013	0.002859821
GO:0043409	negative regulation of MAPK cascade	82	0.302595976	0.0008	0.002313249
GO:0006997	nucleus organization	80	0.302583655	0.0006	0.002177563
GO:0032288	myelin assembly	12	0.302574003	0.0233	0.025417819
GO:0014013	regulation of gliogenesis	66	0.302566203	0.0043	0.006204188
GO:0033108	mitochondrial respiratory chain complex assembly	67	0.302560556	0.007	0.009166016
GO:1901029	negative regulation of mitochondrial outer membrane permeabilization involved in apoptotic signaling pathway	7	0.302555617	0.0136	0.015813319
GO:0002448	mast cell mediated immunity	25	0.302527671	0.0005	0.002177563
GO:0045830	positive regulation of isotype switching	15	0.302513943	< 0.001	< 0.001
GO:0046051	UTP metabolic process	7	0.302482074	0.0254	0.027496891
GO:0007568	aging	175	0.30245854	0.0008	0.002313249
GO:0009205	purine ribonucleoside triphosphate metabolic process	199	0.302419433	0.0037	0.005561344
GO:0009247	glycolipid biosynthetic process	39	0.302418902	0.0021	0.003804235
GO:0006305	DNA alkylation	37	0.302410519	0.0006	0.002177563
GO:0006306	DNA methylation	37	0.302410519	0.0006	0.002177563
GO:0008045	motor neuron axon guidance	7	0.302408531	< 0.001	< 0.001
GO:0006595	polyamine metabolic process	9	0.302388102	0.004	0.005880507
GO:0040018	positive regulation of multicellular organism growth	24	0.302359502	0.0012	0.002741075
GO:0060425	lung morphogenesis	25	0.302352638	0.0068	0.008946408
GO:0007029	endoplasmic reticulum organization	29	0.302329916	0.0008	0.002313249
GO:0060071	Wnt signaling pathway, planar cell polarity pathway	63	0.302322731	0.0008	0.002313249
GO:0034620	cellular response to unfolded protein	91	0.302261445	0.0026	0.004377754
GO:0010565	regulation of cellular ketone metabolic process	104	0.302257202	0.0002	0.001341684
GO:0046849	bone remodeling	42	0.302249188	0.0028	0.004605006
GO:0072177	mesonephric duct development	5	0.302239382	< 0.001	< 0.001
GO:0046034	ATP metabolic process	182	0.302228917	0.0045	0.006417207
GO:0070266	necroptotic process	21	0.302224674	0.0088	0.010992238
GO:0042455	ribonucleoside biosynthetic process	67	0.302203269	0.003	0.004825593
GO:0048246	macrophage chemotaxis	18	0.302187902	0.0002	0.001341684
GO:0070633	transepithelial transport	10	0.302162162	0.0058	0.007856196
GO:0071383	cellular response to steroid hormone stimulus	138	0.302158059	0.0016	0.003223775
GO:0008206	bile acid metabolic process	22	0.302129402	0.0045	0.006417207
GO:0032459	regulation of protein oligomerization	27	0.302083035	0.002	0.003693422
GO:0032205	negative regulation of telomere maintenance	23	0.302075989	0.0001	0.000877824

GOIDlabel	GOName	size	categoryScores	pValPerm	pValPermCorr
GO:0031638	zymogen activation	22	0.302070902	0.008	0.010168329
GO:0046632	alpha-beta T cell differentiation	51	0.302061726	0.0022	0.003910907
GO:0009124	nucleoside monophosphate biosynthetic process	63	0.302028559	0.0027	0.004491543
GO:0006638	neutral lipid metabolic process	61	0.302010676	0.0007	0.002248538
GO:0006639	acylglycerol metabolic process	61	0.302010676	0.0007	0.002248538
GO:0010977	negative regulation of neuron projection development	58	0.301974881	0.0012	0.002741075
GO:0032743	positive regulation of interleukin-2 production	17	0.301960784	0.0015	0.003088691
GO:0048066	developmental pigmentation	24	0.301951952	0.0006	0.002177563
GO:0015701	bicarbonate transport	20	0.301943372	0.0016	0.003223775
GO:0033045	regulation of sister chromatid segregation	43	0.301942474	0.0003	0.001720882
GO:0032652	regulation of interleukin-1 production	45	0.301924782	0.0003	0.001720882
GO:0033048	negative regulation of mitotic sister chromatid segregation	21	0.30189373	< 0.001	< 0.001
GO:0006641	triglyceride metabolic process	53	0.301881936	0.0006	0.002177563
GO:0001704	formation of primary germ layer	60	0.301848992	0.0011	0.002610912
GO:0002703	regulation of leukocyte mediated immunity	95	0.301757095	0.0006	0.002177563
GO:0032612	interleukin-1 production	51	0.301708431	0.0002	0.001341684
GO:0003338	metanephros morphogenesis	17	0.301703384	0.0002	0.001341684
GO:0050702	interleukin-1 beta secretion	24	0.301694552	0.0001	0.000877824
GO:0045987	positive regulation of smooth muscle contraction	18	0.301673102	0.0009	0.002399874
GO:0006888	ER to Golgi vesicle-mediated transport	122	0.301595038	0.0009	0.002399874
GO:0045646	regulation of erythrocyte differentiation	30	0.301578722	0.0012	0.002741075
GO:0033148	positive regulation of intracellular estrogen receptor signaling pathway	8	0.301576577	0.0053	0.007330056
GO:0043029	T cell homeostasis	17	0.301536831	0.004	0.005880507
GO:0001580	detection of chemical stimulus involved in sensory perception of bitter taste	11	0.301532702	0.0152	0.017453982
GO:0008361	regulation of cell size	87	0.301492626	0.0007	0.002248538
GO:0033008	positive regulation of mast cell activation involved in immune response	7	0.301489244	0.0279	0.029969105
GO:0043306	positive regulation of mast cell degranulation	7	0.301489244	0.0279	0.029969105
GO:0071300	cellular response to retinoic acid	28	0.301452473	0.0005	0.002177563
GO:1903900	regulation of viral life cycle	136	0.301421379	0.0009	0.002399874
GO:0002495	antigen processing and presentation of peptide antigen via MHC class II	65	0.301384021	0.0027	0.004491543
GO:0002279	mast cell activation involved in immune response	25	0.301374517	0.0004	0.002007244
GO:0002449	lymphocyte mediated immunity	116	0.3013336	0.0005	0.002177563
GO:1903077	negative regulation of protein localization to plasma membrane	16	0.301303089	0.0042	0.006097175
GO:0048588	developmental cell growth	89	0.301294232	0.0005	0.002177563
GO:0042036	negative regulation of cytokine biosynthetic process	23	0.301281406	0.0002	0.001341684
GO:0002262	myeloid cell homeostasis	93	0.301271779	0.0006	0.002177563
GO:0044546	NLRP3 inflammasome complex assembly	7	0.301268616	0.0031	0.004916949
GO:1900225	regulation of NLRP3 inflammasome complex assembly	7	0.301268616	0.0031	0.004916949
GO:0060586	multicellular organismal iron ion homeostasis	5	0.301261261	0.0161	0.018371585
GO:0002456	T cell mediated immunity	46	0.301259023	0.0021	0.003804235
GO:0070886	positive regulation of calcineurin-NFAT signaling cascade	8	0.301254826	0.0052	0.007215241
GO:0016053	organic acid biosynthetic process	194	0.301249851	0.001	0.002498236
GO:0046394	carboxylic acid biosynthetic process	194	0.301249851	0.001	0.002498236
GO:0070296	sarcoplasmic reticulum calcium ion transport	15	0.301244101	0.0015	0.003088691
GO:0007548	sex differentiation	151	0.301214554	0.0006	0.002177563
GO:0002705	positive regulation of leukocyte mediated immunity	64	0.301202542	0.0013	0.002859821
GO:0002726	positive regulation of T cell cytokine production	10	0.301184041	0.0061	0.008182551
GO:0045737	positive regulation of cyclin-dependent protein serine/threonine kinase activity	16	0.301174389	0.0024	0.004156304
GO:0043392	negative regulation of DNA binding	32	0.301174389	0.0006	0.002177563
GO:0070213	protein auto-ADP-ribosylation	8	0.301158301	0.001	0.002498236
GO:0006312	mitotic recombination	11	0.301134901	< 0.001	< 0.001
GO:0051302	regulation of cell division	89	0.301094674	0.0007	0.002248538
GO:0002719	negative regulation of cytokine production involved in immune response	11	0.301088101	0.0001	0.000877824
GO:0090288	negative regulation of cellular response to growth factor stimulus	72	0.301083226	0.0034	0.005263104
GO:0050709	negative regulation of protein secretion	67	0.301081465	0.0008	0.002313249
GO:0042987	amyloid precursor protein catabolic process	23	0.301068771	0.0069	0.009046225
GO:0050680	negative regulation of epithelial cell proliferation	74	0.301015688	0.0006	0.002177563
GO:0030518	intracellular steroid hormone receptor signaling pathway	80	0.300984556	0.0017	0.003334595
GO:0017015	regulation of transforming growth factor beta receptor signaling pathway	64	0.300953185	0.0012	0.002741075
GO:0061647	histone H3-K9 modification	30	0.300943801	0.0025	0.004279479
GO:0070914	UV-damage excision repair	6	0.300943801	0.0024	0.004156304
GO:0060251	regulation of glial cell proliferation	19	0.300887354	0.0032	0.005044175
GO:0051783	regulation of nuclear division	97	0.300884979	0.0012	0.002741075
GO:0055067	monovalent inorganic cation homeostasis	57	0.300873806	0.0029	0.004714719
GO:0042537	benzene-containing compound metabolic process	14	0.300864129	< 0.001	< 0.001
GO:0046479	glycosphingolipid catabolic process	9	0.300843701	0.0079	0.010077402
GO:0060026	convergent extension	9	0.300843701	0.0006	0.002177563
GO:0021549	cerebellum development	53	0.300842622	0.0022	0.003910907
GO:0045453	bone resorption	27	0.300815101	0.0105	0.012724187
GO:0051224	negative regulation of protein transport	105	0.300731752	0.0008	0.002313249
GO:0051646	mitochondrion localization	25	0.300715573	0.0011	0.002610912
GO:0000086	G2/M transition of mitotic cell cycle	152	0.300699384	0.0004	0.002007244
GO:0051235	maintenance of location	155	0.300688338	0.0009	0.002399874
GO:0018342	protein prenylation	6	0.300686401	0.0221	0.024229021
GO:0097354	prenylation	6	0.300686401	0.0221	0.024229021
GO:0070371	ERK1 and ERK2 cascade	135	0.300670194	0.0004	0.002007244
GO:0006633	fatty acid biosynthetic process	96	0.300664951	0.0007	0.002248538
GO:0033059	cellular pigmentation	32	0.300651544	0.0009	0.002399874
GO:0048739	cardiac muscle fiber development	5	0.300643501	0.0098	0.012008345
GO:0046823	negative regulation of nucleocytoplasmic transport	24	0.300643501	0.0005	0.002177563
GO:0045932	negative regulation of muscle contraction	11	0.300643501	0.0001	0.000877824
GO:0060317	cardiac epithelial to mesenchymal transition	18	0.300600601	0.0009	0.002399874
GO:0031343	positive regulation of cell killing	23	0.300576353	0.006	0.00807902

GOIDlabel	GOName	size	categoryScores	pValPerm	pValPermCorr
GO:0021983	pituitary gland development	22	0.300573301	0.0003	0.001720882
GO:1905269	positive regulation of chromatin organization	62	0.300523104	0.0013	0.002859821
GO:0007050	cell cycle arrest	148	0.300485234	0.0009	0.002399874
GO:0072009	nephron epithelium development	62	0.300464981	0.0012	0.002741075
GO:0051851	modification by host of symbiont morphology or physiology	48	0.300445088	0.0011	0.002610912
GO:0022600	digestive system process	49	0.300443884	0.0003	0.001720882
GO:0009151	purine deoxyribonucleotide metabolic process	8	0.300418275	< 0.001	< 0.001
GO:0009215	purine deoxyribonucleoside triphosphate metabolic process	8	0.300418275	< 0.001	< 0.001
GO:0071674	mononuclear cell migration	34	0.300416383	0.0008	0.002313249
GO:0006541	glutamine metabolic process	13	0.3003861	< 0.001	< 0.001
GO:0090314	positive regulation of protein targeting to membrane	17	0.3003861	0.0027	0.004491543
GO:2000573	positive regulation of DNA biosynthetic process	42	0.300367715	< 0.001	< 0.001
GO:0033151	V(D)J recombination	8	0.300353925	0.0015	0.003088691
GO:1903531	negative regulation of secretion by cell	104	0.300339075	0.0006	0.002177563
GO:0045622	regulation of T-helper cell differentiation	17	0.300310394	0.0006	0.002177563
GO:0043457	regulation of cellular respiration	16	0.300305663	0.0024	0.004156304
GO:0070897	DNA-templated transcriptional preinitiation complex assembly	20	0.30029601	0.0003	0.001720882
GO:0034356	NAD biosynthesis via nicotinamide riboside salvage pathway	11	0.3002457	0.0002	0.001341684
GO:0019184	nonribosomal peptide biosynthetic process	14	0.300220629	0.0112	0.013379119
GO:0036297	interstrand cross-link repair	37	0.300219138	0.001	0.002498236
GO:0046641	positive regulation of alpha-beta T cell proliferation	12	0.3002145	0.0356	0.037550281
GO:2001244	positive regulation of intrinsic apoptotic signaling pathway	40	0.30020592	0.0045	0.006417207
GO:0090175	regulation of establishment of planar polarity	65	0.30010494	0.0009	0.002399874
GO:1904950	negative regulation of establishment of protein localization	107	0.300080588	0.0007	0.002248538
GO:0042632	cholesterol homeostasis	39	0.3000627	0.0027	0.004491543
GO:0055092	sterol homeostasis	39	0.3000627	0.0027	0.004491543
GO:0007249	I-kappaB kinase/NF-kappaB signaling	166	0.300049619	0.0015	0.003088691
GO:0007597	blood coagulation, intrinsic pathway	8	0.300032175	0.0312	0.033261559
GO:0032663	regulation of interleukin-2 production	28	0.300009193	0.0009	0.002399874
GO:0060211	regulation of nuclear-transcribed mRNA poly(A) tail shortening	10	0.29997426	0.0001	0.000877824
GO:0060213	positive regulation of nuclear-transcribed mRNA poly(A) tail shortening	10	0.29997426	0.0001	0.000877824
GO:0071216	cellular response to biotic stimulus	111	0.299971013	0.0009	0.002399874
GO:0050000	chromosome localization	41	0.299959193	0.0004	0.002007244
GO:0051303	establishment of chromosome localization	41	0.299959193	0.0004	0.002007244
GO:1901655	cellular response to ketone	46	0.299921661	0.0006	0.002177563
GO:0009063	cellular amino acid catabolic process	75	0.299898756	0.0014	0.002975058
GO:0010596	negative regulation of endothelial cell migration	33	0.2998557	0.0009	0.002399874
GO:0045747	positive regulation of Notch signaling pathway	28	0.299834528	0.0007	0.002248538
GO:2000116	regulation of cysteine-type endopeptidase activity	137	0.299824329	0.0013	0.002859821
GO:0032757	positive regulation of interleukin-8 production	31	0.299813177	0.0027	0.004491543
GO:0072574	hepatocyte proliferation	12	0.2997426	0.0012	0.002741075
GO:0072575	epithelial cell proliferation involved in liver morphogenesis	12	0.2997426	0.0012	0.002741075
GO:0072576	liver morphogenesis	12	0.2997426	0.0012	0.002741075
GO:0010880	regulation of release of sequestered calcium ion into cytosol by sarcoplasmic reticulum	10	0.2997426	0.0024	0.004156304
GO:0006998	nuclear envelope organization	35	0.299731568	0.0013	0.002859821
GO:0048565	digestive tract development	62	0.299725993	0.0081	0.010256567
GO:0035051	cardiocyte differentiation	70	0.299724214	0.0013	0.002859821
GO:0071636	positive regulation of transforming growth factor beta production	6	0.2996997	0.0108	0.013015961
GO:0070265	necrotic cell death	31	0.299672022	0.0121	0.014285532
GO:1903008	organelle disassembly	57	0.299654542	0.0029	0.004714719
GO:0009799	specification of symmetry	65	0.29958618	0.0001	0.000877824
GO:0009855	determination of bilateral symmetry	65	0.29958618	0.0001	0.000877824
GO:0030431	sleep	16	0.299565637	0.0002	0.001341684
GO:0006448	regulation of translational elongation	11	0.2995437	0.009	0.011215682
GO:0016266	O-glycan processing	29	0.299542893	0.0012	0.002741075
GO:2001241	positive regulation of extrinsic apoptotic signaling pathway in absence of ligand	8	0.299517375	0.0202	0.02235752
GO:0038093	Fc receptor signaling pathway	102	0.299497817	0.0011	0.002610912
GO:0043473	pigmentation	57	0.299496489	0.0015	0.003088691
GO:0006677	glycosylceramide metabolic process	13	0.299495099	0.0052	0.007215241
GO:0042267	natural killer cell mediated cytotoxicity	21	0.299466814	0.0008	0.002313249
GO:1901343	negative regulation of vasculature development	63	0.299401442	0.0013	0.002859821
GO:0046545	development of primary female sexual characteristics	58	0.299392003	0.0008	0.002313249
GO:0050654	chondroitin sulfate proteoglycan metabolic process	20	0.299369369	0.004	0.005880507
GO:0043124	negative regulation of I-kappaB kinase/NF-kappaB signaling	31	0.299314983	0.0009	0.002399874
GO:0051901	positive regulation of mitochondrial depolarization	6	0.299313599	0.025	0.027100697
GO:0086004	regulation of cardiac muscle cell contraction	13	0.299297099	0.0117	0.013882722
GO:0006067	ethanol metabolic process	8	0.299259974	0.0026	0.004377754
GO:0003214	cardiac left ventricle morphogenesis	8	0.299259974	0.0009	0.002399874
GO:1904358	positive regulation of telomere maintenance via telomere lengthening	27	0.299242099	0.0001	0.000877824
GO:0032620	interleukin-17 production	13	0.299237699	0.0019	0.003579279
GO:0051023	regulation of immunoglobulin secretion	12	0.299227799	0.0007	0.002248538
GO:0014909	smooth muscle cell migration	36	0.299220649	0.0007	0.002248538
GO:0097384	cellular lipid biosynthetic process	5	0.299202059	0.0253	0.027397944
GO:0035865	cellular response to potassium ion	5	0.299202059	0.0025	0.004279479
GO:0034380	high-density lipoprotein particle assembly	8	0.299195624	< 0.001	< 0.001
GO:0006244	pyrimidine nucleotide catabolic process	7	0.299172642	0.0025	0.004279479
GO:0009223	pyrimidine deoxyribonucleotide catabolic process	7	0.299172642	0.0025	0.004279479
GO:0042770	signal transduction in response to DNA damage	76	0.299149902	0.0008	0.002313249
GO:1905330	regulation of morphogenesis of an epithelium	101	0.299147521	0.0007	0.002248538
GO:0090087	regulation of peptide transport	96	0.299109824	0.0007	0.002248538
GO:0072520	seminiferous tubule development	7	0.299099099	< 0.001	< 0.001
GO:0060693	regulation of branching involved in salivary gland morphogenesis	5	0.299099099	0.0195	0.021665533
GO:1901186	positive regulation of ERBB signaling pathway	20	0.299073359	0.0051	0.007095023

GOIDlabel	GOName	size	categoryScores	pValPerm	pValPermCorr
GO:0002791	regulation of peptide secretion	94	0.299071716	0.0007	0.002248538
GO:0043631	RNA polyadenylation	29	0.29905472	0.0007	0.002248538
GO:0038202	TORC1 signaling	16	0.299050837	0.0007	0.002248538
GO:0010721	negative regulation of cell development	155	0.299045958	0.0007	0.002248538
GO:0050704	regulation of interleukin-1 secretion	25	0.299037323	0.0001	0.000877824
GO:0032388	positive regulation of intracellular transport	133	0.299035233	0.0007	0.002248538
GO:0030595	leukocyte chemotaxis	94	0.299027903	0.0011	0.002610912
GO:0072073	kidney epithelium development	77	0.299022213	0.0008	0.002313249
GO:0035929	steroid hormone secretion	13	0.299019899	< 0.001	< 0.001
GO:1903018	regulation of glycoprotein metabolic process	34	0.299008252	0.0034	0.005263104
GO:0072577	endothelial cell apoptotic process	30	0.298987559	0.0053	0.007330056
GO:2000194	regulation of female gonad development	6	0.298970399	0.0003	0.001720882
GO:0030968	endoplasmic reticulum unfolded protein response	82	0.298910757	0.004	0.005880507
GO:0032651	regulation of interleukin-1 beta production	37	0.298876483	0.0002	0.001341684
GO:0061035	regulation of cartilage development	31	0.298833396	0.0006	0.002177563
GO:0001522	pseudouridine synthesis	9	0.298784499	0.0147	0.016962357
GO:0090659	walking behavior	18	0.298770199	0.0083	0.010468217
GO:0015874	norepinephrine transport	9	0.298755899	< 0.001	< 0.001
GO:1905461	positive regulation of vascular associated smooth muscle cell apoptotic process	5	0.298738739	0.0026	0.004377754
GO:0072215	regulation of metanephros development	11	0.298701299	0.0013	0.002859821
GO:0038114	interleukin-21-mediated signaling pathway	5	0.298687259	0.0001	0.000877824
GO:0098756	response to interleukin-21	5	0.298687259	0.0001	0.000877824
GO:0098757	cellular response to interleukin-21	5	0.298687259	0.0001	0.000877824
GO:0072210	metanephric nephron development	23	0.298629064	0.0004	0.002007244
GO:0030100	regulation of endocytosis	129	0.298616224	0.0019	0.003579279
GO:0050716	positive regulation of interleukin-1 secretion	17	0.298614581	< 0.001	< 0.001
GO:0001701	in utero embryonic development	200	0.298595882	0.0007	0.002248538
GO:0050770	regulation of axonogenesis	62	0.29858845	0.0021	0.003804235
GO:0048660	regulation of smooth muscle cell proliferation	72	0.298577149	0.0019	0.003579279
GO:0046639	negative regulation of alpha-beta T cell differentiation	13	0.298544699	0.0001	0.000877824
GO:0002709	regulation of T cell mediated immunity	32	0.29854408	0.0025	0.004279479
GO:0007368	determination of left/right symmetry	63	0.298490327	< 0.001	< 0.001
GO:0007044	cell-substrate junction assembly	57	0.298489467	0.0027	0.004491543
GO:0035358	regulation of peroxisome proliferator activated receptor signaling pathway	9	0.298469898	< 0.001	< 0.001
GO:0048008	platelet-derived growth factor receptor signaling pathway	31	0.298468053	0.0031	0.004916949
GO:0038061	NIK/NF-kappaB signaling	116	0.298466693	0.0016	0.003223775
GO:0001776	leukocyte homeostasis	47	0.29845286	0.001	0.002498236
GO:1901223	negative regulation of NIK/NF-kappaB signaling	13	0.298445698	0.0033	0.005149584
GO:1903078	positive regulation of protein localization to plasma membrane	26	0.298435798	0.0008	0.002313249
GO:0071772	response to BMP	72	0.298423423	0.0015	0.003088691
GO:0071773	cellular response to BMP stimulus	72	0.298423423	0.0015	0.003088691
GO:0071679	commissural neuron axon guidance	8	0.298391248	0.0009	0.002399874
GO:0006694	steroid biosynthetic process	98	0.298368923	0.0038	0.005666214
GO:0014808	release of sequestered calcium ion into cytosol by sarcoplasmic reticulum	13	0.298346698	0.0012	0.002741075
GO:1903514	calcium ion transport from endoplasmic reticulum to cytosol	13	0.298346698	0.0012	0.002741075
GO:0090276	regulation of peptide hormone secretion	91	0.298281641	0.0007	0.002248538
GO:0050678	regulation of epithelial cell proliferation	173	0.298279287	0.0006	0.002177563
GO:0045410	positive regulation of interleukin-6 biosynthetic process	10	0.298275418	0.0194	0.021561946
GO:0002639	positive regulation of immunoglobulin production	22	0.298233298	< 0.001	< 0.001
GO:0032845	negative regulation of homeostatic process	102	0.298170439	0.0006	0.002177563
GO:0045787	positive regulation of cell cycle	195	0.298168498	0.0009	0.002399874
GO:0048709	oligodendrocyte differentiation	63	0.298167555	0.0024	0.004156304
GO:0040034	regulation of development, heterochronic	8	0.298166023	0.0004	0.002007244
GO:0071361	cellular response to ethanol	9	0.298126698	0.0006	0.002177563
GO:0019321	pentose metabolic process	8	0.298101673	0.021	0.023166522
GO:0022037	metencephalon development	59	0.298100037	0.0029	0.004714719
GO:0034143	regulation of toll-like receptor 4 signaling pathway	9	0.298098098	0.0004	0.002007244
GO:0043281	regulation of cysteine-type endopeptidase activity involved in apoptotic process	126	0.29809197	0.0016	0.003223775
GO:0032212	positive regulation of telomere maintenance via telomerase	25	0.29809009	0.0001	0.000877824
GO:0003143	embryonic heart tube morphogenesis	37	0.298076455	0.0029	0.004714719
GO:0032507	maintenance of protein location in cell	53	0.298064641	0.0014	0.002975058
GO:0035050	embryonic heart tube development	41	0.298050664	0.0035	0.005358035
GO:0009165	nucleotide biosynthetic process	133	0.298042403	0.001	0.002498236
GO:0035601	protein deacylation	53	0.298040358	0.0026	0.004377754
GO:0045778	positive regulation of ossification	50	0.298033462	< 0.001	< 0.001
GO:1905216	positive regulation of RNA binding	6	0.298026598	0.005	0.006974174
GO:0034587	piRNA metabolic process	6	0.298026598	< 0.001	< 0.001
GO:0031345	negative regulation of cell projection organization	68	0.298008933	0.0015	0.003088691
GO:0044839	cell cycle G2/M phase transition	162	0.298002765	0.0003	0.001720882
GO:1990266	neutrophil migration	38	0.297994987	0.0076	0.009790742
GO:1901184	regulation of ERBB signaling pathway	51	0.29796351	0.0056	0.007655255
GO:2000736	regulation of stem cell differentiation	84	0.297949991	0.0005	0.002177563
GO:0043535	regulation of blood vessel endothelial cell migration	60	0.297949378	0.0013	0.002859821
GO:0050768	negative regulation of neurogenesis	136	0.297940798	0.0008	0.002313249
GO:0048340	paraxial mesoderm morphogenesis	6	0.297940798	0.0004	0.002007244
GO:0034446	substrate adhesion-dependent cell spreading	51	0.297933227	0.0014	0.002975058
GO:0002638	negative regulation of immunoglobulin production	7	0.297885641	< 0.001	< 0.001
GO:0033044	regulation of chromosome organization	101	0.297880908	0.0001	0.000877824
GO:0051100	negative regulation of binding	94	0.297880555	0.0008	0.002313249
GO:0016242	negative regulation of macroautophagy	25	0.297873874	0.0049	0.006860215
GO:0050650	chondroitin sulfate proteoglycan biosynthetic process	14	0.297830484	0.0083	0.010468217
GO:0042063	gliogenesis	166	0.297829154	0.0009	0.002399874
GO:0046209	nitric oxide metabolic process	42	0.297824355	0.0007	0.002248538

GOIDlabel	GOName	size	categoryScores	pValPerm	pValPermCorr
GO:0097711	ciliary basal body docking	66	0.297812098	< 0.001	< 0.001
GO:0050706	regulation of interleukin-1 beta secretion	21	0.297799841	0.0001	0.000877824
GO:0002504	antigen processing and presentation of peptide or polysaccharide antigen via MHC class II	66	0.297788698	0.0029	0.004714719
GO:0003382	epithelial cell morphogenesis	19	0.297785003	0.001	0.002498236
GO:0042761	very long-chain fatty acid biosynthetic process	7	0.297775326	0.0017	0.003334595
GO:0051146	striated muscle cell differentiation	119	0.297753696	0.0017	0.003334595
GO:0032204	regulation of telomere maintenance	55	0.297751258	0.0001	0.000877824
GO:0019886	antigen processing and presentation of exogenous peptide antigen via MHC class II	62	0.297724914	0.0043	0.006204188
GO:0048659	smooth muscle cell proliferation	73	0.297709843	0.0016	0.003223775
GO:0010804	negative regulation of tumor necrosis factor-mediated signaling pathway	12	0.297704848	0.001	0.002498236
GO:0045137	development of primary sexual characteristics	128	0.297689431	0.0006	0.002177563
GO:0030574	collagen catabolic process	17	0.297660686	0.0084	0.010571327
GO:0044243	multicellular organism catabolic process	17	0.297660686	0.0084	0.010571327
GO:0050663	cytokine secretion	117	0.297620698	0.0005	0.002177563
GO:0051289	protein homotetramerization	60	0.297619048	0.0012	0.002741075
GO:0001655	urogenital system development	190	0.297617016	0.0009	0.002399874
GO:0051216	cartilage development	88	0.297601498	0.0005	0.002177563
GO:0046782	regulation of viral transcription	49	0.297591469	0.0018	0.003459409
GO:0032963	collagen metabolic process	50	0.297585586	0.004	0.005880507
GO:0044259	multicellular organismal macromolecule metabolic process	50	0.297585586	0.004	0.005880507
GO:1903364	positive regulation of cellular protein catabolic process	80	0.297580438	0.0007	0.002248538
GO:0010990	regulation of SMAD protein complex assembly	5	0.297554698	0.0279	0.029969105
GO:1902117	positive regulation of organelle assembly	39	0.297548098	0.0035	0.005358035
GO:0019722	calcium-mediated signaling	72	0.297540398	0.0007	0.002248538
GO:0001818	negative regulation of cytokine production	153	0.297472262	0.0006	0.002177563
GO:0033028	myeloid cell apoptotic process	17	0.29746385	0.0017	0.003334595
GO:0010506	regulation of autophagy	197	0.297458009	0.0029	0.004714719
GO:1901293	nucleoside phosphate biosynthetic process	136	0.29745628	0.001	0.002498236
GO:0071577	zinc II ion transmembrane transport	5	0.297451737	0.0245	0.026594873
GO:0043297	apical junction assembly	41	0.297441693	0.0012	0.002741075
GO:0009200	deoxyribonucleoside triphosphate metabolic process	13	0.297435897	0.0011	0.002610912
GO:0044728	DNA methylation or demethylation	47	0.297434212	0.0009	0.002399874
GO:0008406	gonad development	124	0.297382405	0.0006	0.002177563
GO:0090068	positive regulation of cell cycle process	147	0.297374342	0.0016	0.003223775
GO:0099637	neurotransmitter receptor transport	5	0.297348777	0.0006	0.002177563
GO:0065005	protein-lipid complex assembly	16	0.29734556	0.0006	0.002177563
GO:0031146	SCF-dependent proteasomal ubiquitin-dependent protein catabolic process	49	0.297339322	0.001	0.002498236
GO:0002673	regulation of acute inflammatory response	46	0.297336467	0.0055	0.007544447
GO:0010719	negative regulation of epithelial to mesenchymal transition	14	0.297334069	0.0021	0.003804235
GO:0045821	positive regulation of glycolytic process	11	0.297297297	0.024	0.026109935
GO:0051194	positive regulation of cofactor metabolic process	11	0.297297297	0.024	0.026109935
GO:0051197	positive regulation of coenzyme metabolic process	11	0.297297297	0.024	0.026109935
GO:0002825	regulation of T-helper 1 type immune response	12	0.297275847	0.0003	0.001720882
GO:0030509	BMP signaling pathway	68	0.2972708	0.0028	0.004605006
GO:0044784	metaphase/anaphase transition of cell cycle	30	0.297254397	0.0001	0.000877824
GO:0045185	maintenance of protein location	58	0.297252918	0.0013	0.002859821
GO:0030641	regulation of cellular pH	32	0.297240991	0.0087	0.010877987
GO:0051453	regulation of intracellular pH	32	0.297240991	0.0087	0.010877987
GO:0043388	positive regulation of DNA binding	31	0.297239175	0.0009	0.002399874
GO:1903670	regulation of sprouting angiogenesis	40	0.297200772	0.0013	0.002859821
GO:0030004	cellular monovalent inorganic cation homeostasis	39	0.297191697	0.0114	0.01358747
GO:0019370	leukotriene biosynthetic process	14	0.297186983	0.0066	0.008731949
GO:2000269	regulation of fibroblast apoptotic process	12	0.297168597	0.001	0.002498236
GO:1901976	regulation of cell cycle checkpoint	23	0.29715181	0.0001	0.000877824
GO:0000079	regulation of cyclin-dependent protein serine/threonine kinase activity	46	0.297095854	0.0011	0.002610912
GO:0042542	response to hydrogen peroxide	81	0.297093919	0.0027	0.004491543
GO:0002455	humoral immune response mediated by circulating immunoglobulin	24	0.297093522	0.0114	0.01358747
GO:0046883	regulation of hormone secretion	116	0.297066525	0.0006	0.002177563
GO:0002200	somatic diversification of immune receptors	37	0.297053811	0.0001	0.000877824
GO:0055123	digestive system development	70	0.297043574	0.0077	0.009887588
GO:0042762	regulation of sulfur metabolic process	10	0.297014157	0.0005	0.002177563
GO:0070231	T cell apoptotic process	27	0.297001764	0.0044	0.006304227
GO:0000002	mitochondrial genome maintenance	18	0.296996997	0.0096	0.01183839
GO:0090195	chemokine secretion	14	0.29698474	< 0.001	< 0.001
GO:0007039	protein catabolic process in the vacuole	13	0.296980497	0.0102	0.012424427
GO:0008585	female gonad development	55	0.296950977	0.0009	0.002399874
GO:0006476	protein deacetylation	49	0.296945342	0.0024	0.004156304
GO:1901185	negative regulation of ERBB signaling pathway	26	0.296911197	0.0101	0.012328539
GO:0007213	G-protein coupled acetylcholine receptor signaling pathway	7	0.296892811	0.0229	0.025020007
GO:0002706	regulation of lymphocyte mediated immunity	63	0.296851954	0.0008	0.002313249
GO:0007178	transmembrane receptor protein serine/threonine kinase signaling pathway	170	0.296832463	0.0016	0.003223775
GO:0071219	cellular response to molecule of bacterial origin	94	0.296749637	0.0012	0.002741075
GO:0002367	cytokine production involved in immune response	51	0.296747167	0.0007	0.002248538
GO:0070230	positive regulation of lymphocyte apoptotic process	6	0.296739597	0.0167	0.018954426
GO:0072001	renal system development	170	0.296678023	0.0008	0.002313249
GO:0046660	female sex differentiation	65	0.296675577	0.001	0.002498236
GO:0022900	electron transport chain	121	0.296661242	0.0037	0.005561344
GO:0000041	transition metal ion transport	51	0.296626038	0.0025	0.004279479
GO:0007126	meiotic nuclear division	59	0.296625439	0.0014	0.002975058
GO:0033032	regulation of myeloid cell apoptotic process	14	0.296617025	0.0002	0.001341684
GO:0060795	cell fate commitment involved in formation of primary germ layer	16	0.296605534	0.0001	0.000877824
GO:0010633	negative regulation of epithelial cell migration	40	0.296602317	0.0007	0.002248538
GO:0090042	tubulin deacetylation	8	0.296557272	0.0097	0.011922488

GOIDlabel	GOName	size	categoryScores	pValPerm	pValPermCorr
GO:1903671	negative regulation of sprouting angiogenesis	17	0.296555379	0.0026	0.004377754
GO:0016579	protein deubiquitination	170	0.296552351	0.0006	0.002177563
GO:0001738	morphogenesis of a polarized epithelium	81	0.296531452	0.0006	0.002177563
GO:0005996	monosaccharide metabolic process	159	0.296513764	0.0008	0.002313249
GO:0060749	mammary gland alveolus development	11	0.296501697	0.0172	0.019442284
GO:0061377	mammary gland lobule development	11	0.296501697	0.0172	0.019442284
GO:0044346	fibroblast apoptotic process	14	0.296488325	0.0021	0.003804235
GO:0030638	polyketide metabolic process	7	0.296488325	0.0085	0.010671887
GO:0030647	aminoglycoside antibiotic metabolic process	7	0.296488325	0.0085	0.010671887
GO:0044597	daunorubicin metabolic process	7	0.296488325	0.0085	0.010671887
GO:0044598	doxorubicin metabolic process	7	0.296488325	0.0085	0.010671887
GO:0046326	positive regulation of glucose import	18	0.296439296	0.0026	0.004377754
GO:1905508	protein localization to microtubule organizing center	15	0.296422136	0.0006	0.002177563
GO:0098732	macromolecule deacylation	54	0.29639163	0.0029	0.004714719
GO:0072659	protein localization to plasma membrane	133	0.296389623	0.0009	0.002399874
GO:0010894	negative regulation of steroid biosynthetic process	11	0.296384696	0.0136	0.015813319
GO:0045939	negative regulation of steroid metabolic process	11	0.296384696	0.0136	0.015813319
GO:0030865	cortical cytoskeleton organization	22	0.296372996	0.0042	0.006097175
GO:0003157	endocardium development	8	0.296364221	0.0057	0.007745437
GO:1904019	epithelial cell apoptotic process	60	0.296332046	0.0016	0.003223775
GO:0003198	epithelial to mesenchymal transition involved in endocardial cushion formation	12	0.296310596	0.0012	0.002741075
GO:2001236	regulation of extrinsic apoptotic signaling pathway	97	0.296296886	0.0039	0.005778819
GO:0034383	low-density lipoprotein particle clearance	17	0.296252555	0.005	0.006974174
GO:0006576	cellular biogenic amine metabolic process	30	0.296216216	0.0008	0.002313249
GO:0001906	cell killing	59	0.296180442	0.0068	0.008946408
GO:0098813	nuclear chromosome segregation	123	0.296098189	0.0007	0.002248538
GO:0038128	ERBB2 signaling pathway	18	0.296024596	0.0014	0.002975058
GO:0009068	aspartate family amino acid catabolic process	17	0.296010296	0.0041	0.005981983
GO:0043536	positive regulation of blood vessel endothelial cell migration	39	0.296003696	0.0009	0.002399874
GO:1901983	regulation of protein acetylation	41	0.295978906	0.0001	0.000877824
GO:0051099	positive regulation of binding	102	0.295959825	0.0012	0.002741075
GO:0090316	positive regulation of intracellular protein transport	102	0.295937114	0.0008	0.002313249
GO:0032495	response to muramyl dipeptide	9	0.295895896	0.0003	0.001720882
GO:0033002	muscle cell proliferation	105	0.295892627	0.0018	0.003459409
GO:0050673	epithelial cell proliferation	198	0.295888096	0.0006	0.002177563
GO:0032436	positive regulation of proteasomal ubiquitin-dependent protein catabolic process	41	0.295853345	0.0013	0.002859821
GO:0006940	regulation of smooth muscle contraction	28	0.295826439	0.0002	0.001341684
GO:2000392	regulation of lamellipodium morphogenesis	8	0.295817246	0.0285	0.030556948
GO:0048771	tissue remodeling	84	0.295801924	0.0044	0.006304227
GO:0032401	establishment of melanosome localization	11	0.295776296	0.0001	0.000877824
GO:0032402	melanosome transport	11	0.295776296	0.0001	0.000877824
GO:0032000	positive regulation of fatty acid beta-oxidation	6	0.295752896	0.0002	0.001341684
GO:0002762	negative regulation of myeloid leukocyte differentiation	24	0.295731446	0.0005	0.002177563
GO:0045988	negative regulation of striated muscle contraction	5	0.295649936	0.002	0.003693422
GO:0045010	actin nucleation	17	0.295646907	0.0002	0.001341684
GO:0043112	receptor metabolic process	94	0.295632411	0.0026	0.004377754
GO:0061298	retina vasculature development in camera-type eye	12	0.295624196	0.0021	0.003804235
GO:0071375	cellular response to peptide hormone stimulus	154	0.295610824	0.0007	0.002248538
GO:0030308	negative regulation of cell growth	90	0.295566996	0.0008	0.002313249
GO:0042035	regulation of cytokine biosynthetic process	63	0.295552696	0.0012	0.002741075
GO:0031098	stress-activated protein kinase signaling cascade	142	0.295539	0.0007	0.002248538
GO:0050774	negative regulation of dendrite morphogenesis	6	0.295538396	0.0392	0.041055316
GO:0002821	positive regulation of adaptive immune response	57	0.295531622	0.0008	0.002313249
GO:0051149	positive regulation of muscle cell differentiation	43	0.295531412	0.0017	0.003334595
GO:0030307	positive regulation of cell growth	90	0.295512656	0.0007	0.002248538
GO:0002819	regulation of adaptive immune response	85	0.295495495	0.0006	0.002177563
GO:0051438	regulation of ubiquitin-protein transferase activity	27	0.295495495	0.0009	0.002399874
GO:0035791	platelet-derived growth factor receptor-beta signaling pathway	7	0.295458724	0.023	0.025116347
GO:0007163	establishment or maintenance of cell polarity	96	0.295447233	0.0025	0.004279479
GO:0035646	endosome to melanosome transport	7	0.295421953	0.018	0.020203241
GO:0043476	pigment accumulation	7	0.295421953	0.018	0.020203241
GO:0043482	cellular pigment accumulation	7	0.295421953	0.018	0.020203241
GO:0043485	endosome to pigment granule transport	7	0.295421953	0.018	0.020203241
GO:0048757	pigment granule maturation	7	0.295421953	0.018	0.020203241
GO:0007369	gastrulation	95	0.295416921	0.0006	0.002177563
GO:0006120	mitochondrial electron transport, NADH to ubiquinone	39	0.295409695	0.0124	0.014577606
GO:0098779	mitophagy in response to mitochondrial depolarization	11	0.295378495	0.0011	0.002610912
GO:1901222	regulation of NIK/NF-kappaB signaling	72	0.295366795	0.0037	0.005561344
GO:0045429	positive regulation of nitric oxide biosynthetic process	22	0.295366795	0.001	0.002498236
GO:1904407	positive regulation of nitric oxide metabolic process	22	0.295366795	0.001	0.002498236
GO:0043200	response to amino acid	62	0.295362644	0.0004	0.002007244
GO:0061387	regulation of extent of cell growth	44	0.295302445	0.0017	0.003334595
GO:0034331	cell junction maintenance	7	0.295274867	0.0189	0.021076063
GO:0042226	interleukin-6 biosynthetic process	16	0.29527027	0.004	0.005880507
GO:0036315	cellular response to sterol	13	0.295257895	0.0001	0.000877824
GO:0009648	photoperiodism	11	0.295238095	0.0009	0.002399874
GO:2001057	reactive nitrogen species metabolic process	43	0.295160277	0.0005	0.002177563
GO:0007067	mitotic nuclear division	149	0.295115441	0.0011	0.002610912
GO:0033046	negative regulation of sister chromatid segregation	23	0.295114991	< 0.001	< 0.001
GO:2000786	positive regulation of autophagosome assembly	5	0.295083655	0.0086	0.010769857
GO:2001251	negative regulation of chromosome organization	46	0.295081417	< 0.001	< 0.001
GO:0099623	regulation of cardiac muscle cell membrane repolarization	11	0.295074295	0.0004	0.002007244
GO:0010288	response to lead ion	15	0.295066495	0.0073	0.009463568

GOIDlabel	GOName	size	categoryScores	pValPerm	pValPermCorr
GO:0007009	plasma membrane organization	176	0.295058208	0.0014	0.002975058
GO:0055069	zinc ion homeostasis	8	0.295045045	0.0039	0.005778819
GO:0002708	positive regulation of lymphocyte mediated immunity	48	0.29496997	0.0009	0.002399874
GO:1901136	carbohydrate derivative catabolic process	96	0.294964607	0.0008	0.002313249
GO:0035589	G-protein coupled purinergic nucleotide receptor signaling pathway	9	0.294952095	0.0004	0.002007244
GO:0070830	bicellular tight junction assembly	33	0.294949495	0.0012	0.002741075
GO:1901070	guanosine-containing compound biosynthetic process	8	0.29494852	0.0247	0.026793719
GO:0051767	nitric-oxide synthase biosynthetic process	13	0.294941095	0.0006	0.002177563
GO:0051769	regulation of nitric-oxide synthase biosynthetic process	13	0.294941095	0.0006	0.002177563
GO:0060155	platelet dense granule organization	6	0.294937795	< 0.001	< 0.001
GO:0044236	multicellular organism metabolic process	60	0.294907765	0.0054	0.007432873
GO:0046879	hormone secretion	138	0.294876243	0.0007	0.002248538
GO:1901606	alpha-amino acid catabolic process	59	0.294867264	0.0014	0.002975058
GO:0007020	microtubule nucleation	14	0.294851995	0.0138	0.016031243
GO:2000353	positive regulation of endothelial cell apoptotic process	13	0.294802495	0.0233	0.025417819
GO:0032275	luteinizing hormone secretion	7	0.294796838	0.0274	0.029461819
GO:0071827	plasma lipoprotein particle organization	22	0.294793495	0.0002	0.001341684
GO:0098876	vesicle-mediated transport to the plasma membrane	42	0.294778452	0.0026	0.004377754
GO:0045931	positive regulation of mitotic cell cycle	83	0.2947357	0.0011	0.002610912
GO:0001736	establishment of planar polarity	71	0.294730545	0.0006	0.002177563
GO:0007164	establishment of tissue polarity	71	0.294730545	0.0006	0.002177563
GO:0070372	regulation of ERK1 and ERK2 cascade	124	0.294704612	0.0006	0.002177563
GO:0006026	aminoglycan catabolic process	31	0.294665172	0.0005	0.002177563
GO:0006027	glycosaminoglycan catabolic process	31	0.294665172	0.0005	0.002177563
GO:0002701	negative regulation of production of molecular mediator of immune response	17	0.294617306	< 0.001	< 0.001
GO:0007250	activation of NF-kappaB-inducing kinase activity	14	0.29461298	0.0136	0.015813319
GO:0014706	striated muscle tissue development	182	0.294499837	0.0025	0.004279479
GO:0006323	DNA packaging	92	0.294491075	0.0008	0.002313249
GO:0009156	ribonucleoside monophosphate biosynthetic process	56	0.294475087	0.0044	0.006304227
GO:0000280	nuclear division	197	0.294472427	0.0011	0.002610912
GO:0042773	ATP synthesis coupled electron transport	65	0.294469854	0.0116	0.013792313
GO:0042775	mitochondrial ATP synthesis coupled electron transport	65	0.294469854	0.0116	0.013792313
GO:0022904	respiratory electron transport chain	79	0.29443657	0.0093	0.011528671
GO:0034695	response to prostaglandin E	15	0.294431574	0.0001	0.000877824
GO:0043502	regulation of muscle adaptation	44	0.294430794	0.0061	0.008182551
GO:0060711	labyrinthine layer development	26	0.294386694	0.0006	0.002177563
GO:0035710	CD4-positive, alpha-beta T cell activation	50	0.294383526	0.0036	0.005475609
GO:0034629	cellular protein complex localization	9	0.294351494	0.0006	0.002177563
GO:0051055	negative regulation of lipid biosynthetic process	26	0.294307494	0.0063	0.008390784
GO:0003222	ventricular trabecula myocardium morphogenesis	14	0.294300423	0.0038	0.005666214
GO:1901653	cellular response to peptide	185	0.294268322	0.0009	0.002399874
GO:0034101	erythrocyte homeostasis	78	0.294248094	0.0006	0.002177563
GO:0036152	phosphatidylethanolamine acyl-chain remodeling	14	0.294245266	0.0045	0.006417207
GO:0051651	maintenance of location in cell	65	0.294232254	0.0015	0.003088691
GO:0001825	blastocyst formation	25	0.294229086	0.0018	0.003459409
GO:0006513	protein monoubiquitination	40	0.294221364	0.0007	0.002248538
GO:0008593	regulation of Notch signaling pathway	52	0.294218394	0.0007	0.002248538
GO:0048747	muscle fiber development	27	0.294208494	0.0084	0.010571327
GO:0009914	hormone transport	139	0.294199235	0.0008	0.002313249
GO:0007588	excretion	32	0.294192407	0.0016	0.003223775
GO:0070199	establishment of protein localization to chromosome	18	0.294179894	< 0.001	< 0.001
GO:0042180	cellular ketone metabolic process	143	0.294165294	0.0008	0.002313249
GO:0061138	morphogenesis of a branching epithelium	91	0.294154751	0.0029	0.004714719
GO:0034694	response to prostaglandin	17	0.294132788	0.0001	0.000877824
GO:0007281	germ cell development	96	0.294103925	0.0001	0.000877824
GO:0006885	regulation of pH	36	0.294086944	0.0052	0.007215241
GO:0060323	head morphogenesis	20	0.294041184	0.0075	0.00968541
GO:0035176	social behavior	17	0.294011659	0.0002	0.001341684
GO:0051703	intraspecies interaction between organisms	17	0.294011659	0.0002	0.001341684
GO:0071354	cellular response to interleukin-6	21	0.294000123	0.0002	0.001341684
GO:2000021	regulation of ion homeostasis	86	0.293984017	0.0009	0.002399874
GO:0007219	Notch signaling pathway	103	0.29396109	0.0011	0.002610912
GO:0034145	positive regulation of toll-like receptor 4 signaling pathway	5	0.293951094	0.0005	0.002177563
GO:0016445	somatic diversification of immunoglobulins	30	0.293933934	0.0001	0.000877824
GO:1903170	negative regulation of calcium ion transmembrane transport	17	0.293920812	0.0017	0.003334595
GO:0030902	hindbrain development	75	0.293906478	0.003	0.004825593
GO:0060713	labyrinthine layer morphogenesis	9	0.293893894	0.0013	0.002859821
GO:0015807	L-amino acid transport	31	0.293884668	0.0014	0.002975058
GO:0045618	positive regulation of keratinocyte differentiation	7	0.293877551	0.0072	0.009362497
GO:0090092	regulation of transmembrane receptor protein serine/threonine kinase signaling pathway	110	0.293871534	0.0019	0.003579279
GO:0061323	cell proliferation involved in heart morphogenesis	11	0.293857494	0.002	0.003693422
GO:2000136	regulation of cell proliferation involved in heart morphogenesis	11	0.293857494	0.002	0.003693422
GO:0009612	response to mechanical stimulus	114	0.293847231	0.0018	0.003459409
GO:0007585	respiratory gaseous exchange	36	0.293843844	0.0004	0.002007244
GO:0033327	Leydig cell differentiation	10	0.293822394	0.0011	0.002610912
GO:0045844	positive regulation of striated muscle tissue development	33	0.293810694	0.0067	0.008845888
GO:0048636	positive regulation of muscle organ development	33	0.293810694	0.0067	0.008845888
GO:0071318	cellular response to ATP	11	0.293810694	0.0008	0.002313249
GO:0045061	thymic T cell selection	9	0.293808094	0.0004	0.002007244
GO:0006558	L-phenylalanine metabolic process	7	0.293804008	0.0094	0.011616482
GO:0006559	L-phenylalanine catabolic process	7	0.293804008	0.0094	0.011616482
GO:1902221	erythrose 4-phosphate/phosphoenolpyruvate family amino acid metabolic process	7	0.293804008	0.0094	0.011616482
GO:1902222	erythrose 4-phosphate/phosphoenolpyruvate family amino acid catabolic process	7	0.293804008	0.0094	0.011616482

GOIDlabel	GOName	size	categoryScores	pValPerm	pValPermCorr
GO:0090049	regulation of cell migration involved in sprouting angiogenesis	25	0.293796654	0.0017	0.003334595
GO:1903828	negative regulation of cellular protein localization	79	0.293778408	0.0024	0.004156304
GO:1904738	vascular associated smooth muscle cell migration	11	0.293740494	0.0126	0.014782732
GO:1904752	regulation of vascular associated smooth muscle cell migration	11	0.293740494	0.0126	0.014782732
GO:0043122	regulation of I-kappaB kinase/NF-kappaB signaling	150	0.293734878	0.0021	0.003804235
GO:0033363	secretory granule organization	21	0.293730465	0.0002	0.001341684
GO:0002714	positive regulation of B cell mediated immunity	22	0.293717094	0.0002	0.001341684
GO:0002891	positive regulation of immunoglobulin mediated immune response	22	0.293717094	0.0002	0.001341684
GO:1902751	positive regulation of cell cycle G2/M phase transition	14	0.293693694	0.0008	0.002313249
GO:0071230	cellular response to amino acid stimulus	40	0.293635779	0.0002	0.001341684
GO:0060571	morphogenesis of an epithelial fold	8	0.293597169	0.0319	0.033911376
GO:0060326	cell chemotaxis	128	0.29357907	0.0008	0.002313249
GO:0002824	positive regulation of adaptive immune response based on somatic recombination of immune receptors built from immunoglobulin superfamily domains	52	0.293574894	0.0009	0.002399874
GO:0050715	positive regulation of cytokine secretion	71	0.293526927	0.0003	0.001720882
GO:0072330	monocarboxylic acid biosynthetic process	135	0.29351256	0.0013	0.002859821
GO:0014902	myotube differentiation	41	0.29351163	0.0072	0.009362497
GO:0031398	positive regulation of protein ubiquitination	68	0.293470361	0.0004	0.002007244
GO:0061180	mammary gland epithelium development	36	0.293464893	0.0002	0.001341684
GO:0002429	immune response-activating cell surface receptor signaling pathway	191	0.29344977	0.001	0.002498236
GO:0032689	negative regulation of interferon-gamma production	12	0.293436293	0.0007	0.002248538
GO:0038089	positive regulation of cell migration by vascular endothelial growth factor signaling pathway	7	0.293436293	0.0057	0.007745437
GO:0010038	response to metal ion	186	0.293429374	0.0033	0.005149584
GO:0030326	embryonic limb morphogenesis	59	0.293427568	0.0003	0.001720882
GO:0035113	embryonic appendage morphogenesis	59	0.293427568	0.0003	0.001720882
GO:0007160	cell-matrix adhesion	112	0.293397224	0.0021	0.003804235
GO:0003298	physiological muscle hypertrophy	11	0.293342693	0.0003	0.001720882
GO:0003301	physiological cardiac muscle hypertrophy	11	0.293342693	0.0003	0.001720882
GO:0061049	cell growth involved in cardiac muscle cell development	11	0.293342693	0.0003	0.001720882
GO:0031057	negative regulation of histone modification	26	0.293337293	0.0001	0.000877824
GO:0043123	positive regulation of I-kappaB kinase/NF-kappaB signaling	116	0.293287623	0.0024	0.004156304
GO:0042454	ribonucleoside catabolic process	12	0.293286143	0.0036	0.005475609
GO:0031498	chromatin disassembly	12	0.293243243	0.0025	0.004279479
GO:0001657	ureteric bud development	55	0.293206973	0.0003	0.001720882
GO:0072163	mesonephric epithelium development	55	0.293206973	0.0003	0.001720882
GO:0072164	mesonephric tubule development	55	0.293206973	0.0003	0.001720882
GO:0035296	regulation of tube diameter	45	0.293173173	0.0006	0.002177563
GO:0097746	regulation of blood vessel diameter	45	0.293173173	0.0006	0.002177563
GO:0031000	response to caffeine	10	0.293153153	0.0052	0.007215241
GO:0030204	chondroitin sulfate metabolic process	19	0.293124704	0.0037	0.005561344
GO:0060537	muscle tissue development	191	0.293120944	0.0022	0.003910907
GO:0090114	COPII-coated vesicle budding	49	0.293115856	0.0009	0.002399874
GO:0021692	cerebellar Purkinje cell layer morphogenesis	9	0.293064493	0.0042	0.006097175
GO:0035094	response to nicotine	22	0.292979993	0.0008	0.002313249
GO:1902033	regulation of hematopoietic stem cell proliferation	5	0.292972973	0.0021	0.003804235
GO:1904294	positive regulation of ERAD pathway	11	0.292968293	0.0059	0.007964563
GO:0045907	positive regulation of vasoconstriction	12	0.292964393	0.0004	0.002007244
GO:0032206	positive regulation of telomere maintenance	36	0.292935793	0.0001	0.000877824
GO:1902930	regulation of alcohol biosynthetic process	43	0.292897549	0.0014	0.002975058
GO:0019319	hexose biosynthetic process	50	0.292844273	0.0011	0.002610912
GO:0060325	face morphogenesis	19	0.292840209	0.0077	0.009887588
GO:0016233	telomere capping	23	0.292820771	< 0.001	< 0.001
GO:1904029	regulation of cyclin-dependent protein kinase activity	47	0.292795531	0.0014	0.002975058
GO:0030890	positive regulation of B cell proliferation	21	0.292786664	0.0001	0.000877824
GO:0042461	photoreceptor cell development	21	0.292749893	0.0002	0.001341684
GO:0009311	oligosaccharide metabolic process	30	0.292749893	0.0056	0.007655255
GO:0051560	mitochondrial calcium ion homeostasis	14	0.292737636	0.0062	0.008281828
GO:0002790	peptide secretion	111	0.292726704	0.0008	0.002313249
GO:0033210	leptin-mediated signaling pathway	7	0.292700864	0.0001	0.000877824
GO:0036149	phosphatidylinositol acyl-chain remodeling	7	0.292700864	0.0096	0.01183839
GO:0002460	adaptive immune response based on somatic recombination of immune receptors built from immunoglobulin superfamily domains	131	0.292699461	0.0007	0.002248538
GO:0045742	positive regulation of epidermal growth factor receptor signaling pathway	19	0.29267764	0.0049	0.006860215
GO:0021537	telencephalon development	120	0.292666238	0.0005	0.002177563
GO:0014742	positive regulation of muscle hypertrophy	15	0.292664093	0.0086	0.010769857
GO:0006337	nucleosome disassembly	11	0.292664093	0.0018	0.003459409
GO:1902229	regulation of intrinsic apoptotic signaling pathway in response to DNA damage	27	0.292654559	0.0002	0.001341684
GO:1902743	regulation of lamellipodium organization	19	0.292650545	0.0048	0.006748379
GO:0009166	nucleotide catabolic process	34	0.29263381	0.0013	0.002859821
GO:0050710	negative regulation of cytokine secretion	31	0.292622576	0.0008	0.002313249
GO:0035743	CD4-positive, alpha-beta T cell cytokine production	6	0.292621193	0.0162	0.018465856
GO:0032465	regulation of cytokinesis	40	0.292612613	0.0016	0.003223775
GO:0044380	protein localization to cytoskeleton	24	0.292610468	0.0006	0.002177563
GO:0033003	regulation of mast cell activation	22	0.292547093	0.0013	0.002859821
GO:1903975	regulation of glial cell migration	10	0.292535393	0.0083	0.010468217
GO:1902430	negative regulation of beta-amyloid formation	8	0.292535393	0.0026	0.004377754
GO:0007254	JNK cascade	96	0.29253003	0.0007	0.002248538
GO:0046460	neutral lipid biosynthetic process	28	0.292489428	0.001	0.002498236
GO:0046463	acylglycerol biosynthetic process	28	0.292489428	0.001	0.002498236
GO:0072006	nephron development	85	0.292488455	0.0008	0.002313249
GO:0002292	T cell differentiation involved in immune response	33	0.292437892	0.0013	0.002859821
GO:1903305	regulation of regulated secretory pathway	55	0.292392652	0.0008	0.002313249
GO:0007229	integrin-mediated signaling pathway	60	0.292389532	0.0028	0.004605006

GOIDlabel	GOName	size	categoryScores	pValPerm	pValPermCorr
GO:0034656	nucleobase-containing small molecule catabolic process	10	0.292380952	0.0037	0.005561344
GO:0044406	adhesion of symbiont to host	8	0.292374517	0.0019	0.003579279
GO:1903322	positive regulation of protein modification by small protein conjugation or removal	79	0.292361077	0.0004	0.002007244
GO:0010669	epithelial structure maintenance	16	0.29235843	< 0.001	< 0.001
GO:0001822	kidney development	163	0.292343527	0.0008	0.002313249
GO:0006293	nucleotide-excision repair, preincision complex stabilization	15	0.292286572	0.0012	0.002741075
GO:0006295	nucleotide-excision repair, DNA incision, 3'-to lesion	15	0.292286572	0.0012	0.002741075
GO:0043967	histone H4 acetylation	44	0.292242892	0.0013	0.002859821
GO:0048806	genitalia development	22	0.292231192	0.0031	0.004916949
GO:0045992	negative regulation of embryonic development	14	0.292204449	0.0003	0.001720882
GO:0071285	cellular response to lithium ion	6	0.292192192	0.0054	0.007432873
GO:0051893	regulation of focal adhesion assembly	38	0.292189934	0.0034	0.005263104
GO:0090109	regulation of cell-substrate junction assembly	38	0.292189934	0.0034	0.005263104
GO:1903729	regulation of plasma membrane organization	58	0.292171482	0.0015	0.003088691
GO:0045921	positive regulation of exocytosis	37	0.292170162	0.0053	0.007330056
GO:0009581	detection of external stimulus	41	0.292161848	0.0031	0.004916949
GO:1903052	positive regulation of proteolysis involved in cellular protein catabolic process	62	0.292157595	0.0009	0.002399874
GO:0072522	purine-containing compound biosynthetic process	100	0.292110682	0.0023	0.004032435
GO:0019317	fucose catabolic process	5	0.292097812	0.0011	0.002610912
GO:0042354	L-fucose metabolic process	5	0.292097812	0.0011	0.002610912
GO:0042355	L-fucose catabolic process	5	0.292097812	0.0011	0.002610912
GO:0008090	retrograde axonal transport	11	0.292079092	0.0085	0.010671887
GO:0006900	membrane budding	80	0.29207529	0.0009	0.002399874
GO:0006778	porphyrin-containing compound metabolic process	27	0.292063492	0.0104	0.012619802
GO:0051054	positive regulation of DNA metabolic process	135	0.292050145	0.0004	0.002007244
GO:0018242	protein O-linked glycosylation via serine	7	0.292038978	< 0.001	< 0.001
GO:0048634	regulation of muscle organ development	67	0.29203788	0.0098	0.012008345
GO:0071786	endoplasmic reticulum tubular network organization	10	0.291994852	0.0311	0.033166048
GO:0090161	Golgi ribbon formation	8	0.291956242	0.0127	0.014886353
GO:0060348	bone development	98	0.291941796	0.0014	0.002975058
GO:0045926	negative regulation of growth	131	0.291933155	0.0011	0.002610912
GO:0045444	fat cell differentiation	115	0.291932181	0.0006	0.002177563
GO:0042058	regulation of epidermal growth factor receptor signaling pathway	47	0.291930228	0.0079	0.010077402
GO:0048016	inositol phosphate-mediated signaling	21	0.291916406	0.0025	0.004279479
GO:0007007	inner mitochondrial membrane organization	24	0.291891892	0.0064	0.008500875
GO:0046459	short-chain fatty acid metabolic process	10	0.291891892	0.0022	0.003910907
GO:0055002	striated muscle cell development	68	0.291884321	0.0018	0.003459409
GO:0048871	multicellular organismal homeostasis	176	0.291883117	0.0014	0.002975058
GO:0003306	Wnt signaling pathway involved in heart development	5	0.291840412	0.0026	0.004377754
GO:1901992	positive regulation of mitotic cell cycle phase transition	42	0.29181222	0.0021	0.003804235
GO:0022605	oogenesis stage	6	0.291806092	0.0017	0.003334595
GO:0007059	chromosome segregation	153	0.291790951	0.0007	0.002248538
GO:0043401	steroid hormone mediated signaling pathway	102	0.291778333	0.0018	0.003459409
GO:0043403	skeletal muscle tissue regeneration	18	0.291777492	0.0182	0.020388223
GO:0003184	pulmonary valve morphogenesis	11	0.291774892	< 0.001	< 0.001
GO:0046165	alcohol biosynthetic process	78	0.291736792	0.0026	0.004377754
GO:0019318	hexose metabolic process	144	0.291673817	0.0006	0.002177563
GO:0051354	negative regulation of oxidoreductase activity	8	0.291666667	0.0266	0.028683892
GO:0060045	positive regulation of cardiac muscle cell proliferation	17	0.291664774	0.0011	0.002610912
GO:0080111	DNA demethylation	11	0.291657892	0.0044	0.006304227
GO:0019042	viral latency	8	0.291634492	0.0009	0.002399874
GO:0072110	glomerular mesangial cell proliferation	5	0.291634492	0.0013	0.002859821
GO:0002822	regulation of adaptive immune response based on somatic recombination of immune receptors built from immunoglobulin superfamily domains	76	0.291627718	0.0008	0.002313249
GO:2000351	regulation of endothelial cell apoptotic process	29	0.291598988	0.0063	0.008390784
GO:0051091	positive regulation of sequence-specific DNA binding transcription factor activity	135	0.291586825	0.0011	0.002610912
GO:0016137	glycoside metabolic process	13	0.291555292	0.0049	0.006860215
GO:0007256	activation of JNKK activity	5	0.291531532	0.0013	0.002859821
GO:0010968	regulation of microtubule nucleation	5	0.291531532	0.0183	0.020489441
GO:0006687	glycosphingolipid metabolic process	42	0.291493534	0.0046	0.006532041
GO:0000187	activation of MAPK activity	78	0.291456291	0.0006	0.002177563
GO:0010761	fibroblast migration	25	0.291449163	0.0029	0.004714719
GO:0016202	regulation of striated muscle tissue development	66	0.291439491	0.0094	0.011616482
GO:1902186	regulation of viral release from host cell	25	0.291438867	0.004	0.005880507
GO:0031331	positive regulation of cellular catabolic process	141	0.291430032	0.0018	0.003459409
GO:0006751	glutathione catabolic process	5	0.291377091	0.0048	0.006748379
GO:1903320	regulation of protein modification by small protein conjugation or removal	145	0.291354014	0.0008	0.002313249
GO:0042759	long-chain fatty acid biosynthetic process	14	0.29134032	0.0011	0.002610912
GO:0099574	regulation of protein catabolic process at synapse, modulating synaptic transmission	5	0.291325611	0.0041	0.005981983
GO:0031396	regulation of protein ubiquitination	129	0.291299273	0.0009	0.002399874
GO:0043433	negative regulation of sequence-specific DNA binding transcription factor activity	92	0.291295954	0.0001	0.000877824
GO:0048839	inner ear development	92	0.291290359	0.0007	0.002248538
GO:0051705	multi-organism behavior	25	0.291284427	< 0.001	< 0.001
GO:0071241	cellular response to inorganic substance	106	0.291238678	0.0033	0.005149584
GO:0001763	morphogenesis of a branching structure	96	0.291229622	0.002	0.003693422
GO:0032869	cellular response to insulin stimulus	109	0.291209427	0.002	0.003693422
GO:1902992	negative regulation of amyloid precursor protein catabolic process	9	0.291205491	0.0046	0.006532041
GO:1903391	regulation of adherens junction organization	42	0.291187105	0.0031	0.004916949
GO:0060572	morphogenesis of an epithelial bud	5	0.291171171	0.0192	0.021362011
GO:0006525	arginine metabolic process	6	0.291162591	< 0.001	< 0.001
GO:0061098	positive regulation of protein tyrosine kinase activity	21	0.291144205	0.0005	0.002177563
GO:0001890	placenta development	75	0.291136851	0.001	0.002498236
GO:0047496	vesicle transport along microtubule	19	0.291106144	0.0051	0.007095023

GOIDlabel	GOName	size	categoryScores	pValPerm	pValPermCorr
GO:0006470	protein dephosphorylation	164	0.291097718	0.001	0.002498236
GO:0010829	negative regulation of glucose transport	11	0.291096291	0.0002	0.001341684
GO:0060391	positive regulation of SMAD protein import into nucleus	9	0.291091091	0.0072	0.009362497
GO:1904814	regulation of protein localization to chromosome, telomeric region	13	0.291080091	0.0001	0.000877824
GO:1903556	negative regulation of tumor necrosis factor superfamily cytokine production	30	0.291068211	0.0065	0.008621124
GO:1990778	protein localization to cell periphery	148	0.29105708	0.001	0.002498236
GO:1903429	regulation of cell maturation	9	0.291033891	0.0033	0.005149584
GO:0031290	retinal ganglion cell axon guidance	8	0.291023166	< 0.001	< 0.001
GO:0051961	negative regulation of nervous system development	148	0.291017079	0.0007	0.002248538
GO:0009952	anterior/posterior pattern specification	74	0.291008383	0.0007	0.002248538
GO:0030072	peptide hormone secretion	107	0.291001816	0.001	0.002498236
GO:2000352	negative regulation of endothelial cell apoptotic process	15	0.290999571	0.001	0.002498236
GO:0030033	microvillus assembly	8	0.290990991	0.0072	0.009362497
GO:0032411	positive regulation of transporter activity	45	0.290976691	0.0034	0.005263104
GO:0032958	inositol phosphate biosynthetic process	12	0.290969541	0.0006	0.002177563
GO:0001823	mesonephros development	56	0.290949623	0.0002	0.001341684
GO:0002526	acute inflammatory response	75	0.290937795	0.0029	0.004714719
GO:0051983	regulation of chromosome segregation	53	0.290930283	0.0003	0.001720882
GO:0060562	epithelial tube morphogenesis	170	0.29092437	0.0006	0.002177563
GO:0000491	small nucleolar ribonucleoprotein complex assembly	5	0.290913771	0.0066	0.008731949
GO:0032462	regulation of protein homooligomerization	14	0.290899062	0.0031	0.004916949
GO:0007584	response to nutrient	84	0.290853098	0.0011	0.002610912
GO:0001773	myeloid dendritic cell activation	16	0.290846203	0.0012	0.002741075
GO:0071222	cellular response to lipopolysaccharide	88	0.290844741	0.0011	0.002610912
GO:0035855	megakaryocyte development	10	0.290836551	0.0201	0.022258436
GO:0042157	lipoprotein metabolic process	77	0.290815491	0.0031	0.004916949
GO:0032729	positive regulation of interferon-gamma production	32	0.29077381	0.0035	0.005358035
GO:0045665	negative regulation of neuron differentiation	98	0.290767736	0.0008	0.002313249
GO:0044262	cellular carbohydrate metabolic process	148	0.290766635	0.0006	0.002177563
GO:1903069	regulation of ER-associated ubiquitin-dependent protein catabolic process	7	0.290751976	0.0301	0.032169596
GO:0090313	regulation of protein targeting to membrane	18	0.290747891	0.0029	0.004714719
GO:0043583	ear development	105	0.290744622	0.0006	0.002177563
GO:0051403	stress-activated MAPK cascade	136	0.290739269	0.0007	0.002248538
GO:0002440	production of molecular mediator of immune response	98	0.290736217	0.0001	0.000877824
GO:0002093	auditory receptor cell morphogenesis	9	0.290719291	0.0001	0.000877824
GO:0044065	regulation of respiratory system process	7	0.290715205	0.006	0.00807902
GO:0050714	positive regulation of protein secretion	134	0.290687489	0.0002	0.001341684
GO:0090235	regulation of metaphase plate congression	5	0.290656371	0.0221	0.024229021
GO:0048048	embryonic eye morphogenesis	22	0.290604891	0.0023	0.004032435
GO:0002902	regulation of B cell apoptotic process	7	0.290604891	0.0038	0.005666214
GO:0045606	positive regulation of epidermal cell differentiation	10	0.290604891	0.001	0.002498236
GO:0045657	positive regulation of monocyte differentiation	7	0.290604891	0.0063	0.008390784
GO:0003009	skeletal muscle contraction	17	0.290574608	0.0001	0.000877824
GO:0006636	unsaturated fatty acid biosynthetic process	43	0.290568974	0.0013	0.002859821
GO:0042158	lipoprotein biosynthetic process	56	0.290545137	0.0021	0.003804235
GO:0010866	regulation of triglyceride biosynthetic process	14	0.290531348	0.0007	0.002248538
GO:0032868	response to insulin	142	0.290525133	0.0019	0.003579279
GO:0070741	response to interleukin-6	24	0.290508366	0.0002	0.001341684
GO:0002372	myeloid dendritic cell cytokine production	5	0.29045045	0.0269	0.028963288
GO:0002732	positive regulation of dendritic cell cytokine production	5	0.29045045	0.0269	0.028963288
GO:0002733	regulation of myeloid dendritic cell cytokine production	5	0.29045045	0.0269	0.028963288
GO:0002735	positive regulation of myeloid dendritic cell cytokine production	5	0.29045045	0.0269	0.028963288
GO:0019432	triglyceride biosynthetic process	26	0.29040689	0.0012	0.002741075
GO:0015893	drug transport	9	0.29040469	0.0025	0.004279479
GO:1903859	regulation of dendrite extension	5	0.29039897	0.0073	0.009463568
GO:1903861	positive regulation of dendrite extension	5	0.29039897	0.0073	0.009463568
GO:0048872	homeostasis of number of cells	147	0.290361499	0.0006	0.002177563
GO:0045927	positive regulation of growth	147	0.290352743	0.0007	0.002248538
GO:0015850	organic hydroxy compound transport	108	0.290352257	0.0002	0.001341684
GO:0051547	regulation of keratinocyte migration	7	0.29034749	0.0032	0.005044175
GO:0051549	positive regulation of keratinocyte migration	7	0.29034749	0.0032	0.005044175
GO:0046323	glucose import	33	0.29032409	0.0009	0.002399874
GO:0031348	negative regulation of defense response	102	0.290312161	0.0023	0.004032435
GO:0016574	histone ubiquitination	32	0.290267053	0.0006	0.002177563
GO:0010639	negative regulation of organelle organization	178	0.290189869	0.0008	0.002313249
GO:0046321	positive regulation of fatty acid oxidation	8	0.29015444	0.0029	0.004714719
GO:2000241	regulation of reproductive process	62	0.290061029	0.0004	0.002007244
GO:1903265	positive regulation of tumor necrosis factor-mediated signaling pathway	8	0.290057915	0.0027	0.004491543
GO:0036296	response to increased oxygen levels	16	0.290057915	0.0006	0.002177563
GO:0061053	somite development	42	0.290041061	0.0008	0.002313249
GO:1905515	non-motile cilium assembly	34	0.290037096	< 0.001	< 0.001
GO:0090308	regulation of methylation-dependent chromatin silencing	7	0.290016547	0.0042	0.006097175
GO:0046718	viral entry into host cell	74	0.290003131	0.0097	0.011922488
GO:0003272	endocardial cushion formation	15	0.28998713	0.002	0.003693422
GO:0006956	complement activation	33	0.28996529	0.0109	0.013101792
GO:0001654	eye development	185	0.289924519	0.0013	0.002859821
GO:0050855	regulation of B cell receptor signaling pathway	13	0.28989209	0.0033	0.005149584
GO:0051260	protein homooligomerization	193	0.289888704	0.0011	0.002610912
GO:1900087	positive regulation of G1/S transition of mitotic cell cycle	22	0.28984439	0.0035	0.005358035
GO:0045940	positive regulation of steroid metabolic process	15	0.28983269	0.0063	0.008390784
GO:0002062	chondrocyte differentiation	49	0.289816931	0.0008	0.002313249
GO:0034329	cell junction assembly	126	0.289814304	0.0033	0.005149584
GO:0031998	regulation of fatty acid beta-oxidation	12	0.28976834	0.0021	0.003804235

GOIDlabel	GOName	size	categoryScores	pValPerm	pValPermCorr
GO:0051567	histone H3-K9 methylation	24	0.289693265	0.002	0.003693422
GO:0043534	blood vessel endothelial cell migration	74	0.289690076	0.0013	0.002859821
GO:0031952	regulation of protein autophosphorylation	21	0.289624318	0.0023	0.004032435
GO:0050771	negative regulation of axonogenesis	18	0.28961819	0.0023	0.004032435
GO:0050810	regulation of steroid biosynthetic process	55	0.28958933	0.0051	0.007095023
GO:0006144	purine nucleobase metabolic process	10	0.28957529	0.0082	0.010366713
GO:0031333	negative regulation of protein complex assembly	68	0.289567719	0.0009	0.002399874
GO:0008645	hexose transport	56	0.289543115	0.0006	0.002177563
GO:0015749	monosaccharide transport	56	0.289543115	0.0006	0.002177563
GO:1990138	neuron projection extension	61	0.289524653	0.0006	0.002177563
GO:1903076	regulation of protein localization to plasma membrane	53	0.289512154	0.0021	0.003804235
GO:0006939	smooth muscle contraction	52	0.289491139	0.001	0.002498236
GO:0046328	regulation of JNK cascade	83	0.289476051	0.0008	0.002313249
GO:0050772	positive regulation of axonogenesis	32	0.289454633	0.0033	0.005149584
GO:0045080	positive regulation of chemokine biosynthetic process	5	0.289420849	0.024	0.026109935
GO:0051450	myoblast proliferation	8	0.289414414	0.0123	0.014492124
GO:0035107	appendage morphogenesis	70	0.289409818	0.0002	0.001341684
GO:0035108	limb morphogenesis	70	0.289409818	0.0002	0.001341684
GO:0036257	multivesicular body organization	23	0.28940742	0.0043	0.006204188
GO:0036258	multivesicular body assembly	23	0.28940742	0.0043	0.006204188
GO:0060324	face development	24	0.289403689	0.0083	0.010468217
GO:0031670	cellular response to nutrient	21	0.289391432	0.0008	0.002313249
GO:0043901	negative regulation of multi-organism process	78	0.289380589	0.0021	0.003804235
GO:0080154	regulation of fertilization	6	0.289360789	0.0008	0.002313249
GO:1901020	negative regulation of calcium ion transmembrane transporter activity	14	0.289354661	0.0023	0.004032435
GO:0030198	extracellular matrix organization	195	0.289331089	0.005	0.006974174
GO:0043062	extracellular structure organization	195	0.289331089	0.005	0.006974174
GO:0055001	muscle cell development	78	0.289324489	0.0023	0.004032435
GO:1903599	positive regulation of mitophagy	9	0.289317889	0.0076	0.009790742
GO:0032649	regulation of interferon-gamma production	43	0.289305917	0.0024	0.004156304
GO:0016042	lipid catabolic process	172	0.289280476	0.0028	0.004605006
GO:0032365	intracellular lipid transport	26	0.289228789	0.0047	0.006644432
GO:0010811	positive regulation of cell-substrate adhesion	67	0.289225686	0.0009	0.002399874
GO:0002064	epithelial cell development	113	0.289224496	0.0014	0.002975058
GO:0045063	T-helper 1 cell differentiation	13	0.289218889	0.0037	0.005561344
GO:0032846	positive regulation of homeostatic process	116	0.289218036	0.0004	0.002007244
GO:0048168	regulation of neuronal synaptic plasticity	11	0.289200889	0.0017	0.003334595
GO:0035239	tube morphogenesis	190	0.289197318	0.0011	0.002610912
GO:0051155	positive regulation of striated muscle cell differentiation	24	0.289189189	0.0225	0.024620965
GO:0022412	cellular process involved in reproduction in multicellular organism	116	0.289184751	0.0003	0.001720882
GO:0010508	positive regulation of autophagy	70	0.289156095	0.0045	0.006417207
GO:0010447	response to acidic pH	9	0.289146289	0.0015	0.003088691
GO:0031641	regulation of myelination	24	0.289135564	0.0057	0.007745437
GO:0048505	regulation of timing of cell differentiation	7	0.289134032	0.0016	0.003223775
GO:0019369	arachidonic acid metabolic process	19	0.289128226	0.0047	0.006644432
GO:1903523	negative regulation of blood circulation	19	0.289128226	0.0017	0.003334595
GO:1900103	positive regulation of endoplasmic reticulum unfolded protein response	8	0.289124839	0.0267	0.028781986
GO:0006771	riboflavin metabolic process	5	0.289111969	0.0103	0.012519912
GO:0034350	regulation of glial cell apoptotic process	5	0.289111969	0.0266	0.028683892
GO:1901863	positive regulation of muscle tissue development	34	0.289090771	0.008	0.010168329
GO:1901861	regulation of muscle tissue development	67	0.289079698	0.0104	0.012619802
GO:0097529	myeloid leukocyte migration	88	0.289063414	0.001	0.002498236
GO:0031102	neuron projection regeneration	29	0.289051613	0.0109	0.013101792
GO:0043491	protein kinase B signaling	126	0.289042103	0.0008	0.002313249
GO:0032611	interleukin-1 beta production	42	0.289042103	0.0003	0.001720882
GO:0035303	regulation of dephosphorylation	109	0.28901326	0.0005	0.002177563
GO:0006626	protein targeting to mitochondrion	63	0.288999203	0.0011	0.002610912
GO:0051957	positive regulation of amino acid transport	8	0.288996139	0.0047	0.006644432
GO:0006094	gluconeogenesis	49	0.288929162	0.0015	0.003088691
GO:0031937	positive regulation of chromatin silencing	9	0.288888889	0.0018	0.003459409
GO:0006643	membrane lipid metabolic process	127	0.288878079	0.0034	0.005263104
GO:0033004	negative regulation of mast cell activation	8	0.288803089	0.0006	0.002177563
GO:1902100	negative regulation of metaphase/anaphase transition of cell cycle	20	0.288777349	< 0.001	< 0.001
GO:0033273	response to vitamin	45	0.288705849	0.0021	0.003804235
GO:0048260	positive regulation of receptor-mediated endocytosis	29	0.288687702	0.0143	0.016557758
GO:0048207	vesicle targeting, rough ER to cis-Golgi	44	0.288680239	0.001	0.002498236
GO:0048208	COPII vesicle coating	44	0.288680239	0.001	0.002498236
GO:0033690	positive regulation of osteoblast proliferation	6	0.288674389	0.0065	0.008621124
GO:1901021	positive regulation of calcium ion transmembrane transporter activity	12	0.288652939	0.0015	0.003088691
GO:0035627	ceramide transport	10	0.288648649	0.0032	0.005044175
GO:0042451	purine nucleoside biosynthetic process	59	0.288641668	0.0036	0.005475609
GO:0046129	purine ribonucleoside biosynthetic process	59	0.288641668	0.0036	0.005475609
GO:0043367	CD4-positive, alpha-beta T cell differentiation	37	0.288636126	0.0014	0.002975058
GO:1904376	negative regulation of protein localization to cell periphery	17	0.288621395	0.0057	0.007745437
GO:0045663	positive regulation of myoblast differentiation	5	0.288597169	0.0003	0.001720882
GO:0030206	chondroitin sulfate biosynthetic process	13	0.288585289	0.0075	0.00968541
GO:0007045	cell-substrate adherens junction assembly	48	0.288551051	0.0031	0.004916949
GO:0048041	focal adhesion assembly	48	0.288551051	0.0031	0.004916949
GO:0030948	negative regulation of vascular endothelial growth factor receptor signaling pathway	7	0.288545689	0.0457	0.047395622
GO:0032604	granulocyte macrophage colony-stimulating factor production	7	0.288472146	0.0008	0.002313249
GO:0032645	regulation of granulocyte macrophage colony-stimulating factor production	7	0.288472146	0.0008	0.002313249
GO:2000144	positive regulation of DNA-templated transcription, initiation	13	0.288466488	0.002	0.003693422
GO:0043501	skeletal muscle adaptation	9	0.288459888	0.0054	0.007432873

GOIDlabel	GOName	size	categoryScores	pValPerm	pValPermCorr
GO:0098773	skin epidermis development	47	0.28844711	0.0009	0.002399874
GO:0071902	positive regulation of protein serine/threonine kinase activity	166	0.288429393	0.001	0.002498236
GO:1903046	meiotic cell cycle process	62	0.288387927	0.0014	0.002975058
GO:0099625	ventricular cardiac muscle cell membrane repolarization	12	0.288374088	0.0009	0.002399874
GO:0072202	cell differentiation involved in metanephros development	15	0.288356928	0.0005	0.002177563
GO:0090287	regulation of cellular response to growth factor stimulus	132	0.288352638	0.0032	0.005044175
GO:0010675	regulation of cellular carbohydrate metabolic process	74	0.288343942	0.0007	0.002248538
GO:0035459	cargo loading into vesicle	15	0.288322608	0.0066	0.008731949
GO:0050850	positive regulation of calcium-mediated signaling	21	0.288276031	0.0038	0.005666214
GO:0032103	positive regulation of response to external stimulus	154	0.288269903	0.0005	0.002177563
GO:0072075	metanephric mesenchyme development	7	0.288251517	0.0002	0.001341684
GO:0010952	positive regulation of peptidase activity	107	0.288244987	0.0026	0.004377754
GO:0046364	monosaccharide biosynthetic process	54	0.288240622	0.0021	0.003804235
GO:2000543	positive regulation of gastrulation	5	0.288236808	0.0006	0.002177563
GO:0002287	alpha-beta T cell activation involved in immune response	31	0.288221862	0.0013	0.002859821
GO:0002293	alpha-beta T cell differentiation involved in immune response	31	0.288221862	0.0013	0.002859821
GO:0002294	CD4-positive, alpha-beta T cell differentiation involved in immune response	31	0.288221862	0.0013	0.002859821
GO:0042093	T-helper cell differentiation	31	0.288221862	0.0013	0.002859821
GO:1902001	fatty acid transmembrane transport	11	0.288218088	0.0049	0.006860215
GO:0020027	hemoglobin metabolic process	9	0.288202488	0.0028	0.004605006
GO:0035562	negative regulation of chromatin binding	6	0.288202488	0.0083	0.010468217
GO:0031214	biomineral tissue development	70	0.288192682	0.001	0.002498236
GO:0001960	negative regulation of cytokine-mediated signaling pathway	31	0.288172043	0.0002	0.001341684
GO:0036344	platelet morphogenesis	12	0.288138138	0.0125	0.014684332
GO:0033629	negative regulation of cell adhesion mediated by integrin	5	0.288133848	0.0116	0.013792313
GO:0048333	mesodermal cell differentiation	19	0.288112172	0.0004	0.002007244
GO:1903532	positive regulation of secretion by cell	198	0.288098488	0.0004	0.002007244
GO:0072091	regulation of stem cell proliferation	38	0.288078304	0.0013	0.002859821
GO:0031958	corticosteroid receptor signaling pathway	10	0.288056628	0.0001	0.000877824
GO:0019835	cytolysis	13	0.288050688	0.0294	0.031463678
GO:0006749	glutathione metabolic process	35	0.288045597	0.0007	0.002248538
GO:0010543	regulation of platelet activation	14	0.288030888	0.0011	0.002610912
GO:0035357	peroxisome proliferator activated receptor signaling pathway	11	0.288030888	0.0003	0.001720882
GO:0009566	fertilization	63	0.288026802	0.0001	0.000877824
GO:0009583	detection of light stimulus	19	0.287963151	0.0073	0.009463568
GO:0009584	detection of visible light	16	0.287934363	0.004	0.005880507
GO:0070207	protein homotrimerization	18	0.287930788	0.0025	0.004279479
GO:0002250	adaptive immune response	198	0.287928188	0.0009	0.002399874
GO:0001556	oocyte maturation	11	0.287913888	0.0002	0.001341684
GO:0033014	tetrapyrrole biosynthetic process	23	0.287907784	0.0038	0.005666214
GO:0051017	actin filament bundle assembly	76	0.287844612	0.0007	0.002248538
GO:0045833	negative regulation of lipid metabolic process	42	0.287828645	0.0015	0.003088691
GO:0031589	cell-substrate adhesion	176	0.2878276	0.0019	0.003579279
GO:0031294	lymphocyte costimulation	27	0.287821154	0.0031	0.004916949
GO:1900181	negative regulation of protein localization to nucleus	28	0.287819452	0.0007	0.002248538
GO:0070861	regulation of protein exit from endoplasmic reticulum	15	0.287807808	0.0153	0.017559319
GO:0071550	death-inducing signaling complex assembly	8	0.287805663	0.0056	0.007655255
GO:0030010	establishment of cell polarity	67	0.287792697	0.0046	0.006532041
GO:0071499	cellular response to laminar fluid shear stress	6	0.287773488	0.0009	0.002399874
GO:0010975	regulation of neuron projection development	191	0.287731711	0.001	0.002498236
GO:0033119	negative regulation of RNA splicing	18	0.287716288	0.002	0.003693422
GO:0009582	detection of abiotic stimulus	42	0.287675431	0.0019	0.003579279
GO:0006497	protein lipidation	54	0.287673388	0.0029	0.004714719
GO:0046467	membrane lipid biosynthetic process	85	0.287670528	0.0039	0.005778819
GO:0090181	regulation of cholesterol metabolic process	42	0.287626402	0.0015	0.003088691
GO:0036498	IRE1-mediated unfolded protein response	46	0.287611214	0.0066	0.008731949
GO:0048793	pronephros development	6	0.287601888	0.0081	0.010256567
GO:0030260	entry into host cell	80	0.28759009	0.0081	0.010256567
GO:0044409	entry into host	80	0.28759009	0.0081	0.010256567
GO:0051806	entry into cell of other organism involved in symbiotic interaction	80	0.28759009	0.0081	0.010256567
GO:0051828	entry into other organism involved in symbiotic interaction	80	0.28759009	0.0081	0.010256567
GO:0032011	ARF protein signal transduction	7	0.28758963	0.0104	0.012619802
GO:0032012	regulation of ARF protein signal transduction	7	0.28758963	0.0104	0.012619802
GO:0055025	positive regulation of cardiac muscle tissue development	25	0.287516088	0.0068	0.008946408
GO:0007175	negative regulation of epidermal growth factor-activated receptor activity	9	0.287516088	0.0166	0.018864444
GO:1902230	negative regulation of intrinsic apoptotic signaling pathway in response to DNA damage	21	0.287491573	< 0.001	< 0.001
GO:0006509	membrane protein ectodomain proteolysis	31	0.287491178	0.0084	0.010571327
GO:0072511	divalent inorganic cation transport	190	0.287471381	0.0017	0.003334595
GO:0006384	transcription initiation from RNA polymerase III promoter	5	0.287464607	0.0025	0.004279479
GO:0072503	cellular divalent inorganic cation homeostasis	173	0.287458061	0.0012	0.002741075
GO:0042698	ovulation cycle	61	0.287427474	0.0008	0.002313249
GO:0032201	telomere maintenance via semi-conservative replication	20	0.287413127	0.0046	0.006532041
GO:0007596	blood coagulation	151	0.287401877	0.001	0.002498236
GO:1903312	negative regulation of mRNA metabolic process	27	0.287392154	0.0011	0.002610912
GO:0030218	erythrocyte differentiation	71	0.2873892	0.0006	0.002177563
GO:0002260	lymphocyte homeostasis	34	0.287387387	0.0021	0.003804235
GO:0010939	regulation of necrotic cell death	17	0.287379817	0.0268	0.028880014
GO:0022406	membrane docking	95	0.2873481	0.0001	0.000877824
GO:0014002	astrocyte development	22	0.287317187	0.0108	0.013015961
GO:1901386	negative regulation of voltage-gated calcium channel activity	5	0.287310167	0.0027	0.004491543
GO:0031334	positive regulation of protein complex assembly	122	0.287277676	0.0013	0.002859821
GO:0070570	regulation of neuron projection regeneration	13	0.287258687	0.0124	0.014577606
GO:1904816	positive regulation of protein localization to chromosome, telomeric region	11	0.287235287	0.0002	0.001341684

GOIDlabel	GOName	size	categoryScores	pValPerm	pValPermCorr
GO:0016050	vesicle organization	179	0.287229927	0.0026	0.004377754
GO:0015833	peptide transport	123	0.28722939	0.0016	0.003223775
GO:0010770	positive regulation of cell morphogenesis involved in differentiation	67	0.287224111	0.0028	0.004605006
GO:1905049	negative regulation of metallopeptidase activity	5	0.287207207	0.008	0.010168329
GO:0006123	mitochondrial electron transport, cytochrome c to oxygen	13	0.287199287	0.0149	0.017165166
GO:1900024	regulation of substrate adhesion-dependent cell spreading	32	0.28717825	0.0038	0.005666214
GO:0009755	hormone-mediated signaling pathway	122	0.287155305	0.0011	0.002610912
GO:0010043	response to zinc ion	34	0.287152699	0.0049	0.006860215
GO:0006839	mitochondrial transport	158	0.287144649	0.0031	0.004916949
GO:0010876	lipid localization	192	0.287125965	0.0014	0.002975058
GO:0051494	negative regulation of cytoskeleton organization	65	0.287112167	0.0016	0.003223775
GO:0030238	male sex determination	8	0.287065637	0.0013	0.002859821
GO:0030835	negative regulation of actin filament depolymerization	17	0.287031569	0.0007	0.002248538
GO:0001101	response to acid chemical	177	0.287023101	0.0006	0.002177563
GO:1900364	negative regulation of mRNA polyadenylation	5	0.287001287	0.0017	0.003334595
GO:0006874	cellular calcium ion homeostasis	166	0.286991983	0.0011	0.002610912
GO:0001942	hair follicle development	46	0.286990096	0.0008	0.002313249
GO:0007599	hemostasis	154	0.286972873	0.0011	0.002610912
GO:0045839	negative regulation of mitotic nuclear division	28	0.286955323	0.0008	0.002313249
GO:0010613	positive regulation of cardiac muscle hypertrophy	14	0.286927744	0.0079	0.010077402
GO:0022408	negative regulation of cell-cell adhesion	81	0.28692502	0.0005	0.002177563
GO:0002237	response to molecule of bacterial origin	166	0.286923757	0.0006	0.002177563
GO:0052405	negative regulation by host of symbiont molecular function	6	0.286915487	0.0026	0.004377754
GO:0043524	negative regulation of neuron apoptotic process	68	0.286914225	0.0032	0.005044175
GO:0008211	glucocorticoid metabolic process	8	0.286872587	0.0133	0.015521132
GO:0048661	positive regulation of smooth muscle cell proliferation	50	0.286872587	0.0019	0.003579279
GO:0098915	membrane repolarization during ventricular cardiac muscle cell action potential	8	0.286840412	0.0036	0.005475609
GO:0014866	skeletal myofibril assembly	6	0.286829687	0.0214	0.023571068
GO:0035150	regulation of tube size	51	0.28682464	0.0016	0.003223775
GO:0050880	regulation of blood vessel size	51	0.28682464	0.0016	0.003223775
GO:0070509	calcium ion import	70	0.286802721	0.0008	0.002313249
GO:0001774	microglial cell activation	22	0.286802387	0.0114	0.01358747
GO:0070838	divalent metal ion transport	189	0.286790192	0.0017	0.003334595
GO:0036295	cellular response to increased oxygen levels	8	0.286776062	0.0006	0.002177563
GO:0018198	peptidyl-cysteine modification	16	0.286727799	0.003	0.004825593
GO:0051187	cofactor catabolic process	11	0.286720487	0.0008	0.002313249
GO:0009896	positive regulation of catabolic process	177	0.286695897	0.002	0.003693422
GO:1903432	regulation of TORC1 signaling	15	0.286692407	0.0008	0.002313249
GO:0061572	actin filament bundle organization	77	0.286680372	0.0008	0.002313249
GO:0050651	dermatan sulfate proteoglycan biosynthetic process	9	0.286629487	0.0233	0.025417819
GO:0050655	dermatan sulfate proteoglycan metabolic process	9	0.286629487	0.0233	0.025417819
GO:0072529	pyrimidine-containing compound catabolic process	17	0.286622757	0.0009	0.002399874
GO:0001909	leukocyte mediated cytotoxicity	40	0.286615187	0.0071	0.009268394
GO:0009074	aromatic amino acid family catabolic process	10	0.286589447	0.0035	0.005358035
GO:0017014	protein nitrosylation	5	0.286589447	0.0057	0.007745437
GO:0018119	peptidyl-cysteine S-nitrosylation	5	0.286589447	0.0057	0.007745437
GO:1905476	negative regulation of protein localization to membrane	18	0.286572287	0.0053	0.007330056
GO:0015758	glucose transport	53	0.286569049	0.0006	0.002177563
GO:0050707	regulation of cytokine secretion	102	0.286486486	0.0003	0.001720882
GO:0033700	phospholipid efflux	5	0.286486486	0.0035	0.005358035
GO:2001237	negative regulation of extrinsic apoptotic signaling pathway	59	0.286486486	0.0065	0.008621124
GO:0046330	positive regulation of JNK cascade	60	0.286473616	0.0007	0.002248538
GO:0032653	regulation of interleukin-10 production	25	0.286455598	0.004	0.005880507
GO:1903608	protein localization to cytoplasmic stress granule	7	0.286449715	0.0143	0.016557758
GO:2000178	negative regulation of neural precursor cell proliferation	14	0.286431329	0.0009	0.002399874
GO:0070527	platelet aggregation	32	0.28643018	0.0005	0.002177563
GO:0001656	metanephros development	50	0.286409266	0.0024	0.004156304
GO:0046503	glycerolipid catabolic process	27	0.286391153	0.0017	0.003334595
GO:2000643	positive regulation of early endosome to late endosome transport	8	0.286357786	0.0093	0.011528671
GO:2001056	positive regulation of cysteine-type endopeptidase activity	84	0.286354722	0.0031	0.004916949
GO:0051926	negative regulation of calcium ion transport	27	0.286343486	0.0011	0.002610912
GO:0001502	cartilage condensation	7	0.286339401	0.0038	0.005666214
GO:0098743	cell aggregation	7	0.286339401	0.0038	0.005666214
GO:0016486	peptide hormone processing	9	0.286314886	0.0004	0.002007244
GO:0032091	negative regulation of protein binding	56	0.286302629	0.0033	0.005149584
GO:0039702	viral budding via host ESCRT complex	17	0.286289651	0.0026	0.004377754
GO:0060996	dendritic spine development	31	0.286195873	0.0009	0.002399874
GO:0045123	cellular extravasation	29	0.286184707	0.002	0.003693422
GO:0070374	positive regulation of ERK1 and ERK2 cascade	85	0.286168521	0.0004	0.002007244
GO:0006903	vesicle targeting	59	0.28615492	0.0016	0.003223775
GO:0033147	negative regulation of intracellular estrogen receptor signaling pathway	9	0.286114686	0.0045	0.006417207
GO:0046386	deoxyribose phosphate catabolic process	13	0.286050886	0.0016	0.003223775
GO:1904031	positive regulation of cyclin-dependent protein kinase activity	18	0.286043186	0.0046	0.006532041
GO:0060415	muscle tissue morphogenesis	39	0.286024486	0.0015	0.003088691
GO:0048640	negative regulation of developmental growth	42	0.285983943	0.0014	0.002975058
GO:0035909	aorta morphogenesis	20	0.285971686	0.0007	0.002248538
GO:0071539	protein localization to centrosome	14	0.2859533	0.0006	0.002177563
GO:0032092	positive regulation of protein binding	53	0.285942546	0.007	0.009166016
GO:1903955	positive regulation of protein targeting to mitochondrion	23	0.285904538	0.0018	0.003459409
GO:0043393	regulation of protein binding	118	0.285877888	0.004	0.005880507
GO:0034113	heterotypic cell-cell adhesion	27	0.285866819	0.0018	0.003459409
GO:0046626	regulation of insulin receptor signaling pathway	27	0.285857286	0.0011	0.002610912
GO:0033173	calcineurin-NFAT signaling cascade	15	0.285851566	0.0017	0.003334595

GOIDlabel	GOName	size	categoryScores	pValPerm	pValPermCorr
GO:0097720	calcineurin-mediated signaling	15	0.285851566	0.0017	0.003334595
GO:0070302	regulation of stress-activated protein kinase signaling cascade	110	0.285847666	0.001	0.002498236
GO:0006909	phagocytosis	132	0.285833236	0.0022	0.003910907
GO:0046890	regulation of lipid biosynthetic process	111	0.285797767	0.0012	0.002741075
GO:0010921	regulation of phosphatase activity	92	0.285770242	0.0001	0.000877824
GO:0034382	chylomicron remnant clearance	5	0.285765766	0.0005	0.002177563
GO:0071830	triglyceride-rich lipoprotein particle clearance	5	0.285765766	0.0005	0.002177563
GO:0050817	coagulation	154	0.285737686	0.0012	0.002741075
GO:0014850	response to muscle activity	14	0.285732671	0.0242	0.02630058
GO:0016125	sterol metabolic process	91	0.285717114	0.003	0.004825593
GO:0043277	apoptotic cell clearance	23	0.285691903	0.0082	0.010366713
GO:0047484	regulation of response to osmotic stress	6	0.285628486	0.0015	0.003088691
GO:0021548	pons development	6	0.285628486	0.0421	0.043897791
GO:0051654	establishment of mitochondrion localization	11	0.285620686	0.001	0.002498236
GO:0045744	negative regulation of G-protein coupled receptor protein signaling pathway	18	0.285614186	0.0019	0.003579279
GO:0006491	N-glycan processing	6	0.285585586	0.0087	0.010877987
GO:1902932	positive regulation of alcohol biosynthetic process	13	0.285575686	0.001	0.002498236
GO:0007628	adult walking behavior	16	0.285553411	0.0109	0.013101792
GO:0030336	negative regulation of cell migration	142	0.285543894	0.001	0.002498236
GO:0042364	water-soluble vitamin biosynthetic process	6	0.285542686	0.0007	0.002248538
GO:0035269	protein O-linked mannosylation	14	0.285493657	< 0.001	< 0.001
GO:0009260	ribonucleotide biosynthetic process	94	0.285459624	0.0033	0.005149584
GO:0043921	modulation by host of viral transcription	21	0.285371085	0.0058	0.007856196
GO:0052312	modulation of transcription in other organism involved in symbiotic interaction	21	0.285371085	0.0058	0.007856196
GO:0052472	modulation by host of symbiont transcription	21	0.285371085	0.0058	0.007856196
GO:0032872	regulation of stress-activated MAPK cascade	108	0.285368702	0.0011	0.002610912
GO:0001821	histamine secretion	5	0.285353925	0.0009	0.002399874
GO:0071320	cellular response to cAMP	27	0.285352019	0.0002	0.001341684
GO:0009142	nucleoside triphosphate biosynthetic process	45	0.285250965	0.0026	0.004377754
GO:0043279	response to alkaloid	72	0.285199485	0.0004	0.002007244
GO:0008203	cholesterol metabolic process	86	0.285190506	0.0033	0.005149584
GO:0006359	regulation of transcription from RNA polymerase III promoter	16	0.285183398	0.0006	0.002177563
GO:0019471	4-hydroxyproline metabolic process	8	0.28516731	0.0007	0.002248538
GO:0006182	cGMP biosynthetic process	5	0.285148005	0.004	0.005880507
GO:0032927	positive regulation of activin receptor signaling pathway	6	0.285113685	0.0044	0.006304227
GO:0001935	endothelial cell proliferation	84	0.285073849	0.0027	0.004491543
GO:0048844	artery morphogenesis	42	0.285070785	0.0038	0.005666214
GO:1903441	protein localization to ciliary membrane	6	0.285027885	0.0018	0.003459409
GO:0003002	regionalization	136	0.285019684	< 0.001	< 0.001
GO:0032414	positive regulation of ion transmembrane transporter activity	39	0.285014685	0.0037	0.005561344
GO:0001837	epithelial to mesenchymal transition	72	0.285013585	0.0045	0.006417207
GO:0042754	negative regulation of circadian rhythm	8	0.285006435	0.003	0.004825593
GO:0006575	cellular modified amino acid metabolic process	107	0.284992603	0.0029	0.004714719
GO:0035335	peptidyl-tyrosine dephosphorylation	51	0.28496732	0.0008	0.002313249
GO:0090330	regulation of platelet aggregation	8	0.284942085	0.0051	0.007095023
GO:0051608	histamine transport	6	0.284942085	0.0011	0.002610912
GO:0090277	positive regulation of peptide hormone secretion	41	0.284923251	0.0003	0.001720882
GO:0042476	odontogenesis	57	0.284905959	0.0026	0.004377754
GO:0006048	UDP-N-acetylglucosamine biosynthetic process	7	0.284905313	0.0001	0.000877824
GO:0021694	cerebellar Purkinje cell layer formation	7	0.284905313	0.0046	0.006532041
GO:1903829	positive regulation of cellular protein localization	190	0.284901443	0.0009	0.002399874
GO:1901224	positive regulation of NIK/NF-kappaB signaling	52	0.284897535	0.0038	0.005666214
GO:0052548	regulation of endopeptidase activity	194	0.284817365	0.0012	0.002741075
GO:0014855	striated muscle cell proliferation	38	0.284806611	0.0034	0.005263104
GO:0051058	negative regulation of small GTPase mediated signal transduction	27	0.284799085	0.002	0.003693422
GO:0031468	nuclear envelope reassembly	12	0.284791935	0.0013	0.002859821
GO:0022404	molting cycle process	48	0.284786572	0.0012	0.002741075
GO:0022405	hair cycle process	48	0.284786572	0.0012	0.002741075
GO:1904377	positive regulation of protein localization to cell periphery	32	0.284773166	0.0013	0.002859821
GO:0030282	bone mineralization	53	0.284738107	0.0013	0.002859821
GO:0075522	IRES-dependent viral translational initiation	9	0.284713285	0.0008	0.002313249
GO:0072507	divalent inorganic cation homeostasis	182	0.284694585	0.0012	0.002741075
GO:2000647	negative regulation of stem cell proliferation	7	0.284647913	0.0107	0.012912536
GO:0031274	positive regulation of pseudopodium assembly	6	0.284641785	0.006	0.00807902
GO:0045132	meiotic chromosome segregation	27	0.284627485	0.0001	0.000877824
GO:0018394	peptidyl-lysine acetylation	109	0.284616202	0.0011	0.002610912
GO:0046827	positive regulation of protein export from nucleus	15	0.284616045	0.0059	0.007964563
GO:1902043	positive regulation of extrinsic apoptotic signaling pathway via death domain receptors	11	0.284614485	0.0015	0.003088691
GO:0031295	T cell costimulation	26	0.284605485	0.003	0.004825593
GO:0051896	regulation of protein kinase B signaling	111	0.284601204	0.0008	0.002313249
GO:0051592	response to calcium ion	60	0.284590305	0.0026	0.004377754
GO:1903825	organic acid transmembrane transport	45	0.284576005	0.0008	0.002313249
GO:1905039	carboxylic acid transmembrane transport	45	0.284576005	0.0008	0.002313249
GO:0042475	odontogenesis of dentin-containing tooth	36	0.284563135	0.0016	0.003223775
GO:0045165	cell fate commitment	105	0.284562113	0.0006	0.002177563
GO:0043550	regulation of lipid kinase activity	38	0.284535663	0.0009	0.002399874
GO:0044154	histone H3-K14 acetylation	5	0.284530245	< 0.001	< 0.001
GO:0019218	regulation of steroid metabolic process	72	0.284527385	0.0048	0.006748379
GO:0032098	regulation of appetite	18	0.284484484	0.0025	0.004279479
GO:0055074	calcium ion homeostasis	171	0.284467927	0.001	0.002498236
GO:0060088	auditory receptor cell stereocilium organization	7	0.284464056	0.0012	0.002741075
GO:0007033	vacuole organization	116	0.28443616	0.0032	0.005044175
GO:0035883	enteroendocrine cell differentiation	10	0.284427284	0.0007	0.002248538

GOIDlabel	GOName	size	categoryScores	pValPerm	pValPermCorr
GO:1904064	positive regulation of cation transmembrane transport	59	0.284427284	0.0036	0.005475609
GO:0032956	regulation of actin cytoskeleton organization	148	0.284420328	0.0007	0.002248538
GO:1904124	microglial cell migration	5	0.284221364	0.0154	0.017651835
GO:1904139	regulation of microglial cell migration	5	0.284221364	0.0154	0.017651835
GO:0008286	insulin receptor signaling pathway	67	0.28420446	0.0013	0.002859821
GO:0051701	interaction with host	129	0.284173875	0.0037	0.005561344
GO:0017121	phospholipid scrambling	8	0.284169884	0.0013	0.002859821
GO:1901292	nucleoside phosphate catabolic process	39	0.284090684	0.0014	0.002975058
GO:0022612	gland morphogenesis	67	0.284081523	0.0025	0.004279479
GO:0009127	purine nucleoside monophosphate biosynthetic process	46	0.284069162	0.0063	0.008390784
GO:0009168	purine ribonucleoside monophosphate biosynthetic process	46	0.284069162	0.0063	0.008390784
GO:0001936	regulation of endothelial cell proliferation	74	0.284065533	0.0027	0.004491543
GO:0032496	response to lipopolysaccharide	156	0.284052734	0.0006	0.002177563
GO:0010821	regulation of mitochondrion organization	137	0.284042123	0.0037	0.005561344
GO:0044351	macropinocytosis	8	0.284041184	0.0004	0.002007244
GO:0070167	regulation of biomineral tissue development	47	0.283994633	0.0006	0.002177563
GO:0010996	response to auditory stimulus	14	0.283986027	0.001	0.002498236
GO:0017157	regulation of exocytosis	82	0.283968986	0.0021	0.003804235
GO:0021517	ventral spinal cord development	23	0.28396844	0.0002	0.001341684
GO:0046365	monosaccharide catabolic process	35	0.283963964	0.0026	0.004377754
GO:0016525	negative regulation of angiogenesis	58	0.283961301	0.0016	0.003223775
GO:2000181	negative regulation of blood vessel morphogenesis	58	0.283961301	0.0016	0.003223775
GO:0031441	negative regulation of mRNA 3'-end processing	6	0.283912484	0.0033	0.005149584
GO:0009071	serine family amino acid catabolic process	8	0.283912484	0.0002	0.001341684
GO:0090192	regulation of glomerulus development	5	0.283912484	0.0002	0.001341684
GO:0046470	phosphatidylcholine metabolic process	51	0.283882202	0.0028	0.004605006
GO:0033522	histone H2A ubiquitination	19	0.283858294	0.0006	0.002177563
GO:0010971	positive regulation of G2/M transition of mitotic cell cycle	12	0.283826684	0.0019	0.003579279
GO:0034330	cell junction organization	153	0.28381659	0.0021	0.003804235
GO:0050687	negative regulation of defense response to virus	22	0.283807184	0.0142	0.016456919
GO:0031103	axon regeneration	25	0.283799228	0.0109	0.013101792
GO:0046825	regulation of protein export from nucleus	23	0.283733423	0.0031	0.004916949
GO:0034332	adherens junction organization	70	0.283721272	0.001	0.002498236
GO:0043280	positive regulation of cysteine-type endopeptidase activity involved in apoptotic process	77	0.283718598	0.0042	0.006097175
GO:0045076	regulation of interleukin-2 biosynthetic process	11	0.283678484	0.0117	0.013882722
GO:0001708	cell fate specification	35	0.283677147	0.0002	0.001341684
GO:0008344	adult locomotory behavior	39	0.283674884	0.0037	0.005561344
GO:0000422	mitophagy	38	0.283655084	0.006	0.00807902
GO:0061726	mitochondrion disassembly	38	0.283655084	0.006	0.00807902
GO:0008207	C21-steroid hormone metabolic process	21	0.283642826	0.0122	0.014387602
GO:0031109	microtubule polymerization or depolymerization	50	0.28363964	0.0097	0.011922488
GO:0006869	lipid transport	174	0.283624018	0.0017	0.003334595
GO:0032970	regulation of actin filament-based process	164	0.283618985	0.0012	0.002741075
GO:0006816	calcium ion transport	172	0.283610188	0.0017	0.003334595
GO:1901214	regulation of neuron death	157	0.283594422	0.0029	0.004714719
GO:0070536	protein K63-linked deubiquitination	21	0.283593798	0.0004	0.002007244
GO:0060761	negative regulation of response to cytokine stimulus	34	0.283564237	0.0002	0.001341684
GO:1904754	positive regulation of vascular associated smooth muscle cell migration	10	0.283552124	0.0126	0.014782732
GO:0045862	positive regulation of proteolysis	196	0.283542143	0.0026	0.004377754
GO:0007030	Golgi organization	59	0.283532928	0.0041	0.005981983
GO:0048525	negative regulation of viral process	61	0.283532713	0.0029	0.004714719
GO:0006901	vesicle coating	45	0.283506364	0.001	0.002498236
GO:0032835	glomerulus development	37	0.283488121	0.0017	0.003334595
GO:0046466	membrane lipid catabolic process	21	0.283458969	0.024	0.026109935
GO:2000146	negative regulation of cell motility	152	0.283443406	0.0011	0.002610912
GO:0007015	actin filament organization	174	0.283440583	0.0006	0.002177563
GO:0008643	carbohydrate transport	73	0.28343647	0.0008	0.002313249
GO:0060982	coronary artery morphogenesis	7	0.283397683	0.0457	0.047395622
GO:0030220	platelet formation	11	0.283374283	0.0112	0.013379119
GO:0034333	adherens junction assembly	52	0.283367983	0.0019	0.003579279
GO:0050953	sensory perception of light stimulus	89	0.28336587	0.0018	0.003459409
GO:1903358	regulation of Golgi organization	6	0.283354783	0.0062	0.008281828
GO:0032528	microvillus organization	12	0.283354783	0.0076	0.009790742
GO:0051271	negative regulation of cellular component movement	160	0.283344595	0.001	0.002498236
GO:0032387	negative regulation of intracellular transport	55	0.283341523	0.0017	0.003334595
GO:0051985	negative regulation of chromosome segregation	24	0.283333333	< 0.001	< 0.001
GO:0048511	rhythmic process	169	0.283327622	0.001	0.002498236
GO:0010466	negative regulation of peptidase activity	106	0.283324834	0.0005	0.002177563
GO:0072376	protein activation cascade	42	0.283262855	0.0105	0.012724187
GO:0006006	glucose metabolic process	120	0.283236808	0.001	0.002498236
GO:0031338	regulation of vesicle fusion	10	0.283217503	0.0116	0.013792313
GO:0071248	cellular response to metal ion	94	0.28314576	0.0038	0.005666214
GO:0097242	beta-amyloid clearance	16	0.283124196	0.0014	0.002975058
GO:0043090	amino acid import	10	0.283114543	0.0023	0.004032435
GO:0034349	glial cell apoptotic process	9	0.283083083	0.0412	0.043036814
GO:0055093	response to hyperoxia	11	0.283070083	0.0006	0.002177563
GO:0030859	polarized epithelial cell differentiation	11	0.283046683	0.0413	0.043134202
GO:0070262	peptidyl-serine dephosphorylation	7	0.282993197	0.0287	0.030750689
GO:0016573	histone acetylation	99	0.282947883	0.0017	0.003334595
GO:0010575	positive regulation of vascular endothelial growth factor production	14	0.28293804	0.0167	0.018954426
GO:0030225	macrophage differentiation	25	0.282934363	0.005	0.006974174
GO:2000177	regulation of neural precursor cell proliferation	46	0.282916457	0.0004	0.002007244
GO:0008277	regulation of G-protein coupled receptor protein signaling pathway	65	0.282886843	0.0005	0.002177563

GOIDlabel	GOName	size	categoryScores	pValPerm	pValPermCorr
GO:0010951	negative regulation of endopeptidase activity	100	0.282885457	0.0005	0.002177563
GO:0010950	positive regulation of endopeptidase activity	90	0.282842843	0.0034	0.005263104
GO:0007257	activation of JUN kinase activity	17	0.282822318	0.0019	0.003579279
GO:0006921	cellular component disassembly involved in execution phase of apoptosis	24	0.282775633	0.0024	0.004156304
GO:0032048	cardiolipin metabolic process	13	0.282764083	0.0011	0.002610912
GO:0061635	regulation of protein complex stability	6	0.282754183	0.003	0.004825593
GO:0086005	ventricular cardiac muscle cell action potential	14	0.282754183	0.008	0.010168329
GO:0090312	positive regulation of protein deacetylation	13	0.282704683	0.0024	0.004156304
GO:0007204	positive regulation of cytosolic calcium ion concentration	117	0.282687083	0.0006	0.002177563
GO:0032535	regulation of cellular component size	164	0.282641178	0.0007	0.002248538
GO:0043038	amino acid activation	35	0.282625483	0.0052	0.007215241
GO:0035329	hippo signaling	24	0.282582583	0.0024	0.004156304
GO:0002024	diet induced thermogenesis	6	0.282582583	0.02	0.022155397
GO:0021515	cell differentiation in spinal cord	24	0.282571858	0.0008	0.002313249
GO:0010507	negative regulation of autophagy	52	0.282521533	0.0047	0.006644432
GO:0007032	endosome organization	46	0.282519165	0.0086	0.010769857
GO:0008654	phospholipid biosynthetic process	159	0.28250083	0.0029	0.004714719
GO:0072077	renal vesicle morphogenesis	10	0.282496782	0.0077	0.009887588
GO:0072283	metanephric renal vesicle morphogenesis	10	0.282496782	0.0077	0.009887588
GO:0051208	sequestering of calcium ion	50	0.282486486	0.0007	0.002248538
GO:0010460	positive regulation of heart rate	10	0.282471042	0.0105	0.012724187
GO:0048635	negative regulation of muscle organ development	29	0.282465717	0.0134	0.015626386
GO:1904063	negative regulation of cation transmembrane transport	36	0.282446732	0.0009	0.002399874
GO:0040013	negative regulation of locomotion	171	0.282444851	0.0009	0.002399874
GO:0072089	stem cell proliferation	61	0.282439817	0.0015	0.003088691
GO:0002066	columnar/cuboidal epithelial cell development	30	0.282419562	0.0006	0.002177563
GO:0051402	neuron apoptotic process	119	0.282378898	0.0047	0.006644432
GO:0043010	camera-type eye development	162	0.282329949	0.0012	0.002741075
GO:0042311	vasodilation	15	0.282316602	0.0008	0.002313249
GO:0042755	eating behavior	18	0.282310882	0.0041	0.005981983
GO:0060977	coronary vasculature morphogenesis	13	0.282308682	0.0098	0.012008345
GO:0006086	acetyl-CoA biosynthetic process from pyruvate	10	0.282290862	0.0099	0.012121556
GO:0050954	sensory perception of mechanical stimulus	79	0.282289885	0.0011	0.002610912
GO:0018027	peptidyl-lysine dimethylation	11	0.282274482	0.0027	0.004491543
GO:0045746	negative regulation of Notch signaling pathway	20	0.282265122	0.0009	0.002399874
GO:0045017	glycerolipid biosynthetic process	152	0.282224141	0.0019	0.003579279
GO:1904707	positive regulation of vascular smooth muscle cell proliferation	21	0.282220997	0.0016	0.003223775
GO:0050912	detection of chemical stimulus involved in sensory perception of taste	12	0.282196482	0.0188	0.020971889
GO:0006270	DNA replication initiation	18	0.282167882	0.0008	0.002313249
GO:0016241	regulation of macroautophagy	108	0.282160732	0.0032	0.005044175
GO:0033013	tetrapyrrole metabolic process	38	0.282158098	0.0116	0.013792313
GO:0043584	nose development	8	0.282142857	0.0102	0.012424427
GO:0016578	histone deubiquitination	14	0.282092296	0.0001	0.000877824
GO:0007565	female pregnancy	101	0.282052066	0.0014	0.002975058
GO:0030168	platelet activation	65	0.282051282	0.0006	0.002177563
GO:2000027	regulation of organ morphogenesis	143	0.282044082	0.0017	0.003334595
GO:0043255	regulation of carbohydrate biosynthetic process	50	0.282043758	0.0005	0.002177563
GO:0002922	positive regulation of humoral immune response	9	0.282024882	0.0052	0.007215241
GO:0003333	amino acid transmembrane transport	25	0.281997426	0.0011	0.002610912
GO:1903539	protein localization to postsynaptic membrane	5	0.281956242	0.0001	0.000877824
GO:0043405	regulation of MAP kinase activity	175	0.281931237	0.0009	0.002399874
GO:0017158	regulation of calcium ion-dependent exocytosis	27	0.281920015	0.0002	0.001341684
GO:0072384	organelle transport along microtubule	39	0.281892882	0.0056	0.007655255
GO:0044706	multi-multicellular organism process	116	0.28185772	0.001	0.002498236
GO:0016572	histone phosphorylation	16	0.281853282	0.001	0.002498236
GO:0070997	neuron death	175	0.281835632	0.003	0.004825593
GO:0003015	heart process	119	0.28181651	0.001	0.002498236
GO:0043523	regulation of neuron apoptotic process	103	0.281765816	0.0053	0.007330056
GO:0048638	regulation of developmental growth	161	0.281725381	0.0017	0.003334595
GO:0042886	amide transport	141	0.281712716	0.0022	0.003910907
GO:0035672	oligopeptide transmembrane transport	5	0.281698842	0.0028	0.004605006
GO:1901617	organic hydroxy compound biosynthetic process	129	0.281691658	0.0013	0.002859821
GO:0071825	protein-lipid complex subunit organization	25	0.281616474	0.0007	0.002248538
GO:1903587	regulation of blood vessel endothelial cell proliferation involved in sprouting angiogenesis	6	0.281552982	0.0064	0.008500875
GO:0030500	regulation of bone mineralization	37	0.281547184	0.0006	0.002177563
GO:1902652	secondary alcohol metabolic process	88	0.281543232	0.0035	0.005358035
GO:0007601	visual perception	86	0.281524049	0.0017	0.003334595
GO:1990845	adaptive thermogenesis	10	0.281518662	0.0185	0.020677043
GO:0007162	negative regulation of cell adhesion	133	0.281508791	0.0017	0.003334595
GO:0010906	regulation of glucose metabolic process	62	0.281454727	0.0008	0.002313249
GO:0032693	negative regulation of interleukin-10 production	9	0.281424281	0.0145	0.016764967
GO:0042059	negative regulation of epidermal growth factor receptor signaling pathway	23	0.281394438	0.0197	0.021861066
GO:0003417	growth plate cartilage development	5	0.281389961	0.0064	0.008500875
GO:0098911	regulation of ventricular cardiac muscle cell action potential	6	0.281381381	0.0281	0.030173766
GO:0097581	lamellipodium organization	42	0.281375253	0.0015	0.003088691
GO:0018393	internal peptidyl-lysine acetylation	102	0.281353623	0.0013	0.002859821
GO:0019076	viral release from host cell	29	0.281311854	0.0057	0.007745437
GO:0035890	exit from host	29	0.281311854	0.0057	0.007745437
GO:0035891	exit from host cell	29	0.281311854	0.0057	0.007745437
GO:0052126	movement in host environment	29	0.281311854	0.0057	0.007745437
GO:0052192	movement in environment of other organism involved in symbiotic interaction	29	0.281311854	0.0057	0.007745437
GO:0034698	response to gonadotropin	14	0.28130171	0.0057	0.007745437
GO:0048477	oogenesis	35	0.281287001	< 0.001	< 0.001

GOIDlabel	GOName	size	categoryScores	pValPerm	pValPermCorr
GO:0044331	cell-cell adhesion mediated by cadherin	9	0.281252681	0.0005	0.002177563
GO:0035313	wound healing, spreading of epidermal cells	6	0.281209781	0.0267	0.028781986
GO:1903901	negative regulation of viral life cycle	60	0.281175461	0.0035	0.005358035
GO:0001675	acrosome assembly	8	0.281145431	0.0166	0.018864444
GO:1901215	negative regulation of neuron death	102	0.281123981	0.0028	0.004605006
GO:0001845	phagolysosome assembly	7	0.281117853	0.0018	0.003459409
GO:0007043	cell-cell junction assembly	58	0.281112147	0.0075	0.00968541
GO:0050727	regulation of inflammatory response	174	0.281094395	0.0011	0.002610912
GO:0032409	regulation of transporter activity	94	0.281092034	0.0017	0.003334595
GO:0014020	primary neural tube formation	54	0.281066781	0.0006	0.002177563
GO:2000758	positive regulation of peptidyl-lysine acetylation	18	0.281052481	0.0017	0.003334595
GO:0046474	glycerophospholipid biosynthetic process	131	0.281028029	0.0022	0.003910907
GO:0055008	cardiac muscle tissue morphogenesis	37	0.28097673	0.0016	0.003223775
GO:1903510	mucopolysaccharide metabolic process	55	0.280973441	0.0009	0.002399874
GO:0034616	response to laminar fluid shear stress	12	0.280930931	0.0026	0.004377754
GO:0051954	positive regulation of amine transport	17	0.280899387	0.0002	0.001341684
GO:0007263	nitric oxide mediated signal transduction	12	0.280888031	0.003	0.004825593
GO:0071108	protein K48-linked deubiquitination	18	0.280866581	0.001	0.002498236
GO:0030514	negative regulation of BMP signaling pathway	18	0.280866581	0.0096	0.01183839
GO:0006681	galactosylceramide metabolic process	8	0.280855856	0.0078	0.009981807
GO:0019374	galactolipid metabolic process	8	0.280855856	0.0078	0.009981807
GO:0001756	somitogenesis	30	0.280815101	0.0037	0.005561344
GO:0048199	vesicle targeting, to, from or within Golgi	47	0.280812728	0.0009	0.002399874
GO:0030809	negative regulation of nucleotide biosynthetic process	11	0.280800281	0.0026	0.004377754
GO:1900372	negative regulation of purine nucleotide biosynthetic process	11	0.280800281	0.0026	0.004377754
GO:0006362	transcription elongation from RNA polymerase I promoter	21	0.280750138	< 0.001	< 0.001
GO:0007094	mitotic spindle assembly checkpoint	17	0.280747975	< 0.001	< 0.001
GO:0031577	spindle checkpoint	17	0.280747975	< 0.001	< 0.001
GO:0071173	spindle assembly checkpoint	17	0.280747975	< 0.001	< 0.001
GO:0071174	mitotic spindle checkpoint	17	0.280747975	< 0.001	< 0.001
GO:0030262	apoptotic nuclear changes	23	0.280745342	0.0019	0.003579279
GO:0001952	regulation of cell-matrix adhesion	65	0.280744481	0.0035	0.005358035
GO:0001947	heart looping	35	0.280735429	0.0069	0.009046225
GO:0045843	negative regulation of striated muscle tissue development	28	0.280713366	0.0128	0.014984276
GO:1901862	negative regulation of muscle tissue development	28	0.280713366	0.0128	0.014984276
GO:0046390	ribose phosphate biosynthetic process	98	0.280713366	0.0034	0.005263104
GO:0032366	intracellular sterol transport	15	0.280686401	0.0081	0.010256567
GO:0032367	intracellular cholesterol transport	15	0.280686401	0.0081	0.010256567
GO:0002903	negative regulation of B cell apoptotic process	5	0.280669241	0.0026	0.004377754
GO:0051255	spindle midzone assembly	5	0.280669241	0.0176	0.019838132
GO:0035315	hair cell differentiation	19	0.28062047	0.0001	0.000877824
GO:0042491	auditory receptor cell differentiation	19	0.28062047	0.0001	0.000877824
GO:0030201	heparan sulfate proteoglycan metabolic process	10	0.280617761	0.0099	0.012121556
GO:0045933	positive regulation of muscle contraction	26	0.280615781	0.0003	0.001720882
GO:0010769	regulation of cell morphogenesis involved in differentiation	114	0.280597891	0.0025	0.004279479
GO:0006691	leukotriene metabolic process	18	0.280594881	0.0136	0.015813319
GO:0003007	heart morphogenesis	135	0.280585347	0.0011	0.002610912
GO:0002190	cap-independent translational initiation	14	0.280547895	0.0002	0.001341684
GO:0003356	regulation of cilium beat frequency	7	0.280492738	< 0.001	< 0.001
GO:0022617	extracellular matrix disassembly	41	0.280484666	0.0162	0.018465856
GO:0007265	Ras protein signal transduction	177	0.280483389	0.0026	0.004377754
GO:0034111	negative regulation of homotypic cell-cell adhesion	6	0.28048048	0.0192	0.021362011
GO:0071372	cellular response to follicle-stimulating hormone stimulus	9	0.28048048	0.0029	0.004714719
GO:0060712	spongiotrophoblast layer development	8	0.280469755	0.0004	0.002007244
GO:0090207	regulation of triglyceride metabolic process	20	0.28046332	0.0011	0.002610912
GO:0045732	positive regulation of protein catabolic process	125	0.280453024	0.0023	0.004032435
GO:0035590	purinergic nucleotide receptor signaling pathway	13	0.28044748	0.001	0.002498236
GO:0051570	regulation of histone H3-K9 methylation	15	0.280429	0.0033	0.005149584
GO:0090100	positive regulation of transmembrane receptor protein serine/threonine kinase signaling pathway	51	0.280419916	0.0022	0.003910907
GO:0071371	cellular response to gonadotropin stimulus	10	0.2803861	0.002	0.003693422
GO:0051480	regulation of cytosolic calcium ion concentration	130	0.28034848	0.0011	0.002610912
GO:0046887	positive regulation of hormone secretion	60	0.2803432	0.0003	0.001720882
GO:0043406	positive regulation of MAP kinase activity	119	0.280315369	0.0009	0.002399874
GO:0051797	regulation of hair follicle development	10	0.28028314	< 0.001	< 0.001
GO:0002793	positive regulation of peptide secretion	43	0.28027895	0.0002	0.001341684
GO:0001570	vasculogenesis	53	0.280270027	0.0033	0.005149584
GO:0035635	entry of bacterium into host cell	8	0.28024453	0.0101	0.012328539
GO:0042108	positive regulation of cytokine biosynthetic process	38	0.28023437	0.0118	0.013977955
GO:0060261	positive regulation of transcription initiation from RNA polymerase II promoter	9	0.28022308	0.0095	0.011730963
GO:0048644	muscle organ morphogenesis	42	0.28022308	0.0014	0.002975058
GO:0030837	negative regulation of actin filament polymerization	22	0.28021528	0.0008	0.002313249
GO:0060426	lung vasculature development	5	0.28015444	0.0349	0.036866849
GO:0048741	skeletal muscle fiber development	11	0.28012168	0.0112	0.013379119
GO:0001937	negative regulation of endothelial cell proliferation	26	0.28012078	0.0078	0.009981807
GO:0010002	cardioblast differentiation	11	0.28007488	0.012	0.014185865
GO:0021761	limbic system development	52	0.28006633	0.0008	0.002313249
GO:0035970	peptidyl-threonine dephosphorylation	8	0.28005148	0.0181	0.020286898
GO:0072698	protein localization to microtubule cytoskeleton	23	0.280006715	0.0012	0.002741075
GO:0060670	branching involved in labyrinthine layer morphogenesis	5	0.28	0.0037	0.005561344
GO:0007389	pattern specification process	188	0.279976177	< 0.001	< 0.001
GO:0006069	ethanol oxidation	6	0.27996568	0.0026	0.004377754
GO:0001843	neural tube closure	52	0.27995743	0.0006	0.002177563
GO:0060606	tube closure	52	0.27995743	0.0006	0.002177563

GOIDlabel	GOName	size	categoryScores	pValPerm	pValPermCorr
GO:0061351	neural precursor cell proliferation	79	0.279908118	0.0006	0.002177563
GO:0006473	protein acetylation	126	0.279900308	0.0007	0.002248538
GO:0010954	positive regulation of protein processing	17	0.279884927	0.0142	0.016456919
GO:0034381	plasma lipoprotein particle clearance	26	0.27988318	0.0015	0.003088691
GO:0035360	positive regulation of peroxisome proliferator activated receptor signaling pathway	6	0.27987988	< 0.001	< 0.001
GO:1902837	amino acid import into cell	6	0.27987988	0.0014	0.002975058
GO:0045598	regulation of fat cell differentiation	65	0.27985744	0.0006	0.002177563
GO:0035278	miRNA mediated inhibition of translation	13	0.27983368	0.0007	0.002248538
GO:0040033	negative regulation of translation, ncRNA-mediated	13	0.27983368	0.0007	0.002248538
GO:0045974	regulation of translation, ncRNA-mediated	13	0.27983368	0.0007	0.002248538
GO:0010881	regulation of cardiac muscle contraction by regulation of the release of sequestered calcium ion	7	0.279830851	0.0103	0.012519912
GO:0035821	modification of morphology or physiology of other organism	88	0.27981748	0.0036	0.005475609
GO:0043650	dicarboxylic acid biosynthetic process	7	0.27979408	0.0001	0.000877824
GO:0032355	response to estradiol	74	0.279783645	0.0036	0.005475609
GO:0035338	long-chain fatty-acyl-CoA biosynthetic process	10	0.27976834	0.0136	0.015813319
GO:0032785	negative regulation of DNA-templated transcription, elongation	10	0.2797426	0.0111	0.013284563
GO:0010165	response to X-ray	18	0.27973688	0.0003	0.001720882
GO:1904375	regulation of protein localization to cell periphery	60	0.27972115	0.0023	0.004032435
GO:0019081	viral translation	13	0.27971488	0.0016	0.003223775
GO:1900076	regulation of cellular response to insulin stimulus	33	0.27967708	0.001	0.002498236
GO:0042416	dopamine biosynthetic process	7	0.279646994	0.0001	0.000877824
GO:0010463	mesenchymal cell proliferation	25	0.27963964	0.0112	0.013379119
GO:0048596	embryonic camera-type eye morphogenesis	17	0.279582103	0.0027	0.004491543
GO:0035265	organ growth	86	0.279557631	0.0005	0.002177563
GO:0018105	peptidyl-serine phosphorylation	104	0.279539155	0.0007	0.002248538
GO:0007586	digestion	59	0.279523591	0.0003	0.001720882
GO:0051817	modification of morphology or physiology of other organism involved in symbiotic interaction	72	0.279511655	0.0037	0.005561344
GO:0007422	peripheral nervous system development	40	0.279504505	0.0067	0.008845888
GO:0070542	response to fatty acid	46	0.279486319	0.0004	0.002007244
GO:0018209	peptidyl-serine modification	117	0.279486079	0.0007	0.002248538
GO:0070200	establishment of protein localization to telomere	14	0.279481522	< 0.001	< 0.001
GO:0006475	internal protein amino acid acetylation	104	0.279464904	0.0013	0.002859821
GO:0071277	cellular response to calcium ion	33	0.279450879	0.0016	0.003223775
GO:0042659	regulation of cell fate specification	6	0.279450879	< 0.001	< 0.001
GO:0010893	positive regulation of steroid biosynthetic process	11	0.279443079	0.0088	0.010992238
GO:0002223	stimulatory C-type lectin receptor signaling pathway	70	0.279411657	0.0013	0.002859821
GO:0030900	forebrain development	182	0.279395251	0.0006	0.002177563
GO:0048469	cell maturation	78	0.279381579	0.005	0.006974174
GO:0048863	stem cell differentiation	141	0.279381509	0.0007	0.002248538
GO:0032369	negative regulation of lipid transport	13	0.279358479	0.0148	0.017062301
GO:0006577	amino-acid betaine metabolic process	14	0.279297665	< 0.001	< 0.001
GO:0010755	regulation of plasminogen activation	6	0.279279279	0.0075	0.00968541
GO:0032897	negative regulation of viral transcription	15	0.279262119	0.019	0.02117646
GO:0010631	epithelial cell migration	166	0.279231211	0.0014	0.002975058
GO:0048675	axon extension	47	0.27922999	0.001	0.002498236
GO:0010226	response to lithium ion	10	0.279227799	0.0028	0.004605006
GO:0006821	chloride transport	45	0.279216359	0.0017	0.003334595
GO:0009988	cell-cell recognition	34	0.279211144	0.0032	0.005044175
GO:0032331	negative regulation of chondrocyte differentiation	7	0.279205736	0.0067	0.008845888
GO:0060541	respiratory system development	110	0.279103779	0.002	0.003693422
GO:0051261	protein depolymerization	47	0.279093075	0.0021	0.003804235
GO:1903115	regulation of actin filament-based movement	16	0.278973616	0.0166	0.018864444
GO:0007528	neuromuscular junction development	20	0.278970399	0.0039	0.005778819
GO:0018026	peptidyl-lysine monomethylation	6	0.278893179	0.0007	0.002248538
GO:0007623	circadian rhythm	97	0.278860009	0.0012	0.002741075
GO:1901216	positive regulation of neuron death	49	0.278848528	0.011	0.013192134
GO:0060416	response to growth hormone	19	0.278832216	0.0001	0.000877824
GO:0086091	regulation of heart rate by cardiac conduction	13	0.278784279	0.0146	0.016871405
GO:0060306	regulation of membrane repolarization	14	0.278782864	0.0022	0.003910907
GO:0010801	negative regulation of peptidyl-threonine phosphorylation	6	0.278764479	0.0169	0.019147324
GO:0006818	hydrogen transport	75	0.278740455	0.0042	0.006097175
GO:0015992	proton transport	75	0.278740455	0.0042	0.006097175
GO:0043304	regulation of mast cell degranulation	16	0.278716216	0.0036	0.005475609
GO:0090382	phagosome maturation	21	0.27871545	0.0059	0.007964563
GO:0042303	molting cycle	57	0.278710289	0.0017	0.003334595
GO:0042633	hair cycle	57	0.278710289	0.0017	0.003334595
GO:0090257	regulation of muscle system process	104	0.278702604	0.0021	0.003804235
GO:0071621	granulocyte chemotaxis	43	0.278674688	0.0031	0.004916949
GO:0042407	cristae formation	19	0.278642552	0.011	0.013192134
GO:0051238	sequestering of metal ion	53	0.278609067	0.0009	0.002399874
GO:0048013	ephrin receptor signaling pathway	38	0.27860191	0.0002	0.001341684
GO:0030252	growth hormone secretion	9	0.278592879	0.0054	0.007432873
GO:0060123	regulation of growth hormone secretion	9	0.278592879	0.0054	0.007432873
GO:0060047	heart contraction	115	0.278589894	0.0014	0.002975058
GO:0098656	anion transmembrane transport	99	0.278530479	0.0021	0.003804235
GO:0010927	cellular component assembly involved in morphogenesis	49	0.278528091	0.0092	0.011424708
GO:0048738	cardiac muscle tissue development	107	0.278478211	0.0018	0.003459409
GO:0060767	epithelial cell proliferation involved in prostate gland development	6	0.278464178	0.0001	0.000877824
GO:0050829	defense response to Gram-negative bacterium	28	0.278461114	0.0003	0.001720882
GO:0006936	muscle contraction	175	0.27842471	0.001	0.002498236
GO:0071295	cellular response to vitamin	14	0.278341607	0.0006	0.002177563
GO:0008637	apoptotic mitochondrial changes	72	0.278296153	0.004	0.005880507
GO:0090051	negative regulation of cell migration involved in sprouting angiogenesis	12	0.278271128	0.0041	0.005981983

GOIDlabel	GOName	size	categoryScores	pValPerm	pValPermCorr
GO:0070202	regulation of establishment of protein localization to chromosome	10	0.278223938	0.0001	0.000877824
GO:0070203	regulation of establishment of protein localization to telomere	10	0.278223938	0.0001	0.000877824
GO:0048259	regulation of receptor-mediated endocytosis	48	0.278201416	0.0091	0.011322589
GO:0006924	activation-induced cell death of T cells	5	0.278198198	0.0296	0.031661766
GO:0032717	negative regulation of interleukin-8 production	12	0.278185328	0.0008	0.002313249
GO:0010713	negative regulation of collagen metabolic process	5	0.278146718	0.0263	0.02839402
GO:0032966	negative regulation of collagen biosynthetic process	5	0.278146718	0.0263	0.02839402
GO:0044252	negative regulation of multicellular organismal metabolic process	5	0.278146718	0.0263	0.02839402
GO:0060768	regulation of epithelial cell proliferation involved in prostate gland development	5	0.278146718	0.0022	0.003910907
GO:0007494	midgut development	7	0.278139364	0.0399	0.04174043
GO:0008154	actin polymerization or depolymerization	88	0.278100503	0.0005	0.002177563
GO:0022898	regulation of transmembrane transporter activity	88	0.278100503	0.0017	0.003334595
GO:0043113	receptor clustering	20	0.278095238	0.0006	0.002177563
GO:0070535	histone H2A K63-linked ubiquitination	5	0.278095238	0.0062	0.008281828
GO:0003203	endocardial cushion morphogenesis	23	0.278093	0.0023	0.004032435
GO:0015908	fatty acid transport	47	0.278063474	0.0011	0.002610912
GO:0034109	homotypic cell-cell adhesion	42	0.278059692	0.0015	0.003088691
GO:1903147	negative regulation of mitophagy	6	0.278035178	0.0214	0.023571068
GO:0050679	positive regulation of epithelial cell proliferation	98	0.278023796	0.0013	0.002859821
GO:0045724	positive regulation of cilium assembly	16	0.277992278	0.0043	0.006204188
GO:0021543	pallium development	84	0.277927928	0.0006	0.002177563
GO:0014031	mesenchymal cell development	36	0.277913628	0.001	0.002498236
GO:0061371	determination of heart left/right asymmetry	36	0.277906478	0.0079	0.010077402
GO:1903071	positive regulation of ER-associated ubiquitin-dependent protein catabolic process	6	0.277906478	0.0335	0.03551156
GO:0030042	actin filament depolymerization	24	0.277863578	0.0004	0.002007244
GO:0006418	tRNA aminoacylation for protein translation	31	0.27784282	0.0059	0.007964563
GO:0090132	epithelium migration	167	0.277828898	0.0015	0.003088691
GO:0072595	maintenance of protein localization in organelle	23	0.277824408	0.0011	0.002610912
GO:0019932	second-messenger-mediated signaling	115	0.277779643	0.0011	0.002610912
GO:0033081	regulation of T cell differentiation in thymus	8	0.277767053	0.0017	0.003334595
GO:2000398	regulation of thymocyte aggregation	8	0.277767053	0.0017	0.003334595
GO:0070129	regulation of mitochondrial translation	18	0.277763478	0.0169	0.019147324
GO:0006959	humoral immune response	78	0.277741478	0.0127	0.014886353
GO:0042249	establishment of planar polarity of embryonic epithelium	5	0.277734878	0.0007	0.002248538
GO:0007350	blastoderm segmentation	8	0.277734878	0.0001	0.000877824
GO:0030838	positive regulation of actin filament polymerization	38	0.27772133	0.0022	0.003910907
GO:0099518	vesicle cytoskeletal trafficking	22	0.277711478	0.0041	0.005981983
GO:0032613	interleukin-10 production	27	0.277687211	0.0073	0.009463568
GO:0042634	regulation of hair cycle	15	0.277649078	0.0008	0.002313249
GO:0042533	tumor necrosis factor biosynthetic process	18	0.277649078	0.0136	0.015813319
GO:0042534	regulation of tumor necrosis factor biosynthetic process	18	0.277649078	0.0136	0.015813319
GO:0031128	developmental induction	14	0.277642949	0.0015	0.003088691
GO:0032460	negative regulation of protein oligomerization	13	0.277616078	0.0018	0.003459409
GO:0032922	circadian regulation of gene expression	34	0.277583466	0.0023	0.004032435
GO:0035513	oxidative RNA demethylation	5	0.277580438	0.0164	0.018653793
GO:0090075	relaxation of muscle	13	0.277576478	0.0004	0.002007244
GO:0015909	long-chain fatty acid transport	37	0.277567915	0.001	0.002498236
GO:0071971	extracellular exosome assembly	7	0.27755102	0.0098	0.012008345
GO:0007338	single fertilization	48	0.27752574	0.0001	0.000877824
GO:0008064	regulation of actin polymerization or depolymerization	70	0.277517926	0.001	0.002498236
GO:0030832	regulation of actin filament length	70	0.277517926	0.001	0.002498236
GO:0002183	cytoplasmic translational initiation	23	0.27749986	0.0007	0.002248538
GO:0051784	negative regulation of nuclear division	31	0.277494084	0.0013	0.002859821
GO:0045780	positive regulation of bone resorption	7	0.277477477	0.0435	0.045246532
GO:0046852	positive regulation of bone remodeling	7	0.277477477	0.0435	0.045246532
GO:0048736	appendage development	85	0.27745628	0.0005	0.002177563
GO:0060173	limb development	85	0.27745628	0.0005	0.002177563
GO:0038083	peptidyl-tyrosine autophosphorylation	10	0.277425997	0.0425	0.044271412
GO:0072655	establishment of protein localization to mitochondrion	85	0.277416913	0.0013	0.002859821
GO:0045995	regulation of embryonic development	67	0.2773968	0.0006	0.002177563
GO:0032456	endocytic recycling	28	0.277367163	0.0072	0.009362497
GO:2001235	positive regulation of apoptotic signaling pathway	114	0.277346519	0.0026	0.004377754
GO:0061156	pulmonary artery morphogenesis	5	0.277271557	0.0228	0.024919293
GO:0060395	SMAD protein signal transduction	21	0.277269106	0.0002	0.001341684
GO:0051495	positive regulation of cytoskeleton organization	103	0.277267559	0.0031	0.004916949
GO:0045216	cell-cell junction organization	133	0.277252978	0.0021	0.003804235
GO:0006164	purine nucleotide biosynthetic process	89	0.277251891	0.004	0.005880507
GO:0031065	positive regulation of histone deacetylation	11	0.277243477	0.0018	0.003459409
GO:0048592	eye morphogenesis	78	0.277203577	0.0046	0.006532041
GO:0022602	ovulation cycle process	51	0.27716456	0.0008	0.002313249
GO:1901879	regulation of protein depolymerization	35	0.277109763	0.0021	0.003804235
GO:0030032	lamellipodium assembly	32	0.277091377	0.0003	0.001720882
GO:0032874	positive regulation of stress-activated MAPK cascade	76	0.27708799	0.0012	0.002741075
GO:0010976	positive regulation of neuron projection development	114	0.277055251	0.0004	0.002007244
GO:0090200	positive regulation of release of cytochrome c from mitochondria	18	0.277048477	0.016	0.018267288
GO:0043271	negative regulation of ion transport	63	0.277023963	0.0025	0.004279479
GO:0033280	response to vitamin D	15	0.277014157	0.0007	0.002248538
GO:0006665	sphingolipid metabolic process	94	0.276987322	0.0058	0.007856196
GO:0043627	response to estrogen	38	0.276982998	0.0026	0.004377754
GO:0030323	respiratory tube development	101	0.276952483	0.0031	0.004916949
GO:0046148	pigment biosynthetic process	34	0.276909683	0.0014	0.002975058
GO:0006826	iron ion transport	31	0.276887948	0.0033	0.005149584
GO:0051591	response to cAMP	49	0.276883881	0.0002	0.001341684

GOIDlabel	GOName	size	categoryScores	pValPerm	pValPermCorr
GO:0010632	regulation of epithelial cell migration	129	0.276874882	0.0015	0.003088691
GO:0032799	low-density lipoprotein receptor particle metabolic process	10	0.276859717	0.0035	0.005358035
GO:0033006	regulation of mast cell activation involved in immune response	17	0.276826406	0.0023	0.004032435
GO:0002679	respiratory burst involved in defense response	8	0.276801802	0.0035	0.005358035
GO:0035268	protein mannosylation	15	0.276791077	< 0.001	< 0.001
GO:0032725	positive regulation of granulocyte macrophage colony-stimulating factor production	6	0.276791077	0.0026	0.004377754
GO:0034763	negative regulation of transmembrane transport	45	0.276779637	0.0012	0.002741075
GO:0032368	regulation of lipid transport	55	0.276752077	0.0008	0.002313249
GO:0071305	cellular response to vitamin D	11	0.276752077	0.0005	0.002177563
GO:0048332	mesoderm morphogenesis	36	0.276748177	0.0031	0.004916949
GO:0007274	neuromuscular synaptic transmission	5	0.276705277	0.0105	0.012724187
GO:0048562	embryonic organ morphogenesis	144	0.276689189	0.0027	0.004491543
GO:0060349	bone morphogenesis	37	0.276649623	0.0014	0.002975058
GO:0019530	taurine metabolic process	6	0.276576577	< 0.001	< 0.001
GO:0019694	alkanesulfonate metabolic process	6	0.276576577	< 0.001	< 0.001
GO:0002065	columnar/cuboidal epithelial cell differentiation	50	0.276571429	0.0006	0.002177563
GO:0070588	calcium ion transmembrane transport	108	0.27655036	0.002	0.003693422
GO:0050728	negative regulation of inflammatory response	59	0.276539493	0.0015	0.003088691
GO:0032273	positive regulation of protein polymerization	56	0.276526016	0.0031	0.004916949
GO:0060401	cytosolic calcium ion transport	65	0.276507277	0.0019	0.003579279
GO:0035845	photoreceptor cell outer segment organization	7	0.276447876	0.0029	0.004714719
GO:0097553	calcium ion transmembrane import into cytosol	51	0.276336841	0.0009	0.002399874
GO:1902656	calcium ion import into cytosol	51	0.276336841	0.0009	0.002399874
GO:0034767	positive regulation of ion transmembrane transport	65	0.276325116	0.0054	0.007432873
GO:0010771	negative regulation of cell morphogenesis involved in differentiation	31	0.276323328	0.0042	0.006097175
GO:0016197	endosomal transport	185	0.276310133	0.0058	0.007856196
GO:1903747	regulation of establishment of protein localization to mitochondrion	45	0.276293436	0.001	0.002498236
GO:1905247	positive regulation of aspartic-type peptidase activity	5	0.276241956	0.0071	0.009268394
GO:2000727	positive regulation of cardiac muscle cell differentiation	7	0.276227248	0.0431	0.044867086
GO:0033555	multicellular organismal response to stress	32	0.276190476	< 0.001	< 0.001
GO:1904683	regulation of metalloendopeptidase activity	5	0.276190476	0.0283	0.030373173
GO:0030203	glycosaminoglycan metabolic process	78	0.276173976	0.0009	0.002399874
GO:0021915	neural tube development	89	0.276167339	0.0002	0.001341684
GO:0023061	signal release	185	0.276137605	0.0008	0.002313249
GO:0048762	mesenchymal cell differentiation	104	0.276128601	0.0026	0.004377754
GO:0030834	regulation of actin filament depolymerization	22	0.276120276	0.0007	0.002248538
GO:0006482	protein demethylation	17	0.27611477	0.0128	0.014984276
GO:0008214	protein dealkylation	17	0.27611477	0.0128	0.014984276
GO:0016577	histone demethylation	17	0.27611477	0.0128	0.014984276
GO:0030212	hyaluronan metabolic process	17	0.27611477	0.0019	0.003579279
GO:0015696	ammonium transport	42	0.276098548	0.0005	0.002177563
GO:0051875	pigment granule localization	14	0.276098548	0.0008	0.002313249
GO:0003281	ventricular septum development	44	0.276079326	0.0028	0.004605006
GO:0009152	purine ribonucleotide biosynthetic process	85	0.276066318	0.0047	0.006644432
GO:0033622	integrin activation	14	0.27604339	0.0007	0.002248538
GO:0003229	ventricular cardiac muscle tissue development	35	0.276036036	0.0038	0.005666214
GO:0045623	negative regulation of T-helper cell differentiation	10	0.276036036	0.0001	0.000877824
GO:0009264	deoxyribonucleotide catabolic process	12	0.276018876	0.0013	0.002859821
GO:1903551	regulation of extracellular exosome assembly	6	0.275975976	0.0073	0.009463568
GO:0032412	regulation of ion transmembrane transporter activity	84	0.275975976	0.002	0.003693422
GO:0030497	fatty acid elongation	8	0.275965251	0.002	0.003693422
GO:0043542	endothelial cell migration	124	0.275922697	0.0015	0.003088691
GO:0001569	branching involved in blood vessel morphogenesis	13	0.275913276	0.0172	0.019442284
GO:1900424	regulation of defense response to bacterium	10	0.275907336	0.0063	0.008390784
GO:0030516	regulation of axon extension	37	0.275870465	0.0027	0.004491543
GO:0042117	monocyte activation	8	0.275868726	0.0089	0.011106266
GO:0035518	histone H2A monoubiquitination	12	0.275825826	0.0003	0.001720882
GO:0002693	positive regulation of cellular extravasation	9	0.275818676	0.002	0.003693422
GO:0016051	carbohydrate biosynthetic process	108	0.275813909	0.0017	0.003334595
GO:0090130	tissue migration	173	0.275733702	0.002	0.003693422
GO:0043543	protein acylation	149	0.275732684	0.001	0.002498236
GO:1901661	quinone metabolic process	24	0.275729301	0.0035	0.005358035
GO:0007605	sensory perception of sound	69	0.275724171	0.0006	0.002177563
GO:1900223	positive regulation of beta-amyloid clearance	6	0.275718576	0.0001	0.000877824
GO:0016048	detection of temperature stimulus	8	0.275707851	< 0.001	< 0.001
GO:1903522	regulation of blood circulation	111	0.27568727	0.001	0.002498236
GO:0060048	cardiac muscle contraction	59	0.275627686	0.0008	0.002313249
GO:0048149	behavioral response to ethanol	5	0.275624196	0.0003	0.001720882
GO:0001707	mesoderm formation	34	0.275622682	0.0044	0.006304227
GO:0042088	T-helper 1 type immune response	22	0.275570376	0.0013	0.002859821
GO:0032231	regulation of actin filament bundle assembly	51	0.275569687	0.0023	0.004032435
GO:1905207	regulation of cardiocyte differentiation	27	0.275532676	0.0098	0.012008345
GO:0002220	innate immune response activating cell surface receptor signaling pathway	71	0.275508909	0.0015	0.003088691
GO:0010171	body morphogenesis	29	0.275507034	0.011	0.013192134
GO:0045647	negative regulation of erythrocyte differentiation	7	0.275455047	0.0119	0.014078095
GO:0030324	lung development	98	0.275447167	0.003	0.004825593
GO:0071229	cellular response to acid chemical	106	0.275427989	0.0007	0.002248538
GO:0030858	positive regulation of epithelial cell differentiation	29	0.275400524	0.0003	0.001720882
GO:0061515	myeloid cell development	37	0.275383492	0.0067	0.008845888
GO:0021700	developmental maturation	119	0.275379341	0.0059	0.007964563
GO:0001841	neural tube formation	61	0.275257928	0.0002	0.001341684
GO:0070585	protein localization to mitochondrion	87	0.275217089	0.0016	0.003223775
GO:0042339	keratan sulfate metabolic process	19	0.275174423	0.0004	0.002007244

GOIDlabel	GOName	size	categoryScores	pValPerm	pValPermCorr
GO:0070198	protein localization to chromosome, telomeric region	22	0.275172575	< 0.001	< 0.001
GO:0051647	nucleus localization	15	0.275160875	0.0021	0.003804235
GO:0006650	glycerophospholipid metabolic process	185	0.275113569	0.0026	0.004377754
GO:0032720	negative regulation of tumor necrosis factor production	29	0.275098744	0.0094	0.011616482
GO:0045599	negative regulation of fat cell differentiation	22	0.275078975	0.0007	0.002248538
GO:0072348	sulfur compound transport	21	0.275062818	0.0008	0.002313249
GO:1903524	positive regulation of blood circulation	28	0.275059754	0.0018	0.003459409
GO:0050879	multicellular organismal movement	24	0.275010725	0.0003	0.001720882
GO:0050881	musculoskeletal movement	24	0.275010725	0.0003	0.001720882
GO:0070243	regulation of thymocyte apoptotic process	5	0.275006435	0.0016	0.003223775
GO:0035282	segmentation	41	0.274978812	0.001	0.002498236
GO:0035767	endothelial cell chemotaxis	17	0.27496404	0.0007	0.002248538
GO:0046476	glycosylceramide biosynthetic process	5	0.274954955	0.0134	0.015626386
GO:0070304	positive regulation of stress-activated protein kinase signaling cascade	77	0.274950275	0.001	0.002498236
GO:0030534	adult behavior	61	0.274924573	0.001	0.002498236
GO:1901838	positive regulation of transcription of nuclear large rRNA transcript from RNA polymerase I promoter	7	0.274903475	0.009	0.011215682
GO:0032377	regulation of intracellular lipid transport	7	0.274903475	0.0228	0.024919293
GO:0032380	regulation of intracellular sterol transport	7	0.274903475	0.0228	0.024919293
GO:0032383	regulation of intracellular cholesterol transport	7	0.274903475	0.0228	0.024919293
GO:2000679	positive regulation of transcription regulatory region DNA binding	13	0.274883675	0.0015	0.003088691
GO:0042482	positive regulation of odontogenesis	5	0.274851995	0.0119	0.014078095
GO:0006022	aminoglycan metabolic process	80	0.27484556	0.0015	0.003088691
GO:0003337	mesenchymal to epithelial transition involved in metanephros morphogenesis	8	0.2747426	0.0055	0.007544447
GO:0060393	regulation of pathway-restricted SMAD protein phosphorylation	27	0.274731875	0.0069	0.009046225
GO:0071695	anatomical structure maturation	25	0.274728443	0.0082	0.010366713
GO:0008202	steroid metabolic process	160	0.274650901	0.0053	0.007330056
GO:0010919	regulation of inositol phosphate biosynthetic process	7	0.274609303	0.0004	0.002007244
GO:0045217	cell-cell junction maintenance	6	0.274603175	0.0215	0.023664853
GO:1900120	regulation of receptor binding	12	0.274603175	0.0251	0.027199847
GO:0010594	regulation of endothelial cell migration	97	0.274590349	0.0018	0.003459409
GO:0045822	negative regulation of heart contraction	13	0.274547075	0.0004	0.002007244
GO:0010744	positive regulation of macrophage derived foam cell differentiation	5	0.274543115	0.0071	0.009268394
GO:0051693	actin filament capping	14	0.27453576	0.0006	0.002177563
GO:0051963	regulation of synapse assembly	36	0.274524525	< 0.001	< 0.001
GO:0090596	sensory organ morphogenesis	122	0.274521594	0.0046	0.006532041
GO:0050729	positive regulation of inflammatory response	69	0.27451924	0.0008	0.002313249
GO:0051924	regulation of calcium ion transport	97	0.274518701	0.0024	0.004156304
GO:0060485	mesenchyme development	133	0.274493183	0.0032	0.005044175
GO:0043567	regulation of insulin-like growth factor receptor signaling pathway	10	0.274491634	0.0315	0.033525301
GO:0006991	response to sterol depletion	6	0.274474474	0.0087	0.010877987
GO:0060193	positive regulation of lipase activity	36	0.274445874	0.0011	0.002610912
GO:0007041	lysosomal transport	64	0.274432915	0.0075	0.00968541
GO:0097484	dendrite extension	8	0.274420849	0.0011	0.002610912
GO:0007266	Rho protein signal transduction	89	0.274403135	0.003	0.004825593
GO:0048536	spleen development	18	0.274374374	0.0036	0.005475609
GO:0043931	ossification involved in bone maturation	7	0.274351903	0.0142	0.016456919
GO:0031110	regulation of microtubule polymerization or depolymerization	33	0.274349674	0.0096	0.01183839
GO:0048537	mucosal-associated lymphoid tissue development	5	0.274337194	0.021	0.023166522
GO:0048541	Peyer's patch development	5	0.274337194	0.021	0.023166522
GO:0038111	interleukin-7-mediated signaling pathway	11	0.274318474	0.0002	0.001341684
GO:0045947	negative regulation of translational initiation	14	0.274296746	0.0049	0.006860215
GO:0042147	retrograde transport, endosome to Golgi	47	0.274273666	0.004	0.005880507
GO:0003018	vascular process in circulatory system	65	0.274230274	0.0026	0.004377754
GO:0006285	base-excision repair, AP site formation	6	0.274217074	0.0078	0.009981807
GO:0042776	mitochondrial ATP synthesis coupled proton transport	12	0.274174174	0.0252	0.027298929
GO:1904923	regulation of mitophagy in response to mitochondrial depolarization	6	0.274174174	0.0265	0.028585731
GO:0031113	regulation of microtubule polymerization	22	0.274131274	0.011	0.013192134
GO:0043268	positive regulation of potassium ion transport	21	0.274069988	0.0177	0.019919142
GO:0042033	chemokine biosynthetic process	7	0.274057731	0.0111	0.013284563
GO:0045073	regulation of chemokine biosynthetic process	7	0.274057731	0.0111	0.013284563
GO:0050755	chemokine metabolic process	7	0.274057731	0.0111	0.013284563
GO:0050886	endocrine process	39	0.274038874	0.0027	0.004491543
GO:0006047	UDP-N-acetylglucosamine metabolic process	10	0.274028314	0.0002	0.001341684
GO:1900118	negative regulation of execution phase of apoptosis	5	0.273925354	0.0392	0.041055316
GO:0010717	regulation of epithelial to mesenchymal transition	48	0.273895324	0.0122	0.014387602
GO:0010712	regulation of collagen metabolic process	22	0.273850474	0.0048	0.006748379
GO:0044246	regulation of multicellular organismal metabolic process	22	0.273850474	0.0048	0.006748379
GO:0060840	artery development	55	0.273841114	0.0045	0.006417207
GO:2001212	regulation of vasculogenesis	11	0.273827074	0.003	0.004825593
GO:0010510	regulation of acetyl-CoA biosynthetic process from pyruvate	5	0.273822394	0.0022	0.003910907
GO:0050812	regulation of acyl-CoA biosynthetic process	5	0.273822394	0.0022	0.003910907
GO:0046488	phosphatidylinositol metabolic process	93	0.273818519	0.0028	0.004605006
GO:0006383	transcription from RNA polymerase III promoter	35	0.273778268	0.0021	0.003804235
GO:0031529	ruffle organization	28	0.273763559	0.0023	0.004032435
GO:0006308	DNA catabolic process	23	0.273750769	0.007	0.009166016
GO:0045736	negative regulation of cyclin-dependent protein serine/threonine kinase activity	14	0.273745174	0.0194	0.021561946
GO:1904030	negative regulation of cyclin-dependent protein kinase activity	14	0.273745174	0.0194	0.021561946
GO:0001778	plasma membrane repair	6	0.273702274	0.0102	0.012424427
GO:0034308	primary alcohol metabolic process	28	0.273662438	0.0022	0.003910907
GO:0035404	histone-serine phosphorylation	6	0.273659374	0.0017	0.003334595
GO:0032801	receptor catabolic process	19	0.273657116	0.0025	0.004279479
GO:0010257	NADH dehydrogenase complex assembly	48	0.273600386	0.0163	0.018559925
GO:0032981	mitochondrial respiratory chain complex I assembly	48	0.273600386	0.0163	0.018559925

GOIDlabel	GOName	size	categoryScores	pValPerm	pValPermCorr
GO:0097031	mitochondrial respiratory chain complex I biogenesis	48	0.273600386	0.0163	0.018559925
GO:0035850	epithelial cell differentiation involved in kidney development	27	0.273597407	0.0078	0.009981807
GO:0009303	rRNA transcription	22	0.273569674	0.0019	0.003579279
GO:0048701	embryonic cranial skeleton morphogenesis	25	0.273534106	0.0009	0.002399874
GO:0044090	positive regulation of vacuole organization	13	0.273517474	0.0206	0.022756769
GO:0045834	positive regulation of lipid metabolic process	86	0.273463829	0.001	0.002498236
GO:0030007	cellular potassium ion homeostasis	5	0.273462033	0.0341	0.036093556
GO:0072608	interleukin-10 secretion	6	0.273444873	0.0107	0.012912536
GO:0009994	oocyte differentiation	20	0.273436293	0.0004	0.002007244
GO:0002431	Fc receptor mediated stimulatory signaling pathway	41	0.273421854	0.0022	0.003910907
GO:0030321	transepithelial chloride transport	5	0.273410553	0.0147	0.016962357
GO:0016239	positive regulation of macroautophagy	42	0.273408102	0.0055	0.007544447
GO:0010959	regulation of metal ion transport	155	0.27340225	0.0019	0.003579279
GO:2000179	positive regulation of neural precursor cell proliferation	29	0.273350197	0.0001	0.000877824
GO:0051258	protein polymerization	116	0.273330227	0.0017	0.003334595
GO:0048639	positive regulation of developmental growth	89	0.273307015	0.0034	0.005263104
GO:0045666	positive regulation of neuron differentiation	153	0.273300191	0.0006	0.002177563
GO:0097530	granulocyte migration	52	0.273235323	0.0064	0.008500875
GO:0032102	negative regulation of response to external stimulus	154	0.273193602	0.0023	0.004032435
GO:0021602	cranial nerve morphogenesis	6	0.273187473	< 0.001	< 0.001
GO:0000423	macromitophagy	19	0.273155863	0.0068	0.008946408
GO:0055024	regulation of cardiac muscle tissue development	46	0.273107269	0.0092	0.011424708
GO:0001938	positive regulation of endothelial cell proliferation	54	0.273101673	0.0023	0.004032435
GO:1901019	regulation of calcium ion transmembrane transporter activity	28	0.273064902	0.0011	0.002610912
GO:0045806	negative regulation of endocytosis	33	0.273039273	0.0024	0.004156304
GO:0030041	actin filament polymerization	74	0.273035584	0.0005	0.002177563
GO:0046785	microtubule polymerization	35	0.273020776	0.0118	0.013977955
GO:0090481	pyrimidine nucleotide-sugar transmembrane transport	8	0.273005148	0.0068	0.008946408
GO:0006361	transcription initiation from RNA polymerase I promoter	25	0.272988417	0.0001	0.000877824
GO:0035871	protein K11-linked deubiquitination	5	0.272947233	0.0072	0.009362497
GO:0035733	hepatic stellate cell activation	6	0.272930073	0.0216	0.023746214
GO:0061383	trabecula morphogenesis	29	0.272906404	0.0035	0.005358035
GO:0007000	nucleolus organization	6	0.272887173	< 0.001	< 0.001
GO:0030833	regulation of actin filament polymerization	61	0.272861152	0.001	0.002498236
GO:0070979	protein K11-linked ubiquitination	18	0.272858573	0.0053	0.007330056
GO:0042440	pigment metabolic process	41	0.272806605	0.0044	0.006304227
GO:0090102	cochlea development	22	0.272797473	0.0002	0.001341684
GO:0009154	purine ribonucleotide catabolic process	15	0.272741313	0.0079	0.010077402
GO:0009261	ribonucleotide catabolic process	15	0.272741313	0.0079	0.010077402
GO:0071242	cellular response to ammonium ion	28	0.27270638	0.0002	0.001341684
GO:0019371	cyclooxygenase pathway	8	0.272651223	0.0025	0.004279479
GO:0007034	vacuolar transport	69	0.272612986	0.0077	0.009887588
GO:0032008	positive regulation of TOR signaling	21	0.272611387	0.0086	0.010769857
GO:0010762	regulation of fibroblast migration	19	0.272586873	0.0015	0.003088691
GO:0098789	pre-mRNA cleavage required for polyadenylation	7	0.272586873	0.0004	0.002007244
GO:0055023	positive regulation of cardiac muscle tissue growth	19	0.27254623	0.0048	0.006748379
GO:0006183	GTP biosynthetic process	6	0.272543973	0.0374	0.039305772
GO:0006228	UTP biosynthetic process	6	0.272543973	0.0374	0.039305772
GO:0006929	substrate-dependent cell migration	19	0.272519136	0.0114	0.01358747
GO:0002042	cell migration involved in sprouting angiogenesis	33	0.272485472	0.0037	0.005561344
GO:1902017	regulation of cilium assembly	34	0.272473314	0.0005	0.002177563
GO:0060350	endochondral bone morphogenesis	23	0.272452577	0.0011	0.002610912
GO:0070884	regulation of calcineurin-NFAT signaling cascade	13	0.272428472	0.0025	0.004279479
GO:0031346	positive regulation of cell projection organization	170	0.272399122	0.0012	0.002741075
GO:0033627	cell adhesion mediated by integrin	36	0.272386672	0.0007	0.002248538
GO:0051642	centrosome localization	14	0.27238463	0.0046	0.006532041
GO:0007626	locomotory behavior	100	0.272368082	0.0008	0.002313249
GO:0060526	prostate glandular acinus morphogenesis	5	0.272329472	0.0221	0.024229021
GO:0060527	prostate epithelial cord arborization involved in prostate glandular acinus morphogenesis	5	0.272329472	0.0221	0.024229021
GO:0035904	aorta development	34	0.272329472	0.0013	0.002859821
GO:0010634	positive regulation of epithelial cell migration	87	0.272323555	0.0021	0.003804235
GO:0006767	water-soluble vitamin metabolic process	52	0.272309672	0.0112	0.013379119
GO:0031954	positive regulation of protein autophosphorylation	11	0.272282672	0.0018	0.003459409
GO:0060314	regulation of ryanodine-sensitive calcium-release channel activity	8	0.272232947	0.0006	0.002177563
GO:0009084	glutamine family amino acid biosynthetic process	10	0.272226512	0.0001	0.000877824
GO:0031016	pancreas development	39	0.272124872	0.0038	0.005666214
GO:0060052	neurofilament cytoskeleton organization	6	0.272114972	0.0305	0.032586169
GO:0035066	positive regulation of histone acetylation	16	0.272104247	0.0051	0.007095023
GO:0046514	ceramide catabolic process	13	0.272091872	0.0168	0.019054351
GO:0017085	response to insecticide	7	0.272072072	0.008	0.010168329
GO:0031645	negative regulation of neurological system process	8	0.272039897	0.0065	0.008621124
GO:0051209	release of sequestered calcium ion into cytosol	48	0.272034535	0.0009	0.002399874
GO:0051282	regulation of sequestering of calcium ion	48	0.272034535	0.0009	0.002399874
GO:0051283	negative regulation of sequestering of calcium ion	48	0.272034535	0.0009	0.002399874
GO:0055017	cardiac muscle tissue growth	46	0.271999329	0.003	0.004825593
GO:0010835	regulation of protein ADP-ribosylation	7	0.271924986	0.007	0.009166016
GO:0030239	myofibril assembly	25	0.271907336	0.0025	0.004279479
GO:0032272	negative regulation of protein polymerization	28	0.271851443	0.0028	0.004605006
GO:0072531	pyrimidine-containing compound transmembrane transport	11	0.271838072	0.0164	0.018653793
GO:0070106	interleukin-27-mediated signaling pathway	7	0.271814672	0.0001	0.000877824
GO:1905048	regulation of metallopeptidase activity	9	0.271757472	0.002	0.003693422
GO:0006264	mitochondrial DNA replication	8	0.271750322	0.0118	0.013977955
GO:0090218	positive regulation of lipid kinase activity	25	0.2717426	0.0001	0.000877824

GOIDlabel	GOName	size	categoryScores	pValPerm	pValPermCorr
GO:0034764	positive regulation of transmembrane transport	71	0.271687784	0.006	0.00807902
GO:0032147	activation of protein kinase activity	158	0.27165176	0.0022	0.003910907
GO:0043966	histone H3 acetylation	43	0.271617132	0.0022	0.003910907
GO:1905477	positive regulation of protein localization to membrane	61	0.271603688	0.0008	0.002313249
GO:0046889	positive regulation of lipid biosynthetic process	44	0.271533872	0.0019	0.003579279
GO:0043547	positive regulation of GTPase activity	180	0.271425711	0.0056	0.007655255
GO:0060421	positive regulation of heart growth	21	0.271410186	0.0081	0.010256567
GO:0007171	activation of transmembrane receptor protein tyrosine kinase activity	5	0.271402831	0.0107	0.012912536
GO:0007498	mesoderm development	62	0.271366297	0.0002	0.001341684
GO:0038034	signal transduction in absence of ligand	37	0.271348569	0.0106	0.012825859
GO:0097192	extrinsic apoptotic signaling pathway in absence of ligand	37	0.271348569	0.0106	0.012825859
GO:0042742	defense response to bacterium	82	0.271331262	0.0044	0.006304227
GO:2000765	regulation of cytoplasmic translation	13	0.271319671	0.0169	0.019147324
GO:0035308	negative regulation of protein dephosphorylation	30	0.271274131	0.0001	0.000877824
GO:0090208	positive regulation of triglyceride metabolic process	14	0.2712631	0.0017	0.003334595
GO:0050891	multicellular organismal water homeostasis	21	0.271250843	0.0001	0.000877824
GO:0038033	positive regulation of endothelial cell chemotaxis by VEGF-activated vascular endothelial growth factor receptor signaling pathway	5	0.271248391	0.0062	0.008281828
GO:0007292	female gamete generation	54	0.271190238	0.0006	0.002177563
GO:0010569	regulation of double-strand break repair via homologous recombination	24	0.271181896	< 0.001	< 0.001
GO:0051546	keratinocyte migration	9	0.271128271	0.0076	0.009790742
GO:0021533	cell differentiation in hindbrain	9	0.271099671	0.0035	0.005358035
GO:0030728	ovulation	13	0.271082071	0.0101	0.012328539
GO:0042445	hormone metabolic process	89	0.271074285	0.004	0.005880507
GO:1902745	positive regulation of lamellipodium organization	12	0.271042471	0.0043	0.006204188
GO:0016358	dendrite development	85	0.271039443	0.0022	0.003910907
GO:0006474	N-terminal protein amino acid acetylation	12	0.271021021	0.0001	0.000877824
GO:0043039	tRNA aminoacylation	34	0.271004618	0.0062	0.008281828
GO:0034766	negative regulation of ion transmembrane transport	41	0.270992247	0.0014	0.002975058
GO:0071312	cellular response to alkaloid	21	0.270956671	0.0018	0.003459409
GO:2001259	positive regulation of cation channel activity	23	0.270930558	0.0024	0.004156304
GO:2000644	regulation of receptor catabolic process	7	0.270895385	0.0132	0.015415723
GO:0034390	smooth muscle cell apoptotic process	10	0.270836551	0.0069	0.009046225
GO:0034391	regulation of smooth muscle cell apoptotic process	10	0.270836551	0.0069	0.009046225
GO:0019748	secondary metabolic process	35	0.270792425	0.0006	0.002177563
GO:0006563	L-serine metabolic process	9	0.270785071	0.0055	0.007544447
GO:0021952	central nervous system projection neuron axonogenesis	7	0.270785071	0.0014	0.002975058
GO:1904427	positive regulation of calcium ion transmembrane transport	25	0.270754183	0.006	0.00807902
GO:0009304	tRNA transcription	6	0.270742171	0.0147	0.016962357
GO:0015671	oxygen transport	6	0.270742171	0.0154	0.017651835
GO:1902600	hydrogen ion transmembrane transport	63	0.270740128	0.0052	0.007215241
GO:0003416	endochondral bone growth	9	0.270727871	0.0009	0.002399874
GO:0010595	positive regulation of endothelial cell migration	63	0.270703356	0.0021	0.003804235
GO:0043984	histone H4-K16 acetylation	14	0.270693142	0.0059	0.007964563
GO:0016322	neuron remodeling	6	0.270613471	0.0122	0.014387602
GO:0042726	flavin-containing compound metabolic process	6	0.270613471	0.018	0.020203241
GO:0051281	positive regulation of release of sequestered calcium ion into cytosol	16	0.270608108	0.0029	0.004714719
GO:0070936	protein K48-linked ubiquitination	32	0.270608108	0.0068	0.008946408
GO:0006829	zinc II ion transport	9	0.270584871	0.006	0.00807902
GO:0035137	hindlimb morphogenesis	13	0.270567271	0.0001	0.000877824
GO:0014009	glial cell proliferation	28	0.270527671	0.0004	0.002007244
GO:0032271	regulation of protein polymerization	86	0.270470803	0.0028	0.004605006
GO:0098787	mRNA cleavage involved in mRNA processing	8	0.27046332	0.0001	0.000877824
GO:0051573	negative regulation of histone H3-K9 methylation	8	0.27046332	0.0009	0.002399874
GO:0014074	response to purine-containing compound	77	0.270407328	0.0002	0.001341684
GO:0060402	calcium ion transport into cytosol	61	0.270375762	0.0018	0.003459409
GO:0000045	autophagosome assembly	54	0.270365604	0.0037	0.005561344
GO:1904062	regulation of cation transmembrane transport	118	0.270348799	0.0013	0.002859821
GO:0046501	protoporphyrinogen IX metabolic process	7	0.270343813	0.0358	0.037748743
GO:1900363	regulation of mRNA polyadenylation	10	0.27032175	0.0006	0.002177563
GO:0032467	positive regulation of cytokinesis	15	0.27028743	0.0066	0.008731949
GO:0046135	pyrimidine nucleoside catabolic process	10	0.27024453	0.0148	0.017062301
GO:0034754	cellular hormone metabolic process	61	0.270240733	0.0056	0.007655255
GO:0003161	cardiac conduction system development	8	0.270238095	0.0044	0.006304227
GO:0042541	hemoglobin biosynthetic process	7	0.270233499	0.0041	0.005981983
GO:0006750	glutathione biosynthetic process	12	0.27014157	0.0164	0.018653793
GO:0071466	cellular response to xenobiotic stimulus	51	0.270088576	0.0001	0.000877824
GO:0001976	neurological system process involved in regulation of systemic arterial blood pressure	7	0.270086413	0.001	0.002498236
GO:1901381	positive regulation of potassium ion transmembrane transport	18	0.27008437	0.0237	0.025818821
GO:0022029	telencephalon cell migration	27	0.27007007	0.0007	0.002248538
GO:0045333	cellular respiration	129	0.270050782	0.0098	0.012008345
GO:0002566	somatic diversification of immune receptors via somatic mutation	6	0.27001287	0.0049	0.006860215
GO:0033235	positive regulation of protein sumoylation	6	0.27001287	0.0173	0.019544921
GO:0019320	hexose catabolic process	30	0.26993565	0.0022	0.003910907
GO:0050994	regulation of lipid catabolic process	33	0.26989587	0.0022	0.003910907
GO:0010810	regulation of cell-substrate adhesion	113	0.269889865	0.0024	0.004156304
GO:0035902	response to immobilization stress	16	0.269868082	0.0031	0.004916949
GO:1901379	regulation of potassium ion transmembrane transport	32	0.269843951	0.0103	0.012519912
GO:0017156	calcium ion regulated exocytosis	39	0.26978847	0.0007	0.002248538
GO:0048010	vascular endothelial growth factor receptor signaling pathway	54	0.269779303	0.0107	0.012912536
GO:0097164	ammonium ion metabolic process	122	0.269770239	0.0017	0.003334595
GO:0032042	mitochondrial DNA metabolic process	12	0.26971257	0.0175	0.019742875
GO:2001053	regulation of mesenchymal cell apoptotic process	5	0.26970399	0.0006	0.002177563

GOIDlabel	GOName	size	categoryScores	pValPerm	pValPermCorr
GO:2001054	negative regulation of mesenchymal cell apoptotic process	5	0.26970399	0.0006	0.002177563
GO:0046683	response to organophosphorus	68	0.269664623	0.0005	0.002177563
GO:0017145	stem cell division	19	0.269660638	0.0024	0.004156304
GO:0003279	cardiac septum development	61	0.269599342	0.003	0.004825593
GO:0051881	regulation of mitochondrial membrane potential	46	0.269582004	0.0133	0.015521132
GO:0061162	establishment of monopolar cell polarity	9	0.26952667	0.0422	0.043980474
GO:0061339	establishment or maintenance of monopolar cell polarity	9	0.26952667	0.0422	0.043980474
GO:0002467	germinal center formation	9	0.269498069	0.0075	0.00968541
GO:0001829	trophectodermal cell differentiation	6	0.269498069	0.0003	0.001720882
GO:0031032	actomyosin structure organization	88	0.269486369	0.0014	0.002975058
GO:0061037	negative regulation of cartilage development	10	0.269446589	0.0127	0.014886353
GO:0046471	phosphatidylglycerol metabolic process	18	0.269440869	0.003	0.004825593
GO:0003197	endocardial cushion development	28	0.269433719	0.0046	0.006532041
GO:0006882	cellular zinc ion homeostasis	7	0.269424527	0.0091	0.011322589
GO:0051492	regulation of stress fiber assembly	45	0.269400829	0.0025	0.004279479
GO:0070076	histone lysine demethylation	16	0.269385457	0.0142	0.016456919
GO:0072148	epithelial cell fate commitment	8	0.269337194	0.0009	0.002399874
GO:0090670	RNA localization to Cajal body	17	0.269316375	< 0.001	< 0.001
GO:0090671	telomerase RNA localization to Cajal body	17	0.269316375	< 0.001	< 0.001
GO:0090672	telomerase RNA localization	17	0.269316375	< 0.001	< 0.001
GO:1904872	regulation of telomerase RNA localization to Cajal body	17	0.269316375	< 0.001	< 0.001
GO:0032964	collagen biosynthetic process	23	0.269296626	0.005	0.006974174
GO:0070935	3'-UTR-mediated mRNA stabilization	8	0.269272844	0.0126	0.014782732
GO:0016482	cytosolic transport	92	0.269263052	0.0085	0.010671887
GO:0060359	response to ammonium ion	60	0.269210639	0.0001	0.000877824
GO:0071542	dopaminergic neuron differentiation	12	0.269197769	0.0044	0.006304227
GO:0046339	diacylglycerol metabolic process	9	0.269097669	0.0015	0.003088691
GO:0006584	catecholamine metabolic process	27	0.269040469	0.0001	0.000877824
GO:0009712	catechol-containing compound metabolic process	27	0.269040469	0.0001	0.000877824
GO:0032370	positive regulation of lipid transport	34	0.26899084	0.0004	0.002007244
GO:0005976	polysaccharide metabolic process	56	0.268909726	0.0004	0.002007244
GO:0042752	regulation of circadian rhythm	49	0.268893967	0.0026	0.004377754
GO:1904837	beta-catenin-TCF complex assembly	20	0.268893179	0.0011	0.002610912
GO:0043276	anoikis	19	0.26887489	0.011	0.013192134
GO:0030148	sphingolipid biosynthetic process	58	0.268867883	0.0069	0.009046225
GO:0002532	production of molecular mediator involved in inflammatory response	35	0.268836183	0.0013	0.002859821
GO:0051340	regulation of ligase activity	6	0.268811669	0.038	0.03991002
GO:0001662	behavioral fear response	16	0.268806306	< 0.001	< 0.001
GO:0042596	fear response	16	0.268806306	< 0.001	< 0.001
GO:0014904	myotube cell development	12	0.268790219	0.0031	0.004916949
GO:1904796	regulation of core promoter binding	5	0.268777349	0.0011	0.002610912
GO:0060043	regulation of cardiac muscle cell proliferation	26	0.268775369	0.0103	0.012519912
GO:0030901	midbrain development	53	0.268764722	0.0046	0.006532041
GO:0044708	single-organism behavior	185	0.268753696	0.0012	0.002741075
GO:0050996	positive regulation of lipid catabolic process	13	0.268745669	0.008	0.010168329
GO:0006805	xenobiotic metabolic process	47	0.268709439	0.0001	0.000877824
GO:0007618	mating	18	0.268682969	< 0.001	< 0.001
GO:0021510	spinal cord development	54	0.268621002	0.0013	0.002859821
GO:0018345	protein palmitoylation	19	0.268590395	0.0073	0.009463568
GO:0099622	cardiac muscle cell membrane repolarization	16	0.268516731	0.0004	0.002007244
GO:0007409	axonogenesis	182	0.268508069	0.0014	0.002975058
GO:0003151	outflow tract morphogenesis	48	0.268500644	0.0075	0.00968541
GO:0098657	import into cell	38	0.26848879	0.0032	0.005044175
GO:0035588	G-protein coupled purinergic receptor signaling pathway	12	0.268468468	0.0005	0.002177563
GO:0048821	erythrocyte development	18	0.268411268	0.0041	0.005981983
GO:0007416	synapse assembly	57	0.268409763	0.0001	0.000877824
GO:0060433	bronchus development	5	0.268314028	0.0069	0.009046225
GO:0002192	IRES-dependent translational initiation	11	0.268304668	0.0002	0.001341684
GO:1905475	regulation of protein localization to membrane	92	0.268292205	0.0012	0.002741075
GO:0043244	regulation of protein complex disassembly	49	0.268279358	0.004	0.005880507
GO:0055022	negative regulation of cardiac muscle tissue growth	13	0.268211068	0.0212	0.023366932
GO:0061117	negative regulation of heart growth	13	0.268211068	0.0212	0.023366932
GO:0070242	thymocyte apoptotic process	9	0.268211068	0.0107	0.012912536
GO:0051491	positive regulation of filopodium assembly	15	0.268176748	0.0012	0.002741075
GO:0006309	apoptotic DNA fragmentation	17	0.268120221	0.0061	0.008182551
GO:0050999	regulation of nitric-oxide synthase activity	26	0.268102168	0.0031	0.004916949
GO:2000171	negative regulation of dendrite development	9	0.268096668	0.0411	0.042939393
GO:0015849	organic acid transport	138	0.268089829	0.0035	0.005358035
GO:0046942	carboxylic acid transport	138	0.268089829	0.0035	0.005358035
GO:0032515	negative regulation of phosphoprotein phosphatase activity	29	0.268033551	0.0001	0.000877824
GO:0015698	inorganic anion transport	70	0.268019857	0.0034	0.005263104
GO:0007006	mitochondrial membrane organization	76	0.267957055	0.0084	0.010571327
GO:1901880	negative regulation of protein depolymerization	27	0.267944135	0.003	0.004825593
GO:2001222	regulation of neuron migration	18	0.267882168	0.0012	0.002741075
GO:0035482	gastric motility	5	0.267850708	0.0006	0.002177563
GO:0055021	regulation of cardiac muscle tissue growth	33	0.267828868	0.0106	0.012825859
GO:0051668	localization within membrane	40	0.267824968	0.0005	0.002177563
GO:0008016	regulation of heart contraction	97	0.267802412	0.0015	0.003088691
GO:0002040	sprouting angiogenesis	60	0.267790648	0.0023	0.004032435
GO:0055010	ventricular cardiac muscle tissue morphogenesis	30	0.267790648	0.0034	0.005263104
GO:0021885	forebrain cell migration	28	0.267788196	0.0007	0.002248538
GO:0032233	positive regulation of actin filament bundle assembly	31	0.267770997	0.0071	0.009268394
GO:0033198	response to ATP	14	0.267751425	0.0007	0.002248538

GOIDlabel	GOName	size	categoryScores	pValPerm	pValPermCorr
GO:0097531	mast cell migration	6	0.267739168	0.0045	0.006417207
GO:0050995	negative regulation of lipid catabolic process	13	0.267696268	0.002	0.003693422
GO:0008306	associative learning	26	0.267636868	0.0024	0.004156304
GO:0043242	negative regulation of protein complex disassembly	28	0.267622725	0.0029	0.004714719
GO:0035148	tube formation	81	0.26757869	0.0006	0.002177563
GO:1900016	negative regulation of cytokine production involved in inflammatory response	10	0.267567568	0.0012	0.002741075
GO:0060419	heart growth	49	0.267559688	0.0033	0.005149584
GO:0018200	peptidyl-glutamic acid modification	16	0.26748713	0.0034	0.005263104
GO:0032400	melanosome localization	13	0.267419067	0.0018	0.003459409
GO:0060420	regulation of heart growth	35	0.267416805	0.0128	0.014984276
GO:0031272	regulation of pseudopodium assembly	7	0.267402096	0.0124	0.014577606
GO:0009268	response to pH	18	0.267381667	0.0022	0.003910907
GO:0003283	atrial septum development	11	0.267368667	< 0.001	< 0.001
GO:0045792	negative regulation of cell size	7	0.267365325	0.0307	0.03276689
GO:0042462	eye photoreceptor cell development	13	0.267359667	0.0062	0.008281828
GO:2000108	positive regulation of leukocyte apoptotic process	9	0.267353067	0.0044	0.006304227
GO:0019731	antibacterial humoral response	12	0.267310167	0.0052	0.007215241
GO:1904352	positive regulation of protein catabolic process in the vacuole	5	0.267284427	0.0154	0.017651835
GO:0061025	membrane fusion	78	0.267168267	0.0092	0.011424708
GO:0042220	response to cocaine	23	0.267159085	0.0002	0.001341684
GO:0046622	positive regulation of organ growth	28	0.267144696	0.0021	0.003804235
GO:0006941	striated muscle contraction	79	0.267073946	0.0011	0.002610912
GO:0051339	regulation of lyase activity	30	0.267069927	0.0061	0.008182551
GO:1990668	vesicle fusion with endoplasmic reticulum-Golgi intermediate compartment (ERGIC) membrane	5	0.267027027	0.039	0.040872718
GO:0043923	positive regulation by host of viral transcription	16	0.267004505	0.0066	0.008731949
GO:0007530	sex determination	10	0.266949807	0.0054	0.007432873
GO:0006029	proteoglycan metabolic process	41	0.266949179	0.0066	0.008731949
GO:2000251	positive regulation of actin cytoskeleton reorganization	9	0.266895467	0.0021	0.003804235
GO:0060412	ventricular septum morphogenesis	26	0.266884467	0.0007	0.002248538
GO:0098780	response to mitochondrial depolarisation	17	0.266818079	0.0072	0.009362497
GO:0006066	alcohol metabolic process	185	0.266797454	0.0053	0.007330056
GO:0048484	enteric nervous system development	8	0.266763192	0.0452	0.046915244
GO:0097006	regulation of plasma lipoprotein particle levels	43	0.266762444	0.0007	0.002248538
GO:0002686	negative regulation of leukocyte migration	21	0.266752467	0.0004	0.002007244
GO:0006103	2-oxoglutarate metabolic process	11	0.266690067	0.0035	0.005358035
GO:0070534	protein K63-linked ubiquitination	31	0.266683273	0.005	0.006974174
GO:0006040	amino sugar metabolic process	20	0.266602317	0.0004	0.002007244
GO:0006661	phosphatidylinositol biosynthetic process	69	0.266599519	0.0033	0.005149584
GO:0071287	cellular response to manganese ion	7	0.266593124	0.0436	0.045335748
GO:0051897	positive regulation of protein kinase B signaling	79	0.266500497	0.0013	0.002859821
GO:0090103	cochlea morphogenesis	10	0.266486486	0.0026	0.004377754
GO:0035973	aggrephagy	7	0.266482809	0.0041	0.005981983
GO:0015695	organic cation transport	9	0.266380666	0.0356	0.037550281
GO:0060038	cardiac muscle cell proliferation	32	0.266296654	0.0052	0.007215241
GO:0019730	antimicrobial humoral response	25	0.266285714	0.0118	0.013977955
GO:0051606	detection of stimulus	136	0.266282459	0.005	0.006974174
GO:0060525	prostate glandular acinus development	6	0.266280566	0.0085	0.010671887
GO:0032688	negative regulation of interferon-beta production	9	0.266180466	0.0011	0.002610912
GO:0048599	oocyte development	19	0.266151866	0.0003	0.001720882
GO:0043507	positive regulation of JUN kinase activity	35	0.266093032	0.0023	0.004032435
GO:0009069	serine family amino acid metabolic process	34	0.266091301	0.0014	0.002975058
GO:0009410	response to xenobiotic stimulus	55	0.266067626	0.0002	0.001341684
GO:0098751	bone cell development	17	0.266045878	0.0159	0.01817591
GO:0006895	Golgi to endosome transport	10	0.265997426	0.0133	0.015521132
GO:0009110	vitamin biosynthetic process	14	0.265986395	0.0013	0.002859821
GO:0001838	embryonic epithelial tube formation	70	0.265975363	0.0002	0.001341684
GO:0006672	ceramide metabolic process	57	0.265903497	0.0125	0.014684332
GO:0006544	glycine metabolic process	13	0.265894466	0.0109	0.013101792
GO:0002433	immune response-regulating cell surface receptor signaling pathway involved in phagocytosis	40	0.265810811	0.0027	0.004491543
GO:0038094	Fc-gamma receptor signaling pathway	40	0.265810811	0.0027	0.004491543
GO:0038096	Fc-gamma receptor signaling pathway involved in phagocytosis	40	0.265810811	0.0027	0.004491543
GO:1905037	autophagosome organization	57	0.265768024	0.0043	0.006204188
GO:0015718	monocarboxylic acid transport	69	0.26575644	0.0041	0.005981983
GO:0010458	exit from mitosis	23	0.265726596	0.0077	0.009887588
GO:0051561	positive regulation of mitochondrial calcium ion concentration	7	0.265710609	0.0398	0.041649491
GO:0032354	response to follicle-stimulating hormone	11	0.265660466	0.0092	0.011424708
GO:0032881	regulation of polysaccharide metabolic process	24	0.265637066	0.0055	0.007544447
GO:0044321	response to leptin	12	0.265551266	0.0005	0.002177563
GO:0007019	microtubule depolymerization	17	0.265546218	0.0222	0.024321919
GO:0034637	cellular carbohydrate biosynthetic process	40	0.265540541	0.0007	0.002248538
GO:0090280	positive regulation of calcium ion import	25	0.265534106	0.0054	0.007432873
GO:0009064	glutamine family amino acid metabolic process	40	0.26547619	< 0.001	< 0.001
GO:0001702	gastrulation with mouth forming second	13	0.265458865	< 0.001	< 0.001
GO:0090151	establishment of protein localization to mitochondrial membrane	9	0.265408265	0.0093	0.011528671
GO:0032261	purine nucleotide salvage	5	0.265328185	0.0047	0.006644432
GO:0051781	positive regulation of cell division	45	0.265293865	0.0047	0.006644432
GO:0097009	energy homeostasis	20	0.265276705	0.0222	0.024321919
GO:0098868	bone growth	10	0.265276705	0.001	0.002498236
GO:1901985	positive regulation of protein acetylation	23	0.265234178	0.0009	0.002399874
GO:0034405	response to fluid shear stress	22	0.265215865	0.0085	0.010671887
GO:0010822	positive regulation of mitochondrion organization	88	0.26521294	0.0071	0.009268394
GO:0051928	positive regulation of calcium ion transport	47	0.265198938	0.0098	0.012008345
GO:0034982	mitochondrial protein processing	5	0.265122265	0.0068	0.008946408

GOIDlabel	GOName	size	categoryScores	pValPerm	pValPermCorr
GO:0008360	regulation of cell shape	82	0.265081458	0.0021	0.003804235
GO:0098661	inorganic anion transmembrane transport	37	0.265066611	0.0004	0.002007244
GO:0051650	establishment of vesicle localization	126	0.265020122	0.0021	0.003804235
GO:0006937	regulation of muscle contraction	67	0.264972435	0.0006	0.002177563
GO:0072175	epithelial tube formation	71	0.26497	0.0002	0.001341684
GO:0022027	interkinetic nuclear migration	5	0.264967825	0.0008	0.002313249
GO:0001541	ovarian follicle development	35	0.264938408	0.0017	0.003334595
GO:0060041	retina development in camera-type eye	71	0.264897493	0.0006	0.002177563
GO:0090279	regulation of calcium ion import	41	0.264871143	0.0008	0.002313249
GO:0048705	skeletal system morphogenesis	97	0.264819753	0.0011	0.002610912
GO:0016236	macroautophagy	192	0.264785768	0.0074	0.009581512
GO:0009415	response to water	8	0.26476834	0.0025	0.004279479
GO:0060491	regulation of cell projection assembly	85	0.264710425	0.0025	0.004279479
GO:1904888	cranial skeletal system development	33	0.264654265	0.0013	0.002859821
GO:0046697	decidualization	11	0.264654265	0.009	0.011215682
GO:0051181	cofactor transport	16	0.264623552	0.0271	0.029163847
GO:0051024	positive regulation of immunoglobulin secretion	6	0.264607465	0.0002	0.001341684
GO:0090311	regulation of protein deacetylation	21	0.26458295	0.0044	0.006304227
GO:0042471	ear morphogenesis	52	0.264557965	0.0037	0.005561344
GO:0010718	positive regulation of epithelial to mesenchymal transition	28	0.264524729	0.0129	0.015093024
GO:1903319	positive regulation of protein maturation	18	0.264507365	0.0174	0.019643968
GO:0046530	photoreceptor cell differentiation	29	0.264483202	0.0002	0.001341684
GO:0046173	polyol biosynthetic process	28	0.264414414	0.0075	0.00968541
GO:1901626	regulation of postsynaptic membrane organization	6	0.264392964	0.0096	0.01183839
GO:0031365	N-terminal protein amino acid modification	20	0.264337194	0.0015	0.003088691
GO:0033762	response to glucagon	13	0.264330264	0.0001	0.000877824
GO:0061050	regulation of cell growth involved in cardiac muscle cell development	7	0.264313293	0.0126	0.014782732
GO:2001257	regulation of cation channel activity	47	0.264300775	0.0016	0.003223775
GO:0019233	sensory perception of pain	40	0.264298584	0.0001	0.000877824
GO:0010661	positive regulation of muscle cell apoptotic process	10	0.264221364	0.0104	0.012619802
GO:0030149	sphingolipid catabolic process	18	0.264207064	0.0324	0.034402732
GO:0060065	uterus development	10	0.264169884	< 0.001	< 0.001
GO:0032768	regulation of monooxygenase activity	30	0.264161304	0.0021	0.003804235
GO:0043648	dicarboxylic acid metabolic process	60	0.264139854	0.0004	0.002007244
GO:0043247	telomere maintenance in response to DNA damage	6	0.264135564	0.011	0.013192134
GO:0002536	respiratory burst involved in inflammatory response	5	0.264041184	0.0158	0.018074565
GO:0090309	positive regulation of methylation-dependent chromatin silencing	6	0.263878164	0.0076	0.009790742
GO:0060135	maternal process involved in female pregnancy	33	0.263858664	0.0025	0.004279479
GO:0021826	substrate-independent telencephalic tangential migration	5	0.263835264	0.0057	0.007745437
GO:0021830	interneuron migration from the subpallium to the cortex	5	0.263835264	0.0057	0.007745437
GO:0021843	substrate-independent telencephalic tangential interneuron migration	5	0.263835264	0.0057	0.007745437
GO:0031639	plasminogen activation	10	0.263783784	0.0122	0.014387602
GO:1903564	regulation of protein localization to cilium	5	0.263783784	0.03	0.032068098
GO:1903034	regulation of response to wounding	83	0.263732924	0.0081	0.010256567
GO:0030199	collagen fibril organization	28	0.263715757	0.01	0.012232246
GO:0006865	amino acid transport	61	0.263649598	0.0041	0.005981983
GO:0006684	sphingomyelin metabolic process	8	0.263610039	0.0415	0.043328883
GO:0031063	regulation of histone deacetylation	15	0.263543544	0.0027	0.004491543
GO:0006122	mitochondrial electron transport, ubiquinol to cytochrome c	11	0.263531064	0.026	0.02809393
GO:0046884	follicle-stimulating hormone secretion	5	0.263526384	0.0349	0.036866849
GO:0002068	glandular epithelial cell development	11	0.263507664	0.0011	0.002610912
GO:0050690	regulation of defense response to virus by virus	15	0.263423423	0.0169	0.019147324
GO:0097094	craniofacial suture morphogenesis	7	0.263394006	0.0126	0.014782732
GO:0060191	regulation of lipase activity	47	0.263369753	0.001	0.002498236
GO:0009201	ribonucleoside triphosphate biosynthetic process	38	0.263320463	0.0086	0.010769857
GO:0051056	regulation of small GTPase mediated signal transduction	171	0.263308421	0.0027	0.004491543
GO:0035587	purinergic receptor signaling pathway	16	0.263304376	0.0018	0.003459409
GO:0072224	metanephric glomerulus development	9	0.263177463	0.0003	0.001720882
GO:0000271	polysaccharide biosynthetic process	35	0.263136606	0.0004	0.002007244
GO:0052428	modification by host of symbiont molecular function	7	0.263136606	0.0112	0.013379119
GO:0048663	neuron fate commitment	27	0.263091663	0.0044	0.006304227
GO:0035640	exploration behavior	9	0.263091663	0.0001	0.000877824
GO:0034110	regulation of homotypic cell-cell adhesion	12	0.263041613	0.0174	0.019643968
GO:0034393	positive regulation of smooth muscle cell apoptotic process	7	0.263026292	0.0081	0.010256567
GO:0086065	cell communication involved in cardiac conduction	20	0.262985843	0.0106	0.012825859
GO:0043517	positive regulation of DNA damage response, signal transduction by p53 class mediator	5	0.262960103	0.0382	0.040087041
GO:0044264	cellular polysaccharide metabolic process	52	0.262850213	0.0006	0.002177563
GO:0007411	axon guidance	90	0.262845703	0.0007	0.002248538
GO:0097485	neuron projection guidance	90	0.262845703	0.0007	0.002248538
GO:1900746	regulation of vascular endothelial growth factor signaling pathway	10	0.262779923	0.0094	0.011616482
GO:1902547	regulation of cellular response to vascular endothelial growth factor stimulus	10	0.262779923	0.0094	0.011616482
GO:0048679	regulation of axon regeneration	11	0.262758863	0.0163	0.018559925
GO:0015980	energy derivation by oxidation of organic compounds	183	0.262736741	0.0059	0.007964563
GO:0060263	regulation of respiratory burst	7	0.26273212	0.0061	0.008182551
GO:1901077	regulation of relaxation of muscle	5	0.262651223	0.0017	0.003334595
GO:0086011	membrane repolarization during action potential	13	0.262627463	0.0021	0.003804235
GO:0006782	protoporphyrinogen IX biosynthetic process	5	0.262599743	0.0344	0.036368812
GO:0048268	clathrin coat assembly	9	0.262548263	0.0169	0.019147324
GO:0021953	central nervous system neuron differentiation	73	0.262537684	0.0003	0.001720882
GO:0061450	trophoblast cell migration	7	0.262511491	0.0015	0.003088691
GO:1901163	regulation of trophoblast cell migration	7	0.262511491	0.0015	0.003088691
GO:0006919	activation of cysteine-type endopeptidase activity involved in apoptotic process	49	0.262464213	0.0121	0.014285532
GO:0048011	neurotrophin TRK receptor signaling pathway	14	0.262456334	0.0014	0.002975058

GOIDlabel	GOName	size	categoryScores	pValPerm	pValPermCorr
GO:0045600	positive regulation of fat cell differentiation	35	0.262401177	0.0014	0.002975058
GO:0003323	type B pancreatic cell development	8	0.262387387	0.0054	0.007432873
GO:0043266	regulation of potassium ion transport	40	0.262361647	0.0105	0.012724187
GO:0045684	positive regulation of epidermis development	17	0.262336286	0.0005	0.002177563
GO:0036148	phosphatidylglycerol acyl-chain remodeling	6	0.262333762	0.0109	0.013101792
GO:0086064	cell communication by electrical coupling involved in cardiac conduction	8	0.262323037	0.0388	0.040696577
GO:2001135	regulation of endocytic recycling	9	0.262319462	0.013	0.015198863
GO:0032925	regulation of activin receptor signaling pathway	10	0.262290862	0.001	0.002498236
GO:0010635	regulation of mitochondrial fusion	6	0.262162162	0.0021	0.003804235
GO:0034394	protein localization to cell surface	33	0.262142662	0.0012	0.002741075
GO:0060998	regulation of dendritic spine development	22	0.262080262	0.0014	0.002975058
GO:0036151	phosphatidylcholine acyl-chain remodeling	13	0.262033462	0.0097	0.011922488
GO:0003231	cardiac ventricle development	73	0.261959415	0.0016	0.003223775
GO:0046655	folic acid metabolic process	14	0.261941533	0.0081	0.010256567
GO:1901264	carbohydrate derivative transport	39	0.261934462	0.0172	0.019442284
GO:0090174	organelle membrane fusion	38	0.261925083	0.0206	0.022756769
GO:2000008	regulation of protein localization to cell surface	19	0.261721872	0.0041	0.005981983
GO:0032965	regulation of collagen biosynthetic process	19	0.261708325	0.0118	0.013977955
GO:0046578	regulation of Ras protein signal transduction	107	0.261694271	0.0039	0.005778819
GO:0016446	somatic hypermutation of immunoglobulin genes	5	0.261673102	0.0039	0.005778819
GO:0071475	cellular hyperosmotic salinity response	5	0.261673102	0.0061	0.008182551
GO:0008209	androgen metabolic process	18	0.261661662	0.0152	0.017453982
GO:0097011	cellular response to granulocyte macrophage colony-stimulating factor stimulus	5	0.261621622	0.0062	0.008281828
GO:0097012	response to granulocyte macrophage colony-stimulating factor	5	0.261621622	0.0062	0.008281828
GO:0070977	bone maturation	9	0.261575862	0.0077	0.009887588
GO:0051496	positive regulation of stress fiber assembly	26	0.261469161	0.0093	0.011528671
GO:0019674	NAD metabolic process	40	0.261389961	0.0017	0.003334595
GO:0035270	endocrine system development	61	0.261299238	0.0035	0.005358035
GO:0038179	neurotrophin signaling pathway	17	0.261276402	0.0012	0.002741075
GO:1901016	regulation of potassium ion transmembrane transporter activity	20	0.261274131	0.0211	0.023264758
GO:1905168	positive regulation of double-strand break repair via homologous recombination	6	0.261261261	0.0171	0.01934984
GO:0009145	purine nucleoside triphosphate biosynthetic process	37	0.261233434	0.008	0.010168329
GO:0060389	pathway-restricted SMAD protein phosphorylation	30	0.261226941	0.0068	0.008946408
GO:0046580	negative regulation of Ras protein signal transduction	23	0.261171731	0.0055	0.007544447
GO:0090559	regulation of membrane permeability	52	0.261157311	0.0128	0.014984276
GO:0040036	regulation of fibroblast growth factor receptor signaling pathway	11	0.261120861	0.0061	0.008182551
GO:0051043	regulation of membrane protein ectodomain proteolysis	15	0.261106821	0.0092	0.011424708
GO:0010862	positive regulation of pathway-restricted SMAD protein phosphorylation	19	0.261098693	0.0058	0.007856196
GO:0001893	maternal placenta development	16	0.261003861	0.0031	0.004916949
GO:0030038	contractile actin filament bundle assembly	51	0.260968532	0.0016	0.003223775
GO:0043149	stress fiber assembly	51	0.260968532	0.0016	0.003223775
GO:1905288	vascular associated smooth muscle cell apoptotic process	6	0.260918061	0.0093	0.011528671
GO:1905459	regulation of vascular associated smooth muscle cell apoptotic process	6	0.260918061	0.0093	0.011528671
GO:0086013	membrane repolarization during cardiac muscle cell action potential	12	0.260875161	0.0009	0.002399874
GO:2000343	positive regulation of chemokine (C-X-C motif) ligand 2 production	6	0.260875161	0.0004	0.002007244
GO:0050805	negative regulation of synaptic transmission	29	0.260861847	0.0032	0.005044175
GO:0030947	regulation of vascular endothelial growth factor receptor signaling pathway	18	0.260846561	0.0244	0.02650438
GO:0046784	viral mRNA export from host cell nucleus	6	0.260746461	0.0028	0.004605006
GO:1990126	retrograde transport, endosome to plasma membrane	15	0.260729301	0.0019	0.003579279
GO:0048799	animal organ maturation	10	0.260617761	0.0094	0.011616482
GO:0060732	positive regulation of inositol phosphate biosynthetic process	6	0.260617761	0.0008	0.002313249
GO:0086009	membrane repolarization	21	0.260599375	0.0038	0.005666214
GO:2000641	regulation of early endosome to late endosome transport	10	0.260540541	0.0186	0.020774238
GO:0033169	histone H3-K9 demethylation	7	0.260415518	0.026	0.02809393
GO:0010867	positive regulation of triglyceride biosynthetic process	12	0.26038181	0.0006	0.002177563
GO:0002209	behavioral defense response	17	0.260292225	< 0.001	< 0.001
GO:0035305	negative regulation of dephosphorylation	57	0.260159408	0.0003	0.001720882
GO:0098815	modulation of excitatory postsynaptic potential	7	0.260121346	0.0015	0.003088691
GO:0055026	negative regulation of cardiac muscle tissue development	20	0.26009009	0.0114	0.01358747
GO:0010764	negative regulation of fibroblast migration	6	0.26006006	0.001	0.002498236
GO:1902692	regulation of neuroblast proliferation	17	0.260049966	0.0011	0.002610912
GO:0044801	single-organism membrane fusion	59	0.25997426	0.015	0.017255415
GO:0035036	sperm-egg recognition	21	0.259949746	0.001	0.002498236
GO:0051899	membrane depolarization	33	0.25993526	0.0168	0.019054351
GO:1990253	cellular response to leucine starvation	5	0.25992278	< 0.001	< 0.001
GO:0032885	regulation of polysaccharide biosynthetic process	20	0.25990991	0.0032	0.005044175
GO:0070873	regulation of glycogen metabolic process	21	0.259900717	0.0049	0.006860215
GO:0003352	regulation of cilium movement	8	0.259749035	< 0.001	< 0.001
GO:0001573	ganglioside metabolic process	11	0.25974026	0.0026	0.004377754
GO:0007157	heterophilic cell-cell adhesion via plasma membrane cell adhesion molecules	14	0.259735245	0.0037	0.005561344
GO:2000291	regulation of myoblast proliferation	6	0.25967396	0.0381	0.040008453
GO:0051648	vesicle localization	132	0.25965251	0.0023	0.004032435
GO:0010923	negative regulation of phosphatase activity	54	0.259640593	0.0002	0.001341684
GO:0034315	regulation of Arp2/3 complex-mediated actin nucleation	6	0.25963106	0.039	0.040872718
GO:0021511	spinal cord patterning	14	0.259624931	0.0074	0.009581512
GO:0021846	cell proliferation in forebrain	14	0.259624931	0.0008	0.002313249
GO:0051447	negative regulation of meiotic cell cycle	6	0.25958816	0.0012	0.002741075
GO:0019043	establishment of viral latency	7	0.259533002	0.002	0.003693422
GO:0014733	regulation of skeletal muscle adaptation	5	0.25951094	0.0162	0.018465856
GO:0072378	blood coagulation, fibrin clot formation	12	0.259459459	0.0218	0.023945433
GO:0071804	cellular potassium ion transport	51	0.259449365	0.0123	0.014492124
GO:0071805	potassium ion transmembrane transport	51	0.259449365	0.0123	0.014492124
GO:0002832	negative regulation of response to biotic stimulus	34	0.259444318	0.0091	0.011322589

GOIDlabel	GOName	size	categoryScores	pValPerm	pValPermCorr
GO:0060986	endocrine hormone secretion	30	0.259433719	0.0017	0.003334595
GO:0006906	vesicle fusion	36	0.259416559	0.0229	0.025020007
GO:1904181	positive regulation of membrane depolarization	8	0.259395109	0.0443	0.046018566
GO:0046777	protein autophosphorylation	107	0.259372858	0.0009	0.002399874
GO:0021903	rostrocaudal neural tube patterning	5	0.259356499	0.0005	0.002177563
GO:1902476	chloride transmembrane transport	31	0.259343214	0.0023	0.004032435
GO:0060384	innervation	9	0.259316459	0.0006	0.002177563
GO:0071392	cellular response to estradiol stimulus	20	0.259279279	0.0166	0.018864444
GO:0042446	hormone biosynthetic process	35	0.259275602	0.0103	0.012519912
GO:0010544	negative regulation of platelet activation	8	0.259266409	0.0077	0.009887588
GO:2000242	negative regulation of reproductive process	24	0.259255684	0.0013	0.002859821
GO:0002691	regulation of cellular extravasation	12	0.259244959	0.0015	0.003088691
GO:0015732	prostaglandin transport	5	0.259202059	0.0109	0.013101792
GO:0032306	regulation of prostaglandin secretion	5	0.259202059	0.0109	0.013101792
GO:0032310	prostaglandin secretion	5	0.259202059	0.0109	0.013101792
GO:0038066	p38MAPK cascade	26	0.259172359	0.0177	0.019919142
GO:0033692	cellular polysaccharide biosynthetic process	32	0.259137709	0.0003	0.001720882
GO:0090269	fibroblast growth factor production	6	0.259116259	0.0323	0.034307983
GO:0090270	regulation of fibroblast growth factor production	6	0.259116259	0.0323	0.034307983
GO:0006743	ubiquinone metabolic process	12	0.259094809	0.0093	0.011528671
GO:0006744	ubiquinone biosynthetic process	12	0.259094809	0.0093	0.011528671
GO:1901663	quinone biosynthetic process	12	0.259094809	0.0093	0.011528671
GO:0030011	maintenance of cell polarity	8	0.259073359	0.0253	0.027397944
GO:0046320	regulation of fatty acid oxidation	19	0.259066585	0.0004	0.002007244
GO:1901748	leukotriene D4 metabolic process	7	0.259018202	0.0074	0.009581512
GO:1901750	leukotriene D4 biosynthetic process	7	0.259018202	0.0074	0.009581512
GO:0060997	dendritic spine morphogenesis	15	0.259013299	0.0005	0.002177563
GO:0043552	positive regulation of phosphatidylinositol 3-kinase activity	23	0.259000616	< 0.001	< 0.001
GO:0061299	retina vasculature morphogenesis in camera-type eye	6	0.258987559	0.0025	0.004279479
GO:0021702	cerebellar Purkinje cell differentiation	6	0.258987559	0.0063	0.008390784
GO:0016331	morphogenesis of embryonic epithelium	79	0.258987016	0.0005	0.002177563
GO:0010984	regulation of lipoprotein particle clearance	11	0.258944659	0.0063	0.008390784
GO:0015844	monoamine transport	33	0.258921259	< 0.001	< 0.001
GO:0021987	cerebral cortex development	56	0.25886652	0.0008	0.002313249
GO:1904037	positive regulation of epithelial cell apoptotic process	19	0.258863375	0.0219	0.024051129
GO:0035637	multicellular organismal signaling	70	0.258845376	0.0083	0.010468217
GO:1900027	regulation of ruffle assembly	14	0.258779187	0.0172	0.019442284
GO:0015985	energy coupled proton transport, down electrochemical gradient	13	0.258667459	0.0349	0.036866849
GO:0015986	ATP synthesis coupled proton transport	13	0.258667459	0.0349	0.036866849
GO:0051279	regulation of release of sequestered calcium ion into cytosol	28	0.258604523	0.0012	0.002741075
GO:0014066	regulation of phosphatidylinositol 3-kinase signaling	61	0.258590206	0.0012	0.002741075
GO:0006678	glucosylceramide metabolic process	6	0.258515659	0.0098	0.012008345
GO:0055075	potassium ion homeostasis	8	0.258462033	0.0419	0.043717862
GO:0071340	skeletal muscle acetylcholine-gated channel clustering	8	0.258429858	0.001	0.002498236
GO:1903036	positive regulation of response to wounding	33	0.258390858	0.0077	0.009887588
GO:0019226	transmission of nerve impulse	25	0.25834749	0.0117	0.013882722
GO:2000628	regulation of miRNA metabolic process	6	0.258301158	0.0158	0.018074565
GO:0043117	positive regulation of vascular permeability	6	0.258301158	0.0242	0.02630058
GO:0019400	alditol metabolic process	14	0.258227615	0.0044	0.006304227
GO:0016255	attachment of GPI anchor to protein	5	0.258223938	0.0439	0.045625359
GO:0034643	establishment of mitochondrion localization, microtubule-mediated	9	0.258201058	0.0054	0.007432873
GO:0047497	mitochondrion transport along microtubule	9	0.258201058	0.0054	0.007432873
GO:0048593	camera-type eye morphogenesis	62	0.258076971	0.0062	0.008281828
GO:0007631	feeding behavior	45	0.258063778	0.0001	0.000877824
GO:0001974	blood vessel remodeling	27	0.258058058	0.016	0.018267288
GO:0014831	gastro-intestinal system smooth muscle contraction	6	0.258000858	0.0177	0.019919142
GO:1905146	lysosomal protein catabolic process	10	0.257966538	0.041	0.042848967
GO:0043652	engulfment of apoptotic cell	5	0.257966538	0.0187	0.020874952
GO:0046902	regulation of mitochondrial membrane permeability	46	0.257965419	0.0134	0.015626386
GO:0060914	heart formation	14	0.257951829	0.0046	0.006532041
GO:0072087	renal vesicle development	11	0.257915058	0.0037	0.005561344
GO:2000316	regulation of T-helper 17 type immune response	7	0.257915058	0.0046	0.006532041
GO:0061041	regulation of wound healing	71	0.257886055	0.0097	0.011922488
GO:0009235	cobalamin metabolic process	11	0.257844858	0.0149	0.017165166
GO:0050913	sensory perception of bitter taste	15	0.257691978	0.0078	0.009981807
GO:0005977	glycogen metabolic process	44	0.257628408	0.0007	0.002248538
GO:0006073	cellular glucan metabolic process	44	0.257628408	0.0007	0.002248538
GO:0044042	glucan metabolic process	44	0.257628408	0.0007	0.002248538
GO:0050808	synapse organization	123	0.257576043	0.0005	0.002177563
GO:0008544	epidermis development	195	0.257571858	0.0034	0.005263104
GO:0048143	astrocyte activation	8	0.257561133	0.045	0.046730487
GO:0034721	histone H3-K4 demethylation, trimethyl-H3-K4-specific	5	0.257503218	0.0252	0.027298929
GO:0045989	positive regulation of striated muscle contraction	7	0.2574738	0.0061	0.008182551
GO:0002634	regulation of germinal center formation	6	0.257443157	0.0156	0.017858595
GO:0006564	L-serine biosynthetic process	7	0.257400257	0.008	0.010168329
GO:0002544	chronic inflammatory response	8	0.257368082	0.0117	0.013882722
GO:1901894	regulation of calcium-transporting ATPase activity	5	0.257297297	0.0306	0.032676575
GO:0021762	substantia nigra development	32	0.25725547	0.0046	0.006532041
GO:0014033	neural crest cell differentiation	36	0.257250107	0.0009	0.002399874
GO:0008347	glial cell migration	26	0.257241857	0.0007	0.002248538
GO:0071711	basement membrane organization	15	0.257211497	0.0138	0.016031243
GO:1904353	regulation of telomere capping	13	0.257202257	0.0011	0.002610912
GO:0048017	inositol lipid-mediated signaling	87	0.257196112	0.0011	0.002610912

GOIDlabel	GOName	size	categoryScores	pValPerm	pValPermCorr
GO:0042791	5S class rRNA transcription from RNA polymerase III type 1 promoter	5	0.257194337	0.0338	0.035793852
GO:0042797	tRNA transcription from RNA polymerase III promoter	5	0.257194337	0.0338	0.035793852
GO:0003205	cardiac chamber development	89	0.257163102	0.0012	0.002741075
GO:0060563	neuroepithelial cell differentiation	30	0.257134277	0.0001	0.000877824
GO:0010310	regulation of hydrogen peroxide metabolic process	7	0.257069314	0.0105	0.012724187
GO:0098901	regulation of cardiac muscle cell action potential	10	0.257039897	0.0216	0.023746214
GO:0006766	vitamin metabolic process	74	0.256989808	0.0123	0.014492124
GO:0035336	long-chain fatty-acyl-CoA metabolic process	12	0.256971257	0.011	0.013192134
GO:0070293	renal absorption	11	0.256955657	0.0344	0.036368812
GO:0030048	actin filament-based movement	65	0.256893377	0.0011	0.002610912
GO:0032410	negative regulation of transporter activity	30	0.256876877	0.001	0.002498236
GO:0007096	regulation of exit from mitosis	12	0.256864007	0.0191	0.021280472
GO:0031650	regulation of heat generation	8	0.256788932	0.0127	0.014886353
GO:0060124	positive regulation of growth hormone secretion	6	0.256756757	0.0035	0.005358035
GO:0035023	regulation of Rho protein signal transduction	67	0.256747152	0.0036	0.005475609
GO:0045981	positive regulation of nucleotide metabolic process	38	0.256743209	0.0084	0.010571327
GO:1900544	positive regulation of purine nucleotide metabolic process	38	0.256743209	0.0084	0.010571327
GO:0048015	phosphatidylinositol-mediated signaling	86	0.25672982	0.0009	0.002399874
GO:0097178	ruffle assembly	21	0.2567016	0.009	0.011215682
GO:0055117	regulation of cardiac muscle contraction	30	0.256679537	0.0011	0.002610912
GO:0045740	positive regulation of DNA replication	19	0.256628057	0.0072	0.009362497
GO:2001267	regulation of cysteine-type endopeptidase activity involved in apoptotic signaling pathway	11	0.256581257	0.0059	0.007964563
GO:0070828	heterochromatin organization	11	0.256534457	0.0097	0.011922488
GO:0050795	regulation of behavior	31	0.256486902	0.0003	0.001720882
GO:0007613	memory	40	0.256486486	0.0022	0.003910907
GO:0090303	positive regulation of wound healing	28	0.256471778	0.0085	0.010671887
GO:0048521	negative regulation of behavior	10	0.256370656	0.0001	0.000877824
GO:0060339	negative regulation of type I interferon-mediated signaling pathway	6	0.256284856	0.0015	0.003088691
GO:0018243	protein O-linked glycosylation via threonine	5	0.256267696	0.0001	0.000877824
GO:1901889	negative regulation of cell junction assembly	16	0.256241956	0.0126	0.014782732
GO:0018095	protein polyglutamylation	9	0.256170456	0.001	0.002498236
GO:2000651	positive regulation of sodium ion transmembrane transporter activity	6	0.256156156	0.0163	0.018559925
GO:0061337	cardiac conduction	47	0.25609135	0.0094	0.011616482
GO:0034762	regulation of transmembrane transport	181	0.256077704	0.0041	0.005981983
GO:0034765	regulation of ion transmembrane transport	170	0.256058748	0.0046	0.006532041
GO:0072538	T-helper 17 type immune response	10	0.256036036	0.0159	0.01817591
GO:0051205	protein insertion into membrane	25	0.256	0.005	0.006974174
GO:0021536	diencephalon development	36	0.255998856	0.0005	0.002177563
GO:0006857	oligopeptide transport	6	0.255984556	0.0059	0.007964563
GO:0046620	regulation of organ growth	54	0.255970256	0.0043	0.006204188
GO:0045913	positive regulation of carbohydrate metabolic process	41	0.255937471	0.0016	0.003223775
GO:0009125	nucleoside monophosphate catabolic process	5	0.255907336	0.0022	0.003910907
GO:0015919	peroxisomal membrane transport	5	0.255907336	0.0079	0.010077402
GO:0045046	protein import into peroxisome membrane	5	0.255907336	0.0079	0.010077402
GO:0010714	positive regulation of collagen metabolic process	14	0.25583747	0.0081	0.010256567
GO:0032967	positive regulation of collagen biosynthetic process	14	0.25583747	0.0081	0.010256567
GO:0044253	positive regulation of multicellular organismal metabolic process	14	0.25583747	0.0081	0.010256567
GO:0031640	killing of cells of other organism	21	0.255831342	0.0237	0.025818821
GO:0044364	disruption of cells of other organism	21	0.255831342	0.0237	0.025818821
GO:0086003	cardiac muscle cell contraction	29	0.255820352	0.0059	0.007964563
GO:0035428	hexose transmembrane transport	12	0.255770056	0.0107	0.012912536
GO:1904659	glucose transmembrane transport	12	0.255770056	0.0107	0.012912536
GO:0095500	acetylcholine receptor signaling pathway	11	0.255692056	0.0216	0.023746214
GO:1903831	signal transduction involved in cellular response to ammonium ion	11	0.255692056	0.0216	0.023746214
GO:1905144	response to acetylcholine	11	0.255692056	0.0216	0.023746214
GO:1905145	cellular response to acetylcholine	11	0.255692056	0.0216	0.023746214
GO:0046434	organophosphate catabolic process	64	0.255678893	0.0083	0.010468217
GO:0046717	acid secretion	41	0.255629846	0.0041	0.005981983
GO:0046885	regulation of hormone biosynthetic process	9	0.255598456	0.0135	0.015731487
GO:1901526	positive regulation of macromitophagy	5	0.255598456	0.0199	0.022056122
GO:0042472	inner ear morphogenesis	43	0.255580497	0.0076	0.009790742
GO:0070141	response to UV-A	5	0.255392535	0.0338	0.035793852
GO:0033182	regulation of histone ubiquitination	7	0.255341055	0.0002	0.001341684
GO:0051955	regulation of amino acid transport	13	0.255321255	0.0056	0.007655255
GO:0035924	cellular response to vascular endothelial growth factor stimulus	35	0.255311638	0.0081	0.010256567
GO:0060397	JAK-STAT cascade involved in growth hormone signaling pathway	8	0.25530888	0.0003	0.001720882
GO:0005978	glycogen biosynthetic process	26	0.255281655	0.0005	0.002177563
GO:0009250	glucan biosynthetic process	26	0.255281655	0.0005	0.002177563
GO:0042535	positive regulation of tumor necrosis factor biosynthetic process	11	0.255247455	0.0265	0.028585731
GO:0021781	glial cell fate commitment	11	0.255060255	0.001	0.002498236
GO:0018210	peptidyl-threonine modification	43	0.255011822	0.0009	0.002399874
GO:1903169	regulation of calcium ion transmembrane transport	52	0.255009405	0.0026	0.004377754
GO:0010574	regulation of vascular endothelial growth factor production	17	0.25488682	0.0139	0.016141527
GO:0009414	response to water deprivation	5	0.254877735	0.0004	0.002007244
GO:0018107	peptidyl-threonine phosphorylation	38	0.254846576	0.0033	0.005149584
GO:0043270	positive regulation of ion transport	118	0.254841524	0.0049	0.006860215
GO:0046513	ceramide biosynthetic process	35	0.254833609	0.0156	0.017858595
GO:0060159	regulation of dopamine receptor signaling pathway	8	0.254826255	0.0039	0.005778819
GO:1903727	positive regulation of phospholipid metabolic process	30	0.254817675	0.0005	0.002177563
GO:0050885	neuromuscular process controlling balance	24	0.25481553	0.0013	0.002859821
GO:0030916	otic vesicle formation	5	0.254774775	0.0044	0.006304227
GO:0070988	demethylation	35	0.254752712	0.0034	0.005263104
GO:0043586	tongue development	7	0.254752712	0.0039	0.005778819

GOIDlabel	GOName	size	categoryScores	pValPerm	pValPermCorr
GO:0018958	phenol-containing compound metabolic process	46	0.254747916	< 0.001	< 0.001
GO:0010522	regulation of calcium ion transport into cytosol	38	0.254744971	0.0039	0.005778819
GO:0043525	positive regulation of neuron apoptotic process	29	0.254719744	0.0257	0.027793331
GO:0010470	regulation of gastrulation	20	0.254710425	0.0019	0.003579279
GO:0032463	negative regulation of protein homooligomerization	8	0.25453668	0.0073	0.009463568
GO:1902187	negative regulation of viral release from host cell	12	0.254525955	0.015	0.017255415
GO:0006531	aspartate metabolic process	6	0.254525955	0.0152	0.017453982
GO:0061437	renal system vasculature development	17	0.254493149	0.0008	0.002313249
GO:0061440	kidney vasculature development	17	0.254493149	0.0008	0.002313249
GO:0002088	lens development in camera-type eye	40	0.254465894	0.0007	0.002248538
GO:0044359	modulation of molecular function in other organism	8	0.254440154	0.0181	0.020286898
GO:0044362	negative regulation of molecular function in other organism	8	0.254440154	0.0181	0.020286898
GO:0052204	negative regulation of molecular function in other organism involved in symbiotic interaction	8	0.254440154	0.0181	0.020286898
GO:0052205	modulation of molecular function in other organism involved in symbiotic interaction	8	0.254440154	0.0181	0.020286898
GO:0002031	G-protein coupled receptor internalization	6	0.254440154	0.0132	0.015415723
GO:0006658	phosphatidylserine metabolic process	18	0.254397254	0.0329	0.034916187
GO:0001710	mesodermal cell fate commitment	10	0.254362934	< 0.001	< 0.001
GO:0035511	oxidative DNA demethylation	5	0.254362934	0.0167	0.018954426
GO:0015871	choline transport	5	0.254362934	0.0422	0.043980474
GO:0048167	regulation of synaptic plasticity	46	0.254333837	0.0056	0.007655255
GO:0032350	regulation of hormone metabolic process	17	0.254326596	0.0183	0.020489441
GO:0014065	phosphatidylinositol 3-kinase signaling	71	0.254282451	0.0014	0.002975058
GO:0050806	positive regulation of synaptic transmission	50	0.254259974	0.0004	0.002007244
GO:0048702	embryonic neurocranium morphogenesis	5	0.254208494	0.0079	0.010077402
GO:0060411	cardiac septum morphogenesis	43	0.254197719	0.0031	0.004916949
GO:0031649	heat generation	10	0.254182754	0.0168	0.019054351
GO:0009206	purine ribonucleoside triphosphate biosynthetic process	36	0.254168454	0.011	0.013192134
GO:0060343	trabecula formation	11	0.254077454	0.0009	0.002399874
GO:0061384	heart trabecula morphogenesis	23	0.254054054	0.0021	0.003804235
GO:0000737	DNA catabolic process, endonucleolytic	20	0.254015444	0.0092	0.011424708
GO:0007566	embryo implantation	28	0.25394374	0.0042	0.006097175
GO:0043551	regulation of phosphatidylinositol 3-kinase activity	32	0.253925354	0.0011	0.002610912
GO:0090043	regulation of tubulin deacetylation	6	0.253925354	0.0175	0.019742875
GO:0043588	skin development	177	0.253808288	0.0085	0.010671887
GO:0045109	intermediate filament organization	12	0.253796654	0.0174	0.019643968
GO:0009113	purine nucleobase biosynthetic process	5	0.253796654	0.0429	0.044680781
GO:0030540	female genitalia development	9	0.253682254	0.027	0.029066047
GO:0042391	regulation of membrane potential	137	0.253582467	0.0063	0.008390784
GO:0002826	negative regulation of T-helper 1 type immune response	5	0.253539254	0.0002	0.001341684
GO:0072132	mesenchyme morphogenesis	36	0.253503504	0.0062	0.008281828
GO:2001026	regulation of endothelial cell chemotaxis	14	0.253484096	0.0008	0.002313249
GO:0048311	mitochondrion distribution	5	0.253436293	0.0453	0.047003728
GO:0045022	early endosome to late endosome transport	27	0.253396253	0.0281	0.030173766
GO:0035999	tetrahydrofolate interconversion	7	0.253392168	0.0011	0.002610912
GO:0061001	regulation of dendritic spine morphogenesis	11	0.253352053	0.0002	0.001341684
GO:0061036	positive regulation of cartilage development	17	0.253296994	< 0.001	< 0.001
GO:0034163	regulation of toll-like receptor 9 signaling pathway	6	0.253281853	0.0019	0.003579279
GO:0098927	vesicle-mediated transport between endosomal compartments	29	0.253264102	0.0289	0.03094937
GO:0010457	centriole-centriole cohesion	6	0.253238953	0.0137	0.015920879
GO:1900745	positive regulation of p38MAPK cascade	16	0.253217503	0.0399	0.04174043
GO:0046854	phosphatidylinositol phosphorylation	13	0.253182853	0.0147	0.016962357
GO:0006898	receptor-mediated endocytosis	137	0.253176639	0.0082	0.010366713
GO:0031623	receptor internalization	48	0.253126341	0.0068	0.008946408
GO:0010573	vascular endothelial growth factor production	18	0.253067353	0.0081	0.010256567
GO:0051457	maintenance of protein location in nucleus	14	0.253061224	0.0036	0.005475609
GO:0007076	mitotic chromosome condensation	9	0.253053053	0.0075	0.00968541
GO:0046475	glycerophospholipid catabolic process	14	0.252969296	0.0131	0.015312968
GO:0070252	actin-mediated cell contraction	52	0.252955153	0.0015	0.003088691
GO:1905245	regulation of aspartic-type peptidase activity	7	0.252877367	0.0204	0.022555378
GO:0043647	inositol phosphate metabolic process	39	0.252846253	0.0052	0.007215241
GO:0010882	regulation of cardiac muscle contraction by calcium ion signaling	8	0.252767053	0.005	0.006974174
GO:0044539	long-chain fatty acid import	6	0.252767053	0.0459	0.047595298
GO:0050890	cognition	117	0.252685653	0.0024	0.004156304
GO:0050803	regulation of synapse structure or activity	61	0.25267	0.0003	0.001720882
GO:2001169	regulation of ATP biosynthetic process	11	0.252533053	0.0039	0.005778819
GO:0042219	cellular modified amino acid catabolic process	13	0.252430452	0.0082	0.010366713
GO:1903010	regulation of bone development	9	0.252395252	0.0032	0.005044175
GO:0072718	response to cisplatin	8	0.252348777	0.0241	0.026209778
GO:1901642	nucleoside transmembrane transport	6	0.252295152	0.0461	0.047779362
GO:0072216	positive regulation of metanephros development	7	0.252252252	0.0273	0.029359247
GO:0031018	endocrine pancreas development	23	0.252241061	0.0014	0.002975058
GO:0030317	flagellated sperm motility	27	0.252214119	< 0.001	< 0.001
GO:0097722	sperm motility	27	0.252214119	< 0.001	< 0.001
GO:0010107	potassium ion import	11	0.252182052	0.0364	0.038343339
GO:1903651	positive regulation of cytoplasmic transport	10	0.252149292	0.0054	0.007432873
GO:0098974	postsynaptic actin cytoskeleton organization	5	0.252149292	0.001	0.002498236
GO:0042415	norepinephrine metabolic process	7	0.252105166	0.0013	0.002859821
GO:1900046	regulation of hemostasis	40	0.252091377	0.0079	0.010077402
GO:0050807	regulation of synapse organization	60	0.251934792	0.0002	0.001341684
GO:1902307	positive regulation of sodium ion transmembrane transport	10	0.251891892	0.0067	0.008845888
GO:2000341	regulation of chemokine (C-X-C motif) ligand 2 production	8	0.251833977	0.0003	0.001720882
GO:0005979	regulation of glycogen biosynthetic process	17	0.251813158	0.0018	0.003459409
GO:0010962	regulation of glucan biosynthetic process	17	0.251813158	0.0018	0.003459409

GOIDlabel	GOName	size	categoryScores	pValPerm	pValPermCorr
GO:0030511	positive regulation of transforming growth factor beta receptor signaling pathway	16	0.251801802	0.0019	0.003579279
GO:1903846	positive regulation of cellular response to transforming growth factor beta stimulus	16	0.251801802	0.0019	0.003579279
GO:0009134	nucleoside diphosphate catabolic process	5	0.251788932	0.011	0.013192134
GO:0009913	epidermal cell differentiation	141	0.251779439	0.008	0.010168329
GO:0010715	regulation of extracellular matrix disassembly	8	0.251769627	0.023	0.025116347
GO:0030854	positive regulation of granulocyte differentiation	5	0.251737452	0.001	0.002498236
GO:0043497	regulation of protein heterodimerization activity	6	0.251694552	0.0075	0.00968541
GO:0032753	positive regulation of interleukin-4 production	12	0.251694552	0.0025	0.004279479
GO:0046322	negative regulation of fatty acid oxidation	8	0.251640927	< 0.001	< 0.001
GO:0021513	spinal cord dorsal/ventral patterning	13	0.251519652	0.0072	0.009362497
GO:0014874	response to stimulus involved in regulation of muscle adaptation	8	0.251512227	0.0435	0.045246532
GO:0014068	positive regulation of phosphatidylinositol 3-kinase signaling	45	0.251462891	0.0009	0.002399874
GO:0072350	tricarboxylic acid metabolic process	26	0.251460251	0.0124	0.014577606
GO:0015780	nucleotide-sugar transport	9	0.251365651	0.0144	0.016652368
GO:0015781	pyrimidine nucleotide-sugar transport	9	0.251365651	0.0144	0.016652368
GO:0099003	vesicle-mediated transport in synapse	50	0.251330759	0.0035	0.005358035
GO:0042362	fat-soluble vitamin biosynthetic process	8	0.251319176	0.0124	0.014577606
GO:0038007	netrin-activated signaling pathway	8	0.251319176	0.0455	0.047203565
GO:0009225	nucleotide-sugar metabolic process	21	0.25127168	0.0016	0.003223775
GO:0060911	cardiac cell fate commitment	5	0.251222651	0.0207	0.022863276
GO:0070254	mucus secretion	5	0.251119691	0.0007	0.002248538
GO:0050432	catecholamine secretion	20	0.250978121	< 0.001	< 0.001
GO:1904749	regulation of protein localization to nucleolus	7	0.250965251	0.0016	0.003223775
GO:0048483	autonomic nervous system development	22	0.250941851	0.0075	0.00968541
GO:0044130	negative regulation of growth of symbiont in host	5	0.250862291	0.0285	0.030556948
GO:0044146	negative regulation of growth of symbiont involved in interaction with host	5	0.250862291	0.0285	0.030556948
GO:0052173	response to defenses of other organism involved in symbiotic interaction	6	0.250793651	0.0361	0.038033609
GO:0052200	response to host defenses	6	0.250793651	0.0361	0.038033609
GO:0075136	response to host	6	0.250793651	0.0361	0.038033609
GO:0007611	learning or memory	96	0.25079097	0.0029	0.004714719
GO:0071526	semaphorin-plexin signaling pathway	14	0.250744622	0.003	0.004825593
GO:0002580	regulation of antigen processing and presentation of peptide or polysaccharide antigen via MHC class II	5	0.250707851	0.0367	0.038621054
GO:0072044	collecting duct development	5	0.250501931	0.0002	0.001341684
GO:0006754	ATP biosynthetic process	30	0.25049335	0.011	0.013192134
GO:0001659	temperature homeostasis	20	0.25048906	0.0062	0.008281828
GO:0051307	meiotic chromosome separation	7	0.250487222	0.003	0.004825593
GO:0014047	glutamate secretion	10	0.25045045	0.0016	0.003223775
GO:0021794	thalamus development	8	0.2503861	0.0061	0.008182551
GO:2001214	positive regulation of vasculogenesis	8	0.2503861	0.0191	0.021280472
GO:0000186	activation of MAPKK activity	26	0.25035145	0.004	0.005880507
GO:0009226	nucleotide-sugar biosynthetic process	14	0.25032175	0.0004	0.002007244
GO:0000132	establishment of mitotic spindle orientation	13	0.25031185	0.0073	0.009463568
GO:0032892	positive regulation of organic acid transport	15	0.25029601	0.0186	0.020774238
GO:0016101	diterpenoid metabolic process	46	0.250271389	0.0035	0.005358035
GO:0060037	pharyngeal system development	14	0.250174664	0.0038	0.005666214
GO:0099624	atrial cardiac muscle cell membrane repolarization	6	0.25010725	0.0026	0.004377754
GO:0048873	homeostasis of number of cells within a tissue	16	0.25006435	0.0144	0.016652368
GO:1903749	positive regulation of establishment of protein localization to mitochondrion	36	0.24993565	0.0037	0.005561344
GO:0039692	single stranded viral RNA replication via double stranded DNA intermediate	7	0.24993565	0.0135	0.015731487
GO:0034260	negative regulation of GTPase activity	29	0.249891271	0.0118	0.013977955
GO:1904350	regulation of protein catabolic process in the vacuole	9	0.24973545	0.0308	0.032862615
GO:0072012	glomerulus vasculature development	16	0.249726512	0.0006	0.002177563
GO:0048864	stem cell development	35	0.249648833	0.0005	0.002177563
GO:0001754	eye photoreceptor cell differentiation	20	0.2496139	0.0057	0.007745437
GO:1901881	positive regulation of protein depolymerization	10	0.24960103	0.0008	0.002313249
GO:0048813	dendrite morphogenesis	45	0.24950093	0.0038	0.005666214
GO:1901099	negative regulation of signal transduction in absence of ligand	19	0.249488586	0.013	0.015198863
GO:2001240	negative regulation of extrinsic apoptotic signaling pathway in absence of ligand	19	0.249488586	0.013	0.015198863
GO:0050773	regulation of dendrite development	54	0.249478049	0.0089	0.011106266
GO:0035162	embryonic hemopoiesis	12	0.249442299	0.0151	0.01736105
GO:0002067	glandular epithelial cell differentiation	19	0.249244733	0.0047	0.006644432
GO:0035372	protein localization to microtubule	8	0.249227799	0.0018	0.003459409
GO:0007212	dopamine receptor signaling pathway	21	0.249163449	0.0006	0.002177563
GO:1904355	positive regulation of telomere capping	10	0.249009009	0.0029	0.004714719
GO:0090383	phagosome acidification	12	0.248991849	0.0185	0.020677043
GO:0008333	endosome to lysosome transport	33	0.248984049	0.0098	0.012008345
GO:0090153	regulation of sphingolipid biosynthetic process	12	0.248927499	0.0177	0.019919142
GO:1905038	regulation of membrane lipid metabolic process	12	0.248927499	0.0177	0.019919142
GO:2000303	regulation of ceramide biosynthetic process	12	0.248927499	0.0177	0.019919142
GO:0043653	mitochondrial fragmentation involved in apoptotic process	7	0.248906049	0.0078	0.009981807
GO:1904874	positive regulation of telomerase RNA localization to Cajal body	14	0.248887663	< 0.001	< 0.001
GO:0006590	thyroid hormone generation	6	0.248863149	0.0092	0.011424708
GO:0042403	thyroid hormone metabolic process	6	0.248863149	0.0092	0.011424708
GO:0006942	regulation of striated muscle contraction	37	0.248850395	0.0009	0.002399874
GO:0051937	catecholamine transport	27	0.248801182	< 0.001	< 0.001
GO:0015748	organophosphate ester transport	55	0.248732889	0.0137	0.015920879
GO:1990440	positive regulation of transcription from RNA polymerase II promoter in response to endoplasmic reticulum stress	10	0.248648649	0.0236	0.025731873
GO:0006862	nucleotide transport	15	0.248614329	0.039	0.040872718
GO:0006813	potassium ion transport	73	0.248613388	0.0124	0.014577606
GO:1901524	regulation of macromitophagy	10	0.248597169	0.0196	0.021765255
GO:0051445	regulation of meiotic cell cycle	15	0.248597169	0.0113	0.013488462
GO:2001239	regulation of extrinsic apoptotic signaling pathway in absence of ligand	27	0.248562849	0.016	0.018267288

GOIDlabel	GOName	size	categoryScores	pValPerm	pValPermCorr
GO:0048857	neural nucleus development	41	0.2485482	0.0082	0.010366713
GO:0044110	growth involved in symbiotic interaction	6	0.248519949	0.0035	0.005358035
GO:0044116	growth of symbiont involved in interaction with host	6	0.248519949	0.0035	0.005358035
GO:0044117	growth of symbiont in host	6	0.248519949	0.0035	0.005358035
GO:0044126	regulation of growth of symbiont in host	6	0.248519949	0.0035	0.005358035
GO:0044144	modulation of growth of symbiont involved in interaction with host	6	0.248519949	0.0035	0.005358035
GO:0014072	response to isoquinoline alkaloid	19	0.248404796	< 0.001	< 0.001
GO:0043278	response to morphine	19	0.248404796	< 0.001	< 0.001
GO:0008037	cell recognition	57	0.248382217	0.0034	0.005263104
GO:0034219	carbohydrate transmembrane transport	15	0.248339768	0.0096	0.01183839
GO:0001782	B cell homeostasis	20	0.248288288	0.0054	0.007432873
GO:0060021	palate development	38	0.248188038	0.0005	0.002177563
GO:0001578	microtubule bundle formation	39	0.248160248	< 0.001	< 0.001
GO:0010518	positive regulation of phospholipase activity	28	0.248115462	0.0008	0.002313249
GO:0099563	modification of synaptic structure	8	0.248069498	0.0216	0.023746214
GO:0033631	cell-cell adhesion mediated by integrin	10	0.248056628	0.0056	0.007655255
GO:0060900	embryonic camera-type eye formation	7	0.248023534	0.0117	0.013882722
GO:0048557	embryonic digestive tract morphogenesis	7	0.248023534	0.0312	0.033261559
GO:2000648	positive regulation of stem cell proliferation	22	0.247864748	0.0015	0.003088691
GO:0019751	polyol metabolic process	68	0.247796957	0.0076	0.009790742
GO:1900077	negative regulation of cellular response to insulin stimulus	17	0.247785601	0.0031	0.004916949
GO:0006646	phosphatidylethanolamine biosynthetic process	10	0.247747748	0.0019	0.003579279
GO:0050804	modulation of synaptic transmission	133	0.247733233	0.0014	0.002975058
GO:0030949	positive regulation of vascular endothelial growth factor receptor signaling pathway	8	0.247715573	0.0285	0.030556948
GO:0007339	binding of sperm to zona pellucida	17	0.247679612	0.0003	0.001720882
GO:0002090	regulation of receptor internalization	20	0.247541828	0.0067	0.008845888
GO:0007405	neuroblast proliferation	32	0.247369691	0.0015	0.003088691
GO:0060600	dichotomous subdivision of an epithelial terminal unit	5	0.247361647	0.0017	0.003334595
GO:0045773	positive regulation of axon extension	21	0.24734939	0.0244	0.02650438
GO:0050433	regulation of catecholamine secretion	18	0.247290147	< 0.001	< 0.001
GO:0006020	inositol metabolic process	7	0.247288104	0.0335	0.03551156
GO:0032633	interleukin-4 production	17	0.247225377	0.0098	0.012008345
GO:0097194	execution phase of apoptosis	43	0.247200024	0.0037	0.005561344
GO:0006720	isoprenoid metabolic process	64	0.247180663	0.0019	0.003579279
GO:0072539	T-helper 17 cell differentiation	9	0.247132847	0.0152	0.017453982
GO:0045722	positive regulation of gluconeogenesis	11	0.247127647	0.0014	0.002975058
GO:1900744	regulation of p38MAPK cascade	22	0.246998947	0.021	0.023166522
GO:0072015	glomerular visceral epithelial cell development	7	0.246993933	0.0204	0.022555378
GO:0072310	glomerular epithelial cell development	7	0.246993933	0.0204	0.022555378
GO:0050820	positive regulation of coagulation	13	0.246965647	0.0099	0.012121556
GO:0031268	pseudopodium organization	8	0.246943372	0.018	0.020203241
GO:0031269	pseudopodium assembly	8	0.246943372	0.018	0.020203241
GO:0060706	cell differentiation involved in embryonic placenta development	13	0.246926047	0.0039	0.005778819
GO:0030193	regulation of blood coagulation	39	0.246912847	0.0079	0.010077402
GO:0018065	protein-cofactor linkage	10	0.246898327	0.0003	0.001720882
GO:0045006	DNA deamination	5	0.246846847	0.0013	0.002859821
GO:0014032	neural crest cell development	32	0.246742278	0.0018	0.003459409
GO:0036158	outer dynein arm assembly	6	0.246675247	0.0015	0.003088691
GO:0046596	regulation of viral entry into host cell	23	0.246656594	0.0437	0.045424906
GO:0000098	sulfur amino acid catabolic process	9	0.246618047	0.0011	0.002610912
GO:0046621	negative regulation of organ growth	19	0.246589447	0.0141	0.016361854
GO:0006101	citrate metabolic process	24	0.246589447	0.0265	0.028585731
GO:0006112	energy reserve metabolic process	52	0.246554797	0.0007	0.002248538
GO:1902742	apoptotic process involved in development	23	0.246499916	0.0043	0.006204188
GO:0048704	embryonic skeletal system morphogenesis	41	0.246451329	0.0022	0.003910907
GO:0010517	regulation of phospholipase activity	33	0.246394446	0.0014	0.002975058
GO:0006023	aminoglycan biosynthetic process	55	0.246369486	0.0022	0.003910907
GO:0040001	establishment of mitotic spindle localization	15	0.246314886	0.0077	0.009887588
GO:0015865	purine nucleotide transport	11	0.246308646	0.0323	0.034307983
GO:0015868	purine ribonucleotide transport	11	0.246308646	0.0323	0.034307983
GO:0045823	positive regulation of heart contraction	16	0.246235521	0.0096	0.01183839
GO:1902369	negative regulation of RNA catabolic process	8	0.246235521	< 0.001	< 0.001
GO:1902373	negative regulation of mRNA catabolic process	8	0.246235521	< 0.001	< 0.001
GO:0048284	organelle fusion	82	0.246146844	0.0215	0.023664853
GO:0050818	regulation of coagulation	42	0.246111418	0.0102	0.012424427
GO:0030166	proteoglycan biosynthetic process	30	0.246048906	0.0168	0.019054351
GO:0060068	vagina development	6	0.245988846	0.0286	0.030648697
GO:0045091	regulation of single stranded viral RNA replication via double stranded DNA intermediate	6	0.245860146	0.0165	0.018764186
GO:0023058	adaptation of signaling pathway	9	0.245817246	0.024	0.026109935
GO:0072672	neutrophil extravasation	6	0.245817246	0.0218	0.023945433
GO:0038084	vascular endothelial growth factor signaling pathway	23	0.245705333	0.0117	0.013882722
GO:0030104	water homeostasis	25	0.245662806	0.001	0.002498236
GO:0006536	glutamate metabolic process	21	0.24558436	0.0002	0.001341684
GO:0048278	vesicle docking	19	0.245478561	0.0056	0.007655255
GO:0014854	response to inactivity	8	0.24546332	0.0319	0.033911376
GO:0044320	cellular response to leptin stimulus	10	0.245456885	0.0004	0.002007244
GO:0097479	synaptic vesicle localization	47	0.245335305	0.0039	0.005778819
GO:0090231	regulation of spindle checkpoint	10	0.245302445	< 0.001	< 0.001
GO:0090266	regulation of mitotic cell cycle spindle assembly checkpoint	10	0.245302445	< 0.001	< 0.001
GO:1903504	regulation of mitotic spindle checkpoint	10	0.245302445	< 0.001	< 0.001
GO:0034214	protein hexamerization	12	0.245045045	0.0101	0.012328539
GO:0015740	C4-dicarboxylate transport	9	0.245016445	0.0082	0.010366713
GO:0010644	cell communication by electrical coupling	10	0.244942085	0.0464	0.04806684

GOIDlabel	GOName	size	categoryScores	pValPerm	pValPermCorr
GO:1904645	response to beta-amyloid	18	0.244916345	0.0311	0.033166048
GO:2000319	regulation of T-helper 17 cell differentiation	6	0.244873445	0.0022	0.003910907
GO:0042551	neuron maturation	24	0.244830545	0.0172	0.019442284
GO:0048286	lung alveolus development	22	0.244822745	0.0034	0.005263104
GO:0043011	myeloid dendritic cell differentiation	11	0.244764245	0.0034	0.005263104
GO:1902959	regulation of aspartic-type endopeptidase activity involved in amyloid precursor protein catabolic process	6	0.244744745	0.0178	0.020021073
GO:0006688	glycosphingolipid biosynthetic process	12	0.244723295	0.0119	0.014078095
GO:0030397	membrane disassembly	10	0.244684685	0.0026	0.004377754
GO:0051081	nuclear envelope disassembly	10	0.244684685	0.0026	0.004377754
GO:1903725	regulation of phospholipid metabolic process	49	0.244640559	0.0019	0.003579279
GO:0090073	positive regulation of protein homodimerization activity	10	0.244581725	0.038	0.03991002
GO:0071709	membrane assembly	16	0.24456242	0.023	0.025116347
GO:0070050	neuron cellular homeostasis	8	0.24456242	0.0055	0.007544447
GO:0048489	synaptic vesicle transport	45	0.244513085	0.0055	0.007544447
GO:0097480	establishment of synaptic vesicle localization	45	0.244513085	0.0055	0.007544447
GO:0010524	positive regulation of calcium ion transport into cytosol	23	0.244429523	0.0161	0.018371585
GO:0071398	cellular response to fatty acid	30	0.244375804	0.0004	0.002007244
GO:0032232	negative regulation of actin filament bundle assembly	13	0.244371844	0.0032	0.005044175
GO:0051497	negative regulation of stress fiber assembly	13	0.244371844	0.0032	0.005044175
GO:2000060	positive regulation of protein ubiquitination involved in ubiquitin-dependent protein catabolic process	7	0.244346387	0.004	0.005880507
GO:0048103	somatic stem cell division	9	0.244330044	0.0046	0.006532041
GO:1903035	negative regulation of response to wounding	38	0.244327034	0.0149	0.017165166
GO:0045214	sarcomere organization	16	0.244288932	0.0106	0.012825859
GO:0032252	secretory granule localization	6	0.244187044	0.0175	0.019742875
GO:0007202	activation of phospholipase C activity	13	0.244134244	0.0019	0.003579279
GO:0006534	cysteine metabolic process	13	0.244035244	0.0005	0.002177563
GO:1903541	regulation of exosomal secretion	13	0.244035244	0.0162	0.018465856
GO:1903543	positive regulation of exosomal secretion	13	0.244035244	0.0162	0.018465856
GO:0002029	desensitization of G-protein coupled receptor protein signaling pathway	8	0.244015444	0.0184	0.020586938
GO:0022401	negative adaptation of signaling pathway	8	0.244015444	0.0184	0.020586938
GO:0070863	positive regulation of protein exit from endoplasmic reticulum	6	0.243972544	0.0415	0.043328883
GO:0036017	response to erythropoietin	5	0.243809524	0.0196	0.021765255
GO:0072010	glomerular epithelium development	12	0.243779494	0.0296	0.031661766
GO:0006290	pyrimidine dimer repair	7	0.243758044	0.0205	0.022658082
GO:0048645	animal organ formation	29	0.243731416	0.0186	0.020774238
GO:0006655	phosphatidylglycerol biosynthetic process	8	0.243725869	0.0074	0.009581512
GO:0006904	vesicle docking involved in exocytosis	15	0.243723724	0.0037	0.005561344
GO:0046348	amino sugar catabolic process	5	0.243603604	0.0072	0.009362497
GO:0032621	interleukin-18 production	5	0.243603604	0.0005	0.002177563
GO:0032661	regulation of interleukin-18 production	5	0.243603604	0.0005	0.002177563
GO:0009155	purine deoxyribonucleotide catabolic process	5	0.243603604	0.0007	0.002248538
GO:0009217	purine deoxyribonucleoside triphosphate catabolic process	5	0.243603604	0.0007	0.002248538
GO:0006024	glycosaminoglycan biosynthetic process	54	0.243595977	0.0022	0.003910907
GO:0007612	learning	51	0.243591491	0.0037	0.005561344
GO:0032836	glomerular basement membrane development	5	0.243552124	0.0411	0.042939393
GO:0015914	phospholipid transport	39	0.243546844	0.0066	0.008731949
GO:0043981	histone H4-K5 acetylation	13	0.243480843	0.0069	0.009046225
GO:0043982	histone H4-K8 acetylation	13	0.243480843	0.0069	0.009046225
GO:0090110	cargo loading into COPII-coated vesicle	11	0.243453843	0.011	0.013192134
GO:0050832	defense response to fungus	13	0.243401643	0.0322	0.034224583
GO:0032049	cardiolipin biosynthetic process	7	0.243316786	0.0039	0.005778819
GO:0051657	maintenance of organelle location	9	0.243300443	0.0193	0.021465777
GO:0042447	hormone catabolic process	6	0.243243243	0.0071	0.009268394
GO:0007200	phospholipase C-activating G-protein coupled receptor signaling pathway	31	0.24323494	0.0054	0.007432873
GO:0060920	cardiac pacemaker cell differentiation	5	0.243191763	0.0073	0.009463568
GO:0008053	mitochondrial fusion	17	0.243167537	0.0037	0.005561344
GO:1900274	regulation of phospholipase C activity	22	0.243161343	0.0014	0.002975058
GO:0044060	regulation of endocrine process	26	0.243055143	0.0029	0.004714719
GO:0003263	cardioblast proliferation	6	0.243028743	0.0353	0.037246175
GO:0003264	regulation of cardioblast proliferation	6	0.243028743	0.0353	0.037246175
GO:0033630	positive regulation of cell adhesion mediated by integrin	14	0.242967457	0.003	0.004825593
GO:0048169	regulation of long-term neuronal synaptic plasticity	7	0.242875529	0.005	0.006974174
GO:0003309	type B pancreatic cell differentiation	9	0.242871443	0.0024	0.004156304
GO:0050926	regulation of positive chemotaxis	11	0.242868843	0.0169	0.019147324
GO:0034122	negative regulation of toll-like receptor signaling pathway	16	0.242857143	0.0007	0.002248538
GO:0086002	cardiac muscle cell action potential involved in contraction	23	0.242851547	0.012	0.014185865
GO:0050918	positive chemotaxis	28	0.242737636	0.0105	0.012724187
GO:0040019	positive regulation of embryonic development	19	0.242660706	0.0009	0.002399874
GO:0034453	microtubule anchoring	11	0.242658243	0.0035	0.005358035
GO:0001764	neuron migration	65	0.242625483	0.0007	0.002248538
GO:0060291	long-term synaptic potentiation	26	0.242292842	0.0036	0.005475609
GO:0060442	branching involved in prostate gland morphogenesis	7	0.242029785	0.0476	0.049237916
GO:0010452	histone H3-K36 methylation	8	0.241891892	0.0199	0.022056122
GO:0001958	endochondral ossification	14	0.241827542	0.0053	0.007330056
GO:0036075	replacement ossification	14	0.241827542	0.0053	0.007330056
GO:0002043	blood vessel endothelial cell proliferation involved in sprouting angiogenesis	9	0.241784642	0.0062	0.008281828
GO:0051180	vitamin transport	16	0.241666667	0.0342	0.036175369
GO:1901525	negative regulation of macromitophagy	5	0.241595882	0.0383	0.040185364
GO:0006721	terpenoid metabolic process	52	0.241555292	0.0026	0.004377754
GO:0007205	protein kinase C-activating G-protein coupled receptor signaling pathway	9	0.241527242	0.0015	0.003088691
GO:1903649	regulation of cytoplasmic transport	13	0.241520642	0.0158	0.018074565
GO:0010799	regulation of peptidyl-threonine phosphorylation	16	0.241425354	0.0028	0.004605006
GO:0001523	retinoid metabolic process	43	0.241411511	0.0027	0.004491543

GOIDlabel	GOName	size	categoryScores	pValPerm	pValPermCorr
GO:0045730	respiratory burst	17	0.241396018	0.0039	0.005778819
GO:1902305	regulation of sodium ion transmembrane transport	26	0.241362241	0.0054	0.007432873
GO:0046929	negative regulation of neurotransmitter secretion	8	0.241344916	0.0202	0.02235752
GO:0015858	nucleoside transport	14	0.241331127	0.0336	0.03560572
GO:0033628	regulation of cell adhesion mediated by integrin	29	0.241263924	0.0019	0.003579279
GO:0070528	protein kinase C signaling	13	0.241263241	0.0036	0.005475609
GO:0002675	positive regulation of acute inflammatory response	17	0.241229465	0.003	0.004825593
GO:0048012	hepatocyte growth factor receptor signaling pathway	7	0.241220813	0.0142	0.016456919
GO:0021814	cell motility involved in cerebral cortex radial glia guided migration	5	0.241132561	0.0057	0.007745437
GO:0097576	vacuole fusion	28	0.241110498	0.0389	0.040788039
GO:0090140	regulation of mitochondrial fission	17	0.241093194	0.0375	0.039404369
GO:0045869	negative regulation of single stranded viral RNA replication via double stranded DNA intermediate	5	0.241081081	0.0163	0.018559925
GO:0051503	adenine nucleotide transport	10	0.240952381	0.0314	0.033430037
GO:0048706	embryonic skeletal system development	54	0.240898041	0.0052	0.007215241
GO:0044091	membrane biogenesis	20	0.240823681	0.0137	0.015920879
GO:0001539	cilium or flagellum-dependent cell motility	10	0.240772201	< 0.001	< 0.001
GO:0060285	cilium-dependent cell motility	10	0.240772201	< 0.001	< 0.001
GO:0060259	regulation of feeding behavior	12	0.240733591	< 0.001	< 0.001
GO:0042228	interleukin-8 biosynthetic process	7	0.240706012	0.0013	0.002859821
GO:0045414	regulation of interleukin-8 biosynthetic process	7	0.240706012	0.0013	0.002859821
GO:0070306	lens fiber cell differentiation	16	0.240540541	0.0022	0.003910907
GO:2000010	positive regulation of protein localization to cell surface	12	0.24041184	0.0019	0.003579279
GO:0015721	bile acid and bile salt transport	8	0.240379665	0.0108	0.013015961
GO:0032413	negative regulation of ion transmembrane transporter activity	27	0.240316507	0.0014	0.002975058
GO:0051293	establishment of spindle localization	20	0.24029601	0.0097	0.011922488
GO:0051014	actin filament severing	6	0.24028314	0.0098	0.012008345
GO:0043649	dicarboxylic acid catabolic process	11	0.24027144	0.0015	0.003088691
GO:0043114	regulation of vascular permeability	16	0.240267053	0.02	0.022155397
GO:0097061	dendritic spine organization	24	0.24011154	0.0033	0.005149584
GO:0009310	amine catabolic process	14	0.240080897	0.0061	0.008182551
GO:0042402	cellular biogenic amine catabolic process	14	0.240080897	0.0061	0.008182551
GO:0070365	hepatocyte differentiation	5	0.23994852	0.0039	0.005778819
GO:0051489	regulation of filopodium assembly	19	0.239734471	0.0018	0.003459409
GO:0051966	regulation of synaptic transmission, glutamatergic	14	0.239547711	0.0077	0.009887588
GO:0033625	positive regulation of integrin activation	5	0.23953668	0.0003	0.001720882
GO:0097734	extracellular exosome biogenesis	15	0.23950236	0.0223	0.024414688
GO:0002091	negative regulation of receptor internalization	5	0.239485199	0.0144	0.016652368
GO:0009060	aerobic respiration	38	0.239382239	0.0245	0.026594873
GO:0009620	response to fungus	20	0.239369369	0.0119	0.014078095
GO:0071599	otic vesicle development	8	0.239028314	0.0062	0.008281828
GO:0046439	L-cysteine metabolic process	7	0.238940982	0.002	0.003693422
GO:0055119	relaxation of cardiac muscle	10	0.238918919	0.0104	0.012619802
GO:0051645	Golgi localization	8	0.238899614	0.0258	0.02789201
GO:0071850	mitotic cell cycle arrest	12	0.238867439	0.0057	0.007745437
GO:0097352	autophagosome maturation	27	0.238791172	0.0389	0.040788039
GO:0090185	negative regulation of kidney development	6	0.238781639	0.0048	0.006748379
GO:0051589	negative regulation of neurotransmitter transport	10	0.238738739	0.0152	0.017453982
GO:0007341	penetration of zona pellucida	5	0.238712999	0.0113	0.013488462
GO:0071609	chemokine (C-C motif) ligand 5 production	5	0.238661519	0.0242	0.02630058
GO:0042118	endothelial cell activation	7	0.238610039	0.0109	0.013101792
GO:1900272	negative regulation of long-term synaptic potentiation	7	0.238610039	0.0025	0.004279479
GO:0003208	cardiac ventricle morphogenesis	45	0.238592879	0.0036	0.005475609
GO:0021795	cerebral cortex cell migration	20	0.238584299	0.0007	0.002248538
GO:0060074	synapse maturation	7	0.238536496	0.0406	0.042451813
GO:0003206	cardiac chamber morphogenesis	69	0.238483204	0.0018	0.003459409
GO:0030194	positive regulation of blood coagulation	12	0.238481338	0.0097	0.011922488
GO:1900048	positive regulation of hemostasis	12	0.238481338	0.0097	0.011922488
GO:0035272	exocrine system development	32	0.238433076	0.0104	0.012619802
GO:0034141	positive regulation of toll-like receptor 3 signaling pathway	5	0.238404118	0.016	0.018267288
GO:0072567	chemokine (C-X-C motif) ligand 2 production	10	0.238352638	0.0012	0.002741075
GO:0006953	acute-phase response	22	0.238340938	0.0023	0.004032435
GO:0030325	adrenal gland development	15	0.238266838	0.0142	0.016456919
GO:0060413	atrial septum morphogenesis	9	0.238181038	< 0.001	< 0.001
GO:0032780	negative regulation of ATPase activity	10	0.238146718	0.0025	0.004279479
GO:0031114	regulation of microtubule depolymerization	11	0.238118638	0.0401	0.041935888
GO:0010561	negative regulation of glycoprotein biosynthetic process	12	0.237945088	0.0431	0.044867086
GO:0032309	icosanoid secretion	18	0.237923638	0.0032	0.005044175
GO:0071715	icosanoid transport	18	0.237923638	0.0032	0.005044175
GO:1901571	fatty acid derivative transport	18	0.237923638	0.0032	0.005044175
GO:0021955	central nervous system neuron axonogenesis	12	0.237859288	0.0026	0.004377754
GO:0060117	auditory receptor cell development	12	0.237837838	0.0008	0.002313249
GO:0019626	short-chain fatty acid catabolic process	5	0.237683398	0.0298	0.031859654
GO:0061029	eyelid development in camera-type eye	9	0.237637638	0.0182	0.020388223
GO:0010863	positive regulation of phospholipase C activity	21	0.237617209	0.0012	0.002741075
GO:0002092	positive regulation of receptor internalization	14	0.237598823	0.0174	0.019643968
GO:0032793	positive regulation of CREB transcription factor activity	6	0.237580438	0.0112	0.013379119
GO:0097152	mesenchymal cell apoptotic process	6	0.237537538	0.007	0.009166016
GO:0051294	establishment of spindle orientation	16	0.237532175	0.0102	0.012424427
GO:0006107	oxaloacetate metabolic process	5	0.237425997	0.0048	0.006748379
GO:0086014	atrial cardiac muscle cell action potential	10	0.237323037	0.0369	0.038812296
GO:0086026	atrial cardiac muscle cell to AV node cell signaling	10	0.237323037	0.0369	0.038812296
GO:0086066	atrial cardiac muscle cell to AV node cell communication	10	0.237323037	0.0369	0.038812296
GO:0006600	creatine metabolic process	6	0.237237237	0.0333	0.035317174

GOIDlabel	GOName	size	categoryScores	pValPerm	pValPermCorr
GO:0086019	cell-cell signaling involved in cardiac conduction	12	0.237172887	0.0103	0.012519912
GO:0046337	phosphatidylethanolamine metabolic process	16	0.237162162	0.0013	0.002859821
GO:0032957	inositol trisphosphate metabolic process	7	0.23713918	0.0033	0.005149584
GO:0006449	regulation of translational termination	7	0.23713918	0.0395	0.041342339
GO:0032278	positive regulation of gonadotropin secretion	5	0.237117117	0.0473	0.048935536
GO:0034314	Arp2/3 complex-mediated actin nucleation	11	0.237112437	0.0015	0.003088691
GO:0060252	positive regulation of glial cell proliferation	9	0.236979837	0.0089	0.011106266
GO:0030216	keratinocyte differentiation	114	0.236945969	0.0162	0.018465856
GO:0030501	positive regulation of bone mineralization	19	0.236916616	0.0002	0.001341684
GO:0007016	cytoskeletal anchoring at plasma membrane	7	0.236734694	0.0295	0.031565396
GO:0006538	glutamate catabolic process	8	0.236711712	0.0045	0.006417207
GO:0001505	regulation of neurotransmitter levels	80	0.236631274	0.0006	0.002177563
GO:1901018	positive regulation of potassium ion transmembrane transporter activity	10	0.236576577	0.0395	0.041342339
GO:0032673	regulation of interleukin-4 production	16	0.236389961	0.0108	0.013015961
GO:0048009	insulin-like growth factor receptor signaling pathway	20	0.236267696	0.0078	0.009981807
GO:0050927	positive regulation of positive chemotaxis	10	0.236241956	0.0176	0.019838132
GO:0030049	muscle filament sliding	17	0.236111742	< 0.001	< 0.001
GO:0033275	actin-myosin filament sliding	17	0.236111742	< 0.001	< 0.001
GO:1990182	exosomal secretion	14	0.236109579	0.0221	0.024229021
GO:2000649	regulation of sodium ion transmembrane transporter activity	20	0.236087516	0.006	0.00807902
GO:0097035	regulation of membrane lipid distribution	25	0.236036036	0.0043	0.006204188
GO:0035860	glial cell-derived neurotrophic factor receptor signaling pathway	6	0.236036036	0.0003	0.001720882
GO:0002028	regulation of sodium ion transport	38	0.235907336	0.0062	0.008281828
GO:1903977	positive regulation of glial cell migration	6	0.235907336	0.0425	0.044271412
GO:0046579	positive regulation of Ras protein signal transduction	22	0.235860536	0.0009	0.002399874
GO:0042181	ketone biosynthetic process	25	0.235830116	0.0184	0.020586938
GO:0098900	regulation of action potential	17	0.235718071	0.006	0.00807902
GO:0010676	positive regulation of cellular carbohydrate metabolic process	31	0.23571221	0.0007	0.002248538
GO:0007512	adult heart development	10	0.235675676	0.0061	0.008182551
GO:0021984	adenohypophysis development	9	0.235607036	0.044	0.045721833
GO:2001223	negative regulation of neuron migration	7	0.235594778	0.0006	0.002177563
GO:2001028	positive regulation of endothelial cell chemotaxis	11	0.235591436	0.0002	0.001341684
GO:0055012	ventricular cardiac muscle cell differentiation	7	0.235521236	0.0306	0.032676575
GO:0055015	ventricular cardiac muscle cell development	7	0.235521236	0.0306	0.032676575
GO:0051653	spindle localization	21	0.235508978	0.0097	0.011922488
GO:0007026	negative regulation of microtubule depolymerization	10	0.235495495	0.0418	0.043620665
GO:0009190	cyclic nucleotide biosynthetic process	27	0.235321035	0.0069	0.009046225
GO:0052652	cyclic purine nucleotide metabolic process	27	0.235321035	0.0069	0.009046225
GO:0070989	oxidative demethylation	10	0.235315315	0.0013	0.002859821
GO:0048820	hair follicle maturation	7	0.235263835	0.0097	0.011922488
GO:0007431	salivary gland development	25	0.235212355	0.0114	0.01358747
GO:0015988	energy coupled proton transmembrane transport, against electrochemical gradient	11	0.235170235	0.017	0.019243517
GO:0015991	ATP hydrolysis coupled proton transport	11	0.235170235	0.017	0.019243517
GO:0090662	ATP hydrolysis coupled transmembrane transport	11	0.235170235	0.017	0.019243517
GO:1903715	regulation of aerobic respiration	6	0.235135135	0.0448	0.046530378
GO:0086001	cardiac muscle cell action potential	28	0.235043206	0.0164	0.018653793
GO:0072537	fibroblast activation	8	0.235006435	0.0202	0.02235752
GO:0050482	arachidonic acid secretion	13	0.234966835	0.0026	0.004377754
GO:1903963	arachidonate transport	13	0.234966835	0.0026	0.004377754
GO:0044070	regulation of anion transport	39	0.234953635	0.0098	0.012008345
GO:0018146	keratan sulfate biosynthetic process	14	0.234914506	0.0018	0.003459409
GO:0048854	brain morphogenesis	14	0.234877735	0.0167	0.018954426
GO:0007501	mesodermal cell fate specification	6	0.234749035	< 0.001	< 0.001
GO:0072583	clathrin-dependent endocytosis	27	0.234701368	0.0194	0.021561946
GO:0071391	cellular response to estrogen stimulus	10	0.234671815	0.0068	0.008946408
GO:2000300	regulation of synaptic vesicle exocytosis	19	0.23466775	0.009	0.011215682
GO:2000833	positive regulation of steroid hormone secretion	7	0.23463872	< 0.001	< 0.001
GO:0048814	regulation of dendrite morphogenesis	30	0.234448734	0.0117	0.013882722
GO:0006835	dicarboxylic acid transport	35	0.234366612	0.0077	0.009887588
GO:0018149	peptide cross-linking	12	0.234277134	0.0325	0.034497418
GO:0002026	regulation of the force of heart contraction	13	0.234254034	0.0014	0.002975058
GO:2000380	regulation of mesoderm development	7	0.234197463	0.0006	0.002177563
GO:0014029	neural crest formation	6	0.234191334	0.0051	0.007095023
GO:0046068	cGMP metabolic process	9	0.234177034	0.0294	0.031463678
GO:1900047	negative regulation of hemostasis	23	0.234099938	0.008	0.010168329
GO:2000848	positive regulation of corticosteroid hormone secretion	5	0.234028314	0.0046	0.006532041
GO:0007218	neuropeptide signaling pathway	31	0.23399344	< 0.001	< 0.001
GO:0042368	vitamin D biosynthetic process	6	0.233976834	0.0409	0.042751468
GO:0061318	renal filtration cell differentiation	9	0.233919634	0.0226	0.024717659
GO:0072112	glomerular visceral epithelial cell differentiation	9	0.233919634	0.0226	0.024717659
GO:0072311	glomerular epithelial cell differentiation	9	0.233919634	0.0226	0.024717659
GO:0051770	positive regulation of nitric-oxide synthase biosynthetic process	10	0.233899614	0.0007	0.002248538
GO:0030808	regulation of nucleotide biosynthetic process	35	0.233851811	0.0115	0.013693853
GO:1900371	regulation of purine nucleotide biosynthetic process	35	0.233851811	0.0115	0.013693853
GO:0042481	regulation of odontogenesis	11	0.233836434	0.0313	0.033357007
GO:0009143	nucleoside triphosphate catabolic process	10	0.233616474	0.0028	0.004605006
GO:0032695	negative regulation of interleukin-12 production	8	0.233429858	0.0054	0.007432873
GO:2001171	positive regulation of ATP biosynthetic process	8	0.233397683	0.0022	0.003910907
GO:1902108	regulation of mitochondrial membrane permeability involved in apoptotic process	37	0.233350725	0.0197	0.021861066
GO:0010737	protein kinase A signaling	17	0.233340904	0.0148	0.017062301
GO:0006007	glucose catabolic process	19	0.233285917	0.0179	0.020129998
GO:0002534	cytokine production involved in inflammatory response	22	0.233228033	0.0021	0.003804235
GO:1900015	regulation of cytokine production involved in inflammatory response	22	0.233228033	0.0021	0.003804235

GOIDlabel	GOName	size	categoryScores	pValPerm	pValPermCorr
GO:0039694	viral RNA genome replication	15	0.233170313	0.0159	0.01817591
GO:0039703	RNA replication	15	0.233170313	0.0159	0.01817591
GO:2000252	negative regulation of feeding behavior	5	0.233153153	0.0004	0.002007244
GO:0032890	regulation of organic acid transport	25	0.233153153	0.0122	0.014387602
GO:1900044	regulation of protein K63-linked ubiquitination	7	0.23313109	0.0276	0.029671862
GO:0015837	amine transport	34	0.232954804	0.0001	0.000877824
GO:0019048	modulation by virus of host morphology or physiology	25	0.232947233	0.013	0.015198863
GO:0032808	lacrimal gland development	6	0.232861433	0.0076	0.009790742
GO:0050905	neuromuscular process	46	0.232840915	0.0013	0.002859821
GO:0009065	glutamine family amino acid catabolic process	15	0.232792793	0.0041	0.005981983
GO:0034310	primary alcohol catabolic process	5	0.232689833	0.0348	0.036785603
GO:0003215	cardiac right ventricle morphogenesis	11	0.232666433	0.0001	0.000877824
GO:0060174	limb bud formation	6	0.232604033	0.0021	0.003804235
GO:0010812	negative regulation of cell-substrate adhesion	31	0.232515465	0.0244	0.02650438
GO:0042635	positive regulation of hair cycle	7	0.232432432	0.0011	0.002610912
GO:0007379	segment specification	5	0.232432432	0.0004	0.002007244
GO:1902803	regulation of synaptic vesicle transport	25	0.23241184	0.0181	0.020286898
GO:0015846	polyamine transport	6	0.232346632	0.0473	0.048935536
GO:0033632	regulation of cell-cell adhesion mediated by integrin	7	0.232285347	0.0256	0.02770399
GO:0032228	regulation of synaptic transmission, GABAergic	14	0.232101489	0.0005	0.002177563
GO:0090325	regulation of locomotion involved in locomotory behavior	5	0.231814672	0.0156	0.017858595
GO:0035249	synaptic transmission, glutamatergic	17	0.231811644	0.0034	0.005263104
GO:0010765	positive regulation of sodium ion transport	14	0.231788932	0.0076	0.009790742
GO:0071415	cellular response to purine-containing compound	7	0.231770546	0.0214	0.023571068
GO:0030575	nuclear body organization	10	0.231763192	0.0101	0.012328539
GO:0031111	negative regulation of microtubule polymerization or depolymerization	15	0.231574432	0.0433	0.045060565
GO:0051952	regulation of amine transport	31	0.231436044	0.0002	0.001341684
GO:0046847	filopodium assembly	29	0.231331824	0.0031	0.004916949
GO:1904646	cellular response to beta-amyloid	16	0.231081081	0.0361	0.038033609
GO:0034384	high-density lipoprotein particle clearance	6	0.231016731	0.0006	0.002177563
GO:0048384	retinoic acid receptor signaling pathway	18	0.231016731	0.0077	0.009887588
GO:0006071	glycerol metabolic process	12	0.230930931	0.0107	0.012912536
GO:0016339	calcium-dependent cell-cell adhesion via plasma membrane cell adhesion molecules	9	0.230916631	0.0236	0.025731873
GO:0021799	cerebral cortex radially oriented cell migration	12	0.230909481	0.0045	0.006417207
GO:0006171	cAMP biosynthetic process	21	0.230839002	0.015	0.017255415
GO:0030814	regulation of cAMP metabolic process	21	0.230839002	0.015	0.017255415
GO:0030817	regulation of cAMP biosynthetic process	21	0.230839002	0.015	0.017255415
GO:0045761	regulation of adenylate cyclase activity	21	0.230839002	0.015	0.017255415
GO:0036035	osteoclast development	7	0.230630631	0.0257	0.027793331
GO:0008299	isoprenoid biosynthetic process	15	0.230630631	0.0025	0.004279479
GO:0044003	modification by symbiont of host morphology or physiology	32	0.230542149	0.0171	0.01934984
GO:0001941	postsynaptic membrane organization	15	0.230527671	0.0007	0.002248538
GO:0097345	mitochondrial outer membrane permeabilization	33	0.23034983	0.0177	0.019919142
GO:0032352	positive regulation of hormone metabolic process	9	0.23031603	0.0086	0.010769857
GO:1905331	negative regulation of morphogenesis of an epithelium	9	0.23031603	0.024	0.026109935
GO:0043252	sodium-independent organic anion transport	5	0.23016731	0.0285	0.030556948
GO:0003266	regulation of secondary heart field cardioblast proliferation	5	0.23006435	0.0366	0.038528543
GO:0021520	spinal cord motor neuron cell fate specification	5	0.22996139	0.0053	0.007330056
GO:0050892	intestinal absorption	15	0.22992707	0.004	0.005880507
GO:1900452	regulation of long term synaptic depression	5	0.22980695	0.0005	0.002177563
GO:0010907	positive regulation of glucose metabolic process	25	0.229734878	0.0009	0.002399874
GO:0048261	negative regulation of receptor-mediated endocytosis	13	0.22971983	0.0086	0.010769857
GO:0034204	lipid translocation	15	0.22966967	0.0068	0.008946408
GO:0045332	phospholipid translocation	15	0.22966967	0.0068	0.008946408
GO:0060999	positive regulation of dendritic spine development	15	0.22960103	0.0007	0.002248538
GO:0008542	visual learning	17	0.229570747	0.006	0.00807902
GO:0009187	cyclic nucleotide metabolic process	37	0.229545375	0.014	0.01625173
GO:0051798	positive regulation of hair follicle development	6	0.229386529	0.0004	0.002007244
GO:0031663	lipopolysaccharide-mediated signaling pathway	30	0.229377949	0.004	0.005880507
GO:1901317	regulation of flagellated sperm motility	6	0.229300729	0.0019	0.003579279
GO:1902337	regulation of apoptotic process involved in morphogenesis	8	0.229150579	0.0154	0.017651835
GO:1904748	regulation of apoptotic process involved in development	8	0.229150579	0.0154	0.017651835
GO:2000272	negative regulation of receptor activity	26	0.229016929	0.0011	0.002610912
GO:0006814	sodium ion transport	90	0.228986129	0.0038	0.005666214
GO:0007632	visual behavior	18	0.228971829	0.003	0.004825593
GO:0032924	activin receptor signaling pathway	16	0.228844916	0.019	0.02117646
GO:0000266	mitochondrial fission	21	0.228804315	0.0343	0.036275124
GO:1903624	regulation of DNA catabolic process	9	0.228800229	0.0178	0.020021073
GO:0070292	N-acylphosphatidylethanolamine metabolic process	5	0.228777349	0.0033	0.005149584
GO:0001672	regulation of chromatin assembly or disassembly	6	0.228743029	0.0198	0.02196821
GO:1900006	positive regulation of dendrite development	32	0.228684041	0.0038	0.005666214
GO:0061045	negative regulation of wound healing	33	0.228563629	0.015	0.017255415
GO:0050909	sensory perception of taste	21	0.228485628	0.0164	0.018653793
GO:1903392	negative regulation of adherens junction organization	14	0.228258871	0.0263	0.02839402
GO:0003230	cardiac atrium development	19	0.228232744	0.0018	0.003459409
GO:1902686	mitochondrial outer membrane permeabilization involved in programmed cell death	36	0.228178178	0.0196	0.021765255
GO:1900119	positive regulation of execution phase of apoptosis	11	0.228150228	0.0039	0.005778819
GO:0003334	keratinocyte development	6	0.228142428	0.0009	0.002399874
GO:0090030	regulation of steroid hormone biosynthetic process	5	0.228056628	0.0018	0.003459409
GO:0055081	anion homeostasis	20	0.227992278	0.0017	0.003334595
GO:0060561	apoptotic process involved in morphogenesis	20	0.227876448	0.0115	0.013693853
GO:0048665	neuron fate specification	15	0.227627628	0.0292	0.03126539
GO:0099504	synaptic vesicle cycle	45	0.227599028	0.0027	0.004491543

GOIDlabel	GOName	size	categoryScores	pValPerm	pValPermCorr
GO:0003339	regulation of mesenchymal to epithelial transition involved in metanephros morphogenesis	5	0.227593308	0.0023	0.004032435
GO:1903959	regulation of anion transmembrane transport	9	0.227398827	0.0072	0.009362497
GO:0015872	dopamine transport	16	0.227348777	0.0049	0.006860215
GO:0032303	regulation of icosanoid secretion	10	0.227232947	0.0443	0.046018566
GO:0035725	sodium ion transmembrane transport	51	0.227112827	0.004	0.005880507
GO:0048566	embryonic digestive tract development	16	0.22701094	0.0186	0.020774238
GO:1900117	regulation of execution phase of apoptosis	18	0.226969827	0.0069	0.009046225
GO:0009713	catechol-containing compound biosynthetic process	11	0.226933427	< 0.001	< 0.001
GO:0042423	catecholamine biosynthetic process	11	0.226933427	< 0.001	< 0.001
GO:0048703	embryonic viscerocranium morphogenesis	5	0.226924067	0.001	0.002498236
GO:1903793	positive regulation of anion transport	23	0.226769627	0.0108	0.013015961
GO:0009204	deoxyribonucleoside triphosphate catabolic process	7	0.226732855	0.0044	0.006304227
GO:0046519	sphingoid metabolic process	12	0.226726727	0.048	0.049619465
GO:0015810	aspartate transport	6	0.226340626	0.0245	0.026594873
GO:0009249	protein lipoylation	6	0.226211926	0.0102	0.012424427
GO:0061615	glycolytic process through fructose-6-phosphate	18	0.226197626	0.0314	0.033430037
GO:0061620	glycolytic process through glucose-6-phosphate	18	0.226197626	0.0314	0.033430037
GO:0045078	positive regulation of interferon-gamma biosynthetic process	8	0.226190476	0.0167	0.018954426
GO:0035794	positive regulation of mitochondrial membrane permeability	37	0.226185259	0.0215	0.023664853
GO:1905710	positive regulation of membrane permeability	37	0.226185259	0.0215	0.023664853
GO:1902018	negative regulation of cilium assembly	7	0.226181283	0.0213	0.023473094
GO:0010172	embryonic body morphogenesis	8	0.226093951	0.0451	0.046826703
GO:0015682	ferric iron transport	17	0.225755167	0.0314	0.033430037
GO:0033572	transferrin transport	17	0.225755167	0.0314	0.033430037
GO:0072512	trivalent inorganic cation transport	17	0.225755167	0.0314	0.033430037
GO:2000270	negative regulation of fibroblast apoptotic process	7	0.225740026	0.0242	0.02630058
GO:1902915	negative regulation of protein polyubiquitination	6	0.225740026	0.019	0.02117646
GO:1900025	negative regulation of substrate adhesion-dependent cell spreading	7	0.225666483	0.0222	0.024321919
GO:0090649	response to oxygen-glucose deprivation	5	0.225585586	0.0482	0.049810054
GO:0090650	cellular response to oxygen-glucose deprivation	5	0.225585586	0.0482	0.049810054
GO:0007435	salivary gland morphogenesis	23	0.225482625	0.0097	0.011922488
GO:0031664	regulation of lipopolysaccharide-mediated signaling pathway	12	0.225482625	0.0059	0.007964563
GO:0010692	regulation of alkaline phosphatase activity	6	0.225439725	< 0.001	< 0.001
GO:0016079	synaptic vesicle exocytosis	26	0.225373725	0.0073	0.009463568
GO:1900029	positive regulation of ruffle assembly	8	0.22532175	0.0175	0.019742875
GO:0030799	regulation of cyclic nucleotide metabolic process	24	0.225289575	0.0199	0.022056122
GO:0030802	regulation of cyclic nucleotide biosynthetic process	24	0.225289575	0.0199	0.022056122
GO:0031279	regulation of cyclase activity	24	0.225289575	0.0199	0.022056122
GO:0021954	central nervous system neuron development	30	0.225268125	0.0011	0.002610912
GO:0007156	homophilic cell adhesion via plasma membrane adhesion molecules	41	0.225206391	0.0002	0.001341684
GO:0060347	heart trabecula formation	8	0.2251287	0.0006	0.002177563
GO:0006910	phagocytosis, recognition	7	0.225078139	0.0225	0.024620965
GO:0051057	positive regulation of small GTPase mediated signal transduction	26	0.224948025	0.0007	0.002248538
GO:0045745	positive regulation of G-protein coupled receptor protein signaling pathway	16	0.224903475	0.022	0.024152627
GO:0070169	positive regulation of biomineral tissue development	23	0.224777573	0.0003	0.001720882
GO:0015867	ATP transport	8	0.224646075	0.0357	0.037649528
GO:0045945	positive regulation of transcription from RNA polymerase III promoter	5	0.224555985	0.0009	0.002399874
GO:2000826	regulation of heart morphogenesis	24	0.224517375	0.0232	0.025330409
GO:0042572	retinol metabolic process	14	0.224397867	0.0059	0.007964563
GO:0046058	cAMP metabolic process	27	0.224367224	0.0146	0.016871405
GO:0035330	regulation of hippo signaling	9	0.224195624	0.0039	0.005778819
GO:0030195	negative regulation of blood coagulation	22	0.224102024	0.009	0.011215682
GO:0043954	cellular component maintenance	26	0.224047124	0.0107	0.012912536
GO:1902110	positive regulation of mitochondrial membrane permeability involved in apoptotic process	34	0.223945795	0.0223	0.024414688
GO:0007271	synaptic transmission, cholinergic	11	0.223891424	0.0001	0.000877824
GO:1904321	response to forskolin	6	0.223809524	0.0015	0.003088691
GO:1904322	cellular response to forskolin	6	0.223809524	0.0015	0.003088691
GO:0051349	positive regulation of lyase activity	19	0.223328592	0.0182	0.020388223
GO:0035082	axoneme assembly	24	0.223198198	< 0.001	< 0.001
GO:0002052	positive regulation of neuroblast proliferation	11	0.223166023	0.0007	0.002248538
GO:0009070	serine family amino acid biosynthetic process	11	0.223025623	0.0059	0.007964563
GO:1900271	regulation of long-term synaptic potentiation	18	0.222880023	0.0044	0.006304227
GO:0003341	cilium movement	27	0.222794223	< 0.001	< 0.001
GO:0046627	negative regulation of insulin receptor signaling pathway	15	0.222668383	0.0185	0.020677043
GO:0015012	heparan sulfate proteoglycan biosynthetic process	8	0.222490347	0.0387	0.04059837
GO:0007288	sperm axoneme assembly	6	0.222350922	< 0.001	< 0.001
GO:0051000	positive regulation of nitric-oxide synthase activity	10	0.222290862	0.0101	0.012328539
GO:0042745	circadian sleep/wake cycle	13	0.222255222	0.0018	0.003459409
GO:0006775	fat-soluble vitamin metabolic process	20	0.222123552	0.0188	0.020971889
GO:0019935	cyclic-nucleotide-mediated signaling	34	0.221954728	0.0038	0.005666214
GO:0021872	forebrain generation of neurons	28	0.221924986	0.0008	0.002313249
GO:0030836	positive regulation of actin filament depolymerization	8	0.221589447	0.0006	0.002177563
GO:0099601	regulation of neurotransmitter receptor activity	11	0.221528022	0.0053	0.007330056
GO:0099643	signal release from synapse	50	0.221487773	0.0024	0.004156304
GO:0001755	neural crest cell migration	17	0.221379363	0.0109	0.013101792
GO:0051204	protein insertion into mitochondrial membrane	17	0.221303657	0.0188	0.020971889
GO:0009146	purine nucleoside triphosphate catabolic process	6	0.221278421	0.0037	0.005561344
GO:0042723	thiamine-containing compound metabolic process	5	0.221158301	0.0033	0.005149584
GO:0042474	middle ear morphogenesis	10	0.221003861	0.017	0.019243517
GO:0021521	ventral spinal cord interneuron specification	8	0.220978121	0.0288	0.030847462
GO:0060573	cell fate specification involved in pattern specification	8	0.220978121	0.0288	0.030847462
GO:0060445	branching involved in salivary gland morphogenesis	14	0.220849421	0.0395	0.041342339
GO:0021871	forebrain regionalization	9	0.220849421	< 0.001	< 0.001

GOIDlabel	GOName	size	categoryScores	pValPerm	pValPermCorr
GO:0046415	urate metabolic process	8	0.220785071	0.0087	0.010877987
GO:0018410	C-terminal protein amino acid modification	7	0.220702335	0.0002	0.001341684
GO:0071600	otic vesicle morphogenesis	6	0.220634921	0.0092	0.011424708
GO:0010265	SCF complex assembly	5	0.2203861	0.0418	0.043620665
GO:0050819	negative regulation of coagulation	24	0.22035607	0.0124	0.014577606
GO:0010092	specification of animal organ identity	6	0.22033462	0.0015	0.003088691
GO:0001973	adenosine receptor signaling pathway	5	0.22028314	0.0192	0.021362011
GO:0031987	locomotion involved in locomotory behavior	9	0.22013442	0.0014	0.002975058
GO:0006560	proline metabolic process	6	0.22012012	0.0054	0.007432873
GO:0051956	negative regulation of amino acid transport	5	0.21992278	0.0077	0.009887588
GO:0099565	chemical synaptic transmission, postsynaptic	26	0.21991882	0.0006	0.002177563
GO:0051443	positive regulation of ubiquitin-protein transferase activity	14	0.219838206	0.0052	0.007215241
GO:0051458	corticotropin secretion	5	0.21981982	0.0043	0.006204188
GO:0051459	regulation of corticotropin secretion	5	0.21981982	0.0043	0.006204188
GO:0048865	stem cell fate commitment	5	0.21966538	0.0061	0.008182551
GO:0070544	histone H3-K36 demethylation	6	0.21951952	0.0258	0.02789201
GO:0045725	positive regulation of glycogen biosynthetic process	9	0.21950522	0.0002	0.001341684
GO:0051932	synaptic transmission, GABAergic	15	0.219476619	0.0009	0.002399874
GO:0050906	detection of stimulus involved in sensory perception	70	0.219444751	0.0432	0.044963841
GO:0072109	glomerular mesangium development	9	0.219390819	0.0209	0.023072181
GO:0043243	positive regulation of protein complex disassembly	19	0.219345661	0.008	0.010168329
GO:0009395	phospholipid catabolic process	19	0.219305019	0.0408	0.042653937
GO:0010800	positive regulation of peptidyl-threonine phosphorylation	10	0.219021879	0.0006	0.002177563
GO:0048387	negative regulation of retinoic acid receptor signaling pathway	6	0.218833119	0.0176	0.019838132
GO:0075713	establishment of integrated proviral latency	6	0.218532819	0.0054	0.007432873
GO:0021884	forebrain neuron development	13	0.218473418	0.003	0.004825593
GO:0036465	synaptic vesicle recycling	14	0.218459276	0.0193	0.021465777
GO:0033127	regulation of histone phosphorylation	6	0.218404118	0.0111	0.013284563
GO:0006735	NADH regeneration	17	0.218366265	0.0351	0.037053553
GO:0061621	canonical glycolysis	17	0.218366265	0.0351	0.037053553
GO:0061718	glucose catabolic process to pyruvate	17	0.218366265	0.0351	0.037053553
GO:0007622	rhythmic behavior	21	0.217932218	0.0041	0.005981983
GO:0048512	circadian behavior	21	0.217932218	0.0041	0.005981983
GO:1904263	positive regulation of TORC1 signaling	7	0.217907704	0.0106	0.012825859
GO:0007269	neurotransmitter secretion	49	0.217870932	0.0029	0.004714719
GO:1901028	regulation of mitochondrial outer membrane permeabilization involved in apoptotic signaling pathway	27	0.217722484	0.0237	0.025818821
GO:0060192	negative regulation of lipase activity	8	0.217631918	0.0017	0.003334595
GO:0033189	response to vitamin A	6	0.217546118	0.0001	0.000877824
GO:0071071	regulation of phospholipid biosynthetic process	7	0.217172274	0.0019	0.003579279
GO:2000402	negative regulation of lymphocyte migration	7	0.216988417	0.0043	0.006204188
GO:0046834	lipid phosphorylation	18	0.216931217	0.0173	0.019544921
GO:0098742	cell-cell adhesion via plasma-membrane adhesion molecules	84	0.216896488	0.0003	0.001720882
GO:0043249	erythrocyte maturation	8	0.216827542	0.0029	0.004714719
GO:0042095	interferon-gamma biosynthetic process	9	0.216731017	0.0095	0.011730963
GO:0045072	regulation of interferon-gamma biosynthetic process	9	0.216731017	0.0095	0.011730963
GO:0090045	positive regulation of deacetylase activity	5	0.216628057	0.0254	0.027496891
GO:0016114	terpenoid biosynthetic process	8	0.216441441	0.0149	0.017165166
GO:0051683	establishment of Golgi localization	5	0.216216216	0.0359	0.037847924
GO:0006734	NADH metabolic process	20	0.216126126	0.0392	0.041055316
GO:0008300	isoprenoid catabolic process	5	0.216061776	0.038	0.03991002
GO:0006836	neurotransmitter transport	72	0.216030316	0.001	0.002498236
GO:0035116	embryonic hindlimb morphogenesis	10	0.215933076	0.0078	0.009981807
GO:0007187	G-protein coupled receptor signaling pathway, coupled to cyclic nucleotide second messenger	69	0.215899129	0.0016	0.003223775
GO:0009593	detection of chemical stimulus	65	0.215832096	0.0471	0.048744446
GO:0035331	negative regulation of hippo signaling	6	0.215830116	0.0025	0.004279479
GO:0009954	proximal/distal pattern formation	9	0.215587016	0.0004	0.002007244
GO:1901616	organic hydroxy compound catabolic process	24	0.215551266	0.0273	0.029359247
GO:0060039	pericardium development	10	0.215546976	0.0261	0.028197203
GO:0001547	antral ovarian follicle growth	6	0.215529816	< 0.001	< 0.001
GO:1900017	positive regulation of cytokine production involved in inflammatory response	11	0.215514216	0.0102	0.012424427
GO:0034139	regulation of toll-like receptor 3 signaling pathway	6	0.215444015	0.0286	0.030648697
GO:1904778	positive regulation of protein localization to cell cortex	5	0.215289575	0.0316	0.033614889
GO:0071313	cellular response to caffeine	5	0.215289532	0.0461	0.047779362
GO:0060079	excitatory postsynaptic potential	25	0.215258687	0.0002	0.001341684
GO:0034114	regulation of heterotypic cell-cell adhesion	11	0.215186615	0.0064	0.008500875
GO:0032509	endosome transport via multivesicular body sorting pathway	11	0.215186615	0.0072	0.009362497
GO:0003376	sphingosine-1-phosphate signaling pathway	7	0.215113072	0.0014	0.002975058
GO:0030261	chromosome condensation	18	0.215043615	0.0074	0.009581512
GO:0099531	presynaptic process involved in chemical synaptic transmission	53	0.214511547	0.002	0.003693422
GO:1901096	regulation of autophagosome maturation	8	0.214060489	0.0437	0.045424906
GO:0021879	forebrain neuron differentiation	24	0.213610039	0.0008	0.002313249
GO:0008356	asymmetric cell division	8	0.213416988	0.0001	0.000877824
GO:0001508	action potential	47	0.212886443	0.0189	0.021076063
GO:0051895	negative regulation of focal adhesion assembly	13	0.212870013	0.0297	0.0317634
GO:0034115	negative regulation of heterotypic cell-cell adhesion	5	0.212715573	0.0097	0.011922488
GO:0070587	regulation of cell-cell adhesion involved in gastrulation	5	0.212715573	0.0097	0.011922488
GO:0031652	positive regulation of heat generation	6	0.212655513	0.015	0.017255415
GO:0046886	positive regulation of hormone biosynthetic process	6	0.212612613	0.0143	0.016557758
GO:0007188	adenylate cyclase-modulating G-protein coupled receptor signaling pathway	60	0.212522523	0.0014	0.002975058
GO:0021514	ventral spinal cord interneuron differentiation	10	0.211788932	0.0471	0.048744446
GO:0060579	ventral spinal cord interneuron fate commitment	10	0.211788932	0.0471	0.048744446
GO:0060581	cell fate commitment involved in pattern specification	10	0.211788932	0.0471	0.048744446
GO:0010896	regulation of triglyceride catabolic process	5	0.211788932	0.0048	0.006748379

GOIDlabel	GOName	size	categoryScores	pValPerm	pValPermCorr
GO:0090335	regulation of brown fat cell differentiation	9	0.211668812	0.0233	0.025417819
GO:0032332	positive regulation of chondrocyte differentiation	9	0.211554412	0.0017	0.003334595
GO:0090154	positive regulation of sphingolipid biosynthetic process	7	0.211472697	0.0156	0.017858595
GO:2000304	positive regulation of ceramide biosynthetic process	7	0.211472697	0.0156	0.017858595
GO:0048520	positive regulation of behavior	11	0.211419211	0.0004	0.002007244
GO:0046189	phenol-containing compound biosynthetic process	22	0.211395811	< 0.001	< 0.001
GO:0022615	protein to membrane docking	5	0.211274131	0.0181	0.020286898
GO:0006582	melanin metabolic process	12	0.211089661	0.0001	0.000877824
GO:0060452	positive regulation of cardiac muscle contraction	6	0.211025311	0.0255	0.027600459
GO:0060294	cilium movement involved in cell motility	7	0.210663725	< 0.001	< 0.001
GO:0001660	fever generation	5	0.210656371	0.0124	0.014577606
GO:0060042	retina morphogenesis in camera-type eye	25	0.210471042	0.0223	0.024414688
GO:0034720	histone H3-K4 demethylation	7	0.210406325	0.0205	0.022658082
GO:0070286	axonemal dynein complex assembly	13	0.21031581	0.0015	0.003088691
GO:0046487	glyoxylate metabolic process	6	0.20982411	0.0366	0.038528543
GO:0007270	neuron-neuron synaptic transmission	38	0.209821852	0.0011	0.002610912
GO:0061101	neuroendocrine cell differentiation	7	0.209707667	0.0178	0.020021073
GO:0048385	regulation of retinoic acid receptor signaling pathway	12	0.209459459	0.0114	0.01358747
GO:1900273	positive regulation of long-term synaptic potentiation	7	0.209413495	0.043	0.044777614
GO:0001844	protein insertion into mitochondrial membrane involved in apoptotic signaling pathway	16	0.209411197	0.0282	0.030276044
GO:0046469	platelet activating factor metabolic process	6	0.209352209	0.0286	0.030648697
GO:1903792	negative regulation of anion transport	11	0.209289809	0.02	0.022155397
GO:0050873	brown fat cell differentiation	19	0.208832893	0.0113	0.013488462
GO:0032148	activation of protein kinase B activity	14	0.20865968	0.0094	0.011616482
GO:0097062	dendritic spine maintenance	9	0.208608609	0.0316	0.033614889
GO:1902510	regulation of apoptotic DNA fragmentation	8	0.208494208	0.0271	0.029163847
GO:0021801	cerebral cortex radial glia guided migration	7	0.208200037	0.0071	0.009268394
GO:0022030	telencephalon glial cell migration	7	0.208200037	0.0071	0.009268394
GO:0003209	cardiac atrium morphogenesis	17	0.208176243	0.0027	0.004491543
GO:0007193	adenylate cyclase-inhibiting G-protein coupled receptor signaling pathway	30	0.207876448	0.0005	0.002177563
GO:0042573	retinoic acid metabolic process	10	0.207696268	0.0106	0.012825859
GO:0021854	hypothalamus development	10	0.207670528	0.0151	0.01736105
GO:0022410	circadian sleep/wake cycle process	11	0.207511408	0.0016	0.003223775
GO:0050802	circadian sleep/wake cycle, sleep	11	0.207511408	0.0016	0.003223775
GO:1901725	regulation of histone deacetylase activity	6	0.207507508	0.016	0.018267288
GO:2001269	positive regulation of cysteine-type endopeptidase activity involved in apoptotic signaling pathway	6	0.207121407	0.0374	0.039305772
GO:0032770	positive regulation of monooxygenase activity	14	0.207004964	0.0067	0.008845888
GO:0098914	membrane repolarization during atrial cardiac muscle cell action potential	5	0.207001287	0.0112	0.013379119
GO:0070875	positive regulation of glycogen metabolic process	10	0.206924067	0.0009	0.002399874
GO:2000191	regulation of fatty acid transport	13	0.206692407	0.0283	0.030373173
GO:1900449	regulation of glutamate receptor signaling pathway	10	0.206280566	0.0047	0.006644432
GO:0023019	signal transduction involved in regulation of gene expression	9	0.205834406	0.0007	0.002248538
GO:0051482	positive regulation of cytosolic calcium ion concentration involved in phospholipase C-activating G-protein coupled signaling pathway	9	0.205691406	0.0351	0.037053553
GO:0060078	regulation of postsynaptic membrane potential	28	0.20558007	0.0018	0.003459409
GO:0071379	cellular response to prostaglandin stimulus	11	0.205428805	0.0036	0.005475609
GO:1900045	negative regulation of protein K63-linked ubiquitination	5	0.204839125	0.0241	0.026209778
GO:0044550	secondary metabolite biosynthetic process	13	0.204831205	0.0002	0.001341684
GO:0048745	smooth muscle tissue development	11	0.204773605	0.0173	0.019544921
GO:0033561	regulation of water loss via skin	7	0.204081633	0.0147	0.016962357
GO:0090656	t-circle formation	9	0.203517804	0.005	0.006974174
GO:0098885	modification of postsynaptic actin cytoskeleton	5	0.203500644	0.0338	0.035793852
GO:0033598	mammary gland epithelial cell proliferation	13	0.203366003	0.0009	0.002399874
GO:0048021	regulation of melanin biosynthetic process	5	0.203243243	0.0035	0.005358035
GO:1900376	regulation of secondary metabolite biosynthetic process	5	0.203243243	0.0035	0.005358035
GO:0014821	phasic smooth muscle contraction	8	0.203120978	0.0324	0.034402732
GO:1903818	positive regulation of voltage-gated potassium channel activity	6	0.203003003	0.0313	0.033357007
GO:0002739	regulation of cytokine secretion involved in immune response	6	0.202831403	0.0024	0.004156304
GO:0006105	succinate metabolic process	7	0.202390145	0.0316	0.033614889
GO:0050775	positive regulation of dendrite morphogenesis	15	0.202230802	0.0206	0.022756769
GO:0061303	cornea development in camera-type eye	5	0.201956242	0.0177	0.019919142
GO:0070268	cornification	30	0.201818962	0.0453	0.047003728
GO:0046928	regulation of neurotransmitter secretion	30	0.201801802	0.0107	0.012912536
GO:1904668	positive regulation of ubiquitin protein ligase activity	7	0.20150763	0.0185	0.020677043
GO:0035025	positive regulation of Rho protein signal transduction	9	0.201258401	0.0019	0.003579279
GO:0007215	glutamate receptor signaling pathway	18	0.201201201	0.0073	0.009463568
GO:0042737	drug catabolic process	6	0.201115401	0.0001	0.000877824
GO:0007185	transmembrane receptor protein tyrosine phosphatase signaling pathway	5	0.201029601	0.0017	0.003334595
GO:0014046	dopamine secretion	10	0.200900901	0.0048	0.006748379
GO:0014059	regulation of dopamine secretion	10	0.200900901	0.0048	0.006748379
GO:0032736	positive regulation of interleukin-13 production	6	0.200429	0.002	0.003693422
GO:0032530	regulation of microvillus organization	5	0.20030888	0.0124	0.014577606
GO:0051593	response to folic acid	8	0.2002574	0.0382	0.040087041
GO:0001188	RNA polymerase I transcriptional preinitiation complex assembly	7	0.200220629	0.0048	0.006748379
GO:0021756	striatum development	10	0.2	0.0107	0.012912536
GO:0072697	protein localization to cell cortex	8	0.199517375	0.0236	0.025731873
GO:1902414	protein localization to cell junction	6	0.199356499	0.0192	0.021362011
GO:0090520	sphingolipid mediated signaling pathway	8	0.199292149	0.0087	0.010877987
GO:0034638	phosphatidylcholine catabolic process	5	0.198661519	0.018	0.020203241
GO:0090494	dopamine uptake	7	0.198124655	0.002	0.003693422
GO:0035115	embryonic forelimb morphogenesis	13	0.198039798	0.0072	0.009362497
GO:0035136	forelimb morphogenesis	13	0.198039798	0.0072	0.009362497
GO:0003148	outflow tract septum morphogenesis	14	0.198014341	0.0268	0.028880014

GOIDlabel	GOName	size	categoryScores	pValPerm	pValPermCorr
GO:0003407	neural retina development	30	0.197803518	0.0007	0.002248538
GO:0048488	synaptic vesicle endocytosis	10	0.197760618	0.0094	0.011616482
GO:0070307	lens fiber cell development	5	0.197477477	0.0285	0.030556948
GO:0030213	hyaluronan biosynthetic process	6	0.197425997	0.0278	0.029876796
GO:0015800	acidic amino acid transport	16	0.197265122	0.0084	0.010571327
GO:0008038	neuron recognition	12	0.197147147	0.0188	0.020971889
GO:0072079	nephron tubule formation	8	0.196943372	0.0022	0.003910907
GO:0010766	negative regulation of sodium ion transport	9	0.196911197	0.0064	0.008500875
GO:0033599	regulation of mammary gland epithelial cell proliferation	10	0.196833977	0.0011	0.002610912
GO:0001504	neurotransmitter uptake	17	0.196608373	0.0005	0.002177563
GO:0035376	sterol import	5	0.195881596	0.0333	0.035317174
GO:0070508	cholesterol import	5	0.195881596	0.0333	0.035317174
GO:0042438	melanin biosynthetic process	11	0.195858196	0.0001	0.000877824
GO:0032802	low-density lipoprotein particle receptor catabolic process	6	0.195624196	0.0111	0.013284563
GO:0003149	membranous septum morphogenesis	5	0.195469755	0.002	0.003693422
GO:0061436	establishment of skin barrier	6	0.194851995	0.0246	0.026698876
GO:1901978	positive regulation of cell cycle checkpoint	5	0.194285714	0.0006	0.002177563
GO:0019695	choline metabolic process	8	0.193983269	0.0269	0.028963288
GO:0030810	positive regulation of nucleotide biosynthetic process	23	0.193822394	0.0221	0.024229021
GO:1900373	positive regulation of purine nucleotide biosynthetic process	23	0.193822394	0.0221	0.024229021
GO:1904776	regulation of protein localization to cell cortex	6	0.193693694	0.047	0.048672571
GO:0051590	positive regulation of neurotransmitter transport	6	0.193350493	< 0.001	< 0.001
GO:0051588	regulation of neurotransmitter transport	36	0.193157443	0.0064	0.008500875
GO:0002374	cytokine secretion involved in immune response	8	0.193146718	0.0008	0.002313249
GO:0000212	meiotic spindle organization	8	0.192792793	0.0332	0.035228705
GO:0033623	regulation of integrin activation	8	0.192631918	0.001	0.002498236
GO:0007606	sensory perception of chemical stimulus	73	0.192059378	0.0467	0.048369755
GO:1901030	positive regulation of mitochondrial outer membrane permeabilization involved in apoptotic signaling pathway	20	0.191994852	0.0339	0.035893786
GO:0098810	neurotransmitter reuptake	12	0.191848992	0.0005	0.002177563
GO:0010985	negative regulation of lipoprotein particle clearance	5	0.191814672	0.0144	0.016652368
GO:0002138	retinoic acid biosynthetic process	6	0.191205491	0.0366	0.038528543
GO:0016102	diterpenoid biosynthetic process	6	0.191205491	0.0366	0.038528543
GO:0007189	adenylate cyclase-activating G-protein coupled receptor signaling pathway	29	0.190999867	0.0237	0.025818821
GO:0043949	regulation of cAMP-mediated signaling	15	0.190870871	0.0249	0.02700148
GO:0060215	primitive hemopoiesis	5	0.190682111	0.034	0.035993688
GO:0001964	startle response	11	0.18996139	0.0023	0.004032435
GO:0015813	L-glutamate transport	11	0.188604189	0.001	0.002498236
GO:0038042	dimeric G-protein coupled receptor signaling pathway	6	0.188459888	0.0247	0.026793719
GO:0097646	calcitonin family receptor signaling pathway	6	0.188459888	0.0247	0.026793719
GO:0071377	cellular response to glucagon stimulus	7	0.188380217	0.0021	0.003804235
GO:0009820	alkaloid metabolic process	6	0.188331188	0.0043	0.006204188
GO:0044793	negative regulation by host of viral process	5	0.188211068	0.0037	0.005561344
GO:2000738	positive regulation of stem cell differentiation	14	0.188067659	0.0068	0.008946408
GO:0070586	cell-cell adhesion involved in gastrulation	6	0.188030888	0.0139	0.016141527
GO:2000310	regulation of N-methyl-D-aspartate selective glutamate receptor activity	8	0.187548263	0.0015	0.003088691
GO:0043455	regulation of secondary metabolic process	6	0.187044187	0.0111	0.013284563
GO:0099558	maintenance of synapse structure	6	0.186958387	0.0058	0.007856196
GO:1900739	regulation of protein insertion into mitochondrial membrane involved in apoptotic signaling pathway	13	0.186298386	0.0317	0.033710012
GO:1900740	positive regulation of protein insertion into mitochondrial membrane involved in apoptotic signaling pathway	13	0.186298386	0.0317	0.033710012
GO:0036159	inner dynein arm assembly	10	0.186280566	0.0084	0.010571327
GO:0071380	cellular response to prostaglandin E stimulus	9	0.186214786	0.0062	0.008281828
GO:0042749	regulation of circadian sleep/wake cycle	10	0.185456885	0.0031	0.004916949
GO:0045187	regulation of circadian sleep/wake cycle, sleep	10	0.185456885	0.0031	0.004916949
GO:0002551	mast cell chemotaxis	5	0.185379665	0.0483	0.049905302
GO:0003357	noradrenergic neuron differentiation	6	0.185242385	0.008	0.010168329
GO:0051953	negative regulation of amine transport	11	0.185117585	0.0007	0.002248538
GO:0032891	negative regulation of organic acid transport	10	0.184581725	0.0367	0.038621054
GO:0034199	activation of protein kinase A activity	7	0.184482442	0.0036	0.005475609
GO:0043951	negative regulation of cAMP-mediated signaling	7	0.184261813	0.0071	0.009268394
GO:0061003	positive regulation of dendritic spine morphogenesis	6	0.184084084	0.0033	0.005149584
GO:0003207	cardiac chamber formation	9	0.183926784	0.0048	0.006748379
GO:0060004	reflex	10	0.183603604	0.0136	0.015813319
GO:0007616	long-term memory	13	0.183150183	0.0156	0.017858595
GO:0090493	catecholamine uptake	8	0.182046332	0.0004	0.002007244
GO:0033604	negative regulation of catecholamine secretion	7	0.181871668	0.0005	0.002177563
GO:0042753	positive regulation of circadian rhythm	7	0.181724582	0.0083	0.010468217
GO:0007028	cytoplasm organization	5	0.181621622	0.0003	0.001720882
GO:0060219	camera-type eye photoreceptor cell differentiation	7	0.180952381	0.0161	0.018371585
GO:1904396	regulation of neuromuscular junction development	6	0.180952381	0.0012	0.002741075
GO:0060438	trachea development	8	0.180662806	0.0078	0.009981807
GO:0001574	ganglioside biosynthetic process	5	0.180540541	0.0373	0.039220079
GO:0044068	modulation by symbiont of host cellular process	18	0.18012298	0.0325	0.034497418
GO:0060134	prepulse inhibition	6	0.18000858	0.0132	0.015415723
GO:0007413	axonal fasciculation	8	0.17960103	0.0029	0.004714719
GO:2000665	regulation of interleukin-13 secretion	5	0.179047619	0.0238	0.025923329
GO:0021978	telencephalon regionalization	6	0.178592879	0.0009	0.002399874
GO:0044241	lipid digestion	5	0.178120978	0.0026	0.004377754
GO:0010763	positive regulation of fibroblast migration	5	0.177863578	0.0461	0.047779362
GO:0019933	cAMP-mediated signaling	26	0.177833878	0.0245	0.026594873
GO:0060850	regulation of transcription involved in cell fate commitment	11	0.177278577	0.0264	0.028497154
GO:0061548	ganglion development	6	0.176490776	0.0002	0.001341684

GOIDlabel	GOName	size	categoryScores	pValPerm	pValPermCorr
GO:0051583	dopamine uptake involved in synaptic transmission	5	0.176061776	0.0116	0.013792313
GO:0051934	catecholamine uptake involved in synaptic transmission	5	0.176061776	0.0116	0.013792313
GO:0099054	presynapse assembly	10	0.175289575	0.0067	0.008845888
GO:1902306	negative regulation of sodium ion transmembrane transport	8	0.174967825	0.0086	0.010769857
GO:0001963	synaptic transmission, dopaminergic	9	0.172200772	0.0016	0.003223775
GO:0035926	chemokine (C-C motif) ligand 2 secretion	5	0.16993565	0.0064	0.008500875
GO:0006702	androgen biosynthetic process	6	0.169369369	0.0307	0.03276689
GO:0030816	positive regulation of cAMP metabolic process	12	0.169283569	0.0478	0.049420733
GO:0030819	positive regulation of cAMP biosynthetic process	12	0.169283569	0.0478	0.049420733
GO:0045762	positive regulation of adenylate cyclase activity	12	0.169283569	0.0478	0.049420733
GO:0021895	cerebral cortex neuron differentiation	9	0.168911769	0.0013	0.002859821
GO:0032656	regulation of interleukin-13 production	10	0.167078507	0.0308	0.032862615
GO:0072611	interleukin-13 secretion	6	0.166108966	0.0417	0.043530565
GO:0010918	positive regulation of mitochondrial membrane potential	5	0.163346203	0.0463	0.047971045
GO:0042730	fibrinolysis	10	0.163088803	0.0104	0.012619802
GO:0099560	synaptic membrane adhesion	7	0.162934363	0.0307	0.03276689
GO:0045760	positive regulation of action potential	5	0.162213642	0.012	0.014185865
GO:1905606	regulation of presynapse assembly	9	0.161189761	0.0311	0.033166048
GO:0032616	interleukin-13 production	11	0.161109161	0.0463	0.047971045
GO:0031665	negative regulation of lipopolysaccharide-mediated signaling pathway	6	0.16027456	0.0257	0.027793331
GO:0072017	distal tubule development	5	0.16015444	0.0336	0.03560572
GO:0021544	subpallium development	13	0.15974656	0.0054	0.007432873
GO:0021778	oligodendrocyte cell fate specification	5	0.158712999	0.0111	0.013284563
GO:0021779	oligodendrocyte cell fate commitment	5	0.158712999	0.0111	0.013284563
GO:0021780	glial cell fate specification	5	0.158712999	0.0111	0.013284563
GO:0042748	circadian sleep/wake cycle, non-REM sleep	5	0.157683398	0.0003	0.001720882
GO:0045188	regulation of circadian sleep/wake cycle, non-REM sleep	5	0.157683398	0.0003	0.001720882
GO:0019054	modulation by virus of host process	14	0.157676043	0.0423	0.044077485
GO:0051580	regulation of neurotransmitter uptake	7	0.155212355	0.0335	0.03551156
GO:0032754	positive regulation of interleukin-5 production	5	0.152535393	0.002	0.003693422
GO:0007190	activation of adenylate cyclase activity	9	0.149292149	0.0436	0.045335748
GO:0071875	adrenergic receptor signaling pathway	6	0.148691549	0.0055	0.007544447
GO:0042426	choline catabolic process	5	0.147490347	0.0305	0.032586169
GO:2000650	negative regulation of sodium ion transmembrane transporter activity	7	0.142820371	0.018	0.020203241
GO:0048485	sympathetic nervous system development	10	0.136988417	0.0401	0.041935888
GO:0021772	olfactory bulb development	12	0.134813385	0.0031	0.004916949
GO:0021988	olfactory lobe development	12	0.134813385	0.0031	0.004916949
GO:0032225	regulation of synaptic transmission, dopaminergic	6	0.133976834	0.0373	0.039220079
GO:0021889	olfactory bulb interneuron differentiation	5	0.116653797	0.0185	0.020677043
GO:0051967	negative regulation of synaptic transmission, glutamatergic	5	0.10028314	0.0452	0.046915244
GO:0097154	GABAergic neuron differentiation	7	0.097481155	0.0278	0.029876796
GO:0021892	cerebral cortex GABAergic interneuron differentiation	6	0.09021879	0.0293	0.031367193

Reward COR positive

GOIDlabel	GOName	GOID	size	categoryScores	pValZ	pValZCorr	pValPerm	pValPermCorr
GO:0051823	regulation of synapse structural plasticity	51823	6	0.541184041	1.55326E-09	6.88247E-06	< 0.001	< 0.001
GO:0099563	modification of synaptic structure	99563	11	0.441909442	2.65255E-07	0.000587672	< 0.001	< 0.001
GO:0098885	modification of postsynaptic actin cytoskeleton	98885	7	0.396102225	4.08628E-06	0.003668926	< 0.001	< 0.001
